# Comparative transcriptomic analysis of rabbit interscapular brown adipose tissue whitening under physiological conditions

**DOI:** 10.1080/21623945.2022.2111053

**Published:** 2022-08-29

**Authors:** Lei Li, Qian Wan, Qiaoyun Long, Tao Nie, Shiting Zhao, Liufeng Mao, Chuanli Cheng, Chao Zou, Kerry Loomes, Aimin Xu, Liangxue Lai, Xin Liu, Ziyuan Duan, Xiaoyan Hui, Donghai Wu

**Affiliations:** a Key Laboratory of Regenerative Biology, Guangdong Provincial Key Laboratory of Stem Cell and Regenerative Medicine, Guangzhou Institutes of Biomedicine and Health, Chinese Academy of Sciences, Guangzhou, China; b University of Chinese Academy of Sciences, Beijing, China; c Paul C. Lauterbur Research Center for Biomedical Imaging, Shenzhen Institutes of Advanced Technology, Chinese Academy of Sciences, Shenzhen, China; d School of Biomedical Sciences, the Chinese University of Hong Kong, Hong Kong SAR; e China-New Zealand Joint Laboratory on Biomedicine and Health, Chinese Academy of Sciences, Guangzhou, China; f Clinical Department of Guangdong Metabolic Disease Research Center of Integrated Chinese and Western Medicine, the First Affiliated Hospital of Guangdong Pharmaceutical University, Guangzhou, China; g School of Biological Sciences and Maurice Wilkins Centre, University of Auckland, New Zealand; h Department of Medicine, University of Hong Kong, Hong Kong SAR

**Keywords:** Rabbit, iBAT, MRI, whitening, transcriptomic analysis, immune cells

## Abstract

Interscapular brown adipose tissue (iBAT) of both rabbits and humans exhibits a similar whitening phenomenon under physiological conditions. However, a detailed characterization of iBAT whitening in them is still lacking. Here, we chose rabbits as a model to gain a better understanding of the molecular signature changes during the whitening process of iBAT by transcriptomic analysis of rabbit iBAT at day 1, day 14, 1 month and 4 months after birth. We applied non-invasive MRI imaging to monitor the whitening process and correlated these changes with analysis of morphological, histological and molecular features. Principal component analysis (PCA) of differentially expressed genes delineated three major phases for the whitening process as Brown, Transition and Whitened BAT phases. RNA-sequencing data revealed that whitening of iBAT was an orchestrated process where multiple types of cells and tissues participated in a variety of physiological processes including neovascularization, formation of new nervous networks and immune regulation. Several key metabolic and signalling pathways contributed to whitening of iBAT, and immune cells and immune regulation appeared to play an overarching role.

## Introduction

Obesity is a global pandemic and is associated with many metabolic diseases, such as type 2 diabetes, atherosclerosis, cardiovascular diseases, hypertension, fatty liver and some cancers [1]. Notably, China may already have the most obese people in the world according to Chinese criteria [2]. In order to improve quality of life and relieve medical discomfort as well as economic burden, effective ways to prevent and combat obesity for the healthy and sustainable development of human beings are urgently needed.

Activation of brown adipose tissue (BAT) and/or recruitment of beige tissue have been considered as a promising therapeutic strategy to counteract obesity and type 2 diabetes in recent years [3–5]. There is general consensus that two major adipose tissues exist in mammals: BAT and white adipose tissue (WAT). WAT is a major lipid reservoir for excess energy in the form of triglycerides and also acts as an endocrine organ. Beige adipose tissue has been recognized recently as being derived from WAT but behaves metabolically like BAT. Functionally, BAT and beige adipose tissue differ from WAT due to their capacity for uncoupling mitochondrial ATP synthesis from the electron gradient potential to generate heat through the mitochondrial inner membrane protein, uncoupling protein 1 (UCP1) [6]. This process occurs in non-shivering thermogenesis (NST) and is believed to be important for the maintenance of normal body temperature in mammals, such as rodents, hibernating animals and newborn infants [7,8]. Unlike rodents where BAT is present functionally throughout their lifespan, human beings and other animals such as ruminants and rabbits [9–11] are believed to possess functional BAT for only a short period of time after birth whereafter it is replaced by WAT [12]. Thus, historically it has been widely accepted that adults possess little or no metabolically active BAT. However, metabolically active BAT in adult humans was identified in 2009 by positron emission tomography/computed tomography with the glucose analog, F18-fluorodeoxyglucose (FDG-PET/CT) [13–15]. These findings reignited interest in brown fat biology and propelled the idea that BAT may be a promising target to augment energy expenditure and reduce obesity.

Adult human BAT is mainly present in the cervical, supraclavicular, and paravertebral areas, and can be activated by cold exposure [13–16]. Decreased BAT content is associated with accumulation of body fat with age [17]. BAT can also be recruited through repeated stimulation by cold exposure or capsinoid intake, leading to decreased total body fat content in the absence of body weight change [16].

In human infancy, BAT exists principally in the interscapular area, namely iBAT [8,18]. iBAT content is persistent within the first decade but gradually dissipates where it is barely detectable between 30 and 80 years of age [19]. Instead, a fat pad with a phenotype similar to WAT emerges to take its place [20], suggesting that brown adipocytes within iBAT change into white adipocytes with age in humans. A similar whitening phenomenon occurs in rabbits where iBAT is developmentally reprogrammed to a ‘WAT-like’ phenotype after birth [12,21]. However, a detailed molecular description together with a non-invasive live imaging system to monitor the dynamic changes during the process of iBAT whitening is lacking.

Here, we utilized a rabbit animal model, magnetic resonance imaging (MRI) technology, histology and whole-genome RNA-Seq analysis to correlate MRI data with histological and molecular changes to gain insights into the process of iBAT whitening.

## Results

### BAT converts to white-like adipose tissue in rabbits

**Figure 1.**
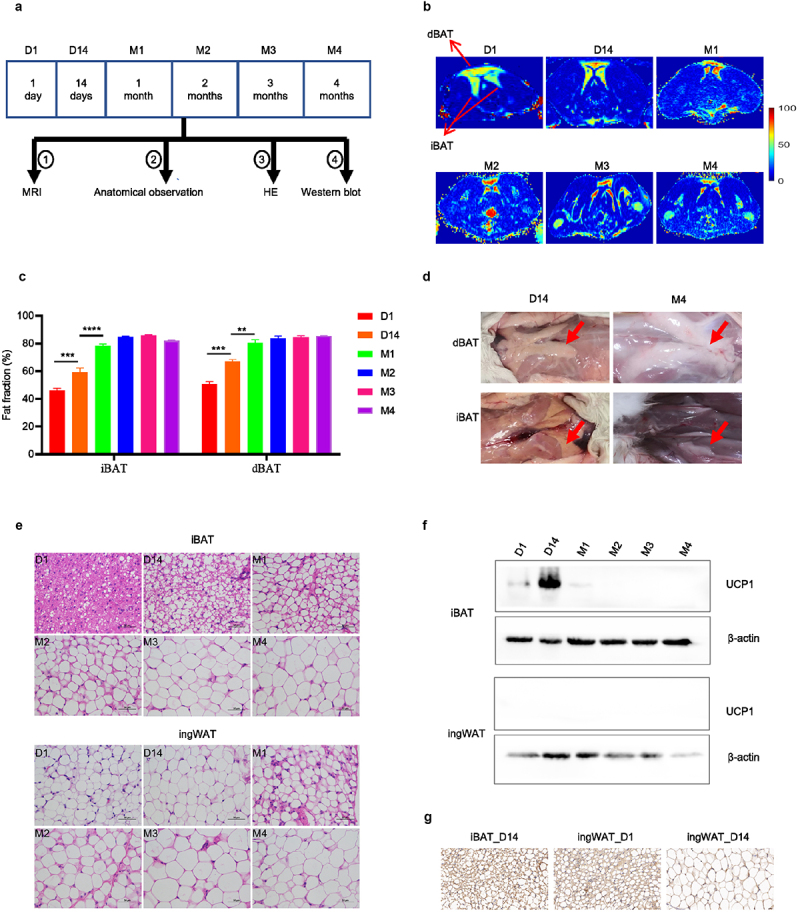
**BAT converts to white-like adipose tissue of rabbits following birth**. (a) Workflow for validation of BAT whitening in rabbits. (b) MRI images of rabbits iBAT and dBAT at different ages. (c) Fat fraction of rabbit iBAT and dBAT. (d) Anatomical images of rabbit dBAT and iBAT at 14 days and 4 months of age. (e) H&E staining results of rabbit iBAT and ingWAT at different ages. Scale bars, 50 µm. (f) UCP1 protein levels of rabbit iBAT and ingWAT at different ages. (g) UCP1 staining of iBAT_D14, ingWAT_D1 and ingWAT_D14 of rabbits. Scale bars, 20 µm. Data represent mean ± SEM, **P < 0.01, ***P < 0.001, ****P < 0.0001.

To gain a better understanding of the whitening process of BAT, we systematically and non-invasively examined the developmental changes of adipose tissues in rabbits using MRI. Three rabbits for each group at 1 day, 14 days, 1 month, 2 months, 3 months and 4 months after birth were selected (Figure 1a). The fat fraction of interscapular, dorsal and cervical adipose tissues increased with age (Figure 1b, 1c, S1A and S1B). Visual inspection after anatomical dissection revealed that the colour of iBATs and dorsal adipose tissues (dBATs) were brown in 14 days old rabbits, and iBATs were darker than dBATs (Figure 1d). However, by 4 months, adipose tissues at the same sites became white in appearance, indicating loss of iBAT content (Figure 1d). This visual change was confirmed by haematoxylin and eosin staining showing that the adipocytes in iBAT and dBAT reprogramed phenotypically from multilocular brown adipocytes to unilocular white-like adipocytes (Figure 1e and S1C). In contrast, adipocytes within the inguinal adipose tissue (ingWAT) depot appeared to be unilocular initially but became larger during the whitening process (Figure 1e).

Corresponding analyses of UCP1 protein expression in iBAT and dBAT showed increased expression from day 1 to 14, followed by a gradual decrease and disappearance at 2, 3 and 4 months of age (Figure 1f and S1D). By comparison, UCP1 protein expression in ingWAT was not detected at any time point by western analysis (Figure 1f). However, multilocular UCP1 positive cells in rabbit ingWAT are detected when immunohistochemical analysis was conducted, which is similar to that of the inguinal adipose tissue of young mice [22,23]. The ingWAT has multilocular UCP1 positive cells on day 1 rabbits and these cells essentially disappeared by day 14 (Figure 1g). Overall, these results show that iBAT and dBAT gradually convert with age to a white-like adipose tissue phenotype in rabbits

### The transcriptional profile of iBAT shifts towards that of WAT during whitening

To delineate the regulatory mechanisms underlying the whitening process of rabbit BAT, we performed whole-genome transcriptomic analysis of rabbit iBAT at different ages. We selected iBAT at day 1, day 14, 1 month and 4 months for RNA sequencing analysis based on UCP1 protein expression content as determined by western blot analysis (Figure 1f). The ingWAT of 4-month-old rabbits was used as a control for iBAT after whitening. Fifteen samples in total were sequenced and 110.05 Gb of clean data were obtained with each sample greater than 6.05 Gb. Average clean reads for iBAT_D1, iBAT_D14, iBAT_M1, iBAT_M4 and ingWAT_M4, 47,034,007.33, 47,921,186.67, 52,221,925.33, 50,757,989.33 and 51,344,360, were generated, respectively, and subsequently mapped to the Oryctolagus Cuniculus reference genome (GCF_000003625.3, https://www.ncbi.nlm.nih.gov/genome/316?genome_assembly_id=203429) with alignment rates ranging from 87.75% to 91.67% (Table S1).

**Figure 2.**
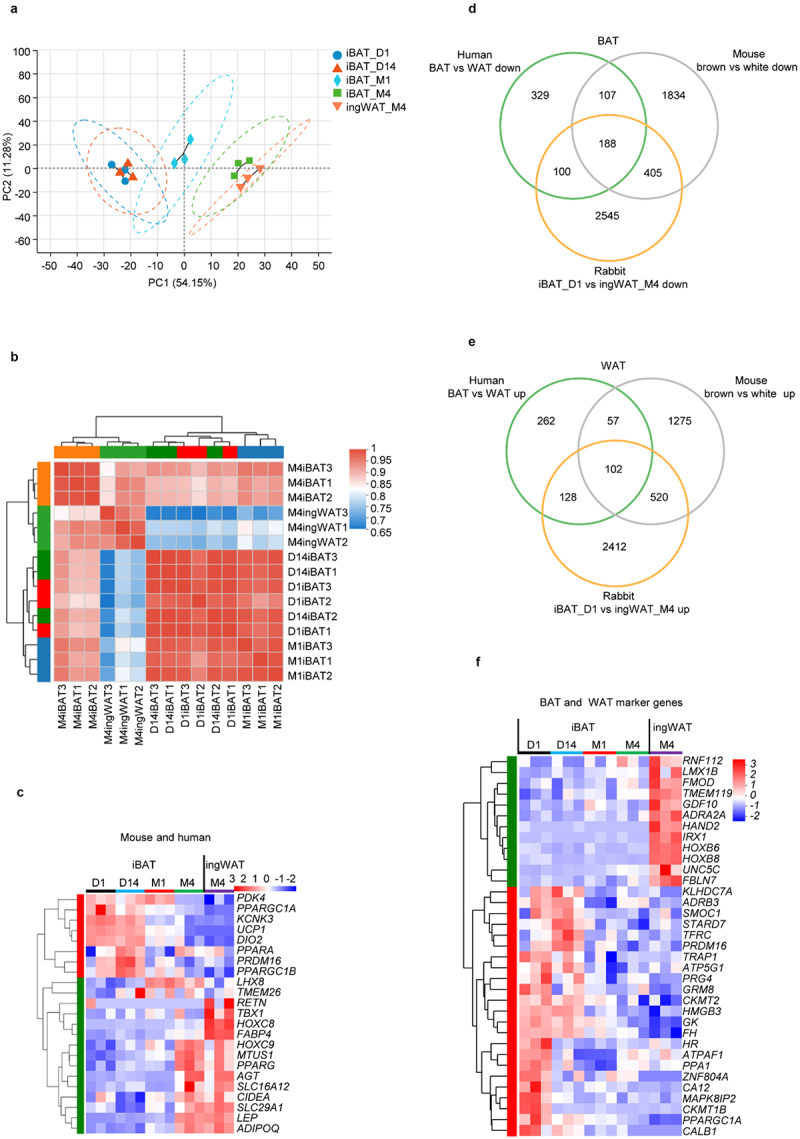
**The transcriptional profile of iBAT shifts towards that of WAT during whitening**. (a) PCA of transcriptomes during the whitening of rabbit iBAT with age, ingWAT as a control after iBAT whitening. (b) Heatmap shows correlation between samples. Pearson correlation coefficient is represented in colour. (c) Heatmap shows expression of known marker genes for BAT and WAT in mice and humans. (d) Venn diagram integrating differentially expressed up-regulated genes in human BAT and WAT, mouse brown and white adipocytes and rabbit iBAT_D1 and ingWAT_M4. (e) Venn diagram integrating differentially expressed down-regulated genes in human BAT and WAT, mouse brown and white adipocytes and rabbit iBAT_D1 and ingWAT_M4. (f) Heatmap shows expression of newly identified marker genes for BAT and WAT.

To compare the time-dependent transcriptional changes of rabbit iBAT globally, we performed PCA of RNA sequencing data obtained from rabbit iBAT_D1, iBAT_D14, iBAT_M1, iBAT_M4 and ingWAT_M4 (Figure 2a). Gene expression analyses indicated that these samples could be divided into three different groups. The first group comprising iBAT_D1 and iBAT_D14 had remarkably similar transcriptomic signatures. The second group includes iBAT_M1 and the third group comprises iBAT_M4 and ingWAT_M4 (Figure 2a). These three groups clustered as distinct phases during the whitening process of iBAT. Thus, iBAT_D1 and iBAT_D14 represent the Brown Phase, while iBAT_M4 represents a Whitened BAT Phase similar to ingWAT_M4. Since iBAT_M1 has a transcriptional profile between Brown Phase and Whitened BAT Phase (Figure 2a), it was defined as a Transition Phase.

Although iBAT_M4 and ingWAT_M4 displayed closely related transcriptomes, they could nevertheless be subdivided into two different groups by PCA (Figure 2a). This analysis indicates that Whitened iBAT and ingWAT are not identical and that fundamental differences may exist between them. Consistent with these results, global correlation analysis of adipose tissue transcriptomes during iBAT whitening revealed that iBAT_D1, iBAT_D14 and iBAT_M1 were tightly correlated (Figure 2b and Table S2). iBAT_M4 was clearly correlated with ingWAT_M4, but its correlation was weaker than that between iBAT_M4 and iBAT_M1 (Figure 2b and Table S2). In contrast, ingWAT_M4 displayed a poor correlation with iBAT_D1, iBAT_D14 and iBAT_M1 (Figure 2b and Table S2). Taken together, these results indicate that rabbit iBAT undergoes a profound transcriptomic shift during whitening.

### Identification of new marker genes for BAT and WAT

We performed a differential gene expression analysis on samples from each group and identified differentially expressed genes (DEGs) between samples from each group (Figure S2A, Table S3 and Table S4). These data show that the number of DEGs in iBAT increased with age during the whitening process (Figure S2A). In addition, the number of DEGs between iBAT and ingWAT decreased with age although iBAT_D1 vs ingWAT_M4 and iBAT_D14 vs ingWAT_M4 had a comparable number of DEGs (Figure S2A). The changes in the number of DEGs also suggest that the expression profile of iBAT becomes closer to that of ingWAT during the process of whitening.

Numerous marker genes have been proposed to differentiate among brown and beige and white adipocytes or brown and white adipose tissues in mice and humans [24–28]. We first examined whether the expression patterns of respective marker genes in rabbits fit those in mice and humans. As shown in Figure 2c, conventional thermogenic marker genes such as *PRDM16, UCP1, PPARGC1A*, and *DIO2* were highly expressed in the Brown phase. Their expression decreased in Transition and Whitened phases. By comparison, white adipocyte marker genes such as *RETN, LEP, AGT, SLC16A12, HOXC8, HOXC9* and *ADIPOQ* exhibited an opposite pattern with increased expression during whitening. Thus, the expression patterns of the above genes in rabbits were consistent with those in mice and humans.

Nevertheless, some BAT and WAT marker genes exhibited unique rabbit expression patterns. For example, mitochondrial tumour suppressor 1 (*MTUS1*) is highly enriched in UCP1-positive human adipocytes and identified as a molecular marker in addition to *UCP1* for assessing thermogenic adipocyte content within human adipose tissues [27]. However, in rabbits, *MTUS1* displayed higher expression in the Whitened phase as compared to the Brown and Transition phases (Figure 2c). Likewise, some other genes which are brown adipocyte markers in mice and humans, such as *CIDEA, SLAC29A1* and *LHX8*, were enriched in the Whitened phase and ingWAT in rabbits (Figure 2c).

Interestingly, *TBX1* and *TMEM26*, which represent beige adipocyte marker genes in mice, exhibited different expression patterns in rabbits. Specifically, *TBX1* was expressed at higher levels in ingWAT as compared to iBAT. By comparison, *TMEM26* displayed a relatively low expression level in ingWAT and in iBAT at day 1, comparable expression levels in Transition and Whitened phases, and a higher expression level in iBAT at day 14 (Figure 2c). The different expression pattern may reflect rabbit specific physiology. Overall, our findings indicate that the majority of marker genes found for BAT and WAT in mice and human also apply to rabbits.

We then determined whether additional BAT and WAT marker genes could be identified from our RNA data and therefore compared DEGs between iBAT_D1 and ingWAT_M4 in rabbits to those of humans BAT and WAT and mouse brown and white adipocytes as reported in BATLAS [29]. By comparing DEGs from all three species, a total of 188 BAT and 102 WAT marker genes were identified using │log_2_FC│≥ 0.5 and Padjust ≤ 0.05 as screening criteria (Figure 2d, 2e, S3A and S3B). This finding suggests the existence of a panel of evolutionarily conserved genes that are fundamental for brown and white adipocyte function. In addition, 100 BAT and 128 WAT marker genes were identified that are shared only by humans and rabbits (Figure 2d, 2e, S3C and S3D).

By definition, BAT marker genes should represent properties characteristic of brown adipose tissue. Consequently, these genes should express at the highest levels in the Brown phase, at a lower level in Transition phase, and at the same or even lower level in the Whitened phase and ingWAT_M4. In contrast, WAT marker genes should express at the highest level in ingWAT_M4 and/or iBAT_M4, and at the lowest level in the Brown phase. As can be seen in Figure S3A-S3D, the expression patterns of some putative BAT and WAT marker genes do not align with the above criteria, and thus visual inspection and manual adjustment were put in place to eliminate these outliers. After manual elimination, only 80 BAT and 99 WAT marker genes were shared among humans, rabbits and mouse (Table 1). Also, 23 BAT (Figure 2f) and 126 WAT marker genes were only shared between humans and rabbits (Table 1).

**Table 1.** Marker genes for BAT and WAT.

	Marker genes shared by humans, rabbits and mouse	Marker genes shared only by humans and rabbits
BAT	*ABHD11, ACAA2, ACAD8, ACADM, ACADS, ACADVL, ACAT1, ACO2, ACOT11, ACOT13, ACSF2, ADCY3, AFG3L2, AHCYL1, AIFM1, AKAP1, AMACR, ATP1A1, ATP5A1, ATP5B, ATP5C1, ATP5F1, ATP5L, ATP5O, BCKDHA, BCKDHB, BDH1, BTG3, CHCHD10, CHCHD3, CIAPIN1, CISD1, CLPB, CLSTN3, CMC2, COMTD1, COQ6, COQ9, COX6A1, CPT1B, CPT2, CRLS1, CS, DECR1, DIO2, DLD, DLST, DNAJA3, DNAJC11, EBF2, ECH1, ECHS1, ECSIT, EHHADH, ESRRA, ESRRG, ETFA, ETFDH, FABP3, FAM162A, FAM210A, FAM63B, FASTKD1, FBP2, FN3K, FURIN, GAPDH, GFM1, GHITM, GLRX5, GOT1, GOT2, GPD2, HADHA, HADHB, HPDL, HSPA9, HSPD1, HSPE1, IDH3A, IDH3B, IDH3G, IMMP1L, IMMT, ISCU, KCNK3, LACE1, LETMD1, LONP1, LRPPRC, LRRC52, LYPLA1, LYRM7, MDH2, MINOS1, MLYCD, MRPL35, MRPL4, MRPL46, MRPL47, MRPS16, MRPS18B, MRPS22, MRPS31, MRPS36, MRPS5, MRPS7, MSI2, MTIF2, MTX2, NDUFA1, NDUFA10, NDUFA11, NDUFA12, NDUFA2, NDUFA3, NDUFA4, NDUFA6, NDUFA8, NDUFA9, NDUFAB1, NDUFAF5, NDUFB2, NDUFB3, NDUFB6, NDUFB8, NDUFB9, NDUFS1, NDUFS2, NDUFS3, NDUFS4, NDUFV2, NFYC, NIPSNAP1, OGDH, OXNAD1, PANK1, PCK1, PDE4D, PDHA1, PDHX, PDSS2, PGAM1, PHB2, PKM, POLDIP2, POMC, POMGNT1, PPARGC1B, PPIF, PPTC7, PTCD3, PTGES2, RDH13, RFK, RTN4IP1, SDHA, SDHB, SDHC, SDHD, SGPL1, SLC25A11, SLC25A20, SLC25A4, SLC25A42, SLC27A2, SLC40A1, SLC4A4, SOD2, SUCLA2, SUCLG1, TBRG4, THEM4, TIMM17A, TIMM44, TIMM50, TMEM38B, TMX2, TOMM40L, TUFM, TXN2, UCP1, UQCRQ, USMG5, VDAC1, VDAC2, VWA8, YBX1*	*ACAA1, ACAD10, ACADSB, ADAL, ADCK3, ADRB3, ALAS1, ALDH5A1, AP5M1, ARL4A, ATP5G1, ATPAF1, AUH, BAG1, BPHL, CA12, CA2, CALB1, CDADC1, CDH19, CHPT1, CKMT1B, CKMT2, CNTN4, COQ2, COX4I1, CPEB3, CTSD, DLEU7, DNAJC27, EPT1, FERMT1, FH, GADD45G, GCSH, GFM2, GK, GRIK3, GRM8, HIBCH, HMGB3, HR, KLHDC7A, LAP3, LDHD, LRRN4, MAB21 L3, MAPK8IP2, MCCC1, MFN2, MPP7, MRPS25, MUT, NNT, PCCB, PDE12, PDE3B, PDE5A, PDLIM5, PEG3, PITRM1, PLP1, PPA1, PPARGC1A, PPIP5K1, PPM1B, PRDM16, PRG4, PRKAR2B, RASGRP3, RNF207, RUFY4, RXRG, S1PR3, SCN7A, SFXN4, SGK1, SIKE1, SIX4, SLC16A11, SLC16A7, SLC22A3, SLC25A3, SLC25A5, SLC4A3, SLC6A6, SLC7A8, SLITRK5, SMOC1, SOX6, ST8SIA4, STARD7, TBX5, TFRC, TNN, TOB1, TRAP1, WARS, WDR12, ZNF804A*
WAT	*A4GALT, ACTN1, ADAM33, ADAMTSL4, ADCYAP1R1, AHNAK, AKAP12, ANPEP, AR, ARID5B, BMP3, C1QTNF1, C1QTNF7, CACHD1, CCDC80, CCND2, CERCAM, COL1A1, COL1A2, COL3A1, COL4A2, COL6A1, COL6A2, COL6A3, COL8A1, CPNE2, CTSK, DBN1, DCLK1, DCN, DDR2, DMRT2, ECM1, EEF2K, EEPD1, EFEMP1, EMX2, ENPP2, FAM114A1, FBLN5, FGFR1, FGFR2, FSTL1, GLT8D2, GNG2, GSN, HOXC6, HTRA1, IGF1, IGSF10, INHBB, ISLR, ITIH5, KCNS3, LAMB2, LASP1, LEP, LMNA, LRIG1, LRIG3, LRP1, MFGE8, MSRB3, MYH10, MYO1D, NOTCH2, NOVA1, NRIP1, NUPR1, PALM, PAM, PCDH7, PHLDB1, PID1, PIK3R1, PLVAP, PLXNA3, PRR16, PRR5, PXDC1, PYGB, QSOX1, RECK, RET, S100A6, SCARB1, SEMA3A, SEMA3C, SFXN3, SHOX2, SLIT3, SPARC, SPON1, TCF7L1, TGFBR3, THBS2, TIMP2, TMEM43, TWIST2, VCAN, WSCD2, ZDHHC8*	*ADA, ADRA2A, AEBP1, ALDOC, ANKS1B, ANXA2, ANXA4, ARHGAP10, ARHGAP21, AXL, BCORL1, BMP4, BST1, BTC, C1R, C1S, C3, CCDC107, CCDC8, CCDC92, CD248, CD276, CDH20, CDON, CDR2L, CLEC3B, COL16A1, CPM, CRIM1, CTIF, CYS1, DDAH2, DHCR24, DKK2, DNM1, DTX4, DZIP1L, EMILIN1, EMP3, ENPP1, FAM110B, FAM180B, FAM46A, FAT1, FBLN1, FBLN2, FBLN7, FBXO27, FMO1, FMOD, GALNT12, GDF10, GLI3, GLIS2, GPC6, GPX8, GRIA3, HAND2, HOXB6, HOXB8, HOXD3, IL17RD, INF2, IQSEC2, IRX1, ITGA11, ITGB1BP1, ITGBL1, ITSN1, KDELR3, KIT, LAPTM4A, LMX1B, LOXL2, LOXL4, LRP5, LUM, MAP1A, MBNL3, METRNL, MFAP4, MLPH, MMP2, MRC2, MYOF, NAT8L, NTRK2, NUMBL, OLFM2, OLFML2B, OLFML3, OSR2, PAPSS1, PARD3B, PCOLCE, PDGFRA, PHLDA3, PI16, PLXNB1, PPL, PQLC1, PRELP, RILPL2, RNASE4, RNF112, S100A10, SCARA5, SLC2A10, SLC31A2, SLC35C1, SLC39A11, SLC6A2, SNTB2, SOD3, SSH3, TIMP3, TLL1, TMEM119, TMEM45A, TNXB, TRIP10, TTPAL, UNC5C, VAT1, WNT9A, WTIP, ZBTB7C, ZDHHC9*

Closer examination of the additional 126 WAT marker genes revealed that only 12 genes, i.e. *RNF112, LMX1B, FMOD, TMEM119, GDF10, ADRA2A, HAND2, IRX1, HOXB6, HOXB8, UNC5C* and *FBLN7*, exhibited an expression pattern characteristic of WAT. These genes were highly expressed in ingWAT_M4 with comparable expression across the three phases of iBAT whitening (figure 2f). Therefore, these genes could serve as bona fide new WAT marker genes that may play an instrumental role in maintaining WAT characteristics. The remaining 114 genes may play a role in promoting the whitening process since their expression levels gradually increased during iBAT whitening and eventually reached levels comparable to those in ingWAT. Similarly, the well-characterized brown fat marker genes, such as *PRDM16* and *PPARGC1A*, as well as several potential new brown fat marker genes, such as *ADRB3, CKMT1B* and *CKMT2*, were present in the 23 BAT marker genes. Therefore, the remaining 18 genes could serve as new potential marker genes for BAT (figure 2f). The function of these newly identified marker genes for BAT or WAT, however, requires further investigation and characterization in terms of their roles in the browning and whitening of adipose tissue as well as adipose biology.

### Mitochondrial content is diminished during iBAT whitening

**Figure 3.**
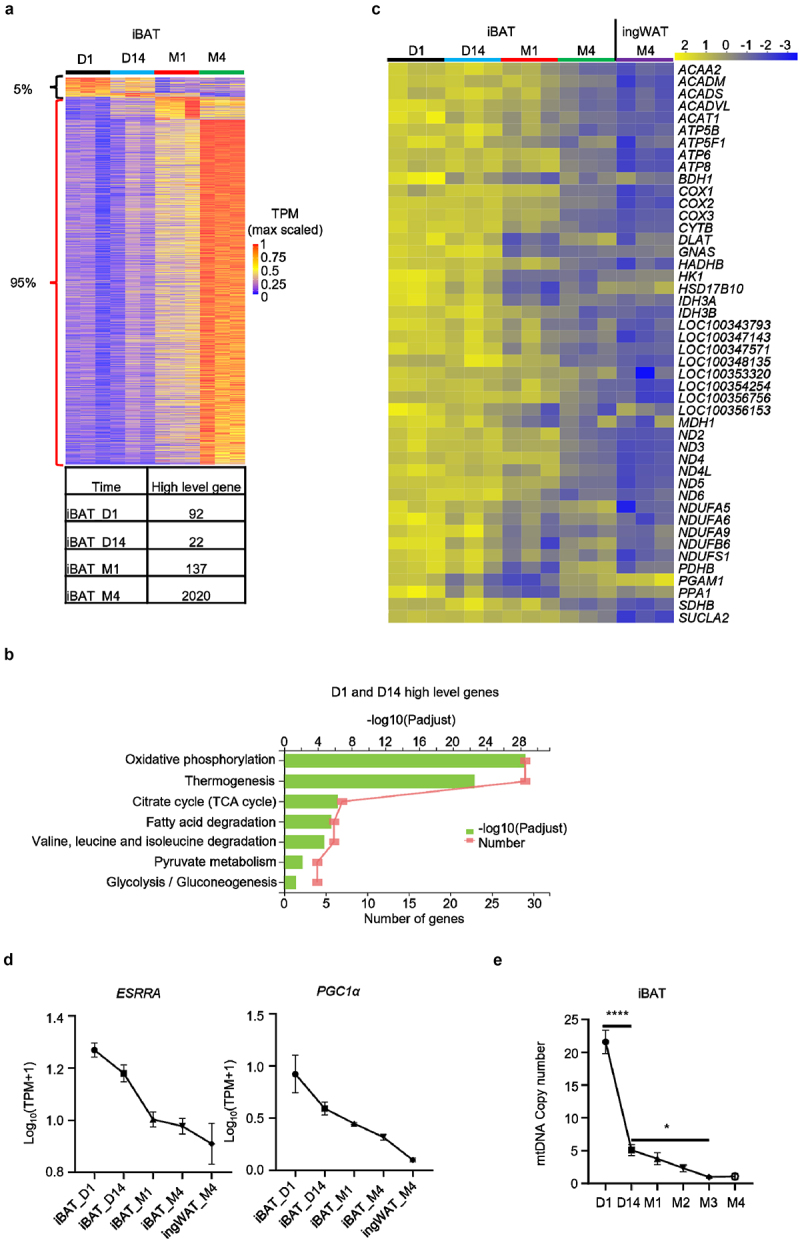
**Mitochondrial content is diminished during iBAT whitening in rabbits**. (a) Time course differential heatmap. (b) KEGG enrichment analysis of highly expressed genes in iBAT_D1 and iBAT_D14 (Brown Phase). Diagram shows significant enrichment of KEGG pathways (Padjust ≤ 0.05). (c) Heatmap shows changes in genes involved in mitochondrial function during rabbit iBAT whitening. (d) Expression changes of *ESRRA* and *PGC1α*. (e) Relative mtDNA copy number of iBAT at 1 day, 14 days, 1 month, 2 months, 3 months and 4 months of age. Data represent mean ± SEM, *P < 0.05, ****P < 0.0001.

We next examined the key biological pathways affected during iBAT whitening in rabbits. Analysis based on DEGs following time course was conducted using maSigPro to catalogue changes in pathways during the whitening process. The largest number of highly expressed gene sets was found in iBAT_M4, comprising 2020 DEGs (Figure 3a). By comparison, iBAT_D1, iBAT_D14, iBAT_M1 displayed 92, 22 and 137 highly expressed DEGs, respectively (Figure 3a). These results showed that approximately 5% of DEGs were down-regulated and about 95% of DEGs were up-regulated during iBAT whitening. Thus, iBAT whitening is likely a reconstruction process rather than a simple decrease in biological functions.

We next carried out a Kyoto Encyclopaedia of Genes and Genomes (KEGG) pathway enrichment analysis of higher expression genes in the Brown, Transition and Whitened BAT phases. KEGG pathway enrichment analysis revealed that genes highly expressed in the Brown Phase were significantly enriched in metabolic pathways related to mitochondrial function (Figure 3b and Table S5). Genes within these pathways were down-regulated during the whitening process (Figure 3c). DEGs with higher expression in the Transition Phase showed that only the Pertussis pathway was significantly enriched when setting Padjust ≤ 0.05. With a more relaxed threshold P ≤ 0.05, 28 pathways including the nod-like receptor signalling pathway, peroxisome function and autophagy were significantly enriched (Figure S4A and Table S5). Finally, 47 KEGG pathways were significantly enriched in the Whitened BAT Phase such as protein processing within the endoplasmic reticulum, endocytosis, lysosome, ubiquitin mediated proteolysis, N-glycan biosynthesis, AMPK and the sphingolipid signalling pathway (Figure S4B and Table S5).

Mitochondrial function is closely tied to mitochondrial content. Adipose tissue mitochondrial homoeostasis is tightly regulated by a balance between mitochondrial biogenesis and degradation [30]. *ESRRA* and *PGC1α* (*PPARGC1A*), two major regulators of mitochondrial biogenesis, were down-regulated during the whitening process (Figure 3d), suggesting mitochondrial biogenesis is decreased. On the other hand, 74 autophagy-related genes were up-regulated (Figure S4C), 34 of them were mitophagy-related (Figure S4D), implying mitochondrial degradation is likely increased during the whitening process. Consistent with these results, mtDNA copy number decreased sharply from day 1 to 14 after birth, followed by a gradual decline and eventual plateau to the same level as ingWAT at 4 months of age (Figure 3e), indicating mitochondrial content is diminished during rabbit iBAT whitening.

### Expression of genes involved in angiogenesis and innervation is augmented during iBAT whitening

**Figure 4.**
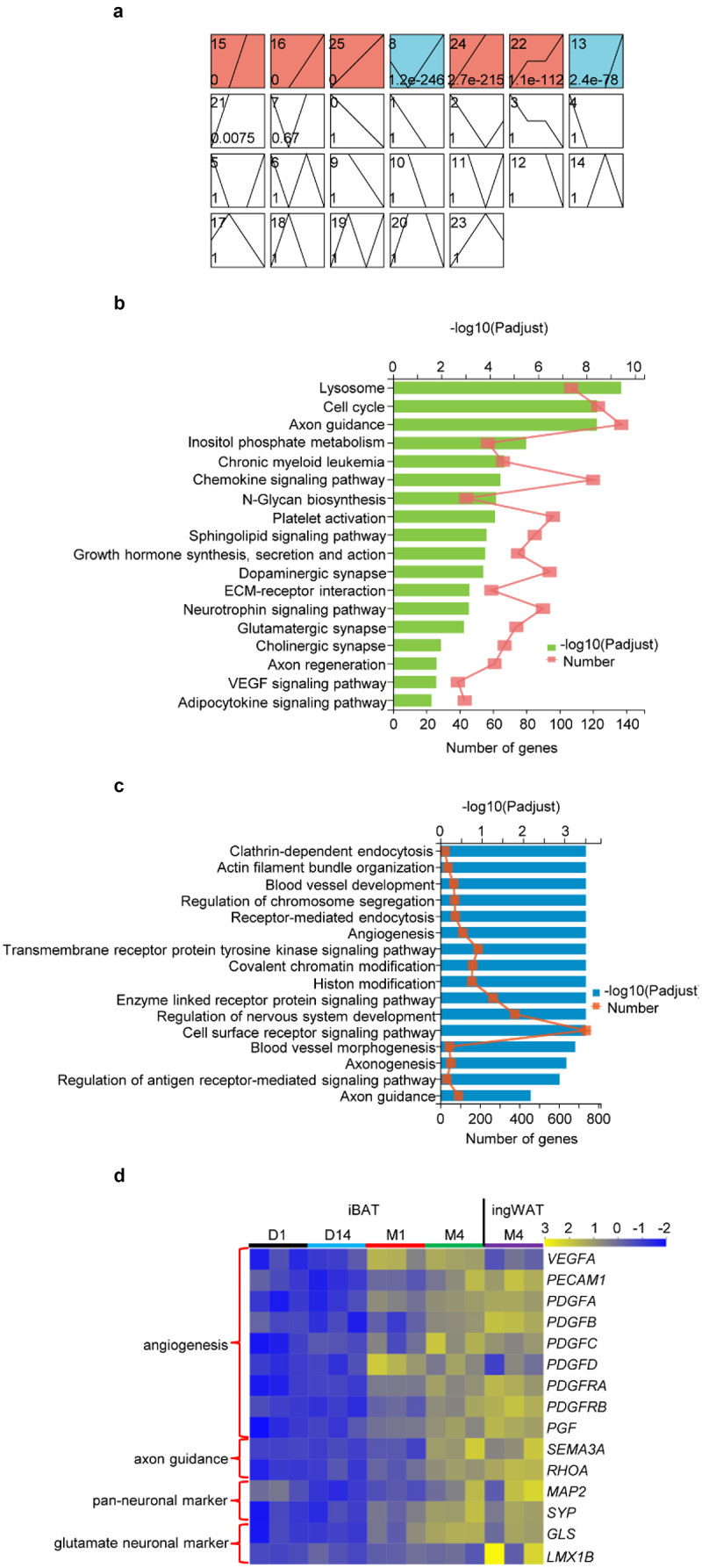
**Expression of genes involved in angiogenesis and innervation is augmented during rabbit iBAT whitening**. (a) Results of temporal expression trend analysis. (b) KEGG enrichment analysis of gene sets including Profiles 15, 16, 25, 8, 24, 22 and 13. Diagram shows significant enrichment of several KEGG pathways (Padjust ≤ 0.05). (c) GO enrichment analysis of gene sets including Profile 15, 16, 25, 8, 24, 22 and 13. Diagram shows significant enrichment of GO pathways (Padjust ≤ 0.05). (d) Heatmap shows expression of marker genes involved in angiogenesis, axon guidance and neuronal markers.

The STEM program was used to classify the 14,899 DEGs into 26 major possible model profiles based on temporal gene expression patterns (Figure 4a). Seven significantly different (P < 0.05) gene expression patterns were identified and defined as Profile 15, 16, 25, 8, 24, 22 and 13 (Figure 4a). A gene set was created for KEGG and Gene Ontology (GO) pathway enrichment analysis utilizing these seven Profiles. KEGG pathway enrichment analyses indicated that 96 pathways were significantly enriched, such as lysosome activation, axon guidance and regeneration, synapses of dopaminergic, glutamatergic and cholinergic nervous systems, platelet activation and the VEGF signalling pathway (Figure 4b and Table S6).

GO enrichment analysis also revealed that 1276 pathways, such as blood vessel development and morphogenesis, angiogenesis, axonogenesis, positive regulation of axon extension and axon guidance (Figure 4c and Table S7), were significantly enriched. DEGs associated with blood vessel development and morphogenesis (Figure S5A), platelet activation (Figure S5B), the VEGF signalling pathway (Figure S5C), angiogenesis (Figure S5D) and axon guidance (Figure S5E) were more abundant in the Transition and Whitened BAT Phases as compared to the Brown Phase. Indeed, the expression of factors that promote vasculogenesis was up-regulated (Figure 4d). On the other hand, the expression of axon guidance cues SEMA3A, which induces RHOA translation [31], pan-neuronal marker genes, as well as glutamate neuronal marker genes, *was* also up-regulated during the whitening process (Figure 4d). These findings indicate an essential role for angiogenesis and neo-vascularization during the whitening process.

### Expression of immune-related genes and infiltration of immune cells are increased during iBAT whitening

Our findings showed that 95% of DEGs were upregulated during iBAT whitening. We believed that the triggering forces for the whitening process of rabbit iBAT must come from the biological processes driven by these upregulated genes. We therefore reasoned that if these up-regulated genes control and co-ordinate the whitening process, then their expression would be relatively stable during the Brown phase (iBAT_D1 and iBAT_D14) and increased during the iBAT_D14 to Transition phase (iBAT_M1). As shown in Figure 4a, the gene expression patterns of profile 15 and profile 16 met the above criteria.

**Figure 5.**
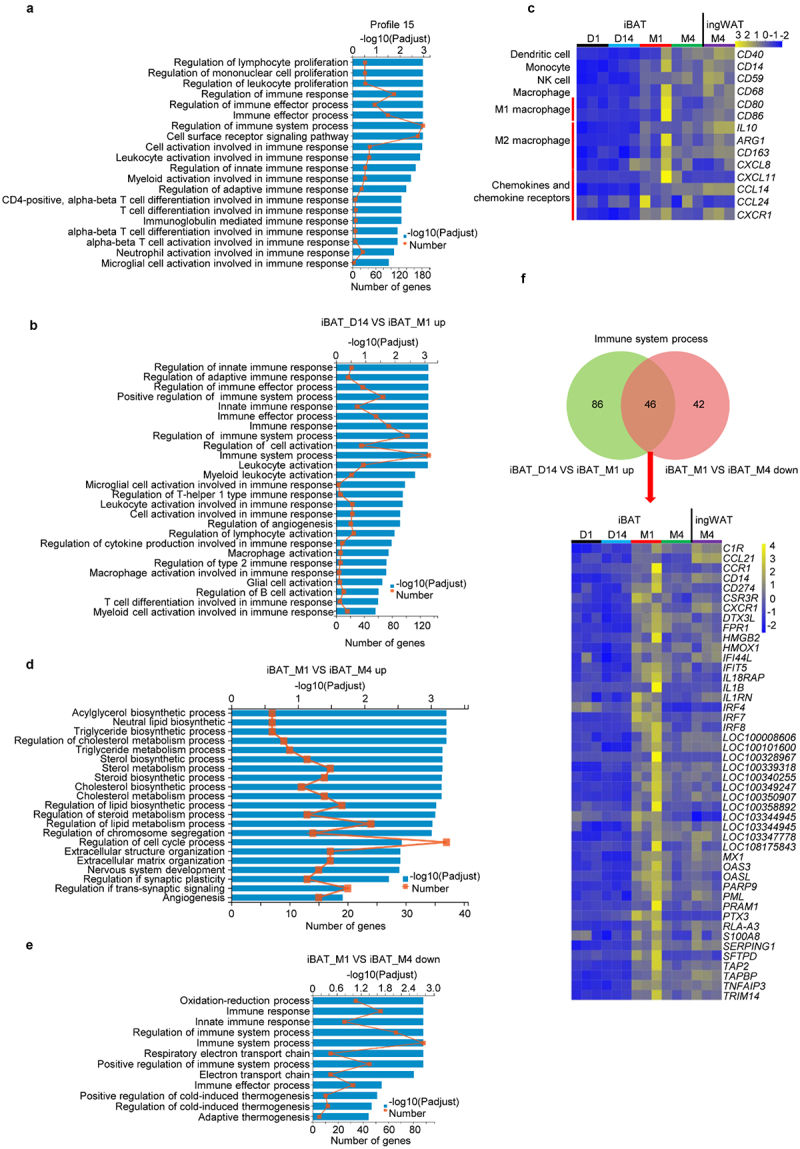
**Expression of immune-related genes and infiltration of immune cells are increased during rabbit iBAT whitening**. (a) GO enrichment analysis of Profile 15. Diagram shows significant enrichment of several GO pathways (Padjust ≤ 0.05). (b) GO enrichment analysis of differentially expressed up-regulated genes between iBAT_D14 and iBAT_M1. Diagram shows significant enrichment of GO pathways (Padjust ≤ 0.05). (c) Heatmap shows expression of marker genes regulating immune function during rabbit iBAT whitening. (d) GO enrichment analysis of differentially expressed up-regulated genes between iBAT_M1 and iBAT_M4. Diagram shows significant enrichment of several GO identified pathways (Padjust ≤ 0.05). (e) GO enrichment analysis of differentially expressed down-regulated genes between iBAT_M1 and iBAT_M4. Diagram shows significant enrichment of GO identified pathways (Padjust ≤ 0.05). (f) Heatmap shows the intersection of up-regulated genes between iBAT_D14 and iBAT_M1 involved in immune system processes and down-regulated genes between iBAT_M1 and iBAT_M4 involved in immune system processes.

GO analysis uncovered 326 and 474 significantly enriched pathways for Profile 15 and Profile 16, respectively (Table S8 and Table S9). For Profile 15, most of the pathways were closely associated with immune processes such as regulation of cell proliferation for lymphocytes, mononuclear cells and leukocytes, regulation of innate and adaptive immune response (Figure 5a and Table S8). For Profile 16, enriched pathways include those for histone modification, chromatin organization, cell cycle processes, protein ubiquitination, covalent chromatin modification and regulation of chromatin modification (Figure S6A and Table S9). Many of these pathways are intimately linked to remodelling of chromatin and epigenetic regulation.

For further verification, GO analysis was performed using DEGs between iBAT_D1 and iBAT_D14, iBAT_D14 and iBAT_M1 as well as iBAT_M1 and iBAT_M4. Up-regulated DEGs between iBAT_D1 and iBAT_D14 were significantly enriched in metabolic pathways related to regulation of T cell, lymphocyte and leukocyte differentiation and activation, and regulation of brown fat cell differentiation (Figure S6B and Table S10). As expected, these pathways were similar to those for Profile 15.

Between iBAT_D14 and iBAT_M1, up-regulated DEGs were also significantly enriched in metabolic pathways related to immune processes, including regulation of innate and adaptive immune responses, and regulation of cell activation comprising leukocyte, myeloid leukocyte, lymphocyte, macrophage and B cell activation (Figure 5b and Table S10). Monocyte marker gene *CD14*, dendritic cell marker gene *CD40*, NK cell marker gene *CD59*, macrophage marker gene *CD68*, M1 macrophage marker genes *CD80* and *CD86*, M2 macrophage marker genes *IL10, CD163* and *ARG1*, were all up-regulated from the Brown to Transition Phases (Figure 5c). These findings suggest an increased number of immune cells by either proliferation or infiltration during iBAT whitening.

The infiltration and passing through tissues of immune cells are coordinated by a large number of chemokines and their receptors [32]. Also, some chemokines and chemokine receptors such as CXCL8, CCL24, CXCL11, CCL14 and CXCR1, are only present in humans and not in mice. We therefore examined the expression of these genes during iBAT whitening. It is intriguing that the expression levels of the chemokines, *CXCL8, CCL24, CXCL11*, and the chemokine receptor, *CXCR1*, were much higher in the Transition phase as compared to the Brown and Whitened BAT phases (Figure 5c). These findings further suggest that the infiltration of immune cells likely increases during iBAT whitening.

The up-regulated DEGs between iBAT_M1 and iBAT_M4 were significantly enriched in metabolic pathways associated with lipogenesis, principally including acylglycerol, neutral lipid, triglyceride and sterol biosynthetic processes (Figure 5d and Table S10). Similar processes were reported for the whitening of murine BAT in response to thermoneutrality, where lipogenesis was prevalent during iBAT whitening [33].

In contrast, down-regulated DEGs between iBAT_D1 and iBAT_D14 were significantly enriched in processes involving oxidoreductase activation as well as fatty acid and lipid metabolism (Figure S6C and Table S10). The down-regulated DEGs between iBAT_D14 and iBAT_M1 were significantly enriched in metabolic pathways involved in mitochondrial function (Figure S6D and Table S10). Unexpectedly, down-regulated DEGs between iBAT_M1 and iBAT_M4 were significantly enriched not only in processes comprising the respiratory electron transport chain, regulation of cold-induced thermogenesis and adaptive thermogenesis, but also in immune response and immune-related processes (Figure 5e and Table S10).

Subsequently, we examined the expression of genes involved in immune regulation between iBAT_D14 and iBAT_M1 as well as between iBAT_M1 and iBAT_M4. Several immune-related genes were initially up-regulated between iBAT_D14 and iBAT_M1 and then down-regulated between iBAT_M1 and iBAT_M4 (figure 5f and Table 2). Taken together, these results indicate that the up-regulation of immune-related genes precedes the up-regulation of lipid synthesis relevant genes and that immune cells are likely the primary determinant driving iBAT whitening.

**Table 2.** Immune-related genes up-regulated between iBAT_D14 and iBAT_M1 and down-regulated between iBAT_M1 and iBAT_M4.

GO_ID	Description	iBAT_D14 VS iBAT_M1 upregulation	iBAT_M1 VS iBAT_M4 downregulation
GO:0006955	immune response	***CD274, LOC100328967, IL1B, LOC100340255, TAPBP, LOC100101600, IL1RN, SFTPD, C1R, RLA-A3, CCR1, IL18RAP, CD14, PTX3, LOC100350907, PARP9, PML, IRF8, IRF7, LOC100349247, IFI44L, CXCR1, HMGB2, TRIM14, OAS3, DTX3L, SERPING1, CCL21, OASL, S100A8, LOC100339318, LOC103347778, DDX58,****LOC100353293, TRIM5, NFKB2, CD247, OAS1, C4A, EIF2AK2, IL27RA, CCL19, LOC100357462, ERAP1, CCL14, CX3CL1, IL10,C7, GRO-A, LOC100352110, TNFAIP8L2, NOTCH2, CTSK, C1RL, LOC100349085, FCER1G, LOC100338822, TIFA,ENPP1, XCR1, C1S, SKAP1, OAS2, IFIH1, IL1R1, LOC100356242, TNF, B2M, TLR5, TLR3, ARG1, TNFSF10, LBP, ALPK1, PTK2B*	***CD274, LOC100328967, IL1B, LOC100340255, TAPBP, LOC100101600, IL1RN, SFTPD, C1R, RLA-A3, CCR1, IL18RAP, CD14, PTX3, LOC100350907, PARP9, PML, IRF8, IRF7, LOC100349247, IFI44L, CXCR1, HMGB2, TRIM14, OAS3, DTX3L, SERPING1, CCL21, OASL, S100A8, LOC100339318, LOC103347778, DDX58,****S100A12, SLAMF1, CR1, THBS1, LOC103348990, PKHD1L1, LTF, COLEC12, GRO-B, MYO1G, LOC100341358, LOC103345076, ALCAM, CLEC4E, S100A9, C5AR1, CD244, PGLYRP1, HMGB3, TNFSF14, CD1D*
GO:0045087	innate immune response	***LOC100340255, CD14, C1R, IRF7, PTX3, PML, LOC100350907, PARP9, HMGB2, TRIM14*****, *DTX3L, SERPING1, S100A8, LOC100101600, DDX58***, *TRIM5, EIF2AK2, C7, LOC100352110, LBP, TNFAIP8L2, C1RL, FCER1G, LOC100353293, IFIH1, TLR5, TLR3, TIFA, ARG1, ALPK1,*	***LOC100340255, CD14, C1R, IRF7, PTX3, PML, LOC100350907, PARP9, HMGB2, TRIM14, DTX3L, SERPING1, S100A8, LOC100101600, DDX58***, *S100A12, LOC103348990, LTF, COLEC12, C1R, HMGB3, S100A9, PGLYRP1, CLEC4E, CD1D*
GO:0002682	regulation of immune system process	***LOC100340255, TBC1D10C, LOC108175843, CCL21, GAB2, LOC100350907, LOC100342826, TNFAIP3, CD274, HMGB2, PRAM1, CSF3R, DDX58, TAP2, IL1B, SERPING1, CCR1, IL18RAP, IRF7, DHX58, DTX3L, C1R, FIGF, FPR1, PARP9, USP18**, GATA3, TRIM5, TAPBPL, LOC100357099, ERAP1, LOC100352110, MSTN, ACVR2A, TYROBP, STAT1, STAT6, CD5L, B2M, LOC100345657, LOC100349257, HERC5, EIF2AK2, LOC100342321, FOXP1, CD38, APOD, LBP, RSAD2, C7, FAXDC2, PTK2B, SPHK1, MZB1, SAMHD1, MITF, MERTK, VCAM1, SKAP1, ADAR, IL1R1, ARG1, RNASEL, SELP, TMEM173, C4A, ZFP36L1, IL18R1, SELL, FAM19A3, LOC100353664, CX3CL1, TNFAIP8L2, CARD11, C1RL, SOX4, ISG15, NFAM1, ENPP3, IL27RA, LPXN, AIF1, P2RX7, LOC108178049, CD247, IL10, SMPD3, FCER1G, PRDM1, C1S, GPX1, NOTCH2, CD83, HLX, LOC100341366, LOC100353293, GRN, IL33, LOC100345918, IL34, RARG, LOC103351157*	***LOC100340255,TBC1D10C, LOC108175843, CCL21, GAB2, LOC100350907, LOC100342826, TNFAIP3, CD274, HMGB2, PRAM1, CSF3R, DDX58, TAP2, IL1B, SERPING1, CCR1, IL18RAP, IRF7, DHX58, DTX3L, C1R, FIGF, FPR1, PARP9, USP18**, LOC100341358, MYC, SIGLEC15, RUNX1, GPLD1, ALOX15, NFKBIA, NR1D1, CR1, COLEC12, PIM1, TMEM64, LOC100342069, SERPINE1, TOX, HMGB3, THBS1, CA2, CD80, MYO1G, SLAMF1, THEMIS2, KLK7, ID2, SCIN, LOC100349447, DUSP1, CLEC4E, MMP12, DOCK8, LOC103348721, DPP4, PDE4D, GLMN, TRIM58, LOC100341926, C5AR1, CD244, ZBTB16, TNFSF14,*
GO:0002376	immune system process	***C1R, CCL21, CCR1, CD14, CD274, CSF3R, CXCR1, DDX58, DTX3L, FPR1, HMGB2, HMOX1, IFI44L, IFIT5, IL18RAP, IL1B, IL1RN, IRF4, IRF7, IRF8, LOC100008606, LOC100101600, LOC100328967, LOC100339318, LOC100340255, LOC100349247, LOC100350907, LOC100358892, LOC103344945, LOC103347778, LOC108175843, MX1, OAS3, OASL, PARP9, PML, PRAM1, PTX3, RLA-A3, S100A8, SERPING1, SFTPD, TAP2, TAPBP, TNFAIP3, TRIM14,****ADAR, ALPK1, APBB1IP, ARG1, B2M, BATF2, C1RL, C1S, C4A, C7, CARD11, CCL14, CCL19, CD247, CD38, CD5L, CHI3L1, CLU, CREG1, CTSK, CX3CL1, EIF2AK2, ENPP1, ENPP3, ERAP1, FCER1G, FOXP1, FZD7, GATA3, GRN, GRO-A, HP, IFIH1, IFIT3, IL10, IL18R1, IL1R1, IL27RA, IL33, ISG15, ITGB7, JAML, LBP, LOC100338822, LOC100341366, LOC100343291, LOC100349085, LOC100352110, LOC100353293, LOC100356242, LOC100357462, LOC103351157, MERTK, NCF4, NFKB2, NOTCH2, OAS1, OAS2, P2RX7, PLEKHO2, PSME1, PTK2B, RAP2B, RNASEL, RSAD2, SAMHD1, SDC2, SELL, SEMA4A, SKAP1, SLC7A7, SOX4, STAT1, STAT2, TIFA, TLR3, TLR5, TMEM173, TNF, TNFAIP8L2, TNFSF10, TRIM5, TYROBP, VCAM1, XCR1, ZFP36L1*	***C1R, CCL21, CCR1, CD14, CD274, CSF3R, CXCR1, DDX58, DTX3L, FPR1, HMGB2, HMOX1, IFI44L, IFIT5, IL18RAP, IL1B, IL1RN, IRF4, IRF7, IRF8, LOC100008606, LOC100101600, LOC100328967, LOC100339318, LOC100340255, LOC100349247, LOC100350907, LOC100358892, LOC103344945, LOC103347778, LOC108175843, MX1, OAS3, OASL, PARP9, PML, PRAM1, PTX3, RLA-A3, S100A8, SERPING1, SFTPD, TAP2, TAPBP, TNFAIP3, TRIM14,****ADGRG3, ALAD, ALCAM, AMPD3, ATP1B1, C5AR1, CD1D, CD244, CD80, CLEC4E, COLEC12, CR1, CTSE, DDIT4, DOCK8, DPP4, GPLD1, GRO-B, HMGB3, ID2, IMPDH1, LOC100341358, LOC100349447, LOC100356702, LOC103345076, LOC103348990, LTF, MYO1G, NDRG1, PDE4D, PGLYRP1, PKHD1L1, S100A11, S100A12, S100A9, SLAMF1, SLC12A2, SUCNR1, THBS1, THEMIS2, TNFSF14, TOX*
GO:0002684	positive regulation of immune system process	***C1R, CCL21, CCR1, CD274, DDX58, DHX58, FIGF, FPR1, GAB2, HMGB2, IL18RAP, IL1B, IRF7, LOC100350907, LOC108175843, PARP9, PRAM1, SERPING1, TAP2***, *ACVR2A, AIF1, ARG1, B2M, C1RL, C1S, C4A, C7, CARD11, CD247, CD38, CD83, CX3CL1, ENPP3, FAM19A3, FAXDC2, FCER1G, FOXP1, GATA3, HLX, IL18R1, IL1R1, IL27RA, IL33, IL34, ISG15, LBP, LOC100341366, LOC100345657, LOC100349257, LOC100352110, MSTN, MZB1, NFAM1, NOTCH2, P2RX7, PTK2B, RSAD2, SELP, SKAP1, SOX4, STAT1, STAT6, TMEM173, TRIM5, TYROBP, VCAM1, ZFP36L1*	***C1R, CCL21, CCR1, CD274, DDX58, DHX58, FIGF, FPR1, GAB2, HMGB2, IL18RAP, IL1B, IRF7, LOC100350907, LOC108175843, PARP9, PRAM1, SERPING1, TAP2***, *C5AR1, CA2, CD244, CD80, CR1, DOCK8, DPP4, GPLD1, HMGB3, ID2, KLK7, LOC100341358, MMP12, MYO1G, PDE4D, RUNX1, SCIN, SERPINE1, SLAMF1, THBS1, THEMIS2, TMEM64, TNFSF14, TOX, TRIM58, ZBTB16*
GO:0002252	immune effector process	***C1R, CXCR1, DDX58, DTX3L, FPR1, IFI44L, IFIT5, IL18RAP, IRF7, LOC100008606, LOC100101600, LOC100350907, LOC103344945, MX1, OAS3, OASL, PARP9, PTX3, SERPING1***, *APBB1IP, ARG1, C1RL, C1S, C4A, C7, CHI3L1, CLU, CREG1, EIF2AK2, ENPP3, FCER1G, FOXP1, GRN, IFIT3, IL18R1, IL33, ISG15, LBP, LOC100341366, LOC100353293, LOC100356242, NOTCH2, OAS1, OAS2, PLEKHO2, PTK2B, RAP2B, RNASEL, RSAD2, SAMHD1, SELL, SEMA4A, STAT1, STAT2, TLR3, TRIM5, TYROBP*	***C1R, CXCR1, DDX58, DTX3L, FPR1, IFI44L, IFIT5, IL18RAP, IRF7, LOC100008606, LOC100101600, LOC100350907, LOC103344945, MX1, OAS3, OASL, PARP9, PTX3, SERPING1***, *ADGRG3, ALAD, C5AR1, CD244, CLEC4E, CR1, DDIT4, LOC100349447, LOC103348990, MYO1G, S100A11, SLAMF1, SUCNR1*

Table shows significantly enriched GO terms relating to various immune processes. Genes in bold are up-regulated between iBAT_D14 and iBAT_M1 and down-regulated between iBAT_M1 and iBAT_M4.

### Identification of potential transcription factors that promote rabbit iBAT whitening

**Figure 6.**
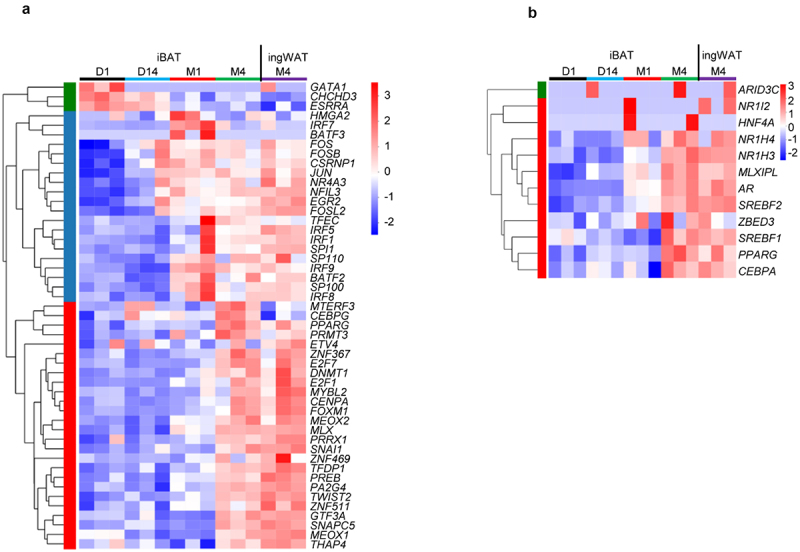
**Identification of putative TFs that promote iBAT whitening in rabbits**. (a) Heatmap shows expression changes of TFs that may promote iBAT whitening. (b) Heatmap shows expression changes of TFs that regulate lipid biosynthesis.

ChIP-X Enrichment Analysis 3 (ChEA3) was used to identify potential transcription factors (TFs) that drive iBAT whitening (https://amp.pharm.mssm.edu/ChEA3, Keenan et al., 2019). We first looked for TFs that control up-regulated and down-regulated DEGs between iBAT_D1 and iBAT_M4, iBAT_D1 and iBAT_14, iBAT_D14 and iBAT_M1 as well as iBAT_M1 and iBAT_M4. The top 10 TFs between each group are listed in Table 1. During iBAT whitening, transcription factors *CHCHD3* and *ESRRA*, which regulate mitochondrial function showed higher expression in the Brown phase as compared to the Transition and Whitened BAT phases (Figure 6a). This finding indicates that mitochondrial function diminishes in the later phases of iBAT whitening.

TFs controlling either up-regulated DEGs between iBAT_D14 and iBAT_M1 or down-regulated DEGs between iBAT_M1 and iBAT_M4, were mostly immune-related (e.g. *BATF2, SP110, SP100, IRF1, IRF7* and *IRF9*) (Figure 6a and Table 3). These results support the notion that TFs regulate these biological processes to execute the reprogramming process of iBAT whitening.

**Table 3.** TFs that may drive iBAT whitening as identified by ChEA3.

	Rank	TFs	Score	Gene number		Rank	TFs	Score	Gene number
iBAT_D1 VS iBAT_M4 up	1	*CENPA*	4	91	iBAT_D14 VS iBAT_M1 up	1	*IRF7*	4	137
	2	*FOXM1*	8.167	167		2	*BATF2*	4.333	135
	3	*TWIST2*	13	84		3	*SP110*	7.333	131
	4	*ZNF367*	16.67	113		4	*SP100*	14	120
	5	*PPARG*	17	395		5	*IRF1*	15.67	246
	6	*PRRX1*	18.33	122		6	*SPI1*	16.83	216
	7	*MEOX2*	27.67	133		7	*TFEC*	17.33	111
	8	*ZNF469*	29	74		8	*IRF9*	18	169
	9	*MEOX1*	29	146		9	*IRF5*	20.67	123
	10	*E2F7*	31.6	176		10	*IRF8*	21.75	127
iBAT_D1 VS iBAT_M4 down	1	*MTERF3*	12.5	135	iBAT_D14 VS iBAT_M1 down	1	*THAP4*	9	125
	2	*CHCHD3*	13	128		2	*PREB*	9.333	105
	3	*PREB*	13	130		3	*MTERF3*	13.5	96
	4	*ZNF511*	14.33	129		4	*ESRRA*	15.2	205
	5	*GTF3A*	20	152		5	*ZNF511*	15.33	99
	6	*SNAPC5*	23.5	89		6	*PRMT3*	21.67	76
	7	*PRMT3*	39.33	85		7	*SNAPC5*	34	64
	8	*GATA1*	44.83	333		8	*NR2F6*	35	123
	9	*CEBPG*	45.25	149		9	*PPARG*	41.2	134
	10	*MLX*	51.33	106		10	*CEBPG*	47	113
iBAT_D1 VS iBAT_D14 up	1	*FOSB*	6.333	66	iBAT_M1 VS iBAT_M4 up	1	*FOXM1*	2.833	119
	2	*EGR2*	9	78		2	*CENPA*	4	79
	3	*CSRNP1*	13.5	32		3	*ZNF367*	7.667	88
	4	*PPARG*	14.6	179		4	*MYBL2*	17.33	173
	5	*JUN*	16.5	125		5	*HMGA2*	19	82
	6	*FOS*	21.6	91		6	*DNMT1*	23.5	70
	7	*NFIL3*	22.33	57		7	*PPARG*	25.4	113
	8	*FOSL2*	27.2	91		8	*PA2G4*	30.5	46
	9	*SNAI1*	27.33	58		9	*E2F7*	31.8	112
	10	*NR4A3*	31.33	70		10	*E2F1*	44	219
iBAT_D1 VS iBAT_D14 down	1	*FOXM1*	3	59	iBAT_M1 VS iBAT_M4 down	1	*BATF2*	6.333	81
	2	*ZNF367*	5	46		2	*IRF7*	7.333	84
	3	*CENPA*	5	39		3	*SP110*	19.67	74
	4	*PA2G4*	19.5	33		4	*SP100*	25.67	66
	5	*HMGA2*	22	44		5	*IRF9*	28.5	111
	6	*MYBL2*	24.33	90		6	*BATF3*	29.67	69
	7	*E2F7*	24.8	59		7	*EGR2*	32.33	97
	8	*TFDP1*	41	56		8	*NFIL3*	37	80
	9	*ETV4*	59	61		9	*FOSB*	40	70
	10	*E2F1*	68	118		10	*IRF1*	40.5	188

Table shows the top 10 TFs that regulate up-regulation or down-regulation of DEGs between iBAT_D1 and iBAT_M4, between iBAT_D1 and iBAT_D14, iBAT_D14 and iBAT_M1 and between iBAT_M1 and iBAT_M4. Score according to Mean RANK (Average integrated ranks across libraries). Gene number means the number of genes regulated by the transcription factor.

Our findings show that iBAT eventually transforms from a thermogenic and energy consuming fat depot to a lipogenic and energy storing depot. The top 12 TFs that control up-regulate genes involved in lipid biosynthesis were also identified (Figure 6b and Table 4). Among them, *MLXIPL, SREBF1, SREBF2, CEBPA* and *PPARG* are well studied and regulate lipid biosynthesis and storage. Taken together, TFs such as *ZNF367, E2F7, TFDP1, FOXM1, TWIST2, ZNF511*, interferon regulatory factors (IRF) family and *AR* appear to facilitate iBAT whitening in rabbits.

**Table 4.** Top 12 TFs that regulate lipid biosynthesis as identified by ChEA3.

Rank	TFs	Score	Gene number
1	*MLXIPL*	4.333	20
2	*PPARG*	6.6	20
3	*SREBF1*	12.17	30
4	*NR1H4*	14.33	15
5	*ZBED3*	21.33	15
6	*NR1I2*	23.25	20
7	*CEBPA*	25	23
8	*HNF4A*	34.67	43
9	*AR*	50.2	28
10	*ARID3C*	52	6
11	*SREBF2*	59.33	43
12	*NR1H3*	64.4	19

Table shows the top 12 TFs that regulate lipid biosynthesis during iBAT whitening in rabbits. Score according to Mean RANK (Average integrated ranks across libraries). Gene number means the number of genes regulated by the transcription factor.

## Discussion

In this study, we established a detection technique to accurately correlate non-invasive MRI measurement with morphological, histological and molecular events during physiological iBAT whitening in rabbits. To the best of our knowledge, MRI imaging coupled with transcriptomic analysis of iBAT whitening under physiological conditions has yet to be reported.

In humans, iBAT is metabolically inactivated with age, resulting in adipocytes that are morphologically indistinguishable from white adipocytes [19,20,34]. Rabbits were used in this work as they are suitable for MRI quantification and are phylogenetically more closely related to humans than rodents. In particular, they resemble the iBAT whitening process in humans where the iBAT depot in newborn rabbits changes to white adipose tissue with age [21]. This phenomenon was confirmed in the present study by MRI, anatomical observations, and histological staining, suggesting that the iBAT depots in both rabbits and humans undergo a similar whitening process postnatally. Thus, our findings demonstrate that the rabbit is a useful animal model for studying iBAT whitening in humans under physiological conditions.

We then performed a detailed transcriptomic analysis of rabbit iBAT whitening. These analyses suggest that iBAT whitening is the result of a combination of multiple factors such as the interaction between adipocytes and immune cells, metabolism reprogramming, transcription and epigenetic modification. Moreover, the RNA-seq data generated in this study provide a valuable resource for understanding the gene regulation underlying iBAT whitening in humans. The identification of specific molecular biomarkers in this process also provides new insights into adipose tissue biology. Our transcriptome data indicate that most of the biomarkers for BAT and WAT in mice and humans are applicable to rabbits. Additionally, we discovered potentially new BAT and WAT marker genes whose biological roles warrant future study.

Adipose tissue is a highly plastic and dynamic tissue. In response to physiological stimulation, significant changes can be induced in its metabolism, structure, and phenotype [35]. WAT can acquire a BAT-like cellular and molecular program in response to various stimuli, such as cold exposure, β-adrenergic receptor agonists or genetic alterations, in a process termed browning [3,36,37]. BAT is present throughout the life of mice and provides resistance to diet-induced obesity through UCP1 [38,39]. Nevertheless, BAT whitening leading to obesity and insulin resistance can also be induced in mice by various interventions, such as high fat diet regimens, thermoneutrality, and genetic manipulation [28,40–43]. Rabbits have a relatively wide thermoneutral zone of 15–25°C [44,45]; however, the cold environment does not prevent rabbit BAT whitening [46], suggesting that thermoneutrality is not the main reason driving iBAT whitening.

Decreased mitochondrial biogenesis and activation of mitochondrial autophagy are also important mechanisms driving BAT whitening in mice [30,47,48]. Under physiological conditions, BAT whitening in rabbits is spontaneous. Our findings suggest that this process involves a decrease in mitochondrial biogenesis and increased autophagy, which may lead to an overall decreased mitochondrial content and number. Recently, BAT-specific deletion of the TFEB gene, a master regulator of lysosomal biogenesis and autophagy, attenuated BAT whitening in mice at thermoneutrality [49]. These results support the idea that inhibiting autophagy and/or promoting mitochondrial biogenesis of brown adipocytes counteracts the process of BAT whiteness [30,48,50].

In mice, vascular rarefaction resulting from chronic high fat feeding regimens is a significant causal factor for BAT whitening. Chronic high fat diet causes BAT whitening, and *Vegfa* expression was significantly down-regulated at 12, 16 and 20 weeks on a high fat diet compared with that of mice on a normal chow diet [51]. Targeted deletion of *Vegfa* in adipose tissue resulted in BAT whitening even in mice fed with a normal chow diet [42]. Denervation of BAT is also reported to cause its whitening [28]. Nevertheless, our RNA seq analysis shows that *VEGFA* expression was in fact upregulated during iBAT whitening in rabbits. KEGG pathway and GO enrichment analysis also found that expression of genes involved in both angiogenesis and innervation was increased during rabbit iBAT whitening, indicating that angiogenesis and innervation are likely boosted, which suggests that the rabbit iBAT whitening process differs from that of mice.

Angiogenesis is also closely related to adipogenesis [52] where the vascular system transports nutrients, oxygen, growth factors, cytokines and hormones required for adipocyte function, growth and survival; the vascular system also controls changes in the microenvironment for adipose tissue [53]. Innervation of sympathetic nerve fibres is also essential for BAT thermogenesis; however, glycinergic stimulation or vagal afferent activation inhibits BAT sympathetic nerve activity and thermogenesis in rats [54,55]. BAT and WAT depots in mice have abundant vascular and nerve distribution as shown by 3D volume fluorescence-imaging [56,57]. Therefore, this imaging technique could be used to determine the distribution of blood vessels and nerves as well as the types of synapses involved during rabbit iBAT whitening in future studies.

An adipose tissue is an extraordinarily heterogeneous organ with mature adipocytes constituting less than 50% of the adipose cell fraction, while the rest is classified as the stromal vascular fraction comprising many different cell types, including various immune cells, endothelial and vascular cells, fibroblasts, and adipose precursor cells [58–60]. Furthermore, mature adipose cells and various other cell types in adipose tissue together orchestrate adipose tissue development and function [61,62]. Our detailed transcriptomic analysis shows that up-regulation of genes involved in immune-related processes precedes the up-regulated expression of genes regulating lipid synthesis, and upregulated expression of marker genes in a variety of immune cells, such as macrophages, monocytes, dendritic cells and NK cells during rabbit iBAT whitening. These findings indicate that immune cells may participate in and orchestrate the whitening process. However, adipocytes themselves as well as adipose-derived stromal cells could also express immune-related genes. Increased IRF7 expression in adipose-derived stromal cells of ageing mice leads to impaired mitochondrial function and BCAA metabolism [63]. Therefore, we cannot rule out that non-immune cells increase the expression of immune-related genes during rabbit iBAT whitening.

The immune system gradually matures during infancy [64] with maturation of immune competence after 1 year in humans, and 8 weeks in rabbits [65]. Therefore, the timing of iBAT whitening appears to be synchronized with the maturation of the immune system, further emphasizing the importance of immune cells in this process. It has been demonstrated that senescent T cells produce and release IFN-γ to inhibit differentiation of preadipocytes from brown adipocytes, thereby contributing to age-induced BAT whitening in mice [66]. Single-cell RNA sequencing (scRNA-seq) and single-nucleus RNA-sequencing (snRNA-seq) technologies have greatly advanced our understanding of the cellular complexity and plasticity of adipose tissue, and can be utilized to identify all major cell types in adipose tissue [67]. Detailed cellular atlases of human and mouse subcutaneous and visceral WAT at single-cell resolution have recently been generated, and subpopulations of various cells, such as adipocytes, adipose stem and progenitor, vascular and immune cells, as well as potential cell–cell interactions have been identified [68]. Using snRNA-seq, it was found that a population of CYP2E1+/ALDH1A1+ adipocytes secrete acetate by paracrine means to suppress adjacent adipocyte thermogenesis [69]. These results indicate that cellular crosstalk between adipocytes themselves, as well as with various other cell types such as immune cells in the adipose microenvironment influences adipose function. Future work may employ scRNA-seq to further pinpoint which specific immune cells play a central role in the whitening process of rabbit iBAT. As a matter of fact, while this manuscript was being prepared, a report was published in Cell Reports where scRNA-seq analysis was used to reveal that brown adipocyte progenitor cells expressing FSTL1 contribute to rabbit iBAT whitening, and their subsequent results elegantly demonstrated that removal of the *Fstl1* or *Fstl1*^+^ progenitors results in BAT paucity in mice [46]. Despite the fact that the iBAT in their article refers to the dBAT depot in our study, we noticed an increased proportion of immune cells such as B cells, T cells and macrophages during the whitening process in rabbits [46], which is consistent with our conclusion that the immune cells and immune regulation may orchestrate the physiological whitening of iBAT in rabbits.

Transcriptional and epigenetic regulation are two of several mechanisms that regulate gene expression to ensure coordinated cellular behaviour and fate determination. In the current study, the discovery of TFs that regulate iBAT whitening reinforces an important role of immune-related genes. In addition, *SREBF1* and *SREBF2*, which encode *SREBP-1a, SREBP-1c*, and *SREBP-2*, respectively, as well as *MLXIPL* (also known as *ChREBP*), are major TFs that drive adipocyte de novo lipogenesis [70]. Their expression was up-regulated during iBAT whitening, suggesting enhanced de novo lipogenesis. Recent studies also show that BAT whitening is enhanced by BAT-specific ChREBP-β overexpression and prevented in ChREBP-deficient mice [33,43]. Whether identified TFs, such as *AR, TWIST2*, and the IRF family promote BAT whitening remain open research avenues. Moreover, epigenetic regulations such as DNA methylation and histone modification alter epigenetic effector activity, thereby altering the chromatin landscape and gene regulation, ultimately affecting adipocyte function [28,71,72]. Our results also show that epigenetic regulations, such as chromatin and histone modification, are enhanced during iBAT whitening.

## Limitations

Current research focuses on bioinformatic analysis to discover potential regulatory mechanisms promoting iBAT whitening in rabbits, and our findings require further experimental validation. Since it is currently difficult to conduct genetic manipulations in rabbits, especially for construction of rabbit models with tissue-specific gene deletions, some verification studies may need to be carried out in mice.

## Conclusion

In summary, we demonstrate that the phenomenon of iBAT whitening occurs in rabbits using MRI imaging together with anatomical and histological observations. Thus, brown adipocytes in BAT change from small multi-locular fat droplets to large white adipocytes-like single-locular fat droplets. The present study provides valuable insights into gene regulation during BAT whitening. A detailed transcriptome analysis was performed to understand the underlying regulatory mechanisms of iBAT whitening in rabbits under physiological conditions. We found that most of the marker genes used to distinguish BAT and WAT in humans and mice can be applied to a rabbit model. New marker genes were also identified and used to discern BAT and WAT. We also observed that iBAT whitening was associated with an up-regulation of approximately 95% of genes, suggesting it is an active process where extensive cellular remodelling or reprogramming takes place. Transcriptomic analyses highlighted that blood vessels, innervation and immune cells within iBAT change dramatically during whitening. Our findings are consistent with a mechanism whereby immune cells and immune regulation orchestrate the whitening process and increase lipid biosynthesis through transcription and epigenetic regulation in adipocytes. The genes and pathways associated with the iBAT whitening revealed here are candidates for subsequent validation studies and could serve as potentially new targets for the prevention and treatment of obesity by reactivating the Whitened BAT.

## Materials and methods

### Animals

Male New England rabbits of different age states were purchased from Guangzhou Bai Yun District Long Gui Xing Ke Animal Farm. Rabbits were housed at a temperature of 22–23°C with free access to food and water. According to the literature [12] and the results of our MRI pilot experiment, rabbit samples were divided into \six groups by the age: 1 day (D1), 14 days (D14), 1 month (M1), 2 months (M2), 3 months (M3) and 4 months (M4). Each group had 3–6 rabbits. iBATs, dBATs and ingWATs were collected, then frozen in liquid nitrogen and stored at −80°C until analysis. All rabbit experiments were approved by the Animal Care and Use Committee of the Guangzhou Institutes of Biomedicine and Health, Chinese Academy of Sciences.

### MRI imaging

MRI scans were completed using a 3.0 T MRI clinical scanner (uMR 790, Shanghai United Imaging healthcare, Shanghai, China) using a 3D 6-echo gradient-echo (GRE) sequence. A 12-channel rat coil and a 12-channel body coil were used to acquire images. Rabbits were anaesthetised with isoflurane (at a flow rate of 0.8 L/min 100% oxygen with 2.5% isoflurane concentration). The imaging parameters were as follows: transverse position, TR = 20.73 ms, TE = 3.80/5.71/7.62/9.53/11.44/13.35 ms, slice thickness = 2 mm, flip angle = 3°, number of averages = 10, bandwidth = 930 Hz/pixel, field of view (FOV) = 200 × 180 mm and pixel resolution = 0.82 × 0.74 mm2. The fat fraction (FF) was calculated as described previously [73].

### Haematoxylin-eosin staining and immunohistochemistry

Brown and white adipose tissues were fixed in 4% formaldehyde overnight at room temperature and embedded in paraffin before sectioning, then cut into 5 μm section with a microtome. Slides were deparaffinized, rehydrated and stained with haematoxylin and eosin and photographed under bright-field microscopy. Alternatively, sections were incubated with UCP1 antibody (MAB6158) followed by DAP chromogenic reaction. Imaging was performed on a slice scanner (Pannoramic MIDI).

### Western blot analysis

Protein lysates were collected from iBAT, dBAT and ingWAT samples derived from D1, D14, M1, M2, M3, and M4 rabbits. Briefly, 200ul RIPA buffer (including PMSF 1:100) was added to 30ug adipose tissue, which was then homogenized. Protein concentration was determined using the BCA method. The primary antibody was a Human/Mouse Ucp1 Antibody (MAB6158) (1:1000) from RD SYSTEMS, and the secondary antibody was anti-mouse IgG, HRP-linked Antibody (#7076) (1:1000) from Cell Signalling Technology. HRP conjugated monoclonal mouse anti-beta actin (KC-5A08) (1:2000) from KangChen as the internal reference antibody.

### Quantitation of relative mitochondrial DNA (mtDNA) copy number

Adipose tissue genomic DNA was extracted using a TIANamp Genomic DNA Kit (DP304-03). mtDNA copy number was determined by qPCR of the mitochondrial encoded gene *ND1* normalized to the nuclear encoded gene, *LPL*. qPCR was performed with 50 ng of DNA using Hieff UNICON® Univeral Blue qPCR SYBR Green Master Mix (11184ES03). qPCR data was analysed by the 2^−∆∆Ct^ method. PCR primers for ND1 amplification were as follows: forward primer 5’-ACCCTAGCAGAAACCAACCG-3’, reverse primer 5’-TCCACATTGAAGCCGGAGAC-3’. Forward primer 5’- TGTATGAGAGTTGGGTGCCG-3’ and reverse primer 5’- AACAGCCAGTCCACCACAAT-3’ were used for LPL amplification.

### RNA sequencing

Rabbit iBAT_D1, iBAT_D14, iBAT_M1, iBAT_M4 and ingWAT_M4 were selected for RNA sequencing. Total RNA was extracted from the adipose tissue using TRIzol® Reagent according to the manufacturer’s instructions (Invitrogen) and genomic DNA was removed using DNase I (TaKara). RNA quality was determined by agarose gel electrophoresis and an Agilent 2100 Bioanalyser. RNA purity and concentration were determined using the ND-2000 (NanoDrop Technologies). Only high-quality RNA samples (OD260/280 = 1.8 ~ 2.2, OD260/230 ≥ 2.0, RIN ≥ 6.5, 28S/18S ≥ 1.0, >1 μg) were used to construct sequencing libraries.

RNA-seq transcriptome libraries were prepared using a TruSeqTM RNA sample preparation kit from Illumina (San Diego, CA) with 1 μg of total RNA. mRNA was isolated according to poly A selection method using oligo(dT) beads and then fragmented by fragmentation buffer. Double-stranded cDNA was then synthesized using a SuperScript double-stranded cDNA synthesis kit (Invitrogen, CA) with random hexamer primers (Illumina). Synthesized cDNA was subjected to end-repair, phosphorylation and ‘A’ base addition according to Illumina’s library construction protocol. Libraries were size selected for cDNA target fragments of 300 bp on 2% Low Range Ultra Agarose followed by PCR amplified using Phusion DNA polymerase (NEB) for 15 PCR cycles. After quantification by TBS380, paired-end RNA-seq sequencing libraries were sequenced with the Illumina NovaSeq 6000 sequencer (2 × 150 bp read length).

Data were analysed on the free online platform of Majorbio Cloud Platform (www.majorbio.com). Methods of analysis are briefly described below.

### Differential expression analysis

The expression level of each transcript was calculated according to the transcripts per million reads (TPM) method. RSEM [74] (http://deweylab.biostat.wisc.edu/rsem/) was used to quantify gene abundance. Differential expression analysis was performed using DESeq2 [75] where Q value ≤ 0.05, and DEGs with Q value ≤ 0.05 and |log2FC| > 1 were considered to be significantly different expressed genes.

### Identification of novel BAT and WAT marker genes

To identify potentially new marker genes for BAT and WAT, we compared the DEGs between iBAT_D1 and ingWAT_M4 in rabbits with those of human BAT and WAT and mouse brown and white adipocytes as reported in BATLAS [29]. A │log2FC│ ≥ 0.5 and Padjust ≤ 0.05 was used as discriminating criteria to filter possible BAT and WAT marker genes. The candidate genes were further examined by inspection and comparison to iBAT_D14, iBAT_M1 and iBAT_M4 before manual elimination of outliers was carried out.

### Gene Ontology and Kyoto Encyclopaedia of Genes and Genomes pathway enrichment analysis

Gene Ontology (GO) and Kyoto Encyclopaedia of Genes and genomes (KEGG) pathway enrichment analysis were performed to identify which DEGs were significantly enriched in GO and KEGG terms with a Bonferroni-corrected P-value (Padjust) ≤ 0.05 compared with the whole-transcriptome background. GO functional enrichment and KEGG pathway analysis were carried out by Goatools (https://github.com/tanghaibao/Goatools) and KOBAS (http://kobas.cbi.pku.edu.cn/home.do) [76].

### Time course differential expression analysis

Time course differential expression analysis was performed according to maSigPro (http://www.bioconductor.org/packages/release/bioc/html/maSigPro.html), which can identify genes with different changes in the whole time-series node samples [77]. The Hclust clustering algorithm was adopted to obtain six clustering numbers.

### Temporal expression trend analysis

The Short Time-series Expression Miner (STEM) program [78] was used to identify temporal expression profiles. The STEM clustering algorithm was used and all parameters set to the default value. The results show that the time-series expression pattern with colour conforms to a significant change trend, while the time-series expression pattern without colour is a statistically non-significant change trend. Profiles with the same colour belong to the same cluster.

### Statistical analysis

Data are expressed as mean ± SEM. GraphPad Prism 8.0 was used for graphing and statistical analysis and one-way ANOVA with multiple comparison adjustment by Tukey’s test was used. P < 0.05 was considered statistically significant.

## Supplementary Material

Supplemental Material

## Data Availability

The raw RNA sequencing data generated in this study have been deposited in the Genome Sequence Archive of the National Genomics Data Center, China National Center for Bioinformation/Beijing Institute of Genomics, Chinese Academy of Sciences with the accession number CRA006767 (https://ngdc.cncb.ac.cn/gsa/s/VsrZ840p). The data supporting the findings of this study are available in this manuscript and its supplementary materials.
