# Supplementary material for: Comparative transcriptomic analysis of rabbit interscapular brown adipose tissue whitening under physiological conditions
Source: Adipocyte. 2022 Aug 29;11(1):529–49. doi: 10.1080/21623945.2022.2111053 (PMC9427046; doi:10.1080/21623945.2022.2111053)
Supplement: Supplemental Material [file KADI_A_2111053_SM2303.zip › Supplementary/4_Supplemental figure legends.doc]

**Supplementary figure legends**

**Figure S1. Rabbit dBAT and cervical brown adipose tissue (cBAT) convert to white-like adipose tissue after birth****.** (A) MRI images of rabbit cBAT at different ages. (B) Fat fraction of rabbit cBAT. (C) H&E staining of rabbit dBAT at different ages. Scale bars, 50 µm. (D) UCP1 protein levels of rabbit dBAT at different ages. Data represent mean ± SEM, ****P<0.0001.

**Figure S2. Statistics of DEGs between comparison groups.** (A) DEGs between iBAT_D1 and iBAT_D14, between iBAT_D1 and iBAT_M1, between iBAT_D1 and iBAT_M4, between iBAT_D1 and ingWAT_M4, between iBAT_D14 and iBAT_M1, between iBAT_D14 and iBAT_M4, between iBAT_D14 and ingWAT_M4, between iBAT_M1 and iBAT_M4, between iBAT_M1 and ingWAT_M4, and between iBAT_M4 and ingWAT_M4.

**Figure S3. BAT and WAT marker genes.** (A-B) Heatmap shows expression of BAT (A) and WAT (B) marker genes jointly shared by humans, rabbits and mouse. (C-D) Heatmap shows expression of BAT (C) and WAT (D) marker genes shared only between humans and rabbits.

**Figure S4. Expression of autophagy-related genes is enhanced during iBAT whitening.** (A) KEGG enrichment analysis of highly expressed genes in iBAT_M1 (Transition Phase). Diagram shows significant enrichment of several KEGG pathways (P ≤ 0.05). (B) KEGG enrichment analysis of highly expressed genes in iBAT_M4 (Whitened BAT Phase). Diagram shows significant enrichment of several KEGG pathways (Padjust ≤ 0.05). (C) Increased expression of autophagy-related genes during rabbit iBAT whitening. The genes in the red box are up-regulated genes. (D) Expression of mitophagy-related genes during rabbit iBAT whitening.

**Figure S5: Gene expression related to angiogenesis and innervation during rabbit iBAT whitening.** Heatmap shows the expression of genes involved in (A) blood vessel development, (B) platelet activation, (C) VEGF signaling pathway, (D) angiogenesis and (E) axon guidance.

**Figure S6: GO enrichment analysis of Profile 16, and GO enrichment analysis of differentially expressed up-regulated or down-regulated genes between iBAT_D1 and iBAT_D14,** **and between iBAT_D14 and iBAT_M1.** (A) GO enrichment analysis of Profile 16. Diagram shows significant enrichment of several GO pathways (Padjust ≤ 0.05). (B) GO enrichment analysis of differentially expressed up-regulated genes between iBAT_D1 and iBAT_D14. Diagram shows significant enrichment of several GO pathways (Padjust ≤ 0.05). (C) GO enrichment analysis of differentially expressed down-regulated genes between iBAT_D1 and iBAT_D14. Diagram shows significant enrichment of several GO pathways (Padjust ≤ 0.05). (D) GO enrichment analysis of differentially expressed up-regulated genes between iBAT_D14 and iBAT_M1. Diagram shows significant enrichment of several GO pathways (Padjust ≤ 0.05).
