# Supplementary material for: Comparative transcriptomic analysis of rabbit interscapular brown adipose tissue whitening under physiological conditions
Source: Adipocyte. 2022 Aug 29;11(1):529–49. doi: 10.1080/21623945.2022.2111053 (PMC9427046; doi:10.1080/21623945.2022.2111053)
Supplement: Supplemental Material [file KADI_A_2111053_SM2303.zip › Supplementary/8_Supplemental table captions.doc]

**Supplementary table captions**

Table S1: Results of mapped reads in rabbit iBAT and ingWAT.

Table S2: Correlation coefficients between samples.

Table S3: DEGs of different comparison groups.

Table S4: Number of DEGs in different comparison groups.

Table S5: Statistical table of KEGG enrichment analysis of highly expressed genes for the Brown Phase, Transition Phase and Whitened BAT Phase.

Table S6: Results of KEGG enrichment analysis for gene set including profile 15, 16, 25, 8, 24, 22 and 13.

Table S7: Results of GO enrichment analysis for gene set including profile 15, 16, 25, 8, 24, 22 and 13.

Table S8: Results of GO enrichment analysis for profile 15.

Table S9: Results of GO enrichment analysis for profile 16.

Table S10: Results of GO enrichment analysis between iBAT_D1 and iBAT_D14, between iBAT_D14 and iBAT_M1, and between iBAT_M1 and iBAT_M4.
