# Supplementary material for: Comparative transcriptomic analysis of rabbit interscapular brown adipose tissue whitening under physiological conditions
Source: Adipocyte. 2022 Aug 29;11(1):529–49. doi: 10.1080/21623945.2022.2111053 (PMC9427046; doi:10.1080/21623945.2022.2111053)
Supplement: Supplemental Material [file KADI_A_2111053_SM2303.zip › Supplementary/Table S10.docx]

**Table S10: Results of GO enrichment analysis between iBAT_D1 and iBAT_D14, between iBAT_D14 and iBAT_M1, and between iBAT_M1 and iBAT_M4.**

iBAT_D1 VS iBAT_D14 up

| go_id | go_type | discription | p_uncorrected | p_corrected |
| --- | --- | --- | --- | --- |
| GO:2000173 | BP | negative regulation of branching morphogenesis of a nerve | 1.73E-07 | 0.00159007 |
| GO:0033366 | BP | protein localization to secretory granule | 8.15E-07 | 0.00159007 |
| GO:0030070 | BP | insulin processing | 8.15E-07 | 0.00159007 |
| GO:0034230 | BP | enkephalin processing | 8.53E-07 | 0.00159007 |
| GO:0016486 | BP | peptide hormone processing | 8.97E-07 | 0.00159007 |
| GO:0051270 | BP | regulation of cellular component movement | 1.04E-06 | 0.00159007 |
| GO:0030155 | BP | regulation of cell adhesion | 1.08E-06 | 0.00159007 |
| GO:0140448 | BP | signaling receptor ligand precursor processing | 1.18E-06 | 0.00159007 |
| GO:0045582 | BP | positive regulation of T cell differentiation | 1.37E-06 | 0.00159007 |
| GO:0007166 | BP | cell surface receptor signaling pathway | 1.43E-06 | 0.00159007 |
| GO:0030334 | BP | regulation of cell migration | 1.45E-06 | 0.00159007 |
| GO:0045597 | BP | positive regulation of cell differentiation | 1.57E-06 | 0.00159007 |
| GO:0001932 | BP | regulation of protein phosphorylation | 1.65E-06 | 0.00159007 |
| GO:0022407 | BP | regulation of cell-cell adhesion | 1.65E-06 | 0.00159007 |
| GO:2000145 | BP | regulation of cell motility | 1.77E-06 | 0.00159007 |
| GO:0043408 | BP | regulation of MAPK cascade | 1.82E-06 | 0.00159007 |
| GO:0008284 | BP | positive regulation of cell population proliferation | 1.83E-06 | 0.00159007 |
| GO:0051094 | BP | positive regulation of developmental process | 1.94E-06 | 0.00159007 |
| GO:0070887 | BP | cellular response to chemical stimulus | 2.09E-06 | 0.00159007 |
| GO:0033993 | BP | response to lipid | 2.16E-06 | 0.00159007 |
| GO:0051240 | BP | positive regulation of multicellular organismal process | 2.19E-06 | 0.00159007 |
| GO:0042325 | BP | regulation of phosphorylation | 2.35E-06 | 0.00159007 |
| GO:2000026 | BP | regulation of multicellular organismal development | 2.45E-06 | 0.00159007 |
| GO:0042221 | BP | response to chemical | 2.48E-06 | 0.00159007 |
| GO:0009967 | BP | positive regulation of signal transduction | 2.48E-06 | 0.00159007 |
| GO:0010817 | BP | regulation of hormone levels | 2.56E-06 | 0.00159007 |
| GO:0009653 | BP | anatomical structure morphogenesis | 2.69E-06 | 0.00159007 |
| GO:0032268 | BP | regulation of cellular protein metabolic process | 2.71E-06 | 0.00159007 |
| GO:0051239 | BP | regulation of multicellular organismal process | 2.73E-06 | 0.00159007 |
| GO:0050793 | BP | regulation of developmental process | 2.75E-06 | 0.00159007 |
| GO:0030336 | BP | negative regulation of cell migration | 2.86E-06 | 0.00159007 |
| GO:0051246 | BP | regulation of protein metabolic process | 2.87E-06 | 0.00159007 |
| GO:0010033 | BP | response to organic substance | 2.87E-06 | 0.00159007 |
| GO:0042127 | BP | regulation of cell population proliferation | 3.08E-06 | 0.001612305 |
| GO:0001934 | BP | positive regulation of protein phosphorylation | 3.17E-06 | 0.001616539 |
| GO:0040012 | BP | regulation of locomotion | 3.25E-06 | 0.001616582 |
| GO:0051271 | BP | negative regulation of cellular component movement | 3.61E-06 | 0.001709594 |
| GO:0070372 | BP | regulation of ERK1 and ERK2 cascade | 3.61E-06 | 0.001709594 |
| GO:1901142 | BP | insulin metabolic process | 3.95E-06 | 0.001755699 |
| GO:0019220 | BP | regulation of phosphate metabolic process | 4.19E-06 | 0.001755699 |
| GO:0048511 | BP | rhythmic process | 4.20E-06 | 0.001755699 |
| GO:0051174 | BP | regulation of phosphorus metabolic process | 4.21E-06 | 0.001755699 |
| GO:0010647 | BP | positive regulation of cell communication | 4.38E-06 | 0.001755699 |
| GO:0023056 | BP | positive regulation of signaling | 4.41E-06 | 0.001755699 |
| GO:0071310 | BP | cellular response to organic substance | 4.44E-06 | 0.001755699 |
| GO:1902533 | BP | positive regulation of intracellular signal transduction | 4.50E-06 | 0.001755699 |
| GO:0048584 | BP | positive regulation of response to stimulus | 4.80E-06 | 0.001805047 |
| GO:0045580 | BP | regulation of T cell differentiation | 4.80E-06 | 0.001805047 |
| GO:0031399 | BP | regulation of protein modification process | 5.05E-06 | 0.001835002 |
| GO:2000146 | BP | negative regulation of cell motility | 5.45E-06 | 0.001938263 |
| GO:2000172 | BP | regulation of branching morphogenesis of a nerve | 5.78E-06 | 0.001983692 |
| GO:1903037 | BP | regulation of leukocyte cell-cell adhesion | 6.14E-06 | 0.002046414 |
| GO:1902107 | BP | positive regulation of leukocyte differentiation | 6.17E-06 | 0.002046414 |
| GO:0042327 | BP | positive regulation of phosphorylation | 6.87E-06 | 0.002211578 |
| GO:0050863 | BP | regulation of T cell activation | 6.89E-06 | 0.002211578 |
| GO:0045621 | BP | positive regulation of lymphocyte differentiation | 7.69E-06 | 0.002431114 |
| GO:0048519 | BP | negative regulation of biological process | 8.35E-06 | 0.002598738 |
| GO:0048583 | BP | regulation of response to stimulus | 8.81E-06 | 0.002650511 |
| GO:0003214 | BP | cardiac left ventricle morphogenesis | 8.92E-06 | 0.002650511 |
| GO:1902531 | BP | regulation of intracellular signal transduction | 9.15E-06 | 0.002678082 |
| GO:0030154 | BP | cell differentiation | 9.56E-06 | 0.002759123 |
| GO:0032496 | BP | response to lipopolysaccharide | 9.95E-06 | 0.002829008 |
| GO:0009966 | BP | regulation of signal transduction | 1.03E-05 | 0.002838578 |
| GO:0050896 | BP | response to stimulus | 1.03E-05 | 0.002838578 |
| GO:0007162 | BP | negative regulation of cell adhesion | 1.19E-05 | 0.003237327 |
| GO:0065007 | BP | biological regulation | 1.25E-05 | 0.003337874 |
| GO:0014066 | BP | regulation of phosphatidylinositol 3-kinase signaling | 1.26E-05 | 0.003337874 |
| GO:0043067 | BP | regulation of programmed cell death | 1.29E-05 | 0.003341592 |
| GO:0010646 | BP | regulation of cell communication | 1.38E-05 | 0.003515988 |
| GO:0045595 | BP | regulation of cell differentiation | 1.43E-05 | 0.003592628 |
| GO:0051272 | BP | positive regulation of cellular component movement | 1.45E-05 | 0.003592628 |
| GO:0048518 | BP | positive regulation of biological process | 1.46E-05 | 0.003592628 |
| GO:0040013 | BP | negative regulation of locomotion | 1.49E-05 | 0.003615629 |
| GO:0007165 | BP | signal transduction | 1.58E-05 | 0.003794142 |
| GO:0042981 | BP | regulation of apoptotic process | 1.66E-05 | 0.003930165 |
| GO:0022409 | BP | positive regulation of cell-cell adhesion | 1.80E-05 | 0.004117885 |
| GO:0007623 | BP | circadian rhythm | 1.93E-05 | 0.004260874 |
| GO:1905114 | BP | cell surface receptor signaling pathway involved in cell-cell signaling | 1.96E-05 | 0.004292251 |
| GO:0014068 | BP | positive regulation of phosphatidylinositol 3-kinase signaling | 1.99E-05 | 0.004310843 |
| GO:0023051 | BP | regulation of signaling | 2.03E-05 | 0.00433202 |
| GO:0048522 | BP | positive regulation of cellular process | 2.16E-05 | 0.004534402 |
| GO:0043410 | BP | positive regulation of MAPK cascade | 2.21E-05 | 0.00458257 |
| GO:0009719 | BP | response to endogenous stimulus | 2.25E-05 | 0.004616541 |
| GO:0032502 | BP | developmental process | 2.40E-05 | 0.004840731 |
| GO:0045785 | BP | positive regulation of cell adhesion | 2.42E-05 | 0.004840731 |
| GO:0050867 | BP | positive regulation of cell activation | 2.43E-05 | 0.004840731 |
| GO:0048869 | BP | cellular developmental process | 2.51E-05 | 0.004946556 |
| GO:0050789 | BP | regulation of biological process | 2.68E-05 | 0.005232975 |
| GO:0010941 | BP | regulation of cell death | 2.74E-05 | 0.005305106 |
| GO:0002237 | BP | response to molecule of bacterial origin | 2.78E-05 | 0.005316277 |
| GO:2000147 | BP | positive regulation of cell motility | 2.86E-05 | 0.00542037 |
| GO:0065009 | BP | regulation of molecular function | 3.27E-05 | 0.006145598 |
| GO:1901700 | BP | response to oxygen-containing compound | 3.45E-05 | 0.006319004 |
| GO:0050870 | BP | positive regulation of T cell activation | 3.45E-05 | 0.006319004 |
| GO:0031401 | BP | positive regulation of protein modification process | 3.46E-05 | 0.006319004 |
| GO:0045859 | BP | regulation of protein kinase activity | 3.56E-05 | 0.006428967 |
| GO:0045937 | BP | positive regulation of phosphate metabolic process | 3.76E-05 | 0.006622252 |
| GO:0010562 | BP | positive regulation of phosphorus metabolic process | 3.76E-05 | 0.006622252 |
| GO:0043549 | BP | regulation of kinase activity | 3.88E-05 | 0.006781086 |
| GO:0030335 | BP | positive regulation of cell migration | 4.04E-05 | 0.006994477 |
| GO:0051247 | BP | positive regulation of protein metabolic process | 4.33E-05 | 0.00742618 |
| GO:0051896 | BP | regulation of protein kinase B signaling | 4.48E-05 | 0.007589709 |
| GO:0051248 | BP | negative regulation of protein metabolic process | 4.50E-05 | 0.007589709 |
| GO:0040017 | BP | positive regulation of locomotion | 4.54E-05 | 0.007589709 |
| GO:0001933 | BP | negative regulation of protein phosphorylation | 4.60E-05 | 0.007638239 |
| GO:0051338 | BP | regulation of transferase activity | 4.73E-05 | 0.007776779 |
| GO:0051251 | BP | positive regulation of lymphocyte activation | 4.97E-05 | 0.00805452 |
| GO:1904752 | BP | regulation of vascular associated smooth muscle cell migration | 5.10E-05 | 0.008129811 |
| GO:0050794 | BP | regulation of cellular process | 5.24E-05 | 0.008269144 |
| GO:0046879 | BP | hormone secretion | 5.27E-05 | 0.008269144 |
| GO:0009605 | BP | response to external stimulus | 5.32E-05 | 0.008275166 |
| GO:0045619 | BP | regulation of lymphocyte differentiation | 5.77E-05 | 0.008909998 |
| GO:0071900 | BP | regulation of protein serine/threonine kinase activity | 6.14E-05 | 0.009406581 |
| GO:0051241 | BP | negative regulation of multicellular organismal process | 6.95E-05 | 0.010569461 |
| GO:0042445 | BP | hormone metabolic process | 7.21E-05 | 0.010868349 |
| GO:0002696 | BP | positive regulation of leukocyte activation | 7.41E-05 | 0.011099037 |
| GO:1903039 | BP | positive regulation of leukocyte cell-cell adhesion | 8.17E-05 | 0.012145128 |
| GO:0043171 | BP | peptide catabolic process | 8.73E-05 | 0.012873983 |
| GO:0032270 | BP | positive regulation of cellular protein metabolic process | 9.29E-05 | 0.013599288 |
| GO:0046626 | BP | regulation of insulin receptor signaling pathway | 9.84E-05 | 0.014198607 |
| GO:0050790 | BP | regulation of catalytic activity | 0.000108352 | 0.015520788 |
| GO:0001649 | BP | osteoblast differentiation | 0.000110403 | 0.015590289 |
| GO:0009914 | BP | hormone transport | 0.000110403 | 0.015590289 |
| GO:0010942 | BP | positive regulation of cell death | 0.000119573 | 0.016766307 |
| GO:1903708 | BP | positive regulation of hemopoiesis | 0.000132528 | 0.018324736 |
| GO:0071495 | BP | cellular response to endogenous stimulus | 0.000135164 | 0.018441528 |
| GO:1902105 | BP | regulation of leukocyte differentiation | 0.000135242 | 0.018441528 |
| GO:0071396 | BP | cellular response to lipid | 0.000141529 | 0.018954097 |
| GO:1901342 | BP | regulation of vasculature development | 0.000141839 | 0.018954097 |
| GO:0051249 | BP | regulation of lymphocyte activation | 0.00014333 | 0.019025601 |
| GO:0071300 | BP | cellular response to retinoic acid | 0.000151581 | 0.019987641 |
| GO:0071496 | BP | cellular response to external stimulus | 0.000156594 | 0.020421891 |
| GO:0042326 | BP | negative regulation of phosphorylation | 0.000158257 | 0.020421891 |
| GO:0022603 | BP | regulation of anatomical structure morphogenesis | 0.000158977 | 0.020421891 |
| GO:0015697 | BP | quaternary ammonium group transport | 0.000162811 | 0.020780256 |
| GO:0033674 | BP | positive regulation of kinase activity | 0.000167128 | 0.02119549 |
| GO:0048523 | BP | negative regulation of cellular process | 0.000170914 | 0.021417003 |
| GO:0032501 | BP | multicellular organismal process | 0.000171026 | 0.021417003 |
| GO:0065008 | BP | regulation of biological quality | 0.000173835 | 0.0216327 |
| GO:0030072 | BP | peptide hormone secretion | 0.000175809 | 0.021742442 |
| GO:0016055 | BP | Wnt signaling pathway | 0.000189557 | 0.023297948 |
| GO:0014070 | BP | response to organic cyclic compound | 0.00019296 | 0.023570725 |
| GO:0051716 | BP | cellular response to stimulus | 0.000195891 | 0.023782896 |
| GO:0043066 | BP | negative regulation of apoptotic process | 0.000216734 | 0.025686826 |
| GO:0048660 | BP | regulation of smooth muscle cell proliferation | 0.000231911 | 0.027266005 |
| GO:0045860 | BP | positive regulation of protein kinase activity | 0.000232797 | 0.027266005 |
| GO:1900076 | BP | regulation of cellular response to insulin stimulus | 0.000242009 | 0.027856886 |
| GO:0051591 | BP | response to cAMP | 0.000242009 | 0.027856886 |
| GO:0051093 | BP | negative regulation of developmental process | 0.000242443 | 0.027856886 |
| GO:0048585 | BP | negative regulation of response to stimulus | 0.000261723 | 0.029608946 |
| GO:0043069 | BP | negative regulation of programmed cell death | 0.000271372 | 0.030370322 |
| GO:0007155 | BP | cell adhesion | 0.000271504 | 0.030370322 |
| GO:1904705 | BP | regulation of vascular associated smooth muscle cell proliferation | 0.000278422 | 0.030798119 |
| GO:0003208 | BP | cardiac ventricle morphogenesis | 0.000278422 | 0.030798119 |
| GO:1901701 | BP | cellular response to oxygen-containing compound | 0.000294861 | 0.032258125 |
| GO:0022610 | BP | biological adhesion | 0.000310974 | 0.033834952 |
| GO:0060548 | BP | negative regulation of cell death | 0.00032039 | 0.034669987 |
| GO:1901343 | BP | negative regulation of vasculature development | 0.000332137 | 0.035746919 |
| GO:0031400 | BP | negative regulation of protein modification process | 0.000375459 | 0.040192262 |
| GO:1900744 | BP | regulation of p38MAPK cascade | 0.000385176 | 0.041011988 |
| GO:0051172 | BP | negative regulation of nitrogen compound metabolic process | 0.000388404 | 0.041135675 |
| GO:0032269 | BP | negative regulation of cellular protein metabolic process | 0.000394866 | 0.041221779 |
| GO:0048646 | BP | anatomical structure formation involved in morphogenesis | 0.000399375 | 0.041221779 |
| GO:0006631 | BP | fatty acid metabolic process | 0.000401337 | 0.041221779 |
| GO:0045936 | BP | negative regulation of phosphate metabolic process | 0.000403509 | 0.041221779 |
| GO:0010563 | BP | negative regulation of phosphorus metabolic process | 0.000403509 | 0.041221779 |
| GO:0071902 | BP | positive regulation of protein serine/threonine kinase activity | 0.000405091 | 0.041221779 |
| GO:0051347 | BP | positive regulation of transferase activity | 0.000414118 | 0.041221779 |
| GO:1903984 | BP | positive regulation of TRAIL-activated apoptotic signaling pathway | 0.00041817 | 0.041221779 |
| GO:0051040 | BP | regulation of calcium-independent cell-cell adhesion | 0.00041817 | 0.041221779 |
| GO:0035799 | BP | ureter maturation | 0.00041817 | 0.041221779 |
| GO:1903121 | BP | regulation of TRAIL-activated apoptotic signaling pathway | 0.00041817 | 0.041221779 |
| GO:0090335 | BP | regulation of brown fat cell differentiation | 0.000422342 | 0.041221779 |
| GO:0032292 | BP | peripheral nervous system axon ensheathment | 0.000422342 | 0.041221779 |
| GO:0022011 | BP | myelination in peripheral nervous system | 0.000422342 | 0.041221779 |
| GO:1904754 | BP | positive regulation of vascular associated smooth muscle cell migration | 0.000442114 | 0.042941091 |
| GO:0046638 | BP | positive regulation of alpha-beta T cell differentiation | 0.000448894 | 0.043195592 |
| GO:0002694 | BP | regulation of leukocyte activation | 0.000449073 | 0.043195592 |
| GO:0070848 | BP | response to growth factor | 0.000453943 | 0.043454145 |
| GO:0001822 | BP | kidney development | 0.000481595 | 0.045880569 |
| GO:0043086 | BP | negative regulation of catalytic activity | 0.00048481 | 0.045966946 |
| GO:0043068 | BP | positive regulation of programmed cell death | 0.000501837 | 0.047355817 |
| GO:0006082 | BP | organic acid metabolic process | 0.000515451 | 0.048411015 |
| GO:0003206 | BP | cardiac chamber morphogenesis | 0.000520239 | 0.04863131 |
| GO:0043372 | BP | positive regulation of CD4-positive, alpha-beta T cell differentiation | 0.00052636 | 0.048728106 |
| GO:0009725 | BP | response to hormone | 0.00054273 | 0.049570184 |
| GO:0005615 | CC | extracellular space | 1.18E-06 | 0.00159007 |
| GO:0042611 | CC | MHC protein complex | 2.98E-06 | 0.001602933 |
| GO:0005576 | CC | extracellular region | 4.16E-06 | 0.001755699 |
| GO:0042613 | CC | MHC class II protein complex | 8.92E-06 | 0.002650511 |
| GO:0062023 | CC | collagen-containing extracellular matrix | 2.05E-05 | 0.00433202 |
| GO:0031012 | CC | extracellular matrix | 4.98E-05 | 0.00805452 |
| GO:0098590 | CC | plasma membrane region | 0.000158977 | 0.020421891 |
| GO:0031045 | CC | dense core granule | 0.000197804 | 0.023869543 |
| GO:0016324 | CC | apical plasma membrane | 0.000208171 | 0.024969206 |
| GO:0043235 | CC | receptor complex | 0.000209936 | 0.025030135 |
| GO:0009986 | CC | cell surface | 0.000243438 | 0.027856886 |
| GO:0035976 | CC | transcription factor AP-1 complex | 0.00041817 | 0.041221779 |
| GO:0031224 | CC | intrinsic component of membrane | 0.000528616 | 0.048728106 |
| GO:0005102 | MF | signaling receptor binding | 1.42E-06 | 0.00159007 |
| GO:0005515 | MF | protein binding | 5.07E-06 | 0.001835002 |
| GO:0050897 | MF | cobalt ion binding | 5.78E-06 | 0.001983692 |
| GO:0042043 | MF | neurexin family protein binding | 1.28E-05 | 0.003341592 |
| GO:0017017 | MF | MAP kinase tyrosine/serine/threonine phosphatase activity | 1.78E-05 | 0.004113259 |
| GO:0033549 | MF | MAP kinase phosphatase activity | 1.78E-05 | 0.004113259 |
| GO:0001664 | MF | G protein-coupled receptor binding | 3.58E-05 | 0.006428967 |
| GO:0043121 | MF | neurotrophin binding | 5.10E-05 | 0.008129811 |
| GO:0030165 | MF | PDZ domain binding | 0.000125077 | 0.017415431 |
| GO:0070412 | MF | R-SMAD binding | 0.000259487 | 0.029523684 |
| GO:0005030 | MF | neurotrophin receptor activity | 0.000280585 | 0.030865852 |
| GO:0032395 | MF | MHC class II receptor activity | 0.00041817 | 0.041221779 |
| GO:0004180 | MF | carboxypeptidase activity | 0.000528177 | 0.048728106 |
| GO:0019838 | MF | growth factor binding | 0.000532648 | 0.048873563 |

iBAT_D1 VS iBAT_D14 down

| go_id | go_type | discription | p_uncorrected | p_corrected |
| --- | --- | --- | --- | --- |
| GO:0009615 | BP | response to virus | 4.52E-07 | 0.002549 |
| GO:0006721 | BP | terpenoid metabolic process | 4.97E-07 | 0.002549 |
| GO:0017144 | BP | drug metabolic process | 5.06E-07 | 0.002549 |
| GO:0051607 | BP | defense response to virus | 5.26E-07 | 0.002549 |
| GO:0006082 | BP | organic acid metabolic process | 9.68E-07 | 0.002549 |
| GO:0051707 | BP | response to other organism | 9.77E-07 | 0.002549 |
| GO:0006631 | BP | fatty acid metabolic process | 1.28E-06 | 0.002549 |
| GO:0032787 | BP | monocarboxylic acid metabolic process | 1.35E-06 | 0.002549 |
| GO:0006629 | BP | lipid metabolic process | 1.39E-06 | 0.002549 |
| GO:0006720 | BP | isoprenoid metabolic process | 1.46E-06 | 0.002549 |
| GO:0019752 | BP | carboxylic acid metabolic process | 1.47E-06 | 0.002549 |
| GO:0043436 | BP | oxoacid metabolic process | 1.54E-06 | 0.002549 |
| GO:0009617 | BP | response to bacterium | 2.09E-06 | 0.002728 |
| GO:0044281 | BP | small molecule metabolic process | 2.23E-06 | 0.002728 |
| GO:0043207 | BP | response to external biotic stimulus | 2.28E-06 | 0.002728 |
| GO:0006775 | BP | fat-soluble vitamin metabolic process | 3.99E-06 | 0.003898 |
| GO:0009607 | BP | response to biotic stimulus | 5.46E-06 | 0.00494 |
| GO:0044255 | BP | cellular lipid metabolic process | 6.96E-06 | 0.006023 |
| GO:0034754 | BP | cellular hormone metabolic process | 1.66E-05 | 0.013049 |
| GO:0016098 | BP | monoterpenoid metabolic process | 2.81E-05 | 0.018656 |
| GO:0006979 | BP | response to oxidative stress | 3.02E-05 | 0.019416 |
| GO:0042445 | BP | hormone metabolic process | 4.08E-05 | 0.025385 |
| GO:0051983 | BP | regulation of chromosome segregation | 5.01E-05 | 0.029966 |
| GO:0022402 | BP | cell cycle process | 6.01E-05 | 0.034183 |
| GO:0002933 | BP | lipid hydroxylation | 6.93E-05 | 0.035522 |
| GO:1901605 | BP | alpha-amino acid metabolic process | 7.00E-05 | 0.035522 |
| GO:0006575 | BP | cellular modified amino acid metabolic process | 7.15E-05 | 0.035522 |
| GO:0001523 | BP | retinoid metabolic process | 7.31E-05 | 0.035522 |
| GO:0008202 | BP | steroid metabolic process | 9.66E-05 | 0.044368 |
| GO:0016101 | BP | diterpenoid metabolic process | 9.80E-05 | 0.044368 |
| GO:0034599 | BP | cellular response to oxidative stress | 0.000101 | 0.04454 |
| GO:0005615 | CC | extracellular space | 3.61E-06 | 0.003898 |
| GO:0005759 | CC | mitochondrial matrix | 7.24E-05 | 0.035522 |
| GO:0016491 | MF | oxidoreductase activity | 2.33E-06 | 0.002728 |
| GO:0016709 | MF | oxidoreductase activity, acting on paired donors, with incorporation or reduction of molecular oxygen, NAD(P)H as one donor, and incorporation of one atom of oxygen | 4.06E-06 | 0.003898 |
| GO:0004497 | MF | monooxygenase activity | 4.11E-06 | 0.003898 |
| GO:0005506 | MF | iron ion binding | 1.70E-05 | 0.013049 |
| GO:0008374 | MF | O-acyltransferase activity | 1.96E-05 | 0.014454 |
| GO:0016416 | MF | O-palmitoyltransferase activity | 2.81E-05 | 0.018656 |
| GO:0004095 | MF | carnitine O-palmitoyltransferase activity | 2.81E-05 | 0.018656 |
| GO:0016705 | MF | oxidoreductase activity, acting on paired donors, with incorporation or reduction of molecular oxygen | 5.12E-05 | 0.029966 |
| GO:0101020 | MF | estrogen 16-alpha-hydroxylase activity | 6.93E-05 | 0.035522 |
| GO:0050221 | MF | prostaglandin-E2 9-reductase activity | 8.20E-05 | 0.03886 |

iBAT_D14 VS iBAT_M1 up

| go_id | go_type | discription | p_uncorrected | p_corrected |
| --- | --- | --- | --- | --- |
| GO:1903900 | BP | regulation of viral life cycle | 3.34E-07 | 0.000744 |
| GO:0009615 | BP | response to virus | 4.14E-07 | 0.000744 |
| GO:0045071 | BP | negative regulation of viral genome replication | 4.44E-07 | 0.000744 |
| GO:0002474 | BP | antigen processing and presentation of peptide antigen via MHC class I | 4.44E-07 | 0.000744 |
| GO:0045069 | BP | regulation of viral genome replication | 4.45E-07 | 0.000744 |
| GO:0045088 | BP | regulation of innate immune response | 4.84E-07 | 0.000744 |
| GO:0048525 | BP | negative regulation of viral process | 5.18E-07 | 0.000744 |
| GO:0002819 | BP | regulation of adaptive immune response | 5.21E-07 | 0.000744 |
| GO:0050792 | BP | regulation of viral process | 5.55E-07 | 0.000744 |
| GO:0002694 | BP | regulation of leukocyte activation | 5.84E-07 | 0.000744 |
| GO:0051607 | BP | defense response to virus | 6.50E-07 | 0.000744 |
| GO:0032020 | BP | ISG15-protein conjugation | 7.21E-07 | 0.000744 |
| GO:0019221 | BP | cytokine-mediated signaling pathway | 7.36E-07 | 0.000744 |
| GO:1903901 | BP | negative regulation of viral life cycle | 7.62E-07 | 0.000744 |
| GO:0060337 | BP | type I interferon signaling pathway | 8.10E-07 | 0.000744 |
| GO:0002697 | BP | regulation of immune effector process | 8.30E-07 | 0.000744 |
| GO:0006954 | BP | inflammatory response | 8.43E-07 | 0.000744 |
| GO:0050778 | BP | positive regulation of immune response | 8.72E-07 | 0.000744 |
| GO:0060759 | BP | regulation of response to cytokine stimulus | 9.08E-07 | 0.000744 |
| GO:1901701 | BP | cellular response to oxygen-containing compound | 9.61E-07 | 0.000744 |
| GO:0043903 | BP | regulation of symbiotic process | 1.04E-06 | 0.000744 |
| GO:0001819 | BP | positive regulation of cytokine production | 1.06E-06 | 0.000744 |
| GO:0002684 | BP | positive regulation of immune system process | 1.08E-06 | 0.000744 |
| GO:0045087 | BP | innate immune response | 1.15E-06 | 0.000744 |
| GO:0051707 | BP | response to other organism | 1.17E-06 | 0.000744 |
| GO:0098542 | BP | defense response to other organism | 1.18E-06 | 0.000744 |
| GO:0002699 | BP | positive regulation of immune effector process | 1.20E-06 | 0.000744 |
| GO:0002831 | BP | regulation of response to biotic stimulus | 1.29E-06 | 0.000774 |
| GO:0001817 | BP | regulation of cytokine production | 1.35E-06 | 0.000774 |
| GO:0050776 | BP | regulation of immune response | 1.36E-06 | 0.000774 |
| GO:0050727 | BP | regulation of inflammatory response | 1.43E-06 | 0.000781 |
| GO:0002252 | BP | immune effector process | 1.49E-06 | 0.000781 |
| GO:0006955 | BP | immune response | 1.54E-06 | 0.000781 |
| GO:0031347 | BP | regulation of defense response | 1.60E-06 | 0.000781 |
| GO:0002682 | BP | regulation of immune system process | 1.76E-06 | 0.000781 |
| GO:0048660 | BP | regulation of smooth muscle cell proliferation | 1.77E-06 | 0.000781 |
| GO:0050777 | BP | negative regulation of immune response | 1.82E-06 | 0.000781 |
| GO:0050865 | BP | regulation of cell activation | 1.84E-06 | 0.000781 |
| GO:0001775 | BP | cell activation | 1.93E-06 | 0.000781 |
| GO:0044419 | BP | interspecies interaction between organisms | 1.95E-06 | 0.000781 |
| GO:0043207 | BP | response to external biotic stimulus | 1.97E-06 | 0.000781 |
| GO:0007166 | BP | cell surface receptor signaling pathway | 2.00E-06 | 0.000781 |
| GO:0006952 | BP | defense response | 2.09E-06 | 0.000781 |
| GO:0002376 | BP | immune system process | 2.10E-06 | 0.000781 |
| GO:0071310 | BP | cellular response to organic substance | 2.21E-06 | 0.000781 |
| GO:0009605 | BP | response to external stimulus | 2.24E-06 | 0.000781 |
| GO:0080134 | BP | regulation of response to stress | 2.36E-06 | 0.000781 |
| GO:0002821 | BP | positive regulation of adaptive immune response | 2.37E-06 | 0.000781 |
| GO:0010647 | BP | positive regulation of cell communication | 2.40E-06 | 0.000781 |
| GO:0045321 | BP | leukocyte activation | 2.42E-06 | 0.000781 |
| GO:0023056 | BP | positive regulation of signaling | 2.45E-06 | 0.000781 |
| GO:0009607 | BP | response to biotic stimulus | 2.55E-06 | 0.000781 |
| GO:0009967 | BP | positive regulation of signal transduction | 2.60E-06 | 0.000786 |
| GO:0042127 | BP | regulation of cell population proliferation | 2.69E-06 | 0.000791 |
| GO:0051240 | BP | positive regulation of multicellular organismal process | 2.71E-06 | 0.000791 |
| GO:0051239 | BP | regulation of multicellular organismal process | 2.74E-06 | 0.000791 |
| GO:0070887 | BP | cellular response to chemical stimulus | 2.84E-06 | 0.000796 |
| GO:0032101 | BP | regulation of response to external stimulus | 2.90E-06 | 0.000802 |
| GO:1901700 | BP | response to oxygen-containing compound | 2.96E-06 | 0.000807 |
| GO:0009966 | BP | regulation of signal transduction | 3.18E-06 | 0.00084 |
| GO:0010033 | BP | response to organic substance | 3.18E-06 | 0.00084 |
| GO:0006950 | BP | response to stress | 3.32E-06 | 0.000858 |
| GO:0050793 | BP | regulation of developmental process | 3.54E-06 | 0.000875 |
| GO:0002683 | BP | negative regulation of immune system process | 3.58E-06 | 0.000875 |
| GO:0032675 | BP | regulation of interleukin-6 production | 3.60E-06 | 0.000875 |
| GO:2000026 | BP | regulation of multicellular organismal development | 3.74E-06 | 0.00088 |
| GO:0048584 | BP | positive regulation of response to stimulus | 3.80E-06 | 0.00088 |
| GO:0048583 | BP | regulation of response to stimulus | 4.12E-06 | 0.000935 |
| GO:0007165 | BP | signal transduction | 4.16E-06 | 0.000935 |
| GO:0050896 | BP | response to stimulus | 4.19E-06 | 0.000935 |
| GO:0042221 | BP | response to chemical | 4.23E-06 | 0.000935 |
| GO:0010646 | BP | regulation of cell communication | 4.37E-06 | 0.000956 |
| GO:0009617 | BP | response to bacterium | 4.77E-06 | 0.001033 |
| GO:0048518 | BP | positive regulation of biological process | 4.86E-06 | 0.00104 |
| GO:0002822 | BP | regulation of adaptive immune response based on somatic recombination of immune receptors built from immunoglobulin superfamily domains | 5.03E-06 | 0.001064 |
| GO:1903706 | BP | regulation of hemopoiesis | 5.15E-06 | 0.001068 |
| GO:0050789 | BP | regulation of biological process | 5.37E-06 | 0.001103 |
| GO:0045637 | BP | regulation of myeloid cell differentiation | 5.46E-06 | 0.00111 |
| GO:0065007 | BP | biological regulation | 5.94E-06 | 0.001188 |
| GO:0048519 | BP | negative regulation of biological process | 5.97E-06 | 0.001188 |
| GO:0023051 | BP | regulation of signaling | 6.12E-06 | 0.001206 |
| GO:0002705 | BP | positive regulation of leukocyte mediated immunity | 6.35E-06 | 0.001206 |
| GO:0001959 | BP | regulation of cytokine-mediated signaling pathway | 6.35E-06 | 0.001206 |
| GO:0051716 | BP | cellular response to stimulus | 6.36E-06 | 0.001206 |
| GO:0014070 | BP | response to organic cyclic compound | 7.18E-06 | 0.001337 |
| GO:0051241 | BP | negative regulation of multicellular organismal process | 7.56E-06 | 0.001393 |
| GO:0034340 | BP | response to type I interferon | 7.97E-06 | 0.00145 |
| GO:0002521 | BP | leukocyte differentiation | 8.01E-06 | 0.00145 |
| GO:0048522 | BP | positive regulation of cellular process | 8.72E-06 | 0.001564 |
| GO:0031349 | BP | positive regulation of defense response | 9.44E-06 | 0.001679 |
| GO:0034097 | BP | response to cytokine | 9.78E-06 | 0.001703 |
| GO:0002824 | BP | positive regulation of adaptive immune response based on somatic recombination of immune receptors built from immunoglobulin superfamily domains | 1.04E-05 | 0.001781 |
| GO:0002700 | BP | regulation of production of molecular mediator of immune response | 1.11E-05 | 0.001873 |
| GO:0008284 | BP | positive regulation of cell population proliferation | 1.13E-05 | 0.001885 |
| GO:0001818 | BP | negative regulation of cytokine production | 1.25E-05 | 0.002063 |
| GO:0002274 | BP | myeloid leukocyte activation | 1.28E-05 | 0.002095 |
| GO:1901699 | BP | cellular response to nitrogen compound | 1.38E-05 | 0.002239 |
| GO:0010604 | BP | positive regulation of macromolecule metabolic process | 1.49E-05 | 0.002392 |
| GO:0071359 | BP | cellular response to dsRNA | 1.56E-05 | 0.002477 |
| GO:0071357 | BP | cellular response to type I interferon | 1.59E-05 | 0.002477 |
| GO:0019885 | BP | antigen processing and presentation of endogenous peptide antigen via MHC class I | 1.59E-05 | 0.002477 |
| GO:0002483 | BP | antigen processing and presentation of endogenous peptide antigen | 1.59E-05 | 0.002477 |
| GO:0032103 | BP | positive regulation of response to external stimulus | 1.64E-05 | 0.002526 |
| GO:0032649 | BP | regulation of interferon-gamma production | 2.02E-05 | 0.003095 |
| GO:0060760 | BP | positive regulation of response to cytokine stimulus | 2.04E-05 | 0.003108 |
| GO:0045824 | BP | negative regulation of innate immune response | 2.12E-05 | 0.003196 |
| GO:0050688 | BP | regulation of defense response to virus | 2.20E-05 | 0.003265 |
| GO:0098586 | BP | cellular response to virus | 2.21E-05 | 0.003265 |
| GO:0009893 | BP | positive regulation of metabolic process | 2.37E-05 | 0.003449 |
| GO:0042832 | BP | defense response to protozoan | 2.37E-05 | 0.003449 |
| GO:1904018 | BP | positive regulation of vasculature development | 2.54E-05 | 0.003658 |
| GO:0050794 | BP | regulation of cellular process | 2.57E-05 | 0.003685 |
| GO:0002702 | BP | positive regulation of production of molecular mediator of immune response | 3.00E-05 | 0.004263 |
| GO:0050678 | BP | regulation of epithelial cell proliferation | 3.03E-05 | 0.004283 |
| GO:0048662 | BP | negative regulation of smooth muscle cell proliferation | 3.16E-05 | 0.004436 |
| GO:0033993 | BP | response to lipid | 3.33E-05 | 0.004622 |
| GO:0002282 | BP | microglial cell activation involved in immune response | 3.45E-05 | 0.004645 |
| GO:0071407 | BP | cellular response to organic cyclic compound | 3.46E-05 | 0.004645 |
| GO:0009892 | BP | negative regulation of metabolic process | 3.48E-05 | 0.004645 |
| GO:0045766 | BP | positive regulation of angiogenesis | 3.54E-05 | 0.004693 |
| GO:0002703 | BP | regulation of leukocyte mediated immunity | 4.15E-05 | 0.005478 |
| GO:0002825 | BP | regulation of T-helper 1 type immune response | 4.19E-05 | 0.005485 |
| GO:0002366 | BP | leukocyte activation involved in immune response | 4.27E-05 | 0.005558 |
| GO:0048523 | BP | negative regulation of cellular process | 4.58E-05 | 0.005883 |
| GO:0002263 | BP | cell activation involved in immune response | 5.33E-05 | 0.00676 |
| GO:0045765 | BP | regulation of angiogenesis | 5.42E-05 | 0.006828 |
| GO:0032642 | BP | regulation of chemokine production | 5.64E-05 | 0.00694 |
| GO:0009143 | BP | nucleoside triphosphate catabolic process | 5.68E-05 | 0.00694 |
| GO:0071360 | BP | cellular response to exogenous dsRNA | 5.68E-05 | 0.00694 |
| GO:0001562 | BP | response to protozoan | 5.68E-05 | 0.00694 |
| GO:0034599 | BP | cellular response to oxidative stress | 6.37E-05 | 0.00774 |
| GO:1901342 | BP | regulation of vasculature development | 7.37E-05 | 0.008837 |
| GO:0002724 | BP | regulation of T cell cytokine production | 7.37E-05 | 0.008837 |
| GO:0050867 | BP | positive regulation of cell activation | 7.45E-05 | 0.008882 |
| GO:0034308 | BP | primary alcohol metabolic process | 7.59E-05 | 0.008991 |
| GO:0014009 | BP | glial cell proliferation | 8.29E-05 | 0.009771 |
| GO:0051249 | BP | regulation of lymphocyte activation | 9.03E-05 | 0.010455 |
| GO:0030098 | BP | lymphocyte differentiation | 0.000111 | 0.012735 |
| GO:0002718 | BP | regulation of cytokine production involved in immune response | 0.000115 | 0.013124 |
| GO:0002726 | BP | positive regulation of T cell cytokine production | 0.000117 | 0.013282 |
| GO:0031348 | BP | negative regulation of defense response | 0.000123 | 0.013835 |
| GO:0032755 | BP | positive regulation of interleukin-6 production | 0.000124 | 0.013835 |
| GO:0030099 | BP | myeloid cell differentiation | 0.000128 | 0.014192 |
| GO:0032481 | BP | positive regulation of type I interferon production | 0.000132 | 0.014502 |
| GO:0010430 | BP | fatty acid omega-oxidation | 0.000135 | 0.014717 |
| GO:0019882 | BP | antigen processing and presentation | 0.000137 | 0.014951 |
| GO:0042981 | BP | regulation of apoptotic process | 0.000146 | 0.015675 |
| GO:0042116 | BP | macrophage activation | 0.000155 | 0.016548 |
| GO:0048002 | BP | antigen processing and presentation of peptide antigen | 0.000156 | 0.016573 |
| GO:0002827 | BP | positive regulation of T-helper 1 type immune response | 0.000162 | 0.016964 |
| GO:0060333 | BP | interferon-gamma-mediated signaling pathway | 0.000162 | 0.016964 |
| GO:0043067 | BP | regulation of programmed cell death | 0.000166 | 0.017254 |
| GO:0032722 | BP | positive regulation of chemokine production | 0.000166 | 0.017254 |
| GO:0071396 | BP | cellular response to lipid | 0.000169 | 0.01737 |
| GO:0048661 | BP | positive regulation of smooth muscle cell proliferation | 0.000169 | 0.01737 |
| GO:0002696 | BP | positive regulation of leukocyte activation | 0.000174 | 0.017752 |
| GO:0062197 | BP | cellular response to chemical stress | 0.000191 | 0.019453 |
| GO:0002828 | BP | regulation of type 2 immune response | 0.000193 | 0.019538 |
| GO:0002711 | BP | positive regulation of T cell mediated immunity | 0.000198 | 0.019937 |
| GO:0002281 | BP | macrophage activation involved in immune response | 0.000201 | 0.020089 |
| GO:1901292 | BP | nucleoside phosphate catabolic process | 0.000219 | 0.021669 |
| GO:0031331 | BP | positive regulation of cellular catabolic process | 0.00022 | 0.021719 |
| GO:0048545 | BP | response to steroid hormone | 0.000231 | 0.022505 |
| GO:0002709 | BP | regulation of T cell mediated immunity | 0.000259 | 0.025172 |
| GO:0050679 | BP | positive regulation of epithelial cell proliferation | 0.000267 | 0.025783 |
| GO:0002720 | BP | positive regulation of cytokine production involved in immune response | 0.000277 | 0.026434 |
| GO:2000106 | BP | regulation of leukocyte apoptotic process | 0.000277 | 0.026434 |
| GO:0061900 | BP | glial cell activation | 0.000288 | 0.027063 |
| GO:0002753 | BP | cytoplasmic pattern recognition receptor signaling pathway | 0.000288 | 0.027063 |
| GO:0032479 | BP | regulation of type I interferon production | 0.000291 | 0.027069 |
| GO:0030217 | BP | T cell differentiation | 0.000291 | 0.027069 |
| GO:0031343 | BP | positive regulation of cell killing | 0.000297 | 0.027471 |
| GO:0050707 | BP | regulation of cytokine secretion | 0.000298 | 0.027471 |
| GO:1902533 | BP | positive regulation of intracellular signal transduction | 0.000302 | 0.02774 |
| GO:0001774 | BP | microglial cell activation | 0.000307 | 0.027819 |
| GO:0019883 | BP | antigen processing and presentation of endogenous antigen | 0.000307 | 0.027819 |
| GO:0002269 | BP | leukocyte activation involved in inflammatory response | 0.000307 | 0.027819 |
| GO:0043331 | BP | response to dsRNA | 0.000325 | 0.028283 |
| GO:0002708 | BP | positive regulation of lymphocyte mediated immunity | 0.000328 | 0.028283 |
| GO:0030522 | BP | intracellular receptor signaling pathway | 0.000328 | 0.028283 |
| GO:0039528 | BP | cytoplasmic pattern recognition receptor signaling pathway in response to virus | 0.000328 | 0.028283 |
| GO:2000556 | BP | positive regulation of T-helper 1 cell cytokine production | 0.000328 | 0.028283 |
| GO:2000554 | BP | regulation of T-helper 1 cell cytokine production | 0.000328 | 0.028283 |
| GO:0061518 | BP | microglial cell proliferation | 0.000328 | 0.028283 |
| GO:0061517 | BP | macrophage proliferation | 0.000328 | 0.028283 |
| GO:0050900 | BP | leukocyte migration | 0.00034 | 0.02917 |
| GO:0031341 | BP | regulation of cell killing | 0.000349 | 0.029797 |
| GO:0001961 | BP | positive regulation of cytokine-mediated signaling pathway | 0.000357 | 0.030361 |
| GO:0002237 | BP | response to molecule of bacterial origin | 0.000361 | 0.030557 |
| GO:0007167 | BP | enzyme linked receptor protein signaling pathway | 0.000362 | 0.030557 |
| GO:0010629 | BP | negative regulation of gene expression | 0.000366 | 0.03077 |
| GO:0071549 | BP | cellular response to dexamethasone stimulus | 0.000374 | 0.031283 |
| GO:0040013 | BP | negative regulation of locomotion | 0.000375 | 0.031283 |
| GO:0009719 | BP | response to endogenous stimulus | 0.000389 | 0.0323 |
| GO:0002706 | BP | regulation of lymphocyte mediated immunity | 0.000408 | 0.033725 |
| GO:0006979 | BP | response to oxidative stress | 0.000416 | 0.034228 |
| GO:0030334 | BP | regulation of cell migration | 0.000448 | 0.03644 |
| GO:0035455 | BP | response to interferon-alpha | 0.000449 | 0.03644 |
| GO:0006067 | BP | ethanol metabolic process | 0.000449 | 0.03644 |
| GO:0071345 | BP | cellular response to cytokine stimulus | 0.00045 | 0.03644 |
| GO:0050864 | BP | regulation of B cell activation | 0.000453 | 0.036505 |
| GO:0008285 | BP | negative regulation of cell population proliferation | 0.000458 | 0.036781 |
| GO:0051094 | BP | positive regulation of developmental process | 0.000474 | 0.037693 |
| GO:0002292 | BP | T cell differentiation involved in immune response | 0.000478 | 0.037693 |
| GO:0071384 | BP | cellular response to corticosteroid stimulus | 0.000493 | 0.03867 |
| GO:0050708 | BP | regulation of protein secretion | 0.000515 | 0.040206 |
| GO:0002791 | BP | regulation of peptide secretion | 0.000523 | 0.040707 |
| GO:0002833 | BP | positive regulation of response to biotic stimulus | 0.000529 | 0.04087 |
| GO:0051384 | BP | response to glucocorticoid | 0.00053 | 0.04087 |
| GO:0022407 | BP | regulation of cell-cell adhesion | 0.000538 | 0.041371 |
| GO:0008406 | BP | gonad development | 0.00058 | 0.044444 |
| GO:0002573 | BP | myeloid leukocyte differentiation | 0.000595 | 0.04519 |
| GO:0002275 | BP | myeloid cell activation involved in immune response | 0.000611 | 0.046291 |
| GO:0030154 | BP | cell differentiation | 0.000626 | 0.046531 |
| GO:0050729 | BP | positive regulation of inflammatory response | 0.000631 | 0.046531 |
| GO:0050901 | BP | leukocyte tethering or rolling | 0.000632 | 0.046531 |
| GO:0045086 | BP | positive regulation of interleukin-2 biosynthetic process | 0.000632 | 0.046531 |
| GO:0002832 | BP | negative regulation of response to biotic stimulus | 0.000635 | 0.046531 |
| GO:0060558 | BP | regulation of calcidiol 1-monooxygenase activity | 0.00064 | 0.046531 |
| GO:0030213 | BP | hyaluronan biosynthetic process | 0.00064 | 0.046531 |
| GO:0010941 | BP | regulation of cell death | 0.000669 | 0.048412 |
| GO:0071385 | BP | cellular response to glucocorticoid stimulus | 0.000671 | 0.048412 |
| GO:0010628 | BP | positive regulation of gene expression | 0.000676 | 0.048593 |
| GO:0071216 | BP | cellular response to biotic stimulus | 0.000695 | 0.049572 |
| GO:1903555 | BP | regulation of tumor necrosis factor superfamily cytokine production | 0.000695 | 0.049572 |
| GO:0042612 | CC | MHC class I protein complex | 2.12E-07 | 0.000744 |
| GO:0009897 | CC | external side of plasma membrane | 6.31E-07 | 0.000744 |
| GO:0005615 | CC | extracellular space | 1.94E-06 | 0.000781 |
| GO:0097629 | CC | extrinsic component of omegasome membrane | 3.45E-05 | 0.004645 |
| GO:0043235 | CC | receptor complex | 0.000228 | 0.022362 |
| GO:0042611 | CC | MHC protein complex | 0.000478 | 0.037693 |
| GO:0004896 | MF | cytokine receptor activity | 9.31E-07 | 0.000744 |
| GO:0140375 | MF | immune receptor activity | 1.08E-06 | 0.000744 |
| GO:0001730 | MF | 2'-5'-oligoadenylate synthetase activity | 1.12E-06 | 0.000744 |
| GO:0005102 | MF | signaling receptor binding | 3.50E-06 | 0.000875 |
| GO:0048018 | MF | receptor ligand activity | 9.83E-06 | 0.001703 |
| GO:0030546 | MF | signaling receptor activator activity | 1.10E-05 | 0.001872 |
| GO:0030545 | MF | receptor regulator activity | 1.14E-05 | 0.001885 |
| GO:0005515 | MF | protein binding | 2.14E-05 | 0.003206 |
| GO:0003725 | MF | double-stranded RNA binding | 3.36E-05 | 0.004622 |
| GO:0043169 | MF | cation binding | 3.37E-05 | 0.004622 |
| GO:0005125 | MF | cytokine activity | 4.94E-05 | 0.006302 |
| GO:0046872 | MF | metal ion binding | 5.59E-05 | 0.00694 |
| GO:0005126 | MF | cytokine receptor binding | 8.52E-05 | 0.009934 |
| GO:0000981 | MF | DNA-binding transcription factor activity, RNA polymerase II-specific | 0.000125 | 0.013907 |
| GO:0043167 | MF | ion binding | 0.000161 | 0.016964 |
| GO:0070851 | MF | growth factor receptor binding | 0.000271 | 0.026091 |
| GO:0003960 | MF | NADPH:quinone reductase activity | 0.000328 | 0.028283 |
| GO:0097677 | MF | STAT family protein binding | 0.000328 | 0.028283 |
| GO:0042296 | MF | ISG15 transferase activity | 0.000328 | 0.028283 |
| GO:0019955 | MF | cytokine binding | 0.000477 | 0.037693 |
| GO:0050786 | MF | RAGE receptor binding | 0.000632 | 0.046531 |
| GO:0050135 | MF | NAD(P)+ nucleosidase activity | 0.00064 | 0.046531 |
| GO:0061809 | MF | NAD+ nucleotidase, cyclic ADP-ribose generating | 0.00064 | 0.046531 |
| GO:0016763 | MF | transferase activity, transferring pentosyl groups | 0.000701 | 0.049862 |

iBAT_D14 VS iBAT_M1 down

| go_id | go_type | discription | p_uncorrected | p_corrected |
| --- | --- | --- | --- | --- |
| GO:0006743 | BP | ubiquinone metabolic process | 5.40E-08 | 0.000304 |
| GO:0006120 | BP | mitochondrial electron transport, NADH to ubiquinone | 1.30E-07 | 0.000304 |
| GO:0072655 | BP | establishment of protein localization to mitochondrion | 1.86E-07 | 0.000304 |
| GO:0017004 | BP | cytochrome complex assembly | 2.12E-07 | 0.000304 |
| GO:0006099 | BP | tricarboxylic acid cycle | 2.69E-07 | 0.000304 |
| GO:1990542 | BP | mitochondrial transmembrane transport | 2.75E-07 | 0.000304 |
| GO:0043648 | BP | dicarboxylic acid metabolic process | 3.57E-07 | 0.000304 |
| GO:0070585 | BP | protein localization to mitochondrion | 3.84E-07 | 0.000304 |
| GO:0006103 | BP | 2-oxoglutarate metabolic process | 3.97E-07 | 0.000304 |
| GO:0006744 | BP | ubiquinone biosynthetic process | 4.11E-07 | 0.000304 |
| GO:1901663 | BP | quinone biosynthetic process | 4.11E-07 | 0.000304 |
| GO:0033108 | BP | mitochondrial respiratory chain complex assembly | 4.13E-07 | 0.000304 |
| GO:0010257 | BP | NADH dehydrogenase complex assembly | 4.19E-07 | 0.000304 |
| GO:0032981 | BP | mitochondrial respiratory chain complex I assembly | 4.19E-07 | 0.000304 |
| GO:0022900 | BP | electron transport chain | 4.74E-07 | 0.000304 |
| GO:0046390 | BP | ribose phosphate biosynthetic process | 5.17E-07 | 0.000304 |
| GO:0007007 | BP | inner mitochondrial membrane organization | 5.51E-07 | 0.000304 |
| GO:0019693 | BP | ribose phosphate metabolic process | 5.60E-07 | 0.000304 |
| GO:0005996 | BP | monosaccharide metabolic process | 5.81E-07 | 0.000304 |
| GO:0016054 | BP | organic acid catabolic process | 6.02E-07 | 0.000304 |
| GO:0046395 | BP | carboxylic acid catabolic process | 6.02E-07 | 0.000304 |
| GO:0006839 | BP | mitochondrial transport | 6.90E-07 | 0.000304 |
| GO:0006163 | BP | purine nucleotide metabolic process | 7.20E-07 | 0.000304 |
| GO:0022904 | BP | respiratory electron transport chain | 7.28E-07 | 0.000304 |
| GO:0006090 | BP | pyruvate metabolic process | 7.28E-07 | 0.000304 |
| GO:1901293 | BP | nucleoside phosphate biosynthetic process | 7.40E-07 | 0.000304 |
| GO:0046034 | BP | ATP metabolic process | 7.48E-07 | 0.000304 |
| GO:0055114 | BP | oxidation-reduction process | 7.73E-07 | 0.000308 |
| GO:0034551 | BP | mitochondrial respiratory chain complex III assembly | 9.01E-07 | 0.00032 |
| GO:0017062 | BP | respiratory chain complex III assembly | 9.01E-07 | 0.00032 |
| GO:0007005 | BP | mitochondrion organization | 9.54E-07 | 0.000327 |
| GO:0032787 | BP | monocarboxylic acid metabolic process | 1.06E-06 | 0.000344 |
| GO:0009259 | BP | ribonucleotide metabolic process | 1.10E-06 | 0.000344 |
| GO:0072329 | BP | monocarboxylic acid catabolic process | 1.10E-06 | 0.000344 |
| GO:0044282 | BP | small molecule catabolic process | 1.16E-06 | 0.000344 |
| GO:0072521 | BP | purine-containing compound metabolic process | 1.17E-06 | 0.000344 |
| GO:0006520 | BP | cellular amino acid metabolic process | 1.18E-06 | 0.000344 |
| GO:0009260 | BP | ribonucleotide biosynthetic process | 1.23E-06 | 0.000344 |
| GO:0006164 | BP | purine nucleotide biosynthetic process | 1.23E-06 | 0.000344 |
| GO:0009062 | BP | fatty acid catabolic process | 1.25E-06 | 0.000344 |
| GO:0006631 | BP | fatty acid metabolic process | 1.26E-06 | 0.000344 |
| GO:0042391 | BP | regulation of membrane potential | 1.35E-06 | 0.000352 |
| GO:0019318 | BP | hexose metabolic process | 1.39E-06 | 0.000352 |
| GO:0009150 | BP | purine ribonucleotide metabolic process | 1.40E-06 | 0.000352 |
| GO:0006091 | BP | generation of precursor metabolites and energy | 1.41E-06 | 0.000352 |
| GO:0006635 | BP | fatty acid beta-oxidation | 1.41E-06 | 0.000352 |
| GO:0006084 | BP | acetyl-CoA metabolic process | 1.42E-06 | 0.000352 |
| GO:0019752 | BP | carboxylic acid metabolic process | 1.45E-06 | 0.000358 |
| GO:0009117 | BP | nucleotide metabolic process | 1.47E-06 | 0.000358 |
| GO:0009165 | BP | nucleotide biosynthetic process | 1.54E-06 | 0.000366 |
| GO:0009152 | BP | purine ribonucleotide biosynthetic process | 1.54E-06 | 0.000366 |
| GO:0055086 | BP | nucleobase-containing small molecule metabolic process | 1.62E-06 | 0.000379 |
| GO:0006753 | BP | nucleoside phosphate metabolic process | 1.73E-06 | 0.000388 |
| GO:0106077 | BP | histone succinylation | 1.74E-06 | 0.000388 |
| GO:0018335 | BP | protein succinylation | 1.74E-06 | 0.000388 |
| GO:0019637 | BP | organophosphate metabolic process | 1.75E-06 | 0.000388 |
| GO:0005975 | BP | carbohydrate metabolic process | 1.77E-06 | 0.000388 |
| GO:0006790 | BP | sulfur compound metabolic process | 1.78E-06 | 0.000388 |
| GO:0055085 | BP | transmembrane transport | 1.81E-06 | 0.000389 |
| GO:0007006 | BP | mitochondrial membrane organization | 1.83E-06 | 0.000389 |
| GO:0044283 | BP | small molecule biosynthetic process | 1.92E-06 | 0.000403 |
| GO:1901135 | BP | carbohydrate derivative metabolic process | 2.04E-06 | 0.00041 |
| GO:0006082 | BP | organic acid metabolic process | 2.36E-06 | 0.000456 |
| GO:0019395 | BP | fatty acid oxidation | 2.40E-06 | 0.000456 |
| GO:0090407 | BP | organophosphate biosynthetic process | 2.44E-06 | 0.000456 |
| GO:0006629 | BP | lipid metabolic process | 2.45E-06 | 0.000456 |
| GO:0043436 | BP | oxoacid metabolic process | 3.06E-06 | 0.00053 |
| GO:0035383 | BP | thioester metabolic process | 3.38E-06 | 0.000556 |
| GO:0006637 | BP | acyl-CoA metabolic process | 3.38E-06 | 0.000556 |
| GO:0045333 | BP | cellular respiration | 3.40E-06 | 0.000556 |
| GO:0044281 | BP | small molecule metabolic process | 3.45E-06 | 0.000559 |
| GO:0006006 | BP | glucose metabolic process | 3.70E-06 | 0.000582 |
| GO:0034440 | BP | lipid oxidation | 3.82E-06 | 0.000594 |
| GO:0008152 | BP | metabolic process | 5.35E-06 | 0.0008 |
| GO:0072522 | BP | purine-containing compound biosynthetic process | 5.73E-06 | 0.000845 |
| GO:0045444 | BP | fat cell differentiation | 5.79E-06 | 0.000848 |
| GO:0044237 | BP | cellular metabolic process | 6.12E-06 | 0.000878 |
| GO:0042407 | BP | cristae formation | 6.13E-06 | 0.000878 |
| GO:0071806 | BP | protein transmembrane transport | 6.62E-06 | 0.000934 |
| GO:0000959 | BP | mitochondrial RNA metabolic process | 6.76E-06 | 0.000948 |
| GO:0006107 | BP | oxaloacetate metabolic process | 7.03E-06 | 0.000973 |
| GO:0006796 | BP | phosphate-containing compound metabolic process | 7.48E-06 | 0.001021 |
| GO:1901661 | BP | quinone metabolic process | 8.51E-06 | 0.001133 |
| GO:0042181 | BP | ketone biosynthetic process | 8.54E-06 | 0.001133 |
| GO:0032868 | BP | response to insulin | 8.60E-06 | 0.001134 |
| GO:0006793 | BP | phosphorus metabolic process | 1.20E-05 | 0.001548 |
| GO:0016052 | BP | carbohydrate catabolic process | 1.26E-05 | 0.001607 |
| GO:0044255 | BP | cellular lipid metabolic process | 1.42E-05 | 0.001786 |
| GO:0042180 | BP | cellular ketone metabolic process | 1.77E-05 | 0.002204 |
| GO:0070887 | BP | cellular response to chemical stimulus | 1.84E-05 | 0.002281 |
| GO:0014074 | BP | response to purine-containing compound | 1.95E-05 | 0.002402 |
| GO:0010821 | BP | regulation of mitochondrion organization | 2.01E-05 | 0.002451 |
| GO:1901700 | BP | response to oxygen-containing compound | 2.17E-05 | 0.002605 |
| GO:0009987 | BP | cellular process | 2.22E-05 | 0.002645 |
| GO:0032869 | BP | cellular response to insulin stimulus | 2.39E-05 | 0.002838 |
| GO:0065002 | BP | intracellular protein transmembrane transport | 2.56E-05 | 0.002976 |
| GO:0065008 | BP | regulation of biological quality | 2.69E-05 | 0.003113 |
| GO:0006811 | BP | ion transport | 3.25E-05 | 0.003721 |
| GO:0070125 | BP | mitochondrial translational elongation | 3.87E-05 | 0.004351 |
| GO:0042773 | BP | ATP synthesis coupled electron transport | 4.04E-05 | 0.004489 |
| GO:0006071 | BP | glycerol metabolic process | 4.04E-05 | 0.004489 |
| GO:0008637 | BP | apoptotic mitochondrial changes | 4.13E-05 | 0.004545 |
| GO:0006096 | BP | glycolytic process | 4.23E-05 | 0.004599 |
| GO:0035384 | BP | thioester biosynthetic process | 4.49E-05 | 0.004815 |
| GO:0071616 | BP | acyl-CoA biosynthetic process | 4.49E-05 | 0.004815 |
| GO:0046031 | BP | ADP metabolic process | 4.50E-05 | 0.004815 |
| GO:0009106 | BP | lipoate metabolic process | 4.80E-05 | 0.00506 |
| GO:0006757 | BP | ATP generation from ADP | 4.84E-05 | 0.005071 |
| GO:0006820 | BP | anion transport | 5.28E-05 | 0.005479 |
| GO:0044743 | BP | protein transmembrane import into intracellular organelle | 5.51E-05 | 0.005681 |
| GO:0072656 | BP | maintenance of protein location in mitochondrion | 5.58E-05 | 0.005681 |
| GO:0006554 | BP | lysine catabolic process | 5.58E-05 | 0.005681 |
| GO:0033865 | BP | nucleoside bisphosphate metabolic process | 5.65E-05 | 0.005681 |
| GO:0033875 | BP | ribonucleoside bisphosphate metabolic process | 5.65E-05 | 0.005681 |
| GO:0034032 | BP | purine nucleoside bisphosphate metabolic process | 5.65E-05 | 0.005681 |
| GO:0046683 | BP | response to organophosphorus | 6.16E-05 | 0.006152 |
| GO:0019400 | BP | alditol metabolic process | 6.36E-05 | 0.006301 |
| GO:0009179 | BP | purine ribonucleoside diphosphate metabolic process | 6.43E-05 | 0.006305 |
| GO:0032543 | BP | mitochondrial translation | 6.72E-05 | 0.006555 |
| GO:0043467 | BP | regulation of generation of precursor metabolites and energy | 6.85E-05 | 0.00665 |
| GO:0046939 | BP | nucleotide phosphorylation | 7.16E-05 | 0.006849 |
| GO:0006165 | BP | nucleoside diphosphate phosphorylation | 7.16E-05 | 0.006849 |
| GO:0009135 | BP | purine nucleoside diphosphate metabolic process | 7.21E-05 | 0.006872 |
| GO:0006085 | BP | acetyl-CoA biosynthetic process | 7.74E-05 | 0.007273 |
| GO:0050873 | BP | brown fat cell differentiation | 8.06E-05 | 0.007494 |
| GO:1901137 | BP | carbohydrate derivative biosynthetic process | 8.22E-05 | 0.007544 |
| GO:0006418 | BP | tRNA aminoacylation for protein translation | 8.26E-05 | 0.007548 |
| GO:0006575 | BP | cellular modified amino acid metabolic process | 8.38E-05 | 0.007622 |
| GO:0008016 | BP | regulation of heart contraction | 8.64E-05 | 0.007782 |
| GO:0009185 | BP | ribonucleoside diphosphate metabolic process | 9.03E-05 | 0.008094 |
| GO:0009058 | BP | biosynthetic process | 0.000103 | 0.009089 |
| GO:0051259 | BP | protein complex oligomerization | 0.000117 | 0.010015 |
| GO:0006399 | BP | tRNA metabolic process | 0.000131 | 0.011028 |
| GO:0006086 | BP | acetyl-CoA biosynthetic process from pyruvate | 0.00014 | 0.011685 |
| GO:0120162 | BP | positive regulation of cold-induced thermogenesis | 0.000147 | 0.012261 |
| GO:0009725 | BP | response to hormone | 0.00015 | 0.012412 |
| GO:0042493 | BP | response to drug | 0.000151 | 0.012445 |
| GO:0043039 | BP | tRNA aminoacylation | 0.000152 | 0.01248 |
| GO:0019725 | BP | cellular homeostasis | 0.000155 | 0.012738 |
| GO:0070584 | BP | mitochondrion morphogenesis | 0.000175 | 0.014161 |
| GO:0043038 | BP | amino acid activation | 0.000175 | 0.014161 |
| GO:0006094 | BP | gluconeogenesis | 0.000188 | 0.015007 |
| GO:0009132 | BP | nucleoside diphosphate metabolic process | 0.000189 | 0.015015 |
| GO:0046364 | BP | monosaccharide biosynthetic process | 0.000194 | 0.015333 |
| GO:0045822 | BP | negative regulation of heart contraction | 0.000197 | 0.015511 |
| GO:0045667 | BP | regulation of osteoblast differentiation | 0.000229 | 0.01786 |
| GO:0006942 | BP | regulation of striated muscle contraction | 0.00023 | 0.017928 |
| GO:0033993 | BP | response to lipid | 0.00027 | 0.020665 |
| GO:1903579 | BP | negative regulation of ATP metabolic process | 0.000271 | 0.020665 |
| GO:0065003 | BP | protein-containing complex assembly | 0.000273 | 0.020695 |
| GO:0019319 | BP | hexose biosynthetic process | 0.000273 | 0.020695 |
| GO:0071453 | BP | cellular response to oxygen levels | 0.000305 | 0.022506 |
| GO:0006553 | BP | lysine metabolic process | 0.000307 | 0.022506 |
| GO:0061732 | BP | mitochondrial acetyl-CoA biosynthetic process from pyruvate | 0.000307 | 0.022506 |
| GO:0052646 | BP | alditol phosphate metabolic process | 0.000307 | 0.022506 |
| GO:0006390 | BP | mitochondrial transcription | 0.000307 | 0.022506 |
| GO:0034214 | BP | protein hexamerization | 0.000307 | 0.022506 |
| GO:0033866 | BP | nucleoside bisphosphate biosynthetic process | 0.000309 | 0.022506 |
| GO:0055117 | BP | regulation of cardiac muscle contraction | 0.000309 | 0.022506 |
| GO:0034030 | BP | ribonucleoside bisphosphate biosynthetic process | 0.000309 | 0.022506 |
| GO:0034033 | BP | purine nucleoside bisphosphate biosynthetic process | 0.000309 | 0.022506 |
| GO:0015711 | BP | organic anion transport | 0.000354 | 0.025518 |
| GO:0043933 | BP | protein-containing complex subunit organization | 0.000397 | 0.028525 |
| GO:0071704 | BP | organic substance metabolic process | 0.000406 | 0.029058 |
| GO:0098656 | BP | anion transmembrane transport | 0.000437 | 0.031218 |
| GO:0046942 | BP | carboxylic acid transport | 0.000445 | 0.031675 |
| GO:0034220 | BP | ion transmembrane transport | 0.000455 | 0.031981 |
| GO:0140212 | BP | regulation of long-chain fatty acid import into cell | 0.000455 | 0.031981 |
| GO:0009437 | BP | carnitine metabolic process | 0.000468 | 0.032712 |
| GO:0015980 | BP | energy derivation by oxidation of organic compounds | 0.00047 | 0.032721 |
| GO:1901605 | BP | alpha-amino acid metabolic process | 0.000474 | 0.032772 |
| GO:0015849 | BP | organic acid transport | 0.000474 | 0.032772 |
| GO:0006122 | BP | mitochondrial electron transport, ubiquinol to cytochrome c | 0.000529 | 0.036174 |
| GO:1901615 | BP | organic hydroxy compound metabolic process | 0.00056 | 0.038208 |
| GO:0016051 | BP | carbohydrate biosynthetic process | 0.000566 | 0.038472 |
| GO:0009060 | BP | aerobic respiration | 0.000618 | 0.041412 |
| GO:0009068 | BP | aspartate family amino acid catabolic process | 0.00062 | 0.041412 |
| GO:0046320 | BP | regulation of fatty acid oxidation | 0.00062 | 0.041412 |
| GO:1903779 | BP | regulation of cardiac conduction | 0.00062 | 0.041412 |
| GO:0070129 | BP | regulation of mitochondrial translation | 0.000641 | 0.042666 |
| GO:1901576 | BP | organic substance biosynthetic process | 0.000695 | 0.045837 |
| GO:0009161 | BP | ribonucleoside monophosphate metabolic process | 0.000702 | 0.046151 |
| GO:0031314 | CC | extrinsic component of mitochondrial inner membrane | 1.55E-08 | 0.000304 |
| GO:0070469 | CC | respirasome | 2.85E-07 | 0.000304 |
| GO:0098803 | CC | respiratory chain complex | 3.62E-07 | 0.000304 |
| GO:0005762 | CC | mitochondrial large ribosomal subunit | 4.57E-07 | 0.000304 |
| GO:0000315 | CC | organellar large ribosomal subunit | 4.57E-07 | 0.000304 |
| GO:0005758 | CC | mitochondrial intermembrane space | 4.78E-07 | 0.000304 |
| GO:0005743 | CC | mitochondrial inner membrane | 5.40E-07 | 0.000304 |
| GO:0005763 | CC | mitochondrial small ribosomal subunit | 5.77E-07 | 0.000304 |
| GO:0000314 | CC | organellar small ribosomal subunit | 5.77E-07 | 0.000304 |
| GO:0030964 | CC | NADH dehydrogenase complex | 6.37E-07 | 0.000304 |
| GO:0005747 | CC | mitochondrial respiratory chain complex I | 6.37E-07 | 0.000304 |
| GO:0045271 | CC | respiratory chain complex I | 6.37E-07 | 0.000304 |
| GO:0005741 | CC | mitochondrial outer membrane | 7.29E-07 | 0.000304 |
| GO:0019866 | CC | organelle inner membrane | 8.94E-07 | 0.00032 |
| GO:0005759 | CC | mitochondrial matrix | 9.96E-07 | 0.000334 |
| GO:1990204 | CC | oxidoreductase complex | 1.01E-06 | 0.000334 |
| GO:0098800 | CC | inner mitochondrial membrane protein complex | 1.17E-06 | 0.000344 |
| GO:0019867 | CC | outer membrane | 1.25E-06 | 0.000344 |
| GO:0031968 | CC | organelle outer membrane | 1.25E-06 | 0.000344 |
| GO:0031966 | CC | mitochondrial membrane | 1.33E-06 | 0.000352 |
| GO:0098798 | CC | mitochondrial protein complex | 1.84E-06 | 0.000389 |
| GO:0031970 | CC | organelle envelope lumen | 2.07E-06 | 0.00041 |
| GO:0031974 | CC | membrane-enclosed lumen | 2.08E-06 | 0.00041 |
| GO:0070013 | CC | intracellular organelle lumen | 2.08E-06 | 0.00041 |
| GO:0043233 | CC | organelle lumen | 2.08E-06 | 0.00041 |
| GO:0005739 | CC | mitochondrion | 2.52E-06 | 0.000461 |
| GO:0045252 | CC | oxoglutarate dehydrogenase complex | 3.22E-06 | 0.000544 |
| GO:0031090 | CC | organelle membrane | 3.62E-06 | 0.000577 |
| GO:0009295 | CC | nucleoid | 4.74E-06 | 0.000715 |
| GO:0042645 | CC | mitochondrial nucleoid | 4.74E-06 | 0.000715 |
| GO:0043231 | CC | intracellular membrane-bounded organelle | 5.91E-06 | 0.000859 |
| GO:0043227 | CC | membrane-bounded organelle | 6.27E-06 | 0.000892 |
| GO:0043229 | CC | intracellular organelle | 7.37E-06 | 0.001012 |
| GO:0043226 | CC | organelle | 8.24E-06 | 0.001116 |
| GO:0045254 | CC | pyruvate dehydrogenase complex | 1.34E-05 | 0.001694 |
| GO:0045240 | CC | dihydrolipoyl dehydrogenase complex | 2.43E-05 | 0.002859 |
| GO:0045239 | CC | tricarboxylic acid cycle enzyme complex | 4.12E-05 | 0.004545 |
| GO:0070069 | CC | cytochrome complex | 7.03E-05 | 0.006795 |
| GO:0031305 | CC | integral component of mitochondrial inner membrane | 8.13E-05 | 0.007494 |
| GO:0031304 | CC | intrinsic component of mitochondrial inner membrane | 8.13E-05 | 0.007494 |
| GO:0001401 | CC | SAM complex | 0.00019 | 0.015035 |
| GO:0098573 | CC | intrinsic component of mitochondrial membrane | 0.000269 | 0.020645 |
| GO:0032592 | CC | integral component of mitochondrial membrane | 0.000269 | 0.020645 |
| GO:0043527 | CC | tRNA methyltransferase complex | 0.000455 | 0.031981 |
| GO:0050136 | MF | NADH dehydrogenase (quinone) activity | 3.10E-07 | 0.000304 |
| GO:0008137 | MF | NADH dehydrogenase (ubiquinone) activity | 3.10E-07 | 0.000304 |
| GO:0009055 | MF | electron transfer activity | 5.13E-07 | 0.000304 |
| GO:0016614 | MF | oxidoreductase activity, acting on CH-OH group of donors | 5.81E-07 | 0.000304 |
| GO:0051540 | MF | metal cluster binding | 6.14E-07 | 0.000304 |
| GO:0051536 | MF | iron-sulfur cluster binding | 6.14E-07 | 0.000304 |
| GO:0016651 | MF | oxidoreductase activity, acting on NAD(P)H | 7.01E-07 | 0.000304 |
| GO:0003954 | MF | NADH dehydrogenase activity | 7.23E-07 | 0.000304 |
| GO:0016829 | MF | lyase activity | 7.48E-07 | 0.000304 |
| GO:0004738 | MF | pyruvate dehydrogenase activity | 9.01E-07 | 0.00032 |
| GO:0034604 | MF | pyruvate dehydrogenase (NAD+) activity | 9.01E-07 | 0.00032 |
| GO:0034603 | MF | pyruvate dehydrogenase [NAD(P)+] activity | 9.01E-07 | 0.00032 |
| GO:0016655 | MF | oxidoreductase activity, acting on NAD(P)H, quinone or similar compound as acceptor | 9.52E-07 | 0.000327 |
| GO:0005215 | MF | transporter activity | 2.03E-06 | 0.00041 |
| GO:0016491 | MF | oxidoreductase activity | 2.25E-06 | 0.00044 |
| GO:0015075 | MF | ion transmembrane transporter activity | 2.50E-06 | 0.000461 |
| GO:0022857 | MF | transmembrane transporter activity | 2.76E-06 | 0.000495 |
| GO:0016616 | MF | oxidoreductase activity, acting on the CH-OH group of donors, NAD or NADP as acceptor | 2.80E-06 | 0.000497 |
| GO:1901265 | MF | nucleoside phosphate binding | 3.09E-06 | 0.00053 |
| GO:0016769 | MF | transferase activity, transferring nitrogenous groups | 3.11E-06 | 0.00053 |
| GO:0036094 | MF | small molecule binding | 3.52E-06 | 0.000566 |
| GO:0015318 | MF | inorganic molecular entity transmembrane transporter activity | 3.72E-06 | 0.000582 |
| GO:0000166 | MF | nucleotide binding | 4.07E-06 | 0.000628 |
| GO:0043168 | MF | anion binding | 4.55E-06 | 0.000697 |
| GO:0003824 | MF | catalytic activity | 5.68E-06 | 0.000845 |
| GO:0050660 | MF | flavin adenine dinucleotide binding | 6.82E-06 | 0.000949 |
| GO:0051539 | MF | 4 iron, 4 sulfur cluster binding | 8.51E-06 | 0.001133 |
| GO:0032555 | MF | purine ribonucleotide binding | 8.85E-06 | 0.00116 |
| GO:0032553 | MF | ribonucleotide binding | 9.44E-06 | 0.001228 |
| GO:0017076 | MF | purine nucleotide binding | 1.23E-05 | 0.001586 |
| GO:0019200 | MF | carbohydrate kinase activity | 1.66E-05 | 0.002077 |
| GO:0004448 | MF | isocitrate dehydrogenase activity | 2.46E-05 | 0.002886 |
| GO:0032559 | MF | adenyl ribonucleotide binding | 2.92E-05 | 0.003363 |
| GO:0016836 | MF | hydro-lyase activity | 3.53E-05 | 0.003998 |
| GO:0030554 | MF | adenyl nucleotide binding | 4.16E-05 | 0.004547 |
| GO:0035639 | MF | purine ribonucleoside triphosphate binding | 4.53E-05 | 0.00482 |
| GO:0140101 | MF | catalytic activity, acting on a tRNA | 4.76E-05 | 0.005046 |
| GO:0051287 | MF | NAD binding | 6.43E-05 | 0.006305 |
| GO:0097367 | MF | carbohydrate derivative binding | 7.30E-05 | 0.006917 |
| GO:0022804 | MF | active transmembrane transporter activity | 7.59E-05 | 0.007164 |
| GO:0008483 | MF | transaminase activity | 8.06E-05 | 0.007494 |
| GO:0015078 | MF | proton transmembrane transporter activity | 8.56E-05 | 0.007749 |
| GO:0008324 | MF | cation transmembrane transporter activity | 9.06E-05 | 0.008094 |
| GO:0051537 | MF | 2 iron, 2 sulfur cluster binding | 0.000101 | 0.008967 |
| GO:0016627 | MF | oxidoreductase activity, acting on the CH-CH group of donors | 0.000104 | 0.009128 |
| GO:0004396 | MF | hexokinase activity | 0.000108 | 0.009468 |
| GO:0016875 | MF | ligase activity, forming carbon-oxygen bonds | 0.000113 | 0.009723 |
| GO:0004812 | MF | aminoacyl-tRNA ligase activity | 0.000113 | 0.009723 |
| GO:0015002 | MF | heme-copper terminal oxidase activity | 0.000131 | 0.011028 |
| GO:0004129 | MF | cytochrome-c oxidase activity | 0.000131 | 0.011028 |
| GO:0016676 | MF | oxidoreductase activity, acting on a heme group of donors, oxygen as acceptor | 0.000131 | 0.011028 |
| GO:0016675 | MF | oxidoreductase activity, acting on a heme group of donors | 0.000131 | 0.011028 |
| GO:0005524 | MF | ATP binding | 0.000162 | 0.013221 |
| GO:0016620 | MF | oxidoreductase activity, acting on the aldehyde or oxo group of donors, NAD or NADP as acceptor | 0.000184 | 0.014845 |
| GO:0004449 | MF | isocitrate dehydrogenase (NAD+) activity | 0.000187 | 0.015005 |
| GO:0015077 | MF | monovalent inorganic cation transmembrane transporter activity | 0.000199 | 0.01561 |
| GO:0016874 | MF | ligase activity | 0.000235 | 0.018203 |
| GO:0016903 | MF | oxidoreductase activity, acting on the aldehyde or oxo group of donors | 0.000312 | 0.022707 |
| GO:0016741 | MF | transferase activity, transferring one-carbon groups | 0.00032 | 0.023162 |
| GO:1901612 | MF | cardiolipin binding | 0.000468 | 0.032712 |
| GO:0016835 | MF | carbon-oxygen lyase activity | 0.000518 | 0.035579 |
| GO:1901611 | MF | phosphatidylglycerol binding | 0.000682 | 0.045125 |
| GO:0008509 | MF | anion transmembrane transporter activity | 0.000743 | 0.048649 |

iBAT_M1 VS iBAT_M4 up

| go_id | go_type | discription | p_uncorrected | p_corrected |
| --- | --- | --- | --- | --- |
| GO:0046463 | BP | acylglycerol biosynthetic process | 6.98E-08 | 0.000585 |
| GO:0046460 | BP | neutral lipid biosynthetic process | 6.98E-08 | 0.000585 |
| GO:0019432 | BP | triglyceride biosynthetic process | 9.06E-08 | 0.000585 |
| GO:0090181 | BP | regulation of cholesterol metabolic process | 1.18E-07 | 0.000585 |
| GO:0006641 | BP | triglyceride metabolic process | 2.33E-07 | 0.00067 |
| GO:0016126 | BP | sterol biosynthetic process | 2.33E-07 | 0.00067 |
| GO:0016125 | BP | sterol metabolic process | 2.35E-07 | 0.00067 |
| GO:0006694 | BP | steroid biosynthetic process | 3.11E-07 | 0.000687 |
| GO:0006695 | BP | cholesterol biosynthetic process | 3.72E-07 | 0.000692 |
| GO:0008203 | BP | cholesterol metabolic process | 3.82E-07 | 0.000692 |
| GO:0030199 | BP | collagen fibril organization | 4.84E-07 | 0.000727 |
| GO:0008202 | BP | steroid metabolic process | 5.11E-07 | 0.000727 |
| GO:0046890 | BP | regulation of lipid biosynthetic process | 6.29E-07 | 0.000835 |
| GO:1902653 | BP | secondary alcohol biosynthetic process | 7.97E-07 | 0.000865 |
| GO:0008610 | BP | lipid biosynthetic process | 8.82E-07 | 0.000865 |
| GO:0019218 | BP | regulation of steroid metabolic process | 8.94E-07 | 0.000865 |
| GO:0006631 | BP | fatty acid metabolic process | 9.72E-07 | 0.000865 |
| GO:1902652 | BP | secondary alcohol metabolic process | 1.02E-06 | 0.000865 |
| GO:0006639 | BP | acylglycerol metabolic process | 1.07E-06 | 0.000865 |
| GO:0006638 | BP | neutral lipid metabolic process | 1.07E-06 | 0.000865 |
| GO:0106118 | BP | regulation of sterol biosynthetic process | 1.09E-06 | 0.000865 |
| GO:0045540 | BP | regulation of cholesterol biosynthetic process | 1.09E-06 | 0.000865 |
| GO:0006066 | BP | alcohol metabolic process | 1.13E-06 | 0.000865 |
| GO:0044255 | BP | cellular lipid metabolic process | 1.17E-06 | 0.000865 |
| GO:0019752 | BP | carboxylic acid metabolic process | 1.25E-06 | 0.000865 |
| GO:0032787 | BP | monocarboxylic acid metabolic process | 1.26E-06 | 0.000865 |
| GO:0043436 | BP | oxoacid metabolic process | 1.57E-06 | 0.00095 |
| GO:1903047 | BP | mitotic cell cycle process | 1.63E-06 | 0.00095 |
| GO:0019216 | BP | regulation of lipid metabolic process | 1.90E-06 | 0.00095 |
| GO:0051988 | BP | regulation of attachment of spindle microtubules to kinetochore | 1.91E-06 | 0.00095 |
| GO:0000281 | BP | mitotic cytokinesis | 1.97E-06 | 0.00095 |
| GO:0022402 | BP | cell cycle process | 2.00E-06 | 0.00095 |
| GO:0044283 | BP | small molecule biosynthetic process | 2.05E-06 | 0.00095 |
| GO:0006082 | BP | organic acid metabolic process | 2.12E-06 | 0.00096 |
| GO:0051983 | BP | regulation of chromosome segregation | 2.22E-06 | 0.000973 |
| GO:0044281 | BP | small molecule metabolic process | 2.41E-06 | 0.000978 |
| GO:1901615 | BP | organic hydroxy compound metabolic process | 2.62E-06 | 0.001033 |
| GO:0006629 | BP | lipid metabolic process | 2.94E-06 | 0.001045 |
| GO:0003433 | BP | chondrocyte development involved in endochondral bone morphogenesis | 2.99E-06 | 0.001046 |
| GO:0042327 | BP | positive regulation of phosphorylation | 3.82E-06 | 0.001171 |
| GO:0051726 | BP | regulation of cell cycle | 4.45E-06 | 0.001223 |
| GO:0018208 | BP | peptidyl-proline modification | 5.32E-06 | 0.001376 |
| GO:0001932 | BP | regulation of protein phosphorylation | 5.53E-06 | 0.001397 |
| GO:0046165 | BP | alcohol biosynthetic process | 5.54E-06 | 0.001397 |
| GO:0045860 | BP | positive regulation of protein kinase activity | 6.04E-06 | 0.001485 |
| GO:0007017 | BP | microtubule-based process | 6.14E-06 | 0.001492 |
| GO:0062012 | BP | regulation of small molecule metabolic process | 8.37E-06 | 0.001946 |
| GO:0051310 | BP | metaphase plate congression | 8.50E-06 | 0.001946 |
| GO:0051301 | BP | cell division | 8.50E-06 | 0.001946 |
| GO:0001934 | BP | positive regulation of protein phosphorylation | 9.13E-06 | 0.002044 |
| GO:0010564 | BP | regulation of cell cycle process | 1.26E-05 | 0.002768 |
| GO:0043062 | BP | extracellular structure organization | 1.35E-05 | 0.002883 |
| GO:0030198 | BP | extracellular matrix organization | 1.35E-05 | 0.002883 |
| GO:0050810 | BP | regulation of steroid biosynthetic process | 1.44E-05 | 0.002989 |
| GO:0044772 | BP | mitotic cell cycle phase transition | 1.45E-05 | 0.002989 |
| GO:0033674 | BP | positive regulation of kinase activity | 1.45E-05 | 0.002989 |
| GO:0007399 | BP | nervous system development | 1.46E-05 | 0.002989 |
| GO:0031401 | BP | positive regulation of protein modification process | 1.49E-05 | 0.003028 |
| GO:0051347 | BP | positive regulation of transferase activity | 1.56E-05 | 0.003086 |
| GO:0006633 | BP | fatty acid biosynthetic process | 1.57E-05 | 0.003086 |
| GO:1901617 | BP | organic hydroxy compound biosynthetic process | 1.58E-05 | 0.003087 |
| GO:0010562 | BP | positive regulation of phosphorus metabolic process | 1.99E-05 | 0.003814 |
| GO:0045937 | BP | positive regulation of phosphate metabolic process | 1.99E-05 | 0.003814 |
| GO:0036155 | BP | acylglycerol acyl-chain remodeling | 2.19E-05 | 0.004161 |
| GO:0048167 | BP | regulation of synaptic plasticity | 2.29E-05 | 0.004299 |
| GO:0065007 | BP | biological regulation | 2.37E-05 | 0.004407 |
| GO:0042325 | BP | regulation of phosphorylation | 2.75E-05 | 0.005009 |
| GO:0000226 | BP | microtubule cytoskeleton organization | 2.77E-05 | 0.005009 |
| GO:0044770 | BP | cell cycle phase transition | 2.91E-05 | 0.005193 |
| GO:0072330 | BP | monocarboxylic acid biosynthetic process | 2.98E-05 | 0.005255 |
| GO:0045859 | BP | regulation of protein kinase activity | 3.11E-05 | 0.0054 |
| GO:0050789 | BP | regulation of biological process | 3.12E-05 | 0.0054 |
| GO:0046394 | BP | carboxylic acid biosynthetic process | 3.17E-05 | 0.0054 |
| GO:0090207 | BP | regulation of triglyceride metabolic process | 3.19E-05 | 0.0054 |
| GO:0016053 | BP | organic acid biosynthetic process | 3.35E-05 | 0.005609 |
| GO:0019217 | BP | regulation of fatty acid metabolic process | 4.00E-05 | 0.006367 |
| GO:0019433 | BP | triglyceride catabolic process | 4.06E-05 | 0.006395 |
| GO:0046464 | BP | acylglycerol catabolic process | 4.15E-05 | 0.006395 |
| GO:0046461 | BP | neutral lipid catabolic process | 4.15E-05 | 0.006395 |
| GO:0048731 | BP | system development | 4.90E-05 | 0.007387 |
| GO:0098813 | BP | nuclear chromosome segregation | 5.98E-05 | 0.008813 |
| GO:0050806 | BP | positive regulation of synaptic transmission | 6.33E-05 | 0.009204 |
| GO:0033865 | BP | nucleoside bisphosphate metabolic process | 7.31E-05 | 0.009915 |
| GO:0033875 | BP | ribonucleoside bisphosphate metabolic process | 7.31E-05 | 0.009915 |
| GO:0034032 | BP | purine nucleoside bisphosphate metabolic process | 7.31E-05 | 0.009915 |
| GO:0031399 | BP | regulation of protein modification process | 7.37E-05 | 0.009915 |
| GO:0002063 | BP | chondrocyte development | 7.98E-05 | 0.010589 |
| GO:0000910 | BP | cytokinesis | 0.000101 | 0.013121 |
| GO:0061640 | BP | cytoskeleton-dependent cytokinesis | 0.000101 | 0.013121 |
| GO:0032502 | BP | developmental process | 0.000107 | 0.013707 |
| GO:0051302 | BP | regulation of cell division | 0.000107 | 0.013714 |
| GO:0010866 | BP | regulation of triglyceride biosynthetic process | 0.000112 | 0.01425 |
| GO:0032465 | BP | regulation of cytokinesis | 0.00012 | 0.014973 |
| GO:0043549 | BP | regulation of kinase activity | 0.000121 | 0.015047 |
| GO:0090276 | BP | regulation of peptide hormone secretion | 0.000123 | 0.015267 |
| GO:0019220 | BP | regulation of phosphate metabolic process | 0.000126 | 0.015546 |
| GO:0051174 | BP | regulation of phosphorus metabolic process | 0.000128 | 0.015575 |
| GO:0000413 | BP | protein peptidyl-prolyl isomerization | 0.00013 | 0.015589 |
| GO:0007080 | BP | mitotic metaphase plate congression | 0.00013 | 0.015589 |
| GO:0007059 | BP | chromosome segregation | 0.000132 | 0.015682 |
| GO:0071900 | BP | regulation of protein serine/threonine kinase activity | 0.000134 | 0.015891 |
| GO:0071902 | BP | positive regulation of protein serine/threonine kinase activity | 0.000136 | 0.016006 |
| GO:0030301 | BP | cholesterol transport | 0.000151 | 0.01743 |
| GO:1902930 | BP | regulation of alcohol biosynthetic process | 0.000151 | 0.01743 |
| GO:0005996 | BP | monosaccharide metabolic process | 0.000156 | 0.017743 |
| GO:1902850 | BP | microtubule cytoskeleton organization involved in mitosis | 0.000163 | 0.018395 |
| GO:0044282 | BP | small molecule catabolic process | 0.000165 | 0.018612 |
| GO:0035383 | BP | thioester metabolic process | 0.000177 | 0.019562 |
| GO:0006637 | BP | acyl-CoA metabolic process | 0.000177 | 0.019562 |
| GO:0097435 | BP | supramolecular fiber organization | 0.000183 | 0.020101 |
| GO:0070328 | BP | triglyceride homeostasis | 0.000186 | 0.020159 |
| GO:0055090 | BP | acylglycerol homeostasis | 0.000186 | 0.020159 |
| GO:0016054 | BP | organic acid catabolic process | 0.000187 | 0.020159 |
| GO:0046395 | BP | carboxylic acid catabolic process | 0.000187 | 0.020159 |
| GO:0099177 | BP | regulation of trans-synaptic signaling | 0.00019 | 0.020303 |
| GO:0001525 | BP | angiogenesis | 0.000199 | 0.021205 |
| GO:0061351 | BP | neural precursor cell proliferation | 0.000205 | 0.02159 |
| GO:0019318 | BP | hexose metabolic process | 0.00023 | 0.023692 |
| GO:0032964 | BP | collagen biosynthetic process | 0.000253 | 0.025858 |
| GO:0051303 | BP | establishment of chromosome localization | 0.000259 | 0.026283 |
| GO:0015918 | BP | sterol transport | 0.000273 | 0.027288 |
| GO:0007088 | BP | regulation of mitotic nuclear division | 0.000281 | 0.027781 |
| GO:0065008 | BP | regulation of biological quality | 0.000282 | 0.027781 |
| GO:0051783 | BP | regulation of nuclear division | 0.000305 | 0.029634 |
| GO:2000816 | BP | negative regulation of mitotic sister chromatid separation | 0.000327 | 0.031169 |
| GO:0046883 | BP | regulation of hormone secretion | 0.000336 | 0.031834 |
| GO:0043408 | BP | regulation of MAPK cascade | 0.000363 | 0.034086 |
| GO:1905819 | BP | negative regulation of chromosome separation | 0.000385 | 0.035823 |
| GO:0032879 | BP | regulation of localization | 0.000404 | 0.037179 |
| GO:0050000 | BP | chromosome localization | 0.000406 | 0.037179 |
| GO:0010965 | BP | regulation of mitotic sister chromatid separation | 0.000406 | 0.037179 |
| GO:0031915 | BP | positive regulation of synaptic plasticity | 0.000412 | 0.037276 |
| GO:0071459 | BP | protein localization to chromosome, centromeric region | 0.000466 | 0.041583 |
| GO:0005975 | BP | carbohydrate metabolic process | 0.000489 | 0.0431 |
| GO:0032147 | BP | activation of protein kinase activity | 0.000492 | 0.043125 |
| GO:0045787 | BP | positive regulation of cell cycle | 0.000514 | 0.04415 |
| GO:0033048 | BP | negative regulation of mitotic sister chromatid segregation | 0.000526 | 0.044747 |
| GO:0006006 | BP | glucose metabolic process | 0.000544 | 0.046104 |
| GO:1903046 | BP | meiotic cell cycle process | 0.000561 | 0.047323 |
| GO:0045922 | BP | negative regulation of fatty acid metabolic process | 0.000569 | 0.047788 |
| GO:0005874 | CC | microtubule | 5.05E-07 | 0.000727 |
| GO:0005876 | CC | spindle microtubule | 4.41E-06 | 0.001223 |
| GO:0099513 | CC | polymeric cytoskeletal fiber | 2.51E-05 | 0.004627 |
| GO:0030496 | CC | midbody | 3.20E-05 | 0.0054 |
| GO:0000776 | CC | kinetochore | 3.47E-05 | 0.005761 |
| GO:0000307 | CC | cyclin-dependent protein kinase holoenzyme complex | 4.18E-05 | 0.006395 |
| GO:0099512 | CC | supramolecular fiber | 6.53E-05 | 0.009285 |
| GO:0099081 | CC | supramolecular polymer | 6.53E-05 | 0.009285 |
| GO:0000777 | CC | condensed chromosome kinetochore | 0.000101 | 0.013121 |
| GO:0000922 | CC | spindle pole | 0.000342 | 0.032244 |
| GO:0099080 | CC | supramolecular complex | 0.000501 | 0.043514 |
| GO:1902554 | CC | serine/threonine protein kinase complex | 0.000503 | 0.043514 |
| GO:1901681 | MF | sulfur compound binding | 3.06E-07 | 0.000687 |
| GO:0016411 | MF | acylglycerol O-acyltransferase activity | 1.18E-06 | 0.000865 |
| GO:0016491 | MF | oxidoreductase activity | 1.62E-06 | 0.00095 |
| GO:0016705 | MF | oxidoreductase activity, acting on paired donors, with incorporation or reduction of molecular oxygen | 2.85E-06 | 0.001045 |
| GO:0043168 | MF | anion binding | 3.62E-06 | 0.001162 |
| GO:0033218 | MF | amide binding | 3.69E-06 | 0.001162 |
| GO:0036094 | MF | small molecule binding | 3.92E-06 | 0.001181 |
| GO:0005506 | MF | iron ion binding | 3.98E-06 | 0.001184 |
| GO:0003824 | MF | catalytic activity | 4.48E-06 | 0.001223 |
| GO:0097367 | MF | carbohydrate derivative binding | 4.74E-06 | 0.001259 |
| GO:0005515 | MF | protein binding | 5.96E-06 | 0.001483 |
| GO:0043167 | MF | ion binding | 7.91E-06 | 0.001875 |
| GO:0008017 | MF | microtubule binding | 1.23E-05 | 0.002711 |
| GO:0016301 | MF | kinase activity | 1.56E-05 | 0.003086 |
| GO:0042802 | MF | identical protein binding | 2.92E-05 | 0.005193 |
| GO:0016645 | MF | oxidoreductase activity, acting on the CH-NH group of donors | 3.67E-05 | 0.006023 |
| GO:0042803 | MF | protein homodimerization activity | 3.69E-05 | 0.006023 |
| GO:0000166 | MF | nucleotide binding | 3.97E-05 | 0.006367 |
| GO:1901265 | MF | nucleoside phosphate binding | 3.98E-05 | 0.006367 |
| GO:0020037 | MF | heme binding | 4.12E-05 | 0.006395 |
| GO:0016853 | MF | isomerase activity | 5.89E-05 | 0.008758 |
| GO:0046906 | MF | tetrapyrrole binding | 6.25E-05 | 0.009147 |
| GO:0015631 | MF | tubulin binding | 6.43E-05 | 0.009278 |
| GO:0004497 | MF | monooxygenase activity | 6.64E-05 | 0.009314 |
| GO:0003777 | MF | microtubule motor activity | 7.07E-05 | 0.009846 |
| GO:0016773 | MF | phosphotransferase activity, alcohol group as acceptor | 7.32E-05 | 0.009915 |
| GO:0003846 | MF | 2-acylglycerol O-acyltransferase activity | 8.59E-05 | 0.01133 |
| GO:0032555 | MF | purine ribonucleotide binding | 0.000113 | 0.01427 |
| GO:0016018 | MF | cyclosporin A binding | 0.00013 | 0.015589 |
| GO:0032553 | MF | ribonucleotide binding | 0.000151 | 0.01743 |
| GO:0017076 | MF | purine nucleotide binding | 0.000154 | 0.017675 |
| GO:0016717 | MF | oxidoreductase activity, acting on paired donors, with oxidation of a pair of donors resulting in the reduction of molecular oxygen to two molecules of water | 0.000173 | 0.019313 |
| GO:0008374 | MF | O-acyltransferase activity | 0.000205 | 0.02159 |
| GO:0008144 | MF | drug binding | 0.000209 | 0.02195 |
| GO:0004672 | MF | protein kinase activity | 0.000263 | 0.026586 |
| GO:0032559 | MF | adenyl ribonucleotide binding | 0.000266 | 0.026716 |
| GO:0005539 | MF | glycosaminoglycan binding | 0.000287 | 0.028155 |
| GO:0120227 | MF | acyl-CoA binding | 0.000302 | 0.029499 |
| GO:0008201 | MF | heparin binding | 0.000313 | 0.030237 |
| GO:0030554 | MF | adenyl nucleotide binding | 0.000371 | 0.034643 |
| GO:0016709 | MF | oxidoreductase activity, acting on paired donors, with incorporation or reduction of molecular oxygen, NAD(P)H as one donor, and incorporation of one atom of oxygen | 0.000407 | 0.037179 |
| GO:0004768 | MF | stearoyl-CoA 9-desaturase activity | 0.000412 | 0.037276 |
| GO:0016829 | MF | lyase activity | 0.000419 | 0.037712 |
| GO:0016405 | MF | CoA-ligase activity | 0.000466 | 0.041583 |
| GO:0019842 | MF | vitamin binding | 0.000489 | 0.0431 |
| GO:0008092 | MF | cytoskeletal protein binding | 0.000507 | 0.043743 |
| GO:0019199 | MF | transmembrane receptor protein kinase activity | 0.000525 | 0.044747 |

iBAT_M1 VS iBAT_M4 down

| go_id | go_type | discription | p_uncorrected | p_corrected |
| --- | --- | --- | --- | --- |
| GO:0055114 | BP | oxidation-reduction process | 6.58E-07 | 0.001784 |
| GO:0006091 | BP | generation of precursor metabolites and energy | 8.56E-07 | 0.001784 |
| GO:0051707 | BP | response to other organism | 9.29E-07 | 0.001784 |
| GO:0006955 | BP | immune response | 1.18E-06 | 0.001784 |
| GO:0045087 | BP | innate immune response | 1.25E-06 | 0.001784 |
| GO:0043207 | BP | response to external biotic stimulus | 1.49E-06 | 0.001784 |
| GO:0006952 | BP | defense response | 1.54E-06 | 0.001784 |
| GO:0042127 | BP | regulation of cell population proliferation | 1.90E-06 | 0.001784 |
| GO:0009607 | BP | response to biotic stimulus | 2.00E-06 | 0.001784 |
| GO:0098542 | BP | defense response to other organism | 2.02E-06 | 0.001784 |
| GO:0002682 | BP | regulation of immune system process | 2.10E-06 | 0.001784 |
| GO:0009968 | BP | negative regulation of signal transduction | 2.11E-06 | 0.001784 |
| GO:0002376 | BP | immune system process | 2.23E-06 | 0.001784 |
| GO:0022904 | BP | respiratory electron transport chain | 2.37E-06 | 0.001784 |
| GO:0009605 | BP | response to external stimulus | 2.42E-06 | 0.001784 |
| GO:0002684 | BP | positive regulation of immune system process | 2.63E-06 | 0.001784 |
| GO:0051240 | BP | positive regulation of multicellular organismal process | 2.85E-06 | 0.001829 |
| GO:0051239 | BP | regulation of multicellular organismal process | 3.32E-06 | 0.002043 |
| GO:1901654 | BP | response to ketone | 3.80E-06 | 0.0021 |
| GO:0050896 | BP | response to stimulus | 4.11E-06 | 0.002156 |
| GO:0010648 | BP | negative regulation of cell communication | 5.69E-06 | 0.002792 |
| GO:0023057 | BP | negative regulation of signaling | 5.75E-06 | 0.002792 |
| GO:0022900 | BP | electron transport chain | 6.60E-06 | 0.003073 |
| GO:0048585 | BP | negative regulation of response to stimulus | 6.64E-06 | 0.003073 |
| GO:0032787 | BP | monocarboxylic acid metabolic process | 7.12E-06 | 0.003221 |
| GO:0048545 | BP | response to steroid hormone | 7.34E-06 | 0.00323 |
| GO:0048583 | BP | regulation of response to stimulus | 7.46E-06 | 0.00323 |
| GO:0065008 | BP | regulation of biological quality | 8.09E-06 | 0.003426 |
| GO:0033993 | BP | response to lipid | 8.80E-06 | 0.003623 |
| GO:0044281 | BP | small molecule metabolic process | 8.92E-06 | 0.003623 |
| GO:0072078 | BP | nephron tubule morphogenesis | 1.04E-05 | 0.004018 |
| GO:0072088 | BP | nephron epithelium morphogenesis | 1.04E-05 | 0.004018 |
| GO:0036293 | BP | response to decreased oxygen levels | 1.05E-05 | 0.004018 |
| GO:0072171 | BP | mesonephric tubule morphogenesis | 1.23E-05 | 0.004623 |
| GO:0061333 | BP | renal tubule morphogenesis | 1.73E-05 | 0.005848 |
| GO:0051094 | BP | positive regulation of developmental process | 2.09E-05 | 0.006823 |
| GO:0009165 | BP | nucleotide biosynthetic process | 2.85E-05 | 0.008741 |
| GO:0008285 | BP | negative regulation of cell population proliferation | 2.96E-05 | 0.008785 |
| GO:0001666 | BP | response to hypoxia | 3.00E-05 | 0.008785 |
| GO:0050801 | BP | ion homeostasis | 3.15E-05 | 0.009078 |
| GO:0070482 | BP | response to oxygen levels | 3.36E-05 | 0.00956 |
| GO:0032501 | BP | multicellular organismal process | 3.84E-05 | 0.010496 |
| GO:0009117 | BP | nucleotide metabolic process | 3.85E-05 | 0.010496 |
| GO:0050793 | BP | regulation of developmental process | 4.00E-05 | 0.010755 |
| GO:1901293 | BP | nucleoside phosphate biosynthetic process | 4.05E-05 | 0.010755 |
| GO:0009628 | BP | response to abiotic stimulus | 4.27E-05 | 0.011177 |
| GO:0008284 | BP | positive regulation of cell population proliferation | 4.62E-05 | 0.011796 |
| GO:0048525 | BP | negative regulation of viral process | 4.87E-05 | 0.012273 |
| GO:0006753 | BP | nucleoside phosphate metabolic process | 5.00E-05 | 0.012433 |
| GO:0010646 | BP | regulation of cell communication | 5.53E-05 | 0.013425 |
| GO:0023051 | BP | regulation of signaling | 5.75E-05 | 0.013655 |
| GO:0098771 | BP | inorganic ion homeostasis | 5.76E-05 | 0.013655 |
| GO:0009966 | BP | regulation of signal transduction | 6.51E-05 | 0.015058 |
| GO:0043066 | BP | negative regulation of apoptotic process | 6.69E-05 | 0.015127 |
| GO:0043067 | BP | regulation of programmed cell death | 7.05E-05 | 0.015776 |
| GO:0048584 | BP | positive regulation of response to stimulus | 7.22E-05 | 0.01597 |
| GO:0060548 | BP | negative regulation of cell death | 7.33E-05 | 0.016048 |
| GO:0043069 | BP | negative regulation of programmed cell death | 7.83E-05 | 0.016118 |
| GO:0006163 | BP | purine nucleotide metabolic process | 8.19E-05 | 0.016647 |
| GO:2000026 | BP | regulation of multicellular organismal development | 8.58E-05 | 0.017222 |
| GO:0009150 | BP | purine ribonucleotide metabolic process | 8.65E-05 | 0.017222 |
| GO:0006950 | BP | response to stress | 9.18E-05 | 0.017624 |
| GO:0010941 | BP | regulation of cell death | 9.29E-05 | 0.017624 |
| GO:0032729 | BP | positive regulation of interferon-gamma production | 9.29E-05 | 0.017624 |
| GO:0050678 | BP | regulation of epithelial cell proliferation | 9.72E-05 | 0.017976 |
| GO:0019752 | BP | carboxylic acid metabolic process | 0.000104 | 0.018798 |
| GO:0002252 | BP | immune effector process | 0.00011 | 0.019519 |
| GO:0001996 | BP | positive regulation of heart rate by epinephrine-norepinephrine | 0.000111 | 0.019519 |
| GO:1903901 | BP | negative regulation of viral life cycle | 0.000119 | 0.020479 |
| GO:0042981 | BP | regulation of apoptotic process | 0.000119 | 0.020479 |
| GO:0009259 | BP | ribonucleotide metabolic process | 0.000132 | 0.022391 |
| GO:0032743 | BP | positive regulation of interleukin-2 production | 0.000136 | 0.022495 |
| GO:0014070 | BP | response to organic cyclic compound | 0.00014 | 0.023061 |
| GO:0055080 | BP | cation homeostasis | 0.000156 | 0.0251 |
| GO:0120162 | BP | positive regulation of cold-induced thermogenesis | 0.000158 | 0.025215 |
| GO:0007166 | BP | cell surface receptor signaling pathway | 0.000164 | 0.025788 |
| GO:0019693 | BP | ribose phosphate metabolic process | 0.000164 | 0.025788 |
| GO:0051345 | BP | positive regulation of hydrolase activity | 0.00017 | 0.026518 |
| GO:0001819 | BP | positive regulation of cytokine production | 0.000178 | 0.027262 |
| GO:0051336 | BP | regulation of hydrolase activity | 0.000178 | 0.027262 |
| GO:0048518 | BP | positive regulation of biological process | 0.000181 | 0.02747 |
| GO:0030278 | BP | regulation of ossification | 0.000183 | 0.027561 |
| GO:0044070 | BP | regulation of anion transport | 0.000195 | 0.029156 |
| GO:0006164 | BP | purine nucleotide biosynthetic process | 0.000198 | 0.02941 |
| GO:0070887 | BP | cellular response to chemical stimulus | 0.000203 | 0.029686 |
| GO:0007155 | BP | cell adhesion | 0.000204 | 0.029686 |
| GO:0046058 | BP | cAMP metabolic process | 0.000213 | 0.030168 |
| GO:0009615 | BP | response to virus | 0.000214 | 0.030168 |
| GO:0009725 | BP | response to hormone | 0.000219 | 0.030533 |
| GO:0010038 | BP | response to metal ion | 0.000221 | 0.030533 |
| GO:0022610 | BP | biological adhesion | 0.000233 | 0.031757 |
| GO:1901135 | BP | carbohydrate derivative metabolic process | 0.000233 | 0.031757 |
| GO:0009791 | BP | post-embryonic development | 0.000249 | 0.033443 |
| GO:0030593 | BP | neutrophil chemotaxis | 0.000255 | 0.033875 |
| GO:0007165 | BP | signal transduction | 0.000261 | 0.034293 |
| GO:0120161 | BP | regulation of cold-induced thermogenesis | 0.000266 | 0.034603 |
| GO:0006885 | BP | regulation of pH | 0.00027 | 0.034893 |
| GO:0048871 | BP | multicellular organismal homeostasis | 0.000285 | 0.035894 |
| GO:0010035 | BP | response to inorganic substance | 0.000296 | 0.036716 |
| GO:0032663 | BP | regulation of interleukin-2 production | 0.000297 | 0.036716 |
| GO:0009152 | BP | purine ribonucleotide biosynthetic process | 0.000303 | 0.037229 |
| GO:0051384 | BP | response to glucocorticoid | 0.000318 | 0.03835 |
| GO:0065007 | BP | biological regulation | 0.000332 | 0.039658 |
| GO:0032649 | BP | regulation of interferon-gamma production | 0.000335 | 0.039658 |
| GO:0032101 | BP | regulation of response to external stimulus | 0.000343 | 0.040388 |
| GO:1901701 | BP | cellular response to oxygen-containing compound | 0.000349 | 0.040626 |
| GO:0008406 | BP | gonad development | 0.000349 | 0.040626 |
| GO:1990845 | BP | adaptive thermogenesis | 0.000354 | 0.041006 |
| GO:0032502 | BP | developmental process | 0.00036 | 0.041006 |
| GO:0052548 | BP | regulation of endopeptidase activity | 0.000362 | 0.041006 |
| GO:2000116 | BP | regulation of cysteine-type endopeptidase activity | 0.000364 | 0.041006 |
| GO:0009187 | BP | cyclic nucleotide metabolic process | 0.000365 | 0.041006 |
| GO:0048008 | BP | platelet-derived growth factor receptor signaling pathway | 0.000365 | 0.041006 |
| GO:0048878 | BP | chemical homeostasis | 0.000379 | 0.042118 |
| GO:0072521 | BP | purine-containing compound metabolic process | 0.000384 | 0.042399 |
| GO:0071396 | BP | cellular response to lipid | 0.000424 | 0.04614 |
| GO:0070469 | CC | respirasome | 3.19E-07 | 0.001784 |
| GO:0005743 | CC | mitochondrial inner membrane | 1.77E-06 | 0.001784 |
| GO:0031966 | CC | mitochondrial membrane | 2.24E-06 | 0.001784 |
| GO:0005615 | CC | extracellular space | 3.59E-06 | 0.002043 |
| GO:0019866 | CC | organelle inner membrane | 1.71E-05 | 0.005848 |
| GO:0031012 | CC | extracellular matrix | 1.79E-05 | 0.005933 |
| GO:0098803 | CC | respiratory chain complex | 2.57E-05 | 0.008125 |
| GO:1990204 | CC | oxidoreductase complex | 0.000118 | 0.020479 |
| GO:0016021 | CC | integral component of membrane | 0.000247 | 0.033422 |
| GO:0005102 | MF | signaling receptor binding | 1.35E-05 | 0.004886 |
| GO:0016491 | MF | oxidoreductase activity | 2.64E-05 | 0.008203 |
| GO:0016655 | MF | oxidoreductase activity, acting on NAD(P)H, quinone or similar compound as acceptor | 6.12E-05 | 0.014327 |
| GO:0050136 | MF | NADH dehydrogenase (quinone) activity | 7.66E-05 | 0.016118 |
| GO:0008137 | MF | NADH dehydrogenase (ubiquinone) activity | 7.66E-05 | 0.016118 |
| GO:0043169 | MF | cation binding | 8.76E-05 | 0.017278 |
| GO:1902936 | MF | phosphatidylinositol bisphosphate binding | 9.14E-05 | 0.017624 |
| GO:0050833 | MF | pyruvate transmembrane transporter activity | 9.60E-05 | 0.017976 |
| GO:0043167 | MF | ion binding | 9.75E-05 | 0.017976 |
| GO:0050543 | MF | icosatetraenoic acid binding | 0.000111 | 0.019519 |
| GO:0003954 | MF | NADH dehydrogenase activity | 0.000133 | 0.022391 |
| GO:0003824 | MF | catalytic activity | 0.000135 | 0.022495 |
| GO:0042287 | MF | MHC protein binding | 0.000207 | 0.02973 |
| GO:0046872 | MF | metal ion binding | 0.000219 | 0.030533 |
| GO:0005546 | MF | phosphatidylinositol-4,5-bisphosphate binding | 0.000255 | 0.033875 |
| GO:0008514 | MF | organic anion transmembrane transporter activity | 0.000262 | 0.034293 |
| GO:0009055 | MF | electron transfer activity | 0.000277 | 0.035618 |
| GO:0140375 | MF | immune receptor activity | 0.000285 | 0.035894 |
| GO:0002020 | MF | protease binding | 0.00031 | 0.037882 |
| GO:1901981 | MF | phosphatidylinositol phosphate binding | 0.000421 | 0.046014 |
