# Supplementary material for: Comparative transcriptomic analysis of rabbit interscapular brown adipose tissue whitening under physiological conditions
Source: Adipocyte. 2022 Aug 29;11(1):529–49. doi: 10.1080/21623945.2022.2111053 (PMC9427046; doi:10.1080/21623945.2022.2111053)
Supplement: Supplemental Material [file KADI_A_2111053_SM2303.zip › Supplementary/Table S1_S3.docx]

**Table S1. Results of mapped reads in rabbit iBAT and ingWAT**

|  | Clean bases | Clean reads | Total mapped | Multiple mapped | Uniquely mapped |
| --- | --- | --- | --- | --- | --- |
| M4ingWAT3 | 6053990054 | 41002130 | 35978237(87.75%) | 1785907(4.36%) | 34192330(83.39%) |
| M4ingWAT2 | 8393543648 | 56986344 | 50456254(88.54%) | 1888355(3.31%) | 48567899(85.23%) |
| M4ingWAT1 | 8231924574 | 56044606 | 49769728(88.8%) | 2207464(3.94%) | 47562264(84.87%) |
| M4iBAT3 | 7927866751 | 53607156 | 47928767(89.41%) | 1758987(3.28%) | 46169780(86.13%) |
| M4iBAT2 | 6802143753 | 46141584 | 41422421(89.77%) | 1479454(3.21%) | 39942967(86.57%) |
| M4iBAT1 | 7759473469 | 52525228 | 46851316(89.2%) | 1570481(2.99%) | 45280835(86.21%) |
| M1iBAT3 | 6733443275 | 45991068 | 41801328(90.89%) | 1682016(3.66%) | 40119312(87.23%) |
| M1iBAT2 | 8326531372 | 56615828 | 51349700(90.7%) | 2110354(3.73%) | 49239346(86.97%) |
| M1iBAT1 | 7928540960 | 54058880 | 49006549(90.65%) | 2033627(3.76%) | 46972922(86.89%) |
| D14iBAT3 | 7381288547 | 50294256 | 46039119(91.54%) | 2749903(5.47%) | 43289216(86.07%) |
| D14iBAT2 | 7142945623 | 48582070 | 44187737(90.95%) | 2484115(5.11%) | 41703622(85.84%) |
| D14iBAT1 | 6615405186 | 44887234 | 41018163(91.38%) | 2545572(5.67%) | 38472591(85.71%) |
| D1iBAT3 | 6840451283 | 46493736 | 42621718(91.67%) | 2755475(5.93%) | 39866243(85.75%) |
| D1iBAT2 | 7223252581 | 49217468 | 44943935(91.32%) | 2611611(5.31%) | 42332324(86.01%) |
| D1iBAT1 | 6693804922 | 45390818 | 41333030(91.06%) | 2355471(5.19%) | 38977559(85.87%) |

**Table S2. Correlation coefficients between samples**

|  | M4iBAT3 | M4iBAT1 | M4iBAT2 | M4ingWAT3 | M4ingWAT1 | M4ingWAT2 | D14iBAT3 | D14iBAT1 | D1iBAT3 | D1iBAT2 | D14iBAT2 | D1iBAT1 | M1iBAT3 | M1iBAT1 | M1iBAT2 |
| --- | --- | --- | --- | --- | --- | --- | --- | --- | --- | --- | --- | --- | --- | --- | --- |
| M4iBAT3 | 1 | 0.9897 | 0.9891 | 0.8511 | 0.9277 | 0.9166 | 0.9396 | 0.936 | 0.932 | 0.9026 | 0.9391 | 0.9287 | 0.9618 | 0.955 | 0.9599 |
| M4iBAT1 | 0.9897 | 1 | 0.9922 | 0.8747 | 0.9547 | 0.9459 | 0.9062 | 0.9035 | 0.8985 | 0.8722 | 0.9113 | 0.8969 | 0.9431 | 0.9199 | 0.9318 |
| M4iBAT2 | 0.9891 | 0.9922 | 1 | 0.8712 | 0.9437 | 0.9459 | 0.9104 | 0.9091 | 0.9032 | 0.8773 | 0.9161 | 0.9038 | 0.9458 | 0.9242 | 0.9377 |
| M4ingWAT3 | 0.8511 | 0.8747 | 0.8712 | 1 | 0.9645 | 0.9415 | 0.697 | 0.6987 | 0.696 | 0.6892 | 0.7146 | 0.7014 | 0.7635 | 0.721 | 0.7401 |
| M4ingWAT1 | 0.9277 | 0.9547 | 0.9437 | 0.9645 | 1 | 0.978 | 0.7962 | 0.7956 | 0.7925 | 0.7804 | 0.812 | 0.794 | 0.8572 | 0.815 | 0.8337 |
| M4ingWAT2 | 0.9166 | 0.9459 | 0.9459 | 0.9415 | 0.978 | 1 | 0.7698 | 0.769 | 0.7639 | 0.75 | 0.7836 | 0.764 | 0.8341 | 0.7891 | 0.8087 |
| D14iBAT3 | 0.9396 | 0.9062 | 0.9104 | 0.697 | 0.7962 | 0.7698 | 1 | 0.9986 | 0.9964 | 0.971 | 0.9961 | 0.9942 | 0.9855 | 0.9835 | 0.9929 |
| D14iBAT1 | 0.936 | 0.9035 | 0.9091 | 0.6987 | 0.7956 | 0.769 | 0.9986 | 1 | 0.9975 | 0.9789 | 0.9971 | 0.997 | 0.9863 | 0.9768 | 0.99 |
| D1iBAT3 | 0.932 | 0.8985 | 0.9032 | 0.696 | 0.7925 | 0.7639 | 0.9964 | 0.9975 | 1 | 0.9839 | 0.9941 | 0.9971 | 0.9824 | 0.9724 | 0.9856 |
| D1iBAT2 | 0.9026 | 0.8722 | 0.8773 | 0.6892 | 0.7804 | 0.75 | 0.971 | 0.9789 | 0.9839 | 1 | 0.977 | 0.9858 | 0.9644 | 0.9292 | 0.9532 |
| D14iBAT2 | 0.9391 | 0.9113 | 0.9161 | 0.7146 | 0.812 | 0.7836 | 0.9961 | 0.9971 | 0.9941 | 0.977 | 1 | 0.9955 | 0.9894 | 0.9719 | 0.9902 |
| D1iBAT1 | 0.9287 | 0.8969 | 0.9038 | 0.7014 | 0.794 | 0.764 | 0.9942 | 0.997 | 0.9971 | 0.9858 | 0.9955 | 1 | 0.9809 | 0.9659 | 0.983 |
| M1iBAT3 | 0.9618 | 0.9431 | 0.9458 | 0.7635 | 0.8572 | 0.8341 | 0.9855 | 0.9863 | 0.9824 | 0.9644 | 0.9894 | 0.9809 | 1 | 0.9737 | 0.9918 |
| M1iBAT1 | 0.955 | 0.9199 | 0.9242 | 0.721 | 0.815 | 0.7891 | 0.9835 | 0.9768 | 0.9724 | 0.9292 | 0.9719 | 0.9659 | 0.9737 | 1 | 0.9914 |
| M1iBAT2 | 0.9599 | 0.9318 | 0.9377 | 0.7401 | 0.8337 | 0.8087 | 0.9929 | 0.99 | 0.9856 | 0.9532 | 0.9902 | 0.983 | 0.9918 | 0.9914 | 1 |

**Table S3.** **DEGs of different comparison groups**

iBAT_D1_VS_iBAT_D14

| Gene name | FC(iBAT_D14/iBAT_D1) | Log2FC(iBAT_D14/iBAT_D1) | Pvalue | Padjust | Significant | Regulate | D1iBAT1 | D1iBAT2 | D1iBAT3 | D14iBAT1 | D14iBAT2 | D14iBAT3 | iBAT_D1 | iBAT_D14 |
| --- | --- | --- | --- | --- | --- | --- | --- | --- | --- | --- | --- | --- | --- | --- |
| ADH1A | 33.386 | 5.061182 | 7.5E-122 | 1.1E-117 | yes | up | 9.29 | 13.89 | 8.71 | 337.12 | 337.2 | 412.88 | 10.63 | 362.4 |
| LOC100352536 | 168.605 | 7.3975 | 2.9E-117 | 2.2E-113 | yes | up | 2.61 | 7.3 | 2.21 | 639.39 | 801.98 | 627.65 | 4.04 | 689.6733 |
| PLXNA4 | 0.058 | -4.10948 | 1.1E-90 | 5.61E-87 | yes | down | 5.72 | 7.1 | 6.19 | 0.39 | 0.4 | 0.38 | 6.336667 | 0.39 |
| LOC100344545 | 52.008 | 5.700664 | 5.03E-77 | 1.92E-73 | yes | up | 0.55 | 0.46 | 0.32 | 22.57 | 25.52 | 21.96 | 0.443333 | 23.35 |
| LOC100352363 | 0.002 | -8.67697 | 8.29E-61 | 2.53E-57 | yes | down | 294.16 | 686.41 | 553.05 | 2.14 | 0.7 | 1.32 | 511.2067 | 1.386667 |
| RPL34 | 43.144 | 5.431078 | 3.51E-53 | 8.94E-50 | yes | up | 0.93 | 0.94 | 0.16 | 105.02 | 109.85 | 84.83 | 0.676667 | 99.9 |
| LBP | 0.156 | -2.68386 | 7.2E-53 | 1.57E-49 | yes | down | 87.15 | 76.54 | 83.19 | 9.88 | 14.21 | 15.32 | 82.29333 | 13.13667 |
| SET | 0.252 | -1.98992 | 2.34E-40 | 4.47E-37 | yes | down | 38.16 | 41.52 | 29.72 | 9.43 | 9.82 | 8.97 | 36.46667 | 9.406667 |
| FABP3 | 0.213 | -2.22896 | 9.96E-39 | 1.69E-35 | yes | down | 1717.61 | 1888.73 | 1516.43 | 392.79 | 434.21 | 301.49 | 1707.59 | 376.1633 |
| PGER5 | 3.332 | 1.736261 | 7.66E-38 | 1.17E-34 | yes | up | 213.95 | 235.44 | 175.62 | 609.31 | 768.98 | 774.51 | 208.3367 | 717.6 |
| IMPDH1 | 3.355 | 1.746173 | 9.31E-36 | 1.29E-32 | yes | up | 86.78 | 103.77 | 80.47 | 266.77 | 353.18 | 325.91 | 90.34 | 315.2867 |
| ACSS3 | 0.219 | -2.19091 | 2.47E-34 | 3.14E-31 | yes | down | 31.97 | 41.32 | 36.82 | 8 | 9.26 | 8.09 | 36.70333 | 8.45 |
| LOC103348029 | 0.088 | -3.50957 | 6.08E-32 | 7.14E-29 | yes | down | 40.84 | 61.22 | 63.14 | 4.52 | 7.18 | 4.75 | 55.06667 | 5.483333 |
| ADORA1 | 34.785 | 5.120408 | 1.56E-31 | 1.7E-28 | yes | up | 0.33 | 0.58 | 0.14 | 6.65 | 12.44 | 16.74 | 0.35 | 11.94333 |
| LPAR6 | 0.23 | -2.1181 | 1.85E-30 | 1.89E-27 | yes | down | 16.42 | 17.38 | 16.47 | 3.9 | 3.85 | 4.26 | 16.75667 | 4.003333 |
| BHMT | 0.049 | -4.36211 | 2.01E-29 | 1.92E-26 | yes | down | 28.03 | 28.35 | 41.67 | 2.51 | 1.43 | 1.06 | 32.68333 | 1.666667 |
| EPHX2 | 2.989 | 1.579612 | 4.49E-29 | 4.03E-26 | yes | up | 101.48 | 103.04 | 90.49 | 245.88 | 363.84 | 312.57 | 98.33667 | 307.43 |
| AHCYL1 | 4.51 | 2.172968 | 7.59E-27 | 6.44E-24 | yes | up | 56.62 | 49.34 | 63.3 | 233.97 | 278.01 | 295.83 | 56.42 | 269.27 |
| LOC100328613 | 0.003 | -8.608 | 9.06E-27 | 7.28E-24 | yes | down | 308.91 | 818.45 | 410.3 | 0.55 | 0.85 | 2.65 | 512.5533 | 1.35 |
| MIF | 0.35 | -1.51484 | 1.8E-26 | 1.37E-23 | yes | down | 400.96 | 424.4 | 393.65 | 129.39 | 166.23 | 147.16 | 406.3367 | 147.5933 |
| KCNQ1 | 3.446 | 1.785113 | 2.31E-26 | 1.68E-23 | yes | up | 0.67 | 0.79 | 0.59 | 1.97 | 2.1 | 1.86 | 0.683333 | 1.976667 |
| ABCD2 | 15.552 | 3.959063 | 8.06E-26 | 5.59E-23 | yes | up | 0.54 | 1.21 | 0.3 | 7.35 | 13.56 | 14.11 | 0.683333 | 11.67333 |
| NPTX1 | 4.098 | 2.034806 | 1.22E-24 | 8.1E-22 | yes | up | 4.53 | 3.19 | 3.52 | 15.49 | 13.67 | 14.91 | 3.746667 | 14.69 |
| LOC100350716 | 0.124 | -3.00898 | 4.44E-24 | 2.82E-21 | yes | down | 18.65 | 14.79 | 12.07 | 1.82 | 2.96 | 1.16 | 15.17 | 1.98 |
| FEZ1 | 4.7 | 2.232564 | 4.84E-24 | 2.95E-21 | yes | up | 13.71 | 16.87 | 12.21 | 56.97 | 63.49 | 86.52 | 14.26333 | 68.99333 |
| LOC100328912 | 5.815 | 2.539827 | 2.32E-23 | 1.35E-20 | yes | up | 77.65 | 74.12 | 90.95 | 330.17 | 545.19 | 605.91 | 80.90667 | 493.7567 |
| LOC100328910 | 11.277 | 3.495362 | 2.38E-23 | 1.35E-20 | yes | up | 9.52 | 4.67 | 4.48 | 50.82 | 59.83 | 102.16 | 6.223333 | 70.93667 |
| PEBP4 | 7.006 | 2.808489 | 3.51E-23 | 1.91E-20 | yes | up | 13.03 | 9.69 | 11.28 | 65.55 | 70.84 | 109.6 | 11.33333 | 81.99667 |
| NCOA4 | 0.439 | -1.18725 | 1.02E-22 | 5.36E-20 | yes | down | 75.2 | 79.24 | 61.31 | 32.62 | 42.58 | 36.37 | 71.91667 | 37.19 |
| GLYAT | 5.944 | 2.571379 | 1.73E-22 | 8.78E-20 | yes | up | 10.35 | 7.08 | 5.08 | 47.4 | 39.79 | 48.13 | 7.503333 | 45.10667 |
| TKT | 3.757 | 1.909568 | 1.9E-22 | 9.34E-20 | yes | up | 46.84 | 36.15 | 38.01 | 119.12 | 171.96 | 184.53 | 40.33333 | 158.5367 |
| NRG4 | 0.21 | -2.25415 | 2E-22 | 9.55E-20 | yes | down | 22.44 | 30.91 | 30.68 | 3.95 | 6.62 | 3.87 | 28.01 | 4.813333 |
| LOC100328937 | 0.078 | -3.68649 | 1.29E-21 | 5.96E-19 | yes | down | 5.43 | 7.66 | 4.94 | 0.31 | 0.8 | 0.36 | 6.01 | 0.49 |
| BSP1 | 0.323 | -1.62911 | 1.41E-21 | 6.34E-19 | yes | down | 509.83 | 538.72 | 433.07 | 179.78 | 169.5 | 141.41 | 493.8733 | 163.5633 |
| FAM222A | 8.578 | 3.10058 | 1.83E-21 | 7.98E-19 | yes | up | 0.68 | 0.48 | 0.68 | 3.92 | 5.71 | 6.95 | 0.613333 | 5.526667 |
| LOC100356386 | 2909.552 | 11.50658 | 2.33E-21 | 9.88E-19 | yes | up | 0 | 0 | 0 | 24.95 | 20.82 | 32.64 | 0 | 26.13667 |
| SLC44A3 | 18.393 | 4.201084 | 2.67E-21 | 1.1E-18 | yes | up | 0.35 | 0.1 | 0.4 | 6.2 | 4.91 | 5.2 | 0.283333 | 5.436667 |
| LOC100345430 | 105.983 | 6.727686 | 5.95E-21 | 2.39E-18 | yes | up | 0 | 0.54 | 0 | 22.13 | 21.74 | 19.2 | 0.18 | 21.02333 |
| FIBIN | 3.298 | 1.721439 | 8.96E-21 | 3.51E-18 | yes | up | 6.12 | 5.03 | 4.6 | 16.79 | 18.92 | 17.93 | 5.25 | 17.88 |
| LOC103350281 | 0.29 | -1.7874 | 1.26E-20 | 4.8E-18 | yes | down | 217.37 | 214.51 | 209.55 | 71.43 | 63.79 | 55.76 | 213.81 | 63.66 |
| LOC100352119 | 4.451 | 2.154002 | 1.54E-20 | 5.74E-18 | yes | up | 4.14 | 3.44 | 2.34 | 13.49 | 18.17 | 14.81 | 3.306667 | 15.49 |
| CTSZ | 0.173 | -2.53346 | 2.47E-20 | 8.98E-18 | yes | down | 176.6 | 311.48 | 284.91 | 37.63 | 61.56 | 42.67 | 257.6633 | 47.28667 |
| SHROOM1 | 5.185 | 2.374352 | 2.85E-20 | 1.01E-17 | yes | up | 1.84 | 2.9 | 1.8 | 11.57 | 9.85 | 13.3 | 2.18 | 11.57333 |
| GLYCTK | 3.804 | 1.927406 | 6.11E-20 | 2.12E-17 | yes | up | 128.27 | 111.81 | 106.02 | 356.36 | 488.77 | 593.05 | 115.3667 | 479.3933 |
| SLC24A3 | 4.825 | 2.270514 | 1.17E-19 | 3.95E-17 | yes | up | 3.25 | 4.64 | 2.85 | 16.83 | 14.54 | 21.65 | 3.58 | 17.67333 |
| LOC100343920 | 10.181 | 3.347841 | 1.93E-19 | 6.4E-17 | yes | up | 1.16 | 0.62 | 0.36 | 5.33 | 8.48 | 8.65 | 0.713333 | 7.486667 |
| LOC100358069 | 0.313 | -1.678 | 3.87E-19 | 1.26E-16 | yes | down | 138.1 | 131.49 | 112.65 | 43.85 | 46.04 | 32.96 | 127.4133 | 40.95 |
| DDHD1 | 0.284 | -1.81632 | 4.71E-19 | 1.5E-16 | yes | down | 17.91 | 26.8 | 17.19 | 4.75 | 6.79 | 5.44 | 20.63333 | 5.66 |
| CDKN3 | 4.845 | 2.276349 | 4.87E-19 | 1.52E-16 | yes | up | 6.91 | 8.63 | 4.63 | 29.52 | 26.31 | 35.29 | 6.723333 | 30.37333 |
| LOC103348133 | 0.262 | -1.93357 | 6.04E-19 | 1.84E-16 | yes | down | 19.07 | 22.31 | 15.42 | 4.71 | 6.78 | 4.39 | 18.93333 | 5.293333 |
| TTC9 | 4.481 | 2.163827 | 1.09E-18 | 3.26E-16 | yes | up | 13.9 | 23.08 | 12.42 | 56.35 | 83.32 | 80.42 | 16.46667 | 73.36333 |
| GPX1 | 0.406 | -1.30013 | 1.88E-18 | 5.51E-16 | yes | down | 495.11 | 556.75 | 478.32 | 197.83 | 257.1 | 191.66 | 510.06 | 215.53 |
| C18H10orf11 | 3.521 | 1.81612 | 2.94E-18 | 8.47E-16 | yes | up | 2.69 | 2.59 | 2.35 | 7.25 | 10.11 | 10.64 | 2.543333 | 9.333333 |
| HSD11B1 | 6.949 | 2.796737 | 4.2E-18 | 1.19E-15 | yes | up | 1.82 | 3.3 | 3.63 | 20.62 | 18.51 | 24.86 | 2.916667 | 21.33 |
| SIDT2 | 3.01 | 1.589929 | 5.62E-18 | 1.56E-15 | yes | up | 10 | 10.12 | 8.59 | 28.2 | 26.34 | 34.77 | 9.57 | 29.77 |
| ASS1 | 0.226 | -2.14696 | 6.73E-18 | 1.84E-15 | yes | down | 12.16 | 13.48 | 13 | 2.87 | 3.89 | 2.41 | 12.88 | 3.056667 |
| HAUS7 | 0.22 | -2.18124 | 1.01E-17 | 2.63E-15 | yes | down | 4.56 | 6.71 | 4.55 | 2.42 | 2.4 | 1.89 | 5.273333 | 2.236667 |
| INSR | 0.444 | -1.17006 | 1.01E-17 | 2.63E-15 | yes | down | 18.27 | 20.7 | 15.46 | 7.99 | 8.59 | 8.39 | 18.14333 | 8.323333 |
| CIB4 | 0.089 | -3.48774 | 1.02E-17 | 2.63E-15 | yes | down | 4.01 | 6.2 | 4.6 | 0.5 | 0.36 | 0.25 | 4.936667 | 0.37 |
| MBOAT1 | 0.19 | -2.39426 | 1.61E-17 | 4.09E-15 | yes | down | 7.17 | 6.29 | 7.66 | 0.97 | 1.65 | 1.68 | 7.04 | 1.433333 |
| LOC103352357 | 6.747 | 2.754186 | 2.55E-17 | 6.37E-15 | yes | up | 0.36 | 0.31 | 0.3 | 2.09 | 1.6 | 3.13 | 0.323333 | 2.273333 |
| LOC100358663 | 0.001 | -10.2339 | 2.77E-17 | 6.82E-15 | yes | down | 10.99 | 12.97 | 8.02 | 0 | 0 | 0 | 10.66 | 0 |
| CKB | 4.101 | 2.036099 | 4.37E-17 | 1.06E-14 | yes | up | 18.58 | 18.53 | 12.82 | 45.46 | 96.68 | 71.69 | 16.64333 | 71.27667 |
| GPD1 | 4.623 | 2.208701 | 5.56E-17 | 1.3E-14 | yes | up | 151.8 | 100.13 | 65.22 | 344.3 | 492.62 | 600.79 | 105.7167 | 479.2367 |
| SERINC2 | 2.929 | 1.550368 | 5.62E-17 | 1.3E-14 | yes | up | 10.34 | 9.31 | 7.81 | 24.77 | 26.52 | 31.42 | 9.153333 | 27.57 |
| LOC100344154 | 1417.599 | 10.46923 | 5.63E-17 | 1.3E-14 | yes | up | 0 | 0 | 0 | 60.19 | 88.79 | 30.24 | 0 | 59.74 |
| NPM1 | 0.381 | -1.39028 | 6.32E-17 | 1.44E-14 | yes | down | 227.51 | 262.02 | 229.64 | 90.53 | 112.25 | 79.78 | 239.7233 | 94.18667 |
| LOC103346372 | 11.674 | 3.545198 | 1.1E-16 | 2.46E-14 | yes | up | 14.98 | 5.48 | 14.97 | 126.07 | 113.41 | 172.11 | 11.81 | 137.1967 |
| PPP1R1A | 0.316 | -1.66079 | 2.08E-16 | 4.59E-14 | yes | down | 142.31 | 163.75 | 96.22 | 44.68 | 46.36 | 37.82 | 134.0933 | 42.95333 |
| TRPM1 | 29.878 | 4.901033 | 3.73E-16 | 8.1E-14 | yes | up | 0.1 | 0.26 | 0.05 | 1.88 | 2.72 | 7.72 | 0.136667 | 4.106667 |
| LOC100338525 | 0.315 | -1.6646 | 3.77E-16 | 8.1E-14 | yes | down | 114.51 | 120.62 | 117.33 | 40.22 | 32.92 | 41.48 | 117.4867 | 38.20667 |
| LOC100351543 | 0.001 | -9.81406 | 5.18E-16 | 1.1E-13 | yes | down | 4.02 | 4.2 | 3.09 | 0 | 0 | 0 | 3.77 | 0 |
| RNPEP | 0.234 | -2.09468 | 5.34E-16 | 1.12E-13 | yes | down | 27.54 | 42.03 | 25.03 | 5.97 | 11.13 | 6.52 | 31.53333 | 7.873333 |
| GSTP1 | 0.469 | -1.09121 | 5.81E-16 | 1.2E-13 | yes | down | 168.26 | 174.82 | 149.39 | 77.48 | 79.93 | 80.89 | 164.1567 | 79.43333 |
| CKMT1B | 0.004 | -8.02802 | 7.61E-16 | 1.55E-13 | yes | down | 2.97 | 13.03 | 10.66 | 0.05 | 0.08 | 0 | 8.886667 | 0.043333 |
| GSTO1 | 0.274 | -1.86593 | 1.01E-15 | 2.02E-13 | yes | down | 174.2 | 310.02 | 195.45 | 57.93 | 68.46 | 65.01 | 226.5567 | 63.8 |
| CTSF | 0.367 | -1.4453 | 1.45E-15 | 2.87E-13 | yes | down | 40.39 | 48.64 | 31.82 | 14.19 | 18.6 | 13.14 | 40.28333 | 15.31 |
| LOC100343425 | 2.719 | 1.443006 | 1.8E-15 | 3.52E-13 | yes | up | 80.61 | 82.18 | 80.87 | 208.26 | 220.72 | 261.11 | 81.22 | 230.03 |
| CPE | 2.699 | 1.432242 | 2.01E-15 | 3.88E-13 | yes | up | 30.07 | 28.46 | 28.6 | 80.57 | 73.33 | 94.06 | 29.04333 | 82.65333 |
| LOC100345217 | 786.56 | 9.619414 | 2.91E-15 | 5.56E-13 | yes | up | 0 | 0 | 0 | 27.47 | 35.66 | 24.13 | 0 | 29.08667 |
| LOC100353463 | 2.938 | 1.554677 | 3.08E-15 | 5.81E-13 | yes | up | 24.82 | 32.31 | 25.29 | 65.81 | 83.22 | 98.12 | 27.47333 | 82.38333 |
| LOC100351248 | 0.001 | -9.97084 | 3.46E-15 | 6.44E-13 | yes | down | 20.66 | 9.51 | 23.36 | 0 | 0 | 0 | 17.84333 | 0 |
| TBC1D10A | 2.871 | 1.521496 | 4.66E-15 | 8.57E-13 | yes | up | 17.62 | 18.52 | 9.87 | 36.81 | 43.98 | 42.75 | 15.33667 | 41.18 |
| OBSL1 | 2.674 | 1.419021 | 6.39E-15 | 1.16E-12 | yes | up | 26.28 | 29.77 | 21.16 | 63.3 | 60.11 | 82.94 | 25.73667 | 68.78333 |
| GRIN2C | 0.135 | -2.88565 | 6.64E-15 | 1.19E-12 | yes | down | 1.71 | 2.36 | 1.84 | 0.25 | 0.35 | 0.25 | 1.97 | 0.283333 |
| SLC35F6 | 0.428 | -1.2232 | 6.85E-15 | 1.21E-12 | yes | down | 13.66 | 14.07 | 13.25 | 5.3 | 8.19 | 5.54 | 13.66 | 6.343333 |
| HMGCS2 | 0.107 | -3.21844 | 6.92E-15 | 1.21E-12 | yes | down | 158.94 | 203.07 | 313.49 | 39.25 | 21.93 | 15.03 | 225.1667 | 25.40333 |
| DFFB | 4.314 | 2.108926 | 7.14E-15 | 1.24E-12 | yes | up | 5.34 | 3.64 | 4.67 | 16.6 | 15.36 | 22.48 | 4.55 | 18.14667 |
| SHMT2 | 0.306 | -1.71063 | 7.63E-15 | 1.31E-12 | yes | down | 18.8 | 22.61 | 16.78 | 4.54 | 8.36 | 5.71 | 19.39667 | 6.203333 |
| CPNE4 | 0.058 | -4.11672 | 9.74E-15 | 1.65E-12 | yes | down | 3.88 | 5.23 | 2.1 | 0.14 | 0.47 | 0.08 | 3.736667 | 0.23 |
| F11R | 2.906 | 1.539131 | 1.13E-14 | 1.89E-12 | yes | up | 23.24 | 26.95 | 28.34 | 73.81 | 76.46 | 88.28 | 26.17667 | 79.51667 |
| LOC103350566 | 5.339 | 2.416675 | 1.58E-14 | 2.62E-12 | yes | up | 2.5 | 1.4 | 3.24 | 11.01 | 15.61 | 14.18 | 2.38 | 13.6 |
| G0S2 | 4.812 | 2.266703 | 2.94E-14 | 4.82E-12 | yes | up | 59.72 | 44.94 | 26.3 | 166.83 | 199.61 | 285.03 | 43.65333 | 217.1567 |
| LOC103346967 | 721.57 | 9.494996 | 3.59E-14 | 5.83E-12 | yes | up | 0 | 0 | 0 | 4.32 | 3.92 | 2.09 | 0 | 3.443333 |
| GFPT1 | 0.391 | -1.35653 | 3.89E-14 | 6.25E-12 | yes | down | 16.1 | 14.89 | 10.38 | 4.04 | 5.3 | 6.1 | 13.79 | 5.146667 |
| HR | 0.115 | -3.11903 | 4.16E-14 | 6.62E-12 | yes | down | 4.28 | 2.19 | 1.45 | 0.21 | 0.44 | 0.28 | 2.64 | 0.31 |
| LOC103349807 | 0.002 | -9.19339 | 5.52E-14 | 8.64E-12 | yes | down | 25.57 | 22.95 | 18.95 | 0 | 0 | 0 | 22.49 | 0 |
| GPAT4 | 0.428 | -1.22581 | 5.54E-14 | 8.64E-12 | yes | down | 271.67 | 347.89 | 285.74 | 138.28 | 142.78 | 128.72 | 301.7667 | 136.5933 |
| CIDEC | 2.374 | 1.247404 | 8.12E-14 | 1.25E-11 | yes | up | 252.79 | 322.75 | 262.24 | 629.11 | 763.04 | 793.54 | 279.26 | 728.5633 |
| KCTD6 | 3.325 | 1.733185 | 8.29E-14 | 1.27E-11 | yes | up | 12.47 | 12.97 | 12.92 | 37.03 | 31.67 | 46.61 | 12.78667 | 38.43667 |
| FMO1 | 9.317 | 3.219883 | 8.46E-14 | 1.28E-11 | yes | up | 0.55 | 0.3 | 0.26 | 2.4 | 3.84 | 4.37 | 0.37 | 3.536667 |
| LOC100350957 | 0.115 | -3.12587 | 1.09E-13 | 1.63E-11 | yes | down | 6.65 | 8.26 | 4.97 | 0.6 | 1.4 | 0.34 | 6.626667 | 0.78 |
| TSKU | 4.092 | 2.032759 | 1.32E-13 | 1.93E-11 | yes | up | 3.11 | 21.09 | 12.96 | 41.27 | 47.97 | 62.32 | 12.38667 | 50.52 |
| GMPR | 0.203 | -2.29712 | 1.45E-13 | 2.11E-11 | yes | down | 488.21 | 895.55 | 491.59 | 93.83 | 206.85 | 106.37 | 625.1167 | 135.6833 |
| LOC100346173 | 75.249 | 6.233602 | 1.69E-13 | 2.44E-11 | yes | up | 0.04 | 0 | 0.06 | 7.41 | 1.21 | 2.98 | 0.033333 | 3.866667 |
| SELENBP1 | 2.855 | 1.513249 | 1.77E-13 | 2.53E-11 | yes | up | 40.41 | 48.93 | 43.15 | 94.08 | 151.8 | 151.42 | 44.16333 | 132.4333 |
| AK2 | 2.722 | 1.444456 | 2.51E-13 | 3.55E-11 | yes | up | 91.96 | 104.89 | 88.07 | 212.89 | 230.42 | 309.33 | 94.97333 | 250.88 |
| BDH1 | 0.487 | -1.03885 | 2.8E-13 | 3.93E-11 | yes | down | 213.64 | 193.34 | 165.86 | 87.58 | 120.21 | 86.39 | 190.9467 | 98.06 |
| C1H11orf80 | 0.131 | -2.93161 | 2.96E-13 | 4.11E-11 | yes | down | 3.45 | 7.18 | 2.6 | 0.44 | 0.53 | 0.81 | 4.41 | 0.593333 |
| MRPL22 | 2.703 | 1.434765 | 3.17E-13 | 4.36E-11 | yes | up | 24.84 | 25.86 | 17.4 | 46.87 | 70.64 | 70.99 | 22.7 | 62.83333 |
| GPLD1 | 0.212 | -2.23673 | 3.7E-13 | 5.05E-11 | yes | down | 2.43 | 2.69 | 2.78 | 0.63 | 0.42 | 0.74 | 2.633333 | 0.596667 |
| IL1RN | 0.149 | -2.74594 | 3.84E-13 | 5.19E-11 | yes | down | 36.87 | 101.32 | 43.36 | 10.76 | 6.77 | 8.82 | 60.51667 | 8.783333 |
| GPR85 | 4.369 | 2.127171 | 4.51E-13 | 6.04E-11 | yes | up | 0.69 | 0.61 | 1.04 | 2.2 | 2.78 | 3.18 | 0.78 | 2.72 |
| CST3 | 4.325 | 2.11267 | 5.52E-13 | 7.32E-11 | yes | up | 286.88 | 305.13 | 484.78 | 1649.69 | 1508.29 | 1758.22 | 358.93 | 1638.733 |
| NFU1 | 0.447 | -1.16036 | 5.82E-13 | 7.66E-11 | yes | down | 47.74 | 58.5 | 44.09 | 22.06 | 25.19 | 22.88 | 50.11 | 23.37667 |
| LGALS12 | 4.569 | 2.191856 | 1.02E-12 | 1.32E-10 | yes | up | 10.45 | 21.77 | 10.3 | 77.67 | 55.77 | 72.49 | 14.17333 | 68.64333 |
| AS3MT | 4.371 | 2.127947 | 1.09E-12 | 1.4E-10 | yes | up | 4.94 | 3.43 | 4.03 | 19.31 | 13.54 | 22.8 | 4.133333 | 18.55 |
| ATXN1 | 0.14 | -2.84091 | 1.34E-12 | 1.7E-10 | yes | down | 9.23 | 19.37 | 4.59 | 1.72 | 2.24 | 1.86 | 11.06333 | 1.94 |
| ADM | 2.753 | 1.461104 | 1.52E-12 | 1.9E-10 | yes | up | 8.85 | 6.92 | 7.56 | 23.12 | 23.25 | 18.82 | 7.776667 | 21.73 |
| AGXT2 | 13.199 | 3.722381 | 1.58E-12 | 1.97E-10 | yes | up | 0.08 | 0.14 | 0.02 | 1.34 | 0.93 | 1.14 | 0.08 | 1.136667 |
| PTPRF | 2.189 | 1.130101 | 1.73E-12 | 2.13E-10 | yes | up | 4.26 | 5.2 | 3.8 | 8.81 | 10.27 | 10.93 | 4.42 | 10.00333 |
| LOC108176558 | 0.142 | -2.81106 | 1.75E-12 | 2.14E-10 | yes | down | 5.05 | 8.54 | 4.46 | 0.67 | 0.9 | 0.59 | 6.016667 | 0.72 |
| GNG10 | 0.476 | -1.07088 | 1.79E-12 | 2.17E-10 | yes | down | 59.41 | 70.66 | 53.74 | 28.52 | 33.84 | 28.25 | 61.27 | 30.20333 |
| CHST2 | 0.241 | -2.05201 | 1.91E-12 | 2.3E-10 | yes | down | 4.54 | 7.5 | 5.36 | 1.13 | 1.97 | 1.31 | 5.8 | 1.47 |
| FAM13C | 0.229 | -2.12501 | 1.96E-12 | 2.33E-10 | yes | down | 8.3 | 8.33 | 9.26 | 2.87 | 1.47 | 1.81 | 8.63 | 2.05 |
| LSM3 | 0.352 | -1.50634 | 2E-12 | 2.37E-10 | yes | down | 33.84 | 28.05 | 29.47 | 8.89 | 13.29 | 11.29 | 30.45333 | 11.15667 |
| S1PR3 | 0.235 | -2.08905 | 2.51E-12 | 2.95E-10 | yes | down | 4.76 | 7.54 | 6.54 | 1.63 | 1.93 | 1.1 | 6.28 | 1.553333 |
| ACOX3 | 2.96 | 1.565585 | 2.64E-12 | 3.08E-10 | yes | up | 8.54 | 10.89 | 7.43 | 28.24 | 25.2 | 27.95 | 8.953333 | 27.13 |
| GFPT2 | 8.335 | 3.059128 | 3.38E-12 | 3.88E-10 | yes | up | 3.47 | 2.19 | 1.5 | 33.37 | 12.98 | 12.64 | 2.386667 | 19.66333 |
| LOC108176200 | 392.488 | 8.616506 | 3.52E-12 | 4.01E-10 | yes | up | 0 | 0 | 0 | 1.79 | 2.13 | 1.77 | 0 | 1.896667 |
| LOC100352749 | 526.025 | 9.038987 | 3.65E-12 | 4.13E-10 | yes | up | 0 | 0 | 0 | 2.21 | 2.64 | 6.34 | 0 | 3.73 |
| LOC103350535 | 3.02 | 1.594319 | 3.75E-12 | 4.21E-10 | yes | up | 7.23 | 6.6 | 4.49 | 18.85 | 20.61 | 16.9 | 6.106667 | 18.78667 |
| CALM3 | 0.439 | -1.18652 | 3.9E-12 | 4.34E-10 | yes | down | 107.74 | 107.62 | 85.54 | 39.34 | 62.86 | 37.88 | 100.3 | 46.69333 |
| ABCA3 | 2.86 | 1.516202 | 4.09E-12 | 4.53E-10 | yes | up | 1.75 | 1.36 | 1.46 | 4.2 | 4.09 | 5.24 | 1.523333 | 4.51 |
| KIAA0408 | 3.702 | 1.888285 | 4.46E-12 | 4.9E-10 | yes | up | 5.97 | 5.22 | 3.12 | 17.21 | 14.72 | 14.17 | 4.77 | 15.36667 |
| ST6GALNAC2 | 3.934 | 1.976109 | 4.88E-12 | 5.32E-10 | yes | up | 1.65 | 1.29 | 0.84 | 4.79 | 5.49 | 5.84 | 1.26 | 5.373333 |
| CNTN1 | 3.071 | 1.618713 | 4.99E-12 | 5.41E-10 | yes | up | 1.21 | 1.01 | 1.24 | 3.04 | 3.85 | 4.29 | 1.153333 | 3.726667 |
| SLC26A10 | 0.202 | -2.31051 | 5.51E-12 | 5.92E-10 | yes | down | 20.78 | 27.65 | 44.23 | 6.51 | 7.67 | 6.03 | 30.88667 | 6.736667 |
| KCNH1 | 0.276 | -1.85685 | 5.87E-12 | 6.26E-10 | yes | down | 7.55 | 8.32 | 4.29 | 2.07 | 2.25 | 1.34 | 6.72 | 1.886667 |
| SLC6A6 | 0.149 | -2.74794 | 5.94E-12 | 6.3E-10 | yes | down | 14.35 | 23.96 | 21.27 | 3.06 | 4.91 | 1.53 | 19.86 | 3.166667 |
| CA12 | 0.072 | -3.80063 | 5.98E-12 | 6.3E-10 | yes | down | 1.22 | 4.38 | 1.46 | 0.13 | 0.36 | 0.22 | 2.353333 | 0.236667 |
| DACT2 | 4.393 | 2.135118 | 6.5E-12 | 6.8E-10 | yes | up | 1.32 | 0.86 | 0.58 | 2.97 | 4.89 | 4.22 | 0.92 | 4.026667 |
| LOC103352391 | 0.338 | -1.56573 | 6.76E-12 | 7.02E-10 | yes | down | 144.95 | 192.77 | 149.66 | 76.38 | 62.43 | 50.94 | 162.46 | 63.25 |
| TPT1 | 0.469 | -1.09369 | 6.85E-12 | 7.06E-10 | yes | down | 1492.88 | 1489.7 | 1229.62 | 716.07 | 765.09 | 559.58 | 1404.067 | 680.2467 |
| CCDC141 | 0.204 | -2.29568 | 8.86E-12 | 9.08E-10 | yes | down | 1.44 | 3.03 | 1.85 | 0.4 | 0.49 | 0.44 | 2.106667 | 0.443333 |
| LOC100008606 | 0.32 | -1.64178 | 9.22E-12 | 9.38E-10 | yes | down | 21.26 | 16.05 | 13.46 | 5.69 | 6 | 4.87 | 16.92333 | 5.52 |
| C8H12orf4 | 0.326 | -1.61511 | 1.3E-11 | 1.3E-09 | yes | down | 5.48 | 7.51 | 5.79 | 1.99 | 2.46 | 2.07 | 6.26 | 2.173333 |
| SNX21 | 2.33 | 1.220635 | 1.33E-11 | 1.32E-09 | yes | up | 16 | 16.32 | 10.63 | 27.25 | 35.54 | 38.41 | 14.31667 | 33.73333 |
| LOC100353046 | 0.003 | -8.25168 | 1.51E-11 | 1.5E-09 | yes | down | 9.16 | 7.52 | 6.57 | 0.08 | 0 | 0 | 7.75 | 0.026667 |
| TFRC | 6.883 | 2.78312 | 1.84E-11 | 1.81E-09 | yes | up | 12.23 | 9.6 | 4.16 | 35.71 | 110.65 | 39.99 | 8.663333 | 62.11667 |
| ADAMTSL3 | 2.417 | 1.273515 | 2.03E-11 | 1.99E-09 | yes | up | 1.47 | 1.35 | 1.37 | 3.64 | 3.47 | 3.62 | 1.396667 | 3.576667 |
| TSPAN5 | 2.525 | 1.33622 | 2.39E-11 | 2.32E-09 | yes | up | 3.23 | 3.26 | 2.04 | 6.97 | 8.24 | 6.97 | 2.843333 | 7.393333 |
| PALD1 | 3.247 | 1.699268 | 2.53E-11 | 2.44E-09 | yes | up | 11.32 | 13.75 | 6.28 | 27.04 | 35.2 | 41.76 | 10.45 | 34.66667 |
| HIF3A | 4.38 | 2.131074 | 2.65E-11 | 2.54E-09 | yes | up | 1.09 | 1.51 | 0.77 | 5.47 | 3.45 | 4.88 | 1.123333 | 4.6 |
| ASPM | 0.188 | -2.41209 | 2.66E-11 | 2.54E-09 | yes | down | 0.86 | 0.63 | 0.68 | 0.09 | 0.21 | 0.16 | 0.723333 | 0.153333 |
| PXMP2 | 2.456 | 1.296363 | 2.88E-11 | 2.73E-09 | yes | up | 8.79 | 7.56 | 8.15 | 21.06 | 22.67 | 23.89 | 8.166667 | 22.54 |
| DSP | 4.341 | 2.118159 | 3.34E-11 | 3.15E-09 | yes | up | 1.67 | 4.27 | 1.96 | 10.73 | 11.37 | 13.35 | 2.633333 | 11.81667 |
| CBR1 | 0.223 | -2.16367 | 4.04E-11 | 3.78E-09 | yes | down | 10.67 | 9.88 | 10.61 | 1.62 | 3.08 | 2.17 | 10.38667 | 2.29 |
| SH3GL2 | 4.879 | 2.286686 | 4.69E-11 | 4.37E-09 | yes | up | 1.63 | 0.89 | 1.35 | 3.98 | 7.49 | 8.43 | 1.29 | 6.633333 |
| SLC40A1 | 0.297 | -1.75111 | 5.27E-11 | 4.88E-09 | yes | down | 44.46 | 50.96 | 68.14 | 18.56 | 17.83 | 14.44 | 54.52 | 16.94333 |
| ANKRD37 | 0.272 | -1.88036 | 5.43E-11 | 4.99E-09 | yes | down | 9.88 | 14.3 | 13.57 | 3.9 | 4.49 | 2.47 | 12.58333 | 3.62 |
| MPV17 | 0.432 | -1.2106 | 5.46E-11 | 4.99E-09 | yes | down | 20.49 | 24.71 | 21.89 | 8.9 | 15.17 | 8.75 | 22.36333 | 10.94 |
| PLEKHA5 | 0.409 | -1.29027 | 6.13E-11 | 5.57E-09 | yes | down | 4.65 | 5.35 | 3.64 | 1.85 | 1.92 | 1.95 | 4.546667 | 1.906667 |
| PDK1 | 0.436 | -1.19905 | 6.59E-11 | 5.95E-09 | yes | down | 43.65 | 59.17 | 49.51 | 22.84 | 25.25 | 20.93 | 50.77667 | 23.00667 |
| KCTD12 | 0.442 | -1.17688 | 7.93E-11 | 7.12E-09 | yes | down | 8.12 | 10.19 | 7.85 | 3.51 | 5.01 | 3.57 | 8.72 | 4.03 |
| GATSL3 | 2.638 | 1.399593 | 1.12E-10 | 9.96E-09 | yes | up | 19.79 | 19.92 | 11.19 | 45.49 | 42.38 | 47.36 | 16.96667 | 45.07667 |
| DSG4 | 12.166 | 3.604739 | 1.29E-10 | 1.13E-08 | yes | up | 0.31 | 0.11 | 0.09 | 2.11 | 1.03 | 2.92 | 0.17 | 2.02 |
| MPP2 | 4.963 | 2.311081 | 1.34E-10 | 1.17E-08 | yes | up | 2.54 | 1.68 | 0.96 | 5.76 | 7.64 | 12.42 | 1.726667 | 8.606667 |
| NCKAP5 | 6.178 | 2.627212 | 1.38E-10 | 1.19E-08 | yes | up | 3.71 | 8.6 | 0.21 | 14.61 | 1.56 | 5.69 | 4.173333 | 7.286667 |
| TMED4 | 2.46 | 1.298751 | 1.38E-10 | 1.19E-08 | yes | up | 58.93 | 61.41 | 64.37 | 157.03 | 138.07 | 176.35 | 61.57 | 157.15 |
| SRXN1 | 0.241 | -2.05352 | 1.58E-10 | 1.36E-08 | yes | down | 20.35 | 41.92 | 24.53 | 5.67 | 9.22 | 6.9 | 28.93333 | 7.263333 |
| LOC100347463 | 3.017 | 1.592931 | 1.6E-10 | 1.36E-08 | yes | up | 10.28 | 9.95 | 9.6 | 34.39 | 23.64 | 34.25 | 9.943333 | 30.76 |
| AKT2 | 2.536 | 1.342432 | 1.63E-10 | 1.38E-08 | yes | up | 80.63 | 60.69 | 40.44 | 128.7 | 184.64 | 175.74 | 60.58667 | 163.0267 |
| DUSP10 | 2.576 | 1.364952 | 1.82E-10 | 1.54E-08 | yes | up | 8.99 | 9.19 | 5.43 | 20.1 | 25.32 | 16.28 | 7.87 | 20.56667 |
| KLF4 | 4.152 | 2.053942 | 1.88E-10 | 1.57E-08 | yes | up | 8.81 | 12.86 | 9.15 | 61.6 | 39.09 | 29.6 | 10.27333 | 43.43 |
| TF | 0.498 | -1.00706 | 1.95E-10 | 1.63E-08 | yes | down | 34.43 | 40.94 | 28.99 | 17.9 | 18.09 | 17.44 | 34.78667 | 17.81 |
| LRP2 | 4.32 | 2.11115 | 2.31E-10 | 1.91E-08 | yes | up | 0.42 | 0.49 | 0.65 | 2.27 | 1.66 | 3.07 | 0.52 | 2.333333 |
| SLC17A9 | 7.421 | 2.891518 | 2.82E-10 | 2.33E-08 | yes | up | 0.4 | 0.26 | 0.19 | 2.28 | 1.72 | 1.87 | 0.283333 | 1.956667 |
| CES3 | 2.776 | 1.472924 | 2.99E-10 | 2.46E-08 | yes | up | 25.58 | 23.77 | 33.86 | 73.06 | 94.39 | 77.88 | 27.73667 | 81.77667 |
| PDP2 | 0.325 | -1.61933 | 3.13E-10 | 2.55E-08 | yes | down | 12.55 | 15.02 | 16.98 | 3.51 | 6.73 | 5.16 | 14.85 | 5.133333 |
| LOC100344556 | 3.648 | 1.867271 | 3.37E-10 | 2.74E-08 | yes | up | 1.64 | 1.32 | 0.78 | 3.48 | 3.37 | 4.92 | 1.246667 | 3.923333 |
| PCBD1 | 2.3 | 1.201893 | 3.5E-10 | 2.82E-08 | yes | up | 15.04 | 13.08 | 11.29 | 25.55 | 37.08 | 31.39 | 13.13667 | 31.34 |
| LOC100344139 | 0.004 | -8.02677 | 3.92E-10 | 3.15E-08 | yes | down | 3.74 | 5.26 | 2.76 | 0 | 0 | 0 | 3.92 | 0 |
| BAIAP2L1 | 2.967 | 1.569011 | 3.95E-10 | 3.16E-08 | yes | up | 3.44 | 5.9 | 3.2 | 12.68 | 12.73 | 12.74 | 4.18 | 12.71667 |
| GLTSCR2 | 0.486 | -1.04005 | 4.29E-10 | 3.41E-08 | yes | down | 40.34 | 40.77 | 39.99 | 17.81 | 23.32 | 20.34 | 40.36667 | 20.49 |
| LOC100343809 | 2.165 | 1.11462 | 4.72E-10 | 3.73E-08 | yes | up | 36.29 | 39.6 | 31.68 | 75.31 | 100.98 | 65.76 | 35.85667 | 80.68333 |
| BCO2 | 2.235 | 1.159985 | 4.78E-10 | 3.76E-08 | yes | up | 13.83 | 14.85 | 15.58 | 27.72 | 36.76 | 37.95 | 14.75333 | 34.14333 |
| RMDN3 | 2.066 | 1.04694 | 5.03E-10 | 3.94E-08 | yes | up | 39.57 | 40.27 | 38.03 | 71.11 | 81.17 | 94.86 | 39.29 | 82.38 |
| UCP2 | 0.46 | -1.12055 | 5.26E-10 | 4.09E-08 | yes | down | 18.21 | 19.11 | 12.98 | 6.98 | 7.95 | 8.63 | 16.76667 | 7.853333 |
| TMEM139 | 15.263 | 3.931966 | 6.13E-10 | 4.75E-08 | yes | up | 0.63 | 0.19 | 0.32 | 5.56 | 2.76 | 8.42 | 0.38 | 5.58 |
| PRR5L | 4.224 | 2.078451 | 6.28E-10 | 4.84E-08 | yes | up | 0.56 | 0.46 | 0.42 | 2.11 | 1.56 | 2.54 | 0.48 | 2.07 |
| DSG1 | 4.628 | 2.210363 | 6.44E-10 | 4.94E-08 | yes | up | 0.28 | 0.53 | 0.25 | 2.06 | 1.87 | 1.91 | 0.353333 | 1.946667 |
| SLC7A6 | 3.376 | 1.755193 | 6.48E-10 | 4.94E-08 | yes | up | 4.26 | 7.22 | 3.17 | 15.71 | 13.09 | 17.44 | 4.883333 | 15.41333 |
| VDR | 0.194 | -2.36743 | 6.53E-10 | 4.96E-08 | yes | down | 8.3 | 5.5 | 3.92 | 0.97 | 1.34 | 1.27 | 5.906667 | 1.193333 |
| CYP27A1 | 0.196 | -2.35077 | 6.65E-10 | 5.03E-08 | yes | down | 77.35 | 140.87 | 80.38 | 12.02 | 36.62 | 13.74 | 99.53333 | 20.79333 |
| MGP | 3.694 | 1.884993 | 7.17E-10 | 5.39E-08 | yes | up | 436.58 | 241.48 | 157.08 | 1173.84 | 976.92 | 916.51 | 278.38 | 1022.423 |
| MOGAT1 | 0.241 | -2.05517 | 7.84E-10 | 5.87E-08 | yes | down | 2.19 | 1.87 | 2.63 | 0.58 | 0.67 | 0.36 | 2.23 | 0.536667 |
| CD96 | 14.865 | 3.893883 | 8.87E-10 | 6.61E-08 | yes | up | 0.3 | 0.06 | 0.07 | 1.03 | 1.37 | 1.85 | 0.143333 | 1.416667 |
| OSBPL11 | 2.391 | 1.257466 | 9.13E-10 | 6.77E-08 | yes | up | 10.21 | 9.19 | 8.6 | 24.36 | 19.15 | 25.37 | 9.333333 | 22.96 |
| LOC100359112 | 3.268 | 1.708373 | 1.11E-09 | 8.14E-08 | yes | up | 1.71 | 1.76 | 1.29 | 5.03 | 5.66 | 5.43 | 1.586667 | 5.373333 |
| LOC108176887 | 307.046 | 8.262312 | 1.11E-09 | 8.14E-08 | yes | up | 0 | 0 | 0 | 28.87 | 14.98 | 7.35 | 0 | 17.06667 |
| ATP5S | 2.158 | 1.109933 | 1.19E-09 | 8.68E-08 | yes | up | 14.91 | 10.98 | 9.11 | 23.52 | 27.34 | 26.82 | 11.66667 | 25.89333 |
| IVD | 0.45 | -1.1523 | 1.29E-09 | 9.3E-08 | yes | down | 121.26 | 158.43 | 142.77 | 54.8 | 81.05 | 64.24 | 140.82 | 66.69667 |
| LOC108178624 | 0.343 | -1.54175 | 1.35E-09 | 9.69E-08 | yes | down | 11.24 | 15.38 | 11.26 | 4.81 | 4.61 | 4.05 | 12.62667 | 4.49 |
| MMP11 | 0.232 | -2.10961 | 1.36E-09 | 9.73E-08 | yes | down | 7.36 | 6.06 | 9.98 | 1.35 | 2.37 | 2.08 | 7.8 | 1.933333 |
| POLR3C | 0.382 | -1.38908 | 1.46E-09 | 1.04E-07 | yes | down | 29.56 | 43.66 | 39.71 | 15.53 | 13.49 | 14.28 | 37.64333 | 14.43333 |
| SAMD15 | 0.085 | -3.55651 | 1.49E-09 | 1.06E-07 | yes | down | 1.25 | 3.56 | 1.25 | 0.06 | 0.27 | 0.24 | 2.02 | 0.19 |
| ACSF2 | 0.38 | -1.39749 | 1.86E-09 | 1.32E-07 | yes | down | 237.09 | 247.04 | 300.26 | 88.64 | 98.12 | 126.72 | 261.4633 | 104.4933 |
| PRKRA | 0.444 | -1.17149 | 1.94E-09 | 1.37E-07 | yes | down | 33.61 | 28.62 | 26.08 | 11.63 | 18.14 | 11.6 | 29.43667 | 13.79 |
| HCAR2 | 7.263 | 2.860658 | 1.95E-09 | 1.37E-07 | yes | up | 0.96 | 0.77 | 0.34 | 7.56 | 3.45 | 3.76 | 0.69 | 4.923333 |
| ME1 | 0.258 | -1.95357 | 2.05E-09 | 1.43E-07 | yes | down | 6.09 | 4.7 | 6.52 | 1 | 2.42 | 1.38 | 5.77 | 1.6 |
| INHBB | 14.188 | 3.826603 | 2.09E-09 | 1.45E-07 | yes | up | 0.22 | 0.23 | 0.12 | 4.93 | 1.84 | 1.14 | 0.19 | 2.636667 |
| MCEE | 2.437 | 1.28526 | 2.12E-09 | 1.46E-07 | yes | up | 30.24 | 20.53 | 16.32 | 51.22 | 55.22 | 62.49 | 22.36333 | 56.31 |
| LOC100342572 | 0.192 | -2.3841 | 2.25E-09 | 1.55E-07 | yes | down | 127.38 | 56.75 | 155.57 | 28 | 25.92 | 15.32 | 113.2333 | 23.08 |
| C7H2orf88 | 3.559 | 1.831406 | 2.4E-09 | 1.64E-07 | yes | up | 4.93 | 5.39 | 4.77 | 10.96 | 23.96 | 28.84 | 5.03 | 21.25333 |
| CCBE1 | 0.173 | -2.52943 | 2.4E-09 | 1.64E-07 | yes | down | 2.59 | 3.53 | 1.6 | 0.65 | 0.48 | 0.22 | 2.573333 | 0.45 |
| NQO1 | 0.447 | -1.16085 | 2.66E-09 | 1.81E-07 | yes | down | 12.45 | 15.34 | 11.79 | 3.8 | 4.44 | 5.08 | 13.19333 | 4.44 |
| SCPEP1 | 0.418 | -1.25696 | 2.8E-09 | 1.88E-07 | yes | down | 27.02 | 35.49 | 25.96 | 10.04 | 17.37 | 11.67 | 29.49 | 13.02667 |
| PDK3 | 0.403 | -1.31082 | 3E-09 | 2E-07 | yes | down | 4.5 | 4.74 | 4.65 | 1.66 | 2.3 | 1.92 | 4.63 | 1.96 |
| GYPA | 0.238 | -2.07153 | 4.2E-09 | 2.77E-07 | yes | down | 5.58 | 11.47 | 7.72 | 1.79 | 1.92 | 1.98 | 8.256667 | 1.896667 |
| ERMP1 | 0.339 | -1.56077 | 4.57E-09 | 2.98E-07 | yes | down | 4.06 | 3.97 | 5.17 | 1.72 | 1.55 | 1.45 | 4.4 | 1.573333 |
| PROM1 | 0.398 | -1.33022 | 5.02E-09 | 3.26E-07 | yes | down | 11.97 | 14.19 | 14.07 | 5.33 | 4.87 | 6.46 | 13.41 | 5.553333 |
| APELA | 0.194 | -2.36723 | 5.07E-09 | 3.26E-07 | yes | down | 2.53 | 2.13 | 2.79 | 0.49 | 0.7 | 0.34 | 2.483333 | 0.51 |
| NPL | 4.805 | 2.264596 | 5.13E-09 | 3.29E-07 | yes | up | 2.12 | 3.36 | 1.29 | 7.78 | 7.17 | 11.71 | 2.256667 | 8.886667 |
| SEC11C | 0.403 | -1.31167 | 5.46E-09 | 3.49E-07 | yes | down | 222.27 | 364.94 | 279.03 | 104.9 | 146.82 | 113.33 | 288.7467 | 121.6833 |
| IL18 | 2.006 | 1.004496 | 5.99E-09 | 3.8E-07 | yes | up | 36.42 | 32.47 | 35.28 | 57.24 | 78.83 | 78.92 | 34.72333 | 71.66333 |
| NMNAT3 | 3.035 | 1.601521 | 6.72E-09 | 4.24E-07 | yes | up | 10.92 | 11.09 | 4.56 | 25.82 | 26.73 | 29.11 | 8.856667 | 27.22 |
| GALE | 3.024 | 1.596474 | 6.99E-09 | 4.39E-07 | yes | up | 3.5 | 4.31 | 4.51 | 8.9 | 16.2 | 14.37 | 4.106667 | 13.15667 |
| SC5D | 2.044 | 1.031235 | 7.14E-09 | 4.47E-07 | yes | up | 6.14 | 7.81 | 5.76 | 12.62 | 14.59 | 14.5 | 6.57 | 13.90333 |
| EDAR | 4.484 | 2.164778 | 7.32E-09 | 4.56E-07 | yes | up | 0.28 | 0.37 | 0.34 | 1.77 | 1.32 | 1.5 | 0.33 | 1.53 |
| LOC103346930 | 21.106 | 4.39956 | 7.78E-09 | 4.81E-07 | yes | up | 0.09 | 0.05 | 0.52 | 4.9 | 7.6 | 3.08 | 0.22 | 5.193333 |
| TREX2 | 0.127 | -2.97205 | 8.51E-09 | 5.24E-07 | yes | down | 3.04 | 4.36 | 3.17 | 0.2 | 0.49 | 0.71 | 3.523333 | 0.466667 |
| FTL | 0.303 | -1.72469 | 8.72E-09 | 5.35E-07 | yes | down | 1094.92 | 2212.67 | 1856.74 | 498.55 | 474 | 653.98 | 1721.443 | 542.1767 |
| SHB | 3.228 | 1.690613 | 8.83E-09 | 5.39E-07 | yes | up | 2.57 | 2.69 | 1.45 | 5.12 | 8.1 | 9 | 2.236667 | 7.406667 |
| ERP27 | 27.891 | 4.801726 | 9.23E-09 | 5.62E-07 | yes | up | 0.04 | 0 | 0.02 | 0.49 | 0.26 | 0.67 | 0.02 | 0.473333 |
| LOC108176650 | 0.261 | -1.93577 | 9.83E-09 | 5.96E-07 | yes | down | 4.18 | 4.93 | 2.42 | 1.18 | 0.68 | 0.72 | 3.843333 | 0.86 |
| LOC100341224 | 0.291 | -1.78286 | 9.97E-09 | 6.02E-07 | yes | down | 5.18 | 3.86 | 3.34 | 1.01 | 1.67 | 1.1 | 4.126667 | 1.26 |
| SLC25A28 | 3.062 | 1.614605 | 1.08E-08 | 6.46E-07 | yes | up | 12.79 | 12.82 | 14.32 | 41.7 | 33.2 | 57.44 | 13.31 | 44.11333 |
| CD2BP2 | 0.491 | -1.02674 | 1.17E-08 | 6.97E-07 | yes | down | 24.27 | 29.08 | 22.58 | 10.87 | 15.79 | 12.26 | 25.31 | 12.97333 |
| LOC103345364 | 3.198 | 1.677353 | 1.2E-08 | 7.13E-07 | yes | up | 6.56 | 10.32 | 5.88 | 25.56 | 28.61 | 20.71 | 7.586667 | 24.96 |
| RFK | 2.486 | 1.313866 | 1.23E-08 | 7.22E-07 | yes | up | 6.49 | 9.12 | 6.58 | 16.9 | 16.8 | 23.23 | 7.396667 | 18.97667 |
| OASL | 0.238 | -2.07103 | 1.28E-08 | 7.48E-07 | yes | down | 2.78 | 2.94 | 2.57 | 0.67 | 0.48 | 0.87 | 2.763333 | 0.673333 |
| GNPAT | 2.064 | 1.045601 | 1.38E-08 | 7.99E-07 | yes | up | 34.63 | 24.97 | 22.07 | 49.48 | 60.04 | 64.67 | 27.22333 | 58.06333 |
| AK4 | 0.372 | -1.42848 | 1.47E-08 | 8.44E-07 | yes | down | 1.17 | 1.24 | 1.05 | 0.42 | 0.51 | 0.41 | 1.153333 | 0.446667 |
| LOC100343872 | 2.666 | 1.414864 | 1.48E-08 | 8.47E-07 | yes | up | 1.95 | 2.1 | 1.63 | 5.25 | 5.28 | 5.11 | 1.893333 | 5.213333 |
| DPP4 | 2.337 | 1.224654 | 1.51E-08 | 8.57E-07 | yes | up | 10.7 | 7.12 | 6.53 | 15.53 | 20.42 | 22.67 | 8.116667 | 19.54 |
| FZD4 | 2.243 | 1.165222 | 1.51E-08 | 8.57E-07 | yes | up | 21.94 | 26.08 | 23.69 | 55.29 | 47.42 | 63.65 | 23.90333 | 55.45333 |
| GLUL | 2.45 | 1.292667 | 1.65E-08 | 9.33E-07 | yes | up | 105.55 | 168.26 | 131.18 | 337.45 | 309.22 | 376.35 | 134.9967 | 341.0067 |
| LOC100356224 | 174.226 | 7.444817 | 1.66E-08 | 9.34E-07 | yes | up | 0 | 0 | 0 | 2.35 | 2.34 | 1.58 | 0 | 2.09 |
| F3 | 3.895 | 1.961493 | 1.86E-08 | 1.04E-06 | yes | up | 24.84 | 35.94 | 13.83 | 132.11 | 73.41 | 82.72 | 24.87 | 96.08 |
| LOC103349243 | 0.006 | -7.40081 | 1.86E-08 | 1.04E-06 | yes | down | 1.22 | 1.65 | 1.24 | 0 | 0 | 0 | 1.37 | 0 |
| CTIF | 2.493 | 1.317925 | 1.88E-08 | 1.04E-06 | yes | up | 4.42 | 5.36 | 2.68 | 8.05 | 9.58 | 12.78 | 4.153333 | 10.13667 |
| C13H1orf105 | 2.743 | 1.455639 | 1.93E-08 | 1.06E-06 | yes | up | 2.29 | 1.97 | 1.57 | 4.8 | 5.32 | 7.39 | 1.943333 | 5.836667 |
| MCM7 | 0.459 | -1.12442 | 2.1E-08 | 1.14E-06 | yes | down | 10.22 | 8.94 | 8.06 | 4.57 | 4.73 | 3.53 | 9.073333 | 4.276667 |
| DERL3 | 0.033 | -4.90827 | 2.1E-08 | 1.14E-06 | yes | down | 2.83 | 4.37 | 3.08 | 0.09 | 0.09 | 0.19 | 3.426667 | 0.123333 |
| LOC100350311 | 0.288 | -1.79417 | 2.16E-08 | 1.17E-06 | yes | down | 79 | 70.63 | 54.01 | 14.98 | 27.58 | 9.23 | 67.88 | 17.26333 |
| LOC100338043 | 2.636 | 1.398245 | 2.18E-08 | 1.18E-06 | yes | up | 11.03 | 14.1 | 12.64 | 35.12 | 27.91 | 39.75 | 12.59 | 34.26 |
| FAM105A | 2.864 | 1.518204 | 2.38E-08 | 1.27E-06 | yes | up | 4.89 | 7.8 | 6.61 | 18.79 | 24.68 | 11.86 | 6.433333 | 18.44333 |
| LRRC8B | 2.503 | 1.323588 | 2.53E-08 | 1.35E-06 | yes | up | 0.62 | 0.55 | 0.57 | 1.37 | 1.61 | 1.57 | 0.58 | 1.516667 |
| GABBR1 | 2.545 | 1.347513 | 2.73E-08 | 1.45E-06 | yes | up | 0.8 | 0.63 | 0.53 | 1.51 | 2.23 | 1.75 | 0.653333 | 1.83 |
| FIGN | 2.38 | 1.251029 | 2.8E-08 | 1.48E-06 | yes | up | 1.61 | 1.42 | 1.46 | 3.07 | 3.12 | 4.53 | 1.496667 | 3.573333 |
| TMEM143 | 2.595 | 1.37573 | 2.82E-08 | 1.49E-06 | yes | up | 11.9 | 6.98 | 9.9 | 22.54 | 25.63 | 29.6 | 9.593333 | 25.92333 |
| ENC1 | 3.073 | 1.619449 | 2.87E-08 | 1.51E-06 | yes | up | 3.05 | 4.05 | 4.3 | 14.49 | 9.77 | 11.06 | 3.8 | 11.77333 |
| BDH2 | 0.338 | -1.56621 | 2.88E-08 | 1.51E-06 | yes | down | 8.82 | 10.68 | 10.46 | 3.82 | 3.31 | 3.4 | 9.986667 | 3.51 |
| LOC100351389 | 2.176 | 1.12186 | 2.94E-08 | 1.53E-06 | yes | up | 6.8 | 8.2 | 6.24 | 15.37 | 17.09 | 14.33 | 7.08 | 15.59667 |
| ENDOG | 2.164 | 1.113466 | 3.04E-08 | 1.58E-06 | yes | up | 6.25 | 5.8 | 5.65 | 12.07 | 14.83 | 12.92 | 5.9 | 13.27333 |
| CTSD | 0.445 | -1.16679 | 3.09E-08 | 1.6E-06 | yes | down | 372.45 | 573.45 | 437.87 | 188.51 | 210.49 | 241.17 | 461.2567 | 213.39 |
| KDR | 0.391 | -1.3544 | 3.09E-08 | 1.6E-06 | yes | down | 40.78 | 68.23 | 58.09 | 21.62 | 28.03 | 18.64 | 55.7 | 22.76333 |
| LOC103349363 | 7.89 | 2.980078 | 3.28E-08 | 1.67E-06 | yes | up | 0.19 | 0.13 | 0.17 | 1.91 | 2.12 | 2.19 | 0.163333 | 2.073333 |
| CHRDL1 | 2.142 | 1.09883 | 3.29E-08 | 1.67E-06 | yes | up | 21.79 | 20.47 | 23.55 | 44.51 | 47.07 | 57.05 | 21.93667 | 49.54333 |
| LOC100345682 | 0.373 | -1.42412 | 3.29E-08 | 1.67E-06 | yes | down | 2.22 | 1.37 | 1.57 | 0.67 | 0.69 | 0.63 | 1.72 | 0.663333 |
| NPR3 | 0.469 | -1.09185 | 3.98E-08 | 2E-06 | yes | down | 37.26 | 55.96 | 48.24 | 18.03 | 26.3 | 25.86 | 47.15333 | 23.39667 |
| PEX16 | 2.16 | 1.110891 | 4.04E-08 | 2.02E-06 | yes | up | 12.53 | 15.34 | 11.01 | 30.16 | 30.61 | 40.15 | 12.96 | 33.64 |
| ABHD5 | 0.407 | -1.2975 | 4.71E-08 | 2.35E-06 | yes | down | 48.11 | 52.34 | 66.79 | 24.45 | 25.31 | 18.59 | 55.74667 | 22.78333 |
| SNN | 0.371 | -1.42908 | 5.25E-08 | 2.6E-06 | yes | down | 4.28 | 6.98 | 4.69 | 1.49 | 1.98 | 2.3 | 5.316667 | 1.923333 |
| SATB2 | 3.731 | 1.899479 | 5.47E-08 | 2.7E-06 | yes | up | 0.4 | 0.45 | 0.34 | 1.6 | 1.31 | 1.9 | 0.396667 | 1.603333 |
| LOC100328967 | 2.033 | 1.023547 | 5.56E-08 | 2.73E-06 | yes | up | 34.65 | 33.45 | 32.82 | 52.48 | 82.71 | 89.76 | 33.64 | 74.98333 |
| ETFBKMT | 0.442 | -1.1764 | 5.58E-08 | 2.73E-06 | yes | down | 12.3 | 16.32 | 15.45 | 6.46 | 8.17 | 7.25 | 14.69 | 7.293333 |
| LOC103346738 | 2.871 | 1.521586 | 5.58E-08 | 2.73E-06 | yes | up | 0.8 | 0.6 | 0.75 | 2.19 | 1.63 | 2.57 | 0.716667 | 2.13 |
| EFS | 2.226 | 1.154136 | 5.62E-08 | 2.74E-06 | yes | up | 2.27 | 1.92 | 1.43 | 4.15 | 5.07 | 3.66 | 1.873333 | 4.293333 |
| SCARA3 | 0.478 | -1.06466 | 5.9E-08 | 2.85E-06 | yes | down | 15.87 | 19.86 | 12.65 | 7.11 | 10.08 | 6.86 | 16.12667 | 8.016667 |
| LOC103349747 | 9.409 | 3.233997 | 6.02E-08 | 2.9E-06 | yes | up | 1.24 | 0.38 | 0.27 | 6.76 | 4.67 | 6.02 | 0.63 | 5.816667 |
| ACAD8 | 0.462 | -1.11377 | 6.45E-08 | 3.08E-06 | yes | down | 22.92 | 32.96 | 27.02 | 11.7 | 13.96 | 14.28 | 27.63333 | 13.31333 |
| MSMO1 | 2.065 | 1.046427 | 6.93E-08 | 3.29E-06 | yes | up | 6.87 | 5.96 | 5.32 | 10.05 | 15.06 | 13.86 | 6.05 | 12.99 |
| ELOVL2 | 2.521 | 1.334052 | 7.44E-08 | 3.51E-06 | yes | up | 4.16 | 7.08 | 5.31 | 13.56 | 13.39 | 16.35 | 5.516667 | 14.43333 |
| ADAM12 | 0.358 | -1.48284 | 7.79E-08 | 3.66E-06 | yes | down | 3.36 | 2.99 | 2.16 | 0.98 | 1.5 | 0.71 | 2.836667 | 1.063333 |
| DACT1 | 0.415 | -1.26781 | 8.05E-08 | 3.76E-06 | yes | down | 3.24 | 3.25 | 2.51 | 0.89 | 1.69 | 1.29 | 3 | 1.29 |
| IL13RA1 | 0.5 | -1.00043 | 8.05E-08 | 3.76E-06 | yes | down | 14.02 | 18.73 | 16.75 | 7.84 | 9.88 | 8.58 | 16.5 | 8.766667 |
| CD74 | 3.513 | 1.812652 | 8.08E-08 | 3.76E-06 | yes | up | 8.4 | 9.16 | 13.97 | 23.57 | 49.97 | 45.08 | 10.51 | 39.54 |
| LOC100346439 | 0.006 | -7.31801 | 8.34E-08 | 3.86E-06 | yes | down | 6.63 | 8.13 | 2.82 | 0 | 0 | 0 | 5.86 | 0 |
| MX1 | 0.493 | -1.01904 | 1E-07 | 4.59E-06 | yes | down | 22.32 | 19.1 | 13.13 | 8.85 | 9.01 | 9.38 | 18.18333 | 9.08 |
| ACSM5 | 0.468 | -1.09638 | 1.23E-07 | 5.56E-06 | yes | down | 25.64 | 20.26 | 21.28 | 8.83 | 14.75 | 9.47 | 22.39333 | 11.01667 |
| OXTR | 16.495 | 4.043954 | 1.26E-07 | 5.67E-06 | yes | up | 0.05 | 0.06 | 0.04 | 1.47 | 0.55 | 0.45 | 0.05 | 0.823333 |
| EXOSC5 | 0.371 | -1.42876 | 1.28E-07 | 5.71E-06 | yes | down | 12.84 | 17.29 | 17.79 | 5.72 | 7.06 | 5.94 | 15.97333 | 6.24 |
| PGPEP1 | 0.453 | -1.14147 | 1.44E-07 | 6.43E-06 | yes | down | 8.68 | 6.33 | 8.05 | 3.3 | 3.65 | 3.92 | 7.686667 | 3.623333 |
| WWOX | 2.117 | 1.081737 | 1.45E-07 | 6.43E-06 | yes | up | 7 | 5.14 | 5.02 | 11.37 | 13.88 | 12.35 | 5.72 | 12.53333 |
| CDH13 | 2.026 | 1.01884 | 1.45E-07 | 6.45E-06 | yes | up | 32.13 | 22.26 | 18.18 | 46.76 | 58.19 | 46.28 | 24.19 | 50.41 |
| LRP1 | 2.259 | 1.175694 | 1.56E-07 | 6.92E-06 | yes | up | 13.75 | 9.89 | 8.36 | 22.68 | 21.96 | 30.41 | 10.66667 | 25.01667 |
| ATP6V1A | 2.103 | 1.072762 | 1.57E-07 | 6.92E-06 | yes | up | 13.02 | 13.43 | 14.92 | 22.66 | 36.41 | 31.78 | 13.79 | 30.28333 |
| KANK4 | 0.307 | -1.70489 | 1.62E-07 | 7.14E-06 | yes | down | 1.42 | 2.32 | 1.55 | 0.41 | 0.7 | 0.67 | 1.763333 | 0.593333 |
| LOC100354804 | 15.049 | 3.911566 | 1.72E-07 | 7.5E-06 | yes | up | 0.33 | 0.78 | 0.07 | 14.48 | 5.94 | 2.27 | 0.393333 | 7.563333 |
| ABCC5 | 0.433 | -1.2061 | 1.87E-07 | 8.11E-06 | yes | down | 8.35 | 13.44 | 5.78 | 3.25 | 4.47 | 2.76 | 9.19 | 3.493333 |
| FURIN | 2.3 | 1.201616 | 2.07E-07 | 8.94E-06 | yes | up | 35.98 | 39.17 | 28.8 | 63.33 | 73.82 | 112.87 | 34.65 | 83.34 |
| CYP4B1 | 0.223 | -2.16468 | 2.12E-07 | 9.14E-06 | yes | down | 13.29 | 10.38 | 10.15 | 3.73 | 3.02 | 1.01 | 11.27333 | 2.586667 |
| AQP7 | 2.178 | 1.123174 | 2.12E-07 | 9.14E-06 | yes | up | 31 | 42.43 | 31.21 | 72.15 | 72.57 | 91.37 | 34.88 | 78.69667 |
| SUN3 | 6.184 | 2.628531 | 2.17E-07 | 9.32E-06 | yes | up | 1.86 | 1.48 | 1.17 | 13.09 | 5.36 | 9.46 | 1.503333 | 9.303333 |
| LDLR | 2.948 | 1.559498 | 2.22E-07 | 9.48E-06 | yes | up | 2.55 | 2.06 | 3.26 | 5.85 | 11.08 | 7.98 | 2.623333 | 8.303333 |
| ZBTB7B | 2.01 | 1.007151 | 2.48E-07 | 1.06E-05 | yes | up | 9.81 | 10.91 | 8.14 | 16.05 | 19.41 | 24.5 | 9.62 | 19.98667 |
| SESN2 | 0.306 | -1.70805 | 2.52E-07 | 1.07E-05 | yes | down | 1.39 | 1.88 | 1.41 | 0.39 | 0.66 | 0.45 | 1.56 | 0.5 |
| RLA-DRB1 | 4.61 | 2.204873 | 2.67E-07 | 1.13E-05 | yes | up | 1.63 | 2.83 | 3.65 | 7.21 | 18.9 | 12.97 | 2.703333 | 13.02667 |
| LOC100351163 | 3.492 | 1.803986 | 2.86E-07 | 1.2E-05 | yes | up | 3.2 | 3.28 | 4.04 | 7.17 | 17.19 | 15.18 | 3.506667 | 13.18 |
| TMEM40 | 0.23 | -2.1193 | 2.87E-07 | 1.2E-05 | yes | down | 3.8 | 2.89 | 1.78 | 0.37 | 1.07 | 0.4 | 2.823333 | 0.613333 |
| THNSL2 | 5.093 | 2.348513 | 2.95E-07 | 1.23E-05 | yes | up | 0.82 | 0.27 | 0.19 | 1.49 | 2.13 | 2.29 | 0.426667 | 1.97 |
| FGL2 | 2.758 | 1.463803 | 3.07E-07 | 1.28E-05 | yes | up | 12.97 | 11.7 | 14.95 | 49.51 | 31.98 | 31.51 | 13.20667 | 37.66667 |
| NEIL2 | 2.457 | 1.296816 | 3.12E-07 | 1.29E-05 | yes | up | 2.22 | 2.29 | 2.2 | 5.6 | 5.29 | 6.17 | 2.236667 | 5.686667 |
| FOSB | 58.209 | 5.863174 | 3.15E-07 | 1.31E-05 | yes | up | 0.26 | 0.22 | 0.09 | 26.1 | 2.09 | 4.21 | 0.19 | 10.8 |
| FRZB | 0.37 | -1.4352 | 3.22E-07 | 1.33E-05 | yes | down | 18.92 | 12.6 | 14.11 | 4.68 | 8.76 | 4.35 | 15.21 | 5.93 |
| SLC25A1 | 2.404 | 1.265236 | 3.23E-07 | 1.33E-05 | yes | up | 22.18 | 27.96 | 32.09 | 54.44 | 79.42 | 75.64 | 27.41 | 69.83333 |
| GNAZ | 0.182 | -2.45731 | 3.25E-07 | 1.33E-05 | yes | down | 2.25 | 4.48 | 1.72 | 0.21 | 0.67 | 0.71 | 2.816667 | 0.53 |
| ATPAF1 | 0.487 | -1.03818 | 3.3E-07 | 1.35E-05 | yes | down | 26.46 | 23.76 | 23.33 | 8.8 | 16.26 | 11.89 | 24.51667 | 12.31667 |
| PDZK1 | 3.323 | 1.732372 | 3.31E-07 | 1.35E-05 | yes | up | 1.87 | 1.86 | 1.74 | 3.34 | 6.31 | 8.21 | 1.823333 | 5.953333 |
| LOC108177279 | 3.201 | 1.678615 | 3.38E-07 | 1.37E-05 | yes | up | 1.9 | 1.9 | 2.5 | 6.05 | 7 | 8.38 | 2.1 | 7.143333 |
| LGALS9 | 0.297 | -1.75292 | 3.42E-07 | 1.38E-05 | yes | down | 18.17 | 38.64 | 36 | 6.88 | 11.06 | 11.41 | 30.93667 | 9.783333 |
| ST6GALNAC3 | 0.256 | -1.96755 | 3.43E-07 | 1.38E-05 | yes | down | 2.12 | 1.59 | 1.47 | 0.59 | 0.27 | 0.43 | 1.726667 | 0.43 |
| PENK | 6.96 | 2.799171 | 3.6E-07 | 1.44E-05 | yes | up | 0.79 | 0.67 | 0.13 | 3.07 | 5.46 | 2.67 | 0.53 | 3.733333 |
| NYNRIN | 0.444 | -1.17252 | 3.64E-07 | 1.45E-05 | yes | down | 2.11 | 2.12 | 2.39 | 0.81 | 1.11 | 1.17 | 2.206667 | 1.03 |
| LOC103349265 | 0.3 | -1.7376 | 3.78E-07 | 1.51E-05 | yes | down | 11.52 | 15.33 | 7.75 | 3.81 | 3.04 | 3.63 | 11.53333 | 3.493333 |
| LOC103349722 | 0.362 | -1.46522 | 3.86E-07 | 1.53E-05 | yes | down | 48.56 | 86.06 | 57.82 | 27.16 | 22.55 | 22.22 | 64.14667 | 23.97667 |
| LOC103352162 | 24.123 | 4.592356 | 4.27E-07 | 1.69E-05 | yes | up | 0.07 | 0.2 | 0 | 2.13 | 2.27 | 1.91 | 0.09 | 2.103333 |
| CEP89 | 0.484 | -1.04543 | 4.28E-07 | 1.69E-05 | yes | down | 3.78 | 3.19 | 3.32 | 1.47 | 2.1 | 1.68 | 3.43 | 1.75 |
| LENG8 | 2.065 | 1.046415 | 4.45E-07 | 1.74E-05 | yes | up | 17.52 | 17.66 | 15.94 | 32.66 | 30.9 | 43.39 | 17.04 | 35.65 |
| POR | 2.543 | 1.346523 | 4.58E-07 | 1.78E-05 | yes | up | 25.18 | 40.46 | 38.9 | 104.12 | 87.73 | 85.7 | 34.84667 | 92.51667 |
| LOC100349056 | 115.635 | 6.853434 | 4.75E-07 | 1.85E-05 | yes | up | 0 | 0 | 0 | 2.53 | 2.5 | 2.54 | 0 | 2.523333 |
| BMP2 | 5.107 | 2.352528 | 4.78E-07 | 1.85E-05 | yes | up | 0.26 | 0.64 | 0.54 | 2.88 | 2.13 | 2.64 | 0.48 | 2.55 |
| EGR1 | 49.508 | 5.629582 | 4.89E-07 | 1.89E-05 | yes | up | 5.58 | 2.09 | 2.06 | 354.62 | 84.54 | 20.29 | 3.243333 | 153.15 |
| COMMD8 | 0.454 | -1.13781 | 4.93E-07 | 1.9E-05 | yes | down | 8.01 | 9.52 | 7.56 | 3.14 | 4.62 | 4.12 | 8.363333 | 3.96 |
| LOC100350916 | 0.335 | -1.57755 | 4.98E-07 | 1.91E-05 | yes | down | 7.8 | 6.18 | 6.15 | 1.64 | 3.09 | 2.33 | 6.71 | 2.353333 |
| FAM19A3 | 0.084 | -3.5714 | 5.1E-07 | 1.95E-05 | yes | down | 0.44 | 0.94 | 0.56 | 0.04 | 0.01 | 0.11 | 0.646667 | 0.053333 |
| TP53I11 | 2.259 | 1.17537 | 5.43E-07 | 2.07E-05 | yes | up | 9.96 | 17.23 | 13.44 | 27.81 | 35.55 | 32.7 | 13.54333 | 32.02 |
| HPCA | 0.007 | -7.12984 | 5.49E-07 | 2.09E-05 | yes | down | 0.47 | 1.42 | 1.34 | 0 | 0 | 0 | 1.076667 | 0 |
| LOC108177122 | 10.115 | 3.338412 | 5.62E-07 | 2.14E-05 | yes | up | 0.14 | 0.22 | 0.55 | 1.18 | 1.8 | 1.58 | 0.303333 | 1.52 |
| EGR2 | 13.843 | 3.791095 | 5.69E-07 | 2.16E-05 | yes | up | 0.38 | 0.14 | 0.11 | 6.15 | 1.27 | 1.08 | 0.21 | 2.833333 |
| HDAC10 | 2.105 | 1.073598 | 5.93E-07 | 2.24E-05 | yes | up | 1.04 | 1.01 | 0.99 | 1.74 | 2.29 | 2.32 | 1.013333 | 2.116667 |
| LOC100358590 | 0.183 | -2.4482 | 6.08E-07 | 2.28E-05 | yes | down | 1.42 | 4.73 | 1.49 | 0.62 | 0.42 | 0.37 | 2.546667 | 0.47 |
| LOC100348521 | 0.006 | -7.36019 | 6.22E-07 | 2.33E-05 | yes | down | 0.07 | 9.87 | 9.08 | 0.06 | 0.06 | 0 | 6.34 | 0.04 |
| CREB5 | 2.585 | 1.370331 | 6.95E-07 | 2.58E-05 | yes | up | 1.55 | 1.06 | 0.74 | 2.84 | 3.78 | 2.14 | 1.116667 | 2.92 |
| MIGA2 | 2.551 | 1.351315 | 7.05E-07 | 2.61E-05 | yes | up | 14.91 | 18.15 | 8.75 | 28.07 | 33.2 | 46.3 | 13.93667 | 35.85667 |
| LOC108178205 | 3.217 | 1.685727 | 7.42E-07 | 2.73E-05 | yes | up | 4.85 | 8.26 | 7 | 26.57 | 14.41 | 24.17 | 6.703333 | 21.71667 |
| ZNF436 | 0.38 | -1.3975 | 7.67E-07 | 2.81E-05 | yes | down | 3.41 | 3.47 | 3.37 | 0.91 | 1.36 | 1.85 | 3.416667 | 1.373333 |
| LOC103345855 | 2.764 | 1.466609 | 7.67E-07 | 2.81E-05 | yes | up | 2.01 | 1.6 | 1.22 | 5.32 | 4.74 | 3.12 | 1.61 | 4.393333 |
| MPI | 0.444 | -1.17127 | 7.72E-07 | 2.82E-05 | yes | down | 20.08 | 14.44 | 16.88 | 7.6 | 10.02 | 6.19 | 17.13333 | 7.936667 |
| LOC103348242 | 6.363 | 2.66967 | 8.02E-07 | 2.92E-05 | yes | up | 2.63 | 9.32 | 5.24 | 19.6 | 31.45 | 61.92 | 5.73 | 37.65667 |
| TNN | 9.344 | 3.224111 | 8.54E-07 | 3.09E-05 | yes | up | 13.07 | 4.22 | 4.54 | 87.34 | 36.22 | 83.24 | 7.276667 | 68.93333 |
| DCDC2B | 4.008 | 2.002992 | 8.59E-07 | 3.1E-05 | yes | up | 0.25 | 0.17 | 0.14 | 0.59 | 0.69 | 0.7 | 0.186667 | 0.66 |
| MAGEL2 | 0.238 | -2.07356 | 8.65E-07 | 3.11E-05 | yes | down | 0.98 | 0.66 | 0.65 | 0.18 | 0.21 | 0.17 | 0.763333 | 0.186667 |
| NGFR | 0.472 | -1.08361 | 8.85E-07 | 3.18E-05 | yes | down | 3.79 | 3.5 | 2.89 | 1.3 | 1.77 | 1.87 | 3.393333 | 1.646667 |
| TMEM37 | 2.255 | 1.17341 | 9.44E-07 | 3.38E-05 | yes | up | 29 | 39.32 | 24.47 | 50.59 | 75.19 | 90.72 | 30.93 | 72.16667 |
| SFRP2 | 0.145 | -2.78339 | 9.6E-07 | 3.43E-05 | yes | down | 1.98 | 1.64 | 3.26 | 0.37 | 0.32 | 0.37 | 2.293333 | 0.353333 |
| MLXIPL | 2.37 | 1.244642 | 9.68E-07 | 3.45E-05 | yes | up | 15.75 | 11.43 | 12.51 | 28.31 | 27.47 | 45.46 | 13.23 | 33.74667 |
| PRKCDBP | 0.298 | -1.74843 | 9.75E-07 | 3.46E-05 | yes | down | 90.17 | 176.24 | 189.37 | 60.74 | 46.84 | 34.61 | 151.9267 | 47.39667 |
| LOC100346046 | 2.533 | 1.340663 | 9.84E-07 | 3.48E-05 | yes | up | 0.59 | 0.62 | 0.53 | 1.71 | 1.59 | 1.28 | 0.58 | 1.526667 |
| LOC100356510 | 0.008 | -6.95899 | 1.03E-06 | 3.63E-05 | yes | down | 0.99 | 1.72 | 0.47 | 0 | 0 | 0 | 1.06 | 0 |
| PSEN2 | 0.472 | -1.08457 | 1.03E-06 | 3.63E-05 | yes | down | 37.91 | 49.77 | 49.04 | 23.11 | 24.22 | 20.43 | 45.57333 | 22.58667 |
| MKI67 | 0.253 | -1.98131 | 1.03E-06 | 3.63E-05 | yes | down | 1.88 | 0.93 | 0.69 | 0.24 | 0.48 | 0.22 | 1.166667 | 0.313333 |
| RPL36AL | 2.12 | 1.084392 | 1.05E-06 | 3.66E-05 | yes | up | 28.18 | 42.89 | 34.9 | 70.37 | 84.82 | 78.47 | 35.32333 | 77.88667 |
| CYR61 | 9.834 | 3.297735 | 1.06E-06 | 3.7E-05 | yes | up | 24.11 | 13.93 | 18.67 | 354.19 | 116.45 | 98.36 | 18.90333 | 189.6667 |
| PHLDA1 | 3.758 | 1.909781 | 1.06E-06 | 3.7E-05 | yes | up | 1.61 | 2.03 | 1.14 | 9.67 | 4.63 | 3.7 | 1.593333 | 6 |
| SPRY1 | 2.864 | 1.518188 | 1.07E-06 | 3.74E-05 | yes | up | 15.18 | 25.73 | 17.75 | 73.71 | 61.33 | 36.23 | 19.55333 | 57.09 |
| GABRD | 0.287 | -1.8001 | 1.11E-06 | 3.86E-05 | yes | down | 27.46 | 79.57 | 42.63 | 12.22 | 14.6 | 18.61 | 49.88667 | 15.14333 |
| METTL21B | 4.318 | 2.110512 | 1.13E-06 | 3.9E-05 | yes | up | 0.69 | 0.76 | 0.54 | 2.66 | 1.67 | 4.33 | 0.663333 | 2.886667 |
| SEMA6D | 0.394 | -1.34531 | 1.23E-06 | 4.24E-05 | yes | down | 2.55 | 4.07 | 3.81 | 1.58 | 1.46 | 1.25 | 3.476667 | 1.43 |
| CHAC1 | 6.214 | 2.635454 | 1.31E-06 | 4.49E-05 | yes | up | 0.35 | 0.27 | 0.1 | 1.9 | 0.96 | 1.56 | 0.24 | 1.473333 |
| ABHD6 | 0.34 | -1.55711 | 1.43E-06 | 4.85E-05 | yes | down | 4.01 | 4.14 | 5.06 | 1.24 | 2.33 | 1.23 | 4.403333 | 1.6 |
| LOC100343231 | 6.611 | 2.724777 | 1.44E-06 | 4.86E-05 | yes | up | 2.94 | 0.37 | 1.05 | 11.66 | 8.01 | 9.42 | 1.453333 | 9.696667 |
| C1QTNF9 | 0.396 | -1.33642 | 1.46E-06 | 4.92E-05 | yes | down | 31.16 | 52.79 | 25.82 | 12.55 | 19.48 | 12.24 | 36.59 | 14.75667 |
| LOC100343018 | 8.165 | 3.0295 | 1.47E-06 | 4.96E-05 | yes | up | 0.43 | 0.32 | 0.15 | 2.51 | 2.71 | 1.96 | 0.3 | 2.393333 |
| MOGAT2 | 0.13 | -2.94779 | 1.49E-06 | 5E-05 | yes | down | 2.55 | 1.12 | 1.97 | 0.48 | 0.11 | 0.16 | 1.88 | 0.25 |
| NR2C2AP | 0.197 | -2.34678 | 1.59E-06 | 5.33E-05 | yes | down | 3.07 | 6.16 | 5.11 | 1.13 | 1.22 | 1.2 | 4.78 | 1.183333 |
| TRIL | 0.214 | -2.2268 | 1.72E-06 | 5.75E-05 | yes | down | 2.99 | 4.04 | 8.92 | 0.82 | 1.85 | 1.12 | 5.316667 | 1.263333 |
| MCP-1 | 0.029 | -5.10055 | 1.81E-06 | 6.02E-05 | yes | down | 3.98 | 13.31 | 6.16 | 0 | 0 | 0.7 | 7.816667 | 0.233333 |
| LOC103350421 | 0.384 | -1.37935 | 1.83E-06 | 6.06E-05 | yes | down | 2.21 | 2.95 | 3.19 | 0.89 | 1.25 | 0.94 | 2.783333 | 1.026667 |
| GMPS | 0.346 | -1.5292 | 1.86E-06 | 6.14E-05 | yes | down | 19.5 | 33.31 | 26.69 | 6.76 | 15.52 | 7.22 | 26.5 | 9.833333 |
| KIAA0232 | 2.155 | 1.107531 | 1.87E-06 | 6.16E-05 | yes | up | 5.1 | 4.43 | 2.66 | 7.2 | 8.58 | 10.4 | 4.063333 | 8.726667 |
| MC5R | 3.29 | 1.717929 | 1.94E-06 | 6.34E-05 | yes | up | 2.39 | 3.05 | 1.49 | 6.3 | 5.28 | 8.12 | 2.31 | 6.566667 |
| ARRB1 | 2.162 | 1.112696 | 2.01E-06 | 6.57E-05 | yes | up | 6.6 | 7.28 | 5.99 | 9.81 | 14.61 | 18.29 | 6.623333 | 14.23667 |
| LOC100338822 | 0.407 | -1.29586 | 2.06E-06 | 6.68E-05 | yes | down | 4.91 | 7.33 | 4.63 | 2.24 | 2.58 | 2.83 | 5.623333 | 2.55 |
| JUN | 4.383 | 2.132012 | 2.29E-06 | 7.32E-05 | yes | up | 24.57 | 19.02 | 17.52 | 162.45 | 50.16 | 53.04 | 20.37 | 88.55 |
| KIF21A | 2.01 | 1.006964 | 2.3E-06 | 7.32E-05 | yes | up | 6.13 | 6.43 | 3.81 | 8.92 | 12.91 | 13.23 | 5.456667 | 11.68667 |
| LOC100359306 | 2.798 | 1.484438 | 2.32E-06 | 7.37E-05 | yes | up | 2.61 | 1.76 | 3.09 | 5.51 | 7.73 | 8.33 | 2.486667 | 7.19 |
| ATF3 | 29.125 | 4.864208 | 2.34E-06 | 7.4E-05 | yes | up | 2.5 | 1.74 | 1.19 | 119.06 | 25.25 | 10.71 | 1.81 | 51.67333 |
| LOC103352377 | 0.148 | -2.76109 | 2.34E-06 | 7.4E-05 | yes | down | 14.63 | 29.42 | 15.86 | 1.69 | 6.21 | 1.45 | 19.97 | 3.116667 |
| ACOT11 | 2.996 | 1.582997 | 2.35E-06 | 7.4E-05 | yes | up | 7.44 | 3.63 | 8.26 | 13.57 | 24.4 | 24.39 | 6.443333 | 20.78667 |
| PPP1R9B | 0.456 | -1.13156 | 2.35E-06 | 7.4E-05 | yes | down | 77.92 | 106.62 | 65.42 | 26.9 | 54.47 | 38.22 | 83.32 | 39.86333 |
| LOC100347623 | 122.72 | 6.939227 | 2.38E-06 | 7.47E-05 | yes | up | 0 | 0 | 0 | 0.61 | 0.76 | 2.26 | 0 | 1.21 |
| FZD8 | 2.218 | 1.149093 | 2.46E-06 | 7.69E-05 | yes | up | 6.7 | 5.89 | 4.63 | 10.48 | 13.65 | 15.18 | 5.74 | 13.10333 |
| SLC38A7 | 0.454 | -1.13923 | 2.47E-06 | 7.71E-05 | yes | down | 2.8 | 2.42 | 2.69 | 1 | 1.34 | 1.36 | 2.636667 | 1.233333 |
| SERPINE2 | 0.323 | -1.62895 | 2.47E-06 | 7.71E-05 | yes | down | 4.7 | 9.67 | 8.9 | 2.77 | 2.78 | 2.39 | 7.756667 | 2.646667 |
| LOC100358241 | 0.296 | -1.7552 | 2.55E-06 | 7.92E-05 | yes | down | 19.72 | 11.66 | 8.91 | 2.54 | 6.25 | 3.58 | 13.43 | 4.123333 |
| LOC100349704 | 0.013 | -6.24112 | 2.75E-06 | 8.5E-05 | yes | down | 68.21 | 73.54 | 1.52 | 1.04 | 0.55 | 0.27 | 47.75667 | 0.62 |
| AHR | 0.473 | -1.08057 | 2.79E-06 | 8.6E-05 | yes | down | 19.37 | 27.1 | 19.08 | 7.94 | 14.83 | 9.79 | 21.85 | 10.85333 |
| ACP6 | 2.029 | 1.020612 | 2.89E-06 | 8.87E-05 | yes | up | 3.52 | 3.48 | 3.66 | 6.55 | 8.21 | 7.86 | 3.553333 | 7.54 |
| KCNJ8 | 0.422 | -1.24543 | 3.07E-06 | 9.37E-05 | yes | down | 12.39 | 16.96 | 16.03 | 4.95 | 9.48 | 5.93 | 15.12667 | 6.786667 |
| LRRC3C | 0.184 | -2.44422 | 3.12E-06 | 9.49E-05 | yes | down | 0.91 | 2.26 | 1.01 | 0.16 | 0.18 | 0.39 | 1.393333 | 0.243333 |
| TMEM268 | 2.69 | 1.427409 | 3.18E-06 | 9.65E-05 | yes | up | 2.33 | 2.78 | 1.69 | 6.59 | 4.28 | 7.55 | 2.266667 | 6.14 |
| SLC6A17 | 0.23 | -2.12082 | 3.21E-06 | 9.73E-05 | yes | down | 0.87 | 1.6 | 1.72 | 0.2 | 0.51 | 0.33 | 1.396667 | 0.346667 |
| DDX56 | 2.133 | 1.092725 | 3.22E-06 | 9.73E-05 | yes | up | 13.52 | 19.76 | 12.6 | 25.25 | 25.91 | 28.02 | 15.29333 | 26.39333 |
| TOP2A | 0.318 | -1.65409 | 3.23E-06 | 9.74E-05 | yes | down | 3.77 | 3.08 | 1.6 | 0.84 | 1.12 | 0.79 | 2.816667 | 0.916667 |
| ANKRD31 | 2.714 | 1.440564 | 3.26E-06 | 9.8E-05 | yes | up | 3.92 | 4.59 | 4.35 | 15.99 | 8.32 | 11.31 | 4.286667 | 11.87333 |
| PAQR4 | 2.243 | 1.165529 | 3.28E-06 | 9.84E-05 | yes | up | 4.09 | 3.5 | 2.9 | 7.23 | 7.16 | 10.19 | 3.496667 | 8.193333 |
| RASD2 | 4.793 | 2.260963 | 3.33E-06 | 9.97E-05 | yes | up | 1.07 | 0.59 | 0.88 | 6.65 | 2.37 | 3.24 | 0.846667 | 4.086667 |
| ACLY | 2.635 | 1.397644 | 3.38E-06 | 0.000101 | yes | up | 33.11 | 21.6 | 42.36 | 70.64 | 114.91 | 89.89 | 32.35667 | 91.81333 |
| OGT | 2.125 | 1.087209 | 3.42E-06 | 0.000102 | yes | up | 26 | 29.28 | 14.66 | 46.11 | 46.45 | 54.83 | 23.31333 | 49.13 |
| LOC103346277 | 3.967 | 1.988111 | 3.42E-06 | 0.000102 | yes | up | 1.08 | 1.52 | 0.64 | 3.9 | 1.82 | 3.71 | 1.08 | 3.143333 |
| LOC100347604 | 0.429 | -1.22137 | 3.53E-06 | 0.000105 | yes | down | 2.17 | 3.24 | 1.93 | 0.88 | 1.22 | 1.12 | 2.446667 | 1.073333 |
| LOC108176834 | 71.751 | 6.164923 | 3.59E-06 | 0.000106 | yes | up | 0 | 0 | 0.33 | 5.11 | 4.01 | 6.13 | 0.11 | 5.083333 |
| TSPAN15 | 0.357 | -1.48504 | 3.88E-06 | 0.000114 | yes | down | 29.47 | 28.74 | 26.85 | 5.09 | 14.15 | 13.04 | 28.35333 | 10.76 |
| PPA1 | 0.452 | -1.14464 | 4.06E-06 | 0.000118 | yes | down | 203.27 | 265.13 | 215.7 | 75.16 | 157.97 | 91.38 | 228.0333 | 108.17 |
| C3 | 2.246 | 1.167661 | 4.06E-06 | 0.000118 | yes | up | 10.59 | 9.5 | 5.94 | 13.74 | 21.2 | 25.2 | 8.676667 | 20.04667 |
| LOC100342322 | 0.037 | -4.75101 | 4.09E-06 | 0.000119 | yes | down | 1.14 | 0.63 | 3.66 | 0.14 | 0 | 0.15 | 1.81 | 0.096667 |
| TP53INP1 | 0.415 | -1.2689 | 4.14E-06 | 0.00012 | yes | down | 20.54 | 19.07 | 29.19 | 10.3 | 7.84 | 9.83 | 22.93333 | 9.323333 |
| SRD5A1 | 2.611 | 1.384408 | 4.33E-06 | 0.000125 | yes | up | 5.76 | 5.7 | 3.31 | 10.11 | 13.45 | 16.44 | 4.923333 | 13.33333 |
| CHAF1B | 3.375 | 1.755101 | 4.47E-06 | 0.000129 | yes | up | 1.31 | 1.32 | 0.72 | 3.31 | 5.31 | 3.22 | 1.116667 | 3.946667 |
| LOC103351528 | 4.139 | 2.049185 | 4.53E-06 | 0.00013 | yes | up | 0.11 | 0.16 | 0.13 | 0.48 | 0.84 | 0.64 | 0.133333 | 0.653333 |
| ALG8 | 0.356 | -1.49089 | 4.57E-06 | 0.000131 | yes | down | 9.26 | 4.93 | 5.44 | 1.61 | 2.6 | 2.98 | 6.543333 | 2.396667 |
| LOC100342112 | 98.947 | 6.628588 | 4.71E-06 | 0.000135 | yes | up | 0 | 0 | 0 | 0.69 | 1.04 | 1.78 | 0 | 1.17 |
| DSG2 | 0.435 | -1.20148 | 4.74E-06 | 0.000135 | yes | down | 3.14 | 4.66 | 2.59 | 1.14 | 1.96 | 1.59 | 3.463333 | 1.563333 |
| MFSD2A | 0.215 | -2.21686 | 4.9E-06 | 0.000139 | yes | down | 2.12 | 8.83 | 4.87 | 0.94 | 1.73 | 0.93 | 5.273333 | 1.2 |
| SDK1 | 2.449 | 1.292009 | 4.94E-06 | 0.00014 | yes | up | 2.91 | 2.21 | 1.63 | 6.67 | 6.26 | 3.94 | 2.25 | 5.623333 |
| FKBP5 | 2.613 | 1.385465 | 4.97E-06 | 0.000141 | yes | up | 8.12 | 9.59 | 10.01 | 31.34 | 16.91 | 26.26 | 9.24 | 24.83667 |
| RLA-DR-ALPHA | 3.598 | 1.847256 | 5E-06 | 0.000141 | yes | up | 3.35 | 6.2 | 7.85 | 12.52 | 29.57 | 25.83 | 5.8 | 22.64 |
| GCG | 8.312 | 3.055209 | 5.25E-06 | 0.000148 | yes | up | 0.43 | 0.71 | 0.06 | 2.46 | 3.05 | 4.82 | 0.4 | 3.443333 |
| KAZALD1 | 2.418 | 1.273902 | 5.36E-06 | 0.00015 | yes | up | 2.77 | 2.08 | 1.93 | 4.25 | 6.46 | 5.75 | 2.26 | 5.486667 |
| TMEM98 | 0.24 | -2.05718 | 5.37E-06 | 0.00015 | yes | down | 0.52 | 0.95 | 1.1 | 0.14 | 0.27 | 0.25 | 0.856667 | 0.22 |
| RFC2 | 0.481 | -1.05698 | 5.44E-06 | 0.000152 | yes | down | 13.72 | 15.33 | 12.15 | 5.1 | 9.33 | 6.3 | 13.73333 | 6.91 |
| SNX33 | 0.418 | -1.25916 | 5.75E-06 | 0.000159 | yes | down | 6.05 | 5.5 | 7.61 | 2.2 | 2.88 | 3.41 | 6.386667 | 2.83 |
| IRF3 | 2.062 | 1.043882 | 5.83E-06 | 0.00016 | yes | up | 11.97 | 13.2 | 8.02 | 14.23 | 14.73 | 13.95 | 11.06333 | 14.30333 |
| ZNF660 | 0.28 | -1.8387 | 6.28E-06 | 0.000172 | yes | down | 1.69 | 1.52 | 2.15 | 0.48 | 0.57 | 0.56 | 1.786667 | 0.536667 |
| LOC108178486 | 0.247 | -2.01882 | 6.31E-06 | 0.000173 | yes | down | 1.48 | 3.29 | 2.11 | 0.4 | 0.3 | 0.78 | 2.293333 | 0.493333 |
| WDR6 | 0.407 | -1.29741 | 6.38E-06 | 0.000174 | yes | down | 2.45 | 2.61 | 1.43 | 0.74 | 0.96 | 1.02 | 2.163333 | 0.906667 |
| HEMGN | 0.155 | -2.68946 | 6.51E-06 | 0.000177 | yes | down | 1.16 | 4.35 | 3.24 | 0.25 | 0.49 | 0.69 | 2.916667 | 0.476667 |
| LOC100348151 | 0.034 | -4.87408 | 6.6E-06 | 0.000179 | yes | down | 2.69 | 0.11 | 2.6 | 0.02 | 0.05 | 0.04 | 1.8 | 0.036667 |
| C18H10orf35 | 5.656 | 2.499824 | 6.69E-06 | 0.000181 | yes | up | 1.31 | 0.97 | 1.13 | 6.71 | 2.15 | 9.63 | 1.136667 | 6.163333 |
| GLDN | 0.283 | -1.82245 | 6.73E-06 | 0.000182 | yes | down | 11.71 | 23.37 | 8.13 | 2.67 | 7.03 | 3.05 | 14.40333 | 4.25 |
| C7H2orf72 | 7.972 | 2.99494 | 6.76E-06 | 0.000182 | yes | up | 0.32 | 0.21 | 0.03 | 1.51 | 1.03 | 1.82 | 0.186667 | 1.453333 |
| LOC100351707 | 0.234 | -2.09409 | 6.87E-06 | 0.000184 | yes | down | 1.62 | 2.45 | 2.01 | 0.73 | 0.36 | 0.38 | 2.026667 | 0.49 |
| SARS2 | 2.139 | 1.096777 | 6.93E-06 | 0.000185 | yes | up | 6.49 | 6.37 | 6.95 | 11.21 | 19.06 | 16.09 | 6.603333 | 15.45333 |
| NUDT16L1 | 0.424 | -1.23813 | 7.01E-06 | 0.000186 | yes | down | 10.16 | 11.66 | 9.2 | 8.3 | 10.98 | 9.74 | 10.34 | 9.673333 |
| OSGIN1 | 2.761 | 1.465337 | 7.02E-06 | 0.000186 | yes | up | 1.43 | 2.12 | 1.68 | 4.02 | 3.35 | 5.21 | 1.743333 | 4.193333 |
| SOCS2 | 0.459 | -1.1246 | 7.15E-06 | 0.000189 | yes | down | 13.84 | 14.8 | 8.5 | 4.67 | 7.64 | 5.06 | 12.38 | 5.79 |
| LOC100009097 | 42.882 | 5.422293 | 7.37E-06 | 0.000194 | yes | up | 0 | 0.12 | 0 | 1.14 | 1.75 | 2.45 | 0.04 | 1.78 |
| LOC100346685 | 614.907 | 9.264225 | 7.38E-06 | 0.000194 | yes | up | 0 | 0 | 0 | 7.01 | 0.52 | 5.06 | 0 | 4.196667 |
| TMEM200A | 0.185 | -2.43158 | 7.6E-06 | 0.0002 | yes | down | 0.82 | 1.1 | 0.59 | 0.22 | 0.11 | 0.16 | 0.836667 | 0.163333 |
| FGF11 | 0.213 | -2.23094 | 8.27E-06 | 0.000216 | yes | down | 0.62 | 0.8 | 0.7 | 0.17 | 0.22 | 0.08 | 0.706667 | 0.156667 |
| HACL1 | 0.491 | -1.02633 | 8.36E-06 | 0.000218 | yes | down | 75.98 | 88.98 | 106.21 | 37.11 | 55.08 | 49.5 | 90.39 | 47.23 |
| IGFBP5 | 2.424 | 1.27737 | 8.4E-06 | 0.000219 | yes | up | 16.78 | 10.08 | 6.73 | 21.17 | 36.86 | 25.7 | 11.19667 | 27.91 |
| LOC108178276 | 0.378 | -1.40217 | 8.43E-06 | 0.000219 | yes | down | 76.95 | 78.27 | 69.78 | 31.19 | 37.62 | 19.61 | 75 | 29.47333 |
| UCP1 | 0.455 | -1.13703 | 8.54E-06 | 0.000221 | yes | down | 4146.2 | 7225.82 | 4811.64 | 1895.23 | 3480.95 | 2355.41 | 5394.553 | 2577.197 |
| LIPA | 2.139 | 1.096908 | 8.85E-06 | 0.000229 | yes | up | 18.92 | 24.97 | 18.87 | 43.45 | 33.52 | 55.53 | 20.92 | 44.16667 |
| MEOX1 | 0.31 | -1.69105 | 8.97E-06 | 0.000231 | yes | down | 2.95 | 2.85 | 2.62 | 0.44 | 1.51 | 0.82 | 2.806667 | 0.923333 |
| TRIM25 | 0.445 | -1.16816 | 9.2E-06 | 0.000237 | yes | down | 3.45 | 4.36 | 3.22 | 2.07 | 1.52 | 1.45 | 3.676667 | 1.68 |
| PIGA | 2.388 | 1.256027 | 9.78E-06 | 0.000251 | yes | up | 1.08 | 1.27 | 0.8 | 2.56 | 2.2 | 2.7 | 1.05 | 2.486667 |
| ABCC9 | 0.4 | -1.32228 | 1.02E-05 | 0.000259 | yes | down | 13.21 | 11.45 | 8.36 | 5.12 | 5.86 | 2.57 | 11.00667 | 4.516667 |
| LBH | 0.338 | -1.56362 | 1.02E-05 | 0.00026 | yes | down | 16.65 | 20.97 | 30.27 | 5.43 | 10.54 | 7.29 | 22.63 | 7.753333 |
| TMTC1 | 2.118 | 1.082486 | 1.05E-05 | 0.000266 | yes | up | 1.86 | 1.39 | 1.48 | 3.75 | 2.82 | 3.7 | 1.576667 | 3.423333 |
| LOC100358947 | 0.124 | -3.01491 | 1.06E-05 | 0.000269 | yes | down | 4.39 | 1.04 | 4.33 | 0.64 | 0.3 | 0.31 | 3.253333 | 0.416667 |
| PER1 | 2.443 | 1.28884 | 1.08E-05 | 0.000273 | yes | up | 10.63 | 14.44 | 6.59 | 33.24 | 18.31 | 28.29 | 10.55333 | 26.61333 |
| CCND3 | 2.511 | 1.328401 | 1.08E-05 | 0.000273 | yes | up | 10.51 | 9 | 7.44 | 14.52 | 22.74 | 34.64 | 8.983333 | 23.96667 |
| LOC100339034 | 0.022 | -5.53371 | 1.08E-05 | 0.000273 | yes | down | 1.02 | 1.03 | 3.33 | 0 | 0.1 | 0 | 1.793333 | 0.033333 |
| ASRGL1 | 0.404 | -1.30802 | 1.1E-05 | 0.000276 | yes | down | 5.23 | 7.15 | 4.89 | 1.9 | 3.2 | 2.18 | 5.756667 | 2.426667 |
| WDR34 | 0.262 | -1.93041 | 1.1E-05 | 0.000276 | yes | down | 2.02 | 2.53 | 3.47 | 0.79 | 0.62 | 0.8 | 2.673333 | 0.736667 |
| HACD2 | 2.081 | 1.057242 | 1.12E-05 | 0.00028 | yes | up | 24.58 | 37.93 | 30.03 | 44.66 | 62.55 | 72.44 | 30.84667 | 59.88333 |
| VWCE | 0.373 | -1.42273 | 1.14E-05 | 0.000284 | yes | down | 1.37 | 1.5 | 1.66 | 0.55 | 0.78 | 0.46 | 1.51 | 0.596667 |
| SEMA3B | 2.79 | 1.480126 | 1.19E-05 | 0.000295 | yes | up | 4.19 | 2.32 | 1.44 | 7.36 | 6.47 | 8.82 | 2.65 | 7.55 |
| DCDC2C | 0.005 | -7.79474 | 1.2E-05 | 0.000298 | yes | down | 0.66 | 42.49 | 4.28 | 0 | 0.13 | 0.06 | 15.81 | 0.063333 |
| BLM | 2.287 | 1.193498 | 1.2E-05 | 0.000298 | yes | up | 2.28 | 1.77 | 1.56 | 10.19 | 7.7 | 13.5 | 1.87 | 10.46333 |
| IL10RA | 3.063 | 1.614784 | 1.26E-05 | 0.000311 | yes | up | 0.69 | 0.44 | 0.34 | 1.74 | 1.67 | 1.17 | 0.49 | 1.526667 |
| SLC30A3 | 0.228 | -2.13109 | 1.26E-05 | 0.000311 | yes | down | 1.11 | 2.22 | 1.1 | 0.29 | 0.56 | 0.42 | 1.476667 | 0.423333 |
| ANGPTL7 | 13.612 | 3.766813 | 1.28E-05 | 0.000314 | yes | up | 0.08 | 0 | 0.09 | 0.58 | 0.57 | 1.21 | 0.056667 | 0.786667 |
| EPSTI1 | 2.625 | 1.392313 | 1.3E-05 | 0.00032 | yes | up | 2.89 | 1.83 | 2.82 | 5.82 | 5.67 | 8.15 | 2.513333 | 6.546667 |
| NR4A1 | 28.34 | 4.824779 | 1.31E-05 | 0.00032 | yes | up | 2.78 | 5.12 | 2.05 | 227.45 | 31.24 | 19.27 | 3.316667 | 92.65333 |
| LOC103346281 | 2.252 | 1.170897 | 1.32E-05 | 0.000321 | yes | up | 1.54 | 2.02 | 1.52 | 3.69 | 3.15 | 4.47 | 1.693333 | 3.77 |
| LAMB3 | 2.493 | 1.317984 | 1.38E-05 | 0.000337 | yes | up | 1.01 | 0.98 | 0.47 | 1.98 | 2.42 | 1.68 | 0.82 | 2.026667 |
| RANBP3L | 4.833 | 2.272907 | 1.4E-05 | 0.000337 | yes | up | 0.14 | 0.14 | 0.32 | 0.77 | 0.97 | 1.14 | 0.2 | 0.96 |
| VIPR1 | 0.2 | -2.32131 | 1.4E-05 | 0.000337 | yes | down | 1.29 | 2.59 | 0.86 | 0.38 | 0.31 | 0.24 | 1.58 | 0.31 |
| LOC108177361 | 9.312 | 3.219054 | 1.41E-05 | 0.000341 | yes | up | 0 | 0.05 | 0.06 | 0.46 | 0.46 | 0.29 | 0.036667 | 0.403333 |
| KLF6 | 3.07 | 1.618062 | 1.42E-05 | 0.000342 | yes | up | 13.73 | 15.4 | 12.36 | 68.21 | 31 | 26.05 | 13.83 | 41.75333 |
| LOC103346972 | 2.161 | 1.111837 | 1.43E-05 | 0.000343 | yes | up | 2.29 | 2.24 | 1.76 | 4.45 | 4.21 | 5.31 | 2.096667 | 4.656667 |
| MDK | 0.263 | -1.92941 | 1.53E-05 | 0.000366 | yes | down | 10.94 | 27.1 | 18.81 | 3.91 | 8.57 | 3.42 | 18.95 | 5.3 |
| ARMC9 | 2.135 | 1.093992 | 1.54E-05 | 0.000368 | yes | up | 2.65 | 3.25 | 2.56 | 5.46 | 5.61 | 7.44 | 2.82 | 6.17 |
| LOC100353320 | 0.463 | -1.11185 | 1.55E-05 | 0.000369 | yes | down | 405.02 | 452.44 | 608.22 | 249.86 | 231.89 | 228.27 | 488.56 | 236.6733 |
| PLK3 | 7.066 | 2.820884 | 1.56E-05 | 0.000371 | yes | up | 0.67 | 0.96 | 0.49 | 10.44 | 2.17 | 1.91 | 0.706667 | 4.84 |
| ANGPTL4 | 2.738 | 1.452912 | 1.57E-05 | 0.000372 | yes | up | 55.04 | 42.34 | 81.17 | 219.56 | 160.08 | 136.46 | 59.51667 | 172.0333 |
| ABCA6 | 2.411 | 1.269717 | 1.64E-05 | 0.000388 | yes | up | 1.15 | 1.12 | 0.74 | 2.59 | 1.73 | 2.82 | 1.003333 | 2.38 |
| CPEB4 | 2.603 | 1.379953 | 1.68E-05 | 0.000397 | yes | up | 10.31 | 12.8 | 5.94 | 30.6 | 16.8 | 24.97 | 9.683333 | 24.12333 |
| LOC100340198 | 0.466 | -1.10081 | 1.71E-05 | 0.000402 | yes | down | 120.61 | 175.85 | 167.77 | 72.25 | 77.16 | 75.71 | 154.7433 | 75.04 |
| SYTL2 | 2.263 | 1.177995 | 1.74E-05 | 0.000408 | yes | up | 1.14 | 0.92 | 0.86 | 2.55 | 2.12 | 1.98 | 0.973333 | 2.216667 |
| HP | 4.299 | 2.104117 | 1.75E-05 | 0.000411 | yes | up | 19.3 | 9.9 | 13.8 | 116.38 | 33.99 | 35.41 | 14.33333 | 61.92667 |
| SLC8A1 | 2.31 | 1.207916 | 1.77E-05 | 0.000414 | yes | up | 0.77 | 0.58 | 0.47 | 1.4 | 1.72 | 1.23 | 0.606667 | 1.45 |
| PDIA5 | 0.462 | -1.11546 | 1.77E-05 | 0.000415 | yes | down | 13.2 | 14.92 | 18.63 | 8.09 | 7.71 | 7.08 | 15.58333 | 7.626667 |
| PIGP | 2.069 | 1.048944 | 1.78E-05 | 0.000416 | yes | up | 24.61 | 18.91 | 11.63 | 35.74 | 35.61 | 31.44 | 18.38333 | 34.26333 |
| NFKBIZ | 8.112 | 3.020009 | 1.8E-05 | 0.000419 | yes | up | 0.51 | 0.89 | 0.35 | 10.08 | 2.15 | 1.5 | 0.583333 | 4.576667 |
| NELL1 | 6.393 | 2.676537 | 1.83E-05 | 0.000425 | yes | up | 0.06 | 0.27 | 0.07 | 0.8 | 0.84 | 1.05 | 0.133333 | 0.896667 |
| JDP2 | 3.212 | 1.683544 | 1.83E-05 | 0.000425 | yes | up | 8.17 | 6.74 | 3.7 | 30.1 | 16.21 | 12.94 | 6.203333 | 19.75 |
| SLC26A6 | 0.368 | -1.44318 | 1.87E-05 | 0.000434 | yes | down | 2.55 | 2.52 | 3.75 | 1.22 | 1.25 | 0.89 | 2.94 | 1.12 |
| LOC103352262 | 11.075 | 3.469228 | 1.91E-05 | 0.000441 | yes | up | 0.06 | 0.06 | 0.04 | 0.64 | 0.34 | 0.96 | 0.053333 | 0.646667 |
| EPHA1 | 6.272 | 2.648985 | 1.93E-05 | 0.000446 | yes | up | 0.04 | 0.12 | 0.11 | 0.59 | 0.58 | 0.62 | 0.09 | 0.596667 |
| PPP1R3E | 2.299 | 1.200823 | 1.94E-05 | 0.000448 | yes | up | 4.51 | 4.72 | 4.77 | 9.68 | 8.61 | 14.85 | 4.666667 | 11.04667 |
| LOC108178928 | 14.308 | 3.838788 | 2.02E-05 | 0.000464 | yes | up | 0.04 | 0.05 | 0 | 0.35 | 0.54 | 0.54 | 0.03 | 0.476667 |
| LOC108176480 | 2.192 | 1.132069 | 2.09E-05 | 0.000479 | yes | up | 1.03 | 1.11 | 0.8 | 1.4 | 2.47 | 2.84 | 0.98 | 2.236667 |
| PLXNB3 | 2.959 | 1.565262 | 2.14E-05 | 0.000487 | yes | up | 0.62 | 1.27 | 0.4 | 2.32 | 2.24 | 2.31 | 0.763333 | 2.29 |
| RBP4 | 0.393 | -1.34557 | 2.17E-05 | 0.000495 | yes | down | 64.82 | 75.27 | 67.81 | 16.01 | 29.84 | 42.45 | 69.3 | 29.43333 |
| IGFBP6 | 0.453 | -1.14293 | 2.33E-05 | 0.000528 | yes | down | 223.15 | 415.79 | 316.91 | 151.37 | 183.28 | 119.51 | 318.6167 | 151.3867 |
| ND1 | 2.167 | 1.115436 | 2.38E-05 | 0.000539 | yes | up | 3389.71 | 2811.48 | 4453.02 | 8712.85 | 7446.45 | 8051.58 | 3551.403 | 8070.293 |
| NDRG1 | 0.412 | -1.2809 | 2.39E-05 | 0.000539 | yes | down | 64.1 | 83.03 | 28.12 | 22.46 | 24 | 26.07 | 58.41667 | 24.17667 |
| LGALS3 | 2.496 | 1.319777 | 2.49E-05 | 0.000562 | yes | up | 24.92 | 15.53 | 11.25 | 54 | 40.23 | 35.11 | 17.23333 | 43.11333 |
| MRPS6 | 0.49 | -1.02806 | 2.5E-05 | 0.000563 | yes | down | 36.37 | 59.83 | 37.81 | 21.15 | 27.36 | 19.92 | 44.67 | 22.81 |
| FDXR | 0.456 | -1.13156 | 2.52E-05 | 0.000566 | yes | down | 19.15 | 18.44 | 24.13 | 8.4 | 10.76 | 10.5 | 20.57333 | 9.886667 |
| PPM1L | 2.028 | 1.019786 | 2.53E-05 | 0.000566 | yes | up | 1.55 | 1.11 | 1.5 | 2.44 | 3.16 | 3.24 | 1.386667 | 2.946667 |
| LOC100354303 | 0.446 | -1.16414 | 2.55E-05 | 0.000571 | yes | down | 20.39 | 27.36 | 26.34 | 10.79 | 10.85 | 12.89 | 24.69667 | 11.51 |
| CTHRC1 | 0.21 | -2.25482 | 2.58E-05 | 0.000575 | yes | down | 11.57 | 11.31 | 4.71 | 1.13 | 3.89 | 1 | 9.196667 | 2.006667 |
| THSD4 | 0.389 | -1.36162 | 2.58E-05 | 0.000575 | yes | down | 1.47 | 1.1 | 1.4 | 0.49 | 0.64 | 0.35 | 1.323333 | 0.493333 |
| HLX | 0.475 | -1.0736 | 2.62E-05 | 0.000582 | yes | down | 6.24 | 9.96 | 8.1 | 3.4 | 4.34 | 4.34 | 8.1 | 4.026667 |
| RADIL | 2.619 | 1.388888 | 2.66E-05 | 0.000588 | yes | up | 0.74 | 0.45 | 0.47 | 1.27 | 1.38 | 1.17 | 0.553333 | 1.273333 |
| CYP4A5 | 0.014 | -6.16242 | 3.03E-05 | 0.000662 | yes | down | 0.34 | 0.37 | 0.34 | 0 | 0 | 0 | 0.35 | 0 |
| MIIP | 2.056 | 1.039999 | 3.14E-05 | 0.000682 | yes | up | 5.73 | 8.05 | 6.42 | 12.14 | 11.51 | 15.39 | 6.733333 | 13.01333 |
| SLC12A2 | 0.493 | -1.02163 | 3.37E-05 | 0.000729 | yes | down | 10.28 | 12.7 | 12.94 | 5.94 | 8.19 | 4.58 | 11.97333 | 6.236667 |
| AVPR1A | 2.326 | 1.217564 | 3.39E-05 | 0.000732 | yes | up | 1.16 | 1.01 | 1.23 | 3.38 | 2.25 | 2.55 | 1.133333 | 2.726667 |
| BAIAP2 | 2.964 | 1.567376 | 3.42E-05 | 0.000737 | yes | up | 1.05 | 1.46 | 1.1 | 2.35 | 3.35 | 5.4 | 1.203333 | 3.7 |
| NAGLU | 2.237 | 1.161735 | 3.55E-05 | 0.000763 | yes | up | 3.7 | 5.28 | 2.55 | 6.6 | 10.52 | 9.58 | 3.843333 | 8.9 |
| CDKL4 | 3.762 | 1.911685 | 3.58E-05 | 0.000768 | yes | up | 0.17 | 0.3 | 0.26 | 1.01 | 0.97 | 0.88 | 0.243333 | 0.953333 |
| LOC108178475 | 0.11 | -3.18034 | 3.62E-05 | 0.000774 | yes | down | 0.32 | 0.3 | 0.28 | 0.05 | 0.04 | 0.01 | 0.3 | 0.033333 |
| MCM2 | 0.313 | -1.67407 | 3.62E-05 | 0.000774 | yes | down | 4.67 | 5.95 | 3.25 | 0.79 | 2.76 | 1.08 | 4.623333 | 1.543333 |
| ADTRP | 0.24 | -2.05907 | 3.73E-05 | 0.000796 | yes | down | 8.18 | 15.79 | 11.56 | 3.94 | 1.07 | 2.49 | 11.84333 | 2.5 |
| PGER8 | 0.355 | -1.49591 | 3.76E-05 | 0.000801 | yes | down | 4.61 | 7.98 | 5.29 | 1.34 | 3.3 | 1.84 | 5.96 | 2.16 |
| RUNDC3A | 0.044 | -4.51299 | 3.78E-05 | 0.000803 | yes | down | 0.44 | 1.09 | 0.59 | 0.03 | 0.07 | 0 | 0.706667 | 0.033333 |
| MCP2 | 0.053 | -4.24166 | 4E-05 | 0.000842 | yes | down | 2.09 | 9.29 | 5.82 | 0.2 | 0 | 0.63 | 5.733333 | 0.276667 |
| IRF9 | 0.478 | -1.06544 | 4.01E-05 | 0.000844 | yes | down | 6.89 | 7.03 | 7.93 | 3.35 | 3.17 | 4.39 | 7.283333 | 3.636667 |
| LOC100347632 | 12.341 | 3.62537 | 4.2E-05 | 0.000876 | yes | up | 0.75 | 0.38 | 0.55 | 4.91 | 11.32 | 0.49 | 0.56 | 5.573333 |
| TTC25 | 2.255 | 1.17292 | 4.31E-05 | 0.000894 | yes | up | 4.99 | 2.75 | 4.74 | 5.53 | 12.33 | 9.34 | 4.16 | 9.066667 |
| MMP16 | 0.496 | -1.01022 | 4.35E-05 | 0.000901 | yes | down | 1.38 | 1.16 | 1.12 | 0.63 | 0.8 | 0.51 | 1.22 | 0.646667 |
| LOC103350763 | 0.038 | -4.71683 | 4.38E-05 | 0.000905 | yes | down | 0.07 | 1.8 | 1.54 | 0.04 | 0.07 | 0.06 | 1.136667 | 0.056667 |
| PLAUR | 4.539 | 2.182216 | 4.61E-05 | 0.00095 | yes | up | 2.5 | 1.14 | 1.9 | 11.46 | 4.89 | 5.24 | 1.846667 | 7.196667 |
| METAP1D | 0.481 | -1.05529 | 4.66E-05 | 0.000959 | yes | down | 5.92 | 7.7 | 5.51 | 2.59 | 3.74 | 3.21 | 6.376667 | 3.18 |
| BTNL2 | 0.064 | -3.95456 | 4.78E-05 | 0.000978 | yes | down | 4.4 | 0.26 | 3.63 | 0.07 | 0.48 | 0.06 | 2.763333 | 0.203333 |
| LOC100348511 | 0.013 | -6.24003 | 4.8E-05 | 0.000981 | yes | down | 25.86 | 1.29 | 33.08 | 0.06 | 0.24 | 0.61 | 20.07667 | 0.303333 |
| FAM180B | 3.715 | 1.893377 | 4.84E-05 | 0.000989 | yes | up | 1.08 | 1.91 | 0.64 | 4.3 | 3.04 | 6.67 | 1.21 | 4.67 |
| SHROOM3 | 0.362 | -1.46706 | 4.93E-05 | 0.001004 | yes | down | 1.25 | 0.86 | 1.21 | 0.29 | 0.63 | 0.38 | 1.106667 | 0.433333 |
| FABP9 | 4.075 | 2.026846 | 4.93E-05 | 0.001004 | yes | up | 0.42 | 0.28 | 0.11 | 0.22 | 3.05 | 1.8 | 0.27 | 1.69 |
| LOC103347060 | 23.105 | 4.530164 | 4.96E-05 | 0.001008 | yes | up | 0.08 | 0.04 | 0 | 0.71 | 1.54 | 0.45 | 0.04 | 0.9 |
| BGN | 0.485 | -1.04458 | 4.98E-05 | 0.001009 | yes | down | 71.49 | 68.97 | 51.27 | 29.26 | 46.65 | 20.98 | 63.91 | 32.29667 |
| ZBED6 | 0.279 | -1.84315 | 5.07E-05 | 0.001025 | yes | down | 2.07 | 1.76 | 1.5 | 0.16 | 0.63 | 0.77 | 1.776667 | 0.52 |
| CCKBR | 0.192 | -2.37979 | 5.09E-05 | 0.001029 | yes | down | 0.5 | 0.67 | 1.5 | 0.19 | 0.14 | 0.23 | 0.89 | 0.186667 |
| C13H1orf162 | 4.736 | 2.243641 | 5.16E-05 | 0.00104 | yes | up | 0.57 | 0.83 | 1.16 | 5.08 | 1.59 | 2.28 | 0.853333 | 2.983333 |
| KNL1 | 0.229 | -2.1287 | 5.17E-05 | 0.001041 | yes | down | 0.33 | 0.35 | 0.14 | 0.05 | 0.08 | 0.06 | 0.273333 | 0.063333 |
| HSPA12A | 2.271 | 1.183146 | 5.22E-05 | 0.00105 | yes | up | 2.49 | 2.18 | 2.37 | 3.99 | 4.08 | 7.9 | 2.346667 | 5.323333 |
| STAG3 | 10.12 | 3.339135 | 5.57E-05 | 0.001111 | yes | up | 0 | 0.09 | 0.02 | 0.39 | 0.34 | 0.45 | 0.036667 | 0.393333 |
| MRAP | 0.283 | -1.81932 | 5.61E-05 | 0.001116 | yes | down | 7.7 | 10.3 | 12.7 | 2.05 | 5.55 | 1.87 | 10.23333 | 3.156667 |
| LOC100357219 | 0.33 | -1.60082 | 5.75E-05 | 0.001139 | yes | down | 70.35 | 53.93 | 44.75 | 30.29 | 14.21 | 11.11 | 56.34333 | 18.53667 |
| TNC | 2.838 | 1.504634 | 5.78E-05 | 0.001143 | yes | up | 2.47 | 2.17 | 1.4 | 4.91 | 9.98 | 3.1 | 2.013333 | 5.996667 |
| MLANA | 0.123 | -3.02435 | 5.8E-05 | 0.001145 | yes | down | 2.89 | 22.5 | 4.56 | 0.98 | 0.8 | 1.6 | 9.983333 | 1.126667 |
| CIDEA | 0.469 | -1.0928 | 5.91E-05 | 0.001167 | yes | down | 2304.52 | 3148.54 | 1333.73 | 893.87 | 1318.27 | 1107.19 | 2262.263 | 1106.443 |
| KLHL6 | 0.324 | -1.62447 | 5.96E-05 | 0.001174 | yes | down | 1.16 | 2.14 | 1.73 | 0.57 | 0.65 | 0.49 | 1.676667 | 0.57 |
| WNT11 | 2.68 | 1.422368 | 6.02E-05 | 0.001184 | yes | up | 1.57 | 1.45 | 0.6 | 2.63 | 3.48 | 3.89 | 1.206667 | 3.333333 |
| PXYLP1 | 2.026 | 1.018528 | 6.21E-05 | 0.001218 | yes | up | 2.28 | 2.39 | 1.28 | 3.44 | 4.98 | 3.96 | 1.983333 | 4.126667 |
| UBR7 | 0.479 | -1.06056 | 6.26E-05 | 0.001225 | yes | down | 20.07 | 24.87 | 30.49 | 11.43 | 14.07 | 11.36 | 25.14333 | 12.28667 |
| AASS | 0.496 | -1.01096 | 6.34E-05 | 0.001239 | yes | down | 6.63 | 6.63 | 7.42 | 2.77 | 3.28 | 4.68 | 6.893333 | 3.576667 |
| LOC100338675 | 2.116 | 1.081231 | 6.36E-05 | 0.001242 | yes | up | 17.79 | 25.2 | 28.96 | 47.91 | 51.92 | 53.26 | 23.98333 | 51.03 |
| RAP1GAP2 | 0.269 | -1.89225 | 6.42E-05 | 0.001251 | yes | down | 3.91 | 4.99 | 1.17 | 0.68 | 1.16 | 0.92 | 3.356667 | 0.92 |
| LOC100009166 | 0.211 | -2.24174 | 6.5E-05 | 0.001264 | yes | down | 2.68 | 4.97 | 6.58 | 0.93 | 1.28 | 0.95 | 4.743333 | 1.053333 |
| CMTR2 | 0.446 | -1.16396 | 6.7E-05 | 0.001296 | yes | down | 1.52 | 1.62 | 1.27 | 0.71 | 0.61 | 0.71 | 1.47 | 0.676667 |
| LOC103351259 | 2.025 | 1.017785 | 6.76E-05 | 0.001307 | yes | up | 10.31 | 11.57 | 10.69 | 22.49 | 15.75 | 24.34 | 10.85667 | 20.86 |
| MAP7 | 0.445 | -1.16908 | 6.78E-05 | 0.001308 | yes | down | 2.84 | 5.28 | 3.36 | 1.53 | 2.25 | 1.56 | 3.826667 | 1.78 |
| HS3ST1 | 2.579 | 1.366606 | 6.8E-05 | 0.001311 | yes | up | 1.3 | 1 | 0.72 | 2.9 | 2.72 | 2.33 | 1.006667 | 2.65 |
| PTX3 | 3.916 | 1.969394 | 6.92E-05 | 0.001328 | yes | up | 0.72 | 0.8 | 1.34 | 5.48 | 2.81 | 1.82 | 0.953333 | 3.37 |
| WHRN | 2.666 | 1.414735 | 7.2E-05 | 0.001374 | yes | up | 1.3 | 1.03 | 0.74 | 3.31 | 1.82 | 3.09 | 1.023333 | 2.74 |
| TIMP3 | 2.147 | 1.102222 | 7.2E-05 | 0.001374 | yes | up | 26.58 | 28.73 | 28.26 | 79.75 | 53.64 | 50.51 | 27.85667 | 61.3 |
| FAXDC2 | 0.431 | -1.21484 | 7.26E-05 | 0.001383 | yes | down | 11.83 | 18.86 | 21.87 | 8.02 | 5.95 | 7.19 | 17.52 | 7.053333 |
| SLC25A25 | 2.803 | 1.486949 | 7.29E-05 | 0.001388 | yes | up | 3.08 | 2.63 | 1.35 | 9.23 | 5.45 | 4.75 | 2.353333 | 6.476667 |
| PCSK7 | 2.206 | 1.141263 | 7.5E-05 | 0.001423 | yes | up | 6.12 | 7.51 | 6.5 | 19.59 | 12.95 | 11.58 | 6.71 | 14.70667 |
| GADD45A | 3.683 | 1.880759 | 7.54E-05 | 0.001429 | yes | up | 16.22 | 9.51 | 14.66 | 91.26 | 32.68 | 26.12 | 13.46333 | 50.02 |
| LOC100347480 | 2.617 | 1.388056 | 7.64E-05 | 0.001443 | yes | up | 9.94 | 27.68 | 11.09 | 41.36 | 44.03 | 44.76 | 16.23667 | 43.38333 |
| IFI44L | 0.458 | -1.12558 | 8.07E-05 | 0.001515 | yes | down | 4.94 | 5.83 | 2.61 | 1.74 | 2.56 | 2.04 | 4.46 | 2.113333 |
| PPP1R9A | 2.517 | 1.331437 | 8.44E-05 | 0.001581 | yes | up | 1.92 | 1.44 | 0.72 | 3.65 | 2.57 | 4.16 | 1.36 | 3.46 |
| LOC103349204 | 0.153 | -2.70968 | 8.65E-05 | 0.001615 | yes | down | 4.19 | 5.97 | 9.33 | 0.46 | 1.41 | 0.83 | 6.496667 | 0.9 |
| CEP76 | 0.48 | -1.05967 | 8.72E-05 | 0.001624 | yes | down | 1.82 | 2.33 | 1.84 | 0.97 | 0.88 | 1.11 | 1.996667 | 0.986667 |
| LOC100359331 | 105.263 | 6.71786 | 9.27E-05 | 0.001702 | yes | up | 0.35 | 0.45 | 0.17 | 44.14 | 58.22 | 0.08 | 0.323333 | 34.14667 |
| IFI44 | 0.474 | -1.07558 | 9.29E-05 | 0.001705 | yes | down | 3.88 | 4.04 | 2.73 | 2.02 | 1.89 | 1.33 | 3.55 | 1.746667 |
| LOC103351904 | 0.474 | -1.077 | 9.32E-05 | 0.001707 | yes | down | 7.67 | 8.18 | 8.83 | 4.54 | 5.39 | 7.44 | 8.226667 | 5.79 |
| ALDH4A1 | 0.425 | -1.23591 | 9.43E-05 | 0.001726 | yes | down | 18.62 | 10.63 | 9.25 | 5.64 | 7.6 | 3.61 | 12.83333 | 5.616667 |
| PPL | 2.26 | 1.176266 | 9.75E-05 | 0.001768 | yes | up | 1.58 | 0.73 | 0.81 | 2.07 | 2.66 | 2.53 | 1.04 | 2.42 |
| KLB | 2.02 | 1.014109 | 9.75E-05 | 0.001768 | yes | up | 5.91 | 7.16 | 8.27 | 14.71 | 12.57 | 17.79 | 7.113333 | 15.02333 |
| LOC100345757 | 63.806 | 5.995613 | 1E-04 | 0.001807 | yes | up | 0 | 0 | 0.06 | 2.13 | 0.04 | 2.62 | 0.02 | 1.596667 |
| LIMD2 | 0.428 | -1.22561 | 0.000102 | 0.001845 | yes | down | 4.39 | 4.83 | 4.57 | 1.69 | 2.23 | 2.23 | 4.596667 | 2.05 |
| DEFB1 | 0.326 | -1.61718 | 0.000103 | 0.001845 | yes | down | 19.13 | 29.71 | 23.17 | 11.4 | 6 | 6.61 | 24.00333 | 8.003333 |
| MET | 0.267 | -1.9028 | 0.000103 | 0.001854 | yes | down | 3.74 | 2.66 | 7.58 | 1.74 | 1.66 | 0.65 | 4.66 | 1.35 |
| CASC1 | 0.45 | -1.15108 | 0.000107 | 0.001916 | yes | down | 2.33 | 2.23 | 1.92 | 0.71 | 0.99 | 1.16 | 2.16 | 0.953333 |
| SDC4 | 3.006 | 1.588014 | 0.000108 | 0.001923 | yes | up | 20.11 | 32.9 | 16.98 | 116.54 | 49.25 | 43.34 | 23.33 | 69.71 |
| HES1 | 3.73 | 1.899009 | 0.000109 | 0.001938 | yes | up | 4.81 | 4.81 | 3.57 | 28.1 | 12.64 | 6.54 | 4.396667 | 15.76 |
| HAS1 | 114.78 | 6.842729 | 0.000111 | 0.001967 | yes | up | 0 | 0 | 0 | 2.2 | 0.21 | 0.12 | 0 | 0.843333 |
| LOC103352280 | 0.458 | -1.12712 | 0.000113 | 0.001996 | yes | down | 1.58 | 1.7 | 1.73 | 0.71 | 0.67 | 0.96 | 1.67 | 0.78 |
| RPS6KA1 | 0.471 | -1.0864 | 0.000115 | 0.002017 | yes | down | 2.98 | 3.39 | 4.07 | 1.47 | 1.82 | 1.89 | 3.48 | 1.726667 |
| LOC108178305 | 4.692 | 2.230349 | 0.000117 | 0.002057 | yes | up | 0.36 | 0.23 | 0.56 | 2.14 | 1.21 | 2.26 | 0.383333 | 1.87 |
| ABCA1 | 0.377 | -1.40772 | 0.000117 | 0.00206 | yes | down | 9.63 | 10.63 | 13.56 | 6.46 | 4.4 | 3.92 | 11.27333 | 4.926667 |
| PGM2 | 0.401 | -1.31799 | 0.00012 | 0.002103 | yes | down | 10.67 | 13.09 | 4.8 | 3.1 | 4.6 | 3.72 | 9.52 | 3.806667 |
| LOC103350039 | 2.008 | 1.005835 | 0.000123 | 0.002137 | yes | up | 0.78 | 1.02 | 0.74 | 1.35 | 2.22 | 1.91 | 0.846667 | 1.826667 |
| ITGBL1 | 3.495 | 1.805325 | 0.000123 | 0.002147 | yes | up | 1.06 | 0.51 | 0.33 | 1.54 | 2.91 | 2.52 | 0.633333 | 2.323333 |
| CSRNP1 | 7.72 | 2.948664 | 0.000125 | 0.002171 | yes | up | 1.83 | 3.05 | 1.71 | 32.25 | 10.8 | 6.17 | 2.196667 | 16.40667 |
| FLYWCH2 | 0.428 | -1.22321 | 0.000126 | 0.002182 | yes | down | 7.32 | 6.61 | 7.01 | 3.29 | 2.68 | 3.26 | 6.98 | 3.076667 |
| GNAT3 | 2.551 | 1.350923 | 0.00013 | 0.00224 | yes | up | 6.95 | 10.77 | 16.33 | 28.88 | 31.86 | 28.13 | 11.35 | 29.62333 |
| NT5C3B | 0.462 | -1.11302 | 0.000135 | 0.002316 | yes | down | 31.38 | 21.16 | 39.14 | 12.83 | 17.22 | 15.28 | 30.56 | 15.11 |
| LOC100341951 | 28.045 | 4.809677 | 0.000135 | 0.002317 | yes | up | 0 | 0.32 | 0 | 2.24 | 3.79 | 2.24 | 0.106667 | 2.756667 |
| CRYGS | 0.277 | -1.85442 | 0.000135 | 0.002324 | yes | down | 8.26 | 19.17 | 9.77 | 5.34 | 3.11 | 2.21 | 12.4 | 3.553333 |
| SIAH2 | 2.129 | 1.090495 | 0.000137 | 0.002342 | yes | up | 37.33 | 50.67 | 50.37 | 130.45 | 86.78 | 86.75 | 46.12333 | 101.3267 |
| HBB2 | 0.425 | -1.23302 | 0.000139 | 0.002358 | yes | down | 7092.06 | 18788.27 | 12438.18 | 5879.16 | 5595.18 | 5414.91 | 12772.84 | 5629.75 |
| BRI3BP | 2.032 | 1.02294 | 0.000139 | 0.002364 | yes | up | 2.6 | 2.48 | 2.87 | 4.01 | 4.97 | 6.72 | 2.65 | 5.233333 |
| LOC103350250 | 0.271 | -1.88111 | 0.00014 | 0.002377 | yes | down | 1.16 | 2.02 | 2.3 | 0.44 | 0.65 | 0.49 | 1.826667 | 0.526667 |
| ADAMTS10 | 0.478 | -1.06593 | 0.000141 | 0.002378 | yes | down | 2.5 | 3.9 | 3.28 | 1.37 | 1.46 | 1.95 | 3.226667 | 1.593333 |
| IFIT3 | 0.485 | -1.04416 | 0.000142 | 0.002405 | yes | down | 6.42 | 6.77 | 4.4 | 3.55 | 2.43 | 2.67 | 5.863333 | 2.883333 |
| DECR2 | 2.082 | 1.058232 | 0.000145 | 0.002448 | yes | up | 11.37 | 11.93 | 12.04 | 19.78 | 27.53 | 22.32 | 11.78 | 23.21 |
| LOC103347534 | 2.19 | 1.130869 | 0.000146 | 0.002452 | yes | up | 8.85 | 8.89 | 11.52 | 19.29 | 22.66 | 25.11 | 9.753333 | 22.35333 |
| CCDC102A | 0.475 | -1.07359 | 0.000152 | 0.002535 | yes | down | 4.64 | 3.33 | 3.93 | 1.63 | 2.52 | 1.77 | 3.966667 | 1.973333 |
| LOC108177718 | 0.337 | -1.57024 | 0.000154 | 0.00256 | yes | down | 1.8 | 2.46 | 2.69 | 0.79 | 0.48 | 1.15 | 2.316667 | 0.806667 |
| MAPK8IP2 | 0.19 | -2.39399 | 0.000154 | 0.00256 | yes | down | 0.59 | 1.58 | 1.52 | 0.11 | 0.3 | 0.34 | 1.23 | 0.25 |
| SCARA5 | 2.155 | 1.107353 | 0.000156 | 0.0026 | yes | up | 9.43 | 4.96 | 3.71 | 13.31 | 13.77 | 12.4 | 6.033333 | 13.16 |
| C11H5orf30 | 0.411 | -1.28281 | 0.000157 | 0.0026 | yes | down | 3.3 | 4.2 | 6.07 | 1.83 | 2.25 | 1.88 | 4.523333 | 1.986667 |
| LY96 | 2.391 | 1.257731 | 0.000158 | 0.002616 | yes | up | 4.98 | 4.85 | 2.58 | 7.25 | 7.99 | 6.48 | 4.136667 | 7.24 |
| TRABD2B | 2.309 | 1.20697 | 0.000159 | 0.002635 | yes | up | 0.63 | 0.39 | 0.44 | 0.79 | 1.37 | 1.3 | 0.486667 | 1.153333 |
| LOC100348276 | 0.249 | -2.00862 | 0.000159 | 0.002635 | yes | down | 8.09 | 3.33 | 11.34 | 1.17 | 0.71 | 1.85 | 7.586667 | 1.243333 |
| LOC100359146 | 5.589 | 2.482643 | 0.000161 | 0.002652 | yes | up | 0.05 | 0.2 | 0.12 | 0.67 | 0.89 | 0.62 | 0.123333 | 0.726667 |
| CHRM4 | 0.051 | -4.29798 | 0.000161 | 0.002657 | yes | down | 0.55 | 0.63 | 1.18 | 0.05 | 0.09 | 0 | 0.786667 | 0.046667 |
| SOD3 | 2.024 | 1.016989 | 0.000162 | 0.002662 | yes | up | 6.39 | 7.09 | 9.85 | 17.07 | 21.06 | 18.44 | 7.776667 | 18.85667 |
| ITIH5 | 2.262 | 1.177662 | 0.000166 | 0.002702 | yes | up | 19.54 | 12.03 | 9 | 18.17 | 43.49 | 35.33 | 13.52333 | 32.33 |
| CHEK1 | 2.175 | 1.120735 | 0.000167 | 0.002715 | yes | up | 2.27 | 2.63 | 2.17 | 5.64 | 4 | 7.23 | 2.356667 | 5.623333 |
| TESMIN | 0.024 | -5.39357 | 0.000171 | 0.002764 | yes | down | 3.4 | 9.5 | 5.54 | 1.59 | 0 | 0 | 6.146667 | 0.53 |
| LOC103349764 | 2.222 | 1.152156 | 0.000174 | 0.002813 | yes | up | 3.11 | 33.03 | 12.73 | 44.9 | 54.56 | 51.42 | 16.29 | 50.29333 |
| LOC100009136 | 0.024 | -5.35595 | 0.000174 | 0.002813 | yes | down | 1.91 | 13.37 | 2.29 | 0 | 0 | 0.41 | 5.856667 | 0.136667 |
| CCNG2 | 0.408 | -1.29202 | 0.000176 | 0.00284 | yes | down | 23.15 | 22.73 | 44.17 | 13.6 | 15.12 | 10.8 | 30.01667 | 13.17333 |
| HEPH | 0.249 | -2.00476 | 0.00018 | 0.002892 | yes | down | 0.7 | 0.37 | 0.76 | 0.09 | 0.2 | 0.2 | 0.61 | 0.163333 |
| SERPINE1 | 10.671 | 3.415676 | 0.000182 | 0.00292 | yes | up | 6.68 | 4.46 | 3.7 | 114.79 | 22.17 | 16.66 | 4.946667 | 51.20667 |
| CXCL12 | 0.406 | -1.30168 | 0.000183 | 0.002928 | yes | down | 21.54 | 31.29 | 45.18 | 12.09 | 20.35 | 10.69 | 32.67 | 14.37667 |
| TRAF3IP2 | 2.318 | 1.213005 | 0.000184 | 0.002943 | yes | up | 3.78 | 4.69 | 3.32 | 10.78 | 7.81 | 6.26 | 3.93 | 8.283333 |
| FAM107A | 2.178 | 1.123332 | 0.000185 | 0.002948 | yes | up | 8.4 | 9.65 | 10.21 | 27.5 | 22.11 | 13.99 | 9.42 | 21.2 |
| ITGA11 | 2.71 | 1.438037 | 0.000186 | 0.002964 | yes | up | 1.24 | 0.9 | 0.39 | 1.56 | 3.22 | 2.2 | 0.843333 | 2.326667 |
| LOC103352172 | 0.021 | -5.60255 | 0.000188 | 0.002978 | yes | down | 1.26 | 0.61 | 0.16 | 0 | 0.04 | 0 | 0.676667 | 0.013333 |
| LETM2 | 3.513 | 1.812616 | 0.000189 | 0.002991 | yes | up | 0.27 | 0.46 | 0.2 | 1.04 | 0.93 | 0.74 | 0.31 | 0.903333 |
| LOC100341303 | 3.628 | 1.85936 | 0.000189 | 0.002991 | yes | up | 0.84 | 0.47 | 1.06 | 2.78 | 3.25 | 3.03 | 0.79 | 3.02 |
| ONECUT1 | 0.068 | -3.87562 | 0.000191 | 0.00301 | yes | down | 0.32 | 0.1 | 0.05 | 0.02 | 0.01 | 0.01 | 0.156667 | 0.013333 |
| CFTR | 4.377 | 2.129923 | 0.000195 | 0.003068 | yes | up | 0.18 | 0.08 | 0.14 | 0.65 | 0.35 | 0.82 | 0.133333 | 0.606667 |
| LOC100353904 | 3.825 | 1.935308 | 0.000197 | 0.003082 | yes | up | 0.25 | 0.33 | 0.19 | 1.01 | 1.32 | 0.69 | 0.256667 | 1.006667 |
| CDT1 | 3.041 | 1.604564 | 0.000197 | 0.003082 | yes | up | 0.72 | 0.98 | 0.45 | 2.56 | 2.34 | 1.74 | 0.716667 | 2.213333 |
| MXI1 | 0.492 | -1.02262 | 0.000197 | 0.003082 | yes | down | 13.32 | 15.4 | 17.35 | 9.39 | 6.71 | 7.28 | 15.35667 | 7.793333 |
| DOCK5 | 2.124 | 1.086695 | 0.000201 | 0.00314 | yes | up | 0.36 | 0.31 | 0.19 | 0.8 | 0.61 | 1.37 | 0.286667 | 0.926667 |
| LOC100345762 | 16.133 | 4.011962 | 0.000208 | 0.003241 | yes | up | 0 | 0.29 | 0 | 1.79 | 1.36 | 2.21 | 0.096667 | 1.786667 |
| SLC24A2 | 0.226 | -2.14299 | 0.000209 | 0.003246 | yes | down | 0.75 | 0.6 | 0.72 | 0.32 | 0.09 | 0.07 | 0.69 | 0.16 |
| ZNF250 | 0.479 | -1.06239 | 0.000212 | 0.003274 | yes | down | 3.93 | 3.62 | 3.55 | 1.87 | 1.48 | 2.13 | 3.7 | 1.826667 |
| BMF | 0.366 | -1.45097 | 0.000213 | 0.003295 | yes | down | 2.11 | 2.96 | 2.94 | 0.96 | 0.45 | 1.55 | 2.67 | 0.986667 |
| LRRN4 | 0.063 | -3.98087 | 0.000218 | 0.003357 | yes | down | 0.04 | 2.24 | 0.99 | 0.02 | 0.11 | 0.09 | 1.09 | 0.073333 |
| ZBED8 | 0.304 | -1.71753 | 0.000224 | 0.003446 | yes | down | 0.97 | 1.02 | 0.64 | 0.2 | 0.24 | 0.47 | 0.876667 | 0.303333 |
| SDSL | 0.339 | -1.56196 | 0.00023 | 0.003516 | yes | down | 4.24 | 6.53 | 9.41 | 1.75 | 3.28 | 2.32 | 6.726667 | 2.45 |
| CA5B | 2.1 | 1.070514 | 0.000232 | 0.003541 | yes | up | 2.63 | 2.24 | 1.33 | 2.88 | 5.36 | 4.17 | 2.066667 | 4.136667 |
| LOC100343111 | 0.057 | -4.12383 | 0.000233 | 0.00355 | yes | down | 0.98 | 0.47 | 0.54 | 0.06 | 0 | 0.11 | 0.663333 | 0.056667 |
| TTC39C | 0.448 | -1.15765 | 0.000233 | 0.00355 | yes | down | 2.46 | 3.2 | 3.49 | 1.13 | 1.49 | 1.75 | 3.05 | 1.456667 |
| KCNE3 | 4.828 | 2.271316 | 0.000236 | 0.003583 | yes | up | 0.28 | 0.29 | 0.21 | 1.86 | 0.96 | 0.98 | 0.26 | 1.266667 |
| FAM84B | 0.41 | -1.28612 | 0.000236 | 0.003589 | yes | down | 1.31 | 1.64 | 2.35 | 0.84 | 0.65 | 0.69 | 1.766667 | 0.726667 |
| LOC103347661 | 2.16 | 1.110845 | 0.000237 | 0.003589 | yes | up | 2.5 | 2.12 | 1.53 | 3.85 | 3.55 | 5.12 | 2.05 | 4.173333 |
| CLDN15 | 0.437 | -1.194 | 0.000238 | 0.0036 | yes | down | 6.72 | 13.62 | 12.77 | 5.36 | 5.83 | 4.12 | 11.03667 | 5.103333 |
| LOC103351001 | 0.407 | -1.29575 | 0.000241 | 0.003643 | yes | down | 0.98 | 1 | 0.96 | 0.41 | 0.44 | 0.39 | 0.98 | 0.413333 |
| C17H14orf37 | 4.216 | 2.075973 | 0.000244 | 0.003684 | yes | up | 0.28 | 3.86 | 0.06 | 8.64 | 4.33 | 7.97 | 1.4 | 6.98 |
| ZFP36 | 10.873 | 3.442714 | 0.000246 | 0.003714 | yes | up | 4.23 | 3.82 | 3.58 | 92.09 | 22.52 | 10.18 | 3.876667 | 41.59667 |
| LOC100354435 | 3.583 | 1.841016 | 0.000247 | 0.003723 | yes | up | 5.07 | 6.91 | 1.45 | 22.1 | 8.11 | 16.64 | 4.476667 | 15.61667 |
| RUFY4 | 2.32 | 1.214004 | 0.000249 | 0.003736 | yes | up | 0.89 | 1 | 0.55 | 2.4 | 2.23 | 2.99 | 0.813333 | 2.54 |
| CLU | 2.724 | 1.445685 | 0.000249 | 0.003736 | yes | up | 14.1 | 7.06 | 4.85 | 21.58 | 36.75 | 14.37 | 8.67 | 24.23333 |
| NFATC2 | 3.075 | 1.620673 | 0.000251 | 0.00375 | yes | up | 0.93 | 0.58 | 0.44 | 3.07 | 1.83 | 1.04 | 0.65 | 1.98 |
| PVR | 2.549 | 1.349937 | 0.000251 | 0.00375 | yes | up | 1.41 | 1.82 | 1.87 | 5.34 | 4.23 | 3.94 | 1.7 | 4.503333 |
| APOLD1 | 12.394 | 3.631558 | 0.000267 | 0.00395 | yes | up | 6.87 | 12.02 | 21.08 | 319.75 | 106.1 | 34.92 | 13.32333 | 153.59 |
| LOC100342240 | 0.485 | -1.0434 | 0.000268 | 0.003957 | yes | down | 3.51 | 5.03 | 3.52 | 1.96 | 2.62 | 1.5 | 4.02 | 2.026667 |
| ENGASE | 2.445 | 1.2899 | 0.000271 | 0.003988 | yes | up | 0.61 | 0.62 | 0.76 | 1.47 | 2.14 | 1.54 | 0.663333 | 1.716667 |
| SEMA5B | 0.214 | -2.22166 | 0.000275 | 0.004042 | yes | down | 0.4 | 0.32 | 0.48 | 0.13 | 0.08 | 0.06 | 0.4 | 0.09 |
| RASSF9 | 0.348 | -1.5218 | 0.000275 | 0.004044 | yes | down | 1.37 | 2.7 | 2.91 | 0.99 | 0.73 | 0.83 | 2.326667 | 0.85 |
| SLC16A2 | 0.35 | -1.51501 | 0.000276 | 0.004051 | yes | down | 9.42 | 24.62 | 6.05 | 3.43 | 5.5 | 5.28 | 13.36333 | 4.736667 |
| DUSP2 | 22.762 | 4.508552 | 0.000279 | 0.004087 | yes | up | 0.08 | 0.07 | 0.07 | 4.46 | 0.31 | 0.14 | 0.073333 | 1.636667 |
| LOC103349164 | 0.077 | -3.70508 | 0.000281 | 0.004096 | yes | down | 0.82 | 1.21 | 1.75 | 0.25 | 0.06 | 0 | 1.26 | 0.103333 |
| ADORA2A | 0.367 | -1.44424 | 0.000284 | 0.004125 | yes | down | 4.57 | 6.22 | 9.52 | 1.82 | 4.03 | 2.15 | 6.77 | 2.666667 |
| SAPCD2 | 0.214 | -2.22745 | 0.000287 | 0.004164 | yes | down | 0.69 | 0.9 | 0.51 | 0.12 | 0.26 | 0.09 | 0.7 | 0.156667 |
| WDSUB1 | 0.355 | -1.49296 | 0.00029 | 0.0042 | yes | down | 1.78 | 2.97 | 2.57 | 0.63 | 1.15 | 0.97 | 2.44 | 0.916667 |
| CCNA2 | 0.147 | -2.76883 | 0.000303 | 0.004357 | yes | down | 1.86 | 1.77 | 0.74 | 0.19 | 0.04 | 0.37 | 1.456667 | 0.2 |
| GRO-A | 4.458 | 2.15625 | 0.000304 | 0.004359 | yes | up | 0.14 | 0.84 | 0.52 | 2.19 | 2.61 | 2.32 | 0.5 | 2.373333 |
| L3MBTL1 | 4.248 | 2.086617 | 0.000315 | 0.004488 | yes | up | 0.12 | 0.2 | 0.07 | 0.37 | 0.46 | 0.58 | 0.13 | 0.47 |
| LOC103346509 | 0.226 | -2.14325 | 0.000316 | 0.004507 | yes | down | 2.26 | 2.86 | 4.18 | 0.58 | 1.01 | 0.6 | 3.1 | 0.73 |
| LOC108176131 | 0.445 | -1.16901 | 0.000321 | 0.004559 | yes | down | 1.47 | 2.12 | 1.84 | 0.94 | 1.13 | 0.53 | 1.81 | 0.866667 |
| LOC103346853 | 3.918 | 1.969996 | 0.000323 | 0.004579 | yes | up | 0.33 | 0.2 | 0.24 | 1.3 | 0.71 | 1.03 | 0.256667 | 1.013333 |
| CSMD2 | 0.265 | -1.91862 | 0.000323 | 0.004585 | yes | down | 0.15 | 0.26 | 0.12 | 0.07 | 0.03 | 0.04 | 0.176667 | 0.046667 |
| KIF2C | 0.169 | -2.56411 | 0.000334 | 0.004704 | yes | down | 1 | 0.96 | 0.43 | 0.1 | 0.29 | 0.04 | 0.796667 | 0.143333 |
| CRYM | 0.31 | -1.69015 | 0.000342 | 0.004803 | yes | down | 16.35 | 70.73 | 64.68 | 19.19 | 16.42 | 13.91 | 50.58667 | 16.50667 |
| LOC100351040 | 0.013 | -6.2416 | 0.000345 | 0.004829 | yes | down | 0.1 | 0.64 | 0.05 | 0 | 0 | 0 | 0.263333 | 0 |
| CD83 | 4.696 | 2.231421 | 0.000348 | 0.004871 | yes | up | 0.24 | 0.22 | 0.11 | 1.21 | 0.68 | 0.8 | 0.19 | 0.896667 |
| LOC100348403 | 0.085 | -3.55794 | 0.000357 | 0.004972 | yes | down | 2.34 | 0.11 | 1.63 | 0.09 | 0.33 | 0.05 | 1.36 | 0.156667 |
| ADCYAP1 | 0.129 | -2.9547 | 0.000359 | 0.00499 | yes | down | 0.51 | 0.93 | 0.27 | 0.02 | 0.07 | 0.13 | 0.57 | 0.073333 |
| LOC100343144 | 2.954 | 1.562536 | 0.000364 | 0.005064 | yes | up | 4.4 | 3.25 | 7.51 | 8.37 | 20.59 | 19.75 | 5.053333 | 16.23667 |
| NIPA1 | 2.255 | 1.173334 | 0.000368 | 0.005094 | yes | up | 0.39 | 0.36 | 0.46 | 0.92 | 0.87 | 1.09 | 0.403333 | 0.96 |
| LOC103346040 | 2.597 | 1.376822 | 0.00037 | 0.005118 | yes | up | 0.52 | 0.45 | 0.57 | 1.34 | 1.07 | 1.64 | 0.513333 | 1.35 |
| LOC103348799 | 0.132 | -2.91997 | 0.000376 | 0.00518 | yes | down | 1.15 | 2.32 | 10.03 | 0.82 | 0.86 | 0.33 | 4.5 | 0.67 |
| CEBPD | 2.877 | 1.524753 | 0.000384 | 0.00528 | yes | up | 11.9 | 14.95 | 10.17 | 58.34 | 32.25 | 16.69 | 12.34 | 35.76 |
| CCDC28A | 0.258 | -1.9546 | 0.000385 | 0.005282 | yes | down | 3.99 | 1.91 | 4.28 | 1.42 | 0.59 | 0.71 | 3.393333 | 0.906667 |
| DFNA5 | 0.456 | -1.13302 | 0.000392 | 0.005363 | yes | down | 3.21 | 2.96 | 1.9 | 0.74 | 1.48 | 1.44 | 2.69 | 1.22 |
| RERGL | 0.376 | -1.41025 | 0.000392 | 0.005363 | yes | down | 5 | 3.75 | 3.08 | 1.71 | 1.03 | 1.77 | 3.943333 | 1.503333 |
| LOC100345696 | 2.173 | 1.119877 | 0.000394 | 0.00537 | yes | up | 6.29 | 4.61 | 2.59 | 7.51 | 10.19 | 9.9 | 4.496667 | 9.2 |
| UBAP1L | 3.577 | 1.838797 | 0.000396 | 0.005385 | yes | up | 0.34 | 1.55 | 0.8 | 2.5 | 3.41 | 4.08 | 0.896667 | 3.33 |
| FOS | 13.826 | 3.789326 | 0.000406 | 0.005482 | yes | up | 17.32 | 4.47 | 3.7 | 277.18 | 80.8 | 36.68 | 8.496667 | 131.5533 |
| BHLHE40 | 2.12 | 1.084265 | 0.000406 | 0.005482 | yes | up | 8.43 | 10.2 | 6.06 | 21.69 | 21.53 | 10.44 | 8.23 | 17.88667 |
| LOC103350987 | 7.314 | 2.870655 | 0.000409 | 0.005515 | yes | up | 0.01 | 0.02 | 0.05 | 0.55 | 0.23 | 0.14 | 0.026667 | 0.306667 |
| LOC103345772 | 2.056 | 1.03986 | 0.00041 | 0.005519 | yes | up | 6.96 | 6.7 | 4.23 | 10.04 | 16.69 | 11.01 | 5.963333 | 12.58 |
| LOC100349875 | 0.336 | -1.57499 | 0.000411 | 0.005519 | yes | down | 1.06 | 1.22 | 1.08 | 0.39 | 0.38 | 0.4 | 1.12 | 0.39 |
| ADAT1 | 0.39 | -1.35767 | 0.000412 | 0.00552 | yes | down | 1.32 | 1.05 | 0.62 | 0.32 | 0.37 | 0.53 | 0.996667 | 0.406667 |
| EDARADD | 6.825 | 2.770883 | 0.000416 | 0.005569 | yes | up | 0.15 | 0.03 | 0.09 | 0.71 | 0.44 | 0.51 | 0.09 | 0.553333 |
| GABRB3 | 0.19 | -2.39317 | 0.000419 | 0.005603 | yes | down | 0.44 | 0.71 | 0.26 | 0.05 | 0.2 | 0.03 | 0.47 | 0.093333 |
| FMO5 | 0.389 | -1.362 | 0.000422 | 0.005631 | yes | down | 2.23 | 1.55 | 2.34 | 0.48 | 0.85 | 1.11 | 2.04 | 0.813333 |
| LOC100344629 | 2.043 | 1.030548 | 0.000426 | 0.005683 | yes | up | 1.22 | 1.12 | 0.7 | 1.72 | 2.43 | 1.99 | 1.013333 | 2.046667 |
| LOC108176823 | 4.745 | 2.246367 | 0.000432 | 0.005756 | yes | up | 0.26 | 0.22 | 0.25 | 2.07 | 0.98 | 0.48 | 0.243333 | 1.176667 |
| SLC25A35 | 2.056 | 1.03993 | 0.000433 | 0.005762 | yes | up | 1.69 | 1.84 | 1.14 | 2.75 | 3.29 | 3.82 | 1.556667 | 3.286667 |
| GREM2 | 0.431 | -1.21359 | 0.000437 | 0.0058 | yes | down | 1.74 | 1.04 | 1.5 | 0.59 | 1.07 | 0.59 | 1.426667 | 0.75 |
| MPZL3 | 2.211 | 1.144787 | 0.00044 | 0.005838 | yes | up | 0.65 | 0.74 | 0.72 | 1.51 | 1.59 | 2.09 | 0.703333 | 1.73 |
| LOC108176676 | 0.354 | -1.50015 | 0.000441 | 0.005842 | yes | down | 2.79 | 3.82 | 2.19 | 1.07 | 0.68 | 1.81 | 2.933333 | 1.186667 |
| LOC108178304 | 0.042 | -4.58189 | 0.000441 | 0.005842 | yes | down | 0.56 | 0.64 | 0.76 | 0 | 0.09 | 0 | 0.653333 | 0.03 |
| ADGRD1 | 2.153 | 1.106024 | 0.000443 | 0.005862 | yes | up | 3.41 | 1.96 | 1.05 | 4.09 | 5.31 | 4.21 | 2.14 | 4.536667 |
| NFE2 | 0.292 | -1.77826 | 0.000455 | 0.005984 | yes | down | 1.67 | 5.07 | 5.16 | 1.53 | 0.87 | 1.14 | 3.966667 | 1.18 |
| ERRFI1 | 19.513 | 4.2864 | 0.000456 | 0.00599 | yes | up | 2.52 | 2.65 | 2.37 | 96.3 | 11.99 | 4.81 | 2.513333 | 37.7 |
| ZCCHC5 | 0.236 | -2.08044 | 0.000462 | 0.006061 | yes | down | 5.75 | 4.35 | 12.08 | 0.52 | 3.66 | 1.81 | 7.393333 | 1.996667 |
| SFRP5 | 2.766 | 1.467825 | 0.000475 | 0.00621 | yes | up | 1.69 | 0.79 | 0.46 | 2.04 | 2.39 | 4.08 | 0.98 | 2.836667 |
| C20H14orf142 | 0.478 | -1.06563 | 0.000478 | 0.006243 | yes | down | 59.98 | 68.68 | 105.99 | 42.85 | 43.13 | 33.64 | 78.21667 | 39.87333 |
| LOC100340865 | 0.107 | -3.21815 | 0.000485 | 0.006312 | yes | down | 26.88 | 4.52 | 2.91 | 1.21 | 1.61 | 0.97 | 11.43667 | 1.263333 |
| ERFE | 7.397 | 2.886968 | 0.000486 | 0.006312 | yes | up | 0.2 | 0.18 | 0.11 | 2.06 | 1.43 | 0.18 | 0.163333 | 1.223333 |
| IER3 | 2.719 | 1.443071 | 0.000486 | 0.006312 | yes | up | 7.54 | 5.15 | 2.55 | 19.23 | 10.8 | 10.84 | 5.08 | 13.62333 |
| LOC100338821 | 0.019 | -5.71063 | 0.000488 | 0.006326 | yes | down | 0.32 | 0.45 | 0.1 | 0 | 0 | 0 | 0.29 | 0 |
| NFIL3 | 2.825 | 1.498211 | 0.000502 | 0.006465 | yes | up | 4.41 | 5.82 | 5.8 | 25.94 | 12.28 | 7.96 | 5.343333 | 15.39333 |
| CADM3 | 2.004 | 1.002881 | 0.000513 | 0.006565 | yes | up | 18.59 | 14.48 | 7.31 | 22.45 | 35.57 | 24.71 | 13.46 | 27.57667 |
| NDC80 | 0.179 | -2.48409 | 0.000516 | 0.006587 | yes | down | 0.96 | 1.01 | 0.38 | 0.22 | 0.16 | 0.05 | 0.783333 | 0.143333 |
| ST13 | 0.487 | -1.03827 | 0.00052 | 0.006643 | yes | down | 85.69 | 87.25 | 75.19 | 58.94 | 32.87 | 31.37 | 82.71 | 41.06 |
| GALM | 0.478 | -1.06367 | 0.000529 | 0.006733 | yes | down | 33.37 | 18.23 | 16.33 | 9.26 | 9.17 | 14.6 | 22.64333 | 11.01 |
| CTPS1 | 2.102 | 1.071646 | 0.000531 | 0.006748 | yes | up | 2.27 | 2.36 | 1.16 | 3.1 | 5.67 | 3.77 | 1.93 | 4.18 |
| CTTNBP2 | 0.396 | -1.33669 | 0.000531 | 0.006748 | yes | down | 0.94 | 0.72 | 1.31 | 0.46 | 0.43 | 0.36 | 0.99 | 0.416667 |
| LOC100339846 | 0.107 | -3.22187 | 0.000531 | 0.00675 | yes | down | 1.09 | 0.12 | 0.11 | 0.19 | 0.01 | 0.17 | 0.44 | 0.123333 |
| CCNB1 | 0.286 | -1.80421 | 0.000546 | 0.006907 | yes | down | 4.56 | 4.16 | 1.59 | 0.72 | 1.73 | 0.59 | 3.436667 | 1.013333 |
| RND3 | 2.39 | 1.256938 | 0.000551 | 0.006959 | yes | up | 10.56 | 7.74 | 4.45 | 26.09 | 16.07 | 11.9 | 7.583333 | 18.02 |
| LOC100343529 | 0.458 | -1.12621 | 0.000555 | 0.007003 | yes | down | 13.58 | 14.57 | 13.87 | 5.56 | 8.74 | 5.8 | 14.00667 | 6.7 |
| TBCCD1 | 0.407 | -1.29675 | 0.000558 | 0.00702 | yes | down | 5.78 | 6.84 | 13 | 3.4 | 4.1 | 4.88 | 8.54 | 4.126667 |
| EML6 | 2.215 | 1.147071 | 0.000571 | 0.007168 | yes | up | 1.14 | 1.23 | 0.48 | 2.07 | 2.7 | 1.63 | 0.95 | 2.133333 |
| SDK2 | 2.27 | 1.182556 | 0.000572 | 0.007176 | yes | up | 2.4 | 3.17 | 3.36 | 8.8 | 3.96 | 7.03 | 2.976667 | 6.596667 |
| LOC103348241 | 13.48 | 3.752787 | 0.000573 | 0.007181 | yes | up | 4 | 5.05 | 8.74 | 118.62 | 7.79 | 122.26 | 5.93 | 82.89 |
| LOC103350057 | 4.523 | 2.177145 | 0.000575 | 0.007192 | yes | up | 0.08 | 0.14 | 0.07 | 0.75 | 1.05 | 0.95 | 0.096667 | 0.916667 |
| LOC108178759 | 0.069 | -3.86287 | 0.000575 | 0.007192 | yes | down | 1.65 | 0.57 | 0.23 | 0.04 | 0.09 | 0.04 | 0.816667 | 0.056667 |
| UGT1-6 | 0.493 | -1.01957 | 0.000584 | 0.007302 | yes | down | 9.54 | 21.09 | 13.06 | 7.47 | 7.03 | 7.04 | 14.56333 | 7.18 |
| SUSD4 | 4.624 | 2.20915 | 0.000589 | 0.00734 | yes | up | 0.06 | 0.13 | 0.11 | 0.48 | 0.43 | 0.52 | 0.1 | 0.476667 |
| LOC103346494 | 27.01 | 4.75544 | 0.000595 | 0.007412 | yes | up | 0 | 0.11 | 0 | 1.37 | 0.4 | 1.48 | 0.036667 | 1.083333 |
| LOC108178072 | 0.319 | -1.65044 | 0.000601 | 0.007469 | yes | down | 3.67 | 6.25 | 3.57 | 1.62 | 0.82 | 1.9 | 4.496667 | 1.446667 |
| LSM11 | 0.463 | -1.11029 | 0.000604 | 0.007503 | yes | down | 0.79 | 0.96 | 0.73 | 0.29 | 0.43 | 0.48 | 0.826667 | 0.4 |
| FAM71F1 | 0.128 | -2.96476 | 0.000611 | 0.007536 | yes | down | 0.67 | 0.48 | 0.68 | 0.07 | 0.14 | 0.03 | 0.61 | 0.08 |
| TMEM53 | 2.347 | 1.231112 | 0.000618 | 0.007612 | yes | up | 1.18 | 1.94 | 0.9 | 2.65 | 3.11 | 3.89 | 1.34 | 3.216667 |
| EPB42 | 0.417 | -1.26193 | 0.000621 | 0.007641 | yes | down | 1.82 | 3.58 | 3.15 | 1.46 | 1.15 | 1.1 | 2.85 | 1.236667 |
| DLX4 | 0.255 | -1.97223 | 0.000631 | 0.007724 | yes | down | 0.57 | 1.09 | 1.06 | 0.26 | 0.26 | 0.24 | 0.906667 | 0.253333 |
| CDK1 | 0.317 | -1.65784 | 0.000638 | 0.007794 | yes | down | 3.87 | 3.96 | 1.17 | 0.71 | 1.33 | 0.68 | 3 | 0.906667 |
| LOC100350920 | 2.318 | 1.212834 | 0.000647 | 0.007895 | yes | up | 2.32 | 0.81 | 1.19 | 5.21 | 2.11 | 2.73 | 1.44 | 3.35 |
| CNTNAP4 | 0.027 | -5.18985 | 0.000648 | 0.007901 | yes | down | 0.07 | 0.12 | 0.22 | 0 | 0.01 | 0 | 0.136667 | 0.003333 |
| LOC100354670 | 0.157 | -2.67459 | 0.000651 | 0.007925 | yes | down | 0.4 | 2.49 | 0.82 | 0.21 | 0.19 | 0.19 | 1.236667 | 0.196667 |
| CA4 | 2.616 | 1.387125 | 0.000681 | 0.008255 | yes | up | 10.88 | 23.6 | 27.41 | 76.75 | 50.34 | 42.73 | 20.63 | 56.60667 |
| CDCA7 | 0.155 | -2.68507 | 0.000683 | 0.008269 | yes | down | 0.73 | 0.63 | 0.3 | 0.05 | 0.18 | 0.04 | 0.553333 | 0.09 |
| LOC100337961 | 3.047 | 1.607413 | 0.000686 | 0.008303 | yes | up | 1.24 | 0.83 | 1.25 | 2.78 | 4.43 | 3.36 | 1.106667 | 3.523333 |
| DPP6 | 5.048 | 2.33584 | 0.000694 | 0.008386 | yes | up | 0.06 | 0.15 | 0.15 | 0.61 | 0.21 | 1.11 | 0.12 | 0.643333 |
| SLC22A4 | 2.556 | 1.353628 | 0.000704 | 0.008471 | yes | up | 0.65 | 1.08 | 1.21 | 2.75 | 2.34 | 2.48 | 0.98 | 2.523333 |
| KNTC1 | 0.392 | -1.35179 | 0.000704 | 0.008471 | yes | down | 0.73 | 0.72 | 0.29 | 0.21 | 0.28 | 0.2 | 0.58 | 0.23 |
| MYOCD | 2.365 | 1.242119 | 0.000713 | 0.00856 | yes | up | 0.39 | 0.32 | 0.22 | 1.09 | 0.88 | 0.53 | 0.31 | 0.833333 |
| PPARGC1A | 0.327 | -1.61362 | 0.000719 | 0.008619 | yes | down | 4.93 | 18.09 | 4.2 | 3.02 | 3.95 | 2.03 | 9.073333 | 3 |
| CFAP126 | 3.254 | 1.702032 | 0.000731 | 0.008716 | yes | up | 0.46 | 0.32 | 0.39 | 0.94 | 1.19 | 1.52 | 0.39 | 1.216667 |
| BMP3 | 2.763 | 1.466118 | 0.000732 | 0.008725 | yes | up | 0.42 | 0.41 | 0.15 | 0.59 | 0.96 | 1.16 | 0.326667 | 0.903333 |
| AHSP | 0.209 | -2.26054 | 0.000735 | 0.008749 | yes | down | 3.23 | 9.22 | 3.9 | 0.94 | 1.62 | 0.97 | 5.45 | 1.176667 |
| DUOX2 | 3.346 | 1.742499 | 0.000747 | 0.00888 | yes | up | 0.44 | 1.03 | 1.33 | 3.16 | 5.69 | 1.45 | 0.933333 | 3.433333 |
| RAP2B | 0.383 | -1.38418 | 0.00075 | 0.008903 | yes | down | 7.66 | 6.51 | 15.32 | 3.76 | 3.93 | 4.42 | 9.83 | 4.036667 |
| SFXN2 | 0.374 | -1.42031 | 0.000762 | 0.009011 | yes | down | 2.23 | 3.67 | 5.26 | 1.49 | 2 | 1.24 | 3.72 | 1.576667 |
| LNX1 | 0.328 | -1.60947 | 0.000764 | 0.009031 | yes | down | 0.93 | 0.95 | 0.96 | 0.35 | 0.25 | 0.31 | 0.946667 | 0.303333 |
| DLGAP5 | 0.249 | -2.00434 | 0.000774 | 0.009115 | yes | down | 0.83 | 0.96 | 0.42 | 0.13 | 0.23 | 0.11 | 0.736667 | 0.156667 |
| SPTBN2 | 2.947 | 1.559157 | 0.000782 | 0.009203 | yes | up | 0.24 | 0.1 | 0.09 | 0.52 | 0.41 | 0.34 | 0.143333 | 0.423333 |
| LOC103348842 | 106.845 | 6.739371 | 0.000801 | 0.009396 | yes | up | 0 | 0 | 0 | 0.02 | 0.82 | 0.02 | 0 | 0.286667 |
| LOC103350794 | 0.472 | -1.08183 | 0.000806 | 0.009435 | yes | down | 5.62 | 6.88 | 7.8 | 2.64 | 3.69 | 3.83 | 6.766667 | 3.386667 |
| CNTFR | 2.367 | 1.242798 | 0.000807 | 0.00944 | yes | up | 2.64 | 2.62 | 0.9 | 4.22 | 4.88 | 5.48 | 2.053333 | 4.86 |
| LOC100343456 | 0.132 | -2.91717 | 0.000815 | 0.009533 | yes | down | 0.47 | 0.94 | 1.11 | 0.04 | 0.08 | 0.22 | 0.84 | 0.113333 |
| AP1G2 | 3.253 | 1.70167 | 0.00084 | 0.009788 | yes | up | 0.35 | 0.26 | 0.2 | 0.83 | 0.64 | 1.11 | 0.27 | 0.86 |
| LOC100347962 | 0.271 | -1.88252 | 0.00084 | 0.009788 | yes | down | 0.3 | 0.28 | 0.31 | 0.04 | 0.1 | 0.11 | 0.296667 | 0.083333 |
| UGDH | 2.193 | 1.132818 | 0.000841 | 0.009788 | yes | up | 11.6 | 11.91 | 11.32 | 39.41 | 20.71 | 17.53 | 11.61 | 25.88333 |
| LOC100349709 | 9.073 | 3.181641 | 0.000843 | 0.009801 | yes | up | 0.05 | 0.03 | 0.01 | 0.43 | 0.05 | 0.35 | 0.03 | 0.276667 |
| PRDM1 | 0.287 | -1.80229 | 0.000864 | 0.010019 | yes | down | 1.1 | 1 | 0.27 | 0.25 | 0.29 | 0.15 | 0.79 | 0.23 |
| LOC103351400 | 0.45 | -1.15154 | 0.00088 | 0.010181 | yes | down | 1.32 | 1.77 | 1.82 | 0.76 | 0.95 | 0.62 | 1.636667 | 0.776667 |
| LOC100350215 | 0.008 | -7.05362 | 0.000885 | 0.010232 | yes | down | 50.24 | 49.4 | 0 | 0.13 | 0.09 | 0.45 | 33.21333 | 0.223333 |
| LOC103348554 | 0.035 | -4.84946 | 0.00089 | 0.010275 | yes | down | 0.17 | 1.73 | 2.1 | 0 | 0.08 | 0.07 | 1.333333 | 0.05 |
| LEP | 16.805 | 4.070815 | 0.000906 | 0.010412 | yes | up | 0.05 | 0 | 0 | 0.34 | 0.16 | 0.64 | 0.016667 | 0.38 |
| ELMOD1 | 0.113 | -3.15085 | 0.000921 | 0.01055 | yes | down | 0.18 | 0.64 | 0.8 | 0.05 | 0.14 | 0.02 | 0.54 | 0.07 |
| YPEL4 | 0.363 | -1.46323 | 0.000927 | 0.010597 | yes | down | 1.03 | 2.03 | 2.16 | 0.48 | 0.77 | 0.72 | 1.74 | 0.656667 |
| MSR1 | 2.147 | 1.102462 | 0.000935 | 0.010684 | yes | up | 0.64 | 0.8 | 0.47 | 1.63 | 1.54 | 1.42 | 0.636667 | 1.53 |
| LOC100337780 | 3.159 | 1.659685 | 0.000944 | 0.010773 | yes | up | 0.29 | 0.27 | 0.19 | 0.97 | 0.62 | 0.8 | 0.25 | 0.796667 |
| LOC103349253 | 0.459 | -1.12199 | 0.000948 | 0.010808 | yes | down | 5.18 | 5.05 | 12.6 | 4.09 | 8.67 | 6.63 | 7.61 | 6.463333 |
| CPS1 | 3.584 | 1.841561 | 0.000949 | 0.010808 | yes | up | 0.14 | 0.05 | 0.12 | 0.24 | 0.46 | 0.42 | 0.103333 | 0.373333 |
| CLIC6 | 46.569 | 5.541297 | 0.000988 | 0.011192 | yes | up | 0 | 0 | 0 | 0.11 | 0.43 | 0.17 | 0 | 0.236667 |
| LOC100343468 | 0.331 | -1.59451 | 0.000991 | 0.011212 | yes | down | 1.25 | 0.89 | 2.07 | 0.33 | 0.67 | 0.51 | 1.403333 | 0.503333 |
| LOC103350884 | 5.106 | 2.352308 | 0.000992 | 0.011213 | yes | up | 0.32 | 0.17 | 0.46 | 2.08 | 1.36 | 1.63 | 0.316667 | 1.69 |
| SLC22A1 | 29.939 | 4.903966 | 0.000994 | 0.011235 | yes | up | 0 | 0 | 0.02 | 0.2 | 0.32 | 0.18 | 0.006667 | 0.233333 |
| ZFAT | 2.316 | 1.211861 | 0.001 | 0.011294 | yes | up | 1.19 | 1.2 | 0.75 | 2.38 | 1.52 | 2.64 | 1.046667 | 2.18 |
| EXD1 | 2.178 | 1.122834 | 0.00102 | 0.011478 | yes | up | 1.59 | 1.57 | 0.89 | 2.39 | 2.47 | 4.09 | 1.35 | 2.983333 |
| HOXB8 | 0.033 | -4.91067 | 0.001037 | 0.011623 | yes | down | 0.4 | 0.34 | 0.19 | 0 | 0.03 | 0 | 0.31 | 0.01 |
| BATF2 | 0.496 | -1.01121 | 0.001047 | 0.011731 | yes | down | 2.3 | 2.2 | 1.96 | 1.25 | 1.11 | 0.95 | 2.153333 | 1.103333 |
| TMEM26 | 4.16 | 2.056592 | 0.001071 | 0.011934 | yes | up | 0.22 | 0.27 | 0.43 | 2.43 | 0.62 | 0.89 | 0.306667 | 1.313333 |
| LCN2 | 0.094 | -3.41389 | 0.001102 | 0.012232 | yes | down | 0.72 | 1.16 | 1.86 | 0.16 | 0.23 | 0 | 1.246667 | 0.13 |
| MAB21L1 | 2.832 | 1.501796 | 0.001113 | 0.012337 | yes | up | 0.4 | 0.4 | 0.5 | 0.72 | 1.27 | 1.9 | 0.433333 | 1.296667 |
| IL7R | 8.102 | 3.018354 | 0.001126 | 0.012457 | yes | up | 0.03 | 0.05 | 0.01 | 0.36 | 0.2 | 0.12 | 0.03 | 0.226667 |
| AGT | 6.371 | 2.671512 | 0.001127 | 0.012457 | yes | up | 0.02 | 0.18 | 0.04 | 0.39 | 0.52 | 0.71 | 0.08 | 0.54 |
| T | 0.019 | -5.74113 | 0.001129 | 0.012458 | yes | down | 0.82 | 0.18 | 0.1 | 0 | 0 | 0 | 0.366667 | 0 |
| SERPINB7 | 2.483 | 1.312367 | 0.001156 | 0.012711 | yes | up | 0.68 | 0.84 | 0.68 | 2.68 | 1.26 | 1.63 | 0.733333 | 1.856667 |
| LOC103346593 | 0.423 | -1.24277 | 0.001165 | 0.012793 | yes | down | 19.32 | 25.73 | 21.63 | 9.2 | 16.33 | 4.38 | 22.22667 | 9.97 |
| USP35 | 0.323 | -1.6315 | 0.001178 | 0.012911 | yes | down | 0.97 | 0.5 | 0.72 | 0.11 | 0.34 | 0.3 | 0.73 | 0.25 |
| LOC103351245 | 8.46 | 3.080608 | 0.001181 | 0.012926 | yes | up | 0.01 | 0.03 | 0.02 | 0.12 | 0.27 | 0.09 | 0.02 | 0.16 |
| CX3CL1 | 2.633 | 1.396631 | 0.001208 | 0.013156 | yes | up | 0.22 | 0.48 | 0.32 | 0.83 | 1.21 | 0.8 | 0.34 | 0.946667 |
| GRO-B | 27.227 | 4.766974 | 0.001209 | 0.013156 | yes | up | 1.24 | 0.86 | 0 | 30.14 | 11.53 | 4.84 | 0.7 | 15.50333 |
| KLHL25 | 2.054 | 1.038396 | 0.001218 | 0.013188 | yes | up | 2.81 | 3.11 | 1.48 | 3.19 | 5.9 | 6.53 | 2.466667 | 5.206667 |
| PALB2 | 2.058 | 1.041451 | 0.001224 | 0.013241 | yes | up | 0.59 | 0.58 | 0.61 | 1.15 | 1.27 | 1.25 | 0.593333 | 1.223333 |
| RET | 3.572 | 1.836589 | 0.001241 | 0.013364 | yes | up | 0.56 | 0.1 | 0.17 | 0.99 | 1.16 | 0.84 | 0.276667 | 0.996667 |
| LOC100345202 | 2.622 | 1.3907 | 0.001243 | 0.013375 | yes | up | 3.7 | 1.67 | 3.86 | 6.19 | 7 | 12.31 | 3.076667 | 8.5 |
| HHEX | 0.451 | -1.14946 | 0.001247 | 0.013399 | yes | down | 3.69 | 3.53 | 5.12 | 2.29 | 1.67 | 1.86 | 4.113333 | 1.94 |
| CATSPER2 | 4.536 | 2.181417 | 0.00127 | 0.013571 | yes | up | 0.32 | 0.12 | 0.08 | 0.56 | 0.47 | 0.79 | 0.173333 | 0.606667 |
| LOC103347584 | 11.717 | 3.550552 | 0.00127 | 0.013571 | yes | up | 0 | 0.49 | 1.02 | 5.81 | 3.17 | 7.23 | 0.503333 | 5.403333 |
| LOC103346060 | 0.27 | -1.89119 | 0.001273 | 0.013585 | yes | down | 0.57 | 0.68 | 0.35 | 0.12 | 0.12 | 0.2 | 0.533333 | 0.146667 |
| ATP1B1 | 0.442 | -1.17632 | 0.001286 | 0.013688 | yes | down | 54.53 | 54.87 | 72.52 | 34.48 | 36.71 | 13.78 | 60.64 | 28.32333 |
| PRC1 | 0.382 | -1.38897 | 0.001301 | 0.013839 | yes | down | 2.72 | 2.38 | 1.18 | 0.88 | 1.15 | 0.42 | 2.093333 | 0.816667 |
| CDKN1A | 2.225 | 1.153819 | 0.001307 | 0.01388 | yes | up | 8.34 | 9.09 | 7.07 | 28.05 | 16.31 | 10.82 | 8.166667 | 18.39333 |
| KIF20B | 0.394 | -1.34277 | 0.001337 | 0.01415 | yes | down | 10.71 | 11.86 | 29.26 | 5.94 | 8.36 | 7.2 | 17.27667 | 7.166667 |
| PAQR9 | 3.321 | 1.731539 | 0.001357 | 0.014328 | yes | up | 0.21 | 0.07 | 0.11 | 0.29 | 0.4 | 0.65 | 0.13 | 0.446667 |
| LOC108176082 | 3.408 | 1.768794 | 0.001368 | 0.014424 | yes | up | 0.46 | 0.62 | 0.34 | 2.5 | 1.37 | 1 | 0.473333 | 1.623333 |
| LOC108177244 | 0.297 | -1.75232 | 0.001378 | 0.014492 | yes | down | 1.92 | 1.91 | 1.73 | 0.43 | 0.77 | 0.51 | 1.853333 | 0.57 |
| HYI | 3.312 | 1.727697 | 0.001385 | 0.014556 | yes | up | 2.09 | 2.34 | 2.1 | 7.35 | 15.02 | 1.99 | 2.176667 | 8.12 |
| TIPARP | 2.499 | 1.321367 | 0.001395 | 0.014625 | yes | up | 4.05 | 5.33 | 4.03 | 19.08 | 7.29 | 7.24 | 4.47 | 11.20333 |
| LOC103348967 | 3.426 | 1.776348 | 0.001404 | 0.014706 | yes | up | 0.36 | 0.41 | 0.19 | 0.86 | 0.91 | 1.61 | 0.32 | 1.126667 |
| TYMS | 0.486 | -1.04161 | 0.001417 | 0.014784 | yes | down | 2.85 | 2.88 | 2.46 | 1.15 | 1.37 | 1.58 | 2.73 | 1.366667 |
| CXCL10 | 6.278 | 2.650247 | 0.001417 | 0.014784 | yes | up | 0.5 | 0.34 | 0.11 | 2.67 | 2.84 | 0.49 | 0.316667 | 2 |
| KIAA0101 | 0.25 | -1.99996 | 0.001417 | 0.014784 | yes | down | 3.63 | 2.26 | 1.66 | 0.87 | 0.99 | 0.34 | 2.516667 | 0.733333 |
| LOC100349447 | 2.667 | 1.415332 | 0.001423 | 0.014833 | yes | up | 27.26 | 52.85 | 62.31 | 202.39 | 127.9 | 66.68 | 47.47333 | 132.3233 |
| ASB18 | 2.654 | 1.407898 | 0.001431 | 0.014887 | yes | up | 0.67 | 0.73 | 0.93 | 1.72 | 1.83 | 2.29 | 0.776667 | 1.946667 |
| LOC108176024 | 2.111 | 1.077817 | 0.001464 | 0.015177 | yes | up | 13.9 | 6.21 | 9.98 | 18.64 | 21.06 | 29.82 | 10.03 | 23.17333 |
| LOC108176252 | 3.725 | 1.897267 | 0.00149 | 0.015378 | yes | up | 0.57 | 0.87 | 0.49 | 2.37 | 1.94 | 3.07 | 0.643333 | 2.46 |
| PRRT4 | 28.275 | 4.821479 | 0.001491 | 0.015385 | yes | up | 0.05 | 0 | 0 | 0.05 | 0.34 | 0.79 | 0.016667 | 0.393333 |
| CCNL1 | 2.447 | 1.290864 | 0.00153 | 0.015623 | yes | up | 14.92 | 28.8 | 12.45 | 77.34 | 38.98 | 34.2 | 18.72333 | 50.17333 |
| SPAG5 | 0.293 | -1.76998 | 0.001558 | 0.015889 | yes | down | 0.79 | 0.68 | 0.31 | 0.18 | 0.25 | 0.09 | 0.593333 | 0.173333 |
| SH2D4A | 2.368 | 1.243754 | 0.001566 | 0.015954 | yes | up | 0.63 | 0.53 | 0.37 | 1.05 | 1.43 | 1.13 | 0.51 | 1.203333 |
| BAG3 | 2.236 | 1.160824 | 0.001585 | 0.016135 | yes | up | 26.75 | 15.41 | 7.33 | 42.44 | 40.03 | 27.98 | 16.49667 | 36.81667 |
| CEACAM1 | 0.201 | -2.31522 | 0.001633 | 0.016501 | yes | down | 0.22 | 2.6 | 2.53 | 0.33 | 0.5 | 0.34 | 1.783333 | 0.39 |
| SAMD13 | 0.341 | -1.55328 | 0.001633 | 0.016501 | yes | down | 4.87 | 4.55 | 2.99 | 2.98 | 1.71 | 3.61 | 4.136667 | 2.766667 |
| ABCA8 | 2.169 | 1.117001 | 0.001648 | 0.01661 | yes | up | 9.58 | 4.59 | 2.89 | 12.42 | 10.62 | 13.76 | 5.686667 | 12.26667 |
| MDFI | 0.311 | -1.68678 | 0.001652 | 0.01662 | yes | down | 2.2 | 2.11 | 3.16 | 0.6 | 0.92 | 1.34 | 2.49 | 0.953333 |
| LOC100343081 | 2.401 | 1.263887 | 0.001654 | 0.01662 | yes | up | 1.32 | 2.69 | 2.69 | 5.57 | 5.21 | 6.46 | 2.233333 | 5.746667 |
| RCOR2 | 2.325 | 1.217414 | 0.001707 | 0.017057 | yes | up | 7.82 | 6.96 | 2.27 | 11.33 | 11.58 | 16.62 | 5.683333 | 13.17667 |
| LOC100339314 | 0.482 | -1.05265 | 0.001707 | 0.017057 | yes | down | 412.07 | 1020.88 | 377.96 | 288.56 | 332.91 | 267.04 | 603.6367 | 296.17 |
| PTS | 0.49 | -1.02961 | 0.001718 | 0.017131 | yes | down | 2.79 | 4.78 | 2.49 | 1.67 | 1.41 | 1.35 | 3.353333 | 1.476667 |
| ALDH1A2 | 2.071 | 1.050082 | 0.001735 | 0.017257 | yes | up | 3.01 | 2.2 | 1.77 | 3.7 | 8.15 | 3.34 | 2.326667 | 5.063333 |
| LOC100352190 | 0.248 | -2.01377 | 0.001735 | 0.017257 | yes | down | 1.7 | 2.92 | 4.53 | 0.64 | 0.46 | 1.27 | 3.05 | 0.79 |
| LOC103347358 | 39.232 | 5.293952 | 0.001742 | 0.017305 | yes | up | 0 | 0 | 0 | 0.35 | 0.23 | 0.41 | 0 | 0.33 |
| RNF24 | 2.373 | 1.246807 | 0.001753 | 0.017379 | yes | up | 2.87 | 3.9 | 1.2 | 4.24 | 6.84 | 7.88 | 2.656667 | 6.32 |
| P4HA3 | 0.328 | -1.60711 | 0.001827 | 0.017942 | yes | down | 2.55 | 1.41 | 1.21 | 0.24 | 0.67 | 0.84 | 1.723333 | 0.583333 |
| GOT1L1 | 0.122 | -3.03907 | 0.00185 | 0.018118 | yes | down | 0.43 | 0.7 | 0.62 | 0.05 | 0.09 | 0.08 | 0.583333 | 0.073333 |
| OAS1 | 0.464 | -1.10907 | 0.001853 | 0.018135 | yes | down | 5.13 | 5.57 | 4.25 | 3.01 | 1.58 | 2.6 | 4.983333 | 2.396667 |
| LOC103349875 | 3.639 | 1.863449 | 0.001897 | 0.018454 | yes | up | 0.22 | 0.23 | 0.22 | 0.7 | 0.65 | 1.2 | 0.223333 | 0.85 |
| LOC108177032 | 2.311 | 1.20855 | 0.001901 | 0.01849 | yes | up | 12.58 | 10.95 | 10.86 | 20.72 | 38.68 | 32.25 | 11.46333 | 30.55 |
| LOC103348798 | 8.199 | 3.035411 | 0.002002 | 0.019293 | yes | up | 0.04 | 0.02 | 0.08 | 0.11 | 0.38 | 0.72 | 0.046667 | 0.403333 |
| SSTR4 | 0.106 | -3.23354 | 0.002004 | 0.019298 | yes | down | 0.25 | 0.44 | 0.28 | 0.02 | 0.09 | 0 | 0.323333 | 0.036667 |
| LOC100347782 | 0.129 | -2.95024 | 0.002017 | 0.019404 | yes | down | 3.13 | 4.31 | 6.75 | 0 | 1.57 | 0.34 | 4.73 | 0.636667 |
| PTGIR | 2.556 | 1.353652 | 0.002021 | 0.019422 | yes | up | 0.34 | 0.37 | 0.17 | 0.86 | 0.62 | 0.79 | 0.293333 | 0.756667 |
| TMEM254 | 0.453 | -1.14265 | 0.002056 | 0.019636 | yes | down | 2.16 | 2.29 | 2.7 | 1.09 | 0.83 | 3.52 | 2.383333 | 1.813333 |
| PURG | 0.463 | -1.10959 | 0.002071 | 0.019752 | yes | down | 2.65 | 2.34 | 2.23 | 0.84 | 1.58 | 1.1 | 2.406667 | 1.173333 |
| DIS3L | 2.656 | 1.409374 | 0.002103 | 0.020007 | yes | up | 0.41 | 0.27 | 0.51 | 0.92 | 0.88 | 1.51 | 0.396667 | 1.103333 |
| KIF23 | 0.265 | -1.9175 | 0.002132 | 0.020206 | yes | down | 1.43 | 0.97 | 0.43 | 0.25 | 0.48 | 0.09 | 0.943333 | 0.273333 |
| SPZ1 | 2.613 | 1.385454 | 0.002165 | 0.020465 | yes | up | 0.75 | 0.53 | 0.62 | 1.45 | 1.68 | 2.16 | 0.633333 | 1.763333 |
| RORC | 4.006 | 2.002278 | 0.002226 | 0.020953 | yes | up | 0.14 | 0.7 | 0.49 | 3.26 | 1.5 | 2.16 | 0.443333 | 2.306667 |
| LOC100351785 | 0.324 | -1.62398 | 0.00227 | 0.021234 | yes | down | 3.36 | 1.01 | 2.44 | 0.36 | 1.06 | 0.92 | 2.27 | 0.78 |
| LOC103346935 | 44.976 | 5.491098 | 0.002276 | 0.021246 | yes | up | 0 | 0 | 0 | 0.53 | 0.09 | 0.29 | 0 | 0.303333 |
| TRPV4 | 3.236 | 1.694178 | 0.002277 | 0.021246 | yes | up | 0.16 | 0.53 | 0.34 | 1.49 | 1.25 | 0.73 | 0.343333 | 1.156667 |
| SLC16A11 | 0.254 | -1.97538 | 0.002283 | 0.021277 | yes | down | 1.59 | 2.81 | 0.57 | 0.32 | 0.34 | 0.61 | 1.656667 | 0.423333 |
| TMEM45B | 5.927 | 2.567385 | 0.002284 | 0.021277 | yes | up | 0.04 | 0.54 | 0.19 | 1.78 | 0.57 | 2.21 | 0.256667 | 1.52 |
| TRPC3 | 0.257 | -1.96194 | 0.002286 | 0.021277 | yes | down | 0.46 | 0.56 | 0.26 | 0.12 | 0.19 | 0.04 | 0.426667 | 0.116667 |
| PGER6 | 0.162 | -2.62267 | 0.002293 | 0.021321 | yes | down | 21.23 | 51.68 | 30.89 | 1.83 | 13.87 | 2.8 | 34.6 | 6.166667 |
| ABCA9 | 3.312 | 1.727617 | 0.002312 | 0.021457 | yes | up | 0.67 | 0.22 | 0.1 | 0.85 | 1.09 | 2.7 | 0.33 | 1.546667 |
| LOC108176045 | 4.79 | 2.259979 | 0.002313 | 0.021457 | yes | up | 0.04 | 0.04 | 0.06 | 0.21 | 0.16 | 0.33 | 0.046667 | 0.233333 |
| BUB1B | 0.273 | -1.87565 | 0.002355 | 0.0218 | yes | down | 1.23 | 0.82 | 0.29 | 0.12 | 0.34 | 0.14 | 0.78 | 0.2 |
| SPRY4 | 7.042 | 2.816062 | 0.002358 | 0.021803 | yes | up | 3.82 | 5.91 | 1.83 | 55.87 | 14.64 | 7.68 | 3.853333 | 26.06333 |
| SLC36A3 | 3.033 | 1.600564 | 0.0024 | 0.022099 | yes | up | 0.31 | 0.3 | 0.25 | 0.93 | 0.83 | 0.88 | 0.286667 | 0.88 |
| FLVCR1 | 2.062 | 1.044121 | 0.002443 | 0.022411 | yes | up | 19.5 | 1.67 | 0.57 | 2.05 | 2.76 | 3.81 | 7.246667 | 2.873333 |
| SPAG16 | 2.718 | 1.442479 | 0.00246 | 0.022511 | yes | up | 1.12 | 1.1 | 1.05 | 3.8 | 1.63 | 3.04 | 1.09 | 2.823333 |
| LOC108178230 | 0.056 | -4.17072 | 0.002465 | 0.022524 | yes | down | 0.55 | 0.33 | 0.13 | 0 | 0.03 | 0.03 | 0.336667 | 0.02 |
| RAD51B | 3.048 | 1.607735 | 0.002474 | 0.022535 | yes | up | 5.49 | 4.68 | 1.48 | 12.64 | 12.51 | 3.21 | 3.883333 | 9.453333 |
| ACOX2 | 2.042 | 1.030294 | 0.002476 | 0.022539 | yes | up | 3.29 | 6.73 | 3.8 | 7.93 | 7.89 | 13.15 | 4.606667 | 9.656667 |
| TSLP | 2.981 | 1.575926 | 0.002479 | 0.022554 | yes | up | 2.03 | 1.99 | 0.4 | 4.32 | 4.35 | 4.41 | 1.473333 | 4.36 |
| LOC103348240 | 10.075 | 3.332671 | 0.002481 | 0.022558 | yes | up | 0 | 0.99 | 0.34 | 0.48 | 5.12 | 8.27 | 0.443333 | 4.623333 |
| CCDC86 | 2.412 | 1.270185 | 0.002487 | 0.022601 | yes | up | 4.42 | 5.3 | 2.44 | 14.66 | 9.29 | 5.34 | 4.053333 | 9.763333 |
| FGFR2 | 2.418 | 1.27394 | 0.002495 | 0.022631 | yes | up | 0.27 | 0.41 | 0.16 | 0.58 | 0.86 | 0.67 | 0.28 | 0.703333 |
| ARID5A | 2.475 | 1.30769 | 0.002495 | 0.022631 | yes | up | 2.52 | 2.66 | 1.86 | 9.45 | 5.32 | 3.1 | 2.346667 | 5.956667 |
| REEP2 | 4.053 | 2.01899 | 0.002523 | 0.022874 | yes | up | 0.21 | 0.16 | 0.18 | 1 | 0.54 | 0.73 | 0.183333 | 0.756667 |
| ZBTB16 | 5.085 | 2.346306 | 0.002529 | 0.022907 | yes | up | 4.55 | 2.46 | 0.52 | 15.12 | 8.99 | 12.62 | 2.51 | 12.24333 |
| KIFC1 | 0.432 | -1.20938 | 0.002535 | 0.022939 | yes | down | 1.16 | 1.36 | 0.76 | 0.35 | 0.73 | 0.4 | 1.093333 | 0.493333 |
| CDC20 | 0.3 | -1.73699 | 0.002574 | 0.023208 | yes | down | 3.25 | 3.17 | 1.08 | 0.55 | 1.42 | 0.35 | 2.5 | 0.773333 |
| ATP5J | 0.427 | -1.22605 | 0.00261 | 0.023467 | yes | down | 377.38 | 154.6 | 100.51 | 80.92 | 128.42 | 58.54 | 210.83 | 89.29333 |
| KIF4A | 0.29 | -1.78566 | 0.002612 | 0.023467 | yes | down | 0.54 | 0.43 | 0.46 | 0.22 | 0.17 | 0.05 | 0.476667 | 0.146667 |
| LOC103352458 | 38.588 | 5.270068 | 0.002614 | 0.023479 | yes | up | 0 | 0 | 0 | 0.27 | 0.19 | 0.58 | 0 | 0.346667 |
| LOC100348165 | 2.403 | 1.264812 | 0.002654 | 0.023737 | yes | up | 2.1 | 1.33 | 1.02 | 4.31 | 2.68 | 2.8 | 1.483333 | 3.263333 |
| LOC100359206 | 0.018 | -5.76399 | 0.002694 | 0.024038 | yes | down | 0.05 | 0.29 | 0.01 | 0 | 0 | 0 | 0.116667 | 0 |
| LOC100347239 | 0.322 | -1.63611 | 0.002699 | 0.024071 | yes | down | 6.67 | 4.87 | 10.21 | 2.37 | 1.94 | 3.09 | 7.25 | 2.466667 |
| PMP22 | 2.2 | 1.13779 | 0.002714 | 0.024174 | yes | up | 72.87 | 42.49 | 18.13 | 59.69 | 104.65 | 112.26 | 44.49667 | 92.2 |
| IRF4 | 3.574 | 1.837603 | 0.002717 | 0.024189 | yes | up | 0.17 | 0.08 | 0.13 | 0.77 | 0.37 | 0.25 | 0.126667 | 0.463333 |
| CGREF1 | 0.199 | -2.32705 | 0.002734 | 0.024313 | yes | down | 1.34 | 1.16 | 0.7 | 0.15 | 0.29 | 0.26 | 1.066667 | 0.233333 |
| PMEPA1 | 2.569 | 1.361159 | 0.002746 | 0.024391 | yes | up | 10.24 | 10.42 | 3.39 | 17.91 | 27.98 | 21.58 | 8.016667 | 22.49 |
| CD247 | 2.704 | 1.435171 | 0.002764 | 0.024491 | yes | up | 1.75 | 2.3 | 2.21 | 4.54 | 3.1 | 7.99 | 2.086667 | 5.21 |
| KRBOX1 | 2.536 | 1.342741 | 0.002773 | 0.024558 | yes | up | 1.18 | 1.44 | 0.9 | 2.68 | 4 | 2.81 | 1.173333 | 3.163333 |
| LOC108175719 | 0.027 | -5.22085 | 0.002795 | 0.024704 | yes | down | 0.58 | 0.78 | 1.24 | 0 | 0 | 0 | 0.866667 | 0 |
| EMID1 | 0.301 | -1.72984 | 0.002809 | 0.024746 | yes | down | 1.76 | 4.16 | 3.65 | 0.66 | 0.84 | 1.18 | 3.19 | 0.893333 |
| LOC103349153 | 14.439 | 3.851899 | 0.00285 | 0.025052 | yes | up | 0.05 | 0.04 | 0.04 | 1.38 | 0.28 | 0.25 | 0.043333 | 0.636667 |
| TTK | 0.279 | -1.83948 | 0.002897 | 0.025377 | yes | down | 0.72 | 0.53 | 0.19 | 0.1 | 0.22 | 0.09 | 0.48 | 0.136667 |
| LOC100347254 | 3.604 | 1.849425 | 0.002918 | 0.025529 | yes | up | 0.64 | 0.63 | 0.12 | 2 | 1.86 | 1.12 | 0.463333 | 1.66 |
| LOC100358230 | 2.337 | 1.22473 | 0.002935 | 0.025611 | yes | up | 3.16 | 2.29 | 0.95 | 5.52 | 4.77 | 5.81 | 2.133333 | 5.366667 |
| SLC25A27 | 2.528 | 1.338121 | 0.002951 | 0.025715 | yes | up | 0.63 | 0.37 | 0.24 | 0.87 | 1.21 | 1.26 | 0.413333 | 1.113333 |
| ORM1 | 43.644 | 5.447699 | 0.002959 | 0.025722 | yes | up | 0 | 0 | 0 | 1.6 | 0.22 | 0.56 | 0 | 0.793333 |
| TRNAA-UGC | 2.366 | 1.24235 | 0.002982 | 0.02588 | yes | up | 0.4 | 0.41 | 0.59 | 1.03 | 1.4 | 0.98 | 0.466667 | 1.136667 |
| MCOLN3 | 0.393 | -1.34797 | 0.00301 | 0.026069 | yes | down | 0.7 | 0.98 | 0.43 | 0.17 | 0.22 | 0.14 | 0.703333 | 0.176667 |
| LOC108176104 | 3.523 | 1.816623 | 0.00302 | 0.026079 | yes | up | 0.9 | 1.39 | 0.54 | 1.16 | 5.38 | 3.85 | 0.943333 | 3.463333 |
| LOC100350949 | 0.003 | -8.34952 | 0.00302 | 0.026079 | yes | down | 531.34 | 0 | 491.72 | 2.1 | 1.1 | 0 | 341.02 | 1.066667 |
| LOC108175916 | 4.109 | 2.038869 | 0.003031 | 0.026145 | yes | up | 0.46 | 0.28 | 0.1 | 1.86 | 1.82 | 1.65 | 0.28 | 1.776667 |
| CENPF | 0.353 | -1.50414 | 0.003052 | 0.026311 | yes | down | 0.67 | 0.53 | 0.22 | 0.22 | 0.2 | 0.08 | 0.473333 | 0.166667 |
| LOC103349339 | 0.022 | -5.48802 | 0.00306 | 0.026351 | yes | down | 0.42 | 0.45 | 0.03 | 0 | 0 | 0 | 0.3 | 0 |
| LOC103350201 | 0.346 | -1.52964 | 0.00306 | 0.026351 | yes | down | 1.39 | 1.75 | 1.73 | 0.36 | 0.35 | 1.06 | 1.623333 | 0.59 |
| LOC100341883 | 0.341 | -1.55276 | 0.003076 | 0.026469 | yes | down | 0.83 | 0.66 | 0.44 | 0.18 | 0.24 | 0.24 | 0.643333 | 0.22 |
| SLITRK6 | 0.484 | -1.04763 | 0.003082 | 0.026492 | yes | down | 0.99 | 1.51 | 1.12 | 0.41 | 0.62 | 0.8 | 1.206667 | 0.61 |
| AQP8 | 0.25 | -1.99847 | 0.00309 | 0.026544 | yes | down | 4.88 | 2.08 | 1.02 | 0.44 | 1.33 | 0.28 | 2.66 | 0.683333 |
| KIAA1211 | 0.42 | -1.25053 | 0.003122 | 0.026774 | yes | down | 4.74 | 5.97 | 1.61 | 1.54 | 1.24 | 2.44 | 4.106667 | 1.74 |
| VIPR2 | 0.307 | -1.70268 | 0.003161 | 0.027036 | yes | down | 0.98 | 0.83 | 0.38 | 0.2 | 0.39 | 0.1 | 0.73 | 0.23 |
| CCNB2 | 0.29 | -1.78698 | 0.003189 | 0.027216 | yes | down | 3.33 | 2.96 | 1.08 | 0.64 | 1.29 | 0.26 | 2.456667 | 0.73 |
| DUSP6 | 2.154 | 1.106891 | 0.003201 | 0.027297 | yes | up | 16.03 | 18.83 | 12.92 | 54.69 | 31.22 | 18.57 | 15.92667 | 34.82667 |
| ZMYND12 | 3.306 | 1.724953 | 0.003211 | 0.027374 | yes | up | 0.34 | 0.41 | 0.44 | 1.52 | 1.15 | 1.44 | 0.396667 | 1.37 |
| TRIM36 | 0.339 | -1.55876 | 0.003253 | 0.027671 | yes | down | 1.5 | 2.53 | 2.89 | 0.98 | 0.29 | 1.07 | 2.306667 | 0.78 |
| TDO2 | 0.028 | -5.14832 | 0.003266 | 0.027717 | yes | down | 0.18 | 0.17 | 0.29 | 0 | 0 | 0 | 0.213333 | 0 |
| KRT23 | 0.19 | -2.39941 | 0.003333 | 0.028188 | yes | down | 35.96 | 118.79 | 25.03 | 13.82 | 4.3 | 14.03 | 59.92667 | 10.71667 |
| LOC100340939 | 0.261 | -1.93557 | 0.003354 | 0.028296 | yes | down | 1.41 | 1.52 | 0.44 | 0.3 | 0.37 | 0.22 | 1.123333 | 0.296667 |
| FAM228B | 34.168 | 5.094562 | 0.003368 | 0.028359 | yes | up | 0 | 0 | 0 | 0.36 | 0.35 | 0.22 | 0 | 0.31 |
| LOC100343913 | 10.093 | 3.335239 | 0.003397 | 0.028571 | yes | up | 0.38 | 0 | 0.08 | 1.89 | 0.55 | 2.07 | 0.153333 | 1.503333 |
| NGB | 0.132 | -2.9218 | 0.003441 | 0.028881 | yes | down | 0.47 | 0.4 | 0.47 | 0.1 | 0.03 | 0.06 | 0.446667 | 0.063333 |
| BCAT1 | 0.066 | -3.92911 | 0.003494 | 0.029262 | yes | down | 0.37 | 0.71 | 0.45 | 0.15 | 0.01 | 0 | 0.51 | 0.053333 |
| RNF212 | 2.436 | 1.284483 | 0.003503 | 0.029306 | yes | up | 1.25 | 1.82 | 1.55 | 3.06 | 3.08 | 5.51 | 1.54 | 3.883333 |
| ARHGAP11A | 0.292 | -1.77427 | 0.003519 | 0.029418 | yes | down | 0.69 | 0.58 | 0.16 | 0.16 | 0.18 | 0.08 | 0.476667 | 0.14 |
| ADAMTS1 | 4.589 | 2.198072 | 0.003544 | 0.029584 | yes | up | 7.05 | 6.73 | 7.57 | 65.09 | 19.19 | 12.56 | 7.116667 | 32.28 |
| MYOC | 3.968 | 1.988473 | 0.00362 | 0.030086 | yes | up | 2.35 | 0.7 | 0.21 | 6.49 | 3.44 | 2.48 | 1.086667 | 4.136667 |
| GABRA2 | 2.546 | 1.348165 | 0.003684 | 0.030499 | yes | up | 9.4 | 10.22 | 6.69 | 18.73 | 33.53 | 19.06 | 8.77 | 23.77333 |
| SAP30 | 0.474 | -1.07585 | 0.003686 | 0.030499 | yes | down | 3.7 | 3.05 | 5.12 | 2.03 | 2.19 | 1.75 | 3.956667 | 1.99 |
| SYP | 0.238 | -2.06965 | 0.003691 | 0.030516 | yes | down | 0.41 | 0.81 | 0.71 | 0.22 | 0.09 | 0.17 | 0.643333 | 0.16 |
| NTNG2 | 2.148 | 1.103143 | 0.003694 | 0.030516 | yes | up | 0.74 | 0.76 | 0.46 | 1.39 | 1.67 | 1.25 | 0.653333 | 1.436667 |
| LOC103347471 | 2.155 | 1.107398 | 0.00372 | 0.030649 | yes | up | 53.67 | 12.84 | 7.23 | 38.64 | 63.49 | 32.06 | 24.58 | 44.73 |
| PBK | 0.245 | -2.03075 | 0.003774 | 0.03101 | yes | down | 0.97 | 0.61 | 0.33 | 0.19 | 0.16 | 0.14 | 0.636667 | 0.163333 |
| LOC100348995 | 0.377 | -1.40917 | 0.003787 | 0.031067 | yes | down | 2.53 | 3.76 | 1.47 | 1.16 | 1.27 | 1.01 | 2.586667 | 1.146667 |
| LOC100350502 | 2.528 | 1.338112 | 0.003849 | 0.031443 | yes | up | 0.49 | 0.48 | 0.32 | 1.15 | 0.92 | 1.23 | 0.43 | 1.1 |
| LOC100351061 | 23.264 | 4.540047 | 0.003902 | 0.031723 | yes | up | 0.03 | 0 | 0 | 0.16 | 0.22 | 0.34 | 0.01 | 0.24 |
| SLC46A3 | 0.371 | -1.43104 | 0.003907 | 0.031741 | yes | down | 0.74 | 0.94 | 1.57 | 0.48 | 0.35 | 0.44 | 1.083333 | 0.423333 |
| BPI | 0.06 | -4.05067 | 0.003925 | 0.031829 | yes | down | 0.23 | 0.52 | 0.42 | 0.07 | 0 | 0 | 0.39 | 0.023333 |
| SERPINA12 | 0.172 | -2.53791 | 0.00393 | 0.03184 | yes | down | 0.41 | 0.57 | 0.38 | 0.07 | 0.11 | 0.06 | 0.453333 | 0.08 |
| LOC100338650 | 0.035 | -4.8377 | 0.003932 | 0.03184 | yes | down | 0.09 | 0.84 | 0.3 | 0.04 | 0 | 0 | 0.41 | 0.013333 |
| ABHD1 | 0.042 | -4.58742 | 0.003933 | 0.03184 | yes | down | 0.28 | 0.26 | 0.53 | 0 | 0.05 | 0 | 0.356667 | 0.016667 |
| TMEM198 | 3.172 | 1.66559 | 0.003939 | 0.03187 | yes | up | 0.6 | 0.78 | 0.21 | 1.4 | 1.57 | 2.67 | 0.53 | 1.88 |
| CPN2 | 5.196 | 2.377493 | 0.003966 | 0.032072 | yes | up | 0.02 | 0.09 | 0.12 | 0.64 | 0.27 | 0.37 | 0.076667 | 0.426667 |
| NTRK2 | 2.68 | 1.422401 | 0.003976 | 0.032121 | yes | up | 0.8 | 0.99 | 0.15 | 1.4 | 1.75 | 2.07 | 0.646667 | 1.74 |
| SLC6A1 | 5.675 | 2.504605 | 0.003998 | 0.032196 | yes | up | 0.14 | 0.03 | 0.02 | 0.21 | 0.36 | 0.5 | 0.063333 | 0.356667 |
| CKAP2L | 0.27 | -1.88879 | 0.003998 | 0.032196 | yes | down | 1.17 | 0.64 | 0.51 | 0.17 | 0.43 | 0.06 | 0.773333 | 0.22 |
| HHIPL2 | 0.114 | -3.13403 | 0.003998 | 0.032196 | yes | down | 0.23 | 0.25 | 0.2 | 0.04 | 0 | 0.04 | 0.226667 | 0.026667 |
| CASP3 | 0.397 | -1.33136 | 0.004044 | 0.032395 | yes | down | 2.71 | 1.86 | 1.04 | 0.62 | 0.8 | 0.89 | 1.87 | 0.77 |
| ITPKA | 0.094 | -3.41437 | 0.004088 | 0.032693 | yes | down | 0.42 | 0.39 | 0.68 | 0.05 | 0.05 | 0.05 | 0.496667 | 0.05 |
| ANLN | 0.266 | -1.90876 | 0.004101 | 0.032778 | yes | down | 1.17 | 0.64 | 0.39 | 0.18 | 0.37 | 0.06 | 0.733333 | 0.203333 |
| LOC100358870 | 0.451 | -1.14874 | 0.004112 | 0.032836 | yes | down | 3.46 | 2.6 | 3.8 | 1.12 | 1.76 | 1.93 | 3.286667 | 1.603333 |
| LOC100352023 | 3.679 | 1.879211 | 0.004163 | 0.033133 | yes | up | 0.18 | 0.21 | 0.07 | 0.57 | 0.61 | 0.52 | 0.153333 | 0.566667 |
| WNT5B | 2.405 | 1.265749 | 0.004181 | 0.033232 | yes | up | 4.32 | 2.39 | 1.2 | 5.9 | 4.97 | 8.46 | 2.636667 | 6.443333 |
| LOC100354425 | 7.612 | 2.928321 | 0.004249 | 0.033683 | yes | up | 0 | 0.1 | 0.08 | 0.78 | 0.25 | 0.49 | 0.06 | 0.506667 |
| ANKEF1 | 2.826 | 1.498802 | 0.004285 | 0.033951 | yes | up | 0.31 | 0.19 | 0.39 | 1.06 | 0.64 | 0.92 | 0.296667 | 0.873333 |
| TNK2 | 2.025 | 1.018271 | 0.004287 | 0.033951 | yes | up | 10.45 | 11 | 3.82 | 10.38 | 21.45 | 20.96 | 8.423333 | 17.59667 |
| LOC103349385 | 8.779 | 3.133983 | 0.004297 | 0.033995 | yes | up | 0.22 | 0.13 | 0.09 | 1.12 | 2 | 0.67 | 0.146667 | 1.263333 |
| NOCT | 2.795 | 1.482828 | 0.004297 | 0.033995 | yes | up | 3.53 | 10.81 | 2.19 | 22.35 | 12.28 | 10.1 | 5.51 | 14.91 |
| UPP1 | 0.398 | -1.32769 | 0.004319 | 0.034108 | yes | down | 7.13 | 3.59 | 3.06 | 1.39 | 3.13 | 1.12 | 4.593333 | 1.88 |
| CTSE | 0.482 | -1.0535 | 0.004321 | 0.034108 | yes | down | 4.84 | 11.23 | 10.41 | 4.53 | 4.35 | 4.47 | 8.826667 | 4.45 |
| BUB1 | 0.303 | -1.72125 | 0.004359 | 0.034289 | yes | down | 0.6 | 0.55 | 0.22 | 0.14 | 0.18 | 0.11 | 0.456667 | 0.143333 |
| FGD4 | 2.224 | 1.153164 | 0.004382 | 0.03443 | yes | up | 1.46 | 1.03 | 0.62 | 3.06 | 1.62 | 1.67 | 1.036667 | 2.116667 |
| LOC100345657 | 0.361 | -1.46959 | 0.004388 | 0.03446 | yes | down | 0.44 | 0.31 | 0.19 | 0.08 | 0.15 | 0.12 | 0.313333 | 0.116667 |
| CORO1A | 0.492 | -1.02406 | 0.004397 | 0.034511 | yes | down | 2.3 | 2.84 | 2.48 | 1.14 | 1.29 | 1.46 | 2.54 | 1.296667 |
| LOC100009135 | 0.041 | -4.61876 | 0.004477 | 0.034994 | yes | down | 0.24 | 8.56 | 2.37 | 0.47 | 0 | 0 | 3.723333 | 0.156667 |
| DPEP1 | 0.264 | -1.91933 | 0.004484 | 0.035035 | yes | down | 0.7 | 1.03 | 0.63 | 0.19 | 0.13 | 0.35 | 0.786667 | 0.223333 |
| RGAG1 | 0.374 | -1.41784 | 0.004521 | 0.035267 | yes | down | 0.88 | 1.12 | 0.3 | 0.21 | 0.41 | 0.26 | 0.766667 | 0.293333 |
| WDHD1 | 0.412 | -1.27788 | 0.004552 | 0.035438 | yes | down | 1.11 | 0.56 | 0.6 | 0.23 | 0.44 | 0.31 | 0.756667 | 0.326667 |
| PCYT1B | 0.371 | -1.43189 | 0.00458 | 0.035599 | yes | down | 0.29 | 0.34 | 0.3 | 0.1 | 0.15 | 0.11 | 0.31 | 0.12 |
| DUSP1 | 6.562 | 2.714237 | 0.004616 | 0.035793 | yes | up | 16.19 | 10.56 | 11.45 | 189.67 | 34.67 | 22.69 | 12.73333 | 82.34333 |
| NDUFA13 | 2.49 | 1.316023 | 0.004703 | 0.036316 | yes | up | 2.11 | 2.76 | 2.95 | 6.99 | 6.19 | 7.05 | 2.606667 | 6.743333 |
| LOC108178005 | 3.347 | 1.742895 | 0.004716 | 0.036373 | yes | up | 0.07 | 0.06 | 0.1 | 0.28 | 0.2 | 0.29 | 0.076667 | 0.256667 |
| RAB40B | 0.085 | -3.55668 | 0.004717 | 0.036373 | yes | down | 0.3 | 0.7 | 1.61 | 0 | 0.27 | 0 | 0.87 | 0.09 |
| FA2H | 2.976 | 1.573274 | 0.004737 | 0.036506 | yes | up | 0.73 | 0.31 | 0.24 | 0.75 | 1.31 | 1.84 | 0.426667 | 1.3 |
| METTL7B | 31.858 | 4.993571 | 0.00476 | 0.036666 | yes | up | 0 | 0 | 0 | 0.2 | 0.2 | 0.29 | 0 | 0.23 |
| SGK2 | 14.454 | 3.853369 | 0.004782 | 0.036776 | yes | up | 0.01 | 0.01 | 0 | 0.12 | 0.09 | 0.13 | 0.006667 | 0.113333 |
| GAREM2 | 0.34 | -1.55629 | 0.004817 | 0.036957 | yes | down | 0.36 | 1.08 | 0.81 | 0.18 | 0.33 | 0.3 | 0.75 | 0.27 |
| LOC103348954 | 0.017 | -5.83742 | 0.004852 | 0.037133 | yes | down | 0 | 0.65 | 0.66 | 0 | 0 | 0 | 0.436667 | 0 |
| RACGAP1 | 0.463 | -1.11024 | 0.004855 | 0.037133 | yes | down | 2.23 | 1.93 | 1.11 | 0.67 | 1.31 | 0.55 | 1.756667 | 0.843333 |
| LOC103350198 | 35.901 | 5.165933 | 0.004906 | 0.037452 | yes | up | 0 | 0 | 0 | 0.28 | 0.08 | 0.39 | 0 | 0.25 |
| LOC100343655 | 0.026 | -5.28916 | 0.004916 | 0.037487 | yes | down | 0.81 | 0.1 | 0.19 | 0 | 0 | 0 | 0.366667 | 0 |
| NEIL3 | 0.197 | -2.34517 | 0.004953 | 0.0377 | yes | down | 0.62 | 0.44 | 0.29 | 0.14 | 0.11 | 0.02 | 0.45 | 0.09 |
| ADAMTSL1 | 2.272 | 1.184184 | 0.005125 | 0.038761 | yes | up | 4.44 | 1.05 | 2.97 | 7.68 | 3.87 | 6.84 | 2.82 | 6.13 |
| GPR88 | 4.199 | 2.070113 | 0.005175 | 0.039018 | yes | up | 0.04 | 0.05 | 0.07 | 0.28 | 0.18 | 0.22 | 0.053333 | 0.226667 |
| LOC103351553 | 5.124 | 2.357346 | 0.005175 | 0.039018 | yes | up | 0.12 | 0.09 | 0.05 | 0.56 | 0.29 | 0.44 | 0.086667 | 0.43 |
| LOC103346024 | 0.387 | -1.37019 | 0.005186 | 0.039061 | yes | down | 0.9 | 0.7 | 0.75 | 0.19 | 0.44 | 0.35 | 0.783333 | 0.326667 |
| BEST4 | 0.168 | -2.57085 | 0.005238 | 0.039394 | yes | down | 0.21 | 0.27 | 0.44 | 0.11 | 0.02 | 0.03 | 0.306667 | 0.053333 |
| PI16 | 0.346 | -1.53071 | 0.005328 | 0.039954 | yes | down | 16.29 | 13.65 | 14.1 | 5.34 | 8.59 | 2.09 | 14.68 | 5.34 |
| CRABP1 | 0.475 | -1.07433 | 0.005334 | 0.039978 | yes | down | 7.22 | 5.62 | 5.62 | 2.12 | 4.42 | 2.63 | 6.153333 | 3.056667 |
| SYT10 | 0.227 | -2.13997 | 0.005677 | 0.042095 | yes | down | 0.53 | 0.5 | 0.43 | 0 | 0.18 | 0.17 | 0.486667 | 0.116667 |
| ANGPT4 | 3.008 | 1.588751 | 0.005682 | 0.042109 | yes | up | 0.34 | 0.27 | 0.14 | 0.99 | 0.36 | 0.89 | 0.25 | 0.746667 |
| LOC103346374 | 10.044 | 3.328229 | 0.005722 | 0.042281 | yes | up | 0.08 | 0.06 | 0 | 0.44 | 0.14 | 0.61 | 0.046667 | 0.396667 |
| MRAP2 | 3.107 | 1.635314 | 0.005739 | 0.042371 | yes | up | 0.35 | 0.25 | 0.2 | 0.73 | 1.09 | 0.75 | 0.266667 | 0.856667 |
| LOC108178071 | 0.162 | -2.62944 | 0.005944 | 0.043441 | yes | down | 0.17 | 0.62 | 0.32 | 0.06 | 0.05 | 0.07 | 0.37 | 0.06 |
| MROH7 | 3.08 | 1.623033 | 0.005985 | 0.043638 | yes | up | 0.13 | 0.09 | 0.12 | 0.32 | 0.31 | 0.31 | 0.113333 | 0.313333 |
| KNSTRN | 0.425 | -1.23377 | 0.006019 | 0.043829 | yes | down | 2.31 | 2.12 | 1.22 | 0.68 | 1.19 | 0.62 | 1.883333 | 0.83 |
| POMC | 0.481 | -1.05579 | 0.00602 | 0.043829 | yes | down | 4.2 | 4.54 | 6.16 | 2.85 | 2.84 | 1.88 | 4.966667 | 2.523333 |
| ZIK1 | 0.459 | -1.12366 | 0.006046 | 0.043978 | yes | down | 0.62 | 1.08 | 0.71 | 0.41 | 0.4 | 0.35 | 0.803333 | 0.386667 |
| LOC108177034 | 0.234 | -2.09254 | 0.006228 | 0.044981 | yes | down | 0.71 | 0.59 | 1.16 | 0.28 | 0.17 | 0.08 | 0.82 | 0.176667 |
| RPS29 | 0.384 | -1.38034 | 0.006241 | 0.045053 | yes | down | 118.95 | 167.72 | 350.86 | 119.51 | 58.79 | 84.01 | 212.51 | 87.43667 |
| CAPS2 | 3.871 | 1.952562 | 0.006299 | 0.045381 | yes | up | 0.13 | 0.09 | 0.05 | 0.43 | 0.23 | 0.38 | 0.09 | 0.346667 |
| SLC2A12 | 2.985 | 1.577663 | 0.006308 | 0.04543 | yes | up | 0.6 | 0.14 | 0.44 | 0.74 | 1.14 | 1.79 | 0.393333 | 1.223333 |
| LOC103348709 | 35.976 | 5.168972 | 0.006367 | 0.04577 | yes | up | 0.02 | 0 | 0 | 0 | 0.56 | 0.21 | 0.006667 | 0.256667 |
| CDHR1 | 0.076 | -3.7127 | 0.006411 | 0.04595 | yes | down | 9.03 | 10.92 | 0.16 | 0.34 | 0.2 | 0.93 | 6.703333 | 0.49 |
| LOC108177737 | 10.133 | 3.341034 | 0.006453 | 0.04621 | yes | up | 0.03 | 0.03 | 0.02 | 0.11 | 0.47 | 0.27 | 0.026667 | 0.283333 |
| RIC3 | 2.11 | 1.077382 | 0.00646 | 0.046238 | yes | up | 1.91 | 4.57 | 1.81 | 3.43 | 7.78 | 7.16 | 2.763333 | 6.123333 |
| ZNF691 | 0.411 | -1.28288 | 0.006466 | 0.046256 | yes | down | 1.94 | 2.12 | 2.78 | 0.65 | 0.79 | 1.49 | 2.28 | 0.976667 |
| IL17RE | 6.801 | 2.765835 | 0.006486 | 0.046335 | yes | up | 0.02 | 0.07 | 0.02 | 0.28 | 0.23 | 0.25 | 0.036667 | 0.253333 |
| VIL1 | 0.446 | -1.16389 | 0.006547 | 0.046751 | yes | down | 1.68 | 2.86 | 2.61 | 1.1 | 0.72 | 1.52 | 2.383333 | 1.113333 |
| POLE2 | 0.183 | -2.45308 | 0.006582 | 0.046917 | yes | down | 0.52 | 0.47 | 0.41 | 0.12 | 0.04 | 0.1 | 0.466667 | 0.086667 |
| LOC100338274 | 0.102 | -3.29579 | 0.006602 | 0.047015 | yes | down | 1.67 | 0.21 | 0.05 | 0.07 | 0.13 | 0 | 0.643333 | 0.066667 |
| LOC103348410 | 0.427 | -1.22786 | 0.006614 | 0.047033 | yes | down | 0.84 | 1.06 | 0.49 | 0.41 | 0.35 | 0.32 | 0.796667 | 0.36 |
| IL18BP | 2.172 | 1.118721 | 0.006646 | 0.047218 | yes | up | 1.35 | 1.42 | 1.45 | 4.16 | 2.62 | 3.07 | 1.406667 | 3.283333 |
| ATP10A | 0.498 | -1.00582 | 0.006681 | 0.047378 | yes | down | 1.7 | 2.58 | 1.24 | 1.26 | 0.87 | 0.64 | 1.84 | 0.923333 |
| SGO2 | 0.241 | -2.0552 | 0.006731 | 0.047658 | yes | down | 0.31 | 0.5 | 0.2 | 0.04 | 0.18 | 0.04 | 0.336667 | 0.086667 |
| ADGRL1 | 0.445 | -1.1686 | 0.006793 | 0.047948 | yes | down | 0.48 | 0.65 | 0.29 | 0.13 | 0.24 | 0.28 | 0.473333 | 0.216667 |
| LOC103351786 | 0.272 | -1.8764 | 0.006797 | 0.04795 | yes | down | 1.66 | 0.5 | 0.36 | 0.23 | 0.23 | 0.23 | 0.84 | 0.23 |
| LOC100338913 | 3.155 | 1.657529 | 0.006845 | 0.04818 | yes | up | 0.24 | 1.02 | 0.49 | 2.03 | 2.07 | 1.47 | 0.583333 | 1.856667 |
| VCAM1 | 0.477 | -1.06727 | 0.006904 | 0.048531 | yes | down | 2.4 | 5.53 | 4.11 | 1.12 | 3.04 | 1.87 | 4.013333 | 2.01 |
| ZCWPW1 | 3.16 | 1.660103 | 0.006994 | 0.049022 | yes | up | 0.27 | 0.17 | 0.14 | 0.54 | 0.42 | 0.74 | 0.193333 | 0.566667 |
| ADGRB2 | 2.304 | 1.203979 | 0.007005 | 0.049054 | yes | up | 0.49 | 0.24 | 0.18 | 0.75 | 0.72 | 0.66 | 0.303333 | 0.71 |
| RAB27B | 0.302 | -1.72787 | 0.007046 | 0.049198 | yes | down | 0.2 | 0.15 | 0.14 | 0.07 | 0.05 | 0.03 | 0.163333 | 0.05 |
| TSPAN33 | 2.713 | 1.439668 | 0.007048 | 0.049198 | yes | up | 0.21 | 0.41 | 0.31 | 0.95 | 0.75 | 0.93 | 0.31 | 0.876667 |
| SHANK2 | 9.612 | 3.26485 | 0.007058 | 0.049251 | yes | up | 0.01 | 0 | 0.02 | 0.2 | 0.03 | 0.08 | 0.01 | 0.103333 |
| SERF1A | 2.322 | 1.215388 | 0.007076 | 0.04935 | yes | up | 4.63 | 2.5 | 2.06 | 6.39 | 7.92 | 7.3 | 3.063333 | 7.203333 |

iBAT_D1_VS_iBAT_M1

| Gene name | FC(iBAT_M1/iBAT_D1) | Log2FC(iBAT_M1/iBAT_D1) | Pvalue | Padjust | Significant | Regulate | D1iBAT1 | D1iBAT2 | D1iBAT3 | M1iBAT1 | M1iBAT2 | M1iBAT3 | iBAT_D1 | iBAT_M1 |
| --- | --- | --- | --- | --- | --- | --- | --- | --- | --- | --- | --- | --- | --- | --- |
| ADH1A | 30.958 | 4.952246 | 2.1E-116 | 3.4E-112 | yes | up | 9.29 | 13.89 | 8.71 | 526.94 | 552.75 | 478.96 | 10.63 | 519.55 |
| KCNK3 | 0.022 | -5.53752 | 1.6E-108 | 1.3E-104 | yes | down | 95.58 | 118.79 | 82.3 | 2.68 | 4.37 | 3.04 | 98.89 | 3.363333 |
| HP | 87.296 | 6.44784 | 7.57E-83 | 4.04E-79 | yes | up | 19.3 | 9.9 | 13.8 | 1542.44 | 1538.42 | 3022.6 | 14.33333 | 2034.487 |
| LOC100328910 | 110.266 | 6.784848 | 3.71E-74 | 1.48E-70 | yes | up | 9.52 | 4.67 | 4.48 | 867.44 | 722.46 | 1669.67 | 6.223333 | 1086.523 |
| HIGD1A | 0.122 | -3.0368 | 1.22E-64 | 3.91E-61 | yes | down | 105.55 | 89.01 | 101.38 | 19.83 | 18.21 | 19.05 | 98.64667 | 19.03 |
| UCP1 | 0.015 | -6.07147 | 4.73E-61 | 1.26E-57 | yes | down | 4146.2 | 7225.82 | 4811.64 | 78.55 | 187.12 | 112.87 | 5394.553 | 126.18 |
| HK1 | 0.141 | -2.82756 | 5.91E-61 | 1.35E-57 | yes | down | 86.26 | 101.96 | 97.03 | 21.79 | 21.69 | 21.08 | 95.08333 | 21.52 |
| ERRFI1 | 7.712 | 2.947033 | 5.51E-58 | 1.1E-54 | yes | up | 2.52 | 2.65 | 2.37 | 26.08 | 23.32 | 36.67 | 2.513333 | 28.69 |
| CYP2A11 | 65.804 | 6.040097 | 4.56E-56 | 8.1E-53 | yes | up | 3.17 | 2.26 | 1.24 | 128.53 | 254.65 | 308.49 | 2.223333 | 230.5567 |
| ACAD9 | 0.187 | -2.41963 | 6.42E-55 | 1.03E-51 | yes | down | 22.49 | 24.34 | 20.79 | 6.85 | 6.82 | 6.86 | 22.54 | 6.843333 |
| PPA1 | 0.165 | -2.5982 | 9.05E-55 | 1.32E-51 | yes | down | 203.27 | 265.13 | 215.7 | 59.69 | 55.39 | 66.77 | 228.0333 | 60.61667 |
| COQ3 | 0.084 | -3.57106 | 2.6E-54 | 3.46E-51 | yes | down | 11.51 | 12.02 | 10.3 | 1.37 | 1.39 | 1.84 | 11.27667 | 1.533333 |
| GPT2 | 0.278 | -1.84569 | 4.42E-53 | 5.43E-50 | yes | down | 71.29 | 75.71 | 57.82 | 29.38 | 31.04 | 30.33 | 68.27333 | 30.25 |
| LOC100359331 | 322.265 | 8.332104 | 2.28E-52 | 2.6E-49 | yes | up | 0.35 | 0.45 | 0.17 | 86.42 | 173.89 | 222.83 | 0.323333 | 161.0467 |
| LOC103348029 | 0.042 | -4.58187 | 2.28E-50 | 2.43E-47 | yes | down | 40.84 | 61.22 | 63.14 | 4.24 | 3.05 | 4.65 | 55.06667 | 3.98 |
| SEMA7A | 0.218 | -2.20082 | 4.77E-50 | 4.77E-47 | yes | down | 216.83 | 203.56 | 147.66 | 69.01 | 56.6 | 70.21 | 189.35 | 65.27333 |
| HSPA9 | 0.18 | -2.47477 | 9.58E-50 | 9.02E-47 | yes | down | 263.87 | 273.67 | 278.08 | 76.76 | 83.13 | 75.95 | 271.8733 | 78.61333 |
| GMPR | 0.054 | -4.21187 | 3.58E-49 | 3.18E-46 | yes | down | 488.21 | 895.55 | 491.59 | 41.97 | 58.43 | 60.55 | 625.1167 | 53.65 |
| ATPAF1 | 0.239 | -2.06627 | 5.17E-47 | 4.36E-44 | yes | down | 26.46 | 23.76 | 23.33 | 9.26 | 9.02 | 9 | 24.51667 | 9.093333 |
| PPIF | 0.121 | -3.04706 | 3.83E-46 | 3.07E-43 | yes | down | 82.23 | 104.32 | 91.24 | 15.78 | 15.66 | 22.9 | 92.59667 | 18.11333 |
| IRF4 | 120.14 | 6.908567 | 9.84E-46 | 7.5E-43 | yes | up | 0.17 | 0.08 | 0.13 | 11.74 | 18.56 | 39.75 | 0.126667 | 23.35 |
| BDH1 | 0.189 | -2.4072 | 1.37E-44 | 9.98E-42 | yes | down | 213.64 | 193.34 | 165.86 | 52.48 | 67.38 | 68.45 | 190.9467 | 62.77 |
| ECM2 | 3.619 | 1.85552 | 7.04E-44 | 4.89E-41 | yes | up | 25.41 | 26.64 | 18.49 | 140.59 | 128.63 | 130.2 | 23.51333 | 133.14 |
| GLYAT | 11.729 | 3.552026 | 8.2E-44 | 5.46E-41 | yes | up | 10.35 | 7.08 | 5.08 | 120.14 | 152.2 | 136.68 | 7.503333 | 136.34 |
| MRPS5 | 0.206 | -2.2791 | 1.65E-42 | 1.06E-39 | yes | down | 40.84 | 47.19 | 41.65 | 13.13 | 13.75 | 16.84 | 43.22667 | 14.57333 |
| TNFAIP3 | 6.123 | 2.614156 | 6.42E-42 | 3.95E-39 | yes | up | 3.86 | 3.26 | 3.73 | 39.99 | 30.37 | 36.22 | 3.616667 | 35.52667 |
| LOC100358417 | 0.138 | -2.86026 | 7.35E-40 | 4.35E-37 | yes | down | 14.23 | 11.87 | 9.54 | 3.02 | 2.6 | 2.3 | 11.88 | 2.64 |
| LOC100350716 | 0.097 | -3.36869 | 1.14E-39 | 6.49E-37 | yes | down | 18.65 | 14.79 | 12.07 | 2.98 | 1.6 | 2.42 | 15.17 | 2.333333 |
| MED9 | 0.092 | -3.44327 | 4.34E-39 | 2.39E-36 | yes | down | 17.28 | 18.88 | 12.39 | 1.78 | 2.33 | 3.01 | 16.18333 | 2.373333 |
| LOC100344545 | 20.886 | 4.38449 | 2.41E-38 | 1.28E-35 | yes | up | 0.55 | 0.46 | 0.32 | 14.53 | 12.36 | 16.65 | 0.443333 | 14.51333 |
| SHMT2 | 0.169 | -2.56221 | 4.46E-38 | 2.3E-35 | yes | down | 18.8 | 22.61 | 16.78 | 6.01 | 4.91 | 4.73 | 19.39667 | 5.216667 |
| TIMM8A | 0.206 | -2.28095 | 9.94E-38 | 4.97E-35 | yes | down | 27.01 | 32.41 | 25.55 | 9.17 | 9.17 | 9.52 | 28.32333 | 9.286667 |
| ID3 | 0.19 | -2.39228 | 1.31E-37 | 6.33E-35 | yes | down | 491.74 | 527.12 | 400.77 | 114.38 | 137.78 | 179.38 | 473.21 | 143.8467 |
| PLXNA4 | 0.095 | -3.39818 | 3.1E-37 | 1.46E-34 | yes | down | 5.72 | 7.1 | 6.19 | 1.13 | 0.98 | 0.72 | 6.336667 | 0.943333 |
| SATB2 | 9.872 | 3.303358 | 1.11E-36 | 5.08E-34 | yes | up | 0.4 | 0.45 | 0.34 | 5.87 | 6.67 | 7.12 | 0.396667 | 6.553333 |
| MC5R | 32.956 | 5.042468 | 2.2E-35 | 9.77E-33 | yes | up | 2.39 | 3.05 | 1.49 | 39.26 | 93.46 | 81.54 | 2.31 | 71.42 |
| PTX3 | 145.729 | 7.187145 | 2.89E-34 | 1.25E-31 | yes | up | 0.72 | 0.8 | 1.34 | 182.48 | 55.43 | 388.45 | 0.953333 | 208.7867 |
| LOC100328613 | 0.049 | -4.35719 | 1.37E-33 | 5.77E-31 | yes | down | 308.91 | 818.45 | 410.3 | 37.88 | 46.66 | 33.96 | 512.5533 | 39.5 |
| LOC100328937 | 0.026 | -5.2551 | 1.72E-33 | 7.05E-31 | yes | down | 5.43 | 7.66 | 4.94 | 0.13 | 0.34 | 0.29 | 6.01 | 0.253333 |
| ACSF2 | 0.132 | -2.92333 | 2.53E-32 | 1.01E-29 | yes | down | 237.09 | 247.04 | 300.26 | 44.25 | 58.81 | 66.59 | 261.4633 | 56.55 |
| ITPK1 | 0.279 | -1.84231 | 3.44E-32 | 1.33E-29 | yes | down | 33.15 | 38.95 | 30.55 | 14.03 | 14.18 | 17.92 | 34.21667 | 15.37667 |
| EGLN3 | 0.074 | -3.75548 | 3.51E-32 | 1.33E-29 | yes | down | 41.25 | 80.09 | 65.01 | 5.63 | 8.69 | 7.91 | 62.11667 | 7.41 |
| ALOX15 | 0.01 | -6.5998 | 8.3E-32 | 3.09E-29 | yes | down | 24.96 | 66.63 | 52.88 | 1.58 | 0.24 | 0.67 | 48.15667 | 0.83 |
| LOC100350311 | 0.084 | -3.56783 | 1.74E-31 | 6.33E-29 | yes | down | 79 | 70.63 | 54.01 | 11.35 | 4.95 | 7.78 | 67.88 | 8.026667 |
| LRPPRC | 0.31 | -1.68991 | 1.82E-31 | 6.46E-29 | yes | down | 57.93 | 50.28 | 41.92 | 23.71 | 27.81 | 26.84 | 50.04333 | 26.12 |
| SLC25A28 | 5.145 | 2.363072 | 1.94E-31 | 6.75E-29 | yes | up | 12.79 | 12.82 | 14.32 | 82.95 | 97.59 | 99.4 | 13.31 | 93.31333 |
| MRPS18B | 0.212 | -2.2375 | 2.22E-31 | 7.54E-29 | yes | down | 72.24 | 72.41 | 64.77 | 20.33 | 27.38 | 22.93 | 69.80667 | 23.54667 |
| NCOA4 | 0.435 | -1.19968 | 1.36E-30 | 4.54E-28 | yes | down | 75.2 | 79.24 | 61.31 | 54.77 | 52.42 | 57.28 | 71.91667 | 54.82333 |
| NR4A1 | 13.814 | 3.788032 | 2.49E-30 | 8.12E-28 | yes | up | 2.78 | 5.12 | 2.05 | 50.21 | 68.83 | 100.21 | 3.316667 | 73.08333 |
| GSN | 5.08 | 2.344967 | 2.54E-30 | 8.13E-28 | yes | up | 138.85 | 112.15 | 77.06 | 761.48 | 830.5 | 1043.04 | 109.3533 | 878.34 |
| PRELID3B | 0.169 | -2.565 | 2.19E-29 | 6.88E-27 | yes | down | 105.59 | 158.26 | 124.63 | 45.89 | 36.8 | 30.65 | 129.4933 | 37.78 |
| LIFR | 2.66 | 1.411552 | 2.39E-29 | 7.36E-27 | yes | up | 9.05 | 10.36 | 8.21 | 39.31 | 35.89 | 40.84 | 9.206667 | 38.68 |
| QPCT | 0.213 | -2.22849 | 3.51E-29 | 1.06E-26 | yes | down | 361.16 | 337.63 | 321.24 | 92.04 | 109.99 | 149.03 | 340.01 | 117.02 |
| LOC100359112 | 9.618 | 3.265743 | 3.86E-29 | 1.14E-26 | yes | up | 1.71 | 1.76 | 1.29 | 27.86 | 17.26 | 28.56 | 1.586667 | 24.56 |
| MRPL18 | 0.305 | -1.71154 | 4.97E-29 | 1.45E-26 | yes | down | 45.02 | 46.12 | 37.83 | 21.26 | 22.76 | 19.05 | 42.99 | 21.02333 |
| HAUS7 | 0.115 | -3.12654 | 1.66E-28 | 4.74E-26 | yes | down | 4.56 | 6.71 | 4.55 | 1.34 | 1.51 | 1.14 | 5.273333 | 1.33 |
| SNX21 | 3.089 | 1.626948 | 2.19E-28 | 6.15E-26 | yes | up | 16 | 16.32 | 10.63 | 40.1 | 44.23 | 43.21 | 14.31667 | 42.51333 |
| ACSL1 | 0.288 | -1.79354 | 2.71E-28 | 7.47E-26 | yes | down | 215.22 | 207.47 | 178.33 | 86.08 | 99 | 80.53 | 200.34 | 88.53667 |
| LOC108178205 | 7.17 | 2.841879 | 9.53E-28 | 2.58E-25 | yes | up | 4.85 | 8.26 | 7 | 72.49 | 64.55 | 91.64 | 6.703333 | 76.22667 |
| ATP5F1 | 0.384 | -1.37943 | 1.57E-27 | 4.17E-25 | yes | down | 575.7 | 583.25 | 496.33 | 296.61 | 330.37 | 378.63 | 551.76 | 335.2033 |
| CD247 | 8.758 | 3.130598 | 1.82E-27 | 4.76E-25 | yes | up | 1.75 | 2.3 | 2.21 | 28.89 | 22.71 | 26.08 | 2.086667 | 25.89333 |
| GFM1 | 0.251 | -1.99678 | 1.87E-27 | 4.82E-25 | yes | down | 49.56 | 41.64 | 32.97 | 16.6 | 17.44 | 14.35 | 41.39 | 16.13 |
| PARN | 0.289 | -1.79144 | 1.99E-27 | 5.04E-25 | yes | down | 22.76 | 27.32 | 24.43 | 11.32 | 12.48 | 12.71 | 24.83667 | 12.17 |
| FABP3 | 0.044 | -4.51219 | 6.55E-27 | 1.64E-24 | yes | down | 1717.61 | 1888.73 | 1516.43 | 49.14 | 180.84 | 124.1 | 1707.59 | 118.0267 |
| BAG1 | 0.417 | -1.26036 | 7.87E-27 | 1.94E-24 | yes | down | 88.66 | 91.59 | 72.26 | 56.62 | 59.87 | 55.08 | 84.17 | 57.19 |
| HSPD1 | 0.255 | -1.97103 | 9.98E-27 | 2.42E-24 | yes | down | 291.93 | 271.12 | 281.26 | 118.88 | 124.77 | 100.73 | 281.4367 | 114.7933 |
| CDKN3 | 5.884 | 2.556703 | 1.18E-26 | 2.82E-24 | yes | up | 6.91 | 8.63 | 4.63 | 67.32 | 59.52 | 83.5 | 6.723333 | 70.11333 |
| SERPINE1 | 7.101 | 2.828061 | 1.34E-26 | 3.14E-24 | yes | up | 6.68 | 4.46 | 3.7 | 44.43 | 49.39 | 73.89 | 4.946667 | 55.90333 |
| N4BP2L1 | 4.313 | 2.108665 | 1.44E-26 | 3.33E-24 | yes | up | 3.64 | 5.29 | 4.44 | 28.8 | 21.38 | 30.58 | 4.456667 | 26.92 |
| HMGCS2 | 0.036 | -4.81182 | 1.7E-26 | 3.88E-24 | yes | down | 158.94 | 203.07 | 313.49 | 6.38 | 13.21 | 20.34 | 225.1667 | 13.31 |
| ATF3 | 10.187 | 3.348723 | 2.55E-26 | 5.75E-24 | yes | up | 2.5 | 1.74 | 1.19 | 39.44 | 22.72 | 26.56 | 1.81 | 29.57333 |
| MDH1 | 0.257 | -1.96167 | 2.59E-26 | 5.76E-24 | yes | down | 633.87 | 643.26 | 589 | 204.48 | 252.55 | 312.1 | 622.0433 | 256.3767 |
| DMBT1 | 0.016 | -6.01135 | 2.76E-26 | 6.04E-24 | yes | down | 8.34 | 7.09 | 5.29 | 0.05 | 0.07 | 0.29 | 6.906667 | 0.136667 |
| MIF | 0.223 | -2.16292 | 2.91E-26 | 6.28E-24 | yes | down | 400.96 | 424.4 | 393.65 | 111.33 | 147.59 | 176.81 | 406.3367 | 145.2433 |
| PHLDA1 | 5.6 | 2.485463 | 3.87E-26 | 8.26E-24 | yes | up | 1.61 | 2.03 | 1.14 | 12.48 | 13.4 | 18.3 | 1.593333 | 14.72667 |
| ADH2-2 | 17.359 | 4.11764 | 5.87E-26 | 1.23E-23 | yes | up | 1.39 | 0.42 | 0.87 | 20.15 | 25.3 | 28.87 | 0.893333 | 24.77333 |
| PDE1A | 4.361 | 2.12462 | 6.02E-26 | 1.25E-23 | yes | up | 5.74 | 9.22 | 3.65 | 20.75 | 15.78 | 25.46 | 6.203333 | 20.66333 |
| GFPT2 | 8.518 | 3.090525 | 6.42E-26 | 1.32E-23 | yes | up | 3.47 | 2.19 | 1.5 | 38.87 | 26.07 | 33.3 | 2.386667 | 32.74667 |
| GNAS | 0.135 | -2.88537 | 6.91E-26 | 1.4E-23 | yes | down | 4031.76 | 4571.39 | 5277.49 | 913.76 | 1261.56 | 826.04 | 4626.88 | 1000.453 |
| CYR61 | 5.892 | 2.558754 | 9.48E-26 | 1.9E-23 | yes | up | 24.11 | 13.93 | 18.67 | 191.3 | 177.29 | 175.37 | 18.90333 | 181.32 |
| MRPL46 | 0.291 | -1.78246 | 1.5E-25 | 2.96E-23 | yes | down | 13.6 | 14.5 | 12.45 | 6.32 | 5.49 | 7.13 | 13.51667 | 6.313333 |
| IDH3A | 0.105 | -3.2523 | 2.6E-25 | 5.07E-23 | yes | down | 406.87 | 575.78 | 477.51 | 52.49 | 109.1 | 80.41 | 486.72 | 80.66667 |
| JUN | 4.174 | 2.061388 | 9.83E-25 | 1.89E-22 | yes | up | 24.57 | 19.02 | 17.52 | 110.07 | 143.19 | 152.37 | 20.37 | 135.21 |
| DLAT | 0.221 | -2.17802 | 1.3E-24 | 2.48E-22 | yes | down | 48.28 | 37.06 | 39.02 | 14.6 | 16.71 | 12.45 | 41.45333 | 14.58667 |
| LOC100343425 | 3.694 | 1.88527 | 1.47E-24 | 2.77E-22 | yes | up | 80.61 | 82.18 | 80.87 | 476.94 | 351.17 | 528.76 | 81.22 | 452.29 |
| CPNE4 | 0.041 | -4.59817 | 5.09E-24 | 9.47E-22 | yes | down | 3.88 | 5.23 | 2.1 | 0.19 | 0.16 | 0.4 | 3.736667 | 0.25 |
| IGFBP5 | 9.279 | 3.213923 | 5.74E-24 | 1.06E-21 | yes | up | 16.78 | 10.08 | 6.73 | 131.31 | 140.38 | 219.82 | 11.19667 | 163.8367 |
| LOC100345097 | 0 | -12.2529 | 6.14E-24 | 1.12E-21 | yes | down | 88.41 | 134.84 | 112.91 | 0 | 0 | 0 | 112.0533 | 0 |
| FMO1 | 14.667 | 3.874508 | 7.47E-24 | 1.34E-21 | yes | up | 0.55 | 0.3 | 0.26 | 7.1 | 8.19 | 10.47 | 0.37 | 8.586667 |
| LGALS12 | 8.496 | 3.086775 | 8.21E-24 | 1.46E-21 | yes | up | 10.45 | 21.77 | 10.3 | 116.72 | 170.1 | 153.24 | 14.17333 | 146.6867 |
| PRKRA | 0.278 | -1.84467 | 9.98E-24 | 1.75E-21 | yes | down | 33.61 | 28.62 | 26.08 | 10.97 | 14.05 | 14.2 | 29.43667 | 13.07333 |
| LOC103347528 | 0.302 | -1.7296 | 1.03E-23 | 1.78E-21 | yes | down | 36.73 | 37.01 | 32.93 | 17.07 | 17.69 | 16.39 | 35.55667 | 17.05 |
| HSD17B10 | 0.266 | -1.9091 | 1.73E-23 | 2.98E-21 | yes | down | 219.08 | 248.75 | 241.03 | 87.78 | 110.58 | 104.46 | 236.2867 | 100.94 |
| CIB4 | 0.081 | -3.63258 | 2.26E-23 | 3.84E-21 | yes | down | 4.01 | 6.2 | 4.6 | 0.51 | 0.55 | 0.41 | 4.936667 | 0.49 |
| PRR5L | 8.215 | 3.038226 | 2.53E-23 | 4.26E-21 | yes | up | 0.56 | 0.46 | 0.42 | 4.68 | 5.92 | 8.82 | 0.48 | 6.473333 |
| KIFC3 | 0.201 | -2.31817 | 4.79E-23 | 7.97E-21 | yes | down | 6.07 | 7.97 | 5.47 | 2.38 | 1.73 | 1.92 | 6.503333 | 2.01 |
| TFB2M | 0.296 | -1.7568 | 6.89E-23 | 1.14E-20 | yes | down | 38.79 | 42.95 | 39.91 | 17.29 | 17.97 | 23.11 | 40.55 | 19.45667 |
| FOSB | 32.399 | 5.017858 | 1.31E-22 | 2.15E-20 | yes | up | 0.26 | 0.22 | 0.09 | 15.71 | 7.24 | 6.69 | 0.19 | 9.88 |
| GMPS | 0.185 | -2.43549 | 1.92E-22 | 3.11E-20 | yes | down | 19.5 | 33.31 | 26.69 | 7.28 | 7.84 | 8.69 | 26.5 | 7.936667 |
| LOC100347632 | 43.328 | 5.43722 | 2.72E-22 | 4.36E-20 | yes | up | 0.75 | 0.38 | 0.55 | 15.72 | 28.23 | 38.61 | 0.56 | 27.52 |
| SOD2 | 0.417 | -1.26242 | 2.78E-22 | 4.4E-20 | yes | down | 338.17 | 351.73 | 300.93 | 198.06 | 211.74 | 252.23 | 330.2767 | 220.6767 |
| ND5 | 0.379 | -1.39923 | 3.14E-22 | 4.92E-20 | yes | down | 63007.72 | 62554.36 | 62343.01 | 38445.16 | 42171.02 | 38570.36 | 62635.03 | 39728.85 |
| MRPS23 | 0.384 | -1.38068 | 4.29E-22 | 6.66E-20 | yes | down | 52.21 | 55.15 | 40.33 | 29.57 | 28.74 | 31.67 | 49.23 | 29.99333 |
| CEBPD | 6.392 | 2.676379 | 5.43E-22 | 8.35E-20 | yes | up | 11.9 | 14.95 | 10.17 | 136.44 | 81.06 | 161.86 | 12.34 | 126.4533 |
| RTP4 | 6.252 | 2.644299 | 5.82E-22 | 8.87E-20 | yes | up | 11.55 | 9.99 | 7.72 | 111.12 | 105.79 | 66.51 | 9.753333 | 94.47333 |
| PLA2G16 | 2.415 | 1.271946 | 7.75E-22 | 1.17E-19 | yes | up | 252.49 | 251.54 | 237.93 | 886.58 | 903.94 | 1077.42 | 247.32 | 955.98 |
| MRPL2 | 0.238 | -2.07305 | 7.97E-22 | 1.19E-19 | yes | down | 52.98 | 40.55 | 51.1 | 17.31 | 18.41 | 19.66 | 48.21 | 18.46 |
| NRG4 | 0.164 | -2.60496 | 9.56E-22 | 1.42E-19 | yes | down | 22.44 | 30.91 | 30.68 | 3.23 | 3.14 | 5.99 | 28.01 | 4.12 |
| MRPS14 | 0.409 | -1.29081 | 1.25E-21 | 1.83E-19 | yes | down | 118.13 | 114.86 | 94.28 | 68.42 | 71.54 | 70.43 | 109.09 | 70.13 |
| IARS2 | 0.322 | -1.6352 | 1.49E-21 | 2.16E-19 | yes | down | 41.07 | 33.96 | 37.66 | 20.93 | 18.25 | 22.13 | 37.56333 | 20.43667 |
| LOC100346173 | 101.344 | 6.663113 | 1.55E-21 | 2.24E-19 | yes | up | 0.04 | 0 | 0.06 | 4.91 | 8.85 | 8.04 | 0.033333 | 7.266667 |
| LOC100343920 | 14.582 | 3.866162 | 1.74E-21 | 2.48E-19 | yes | up | 1.16 | 0.62 | 0.36 | 16.32 | 11.89 | 21.28 | 0.713333 | 16.49667 |
| SUPV3L1 | 0.375 | -1.41317 | 1.95E-21 | 2.75E-19 | yes | down | 17.07 | 19.66 | 15.98 | 10.62 | 10.83 | 10.94 | 17.57 | 10.79667 |
| GFM2 | 0.354 | -1.4986 | 2.62E-21 | 3.67E-19 | yes | down | 18.15 | 15.33 | 12.24 | 8.95 | 7.82 | 9.06 | 15.24 | 8.61 |
| ABR | 2.621 | 1.390235 | 2.72E-21 | 3.79E-19 | yes | up | 5.33 | 4.72 | 5.57 | 24.37 | 20.44 | 24.1 | 5.206667 | 22.97 |
| TXN2 | 0.283 | -1.82252 | 2.94E-21 | 4.06E-19 | yes | down | 160.26 | 164.68 | 134.74 | 55.92 | 64.48 | 82.51 | 153.2267 | 67.63667 |
| PPP1R3E | 6.499 | 2.700304 | 4.29E-21 | 5.87E-19 | yes | up | 4.51 | 4.72 | 4.77 | 30.54 | 53.85 | 61.58 | 4.666667 | 48.65667 |
| FFAR2 | 0.108 | -3.20802 | 4.35E-21 | 5.89E-19 | yes | down | 23.76 | 28.54 | 25.67 | 2.71 | 4.35 | 7.45 | 25.99 | 4.836667 |
| SH3D19 | 2.994 | 1.582225 | 4.75E-21 | 6.38E-19 | yes | up | 6.07 | 5.65 | 5.71 | 26.57 | 23.61 | 32.67 | 5.81 | 27.61667 |
| CPE | 3.668 | 1.874997 | 4.93E-21 | 6.57E-19 | yes | up | 30.07 | 28.46 | 28.6 | 177.6 | 135.16 | 214.17 | 29.04333 | 175.6433 |
| RNPEP | 0.187 | -2.41715 | 5.52E-21 | 7.29E-19 | yes | down | 27.54 | 42.03 | 25.03 | 9.08 | 7.49 | 12.36 | 31.53333 | 9.643333 |
| GPAM | 0.397 | -1.33248 | 7.15E-21 | 9.37E-19 | yes | down | 16.27 | 13.43 | 12.53 | 8.51 | 8.9 | 9.38 | 14.07667 | 8.93 |
| LYPLA1 | 0.43 | -1.21807 | 7.57E-21 | 9.85E-19 | yes | down | 20.89 | 20.66 | 15.98 | 12.63 | 13.23 | 13.45 | 19.17667 | 13.10333 |
| LOC103351664 | 0.348 | -1.52087 | 8.27E-21 | 1.07E-18 | yes | down | 25.91 | 23.37 | 20.65 | 13.81 | 12.63 | 12.2 | 23.31 | 12.88 |
| DLST | 0.349 | -1.51918 | 8.53E-21 | 1.09E-18 | yes | down | 281.34 | 270.81 | 265.83 | 131.71 | 162.73 | 163.43 | 272.66 | 152.6233 |
| SEMA4B | 6.509 | 2.702446 | 1.18E-20 | 1.49E-18 | yes | up | 3.11 | 2.39 | 3.5 | 27.62 | 25.45 | 42.95 | 3 | 32.00667 |
| MRPL1 | 0.421 | -1.24683 | 1.33E-20 | 1.67E-18 | yes | down | 23.68 | 27.8 | 22.57 | 13.91 | 13.77 | 14.36 | 24.68333 | 14.01333 |
| MPV17 | 0.31 | -1.69145 | 1.59E-20 | 1.99E-18 | yes | down | 20.49 | 24.71 | 21.89 | 12.79 | 11.74 | 14.17 | 22.36333 | 12.9 |
| AGXT2 | 26.308 | 4.717409 | 1.64E-20 | 2.04E-18 | yes | up | 0.08 | 0.14 | 0.02 | 2.28 | 3.92 | 3.09 | 0.08 | 3.096667 |
| LOC100358333 | 8.541 | 3.094434 | 1.98E-20 | 2.44E-18 | yes | up | 2.41 | 4.19 | 5.27 | 21.35 | 31.78 | 21.36 | 3.956667 | 24.83 |
| TIMM10 | 0.351 | -1.51059 | 2.22E-20 | 2.71E-18 | yes | down | 78.72 | 78.77 | 78.78 | 43.73 | 43.46 | 45.5 | 78.75667 | 44.23 |
| AARS2 | 0.297 | -1.75143 | 2.94E-20 | 3.57E-18 | yes | down | 6.87 | 6.89 | 6.61 | 2.99 | 3.39 | 3 | 6.79 | 3.126667 |
| EHD3 | 0.245 | -2.02819 | 3.29E-20 | 3.96E-18 | yes | down | 14.98 | 20.07 | 19.03 | 7.15 | 7.3 | 8.14 | 18.02667 | 7.53 |
| SLC4A5 | 22.334 | 4.481177 | 3.38E-20 | 4.03E-18 | yes | up | 0.03 | 0.05 | 0.07 | 2.06 | 1.6 | 2.04 | 0.05 | 1.9 |
| DNAJC11 | 0.442 | -1.17858 | 3.49E-20 | 4.13E-18 | yes | down | 30.36 | 34.91 | 27.85 | 22.44 | 20.58 | 22.9 | 31.04 | 21.97333 |
| ADM | 7.654 | 2.936238 | 4.55E-20 | 5.35E-18 | yes | up | 8.85 | 6.92 | 7.56 | 56.3 | 92.99 | 135.49 | 7.776667 | 94.92667 |
| LOC100338849 | 0.204 | -2.29121 | 4.88E-20 | 5.7E-18 | yes | down | 7.49 | 6.55 | 6.37 | 2.45 | 2.11 | 2.04 | 6.803333 | 2.2 |
| KLF15 | 5.439 | 2.443274 | 5.09E-20 | 5.89E-18 | yes | up | 2.19 | 1.1 | 2.4 | 12.56 | 9.16 | 9.56 | 1.896667 | 10.42667 |
| COX2 | 0.306 | -1.70981 | 5.58E-20 | 6.42E-18 | yes | down | 67579.39 | 75887.18 | 77144.5 | 31781.27 | 37955.82 | 38572.65 | 73537.02 | 36103.25 |
| PM20D2 | 5.844 | 2.54697 | 5.72E-20 | 6.54E-18 | yes | up | 6.5 | 3.87 | 3.91 | 30.95 | 49.03 | 45.6 | 4.76 | 41.86 |
| AIFM1 | 0.333 | -1.58679 | 6.87E-20 | 7.79E-18 | yes | down | 84.09 | 89 | 76.22 | 39.62 | 50.41 | 42.18 | 83.10333 | 44.07 |
| LOC100347463 | 4.612 | 2.205367 | 7.6E-20 | 8.56E-18 | yes | up | 10.28 | 9.95 | 9.6 | 64.16 | 57.34 | 101.34 | 9.943333 | 74.28 |
| MEOX1 | 0.181 | -2.46343 | 8.49E-20 | 9.49E-18 | yes | down | 2.95 | 2.85 | 2.62 | 0.69 | 0.87 | 0.89 | 2.806667 | 0.816667 |
| MAMLD1 | 3.807 | 1.928499 | 1.34E-19 | 1.49E-17 | yes | up | 1.4 | 1.69 | 1.3 | 10.04 | 8.34 | 7.79 | 1.463333 | 8.723333 |
| LOC100338010 | 0.366 | -1.44877 | 1.45E-19 | 1.59E-17 | yes | down | 29.92 | 28.02 | 24.6 | 16.67 | 13.86 | 15.79 | 27.51333 | 15.44 |
| GALM | 0.144 | -2.79242 | 1.45E-19 | 1.59E-17 | yes | down | 33.37 | 18.23 | 16.33 | 4.13 | 5.92 | 5.38 | 22.64333 | 5.143333 |
| EPRS | 0.409 | -1.29059 | 1.76E-19 | 1.92E-17 | yes | down | 36.18 | 29.13 | 26.77 | 18.97 | 17.03 | 16.8 | 30.69333 | 17.6 |
| DGAT2 | 0.248 | -2.0141 | 1.9E-19 | 2.06E-17 | yes | down | 532.47 | 697.87 | 458.33 | 249.03 | 317.05 | 373.26 | 562.89 | 313.1133 |
| LOC100357740 | 2.749 | 1.459097 | 2.24E-19 | 2.4E-17 | yes | up | 1.79 | 1.9 | 1.52 | 7.63 | 7.83 | 6.89 | 1.736667 | 7.45 |
| GPR137B | 2.703 | 1.434333 | 2.43E-19 | 2.59E-17 | yes | up | 2.58 | 2.42 | 2.06 | 9.43 | 8.34 | 11.74 | 2.353333 | 9.836667 |
| CABLES1 | 4.29 | 2.10089 | 2.5E-19 | 2.63E-17 | yes | up | 2.88 | 2.89 | 2.66 | 15.56 | 13.91 | 24.2 | 2.81 | 17.89 |
| DSE | 3.618 | 1.855223 | 2.51E-19 | 2.63E-17 | yes | up | 2.72 | 2.87 | 2.81 | 18.76 | 13.35 | 15.06 | 2.8 | 15.72333 |
| CIAPIN1 | 0.245 | -2.02959 | 2.52E-19 | 2.63E-17 | yes | down | 55.21 | 68.41 | 66.59 | 20.63 | 23.98 | 30.86 | 63.40333 | 25.15667 |
| NFU1 | 0.357 | -1.48775 | 3.14E-19 | 3.26E-17 | yes | down | 47.74 | 58.5 | 44.09 | 26.52 | 28.97 | 31.54 | 50.11 | 29.01 |
| OASL | 11.589 | 3.534701 | 3.21E-19 | 3.31E-17 | yes | up | 2.78 | 2.94 | 2.57 | 74.46 | 51.84 | 25.76 | 2.763333 | 50.68667 |
| LENG8 | 2.979 | 1.574739 | 3.44E-19 | 3.53E-17 | yes | up | 17.52 | 17.66 | 15.94 | 84.15 | 83.97 | 65.77 | 17.04 | 77.96333 |
| HDAC10 | 3.26 | 1.70476 | 3.96E-19 | 4.02E-17 | yes | up | 1.04 | 1.01 | 0.99 | 4.54 | 3.42 | 3.51 | 1.013333 | 3.823333 |
| HSDL2 | 0.311 | -1.6831 | 3.98E-19 | 4.02E-17 | yes | down | 224.46 | 180.35 | 180.2 | 80.09 | 103.02 | 110.68 | 195.0033 | 97.93 |
| SIAH2 | 6.009 | 2.587221 | 4.07E-19 | 4.09E-17 | yes | up | 37.33 | 50.67 | 50.37 | 334.8 | 390.1 | 629.99 | 46.12333 | 451.63 |
| CSRNP1 | 6.014 | 2.58831 | 4.84E-19 | 4.84E-17 | yes | up | 1.83 | 3.05 | 1.71 | 22.87 | 14.99 | 24.37 | 2.196667 | 20.74333 |
| C18H10orf2 | 0.323 | -1.63038 | 6.29E-19 | 6.25E-17 | yes | down | 8.6 | 8.07 | 8.59 | 4.26 | 4.05 | 4.84 | 8.42 | 4.383333 |
| IVD | 0.245 | -2.02832 | 7.97E-19 | 7.87E-17 | yes | down | 121.26 | 158.43 | 142.77 | 45.36 | 64.64 | 55.77 | 140.82 | 55.25667 |
| KCNH1 | 0.245 | -2.02742 | 9.09E-19 | 8.92E-17 | yes | down | 7.55 | 8.32 | 4.29 | 2.41 | 2.56 | 2.79 | 6.72 | 2.586667 |
| BMP4 | 4.013 | 2.004788 | 9.28E-19 | 9.04E-17 | yes | up | 4.26 | 3.79 | 4.21 | 23.48 | 24.69 | 31.12 | 4.086667 | 26.43 |
| PTCD3 | 0.433 | -1.20784 | 1E-18 | 9.73E-17 | yes | down | 23.71 | 27.37 | 20.52 | 15.1 | 13.89 | 15.98 | 23.86667 | 14.99 |
| FOXO3 | 2.561 | 1.356916 | 1.14E-18 | 1.09E-16 | yes | up | 6.08 | 5.67 | 4.14 | 23.78 | 19.34 | 21.5 | 5.296667 | 21.54 |
| FARSB | 0.382 | -1.38661 | 1.14E-18 | 1.09E-16 | yes | down | 13.8 | 13.53 | 12.2 | 7.63 | 7.61 | 9.01 | 13.17667 | 8.083333 |
| PENK | 17.201 | 4.104425 | 1.49E-18 | 1.42E-16 | yes | up | 0.79 | 0.67 | 0.13 | 11.56 | 12.97 | 17.8 | 0.53 | 14.11 |
| PARP8 | 3.886 | 1.958375 | 1.55E-18 | 1.46E-16 | yes | up | 0.82 | 0.91 | 0.77 | 4.55 | 5.99 | 4.98 | 0.833333 | 5.173333 |
| PHOSPHO1 | 0.086 | -3.53127 | 1.55E-18 | 1.46E-16 | yes | down | 36.4 | 49.82 | 25.31 | 4.02 | 7.39 | 2.31 | 37.17667 | 4.573333 |
| TIMM8B | 0.295 | -1.76176 | 1.9E-18 | 1.78E-16 | yes | down | 162.93 | 208.3 | 187.76 | 80.6 | 80.21 | 104 | 186.33 | 88.27 |
| PDHB | 0.329 | -1.60475 | 1.93E-18 | 1.8E-16 | yes | down | 252.08 | 225.67 | 217.11 | 107.94 | 140.36 | 115.67 | 231.62 | 121.3233 |
| MRPL51 | 0.374 | -1.41742 | 2.21E-18 | 2.04E-16 | yes | down | 78.01 | 80.35 | 69.64 | 47.93 | 43.68 | 42.24 | 76 | 44.61667 |
| LOC108176823 | 8.878 | 3.150244 | 2.36E-18 | 2.17E-16 | yes | up | 0.26 | 0.22 | 0.25 | 3.41 | 3.28 | 3.74 | 0.243333 | 3.476667 |
| MRPS27 | 0.343 | -1.5454 | 2.57E-18 | 2.35E-16 | yes | down | 13.3 | 14.87 | 14.17 | 7.46 | 7.45 | 8.47 | 14.11333 | 7.793333 |
| FAM96A | 0.365 | -1.45248 | 2.87E-18 | 2.6E-16 | yes | down | 59.02 | 56.54 | 46.08 | 35.9 | 26.43 | 31.84 | 53.88 | 31.39 |
| ADAMTSL3 | 3.892 | 1.960538 | 2.97E-18 | 2.68E-16 | yes | up | 1.47 | 1.35 | 1.37 | 9.77 | 6.93 | 12.07 | 1.396667 | 9.59 |
| PHB2 | 0.343 | -1.5424 | 3E-18 | 2.7E-16 | yes | down | 212.4 | 219.22 | 199.79 | 97.41 | 107.48 | 143.86 | 210.47 | 116.25 |
| SET | 0.443 | -1.17414 | 3.63E-18 | 3.24E-16 | yes | down | 38.16 | 41.52 | 29.72 | 24.86 | 22.51 | 27.67 | 36.46667 | 25.01333 |
| PPP1R9B | 0.309 | -1.69278 | 3.75E-18 | 3.33E-16 | yes | down | 77.92 | 106.62 | 65.42 | 44.85 | 39.55 | 38.11 | 83.32 | 40.83667 |
| CKMT1B | 0.004 | -7.86794 | 4.25E-18 | 3.76E-16 | yes | down | 2.97 | 13.03 | 10.66 | 0.05 | 0.2 | 0 | 8.886667 | 0.083333 |
| IRF7 | 33.913 | 5.08377 | 4.77E-18 | 4.19E-16 | yes | up | 2.23 | 2.28 | 2.67 | 273.4 | 70.67 | 47.95 | 2.393333 | 130.6733 |
| GPR88 | 27.163 | 4.763549 | 4.84E-18 | 4.23E-16 | yes | up | 0.04 | 0.05 | 0.07 | 1.81 | 1.86 | 3.18 | 0.053333 | 2.283333 |
| ETFBKMT | 0.259 | -1.95162 | 5.32E-18 | 4.62E-16 | yes | down | 12.3 | 16.32 | 15.45 | 6.45 | 5.9 | 5.32 | 14.69 | 5.89 |
| GALK1 | 0.234 | -2.09567 | 6.17E-18 | 5.33E-16 | yes | down | 18.65 | 21.94 | 20.98 | 8.57 | 5.74 | 9.03 | 20.52333 | 7.78 |
| PGER5 | 2.287 | 1.19338 | 6.22E-18 | 5.35E-16 | yes | up | 213.95 | 235.44 | 175.62 | 688.88 | 798.04 | 781.76 | 208.3367 | 756.2267 |
| SLC41A1 | 2.258 | 1.175037 | 6.66E-18 | 5.69E-16 | yes | up | 16.24 | 14.61 | 12.48 | 53.73 | 52.58 | 49.72 | 14.44333 | 52.01 |
| MRPL39 | 0.329 | -1.60274 | 8.97E-18 | 7.63E-16 | yes | down | 119.67 | 117.28 | 107.33 | 50.24 | 68.94 | 60.32 | 114.76 | 59.83333 |
| PDSS2 | 0.329 | -1.60314 | 1.01E-17 | 8.54E-16 | yes | down | 10.56 | 9.68 | 9.01 | 4.72 | 5.56 | 5.09 | 9.75 | 5.123333 |
| TOMM5 | 0.418 | -1.25748 | 1.14E-17 | 9.56E-16 | yes | down | 80.41 | 73.87 | 72.89 | 50.83 | 47.43 | 53.66 | 75.72333 | 50.64 |
| LOC108176503 | 0.277 | -1.85256 | 1.29E-17 | 1.08E-15 | yes | down | 12.41 | 16.05 | 15.81 | 4.99 | 3.21 | 6.07 | 14.75667 | 4.756667 |
| HMGB3 | 0.231 | -2.1154 | 1.54E-17 | 1.29E-15 | yes | down | 60.11 | 72.68 | 52.81 | 23.22 | 18.5 | 11.32 | 61.86667 | 17.68 |
| SLIT3 | 2.901 | 1.536663 | 1.79E-17 | 1.48E-15 | yes | up | 2.84 | 3.93 | 2.46 | 13.92 | 13.42 | 15.33 | 3.076667 | 14.22333 |
| APLN | 0.062 | -4.00647 | 1.81E-17 | 1.49E-15 | yes | down | 5.15 | 4.22 | 1.68 | 0.47 | 0.34 | 0.25 | 3.683333 | 0.353333 |
| GOT2 | 0.25 | -2.00224 | 2E-17 | 1.64E-15 | yes | down | 123.77 | 87.1 | 87.13 | 37.4 | 47.23 | 33.17 | 99.33333 | 39.26667 |
| ABHD11 | 0.208 | -2.2646 | 2E-17 | 1.64E-15 | yes | down | 13.66 | 11.21 | 12.19 | 3.65 | 4.74 | 3.42 | 12.35333 | 3.936667 |
| ZFP36 | 5.341 | 2.416982 | 2.15E-17 | 1.75E-15 | yes | up | 4.23 | 3.82 | 3.58 | 45.23 | 25.53 | 28.56 | 3.876667 | 33.10667 |
| TBRG4 | 0.409 | -1.29012 | 2.19E-17 | 1.77E-15 | yes | down | 39.02 | 33.62 | 31.79 | 20.33 | 23.68 | 24.62 | 34.81 | 22.87667 |
| NDUFA9 | 0.348 | -1.52389 | 2.47E-17 | 1.99E-15 | yes | down | 131.15 | 128.38 | 115.41 | 62.02 | 80.35 | 65.22 | 124.98 | 69.19667 |
| ADCY10 | 5.627 | 2.492289 | 2.68E-17 | 2.14E-15 | yes | up | 0.4 | 0.48 | 0.37 | 3.24 | 3.14 | 5.12 | 0.416667 | 3.833333 |
| CRAT | 0.281 | -1.83141 | 2.9E-17 | 2.3E-15 | yes | down | 122.83 | 120.22 | 108.75 | 38 | 59.52 | 60.68 | 117.2667 | 52.73333 |
| KLF9 | 3.824 | 1.935064 | 3.2E-17 | 2.53E-15 | yes | up | 11.13 | 11.33 | 12.37 | 69.43 | 56.35 | 96.16 | 11.61 | 73.98 |
| MMACHC | 0.277 | -1.85012 | 3.25E-17 | 2.56E-15 | yes | down | 12.27 | 13.31 | 10.49 | 4.56 | 5.61 | 5.77 | 12.02333 | 5.313333 |
| PGAM1 | 0.259 | -1.94965 | 3.66E-17 | 2.87E-15 | yes | down | 148.36 | 172.57 | 169.71 | 85.91 | 59.11 | 59.47 | 163.5467 | 68.16333 |
| MIPEP | 0.324 | -1.6268 | 4.17E-17 | 3.25E-15 | yes | down | 22.42 | 25.39 | 23.11 | 13.96 | 12.05 | 10.75 | 23.64 | 12.25333 |
| LOC108177974 | 16.34 | 4.030339 | 4.25E-17 | 3.3E-15 | yes | up | 48.02 | 194.62 | 54.88 | 2350.55 | 2144.07 | 3123.81 | 99.17333 | 2539.477 |
| ATP1B1 | 0.236 | -2.08186 | 4.97E-17 | 3.84E-15 | yes | down | 54.53 | 54.87 | 72.52 | 23.34 | 23.36 | 23.1 | 60.64 | 23.26667 |
| LOC103346593 | 0.123 | -3.02725 | 5.32E-17 | 4.09E-15 | yes | down | 19.32 | 25.73 | 21.63 | 5.33 | 2.12 | 5.85 | 22.22667 | 4.433333 |
| MGP | 6.079 | 2.603928 | 6.27E-17 | 4.8E-15 | yes | up | 436.58 | 241.48 | 157.08 | 2387.31 | 2408.5 | 3041.22 | 278.38 | 2612.343 |
| MRPS22 | 0.388 | -1.36562 | 6.53E-17 | 4.98E-15 | yes | down | 37.1 | 47.28 | 35.17 | 24.15 | 24.09 | 28.37 | 39.85 | 25.53667 |
| LOC103352357 | 4.106 | 2.037907 | 6.8E-17 | 5.15E-15 | yes | up | 0.36 | 0.31 | 0.3 | 1.99 | 2.64 | 2.41 | 0.323333 | 2.346667 |
| MPI | 0.178 | -2.48968 | 7.25E-17 | 5.47E-15 | yes | down | 20.08 | 14.44 | 16.88 | 4.38 | 6.3 | 3.81 | 17.13333 | 4.83 |
| LOC108176558 | 0.073 | -3.77111 | 7.84E-17 | 5.86E-15 | yes | down | 5.05 | 8.54 | 4.46 | 0.38 | 0.53 | 0.84 | 6.016667 | 0.583333 |
| SEC11C | 0.225 | -2.15005 | 7.84E-17 | 5.86E-15 | yes | down | 222.27 | 364.94 | 279.03 | 83.59 | 101.45 | 128.85 | 288.7467 | 104.63 |
| LOC103346040 | 8.482 | 3.084373 | 8.11E-17 | 6.03E-15 | yes | up | 0.52 | 0.45 | 0.57 | 4.68 | 9.55 | 8.73 | 0.513333 | 7.653333 |
| IL6R | 5.073 | 2.342721 | 8.52E-17 | 6.31E-15 | yes | up | 0.64 | 0.86 | 0.71 | 7.5 | 4.55 | 5.94 | 0.736667 | 5.996667 |
| ABCA6 | 7.24 | 2.855956 | 9.56E-17 | 7.05E-15 | yes | up | 1.15 | 1.12 | 0.74 | 17.01 | 6.93 | 9.92 | 1.003333 | 11.28667 |
| KLF6 | 2.4 | 1.263108 | 1.31E-16 | 9.61E-15 | yes | up | 13.73 | 15.4 | 12.36 | 53.55 | 56.28 | 49.07 | 13.83 | 52.96667 |
| LRP1 | 2.894 | 1.533256 | 1.54E-16 | 1.13E-14 | yes | up | 13.75 | 9.89 | 8.36 | 50.21 | 48.6 | 49.11 | 10.66667 | 49.30667 |
| SRPX | 4.921 | 2.298856 | 1.62E-16 | 1.18E-14 | yes | up | 6.84 | 5.65 | 3.1 | 34.51 | 35.66 | 50.56 | 5.196667 | 40.24333 |
| LOC100346685 | 1027.584 | 10.00504 | 1.69E-16 | 1.22E-14 | yes | up | 0 | 0 | 0 | 9.75 | 11.59 | 11.56 | 0 | 10.96667 |
| CHCHD5 | 0.28 | -1.83889 | 1.87E-16 | 1.34E-14 | yes | down | 23.31 | 27.22 | 23.37 | 10.23 | 12.04 | 10.6 | 24.63333 | 10.95667 |
| LOC100355645 | 0.425 | -1.23491 | 2.01E-16 | 1.44E-14 | yes | down | 22.39 | 22.75 | 19.75 | 13.27 | 14.5 | 16.37 | 21.63 | 14.71333 |
| FIGN | 3.088 | 1.626801 | 2.03E-16 | 1.45E-14 | yes | up | 1.61 | 1.42 | 1.46 | 9.04 | 6.71 | 7.1 | 1.496667 | 7.616667 |
| C7 | 11.467 | 3.51939 | 2.09E-16 | 1.49E-14 | yes | up | 3.05 | 1.65 | 0.78 | 44.4 | 22.93 | 28.41 | 1.826667 | 31.91333 |
| MARS2 | 0.133 | -2.90793 | 2.26E-16 | 1.6E-14 | yes | down | 4.29 | 5.38 | 2.14 | 0.85 | 0.83 | 0.77 | 3.936667 | 0.816667 |
| TRIM25 | 3.508 | 1.810663 | 2.48E-16 | 1.75E-14 | yes | up | 3.45 | 4.36 | 3.22 | 24.61 | 19.73 | 17.24 | 3.676667 | 20.52667 |
| ETFDH | 0.328 | -1.60757 | 2.51E-16 | 1.76E-14 | yes | down | 239.9 | 219.11 | 179.47 | 93.57 | 124.24 | 139.77 | 212.8267 | 119.1933 |
| ARHGEF37 | 0.33 | -1.60128 | 2.67E-16 | 1.86E-14 | yes | down | 16.35 | 23.27 | 15.77 | 9.12 | 9.79 | 10.09 | 18.46333 | 9.666667 |
| PRDX3 | 0.402 | -1.31402 | 2.76E-16 | 1.92E-14 | yes | down | 317.34 | 302.63 | 289.05 | 175.95 | 209.08 | 189.38 | 303.0067 | 191.47 |
| TMEM177 | 0.268 | -1.89721 | 2.78E-16 | 1.92E-14 | yes | down | 5.1 | 6.11 | 4.52 | 2.32 | 2.3 | 2.07 | 5.243333 | 2.23 |
| SAT1 | 3.284 | 1.715432 | 3.38E-16 | 2.33E-14 | yes | up | 112.09 | 172.52 | 143.21 | 699.74 | 664.87 | 810.96 | 142.6067 | 725.19 |
| LOC100338043 | 4.061 | 2.021787 | 3.42E-16 | 2.35E-14 | yes | up | 11.03 | 14.1 | 12.64 | 77.65 | 62.73 | 108.49 | 12.59 | 82.95667 |
| FBN2 | 0.215 | -2.22074 | 3.57E-16 | 2.44E-14 | yes | down | 1.2 | 1.05 | 0.68 | 0.37 | 0.28 | 0.34 | 0.976667 | 0.33 |
| LOC100345038 | 2.713 | 1.439932 | 3.84E-16 | 2.61E-14 | yes | up | 2.29 | 1.91 | 1.47 | 7.98 | 7.81 | 8.97 | 1.89 | 8.253333 |
| SGIP1 | 5.098 | 2.350035 | 3.95E-16 | 2.68E-14 | yes | up | 0.31 | 0.44 | 0.3 | 2.97 | 2.94 | 3.3 | 0.35 | 3.07 |
| MRPS12 | 0.39 | -1.35731 | 4E-16 | 2.7E-14 | yes | down | 31.94 | 34.57 | 30.78 | 21.17 | 21.22 | 20.64 | 32.43 | 21.01 |
| SNRNP70 | 2.764 | 1.466772 | 4.02E-16 | 2.7E-14 | yes | up | 39.44 | 42.3 | 34.87 | 125.25 | 163.05 | 155.68 | 38.87 | 147.9933 |
| LOC103346281 | 0.006 | -7.35735 | 4.6E-16 | 3.08E-14 | yes | down | 1.54 | 2.02 | 1.52 | 0 | 0.01 | 0.02 | 1.693333 | 0.01 |
| ANKRD37 | 0.196 | -2.34923 | 4.95E-16 | 3.3E-14 | yes | down | 9.88 | 14.3 | 13.57 | 3.52 | 3.7 | 3.54 | 12.58333 | 3.586667 |
| DNAJA3 | 0.431 | -1.21488 | 6.16E-16 | 4.09E-14 | yes | down | 50.05 | 46.79 | 40.98 | 27.59 | 33.68 | 33.79 | 45.94 | 31.68667 |
| F11R | 3.02 | 1.594506 | 6.55E-16 | 4.33E-14 | yes | up | 23.24 | 26.95 | 28.34 | 140.46 | 113.2 | 130.15 | 26.17667 | 127.9367 |
| CHRDL1 | 2.751 | 1.459701 | 6.68E-16 | 4.4E-14 | yes | up | 21.79 | 20.47 | 23.55 | 103.95 | 95.99 | 101.35 | 21.93667 | 100.43 |
| LRRN3 | 12.518 | 3.645892 | 6.71E-16 | 4.4E-14 | yes | up | 0.15 | 0.14 | 0.08 | 2.72 | 1.56 | 3.17 | 0.123333 | 2.483333 |
| ARID5B | 3.608 | 1.851255 | 7.06E-16 | 4.61E-14 | yes | up | 4.86 | 3.02 | 2.92 | 19.27 | 18.4 | 24.97 | 3.6 | 20.88 |
| KIF20B | 0.067 | -3.90558 | 7.11E-16 | 4.62E-14 | yes | down | 10.71 | 11.86 | 29.26 | 1.42 | 1.79 | 1.32 | 17.27667 | 1.51 |
| LHX8 | 5.774 | 2.529679 | 8.44E-16 | 5.47E-14 | yes | up | 1.2 | 3.57 | 2.63 | 20.45 | 14.77 | 17.05 | 2.466667 | 17.42333 |
| GALNT12 | 2.891 | 1.53152 | 8.51E-16 | 5.49E-14 | yes | up | 3.76 | 3.91 | 2.61 | 15.67 | 14.9 | 16.74 | 3.426667 | 15.77 |
| CTSZ | 0.125 | -3.00382 | 9.15E-16 | 5.88E-14 | yes | down | 176.6 | 311.48 | 284.91 | 78.28 | 33.84 | 44.21 | 257.6633 | 52.11 |
| DUSP1 | 4.719 | 2.238633 | 9.85E-16 | 6.3E-14 | yes | up | 16.19 | 10.56 | 11.45 | 77.57 | 79.4 | 137.19 | 12.73333 | 98.05333 |
| PRSS23 | 2.837 | 1.50427 | 1E-15 | 6.36E-14 | yes | up | 22.85 | 18.62 | 15.29 | 71.19 | 83.1 | 102.15 | 18.92 | 85.48 |
| COQ5 | 0.442 | -1.17634 | 1E-15 | 6.36E-14 | yes | down | 38.3 | 32.31 | 30.25 | 19.33 | 19.88 | 19.63 | 33.62 | 19.61333 |
| RDH13 | 0.272 | -1.87646 | 1.08E-15 | 6.82E-14 | yes | down | 9.79 | 7.47 | 8.3 | 3.79 | 3.56 | 3.81 | 8.52 | 3.72 |
| FNBP4 | 2.411 | 1.269339 | 1.13E-15 | 7.1E-14 | yes | up | 4.42 | 4.03 | 2.95 | 14.12 | 13.48 | 15.48 | 3.8 | 14.36 |
| SLC35F6 | 0.357 | -1.48791 | 1.14E-15 | 7.14E-14 | yes | down | 13.66 | 14.07 | 13.25 | 8.74 | 5.56 | 8.03 | 13.66 | 7.443333 |
| NPC1 | 3.218 | 1.686355 | 1.43E-15 | 8.92E-14 | yes | up | 5.67 | 6.33 | 6.2 | 34.49 | 23.75 | 35.47 | 6.066667 | 31.23667 |
| CYP27A1 | 0.213 | -2.2311 | 1.82E-15 | 1.13E-13 | yes | down | 77.35 | 140.87 | 80.38 | 38.24 | 25.05 | 37.73 | 99.53333 | 33.67333 |
| LOC100351828 | 0.389 | -1.363 | 1.92E-15 | 1.19E-13 | yes | down | 21.4 | 18.65 | 14.58 | 10.37 | 11.2 | 12.39 | 18.21 | 11.32 |
| CLUH | 0.306 | -1.7097 | 2.16E-15 | 1.34E-13 | yes | down | 49.63 | 61.19 | 56.17 | 24.77 | 32.12 | 25.11 | 55.66333 | 27.33333 |
| OGT | 3.431 | 1.778688 | 2.27E-15 | 1.4E-13 | yes | up | 26 | 29.28 | 14.66 | 116.49 | 118 | 132.5 | 23.31333 | 122.33 |
| MGEA5 | 2.294 | 1.198144 | 2.37E-15 | 1.45E-13 | yes | up | 16.79 | 14.59 | 11.67 | 52.39 | 44.61 | 61.02 | 14.35 | 52.67333 |
| MXRA7 | 0.206 | -2.27928 | 3.13E-15 | 1.91E-13 | yes | down | 45.76 | 30.29 | 27.72 | 7.75 | 13.6 | 11.81 | 34.59 | 11.05333 |
| MARC2 | 2.506 | 1.325569 | 3.73E-15 | 2.27E-13 | yes | up | 19.65 | 19.49 | 13.11 | 46.14 | 54.62 | 67.94 | 17.41667 | 56.23333 |
| BOLA3 | 0.311 | -1.68656 | 4.72E-15 | 2.86E-13 | yes | down | 98.4 | 101.25 | 91.4 | 45.28 | 52.13 | 42.6 | 97.01667 | 46.67 |
| NSA2 | 0.473 | -1.07951 | 5E-15 | 3.01E-13 | yes | down | 47.75 | 50.14 | 43.35 | 35.22 | 32.04 | 39.79 | 47.08 | 35.68333 |
| C1QTNF7 | 5.266 | 2.39674 | 5.13E-15 | 3.09E-13 | yes | up | 1.16 | 0.9 | 0.72 | 6.84 | 8.36 | 6.6 | 0.926667 | 7.266667 |
| HR | 0.066 | -3.92133 | 5.74E-15 | 3.44E-13 | yes | down | 4.28 | 2.19 | 1.45 | 0.25 | 0.4 | 0.15 | 2.64 | 0.266667 |
| GOT1 | 0.224 | -2.15843 | 5.85E-15 | 3.49E-13 | yes | down | 116.87 | 61.3 | 66.23 | 24.1 | 31.38 | 31.33 | 81.46667 | 28.93667 |
| PLTP | 3.81 | 1.929835 | 6.08E-15 | 3.62E-13 | yes | up | 15.55 | 17.47 | 15.81 | 126.23 | 67.94 | 109.15 | 16.27667 | 101.1067 |
| PHF8 | 2.029 | 1.020999 | 6.44E-15 | 3.82E-13 | yes | up | 3.07 | 3.34 | 2.7 | 9.85 | 9.47 | 10.55 | 3.036667 | 9.956667 |
| LOC100343557 | 4.197 | 2.069265 | 6.63E-15 | 3.91E-13 | yes | up | 154.09 | 171.98 | 196.99 | 741.86 | 1030.82 | 1258.37 | 174.3533 | 1010.35 |
| EGR1 | 9.455 | 3.241116 | 7.22E-15 | 4.22E-13 | yes | up | 5.58 | 2.09 | 2.06 | 66.5 | 38.42 | 38.27 | 3.243333 | 47.73 |
| LOC103351259 | 2.293 | 1.19738 | 7.23E-15 | 4.22E-13 | yes | up | 10.31 | 11.57 | 10.69 | 33.73 | 29.08 | 28.89 | 10.85667 | 30.56667 |
| FYN | 2.053 | 1.038026 | 7.24E-15 | 4.22E-13 | yes | up | 11.09 | 10.07 | 8.42 | 33.99 | 30.81 | 31.94 | 9.86 | 32.24667 |
| MTX2 | 0.408 | -1.29318 | 7.71E-15 | 4.49E-13 | yes | down | 25.33 | 29.6 | 22.64 | 15.29 | 16.46 | 18.8 | 25.85667 | 16.85 |
| LOC100353463 | 2.66 | 1.411262 | 7.84E-15 | 4.54E-13 | yes | up | 24.82 | 32.31 | 25.29 | 103.5 | 123.63 | 116.89 | 27.47333 | 114.6733 |
| ABCD3 | 0.417 | -1.26327 | 7.99E-15 | 4.61E-13 | yes | down | 17.14 | 16.49 | 17.08 | 10.74 | 10.73 | 12.8 | 16.90333 | 11.42333 |
| IPO7 | 0.44 | -1.1846 | 8.59E-15 | 4.94E-13 | yes | down | 31.69 | 24.54 | 23.5 | 18.27 | 18.39 | 19.14 | 26.57667 | 18.6 |
| EFS | 2.764 | 1.466618 | 9.39E-15 | 5.38E-13 | yes | up | 2.27 | 1.92 | 1.43 | 8.27 | 7.35 | 8.99 | 1.873333 | 8.203333 |
| ST6GALNAC6 | 3.267 | 1.708185 | 9.59E-15 | 5.48E-13 | yes | up | 12.73 | 8.01 | 9.83 | 48.13 | 51.06 | 61.19 | 10.19 | 53.46 |
| SLC25A11 | 0.33 | -1.59912 | 1.12E-14 | 6.35E-13 | yes | down | 90.59 | 64.45 | 62.74 | 34.44 | 43 | 36.55 | 72.59333 | 37.99667 |
| ISOC2 | 0.333 | -1.5871 | 1.15E-14 | 6.51E-13 | yes | down | 87.07 | 81.39 | 97.5 | 42.41 | 47.92 | 51.75 | 88.65333 | 47.36 |
| TCP1 | 0.496 | -1.01188 | 1.21E-14 | 6.85E-13 | yes | down | 103.83 | 102.59 | 91 | 71.13 | 75.43 | 89.92 | 99.14 | 78.82667 |
| LOC100341175 | 0.369 | -1.43686 | 1.29E-14 | 7.26E-13 | yes | down | 690.28 | 539.94 | 592.45 | 321.05 | 395.14 | 359.13 | 607.5567 | 358.44 |
| LOC100346046 | 6.372 | 2.671679 | 1.38E-14 | 7.77E-13 | yes | up | 0.59 | 0.62 | 0.53 | 3.58 | 5.98 | 8.33 | 0.58 | 5.963333 |
| TSFM | 0.322 | -1.63528 | 1.52E-14 | 8.52E-13 | yes | down | 63.16 | 67.86 | 62.5 | 27.94 | 38.23 | 28.21 | 64.50667 | 31.46 |
| SLC6A6 | 0.166 | -2.59324 | 1.56E-14 | 8.64E-13 | yes | down | 14.35 | 23.96 | 21.27 | 6.42 | 3.41 | 5.46 | 19.86 | 5.096667 |
| NSUN2 | 0.496 | -1.01273 | 1.66E-14 | 9.21E-13 | yes | down | 22.13 | 22.45 | 19.65 | 15.87 | 16.23 | 18.92 | 21.41 | 17.00667 |
| AHCY | 0.488 | -1.03439 | 1.69E-14 | 9.28E-13 | yes | down | 23.98 | 22.59 | 18.28 | 17.95 | 15.24 | 17.24 | 21.61667 | 16.81 |
| POLDIP2 | 0.437 | -1.19494 | 1.69E-14 | 9.28E-13 | yes | down | 73.48 | 79.03 | 74.97 | 47.96 | 52.81 | 59.1 | 75.82667 | 53.29 |
| LOC100358789 | 0.248 | -2.01224 | 1.7E-14 | 9.29E-13 | yes | down | 49.9 | 35.68 | 37.88 | 13.89 | 20.74 | 13.91 | 41.15333 | 16.18 |
| APOD | 3.729 | 1.898677 | 1.77E-14 | 9.67E-13 | yes | up | 106.66 | 169.18 | 115.38 | 608.77 | 739.71 | 981.09 | 130.4067 | 776.5233 |
| TCTN2 | 0.409 | -1.2899 | 1.82E-14 | 9.89E-13 | yes | down | 13.5 | 11.54 | 10.92 | 7.56 | 7.09 | 8.89 | 11.98667 | 7.846667 |
| ABCD2 | 20.909 | 4.386027 | 2.15E-14 | 1.16E-12 | yes | up | 0.54 | 1.21 | 0.3 | 7.29 | 24.37 | 30.84 | 0.683333 | 20.83333 |
| AEBP1 | 2.913 | 1.542518 | 2.29E-14 | 1.24E-12 | yes | up | 12.37 | 8.32 | 7.37 | 44.32 | 40.98 | 44.5 | 9.353333 | 43.26667 |
| PNPT1 | 0.31 | -1.68782 | 2.32E-14 | 1.25E-12 | yes | down | 11.78 | 13.53 | 13.86 | 6.56 | 6.48 | 5.67 | 13.05667 | 6.236667 |
| BPNT1 | 0.461 | -1.11603 | 2.51E-14 | 1.35E-12 | yes | down | 25.71 | 27.47 | 24.93 | 20.15 | 18.01 | 19.64 | 26.03667 | 19.26667 |
| NOTCH2 | 2.655 | 1.408751 | 2.53E-14 | 1.35E-12 | yes | up | 3.58 | 2.8 | 2.02 | 11.66 | 10.4 | 12.51 | 2.8 | 11.52333 |
| SREBF1 | 0.172 | -2.54029 | 3.2E-14 | 1.71E-12 | yes | down | 19.94 | 32.75 | 21.03 | 4.37 | 8.97 | 6.74 | 24.57333 | 6.693333 |
| SLIRP | 0.324 | -1.62615 | 3.37E-14 | 1.79E-12 | yes | down | 227.52 | 220.75 | 216.64 | 90.17 | 116.92 | 136.72 | 221.6367 | 114.6033 |
| RBP4 | 0.293 | -1.76993 | 3.64E-14 | 1.93E-12 | yes | down | 64.82 | 75.27 | 67.81 | 23.71 | 35.98 | 39.15 | 69.3 | 32.94667 |
| SLC25A13 | 0.41 | -1.28623 | 3.7E-14 | 1.95E-12 | yes | down | 21.37 | 21.79 | 21.58 | 12.45 | 14.84 | 14.98 | 21.58 | 14.09 |
| FAM84B | 8.644 | 3.111736 | 3.81E-14 | 2E-12 | yes | up | 1.31 | 1.64 | 2.35 | 13.41 | 20.11 | 36.79 | 1.766667 | 23.43667 |
| CHCHD3 | 0.203 | -2.30325 | 3.83E-14 | 2.01E-12 | yes | down | 229.83 | 357.33 | 292.38 | 69.09 | 126 | 89.39 | 293.18 | 94.82667 |
| SPATA6L | 5.27 | 2.397831 | 3.94E-14 | 2.06E-12 | yes | up | 1.24 | 1.47 | 1.1 | 4.87 | 6.3 | 5.56 | 1.27 | 5.576667 |
| TNC | 4.105 | 2.037497 | 4.22E-14 | 2.2E-12 | yes | up | 2.47 | 2.17 | 1.4 | 14.19 | 8.88 | 16.2 | 2.013333 | 13.09 |
| MRPL50 | 0.314 | -1.67111 | 4.43E-14 | 2.3E-12 | yes | down | 14.48 | 17.62 | 13.24 | 8.25 | 6.65 | 7.71 | 15.11333 | 7.536667 |
| LRWD1 | 0.307 | -1.70488 | 4.55E-14 | 2.35E-12 | yes | down | 5.6 | 5.84 | 5.43 | 2.35 | 2.84 | 3.1 | 5.623333 | 2.763333 |
| LGMN | 2.07 | 1.049862 | 4.7E-14 | 2.41E-12 | yes | up | 51.72 | 61.16 | 44.84 | 174.63 | 152.95 | 194.12 | 52.57333 | 173.9 |
| SLC4A4 | 0.367 | -1.44741 | 4.7E-14 | 2.41E-12 | yes | down | 22.45 | 28.8 | 23.72 | 13.34 | 12.8 | 18.3 | 24.99 | 14.81333 |
| PER1 | 3.85 | 1.944818 | 4.71E-14 | 2.41E-12 | yes | up | 10.63 | 14.44 | 6.59 | 63.99 | 57.05 | 58.56 | 10.55333 | 59.86667 |
| FGL2 | 4.837 | 2.274022 | 4.73E-14 | 2.42E-12 | yes | up | 12.97 | 11.7 | 14.95 | 138.98 | 67.22 | 106.4 | 13.20667 | 104.2 |
| MOGAT2 | 0.02 | -5.64484 | 5.24E-14 | 2.67E-12 | yes | down | 2.55 | 1.12 | 1.97 | 0 | 0.11 | 0.1 | 1.88 | 0.07 |
| FAM65C | 9.799 | 3.292677 | 5.27E-14 | 2.68E-12 | yes | up | 0.18 | 0.07 | 0.14 | 1.7 | 2.2 | 2.27 | 0.13 | 2.056667 |
| MOGAT1 | 0.195 | -2.36166 | 5.36E-14 | 2.71E-12 | yes | down | 2.19 | 1.87 | 2.63 | 0.61 | 0.75 | 0.62 | 2.23 | 0.66 |
| LOC103345217 | 0.039 | -4.68473 | 5.44E-14 | 2.74E-12 | yes | down | 6.05 | 9.34 | 3.96 | 0.42 | 0.62 | 0.09 | 6.45 | 0.376667 |
| PTPA | 0.411 | -1.28384 | 5.62E-14 | 2.83E-12 | yes | down | 29.02 | 30.19 | 22.49 | 19.7 | 16.41 | 17.04 | 27.23333 | 17.71667 |
| CCBE1 | 0.203 | -2.29736 | 5.75E-14 | 2.88E-12 | yes | down | 2.59 | 3.53 | 1.6 | 0.87 | 0.78 | 0.82 | 2.573333 | 0.823333 |
| PHB | 0.365 | -1.45208 | 5.98E-14 | 2.99E-12 | yes | down | 219.4 | 210.39 | 233.74 | 126.3 | 145.86 | 123.73 | 221.1767 | 131.9633 |
| METAP1D | 0.185 | -2.43461 | 6.14E-14 | 3.06E-12 | yes | down | 5.92 | 7.7 | 5.51 | 1.94 | 2.2 | 1.4 | 6.376667 | 1.846667 |
| LOC100353320 | 0.195 | -2.35854 | 6.62E-14 | 3.29E-12 | yes | down | 405.02 | 452.44 | 608.22 | 117.4 | 190.93 | 152.19 | 488.56 | 153.5067 |
| HAS2 | 7.056 | 2.818805 | 6.86E-14 | 3.39E-12 | yes | up | 0.43 | 0.34 | 0.25 | 3.86 | 2.8 | 3.76 | 0.34 | 3.473333 |
| PTGES2 | 0.236 | -2.08057 | 7.04E-14 | 3.48E-12 | yes | down | 66.13 | 63.75 | 72.29 | 25.45 | 31.9 | 19.52 | 67.39 | 25.62333 |
| OLFML3 | 3.574 | 1.837484 | 7.14E-14 | 3.51E-12 | yes | up | 11.97 | 10.97 | 5.82 | 60.17 | 46.5 | 54.56 | 9.586667 | 53.74333 |
| TIMM17A | 0.301 | -1.73295 | 8.16E-14 | 4E-12 | yes | down | 97.33 | 156.79 | 103.4 | 52.36 | 50.45 | 68.54 | 119.1733 | 57.11667 |
| EIF2A | 0.437 | -1.19389 | 8.48E-14 | 4.14E-12 | yes | down | 16.77 | 18.12 | 15.03 | 12.78 | 9.79 | 12.33 | 16.64 | 11.63333 |
| SSPO | 7.801 | 2.96363 | 8.49E-14 | 4.14E-12 | yes | up | 0.12 | 0.07 | 0.04 | 1.01 | 0.69 | 0.87 | 0.076667 | 0.856667 |
| C13H1orf162 | 21.952 | 4.456297 | 8.72E-14 | 4.24E-12 | yes | up | 0.57 | 0.83 | 1.16 | 43.29 | 7.81 | 14.37 | 0.853333 | 21.82333 |
| GLRX | 0.469 | -1.09322 | 8.81E-14 | 4.27E-12 | yes | down | 61.42 | 52.21 | 51.85 | 42.47 | 40.36 | 41.68 | 55.16 | 41.50333 |
| ACADVL | 0.267 | -1.90354 | 8.86E-14 | 4.28E-12 | yes | down | 786.87 | 941.08 | 939.41 | 283.82 | 464.34 | 400.23 | 889.12 | 382.7967 |
| LOC100352100 | 0.405 | -1.30307 | 9E-14 | 4.33E-12 | yes | down | 109.67 | 89.87 | 95.71 | 57.82 | 66.13 | 67.16 | 98.41667 | 63.70333 |
| TIMM44 | 0.311 | -1.68633 | 9.24E-14 | 4.44E-12 | yes | down | 55.27 | 62.39 | 49.71 | 23.03 | 34.33 | 25.11 | 55.79 | 27.49 |
| CHCHD2 | 0.204 | -2.29041 | 9.49E-14 | 4.55E-12 | yes | down | 981.03 | 591.85 | 1043.5 | 220.56 | 304.55 | 332.19 | 872.1267 | 285.7667 |
| VWA8 | 0.44 | -1.18322 | 9.61E-14 | 4.59E-12 | yes | down | 10.94 | 10.39 | 10.44 | 8.78 | 7.08 | 8.23 | 10.59 | 8.03 |
| TIMM50 | 0.354 | -1.49921 | 1.16E-13 | 5.48E-12 | yes | down | 42.8 | 42.5 | 46.2 | 22.37 | 27.03 | 25.38 | 43.83333 | 24.92667 |
| ADAMTS4 | 7.442 | 2.8957 | 1.43E-13 | 6.74E-12 | yes | up | 0.74 | 0.96 | 0.78 | 5.17 | 10.42 | 14.13 | 0.826667 | 9.906667 |
| CEL | 0.022 | -5.49961 | 1.54E-13 | 7.23E-12 | yes | down | 1.77 | 6.37 | 7.31 | 0.26 | 0.25 | 0.04 | 5.15 | 0.183333 |
| CECR5 | 0.433 | -1.20608 | 1.68E-13 | 7.86E-12 | yes | down | 8.66 | 8.19 | 7.56 | 4.85 | 4.69 | 4.47 | 8.136667 | 4.67 |
| DIO2 | 0.176 | -2.50477 | 1.7E-13 | 7.94E-12 | yes | down | 5.85 | 6.29 | 7.31 | 1.84 | 3.18 | 1.82 | 6.483333 | 2.28 |
| ERP27 | 55.251 | 5.78794 | 1.74E-13 | 8.09E-12 | yes | up | 0.04 | 0 | 0.02 | 0.97 | 1.15 | 2.36 | 0.02 | 1.493333 |
| EZH1 | 2.31 | 1.207993 | 2E-13 | 9.28E-12 | yes | up | 11.17 | 10.53 | 11.64 | 39.77 | 37.05 | 46.15 | 11.11333 | 40.99 |
| TGFBR3 | 3.653 | 1.869161 | 2.21E-13 | 1.02E-11 | yes | up | 10.57 | 10.99 | 13.76 | 66.43 | 56.35 | 90.46 | 11.77333 | 71.08 |
| NDUFAF6 | 0.402 | -1.31629 | 2.28E-13 | 1.06E-11 | yes | down | 20.72 | 20.3 | 19.11 | 10.27 | 12.31 | 13.9 | 20.04333 | 12.16 |
| LOC103350057 | 12.478 | 3.641263 | 2.3E-13 | 1.06E-11 | yes | up | 0.08 | 0.14 | 0.07 | 3.12 | 4.6 | 2.51 | 0.096667 | 3.41 |
| IRF9 | 4.366 | 2.126375 | 2.41E-13 | 1.11E-11 | yes | up | 6.89 | 7.03 | 7.93 | 65.61 | 52.24 | 36.54 | 7.283333 | 51.46333 |
| GABBR1 | 3.863 | 1.949722 | 2.62E-13 | 1.2E-11 | yes | up | 0.8 | 0.63 | 0.53 | 5.11 | 2.99 | 5.53 | 0.653333 | 4.543333 |
| NOA1 | 0.36 | -1.47312 | 2.72E-13 | 1.24E-11 | yes | down | 14.92 | 14.82 | 13.26 | 6.7 | 8.18 | 9.76 | 14.33333 | 8.213333 |
| HS3ST1 | 6.069 | 2.601497 | 2.75E-13 | 1.25E-11 | yes | up | 1.3 | 1 | 0.72 | 11.56 | 6.26 | 11.59 | 1.006667 | 9.803333 |
| PNISR | 2.078 | 1.055134 | 2.88E-13 | 1.31E-11 | yes | up | 12.49 | 13.31 | 11.06 | 38.83 | 39.39 | 36.72 | 12.28667 | 38.31333 |
| SPON1 | 2.492 | 1.317146 | 2.88E-13 | 1.31E-11 | yes | up | 9.05 | 8.88 | 8.35 | 31.79 | 29.92 | 43.95 | 8.76 | 35.22 |
| LOC108175430 | 0.373 | -1.42324 | 3.11E-13 | 1.4E-11 | yes | down | 34.17 | 35.23 | 34.47 | 17.64 | 22.41 | 21.93 | 34.62333 | 20.66 |
| PPTC7 | 0.443 | -1.17353 | 3.19E-13 | 1.44E-11 | yes | down | 11.49 | 12.25 | 9.6 | 8.56 | 7.87 | 7.06 | 11.11333 | 7.83 |
| AFG3L2 | 0.381 | -1.39108 | 3.26E-13 | 1.46E-11 | yes | down | 41.84 | 47.4 | 40.3 | 21 | 27.96 | 30.22 | 43.18 | 26.39333 |
| ANG | 8.13 | 3.023182 | 3.27E-13 | 1.47E-11 | yes | up | 2.77 | 4.79 | 3.2 | 65.45 | 26.54 | 46.9 | 3.586667 | 46.29667 |
| ECSIT | 0.434 | -1.20368 | 3.85E-13 | 1.72E-11 | yes | down | 39.67 | 39.87 | 38.48 | 25.43 | 28.96 | 26.99 | 39.34 | 27.12667 |
| TFAM | 0.391 | -1.35306 | 3.91E-13 | 1.74E-11 | yes | down | 10.7 | 12.49 | 8.31 | 6.23 | 6 | 7.41 | 10.5 | 6.546667 |
| LPAR6 | 0.265 | -1.91447 | 3.97E-13 | 1.76E-11 | yes | down | 16.42 | 17.38 | 16.47 | 9.63 | 5.25 | 6.57 | 16.75667 | 7.15 |
| PARL | 0.46 | -1.12153 | 3.98E-13 | 1.76E-11 | yes | down | 36.75 | 40.16 | 35.57 | 27.06 | 28.5 | 26.94 | 37.49333 | 27.5 |
| LOC100337961 | 8.953 | 3.162397 | 4.32E-13 | 1.91E-11 | yes | up | 1.24 | 0.83 | 1.25 | 15.99 | 10.55 | 21.37 | 1.106667 | 15.97 |
| AKAP1 | 0.311 | -1.6856 | 4.52E-13 | 1.99E-11 | yes | down | 45.33 | 71.22 | 45.91 | 22.09 | 28.07 | 28.55 | 54.15333 | 26.23667 |
| LUC7L3 | 2.249 | 1.169113 | 4.56E-13 | 2.01E-11 | yes | up | 16.64 | 17.35 | 14.89 | 42.46 | 52.03 | 56.35 | 16.29333 | 50.28 |
| CA5B | 4.054 | 2.019518 | 4.95E-13 | 2.17E-11 | yes | up | 2.63 | 2.24 | 1.33 | 10.02 | 12.81 | 14.06 | 2.066667 | 12.29667 |
| LOC100356572 | 0.231 | -2.1146 | 5.28E-13 | 2.31E-11 | yes | down | 159.1 | 119.94 | 131.71 | 69.31 | 46.91 | 35.1 | 136.9167 | 50.44 |
| SRXN1 | 0.184 | -2.44101 | 5.51E-13 | 2.4E-11 | yes | down | 20.35 | 41.92 | 24.53 | 7.93 | 9.59 | 7.76 | 28.93333 | 8.426667 |
| CCDC71L | 6.756 | 2.756122 | 5.61E-13 | 2.44E-11 | yes | up | 1.16 | 1.18 | 0.69 | 7.22 | 7.89 | 17.62 | 1.01 | 10.91 |
| MRPL43 | 0.399 | -1.32693 | 5.71E-13 | 2.47E-11 | yes | down | 26.55 | 25.99 | 26.89 | 16.29 | 16.33 | 18.29 | 26.47667 | 16.97 |
| LOC100356693 | 0.383 | -1.38557 | 6.21E-13 | 2.68E-11 | yes | down | 55.32 | 75.82 | 61.39 | 36.43 | 37.76 | 44.1 | 64.17667 | 39.43 |
| BEND7 | 3.515 | 1.813364 | 6.43E-13 | 2.77E-11 | yes | up | 3.28 | 3.64 | 3.6 | 16.88 | 17.1 | 27.66 | 3.506667 | 20.54667 |
| HINT1 | 0.403 | -1.30985 | 6.8E-13 | 2.92E-11 | yes | down | 312.83 | 316.03 | 315.07 | 164.33 | 204.01 | 228.01 | 314.6433 | 198.7833 |
| NDUFA6 | 0.357 | -1.48683 | 7.11E-13 | 3.04E-11 | yes | down | 513.36 | 593.03 | 536.36 | 265.95 | 362.96 | 302.3 | 547.5833 | 310.4033 |
| FAM162A | 0.383 | -1.3854 | 7.31E-13 | 3.12E-11 | yes | down | 62.18 | 55.55 | 55.9 | 28.62 | 38.18 | 39.9 | 57.87667 | 35.56667 |
| DGAT1 | 0.431 | -1.21279 | 8.47E-13 | 3.6E-11 | yes | down | 48.09 | 55.25 | 53.97 | 31.96 | 33.89 | 38.3 | 52.43667 | 34.71667 |
| ITGBL1 | 10.379 | 3.375595 | 8.81E-13 | 3.74E-11 | yes | up | 1.06 | 0.51 | 0.33 | 8.26 | 14.39 | 12.65 | 0.633333 | 11.76667 |
| CACYBP | 0.491 | -1.02524 | 9.55E-13 | 4.04E-11 | yes | down | 76.46 | 71.44 | 53.39 | 47.63 | 51.71 | 53.69 | 67.09667 | 51.01 |
| MYBBP1A | 0.409 | -1.28807 | 1.03E-12 | 4.33E-11 | yes | down | 15.06 | 17.19 | 11.52 | 11.64 | 9.1 | 10 | 14.59 | 10.24667 |
| ANTXR2 | 3.235 | 1.693912 | 1.05E-12 | 4.42E-11 | yes | up | 18.88 | 24.43 | 22.62 | 88.25 | 110.82 | 149.64 | 21.97667 | 116.2367 |
| NDUFC2 | 0.356 | -1.48926 | 1.08E-12 | 4.54E-11 | yes | down | 495.33 | 620.83 | 501.21 | 264.4 | 357.74 | 290.86 | 539.1233 | 304.3333 |
| NDEL1 | 2.17 | 1.117618 | 1.09E-12 | 4.58E-11 | yes | up | 13.4 | 14.34 | 11.03 | 44.32 | 36.39 | 50.62 | 12.92333 | 43.77667 |
| RORC | 19.869 | 4.312468 | 1.16E-12 | 4.84E-11 | yes | up | 0.14 | 0.7 | 0.49 | 10.87 | 11.32 | 26.9 | 0.443333 | 16.36333 |
| TREX2 | 0.115 | -3.12225 | 1.19E-12 | 4.95E-11 | yes | down | 3.04 | 4.36 | 3.17 | 0.73 | 0.55 | 0.65 | 3.523333 | 0.643333 |
| TMEM100 | 2.142 | 1.099063 | 1.26E-12 | 5.23E-11 | yes | up | 10.16 | 11.64 | 9.42 | 34.12 | 33.44 | 39.47 | 10.40667 | 35.67667 |
| EXTL1 | 0.265 | -1.91355 | 1.28E-12 | 5.31E-11 | yes | down | 8.74 | 13.22 | 7.34 | 4.66 | 4.31 | 3.36 | 9.766667 | 4.11 |
| DCPS | 501.916 | 8.971301 | 1.28E-12 | 5.31E-11 | yes | up | 0 | 0 | 0 | 9.69 | 8.15 | 5.47 | 0 | 7.77 |
| AVPR1A | 3.648 | 1.866942 | 1.3E-12 | 5.37E-11 | yes | up | 1.16 | 1.01 | 1.23 | 5.63 | 5.81 | 8.76 | 1.133333 | 6.733333 |
| PIGV | 2.193 | 1.133107 | 1.35E-12 | 5.52E-11 | yes | up | 3.84 | 4.02 | 3.16 | 11.84 | 13.03 | 13.58 | 3.673333 | 12.81667 |
| ACAD8 | 0.335 | -1.57689 | 1.37E-12 | 5.6E-11 | yes | down | 22.92 | 32.96 | 27.02 | 13.56 | 16.16 | 14.8 | 27.63333 | 14.84 |
| ANKEF1 | 6.913 | 2.789224 | 1.53E-12 | 6.24E-11 | yes | up | 0.31 | 0.19 | 0.39 | 3.41 | 3.13 | 3.46 | 0.296667 | 3.333333 |
| SLC25A42 | 0.15 | -2.73759 | 1.58E-12 | 6.42E-11 | yes | down | 42.47 | 39.37 | 31.24 | 5.1 | 10.6 | 11.03 | 37.69333 | 8.91 |
| RNASE4 | 2.476 | 1.307956 | 1.66E-12 | 6.7E-11 | yes | up | 80.65 | 61.47 | 56.73 | 230.21 | 239.53 | 319.26 | 66.28333 | 263 |
| PDSS1 | 0.33 | -1.5989 | 1.7E-12 | 6.85E-11 | yes | down | 9.03 | 7.99 | 5.81 | 4 | 3.73 | 3.84 | 7.61 | 3.856667 |
| LOC103345926 | 5.132 | 2.359469 | 1.71E-12 | 6.87E-11 | yes | up | 104.09 | 58.64 | 45.73 | 523.09 | 365.31 | 789.36 | 69.48667 | 559.2533 |
| SMIM4 | 0.317 | -1.65624 | 1.85E-12 | 7.45E-11 | yes | down | 30.26 | 36.22 | 39.74 | 13.21 | 16.51 | 16.72 | 35.40667 | 15.48 |
| LOC100343802 | 5.069 | 2.341573 | 1.9E-12 | 7.6E-11 | yes | up | 179.99 | 300.58 | 118.51 | 1118.8 | 1634.71 | 1982.51 | 199.6933 | 1578.673 |
| RBM3 | 0.415 | -1.26915 | 1.94E-12 | 7.77E-11 | yes | down | 166.91 | 235.97 | 165.1 | 99.16 | 89.89 | 114.81 | 189.3267 | 101.2867 |
| GCG | 37.706 | 5.236728 | 1.99E-12 | 7.93E-11 | yes | up | 0.43 | 0.71 | 0.06 | 13.44 | 11.85 | 48.64 | 0.4 | 24.64333 |
| ZNF281 | 2.593 | 1.374389 | 2.14E-12 | 8.51E-11 | yes | up | 2.02 | 2.07 | 2.39 | 8.94 | 7.49 | 9.5 | 2.16 | 8.643333 |
| HYPK | 0.42 | -1.25148 | 2.19E-12 | 8.68E-11 | yes | down | 199.91 | 162.65 | 148.09 | 95.35 | 108.17 | 83.54 | 170.2167 | 95.68667 |
| PPP1R9A | 5.698 | 2.510529 | 2.4E-12 | 9.48E-11 | yes | up | 1.92 | 1.44 | 0.72 | 8.26 | 11.43 | 16.44 | 1.36 | 12.04333 |
| TNRC6A | 2.128 | 1.089819 | 2.45E-12 | 9.65E-11 | yes | up | 6.59 | 6.48 | 5.69 | 24.16 | 18.87 | 28.13 | 6.253333 | 23.72 |
| TRIP10 | 2.198 | 1.136048 | 2.64E-12 | 1.04E-10 | yes | up | 20.19 | 20.07 | 14.15 | 52.91 | 58.18 | 60.52 | 18.13667 | 57.20333 |
| INMT | 16.763 | 4.067212 | 2.79E-12 | 1.1E-10 | yes | up | 0.62 | 0.54 | 0.08 | 13.29 | 5.48 | 13.6 | 0.413333 | 10.79 |
| ARHGAP36 | 0.02 | -5.61478 | 2.98E-12 | 1.17E-10 | yes | down | 17.39 | 2.5 | 1.46 | 0.19 | 0.24 | 0.22 | 7.116667 | 0.216667 |
| LOC100344750 | 2.119 | 1.083308 | 3.07E-12 | 1.2E-10 | yes | up | 3.69 | 3.16 | 2.68 | 9.91 | 9.71 | 11.56 | 3.176667 | 10.39333 |
| AHR | 0.404 | -1.30648 | 3.12E-12 | 1.21E-10 | yes | down | 19.37 | 27.1 | 19.08 | 14.41 | 12.29 | 15.73 | 21.85 | 14.14333 |
| LOC108175423 | 0.284 | -1.81415 | 3.34E-12 | 1.3E-10 | yes | down | 20.4 | 26.1 | 24.22 | 10.11 | 12.45 | 9.44 | 23.57333 | 10.66667 |
| MAOA | 2.746 | 1.457592 | 3.38E-12 | 1.31E-10 | yes | up | 4.8 | 5.27 | 5.36 | 21.85 | 18.64 | 28.34 | 5.143333 | 22.94333 |
| LOC100344556 | 3.913 | 1.968382 | 3.54E-12 | 1.37E-10 | yes | up | 1.64 | 1.32 | 0.78 | 6.46 | 6.99 | 5.96 | 1.246667 | 6.47 |
| NDUFA12 | 0.401 | -1.3172 | 3.68E-12 | 1.42E-10 | yes | down | 383.08 | 307.07 | 264.11 | 177.41 | 229.79 | 206.2 | 318.0867 | 204.4667 |
| NFS1 | 0.335 | -1.57935 | 3.69E-12 | 1.42E-10 | yes | down | 43.9 | 54.37 | 45.44 | 21.2 | 23.2 | 32.18 | 47.90333 | 25.52667 |
| NDUFS1 | 0.356 | -1.49132 | 3.77E-12 | 1.45E-10 | yes | down | 191.76 | 177.2 | 163.09 | 86.22 | 122.64 | 87.81 | 177.35 | 98.89 |
| DFFB | 3.748 | 1.906006 | 3.79E-12 | 1.45E-10 | yes | up | 5.34 | 3.64 | 4.67 | 20.22 | 24.8 | 33.98 | 4.55 | 26.33333 |
| NCOA1 | 0.493 | -1.01968 | 3.81E-12 | 1.45E-10 | yes | down | 13.98 | 17.83 | 13.17 | 11.82 | 11.43 | 12.12 | 14.99333 | 11.79 |
| LOC103344988 | 0.359 | -1.4774 | 3.87E-12 | 1.47E-10 | yes | down | 294.23 | 283.59 | 210.23 | 122.45 | 130.73 | 201.15 | 262.6833 | 151.4433 |
| RBPMS2 | 2.496 | 1.319605 | 3.91E-12 | 1.48E-10 | yes | up | 5.52 | 6.06 | 6.05 | 25.83 | 21.7 | 24.89 | 5.876667 | 24.14 |
| KAZALD1 | 3.52 | 1.815664 | 3.98E-12 | 1.51E-10 | yes | up | 2.77 | 2.08 | 1.93 | 10.59 | 13.65 | 12.71 | 2.26 | 12.31667 |
| SLC25A15 | 0.357 | -1.48788 | 4.12E-12 | 1.56E-10 | yes | down | 11.04 | 8.12 | 8.39 | 4.41 | 5.26 | 6.08 | 9.183333 | 5.25 |
| LIAS | 0.498 | -1.00506 | 4.13E-12 | 1.56E-10 | yes | down | 16.81 | 17.5 | 14.18 | 12.15 | 13.37 | 13.41 | 16.16333 | 12.97667 |
| SAMM50 | 0.333 | -1.58532 | 4.52E-12 | 1.7E-10 | yes | down | 117.8 | 111.39 | 100.02 | 48.43 | 73.32 | 52.14 | 109.7367 | 57.96333 |
| NTMT1 | 0.383 | -1.38497 | 4.56E-12 | 1.71E-10 | yes | down | 63.05 | 56.56 | 48.6 | 29.69 | 38.92 | 33.09 | 56.07 | 33.9 |
| HRASLS5 | 5.882 | 2.556419 | 4.62E-12 | 1.73E-10 | yes | up | 0.74 | 2.35 | 1.22 | 14.61 | 15.37 | 25.72 | 1.436667 | 18.56667 |
| GNAZ | 0.131 | -2.93459 | 4.77E-12 | 1.78E-10 | yes | down | 2.25 | 4.48 | 1.72 | 0.49 | 0.54 | 0.71 | 2.816667 | 0.58 |
| ABHD18 | 0.383 | -1.38319 | 5E-12 | 1.87E-10 | yes | down | 12.86 | 9.73 | 8.95 | 5.78 | 6.88 | 5.59 | 10.51333 | 6.083333 |
| LAMTOR5 | 0.499 | -1.00336 | 5.13E-12 | 1.91E-10 | yes | down | 125.41 | 103.68 | 94.61 | 88.67 | 77 | 93.98 | 107.9 | 86.55 |
| TRPM1 | 14.465 | 3.854525 | 5.22E-12 | 1.94E-10 | yes | up | 0.1 | 0.26 | 0.05 | 1.95 | 2.75 | 4.57 | 0.136667 | 3.09 |
| DNAJA2 | 0.492 | -1.02448 | 5.66E-12 | 2.09E-10 | yes | down | 40.1 | 45.35 | 39.05 | 29.65 | 31.29 | 37.38 | 41.5 | 32.77333 |
| COMTD1 | 0.273 | -1.87137 | 6.19E-12 | 2.29E-10 | yes | down | 8.26 | 9.28 | 7.06 | 3.17 | 3.83 | 3.68 | 8.2 | 3.56 |
| UBE2M | 0.467 | -1.09997 | 6.25E-12 | 2.3E-10 | yes | down | 56.56 | 50.68 | 44.22 | 32.8 | 40.17 | 39.71 | 50.48667 | 37.56 |
| GAB2 | 3.341 | 1.740199 | 6.34E-12 | 2.33E-10 | yes | up | 4.95 | 5.63 | 7.18 | 30.28 | 29.86 | 36.88 | 5.92 | 32.34 |
| UBA7 | 6.621 | 2.726953 | 6.65E-12 | 2.44E-10 | yes | up | 1.94 | 1.81 | 1.41 | 26.67 | 17.65 | 9.18 | 1.72 | 17.83333 |
| NDUFS3 | 0.395 | -1.34187 | 6.85E-12 | 2.51E-10 | yes | down | 380.65 | 377.25 | 345.37 | 189.45 | 271 | 232.34 | 367.7567 | 230.93 |
| TTC27 | 0.426 | -1.2309 | 6.95E-12 | 2.54E-10 | yes | down | 6.35 | 6.84 | 5.63 | 4.06 | 3.62 | 4.88 | 6.273333 | 4.186667 |
| LRRK2 | 2.308 | 1.206406 | 7.04E-12 | 2.57E-10 | yes | up | 1.26 | 1.1 | 1.03 | 4.49 | 3.7 | 4.39 | 1.13 | 4.193333 |
| MFN2 | 0.454 | -1.13821 | 7.38E-12 | 2.68E-10 | yes | down | 71.86 | 60.51 | 61.18 | 44.08 | 51.23 | 39.8 | 64.51667 | 45.03667 |
| SMIM12 | 0.492 | -1.02411 | 7.76E-12 | 2.81E-10 | yes | down | 39.62 | 40.12 | 31.04 | 27.25 | 26.26 | 33.37 | 36.92667 | 28.96 |
| LOC100352673 | 142.488 | 7.154696 | 8.26E-12 | 2.99E-10 | yes | up | 0 | 0.2 | 0 | 6.25 | 17.03 | 25.32 | 0.066667 | 16.2 |
| BPGM | 0.244 | -2.03583 | 9.37E-12 | 3.38E-10 | yes | down | 10 | 17.26 | 15.08 | 4.85 | 5.5 | 6.37 | 14.11333 | 5.573333 |
| LOC100355134 | 0.197 | -2.34687 | 9.88E-12 | 3.55E-10 | yes | down | 5.36 | 4.07 | 2.44 | 1.31 | 1.08 | 1.26 | 3.956667 | 1.216667 |
| HPSE2 | 26.414 | 4.723243 | 1.13E-11 | 4.04E-10 | yes | up | 8.83 | 0.01 | 0.02 | 3.37 | 3.05 | 5.59 | 2.953333 | 4.003333 |
| LYRM2 | 0.384 | -1.37996 | 1.19E-11 | 4.23E-10 | yes | down | 9.23 | 9.74 | 8.04 | 5.44 | 5.62 | 6.57 | 9.003333 | 5.876667 |
| DPT | 3.696 | 1.885984 | 1.21E-11 | 4.31E-10 | yes | up | 54.92 | 35.01 | 24.21 | 230.25 | 260.99 | 183.13 | 38.04667 | 224.79 |
| LOC100341224 | 0.213 | -2.23345 | 1.27E-11 | 4.5E-10 | yes | down | 5.18 | 3.86 | 3.34 | 1.23 | 1.16 | 1.91 | 4.126667 | 1.433333 |
| SLC22A3 | 0.223 | -2.162 | 1.39E-11 | 4.94E-10 | yes | down | 17.66 | 29.1 | 18.68 | 4.89 | 7.22 | 10.25 | 21.81333 | 7.453333 |
| ZDHHC20 | 2.072 | 1.051195 | 1.41E-11 | 5E-10 | yes | up | 3.71 | 3.33 | 2.84 | 10.48 | 10.25 | 12.79 | 3.293333 | 11.17333 |
| NDUFAF1 | 0.47 | -1.09055 | 1.57E-11 | 5.5E-10 | yes | down | 27.48 | 25.03 | 19.97 | 15.25 | 16.3 | 20.1 | 24.16 | 17.21667 |
| ISM1 | 19.646 | 4.29613 | 1.64E-11 | 5.74E-10 | yes | up | 0.08 | 0.1 | 0.07 | 3.94 | 1.37 | 2.62 | 0.083333 | 2.643333 |
| IFRD2 | 0.401 | -1.31794 | 1.65E-11 | 5.77E-10 | yes | down | 31.81 | 30.47 | 22.67 | 14.06 | 17.29 | 21.69 | 28.31667 | 17.68 |
| FRMD6 | 2.226 | 1.154693 | 1.66E-11 | 5.79E-10 | yes | up | 3.65 | 3.53 | 2.41 | 12.25 | 10.5 | 11.07 | 3.196667 | 11.27333 |
| PAICS | 0.44 | -1.18547 | 1.73E-11 | 6.01E-10 | yes | down | 13.15 | 11.42 | 9.28 | 7.85 | 7.39 | 8.43 | 11.28333 | 7.89 |
| PMPCB | 0.47 | -1.08957 | 1.78E-11 | 6.17E-10 | yes | down | 83.05 | 91.66 | 82.93 | 56.87 | 68.54 | 68.86 | 85.88 | 64.75667 |
| MAP7 | 0.244 | -2.03516 | 1.83E-11 | 6.36E-10 | yes | down | 2.84 | 5.28 | 3.36 | 1.58 | 1.44 | 1.45 | 3.826667 | 1.49 |
| CUL9 | 2.693 | 1.429158 | 1.87E-11 | 6.49E-10 | yes | up | 3.17 | 3.83 | 2.45 | 14.25 | 14.64 | 11.33 | 3.15 | 13.40667 |
| CLU | 7.89 | 2.979956 | 2E-11 | 6.91E-10 | yes | up | 14.1 | 7.06 | 4.85 | 57.98 | 96.79 | 168.22 | 8.67 | 107.6633 |
| CBX7 | 2.623 | 1.390985 | 2.07E-11 | 7.12E-10 | yes | up | 3.2 | 4.12 | 3.29 | 13.41 | 13.66 | 18.3 | 3.536667 | 15.12333 |
| REC8 | 4.285 | 2.099168 | 2.21E-11 | 7.59E-10 | yes | up | 0.68 | 0.87 | 0.71 | 6.04 | 6.23 | 4.46 | 0.753333 | 5.576667 |
| GFPT1 | 0.415 | -1.26784 | 2.22E-11 | 7.6E-10 | yes | down | 16.1 | 14.89 | 10.38 | 8.29 | 8.93 | 7.51 | 13.79 | 8.243333 |
| IFI44 | 5.316 | 2.410419 | 2.26E-11 | 7.71E-10 | yes | up | 3.88 | 4.04 | 2.73 | 40.45 | 32.56 | 16.82 | 3.55 | 29.94333 |
| LOC100356833 | 0.338 | -1.56398 | 2.26E-11 | 7.71E-10 | yes | down | 7.95 | 8.16 | 5.88 | 4.76 | 3.86 | 3.12 | 7.33 | 3.913333 |
| PDK1 | 0.327 | -1.61193 | 2.27E-11 | 7.71E-10 | yes | down | 43.65 | 59.17 | 49.51 | 19.89 | 28.68 | 31.69 | 50.77667 | 26.75333 |
| LOC100356413 | 0.452 | -1.1467 | 2.33E-11 | 7.92E-10 | yes | down | 47.55 | 58.13 | 49.53 | 37.02 | 34.85 | 35.07 | 51.73667 | 35.64667 |
| MRPS35 | 0.381 | -1.39371 | 2.48E-11 | 8.39E-10 | yes | down | 48.58 | 55.93 | 57.28 | 28.79 | 32.03 | 38.7 | 53.93 | 33.17333 |
| TRIM8 | 2.353 | 1.234276 | 2.56E-11 | 8.65E-10 | yes | up | 5.23 | 4.29 | 4.19 | 15.9 | 15.14 | 20.78 | 4.57 | 17.27333 |
| YBX2 | 0.223 | -2.16273 | 2.68E-11 | 9.03E-10 | yes | down | 7.76 | 6.5 | 8.33 | 3.21 | 2.62 | 1.94 | 7.53 | 2.59 |
| GPLD1 | 0.24 | -2.05728 | 2.68E-11 | 9.03E-10 | yes | down | 2.43 | 2.69 | 2.78 | 1.22 | 0.76 | 1.25 | 2.633333 | 1.076667 |
| INSIG2 | 0.442 | -1.17902 | 2.71E-11 | 9.09E-10 | yes | down | 13.34 | 17.74 | 13.17 | 10.58 | 9.47 | 11.22 | 14.75 | 10.42333 |
| CA8 | 0.155 | -2.68937 | 2.73E-11 | 9.17E-10 | yes | down | 6.19 | 7.74 | 9.21 | 2.57 | 1.31 | 1.97 | 7.713333 | 1.95 |
| ACSM5 | 0.328 | -1.60675 | 2.78E-11 | 9.29E-10 | yes | down | 25.64 | 20.26 | 21.28 | 12.67 | 13.39 | 9.05 | 22.39333 | 11.70333 |
| KDR | 0.266 | -1.91218 | 2.78E-11 | 9.29E-10 | yes | down | 40.78 | 68.23 | 58.09 | 18.44 | 28.53 | 22.46 | 55.7 | 23.14333 |
| BMP2 | 8.51 | 3.089165 | 2.98E-11 | 9.92E-10 | yes | up | 0.26 | 0.64 | 0.54 | 6.38 | 4.59 | 9.15 | 0.48 | 6.706667 |
| MRPL47 | 0.487 | -1.03792 | 3.01E-11 | 1E-09 | yes | down | 41.93 | 41.45 | 38.15 | 28.74 | 31.98 | 31.96 | 40.51 | 30.89333 |
| CH25H | 8.883 | 3.15111 | 3.17E-11 | 1.05E-09 | yes | up | 0.29 | 0.62 | 0.27 | 4 | 4.42 | 8.48 | 0.393333 | 5.633333 |
| MAPK8IP3 | 3.117 | 1.640223 | 3.26E-11 | 1.08E-09 | yes | up | 4.68 | 6.6 | 4.75 | 24.06 | 30.54 | 22.73 | 5.343333 | 25.77667 |
| DNAJB12 | 0.446 | -1.16636 | 3.32E-11 | 1.09E-09 | yes | down | 18.72 | 21.85 | 13.02 | 12.62 | 12.48 | 13.29 | 17.86333 | 12.79667 |
| LOC100356242 | 28.204 | 4.817836 | 3.34E-11 | 1.1E-09 | yes | up | 0.64 | 0.49 | 0.29 | 42.98 | 15.59 | 3.86 | 0.473333 | 20.81 |
| LOC108176462 | 0.041 | -4.61919 | 3.47E-11 | 1.14E-09 | yes | down | 3.89 | 2.83 | 3.36 | 0.31 | 0.16 | 0.2 | 3.36 | 0.223333 |
| RBM33 | 2.071 | 1.050662 | 3.67E-11 | 1.2E-09 | yes | up | 3.44 | 3.42 | 2.99 | 9.38 | 11.48 | 11.75 | 3.283333 | 10.87 |
| KLF7 | 2.378 | 1.249914 | 3.75E-11 | 1.22E-09 | yes | up | 2.24 | 2.84 | 2.13 | 10.51 | 8 | 8.91 | 2.403333 | 9.14 |
| IDH3B | 0.406 | -1.29935 | 3.8E-11 | 1.24E-09 | yes | down | 412.69 | 458.76 | 468.68 | 260.29 | 302.59 | 354.89 | 446.71 | 305.9233 |
| TIMP3 | 2.26 | 1.17664 | 3.92E-11 | 1.27E-09 | yes | up | 26.58 | 28.73 | 28.26 | 101.82 | 90.83 | 110.54 | 27.85667 | 101.0633 |
| LOC103351083 | 3.752 | 1.907694 | 3.94E-11 | 1.28E-09 | yes | up | 0.75 | 3.33 | 1.53 | 12.01 | 7.31 | 5.69 | 1.87 | 8.336667 |
| C18H10orf11 | 2.522 | 1.334847 | 3.95E-11 | 1.28E-09 | yes | up | 2.69 | 2.59 | 2.35 | 11.57 | 9.67 | 9.68 | 2.543333 | 10.30667 |
| LOC100357095 | 347.356 | 8.440272 | 4.1E-11 | 1.32E-09 | yes | up | 0 | 0 | 0 | 9.11 | 5.48 | 5.72 | 0 | 6.77 |
| MOGS | 2.037 | 1.026144 | 4.22E-11 | 1.36E-09 | yes | up | 6.19 | 5.52 | 5.42 | 18.79 | 16.19 | 19.53 | 5.71 | 18.17 |
| NDUFAB1 | 0.496 | -1.01064 | 4.23E-11 | 1.36E-09 | yes | down | 297.78 | 292.35 | 255.72 | 208.29 | 244.01 | 213.51 | 281.95 | 221.9367 |
| MRPS28 | 0.364 | -1.45811 | 4.31E-11 | 1.38E-09 | yes | down | 37.53 | 40.03 | 25.7 | 17.66 | 20.82 | 20.61 | 34.42 | 19.69667 |
| KYAT3 | 0.435 | -1.1999 | 4.32E-11 | 1.38E-09 | yes | down | 56.1 | 52.77 | 54.37 | 35.75 | 42.42 | 35.93 | 54.41333 | 38.03333 |
| FAM13C | 0.235 | -2.09018 | 4.4E-11 | 1.41E-09 | yes | down | 8.3 | 8.33 | 9.26 | 2.09 | 4.08 | 3.18 | 8.63 | 3.116667 |
| CHP1 | 0.425 | -1.23589 | 4.69E-11 | 1.49E-09 | yes | down | 41.43 | 46.82 | 38.7 | 26.93 | 24.09 | 35.62 | 42.31667 | 28.88 |
| LOC100357567 | 0.413 | -1.2751 | 4.72E-11 | 1.5E-09 | yes | down | 27.15 | 34.14 | 21.9 | 19.99 | 17.59 | 16.82 | 27.73 | 18.13333 |
| CYTB | 0.312 | -1.68176 | 4.85E-11 | 1.54E-09 | yes | down | 20607.73 | 18026.4 | 22767.76 | 8584.96 | 12681.94 | 9498.33 | 20467.3 | 10255.08 |
| TMEM159 | 5.81 | 2.538585 | 5.21E-11 | 1.65E-09 | yes | up | 17.16 | 19.89 | 31.07 | 202.51 | 135.36 | 322.83 | 22.70667 | 220.2333 |
| DACT2 | 4.81 | 2.265985 | 5.29E-11 | 1.67E-09 | yes | up | 1.32 | 0.86 | 0.58 | 8.16 | 5.69 | 5.41 | 0.92 | 6.42 |
| PPL | 4.025 | 2.00909 | 5.39E-11 | 1.7E-09 | yes | up | 1.58 | 0.73 | 0.81 | 6.14 | 6.83 | 6.92 | 1.04 | 6.63 |
| GLUL | 3.017 | 1.593207 | 5.39E-11 | 1.7E-09 | yes | up | 105.55 | 168.26 | 131.18 | 524.26 | 656.45 | 766.34 | 134.9967 | 649.0167 |
| PIK3R1 | 2.55 | 1.350779 | 5.92E-11 | 1.86E-09 | yes | up | 7.22 | 6.03 | 4.31 | 18.9 | 24.08 | 27.52 | 5.853333 | 23.5 |
| LOC103345437 | 0.324 | -1.62452 | 5.97E-11 | 1.87E-09 | yes | down | 49.27 | 51.07 | 58.21 | 27.69 | 33.7 | 24.56 | 52.85 | 28.65 |
| ATP5L | 0.385 | -1.37711 | 6E-11 | 1.87E-09 | yes | down | 976.85 | 993.97 | 879.49 | 434.86 | 658.95 | 651.8 | 950.1033 | 581.87 |
| MRPL37 | 0.38 | -1.39486 | 6.13E-11 | 1.91E-09 | yes | down | 102.92 | 141.19 | 117.04 | 60.49 | 74.03 | 86.36 | 120.3833 | 73.62667 |
| TEFM | 0.352 | -1.50797 | 6.55E-11 | 2.04E-09 | yes | down | 8.45 | 10.96 | 9.47 | 4.71 | 5.7 | 5.9 | 9.626667 | 5.436667 |
| RCC1L | 0.278 | -1.84846 | 6.74E-11 | 2.09E-09 | yes | down | 24.53 | 23.92 | 21.98 | 13.54 | 10.99 | 7.29 | 23.47667 | 10.60667 |
| LOC100348091 | 0.337 | -1.56977 | 6.74E-11 | 2.09E-09 | yes | down | 8.47 | 11.38 | 8.22 | 5.27 | 4.21 | 6.35 | 9.356667 | 5.276667 |
| HSPBP1 | 0.387 | -1.3701 | 6.88E-11 | 2.12E-09 | yes | down | 8.77 | 8.43 | 6.28 | 5.03 | 4.79 | 4.54 | 7.826667 | 4.786667 |
| PMEPA1 | 0.097 | -3.36799 | 6.88E-11 | 2.12E-09 | yes | down | 10.24 | 10.42 | 3.39 | 0.95 | 0.89 | 1.16 | 8.016667 | 1 |
| ABCA8 | 5.125 | 2.357558 | 7.69E-11 | 2.36E-09 | yes | up | 9.58 | 4.59 | 2.89 | 43.22 | 41.81 | 49.98 | 5.686667 | 45.00333 |
| TECR | 0.305 | -1.71542 | 8.15E-11 | 2.5E-09 | yes | down | 161.52 | 137.52 | 183.97 | 72.17 | 91.8 | 71.23 | 161.0033 | 78.4 |
| QARS | 0.49 | -1.02959 | 8.3E-11 | 2.54E-09 | yes | down | 49.31 | 59.27 | 50.65 | 37.87 | 42.07 | 45 | 53.07667 | 41.64667 |
| ACSS3 | 0.443 | -1.1741 | 8.62E-11 | 2.64E-09 | yes | down | 31.97 | 41.32 | 36.82 | 26.18 | 26.38 | 25.89 | 36.70333 | 26.15 |
| ENG | 0.275 | -1.86116 | 8.67E-11 | 2.65E-09 | yes | down | 71.09 | 105.44 | 123.35 | 46.32 | 38.48 | 50.57 | 99.96 | 45.12333 |
| EIF4EBP1 | 0.396 | -1.33479 | 8.93E-11 | 2.72E-09 | yes | down | 58.67 | 77.69 | 63.99 | 35.69 | 42.37 | 49.47 | 66.78333 | 42.51 |
| LOC100345918 | 14.256 | 3.833519 | 9.12E-11 | 2.77E-09 | yes | up | 2.45 | 1.76 | 1 | 67.86 | 36.06 | 10.62 | 1.736667 | 38.18 |
| GTPBP6 | 0.431 | -1.21415 | 9.5E-11 | 2.88E-09 | yes | down | 35.95 | 35.53 | 33.63 | 21.09 | 27.56 | 23.57 | 35.03667 | 24.07333 |
| GADD45A | 3.467 | 1.793562 | 9.5E-11 | 2.88E-09 | yes | up | 16.22 | 9.51 | 14.66 | 63.19 | 77.5 | 85.61 | 13.46333 | 75.43333 |
| DCDC2B | 7.576 | 2.921458 | 9.53E-11 | 2.88E-09 | yes | up | 0.25 | 0.17 | 0.14 | 1.13 | 2.3 | 2.04 | 0.186667 | 1.823333 |
| LOC100347175 | 0.435 | -1.20238 | 1.02E-10 | 3.06E-09 | yes | down | 47.54 | 48.45 | 50.93 | 30.8 | 36.05 | 35.89 | 48.97333 | 34.24667 |
| HSF4 | 15.2 | 3.925967 | 1.08E-10 | 3.26E-09 | yes | up | 0.18 | 0.07 | 0.07 | 2 | 2.52 | 3.72 | 0.106667 | 2.746667 |
| STIP1 | 0.434 | -1.20398 | 1.19E-10 | 3.56E-09 | yes | down | 48.61 | 41.44 | 37.43 | 32.94 | 29.23 | 25.03 | 42.49333 | 29.06667 |
| MMP28 | 3.342 | 1.740816 | 1.21E-10 | 3.63E-09 | yes | up | 4.19 | 3.05 | 3.64 | 14.36 | 20.26 | 27.96 | 3.626667 | 20.86 |
| MITF | 2.583 | 1.368937 | 1.24E-10 | 3.7E-09 | yes | up | 3.72 | 2.53 | 2.49 | 11.41 | 12.27 | 12.73 | 2.913333 | 12.13667 |
| MTX1 | 0.337 | -1.57132 | 1.29E-10 | 3.85E-09 | yes | down | 35.53 | 47.75 | 45.49 | 20.34 | 26.9 | 22.41 | 42.92333 | 23.21667 |
| AKAP7 | 3.153 | 1.656837 | 1.32E-10 | 3.93E-09 | yes | up | 6.19 | 5.65 | 4.59 | 25.51 | 17.01 | 29.79 | 5.476667 | 24.10333 |
| GNG2 | 2.957 | 1.564178 | 1.36E-10 | 4.02E-09 | yes | up | 5.73 | 7.45 | 5.36 | 34.94 | 20.06 | 33.26 | 6.18 | 29.42 |
| NR4A3 | 11.202 | 3.485669 | 1.46E-10 | 4.33E-09 | yes | up | 0.11 | 0.62 | 0.16 | 2.66 | 3.89 | 5.2 | 0.296667 | 3.916667 |
| PDP2 | 0.311 | -1.68651 | 1.51E-10 | 4.47E-09 | yes | down | 12.55 | 15.02 | 16.98 | 6.61 | 8.52 | 6.61 | 14.85 | 7.246667 |
| EMC6 | 0.484 | -1.04736 | 1.52E-10 | 4.47E-09 | yes | down | 60.62 | 59.15 | 55.96 | 42.41 | 39.74 | 51.48 | 58.57667 | 44.54333 |
| LOC103345923 | 0.415 | -1.26794 | 1.55E-10 | 4.55E-09 | yes | down | 146.25 | 151.45 | 130.05 | 72.18 | 98.05 | 114.33 | 142.5833 | 94.85333 |
| FZD6 | 0.376 | -1.41194 | 1.55E-10 | 4.56E-09 | yes | down | 10.23 | 13.13 | 12.31 | 7.98 | 7.45 | 6.81 | 11.89 | 7.413333 |
| NEDD4L | 0.259 | -1.95112 | 1.6E-10 | 4.69E-09 | yes | down | 8.05 | 6.55 | 3.09 | 2.26 | 2.48 | 2.28 | 5.896667 | 2.34 |
| LOC103351504 | 0.282 | -1.82448 | 1.61E-10 | 4.69E-09 | yes | down | 8.11 | 12.59 | 10.98 | 3.24 | 4.58 | 4.71 | 10.56 | 4.176667 |
| FBXO38 | 2.089 | 1.06249 | 1.61E-10 | 4.71E-09 | yes | up | 6.95 | 7.26 | 6.73 | 20.42 | 19.39 | 26.55 | 6.98 | 22.12 |
| NDUFB6 | 0.399 | -1.32472 | 1.65E-10 | 4.79E-09 | yes | down | 335.18 | 334.89 | 335.58 | 137.44 | 214.98 | 188.92 | 335.2167 | 180.4467 |
| COQ4 | 0.275 | -1.86129 | 1.75E-10 | 5.08E-09 | yes | down | 7.4 | 7.12 | 6.39 | 3.62 | 3.19 | 2.29 | 6.97 | 3.033333 |
| LOC103347061 | 0.309 | -1.69551 | 1.75E-10 | 5.08E-09 | yes | down | 30.7 | 28.48 | 24.62 | 14.82 | 14.44 | 11.26 | 27.93333 | 13.50667 |
| ACAT1 | 0.328 | -1.60729 | 1.76E-10 | 5.1E-09 | yes | down | 443.03 | 336.09 | 383.83 | 161.67 | 256.25 | 190.65 | 387.65 | 202.8567 |
| COQ7 | 0.45 | -1.15296 | 1.79E-10 | 5.17E-09 | yes | down | 44.92 | 39.57 | 36.99 | 29.88 | 30.53 | 26.25 | 40.49333 | 28.88667 |
| TSPAN33 | 8.307 | 3.054391 | 1.9E-10 | 5.46E-09 | yes | up | 0.21 | 0.41 | 0.31 | 3.15 | 3.4 | 6.11 | 0.31 | 4.22 |
| MTO1 | 0.45 | -1.15083 | 1.91E-10 | 5.5E-09 | yes | down | 8.51 | 7.95 | 5.98 | 4.61 | 6.03 | 5.9 | 7.48 | 5.513333 |
| CBLB | 2.224 | 1.153441 | 2E-10 | 5.76E-09 | yes | up | 3.54 | 4.42 | 3.77 | 14.71 | 12.16 | 16.49 | 3.91 | 14.45333 |
| RECQL5 | 2.001 | 1.000925 | 2.11E-10 | 6.04E-09 | yes | up | 3.21 | 3.3 | 2.84 | 9.79 | 10.09 | 9.6 | 3.116667 | 9.826667 |
| MRPS10 | 0.42 | -1.25064 | 2.33E-10 | 6.65E-09 | yes | down | 30.11 | 37.56 | 33.05 | 21.8 | 18.1 | 15.97 | 33.57333 | 18.62333 |
| ARHGAP1 | 2.05 | 1.035305 | 2.48E-10 | 7.08E-09 | yes | up | 7.74 | 7.6 | 6.43 | 23.21 | 19.74 | 28.69 | 7.256667 | 23.88 |
| TSC22D3 | 2.675 | 1.419334 | 2.56E-10 | 7.29E-09 | yes | up | 33.59 | 39.23 | 29.06 | 186.69 | 111.25 | 137.32 | 33.96 | 145.0867 |
| NUDT4 | 2.264 | 1.178738 | 2.61E-10 | 7.4E-09 | yes | up | 15.04 | 12.1 | 11.98 | 40.13 | 45.72 | 54.7 | 13.04 | 46.85 |
| ND6 | 0.213 | -2.23037 | 2.75E-10 | 7.77E-09 | yes | down | 8343.68 | 5444.94 | 4871.46 | 968.47 | 2443.26 | 2982.3 | 6220.027 | 2131.343 |
| ADAMTS1 | 3.846 | 1.943308 | 2.8E-10 | 7.9E-09 | yes | up | 7.05 | 6.73 | 7.57 | 29.47 | 37.15 | 64.73 | 7.116667 | 43.78333 |
| NDUFV2 | 0.461 | -1.11609 | 2.85E-10 | 8.02E-09 | yes | down | 211.27 | 204.5 | 149.03 | 112.44 | 146.97 | 150.09 | 188.2667 | 136.5 |
| ND3 | 0.37 | -1.43595 | 2.94E-10 | 8.25E-09 | yes | down | 27501.35 | 42960.9 | 30782.62 | 20418.57 | 20002.31 | 18339.04 | 33748.29 | 19586.64 |
| SESTD1 | 2.406 | 1.26661 | 2.96E-10 | 8.28E-09 | yes | up | 6.41 | 7.23 | 8.5 | 25.21 | 20.43 | 25.52 | 7.38 | 23.72 |
| KBTBD11 | 3.814 | 1.931459 | 2.98E-10 | 8.32E-09 | yes | up | 1.26 | 1.51 | 1.07 | 5.21 | 8.13 | 9.8 | 1.28 | 7.713333 |
| LOC100354804 | 12.839 | 3.682478 | 3.04E-10 | 8.46E-09 | yes | up | 0.33 | 0.78 | 0.07 | 13.07 | 5.83 | 7.82 | 0.393333 | 8.906667 |
| TRIM36 | 0.176 | -2.50685 | 3.04E-10 | 8.46E-09 | yes | down | 1.5 | 2.53 | 2.89 | 0.74 | 0.69 | 0.51 | 2.306667 | 0.646667 |
| KLHL42 | 2.326 | 1.217998 | 3.13E-10 | 8.68E-09 | yes | up | 1.76 | 1.55 | 1.23 | 5.84 | 4.65 | 5.85 | 1.513333 | 5.446667 |
| LOC100353330 | 0.321 | -1.63754 | 3.18E-10 | 8.81E-09 | yes | down | 48.51 | 56.83 | 52.92 | 15.56 | 16.36 | 11.92 | 52.75333 | 14.61333 |
| DARS | 0.492 | -1.02371 | 3.32E-10 | 9.19E-09 | yes | down | 38.56 | 33.48 | 24.63 | 23.23 | 25.52 | 25.74 | 32.22333 | 24.83 |
| PHKA2 | 3.217 | 1.685669 | 3.43E-10 | 9.47E-09 | yes | up | 1.89 | 2.16 | 1.42 | 6.47 | 9.95 | 11.49 | 1.823333 | 9.303333 |
| TMEM40 | 0.189 | -2.40471 | 3.67E-10 | 1.01E-08 | yes | down | 3.8 | 2.89 | 1.78 | 1.62 | 1.48 | 1.88 | 2.823333 | 1.66 |
| LGALS8 | 2.253 | 1.171828 | 3.82E-10 | 1.05E-08 | yes | up | 5.1 | 4.27 | 4.36 | 16.69 | 11.53 | 15.51 | 4.576667 | 14.57667 |
| BCCIP | 0.499 | -1.00401 | 3.85E-10 | 1.06E-08 | yes | down | 41.97 | 43.99 | 42.97 | 33.31 | 30.81 | 37.44 | 42.97667 | 33.85333 |
| LOC100009551 | 4.733 | 2.242873 | 3.9E-10 | 1.07E-08 | yes | up | 7.13 | 14.39 | 10.17 | 67.93 | 50.35 | 115.79 | 10.56333 | 78.02333 |
| LOC100352119 | 9.347 | 3.224428 | 3.93E-10 | 1.08E-08 | yes | up | 4.14 | 3.44 | 2.34 | 15.84 | 57.77 | 78.31 | 3.306667 | 50.64 |
| LOC103347403 | 8.127 | 3.022664 | 3.96E-10 | 1.08E-08 | yes | up | 0.79 | 1.08 | 0.49 | 14.16 | 10.91 | 5.31 | 0.786667 | 10.12667 |
| MX1 | 4.935 | 2.302989 | 4.01E-10 | 1.09E-08 | yes | up | 22.32 | 19.1 | 13.13 | 190.01 | 159.64 | 72.62 | 18.18333 | 140.7567 |
| BCL2L11 | 3.676 | 1.878108 | 4.08E-10 | 1.11E-08 | yes | up | 2.24 | 1.57 | 2.09 | 15.12 | 8.25 | 11.79 | 1.966667 | 11.72 |
| MRPL19 | 0.486 | -1.04189 | 4.13E-10 | 1.12E-08 | yes | down | 36.8 | 38.07 | 35.68 | 25.8 | 31.19 | 27.44 | 36.85 | 28.14333 |
| LOC100009451 | 21.657 | 4.436739 | 4.22E-10 | 1.15E-08 | yes | up | 1.03 | 0.55 | 0.26 | 38.34 | 17.08 | 5.29 | 0.613333 | 20.23667 |
| ALKBH7 | 0.287 | -1.80202 | 4.38E-10 | 1.19E-08 | yes | down | 12.48 | 16.4 | 14.73 | 5.68 | 8.15 | 6.13 | 14.53667 | 6.653333 |
| HAL | 36.742 | 5.199359 | 4.48E-10 | 1.21E-08 | yes | up | 0.02 | 0.02 | 0.05 | 1.34 | 1.25 | 2.77 | 0.03 | 1.786667 |
| WNT9A | 2.497 | 1.320106 | 4.59E-10 | 1.24E-08 | yes | up | 1.43 | 1.15 | 0.93 | 4.14 | 4.85 | 4.95 | 1.17 | 4.646667 |
| MTERF4 | 0.437 | -1.19555 | 4.65E-10 | 1.25E-08 | yes | down | 17.53 | 14.97 | 12.81 | 9.56 | 10.13 | 11.31 | 15.10333 | 10.33333 |
| ADNP2 | 2.352 | 1.233585 | 4.7E-10 | 1.26E-08 | yes | up | 10.67 | 15.27 | 9.24 | 38.83 | 43.15 | 45.92 | 11.72667 | 42.63333 |
| ZFAND4 | 0.388 | -1.36511 | 5.62E-10 | 1.5E-08 | yes | down | 6.34 | 7.66 | 6.65 | 2.93 | 2.97 | 3.8 | 6.883333 | 3.233333 |
| LOC108176480 | 3.22 | 1.687087 | 5.8E-10 | 1.55E-08 | yes | up | 1.03 | 1.11 | 0.8 | 3.42 | 5.26 | 6.4 | 0.98 | 5.026667 |
| FAM126A | 3.829 | 1.936925 | 5.87E-10 | 1.56E-08 | yes | up | 1.77 | 1.04 | 0.85 | 5.27 | 7.62 | 8.48 | 1.22 | 7.123333 |
| ANGPTL4 | 5.528 | 2.46687 | 6.17E-10 | 1.64E-08 | yes | up | 55.04 | 42.34 | 81.17 | 308.46 | 582.3 | 738.74 | 59.51667 | 543.1667 |
| ESRRA | 0.331 | -1.59724 | 6.22E-10 | 1.65E-08 | yes | down | 16.94 | 20 | 16.06 | 8.14 | 10.41 | 8.8 | 17.66667 | 9.116667 |
| DSG4 | 14.878 | 3.895075 | 6.32E-10 | 1.67E-08 | yes | up | 0.31 | 0.11 | 0.09 | 5.68 | 1.41 | 4.77 | 0.17 | 3.953333 |
| ATG4D | 0.399 | -1.32448 | 6.39E-10 | 1.69E-08 | yes | down | 10.81 | 11.87 | 11.74 | 7.3 | 7.63 | 7.12 | 11.47333 | 7.35 |
| PTPN5 | 14.361 | 3.844107 | 6.58E-10 | 1.74E-08 | yes | up | 0.11 | 0.23 | 0.26 | 2.69 | 4.13 | 7.75 | 0.2 | 4.856667 |
| CPEB1 | 6.175 | 2.626432 | 7.18E-10 | 1.88E-08 | yes | up | 0.45 | 0.36 | 0.25 | 2.15 | 3.23 | 4.8 | 0.353333 | 3.393333 |
| LOC100354761 | 0.478 | -1.06541 | 7.38E-10 | 1.93E-08 | yes | down | 31.05 | 31.34 | 22.61 | 20.19 | 24.08 | 23.37 | 28.33333 | 22.54667 |
| WDR62 | 3.359 | 1.748182 | 7.39E-10 | 1.93E-08 | yes | up | 1.12 | 1.04 | 0.91 | 4.38 | 7.06 | 4.93 | 1.023333 | 5.456667 |
| LOC100356803 | 0.461 | -1.11749 | 7.53E-10 | 1.96E-08 | yes | down | 508.43 | 510.32 | 496.57 | 311.83 | 406.95 | 391.84 | 505.1067 | 370.2067 |
| FTSJ3 | 0.428 | -1.22302 | 7.59E-10 | 1.97E-08 | yes | down | 13.21 | 13.83 | 11.57 | 10.07 | 6.61 | 9.91 | 12.87 | 8.863333 |
| ALAS1 | 0.403 | -1.31029 | 7.72E-10 | 2E-08 | yes | down | 55.44 | 50.22 | 51.5 | 26.59 | 39.05 | 35.91 | 52.38667 | 33.85 |
| LOC100346269 | 2.08 | 1.056797 | 8.01E-10 | 2.07E-08 | yes | up | 1.3 | 1.04 | 1.1 | 3.09 | 2.53 | 3.3 | 1.146667 | 2.973333 |
| ZNF446 | 2.166 | 1.115039 | 8.04E-10 | 2.08E-08 | yes | up | 2.55 | 3.34 | 2.57 | 9.73 | 8.21 | 9.46 | 2.82 | 9.133333 |
| ADAMTSL1 | 6.114 | 2.612128 | 8.1E-10 | 2.09E-08 | yes | up | 4.44 | 1.05 | 2.97 | 22.5 | 29.71 | 12.33 | 2.82 | 21.51333 |
| LOC100356626 | 4.323 | 2.111913 | 8.4E-10 | 2.16E-08 | yes | up | 1.93 | 1.04 | 0.74 | 6.19 | 9.21 | 9.64 | 1.236667 | 8.346667 |
| ANGPTL7 | 35.65 | 5.155831 | 8.71E-10 | 2.24E-08 | yes | up | 0.08 | 0 | 0.09 | 4.12 | 1.28 | 4.42 | 0.056667 | 3.273333 |
| MFSD2A | 0.085 | -3.55373 | 8.74E-10 | 2.24E-08 | yes | down | 2.12 | 8.83 | 4.87 | 0.38 | 1.03 | 0.74 | 5.273333 | 0.716667 |
| CAMKK2 | 2.321 | 1.214535 | 8.95E-10 | 2.29E-08 | yes | up | 2.79 | 2.67 | 2.08 | 7.76 | 8.88 | 11.28 | 2.513333 | 9.306667 |
| LOC100341136 | 8.65 | 3.112648 | 9.01E-10 | 2.3E-08 | yes | up | 0.35 | 0.71 | 0.8 | 13.64 | 5.46 | 4.7 | 0.62 | 7.933333 |
| THBS1 | 5.496 | 2.458353 | 9.03E-10 | 2.3E-08 | yes | up | 17.43 | 30.85 | 26.67 | 336.28 | 112.43 | 222.4 | 24.98333 | 223.7033 |
| CATSPER2 | 9.032 | 3.175082 | 9.52E-10 | 2.42E-08 | yes | up | 0.32 | 0.12 | 0.08 | 1.79 | 1.69 | 2.11 | 0.173333 | 1.863333 |
| LOC100358060 | 3.144 | 1.652564 | 9.65E-10 | 2.45E-08 | yes | up | 8.32 | 7.91 | 11.51 | 45.47 | 40.69 | 57.05 | 9.246667 | 47.73667 |
| ITGA11 | 4.787 | 2.259181 | 9.88E-10 | 2.5E-08 | yes | up | 1.24 | 0.9 | 0.39 | 5.34 | 7.2 | 6.12 | 0.843333 | 6.22 |
| FAM107A | 3.411 | 1.770282 | 1.04E-09 | 2.64E-08 | yes | up | 8.4 | 9.65 | 10.21 | 65.16 | 32.8 | 57.21 | 9.42 | 51.72333 |
| B3GNT4 | 4.335 | 2.115975 | 1.05E-09 | 2.66E-08 | yes | up | 0.85 | 0.85 | 0.49 | 4.59 | 4.8 | 5.57 | 0.73 | 4.986667 |
| MRPS16 | 0.407 | -1.298 | 1.06E-09 | 2.66E-08 | yes | down | 81.24 | 67.31 | 83.83 | 49.32 | 45.09 | 58.35 | 77.46 | 50.92 |
| CDH18 | 7.473 | 2.901631 | 1.1E-09 | 2.76E-08 | yes | up | 0.39 | 0.43 | 0.21 | 3.83 | 4.78 | 2.58 | 0.343333 | 3.73 |
| LOC100009247 | 0.444 | -1.17258 | 1.1E-09 | 2.76E-08 | yes | down | 20.01 | 23.13 | 15.86 | 12.7 | 15.61 | 13.79 | 19.66667 | 14.03333 |
| HLF | 3.188 | 1.67282 | 1.12E-09 | 2.8E-08 | yes | up | 1.98 | 2.13 | 1.28 | 6.96 | 9.44 | 11.38 | 1.796667 | 9.26 |
| LOC100345961 | 0.358 | -1.48287 | 1.12E-09 | 2.8E-08 | yes | down | 6.66 | 7.55 | 5.06 | 3.13 | 3.12 | 4.8 | 6.423333 | 3.683333 |
| GLDN | 0.155 | -2.68553 | 1.15E-09 | 2.88E-08 | yes | down | 11.71 | 23.37 | 8.13 | 4.98 | 1.85 | 3.75 | 14.40333 | 3.526667 |
| MTMR9 | 0.463 | -1.11246 | 1.18E-09 | 2.95E-08 | yes | down | 2.53 | 2.27 | 2.02 | 1.8 | 1.52 | 1.71 | 2.273333 | 1.676667 |
| CINP | 0.439 | -1.18641 | 1.22E-09 | 3.03E-08 | yes | down | 11.43 | 10.93 | 9.33 | 6.65 | 7.8 | 9.64 | 10.56333 | 8.03 |
| LOC108176153 | 0.27 | -1.88711 | 1.27E-09 | 3.16E-08 | yes | down | 57.78 | 70.26 | 78.62 | 21.09 | 31.99 | 37.37 | 68.88667 | 30.15 |
| PALD1 | 3.331 | 1.735798 | 1.28E-09 | 3.18E-08 | yes | up | 11.32 | 13.75 | 6.28 | 40.04 | 61.74 | 64.1 | 10.45 | 55.29333 |
| KIAA1456 | 2.903 | 1.53766 | 1.29E-09 | 3.19E-08 | yes | up | 2.03 | 1.41 | 1.92 | 7.17 | 5.82 | 7.78 | 1.786667 | 6.923333 |
| CD82 | 2.432 | 1.282156 | 1.3E-09 | 3.22E-08 | yes | up | 15.82 | 19.84 | 13.64 | 52.26 | 68.21 | 68.99 | 16.43333 | 63.15333 |
| FASTKD2 | 0.331 | -1.59594 | 1.3E-09 | 3.22E-08 | yes | down | 4.02 | 5 | 3.99 | 2.23 | 1.64 | 2.96 | 4.336667 | 2.276667 |
| MRPL38 | 0.342 | -1.5481 | 1.35E-09 | 3.34E-08 | yes | down | 30.09 | 26.72 | 29.86 | 12.33 | 14.31 | 21.45 | 28.89 | 16.03 |
| ELL2 | 2.454 | 1.295059 | 1.38E-09 | 3.41E-08 | yes | up | 4.22 | 5.71 | 5.46 | 20.07 | 17.75 | 18.78 | 5.13 | 18.86667 |
| SDK1 | 3.924 | 1.972196 | 1.39E-09 | 3.42E-08 | yes | up | 2.91 | 2.21 | 1.63 | 20.13 | 9.97 | 11.74 | 2.25 | 13.94667 |
| LMOD1 | 2.984 | 1.577145 | 1.4E-09 | 3.43E-08 | yes | up | 5.63 | 3.44 | 2.76 | 17.9 | 16.54 | 21.41 | 3.943333 | 18.61667 |
| SHROOM1 | 3.097 | 1.630742 | 1.41E-09 | 3.47E-08 | yes | up | 1.84 | 2.9 | 1.8 | 8.45 | 11.06 | 12.8 | 2.18 | 10.77 |
| ATP5A1 | 0.387 | -1.36832 | 1.44E-09 | 3.51E-08 | yes | down | 1403.47 | 1568.38 | 1548.89 | 762.33 | 1130.58 | 914.94 | 1506.913 | 935.95 |
| SORBS1 | 2.232 | 1.15804 | 1.45E-09 | 3.53E-08 | yes | up | 64.03 | 81.31 | 47.95 | 191.91 | 191.75 | 207.81 | 64.43 | 197.1567 |
| C1H9orf153 | 0.295 | -1.75999 | 1.46E-09 | 3.56E-08 | yes | down | 3.49 | 5.66 | 4.54 | 2.16 | 1.77 | 2.6 | 4.563333 | 2.176667 |
| IGFBP6 | 0.328 | -1.60671 | 1.52E-09 | 3.69E-08 | yes | down | 223.15 | 415.79 | 316.91 | 159.46 | 150.62 | 194.48 | 318.6167 | 168.1867 |
| ENO1 | 0.466 | -1.10031 | 1.54E-09 | 3.73E-08 | yes | down | 280.72 | 319.28 | 277.13 | 203.98 | 248.23 | 200.18 | 292.3767 | 217.4633 |
| LOC108175456 | 0.004 | -8.00372 | 1.54E-09 | 3.73E-08 | yes | down | 0.95 | 1.09 | 1.1 | 0 | 0 | 0 | 1.046667 | 0 |
| FH | 0.362 | -1.46458 | 1.56E-09 | 3.77E-08 | yes | down | 145.51 | 138.28 | 143.65 | 73.14 | 104.52 | 69.44 | 142.48 | 82.36667 |
| APCDD1 | 6.063 | 2.599974 | 1.59E-09 | 3.85E-08 | yes | up | 3.33 | 1.47 | 4.15 | 26.38 | 22.49 | 41.35 | 2.983333 | 30.07333 |
| SLC25A12 | 0.289 | -1.79255 | 1.67E-09 | 4.03E-08 | yes | down | 21.97 | 16.5 | 11.7 | 5.63 | 9.84 | 7.21 | 16.72333 | 7.56 |
| COX3 | 0.421 | -1.24807 | 1.75E-09 | 4.21E-08 | yes | down | 64268.61 | 68943.2 | 77249 | 46936.04 | 51062.66 | 44483.44 | 70153.6 | 47494.05 |
| BDKRB2 | 2.961 | 1.566289 | 1.78E-09 | 4.27E-08 | yes | up | 0.75 | 0.77 | 0.57 | 3.22 | 2.72 | 3.73 | 0.696667 | 3.223333 |
| LOC100328948 | 6.228 | 2.638803 | 1.81E-09 | 4.32E-08 | yes | up | 1.54 | 1.06 | 2.2 | 11.21 | 15.44 | 25.33 | 1.6 | 17.32667 |
| CTNNBL1 | 0.444 | -1.17121 | 1.87E-09 | 4.45E-08 | yes | down | 64.28 | 76.11 | 58.52 | 37.82 | 51.35 | 48.35 | 66.30333 | 45.84 |
| TIPARP | 2.169 | 1.117192 | 1.94E-09 | 4.62E-08 | yes | up | 4.05 | 5.33 | 4.03 | 13.85 | 15.4 | 17.35 | 4.47 | 15.53333 |
| POLR3C | 0.313 | -1.675 | 2.1E-09 | 4.97E-08 | yes | down | 29.56 | 43.66 | 39.71 | 13.57 | 18.39 | 24.49 | 37.64333 | 18.81667 |
| SLC35A4 | 0.477 | -1.06871 | 2.14E-09 | 5.07E-08 | yes | down | 17.77 | 19.93 | 19.56 | 13.93 | 12.87 | 15.17 | 19.08667 | 13.99 |
| TCF7L2 | 2.301 | 1.202433 | 2.22E-09 | 5.25E-08 | yes | up | 5 | 3.96 | 3.69 | 15.86 | 11.63 | 17.94 | 4.216667 | 15.14333 |
| SCTR | 0.13 | -2.94836 | 2.27E-09 | 5.36E-08 | yes | down | 8.65 | 12.65 | 5.89 | 0.57 | 2.76 | 2.81 | 9.063333 | 2.046667 |
| LOC100343982 | 0.419 | -1.25409 | 2.33E-09 | 5.48E-08 | yes | down | 1355.42 | 1545.25 | 1393.32 | 742 | 1085.98 | 1038.58 | 1431.33 | 955.52 |
| AGTR2 | 0.133 | -2.90957 | 2.38E-09 | 5.59E-08 | yes | down | 1.63 | 1.31 | 0.69 | 0.25 | 0.27 | 0.22 | 1.21 | 0.246667 |
| TTC25 | 0.124 | -3.00678 | 2.43E-09 | 5.7E-08 | yes | down | 4.99 | 2.75 | 4.74 | 0.35 | 1.25 | 0.7 | 4.16 | 0.766667 |
| FASTKD3 | 0.304 | -1.71599 | 2.48E-09 | 5.8E-08 | yes | down | 3.44 | 4.65 | 4.46 | 1.8 | 2.2 | 1.98 | 4.183333 | 1.993333 |
| PPARGC1A | 0.128 | -2.96276 | 2.51E-09 | 5.88E-08 | yes | down | 4.93 | 18.09 | 4.2 | 1.84 | 1.71 | 1.89 | 9.073333 | 1.813333 |
| FDXR | 0.297 | -1.75139 | 2.65E-09 | 6.17E-08 | yes | down | 19.15 | 18.44 | 24.13 | 8.28 | 10.65 | 10.77 | 20.57333 | 9.9 |
| DCN | 2.828 | 1.499951 | 2.79E-09 | 6.48E-08 | yes | up | 288.23 | 193.71 | 126.3 | 885.38 | 782.89 | 1016.28 | 202.7467 | 894.85 |
| RECQL | 2.295 | 1.19856 | 2.81E-09 | 6.52E-08 | yes | up | 2.75 | 2.74 | 2.57 | 9.38 | 7.22 | 8.82 | 2.686667 | 8.473333 |
| SLC7A6 | 3.718 | 1.894378 | 3.17E-09 | 7.31E-08 | yes | up | 4.26 | 7.22 | 3.17 | 28.21 | 18.77 | 33.29 | 4.883333 | 26.75667 |
| TMEM120B | 0.318 | -1.65149 | 3.28E-09 | 7.54E-08 | yes | down | 12.37 | 17.36 | 9.27 | 6.83 | 6.54 | 6.29 | 13 | 6.553333 |
| RGAG1 | 0.09 | -3.46768 | 3.29E-09 | 7.55E-08 | yes | down | 0.88 | 1.12 | 0.3 | 0.09 | 0.09 | 0.14 | 0.766667 | 0.106667 |
| LOC103350464 | 0.284 | -1.81803 | 3.35E-09 | 7.67E-08 | yes | down | 7.06 | 9.49 | 8.93 | 4.41 | 3.16 | 4.1 | 8.493333 | 3.89 |
| ABCB8 | 0.256 | -1.96747 | 3.39E-09 | 7.75E-08 | yes | down | 6.96 | 3.19 | 3.86 | 1.93 | 2.03 | 1.72 | 4.67 | 1.893333 |
| NAALADL2 | 2.993 | 1.581749 | 3.77E-09 | 8.58E-08 | yes | up | 0.37 | 0.4 | 0.27 | 1.31 | 1.76 | 1.84 | 0.346667 | 1.636667 |
| LOC103347346 | 6.652 | 2.733834 | 3.78E-09 | 8.6E-08 | yes | up | 2.37 | 3.05 | 0.87 | 25.96 | 19.76 | 18.84 | 2.096667 | 21.52 |
| SEPT10 | 0.427 | -1.22643 | 3.97E-09 | 8.99E-08 | yes | down | 133.79 | 198.65 | 159.43 | 105.23 | 105.56 | 143.61 | 163.9567 | 118.1333 |
| AFF1 | 3.442 | 1.783366 | 4.01E-09 | 9.08E-08 | yes | up | 2.97 | 1.96 | 1.61 | 15.97 | 8.09 | 11.71 | 2.18 | 11.92333 |
| NFIL3 | 4.412 | 2.141305 | 4.08E-09 | 9.21E-08 | yes | up | 4.41 | 5.82 | 5.8 | 24.74 | 29.71 | 61.49 | 5.343333 | 38.64667 |
| FHL5 | 2.762 | 1.465967 | 4.11E-09 | 9.26E-08 | yes | up | 2.81 | 1.95 | 1.94 | 8.02 | 9.06 | 12.57 | 2.233333 | 9.883333 |
| ALDH1A2 | 3.007 | 1.588374 | 4.17E-09 | 9.4E-08 | yes | up | 3.01 | 2.2 | 1.77 | 13.2 | 7.79 | 12.45 | 2.326667 | 11.14667 |
| GPRIN3 | 3.562 | 1.832822 | 4.24E-09 | 9.51E-08 | yes | up | 0.44 | 0.22 | 0.32 | 1.69 | 1.78 | 2.16 | 0.326667 | 1.876667 |
| HIBCH | 0.44 | -1.18568 | 4.26E-09 | 9.55E-08 | yes | down | 53.89 | 61.46 | 46.48 | 29.14 | 41.89 | 39.44 | 53.94333 | 36.82333 |
| ADRB3 | 0.187 | -2.41904 | 4.3E-09 | 9.61E-08 | yes | down | 9.11 | 12.19 | 6.46 | 1.67 | 2.54 | 2.65 | 9.253333 | 2.286667 |
| RASGEF1B | 2.981 | 1.575749 | 4.32E-09 | 9.64E-08 | yes | up | 5.42 | 7.5 | 4.63 | 20.01 | 32.56 | 30.34 | 5.85 | 27.63667 |
| NARF | 0.393 | -1.34611 | 4.38E-09 | 9.76E-08 | yes | down | 7.24 | 7.73 | 6.67 | 3.5 | 5.61 | 4.85 | 7.213333 | 4.653333 |
| ZBTB38 | 2.137 | 1.095673 | 4.39E-09 | 9.76E-08 | yes | up | 7.28 | 10.13 | 4.94 | 19.88 | 17.32 | 24.4 | 7.45 | 20.53333 |
| KLHL4 | 0.391 | -1.35567 | 4.57E-09 | 1.01E-07 | yes | down | 2.23 | 2.86 | 2.25 | 1.5 | 1.54 | 1.56 | 2.446667 | 1.533333 |
| EEF2KMT | 0.131 | -2.92947 | 4.58E-09 | 1.01E-07 | yes | down | 2.43 | 3.95 | 2.5 | 0.82 | 0.63 | 0.37 | 2.96 | 0.606667 |
| CCDC190 | 5.6 | 2.485517 | 4.67E-09 | 1.03E-07 | yes | up | 0.53 | 0.77 | 0.53 | 5.65 | 6.28 | 10.47 | 0.61 | 7.466667 |
| AK4 | 0.3 | -1.73543 | 4.69E-09 | 1.03E-07 | yes | down | 1.17 | 1.24 | 1.05 | 0.41 | 0.66 | 0.59 | 1.153333 | 0.553333 |
| LOC100347571 | 0.326 | -1.6189 | 4.89E-09 | 1.07E-07 | yes | down | 529.32 | 497.38 | 593.46 | 242.76 | 369.7 | 233.2 | 540.0533 | 281.8867 |
| GRWD1 | 0.423 | -1.2427 | 4.9E-09 | 1.08E-07 | yes | down | 6.82 | 7.06 | 6.73 | 5.04 | 3.79 | 5.22 | 6.87 | 4.683333 |
| ABI3BP | 3.825 | 1.935561 | 4.93E-09 | 1.08E-07 | yes | up | 5.35 | 3.23 | 1.83 | 22.2 | 19.17 | 19.91 | 3.47 | 20.42667 |
| MRPS25 | 0.47 | -1.09035 | 5.26E-09 | 1.15E-07 | yes | down | 63.75 | 81.44 | 60.14 | 37.38 | 44.77 | 49.42 | 68.44333 | 43.85667 |
| USP11 | 0.318 | -1.65111 | 5.39E-09 | 1.18E-07 | yes | down | 18.26 | 10.65 | 8.01 | 6.02 | 6.26 | 5.96 | 12.30667 | 6.08 |
| ERAL1 | 0.498 | -1.00676 | 5.43E-09 | 1.18E-07 | yes | down | 11.67 | 10.89 | 9.17 | 7.58 | 8.49 | 9.11 | 10.57667 | 8.393333 |
| PIGA | 3.147 | 1.653952 | 5.61E-09 | 1.22E-07 | yes | up | 1.08 | 1.27 | 0.8 | 6.45 | 3.99 | 4.83 | 1.05 | 5.09 |
| FBL | 0.466 | -1.10151 | 5.61E-09 | 1.22E-07 | yes | down | 22.86 | 24.7 | 16.99 | 15.17 | 13.76 | 19 | 21.51667 | 15.97667 |
| SAR1B | 0.494 | -1.01686 | 5.74E-09 | 1.24E-07 | yes | down | 27.9 | 21.15 | 20.96 | 16.41 | 19.78 | 20.52 | 23.33667 | 18.90333 |
| FAM13A | 3.951 | 1.982371 | 5.75E-09 | 1.25E-07 | yes | up | 33.78 | 32.99 | 55.62 | 214.49 | 286.24 | 341.86 | 40.79667 | 280.8633 |
| FIGF | 4.811 | 2.266404 | 5.84E-09 | 1.26E-07 | yes | up | 1.2 | 0.79 | 0.43 | 4.69 | 5.57 | 8.05 | 0.806667 | 6.103333 |
| CXCL12 | 0.217 | -2.20548 | 5.86E-09 | 1.27E-07 | yes | down | 21.54 | 31.29 | 45.18 | 11.98 | 7.92 | 15.53 | 32.67 | 11.81 |
| PCF11 | 2.48 | 1.310085 | 5.91E-09 | 1.27E-07 | yes | up | 5.48 | 5.86 | 3.5 | 14.93 | 17.62 | 20.63 | 4.946667 | 17.72667 |
| BTBD3 | 2.276 | 1.186508 | 6.26E-09 | 1.35E-07 | yes | up | 3.52 | 3.8 | 4.37 | 14.21 | 13.95 | 14.22 | 3.896667 | 14.12667 |
| CIB2 | 0.237 | -2.07709 | 6.3E-09 | 1.35E-07 | yes | down | 167.87 | 180.08 | 238.74 | 56.56 | 108 | 59.08 | 195.5633 | 74.54667 |
| LOC100353245 | 4.375 | 2.129146 | 6.38E-09 | 1.37E-07 | yes | up | 133.22 | 93.19 | 83.28 | 1020.11 | 696.89 | 393.51 | 103.23 | 703.5033 |
| PRPSAP2 | 2.386 | 1.254811 | 6.53E-09 | 1.4E-07 | yes | up | 3.9 | 3.57 | 3.33 | 11.55 | 14.47 | 15.8 | 3.6 | 13.94 |
| CPM | 2.917 | 1.54439 | 6.59E-09 | 1.41E-07 | yes | up | 2.17 | 1.45 | 2.1 | 8.74 | 8.06 | 10.24 | 1.906667 | 9.013333 |
| MRTO4 | 0.462 | -1.11497 | 6.67E-09 | 1.42E-07 | yes | down | 10.87 | 12.73 | 9.39 | 8.19 | 6.71 | 9.5 | 10.99667 | 8.133333 |
| CA12 | 0.107 | -3.21877 | 6.83E-09 | 1.45E-07 | yes | down | 1.22 | 4.38 | 1.46 | 0.27 | 0.53 | 0.42 | 2.353333 | 0.406667 |
| STYXL1 | 0.36 | -1.47441 | 7.78E-09 | 1.65E-07 | yes | down | 6.45 | 7.63 | 5.78 | 4.27 | 3.46 | 4.09 | 6.62 | 3.94 |
| HBB2 | 0.235 | -2.08897 | 7.81E-09 | 1.65E-07 | yes | down | 7092.06 | 18788.27 | 12438.18 | 5865.55 | 3696.41 | 4926.74 | 12772.84 | 4829.567 |
| LDLRAD3 | 3.063 | 1.615154 | 7.85E-09 | 1.66E-07 | yes | up | 3.34 | 3.37 | 3.28 | 11.98 | 14.17 | 23.46 | 3.33 | 16.53667 |
| MRPS30 | 0.402 | -1.31499 | 7.87E-09 | 1.66E-07 | yes | down | 17.03 | 20.14 | 19.56 | 10.32 | 11.24 | 15.4 | 18.91 | 12.32 |
| ULK1 | 2.482 | 1.311347 | 7.92E-09 | 1.67E-07 | yes | up | 9 | 8.92 | 9.32 | 27.71 | 39.31 | 43.58 | 9.08 | 36.86667 |
| NDN | 0.366 | -1.4511 | 8.01E-09 | 1.69E-07 | yes | down | 49 | 29.39 | 27.17 | 18.12 | 18.67 | 24.54 | 35.18667 | 20.44333 |
| SF3B5 | 0.448 | -1.15785 | 8.07E-09 | 1.7E-07 | yes | down | 47.95 | 52.72 | 39.91 | 39.66 | 27.77 | 32.56 | 46.86 | 33.33 |
| LOC100343872 | 3.611 | 1.852303 | 8.1E-09 | 1.7E-07 | yes | up | 1.95 | 2.1 | 1.63 | 11.56 | 7.08 | 14.46 | 1.893333 | 11.03333 |
| RGS20 | 4.66 | 2.220288 | 8.36E-09 | 1.75E-07 | yes | up | 10.39 | 15.98 | 8.68 | 25.83 | 21.72 | 40.26 | 11.68333 | 29.27 |
| NDUFAF7 | 0.326 | -1.61585 | 8.49E-09 | 1.78E-07 | yes | down | 10.83 | 10.46 | 7.72 | 3.5 | 5.54 | 6.67 | 9.67 | 5.236667 |
| MRPS36 | 0.449 | -1.1558 | 8.62E-09 | 1.8E-07 | yes | down | 71.79 | 54.1 | 51.75 | 35.37 | 44.89 | 49.46 | 59.21333 | 43.24 |
| LOC103347486 | 2.465 | 1.30167 | 8.62E-09 | 1.8E-07 | yes | up | 48.39 | 68.05 | 58.48 | 278.05 | 205.21 | 210.18 | 58.30667 | 231.1467 |
| CLK1 | 2.375 | 1.247702 | 8.86E-09 | 1.85E-07 | yes | up | 56.58 | 60.84 | 41.37 | 167.54 | 180.46 | 245.08 | 52.93 | 197.6933 |
| GRO-A | 11.136 | 3.477165 | 9.01E-09 | 1.87E-07 | yes | up | 0.14 | 0.84 | 0.52 | 11.67 | 4.51 | 9.67 | 0.5 | 8.616667 |
| MYOCD | 3.553 | 1.82899 | 9.17E-09 | 1.9E-07 | yes | up | 0.39 | 0.32 | 0.22 | 1.27 | 1.73 | 2.29 | 0.31 | 1.763333 |
| SMYD5 | 0.457 | -1.13007 | 9.26E-09 | 1.92E-07 | yes | down | 5.63 | 6.53 | 4.55 | 4.35 | 3.56 | 4.26 | 5.57 | 4.056667 |
| MTCH2 | 0.455 | -1.13676 | 9.29E-09 | 1.92E-07 | yes | down | 199.74 | 183.79 | 161.74 | 103.31 | 152.26 | 142.82 | 181.7567 | 132.7967 |
| ALDH4A1 | 0.315 | -1.66867 | 9.34E-09 | 1.93E-07 | yes | down | 18.62 | 10.63 | 9.25 | 6.72 | 6.86 | 5.43 | 12.83333 | 6.336667 |
| NDUFB4 | 0.463 | -1.1109 | 9.36E-09 | 1.93E-07 | yes | down | 467.2 | 507.4 | 439.22 | 279.53 | 391.98 | 365.38 | 471.2733 | 345.63 |
| CLK4 | 2.156 | 1.108291 | 9.69E-09 | 1.99E-07 | yes | up | 5.89 | 5.53 | 4.3 | 15.21 | 18.56 | 20.66 | 5.24 | 18.14333 |
| TMEM223 | 0.374 | -1.41967 | 9.98E-09 | 2.05E-07 | yes | down | 17.62 | 19.16 | 17.82 | 9.16 | 11.38 | 12.15 | 18.2 | 10.89667 |
| PRKAR2B | 0.445 | -1.16751 | 1E-08 | 2.05E-07 | yes | down | 237.03 | 243.8 | 261.67 | 145.61 | 180.27 | 206.46 | 247.5 | 177.4467 |
| ABCC9 | 0.332 | -1.59154 | 1.03E-08 | 2.11E-07 | yes | down | 13.21 | 11.45 | 8.36 | 4.04 | 7.43 | 5.84 | 11.00667 | 5.77 |
| KIAA0391 | 0.458 | -1.12704 | 1.04E-08 | 2.13E-07 | yes | down | 8.5 | 9.92 | 6.56 | 5.52 | 6.35 | 6.61 | 8.326667 | 6.16 |
| APOO | 0.464 | -1.1068 | 1.04E-08 | 2.13E-07 | yes | down | 48.26 | 39.46 | 30.79 | 25.81 | 30.82 | 29.91 | 39.50333 | 28.84667 |
| EPHX1 | 2.737 | 1.452394 | 1.05E-08 | 2.14E-07 | yes | up | 37.48 | 56.84 | 51.44 | 171.53 | 221.26 | 256.65 | 48.58667 | 216.48 |
| OSBPL5 | 2.172 | 1.118903 | 1.12E-08 | 2.27E-07 | yes | up | 6.51 | 5.3 | 4.45 | 18.3 | 16.78 | 21.15 | 5.42 | 18.74333 |
| MOCS1 | 0.486 | -1.04062 | 1.17E-08 | 2.37E-07 | yes | down | 16.6 | 15.85 | 16.36 | 11.88 | 11.2 | 12.9 | 16.27 | 11.99333 |
| ADAMTS16 | 5.562 | 2.475654 | 1.18E-08 | 2.39E-07 | yes | up | 0.2 | 0.25 | 0.24 | 2.21 | 2.58 | 1.31 | 0.23 | 2.033333 |
| APOE | 5.16 | 2.367406 | 1.27E-08 | 2.57E-07 | yes | up | 31.49 | 17.76 | 12.08 | 239.02 | 124.64 | 127.72 | 20.44333 | 163.7933 |
| ANKS1A | 2.805 | 1.488065 | 1.27E-08 | 2.57E-07 | yes | up | 14.98 | 16.7 | 21.5 | 66.64 | 83.83 | 93.85 | 17.72667 | 81.44 |
| LOC103348241 | 7.917 | 2.984865 | 1.32E-08 | 2.65E-07 | yes | up | 4 | 5.05 | 8.74 | 109.81 | 31.51 | 94.89 | 5.93 | 78.73667 |
| ARMC6 | 0.247 | -2.01513 | 1.33E-08 | 2.68E-07 | yes | down | 3.93 | 3.17 | 4.19 | 1.6 | 1.65 | 1.36 | 3.763333 | 1.536667 |
| MRPS7 | 0.485 | -1.04324 | 1.4E-08 | 2.8E-07 | yes | down | 53.86 | 53.12 | 53.34 | 37.93 | 47.59 | 39.29 | 53.44 | 41.60333 |
| SMARCD2 | 2.092 | 1.064933 | 1.43E-08 | 2.86E-07 | yes | up | 11.89 | 14.22 | 12.09 | 39.32 | 33.11 | 50.25 | 12.73333 | 40.89333 |
| ST8SIA5 | 0.211 | -2.24455 | 1.43E-08 | 2.86E-07 | yes | down | 3.21 | 2.33 | 3.27 | 0.52 | 1.18 | 1.32 | 2.936667 | 1.006667 |
| LOC100353151 | 0.42 | -1.25086 | 1.45E-08 | 2.89E-07 | yes | down | 65.02 | 63.08 | 73.4 | 39.02 | 51.15 | 45.1 | 67.16667 | 45.09 |
| COG6 | 0.449 | -1.15561 | 1.47E-08 | 2.93E-07 | yes | down | 9.34 | 10.04 | 10.2 | 7.24 | 5.48 | 7.98 | 9.86 | 6.9 |
| RADIL | 3.65 | 1.867962 | 1.48E-08 | 2.94E-07 | yes | up | 0.74 | 0.45 | 0.47 | 2.18 | 2.42 | 3.4 | 0.553333 | 2.666667 |
| YPEL5 | 2.027 | 1.019112 | 1.5E-08 | 2.98E-07 | yes | up | 9.27 | 10.21 | 8.73 | 31.94 | 24.52 | 34.62 | 9.403333 | 30.36 |
| COMMD8 | 0.358 | -1.48156 | 1.5E-08 | 2.98E-07 | yes | down | 8.01 | 9.52 | 7.56 | 6.01 | 4.16 | 4.15 | 8.363333 | 4.773333 |
| MYC | 2.822 | 1.49661 | 1.51E-08 | 2.99E-07 | yes | up | 6.77 | 6.29 | 5.66 | 27.25 | 18.71 | 35.64 | 6.24 | 27.2 |
| GPATCH4 | 0.357 | -1.48799 | 1.52E-08 | 3.01E-07 | yes | down | 6.12 | 8.93 | 5.61 | 4.13 | 3.14 | 4.47 | 6.886667 | 3.913333 |
| DRAP1 | 0.47 | -1.08914 | 1.52E-08 | 3.01E-07 | yes | down | 51.07 | 56.55 | 53.68 | 35.29 | 40.42 | 45.96 | 53.76667 | 40.55667 |
| SNRNP25 | 0.43 | -1.21624 | 1.54E-08 | 3.05E-07 | yes | down | 31.13 | 27.41 | 25.95 | 15.27 | 17.94 | 23.72 | 28.16333 | 18.97667 |
| PPAT | 0.232 | -2.10603 | 1.56E-08 | 3.08E-07 | yes | down | 2.15 | 1.93 | 1.6 | 0.85 | 0.79 | 0.44 | 1.893333 | 0.693333 |
| SNCA | 0.241 | -2.05285 | 1.76E-08 | 3.45E-07 | yes | down | 25.06 | 61.62 | 44.5 | 15.59 | 12.81 | 20.26 | 43.72667 | 16.22 |
| ACVR2A | 3.335 | 1.737566 | 1.76E-08 | 3.45E-07 | yes | up | 1.74 | 1.02 | 1.34 | 5.25 | 8.03 | 8.65 | 1.366667 | 7.31 |
| NTRK2 | 7.117 | 2.831181 | 1.76E-08 | 3.46E-07 | yes | up | 0.8 | 0.99 | 0.15 | 6.39 | 7.14 | 7.74 | 0.646667 | 7.09 |
| PRELID1 | 0.488 | -1.03466 | 1.84E-08 | 3.61E-07 | yes | down | 54.05 | 70.34 | 51.99 | 51.23 | 39.33 | 46.98 | 58.79333 | 45.84667 |
| TOMM6 | 0.497 | -1.00888 | 1.85E-08 | 3.63E-07 | yes | down | 195.07 | 256.37 | 189.53 | 129.37 | 143.5 | 175.95 | 213.6567 | 149.6067 |
| PDE7A | 5.076 | 2.343593 | 1.89E-08 | 3.69E-07 | yes | up | 5.99 | 2.08 | 2.23 | 19.2 | 25.41 | 31.19 | 3.433333 | 25.26667 |
| RAP1GAP2 | 0.104 | -3.25862 | 1.89E-08 | 3.7E-07 | yes | down | 3.91 | 4.99 | 1.17 | 0.81 | 0.27 | 0.6 | 3.356667 | 0.56 |
| ATP5C1 | 0.472 | -1.08304 | 1.93E-08 | 3.76E-07 | yes | down | 524.06 | 418.33 | 340.07 | 266.93 | 358.67 | 330.99 | 427.4867 | 318.8633 |
| MSANTD2 | 2.136 | 1.09504 | 1.98E-08 | 3.86E-07 | yes | up | 6.18 | 8.84 | 5.62 | 19.47 | 18.77 | 22.42 | 6.88 | 20.22 |
| LOC100343402 | 0.485 | -1.04463 | 2.03E-08 | 3.93E-07 | yes | down | 69.98 | 79.7 | 54.88 | 49.26 | 54.13 | 52.54 | 68.18667 | 51.97667 |
| TTPA | 5.118 | 2.355508 | 2.18E-08 | 4.22E-07 | yes | up | 0.32 | 0.53 | 0.79 | 4.86 | 5.09 | 5.35 | 0.546667 | 5.1 |
| SH3GLB2 | 0.396 | -1.33726 | 2.18E-08 | 4.22E-07 | yes | down | 10.78 | 16.68 | 12.82 | 8.18 | 7.69 | 9.8 | 13.42667 | 8.556667 |
| PDGFD | 3.214 | 1.684234 | 2.24E-08 | 4.32E-07 | yes | up | 22.27 | 18.02 | 21.84 | 73.02 | 105.74 | 140.97 | 20.71 | 106.5767 |
| MSANTD4 | 0.421 | -1.24781 | 2.26E-08 | 4.36E-07 | yes | down | 5.84 | 7.63 | 5.09 | 3.73 | 3.68 | 5.09 | 6.186667 | 4.166667 |
| NDUFA2 | 0.421 | -1.24807 | 2.28E-08 | 4.39E-07 | yes | down | 298.09 | 384.74 | 340.55 | 190.3 | 262.02 | 235.23 | 341.1267 | 229.1833 |
| SPRY1 | 2.855 | 1.513506 | 2.38E-08 | 4.58E-07 | yes | up | 15.18 | 25.73 | 17.75 | 68.05 | 81.55 | 113.88 | 19.55333 | 87.82667 |
| BCKDHB | 0.494 | -1.01693 | 2.45E-08 | 4.7E-07 | yes | down | 37.04 | 35.61 | 34.46 | 23.73 | 27.56 | 33.86 | 35.70333 | 28.38333 |
| PLA2R1 | 4.629 | 2.210754 | 2.46E-08 | 4.71E-07 | yes | up | 1 | 0.41 | 0.61 | 6.06 | 2.73 | 5.39 | 0.673333 | 4.726667 |
| IFIH1 | 6.814 | 2.768498 | 2.49E-08 | 4.76E-07 | yes | up | 0.88 | 0.78 | 0.61 | 14.16 | 5.91 | 3.57 | 0.756667 | 7.88 |
| LOC100345822 | 0.445 | -1.16855 | 2.56E-08 | 4.89E-07 | yes | down | 13.18 | 13.59 | 14.29 | 9.55 | 8.59 | 11.44 | 13.68667 | 9.86 |
| ADSL | 0.429 | -1.21958 | 2.6E-08 | 4.95E-07 | yes | down | 27.96 | 20.54 | 14.99 | 14.26 | 13.85 | 12.28 | 21.16333 | 13.46333 |
| ARL6IP4 | 0.334 | -1.5807 | 2.66E-08 | 5.06E-07 | yes | down | 39.35 | 29.59 | 25.38 | 13.27 | 19.8 | 15.43 | 31.44 | 16.16667 |
| SAMD15 | 0.112 | -3.15846 | 2.68E-08 | 5.09E-07 | yes | down | 1.25 | 3.56 | 1.25 | 0.52 | 0.35 | 0.34 | 2.02 | 0.403333 |
| ATXN7L2 | 0.232 | -2.10854 | 2.75E-08 | 5.21E-07 | yes | down | 4.49 | 3.68 | 1.88 | 2.12 | 2.5 | 2.33 | 3.35 | 2.316667 |
| LIPA | 2.534 | 1.341232 | 2.75E-08 | 5.21E-07 | yes | up | 18.92 | 24.97 | 18.87 | 106.98 | 69.53 | 70.23 | 20.92 | 82.24667 |
| TP53INP1 | 3.97 | 1.989001 | 2.92E-08 | 5.52E-07 | yes | up | 20.54 | 19.07 | 29.19 | 74.27 | 131.09 | 176.05 | 22.93333 | 127.1367 |
| KCTD7 | 4.473 | 2.161249 | 2.98E-08 | 5.61E-07 | yes | up | 2.47 | 1.54 | 3.77 | 20.38 | 19.8 | 16.25 | 2.593333 | 18.81 |
| IGSF8 | 3.021 | 1.59482 | 2.98E-08 | 5.61E-07 | yes | up | 4.85 | 3.65 | 3.63 | 14.58 | 16.68 | 27.74 | 4.043333 | 19.66667 |
| SDHA | 0.4 | -1.32314 | 3E-08 | 5.64E-07 | yes | down | 706.37 | 607.87 | 678.16 | 375.77 | 544.28 | 389.3 | 664.1333 | 436.45 |
| LOC108178771 | 338.296 | 8.402141 | 3.07E-08 | 5.76E-07 | yes | up | 0 | 0.01 | 0 | 0.47 | 10.8 | 8.66 | 0.003333 | 6.643333 |
| BTNL2 | 0.008 | -7.02229 | 3.2E-08 | 5.99E-07 | yes | down | 4.4 | 0.26 | 3.63 | 0 | 0.03 | 0.08 | 2.763333 | 0.036667 |
| DTX3 | 2.692 | 1.428687 | 3.5E-08 | 6.54E-07 | yes | up | 4.14 | 5.44 | 2.51 | 16.97 | 15.16 | 19.2 | 4.03 | 17.11 |
| TMEM268 | 3.389 | 1.760705 | 3.52E-08 | 6.57E-07 | yes | up | 2.33 | 2.78 | 1.69 | 7.51 | 13.59 | 15.21 | 2.266667 | 12.10333 |
| PC | 0.347 | -1.52892 | 3.57E-08 | 6.65E-07 | yes | down | 10.82 | 13.38 | 14.58 | 6.41 | 8.54 | 6.72 | 12.92667 | 7.223333 |
| GATSL2 | 0.324 | -1.62694 | 3.71E-08 | 6.88E-07 | yes | down | 11.6 | 9.31 | 8.54 | 3.66 | 6.58 | 4.85 | 9.816667 | 5.03 |
| IL1R1 | 2.052 | 1.037035 | 3.71E-08 | 6.88E-07 | yes | up | 5.02 | 5.05 | 4.35 | 12.88 | 16.33 | 18.58 | 4.806667 | 15.93 |
| IGFBP2 | 2.33 | 1.220229 | 3.77E-08 | 6.98E-07 | yes | up | 32.61 | 23.84 | 16.47 | 86.3 | 90.77 | 92.51 | 24.30667 | 89.86 |
| C1R | 8.335 | 3.059144 | 3.82E-08 | 7.06E-07 | yes | up | 23.71 | 12.8 | 10.39 | 413.54 | 89.3 | 111.21 | 15.63333 | 204.6833 |
| EPSTI1 | 5.608 | 2.487443 | 3.86E-08 | 7.12E-07 | yes | up | 2.89 | 1.83 | 2.82 | 39.18 | 12.24 | 18.08 | 2.513333 | 23.16667 |
| TMEM147 | 0.469 | -1.0925 | 3.93E-08 | 7.24E-07 | yes | down | 60.62 | 55.52 | 59.73 | 37.14 | 44.26 | 51.34 | 58.62333 | 44.24667 |
| MRPL41 | 0.418 | -1.25789 | 4.08E-08 | 7.48E-07 | yes | down | 47.95 | 44.47 | 50.79 | 30.16 | 27.7 | 38.83 | 47.73667 | 32.23 |
| ARRB1 | 2.657 | 1.409778 | 4.08E-08 | 7.48E-07 | yes | up | 6.6 | 7.28 | 5.99 | 18.93 | 31.29 | 32.62 | 6.623333 | 27.61333 |
| NCKAP5 | 4.671 | 2.223772 | 4.16E-08 | 7.61E-07 | yes | up | 3.71 | 8.6 | 0.21 | 6.96 | 10.54 | 1.5 | 4.173333 | 6.333333 |
| SLC25A39 | 0.46 | -1.12038 | 4.29E-08 | 7.83E-07 | yes | down | 48.06 | 50.61 | 56.4 | 34.17 | 37.18 | 44.16 | 51.69 | 38.50333 |
| NUPR1 | 3.054 | 1.610933 | 4.42E-08 | 8.05E-07 | yes | up | 16.17 | 17.06 | 19.28 | 66.57 | 71.99 | 122.89 | 17.50333 | 87.15 |
| IL10RA | 13.191 | 3.721444 | 4.42E-08 | 8.05E-07 | yes | up | 0.69 | 0.44 | 0.34 | 22.49 | 3.25 | 4.84 | 0.49 | 10.19333 |
| CD5L | 29.147 | 4.86529 | 4.62E-08 | 8.37E-07 | yes | up | 0.14 | 0.25 | 0.06 | 13.2 | 3.01 | 4.38 | 0.15 | 6.863333 |
| SDC2 | 2.592 | 1.373919 | 4.82E-08 | 8.71E-07 | yes | up | 13.01 | 14.83 | 15.59 | 44.6 | 63.04 | 74.83 | 14.47667 | 60.82333 |
| FCGRT | 2.065 | 1.046108 | 4.92E-08 | 8.89E-07 | yes | up | 55.7 | 60.32 | 53.61 | 222.68 | 173.7 | 162.35 | 56.54333 | 186.2433 |
| ALAD | 2.698 | 1.43197 | 4.99E-08 | 9.01E-07 | yes | up | 35.45 | 55.3 | 37.53 | 135.15 | 190.21 | 227.55 | 42.76 | 184.3033 |
| CERK | 0.348 | -1.52422 | 5.04E-08 | 9.08E-07 | yes | down | 36.53 | 39.63 | 17.13 | 14.5 | 17.12 | 19.07 | 31.09667 | 16.89667 |
| LOC100357076 | 0.454 | -1.13978 | 5.11E-08 | 9.21E-07 | yes | down | 2.74 | 2.71 | 1.9 | 1.74 | 1.44 | 2.3 | 2.45 | 1.826667 |
| SPTA1 | 0.122 | -3.02939 | 5.13E-08 | 9.22E-07 | yes | down | 0.83 | 2.42 | 0.76 | 0.45 | 0.42 | 0.56 | 1.336667 | 0.476667 |
| GPATCH2 | 2.15 | 1.104367 | 5.18E-08 | 9.31E-07 | yes | up | 3.57 | 2.97 | 1.92 | 12.19 | 10.86 | 13.11 | 2.82 | 12.05333 |
| CTSF | 0.413 | -1.2764 | 5.19E-08 | 9.31E-07 | yes | down | 40.39 | 48.64 | 31.82 | 19.33 | 28.76 | 31.09 | 40.28333 | 26.39333 |
| RBKS | 0.356 | -1.49187 | 5.32E-08 | 9.52E-07 | yes | down | 7.38 | 9.02 | 4.55 | 6.94 | 4.9 | 3.93 | 6.983333 | 5.256667 |
| STAC | 2.902 | 1.537052 | 5.41E-08 | 9.66E-07 | yes | up | 1.55 | 1.22 | 0.79 | 5.38 | 4.49 | 6.51 | 1.186667 | 5.46 |
| ZBTB16 | 8.247 | 3.043947 | 5.46E-08 | 9.75E-07 | yes | up | 4.55 | 2.46 | 0.52 | 39.43 | 24.05 | 31.16 | 2.51 | 31.54667 |
| TXNRD2 | 0.362 | -1.46616 | 5.48E-08 | 9.76E-07 | yes | down | 9.85 | 10.63 | 11.03 | 5.45 | 7.53 | 5.27 | 10.50333 | 6.083333 |
| KCNE3 | 6.394 | 2.676828 | 5.51E-08 | 9.81E-07 | yes | up | 0.28 | 0.29 | 0.21 | 2.51 | 2.51 | 3.08 | 0.26 | 2.7 |
| PRRG4 | 0.329 | -1.60492 | 5.54E-08 | 9.84E-07 | yes | down | 7.14 | 7.8 | 3.46 | 2.7 | 2.8 | 3.89 | 6.133333 | 3.13 |
| DEF8 | 2.06 | 1.042636 | 5.57E-08 | 9.88E-07 | yes | up | 8.35 | 9.06 | 8.04 | 32.28 | 29.52 | 24.15 | 8.483333 | 28.65 |
| LETM2 | 5.384 | 2.428657 | 5.64E-08 | 1E-06 | yes | up | 0.27 | 0.46 | 0.2 | 2.04 | 2.83 | 2.41 | 0.31 | 2.426667 |
| LOC103347778 | 271.043 | 8.08238 | 5.85E-08 | 1.04E-06 | yes | up | 0 | 0 | 0.34 | 119.68 | 7.25 | 16.14 | 0.113333 | 47.69 |
| HHIP | 8.248 | 3.043961 | 6E-08 | 1.06E-06 | yes | up | 0.2 | 0.17 | 0.02 | 1.88 | 1.65 | 2.42 | 0.13 | 1.983333 |
| C1S | 8.441 | 3.077461 | 6.01E-08 | 1.06E-06 | yes | up | 11.85 | 9.65 | 6.48 | 258.33 | 66.12 | 46.7 | 9.326667 | 123.7167 |
| MSTO1 | 0.391 | -1.3562 | 6.03E-08 | 1.06E-06 | yes | down | 7.5 | 7.17 | 8.71 | 4.4 | 4.35 | 5.86 | 7.793333 | 4.87 |
| DOLK | 0.46 | -1.12146 | 6.08E-08 | 1.07E-06 | yes | down | 19.56 | 21.51 | 17.71 | 11.19 | 14.72 | 17.4 | 19.59333 | 14.43667 |
| AHNAK | 2.177 | 1.122082 | 6.22E-08 | 1.09E-06 | yes | up | 62.52 | 46.41 | 42.63 | 191.52 | 187.71 | 149.76 | 50.52 | 176.33 |
| MTFP1 | 0.324 | -1.6263 | 6.34E-08 | 1.11E-06 | yes | down | 18.64 | 16.63 | 16.77 | 9.49 | 11 | 6.06 | 17.34667 | 8.85 |
| LOC103352377 | 0.127 | -2.97958 | 6.34E-08 | 1.11E-06 | yes | down | 14.63 | 29.42 | 15.86 | 1.76 | 5.04 | 5.26 | 19.97 | 4.02 |
| GK | 0.338 | -1.56485 | 6.37E-08 | 1.11E-06 | yes | down | 28.02 | 41.45 | 27.18 | 10.11 | 14.8 | 18.91 | 32.21667 | 14.60667 |
| LOC100338456 | 0.149 | -2.74507 | 6.56E-08 | 1.14E-06 | yes | down | 8.24 | 6.68 | 6.21 | 0.66 | 0.54 | 2.78 | 7.043333 | 1.326667 |
| TRA2A | 2.068 | 1.048217 | 6.59E-08 | 1.15E-06 | yes | up | 29.36 | 36.03 | 22.69 | 79.98 | 69.65 | 84.99 | 29.36 | 78.20667 |
| CD2BP2 | 0.491 | -1.0255 | 6.73E-08 | 1.17E-06 | yes | down | 24.27 | 29.08 | 22.58 | 22.65 | 16.37 | 20.39 | 25.31 | 19.80333 |
| ATP5B | 0.427 | -1.2268 | 6.83E-08 | 1.19E-06 | yes | down | 2240.99 | 2222.67 | 2497.12 | 1442.05 | 1902.88 | 1432.62 | 2320.26 | 1592.517 |
| ECHDC1 | 0.372 | -1.42848 | 6.86E-08 | 1.19E-06 | yes | down | 21.5 | 13.48 | 14.9 | 11.24 | 9.23 | 7.61 | 16.62667 | 9.36 |
| MRPL35 | 0.466 | -1.10196 | 6.91E-08 | 1.2E-06 | yes | down | 58.66 | 64.51 | 52.44 | 35.59 | 47.99 | 47.47 | 58.53667 | 43.68333 |
| ACAD10 | 0.4 | -1.32133 | 6.95E-08 | 1.2E-06 | yes | down | 14.84 | 14.76 | 17.36 | 9.32 | 11.98 | 9.05 | 15.65333 | 10.11667 |
| ND4L | 0.311 | -1.68706 | 7.14E-08 | 1.23E-06 | yes | down | 17325.17 | 20918.76 | 16409.85 | 12661.41 | 7484.3 | 6137.8 | 18217.93 | 8761.17 |
| MET | 0.185 | -2.43627 | 7.26E-08 | 1.25E-06 | yes | down | 3.74 | 2.66 | 7.58 | 1.61 | 1.31 | 1.41 | 4.66 | 1.443333 |
| HYAL1 | 2.013 | 1.009175 | 7.44E-08 | 1.28E-06 | yes | up | 8.38 | 8.48 | 7.56 | 21.65 | 28.86 | 24.64 | 8.14 | 25.05 |
| LOC108178279 | 2.986 | 1.577982 | 7.51E-08 | 1.29E-06 | yes | up | 2.15 | 1.85 | 1.52 | 10.68 | 7.39 | 8.09 | 1.84 | 8.72 |
| ADRB2 | 4.091 | 2.032454 | 7.62E-08 | 1.31E-06 | yes | up | 4.55 | 4.29 | 5.44 | 17.55 | 29.2 | 49.37 | 4.76 | 32.04 |
| CITED2 | 3.386 | 1.759792 | 7.63E-08 | 1.31E-06 | yes | up | 18.58 | 8 | 11.97 | 56.33 | 72.61 | 80.07 | 12.85 | 69.67 |
| GHITM | 0.268 | -1.89819 | 7.71E-08 | 1.32E-06 | yes | down | 377.1 | 469.36 | 175.99 | 108.16 | 112.57 | 209.54 | 340.8167 | 143.4233 |
| EIF4EBP2 | 2.006 | 1.004333 | 7.72E-08 | 1.32E-06 | yes | up | 96.1 | 103.53 | 99.25 | 277.71 | 283.55 | 394.01 | 99.62667 | 318.4233 |
| SAMHD1 | 4.565 | 2.190551 | 7.81E-08 | 1.33E-06 | yes | up | 7.85 | 6.38 | 4.81 | 73.86 | 22.2 | 39.04 | 6.346667 | 45.03333 |
| MECR | 0.452 | -1.147 | 8.03E-08 | 1.37E-06 | yes | down | 40.9 | 38.77 | 43.4 | 25.13 | 32.59 | 34.82 | 41.02333 | 30.84667 |
| UBE2L6 | 6.506 | 2.701742 | 8.25E-08 | 1.41E-06 | yes | up | 3.58 | 2.19 | 2.53 | 52.09 | 19.6 | 14.26 | 2.766667 | 28.65 |
| NOL10 | 0.481 | -1.0571 | 8.28E-08 | 1.41E-06 | yes | down | 5.35 | 5 | 3.75 | 3.6 | 2.54 | 3.72 | 4.7 | 3.286667 |
| SNED1 | 2.467 | 1.302633 | 8.39E-08 | 1.43E-06 | yes | up | 4.67 | 3.94 | 2.68 | 23.59 | 24.79 | 17 | 3.763333 | 21.79333 |
| IDH2 | 0.364 | -1.45737 | 8.47E-08 | 1.44E-06 | yes | down | 460.51 | 232.15 | 303.35 | 192.16 | 207.04 | 182.79 | 332.0033 | 193.9967 |
| RARRES1 | 5.971 | 2.577994 | 8.62E-08 | 1.46E-06 | yes | up | 0.91 | 0.88 | 0.49 | 8.97 | 3.96 | 8.68 | 0.76 | 7.203333 |
| NDUFS4 | 0.452 | -1.1448 | 8.62E-08 | 1.46E-06 | yes | down | 267.73 | 265.82 | 268.08 | 149.07 | 220.85 | 205.81 | 267.21 | 191.91 |
| CNNM4 | 3.147 | 1.653875 | 8.63E-08 | 1.46E-06 | yes | up | 1.54 | 0.9 | 1.51 | 6.32 | 7.4 | 6.35 | 1.316667 | 6.69 |
| LOC100354435 | 8.459 | 3.080449 | 8.64E-08 | 1.46E-06 | yes | up | 5.07 | 6.91 | 1.45 | 98.91 | 51.44 | 24.83 | 4.476667 | 58.39333 |
| CLPTM1 | 0.496 | -1.01153 | 8.66E-08 | 1.46E-06 | yes | down | 43.48 | 49.17 | 49.13 | 38.73 | 37.41 | 34.54 | 47.26 | 36.89333 |
| TMEM64 | 2.368 | 1.243824 | 8.71E-08 | 1.47E-06 | yes | up | 7.83 | 4.98 | 7.18 | 25.42 | 20.87 | 27.24 | 6.663333 | 24.51 |
| SH3BP2 | 2.865 | 1.518567 | 8.91E-08 | 1.5E-06 | yes | up | 3.35 | 4.63 | 3.28 | 21.52 | 11.81 | 18.42 | 3.753333 | 17.25 |
| NEK3 | 2.569 | 1.361304 | 8.97E-08 | 1.51E-06 | yes | up | 1.35 | 1.66 | 1.22 | 5.72 | 4.84 | 5.11 | 1.41 | 5.223333 |
| SFRP5 | 5.131 | 2.359247 | 9.4E-08 | 1.58E-06 | yes | up | 1.69 | 0.79 | 0.46 | 4.71 | 8.68 | 11.11 | 0.98 | 8.166667 |
| LOC100348511 | 0.03 | -5.08309 | 9.43E-08 | 1.58E-06 | yes | down | 25.86 | 1.29 | 33.08 | 0.95 | 1.01 | 1 | 20.07667 | 0.986667 |
| LOC100351224 | 0.247 | -2.01801 | 9.45E-08 | 1.58E-06 | yes | down | 1.57 | 1.5 | 1.19 | 0.61 | 0.61 | 0.43 | 1.42 | 0.55 |
| LRRC3C | 0.149 | -2.74648 | 9.48E-08 | 1.59E-06 | yes | down | 0.91 | 2.26 | 1.01 | 0.22 | 0.47 | 0.26 | 1.393333 | 0.316667 |
| GCHFR | 0.281 | -1.83349 | 9.91E-08 | 1.66E-06 | yes | down | 9.83 | 18.69 | 15.99 | 5.5 | 7.04 | 7.66 | 14.83667 | 6.733333 |
| DHRS11 | 0.299 | -1.73994 | 1.01E-07 | 1.69E-06 | yes | down | 107.95 | 111.63 | 113.85 | 58.33 | 68.56 | 31.66 | 111.1433 | 52.85 |
| ASB1 | 0.488 | -1.03628 | 1.02E-07 | 1.69E-06 | yes | down | 17.36 | 20.19 | 19.4 | 13.65 | 14.57 | 16.55 | 18.98333 | 14.92333 |
| TST | 3.842 | 1.942008 | 1.08E-07 | 1.79E-06 | yes | up | 14.48 | 7.95 | 8.41 | 40.14 | 55.68 | 96.02 | 10.28 | 63.94667 |
| KCTD6 | 2.922 | 1.546847 | 1.09E-07 | 1.81E-06 | yes | up | 12.47 | 12.97 | 12.92 | 31.02 | 53.84 | 63.71 | 12.78667 | 49.52333 |
| GSE1 | 0.494 | -1.01637 | 1.09E-07 | 1.81E-06 | yes | down | 11.38 | 13.24 | 8.22 | 8.52 | 7.29 | 10.06 | 10.94667 | 8.623333 |
| CPLX1 | 0.039 | -4.66873 | 1.1E-07 | 1.82E-06 | yes | down | 1.19 | 2.05 | 3.23 | 0.12 | 0.28 | 0 | 2.156667 | 0.133333 |
| CPXM1 | 5.441 | 2.443837 | 1.1E-07 | 1.82E-06 | yes | up | 1.36 | 1.17 | 0.32 | 7.68 | 9.55 | 6.5 | 0.95 | 7.91 |
| TLR5 | 7.132 | 2.83435 | 1.12E-07 | 1.84E-06 | yes | up | 0.15 | 0.12 | 0.07 | 1.68 | 0.93 | 1.16 | 0.113333 | 1.256667 |
| B3GNT7 | 0.308 | -1.70082 | 1.12E-07 | 1.85E-06 | yes | down | 2.51 | 2.56 | 2.33 | 1.17 | 1.48 | 0.96 | 2.466667 | 1.203333 |
| NOVA1 | 2.801 | 1.485759 | 1.16E-07 | 1.91E-06 | yes | up | 2.11 | 2.41 | 1.28 | 10.26 | 7.24 | 8.02 | 1.933333 | 8.506667 |
| IQUB | 0.406 | -1.30072 | 1.17E-07 | 1.93E-06 | yes | down | 3.89 | 3.35 | 4.4 | 2.33 | 2.82 | 2.56 | 3.88 | 2.57 |
| ME1 | 0.207 | -2.27058 | 1.18E-07 | 1.94E-06 | yes | down | 6.09 | 4.7 | 6.52 | 0.89 | 2.53 | 2.39 | 5.77 | 1.936667 |
| RIDA | 0.402 | -1.31398 | 1.19E-07 | 1.95E-06 | yes | down | 17.33 | 17.64 | 20.59 | 10.49 | 12.64 | 13.02 | 18.52 | 12.05 |
| AGBL2 | 2.006 | 1.004602 | 1.2E-07 | 1.97E-06 | yes | up | 2.75 | 3.2 | 1.75 | 8.67 | 8.38 | 8.7 | 2.566667 | 8.583333 |
| G0S2 | 0.233 | -2.10435 | 1.23E-07 | 2.01E-06 | yes | down | 59.72 | 44.94 | 26.3 | 7.82 | 15.95 | 20.97 | 43.65333 | 14.91333 |
| IRGM | 153.621 | 7.263234 | 1.23E-07 | 2.02E-06 | yes | up | 0.05 | 0.08 | 0.1 | 84.72 | 1.68 | 3.58 | 0.076667 | 29.99333 |
| REPS1 | 2.258 | 1.175084 | 1.24E-07 | 2.03E-06 | yes | up | 5.53 | 4.8 | 4.75 | 16.46 | 15.02 | 23.54 | 5.026667 | 18.34 |
| SMUG1 | 2.098 | 1.068786 | 1.26E-07 | 2.05E-06 | yes | up | 8.3 | 10.48 | 7.25 | 20.33 | 24.15 | 18.94 | 8.676667 | 21.14 |
| ECH1 | 0.373 | -1.42082 | 1.26E-07 | 2.06E-06 | yes | down | 423.64 | 402.33 | 538.56 | 229 | 343.87 | 268.1 | 454.8433 | 280.3233 |
| LOC100345779 | 9.771 | 3.288437 | 1.28E-07 | 2.08E-06 | yes | up | 0.56 | 0.3 | 0.19 | 11.17 | 3.78 | 2.15 | 0.35 | 5.7 |
| ASNS | 0.28 | -1.8387 | 1.29E-07 | 2.1E-06 | yes | down | 7.32 | 11.17 | 13.68 | 5.33 | 5.03 | 4.31 | 10.72333 | 4.89 |
| PDGFRA | 2.611 | 1.384608 | 1.3E-07 | 2.1E-06 | yes | up | 4.8 | 2.7 | 2.37 | 13.39 | 13.8 | 13.46 | 3.29 | 13.55 |
| GTF3A | 0.487 | -1.03674 | 1.37E-07 | 2.22E-06 | yes | down | 7.6 | 7.53 | 7.33 | 5.78 | 6.03 | 6.19 | 7.486667 | 6 |
| IFFO1 | 2.375 | 1.247802 | 1.39E-07 | 2.24E-06 | yes | up | 8.43 | 5.99 | 5.43 | 26.88 | 21.73 | 32.2 | 6.616667 | 26.93667 |
| P2RX4 | 2.779 | 1.474768 | 1.41E-07 | 2.27E-06 | yes | up | 3.25 | 4.99 | 3.06 | 20.43 | 13.11 | 16.5 | 3.766667 | 16.68 |
| SLC38A9 | 2.201 | 1.138357 | 1.41E-07 | 2.28E-06 | yes | up | 0.89 | 0.82 | 0.7 | 2.91 | 2.4 | 2.8 | 0.803333 | 2.703333 |
| DDX21 | 0.418 | -1.25987 | 1.43E-07 | 2.3E-06 | yes | down | 13.41 | 16.73 | 11.88 | 11.32 | 6.51 | 10.15 | 14.00667 | 9.326667 |
| LOC100358008 | 0.409 | -1.28856 | 1.5E-07 | 2.4E-06 | yes | down | 22.28 | 22.39 | 24.21 | 13.32 | 15.44 | 16.58 | 22.96 | 15.11333 |
| AR | 4.58 | 2.195393 | 1.5E-07 | 2.41E-06 | yes | up | 0.53 | 0.43 | 0.16 | 2.41 | 2.58 | 2.99 | 0.373333 | 2.66 |
| MED4 | 0.44 | -1.18553 | 1.52E-07 | 2.44E-06 | yes | down | 31.04 | 37.12 | 40.54 | 24.13 | 24.12 | 28.89 | 36.23333 | 25.71333 |
| NDUFA5 | 0.416 | -1.26429 | 1.54E-07 | 2.46E-06 | yes | down | 181 | 161 | 210.02 | 95.82 | 107.15 | 89.75 | 184.0067 | 97.57333 |
| FNDC5 | 0.084 | -3.56988 | 1.54E-07 | 2.47E-06 | yes | down | 55.24 | 10.26 | 7.65 | 2.05 | 4.04 | 3.53 | 24.38333 | 3.206667 |
| NUP35 | 0.362 | -1.46483 | 1.6E-07 | 2.55E-06 | yes | down | 7.83 | 12.17 | 7 | 4.51 | 5.77 | 5.2 | 9 | 5.16 |
| IMP4 | 0.497 | -1.00908 | 1.66E-07 | 2.64E-06 | yes | down | 15.62 | 16.96 | 16.86 | 13.27 | 11.01 | 15.49 | 16.48 | 13.25667 |
| PCOLCE | 3.366 | 1.751181 | 1.66E-07 | 2.64E-06 | yes | up | 35.78 | 20.73 | 15.56 | 149.83 | 138.41 | 88.97 | 24.02333 | 125.7367 |
| SAV1 | 0.497 | -1.00875 | 1.67E-07 | 2.65E-06 | yes | down | 16.55 | 21.77 | 18.51 | 15.35 | 12.96 | 17.29 | 18.94333 | 15.2 |
| EFNB2 | 0.458 | -1.12802 | 1.68E-07 | 2.67E-06 | yes | down | 10.56 | 14.1 | 12.82 | 8.4 | 9.95 | 9.18 | 12.49333 | 9.176667 |
| LOC100345657 | 11.336 | 3.502836 | 1.71E-07 | 2.7E-06 | yes | up | 0.44 | 0.31 | 0.19 | 11.75 | 3.15 | 1.64 | 0.313333 | 5.513333 |
| CLPB | 0.492 | -1.0242 | 1.77E-07 | 2.8E-06 | yes | down | 12 | 10.44 | 9.14 | 8.04 | 9.37 | 7.24 | 10.52667 | 8.216667 |
| MRPS31 | 0.371 | -1.42973 | 1.79E-07 | 2.82E-06 | yes | down | 14.56 | 17.07 | 11.03 | 10.15 | 6.26 | 9.03 | 14.22 | 8.48 |
| LOC100359087 | 5.684 | 2.506906 | 1.8E-07 | 2.82E-06 | yes | up | 1.28 | 0.62 | 0.28 | 7.65 | 7.63 | 6.86 | 0.726667 | 7.38 |
| IRF2BP2 | 2.291 | 1.19619 | 1.83E-07 | 2.87E-06 | yes | up | 14.51 | 19.8 | 17.13 | 47.85 | 62.52 | 71.59 | 17.14667 | 60.65333 |
| LOC100342826 | 5.652 | 2.498867 | 1.91E-07 | 2.99E-06 | yes | up | 5.19 | 3.49 | 2.98 | 55.41 | 35.25 | 13.51 | 3.886667 | 34.72333 |
| MRPL11 | 0.476 | -1.07122 | 1.95E-07 | 3.04E-06 | yes | down | 45.13 | 54.3 | 51.07 | 33.8 | 40.78 | 40.4 | 50.16667 | 38.32667 |
| LOC103349164 | 0.006 | -7.50512 | 1.95E-07 | 3.05E-06 | yes | down | 0.82 | 1.21 | 1.75 | 0 | 0 | 0 | 1.26 | 0 |
| FAM210A | 0.485 | -1.04403 | 1.96E-07 | 3.06E-06 | yes | down | 14.27 | 10.67 | 9.48 | 7.59 | 9.65 | 9.43 | 11.47333 | 8.89 |
| LOC103345364 | 3.211 | 1.68284 | 2.04E-07 | 3.17E-06 | yes | up | 6.56 | 10.32 | 5.88 | 48.01 | 30.66 | 36.95 | 7.586667 | 38.54 |
| NFYC | 0.458 | -1.12613 | 2.04E-07 | 3.17E-06 | yes | down | 20.95 | 27.04 | 25.46 | 20.95 | 16.23 | 18.26 | 24.48333 | 18.48 |
| CREB5 | 2.249 | 1.169396 | 2.07E-07 | 3.2E-06 | yes | up | 1.55 | 1.06 | 0.74 | 3.76 | 4.22 | 3.79 | 1.116667 | 3.923333 |
| FGFR4 | 0.351 | -1.51209 | 2.08E-07 | 3.22E-06 | yes | down | 6.62 | 4.17 | 3.85 | 2.18 | 2.33 | 3.51 | 4.88 | 2.673333 |
| CP | 14.133 | 3.820949 | 2.09E-07 | 3.24E-06 | yes | up | 0.61 | 0.22 | 0.33 | 18.47 | 2.22 | 4.73 | 0.386667 | 8.473333 |
| OBFC1 | 2.121 | 1.084719 | 2.16E-07 | 3.33E-06 | yes | up | 2.95 | 2.32 | 2.72 | 8.64 | 8.39 | 10.03 | 2.663333 | 9.02 |
| YWHAG | 0.483 | -1.05043 | 2.17E-07 | 3.35E-06 | yes | down | 59.49 | 52.03 | 32.73 | 33.19 | 38.61 | 37.74 | 48.08333 | 36.51333 |
| SLC8A1 | 2.557 | 1.354635 | 2.18E-07 | 3.35E-06 | yes | up | 0.77 | 0.58 | 0.47 | 2.71 | 2.29 | 2.44 | 0.606667 | 2.48 |
| TMEM37 | 0.231 | -2.11458 | 2.18E-07 | 3.36E-06 | yes | down | 29 | 39.32 | 24.47 | 4.93 | 13.38 | 15.79 | 30.93 | 11.36667 |
| MGLL | 0.33 | -1.60082 | 2.2E-07 | 3.37E-06 | yes | down | 188.45 | 249.64 | 291.63 | 96.86 | 163.74 | 128.55 | 243.24 | 129.7167 |
| DUSP10 | 2.586 | 1.370478 | 2.29E-07 | 3.51E-06 | yes | up | 8.99 | 9.19 | 5.43 | 22.36 | 35.09 | 37.92 | 7.87 | 31.79 |
| MCAM | 0.486 | -1.04144 | 2.32E-07 | 3.55E-06 | yes | down | 86.46 | 101.36 | 85.87 | 50.99 | 69.33 | 83.89 | 91.23 | 68.07 |
| NPL | 3.234 | 1.693287 | 2.34E-07 | 3.57E-06 | yes | up | 2.12 | 3.36 | 1.29 | 8.03 | 7.54 | 9.21 | 2.256667 | 8.26 |
| C19H17orf50 | 3.032 | 1.600125 | 2.35E-07 | 3.58E-06 | yes | up | 3.07 | 4.93 | 3.54 | 12.25 | 15.75 | 25.39 | 3.846667 | 17.79667 |
| ATXN1 | 0.199 | -2.33203 | 2.36E-07 | 3.59E-06 | yes | down | 9.23 | 19.37 | 4.59 | 5.05 | 4.6 | 3.06 | 11.06333 | 4.236667 |
| EGR2 | 6.805 | 2.766588 | 2.37E-07 | 3.61E-06 | yes | up | 0.38 | 0.14 | 0.11 | 2.28 | 1.74 | 2.71 | 0.21 | 2.243333 |
| SMAD7 | 0.345 | -1.53327 | 2.41E-07 | 3.67E-06 | yes | down | 13.64 | 14.7 | 9.85 | 5 | 5.75 | 10.2 | 12.73 | 6.983333 |
| YIF1B | 0.486 | -1.04156 | 2.45E-07 | 3.71E-06 | yes | down | 20.59 | 23.5 | 20.8 | 18.76 | 16.35 | 15.15 | 21.63 | 16.75333 |
| EXOSC5 | 0.364 | -1.45915 | 2.46E-07 | 3.72E-06 | yes | down | 12.84 | 17.29 | 17.79 | 9.88 | 9.66 | 8.53 | 15.97333 | 9.356667 |
| RMDN1 | 0.373 | -1.42465 | 2.51E-07 | 3.79E-06 | yes | down | 7.31 | 8.71 | 5.86 | 3.5 | 4.86 | 4.58 | 7.293333 | 4.313333 |
| NDUFAF4 | 0.343 | -1.54531 | 2.57E-07 | 3.88E-06 | yes | down | 6.21 | 7.69 | 3.53 | 3.06 | 3.2 | 3.09 | 5.81 | 3.116667 |
| PRELID3A | 5.66 | 2.500796 | 2.61E-07 | 3.93E-06 | yes | up | 0.41 | 0.33 | 0.33 | 1.51 | 3.33 | 4.23 | 0.356667 | 3.023333 |
| TXNIP | 2.815 | 1.493382 | 2.62E-07 | 3.95E-06 | yes | up | 439.56 | 565.16 | 655.88 | 2142.29 | 2049.8 | 3488.18 | 553.5333 | 2560.09 |
| CMTM3 | 2.017 | 1.012533 | 2.99E-07 | 4.48E-06 | yes | up | 8.45 | 8.28 | 5.85 | 26.57 | 19.59 | 26.63 | 7.526667 | 24.26333 |
| LOC103345082 | 0.251 | -1.99297 | 3.03E-07 | 4.53E-06 | yes | down | 9.05 | 10.1 | 5.15 | 3.92 | 2.92 | 1.99 | 8.1 | 2.943333 |
| NDUFAF5 | 0.456 | -1.13145 | 3.11E-07 | 4.65E-06 | yes | down | 8.62 | 9.5 | 9.64 | 5.88 | 6.8 | 7.43 | 9.253333 | 6.703333 |
| TRIL | 0.175 | -2.51781 | 3.15E-07 | 4.71E-06 | yes | down | 2.99 | 4.04 | 8.92 | 1.28 | 1.31 | 2.13 | 5.316667 | 1.573333 |
| SLC25A27 | 3.701 | 1.888051 | 3.16E-07 | 4.71E-06 | yes | up | 0.63 | 0.37 | 0.24 | 2 | 2.24 | 2.54 | 0.413333 | 2.26 |
| SOWAHC | 0.234 | -2.09847 | 3.23E-07 | 4.81E-06 | yes | down | 20.97 | 41.69 | 11.36 | 7.09 | 7.62 | 11.64 | 24.67333 | 8.783333 |
| TUFM | 0.431 | -1.21465 | 3.24E-07 | 4.82E-06 | yes | down | 148.51 | 160.02 | 158.87 | 92.18 | 135.86 | 98.95 | 155.8 | 108.9967 |
| LOC103351346 | 2.563 | 1.357726 | 3.42E-07 | 5.08E-06 | yes | up | 1.1 | 1 | 1.09 | 3.7 | 4.49 | 5.06 | 1.063333 | 4.416667 |
| CHCHD4 | 0.491 | -1.02717 | 3.46E-07 | 5.13E-06 | yes | down | 11.89 | 14.07 | 10.21 | 9.21 | 7.14 | 9.59 | 12.05667 | 8.646667 |
| PAK1IP1 | 0.419 | -1.25535 | 3.49E-07 | 5.16E-06 | yes | down | 6.61 | 6.9 | 7.13 | 5.16 | 4.51 | 4.2 | 6.88 | 4.623333 |
| CC2D2A | 2.003 | 1.002494 | 3.59E-07 | 5.31E-06 | yes | up | 5.59 | 4.49 | 2.03 | 9.08 | 8.42 | 11.71 | 4.036667 | 9.736667 |
| LOC100350957 | 0.214 | -2.22619 | 3.69E-07 | 5.44E-06 | yes | down | 6.65 | 8.26 | 4.97 | 2.06 | 3.49 | 1.51 | 6.626667 | 2.353333 |
| IFI44L | 4.354 | 2.122348 | 3.71E-07 | 5.47E-06 | yes | up | 4.94 | 5.83 | 2.61 | 47.3 | 33.87 | 16.43 | 4.46 | 32.53333 |
| U2AF2 | 0.407 | -1.2973 | 3.73E-07 | 5.49E-06 | yes | down | 7.45 | 5.97 | 6.11 | 4.3 | 3.57 | 4.9 | 6.51 | 4.256667 |
| CD96 | 29.105 | 4.863197 | 3.76E-07 | 5.53E-06 | yes | up | 0.3 | 0.06 | 0.07 | 12.02 | 1 | 2.24 | 0.143333 | 5.086667 |
| LOC100359306 | 8.817 | 3.140319 | 3.9E-07 | 5.74E-06 | yes | up | 2.61 | 1.76 | 3.09 | 71.62 | 12.99 | 18.3 | 2.486667 | 34.30333 |
| C17H15orf41 | 0.283 | -1.82294 | 3.94E-07 | 5.79E-06 | yes | down | 3.15 | 2.35 | 2.53 | 1.62 | 1.11 | 0.87 | 2.676667 | 1.2 |
| ZDHHC14 | 3.197 | 1.676635 | 3.96E-07 | 5.8E-06 | yes | up | 1.81 | 1.75 | 1.37 | 9.89 | 5.8 | 5.53 | 1.643333 | 7.073333 |
| CCL14 | 2.424 | 1.277233 | 3.96E-07 | 5.8E-06 | yes | up | 13.15 | 15.26 | 9.48 | 54.69 | 38.98 | 51.79 | 12.63 | 48.48667 |
| WSCD2 | 5.208 | 2.380592 | 4.07E-07 | 5.96E-06 | yes | up | 0.19 | 0.15 | 0.09 | 1.08 | 0.96 | 1.35 | 0.143333 | 1.13 |
| SPATA6 | 2.23 | 1.156783 | 4.12E-07 | 6.02E-06 | yes | up | 1.58 | 1.44 | 0.95 | 4.68 | 6.43 | 6.21 | 1.323333 | 5.773333 |
| HPRT1 | 0.463 | -1.11113 | 4.16E-07 | 6.07E-06 | yes | down | 23.09 | 19.86 | 13.58 | 15.52 | 11.23 | 14.64 | 18.84333 | 13.79667 |
| JADE2 | 2.894 | 1.533094 | 4.22E-07 | 6.16E-06 | yes | up | 1.12 | 1.72 | 1.5 | 8.59 | 4.78 | 7.02 | 1.446667 | 6.796667 |
| RGS3 | 3.131 | 1.646467 | 4.29E-07 | 6.25E-06 | yes | up | 40.14 | 52.18 | 54.23 | 207.5 | 266.68 | 489.64 | 48.85 | 321.2733 |
| LOC100351672 | 3.61 | 1.851889 | 4.33E-07 | 6.3E-06 | yes | up | 10.24 | 11.94 | 18.64 | 59.44 | 70.4 | 114.91 | 13.60667 | 81.58333 |
| HSD11B2 | 0.198 | -2.33779 | 4.38E-07 | 6.36E-06 | yes | down | 2.17 | 3.06 | 1.41 | 0.56 | 0.59 | 0.94 | 2.213333 | 0.696667 |
| BCL6 | 2.078 | 1.055064 | 4.56E-07 | 6.61E-06 | yes | up | 8.46 | 6.06 | 6.54 | 19.84 | 22.58 | 23.42 | 7.02 | 21.94667 |
| MKRN3 | 6.342 | 2.664858 | 4.6E-07 | 6.67E-06 | yes | up | 0.2 | 0.1 | 0.27 | 2.46 | 1.78 | 1.73 | 0.19 | 1.99 |
| SLC25A20 | 0.441 | -1.18192 | 4.62E-07 | 6.69E-06 | yes | down | 255.75 | 316.72 | 307.15 | 165.04 | 241.21 | 218.68 | 293.2067 | 208.31 |
| LOC108176880 | 9.329 | 3.221704 | 4.69E-07 | 6.77E-06 | yes | up | 0.27 | 0.42 | 0.47 | 3.39 | 7.58 | 4.97 | 0.386667 | 5.313333 |
| OXNAD1 | 0.399 | -1.32523 | 4.74E-07 | 6.84E-06 | yes | down | 17.66 | 21.76 | 24.43 | 13.45 | 15.27 | 12.05 | 21.28333 | 13.59 |
| HSPA13 | 0.361 | -1.47098 | 4.98E-07 | 7.17E-06 | yes | down | 6.6 | 6.97 | 8.69 | 5.46 | 3.16 | 4.5 | 7.42 | 4.373333 |
| ABT1 | 0.437 | -1.19462 | 5.02E-07 | 7.21E-06 | yes | down | 8.69 | 10.61 | 6.51 | 6.77 | 4.7 | 6.44 | 8.603333 | 5.97 |
| LOC100349113 | 3.396 | 1.763903 | 5.13E-07 | 7.37E-06 | yes | up | 63.44 | 129.22 | 83.67 | 310.04 | 582.3 | 606.98 | 92.11 | 499.7733 |
| TRAP1 | 0.369 | -1.43956 | 5.17E-07 | 7.42E-06 | yes | down | 93.6 | 90.99 | 109.75 | 44.33 | 75.16 | 54.79 | 98.11333 | 58.09333 |
| PALMD | 0.397 | -1.33186 | 5.19E-07 | 7.43E-06 | yes | down | 15.78 | 20.06 | 12.17 | 7.12 | 10.25 | 12.98 | 16.00333 | 10.11667 |
| CHST2 | 0.373 | -1.42143 | 5.26E-07 | 7.52E-06 | yes | down | 4.54 | 7.5 | 5.36 | 2.79 | 3.69 | 3.95 | 5.8 | 3.476667 |
| PTGER4 | 2.114 | 1.079805 | 5.27E-07 | 7.53E-06 | yes | up | 2 | 2.02 | 2.23 | 6.87 | 7.22 | 7.21 | 2.083333 | 7.1 |
| LOC100358984 | 0.474 | -1.07755 | 5.3E-07 | 7.56E-06 | yes | down | 10.87 | 11.78 | 12.55 | 10.21 | 8.34 | 8.06 | 11.73333 | 8.87 |
| SEMA3B | 3.194 | 1.675502 | 5.52E-07 | 7.85E-06 | yes | up | 4.19 | 2.32 | 1.44 | 12.05 | 12.81 | 13.53 | 2.65 | 12.79667 |
| LOC100346827 | 0.322 | -1.63483 | 5.52E-07 | 7.85E-06 | yes | down | 10.94 | 15.24 | 9.21 | 5.77 | 6.55 | 8.94 | 11.79667 | 7.086667 |
| POP5 | 0.477 | -1.067 | 5.63E-07 | 8E-06 | yes | down | 15.6 | 13.88 | 11.33 | 8.36 | 10.87 | 11.78 | 13.60333 | 10.33667 |
| NCEH1 | 0.377 | -1.40608 | 5.69E-07 | 8.06E-06 | yes | down | 5.53 | 4.96 | 7.34 | 3.89 | 3.21 | 3.93 | 5.943333 | 3.676667 |
| MYO19 | 0.385 | -1.37526 | 5.76E-07 | 8.15E-06 | yes | down | 8.93 | 13.37 | 7.31 | 5.81 | 7.19 | 5.08 | 9.87 | 6.026667 |
| ISG15 | 8.006 | 3.001039 | 5.82E-07 | 8.21E-06 | yes | up | 10.83 | 4.85 | 4.18 | 124.51 | 94.23 | 23.48 | 6.62 | 80.74 |
| LOC100347143 | 0.461 | -1.11618 | 5.99E-07 | 8.4E-06 | yes | down | 493.49 | 493.04 | 467.93 | 296.83 | 445.43 | 326.72 | 484.82 | 356.3267 |
| LOC100348403 | 0.006 | -7.45729 | 5.99E-07 | 8.41E-06 | yes | down | 2.34 | 0.11 | 1.63 | 0.02 | 0.02 | 0 | 1.36 | 0.013333 |
| LOC100343793 | 0.465 | -1.10435 | 6.01E-07 | 8.42E-06 | yes | down | 2048.69 | 2190.49 | 2234.3 | 1348.14 | 1906.12 | 1538.53 | 2157.827 | 1597.597 |
| KAT2A | 2.688 | 1.42643 | 6.02E-07 | 8.42E-06 | yes | up | 7.23 | 5.62 | 4.08 | 16.8 | 29.18 | 28.47 | 5.643333 | 24.81667 |
| MAPK8IP1 | 0.416 | -1.26646 | 6.07E-07 | 8.49E-06 | yes | down | 12.87 | 14.85 | 10.19 | 6.06 | 9.81 | 9.11 | 12.63667 | 8.326667 |
| SLC6A17 | 0.157 | -2.67105 | 6.16E-07 | 8.6E-06 | yes | down | 0.87 | 1.6 | 1.72 | 0.19 | 0.53 | 0.35 | 1.396667 | 0.356667 |
| GEMIN4 | 0.421 | -1.24669 | 6.2E-07 | 8.64E-06 | yes | down | 3.69 | 3.71 | 2.78 | 1.74 | 2.55 | 2.52 | 3.393333 | 2.27 |
| QRSL1 | 0.468 | -1.09526 | 6.2E-07 | 8.64E-06 | yes | down | 7.31 | 6.45 | 4.94 | 4.28 | 4.21 | 5.45 | 6.233333 | 4.646667 |
| PAM16 | 0.346 | -1.52938 | 6.41E-07 | 8.92E-06 | yes | down | 38.14 | 73.09 | 53.23 | 30.02 | 28.2 | 28.34 | 54.82 | 28.85333 |
| B3GALNT2 | 2.668 | 1.41586 | 6.44E-07 | 8.96E-06 | yes | up | 10 | 5.15 | 4.86 | 27.51 | 25.83 | 31.81 | 6.67 | 28.38333 |
| MDM4 | 2.319 | 1.213421 | 6.5E-07 | 9.03E-06 | yes | up | 7.1 | 8 | 3.73 | 20.74 | 21.24 | 28.1 | 6.276667 | 23.36 |
| MLXIPL | 2.584 | 1.369663 | 6.52E-07 | 9.05E-06 | yes | up | 15.75 | 11.43 | 12.51 | 37.44 | 66.44 | 55.6 | 13.23 | 53.16 |
| USP18 | 6.972 | 2.80157 | 6.54E-07 | 9.07E-06 | yes | up | 1.44 | 0.91 | 0.49 | 13.7 | 10.11 | 3.67 | 0.946667 | 9.16 |
| CX3CL1 | 12.094 | 3.596239 | 6.7E-07 | 9.27E-06 | yes | up | 0.22 | 0.48 | 0.32 | 14.56 | 2.17 | 2.8 | 0.34 | 6.51 |
| IMMT | 0.477 | -1.06742 | 6.95E-07 | 9.59E-06 | yes | down | 146.73 | 136.6 | 125.74 | 80.6 | 127.83 | 120 | 136.3567 | 109.4767 |
| ODC1 | 0.326 | -1.61526 | 7.11E-07 | 9.81E-06 | yes | down | 78.18 | 183.49 | 105.57 | 55.64 | 55.96 | 80.48 | 122.4133 | 64.02667 |
| TMED8 | 2.142 | 1.098849 | 7.13E-07 | 9.82E-06 | yes | up | 2.96 | 3.31 | 3.73 | 11.27 | 9.63 | 9.42 | 3.333333 | 10.10667 |
| IGF2R | 0.396 | -1.33509 | 7.21E-07 | 9.92E-06 | yes | down | 17.44 | 16.22 | 14.99 | 13.62 | 9.94 | 7.16 | 16.21667 | 10.24 |
| OPN3 | 3.713 | 1.892425 | 7.31E-07 | 1E-05 | yes | up | 1.21 | 0.74 | 0.48 | 3.13 | 4.77 | 5.38 | 0.81 | 4.426667 |
| LOC100352395 | 2.831 | 1.501544 | 7.36E-07 | 1.01E-05 | yes | up | 0.49 | 0.47 | 0.36 | 1.47 | 2.09 | 2.35 | 0.44 | 1.97 |
| LOC108176449 | 11.442 | 3.516254 | 7.43E-07 | 1.02E-05 | yes | up | 0.01 | 0.16 | 0.04 | 1.11 | 1.23 | 2.31 | 0.07 | 1.55 |
| TLN2 | 0.467 | -1.09875 | 7.51E-07 | 1.03E-05 | yes | down | 5.51 | 3.93 | 3.08 | 2.84 | 3.43 | 3.36 | 4.173333 | 3.21 |
| RSAD2 | 6.225 | 2.637976 | 7.52E-07 | 1.03E-05 | yes | up | 2.37 | 1.34 | 0.65 | 21.82 | 13.39 | 6.35 | 1.453333 | 13.85333 |
| HEY1 | 0.428 | -1.2247 | 7.55E-07 | 1.03E-05 | yes | down | 8.92 | 14.47 | 9.62 | 6.82 | 6.92 | 8.43 | 11.00333 | 7.39 |
| CAD | 0.458 | -1.12699 | 7.76E-07 | 1.06E-05 | yes | down | 2.1 | 2.63 | 2.44 | 1.88 | 1.72 | 1.57 | 2.39 | 1.723333 |
| BYSL | 0.39 | -1.35719 | 7.88E-07 | 1.07E-05 | yes | down | 4.65 | 4.77 | 3.08 | 2.93 | 1.97 | 2.85 | 4.166667 | 2.583333 |
| LOC100328912 | 3.118 | 1.640656 | 7.97E-07 | 1.08E-05 | yes | up | 77.65 | 74.12 | 90.95 | 238.28 | 412.01 | 573.51 | 80.90667 | 407.9333 |
| ACO2 | 0.426 | -1.23125 | 8.2E-07 | 1.11E-05 | yes | down | 773.84 | 692.34 | 801.18 | 509.99 | 629.48 | 407.37 | 755.7867 | 515.6133 |
| NKTR | 2.336 | 1.22421 | 8.23E-07 | 1.12E-05 | yes | up | 6.45 | 7.53 | 3.48 | 19.44 | 22.47 | 20.59 | 5.82 | 20.83333 |
| FRZB | 0.408 | -1.29382 | 8.33E-07 | 1.13E-05 | yes | down | 18.92 | 12.6 | 14.11 | 7.42 | 11 | 11.35 | 15.21 | 9.923333 |
| LOC100346985 | 10.546 | 3.398674 | 8.56E-07 | 1.16E-05 | yes | up | 1.73 | 0.86 | 0.84 | 43.43 | 8.05 | 6.31 | 1.143333 | 19.26333 |
| COQ9 | 0.486 | -1.04095 | 8.71E-07 | 1.18E-05 | yes | down | 140.06 | 112.04 | 100.98 | 76.17 | 108.45 | 86.29 | 117.6933 | 90.30333 |
| CTSD | 0.481 | -1.05602 | 8.79E-07 | 1.19E-05 | yes | down | 372.45 | 573.45 | 437.87 | 398.85 | 317.64 | 350.93 | 461.2567 | 355.8067 |
| HOGA1 | 0.404 | -1.30663 | 8.88E-07 | 1.2E-05 | yes | down | 13.11 | 12.3 | 12.86 | 10.27 | 7.84 | 6.29 | 12.75667 | 8.133333 |
| RGS2 | 3.975 | 1.99079 | 8.91E-07 | 1.2E-05 | yes | up | 9.75 | 9.16 | 8.46 | 84.48 | 24.89 | 66.45 | 9.123333 | 58.60667 |
| MBD4 | 0.437 | -1.19556 | 8.95E-07 | 1.2E-05 | yes | down | 4.17 | 5.1 | 3.08 | 2.85 | 2.39 | 3.34 | 4.116667 | 2.86 |
| ACKR1 | 2.408 | 1.267553 | 9.12E-07 | 1.22E-05 | yes | up | 3.28 | 3.02 | 2.03 | 9.63 | 8.08 | 14.33 | 2.776667 | 10.68 |
| JAGN1 | 0.457 | -1.12955 | 9.15E-07 | 1.23E-05 | yes | down | 37.14 | 34.52 | 34.21 | 24.16 | 25.18 | 37.9 | 35.29 | 29.08 |
| LOC108178305 | 5.948 | 2.572464 | 9.32E-07 | 1.25E-05 | yes | up | 0.36 | 0.23 | 0.56 | 2.85 | 4.72 | 3.79 | 0.383333 | 3.786667 |
| LOC100355689 | 10.599 | 3.405919 | 9.65E-07 | 1.29E-05 | yes | up | 0.04 | 0.06 | 0.04 | 0.39 | 0.79 | 1.1 | 0.046667 | 0.76 |
| CDKN1A | 2.338 | 1.225227 | 9.86E-07 | 1.32E-05 | yes | up | 8.34 | 9.09 | 7.07 | 38.02 | 20.9 | 32.86 | 8.166667 | 30.59333 |
| PDE4D | 0.347 | -1.52734 | 1.04E-06 | 1.38E-05 | yes | down | 17.34 | 26.12 | 9.41 | 10.81 | 15.62 | 22.39 | 17.62333 | 16.27333 |
| DENND5B | 0.404 | -1.30693 | 1.04E-06 | 1.38E-05 | yes | down | 2.11 | 1.43 | 1.26 | 0.96 | 1.17 | 0.93 | 1.6 | 1.02 |
| SSH2 | 2.394 | 1.259615 | 1.08E-06 | 1.43E-05 | yes | up | 4.96 | 2.86 | 2.75 | 15.76 | 11.55 | 15.51 | 3.523333 | 14.27333 |
| PLA2G12A | 0.29 | -1.78778 | 1.08E-06 | 1.44E-05 | yes | down | 43.19 | 20.98 | 14.63 | 10.68 | 11 | 13.82 | 26.26667 | 11.83333 |
| DDIT3 | 2.262 | 1.177411 | 1.09E-06 | 1.44E-05 | yes | up | 6.31 | 8.23 | 5.89 | 18.3 | 22.11 | 29.65 | 6.81 | 23.35333 |
| ELAC2 | 0.456 | -1.13375 | 1.1E-06 | 1.46E-05 | yes | down | 9.49 | 11.8 | 10.55 | 6.65 | 8.85 | 8.16 | 10.61333 | 7.886667 |
| LOC100343529 | 0.212 | -2.23816 | 1.11E-06 | 1.47E-05 | yes | down | 13.58 | 14.57 | 13.87 | 2.3 | 5.76 | 6.43 | 14.00667 | 4.83 |
| TRAM1L1 | 0.171 | -2.54919 | 1.11E-06 | 1.47E-05 | yes | down | 0.88 | 1.12 | 0.69 | 0.23 | 0.21 | 0.29 | 0.896667 | 0.243333 |
| LOC100340875 | 0.338 | -1.56433 | 1.12E-06 | 1.48E-05 | yes | down | 42.8 | 51.44 | 52.33 | 23.39 | 34.55 | 20.64 | 48.85667 | 26.19333 |
| SLC30A2 | 102.74 | 6.682856 | 1.13E-06 | 1.48E-05 | yes | up | 0 | 0 | 0.01 | 1.48 | 0.65 | 0.55 | 0.003333 | 0.893333 |
| SERPING1 | 6.785 | 2.762346 | 1.14E-06 | 1.5E-05 | yes | up | 20.57 | 19.74 | 14.09 | 411.85 | 76.94 | 95.45 | 18.13333 | 194.7467 |
| LOC103347367 | 0.342 | -1.54924 | 1.16E-06 | 1.53E-05 | yes | down | 58.55 | 73.31 | 76.2 | 42.41 | 45.61 | 25.47 | 69.35333 | 37.83 |
| MCFD2 | 2.438 | 1.285577 | 1.16E-06 | 1.53E-05 | yes | up | 28.45 | 29.95 | 29.49 | 92.93 | 88.98 | 166.39 | 29.29667 | 116.1 |
| PPP1R13L | 0.265 | -1.91326 | 1.18E-06 | 1.55E-05 | yes | down | 1.58 | 1.75 | 1.66 | 0.89 | 0.67 | 0.56 | 1.663333 | 0.706667 |
| LOC103350250 | 0.17 | -2.56047 | 1.2E-06 | 1.57E-05 | yes | down | 1.16 | 2.02 | 2.3 | 0.41 | 0.48 | 0.65 | 1.826667 | 0.513333 |
| CCDC58 | 0.373 | -1.42261 | 1.22E-06 | 1.6E-05 | yes | down | 47.25 | 27.76 | 28.53 | 23.13 | 17.24 | 16.34 | 34.51333 | 18.90333 |
| KLF4 | 2.63 | 1.394999 | 1.24E-06 | 1.62E-05 | yes | up | 8.81 | 12.86 | 9.15 | 55.93 | 28.86 | 45.4 | 10.27333 | 43.39667 |
| LOC103346740 | 0.288 | -1.79515 | 1.25E-06 | 1.63E-05 | yes | down | 114.36 | 167.55 | 160.17 | 68.51 | 80.06 | 50.35 | 147.36 | 66.30667 |
| ATP5G1 | 0.284 | -1.81449 | 1.27E-06 | 1.66E-05 | yes | down | 427.15 | 234.16 | 173.7 | 81.43 | 164.54 | 126.59 | 278.3367 | 124.1867 |
| FAS | 2.249 | 1.169461 | 1.29E-06 | 1.68E-05 | yes | up | 3.15 | 3.38 | 2.48 | 11.31 | 7.82 | 8.94 | 3.003333 | 9.356667 |
| LOC103349229 | 3.524 | 1.817126 | 1.3E-06 | 1.69E-05 | yes | up | 0.53 | 0.58 | 0.87 | 4.59 | 3.63 | 3.91 | 0.66 | 4.043333 |
| GYPA | 0.256 | -1.96391 | 1.32E-06 | 1.71E-05 | yes | down | 5.58 | 11.47 | 7.72 | 2.99 | 2.55 | 4.43 | 8.256667 | 3.323333 |
| LOC100339034 | 0.029 | -5.10836 | 1.32E-06 | 1.72E-05 | yes | down | 1.02 | 1.03 | 3.33 | 0.15 | 0.18 | 0 | 1.793333 | 0.11 |
| CMPK2 | 3.08 | 1.623086 | 1.41E-06 | 1.83E-05 | yes | up | 1.77 | 2.73 | 2.48 | 15.35 | 10.35 | 16.57 | 2.326667 | 14.09 |
| LOC103345230 | 3.152 | 1.656061 | 1.43E-06 | 1.85E-05 | yes | up | 0.79 | 1.42 | 1.41 | 4.87 | 6.03 | 6.9 | 1.206667 | 5.933333 |
| GRPEL2 | 0.443 | -1.17501 | 1.44E-06 | 1.85E-05 | yes | down | 4.3 | 3.85 | 2.64 | 2.84 | 2.22 | 2.11 | 3.596667 | 2.39 |
| FAM160B1 | 2.069 | 1.04906 | 1.46E-06 | 1.89E-05 | yes | up | 5.98 | 5.52 | 5.78 | 15.01 | 18.89 | 23.83 | 5.76 | 19.24333 |
| BARD1 | 2.221 | 1.150912 | 1.5E-06 | 1.93E-05 | yes | up | 1.67 | 1.3 | 1.17 | 4 | 4.83 | 5.65 | 1.38 | 4.826667 |
| NELL1 | 9.447 | 3.239875 | 1.51E-06 | 1.94E-05 | yes | up | 0.06 | 0.27 | 0.07 | 1.28 | 2.77 | 2.04 | 0.133333 | 2.03 |
| LOC100348151 | 0.017 | -5.90347 | 1.52E-06 | 1.96E-05 | yes | down | 2.69 | 0.11 | 2.6 | 0.06 | 0.04 | 0.02 | 1.8 | 0.04 |
| TPRA1 | 0.417 | -1.26314 | 1.54E-06 | 1.98E-05 | yes | down | 20.95 | 27.33 | 30.37 | 16.42 | 15.59 | 21.71 | 26.21667 | 17.90667 |
| ACBD6 | 0.432 | -1.21062 | 1.6E-06 | 2.04E-05 | yes | down | 5.72 | 5.26 | 4.38 | 3.93 | 2.91 | 3.74 | 5.12 | 3.526667 |
| HADHB | 0.346 | -1.53199 | 1.6E-06 | 2.05E-05 | yes | down | 1037.1 | 975.66 | 1224.12 | 508.96 | 837.75 | 444.31 | 1078.96 | 597.0067 |
| COQ2 | 0.437 | -1.19291 | 1.62E-06 | 2.07E-05 | yes | down | 13.92 | 9.67 | 9.89 | 6.53 | 8.55 | 8.32 | 11.16 | 7.8 |
| YEATS4 | 0.388 | -1.36624 | 1.62E-06 | 2.07E-05 | yes | down | 11.16 | 7.06 | 10.46 | 7.84 | 6.01 | 6.72 | 9.56 | 6.856667 |
| SLC15A3 | 5.99 | 2.582444 | 1.65E-06 | 2.1E-05 | yes | up | 1.22 | 1.6 | 1.37 | 25.73 | 6.46 | 8.13 | 1.396667 | 13.44 |
| SELP | 3.867 | 1.951289 | 1.69E-06 | 2.15E-05 | yes | up | 0.49 | 0.36 | 0.33 | 1.63 | 2.32 | 3.06 | 0.393333 | 2.336667 |
| LOC103347841 | 4.296 | 2.103079 | 1.7E-06 | 2.16E-05 | yes | up | 0.17 | 0.11 | 0.08 | 0.94 | 0.73 | 0.8 | 0.12 | 0.823333 |
| NUBPL | 0.298 | -1.746 | 1.71E-06 | 2.17E-05 | yes | down | 5.85 | 4.66 | 7.53 | 2.73 | 2.68 | 2.89 | 6.013333 | 2.766667 |
| DCTPP1 | 0.332 | -1.59252 | 1.77E-06 | 2.25E-05 | yes | down | 7.45 | 9.5 | 6.13 | 3.41 | 4.37 | 4.34 | 7.693333 | 4.04 |
| NDUFA10 | 0.426 | -1.22975 | 1.78E-06 | 2.25E-05 | yes | down | 264.6 | 282.52 | 335.43 | 177.27 | 246.92 | 183.18 | 294.1833 | 202.4567 |
| F3 | 3.015 | 1.592088 | 1.81E-06 | 2.29E-05 | yes | up | 24.84 | 35.94 | 13.83 | 82.1 | 126.99 | 142.71 | 24.87 | 117.2667 |
| FBXL4 | 2.193 | 1.133176 | 1.83E-06 | 2.32E-05 | yes | up | 3.94 | 3.62 | 4.03 | 11.01 | 14.85 | 14.92 | 3.863333 | 13.59333 |
| PTRH2 | 0.442 | -1.17783 | 1.87E-06 | 2.36E-05 | yes | down | 13.98 | 17.29 | 13.84 | 10.96 | 8.6 | 13.12 | 15.03667 | 10.89333 |
| LOC100345689 | 8.872 | 3.14934 | 1.93E-06 | 2.43E-05 | yes | up | 0.17 | 0.1 | 0.07 | 2.21 | 1.46 | 1.1 | 0.113333 | 1.59 |
| FDFT1 | 2.103 | 1.072405 | 1.93E-06 | 2.43E-05 | yes | up | 29.22 | 24.14 | 29.72 | 75.74 | 94.17 | 111.97 | 27.69333 | 93.96 |
| BMPER | 3.617 | 1.854967 | 1.96E-06 | 2.47E-05 | yes | up | 0.43 | 0.44 | 0.37 | 1.56 | 2.89 | 2.67 | 0.413333 | 2.373333 |
| IKZF2 | 3.413 | 1.771052 | 1.97E-06 | 2.47E-05 | yes | up | 0.63 | 0.4 | 0.87 | 3.31 | 3.98 | 3.39 | 0.633333 | 3.56 |
| SETD4 | 2.294 | 1.197772 | 1.97E-06 | 2.48E-05 | yes | up | 3.13 | 2.89 | 2.82 | 10.53 | 6.81 | 12.12 | 2.946667 | 9.82 |
| LOC108178304 | 0.008 | -6.97808 | 1.98E-06 | 2.48E-05 | yes | down | 0.56 | 0.64 | 0.76 | 0 | 0 | 0 | 0.653333 | 0 |
| DHX58 | 2.96 | 1.565775 | 2.08E-06 | 2.6E-05 | yes | up | 5.08 | 4.99 | 4.03 | 25.57 | 27.04 | 13.06 | 4.7 | 21.89 |
| MST1R | 2.405 | 1.26619 | 2.08E-06 | 2.6E-05 | yes | up | 3.12 | 3.73 | 1.75 | 8.38 | 10.3 | 13.09 | 2.866667 | 10.59 |
| CD83 | 21.387 | 4.418664 | 2.11E-06 | 2.64E-05 | yes | up | 0.24 | 0.22 | 0.11 | 15.7 | 1.11 | 2.31 | 0.19 | 6.373333 |
| ND2 | 0.413 | -1.27641 | 2.17E-06 | 2.7E-05 | yes | down | 16686.18 | 22555.63 | 16117.67 | 16566.06 | 10186.26 | 9553.46 | 18453.16 | 12101.93 |
| TRIM37 | 0.416 | -1.26699 | 2.2E-06 | 2.74E-05 | yes | down | 540.56 | 593.9 | 624.69 | 306.65 | 487.77 | 332.46 | 586.3833 | 375.6267 |
| S100B | 3.987 | 1.995124 | 2.21E-06 | 2.75E-05 | yes | up | 12.48 | 9.86 | 3.63 | 44.86 | 48.55 | 75.27 | 8.656667 | 56.22667 |
| CHAC2 | 0.117 | -3.10022 | 2.21E-06 | 2.75E-05 | yes | down | 1.2 | 1.06 | 0.96 | 0.3 | 0.19 | 0.09 | 1.073333 | 0.193333 |
| PDK3 | 0.315 | -1.66814 | 2.27E-06 | 2.81E-05 | yes | down | 4.5 | 4.74 | 4.65 | 3.52 | 1.68 | 1.82 | 4.63 | 2.34 |
| LOC108177747 | 27.517 | 4.78225 | 2.28E-06 | 2.83E-05 | yes | up | 0 | 0.02 | 0.01 | 0.58 | 0.43 | 0.54 | 0.01 | 0.516667 |
| APTX | 0.434 | -1.20268 | 2.29E-06 | 2.83E-05 | yes | down | 16.94 | 23.65 | 18.48 | 10.25 | 13.77 | 14.94 | 19.69 | 12.98667 |
| LOC100359197 | 20.057 | 4.326016 | 2.35E-06 | 2.91E-05 | yes | up | 0.06 | 0.16 | 0.14 | 7.58 | 1.71 | 2.47 | 0.12 | 3.92 |
| ACSL4 | 2.036 | 1.025553 | 2.35E-06 | 2.91E-05 | yes | up | 12.6 | 10.03 | 10.72 | 38.63 | 26.83 | 43.88 | 11.11667 | 36.44667 |
| NID2 | 0.493 | -1.02046 | 2.39E-06 | 2.95E-05 | yes | down | 24.9 | 32.69 | 24.39 | 16.66 | 23.7 | 24.51 | 27.32667 | 21.62333 |
| DDX56 | 2.059 | 1.041664 | 2.48E-06 | 3.06E-05 | yes | up | 13.52 | 19.76 | 12.6 | 30.12 | 28.13 | 40.31 | 15.29333 | 32.85333 |
| SYTL2 | 2.295 | 1.198695 | 2.5E-06 | 3.07E-05 | yes | up | 1.14 | 0.92 | 0.86 | 3.32 | 2.98 | 4.42 | 0.973333 | 3.573333 |
| FHDC1 | 3.32 | 1.73132 | 2.52E-06 | 3.09E-05 | yes | up | 0.33 | 0.27 | 0.19 | 1.51 | 1.27 | 1.34 | 0.263333 | 1.373333 |
| LOC103346635 | 5.441 | 2.443747 | 2.56E-06 | 3.12E-05 | yes | up | 0.22 | 0.28 | 0.35 | 1.71 | 2.17 | 2.78 | 0.283333 | 2.22 |
| LOC100348760 | 0.355 | -1.49342 | 2.68E-06 | 3.27E-05 | yes | down | 393.64 | 190.53 | 212.14 | 121.02 | 185.39 | 138.4 | 265.4367 | 148.27 |
| SEPP1 | 2.021 | 1.015337 | 2.7E-06 | 3.29E-05 | yes | up | 350.81 | 433.9 | 432.47 | 1225.73 | 1117.33 | 1650.03 | 405.7267 | 1331.03 |
| LOC103345855 | 4.05 | 2.017861 | 2.71E-06 | 3.31E-05 | yes | up | 2.01 | 1.6 | 1.22 | 16.76 | 9.59 | 5.78 | 1.61 | 10.71 |
| HPCA | 0.009 | -6.73111 | 2.75E-06 | 3.35E-05 | yes | down | 0.47 | 1.42 | 1.34 | 0.11 | 0 | 0 | 1.076667 | 0.036667 |
| HEMGN | 0.127 | -2.97658 | 2.8E-06 | 3.41E-05 | yes | down | 1.16 | 4.35 | 3.24 | 0.82 | 0.3 | 0.71 | 2.916667 | 0.61 |
| SEMA4C | 2.087 | 1.061541 | 2.83E-06 | 3.44E-05 | yes | up | 4.88 | 4.41 | 4.93 | 12.94 | 17.59 | 17.29 | 4.74 | 15.94 |
| ARID5A | 2.413 | 1.270924 | 2.84E-06 | 3.45E-05 | yes | up | 2.52 | 2.66 | 1.86 | 10.88 | 6.72 | 9.34 | 2.346667 | 8.98 |
| ADPRHL2 | 0.425 | -1.2342 | 2.9E-06 | 3.51E-05 | yes | down | 12.42 | 16.94 | 12.4 | 7.18 | 9.09 | 12.55 | 13.92 | 9.606667 |
| FMO4 | 2.408 | 1.268134 | 2.94E-06 | 3.55E-05 | yes | up | 5.92 | 7.35 | 5.44 | 18.94 | 19.19 | 34.41 | 6.236667 | 24.18 |
| MC2R | 136.476 | 7.092501 | 3.06E-06 | 3.68E-05 | yes | up | 0 | 0 | 0 | 0.2 | 0.9 | 0.9 | 0 | 0.666667 |
| OXCT1 | 0.386 | -1.37212 | 3.09E-06 | 3.72E-05 | yes | down | 116.21 | 82.15 | 102.41 | 63.59 | 78.31 | 43.54 | 100.2567 | 61.81333 |
| PLXDC2 | 2.399 | 1.262479 | 3.14E-06 | 3.78E-05 | yes | up | 3.56 | 2.61 | 2.03 | 8.43 | 8.87 | 14.01 | 2.733333 | 10.43667 |
| ADCYAP1 | 0.026 | -5.24431 | 3.15E-06 | 3.78E-05 | yes | down | 0.51 | 0.93 | 0.27 | 0.04 | 0.02 | 0 | 0.57 | 0.02 |
| SYCE1L | 2.479 | 1.309539 | 3.16E-06 | 3.79E-05 | yes | up | 0.56 | 0.85 | 0.47 | 2.28 | 2.62 | 2.45 | 0.626667 | 2.45 |
| AUTS2 | 2.293 | 1.19752 | 3.18E-06 | 3.82E-05 | yes | up | 1.39 | 2.09 | 1.21 | 5.4 | 8.1 | 7.01 | 1.563333 | 6.836667 |
| S1PR3 | 0.311 | -1.68461 | 3.2E-06 | 3.83E-05 | yes | down | 4.76 | 7.54 | 6.54 | 1.86 | 3.78 | 3.83 | 6.28 | 3.156667 |
| NRIP1 | 2.306 | 1.20556 | 3.22E-06 | 3.85E-05 | yes | up | 2.89 | 1.8 | 1.88 | 6.62 | 9.42 | 8.06 | 2.19 | 8.033333 |
| TMEM120A | 0.227 | -2.13693 | 3.33E-06 | 3.99E-05 | yes | down | 23.19 | 21.48 | 18.04 | 8.18 | 12.41 | 3.89 | 20.90333 | 8.16 |
| SCARA5 | 2.776 | 1.472804 | 3.34E-06 | 3.99E-05 | yes | up | 9.43 | 4.96 | 3.71 | 27.46 | 21.4 | 30.2 | 6.033333 | 26.35333 |
| LOC100343635 | 0.115 | -3.12196 | 3.49E-06 | 4.15E-05 | yes | down | 0.72 | 0.46 | 0.4 | 0.04 | 0.16 | 0.08 | 0.526667 | 0.093333 |
| LOC103346853 | 4.595 | 2.199953 | 3.5E-06 | 4.16E-05 | yes | up | 0.33 | 0.2 | 0.24 | 1.82 | 1.49 | 2.32 | 0.256667 | 1.876667 |
| TRNAA-UGC | 3.708 | 1.890562 | 3.54E-06 | 4.2E-05 | yes | up | 0.4 | 0.41 | 0.59 | 3.02 | 2.51 | 2.26 | 0.466667 | 2.596667 |
| IFIT5 | 5.68 | 2.505804 | 3.54E-06 | 4.2E-05 | yes | up | 2.49 | 2.11 | 1.28 | 31.54 | 14.18 | 5.82 | 1.96 | 17.18 |
| LOC103346060 | 0.127 | -2.9806 | 3.58E-06 | 4.24E-05 | yes | down | 0.57 | 0.68 | 0.35 | 0.15 | 0.07 | 0.09 | 0.533333 | 0.103333 |
| ALG8 | 0.345 | -1.53548 | 3.59E-06 | 4.24E-05 | yes | down | 9.26 | 4.93 | 5.44 | 4.37 | 2.68 | 3.91 | 6.543333 | 3.653333 |
| LOC103348123 | 0.098 | -3.34381 | 3.61E-06 | 4.27E-05 | yes | down | 0.86 | 1.2 | 0.85 | 0.19 | 0.08 | 0.21 | 0.97 | 0.16 |
| PDHX | 0.449 | -1.15476 | 3.63E-06 | 4.28E-05 | yes | down | 34.82 | 24.72 | 17.92 | 15.68 | 20.77 | 18.16 | 25.82 | 18.20333 |
| C4A | 13.651 | 3.770884 | 3.72E-06 | 4.39E-05 | yes | up | 12.37 | 8.2 | 3.75 | 341.65 | 88.81 | 83.54 | 8.106667 | 171.3333 |
| KCNH2 | 0.234 | -2.09294 | 3.73E-06 | 4.4E-05 | yes | down | 1.4 | 0.85 | 0.84 | 0.29 | 0.23 | 0.42 | 1.03 | 0.313333 |
| DCAF16 | 2.347 | 1.230968 | 3.77E-06 | 4.44E-05 | yes | up | 2.78 | 3.62 | 1.79 | 9.78 | 8.43 | 12.22 | 2.73 | 10.14333 |
| C5AR1 | 8.541 | 3.094474 | 3.86E-06 | 4.54E-05 | yes | up | 2.34 | 2.83 | 2.08 | 72.34 | 7.82 | 18.5 | 2.416667 | 32.88667 |
| B3GALNT1 | 2.479 | 1.309966 | 4.01E-06 | 4.71E-05 | yes | up | 0.91 | 1.2 | 1 | 4.31 | 3.54 | 4.42 | 1.036667 | 4.09 |
| LOC100352190 | 0.101 | -3.30318 | 4.06E-06 | 4.77E-05 | yes | down | 1.7 | 2.92 | 4.53 | 0.76 | 0.29 | 0.4 | 3.05 | 0.483333 |
| MRRF | 0.439 | -1.18814 | 4.07E-06 | 4.78E-05 | yes | down | 14.35 | 14.24 | 13.43 | 6.51 | 10.18 | 12.2 | 14.00667 | 9.63 |
| NUDT16L1 | 0.379 | -1.39802 | 4.08E-06 | 4.78E-05 | yes | down | 10.16 | 11.66 | 9.2 | 10.86 | 12.87 | 17.49 | 10.34 | 13.74 |
| CMC2 | 0.467 | -1.09881 | 4.19E-06 | 4.9E-05 | yes | down | 19.82 | 18.62 | 9.92 | 12.73 | 12.1 | 14.23 | 16.12 | 13.02 |
| ERN1 | 2.349 | 1.232097 | 4.19E-06 | 4.9E-05 | yes | up | 0.69 | 0.66 | 0.58 | 3.04 | 1.9 | 2.31 | 0.643333 | 2.416667 |
| ACAA2 | 0.432 | -1.20968 | 4.33E-06 | 5.04E-05 | yes | down | 1237.23 | 1235.06 | 1573.12 | 732.92 | 1104.79 | 997.28 | 1348.47 | 944.9967 |
| LOC103349237 | 0.038 | -4.72328 | 4.41E-06 | 5.13E-05 | yes | down | 9.56 | 2.55 | 0.47 | 0.04 | 0.32 | 0.37 | 4.193333 | 0.243333 |
| MCCC1 | 0.405 | -1.30332 | 4.42E-06 | 5.14E-05 | yes | down | 14.32 | 12.59 | 16.18 | 6.97 | 9.27 | 12.2 | 14.36333 | 9.48 |
| C14H3orf58 | 2.57 | 1.361542 | 4.5E-06 | 5.22E-05 | yes | up | 2.3 | 1.98 | 1.97 | 7.1 | 6.28 | 12.64 | 2.083333 | 8.673333 |
| MCP-1 | 0.064 | -3.96264 | 4.52E-06 | 5.24E-05 | yes | down | 3.98 | 13.31 | 6.16 | 1.5 | 0.35 | 2.1 | 7.816667 | 1.316667 |
| LOC103345308 | 0.48 | -1.05908 | 4.53E-06 | 5.25E-05 | yes | down | 10.77 | 10.65 | 9.5 | 9.23 | 5.96 | 8.63 | 10.30667 | 7.94 |
| ATG14 | 2.092 | 1.064767 | 4.58E-06 | 5.29E-05 | yes | up | 2.72 | 3.15 | 3.02 | 7.98 | 10.13 | 11.93 | 2.963333 | 10.01333 |
| LOC108178465 | 0.165 | -2.60217 | 4.64E-06 | 5.36E-05 | yes | down | 23.45 | 11.74 | 10.98 | 6.09 | 4.34 | 1.24 | 15.39 | 3.89 |
| RWDD3 | 2.475 | 1.307423 | 4.72E-06 | 5.45E-05 | yes | up | 1.59 | 1.1 | 0.94 | 3.81 | 3.67 | 4.14 | 1.21 | 3.873333 |
| SLC4A1 | 0.075 | -3.72998 | 4.73E-06 | 5.45E-05 | yes | down | 0.16 | 0.93 | 0.83 | 0.08 | 0.05 | 0.13 | 0.64 | 0.086667 |
| RLA-A3 | 6.482 | 2.696495 | 4.79E-06 | 5.51E-05 | yes | up | 29.24 | 33.95 | 33.97 | 729.7 | 113.58 | 123.93 | 32.38667 | 322.4033 |
| RARG | 2.083 | 1.058332 | 4.8E-06 | 5.52E-05 | yes | up | 4.4 | 4.97 | 5.17 | 13.44 | 14.44 | 20.74 | 4.846667 | 16.20667 |
| ABCC5 | 3.057 | 1.61199 | 4.83E-06 | 5.54E-05 | yes | up | 8.35 | 13.44 | 5.78 | 22.8 | 32.65 | 53.1 | 9.19 | 36.18333 |
| LOC108177431 | 47.791 | 5.578677 | 4.86E-06 | 5.57E-05 | yes | up | 3 | 0 | 3.42 | 142.68 | 145.46 | 222.07 | 2.14 | 170.07 |
| OXA1L | 0.427 | -1.22734 | 4.9E-06 | 5.61E-05 | yes | down | 66.96 | 77.86 | 65.81 | 32.15 | 55.16 | 58.58 | 70.21 | 48.63 |
| LOC100345696 | 2.709 | 1.438003 | 5.01E-06 | 5.73E-05 | yes | up | 6.29 | 4.61 | 2.59 | 17.1 | 17.73 | 18.13 | 4.496667 | 17.65333 |
| CNNM3 | 2.142 | 1.099223 | 5.06E-06 | 5.76E-05 | yes | up | 7.64 | 7.51 | 7.96 | 20.68 | 30.3 | 26.08 | 7.703333 | 25.68667 |
| TRIM14 | 12.558 | 3.650529 | 5.13E-06 | 5.85E-05 | yes | up | 0.08 | 0.14 | 0.01 | 2.8 | 0.86 | 0.88 | 0.076667 | 1.513333 |
| LOC100358198 | 0.171 | -2.54868 | 5.32E-06 | 6.04E-05 | yes | down | 0.54 | 1.84 | 0.79 | 0.33 | 0.2 | 0.33 | 1.056667 | 0.286667 |
| RARRES2 | 2.423 | 1.276635 | 5.39E-06 | 6.11E-05 | yes | up | 84.95 | 143.84 | 88.18 | 355.58 | 311.68 | 547.75 | 105.6567 | 405.0033 |
| MYH3 | 0.004 | -8.09795 | 5.44E-06 | 6.17E-05 | yes | down | 86.32 | 12.24 | 0.45 | 0.01 | 0.32 | 0.21 | 33.00333 | 0.18 |
| PARP12 | 3.863 | 1.949755 | 5.49E-06 | 6.22E-05 | yes | up | 2.85 | 2.72 | 2.06 | 26.69 | 9.98 | 9.94 | 2.543333 | 15.53667 |
| EPT1 | 0.341 | -1.55403 | 5.5E-06 | 6.22E-05 | yes | down | 2.01 | 1.7 | 1 | 0.96 | 0.76 | 0.81 | 1.57 | 0.843333 |
| LOC100345682 | 0.392 | -1.35109 | 5.84E-06 | 6.58E-05 | yes | down | 2.22 | 1.37 | 1.57 | 1.05 | 1.28 | 0.88 | 1.72 | 1.07 |
| LOC103350535 | 2.45 | 1.293005 | 5.87E-06 | 6.61E-05 | yes | up | 7.23 | 6.6 | 4.49 | 18.44 | 21.54 | 31.04 | 6.106667 | 23.67333 |
| RPP40 | 0.338 | -1.56695 | 5.88E-06 | 6.62E-05 | yes | down | 29.46 | 27.9 | 22.56 | 7.92 | 20.08 | 13.75 | 26.64 | 13.91667 |
| LRRC8B | 2.608 | 1.383007 | 5.89E-06 | 6.62E-05 | yes | up | 0.62 | 0.55 | 0.57 | 2.79 | 1.65 | 2.94 | 0.58 | 2.46 |
| LOC100348443 | 102.302 | 6.676694 | 5.89E-06 | 6.62E-05 | yes | up | 0 | 0 | 0 | 1.11 | 0.35 | 0.73 | 0 | 0.73 |
| HMGB2 | 2.222 | 1.15197 | 5.92E-06 | 6.65E-05 | yes | up | 30.27 | 31.76 | 39.7 | 110.97 | 103.04 | 155.54 | 33.91 | 123.1833 |
| SRRM2 | 2.074 | 1.052241 | 5.95E-06 | 6.68E-05 | yes | up | 25.89 | 25.62 | 14.14 | 74.44 | 76.25 | 60.39 | 21.88333 | 70.36 |
| LOC108178220 | 4.68 | 2.22657 | 6.08E-06 | 6.82E-05 | yes | up | 0.19 | 0.35 | 0.15 | 1.98 | 1.38 | 1.78 | 0.23 | 1.713333 |
| HIF3A | 4.756 | 2.249896 | 6.26E-06 | 7.01E-05 | yes | up | 1.09 | 1.51 | 0.77 | 15.2 | 4.98 | 8.09 | 1.123333 | 9.423333 |
| MAPK8IP2 | 0.106 | -3.23136 | 6.29E-06 | 7.02E-05 | yes | down | 0.59 | 1.58 | 1.52 | 0.36 | 0.18 | 0.1 | 1.23 | 0.213333 |
| CCDC114 | 7.78 | 2.959768 | 6.37E-06 | 7.1E-05 | yes | up | 0.94 | 0.89 | 0.04 | 3.05 | 2.1 | 1.32 | 0.623333 | 2.156667 |
| EPYC | 9.46 | 3.241873 | 6.37E-06 | 7.1E-05 | yes | up | 0.23 | 0.31 | 0.07 | 1.44 | 2.52 | 4.89 | 0.203333 | 2.95 |
| PPM1H | 0.47 | -1.09018 | 6.43E-06 | 7.16E-05 | yes | down | 4.53 | 5.44 | 4.82 | 4.76 | 3.09 | 3.31 | 4.93 | 3.72 |
| YARS2 | 0.469 | -1.09166 | 6.46E-06 | 7.19E-05 | yes | down | 13.2 | 15.94 | 13.54 | 11 | 7.8 | 13.59 | 14.22667 | 10.79667 |
| LOC100351933 | 0.007 | -7.10193 | 6.49E-06 | 7.22E-05 | yes | down | 12.11 | 0.69 | 28.69 | 0 | 0 | 0.59 | 13.83 | 0.196667 |
| OAS3 | 7.358 | 2.879405 | 6.55E-06 | 7.27E-05 | yes | up | 0.9 | 0.44 | 0.41 | 13.12 | 4.99 | 2 | 0.583333 | 6.703333 |
| DTX3L | 3.867 | 1.951058 | 6.62E-06 | 7.34E-05 | yes | up | 2.03 | 2.08 | 1.86 | 19.69 | 10.53 | 5.87 | 1.99 | 12.03 |
| ABCA9 | 6.277 | 2.650165 | 6.63E-06 | 7.34E-05 | yes | up | 0.67 | 0.22 | 0.1 | 3.23 | 3.02 | 2.64 | 0.33 | 2.963333 |
| LOC100358368 | 0.367 | -1.44716 | 6.63E-06 | 7.34E-05 | yes | down | 642.79 | 321.97 | 309.65 | 184.63 | 299.54 | 248.86 | 424.8033 | 244.3433 |
| MPC1 | 0.395 | -1.3408 | 6.7E-06 | 7.41E-05 | yes | down | 432.73 | 323.74 | 304.46 | 148.34 | 294.83 | 194.63 | 353.6433 | 212.6 |
| HADHA | 0.36 | -1.47308 | 6.76E-06 | 7.47E-05 | yes | down | 601.24 | 519.6 | 629.27 | 300.03 | 477.04 | 228.94 | 583.37 | 335.3367 |
| LOC103349284 | 93.37 | 6.544886 | 6.78E-06 | 7.48E-05 | yes | up | 0 | 0 | 0 | 1.52 | 0.68 | 1.24 | 0 | 1.146667 |
| TSPO2 | 0.122 | -3.03089 | 6.79E-06 | 7.49E-05 | yes | down | 9.81 | 12.06 | 16.47 | 0.35 | 0.8 | 4.32 | 12.78 | 1.823333 |
| SYTL4 | 2.144 | 1.100315 | 6.83E-06 | 7.52E-05 | yes | up | 2.49 | 1.61 | 1.09 | 5.43 | 5.35 | 6.53 | 1.73 | 5.77 |
| ADGRG6 | 2.36 | 1.238582 | 7.1E-06 | 7.8E-05 | yes | up | 1.94 | 1.74 | 1 | 4.68 | 5.07 | 8.26 | 1.56 | 6.003333 |
| IDH3G | 0.47 | -1.08799 | 7.12E-06 | 7.81E-05 | yes | down | 365.31 | 361.58 | 397.81 | 249.54 | 350.51 | 243.54 | 374.9 | 281.1967 |
| LOC103350987 | 7.694 | 2.943696 | 7.46E-06 | 8.15E-05 | yes | up | 0.01 | 0.02 | 0.05 | 0.48 | 0.46 | 0.5 | 0.026667 | 0.48 |
| CAPN8 | 8.26 | 3.046108 | 7.46E-06 | 8.15E-05 | yes | up | 0.48 | 1.02 | 0.21 | 2.27 | 12.35 | 7.18 | 0.57 | 7.266667 |
| LOC108175449 | 5.149 | 2.364423 | 7.47E-06 | 8.15E-05 | yes | up | 10.31 | 0.54 | 5 | 31.66 | 40.78 | 38.9 | 5.283333 | 37.11333 |
| PSEN2 | 0.469 | -1.09196 | 7.47E-06 | 8.15E-05 | yes | down | 37.91 | 49.77 | 49.04 | 42.8 | 29.31 | 36.56 | 45.57333 | 36.22333 |
| GOT1L1 | 0.009 | -6.76624 | 7.53E-06 | 8.21E-05 | yes | down | 0.43 | 0.7 | 0.62 | 0 | 0 | 0 | 0.583333 | 0 |
| FLT1 | 0.365 | -1.45524 | 7.64E-06 | 8.33E-05 | yes | down | 11.17 | 18.47 | 22.73 | 11.03 | 9.25 | 11.16 | 17.45667 | 10.48 |
| FICD | 0.417 | -1.2635 | 7.9E-06 | 8.57E-05 | yes | down | 2.59 | 2.84 | 1.81 | 1.74 | 1.2 | 1.87 | 2.413333 | 1.603333 |
| SDC4 | 2.196 | 1.135139 | 7.95E-06 | 8.62E-05 | yes | up | 20.11 | 32.9 | 16.98 | 84.98 | 65.38 | 93.82 | 23.33 | 81.39333 |
| RBMS3 | 2.005 | 1.003796 | 8.07E-06 | 8.75E-05 | yes | up | 1.16 | 1.47 | 0.77 | 3.53 | 3.2 | 4.29 | 1.133333 | 3.673333 |
| TOB1 | 0.408 | -1.29391 | 8.17E-06 | 8.85E-05 | yes | down | 22.47 | 22.86 | 10.41 | 11.79 | 10.25 | 14.05 | 18.58 | 12.03 |
| SLC24A2 | 0.306 | -1.71026 | 8.28E-06 | 8.95E-05 | yes | down | 0.75 | 0.6 | 0.72 | 0.2 | 0.36 | 0.48 | 0.69 | 0.346667 |
| SCEL | 5.191 | 2.375993 | 8.35E-06 | 9E-05 | yes | up | 0.34 | 0.17 | 0.09 | 1.9 | 1.63 | 1.15 | 0.2 | 1.56 |
| TIAM1 | 2.467 | 1.302583 | 8.4E-06 | 9.05E-05 | yes | up | 0.4 | 0.36 | 0.28 | 1.68 | 1.21 | 1.21 | 0.346667 | 1.366667 |
| EFEMP1 | 2.304 | 1.204225 | 8.48E-06 | 9.12E-05 | yes | up | 43.89 | 28.81 | 18.27 | 118.08 | 97.91 | 112.42 | 30.32333 | 109.47 |
| RUNX2 | 4.808 | 2.265537 | 8.5E-06 | 9.14E-05 | yes | up | 0.19 | 0.14 | 0.1 | 1.65 | 0.71 | 0.98 | 0.143333 | 1.113333 |
| LOC103351328 | 0.112 | -3.16034 | 8.51E-06 | 9.14E-05 | yes | down | 1.56 | 1 | 1.89 | 0.29 | 0.23 | 0.3 | 1.483333 | 0.273333 |
| LOC100339259 | 0.336 | -1.57241 | 8.52E-06 | 9.14E-05 | yes | down | 47.81 | 38.66 | 39.9 | 18.76 | 32.72 | 14.39 | 42.12333 | 21.95667 |
| PARD3B | 2.482 | 1.311672 | 8.56E-06 | 9.18E-05 | yes | up | 6.52 | 6.22 | 4.16 | 9.26 | 46.04 | 106.53 | 5.633333 | 53.94333 |
| LOC100346271 | 2.433 | 1.282733 | 8.66E-06 | 9.25E-05 | yes | up | 0.81 | 0.71 | 0.51 | 2.39 | 2.38 | 2.47 | 0.676667 | 2.413333 |
| WHRN | 2.64 | 1.400805 | 8.67E-06 | 9.26E-05 | yes | up | 1.3 | 1.03 | 0.74 | 3.09 | 4.36 | 5.43 | 1.023333 | 4.293333 |
| XPO1 | 2.104 | 1.073184 | 8.93E-06 | 9.54E-05 | yes | up | 17.98 | 14.62 | 11.87 | 38.36 | 42.12 | 69.06 | 14.82333 | 49.84667 |
| LOC103349362 | 236.141 | 7.883505 | 8.99E-06 | 9.59E-05 | yes | up | 0 | 0 | 0 | 0.13 | 6.55 | 6.66 | 0 | 4.446667 |
| PRRT4 | 49.387 | 5.626068 | 9.12E-06 | 9.72E-05 | yes | up | 0.05 | 0 | 0 | 0.47 | 1.17 | 1.26 | 0.016667 | 0.966667 |
| DSG1 | 5.171 | 2.37048 | 9.13E-06 | 9.72E-05 | yes | up | 0.28 | 0.53 | 0.25 | 6.05 | 1.59 | 4.27 | 0.353333 | 3.97 |
| PPCDC | 2.019 | 1.013846 | 9.14E-06 | 9.72E-05 | yes | up | 5.65 | 4.22 | 4.03 | 8.01 | 10.43 | 10.62 | 4.633333 | 9.686667 |
| LOC100356339 | 94.649 | 6.564511 | 9.36E-06 | 9.93E-05 | yes | up | 0 | 0 | 0 | 1.01 | 0.63 | 1.85 | 0 | 1.163333 |
| ABCA3 | 2.245 | 1.16655 | 9.36E-06 | 9.93E-05 | yes | up | 1.75 | 1.36 | 1.46 | 6.61 | 5.55 | 4.25 | 1.523333 | 5.47 |
| GDE1 | 0.456 | -1.13418 | 9.48E-06 | 0.0001 | yes | down | 8.39 | 10.55 | 9.93 | 7.25 | 5.63 | 9.13 | 9.623333 | 7.336667 |
| ABCF2 | 0.499 | -1.0039 | 9.56E-06 | 0.000101 | yes | down | 28.68 | 18.28 | 15.82 | 16.3 | 16.8 | 16.3 | 20.92667 | 16.46667 |
| FGD4 | 2.498 | 1.320872 | 9.6E-06 | 0.000101 | yes | up | 1.46 | 1.03 | 0.62 | 4.85 | 3.2 | 3.5 | 1.036667 | 3.85 |
| GRIN3A | 2.56 | 1.355917 | 9.62E-06 | 0.000102 | yes | up | 0.56 | 0.29 | 0.34 | 1.71 | 1.51 | 1.64 | 0.396667 | 1.62 |
| LOC100337966 | 0.46 | -1.11994 | 9.7E-06 | 0.000102 | yes | down | 635.09 | 603.45 | 680.17 | 352.85 | 613.16 | 469.13 | 639.57 | 478.38 |
| SAMD9L | 6.618 | 2.726488 | 9.96E-06 | 0.000105 | yes | up | 1.61 | 1.57 | 1.33 | 33.81 | 8.94 | 4.6 | 1.503333 | 15.78333 |
| LOC108175950 | 0.216 | -2.2121 | 9.97E-06 | 0.000105 | yes | down | 1.82 | 4.67 | 5.3 | 1.32 | 1.13 | 1.29 | 3.93 | 1.246667 |
| FAM46A | 2.406 | 1.266461 | 1E-05 | 0.000106 | yes | up | 3.7 | 3.1 | 1.58 | 10.98 | 11.15 | 9.17 | 2.793333 | 10.43333 |
| LOC103349801 | 0.426 | -1.23089 | 1.02E-05 | 0.000107 | yes | down | 154.27 | 189.95 | 176.76 | 87.53 | 147.26 | 104.41 | 173.66 | 113.0667 |
| GRSF1 | 0.46 | -1.11968 | 1.02E-05 | 0.000107 | yes | down | 34.86 | 21.49 | 25.59 | 14.7 | 20.79 | 23.33 | 27.31333 | 19.60667 |
| MMP16 | 0.414 | -1.27254 | 1.03E-05 | 0.000108 | yes | down | 1.38 | 1.16 | 1.12 | 0.57 | 0.94 | 0.92 | 1.22 | 0.81 |
| RTN4 | 0.488 | -1.03484 | 1.03E-05 | 0.000108 | yes | down | 322.96 | 229.79 | 175.07 | 146.28 | 225.56 | 222.55 | 242.6067 | 198.13 |
| ANGPT4 | 3.6 | 1.848136 | 1.04E-05 | 0.000109 | yes | up | 0.34 | 0.27 | 0.14 | 1.26 | 1.35 | 1.68 | 0.25 | 1.43 |
| TMEM205 | 0.397 | -1.33364 | 1.05E-05 | 0.000109 | yes | down | 53.82 | 79.62 | 92.79 | 44.31 | 43.66 | 58.76 | 75.41 | 48.91 |
| SIRPA | 2.718 | 1.442487 | 1.05E-05 | 0.00011 | yes | up | 8.52 | 7.97 | 5.36 | 46.92 | 19.99 | 27.25 | 7.283333 | 31.38667 |
| PIM1 | 2.535 | 1.342241 | 1.07E-05 | 0.000112 | yes | up | 6.67 | 6.21 | 10.16 | 29.13 | 29.13 | 38.03 | 7.68 | 32.09667 |
| LRRC58 | 2.105 | 1.073934 | 1.09E-05 | 0.000113 | yes | up | 6.44 | 10.41 | 6.93 | 25.53 | 21.47 | 33.47 | 7.926667 | 26.82333 |
| LOC100353747 | 0.008 | -7.03014 | 1.09E-05 | 0.000113 | yes | down | 0.32 | 0.49 | 1.09 | 0 | 0 | 0 | 0.633333 | 0 |
| MYO7A | 3.476 | 1.797447 | 1.09E-05 | 0.000114 | yes | up | 0.66 | 0.46 | 0.28 | 3.61 | 1.58 | 2.51 | 0.466667 | 2.566667 |
| MOCOS | 2.701 | 1.43361 | 1.1E-05 | 0.000115 | yes | up | 1.23 | 1.45 | 1.44 | 7.6 | 4.02 | 6.44 | 1.373333 | 6.02 |
| PIK3IP1 | 3.354 | 1.745966 | 1.11E-05 | 0.000115 | yes | up | 4.99 | 4.59 | 9.28 | 25.98 | 33.86 | 45.63 | 6.286667 | 35.15667 |
| LOC108176325 | 0.468 | -1.09442 | 1.12E-05 | 0.000116 | yes | down | 105.32 | 84.62 | 66.57 | 47.4 | 60.49 | 81.57 | 85.50333 | 63.15333 |
| LOC103348967 | 4.167 | 2.058889 | 1.14E-05 | 0.000118 | yes | up | 0.36 | 0.41 | 0.19 | 2.34 | 2.05 | 1.93 | 0.32 | 2.106667 |
| DMTF1 | 2.133 | 1.092899 | 1.14E-05 | 0.000118 | yes | up | 4.66 | 6.14 | 5.6 | 21.86 | 14.65 | 18.62 | 5.466667 | 18.37667 |
| POMC | 0.277 | -1.85188 | 1.15E-05 | 0.000119 | yes | down | 4.2 | 4.54 | 6.16 | 2.32 | 2.67 | 1.66 | 4.966667 | 2.216667 |
| BIRC3 | 7.726 | 2.949742 | 1.15E-05 | 0.000119 | yes | up | 1.23 | 1.66 | 1.99 | 46.55 | 8.27 | 6.63 | 1.626667 | 20.48333 |
| DTHD1 | 53.042 | 5.729073 | 1.16E-05 | 0.000119 | yes | up | 0.01 | 0.01 | 0.01 | 1.99 | 0.33 | 0.12 | 0.01 | 0.813333 |
| CST3 | 2.759 | 1.463908 | 1.16E-05 | 0.00012 | yes | up | 286.88 | 305.13 | 484.78 | 2059.46 | 1427.53 | 1395.75 | 358.93 | 1627.58 |
| HSF5 | 0.278 | -1.84698 | 1.16E-05 | 0.00012 | yes | down | 2.53 | 3.5 | 1.72 | 1.43 | 1.25 | 0.71 | 2.583333 | 1.13 |
| NUDCD1 | 0.474 | -1.07609 | 1.18E-05 | 0.000121 | yes | down | 2.42 | 2.62 | 2.62 | 1.71 | 1.87 | 2.3 | 2.553333 | 1.96 |
| LOC100353259 | 108.221 | 6.757838 | 1.19E-05 | 0.000122 | yes | up | 0 | 0 | 0 | 0.79 | 2.35 | 3.96 | 0 | 2.366667 |
| PDZK1 | 0.327 | -1.6117 | 1.21E-05 | 0.000124 | yes | down | 1.87 | 1.86 | 1.74 | 1.21 | 0.87 | 0.7 | 1.823333 | 0.926667 |
| KHDRBS3 | 0.349 | -1.51966 | 1.22E-05 | 0.000125 | yes | down | 2.08 | 2.75 | 2.59 | 1.13 | 1.83 | 1.18 | 2.473333 | 1.38 |
| LOC103348530 | 2.726 | 1.446546 | 1.23E-05 | 0.000126 | yes | up | 2.31 | 2.35 | 2.17 | 6.64 | 10.18 | 5.94 | 2.276667 | 7.586667 |
| JDP2 | 2.482 | 1.311675 | 1.23E-05 | 0.000126 | yes | up | 8.17 | 6.74 | 3.7 | 29.66 | 19.56 | 23.08 | 6.203333 | 24.1 |
| AS3MT | 2.514 | 1.329928 | 1.24E-05 | 0.000126 | yes | up | 4.94 | 3.43 | 4.03 | 17.17 | 20.12 | 12.67 | 4.133333 | 16.65333 |
| MPZL3 | 2.332 | 1.221654 | 1.24E-05 | 0.000127 | yes | up | 0.65 | 0.74 | 0.72 | 2.63 | 2.72 | 2.88 | 0.703333 | 2.743333 |
| SP110 | 5.103 | 2.3514 | 1.24E-05 | 0.000127 | yes | up | 0.64 | 0.8 | 1.28 | 5.14 | 3.05 | 2.67 | 0.906667 | 3.62 |
| IGF1 | 2.23 | 1.15719 | 1.28E-05 | 0.00013 | yes | up | 15.53 | 12.79 | 11.65 | 31.45 | 55.15 | 57.01 | 13.32333 | 47.87 |
| CSMD2 | 0.185 | -2.43247 | 1.28E-05 | 0.00013 | yes | down | 0.15 | 0.26 | 0.12 | 0.07 | 0.03 | 0.06 | 0.176667 | 0.053333 |
| LOC103347240 | 5.839 | 2.545717 | 1.32E-05 | 0.000134 | yes | up | 1.25 | 0.86 | 0.87 | 16.15 | 8.68 | 2.59 | 0.993333 | 9.14 |
| APH1B | 2.028 | 1.019736 | 1.33E-05 | 0.000135 | yes | up | 10.3 | 14.69 | 13.96 | 45.25 | 34.91 | 47.62 | 12.98333 | 42.59333 |
| LOC100356870 | 84.711 | 6.404472 | 1.33E-05 | 0.000135 | yes | up | 0 | 0 | 0 | 2.05 | 3.37 | 2.08 | 0 | 2.5 |
| TRAF3IP2 | 2.296 | 1.198852 | 1.36E-05 | 0.000138 | yes | up | 3.78 | 4.69 | 3.32 | 14.55 | 9.45 | 11.52 | 3.93 | 11.84 |
| TXNRD3 | 0.337 | -1.57107 | 1.4E-05 | 0.000141 | yes | down | 2.63 | 3.81 | 3.31 | 1.53 | 2.33 | 1.46 | 3.25 | 1.773333 |
| APOLD1 | 3.564 | 1.833481 | 1.4E-05 | 0.000141 | yes | up | 6.87 | 12.02 | 21.08 | 59.85 | 75.43 | 90.99 | 13.32333 | 75.42333 |
| LOC100356907 | 2.261 | 1.177266 | 1.41E-05 | 0.000142 | yes | up | 19.51 | 22.42 | 18.39 | 48.34 | 70.86 | 100.33 | 20.10667 | 73.17667 |
| RENBP | 3.422 | 1.774913 | 1.43E-05 | 0.000144 | yes | up | 3.02 | 4.98 | 2.19 | 26.72 | 13.02 | 12.26 | 3.396667 | 17.33333 |
| FAM63A | 2.075 | 1.053085 | 1.48E-05 | 0.000148 | yes | up | 3.46 | 3.18 | 3.72 | 9.22 | 11.12 | 13.74 | 3.453333 | 11.36 |
| TENM4 | 2.516 | 1.33092 | 1.5E-05 | 0.00015 | yes | up | 1 | 1.13 | 1.24 | 4.41 | 3.26 | 5.27 | 1.123333 | 4.313333 |
| LOC103350962 | 37.633 | 5.23394 | 1.51E-05 | 0.000151 | yes | up | 0 | 0.03 | 0.04 | 1.65 | 0.88 | 1.75 | 0.023333 | 1.426667 |
| COL25A1 | 7.053 | 2.818243 | 1.52E-05 | 0.000151 | yes | up | 0.2 | 0.25 | 0.06 | 3.36 | 1.09 | 1.07 | 0.17 | 1.84 |
| C1H11orf1 | 2.554 | 1.352709 | 1.52E-05 | 0.000152 | yes | up | 1.07 | 0.55 | 0.66 | 3.23 | 3.05 | 2.93 | 0.76 | 3.07 |
| LOC103347661 | 2.226 | 1.15419 | 1.55E-05 | 0.000154 | yes | up | 2.5 | 2.12 | 1.53 | 7 | 6.39 | 7.77 | 2.05 | 7.053333 |
| ZC3H8 | 0.411 | -1.28191 | 1.56E-05 | 0.000155 | yes | down | 4.24 | 3.93 | 4.27 | 2.15 | 3.07 | 2.86 | 4.146667 | 2.693333 |
| LOC103348133 | 0.227 | -2.14176 | 1.56E-05 | 0.000155 | yes | down | 19.07 | 22.31 | 15.42 | 2.86 | 4.19 | 13.66 | 18.93333 | 6.903333 |
| SUN2 | 2.239 | 1.162723 | 1.63E-05 | 0.000162 | yes | up | 17.46 | 16.31 | 14.2 | 39.29 | 52.12 | 81.08 | 15.99 | 57.49667 |
| FAM110C | 2.344 | 1.229195 | 1.67E-05 | 0.000166 | yes | up | 1.61 | 1.77 | 1.71 | 4.44 | 5.94 | 8 | 1.696667 | 6.126667 |
| MTRF1 | 0.385 | -1.3755 | 1.72E-05 | 0.00017 | yes | down | 3.49 | 3.15 | 3.27 | 2.53 | 1.68 | 1.84 | 3.303333 | 2.016667 |
| CCSAP | 2.611 | 1.38457 | 1.73E-05 | 0.000171 | yes | up | 0.42 | 0.38 | 0.25 | 1.46 | 1.24 | 1.65 | 0.35 | 1.45 |
| COLEC12 | 2.105 | 1.074131 | 1.75E-05 | 0.000172 | yes | up | 14.4 | 10.35 | 7.96 | 35.67 | 27.45 | 46.48 | 10.90333 | 36.53333 |
| MED29 | 0.491 | -1.02494 | 1.76E-05 | 0.000173 | yes | down | 43.24 | 43.79 | 44.82 | 26.41 | 36.16 | 41.64 | 43.95 | 34.73667 |
| IFIT3 | 4.556 | 2.187782 | 1.79E-05 | 0.000175 | yes | up | 6.42 | 6.77 | 4.4 | 72.62 | 38.41 | 14.36 | 5.863333 | 41.79667 |
| SLC35G2 | 0.396 | -1.33798 | 1.79E-05 | 0.000176 | yes | down | 4.03 | 5.06 | 4.3 | 2.05 | 3.45 | 2.99 | 4.463333 | 2.83 |
| LOC103346930 | 16.682 | 4.060186 | 1.79E-05 | 0.000176 | yes | up | 0.09 | 0.05 | 0.52 | 1.27 | 5.76 | 11.88 | 0.22 | 6.303333 |
| LOC108178475 | 0.109 | -3.19956 | 1.8E-05 | 0.000177 | yes | down | 0.32 | 0.3 | 0.28 | 0.05 | 0.09 | 0.02 | 0.3 | 0.053333 |
| LOC100339982 | 2.784 | 1.477254 | 1.83E-05 | 0.000179 | yes | up | 1.27 | 1.37 | 1.21 | 6.41 | 7.08 | 4.48 | 1.283333 | 5.99 |
| ACKR2 | 3.014 | 1.591545 | 1.86E-05 | 0.000182 | yes | up | 1.05 | 0.78 | 0.49 | 4.76 | 3.25 | 2.76 | 0.773333 | 3.59 |
| RRP12 | 0.44 | -1.18288 | 1.86E-05 | 0.000182 | yes | down | 5.26 | 4.46 | 3.77 | 3.85 | 3.3 | 2.23 | 4.496667 | 3.126667 |
| F10 | 0.07 | -3.84259 | 1.87E-05 | 0.000182 | yes | down | 0.55 | 0.77 | 1.17 | 0.16 | 0.08 | 0.05 | 0.83 | 0.096667 |
| SFXN5 | 0.469 | -1.09188 | 1.87E-05 | 0.000183 | yes | down | 1.84 | 2.11 | 1.94 | 1.25 | 1.47 | 1.74 | 1.963333 | 1.486667 |
| SDSL | 0.138 | -2.85468 | 1.88E-05 | 0.000183 | yes | down | 4.24 | 6.53 | 9.41 | 1.86 | 2.42 | 0.4 | 6.726667 | 1.56 |
| NFKBIZ | 4.846 | 2.27678 | 1.9E-05 | 0.000185 | yes | up | 0.51 | 0.89 | 0.35 | 7.22 | 2.78 | 3.32 | 0.583333 | 4.44 |
| DMGDH | 0.194 | -2.36286 | 1.92E-05 | 0.000187 | yes | down | 232.71 | 120.15 | 133.51 | 103.7 | 26.58 | 22.43 | 162.1233 | 50.90333 |
| IRF3 | 2.094 | 1.066348 | 1.92E-05 | 0.000187 | yes | up | 11.97 | 13.2 | 8.02 | 23.46 | 29.29 | 26.55 | 11.06333 | 26.43333 |
| LOC100348521 | 0.023 | -5.41674 | 1.93E-05 | 0.000188 | yes | down | 0.07 | 9.87 | 9.08 | 0.06 | 0.18 | 0.53 | 6.34 | 0.256667 |
| ARL4C | 4.988 | 2.318416 | 1.94E-05 | 0.000188 | yes | up | 0.26 | 0.23 | 0.26 | 3.25 | 1.01 | 1.76 | 0.25 | 2.006667 |
| FMO2 | 3.997 | 1.998806 | 1.95E-05 | 0.000189 | yes | up | 3.57 | 1.62 | 2.28 | 5.77 | 14.34 | 24.27 | 2.49 | 14.79333 |
| PIGBOS1 | 0.498 | -1.0053 | 1.99E-05 | 0.000193 | yes | down | 16.13 | 19.03 | 15.52 | 11.2 | 14.55 | 14.26 | 16.89333 | 13.33667 |
| MCP2 | 0.087 | -3.52724 | 2E-05 | 0.000194 | yes | down | 2.09 | 9.29 | 5.82 | 0.86 | 0.55 | 0.94 | 5.733333 | 0.783333 |
| RBBP8 | 0.44 | -1.18576 | 2.04E-05 | 0.000197 | yes | down | 2.06 | 2.71 | 1.85 | 1.89 | 1.36 | 1.43 | 2.206667 | 1.56 |
| ITGB4 | 2.728 | 1.447754 | 2.11E-05 | 0.000203 | yes | up | 2.53 | 1.44 | 1.15 | 5.09 | 7.85 | 11.16 | 1.706667 | 8.033333 |
| LOC100346376 | 76.59 | 6.259092 | 2.15E-05 | 0.000207 | yes | up | 0 | 0 | 0 | 1.31 | 1.77 | 2.2 | 0 | 1.76 |
| TRAF4 | 0.415 | -1.26729 | 2.15E-05 | 0.000207 | yes | down | 16.86 | 20.5 | 11.51 | 7.66 | 11.7 | 14.33 | 16.29 | 11.23 |
| AOX3 | 5.532 | 2.467704 | 2.16E-05 | 0.000208 | yes | up | 0.41 | 2.29 | 0.49 | 7.22 | 15.04 | 14.74 | 1.063333 | 12.33333 |
| PERM1 | 0.154 | -2.70323 | 2.16E-05 | 0.000208 | yes | down | 6.74 | 3.6 | 1.91 | 0.47 | 1.88 | 0.51 | 4.083333 | 0.953333 |
| DNAJC28 | 0.406 | -1.30185 | 2.25E-05 | 0.000216 | yes | down | 2.19 | 1.72 | 1.27 | 1.14 | 1.14 | 1.02 | 1.726667 | 1.1 |
| LOC100356891 | 0.457 | -1.13049 | 2.25E-05 | 0.000216 | yes | down | 22.66 | 23.98 | 22.49 | 14.16 | 16.71 | 19.86 | 23.04333 | 16.91 |
| LOC100354303 | 0.413 | -1.27618 | 2.25E-05 | 0.000216 | yes | down | 20.39 | 27.36 | 26.34 | 12.71 | 16.45 | 20.64 | 24.69667 | 16.6 |
| SH3TC2 | 3.75 | 1.906917 | 2.29E-05 | 0.000219 | yes | up | 0.84 | 0.94 | 0.23 | 2.74 | 3.57 | 4.84 | 0.67 | 3.716667 |
| NIPSNAP1 | 0.232 | -2.10692 | 2.3E-05 | 0.00022 | yes | down | 50.9 | 56.56 | 101.52 | 17.06 | 46.96 | 18.09 | 69.66 | 27.37 |
| SLC46A3 | 3.198 | 1.677012 | 2.31E-05 | 0.000221 | yes | up | 0.74 | 0.94 | 1.57 | 4.94 | 5.17 | 7.39 | 1.083333 | 5.833333 |
| ECI1 | 0.454 | -1.13852 | 2.32E-05 | 0.000221 | yes | down | 187.4 | 235.64 | 250.07 | 131.33 | 199.89 | 158.52 | 224.37 | 163.2467 |
| LOC103350039 | 5.656 | 2.499733 | 2.32E-05 | 0.000222 | yes | up | 0.78 | 1.02 | 0.74 | 21.58 | 1.57 | 5.95 | 0.846667 | 9.7 |
| GALNT15 | 2.434 | 1.283231 | 2.34E-05 | 0.000223 | yes | up | 4.74 | 5.57 | 4.73 | 26.13 | 11.94 | 20.99 | 5.013333 | 19.68667 |
| PARS2 | 0.419 | -1.25535 | 2.34E-05 | 0.000223 | yes | down | 2.94 | 2.87 | 2.59 | 2.02 | 1.01 | 1.59 | 2.8 | 1.54 |
| STEAP2 | 0.303 | -1.72248 | 2.37E-05 | 0.000225 | yes | down | 0.83 | 1.09 | 1.35 | 0.39 | 0.58 | 0.54 | 1.09 | 0.503333 |
| PLEKHF1 | 2.762 | 1.465471 | 2.38E-05 | 0.000226 | yes | up | 11.31 | 21.37 | 16.88 | 50.42 | 66.99 | 104.9 | 16.52 | 74.10333 |
| SOSTDC1 | 0.115 | -3.122 | 2.4E-05 | 0.000228 | yes | down | 1.49 | 7.84 | 2.73 | 0.71 | 1.79 | 0.28 | 4.02 | 0.926667 |
| LOC103346901 | 89.456 | 6.483103 | 2.41E-05 | 0.000229 | yes | up | 0.21 | 0.94 | 0.87 | 251.62 | 19.17 | 7.1 | 0.673333 | 92.63 |
| LOC100341914 | 5.114 | 2.354573 | 2.42E-05 | 0.000229 | yes | up | 0.27 | 0.29 | 0.19 | 2.02 | 1.11 | 3.06 | 0.25 | 2.063333 |
| DCAF4 | 2.13 | 1.091136 | 2.43E-05 | 0.00023 | yes | up | 1.68 | 1.94 | 1.66 | 5.61 | 5.38 | 7.08 | 1.76 | 6.023333 |
| CGNL1 | 2.213 | 1.14596 | 2.43E-05 | 0.00023 | yes | up | 3.98 | 4.78 | 2.07 | 13.9 | 12.1 | 11.49 | 3.61 | 12.49667 |
| INSIG1 | 4.483 | 2.16442 | 2.44E-05 | 0.00023 | yes | up | 6.85 | 8.42 | 23.42 | 83.29 | 73.52 | 135.47 | 12.89667 | 97.42667 |
| LOC100337920 | 2.078 | 1.055367 | 2.51E-05 | 0.000236 | yes | up | 0.69 | 0.52 | 0.58 | 1.96 | 1.92 | 2.09 | 0.596667 | 1.99 |
| VASN | 2.029 | 1.021041 | 2.52E-05 | 0.000237 | yes | up | 2.07 | 1.42 | 1.69 | 5.61 | 5.3 | 5.92 | 1.726667 | 5.61 |
| LOC103346277 | 5.65 | 2.498362 | 2.54E-05 | 0.000239 | yes | up | 1.08 | 1.52 | 0.64 | 14.16 | 3.25 | 5.2 | 1.08 | 7.536667 |
| PPIL1 | 0.454 | -1.1396 | 2.55E-05 | 0.000239 | yes | down | 4.32 | 5.23 | 4.56 | 4.17 | 2.77 | 3.34 | 4.703333 | 3.426667 |
| LOC103351338 | 60.097 | 5.909231 | 2.57E-05 | 0.000242 | yes | up | 0 | 0.11 | 0 | 2 | 4.41 | 4.55 | 0.036667 | 3.653333 |
| SH3RF2 | 0.16 | -2.6433 | 2.6E-05 | 0.000244 | yes | down | 1.75 | 2.21 | 0.37 | 0.49 | 0.36 | 0.27 | 1.443333 | 0.373333 |
| LOC103345755 | 15.709 | 3.973555 | 2.62E-05 | 0.000246 | yes | up | 0.03 | 0.02 | 0.01 | 0.38 | 0.53 | 0.59 | 0.02 | 0.5 |
| LOC100328967 | 13.587 | 3.764176 | 2.64E-05 | 0.000247 | yes | up | 34.65 | 33.45 | 32.82 | 1876.93 | 375.33 | 236.23 | 33.64 | 829.4967 |
| RPL34 | 59.41 | 5.892636 | 2.66E-05 | 0.000248 | yes | up | 0.93 | 0.94 | 0.16 | 1.01 | 376.46 | 262.18 | 0.676667 | 213.2167 |
| DLD | 0.469 | -1.09178 | 2.66E-05 | 0.000248 | yes | down | 144.02 | 111.29 | 153.8 | 92.02 | 123.81 | 93.65 | 136.37 | 103.16 |
| LOC108177708 | 0.241 | -2.05235 | 2.67E-05 | 0.000249 | yes | down | 83.91 | 61.69 | 18.14 | 23.75 | 18.78 | 17.05 | 54.58 | 19.86 |
| CRLS1 | 0.453 | -1.14345 | 2.68E-05 | 0.000249 | yes | down | 58.82 | 80.95 | 73.74 | 36.47 | 59.34 | 58.29 | 71.17 | 51.36667 |
| LOC103350570 | 31.932 | 4.996951 | 2.68E-05 | 0.00025 | yes | up | 0.01 | 0.02 | 0 | 0.14 | 0.48 | 0.81 | 0.01 | 0.476667 |
| ANKRD44 | 2.554 | 1.352867 | 2.69E-05 | 0.00025 | yes | up | 1.78 | 1.69 | 1.01 | 8.17 | 4.39 | 4.98 | 1.493333 | 5.846667 |
| CKMT2 | 0.198 | -2.33601 | 2.74E-05 | 0.000255 | yes | down | 192.49 | 35.04 | 54.08 | 15.96 | 33.31 | 21.91 | 93.87 | 23.72667 |
| MEP1B | 7.014 | 2.810193 | 2.75E-05 | 0.000255 | yes | up | 0.1 | 0.09 | 0.04 | 0.82 | 0.99 | 0.71 | 0.076667 | 0.84 |
| IGSF22 | 2.987 | 1.578779 | 2.77E-05 | 0.000258 | yes | up | 0.59 | 1.28 | 1.06 | 3.41 | 3.44 | 5.36 | 0.976667 | 4.07 |
| EXOSC4 | 0.448 | -1.159 | 2.82E-05 | 0.000261 | yes | down | 7.06 | 5.48 | 7.36 | 4.61 | 4.48 | 5.21 | 6.633333 | 4.766667 |
| BOC | 2.233 | 1.158848 | 2.83E-05 | 0.000262 | yes | up | 4.01 | 3.24 | 2.42 | 8.05 | 10.63 | 15.57 | 3.223333 | 11.41667 |
| AIF1L | 0.427 | -1.22801 | 2.83E-05 | 0.000263 | yes | down | 16.62 | 14.95 | 19 | 7.87 | 13.08 | 14.11 | 16.85667 | 11.68667 |
| BTG1 | 2.529 | 1.338347 | 2.85E-05 | 0.000264 | yes | up | 46.34 | 84.46 | 78.13 | 230.88 | 243.97 | 388.59 | 69.64333 | 287.8133 |
| LOC100353469 | 0.459 | -1.12444 | 2.85E-05 | 0.000264 | yes | down | 26.91 | 17.97 | 19.86 | 14.94 | 14.95 | 17.28 | 21.58 | 15.72333 |
| ASRGL1 | 0.413 | -1.27595 | 2.87E-05 | 0.000265 | yes | down | 5.23 | 7.15 | 4.89 | 2.82 | 4.08 | 3.93 | 5.756667 | 3.61 |
| NT5C3B | 0.339 | -1.56114 | 2.88E-05 | 0.000266 | yes | down | 31.38 | 21.16 | 39.14 | 14.02 | 22.7 | 13.59 | 30.56 | 16.77 |
| PHKG1 | 0.376 | -1.41161 | 2.88E-05 | 0.000266 | yes | down | 77.3 | 40.37 | 64.92 | 25.65 | 44.78 | 33.48 | 60.86333 | 34.63667 |
| TOMM22 | 0.397 | -1.33161 | 2.9E-05 | 0.000267 | yes | down | 31.18 | 56.22 | 47.96 | 22.63 | 27.93 | 36.57 | 45.12 | 29.04333 |
| LOC108178624 | 0.471 | -1.08576 | 2.91E-05 | 0.000269 | yes | down | 11.24 | 15.38 | 11.26 | 8.07 | 21.89 | 11.19 | 12.62667 | 13.71667 |
| LGI4 | 2.988 | 1.579236 | 2.92E-05 | 0.000269 | yes | up | 1 | 0.73 | 0.41 | 2.69 | 3.06 | 4.33 | 0.713333 | 3.36 |
| LOC108176506 | 0.121 | -3.04205 | 3.03E-05 | 0.000279 | yes | down | 5.26 | 3.92 | 2.44 | 0.11 | 0.47 | 0.46 | 3.873333 | 0.346667 |
| C8H12orf40 | 81.174 | 6.34295 | 3.06E-05 | 0.000281 | yes | up | 0 | 0.02 | 0 | 0.44 | 2.3 | 0.61 | 0.006667 | 1.116667 |
| MAGEL2 | 0.324 | -1.62641 | 3.11E-05 | 0.000285 | yes | down | 0.98 | 0.66 | 0.65 | 0.32 | 0.36 | 0.51 | 0.763333 | 0.396667 |
| SMOC1 | 0.371 | -1.42962 | 3.11E-05 | 0.000286 | yes | down | 27.17 | 39.52 | 12.34 | 15.82 | 16.33 | 18.31 | 26.34333 | 16.82 |
| FAM19A3 | 2.757 | 1.463071 | 3.12E-05 | 0.000287 | yes | up | 0.44 | 0.94 | 0.56 | 2.88 | 2.46 | 3.36 | 0.646667 | 2.9 |
| PLK3 | 2.688 | 1.426744 | 3.15E-05 | 0.000289 | yes | up | 0.67 | 0.96 | 0.49 | 2.8 | 2.77 | 3.44 | 0.706667 | 3.003333 |
| LUZP2 | 13.119 | 3.713562 | 3.19E-05 | 0.000292 | yes | up | 0.02 | 0.02 | 0.01 | 0.43 | 0.22 | 0.34 | 0.016667 | 0.33 |
| STRC | 5.372 | 2.425338 | 3.23E-05 | 0.000295 | yes | up | 0.04 | 0.08 | 0.05 | 0.42 | 0.47 | 0.58 | 0.056667 | 0.49 |
| DPF3 | 2.196 | 1.134864 | 3.25E-05 | 0.000297 | yes | up | 1.18 | 0.8 | 0.68 | 2.91 | 3.4 | 2.77 | 0.886667 | 3.026667 |
| IKBKE | 4.77 | 2.254111 | 3.28E-05 | 0.000299 | yes | up | 1.34 | 1.57 | 0.76 | 17.07 | 5.58 | 4.84 | 1.223333 | 9.163333 |
| ELN | 0.45 | -1.15099 | 3.28E-05 | 0.000299 | yes | down | 179.23 | 182 | 134.44 | 90.77 | 89.66 | 178.83 | 165.2233 | 119.7533 |
| VAPB | 0.489 | -1.03279 | 3.33E-05 | 0.000303 | yes | down | 24.41 | 21.61 | 14.57 | 12.93 | 19.31 | 14.89 | 20.19667 | 15.71 |
| SLC24A3 | 2.054 | 1.038398 | 3.36E-05 | 0.000306 | yes | up | 3.25 | 4.64 | 2.85 | 9.7 | 10.45 | 15.12 | 3.58 | 11.75667 |
| MMP11 | 2.347 | 1.230983 | 3.37E-05 | 0.000306 | yes | up | 7.36 | 6.06 | 9.98 | 29.12 | 26.25 | 34.83 | 7.8 | 30.06667 |
| FAM227B | 70.487 | 6.139295 | 3.41E-05 | 0.000309 | yes | up | 0 | 0 | 0 | 3.41 | 3.83 | 3.17 | 0 | 3.47 |
| BAIAP2 | 2.551 | 1.350913 | 3.42E-05 | 0.00031 | yes | up | 1.05 | 1.46 | 1.1 | 3.67 | 4.96 | 6.14 | 1.203333 | 4.923333 |
| PGGHG | 2.095 | 1.067067 | 3.44E-05 | 0.000311 | yes | up | 2.87 | 4.2 | 3.4 | 9.92 | 11.89 | 13.37 | 3.49 | 11.72667 |
| PRG4 | 0.147 | -2.76662 | 3.45E-05 | 0.000312 | yes | down | 20.97 | 6.57 | 4.12 | 0.65 | 3.1 | 3.3 | 10.55333 | 2.35 |
| FOSL2 | 2.551 | 1.351217 | 3.49E-05 | 0.000315 | yes | up | 4.35 | 6.07 | 6.6 | 31.76 | 16.05 | 22.04 | 5.673333 | 23.28333 |
| NRIP3 | 0.292 | -1.774 | 3.51E-05 | 0.000317 | yes | down | 0.9 | 1.62 | 0.68 | 0.42 | 0.45 | 0.6 | 1.066667 | 0.49 |
| ADORA2A | 0.322 | -1.63705 | 3.56E-05 | 0.00032 | yes | down | 4.57 | 6.22 | 9.52 | 3.75 | 2.62 | 4.4 | 6.77 | 3.59 |
| ALDH2 | 2.164 | 1.113926 | 3.56E-05 | 0.000321 | yes | up | 48.4 | 27.58 | 35.87 | 102.02 | 128.18 | 159.03 | 37.28333 | 129.7433 |
| PPP1R3B | 0.332 | -1.59262 | 3.59E-05 | 0.000323 | yes | down | 7.29 | 14.32 | 4.38 | 4.05 | 5.08 | 4.33 | 8.663333 | 4.486667 |
| LOC100349551 | 0.378 | -1.40327 | 3.59E-05 | 0.000323 | yes | down | 754.82 | 404.15 | 409.01 | 248.99 | 430.94 | 247.82 | 522.66 | 309.25 |
| TMPRSS13 | 4.327 | 2.113401 | 3.6E-05 | 0.000324 | yes | up | 0.4 | 0.12 | 0.24 | 2.13 | 1.33 | 1.86 | 0.253333 | 1.773333 |
| LOC108177287 | 0.199 | -2.33233 | 3.61E-05 | 0.000324 | yes | down | 14.6 | 11.13 | 21.94 | 2.22 | 7.12 | 6.05 | 15.89 | 5.13 |
| CES4A | 0.349 | -1.51835 | 3.61E-05 | 0.000324 | yes | down | 3.02 | 3.3 | 2.35 | 1.48 | 2.12 | 1.1 | 2.89 | 1.566667 |
| UMPS | 0.382 | -1.38728 | 3.61E-05 | 0.000324 | yes | down | 7.09 | 11.93 | 5.64 | 6.15 | 3.79 | 4.73 | 8.22 | 4.89 |
| LOC103349747 | 6.458 | 2.691047 | 3.63E-05 | 0.000325 | yes | up | 1.24 | 0.38 | 0.27 | 6.53 | 7.46 | 5.6 | 0.63 | 6.53 |
| MRAP | 0.189 | -2.40541 | 3.73E-05 | 0.000333 | yes | down | 7.7 | 10.3 | 12.7 | 1.54 | 1.74 | 6.46 | 10.23333 | 3.246667 |
| ITPRIP | 2.624 | 1.391687 | 3.86E-05 | 0.000344 | yes | up | 1.7 | 2.86 | 3 | 7.39 | 9.89 | 12.44 | 2.52 | 9.906667 |
| LOC100338527 | 0.435 | -1.20104 | 3.91E-05 | 0.000348 | yes | down | 25.1 | 16.15 | 12.11 | 9.23 | 13.78 | 12.99 | 17.78667 | 12 |
| CCL19 | 8.597 | 3.103916 | 3.92E-05 | 0.000349 | yes | up | 0.96 | 0.54 | 0.93 | 21.95 | 4.3 | 7.29 | 0.81 | 11.18 |
| SP100 | 4.111 | 2.039479 | 3.94E-05 | 0.00035 | yes | up | 1.8 | 2.28 | 1.46 | 20.98 | 8.76 | 5.94 | 1.846667 | 11.89333 |
| LOC100345833 | 0.348 | -1.52325 | 3.95E-05 | 0.000351 | yes | down | 2.94 | 2.57 | 3.52 | 1.37 | 1.83 | 1.92 | 3.01 | 1.706667 |
| TAP2 | 7.663 | 2.937984 | 3.98E-05 | 0.000353 | yes | up | 1.49 | 2.09 | 2.01 | 58.13 | 7.55 | 7.81 | 1.863333 | 24.49667 |
| PSTK | 3.006 | 1.588062 | 3.98E-05 | 0.000353 | yes | up | 7.92 | 4.33 | 8.62 | 14.7 | 16.13 | 20.59 | 6.956667 | 17.14 |
| XAF1 | 5.645 | 2.49696 | 4.01E-05 | 0.000355 | yes | up | 3.8 | 3.63 | 2.59 | 59.41 | 19.75 | 9.51 | 3.34 | 29.55667 |
| VEGFA | 2.115 | 1.080663 | 4.02E-05 | 0.000356 | yes | up | 48.02 | 72.53 | 43.47 | 117.52 | 184.09 | 191.98 | 54.67333 | 164.53 |
| LOC100352738 | 0.471 | -1.08617 | 4.02E-05 | 0.000356 | yes | down | 43.76 | 35.8 | 25.59 | 19.07 | 26.02 | 33.38 | 35.05 | 26.15667 |
| P2RX7 | 2.865 | 1.518377 | 4.14E-05 | 0.000366 | yes | up | 0.57 | 0.43 | 0.24 | 2.25 | 1.58 | 1.71 | 0.413333 | 1.846667 |
| KIAA1551 | 2.136 | 1.094888 | 4.14E-05 | 0.000366 | yes | up | 4.16 | 3.13 | 4.1 | 11.07 | 9.09 | 8.21 | 3.796667 | 9.456667 |
| ADCK2 | 2.611 | 1.384492 | 4.19E-05 | 0.000369 | yes | up | 0.68 | 0.5 | 0.43 | 2.29 | 1.98 | 2.34 | 0.536667 | 2.203333 |
| NDUFA8 | 0.452 | -1.14681 | 4.23E-05 | 0.000373 | yes | down | 175.54 | 197.71 | 130.91 | 76.76 | 149.65 | 132.67 | 168.0533 | 119.6933 |
| GRIN2C | 0.064 | -3.96169 | 4.25E-05 | 0.000375 | yes | down | 1.71 | 2.36 | 1.84 | 0.03 | 0.65 | 0.03 | 1.97 | 0.236667 |
| MRPL4 | 0.383 | -1.38367 | 4.26E-05 | 0.000375 | yes | down | 17.27 | 16.69 | 24.72 | 11.1 | 12.85 | 13 | 19.56 | 12.31667 |
| LOC103352131 | 2.997 | 1.583369 | 4.26E-05 | 0.000375 | yes | up | 2.39 | 1.65 | 1.6 | 8.15 | 9.15 | 10.87 | 1.88 | 9.39 |
| P4HA3 | 0.245 | -2.02707 | 4.27E-05 | 0.000376 | yes | down | 2.55 | 1.41 | 1.21 | 0.69 | 0.39 | 0.94 | 1.723333 | 0.673333 |
| RTN4IP1 | 0.472 | -1.08231 | 4.31E-05 | 0.000379 | yes | down | 13.16 | 11 | 9.43 | 7.86 | 10.41 | 6.72 | 11.19667 | 8.33 |
| RGS6 | 0.433 | -1.20666 | 4.32E-05 | 0.00038 | yes | down | 447.46 | 504.63 | 501.41 | 256.73 | 450.69 | 268.76 | 484.5 | 325.3933 |
| LOC103352078 | 3.48 | 1.799097 | 4.32E-05 | 0.00038 | yes | up | 0.29 | 0.4 | 0.27 | 1.29 | 1.5 | 2.56 | 0.32 | 1.783333 |
| DOCK5 | 2.641 | 1.401019 | 4.36E-05 | 0.000382 | yes | up | 0.36 | 0.31 | 0.19 | 3.1 | 2.07 | 2.67 | 0.286667 | 2.613333 |
| LYRM7 | 0.417 | -1.26092 | 4.53E-05 | 0.000395 | yes | down | 11.81 | 9.06 | 5.3 | 4.71 | 5.79 | 4.91 | 8.723333 | 5.136667 |
| LOC103351480 | 2.282 | 1.190135 | 4.57E-05 | 0.000398 | yes | up | 2.18 | 3.09 | 2.51 | 10.56 | 8.35 | 9.62 | 2.593333 | 9.51 |
| LOC103350544 | 16.122 | 4.010936 | 4.57E-05 | 0.000398 | yes | up | 0.02 | 0.04 | 0 | 0.45 | 0.41 | 0.61 | 0.02 | 0.49 |
| SH3BGRL2 | 2.703 | 1.434471 | 4.59E-05 | 0.000399 | yes | up | 0.46 | 0.44 | 0.29 | 1.64 | 2.13 | 1.77 | 0.396667 | 1.846667 |
| LOC108176082 | 4.381 | 2.131276 | 4.65E-05 | 0.000403 | yes | up | 0.46 | 0.62 | 0.34 | 2.08 | 4.76 | 2.91 | 0.473333 | 3.25 |
| MCM2 | 0.316 | -1.66234 | 4.69E-05 | 0.000407 | yes | down | 4.67 | 5.95 | 3.25 | 3.63 | 1.93 | 1.36 | 4.623333 | 2.306667 |
| LOC103345438 | 6.571 | 2.716199 | 4.7E-05 | 0.000408 | yes | up | 0.09 | 0.13 | 0.1 | 1.3 | 1.19 | 0.87 | 0.106667 | 1.12 |
| LCAT | 97.42 | 6.606149 | 4.89E-05 | 0.000423 | yes | up | 0 | 0 | 0 | 0.3 | 1.38 | 2.29 | 0 | 1.323333 |
| LRRC3 | 2.404 | 1.265396 | 4.89E-05 | 0.000423 | yes | up | 1.35 | 1.56 | 1.51 | 4.68 | 5.33 | 7.21 | 1.473333 | 5.74 |
| LOC100344012 | 14.407 | 3.84872 | 4.94E-05 | 0.000426 | yes | up | 0.12 | 0.14 | 0.08 | 6.04 | 0.98 | 0.78 | 0.113333 | 2.6 |
| PTGS2 | 2.537 | 1.342844 | 4.95E-05 | 0.000426 | yes | up | 0.67 | 0.86 | 1.06 | 3.07 | 3.46 | 4.22 | 0.863333 | 3.583333 |
| SLC25A10 | 0.476 | -1.07086 | 4.96E-05 | 0.000427 | yes | down | 15.58 | 16.3 | 20.56 | 13.15 | 11.49 | 15.14 | 17.48 | 13.26 |
| PLEK2 | 0.127 | -2.98275 | 5.01E-05 | 0.000431 | yes | down | 2.43 | 2 | 0.34 | 0.33 | 0.2 | 0.46 | 1.59 | 0.33 |
| LOC100348124 | 0.36 | -1.47201 | 5.02E-05 | 0.000431 | yes | down | 110.38 | 80.42 | 82.83 | 35.49 | 80.64 | 39.18 | 91.21 | 51.77 |
| PGF | 4.327 | 2.113518 | 5.03E-05 | 0.000432 | yes | up | 1.8 | 1.1 | 0.29 | 7.34 | 7.15 | 6.47 | 1.063333 | 6.986667 |
| CHRM4 | 0.022 | -5.47426 | 5.07E-05 | 0.000435 | yes | down | 0.55 | 0.63 | 1.18 | 0.08 | 0 | 0 | 0.786667 | 0.026667 |
| TRUB2 | 0.466 | -1.10294 | 5.08E-05 | 0.000435 | yes | down | 2.79 | 4.04 | 2.82 | 2.84 | 2.08 | 2.25 | 3.216667 | 2.39 |
| ZSWIM7 | 2.701 | 1.433258 | 5.26E-05 | 0.000449 | yes | up | 3.05 | 1.71 | 2.21 | 8.73 | 9.37 | 8.74 | 2.323333 | 8.946667 |
| TSKU | 2.297 | 1.199885 | 5.27E-05 | 0.000449 | yes | up | 3.11 | 21.09 | 12.96 | 25.62 | 44.48 | 48.05 | 12.38667 | 39.38333 |
| OXTR | 8.901 | 3.154042 | 5.34E-05 | 0.000455 | yes | up | 0.05 | 0.06 | 0.04 | 0.81 | 0.31 | 1.04 | 0.05 | 0.72 |
| FAM71F1 | 0.088 | -3.50968 | 5.34E-05 | 0.000455 | yes | down | 0.67 | 0.48 | 0.68 | 0.03 | 0.1 | 0.13 | 0.61 | 0.086667 |
| FLYWCH2 | 0.421 | -1.24761 | 5.53E-05 | 0.00047 | yes | down | 7.32 | 6.61 | 7.01 | 5.07 | 3.55 | 5.58 | 6.98 | 4.733333 |
| COQ6 | 0.49 | -1.03005 | 5.63E-05 | 0.000478 | yes | down | 30.22 | 29.34 | 27.44 | 19.75 | 29.19 | 18.78 | 29 | 22.57333 |
| KLHL6 | 0.325 | -1.62085 | 5.65E-05 | 0.000479 | yes | down | 1.16 | 2.14 | 1.73 | 1.03 | 0.77 | 0.83 | 1.676667 | 0.876667 |
| DCLK2 | 2.16 | 1.110749 | 5.76E-05 | 0.000488 | yes | up | 0.8 | 0.62 | 0.75 | 1.81 | 2.34 | 2.18 | 0.723333 | 2.11 |
| MTERF2 | 0.499 | -1.00337 | 5.82E-05 | 0.000493 | yes | down | 5.95 | 4.24 | 4.87 | 3.47 | 4.92 | 4.38 | 5.02 | 4.256667 |
| HSF2BP | 0.309 | -1.69531 | 5.87E-05 | 0.000496 | yes | down | 2 | 2.27 | 1.77 | 1.21 | 0.72 | 1.04 | 2.013333 | 0.99 |
| TSEN2 | 0.394 | -1.34321 | 5.93E-05 | 0.0005 | yes | down | 4.53 | 6.86 | 3.09 | 2.94 | 2.43 | 3.96 | 4.826667 | 3.11 |
| LOC100353233 | 0.496 | -1.01175 | 5.95E-05 | 0.000501 | yes | down | 28.77 | 33.32 | 27.14 | 16.98 | 26.43 | 27.22 | 29.74333 | 23.54333 |
| VDAC3 | 0.433 | -1.20719 | 5.95E-05 | 0.000501 | yes | down | 205.13 | 130.52 | 89.17 | 85.01 | 111.19 | 99.04 | 141.6067 | 98.41333 |
| LOC100009136 | 0.026 | -5.29096 | 5.98E-05 | 0.000503 | yes | down | 1.91 | 13.37 | 2.29 | 0 | 0.42 | 0.27 | 5.856667 | 0.23 |
| SLC25A5 | 0.442 | -1.17756 | 6.05E-05 | 0.000508 | yes | down | 701.85 | 505.62 | 910.98 | 467.44 | 500.07 | 567.55 | 706.15 | 511.6867 |
| DFNA5 | 0.391 | -1.35589 | 6.16E-05 | 0.000517 | yes | down | 3.21 | 2.96 | 1.9 | 2.12 | 1.46 | 1.25 | 2.69 | 1.61 |
| CTTNBP2 | 0.25 | -1.99754 | 6.2E-05 | 0.00052 | yes | down | 0.94 | 0.72 | 1.31 | 0.21 | 0.53 | 0.49 | 0.99 | 0.41 |
| TPD52L1 | 0.493 | -1.02138 | 6.31E-05 | 0.000527 | yes | down | 21.54 | 14.67 | 19.11 | 12.54 | 15.78 | 15.97 | 18.44 | 14.76333 |
| LOC108176674 | 6.111 | 2.611429 | 6.51E-05 | 0.000542 | yes | up | 0.12 | 0.13 | 0.04 | 0.81 | 0.91 | 1.11 | 0.096667 | 0.943333 |
| SLC19A1 | 0.256 | -1.96391 | 6.51E-05 | 0.000543 | yes | down | 3.24 | 4.09 | 1.35 | 0.96 | 0.7 | 1.91 | 2.893333 | 1.19 |
| FOS | 4.828 | 2.271367 | 6.83E-05 | 0.000567 | yes | up | 17.32 | 4.47 | 3.7 | 94.77 | 55.35 | 70.03 | 8.496667 | 73.38333 |
| SOX5 | 2.085 | 1.060037 | 6.88E-05 | 0.000571 | yes | up | 2.29 | 3.5 | 1.81 | 6.65 | 8.13 | 9.98 | 2.533333 | 8.253333 |
| TMEM140 | 4.258 | 2.090189 | 6.89E-05 | 0.000571 | yes | up | 9.29 | 17.1 | 42.38 | 133.07 | 174.9 | 187.83 | 22.92333 | 165.2667 |
| LOC100346411 | 0.477 | -1.06944 | 6.99E-05 | 0.000578 | yes | down | 1168.72 | 1214.95 | 1181.15 | 635.7 | 1168.83 | 887.67 | 1188.273 | 897.4 |
| NPTXR | 2.188 | 1.129592 | 7.02E-05 | 0.000581 | yes | up | 1.75 | 1.38 | 1.55 | 7.01 | 4.92 | 4.98 | 1.56 | 5.636667 |
| LOC100343081 | 2.992 | 1.581036 | 7.08E-05 | 0.000585 | yes | up | 1.32 | 2.69 | 2.69 | 10.66 | 9 | 8.04 | 2.233333 | 9.233333 |
| CNTFR | 3.512 | 1.812329 | 7.1E-05 | 0.000587 | yes | up | 2.64 | 2.62 | 0.9 | 6.92 | 14.92 | 10.42 | 2.053333 | 10.75333 |
| CPEB4 | 2.111 | 1.07798 | 7.16E-05 | 0.00059 | yes | up | 10.31 | 12.8 | 5.94 | 24.92 | 32.56 | 35.46 | 9.683333 | 30.98 |
| LIMK2 | 2.427 | 1.279098 | 7.17E-05 | 0.000591 | yes | up | 5.27 | 7.58 | 4.32 | 13.85 | 27.77 | 23.24 | 5.723333 | 21.62 |
| DLGAP2 | 0.068 | -3.88214 | 7.25E-05 | 0.000597 | yes | down | 0.1 | 0.44 | 0.46 | 0.01 | 0.05 | 0.05 | 0.333333 | 0.036667 |
| GAREM2 | 0.186 | -2.42918 | 7.25E-05 | 0.000597 | yes | down | 0.36 | 1.08 | 0.81 | 0.19 | 0.17 | 0.33 | 0.75 | 0.23 |
| ADTRP | 0.311 | -1.68598 | 7.29E-05 | 0.0006 | yes | down | 8.18 | 15.79 | 11.56 | 2.85 | 7.09 | 6.89 | 11.84333 | 5.61 |
| UTP3 | 0.485 | -1.04481 | 7.36E-05 | 0.000605 | yes | down | 4.55 | 4.55 | 3.07 | 3.8 | 2.35 | 3.21 | 4.056667 | 3.12 |
| ZNF503 | 2.338 | 1.225373 | 7.37E-05 | 0.000606 | yes | up | 3.58 | 1.74 | 1.86 | 7.82 | 8.89 | 9.87 | 2.393333 | 8.86 |
| LOC100348238 | 3.731 | 1.899587 | 7.39E-05 | 0.000607 | yes | up | 0.14 | 0.21 | 0.08 | 0.87 | 0.95 | 0.69 | 0.143333 | 0.836667 |
| NFKBIA | 2.03 | 1.021368 | 7.4E-05 | 0.000607 | yes | up | 23.84 | 38.62 | 31.45 | 113.93 | 78.4 | 115.74 | 31.30333 | 102.69 |
| KEL | 0.128 | -2.96933 | 7.4E-05 | 0.000607 | yes | down | 0.27 | 0.94 | 0.64 | 0.13 | 0.12 | 0.15 | 0.616667 | 0.133333 |
| PARP9 | 3.428 | 1.777403 | 7.4E-05 | 0.000607 | yes | up | 2.26 | 2.08 | 2.2 | 21.05 | 10.41 | 5.62 | 2.18 | 12.36 |
| LOC103348580 | 61.791 | 5.949318 | 7.46E-05 | 0.00061 | yes | up | 0.06 | 0.07 | 0 | 0.26 | 6.4 | 4.86 | 0.043333 | 3.84 |
| CYP4A5 | 0.018 | -5.76407 | 7.47E-05 | 0.000611 | yes | down | 0.34 | 0.37 | 0.34 | 0.02 | 0 | 0 | 0.35 | 0.006667 |
| ACADM | 0.425 | -1.23328 | 7.55E-05 | 0.000617 | yes | down | 333.02 | 302.41 | 405.62 | 157.18 | 234.73 | 331.68 | 347.0167 | 241.1967 |
| LOC100345095 | 0.053 | -4.22464 | 7.57E-05 | 0.000618 | yes | down | 1.25 | 0.77 | 4.61 | 0.29 | 0 | 0.34 | 2.21 | 0.21 |
| ANKFN1 | 10.542 | 3.398111 | 7.68E-05 | 0.000626 | yes | up | 0.02 | 0.02 | 0 | 0.25 | 0.15 | 0.25 | 0.013333 | 0.216667 |
| RCOR2 | 0.288 | -1.79657 | 7.69E-05 | 0.000627 | yes | down | 7.82 | 6.96 | 2.27 | 2.21 | 2.77 | 2.58 | 5.683333 | 2.52 |
| C12H6orf203 | 0.477 | -1.06741 | 7.81E-05 | 0.000635 | yes | down | 15.79 | 12.92 | 11.68 | 7.98 | 9.38 | 13.62 | 13.46333 | 10.32667 |
| CD46 | 19.503 | 4.285656 | 8.01E-05 | 0.000651 | yes | up | 0.11 | 0.05 | 0 | 2.14 | 1.46 | 1.32 | 0.053333 | 1.64 |
| LOC100349976 | 0.44 | -1.1836 | 8.07E-05 | 0.000654 | yes | down | 3.56 | 4.5 | 3.93 | 3.49 | 2.45 | 2.53 | 3.996667 | 2.823333 |
| LOC100008606 | 4.809 | 2.265591 | 8.16E-05 | 0.000661 | yes | up | 21.26 | 16.05 | 13.46 | 228.7 | 87.14 | 36.6 | 16.92333 | 117.48 |
| CNTN1 | 2.388 | 1.255794 | 8.24E-05 | 0.000666 | yes | up | 1.21 | 1.01 | 1.24 | 6.14 | 3.64 | 3.57 | 1.153333 | 4.45 |
| LOC100343291 | 2.444 | 1.289226 | 8.34E-05 | 0.000674 | yes | up | 0.59 | 0.83 | 0.65 | 3.37 | 2.09 | 2.66 | 0.69 | 2.706667 |
| LOC100352411 | 0.415 | -1.27021 | 8.48E-05 | 0.000684 | yes | down | 3.22 | 4.41 | 3.69 | 2.91 | 1.65 | 3.04 | 3.773333 | 2.533333 |
| LDHA | 0.313 | -1.6757 | 8.53E-05 | 0.000688 | yes | down | 541.26 | 375.57 | 361.73 | 114.98 | 352.53 | 153.07 | 426.1867 | 206.86 |
| CYP4B1 | 0.367 | -1.44655 | 8.56E-05 | 0.000691 | yes | down | 13.29 | 10.38 | 10.15 | 7.05 | 3.31 | 9.77 | 11.27333 | 6.71 |
| TMEM86A | 2.804 | 1.487605 | 8.75E-05 | 0.000704 | yes | up | 3.7 | 5.6 | 6.47 | 18.11 | 17.8 | 36.16 | 5.256667 | 24.02333 |
| LOC100353852 | 3.19 | 1.673619 | 8.79E-05 | 0.000707 | yes | up | 1.83 | 2.86 | 1.64 | 17.56 | 5.8 | 8.78 | 2.11 | 10.71333 |
| LOC100340255 | 4.571 | 2.192373 | 8.97E-05 | 0.000719 | yes | up | 1.92 | 1.65 | 1.44 | 23.65 | 9.06 | 3.94 | 1.67 | 12.21667 |
| LOC108177122 | 10.445 | 3.38479 | 9.08E-05 | 0.000726 | yes | up | 0.14 | 0.22 | 0.55 | 4.57 | 3.9 | 0.72 | 0.303333 | 3.063333 |
| TRIB3 | 0.257 | -1.96128 | 9.1E-05 | 0.000728 | yes | down | 4.41 | 5.91 | 1.37 | 1.38 | 1.41 | 1.86 | 3.896667 | 1.55 |
| BEST4 | 0.049 | -4.35933 | 9.19E-05 | 0.000733 | yes | down | 0.21 | 0.27 | 0.44 | 0 | 0.05 | 0.02 | 0.306667 | 0.023333 |
| ENC1 | 2.108 | 1.075975 | 9.41E-05 | 0.000748 | yes | up | 3.05 | 4.05 | 4.3 | 12.39 | 9.28 | 15.16 | 3.8 | 12.27667 |
| FZD1 | 2.053 | 1.037417 | 9.52E-05 | 0.000757 | yes | up | 2.56 | 3.12 | 2.56 | 7.32 | 8.68 | 11.21 | 2.746667 | 9.07 |
| GPR153 | 0.313 | -1.67666 | 9.6E-05 | 0.000762 | yes | down | 6.01 | 10 | 2.6 | 2.37 | 4.07 | 5.21 | 6.203333 | 3.883333 |
| SMPD3 | 56.089 | 5.809658 | 9.82E-05 | 0.000779 | yes | up | 0.08 | 0.04 | 0.01 | 0.13 | 2.7 | 10.17 | 0.043333 | 4.333333 |
| CLSTN3 | 0.397 | -1.3334 | 9.85E-05 | 0.00078 | yes | down | 9.34 | 13.12 | 13.51 | 8.52 | 9.23 | 3.96 | 11.99 | 7.236667 |
| LOC100339318 | 7.037 | 2.814959 | 9.86E-05 | 0.00078 | yes | up | 1.91 | 2.25 | 3.69 | 84.99 | 8.61 | 20.79 | 2.616667 | 38.13 |
| IL10 | 6.814 | 2.768448 | 0.000101 | 0.0008 | yes | up | 0.47 | 0.37 | 0.05 | 2.32 | 2.24 | 3.99 | 0.296667 | 2.85 |
| DLX4 | 0.197 | -2.34324 | 0.000102 | 0.000802 | yes | down | 0.57 | 1.09 | 1.06 | 0.31 | 0.35 | 0.21 | 0.906667 | 0.29 |
| HSPA4L | 0.409 | -1.2881 | 0.000102 | 0.000802 | yes | down | 0.72 | 1.01 | 0.76 | 0.54 | 0.51 | 0.6 | 0.83 | 0.55 |
| ALDOB | 0.272 | -1.88011 | 0.000102 | 0.000807 | yes | down | 1.33 | 2.63 | 3.09 | 0.95 | 0.81 | 1.43 | 2.35 | 1.063333 |
| LOC103345896 | 7.147 | 2.83731 | 0.000103 | 0.00081 | yes | up | 0.13 | 0.05 | 0.08 | 1.61 | 0.68 | 0.61 | 0.086667 | 0.966667 |
| ARG1 | 89.279 | 6.480241 | 0.000104 | 0.000815 | yes | up | 0.05 | 0 | 0 | 5.68 | 0.25 | 1.09 | 0.016667 | 2.34 |
| SGMS2 | 2.274 | 1.185157 | 0.000105 | 0.000827 | yes | up | 1.37 | 0.7 | 0.78 | 3.84 | 3 | 3.42 | 0.95 | 3.42 |
| TLL1 | 4.693 | 2.230366 | 0.000106 | 0.00083 | yes | up | 0.17 | 0.24 | 0.13 | 0.95 | 2 | 1.01 | 0.18 | 1.32 |
| CCDC30 | 3.929 | 1.974317 | 0.000106 | 0.000832 | yes | up | 0.25 | 0.16 | 0.17 | 1.24 | 1.28 | 1.25 | 0.193333 | 1.256667 |
| LOC100355902 | 0.397 | -1.33423 | 0.000111 | 0.000867 | yes | down | 0.93 | 1 | 0.73 | 0.36 | 0.67 | 0.65 | 0.886667 | 0.56 |
| DCLK1 | 2.342 | 1.227588 | 0.000111 | 0.000867 | yes | up | 3.22 | 1.8 | 1.23 | 8.31 | 6.16 | 8.1 | 2.083333 | 7.523333 |
| C2CD2 | 0.458 | -1.12633 | 0.000111 | 0.000867 | yes | down | 6.26 | 9.75 | 4.5 | 5.35 | 4.86 | 4.54 | 6.836667 | 4.916667 |
| TUBGCP5 | 0.454 | -1.13854 | 0.000111 | 0.000869 | yes | down | 1.94 | 2.39 | 1.77 | 1.77 | 1.47 | 1.14 | 2.033333 | 1.46 |
| LOC100346342 | 3.02 | 1.594736 | 0.000111 | 0.000869 | yes | up | 0.38 | 0.42 | 0.18 | 1.07 | 1.34 | 2.22 | 0.326667 | 1.543333 |
| LOC100340198 | 0.441 | -1.18138 | 0.000113 | 0.00088 | yes | down | 120.61 | 175.85 | 167.77 | 79.05 | 120.22 | 128.33 | 154.7433 | 109.2 |
| C14H21orf91 | 2.038 | 1.02729 | 0.000115 | 0.000896 | yes | up | 1.83 | 1.27 | 1.01 | 5.34 | 3.67 | 4.29 | 1.37 | 4.433333 |
| OSGIN1 | 2.628 | 1.393879 | 0.000116 | 0.0009 | yes | up | 1.43 | 2.12 | 1.68 | 3.91 | 7.19 | 4.41 | 1.743333 | 5.17 |
| OSR1 | 2.575 | 1.364388 | 0.000117 | 0.000909 | yes | up | 3.56 | 3.01 | 2.21 | 11.03 | 8.77 | 18.48 | 2.926667 | 12.76 |
| TTC8 | 0.354 | -1.49893 | 0.000118 | 0.000913 | yes | down | 2.52 | 2.44 | 1.23 | 1.37 | 1.08 | 0.95 | 2.063333 | 1.133333 |
| CRYGS | 0.295 | -1.76107 | 0.000118 | 0.000917 | yes | down | 8.26 | 19.17 | 9.77 | 4.22 | 5.12 | 8.64 | 12.4 | 5.993333 |
| LIN7A | 4.238 | 2.083352 | 0.000119 | 0.000923 | yes | up | 0.11 | 0.07 | 0.07 | 0.72 | 0.35 | 0.64 | 0.083333 | 0.57 |
| CRTAC1 | 2.045 | 1.031788 | 0.00012 | 0.000925 | yes | up | 1.68 | 1.8 | 1.7 | 6.84 | 5.35 | 5.43 | 1.726667 | 5.873333 |
| TESMIN | 0.045 | -4.48535 | 0.000121 | 0.000934 | yes | down | 3.4 | 9.5 | 5.54 | 1.34 | 0 | 0.02 | 6.146667 | 0.453333 |
| LOC100351437 | 2.509 | 1.327269 | 0.000122 | 0.00094 | yes | up | 1.07 | 1.1 | 0.87 | 5.9 | 3.58 | 2.64 | 1.013333 | 4.04 |
| ADRA1B | 0.302 | -1.72527 | 0.000123 | 0.000944 | yes | down | 1.84 | 2.21 | 2.34 | 1.33 | 1.14 | 0.6 | 2.13 | 1.023333 |
| HOXA6 | 2.816 | 1.493561 | 0.000123 | 0.000947 | yes | up | 2.42 | 2.22 | 4.35 | 10.99 | 14.75 | 16.19 | 2.996667 | 13.97667 |
| SPSB1 | 0.483 | -1.04959 | 0.000123 | 0.000947 | yes | down | 17.7 | 12.43 | 8.96 | 8.18 | 10.3 | 11.25 | 13.03 | 9.91 |
| SLC19A2 | 0.325 | -1.62208 | 0.000124 | 0.000953 | yes | down | 0.94 | 1.02 | 1.1 | 0.44 | 0.53 | 0.64 | 1.02 | 0.536667 |
| CD14 | 5.162 | 2.367897 | 0.000124 | 0.000953 | yes | up | 3.67 | 4.34 | 3.73 | 69.01 | 11.85 | 16.21 | 3.913333 | 32.35667 |
| MYL10 | 0.012 | -6.35832 | 0.000124 | 0.000954 | yes | down | 12.46 | 2.46 | 0.27 | 0 | 0.26 | 0 | 5.063333 | 0.086667 |
| CMTM8 | 0.29 | -1.78538 | 0.000126 | 0.000963 | yes | down | 3.68 | 8.1 | 8.34 | 2.93 | 3.11 | 3.89 | 6.706667 | 3.31 |
| PXMP2 | 2.335 | 1.223261 | 0.000126 | 0.000963 | yes | up | 8.79 | 7.56 | 8.15 | 20.78 | 31.06 | 45.7 | 8.166667 | 32.51333 |
| LOC108178915 | 16.032 | 4.002927 | 0.00013 | 0.000997 | yes | up | 0.12 | 0.45 | 0 | 1.7 | 6.18 | 5 | 0.19 | 4.293333 |
| IFI35 | 4.017 | 2.006134 | 0.000131 | 0.000997 | yes | up | 2.24 | 1.25 | 2.71 | 18.76 | 14.76 | 6.81 | 2.066667 | 13.44333 |
| LOC103348799 | 0.088 | -3.50094 | 0.000134 | 0.001024 | yes | down | 1.15 | 2.32 | 10.03 | 0.41 | 0.4 | 7.88 | 4.5 | 2.896667 |
| NSUN4 | 0.461 | -1.11708 | 0.000135 | 0.001027 | yes | down | 15.08 | 13.04 | 12.21 | 6.8 | 7.38 | 14.27 | 13.44333 | 9.483333 |
| THY1 | 2.133 | 1.093129 | 0.000135 | 0.001029 | yes | up | 18.43 | 20.09 | 8.34 | 46.49 | 53.24 | 56.08 | 15.62 | 51.93667 |
| KIF3A | 2.092 | 1.064774 | 0.000135 | 0.001029 | yes | up | 2.6 | 2.18 | 1.41 | 4.81 | 6.12 | 5.66 | 2.063333 | 5.53 |
| RGS4 | 2.929 | 1.550363 | 0.000136 | 0.001033 | yes | up | 2.75 | 2.99 | 2.08 | 7.92 | 14.54 | 13.77 | 2.606667 | 12.07667 |
| LOC100345021 | 0.487 | -1.0393 | 0.000137 | 0.001042 | yes | down | 45.59 | 56.94 | 37.85 | 23.08 | 41.01 | 41.65 | 46.79333 | 35.24667 |
| CXCR1 | 3.213 | 1.683738 | 0.000138 | 0.001048 | yes | up | 0.44 | 0.54 | 0.66 | 3.79 | 2.18 | 2.55 | 0.546667 | 2.84 |
| CSF1 | 2.716 | 1.441444 | 0.00014 | 0.001062 | yes | up | 3.24 | 3.13 | 2.61 | 21.11 | 8.86 | 8.89 | 2.993333 | 12.95333 |
| FAH | 0.455 | -1.13508 | 0.000147 | 0.00111 | yes | down | 36.16 | 62.75 | 52.83 | 28.33 | 38.84 | 44.29 | 50.58 | 37.15333 |
| INHA | 2.691 | 1.428288 | 0.000148 | 0.001118 | yes | up | 1.85 | 1.06 | 1.45 | 4.54 | 7.03 | 7.47 | 1.453333 | 6.346667 |
| HYI | 3.916 | 1.969263 | 0.000149 | 0.001125 | yes | up | 2.09 | 2.34 | 2.1 | 15.53 | 17.7 | 5.12 | 2.176667 | 12.78333 |
| NAAA | 2.337 | 1.224823 | 0.00015 | 0.001131 | yes | up | 3.19 | 4.34 | 3.53 | 15.78 | 8.5 | 17.6 | 3.686667 | 13.96 |
| GTPBP8 | 0.488 | -1.03502 | 0.000151 | 0.001137 | yes | down | 8.57 | 7.29 | 8.24 | 5.24 | 5.59 | 7.82 | 8.033333 | 6.216667 |
| TMEM88 | 0.45 | -1.15044 | 0.000153 | 0.001146 | yes | down | 34.02 | 42.12 | 48.09 | 22.31 | 29.46 | 39.4 | 41.41 | 30.39 |
| LOC103346332 | 2.188 | 1.129733 | 0.000153 | 0.001149 | yes | up | 16.88 | 15.51 | 9.82 | 57.39 | 32.47 | 56.08 | 14.07 | 48.64667 |
| ARSE | 2.182 | 1.125602 | 0.000154 | 0.001155 | yes | up | 2.54 | 1.67 | 1.25 | 6.98 | 7.94 | 4.8 | 1.82 | 6.573333 |
| ZNF593 | 0.327 | -1.61158 | 0.000155 | 0.001165 | yes | down | 3.6 | 4.97 | 4.38 | 2.38 | 2.4 | 1.88 | 4.316667 | 2.22 |
| ADAR | 2.011 | 1.008257 | 0.000156 | 0.001171 | yes | up | 16.55 | 16.52 | 13.45 | 65.4 | 48.41 | 34.43 | 15.50667 | 49.41333 |
| MIEF2 | 0.392 | -1.35215 | 0.000158 | 0.001185 | yes | down | 7.33 | 4.01 | 3.32 | 2.39 | 2.75 | 3.73 | 4.886667 | 2.956667 |
| IFIT2 | 6.641 | 2.731292 | 0.00016 | 0.001194 | yes | up | 2.13 | 1.1 | 0.91 | 30.19 | 9.84 | 2.73 | 1.38 | 14.25333 |
| NFKB2 | 2.044 | 1.031495 | 0.000161 | 0.001205 | yes | up | 5.81 | 5.64 | 4.61 | 23.93 | 14.28 | 14.02 | 5.353333 | 17.41 |
| LOC103346372 | 4.909 | 2.295287 | 0.000161 | 0.001205 | yes | up | 14.98 | 5.48 | 14.97 | 40.05 | 131.29 | 79.93 | 11.81 | 83.75667 |
| METTL1 | 0.409 | -1.2909 | 0.000162 | 0.001212 | yes | down | 2.7 | 3.97 | 2.79 | 2.63 | 1.61 | 1.96 | 3.153333 | 2.066667 |
| TBC1D30 | 0.421 | -1.24888 | 0.000163 | 0.001213 | yes | down | 0.74 | 0.94 | 1 | 0.51 | 0.58 | 0.67 | 0.893333 | 0.586667 |
| MPPE1 | 0.489 | -1.03301 | 0.000163 | 0.001214 | yes | down | 17.59 | 23.28 | 28.27 | 17.01 | 18.32 | 19.82 | 23.04667 | 18.38333 |
| LOC103348003 | 3.571 | 1.83647 | 0.000165 | 0.001228 | yes | up | 1.06 | 0.97 | 0.84 | 5.04 | 6.79 | 4.27 | 0.956667 | 5.366667 |
| LOC100345975 | 2.099 | 1.069918 | 0.000168 | 0.001248 | yes | up | 1.13 | 2.15 | 4.2 | 3.71 | 4.14 | 5.35 | 2.493333 | 4.4 |
| LOC100008880 | 852.674 | 9.735851 | 0.000168 | 0.001251 | yes | up | 0 | 0 | 0 | 1.6 | 19.41 | 182.15 | 0 | 67.72 |
| FLAD1 | 0.49 | -1.02961 | 0.000169 | 0.001252 | yes | down | 4 | 6.16 | 4.68 | 3.76 | 3.47 | 3.29 | 4.946667 | 3.506667 |
| LOC103351715 | 142.149 | 7.151255 | 0.000169 | 0.001256 | yes | up | 0 | 0 | 0 | 8.82 | 0.66 | 0.29 | 0 | 3.256667 |
| PTPRD | 3.225 | 1.689487 | 0.00017 | 0.001262 | yes | up | 1.24 | 1.18 | 0.35 | 4.09 | 3.02 | 7.05 | 0.923333 | 4.72 |
| EFCAB2 | 2.349 | 1.232198 | 0.00017 | 0.001262 | yes | up | 3.47 | 2.26 | 1.66 | 18.89 | 27.76 | 32.15 | 2.463333 | 26.26667 |
| SUSD4 | 5.867 | 2.552743 | 0.000173 | 0.001276 | yes | up | 0.06 | 0.13 | 0.11 | 0.56 | 0.89 | 1.4 | 0.1 | 0.95 |
| GFRA1 | 2.222 | 1.151676 | 0.000173 | 0.001276 | yes | up | 0.58 | 0.34 | 0.31 | 1.54 | 1.26 | 1.66 | 0.41 | 1.486667 |
| LOC103345511 | 2.385 | 1.253734 | 0.000173 | 0.001277 | yes | up | 5.52 | 7.53 | 2.6 | 10.25 | 11.23 | 14.88 | 5.216667 | 12.12 |
| SERPINA12 | 0.049 | -4.34296 | 0.000175 | 0.001293 | yes | down | 0.41 | 0.57 | 0.38 | 0.07 | 0.03 | 0 | 0.453333 | 0.033333 |
| LOC100009483 | 4.196 | 2.069042 | 0.000175 | 0.001294 | yes | up | 1.1 | 0.66 | 0.39 | 2.62 | 6.02 | 5.34 | 0.716667 | 4.66 |
| SLC35G1 | 0.451 | -1.15029 | 0.000177 | 0.0013 | yes | down | 1.69 | 1.63 | 1.43 | 1.26 | 0.86 | 1.31 | 1.583333 | 1.143333 |
| ARSJ | 2.772 | 1.470822 | 0.000181 | 0.001331 | yes | up | 1.12 | 1.94 | 2.13 | 14.79 | 9.66 | 6.73 | 1.73 | 10.39333 |
| SOD3 | 2.559 | 1.355347 | 0.000183 | 0.001341 | yes | up | 6.39 | 7.09 | 9.85 | 23.14 | 44.01 | 29.34 | 7.776667 | 32.16333 |
| LOC100349875 | 0.334 | -1.58217 | 0.000184 | 0.001346 | yes | down | 1.06 | 1.22 | 1.08 | 0.55 | 0.5 | 0.77 | 1.12 | 0.606667 |
| ABCC10 | 2.051 | 1.036107 | 0.000186 | 0.001362 | yes | up | 1.52 | 1.99 | 1.47 | 4.22 | 5.72 | 6.39 | 1.66 | 5.443333 |
| FST | 2.764 | 1.466846 | 0.000186 | 0.001363 | yes | up | 7.16 | 4.27 | 10.13 | 25.15 | 27.2 | 45.17 | 7.186667 | 32.50667 |
| MAB21L1 | 4.1 | 2.03553 | 0.000187 | 0.00137 | yes | up | 0.4 | 0.4 | 0.5 | 4.77 | 2.4 | 1.41 | 0.433333 | 2.86 |
| LSM4 | 2.467 | 1.302753 | 0.000189 | 0.001381 | yes | up | 14.75 | 15.79 | 13.73 | 75.41 | 43.18 | 33.54 | 14.75667 | 50.71 |
| FKBP5 | 2.59 | 1.372695 | 0.000196 | 0.00143 | yes | up | 8.12 | 9.59 | 10.01 | 61.06 | 22.62 | 34.14 | 9.24 | 39.27333 |
| GRO-B | 23.548 | 4.55751 | 0.000198 | 0.001438 | yes | up | 1.24 | 0.86 | 0 | 26.84 | 19.17 | 19.78 | 0.7 | 21.93 |
| CD274 | 4.35 | 2.121101 | 0.000201 | 0.001461 | yes | up | 0.69 | 1.82 | 0.51 | 11.47 | 4.04 | 4.17 | 1.006667 | 6.56 |
| SLC1A5 | 0.388 | -1.36489 | 0.000202 | 0.00147 | yes | down | 38.76 | 40.94 | 23 | 10.52 | 22.16 | 28.36 | 34.23333 | 20.34667 |
| LOC103346166 | 0.054 | -4.21735 | 0.000203 | 0.001476 | yes | down | 0.26 | 1.34 | 1.05 | 0 | 0.13 | 0.11 | 0.883333 | 0.08 |
| THEMIS2 | 2.674 | 1.419155 | 0.000204 | 0.001479 | yes | up | 1.76 | 3.35 | 1.85 | 11.93 | 6.76 | 13.51 | 2.32 | 10.73333 |
| SLC30A3 | 0.272 | -1.88067 | 0.000206 | 0.001493 | yes | down | 1.11 | 2.22 | 1.1 | 0.57 | 1.79 | 1.59 | 1.476667 | 1.316667 |
| PLPP6 | 2.143 | 1.099606 | 0.000208 | 0.001502 | yes | up | 0.83 | 0.93 | 0.72 | 3.08 | 2.41 | 2.95 | 0.826667 | 2.813333 |
| GDF9 | 2.961 | 1.56602 | 0.000211 | 0.001525 | yes | up | 0.71 | 0.79 | 0.77 | 2.84 | 3.93 | 5.2 | 0.756667 | 3.99 |
| AIM1 | 2.226 | 1.154606 | 0.000212 | 0.001526 | yes | up | 3.15 | 3.37 | 1.92 | 13.76 | 8.26 | 7.17 | 2.813333 | 9.73 |
| HTR7 | 2.951 | 1.561301 | 0.000213 | 0.001532 | yes | up | 0.57 | 0.49 | 0.25 | 2.88 | 1.25 | 2.24 | 0.436667 | 2.123333 |
| DSG2 | 0.48 | -1.05974 | 0.000215 | 0.001543 | yes | down | 3.14 | 4.66 | 2.59 | 3.15 | 2.62 | 2.07 | 3.463333 | 2.613333 |
| CD38 | 4.781 | 2.257172 | 0.000217 | 0.001556 | yes | up | 0.84 | 0.59 | 0.65 | 11.56 | 2.42 | 2.93 | 0.693333 | 5.636667 |
| CNTNAP4 | 0.019 | -5.75428 | 0.000218 | 0.001562 | yes | down | 0.07 | 0.12 | 0.22 | 0.01 | 0 | 0 | 0.136667 | 0.003333 |
| LRIG1 | 2.184 | 1.126977 | 0.000219 | 0.001568 | yes | up | 8.37 | 4.63 | 4.65 | 15.33 | 18.45 | 27.71 | 5.883333 | 20.49667 |
| AVP | 0.24 | -2.05629 | 0.000219 | 0.001569 | yes | down | 10.55 | 17.95 | 26.7 | 2.96 | 8.62 | 9.93 | 18.4 | 7.17 |
| WDR6 | 0.418 | -1.25887 | 0.00022 | 0.001575 | yes | down | 2.45 | 2.61 | 1.43 | 1.48 | 1.73 | 1.02 | 2.163333 | 1.41 |
| KRT23 | 0.198 | -2.3364 | 0.000222 | 0.001584 | yes | down | 35.96 | 118.79 | 25.03 | 29.41 | 5.38 | 20.25 | 59.92667 | 18.34667 |
| PKHD1L1 | 4.759 | 2.250809 | 0.000223 | 0.001592 | yes | up | 0.02 | 0.04 | 0.01 | 0.16 | 0.17 | 0.2 | 0.023333 | 0.176667 |
| ID1 | 0.403 | -1.31229 | 0.000224 | 0.001597 | yes | down | 43.2 | 53.86 | 39.08 | 37.11 | 14.26 | 36.93 | 45.38 | 29.43333 |
| KDSR | 0.496 | -1.01172 | 0.000225 | 0.001603 | yes | down | 7.87 | 11.95 | 11.38 | 6.78 | 8 | 10.41 | 10.4 | 8.396667 |
| LOC100356618 | 0.43 | -1.21671 | 0.000227 | 0.001618 | yes | down | 6.92 | 10.92 | 5.59 | 4.73 | 4.89 | 7.23 | 7.81 | 5.616667 |
| MRM2 | 0.494 | -1.01664 | 0.000229 | 0.001627 | yes | down | 5.94 | 6.76 | 6.63 | 4.8 | 4.25 | 6.45 | 6.443333 | 5.166667 |
| VCAM1 | 3.213 | 1.683935 | 0.000232 | 0.001649 | yes | up | 2.4 | 5.53 | 4.11 | 33.91 | 10.95 | 17.21 | 4.013333 | 20.69 |
| RAD51B | 3.179 | 1.668772 | 0.000233 | 0.001654 | yes | up | 5.49 | 4.68 | 1.48 | 9.96 | 11.78 | 21.7 | 3.883333 | 14.48 |
| DDN | 0.024 | -5.39065 | 0.000236 | 0.001673 | yes | down | 1.15 | 0.18 | 0.1 | 0 | 0 | 0.06 | 0.476667 | 0.02 |
| LOC103346516 | 2.006 | 1.004216 | 0.000239 | 0.001687 | yes | up | 2.96 | 1.07 | 2.66 | 6.61 | 6.57 | 4.65 | 2.23 | 5.943333 |
| PLEKHB1 | 2.756 | 1.462728 | 0.00024 | 0.001693 | yes | up | 5.13 | 3.12 | 1.75 | 11.26 | 11.08 | 21.61 | 3.333333 | 14.65 |
| PCK1 | 0.42 | -1.25276 | 0.000242 | 0.001705 | yes | down | 898.83 | 1267.15 | 675.53 | 539.27 | 380.08 | 979.58 | 947.17 | 632.9767 |
| LOC100342521 | 0.014 | -6.18127 | 0.000242 | 0.001706 | yes | down | 0.16 | 0.43 | 0.23 | 0 | 0 | 0 | 0.273333 | 0 |
| ALCAM | 2.342 | 1.227791 | 0.000242 | 0.001709 | yes | up | 0.74 | 0.93 | 0.42 | 2.83 | 1.88 | 3.14 | 0.696667 | 2.616667 |
| LOC103345076 | 10.408 | 3.37969 | 0.000247 | 0.001738 | yes | up | 0.49 | 0.21 | 0.9 | 12.56 | 7.05 | 1.99 | 0.533333 | 7.2 |
| TYROBP | 4.775 | 2.255501 | 0.000248 | 0.001746 | yes | up | 6.2 | 7.94 | 7.45 | 116.89 | 20.37 | 28.37 | 7.196667 | 55.21 |
| CNTNAP5 | 0.496 | -1.012 | 0.000249 | 0.001752 | yes | down | 7.77 | 7.24 | 7.09 | 4.47 | 6.86 | 6.21 | 7.366667 | 5.846667 |
| MDH2 | 0.477 | -1.06677 | 0.00025 | 0.001756 | yes | down | 465.74 | 340.92 | 420.39 | 289.38 | 409.67 | 234 | 409.0167 | 311.0167 |
| CAMK1G | 0.013 | -6.23145 | 0.00025 | 0.001756 | yes | down | 0.18 | 0.1 | 0.27 | 0 | 0 | 0 | 0.183333 | 0 |
| KLHL21 | 2.119 | 1.083668 | 0.000253 | 0.001776 | yes | up | 9.9 | 10.93 | 14 | 29.03 | 40.64 | 51.15 | 11.61 | 40.27333 |
| LOC100349085 | 23.93 | 4.580723 | 0.000259 | 0.001815 | yes | up | 9.46 | 24.35 | 11 | 1486.61 | 40.35 | 171.62 | 14.93667 | 566.1933 |
| CLIP3 | 2.254 | 1.172574 | 0.000263 | 0.001836 | yes | up | 3.78 | 3.23 | 3.02 | 9.7 | 12.17 | 12.93 | 3.343333 | 11.6 |
| DHTKD1 | 2.186 | 1.128487 | 0.000263 | 0.001837 | yes | up | 1.7 | 1.28 | 1.72 | 4 | 5.57 | 7.13 | 1.566667 | 5.566667 |
| SLC26A10 | 0.305 | -1.71453 | 0.000264 | 0.001842 | yes | down | 20.78 | 27.65 | 44.23 | 8.29 | 22.67 | 15.49 | 30.88667 | 15.48333 |
| LOC103350198 | 55.731 | 5.800402 | 0.000275 | 0.001905 | yes | up | 0 | 0 | 0 | 0.58 | 0.8 | 0.41 | 0 | 0.596667 |
| MS4A15 | 99.646 | 6.638738 | 0.000275 | 0.001908 | yes | up | 0 | 0 | 0 | 0.09 | 1.71 | 0.5 | 0 | 0.766667 |
| LOC103347176 | 129.275 | 7.014298 | 0.000277 | 0.001915 | yes | up | 0 | 0 | 0 | 36.49 | 1.06 | 3.17 | 0 | 13.57333 |
| RPS19BP1 | 0.495 | -1.01406 | 0.000278 | 0.001924 | yes | down | 15.47 | 19.19 | 13.25 | 10.57 | 13.02 | 14.01 | 15.97 | 12.53333 |
| RBBP9 | 0.454 | -1.13812 | 0.000279 | 0.001926 | yes | down | 3.15 | 3.52 | 2.94 | 3.21 | 1.65 | 2.13 | 3.203333 | 2.33 |
| FOXP1 | 2.007 | 1.00469 | 0.000281 | 0.001941 | yes | up | 6.33 | 4.19 | 3.98 | 11.45 | 12.73 | 16.88 | 4.833333 | 13.68667 |
| NGB | 0.036 | -4.81254 | 0.000284 | 0.001961 | yes | down | 0.47 | 0.4 | 0.47 | 0.03 | 0 | 0.04 | 0.446667 | 0.023333 |
| LAMA2 | 2.392 | 1.258459 | 0.000286 | 0.001974 | yes | up | 10.41 | 4.87 | 3.73 | 20.3 | 22.09 | 29.07 | 6.336667 | 23.82 |
| LOC108176650 | 0.436 | -1.19808 | 0.00029 | 0.001997 | yes | down | 4.18 | 4.93 | 2.42 | 2.22 | 1.78 | 3.43 | 3.843333 | 2.476667 |
| LOC108177101 | 25.887 | 4.694165 | 0.000291 | 0.002 | yes | up | 0.08 | 0 | 0.08 | 1.26 | 2.77 | 3.59 | 0.053333 | 2.54 |
| PTPMT1 | 0.471 | -1.08644 | 0.000298 | 0.002043 | yes | down | 5.49 | 8.11 | 5.58 | 4.62 | 4.48 | 5.34 | 6.393333 | 4.813333 |
| TLR3 | 3.086 | 1.625654 | 0.000303 | 0.002078 | yes | up | 0.73 | 0.54 | 0.62 | 4.71 | 2.94 | 1.65 | 0.63 | 3.1 |
| GFOD1 | 0.488 | -1.03364 | 0.000309 | 0.002114 | yes | down | 9.56 | 19.2 | 9.41 | 8.85 | 9.86 | 10.78 | 12.72333 | 9.83 |
| C1QTNF2 | 2.472 | 1.30558 | 0.000309 | 0.002115 | yes | up | 3.65 | 2.13 | 1.4 | 7.26 | 9.64 | 9.94 | 2.393333 | 8.946667 |
| LOC100339781 | 39.119 | 5.289785 | 0.000322 | 0.002197 | yes | up | 0 | 0 | 0.05 | 0.76 | 1.42 | 0.71 | 0.016667 | 0.963333 |
| LEP | 32.537 | 5.024016 | 0.000322 | 0.002197 | yes | up | 0.05 | 0 | 0 | 0.12 | 1.53 | 0.87 | 0.016667 | 0.84 |
| LOC103347627 | 21.8 | 4.446274 | 0.000325 | 0.002211 | yes | up | 0.2 | 0.54 | 0.17 | 24.35 | 3.18 | 1.92 | 0.303333 | 9.816667 |
| ADAM12 | 0.296 | -1.75393 | 0.000328 | 0.002233 | yes | down | 3.36 | 2.99 | 2.16 | 0.45 | 1.88 | 1.66 | 2.836667 | 1.33 |
| LOC103349875 | 3.681 | 1.879969 | 0.00033 | 0.002244 | yes | up | 0.22 | 0.23 | 0.22 | 1.4 | 1.28 | 1.32 | 0.223333 | 1.333333 |
| LOC108178230 | 0.021 | -5.60145 | 0.000332 | 0.002255 | yes | down | 0.55 | 0.33 | 0.13 | 0 | 0.03 | 0 | 0.336667 | 0.01 |
| PFKFB1 | 2.069 | 1.048971 | 0.000334 | 0.002265 | yes | up | 21.68 | 12.53 | 13.2 | 35.93 | 50.82 | 63.52 | 15.80333 | 50.09 |
| RHOU | 2.088 | 1.062003 | 0.000334 | 0.002267 | yes | up | 2.95 | 1.82 | 1.72 | 5.65 | 6.62 | 9.36 | 2.163333 | 7.21 |
| WISP1 | 0.324 | -1.62767 | 0.000337 | 0.00228 | yes | down | 1 | 0.79 | 0.51 | 0.28 | 0.3 | 0.62 | 0.766667 | 0.4 |
| NIP7 | 0.498 | -1.00666 | 0.000341 | 0.002305 | yes | down | 7.19 | 8.69 | 4.72 | 5.66 | 4.14 | 5.25 | 6.866667 | 5.016667 |
| NR2C2AP | 0.329 | -1.60555 | 0.000341 | 0.002305 | yes | down | 3.07 | 6.16 | 5.11 | 2.8 | 2.54 | 2.16 | 4.78 | 2.5 |
| SAMD9 | 3.266 | 1.707436 | 0.000346 | 0.002331 | yes | up | 1.2 | 0.87 | 0.85 | 8.7 | 4.07 | 2.32 | 0.973333 | 5.03 |
| LOC100337780 | 0.055 | -4.19663 | 0.000347 | 0.002338 | yes | down | 0.29 | 0.27 | 0.19 | 0.04 | 0.02 | 0 | 0.25 | 0.02 |
| MEIOB | 0.254 | -1.9795 | 0.000349 | 0.00235 | yes | down | 0.94 | 1.03 | 0.79 | 0.31 | 0.4 | 0.36 | 0.92 | 0.356667 |
| TRNAF-GAA | 3.256 | 1.70297 | 0.00035 | 0.002353 | yes | up | 0.42 | 0.22 | 0.13 | 2.18 | 1.18 | 2.16 | 0.256667 | 1.84 |
| LOC108178759 | 0.07 | -3.83706 | 0.000352 | 0.002365 | yes | down | 1.65 | 0.57 | 0.23 | 0.12 | 0.04 | 0.1 | 0.816667 | 0.086667 |
| LOC100356985 | 6.214 | 2.635538 | 0.000353 | 0.002369 | yes | up | 0.91 | 1.21 | 0.66 | 2.06 | 8.16 | 18.29 | 0.926667 | 9.503333 |
| PAK5 | 79.609 | 6.314866 | 0.000359 | 0.002406 | yes | up | 0.03 | 0 | 0 | 2.84 | 0.2 | 0.03 | 0.01 | 1.023333 |
| LOC100355679 | 0.015 | -6.05038 | 0.000361 | 0.002421 | yes | down | 0.64 | 3.07 | 0.92 | 0 | 0 | 0 | 1.543333 | 0 |
| JAML | 5.138 | 2.36127 | 0.000362 | 0.002422 | yes | up | 0.47 | 0.48 | 0.45 | 7.75 | 2.06 | 2.5 | 0.466667 | 4.103333 |
| ABHD1 | 0.014 | -6.11477 | 0.000362 | 0.002423 | yes | down | 0.28 | 0.26 | 0.53 | 0 | 0 | 0 | 0.356667 | 0 |
| TTC23L | 2.997 | 1.583594 | 0.000366 | 0.002447 | yes | up | 1.11 | 0.6 | 0.4 | 2.95 | 3.66 | 2.93 | 0.703333 | 3.18 |
| CMSS1 | 0.411 | -1.28107 | 0.000367 | 0.002453 | yes | down | 4.94 | 2.52 | 2.72 | 2.32 | 2.06 | 2.03 | 3.393333 | 2.136667 |
| LOC108176084 | 8.304 | 3.053858 | 0.000368 | 0.002459 | yes | up | 0.18 | 0.06 | 0.03 | 1.56 | 1.05 | 0.77 | 0.09 | 1.126667 |
| TRIM10 | 0.163 | -2.62039 | 0.000369 | 0.002463 | yes | down | 0.36 | 0.43 | 0.38 | 0.12 | 0.08 | 0.13 | 0.39 | 0.11 |
| BTBD11 | 2.757 | 1.462936 | 0.000369 | 0.002464 | yes | up | 0.39 | 0.52 | 0.34 | 4.71 | 1.16 | 3.63 | 0.416667 | 3.166667 |
| SSTR4 | 0.09 | -3.4813 | 0.00037 | 0.002464 | yes | down | 0.25 | 0.44 | 0.28 | 0.02 | 0.02 | 0.11 | 0.323333 | 0.05 |
| PARP4 | 2.231 | 1.157478 | 0.000372 | 0.002479 | yes | up | 3.35 | 1.81 | 1.74 | 10.71 | 6.14 | 7.35 | 2.3 | 8.066667 |
| LOC100344620 | 3.222 | 1.68794 | 0.000373 | 0.002482 | yes | up | 1.33 | 2.54 | 2.52 | 16.58 | 7.28 | 9.73 | 2.13 | 11.19667 |
| LOC108178313 | 4.433 | 2.148123 | 0.000375 | 0.002493 | yes | up | 0.91 | 1.75 | 1.97 | 5.83 | 5.87 | 11.76 | 1.543333 | 7.82 |
| RAPGEF4 | 0.404 | -1.30873 | 0.000377 | 0.002506 | yes | down | 1.44 | 1.58 | 2.38 | 1.22 | 1.04 | 1.43 | 1.8 | 1.23 |
| LOC100349768 | 0.348 | -1.52249 | 0.000386 | 0.002561 | yes | down | 986.6 | 374.03 | 287.72 | 210.37 | 368.79 | 307.95 | 549.45 | 295.7033 |
| DRD1 | 4.542 | 2.183244 | 0.00039 | 0.002582 | yes | up | 0.29 | 0.06 | 0.08 | 1.4 | 0.45 | 1.01 | 0.143333 | 0.953333 |
| TLDC2 | 4.425 | 2.145795 | 0.000391 | 0.002591 | yes | up | 0.18 | 0.41 | 0.31 | 3.34 | 1.33 | 1.69 | 0.3 | 2.12 |
| LOC100354703 | 0.364 | -1.45698 | 0.000394 | 0.002611 | yes | down | 0.98 | 1.32 | 0.92 | 0.67 | 0.69 | 0.48 | 1.073333 | 0.613333 |
| MYL4 | 0.03 | -5.05609 | 0.000396 | 0.002619 | yes | down | 56.03 | 5.65 | 1.32 | 0.22 | 1.24 | 1.39 | 21 | 0.95 |
| LOC103351786 | 0.133 | -2.90787 | 0.000399 | 0.002637 | yes | down | 1.66 | 0.5 | 0.36 | 0.07 | 0.22 | 0.24 | 0.84 | 0.176667 |
| LOC100355262 | 0.282 | -1.8267 | 0.000401 | 0.002649 | yes | down | 186.94 | 116.68 | 121.75 | 29.94 | 118.99 | 36.58 | 141.79 | 61.83667 |
| TRIM69 | 93.281 | 6.54351 | 0.000401 | 0.002649 | yes | up | 0 | 0 | 0 | 2.09 | 0.21 | 0.19 | 0 | 0.83 |
| PEBP4 | 2.868 | 1.520153 | 0.000407 | 0.002686 | yes | up | 13.03 | 9.69 | 11.28 | 31.87 | 81.19 | 40.7 | 11.33333 | 51.25333 |
| LOC108176983 | 2.566 | 1.359598 | 0.000408 | 0.002693 | yes | up | 1.19 | 0.46 | 0.55 | 3.36 | 2.66 | 3.54 | 0.733333 | 3.186667 |
| WDR54 | 0.485 | -1.04293 | 0.00041 | 0.002702 | yes | down | 4.49 | 6.03 | 4.25 | 4.12 | 3.14 | 4.2 | 4.923333 | 3.82 |
| LOC100353185 | 0.428 | -1.22363 | 0.000416 | 0.002737 | yes | down | 14.76 | 24.16 | 15.71 | 10.36 | 8.59 | 18.76 | 18.21 | 12.57 |
| PDE12 | 0.466 | -1.10088 | 0.000421 | 0.00277 | yes | down | 3.1 | 3.03 | 2.22 | 2.89 | 1.57 | 1.71 | 2.783333 | 2.056667 |
| PRKCDBP | 0.342 | -1.54611 | 0.000425 | 0.00279 | yes | down | 90.17 | 176.24 | 189.37 | 43.88 | 85.91 | 127.3 | 151.9267 | 85.69667 |
| PNP | 2.082 | 1.058263 | 0.000429 | 0.002815 | yes | up | 8.98 | 11.15 | 10.3 | 47.28 | 26.64 | 34.01 | 10.14333 | 35.97667 |
| LOC103351959 | 3.783 | 1.919476 | 0.000429 | 0.002818 | yes | up | 0.08 | 0.2 | 0.15 | 0.89 | 0.6 | 0.68 | 0.143333 | 0.723333 |
| LOC100338274 | 0.037 | -4.76574 | 0.000432 | 0.002832 | yes | down | 1.67 | 0.21 | 0.05 | 0 | 0.08 | 0.03 | 0.643333 | 0.036667 |
| POSTN | 0.379 | -1.40033 | 0.000437 | 0.002856 | yes | down | 192.67 | 126.64 | 76.45 | 97.63 | 93.14 | 42.33 | 131.92 | 77.7 |
| ISLR | 2.13 | 1.090675 | 0.000438 | 0.00286 | yes | up | 8.87 | 4.38 | 3.92 | 18.19 | 18 | 21.43 | 5.723333 | 19.20667 |
| TNFAIP8 | 2.168 | 1.116067 | 0.000438 | 0.00286 | yes | up | 5.46 | 10.49 | 7.58 | 31.06 | 19.48 | 31.78 | 7.843333 | 27.44 |
| LOC100356330 | 2.248 | 1.168411 | 0.000444 | 0.002888 | yes | up | 1.54 | 2.52 | 1.53 | 5.27 | 5.79 | 8.34 | 1.863333 | 6.466667 |
| FGF14 | 4.586 | 2.197347 | 0.000446 | 0.002897 | yes | up | 0.08 | 0.06 | 0.03 | 0.27 | 0.49 | 0.54 | 0.056667 | 0.433333 |
| BLVRA | 2.304 | 1.203868 | 0.000447 | 0.002903 | yes | up | 2.18 | 1.33 | 1.65 | 6.66 | 5.24 | 7.17 | 1.72 | 6.356667 |
| VNN1 | 3.071 | 1.618844 | 0.000448 | 0.002909 | yes | up | 0.51 | 0.34 | 0.35 | 1.57 | 1.97 | 2.35 | 0.4 | 1.963333 |
| MAML3 | 2.024 | 1.017541 | 0.000457 | 0.002964 | yes | up | 0.82 | 0.63 | 0.5 | 1.59 | 2.09 | 2.59 | 0.65 | 2.09 |
| MAP2K3 | 0.446 | -1.16599 | 0.00046 | 0.002982 | yes | down | 39.49 | 39.52 | 16.03 | 18.88 | 26.71 | 18.81 | 31.68 | 21.46667 |
| LOC100350949 | 0 | -13.6738 | 0.000465 | 0.003011 | yes | down | 531.34 | 0 | 491.72 | 0 | 0 | 0 | 341.02 | 0 |
| BHLHE22 | 5.736 | 2.520062 | 0.000475 | 0.003067 | yes | up | 0.03 | 0.07 | 0.05 | 0.57 | 0.35 | 0.51 | 0.05 | 0.476667 |
| LOC100345090 | 0.353 | -1.50235 | 0.000485 | 0.003127 | yes | down | 0.77 | 0.54 | 0.6 | 0.37 | 0.26 | 0.46 | 0.636667 | 0.363333 |
| FKBP3 | 0.426 | -1.23213 | 0.000486 | 0.003129 | yes | down | 115.47 | 56.22 | 48.78 | 40.74 | 62.38 | 42.23 | 73.49 | 48.45 |
| STC2 | 0.414 | -1.27348 | 0.000489 | 0.003146 | yes | down | 1.7 | 1.41 | 0.85 | 0.9 | 0.61 | 1.09 | 1.32 | 0.866667 |
| CCDC122 | 4.22 | 2.077336 | 0.000491 | 0.003156 | yes | up | 0.63 | 0.42 | 0.67 | 5.18 | 4.59 | 1.72 | 0.573333 | 3.83 |
| REEP4 | 0.427 | -1.22881 | 0.000495 | 0.003177 | yes | down | 3.46 | 2.99 | 3.14 | 2.27 | 2.38 | 1.85 | 3.196667 | 2.166667 |
| PIK3CD | 4.92 | 2.298547 | 0.000502 | 0.003218 | yes | up | 0.39 | 0.59 | 0.44 | 7.98 | 1.65 | 1.51 | 0.473333 | 3.713333 |
| FAM107B | 2.335 | 1.223201 | 0.000504 | 0.00323 | yes | up | 1.91 | 1.99 | 1.67 | 9.72 | 4.09 | 7.24 | 1.856667 | 7.016667 |
| RND3 | 2.07 | 1.049578 | 0.000506 | 0.003242 | yes | up | 10.56 | 7.74 | 4.45 | 21.76 | 20.06 | 32.36 | 7.583333 | 24.72667 |
| LOC103347586 | 3.816 | 1.93207 | 0.000507 | 0.003246 | yes | up | 16.18 | 4.47 | 2.55 | 47.37 | 56.83 | 70.44 | 7.733333 | 58.21333 |
| GGT5 | 2.928 | 1.54979 | 0.000507 | 0.003249 | yes | up | 0.43 | 0.48 | 0.45 | 3.15 | 1.47 | 1.78 | 0.453333 | 2.133333 |
| DENND1C | 4.688 | 2.228994 | 0.000508 | 0.003249 | yes | up | 0.56 | 0.54 | 0.47 | 6.44 | 3.05 | 1.93 | 0.523333 | 3.806667 |
| TTC9 | 2.085 | 1.06039 | 0.000508 | 0.003249 | yes | up | 13.9 | 23.08 | 12.42 | 42.99 | 39.97 | 72.91 | 16.46667 | 51.95667 |
| IGSF1 | 0.129 | -2.95147 | 0.000508 | 0.003249 | yes | down | 2.31 | 0.55 | 0.42 | 0.37 | 0.07 | 0.09 | 1.093333 | 0.176667 |
| EML6 | 2.331 | 1.22072 | 0.000512 | 0.003273 | yes | up | 1.14 | 1.23 | 0.48 | 2.73 | 4 | 3.56 | 0.95 | 3.43 |
| LOC103348951 | 2 | 1.000352 | 0.000516 | 0.003291 | yes | up | 8.34 | 5.48 | 3.43 | 11.97 | 13.58 | 14.99 | 5.75 | 13.51333 |
| LOC103349335 | 0.071 | -3.81899 | 0.000516 | 0.003294 | yes | down | 0.79 | 2.13 | 2.61 | 0.22 | 0 | 0.4 | 1.843333 | 0.206667 |
| SSBP2 | 2.283 | 1.190937 | 0.000518 | 0.003298 | yes | up | 7.36 | 9.52 | 3.68 | 18.92 | 21.65 | 33.28 | 6.853333 | 24.61667 |
| LOC108176201 | 4.193 | 2.068132 | 0.000522 | 0.003321 | yes | up | 0.18 | 0.37 | 0.36 | 3.05 | 1.52 | 3.34 | 0.303333 | 2.636667 |
| FGF11 | 0.26 | -1.94373 | 0.000522 | 0.003321 | yes | down | 0.62 | 0.8 | 0.7 | 0.11 | 0.38 | 0.39 | 0.706667 | 0.293333 |
| LOC100355766 | 33.654 | 5.072709 | 0.000525 | 0.003335 | yes | up | 0 | 0.04 | 0.04 | 3.45 | 0.34 | 0.91 | 0.026667 | 1.566667 |
| PLLP | 2.941 | 1.556349 | 0.000531 | 0.003367 | yes | up | 1.02 | 0.84 | 0.63 | 2.91 | 3.17 | 5.63 | 0.83 | 3.903333 |
| CCR1 | 10.256 | 3.358412 | 0.000533 | 0.003378 | yes | up | 0.14 | 0.17 | 0.17 | 5.68 | 0.77 | 0.63 | 0.16 | 2.36 |
| SATL1 | 21.536 | 4.428668 | 0.000542 | 0.00343 | yes | up | 0.07 | 0 | 0 | 0.43 | 0.4 | 1.35 | 0.023333 | 0.726667 |
| IL7R | 17.918 | 4.163357 | 0.000544 | 0.003436 | yes | up | 0.03 | 0.05 | 0.01 | 1.96 | 0.09 | 0.3 | 0.03 | 0.783333 |
| GABRD | 0.334 | -1.58334 | 0.000549 | 0.003466 | yes | down | 27.46 | 79.57 | 42.63 | 14.54 | 36.58 | 27.84 | 49.88667 | 26.32 |
| DIXDC1 | 2.563 | 1.35794 | 0.000553 | 0.003487 | yes | up | 1.37 | 0.69 | 0.31 | 3.83 | 6.16 | 6.55 | 0.79 | 5.513333 |
| NEGR1 | 2.766 | 1.467744 | 0.000553 | 0.003489 | yes | up | 0.34 | 0.36 | 0.32 | 1.76 | 1.38 | 1.35 | 0.34 | 1.496667 |
| LOC103350952 | 0.385 | -1.37787 | 0.000558 | 0.003517 | yes | down | 1.13 | 1.02 | 0.66 | 0.78 | 0.38 | 0.55 | 0.936667 | 0.57 |
| CEP112 | 2.296 | 1.198857 | 0.000561 | 0.003533 | yes | up | 0.5 | 0.43 | 0.33 | 1.71 | 1.55 | 1.35 | 0.42 | 1.536667 |
| SGTB | 2.879 | 1.525784 | 0.000562 | 0.00354 | yes | up | 6.81 | 2.41 | 1.84 | 9.53 | 10.78 | 9.08 | 3.686667 | 9.796667 |
| TRMT61A | 0.457 | -1.13052 | 0.000565 | 0.003552 | yes | down | 3.83 | 5.43 | 2.05 | 3.04 | 2.45 | 2.6 | 3.77 | 2.696667 |
| CA13 | 0.498 | -1.00598 | 0.000568 | 0.003569 | yes | down | 13.33 | 15.25 | 19.25 | 11.16 | 14.61 | 13.75 | 15.94333 | 13.17333 |
| TBCCD1 | 0.367 | -1.44615 | 0.000574 | 0.003602 | yes | down | 5.78 | 6.84 | 13 | 4.18 | 4.91 | 7.47 | 8.54 | 5.52 |
| PGM2 | 0.409 | -1.28877 | 0.000575 | 0.003606 | yes | down | 10.67 | 13.09 | 4.8 | 7.37 | 5.25 | 5.07 | 9.52 | 5.896667 |
| ACAP1 | 4.385 | 2.132655 | 0.000579 | 0.003628 | yes | up | 0.27 | 0.35 | 0.26 | 3.71 | 1.23 | 1.1 | 0.293333 | 2.013333 |
| CISH | 2.189 | 1.130228 | 0.000584 | 0.003653 | yes | up | 32.58 | 32.26 | 36.82 | 66.63 | 99.32 | 168.21 | 33.88667 | 111.3867 |
| LOC100338650 | 0.012 | -6.35956 | 0.000584 | 0.003653 | yes | down | 0.09 | 0.84 | 0.3 | 0 | 0 | 0 | 0.41 | 0 |
| PSMG4 | 0.48 | -1.05822 | 0.000586 | 0.003666 | yes | down | 36.04 | 21.61 | 22.99 | 16.74 | 22.5 | 21.66 | 26.88 | 20.3 |
| SERPINF1 | 2.205 | 1.140519 | 0.000587 | 0.003669 | yes | up | 90.88 | 52.9 | 32.8 | 162.65 | 235.95 | 211.55 | 58.86 | 203.3833 |
| NEIL2 | 2.177 | 1.122387 | 0.000589 | 0.00368 | yes | up | 2.22 | 2.29 | 2.2 | 5.48 | 7.36 | 10.79 | 2.236667 | 7.876667 |
| AP1B1 | 0.465 | -1.10513 | 0.000592 | 0.003694 | yes | down | 21.38 | 19.46 | 25.16 | 24.68 | 16.41 | 10.93 | 22 | 17.34 |
| AOX1 | 3.109 | 1.636248 | 0.0006 | 0.003741 | yes | up | 4.17 | 12.97 | 14.87 | 43.93 | 46.35 | 77.76 | 10.67 | 56.01333 |
| LOC100354991 | 2.391 | 1.257886 | 0.000605 | 0.003766 | yes | up | 5.84 | 9.83 | 8.66 | 29.06 | 15.79 | 15.76 | 8.11 | 20.20333 |
| NMNAT2 | 2.181 | 1.125139 | 0.000605 | 0.00377 | yes | up | 1.49 | 1.33 | 0.78 | 4.92 | 3.63 | 3.95 | 1.2 | 4.166667 |
| ANKRD61 | 6.542 | 2.709751 | 0.000609 | 0.003786 | yes | up | 0.11 | 0.07 | 0.07 | 0.47 | 1.2 | 0.93 | 0.083333 | 0.866667 |
| NRK | 0.237 | -2.07503 | 0.00061 | 0.003791 | yes | down | 12.2 | 3.75 | 1.4 | 1.95 | 2.53 | 1.74 | 5.783333 | 2.073333 |
| CTHRC1 | 0.345 | -1.5346 | 0.000611 | 0.003797 | yes | down | 11.57 | 11.31 | 4.71 | 2.95 | 6.05 | 5.78 | 9.196667 | 4.926667 |
| LOC100344884 | 0.015 | -6.08965 | 0.000612 | 0.003797 | yes | down | 0.42 | 0.18 | 0.14 | 0 | 0 | 0 | 0.246667 | 0 |
| INHBB | 4.064 | 2.022772 | 0.000617 | 0.003822 | yes | up | 0.22 | 0.23 | 0.12 | 1 | 1.72 | 0.85 | 0.19 | 1.19 |
| NABP1 | 2.439 | 1.286207 | 0.00062 | 0.00384 | yes | up | 2.63 | 1.4 | 0.89 | 6.95 | 4.33 | 4.52 | 1.64 | 5.266667 |
| DECR2 | 2.165 | 1.114283 | 0.000623 | 0.003853 | yes | up | 11.37 | 11.93 | 12.04 | 18.68 | 33.9 | 36.82 | 11.78 | 29.8 |
| VIT | 3.275 | 1.711598 | 0.000623 | 0.003854 | yes | up | 1.3 | 0.7 | 0.29 | 4.04 | 2.68 | 4.86 | 0.763333 | 3.86 |
| CADM2 | 3.871 | 1.952522 | 0.000624 | 0.003855 | yes | up | 0.3 | 0.08 | 0.07 | 0.83 | 0.82 | 1.01 | 0.15 | 0.886667 |
| TRIM5 | 2.667 | 1.415005 | 0.000625 | 0.003862 | yes | up | 3.98 | 3.01 | 3.06 | 21.1 | 9.87 | 8.94 | 3.35 | 13.30333 |
| HOXA1 | 2.043 | 1.030741 | 0.000629 | 0.003883 | yes | up | 1.41 | 1.7 | 1.6 | 5.16 | 4.17 | 4.73 | 1.57 | 4.686667 |
| C7H2orf72 | 5.577 | 2.479439 | 0.000632 | 0.003901 | yes | up | 0.32 | 0.21 | 0.03 | 1.57 | 1.12 | 2.12 | 0.186667 | 1.603333 |
| NNT | 0.49 | -1.02959 | 0.000637 | 0.003929 | yes | down | 104.07 | 56.48 | 50.51 | 46.24 | 67.91 | 51.76 | 70.35333 | 55.30333 |
| OAS2 | 34.311 | 5.100595 | 0.00064 | 0.003946 | yes | up | 0.53 | 0.13 | 0.05 | 32.84 | 2.75 | 1.13 | 0.236667 | 12.24 |
| LRP3 | 0.498 | -1.00451 | 0.000645 | 0.00397 | yes | down | 30.83 | 32.69 | 21.93 | 13.27 | 23.12 | 28.41 | 28.48333 | 21.6 |
| LOC108178714 | 3.258 | 1.703799 | 0.000647 | 0.00398 | yes | up | 0.77 | 1.26 | 0.36 | 2.83 | 3.59 | 4.64 | 0.796667 | 3.686667 |
| TMEM144 | 5.749 | 2.523347 | 0.000649 | 0.003984 | yes | up | 0.45 | 0.3 | 0.51 | 8.37 | 1.42 | 1.53 | 0.42 | 3.773333 |
| LOC103346185 | 33.37 | 5.06047 | 0.000653 | 0.004006 | yes | up | 0 | 0 | 0.05 | 1.18 | 1.23 | 0.68 | 0.016667 | 1.03 |
| ALG13 | 2.171 | 1.118297 | 0.000655 | 0.004014 | yes | up | 1.15 | 1.06 | 0.54 | 2.53 | 6.83 | 6.18 | 0.916667 | 5.18 |
| MAGI2 | 2.743 | 1.455841 | 0.000658 | 0.004031 | yes | up | 0.34 | 0.19 | 0.15 | 1.12 | 0.72 | 1.09 | 0.226667 | 0.976667 |
| LOC108176666 | 5.391 | 2.430466 | 0.000669 | 0.004093 | yes | up | 0.7 | 0.23 | 0.32 | 4.75 | 3.16 | 2.33 | 0.416667 | 3.413333 |
| GAL3ST4 | 2.492 | 1.317311 | 0.000679 | 0.004148 | yes | up | 1.91 | 1.16 | 0.82 | 3.7 | 5.88 | 5.74 | 1.296667 | 5.106667 |
| EMILIN2 | 2.074 | 1.052164 | 0.000679 | 0.00415 | yes | up | 9.76 | 6.95 | 5.17 | 33.24 | 17.01 | 20.9 | 7.293333 | 23.71667 |
| T | 0.013 | -6.30689 | 0.000681 | 0.004158 | yes | down | 0.82 | 0.18 | 0.1 | 0 | 0 | 0 | 0.366667 | 0 |
| EPB42 | 0.403 | -1.31205 | 0.000681 | 0.004161 | yes | down | 1.82 | 3.58 | 3.15 | 2.24 | 1.33 | 2.05 | 2.85 | 1.873333 |
| MB | 0.259 | -1.94722 | 0.000684 | 0.004174 | yes | down | 372.41 | 77.37 | 64.17 | 46.54 | 61.85 | 84.63 | 171.3167 | 64.34 |
| ST8SIA3 | 5.022 | 2.328206 | 0.000687 | 0.004189 | yes | up | 0.12 | 0.05 | 0.02 | 0.27 | 0.46 | 0.74 | 0.063333 | 0.49 |
| SORL1 | 2.349 | 1.232141 | 0.000692 | 0.004222 | yes | up | 6.89 | 11.01 | 6.92 | 17.9 | 27.51 | 50.62 | 8.273333 | 32.01 |
| ST6GALNAC3 | 0.473 | -1.08025 | 0.000693 | 0.004225 | yes | down | 2.12 | 1.59 | 1.47 | 1.04 | 1.36 | 1.24 | 1.726667 | 1.213333 |
| LOC103351933 | 2.56 | 1.355929 | 0.000694 | 0.004231 | yes | up | 1.1 | 1.69 | 1.03 | 3.58 | 4.91 | 7.77 | 1.273333 | 5.42 |
| LOC100352436 | 5.028 | 2.329868 | 0.000695 | 0.004234 | yes | up | 0.38 | 0.51 | 0.45 | 7.04 | 0.9 | 3.22 | 0.446667 | 3.72 |
| LOC100008980 | 0.014 | -6.19903 | 0.000696 | 0.004234 | yes | down | 0.95 | 0.35 | 0.18 | 0 | 0 | 0 | 0.493333 | 0 |
| LOC103346136 | 4.088 | 2.0313 | 0.000699 | 0.004251 | yes | up | 0.36 | 1.36 | 0.45 | 4.39 | 5.63 | 3.37 | 0.723333 | 4.463333 |
| C1RL | 12.375 | 3.62935 | 0.000701 | 0.004258 | yes | up | 0.18 | 0.08 | 0.05 | 4.86 | 0.51 | 0.54 | 0.103333 | 1.97 |
| TSC22D1 | 2.025 | 1.018081 | 0.000702 | 0.004261 | yes | up | 52.43 | 48.81 | 87.94 | 184.43 | 236.42 | 261.12 | 63.06 | 227.3233 |
| LOC100351810 | 92.775 | 6.535671 | 0.000704 | 0.004273 | yes | up | 0 | 0 | 0 | 36.26 | 5.3 | 1.43 | 0 | 14.33 |
| NWD2 | 0.434 | -1.20551 | 0.000727 | 0.004386 | yes | down | 3.04 | 7.33 | 1.81 | 2.74 | 3.51 | 4.35 | 4.06 | 3.533333 |
| LOC108178964 | 2.095 | 1.06721 | 0.000728 | 0.004391 | yes | up | 1.76 | 3.74 | 2.17 | 8.63 | 9.01 | 7.96 | 2.556667 | 8.533333 |
| RNF19B | 3.109 | 1.636519 | 0.000733 | 0.00442 | yes | up | 9.12 | 9.9 | 8.88 | 90.53 | 26.87 | 27.93 | 9.3 | 48.44333 |
| PRRT1 | 10.09 | 3.334785 | 0.000744 | 0.00448 | yes | up | 0 | 0.04 | 0.06 | 0.43 | 0.71 | 0.63 | 0.033333 | 0.59 |
| LOC100349198 | 2.022 | 1.015679 | 0.00075 | 0.004512 | yes | up | 0.67 | 0.75 | 0.66 | 2.52 | 1.73 | 2.46 | 0.693333 | 2.236667 |
| GLIPR1 | 3.265 | 1.707012 | 0.000756 | 0.004543 | yes | up | 4.32 | 3.88 | 2.67 | 32.94 | 9.88 | 13.34 | 3.623333 | 18.72 |
| ENPP3 | 4.79 | 2.259904 | 0.00076 | 0.004569 | yes | up | 1.82 | 1.34 | 0.31 | 2.63 | 9.21 | 13.82 | 1.156667 | 8.553333 |
| RUNDC3A | 0.057 | -4.13644 | 0.000762 | 0.004573 | yes | down | 0.44 | 1.09 | 0.59 | 0.19 | 0 | 0 | 0.706667 | 0.063333 |
| SLC7A8 | 2.759 | 1.464356 | 0.000764 | 0.004589 | yes | up | 9.11 | 8 | 2.69 | 37.75 | 15.91 | 31.28 | 6.6 | 28.31333 |
| EDARADD | 10.221 | 3.3535 | 0.000767 | 0.004602 | yes | up | 0.15 | 0.03 | 0.09 | 2.03 | 0.23 | 1.76 | 0.09 | 1.34 |
| LOC108178822 | 2.066 | 1.04703 | 0.000768 | 0.004606 | yes | up | 0.88 | 0.99 | 1.3 | 3.06 | 3.43 | 4.24 | 1.056667 | 3.576667 |
| LOC108178548 | 3.348 | 1.743372 | 0.000774 | 0.004629 | yes | up | 0.34 | 0.21 | 0.16 | 0.76 | 1.69 | 1.28 | 0.236667 | 1.243333 |
| LOC103345689 | 2.218 | 1.14927 | 0.000777 | 0.004645 | yes | up | 0.81 | 1.01 | 0.67 | 3.05 | 2.65 | 3.67 | 0.83 | 3.123333 |
| TMEM53 | 2.271 | 1.183579 | 0.000784 | 0.004683 | yes | up | 1.18 | 1.94 | 0.9 | 5.45 | 4.01 | 4.99 | 1.34 | 4.816667 |
| ITIH3 | 5.762 | 2.526561 | 0.000786 | 0.004691 | yes | up | 0.17 | 0.19 | 0.07 | 1.31 | 2.06 | 0.46 | 0.143333 | 1.276667 |
| WDR34 | 0.389 | -1.36177 | 0.000789 | 0.004705 | yes | down | 2.02 | 2.53 | 3.47 | 1.49 | 1.67 | 1.97 | 2.673333 | 1.71 |
| USP54 | 2.211 | 1.144853 | 0.000789 | 0.004705 | yes | up | 2.27 | 2.41 | 2.46 | 11.22 | 4.46 | 9.68 | 2.38 | 8.453333 |
| GNGT2 | 0.486 | -1.0409 | 0.00079 | 0.004711 | yes | down | 11.36 | 14.09 | 15.91 | 9.08 | 9.61 | 14.21 | 13.78667 | 10.96667 |
| LOC108176611 | 2.796 | 1.483329 | 0.000792 | 0.004721 | yes | up | 1.57 | 1.14 | 0.95 | 4.68 | 4.25 | 7.38 | 1.22 | 5.436667 |
| PMP22 | 2.676 | 1.420314 | 0.000793 | 0.004721 | yes | up | 72.87 | 42.49 | 18.13 | 140.2 | 149.83 | 268.39 | 44.49667 | 186.14 |
| NUAK2 | 4.218 | 2.076601 | 0.0008 | 0.00476 | yes | up | 0.41 | 0.3 | 0.22 | 3.69 | 0.91 | 2.22 | 0.31 | 2.273333 |
| SHISA3 | 3.931 | 1.974886 | 0.000802 | 0.00477 | yes | up | 0.53 | 0.47 | 0.15 | 1.86 | 1.39 | 3.84 | 0.383333 | 2.363333 |
| LOC103346732 | 2.019 | 1.01368 | 0.000807 | 0.004794 | yes | up | 36.24 | 15.3 | 28.26 | 54.04 | 114.51 | 105.39 | 26.6 | 91.31333 |
| HDC | 0.111 | -3.16904 | 0.000828 | 0.004909 | yes | down | 0.37 | 0.2 | 0.37 | 0.09 | 0.02 | 0.06 | 0.313333 | 0.056667 |
| LOC103351035 | 3.653 | 1.868973 | 0.000829 | 0.00491 | yes | up | 1.44 | 2.7 | 1.95 | 21.02 | 4.45 | 7.05 | 2.03 | 10.84 |
| LOC100345907 | 16.103 | 4.009245 | 0.000836 | 0.004944 | yes | up | 0 | 0.05 | 0.11 | 2.01 | 0.62 | 1.31 | 0.053333 | 1.313333 |
| LOC103349808 | 0.023 | -5.41387 | 0.000838 | 0.004954 | yes | down | 3.16 | 0.71 | 1.69 | 0 | 0 | 0.23 | 1.853333 | 0.076667 |
| LOC103351156 | 6.863 | 2.778782 | 0.00084 | 0.004957 | yes | up | 0.05 | 0.21 | 0.08 | 1.02 | 1.19 | 1.56 | 0.113333 | 1.256667 |
| CCDC86 | 0.405 | -1.30299 | 0.00084 | 0.004957 | yes | down | 4.42 | 5.3 | 2.44 | 3.49 | 1.91 | 2.32 | 4.053333 | 2.573333 |
| LOC103352375 | 6.099 | 2.608588 | 0.00084 | 0.004957 | yes | up | 0.18 | 0.25 | 0.13 | 1.83 | 2.5 | 1.28 | 0.186667 | 1.87 |
| TENM1 | 3.792 | 1.923035 | 0.000857 | 0.005045 | yes | up | 0.05 | 0.04 | 0.03 | 0.28 | 0.25 | 0.21 | 0.04 | 0.246667 |
| LOC100351040 | 0.017 | -5.83971 | 0.00087 | 0.005111 | yes | down | 0.1 | 0.64 | 0.05 | 0 | 0 | 0.02 | 0.263333 | 0.006667 |
| CEACAM1 | 0.128 | -2.96405 | 0.000877 | 0.005149 | yes | down | 0.22 | 2.6 | 2.53 | 0.68 | 0.3 | 0.15 | 1.783333 | 0.376667 |
| LOC100349542 | 2.498 | 1.321013 | 0.000879 | 0.005159 | yes | up | 1.68 | 1.52 | 0.72 | 4.38 | 4.01 | 7.43 | 1.306667 | 5.273333 |
| LOC100342321 | 45.467 | 5.506737 | 0.000882 | 0.005171 | yes | up | 0.36 | 0.1 | 0 | 25.23 | 4.96 | 1.16 | 0.153333 | 10.45 |
| MT3 | 0.47 | -1.08966 | 0.000902 | 0.005277 | yes | down | 70.08 | 41.41 | 37.82 | 41.73 | 26.7 | 41.14 | 49.77 | 36.52333 |
| FOLR2 | 3.602 | 1.848711 | 0.000902 | 0.005277 | yes | up | 8.68 | 7.47 | 5.45 | 75.04 | 15.81 | 22.38 | 7.2 | 37.74333 |
| MECP2 | 3.277 | 1.712181 | 0.000911 | 0.005324 | yes | up | 0.78 | 0.6 | 1.61 | 5.03 | 4.61 | 6.77 | 0.996667 | 5.47 |
| CLSTN2 | 0.283 | -1.82148 | 0.000913 | 0.005331 | yes | down | 0.36 | 0.3 | 0.51 | 0.25 | 0.07 | 0.23 | 0.39 | 0.183333 |
| LOC100347041 | 8.051 | 3.009137 | 0.00092 | 0.005366 | yes | up | 0.12 | 0.2 | 0.29 | 5.58 | 1.02 | 1.5 | 0.203333 | 2.7 |
| LOC108178314 | 4.835 | 2.27355 | 0.00092 | 0.005366 | yes | up | 0.45 | 1.03 | 0.29 | 3.87 | 3.26 | 6.5 | 0.59 | 4.543333 |
| STAT2 | 3.485 | 1.801319 | 0.000931 | 0.005425 | yes | up | 4.23 | 3.43 | 3.93 | 42.45 | 13.6 | 10 | 3.863333 | 22.01667 |
| LGALS3 | 3.286 | 1.716333 | 0.000939 | 0.005463 | yes | up | 24.92 | 15.53 | 11.25 | 163.78 | 39.22 | 62.82 | 17.23333 | 88.60667 |
| MYH13 | 0.016 | -5.95886 | 0.000939 | 0.005463 | yes | down | 247.41 | 46.22 | 8.12 | 0.01 | 3.85 | 1.06 | 100.5833 | 1.64 |
| SH3PXD2B | 2.193 | 1.133179 | 0.000941 | 0.005468 | yes | up | 1.07 | 0.83 | 0.52 | 1.91 | 3.47 | 2.9 | 0.806667 | 2.76 |
| LOC103351157 | 6.076 | 2.603093 | 0.000946 | 0.005487 | yes | up | 0.08 | 0.06 | 0.01 | 0.38 | 0.59 | 0.44 | 0.05 | 0.47 |
| LOC100351490 | 2.01 | 1.007393 | 0.000946 | 0.005488 | yes | up | 3.05 | 4.78 | 3.82 | 8.59 | 12.47 | 8.74 | 3.883333 | 9.933333 |
| SAAL1 | 0.475 | -1.07358 | 0.000953 | 0.005517 | yes | down | 2.59 | 3.01 | 2.65 | 2.54 | 1.84 | 1.91 | 2.75 | 2.096667 |
| LOC103346986 | 4.232 | 2.081195 | 0.000961 | 0.005553 | yes | up | 0.32 | 0.2 | 0.14 | 0.78 | 1.92 | 1.91 | 0.22 | 1.536667 |
| LOC100338442 | 6.213 | 2.635382 | 0.000961 | 0.005557 | yes | up | 0.39 | 0.16 | 0.2 | 1.5 | 2.12 | 3.73 | 0.25 | 2.45 |
| LOC100355380 | 17.695 | 4.145249 | 0.000965 | 0.005574 | yes | up | 0.07 | 0.06 | 0.15 | 7.2 | 0.16 | 0.78 | 0.093333 | 2.713333 |
| LOC108178426 | 45.519 | 5.508389 | 0.00098 | 0.00565 | yes | up | 0 | 0 | 0 | 0.73 | 0.73 | 1.61 | 0 | 1.023333 |
| EPB41L4B | 0.427 | -1.22763 | 0.000988 | 0.005692 | yes | down | 10.86 | 7.23 | 9.09 | 3.17 | 7.9 | 5.35 | 9.06 | 5.473333 |
| LIX1 | 2.98 | 1.575196 | 0.000988 | 0.005692 | yes | up | 2.07 | 2.46 | 0.83 | 4.38 | 7.68 | 13.02 | 1.786667 | 8.36 |
| TAPBP | 3.072 | 1.619082 | 0.00099 | 0.005699 | yes | up | 3.28 | 2.92 | 4.03 | 30.96 | 10.45 | 9.71 | 3.41 | 17.04 |
| ARL4D | 2.338 | 1.225238 | 0.000999 | 0.005749 | yes | up | 3.2 | 4.08 | 6.16 | 14.39 | 15.64 | 21.99 | 4.48 | 17.34 |
| DDX58 | 2.706 | 1.436101 | 0.001 | 0.005752 | yes | up | 12.79 | 6.67 | 12.6 | 75.89 | 35.69 | 28.94 | 10.68667 | 46.84 |
| PVALB | 132.011 | 7.044519 | 0.001023 | 0.005863 | yes | up | 1.37 | 0.13 | 0 | 0.88 | 235.94 | 44.87 | 0.5 | 93.89667 |
| PAQR8 | 4.401 | 2.137923 | 0.001032 | 0.005912 | yes | up | 0.15 | 0.18 | 0.03 | 1.09 | 0.47 | 0.93 | 0.12 | 0.83 |
| CLVS1 | 2.638 | 1.399623 | 0.001033 | 0.005918 | yes | up | 0.76 | 0.97 | 0.92 | 2.2 | 3.39 | 5.8 | 0.883333 | 3.796667 |
| LOC108177987 | 13.537 | 3.758805 | 0.001043 | 0.005969 | yes | up | 0.01 | 0.18 | 0.08 | 4.4 | 0.3 | 0.47 | 0.09 | 1.723333 |
| RTL1 | 0.022 | -5.53717 | 0.001054 | 0.006019 | yes | down | 2.54 | 0.21 | 0.04 | 0 | 0.04 | 0 | 0.93 | 0.013333 |
| VSIG4 | 4.339 | 2.117308 | 0.001072 | 0.00611 | yes | up | 6.61 | 5.99 | 4.15 | 83.17 | 9.94 | 16.95 | 5.583333 | 36.68667 |
| SYT12 | 0.356 | -1.49038 | 0.00108 | 0.006147 | yes | down | 0.52 | 0.91 | 0.69 | 0.31 | 0.4 | 0.51 | 0.706667 | 0.406667 |
| NOS2 | 2.8 | 1.485645 | 0.001082 | 0.006157 | yes | up | 0.34 | 0.15 | 0.2 | 0.99 | 0.92 | 1.23 | 0.23 | 1.046667 |
| GAREM1 | 2.395 | 1.259986 | 0.001083 | 0.00616 | yes | up | 1.29 | 0.7 | 1.74 | 4.9 | 4.8 | 5.11 | 1.243333 | 4.936667 |
| SEMA4F | 2.17 | 1.117389 | 0.001091 | 0.006198 | yes | up | 0.67 | 0.58 | 0.42 | 1.89 | 2.37 | 1.53 | 0.556667 | 1.93 |
| PRKACA | 0.381 | -1.39073 | 0.001122 | 0.006341 | yes | down | 40.95 | 34.8 | 19.03 | 19.56 | 18.72 | 19.55 | 31.59333 | 19.27667 |
| LAPTM5 | 4.116 | 2.041264 | 0.001127 | 0.006367 | yes | up | 7.48 | 6.61 | 6.32 | 98.94 | 13.39 | 22.4 | 6.803333 | 44.91 |
| CNTN3 | 4.984 | 2.317381 | 0.001128 | 0.00637 | yes | up | 0.1 | 0.02 | 0.04 | 0.4 | 0.48 | 0.39 | 0.053333 | 0.423333 |
| ATG16L2 | 3.249 | 1.700129 | 0.00113 | 0.00638 | yes | up | 0.84 | 0.47 | 0.46 | 4.83 | 2.3 | 1.96 | 0.59 | 3.03 |
| LOC108178917 | 2.669 | 1.416409 | 0.001139 | 0.006425 | yes | up | 0.41 | 0.32 | 0.43 | 1.94 | 1.82 | 1.85 | 0.386667 | 1.87 |
| ATP2A2 | 0.324 | -1.62697 | 0.001149 | 0.006481 | yes | down | 117.72 | 42.21 | 23.26 | 29.23 | 24.78 | 41.37 | 61.06333 | 31.79333 |
| CADM3 | 2.022 | 1.015744 | 0.00116 | 0.006534 | yes | up | 18.59 | 14.48 | 7.31 | 50.54 | 39.52 | 37.1 | 13.46 | 42.38667 |
| RHAG | 0.016 | -5.92431 | 0.001169 | 0.006571 | yes | down | 0.12 | 0.26 | 0.43 | 0 | 0 | 0 | 0.27 | 0 |
| LOC103349017 | 18.831 | 4.235004 | 0.001171 | 0.006576 | yes | up | 0.07 | 0 | 0.04 | 0.83 | 1.54 | 1.25 | 0.036667 | 1.206667 |
| LOC100347623 | 50.599 | 5.661033 | 0.001187 | 0.006653 | yes | up | 0 | 0 | 0 | 0.61 | 1.33 | 0.32 | 0 | 0.753333 |
| FFAR4 | 0.127 | -2.97576 | 0.00121 | 0.006768 | yes | down | 0.39 | 0.9 | 0.07 | 0.12 | 0.05 | 0.09 | 0.453333 | 0.086667 |
| PSD4 | 2.563 | 1.357825 | 0.001215 | 0.006789 | yes | up | 1.14 | 1.12 | 0.89 | 7.17 | 2.76 | 2.97 | 1.05 | 4.3 |
| SHROOM3 | 0.452 | -1.14546 | 0.00122 | 0.006812 | yes | down | 1.25 | 0.86 | 1.21 | 0.6 | 0.86 | 1.14 | 1.106667 | 0.866667 |
| AP2S1 | 0.495 | -1.01537 | 0.001227 | 0.006849 | yes | down | 45.66 | 55.49 | 57.35 | 49.72 | 41.14 | 33.22 | 52.83333 | 41.36 |
| ZNF74 | 2.44 | 1.286596 | 0.001228 | 0.006854 | yes | up | 2.26 | 1.02 | 1.74 | 4.26 | 7.22 | 7.87 | 1.673333 | 6.45 |
| MRPL36 | 0.386 | -1.37342 | 0.001237 | 0.006897 | yes | down | 2.3 | 2.31 | 2.44 | 1.19 | 1.23 | 2.03 | 2.35 | 1.483333 |
| LOC108176834 | 42.876 | 5.422103 | 0.001242 | 0.006923 | yes | up | 0 | 0 | 0.33 | 11.59 | 3.14 | 0.96 | 0.11 | 5.23 |
| LOC108176185 | 3.484 | 1.800613 | 0.001254 | 0.006979 | yes | up | 1.29 | 1.87 | 1.97 | 5.15 | 7.42 | 11.64 | 1.71 | 8.07 |
| PPFIA4 | 0.089 | -3.49598 | 0.001271 | 0.007059 | yes | down | 3.01 | 0.37 | 0.17 | 0.05 | 0.36 | 0.08 | 1.183333 | 0.163333 |
| LOC100352996 | 64.812 | 6.018192 | 0.001284 | 0.007117 | yes | up | 0.31 | 0 | 0 | 28.48 | 2.23 | 0.77 | 0.103333 | 10.49333 |
| GDPD5 | 2.262 | 1.177711 | 0.001287 | 0.007127 | yes | up | 2.1 | 1.97 | 1.1 | 3.9 | 7.92 | 6.46 | 1.723333 | 6.093333 |
| C1H11orf80 | 0.337 | -1.56758 | 0.001288 | 0.007131 | yes | down | 3.45 | 7.18 | 2.6 | 1.22 | 3.12 | 3.01 | 4.41 | 2.45 |
| ANKRD33B | 2.246 | 1.167203 | 0.00129 | 0.007135 | yes | up | 0.49 | 0.73 | 0.24 | 1.8 | 1.55 | 1.82 | 0.486667 | 1.723333 |
| RNASE1 | 5.093 | 2.348479 | 0.001293 | 0.007147 | yes | up | 0.57 | 0.41 | 0.11 | 1.91 | 3.14 | 4.21 | 0.363333 | 3.086667 |
| CDRT1 | 2.688 | 1.426445 | 0.001298 | 0.007167 | yes | up | 0.28 | 0.29 | 0.35 | 0.92 | 1.89 | 1.55 | 0.306667 | 1.453333 |
| CACNB4 | 0.385 | -1.37615 | 0.001307 | 0.007204 | yes | down | 1.01 | 1.89 | 0.7 | 1.21 | 3.72 | 1.48 | 1.2 | 2.136667 |
| LOC108176187 | 18.627 | 4.219334 | 0.001316 | 0.007248 | yes | up | 0 | 0.03 | 0.03 | 0.81 | 0.52 | 0.44 | 0.02 | 0.59 |
| GGH | 2.307 | 1.205813 | 0.001326 | 0.007289 | yes | up | 2.15 | 1.95 | 2.12 | 11.33 | 5.34 | 6.24 | 2.073333 | 7.636667 |
| LOC103345623 | 3.8 | 1.925982 | 0.001336 | 0.00733 | yes | up | 0.26 | 0.67 | 0.26 | 2.44 | 2.05 | 3.22 | 0.396667 | 2.57 |
| LOC108175719 | 0.018 | -5.78522 | 0.001343 | 0.00736 | yes | down | 0.58 | 0.78 | 1.24 | 0 | 0 | 0 | 0.866667 | 0 |
| AMHR2 | 8.285 | 3.050519 | 0.001352 | 0.007398 | yes | up | 0.03 | 0.05 | 0.04 | 0.26 | 0.54 | 0.51 | 0.04 | 0.436667 |
| PRX | 2.199 | 1.136692 | 0.001364 | 0.007455 | yes | up | 7 | 4.06 | 2.41 | 12.15 | 14.72 | 19 | 4.49 | 15.29 |
| LOC103348721 | 12.393 | 3.631397 | 0.001367 | 0.007464 | yes | up | 0.12 | 0.11 | 0.3 | 0.97 | 2.8 | 7.57 | 0.176667 | 3.78 |
| ANKRD45 | 3.806 | 1.928219 | 0.001367 | 0.007464 | yes | up | 0.31 | 0.35 | 0.24 | 2.61 | 1.55 | 1.09 | 0.3 | 1.75 |
| CCDC192 | 2.383 | 1.252985 | 0.001369 | 0.007469 | yes | up | 1.31 | 1.79 | 1.4 | 4.52 | 4.73 | 8.4 | 1.5 | 5.883333 |
| RXFP1 | 4.308 | 2.10711 | 0.001369 | 0.007469 | yes | up | 0.09 | 0.12 | 0.13 | 0.79 | 1.03 | 0.56 | 0.113333 | 0.793333 |
| NFKBID | 2.032 | 1.023029 | 0.001384 | 0.007542 | yes | up | 1.03 | 1 | 0.78 | 3.3 | 2.97 | 2.82 | 0.936667 | 3.03 |
| GGT6 | 0.266 | -1.91103 | 0.001388 | 0.007561 | yes | down | 1.37 | 1.13 | 3.69 | 0.95 | 0.5 | 0.94 | 2.063333 | 0.796667 |
| LOC108175795 | 4.954 | 2.308544 | 0.001395 | 0.007586 | yes | up | 0.31 | 0.12 | 0.19 | 1.18 | 1.18 | 2.68 | 0.206667 | 1.68 |
| CLEC4F | 3.549 | 1.827567 | 0.001395 | 0.007586 | yes | up | 2.72 | 1.83 | 1.11 | 19.06 | 7.45 | 4.65 | 1.886667 | 10.38667 |
| LOC108176740 | 24.46 | 4.612336 | 0.001399 | 0.007603 | yes | up | 0 | 0.11 | 0 | 0.68 | 0.96 | 2.87 | 0.036667 | 1.503333 |
| LOC108175473 | 2.203 | 1.139622 | 0.001404 | 0.007619 | yes | up | 0.65 | 1.14 | 0.69 | 2.43 | 2.64 | 3.64 | 0.826667 | 2.903333 |
| SLC44A3 | 6.332 | 2.662753 | 0.001404 | 0.007619 | yes | up | 0.35 | 0.1 | 0.4 | 5.64 | 2.28 | 0.78 | 0.283333 | 2.9 |
| CD37 | 5.255 | 2.393661 | 0.001407 | 0.00763 | yes | up | 1.12 | 1.35 | 1.57 | 25.69 | 3.29 | 5.27 | 1.346667 | 11.41667 |
| CPS1 | 4.545 | 2.184289 | 0.001429 | 0.007737 | yes | up | 0.14 | 0.05 | 0.12 | 0.52 | 1.22 | 0.42 | 0.103333 | 0.72 |
| C9 | 0.27 | -1.88801 | 0.001443 | 0.007802 | yes | down | 2.16 | 3.47 | 2.72 | 3.58 | 1.27 | 1.9 | 2.783333 | 2.25 |
| FMNL1 | 4.955 | 2.308853 | 0.001446 | 0.00781 | yes | up | 1.1 | 0.73 | 1.03 | 17.82 | 2.27 | 3.11 | 0.953333 | 7.733333 |
| LOC108177279 | 2.627 | 1.39326 | 0.001455 | 0.007854 | yes | up | 1.9 | 1.9 | 2.5 | 5.3 | 9.33 | 12.7 | 2.1 | 9.11 |
| LOC108176099 | 8.719 | 3.124205 | 0.001459 | 0.007873 | yes | up | 0.07 | 0.12 | 0 | 1.28 | 0.63 | 0.88 | 0.063333 | 0.93 |
| EGFL8 | 2.316 | 1.211805 | 0.001465 | 0.007894 | yes | up | 1.43 | 1.95 | 1.34 | 3.86 | 4.99 | 8.77 | 1.573333 | 5.873333 |
| ASPN | 0.467 | -1.09874 | 0.001466 | 0.007894 | yes | down | 63.61 | 42.68 | 27.27 | 22.17 | 40.66 | 25.2 | 44.52 | 29.34333 |
| PALM2 | 0.461 | -1.11587 | 0.001491 | 0.00802 | yes | down | 1.5 | 3.78 | 2.4 | 1.99 | 1.97 | 1.78 | 2.56 | 1.913333 |
| ZMYND12 | 3.363 | 1.749655 | 0.0015 | 0.008056 | yes | up | 0.34 | 0.41 | 0.44 | 1.91 | 2.01 | 2.62 | 0.396667 | 2.18 |
| ALPK1 | 2.992 | 1.58118 | 0.001504 | 0.00807 | yes | up | 0.39 | 0.52 | 0.37 | 3.42 | 1.03 | 1.68 | 0.426667 | 2.043333 |
| PTGIR | 2.547 | 1.348639 | 0.001521 | 0.008146 | yes | up | 0.34 | 0.37 | 0.17 | 1.44 | 1.06 | 1.03 | 0.293333 | 1.176667 |
| LOC103345531 | 36.092 | 5.173616 | 0.001522 | 0.008146 | yes | up | 0 | 0.03 | 0.02 | 1.01 | 1.89 | 0.03 | 0.016667 | 0.976667 |
| FCER1G | 4.814 | 2.26712 | 0.001523 | 0.008151 | yes | up | 7.84 | 10.24 | 9.88 | 166.81 | 20.85 | 27.48 | 9.32 | 71.71333 |
| KIFC2 | 2.29 | 1.195537 | 0.001526 | 0.008162 | yes | up | 1.1 | 0.79 | 0.78 | 2.8 | 3.36 | 3.59 | 0.89 | 3.25 |
| TMEM42 | 2.303 | 1.203676 | 0.001527 | 0.008169 | yes | up | 4.08 | 5.42 | 5.99 | 12.07 | 14.8 | 25.65 | 5.163333 | 17.50667 |
| URB2 | 0.446 | -1.16649 | 0.001539 | 0.00822 | yes | down | 1 | 1.11 | 0.68 | 0.91 | 0.46 | 0.59 | 0.93 | 0.653333 |
| LOC100350196 | 0.02 | -5.67686 | 0.001549 | 0.00827 | yes | down | 0.27 | 0.45 | 0.24 | 0 | 0 | 0 | 0.32 | 0 |
| FPR1 | 8.796 | 3.136788 | 0.001556 | 0.008295 | yes | up | 0.53 | 0.24 | 0.37 | 13.4 | 0.87 | 1.84 | 0.38 | 5.37 |
| DNAAF2 | 0.491 | -1.02495 | 0.001556 | 0.008295 | yes | down | 4.97 | 4.85 | 4.66 | 6.48 | 3.54 | 3.31 | 4.826667 | 4.443333 |
| STAT1 | 6.51 | 2.702698 | 0.001557 | 0.008295 | yes | up | 12.57 | 7.67 | 6.97 | 199.44 | 50.6 | 29.6 | 9.07 | 93.21333 |
| CD1D | 2.444 | 1.289295 | 0.001587 | 0.00844 | yes | up | 10.66 | 11.42 | 22.03 | 81.07 | 41.27 | 66.25 | 14.70333 | 62.86333 |
| RUNX1 | 6.876 | 2.781667 | 0.001589 | 0.008445 | yes | up | 0.14 | 0.26 | 0.04 | 2.51 | 0.59 | 1.66 | 0.146667 | 1.586667 |
| ARL9 | 3.487 | 1.80193 | 0.001599 | 0.008493 | yes | up | 1.16 | 0.45 | 0.48 | 5.2 | 2.33 | 2.95 | 0.696667 | 3.493333 |
| FBXO10 | 0.365 | -1.45265 | 0.001619 | 0.008583 | yes | down | 6.56 | 2.48 | 3.54 | 1.56 | 1.91 | 3.44 | 4.193333 | 2.303333 |
| MBOAT2 | 0.32 | -1.64474 | 0.001622 | 0.008593 | yes | down | 3.91 | 1.79 | 0.87 | 0.74 | 1.23 | 1.53 | 2.19 | 1.166667 |
| LOC100344629 | 3.899 | 1.962931 | 0.001627 | 0.008613 | yes | up | 1.22 | 1.12 | 0.7 | 12.33 | 2.64 | 2.89 | 1.013333 | 5.953333 |
| C18H10orf35 | 6.816 | 2.768896 | 0.001627 | 0.008613 | yes | up | 1.31 | 0.97 | 1.13 | 2.31 | 3.62 | 29.9 | 1.136667 | 11.94333 |
| SLITRK3 | 9.815 | 3.294981 | 0.001628 | 0.008614 | yes | up | 0 | 0.02 | 0.02 | 0.19 | 0.19 | 0.2 | 0.013333 | 0.193333 |
| PKDREJ | 0.436 | -1.19767 | 0.001643 | 0.008683 | yes | down | 0.53 | 0.47 | 0.35 | 0.34 | 0.34 | 0.23 | 0.45 | 0.303333 |
| MYPOP | 0.336 | -1.57517 | 0.00165 | 0.008714 | yes | down | 1.34 | 1.75 | 1.05 | 0.46 | 0.76 | 1.01 | 1.38 | 0.743333 |
| LOC100340040 | 11.156 | 3.479801 | 0.001652 | 0.008718 | yes | up | 0.2 | 0 | 0.03 | 0.76 | 2.04 | 0.74 | 0.076667 | 1.18 |
| N6AMT1 | 3.017 | 1.593165 | 0.001677 | 0.008835 | yes | up | 1.76 | 1.07 | 2.38 | 7.67 | 5.75 | 12.48 | 1.736667 | 8.633333 |
| CASP7 | 2.457 | 1.297155 | 0.001713 | 0.008994 | yes | up | 3.92 | 4.85 | 3.71 | 27.51 | 9.64 | 11.89 | 4.16 | 16.34667 |
| RASSF9 | 0.368 | -1.44144 | 0.001718 | 0.009021 | yes | down | 1.37 | 2.7 | 2.91 | 1.52 | 1.7 | 0.94 | 2.326667 | 1.386667 |
| PSD2 | 0.089 | -3.48347 | 0.001719 | 0.009021 | yes | down | 0.15 | 0.14 | 0.13 | 0.05 | 0.01 | 0 | 0.14 | 0.02 |
| DUSP4 | 0.288 | -1.79606 | 0.001723 | 0.009035 | yes | down | 3.7 | 8.9 | 1.59 | 1.7 | 1.62 | 3.05 | 4.73 | 2.123333 |
| FA2H | 3.249 | 1.699883 | 0.001742 | 0.009126 | yes | up | 0.73 | 0.31 | 0.24 | 3.31 | 1.42 | 2.58 | 0.426667 | 2.436667 |
| PDXP | 0.499 | -1.00298 | 0.001742 | 0.009126 | yes | down | 3.78 | 3.27 | 3.26 | 2.5 | 2.12 | 3.75 | 3.436667 | 2.79 |
| NSUN3 | 0.414 | -1.27219 | 0.001747 | 0.009144 | yes | down | 0.85 | 0.97 | 1.11 | 0.51 | 0.7 | 0.76 | 0.976667 | 0.656667 |
| LOC100355643 | 0.351 | -1.50874 | 0.001762 | 0.009217 | yes | down | 3.41 | 2.67 | 2.27 | 2.39 | 1.19 | 1.03 | 2.783333 | 1.536667 |
| MROH7 | 4.082 | 2.029403 | 0.001764 | 0.009223 | yes | up | 0.13 | 0.09 | 0.12 | 0.43 | 1.06 | 0.72 | 0.113333 | 0.736667 |
| LOC100352631 | 0.196 | -2.34774 | 0.001768 | 0.009239 | yes | down | 0.68 | 0.79 | 0.48 | 0.17 | 0.26 | 0.17 | 0.65 | 0.2 |
| ST13 | 0.104 | -3.26237 | 0.001794 | 0.009352 | yes | down | 85.69 | 87.25 | 75.19 | 35.03 | 6.07 | 10.24 | 82.71 | 17.11333 |
| PRAM1 | 9.462 | 3.242082 | 0.0018 | 0.00938 | yes | up | 0.1 | 0.06 | 0.09 | 3.11 | 0.3 | 0.38 | 0.083333 | 1.263333 |
| C11H5orf30 | 0.458 | -1.12688 | 0.001819 | 0.009467 | yes | down | 3.3 | 4.2 | 6.07 | 2.93 | 3.72 | 3.56 | 4.523333 | 3.403333 |
| LOC100339055 | 2.404 | 1.265491 | 0.001828 | 0.009504 | yes | up | 0.64 | 0.89 | 0.46 | 1.56 | 2.9 | 2.96 | 0.663333 | 2.473333 |
| FHL1 | 0.449 | -1.15476 | 0.001829 | 0.009511 | yes | down | 1585.86 | 934.61 | 506.07 | 464.35 | 748.82 | 885.11 | 1008.847 | 699.4267 |
| CTPS1 | 0.321 | -1.64025 | 0.001832 | 0.009521 | yes | down | 2.27 | 2.36 | 1.16 | 1.68 | 0.59 | 0.63 | 1.93 | 0.966667 |
| SLC25A4 | 0.381 | -1.39367 | 0.001853 | 0.009614 | yes | down | 652.87 | 244 | 177.25 | 138.93 | 254.77 | 237.22 | 358.04 | 210.3067 |
| SELPLG | 4.485 | 2.165255 | 0.001861 | 0.009649 | yes | up | 1.43 | 1.25 | 1.19 | 20.94 | 2.73 | 4.07 | 1.29 | 9.246667 |
| CAMK2N1 | 2.02 | 1.014383 | 0.00187 | 0.009692 | yes | up | 16 | 21.95 | 5.58 | 11.04 | 21.47 | 37.28 | 14.51 | 23.26333 |
| RDH8 | 4.318 | 2.110344 | 0.001895 | 0.009795 | yes | up | 0.58 | 0.42 | 0.48 | 1.04 | 4.85 | 4.21 | 0.493333 | 3.366667 |
| GRM8 | 0.257 | -1.96036 | 0.001938 | 0.009983 | yes | down | 0.29 | 0.25 | 0.36 | 0.1 | 0.14 | 0.14 | 0.3 | 0.126667 |
| COL1A2 | 2.275 | 1.185568 | 0.001957 | 0.010053 | yes | up | 460.78 | 250.11 | 129.76 | 1005.36 | 1129.82 | 811.7 | 280.2167 | 982.2933 |
| THNSL2 | 4.116 | 2.041138 | 0.001963 | 0.010081 | yes | up | 0.82 | 0.27 | 0.19 | 1.28 | 1.98 | 3.43 | 0.426667 | 2.23 |
| CCNL1 | 2.011 | 1.007648 | 0.001975 | 0.01013 | yes | up | 14.92 | 28.8 | 12.45 | 55.18 | 54.74 | 70.81 | 18.72333 | 60.24333 |
| ADH2-1 | 2.884 | 1.528195 | 0.001997 | 0.010235 | yes | up | 2.3 | 2.25 | 4.08 | 6.71 | 14.43 | 20.2 | 2.876667 | 13.78 |
| LOC100349257 | 14.072 | 3.814798 | 0.001998 | 0.010235 | yes | up | 1.39 | 1.08 | 0.94 | 66.56 | 5.33 | 3.04 | 1.136667 | 24.97667 |
| CNTNAP1 | 2.895 | 1.533454 | 0.002001 | 0.01024 | yes | up | 0.3 | 0.14 | 0.1 | 0.78 | 0.85 | 0.85 | 0.18 | 0.826667 |
| BATF2 | 2.77 | 1.470019 | 0.00201 | 0.010278 | yes | up | 2.3 | 2.2 | 1.96 | 17.02 | 5.58 | 5.95 | 2.153333 | 9.516667 |
| CYP2B4 | 5.566 | 2.476719 | 0.002019 | 0.01032 | yes | up | 0.9 | 5.5 | 0.24 | 15.49 | 20.75 | 19.36 | 2.213333 | 18.53333 |
| CD68 | 15.023 | 3.909061 | 0.002042 | 0.010421 | yes | up | 1.37 | 1.26 | 1.22 | 81.43 | 2.91 | 7.06 | 1.283333 | 30.46667 |
| LOC103350794 | 0.452 | -1.14407 | 0.002044 | 0.010427 | yes | down | 5.62 | 6.88 | 7.8 | 4.89 | 3.34 | 6.94 | 6.766667 | 5.056667 |
| RELB | 4.279 | 2.097199 | 0.002052 | 0.010455 | yes | up | 0.8 | 0.39 | 0.6 | 7.57 | 2.37 | 2.27 | 0.596667 | 4.07 |
| MPZ | 2.76 | 1.464439 | 0.002055 | 0.010468 | yes | up | 145.88 | 93.83 | 33.01 | 226.33 | 355.38 | 587.05 | 90.90667 | 389.5867 |
| LOC103348916 | 3.077 | 1.621503 | 0.002077 | 0.010571 | yes | up | 1.15 | 0.51 | 1.41 | 4.44 | 5.39 | 6.38 | 1.023333 | 5.403333 |
| LOC100349247 | 6.607 | 2.724057 | 0.002087 | 0.010615 | yes | up | 12.75 | 14.05 | 15.68 | 337.25 | 44.29 | 62.56 | 14.16 | 148.0333 |
| CD80 | 6.292 | 2.653544 | 0.002106 | 0.010701 | yes | up | 0.17 | 0.41 | 0.16 | 4.9 | 0.6 | 1.08 | 0.246667 | 2.193333 |
| GJA3 | 17.364 | 4.117997 | 0.002122 | 0.010765 | yes | up | 0.02 | 0.04 | 0.04 | 2.64 | 0.19 | 0.13 | 0.033333 | 0.986667 |
| CHRNA1 | 0.273 | -1.87534 | 0.002136 | 0.010828 | yes | down | 5.24 | 3.62 | 1 | 0.68 | 1.73 | 1.71 | 3.286667 | 1.373333 |
| CYTL1 | 6.406 | 2.679434 | 0.002142 | 0.010848 | yes | up | 0.18 | 0.24 | 0.15 | 1.45 | 1.53 | 2.97 | 0.19 | 1.983333 |
| VWA3A | 2.311 | 1.208804 | 0.002148 | 0.010874 | yes | up | 0.44 | 0.94 | 0.62 | 2.12 | 2.25 | 3.36 | 0.666667 | 2.576667 |
| SPTBN5 | 4.579 | 2.194936 | 0.002151 | 0.010884 | yes | up | 0.11 | 0.07 | 0.03 | 0.28 | 0.57 | 0.6 | 0.07 | 0.483333 |
| LOC100351519 | 0.268 | -1.8987 | 0.002167 | 0.010942 | yes | down | 64.21 | 46.23 | 46.61 | 13.57 | 45.27 | 6.29 | 52.35 | 21.71 |
| CCDC68 | 0.321 | -1.64043 | 0.002167 | 0.010942 | yes | down | 1.86 | 1.1 | 2.59 | 0.71 | 0.89 | 1.36 | 1.85 | 0.986667 |
| RGS1 | 13.014 | 3.70194 | 0.002169 | 0.010947 | yes | up | 0.05 | 0.15 | 0.05 | 3.9 | 0.49 | 0.68 | 0.083333 | 1.69 |
| MDFI | 0.267 | -1.90245 | 0.00217 | 0.010949 | yes | down | 2.2 | 2.11 | 3.16 | 0.6 | 1 | 1.78 | 2.49 | 1.126667 |
| HERC5 | 3.259 | 1.704465 | 0.002176 | 0.010979 | yes | up | 0.82 | 1.21 | 1.12 | 8.49 | 3.45 | 2.76 | 1.05 | 4.9 |
| LOC100353012 | 65.166 | 6.026039 | 0.002202 | 0.0111 | yes | up | 0 | 0 | 0 | 0.26 | 0.01 | 0.35 | 0 | 0.206667 |
| LOC103347389 | 50.195 | 5.649458 | 0.002204 | 0.011107 | yes | up | 0 | 0 | 0.07 | 18.23 | 0.68 | 2.66 | 0.023333 | 7.19 |
| PDE6H | 3.292 | 1.718938 | 0.002211 | 0.011138 | yes | up | 0.34 | 0.34 | 0.2 | 1.39 | 1.68 | 1.74 | 0.293333 | 1.603333 |
| CPXM2 | 3.664 | 1.873327 | 0.002234 | 0.011235 | yes | up | 0.57 | 0.3 | 0.06 | 1 | 1.76 | 1.94 | 0.31 | 1.566667 |
| LOC103349204 | 0.192 | -2.37941 | 0.002239 | 0.011257 | yes | down | 4.19 | 5.97 | 9.33 | 3.73 | 0.44 | 1.45 | 6.496667 | 1.873333 |
| MNDA | 2.235 | 1.160542 | 0.002244 | 0.011272 | yes | up | 10.81 | 21.89 | 10.88 | 79.85 | 40.71 | 29.54 | 14.52667 | 50.03333 |
| RSPH14 | 0.096 | -3.37477 | 0.002247 | 0.011282 | yes | down | 1.52 | 1.18 | 0.91 | 0.27 | 0.32 | 0 | 1.203333 | 0.196667 |
| KIAA1211L | 2.877 | 1.524567 | 0.002282 | 0.011445 | yes | up | 0.71 | 0.53 | 0.33 | 3.87 | 1.36 | 1.92 | 0.523333 | 2.383333 |
| RBM43 | 2.047 | 1.033426 | 0.00232 | 0.011621 | yes | up | 1.31 | 1.25 | 1.05 | 5.34 | 3.03 | 3.61 | 1.203333 | 3.993333 |
| DUSP2 | 8.651 | 3.112946 | 0.002324 | 0.011639 | yes | up | 0.08 | 0.07 | 0.07 | 1.98 | 0.58 | 0.51 | 0.073333 | 1.023333 |
| GBAS | 0.474 | -1.07666 | 0.002329 | 0.011658 | yes | down | 102.83 | 50.86 | 34.49 | 37.96 | 48.25 | 52.93 | 62.72667 | 46.38 |
| LOC103345817 | 13.921 | 3.799153 | 0.002355 | 0.011766 | yes | up | 0 | 0.1 | 0 | 0.83 | 0.76 | 0.58 | 0.033333 | 0.723333 |
| GJB3 | 0.385 | -1.37576 | 0.002375 | 0.011849 | yes | down | 7.24 | 8.66 | 16.71 | 4.77 | 6.71 | 9.59 | 10.87 | 7.023333 |
| SCN7A | 2.144 | 1.100205 | 0.002384 | 0.011868 | yes | up | 1.94 | 2.87 | 0.9 | 5.7 | 5.35 | 8.16 | 1.903333 | 6.403333 |
| ENHO | 2.821 | 1.496211 | 0.002397 | 0.011919 | yes | up | 2.24 | 1.17 | 1.11 | 4.9 | 5.43 | 9.98 | 1.506667 | 6.77 |
| SOCS3 | 2.79 | 1.480052 | 0.002422 | 0.012031 | yes | up | 2.7 | 1.38 | 1.3 | 10.09 | 3.58 | 10.3 | 1.793333 | 7.99 |
| SLC2A4 | 0.446 | -1.16641 | 0.002422 | 0.012031 | yes | down | 21.66 | 19.3 | 23.74 | 8.42 | 24.99 | 16.37 | 21.56667 | 16.59333 |
| CTSK | 3.098 | 1.631537 | 0.002425 | 0.012042 | yes | up | 33.86 | 34.44 | 20.66 | 290.4 | 53.45 | 90.47 | 29.65333 | 144.7733 |
| BAALC | 0.322 | -1.63689 | 0.002435 | 0.012074 | yes | down | 0.74 | 0.49 | 0.84 | 0.46 | 0.29 | 0.32 | 0.69 | 0.356667 |
| SNCG | 2.093 | 1.065631 | 0.002435 | 0.012074 | yes | up | 15.48 | 12.76 | 14.11 | 27.01 | 53.14 | 62.04 | 14.11667 | 47.39667 |
| LOC103349365 | 5.236 | 2.388556 | 0.00244 | 0.012091 | yes | up | 0.07 | 0.62 | 0.28 | 2.51 | 3.23 | 2.18 | 0.323333 | 2.64 |
| LOC103347895 | 3.542 | 1.824555 | 0.002452 | 0.012133 | yes | up | 0.12 | 0.09 | 0.04 | 0.56 | 0.44 | 0.35 | 0.083333 | 0.45 |
| LOC108176569 | 16.293 | 4.026153 | 0.002452 | 0.012133 | yes | up | 0.04 | 0.02 | 0 | 0.04 | 0.86 | 1.19 | 0.02 | 0.696667 |
| KCNA4 | 0.016 | -6.0074 | 0.002455 | 0.012143 | yes | down | 0.17 | 0.12 | 0.01 | 0 | 0 | 0 | 0.1 | 0 |
| LOC100350373 | 28.816 | 4.848821 | 0.002478 | 0.012247 | yes | up | 0 | 0.04 | 0 | 0.29 | 0.61 | 1.31 | 0.013333 | 0.736667 |
| CST6 | 3.184 | 1.670923 | 0.00248 | 0.012254 | yes | up | 4.74 | 3.1 | 1.54 | 19.42 | 17.51 | 8.45 | 3.126667 | 15.12667 |
| ADAM23 | 4.358 | 2.123828 | 0.002485 | 0.012265 | yes | up | 0.87 | 0.26 | 0.1 | 1.46 | 3.79 | 4.15 | 0.41 | 3.133333 |
| SKP2 | 0.498 | -1.0059 | 0.002502 | 0.012342 | yes | down | 3.07 | 2.19 | 1.48 | 1.64 | 1.9 | 2.01 | 2.246667 | 1.85 |
| LOC103350037 | 4.555 | 2.187364 | 0.002532 | 0.01245 | yes | up | 0.66 | 0.69 | 0.34 | 8.7 | 1.36 | 1.99 | 0.563333 | 4.016667 |
| KIAA1107 | 2.177 | 1.122178 | 0.002533 | 0.01245 | yes | up | 0.23 | 0.42 | 0.3 | 1.13 | 1.25 | 0.91 | 0.316667 | 1.096667 |
| LOC100009135 | 0.04 | -4.64342 | 0.002545 | 0.012506 | yes | down | 0.24 | 8.56 | 2.37 | 0.21 | 0 | 0.54 | 3.723333 | 0.25 |
| CSK | 2.086 | 1.060993 | 0.002549 | 0.012519 | yes | up | 15 | 16 | 12.43 | 72.92 | 29.74 | 42.29 | 14.47667 | 48.31667 |
| LOC100356524 | 7.438 | 2.894837 | 0.002571 | 0.01261 | yes | up | 0 | 0.16 | 0.07 | 0.75 | 1.08 | 0.94 | 0.076667 | 0.923333 |
| LOC108176404 | 64.709 | 6.015896 | 0.002572 | 0.01261 | yes | up | 0 | 0 | 0 | 15.29 | 1.38 | 0.92 | 0 | 5.863333 |
| LOC103347127 | 0.243 | -2.04261 | 0.002591 | 0.012683 | yes | down | 10.79 | 11.98 | 5.53 | 2.43 | 5.29 | 2.67 | 9.433333 | 3.463333 |
| GFRA3 | 2.993 | 1.581805 | 0.002623 | 0.012817 | yes | up | 0.29 | 0.31 | 0.2 | 1.54 | 0.88 | 1.38 | 0.266667 | 1.266667 |
| PLEKHH2 | 2.942 | 1.556724 | 0.002637 | 0.012877 | yes | up | 0.68 | 0.32 | 0.75 | 4.53 | 1.73 | 1.93 | 0.583333 | 2.73 |
| CYTIP | 4.985 | 2.317671 | 0.002648 | 0.012919 | yes | up | 0.57 | 0.53 | 0.59 | 9.88 | 0.96 | 2.71 | 0.563333 | 4.516667 |
| LOC108176066 | 17.884 | 4.160613 | 0.002658 | 0.01296 | yes | up | 0 | 0.05 | 0.05 | 0.4 | 1.29 | 1.19 | 0.033333 | 0.96 |
| PODN | 2.16 | 1.110788 | 0.002659 | 0.01296 | yes | up | 5.16 | 11.43 | 4.06 | 19.65 | 21.19 | 29.46 | 6.883333 | 23.43333 |
| LOC103345165 | 6.894 | 2.785413 | 0.002663 | 0.012976 | yes | up | 0.11 | 0.26 | 0.2 | 0.52 | 2.45 | 3.5 | 0.19 | 2.156667 |
| LOC100009166 | 0.272 | -1.88086 | 0.002685 | 0.013058 | yes | down | 2.68 | 4.97 | 6.58 | 2.13 | 0.96 | 3.3 | 4.743333 | 2.13 |
| SYTL3 | 11.731 | 3.552206 | 0.002686 | 0.013062 | yes | up | 0.04 | 0.04 | 0.03 | 1.41 | 0.25 | 0.13 | 0.036667 | 0.596667 |
| FUCA2 | 2.014 | 1.010128 | 0.002703 | 0.013135 | yes | up | 1.38 | 0.84 | 0.68 | 3.85 | 2.75 | 2.59 | 0.966667 | 3.063333 |
| LOC100355114 | 0.474 | -1.07793 | 0.002748 | 0.013337 | yes | down | 4.46 | 2.86 | 2.53 | 3.5 | 1.83 | 2.09 | 3.283333 | 2.473333 |
| HSPA12A | 2.716 | 1.441244 | 0.002752 | 0.013347 | yes | up | 2.49 | 2.18 | 2.37 | 3.13 | 9.91 | 16.15 | 2.346667 | 9.73 |
| TCTEX1D2 | 0.49 | -1.03047 | 0.002789 | 0.013512 | yes | down | 4.94 | 6.21 | 3.92 | 4.86 | 3.52 | 3.11 | 5.023333 | 3.83 |
| LOC108176170 | 95.623 | 6.579287 | 0.00281 | 0.013596 | yes | up | 0 | 0 | 0 | 7.92 | 0.1 | 0.25 | 0 | 2.756667 |
| LOC100348108 | 4.518 | 2.175738 | 0.002826 | 0.013658 | yes | up | 1.07 | 1.34 | 1.21 | 20.3 | 2.59 | 3.32 | 1.206667 | 8.736667 |
| MAFF | 3.41 | 1.769717 | 0.002844 | 0.013735 | yes | up | 1.61 | 1.27 | 1.04 | 7.17 | 4.84 | 9.26 | 1.306667 | 7.09 |
| DCSTAMP | 5.189 | 2.375522 | 0.002864 | 0.013812 | yes | up | 0.21 | 0.28 | 0.06 | 3.06 | 0.64 | 0.75 | 0.183333 | 1.483333 |
| LOC100346219 | 13.247 | 3.72756 | 0.002868 | 0.013823 | yes | up | 0.15 | 0.53 | 0 | 10.43 | 4.76 | 0.5 | 0.226667 | 5.23 |
| LOC103351479 | 2.224 | 1.15298 | 0.002881 | 0.013871 | yes | up | 1.19 | 1.82 | 1.02 | 5.95 | 4.1 | 4.28 | 1.343333 | 4.776667 |
| L3MBTL1 | 3.642 | 1.864602 | 0.002908 | 0.013986 | yes | up | 0.12 | 0.2 | 0.07 | 0.46 | 0.91 | 0.58 | 0.13 | 0.65 |
| WT1 | 11.738 | 3.553153 | 0.002913 | 0.014 | yes | up | 0.15 | 0.08 | 0 | 0.52 | 0.36 | 3.53 | 0.076667 | 1.47 |
| LOC103352041 | 6.892 | 2.784857 | 0.002966 | 0.014232 | yes | up | 0.02 | 0.06 | 0.04 | 0.29 | 0.48 | 0.63 | 0.04 | 0.466667 |
| TMEM189 | 0.493 | -1.02077 | 0.002967 | 0.014234 | yes | down | 15.47 | 17.62 | 15.68 | 11.75 | 13.53 | 12.68 | 16.25667 | 12.65333 |
| LDLR | 2.06 | 1.042927 | 0.00297 | 0.01424 | yes | up | 2.55 | 2.06 | 3.26 | 8.43 | 11.1 | 6.7 | 2.623333 | 8.743333 |
| LOC100340132 | 0.498 | -1.0055 | 0.002979 | 0.014278 | yes | down | 6.56 | 7.62 | 5.71 | 5.74 | 5.99 | 3.73 | 6.63 | 5.153333 |
| OSBPL7 | 0.461 | -1.11829 | 0.002979 | 0.014278 | yes | down | 10.5 | 12.66 | 12.77 | 7.82 | 12.81 | 5.37 | 11.97667 | 8.666667 |
| TRNAK-CUU | 0.204 | -2.2936 | 0.002987 | 0.014311 | yes | down | 1.49 | 2.29 | 2.3 | 0.96 | 0.53 | 0.52 | 2.026667 | 0.67 |
| RSAD1 | 0.233 | -2.10045 | 0.003005 | 0.01439 | yes | down | 1.72 | 1.88 | 1.04 | 0.13 | 0.97 | 0.59 | 1.546667 | 0.563333 |
| LOC100351957 | 25.218 | 4.656354 | 0.003013 | 0.014426 | yes | up | 0 | 0.07 | 0 | 0.56 | 1.01 | 1.51 | 0.023333 | 1.026667 |
| LOC100339205 | 4.916 | 2.297356 | 0.003025 | 0.014474 | yes | up | 0.26 | 0.42 | 0.1 | 1.17 | 1.49 | 3.31 | 0.26 | 1.99 |
| PITPNC1 | 0.49 | -1.03021 | 0.003031 | 0.014496 | yes | down | 5.83 | 6.02 | 8.46 | 4.77 | 5.21 | 6.29 | 6.77 | 5.423333 |
| LOC103349053 | 2.258 | 1.174754 | 0.003033 | 0.014503 | yes | up | 7.37 | 16.22 | 10.61 | 39.28 | 38.3 | 45.62 | 11.4 | 41.06667 |
| LOC108176107 | 0.386 | -1.37507 | 0.003035 | 0.014505 | yes | down | 4.09 | 2.61 | 2.23 | 0.85 | 1.79 | 2.86 | 2.976667 | 1.833333 |
| MCOLN2 | 18.227 | 4.187967 | 0.003052 | 0.014578 | yes | up | 0.07 | 0.06 | 0.02 | 4.01 | 0.04 | 0.28 | 0.05 | 1.443333 |
| MUM1L1 | 0.158 | -2.66108 | 0.003126 | 0.014862 | yes | down | 2.26 | 0.42 | 0.16 | 0.14 | 0.33 | 0.2 | 0.946667 | 0.223333 |
| LOC100351707 | 0.351 | -1.51072 | 0.003149 | 0.014958 | yes | down | 1.62 | 2.45 | 2.01 | 0.56 | 1.73 | 1.11 | 2.026667 | 1.133333 |
| LOC103347164 | 26.926 | 4.750903 | 0.003154 | 0.014974 | yes | up | 0 | 0.02 | 0 | 0.09 | 0.34 | 0.29 | 0.006667 | 0.24 |
| CSF1R | 2.963 | 1.566817 | 0.003156 | 0.014982 | yes | up | 6.51 | 5.55 | 4.77 | 52.5 | 10.34 | 16.79 | 5.61 | 26.54333 |
| LOC103350467 | 0.456 | -1.1336 | 0.003161 | 0.015 | yes | down | 2.64 | 2.42 | 2.47 | 1.09 | 2.27 | 2.1 | 2.51 | 1.82 |
| LOC103346247 | 7.018 | 2.81108 | 0.003174 | 0.015041 | yes | up | 0.15 | 0.18 | 0.3 | 5.27 | 0.84 | 1.13 | 0.21 | 2.413333 |
| ITGA9 | 0.476 | -1.07105 | 0.003181 | 0.015061 | yes | down | 4.45 | 3.24 | 3.02 | 3.07 | 3.1 | 1.84 | 3.57 | 2.67 |
| SFXN2 | 0.386 | -1.37434 | 0.003215 | 0.015201 | yes | down | 2.23 | 3.67 | 5.26 | 1.78 | 1.66 | 3.13 | 3.72 | 2.19 |
| LOC103351553 | 4.984 | 2.317253 | 0.003215 | 0.015201 | yes | up | 0.12 | 0.09 | 0.05 | 0.78 | 0.62 | 0.58 | 0.086667 | 0.66 |
| AZGP1 | 2.423 | 1.276525 | 0.003218 | 0.015206 | yes | up | 1.24 | 0.94 | 0.85 | 4.85 | 2.67 | 3.51 | 1.01 | 3.676667 |
| CCL2 | 4.945 | 2.305841 | 0.003236 | 0.015272 | yes | up | 8.49 | 2.83 | 0.61 | 48.31 | 13.14 | 24.86 | 3.976667 | 28.77 |
| PLCH1 | 3.695 | 1.885432 | 0.003279 | 0.015449 | yes | up | 0.06 | 0.03 | 0.04 | 0.24 | 0.27 | 0.31 | 0.043333 | 0.273333 |
| ADGRL1 | 0.382 | -1.38668 | 0.003279 | 0.015449 | yes | down | 0.48 | 0.65 | 0.29 | 0.3 | 0.36 | 0.18 | 0.473333 | 0.28 |
| ADAMTS14 | 0.288 | -1.79363 | 0.00328 | 0.015449 | yes | down | 0.28 | 0.26 | 0.45 | 0.21 | 0.16 | 0.09 | 0.33 | 0.153333 |
| DCDC2C | 0.064 | -3.96309 | 0.003322 | 0.015621 | yes | down | 0.66 | 42.49 | 4.28 | 1.1 | 1.16 | 2.91 | 15.81 | 1.723333 |
| PSME1 | 2.967 | 1.568823 | 0.003323 | 0.015621 | yes | up | 26.57 | 19.83 | 13.77 | 194.49 | 46.7 | 49.79 | 20.05667 | 96.99333 |
| GABRB3 | 0.195 | -2.35753 | 0.003324 | 0.015622 | yes | down | 0.44 | 0.71 | 0.26 | 0.05 | 0.06 | 0.34 | 0.47 | 0.15 |
| TP53I3 | 2.188 | 1.129876 | 0.003333 | 0.015656 | yes | up | 1.14 | 1.99 | 1.44 | 5 | 4.19 | 6.99 | 1.523333 | 5.393333 |
| LOC103352342 | 5.729 | 2.518338 | 0.003345 | 0.015708 | yes | up | 0.02 | 0.01 | 0.03 | 0.15 | 0.23 | 0.18 | 0.02 | 0.186667 |
| MAGED1 | 0.421 | -1.24886 | 0.003352 | 0.015734 | yes | down | 19.14 | 8.43 | 5.59 | 6.24 | 8.95 | 6.31 | 11.05333 | 7.166667 |
| WNT11 | 2.436 | 1.284627 | 0.003355 | 0.01574 | yes | up | 1.57 | 1.45 | 0.6 | 3.11 | 4.19 | 7.52 | 1.206667 | 4.94 |
| LOC103351558 | 2.356 | 1.236481 | 0.003364 | 0.01577 | yes | up | 1.44 | 1.57 | 1.03 | 2.71 | 6.26 | 5.77 | 1.346667 | 4.913333 |
| LCN2 | 0.136 | -2.88246 | 0.003448 | 0.016105 | yes | down | 0.72 | 1.16 | 1.86 | 0.58 | 0.07 | 0.18 | 1.246667 | 0.276667 |
| TM7SF2 | 2.331 | 1.221194 | 0.003535 | 0.016443 | yes | up | 2.49 | 1.77 | 0.85 | 4.09 | 6.77 | 6.4 | 1.703333 | 5.753333 |
| MAPT | 4.288 | 2.100168 | 0.003584 | 0.016647 | yes | up | 3.17 | 0.36 | 0.46 | 3.34 | 10.51 | 6.73 | 1.33 | 6.86 |
| CSF2RB | 4.633 | 2.211868 | 0.003595 | 0.016681 | yes | up | 0.44 | 0.85 | 0.72 | 11.47 | 1.28 | 2.12 | 0.67 | 4.956667 |
| FSCN1 | 0.494 | -1.01795 | 0.003601 | 0.016698 | yes | down | 29.59 | 28.32 | 22.93 | 11.39 | 29.16 | 22.76 | 26.94667 | 21.10333 |
| LOC100347782 | 0.263 | -1.92752 | 0.003601 | 0.016698 | yes | down | 3.13 | 4.31 | 6.75 | 1.86 | 1.99 | 2.38 | 4.73 | 2.076667 |
| ERAP1 | 2.457 | 1.297033 | 0.003623 | 0.01679 | yes | up | 7.81 | 14.6 | 12.28 | 45.95 | 19.06 | 17.23 | 11.56333 | 27.41333 |
| LOC100356308 | 16.742 | 4.065432 | 0.003624 | 0.01679 | yes | up | 0.08 | 0 | 0 | 0.68 | 0.45 | 0.95 | 0.026667 | 0.693333 |
| LOC108176233 | 25.574 | 4.676581 | 0.003632 | 0.016814 | yes | up | 0.18 | 0 | 0 | 1.27 | 0.39 | 5.23 | 0.06 | 2.296667 |
| LOC100352215 | 23.1 | 4.529827 | 0.003639 | 0.016838 | yes | up | 0 | 0.02 | 0 | 0.42 | 0.31 | 0.23 | 0.006667 | 0.32 |
| LOC103345465 | 2.516 | 1.3314 | 0.003641 | 0.01684 | yes | up | 0.36 | 0.41 | 0.41 | 1.66 | 1.26 | 1.55 | 0.393333 | 1.49 |
| LOC108176017 | 0.119 | -3.0717 | 0.003663 | 0.016926 | yes | down | 8.01 | 0.96 | 2.34 | 0.08 | 0.43 | 1.55 | 3.77 | 0.686667 |
| AHSP | 0.241 | -2.05326 | 0.003667 | 0.016937 | yes | down | 3.23 | 9.22 | 3.9 | 1.52 | 1.18 | 3.7 | 5.45 | 2.133333 |
| LOC100356012 | 2.303 | 1.203278 | 0.003698 | 0.017062 | yes | up | 3.93 | 7.56 | 3.95 | 30.7 | 13.25 | 13.74 | 5.146667 | 19.23 |
| TCEAL7 | 0.029 | -5.12126 | 0.003747 | 0.017262 | yes | down | 93.55 | 13.97 | 0.54 | 0.09 | 2.58 | 2 | 36.02 | 1.556667 |
| SLC16A2 | 0.411 | -1.28382 | 0.003754 | 0.017291 | yes | down | 9.42 | 24.62 | 6.05 | 7.25 | 8.05 | 10.13 | 13.36333 | 8.476667 |
| TMEM38B | 0.336 | -1.57411 | 0.003756 | 0.017294 | yes | down | 10.58 | 4.77 | 2.91 | 1.61 | 4.23 | 3.62 | 6.086667 | 3.153333 |
| PARM1 | 2.047 | 1.033862 | 0.003773 | 0.017352 | yes | up | 1.72 | 1.52 | 1.33 | 6.11 | 2.59 | 6.4 | 1.523333 | 5.033333 |
| LOC100358140 | 0.436 | -1.1972 | 0.003783 | 0.01738 | yes | down | 5.71 | 8.2 | 9.04 | 5.89 | 4.19 | 6.22 | 7.65 | 5.433333 |
| LOC103352262 | 6.401 | 2.678285 | 0.003784 | 0.01738 | yes | up | 0.06 | 0.06 | 0.04 | 0.4 | 0.98 | 0.33 | 0.053333 | 0.57 |
| CLEC2L | 0.126 | -2.98967 | 0.003793 | 0.017414 | yes | down | 1.35 | 0.53 | 0.39 | 0.06 | 0.23 | 0.15 | 0.756667 | 0.146667 |
| FRMPD4 | 0.326 | -1.61919 | 0.003799 | 0.017432 | yes | down | 0.16 | 0.23 | 0.22 | 0.08 | 0.08 | 0.17 | 0.203333 | 0.11 |
| LOC100341993 | 0.361 | -1.47123 | 0.003808 | 0.017454 | yes | down | 84.08 | 75.38 | 67.81 | 14.92 | 72.52 | 41.87 | 75.75667 | 43.10333 |
| LOC100354254 | 0.44 | -1.18369 | 0.003814 | 0.017471 | yes | down | 875.68 | 1200.27 | 1287.57 | 606.55 | 1230.44 | 495.32 | 1121.173 | 777.4367 |
| ENDOV | 0.427 | -1.22682 | 0.003816 | 0.017477 | yes | down | 5.24 | 2.92 | 4.81 | 3.27 | 2.38 | 3.33 | 4.323333 | 2.993333 |
| LEPR | 5.363 | 2.422942 | 0.003821 | 0.017489 | yes | up | 0.04 | 0.03 | 0.01 | 0.17 | 0.19 | 0.22 | 0.026667 | 0.193333 |
| SLC48A1 | 2.133 | 1.092928 | 0.003845 | 0.017583 | yes | up | 9.65 | 4.93 | 6.34 | 13.25 | 21.33 | 28.47 | 6.973333 | 21.01667 |
| LOC108178580 | 14.604 | 3.868326 | 0.003864 | 0.017655 | yes | up | 0 | 0.08 | 0.08 | 1.07 | 1.56 | 1.26 | 0.053333 | 1.296667 |
| ATPIF1 | 0.448 | -1.15902 | 0.003872 | 0.017683 | yes | down | 425.19 | 173.81 | 131.06 | 126.41 | 191.77 | 183.42 | 243.3533 | 167.2 |
| COL28A1 | 2.055 | 1.038907 | 0.003891 | 0.017751 | yes | up | 1.35 | 1.42 | 0.66 | 2.44 | 3.61 | 5.07 | 1.143333 | 3.706667 |
| EDAR | 0.231 | -2.11128 | 0.003892 | 0.017751 | yes | down | 0.28 | 0.37 | 0.34 | 0.05 | 0.11 | 0.22 | 0.33 | 0.126667 |
| LOC100345766 | 11.527 | 3.526992 | 0.003923 | 0.017849 | yes | up | 1.01 | 0 | 0 | 6.84 | 7.09 | 3.95 | 0.336667 | 5.96 |
| LOC108176811 | 6.047 | 2.5962 | 0.003942 | 0.017912 | yes | up | 0.15 | 0.35 | 0 | 1.58 | 1.66 | 2.03 | 0.166667 | 1.756667 |
| MREG | 3.834 | 1.938975 | 0.003945 | 0.017916 | yes | up | 0.14 | 0.11 | 0.16 | 1.16 | 0.82 | 0.51 | 0.136667 | 0.83 |
| SLC25A33 | 0.402 | -1.31569 | 0.003962 | 0.017977 | yes | down | 41.83 | 32.23 | 17.29 | 8.85 | 17.09 | 32.07 | 30.45 | 19.33667 |
| IL21R | 57.195 | 5.837815 | 0.003994 | 0.01811 | yes | up | 0 | 0 | 0 | 1.26 | 0.06 | 0.18 | 0 | 0.5 |
| HSD11B1 | 2.141 | 1.098517 | 0.004002 | 0.018137 | yes | up | 1.82 | 3.3 | 3.63 | 13.22 | 8.1 | 9.33 | 2.916667 | 10.21667 |
| LAMB3 | 2.054 | 1.038691 | 0.004021 | 0.018208 | yes | up | 1.01 | 0.98 | 0.47 | 2.06 | 3.44 | 2.41 | 0.82 | 2.636667 |
| C13H1orf210 | 0.317 | -1.65539 | 0.004023 | 0.01821 | yes | down | 1.15 | 1.71 | 2.31 | 0.59 | 0.99 | 1.19 | 1.723333 | 0.923333 |
| LOC100347604 | 0.474 | -1.07582 | 0.004026 | 0.018217 | yes | down | 2.17 | 3.24 | 1.93 | 2.67 | 1.23 | 1.57 | 2.446667 | 1.823333 |
| LOC108176853 | 34.339 | 5.101761 | 0.004087 | 0.01846 | yes | up | 0 | 0 | 0 | 0.07 | 0.08 | 0.14 | 0 | 0.096667 |
| ABCC4 | 0.384 | -1.38067 | 0.004105 | 0.018527 | yes | down | 2.66 | 4.36 | 2.1 | 2.97 | 0.75 | 1.87 | 3.04 | 1.863333 |
| BZW2 | 0.436 | -1.19891 | 0.004114 | 0.018566 | yes | down | 72.55 | 36.8 | 19.01 | 19.62 | 39.88 | 33.33 | 42.78667 | 30.94333 |
| LOC100343903 | 3.737 | 1.901927 | 0.004123 | 0.018584 | yes | up | 0.08 | 0.17 | 0.12 | 0.65 | 0.65 | 0.96 | 0.123333 | 0.753333 |
| LOC103346963 | 3.097 | 1.631074 | 0.004133 | 0.018607 | yes | up | 2.04 | 1.34 | 1.2 | 10 | 3.08 | 9.61 | 1.526667 | 7.563333 |
| ICAM1 | 3.759 | 1.910274 | 0.004143 | 0.018643 | yes | up | 2.87 | 2.11 | 2.54 | 32.67 | 4.2 | 6.62 | 2.506667 | 14.49667 |
| KCTD14 | 0.164 | -2.61172 | 0.004178 | 0.018777 | yes | down | 0.73 | 0.97 | 0.44 | 0.2 | 0.27 | 0.17 | 0.713333 | 0.213333 |
| LOC103346967 | 53.17 | 5.732553 | 0.004184 | 0.018791 | yes | up | 0 | 0 | 0 | 0.62 | 0.04 | 0.57 | 0 | 0.41 |
| AXIN2 | 0.462 | -1.11339 | 0.004229 | 0.018966 | yes | down | 3.8 | 3.2 | 1.77 | 1.59 | 2.45 | 2.58 | 2.923333 | 2.206667 |
| AKNA | 4.678 | 2.22588 | 0.004245 | 0.019033 | yes | up | 0.55 | 0.41 | 0.4 | 6.84 | 0.81 | 0.86 | 0.453333 | 2.836667 |
| LOC100345438 | 0.37 | -1.43277 | 0.004261 | 0.019081 | yes | down | 5.11 | 3.12 | 2.05 | 1.08 | 2.16 | 2.81 | 3.426667 | 2.016667 |
| LOC103347640 | 22.677 | 4.503143 | 0.004276 | 0.01913 | yes | up | 0.03 | 0 | 0 | 0.24 | 0.24 | 0.48 | 0.01 | 0.32 |
| DPYSL5 | 0.1 | -3.32426 | 0.00428 | 0.019139 | yes | down | 0.28 | 0.14 | 0.32 | 0.06 | 0.03 | 0.04 | 0.246667 | 0.043333 |
| LGALS3BP | 2.087 | 1.061186 | 0.004283 | 0.019148 | yes | up | 23.89 | 27.99 | 24.68 | 116.2 | 94.25 | 43.07 | 25.52 | 84.50667 |
| GPR1 | 3.748 | 1.906 | 0.004296 | 0.01919 | yes | up | 0.97 | 0.43 | 0.12 | 4.57 | 1.74 | 2.31 | 0.506667 | 2.873333 |
| FHIT | 2.472 | 1.30597 | 0.004303 | 0.019216 | yes | up | 1.28 | 1.97 | 0.93 | 4.27 | 6.95 | 4.82 | 1.393333 | 5.346667 |
| LOC100358544 | 0.315 | -1.66814 | 0.004312 | 0.01924 | yes | down | 0.94 | 0.36 | 0.41 | 0.34 | 0.37 | 0.2 | 0.57 | 0.303333 |
| LRFN1 | 0.236 | -2.08529 | 0.004312 | 0.01924 | yes | down | 0.38 | 0.35 | 0.22 | 0.08 | 0.17 | 0.1 | 0.316667 | 0.116667 |
| LOC108176622 | 3.967 | 1.988151 | 0.004333 | 0.019304 | yes | up | 0.79 | 0.95 | 0.15 | 3.83 | 3.46 | 3.71 | 0.63 | 3.666667 |
| SH3GL2 | 2.158 | 1.109949 | 0.004334 | 0.019304 | yes | up | 1.63 | 0.89 | 1.35 | 3.9 | 5.87 | 3.55 | 1.29 | 4.44 |
| LOC108178561 | 2.714 | 1.440544 | 0.004338 | 0.019315 | yes | up | 0.49 | 0.49 | 0.27 | 1.71 | 1.75 | 1.91 | 0.416667 | 1.79 |
| COL9A1 | 0.033 | -4.91421 | 0.004391 | 0.019512 | yes | down | 0.16 | 0.07 | 0.28 | 0 | 0.02 | 0 | 0.17 | 0.006667 |
| FAM20A | 0.417 | -1.26142 | 0.004416 | 0.0196 | yes | down | 0.79 | 1.01 | 0.84 | 0.53 | 0.8 | 0.41 | 0.88 | 0.58 |
| IGF2BP3 | 0.447 | -1.16115 | 0.004417 | 0.0196 | yes | down | 3.88 | 3.03 | 2.82 | 3.78 | 1.4 | 1.77 | 3.243333 | 2.316667 |
| MANBA | 2.354 | 1.235079 | 0.004436 | 0.019666 | yes | up | 0.45 | 0.51 | 0.14 | 1.36 | 1.58 | 1.38 | 0.366667 | 1.44 |
| STAG3 | 6.742 | 2.753176 | 0.004441 | 0.019686 | yes | up | 0 | 0.09 | 0.02 | 0.38 | 0.47 | 0.32 | 0.036667 | 0.39 |
| LOC103350944 | 3.079 | 1.622486 | 0.004451 | 0.019715 | yes | up | 1.9 | 3.16 | 1.09 | 7.24 | 5.99 | 17 | 2.05 | 10.07667 |
| LOC103346936 | 0.377 | -1.40701 | 0.004452 | 0.019715 | yes | down | 3.29 | 2.96 | 4.41 | 3.29 | 2.98 | 3.32 | 3.553333 | 3.196667 |
| LOC108175843 | 40.975 | 5.356661 | 0.004466 | 0.019764 | yes | up | 0.16 | 0 | 0 | 8.67 | 0.28 | 1.07 | 0.053333 | 3.34 |
| LOC103350919 | 53.742 | 5.747974 | 0.004466 | 0.019764 | yes | up | 0 | 0 | 0 | 0.01 | 0.37 | 0.22 | 0 | 0.2 |
| ADAMTS17 | 0.488 | -1.03369 | 0.004519 | 0.019949 | yes | down | 3.35 | 3.4 | 1.34 | 1.59 | 2.42 | 2.12 | 2.696667 | 2.043333 |
| DACH2 | 3.611 | 1.852449 | 0.00455 | 0.020054 | yes | up | 0.24 | 0.4 | 0.04 | 0.93 | 1.89 | 1.04 | 0.226667 | 1.286667 |
| ACP6 | 0.482 | -1.05276 | 0.004628 | 0.020368 | yes | down | 3.52 | 3.48 | 3.66 | 2.36 | 3.79 | 2.02 | 3.553333 | 2.723333 |
| BHMT | 0.302 | -1.72844 | 0.004639 | 0.020391 | yes | down | 28.03 | 28.35 | 41.67 | 7.01 | 6.66 | 36.28 | 32.68333 | 16.65 |
| LOC100348853 | 0.456 | -1.13386 | 0.004704 | 0.020626 | yes | down | 17.7 | 18.63 | 7.17 | 12.61 | 10.57 | 6.74 | 14.5 | 9.973333 |
| OMD | 3.064 | 1.615385 | 0.00475 | 0.020801 | yes | up | 0.17 | 0.26 | 0.28 | 1.68 | 0.69 | 1.2 | 0.236667 | 1.19 |
| SYNC | 0.225 | -2.15414 | 0.004754 | 0.020811 | yes | down | 4.66 | 1.64 | 0.52 | 0.34 | 0.84 | 1.18 | 2.273333 | 0.786667 |
| GPR21 | 2.741 | 1.454666 | 0.004759 | 0.020827 | yes | up | 0.36 | 0.22 | 0.19 | 1.47 | 0.98 | 0.9 | 0.256667 | 1.116667 |
| GADD45GIP1 | 0.472 | -1.08233 | 0.004778 | 0.020899 | yes | down | 19.37 | 9 | 12.53 | 11.07 | 7.49 | 12.34 | 13.63333 | 10.3 |
| KCNK5 | 2.103 | 1.072401 | 0.004784 | 0.020917 | yes | up | 1.25 | 0.67 | 1.29 | 3.67 | 2.97 | 3.67 | 1.07 | 3.436667 |
| RASL10B | 0.375 | -1.41515 | 0.004797 | 0.020968 | yes | down | 1.17 | 0.9 | 0.64 | 0.7 | 0.26 | 0.67 | 0.903333 | 0.543333 |
| THSD7A | 2.121 | 1.084747 | 0.004814 | 0.021024 | yes | up | 0.19 | 0.21 | 0.13 | 0.63 | 0.58 | 0.57 | 0.176667 | 0.593333 |
| LOC103345487 | 5.212 | 2.381818 | 0.004869 | 0.021216 | yes | up | 0.89 | 1.51 | 1.02 | 21.81 | 3.12 | 3.4 | 1.14 | 9.443333 |
| LOC103344972 | 2.615 | 1.387077 | 0.004896 | 0.021317 | yes | up | 20.87 | 17.83 | 5.14 | 81.53 | 36.46 | 56.98 | 14.61333 | 58.32333 |
| TMPRSS4 | 4.149 | 2.052742 | 0.004913 | 0.021368 | yes | up | 0.26 | 0.16 | 0.55 | 2.37 | 1.77 | 2.34 | 0.323333 | 2.16 |
| LOC100339419 | 2.782 | 1.476307 | 0.004954 | 0.021504 | yes | up | 0.12 | 0.17 | 0.12 | 0.5 | 0.55 | 0.76 | 0.136667 | 0.603333 |
| MYO1G | 4.714 | 2.236825 | 0.004965 | 0.021547 | yes | up | 0.69 | 0.8 | 0.37 | 14.13 | 1.32 | 1.43 | 0.62 | 5.626667 |
| SFTPD | 4.621 | 2.208073 | 0.004973 | 0.021558 | yes | up | 0.42 | 0.25 | 0.24 | 3.6 | 1.01 | 2.08 | 0.303333 | 2.23 |
| FABP9 | 2.064 | 1.045346 | 0.004994 | 0.021639 | yes | up | 0.42 | 0.28 | 0.11 | 0.43 | 0.85 | 1.23 | 0.27 | 0.836667 |
| LOC100341883 | 0.362 | -1.46681 | 0.005003 | 0.021667 | yes | down | 0.83 | 0.66 | 0.44 | 0.49 | 0.34 | 0.27 | 0.643333 | 0.366667 |
| LOC103349265 | 0.493 | -1.01986 | 0.005055 | 0.021872 | yes | down | 11.52 | 15.33 | 7.75 | 10.44 | 5.59 | 11.08 | 11.53333 | 9.036667 |
| LOC100347711 | 3.698 | 1.88666 | 0.005059 | 0.021886 | yes | up | 1.86 | 2.11 | 1.71 | 20.03 | 2.86 | 7.64 | 1.893333 | 10.17667 |
| SMOC2 | 0.416 | -1.2667 | 0.005073 | 0.02194 | yes | down | 10.22 | 9.56 | 10.8 | 2.6 | 8.71 | 9.22 | 10.19333 | 6.843333 |
| GDPD1 | 0.318 | -1.65235 | 0.005087 | 0.021985 | yes | down | 1.42 | 0.87 | 2.37 | 0.56 | 0.82 | 1.18 | 1.553333 | 0.853333 |
| LOC103347026 | 0.335 | -1.57757 | 0.005102 | 0.022041 | yes | down | 0.21 | 0.17 | 0.21 | 0.11 | 0.09 | 0.12 | 0.196667 | 0.106667 |
| CDHR1 | 0.09 | -3.4758 | 0.005108 | 0.022059 | yes | down | 9.03 | 10.92 | 0.16 | 0.93 | 0.79 | 1.01 | 6.703333 | 0.91 |
| LOC103345743 | 33.744 | 5.076546 | 0.005129 | 0.022143 | yes | up | 0 | 0 | 0 | 0.34 | 0.13 | 0.34 | 0 | 0.27 |
| LOC100342853 | 0.203 | -2.30325 | 0.0052 | 0.022413 | yes | down | 1.19 | 3.61 | 3.56 | 0.4 | 1.19 | 1.19 | 2.786667 | 0.926667 |
| LOC103348640 | 10.504 | 3.392865 | 0.005258 | 0.022629 | yes | up | 0 | 0.03 | 0.02 | 0.21 | 0.38 | 0.29 | 0.016667 | 0.293333 |
| LOC103347194 | 0.097 | -3.36614 | 0.005263 | 0.022642 | yes | down | 0.38 | 1.43 | 0.37 | 0 | 0.1 | 0.26 | 0.726667 | 0.12 |
| LOC103351001 | 0.344 | -1.54056 | 0.005292 | 0.022751 | yes | down | 0.98 | 1 | 0.96 | 0.19 | 0.67 | 0.76 | 0.98 | 0.54 |
| TMEM200A | 0.385 | -1.37535 | 0.005327 | 0.022887 | yes | down | 0.82 | 1.1 | 0.59 | 0.38 | 0.4 | 0.69 | 0.836667 | 0.49 |
| SLC16A4 | 3.126 | 1.64416 | 0.005335 | 0.022916 | yes | up | 0.24 | 0.61 | 0.1 | 1.05 | 1.44 | 1.69 | 0.316667 | 1.393333 |
| ITGB8 | 2.149 | 1.103496 | 0.005357 | 0.022993 | yes | up | 0.5 | 0.44 | 0.22 | 0.99 | 1.16 | 1.66 | 0.386667 | 1.27 |
| DPF1 | 0.14 | -2.83914 | 0.00537 | 0.02303 | yes | down | 0.53 | 0.67 | 0.23 | 0.1 | 0.1 | 0.13 | 0.476667 | 0.11 |
| KLHDC7A | 0.072 | -3.80062 | 0.005374 | 0.023041 | yes | down | 0.1 | 0.12 | 0.02 | 0.01 | 0 | 0.02 | 0.08 | 0.01 |
| ERMAP | 0.088 | -3.50743 | 0.005379 | 0.023054 | yes | down | 0.1 | 0.19 | 0.1 | 0.02 | 0.02 | 0.02 | 0.13 | 0.02 |
| LOC103351044 | 7.162 | 2.840282 | 0.005397 | 0.023125 | yes | up | 1.79 | 1.35 | 1.31 | 17 | 14.28 | 6.72 | 1.483333 | 12.66667 |
| LOC103347072 | 66.767 | 6.061054 | 0.005413 | 0.023177 | yes | up | 0 | 0 | 0 | 14.36 | 0.35 | 0.99 | 0 | 5.233333 |
| TMEM55A | 3.561 | 1.832217 | 0.005447 | 0.023317 | yes | up | 0.24 | 0.16 | 0.12 | 0.9 | 0.75 | 1.26 | 0.173333 | 0.97 |
| TENM2 | 3.373 | 1.753972 | 0.005449 | 0.02332 | yes | up | 0.05 | 0.09 | 0.03 | 0.2 | 0.42 | 0.28 | 0.056667 | 0.3 |
| LOC108175810 | 6.828 | 2.771542 | 0.005458 | 0.023343 | yes | up | 0 | 0.46 | 0.73 | 2.42 | 5.75 | 5.45 | 0.396667 | 4.54 |
| LOC103348324 | 6.798 | 2.765084 | 0.005463 | 0.023358 | yes | up | 0.2 | 0.06 | 0.06 | 0.9 | 1.14 | 1.29 | 0.106667 | 1.11 |
| LOC100348165 | 2.31 | 1.207603 | 0.005488 | 0.023421 | yes | up | 2.1 | 1.33 | 1.02 | 3.64 | 5.21 | 4.45 | 1.483333 | 4.433333 |
| TKTL1 | 0.038 | -4.73143 | 0.005504 | 0.023485 | yes | down | 0.08 | 0.19 | 0.18 | 0 | 0 | 0.03 | 0.15 | 0.01 |
| GDF5 | 5.204 | 2.37958 | 0.005511 | 0.023508 | yes | up | 0.22 | 0.12 | 0.07 | 0.51 | 1.38 | 1.44 | 0.136667 | 1.11 |
| MGAT5B | 0.063 | -3.99626 | 0.005566 | 0.023712 | yes | down | 0.67 | 0.61 | 0.36 | 0.08 | 0.08 | 0 | 0.546667 | 0.053333 |
| LOC103345854 | 0.023 | -5.44482 | 0.005608 | 0.023872 | yes | down | 0.31 | 1.07 | 1.1 | 0 | 0 | 0 | 0.826667 | 0 |
| LOC108175435 | 4.496 | 2.16868 | 0.005614 | 0.023878 | yes | up | 0.13 | 0.08 | 0.02 | 0.54 | 0.5 | 0.52 | 0.076667 | 0.52 |
| UGT8 | 2.85 | 1.51103 | 0.005664 | 0.024072 | yes | up | 1.13 | 0.7 | 0.29 | 1.7 | 2.75 | 5 | 0.706667 | 3.15 |
| LOC103350144 | 0.397 | -1.33307 | 0.005697 | 0.0242 | yes | down | 1.88 | 4.6 | 2.19 | 2.35 | 1.41 | 2.27 | 2.89 | 2.01 |
| LOC108175916 | 0.023 | -5.42268 | 0.005725 | 0.024299 | yes | down | 0.46 | 0.28 | 0.1 | 0 | 0 | 0 | 0.28 | 0 |
| SFRP2 | 0.358 | -1.48306 | 0.005755 | 0.024405 | yes | down | 1.98 | 1.64 | 3.26 | 1.93 | 1.03 | 1.09 | 2.293333 | 1.35 |
| LOC103350920 | 25.17 | 4.653613 | 0.005776 | 0.024481 | yes | up | 0 | 0.06 | 0 | 0.06 | 1.51 | 1.15 | 0.02 | 0.906667 |
| LOC103346383 | 3.66 | 1.871826 | 0.005778 | 0.024481 | yes | up | 0.45 | 0.2 | 0.39 | 1.69 | 2.09 | 2.31 | 0.346667 | 2.03 |
| TPGS1 | 0.477 | -1.06876 | 0.00581 | 0.024588 | yes | down | 3.66 | 4.42 | 5.52 | 3.12 | 2.99 | 4.53 | 4.533333 | 3.546667 |
| HSPB3 | 0.105 | -3.2526 | 0.005852 | 0.02474 | yes | down | 23 | 3.68 | 0.54 | 0.19 | 2.9 | 1.08 | 9.073333 | 1.39 |
| MYLK3 | 0.116 | -3.10985 | 0.005855 | 0.02474 | yes | down | 22.25 | 3.79 | 1.47 | 0.67 | 2.7 | 1.42 | 9.17 | 1.596667 |
| CASQ2 | 0.092 | -3.44166 | 0.00586 | 0.024753 | yes | down | 91.96 | 13.65 | 2.29 | 2.81 | 4.55 | 7.72 | 35.96667 | 5.026667 |
| DRAM1 | 2.779 | 1.474663 | 0.005865 | 0.024761 | yes | up | 1.99 | 2.79 | 1.98 | 19.11 | 4.09 | 7.75 | 2.253333 | 10.31667 |
| MERTK | 2.565 | 1.358716 | 0.005887 | 0.024847 | yes | up | 1.86 | 2.94 | 2.82 | 18.99 | 4.95 | 7.86 | 2.54 | 10.6 |
| LOC103352519 | 0.341 | -1.5502 | 0.00589 | 0.024853 | yes | down | 0.44 | 0.99 | 0.58 | 0.23 | 0.43 | 0.45 | 0.67 | 0.37 |
| FGD2 | 8.61 | 3.105992 | 0.005899 | 0.024873 | yes | up | 0.09 | 0.04 | 0.1 | 2.75 | 0.25 | 0.26 | 0.076667 | 1.086667 |
| LOC100358379 | 15.168 | 3.922912 | 0.005916 | 0.024934 | yes | up | 0 | 0.09 | 0.26 | 7.58 | 0.64 | 0.93 | 0.116667 | 3.05 |
| LOC108176236 | 22.38 | 4.484162 | 0.005922 | 0.024937 | yes | up | 0.12 | 0.11 | 0.09 | 0 | 4.28 | 8.05 | 0.106667 | 4.11 |
| GRTP1 | 0.427 | -1.22832 | 0.005925 | 0.024937 | yes | down | 4.39 | 2.7 | 1.49 | 1.83 | 2.17 | 1.93 | 2.86 | 1.976667 |
| PMP2 | 2.533 | 1.340861 | 0.005928 | 0.024943 | yes | up | 9.32 | 6.51 | 1.92 | 15.95 | 20.77 | 32.88 | 5.916667 | 23.2 |
| ALKBH8 | 0.475 | -1.07544 | 0.005935 | 0.024966 | yes | down | 1.34 | 0.98 | 1.17 | 1.07 | 0.54 | 0.84 | 1.163333 | 0.816667 |
| PAPLN | 2.672 | 1.418 | 0.005944 | 0.024989 | yes | up | 0.73 | 0.26 | 0.19 | 1.82 | 1.69 | 1.38 | 0.393333 | 1.63 |
| ARPC1B | 2.069 | 1.048852 | 0.005954 | 0.025024 | yes | up | 23.15 | 27.8 | 21.49 | 129.79 | 47.97 | 60.54 | 24.14667 | 79.43333 |
| NAP1L5 | 41.486 | 5.374552 | 0.005968 | 0.025072 | yes | up | 0 | 0 | 0 | 0.04 | 0.4 | 0.26 | 0 | 0.233333 |
| PLEKHA6 | 2.125 | 1.087306 | 0.005978 | 0.025076 | yes | up | 0.28 | 0.17 | 0.24 | 0.72 | 0.95 | 0.67 | 0.23 | 0.78 |
| MOB3C | 2.011 | 1.007967 | 0.00598 | 0.025076 | yes | up | 3.21 | 3.4 | 2.59 | 14.77 | 6.75 | 7.44 | 3.066667 | 9.653333 |
| LOC100348203 | 18.151 | 4.182002 | 0.005981 | 0.025076 | yes | up | 0 | 0.02 | 0.02 | 0.94 | 0.18 | 0.17 | 0.013333 | 0.43 |
| C1QC | 6.042 | 2.595034 | 0.005986 | 0.025087 | yes | up | 35.84 | 27.89 | 25.88 | 662.2 | 73.36 | 139.54 | 29.87 | 291.7 |
| LOC100344547 | 0.473 | -1.08045 | 0.005989 | 0.025087 | yes | down | 1.81 | 1.65 | 1.71 | 1.16 | 1.52 | 1.22 | 1.723333 | 1.3 |
| CMBL | 0.415 | -1.26875 | 0.006024 | 0.025212 | yes | down | 28.08 | 12.93 | 11.74 | 6.48 | 17.16 | 10.77 | 17.58333 | 11.47 |
| SCUBE1 | 0.388 | -1.36472 | 0.006096 | 0.025472 | yes | down | 1.76 | 2.28 | 0.69 | 0.72 | 0.45 | 0.91 | 1.576667 | 0.693333 |
| ZNF135 | 2.315 | 1.211258 | 0.006113 | 0.025538 | yes | up | 0.6 | 0.66 | 0.47 | 3.31 | 1.48 | 2.12 | 0.576667 | 2.303333 |
| RBM47 | 7.347 | 2.877205 | 0.006145 | 0.025644 | yes | up | 0.04 | 0.1 | 0.11 | 2.52 | 0.15 | 0.38 | 0.083333 | 1.016667 |
| LOC108178461 | 2.42 | 1.274944 | 0.006196 | 0.025831 | yes | up | 0.57 | 0.37 | 0.65 | 2.36 | 1.84 | 3.23 | 0.53 | 2.476667 |
| PFAS | 0.408 | -1.29319 | 0.006199 | 0.025838 | yes | down | 0.62 | 0.76 | 0.65 | 0.71 | 0.26 | 0.35 | 0.676667 | 0.44 |
| LOC103346647 | 2.526 | 1.336898 | 0.006244 | 0.02601 | yes | up | 0.99 | 0.3 | 0.73 | 2.9 | 2.81 | 2.59 | 0.673333 | 2.766667 |
| LOC108177611 | 7.052 | 2.817954 | 0.006254 | 0.026046 | yes | up | 0.32 | 0.43 | 0.14 | 1.29 | 2.95 | 5.48 | 0.296667 | 3.24 |
| LOC103349153 | 9.538 | 3.25374 | 0.006284 | 0.026155 | yes | up | 0.05 | 0.04 | 0.04 | 0.96 | 0.61 | 0.44 | 0.043333 | 0.67 |
| NPTX1 | 2.46 | 1.298888 | 0.006312 | 0.026258 | yes | up | 4.53 | 3.19 | 3.52 | 5.29 | 15.35 | 22.83 | 3.746667 | 14.49 |
| CXCL16 | 3.682 | 1.880357 | 0.006338 | 0.026347 | yes | up | 5.25 | 4.66 | 4.73 | 66.05 | 8 | 9.35 | 4.88 | 27.8 |
| LOC103346478 | 0.442 | -1.1791 | 0.006369 | 0.026462 | yes | down | 0.42 | 0.43 | 0.46 | 0.32 | 0.35 | 0.25 | 0.436667 | 0.306667 |
| PRG2 | 0.026 | -5.26203 | 0.006406 | 0.026573 | yes | down | 0.45 | 0.33 | 0.39 | 0 | 0 | 0 | 0.39 | 0 |
| NFE2 | 0.387 | -1.3693 | 0.006438 | 0.026665 | yes | down | 1.67 | 5.07 | 5.16 | 2.72 | 1.91 | 2.91 | 3.966667 | 2.513333 |
| DNAJC24 | 0.421 | -1.24872 | 0.00645 | 0.026703 | yes | down | 5.45 | 4.1 | 4.48 | 5.4 | 1.6 | 2.2 | 4.676667 | 3.066667 |
| PTPRU | 4.717 | 2.23779 | 0.006488 | 0.02685 | yes | up | 0.1 | 0.01 | 0.03 | 0.34 | 0.33 | 0.39 | 0.046667 | 0.353333 |
| KCNB2 | 6.965 | 2.800119 | 0.006489 | 0.026851 | yes | up | 0.04 | 0.05 | 0 | 0.2 | 0.41 | 0.47 | 0.03 | 0.36 |
| IRF8 | 3.901 | 1.963671 | 0.00652 | 0.026958 | yes | up | 0.86 | 2.33 | 2.1 | 22.92 | 7.88 | 2.52 | 1.763333 | 11.10667 |
| C2H16orf91 | 0.115 | -3.11423 | 0.006554 | 0.027081 | yes | down | 1.45 | 1.94 | 1.64 | 0.21 | 0.43 | 0.28 | 1.676667 | 0.306667 |
| COL3A1 | 2.101 | 1.07085 | 0.006628 | 0.027331 | yes | up | 1067.4 | 563.07 | 308.18 | 1946.26 | 2554.37 | 1941.32 | 646.2167 | 2147.317 |
| CDKL2 | 3.3 | 1.722496 | 0.006649 | 0.027406 | yes | up | 0.14 | 0.11 | 0.04 | 0.57 | 0.74 | 0.41 | 0.096667 | 0.573333 |
| CARMIL2 | 4.246 | 2.086263 | 0.0067 | 0.027567 | yes | up | 0.15 | 0.11 | 0.11 | 1.69 | 0.44 | 0.31 | 0.123333 | 0.813333 |
| LOC103345474 | 2.803 | 1.487203 | 0.0067 | 0.027567 | yes | up | 0.53 | 0.33 | 0.45 | 2.76 | 1.27 | 1.89 | 0.436667 | 1.973333 |
| ENPP6 | 0.279 | -1.84067 | 0.006726 | 0.027666 | yes | down | 2.63 | 2.23 | 0.31 | 0.57 | 1.12 | 0.69 | 1.723333 | 0.793333 |
| LOC108175803 | 0.078 | -3.6757 | 0.006732 | 0.027682 | yes | down | 0.41 | 0.07 | 0.06 | 0.02 | 0 | 0.05 | 0.18 | 0.023333 |
| LOC100347161 | 0.062 | -4.00781 | 0.00679 | 0.027899 | yes | down | 0.56 | 1.51 | 0.72 | 0 | 0.13 | 0.16 | 0.93 | 0.096667 |
| COL1A1 | 2.052 | 1.03716 | 0.006848 | 0.028113 | yes | up | 737.6 | 405.68 | 247 | 1575.27 | 1852.87 | 1042.86 | 463.4267 | 1490.333 |
| LOC108177991 | 9.961 | 3.316283 | 0.006849 | 0.028113 | yes | up | 0.02 | 0.01 | 0 | 0.3 | 0.29 | 0.7 | 0.01 | 0.43 |
| BAIAP2L1 | 2.391 | 1.25776 | 0.006898 | 0.028283 | yes | up | 3.44 | 5.9 | 3.2 | 6.45 | 21.5 | 19.43 | 4.18 | 15.79333 |
| LOC100344152 | 0.367 | -1.44804 | 0.006918 | 0.028359 | yes | down | 2.21 | 1.71 | 1.84 | 0.57 | 1.58 | 1.24 | 1.92 | 1.13 |
| DOK5 | 0.199 | -2.33083 | 0.006924 | 0.028369 | yes | down | 18.24 | 3.67 | 2.94 | 0.4 | 3.83 | 3 | 8.283333 | 2.41 |
| LOC100346229 | 2.353 | 1.234257 | 0.006965 | 0.028506 | yes | up | 1.17 | 0.35 | 0.65 | 2.3 | 2.86 | 2.94 | 0.723333 | 2.7 |
| CARD11 | 14.971 | 3.904099 | 0.007011 | 0.028626 | yes | up | 0.02 | 0.03 | 0.01 | 1.29 | 0.09 | 0.06 | 0.02 | 0.48 |
| RPS29 | 0.34 | -1.55707 | 0.007012 | 0.028626 | yes | down | 118.95 | 167.72 | 350.86 | 127.19 | 159.2 | 61.3 | 212.51 | 115.8967 |
| LOC103345532 | 3.176 | 1.667229 | 0.00702 | 0.028655 | yes | up | 0.35 | 0.28 | 0.26 | 1.33 | 1.19 | 2.03 | 0.296667 | 1.516667 |
| CXCL8 | 4.888 | 2.289217 | 0.007033 | 0.0287 | yes | up | 0.13 | 0.2 | 0.07 | 1.62 | 0.59 | 0.9 | 0.133333 | 1.036667 |
| LOC100345908 | 2.574 | 1.364036 | 0.007084 | 0.028852 | yes | up | 7.57 | 5.45 | 12.66 | 15.55 | 34.17 | 57.22 | 8.56 | 35.64667 |
| LOC108178511 | 11.111 | 3.473856 | 0.007099 | 0.028894 | yes | up | 0 | 0.11 | 0.05 | 0.45 | 0.84 | 1.78 | 0.053333 | 1.023333 |
| LOC103349679 | 2.432 | 1.282076 | 0.007107 | 0.028907 | yes | up | 0.74 | 0.54 | 0.65 | 2.38 | 1.68 | 1.96 | 0.643333 | 2.006667 |
| LOC100359146 | 3.766 | 1.91295 | 0.007117 | 0.028924 | yes | up | 0.05 | 0.2 | 0.12 | 0.84 | 0.71 | 0.68 | 0.123333 | 0.743333 |
| PRKG2 | 5.505 | 2.460659 | 0.007121 | 0.028933 | yes | up | 0.02 | 0.09 | 0.03 | 0.59 | 0.17 | 0.32 | 0.046667 | 0.36 |
| MYOF | 2.575 | 1.36468 | 0.007163 | 0.029087 | yes | up | 2.07 | 1.25 | 0.97 | 10.75 | 3.15 | 3.48 | 1.43 | 5.793333 |
| LOC100341951 | 23.658 | 4.564253 | 0.007172 | 0.02912 | yes | up | 0 | 0.32 | 0 | 0.14 | 6.27 | 3.91 | 0.106667 | 3.44 |
| LOC100340032 | 2.116 | 1.081509 | 0.007178 | 0.029133 | yes | up | 1.77 | 0.7 | 0.67 | 2.84 | 4.21 | 3.62 | 1.046667 | 3.556667 |
| IRF6 | 0.324 | -1.62649 | 0.007223 | 0.029301 | yes | down | 0.53 | 0.28 | 0.3 | 0.16 | 0.24 | 0.16 | 0.37 | 0.186667 |
| NTN4 | 2.049 | 1.034637 | 0.007236 | 0.029347 | yes | up | 6.37 | 3.81 | 2 | 9.69 | 15.23 | 13.24 | 4.06 | 12.72 |
| CALB1 | 0.07 | -3.84551 | 0.007243 | 0.029369 | yes | down | 0.07 | 0.39 | 0.43 | 0.03 | 0.06 | 0 | 0.296667 | 0.03 |
| LOC100346033 | 3.351 | 1.744685 | 0.007263 | 0.029444 | yes | up | 0.1 | 0.16 | 0.09 | 0.56 | 0.65 | 0.81 | 0.116667 | 0.673333 |
| LOC108176463 | 3.457 | 1.789616 | 0.007304 | 0.029579 | yes | up | 0.42 | 0.62 | 0.3 | 4.83 | 1.09 | 1.43 | 0.446667 | 2.45 |
| LOC108177064 | 34.705 | 5.117054 | 0.007333 | 0.029659 | yes | up | 0 | 0 | 0 | 0.63 | 1.79 | 2.78 | 0 | 1.733333 |
| WDR4 | 0.424 | -1.23924 | 0.007351 | 0.029716 | yes | down | 1.84 | 2.14 | 1.87 | 1.93 | 1.06 | 0.92 | 1.95 | 1.303333 |
| B2M | 5.875 | 2.5547 | 0.007388 | 0.029829 | yes | up | 116.12 | 128.13 | 113.81 | 2887.63 | 387.76 | 485.36 | 119.3533 | 1253.583 |
| LOC108178576 | 0.053 | -4.24066 | 0.007431 | 0.029986 | yes | down | 3.61 | 9.13 | 3.01 | 1.24 | 0 | 0 | 5.25 | 0.413333 |
| LOC108177881 | 12.69 | 3.665638 | 0.007441 | 0.030021 | yes | up | 0.05 | 0 | 0.05 | 0.87 | 0.53 | 0.61 | 0.033333 | 0.67 |
| SLCO2B1 | 2.108 | 1.075834 | 0.007493 | 0.03017 | yes | up | 0.5 | 0.69 | 0.77 | 1.69 | 2.88 | 2.07 | 0.653333 | 2.213333 |
| BNC2 | 2.029 | 1.020476 | 0.007547 | 0.030362 | yes | up | 0.75 | 0.49 | 0.28 | 1.49 | 2.07 | 2.18 | 0.506667 | 1.913333 |
| FAM46B | 10.15 | 3.343478 | 0.00762 | 0.030627 | yes | up | 0.02 | 0.04 | 0 | 0.24 | 0.26 | 0.58 | 0.02 | 0.36 |
| TECTB | 0.049 | -4.35574 | 0.00763 | 0.030649 | yes | down | 0.68 | 0.25 | 0.1 | 0 | 0.07 | 0 | 0.343333 | 0.023333 |
| PRDM8 | 2.254 | 1.172282 | 0.007717 | 0.030892 | yes | up | 0.6 | 0.46 | 0.24 | 1.3 | 1.6 | 1.06 | 0.433333 | 1.32 |
| LOC100350916 | 0.202 | -2.30829 | 0.007763 | 0.031035 | yes | down | 7.8 | 6.18 | 6.15 | 3.07 | 3.19 | 0.08 | 6.71 | 2.113333 |
| LSAMP | 2.234 | 1.159398 | 0.007786 | 0.031111 | yes | up | 0.96 | 0.27 | 0.13 | 0.96 | 1 | 0.81 | 0.453333 | 0.923333 |
| TREX1 | 2.41 | 1.269074 | 0.007885 | 0.031447 | yes | up | 1.95 | 1.52 | 1.89 | 10.77 | 5.63 | 4.15 | 1.786667 | 6.85 |
| GINS2 | 2.433 | 1.282458 | 0.007897 | 0.031485 | yes | up | 2.23 | 1.6 | 0.72 | 4.87 | 6.2 | 6.09 | 1.516667 | 5.72 |
| TACSTD2 | 5.482 | 2.454792 | 0.008022 | 0.031912 | yes | up | 0.05 | 0.07 | 0.04 | 0.68 | 0.25 | 0.48 | 0.053333 | 0.47 |
| LOC103346726 | 2.087 | 1.06134 | 0.008025 | 0.031914 | yes | up | 2.38 | 1.33 | 1.07 | 5.02 | 6.81 | 3.46 | 1.593333 | 5.096667 |
| LOC103347003 | 5.045 | 2.334861 | 0.008034 | 0.031923 | yes | up | 0.48 | 0.38 | 0.4 | 6.23 | 3.45 | 0.28 | 0.42 | 3.32 |
| KANK4 | 0.487 | -1.03925 | 0.008126 | 0.032197 | yes | down | 1.42 | 2.32 | 1.55 | 1.15 | 0.87 | 2.1 | 1.763333 | 1.373333 |
| SYCE3 | 0.277 | -1.85343 | 0.008137 | 0.032225 | yes | down | 0.69 | 1.52 | 2.36 | 3.03 | 10.9 | 1.33 | 1.523333 | 5.086667 |
| SNPH | 0.356 | -1.49118 | 0.008143 | 0.032235 | yes | down | 0.49 | 1.24 | 0.3 | 0.29 | 0.45 | 0.38 | 0.676667 | 0.373333 |
| LOC103348466 | 6.288 | 2.652662 | 0.008292 | 0.032784 | yes | up | 0.08 | 0.04 | 0.07 | 0.54 | 0.78 | 0.5 | 0.063333 | 0.606667 |
| PTPRT | 2.128 | 1.089569 | 0.008358 | 0.032996 | yes | up | 0.28 | 0.41 | 0.5 | 1.19 | 1.89 | 1.02 | 0.396667 | 1.366667 |
| LOC103352261 | 2.309 | 1.206976 | 0.008364 | 0.03301 | yes | up | 7.99 | 5.04 | 6.34 | 41.45 | 11.58 | 19 | 6.456667 | 24.01 |
| GRB14 | 2.015 | 1.010634 | 0.008397 | 0.033098 | yes | up | 1.91 | 0.93 | 1.06 | 3.34 | 4.31 | 4.32 | 1.3 | 3.99 |
| DEF6 | 4.194 | 2.068401 | 0.008408 | 0.033127 | yes | up | 0.35 | 0.49 | 0.3 | 5.51 | 0.73 | 1.32 | 0.38 | 2.52 |
| TECRL | 0.024 | -5.38023 | 0.00842 | 0.033164 | yes | down | 18.04 | 2.07 | 0.17 | 0 | 0.07 | 0.74 | 6.76 | 0.27 |
| RANBP3L | 3.471 | 1.795498 | 0.008436 | 0.033221 | yes | up | 0.14 | 0.14 | 0.32 | 0.52 | 0.95 | 1.66 | 0.2 | 1.043333 |
| PRSS12 | 0.437 | -1.19471 | 0.008493 | 0.033403 | yes | down | 2.29 | 3.15 | 1.3 | 2.48 | 1.12 | 0.99 | 2.246667 | 1.53 |
| LOC100355410 | 6.864 | 2.779027 | 0.008551 | 0.033578 | yes | up | 0 | 0.09 | 0.07 | 0.32 | 0.94 | 0.49 | 0.053333 | 0.583333 |
| TTYH1 | 2.898 | 1.534918 | 0.008552 | 0.033578 | yes | up | 0.42 | 0.5 | 0.37 | 1.69 | 2.22 | 3 | 0.43 | 2.303333 |
| NCF4 | 4.321 | 2.11122 | 0.008581 | 0.033666 | yes | up | 0.92 | 0.87 | 0.45 | 12.27 | 1.34 | 1.73 | 0.746667 | 5.113333 |
| TMEM198 | 2.709 | 1.437827 | 0.008638 | 0.033882 | yes | up | 0.6 | 0.78 | 0.21 | 2.72 | 3.1 | 2.14 | 0.53 | 2.653333 |
| SORCS1 | 2.85 | 1.510971 | 0.008654 | 0.033936 | yes | up | 0.24 | 0.2 | 0.05 | 0.56 | 0.59 | 1.05 | 0.163333 | 0.733333 |
| LOC100351389 | 2.193 | 1.132784 | 0.008679 | 0.03402 | yes | up | 6.8 | 8.2 | 6.24 | 47.16 | 15.82 | 18.03 | 7.08 | 27.00333 |
| DNAH3 | 21.28 | 4.411402 | 0.008779 | 0.034302 | yes | up | 0 | 0 | 0 | 0.06 | 0.02 | 0.07 | 0 | 0.05 |
| LOC100352468 | 6.068 | 2.601328 | 0.008797 | 0.034353 | yes | up | 0.05 | 0.25 | 0.04 | 0.55 | 1.51 | 1.71 | 0.113333 | 1.256667 |
| ADCY2 | 0.455 | -1.13645 | 0.008828 | 0.034443 | yes | down | 19.83 | 13.36 | 7.68 | 4.5 | 11.67 | 12.9 | 13.62333 | 9.69 |
| TXK | 66.324 | 6.051458 | 0.008833 | 0.034455 | yes | up | 0 | 0 | 0 | 1.06 | 0.02 | 0.02 | 0 | 0.366667 |
| MICAL1 | 2.084 | 1.059348 | 0.008881 | 0.034615 | yes | up | 2.7 | 2.44 | 1.34 | 10.96 | 4.77 | 6.54 | 2.16 | 7.423333 |
| LOC100356714 | 22.051 | 4.462746 | 0.008895 | 0.03466 | yes | up | 0 | 0 | 0.01 | 1.88 | 0.4 | 0.3 | 0.003333 | 0.86 |
| SPTLC3 | 2.365 | 1.241707 | 0.008937 | 0.034806 | yes | up | 0.45 | 0.29 | 0.11 | 0.97 | 0.9 | 1.27 | 0.283333 | 1.046667 |
| TNNI3K | 7.262 | 2.860449 | 0.008946 | 0.034833 | yes | up | 0.02 | 0.05 | 0.02 | 0.39 | 0.2 | 0.22 | 0.03 | 0.27 |
| MYOC | 4.125 | 2.044479 | 0.008965 | 0.034901 | yes | up | 2.35 | 0.7 | 0.21 | 2.45 | 9.98 | 7.85 | 1.086667 | 6.76 |
| ABCG4 | 5.59 | 2.482738 | 0.009071 | 0.035235 | yes | up | 0.04 | 0.61 | 0.02 | 1.87 | 1.75 | 2.27 | 0.223333 | 1.963333 |
| LOC103345542 | 5.211 | 2.381621 | 0.009075 | 0.035242 | yes | up | 0.53 | 0.09 | 0.04 | 1.03 | 2.17 | 1.59 | 0.22 | 1.596667 |
| ANLN | 0.396 | -1.33598 | 0.009093 | 0.035302 | yes | down | 1.17 | 0.64 | 0.39 | 0.48 | 0.33 | 0.55 | 0.733333 | 0.453333 |
| NLRP3 | 3.43 | 1.778346 | 0.009099 | 0.035317 | yes | up | 0.56 | 0.62 | 0.46 | 6.44 | 0.98 | 1.36 | 0.546667 | 2.926667 |
| C1QTNF3 | 0.249 | -2.00803 | 0.009104 | 0.03532 | yes | down | 6.91 | 1.92 | 1.17 | 0.31 | 1.89 | 1.61 | 3.333333 | 1.27 |
| LOC100341315 | 2.982 | 1.576425 | 0.009122 | 0.035366 | yes | up | 0.13 | 0.44 | 0.19 | 1.35 | 0.91 | 1.39 | 0.253333 | 1.216667 |
| LOC103352144 | 18.628 | 4.219409 | 0.009208 | 0.035665 | yes | up | 0 | 0.03 | 0 | 0.36 | 0.21 | 0.41 | 0.01 | 0.326667 |
| LOC100345155 | 0.055 | -4.18926 | 0.009217 | 0.035691 | yes | down | 0.35 | 0.67 | 0 | 0.03 | 0.03 | 0.03 | 0.34 | 0.03 |
| CCL7 | 4.898 | 2.292222 | 0.009263 | 0.035816 | yes | up | 3.68 | 2.25 | 0.03 | 26.1 | 6.56 | 9.57 | 1.986667 | 14.07667 |
| TMEM163 | 32.138 | 5.006187 | 0.009318 | 0.03597 | yes | up | 0 | 0.03 | 0 | 1.58 | 0.06 | 0.16 | 0.01 | 0.6 |
| ANKRD16 | 2.015 | 1.011045 | 0.009354 | 0.036074 | yes | up | 1.41 | 1.15 | 1.13 | 3.51 | 5.71 | 5.78 | 1.23 | 5 |
| PTK2B | 4.105 | 2.037322 | 0.009455 | 0.036407 | yes | up | 0.48 | 0.42 | 0.4 | 7.04 | 0.87 | 0.97 | 0.433333 | 2.96 |
| PREPL | 0.351 | -1.50949 | 0.009565 | 0.036789 | yes | down | 20.4 | 5.38 | 2.75 | 5.06 | 5.14 | 5.05 | 9.51 | 5.083333 |
| C19H17orf58 | 2.689 | 1.427088 | 0.009587 | 0.036865 | yes | up | 1.98 | 1.17 | 3.19 | 5.61 | 10.49 | 12.18 | 2.113333 | 9.426667 |
| LOC100346308 | 3.276 | 1.711824 | 0.009624 | 0.036982 | yes | up | 0.88 | 0.29 | 0.24 | 1.31 | 3.01 | 2.84 | 0.47 | 2.386667 |
| TUBA4A | 0.32 | -1.64282 | 0.009715 | 0.037299 | yes | down | 62.89 | 15.33 | 6.89 | 15.64 | 14.67 | 10.77 | 28.37 | 13.69333 |
| LOC103347986 | 4.522 | 2.176906 | 0.009744 | 0.037368 | yes | up | 0.54 | 1.02 | 0.64 | 6.71 | 3.09 | 1.44 | 0.733333 | 3.746667 |
| TM6SF1 | 2.159 | 1.110434 | 0.009745 | 0.037368 | yes | up | 4.34 | 2.24 | 0.99 | 7.04 | 5.76 | 7.81 | 2.523333 | 6.87 |
| LOC100349989 | 3.347 | 1.743076 | 0.009748 | 0.037368 | yes | up | 0.12 | 0.1 | 0.05 | 0.55 | 0.44 | 0.41 | 0.09 | 0.466667 |
| LOC103351303 | 28.062 | 4.810562 | 0.009749 | 0.037368 | yes | up | 0 | 0 | 0 | 0.05 | 0.06 | 0.1 | 0 | 0.07 |
| LOC100340985 | 0.092 | -3.44586 | 0.009832 | 0.037608 | yes | down | 0.65 | 0.39 | 0.55 | 0.18 | 0.08 | 0 | 0.53 | 0.086667 |
| ZAR1L | 5.474 | 2.452559 | 0.009871 | 0.037742 | yes | up | 0.2 | 0.15 | 0 | 0.85 | 0.7 | 1.41 | 0.116667 | 0.986667 |
| IPPK | 2.297 | 1.199947 | 0.009919 | 0.037884 | yes | up | 4.21 | 5.14 | 2.58 | 4.45 | 10.26 | 8.81 | 3.976667 | 7.84 |
| STX11 | 9.48 | 3.244842 | 0.009921 | 0.037884 | yes | up | 0.02 | 0.04 | 0.05 | 1.47 | 0.15 | 0.15 | 0.036667 | 0.59 |
| P2RX2 | 14.038 | 3.811317 | 0.009944 | 0.037951 | yes | up | 0.08 | 0 | 0 | 0.45 | 0.79 | 0.38 | 0.026667 | 0.54 |
| LOC100346803 | 2.254 | 1.172727 | 0.009946 | 0.037951 | yes | up | 0.51 | 0.69 | 0.46 | 1.79 | 1.99 | 1 | 0.553333 | 1.593333 |
| IGF2BP2 | 0.368 | -1.44072 | 0.009987 | 0.038075 | yes | down | 4.58 | 1.34 | 0.92 | 1.36 | 1.24 | 1.28 | 2.28 | 1.293333 |
| LOC108176045 | 0.028 | -5.16011 | 0.009987 | 0.038075 | yes | down | 0.04 | 0.04 | 0.06 | 0 | 0 | 0 | 0.046667 | 0 |
| EPHA6 | 2.493 | 1.317973 | 0.009989 | 0.038075 | yes | up | 0.76 | 0.7 | 0.62 | 0.82 | 1.76 | 2.58 | 0.693333 | 1.72 |
| LOC103349403 | 2.2 | 1.137395 | 0.00999 | 0.038075 | yes | up | 0.37 | 0.61 | 0.36 | 1.13 | 1.81 | 1.74 | 0.446667 | 1.56 |
| LONP1 | 0.337 | -1.57091 | 0.010004 | 0.03811 | yes | down | 11.31 | 11.39 | 6.17 | 3.88 | 4.88 | 6.41 | 9.623333 | 5.056667 |
| TRPC3 | 0.3 | -1.7367 | 0.01006 | 0.038286 | yes | down | 0.46 | 0.56 | 0.26 | 0.06 | 0.24 | 0.32 | 0.426667 | 0.206667 |
| ASPM | 0.405 | -1.30531 | 0.010069 | 0.038313 | yes | down | 0.86 | 0.63 | 0.68 | 0.75 | 0.4 | 0.25 | 0.723333 | 0.466667 |
| DNAH2 | 0.095 | -3.40303 | 0.010307 | 0.039107 | yes | down | 0.05 | 0.04 | 0.01 | 0 | 0.01 | 0 | 0.033333 | 0.003333 |
| CD209 | 2.054 | 1.038476 | 0.010325 | 0.039146 | yes | up | 4.74 | 2.88 | 1.58 | 7.76 | 8.48 | 13.43 | 3.066667 | 9.89 |
| FERMT3 | 4.929 | 2.301349 | 0.010332 | 0.039163 | yes | up | 0.58 | 0.46 | 0.5 | 9.18 | 1.11 | 1.83 | 0.513333 | 4.04 |
| IGSF11 | 2.674 | 1.418785 | 0.01034 | 0.039185 | yes | up | 0.19 | 0.17 | 0.15 | 0.6 | 0.55 | 1.04 | 0.17 | 0.73 |
| LOC108176881 | 5.569 | 2.477393 | 0.010383 | 0.03932 | yes | up | 0 | 0.13 | 0.06 | 0.4 | 0.76 | 0.72 | 0.063333 | 0.626667 |
| DQX1 | 3.298 | 1.721726 | 0.010402 | 0.039367 | yes | up | 0.91 | 0.7 | 0.24 | 1.78 | 2.58 | 4.88 | 0.616667 | 3.08 |
| CCL22 | 15.445 | 3.949103 | 0.010403 | 0.039367 | yes | up | 0 | 0.03 | 0.05 | 1.8 | 0.09 | 0.29 | 0.026667 | 0.726667 |
| LOC108176507 | 6.477 | 2.695398 | 0.010508 | 0.039709 | yes | up | 0.02 | 0.04 | 0.01 | 0.36 | 0.16 | 0.19 | 0.023333 | 0.236667 |
| P2RY8 | 14.213 | 3.829127 | 0.010544 | 0.039815 | yes | up | 0 | 0.06 | 0 | 0.94 | 0.14 | 0.28 | 0.02 | 0.453333 |
| RAD9B | 3.414 | 1.771423 | 0.010591 | 0.039973 | yes | up | 0.21 | 0.16 | 0.07 | 0.35 | 1.05 | 1.2 | 0.146667 | 0.866667 |
| LOC100358934 | 12.431 | 3.635871 | 0.010626 | 0.04007 | yes | up | 0 | 0.06 | 0.06 | 1.29 | 0.48 | 0.67 | 0.04 | 0.813333 |
| PTGES | 3.328 | 1.734749 | 0.010661 | 0.040181 | yes | up | 0.76 | 0.62 | 0.37 | 4.84 | 1.92 | 2.34 | 0.583333 | 3.033333 |
| LOC103351528 | 2.794 | 1.482162 | 0.010705 | 0.040314 | yes | up | 0.11 | 0.16 | 0.13 | 0.99 | 0.39 | 0.55 | 0.133333 | 0.643333 |
| LOC108176812 | 18.622 | 4.218975 | 0.010787 | 0.040582 | yes | up | 0 | 0.02 | 0 | 0.09 | 0.23 | 0.17 | 0.006667 | 0.163333 |
| LOC103350364 | 4.729 | 2.241401 | 0.010816 | 0.04065 | yes | up | 0.06 | 0.08 | 0.05 | 0.52 | 0.44 | 0.42 | 0.063333 | 0.46 |
| CCDC17 | 2.373 | 1.246726 | 0.010825 | 0.040676 | yes | up | 0.33 | 0.3 | 0.18 | 1.08 | 1.04 | 0.87 | 0.27 | 0.996667 |
| SCML2 | 0.286 | -1.80358 | 0.010845 | 0.040732 | yes | down | 0.42 | 0.73 | 0.59 | 0.15 | 0.42 | 0.23 | 0.58 | 0.266667 |
| TNFRSF9 | 9.408 | 3.233896 | 0.010917 | 0.040935 | yes | up | 0.06 | 0.05 | 0 | 1.04 | 0.29 | 0.23 | 0.036667 | 0.52 |
| LOC103345190 | 27.262 | 4.7688 | 0.01093 | 0.040966 | yes | up | 0 | 0 | 0 | 0.29 | 0.23 | 0.18 | 0 | 0.233333 |
| GRIA3 | 2.596 | 1.376133 | 0.010936 | 0.040978 | yes | up | 0.58 | 0.37 | 0.18 | 0.84 | 2 | 1.57 | 0.376667 | 1.47 |
| LOC103351304 | 2.506 | 1.325652 | 0.010981 | 0.041127 | yes | up | 0.23 | 0.31 | 0.13 | 0.93 | 0.93 | 0.78 | 0.223333 | 0.88 |
| EFR3B | 0.489 | -1.0309 | 0.011017 | 0.041241 | yes | down | 1.98 | 1.1 | 1.21 | 0.55 | 1.18 | 1.19 | 1.43 | 0.973333 |
| LOC103351689 | 10.191 | 3.349181 | 0.011055 | 0.041345 | yes | up | 0.06 | 0.1 | 0 | 0.3 | 0.9 | 1.27 | 0.053333 | 0.823333 |
| CAMP | 0.144 | -2.79485 | 0.01115 | 0.041661 | yes | down | 0.37 | 1.28 | 1.72 | 0.15 | 0.1 | 0.2 | 1.123333 | 0.15 |
| SEPT1 | 13.072 | 3.708391 | 0.011253 | 0.041963 | yes | up | 0.14 | 0.06 | 0.06 | 4.74 | 0.44 | 0.16 | 0.086667 | 1.78 |
| LOC103349490 | 0.092 | -3.44462 | 0.011362 | 0.042344 | yes | down | 0.62 | 0.67 | 1.17 | 0 | 0.11 | 0.29 | 0.82 | 0.133333 |
| PDE1C | 3.102 | 1.633047 | 0.011385 | 0.042402 | yes | up | 0.14 | 0.1 | 0.02 | 0.42 | 0.38 | 0.55 | 0.086667 | 0.45 |
| LRRC36 | 2.375 | 1.24785 | 0.011405 | 0.042448 | yes | up | 0.89 | 0.46 | 0.57 | 1.35 | 3.12 | 2.79 | 0.64 | 2.42 |
| ANKS1B | 3.187 | 1.672186 | 0.011406 | 0.042448 | yes | up | 0.06 | 1.19 | 0.03 | 0.59 | 0.68 | 1.09 | 0.426667 | 0.786667 |
| C5H16orf46 | 2.588 | 1.371979 | 0.011515 | 0.042804 | yes | up | 0.53 | 0.93 | 0.46 | 3.14 | 2.96 | 1.62 | 0.64 | 2.573333 |
| ST8SIA2 | 0.21 | -2.25452 | 0.011632 | 0.043161 | yes | down | 0.15 | 0.19 | 0.11 | 0.01 | 0.07 | 0.07 | 0.15 | 0.05 |
| NACC1 | 0.338 | -1.56625 | 0.01166 | 0.043244 | yes | down | 2.19 | 3.4 | 1.7 | 1.88 | 1.22 | 0.72 | 2.43 | 1.273333 |
| GMFG | 3.189 | 1.673025 | 0.011756 | 0.043519 | yes | up | 5.19 | 5.09 | 3.82 | 49.52 | 9.15 | 12.09 | 4.7 | 23.58667 |
| EGR3 | 4.293 | 2.101972 | 0.011768 | 0.043553 | yes | up | 0.15 | 0.04 | 0.03 | 0.74 | 0.35 | 0.37 | 0.073333 | 0.486667 |
| CSF2RA | 9.576 | 3.259404 | 0.011898 | 0.043962 | yes | up | 1.25 | 1.64 | 0.76 | 48.71 | 1.46 | 4.6 | 1.216667 | 18.25667 |
| ATP1A4 | 50.888 | 5.669253 | 0.01191 | 0.043986 | yes | up | 0 | 0 | 0 | 0.95 | 0.02 | 0.06 | 0 | 0.343333 |
| TTC7A | 2.167 | 1.116023 | 0.011949 | 0.04411 | yes | up | 1.67 | 1.6 | 1.32 | 9.4 | 2.86 | 3.96 | 1.53 | 5.406667 |
| ADPRHL1 | 0.111 | -3.17252 | 0.011966 | 0.044141 | yes | down | 19.45 | 2.49 | 2.65 | 0.15 | 2.1 | 1.97 | 8.196667 | 1.406667 |
| LOC108176869 | 2.308 | 1.206775 | 0.01198 | 0.044167 | yes | up | 0.32 | 0.24 | 0.1 | 0.73 | 0.79 | 0.84 | 0.22 | 0.786667 |
| XKR6 | 3.441 | 1.782836 | 0.011981 | 0.044167 | yes | up | 0.02 | 0.02 | 0.03 | 0.13 | 0.12 | 0.08 | 0.023333 | 0.11 |
| LOC100344252 | 6.087 | 2.605702 | 0.012006 | 0.04424 | yes | up | 0.11 | 0.17 | 0.14 | 3.49 | 0.64 | 0.41 | 0.14 | 1.513333 |
| LOC100348315 | 49.524 | 5.630067 | 0.012007 | 0.04424 | yes | up | 0 | 0 | 0.03 | 2.38 | 0.12 | 0 | 0.01 | 0.833333 |
| LOC100349217 | 0.161 | -2.63922 | 0.012088 | 0.044492 | yes | down | 0.25 | 0.59 | 0.62 | 0.09 | 0.04 | 0.26 | 0.486667 | 0.13 |
| RRS1 | 0.405 | -1.30387 | 0.012092 | 0.044496 | yes | down | 3.97 | 6.01 | 1.69 | 4.17 | 1.7 | 1.71 | 3.89 | 2.526667 |
| SLC9B1 | 0.371 | -1.43155 | 0.012141 | 0.044613 | yes | down | 0.63 | 1.76 | 1 | 0.81 | 0.73 | 0.64 | 1.13 | 0.726667 |
| SEMA5B | 0.439 | -1.18855 | 0.012169 | 0.044652 | yes | down | 0.4 | 0.32 | 0.48 | 0.32 | 0.26 | 0.28 | 0.4 | 0.286667 |
| LOC103350261 | 0.405 | -1.30315 | 0.012171 | 0.044652 | yes | down | 0.5 | 0.47 | 0.39 | 0.33 | 0.32 | 0.21 | 0.453333 | 0.286667 |
| LOC100009134 | 0.035 | -4.82247 | 0.012174 | 0.044652 | yes | down | 0.25 | 3.57 | 0.85 | 0.22 | 0 | 0 | 1.556667 | 0.073333 |
| MAP4K1 | 6.513 | 2.703275 | 0.012185 | 0.044654 | yes | up | 0.14 | 0.16 | 0.23 | 4.76 | 0.37 | 0.47 | 0.176667 | 1.866667 |
| GALNTL5 | 0.195 | -2.35595 | 0.012195 | 0.044661 | yes | down | 0.58 | 0.27 | 0.79 | 0.14 | 0.35 | 0.02 | 0.546667 | 0.17 |
| KIAA1257 | 0.201 | -2.31119 | 0.01226 | 0.044834 | yes | down | 0.24 | 0.19 | 0.63 | 0.06 | 0.23 | 0.05 | 0.353333 | 0.113333 |
| TDRD12 | 0.493 | -1.02144 | 0.012344 | 0.045089 | yes | down | 1.46 | 0.82 | 0.59 | 0.8 | 0.78 | 0.54 | 0.956667 | 0.706667 |
| KCNH4 | 4.373 | 2.128693 | 0.012359 | 0.04512 | yes | up | 0.09 | 0.38 | 0.28 | 4.71 | 0.91 | 1.37 | 0.25 | 2.33 |
| LOC103349932 | 26.846 | 4.746618 | 0.012381 | 0.045145 | yes | up | 0 | 0 | 0.04 | 0.04 | 0.51 | 1.35 | 0.013333 | 0.633333 |
| LOC108178658 | 5.396 | 2.43183 | 0.012381 | 0.045145 | yes | up | 0.13 | 0.12 | 0.03 | 0.41 | 1.33 | 0.47 | 0.093333 | 0.736667 |
| PPBP | 0.065 | -3.94091 | 0.01241 | 0.045239 | yes | down | 1.77 | 2.83 | 1.8 | 0.62 | 0 | 0 | 2.133333 | 0.206667 |
| FAHD1 | 0.451 | -1.14927 | 0.012416 | 0.045249 | yes | down | 6.4 | 4.16 | 6.26 | 2.26 | 4.28 | 5.88 | 5.606667 | 4.14 |
| IL20RB | 7.757 | 2.955481 | 0.012454 | 0.045358 | yes | up | 0.02 | 0.02 | 0.02 | 0.25 | 0.25 | 0.28 | 0.02 | 0.26 |
| LOC100347480 | 2.031 | 1.022159 | 0.012525 | 0.045573 | yes | up | 9.94 | 27.68 | 11.09 | 46.3 | 39.01 | 72.13 | 16.23667 | 52.48 |
| PDLIM5 | 0.381 | -1.39056 | 0.012571 | 0.045727 | yes | down | 134.61 | 25.17 | 9.45 | 12.1 | 31.13 | 25.44 | 56.41 | 22.89 |
| LOC108175489 | 19.798 | 4.307313 | 0.012573 | 0.045727 | yes | up | 0 | 0 | 0.13 | 3.1 | 1.04 | 1.62 | 0.043333 | 1.92 |
| CA1 | 0.482 | -1.05337 | 0.012594 | 0.045773 | yes | down | 3.71 | 7.57 | 5.21 | 4.01 | 3.1 | 5.52 | 5.496667 | 4.21 |
| ZNF483 | 8.138 | 3.024632 | 0.012711 | 0.046124 | yes | up | 0 | 0.02 | 0.03 | 0.05 | 0.25 | 0.29 | 0.016667 | 0.196667 |
| TFAP2A | 4.706 | 2.234396 | 0.01291 | 0.046711 | yes | up | 0.19 | 0.09 | 0.21 | 0.47 | 0.33 | 2.88 | 0.163333 | 1.226667 |
| AIF1 | 3.823 | 1.934592 | 0.012945 | 0.046801 | yes | up | 8.62 | 7.79 | 5.58 | 105.24 | 9.36 | 17.37 | 7.33 | 43.99 |
| PLCXD1 | 2.226 | 1.154443 | 0.012998 | 0.046933 | yes | up | 0.35 | 0.43 | 0.76 | 1.72 | 1.5 | 2.22 | 0.513333 | 1.813333 |
| ADAM18 | 5.855 | 2.549676 | 0.01301 | 0.046955 | yes | up | 0.05 | 0.13 | 0.02 | 0.6 | 0.48 | 0.5 | 0.066667 | 0.526667 |
| DACH1 | 0.481 | -1.05528 | 0.013062 | 0.047089 | yes | down | 2.51 | 2.62 | 5.37 | 2.12 | 3.17 | 3.12 | 3.5 | 2.803333 |
| EDN3 | 0.092 | -3.43616 | 0.013084 | 0.047138 | yes | down | 1.3 | 0.09 | 0.12 | 0 | 0.14 | 0.08 | 0.503333 | 0.073333 |
| SLC2A12 | 2.704 | 1.435232 | 0.013084 | 0.047138 | yes | up | 0.6 | 0.14 | 0.44 | 1.25 | 1.9 | 1.98 | 0.393333 | 1.71 |
| NFAM1 | 4.557 | 2.188062 | 0.013134 | 0.047274 | yes | up | 0.21 | 0.21 | 0.15 | 3.41 | 0.23 | 0.5 | 0.19 | 1.38 |
| FAM81A | 2.048 | 1.034031 | 0.013134 | 0.047274 | yes | up | 0.61 | 0.39 | 0.23 | 1.44 | 1.31 | 1.39 | 0.41 | 1.38 |
| ACSL5 | 2.433 | 1.282497 | 0.013165 | 0.047345 | yes | up | 1.77 | 2.23 | 1.74 | 14.18 | 3.1 | 5.59 | 1.913333 | 7.623333 |
| BBOX1 | 0.449 | -1.15416 | 0.013369 | 0.048024 | yes | down | 1.07 | 1.45 | 1.26 | 0.87 | 0.55 | 1.33 | 1.26 | 0.916667 |
| DHDH | 0.356 | -1.48953 | 0.01338 | 0.048051 | yes | down | 1.3 | 1.12 | 1.06 | 0.41 | 0.82 | 0.75 | 1.16 | 0.66 |
| LOC108177736 | 0.145 | -2.78339 | 0.01352 | 0.048439 | yes | down | 0.16 | 0.25 | 0.13 | 0.08 | 0 | 0.04 | 0.18 | 0.04 |
| MPP7 | 0.418 | -1.25766 | 0.013651 | 0.048807 | yes | down | 4.1 | 1.65 | 0.85 | 1.36 | 1.23 | 1.73 | 2.2 | 1.44 |
| LOC100343999 | 2.188 | 1.129554 | 0.013659 | 0.048823 | yes | up | 0.59 | 0.48 | 0.95 | 3.12 | 1.97 | 2.26 | 0.673333 | 2.45 |
| LOC100343264 | 10.942 | 3.451826 | 0.013741 | 0.049073 | yes | up | 0.01 | 0.01 | 0 | 0.15 | 0.11 | 0.11 | 0.006667 | 0.123333 |
| ZKSCAN4 | 2.148 | 1.102791 | 0.013764 | 0.049144 | yes | up | 0.27 | 0.5 | 0.54 | 1.31 | 1.43 | 1.92 | 0.436667 | 1.553333 |
| VIL1 | 0.459 | -1.12431 | 0.013804 | 0.049264 | yes | down | 1.68 | 2.86 | 2.61 | 3.28 | 1.58 | 1.05 | 2.383333 | 1.97 |
| LOC100338379 | 0.028 | -5.17895 | 0.013852 | 0.049412 | yes | down | 0.05 | 0.06 | 0.16 | 0 | 0 | 0 | 0.09 | 0 |
| LOC108177420 | 17.75 | 4.149721 | 0.013869 | 0.049462 | yes | up | 0.12 | 0 | 0 | 0.43 | 0.77 | 1.32 | 0.04 | 0.84 |
| RWDD2A | 2.229 | 1.156545 | 0.01395 | 0.049662 | yes | up | 1.13 | 1.13 | 0.51 | 2.35 | 3.07 | 4.21 | 0.923333 | 3.21 |
| ZBTB32 | 13.908 | 3.797825 | 0.014022 | 0.049855 | yes | up | 0 | 0.06 | 0.03 | 1.72 | 0.09 | 0.28 | 0.03 | 0.696667 |
| NEURL2 | 0.294 | -1.76533 | 0.014022 | 0.049855 | yes | down | 7.84 | 3.03 | 1.85 | 0.67 | 3.01 | 2.04 | 4.24 | 1.906667 |
| MZB1 | 29.014 | 4.858667 | 0.014041 | 0.049889 | yes | up | 0 | 0 | 0.08 | 3.67 | 0.42 | 0.11 | 0.026667 | 1.4 |

iBAT_D1_VS_iBAT_M4

| Gene name | FC(iBAT_M4/iBAT_D1) | Log2FC(iBAT_M4/iBAT_D1) | Pvalue | Padjust | Significant | Regulate | D1iBAT1 | D1iBAT2 | D1iBAT3 | M4iBAT1 | M4iBAT2 | M4iBAT3 | iBAT_D1 | iBAT_M4 |
| --- | --- | --- | --- | --- | --- | --- | --- | --- | --- | --- | --- | --- | --- | --- |
| LOC100328910 | 250.139 | 7.966589 | 7.5E-183 | 1.2E-178 | yes | up | 9.52 | 4.67 | 4.48 | 3518.64 | 4070.66 | 3360.8 | 6.223333 | 3650.033 |
| LOC100350311 | 0.044 | -4.52198 | 7.4E-181 | 6.1E-177 | yes | down | 79 | 70.63 | 54.01 | 7.61 | 6.37 | 5.81 | 67.88 | 6.596667 |
| SNCG | 76.406 | 6.255619 | 2.1E-178 | 1.2E-174 | yes | up | 15.48 | 12.76 | 14.11 | 3445.74 | 2070.04 | 2401.75 | 14.11667 | 2639.177 |
| GPAT4 | 0.043 | -4.53743 | 1.3E-157 | 5.5E-154 | yes | down | 271.67 | 347.89 | 285.74 | 33.39 | 28.38 | 34.76 | 301.7667 | 32.17667 |
| MC5R | 119.506 | 6.900938 | 4.1E-124 | 1.3E-120 | yes | up | 2.39 | 3.05 | 1.49 | 269.99 | 409.68 | 375.87 | 2.31 | 351.8467 |
| ND5 | 0.103 | -3.27347 | 9.9E-116 | 2.7E-112 | yes | down | 63007.72 | 62554.36 | 62343.01 | 15982.22 | 15887.78 | 17702.12 | 62635.03 | 16524.04 |
| LOC100346046 | 851.763 | 9.734309 | 3.1E-112 | 7.3E-109 | yes | up | 0.59 | 0.62 | 0.53 | 1681.04 | 423.03 | 1524.45 | 0.58 | 1209.507 |
| MRPL39 | 0.153 | -2.71117 | 1E-109 | 2.2E-106 | yes | down | 119.67 | 117.28 | 107.33 | 44.75 | 40.73 | 40.73 | 114.76 | 42.07 |
| LOC100350716 | 0.034 | -4.87913 | 5.6E-99 | 1.02E-95 | yes | down | 18.65 | 14.79 | 12.07 | 1.6 | 1.2 | 0.92 | 15.17 | 1.24 |
| LOC100009551 | 49.728 | 5.635982 | 1.5E-90 | 2.48E-87 | yes | up | 7.13 | 14.39 | 10.17 | 1534.89 | 923.33 | 1170.32 | 10.56333 | 1209.513 |
| KCNH1 | 0.025 | -5.34335 | 3.46E-90 | 5.22E-87 | yes | down | 7.55 | 8.32 | 4.29 | 0.4 | 0.36 | 0.42 | 6.72 | 0.393333 |
| PPIF | 0.065 | -3.95431 | 1.14E-89 | 1.57E-86 | yes | down | 82.23 | 104.32 | 91.24 | 14.16 | 12.75 | 16.4 | 92.59667 | 14.43667 |
| NDUFA9 | 0.223 | -2.16266 | 3.5E-89 | 4.46E-86 | yes | down | 131.15 | 128.38 | 115.41 | 72.58 | 61.96 | 67.14 | 124.98 | 67.22667 |
| CRAT | 0.209 | -2.25747 | 4.12E-89 | 4.87E-86 | yes | down | 122.83 | 120.22 | 108.75 | 64.69 | 55.93 | 55.62 | 117.2667 | 58.74667 |
| PDK1 | 0.054 | -4.20179 | 3.88E-87 | 4.29E-84 | yes | down | 43.65 | 59.17 | 49.51 | 6.26 | 5.63 | 7.65 | 50.77667 | 6.513333 |
| QPCT | 0.033 | -4.90883 | 1.09E-85 | 1.13E-82 | yes | down | 361.16 | 337.63 | 321.24 | 22.38 | 22.08 | 37.19 | 340.01 | 27.21667 |
| MIF | 0.154 | -2.69938 | 1.77E-85 | 1.73E-82 | yes | down | 400.96 | 424.4 | 393.65 | 161.11 | 149.84 | 145.86 | 406.3367 | 152.27 |
| PRR5L | 29.092 | 4.862531 | 3.31E-83 | 3.04E-80 | yes | up | 0.56 | 0.46 | 0.42 | 30.96 | 40.68 | 28.17 | 0.48 | 33.27 |
| CST6 | 49.977 | 5.643199 | 8.47E-83 | 7.38E-80 | yes | up | 4.74 | 3.1 | 1.54 | 340.08 | 369.52 | 391.59 | 3.126667 | 367.0633 |
| HMGB3 | 0.096 | -3.37977 | 8.94E-81 | 7.4E-78 | yes | down | 60.11 | 72.68 | 52.81 | 11.57 | 14.29 | 11.46 | 61.86667 | 12.44 |
| ACAD9 | 0.145 | -2.78654 | 1.96E-79 | 1.54E-76 | yes | down | 22.49 | 24.34 | 20.79 | 7.31 | 7.13 | 7.3 | 22.54 | 7.246667 |
| NDUFS3 | 0.227 | -2.14105 | 4.79E-79 | 3.61E-76 | yes | down | 380.65 | 377.25 | 345.37 | 207.19 | 194.61 | 201.86 | 367.7567 | 201.22 |
| ARRB1 | 7.22 | 2.851978 | 2.43E-76 | 1.75E-73 | yes | up | 6.6 | 7.28 | 5.99 | 119.63 | 88.77 | 119.56 | 6.623333 | 109.32 |
| MRPS18B | 0.149 | -2.74597 | 1.63E-75 | 1.13E-72 | yes | down | 72.24 | 72.41 | 64.77 | 25.63 | 26.62 | 22.94 | 69.80667 | 25.06333 |
| LOC100359087 | 61.699 | 5.947165 | 6.5E-72 | 4.31E-69 | yes | up | 1.28 | 0.62 | 0.28 | 84.97 | 90.12 | 91.84 | 0.726667 | 88.97667 |
| LOC103348029 | 0.007 | -7.18073 | 1.82E-69 | 1.16E-66 | yes | down | 40.84 | 61.22 | 63.14 | 0.93 | 1.1 | 1.3 | 55.06667 | 1.11 |
| HK1 | 0.095 | -3.40351 | 2.37E-67 | 1.45E-64 | yes | down | 86.26 | 101.96 | 97.03 | 25.43 | 16.98 | 23.11 | 95.08333 | 21.84 |
| ND6 | 0.051 | -4.28215 | 2.29E-66 | 1.35E-63 | yes | down | 8343.68 | 5444.94 | 4871.46 | 936.7 | 914.38 | 463.62 | 6220.027 | 771.5667 |
| LOC100347143 | 0.232 | -2.1067 | 6.86E-66 | 3.92E-63 | yes | down | 493.49 | 493.04 | 467.93 | 284.64 | 250.96 | 279.66 | 484.82 | 271.7533 |
| COX2 | 0.094 | -3.40604 | 1.09E-65 | 6.03E-63 | yes | down | 67579.39 | 75887.18 | 77144.5 | 14877.61 | 17603.19 | 18024.67 | 73537.02 | 16835.16 |
| BDH1 | 0.158 | -2.66204 | 4.38E-65 | 2.34E-62 | yes | down | 213.64 | 193.34 | 165.86 | 78.91 | 62.5 | 91.34 | 190.9467 | 77.58333 |
| MPC1 | 0.158 | -2.66506 | 3.18E-64 | 1.65E-61 | yes | down | 432.73 | 323.74 | 304.46 | 141.56 | 131.43 | 126.7 | 353.6433 | 133.23 |
| CYP2A11 | 95.26 | 6.573799 | 1.16E-63 | 5.83E-61 | yes | up | 3.17 | 2.26 | 1.24 | 818.14 | 414.89 | 293.83 | 2.223333 | 508.9533 |
| IDH3A | 0.093 | -3.42839 | 1.74E-63 | 8.48E-61 | yes | down | 406.87 | 575.78 | 477.51 | 99.18 | 107.64 | 117.7 | 486.72 | 108.1733 |
| HSPA9 | 0.134 | -2.90003 | 2.53E-60 | 1.2E-57 | yes | down | 263.87 | 273.67 | 278.08 | 86.76 | 77.01 | 100.47 | 271.8733 | 88.08 |
| PCK2 | 12.987 | 3.69901 | 3.12E-60 | 1.43E-57 | yes | up | 19.27 | 32.69 | 19.94 | 892.11 | 671.73 | 710.56 | 23.96667 | 758.1333 |
| ND4L | 0.115 | -3.12401 | 3.84E-60 | 1.72E-57 | yes | down | 17325.17 | 20918.76 | 16409.85 | 4643.3 | 4910.35 | 6162.44 | 18217.93 | 5238.697 |
| HADHB | 0.059 | -4.08317 | 1.49E-59 | 6.5E-57 | yes | down | 1037.1 | 975.66 | 1224.12 | 127.97 | 144.98 | 194.96 | 1078.96 | 155.97 |
| APOE | 42.942 | 5.424313 | 2.71E-59 | 1.15E-56 | yes | up | 31.49 | 17.76 | 12.08 | 1839.82 | 2731.43 | 1839.31 | 20.44333 | 2136.853 |
| HIGD1A | 0.126 | -2.9863 | 6.15E-59 | 2.55E-56 | yes | down | 105.55 | 89.01 | 101.38 | 28.31 | 30.56 | 30.53 | 98.64667 | 29.8 |
| GNAS | 0.1 | -3.3291 | 9.63E-59 | 3.89E-56 | yes | down | 4031.76 | 4571.39 | 5277.49 | 1240.08 | 1064.67 | 1018.94 | 4626.88 | 1107.897 |
| SLC4A4 | 0.063 | -3.97912 | 2.78E-58 | 1.1E-55 | yes | down | 22.45 | 28.8 | 23.72 | 4.4 | 2.49 | 4.54 | 24.99 | 3.81 |
| TRABD2B | 16.858 | 4.075336 | 1.25E-56 | 4.8E-54 | yes | up | 0.63 | 0.39 | 0.44 | 16.24 | 19.69 | 21.12 | 0.486667 | 19.01667 |
| AIFM1 | 0.212 | -2.23864 | 4.41E-56 | 1.66E-53 | yes | down | 84.09 | 89 | 76.22 | 47.26 | 35.28 | 44.83 | 83.10333 | 42.45667 |
| COX3 | 0.12 | -3.06261 | 2.85E-55 | 1.05E-52 | yes | down | 64268.61 | 68943.2 | 77249 | 19581.63 | 20356.52 | 21467.15 | 70153.6 | 20468.43 |
| NDUFS1 | 0.235 | -2.0898 | 4.1E-55 | 1.48E-52 | yes | down | 191.76 | 177.2 | 163.09 | 108.21 | 83.32 | 105.07 | 177.35 | 98.86667 |
| HP | 72.079 | 6.171501 | 5.05E-55 | 1.78E-52 | yes | up | 19.3 | 9.9 | 13.8 | 1265.74 | 3076.44 | 3076.01 | 14.33333 | 2472.73 |
| ABCD2 | 53.482 | 5.740983 | 3.21E-54 | 1.11E-51 | yes | up | 0.54 | 1.21 | 0.3 | 112.53 | 62.87 | 76.14 | 0.683333 | 83.84667 |
| LOC100343920 | 30.397 | 4.925861 | 7.66E-54 | 2.59E-51 | yes | up | 1.16 | 0.62 | 0.36 | 59.21 | 46.46 | 49.99 | 0.713333 | 51.88667 |
| ADAMTS18 | 8.818 | 3.140409 | 1.44E-53 | 4.78E-51 | yes | up | 0.53 | 0.63 | 0.43 | 12.86 | 11.05 | 10.08 | 0.53 | 11.33 |
| PHB2 | 0.29 | -1.78472 | 3.69E-53 | 1.2E-50 | yes | down | 212.4 | 219.22 | 199.79 | 154.33 | 141.67 | 145.78 | 210.47 | 147.26 |
| GRIK3 | 0.035 | -4.82489 | 1.91E-52 | 6.08E-50 | yes | down | 1.95 | 1.54 | 1.44 | 0.12 | 0.13 | 0.17 | 1.643333 | 0.14 |
| LOC100350787 | 0.242 | -2.04437 | 1.14E-51 | 3.56E-49 | yes | down | 756.27 | 750.88 | 651.09 | 423.81 | 369.19 | 467.77 | 719.4133 | 420.2567 |
| HSDL2 | 0.218 | -2.19512 | 2.28E-51 | 6.98E-49 | yes | down | 224.46 | 180.35 | 180.2 | 103.83 | 104.69 | 99.62 | 195.0033 | 102.7133 |
| LRPPRC | 0.261 | -1.93649 | 3.55E-51 | 1.07E-48 | yes | down | 57.93 | 50.28 | 41.92 | 34.55 | 35.18 | 36.28 | 50.04333 | 35.33667 |
| SAMM50 | 0.273 | -1.87497 | 8.19E-51 | 2.42E-48 | yes | down | 117.8 | 111.39 | 100.02 | 79.17 | 62.77 | 74.47 | 109.7367 | 72.13667 |
| LOC100009353 | 4.194 | 2.068321 | 9.15E-51 | 2.66E-48 | yes | up | 91.94 | 85.06 | 64.44 | 808.43 | 733.08 | 858.3 | 80.48 | 799.9367 |
| TM7SF2 | 23.715 | 4.567739 | 1.33E-50 | 3.81E-48 | yes | up | 2.49 | 1.77 | 0.85 | 112.49 | 87.66 | 68.73 | 1.703333 | 89.62667 |
| RDH8 | 30.486 | 4.930096 | 1.7E-50 | 4.76E-48 | yes | up | 0.58 | 0.42 | 0.48 | 32.65 | 33 | 41.33 | 0.493333 | 35.66 |
| ST13 | 0.292 | -1.77466 | 3.32E-50 | 9.15E-48 | yes | down | 85.69 | 87.25 | 75.19 | 62.68 | 50.61 | 62.82 | 82.71 | 58.70333 |
| IL1RN | 0.014 | -6.15792 | 4.49E-50 | 1.22E-47 | yes | down | 36.87 | 101.32 | 43.36 | 1.68 | 1.53 | 2.65 | 60.51667 | 1.953333 |
| LOC103348133 | 0.021 | -5.54804 | 5.83E-50 | 1.56E-47 | yes | down | 19.07 | 22.31 | 15.42 | 1.17 | 1.27 | 0.39 | 18.93333 | 0.943333 |
| APLP2 | 3.907 | 1.966089 | 8.92E-50 | 2.34E-47 | yes | up | 79.84 | 88.01 | 74.71 | 733.91 | 745.47 | 790.46 | 80.85333 | 756.6133 |
| ACADVL | 0.089 | -3.49367 | 1.91E-49 | 4.94E-47 | yes | down | 786.87 | 941.08 | 939.41 | 174.28 | 157.85 | 242.58 | 889.12 | 191.57 |
| AQP8 | 126.26 | 6.980251 | 3.52E-49 | 8.97E-47 | yes | up | 4.88 | 2.08 | 1.02 | 748.58 | 468.03 | 1121.34 | 2.66 | 779.3167 |
| MTCH2 | 0.292 | -1.77581 | 6.67E-49 | 1.67E-46 | yes | down | 199.74 | 183.79 | 161.74 | 129.16 | 131.95 | 124.44 | 181.7567 | 128.5167 |
| TIMM44 | 0.262 | -1.93073 | 7.95E-49 | 1.96E-46 | yes | down | 55.27 | 62.39 | 49.71 | 39.74 | 33.21 | 32.7 | 55.79 | 35.21667 |
| ITIH3 | 101.154 | 6.660411 | 2.24E-48 | 5.44E-46 | yes | up | 0.17 | 0.19 | 0.07 | 23.91 | 46.14 | 32.33 | 0.143333 | 34.12667 |
| GLRX | 0.179 | -2.47994 | 4.32E-48 | 1.04E-45 | yes | down | 61.42 | 52.21 | 51.85 | 25.47 | 26.19 | 20.95 | 55.16 | 24.20333 |
| FADS3 | 4.731 | 2.242175 | 5.26E-48 | 1.24E-45 | yes | up | 24.26 | 22.47 | 23.95 | 269.23 | 265.3 | 273.61 | 23.56 | 269.38 |
| COL5A3 | 11.153 | 3.479375 | 8.16E-48 | 1.9E-45 | yes | up | 6.87 | 5.19 | 3.32 | 147.97 | 107.04 | 151.46 | 5.126667 | 135.49 |
| LOC100347571 | 0.15 | -2.7338 | 1.73E-47 | 3.99E-45 | yes | down | 529.32 | 497.38 | 593.46 | 220.17 | 173.17 | 201.23 | 540.0533 | 198.19 |
| SUCLG1 | 0.329 | -1.60338 | 5.48E-46 | 1.24E-43 | yes | down | 448.69 | 450.62 | 416.1 | 379.38 | 324.01 | 339.9 | 438.47 | 347.7633 |
| ND4 | 0.113 | -3.14638 | 6.08E-46 | 1.36E-43 | yes | down | 12347.23 | 16522.07 | 14129.99 | 3334.01 | 3704.94 | 4650.12 | 14333.1 | 3896.357 |
| DNAJA3 | 0.328 | -1.61037 | 6.28E-46 | 1.39E-43 | yes | down | 50.05 | 46.79 | 40.98 | 37.5 | 33.4 | 37.31 | 45.94 | 36.07 |
| MRPS5 | 0.216 | -2.20802 | 1.37E-45 | 2.98E-43 | yes | down | 40.84 | 47.19 | 41.65 | 23.04 | 23.34 | 21.25 | 43.22667 | 22.54333 |
| GABBR1 | 6.439 | 2.686818 | 9.28E-45 | 2E-42 | yes | up | 0.8 | 0.63 | 0.53 | 12.51 | 9.86 | 11.27 | 0.653333 | 11.21333 |
| GFM1 | 0.235 | -2.08686 | 1.02E-44 | 2.17E-42 | yes | down | 49.56 | 41.64 | 32.97 | 23.52 | 22.5 | 24.81 | 41.39 | 23.61 |
| GMPR | 0.008 | -7.04549 | 1.19E-44 | 2.49E-42 | yes | down | 488.21 | 895.55 | 491.59 | 3.54 | 13.4 | 16.08 | 625.1167 | 11.00667 |
| GPLD1 | 0.035 | -4.8474 | 3.26E-44 | 6.76E-42 | yes | down | 2.43 | 2.69 | 2.78 | 0.23 | 0.24 | 0.18 | 2.633333 | 0.216667 |
| PPA1 | 0.209 | -2.26019 | 4.67E-44 | 9.55E-42 | yes | down | 203.27 | 265.13 | 215.7 | 125.88 | 108.19 | 107.94 | 228.0333 | 114.0033 |
| GSN | 5.673 | 2.50421 | 6.69E-44 | 1.35E-41 | yes | up | 138.85 | 112.15 | 77.06 | 1517.61 | 1397.54 | 1493.06 | 109.3533 | 1469.403 |
| PPA2 | 0.249 | -2.00563 | 1.26E-43 | 2.5E-41 | yes | down | 70.64 | 80.18 | 71.6 | 49.12 | 41.74 | 43.67 | 74.14 | 44.84333 |
| PDK4 | 0.079 | -3.66425 | 3.55E-43 | 7E-41 | yes | down | 452.22 | 343.59 | 563.72 | 82.81 | 87.36 | 90.05 | 453.1767 | 86.74 |
| ND2 | 0.091 | -3.45244 | 6.23E-43 | 1.21E-40 | yes | down | 16686.18 | 22555.63 | 16117.67 | 3159.24 | 3684.37 | 5237.32 | 18453.16 | 4026.977 |
| FMO1 | 78.33 | 6.291489 | 1.38E-42 | 2.66E-40 | yes | up | 0.55 | 0.3 | 0.26 | 52.89 | 43.83 | 109.48 | 0.37 | 68.73333 |
| ATP5A1 | 0.204 | -2.2956 | 1.89E-42 | 3.6E-40 | yes | down | 1403.47 | 1568.38 | 1548.89 | 841.67 | 633.31 | 763.74 | 1506.913 | 746.24 |
| ETFDH | 0.227 | -2.1373 | 2.03E-42 | 3.83E-40 | yes | down | 239.9 | 219.11 | 179.47 | 95.88 | 101.6 | 140.36 | 212.8267 | 112.6133 |
| C19H17orf50 | 5.654 | 2.499333 | 2.29E-42 | 4.23E-40 | yes | up | 3.07 | 4.93 | 3.54 | 53.64 | 40.77 | 51.17 | 3.846667 | 48.52667 |
| ADORA1 | 28.849 | 4.850459 | 2.3E-42 | 4.23E-40 | yes | up | 0.33 | 0.58 | 0.14 | 21.49 | 19.82 | 25.44 | 0.35 | 22.25 |
| LOC100341993 | 0.231 | -2.11273 | 3.4E-42 | 6.19E-40 | yes | down | 84.08 | 75.38 | 67.81 | 46.95 | 36.26 | 45.02 | 75.75667 | 42.74333 |
| DHRS11 | 0.151 | -2.72829 | 5.86E-42 | 1.05E-39 | yes | down | 107.95 | 111.63 | 113.85 | 42.07 | 32.1 | 47.94 | 111.1433 | 40.70333 |
| LOC100343982 | 0.25 | -1.99928 | 7.47E-42 | 1.33E-39 | yes | down | 1355.42 | 1545.25 | 1393.32 | 963.09 | 857 | 798.97 | 1431.33 | 873.02 |
| IMMT | 0.298 | -1.74877 | 9.85E-42 | 1.74E-39 | yes | down | 146.73 | 136.6 | 125.74 | 100.94 | 104.43 | 103.03 | 136.3567 | 102.8 |
| ATP6 | 0.128 | -2.96333 | 9.97E-42 | 1.74E-39 | yes | down | 30410.82 | 32018.67 | 37755.09 | 9492.52 | 10250.74 | 11643.64 | 33394.86 | 10462.3 |
| DLST | 0.234 | -2.09254 | 1.32E-41 | 2.27E-39 | yes | down | 281.34 | 270.81 | 265.83 | 186.69 | 131.46 | 146.96 | 272.66 | 155.0367 |
| CIAPIN1 | 0.156 | -2.67578 | 1.54E-41 | 2.62E-39 | yes | down | 55.21 | 68.41 | 66.59 | 23.3 | 23.1 | 25.72 | 63.40333 | 24.04 |
| TUFM | 0.236 | -2.08086 | 1.77E-41 | 3E-39 | yes | down | 148.51 | 160.02 | 158.87 | 101.21 | 83.76 | 86.9 | 155.8 | 90.62333 |
| HYAL1 | 4.779 | 2.256803 | 2.5E-41 | 4.18E-39 | yes | up | 8.38 | 8.48 | 7.56 | 90.2 | 71.71 | 94.04 | 8.14 | 85.31667 |
| PDHA1 | 0.257 | -1.96056 | 2.72E-41 | 4.5E-39 | yes | down | 187.67 | 180.23 | 175.65 | 118.56 | 97.65 | 122.44 | 181.1833 | 112.8833 |
| NDUFA6 | 0.254 | -1.97508 | 6.45E-41 | 1.06E-38 | yes | down | 513.36 | 593.03 | 536.36 | 357.48 | 327.22 | 334.23 | 547.5833 | 339.6433 |
| PRDX3 | 0.273 | -1.8706 | 1.12E-40 | 1.83E-38 | yes | down | 317.34 | 302.63 | 289.05 | 206.2 | 188.72 | 220.08 | 303.0067 | 205 |
| ACADM | 0.101 | -3.31177 | 1.81E-40 | 2.91E-38 | yes | down | 333.02 | 302.41 | 405.62 | 74.38 | 80.29 | 100.43 | 347.0167 | 85.03333 |
| ATP5L | 0.311 | -1.68581 | 2.38E-40 | 3.79E-38 | yes | down | 976.85 | 993.97 | 879.49 | 829.41 | 693.88 | 660.91 | 950.1033 | 728.0667 |
| INMT | 43.231 | 5.433995 | 2.4E-40 | 3.79E-38 | yes | up | 0.62 | 0.54 | 0.08 | 46.91 | 31.38 | 46.91 | 0.413333 | 41.73333 |
| FBN2 | 0.056 | -4.16469 | 2.71E-40 | 4.23E-38 | yes | down | 1.2 | 1.05 | 0.68 | 0.13 | 0.11 | 0.14 | 0.976667 | 0.126667 |
| GPD1 | 9.581 | 3.26014 | 1.02E-39 | 1.58E-37 | yes | up | 151.8 | 100.13 | 65.22 | 2850.45 | 2119.34 | 2316.5 | 105.7167 | 2428.763 |
| ADAMTS16 | 25.537 | 4.674508 | 1.05E-39 | 1.61E-37 | yes | up | 0.2 | 0.25 | 0.24 | 10.49 | 11.84 | 18.91 | 0.23 | 13.74667 |
| ADH1A | 12.776 | 3.675318 | 1.46E-39 | 2.21E-37 | yes | up | 9.29 | 13.89 | 8.71 | 284.6 | 241.67 | 424.92 | 10.63 | 317.0633 |
| KLF6 | 2.844 | 1.508007 | 4.31E-39 | 6.48E-37 | yes | up | 13.73 | 15.4 | 12.36 | 100.09 | 85.37 | 91.48 | 13.83 | 92.31333 |
| IGSF8 | 7.036 | 2.814675 | 9.9E-39 | 1.48E-36 | yes | up | 4.85 | 3.65 | 3.63 | 60.98 | 78.3 | 64.02 | 4.043333 | 67.76667 |
| SLC35F6 | 0.234 | -2.09755 | 2.01E-38 | 2.97E-36 | yes | down | 13.66 | 14.07 | 13.25 | 8.94 | 5.04 | 7.19 | 13.66 | 7.056667 |
| SDHB | 0.263 | -1.92939 | 2.76E-38 | 4.04E-36 | yes | down | 455.21 | 443.04 | 464.43 | 306.98 | 267.46 | 295.63 | 454.2267 | 290.0233 |
| TBRG4 | 0.197 | -2.34721 | 4.38E-38 | 6.37E-36 | yes | down | 39.02 | 33.62 | 31.79 | 14.38 | 15.24 | 18.51 | 34.81 | 16.04333 |
| LOC100328948 | 34.655 | 5.114977 | 4.9E-38 | 7.06E-36 | yes | up | 1.54 | 1.06 | 2.2 | 177.83 | 106.24 | 148.22 | 1.6 | 144.0967 |
| PRELID3B | 0.137 | -2.86296 | 7.61E-38 | 1.09E-35 | yes | down | 105.59 | 158.26 | 124.63 | 48.22 | 49.24 | 39.09 | 129.4933 | 45.51667 |
| GLDN | 0.017 | -5.8458 | 9.87E-38 | 1.4E-35 | yes | down | 11.71 | 23.37 | 8.13 | 0.32 | 0.88 | 0.54 | 14.40333 | 0.58 |
| MIPEP | 0.205 | -2.28308 | 3.03E-37 | 4.26E-35 | yes | down | 22.42 | 25.39 | 23.11 | 10.7 | 12.1 | 12.22 | 23.64 | 11.67333 |
| LEP | 2622.059 | 11.35648 | 8.14E-37 | 1.13E-34 | yes | up | 0.05 | 0 | 0 | 75.37 | 130.73 | 82.93 | 0.016667 | 96.34333 |
| RAB27A | 7.22 | 2.852005 | 8.68E-37 | 1.2E-34 | yes | up | 3.85 | 3.51 | 4.25 | 59.6 | 71.04 | 74.18 | 3.87 | 68.27333 |
| LPL | 3.544 | 1.825236 | 1.1E-36 | 1.5E-34 | yes | up | 976.79 | 1172.04 | 962.92 | 8689.72 | 8316.34 | 9381.38 | 1037.25 | 8795.813 |
| ATP5F1 | 0.232 | -2.1106 | 2.02E-36 | 2.74E-34 | yes | down | 575.7 | 583.25 | 496.33 | 343.75 | 327.23 | 245.73 | 551.76 | 305.57 |
| CYTB | 0.116 | -3.10522 | 3.13E-36 | 4.22E-34 | yes | down | 20607.73 | 18026.4 | 22767.76 | 4859.1 | 5264.82 | 7198.4 | 20467.3 | 5774.107 |
| MPI | 0.141 | -2.82124 | 4.73E-36 | 6.31E-34 | yes | down | 20.08 | 14.44 | 16.88 | 7.04 | 5.99 | 5 | 17.13333 | 6.01 |
| CA5B | 9.17 | 3.196908 | 5.79E-36 | 7.68E-34 | yes | up | 2.63 | 2.24 | 1.33 | 35.36 | 44.61 | 41.59 | 2.066667 | 40.52 |
| CHCHD3 | 0.161 | -2.63656 | 1.31E-35 | 1.72E-33 | yes | down | 229.83 | 357.33 | 292.38 | 121.12 | 105.98 | 115.58 | 293.18 | 114.2267 |
| ECH1 | 0.144 | -2.79757 | 1.55E-35 | 2.03E-33 | yes | down | 423.64 | 402.33 | 538.56 | 171.61 | 137.92 | 182.39 | 454.8433 | 163.9733 |
| CHCHD10 | 0.127 | -2.98207 | 4.52E-35 | 5.85E-33 | yes | down | 84 | 85.2 | 112.75 | 28.61 | 32.33 | 28.63 | 93.98333 | 29.85667 |
| LOC103346593 | 0.052 | -4.27514 | 5.06E-35 | 6.5E-33 | yes | down | 19.32 | 25.73 | 21.63 | 2.38 | 4.17 | 1.72 | 22.22667 | 2.756667 |
| ACSS2 | 13.932 | 3.800337 | 5.55E-35 | 7.07E-33 | yes | up | 19.89 | 14.77 | 18.46 | 853.64 | 347.55 | 621.29 | 17.70667 | 607.4933 |
| ATP6V1E1 | 0.353 | -1.50294 | 5.82E-35 | 7.36E-33 | yes | down | 169.73 | 185.27 | 155.11 | 151.15 | 131.06 | 151.07 | 170.0367 | 144.4267 |
| ACAT1 | 0.176 | -2.51029 | 5.89E-35 | 7.39E-33 | yes | down | 443.03 | 336.09 | 383.83 | 149.32 | 150.12 | 191.94 | 387.65 | 163.7933 |
| AFG3L2 | 0.256 | -1.96602 | 1.69E-34 | 2.11E-32 | yes | down | 41.84 | 47.4 | 40.3 | 24.49 | 26.32 | 28.68 | 43.18 | 26.49667 |
| NPC2 | 3.957 | 1.984542 | 5.69E-34 | 7.04E-32 | yes | up | 32.93 | 35.08 | 35.72 | 329.96 | 326.49 | 338.08 | 34.57667 | 331.51 |
| LOC100355623 | 3.454 | 1.788418 | 6.97E-34 | 8.55E-32 | yes | up | 4.04 | 3.75 | 2.84 | 31.32 | 27.61 | 29.61 | 3.543333 | 29.51333 |
| LOC100358417 | 0.13 | -2.94088 | 7.6E-34 | 9.26E-32 | yes | down | 14.23 | 11.87 | 9.54 | 3.77 | 3 | 5.02 | 11.88 | 3.93 |
| ATP5B | 0.188 | -2.41327 | 8.55E-34 | 1.03E-31 | yes | down | 2240.99 | 2222.67 | 2497.12 | 1057.96 | 895.79 | 1222.51 | 2320.26 | 1058.753 |
| LOC100343793 | 0.259 | -1.94819 | 9.33E-34 | 1.12E-31 | yes | down | 2048.69 | 2190.49 | 2234.3 | 1531.06 | 1226.48 | 1363.03 | 2157.827 | 1373.523 |
| LOC100339305 | 68.744 | 6.103153 | 1.19E-33 | 1.42E-31 | yes | up | 0.88 | 0.21 | 0.16 | 65.59 | 71.52 | 61.63 | 0.416667 | 66.24667 |
| TFB2M | 0.211 | -2.24459 | 1.37E-33 | 1.62E-31 | yes | down | 38.79 | 42.95 | 39.91 | 19.02 | 22.9 | 20.38 | 40.55 | 20.76667 |
| LOC100328613 | 0.035 | -4.85537 | 1.49E-33 | 1.75E-31 | yes | down | 308.91 | 818.45 | 410.3 | 31.54 | 32.21 | 62 | 512.5533 | 41.91667 |
| NRIP1 | 5.667 | 2.502474 | 1.53E-33 | 1.78E-31 | yes | up | 2.89 | 1.8 | 1.88 | 29.44 | 30.83 | 28.57 | 2.19 | 29.61333 |
| ITPK1 | 0.264 | -1.91978 | 1.54E-33 | 1.78E-31 | yes | down | 33.15 | 38.95 | 30.55 | 21.07 | 19.98 | 24.23 | 34.21667 | 21.76 |
| RBM3 | 0.204 | -2.29622 | 1.8E-33 | 2.07E-31 | yes | down | 166.91 | 235.97 | 165.1 | 76.13 | 75.96 | 58.5 | 189.3267 | 70.19667 |
| IGF1 | 4.149 | 2.052835 | 1.86E-33 | 2.12E-31 | yes | up | 15.53 | 12.79 | 11.65 | 123.5 | 145.41 | 126.21 | 13.32333 | 131.7067 |
| ID3 | 0.152 | -2.71712 | 1.99E-33 | 2.25E-31 | yes | down | 491.74 | 527.12 | 400.77 | 166.34 | 127.43 | 224.63 | 473.21 | 172.8 |
| CLPX | 0.315 | -1.66763 | 2.03E-33 | 2.29E-31 | yes | down | 55.53 | 59.94 | 44.98 | 37.78 | 37.12 | 40.66 | 53.48333 | 38.52 |
| CKB | 7.551 | 2.916733 | 2.5E-33 | 2.8E-31 | yes | up | 18.58 | 18.53 | 12.82 | 428.3 | 238.37 | 242.89 | 16.64333 | 303.1867 |
| TBC1D9B | 2.514 | 1.329798 | 2.87E-33 | 3.2E-31 | yes | up | 13.44 | 13.17 | 10.56 | 75.76 | 72.66 | 72.5 | 12.39 | 73.64 |
| IGF2BP3 | 0.122 | -3.03301 | 3.22E-33 | 3.55E-31 | yes | down | 3.88 | 3.03 | 2.82 | 1.11 | 0.99 | 0.76 | 3.243333 | 0.953333 |
| SIRPA | 3.94 | 1.978086 | 3.81E-33 | 4.18E-31 | yes | up | 8.52 | 7.97 | 5.36 | 67.2 | 70.47 | 66.16 | 7.283333 | 67.94333 |
| MRPS22 | 0.267 | -1.90275 | 4.48E-33 | 4.88E-31 | yes | down | 37.1 | 47.28 | 35.17 | 27.92 | 24.11 | 26.45 | 39.85 | 26.16 |
| EPYC | 34.513 | 5.109085 | 6.03E-33 | 6.53E-31 | yes | up | 0.23 | 0.31 | 0.07 | 16.91 | 16.14 | 14.66 | 0.203333 | 15.90333 |
| PDHB | 0.341 | -1.55374 | 1.33E-32 | 1.43E-30 | yes | down | 252.08 | 225.67 | 217.11 | 198.48 | 194.8 | 176.35 | 231.62 | 189.8767 |
| DHCR24 | 5.225 | 2.385419 | 1.35E-32 | 1.44E-30 | yes | up | 4.19 | 2.92 | 2.85 | 49.73 | 34.43 | 41 | 3.32 | 41.72 |
| GMPS | 0.131 | -2.92931 | 1.99E-32 | 2.12E-30 | yes | down | 19.5 | 33.31 | 26.69 | 8.98 | 7.24 | 9.06 | 26.5 | 8.426667 |
| EBP | 7.807 | 2.964779 | 5.25E-32 | 5.54E-30 | yes | up | 24.39 | 25.94 | 23.97 | 630.56 | 304.05 | 483.85 | 24.76667 | 472.82 |
| PLA2G16 | 3.133 | 1.647321 | 5.84E-32 | 6.12E-30 | yes | up | 252.49 | 251.54 | 237.93 | 1819.39 | 1768.66 | 2013.9 | 247.32 | 1867.317 |
| SHMT2 | 0.234 | -2.09248 | 6.23E-32 | 6.49E-30 | yes | down | 18.8 | 22.61 | 16.78 | 13.16 | 9.97 | 9.72 | 19.39667 | 10.95 |
| ND3 | 0.114 | -3.13119 | 7E-32 | 7.24E-30 | yes | down | 27501.35 | 42960.9 | 30782.62 | 7487.68 | 9202.21 | 11850.8 | 33748.29 | 9513.563 |
| SDHA | 0.254 | -1.9788 | 7.57E-32 | 7.77E-30 | yes | down | 706.37 | 607.87 | 678.16 | 420.16 | 383.85 | 455.69 | 664.1333 | 419.9 |
| NDUFB6 | 0.342 | -1.54658 | 7.6E-32 | 7.77E-30 | yes | down | 335.18 | 334.89 | 335.58 | 250.53 | 230.39 | 211.02 | 335.2167 | 230.6467 |
| ALDH5A1 | 0.266 | -1.91128 | 8.91E-32 | 9.05E-30 | yes | down | 22.81 | 27.52 | 25.12 | 20.84 | 17.12 | 16.36 | 25.15 | 18.10667 |
| HSD17B10 | 0.23 | -2.12328 | 9.74E-32 | 9.83E-30 | yes | down | 219.08 | 248.75 | 241.03 | 157.71 | 109.27 | 130.03 | 236.2867 | 132.3367 |
| LOC100346411 | 0.277 | -1.85184 | 1.32E-31 | 1.33E-29 | yes | down | 1168.72 | 1214.95 | 1181.15 | 941.13 | 677.13 | 804.33 | 1188.273 | 807.53 |
| LOC103344988 | 0.141 | -2.824 | 1.39E-31 | 1.39E-29 | yes | down | 294.23 | 283.59 | 210.23 | 109.53 | 57.44 | 105.24 | 262.6833 | 90.73667 |
| CTNNBL1 | 0.299 | -1.74282 | 2.09E-31 | 2.07E-29 | yes | down | 64.28 | 76.11 | 58.52 | 53.25 | 40.89 | 49.26 | 66.30333 | 47.8 |
| VDAC2 | 0.327 | -1.61415 | 2.63E-31 | 2.6E-29 | yes | down | 287.01 | 266.32 | 224.84 | 209.94 | 217.68 | 180.19 | 259.39 | 202.6033 |
| MTFP1 | 0.126 | -2.99385 | 3.46E-31 | 3.39E-29 | yes | down | 18.64 | 16.63 | 16.77 | 6.38 | 3.38 | 5.95 | 17.34667 | 5.236667 |
| NDUFC2 | 0.273 | -1.8707 | 3.64E-31 | 3.54E-29 | yes | down | 495.33 | 620.83 | 501.21 | 394.81 | 307.06 | 373.37 | 539.1233 | 358.4133 |
| LOC100355688 | 0.293 | -1.77326 | 4.75E-31 | 4.6E-29 | yes | down | 50.65 | 54.94 | 42.27 | 33.11 | 36.66 | 34.13 | 49.28667 | 34.63333 |
| PARN | 0.283 | -1.82363 | 8.95E-31 | 8.62E-29 | yes | down | 22.76 | 27.32 | 24.43 | 19.65 | 15.8 | 19.61 | 24.83667 | 18.35333 |
| RNPEP | 0.144 | -2.79444 | 9.03E-31 | 8.64E-29 | yes | down | 27.54 | 42.03 | 25.03 | 14.11 | 10.67 | 9.09 | 31.53333 | 11.29 |
| COQ9 | 0.284 | -1.81551 | 1.65E-30 | 1.57E-28 | yes | down | 140.06 | 112.04 | 100.98 | 90.01 | 81.31 | 68.93 | 117.6933 | 80.08333 |
| LOC100355645 | 0.318 | -1.6543 | 1.68E-30 | 1.59E-28 | yes | down | 22.39 | 22.75 | 19.75 | 18.03 | 16.63 | 14.96 | 21.63 | 16.54 |
| NAT8L | 6.118 | 2.61301 | 1.72E-30 | 1.62E-28 | yes | up | 2.03 | 1.84 | 2.04 | 26.58 | 23.48 | 32.45 | 1.97 | 27.50333 |
| SEMA7A | 0.325 | -1.62137 | 1.96E-30 | 1.83E-28 | yes | down | 216.83 | 203.56 | 147.66 | 169.06 | 130.45 | 142.94 | 189.35 | 147.4833 |
| MRPL18 | 0.337 | -1.56803 | 2.73E-30 | 2.54E-28 | yes | down | 45.02 | 46.12 | 37.83 | 37.09 | 28.28 | 35.57 | 42.99 | 33.64667 |
| ALDH2 | 6.067 | 2.60107 | 3.19E-30 | 2.95E-28 | yes | up | 48.4 | 27.58 | 35.87 | 579.19 | 507.22 | 549.66 | 37.28333 | 545.3567 |
| TXN2 | 0.193 | -2.37442 | 3.57E-30 | 3.29E-28 | yes | down | 160.26 | 164.68 | 134.74 | 94.56 | 58.41 | 61.38 | 153.2267 | 71.45 |
| SEC11C | 0.17 | -2.5529 | 3.74E-30 | 3.42E-28 | yes | down | 222.27 | 364.94 | 279.03 | 122.5 | 115.85 | 119.38 | 288.7467 | 119.2433 |
| RNF115 | 0.235 | -2.08935 | 4.89E-30 | 4.45E-28 | yes | down | 59.24 | 65.52 | 71.56 | 33.64 | 31.09 | 35.99 | 65.44 | 33.57333 |
| MRPS35 | 0.221 | -2.17616 | 5.51E-30 | 4.99E-28 | yes | down | 48.58 | 55.93 | 57.28 | 29.56 | 28.62 | 28.86 | 53.93 | 29.01333 |
| MTCH1 | 0.333 | -1.5877 | 6.87E-30 | 6.18E-28 | yes | down | 257.13 | 311.36 | 260.11 | 224.96 | 214.87 | 212.96 | 276.2 | 217.5967 |
| IDH3B | 0.178 | -2.48901 | 7.47E-30 | 6.69E-28 | yes | down | 412.69 | 458.76 | 468.68 | 187.64 | 160.33 | 246.72 | 446.71 | 198.23 |
| UBXN1 | 0.391 | -1.35379 | 7.58E-30 | 6.75E-28 | yes | down | 103.61 | 99.61 | 89.88 | 95.01 | 89.53 | 96.37 | 97.7 | 93.63667 |
| PTGES2 | 0.176 | -2.50638 | 7.79E-30 | 6.9E-28 | yes | down | 66.13 | 63.75 | 72.29 | 33.82 | 22.59 | 30.92 | 67.39 | 29.11 |
| ELN | 0.138 | -2.85818 | 8.24E-30 | 7.26E-28 | yes | down | 179.23 | 182 | 134.44 | 73.34 | 34.34 | 58.47 | 165.2233 | 55.38333 |
| LOC100353463 | 6.995 | 2.806365 | 8.95E-30 | 7.84E-28 | yes | up | 24.82 | 32.31 | 25.29 | 368.89 | 424.64 | 584.52 | 27.47333 | 459.35 |
| LOC108177279 | 11.768 | 3.55674 | 9.06E-30 | 7.89E-28 | yes | up | 1.9 | 1.9 | 2.5 | 58.39 | 46.04 | 78.73 | 2.1 | 61.05333 |
| NDUFAB1 | 0.362 | -1.46685 | 1.37E-29 | 1.19E-27 | yes | down | 297.78 | 292.35 | 255.72 | 286.61 | 211.58 | 243.46 | 281.95 | 247.2167 |
| EEPD1 | 4.676 | 2.225319 | 1.82E-29 | 1.57E-27 | yes | up | 9.81 | 12.18 | 8.1 | 135.03 | 86.33 | 118.71 | 10.03 | 113.3567 |
| PHKA2 | 7.289 | 2.865707 | 2.46E-29 | 2.11E-27 | yes | up | 1.89 | 2.16 | 1.42 | 30.6 | 22.93 | 40.36 | 1.823333 | 31.29667 |
| PPP1R3E | 3.813 | 1.931105 | 3.69E-29 | 3.15E-27 | yes | up | 4.51 | 4.72 | 4.77 | 42.65 | 42.68 | 43.38 | 4.666667 | 42.90333 |
| MPC2 | 0.245 | -2.02709 | 3.93E-29 | 3.32E-27 | yes | down | 348.32 | 346.18 | 389.13 | 223.16 | 221.15 | 203.57 | 361.21 | 215.96 |
| DLAT | 0.263 | -1.92522 | 3.93E-29 | 3.32E-27 | yes | down | 48.28 | 37.06 | 39.02 | 30.95 | 24.83 | 23.25 | 41.45333 | 26.34333 |
| IDH3G | 0.227 | -2.13771 | 7.29E-29 | 6.13E-27 | yes | down | 365.31 | 361.58 | 397.81 | 217.79 | 168.89 | 231.08 | 374.9 | 205.92 |
| ATP8 | 0.161 | -2.632 | 8.75E-29 | 7.32E-27 | yes | down | 173724.6 | 147645.2 | 203919.5 | 68818.54 | 75201.95 | 86905.65 | 175096.4 | 76975.38 |
| PXMP2 | 3.658 | 1.870899 | 8.92E-29 | 7.43E-27 | yes | up | 8.79 | 7.56 | 8.15 | 79.36 | 72.67 | 77.46 | 8.166667 | 76.49667 |
| GOT2 | 0.218 | -2.19649 | 1.49E-28 | 1.24E-26 | yes | down | 123.77 | 87.1 | 87.13 | 49.4 | 57.86 | 47.83 | 99.33333 | 51.69667 |
| COQ7 | 0.308 | -1.70075 | 1.84E-28 | 1.51E-26 | yes | down | 44.92 | 39.57 | 36.99 | 34.07 | 27.37 | 28.82 | 40.49333 | 30.08667 |
| FAM149A | 5.859 | 2.550742 | 2.84E-28 | 2.33E-26 | yes | up | 3.42 | 3.25 | 2.36 | 37.22 | 52.66 | 44.26 | 3.01 | 44.71333 |
| FAM126A | 6.883 | 2.783136 | 3E-28 | 2.44E-26 | yes | up | 1.77 | 1.04 | 0.85 | 20.1 | 19.16 | 18.75 | 1.22 | 19.33667 |
| NDUFA12 | 0.299 | -1.74278 | 4.69E-28 | 3.8E-26 | yes | down | 383.08 | 307.07 | 264.11 | 268.78 | 223.61 | 203.77 | 318.0867 | 232.0533 |
| ISCU | 0.393 | -1.34891 | 4.75E-28 | 3.84E-26 | yes | down | 234.94 | 230.8 | 218.68 | 224.59 | 201.67 | 221.84 | 228.14 | 216.0333 |
| PALD1 | 6.806 | 2.766876 | 4.89E-28 | 3.93E-26 | yes | up | 11.32 | 13.75 | 6.28 | 162.11 | 147.9 | 201.62 | 10.45 | 170.5433 |
| ACOT13 | 0.238 | -2.07114 | 4.96E-28 | 3.97E-26 | yes | down | 121.21 | 118.48 | 105.96 | 60.89 | 58.02 | 79.73 | 115.2167 | 66.21333 |
| ETFBKMT | 0.143 | -2.80854 | 5.59E-28 | 4.45E-26 | yes | down | 12.3 | 16.32 | 15.45 | 5.17 | 3.79 | 3.6 | 14.69 | 4.186667 |
| ALOX15 | 0 | -13.6403 | 6.22E-28 | 4.93E-26 | yes | down | 24.96 | 66.63 | 52.88 | 0.03 | 0 | 0 | 48.15667 | 0.01 |
| MRPL1 | 0.365 | -1.4528 | 6.85E-28 | 5.4E-26 | yes | down | 23.68 | 27.8 | 22.57 | 20.09 | 17.89 | 17.35 | 24.68333 | 18.44333 |
| MRPL46 | 0.291 | -1.77931 | 8.85E-28 | 6.95E-26 | yes | down | 13.6 | 14.5 | 12.45 | 9.24 | 9.21 | 9.93 | 13.51667 | 9.46 |
| GATSL2 | 0.191 | -2.39005 | 9.56E-28 | 7.45E-26 | yes | down | 11.6 | 9.31 | 8.54 | 3.94 | 4.54 | 4.93 | 9.816667 | 4.47 |
| PGER5 | 3.067 | 1.616645 | 9.59E-28 | 7.45E-26 | yes | up | 213.95 | 235.44 | 175.62 | 1417.76 | 1494.18 | 1662.42 | 208.3367 | 1524.787 |
| LOC103345855 | 4.534 | 2.180746 | 9.63E-28 | 7.45E-26 | yes | up | 2.01 | 1.6 | 1.22 | 20.92 | 16.02 | 15.82 | 1.61 | 17.58667 |
| TIMM10 | 0.293 | -1.77278 | 9.7E-28 | 7.47E-26 | yes | down | 78.72 | 78.77 | 78.78 | 62.41 | 50.26 | 53.72 | 78.75667 | 55.46333 |
| RDH13 | 0.101 | -3.30083 | 1.14E-27 | 8.74E-26 | yes | down | 9.79 | 7.47 | 8.3 | 2.91 | 1.71 | 1.54 | 8.52 | 2.053333 |
| PTPRF | 3.297 | 1.721141 | 1.18E-27 | 9.01E-26 | yes | up | 4.26 | 5.2 | 3.8 | 35.36 | 31.01 | 38.28 | 4.42 | 34.88333 |
| SSPO | 24.007 | 4.58538 | 1.36E-27 | 1.03E-25 | yes | up | 0.12 | 0.07 | 0.04 | 3.79 | 2.51 | 6.22 | 0.076667 | 4.173333 |
| ACAA2 | 0.152 | -2.7138 | 1.76E-27 | 1.33E-25 | yes | down | 1237.23 | 1235.06 | 1573.12 | 448.63 | 442.11 | 612.65 | 1348.47 | 501.13 |
| COMTD1 | 0.076 | -3.72105 | 1.84E-27 | 1.39E-25 | yes | down | 8.26 | 9.28 | 7.06 | 1.08 | 1.74 | 1.65 | 8.2 | 1.49 |
| CGRRF1 | 3.903 | 1.964501 | 2.51E-27 | 1.88E-25 | yes | up | 1.27 | 1.47 | 1.32 | 14.09 | 15.69 | 13.78 | 1.353333 | 14.52 |
| ACAD8 | 0.204 | -2.29333 | 2.92E-27 | 2.18E-25 | yes | down | 22.92 | 32.96 | 27.02 | 13.14 | 13.12 | 14.51 | 27.63333 | 13.59 |
| NSUN4 | 0.308 | -1.69983 | 3.83E-27 | 2.85E-25 | yes | down | 15.08 | 13.04 | 12.21 | 10.04 | 10 | 11.4 | 13.44333 | 10.48 |
| DDR2 | 2.56 | 1.356201 | 5.3E-27 | 3.92E-25 | yes | up | 5.25 | 4.49 | 3.84 | 30.42 | 25.89 | 26.48 | 4.526667 | 27.59667 |
| FFAR2 | 0.066 | -3.91183 | 5.34E-27 | 3.93E-25 | yes | down | 23.76 | 28.54 | 25.67 | 3.3 | 2.63 | 6.66 | 25.99 | 4.196667 |
| KLB | 9.231 | 3.206543 | 5.9E-27 | 4.32E-25 | yes | up | 5.91 | 7.16 | 8.27 | 112.87 | 170.66 | 192.27 | 7.113333 | 158.6 |
| CLU | 11.564 | 3.53154 | 1.1E-26 | 8.04E-25 | yes | up | 14.1 | 7.06 | 4.85 | 229.37 | 254.35 | 211.56 | 8.67 | 231.76 |
| COQ3 | 0.114 | -3.13357 | 1.17E-26 | 8.48E-25 | yes | down | 11.51 | 12.02 | 10.3 | 2.29 | 4.2 | 2.71 | 11.27667 | 3.066667 |
| TSFM | 0.279 | -1.84338 | 1.31E-26 | 9.5E-25 | yes | down | 63.16 | 67.86 | 62.5 | 47.78 | 33.38 | 43.09 | 64.50667 | 41.41667 |
| FAM162A | 0.335 | -1.57565 | 1.33E-26 | 9.6E-25 | yes | down | 62.18 | 55.55 | 55.9 | 50.25 | 47.73 | 42.58 | 57.87667 | 46.85333 |
| LRRC8B | 7.979 | 2.996264 | 1.34E-26 | 9.62E-25 | yes | up | 0.62 | 0.55 | 0.57 | 11.03 | 8.02 | 14.52 | 0.58 | 11.19 |
| PMPCB | 0.364 | -1.45888 | 1.35E-26 | 9.63E-25 | yes | down | 83.05 | 91.66 | 82.93 | 81.54 | 74.76 | 69.93 | 85.88 | 75.41 |
| ADCK3 | 0.182 | -2.45971 | 1.45E-26 | 1.03E-24 | yes | down | 320.57 | 351.02 | 314.19 | 117.4 | 122.75 | 184.25 | 328.5933 | 141.4667 |
| SPATA6 | 5.354 | 2.420516 | 1.51E-26 | 1.07E-24 | yes | up | 1.58 | 1.44 | 0.95 | 12.99 | 16.14 | 15.15 | 1.323333 | 14.76 |
| IL18 | 3.489 | 1.802731 | 1.67E-26 | 1.18E-24 | yes | up | 36.42 | 32.47 | 35.28 | 288.39 | 257.81 | 322.3 | 34.72333 | 289.5 |
| ADAMTS15 | 5.058 | 2.338507 | 1.97E-26 | 1.38E-24 | yes | up | 2.16 | 2.66 | 2.33 | 30.75 | 30.41 | 22.24 | 2.383333 | 27.8 |
| DEFB1 | 11.158 | 3.479959 | 1.99E-26 | 1.39E-24 | yes | up | 19.13 | 29.71 | 23.17 | 470.86 | 578.58 | 912.09 | 24.00333 | 653.8433 |
| MFN2 | 0.38 | -1.397 | 2.65E-26 | 1.85E-24 | yes | down | 71.86 | 60.51 | 61.18 | 64.03 | 51.98 | 65.94 | 64.51667 | 60.65 |
| HSPA12A | 4.472 | 2.160896 | 2.75E-26 | 1.91E-24 | yes | up | 2.49 | 2.18 | 2.37 | 19.94 | 24.13 | 26.97 | 2.346667 | 23.68 |
| CTSZ | 0.131 | -2.93254 | 3.03E-26 | 2.09E-24 | yes | down | 176.6 | 311.48 | 284.91 | 85.1 | 89.78 | 71.65 | 257.6633 | 82.17667 |
| COQ5 | 0.338 | -1.56617 | 3.44E-26 | 2.37E-24 | yes | down | 38.3 | 32.31 | 30.25 | 17.85 | 17.89 | 18.81 | 33.62 | 18.18333 |
| MRPS12 | 0.279 | -1.84158 | 5.34E-26 | 3.66E-24 | yes | down | 31.94 | 34.57 | 30.78 | 20.59 | 21.1 | 22.34 | 32.43 | 21.34333 |
| NDUFB5 | 0.38 | -1.39782 | 7.03E-26 | 4.79E-24 | yes | down | 172.21 | 169.03 | 143.2 | 163.19 | 150.09 | 128.96 | 161.48 | 147.4133 |
| CYP4B1 | 0.189 | -2.40277 | 8.16E-26 | 5.54E-24 | yes | down | 13.29 | 10.38 | 10.15 | 4.26 | 5.24 | 5.77 | 11.27333 | 5.09 |
| MDH2 | 0.261 | -1.9377 | 8.93E-26 | 6.04E-24 | yes | down | 465.74 | 340.92 | 420.39 | 276.01 | 248.94 | 252.14 | 409.0167 | 259.03 |
| ACO2 | 0.227 | -2.14141 | 9.67E-26 | 6.51E-24 | yes | down | 773.84 | 692.34 | 801.18 | 380.38 | 375.93 | 486.5 | 755.7867 | 414.27 |
| GK | 0.097 | -3.36891 | 1.07E-25 | 7.15E-24 | yes | down | 28.02 | 41.45 | 27.18 | 5.02 | 4.39 | 7.86 | 32.21667 | 5.756667 |
| KARS | 0.488 | -1.03621 | 1.92E-25 | 1.28E-23 | yes | down | 121.43 | 115.79 | 99.06 | 140.69 | 125.68 | 123.17 | 112.0933 | 129.8467 |
| PTCD3 | 0.332 | -1.59261 | 2.12E-25 | 1.41E-23 | yes | down | 23.71 | 27.37 | 20.52 | 17.38 | 18.55 | 16.42 | 23.86667 | 17.45 |
| LOC103352391 | 0.312 | -1.67838 | 2.25E-25 | 1.49E-23 | yes | down | 144.95 | 192.77 | 149.66 | 126.21 | 124.24 | 147.57 | 162.46 | 132.6733 |
| ATP1B1 | 0.088 | -3.51065 | 2.36E-25 | 1.55E-23 | yes | down | 54.53 | 54.87 | 72.52 | 9.93 | 18.27 | 10.52 | 60.64 | 12.90667 |
| CNTN1 | 6.721 | 2.748636 | 3.04E-25 | 2E-23 | yes | up | 1.21 | 1.01 | 1.24 | 15 | 17.9 | 22.98 | 1.153333 | 18.62667 |
| LOC100345097 | 0 | -12.5265 | 4.27E-25 | 2.8E-23 | yes | down | 88.41 | 134.84 | 112.91 | 0 | 0 | 0 | 112.0533 | 0 |
| TKT | 5.154 | 2.365594 | 4.47E-25 | 2.91E-23 | yes | up | 46.84 | 36.15 | 38.01 | 645.66 | 353.31 | 518.57 | 40.33333 | 505.8467 |
| LOC100339133 | 0.476 | -1.07204 | 4.51E-25 | 2.93E-23 | yes | down | 315.61 | 315.88 | 269.75 | 379.28 | 329.99 | 325.24 | 300.4133 | 344.8367 |
| MDH1 | 0.212 | -2.241 | 4.68E-25 | 3.03E-23 | yes | down | 633.87 | 643.26 | 589 | 432.34 | 231.73 | 294.52 | 622.0433 | 319.53 |
| IARS2 | 0.314 | -1.67142 | 7.51E-25 | 4.84E-23 | yes | down | 41.07 | 33.96 | 37.66 | 27.13 | 27.38 | 26.58 | 37.56333 | 27.03 |
| ATP5J2 | 0.367 | -1.44607 | 7.81E-25 | 5.01E-23 | yes | down | 789.29 | 817.51 | 761.93 | 789.75 | 606.33 | 729.49 | 789.5767 | 708.5233 |
| RCC1L | 0.235 | -2.08798 | 9.17E-25 | 5.87E-23 | yes | down | 24.53 | 23.92 | 21.98 | 16.68 | 10.16 | 14.55 | 23.47667 | 13.79667 |
| NDUFA10 | 0.234 | -2.0981 | 9.92E-25 | 6.32E-23 | yes | down | 264.6 | 282.52 | 335.43 | 176.19 | 155.04 | 172.06 | 294.1833 | 167.7633 |
| FAM213A | 3.954 | 1.983411 | 1.02E-24 | 6.48E-23 | yes | up | 77.93 | 60.69 | 64.14 | 567.91 | 620.47 | 732.24 | 67.58667 | 640.2067 |
| LOC100337966 | 0.314 | -1.67183 | 1.22E-24 | 7.7E-23 | yes | down | 635.09 | 603.45 | 680.17 | 511.39 | 444.31 | 481.23 | 639.57 | 478.9767 |
| SGPL1 | 0.364 | -1.45791 | 1.41E-24 | 8.86E-23 | yes | down | 12.49 | 12.98 | 9.67 | 10.79 | 8.9 | 10.88 | 11.71333 | 10.19 |
| C20H14orf142 | 0.103 | -3.27382 | 1.42E-24 | 8.93E-23 | yes | down | 59.98 | 68.68 | 105.99 | 18.09 | 21.24 | 20.7 | 78.21667 | 20.01 |
| MRPL37 | 0.244 | -2.03623 | 1.47E-24 | 9.19E-23 | yes | down | 102.92 | 141.19 | 117.04 | 81.67 | 71.73 | 59.83 | 120.3833 | 71.07667 |
| LOC100338015 | 3.45 | 1.786691 | 1.75E-24 | 1.09E-22 | yes | up | 34.36 | 28.22 | 24.3 | 290.86 | 234.19 | 203.61 | 28.96 | 242.8867 |
| GFPT2 | 9.264 | 3.211655 | 1.9E-24 | 1.18E-22 | yes | up | 3.47 | 2.19 | 1.5 | 39.11 | 53.67 | 62.5 | 2.386667 | 51.76 |
| GOT1 | 0.117 | -3.09157 | 2.34E-24 | 1.44E-22 | yes | down | 116.87 | 61.3 | 66.23 | 21.89 | 28.76 | 17.42 | 81.46667 | 22.69 |
| SUPV3L1 | 0.298 | -1.74706 | 2.74E-24 | 1.68E-22 | yes | down | 17.07 | 19.66 | 15.98 | 12.37 | 13.95 | 11.94 | 17.57 | 12.75333 |
| LOC100341175 | 0.137 | -2.86948 | 3.3E-24 | 2.03E-22 | yes | down | 690.28 | 539.94 | 592.45 | 132.59 | 218.1 | 244.21 | 607.5567 | 198.3 |
| RASGRF2 | 4.831 | 2.272184 | 4.1E-24 | 2.5E-22 | yes | up | 0.64 | 0.86 | 0.82 | 10.14 | 7.73 | 9.26 | 0.773333 | 9.043333 |
| ATPAF1 | 0.273 | -1.87439 | 4.14E-24 | 2.52E-22 | yes | down | 26.46 | 23.76 | 23.33 | 15.22 | 18.18 | 14.03 | 24.51667 | 15.81 |
| NFS1 | 0.307 | -1.70231 | 4.75E-24 | 2.88E-22 | yes | down | 43.9 | 54.37 | 45.44 | 37.3 | 35.63 | 33.33 | 47.90333 | 35.42 |
| ECI1 | 0.222 | -2.17025 | 4.95E-24 | 2.99E-22 | yes | down | 187.4 | 235.64 | 250.07 | 120.1 | 119.69 | 123.39 | 224.37 | 121.06 |
| GGA2 | 4.8 | 2.262949 | 6.27E-24 | 3.77E-22 | yes | up | 15.03 | 18.41 | 10.36 | 193.93 | 126.66 | 183.6 | 14.6 | 168.0633 |
| ECSIT | 0.366 | -1.44896 | 6.28E-24 | 3.77E-22 | yes | down | 39.67 | 39.87 | 38.48 | 38.28 | 31.39 | 33.54 | 39.34 | 34.40333 |
| ITGA7 | 10.981 | 3.456891 | 8.23E-24 | 4.92E-22 | yes | up | 25.86 | 11.07 | 8.3 | 421.7 | 360.25 | 368.75 | 15.07667 | 383.5667 |
| TIMM17A | 0.216 | -2.2101 | 8.81E-24 | 5.25E-22 | yes | down | 97.33 | 156.79 | 103.4 | 63.81 | 64.15 | 57.33 | 119.1733 | 61.76333 |
| SNRPB2 | 0.35 | -1.51663 | 9.78E-24 | 5.81E-22 | yes | down | 64.6 | 71.04 | 60.19 | 53.08 | 52.51 | 59.2 | 65.27667 | 54.93 |
| NDUFS2 | 0.405 | -1.30553 | 1.03E-23 | 6.08E-22 | yes | down | 328.36 | 290.22 | 295.94 | 319.75 | 281.02 | 292.72 | 304.84 | 297.83 |
| LOC100348135 | 0.239 | -2.0653 | 1.04E-23 | 6.13E-22 | yes | down | 24.08 | 24.57 | 26.24 | 15 | 14.89 | 13.41 | 24.96333 | 14.43333 |
| LRRK2 | 2.977 | 1.573931 | 1.17E-23 | 6.87E-22 | yes | up | 1.26 | 1.1 | 1.03 | 8.62 | 7.6 | 7.52 | 1.13 | 7.913333 |
| LOC108176503 | 0.267 | -1.90767 | 1.24E-23 | 7.25E-22 | yes | down | 12.41 | 16.05 | 15.81 | 6.78 | 5.84 | 7.61 | 14.75667 | 6.743333 |
| PLP2 | 4.54 | 2.182775 | 1.25E-23 | 7.28E-22 | yes | up | 14.4 | 10.49 | 8.11 | 125.35 | 110.5 | 122.88 | 11 | 119.5767 |
| ATP5H | 0.363 | -1.46092 | 1.29E-23 | 7.52E-22 | yes | down | 712.84 | 816.15 | 751.73 | 694.8 | 636.67 | 680.61 | 760.24 | 670.6933 |
| C12H6orf89 | 0.331 | -1.59633 | 1.71E-23 | 9.93E-22 | yes | down | 67.49 | 79.82 | 74.57 | 73.85 | 68.06 | 75.95 | 73.96 | 72.62 |
| SRPX | 6.28 | 2.650877 | 2.09E-23 | 1.21E-21 | yes | up | 6.84 | 5.65 | 3.1 | 69.52 | 87.1 | 72.66 | 5.196667 | 76.42667 |
| PREX2 | 0.368 | -1.44157 | 2.18E-23 | 1.25E-21 | yes | down | 10.06 | 10.57 | 10.06 | 9.66 | 9.23 | 8.36 | 10.23 | 9.083333 |
| NFU1 | 0.34 | -1.55814 | 2.27E-23 | 1.3E-21 | yes | down | 47.74 | 58.5 | 44.09 | 45.93 | 39.52 | 38.44 | 50.11 | 41.29667 |
| LOC100341136 | 7.997 | 2.999431 | 2.51E-23 | 1.43E-21 | yes | up | 0.35 | 0.71 | 0.8 | 12.79 | 11.17 | 10.16 | 0.62 | 11.37333 |
| LOC100345961 | 0.273 | -1.87549 | 2.92E-23 | 1.66E-21 | yes | down | 6.66 | 7.55 | 5.06 | 4.46 | 4.11 | 4 | 6.423333 | 4.19 |
| CPEB1 | 9.796 | 3.292209 | 3.25E-23 | 1.85E-21 | yes | up | 0.45 | 0.36 | 0.25 | 6.18 | 8.52 | 9.13 | 0.353333 | 7.943333 |
| LOC103352280 | 0.18 | -2.47215 | 3.66E-23 | 2.07E-21 | yes | down | 1.58 | 1.7 | 1.73 | 0.81 | 0.74 | 0.76 | 1.67 | 0.77 |
| RNF152 | 0.257 | -1.96143 | 4.13E-23 | 2.32E-21 | yes | down | 10.5 | 11.15 | 8.44 | 7.3 | 4.45 | 6.82 | 10.03 | 6.19 |
| LOC100343510 | 4.979 | 2.315821 | 4.24E-23 | 2.38E-21 | yes | up | 4.34 | 3.84 | 2.97 | 46.88 | 27.79 | 39.79 | 3.716667 | 38.15333 |
| LOC100009483 | 14.017 | 3.809141 | 4.31E-23 | 2.41E-21 | yes | up | 1.1 | 0.66 | 0.39 | 26.62 | 17.42 | 25.55 | 0.716667 | 23.19667 |
| ADAMTSL3 | 3.863 | 1.949606 | 4.6E-23 | 2.56E-21 | yes | up | 1.47 | 1.35 | 1.37 | 11.89 | 12.56 | 14.97 | 1.396667 | 13.14 |
| MRPL2 | 0.196 | -2.34827 | 5.21E-23 | 2.9E-21 | yes | down | 52.98 | 40.55 | 51.1 | 22.69 | 18.84 | 27.48 | 48.21 | 23.00333 |
| NDUFB4 | 0.38 | -1.39698 | 5.57E-23 | 3.09E-21 | yes | down | 467.2 | 507.4 | 439.22 | 492.61 | 428.51 | 388.8 | 471.2733 | 436.64 |
| CIB4 | 0.013 | -6.22148 | 6.22E-23 | 3.43E-21 | yes | down | 4.01 | 6.2 | 4.6 | 0 | 0.13 | 0.32 | 4.936667 | 0.15 |
| SLC7A6 | 7.277 | 2.863316 | 7.76E-23 | 4.27E-21 | yes | up | 4.26 | 7.22 | 3.17 | 65.47 | 87.48 | 72.37 | 4.883333 | 75.10667 |
| MRPL43 | 0.293 | -1.76937 | 8.2E-23 | 4.5E-21 | yes | down | 26.55 | 25.99 | 26.89 | 20.12 | 18.22 | 18.18 | 26.47667 | 18.84 |
| LOC103347528 | 0.31 | -1.68962 | 8.66E-23 | 4.73E-21 | yes | down | 36.73 | 37.01 | 32.93 | 25.98 | 24.02 | 29.71 | 35.55667 | 26.57 |
| PRKD3 | 0.396 | -1.33648 | 9.42E-23 | 5.13E-21 | yes | down | 30.27 | 26.42 | 25.62 | 26.67 | 23.83 | 27.81 | 27.43667 | 26.10333 |
| HACL1 | 0.215 | -2.21839 | 1.13E-22 | 6.12E-21 | yes | down | 75.98 | 88.98 | 106.21 | 49.73 | 47.72 | 45.77 | 90.39 | 47.74 |
| FIGN | 3.764 | 1.912163 | 1.14E-22 | 6.16E-21 | yes | up | 1.61 | 1.42 | 1.46 | 13.14 | 10.34 | 16.19 | 1.496667 | 13.22333 |
| VWA8 | 0.338 | -1.56609 | 1.86E-22 | 1.01E-20 | yes | down | 10.94 | 10.39 | 10.44 | 8.78 | 9.72 | 9.34 | 10.59 | 9.28 |
| LIAS | 0.36 | -1.47258 | 1.96E-22 | 1.05E-20 | yes | down | 16.81 | 17.5 | 14.18 | 12.93 | 13.65 | 14.67 | 16.16333 | 13.75 |
| PLTP | 4.055 | 2.01983 | 2.13E-22 | 1.14E-20 | yes | up | 15.55 | 17.47 | 15.81 | 132.48 | 175.54 | 161.55 | 16.27667 | 156.5233 |
| TPT1 | 0.426 | -1.23256 | 2.33E-22 | 1.25E-20 | yes | down | 1492.88 | 1489.7 | 1229.62 | 1469.42 | 1518.6 | 1306.37 | 1404.067 | 1431.463 |
| CD248 | 3.149 | 1.655077 | 2.64E-22 | 1.41E-20 | yes | up | 6.59 | 4.97 | 5.15 | 42.35 | 41.44 | 41.51 | 5.57 | 41.76667 |
| PXDN | 2.676 | 1.419953 | 2.89E-22 | 1.53E-20 | yes | up | 21.92 | 17.39 | 17.95 | 133.91 | 111.66 | 120.31 | 19.08667 | 121.96 |
| MSMO1 | 6.603 | 2.723096 | 3.3E-22 | 1.75E-20 | yes | up | 6.87 | 5.96 | 5.32 | 122.35 | 57.07 | 110.39 | 6.05 | 96.60333 |
| GLYAT | 6.672 | 2.738152 | 3.48E-22 | 1.83E-20 | yes | up | 10.35 | 7.08 | 5.08 | 93.89 | 113.99 | 138.96 | 7.503333 | 115.6133 |
| LOC100345019 | 3.459 | 1.790314 | 3.79E-22 | 1.99E-20 | yes | up | 12.11 | 9.72 | 9.29 | 92.08 | 70.91 | 96.3 | 10.37333 | 86.43 |
| SLC25A11 | 0.27 | -1.88833 | 4.31E-22 | 2.26E-20 | yes | down | 90.59 | 64.45 | 62.74 | 47.2 | 51.82 | 41.52 | 72.59333 | 46.84667 |
| MARC2 | 2.842 | 1.506846 | 4.88E-22 | 2.55E-20 | yes | up | 19.65 | 19.49 | 13.11 | 115.78 | 111.54 | 91.35 | 17.41667 | 106.2233 |
| PDE3B | 0.37 | -1.43455 | 5E-22 | 2.6E-20 | yes | down | 31.76 | 38.13 | 33.91 | 31.75 | 28.15 | 30.12 | 34.6 | 30.00667 |
| DCLK2 | 4.827 | 2.271254 | 5.01E-22 | 2.6E-20 | yes | up | 0.8 | 0.62 | 0.75 | 8.14 | 5.68 | 7.78 | 0.723333 | 7.2 |
| PHOSPHO1 | 0.048 | -4.39073 | 5.65E-22 | 2.92E-20 | yes | down | 36.4 | 49.82 | 25.31 | 2.34 | 7.41 | 2.88 | 37.17667 | 4.21 |
| MRPS30 | 0.286 | -1.80603 | 5.82E-22 | 3E-20 | yes | down | 17.03 | 20.14 | 19.56 | 13.75 | 12.47 | 13.11 | 18.91 | 13.11 |
| LOC103350039 | 4.247 | 2.086361 | 6.01E-22 | 3.09E-20 | yes | up | 0.78 | 1.02 | 0.74 | 13.18 | 9.79 | 10.01 | 0.846667 | 10.99333 |
| TSKU | 4.925 | 2.300247 | 6.06E-22 | 3.11E-20 | yes | up | 3.11 | 21.09 | 12.96 | 108.48 | 38.14 | 34.31 | 12.38667 | 60.31 |
| MRPL16 | 0.408 | -1.29366 | 6.09E-22 | 3.11E-20 | yes | down | 66.25 | 71.05 | 59.96 | 63.82 | 59.74 | 68.56 | 65.75333 | 64.04 |
| ACADS | 0.208 | -2.26197 | 7.4E-22 | 3.77E-20 | yes | down | 299.48 | 290.92 | 395.04 | 161.68 | 149.26 | 179.79 | 328.48 | 163.5767 |
| POLDIP2 | 0.357 | -1.48509 | 8.91E-22 | 4.53E-20 | yes | down | 73.48 | 79.03 | 74.97 | 62.7 | 65.04 | 68.04 | 75.82667 | 65.26 |
| COQ6 | 0.356 | -1.48829 | 9.3E-22 | 4.71E-20 | yes | down | 30.22 | 29.34 | 27.44 | 24.13 | 23.25 | 27.18 | 29 | 24.85333 |
| GRIN2C | 0.023 | -5.4593 | 9.59E-22 | 4.84E-20 | yes | down | 1.71 | 2.36 | 1.84 | 0.13 | 0.13 | 0 | 1.97 | 0.086667 |
| MECR | 0.309 | -1.69609 | 9.65E-22 | 4.86E-20 | yes | down | 40.9 | 38.77 | 43.4 | 34.94 | 29.66 | 32.17 | 41.02333 | 32.25667 |
| SMARCA2 | 2.89 | 1.530984 | 9.83E-22 | 4.93E-20 | yes | up | 18.77 | 15.31 | 16.09 | 105.35 | 118.05 | 117.13 | 16.72333 | 113.51 |
| LGMN | 2.343 | 1.228299 | 1.23E-21 | 6.15E-20 | yes | up | 51.72 | 61.16 | 44.84 | 301.81 | 287.2 | 295.33 | 52.57333 | 294.78 |
| OXNAD1 | 0.208 | -2.2627 | 1.25E-21 | 6.22E-20 | yes | down | 17.66 | 21.76 | 24.43 | 10.97 | 10.3 | 10.57 | 21.28333 | 10.61333 |
| SLC25A20 | 0.304 | -1.71674 | 1.44E-21 | 7.14E-20 | yes | down | 255.75 | 316.72 | 307.15 | 228.29 | 198.38 | 223.13 | 293.2067 | 216.6 |
| POLR3C | 0.227 | -2.13651 | 1.6E-21 | 7.95E-20 | yes | down | 29.56 | 43.66 | 39.71 | 23.69 | 17.51 | 20.81 | 37.64333 | 20.67 |
| IVNS1ABP | 0.193 | -2.37521 | 1.63E-21 | 8.06E-20 | yes | down | 100.61 | 164.78 | 94.97 | 49.77 | 50.5 | 66.25 | 120.12 | 55.50667 |
| S100B | 13.786 | 3.785172 | 1.63E-21 | 8.06E-20 | yes | up | 12.48 | 9.86 | 3.63 | 312.54 | 354.23 | 191.97 | 8.656667 | 286.2467 |
| BCO2 | 2.839 | 1.505616 | 1.8E-21 | 8.83E-20 | yes | up | 13.83 | 14.85 | 15.58 | 100.77 | 104.06 | 93.79 | 14.75333 | 99.54 |
| COX1 | 0.193 | -2.37496 | 1.84E-21 | 9.03E-20 | yes | down | 29853.53 | 22157.19 | 34005.72 | 12894.12 | 13430.43 | 13536.51 | 28672.15 | 13287.02 |
| TSHZ2 | 4.081 | 2.029002 | 1.88E-21 | 9.17E-20 | yes | up | 1.39 | 1.6 | 1.17 | 17.13 | 10.19 | 13.98 | 1.386667 | 13.76667 |
| LOC100340645 | 0.432 | -1.21041 | 2.11E-21 | 1.03E-19 | yes | down | 1430.86 | 1181.06 | 1093.36 | 1447.61 | 1237.04 | 1206.12 | 1235.093 | 1296.923 |
| EGR1 | 10.41 | 3.379927 | 2.24E-21 | 1.09E-19 | yes | up | 5.58 | 2.09 | 2.06 | 92.41 | 68.26 | 78.01 | 3.243333 | 79.56 |
| MRPS10 | 0.31 | -1.68734 | 2.5E-21 | 1.21E-19 | yes | down | 30.11 | 37.56 | 33.05 | 20.87 | 16.75 | 22.89 | 33.57333 | 20.17 |
| HSPD1 | 0.348 | -1.52137 | 3.43E-21 | 1.66E-19 | yes | down | 291.93 | 271.12 | 281.26 | 249.76 | 201.02 | 258.6 | 281.4367 | 236.46 |
| LOC100344910 | 0.308 | -1.69915 | 4E-21 | 1.92E-19 | yes | down | 506.18 | 507.63 | 540.82 | 367.72 | 417.58 | 372.42 | 518.21 | 385.9067 |
| SLC25A13 | 0.335 | -1.57734 | 4.59E-21 | 2.2E-19 | yes | down | 21.37 | 21.79 | 21.58 | 19.77 | 14.41 | 18.27 | 21.58 | 17.48333 |
| CNIH1 | 2.066 | 1.046885 | 5.57E-21 | 2.67E-19 | yes | up | 9.64 | 9.44 | 8.13 | 46.16 | 42.71 | 45.86 | 9.07 | 44.91 |
| PGAM1 | 0.331 | -1.59534 | 5.62E-21 | 2.68E-19 | yes | down | 148.36 | 172.57 | 169.71 | 148.31 | 122.08 | 124.38 | 163.5467 | 131.59 |
| LOC100348443 | 3060.68 | 11.57964 | 6E-21 | 2.85E-19 | yes | up | 0 | 0 | 0 | 49.36 | 18.95 | 27.88 | 0 | 32.06333 |
| PHB | 0.338 | -1.56467 | 6.35E-21 | 3.01E-19 | yes | down | 219.4 | 210.39 | 233.74 | 213.7 | 160.46 | 180.07 | 221.1767 | 184.7433 |
| NDUFA4 | 0.397 | -1.33261 | 6.88E-21 | 3.26E-19 | yes | down | 1073.81 | 1124.88 | 1040.87 | 1030.2 | 1082.81 | 1012.86 | 1079.853 | 1041.957 |
| MRPL38 | 0.291 | -1.78035 | 7.59E-21 | 3.58E-19 | yes | down | 30.09 | 26.72 | 29.86 | 19.85 | 19.37 | 21.86 | 28.89 | 20.36 |
| BNIP3 | 0.395 | -1.33905 | 7.63E-21 | 3.59E-19 | yes | down | 192.61 | 176.19 | 159.78 | 153.62 | 164.93 | 174.94 | 176.1933 | 164.4967 |
| NDUFS6 | 0.357 | -1.48447 | 8.11E-21 | 3.81E-19 | yes | down | 368.05 | 350.18 | 388.64 | 355.68 | 311.03 | 334.54 | 368.9567 | 333.75 |
| LAMA4 | 2.985 | 1.577729 | 8.15E-21 | 3.81E-19 | yes | up | 19.72 | 20.56 | 13.33 | 124.76 | 140.47 | 120.07 | 17.87 | 128.4333 |
| HR | 0.068 | -3.87399 | 9.67E-21 | 4.51E-19 | yes | down | 4.28 | 2.19 | 1.45 | 0.35 | 0.39 | 0.52 | 2.64 | 0.42 |
| LOC100353320 | 0.093 | -3.43433 | 9.86E-21 | 4.59E-19 | yes | down | 405.02 | 452.44 | 608.22 | 136.59 | 57.78 | 146.75 | 488.56 | 113.7067 |
| CIB2 | 0.204 | -2.29367 | 1.22E-20 | 5.66E-19 | yes | down | 167.87 | 180.08 | 238.74 | 97 | 93.68 | 103.28 | 195.5633 | 97.98667 |
| ABR | 2.879 | 1.525676 | 1.22E-20 | 5.66E-19 | yes | up | 5.33 | 4.72 | 5.57 | 34.15 | 28.25 | 35.17 | 5.206667 | 32.52333 |
| ARL6IP1 | 3.574 | 1.837681 | 1.3E-20 | 6.02E-19 | yes | up | 40.93 | 47.29 | 42.48 | 492.27 | 326.02 | 318.22 | 43.56667 | 378.8367 |
| AVP | 0.021 | -5.54378 | 1.33E-20 | 6.14E-19 | yes | down | 10.55 | 17.95 | 26.7 | 0.42 | 0.89 | 1.02 | 18.4 | 0.776667 |
| PPP1R9B | 0.31 | -1.68785 | 1.48E-20 | 6.81E-19 | yes | down | 77.92 | 106.62 | 65.42 | 61.91 | 57.35 | 65.54 | 83.32 | 61.6 |
| NDUFV2 | 0.397 | -1.33268 | 1.56E-20 | 7.12E-19 | yes | down | 211.27 | 204.5 | 149.03 | 187.34 | 184.57 | 159.83 | 188.2667 | 177.2467 |
| ECM2 | 2.724 | 1.445551 | 1.91E-20 | 8.73E-19 | yes | up | 25.41 | 26.64 | 18.49 | 144.62 | 150.89 | 167.79 | 23.51333 | 154.4333 |
| TRAP1 | 0.267 | -1.90525 | 1.97E-20 | 8.96E-19 | yes | down | 93.6 | 90.99 | 109.75 | 63.78 | 65.45 | 62.35 | 98.11333 | 63.86 |
| APMAP | 2.422 | 1.275935 | 1.99E-20 | 9.05E-19 | yes | up | 15.82 | 18.6 | 14.97 | 98.73 | 96.8 | 91.96 | 16.46333 | 95.83 |
| KIRREL | 2.809 | 1.490303 | 2.19E-20 | 9.9E-19 | yes | up | 3.78 | 3.91 | 2.7 | 27.16 | 18.38 | 23.54 | 3.463333 | 23.02667 |
| POLN | 22.998 | 4.523429 | 2.35E-20 | 1.06E-18 | yes | up | 0.13 | 0.11 | 0.07 | 23.09 | 8.75 | 10.79 | 0.103333 | 14.21 |
| EPHX1 | 4.99 | 2.319081 | 2.36E-20 | 1.06E-18 | yes | up | 37.48 | 56.84 | 51.44 | 501.44 | 579.29 | 673.66 | 48.58667 | 584.7967 |
| LOC100359112 | 8.366 | 3.06451 | 3.41E-20 | 1.53E-18 | yes | up | 1.71 | 1.76 | 1.29 | 22.34 | 31.6 | 41.11 | 1.586667 | 31.68333 |
| HAUS7 | 0.165 | -2.59977 | 4.15E-20 | 1.86E-18 | yes | down | 4.56 | 6.71 | 4.55 | 3.42 | 3.8 | 2.92 | 5.273333 | 3.38 |
| MTUS2 | 7.872 | 2.9767 | 4.26E-20 | 1.9E-18 | yes | up | 0.73 | 1.61 | 0.72 | 16.58 | 20.52 | 18.42 | 1.02 | 18.50667 |
| PARL | 0.376 | -1.41311 | 4.3E-20 | 1.92E-18 | yes | down | 36.75 | 40.16 | 35.57 | 39.97 | 30.26 | 31.92 | 37.49333 | 34.05 |
| LOC103349801 | 0.276 | -1.85584 | 4.42E-20 | 1.96E-18 | yes | down | 154.27 | 189.95 | 176.76 | 141.1 | 109.61 | 94.84 | 173.66 | 115.1833 |
| ZDHHC20 | 2.437 | 1.284964 | 4.86E-20 | 2.15E-18 | yes | up | 3.71 | 3.33 | 2.84 | 19.97 | 19.56 | 18.82 | 3.293333 | 19.45 |
| GALNT12 | 3.711 | 1.891887 | 5.09E-20 | 2.25E-18 | yes | up | 3.76 | 3.91 | 2.61 | 27.84 | 30.54 | 32.59 | 3.426667 | 30.32333 |
| TNN | 0.036 | -4.80911 | 5.2E-20 | 2.29E-18 | yes | down | 13.07 | 4.22 | 4.54 | 0.92 | 0.25 | 0.63 | 7.276667 | 0.6 |
| ISCA1 | 0.353 | -1.50071 | 5.55E-20 | 2.44E-18 | yes | down | 74.9 | 79.36 | 69.24 | 56.82 | 67.64 | 64.66 | 74.5 | 63.04 |
| FABP3 | 0.009 | -6.75913 | 5.88E-20 | 2.57E-18 | yes | down | 1717.61 | 1888.73 | 1516.43 | 9.28 | 60.7 | 40.76 | 1707.59 | 36.91333 |
| SUCLA2 | 0.181 | -2.46634 | 6.49E-20 | 2.83E-18 | yes | down | 408.77 | 464 | 396.18 | 130.98 | 170.39 | 247.99 | 422.9833 | 183.12 |
| PATL1 | 0.417 | -1.26321 | 6.95E-20 | 3.03E-18 | yes | down | 13.95 | 16.07 | 12.75 | 14.91 | 12.89 | 14.84 | 14.25667 | 14.21333 |
| MRPL35 | 0.394 | -1.34371 | 7.73E-20 | 3.36E-18 | yes | down | 58.66 | 64.51 | 52.44 | 56.86 | 52.73 | 57.99 | 58.53667 | 55.86 |
| CPNE4 | 0.055 | -4.19253 | 8.23E-20 | 3.57E-18 | yes | down | 3.88 | 5.23 | 2.1 | 0.67 | 0.18 | 0.6 | 3.736667 | 0.483333 |
| SEPT10 | 0.304 | -1.71999 | 8.46E-20 | 3.66E-18 | yes | down | 133.79 | 198.65 | 159.43 | 114.13 | 114.36 | 127.48 | 163.9567 | 118.6567 |
| ENO1 | 0.364 | -1.45671 | 8.69E-20 | 3.75E-18 | yes | down | 280.72 | 319.28 | 277.13 | 255.04 | 224.54 | 290.44 | 292.3767 | 256.6733 |
| ESRRA | 0.2 | -2.31999 | 8.92E-20 | 3.84E-18 | yes | down | 16.94 | 20 | 16.06 | 7.72 | 9.87 | 8.01 | 17.66667 | 8.533333 |
| LOC100359306 | 6.177 | 2.626929 | 9.56E-20 | 4.1E-18 | yes | up | 2.61 | 1.76 | 3.09 | 42.46 | 37.65 | 30.92 | 2.486667 | 37.01 |
| ALDH1L1 | 40.256 | 5.331129 | 1.12E-19 | 4.78E-18 | yes | up | 0.9 | 0.14 | 0.17 | 56.45 | 32.95 | 23.58 | 0.403333 | 37.66 |
| SLC25A42 | 0.12 | -3.0591 | 1.18E-19 | 5.02E-18 | yes | down | 42.47 | 39.37 | 31.24 | 8.16 | 11.95 | 12.95 | 37.69333 | 11.02 |
| MCOLN3 | 16.507 | 4.04499 | 1.21E-19 | 5.13E-18 | yes | up | 0.7 | 0.98 | 0.43 | 49.31 | 13.56 | 20.3 | 0.703333 | 27.72333 |
| SLC16A1 | 2.634 | 1.397341 | 1.28E-19 | 5.44E-18 | yes | up | 28.64 | 25.45 | 25.14 | 182.85 | 174.93 | 144.74 | 26.41 | 167.5067 |
| LOC100346670 | 0.33 | -1.59735 | 1.43E-19 | 6.07E-18 | yes | down | 1374.26 | 1549.24 | 1611.56 | 1284.35 | 1183.89 | 1223.98 | 1511.687 | 1230.74 |
| APOOL | 0.353 | -1.50183 | 1.5E-19 | 6.35E-18 | yes | down | 36.05 | 41.14 | 32.46 | 36.14 | 30.67 | 26.52 | 36.55 | 31.11 |
| CPNE3 | 2.041 | 1.029416 | 1.53E-19 | 6.43E-18 | yes | up | 9.54 | 8.52 | 7.65 | 39.82 | 32.41 | 38.94 | 8.57 | 37.05667 |
| PIPOX | 19.958 | 4.318871 | 1.7E-19 | 7.15E-18 | yes | up | 1.73 | 0.35 | 0.54 | 40.93 | 29.93 | 51.05 | 0.873333 | 40.63667 |
| ADRB1 | 0.234 | -2.09332 | 1.87E-19 | 7.84E-18 | yes | down | 3.98 | 4.72 | 4.32 | 2.91 | 2.42 | 2.06 | 4.34 | 2.463333 |
| ATP5C1 | 0.407 | -1.29568 | 2.1E-19 | 8.79E-18 | yes | down | 524.06 | 418.33 | 340.07 | 454.83 | 394.72 | 386.16 | 427.4867 | 411.9033 |
| LOC103345437 | 0.273 | -1.87373 | 2.27E-19 | 9.48E-18 | yes | down | 49.27 | 51.07 | 58.21 | 40.16 | 32.37 | 40.37 | 52.85 | 37.63333 |
| NDUFB8 | 0.416 | -1.26599 | 2.31E-19 | 9.6E-18 | yes | down | 396.86 | 388.1 | 385.57 | 438.99 | 382.52 | 361.74 | 390.1767 | 394.4167 |
| YKT6 | 0.39 | -1.35863 | 2.55E-19 | 1.06E-17 | yes | down | 33.86 | 37.17 | 35.54 | 30.96 | 30.34 | 31.76 | 35.52333 | 31.02 |
| CLSTN2 | 0.019 | -5.7556 | 3E-19 | 1.24E-17 | yes | down | 0.36 | 0.3 | 0.51 | 0.01 | 0.02 | 0.02 | 0.39 | 0.016667 |
| MID2 | 2.118 | 1.083033 | 3.02E-19 | 1.25E-17 | yes | up | 4.6 | 4.47 | 4.14 | 23.4 | 20.15 | 20.98 | 4.403333 | 21.51 |
| PDSS2 | 0.368 | -1.4435 | 3.51E-19 | 1.45E-17 | yes | down | 10.56 | 9.68 | 9.01 | 8.56 | 8.36 | 8.91 | 9.75 | 8.61 |
| CD5L | 59.178 | 5.886978 | 3.61E-19 | 1.48E-17 | yes | up | 0.14 | 0.25 | 0.06 | 24.16 | 13.57 | 26.18 | 0.15 | 21.30333 |
| C18H10orf2 | 0.266 | -1.90834 | 3.67E-19 | 1.5E-17 | yes | down | 8.6 | 8.07 | 8.59 | 5.25 | 4.56 | 6.44 | 8.42 | 5.416667 |
| C7 | 16.287 | 4.025646 | 3.87E-19 | 1.58E-17 | yes | up | 3.05 | 1.65 | 0.78 | 51.95 | 100.68 | 51.76 | 1.826667 | 68.13 |
| PANK3 | 3.24 | 1.695981 | 3.98E-19 | 1.62E-17 | yes | up | 2.82 | 3.37 | 1.95 | 19.92 | 20.33 | 22.15 | 2.713333 | 20.8 |
| NARF | 0.389 | -1.36257 | 4.07E-19 | 1.66E-17 | yes | down | 7.24 | 7.73 | 6.67 | 7.77 | 5.8 | 6.72 | 7.213333 | 6.763333 |
| STARD7 | 0.341 | -1.55371 | 5.49E-19 | 2.23E-17 | yes | down | 55 | 51.98 | 50.13 | 37.67 | 42.28 | 47.91 | 52.37 | 42.62 |
| ADSL | 0.267 | -1.90776 | 5.68E-19 | 2.3E-17 | yes | down | 27.96 | 20.54 | 14.99 | 13.81 | 13.46 | 11.82 | 21.16333 | 13.03 |
| LOC100347623 | 1679.205 | 10.71356 | 5.72E-19 | 2.31E-17 | yes | up | 0 | 0 | 0 | 36.23 | 43.5 | 35.84 | 0 | 38.52333 |
| ST6GALNAC6 | 3.569 | 1.835588 | 5.77E-19 | 2.33E-17 | yes | up | 12.73 | 8.01 | 9.83 | 94.99 | 76.93 | 88 | 10.19 | 86.64 |
| PENK | 37.506 | 5.229055 | 7.81E-19 | 3.14E-17 | yes | up | 0.79 | 0.67 | 0.13 | 22.31 | 37.34 | 76.48 | 0.53 | 45.37667 |
| RPS6KA6 | 2.961 | 1.566118 | 8.07E-19 | 3.24E-17 | yes | up | 0.87 | 1.16 | 0.93 | 6.69 | 5.26 | 7.18 | 0.986667 | 6.376667 |
| LOC100356833 | 0.219 | -2.19422 | 8.2E-19 | 3.28E-17 | yes | down | 7.95 | 8.16 | 5.88 | 2.89 | 3.84 | 4.64 | 7.33 | 3.79 |
| BTC | 13.272 | 3.730272 | 8.4E-19 | 3.35E-17 | yes | up | 0.28 | 0.37 | 0.35 | 6.77 | 12.52 | 12.35 | 0.333333 | 10.54667 |
| MRPS23 | 0.35 | -1.51493 | 8.53E-19 | 3.4E-17 | yes | down | 52.21 | 55.15 | 40.33 | 50.92 | 35.9 | 37.67 | 49.23 | 41.49667 |
| CATSPER1 | 14.376 | 3.845577 | 9.79E-19 | 3.89E-17 | yes | up | 0.54 | 0.23 | 0.15 | 12.48 | 6.83 | 11.07 | 0.306667 | 10.12667 |
| MRPL19 | 0.425 | -1.23413 | 1.29E-18 | 5.1E-17 | yes | down | 36.8 | 38.07 | 35.68 | 36.69 | 28.41 | 34.72 | 36.85 | 33.27333 |
| BPGM | 0.158 | -2.66391 | 1.3E-18 | 5.13E-17 | yes | down | 10 | 17.26 | 15.08 | 4.84 | 6.12 | 5.21 | 14.11333 | 5.39 |
| FMO2 | 6.068 | 2.601126 | 1.32E-18 | 5.21E-17 | yes | up | 3.57 | 1.62 | 2.28 | 27.18 | 21.18 | 27.06 | 2.49 | 25.14 |
| ZFAND4 | 0.249 | -2.00299 | 1.33E-18 | 5.22E-17 | yes | down | 6.34 | 7.66 | 6.65 | 3.04 | 3.81 | 3.51 | 6.883333 | 3.453333 |
| METTL17 | 0.364 | -1.45681 | 1.38E-18 | 5.43E-17 | yes | down | 16.96 | 21.01 | 13.25 | 16.32 | 15.2 | 17.11 | 17.07333 | 16.21 |
| SDK1 | 4.01 | 2.003433 | 1.45E-18 | 5.69E-17 | yes | up | 2.91 | 2.21 | 1.63 | 24.54 | 22.74 | 17.13 | 2.25 | 21.47 |
| PRRT4 | 627.096 | 9.292543 | 1.61E-18 | 6.31E-17 | yes | up | 0.05 | 0 | 0 | 14.16 | 22.03 | 17.84 | 0.016667 | 18.01 |
| FAM65B | 0.349 | -1.5192 | 1.98E-18 | 7.72E-17 | yes | down | 10.61 | 13.67 | 11.01 | 11.05 | 8.2 | 10.15 | 11.76333 | 9.8 |
| CS | 0.355 | -1.49336 | 2.03E-18 | 7.9E-17 | yes | down | 605.22 | 598.42 | 629.22 | 526.66 | 462.83 | 586.12 | 610.9533 | 525.2033 |
| LOC103345217 | 0.04 | -4.62931 | 2.1E-18 | 8.15E-17 | yes | down | 6.05 | 9.34 | 3.96 | 1.02 | 0.4 | 0.45 | 6.45 | 0.623333 |
| SLC25A12 | 0.178 | -2.49319 | 2.23E-18 | 8.63E-17 | yes | down | 21.97 | 16.5 | 11.7 | 6.6 | 9.08 | 5.28 | 16.72333 | 6.986667 |
| MRPL49 | 0.43 | -1.21731 | 2.31E-18 | 8.91E-17 | yes | down | 56.57 | 60.03 | 56.79 | 66.31 | 57.23 | 56.86 | 57.79667 | 60.13333 |
| SLIRP | 0.309 | -1.69226 | 2.71E-18 | 1.04E-16 | yes | down | 227.52 | 220.75 | 216.64 | 185.47 | 174.75 | 149.56 | 221.6367 | 169.9267 |
| AP1G2 | 8.267 | 3.047366 | 3.45E-18 | 1.32E-16 | yes | up | 0.35 | 0.26 | 0.2 | 5.09 | 4.12 | 5.04 | 0.27 | 4.75 |
| LOC100356756 | 0.114 | -3.13052 | 3.6E-18 | 1.38E-16 | yes | down | 993.67 | 866.09 | 977.06 | 141.5 | 267.63 | 385.67 | 945.6067 | 264.9333 |
| CA12 | 0.03 | -5.03967 | 3.77E-18 | 1.44E-16 | yes | down | 1.22 | 4.38 | 1.46 | 0.18 | 0.12 | 0.34 | 2.353333 | 0.213333 |
| PSMD8 | 0.412 | -1.27776 | 4.21E-18 | 1.61E-16 | yes | down | 103.13 | 119.38 | 106.71 | 123.51 | 100.65 | 100.65 | 109.74 | 108.27 |
| APOO | 0.338 | -1.56618 | 4.23E-18 | 1.61E-16 | yes | down | 48.26 | 39.46 | 30.79 | 32.17 | 32.31 | 30.23 | 39.50333 | 31.57 |
| SH3PXD2B | 4.72 | 2.238798 | 4.29E-18 | 1.63E-16 | yes | up | 1.07 | 0.83 | 0.52 | 9.51 | 7.1 | 10.31 | 0.806667 | 8.973333 |
| GREM2 | 4.814 | 2.267318 | 4.55E-18 | 1.72E-16 | yes | up | 1.74 | 1.04 | 1.5 | 19.27 | 18.5 | 14.36 | 1.426667 | 17.37667 |
| NPC1 | 2.969 | 1.570214 | 4.58E-18 | 1.73E-16 | yes | up | 5.67 | 6.33 | 6.2 | 44.02 | 46.61 | 39.38 | 6.066667 | 43.33667 |
| FABP9 | 18.752 | 4.228953 | 4.83E-18 | 1.82E-16 | yes | up | 0.42 | 0.28 | 0.11 | 34.93 | 12.76 | 8.98 | 0.27 | 18.89 |
| MRPL51 | 0.432 | -1.21137 | 5.59E-18 | 2.1E-16 | yes | down | 78.01 | 80.35 | 69.64 | 88.56 | 73.02 | 75.54 | 76 | 79.04 |
| LOC103345308 | 0.339 | -1.56169 | 5.74E-18 | 2.16E-16 | yes | down | 10.77 | 10.65 | 9.5 | 9.49 | 7.72 | 8.05 | 10.30667 | 8.42 |
| MTHFD1 | 0.358 | -1.4833 | 7.27E-18 | 2.72E-16 | yes | down | 37.09 | 39.64 | 39.76 | 32.47 | 35.57 | 32.51 | 38.83 | 33.51667 |
| NSUN2 | 0.47 | -1.08825 | 8.06E-18 | 3.01E-16 | yes | down | 22.13 | 22.45 | 19.65 | 24.28 | 23.89 | 24.37 | 21.41 | 24.18 |
| LGALS12 | 6.517 | 2.704251 | 8.28E-18 | 3.09E-16 | yes | up | 10.45 | 21.77 | 10.3 | 127.75 | 147.66 | 217.43 | 14.17333 | 164.28 |
| MYO19 | 0.247 | -2.01895 | 8.66E-18 | 3.22E-16 | yes | down | 8.93 | 13.37 | 7.31 | 7 | 4.86 | 5.84 | 9.87 | 5.9 |
| CTSE | 0.06 | -4.04957 | 8.82E-18 | 3.28E-16 | yes | down | 4.84 | 11.23 | 10.41 | 0.81 | 1.2 | 1.84 | 8.826667 | 1.283333 |
| PNPT1 | 0.309 | -1.69435 | 9.04E-18 | 3.35E-16 | yes | down | 11.78 | 13.53 | 13.86 | 11.07 | 8.51 | 11.42 | 13.05667 | 10.33333 |
| SPATA6L | 6.084 | 2.60496 | 9.17E-18 | 3.39E-16 | yes | up | 1.24 | 1.47 | 1.1 | 9.9 | 6.48 | 9.61 | 1.27 | 8.663333 |
| KLHDC2 | 0.477 | -1.06742 | 9.83E-18 | 3.62E-16 | yes | down | 34.33 | 31.15 | 26.86 | 35.78 | 34.47 | 35.29 | 30.78 | 35.18 |
| PDE4D | 0.156 | -2.68401 | 9.9E-18 | 3.65E-16 | yes | down | 17.34 | 26.12 | 9.41 | 13.73 | 5.57 | 15.47 | 17.62333 | 11.59 |
| PRRG4 | 0.145 | -2.79019 | 1.06E-17 | 3.89E-16 | yes | down | 7.14 | 7.8 | 3.46 | 1.83 | 1.64 | 2.77 | 6.133333 | 2.08 |
| MPZL2 | 2.508 | 1.3267 | 1.09E-17 | 3.99E-16 | yes | up | 9.07 | 10.57 | 7.84 | 58.46 | 56.91 | 48.98 | 9.16 | 54.78333 |
| SORD | 2.696 | 1.431062 | 1.16E-17 | 4.26E-16 | yes | up | 3.7 | 4.01 | 3.54 | 25.68 | 25.16 | 22.14 | 3.75 | 24.32667 |
| GALK1 | 0.197 | -2.34594 | 1.19E-17 | 4.33E-16 | yes | down | 18.65 | 21.94 | 20.98 | 12.83 | 10.12 | 6.59 | 20.52333 | 9.846667 |
| RASD2 | 5.753 | 2.524278 | 1.21E-17 | 4.4E-16 | yes | up | 1.07 | 0.59 | 0.88 | 13.91 | 9.71 | 11.69 | 0.846667 | 11.77 |
| TSC22D3 | 2.314 | 1.210604 | 1.24E-17 | 4.5E-16 | yes | up | 33.59 | 39.23 | 29.06 | 182.51 | 192.4 | 187.36 | 33.96 | 187.4233 |
| LOC100328912 | 3.609 | 1.851529 | 1.46E-17 | 5.28E-16 | yes | up | 77.65 | 74.12 | 90.95 | 690.19 | 631.99 | 804.61 | 80.90667 | 708.93 |
| EPHA6 | 5.77 | 2.528655 | 1.47E-17 | 5.31E-16 | yes | up | 0.76 | 0.7 | 0.62 | 8.42 | 12.25 | 7.19 | 0.693333 | 9.286667 |
| TENM2 | 20.678 | 4.369994 | 1.51E-17 | 5.44E-16 | yes | up | 0.05 | 0.09 | 0.03 | 1.83 | 2.12 | 4.21 | 0.056667 | 2.72 |
| FZD4 | 4.159 | 2.056313 | 1.52E-17 | 5.48E-16 | yes | up | 21.94 | 26.08 | 23.69 | 188.06 | 292.55 | 232.19 | 23.90333 | 237.6 |
| NDUFAF6 | 0.358 | -1.4808 | 1.64E-17 | 5.9E-16 | yes | down | 20.72 | 20.3 | 19.11 | 20.5 | 17.75 | 14.28 | 20.04333 | 17.51 |
| FURIN | 0.408 | -1.29315 | 1.68E-17 | 6.03E-16 | yes | down | 35.98 | 39.17 | 28.8 | 31.52 | 32.33 | 36.82 | 34.65 | 33.55667 |
| OGDHL | 6.205 | 2.633438 | 1.88E-17 | 6.73E-16 | yes | up | 1.88 | 2.93 | 3.52 | 41.53 | 48.71 | 36.05 | 2.776667 | 42.09667 |
| PPARGC1A | 0.052 | -4.26643 | 2.05E-17 | 7.3E-16 | yes | down | 4.93 | 18.09 | 4.2 | 0.88 | 1.07 | 1.32 | 9.073333 | 1.09 |
| ADM | 4.447 | 2.152758 | 2.08E-17 | 7.4E-16 | yes | up | 8.85 | 6.92 | 7.56 | 66.39 | 105.21 | 75.3 | 7.776667 | 82.3 |
| LOC100345908 | 0.05 | -4.33431 | 2.18E-17 | 7.74E-16 | yes | down | 7.57 | 5.45 | 12.66 | 0.55 | 1.2 | 1.24 | 8.56 | 0.996667 |
| MOSPD1 | 0.38 | -1.39525 | 2.23E-17 | 7.92E-16 | yes | down | 11.28 | 12.2 | 9.16 | 10.86 | 10.25 | 8.62 | 10.88 | 9.91 |
| STX2 | 2.417 | 1.273158 | 2.27E-17 | 8.02E-16 | yes | up | 8.74 | 10.85 | 7.75 | 56.44 | 50.48 | 56.65 | 9.113333 | 54.52333 |
| MLXIPL | 3.586 | 1.842511 | 2.72E-17 | 9.59E-16 | yes | up | 15.75 | 11.43 | 12.51 | 131.73 | 87.05 | 126.82 | 13.23 | 115.2 |
| PSEN2 | 0.275 | -1.86182 | 2.99E-17 | 1.05E-15 | yes | down | 37.91 | 49.77 | 49.04 | 33.53 | 31.39 | 29.52 | 45.57333 | 31.48 |
| AOX3 | 20.026 | 4.323832 | 3.13E-17 | 1.1E-15 | yes | up | 0.41 | 2.29 | 0.49 | 39.48 | 45.49 | 55.76 | 1.063333 | 46.91 |
| MRPL47 | 0.375 | -1.41515 | 3.38E-17 | 1.19E-15 | yes | down | 41.93 | 41.45 | 38.15 | 34.97 | 37.67 | 35.03 | 40.51 | 35.89 |
| LIPA | 4.966 | 2.312228 | 3.4E-17 | 1.19E-15 | yes | up | 18.92 | 24.97 | 18.87 | 160.23 | 266.37 | 279.11 | 20.92 | 235.2367 |
| SLC25A1 | 7.034 | 2.814375 | 3.48E-17 | 1.22E-15 | yes | up | 22.18 | 27.96 | 32.09 | 579.64 | 266.95 | 588.62 | 27.41 | 478.4033 |
| NCOA4 | 0.426 | -1.22948 | 3.57E-17 | 1.24E-15 | yes | down | 75.2 | 79.24 | 61.31 | 86.47 | 69.63 | 80.72 | 71.91667 | 78.94 |
| ABHD18 | 0.351 | -1.51227 | 3.96E-17 | 1.38E-15 | yes | down | 12.86 | 9.73 | 8.95 | 9.87 | 7.04 | 8.77 | 10.51333 | 8.56 |
| DLD | 0.275 | -1.86273 | 4.11E-17 | 1.43E-15 | yes | down | 144.02 | 111.29 | 153.8 | 93.15 | 81.59 | 99.36 | 136.37 | 91.36667 |
| AARS2 | 0.353 | -1.50113 | 4.32E-17 | 1.5E-15 | yes | down | 6.87 | 6.89 | 6.61 | 6.71 | 4.61 | 5.79 | 6.79 | 5.703333 |
| MAB21L1 | 5.96 | 2.575314 | 4.33E-17 | 1.5E-15 | yes | up | 0.4 | 0.4 | 0.5 | 5.71 | 6.13 | 6.92 | 0.433333 | 6.253333 |
| MRPS27 | 0.307 | -1.70479 | 4.5E-17 | 1.55E-15 | yes | down | 13.3 | 14.87 | 14.17 | 9.67 | 9.52 | 12.13 | 14.11333 | 10.44 |
| C1QC | 2.36 | 1.238633 | 4.53E-17 | 1.56E-15 | yes | up | 35.84 | 27.89 | 25.88 | 171.66 | 171.5 | 158.29 | 29.87 | 167.15 |
| COQ4 | 0.255 | -1.97145 | 5.01E-17 | 1.72E-15 | yes | down | 7.4 | 7.12 | 6.39 | 5.19 | 3.57 | 4.11 | 6.97 | 4.29 |
| NAV3 | 3.163 | 1.661483 | 5.05E-17 | 1.73E-15 | yes | up | 0.75 | 1.1 | 0.95 | 7.99 | 6.88 | 6.48 | 0.933333 | 7.116667 |
| LOC100354254 | 0.11 | -3.18462 | 5.19E-17 | 1.78E-15 | yes | down | 875.68 | 1200.27 | 1287.57 | 184.2 | 256.4 | 471.84 | 1121.173 | 304.1467 |
| ITGBL1 | 18.158 | 4.182568 | 5.37E-17 | 1.84E-15 | yes | up | 1.06 | 0.51 | 0.33 | 18.5 | 43.13 | 21.17 | 0.633333 | 27.6 |
| ORAI2 | 3.584 | 1.84156 | 5.78E-17 | 1.97E-15 | yes | up | 3.12 | 3.94 | 2.38 | 28.31 | 32.82 | 30.45 | 3.146667 | 30.52667 |
| CPT2 | 0.34 | -1.55557 | 6.09E-17 | 2.07E-15 | yes | down | 53.97 | 69.28 | 60.92 | 51.27 | 44.11 | 56.05 | 61.39 | 50.47667 |
| DPT | 8.251 | 3.04455 | 6.69E-17 | 2.27E-15 | yes | up | 54.92 | 35.01 | 24.21 | 441.1 | 900.48 | 874.53 | 38.04667 | 738.7033 |
| SOD2 | 0.318 | -1.65431 | 6.97E-17 | 2.36E-15 | yes | down | 338.17 | 351.73 | 300.93 | 202.32 | 268.22 | 279.72 | 330.2767 | 250.0867 |
| MRPS36 | 0.352 | -1.50519 | 8.1E-17 | 2.74E-15 | yes | down | 71.79 | 54.1 | 51.75 | 54.31 | 52.96 | 42.47 | 59.21333 | 49.91333 |
| CRYGS | 0.048 | -4.3942 | 1.05E-16 | 3.54E-15 | yes | down | 8.26 | 19.17 | 9.77 | 0.74 | 1.43 | 2.01 | 12.4 | 1.393333 |
| TOMM6 | 0.389 | -1.36177 | 1.16E-16 | 3.92E-15 | yes | down | 195.07 | 256.37 | 189.53 | 171.15 | 133.11 | 167.2 | 213.6567 | 157.1533 |
| FH | 0.206 | -2.27658 | 1.17E-16 | 3.92E-15 | yes | down | 145.51 | 138.28 | 143.65 | 47.61 | 78.82 | 84.52 | 142.48 | 70.31667 |
| CHRDL1 | 3.165 | 1.662406 | 1.28E-16 | 4.3E-15 | yes | up | 21.79 | 20.47 | 23.55 | 168.67 | 147.19 | 194.01 | 21.93667 | 169.9567 |
| PDK2 | 0.271 | -1.88347 | 1.31E-16 | 4.37E-15 | yes | down | 54.55 | 57.21 | 51.94 | 30.9 | 44.65 | 30.54 | 54.56667 | 35.36333 |
| TMBIM1 | 2.995 | 1.58239 | 1.38E-16 | 4.6E-15 | yes | up | 33.35 | 25.32 | 19.47 | 173.01 | 189.21 | 191.65 | 26.04667 | 184.6233 |
| LOC108176153 | 0.167 | -2.58156 | 1.38E-16 | 4.61E-15 | yes | down | 57.78 | 70.26 | 78.62 | 20.8 | 28.86 | 34.87 | 68.88667 | 28.17667 |
| IDH2 | 0.147 | -2.76483 | 1.41E-16 | 4.69E-15 | yes | down | 460.51 | 232.15 | 303.35 | 94.48 | 160.68 | 94.3 | 332.0033 | 116.4867 |
| LRP1 | 3.268 | 1.708212 | 1.53E-16 | 5.07E-15 | yes | up | 13.75 | 9.89 | 8.36 | 89.11 | 66.96 | 91.23 | 10.66667 | 82.43333 |
| DAP3 | 0.454 | -1.13784 | 1.67E-16 | 5.53E-15 | yes | down | 21.95 | 23.88 | 20.84 | 26.17 | 23.58 | 23.26 | 22.22333 | 24.33667 |
| MRPS24 | 0.358 | -1.48145 | 1.92E-16 | 6.35E-15 | yes | down | 56.45 | 55.03 | 48.92 | 43.59 | 52.1 | 42.39 | 53.46667 | 46.02667 |
| ACOX2 | 5.658 | 2.50034 | 1.94E-16 | 6.39E-15 | yes | up | 3.29 | 6.73 | 3.8 | 51.01 | 67.94 | 66.28 | 4.606667 | 61.74333 |
| MFSD2A | 0.02 | -5.64959 | 1.99E-16 | 6.55E-15 | yes | down | 2.12 | 8.83 | 4.87 | 0.16 | 0.52 | 0.08 | 5.273333 | 0.253333 |
| LOC100358789 | 0.236 | -2.0824 | 2.02E-16 | 6.62E-15 | yes | down | 49.9 | 35.68 | 37.88 | 23.07 | 29.15 | 17.5 | 41.15333 | 23.24 |
| CRABP2 | 4.13 | 2.045985 | 2.05E-16 | 6.69E-15 | yes | up | 5.89 | 3.7 | 3.29 | 40.84 | 46.4 | 38.92 | 4.293333 | 42.05333 |
| HSF4 | 19.271 | 4.268324 | 2.19E-16 | 7.14E-15 | yes | up | 0.18 | 0.07 | 0.07 | 5.23 | 5.06 | 5.24 | 0.106667 | 5.176667 |
| GTPBP6 | 0.388 | -1.36521 | 2.45E-16 | 8E-15 | yes | down | 35.95 | 35.53 | 33.63 | 35.32 | 26.78 | 36.36 | 35.03667 | 32.82 |
| ETFB | 0.368 | -1.44396 | 2.58E-16 | 8.39E-15 | yes | down | 254.95 | 307.48 | 310.64 | 249.26 | 232.94 | 256.37 | 291.0233 | 246.19 |
| MMACHC | 0.223 | -2.16229 | 2.63E-16 | 8.53E-15 | yes | down | 12.27 | 13.31 | 10.49 | 4.95 | 6.36 | 7.92 | 12.02333 | 6.41 |
| EGLN3 | 0.118 | -3.08451 | 2.66E-16 | 8.6E-15 | yes | down | 41.25 | 80.09 | 65.01 | 25.89 | 9.31 | 18.91 | 62.11667 | 18.03667 |
| MRPS7 | 0.402 | -1.31573 | 2.69E-16 | 8.69E-15 | yes | down | 53.86 | 53.12 | 53.34 | 64.07 | 50.62 | 45.97 | 53.44 | 53.55333 |
| CEL | 0.001 | -10.8377 | 3.05E-16 | 9.82E-15 | yes | down | 1.77 | 6.37 | 7.31 | 0 | 0 | 0 | 5.15 | 0 |
| QARS | 0.411 | -1.28265 | 3.07E-16 | 9.84E-15 | yes | down | 49.31 | 59.27 | 50.65 | 61.33 | 46.26 | 50.6 | 53.07667 | 52.73 |
| SOD3 | 6.757 | 2.756432 | 3.09E-16 | 9.89E-15 | yes | up | 6.39 | 7.09 | 9.85 | 87.24 | 154.17 | 140.17 | 7.776667 | 127.1933 |
| LOC100357711 | 3.787 | 1.920965 | 3.28E-16 | 1.05E-14 | yes | up | 4.54 | 4.46 | 2.84 | 33.66 | 41.45 | 31.87 | 3.946667 | 35.66 |
| TRIM37 | 0.342 | -1.54679 | 3.51E-16 | 1.12E-14 | yes | down | 540.56 | 593.9 | 624.69 | 522.1 | 405.17 | 403.76 | 586.3833 | 443.6767 |
| FAM96A | 0.389 | -1.36179 | 3.56E-16 | 1.13E-14 | yes | down | 59.02 | 56.54 | 46.08 | 44.98 | 52.61 | 53.04 | 53.88 | 50.21 |
| FGFR4 | 0.167 | -2.58528 | 3.77E-16 | 1.2E-14 | yes | down | 6.62 | 4.17 | 3.85 | 1.39 | 2.22 | 2.13 | 4.88 | 1.913333 |
| LOC100343809 | 2.629 | 1.394254 | 4.07E-16 | 1.29E-14 | yes | up | 36.29 | 39.6 | 31.68 | 227.32 | 253.33 | 197.01 | 35.85667 | 225.8867 |
| TIMM8A | 0.221 | -2.18038 | 4.32E-16 | 1.37E-14 | yes | down | 27.01 | 32.41 | 25.55 | 10.74 | 17.84 | 16.08 | 28.32333 | 14.88667 |
| MRM3 | 0.369 | -1.43667 | 4.43E-16 | 1.4E-14 | yes | down | 9.68 | 10.76 | 10.35 | 9.93 | 7.99 | 9.62 | 10.26333 | 9.18 |
| RALGPS2 | 0.247 | -2.01798 | 4.49E-16 | 1.42E-14 | yes | down | 3.66 | 4.16 | 2.1 | 1.74 | 1.93 | 2.05 | 3.306667 | 1.906667 |
| LOC108178113 | 0.356 | -1.48894 | 4.59E-16 | 1.44E-14 | yes | down | 2307.77 | 2401.5 | 2625.26 | 2294.87 | 2073.17 | 2314.43 | 2444.843 | 2227.49 |
| SLC25A47 | 0.173 | -2.53323 | 5.07E-16 | 1.59E-14 | yes | down | 9.31 | 8.83 | 4.21 | 2.47 | 3.12 | 2.34 | 7.45 | 2.643333 |
| DHRS7 | 3.732 | 1.900035 | 5.24E-16 | 1.64E-14 | yes | up | 30.57 | 22.55 | 27.94 | 215.8 | 283.4 | 225.49 | 27.02 | 241.5633 |
| CCDC58 | 0.294 | -1.76646 | 5.74E-16 | 1.79E-14 | yes | down | 47.25 | 27.76 | 28.53 | 22.61 | 25.15 | 27.61 | 34.51333 | 25.12333 |
| RGS6 | 0.342 | -1.54718 | 5.78E-16 | 1.8E-14 | yes | down | 447.46 | 504.63 | 501.41 | 473.36 | 343.74 | 337.49 | 484.5 | 384.8633 |
| CCND3 | 3.616 | 1.854524 | 5.97E-16 | 1.86E-14 | yes | up | 10.51 | 9 | 7.44 | 55.82 | 75.86 | 88.46 | 8.983333 | 73.38 |
| OXA1L | 0.354 | -1.49773 | 6.04E-16 | 1.88E-14 | yes | down | 66.96 | 77.86 | 65.81 | 54.28 | 62.04 | 65.01 | 70.21 | 60.44333 |
| PNPO | 0.241 | -2.05028 | 6.06E-16 | 1.88E-14 | yes | down | 16.93 | 21.07 | 14.87 | 8 | 9.14 | 13.21 | 17.62333 | 10.11667 |
| TENM4 | 4.444 | 2.151753 | 6.43E-16 | 1.99E-14 | yes | up | 1 | 1.13 | 1.24 | 9.55 | 10.98 | 7.92 | 1.123333 | 9.483333 |
| NDUFA2 | 0.354 | -1.49934 | 6.54E-16 | 2.02E-14 | yes | down | 298.09 | 384.74 | 340.55 | 334.56 | 284.96 | 263.51 | 341.1267 | 294.3433 |
| MYO5B | 0.076 | -3.71826 | 6.76E-16 | 2.08E-14 | yes | down | 1.22 | 1.85 | 1.89 | 0.21 | 0.2 | 0.51 | 1.653333 | 0.306667 |
| MAOB | 2.658 | 1.410246 | 7.23E-16 | 2.23E-14 | yes | up | 5.69 | 7.29 | 5.5 | 46.12 | 34.08 | 38.18 | 6.16 | 39.46 |
| PDK3 | 0.329 | -1.60496 | 7.29E-16 | 2.24E-14 | yes | down | 4.5 | 4.74 | 4.65 | 4.02 | 3.53 | 3.5 | 4.63 | 3.683333 |
| DDAH2 | 2.452 | 1.293982 | 7.87E-16 | 2.41E-14 | yes | up | 25.4 | 24.05 | 15.6 | 134.33 | 118.35 | 126.19 | 21.68333 | 126.29 |
| IGFBP5 | 5.197 | 2.377762 | 8.06E-16 | 2.47E-14 | yes | up | 16.78 | 10.08 | 6.73 | 116.36 | 147.92 | 143 | 11.19667 | 135.76 |
| MNDA | 2.3 | 1.201676 | 9.12E-16 | 2.79E-14 | yes | up | 10.81 | 21.89 | 10.88 | 92.22 | 59.78 | 66.64 | 14.52667 | 72.88 |
| MBOAT1 | 0.244 | -2.03674 | 9.24E-16 | 2.82E-14 | yes | down | 7.17 | 6.29 | 7.66 | 3.7 | 4.57 | 3.99 | 7.04 | 4.086667 |
| CERK | 0.208 | -2.26485 | 9.37E-16 | 2.85E-14 | yes | down | 36.53 | 39.63 | 17.13 | 17.85 | 11.68 | 16.22 | 31.09667 | 15.25 |
| SLC24A2 | 3.386 | 1.7597 | 9.49E-16 | 2.88E-14 | yes | up | 0.75 | 0.6 | 0.72 | 5.79 | 6.19 | 4.9 | 0.69 | 5.626667 |
| TOMM5 | 0.444 | -1.17083 | 9.53E-16 | 2.89E-14 | yes | down | 80.41 | 73.87 | 72.89 | 83.38 | 81.42 | 79.36 | 75.72333 | 81.38667 |
| MFN1 | 0.363 | -1.46309 | 9.89E-16 | 2.99E-14 | yes | down | 33.55 | 29.39 | 28.92 | 19.35 | 24.66 | 23.72 | 30.62 | 22.57667 |
| CPXM1 | 8.753 | 3.129777 | 1.01E-15 | 3.04E-14 | yes | up | 1.36 | 1.17 | 0.32 | 22.85 | 17.11 | 18.13 | 0.95 | 19.36333 |
| G0S2 | 6.031 | 2.592345 | 1.12E-15 | 3.37E-14 | yes | up | 59.72 | 44.94 | 26.3 | 444.59 | 423.38 | 849.56 | 43.65333 | 572.51 |
| IMP4 | 0.36 | -1.47357 | 1.13E-15 | 3.41E-14 | yes | down | 15.62 | 16.96 | 16.86 | 14.42 | 12.77 | 15.87 | 16.48 | 14.35333 |
| FAM92A1 | 2.232 | 1.15809 | 1.23E-15 | 3.71E-14 | yes | up | 5.85 | 6.86 | 4.96 | 35.22 | 36.5 | 36.99 | 5.89 | 36.23667 |
| MCFD2 | 2.227 | 1.154909 | 1.34E-15 | 4.02E-14 | yes | up | 28.45 | 29.95 | 29.49 | 164.48 | 150.08 | 158.71 | 29.29667 | 157.7567 |
| LOC100356693 | 0.327 | -1.61234 | 1.35E-15 | 4.04E-14 | yes | down | 55.32 | 75.82 | 61.39 | 51.93 | 55.07 | 44.92 | 64.17667 | 50.64 |
| DUSP10 | 2.706 | 1.436406 | 1.36E-15 | 4.06E-14 | yes | up | 8.99 | 9.19 | 5.43 | 49.9 | 43.51 | 50.01 | 7.87 | 47.80667 |
| DECR1 | 0.285 | -1.81206 | 1.36E-15 | 4.07E-14 | yes | down | 424.16 | 560.36 | 554.25 | 333.46 | 306.98 | 415.35 | 512.9233 | 351.93 |
| LOC100354074 | 0.253 | -1.98466 | 1.44E-15 | 4.27E-14 | yes | down | 48.81 | 52.03 | 48.45 | 25.81 | 24.76 | 40.36 | 49.76333 | 30.31 |
| BAIAP2L1 | 4.792 | 2.26077 | 1.44E-15 | 4.27E-14 | yes | up | 3.44 | 5.9 | 3.2 | 38.78 | 46.97 | 56.5 | 4.18 | 47.41667 |
| MT3 | 0.14 | -2.84079 | 1.54E-15 | 4.58E-14 | yes | down | 70.08 | 41.41 | 37.82 | 20.1 | 18.75 | 11.79 | 49.77 | 16.88 |
| GSS | 2.587 | 1.371537 | 1.61E-15 | 4.76E-14 | yes | up | 20.64 | 19.13 | 19.02 | 148.41 | 99.53 | 121.21 | 19.59667 | 123.05 |
| ERAL1 | 0.398 | -1.32883 | 1.75E-15 | 5.16E-14 | yes | down | 11.67 | 10.89 | 9.17 | 9.98 | 9.48 | 10.78 | 10.57667 | 10.08 |
| KDELC2 | 2.432 | 1.282049 | 1.75E-15 | 5.17E-14 | yes | up | 6.74 | 5.3 | 4.67 | 28.15 | 22.37 | 26.52 | 5.57 | 25.68 |
| ELAC2 | 0.339 | -1.56271 | 1.98E-15 | 5.82E-14 | yes | down | 9.49 | 11.8 | 10.55 | 9.14 | 9.16 | 9.17 | 10.61333 | 9.156667 |
| AR | 7.794 | 2.962422 | 2.08E-15 | 6.13E-14 | yes | up | 0.53 | 0.43 | 0.16 | 8.47 | 5.46 | 6.59 | 0.373333 | 6.84 |
| CCT3 | 0.49 | -1.02808 | 2.1E-15 | 6.17E-14 | yes | down | 61.64 | 64.77 | 46.31 | 67.56 | 64.68 | 69.67 | 57.57333 | 67.30333 |
| PRPSAP2 | 2.683 | 1.423787 | 2.44E-15 | 7.15E-14 | yes | up | 3.9 | 3.57 | 3.33 | 27.51 | 20.89 | 22.31 | 3.6 | 23.57 |
| SLC16A7 | 0.09 | -3.47296 | 2.47E-15 | 7.23E-14 | yes | down | 12.47 | 11.44 | 24.03 | 2.79 | 2.07 | 5.29 | 15.98 | 3.383333 |
| SMIM4 | 0.297 | -1.74904 | 2.61E-15 | 7.62E-14 | yes | down | 30.26 | 36.22 | 39.74 | 25.55 | 23.82 | 26.65 | 35.40667 | 25.34 |
| NDUFS4 | 0.42 | -1.24992 | 2.77E-15 | 8.08E-14 | yes | down | 267.73 | 265.82 | 268.08 | 301.3 | 271.48 | 240.7 | 267.21 | 271.16 |
| LOC100345021 | 0.195 | -2.35901 | 2.81E-15 | 8.17E-14 | yes | down | 45.59 | 56.94 | 37.85 | 25.59 | 12.31 | 26.96 | 46.79333 | 21.62 |
| AGTR2 | 0.049 | -4.34517 | 2.83E-15 | 8.21E-14 | yes | down | 1.63 | 1.31 | 0.69 | 0.13 | 0.14 | 0.15 | 1.21 | 0.14 |
| CKMT1B | 0.001 | -10.3407 | 3.05E-15 | 8.86E-14 | yes | down | 2.97 | 13.03 | 10.66 | 0.05 | 0 | 0 | 8.886667 | 0.016667 |
| IGFBP2 | 3.274 | 1.710887 | 3.06E-15 | 8.87E-14 | yes | up | 32.61 | 23.84 | 16.47 | 195.36 | 168.51 | 206.44 | 24.30667 | 190.1033 |
| LOC103348101 | 0.277 | -1.85014 | 3.23E-15 | 9.34E-14 | yes | down | 4.14 | 4.89 | 4.17 | 3.7 | 1.98 | 2.44 | 4.4 | 2.706667 |
| RFWD2 | 0.356 | -1.48902 | 3.55E-15 | 1.03E-13 | yes | down | 19.8 | 26.73 | 22.58 | 20.56 | 20.17 | 18.87 | 23.03667 | 19.86667 |
| CXCL10 | 27.69 | 4.791298 | 4.08E-15 | 1.18E-13 | yes | up | 0.5 | 0.34 | 0.11 | 16.17 | 11.71 | 33.44 | 0.316667 | 20.44 |
| STYXL1 | 0.208 | -2.26582 | 4.29E-15 | 1.23E-13 | yes | down | 6.45 | 7.63 | 5.78 | 3.13 | 2.81 | 4.14 | 6.62 | 3.36 |
| FOSB | 15.333 | 3.93853 | 4.31E-15 | 1.24E-13 | yes | up | 0.26 | 0.22 | 0.09 | 4.06 | 9 | 7.3 | 0.19 | 6.786667 |
| C3 | 3.443 | 1.783854 | 4.9E-15 | 1.41E-13 | yes | up | 10.59 | 9.5 | 5.94 | 59.72 | 77.51 | 73.04 | 8.676667 | 70.09 |
| COX6A1 | 0.261 | -1.93929 | 5.02E-15 | 1.43E-13 | yes | down | 2349.02 | 3093.1 | 3630.38 | 2187.05 | 1845.83 | 1852.01 | 3024.167 | 1961.63 |
| PIGV | 2.443 | 1.288807 | 5.52E-15 | 1.58E-13 | yes | up | 3.84 | 4.02 | 3.16 | 22.66 | 18.15 | 22.97 | 3.673333 | 21.26 |
| ASB1 | 0.37 | -1.43297 | 5.59E-15 | 1.59E-13 | yes | down | 17.36 | 20.19 | 19.4 | 18.47 | 15.84 | 16.86 | 18.98333 | 17.05667 |
| SNX24 | 0.287 | -1.80339 | 5.76E-15 | 1.64E-13 | yes | down | 15.26 | 11.65 | 15.63 | 10.05 | 9.9 | 9.69 | 14.18 | 9.88 |
| HBQ1 | 4.377 | 2.130067 | 6.27E-15 | 1.78E-13 | yes | up | 8.59 | 14.18 | 22.47 | 59.8 | 41.7 | 34.89 | 15.08 | 45.46333 |
| ANKRD37 | 0.233 | -2.10045 | 7E-15 | 1.98E-13 | yes | down | 9.88 | 14.3 | 13.57 | 9.1 | 5.58 | 6.09 | 12.58333 | 6.923333 |
| LOC100342572 | 8.181 | 3.03219 | 7.01E-15 | 1.99E-13 | yes | up | 127.38 | 56.75 | 155.57 | 2340.98 | 2664.43 | 1896.7 | 113.2333 | 2300.703 |
| FDXR | 0.231 | -2.11502 | 8.18E-15 | 2.31E-13 | yes | down | 19.15 | 18.44 | 24.13 | 13.59 | 10.5 | 10.97 | 20.57333 | 11.68667 |
| RPP40 | 0.36 | -1.47558 | 8.19E-15 | 2.31E-13 | yes | down | 29.46 | 27.9 | 22.56 | 28.26 | 17.29 | 23.88 | 26.64 | 23.14333 |
| PSMA2 | 0.499 | -1.00282 | 8.4E-15 | 2.37E-13 | yes | down | 123.34 | 105.06 | 95.66 | 130.86 | 124.89 | 132.6 | 108.02 | 129.45 |
| LOC108176650 | 0.21 | -2.25114 | 8.46E-15 | 2.38E-13 | yes | down | 4.18 | 4.93 | 2.42 | 1.24 | 1.68 | 1.4 | 3.843333 | 1.44 |
| LOC100351248 | 0.001 | -9.84745 | 8.67E-15 | 2.43E-13 | yes | down | 20.66 | 9.51 | 23.36 | 0 | 0.15 | 0 | 17.84333 | 0.05 |
| LOC100358208 | 0.469 | -1.09131 | 9.29E-15 | 2.6E-13 | yes | down | 706.5 | 701.87 | 692.76 | 868.54 | 796.34 | 748.62 | 700.3767 | 804.5 |
| HADHA | 0.188 | -2.41086 | 9.81E-15 | 2.74E-13 | yes | down | 601.24 | 519.6 | 629.27 | 254.33 | 164.82 | 381.33 | 583.37 | 266.8267 |
| FARSB | 0.444 | -1.17028 | 9.82E-15 | 2.74E-13 | yes | down | 13.8 | 13.53 | 12.2 | 14.32 | 12.96 | 14.96 | 13.17667 | 14.08 |
| EPRS | 0.476 | -1.07102 | 9.84E-15 | 2.74E-13 | yes | down | 36.18 | 29.13 | 26.77 | 34.58 | 32.81 | 35.34 | 30.69333 | 34.24333 |
| OLFML3 | 3.628 | 1.859039 | 9.87E-15 | 2.75E-13 | yes | up | 11.97 | 10.97 | 5.82 | 78.88 | 74.73 | 91.8 | 9.586667 | 81.80333 |
| SOWAHC | 0.128 | -2.9709 | 1.03E-14 | 2.86E-13 | yes | down | 20.97 | 41.69 | 11.36 | 7.9 | 5.87 | 8.27 | 24.67333 | 7.346667 |
| ALKBH7 | 0.266 | -1.90922 | 1.11E-14 | 3.07E-13 | yes | down | 12.48 | 16.4 | 14.73 | 10.65 | 9.77 | 7.8 | 14.53667 | 9.406667 |
| HTRA1 | 2.691 | 1.428008 | 1.11E-14 | 3.08E-13 | yes | up | 124.08 | 109.58 | 107.47 | 806.23 | 816.2 | 585.01 | 113.71 | 735.8133 |
| TMEM164 | 2.534 | 1.341296 | 1.14E-14 | 3.16E-13 | yes | up | 3.88 | 2.97 | 2.45 | 18.7 | 16.6 | 18.4 | 3.1 | 17.9 |
| LOC108178477 | 0.22 | -2.18297 | 1.16E-14 | 3.21E-13 | yes | down | 2.4 | 2.37 | 1.59 | 1.42 | 1.1 | 0.84 | 2.12 | 1.12 |
| LOC100338849 | 0.324 | -1.62653 | 1.2E-14 | 3.31E-13 | yes | down | 7.49 | 6.55 | 6.37 | 5.26 | 5.35 | 5.27 | 6.803333 | 5.293333 |
| LOC100353508 | 2.249 | 1.169371 | 1.25E-14 | 3.43E-13 | yes | up | 17.22 | 13.74 | 10.29 | 76.99 | 71.57 | 70.67 | 13.75 | 73.07667 |
| NDUFB11 | 0.464 | -1.10773 | 1.31E-14 | 3.58E-13 | yes | down | 187.36 | 204.08 | 193.27 | 230.06 | 215.52 | 212.47 | 194.9033 | 219.35 |
| SEMA3A | 8.52 | 3.090783 | 1.54E-14 | 4.2E-13 | yes | up | 0.5 | 0.42 | 0.4 | 14.14 | 5.01 | 5.75 | 0.44 | 8.3 |
| LOC100346685 | 981.461 | 9.938788 | 1.58E-14 | 4.32E-13 | yes | up | 0 | 0 | 0 | 27.89 | 9.79 | 9.02 | 0 | 15.56667 |
| CXCL12 | 0.156 | -2.6834 | 1.66E-14 | 4.53E-13 | yes | down | 21.54 | 31.29 | 45.18 | 10.49 | 13.03 | 14.05 | 32.67 | 12.52333 |
| GPD2 | 0.101 | -3.31103 | 1.78E-14 | 4.85E-13 | yes | down | 103.06 | 78.28 | 108.95 | 14.7 | 16 | 40.98 | 96.76333 | 23.89333 |
| ADRB3 | 0.319 | -1.64968 | 1.87E-14 | 5.09E-13 | yes | down | 9.11 | 12.19 | 6.46 | 7.09 | 6.13 | 6.06 | 9.253333 | 6.426667 |
| LOC103349531 | 143.431 | 7.164217 | 2.2E-14 | 5.96E-13 | yes | up | 0.03 | 0.02 | 0 | 4.2 | 3.69 | 7.26 | 0.016667 | 5.05 |
| C8H12orf40 | 771.785 | 9.592055 | 2.36E-14 | 6.4E-13 | yes | up | 0 | 0.02 | 0 | 27.41 | 13.14 | 9.3 | 0.006667 | 16.61667 |
| SAV1 | 0.382 | -1.38673 | 2.6E-14 | 7.02E-13 | yes | down | 16.55 | 21.77 | 18.51 | 18.64 | 17.84 | 16.11 | 18.94333 | 17.53 |
| NDUFA11 | 0.43 | -1.21852 | 2.69E-14 | 7.25E-13 | yes | down | 105.4 | 128.91 | 98.7 | 143.78 | 100.3 | 122.34 | 111.0033 | 122.14 |
| OPN3 | 4.811 | 2.266207 | 2.8E-14 | 7.55E-13 | yes | up | 1.21 | 0.74 | 0.48 | 8.67 | 8.1 | 8.61 | 0.81 | 8.46 |
| MAPKAPK3 | 0.412 | -1.27955 | 3.14E-14 | 8.43E-13 | yes | down | 13.82 | 15.06 | 13.73 | 13.11 | 13.1 | 15.32 | 14.20333 | 13.84333 |
| DUSP4 | 0.045 | -4.47588 | 3.23E-14 | 8.67E-13 | yes | down | 3.7 | 8.9 | 1.59 | 0.63 | 0.34 | 0.51 | 4.73 | 0.493333 |
| LOC100338010 | 0.476 | -1.07157 | 3.38E-14 | 9.05E-13 | yes | down | 29.92 | 28.02 | 24.6 | 32.98 | 28.91 | 33.42 | 27.51333 | 31.77 |
| MICU3 | 0.274 | -1.86917 | 3.39E-14 | 9.07E-13 | yes | down | 6.37 | 9.69 | 8.19 | 4.51 | 4.99 | 6.01 | 8.083333 | 5.17 |
| FAM105A | 0.124 | -3.01137 | 3.41E-14 | 9.11E-13 | yes | down | 4.89 | 7.8 | 6.61 | 1.63 | 1.19 | 3.19 | 6.433333 | 2.003333 |
| BMPER | 5.131 | 2.359244 | 3.65E-14 | 9.73E-13 | yes | up | 0.43 | 0.44 | 0.37 | 4.52 | 6.04 | 4.55 | 0.413333 | 5.036667 |
| TFAM | 0.354 | -1.49663 | 3.76E-14 | 1E-12 | yes | down | 10.7 | 12.49 | 8.31 | 8.16 | 9.47 | 8.9 | 10.5 | 8.843333 |
| RDH5 | 10.158 | 3.344536 | 3.79E-14 | 1.01E-12 | yes | up | 0.51 | 0.24 | 0.21 | 7.36 | 11.43 | 8.23 | 0.32 | 9.006667 |
| CHCHD2 | 0.203 | -2.3019 | 3.8E-14 | 1.01E-12 | yes | down | 981.03 | 591.85 | 1043.5 | 395.68 | 521.84 | 374.53 | 872.1267 | 430.6833 |
| SNCA | 0.077 | -3.69092 | 4E-14 | 1.06E-12 | yes | down | 25.06 | 61.62 | 44.5 | 3.34 | 8.35 | 12.1 | 43.72667 | 7.93 |
| ARHGEF37 | 0.376 | -1.4094 | 4.03E-14 | 1.07E-12 | yes | down | 16.35 | 23.27 | 15.77 | 17.69 | 15.08 | 16.81 | 18.46333 | 16.52667 |
| PDSS1 | 0.302 | -1.72674 | 4.08E-14 | 1.08E-12 | yes | down | 9.03 | 7.99 | 5.81 | 4.93 | 5.6 | 5.3 | 7.61 | 5.276667 |
| MED4 | 0.306 | -1.70801 | 4.16E-14 | 1.1E-12 | yes | down | 31.04 | 37.12 | 40.54 | 26.39 | 25.67 | 29.27 | 36.23333 | 27.11 |
| ITGA11 | 7.025 | 2.812491 | 4.23E-14 | 1.11E-12 | yes | up | 1.24 | 0.9 | 0.39 | 12.13 | 10.89 | 18.13 | 0.843333 | 13.71667 |
| SGMS2 | 4.217 | 2.076293 | 4.25E-14 | 1.12E-12 | yes | up | 1.37 | 0.7 | 0.78 | 10.79 | 7.94 | 9.66 | 0.95 | 9.463333 |
| GDF5 | 19.826 | 4.30929 | 4.5E-14 | 1.18E-12 | yes | up | 0.22 | 0.12 | 0.07 | 6.96 | 5.22 | 6.92 | 0.136667 | 6.366667 |
| SNRNP25 | 0.417 | -1.26293 | 4.53E-14 | 1.18E-12 | yes | down | 31.13 | 27.41 | 25.95 | 30.05 | 27.46 | 25.71 | 28.16333 | 27.74 |
| LOC100353574 | 5.224 | 2.385279 | 4.58E-14 | 1.2E-12 | yes | up | 10.95 | 10.6 | 5.94 | 56.8 | 54.96 | 106.69 | 9.163333 | 72.81667 |
| MCRS1 | 0.475 | -1.07343 | 4.6E-14 | 1.2E-12 | yes | down | 31.08 | 35.35 | 30.97 | 39.67 | 30.73 | 36.22 | 32.46667 | 35.54 |
| MAP7 | 0.182 | -2.45633 | 4.71E-14 | 1.23E-12 | yes | down | 2.84 | 5.28 | 3.36 | 2.25 | 1.21 | 1.6 | 3.826667 | 1.686667 |
| BCL6 | 4.086 | 2.030638 | 5.06E-14 | 1.31E-12 | yes | up | 8.46 | 6.06 | 6.54 | 45.08 | 48.78 | 82.13 | 7.02 | 58.66333 |
| MPPE1 | 0.24 | -2.0574 | 5.06E-14 | 1.31E-12 | yes | down | 17.59 | 23.28 | 28.27 | 13.24 | 14.67 | 11.56 | 23.04667 | 13.15667 |
| MRPS25 | 0.395 | -1.34091 | 5.23E-14 | 1.35E-12 | yes | down | 63.75 | 81.44 | 60.14 | 68.01 | 61.46 | 52.26 | 68.44333 | 60.57667 |
| LOC108176558 | 0.084 | -3.56647 | 5.33E-14 | 1.38E-12 | yes | down | 5.05 | 8.54 | 4.46 | 0.44 | 1.08 | 1.39 | 6.016667 | 0.97 |
| DNAJC11 | 0.444 | -1.17271 | 5.34E-14 | 1.38E-12 | yes | down | 30.36 | 34.91 | 27.85 | 30.65 | 33.06 | 35.09 | 31.04 | 32.93333 |
| CCDC190 | 7.698 | 2.944479 | 5.48E-14 | 1.41E-12 | yes | up | 0.53 | 0.77 | 0.53 | 13.75 | 11.18 | 19.43 | 0.61 | 14.78667 |
| LOC100356940 | 4.701 | 2.233069 | 5.71E-14 | 1.47E-12 | yes | up | 1.55 | 1.64 | 0.79 | 24.48 | 25.08 | 11.86 | 1.326667 | 20.47333 |
| LOC100358198 | 0.038 | -4.71362 | 5.95E-14 | 1.53E-12 | yes | down | 0.54 | 1.84 | 0.79 | 0.08 | 0.09 | 0.11 | 1.056667 | 0.093333 |
| BOLA3 | 0.423 | -1.24119 | 6.28E-14 | 1.61E-12 | yes | down | 98.4 | 101.25 | 91.4 | 114.48 | 87.3 | 97.49 | 97.01667 | 99.75667 |
| RASSF2 | 3.188 | 1.672794 | 6.53E-14 | 1.67E-12 | yes | up | 3.65 | 3.11 | 2.03 | 27.21 | 20.15 | 18.49 | 2.93 | 21.95 |
| PSMB1 | 0.498 | -1.00719 | 6.59E-14 | 1.68E-12 | yes | down | 140.09 | 135.92 | 113.35 | 176.07 | 154.79 | 135.83 | 129.7867 | 155.5633 |
| DACT2 | 4.269 | 2.093868 | 6.63E-14 | 1.69E-12 | yes | up | 1.32 | 0.86 | 0.58 | 7.93 | 8.07 | 8.96 | 0.92 | 8.32 |
| ABCA6 | 3.188 | 1.672455 | 7E-14 | 1.78E-12 | yes | up | 1.15 | 1.12 | 0.74 | 8.65 | 8.21 | 5.85 | 1.003333 | 7.57 |
| MTX1 | 0.326 | -1.61561 | 7.28E-14 | 1.85E-12 | yes | down | 35.53 | 47.75 | 45.49 | 40.12 | 29.1 | 33.34 | 42.92333 | 34.18667 |
| OLFML1 | 5.882 | 2.556241 | 7.67E-14 | 1.95E-12 | yes | up | 6.54 | 3.75 | 2.34 | 49.21 | 72.65 | 51.05 | 4.21 | 57.63667 |
| LOC108177262 | 0.349 | -1.52075 | 8.14E-14 | 2.06E-12 | yes | down | 1668.78 | 1675.01 | 2020.05 | 1714.22 | 1512.29 | 1409.53 | 1787.947 | 1545.347 |
| RASGRP2 | 3.63 | 1.85994 | 8.18E-14 | 2.07E-12 | yes | up | 2.32 | 4.94 | 2.96 | 36.59 | 36.79 | 32.49 | 3.406667 | 35.29 |
| PRKRA | 0.345 | -1.53483 | 8.42E-14 | 2.13E-12 | yes | down | 33.61 | 28.62 | 26.08 | 24.67 | 28.71 | 20.03 | 29.43667 | 24.47 |
| SQLE | 3.115 | 1.639108 | 8.63E-14 | 2.18E-12 | yes | up | 1.58 | 1.9 | 1.2 | 12.66 | 12.05 | 10.11 | 1.56 | 11.60667 |
| SFXN1 | 4.082 | 2.029299 | 8.86E-14 | 2.23E-12 | yes | up | 4.15 | 6.84 | 4.23 | 51.41 | 58.06 | 37.35 | 5.073333 | 48.94 |
| TST | 4.045 | 2.016031 | 8.88E-14 | 2.23E-12 | yes | up | 14.48 | 7.95 | 8.41 | 127.64 | 78.7 | 93.66 | 10.28 | 100 |
| GFM2 | 0.327 | -1.61115 | 8.88E-14 | 2.23E-12 | yes | down | 18.15 | 15.33 | 12.24 | 14.22 | 13.04 | 8.99 | 15.24 | 12.08333 |
| NFYC | 0.365 | -1.45421 | 9.06E-14 | 2.26E-12 | yes | down | 20.95 | 27.04 | 25.46 | 23.62 | 21.08 | 22.78 | 24.48333 | 22.49333 |
| GPX3 | 0.135 | -2.88858 | 9.54E-14 | 2.38E-12 | yes | down | 478.69 | 471.59 | 611.6 | 99.11 | 261.7 | 142.24 | 520.6267 | 167.6833 |
| MYBBP1A | 0.4 | -1.32342 | 9.85E-14 | 2.45E-12 | yes | down | 15.06 | 17.19 | 11.52 | 14.86 | 14.15 | 18.27 | 14.59 | 15.76 |
| RNF19B | 2.664 | 1.413549 | 9.9E-14 | 2.46E-12 | yes | up | 9.12 | 9.9 | 8.88 | 63.21 | 70.33 | 55.07 | 9.3 | 62.87 |
| CAPN8 | 12.789 | 3.67685 | 1.07E-13 | 2.66E-12 | yes | up | 0.48 | 1.02 | 0.21 | 13.9 | 7.29 | 21.69 | 0.57 | 14.29333 |
| TSPO | 2.366 | 1.242203 | 1.1E-13 | 2.73E-12 | yes | up | 80.68 | 88.65 | 84.15 | 564.59 | 416.91 | 481.73 | 84.49333 | 487.7433 |
| EPB42 | 0.07 | -3.83638 | 1.12E-13 | 2.78E-12 | yes | down | 1.82 | 3.58 | 3.15 | 0.25 | 0.37 | 0.83 | 2.85 | 0.483333 |
| HBB2 | 0.069 | -3.85573 | 1.14E-13 | 2.83E-12 | yes | down | 7092.06 | 18788.27 | 12438.18 | 808.83 | 1950.5 | 3563.21 | 12772.84 | 2107.513 |
| CLUH | 0.306 | -1.70727 | 1.16E-13 | 2.86E-12 | yes | down | 49.63 | 61.19 | 56.17 | 39.5 | 32.03 | 51.59 | 55.66333 | 41.04 |
| BCKDK | 0.474 | -1.0773 | 1.22E-13 | 3E-12 | yes | down | 47.17 | 44.86 | 45.18 | 55.09 | 52.82 | 49.28 | 45.73667 | 52.39667 |
| LOC108177673 | 5.5 | 2.459321 | 1.26E-13 | 3.09E-12 | yes | up | 1.62 | 1.04 | 1.24 | 15.89 | 11.34 | 20.22 | 1.3 | 15.81667 |
| WSCD2 | 8.128 | 3.022825 | 1.55E-13 | 3.81E-12 | yes | up | 0.19 | 0.15 | 0.09 | 3.08 | 2.17 | 2.61 | 0.143333 | 2.62 |
| CREB5 | 3.567 | 1.83473 | 1.59E-13 | 3.91E-12 | yes | up | 1.55 | 1.06 | 0.74 | 7.92 | 8.54 | 11.32 | 1.116667 | 9.26 |
| LOC100356012 | 4.451 | 2.15427 | 1.61E-13 | 3.93E-12 | yes | up | 3.93 | 7.56 | 3.95 | 53.92 | 64.38 | 46.4 | 5.146667 | 54.9 |
| ATP5G1 | 0.186 | -2.42571 | 1.61E-13 | 3.93E-12 | yes | down | 427.15 | 234.16 | 173.7 | 128.65 | 151.32 | 93.97 | 278.3367 | 124.6467 |
| ABCC9 | 0.396 | -1.33659 | 1.63E-13 | 3.98E-12 | yes | down | 13.21 | 11.45 | 8.36 | 10.7 | 11.26 | 9.17 | 11.00667 | 10.37667 |
| LOC108178465 | 0.179 | -2.48528 | 1.85E-13 | 4.51E-12 | yes | down | 23.45 | 11.74 | 10.98 | 6.45 | 7.29 | 5.81 | 15.39 | 6.516667 |
| NDUFAF5 | 0.333 | -1.58835 | 1.93E-13 | 4.68E-12 | yes | down | 8.62 | 9.5 | 9.64 | 6.33 | 7.25 | 6.59 | 9.253333 | 6.723333 |
| SERPINF1 | 5.381 | 2.427919 | 2.02E-13 | 4.89E-12 | yes | up | 90.88 | 52.9 | 32.8 | 600.69 | 878.44 | 761.43 | 58.86 | 746.8533 |
| HLF | 3.184 | 1.670982 | 2.24E-13 | 5.41E-12 | yes | up | 1.98 | 2.13 | 1.28 | 15.56 | 14.48 | 11.61 | 1.796667 | 13.88333 |
| LOC103347061 | 0.312 | -1.67955 | 2.33E-13 | 5.64E-12 | yes | down | 30.7 | 28.48 | 24.62 | 24.73 | 17 | 21.81 | 27.93333 | 21.18 |
| MTG2 | 0.471 | -1.08601 | 2.35E-13 | 5.67E-12 | yes | down | 13.39 | 14.94 | 12.76 | 15.2 | 12.52 | 14.09 | 13.69667 | 13.93667 |
| AEBP1 | 3.458 | 1.789748 | 2.43E-13 | 5.86E-12 | yes | up | 12.37 | 8.32 | 7.37 | 61.83 | 86.12 | 78.6 | 9.353333 | 75.51667 |
| NAA40 | 0.376 | -1.4107 | 2.6E-13 | 6.26E-12 | yes | down | 6.49 | 7.57 | 4.71 | 5.81 | 5.68 | 5.3 | 6.256667 | 5.596667 |
| MRPS31 | 0.296 | -1.7553 | 2.63E-13 | 6.3E-12 | yes | down | 14.56 | 17.07 | 11.03 | 12.23 | 9.6 | 8.73 | 14.22 | 10.18667 |
| SFRP5 | 17.378 | 4.119172 | 2.63E-13 | 6.3E-12 | yes | up | 1.69 | 0.79 | 0.46 | 35.35 | 74.29 | 13.88 | 0.98 | 41.17333 |
| TIMM50 | 0.329 | -1.60328 | 2.74E-13 | 6.55E-12 | yes | down | 42.8 | 42.5 | 46.2 | 44.13 | 33.76 | 27.81 | 43.83333 | 35.23333 |
| MRPS15 | 0.494 | -1.0172 | 2.74E-13 | 6.55E-12 | yes | down | 60.74 | 57.12 | 56.23 | 69.47 | 66.76 | 65.3 | 58.03 | 67.17667 |
| CTSH | 3.046 | 1.607146 | 3.14E-13 | 7.5E-12 | yes | up | 34.16 | 42.38 | 38.52 | 223.03 | 294.45 | 252.73 | 38.35333 | 256.7367 |
| PFKFB3 | 3.776 | 1.917003 | 3.19E-13 | 7.59E-12 | yes | up | 46.8 | 58.77 | 41.6 | 424.1 | 291.48 | 572.38 | 49.05667 | 429.32 |
| MTMR1 | 0.497 | -1.00837 | 3.44E-13 | 8.17E-12 | yes | down | 10.45 | 10.35 | 8.79 | 11.33 | 11.83 | 12.03 | 9.863333 | 11.73 |
| PAM | 2.11 | 1.076988 | 3.88E-13 | 9.21E-12 | yes | up | 28.3 | 24.05 | 18.38 | 117.08 | 117.01 | 119.37 | 23.57667 | 117.82 |
| ABCA3 | 3.47 | 1.795072 | 3.99E-13 | 9.47E-12 | yes | up | 1.75 | 1.36 | 1.46 | 14.81 | 8.84 | 14.83 | 1.523333 | 12.82667 |
| AHNAK | 2.502 | 1.32294 | 4.07E-13 | 9.64E-12 | yes | up | 62.52 | 46.41 | 42.63 | 287.79 | 269.6 | 342.47 | 50.52 | 299.9533 |
| SRXN1 | 0.21 | -2.25008 | 4.35E-13 | 1.03E-11 | yes | down | 20.35 | 41.92 | 24.53 | 16.22 | 12.34 | 15.47 | 28.93333 | 14.67667 |
| GRIA3 | 6.475 | 2.694893 | 4.48E-13 | 1.06E-11 | yes | up | 0.58 | 0.37 | 0.18 | 6.43 | 4.75 | 5.53 | 0.376667 | 5.57 |
| CD82 | 2.777 | 1.473327 | 4.49E-13 | 1.06E-11 | yes | up | 15.82 | 19.84 | 13.64 | 139.63 | 94.21 | 94.66 | 16.43333 | 109.5 |
| TIMM8B | 0.364 | -1.45907 | 4.6E-13 | 1.08E-11 | yes | down | 162.93 | 208.3 | 187.76 | 172.66 | 177.96 | 145.57 | 186.33 | 165.3967 |
| SDHD | 0.337 | -1.56858 | 5.29E-13 | 1.24E-11 | yes | down | 360.27 | 231.37 | 192.57 | 229.13 | 204.44 | 192.28 | 261.4033 | 208.6167 |
| FTSJ3 | 0.477 | -1.06652 | 5.29E-13 | 1.24E-11 | yes | down | 13.21 | 13.83 | 11.57 | 13.99 | 14.52 | 15.61 | 12.87 | 14.70667 |
| EIF2A | 0.493 | -1.01953 | 6.06E-13 | 1.42E-11 | yes | down | 16.77 | 18.12 | 15.03 | 20.14 | 20.24 | 18.45 | 16.64 | 19.61 |
| LOC100357740 | 2.116 | 1.081532 | 6.37E-13 | 1.49E-11 | yes | up | 1.79 | 1.9 | 1.52 | 9.04 | 7.84 | 9.37 | 1.736667 | 8.75 |
| CCNI | 0.386 | -1.37319 | 6.51E-13 | 1.52E-11 | yes | down | 162.35 | 216.66 | 173.24 | 153.41 | 173.72 | 183.89 | 184.0833 | 170.34 |
| FASTKD2 | 0.354 | -1.49951 | 6.54E-13 | 1.52E-11 | yes | down | 4.02 | 5 | 3.99 | 4.38 | 3.35 | 3.24 | 4.336667 | 3.656667 |
| LETMD1 | 0.383 | -1.38324 | 6.7E-13 | 1.56E-11 | yes | down | 50.31 | 64.01 | 58.58 | 54.03 | 48.62 | 58.98 | 57.63333 | 53.87667 |
| LIFR | 2.294 | 1.197843 | 6.98E-13 | 1.62E-11 | yes | up | 9.05 | 10.36 | 8.21 | 50.21 | 42.02 | 58.02 | 9.206667 | 50.08333 |
| GRK3 | 2.2 | 1.137745 | 7.28E-13 | 1.69E-11 | yes | up | 10.76 | 11.61 | 10.48 | 58.81 | 62.51 | 62.39 | 10.95 | 61.23667 |
| LOC103350250 | 0.051 | -4.30061 | 7.41E-13 | 1.72E-11 | yes | down | 1.16 | 2.02 | 2.3 | 0.27 | 0.19 | 0.22 | 1.826667 | 0.226667 |
| C19H17orf53 | 0.268 | -1.90139 | 7.88E-13 | 1.82E-11 | yes | down | 2.71 | 3.13 | 2.49 | 1.86 | 2.08 | 1.42 | 2.776667 | 1.786667 |
| CCKBR | 0.001 | -9.60182 | 8.06E-13 | 1.86E-11 | yes | down | 0.5 | 0.67 | 1.5 | 0 | 0 | 0 | 0.89 | 0 |
| DNAJA2 | 0.457 | -1.13014 | 8.72E-13 | 2.01E-11 | yes | down | 40.1 | 45.35 | 39.05 | 43.89 | 49.19 | 43.35 | 41.5 | 45.47667 |
| VIL1 | 0.091 | -3.46317 | 9.13E-13 | 2.1E-11 | yes | down | 1.68 | 2.86 | 2.61 | 0.49 | 0.26 | 0.35 | 2.383333 | 0.366667 |
| ANXA2 | 3.49 | 1.803182 | 9.67E-13 | 2.22E-11 | yes | up | 168.69 | 114.45 | 75.7 | 1201.01 | 825.62 | 930.77 | 119.6133 | 985.8 |
| CREB3L1 | 3.733 | 1.900349 | 9.73E-13 | 2.23E-11 | yes | up | 2.04 | 1.5 | 1 | 12.27 | 14.34 | 13.21 | 1.513333 | 13.27333 |
| CSF1 | 2.148 | 1.103039 | 9.77E-13 | 2.24E-11 | yes | up | 3.24 | 3.13 | 2.61 | 17.05 | 13.28 | 15.98 | 2.993333 | 15.43667 |
| TRIM77 | 10.84 | 3.438345 | 9.92E-13 | 2.27E-11 | yes | up | 0.06 | 0.26 | 0.28 | 5.72 | 5.42 | 5.14 | 0.2 | 5.426667 |
| CTSB | 2.322 | 1.215371 | 1.02E-12 | 2.34E-11 | yes | up | 200.19 | 227.81 | 213.07 | 1105.19 | 1199.33 | 1280.5 | 213.69 | 1195.007 |
| SLC40A1 | 0.237 | -2.07555 | 1.03E-12 | 2.35E-11 | yes | down | 44.46 | 50.96 | 68.14 | 30.21 | 25.34 | 39.74 | 54.52 | 31.76333 |
| EPB41L3 | 4.386 | 2.132892 | 1.12E-12 | 2.55E-11 | yes | up | 2.57 | 1.47 | 1.02 | 20 | 17.9 | 14.92 | 1.686667 | 17.60667 |
| ALDH1A1 | 4.334 | 2.115829 | 1.12E-12 | 2.56E-11 | yes | up | 115.2 | 77.84 | 55.9 | 628.01 | 869.95 | 1110.68 | 82.98 | 869.5467 |
| LOC100353151 | 0.362 | -1.46476 | 1.14E-12 | 2.6E-11 | yes | down | 65.02 | 63.08 | 73.4 | 73.83 | 51.41 | 51.06 | 67.16667 | 58.76667 |
| GHITM | 0.174 | -2.52467 | 1.21E-12 | 2.75E-11 | yes | down | 377.1 | 469.36 | 175.99 | 158.7 | 81.18 | 176.62 | 340.8167 | 138.8333 |
| NWD2 | 0.116 | -3.10591 | 1.23E-12 | 2.78E-11 | yes | down | 3.04 | 7.33 | 1.81 | 3.83 | 1.23 | 3.39 | 4.06 | 2.816667 |
| OSBPL5 | 2.578 | 1.366255 | 1.23E-12 | 2.78E-11 | yes | up | 6.51 | 5.3 | 4.45 | 33.34 | 30.19 | 36.58 | 5.42 | 33.37 |
| CTSD | 0.347 | -1.52837 | 1.23E-12 | 2.78E-11 | yes | down | 372.45 | 573.45 | 437.87 | 365.57 | 357.63 | 431.36 | 461.2567 | 384.8533 |
| HINT1 | 0.43 | -1.21717 | 1.38E-12 | 3.11E-11 | yes | down | 312.83 | 316.03 | 315.07 | 394.33 | 306.06 | 280.89 | 314.6433 | 327.0933 |
| FUS | 0.457 | -1.13027 | 1.39E-12 | 3.13E-11 | yes | down | 66.7 | 77.42 | 54.45 | 65.52 | 55.81 | 70.86 | 66.19 | 64.06333 |
| NOVA1 | 3.339 | 1.739541 | 1.46E-12 | 3.29E-11 | yes | up | 2.11 | 2.41 | 1.28 | 17.35 | 12.18 | 15.13 | 1.933333 | 14.88667 |
| LOC103350570 | 79.828 | 6.318824 | 1.47E-12 | 3.31E-11 | yes | up | 0.01 | 0.02 | 0 | 2 | 1.72 | 1.65 | 0.01 | 1.79 |
| LOC103349390 | 0.046 | -4.43046 | 1.52E-12 | 3.42E-11 | yes | down | 2.07 | 4.58 | 1.21 | 0.42 | 0.27 | 0.16 | 2.62 | 0.283333 |
| HEY1 | 0.309 | -1.69359 | 1.56E-12 | 3.5E-11 | yes | down | 8.92 | 14.47 | 9.62 | 8.71 | 8.16 | 7.83 | 11.00333 | 8.233333 |
| PRAP1 | 20.198 | 4.336175 | 1.57E-12 | 3.5E-11 | yes | up | 1.06 | 0.94 | 0.72 | 41.29 | 53 | 34.75 | 0.906667 | 43.01333 |
| NDUFA5 | 0.267 | -1.90488 | 1.64E-12 | 3.66E-11 | yes | down | 181 | 161 | 210.02 | 148.6 | 124.21 | 111.61 | 184.0067 | 128.14 |
| U2AF2 | 0.308 | -1.69753 | 1.64E-12 | 3.66E-11 | yes | down | 7.45 | 5.97 | 6.11 | 4.89 | 4.63 | 4.98 | 6.51 | 4.833333 |
| LOC100358318 | 0.344 | -1.53813 | 1.68E-12 | 3.75E-11 | yes | down | 38.06 | 43.14 | 50.87 | 39.1 | 33.88 | 38.15 | 44.02333 | 37.04333 |
| NPM1 | 0.446 | -1.16593 | 1.7E-12 | 3.78E-11 | yes | down | 227.51 | 262.02 | 229.64 | 284.01 | 268.92 | 216.64 | 239.7233 | 256.5233 |
| ATP5O | 0.455 | -1.135 | 1.76E-12 | 3.9E-11 | yes | down | 1021.47 | 1334.39 | 1077.47 | 1312.11 | 1178.59 | 1301.97 | 1144.443 | 1264.223 |
| SATB2 | 7.109 | 2.829573 | 1.79E-12 | 3.96E-11 | yes | up | 0.4 | 0.45 | 0.34 | 6.37 | 4.41 | 10.85 | 0.396667 | 7.21 |
| NDUFB9 | 0.499 | -1.00326 | 1.82E-12 | 4.02E-11 | yes | down | 471.98 | 533.77 | 492.27 | 610.54 | 533.62 | 534.92 | 499.34 | 559.6933 |
| HPSE2 | 37.308 | 5.22143 | 1.95E-12 | 4.31E-11 | yes | up | 8.83 | 0.01 | 0.02 | 26.92 | 6.85 | 31.53 | 2.953333 | 21.76667 |
| WDR45 | 0.474 | -1.0771 | 2.11E-12 | 4.65E-11 | yes | down | 25.92 | 26.53 | 23.73 | 31.85 | 28.93 | 25.95 | 25.39333 | 28.91 |
| NPC1L1 | 30.262 | 4.919425 | 2.17E-12 | 4.77E-11 | yes | up | 0.02 | 0.07 | 0.01 | 2.91 | 2.36 | 1.93 | 0.033333 | 2.4 |
| PDPR | 0.4 | -1.32171 | 2.17E-12 | 4.77E-11 | yes | down | 13.56 | 15.55 | 15.93 | 15.37 | 13.49 | 15.01 | 15.01333 | 14.62333 |
| SERINC2 | 3.242 | 1.697095 | 2.19E-12 | 4.81E-11 | yes | up | 10.34 | 9.31 | 7.81 | 66.33 | 54.51 | 92.18 | 9.153333 | 71.00667 |
| MMP28 | 4.159 | 2.056076 | 2.37E-12 | 5.19E-11 | yes | up | 4.19 | 3.05 | 3.64 | 49.72 | 22.14 | 42.93 | 3.626667 | 38.26333 |
| ADCY6 | 2.414 | 1.271555 | 2.41E-12 | 5.27E-11 | yes | up | 14.14 | 13.68 | 11.47 | 79.2 | 58.41 | 89.85 | 13.09667 | 75.82 |
| CECR5 | 0.325 | -1.62145 | 2.45E-12 | 5.35E-11 | yes | down | 8.66 | 8.19 | 7.56 | 6.84 | 3.83 | 4.07 | 8.136667 | 4.913333 |
| GLE1 | 0.434 | -1.20427 | 2.66E-12 | 5.8E-11 | yes | down | 13.79 | 16.28 | 14.85 | 17.87 | 13.53 | 15.82 | 14.97333 | 15.74 |
| LOC100343402 | 0.44 | -1.18395 | 2.68E-12 | 5.84E-11 | yes | down | 69.98 | 79.7 | 54.88 | 79.46 | 68.78 | 69.45 | 68.18667 | 72.56333 |
| SLC12A2 | 0.285 | -1.81019 | 2.73E-12 | 5.94E-11 | yes | down | 10.28 | 12.7 | 12.94 | 7.27 | 10.29 | 7.14 | 11.97333 | 8.233333 |
| LOC108175950 | 0.046 | -4.4443 | 2.76E-12 | 6E-11 | yes | down | 1.82 | 4.67 | 5.3 | 0.35 | 0.16 | 0.6 | 3.93 | 0.37 |
| BCAM | 2.879 | 1.525478 | 2.81E-12 | 6.09E-11 | yes | up | 42.32 | 41.74 | 44.45 | 248.04 | 289.05 | 354.24 | 42.83667 | 297.11 |
| THNSL2 | 10.343 | 3.370554 | 2.82E-12 | 6.12E-11 | yes | up | 0.82 | 0.27 | 0.19 | 9.08 | 10.18 | 6.13 | 0.426667 | 8.463333 |
| CHCHD5 | 0.332 | -1.59281 | 2.85E-12 | 6.17E-11 | yes | down | 23.31 | 27.22 | 23.37 | 25.62 | 17.46 | 16.6 | 24.63333 | 19.89333 |
| PAPSS2 | 3.511 | 1.811711 | 2.9E-12 | 6.26E-11 | yes | up | 1.19 | 1.23 | 0.8 | 7.71 | 6.84 | 10.43 | 1.073333 | 8.326667 |
| LOC100341224 | 0.194 | -2.36485 | 2.9E-12 | 6.27E-11 | yes | down | 5.18 | 3.86 | 3.34 | 2.72 | 1.71 | 1.32 | 4.126667 | 1.916667 |
| AQP7 | 2.544 | 1.346951 | 2.93E-12 | 6.32E-11 | yes | up | 31 | 42.43 | 31.21 | 230.17 | 189.22 | 233.85 | 34.88 | 217.7467 |
| YARS2 | 0.432 | -1.21152 | 2.95E-12 | 6.36E-11 | yes | down | 13.2 | 15.94 | 13.54 | 15.28 | 14.78 | 14.43 | 14.22667 | 14.83 |
| MRRF | 0.422 | -1.24428 | 3.01E-12 | 6.48E-11 | yes | down | 14.35 | 14.24 | 13.43 | 13.04 | 14.11 | 15.82 | 14.00667 | 14.32333 |
| CCDC141 | 0.187 | -2.41917 | 3.04E-12 | 6.53E-11 | yes | down | 1.44 | 3.03 | 1.85 | 1.59 | 1.1 | 0.89 | 2.106667 | 1.193333 |
| ARHGAP21 | 2.649 | 1.405708 | 3.15E-12 | 6.75E-11 | yes | up | 5.09 | 4.27 | 3.68 | 24.45 | 18.44 | 28.41 | 4.346667 | 23.76667 |
| RSPO3 | 19.375 | 4.276111 | 3.55E-12 | 7.6E-11 | yes | up | 1.66 | 0.28 | 0.2 | 27.05 | 35.76 | 17.37 | 0.713333 | 26.72667 |
| LDLR | 5.823 | 2.541811 | 3.6E-12 | 7.7E-11 | yes | up | 2.55 | 2.06 | 3.26 | 37.43 | 22.29 | 53.01 | 2.623333 | 37.57667 |
| SLC25A33 | 0.197 | -2.34331 | 3.74E-12 | 7.98E-11 | yes | down | 41.83 | 32.23 | 17.29 | 10.17 | 13.82 | 17.95 | 30.45 | 13.98 |
| ACKR2 | 4.269 | 2.093847 | 3.78E-12 | 8.05E-11 | yes | up | 1.05 | 0.78 | 0.49 | 6.8 | 8.3 | 7.63 | 0.773333 | 7.576667 |
| GDPGP1 | 0.445 | -1.16904 | 3.79E-12 | 8.06E-11 | yes | down | 5.05 | 5.21 | 3.73 | 5.13 | 4.39 | 5.09 | 4.663333 | 4.87 |
| LOC100342874 | 0.454 | -1.13859 | 4.48E-12 | 9.51E-11 | yes | down | 77.16 | 99.38 | 80.85 | 105.34 | 90.94 | 88.78 | 85.79667 | 95.02 |
| CTSA | 0.48 | -1.05995 | 4.54E-12 | 9.64E-11 | yes | down | 53.7 | 67.02 | 51.56 | 65.38 | 63.42 | 69.39 | 57.42667 | 66.06333 |
| MRPS28 | 0.386 | -1.37278 | 4.59E-12 | 9.73E-11 | yes | down | 37.53 | 40.03 | 25.7 | 32.82 | 29.17 | 33.53 | 34.42 | 31.84 |
| LOC108176754 | 0.13 | -2.94749 | 4.7E-12 | 9.94E-11 | yes | down | 8.66 | 10.49 | 6.82 | 4.29 | 1.06 | 2.85 | 8.656667 | 2.733333 |
| LOC100358947 | 0.001 | -9.44959 | 4.78E-12 | 1.01E-10 | yes | down | 4.39 | 1.04 | 4.33 | 0 | 0 | 0 | 3.253333 | 0 |
| SAA3 | 7.79 | 2.961534 | 4.95E-12 | 1.04E-10 | yes | up | 3.35 | 4.54 | 1.71 | 59.66 | 42.11 | 79.39 | 3.2 | 60.38667 |
| FNDC3A | 2.272 | 1.184164 | 5.03E-12 | 1.06E-10 | yes | up | 5.85 | 5.9 | 5.03 | 37.41 | 26.47 | 25.76 | 5.593333 | 29.88 |
| PIK3R1 | 3.948 | 1.981167 | 5.25E-12 | 1.1E-10 | yes | up | 7.22 | 6.03 | 4.31 | 37.39 | 70.77 | 52.3 | 5.853333 | 53.48667 |
| MMADHC | 0.423 | -1.24186 | 5.67E-12 | 1.19E-10 | yes | down | 40.39 | 40.23 | 38.64 | 40.76 | 45.4 | 34.74 | 39.75333 | 40.3 |
| EDIL3 | 6.519 | 2.704752 | 5.67E-12 | 1.19E-10 | yes | up | 0.36 | 0.18 | 0.17 | 4.83 | 3.58 | 2.63 | 0.236667 | 3.68 |
| FCGRT | 2.151 | 1.104748 | 5.77E-12 | 1.21E-10 | yes | up | 55.7 | 60.32 | 53.61 | 268.85 | 307.66 | 299.26 | 56.54333 | 291.9233 |
| CAMK1 | 2.623 | 1.391338 | 5.89E-12 | 1.23E-10 | yes | up | 12.21 | 9.25 | 6.41 | 61.01 | 60.51 | 56.43 | 9.29 | 59.31667 |
| RANBP3L | 11.171 | 3.481724 | 6.29E-12 | 1.31E-10 | yes | up | 0.14 | 0.14 | 0.32 | 7.76 | 3.4 | 4.09 | 0.2 | 5.083333 |
| LOC100351828 | 0.473 | -1.08121 | 6.31E-12 | 1.32E-10 | yes | down | 21.4 | 18.65 | 14.58 | 23.34 | 18.75 | 20.24 | 18.21 | 20.77667 |
| LOC100347463 | 2.327 | 1.218658 | 6.36E-12 | 1.33E-10 | yes | up | 10.28 | 9.95 | 9.6 | 51.59 | 54.85 | 60.57 | 9.943333 | 55.67 |
| CDKN3 | 4.441 | 2.15081 | 6.66E-12 | 1.39E-10 | yes | up | 6.91 | 8.63 | 4.63 | 53.5 | 85.02 | 78.91 | 6.723333 | 72.47667 |
| HEPH | 0.024 | -5.36369 | 6.68E-12 | 1.39E-10 | yes | down | 0.7 | 0.37 | 0.76 | 0.06 | 0.05 | 0 | 0.61 | 0.036667 |
| ANKS1B | 11.905 | 3.573518 | 7.03E-12 | 1.46E-10 | yes | up | 0.06 | 1.19 | 0.03 | 1.89 | 2.07 | 3.49 | 0.426667 | 2.483333 |
| LRRC75A | 3.41 | 1.769924 | 7.31E-12 | 1.52E-10 | yes | up | 2.92 | 2 | 2.04 | 18.47 | 22 | 16.17 | 2.32 | 18.88 |
| MRPL42 | 0.415 | -1.26972 | 7.74E-12 | 1.6E-10 | yes | down | 26.31 | 29.35 | 29.6 | 31.26 | 25.84 | 29.09 | 28.42 | 28.73 |
| ANKRD31 | 0.278 | -1.84495 | 7.96E-12 | 1.64E-10 | yes | down | 3.92 | 4.59 | 4.35 | 2.71 | 2.32 | 3.9 | 4.286667 | 2.976667 |
| PTGER4 | 2.69 | 1.427599 | 8.25E-12 | 1.7E-10 | yes | up | 2 | 2.02 | 2.23 | 15.75 | 13.07 | 12.02 | 2.083333 | 13.61333 |
| STARD4 | 5.918 | 2.565208 | 8.29E-12 | 1.71E-10 | yes | up | 0.99 | 1.46 | 1.52 | 26.08 | 9.99 | 21.82 | 1.323333 | 19.29667 |
| VWCE | 0.242 | -2.04623 | 8.97E-12 | 1.84E-10 | yes | down | 1.37 | 1.5 | 1.66 | 0.88 | 0.85 | 1.01 | 1.51 | 0.913333 |
| PSMC3 | 0.49 | -1.02785 | 9.37E-12 | 1.93E-10 | yes | down | 68.69 | 78.69 | 66.28 | 92.38 | 85.96 | 73.49 | 71.22 | 83.94333 |
| UCP2 | 0.465 | -1.10536 | 9.91E-12 | 2.03E-10 | yes | down | 18.21 | 19.11 | 12.98 | 21.04 | 17.69 | 16.56 | 16.76667 | 18.43 |
| NSA2 | 0.462 | -1.11253 | 1.04E-11 | 2.12E-10 | yes | down | 47.75 | 50.14 | 43.35 | 49.77 | 48.06 | 59.31 | 47.08 | 52.38 |
| CLPB | 0.463 | -1.11122 | 1.04E-11 | 2.14E-10 | yes | down | 12 | 10.44 | 9.14 | 12.89 | 9.61 | 12.59 | 10.52667 | 11.69667 |
| LOC103352131 | 4.702 | 2.233306 | 1.09E-11 | 2.22E-10 | yes | up | 2.39 | 1.65 | 1.6 | 26.32 | 19.99 | 21.4 | 1.88 | 22.57 |
| FAM210A | 0.388 | -1.36431 | 1.11E-11 | 2.27E-10 | yes | down | 14.27 | 10.67 | 9.48 | 9.38 | 11.11 | 11.55 | 11.47333 | 10.68 |
| RASGEF1B | 3.102 | 1.633203 | 1.13E-11 | 2.31E-10 | yes | up | 5.42 | 7.5 | 4.63 | 47.62 | 31.65 | 51.11 | 5.85 | 43.46 |
| ACYP1 | 3.373 | 1.753986 | 1.14E-11 | 2.31E-10 | yes | up | 14.11 | 10.41 | 9.26 | 67.1 | 75.75 | 95.15 | 11.26 | 79.33333 |
| CCND2 | 3.153 | 1.656556 | 1.17E-11 | 2.37E-10 | yes | up | 4.13 | 3.32 | 2.56 | 20.81 | 22.17 | 31.71 | 3.336667 | 24.89667 |
| LMO1 | 6.522 | 2.705365 | 1.28E-11 | 2.59E-10 | yes | up | 1.92 | 3.36 | 2.78 | 28.82 | 51.62 | 46.25 | 2.686667 | 42.23 |
| NOTCH2 | 2.935 | 1.553245 | 1.32E-11 | 2.67E-10 | yes | up | 3.58 | 2.8 | 2.02 | 18.08 | 15.59 | 23.44 | 2.8 | 19.03667 |
| PROS1 | 0.359 | -1.47604 | 1.37E-11 | 2.76E-10 | yes | down | 13.26 | 14.39 | 12.41 | 13.63 | 12.61 | 8.48 | 13.35333 | 11.57333 |
| NDUFA8 | 0.42 | -1.2528 | 1.39E-11 | 2.8E-10 | yes | down | 175.54 | 197.71 | 130.91 | 214.23 | 148.08 | 146.53 | 168.0533 | 169.6133 |
| TRAK1 | 2.622 | 1.390461 | 1.45E-11 | 2.91E-10 | yes | up | 20.93 | 17.6 | 13.8 | 104.82 | 71.53 | 118.76 | 17.44333 | 98.37 |
| ACACA | 5.014 | 2.325834 | 1.47E-11 | 2.95E-10 | yes | up | 3.28 | 2.9 | 3.33 | 57.19 | 19.37 | 41.09 | 3.17 | 39.21667 |
| C9H3orf18 | 0.327 | -1.61362 | 1.56E-11 | 3.13E-10 | yes | down | 18.46 | 23.65 | 15.08 | 11.14 | 15.99 | 18.29 | 19.06333 | 15.14 |
| ISOC2 | 0.299 | -1.74224 | 1.56E-11 | 3.13E-10 | yes | down | 87.07 | 81.39 | 97.5 | 84.95 | 63.19 | 45.61 | 88.65333 | 64.58333 |
| MTMR4 | 2.36 | 1.238571 | 1.66E-11 | 3.32E-10 | yes | up | 3.02 | 2.65 | 2.93 | 16.68 | 14.19 | 17.92 | 2.866667 | 16.26333 |
| UBE2M | 0.489 | -1.03102 | 1.74E-11 | 3.47E-10 | yes | down | 56.56 | 50.68 | 44.22 | 60.32 | 64 | 52.8 | 50.48667 | 59.04 |
| OXCT1 | 0.403 | -1.31089 | 1.83E-11 | 3.64E-10 | yes | down | 116.21 | 82.15 | 102.41 | 97.48 | 98.54 | 96.22 | 100.2567 | 97.41333 |
| ITM2B | 0.433 | -1.20699 | 1.84E-11 | 3.67E-10 | yes | down | 599.43 | 803.13 | 683.63 | 719.22 | 749.88 | 710.68 | 695.3967 | 726.5933 |
| LGALS9 | 0.173 | -2.53047 | 1.88E-11 | 3.74E-10 | yes | down | 18.17 | 38.64 | 36 | 16.92 | 8.35 | 14.64 | 30.93667 | 13.30333 |
| NEK3 | 3.08 | 1.622977 | 1.92E-11 | 3.82E-10 | yes | up | 1.35 | 1.66 | 1.22 | 9.17 | 8.44 | 10.41 | 1.41 | 9.34 |
| LOC108176315 | 0.374 | -1.41901 | 1.97E-11 | 3.9E-10 | yes | down | 13.5 | 13 | 11.1 | 10.81 | 10.51 | 14.06 | 12.53333 | 11.79333 |
| FHL5 | 2.697 | 1.431524 | 2E-11 | 3.96E-10 | yes | up | 2.81 | 1.95 | 1.94 | 16.25 | 11.79 | 15.08 | 2.233333 | 14.37333 |
| HAS2 | 5.262 | 2.395567 | 2.01E-11 | 3.97E-10 | yes | up | 0.43 | 0.34 | 0.25 | 4.34 | 3.99 | 3.58 | 0.34 | 3.97 |
| NDUFB3 | 0.457 | -1.13074 | 2.02E-11 | 3.99E-10 | yes | down | 202.77 | 144.26 | 137.55 | 179.63 | 167.42 | 181.65 | 161.5267 | 176.2333 |
| PPTC7 | 0.339 | -1.56247 | 2.03E-11 | 4E-10 | yes | down | 11.49 | 12.25 | 9.6 | 7.37 | 7.87 | 11.62 | 11.11333 | 8.953333 |
| LOXL3 | 2.124 | 1.086721 | 2.06E-11 | 4.06E-10 | yes | up | 3.46 | 3.48 | 2.86 | 17.94 | 16.38 | 16.32 | 3.266667 | 16.88 |
| LOC100347175 | 0.447 | -1.16308 | 2.09E-11 | 4.12E-10 | yes | down | 47.54 | 48.45 | 50.93 | 53.35 | 54.79 | 50.76 | 48.97333 | 52.96667 |
| TIMM9 | 0.217 | -2.20429 | 2.1E-11 | 4.13E-10 | yes | down | 39.29 | 47.99 | 33.41 | 16.05 | 16.65 | 31.36 | 40.23 | 21.35333 |
| SLC26A10 | 0.16 | -2.64619 | 2.16E-11 | 4.23E-10 | yes | down | 20.78 | 27.65 | 44.23 | 19.05 | 9.21 | 10.1 | 30.88667 | 12.78667 |
| LOC108176462 | 0.025 | -5.31562 | 2.18E-11 | 4.27E-10 | yes | down | 3.89 | 2.83 | 3.36 | 0.1 | 0 | 0.51 | 3.36 | 0.203333 |
| MRPL13 | 0.435 | -1.20241 | 2.18E-11 | 4.27E-10 | yes | down | 29.8 | 32.08 | 26.33 | 38.61 | 26.4 | 30.75 | 29.40333 | 31.92 |
| IL10RA | 4.365 | 2.125978 | 2.2E-11 | 4.3E-10 | yes | up | 0.69 | 0.44 | 0.34 | 6.25 | 4.85 | 4.15 | 0.49 | 5.083333 |
| NR4A1 | 5.368 | 2.424337 | 2.25E-11 | 4.4E-10 | yes | up | 2.78 | 5.12 | 2.05 | 24.64 | 44.86 | 56.49 | 3.316667 | 41.99667 |
| SLC6A17 | 0.049 | -4.36154 | 2.3E-11 | 4.49E-10 | yes | down | 0.87 | 1.6 | 1.72 | 0.19 | 0.02 | 0.29 | 1.396667 | 0.166667 |
| ATG4D | 0.335 | -1.57731 | 2.3E-11 | 4.49E-10 | yes | down | 10.81 | 11.87 | 11.74 | 9.11 | 7.67 | 11.16 | 11.47333 | 9.313333 |
| LOC100358892 | 0.222 | -2.17104 | 2.31E-11 | 4.5E-10 | yes | down | 16.64 | 17.31 | 27.03 | 10.22 | 9.49 | 13.68 | 20.32667 | 11.13 |
| TEFM | 0.375 | -1.41324 | 2.36E-11 | 4.59E-10 | yes | down | 8.45 | 10.96 | 9.47 | 8.38 | 8.63 | 9.13 | 9.626667 | 8.713333 |
| NIPSNAP1 | 0.201 | -2.31164 | 2.4E-11 | 4.66E-10 | yes | down | 50.9 | 56.56 | 101.52 | 47.67 | 27.62 | 30.85 | 69.66 | 35.38 |
| CYP2B4 | 168.614 | 7.397581 | 2.49E-11 | 4.83E-10 | yes | up | 0.9 | 5.5 | 0.24 | 510.43 | 893.72 | 1084.18 | 2.213333 | 829.4433 |
| FAM180B | 7.617 | 2.929246 | 2.56E-11 | 4.96E-10 | yes | up | 1.08 | 1.91 | 0.64 | 14.2 | 29.76 | 21.42 | 1.21 | 21.79333 |
| TMEM62 | 0.454 | -1.13827 | 2.61E-11 | 5.04E-10 | yes | down | 6.31 | 6.94 | 6.22 | 7.04 | 6.62 | 6.91 | 6.49 | 6.856667 |
| FBLN5 | 0.484 | -1.0474 | 2.61E-11 | 5.04E-10 | yes | down | 18.66 | 15.94 | 12.59 | 20.52 | 15.83 | 18.24 | 15.73 | 18.19667 |
| ABAT | 5.631 | 2.493297 | 2.65E-11 | 5.11E-10 | yes | up | 0.47 | 0.24 | 0.43 | 6.86 | 4.88 | 3.94 | 0.38 | 5.226667 |
| CRYM | 0.079 | -3.65304 | 2.73E-11 | 5.26E-10 | yes | down | 16.35 | 70.73 | 64.68 | 5.31 | 11.66 | 12.15 | 50.58667 | 9.706667 |
| YPEL4 | 0.109 | -3.20243 | 2.81E-11 | 5.41E-10 | yes | down | 1.03 | 2.03 | 2.16 | 0.51 | 0.38 | 0.53 | 1.74 | 0.473333 |
| PLXNA4 | 0.15 | -2.73258 | 2.85E-11 | 5.48E-10 | yes | down | 5.72 | 7.1 | 6.19 | 4.31 | 1.23 | 1.6 | 6.336667 | 2.38 |
| MUL1 | 0.462 | -1.11314 | 2.92E-11 | 5.6E-10 | yes | down | 12.39 | 15.29 | 12.73 | 14.96 | 14.75 | 15.26 | 13.47 | 14.99 |
| CLIC1 | 2.067 | 1.04721 | 2.95E-11 | 5.66E-10 | yes | up | 55.45 | 63.41 | 50.65 | 265.4 | 282.13 | 232.9 | 56.50333 | 260.1433 |
| COX4I1 | 0.454 | -1.13997 | 2.96E-11 | 5.68E-10 | yes | down | 2435.24 | 2674.56 | 2808.35 | 3232.93 | 2678.02 | 2872.07 | 2639.383 | 2927.673 |
| KIFC3 | 0.373 | -1.4225 | 3.04E-11 | 5.8E-10 | yes | down | 6.07 | 7.97 | 5.47 | 7.18 | 4.7 | 5.27 | 6.503333 | 5.716667 |
| CPQ | 2.265 | 1.179349 | 3.09E-11 | 5.88E-10 | yes | up | 9 | 8.45 | 6.78 | 39.56 | 46.97 | 44.04 | 8.076667 | 43.52333 |
| FASTKD3 | 0.267 | -1.90741 | 3.09E-11 | 5.89E-10 | yes | down | 3.44 | 4.65 | 4.46 | 2.53 | 2.05 | 3.11 | 4.183333 | 2.563333 |
| LOC103351933 | 4.163 | 2.057471 | 3.12E-11 | 5.94E-10 | yes | up | 1.1 | 1.69 | 1.03 | 11.73 | 15.88 | 10.18 | 1.273333 | 12.59667 |
| ACSS3 | 0.431 | -1.2143 | 3.25E-11 | 6.18E-10 | yes | down | 31.97 | 41.32 | 36.82 | 43.42 | 37.28 | 33.66 | 36.70333 | 38.12 |
| F11R | 2.385 | 1.253788 | 3.37E-11 | 6.39E-10 | yes | up | 23.24 | 26.95 | 28.34 | 172.7 | 134.53 | 149.04 | 26.17667 | 152.09 |
| ZDHHC15 | 3.459 | 1.790483 | 3.38E-11 | 6.4E-10 | yes | up | 0.8 | 0.82 | 0.62 | 7 | 4.19 | 7.38 | 0.746667 | 6.19 |
| LOC100339431 | 0.429 | -1.22227 | 3.52E-11 | 6.67E-10 | yes | down | 658.96 | 665.88 | 729.54 | 804.95 | 716.06 | 662.54 | 684.7933 | 727.85 |
| CDK5RAP1 | 0.346 | -1.52987 | 3.6E-11 | 6.81E-10 | yes | down | 10.55 | 9.03 | 10.12 | 10.43 | 6.32 | 8.38 | 9.9 | 8.376667 |
| ARSE | 2.795 | 1.483024 | 3.64E-11 | 6.87E-10 | yes | up | 2.54 | 1.67 | 1.25 | 13.68 | 13.02 | 12.24 | 1.82 | 12.98 |
| GAL3ST4 | 3.978 | 1.991892 | 3.69E-11 | 6.96E-10 | yes | up | 1.91 | 1.16 | 0.82 | 12.53 | 12.87 | 11.42 | 1.296667 | 12.27333 |
| MAP6D1 | 3.189 | 1.672981 | 3.73E-11 | 7.02E-10 | yes | up | 0.99 | 0.92 | 0.75 | 5.98 | 7.41 | 7.82 | 0.886667 | 7.07 |
| PLOD2 | 3.112 | 1.637908 | 3.74E-11 | 7.04E-10 | yes | up | 2.95 | 2.17 | 2.97 | 18.18 | 22.4 | 20.19 | 2.696667 | 20.25667 |
| PDP2 | 0.35 | -1.51312 | 3.88E-11 | 7.29E-10 | yes | down | 12.55 | 15.02 | 16.98 | 15.23 | 10.65 | 11.99 | 14.85 | 12.62333 |
| COL15A1 | 3.016 | 1.592406 | 4.09E-11 | 7.67E-10 | yes | up | 34.85 | 25.55 | 22.08 | 233.88 | 220.09 | 139.14 | 27.49333 | 197.7033 |
| ATP5E | 0.457 | -1.12818 | 4.18E-11 | 7.83E-10 | yes | down | 1756.05 | 1946.42 | 1967.74 | 2273.88 | 2136.25 | 1956.72 | 1890.07 | 2122.283 |
| DSP | 4.548 | 2.185131 | 4.22E-11 | 7.9E-10 | yes | up | 1.67 | 4.27 | 1.96 | 33.44 | 23.66 | 29.24 | 2.633333 | 28.78 |
| SLC35G1 | 0.247 | -2.01848 | 4.61E-11 | 8.61E-10 | yes | down | 1.69 | 1.63 | 1.43 | 0.9 | 0.8 | 1.1 | 1.583333 | 0.933333 |
| NDUFA7 | 0.402 | -1.31415 | 4.7E-11 | 8.76E-10 | yes | down | 317.19 | 267.5 | 333.93 | 352.9 | 291.34 | 280.12 | 306.2067 | 308.12 |
| MSTO1 | 0.357 | -1.48548 | 4.72E-11 | 8.79E-10 | yes | down | 7.5 | 7.17 | 8.71 | 7.17 | 5.99 | 6.86 | 7.793333 | 6.673333 |
| DNAJC25 | 0.331 | -1.59317 | 4.74E-11 | 8.8E-10 | yes | down | 14.28 | 21.74 | 15.45 | 12.07 | 10.76 | 15.89 | 17.15667 | 12.90667 |
| STK17B | 0.376 | -1.4129 | 4.9E-11 | 9.1E-10 | yes | down | 13.19 | 19.27 | 17.18 | 15.57 | 13.56 | 15.59 | 16.54667 | 14.90667 |
| TLR3 | 5.163 | 2.368141 | 5.02E-11 | 9.31E-10 | yes | up | 0.73 | 0.54 | 0.62 | 4.87 | 8.42 | 9.93 | 0.63 | 7.74 |
| MFSD8 | 0.439 | -1.18683 | 5.05E-11 | 9.36E-10 | yes | down | 4.87 | 4.87 | 4.01 | 5.8 | 4.05 | 4.84 | 4.583333 | 4.896667 |
| CA8 | 0.163 | -2.61693 | 5.29E-11 | 9.79E-10 | yes | down | 6.19 | 7.74 | 9.21 | 3.83 | 1.71 | 3.77 | 7.713333 | 3.103333 |
| LOC100009097 | 152.611 | 7.253718 | 5.5E-11 | 1.02E-09 | yes | up | 0 | 0.12 | 0 | 10.21 | 13.07 | 18.38 | 0.04 | 13.88667 |
| THY1 | 3.571 | 1.836249 | 5.54E-11 | 1.02E-09 | yes | up | 18.43 | 20.09 | 8.34 | 132.92 | 111.38 | 148.25 | 15.62 | 130.85 |
| RRAGB | 0.261 | -1.93545 | 5.76E-11 | 1.06E-09 | yes | down | 2.52 | 2.07 | 1.74 | 1.45 | 1.23 | 1.28 | 2.11 | 1.32 |
| ATP1A1 | 0.433 | -1.20717 | 5.98E-11 | 1.1E-09 | yes | down | 139.13 | 118.67 | 126.72 | 123.23 | 122.72 | 155.1 | 128.1733 | 133.6833 |
| LPAR6 | 0.407 | -1.29579 | 6.03E-11 | 1.11E-09 | yes | down | 16.42 | 17.38 | 16.47 | 21.01 | 15.15 | 13.57 | 16.75667 | 16.57667 |
| USP19 | 0.457 | -1.12925 | 6.2E-11 | 1.14E-09 | yes | down | 26.46 | 33.11 | 22.02 | 29.23 | 26.46 | 33.18 | 27.19667 | 29.62333 |
| LOC100340711 | 3.247 | 1.699127 | 6.43E-11 | 1.18E-09 | yes | up | 4.95 | 8.65 | 6.87 | 57.55 | 57.56 | 45.17 | 6.823333 | 53.42667 |
| MTO1 | 0.39 | -1.35744 | 6.67E-11 | 1.22E-09 | yes | down | 8.51 | 7.95 | 5.98 | 10.03 | 7.08 | 7.75 | 7.48 | 8.286667 |
| TRAM1L1 | 0.059 | -4.0729 | 6.68E-11 | 1.22E-09 | yes | down | 0.88 | 1.12 | 0.69 | 0.17 | 0.11 | 0.1 | 0.896667 | 0.126667 |
| LAMTOR5 | 0.486 | -1.04152 | 6.74E-11 | 1.23E-09 | yes | down | 125.41 | 103.68 | 94.61 | 143.71 | 124.06 | 107.94 | 107.9 | 125.2367 |
| AMPD2 | 0.369 | -1.43885 | 7.1E-11 | 1.29E-09 | yes | down | 8.94 | 9.2 | 11.02 | 10.38 | 7.77 | 8.28 | 9.72 | 8.81 |
| LOC100358143 | 19.939 | 4.317518 | 7.17E-11 | 1.31E-09 | yes | up | 0 | 0.27 | 0.16 | 6.39 | 5.48 | 7.22 | 0.143333 | 6.363333 |
| MMP2 | 2.408 | 1.267832 | 7.52E-11 | 1.37E-09 | yes | up | 35.66 | 38.13 | 23.28 | 163.36 | 192.96 | 195.14 | 32.35667 | 183.82 |
| MGP | 4.245 | 2.085615 | 7.68E-11 | 1.4E-09 | yes | up | 436.58 | 241.48 | 157.08 | 2457.56 | 3214.64 | 2616.49 | 278.38 | 2762.897 |
| PRLR | 0.261 | -1.93582 | 8.29E-11 | 1.5E-09 | yes | down | 18.53 | 23.67 | 34.36 | 16.42 | 13.29 | 19.03 | 25.52 | 16.24667 |
| INSIG2 | 0.457 | -1.12853 | 8.4E-11 | 1.52E-09 | yes | down | 13.34 | 17.74 | 13.17 | 17.84 | 14.29 | 16.41 | 14.75 | 16.18 |
| RNF212 | 5.571 | 2.477858 | 8.48E-11 | 1.54E-09 | yes | up | 1.25 | 1.82 | 1.55 | 28.24 | 12.79 | 22.54 | 1.54 | 21.19 |
| LOC103346740 | 0.255 | -1.96964 | 8.63E-11 | 1.56E-09 | yes | down | 114.36 | 167.55 | 160.17 | 106.29 | 88.73 | 96.47 | 147.36 | 97.16333 |
| PPP1R9A | 3.893 | 1.960733 | 8.63E-11 | 1.56E-09 | yes | up | 1.92 | 1.44 | 0.72 | 14.32 | 10.03 | 13.04 | 1.36 | 12.46333 |
| LOC100337961 | 8.88 | 3.15059 | 8.73E-11 | 1.58E-09 | yes | up | 1.24 | 0.83 | 1.25 | 13.33 | 28.32 | 28.52 | 1.106667 | 23.39 |
| DMGDH | 0.01 | -6.66726 | 8.76E-11 | 1.58E-09 | yes | down | 232.71 | 120.15 | 133.51 | 3.26 | 0.86 | 9.28 | 162.1233 | 4.466667 |
| GDF9 | 5.104 | 2.351576 | 9.68E-11 | 1.74E-09 | yes | up | 0.71 | 0.79 | 0.77 | 10.78 | 6.8 | 12.17 | 0.756667 | 9.916667 |
| LOC103349113 | 0.315 | -1.66867 | 9.84E-11 | 1.77E-09 | yes | down | 17.49 | 29.77 | 16.38 | 14.88 | 17.3 | 15.34 | 21.21333 | 15.84 |
| NCOA1 | 0.498 | -1.00557 | 1.05E-10 | 1.89E-09 | yes | down | 13.98 | 17.83 | 13.17 | 18.53 | 16.06 | 19.39 | 14.99333 | 17.99333 |
| SYN2 | 0.289 | -1.79157 | 1.08E-10 | 1.94E-09 | yes | down | 7.48 | 7.78 | 9.84 | 7.73 | 4.86 | 5.99 | 8.366667 | 6.193333 |
| GNAZ | 0.129 | -2.95098 | 1.13E-10 | 2.01E-09 | yes | down | 2.25 | 4.48 | 1.72 | 0.58 | 1.22 | 0.74 | 2.816667 | 0.846667 |
| CCBE1 | 0.17 | -2.55255 | 1.21E-10 | 2.15E-09 | yes | down | 2.59 | 3.53 | 1.6 | 1.27 | 0.51 | 1.34 | 2.573333 | 1.04 |
| INHBB | 8.127 | 3.022778 | 1.26E-10 | 2.25E-09 | yes | up | 0.22 | 0.23 | 0.12 | 2.74 | 4.51 | 3.53 | 0.19 | 3.593333 |
| TRMT10C | 0.429 | -1.22021 | 1.3E-10 | 2.31E-09 | yes | down | 10.38 | 11.8 | 8.08 | 9.33 | 9.74 | 11.25 | 10.08667 | 10.10667 |
| TRIO | 2.154 | 1.106884 | 1.3E-10 | 2.31E-09 | yes | up | 6.23 | 5.74 | 4.2 | 28.18 | 26.92 | 34.97 | 5.39 | 30.02333 |
| PLIN2 | 0.376 | -1.41075 | 1.31E-10 | 2.32E-09 | yes | down | 55.13 | 86.3 | 68.76 | 62.56 | 61.31 | 68.44 | 70.06333 | 64.10333 |
| LOC100348091 | 0.309 | -1.69609 | 1.31E-10 | 2.32E-09 | yes | down | 8.47 | 11.38 | 8.22 | 5.95 | 8.78 | 8.52 | 9.356667 | 7.75 |
| BNC2 | 4.446 | 2.152642 | 1.31E-10 | 2.33E-09 | yes | up | 0.75 | 0.49 | 0.28 | 4.45 | 5.47 | 4.94 | 0.506667 | 4.953333 |
| PAX8 | 300.072 | 8.229164 | 1.33E-10 | 2.36E-09 | yes | up | 0 | 0 | 0 | 3.41 | 4.61 | 7.39 | 0 | 5.136667 |
| NARS2 | 0.4 | -1.32232 | 1.35E-10 | 2.39E-09 | yes | down | 8.19 | 11.14 | 7.48 | 7.84 | 6.75 | 9.1 | 8.936667 | 7.896667 |
| LRRC3 | 4.117 | 2.041642 | 1.38E-10 | 2.44E-09 | yes | up | 1.35 | 1.56 | 1.51 | 12.52 | 18.72 | 12.51 | 1.473333 | 14.58333 |
| DUS2 | 0.49 | -1.02965 | 1.41E-10 | 2.48E-09 | yes | down | 6.9 | 5.58 | 5.44 | 8.48 | 6.42 | 8.88 | 5.973333 | 7.926667 |
| EPB41L2 | 2.2 | 1.137381 | 1.42E-10 | 2.51E-09 | yes | up | 20.7 | 18.8 | 12.86 | 95.62 | 75.29 | 100.07 | 17.45333 | 90.32667 |
| LOXHD1 | 18.203 | 4.186132 | 1.45E-10 | 2.56E-09 | yes | up | 4.24 | 4.61 | 1.8 | 63.71 | 22.8 | 45.97 | 3.55 | 44.16 |
| RTN4IP1 | 0.273 | -1.87062 | 1.46E-10 | 2.56E-09 | yes | down | 13.16 | 11 | 9.43 | 6.65 | 5.12 | 10.17 | 11.19667 | 7.313333 |
| LOC100354142 | 0.459 | -1.1239 | 1.56E-10 | 2.73E-09 | yes | down | 25.27 | 32.14 | 27.21 | 32.27 | 28.57 | 26.62 | 28.20667 | 29.15333 |
| ADAMTS4 | 3.493 | 1.804291 | 1.6E-10 | 2.8E-09 | yes | up | 0.74 | 0.96 | 0.78 | 8.35 | 7.46 | 5.08 | 0.826667 | 6.963333 |
| LOC100344556 | 3.584 | 1.841559 | 1.7E-10 | 2.98E-09 | yes | up | 1.64 | 1.32 | 0.78 | 10.36 | 7.26 | 10.35 | 1.246667 | 9.323333 |
| GPAT3 | 8.234 | 3.041591 | 1.71E-10 | 2.99E-09 | yes | up | 7.82 | 5 | 1.25 | 80.73 | 102.88 | 80.48 | 4.69 | 88.03 |
| LOC103346040 | 5.852 | 2.549015 | 1.82E-10 | 3.17E-09 | yes | up | 0.52 | 0.45 | 0.57 | 4.2 | 7.05 | 9.94 | 0.513333 | 7.063333 |
| MTG1 | 0.441 | -1.18101 | 1.84E-10 | 3.21E-09 | yes | down | 20.18 | 22.5 | 22.2 | 25.62 | 20.71 | 22.3 | 21.62667 | 22.87667 |
| GABARAPL2 | 0.434 | -1.20307 | 1.91E-10 | 3.33E-09 | yes | down | 129.69 | 154.63 | 129.63 | 123.52 | 153.55 | 151.33 | 137.9833 | 142.8 |
| TANC2 | 2.46 | 1.298478 | 2.05E-10 | 3.56E-09 | yes | up | 1.27 | 1.26 | 0.74 | 6.94 | 5.36 | 6.28 | 1.09 | 6.193333 |
| SCUBE1 | 0.076 | -3.7255 | 2.08E-10 | 3.6E-09 | yes | down | 1.76 | 2.28 | 0.69 | 0.18 | 0.52 | 0.1 | 1.576667 | 0.266667 |
| DOCK8 | 0.379 | -1.3982 | 2.25E-10 | 3.9E-09 | yes | down | 2.93 | 3.33 | 2.91 | 3.6 | 2.14 | 2.71 | 3.056667 | 2.816667 |
| LOC100356413 | 0.472 | -1.08204 | 2.37E-10 | 4.1E-09 | yes | down | 47.55 | 58.13 | 49.53 | 62.29 | 50.23 | 61.48 | 51.73667 | 58 |
| LOC100341581 | 6.314 | 2.658443 | 2.45E-10 | 4.24E-09 | yes | up | 0.18 | 0.43 | 0.15 | 3.69 | 3.43 | 4.18 | 0.253333 | 3.766667 |
| GNE | 2.127 | 1.089029 | 2.5E-10 | 4.31E-09 | yes | up | 4.18 | 3.49 | 2.61 | 18.12 | 17.27 | 18.07 | 3.426667 | 17.82 |
| ETFA | 0.396 | -1.33586 | 2.58E-10 | 4.44E-09 | yes | down | 420.31 | 419.64 | 473.49 | 421.08 | 347.07 | 501.88 | 437.8133 | 423.3433 |
| COL23A1 | 3.368 | 1.751908 | 2.58E-10 | 4.45E-09 | yes | up | 0.69 | 0.65 | 0.53 | 5.12 | 5.5 | 4.48 | 0.623333 | 5.033333 |
| ZNF629 | 0.475 | -1.07342 | 2.61E-10 | 4.5E-09 | yes | down | 3.19 | 3.61 | 2.76 | 4.46 | 3.06 | 3.7 | 3.186667 | 3.74 |
| ASIC2 | 0.011 | -6.51904 | 2.68E-10 | 4.61E-09 | yes | down | 1.08 | 2.17 | 0.58 | 0.07 | 0 | 0.03 | 1.276667 | 0.033333 |
| SAR1B | 0.434 | -1.20368 | 2.75E-10 | 4.73E-09 | yes | down | 27.9 | 21.15 | 20.96 | 20.93 | 25.51 | 19.95 | 23.33667 | 22.13 |
| SLC25A26 | 0.407 | -1.29835 | 2.75E-10 | 4.73E-09 | yes | down | 6.79 | 7.26 | 6.79 | 8.23 | 6.54 | 6.91 | 6.946667 | 7.226667 |
| TMEM177 | 0.275 | -1.86264 | 2.79E-10 | 4.79E-09 | yes | down | 5.1 | 6.11 | 4.52 | 3.15 | 2.5 | 4.79 | 5.243333 | 3.48 |
| ELOVL1 | 2.014 | 1.010417 | 2.82E-10 | 4.82E-09 | yes | up | 11.14 | 11.18 | 9.34 | 60.37 | 49.21 | 45.15 | 10.55333 | 51.57667 |
| ADAM23 | 11.948 | 3.578725 | 2.87E-10 | 4.9E-09 | yes | up | 0.87 | 0.26 | 0.1 | 11.9 | 16.07 | 12.9 | 0.41 | 13.62333 |
| B3GNT7 | 0.248 | -2.01323 | 2.87E-10 | 4.9E-09 | yes | down | 2.51 | 2.56 | 2.33 | 1.36 | 1.09 | 1.96 | 2.466667 | 1.47 |
| RPL9 | 2.472 | 1.305946 | 2.88E-10 | 4.91E-09 | yes | up | 55.43 | 80.64 | 51.21 | 180.64 | 191.76 | 150.78 | 62.42667 | 174.3933 |
| ANKEF1 | 8.409 | 3.07185 | 2.97E-10 | 5.06E-09 | yes | up | 0.31 | 0.19 | 0.39 | 9.17 | 3.27 | 6.1 | 0.296667 | 6.18 |
| STUM | 2.893 | 1.53266 | 2.99E-10 | 5.09E-09 | yes | up | 0.46 | 0.7 | 0.55 | 4.59 | 3.76 | 3.59 | 0.57 | 3.98 |
| DDX21 | 0.413 | -1.27426 | 3.17E-10 | 5.39E-09 | yes | down | 13.41 | 16.73 | 11.88 | 11.65 | 14.35 | 15.31 | 14.00667 | 13.77 |
| PARM1 | 2.07 | 1.04993 | 3.19E-10 | 5.42E-09 | yes | up | 1.72 | 1.52 | 1.33 | 7.82 | 7.12 | 7.7 | 1.523333 | 7.546667 |
| PLA2G4B | 2.598 | 1.377293 | 3.28E-10 | 5.55E-09 | yes | up | 2.1 | 2.36 | 2.04 | 16.72 | 9.06 | 13.35 | 2.166667 | 13.04333 |
| PHLDB2 | 2.094 | 1.066453 | 3.29E-10 | 5.57E-09 | yes | up | 5.78 | 5.99 | 4.78 | 28.02 | 23.22 | 32.51 | 5.516667 | 27.91667 |
| LOC100340040 | 25.581 | 4.677012 | 3.3E-10 | 5.57E-09 | yes | up | 0.2 | 0 | 0.03 | 3.82 | 4.65 | 4.31 | 0.076667 | 4.26 |
| ARHGAP1 | 2.311 | 1.208637 | 3.3E-10 | 5.57E-09 | yes | up | 7.74 | 7.6 | 6.43 | 39.63 | 32.17 | 48.78 | 7.256667 | 40.19333 |
| EXOSC5 | 0.318 | -1.65512 | 3.31E-10 | 5.58E-09 | yes | down | 12.84 | 17.29 | 17.79 | 12.84 | 11.06 | 13.27 | 15.97333 | 12.39 |
| FGF14 | 13.871 | 3.79402 | 3.32E-10 | 5.6E-09 | yes | up | 0.08 | 0.06 | 0.03 | 1.17 | 3.14 | 1.44 | 0.056667 | 1.916667 |
| PRKAG2 | 0.433 | -1.20665 | 3.36E-10 | 5.66E-09 | yes | down | 20.18 | 25.56 | 13.67 | 18.79 | 18.03 | 20.69 | 19.80333 | 19.17 |
| LOC100346173 | 31.121 | 4.959838 | 3.39E-10 | 5.7E-09 | yes | up | 0.04 | 0 | 0.06 | 4.28 | 1.99 | 2.18 | 0.033333 | 2.816667 |
| TMEM223 | 0.289 | -1.78895 | 3.45E-10 | 5.78E-09 | yes | down | 17.62 | 19.16 | 17.82 | 10.17 | 15.6 | 12.33 | 18.2 | 12.7 |
| CBR4 | 0.438 | -1.19076 | 3.65E-10 | 6.12E-09 | yes | down | 6.14 | 5.66 | 5.99 | 6.25 | 7.3 | 4.72 | 5.93 | 6.09 |
| MRPL4 | 0.226 | -2.14389 | 3.74E-10 | 6.27E-09 | yes | down | 17.27 | 16.69 | 24.72 | 13.76 | 9.54 | 9.68 | 19.56 | 10.99333 |
| GPR89B | 0.493 | -1.02143 | 3.81E-10 | 6.36E-09 | yes | down | 19.89 | 22.59 | 21.81 | 27.12 | 24.23 | 22.33 | 21.43 | 24.56 |
| SLC35A4 | 0.488 | -1.03375 | 3.82E-10 | 6.38E-09 | yes | down | 17.77 | 19.93 | 19.56 | 22.65 | 20.96 | 18.93 | 19.08667 | 20.84667 |
| SLC24A3 | 2.598 | 1.377448 | 3.95E-10 | 6.59E-09 | yes | up | 3.25 | 4.64 | 2.85 | 20.5 | 20.71 | 25.31 | 3.58 | 22.17333 |
| GYPA | 0.061 | -4.03247 | 4.05E-10 | 6.73E-09 | yes | down | 5.58 | 11.47 | 7.72 | 0.29 | 0.76 | 2.26 | 8.256667 | 1.103333 |
| CDRT1 | 4.602 | 2.202276 | 4.05E-10 | 6.73E-09 | yes | up | 0.28 | 0.29 | 0.35 | 3.55 | 3.32 | 4.5 | 0.306667 | 3.79 |
| ACSF2 | 0.396 | -1.33486 | 4.11E-10 | 6.82E-09 | yes | down | 237.09 | 247.04 | 300.26 | 297.39 | 236.69 | 231.26 | 261.4633 | 255.1133 |
| OGDH | 0.405 | -1.30237 | 4.23E-10 | 7.02E-09 | yes | down | 219.25 | 260.52 | 293.33 | 262.22 | 246.96 | 251.97 | 257.7 | 253.7167 |
| LOC100352363 | 0.241 | -2.0525 | 4.27E-10 | 7.07E-09 | yes | down | 294.16 | 686.41 | 553.05 | 309.92 | 301.86 | 304.68 | 511.2067 | 305.4867 |
| SMAD7 | 0.367 | -1.44495 | 4.55E-10 | 7.51E-09 | yes | down | 13.64 | 14.7 | 9.85 | 9.41 | 9.34 | 12.93 | 12.73 | 10.56 |
| MRPL3 | 0.484 | -1.04711 | 4.55E-10 | 7.51E-09 | yes | down | 28.36 | 33.03 | 30.69 | 35.25 | 34.43 | 38.62 | 30.69333 | 36.1 |
| LOC108178409 | 0.276 | -1.85667 | 4.68E-10 | 7.7E-09 | yes | down | 5.3 | 8.06 | 6.47 | 5.52 | 4.41 | 3.37 | 6.61 | 4.433333 |
| PFDN6 | 0.431 | -1.2145 | 4.69E-10 | 7.72E-09 | yes | down | 60.92 | 57.46 | 47.54 | 61.94 | 41.61 | 62.81 | 55.30667 | 55.45333 |
| PDIA6 | 2.127 | 1.089021 | 4.89E-10 | 8.03E-09 | yes | up | 56.55 | 47.95 | 33.92 | 232.96 | 246.45 | 218.5 | 46.14 | 232.6367 |
| LOC108178466 | 31.18 | 4.962528 | 4.98E-10 | 8.17E-09 | yes | up | 0.04 | 0.06 | 0 | 3.45 | 1.76 | 2.03 | 0.033333 | 2.413333 |
| NCEH1 | 0.28 | -1.83549 | 5.09E-10 | 8.35E-09 | yes | down | 5.53 | 4.96 | 7.34 | 4.45 | 4.6 | 3.2 | 5.943333 | 4.083333 |
| MEIS3 | 3.033 | 1.600563 | 5.1E-10 | 8.35E-09 | yes | up | 0.95 | 1.12 | 0.84 | 8.85 | 5.51 | 6 | 0.97 | 6.786667 |
| KAZALD1 | 3.611 | 1.852376 | 5.23E-10 | 8.57E-09 | yes | up | 2.77 | 2.08 | 1.93 | 15.43 | 21.74 | 19.96 | 2.26 | 19.04333 |
| TMEM40 | 0.169 | -2.56731 | 5.31E-10 | 8.68E-09 | yes | down | 3.8 | 2.89 | 1.78 | 1.62 | 0.64 | 1.9 | 2.823333 | 1.386667 |
| NDUFAF1 | 0.453 | -1.14266 | 5.48E-10 | 8.94E-09 | yes | down | 27.48 | 25.03 | 19.97 | 22.01 | 26.63 | 26.22 | 24.16 | 24.95333 |
| MLYCD | 0.487 | -1.03799 | 5.6E-10 | 9.12E-09 | yes | down | 27.46 | 21.06 | 20.94 | 26.51 | 23.92 | 26.98 | 23.15333 | 25.80333 |
| CIDEC | 2.222 | 1.151625 | 5.71E-10 | 9.29E-09 | yes | up | 252.79 | 322.75 | 262.24 | 1570.16 | 1298.35 | 1773.5 | 279.26 | 1547.337 |
| BHMT2 | 0.087 | -3.52377 | 5.71E-10 | 9.29E-09 | yes | down | 31.13 | 38.13 | 40.26 | 4.99 | 1.97 | 17.32 | 36.50667 | 8.093333 |
| NFE2 | 0.074 | -3.76243 | 5.77E-10 | 9.38E-09 | yes | down | 1.67 | 5.07 | 5.16 | 0.34 | 0.62 | 1.07 | 3.966667 | 0.676667 |
| NCKAP5 | 4.485 | 2.165022 | 6.44E-10 | 1.04E-08 | yes | up | 3.71 | 8.6 | 0.21 | 23.96 | 16.94 | 20.88 | 4.173333 | 20.59333 |
| PAFAH2 | 2.206 | 1.141752 | 6.59E-10 | 1.06E-08 | yes | up | 5.03 | 4.58 | 3.27 | 20.11 | 20.47 | 24.66 | 4.293333 | 21.74667 |
| ERRFI1 | 2.527 | 1.337258 | 6.74E-10 | 1.09E-08 | yes | up | 2.52 | 2.65 | 2.37 | 11.22 | 12.78 | 14.35 | 2.513333 | 12.78333 |
| PFKFB1 | 2.646 | 1.403751 | 6.74E-10 | 1.09E-08 | yes | up | 21.68 | 12.53 | 13.2 | 94.47 | 95.44 | 94.88 | 15.80333 | 94.93 |
| LOC100348511 | 0.014 | -6.17221 | 6.87E-10 | 1.11E-08 | yes | down | 25.86 | 1.29 | 33.08 | 0.96 | 0.28 | 1.01 | 20.07667 | 0.75 |
| PPARGC1B | 0.301 | -1.73422 | 7.04E-10 | 1.13E-08 | yes | down | 11.81 | 11.73 | 7.68 | 6.14 | 5.45 | 10.48 | 10.40667 | 7.356667 |
| ATP6V0E2 | 10.59 | 3.404662 | 7.21E-10 | 1.16E-08 | yes | up | 2.46 | 1.13 | 3.47 | 60.61 | 24.18 | 103.82 | 2.353333 | 62.87 |
| KIF21B | 0.125 | -2.99679 | 7.35E-10 | 1.18E-08 | yes | down | 1.11 | 1.98 | 0.46 | 0.46 | 0.25 | 0.34 | 1.183333 | 0.35 |
| GALE | 3.004 | 1.586826 | 7.35E-10 | 1.18E-08 | yes | up | 3.5 | 4.31 | 4.51 | 27.19 | 26.71 | 35.87 | 4.106667 | 29.92333 |
| GCNT4 | 332.504 | 8.377229 | 7.84E-10 | 1.25E-08 | yes | up | 0 | 0 | 0 | 4.24 | 0.97 | 4.13 | 0 | 3.113333 |
| OSBPL7 | 0.345 | -1.53434 | 8E-10 | 1.28E-08 | yes | down | 10.5 | 12.66 | 12.77 | 10.3 | 7.34 | 11.9 | 11.97667 | 9.846667 |
| KIF20B | 0.109 | -3.19587 | 8.05E-10 | 1.28E-08 | yes | down | 10.71 | 11.86 | 29.26 | 4.31 | 3.88 | 1.65 | 17.27667 | 3.28 |
| ZDHHC14 | 2.552 | 1.351686 | 8.25E-10 | 1.31E-08 | yes | up | 1.81 | 1.75 | 1.37 | 10.83 | 9.64 | 7.69 | 1.643333 | 9.386667 |
| KIAA1211 | 0.166 | -2.59095 | 8.44E-10 | 1.34E-08 | yes | down | 4.74 | 5.97 | 1.61 | 1.97 | 0.98 | 1.39 | 4.106667 | 1.446667 |
| BCKDHA | 0.39 | -1.35774 | 8.74E-10 | 1.39E-08 | yes | down | 123.04 | 141.92 | 167.47 | 145.38 | 123.2 | 144.41 | 144.1433 | 137.6633 |
| PRKAG1 | 0.49 | -1.02987 | 8.74E-10 | 1.39E-08 | yes | down | 36.99 | 30.19 | 33.21 | 40.41 | 39.84 | 38.37 | 33.46333 | 39.54 |
| C1H11orf1 | 3.895 | 1.961627 | 9.03E-10 | 1.43E-08 | yes | up | 1.07 | 0.55 | 0.66 | 8.15 | 5.37 | 8.73 | 0.76 | 7.416667 |
| TPM4 | 2.262 | 1.177331 | 9.22E-10 | 1.46E-08 | yes | up | 31.15 | 43.94 | 32.45 | 195.05 | 210.72 | 177.55 | 35.84667 | 194.44 |
| MBNL3 | 5.148 | 2.363929 | 9.89E-10 | 1.56E-08 | yes | up | 0.3 | 0.15 | 0.17 | 3.57 | 1.68 | 2.93 | 0.206667 | 2.726667 |
| OSGEPL1 | 0.478 | -1.0661 | 1.01E-09 | 1.59E-08 | yes | down | 11.16 | 12.22 | 9.65 | 12.49 | 12.95 | 10.71 | 11.01 | 12.05 |
| MRPL11 | 0.463 | -1.1108 | 1.02E-09 | 1.61E-08 | yes | down | 45.13 | 54.3 | 51.07 | 58.96 | 52.43 | 58.03 | 50.16667 | 56.47333 |
| CCL14 | 2.603 | 1.380444 | 1.12E-09 | 1.76E-08 | yes | up | 13.15 | 15.26 | 9.48 | 74.37 | 81.18 | 81.09 | 12.63 | 78.88 |
| AKR1C5 | 86.194 | 6.429524 | 1.13E-09 | 1.78E-08 | yes | up | 0.23 | 0 | 0 | 32.38 | 6.73 | 6.39 | 0.076667 | 15.16667 |
| LDHB | 0.459 | -1.12288 | 1.16E-09 | 1.82E-08 | yes | down | 548.65 | 380.67 | 369.34 | 509.93 | 394.69 | 501.48 | 432.8867 | 468.7 |
| HMGN5 | 2.978 | 1.574483 | 1.2E-09 | 1.87E-08 | yes | up | 2.57 | 1.97 | 1.95 | 20.16 | 13.7 | 12.8 | 2.163333 | 15.55333 |
| LRRC8D | 0.402 | -1.31306 | 1.21E-09 | 1.89E-08 | yes | down | 7.43 | 8.75 | 9.08 | 8.9 | 8.59 | 7.22 | 8.42 | 8.236667 |
| SLC19A2 | 0.15 | -2.73863 | 1.22E-09 | 1.91E-08 | yes | down | 0.94 | 1.02 | 1.1 | 0.38 | 0.3 | 0.44 | 1.02 | 0.373333 |
| HTRA2 | 0.498 | -1.00599 | 1.41E-09 | 2.19E-08 | yes | down | 23.11 | 27.92 | 24.39 | 33.76 | 26.22 | 30.43 | 25.14 | 30.13667 |
| OAF | 3.193 | 1.674794 | 1.45E-09 | 2.25E-08 | yes | up | 5.61 | 5.06 | 4.24 | 35.72 | 37.57 | 41.2 | 4.97 | 38.16333 |
| CPLX1 | 0.007 | -7.20773 | 1.46E-09 | 2.27E-08 | yes | down | 1.19 | 2.05 | 3.23 | 0 | 0.11 | 0 | 2.156667 | 0.036667 |
| HMGCS2 | 0.028 | -5.15585 | 1.49E-09 | 2.31E-08 | yes | down | 158.94 | 203.07 | 313.49 | 24.34 | 20.59 | 2.92 | 225.1667 | 15.95 |
| C18H10orf11 | 3.149 | 1.654847 | 1.5E-09 | 2.33E-08 | yes | up | 2.69 | 2.59 | 2.35 | 14.75 | 17.81 | 24.88 | 2.543333 | 19.14667 |
| TRPM1 | 20.506 | 4.357946 | 1.54E-09 | 2.38E-08 | yes | up | 0.1 | 0.26 | 0.05 | 1.5 | 6.82 | 10.69 | 0.136667 | 6.336667 |
| HSD17B12 | 0.274 | -1.86942 | 1.57E-09 | 2.43E-08 | yes | down | 46.73 | 103.63 | 82.34 | 51.37 | 51.9 | 40.28 | 77.56667 | 47.85 |
| USP11 | 0.328 | -1.60778 | 1.58E-09 | 2.43E-08 | yes | down | 18.26 | 10.65 | 8.01 | 10.05 | 9.68 | 9.08 | 12.30667 | 9.603333 |
| RILPL2 | 2.222 | 1.151618 | 1.59E-09 | 2.45E-08 | yes | up | 20.22 | 27.59 | 17.2 | 127.09 | 109.91 | 110.12 | 21.67 | 115.7067 |
| CPE | 2.126 | 1.08848 | 1.61E-09 | 2.49E-08 | yes | up | 30.07 | 28.46 | 28.6 | 139.8 | 138.24 | 178 | 29.04333 | 152.0133 |
| BAG1 | 0.445 | -1.16844 | 1.65E-09 | 2.54E-08 | yes | down | 88.66 | 91.59 | 72.26 | 83.97 | 98.34 | 70.15 | 84.17 | 84.15333 |
| LOC103347892 | 0.475 | -1.07486 | 1.67E-09 | 2.56E-08 | yes | down | 82.02 | 99.86 | 94.15 | 111.71 | 106.25 | 102.53 | 92.01 | 106.83 |
| ANK2 | 3.292 | 1.719128 | 1.68E-09 | 2.58E-08 | yes | up | 15.29 | 9.95 | 7.56 | 76.38 | 126.38 | 92.57 | 10.93333 | 98.44333 |
| NUDT4 | 2.01 | 1.007104 | 1.82E-09 | 2.79E-08 | yes | up | 15.04 | 12.1 | 11.98 | 63.18 | 68.86 | 58.23 | 13.04 | 63.42333 |
| BANP | 2.085 | 1.060323 | 1.85E-09 | 2.84E-08 | yes | up | 3.13 | 3.26 | 2.7 | 16.71 | 14.41 | 14.65 | 3.03 | 15.25667 |
| FRZB | 0.268 | -1.90034 | 1.87E-09 | 2.86E-08 | yes | down | 18.92 | 12.6 | 14.11 | 11.51 | 12.04 | 5.85 | 15.21 | 9.8 |
| ODC1 | 0.279 | -1.84362 | 1.89E-09 | 2.89E-08 | yes | down | 78.18 | 183.49 | 105.57 | 90.6 | 68.93 | 86.49 | 122.4133 | 82.00667 |
| LOC100346342 | 3.976 | 1.991393 | 1.95E-09 | 2.97E-08 | yes | up | 0.38 | 0.42 | 0.18 | 2.99 | 3.2 | 2.34 | 0.326667 | 2.843333 |
| EFEMP1 | 3.216 | 1.685147 | 1.99E-09 | 3.04E-08 | yes | up | 43.89 | 28.81 | 18.27 | 210.64 | 202.27 | 271.8 | 30.32333 | 228.2367 |
| LOC108178244 | 2.452 | 1.294058 | 2.01E-09 | 3.05E-08 | yes | up | 45.72 | 37.38 | 37.94 | 309.38 | 183.28 | 256.87 | 40.34667 | 249.8433 |
| LOC108176325 | 0.441 | -1.18033 | 2.01E-09 | 3.06E-08 | yes | down | 105.32 | 84.62 | 66.57 | 89.87 | 97.88 | 84.57 | 85.50333 | 90.77333 |
| EXTL1 | 0.143 | -2.81031 | 2.02E-09 | 3.07E-08 | yes | down | 8.74 | 13.22 | 7.34 | 3.85 | 1.1 | 5.12 | 9.766667 | 3.356667 |
| TREX2 | 0.092 | -3.44977 | 2.06E-09 | 3.12E-08 | yes | down | 3.04 | 4.36 | 3.17 | 0.87 | 1.33 | 0.19 | 3.523333 | 0.796667 |
| HMGCR | 2.238 | 1.161909 | 2.06E-09 | 3.12E-08 | yes | up | 3.84 | 4.57 | 3.32 | 26.31 | 16.77 | 20.13 | 3.91 | 21.07 |
| NTRK2 | 7.496 | 2.906184 | 2.06E-09 | 3.13E-08 | yes | up | 0.8 | 0.99 | 0.15 | 12.32 | 11.86 | 9.41 | 0.646667 | 11.19667 |
| LOC100354761 | 0.483 | -1.04847 | 2.1E-09 | 3.18E-08 | yes | down | 31.05 | 31.34 | 22.61 | 32.1 | 35.3 | 35.37 | 28.33333 | 34.25667 |
| RAP1GAP2 | 0.075 | -3.72888 | 2.11E-09 | 3.19E-08 | yes | down | 3.91 | 4.99 | 1.17 | 0.84 | 0.17 | 0.8 | 3.356667 | 0.603333 |
| CDKL2 | 8.852 | 3.146025 | 2.15E-09 | 3.24E-08 | yes | up | 0.14 | 0.11 | 0.04 | 1.55 | 2.5 | 2.25 | 0.096667 | 2.1 |
| LOC103345926 | 3.092 | 1.628581 | 2.17E-09 | 3.28E-08 | yes | up | 104.09 | 58.64 | 45.73 | 479.07 | 546.07 | 490.24 | 69.48667 | 505.1267 |
| MMD | 3.288 | 1.717124 | 2.18E-09 | 3.29E-08 | yes | up | 6.84 | 10.08 | 7.42 | 76 | 74.75 | 41.16 | 8.113333 | 63.97 |
| PSD4 | 2.317 | 1.21234 | 2.21E-09 | 3.32E-08 | yes | up | 1.14 | 1.12 | 0.89 | 5.68 | 5.14 | 6.45 | 1.05 | 5.756667 |
| CYP2C30 | 435.881 | 8.76779 | 2.23E-09 | 3.36E-08 | yes | up | 0 | 0 | 0 | 22.95 | 1.07 | 10.84 | 0 | 11.62 |
| TMEM208 | 0.447 | -1.16019 | 2.26E-09 | 3.4E-08 | yes | down | 28.79 | 35.51 | 32.2 | 39.3 | 32.1 | 33.26 | 32.16667 | 34.88667 |
| WISP1 | 0.212 | -2.23887 | 2.34E-09 | 3.51E-08 | yes | down | 1 | 0.79 | 0.51 | 0.41 | 0.44 | 0.31 | 0.766667 | 0.386667 |
| LOC100343425 | 2.043 | 1.030457 | 2.34E-09 | 3.51E-08 | yes | up | 80.61 | 82.18 | 80.87 | 391.31 | 366.36 | 481.91 | 81.22 | 413.1933 |
| RASD1 | 0.168 | -2.57469 | 2.45E-09 | 3.68E-08 | yes | down | 7.67 | 11.7 | 14.66 | 2.76 | 4.5 | 6.67 | 11.34333 | 4.643333 |
| MSANTD4 | 0.371 | -1.43133 | 2.46E-09 | 3.69E-08 | yes | down | 5.84 | 7.63 | 5.09 | 7.28 | 4.39 | 4.88 | 6.186667 | 5.516667 |
| IMPDH1 | 0.383 | -1.38528 | 2.5E-09 | 3.74E-08 | yes | down | 86.78 | 103.77 | 80.47 | 71.48 | 68.21 | 108.49 | 90.34 | 82.72667 |
| MRPS16 | 0.395 | -1.34049 | 2.57E-09 | 3.84E-08 | yes | down | 81.24 | 67.31 | 83.83 | 93.82 | 67.34 | 64.34 | 77.46 | 75.16667 |
| CCSAP | 3.203 | 1.679457 | 2.57E-09 | 3.84E-08 | yes | up | 0.42 | 0.38 | 0.25 | 2.85 | 2.5 | 2.61 | 0.35 | 2.653333 |
| WDR46 | 0.489 | -1.03188 | 2.67E-09 | 3.99E-08 | yes | down | 8.54 | 7.96 | 5.87 | 8.86 | 8.4 | 8.82 | 7.456667 | 8.693333 |
| ITIH5 | 2.763 | 1.466059 | 2.78E-09 | 4.15E-08 | yes | up | 19.54 | 12.03 | 9 | 93.37 | 83.02 | 93.61 | 13.52333 | 90 |
| MRPL41 | 0.414 | -1.27243 | 2.92E-09 | 4.35E-08 | yes | down | 47.95 | 44.47 | 50.79 | 57.47 | 43.94 | 44.14 | 47.73667 | 48.51667 |
| LOC100339034 | 0.003 | -8.20608 | 2.94E-09 | 4.37E-08 | yes | down | 1.02 | 1.03 | 3.33 | 0 | 0 | 0 | 1.793333 | 0 |
| EFHD1 | 2.638 | 1.399523 | 2.97E-09 | 4.42E-08 | yes | up | 3.77 | 3.11 | 2.04 | 20.4 | 19.12 | 16.67 | 2.973333 | 18.73 |
| NGFR | 0.346 | -1.53321 | 3.07E-09 | 4.55E-08 | yes | down | 3.79 | 3.5 | 2.89 | 3.75 | 2.18 | 2.22 | 3.393333 | 2.716667 |
| LOC100338525 | 0.43 | -1.2179 | 3.09E-09 | 4.58E-08 | yes | down | 114.51 | 120.62 | 117.33 | 139.45 | 134.01 | 94.53 | 117.4867 | 122.6633 |
| THBS2 | 2.231 | 1.157981 | 3.1E-09 | 4.59E-08 | yes | up | 7.52 | 5.36 | 4.22 | 31.77 | 29.06 | 30.53 | 5.7 | 30.45333 |
| N4BP2L1 | 2.103 | 1.07273 | 3.22E-09 | 4.77E-08 | yes | up | 3.64 | 5.29 | 4.44 | 19.76 | 18.65 | 19.93 | 4.456667 | 19.44667 |
| HIPK2 | 2.248 | 1.168708 | 3.25E-09 | 4.8E-08 | yes | up | 2.13 | 1.7 | 1.42 | 8.65 | 8.53 | 10.8 | 1.75 | 9.326667 |
| L3MBTL1 | 7.829 | 2.968817 | 3.27E-09 | 4.83E-08 | yes | up | 0.12 | 0.2 | 0.07 | 1.84 | 1.75 | 2.71 | 0.13 | 2.1 |
| AHR | 0.3 | -1.73923 | 3.32E-09 | 4.89E-08 | yes | down | 19.37 | 27.1 | 19.08 | 15.14 | 10.23 | 21.73 | 21.85 | 15.7 |
| ANKRD29 | 2.555 | 1.35305 | 3.37E-09 | 4.97E-08 | yes | up | 2.32 | 1.78 | 1.49 | 10.35 | 12.5 | 10.86 | 1.863333 | 11.23667 |
| EXTL3 | 2.003 | 1.002097 | 3.46E-09 | 5.09E-08 | yes | up | 3.49 | 2.79 | 2.21 | 14.73 | 11.92 | 15 | 2.83 | 13.88333 |
| PHF19 | 2.613 | 1.385579 | 3.51E-09 | 5.16E-08 | yes | up | 3.22 | 2.74 | 1.68 | 17.44 | 18.91 | 13.69 | 2.546667 | 16.68 |
| CX3CL1 | 4.514 | 2.174439 | 3.6E-09 | 5.3E-08 | yes | up | 0.22 | 0.48 | 0.32 | 3.79 | 4.05 | 3.06 | 0.34 | 3.633333 |
| SLC7A10 | 4.021 | 2.007458 | 3.63E-09 | 5.33E-08 | yes | up | 1.71 | 2.32 | 1 | 16.06 | 26.03 | 15.48 | 1.676667 | 19.19 |
| NAMPT | 0.27 | -1.89158 | 3.67E-09 | 5.39E-08 | yes | down | 26.15 | 30.82 | 45.95 | 21 | 19.17 | 28.25 | 34.30667 | 22.80667 |
| PRKD2 | 2.479 | 1.31 | 4.08E-09 | 5.98E-08 | yes | up | 2.51 | 2.67 | 2.07 | 16.42 | 13.94 | 12.04 | 2.416667 | 14.13333 |
| LOC100328967 | 2.583 | 1.36883 | 4.26E-09 | 6.23E-08 | yes | up | 34.65 | 33.45 | 32.82 | 170.35 | 286.5 | 211.02 | 33.64 | 222.6233 |
| LOC103346934 | 0.177 | -2.50134 | 4.34E-09 | 6.33E-08 | yes | down | 2.88 | 3.25 | 1.1 | 1.21 | 0.94 | 1 | 2.41 | 1.05 |
| TMEM201 | 0.456 | -1.13335 | 4.36E-09 | 6.35E-08 | yes | down | 4.03 | 3.24 | 3.36 | 3.53 | 3.77 | 3.79 | 3.543333 | 3.696667 |
| MRPL44 | 0.471 | -1.0853 | 4.45E-09 | 6.48E-08 | yes | down | 23.4 | 30.39 | 25.65 | 30.25 | 29.05 | 31.65 | 26.48 | 30.31667 |
| STAT5B | 2.104 | 1.073112 | 4.49E-09 | 6.54E-08 | yes | up | 10.26 | 10.59 | 9.02 | 42.94 | 51.01 | 56.61 | 9.956667 | 50.18667 |
| ADCY5 | 3.103 | 1.633759 | 4.58E-09 | 6.67E-08 | yes | up | 1.07 | 1.16 | 0.81 | 7.38 | 5.6 | 9.47 | 1.013333 | 7.483333 |
| RECQL | 2.472 | 1.305441 | 4.63E-09 | 6.72E-08 | yes | up | 2.75 | 2.74 | 2.57 | 16.01 | 10.32 | 13.59 | 2.686667 | 13.30667 |
| CLCN5 | 2.696 | 1.430629 | 4.75E-09 | 6.88E-08 | yes | up | 1.44 | 0.94 | 0.72 | 6.46 | 6.74 | 6.6 | 1.033333 | 6.6 |
| THRSP | 2.208 | 1.142726 | 4.83E-09 | 6.99E-08 | yes | up | 329.53 | 356.96 | 338.55 | 2004.82 | 1174.32 | 1378.17 | 341.68 | 1519.103 |
| NRG4 | 0.192 | -2.38129 | 4.88E-09 | 7.06E-08 | yes | down | 22.44 | 30.91 | 30.68 | 4.83 | 5.11 | 15.22 | 28.01 | 8.386667 |
| SELENBP1 | 2.68 | 1.422005 | 4.98E-09 | 7.18E-08 | yes | up | 40.41 | 48.93 | 43.15 | 247.05 | 361.85 | 241.48 | 44.16333 | 283.46 |
| VNN1 | 9.825 | 3.296451 | 5.03E-09 | 7.24E-08 | yes | up | 0.51 | 0.34 | 0.35 | 5.37 | 16.23 | 6.13 | 0.4 | 9.243333 |
| KIT | 3.691 | 1.884051 | 5.18E-09 | 7.44E-08 | yes | up | 2.09 | 2.04 | 2.12 | 28.3 | 15.33 | 11.44 | 2.083333 | 18.35667 |
| NUDT19 | 0.475 | -1.07405 | 5.31E-09 | 7.62E-08 | yes | down | 9.1 | 10.2 | 9.51 | 11.77 | 10.65 | 11.27 | 9.603333 | 11.23 |
| LOC100352100 | 0.47 | -1.0904 | 5.33E-09 | 7.65E-08 | yes | down | 109.67 | 89.87 | 95.71 | 129.46 | 88.9 | 119.03 | 98.41667 | 112.4633 |
| LCAT | 187.253 | 7.548844 | 5.38E-09 | 7.71E-08 | yes | up | 0 | 0 | 0 | 4.39 | 4.38 | 2.65 | 0 | 3.806667 |
| COL27A1 | 4.476 | 2.162284 | 5.46E-09 | 7.81E-08 | yes | up | 2.18 | 1.52 | 0.97 | 23.19 | 9.15 | 17.34 | 1.556667 | 16.56 |
| BAIAP2 | 2.939 | 1.555439 | 5.61E-09 | 8.01E-08 | yes | up | 1.05 | 1.46 | 1.1 | 7.83 | 8.25 | 9.32 | 1.203333 | 8.466667 |
| ME3 | 0.147 | -2.76174 | 5.63E-09 | 8.03E-08 | yes | down | 9.6 | 4.83 | 11.71 | 0.95 | 2.2 | 2.44 | 8.713333 | 1.863333 |
| MEP1B | 12.238 | 3.613275 | 5.65E-09 | 8.05E-08 | yes | up | 0.1 | 0.09 | 0.04 | 3.26 | 1.46 | 1.96 | 0.076667 | 2.226667 |
| MET | 0.157 | -2.66894 | 5.67E-09 | 8.07E-08 | yes | down | 3.74 | 2.66 | 7.58 | 1.97 | 2.24 | 1.32 | 4.66 | 1.843333 |
| ND1 | 0.224 | -2.15884 | 5.8E-09 | 8.24E-08 | yes | down | 3389.71 | 2811.48 | 4453.02 | 1309.84 | 1569.25 | 2954.95 | 3551.403 | 1944.68 |
| NFXL1 | 2.017 | 1.012042 | 5.81E-09 | 8.25E-08 | yes | up | 2.15 | 2.06 | 1.55 | 9.2 | 8.97 | 9.35 | 1.92 | 9.173333 |
| ADCYAP1R1 | 4.288 | 2.100368 | 5.81E-09 | 8.25E-08 | yes | up | 0.48 | 0.45 | 0.35 | 6.9 | 3.56 | 2.84 | 0.426667 | 4.433333 |
| TBC1D15 | 0.467 | -1.09925 | 6.04E-09 | 8.55E-08 | yes | down | 12.7 | 13.07 | 9.58 | 11.24 | 13.46 | 14.42 | 11.78333 | 13.04 |
| ASUN | 2.079 | 1.055549 | 6.04E-09 | 8.55E-08 | yes | up | 14.54 | 10.61 | 9.38 | 54.8 | 62.85 | 58.94 | 11.51 | 58.86333 |
| SPTA1 | 0.055 | -4.18624 | 6.21E-09 | 8.77E-08 | yes | down | 0.83 | 2.42 | 0.76 | 0.01 | 0.51 | 0.3 | 1.336667 | 0.273333 |
| PRKCZ | 5.937 | 2.56963 | 6.31E-09 | 8.91E-08 | yes | up | 1.75 | 2.1 | 1.33 | 24.47 | 29.43 | 20.09 | 1.726667 | 24.66333 |
| ANKS6 | 2.217 | 1.148396 | 6.58E-09 | 9.27E-08 | yes | up | 1.38 | 1.5 | 1.17 | 6.87 | 7.29 | 7.25 | 1.35 | 7.136667 |
| TROAP | 9.173 | 3.19739 | 6.74E-09 | 9.49E-08 | yes | up | 0.4 | 0.46 | 0.16 | 8.79 | 9.05 | 2.83 | 0.34 | 6.89 |
| RWDD3 | 2.626 | 1.392706 | 6.8E-09 | 9.57E-08 | yes | up | 1.59 | 1.1 | 0.94 | 7.45 | 8.13 | 5.86 | 1.21 | 7.146667 |
| ADRA1A | 0.197 | -2.34221 | 6.83E-09 | 9.6E-08 | yes | down | 13.84 | 16.71 | 14 | 4.67 | 2.66 | 9.6 | 14.85 | 5.643333 |
| DSG2 | 0.202 | -2.3109 | 6.87E-09 | 9.65E-08 | yes | down | 3.14 | 4.66 | 2.59 | 1.59 | 0.84 | 2.55 | 3.463333 | 1.66 |
| C1S | 3.736 | 1.90133 | 7.02E-09 | 9.86E-08 | yes | up | 11.85 | 9.65 | 6.48 | 52.25 | 75.98 | 115.44 | 9.326667 | 81.22333 |
| LOC103349956 | 0.172 | -2.53567 | 7.22E-09 | 1.01E-07 | yes | down | 7.95 | 3.72 | 4.29 | 2.04 | 1.15 | 2.35 | 5.32 | 1.846667 |
| TRIM36 | 0.134 | -2.9043 | 7.54E-09 | 1.05E-07 | yes | down | 1.5 | 2.53 | 2.89 | 0.86 | 0.29 | 1.06 | 2.306667 | 0.736667 |
| PRKCDBP | 0.215 | -2.21746 | 7.56E-09 | 1.06E-07 | yes | down | 90.17 | 176.24 | 189.37 | 122.34 | 66.13 | 56.46 | 151.9267 | 81.64333 |
| POSTN | 0.299 | -1.73975 | 7.7E-09 | 1.07E-07 | yes | down | 192.67 | 126.64 | 76.45 | 74.52 | 99.56 | 102.2 | 131.92 | 92.09333 |
| LOC108178279 | 2.758 | 1.463854 | 7.74E-09 | 1.08E-07 | yes | up | 2.15 | 1.85 | 1.52 | 11.92 | 13 | 11.4 | 1.84 | 12.10667 |
| MTUS1 | 2.904 | 1.538145 | 7.81E-09 | 1.09E-07 | yes | up | 15.98 | 12.45 | 14.62 | 78.89 | 116.26 | 94.22 | 14.35 | 96.45667 |
| TMEM200A | 0.142 | -2.81619 | 7.93E-09 | 1.1E-07 | yes | down | 0.82 | 1.1 | 0.59 | 0.45 | 0.18 | 0.37 | 0.836667 | 0.333333 |
| LOC103347198 | 14.055 | 3.813015 | 7.96E-09 | 1.11E-07 | yes | up | 0.09 | 0.4 | 0.23 | 9.64 | 5.85 | 9.42 | 0.24 | 8.303333 |
| LSG1 | 0.404 | -1.30759 | 8.01E-09 | 1.11E-07 | yes | down | 10.28 | 14.88 | 11.65 | 13.86 | 9.46 | 12.99 | 12.27 | 12.10333 |
| ACAA1 | 0.264 | -1.92243 | 8.02E-09 | 1.11E-07 | yes | down | 40.11 | 43.56 | 75.27 | 35.79 | 36.82 | 31.5 | 52.98 | 34.70333 |
| DCN | 3.021 | 1.594959 | 8.05E-09 | 1.12E-07 | yes | up | 288.23 | 193.71 | 126.3 | 1152.66 | 1532.22 | 1540.79 | 202.7467 | 1408.557 |
| SLC2A12 | 7.354 | 2.878471 | 8.07E-09 | 1.12E-07 | yes | up | 0.6 | 0.14 | 0.44 | 5.4 | 8.86 | 6.44 | 0.393333 | 6.9 |
| LOC100353012 | 175.651 | 7.45657 | 8.08E-09 | 1.12E-07 | yes | up | 0 | 0 | 0 | 1 | 0.54 | 0.85 | 0 | 0.796667 |
| GLRX3 | 0.479 | -1.0622 | 8.16E-09 | 1.13E-07 | yes | down | 90.69 | 118.27 | 98.23 | 123.79 | 126.52 | 104.79 | 102.3967 | 118.3667 |
| GRWD1 | 0.411 | -1.28277 | 8.31E-09 | 1.15E-07 | yes | down | 6.82 | 7.06 | 6.73 | 6.54 | 5.7 | 8.17 | 6.87 | 6.803333 |
| LOC108176107 | 0.324 | -1.62612 | 8.41E-09 | 1.16E-07 | yes | down | 4.09 | 2.61 | 2.23 | 2.36 | 2.39 | 2.12 | 2.976667 | 2.29 |
| RARRES1 | 5.832 | 2.543893 | 8.45E-09 | 1.16E-07 | yes | up | 0.91 | 0.88 | 0.49 | 11.88 | 6.33 | 13.46 | 0.76 | 10.55667 |
| MAPK8IP2 | 0.05 | -4.3295 | 8.54E-09 | 1.18E-07 | yes | down | 0.59 | 1.58 | 1.52 | 0.17 | 0.25 | 0.03 | 1.23 | 0.15 |
| LOC100352044 | 2.478 | 1.309088 | 8.72E-09 | 1.2E-07 | yes | up | 33.84 | 45.18 | 36.15 | 181.87 | 240.88 | 261.07 | 38.39 | 227.94 |
| TPRA1 | 0.365 | -1.45312 | 8.79E-09 | 1.21E-07 | yes | down | 20.95 | 27.33 | 30.37 | 26.46 | 23.75 | 19.8 | 26.21667 | 23.33667 |
| CASC1 | 0.287 | -1.79949 | 8.98E-09 | 1.23E-07 | yes | down | 2.33 | 2.23 | 1.92 | 1.27 | 1.18 | 1.89 | 2.16 | 1.446667 |
| TTC37 | 2.905 | 1.538385 | 9.04E-09 | 1.24E-07 | yes | up | 4.24 | 3.41 | 3.63 | 26.34 | 14.44 | 22.6 | 3.76 | 21.12667 |
| NEU1 | 0.47 | -1.08893 | 9.46E-09 | 1.29E-07 | yes | down | 15.64 | 17.33 | 16.67 | 16.49 | 17.64 | 18.89 | 16.54667 | 17.67333 |
| PTEN | 2.125 | 1.087234 | 9.49E-09 | 1.3E-07 | yes | up | 9.65 | 9.08 | 8.73 | 44.55 | 54.26 | 40.54 | 9.153333 | 46.45 |
| HPRT1 | 0.424 | -1.23846 | 9.5E-09 | 1.3E-07 | yes | down | 23.09 | 19.86 | 13.58 | 23.31 | 15.27 | 18.64 | 18.84333 | 19.07333 |
| RARRES2 | 2.988 | 1.579209 | 9.78E-09 | 1.33E-07 | yes | up | 84.95 | 143.84 | 88.18 | 625.98 | 678.41 | 947.5 | 105.6567 | 750.63 |
| SYT10 | 0.006 | -7.32698 | 9.79E-09 | 1.33E-07 | yes | down | 0.53 | 0.5 | 0.43 | 0 | 0 | 0.02 | 0.486667 | 0.006667 |
| LOC103346787 | 0.308 | -1.70093 | 9.95E-09 | 1.35E-07 | yes | down | 17.31 | 28.98 | 23.86 | 17.81 | 14.71 | 20.12 | 23.38333 | 17.54667 |
| NFIL3 | 3.068 | 1.617498 | 1.03E-08 | 1.41E-07 | yes | up | 4.41 | 5.82 | 5.8 | 44.06 | 47.46 | 27.9 | 5.343333 | 39.80667 |
| KLF15 | 2.903 | 1.537543 | 1.04E-08 | 1.41E-07 | yes | up | 2.19 | 1.1 | 2.4 | 14.8 | 8.36 | 8.66 | 1.896667 | 10.60667 |
| LOC100346271 | 3.191 | 1.674045 | 1.04E-08 | 1.41E-07 | yes | up | 0.81 | 0.71 | 0.51 | 5.44 | 5.38 | 3.88 | 0.676667 | 4.9 |
| FAM124A | 0.285 | -1.80936 | 1.06E-08 | 1.43E-07 | yes | down | 6.56 | 7.38 | 6.15 | 3.71 | 3.18 | 6.83 | 6.696667 | 4.573333 |
| LSAMP | 7.863 | 2.975082 | 1.06E-08 | 1.43E-07 | yes | up | 0.96 | 0.27 | 0.13 | 8.98 | 3.61 | 2.45 | 0.453333 | 5.013333 |
| CPT1B | 0.279 | -1.84042 | 1.14E-08 | 1.54E-07 | yes | down | 127.28 | 107.72 | 92.56 | 68.77 | 39.99 | 107.13 | 109.1867 | 71.96333 |
| NEGR1 | 4.076 | 2.027049 | 1.14E-08 | 1.54E-07 | yes | up | 0.34 | 0.36 | 0.32 | 3.58 | 2.95 | 3.45 | 0.34 | 3.326667 |
| GPC3 | 0.316 | -1.66144 | 1.17E-08 | 1.57E-07 | yes | down | 16.43 | 16.09 | 13.54 | 18.23 | 8.66 | 8.56 | 15.35333 | 11.81667 |
| PPAT | 0.22 | -2.18637 | 1.17E-08 | 1.57E-07 | yes | down | 2.15 | 1.93 | 1.6 | 0.59 | 1.04 | 1.33 | 1.893333 | 0.986667 |
| LOC100351134 | 3.069 | 1.617818 | 1.18E-08 | 1.58E-07 | yes | up | 0.75 | 0.75 | 0.61 | 5.94 | 5.17 | 4.22 | 0.703333 | 5.11 |
| SIK3 | 0.435 | -1.20137 | 1.19E-08 | 1.6E-07 | yes | down | 9.9 | 12.25 | 13.4 | 14.25 | 11.07 | 12.41 | 11.85 | 12.57667 |
| LOC103347134 | 0.497 | -1.00919 | 1.2E-08 | 1.61E-07 | yes | down | 14.46 | 14.73 | 10.75 | 14.09 | 15.6 | 17.43 | 13.31333 | 15.70667 |
| TINAGL1 | 2.41 | 1.269193 | 1.21E-08 | 1.62E-07 | yes | up | 67.05 | 107.6 | 72.36 | 465.21 | 524.8 | 433.48 | 82.33667 | 474.4967 |
| DYNLRB2 | 0.38 | -1.39431 | 1.21E-08 | 1.63E-07 | yes | down | 17.17 | 21.7 | 17.15 | 15.79 | 15.26 | 18.3 | 18.67333 | 16.45 |
| NFX1 | 0.436 | -1.19814 | 1.22E-08 | 1.64E-07 | yes | down | 8.07 | 12.36 | 8.46 | 9.98 | 9.48 | 10.69 | 9.63 | 10.05 |
| SORT1 | 2.756 | 1.462782 | 1.24E-08 | 1.66E-07 | yes | up | 11 | 7.18 | 5.04 | 45.73 | 48.25 | 56.63 | 7.74 | 50.20333 |
| UNC119 | 2.113 | 1.079278 | 1.26E-08 | 1.69E-07 | yes | up | 6.52 | 7.51 | 5.92 | 37.16 | 31.81 | 32.47 | 6.65 | 33.81333 |
| HSPBP1 | 0.467 | -1.0983 | 1.31E-08 | 1.75E-07 | yes | down | 8.77 | 8.43 | 6.28 | 8.85 | 9.17 | 8.16 | 7.826667 | 8.726667 |
| MCP-1 | 0.004 | -7.79709 | 1.34E-08 | 1.79E-07 | yes | down | 3.98 | 13.31 | 6.16 | 0.25 | 0 | 0 | 7.816667 | 0.083333 |
| FLCN | 0.444 | -1.17191 | 1.36E-08 | 1.81E-07 | yes | down | 10.28 | 13.03 | 12.53 | 12.81 | 11.6 | 14.06 | 11.94667 | 12.82333 |
| HES1 | 2.54 | 1.34465 | 1.38E-08 | 1.83E-07 | yes | up | 4.81 | 4.81 | 3.57 | 22.72 | 24.78 | 30.49 | 4.396667 | 25.99667 |
| LOC103347367 | 0.396 | -1.33624 | 1.45E-08 | 1.93E-07 | yes | down | 58.55 | 73.31 | 76.2 | 80.11 | 52.52 | 69.84 | 69.35333 | 67.49 |
| AGT | 149.832 | 7.227205 | 1.47E-08 | 1.95E-07 | yes | up | 0.02 | 0.18 | 0.04 | 43.88 | 41.67 | 2.79 | 0.08 | 29.44667 |
| UCK2 | 0.385 | -1.37863 | 1.54E-08 | 2.04E-07 | yes | down | 8.44 | 6.64 | 7.47 | 6.43 | 5.4 | 8.53 | 7.516667 | 6.786667 |
| LOC103346901 | 17.641 | 4.140838 | 1.55E-08 | 2.05E-07 | yes | up | 0.21 | 0.94 | 0.87 | 18.05 | 29.02 | 37.64 | 0.673333 | 28.23667 |
| WWOX | 2.176 | 1.121562 | 1.56E-08 | 2.06E-07 | yes | up | 7 | 5.14 | 5.02 | 29.38 | 28.84 | 31.31 | 5.72 | 29.84333 |
| GADD45A | 2.836 | 1.503757 | 1.56E-08 | 2.06E-07 | yes | up | 16.22 | 9.51 | 14.66 | 85.39 | 94.18 | 97.86 | 13.46333 | 92.47667 |
| G6PD | 4.865 | 2.282581 | 1.57E-08 | 2.07E-07 | yes | up | 5.1 | 6.92 | 7.69 | 104.85 | 34.18 | 87.63 | 6.57 | 75.55333 |
| LOC100358489 | 154.565 | 7.272066 | 1.58E-08 | 2.09E-07 | yes | up | 0 | 0 | 0 | 2.3 | 2.22 | 2.03 | 0 | 2.183333 |
| ITPR2 | 2.017 | 1.012005 | 1.61E-08 | 2.12E-07 | yes | up | 7.03 | 7.69 | 7.4 | 44.55 | 30.55 | 32.61 | 7.373333 | 35.90333 |
| LOC100338043 | 2.229 | 1.156548 | 1.62E-08 | 2.14E-07 | yes | up | 11.03 | 14.1 | 12.64 | 63.13 | 67.62 | 72.79 | 12.59 | 67.84667 |
| SH3RF2 | 0.096 | -3.38701 | 1.69E-08 | 2.22E-07 | yes | down | 1.75 | 2.21 | 0.37 | 0.33 | 0.34 | 0.33 | 1.443333 | 0.333333 |
| HEMGN | 0.071 | -3.8183 | 1.75E-08 | 2.3E-07 | yes | down | 1.16 | 4.35 | 3.24 | 0.17 | 0.52 | 0.8 | 2.916667 | 0.496667 |
| IGIP | 2.256 | 1.17356 | 1.81E-08 | 2.37E-07 | yes | up | 3.22 | 3.49 | 2.01 | 15.3 | 16.05 | 15.17 | 2.906667 | 15.50667 |
| CATSPER2 | 9.36 | 3.226497 | 1.84E-08 | 2.42E-07 | yes | up | 0.32 | 0.12 | 0.08 | 1.86 | 2.49 | 4.05 | 0.173333 | 2.8 |
| TOMM22 | 0.321 | -1.63936 | 1.86E-08 | 2.43E-07 | yes | down | 31.18 | 56.22 | 47.96 | 32.66 | 34.12 | 39.05 | 45.12 | 35.27667 |
| FAU | 0.47 | -1.08973 | 1.87E-08 | 2.45E-07 | yes | down | 913.32 | 1171.11 | 1152.27 | 1355.57 | 1149.32 | 1223.71 | 1078.9 | 1242.867 |
| ENPP1 | 3.213 | 1.68376 | 1.89E-08 | 2.47E-07 | yes | up | 5.01 | 4.28 | 2.72 | 27.34 | 40.36 | 22.8 | 4.003333 | 30.16667 |
| TNNI3K | 30.77 | 4.94346 | 1.92E-08 | 2.51E-07 | yes | up | 0.02 | 0.05 | 0.02 | 2.63 | 1.05 | 1.78 | 0.03 | 1.82 |
| SH3GL2 | 3.542 | 1.824388 | 1.97E-08 | 2.57E-07 | yes | up | 1.63 | 0.89 | 1.35 | 9.1 | 12.99 | 10.75 | 1.29 | 10.94667 |
| HAVCR1 | 8.558 | 3.097292 | 1.98E-08 | 2.58E-07 | yes | up | 0.18 | 0.78 | 0.19 | 6.96 | 6.35 | 11.23 | 0.383333 | 8.18 |
| C17H15orf41 | 0.363 | -1.46148 | 1.99E-08 | 2.59E-07 | yes | down | 3.15 | 2.35 | 2.53 | 2.39 | 2.46 | 2.13 | 2.676667 | 2.326667 |
| LOC100351224 | 0.289 | -1.79016 | 2.01E-08 | 2.62E-07 | yes | down | 1.57 | 1.5 | 1.19 | 1.01 | 1 | 0.93 | 1.42 | 0.98 |
| SLC6A6 | 0.18 | -2.47756 | 2.02E-08 | 2.63E-07 | yes | down | 14.35 | 23.96 | 21.27 | 15.76 | 4.49 | 5.31 | 19.86 | 8.52 |
| DGAT2 | 2.167 | 1.11583 | 2.02E-08 | 2.63E-07 | yes | up | 532.47 | 697.87 | 458.33 | 4291.84 | 3444.41 | 3236.17 | 562.89 | 3657.473 |
| AKAP12 | 2.419 | 1.27456 | 2.03E-08 | 2.63E-07 | yes | up | 12.49 | 11.15 | 6.59 | 59.13 | 46.25 | 66.81 | 10.07667 | 57.39667 |
| DENND5B | 0.332 | -1.59015 | 2.1E-08 | 2.72E-07 | yes | down | 2.11 | 1.43 | 1.26 | 1.02 | 1.51 | 1.24 | 1.6 | 1.256667 |
| ARHGAP36 | 0.003 | -8.48638 | 2.1E-08 | 2.72E-07 | yes | down | 17.39 | 2.5 | 1.46 | 0.03 | 0.11 | 0 | 7.116667 | 0.046667 |
| LOC103349722 | 0.356 | -1.49155 | 2.15E-08 | 2.78E-07 | yes | down | 48.56 | 86.06 | 57.82 | 64.98 | 50.32 | 52.26 | 64.14667 | 55.85333 |
| ADAMTS2 | 2.046 | 1.033123 | 2.16E-08 | 2.79E-07 | yes | up | 8.44 | 6.2 | 5.16 | 30.71 | 31.81 | 33 | 6.6 | 31.84 |
| LOC108176569 | 40.307 | 5.332956 | 2.17E-08 | 2.79E-07 | yes | up | 0.04 | 0.02 | 0 | 5.45 | 1.97 | 1.1 | 0.02 | 2.84 |
| PCF11 | 2.047 | 1.033621 | 2.17E-08 | 2.8E-07 | yes | up | 5.48 | 5.86 | 3.5 | 32.99 | 31.68 | 24.37 | 4.946667 | 29.68 |
| NPTX1 | 3.51 | 1.811346 | 2.18E-08 | 2.8E-07 | yes | up | 4.53 | 3.19 | 3.52 | 40.6 | 15.86 | 32.87 | 3.746667 | 29.77667 |
| LOC103349164 | 0.005 | -7.77821 | 2.26E-08 | 2.9E-07 | yes | down | 0.82 | 1.21 | 1.75 | 0 | 0 | 0 | 1.26 | 0 |
| SLC38A1 | 0.397 | -1.33323 | 2.27E-08 | 2.92E-07 | yes | down | 9.04 | 8.02 | 5.68 | 7.48 | 8.14 | 6.88 | 7.58 | 7.5 |
| FTH1 | 2.044 | 1.031584 | 2.28E-08 | 2.92E-07 | yes | up | 1091.47 | 1281.14 | 1048.35 | 5085.85 | 6416.89 | 5304.04 | 1140.32 | 5602.26 |
| PSRC1 | 5.043 | 2.334204 | 2.29E-08 | 2.94E-07 | yes | up | 0.66 | 0.71 | 0.67 | 12.25 | 8.01 | 5.09 | 0.68 | 8.45 |
| LOC103345923 | 2.089 | 1.062771 | 2.32E-08 | 2.97E-07 | yes | up | 146.25 | 151.45 | 130.05 | 601.22 | 814.47 | 715.93 | 142.5833 | 710.54 |
| LOC108176678 | 9.048 | 3.177607 | 2.35E-08 | 3.01E-07 | yes | up | 0.05 | 0.23 | 0.09 | 2.58 | 2.44 | 3.05 | 0.123333 | 2.69 |
| RPS29 | 0.138 | -2.853 | 2.35E-08 | 3.01E-07 | yes | down | 118.95 | 167.72 | 350.86 | 99.29 | 83.59 | 53.73 | 212.51 | 78.87 |
| MSRB1 | 2.136 | 1.094946 | 2.37E-08 | 3.03E-07 | yes | up | 82.28 | 63.3 | 61.43 | 446.1 | 341.52 | 286.17 | 69.00333 | 357.93 |
| UGT1-6 | 3.391 | 1.76172 | 2.4E-08 | 3.06E-07 | yes | up | 9.54 | 21.09 | 13.06 | 87.67 | 112.21 | 118.72 | 14.56333 | 106.2 |
| NDUFAF7 | 0.424 | -1.2371 | 2.44E-08 | 3.11E-07 | yes | down | 10.83 | 10.46 | 7.72 | 12.16 | 9.96 | 7.7 | 9.67 | 9.94 |
| LOC100354303 | 0.32 | -1.64208 | 2.45E-08 | 3.13E-07 | yes | down | 20.39 | 27.36 | 26.34 | 15.78 | 21.82 | 20.44 | 24.69667 | 19.34667 |
| DTL | 3.265 | 1.706938 | 2.45E-08 | 3.13E-07 | yes | up | 0.86 | 1.12 | 0.62 | 9.61 | 5.62 | 5.97 | 0.866667 | 7.066667 |
| PPP1R13L | 0.273 | -1.871 | 2.48E-08 | 3.16E-07 | yes | down | 1.58 | 1.75 | 1.66 | 1.18 | 0.95 | 1.17 | 1.663333 | 1.1 |
| LOC103347974 | 0.184 | -2.44209 | 2.54E-08 | 3.23E-07 | yes | down | 2.16 | 2.25 | 2.4 | 0.8 | 0.91 | 1.28 | 2.27 | 0.996667 |
| F10 | 0.005 | -7.75776 | 2.68E-08 | 3.41E-07 | yes | down | 0.55 | 0.77 | 1.17 | 0 | 0 | 0 | 0.83 | 0 |
| ID2 | 0.246 | -2.02194 | 2.72E-08 | 3.45E-07 | yes | down | 4.77 | 5.99 | 5.61 | 3.48 | 2.16 | 4.16 | 5.456667 | 3.266667 |
| SPSB1 | 0.332 | -1.59198 | 2.73E-08 | 3.46E-07 | yes | down | 17.7 | 12.43 | 8.96 | 8.51 | 9.69 | 12.35 | 13.03 | 10.18333 |
| LOC103348123 | 0.027 | -5.23756 | 2.78E-08 | 3.51E-07 | yes | down | 0.86 | 1.2 | 0.85 | 0.59 | 0 | 0.13 | 0.97 | 0.24 |
| SC5D | 2.082 | 1.057903 | 2.86E-08 | 3.62E-07 | yes | up | 6.14 | 7.81 | 5.76 | 40.09 | 29.97 | 28.88 | 6.57 | 32.98 |
| RABGGTB | 0.481 | -1.05692 | 2.9E-08 | 3.65E-07 | yes | down | 31.67 | 22.46 | 24.32 | 28.84 | 29.99 | 27.85 | 26.15 | 28.89333 |
| CDO1 | 2.181 | 1.125166 | 2.96E-08 | 3.73E-07 | yes | up | 151.8 | 221.26 | 128.18 | 926.52 | 902.8 | 779.3 | 167.08 | 869.54 |
| ADGRE5 | 2.479 | 1.310039 | 2.97E-08 | 3.74E-07 | yes | up | 2.95 | 3.81 | 3.69 | 22.26 | 15.42 | 15.22 | 3.483333 | 17.63333 |
| LOC100355548 | 0.403 | -1.3095 | 3.03E-08 | 3.81E-07 | yes | down | 33.19 | 38.56 | 33.01 | 28.28 | 34.5 | 39.41 | 34.92 | 34.06333 |
| LOC103349204 | 0.024 | -5.41017 | 3.08E-08 | 3.87E-07 | yes | down | 4.19 | 5.97 | 9.33 | 0.29 | 0.96 | 0 | 6.496667 | 0.416667 |
| DARS | 0.465 | -1.10547 | 3.2E-08 | 4.01E-07 | yes | down | 38.56 | 33.48 | 24.63 | 34.42 | 41.06 | 30.3 | 32.22333 | 35.26 |
| LOC100358060 | 3.304 | 1.724353 | 3.21E-08 | 4.02E-07 | yes | up | 8.32 | 7.91 | 11.51 | 55.04 | 88.69 | 78.76 | 9.246667 | 74.16333 |
| PXDNL | 5.379 | 2.427273 | 3.24E-08 | 4.06E-07 | yes | up | 0.1 | 0.16 | 0.24 | 1.96 | 2.05 | 2.53 | 0.166667 | 2.18 |
| FLOT2 | 2.506 | 1.325366 | 3.27E-08 | 4.09E-07 | yes | up | 35.15 | 22.3 | 16.55 | 147.15 | 155.63 | 138.43 | 24.66667 | 147.07 |
| HS3ST1 | 3.137 | 1.64925 | 3.32E-08 | 4.15E-07 | yes | up | 1.3 | 1 | 0.72 | 7.44 | 6.82 | 8.38 | 1.006667 | 7.546667 |
| TXNRD2 | 0.476 | -1.07176 | 3.33E-08 | 4.16E-07 | yes | down | 9.85 | 10.63 | 11.03 | 12.5 | 11.94 | 11.9 | 10.50333 | 12.11333 |
| AIFM2 | 0.492 | -1.02236 | 3.42E-08 | 4.26E-07 | yes | down | 349.06 | 407.62 | 427.71 | 503.66 | 410.11 | 485.44 | 394.7967 | 466.4033 |
| PCOLCE | 3.105 | 1.634622 | 3.42E-08 | 4.27E-07 | yes | up | 35.78 | 20.73 | 15.56 | 147.54 | 203.71 | 172.88 | 24.02333 | 174.71 |
| CDH23 | 0.348 | -1.52313 | 3.55E-08 | 4.41E-07 | yes | down | 2.79 | 2.04 | 1.92 | 2.29 | 1.22 | 2.47 | 2.25 | 1.993333 |
| KIF3A | 2.437 | 1.285288 | 3.58E-08 | 4.45E-07 | yes | up | 2.6 | 2.18 | 1.41 | 10.99 | 11.24 | 9.16 | 2.063333 | 10.46333 |
| CBS | 5.261 | 2.395347 | 3.62E-08 | 4.49E-07 | yes | up | 5.78 | 1.73 | 1.82 | 32.42 | 41.97 | 39.4 | 3.11 | 37.93 |
| LOC103352377 | 0.146 | -2.78068 | 3.63E-08 | 4.5E-07 | yes | down | 14.63 | 29.42 | 15.86 | 11.45 | 6.3 | 3.72 | 19.97 | 7.156667 |
| PTPRR | 0.195 | -2.35587 | 3.65E-08 | 4.53E-07 | yes | down | 1.16 | 1.78 | 1.53 | 0.6 | 0.99 | 0.53 | 1.49 | 0.706667 |
| ID1 | 0.315 | -1.66542 | 3.67E-08 | 4.55E-07 | yes | down | 43.2 | 53.86 | 39.08 | 22.65 | 33.43 | 46.01 | 45.38 | 34.03 |
| VDAC3 | 0.319 | -1.64722 | 3.83E-08 | 4.75E-07 | yes | down | 205.13 | 130.52 | 89.17 | 102.84 | 119.74 | 102.4 | 141.6067 | 108.3267 |
| TLR5 | 7.013 | 2.810055 | 3.84E-08 | 4.75E-07 | yes | up | 0.15 | 0.12 | 0.07 | 1.81 | 2.36 | 1.37 | 0.113333 | 1.846667 |
| MPV17 | 0.496 | -1.01229 | 3.87E-08 | 4.78E-07 | yes | down | 20.49 | 24.71 | 21.89 | 33.38 | 31.56 | 25.96 | 22.36333 | 30.3 |
| BTNL9 | 0.433 | -1.2063 | 4.05E-08 | 5.01E-07 | yes | down | 74.79 | 92.12 | 89.49 | 79.21 | 104.89 | 76.26 | 85.46667 | 86.78667 |
| VASN | 2.429 | 1.280453 | 4.09E-08 | 5.04E-07 | yes | up | 2.07 | 1.42 | 1.69 | 10.03 | 9.27 | 10.89 | 1.726667 | 10.06333 |
| NKTR | 2.519 | 1.333049 | 4.11E-08 | 5.07E-07 | yes | up | 6.45 | 7.53 | 3.48 | 38.78 | 28.38 | 35.02 | 5.82 | 34.06 |
| PRG4 | 0.135 | -2.88781 | 4.27E-08 | 5.25E-07 | yes | down | 20.97 | 6.57 | 4.12 | 3.47 | 3.95 | 2.27 | 10.55333 | 3.23 |
| SLC9C2 | 6.654 | 2.734318 | 4.31E-08 | 5.3E-07 | yes | up | 0.05 | 0.07 | 0.04 | 0.92 | 0.86 | 0.81 | 0.053333 | 0.863333 |
| LOC100343913 | 25.55 | 4.675265 | 4.35E-08 | 5.35E-07 | yes | up | 0.38 | 0 | 0.08 | 6.59 | 12.01 | 8.39 | 0.153333 | 8.996667 |
| ADRA1B | 0.145 | -2.78158 | 4.39E-08 | 5.39E-07 | yes | down | 1.84 | 2.21 | 2.34 | 1.34 | 0.43 | 0.59 | 2.13 | 0.786667 |
| NDRG1 | 0.3 | -1.73687 | 4.6E-08 | 5.65E-07 | yes | down | 64.1 | 83.03 | 28.12 | 37.88 | 40.92 | 43.39 | 58.41667 | 40.73 |
| PITRM1 | 0.477 | -1.0676 | 4.64E-08 | 5.69E-07 | yes | down | 19.37 | 18.71 | 17.31 | 17.6 | 21.8 | 23.77 | 18.46333 | 21.05667 |
| MRPL50 | 0.47 | -1.08934 | 4.66E-08 | 5.71E-07 | yes | down | 14.48 | 17.62 | 13.24 | 16.73 | 15.79 | 18.42 | 15.11333 | 16.98 |
| FER | 2.23 | 1.157323 | 4.68E-08 | 5.72E-07 | yes | up | 2.15 | 1.56 | 1.43 | 8.14 | 6.89 | 10.06 | 1.713333 | 8.363333 |
| EMILIN1 | 2.333 | 1.222155 | 4.75E-08 | 5.8E-07 | yes | up | 2.58 | 2.53 | 1.53 | 15.21 | 10.36 | 11.32 | 2.213333 | 12.29667 |
| C12H6orf136 | 0.455 | -1.13494 | 4.75E-08 | 5.81E-07 | yes | down | 16.09 | 13.57 | 14.81 | 15.48 | 17.93 | 15.41 | 14.82333 | 16.27333 |
| DOC2B | 4.298 | 2.103522 | 4.76E-08 | 5.81E-07 | yes | up | 1.37 | 0.69 | 1.39 | 18.56 | 10.88 | 8.86 | 1.15 | 12.76667 |
| LOC100346561 | 5.17 | 2.37009 | 4.82E-08 | 5.87E-07 | yes | up | 5.74 | 4.89 | 8.31 | 131.88 | 35.39 | 79.38 | 6.313333 | 82.21667 |
| ARAP2 | 2.998 | 1.583825 | 4.82E-08 | 5.88E-07 | yes | up | 0.56 | 0.54 | 0.82 | 5.65 | 4.17 | 4.44 | 0.64 | 4.753333 |
| MOGAT1 | 0.257 | -1.96279 | 4.93E-08 | 6E-07 | yes | down | 2.19 | 1.87 | 2.63 | 1.67 | 0.9 | 2.12 | 2.23 | 1.563333 |
| LOC103349247 | 5.123 | 2.356929 | 4.99E-08 | 6.07E-07 | yes | up | 0.64 | 0.57 | 0.9 | 8.79 | 7.89 | 6.73 | 0.703333 | 7.803333 |
| SMOC1 | 0.257 | -1.95777 | 5.01E-08 | 6.08E-07 | yes | down | 27.17 | 39.52 | 12.34 | 12.16 | 17.4 | 16.11 | 26.34333 | 15.22333 |
| LOC100351302 | 3.625 | 1.857871 | 5.03E-08 | 6.1E-07 | yes | up | 1.71 | 1.09 | 0.93 | 12.6 | 6.3 | 12.53 | 1.243333 | 10.47667 |
| LOC100344545 | 18.103 | 4.178148 | 5.04E-08 | 6.1E-07 | yes | up | 0.55 | 0.46 | 0.32 | 2.29 | 24.85 | 27.74 | 0.443333 | 18.29333 |
| ROBO1 | 3.054 | 1.610888 | 5.08E-08 | 6.14E-07 | yes | up | 2.39 | 3.37 | 1.74 | 6.02 | 7.95 | 6.51 | 2.5 | 6.826667 |
| SHROOM1 | 3.888 | 1.959041 | 5.17E-08 | 6.24E-07 | yes | up | 1.84 | 2.9 | 1.8 | 16.28 | 13.64 | 30.63 | 2.18 | 20.18333 |
| ATP6V1B2 | 0.495 | -1.01413 | 5.27E-08 | 6.36E-07 | yes | down | 28.46 | 40 | 32.89 | 44.37 | 36.43 | 37.89 | 33.78333 | 39.56333 |
| HIGD2A | 0.433 | -1.20786 | 5.36E-08 | 6.46E-07 | yes | down | 44.2 | 53.4 | 37.24 | 51.58 | 35.41 | 54.11 | 44.94667 | 47.03333 |
| ERN2 | 12.118 | 3.599087 | 5.53E-08 | 6.67E-07 | yes | up | 0.14 | 0.09 | 0.17 | 2.99 | 1.61 | 7.03 | 0.133333 | 3.876667 |
| CA1 | 0.12 | -3.06464 | 5.7E-08 | 6.86E-07 | yes | down | 3.71 | 7.57 | 5.21 | 0.69 | 1.38 | 2.58 | 5.496667 | 1.55 |
| LOC100351437 | 2.667 | 1.415466 | 5.75E-08 | 6.91E-07 | yes | up | 1.07 | 1.1 | 0.87 | 6.73 | 4.5 | 8.24 | 1.013333 | 6.49 |
| HSPA5 | 2.264 | 1.178887 | 5.83E-08 | 7.01E-07 | yes | up | 31.65 | 38.56 | 19.91 | 186.86 | 159.39 | 137.22 | 30.04 | 161.1567 |
| SLC25A28 | 2.291 | 1.195694 | 5.92E-08 | 7.11E-07 | yes | up | 12.79 | 12.82 | 14.32 | 61.45 | 64.74 | 81.29 | 13.31 | 69.16 |
| NUDT5 | 0.443 | -1.17465 | 5.95E-08 | 7.14E-07 | yes | down | 24.41 | 32.94 | 25.92 | 36.2 | 28.42 | 25.56 | 27.75667 | 30.06 |
| CCDC122 | 5.175 | 2.371427 | 5.97E-08 | 7.16E-07 | yes | up | 0.63 | 0.42 | 0.67 | 5.86 | 8.12 | 5.72 | 0.573333 | 6.566667 |
| ADCY2 | 0.124 | -3.01312 | 6.09E-08 | 7.3E-07 | yes | down | 19.83 | 13.36 | 7.68 | 1.51 | 7.48 | 2.54 | 13.62333 | 3.843333 |
| RORC | 39.797 | 5.314571 | 6.1E-08 | 7.3E-07 | yes | up | 0.14 | 0.7 | 0.49 | 30.23 | 14.69 | 89.7 | 0.443333 | 44.87333 |
| ZNF330 | 0.334 | -1.58248 | 6.11E-08 | 7.31E-07 | yes | down | 25.37 | 22.47 | 35.24 | 19.91 | 24.32 | 24.83 | 27.69333 | 23.02 |
| NELL2 | 6.554 | 2.712296 | 6.28E-08 | 7.5E-07 | yes | up | 0.3 | 0.42 | 0.25 | 3.86 | 2.83 | 8.29 | 0.323333 | 4.993333 |
| LOC103348990 | 0.104 | -3.26924 | 6.39E-08 | 7.63E-07 | yes | down | 2.09 | 2.21 | 2.27 | 0.79 | 0.67 | 0.2 | 2.19 | 0.553333 |
| COL6A1 | 2.584 | 1.369607 | 6.6E-08 | 7.87E-07 | yes | up | 180.58 | 106.2 | 91.41 | 784.05 | 679.92 | 694.64 | 126.0633 | 719.5367 |
| SH2D4A | 3.478 | 1.798309 | 6.8E-08 | 8.1E-07 | yes | up | 0.63 | 0.53 | 0.37 | 4.28 | 3.69 | 4.57 | 0.51 | 4.18 |
| VGLL3 | 2.471 | 1.305193 | 7.12E-08 | 8.46E-07 | yes | up | 1.17 | 0.91 | 0.73 | 6.13 | 4.16 | 6.28 | 0.936667 | 5.523333 |
| NFIB | 2.143 | 1.099822 | 7.13E-08 | 8.47E-07 | yes | up | 11.07 | 10.15 | 12.61 | 59.97 | 52.66 | 55.36 | 11.27667 | 55.99667 |
| ACE | 0.351 | -1.51003 | 7.36E-08 | 8.73E-07 | yes | down | 22.65 | 28.44 | 28.65 | 28.17 | 14.88 | 25 | 26.58 | 22.68333 |
| SPRYD3 | 0.426 | -1.2323 | 7.56E-08 | 8.97E-07 | yes | down | 85.28 | 59.45 | 44.27 | 75.81 | 62.13 | 77.46 | 63 | 71.8 |
| NTMT1 | 0.372 | -1.426 | 7.69E-08 | 9.12E-07 | yes | down | 63.05 | 56.56 | 48.6 | 44.05 | 66.22 | 39.32 | 56.07 | 49.86333 |
| SAMHD1 | 2.529 | 1.338445 | 7.76E-08 | 9.2E-07 | yes | up | 7.85 | 6.38 | 4.81 | 29.23 | 35.24 | 47.27 | 6.346667 | 37.24667 |
| LOC108177453 | 2.118 | 1.082735 | 7.77E-08 | 9.2E-07 | yes | up | 637.15 | 802.61 | 620.47 | 3707.82 | 4026.42 | 2875.33 | 686.7433 | 3536.523 |
| WARS2 | 0.447 | -1.16216 | 7.81E-08 | 9.24E-07 | yes | down | 3.6 | 4.16 | 3.61 | 4.06 | 4.34 | 4.1 | 3.79 | 4.166667 |
| FRMD6 | 2.054 | 1.038542 | 8.14E-08 | 9.61E-07 | yes | up | 3.65 | 3.53 | 2.41 | 16.77 | 17.01 | 13.02 | 3.196667 | 15.6 |
| TAP1 | 2.58 | 1.367468 | 8.4E-08 | 9.9E-07 | yes | up | 0.8 | 0.96 | 0.81 | 5.62 | 5.29 | 5.09 | 0.856667 | 5.333333 |
| MTRF1 | 0.334 | -1.58265 | 8.46E-08 | 9.96E-07 | yes | down | 3.49 | 3.15 | 3.27 | 2.6 | 2.07 | 3.11 | 3.303333 | 2.593333 |
| KRT23 | 0.002 | -8.95271 | 8.53E-08 | 1E-06 | yes | down | 35.96 | 118.79 | 25.03 | 0.04 | 0 | 0.74 | 59.92667 | 0.26 |
| MAP3K6 | 2.493 | 1.318147 | 8.53E-08 | 1E-06 | yes | up | 1.05 | 0.96 | 1.18 | 7.59 | 5.29 | 6.55 | 1.063333 | 6.476667 |
| DKK3 | 0.293 | -1.7709 | 8.7E-08 | 1.02E-06 | yes | down | 3.45 | 2.53 | 1.83 | 1.86 | 2.19 | 1.37 | 2.603333 | 1.806667 |
| SLC4A1 | 0.051 | -4.29715 | 8.95E-08 | 1.05E-06 | yes | down | 0.16 | 0.93 | 0.83 | 0.07 | 0.05 | 0.13 | 0.64 | 0.083333 |
| RBKS | 0.405 | -1.30429 | 9.28E-08 | 1.09E-06 | yes | down | 7.38 | 9.02 | 4.55 | 5.3 | 9.01 | 14.72 | 6.983333 | 9.676667 |
| LOC100352190 | 0.037 | -4.7385 | 9.57E-08 | 1.12E-06 | yes | down | 1.7 | 2.92 | 4.53 | 0.64 | 0.21 | 0 | 3.05 | 0.283333 |
| GAREM2 | 0.103 | -3.28095 | 9.93E-08 | 1.16E-06 | yes | down | 0.36 | 1.08 | 0.81 | 0.12 | 0.2 | 0.24 | 0.75 | 0.186667 |
| GALNTL5 | 0.045 | -4.48132 | 1.04E-07 | 1.21E-06 | yes | down | 0.58 | 0.27 | 0.79 | 0.05 | 0.06 | 0.07 | 0.546667 | 0.06 |
| ERP27 | 25.97 | 4.698786 | 1.1E-07 | 1.28E-06 | yes | up | 0.04 | 0 | 0.02 | 0.47 | 1.57 | 1.01 | 0.02 | 1.016667 |
| FKBP14 | 2.48 | 1.31021 | 1.12E-07 | 1.3E-06 | yes | up | 3.57 | 3.23 | 3.09 | 26.46 | 19.77 | 18.91 | 3.296667 | 21.71333 |
| THEM4 | 0.347 | -1.52859 | 1.15E-07 | 1.33E-06 | yes | down | 15.84 | 23.49 | 20.59 | 15.89 | 22.17 | 14.81 | 19.97333 | 17.62333 |
| KBTBD11 | 2.894 | 1.533087 | 1.18E-07 | 1.37E-06 | yes | up | 1.26 | 1.51 | 1.07 | 6.71 | 8.58 | 10.76 | 1.28 | 8.683333 |
| INO80C | 0.496 | -1.01118 | 1.2E-07 | 1.39E-06 | yes | down | 26.4 | 35.29 | 26.42 | 34.66 | 35.19 | 35.45 | 29.37 | 35.1 |
| LOC103349888 | 3.098 | 1.631362 | 1.2E-07 | 1.39E-06 | yes | up | 2.03 | 1.52 | 1.41 | 9.32 | 7.79 | 5.82 | 1.653333 | 7.643333 |
| COL6A3 | 2.703 | 1.434319 | 1.21E-07 | 1.4E-06 | yes | up | 67.97 | 36.66 | 28.74 | 345.66 | 212.15 | 274.47 | 44.45667 | 277.4267 |
| SUN3 | 4.521 | 2.176688 | 1.22E-07 | 1.4E-06 | yes | up | 1.86 | 1.48 | 1.17 | 18.25 | 11.35 | 19.44 | 1.503333 | 16.34667 |
| TMEM161B | 0.359 | -1.47595 | 1.23E-07 | 1.42E-06 | yes | down | 5.5 | 7.18 | 6.73 | 4.9 | 5.02 | 7.1 | 6.47 | 5.673333 |
| AACS | 3.877 | 1.954774 | 1.23E-07 | 1.42E-06 | yes | up | 3.85 | 4.04 | 3.22 | 41.57 | 15.67 | 46.7 | 3.703333 | 34.64667 |
| NXT2 | 0.361 | -1.46961 | 1.24E-07 | 1.42E-06 | yes | down | 4.98 | 5.32 | 4.35 | 3.3 | 3.67 | 2.62 | 4.883333 | 3.196667 |
| RMDN1 | 0.415 | -1.2678 | 1.29E-07 | 1.48E-06 | yes | down | 7.31 | 8.71 | 5.86 | 8.3 | 7.39 | 6.12 | 7.293333 | 7.27 |
| PCDH18 | 3.749 | 1.906412 | 1.3E-07 | 1.49E-06 | yes | up | 0.97 | 0.63 | 0.31 | 6.27 | 4.07 | 6.48 | 0.636667 | 5.606667 |
| CLPP | 0.416 | -1.26591 | 1.3E-07 | 1.49E-06 | yes | down | 15.47 | 21.49 | 18.95 | 19.79 | 17.52 | 18.64 | 18.63667 | 18.65 |
| ACP6 | 0.384 | -1.38191 | 1.35E-07 | 1.54E-06 | yes | down | 3.52 | 3.48 | 3.66 | 3.91 | 2.8 | 3.23 | 3.553333 | 3.313333 |
| TMSB10 | 2.022 | 1.015931 | 1.37E-07 | 1.56E-06 | yes | up | 955.87 | 1223.37 | 928.2 | 5240.4 | 5734.81 | 4381.77 | 1035.813 | 5118.993 |
| LOC100341772 | 0.209 | -2.25628 | 1.37E-07 | 1.57E-06 | yes | down | 10.54 | 12.15 | 11.65 | 10.11 | 5.07 | 2.55 | 11.44667 | 5.91 |
| MC2R | 132.929 | 7.054509 | 1.42E-07 | 1.62E-06 | yes | up | 0 | 0 | 0 | 0.78 | 1.3 | 0.81 | 0 | 0.963333 |
| RPP30 | 0.47 | -1.08834 | 1.43E-07 | 1.63E-06 | yes | down | 16.67 | 20.31 | 19.11 | 22.03 | 21.5 | 20.48 | 18.69667 | 21.33667 |
| NUDT16L1 | 0.398 | -1.33057 | 1.44E-07 | 1.63E-06 | yes | down | 10.16 | 11.66 | 9.2 | 24.12 | 20.23 | 21.21 | 10.34 | 21.85333 |
| S100A6 | 2.828 | 1.499787 | 1.45E-07 | 1.65E-06 | yes | up | 120.5 | 133.84 | 54.64 | 675.23 | 753.03 | 655.8 | 102.9933 | 694.6867 |
| UBR7 | 0.358 | -1.48221 | 1.46E-07 | 1.65E-06 | yes | down | 20.07 | 24.87 | 30.49 | 19.11 | 21.77 | 20 | 25.14333 | 20.29333 |
| PCCB | 0.452 | -1.14443 | 1.46E-07 | 1.66E-06 | yes | down | 59.62 | 69.77 | 78.15 | 81.84 | 65.1 | 82.46 | 69.18 | 76.46667 |
| TERF1 | 2.131 | 1.09138 | 1.48E-07 | 1.68E-06 | yes | up | 5.73 | 4.43 | 4.54 | 28.08 | 28.17 | 20.85 | 4.9 | 25.7 |
| SCTR | 0.241 | -2.05313 | 1.49E-07 | 1.69E-06 | yes | down | 8.65 | 12.65 | 5.89 | 3.67 | 2.46 | 5.72 | 9.063333 | 3.95 |
| ADAMTS17 | 0.293 | -1.77152 | 1.5E-07 | 1.69E-06 | yes | down | 3.35 | 3.4 | 1.34 | 1.71 | 1.69 | 2.11 | 2.696667 | 1.836667 |
| DDAH1 | 0.141 | -2.82934 | 1.51E-07 | 1.71E-06 | yes | down | 14.17 | 4.66 | 4.03 | 2.13 | 4.42 | 1.41 | 7.62 | 2.653333 |
| D2HGDH | 0.469 | -1.09342 | 1.51E-07 | 1.71E-06 | yes | down | 14.74 | 14.96 | 18.09 | 18.87 | 15.51 | 17.66 | 15.93 | 17.34667 |
| KAT2A | 2.352 | 1.233791 | 1.52E-07 | 1.71E-06 | yes | up | 7.23 | 5.62 | 4.08 | 33.6 | 25.3 | 38.56 | 5.643333 | 32.48667 |
| SPAG4 | 0.138 | -2.86023 | 1.53E-07 | 1.73E-06 | yes | down | 1.15 | 1.6 | 1.95 | 0.61 | 0.52 | 0.47 | 1.566667 | 0.533333 |
| LOC100345427 | 3.377 | 1.755855 | 1.58E-07 | 1.79E-06 | yes | up | 1.07 | 1.26 | 1.03 | 13.21 | 7.23 | 6.83 | 1.12 | 9.09 |
| MKRN1 | 0.459 | -1.12491 | 1.64E-07 | 1.85E-06 | yes | down | 23.33 | 34.17 | 30.44 | 34.44 | 27.06 | 33.54 | 29.31333 | 31.68 |
| PPP1R1A | 0.28 | -1.83549 | 1.67E-07 | 1.88E-06 | yes | down | 142.31 | 163.75 | 96.22 | 47.6 | 113.53 | 91.82 | 134.0933 | 84.31667 |
| TGFBR1 | 2.543 | 1.346699 | 1.67E-07 | 1.88E-06 | yes | up | 10.71 | 10.51 | 6.59 | 43.62 | 69.81 | 52.38 | 9.27 | 55.27 |
| LOC108175430 | 0.375 | -1.41378 | 1.71E-07 | 1.92E-06 | yes | down | 34.17 | 35.23 | 34.47 | 25.44 | 41.3 | 26.69 | 34.62333 | 31.14333 |
| JAGN1 | 0.414 | -1.27281 | 1.71E-07 | 1.92E-06 | yes | down | 37.14 | 34.52 | 34.21 | 33.55 | 33.46 | 49.54 | 35.29 | 38.85 |
| LOC100349037 | 0.245 | -2.0313 | 1.73E-07 | 1.93E-06 | yes | down | 37.29 | 40.41 | 32.28 | 34.73 | 21.25 | 9.54 | 36.66 | 21.84 |
| HHIP | 8.472 | 3.082765 | 1.74E-07 | 1.95E-06 | yes | up | 0.2 | 0.17 | 0.02 | 3.01 | 4.38 | 1.87 | 0.13 | 3.086667 |
| CSMD2 | 0.15 | -2.73218 | 1.77E-07 | 1.97E-06 | yes | down | 0.15 | 0.26 | 0.12 | 0.08 | 0.03 | 0.08 | 0.176667 | 0.063333 |
| TMEM132A | 2.613 | 1.38583 | 1.77E-07 | 1.98E-06 | yes | up | 1.69 | 1.48 | 1.54 | 13.69 | 7.91 | 8.35 | 1.57 | 9.983333 |
| FAM49B | 2.272 | 1.183903 | 1.77E-07 | 1.98E-06 | yes | up | 7.22 | 6.06 | 3.77 | 27.32 | 29.68 | 32.9 | 5.683333 | 29.96667 |
| ADAMTS12 | 2.753 | 1.46114 | 1.78E-07 | 1.99E-06 | yes | up | 7.13 | 6.91 | 4.32 | 57.64 | 35.55 | 29.57 | 6.12 | 40.92 |
| BTNL10 | 0.1 | -3.31911 | 1.79E-07 | 1.99E-06 | yes | down | 1.15 | 4.98 | 2.82 | 0.42 | 0.56 | 1.57 | 2.983333 | 0.85 |
| SNAI2 | 2.375 | 1.247959 | 1.84E-07 | 2.04E-06 | yes | up | 4.32 | 3.66 | 2.35 | 22.86 | 18.88 | 15.7 | 3.443333 | 19.14667 |
| BST1 | 2.819 | 1.49518 | 1.85E-07 | 2.06E-06 | yes | up | 5.54 | 5.58 | 2.71 | 27.04 | 26.89 | 37.19 | 4.61 | 30.37333 |
| ALAS2 | 0.141 | -2.82758 | 1.88E-07 | 2.09E-06 | yes | down | 26.38 | 65.65 | 70.75 | 7.56 | 16.57 | 31.58 | 54.26 | 18.57 |
| ASAP3 | 0.454 | -1.14036 | 1.92E-07 | 2.13E-06 | yes | down | 12.31 | 12.05 | 7.07 | 10.44 | 10.33 | 12.85 | 10.47667 | 11.20667 |
| TMEM147 | 0.483 | -1.04939 | 1.93E-07 | 2.14E-06 | yes | down | 60.62 | 55.52 | 59.73 | 73.71 | 76.11 | 56.35 | 58.62333 | 68.72333 |
| FHL1 | 0.301 | -1.73088 | 1.96E-07 | 2.17E-06 | yes | down | 1585.86 | 934.61 | 506.07 | 591.69 | 738.28 | 761.26 | 1008.847 | 697.0767 |
| COL4A1 | 2.208 | 1.143058 | 1.99E-07 | 2.21E-06 | yes | up | 252.86 | 232.06 | 257.25 | 1796.38 | 1327.47 | 1071.68 | 247.39 | 1398.51 |
| FAS | 2.457 | 1.29699 | 2E-07 | 2.21E-06 | yes | up | 3.15 | 3.38 | 2.48 | 14.62 | 13.86 | 18.16 | 3.003333 | 15.54667 |
| SLC6A8 | 0.321 | -1.64022 | 2.02E-07 | 2.23E-06 | yes | down | 42.81 | 56.33 | 45.92 | 23.22 | 45.97 | 38.78 | 48.35333 | 35.99 |
| BHLHE22 | 9.26 | 3.211087 | 2.04E-07 | 2.26E-06 | yes | up | 0.03 | 0.07 | 0.05 | 1.39 | 1.17 | 0.91 | 0.05 | 1.156667 |
| LOC100342335 | 2.006 | 1.004176 | 2.07E-07 | 2.29E-06 | yes | up | 4.43 | 5.49 | 4.53 | 14.38 | 13.28 | 13.7 | 4.816667 | 13.78667 |
| NUFIP2 | 2.012 | 1.008941 | 2.1E-07 | 2.32E-06 | yes | up | 5.67 | 6.17 | 3.56 | 22.92 | 23.73 | 26.44 | 5.133333 | 24.36333 |
| CARMIL3 | 22.02 | 4.460734 | 2.12E-07 | 2.33E-06 | yes | up | 0.01 | 0.03 | 0.01 | 1.07 | 0.76 | 0.89 | 0.016667 | 0.906667 |
| LOC100339756 | 0.492 | -1.02223 | 2.13E-07 | 2.35E-06 | yes | down | 18.07 | 15.87 | 18.65 | 23.42 | 21.46 | 19.72 | 17.53 | 21.53333 |
| BDNF | 4.707 | 2.234856 | 2.14E-07 | 2.35E-06 | yes | up | 0.38 | 0.35 | 0.24 | 2.37 | 3.1 | 5.31 | 0.323333 | 3.593333 |
| ADIPOQ | 2.722 | 1.444554 | 2.17E-07 | 2.39E-06 | yes | up | 187.79 | 271.95 | 208.9 | 1375.9 | 1883.85 | 1088.34 | 222.88 | 1449.363 |
| CCDC59 | 0.492 | -1.02305 | 2.18E-07 | 2.39E-06 | yes | down | 11.32 | 12.47 | 9.78 | 13.03 | 13.28 | 14.28 | 11.19 | 13.53 |
| CD38 | 2.614 | 1.386393 | 2.25E-07 | 2.46E-06 | yes | up | 0.84 | 0.59 | 0.65 | 4.68 | 3.54 | 5.05 | 0.693333 | 4.423333 |
| SEMA4F | 2.959 | 1.565068 | 2.28E-07 | 2.5E-06 | yes | up | 0.67 | 0.58 | 0.42 | 5.29 | 3.22 | 3.71 | 0.556667 | 4.073333 |
| LOC103352286 | 6.542 | 2.709712 | 2.34E-07 | 2.55E-06 | yes | up | 0.15 | 0.55 | 0.15 | 4.34 | 4.96 | 3.99 | 0.283333 | 4.43 |
| KCNK3 | 0.014 | -6.13167 | 2.34E-07 | 2.55E-06 | yes | down | 95.58 | 118.79 | 82.3 | 0.93 | 0.33 | 8.74 | 98.89 | 3.333333 |
| ENOSF1 | 3.221 | 1.687422 | 2.34E-07 | 2.55E-06 | yes | up | 2.84 | 3.71 | 1.9 | 30.04 | 14.42 | 20.83 | 2.816667 | 21.76333 |
| ZBTB1 | 2.021 | 1.014935 | 2.34E-07 | 2.55E-06 | yes | up | 2.66 | 2.66 | 2.15 | 13.55 | 10.16 | 10.93 | 2.49 | 11.54667 |
| CAPN5 | 2.356 | 1.236185 | 2.36E-07 | 2.57E-06 | yes | up | 2.1 | 1.76 | 1.11 | 9.01 | 10 | 8.63 | 1.656667 | 9.213333 |
| FAM46A | 2.521 | 1.333849 | 2.39E-07 | 2.6E-06 | yes | up | 3.7 | 3.1 | 1.58 | 17.62 | 15.15 | 16.64 | 2.793333 | 16.47 |
| C1H11orf80 | 0.156 | -2.68096 | 2.41E-07 | 2.62E-06 | yes | down | 3.45 | 7.18 | 2.6 | 0.74 | 1.42 | 2.74 | 4.41 | 1.633333 |
| CDKN1A | 3.679 | 1.879505 | 2.45E-07 | 2.66E-06 | yes | up | 8.34 | 9.09 | 7.07 | 88.37 | 32.82 | 97.14 | 8.166667 | 72.77667 |
| M1AP | 112.818 | 6.817858 | 2.57E-07 | 2.78E-06 | yes | up | 0 | 0 | 0 | 1.51 | 1.21 | 1.41 | 0 | 1.376667 |
| RGAG1 | 0.168 | -2.57448 | 2.57E-07 | 2.78E-06 | yes | down | 0.88 | 1.12 | 0.3 | 0.32 | 0.25 | 0.33 | 0.766667 | 0.3 |
| MSANTD2 | 2.058 | 1.041229 | 2.64E-07 | 2.85E-06 | yes | up | 6.18 | 8.84 | 5.62 | 28.82 | 26.32 | 30.05 | 6.88 | 28.39667 |
| LOC100358239 | 0.483 | -1.05097 | 2.73E-07 | 2.94E-06 | yes | down | 52.61 | 76.25 | 64.08 | 79.4 | 76.07 | 70.08 | 64.31333 | 75.18333 |
| PLEKHG3 | 2.115 | 1.08094 | 2.79E-07 | 3E-06 | yes | up | 3.53 | 4.13 | 2.17 | 17.19 | 14.1 | 18.03 | 3.276667 | 16.44 |
| LOC100353330 | 0.312 | -1.68183 | 2.88E-07 | 3.09E-06 | yes | down | 48.51 | 56.83 | 52.92 | 16.57 | 51.33 | 36.04 | 52.75333 | 34.64667 |
| PABPC4 | 0.442 | -1.17755 | 2.9E-07 | 3.11E-06 | yes | down | 74.06 | 45.8 | 40.4 | 55.81 | 60.13 | 51.27 | 53.42 | 55.73667 |
| LOC100009136 | 0.004 | -8.0167 | 2.94E-07 | 3.14E-06 | yes | down | 1.91 | 13.37 | 2.29 | 0 | 0 | 0 | 5.856667 | 0 |
| GPC6 | 2.634 | 1.397492 | 2.98E-07 | 3.19E-06 | yes | up | 1.12 | 0.92 | 0.57 | 5.87 | 4.09 | 6.33 | 0.87 | 5.43 |
| ABHD11 | 0.349 | -1.51887 | 3.08E-07 | 3.28E-06 | yes | down | 13.66 | 11.21 | 12.19 | 14.33 | 8.52 | 6.9 | 12.35333 | 9.916667 |
| RSL24D1 | 0.484 | -1.04724 | 3.11E-07 | 3.31E-06 | yes | down | 74.43 | 64.75 | 44.06 | 65.56 | 77.95 | 66.61 | 61.08 | 70.04 |
| NEURL2 | 0.119 | -3.07322 | 3.17E-07 | 3.37E-06 | yes | down | 7.84 | 3.03 | 1.85 | 1.52 | 1.15 | 0.86 | 4.24 | 1.176667 |
| QTRT1 | 0.397 | -1.33263 | 3.17E-07 | 3.37E-06 | yes | down | 5.82 | 8.04 | 6.16 | 7.19 | 5.37 | 6.66 | 6.673333 | 6.406667 |
| C7H2orf47 | 0.458 | -1.12794 | 3.19E-07 | 3.39E-06 | yes | down | 11.17 | 14.25 | 11.32 | 14.21 | 14.34 | 13.94 | 12.24667 | 14.16333 |
| RUNDC3A | 0.041 | -4.60812 | 3.27E-07 | 3.47E-06 | yes | down | 0.44 | 1.09 | 0.59 | 0.08 | 0.09 | 0.04 | 0.706667 | 0.07 |
| MCP2 | 0.048 | -4.38933 | 3.49E-07 | 3.7E-06 | yes | down | 2.09 | 9.29 | 5.82 | 0.42 | 0.53 | 0.97 | 5.733333 | 0.64 |
| LOC100349551 | 0.289 | -1.79008 | 3.49E-07 | 3.7E-06 | yes | down | 754.82 | 404.15 | 409.01 | 279.1 | 519.11 | 268.02 | 522.66 | 355.41 |
| MRC2 | 2.763 | 1.466429 | 3.53E-07 | 3.74E-06 | yes | up | 6.27 | 4.39 | 2.34 | 27.35 | 27.65 | 28.89 | 4.333333 | 27.96333 |
| ZFP36 | 2.675 | 1.419585 | 3.64E-07 | 3.86E-06 | yes | up | 4.23 | 3.82 | 3.58 | 20.22 | 21.36 | 32.92 | 3.876667 | 24.83333 |
| RBP4 | 4.014 | 2.005014 | 3.64E-07 | 3.86E-06 | yes | up | 64.82 | 75.27 | 67.81 | 379.22 | 481.12 | 1119.11 | 69.3 | 659.8167 |
| CYGB | 2.15 | 1.104137 | 3.66E-07 | 3.87E-06 | yes | up | 4.35 | 5.89 | 5.19 | 28.7 | 27.11 | 22.06 | 5.143333 | 25.95667 |
| GRIN3A | 3.07 | 1.618149 | 3.81E-07 | 4.02E-06 | yes | up | 0.56 | 0.29 | 0.34 | 2.54 | 3.39 | 2.74 | 0.396667 | 2.89 |
| CDH13 | 2.073 | 1.052056 | 3.9E-07 | 4.11E-06 | yes | up | 32.13 | 22.26 | 18.18 | 140.12 | 115.75 | 102.52 | 24.19 | 119.4633 |
| CA2 | 0.363 | -1.46314 | 3.94E-07 | 4.15E-06 | yes | down | 13.37 | 16.11 | 18.87 | 19.55 | 11.96 | 12.45 | 16.11667 | 14.65333 |
| CLEC4G | 3.697 | 1.886301 | 3.98E-07 | 4.19E-06 | yes | up | 1.16 | 0.55 | 0.76 | 6.6 | 6.98 | 8.15 | 0.823333 | 7.243333 |
| SLIT3 | 2.056 | 1.040074 | 4.25E-07 | 4.46E-06 | yes | up | 2.84 | 3.93 | 2.46 | 17.18 | 12.05 | 16.26 | 3.076667 | 15.16333 |
| RPL18A | 0.47 | -1.08804 | 4.26E-07 | 4.47E-06 | yes | down | 312.06 | 367.41 | 387.28 | 509.02 | 371.67 | 355.4 | 355.5833 | 412.03 |
| GBAS | 0.264 | -1.92314 | 4.33E-07 | 4.53E-06 | yes | down | 102.83 | 50.86 | 34.49 | 36.24 | 50.95 | 28.2 | 62.72667 | 38.46333 |
| SLC15A3 | 2.76 | 1.464851 | 4.37E-07 | 4.57E-06 | yes | up | 1.22 | 1.6 | 1.37 | 8.86 | 11.11 | 7.82 | 1.396667 | 9.263333 |
| SAMD10 | 5.904 | 2.561667 | 4.46E-07 | 4.66E-06 | yes | up | 0.25 | 0.33 | 0.26 | 3.94 | 3.9 | 4.16 | 0.28 | 4 |
| ESPL1 | 3.442 | 1.783404 | 4.5E-07 | 4.7E-06 | yes | up | 0.31 | 0.18 | 0.2 | 2.38 | 1.87 | 1.39 | 0.23 | 1.88 |
| ADORA2A | 0.232 | -2.10906 | 4.52E-07 | 4.72E-06 | yes | down | 4.57 | 6.22 | 9.52 | 5.52 | 2.94 | 2.68 | 6.77 | 3.713333 |
| TSPAN8 | 0.057 | -4.12898 | 4.58E-07 | 4.78E-06 | yes | down | 0.39 | 1.91 | 0.92 | 0.06 | 0.09 | 0.29 | 1.073333 | 0.146667 |
| HOXA1 | 2.382 | 1.252409 | 4.59E-07 | 4.78E-06 | yes | up | 1.41 | 1.7 | 1.6 | 10.96 | 10.4 | 8.74 | 1.57 | 10.03333 |
| ZNF862 | 2.582 | 1.368704 | 4.62E-07 | 4.81E-06 | yes | up | 2.41 | 1.77 | 1.28 | 7.78 | 6.99 | 10.42 | 1.82 | 8.396667 |
| LSS | 3.547 | 1.826735 | 4.62E-07 | 4.81E-06 | yes | up | 6.13 | 6.47 | 5.08 | 74.43 | 22.54 | 54.44 | 5.893333 | 50.47 |
| IL17RE | 17.521 | 4.131047 | 4.63E-07 | 4.81E-06 | yes | up | 0.02 | 0.07 | 0.02 | 1.92 | 1.43 | 1.16 | 0.036667 | 1.503333 |
| SUCNR1 | 0.29 | -1.78762 | 4.63E-07 | 4.81E-06 | yes | down | 15.06 | 26.94 | 27.05 | 24.91 | 12.26 | 12.63 | 23.01667 | 16.6 |
| CARMIL1 | 2.228 | 1.155934 | 4.93E-07 | 5.12E-06 | yes | up | 0.83 | 0.88 | 0.79 | 4.48 | 3.88 | 5.12 | 0.833333 | 4.493333 |
| SKA3 | 2.561 | 1.356966 | 4.95E-07 | 5.13E-06 | yes | up | 0.6 | 0.77 | 0.36 | 3.96 | 3.51 | 2.33 | 0.576667 | 3.266667 |
| ABI3BP | 3.679 | 1.879158 | 5.04E-07 | 5.22E-06 | yes | up | 5.35 | 3.23 | 1.83 | 21.11 | 27.2 | 37.67 | 3.47 | 28.66 |
| HPCA | 0.008 | -7.00481 | 5.05E-07 | 5.23E-06 | yes | down | 0.47 | 1.42 | 1.34 | 0 | 0.06 | 0 | 1.076667 | 0.02 |
| YAE1D1 | 0.493 | -1.01995 | 5.2E-07 | 5.37E-06 | yes | down | 13.45 | 14.18 | 14.22 | 18.55 | 16.22 | 15.32 | 13.95 | 16.69667 |
| LOC100353852 | 2.445 | 1.289632 | 5.21E-07 | 5.37E-06 | yes | up | 1.83 | 2.86 | 1.64 | 15.13 | 12.1 | 9.86 | 2.11 | 12.36333 |
| RELB | 4.27 | 2.094145 | 5.36E-07 | 5.52E-06 | yes | up | 0.8 | 0.39 | 0.6 | 6.4 | 5.61 | 6.36 | 0.596667 | 6.123333 |
| LOC108175364 | 2.43 | 1.280785 | 5.39E-07 | 5.55E-06 | yes | up | 3.9 | 3.61 | 2.8 | 21.74 | 20.47 | 28.61 | 3.436667 | 23.60667 |
| C1H9orf153 | 0.322 | -1.63602 | 5.46E-07 | 5.62E-06 | yes | down | 3.49 | 5.66 | 4.54 | 3.81 | 2.32 | 4.54 | 4.563333 | 3.556667 |
| LONRF1 | 2.524 | 1.335524 | 5.48E-07 | 5.63E-06 | yes | up | 2.7 | 2.5 | 1.61 | 11.66 | 16.64 | 12.15 | 2.27 | 13.48333 |
| TBCCD1 | 0.244 | -2.03672 | 5.49E-07 | 5.64E-06 | yes | down | 5.78 | 6.84 | 13 | 5.03 | 4.09 | 7.1 | 8.54 | 5.406667 |
| RUNX2 | 4.881 | 2.287217 | 5.69E-07 | 5.84E-06 | yes | up | 0.19 | 0.14 | 0.1 | 2.52 | 1.38 | 1.19 | 0.143333 | 1.696667 |
| LOC100351163 | 3.54 | 1.823886 | 6.32E-07 | 6.45E-06 | yes | up | 3.2 | 3.28 | 4.04 | 41.21 | 32.77 | 18.31 | 3.506667 | 30.76333 |
| MFAP5 | 2.432 | 1.2824 | 6.42E-07 | 6.55E-06 | yes | up | 79.78 | 51.6 | 34.3 | 303.78 | 314.31 | 324.46 | 55.22667 | 314.1833 |
| LOC108176869 | 4.356 | 2.123007 | 6.45E-07 | 6.57E-06 | yes | up | 0.32 | 0.24 | 0.1 | 1.9 | 2.57 | 2.15 | 0.22 | 2.206667 |
| SLC3A2 | 0.474 | -1.078 | 6.52E-07 | 6.64E-06 | yes | down | 25.61 | 38.63 | 22.66 | 31.83 | 34.48 | 31.56 | 28.96667 | 32.62333 |
| DCDC2C | 0.005 | -7.51766 | 6.53E-07 | 6.65E-06 | yes | down | 0.66 | 42.49 | 4.28 | 0.04 | 0.25 | 0.16 | 15.81 | 0.15 |
| IRF3 | 2.109 | 1.076689 | 6.62E-07 | 6.72E-06 | yes | up | 11.97 | 13.2 | 8.02 | 48.4 | 39.86 | 51.39 | 11.06333 | 46.55 |
| FABP7 | 23.809 | 4.57343 | 6.63E-07 | 6.72E-06 | yes | up | 6.75 | 4.6 | 12.19 | 907.09 | 89.54 | 458.69 | 7.846667 | 485.1067 |
| TRIM8 | 2.144 | 1.10021 | 6.66E-07 | 6.75E-06 | yes | up | 5.23 | 4.29 | 4.19 | 20.87 | 28.02 | 21.16 | 4.57 | 23.35 |
| WFS1 | 0.325 | -1.61986 | 6.67E-07 | 6.76E-06 | yes | down | 7.77 | 5.33 | 8.26 | 7.68 | 4.17 | 5.28 | 7.12 | 5.71 |
| LOC100353349 | 0.318 | -1.65357 | 6.7E-07 | 6.78E-06 | yes | down | 6.02 | 4.7 | 2.53 | 2.67 | 4.1 | 3.04 | 4.416667 | 3.27 |
| LOC100343468 | 0.184 | -2.44517 | 6.7E-07 | 6.78E-06 | yes | down | 1.25 | 0.89 | 2.07 | 0.54 | 0.56 | 0.81 | 1.403333 | 0.636667 |
| MAN2B2 | 2.771 | 1.470588 | 6.71E-07 | 6.78E-06 | yes | up | 3.69 | 2.97 | 3.09 | 22.97 | 17.62 | 24.68 | 3.25 | 21.75667 |
| MAMLD1 | 2.622 | 1.390713 | 6.73E-07 | 6.8E-06 | yes | up | 1.4 | 1.69 | 1.3 | 8.86 | 6.55 | 12.41 | 1.463333 | 9.273333 |
| FAM3D | 29.313 | 4.873466 | 7.1E-07 | 7.17E-06 | yes | up | 0.03 | 0.15 | 0 | 6.44 | 3.53 | 4.24 | 0.06 | 4.736667 |
| C18H10orf35 | 4.019 | 2.006672 | 7.29E-07 | 7.35E-06 | yes | up | 1.31 | 0.97 | 1.13 | 14.15 | 6.93 | 11.41 | 1.136667 | 10.83 |
| SMAD6 | 0.357 | -1.48526 | 7.35E-07 | 7.41E-06 | yes | down | 3.01 | 3.5 | 4.28 | 3.74 | 2.66 | 3.07 | 3.596667 | 3.156667 |
| TMEM170B | 2.147 | 1.102091 | 7.36E-07 | 7.41E-06 | yes | up | 4.54 | 3.38 | 2.71 | 18.41 | 21.31 | 19.66 | 3.543333 | 19.79333 |
| TMTC1 | 2.262 | 1.177886 | 7.41E-07 | 7.46E-06 | yes | up | 1.86 | 1.39 | 1.48 | 8.34 | 6.76 | 10.58 | 1.576667 | 8.56 |
| PDGFRA | 2.66 | 1.411278 | 7.82E-07 | 7.84E-06 | yes | up | 4.8 | 2.7 | 2.37 | 19.98 | 16.68 | 25.67 | 3.29 | 20.77667 |
| CD83 | 9.418 | 3.235482 | 7.87E-07 | 7.89E-06 | yes | up | 0.24 | 0.22 | 0.11 | 8.06 | 2.73 | 2.2 | 0.19 | 4.33 |
| WEE1 | 3.17 | 1.664273 | 7.9E-07 | 7.91E-06 | yes | up | 2.25 | 2.26 | 0.91 | 11 | 15.09 | 13.95 | 1.806667 | 13.34667 |
| MRPL48 | 0.385 | -1.37877 | 7.91E-07 | 7.91E-06 | yes | down | 25.7 | 16.58 | 16.71 | 15.83 | 16.58 | 23.15 | 19.66333 | 18.52 |
| CNBD2 | 0.209 | -2.26148 | 7.93E-07 | 7.93E-06 | yes | down | 0.97 | 1.36 | 1.32 | 0.75 | 0.49 | 0.61 | 1.216667 | 0.616667 |
| PRDM16 | 0.206 | -2.2775 | 7.99E-07 | 7.99E-06 | yes | down | 1.34 | 1.42 | 0.65 | 0.69 | 0.37 | 0.6 | 1.136667 | 0.553333 |
| NT5C3B | 0.322 | -1.63435 | 8.05E-07 | 8.04E-06 | yes | down | 31.38 | 21.16 | 39.14 | 31.46 | 23.71 | 18 | 30.56 | 24.39 |
| LOC108178025 | 3.39 | 1.761139 | 8.05E-07 | 8.04E-06 | yes | up | 1.64 | 1.7 | 1.46 | 10.18 | 11.44 | 17.33 | 1.6 | 12.98333 |
| MRPS26 | 0.467 | -1.09849 | 8.16E-07 | 8.14E-06 | yes | down | 34.16 | 40.99 | 40.42 | 39.18 | 48.56 | 42.85 | 38.52333 | 43.53 |
| ADAMTS7 | 2.139 | 1.096602 | 8.19E-07 | 8.16E-06 | yes | up | 2.07 | 2.03 | 1.8 | 13.38 | 9.22 | 7.93 | 1.966667 | 10.17667 |
| WDR54 | 0.325 | -1.62212 | 8.26E-07 | 8.22E-06 | yes | down | 4.49 | 6.03 | 4.25 | 5.16 | 3.55 | 2.6 | 4.923333 | 3.77 |
| ARL6IP4 | 0.377 | -1.40613 | 8.32E-07 | 8.28E-06 | yes | down | 39.35 | 29.59 | 25.38 | 29.46 | 18.93 | 35.2 | 31.44 | 27.86333 |
| FGL2 | 2.514 | 1.329709 | 8.39E-07 | 8.34E-06 | yes | up | 12.97 | 11.7 | 14.95 | 60.99 | 91.8 | 87.42 | 13.20667 | 80.07 |
| UCP1 | 0.002 | -9.33964 | 8.77E-07 | 8.71E-06 | yes | down | 4146.2 | 7225.82 | 4811.64 | 0.1 | 0.67 | 58.09 | 5394.553 | 19.62 |
| ATXN1 | 0.222 | -2.17249 | 8.86E-07 | 8.78E-06 | yes | down | 9.23 | 19.37 | 4.59 | 7.76 | 4.82 | 7.2 | 11.06333 | 6.593333 |
| GADD45G | 0.249 | -2.00816 | 8.89E-07 | 8.81E-06 | yes | down | 30.19 | 52 | 55.04 | 16.08 | 37.17 | 29.33 | 45.74333 | 27.52667 |
| NDUFAF4 | 0.333 | -1.58734 | 8.93E-07 | 8.84E-06 | yes | down | 6.21 | 7.69 | 3.53 | 3.6 | 4.54 | 5.48 | 5.81 | 4.54 |
| LOC103346738 | 2.429 | 1.280222 | 8.97E-07 | 8.88E-06 | yes | up | 0.8 | 0.6 | 0.75 | 5.59 | 3.16 | 4.03 | 0.716667 | 4.26 |
| MARS2 | 0.239 | -2.06349 | 9.01E-07 | 8.91E-06 | yes | down | 4.29 | 5.38 | 2.14 | 1.34 | 2.01 | 3.2 | 3.936667 | 2.183333 |
| BCAT2 | 4.25 | 2.087377 | 9.02E-07 | 8.91E-06 | yes | up | 8.53 | 3.81 | 3.2 | 42.73 | 76.48 | 34.24 | 5.18 | 51.15 |
| LOC100352781 | 0.003 | -8.55166 | 9.03E-07 | 8.91E-06 | yes | down | 0.23 | 13.67 | 1.69 | 0 | 0 | 0.01 | 5.196667 | 0.003333 |
| LOC103350429 | 118.796 | 6.892344 | 9.03E-07 | 8.91E-06 | yes | up | 0 | 0 | 0 | 2.01 | 0.75 | 2.4 | 0 | 1.72 |
| GPR85 | 4.147 | 2.051906 | 9.08E-07 | 8.95E-06 | yes | up | 0.69 | 0.61 | 1.04 | 9.43 | 4.58 | 5.76 | 0.78 | 6.59 |
| NEURL1B | 3.021 | 1.594892 | 9.25E-07 | 9.11E-06 | yes | up | 2.46 | 2.95 | 2.03 | 27.93 | 13.08 | 13.62 | 2.48 | 18.21 |
| KEL | 0.02 | -5.66978 | 9.27E-07 | 9.13E-06 | yes | down | 0.27 | 0.94 | 0.64 | 0 | 0 | 0.09 | 0.616667 | 0.03 |
| DCLK1 | 2.728 | 1.44776 | 9.31E-07 | 9.15E-06 | yes | up | 3.22 | 1.8 | 1.23 | 13.68 | 12.4 | 13.5 | 2.083333 | 13.19333 |
| LOC103346861 | 2.324 | 1.216447 | 9.52E-07 | 9.35E-06 | yes | up | 3.65 | 2.41 | 3.41 | 22.62 | 12.17 | 14.59 | 3.156667 | 16.46 |
| LOC100340875 | 0.478 | -1.06613 | 9.8E-07 | 9.59E-06 | yes | down | 42.8 | 51.44 | 52.33 | 57.82 | 54.63 | 59.04 | 48.85667 | 57.16333 |
| ACAD10 | 0.41 | -1.28795 | 9.91E-07 | 9.68E-06 | yes | down | 14.84 | 14.76 | 17.36 | 15.75 | 11.3 | 19.9 | 15.65333 | 15.65 |
| LOC100346229 | 4.501 | 2.17021 | 1E-06 | 9.75E-06 | yes | up | 1.17 | 0.35 | 0.65 | 10.45 | 7.8 | 5.2 | 0.723333 | 7.816667 |
| TEK | 0.402 | -1.31459 | 1.01E-06 | 9.81E-06 | yes | down | 33.19 | 48.8 | 52.4 | 49.58 | 46.9 | 35.32 | 44.79667 | 43.93333 |
| LOC108176017 | 0.02 | -5.62433 | 1.01E-06 | 9.83E-06 | yes | down | 8.01 | 0.96 | 2.34 | 0.38 | 0 | 0.12 | 3.77 | 0.166667 |
| B3GALNT1 | 3.749 | 1.906489 | 1.03E-06 | 1E-05 | yes | up | 0.91 | 1.2 | 1 | 5.61 | 9.68 | 12.07 | 1.036667 | 9.12 |
| PARP3 | 2.188 | 1.129474 | 1.03E-06 | 1.01E-05 | yes | up | 3.62 | 3.15 | 2.44 | 16.83 | 12.29 | 18.99 | 3.07 | 16.03667 |
| LACE1 | 0.455 | -1.13599 | 1.08E-06 | 1.05E-05 | yes | down | 11.58 | 16.08 | 11.42 | 14.32 | 11.45 | 17.17 | 13.02667 | 14.31333 |
| MLH3 | 2.049 | 1.034949 | 1.1E-06 | 1.06E-05 | yes | up | 0.88 | 1.11 | 0.96 | 5.13 | 5.13 | 4.43 | 0.983333 | 4.896667 |
| ELOVL2 | 0.247 | -2.0201 | 1.1E-06 | 1.07E-05 | yes | down | 4.16 | 7.08 | 5.31 | 3.83 | 4.48 | 1.52 | 5.516667 | 3.276667 |
| NAALADL2 | 2.632 | 1.396274 | 1.12E-06 | 1.08E-05 | yes | up | 0.37 | 0.4 | 0.27 | 1.88 | 2.66 | 1.92 | 0.346667 | 2.153333 |
| LOC103348967 | 5.02 | 2.327767 | 1.12E-06 | 1.08E-05 | yes | up | 0.36 | 0.41 | 0.19 | 2.83 | 3.76 | 4.92 | 0.32 | 3.836667 |
| CCDC30 | 4.909 | 2.295412 | 1.15E-06 | 1.11E-05 | yes | up | 0.25 | 0.16 | 0.17 | 2.11 | 2.08 | 2.68 | 0.193333 | 2.29 |
| TMEM120A | 0.387 | -1.36884 | 1.17E-06 | 1.12E-05 | yes | down | 23.19 | 21.48 | 18.04 | 17.54 | 21.62 | 27.13 | 20.90333 | 22.09667 |
| LIMA1 | 2.079 | 1.055792 | 1.17E-06 | 1.13E-05 | yes | up | 8.04 | 6.12 | 4.67 | 29.83 | 26.68 | 36.1 | 6.276667 | 30.87 |
| LTBP2 | 0.363 | -1.46208 | 1.18E-06 | 1.13E-05 | yes | down | 1.1 | 2.05 | 1.09 | 1.77 | 1.13 | 1.34 | 1.413333 | 1.413333 |
| LOC103347657 | 8.295 | 3.052273 | 1.18E-06 | 1.14E-05 | yes | up | 2.31 | 0.59 | 0.57 | 23.07 | 28.27 | 16 | 1.156667 | 22.44667 |
| LOC103348799 | 0.054 | -4.20904 | 1.18E-06 | 1.14E-05 | yes | down | 1.15 | 2.32 | 10.03 | 1.08 | 0.41 | 0.45 | 4.5 | 0.646667 |
| LOC100338714 | 0.381 | -1.3924 | 1.19E-06 | 1.15E-05 | yes | down | 396.41 | 708.29 | 660.4 | 596.32 | 562.28 | 538.88 | 588.3667 | 565.8267 |
| CSPG4 | 2.456 | 1.296461 | 1.2E-06 | 1.16E-05 | yes | up | 1.74 | 1.01 | 1.41 | 10.29 | 7.63 | 6.9 | 1.386667 | 8.273333 |
| IL1RAPL1 | 4.787 | 2.259271 | 1.2E-06 | 1.16E-05 | yes | up | 0.05 | 0.11 | 0.15 | 1.34 | 1.23 | 0.98 | 0.103333 | 1.183333 |
| DIS3L | 3.694 | 1.885256 | 1.21E-06 | 1.16E-05 | yes | up | 0.41 | 0.27 | 0.51 | 4.67 | 2.83 | 3.28 | 0.396667 | 3.593333 |
| KANK1 | 2.667 | 1.415182 | 1.23E-06 | 1.18E-05 | yes | up | 4.37 | 3.03 | 3.83 | 23.68 | 16.57 | 32.04 | 3.743333 | 24.09667 |
| LOC100343291 | 2.666 | 1.414896 | 1.28E-06 | 1.23E-05 | yes | up | 0.59 | 0.83 | 0.65 | 3.88 | 4.96 | 4.36 | 0.69 | 4.4 |
| ADCYAP1 | 0.015 | -6.04934 | 1.29E-06 | 1.23E-05 | yes | down | 0.51 | 0.93 | 0.27 | 0 | 0.06 | 0 | 0.57 | 0.02 |
| PRICKLE2 | 2.118 | 1.082771 | 1.31E-06 | 1.25E-05 | yes | up | 0.66 | 0.68 | 0.4 | 3.21 | 2.81 | 2.72 | 0.58 | 2.913333 |
| ACAT2 | 3.654 | 1.869504 | 1.32E-06 | 1.26E-05 | yes | up | 21.02 | 27.61 | 27.62 | 396.95 | 125.08 | 173.31 | 25.41667 | 231.78 |
| POLR3G | 3.59 | 1.844051 | 1.33E-06 | 1.27E-05 | yes | up | 0.31 | 0.22 | 0.28 | 2.34 | 2.25 | 2.43 | 0.27 | 2.34 |
| ARSK | 3.049 | 1.608411 | 1.37E-06 | 1.3E-05 | yes | up | 1.3 | 2.07 | 0.95 | 9.91 | 11.32 | 9.08 | 1.44 | 10.10333 |
| METAP1D | 0.406 | -1.30219 | 1.37E-06 | 1.3E-05 | yes | down | 5.92 | 7.7 | 5.51 | 6.99 | 6.89 | 4.71 | 6.376667 | 6.196667 |
| CSK | 2.196 | 1.134758 | 1.39E-06 | 1.32E-05 | yes | up | 15 | 16 | 12.43 | 92.01 | 53.42 | 87.63 | 14.47667 | 77.68667 |
| GPATCH4 | 0.372 | -1.42635 | 1.39E-06 | 1.32E-05 | yes | down | 6.12 | 8.93 | 5.61 | 4.58 | 6.59 | 7.16 | 6.886667 | 6.11 |
| NDN | 0.441 | -1.18249 | 1.46E-06 | 1.38E-05 | yes | down | 49 | 29.39 | 27.17 | 44.34 | 29.66 | 37.29 | 35.18667 | 37.09667 |
| NOX5 | 8.368 | 3.064879 | 1.56E-06 | 1.47E-05 | yes | up | 0.44 | 0.33 | 0.22 | 5.77 | 1.92 | 10.28 | 0.33 | 5.99 |
| LOC108176476 | 5.99 | 2.582474 | 1.57E-06 | 1.48E-05 | yes | up | 0.08 | 0.16 | 0.03 | 1.1 | 1.3 | 1.42 | 0.09 | 1.273333 |
| KIF14 | 6.872 | 2.780727 | 1.58E-06 | 1.48E-05 | yes | up | 0.34 | 0.21 | 0.07 | 4.05 | 4.24 | 1.33 | 0.206667 | 3.206667 |
| AHCYL2 | 2.196 | 1.134624 | 1.59E-06 | 1.49E-05 | yes | up | 3.38 | 2.38 | 1.66 | 13.65 | 12.46 | 12.4 | 2.473333 | 12.83667 |
| CPM | 2.769 | 1.469164 | 1.65E-06 | 1.55E-05 | yes | up | 2.17 | 1.45 | 2.1 | 17.82 | 10.28 | 10.75 | 1.906667 | 12.95 |
| ATF6 | 0.461 | -1.11628 | 1.69E-06 | 1.58E-05 | yes | down | 8.85 | 11.33 | 11.6 | 10.84 | 11.51 | 13.17 | 10.59333 | 11.84 |
| LOC100339982 | 2.438 | 1.285487 | 1.69E-06 | 1.58E-05 | yes | up | 1.27 | 1.37 | 1.21 | 7.43 | 6.51 | 8.58 | 1.283333 | 7.506667 |
| COL6A2 | 2.556 | 1.353812 | 1.71E-06 | 1.59E-05 | yes | up | 117.98 | 72.97 | 56.46 | 601.53 | 620.21 | 315.49 | 82.47 | 512.41 |
| LOC100347331 | 5.277 | 2.399614 | 1.72E-06 | 1.6E-05 | yes | up | 0.4 | 0.31 | 0.4 | 4.06 | 5.05 | 4.93 | 0.37 | 4.68 |
| PRUNE2 | 17.254 | 4.108856 | 1.73E-06 | 1.61E-05 | yes | up | 0.78 | 0.47 | 0.13 | 22.58 | 5.17 | 27.95 | 0.46 | 18.56667 |
| MITF | 2.133 | 1.093208 | 1.74E-06 | 1.62E-05 | yes | up | 3.72 | 2.53 | 2.49 | 18.76 | 12.47 | 14.31 | 2.913333 | 15.18 |
| SDC2 | 2.378 | 1.249556 | 1.78E-06 | 1.66E-05 | yes | up | 13.01 | 14.83 | 15.59 | 62.52 | 86.23 | 99.81 | 14.47667 | 82.85333 |
| GCHFR | 0.298 | -1.74537 | 1.83E-06 | 1.7E-05 | yes | down | 9.83 | 18.69 | 15.99 | 13.41 | 12.06 | 7.02 | 14.83667 | 10.83 |
| GNPTAB | 2.882 | 1.527163 | 1.84E-06 | 1.7E-05 | yes | up | 2.84 | 3.32 | 2.03 | 11.48 | 20.63 | 23.2 | 2.73 | 18.43667 |
| PIP5KL1 | 3.178 | 1.668026 | 1.87E-06 | 1.73E-05 | yes | up | 1.19 | 0.86 | 0.4 | 7.78 | 5.57 | 5.83 | 0.816667 | 6.393333 |
| LOXL2 | 2.583 | 1.3689 | 1.89E-06 | 1.75E-05 | yes | up | 42.65 | 26.3 | 21.02 | 172.75 | 231.53 | 143.58 | 29.99 | 182.62 |
| BZW2 | 0.286 | -1.80548 | 1.9E-06 | 1.76E-05 | yes | down | 72.55 | 36.8 | 19.01 | 26.53 | 36.66 | 25 | 42.78667 | 29.39667 |
| YEATS4 | 0.422 | -1.24417 | 1.96E-06 | 1.81E-05 | yes | down | 11.16 | 7.06 | 10.46 | 9.48 | 10.84 | 9.61 | 9.56 | 9.976667 |
| TGFBR3 | 2.09 | 1.063503 | 1.96E-06 | 1.81E-05 | yes | up | 10.57 | 10.99 | 13.76 | 58.47 | 59.83 | 62.15 | 11.77333 | 60.15 |
| DAAM2 | 2.565 | 1.358833 | 2E-06 | 1.84E-05 | yes | up | 4.25 | 7.44 | 3.26 | 34.87 | 23.25 | 31.68 | 4.983333 | 29.93333 |
| PGBD2 | 2.056 | 1.039857 | 2.04E-06 | 1.88E-05 | yes | up | 2.64 | 3.13 | 2.11 | 11.88 | 10.54 | 13.68 | 2.626667 | 12.03333 |
| JUN | 2.706 | 1.436229 | 2.04E-06 | 1.88E-05 | yes | up | 24.57 | 19.02 | 17.52 | 100.4 | 103.69 | 190.35 | 20.37 | 131.48 |
| MMP16 | 0.481 | -1.0563 | 2.08E-06 | 1.91E-05 | yes | down | 1.38 | 1.16 | 1.12 | 1.77 | 1.25 | 1.21 | 1.22 | 1.41 |
| HOGA1 | 0.478 | -1.06561 | 2.1E-06 | 1.93E-05 | yes | down | 13.11 | 12.3 | 12.86 | 14.44 | 11.11 | 17.37 | 12.75667 | 14.30667 |
| IMMP2L | 0.43 | -1.21916 | 2.11E-06 | 1.94E-05 | yes | down | 7.57 | 6.67 | 5.11 | 5.61 | 5.51 | 7.63 | 6.45 | 6.25 |
| SLC25A5 | 0.386 | -1.37327 | 2.12E-06 | 1.95E-05 | yes | down | 701.85 | 505.62 | 910.98 | 788.94 | 646.4 | 588.22 | 706.15 | 674.52 |
| LOC103346986 | 4.744 | 2.246236 | 2.14E-06 | 1.96E-05 | yes | up | 0.32 | 0.2 | 0.14 | 3.07 | 2.52 | 2.22 | 0.22 | 2.603333 |
| IL1R1 | 2.799 | 1.484844 | 2.16E-06 | 1.98E-05 | yes | up | 5.02 | 5.05 | 4.35 | 25.2 | 46.56 | 23.01 | 4.806667 | 31.59 |
| ZNF70 | 0.481 | -1.056 | 2.17E-06 | 1.98E-05 | yes | down | 2.23 | 2.82 | 2.47 | 2.74 | 2.86 | 3.11 | 2.506667 | 2.903333 |
| PTPN1 | 0.47 | -1.08878 | 2.17E-06 | 1.98E-05 | yes | down | 8.69 | 12.77 | 6.87 | 11.3 | 9.01 | 11.22 | 9.443333 | 10.51 |
| MME | 3.191 | 1.673785 | 2.18E-06 | 1.99E-05 | yes | up | 24.67 | 11.12 | 14.02 | 145.01 | 156.48 | 77.28 | 16.60333 | 126.2567 |
| PCBD1 | 2.033 | 1.023576 | 2.18E-06 | 1.99E-05 | yes | up | 15.04 | 13.08 | 11.29 | 70.03 | 49.05 | 73.33 | 13.13667 | 64.13667 |
| DLGAP2 | 0.014 | -6.15789 | 2.19E-06 | 1.99E-05 | yes | down | 0.1 | 0.44 | 0.46 | 0 | 0.02 | 0.02 | 0.333333 | 0.013333 |
| CD96 | 8.983 | 3.167178 | 2.2E-06 | 2E-05 | yes | up | 0.3 | 0.06 | 0.07 | 2.9 | 1.57 | 2.02 | 0.143333 | 2.163333 |
| KDR | 0.41 | -1.28777 | 2.22E-06 | 2.01E-05 | yes | down | 40.78 | 68.23 | 58.09 | 74.48 | 42.18 | 49.04 | 55.7 | 55.23333 |
| COL7A1 | 0.423 | -1.24203 | 2.25E-06 | 2.04E-05 | yes | down | 0.63 | 0.61 | 0.63 | 0.71 | 0.49 | 0.69 | 0.623333 | 0.63 |
| CADM3 | 2.51 | 1.327866 | 2.27E-06 | 2.06E-05 | yes | up | 18.59 | 14.48 | 7.31 | 75.89 | 81.62 | 81.31 | 13.46 | 79.60667 |
| KIAA2022 | 0.045 | -4.48388 | 2.28E-06 | 2.06E-05 | yes | down | 0.09 | 0.12 | 0.45 | 0.02 | 0.01 | 0.05 | 0.22 | 0.026667 |
| DOCK5 | 2.443 | 1.288389 | 2.29E-06 | 2.07E-05 | yes | up | 0.36 | 0.31 | 0.19 | 3.25 | 2.74 | 4.08 | 0.286667 | 3.356667 |
| LOC103349679 | 4.211 | 2.073994 | 2.29E-06 | 2.08E-05 | yes | up | 0.74 | 0.54 | 0.65 | 7.28 | 3.33 | 4.27 | 0.643333 | 4.96 |
| UGDH | 2.3 | 1.201518 | 2.3E-06 | 2.08E-05 | yes | up | 11.6 | 11.91 | 11.32 | 51.07 | 55.63 | 85.48 | 11.61 | 64.06 |
| CDK15 | 4.998 | 2.321477 | 2.31E-06 | 2.09E-05 | yes | up | 0.17 | 0.21 | 0.07 | 1.31 | 1.65 | 2.3 | 0.15 | 1.753333 |
| TRUB2 | 0.378 | -1.40169 | 2.44E-06 | 2.2E-05 | yes | down | 2.79 | 4.04 | 2.82 | 2.36 | 2.64 | 3.7 | 3.216667 | 2.9 |
| DOK5 | 0.093 | -3.42656 | 2.44E-06 | 2.2E-05 | yes | down | 18.24 | 3.67 | 2.94 | 1.21 | 2.68 | 1.56 | 8.283333 | 1.816667 |
| NHSL2 | 2.296 | 1.198813 | 2.44E-06 | 2.2E-05 | yes | up | 1.65 | 1.72 | 1.88 | 13.98 | 9.59 | 7.88 | 1.75 | 10.48333 |
| ALOX5 | 2.929 | 1.550631 | 2.49E-06 | 2.24E-05 | yes | up | 2.25 | 1.64 | 2.63 | 15.28 | 19.38 | 11.86 | 2.173333 | 15.50667 |
| CHRM4 | 0.01 | -6.61306 | 2.54E-06 | 2.28E-05 | yes | down | 0.55 | 0.63 | 1.18 | 0.06 | 0 | 0 | 0.786667 | 0.02 |
| PAM16 | 0.364 | -1.4564 | 2.6E-06 | 2.32E-05 | yes | down | 38.14 | 73.09 | 53.23 | 54.22 | 41.17 | 36.63 | 54.82 | 44.00667 |
| TMEM233 | 38.382 | 5.262341 | 2.61E-06 | 2.33E-05 | yes | up | 1.41 | 0.18 | 0.05 | 45.21 | 46.88 | 61.61 | 0.546667 | 51.23333 |
| HS3ST6 | 9.182 | 3.198852 | 2.64E-06 | 2.35E-05 | yes | up | 0.03 | 0.14 | 0.11 | 3.02 | 1.31 | 1.91 | 0.093333 | 2.08 |
| LOC100338274 | 0.008 | -7.02464 | 2.64E-06 | 2.35E-05 | yes | down | 1.67 | 0.21 | 0.05 | 0 | 0.02 | 0.02 | 0.643333 | 0.013333 |
| NR2F1 | 2.566 | 1.359382 | 2.65E-06 | 2.36E-05 | yes | up | 2.4 | 3.4 | 2.82 | 15.55 | 18.39 | 19.31 | 2.873333 | 17.75 |
| LOC103350713 | 0.484 | -1.04561 | 2.72E-06 | 2.42E-05 | yes | down | 46.46 | 56.95 | 57.03 | 71.62 | 68.66 | 57.82 | 53.48 | 66.03333 |
| KLHL42 | 2.379 | 1.250148 | 2.74E-06 | 2.44E-05 | yes | up | 1.76 | 1.55 | 1.23 | 6.75 | 7.64 | 10.93 | 1.513333 | 8.44 |
| RBM47 | 8.426 | 3.074898 | 2.74E-06 | 2.44E-05 | yes | up | 0.04 | 0.1 | 0.11 | 1.54 | 0.83 | 2.9 | 0.083333 | 1.756667 |
| LOC100340198 | 0.414 | -1.2733 | 2.75E-06 | 2.44E-05 | yes | down | 120.61 | 175.85 | 167.77 | 201.8 | 144.41 | 137.16 | 154.7433 | 161.1233 |
| TMEM100 | 2.458 | 1.297312 | 2.8E-06 | 2.49E-05 | yes | up | 10.16 | 11.64 | 9.42 | 51.63 | 82.23 | 48.61 | 10.40667 | 60.82333 |
| IGF2BP2 | 0.126 | -2.98821 | 2.8E-06 | 2.49E-05 | yes | down | 4.58 | 1.34 | 0.92 | 0.46 | 1.1 | 0.43 | 2.28 | 0.663333 |
| PIGL | 2.591 | 1.373643 | 2.81E-06 | 2.49E-05 | yes | up | 1.1 | 1.08 | 0.99 | 7 | 4.73 | 5.74 | 1.056667 | 5.823333 |
| TNK2 | 3.201 | 1.678528 | 2.83E-06 | 2.51E-05 | yes | up | 10.45 | 11 | 3.82 | 55.22 | 49.72 | 83.74 | 8.423333 | 62.89333 |
| FDFT1 | 2.099 | 1.069542 | 2.83E-06 | 2.51E-05 | yes | up | 29.22 | 24.14 | 29.72 | 159.1 | 102.71 | 157.19 | 27.69333 | 139.6667 |
| DBN1 | 2.377 | 1.249052 | 2.84E-06 | 2.51E-05 | yes | up | 12.56 | 8.45 | 5.23 | 48.51 | 44.76 | 50.29 | 8.746667 | 47.85333 |
| SH3BGRL2 | 3.455 | 1.788712 | 2.84E-06 | 2.51E-05 | yes | up | 0.46 | 0.44 | 0.29 | 4.98 | 3.49 | 2.08 | 0.396667 | 3.516667 |
| ACVR2A | 2.453 | 1.294472 | 2.84E-06 | 2.51E-05 | yes | up | 1.74 | 1.02 | 1.34 | 10.22 | 7.69 | 6.46 | 1.366667 | 8.123333 |
| TPD52L1 | 0.2 | -2.31857 | 2.88E-06 | 2.54E-05 | yes | down | 21.54 | 14.67 | 19.11 | 2.29 | 11.6 | 10.39 | 18.44 | 8.093333 |
| FAM107A | 2.338 | 1.224995 | 2.94E-06 | 2.59E-05 | yes | up | 8.4 | 9.65 | 10.21 | 41.47 | 63.18 | 53.33 | 9.42 | 52.66 |
| MMRN2 | 2.338 | 1.225066 | 2.94E-06 | 2.59E-05 | yes | up | 5.9 | 7.99 | 8.65 | 55.72 | 36.52 | 37.29 | 7.513333 | 43.17667 |
| CD209 | 3.243 | 1.697249 | 2.95E-06 | 2.59E-05 | yes | up | 4.74 | 2.88 | 1.58 | 22.24 | 28.07 | 19.39 | 3.066667 | 23.23333 |
| LOC100346827 | 0.413 | -1.2757 | 3E-06 | 2.63E-05 | yes | down | 10.94 | 15.24 | 9.21 | 11.52 | 10.58 | 13.9 | 11.79667 | 12 |
| TRPT1 | 0.495 | -1.01562 | 3.07E-06 | 2.69E-05 | yes | down | 14.59 | 16.99 | 17.47 | 22.44 | 15.86 | 18.9 | 16.35 | 19.06667 |
| LOC108177728 | 77.282 | 6.272065 | 3.11E-06 | 2.72E-05 | yes | up | 0.19 | 0 | 0 | 4.21 | 9.31 | 7.66 | 0.063333 | 7.06 |
| PAK1IP1 | 0.463 | -1.11053 | 3.16E-06 | 2.76E-05 | yes | down | 6.61 | 6.9 | 7.13 | 6.86 | 8.25 | 7.96 | 6.88 | 7.69 |
| SLC27A2 | 0.012 | -6.39567 | 3.2E-06 | 2.79E-05 | yes | down | 0.43 | 0.14 | 1.17 | 0 | 0.03 | 0.03 | 0.58 | 0.02 |
| LOC100349768 | 0.269 | -1.8921 | 3.24E-06 | 2.83E-05 | yes | down | 986.6 | 374.03 | 287.72 | 300.69 | 425.97 | 303.46 | 549.45 | 343.3733 |
| SLC17A9 | 4.056 | 2.01995 | 3.31E-06 | 2.88E-05 | yes | up | 0.4 | 0.26 | 0.19 | 2.51 | 2.42 | 2.8 | 0.283333 | 2.576667 |
| EEF2KMT | 0.206 | -2.28059 | 3.35E-06 | 2.91E-05 | yes | down | 2.43 | 3.95 | 2.5 | 2.22 | 0.65 | 1.57 | 2.96 | 1.48 |
| LACTB2 | 0.457 | -1.13021 | 3.38E-06 | 2.94E-05 | yes | down | 32.21 | 26.49 | 36.28 | 32.82 | 35.81 | 38.9 | 31.66 | 35.84333 |
| ADGRG6 | 2.198 | 1.135923 | 3.44E-06 | 2.98E-05 | yes | up | 1.94 | 1.74 | 1 | 9 | 6.47 | 7.74 | 1.56 | 7.736667 |
| CD74 | 2.901 | 1.536465 | 3.45E-06 | 2.99E-05 | yes | up | 8.4 | 9.16 | 13.97 | 101.97 | 56.26 | 70.47 | 10.51 | 76.23333 |
| ENPP2 | 2.536 | 1.342542 | 3.53E-06 | 3.06E-05 | yes | up | 3.03 | 2.07 | 1.57 | 12.33 | 16.81 | 11.36 | 2.223333 | 13.5 |
| LOC100355134 | 0.356 | -1.49045 | 3.54E-06 | 3.07E-05 | yes | down | 5.36 | 4.07 | 2.44 | 2.81 | 3.58 | 3.51 | 3.956667 | 3.3 |
| LOC100350701 | 2.041 | 1.029038 | 3.55E-06 | 3.07E-05 | yes | up | 9.78 | 11.93 | 10.05 | 53.74 | 49.97 | 59.79 | 10.58667 | 54.5 |
| MRM2 | 0.466 | -1.10172 | 3.57E-06 | 3.08E-05 | yes | down | 5.94 | 6.76 | 6.63 | 7.38 | 6.99 | 7.44 | 6.443333 | 7.27 |
| PDHX | 0.499 | -1.00198 | 3.6E-06 | 3.1E-05 | yes | down | 34.82 | 24.72 | 17.92 | 30.48 | 29.74 | 31.18 | 25.82 | 30.46667 |
| LOC103348580 | 120.281 | 6.91027 | 3.61E-06 | 3.11E-05 | yes | up | 0.06 | 0.07 | 0 | 0.31 | 11.78 | 21.05 | 0.043333 | 11.04667 |
| NR4A3 | 7.238 | 2.855624 | 3.64E-06 | 3.14E-05 | yes | up | 0.11 | 0.62 | 0.16 | 2.02 | 6.6 | 3.83 | 0.296667 | 4.15 |
| LDLRAD3 | 2.657 | 1.409878 | 3.65E-06 | 3.14E-05 | yes | up | 3.34 | 3.37 | 3.28 | 15.08 | 18.57 | 29.86 | 3.33 | 21.17 |
| PXMP4 | 2.393 | 1.258724 | 3.75E-06 | 3.22E-05 | yes | up | 3.64 | 3.29 | 3.83 | 24.98 | 14.25 | 23.65 | 3.586667 | 20.96 |
| MYOF | 2.339 | 1.225979 | 3.77E-06 | 3.24E-05 | yes | up | 2.07 | 1.25 | 0.97 | 8.32 | 6.79 | 8.62 | 1.43 | 7.91 |
| ADAMTS5 | 2.315 | 1.211074 | 3.86E-06 | 3.31E-05 | yes | up | 5.9 | 6.4 | 8.62 | 51.38 | 37.62 | 36.26 | 6.973333 | 41.75333 |
| LOC103346472 | 209.69 | 7.712111 | 3.89E-06 | 3.34E-05 | yes | up | 0 | 0 | 0 | 2.06 | 0.09 | 3.62 | 0 | 1.923333 |
| FASTKD5 | 0.42 | -1.25124 | 3.9E-06 | 3.34E-05 | yes | down | 4.45 | 5.6 | 3.12 | 3.55 | 4.17 | 5.21 | 4.39 | 4.31 |
| UTP3 | 0.482 | -1.05178 | 3.96E-06 | 3.39E-05 | yes | down | 4.55 | 4.55 | 3.07 | 5.45 | 3.63 | 4.95 | 4.056667 | 4.676667 |
| CLTCL1 | 3.457 | 1.789485 | 3.97E-06 | 3.4E-05 | yes | up | 6.14 | 2.86 | 1.68 | 25.38 | 31.69 | 28.66 | 3.56 | 28.57667 |
| SGK2 | 46.142 | 5.528018 | 4.02E-06 | 3.43E-05 | yes | up | 0.01 | 0.01 | 0 | 0.67 | 0.54 | 1.35 | 0.006667 | 0.853333 |
| ADGRF5 | 0.458 | -1.12774 | 4.05E-06 | 3.46E-05 | yes | down | 41 | 60.7 | 63.52 | 76.62 | 58.55 | 62.82 | 55.07333 | 65.99667 |
| PPFIA4 | 0.037 | -4.76255 | 4.06E-06 | 3.47E-05 | yes | down | 3.01 | 0.37 | 0.17 | 0.11 | 0.18 | 0.02 | 1.183333 | 0.103333 |
| LOC103351328 | 0.093 | -3.43433 | 4.07E-06 | 3.47E-05 | yes | down | 1.56 | 1 | 1.89 | 0.15 | 0.25 | 0.6 | 1.483333 | 0.333333 |
| LOC103346166 | 0.009 | -6.72665 | 4.07E-06 | 3.47E-05 | yes | down | 0.26 | 1.34 | 1.05 | 0.06 | 0 | 0 | 0.883333 | 0.02 |
| MYH13 | 0.006 | -7.4041 | 4.11E-06 | 3.49E-05 | yes | down | 247.41 | 46.22 | 8.12 | 0.06 | 3.19 | 0.12 | 100.5833 | 1.123333 |
| ABCC5 | 0.469 | -1.09286 | 4.22E-06 | 3.59E-05 | yes | down | 8.35 | 13.44 | 5.78 | 9.68 | 7.04 | 9.22 | 9.19 | 8.646667 |
| FAM134B | 0.194 | -2.36395 | 4.26E-06 | 3.62E-05 | yes | down | 23.14 | 8.44 | 6.91 | 2.95 | 8.22 | 5.83 | 12.83 | 5.666667 |
| GTF2B | 0.42 | -1.25311 | 4.28E-06 | 3.64E-05 | yes | down | 26.62 | 23.18 | 35.51 | 28.3 | 27.98 | 31.56 | 28.43667 | 29.28 |
| SELP | 2.903 | 1.537617 | 4.37E-06 | 3.71E-05 | yes | up | 0.49 | 0.36 | 0.33 | 2.67 | 2.45 | 2.71 | 0.393333 | 2.61 |
| PGF | 5.177 | 2.372132 | 4.38E-06 | 3.71E-05 | yes | up | 1.8 | 1.1 | 0.29 | 9.76 | 15.85 | 11.84 | 1.063333 | 12.48333 |
| TLDC1 | 0.444 | -1.17048 | 4.43E-06 | 3.75E-05 | yes | down | 3.18 | 3.07 | 2.3 | 4.81 | 2.66 | 3.6 | 2.85 | 3.69 |
| WNK4 | 0.45 | -1.1508 | 4.48E-06 | 3.79E-05 | yes | down | 1.93 | 2.46 | 1.8 | 2.92 | 1.69 | 2.26 | 2.063333 | 2.29 |
| PHGDH | 2.212 | 1.145559 | 4.51E-06 | 3.81E-05 | yes | up | 32.74 | 56.37 | 41.08 | 274.96 | 238.94 | 188.16 | 43.39667 | 234.02 |
| SREBF2 | 2.56 | 1.356264 | 4.58E-06 | 3.86E-05 | yes | up | 5.87 | 3.5 | 2.55 | 31.91 | 19.31 | 21.34 | 3.973333 | 24.18667 |
| SEMA3C | 2.495 | 1.318813 | 4.59E-06 | 3.87E-05 | yes | up | 9.38 | 5.95 | 3.99 | 32.55 | 43.39 | 36.11 | 6.44 | 37.35 |
| LOC100353654 | 0.401 | -1.31797 | 4.63E-06 | 3.89E-05 | yes | down | 73.68 | 56.8 | 57.62 | 42.78 | 86.7 | 60.34 | 62.7 | 63.27333 |
| GDE1 | 0.457 | -1.12941 | 4.64E-06 | 3.9E-05 | yes | down | 8.39 | 10.55 | 9.93 | 13.42 | 8.6 | 10.89 | 9.623333 | 10.97 |
| WSCD1 | 2.124 | 1.086583 | 4.75E-06 | 3.98E-05 | yes | up | 4.56 | 4.31 | 3.5 | 21.2 | 25.46 | 16.06 | 4.123333 | 20.90667 |
| LRRN4 | 0.019 | -5.74829 | 4.77E-06 | 3.99E-05 | yes | down | 0.04 | 2.24 | 0.99 | 0 | 0.05 | 0.09 | 1.09 | 0.046667 |
| DCHS2 | 3.913 | 1.968327 | 4.77E-06 | 4E-05 | yes | up | 0.21 | 0.08 | 0.09 | 0.95 | 1.38 | 1.18 | 0.126667 | 1.17 |
| LOC103351772 | 0.137 | -2.87116 | 4.78E-06 | 4E-05 | yes | down | 64.03 | 78.18 | 119.96 | 35.6 | 16.68 | 51.18 | 87.39 | 34.48667 |
| SLC16A11 | 0.098 | -3.35161 | 4.82E-06 | 4.03E-05 | yes | down | 1.59 | 2.81 | 0.57 | 0.68 | 0.37 | 0.14 | 1.656667 | 0.396667 |
| TSPO2 | 0.068 | -3.87362 | 4.82E-06 | 4.03E-05 | yes | down | 9.81 | 12.06 | 16.47 | 0 | 4.86 | 10.66 | 12.78 | 5.173333 |
| TLR9 | 0.43 | -1.21868 | 4.88E-06 | 4.07E-05 | yes | down | 48.39 | 28.25 | 32.83 | 46.47 | 38.38 | 27.83 | 36.49 | 37.56 |
| NOSIP | 0.464 | -1.10681 | 4.94E-06 | 4.12E-05 | yes | down | 7.68 | 7.21 | 6.4 | 7.08 | 7.71 | 8.87 | 7.096667 | 7.886667 |
| ADCY10 | 2.747 | 1.457846 | 5.11E-06 | 4.26E-05 | yes | up | 0.4 | 0.48 | 0.37 | 2.59 | 2.16 | 3.69 | 0.416667 | 2.813333 |
| NOS2 | 4.665 | 2.222001 | 5.12E-06 | 4.26E-05 | yes | up | 0.34 | 0.15 | 0.2 | 3.91 | 1.5 | 2.63 | 0.23 | 2.68 |
| LOC103346930 | 22.411 | 4.486166 | 5.31E-06 | 4.41E-05 | yes | up | 0.09 | 0.05 | 0.52 | 8.81 | 4.26 | 31.22 | 0.22 | 14.76333 |
| KCNH2 | 0.183 | -2.4471 | 5.49E-06 | 4.56E-05 | yes | down | 1.4 | 0.85 | 0.84 | 0.24 | 0.42 | 0.57 | 1.03 | 0.41 |
| PLCG2 | 2.236 | 1.160645 | 5.53E-06 | 4.59E-05 | yes | up | 0.67 | 0.79 | 0.68 | 3.21 | 4.55 | 3.68 | 0.713333 | 3.813333 |
| LOC100343872 | 2.291 | 1.195713 | 5.69E-06 | 4.71E-05 | yes | up | 1.95 | 2.1 | 1.63 | 9.03 | 10.14 | 11.93 | 1.893333 | 10.36667 |
| QRSL1 | 0.431 | -1.21581 | 5.7E-06 | 4.71E-05 | yes | down | 7.31 | 6.45 | 4.94 | 6.26 | 7.93 | 4.94 | 6.233333 | 6.376667 |
| PON3 | 3.636 | 1.862387 | 5.78E-06 | 4.78E-05 | yes | up | 2.3 | 1.79 | 1.53 | 18.11 | 21.45 | 9.21 | 1.873333 | 16.25667 |
| C1R | 2.94 | 1.556002 | 5.86E-06 | 4.83E-05 | yes | up | 23.71 | 12.8 | 10.39 | 84.07 | 89.19 | 150.18 | 15.63333 | 107.8133 |
| MOCS2 | 0.457 | -1.13065 | 6.1E-06 | 5.03E-05 | yes | down | 47.31 | 50.18 | 57.69 | 42.45 | 44.17 | 69.63 | 51.72667 | 52.08333 |
| ZNF655 | 0.445 | -1.16911 | 6.14E-06 | 5.06E-05 | yes | down | 1.96 | 1.81 | 2.14 | 2.61 | 1.93 | 1.87 | 1.97 | 2.136667 |
| ACSS1 | 0.229 | -2.12509 | 6.21E-06 | 5.11E-05 | yes | down | 83.24 | 20.12 | 23.21 | 26.42 | 23.38 | 18.21 | 42.19 | 22.67 |
| LOC100343299 | 0.151 | -2.72647 | 6.24E-06 | 5.13E-05 | yes | down | 23.64 | 33.37 | 101.68 | 16.03 | 27.05 | 18.24 | 52.89667 | 20.44 |
| NMNAT2 | 2.656 | 1.40939 | 6.28E-06 | 5.17E-05 | yes | up | 1.49 | 1.33 | 0.78 | 10.01 | 6.13 | 7.05 | 1.2 | 7.73 |
| TMEM53 | 2.909 | 1.540415 | 6.33E-06 | 5.2E-05 | yes | up | 1.18 | 1.94 | 0.9 | 7.7 | 10.01 | 9.9 | 1.34 | 9.203333 |
| FAM13C | 0.409 | -1.29008 | 6.36E-06 | 5.22E-05 | yes | down | 8.3 | 8.33 | 9.26 | 11.95 | 7.22 | 5.82 | 8.63 | 8.33 |
| BEST4 | 0.031 | -5.03266 | 6.39E-06 | 5.24E-05 | yes | down | 0.21 | 0.27 | 0.44 | 0.02 | 0.05 | 0 | 0.306667 | 0.023333 |
| TRIP10 | 2.072 | 1.050923 | 6.55E-06 | 5.36E-05 | yes | up | 20.19 | 20.07 | 14.15 | 70.81 | 96.7 | 80.93 | 18.13667 | 82.81333 |
| S100A9 | 0.133 | -2.90847 | 6.61E-06 | 5.4E-05 | yes | down | 10.75 | 49.85 | 62.01 | 8.29 | 10.78 | 21.97 | 40.87 | 13.68 |
| PLEK2 | 0.11 | -3.18316 | 6.63E-06 | 5.41E-05 | yes | down | 2.43 | 2 | 0.34 | 0.33 | 0.63 | 0.36 | 1.59 | 0.44 |
| SMTN | 2.473 | 1.30602 | 6.65E-06 | 5.43E-05 | yes | up | 16.91 | 14.37 | 10.73 | 117.82 | 65.3 | 59.28 | 14.00333 | 80.8 |
| SLC25A25 | 2.569 | 1.361289 | 6.68E-06 | 5.45E-05 | yes | up | 3.08 | 2.63 | 1.35 | 14.03 | 16.51 | 11.23 | 2.353333 | 13.92333 |
| LOC100353245 | 2.097 | 1.068497 | 6.73E-06 | 5.49E-05 | yes | up | 133.22 | 93.19 | 83.28 | 413.8 | 547.02 | 586.54 | 103.23 | 515.7867 |
| MYLK3 | 0.037 | -4.77563 | 6.84E-06 | 5.57E-05 | yes | down | 22.25 | 3.79 | 1.47 | 0.69 | 1.19 | 0.45 | 9.17 | 0.776667 |
| LOC103348324 | 13.68 | 3.77397 | 7E-06 | 5.7E-05 | yes | up | 0.2 | 0.06 | 0.06 | 4.17 | 2.57 | 3.4 | 0.106667 | 3.38 |
| OGT | 2.08 | 1.056824 | 7.02E-06 | 5.71E-05 | yes | up | 26 | 29.28 | 14.66 | 127.72 | 91.68 | 116.97 | 23.31333 | 112.1233 |
| PLEKHF1 | 2.427 | 1.279113 | 7.03E-06 | 5.72E-05 | yes | up | 11.31 | 21.37 | 16.88 | 103.78 | 107.61 | 80.09 | 16.52 | 97.16 |
| RTN4 | 0.499 | -1.00282 | 7.09E-06 | 5.77E-05 | yes | down | 322.96 | 229.79 | 175.07 | 266.9 | 352.51 | 333.61 | 242.6067 | 317.6733 |
| LOC100344478 | 0.469 | -1.09313 | 7.1E-06 | 5.77E-05 | yes | down | 9.59 | 11.4 | 9.31 | 11.79 | 12.58 | 9.62 | 10.1 | 11.33 |
| TMEM205 | 0.413 | -1.27538 | 7.15E-06 | 5.8E-05 | yes | down | 53.82 | 79.62 | 92.79 | 85.71 | 72.96 | 72.31 | 75.41 | 76.99333 |
| LOC100342182 | 0.009 | -6.83303 | 7.15E-06 | 5.8E-05 | yes | down | 2.15 | 1.42 | 3.12 | 0 | 0 | 0 | 2.23 | 0 |
| LOC103346060 | 0.192 | -2.37797 | 7.16E-06 | 5.81E-05 | yes | down | 0.57 | 0.68 | 0.35 | 0.25 | 0.29 | 0.19 | 0.533333 | 0.243333 |
| TSEN54 | 0.49 | -1.0277 | 7.17E-06 | 5.81E-05 | yes | down | 6.01 | 7.54 | 6.79 | 6.17 | 6.78 | 8.39 | 6.78 | 7.113333 |
| LOC103347778 | 64.661 | 6.014823 | 7.2E-06 | 5.83E-05 | yes | up | 0 | 0 | 0.34 | 13.85 | 25.89 | 27.41 | 0.113333 | 22.38333 |
| LUM | 2.265 | 1.179289 | 7.37E-06 | 5.96E-05 | yes | up | 134.46 | 106.38 | 57.74 | 474.39 | 524.62 | 582.63 | 99.52667 | 527.2133 |
| CLCN2 | 0.495 | -1.01353 | 7.39E-06 | 5.97E-05 | yes | down | 1.97 | 2.15 | 1.94 | 2.59 | 2.39 | 2.26 | 2.02 | 2.413333 |
| FAT3 | 17.973 | 4.167788 | 7.49E-06 | 6.05E-05 | yes | up | 0.01 | 0 | 0 | 46.78 | 0.15 | 0.12 | 0.003333 | 15.68333 |
| LOC100352631 | 0.038 | -4.72994 | 7.65E-06 | 6.18E-05 | yes | down | 0.68 | 0.79 | 0.48 | 0.05 | 0.06 | 0.05 | 0.65 | 0.053333 |
| LRRC2 | 0.253 | -1.98292 | 7.87E-06 | 6.34E-05 | yes | down | 10.17 | 5.52 | 6.8 | 3.18 | 7.23 | 3.07 | 7.496667 | 4.493333 |
| MYOCD | 2.619 | 1.388866 | 7.93E-06 | 6.38E-05 | yes | up | 0.39 | 0.32 | 0.22 | 1.62 | 1.63 | 2.4 | 0.31 | 1.883333 |
| DMBT1 | 0.088 | -3.50237 | 8.03E-06 | 6.46E-05 | yes | down | 8.34 | 7.09 | 5.29 | 2.75 | 0.36 | 0.12 | 6.906667 | 1.076667 |
| TAF1D | 0.375 | -1.41542 | 8.05E-06 | 6.47E-05 | yes | down | 53.96 | 55.86 | 74.55 | 65.84 | 41.04 | 34.6 | 61.45667 | 47.16 |
| CENPU | 2.275 | 1.186058 | 8.1E-06 | 6.5E-05 | yes | up | 8.52 | 8.79 | 4.4 | 39.8 | 39.35 | 44.97 | 7.236667 | 41.37333 |
| P4HA3 | 0.24 | -2.06107 | 8.13E-06 | 6.52E-05 | yes | down | 2.55 | 1.41 | 1.21 | 0.77 | 0.75 | 1.42 | 1.723333 | 0.98 |
| TMEM74B | 0.29 | -1.7868 | 8.27E-06 | 6.62E-05 | yes | down | 0.18 | 0.18 | 0.16 | 0.14 | 0.08 | 0.13 | 0.173333 | 0.116667 |
| STEAP1 | 3.45 | 1.786688 | 8.47E-06 | 6.77E-05 | yes | up | 1.02 | 2.18 | 2.29 | 14.49 | 17.2 | 13.65 | 1.83 | 15.11333 |
| TUB | 2.22 | 1.1508 | 8.53E-06 | 6.81E-05 | yes | up | 1.55 | 1.02 | 0.83 | 5.73 | 4.82 | 7.15 | 1.133333 | 5.9 |
| RAPH1 | 2.083 | 1.05856 | 8.68E-06 | 6.92E-05 | yes | up | 5.71 | 3.6 | 2.28 | 20.17 | 17.06 | 20.26 | 3.863333 | 19.16333 |
| MIEF2 | 0.366 | -1.45187 | 8.85E-06 | 7.06E-05 | yes | down | 7.33 | 4.01 | 3.32 | 3.86 | 4.07 | 4.56 | 4.886667 | 4.163333 |
| SYTL4 | 2.308 | 1.206455 | 8.88E-06 | 7.08E-05 | yes | up | 2.49 | 1.61 | 1.09 | 10.45 | 7.22 | 10.42 | 1.73 | 9.363333 |
| LOC103349307 | 0.11 | -3.18318 | 8.88E-06 | 7.08E-05 | yes | down | 1.8 | 2.99 | 3.25 | 0.22 | 0.69 | 1.06 | 2.68 | 0.656667 |
| GGT5 | 2.618 | 1.38834 | 9.12E-06 | 7.26E-05 | yes | up | 0.43 | 0.48 | 0.45 | 3.31 | 2.53 | 2.77 | 0.453333 | 2.87 |
| TRIM10 | 0.067 | -3.90033 | 9.19E-06 | 7.31E-05 | yes | down | 0.36 | 0.43 | 0.38 | 0.04 | 0.04 | 0.12 | 0.39 | 0.066667 |
| ARSG | 2.61 | 1.383812 | 9.49E-06 | 7.52E-05 | yes | up | 0.51 | 0.61 | 0.47 | 3.91 | 2.66 | 3.67 | 0.53 | 3.413333 |
| LOC108178230 | 0.009 | -6.83651 | 9.61E-06 | 7.6E-05 | yes | down | 0.55 | 0.33 | 0.13 | 0 | 0 | 0 | 0.336667 | 0 |
| FPGS | 0.48 | -1.05999 | 9.66E-06 | 7.64E-05 | yes | down | 8.07 | 9.82 | 8.14 | 9.61 | 7.84 | 12.42 | 8.676667 | 9.956667 |
| TRIM16 | 2.061 | 1.043617 | 9.76E-06 | 7.71E-05 | yes | up | 15.52 | 16.78 | 11.22 | 60.53 | 52.67 | 79.67 | 14.50667 | 64.29 |
| LRRC3C | 0.273 | -1.87378 | 9.8E-06 | 7.74E-05 | yes | down | 0.91 | 2.26 | 1.01 | 0.68 | 0.62 | 1.54 | 1.393333 | 0.946667 |
| GUCY1B3 | 2.104 | 1.073093 | 9.86E-06 | 7.78E-05 | yes | up | 4.01 | 3.43 | 2.76 | 23.49 | 13.13 | 15.13 | 3.4 | 17.25 |
| SVEP1 | 2.058 | 1.040894 | 9.88E-06 | 7.8E-05 | yes | up | 6.24 | 4.54 | 4.3 | 27.6 | 17.21 | 29.49 | 5.026667 | 24.76667 |
| LOC108176138 | 2.192 | 1.132071 | 9.93E-06 | 7.83E-05 | yes | up | 2.55 | 4.14 | 3.28 | 17.25 | 16.13 | 19.34 | 3.323333 | 17.57333 |
| VDAC1 | 0.486 | -1.04078 | 9.99E-06 | 7.87E-05 | yes | down | 377.7 | 236.89 | 227.28 | 329.53 | 386.7 | 369.18 | 280.6233 | 361.8033 |
| SHROOM3 | 0.36 | -1.4734 | 1.01E-05 | 7.94E-05 | yes | down | 1.25 | 0.86 | 1.21 | 1.4 | 0.84 | 0.82 | 1.106667 | 1.02 |
| PERM1 | 0.133 | -2.90586 | 1.02E-05 | 8.04E-05 | yes | down | 6.74 | 3.6 | 1.91 | 0.37 | 2.52 | 0.8 | 4.083333 | 1.23 |
| LOC100343274 | 0.49 | -1.03017 | 1.03E-05 | 8.06E-05 | yes | down | 5.08 | 7.42 | 5.41 | 8 | 6.29 | 6.88 | 5.97 | 7.056667 |
| LOC103344972 | 3.623 | 1.856986 | 1.03E-05 | 8.13E-05 | yes | up | 20.87 | 17.83 | 5.14 | 111.83 | 142.74 | 111.96 | 14.61333 | 122.1767 |
| MMP11 | 0.358 | -1.48142 | 1.04E-05 | 8.18E-05 | yes | down | 7.36 | 6.06 | 9.98 | 8.47 | 5.01 | 7.28 | 7.8 | 6.92 |
| LOC100358731 | 18.545 | 4.212985 | 1.05E-05 | 8.22E-05 | yes | up | 0 | 0.04 | 0.16 | 5.47 | 2.03 | 1.8 | 0.066667 | 3.1 |
| LOC100348124 | 0.335 | -1.57873 | 1.05E-05 | 8.22E-05 | yes | down | 110.38 | 80.42 | 82.83 | 57.19 | 113.25 | 46.54 | 91.21 | 72.32667 |
| TMEM251 | 0.472 | -1.08228 | 1.05E-05 | 8.25E-05 | yes | down | 14.17 | 19.38 | 17.15 | 18.68 | 16.1 | 20.41 | 16.9 | 18.39667 |
| LOC108176506 | 0.112 | -3.15736 | 1.07E-05 | 8.34E-05 | yes | down | 5.26 | 3.92 | 2.44 | 0.54 | 0.12 | 0.27 | 3.873333 | 0.31 |
| LRRN4CL | 150.992 | 7.23833 | 1.07E-05 | 8.38E-05 | yes | up | 0 | 0 | 0 | 3.11 | 2.73 | 0.17 | 0 | 2.003333 |
| SSH3 | 2.007 | 1.005125 | 1.08E-05 | 8.45E-05 | yes | up | 2.52 | 2.1 | 2.1 | 14.11 | 9.98 | 10.68 | 2.24 | 11.59 |
| DNAJC5B | 71.316 | 6.156144 | 1.09E-05 | 8.49E-05 | yes | up | 0 | 0 | 0 | 0.86 | 0.68 | 0.77 | 0 | 0.77 |
| LOC100338675 | 0.203 | -2.30311 | 1.11E-05 | 8.63E-05 | yes | down | 17.79 | 25.2 | 28.96 | 4.69 | 3.66 | 20.67 | 23.98333 | 9.673333 |
| DDO | 0.39 | -1.3577 | 1.12E-05 | 8.72E-05 | yes | down | 5.66 | 7.33 | 7.22 | 6.29 | 4.03 | 7.85 | 6.736667 | 6.056667 |
| AQP4 | 17.831 | 4.156308 | 1.13E-05 | 8.77E-05 | yes | up | 6.11 | 0.94 | 0.78 | 139.5 | 79.99 | 103.53 | 2.61 | 107.6733 |
| KCNC4 | 0.037 | -4.76768 | 1.15E-05 | 8.94E-05 | yes | down | 6.27 | 6.37 | 3.23 | 0.1 | 1.68 | 0.22 | 5.29 | 0.666667 |
| CYP27A1 | 0.334 | -1.58305 | 1.17E-05 | 9.08E-05 | yes | down | 77.35 | 140.87 | 80.38 | 45.66 | 94.29 | 93.52 | 99.53333 | 77.82333 |
| NUPR1 | 2.28 | 1.188919 | 1.2E-05 | 9.29E-05 | yes | up | 16.17 | 17.06 | 19.28 | 75.55 | 108.69 | 105.73 | 17.50333 | 96.65667 |
| CNTNAP1 | 4.878 | 2.286209 | 1.2E-05 | 9.29E-05 | yes | up | 0.3 | 0.14 | 0.1 | 2.81 | 1.11 | 2.42 | 0.18 | 2.113333 |
| RHOU | 2.353 | 1.234713 | 1.2E-05 | 9.32E-05 | yes | up | 2.95 | 1.82 | 1.72 | 13.55 | 13.77 | 9.05 | 2.163333 | 12.12333 |
| ADIPOR1 | 0.481 | -1.05496 | 1.21E-05 | 9.32E-05 | yes | down | 62.72 | 94.12 | 89.85 | 102.64 | 102.53 | 83.56 | 82.23 | 96.24333 |
| ECHDC2 | 0.406 | -1.29946 | 1.21E-05 | 9.37E-05 | yes | down | 23.62 | 47.51 | 32.93 | 35.48 | 27.26 | 39.63 | 34.68667 | 34.12333 |
| LOC103350794 | 0.295 | -1.76312 | 1.22E-05 | 9.42E-05 | yes | down | 5.62 | 6.88 | 7.8 | 5.65 | 2.6 | 6.52 | 6.766667 | 4.923333 |
| ZC3H8 | 0.48 | -1.05759 | 1.25E-05 | 9.61E-05 | yes | down | 4.24 | 3.93 | 4.27 | 4.74 | 4.8 | 4.93 | 4.146667 | 4.823333 |
| ASPA | 2.031 | 1.022489 | 1.25E-05 | 9.65E-05 | yes | up | 30.82 | 36.05 | 34.49 | 119.4 | 179.3 | 147.37 | 33.78667 | 148.69 |
| PRKCD | 2.021 | 1.015131 | 1.27E-05 | 9.76E-05 | yes | up | 4.41 | 4.89 | 3.62 | 18.62 | 16.88 | 25.06 | 4.306667 | 20.18667 |
| LOC100348238 | 4.041 | 2.014707 | 1.28E-05 | 9.82E-05 | yes | up | 0.14 | 0.21 | 0.08 | 1.37 | 1 | 1.77 | 0.143333 | 1.38 |
| SSTR4 | 0.036 | -4.78518 | 1.29E-05 | 9.9E-05 | yes | down | 0.25 | 0.44 | 0.28 | 0 | 0.03 | 0.05 | 0.323333 | 0.026667 |
| LOC103346967 | 179.126 | 7.484831 | 1.31E-05 | 0.000101 | yes | up | 0 | 0 | 0 | 4.24 | 1.22 | 0.1 | 0 | 1.853333 |
| APELA | 0.329 | -1.60194 | 1.31E-05 | 0.000101 | yes | down | 2.53 | 2.13 | 2.79 | 1.42 | 2.28 | 2.18 | 2.483333 | 1.96 |
| LGALS1 | 2.909 | 1.540753 | 1.32E-05 | 0.000101 | yes | up | 390.45 | 234.52 | 130.52 | 2052.8 | 1920.25 | 1189.55 | 251.83 | 1720.867 |
| FLT1 | 0.388 | -1.36682 | 1.33E-05 | 0.000102 | yes | down | 11.17 | 18.47 | 22.73 | 17.26 | 16.3 | 16.43 | 17.45667 | 16.66333 |
| PAMR1 | 0.455 | -1.13547 | 1.35E-05 | 0.000103 | yes | down | 6.3 | 4.32 | 3.11 | 5.76 | 4.24 | 4.86 | 4.576667 | 4.953333 |
| BCL6B | 2.242 | 1.164678 | 1.36E-05 | 0.000104 | yes | up | 5.6 | 7.89 | 7.84 | 48.62 | 39.13 | 29.21 | 7.11 | 38.98667 |
| TLL1 | 4.581 | 2.195575 | 1.36E-05 | 0.000104 | yes | up | 0.17 | 0.24 | 0.13 | 2.58 | 1.15 | 2.21 | 0.18 | 1.98 |
| PTGIR | 3.021 | 1.59485 | 1.38E-05 | 0.000106 | yes | up | 0.34 | 0.37 | 0.17 | 2.52 | 1.96 | 1.83 | 0.293333 | 2.103333 |
| PM20D2 | 2.187 | 1.129147 | 1.38E-05 | 0.000106 | yes | up | 6.5 | 3.87 | 3.91 | 32.07 | 22.17 | 18.89 | 4.76 | 24.37667 |
| CEP112 | 2.602 | 1.379438 | 1.41E-05 | 0.000107 | yes | up | 0.5 | 0.43 | 0.33 | 2.38 | 2.63 | 2.86 | 0.42 | 2.623333 |
| TMEM245 | 2.182 | 1.125423 | 1.43E-05 | 0.000108 | yes | up | 8.28 | 4.77 | 3.94 | 30.62 | 31.03 | 25.35 | 5.663333 | 29 |
| SLC25A35 | 2.255 | 1.173431 | 1.43E-05 | 0.000108 | yes | up | 1.69 | 1.84 | 1.14 | 10.04 | 8.2 | 6.95 | 1.556667 | 8.396667 |
| LOC103350198 | 68.831 | 6.104995 | 1.44E-05 | 0.000109 | yes | up | 0 | 0 | 0 | 1.11 | 1.14 | 1.12 | 0 | 1.123333 |
| FN3K | 0.426 | -1.22992 | 1.46E-05 | 0.00011 | yes | down | 11.2 | 14.29 | 17.6 | 17.43 | 13.67 | 12.82 | 14.36333 | 14.64 |
| DLG4 | 2.368 | 1.243942 | 1.46E-05 | 0.000111 | yes | up | 0.82 | 0.85 | 0.68 | 5.89 | 3.66 | 4.11 | 0.783333 | 4.553333 |
| LOC108178580 | 36.932 | 5.206797 | 1.46E-05 | 0.000111 | yes | up | 0 | 0.08 | 0.08 | 2.97 | 5.19 | 6.6 | 0.053333 | 4.92 |
| TMED3 | 2.101 | 1.07083 | 1.47E-05 | 0.000111 | yes | up | 22.53 | 19.29 | 12.39 | 82.11 | 104.3 | 77.5 | 18.07 | 87.97 |
| ELMOD1 | 0.063 | -3.99365 | 1.53E-05 | 0.000116 | yes | down | 0.18 | 0.64 | 0.8 | 0.08 | 0.03 | 0.15 | 0.54 | 0.086667 |
| LOC100125995 | 3.872 | 1.953168 | 1.54E-05 | 0.000116 | yes | up | 0.32 | 0.6 | 0.23 | 3.78 | 4 | 2.74 | 0.383333 | 3.506667 |
| LOC100357419 | 17.142 | 4.099494 | 1.54E-05 | 0.000117 | yes | up | 0.09 | 0.03 | 0.05 | 5.48 | 0.18 | 2.51 | 0.056667 | 2.723333 |
| FAM35A | 2.196 | 1.134687 | 1.56E-05 | 0.000117 | yes | up | 1.68 | 1.02 | 1.04 | 6.05 | 5.42 | 6.3 | 1.246667 | 5.923333 |
| COL5A2 | 2.045 | 1.032204 | 1.56E-05 | 0.000118 | yes | up | 76.77 | 51.22 | 35.48 | 305.94 | 240.76 | 244.55 | 54.49 | 263.75 |
| TCAIM | 2.166 | 1.115183 | 1.59E-05 | 0.00012 | yes | up | 1.86 | 1.52 | 1.62 | 9.68 | 10.04 | 6.48 | 1.666667 | 8.733333 |
| KCNB2 | 14.61 | 3.868899 | 1.61E-05 | 0.000121 | yes | up | 0.04 | 0.05 | 0 | 1.11 | 0.44 | 1.68 | 0.03 | 1.076667 |
| CSTF3 | 2.005 | 1.003903 | 1.62E-05 | 0.000122 | yes | up | 8.49 | 9.28 | 6.6 | 43.53 | 29.75 | 31.42 | 8.123333 | 34.9 |
| FAM174A | 0.468 | -1.09565 | 1.62E-05 | 0.000122 | yes | down | 26.46 | 17.92 | 17.34 | 21.44 | 27.89 | 19.52 | 20.57333 | 22.95 |
| ENG | 0.401 | -1.31877 | 1.64E-05 | 0.000123 | yes | down | 71.09 | 105.44 | 123.35 | 130.08 | 91.8 | 75.55 | 99.96 | 99.14333 |
| SLCO2A1 | 0.347 | -1.52693 | 1.64E-05 | 0.000123 | yes | down | 2.21 | 1.64 | 2.31 | 1.25 | 2.19 | 1.71 | 2.053333 | 1.716667 |
| EGR2 | 4.654 | 2.218546 | 1.64E-05 | 0.000123 | yes | up | 0.38 | 0.14 | 0.11 | 2.64 | 2.17 | 2.05 | 0.21 | 2.286667 |
| MMRN1 | 0.413 | -1.27455 | 1.65E-05 | 0.000124 | yes | down | 7.28 | 6.09 | 3.55 | 7.38 | 5.36 | 3.86 | 5.64 | 5.533333 |
| ARHGAP22 | 4.249 | 2.087217 | 1.67E-05 | 0.000125 | yes | up | 0.54 | 0.43 | 0.13 | 3.54 | 5.55 | 4.58 | 0.366667 | 4.556667 |
| KANK4 | 0.271 | -1.88419 | 1.68E-05 | 0.000126 | yes | down | 1.42 | 2.32 | 1.55 | 0.82 | 1.81 | 0.76 | 1.763333 | 1.13 |
| C2H2orf73 | 0.296 | -1.75487 | 1.71E-05 | 0.000128 | yes | down | 2.95 | 2.23 | 3.03 | 1.85 | 1.57 | 2.53 | 2.736667 | 1.983333 |
| BHMT | 0.004 | -8.10056 | 1.72E-05 | 0.000128 | yes | down | 28.03 | 28.35 | 41.67 | 0 | 0 | 0.86 | 32.68333 | 0.286667 |
| ADAM18 | 13.128 | 3.714544 | 1.75E-05 | 0.00013 | yes | up | 0.05 | 0.13 | 0.02 | 2.58 | 1.17 | 1.54 | 0.066667 | 1.763333 |
| LOC100358544 | 0.094 | -3.41717 | 1.75E-05 | 0.000131 | yes | down | 0.94 | 0.36 | 0.41 | 0.15 | 0.05 | 0.25 | 0.57 | 0.15 |
| PCK1 | 0.282 | -1.82423 | 1.75E-05 | 0.000131 | yes | down | 898.83 | 1267.15 | 675.53 | 261.11 | 812.71 | 785.75 | 947.17 | 619.8567 |
| DDN | 0.013 | -6.27186 | 1.77E-05 | 0.000132 | yes | down | 1.15 | 0.18 | 0.1 | 0 | 0.04 | 0 | 0.476667 | 0.013333 |
| ZFAT | 2.679 | 1.421925 | 1.82E-05 | 0.000135 | yes | up | 1.19 | 1.2 | 0.75 | 7.52 | 5.61 | 3.77 | 1.046667 | 5.633333 |
| LOC103352092 | 3.614 | 1.853556 | 1.93E-05 | 0.000143 | yes | up | 0.32 | 0.16 | 0.25 | 2.86 | 2 | 1.49 | 0.243333 | 2.116667 |
| FAM20A | 0.362 | -1.46699 | 1.93E-05 | 0.000143 | yes | down | 0.79 | 1.01 | 0.84 | 0.89 | 0.68 | 0.74 | 0.88 | 0.77 |
| MID1IP1 | 3.556 | 1.830432 | 1.93E-05 | 0.000143 | yes | up | 3.6 | 4.08 | 4.8 | 44.61 | 14.44 | 49.15 | 4.16 | 36.06667 |
| SLC25A4 | 0.278 | -1.84632 | 1.97E-05 | 0.000146 | yes | down | 652.87 | 244 | 177.25 | 193.22 | 305.01 | 192.46 | 358.04 | 230.23 |
| FST | 2.705 | 1.435462 | 2.03E-05 | 0.00015 | yes | up | 7.16 | 4.27 | 10.13 | 50.36 | 49.24 | 40.26 | 7.186667 | 46.62 |
| TESMIN | 0.025 | -5.33342 | 2.13E-05 | 0.000157 | yes | down | 3.4 | 9.5 | 5.54 | 2 | 2.76 | 0 | 6.146667 | 1.586667 |
| ARHGEF28 | 0.356 | -1.48877 | 2.14E-05 | 0.000157 | yes | down | 2.94 | 4.75 | 5.08 | 5.07 | 3.1 | 2.47 | 4.256667 | 3.546667 |
| PIK3R6 | 3.183 | 1.670409 | 2.15E-05 | 0.000158 | yes | up | 0.63 | 0.79 | 0.47 | 7.25 | 3.58 | 3.79 | 0.63 | 4.873333 |
| SLC30A7 | 0.49 | -1.0303 | 2.15E-05 | 0.000158 | yes | down | 5 | 7.71 | 3.87 | 6.29 | 6.96 | 5.42 | 5.526667 | 6.223333 |
| TOB1 | 0.375 | -1.41456 | 2.21E-05 | 0.000162 | yes | down | 22.47 | 22.86 | 10.41 | 13.27 | 21.83 | 15.86 | 18.58 | 16.98667 |
| LOC100349105 | 0.447 | -1.16048 | 2.22E-05 | 0.000163 | yes | down | 38.27 | 48.93 | 54.28 | 44.46 | 56.18 | 54.01 | 47.16 | 51.55 |
| LOC100348760 | 0.317 | -1.65765 | 2.23E-05 | 0.000164 | yes | down | 393.64 | 190.53 | 212.14 | 144 | 304.72 | 143.88 | 265.4367 | 197.5333 |
| LNP1 | 12.126 | 3.600017 | 2.26E-05 | 0.000165 | yes | up | 0.26 | 0.24 | 0.25 | 21.77 | 2.11 | 6.33 | 0.25 | 10.07 |
| LOC108176227 | 0.101 | -3.30906 | 2.26E-05 | 0.000166 | yes | down | 6.84 | 3.39 | 0.98 | 0.37 | 0.27 | 1.94 | 3.736667 | 0.86 |
| LOC103351480 | 2.283 | 1.191014 | 2.29E-05 | 0.000167 | yes | up | 2.18 | 3.09 | 2.51 | 16.29 | 14.7 | 12.12 | 2.593333 | 14.37 |
| LOC100358539 | 3.675 | 1.877849 | 2.3E-05 | 0.000168 | yes | up | 0.68 | 0.81 | 0.72 | 4.56 | 4.21 | 10.44 | 0.736667 | 6.403333 |
| SLC22A4 | 0.124 | -3.01603 | 2.3E-05 | 0.000168 | yes | down | 0.65 | 1.08 | 1.21 | 0.09 | 0.35 | 0.41 | 0.98 | 0.283333 |
| C19H17orf58 | 4.282 | 2.098433 | 2.33E-05 | 0.00017 | yes | up | 1.98 | 1.17 | 3.19 | 14.69 | 27.24 | 24.98 | 2.113333 | 22.30333 |
| RDH11 | 2.039 | 1.027858 | 2.38E-05 | 0.000173 | yes | up | 2.91 | 3.04 | 3.33 | 16.79 | 11.79 | 16.76 | 3.093333 | 15.11333 |
| LOC100355643 | 0.332 | -1.58884 | 2.39E-05 | 0.000174 | yes | down | 3.41 | 2.67 | 2.27 | 2.17 | 2.71 | 1.75 | 2.783333 | 2.21 |
| SPACA9 | 0.131 | -2.92873 | 2.4E-05 | 0.000174 | yes | down | 0.69 | 1.23 | 1.35 | 0.76 | 0.12 | 0.27 | 1.09 | 0.383333 |
| TNFAIP8L3 | 0.243 | -2.04284 | 2.4E-05 | 0.000174 | yes | down | 1.12 | 1.43 | 0.68 | 0.74 | 0.44 | 0.68 | 1.076667 | 0.62 |
| CRTAP | 2.031 | 1.022206 | 2.45E-05 | 0.000178 | yes | up | 13.06 | 12.5 | 16.16 | 61.81 | 74.55 | 69.82 | 13.90667 | 68.72667 |
| ZNF496 | 0.457 | -1.13027 | 2.53E-05 | 0.000183 | yes | down | 2.17 | 2.68 | 2.81 | 2.8 | 3.08 | 2.93 | 2.553333 | 2.936667 |
| TTC39C | 0.389 | -1.362 | 2.56E-05 | 0.000185 | yes | down | 2.46 | 3.2 | 3.49 | 3.37 | 3.37 | 2.21 | 3.05 | 2.983333 |
| LOC108177708 | 0.234 | -2.09486 | 2.6E-05 | 0.000188 | yes | down | 83.91 | 61.69 | 18.14 | 19.24 | 32.48 | 36.8 | 54.58 | 29.50667 |
| ANKRD45 | 3.723 | 1.89663 | 2.6E-05 | 0.000188 | yes | up | 0.31 | 0.35 | 0.24 | 2.56 | 2.56 | 3.09 | 0.3 | 2.736667 |
| LOC100345095 | 0.092 | -3.4414 | 2.61E-05 | 0.000189 | yes | down | 1.25 | 0.77 | 4.61 | 0.23 | 0.62 | 0.68 | 2.21 | 0.51 |
| LOC100355311 | 0.483 | -1.05035 | 2.64E-05 | 0.00019 | yes | down | 5.84 | 4.54 | 4.17 | 5.92 | 4.58 | 6.28 | 4.85 | 5.593333 |
| MYL4 | 0.02 | -5.65022 | 2.64E-05 | 0.00019 | yes | down | 56.03 | 5.65 | 1.32 | 0.59 | 1.36 | 0.85 | 21 | 0.933333 |
| CCL19 | 4.526 | 2.178117 | 2.65E-05 | 0.000191 | yes | up | 0.96 | 0.54 | 0.93 | 7.82 | 9.77 | 8.97 | 0.81 | 8.853333 |
| LOC103352519 | 0.2 | -2.31942 | 2.66E-05 | 0.000192 | yes | down | 0.44 | 0.99 | 0.58 | 0.33 | 0.21 | 0.42 | 0.67 | 0.32 |
| SLC8B1 | 2.141 | 1.097966 | 2.67E-05 | 0.000192 | yes | up | 3.77 | 3.7 | 2.11 | 17.04 | 19.67 | 15.79 | 3.193333 | 17.5 |
| FAM120C | 2.019 | 1.013453 | 2.68E-05 | 0.000193 | yes | up | 1.25 | 0.78 | 0.97 | 4.68 | 3.93 | 5.24 | 1 | 4.616667 |
| CCDC71L | 2.822 | 1.496528 | 2.68E-05 | 0.000193 | yes | up | 1.16 | 1.18 | 0.69 | 9.53 | 6.97 | 3.91 | 1.01 | 6.803333 |
| SOX9 | 4.177 | 2.062338 | 2.73E-05 | 0.000196 | yes | up | 0.18 | 0.2 | 0.08 | 1.38 | 1.34 | 1.77 | 0.153333 | 1.496667 |
| LOC100339259 | 0.405 | -1.30313 | 2.74E-05 | 0.000197 | yes | down | 47.81 | 38.66 | 39.9 | 61.92 | 24.19 | 37.1 | 42.12333 | 41.07 |
| PRR13 | 2.248 | 1.168692 | 2.75E-05 | 0.000197 | yes | up | 4.83 | 2.67 | 2.65 | 19.71 | 17.46 | 17.56 | 3.383333 | 18.24333 |
| SLC22A23 | 0.429 | -1.22161 | 2.77E-05 | 0.000198 | yes | down | 3.07 | 2.01 | 2.14 | 2.28 | 1.94 | 2.71 | 2.406667 | 2.31 |
| EFCC1 | 3.43 | 1.778026 | 2.79E-05 | 0.0002 | yes | up | 1.1 | 0.98 | 0.95 | 7.6 | 4.31 | 13.25 | 1.01 | 8.386667 |
| AKAP1 | 0.431 | -1.2152 | 2.85E-05 | 0.000204 | yes | down | 45.33 | 71.22 | 45.91 | 47.23 | 43.14 | 75.04 | 54.15333 | 55.13667 |
| SEMA3B | 2.635 | 1.397559 | 2.85E-05 | 0.000204 | yes | up | 4.19 | 2.32 | 1.44 | 14.47 | 17.47 | 15.53 | 2.65 | 15.82333 |
| RGS4 | 3.602 | 1.84888 | 2.86E-05 | 0.000204 | yes | up | 2.75 | 2.99 | 2.08 | 25.79 | 11.4 | 31.7 | 2.606667 | 22.96333 |
| PROX1 | 0.223 | -2.1668 | 2.87E-05 | 0.000205 | yes | down | 0.99 | 0.43 | 0.27 | 0.27 | 0.37 | 0.22 | 0.563333 | 0.286667 |
| AK4 | 0.426 | -1.23255 | 2.94E-05 | 0.00021 | yes | down | 1.17 | 1.24 | 1.05 | 1.07 | 1.54 | 0.91 | 1.153333 | 1.173333 |
| LOC103346732 | 2.235 | 1.160374 | 3.04E-05 | 0.000216 | yes | up | 36.24 | 15.3 | 28.26 | 289.42 | 130.97 | 231.34 | 26.6 | 217.2433 |
| PSPH | 2.017 | 1.012487 | 3.12E-05 | 0.000221 | yes | up | 4.49 | 4.29 | 2.75 | 17.56 | 15.89 | 21.47 | 3.843333 | 18.30667 |
| TRNAK-CUU | 0.084 | -3.56697 | 3.16E-05 | 0.000224 | yes | down | 1.49 | 2.29 | 2.3 | 0.54 | 0.17 | 0.52 | 2.026667 | 0.41 |
| STC2 | 0.33 | -1.60063 | 3.2E-05 | 0.000227 | yes | down | 1.7 | 1.41 | 0.85 | 0.87 | 0.77 | 1.43 | 1.32 | 1.023333 |
| CDKN2C | 2.086 | 1.060848 | 3.23E-05 | 0.000228 | yes | up | 13.67 | 10.3 | 14.63 | 74.22 | 57.94 | 46.33 | 12.86667 | 59.49667 |
| IKBKE | 2.264 | 1.179053 | 3.28E-05 | 0.000231 | yes | up | 1.34 | 1.57 | 0.76 | 7.86 | 5.95 | 5.87 | 1.223333 | 6.56 |
| TADA3 | 2.031 | 1.021967 | 3.31E-05 | 0.000234 | yes | up | 12.08 | 9.47 | 6.37 | 47.55 | 38.56 | 46.47 | 9.306667 | 44.19333 |
| LOC108178461 | 3.424 | 1.775501 | 3.33E-05 | 0.000235 | yes | up | 0.57 | 0.37 | 0.65 | 6.11 | 6.01 | 3.91 | 0.53 | 5.343333 |
| ISLR | 2.45 | 1.29295 | 3.35E-05 | 0.000236 | yes | up | 8.87 | 4.38 | 3.92 | 28.97 | 34.7 | 35.19 | 5.723333 | 32.95333 |
| REM1 | 2.523 | 1.334975 | 3.37E-05 | 0.000237 | yes | up | 1.86 | 1.86 | 2.26 | 15.32 | 7.75 | 9.17 | 1.993333 | 10.74667 |
| SYNPO2 | 0.396 | -1.33699 | 3.42E-05 | 0.00024 | yes | down | 14.62 | 6.33 | 6.57 | 7.82 | 8.45 | 9.39 | 9.173333 | 8.553333 |
| RGS10 | 0.305 | -1.71363 | 3.46E-05 | 0.000243 | yes | down | 11.37 | 22.63 | 7.18 | 9.61 | 11.2 | 8.71 | 13.72667 | 9.84 |
| LOC103347318 | 2.407 | 1.267347 | 3.48E-05 | 0.000245 | yes | up | 8.12 | 4.68 | 4.73 | 31.69 | 38.19 | 30.48 | 5.843333 | 33.45333 |
| LOC103350987 | 6.703 | 2.744851 | 3.51E-05 | 0.000246 | yes | up | 0.01 | 0.02 | 0.05 | 0.79 | 0.7 | 0.47 | 0.026667 | 0.653333 |
| LOC103345165 | 7.684 | 2.941896 | 3.52E-05 | 0.000247 | yes | up | 0.11 | 0.26 | 0.2 | 2.57 | 2.75 | 5.37 | 0.19 | 3.563333 |
| MLPH | 43.353 | 5.438076 | 3.67E-05 | 0.000257 | yes | up | 0 | 0.05 | 0.04 | 0.85 | 4.17 | 5.04 | 0.03 | 3.353333 |
| CENPV | 0.459 | -1.12422 | 3.67E-05 | 0.000257 | yes | down | 6.83 | 7.31 | 7.2 | 6.5 | 7.77 | 8.51 | 7.113333 | 7.593333 |
| AHSP | 0.17 | -2.55864 | 3.71E-05 | 0.000259 | yes | down | 3.23 | 9.22 | 3.9 | 2.3 | 1.72 | 2.67 | 5.45 | 2.23 |
| DCTPP1 | 0.436 | -1.19796 | 3.76E-05 | 0.000262 | yes | down | 7.45 | 9.5 | 6.13 | 9.67 | 6.99 | 7.58 | 7.693333 | 8.08 |
| LOC108178915 | 14.226 | 3.830421 | 3.79E-05 | 0.000264 | yes | up | 0.12 | 0.45 | 0 | 9.05 | 5.49 | 3.14 | 0.19 | 5.893333 |
| FAM96B | 0.471 | -1.08697 | 3.91E-05 | 0.000272 | yes | down | 67.09 | 48.35 | 33.85 | 56.49 | 65.28 | 45.18 | 49.76333 | 55.65 |
| PHKG1 | 0.069 | -3.86088 | 3.96E-05 | 0.000275 | yes | down | 77.3 | 40.37 | 64.92 | 1.19 | 12.5 | 14.68 | 60.86333 | 9.456667 |
| ABCC2 | 3.465 | 1.792938 | 3.97E-05 | 0.000276 | yes | up | 0.12 | 0.14 | 0.09 | 0.87 | 1.06 | 0.94 | 0.116667 | 0.956667 |
| LOC103351786 | 0.079 | -3.66803 | 4.04E-05 | 0.00028 | yes | down | 1.66 | 0.5 | 0.36 | 0.13 | 0.3 | 0.03 | 0.84 | 0.153333 |
| AVPR1A | 2.912 | 1.542159 | 4.09E-05 | 0.000283 | yes | up | 1.16 | 1.01 | 1.23 | 13.57 | 5.58 | 5.37 | 1.133333 | 8.173333 |
| ZNF672 | 0.384 | -1.38144 | 4.13E-05 | 0.000286 | yes | down | 2.64 | 1.94 | 3.25 | 2.04 | 2.85 | 2.33 | 2.61 | 2.406667 |
| GGT6 | 0.145 | -2.78514 | 4.15E-05 | 0.000287 | yes | down | 1.37 | 1.13 | 3.69 | 0.3 | 0.44 | 1.14 | 2.063333 | 0.626667 |
| TMEM186 | 0.477 | -1.06727 | 4.15E-05 | 0.000287 | yes | down | 11.22 | 6.66 | 8.52 | 10.65 | 8.84 | 10.9 | 8.8 | 10.13 |
| LOC103350057 | 8.668 | 3.115699 | 4.18E-05 | 0.000288 | yes | up | 0.08 | 0.14 | 0.07 | 10.89 | 1.94 | 5.4 | 0.096667 | 6.076667 |
| LOC100351488 | 2.149 | 1.103543 | 4.25E-05 | 0.000292 | yes | up | 14.11 | 15.56 | 13.33 | 112.88 | 73.97 | 61.75 | 14.33333 | 82.86667 |
| SH3PXD2A | 2.175 | 1.120998 | 4.27E-05 | 0.000293 | yes | up | 5.89 | 3.73 | 3.28 | 26.53 | 15 | 25.57 | 4.3 | 22.36667 |
| TMEM43 | 2.34 | 1.226585 | 4.27E-05 | 0.000293 | yes | up | 20.73 | 22.92 | 18.77 | 140.47 | 63.87 | 149.43 | 20.80667 | 117.9233 |
| NR2C2AP | 0.288 | -1.79408 | 4.32E-05 | 0.000297 | yes | down | 3.07 | 6.16 | 5.11 | 3.94 | 3.16 | 5.15 | 4.78 | 4.083333 |
| LOC100352738 | 0.482 | -1.05262 | 4.38E-05 | 0.000301 | yes | down | 43.76 | 35.8 | 25.59 | 39.36 | 49.54 | 31.27 | 35.05 | 40.05667 |
| NUP35 | 0.473 | -1.0802 | 4.44E-05 | 0.000304 | yes | down | 7.83 | 12.17 | 7 | 9.76 | 11.52 | 9.1 | 9 | 10.12667 |
| TCTEX1D2 | 0.36 | -1.47518 | 4.47E-05 | 0.000305 | yes | down | 4.94 | 6.21 | 3.92 | 3.4 | 3.65 | 5.81 | 5.023333 | 4.286667 |
| LOC100009591 | 3.676 | 1.877944 | 4.48E-05 | 0.000306 | yes | up | 0.2 | 0.12 | 0.08 | 5.26 | 1.56 | 2.1 | 0.133333 | 2.973333 |
| RIN3 | 2.175 | 1.120739 | 4.51E-05 | 0.000308 | yes | up | 2.95 | 3.39 | 2.45 | 21.22 | 14.2 | 10.78 | 2.93 | 15.4 |
| LOC100343529 | 0.31 | -1.69059 | 4.62E-05 | 0.000315 | yes | down | 13.58 | 14.57 | 13.87 | 17.17 | 9.03 | 6.05 | 14.00667 | 10.75 |
| GPR88 | 6.817 | 2.769175 | 4.65E-05 | 0.000317 | yes | up | 0.04 | 0.05 | 0.07 | 1.39 | 0.63 | 0.57 | 0.053333 | 0.863333 |
| LOC100355256 | 0.441 | -1.1805 | 4.75E-05 | 0.000323 | yes | down | 9.66 | 9.37 | 8.09 | 9.58 | 6.57 | 9.43 | 9.04 | 8.526667 |
| NYAP1 | 3.432 | 1.779087 | 4.75E-05 | 0.000323 | yes | up | 0.15 | 0.16 | 0.13 | 1.37 | 1.14 | 1.03 | 0.146667 | 1.18 |
| LOC103348530 | 2.264 | 1.178687 | 4.75E-05 | 0.000323 | yes | up | 2.31 | 2.35 | 2.17 | 15.17 | 7.38 | 7.14 | 2.276667 | 9.896667 |
| SLC25A27 | 2.87 | 1.520826 | 4.75E-05 | 0.000323 | yes | up | 0.63 | 0.37 | 0.24 | 3.14 | 2.34 | 2.62 | 0.413333 | 2.7 |
| OSGIN1 | 2.268 | 1.181242 | 4.78E-05 | 0.000325 | yes | up | 1.43 | 2.12 | 1.68 | 8.91 | 5.6 | 10.27 | 1.743333 | 8.26 |
| GRO-B | 8.935 | 3.159456 | 4.84E-05 | 0.000328 | yes | up | 1.24 | 0.86 | 0 | 9.26 | 15.91 | 14.33 | 0.7 | 13.16667 |
| LOC108176489 | 4.869 | 2.28373 | 4.84E-05 | 0.000328 | yes | up | 0.17 | 0.26 | 0.08 | 2.89 | 1.5 | 1.5 | 0.17 | 1.963333 |
| LOC103349764 | 2.43 | 1.280738 | 4.91E-05 | 0.000332 | yes | up | 3.11 | 33.03 | 12.73 | 39.26 | 85.89 | 95.26 | 16.29 | 73.47 |
| COQ2 | 0.467 | -1.09904 | 4.92E-05 | 0.000333 | yes | down | 13.92 | 9.67 | 9.89 | 9.74 | 13.46 | 14.47 | 11.16 | 12.55667 |
| NEDD4L | 0.446 | -1.16621 | 4.94E-05 | 0.000333 | yes | down | 8.05 | 6.55 | 3.09 | 6.1 | 6.18 | 5.93 | 5.896667 | 6.07 |
| LOC100356618 | 0.4 | -1.32364 | 4.94E-05 | 0.000334 | yes | down | 6.92 | 10.92 | 5.59 | 9.89 | 7.56 | 5.78 | 7.81 | 7.743333 |
| ADGRL1 | 0.331 | -1.59597 | 5.06E-05 | 0.000341 | yes | down | 0.48 | 0.65 | 0.29 | 0.49 | 0.31 | 0.32 | 0.473333 | 0.373333 |
| FOXM1 | 5.178 | 2.372527 | 5.15E-05 | 0.000347 | yes | up | 0.63 | 0.68 | 0.27 | 7.48 | 9.75 | 1.89 | 0.526667 | 6.373333 |
| LOC100351040 | 0.007 | -7.07595 | 5.17E-05 | 0.000348 | yes | down | 0.1 | 0.64 | 0.05 | 0 | 0 | 0 | 0.263333 | 0 |
| BIRC3 | 2.276 | 1.186429 | 5.2E-05 | 0.000349 | yes | up | 1.23 | 1.66 | 1.99 | 8.77 | 7.76 | 10.64 | 1.626667 | 9.056667 |
| GGACT | 0.332 | -1.59183 | 5.25E-05 | 0.000352 | yes | down | 2.7 | 2.18 | 2.83 | 2.61 | 1.35 | 2.2 | 2.57 | 2.053333 |
| RLA-DRB1 | 3.104 | 1.633961 | 5.29E-05 | 0.000355 | yes | up | 1.63 | 2.83 | 3.65 | 25.34 | 14.07 | 21.87 | 2.703333 | 20.42667 |
| ADPRHL1 | 0.041 | -4.61301 | 5.6E-05 | 0.000374 | yes | down | 19.45 | 2.49 | 2.65 | 0.83 | 1.32 | 0.22 | 8.196667 | 0.79 |
| DNER | 0.071 | -3.81107 | 5.6E-05 | 0.000374 | yes | down | 0.39 | 0.2 | 0.24 | 0.07 | 0 | 0.07 | 0.276667 | 0.046667 |
| SDC1 | 0.367 | -1.44453 | 5.6E-05 | 0.000374 | yes | down | 8.11 | 12.59 | 7.75 | 6.15 | 6.19 | 12.5 | 9.483333 | 8.28 |
| WNT9A | 2.058 | 1.041109 | 5.69E-05 | 0.00038 | yes | up | 1.43 | 1.15 | 0.93 | 4.93 | 5.07 | 7.16 | 1.17 | 5.72 |
| LOC100358368 | 0.324 | -1.62394 | 5.7E-05 | 0.00038 | yes | down | 642.79 | 321.97 | 309.65 | 232.34 | 509.23 | 223.85 | 424.8033 | 321.8067 |
| DOK4 | 2.127 | 1.088959 | 5.74E-05 | 0.000382 | yes | up | 5.46 | 5.54 | 4.14 | 35.25 | 24.68 | 17.69 | 5.046667 | 25.87333 |
| MAPK8IP3 | 2.289 | 1.194624 | 5.88E-05 | 0.00039 | yes | up | 4.68 | 6.6 | 4.75 | 38.15 | 18.57 | 34.02 | 5.343333 | 30.24667 |
| SCARA5 | 2.4 | 1.263009 | 5.94E-05 | 0.000394 | yes | up | 9.43 | 4.96 | 3.71 | 29.71 | 33.68 | 38.19 | 6.033333 | 33.86 |
| EXD1 | 2.074 | 1.052431 | 5.96E-05 | 0.000395 | yes | up | 1.59 | 1.57 | 0.89 | 6.72 | 7.38 | 6.03 | 1.35 | 6.71 |
| MTERF2 | 0.471 | -1.08618 | 5.98E-05 | 0.000397 | yes | down | 5.95 | 4.24 | 4.87 | 6.46 | 5.69 | 6.99 | 5.02 | 6.38 |
| CHI3L1 | 6.96 | 2.79915 | 6.1E-05 | 0.000404 | yes | up | 0.43 | 1.45 | 1.34 | 19.94 | 30.78 | 3.92 | 1.073333 | 18.21333 |
| LOC100349989 | 5.855 | 2.549644 | 6.16E-05 | 0.000408 | yes | up | 0.12 | 0.1 | 0.05 | 1.14 | 0.76 | 1.76 | 0.09 | 1.22 |
| TRUB1 | 0.417 | -1.26332 | 6.19E-05 | 0.000409 | yes | down | 3.97 | 5.64 | 6.18 | 4.31 | 5.56 | 5.9 | 5.263333 | 5.256667 |
| NLGN2 | 2.014 | 1.009862 | 6.21E-05 | 0.00041 | yes | up | 1.2 | 0.92 | 0.79 | 5.99 | 4.07 | 4.05 | 0.97 | 4.703333 |
| TYMS | 2.352 | 1.233723 | 6.34E-05 | 0.000418 | yes | up | 2.85 | 2.88 | 2.46 | 17.45 | 18.6 | 10.24 | 2.73 | 15.43 |
| ADA | 2.784 | 1.477332 | 6.35E-05 | 0.000419 | yes | up | 3.24 | 2.53 | 2.29 | 17.4 | 25.31 | 10.95 | 2.686667 | 17.88667 |
| ARHGAP18 | 2.278 | 1.187859 | 6.4E-05 | 0.000422 | yes | up | 5.39 | 4.37 | 2.23 | 18.35 | 19.54 | 25.59 | 3.996667 | 21.16 |
| CGNL1 | 2.828 | 1.499678 | 6.44E-05 | 0.000424 | yes | up | 3.98 | 4.78 | 2.07 | 13.6 | 26.39 | 30.92 | 3.61 | 23.63667 |
| HSF5 | 0.334 | -1.58001 | 6.47E-05 | 0.000426 | yes | down | 2.53 | 3.5 | 1.72 | 1.34 | 1.58 | 2.94 | 2.583333 | 1.953333 |
| RADIL | 2.756 | 1.462809 | 6.48E-05 | 0.000426 | yes | up | 0.74 | 0.45 | 0.47 | 2.83 | 2.43 | 3.56 | 0.553333 | 2.94 |
| TFRC | 0.348 | -1.52431 | 6.48E-05 | 0.000426 | yes | down | 12.23 | 9.6 | 4.16 | 5.72 | 9.62 | 5.5 | 8.663333 | 6.946667 |
| PHTF2 | 2.818 | 1.494432 | 6.48E-05 | 0.000426 | yes | up | 6.74 | 3.25 | 1.83 | 28.27 | 23.53 | 26.24 | 3.94 | 26.01333 |
| ZNF593 | 0.315 | -1.66806 | 6.51E-05 | 0.000427 | yes | down | 3.6 | 4.97 | 4.38 | 2.65 | 4.07 | 3.63 | 4.316667 | 3.45 |
| LOC108176024 | 0.36 | -1.4749 | 6.67E-05 | 0.000437 | yes | down | 13.9 | 6.21 | 9.98 | 7.87 | 7.24 | 11.04 | 10.03 | 8.716667 |
| PARS2 | 0.443 | -1.1731 | 6.72E-05 | 0.00044 | yes | down | 2.94 | 2.87 | 2.59 | 1.94 | 2.15 | 3.38 | 2.8 | 2.49 |
| CYP4A5 | 0.028 | -5.15376 | 6.88E-05 | 0.00045 | yes | down | 0.34 | 0.37 | 0.34 | 0 | 0 | 0.06 | 0.35 | 0.02 |
| SGK1 | 0.319 | -1.64933 | 6.91E-05 | 0.000451 | yes | down | 45.68 | 75.05 | 62.16 | 83.76 | 23.79 | 37.67 | 60.96333 | 48.40667 |
| LOC103347586 | 6.999 | 2.807055 | 6.94E-05 | 0.000453 | yes | up | 16.18 | 4.47 | 2.55 | 249.42 | 84.13 | 75.27 | 7.733333 | 136.2733 |
| ERN1 | 2.119 | 1.083115 | 7.29E-05 | 0.000474 | yes | up | 0.69 | 0.66 | 0.58 | 2.53 | 3.69 | 3.5 | 0.643333 | 3.24 |
| KPNA2 | 2.231 | 1.157501 | 7.35E-05 | 0.000478 | yes | up | 10.2 | 7.68 | 5.08 | 36.76 | 51.47 | 31.87 | 7.653333 | 40.03333 |
| PSD2 | 0.076 | -3.71015 | 7.46E-05 | 0.000485 | yes | down | 0.15 | 0.14 | 0.13 | 0.03 | 0.03 | 0.01 | 0.14 | 0.023333 |
| ADCY3 | 0.428 | -1.22355 | 7.57E-05 | 0.000491 | yes | down | 20.21 | 35.2 | 27.59 | 21.11 | 35.96 | 27.98 | 27.66667 | 28.35 |
| RACGAP1 | 3.105 | 1.63477 | 7.61E-05 | 0.000493 | yes | up | 2.23 | 1.93 | 1.11 | 15.14 | 17.19 | 6.12 | 1.756667 | 12.81667 |
| MEIOB | 0.221 | -2.17476 | 7.9E-05 | 0.000511 | yes | down | 0.94 | 1.03 | 0.79 | 0.5 | 0.28 | 0.5 | 0.92 | 0.426667 |
| COL12A1 | 0.425 | -1.23553 | 8.05E-05 | 0.00052 | yes | down | 16.87 | 13.61 | 6.25 | 12.31 | 9.25 | 14.66 | 12.24333 | 12.07333 |
| UBALD1 | 0.39 | -1.35799 | 8.09E-05 | 0.000522 | yes | down | 9.35 | 15.15 | 15.23 | 12.32 | 13.06 | 20.16 | 13.24333 | 15.18 |
| DGUOK | 0.427 | -1.22643 | 8.13E-05 | 0.000525 | yes | down | 5.05 | 6.56 | 6.13 | 8.54 | 4.67 | 6.76 | 5.913333 | 6.656667 |
| GALM | 0.383 | -1.38367 | 8.14E-05 | 0.000525 | yes | down | 33.37 | 18.23 | 16.33 | 29.01 | 11.86 | 21.66 | 22.64333 | 20.84333 |
| LOC103346296 | 120.639 | 6.914551 | 8.17E-05 | 0.000527 | yes | up | 0.04 | 0 | 0 | 8.07 | 0.04 | 2.02 | 0.013333 | 3.376667 |
| IFIH1 | 2.774 | 1.471964 | 8.28E-05 | 0.000533 | yes | up | 0.88 | 0.78 | 0.61 | 4.19 | 3.81 | 7.75 | 0.756667 | 5.25 |
| TLDC2 | 3.78 | 1.918447 | 8.51E-05 | 0.000547 | yes | up | 0.18 | 0.41 | 0.31 | 2.24 | 3.14 | 2.84 | 0.3 | 2.74 |
| LOC100347628 | 0.198 | -2.33311 | 8.57E-05 | 0.000551 | yes | down | 1.94 | 2.35 | 4.04 | 2.31 | 0.48 | 1.41 | 2.776667 | 1.4 |
| TTC4 | 0.458 | -1.12529 | 8.69E-05 | 0.000558 | yes | down | 3.9 | 4.9 | 3.14 | 3.4 | 4.27 | 5.29 | 3.98 | 4.32 |
| CRABP1 | 0.398 | -1.32749 | 8.91E-05 | 0.000571 | yes | down | 7.22 | 5.62 | 5.62 | 6.82 | 4.23 | 6.73 | 6.153333 | 5.926667 |
| EMC9 | 0.481 | -1.05478 | 8.99E-05 | 0.000576 | yes | down | 43.19 | 23.82 | 22.25 | 35.89 | 32.45 | 29.06 | 29.75333 | 32.46667 |
| C4A | 3.763 | 1.911842 | 9E-05 | 0.000577 | yes | up | 12.37 | 8.2 | 3.75 | 54.14 | 34.71 | 123.57 | 8.106667 | 70.80667 |
| CACHD1 | 2.436 | 1.284422 | 9.06E-05 | 0.00058 | yes | up | 2.75 | 3.74 | 1.99 | 6.33 | 4.34 | 3.53 | 2.826667 | 4.733333 |
| SHISA3 | 3.766 | 1.912851 | 9.1E-05 | 0.000582 | yes | up | 0.53 | 0.47 | 0.15 | 3.75 | 3.99 | 2.29 | 0.383333 | 3.343333 |
| SAMD15 | 0.293 | -1.77114 | 9.18E-05 | 0.000586 | yes | down | 1.25 | 3.56 | 1.25 | 1.34 | 1.24 | 1.55 | 2.02 | 1.376667 |
| STAC | 2.552 | 1.351448 | 9.19E-05 | 0.000587 | yes | up | 1.55 | 1.22 | 0.79 | 10.54 | 4.96 | 6.29 | 1.186667 | 7.263333 |
| LOC103351679 | 4.296 | 2.102869 | 9.2E-05 | 0.000587 | yes | up | 0.33 | 0.29 | 0.31 | 1.82 | 2.26 | 3.49 | 0.31 | 2.523333 |
| CCDC17 | 3.198 | 1.67737 | 9.23E-05 | 0.000588 | yes | up | 0.33 | 0.3 | 0.18 | 2.51 | 2.04 | 1.68 | 0.27 | 2.076667 |
| LOC108176749 | 11.088 | 3.470978 | 9.24E-05 | 0.000589 | yes | up | 0.13 | 0.02 | 0 | 1.72 | 1.08 | 0.98 | 0.05 | 1.26 |
| SLC46A2 | 4.937 | 2.303586 | 9.27E-05 | 0.00059 | yes | up | 0.22 | 0.15 | 0.1 | 1.74 | 1.12 | 2.66 | 0.156667 | 1.84 |
| LOC100354804 | 6.476 | 2.695095 | 9.29E-05 | 0.000591 | yes | up | 0.33 | 0.78 | 0.07 | 4.34 | 6.84 | 5.33 | 0.393333 | 5.503333 |
| LOC103346188 | 3.855 | 1.946787 | 9.42E-05 | 0.000598 | yes | up | 0.76 | 1.29 | 0.58 | 2.23 | 3.17 | 2.34 | 0.876667 | 2.58 |
| USH2A | 5.966 | 2.576784 | 9.44E-05 | 0.000599 | yes | up | 3.85 | 44.87 | 8.24 | 2.52 | 1.03 | 2.57 | 18.98667 | 2.04 |
| LNX2 | 2.216 | 1.147658 | 9.48E-05 | 0.000602 | yes | up | 1.46 | 0.99 | 0.96 | 5.41 | 4.89 | 7.88 | 1.136667 | 6.06 |
| MMP19 | 2.14 | 1.097754 | 9.49E-05 | 0.000602 | yes | up | 2.5 | 2.15 | 1.73 | 12.01 | 12.19 | 7.41 | 2.126667 | 10.53667 |
| PARD6B | 0.316 | -1.66082 | 9.58E-05 | 0.000607 | yes | down | 0.85 | 0.94 | 0.81 | 0.77 | 0.36 | 0.86 | 0.866667 | 0.663333 |
| LOC108176507 | 10.453 | 3.385837 | 9.64E-05 | 0.000611 | yes | up | 0.02 | 0.04 | 0.01 | 0.62 | 0.62 | 0.49 | 0.023333 | 0.576667 |
| WIPI1 | 0.422 | -1.24544 | 9.76E-05 | 0.000618 | yes | down | 18.41 | 11.25 | 6.59 | 11.43 | 13.98 | 9.77 | 12.08333 | 11.72667 |
| TMCC2 | 0.343 | -1.54247 | 9.77E-05 | 0.000618 | yes | down | 2.7 | 3.9 | 4.96 | 4.14 | 2.56 | 2.07 | 3.853333 | 2.923333 |
| SLC7A8 | 0.321 | -1.63823 | 9.78E-05 | 0.000618 | yes | down | 9.11 | 8 | 2.69 | 4.82 | 3.21 | 6.69 | 6.6 | 4.906667 |
| AMPD3 | 0.409 | -1.29013 | 9.91E-05 | 0.000626 | yes | down | 1.9 | 1.35 | 0.86 | 1.12 | 1.38 | 1.41 | 1.37 | 1.303333 |
| KATNAL1 | 2.292 | 1.196494 | 9.95E-05 | 0.000628 | yes | up | 0.53 | 0.97 | 0.47 | 3.37 | 3.23 | 3.32 | 0.656667 | 3.306667 |
| LOC100352119 | 3.352 | 1.74497 | 9.98E-05 | 0.000629 | yes | up | 4.14 | 3.44 | 2.34 | 48.8 | 19.44 | 13.91 | 3.306667 | 27.38333 |
| LOC100338194 | 5.001 | 2.322125 | 0.0001 | 0.00063 | yes | up | 0.67 | 0.24 | 0.88 | 9.16 | 7.98 | 4.31 | 0.596667 | 7.15 |
| LHX8 | 2.586 | 1.370851 | 0.000102 | 0.000639 | yes | up | 1.2 | 3.57 | 2.63 | 14 | 8.29 | 14.26 | 2.466667 | 12.18333 |
| TMEM256 | 0.472 | -1.08218 | 0.000102 | 0.00064 | yes | down | 63.39 | 78.49 | 56.54 | 54.35 | 87.4 | 83.65 | 66.14 | 75.13333 |
| FAM117B | 0.429 | -1.21998 | 0.000102 | 0.000643 | yes | down | 2.54 | 3.27 | 3.99 | 3.95 | 2.56 | 3.83 | 3.266667 | 3.446667 |
| MARCO | 0.063 | -3.98549 | 0.000104 | 0.000651 | yes | down | 0.4 | 0.57 | 0.26 | 0.1 | 0.05 | 0.05 | 0.41 | 0.066667 |
| N4BP3 | 2.31 | 1.207847 | 0.000104 | 0.000651 | yes | up | 5.17 | 6.26 | 6.98 | 43.52 | 28.56 | 23.37 | 6.136667 | 31.81667 |
| GAS7 | 2.21 | 1.143832 | 0.000105 | 0.000657 | yes | up | 6.78 | 3.18 | 2.39 | 20.37 | 16.83 | 19.66 | 4.116667 | 18.95333 |
| SLC16A10 | 0.293 | -1.77222 | 0.000105 | 0.000657 | yes | down | 1.95 | 1.5 | 1.09 | 0.65 | 1.57 | 0.9 | 1.513333 | 1.04 |
| MS4A15 | 53.829 | 5.750325 | 0.000106 | 0.000661 | yes | up | 0 | 0 | 0 | 0.59 | 0.69 | 0.84 | 0 | 0.706667 |
| LOC100341595 | 26.976 | 4.753608 | 0.000107 | 0.000666 | yes | up | 0 | 0.11 | 0 | 2.55 | 1.77 | 3.05 | 0.036667 | 2.456667 |
| HPDL | 0.183 | -2.45078 | 0.000107 | 0.000667 | yes | down | 0.84 | 1.19 | 0.54 | 0.56 | 0.12 | 0.44 | 0.856667 | 0.373333 |
| FLYWCH1 | 2.019 | 1.013781 | 0.000107 | 0.000668 | yes | up | 0.9 | 0.68 | 0.97 | 5.06 | 3.26 | 3.73 | 0.85 | 4.016667 |
| CHAC2 | 0.252 | -1.99051 | 0.000107 | 0.000669 | yes | down | 1.2 | 1.06 | 0.96 | 0.6 | 0.91 | 0.43 | 1.073333 | 0.646667 |
| NNT | 0.487 | -1.03723 | 0.000108 | 0.000671 | yes | down | 104.07 | 56.48 | 50.51 | 78.37 | 88.35 | 81.09 | 70.35333 | 82.60333 |
| SYCE3 | 0.198 | -2.33641 | 0.000108 | 0.000673 | yes | down | 0.69 | 1.52 | 2.36 | 0.43 | 0.14 | 2.25 | 1.523333 | 0.94 |
| LOC108176518 | 0.46 | -1.11958 | 0.000108 | 0.000673 | yes | down | 3.86 | 2.94 | 3.21 | 5.06 | 2.1 | 2.8 | 3.336667 | 3.32 |
| ADGRD1 | 2.347 | 1.231041 | 0.000109 | 0.000676 | yes | up | 3.41 | 1.96 | 1.05 | 11.77 | 9.32 | 11.43 | 2.14 | 10.84 |
| MCAT | 0.465 | -1.10572 | 0.000109 | 0.000676 | yes | down | 4.33 | 4.44 | 4.94 | 4.91 | 3.8 | 6.1 | 4.57 | 4.936667 |
| APBB1IP | 2.203 | 1.139181 | 0.000111 | 0.000685 | yes | up | 1.22 | 1.09 | 0.94 | 5.75 | 4.79 | 6.59 | 1.083333 | 5.71 |
| PRR16 | 11.998 | 3.584751 | 0.000111 | 0.000687 | yes | up | 0.23 | 0.02 | 0 | 2.9 | 1.38 | 2.6 | 0.083333 | 2.293333 |
| RYR2 | 3.106 | 1.635201 | 0.000111 | 0.000687 | yes | up | 0.24 | 0.14 | 0.11 | 1.93 | 0.73 | 1.02 | 0.163333 | 1.226667 |
| ABHD14B | 2.171 | 1.118508 | 0.000111 | 0.000687 | yes | up | 7.26 | 6.39 | 6.36 | 41.53 | 24.39 | 38.01 | 6.67 | 34.64333 |
| LOC100345682 | 0.479 | -1.0633 | 0.000115 | 0.000707 | yes | down | 2.22 | 1.37 | 1.57 | 2.64 | 1.56 | 1.77 | 1.72 | 1.99 |
| IRAK2 | 2.083 | 1.058418 | 0.000116 | 0.000714 | yes | up | 1.32 | 2.04 | 1.83 | 8.83 | 10.08 | 7.48 | 1.73 | 8.796667 |
| LOC103350920 | 31.236 | 4.965137 | 0.000117 | 0.000722 | yes | up | 0 | 0.06 | 0 | 3.44 | 0.94 | 1.28 | 0.02 | 1.886667 |
| LOC103348641 | 0.458 | -1.12518 | 0.000118 | 0.000724 | yes | down | 5.41 | 7.77 | 7.03 | 7.58 | 4.51 | 6.29 | 6.736667 | 6.126667 |
| LOC100350390 | 0.384 | -1.38187 | 0.000118 | 0.000725 | yes | down | 1.11 | 1.04 | 1.25 | 1.06 | 0.88 | 1.21 | 1.133333 | 1.05 |
| LOC108175372 | 4.238 | 2.083483 | 0.000119 | 0.00073 | yes | up | 0.93 | 0.84 | 0.35 | 5.89 | 10.51 | 7.74 | 0.706667 | 8.046667 |
| NRL | 6.049 | 2.596579 | 0.000119 | 0.000734 | yes | up | 0.08 | 0.13 | 0.08 | 1.35 | 0.94 | 1.39 | 0.096667 | 1.226667 |
| LGALS3 | 2.738 | 1.452872 | 0.000121 | 0.000742 | yes | up | 24.92 | 15.53 | 11.25 | 65.73 | 123.95 | 141.05 | 17.23333 | 110.2433 |
| MKI67 | 3.7 | 1.88767 | 0.000121 | 0.000745 | yes | up | 1.88 | 0.93 | 0.69 | 14.43 | 12.12 | 3.52 | 1.166667 | 10.02333 |
| FGF1 | 3.176 | 1.667399 | 0.000122 | 0.000749 | yes | up | 5.77 | 3.23 | 1.79 | 21.24 | 18.42 | 30.92 | 3.596667 | 23.52667 |
| LOC103348853 | 16.943 | 4.082581 | 0.000122 | 0.00075 | yes | up | 0 | 0.07 | 0.03 | 1.45 | 0.97 | 1.79 | 0.033333 | 1.403333 |
| TBC1D30 | 0.306 | -1.70799 | 0.000123 | 0.000751 | yes | down | 0.74 | 0.94 | 1 | 0.7 | 0.31 | 0.92 | 0.893333 | 0.643333 |
| LOC108178220 | 3.846 | 1.943237 | 0.000124 | 0.000757 | yes | up | 0.19 | 0.35 | 0.15 | 2.95 | 1.75 | 1.69 | 0.23 | 2.13 |
| FRRS1 | 0.473 | -1.08069 | 0.000124 | 0.000759 | yes | down | 8.22 | 7.86 | 7.78 | 9.43 | 5.86 | 11.84 | 7.953333 | 9.043333 |
| P2RY14 | 0.459 | -1.12459 | 0.000126 | 0.000772 | yes | down | 2.53 | 2.69 | 3.62 | 3.51 | 3.26 | 3.11 | 2.946667 | 3.293333 |
| B3GALNT2 | 2.248 | 1.168503 | 0.000127 | 0.000773 | yes | up | 10 | 5.15 | 4.86 | 31.83 | 42.19 | 32.5 | 6.67 | 35.50667 |
| EHF | 27.963 | 4.805432 | 0.000127 | 0.000774 | yes | up | 0 | 0.02 | 0 | 0.48 | 0.57 | 0.26 | 0.006667 | 0.436667 |
| CKAP2 | 2.518 | 1.332378 | 0.000127 | 0.000775 | yes | up | 1.68 | 1.72 | 0.89 | 10.21 | 9.89 | 5.38 | 1.43 | 8.493333 |
| FAM222A | 0.288 | -1.79757 | 0.000128 | 0.000778 | yes | down | 0.68 | 0.48 | 0.68 | 0.42 | 0.43 | 0.43 | 0.613333 | 0.426667 |
| LOC108177116 | 0.248 | -2.0101 | 0.000128 | 0.00078 | yes | down | 0.68 | 1.59 | 0.99 | 0.57 | 0.96 | 0.52 | 1.086667 | 0.683333 |
| ARMC6 | 0.437 | -1.19436 | 0.000129 | 0.000784 | yes | down | 3.93 | 3.17 | 4.19 | 4.5 | 2.77 | 4.23 | 3.763333 | 3.833333 |
| LOC100346306 | 77.947 | 6.284426 | 0.000133 | 0.000806 | yes | up | 0 | 0 | 0 | 6.05 | 4.08 | 30.45 | 0 | 13.52667 |
| NSUN3 | 0.382 | -1.38972 | 0.000133 | 0.000809 | yes | down | 0.85 | 0.97 | 1.11 | 1.03 | 0.78 | 0.92 | 0.976667 | 0.91 |
| ADORA2B | 2.624 | 1.391787 | 0.000133 | 0.000809 | yes | up | 0.46 | 0.76 | 0.57 | 4.29 | 3.44 | 3.68 | 0.596667 | 3.803333 |
| DTWD1 | 2.215 | 1.147423 | 0.000133 | 0.000809 | yes | up | 6.65 | 6.31 | 4.85 | 32 | 20.32 | 23.27 | 5.936667 | 25.19667 |
| LOC100338650 | 0.01 | -6.63439 | 0.000134 | 0.000815 | yes | down | 0.09 | 0.84 | 0.3 | 0 | 0 | 0 | 0.41 | 0 |
| LOC108178205 | 4.349 | 2.120838 | 0.000134 | 0.000815 | yes | up | 4.85 | 8.26 | 7 | 84.53 | 14.88 | 99.06 | 6.703333 | 66.15667 |
| PDCD2L | 0.49 | -1.0284 | 0.000135 | 0.00082 | yes | down | 4.71 | 5.58 | 3.39 | 4.82 | 5.89 | 5.55 | 4.56 | 5.42 |
| PRDM10 | 2.093 | 1.065507 | 0.000138 | 0.000832 | yes | up | 0.56 | 0.49 | 0.49 | 3.47 | 2.02 | 2.32 | 0.513333 | 2.603333 |
| DDHD1 | 0.483 | -1.04912 | 0.000138 | 0.000833 | yes | down | 17.91 | 26.8 | 17.19 | 18.07 | 24.96 | 15.86 | 20.63333 | 19.63 |
| PDE6H | 4.111 | 2.039604 | 0.000139 | 0.000841 | yes | up | 0.34 | 0.34 | 0.2 | 4.37 | 3.08 | 2.42 | 0.293333 | 3.29 |
| HYLS1 | 2.137 | 1.095471 | 0.000139 | 0.000841 | yes | up | 1.8 | 1.69 | 1.47 | 8.85 | 11.96 | 6.93 | 1.653333 | 9.246667 |
| ADAMTS1 | 2.023 | 1.016417 | 0.000141 | 0.000852 | yes | up | 7.05 | 6.73 | 7.57 | 33.19 | 23.97 | 45.63 | 7.116667 | 34.26333 |
| LOC108178630 | 0.081 | -3.63362 | 0.000141 | 0.000853 | yes | down | 3.04 | 4.53 | 3 | 1.03 | 0.55 | 1 | 3.523333 | 0.86 |
| LOC100339055 | 2.736 | 1.452251 | 0.000142 | 0.000854 | yes | up | 0.64 | 0.89 | 0.46 | 5.1 | 4.96 | 2.56 | 0.663333 | 4.206667 |
| LOC108177483 | 60.081 | 5.908825 | 0.000145 | 0.000873 | yes | up | 0 | 0 | 0 | 11.26 | 3.87 | 3.37 | 0 | 6.166667 |
| LOC103349265 | 0.39 | -1.35715 | 0.000147 | 0.000879 | yes | down | 11.52 | 15.33 | 7.75 | 14.26 | 6.43 | 11.79 | 11.53333 | 10.82667 |
| ZSWIM7 | 2.453 | 1.294613 | 0.000147 | 0.00088 | yes | up | 3.05 | 1.71 | 2.21 | 14.77 | 10.91 | 13.17 | 2.323333 | 12.95 |
| VASH1 | 2.013 | 1.009243 | 0.000147 | 0.00088 | yes | up | 8.24 | 5.58 | 4.17 | 36.76 | 28.16 | 21.37 | 5.996667 | 28.76333 |
| FGFR2 | 3.927 | 1.973296 | 0.000147 | 0.000881 | yes | up | 0.27 | 0.41 | 0.16 | 2.32 | 1.38 | 4.65 | 0.28 | 2.783333 |
| PTPMT1 | 0.461 | -1.11577 | 0.000148 | 0.000884 | yes | down | 5.49 | 8.11 | 5.58 | 7.56 | 7.7 | 6.06 | 6.393333 | 7.106667 |
| LOC100341622 | 0.389 | -1.36306 | 0.000148 | 0.000886 | yes | down | 5.39 | 5.4 | 6.84 | 4.24 | 4.94 | 7.52 | 5.876667 | 5.566667 |
| ARL4C | 4.385 | 2.132505 | 0.000148 | 0.000887 | yes | up | 0.26 | 0.23 | 0.26 | 1.93 | 4.47 | 1.38 | 0.25 | 2.593333 |
| SYTL2 | 3.295 | 1.720117 | 0.000149 | 0.000888 | yes | up | 1.14 | 0.92 | 0.86 | 7.63 | 3.24 | 12.48 | 0.973333 | 7.783333 |
| CYTIP | 2.554 | 1.352876 | 0.000149 | 0.000891 | yes | up | 0.57 | 0.53 | 0.59 | 4.18 | 3.5 | 2.82 | 0.563333 | 3.5 |
| TECRL | 0.003 | -8.57101 | 0.000151 | 0.000899 | yes | down | 18.04 | 2.07 | 0.17 | 0 | 0.13 | 0 | 6.76 | 0.043333 |
| LONP1 | 0.198 | -2.3345 | 0.000154 | 0.000919 | yes | down | 11.31 | 11.39 | 6.17 | 4.81 | 5.11 | 4.04 | 9.623333 | 4.653333 |
| MMP12 | 0.022 | -5.50699 | 0.000155 | 0.000924 | yes | down | 0.35 | 1.26 | 0.15 | 0 | 0.09 | 0 | 0.586667 | 0.03 |
| HNRNPA0 | 0.473 | -1.081 | 0.000156 | 0.000927 | yes | down | 4.12 | 4.82 | 4.53 | 3.79 | 6.07 | 5.39 | 4.49 | 5.083333 |
| LOC100340053 | 0.244 | -2.03455 | 0.000157 | 0.000934 | yes | down | 0.74 | 0.71 | 0.74 | 0.42 | 0.45 | 0.47 | 0.73 | 0.446667 |
| HHAT | 2.847 | 1.509397 | 0.000158 | 0.00094 | yes | up | 0.56 | 0.52 | 0.23 | 3.74 | 2.59 | 2.53 | 0.436667 | 2.953333 |
| FXN | 0.433 | -1.20707 | 0.000159 | 0.000942 | yes | down | 20.56 | 20.97 | 17.92 | 16.13 | 13.98 | 29.32 | 19.81667 | 19.81 |
| SLITRK2 | 0.409 | -1.28958 | 0.000159 | 0.000944 | yes | down | 0.4 | 0.59 | 0.3 | 0.48 | 0.37 | 0.41 | 0.43 | 0.42 |
| EHD3 | 0.466 | -1.1017 | 0.000161 | 0.000955 | yes | down | 14.98 | 20.07 | 19.03 | 25.22 | 12.99 | 25.84 | 18.02667 | 21.35 |
| PGER6 | 0.222 | -2.17326 | 0.000161 | 0.000955 | yes | down | 21.23 | 51.68 | 30.89 | 10.88 | 7.16 | 37.06 | 34.6 | 18.36667 |
| NYX | 8.416 | 3.073134 | 0.000162 | 0.000958 | yes | up | 0.05 | 0 | 0.03 | 0.72 | 0.49 | 0.44 | 0.026667 | 0.55 |
| GABRB2 | 62.523 | 5.966309 | 0.000163 | 0.000962 | yes | up | 0 | 0 | 0 | 6.48 | 1.12 | 4.35 | 0 | 3.983333 |
| TRIM58 | 0.148 | -2.76047 | 0.000163 | 0.000962 | yes | down | 1.39 | 4.28 | 4.16 | 0.39 | 0.88 | 2.49 | 3.276667 | 1.253333 |
| KIFC2 | 2.461 | 1.299085 | 0.000163 | 0.000962 | yes | up | 1.1 | 0.79 | 0.78 | 5.71 | 4.54 | 5.59 | 0.89 | 5.28 |
| T | 0.01 | -6.57956 | 0.000163 | 0.000965 | yes | down | 0.82 | 0.18 | 0.1 | 0 | 0 | 0 | 0.366667 | 0 |
| RSPH14 | 0.048 | -4.37483 | 0.000165 | 0.000971 | yes | down | 1.52 | 1.18 | 0.91 | 0.07 | 0 | 0.24 | 1.203333 | 0.103333 |
| LOC100341951 | 35.114 | 5.13397 | 0.000165 | 0.000972 | yes | up | 0 | 0.32 | 0 | 17.8 | 4.03 | 3.97 | 0.106667 | 8.6 |
| LOC103350919 | 56.523 | 5.820772 | 0.000167 | 0.000982 | yes | up | 0 | 0 | 0 | 0.42 | 0.5 | 0.14 | 0 | 0.353333 |
| RELN | 0.435 | -1.1993 | 0.000167 | 0.000982 | yes | down | 0.7 | 0.52 | 0.31 | 0.48 | 0.52 | 0.56 | 0.51 | 0.52 |
| CENPF | 3.919 | 1.970497 | 0.000167 | 0.000983 | yes | up | 0.67 | 0.53 | 0.22 | 6.3 | 5.25 | 1.5 | 0.473333 | 4.35 |
| LOC100348309 | 2.006 | 1.004033 | 0.000169 | 0.000991 | yes | up | 1.86 | 1.09 | 0.99 | 5.69 | 6.71 | 6.3 | 1.313333 | 6.233333 |
| LOC100341660 | 0.015 | -6.10461 | 0.000172 | 0.001012 | yes | down | 0.29 | 0.44 | 0.23 | 0 | 0 | 0 | 0.32 | 0 |
| SRGN | 0.488 | -1.03479 | 0.000174 | 0.001022 | yes | down | 27.12 | 40.47 | 43.59 | 52.02 | 44.54 | 36.5 | 37.06 | 44.35333 |
| PCDHB1 | 15.316 | 3.936993 | 0.000174 | 0.001022 | yes | up | 0 | 0.06 | 0.06 | 3.09 | 0.39 | 1.08 | 0.04 | 1.52 |
| SH3TC2 | 3.308 | 1.725911 | 0.000175 | 0.001026 | yes | up | 0.84 | 0.94 | 0.23 | 6.88 | 4.87 | 3 | 0.67 | 4.916667 |
| TCEAL7 | 0.019 | -5.70649 | 0.000176 | 0.00103 | yes | down | 93.55 | 13.97 | 0.54 | 1.08 | 2.83 | 0.74 | 36.02 | 1.55 |
| LOC108176983 | 2.539 | 1.344394 | 0.000176 | 0.001031 | yes | up | 1.19 | 0.46 | 0.55 | 4.92 | 4.14 | 5.35 | 0.733333 | 4.803333 |
| LOC100343557 | 0.444 | -1.16998 | 0.000176 | 0.001031 | yes | down | 154.09 | 171.98 | 196.99 | 133.87 | 168.83 | 266.34 | 174.3533 | 189.68 |
| WDR12 | 0.496 | -1.01273 | 0.000177 | 0.001035 | yes | down | 4.95 | 5.4 | 4.23 | 4.43 | 6.86 | 5.89 | 4.86 | 5.726667 |
| SERPINB5 | 0.217 | -2.20119 | 0.000177 | 0.001036 | yes | down | 0.61 | 1.14 | 1.32 | 0.71 | 0.25 | 0.72 | 1.023333 | 0.56 |
| DLEU7 | 0.088 | -3.51184 | 0.000178 | 0.001041 | yes | down | 0.98 | 1.43 | 0.42 | 0.47 | 0.06 | 0.06 | 0.943333 | 0.196667 |
| LRRN1 | 3.843 | 1.942362 | 0.000179 | 0.001048 | yes | up | 0.4 | 0.11 | 0.15 | 1.71 | 2.39 | 1.87 | 0.22 | 1.99 |
| RNF148 | 0.111 | -3.17693 | 0.00018 | 0.00105 | yes | down | 1.03 | 0.79 | 1.02 | 0.29 | 0.1 | 0.37 | 0.946667 | 0.253333 |
| ZDHHC23 | 2.966 | 1.568373 | 0.000181 | 0.001053 | yes | up | 0.19 | 0.25 | 0.1 | 1.67 | 0.94 | 1.15 | 0.18 | 1.253333 |
| GOT1L1 | 0.108 | -3.20638 | 0.000181 | 0.001055 | yes | down | 0.43 | 0.7 | 0.62 | 0.23 | 0.06 | 0.17 | 0.583333 | 0.153333 |
| GNGT2 | 0.477 | -1.06867 | 0.000181 | 0.001055 | yes | down | 11.36 | 14.09 | 15.91 | 20.45 | 14.17 | 14.25 | 13.78667 | 16.29 |
| TCF19 | 3.555 | 1.829744 | 0.000182 | 0.001058 | yes | up | 0.91 | 0.75 | 0.52 | 6.78 | 8.72 | 2.83 | 0.726667 | 6.11 |
| KIF23 | 4.012 | 2.004144 | 0.00019 | 0.001104 | yes | up | 1.43 | 0.97 | 0.43 | 9.7 | 13.22 | 3.37 | 0.943333 | 8.763333 |
| CNTN2 | 2.08 | 1.056666 | 0.000191 | 0.001109 | yes | up | 1.41 | 1.26 | 1.69 | 8.69 | 8.15 | 5.35 | 1.453333 | 7.396667 |
| LXN | 2.333 | 1.221962 | 0.000192 | 0.001111 | yes | up | 19.22 | 18.95 | 8.13 | 116.1 | 61.29 | 87.16 | 15.43333 | 88.18333 |
| LOC108177072 | 24.274 | 4.601366 | 0.000194 | 0.001123 | yes | up | 0 | 0.09 | 0 | 2.4 | 1.5 | 2.43 | 0.03 | 2.11 |
| HDC | 0.065 | -3.94216 | 0.000194 | 0.001125 | yes | down | 0.37 | 0.2 | 0.37 | 0.03 | 0 | 0.12 | 0.313333 | 0.05 |
| LOC100355410 | 9.647 | 3.270053 | 0.000196 | 0.001135 | yes | up | 0 | 0.09 | 0.07 | 0.94 | 0.91 | 1.88 | 0.053333 | 1.243333 |
| LOC103346609 | 0.26 | -1.9427 | 0.000199 | 0.001147 | yes | down | 0.67 | 0.69 | 0.87 | 0.85 | 0.17 | 0.61 | 0.743333 | 0.543333 |
| NTN4 | 2.575 | 1.364792 | 0.000205 | 0.001181 | yes | up | 6.37 | 3.81 | 2 | 19.28 | 28.71 | 24.09 | 4.06 | 24.02667 |
| LOC100338511 | 0.33 | -1.59924 | 0.000206 | 0.001187 | yes | down | 14.61 | 16.92 | 18.37 | 15.97 | 5.35 | 19.29 | 16.63333 | 13.53667 |
| IMPG2 | 12.557 | 3.650453 | 0.000207 | 0.001188 | yes | up | 0 | 0.03 | 0.02 | 0.71 | 0.33 | 0.56 | 0.016667 | 0.533333 |
| ISM1 | 5.993 | 2.583343 | 0.000213 | 0.00122 | yes | up | 0.08 | 0.1 | 0.07 | 1.49 | 1.54 | 0.58 | 0.083333 | 1.203333 |
| COL1A2 | 2.713 | 1.440117 | 0.000216 | 0.001236 | yes | up | 460.78 | 250.11 | 129.76 | 1292.12 | 2024.63 | 1933.53 | 280.2167 | 1750.093 |
| LOC100347161 | 0.014 | -6.15582 | 0.000217 | 0.001244 | yes | down | 0.56 | 1.51 | 0.72 | 0 | 0 | 0 | 0.93 | 0 |
| MYH3 | 0.001 | -9.98544 | 0.000218 | 0.001246 | yes | down | 86.32 | 12.24 | 0.45 | 0 | 0.21 | 0 | 33.00333 | 0.07 |
| COL3A1 | 2.685 | 1.424704 | 0.000219 | 0.001255 | yes | up | 1067.4 | 563.07 | 308.18 | 3075.48 | 4370.26 | 4242.49 | 646.2167 | 3896.077 |
| LOC103348061 | 62.227 | 5.959469 | 0.000221 | 0.001261 | yes | up | 0 | 0 | 0 | 0.48 | 0.23 | 1.19 | 0 | 0.633333 |
| LOC103351479 | 2.386 | 1.254615 | 0.000222 | 0.001269 | yes | up | 1.19 | 1.82 | 1.02 | 9.65 | 6.66 | 7.25 | 1.343333 | 7.853333 |
| P3H4 | 2.103 | 1.072502 | 0.000223 | 0.001274 | yes | up | 10.68 | 6.92 | 4.24 | 36.59 | 31.45 | 40.18 | 7.28 | 36.07333 |
| GSTO1 | 0.326 | -1.61562 | 0.000226 | 0.001286 | yes | down | 174.2 | 310.02 | 195.45 | 154.7 | 79.21 | 299.24 | 226.5567 | 177.7167 |
| LOC100342853 | 0.141 | -2.8242 | 0.000226 | 0.001286 | yes | down | 1.19 | 3.61 | 3.56 | 0.9 | 1.34 | 0.69 | 2.786667 | 0.976667 |
| ABCB8 | 0.456 | -1.13274 | 0.000231 | 0.001315 | yes | down | 6.96 | 3.19 | 3.86 | 5.97 | 4.28 | 5.05 | 4.67 | 5.1 |
| CKS1B | 2.244 | 1.166031 | 0.000232 | 0.001319 | yes | up | 11.84 | 10.95 | 7.76 | 64.7 | 66.73 | 33.74 | 10.18333 | 55.05667 |
| ZC3H12A | 2.38 | 1.25113 | 0.000234 | 0.001331 | yes | up | 1.67 | 1.89 | 1 | 9.07 | 14.29 | 7.16 | 1.52 | 10.17333 |
| NGB | 0.06 | -4.04831 | 0.000236 | 0.001339 | yes | down | 0.47 | 0.4 | 0.47 | 0 | 0.17 | 0.08 | 0.446667 | 0.083333 |
| CERCAM | 2.242 | 1.164587 | 0.000236 | 0.00134 | yes | up | 4.55 | 3.82 | 1.84 | 19.96 | 12.45 | 19.43 | 3.403333 | 17.28 |
| RPL34 | 26.794 | 4.743824 | 0.000237 | 0.001342 | yes | up | 0.93 | 0.94 | 0.16 | 1.05 | 207.24 | 219.8 | 0.676667 | 142.6967 |
| CNTFR | 4.304 | 2.105788 | 0.000238 | 0.001345 | yes | up | 2.64 | 2.62 | 0.9 | 6.93 | 32.58 | 18.74 | 2.053333 | 19.41667 |
| HOXB8 | 0.051 | -4.28919 | 0.000238 | 0.001349 | yes | down | 0.4 | 0.34 | 0.19 | 0.04 | 0 | 0.07 | 0.31 | 0.036667 |
| LOC100344884 | 34.455 | 5.106653 | 0.000239 | 0.001353 | yes | up | 0.42 | 0.18 | 0.14 | 38.14 | 0.44 | 24.14 | 0.246667 | 20.90667 |
| NINJ1 | 0.437 | -1.19537 | 0.000241 | 0.00136 | yes | down | 15.68 | 21.82 | 22.06 | 22.69 | 24.57 | 12.92 | 19.85333 | 20.06 |
| PI15 | 0.239 | -2.06654 | 0.000241 | 0.00136 | yes | down | 1.9 | 1.14 | 0.54 | 0.71 | 0.6 | 0.68 | 1.193333 | 0.663333 |
| LOC103345420 | 3.634 | 1.861438 | 0.000241 | 0.001361 | yes | up | 41.62 | 14.74 | 6.74 | 157.41 | 185.9 | 183.33 | 21.03333 | 175.5467 |
| ASB4 | 0.182 | -2.46161 | 0.000241 | 0.001362 | yes | down | 7.41 | 1.08 | 1.19 | 1.14 | 1.72 | 1.16 | 3.226667 | 1.34 |
| NAPEPLD | 0.465 | -1.10328 | 0.000241 | 0.001362 | yes | down | 3.32 | 2.19 | 1.98 | 2.81 | 3.35 | 2.17 | 2.496667 | 2.776667 |
| CEP290 | 2.103 | 1.072247 | 0.000244 | 0.001372 | yes | up | 0.34 | 0.33 | 0.38 | 2.14 | 1.31 | 1.91 | 0.35 | 1.786667 |
| RASL11A | 2.765 | 1.467291 | 0.000245 | 0.00138 | yes | up | 1.29 | 1.12 | 1.67 | 8.86 | 6.84 | 11.91 | 1.36 | 9.203333 |
| LOC103348036 | 10.441 | 3.384169 | 0.000247 | 0.00139 | yes | up | 0.03 | 0.06 | 0.06 | 2.02 | 0.81 | 0.9 | 0.05 | 1.243333 |
| LOC100342322 | 0.154 | -2.69621 | 0.000248 | 0.001398 | yes | down | 1.14 | 0.63 | 3.66 | 3.4 | 2.5 | 1.69 | 1.81 | 2.53 |
| LOC100328937 | 0.148 | -2.75385 | 0.000249 | 0.001401 | yes | down | 5.43 | 7.66 | 4.94 | 4.61 | 0.18 | 1.72 | 6.01 | 2.17 |
| ENHO | 2.76 | 1.464478 | 0.000255 | 0.001432 | yes | up | 2.24 | 1.17 | 1.11 | 9.88 | 8.47 | 11.27 | 1.506667 | 9.873333 |
| CFTR | 0.063 | -3.99626 | 0.000255 | 0.001432 | yes | down | 0.18 | 0.08 | 0.14 | 0.03 | 0 | 0.03 | 0.133333 | 0.02 |
| CDHR5 | 23.377 | 4.547003 | 0.000258 | 0.001447 | yes | up | 0 | 0.13 | 0 | 2.77 | 1.72 | 2.7 | 0.043333 | 2.396667 |
| HBEGF | 0.493 | -1.01902 | 0.00026 | 0.001454 | yes | down | 3.09 | 3.35 | 3.62 | 3.44 | 4.63 | 3.91 | 3.353333 | 3.993333 |
| CENPI | 2.745 | 1.456986 | 0.00026 | 0.001457 | yes | up | 0.52 | 0.59 | 0.24 | 3.18 | 3.4 | 2.14 | 0.45 | 2.906667 |
| ONECUT1 | 0.101 | -3.30437 | 0.000261 | 0.001458 | yes | down | 0.32 | 0.1 | 0.05 | 0.06 | 0.01 | 0.05 | 0.156667 | 0.04 |
| LOC108176398 | 6.856 | 2.777374 | 0.000261 | 0.001463 | yes | up | 0.05 | 0.23 | 0.04 | 1.44 | 1.22 | 2.68 | 0.106667 | 1.78 |
| ZNF853 | 0.456 | -1.13358 | 0.000266 | 0.001486 | yes | down | 4.06 | 2.25 | 2.17 | 2.92 | 2.98 | 3.34 | 2.826667 | 3.08 |
| LOC103352496 | 2.721 | 1.44406 | 0.000266 | 0.001486 | yes | up | 0.7 | 1.74 | 1.02 | 7.03 | 5.98 | 12.07 | 1.153333 | 8.36 |
| LOC108178241 | 4.895 | 2.291355 | 0.000268 | 0.001492 | yes | up | 0.07 | 0.28 | 0.16 | 2.02 | 2.5 | 2.85 | 0.17 | 2.456667 |
| MXRA7 | 0.297 | -1.74952 | 0.000268 | 0.001493 | yes | down | 45.76 | 30.29 | 27.72 | 29.09 | 36.2 | 7.68 | 34.59 | 24.32333 |
| S100A8 | 0.227 | -2.13639 | 0.000273 | 0.001518 | yes | down | 9.93 | 43 | 38.79 | 12.83 | 13.12 | 26.03 | 30.57333 | 17.32667 |
| PRC1 | 3.282 | 1.714779 | 0.000275 | 0.001528 | yes | up | 2.72 | 2.38 | 1.18 | 20.13 | 22.02 | 6.28 | 2.093333 | 16.14333 |
| GRM8 | 0.202 | -2.30953 | 0.000277 | 0.001538 | yes | down | 0.29 | 0.25 | 0.36 | 0.22 | 0.13 | 0.1 | 0.3 | 0.15 |
| RHAG | 0.014 | -6.19731 | 0.000282 | 0.001564 | yes | down | 0.12 | 0.26 | 0.43 | 0 | 0 | 0 | 0.27 | 0 |
| LOC100345696 | 2.288 | 1.19413 | 0.000285 | 0.001579 | yes | up | 6.29 | 4.61 | 2.59 | 20.63 | 21.47 | 26.61 | 4.496667 | 22.90333 |
| C15H4orf32 | 2.832 | 1.50168 | 0.000285 | 0.00158 | yes | up | 1.76 | 2.07 | 3.09 | 15.95 | 10.61 | 21.97 | 2.306667 | 16.17667 |
| PLA2G12A | 0.324 | -1.62404 | 0.000285 | 0.00158 | yes | down | 43.19 | 20.98 | 14.63 | 16.67 | 30.29 | 11.88 | 26.26667 | 19.61333 |
| CNTNAP5 | 0.494 | -1.01749 | 0.000286 | 0.001583 | yes | down | 7.77 | 7.24 | 7.09 | 6.86 | 10.76 | 8.5 | 7.366667 | 8.706667 |
| STRC | 4.849 | 2.277617 | 0.000289 | 0.001597 | yes | up | 0.04 | 0.08 | 0.05 | 0.63 | 0.42 | 0.95 | 0.056667 | 0.666667 |
| INCENP | 2.424 | 1.277247 | 0.000289 | 0.001599 | yes | up | 0.96 | 0.79 | 0.68 | 5.86 | 5.6 | 2.65 | 0.81 | 4.703333 |
| GDPD1 | 0.253 | -1.98399 | 0.000291 | 0.001605 | yes | down | 1.42 | 0.87 | 2.37 | 0.94 | 1.19 | 0.74 | 1.553333 | 0.956667 |
| LOC108178624 | 0.463 | -1.11226 | 0.000291 | 0.001605 | yes | down | 11.24 | 15.38 | 11.26 | 19.57 | 13.7 | 9.2 | 12.62667 | 14.15667 |
| LCK | 5.072 | 2.342565 | 0.000292 | 0.001613 | yes | up | 0.12 | 0.08 | 0.13 | 1.67 | 1.18 | 1.17 | 0.11 | 1.34 |
| SETD6 | 0.473 | -1.0798 | 0.000293 | 0.001613 | yes | down | 2.45 | 1.85 | 2.4 | 2.84 | 2.13 | 2.9 | 2.233333 | 2.623333 |
| DUSP14 | 0.387 | -1.36871 | 0.0003 | 0.001652 | yes | down | 3.69 | 6.35 | 2.35 | 4.41 | 3.75 | 3.18 | 4.13 | 3.78 |
| LOC100346439 | 0.081 | -3.62946 | 0.0003 | 0.001652 | yes | down | 6.63 | 8.13 | 2.82 | 0.52 | 2.84 | 0 | 5.86 | 1.12 |
| SGCB | 2.016 | 1.011369 | 0.000301 | 0.001653 | yes | up | 19.34 | 8.98 | 13.45 | 70.41 | 70.46 | 64.87 | 13.92333 | 68.58 |
| WNT2B | 0.417 | -1.26106 | 0.000306 | 0.001678 | yes | down | 1.49 | 1.46 | 0.68 | 1.26 | 0.79 | 1.5 | 1.21 | 1.183333 |
| ABHD5 | 0.494 | -1.01712 | 0.000307 | 0.001687 | yes | down | 48.11 | 52.34 | 66.79 | 74.93 | 78.49 | 47.27 | 55.74667 | 66.89667 |
| CLVS1 | 2.386 | 1.254669 | 0.00031 | 0.001702 | yes | up | 0.76 | 0.97 | 0.92 | 3.98 | 6.55 | 4.63 | 0.883333 | 5.053333 |
| LPAR1 | 2.492 | 1.317289 | 0.000311 | 0.001705 | yes | up | 5.58 | 3.03 | 1.81 | 16.51 | 19.19 | 24.25 | 3.473333 | 19.98333 |
| LOC103347537 | 0.092 | -3.43859 | 0.000312 | 0.001708 | yes | down | 2.72 | 1.64 | 0.78 | 0.37 | 0.39 | 0.35 | 1.713333 | 0.37 |
| SAMD9L | 2.228 | 1.15564 | 0.000313 | 0.001715 | yes | up | 1.61 | 1.57 | 1.33 | 8.9 | 4.54 | 10.74 | 1.503333 | 8.06 |
| DOK3 | 0.298 | -1.74826 | 0.000315 | 0.001721 | yes | down | 0.61 | 1.27 | 0.69 | 0.55 | 0.66 | 0.62 | 0.856667 | 0.61 |
| DCDC2B | 3.457 | 1.789539 | 0.000315 | 0.001722 | yes | up | 0.25 | 0.17 | 0.14 | 2.12 | 0.84 | 1.05 | 0.186667 | 1.336667 |
| GNAT3 | 0.073 | -3.77987 | 0.000316 | 0.001727 | yes | down | 6.95 | 10.77 | 16.33 | 2.36 | 4.06 | 0.72 | 11.35 | 2.38 |
| LOC100358140 | 0.386 | -1.374 | 0.000316 | 0.001727 | yes | down | 5.71 | 8.2 | 9.04 | 7.07 | 6.84 | 7.86 | 7.65 | 7.256667 |
| NKAIN1 | 5.063 | 2.339981 | 0.000317 | 0.001732 | yes | up | 0.18 | 0.12 | 0.04 | 1.75 | 1.25 | 1.01 | 0.113333 | 1.336667 |
| LOC100355642 | 0.102 | -3.29101 | 0.000318 | 0.001734 | yes | down | 1.59 | 1.42 | 4.12 | 0.32 | 0.04 | 1.21 | 2.376667 | 0.523333 |
| CCDC68 | 0.247 | -2.01911 | 0.000318 | 0.001737 | yes | down | 1.86 | 1.1 | 2.59 | 1.95 | 0.77 | 0.78 | 1.85 | 1.166667 |
| LOC108175719 | 0.015 | -6.05808 | 0.000322 | 0.001759 | yes | down | 0.58 | 0.78 | 1.24 | 0 | 0 | 0 | 0.866667 | 0 |
| LOC100343231 | 4.979 | 2.315796 | 0.000323 | 0.001762 | yes | up | 2.94 | 0.37 | 1.05 | 9.77 | 17.69 | 23.56 | 1.453333 | 17.00667 |
| RCOR2 | 0.323 | -1.6301 | 0.000326 | 0.001773 | yes | down | 7.82 | 6.96 | 2.27 | 3.12 | 4.45 | 5.12 | 5.683333 | 4.23 |
| BSP1 | 0.433 | -1.20638 | 0.000327 | 0.001777 | yes | down | 509.83 | 538.72 | 433.07 | 888.74 | 379.63 | 346.48 | 493.8733 | 538.2833 |
| CHST2 | 0.384 | -1.38059 | 0.000331 | 0.001795 | yes | down | 4.54 | 7.5 | 5.36 | 6.58 | 6.81 | 2.73 | 5.8 | 5.373333 |
| QSOX1 | 2.013 | 1.00911 | 0.000332 | 0.001801 | yes | up | 4 | 3.57 | 2.86 | 12.93 | 21.77 | 14.84 | 3.476667 | 16.51333 |
| THSD4 | 0.289 | -1.78847 | 0.000334 | 0.001813 | yes | down | 1.47 | 1.1 | 1.4 | 0.32 | 1.26 | 0.9 | 1.323333 | 0.826667 |
| ZNF385A | 3.329 | 1.735069 | 0.000336 | 0.001823 | yes | up | 9.94 | 2.71 | 2.88 | 28.9 | 51.62 | 39.18 | 5.176667 | 39.9 |
| LOC108178304 | 0.057 | -4.12897 | 0.000337 | 0.001828 | yes | down | 0.56 | 0.64 | 0.76 | 0.28 | 0 | 0 | 0.653333 | 0.093333 |
| METTL1 | 0.487 | -1.03725 | 0.000339 | 0.001835 | yes | down | 2.7 | 3.97 | 2.79 | 3.91 | 2.98 | 4.19 | 3.153333 | 3.693333 |
| LOC103352375 | 6.253 | 2.64461 | 0.000339 | 0.001838 | yes | up | 0.18 | 0.25 | 0.13 | 3.59 | 2.92 | 1.9 | 0.186667 | 2.803333 |
| GINS2 | 2.869 | 1.520307 | 0.00034 | 0.001842 | yes | up | 2.23 | 1.6 | 0.72 | 10.35 | 10.6 | 9.68 | 1.516667 | 10.21 |
| LOC103347756 | 49.169 | 5.619679 | 0.000343 | 0.001851 | yes | up | 0 | 0 | 0 | 4.74 | 11.26 | 7.27 | 0 | 7.756667 |
| KHDRBS3 | 0.439 | -1.18871 | 0.000343 | 0.001851 | yes | down | 2.08 | 2.75 | 2.59 | 3.34 | 2.91 | 1.67 | 2.473333 | 2.64 |
| WDR62 | 2.536 | 1.342756 | 0.000343 | 0.001851 | yes | up | 1.12 | 1.04 | 0.91 | 10.26 | 5.19 | 3.54 | 1.023333 | 6.33 |
| SYCE1L | 2.09 | 1.063177 | 0.000346 | 0.001866 | yes | up | 0.56 | 0.85 | 0.47 | 4.05 | 2.49 | 2.85 | 0.626667 | 3.13 |
| CCL21 | 0.427 | -1.22866 | 0.000347 | 0.001869 | yes | down | 29.27 | 29.12 | 11.24 | 25.16 | 25.89 | 18.53 | 23.21 | 23.19333 |
| CORO2A | 4.476 | 2.162303 | 0.000348 | 0.001872 | yes | up | 0.12 | 0.4 | 0.06 | 1.68 | 1.88 | 2.49 | 0.193333 | 2.016667 |
| COL6A6 | 3.147 | 1.653828 | 0.000348 | 0.001873 | yes | up | 2.03 | 1.45 | 0.38 | 10.96 | 10.14 | 6.9 | 1.286667 | 9.333333 |
| SLC26A6 | 0.46 | -1.12031 | 0.00035 | 0.001884 | yes | down | 2.55 | 2.52 | 3.75 | 4.27 | 3.25 | 3.31 | 2.94 | 3.61 |
| LOC108176099 | 9.008 | 3.171143 | 0.000357 | 0.001918 | yes | up | 0.07 | 0.12 | 0 | 1.27 | 1.23 | 1.84 | 0.063333 | 1.446667 |
| LOC100350949 | 0 | -13.9464 | 0.000357 | 0.001918 | yes | down | 531.34 | 0 | 491.72 | 0 | 0 | 0 | 341.02 | 0 |
| SLC20A1 | 2.369 | 1.244204 | 0.000358 | 0.001918 | yes | up | 7.34 | 10.54 | 4.62 | 26.29 | 31.3 | 51.91 | 7.5 | 36.5 |
| LOC100349217 | 0.049 | -4.34405 | 0.000359 | 0.001926 | yes | down | 0.25 | 0.59 | 0.62 | 0.04 | 0.12 | 0 | 0.486667 | 0.053333 |
| KCNK5 | 2.613 | 1.385551 | 0.00036 | 0.001933 | yes | up | 1.25 | 0.67 | 1.29 | 7.18 | 4.56 | 8.27 | 1.07 | 6.67 |
| FAM227B | 98.693 | 6.624881 | 0.000363 | 0.001944 | yes | up | 0 | 0 | 0 | 0.04 | 6.68 | 15.21 | 0 | 7.31 |
| SLC16A6 | 0.248 | -2.01155 | 0.000366 | 0.00196 | yes | down | 0.56 | 0.35 | 0.26 | 0.2 | 0.17 | 0.31 | 0.39 | 0.226667 |
| SIX1 | 2.005 | 1.003909 | 0.00037 | 0.00198 | yes | up | 9.99 | 6.13 | 5.28 | 27.43 | 32.8 | 39.84 | 7.133333 | 33.35667 |
| LOC103350977 | 0.08 | -3.63687 | 0.000371 | 0.001985 | yes | down | 0.47 | 1.52 | 0.64 | 0.09 | 0.35 | 0.1 | 0.876667 | 0.18 |
| RAD51C | 2.33 | 1.220071 | 0.000379 | 0.002018 | yes | up | 1.5 | 1.2 | 1.31 | 8.7 | 3.97 | 6.82 | 1.336667 | 6.496667 |
| LOC100344620 | 2.423 | 1.276858 | 0.00038 | 0.002021 | yes | up | 1.33 | 2.54 | 2.52 | 12.89 | 12.77 | 12.36 | 2.13 | 12.67333 |
| LOC100350916 | 0.099 | -3.34321 | 0.000381 | 0.002027 | yes | down | 7.8 | 6.18 | 6.15 | 4.64 | 0.19 | 0.25 | 6.71 | 1.693333 |
| AKAP5 | 2.61 | 1.384004 | 0.000383 | 0.002034 | yes | up | 0.32 | 0.47 | 0.3 | 2.31 | 2.81 | 1.6 | 0.363333 | 2.24 |
| PRCP | 2.067 | 1.047225 | 0.000383 | 0.002036 | yes | up | 15.69 | 16.82 | 20.54 | 117.44 | 94.9 | 59.73 | 17.68333 | 90.69 |
| COL9A1 | 0.014 | -6.14921 | 0.000384 | 0.00204 | yes | down | 0.16 | 0.07 | 0.28 | 0 | 0 | 0 | 0.17 | 0 |
| TOP2A | 3.288 | 1.717274 | 0.000386 | 0.002047 | yes | up | 3.77 | 3.08 | 1.6 | 28.26 | 32.88 | 7.39 | 2.816667 | 22.84333 |
| RAD54L | 2.946 | 1.558719 | 0.0004 | 0.002119 | yes | up | 0.45 | 0.37 | 0.35 | 3.21 | 3.25 | 1.69 | 0.39 | 2.716667 |
| COASY | 0.494 | -1.01769 | 0.0004 | 0.00212 | yes | down | 16.49 | 18.78 | 19.4 | 21.7 | 14.62 | 29.07 | 18.22333 | 21.79667 |
| MAD2L1 | 2.38 | 1.250739 | 0.000401 | 0.002122 | yes | up | 3.67 | 4.12 | 2.43 | 21.55 | 24.68 | 11.47 | 3.406667 | 19.23333 |
| LOC103348323 | 0.089 | -3.4961 | 0.000402 | 0.002128 | yes | down | 0.91 | 0.99 | 0.71 | 0.11 | 0.12 | 0.32 | 0.87 | 0.183333 |
| CTPS1 | 0.432 | -1.21128 | 0.000407 | 0.002148 | yes | down | 2.27 | 2.36 | 1.16 | 1.65 | 2.2 | 2.01 | 1.93 | 1.953333 |
| LOC100342069 | 0.125 | -2.99645 | 0.000407 | 0.00215 | yes | down | 15.36 | 40.43 | 22.38 | 3.33 | 3.25 | 17.67 | 26.05667 | 8.083333 |
| INSIG1 | 7.125 | 2.832954 | 0.000409 | 0.002158 | yes | up | 6.85 | 8.42 | 23.42 | 254.44 | 105.32 | 324.82 | 12.89667 | 228.1933 |
| LOC103346635 | 3.618 | 1.855216 | 0.00041 | 0.002162 | yes | up | 0.22 | 0.28 | 0.35 | 2.61 | 1.33 | 2.56 | 0.283333 | 2.166667 |
| SAMD13 | 0.334 | -1.57995 | 0.00041 | 0.002163 | yes | down | 4.87 | 4.55 | 2.99 | 2.72 | 7.57 | 1.86 | 4.136667 | 4.05 |
| LOC100344629 | 2.229 | 1.156601 | 0.000412 | 0.002171 | yes | up | 1.22 | 1.12 | 0.7 | 8.04 | 4.79 | 4.42 | 1.013333 | 5.75 |
| SIM2 | 0.321 | -1.63774 | 0.000412 | 0.002175 | yes | down | 2.1 | 2.61 | 0.5 | 1.49 | 1.13 | 1.16 | 1.736667 | 1.26 |
| NCAPG2 | 2.444 | 1.289104 | 0.000415 | 0.002187 | yes | up | 11.15 | 9.42 | 6.48 | 43.64 | 43.32 | 34.43 | 9.016667 | 40.46333 |
| HSPA13 | 0.497 | -1.00932 | 0.000415 | 0.002188 | yes | down | 6.6 | 6.97 | 8.69 | 7.58 | 11.24 | 8.05 | 7.42 | 8.956667 |
| FAM71F1 | 0.193 | -2.37519 | 0.000418 | 0.002201 | yes | down | 0.67 | 0.48 | 0.68 | 0.32 | 0.15 | 0.39 | 0.61 | 0.286667 |
| IQGAP3 | 3.876 | 1.954559 | 0.00042 | 0.00221 | yes | up | 0.97 | 0.73 | 0.3 | 8.16 | 8.11 | 1.92 | 0.666667 | 6.063333 |
| LOC100346169 | 0.409 | -1.28842 | 0.00042 | 0.002211 | yes | down | 29.14 | 48.9 | 34.35 | 20.73 | 44.35 | 44.13 | 37.46333 | 36.40333 |
| ESM1 | 2.755 | 1.462245 | 0.000422 | 0.002216 | yes | up | 1.16 | 0.86 | 0.39 | 4.65 | 6.59 | 4.19 | 0.803333 | 5.143333 |
| TUBB1 | 0.181 | -2.46206 | 0.000422 | 0.002217 | yes | down | 0.28 | 0.49 | 0.45 | 0.11 | 0.1 | 0.33 | 0.406667 | 0.18 |
| KCNE3 | 4.354 | 2.122283 | 0.000426 | 0.002238 | yes | up | 0.28 | 0.29 | 0.21 | 4.31 | 2.33 | 1.65 | 0.26 | 2.763333 |
| ST3GAL6 | 0.466 | -1.10191 | 0.000427 | 0.00224 | yes | down | 17.71 | 21.73 | 30.8 | 23.8 | 25.04 | 30.7 | 23.41333 | 26.51333 |
| LOC103351558 | 2.197 | 1.135275 | 0.000427 | 0.002241 | yes | up | 1.44 | 1.57 | 1.03 | 9.65 | 6.07 | 5.29 | 1.346667 | 7.003333 |
| LOC103349541 | 0.419 | -1.25467 | 0.000427 | 0.002241 | yes | down | 2.16 | 2.39 | 1.53 | 1.96 | 2.26 | 2.5 | 2.026667 | 2.24 |
| LOC108176529 | 3.104 | 1.634123 | 0.000429 | 0.002246 | yes | up | 0.44 | 0.61 | 0.6 | 5.1 | 4.99 | 2.35 | 0.55 | 4.146667 |
| MPZL3 | 2.208 | 1.142814 | 0.000429 | 0.002249 | yes | up | 0.65 | 0.74 | 0.72 | 3.8 | 2.83 | 5.18 | 0.703333 | 3.936667 |
| THBS1 | 0.469 | -1.09248 | 0.00043 | 0.002254 | yes | down | 17.43 | 30.85 | 26.67 | 23.64 | 23.77 | 37.52 | 24.98333 | 28.31 |
| P4HA2 | 2.129 | 1.090485 | 0.000431 | 0.002256 | yes | up | 17.19 | 11.15 | 6.59 | 50.47 | 53.47 | 69.6 | 11.64333 | 57.84667 |
| LOC103350281 | 0.419 | -1.25599 | 0.000432 | 0.002258 | yes | down | 217.37 | 214.51 | 209.55 | 379.85 | 138.05 | 152.85 | 213.81 | 223.5833 |
| NT5M | 0.369 | -1.43745 | 0.000438 | 0.002286 | yes | down | 3.26 | 2.04 | 2.28 | 2.34 | 2.12 | 3.13 | 2.526667 | 2.53 |
| CD3EAP | 0.346 | -1.52921 | 0.000439 | 0.002293 | yes | down | 1.05 | 2 | 1.53 | 1.43 | 1.15 | 1.28 | 1.526667 | 1.286667 |
| UBASH3B | 0.485 | -1.04351 | 0.000445 | 0.002318 | yes | down | 1.24 | 1.28 | 1.59 | 1.56 | 1.7 | 1.49 | 1.37 | 1.583333 |
| CES4A | 0.435 | -1.20009 | 0.000448 | 0.002332 | yes | down | 3.02 | 3.3 | 2.35 | 2.65 | 2.07 | 4.2 | 2.89 | 2.973333 |
| C1RL | 7.912 | 2.984057 | 0.00045 | 0.002342 | yes | up | 0.18 | 0.08 | 0.05 | 0.6 | 2.79 | 2.16 | 0.103333 | 1.85 |
| PTX3 | 0.265 | -1.91375 | 0.000455 | 0.002369 | yes | down | 0.72 | 0.8 | 1.34 | 0.82 | 0.37 | 0.53 | 0.953333 | 0.573333 |
| GNRH1 | 37.181 | 5.216513 | 0.000456 | 0.002373 | yes | up | 0 | 0.05 | 0 | 2.84 | 2.82 | 0.77 | 0.016667 | 2.143333 |
| ABCA8 | 2.381 | 1.251436 | 0.000459 | 0.002383 | yes | up | 9.58 | 4.59 | 2.89 | 31.23 | 28.26 | 34.19 | 5.686667 | 31.22667 |
| SCARB1 | 2.369 | 1.244433 | 0.000464 | 0.002406 | yes | up | 18.9 | 18.04 | 17.07 | 178.37 | 70.78 | 65.82 | 18.00333 | 104.99 |
| LOC100303760 | 0.006 | -7.45497 | 0.000466 | 0.002414 | yes | down | 505.1 | 49.49 | 7.22 | 0.01 | 6.72 | 0.25 | 187.27 | 2.326667 |
| PGM2 | 0.429 | -1.22095 | 0.000469 | 0.002429 | yes | down | 10.67 | 13.09 | 4.8 | 11.62 | 7.48 | 10.43 | 9.52 | 9.843333 |
| ABTB1 | 0.498 | -1.00434 | 0.000471 | 0.002439 | yes | down | 27.28 | 40.38 | 37.64 | 58.86 | 39.14 | 31.7 | 35.1 | 43.23333 |
| IL18BP | 2.339 | 1.225839 | 0.000477 | 0.002466 | yes | up | 1.35 | 1.42 | 1.45 | 7.81 | 6.23 | 7.81 | 1.406667 | 7.283333 |
| P3H3 | 2.194 | 1.133752 | 0.000481 | 0.002485 | yes | up | 3.57 | 2.93 | 1.65 | 14.87 | 16.56 | 15.4 | 2.716667 | 15.61 |
| LOC103350989 | 0.496 | -1.01265 | 0.000481 | 0.002486 | yes | down | 3.18 | 3.13 | 2.81 | 4.58 | 3.13 | 5.26 | 3.04 | 4.323333 |
| GATM | 0.237 | -2.0744 | 0.000484 | 0.002499 | yes | down | 10.1 | 4.25 | 1.48 | 2.01 | 4.18 | 2.17 | 5.276667 | 2.786667 |
| PNPLA7 | 2.049 | 1.034717 | 0.000487 | 0.002511 | yes | up | 0.77 | 0.6 | 0.37 | 2.42 | 1.73 | 3.27 | 0.58 | 2.473333 |
| IRS2 | 0.269 | -1.89589 | 0.00049 | 0.002525 | yes | down | 4.04 | 5.58 | 4.98 | 3.11 | 1.19 | 5.26 | 4.866667 | 3.186667 |
| CXCL9 | 43.128 | 5.430537 | 0.000493 | 0.002539 | yes | up | 0.02 | 0 | 0 | 0.41 | 0.31 | 1.74 | 0.006667 | 0.82 |
| RSAD1 | 0.325 | -1.6202 | 0.000498 | 0.002559 | yes | down | 1.72 | 1.88 | 1.04 | 1 | 0.76 | 1.81 | 1.546667 | 1.19 |
| TKTL1 | 0.016 | -5.96691 | 0.000504 | 0.00259 | yes | down | 0.08 | 0.19 | 0.18 | 0 | 0 | 0 | 0.15 | 0 |
| RGL1 | 2.121 | 1.085079 | 0.000508 | 0.002606 | yes | up | 5.5 | 5.59 | 5.85 | 33.11 | 20.43 | 31.46 | 5.646667 | 28.33333 |
| ZBTB43 | 0.47 | -1.08779 | 0.00051 | 0.002616 | yes | down | 2.49 | 2.77 | 1.51 | 2.07 | 1.98 | 3.51 | 2.256667 | 2.52 |
| FAXDC2 | 0.464 | -1.10883 | 0.000516 | 0.002641 | yes | down | 11.83 | 18.86 | 21.87 | 25.93 | 13.4 | 21.61 | 17.52 | 20.31333 |
| UBE2D1 | 0.427 | -1.22925 | 0.000516 | 0.002642 | yes | down | 14.54 | 8.02 | 13.73 | 10.16 | 16.27 | 10.95 | 12.09667 | 12.46 |
| KIF20A | 3.037 | 1.602702 | 0.000518 | 0.002653 | yes | up | 1.73 | 1.4 | 0.82 | 11.36 | 12.94 | 3.83 | 1.316667 | 9.376667 |
| LOC108177736 | 0.088 | -3.49991 | 0.000529 | 0.002704 | yes | down | 0.16 | 0.25 | 0.13 | 0.02 | 0.07 | 0.02 | 0.18 | 0.036667 |
| KIF18B | 5.135 | 2.360449 | 0.000529 | 0.002704 | yes | up | 0.32 | 0.32 | 0.1 | 4.11 | 4.01 | 0.72 | 0.246667 | 2.946667 |
| STMN1 | 3.124 | 1.643625 | 0.000531 | 0.002711 | yes | up | 11.06 | 18.81 | 5.47 | 119.28 | 100.42 | 41.16 | 11.78 | 86.95333 |
| COL1A1 | 2.324 | 1.21648 | 0.000532 | 0.002715 | yes | up | 737.6 | 405.68 | 247 | 1988.96 | 2772.57 | 2861.96 | 463.4267 | 2541.163 |
| SCN5A | 0.056 | -4.16232 | 0.000538 | 0.002743 | yes | down | 0.25 | 0.47 | 0.02 | 0.05 | 0 | 0.05 | 0.246667 | 0.033333 |
| BPI | 0.065 | -3.94266 | 0.000541 | 0.002757 | yes | down | 0.23 | 0.52 | 0.42 | 0 | 0.05 | 0.13 | 0.39 | 0.06 |
| LOC100358160 | 47.472 | 5.569003 | 0.000543 | 0.002764 | yes | up | 0 | 0 | 0 | 2.51 | 0.85 | 3.07 | 0 | 2.143333 |
| CKS2 | 2.827 | 1.499092 | 0.000548 | 0.002784 | yes | up | 11.02 | 12.54 | 4.18 | 70.98 | 76.94 | 36 | 9.246667 | 61.30667 |
| LOC103349339 | 0.012 | -6.32449 | 0.000551 | 0.002798 | yes | down | 0.42 | 0.45 | 0.03 | 0 | 0 | 0 | 0.3 | 0 |
| LOC103348003 | 4.565 | 2.190761 | 0.000552 | 0.002803 | yes | up | 1.06 | 0.97 | 0.84 | 7.07 | 4.82 | 19.47 | 0.956667 | 10.45333 |
| GABRD | 2.358 | 1.237395 | 0.000554 | 0.002808 | yes | up | 27.46 | 79.57 | 42.63 | 259.1 | 273.35 | 328.98 | 49.88667 | 287.1433 |
| TRIL | 0.29 | -1.78644 | 0.000565 | 0.002857 | yes | down | 2.99 | 4.04 | 8.92 | 6.4 | 2.2 | 3.31 | 5.316667 | 3.97 |
| ABCA9 | 4.05 | 2.017998 | 0.000568 | 0.00287 | yes | up | 0.67 | 0.22 | 0.1 | 3.41 | 3.4 | 2.2 | 0.33 | 3.003333 |
| PGLYRP1 | 0.18 | -2.47568 | 0.000571 | 0.002884 | yes | down | 1.09 | 3.65 | 6.41 | 1.31 | 1.41 | 2.35 | 3.716667 | 1.69 |
| PELI3 | 0.181 | -2.46513 | 0.000573 | 0.002894 | yes | down | 0.31 | 0.56 | 0.44 | 0.09 | 0.3 | 0.18 | 0.436667 | 0.19 |
| LOC100354670 | 0.167 | -2.58115 | 0.000574 | 0.002897 | yes | down | 0.4 | 2.49 | 0.82 | 0.42 | 0.66 | 0.41 | 1.236667 | 0.496667 |
| LOC103351304 | 3.215 | 1.684615 | 0.000574 | 0.002897 | yes | up | 0.23 | 0.31 | 0.13 | 1.72 | 2.08 | 1.32 | 0.223333 | 1.706667 |
| DIAPH3 | 3.457 | 1.789612 | 0.000579 | 0.002918 | yes | up | 0.37 | 0.44 | 0.17 | 2.64 | 4.03 | 1.28 | 0.326667 | 2.65 |
| SERPINE2 | 0.417 | -1.26197 | 0.000586 | 0.002952 | yes | down | 4.7 | 9.67 | 8.9 | 10.78 | 7.2 | 5.98 | 7.756667 | 7.986667 |
| FAM185A | 0.472 | -1.08418 | 0.000587 | 0.002952 | yes | down | 2.29 | 1.93 | 2.47 | 2 | 2.53 | 3.08 | 2.23 | 2.536667 |
| RLA-DR-ALPHA | 2.661 | 1.411757 | 0.000588 | 0.002956 | yes | up | 3.35 | 6.2 | 7.85 | 56.6 | 29.5 | 30.16 | 5.8 | 38.75333 |
| COL9A2 | 0.13 | -2.94386 | 0.000594 | 0.002985 | yes | down | 0.26 | 0.39 | 0.37 | 0.08 | 0.13 | 0.11 | 0.34 | 0.106667 |
| C1QTNF3 | 0.16 | -2.64482 | 0.000595 | 0.002986 | yes | down | 6.91 | 1.92 | 1.17 | 0.71 | 2.51 | 0.41 | 3.333333 | 1.21 |
| SEMA6D | 0.412 | -1.28025 | 0.000598 | 0.002998 | yes | down | 2.55 | 4.07 | 3.81 | 5.71 | 2.47 | 2.43 | 3.476667 | 3.536667 |
| LOC103346094 | 46.3 | 5.53294 | 0.000598 | 0.002998 | yes | up | 0 | 0 | 0 | 0.53 | 0.14 | 0.39 | 0 | 0.353333 |
| SCEL | 3.712 | 1.892365 | 0.000604 | 0.003024 | yes | up | 0.34 | 0.17 | 0.09 | 2.58 | 1.17 | 1.42 | 0.2 | 1.723333 |
| KIF2C | 3.33 | 1.735318 | 0.000605 | 0.00303 | yes | up | 1 | 0.96 | 0.43 | 7.64 | 8.27 | 2.65 | 0.796667 | 6.186667 |
| LOC108176412 | 5.608 | 2.48753 | 0.000608 | 0.003039 | yes | up | 0.14 | 0.06 | 0.09 | 1.53 | 1.05 | 1.39 | 0.096667 | 1.323333 |
| TPGS1 | 0.436 | -1.19868 | 0.000619 | 0.003091 | yes | down | 3.66 | 4.42 | 5.52 | 5.28 | 4.19 | 5.1 | 4.533333 | 4.856667 |
| WDR4 | 0.42 | -1.25247 | 0.000622 | 0.0031 | yes | down | 1.84 | 2.14 | 1.87 | 2.01 | 1.52 | 2.36 | 1.95 | 1.963333 |
| CCDC114 | 4.801 | 2.263434 | 0.000622 | 0.003101 | yes | up | 0.94 | 0.89 | 0.04 | 0.98 | 1.12 | 2.28 | 0.623333 | 1.46 |
| KNSTRN | 2.949 | 1.56029 | 0.000623 | 0.003101 | yes | up | 2.31 | 2.12 | 1.22 | 14.85 | 18.1 | 6.04 | 1.883333 | 12.99667 |
| DTHD1 | 12.796 | 3.677574 | 0.000628 | 0.003125 | yes | up | 0.01 | 0.01 | 0.01 | 0.37 | 0.19 | 0.34 | 0.01 | 0.3 |
| PYCR1 | 2.817 | 1.49433 | 0.000628 | 0.003125 | yes | up | 2.05 | 1.53 | 0.51 | 11.38 | 7.6 | 10.22 | 1.363333 | 9.733333 |
| CIT | 3.903 | 1.964454 | 0.000634 | 0.003151 | yes | up | 0.28 | 0.18 | 0.09 | 2.61 | 1.92 | 0.59 | 0.183333 | 1.706667 |
| MRPS17 | 0.498 | -1.00616 | 0.000641 | 0.003184 | yes | down | 32.18 | 25.68 | 28.42 | 25.12 | 41.55 | 37.17 | 28.76 | 34.61333 |
| LOC103347207 | 4.418 | 2.143441 | 0.000642 | 0.003188 | yes | up | 0.15 | 0.19 | 0.11 | 2.65 | 1.14 | 1.08 | 0.15 | 1.623333 |
| LOC100340458 | 4.772 | 2.254537 | 0.000659 | 0.003265 | yes | up | 0.89 | 0.33 | 0.09 | 4.37 | 1.91 | 8.93 | 0.436667 | 5.07 |
| LOC103351553 | 6.806 | 2.766723 | 0.000664 | 0.003289 | yes | up | 0.12 | 0.09 | 0.05 | 1.54 | 0.57 | 1.93 | 0.086667 | 1.346667 |
| LOC108176477 | 2.195 | 1.13415 | 0.000666 | 0.003297 | yes | up | 1.15 | 0.91 | 0.79 | 4.44 | 3.5 | 5.65 | 0.95 | 4.53 |
| COL21A1 | 3.181 | 1.669471 | 0.000667 | 0.003301 | yes | up | 2.53 | 1.06 | 0.61 | 10.23 | 11.26 | 7.98 | 1.4 | 9.823333 |
| FXYD3 | 19.313 | 4.271464 | 0.000678 | 0.003346 | yes | up | 0.07 | 0.07 | 0 | 2.88 | 1.53 | 1.91 | 0.046667 | 2.106667 |
| KCNA4 | 0.013 | -6.28097 | 0.000683 | 0.003366 | yes | down | 0.17 | 0.12 | 0.01 | 0 | 0 | 0 | 0.1 | 0 |
| CES3 | 0.403 | -1.31105 | 0.000683 | 0.003366 | yes | down | 25.58 | 23.77 | 33.86 | 25.12 | 15.15 | 42.14 | 27.73667 | 27.47 |
| DTX3 | 2.027 | 1.019616 | 0.000684 | 0.003367 | yes | up | 4.14 | 5.44 | 2.51 | 15.13 | 20.61 | 21.7 | 4.03 | 19.14667 |
| RTL1 | 0.037 | -4.73725 | 0.000689 | 0.00339 | yes | down | 2.54 | 0.21 | 0.04 | 0.03 | 0.09 | 0 | 0.93 | 0.04 |
| RIMS4 | 3.338 | 1.739024 | 0.00069 | 0.003395 | yes | up | 0.12 | 0.12 | 0.07 | 0.63 | 0.77 | 1.03 | 0.103333 | 0.81 |
| COQ10A | 0.334 | -1.5831 | 0.000691 | 0.003397 | yes | down | 20.64 | 8.46 | 6.37 | 4.68 | 12.86 | 7.09 | 11.82333 | 8.21 |
| LOC103345583 | 0.018 | -5.79475 | 0.000692 | 0.003402 | yes | down | 0.33 | 0.35 | 0.19 | 0 | 0 | 0 | 0.29 | 0 |
| LOC103345827 | 47.287 | 5.563384 | 0.000696 | 0.003418 | yes | up | 0 | 0 | 0 | 0.4 | 0.11 | 0.46 | 0 | 0.323333 |
| KIFC1 | 3.14 | 1.650818 | 0.000699 | 0.003431 | yes | up | 1.16 | 1.36 | 0.76 | 10.33 | 11.2 | 2.96 | 1.093333 | 8.163333 |
| ADAL | 0.488 | -1.03492 | 0.000699 | 0.003432 | yes | down | 2.08 | 1.92 | 2 | 2.09 | 2.37 | 2.45 | 2 | 2.303333 |
| LOC108177032 | 2.619 | 1.389093 | 0.0007 | 0.003435 | yes | up | 12.58 | 10.95 | 10.86 | 80.07 | 101.53 | 49.83 | 11.46333 | 77.14333 |
| TMEM56 | 2.539 | 1.344303 | 0.000708 | 0.003473 | yes | up | 0.52 | 0.4 | 0.91 | 4.74 | 3.28 | 3.97 | 0.61 | 3.996667 |
| NCKIPSD | 0.342 | -1.54748 | 0.000713 | 0.003492 | yes | down | 2.71 | 3.9 | 6.72 | 5.21 | 3.27 | 3.03 | 4.443333 | 3.836667 |
| LOC100350067 | 0.06 | -4.06486 | 0.000719 | 0.003521 | yes | down | 0.42 | 0.5 | 0.63 | 0 | 0.16 | 0.07 | 0.516667 | 0.076667 |
| TMEM198 | 3.256 | 1.70318 | 0.00072 | 0.003525 | yes | up | 0.6 | 0.78 | 0.21 | 3.65 | 4.03 | 6.73 | 0.53 | 4.803333 |
| LOC100347254 | 4.696 | 2.231472 | 0.000721 | 0.003526 | yes | up | 0.64 | 0.63 | 0.12 | 3.06 | 3.81 | 8.1 | 0.463333 | 4.99 |
| SMIM5 | 0.108 | -3.20825 | 0.000723 | 0.003537 | yes | down | 1.1 | 0.72 | 0.15 | 0.11 | 0.23 | 0.15 | 0.656667 | 0.163333 |
| LOC100359197 | 7.95 | 2.991005 | 0.000725 | 0.003545 | yes | up | 0.06 | 0.16 | 0.14 | 1.1 | 3.01 | 2.78 | 0.12 | 2.296667 |
| LOC108175449 | 3.796 | 1.924495 | 0.000726 | 0.003546 | yes | up | 10.31 | 0.54 | 5 | 41.57 | 35.17 | 46.24 | 5.283333 | 40.99333 |
| PLXNA1 | 2.001 | 1.000969 | 0.000735 | 0.003586 | yes | up | 4.24 | 3.24 | 1.97 | 15.98 | 10.23 | 18.49 | 3.15 | 14.9 |
| PRX | 0.352 | -1.50546 | 0.000738 | 0.003597 | yes | down | 7 | 4.06 | 2.41 | 3.22 | 5.56 | 2.14 | 4.49 | 3.64 |
| LOC103349409 | 22.751 | 4.507856 | 0.000745 | 0.003623 | yes | up | 0.11 | 0.1 | 0 | 5.6 | 0.16 | 6.18 | 0.07 | 3.98 |
| LRRC36 | 2.594 | 1.375068 | 0.000747 | 0.003635 | yes | up | 0.89 | 0.46 | 0.57 | 4.43 | 2.63 | 4.84 | 0.64 | 3.966667 |
| RAB11FIP4 | 0.255 | -1.97279 | 0.000751 | 0.003653 | yes | down | 2.56 | 1.19 | 0.77 | 0.51 | 0.81 | 1.32 | 1.506667 | 0.88 |
| AURKA | 3.46 | 1.790809 | 0.000753 | 0.003659 | yes | up | 0.65 | 0.89 | 0.39 | 6.7 | 6.9 | 2.2 | 0.643333 | 5.266667 |
| ABLIM2 | 0.117 | -3.093 | 0.000757 | 0.003678 | yes | down | 11.01 | 2.9 | 1.89 | 0.51 | 3.8 | 1.15 | 5.266667 | 1.82 |
| CAMKK1 | 2.158 | 1.109869 | 0.000772 | 0.003741 | yes | up | 0.61 | 0.47 | 0.3 | 2.48 | 2.27 | 2.38 | 0.46 | 2.376667 |
| SEMA3G | 0.417 | -1.26334 | 0.000782 | 0.003785 | yes | down | 17.89 | 24.41 | 34.07 | 36.57 | 26.98 | 15.65 | 25.45667 | 26.4 |
| CNTNAP4 | 0.045 | -4.47902 | 0.000783 | 0.003788 | yes | down | 0.07 | 0.12 | 0.22 | 0 | 0.05 | 0 | 0.136667 | 0.016667 |
| LOC103347895 | 4.457 | 2.156104 | 0.000785 | 0.003796 | yes | up | 0.12 | 0.09 | 0.04 | 0.8 | 0.44 | 1.33 | 0.083333 | 0.856667 |
| SESN2 | 0.406 | -1.29984 | 0.000788 | 0.003811 | yes | down | 1.39 | 1.88 | 1.41 | 2.33 | 1.44 | 0.88 | 1.56 | 1.55 |
| LOC108178146 | 5.355 | 2.42087 | 0.000796 | 0.003842 | yes | up | 0.41 | 0.38 | 0.11 | 7.47 | 4.33 | 3.61 | 0.3 | 5.136667 |
| LOC100344797 | 2.051 | 1.036395 | 0.000798 | 0.003849 | yes | up | 4.1 | 2.51 | 2.32 | 11.85 | 17.93 | 12.24 | 2.976667 | 14.00667 |
| LMNB1 | 2.828 | 1.499653 | 0.000799 | 0.003857 | yes | up | 5.99 | 4.46 | 2.48 | 32.63 | 41.87 | 12.56 | 4.31 | 29.02 |
| GALNT10 | 2.05 | 1.035896 | 0.000811 | 0.0039 | yes | up | 1.99 | 1.6 | 1.22 | 11.89 | 6.35 | 5.53 | 1.603333 | 7.923333 |
| GLIS3 | 0.351 | -1.51053 | 0.000811 | 0.0039 | yes | down | 0.37 | 0.54 | 0.27 | 0.22 | 0.36 | 0.39 | 0.393333 | 0.323333 |
| ATP10A | 0.384 | -1.38084 | 0.000819 | 0.003936 | yes | down | 1.7 | 2.58 | 1.24 | 1.55 | 0.93 | 2.53 | 1.84 | 1.67 |
| CCNA2 | 3.237 | 1.69461 | 0.000837 | 0.004015 | yes | up | 1.86 | 1.77 | 0.74 | 13.11 | 14.87 | 5.12 | 1.456667 | 11.03333 |
| LOC103350239 | 2.071 | 1.050452 | 0.000846 | 0.004054 | yes | up | 1.37 | 1.21 | 1.07 | 7.9 | 6.13 | 4.22 | 1.216667 | 6.083333 |
| SPX | 12.07 | 3.59331 | 0.000849 | 0.004068 | yes | up | 0.03 | 0.03 | 0.03 | 0.71 | 1 | 0.65 | 0.03 | 0.786667 |
| LOC100349542 | 0.351 | -1.51059 | 0.000852 | 0.00408 | yes | down | 1.68 | 1.52 | 0.72 | 1.84 | 0.82 | 0.63 | 1.306667 | 1.096667 |
| ASB18 | 2.734 | 1.451158 | 0.000867 | 0.004147 | yes | up | 0.67 | 0.73 | 0.93 | 6.42 | 3.66 | 4.17 | 0.776667 | 4.75 |
| OAS2 | 7.111 | 2.830087 | 0.000872 | 0.004169 | yes | up | 0.53 | 0.13 | 0.05 | 2.09 | 1.5 | 7.8 | 0.236667 | 3.796667 |
| DIO2 | 0.103 | -3.28096 | 0.000874 | 0.004175 | yes | down | 5.85 | 6.29 | 7.31 | 0.96 | 0.15 | 3.43 | 6.483333 | 1.513333 |
| LOC103352202 | 2.943 | 1.557437 | 0.00088 | 0.0042 | yes | up | 0.3 | 0.25 | 0.26 | 2.51 | 1.29 | 1.9 | 0.27 | 1.9 |
| POMC | 0.361 | -1.46855 | 0.000886 | 0.004224 | yes | down | 4.2 | 4.54 | 6.16 | 6.9 | 3.91 | 2.61 | 4.966667 | 4.473333 |
| LOC108177737 | 12.384 | 3.630437 | 0.000894 | 0.004256 | yes | up | 0.03 | 0.03 | 0.02 | 1.13 | 0.48 | 0.76 | 0.026667 | 0.79 |
| FMO5 | 0.452 | -1.14431 | 0.000894 | 0.004259 | yes | down | 2.23 | 1.55 | 2.34 | 2.68 | 1.8 | 2.22 | 2.04 | 2.233333 |
| TNFSF13B | 6.74 | 2.752814 | 0.000899 | 0.004279 | yes | up | 0 | 0.21 | 0.2 | 1.82 | 3.07 | 1.86 | 0.136667 | 2.25 |
| VWA3A | 2.156 | 1.108097 | 0.000905 | 0.004303 | yes | up | 0.44 | 0.94 | 0.62 | 3.6 | 3.42 | 3.31 | 0.666667 | 3.443333 |
| CRISPLD2 | 2.628 | 1.393832 | 0.000909 | 0.004316 | yes | up | 0.85 | 2.61 | 1.84 | 12.03 | 12.4 | 9.47 | 1.766667 | 11.3 |
| LOC103348702 | 6.749 | 2.754621 | 0.000923 | 0.004377 | yes | up | 0.26 | 0.14 | 0 | 1.27 | 2.97 | 1.96 | 0.133333 | 2.066667 |
| FBXO32 | 0.301 | -1.73015 | 0.000924 | 0.004377 | yes | down | 10.96 | 5.59 | 18.4 | 7 | 13.94 | 5.09 | 11.65 | 8.676667 |
| LOC100358277 | 48.624 | 5.603608 | 0.000928 | 0.004394 | yes | up | 0 | 0 | 0 | 6.52 | 1.01 | 2.2 | 0 | 3.243333 |
| LOC108178759 | 0.086 | -3.53838 | 0.00093 | 0.004403 | yes | down | 1.65 | 0.57 | 0.23 | 0.05 | 0.06 | 0.37 | 0.816667 | 0.16 |
| LOC103349960 | 28.297 | 4.822558 | 0.000935 | 0.004426 | yes | up | 0 | 0.05 | 0 | 1.69 | 0.99 | 0.77 | 0.016667 | 1.15 |
| TP53I3 | 2.131 | 1.091851 | 0.000956 | 0.00452 | yes | up | 1.14 | 1.99 | 1.44 | 7.79 | 7.41 | 8.31 | 1.523333 | 7.836667 |
| LOC100349005 | 2.291 | 1.196278 | 0.000962 | 0.004545 | yes | up | 3.55 | 1.4 | 2.64 | 14.79 | 10.77 | 16.53 | 2.53 | 14.03 |
| ATF3 | 7.839 | 2.970718 | 0.000978 | 0.004616 | yes | up | 2.5 | 1.74 | 1.19 | 6.68 | 25.67 | 67.62 | 1.81 | 33.32333 |
| LOC103347553 | 8.872 | 3.149236 | 0.000982 | 0.004629 | yes | up | 0.36 | 0.11 | 0.32 | 6.53 | 1.57 | 8.89 | 0.263333 | 5.663333 |
| LOC103350962 | 21.289 | 4.412059 | 0.000989 | 0.004661 | yes | up | 0 | 0.03 | 0.04 | 1.56 | 1.78 | 0.38 | 0.023333 | 1.24 |
| LOC103345511 | 2.109 | 1.076561 | 0.000992 | 0.004672 | yes | up | 5.52 | 7.53 | 2.6 | 18.77 | 15.94 | 20.34 | 5.216667 | 18.35 |
| TMEM200B | 0.48 | -1.05884 | 0.000995 | 0.004682 | yes | down | 1.99 | 1.48 | 1.13 | 2.07 | 1.64 | 1.51 | 1.533333 | 1.74 |
| LOC103346936 | 0.287 | -1.80195 | 0.000995 | 0.004684 | yes | down | 3.29 | 2.96 | 4.41 | 2.93 | 2.4 | 5.88 | 3.553333 | 3.736667 |
| LOC108177034 | 0.2 | -2.32202 | 0.000998 | 0.004697 | yes | down | 0.71 | 0.59 | 1.16 | 0.29 | 0.23 | 0.77 | 0.82 | 0.43 |
| UBE2L6 | 2.24 | 1.163671 | 0.001032 | 0.004836 | yes | up | 3.58 | 2.19 | 2.53 | 13.49 | 10.36 | 20.92 | 2.766667 | 14.92333 |
| LOC100355689 | 5.137 | 2.360872 | 0.001037 | 0.004854 | yes | up | 0.04 | 0.06 | 0.04 | 0.37 | 0.42 | 0.88 | 0.046667 | 0.556667 |
| ATXN7L2 | 0.347 | -1.52783 | 0.001057 | 0.004934 | yes | down | 4.49 | 3.68 | 1.88 | 2.82 | 2.83 | 4.77 | 3.35 | 3.473333 |
| LOC100343802 | 0.432 | -1.20957 | 0.001057 | 0.004935 | yes | down | 179.99 | 300.58 | 118.51 | 206.55 | 128.88 | 287.36 | 199.6933 | 207.5967 |
| LOC103346165 | 2.696 | 1.430956 | 0.001059 | 0.004937 | yes | up | 1.99 | 0.61 | 0.21 | 3.88 | 3.53 | 3.57 | 0.936667 | 3.66 |
| CSRP2 | 2.091 | 1.064441 | 0.001065 | 0.004964 | yes | up | 18.2 | 18.31 | 14.65 | 63.56 | 63.59 | 128.5 | 17.05333 | 85.21667 |
| REL | 2.34 | 1.226443 | 0.001071 | 0.004985 | yes | up | 0.23 | 0.25 | 0.21 | 1.41 | 1.49 | 1.02 | 0.23 | 1.306667 |
| TIMP4 | 0.469 | -1.09387 | 0.001075 | 0.005005 | yes | down | 27.46 | 41.78 | 27.32 | 34.7 | 60.94 | 40.21 | 32.18667 | 45.28333 |
| LOC100359118 | 0.499 | -1.00423 | 0.001085 | 0.005045 | yes | down | 18.96 | 22.06 | 26.76 | 34.97 | 29.89 | 18.29 | 22.59333 | 27.71667 |
| LOC103345512 | 3.036 | 1.602284 | 0.001094 | 0.005084 | yes | up | 0.26 | 0.26 | 0.22 | 2.07 | 1.18 | 2.14 | 0.246667 | 1.796667 |
| SHCBP1L | 0.098 | -3.35825 | 0.001094 | 0.005084 | yes | down | 2.4 | 0.43 | 1.98 | 0.15 | 0.05 | 0.87 | 1.603333 | 0.356667 |
| LOC103346937 | 0.266 | -1.91206 | 0.001107 | 0.005138 | yes | down | 1.4 | 0.71 | 0.84 | 0.88 | 0.44 | 0.57 | 0.983333 | 0.63 |
| TRIM46 | 3.262 | 1.705709 | 0.001115 | 0.005168 | yes | up | 0.2 | 0.13 | 0.14 | 1.55 | 0.76 | 1.14 | 0.156667 | 1.15 |
| SNAI1 | 4.507 | 2.172085 | 0.001115 | 0.005168 | yes | up | 0.71 | 0.26 | 0.16 | 6.44 | 3.3 | 2.25 | 0.376667 | 3.996667 |
| LOC100349957 | 113.911 | 6.83176 | 0.001117 | 0.005177 | yes | up | 0.03 | 0 | 0 | 3.57 | 0.08 | 5.4 | 0.01 | 3.016667 |
| LOC108175448 | 0.18 | -2.47769 | 0.001119 | 0.005177 | yes | down | 1.15 | 1.97 | 3.52 | 2.05 | 0.69 | 0.2 | 2.213333 | 0.98 |
| LOC108178799 | 9.531 | 3.25266 | 0.001128 | 0.005213 | yes | up | 0.13 | 0.03 | 0 | 0.96 | 0.9 | 1.61 | 0.053333 | 1.156667 |
| LOC108178573 | 0.014 | -6.12064 | 0.001138 | 0.005255 | yes | down | 0.13 | 1 | 0.17 | 0 | 0 | 0 | 0.433333 | 0 |
| PSMC3IP | 2.996 | 1.582824 | 0.001139 | 0.005256 | yes | up | 0.76 | 0.42 | 0.26 | 3.85 | 3.93 | 2.23 | 0.48 | 3.336667 |
| HK2 | 2.203 | 1.1392 | 0.001142 | 0.005267 | yes | up | 7.25 | 7.65 | 2.65 | 31.19 | 21.98 | 36.44 | 5.85 | 29.87 |
| LOC108177268 | 0.387 | -1.36883 | 0.001149 | 0.005294 | yes | down | 2.15 | 2.31 | 1.86 | 1.62 | 1.58 | 2.15 | 2.106667 | 1.783333 |
| LOC103351959 | 3.367 | 1.751445 | 0.001156 | 0.005319 | yes | up | 0.08 | 0.2 | 0.15 | 1.3 | 0.77 | 1.03 | 0.143333 | 1.033333 |
| LOC103348254 | 18.223 | 4.18769 | 0.001165 | 0.00536 | yes | up | 0 | 0.26 | 0 | 2.21 | 7.41 | 0.77 | 0.086667 | 3.463333 |
| LOC100346308 | 5.206 | 2.380065 | 0.001172 | 0.005386 | yes | up | 0.88 | 0.29 | 0.24 | 12.1 | 2.43 | 3.16 | 0.47 | 5.896667 |
| LOC103347329 | 0.156 | -2.68139 | 0.001173 | 0.005387 | yes | down | 13.24 | 2.85 | 1.45 | 0.95 | 3.98 | 1.2 | 5.846667 | 2.043333 |
| LHX6 | 2.36 | 1.238748 | 0.001177 | 0.005404 | yes | up | 1.24 | 1.14 | 1.12 | 11.2 | 4.39 | 4.68 | 1.166667 | 6.756667 |
| FAM81B | 0.024 | -5.38717 | 0.001179 | 0.005412 | yes | down | 0.64 | 0.31 | 0.07 | 0 | 0 | 0.05 | 0.34 | 0.016667 |
| LOC100356929 | 2.199 | 1.136864 | 0.001182 | 0.005428 | yes | up | 1.78 | 0.71 | 1.27 | 8.31 | 5.91 | 6.04 | 1.253333 | 6.753333 |
| LOC103348554 | 0.087 | -3.51608 | 0.001191 | 0.005466 | yes | down | 0.17 | 1.73 | 2.1 | 0.4 | 0.1 | 0.37 | 1.333333 | 0.29 |
| SEZ6L2 | 9.48 | 3.24484 | 0.001195 | 0.005479 | yes | up | 0.19 | 0.05 | 0.02 | 4.85 | 0.55 | 0.47 | 0.086667 | 1.956667 |
| MAPKBP1 | 2.151 | 1.104744 | 0.001198 | 0.005491 | yes | up | 2.95 | 2.27 | 2.57 | 16.54 | 7.07 | 17.15 | 2.596667 | 13.58667 |
| LOC103347584 | 10.715 | 3.42154 | 0.001203 | 0.005513 | yes | up | 0 | 0.49 | 1.02 | 11.64 | 7.26 | 17.17 | 0.503333 | 12.02333 |
| ADAMTS20 | 0.199 | -2.33209 | 0.001205 | 0.005519 | yes | down | 0.82 | 0.43 | 0.08 | 0.15 | 0.23 | 0.22 | 0.443333 | 0.2 |
| LOC108175489 | 25.844 | 4.691748 | 0.001208 | 0.005528 | yes | up | 0 | 0 | 0.13 | 2.77 | 4.16 | 2.87 | 0.043333 | 3.266667 |
| PPBP | 0.03 | -5.06844 | 0.001214 | 0.005552 | yes | down | 1.77 | 2.83 | 1.8 | 0 | 0.46 | 0 | 2.133333 | 0.153333 |
| ZNF248 | 2.02 | 1.014444 | 0.001214 | 0.005552 | yes | up | 0.55 | 0.51 | 0.79 | 2.83 | 2.61 | 2.56 | 0.616667 | 2.666667 |
| LOC103347471 | 2.48 | 1.310342 | 0.001217 | 0.005562 | yes | up | 53.67 | 12.84 | 7.23 | 97.37 | 68.94 | 85.93 | 24.58 | 84.08 |
| SLC8A1 | 2.025 | 1.017833 | 0.001221 | 0.00557 | yes | up | 0.77 | 0.58 | 0.47 | 3.59 | 1.89 | 3.36 | 0.606667 | 2.946667 |
| LOC100343177 | 2.986 | 1.578263 | 0.001223 | 0.005578 | yes | up | 0.35 | 0.32 | 0.36 | 2.26 | 1.32 | 3.82 | 0.343333 | 2.466667 |
| FBXO30 | 0.468 | -1.09406 | 0.001226 | 0.005589 | yes | down | 6.43 | 5.27 | 10.51 | 8.8 | 7.94 | 9.02 | 7.403333 | 8.586667 |
| LOC103350566 | 0.266 | -1.91223 | 0.001229 | 0.005599 | yes | down | 2.5 | 1.4 | 3.24 | 3.02 | 0.46 | 1.26 | 2.38 | 1.58 |
| SUV39H1 | 2.409 | 1.268374 | 0.001237 | 0.005632 | yes | up | 0.49 | 0.5 | 0.47 | 2.82 | 3.71 | 1.91 | 0.486667 | 2.813333 |
| OTUD7A | 3.822 | 1.934341 | 0.001237 | 0.005632 | yes | up | 0.31 | 0.39 | 0.03 | 3.16 | 2.59 | 2.96 | 0.243333 | 2.903333 |
| VSTM4 | 0.491 | -1.02709 | 0.001245 | 0.005668 | yes | down | 20.09 | 28.17 | 30.79 | 29.34 | 24.14 | 41.63 | 26.35 | 31.70333 |
| NOSTRIN | 0.367 | -1.4469 | 0.001246 | 0.00567 | yes | down | 2.19 | 4.95 | 3.81 | 4.64 | 3.28 | 1.9 | 3.65 | 3.273333 |
| DLGAP5 | 3.339 | 1.739387 | 0.001248 | 0.005674 | yes | up | 0.83 | 0.96 | 0.42 | 6.58 | 8.17 | 2.32 | 0.736667 | 5.69 |
| CEP55 | 4.466 | 2.159109 | 0.001253 | 0.005698 | yes | up | 0.73 | 0.69 | 0.23 | 5.57 | 9.86 | 1.51 | 0.55 | 5.646667 |
| SLC16A2 | 0.387 | -1.36833 | 0.001265 | 0.005743 | yes | down | 9.42 | 24.62 | 6.05 | 12.26 | 12.65 | 10.76 | 13.36333 | 11.89 |
| LOC103346823 | 2.547 | 1.348688 | 0.001268 | 0.005755 | yes | up | 1.09 | 0.42 | 0.37 | 3.24 | 2.55 | 2.67 | 0.626667 | 2.82 |
| NEK2 | 4.148 | 2.052292 | 0.00128 | 0.005806 | yes | up | 1.15 | 1.33 | 0.34 | 10.43 | 13.89 | 2.68 | 0.94 | 9 |
| LOC100343607 | 36.65 | 5.195726 | 0.001281 | 0.005812 | yes | up | 0 | 0.05 | 0.09 | 6.82 | 0 | 7.11 | 0.046667 | 4.643333 |
| JAML | 2.571 | 1.362541 | 0.001283 | 0.00582 | yes | up | 0.47 | 0.48 | 0.45 | 4.23 | 2.45 | 4.06 | 0.466667 | 3.58 |
| ULK4 | 2.23 | 1.157218 | 0.001293 | 0.005858 | yes | up | 2.96 | 5.4 | 2.15 | 19.13 | 21.78 | 11.54 | 3.503333 | 17.48333 |
| SPAG5 | 3.517 | 1.814177 | 0.001294 | 0.005861 | yes | up | 0.79 | 0.68 | 0.31 | 6.04 | 6.96 | 1.56 | 0.593333 | 4.853333 |
| LOC100343032 | 0.349 | -1.51783 | 0.0013 | 0.005888 | yes | down | 2.67 | 3.36 | 3.36 | 3.08 | 3.13 | 1.92 | 3.13 | 2.71 |
| TPX2 | 2.285 | 1.192342 | 0.00133 | 0.006011 | yes | up | 2.74 | 2.86 | 1.66 | 14.19 | 17.9 | 7.04 | 2.42 | 13.04333 |
| ACLY | 2.721 | 1.44393 | 0.001338 | 0.006043 | yes | up | 33.11 | 21.6 | 42.36 | 323.58 | 86.85 | 241.97 | 32.35667 | 217.4667 |
| ADAM19 | 2.048 | 1.033906 | 0.001342 | 0.00606 | yes | up | 3.97 | 3.22 | 1.78 | 17.35 | 9.08 | 11.86 | 2.99 | 12.76333 |
| FNDC5 | 0.112 | -3.15993 | 0.001348 | 0.00608 | yes | down | 55.24 | 10.26 | 7.65 | 4.17 | 10.67 | 2.31 | 24.38333 | 5.716667 |
| ENTPD3 | 0.448 | -1.15731 | 0.001362 | 0.00613 | yes | down | 1.94 | 3.07 | 3.92 | 4.19 | 3.58 | 4.08 | 2.976667 | 3.95 |
| NALCN | 17.29 | 4.111851 | 0.001362 | 0.00613 | yes | up | 0.02 | 0 | 0 | 0.23 | 0.24 | 0.27 | 0.006667 | 0.246667 |
| LOC103350682 | 0.118 | -3.08416 | 0.001369 | 0.006157 | yes | down | 0.45 | 0.37 | 0.25 | 0.15 | 0.11 | 0.05 | 0.356667 | 0.103333 |
| LOC103350917 | 28.457 | 4.830722 | 0.001373 | 0.00617 | yes | up | 0.03 | 0 | 0 | 1.25 | 0.48 | 0.38 | 0.01 | 0.703333 |
| LOC108177747 | 12.51 | 3.645031 | 0.001377 | 0.006185 | yes | up | 0 | 0.02 | 0.01 | 0.38 | 0.2 | 0.47 | 0.01 | 0.35 |
| ADGRG2 | 2.305 | 1.204906 | 0.001385 | 0.006213 | yes | up | 0.57 | 0.38 | 0.18 | 2.21 | 1.96 | 1.94 | 0.376667 | 2.036667 |
| ATAD2 | 2.149 | 1.103787 | 0.001389 | 0.006232 | yes | up | 1.65 | 1.17 | 0.68 | 7.34 | 6.53 | 3.94 | 1.166667 | 5.936667 |
| ST8SIA5 | 0.452 | -1.14715 | 0.001396 | 0.006258 | yes | down | 3.21 | 2.33 | 3.27 | 2.57 | 4.66 | 2.32 | 2.936667 | 3.183333 |
| SERPINE1 | 2.302 | 1.20282 | 0.0014 | 0.006276 | yes | up | 6.68 | 4.46 | 3.7 | 27.64 | 38.54 | 14.44 | 4.946667 | 26.87333 |
| KIF15 | 2.888 | 1.530001 | 0.00142 | 0.006355 | yes | up | 0.91 | 0.69 | 0.37 | 5.49 | 6.01 | 1.9 | 0.656667 | 4.466667 |
| RGS17 | 34.709 | 5.117222 | 0.001428 | 0.006387 | yes | up | 0 | 0 | 0 | 0.45 | 0.37 | 0.4 | 0 | 0.406667 |
| LOC103346185 | 24.78 | 4.631119 | 0.001429 | 0.006391 | yes | up | 0 | 0 | 0.05 | 1.43 | 0.92 | 1.18 | 0.016667 | 1.176667 |
| PRICKLE3 | 2.64 | 1.400745 | 0.001451 | 0.006474 | yes | up | 0.69 | 0.63 | 0.8 | 5.24 | 3.44 | 4.99 | 0.706667 | 4.556667 |
| ACKR4 | 0.428 | -1.22392 | 0.001456 | 0.006489 | yes | down | 0.93 | 0.68 | 0.65 | 0.97 | 0.74 | 0.62 | 0.753333 | 0.776667 |
| CPS1 | 8.402 | 3.070802 | 0.001459 | 0.006504 | yes | up | 0.14 | 0.05 | 0.12 | 4.1 | 2.1 | 0.09 | 0.103333 | 2.096667 |
| DDX28 | 0.412 | -1.28066 | 0.001461 | 0.006511 | yes | down | 2.63 | 4.04 | 2.96 | 3.75 | 1.97 | 3.9 | 3.21 | 3.206667 |
| LOC100348940 | 62.692 | 5.970202 | 0.001511 | 0.00671 | yes | up | 0 | 0 | 0 | 1.61 | 1.09 | 0.04 | 0 | 0.913333 |
| LOC108176538 | 2.427 | 1.279231 | 0.001515 | 0.006728 | yes | up | 1.94 | 2.77 | 0.49 | 9.19 | 10.31 | 9.06 | 1.733333 | 9.52 |
| GATA2 | 0.5 | -1.00084 | 0.001543 | 0.006837 | yes | down | 4.49 | 6.86 | 8.54 | 9.3 | 7.8 | 7.29 | 6.63 | 8.13 |
| CASQ2 | 0.054 | -4.19934 | 0.001543 | 0.006838 | yes | down | 91.96 | 13.65 | 2.29 | 1.57 | 7.53 | 3.79 | 35.96667 | 4.296667 |
| CEBPB | 0.463 | -1.11143 | 0.001553 | 0.006874 | yes | down | 19.31 | 23.46 | 19.65 | 12.76 | 25.53 | 31.06 | 20.80667 | 23.11667 |
| WNT10B | 2.771 | 1.470302 | 0.00157 | 0.006945 | yes | up | 0.5 | 0.38 | 0.25 | 2.35 | 1.83 | 3.23 | 0.376667 | 2.47 |
| ADGRB2 | 2.835 | 1.503505 | 0.001584 | 0.007004 | yes | up | 0.49 | 0.24 | 0.18 | 1.98 | 2.7 | 1.37 | 0.303333 | 2.016667 |
| RHOV | 7.993 | 2.998796 | 0.001587 | 0.007018 | yes | up | 0.04 | 0.21 | 0.07 | 0.99 | 1.11 | 3.94 | 0.106667 | 2.013333 |
| STK31 | 24.363 | 4.606642 | 0.001593 | 0.00704 | yes | up | 0 | 0 | 0.01 | 0.32 | 0.26 | 0.39 | 0.003333 | 0.323333 |
| LOC100348739 | 2.058 | 1.04134 | 0.001602 | 0.007073 | yes | up | 2.05 | 3.75 | 1.83 | 12.92 | 10.52 | 14.03 | 2.543333 | 12.49 |
| EPHA1 | 4.055 | 2.019846 | 0.001602 | 0.007073 | yes | up | 0.04 | 0.12 | 0.11 | 0.97 | 0.7 | 1.02 | 0.09 | 0.896667 |
| RIMS1 | 4.486 | 2.165534 | 0.001608 | 0.007089 | yes | up | 0.11 | 0.08 | 0.01 | 0.62 | 0.38 | 1.01 | 0.066667 | 0.67 |
| COL8A1 | 0.426 | -1.23089 | 0.001639 | 0.007216 | yes | down | 4.26 | 2.93 | 1.31 | 2.29 | 2.97 | 3.06 | 2.833333 | 2.773333 |
| LOC103346136 | 3.678 | 1.879054 | 0.001647 | 0.007243 | yes | up | 0.36 | 1.36 | 0.45 | 8.86 | 3.99 | 8.54 | 0.723333 | 7.13 |
| TLR6 | 2.316 | 1.211521 | 0.001659 | 0.00729 | yes | up | 0.68 | 0.57 | 0.3 | 2.86 | 1.92 | 3.52 | 0.516667 | 2.766667 |
| IL15RA | 2.752 | 1.46038 | 0.001669 | 0.007326 | yes | up | 1.12 | 2.5 | 0.67 | 9.66 | 6.75 | 11.84 | 1.43 | 9.416667 |
| LOC103351303 | 36.534 | 5.191161 | 0.001681 | 0.007372 | yes | up | 0 | 0 | 0 | 0.2 | 0.13 | 0.07 | 0 | 0.133333 |
| LOC103349362 | 394.769 | 8.624864 | 0.001686 | 0.007392 | yes | up | 0 | 0 | 0 | 0.1 | 15.31 | 17.19 | 0 | 10.86667 |
| MFAP3L | 2.823 | 1.49742 | 0.00169 | 0.007406 | yes | up | 1.63 | 1.31 | 4.23 | 15.79 | 16.82 | 18.51 | 2.39 | 17.04 |
| ZSCAN23 | 0.421 | -1.24882 | 0.00169 | 0.007409 | yes | down | 1.03 | 1.31 | 1.13 | 1.72 | 0.64 | 1.3 | 1.156667 | 1.22 |
| HSPB3 | 0.087 | -3.52171 | 0.001698 | 0.007438 | yes | down | 23 | 3.68 | 0.54 | 1.38 | 3.64 | 0.24 | 9.073333 | 1.753333 |
| RET | 3.806 | 1.928106 | 0.001702 | 0.007448 | yes | up | 0.56 | 0.1 | 0.17 | 1.91 | 1.9 | 3.55 | 0.276667 | 2.453333 |
| LOC100341069 | 0.167 | -2.57779 | 0.001717 | 0.007505 | yes | down | 0.74 | 0.89 | 0.61 | 0.16 | 0.15 | 0.33 | 0.746667 | 0.213333 |
| LOC100342534 | 0.396 | -1.33738 | 0.001723 | 0.007526 | yes | down | 16.52 | 8.82 | 12.29 | 12.83 | 8.19 | 15.3 | 12.54333 | 12.10667 |
| LOC108175809 | 3.177 | 1.66775 | 0.001724 | 0.007529 | yes | up | 0.25 | 0.28 | 0.44 | 3.22 | 1.93 | 2.47 | 0.323333 | 2.54 |
| NLRC5 | 5.73 | 2.518568 | 0.001727 | 0.007532 | yes | up | 0.05 | 0.07 | 0 | 0.46 | 0.4 | 0.72 | 0.04 | 0.526667 |
| NXPH2 | 2.378 | 1.249776 | 0.001748 | 0.007608 | yes | up | 0.62 | 0.44 | 0.38 | 3.92 | 2.17 | 2.16 | 0.48 | 2.75 |
| LOC100347480 | 0.27 | -1.88945 | 0.001752 | 0.007621 | yes | down | 9.94 | 27.68 | 11.09 | 4.37 | 5.94 | 20.53 | 16.23667 | 10.28 |
| SAPCD1 | 2.739 | 1.453805 | 0.001761 | 0.007661 | yes | up | 1.82 | 1.45 | 0.52 | 7.67 | 9.91 | 6.65 | 1.263333 | 8.076667 |
| PLXDC1 | 3.419 | 1.773579 | 0.001764 | 0.00767 | yes | up | 2.58 | 1.43 | 0.46 | 7.14 | 17.12 | 10.9 | 1.49 | 11.72 |
| BARX1 | 0.079 | -3.66344 | 0.001767 | 0.00768 | yes | down | 0.4 | 0.47 | 1.18 | 0.07 | 0 | 0.33 | 0.683333 | 0.133333 |
| LOC103347227 | 0.483 | -1.05075 | 0.001769 | 0.00769 | yes | down | 13.13 | 13.32 | 21.17 | 20.29 | 19.85 | 14.68 | 15.87333 | 18.27333 |
| PERP | 0.066 | -3.93116 | 0.001772 | 0.007698 | yes | down | 2.86 | 0.54 | 0.09 | 0.04 | 0.43 | 0.04 | 1.163333 | 0.17 |
| JCHAIN | 3.085 | 1.625344 | 0.001774 | 0.007704 | yes | up | 2.16 | 0.61 | 0.64 | 5.87 | 9.57 | 9.33 | 1.136667 | 8.256667 |
| PGR | 3.492 | 1.804152 | 0.001776 | 0.007709 | yes | up | 0.09 | 0.13 | 0.15 | 1.51 | 0.76 | 0.98 | 0.123333 | 1.083333 |
| PSTK | 2.354 | 1.234987 | 0.001779 | 0.007717 | yes | up | 7.92 | 4.33 | 8.62 | 22.9 | 24.4 | 24.57 | 6.956667 | 23.95667 |
| LOC100353731 | 0.353 | -1.50247 | 0.001798 | 0.007787 | yes | down | 61.24 | 87.05 | 45.43 | 28.98 | 33.28 | 100.01 | 64.57333 | 54.09 |
| ALOX12 | 4.021 | 2.007588 | 0.00182 | 0.007866 | yes | up | 0.1 | 0.11 | 0.21 | 1 | 1.07 | 1.8 | 0.14 | 1.29 |
| SLC1A2 | 0.021 | -5.60493 | 0.001821 | 0.007867 | yes | down | 0.01 | 0.29 | 0.06 | 0 | 0 | 0.02 | 0.12 | 0.006667 |
| STRIP2 | 8.591 | 3.102828 | 0.001827 | 0.007887 | yes | up | 2.15 | 0.32 | 0.17 | 20.35 | 11.75 | 16.79 | 0.88 | 16.29667 |
| DERL3 | 0.196 | -2.34877 | 0.00183 | 0.007893 | yes | down | 2.83 | 4.37 | 3.08 | 1.14 | 3.29 | 0.33 | 3.426667 | 1.586667 |
| LOC108175803 | 0.045 | -4.46531 | 0.001834 | 0.007906 | yes | down | 0.41 | 0.07 | 0.06 | 0.05 | 0 | 0 | 0.18 | 0.016667 |
| LOC108178184 | 11.88 | 3.570521 | 0.001853 | 0.007979 | yes | up | 0.03 | 0.01 | 0 | 0.5 | 0.23 | 0.36 | 0.013333 | 0.363333 |
| IRX3 | 0.295 | -1.75996 | 0.001855 | 0.007979 | yes | down | 3.89 | 1.81 | 2.68 | 1.57 | 1.23 | 3.13 | 2.793333 | 1.976667 |
| NUF2 | 3.391 | 1.761767 | 0.001869 | 0.008026 | yes | up | 0.82 | 0.48 | 0.25 | 4.38 | 6.31 | 1.59 | 0.516667 | 4.093333 |
| ABCB9 | 0.311 | -1.6835 | 0.001885 | 0.008087 | yes | down | 0.47 | 0.33 | 0.39 | 0.31 | 0.4 | 0.19 | 0.396667 | 0.3 |
| LOC100009134 | 0.015 | -6.05842 | 0.0019 | 0.008145 | yes | down | 0.25 | 3.57 | 0.85 | 0 | 0 | 0 | 1.556667 | 0 |
| CST3 | 2.139 | 1.096917 | 0.001903 | 0.008156 | yes | up | 286.88 | 305.13 | 484.78 | 1317.18 | 2066.1 | 2320.48 | 358.93 | 1901.253 |
| DHX33 | 0.489 | -1.03214 | 0.001906 | 0.008161 | yes | down | 1.67 | 2.14 | 1.84 | 2.12 | 1.57 | 2.96 | 1.883333 | 2.216667 |
| CCL7 | 6.016 | 2.588687 | 0.001913 | 0.008191 | yes | up | 3.68 | 2.25 | 0.03 | 8.97 | 32.41 | 29.42 | 1.986667 | 23.6 |
| MUC19 | 8.33 | 3.058339 | 0.001916 | 0.0082 | yes | up | 0.01 | 1.84 | 1.82 | 22.46 | 20.28 | 19.21 | 1.223333 | 20.65 |
| LOC108177440 | 0.406 | -1.29894 | 0.001918 | 0.008206 | yes | down | 1.36 | 2.26 | 2.24 | 2.44 | 1.39 | 2.02 | 1.953333 | 1.95 |
| LOC103346025 | 12.649 | 3.660991 | 0.001926 | 0.008237 | yes | up | 0 | 0.12 | 0 | 1.38 | 0.89 | 0.86 | 0.04 | 1.043333 |
| GAREM1 | 2.266 | 1.180345 | 0.001938 | 0.008286 | yes | up | 1.29 | 0.7 | 1.74 | 6.73 | 6.15 | 8.04 | 1.243333 | 6.973333 |
| TTC8 | 0.477 | -1.06846 | 0.001941 | 0.008292 | yes | down | 2.52 | 2.44 | 1.23 | 2.51 | 2.57 | 1.86 | 2.063333 | 2.313333 |
| LOC100346672 | 11.173 | 3.481994 | 0.001943 | 0.008298 | yes | up | 0 | 0.04 | 0.01 | 0.37 | 0.16 | 0.8 | 0.016667 | 0.443333 |
| PRG2 | 0.022 | -5.53494 | 0.001948 | 0.008312 | yes | down | 0.45 | 0.33 | 0.39 | 0 | 0 | 0 | 0.39 | 0 |
| TMEM37 | 0.46 | -1.12017 | 0.001956 | 0.008341 | yes | down | 29 | 39.32 | 24.47 | 23.66 | 23.81 | 53.76 | 30.93 | 33.74333 |
| TBX6 | 2.218 | 1.149344 | 0.00196 | 0.008359 | yes | up | 0.46 | 0.37 | 0.23 | 1.99 | 1.75 | 1.64 | 0.353333 | 1.793333 |
| NIPAL2 | 0.489 | -1.03175 | 0.001991 | 0.008473 | yes | down | 7.8 | 5.37 | 3.68 | 8.83 | 6.76 | 4.09 | 5.616667 | 6.56 |
| SLC16A14 | 5.656 | 2.499686 | 0.001992 | 0.008474 | yes | up | 0.05 | 0.06 | 0.03 | 0.45 | 1.09 | 0.29 | 0.046667 | 0.61 |
| LOC100352425 | 0.052 | -4.25931 | 0.002006 | 0.008531 | yes | down | 0.66 | 0.51 | 0.65 | 0.11 | 0.12 | 0 | 0.606667 | 0.076667 |
| CENPE | 2.937 | 1.554332 | 0.002025 | 0.0086 | yes | up | 0.16 | 0.18 | 0.22 | 2.07 | 1.42 | 0.55 | 0.186667 | 1.346667 |
| LOC103349747 | 4.227 | 2.079656 | 0.002027 | 0.008609 | yes | up | 1.24 | 0.38 | 0.27 | 5.27 | 4.97 | 11.4 | 0.63 | 7.213333 |
| LOC103352014 | 3.254 | 1.702026 | 0.002042 | 0.008668 | yes | up | 0.37 | 0.63 | 0.67 | 2.92 | 6.42 | 3.67 | 0.556667 | 4.336667 |
| KCP | 0.277 | -1.85171 | 0.002045 | 0.008676 | yes | down | 0.26 | 0.28 | 0.21 | 0.31 | 0.1 | 0.1 | 0.25 | 0.17 |
| SLC19A3 | 28.177 | 4.816468 | 0.002052 | 0.008704 | yes | up | 0.02 | 0 | 0 | 0.16 | 0.62 | 0.7 | 0.006667 | 0.493333 |
| CLEC2L | 0.149 | -2.7445 | 0.002064 | 0.008741 | yes | down | 1.35 | 0.53 | 0.39 | 0.31 | 0.33 | 0.15 | 0.756667 | 0.263333 |
| TMEM38B | 0.297 | -1.75233 | 0.002075 | 0.008774 | yes | down | 10.58 | 4.77 | 2.91 | 2.51 | 7.17 | 2.72 | 6.086667 | 4.133333 |
| MAB21L3 | 0.244 | -2.03494 | 0.002108 | 0.008908 | yes | down | 0.99 | 0.35 | 0.73 | 0.22 | 0.53 | 0.47 | 0.69 | 0.406667 |
| TMEM200C | 0.129 | -2.95065 | 0.002113 | 0.008918 | yes | down | 0.19 | 0.32 | 0.07 | 0.1 | 0.06 | 0.02 | 0.193333 | 0.06 |
| GPR1 | 3.339 | 1.739442 | 0.002115 | 0.008923 | yes | up | 0.97 | 0.43 | 0.12 | 3.71 | 4.43 | 3.41 | 0.506667 | 3.85 |
| CAPN12 | 8.295 | 3.052176 | 0.002128 | 0.008966 | yes | up | 0.78 | 0.02 | 0 | 7.59 | 5.92 | 3.69 | 0.266667 | 5.733333 |
| LOC103350276 | 0.226 | -2.14411 | 0.00213 | 0.008975 | yes | down | 7.97 | 2.27 | 0.92 | 1.43 | 2.15 | 2.19 | 3.72 | 1.923333 |
| TNNT2 | 0.05 | -4.3316 | 0.002131 | 0.008978 | yes | down | 9.24 | 1.15 | 0.24 | 0.11 | 1.31 | 0 | 3.543333 | 0.473333 |
| LOC100352749 | 307.89 | 8.266272 | 0.002133 | 0.008984 | yes | up | 0 | 0 | 0 | 0.05 | 7.54 | 7.16 | 0 | 4.916667 |
| CHKA | 3.019 | 1.594031 | 0.002148 | 0.009043 | yes | up | 0.6 | 0.52 | 0.45 | 1.52 | 1.88 | 2.06 | 0.523333 | 1.82 |
| NUBPL | 0.448 | -1.15996 | 0.002159 | 0.009083 | yes | down | 5.85 | 4.66 | 7.53 | 8.69 | 6.5 | 4.59 | 6.013333 | 6.593333 |
| CRLS1 | 0.427 | -1.22856 | 0.002179 | 0.009156 | yes | down | 58.82 | 80.95 | 73.74 | 81 | 30.97 | 107.7 | 71.17 | 73.22333 |
| CKAP2L | 2.729 | 1.448145 | 0.002181 | 0.00916 | yes | up | 1.17 | 0.64 | 0.51 | 6.18 | 6.44 | 2.36 | 0.773333 | 4.993333 |
| CENPA | 4.021 | 2.007495 | 0.002186 | 0.009178 | yes | up | 1.79 | 1.69 | 0.56 | 13.49 | 19.7 | 2.42 | 1.346667 | 11.87 |
| LOC100358590 | 0.372 | -1.42533 | 0.002214 | 0.009272 | yes | down | 1.42 | 4.73 | 1.49 | 2.42 | 2.56 | 1.71 | 2.546667 | 2.23 |
| KIAA0895 | 3.48 | 1.799109 | 0.002224 | 0.009307 | yes | up | 0.12 | 0.09 | 0.05 | 0.86 | 0.67 | 0.57 | 0.086667 | 0.7 |
| TWIST2 | 2.805 | 1.488048 | 0.002226 | 0.009316 | yes | up | 1.73 | 1.32 | 0.68 | 8.82 | 7.38 | 8.54 | 1.243333 | 8.246667 |
| LOC108177718 | 0.472 | -1.08268 | 0.002233 | 0.009342 | yes | down | 1.8 | 2.46 | 2.69 | 3.66 | 2.63 | 1.78 | 2.316667 | 2.69 |
| USP35 | 0.388 | -1.36446 | 0.002254 | 0.009417 | yes | down | 0.97 | 0.5 | 0.72 | 0.86 | 0.8 | 0.4 | 0.73 | 0.686667 |
| CDHR4 | 5.946 | 2.571932 | 0.002254 | 0.009417 | yes | up | 0.09 | 0.02 | 0.06 | 1.23 | 0.78 | 0.48 | 0.056667 | 0.83 |
| PTGER2 | 0.493 | -1.02112 | 0.002279 | 0.0095 | yes | down | 5.99 | 9.16 | 9.28 | 11.72 | 11.46 | 6.23 | 8.143333 | 9.803333 |
| MRAP | 0.303 | -1.72241 | 0.00229 | 0.009537 | yes | down | 7.7 | 10.3 | 12.7 | 8.06 | 12.37 | 2.34 | 10.23333 | 7.59 |
| LOC103351056 | 0.368 | -1.4413 | 0.00229 | 0.009537 | yes | down | 0.76 | 0.65 | 1.26 | 0.64 | 0.92 | 1.42 | 0.89 | 0.993333 |
| PODN | 2.213 | 1.145954 | 0.002304 | 0.009585 | yes | up | 5.16 | 11.43 | 4.06 | 28.43 | 43.22 | 35.17 | 6.883333 | 35.60667 |
| CPVL | 0.278 | -1.84757 | 0.002327 | 0.009669 | yes | down | 0.71 | 0.77 | 0.53 | 0.35 | 0.79 | 0.3 | 0.67 | 0.48 |
| LOC103348108 | 0.482 | -1.05351 | 0.002351 | 0.009758 | yes | down | 0.83 | 1.14 | 0.69 | 1.51 | 0.76 | 1.42 | 0.886667 | 1.23 |
| LOC103349284 | 32.973 | 5.043199 | 0.002358 | 0.009782 | yes | up | 0 | 0 | 0 | 0.44 | 0.59 | 0.77 | 0 | 0.6 |
| LOC100355766 | 16.142 | 4.012764 | 0.00236 | 0.009787 | yes | up | 0 | 0.04 | 0.04 | 0.74 | 1.73 | 0.89 | 0.026667 | 1.12 |
| LOC100351672 | 2.158 | 1.109582 | 0.002368 | 0.009818 | yes | up | 10.24 | 11.94 | 18.64 | 108.03 | 50.92 | 62.32 | 13.60667 | 73.75667 |
| LOC100009451 | 4.259 | 2.090469 | 0.002376 | 0.009844 | yes | up | 1.03 | 0.55 | 0.26 | 4.17 | 2.56 | 11.43 | 0.613333 | 6.053333 |
| RIC3 | 2.158 | 1.109401 | 0.002393 | 0.0099 | yes | up | 1.91 | 4.57 | 1.81 | 17.65 | 10.92 | 14.11 | 2.763333 | 14.22667 |
| TSLP | 3.153 | 1.656865 | 0.002424 | 0.010013 | yes | up | 2.03 | 1.99 | 0.4 | 9.54 | 8.19 | 14.51 | 1.473333 | 10.74667 |
| LOC100345090 | 0.432 | -1.20954 | 0.002427 | 0.010025 | yes | down | 0.77 | 0.54 | 0.6 | 0.52 | 0.63 | 0.82 | 0.636667 | 0.656667 |
| COL13A1 | 0.33 | -1.60134 | 0.002432 | 0.010042 | yes | down | 0.69 | 0.78 | 1.12 | 0.77 | 0.83 | 0.47 | 0.863333 | 0.69 |
| LOC103349438 | 5.881 | 2.556125 | 0.002438 | 0.010061 | yes | up | 0.15 | 0.03 | 0.02 | 0.58 | 1.19 | 0.8 | 0.066667 | 0.856667 |
| RHNO1 | 2.176 | 1.121758 | 0.002465 | 0.010166 | yes | up | 1.86 | 1.2 | 1.28 | 8.52 | 8.13 | 5.7 | 1.446667 | 7.45 |
| LOC108176025 | 0.062 | -4.00768 | 0.002469 | 0.010178 | yes | down | 1.54 | 0.12 | 0.06 | 0.04 | 0.12 | 0.1 | 0.573333 | 0.086667 |
| KRT8 | 0.397 | -1.33123 | 0.002475 | 0.010201 | yes | down | 2.58 | 1.89 | 0.91 | 2.15 | 1.27 | 1.61 | 1.793333 | 1.676667 |
| MAL | 0.37 | -1.43263 | 0.002478 | 0.010209 | yes | down | 3.8 | 1.53 | 4.58 | 3.86 | 3.54 | 1.94 | 3.303333 | 3.113333 |
| LOC100338996 | 19.383 | 4.276738 | 0.002479 | 0.010209 | yes | up | 0 | 0.7 | 0.03 | 13.76 | 10.08 | 3.18 | 0.243333 | 9.006667 |
| KCNN3 | 2.214 | 1.146919 | 0.002499 | 0.010273 | yes | up | 0.81 | 0.49 | 0.23 | 2.47 | 2.17 | 2.86 | 0.51 | 2.5 |
| EDARADD | 6.064 | 2.600381 | 0.002503 | 0.010287 | yes | up | 0.15 | 0.03 | 0.09 | 0.62 | 0.89 | 1.93 | 0.09 | 1.146667 |
| ADTRP | 2.155 | 1.107949 | 0.002508 | 0.010301 | yes | up | 8.18 | 15.79 | 11.56 | 45.55 | 44.1 | 87.45 | 11.84333 | 59.03333 |
| DNA2 | 4.715 | 2.237123 | 0.002509 | 0.010304 | yes | up | 0.05 | 0.07 | 0.05 | 0.92 | 0.78 | 0.26 | 0.056667 | 0.653333 |
| PDE9A | 0.348 | -1.52173 | 0.002515 | 0.010324 | yes | down | 2.45 | 4.17 | 5.79 | 5.26 | 2.67 | 2.96 | 4.136667 | 3.63 |
| NCAPG | 3.221 | 1.687599 | 0.002565 | 0.010512 | yes | up | 0.64 | 0.38 | 0.25 | 3.53 | 4.76 | 1.25 | 0.423333 | 3.18 |
| LOC100344587 | 0.283 | -1.81904 | 0.002567 | 0.010514 | yes | down | 0.61 | 1.6 | 1.74 | 0.74 | 1.18 | 0.79 | 1.316667 | 0.903333 |
| SRRM5 | 3.574 | 1.837378 | 0.00257 | 0.010526 | yes | up | 0.08 | 0.05 | 0.09 | 0.96 | 0.45 | 0.47 | 0.073333 | 0.626667 |
| LOC100339548 | 0.243 | -2.04036 | 0.002591 | 0.010596 | yes | down | 0.33 | 0.61 | 0.41 | 0.13 | 0.32 | 0.32 | 0.45 | 0.256667 |
| LOC108176082 | 2.437 | 1.285035 | 0.002599 | 0.010629 | yes | up | 0.46 | 0.62 | 0.34 | 3.4 | 2.31 | 2.58 | 0.473333 | 2.763333 |
| IRF4 | 2.945 | 1.558173 | 0.002601 | 0.010635 | yes | up | 0.17 | 0.08 | 0.13 | 0.67 | 1.25 | 0.74 | 0.126667 | 0.886667 |
| CDC20 | 3.045 | 1.60634 | 0.002619 | 0.010698 | yes | up | 3.25 | 3.17 | 1.08 | 22.04 | 24.58 | 6.54 | 2.5 | 17.72 |
| PRR11 | 4.352 | 2.121652 | 0.002656 | 0.01083 | yes | up | 0.2 | 0.29 | 0.08 | 2.78 | 2.56 | 0.48 | 0.19 | 1.94 |
| S100A12 | 0.199 | -2.33074 | 0.002659 | 0.010841 | yes | down | 2.98 | 20.81 | 24.55 | 3.6 | 7.98 | 12.5 | 16.11333 | 8.026667 |
| CALB1 | 0.039 | -4.69186 | 0.002663 | 0.010854 | yes | down | 0.07 | 0.39 | 0.43 | 0 | 0 | 0.08 | 0.296667 | 0.026667 |
| LOC100347994 | 6.8 | 2.765497 | 0.002668 | 0.01087 | yes | up | 0.63 | 0.02 | 0.05 | 5.95 | 2.13 | 3.19 | 0.233333 | 3.756667 |
| CHRD | 3.01 | 1.589672 | 0.002681 | 0.010916 | yes | up | 1.12 | 0.35 | 0.29 | 3.13 | 3.91 | 5.36 | 0.586667 | 4.133333 |
| FAM184A | 0.3 | -1.73917 | 0.002685 | 0.010929 | yes | down | 0.47 | 0.31 | 0.22 | 0.17 | 0.33 | 0.18 | 0.333333 | 0.226667 |
| LOC108176449 | 6.719 | 2.74817 | 0.002695 | 0.01096 | yes | up | 0.01 | 0.16 | 0.04 | 2.38 | 0.43 | 1.31 | 0.07 | 1.373333 |
| NTNG2 | 2.171 | 1.1184 | 0.002767 | 0.011219 | yes | up | 0.74 | 0.76 | 0.46 | 3.01 | 2.88 | 4.11 | 0.653333 | 3.333333 |
| TSTD1 | 0.407 | -1.29845 | 0.002792 | 0.011309 | yes | down | 4.89 | 6.67 | 8.54 | 8.81 | 5.64 | 6.09 | 6.7 | 6.846667 |
| SLCO5A1 | 0.479 | -1.06054 | 0.002795 | 0.011317 | yes | down | 2.89 | 1.7 | 3.55 | 4.12 | 2.97 | 2.6 | 2.713333 | 3.23 |
| FYB | 3.325 | 1.733253 | 0.002809 | 0.011369 | yes | up | 0.54 | 0.43 | 0.31 | 2.02 | 1.63 | 6.04 | 0.426667 | 3.23 |
| RNF207 | 0.139 | -2.8484 | 0.002825 | 0.011432 | yes | down | 0.57 | 0.12 | 0.21 | 0.1 | 0.07 | 0.13 | 0.3 | 0.1 |
| APOC3 | 41.479 | 5.374295 | 0.002834 | 0.011465 | yes | up | 0 | 0 | 0 | 2.35 | 4.64 | 0.53 | 0 | 2.506667 |
| LOC108176666 | 4.039 | 2.014107 | 0.00287 | 0.011588 | yes | up | 0.7 | 0.23 | 0.32 | 2.59 | 4.76 | 3.4 | 0.416667 | 3.583333 |
| LOC103349294 | 10.499 | 3.392173 | 0.002874 | 0.011594 | yes | up | 0.11 | 0.15 | 0 | 3.52 | 0.45 | 1.77 | 0.086667 | 1.913333 |
| LOC100359245 | 2.348 | 1.231411 | 0.002877 | 0.011598 | yes | up | 103.17 | 47.73 | 45.98 | 434.57 | 478.65 | 185.53 | 65.62667 | 366.25 |
| PRDM8 | 2.244 | 1.166065 | 0.002877 | 0.011598 | yes | up | 0.6 | 0.46 | 0.24 | 2.47 | 1.62 | 1.72 | 0.433333 | 1.936667 |
| BUB1 | 3.197 | 1.6765 | 0.002883 | 0.011619 | yes | up | 0.6 | 0.55 | 0.22 | 4.74 | 4.33 | 1.26 | 0.456667 | 3.443333 |
| LOC108176480 | 2.056 | 1.039535 | 0.002901 | 0.011684 | yes | up | 1.03 | 1.11 | 0.8 | 4.84 | 2.64 | 7.15 | 0.98 | 4.876667 |
| LOC103350873 | 0.031 | -5.01225 | 0.002916 | 0.011735 | yes | down | 2.55 | 0.48 | 0 | 0 | 0.08 | 0.12 | 1.01 | 0.066667 |
| PRR32 | 0.008 | -6.9523 | 0.002937 | 0.011814 | yes | down | 25.83 | 4.52 | 0.36 | 0 | 0.54 | 0 | 10.23667 | 0.18 |
| TENM1 | 4.954 | 2.308514 | 0.002954 | 0.011872 | yes | up | 0.05 | 0.04 | 0.03 | 1.02 | 0.27 | 0.18 | 0.04 | 0.49 |
| LOC108178891 | 16.045 | 4.004087 | 0.002979 | 0.011961 | yes | up | 0 | 0.63 | 0 | 4.31 | 3.57 | 6.2 | 0.21 | 4.693333 |
| DNHD1 | 3.46 | 1.790628 | 0.002994 | 0.012011 | yes | up | 0.05 | 0.03 | 0.01 | 0.34 | 0.15 | 0.28 | 0.03 | 0.256667 |
| LOC100337703 | 0.355 | -1.4945 | 0.003043 | 0.012195 | yes | down | 55.28 | 18.25 | 27.34 | 17.51 | 47.2 | 19.06 | 33.62333 | 27.92333 |
| C1QTNF2 | 2.002 | 1.001093 | 0.003047 | 0.012204 | yes | up | 3.65 | 2.13 | 1.4 | 10.6 | 11.84 | 11.19 | 2.393333 | 11.21 |
| CAMP | 0.079 | -3.66062 | 0.003054 | 0.012227 | yes | down | 0.37 | 1.28 | 1.72 | 0.07 | 0 | 0.22 | 1.123333 | 0.096667 |
| KNL1 | 3.128 | 1.645401 | 0.003064 | 0.01225 | yes | up | 0.33 | 0.35 | 0.14 | 2.24 | 3 | 0.69 | 0.273333 | 1.976667 |
| LOC100356702 | 0.238 | -2.07044 | 0.00308 | 0.012305 | yes | down | 0.27 | 0.23 | 0.16 | 0.18 | 0.09 | 0.11 | 0.22 | 0.126667 |
| LOC108177137 | 6.004 | 2.585992 | 0.003122 | 0.012454 | yes | up | 0.1 | 0 | 0.22 | 1.87 | 1.67 | 2.4 | 0.106667 | 1.98 |
| PODXL2 | 0.295 | -1.76256 | 0.003124 | 0.012458 | yes | down | 2.18 | 3.03 | 1.37 | 2.29 | 0.62 | 0.97 | 2.193333 | 1.293333 |
| MANBA | 2.275 | 1.186102 | 0.003134 | 0.01249 | yes | up | 0.45 | 0.51 | 0.14 | 2.23 | 2.01 | 2.15 | 0.366667 | 2.13 |
| NFKBIZ | 2.66 | 1.411354 | 0.003145 | 0.012529 | yes | up | 0.51 | 0.89 | 0.35 | 2.97 | 2.74 | 5.24 | 0.583333 | 3.65 |
| FAM111A | 2.058 | 1.041201 | 0.003154 | 0.012562 | yes | up | 3.17 | 1.81 | 1.36 | 13.59 | 10.66 | 6.35 | 2.113333 | 10.2 |
| KIAA1524 | 2.56 | 1.356143 | 0.003159 | 0.012576 | yes | up | 0.27 | 0.29 | 0.15 | 1.55 | 1.59 | 1.14 | 0.236667 | 1.426667 |
| PLEKHA6 | 2.116 | 1.081122 | 0.003169 | 0.012612 | yes | up | 0.28 | 0.17 | 0.24 | 1.08 | 1.4 | 1.02 | 0.23 | 1.166667 |
| LMX1A | 2.831 | 1.501242 | 0.003179 | 0.012642 | yes | up | 0.31 | 0.17 | 0.1 | 1.26 | 1.14 | 1.39 | 0.193333 | 1.263333 |
| CYSLTR1 | 4.754 | 2.249075 | 0.003191 | 0.012687 | yes | up | 0.12 | 0.05 | 0.02 | 0.67 | 0.69 | 0.71 | 0.063333 | 0.69 |
| AZIN2 | 2.293 | 1.197313 | 0.003192 | 0.012687 | yes | up | 0.52 | 0.4 | 1.19 | 2.42 | 2.51 | 2.52 | 0.703333 | 2.483333 |
| FAM47E | 0.022 | -5.4993 | 0.003245 | 0.012878 | yes | down | 0.17 | 0.22 | 0.06 | 0 | 0 | 0 | 0.15 | 0 |
| WNT16 | 0.02 | -5.62353 | 0.003257 | 0.01292 | yes | down | 0.48 | 0.03 | 0.02 | 0 | 0.02 | 0 | 0.176667 | 0.006667 |
| ARHGAP33 | 2.351 | 1.233215 | 0.003258 | 0.01292 | yes | up | 0.33 | 0.32 | 0.23 | 2.33 | 1.64 | 1.03 | 0.293333 | 1.666667 |
| SLITRK3 | 8.839 | 3.143836 | 0.003258 | 0.01292 | yes | up | 0 | 0.02 | 0.02 | 0.22 | 0.16 | 0.39 | 0.013333 | 0.256667 |
| GRO-A | 5.631 | 2.493394 | 0.00326 | 0.012921 | yes | up | 0.14 | 0.84 | 0.52 | 1.68 | 8.64 | 7.91 | 0.5 | 6.076667 |
| LOC103347194 | 0.151 | -2.72716 | 0.003268 | 0.012951 | yes | down | 0.38 | 1.43 | 0.37 | 0.45 | 0.15 | 0.18 | 0.726667 | 0.26 |
| SLC16A4 | 3.024 | 1.596284 | 0.003273 | 0.012967 | yes | up | 0.24 | 0.61 | 0.1 | 2.08 | 1.89 | 2.14 | 0.316667 | 2.036667 |
| SFXN2 | 0.416 | -1.26362 | 0.003275 | 0.012972 | yes | down | 2.23 | 3.67 | 5.26 | 5.76 | 3.95 | 2.46 | 3.72 | 4.056667 |
| ENPP3 | 2.752 | 1.460633 | 0.003328 | 0.013173 | yes | up | 1.82 | 1.34 | 0.31 | 8.17 | 5.6 | 8.36 | 1.156667 | 7.376667 |
| LOC103351576 | 5.173 | 2.370949 | 0.003332 | 0.013186 | yes | up | 0.26 | 0.11 | 0.24 | 3.93 | 2.75 | 0.45 | 0.203333 | 2.376667 |
| ECT2 | 3.003 | 1.586378 | 0.003345 | 0.013229 | yes | up | 0.67 | 0.34 | 0.2 | 3.36 | 3.86 | 1.27 | 0.403333 | 2.83 |
| LOC103349420 | 5.834 | 2.544608 | 0.003372 | 0.013316 | yes | up | 0.44 | 1.02 | 0.37 | 9.06 | 10.33 | 11.34 | 0.61 | 10.24333 |
| LOC100340032 | 2.242 | 1.164781 | 0.003373 | 0.013316 | yes | up | 1.77 | 0.7 | 0.67 | 7.6 | 4.52 | 5.07 | 1.046667 | 5.73 |
| LOC100349270 | 30.076 | 4.910556 | 0.0034 | 0.013415 | yes | up | 0 | 0 | 0 | 0.17 | 0.19 | 0.12 | 0 | 0.16 |
| LOC100345907 | 11.393 | 3.510014 | 0.003425 | 0.013502 | yes | up | 0 | 0.05 | 0.11 | 0.69 | 1.95 | 1.5 | 0.053333 | 1.38 |
| CCDC142 | 2.02 | 1.014236 | 0.003426 | 0.013502 | yes | up | 0.41 | 0.42 | 0.41 | 2.16 | 1.6 | 1.84 | 0.413333 | 1.866667 |
| LOC103352144 | 23.86 | 4.576495 | 0.003435 | 0.01353 | yes | up | 0 | 0.03 | 0 | 1.18 | 0.38 | 0.34 | 0.01 | 0.633333 |
| SHCBP1 | 3.138 | 1.649963 | 0.003482 | 0.013695 | yes | up | 0.96 | 0.58 | 0.36 | 5.09 | 7.04 | 1.8 | 0.633333 | 4.643333 |
| EML6 | 2.136 | 1.094964 | 0.003496 | 0.013742 | yes | up | 1.14 | 1.23 | 0.48 | 5.95 | 2.89 | 5.46 | 0.95 | 4.766667 |
| LOC108178305 | 2.833 | 1.502326 | 0.003502 | 0.013763 | yes | up | 0.36 | 0.23 | 0.56 | 3.48 | 2.39 | 2.42 | 0.383333 | 2.763333 |
| UBE2C | 3.391 | 1.761711 | 0.003503 | 0.013765 | yes | up | 4.54 | 3.02 | 1.13 | 29.31 | 31.25 | 8.15 | 2.896667 | 22.90333 |
| IGSF10 | 2.211 | 1.144979 | 0.003548 | 0.013919 | yes | up | 1.45 | 1.28 | 0.45 | 5.34 | 3.84 | 7.16 | 1.06 | 5.446667 |
| TMEM86A | 0.485 | -1.04319 | 0.003552 | 0.013931 | yes | down | 3.7 | 5.6 | 6.47 | 8.75 | 4.75 | 5.29 | 5.256667 | 6.263333 |
| KIF11 | 2.854 | 1.513052 | 0.003584 | 0.014038 | yes | up | 1.01 | 1.02 | 0.36 | 5.79 | 7.85 | 2.17 | 0.796667 | 5.27 |
| RIIAD1 | 0.224 | -2.15617 | 0.00359 | 0.014053 | yes | down | 2.39 | 4.06 | 3.01 | 1.38 | 2.78 | 0.84 | 3.153333 | 1.666667 |
| LOC100352436 | 2.434 | 1.283303 | 0.003609 | 0.014125 | yes | up | 0.38 | 0.51 | 0.45 | 2.55 | 1.7 | 3.8 | 0.446667 | 2.683333 |
| LOC103349017 | 13.308 | 3.734197 | 0.003615 | 0.014141 | yes | up | 0.07 | 0 | 0.04 | 1.53 | 1.18 | 1.13 | 0.036667 | 1.28 |
| LOC108176197 | 0.206 | -2.27682 | 0.003652 | 0.014274 | yes | down | 0.46 | 0.52 | 0.54 | 0.19 | 0.27 | 0.3 | 0.506667 | 0.253333 |
| KIAA1324 | 4.346 | 2.119602 | 0.003656 | 0.014282 | yes | up | 0.37 | 0.17 | 0.17 | 1.86 | 0.56 | 4.16 | 0.236667 | 2.193333 |
| LOC100008826 | 3.54 | 1.823869 | 0.003673 | 0.014335 | yes | up | 1.55 | 0.56 | 0.27 | 6.63 | 5.63 | 7.26 | 0.793333 | 6.506667 |
| CSPG5 | 2.161 | 1.111816 | 0.003717 | 0.014479 | yes | up | 1.99 | 1.81 | 1.31 | 6.09 | 12.34 | 7.86 | 1.703333 | 8.763333 |
| MYBL1 | 2.96 | 1.56543 | 0.003734 | 0.014541 | yes | up | 0.31 | 0.15 | 0.13 | 1.28 | 2 | 0.77 | 0.196667 | 1.35 |
| MAPT | 7.594 | 2.92482 | 0.003787 | 0.014739 | yes | up | 3.17 | 0.36 | 0.46 | 13.18 | 9.64 | 34.74 | 1.33 | 19.18667 |
| LOC103349229 | 2.164 | 1.11357 | 0.003794 | 0.014756 | yes | up | 0.53 | 0.58 | 0.87 | 4.03 | 4.11 | 5.28 | 0.66 | 4.473333 |
| DSC2 | 2.54 | 1.344545 | 0.003798 | 0.014769 | yes | up | 0.47 | 0.93 | 0.38 | 4.91 | 1.69 | 4.25 | 0.593333 | 3.616667 |
| PLA2G10 | 0.266 | -1.91048 | 0.003817 | 0.014826 | yes | down | 0.85 | 2.37 | 0.87 | 0.83 | 0.72 | 1 | 1.363333 | 0.85 |
| ANG | 2.782 | 1.476309 | 0.003824 | 0.014847 | yes | up | 2.77 | 4.79 | 3.2 | 12.07 | 21.15 | 38.13 | 3.586667 | 23.78333 |
| LOC100354784 | 5.356 | 2.421246 | 0.003831 | 0.014869 | yes | up | 0.25 | 0.9 | 0.43 | 7.95 | 6.21 | 6.84 | 0.526667 | 7 |
| SMC2 | 2.032 | 1.022765 | 0.003844 | 0.014912 | yes | up | 1.72 | 1.17 | 0.74 | 6.44 | 7.34 | 3.42 | 1.21 | 5.733333 |
| KRT18 | 0.077 | -3.69563 | 0.003931 | 0.015203 | yes | down | 0.48 | 0.26 | 0.21 | 0.06 | 0 | 0.11 | 0.316667 | 0.056667 |
| EPHB1 | 4.13 | 2.046205 | 0.003933 | 0.015209 | yes | up | 0.16 | 0.07 | 0.04 | 1.16 | 0.33 | 1.13 | 0.09 | 0.873333 |
| LOC103345572 | 9.752 | 3.285674 | 0.003937 | 0.015217 | yes | up | 0.08 | 0.07 | 0.07 | 1.24 | 2.46 | 1.39 | 0.073333 | 1.696667 |
| LIN7A | 3.338 | 1.738864 | 0.003944 | 0.015239 | yes | up | 0.11 | 0.07 | 0.07 | 1.1 | 0.32 | 0.62 | 0.083333 | 0.68 |
| POLQ | 4.559 | 2.188672 | 0.004064 | 0.015661 | yes | up | 0.08 | 0.03 | 0.02 | 0.68 | 0.57 | 0.16 | 0.043333 | 0.47 |
| CDHR1 | 0.073 | -3.77155 | 0.004088 | 0.015746 | yes | down | 9.03 | 10.92 | 0.16 | 0.51 | 1.67 | 1.07 | 6.703333 | 1.083333 |
| LOC103350094 | 3.567 | 1.834724 | 0.004097 | 0.015775 | yes | up | 0.16 | 0.33 | 0.54 | 3.89 | 3.23 | 2.06 | 0.343333 | 3.06 |
| LOC100340985 | 0.079 | -3.65824 | 0.004113 | 0.015823 | yes | down | 0.65 | 0.39 | 0.55 | 0.1 | 0.21 | 0 | 0.53 | 0.103333 |
| MND1 | 3.981 | 1.993144 | 0.004158 | 0.015988 | yes | up | 0.57 | 0.4 | 0.38 | 4.24 | 5.43 | 2.45 | 0.45 | 4.04 |
| ST3GAL5 | 0.494 | -1.01786 | 0.004168 | 0.01602 | yes | down | 2.46 | 2.33 | 2.45 | 2.01 | 3.35 | 3.36 | 2.413333 | 2.906667 |
| BMP2 | 3.43 | 1.778253 | 0.004193 | 0.016102 | yes | up | 0.26 | 0.64 | 0.54 | 2.27 | 2.87 | 6.8 | 0.48 | 3.98 |
| DYRK1B | 0.499 | -1.0028 | 0.004214 | 0.016168 | yes | down | 12.77 | 5.81 | 6.01 | 11.8 | 12.95 | 7.99 | 8.196667 | 10.91333 |
| ALOX12B | 0.023 | -5.41296 | 0.004228 | 0.016215 | yes | down | 0.05 | 0.15 | 0.12 | 0 | 0 | 0 | 0.106667 | 0 |
| TLX3 | 0.055 | -4.17997 | 0.004246 | 0.016277 | yes | down | 0.12 | 0.32 | 0.57 | 0 | 0 | 0.14 | 0.336667 | 0.046667 |
| GDF11 | 2.098 | 1.0691 | 0.004292 | 0.016438 | yes | up | 1.23 | 0.66 | 0.51 | 5.62 | 3.28 | 3.4 | 0.8 | 4.1 |
| FGF7 | 3.81 | 1.92976 | 0.00431 | 0.016503 | yes | up | 0.3 | 0.31 | 0.14 | 3.54 | 2.58 | 1.65 | 0.25 | 2.59 |
| HCAR2 | 3.517 | 1.814164 | 0.004312 | 0.016507 | yes | up | 0.96 | 0.77 | 0.34 | 4.29 | 2.07 | 10.63 | 0.69 | 5.663333 |
| RASSF9 | 0.429 | -1.22238 | 0.00435 | 0.016647 | yes | down | 1.37 | 2.7 | 2.91 | 3.65 | 1.89 | 1.91 | 2.326667 | 2.483333 |
| MAP3K5 | 2.102 | 1.07143 | 0.004368 | 0.016703 | yes | up | 1.76 | 2.1 | 3.16 | 8.41 | 11.87 | 15.8 | 2.34 | 12.02667 |
| FANCI | 2.369 | 1.244381 | 0.00437 | 0.016703 | yes | up | 0.7 | 0.65 | 0.29 | 4.13 | 3.35 | 1.7 | 0.546667 | 3.06 |
| MAGEE2 | 3.319 | 1.73069 | 0.00437 | 0.016703 | yes | up | 0.22 | 0.2 | 0.28 | 2.26 | 1.07 | 2.3 | 0.233333 | 1.876667 |
| CDH18 | 2.743 | 1.455752 | 0.004395 | 0.016789 | yes | up | 0.39 | 0.43 | 0.21 | 2.98 | 1.51 | 2.16 | 0.343333 | 2.216667 |
| LOC108178976 | 3.609 | 1.851502 | 0.004402 | 0.016811 | yes | up | 0.65 | 0.37 | 0.35 | 1.85 | 6.33 | 3.39 | 0.456667 | 3.856667 |
| SLC10A6 | 0.464 | -1.10791 | 0.004404 | 0.016815 | yes | down | 1.84 | 2.54 | 2.51 | 3.38 | 2.95 | 1.44 | 2.296667 | 2.59 |
| MOG | 0.485 | -1.04407 | 0.004405 | 0.016815 | yes | down | 0.62 | 0.83 | 0.56 | 0.81 | 0.86 | 0.68 | 0.67 | 0.783333 |
| ONECUT2 | 0.335 | -1.57897 | 0.004473 | 0.017048 | yes | down | 0.06 | 0.07 | 0.07 | 0.06 | 0.04 | 0.07 | 0.066667 | 0.056667 |
| IL10 | 4.421 | 2.144488 | 0.004474 | 0.017048 | yes | up | 0.47 | 0.37 | 0.05 | 2.65 | 4.15 | 1.99 | 0.296667 | 2.93 |
| LOC103348900 | 3.37 | 1.752605 | 0.004501 | 0.017125 | yes | up | 0.25 | 0.05 | 0.05 | 1.01 | 0.77 | 1 | 0.116667 | 0.926667 |
| USP2 | 0.249 | -2.00689 | 0.004509 | 0.01715 | yes | down | 9.88 | 2.47 | 1.03 | 1.43 | 3.94 | 1.43 | 4.46 | 2.266667 |
| ZWILCH | 2.322 | 1.215419 | 0.004515 | 0.017167 | yes | up | 1.17 | 0.75 | 0.52 | 4.72 | 5.25 | 2.16 | 0.813333 | 4.043333 |
| LOC100356209 | 0.491 | -1.02661 | 0.004556 | 0.01731 | yes | down | 0.76 | 1.02 | 0.59 | 1.27 | 0.76 | 0.76 | 0.79 | 0.93 |
| CMPK2 | 2.253 | 1.172067 | 0.004582 | 0.017393 | yes | up | 1.77 | 2.73 | 2.48 | 20.38 | 6.54 | 16.44 | 2.326667 | 14.45333 |
| TCTEX1D4 | 0.34 | -1.55841 | 0.004589 | 0.017414 | yes | down | 0.97 | 0.63 | 0.85 | 0.75 | 0.53 | 0.69 | 0.816667 | 0.656667 |
| CXCR4 | 2.337 | 1.224529 | 0.004625 | 0.017544 | yes | up | 3.75 | 1.84 | 1.52 | 10.17 | 9.33 | 19.5 | 2.37 | 13 |
| LOC103352505 | 0.206 | -2.28132 | 0.004627 | 0.017546 | yes | down | 0.31 | 0.53 | 0.17 | 0.05 | 0.25 | 0.18 | 0.336667 | 0.16 |
| AGXT2 | 3.265 | 1.706868 | 0.004631 | 0.01756 | yes | up | 0.08 | 0.14 | 0.02 | 0.5 | 0.61 | 0.46 | 0.08 | 0.523333 |
| LOC103350471 | 2.094 | 1.066438 | 0.004641 | 0.017594 | yes | up | 1.72 | 0.68 | 1.13 | 7.87 | 4.36 | 5.12 | 1.176667 | 5.783333 |
| CDK1 | 3.188 | 1.672472 | 0.004655 | 0.017638 | yes | up | 3.87 | 3.96 | 1.17 | 22.08 | 36.3 | 7.06 | 3 | 21.81333 |
| CP | 2.49 | 1.316093 | 0.00469 | 0.017756 | yes | up | 0.61 | 0.22 | 0.33 | 2.49 | 2.62 | 1.71 | 0.386667 | 2.273333 |
| LOC100350375 | 0.247 | -2.01484 | 0.004693 | 0.017761 | yes | down | 0.91 | 0.63 | 0.4 | 0.14 | 0.23 | 0.37 | 0.646667 | 0.246667 |
| LOC100339846 | 0.31 | -1.69055 | 0.004727 | 0.017868 | yes | down | 1.09 | 0.12 | 0.11 | 1.28 | 0.31 | 0.79 | 0.44 | 0.793333 |
| CAMKMT | 2.612 | 1.384947 | 0.004739 | 0.01791 | yes | up | 0.76 | 1.19 | 0.49 | 3.22 | 7.41 | 4.22 | 0.813333 | 4.95 |
| DEPDC1B | 4.015 | 2.005486 | 0.004744 | 0.017927 | yes | up | 0.19 | 0.23 | 0.09 | 1.89 | 2.32 | 0.5 | 0.17 | 1.57 |
| LOC100356864 | 0.305 | -1.71346 | 0.004748 | 0.017939 | yes | down | 7.6 | 7.2 | 9.28 | 8.25 | 1.51 | 8.5 | 8.026667 | 6.086667 |
| WDR34 | 0.453 | -1.14138 | 0.004764 | 0.017991 | yes | down | 2.02 | 2.53 | 3.47 | 3.96 | 2.93 | 2.18 | 2.673333 | 3.023333 |
| SMAD9 | 0.434 | -1.20409 | 0.004772 | 0.018016 | yes | down | 0.51 | 0.38 | 0.28 | 0.33 | 0.48 | 0.39 | 0.39 | 0.4 |
| KLHL6 | 0.469 | -1.09116 | 0.004783 | 0.018047 | yes | down | 1.16 | 2.14 | 1.73 | 2.34 | 1.28 | 2.14 | 1.676667 | 1.92 |
| SEMA5B | 0.32 | -1.64296 | 0.004802 | 0.018114 | yes | down | 0.4 | 0.32 | 0.48 | 0.46 | 0.37 | 0.13 | 0.4 | 0.32 |
| SLC4A5 | 5.266 | 2.396612 | 0.004815 | 0.018148 | yes | up | 0.03 | 0.05 | 0.07 | 0.84 | 0.15 | 1.03 | 0.05 | 0.673333 |
| RAPGEF4 | 0.495 | -1.01569 | 0.00484 | 0.018236 | yes | down | 1.44 | 1.58 | 2.38 | 2.18 | 2.57 | 1.76 | 1.8 | 2.17 |
| CD300LG | 0.484 | -1.04746 | 0.004856 | 0.018287 | yes | down | 83.42 | 123.06 | 143.67 | 207.28 | 129.78 | 77.65 | 116.7167 | 138.2367 |
| PLXNA3 | 2.303 | 1.203491 | 0.004863 | 0.018304 | yes | up | 0.67 | 0.31 | 0.18 | 2.4 | 1.76 | 2.1 | 0.386667 | 2.086667 |
| NTF3 | 0.479 | -1.0611 | 0.004864 | 0.018304 | yes | down | 1.88 | 1.75 | 1.37 | 2.22 | 1.46 | 1.89 | 1.666667 | 1.856667 |
| LOC103350087 | 0.134 | -2.89585 | 0.004873 | 0.018308 | yes | down | 0.19 | 0.64 | 0.4 | 0.06 | 0.12 | 0.22 | 0.41 | 0.133333 |
| GFRA3 | 2.753 | 1.460768 | 0.004911 | 0.018443 | yes | up | 0.29 | 0.31 | 0.2 | 1.28 | 1.7 | 2.2 | 0.266667 | 1.726667 |
| KALRN | 0.422 | -1.24424 | 0.004921 | 0.018477 | yes | down | 2.09 | 0.9 | 4.44 | 2.75 | 1.73 | 1.33 | 2.476667 | 1.936667 |
| SIGLEC15 | 0.287 | -1.799 | 0.004926 | 0.018492 | yes | down | 0.64 | 0.36 | 0.54 | 0.3 | 0.12 | 0.63 | 0.513333 | 0.35 |
| AKNA | 2.127 | 1.089114 | 0.004928 | 0.018495 | yes | up | 0.55 | 0.41 | 0.4 | 3.15 | 2.06 | 1.34 | 0.453333 | 2.183333 |
| HJURP | 2.698 | 1.431806 | 0.004944 | 0.018546 | yes | up | 0.46 | 0.58 | 0.17 | 3.67 | 2.51 | 1.13 | 0.403333 | 2.436667 |
| LOC103349621 | 3.568 | 1.835147 | 0.004958 | 0.01859 | yes | up | 0.21 | 0.1 | 0.12 | 1.12 | 1.2 | 1.21 | 0.143333 | 1.176667 |
| CLSPN | 3.414 | 1.771431 | 0.004971 | 0.018622 | yes | up | 0.32 | 0.19 | 0.07 | 1.74 | 2.22 | 0.65 | 0.193333 | 1.536667 |
| ANXA4 | 2.084 | 1.059128 | 0.004999 | 0.018711 | yes | up | 7.72 | 4.53 | 2.34 | 21.3 | 25.06 | 24.94 | 4.863333 | 23.76667 |
| ZNF74 | 2.007 | 1.004793 | 0.005 | 0.018711 | yes | up | 2.26 | 1.02 | 1.74 | 10.39 | 7.1 | 6.31 | 1.673333 | 7.933333 |
| LOC100357628 | 24.645 | 4.623222 | 0.005038 | 0.018818 | yes | up | 0.08 | 0.07 | 0 | 8.01 | 0.61 | 0.27 | 0.05 | 2.963333 |
| LOC108177741 | 2.877 | 1.524664 | 0.005063 | 0.018899 | yes | up | 0.62 | 0.17 | 0.27 | 2.87 | 1.7 | 2.83 | 0.353333 | 2.466667 |
| TMEM246 | 0.461 | -1.1168 | 0.005068 | 0.018914 | yes | down | 2.15 | 1.4 | 2.26 | 2.8 | 1.56 | 2.17 | 1.936667 | 2.176667 |
| LOC103348750 | 0.302 | -1.7258 | 0.005071 | 0.018916 | yes | down | 1.17 | 3.33 | 1.44 | 1.87 | 1.77 | 1.15 | 1.98 | 1.596667 |
| CNGB1 | 0.256 | -1.96327 | 0.005077 | 0.018932 | yes | down | 0.19 | 0.18 | 0.27 | 0.05 | 0.13 | 0.22 | 0.213333 | 0.133333 |
| GRP | 0.013 | -6.22031 | 0.005087 | 0.018963 | yes | down | 0 | 1.85 | 1.13 | 0 | 0 | 0 | 0.993333 | 0 |
| LOC103348513 | 6.485 | 2.697096 | 0.005098 | 0.019 | yes | up | 0 | 0.14 | 0.07 | 1.6 | 0.85 | 0.91 | 0.07 | 1.12 |
| KIAA0101 | 3.208 | 1.681498 | 0.005134 | 0.019114 | yes | up | 3.63 | 2.26 | 1.66 | 25.35 | 26.43 | 5.43 | 2.516667 | 19.07 |
| KLHL29 | 2.025 | 1.01757 | 0.005139 | 0.019131 | yes | up | 0.63 | 0.54 | 0.3 | 2.93 | 1.6 | 2.54 | 0.49 | 2.356667 |
| LOC108177021 | 10.885 | 3.444236 | 0.005149 | 0.019162 | yes | up | 0.01 | 0.02 | 0 | 0.58 | 0.2 | 0.17 | 0.01 | 0.316667 |
| LOC100356339 | 66.987 | 6.065817 | 0.005189 | 0.0193 | yes | up | 0 | 0 | 0 | 0 | 1.65 | 1.87 | 0 | 1.173333 |
| TLR2 | 0.399 | -1.32525 | 0.005199 | 0.019334 | yes | down | 0.83 | 0.81 | 1.52 | 1.63 | 0.56 | 1.42 | 1.053333 | 1.203333 |
| LOC103348495 | 0.16 | -2.64319 | 0.00521 | 0.019357 | yes | down | 0.76 | 1.03 | 0.94 | 0.31 | 0.33 | 0.45 | 0.91 | 0.363333 |
| RAB15 | 2.065 | 1.046197 | 0.005224 | 0.019405 | yes | up | 0.74 | 1.06 | 0.36 | 3.6 | 3.51 | 3.35 | 0.72 | 3.486667 |
| SGIP1 | 3.204 | 1.679698 | 0.00523 | 0.01942 | yes | up | 0.31 | 0.44 | 0.3 | 2.2 | 1.18 | 5.58 | 0.35 | 2.986667 |
| MAGEF1 | 0.146 | -2.77819 | 0.00527 | 0.019553 | yes | down | 1.39 | 0.29 | 0.54 | 0.15 | 0.32 | 0.22 | 0.74 | 0.23 |
| ALX3 | 5.356 | 2.421083 | 0.005288 | 0.019605 | yes | up | 0.12 | 0.09 | 0 | 1.16 | 0.59 | 0.81 | 0.07 | 0.853333 |
| LOC108176880 | 3.356 | 1.746548 | 0.005332 | 0.019738 | yes | up | 0.27 | 0.42 | 0.47 | 5.31 | 3.6 | 3.82 | 0.386667 | 4.243333 |
| LOC100008885 | 0.023 | -5.41968 | 0.005344 | 0.01978 | yes | down | 0.36 | 0.13 | 0.07 | 0 | 0 | 0 | 0.186667 | 0 |
| KCNJ9 | 19.509 | 4.286057 | 0.005363 | 0.019839 | yes | up | 0.02 | 0 | 0 | 0.36 | 0.15 | 0.27 | 0.006667 | 0.26 |
| SLC9B1 | 0.32 | -1.64611 | 0.005408 | 0.01999 | yes | down | 0.63 | 1.76 | 1 | 1.58 | 0.57 | 0.87 | 1.13 | 1.006667 |
| LOC108177684 | 0.05 | -4.32531 | 0.005416 | 0.020014 | yes | down | 6.54 | 0.88 | 0 | 0.15 | 0.32 | 0.14 | 2.473333 | 0.203333 |
| ZMYND12 | 2.779 | 1.474508 | 0.005437 | 0.020079 | yes | up | 0.34 | 0.41 | 0.44 | 2.89 | 2.36 | 2.86 | 0.396667 | 2.703333 |
| CFP | 2.085 | 1.059782 | 0.005465 | 0.020155 | yes | up | 0.83 | 0.66 | 0.96 | 3.46 | 4.75 | 4.69 | 0.816667 | 4.3 |
| MCM10 | 3.926 | 1.973102 | 0.005475 | 0.020173 | yes | up | 0.11 | 0.11 | 0.06 | 0.9 | 1.35 | 0.32 | 0.093333 | 0.856667 |
| C2H2orf40 | 8.755 | 3.130155 | 0.005505 | 0.020258 | yes | up | 0.12 | 0.06 | 0.05 | 0.53 | 2.26 | 1.96 | 0.076667 | 1.583333 |
| LOC108175394 | 2.53 | 1.339365 | 0.005515 | 0.020288 | yes | up | 0.55 | 0.3 | 0.4 | 6.78 | 1.88 | 5.71 | 0.416667 | 4.79 |
| LOC108176381 | 3.335 | 1.737684 | 0.005525 | 0.020317 | yes | up | 2.17 | 2.38 | 0.47 | 20.62 | 5.49 | 12.25 | 1.673333 | 12.78667 |
| LOC108177184 | 0.405 | -1.30522 | 0.005538 | 0.02036 | yes | down | 17.41 | 55.52 | 20.3 | 37.78 | 22.05 | 31.75 | 31.07667 | 30.52667 |
| SLC43A2 | 0.401 | -1.31888 | 0.005543 | 0.020375 | yes | down | 7.47 | 3.52 | 1.88 | 6.8 | 4.93 | 2.8 | 4.29 | 4.843333 |
| SLC25A37 | 2.1 | 1.070473 | 0.005587 | 0.020513 | yes | up | 5.31 | 5.73 | 5.51 | 11.77 | 26.88 | 36.77 | 5.516667 | 25.14 |
| ATP2A2 | 0.361 | -1.47107 | 0.005588 | 0.020513 | yes | down | 117.72 | 42.21 | 23.26 | 38.6 | 77.18 | 39.79 | 61.06333 | 51.85667 |
| CCNB2 | 2.829 | 1.500279 | 0.005592 | 0.020517 | yes | up | 3.33 | 2.96 | 1.08 | 18.53 | 23.79 | 6.16 | 2.456667 | 16.16 |
| HIVEP3 | 2.325 | 1.217079 | 0.005634 | 0.020659 | yes | up | 0.13 | 0.07 | 0.09 | 0.53 | 0.37 | 0.7 | 0.096667 | 0.533333 |
| ATG16L2 | 2.223 | 1.152268 | 0.005644 | 0.020684 | yes | up | 0.84 | 0.47 | 0.46 | 3.81 | 2.8 | 2.62 | 0.59 | 3.076667 |
| LOC108175795 | 4.047 | 2.016933 | 0.005647 | 0.020688 | yes | up | 0.31 | 0.12 | 0.19 | 2.49 | 0.93 | 2.72 | 0.206667 | 2.046667 |
| MBP | 0.491 | -1.02489 | 0.00565 | 0.020692 | yes | down | 24.66 | 18.42 | 8.91 | 9.68 | 22.42 | 15.1 | 17.33 | 15.73333 |
| LOC100344189 | 2.242 | 1.164858 | 0.005684 | 0.020799 | yes | up | 1.15 | 1.33 | 2.43 | 12.41 | 7.04 | 6.62 | 1.636667 | 8.69 |
| B4GALNT3 | 0.165 | -2.60118 | 0.005756 | 0.021025 | yes | down | 0.42 | 0.1 | 0.07 | 0.04 | 0.13 | 0.04 | 0.196667 | 0.07 |
| CCL5 | 19.63 | 4.294955 | 0.005764 | 0.02105 | yes | up | 0.1 | 0 | 0 | 1.39 | 0.87 | 2.25 | 0.033333 | 1.503333 |
| KCTD14 | 0.226 | -2.14589 | 0.005819 | 0.021219 | yes | down | 0.73 | 0.97 | 0.44 | 0.44 | 0.38 | 0.37 | 0.713333 | 0.396667 |
| CFAP57 | 3.66 | 1.871689 | 0.005833 | 0.021261 | yes | up | 0.05 | 0.09 | 0.04 | 0.51 | 0.49 | 0.57 | 0.06 | 0.523333 |
| TRMT61B | 0.396 | -1.3354 | 0.005836 | 0.021261 | yes | down | 1.35 | 0.74 | 0.75 | 1.13 | 0.96 | 0.6 | 0.946667 | 0.896667 |
| LOC103346561 | 2.558 | 1.355249 | 0.005852 | 0.021307 | yes | up | 1.53 | 0.74 | 0.61 | 6.05 | 6 | 3.05 | 0.96 | 5.033333 |
| LOC100353915 | 36.088 | 5.173435 | 0.005861 | 0.021329 | yes | up | 0 | 0 | 0 | 2.29 | 3.78 | 0.39 | 0 | 2.153333 |
| LOC100338113 | 4.529 | 2.179121 | 0.005867 | 0.021343 | yes | up | 0.14 | 0.07 | 0.12 | 2.21 | 1.03 | 0.46 | 0.11 | 1.233333 |
| LMCD1 | 0.343 | -1.54416 | 0.005881 | 0.021382 | yes | down | 71.27 | 17.77 | 10.69 | 25.15 | 31.25 | 21.96 | 33.24333 | 26.12 |
| LOC108175812 | 5.249 | 2.392112 | 0.005915 | 0.021483 | yes | up | 0.31 | 0.29 | 0 | 2.16 | 1.36 | 1.94 | 0.2 | 1.82 |
| DENND1C | 2.183 | 1.126285 | 0.00594 | 0.021563 | yes | up | 0.56 | 0.54 | 0.47 | 3.89 | 3.23 | 2.02 | 0.523333 | 3.046667 |
| CCNF | 3.118 | 1.640592 | 0.005962 | 0.021629 | yes | up | 0.53 | 0.5 | 0.19 | 4.7 | 3.27 | 1.01 | 0.406667 | 2.993333 |
| NMB | 4.962 | 2.310921 | 0.00597 | 0.021654 | yes | up | 0.17 | 0.5 | 0.44 | 4.34 | 4.56 | 4.65 | 0.37 | 4.516667 |
| LOC103351799 | 0.087 | -3.52864 | 0.006001 | 0.02174 | yes | down | 0.23 | 0.21 | 0.14 | 0.08 | 0.04 | 0 | 0.193333 | 0.04 |
| LOC103346726 | 2.094 | 1.066203 | 0.006044 | 0.021877 | yes | up | 2.38 | 1.33 | 1.07 | 9.43 | 4.37 | 9.76 | 1.593333 | 7.853333 |
| CHDH | 0.314 | -1.66923 | 0.00606 | 0.021929 | yes | down | 1.68 | 0.44 | 0.31 | 0.67 | 0.59 | 0.51 | 0.81 | 0.59 |
| AMIGO1 | 0.447 | -1.16142 | 0.00617 | 0.022257 | yes | down | 2.37 | 1.32 | 0.91 | 1.07 | 2.24 | 1.44 | 1.533333 | 1.583333 |
| LOC100346502 | 0.042 | -4.57271 | 0.006185 | 0.022302 | yes | down | 0.14 | 0.1 | 0.12 | 0.03 | 0 | 0 | 0.12 | 0.01 |
| LOC103347314 | 5.414 | 2.436652 | 0.006192 | 0.022323 | yes | up | 0.05 | 0.07 | 0.02 | 0.65 | 0.53 | 0.76 | 0.046667 | 0.646667 |
| LOC108176074 | 0.17 | -2.5577 | 0.006208 | 0.022367 | yes | down | 1.1 | 2.57 | 1.51 | 0.8 | 0.57 | 0.78 | 1.726667 | 0.716667 |
| LOC100346985 | 2.122 | 1.085111 | 0.006228 | 0.022424 | yes | up | 1.73 | 0.86 | 0.84 | 5.76 | 3.86 | 7.67 | 1.143333 | 5.763333 |
| MAGED1 | 0.483 | -1.04883 | 0.006246 | 0.022478 | yes | down | 19.14 | 8.43 | 5.59 | 12.7 | 11.48 | 12.95 | 11.05333 | 12.37667 |
| LOC108178044 | 0.036 | -4.79922 | 0.006257 | 0.022515 | yes | down | 0.1 | 0.04 | 0.21 | 0.03 | 0 | 0 | 0.116667 | 0.01 |
| SLC16A3 | 0.183 | -2.45076 | 0.006315 | 0.022701 | yes | down | 8.02 | 1.79 | 1 | 0.64 | 3.24 | 0.51 | 3.603333 | 1.463333 |
| FHDC1 | 2.493 | 1.318157 | 0.006337 | 0.022765 | yes | up | 0.33 | 0.27 | 0.19 | 1.29 | 1 | 2.33 | 0.263333 | 1.54 |
| LOC100352785 | 2.124 | 1.086949 | 0.006357 | 0.022822 | yes | up | 1.34 | 1.28 | 0.63 | 5.84 | 6.73 | 3.52 | 1.083333 | 5.363333 |
| MYOM3 | 0.028 | -5.13622 | 0.006366 | 0.022851 | yes | down | 45.84 | 4.88 | 0.67 | 0.07 | 2.97 | 0.09 | 17.13 | 1.043333 |
| LOC108177094 | 4.112 | 2.039742 | 0.006368 | 0.022855 | yes | up | 0.11 | 0.39 | 0.34 | 4.2 | 0.92 | 3.75 | 0.28 | 2.956667 |
| PRIMPOL | 3.28 | 1.713737 | 0.006379 | 0.022881 | yes | up | 0.49 | 0.5 | 0.18 | 3.75 | 4.25 | 1.38 | 0.39 | 3.126667 |
| RASEF | 6.435 | 2.686048 | 0.006452 | 0.023125 | yes | up | 0.04 | 0.03 | 0.01 | 0.51 | 0.11 | 0.63 | 0.026667 | 0.416667 |
| COL25A1 | 3.621 | 1.856487 | 0.006455 | 0.02313 | yes | up | 0.2 | 0.25 | 0.06 | 1.29 | 0.5 | 2.39 | 0.17 | 1.393333 |
| CFB | 3.599 | 1.847653 | 0.006465 | 0.023161 | yes | up | 2.11 | 0.74 | 0.26 | 4.5 | 5.72 | 15.11 | 1.036667 | 8.443333 |
| LOC103352262 | 4.494 | 2.168075 | 0.006555 | 0.02346 | yes | up | 0.06 | 0.06 | 0.04 | 0.74 | 0.47 | 0.68 | 0.053333 | 0.63 |
| CDH24 | 5.067 | 2.341037 | 0.006586 | 0.023549 | yes | up | 0.02 | 0.1 | 0.03 | 0.35 | 0.72 | 0.7 | 0.05 | 0.59 |
| CHRNA7 | 3.214 | 1.684533 | 0.006617 | 0.02363 | yes | up | 0.15 | 0.14 | 0.24 | 1.98 | 1.24 | 1.05 | 0.176667 | 1.423333 |
| SLC44A3 | 5.416 | 2.437191 | 0.006648 | 0.023721 | yes | up | 0.35 | 0.1 | 0.4 | 6.83 | 4.31 | 0.43 | 0.283333 | 3.856667 |
| SLC19A1 | 2.152 | 1.105693 | 0.006666 | 0.023773 | yes | up | 3.24 | 4.09 | 1.35 | 15 | 10.55 | 22.92 | 2.893333 | 16.15667 |
| LOC108176634 | 0.286 | -1.80567 | 0.006676 | 0.023784 | yes | down | 4.98 | 13.86 | 3.01 | 8.24 | 2.11 | 4.62 | 7.283333 | 4.99 |
| TMEM266 | 0.258 | -1.9547 | 0.006684 | 0.023805 | yes | down | 1.79 | 4.71 | 1.95 | 1.23 | 1.28 | 0.75 | 2.816667 | 1.086667 |
| EDN3 | 0.055 | -4.19299 | 0.006701 | 0.023856 | yes | down | 1.3 | 0.09 | 0.12 | 0 | 0.18 | 0 | 0.503333 | 0.06 |
| LOC103352078 | 2.293 | 1.197175 | 0.006723 | 0.023932 | yes | up | 0.29 | 0.4 | 0.27 | 2.15 | 1.06 | 2.07 | 0.32 | 1.76 |
| LOC100342660 | 0.235 | -2.08641 | 0.006771 | 0.024079 | yes | down | 0.16 | 0.35 | 0.23 | 0.6 | 0.09 | 0.12 | 0.246667 | 0.27 |
| HRH1 | 0.429 | -1.21943 | 0.006788 | 0.02412 | yes | down | 1.01 | 0.98 | 0.45 | 0.88 | 0.54 | 1.05 | 0.813333 | 0.823333 |
| DUS3L | 0.329 | -1.60501 | 0.00681 | 0.024177 | yes | down | 1.8 | 1.27 | 1.2 | 0.79 | 1.59 | 0.96 | 1.423333 | 1.113333 |
| CPN2 | 0.023 | -5.43131 | 0.00682 | 0.024196 | yes | down | 0.02 | 0.09 | 0.12 | 0 | 0 | 0 | 0.076667 | 0 |
| CD1B | 17.715 | 4.146888 | 0.006856 | 0.024316 | yes | up | 0.07 | 0 | 0 | 0.44 | 0.46 | 0.4 | 0.023333 | 0.433333 |
| DDX25 | 0.042 | -4.57081 | 0.006906 | 0.024472 | yes | down | 0.11 | 0.16 | 0.19 | 0.05 | 0 | 0 | 0.153333 | 0.016667 |
| GSTM2 | 2.877 | 1.524367 | 0.00693 | 0.024539 | yes | up | 23.03 | 5.78 | 3.57 | 69.82 | 91.47 | 51.99 | 10.79333 | 71.09333 |
| SEC16B | 2.484 | 1.312653 | 0.006933 | 0.024544 | yes | up | 0.14 | 0.13 | 0.16 | 0.94 | 0.89 | 0.74 | 0.143333 | 0.856667 |
| ARHGEF39 | 3.427 | 1.777024 | 0.006945 | 0.024583 | yes | up | 0.52 | 0.41 | 0.23 | 4.45 | 3.73 | 1.17 | 0.386667 | 3.116667 |
| PARD6A | 0.498 | -1.0049 | 0.006973 | 0.024678 | yes | down | 2.1 | 2.55 | 1.51 | 2.54 | 2.85 | 1.93 | 2.053333 | 2.44 |
| ANGPTL7 | 7.414 | 2.890212 | 0.006982 | 0.024702 | yes | up | 0.08 | 0 | 0.09 | 0.58 | 1.88 | 0.57 | 0.056667 | 1.01 |
| LOC100348097 | 3.502 | 1.808023 | 0.007008 | 0.024789 | yes | up | 0.06 | 0.34 | 0.29 | 2.13 | 0.94 | 2.93 | 0.23 | 2 |
| SLC41A3 | 3.574 | 1.837651 | 0.007014 | 0.024795 | yes | up | 1.88 | 0.59 | 0.44 | 11.9 | 10.45 | 2.2 | 0.97 | 8.183333 |
| DIXDC1 | 2.039 | 1.027751 | 0.007014 | 0.024795 | yes | up | 1.37 | 0.69 | 0.31 | 8.73 | 3.91 | 11.65 | 0.79 | 8.096667 |
| SGO2 | 2.811 | 1.49103 | 0.00705 | 0.024896 | yes | up | 0.31 | 0.5 | 0.2 | 3.06 | 2.74 | 0.87 | 0.336667 | 2.223333 |
| SPATA5 | 0.492 | -1.02218 | 0.00705 | 0.024896 | yes | down | 1.17 | 0.73 | 1.16 | 1.02 | 1.41 | 1.21 | 1.02 | 1.213333 |
| VIPR2 | 0.359 | -1.47687 | 0.00706 | 0.024909 | yes | down | 0.98 | 0.83 | 0.38 | 0.41 | 0.41 | 0.94 | 0.73 | 0.586667 |
| GPR153 | 0.488 | -1.03584 | 0.007129 | 0.025138 | yes | down | 6.01 | 10 | 2.6 | 6.96 | 7.55 | 6.29 | 6.203333 | 6.933333 |
| LOC103349335 | 0.181 | -2.46471 | 0.007156 | 0.025227 | yes | down | 0.79 | 2.13 | 2.61 | 0.63 | 1.44 | 0.08 | 1.843333 | 0.716667 |
| C13H1orf162 | 2.461 | 1.299165 | 0.007198 | 0.025343 | yes | up | 0.57 | 0.83 | 1.16 | 5.1 | 4.69 | 3.58 | 0.853333 | 4.456667 |
| ACER3 | 0.445 | -1.16752 | 0.007224 | 0.025423 | yes | down | 2.81 | 1.75 | 2.01 | 1.99 | 2.04 | 2.96 | 2.19 | 2.33 |
| LOC100343263 | 11.511 | 3.524909 | 0.007307 | 0.025679 | yes | up | 0 | 0.04 | 0.04 | 0.87 | 0.7 | 0.66 | 0.026667 | 0.743333 |
| STIL | 4.122 | 2.043484 | 0.00732 | 0.025712 | yes | up | 0.16 | 0.09 | 0.04 | 1.43 | 1.14 | 0.26 | 0.096667 | 0.943333 |
| ZNF367 | 2.55 | 1.350493 | 0.007434 | 0.026069 | yes | up | 1.11 | 0.62 | 0.32 | 3.47 | 6.07 | 2.5 | 0.683333 | 4.013333 |
| PTX4 | 0.291 | -1.78321 | 0.007447 | 0.026104 | yes | down | 0.75 | 1.22 | 0.4 | 0.23 | 0.6 | 0.76 | 0.79 | 0.53 |
| LOC103348128 | 0.09 | -3.47185 | 0.007484 | 0.026223 | yes | down | 0.32 | 0.25 | 0.2 | 0.05 | 0 | 0.11 | 0.256667 | 0.053333 |
| LOC103350737 | 0.047 | -4.41753 | 0.007502 | 0.026273 | yes | down | 4.84 | 0.96 | 0.23 | 0.02 | 0 | 0.32 | 2.01 | 0.113333 |
| SLC38A5 | 2.395 | 1.25978 | 0.007563 | 0.026454 | yes | up | 1.07 | 1.09 | 0.32 | 3.54 | 4.49 | 5.69 | 0.826667 | 4.573333 |
| KCNJ5 | 39.687 | 5.310611 | 0.007578 | 0.026494 | yes | up | 0 | 0 | 0.01 | 0.36 | 0 | 0.87 | 0.003333 | 0.41 |
| SPDL1 | 2.669 | 1.416261 | 0.007611 | 0.026592 | yes | up | 0.47 | 0.3 | 0.29 | 2.72 | 2.97 | 1.07 | 0.353333 | 2.253333 |
| LOC103349209 | 0.158 | -2.66392 | 0.007747 | 0.027007 | yes | down | 0.2 | 0.1 | 0.11 | 0.05 | 0.02 | 0.09 | 0.136667 | 0.053333 |
| FAM227A | 28.162 | 4.815696 | 0.007747 | 0.027007 | yes | up | 0 | 0 | 0 | 0.09 | 0.21 | 0.32 | 0 | 0.206667 |
| LOC103351410 | 3.108 | 1.635815 | 0.007789 | 0.027142 | yes | up | 0.08 | 0.21 | 0.06 | 1.52 | 0.47 | 0.71 | 0.116667 | 0.9 |
| CCNB1 | 2.755 | 1.462264 | 0.007798 | 0.027165 | yes | up | 4.56 | 4.16 | 1.59 | 25.82 | 32.84 | 7.48 | 3.436667 | 22.04667 |
| LOC103347295 | 4.154 | 2.054559 | 0.007884 | 0.027433 | yes | up | 0.03 | 0.25 | 0.09 | 1.33 | 1.29 | 1.01 | 0.123333 | 1.21 |
| LCA5L | 6.48 | 2.696048 | 0.007884 | 0.027433 | yes | up | 0.01 | 0.05 | 0.02 | 0.67 | 0.15 | 1.03 | 0.026667 | 0.616667 |
| CARMIL2 | 2.501 | 1.322658 | 0.007894 | 0.027457 | yes | up | 0.15 | 0.11 | 0.11 | 0.91 | 0.49 | 0.78 | 0.123333 | 0.726667 |
| LOC100346219 | 10.965 | 3.454827 | 0.007895 | 0.027457 | yes | up | 0.15 | 0.53 | 0 | 11.35 | 0.16 | 8.89 | 0.226667 | 6.8 |
| ACADL | 0.496 | -1.01111 | 0.007949 | 0.0276 | yes | down | 723.7 | 460.67 | 936.24 | 779.48 | 572.27 | 1246.79 | 706.87 | 866.18 |
| AURKB | 3.228 | 1.690788 | 0.007988 | 0.027716 | yes | up | 1.57 | 1.22 | 0.49 | 9.03 | 12.96 | 2.71 | 1.093333 | 8.233333 |
| LOC100353131 | 0.442 | -1.17836 | 0.007995 | 0.027735 | yes | down | 0.87 | 1.83 | 1.4 | 1.25 | 1.28 | 1.82 | 1.366667 | 1.45 |
| LOC100355990 | 2.115 | 1.080616 | 0.008007 | 0.027767 | yes | up | 0.8 | 1.14 | 0.98 | 6.64 | 4.32 | 3.21 | 0.973333 | 4.723333 |
| ADGRG3 | 0.11 | -3.18991 | 0.008021 | 0.027809 | yes | down | 0.22 | 0.11 | 0.05 | 0.05 | 0.03 | 0.02 | 0.126667 | 0.033333 |
| SWSAP1 | 0.475 | -1.07422 | 0.008032 | 0.027829 | yes | down | 1.45 | 1.43 | 0.81 | 1.38 | 1.22 | 1.56 | 1.23 | 1.386667 |
| LURAP1L | 2.047 | 1.033637 | 0.008034 | 0.027829 | yes | up | 1.2 | 0.64 | 0.55 | 3.79 | 3.52 | 4.24 | 0.796667 | 3.85 |
| PLK3 | 2.027 | 1.019441 | 0.008061 | 0.027913 | yes | up | 0.67 | 0.96 | 0.49 | 2.75 | 3.13 | 4.25 | 0.706667 | 3.376667 |
| CHN2 | 0.457 | -1.12816 | 0.008075 | 0.027951 | yes | down | 1.41 | 2.45 | 2.46 | 3.26 | 2.59 | 1.27 | 2.106667 | 2.373333 |
| PHLDA3 | 2.447 | 1.290786 | 0.008105 | 0.028035 | yes | up | 11.29 | 3.49 | 2.32 | 29.92 | 34.84 | 30.93 | 5.7 | 31.89667 |
| FHL2 | 0.266 | -1.90892 | 0.008171 | 0.028241 | yes | down | 11.33 | 3.24 | 1.01 | 2.94 | 4.46 | 1.96 | 5.193333 | 3.12 |
| FSIP1 | 27.732 | 4.793489 | 0.008177 | 0.028251 | yes | up | 0 | 0 | 0 | 0.13 | 0.45 | 0.31 | 0 | 0.296667 |
| LAMB3 | 2.763 | 1.466466 | 0.008178 | 0.028251 | yes | up | 1.01 | 0.98 | 0.47 | 3.8 | 2.26 | 10.26 | 0.82 | 5.44 |
| LOC103346935 | 25.701 | 4.683748 | 0.008295 | 0.028615 | yes | up | 0 | 0 | 0 | 0.37 | 0.33 | 0.6 | 0 | 0.433333 |
| PMEPA1 | 0.408 | -1.29497 | 0.008332 | 0.028724 | yes | down | 10.24 | 10.42 | 3.39 | 5.59 | 5.15 | 9.55 | 8.016667 | 6.763333 |
| LOC103346474 | 0.496 | -1.01176 | 0.008389 | 0.028891 | yes | down | 12.08 | 14.84 | 12.12 | 23.76 | 10.4 | 13.63 | 13.01333 | 15.93 |
| LOC103351270 | 3.535 | 1.821809 | 0.008415 | 0.028969 | yes | up | 0.02 | 0.03 | 0.03 | 0.18 | 0.27 | 0.29 | 0.026667 | 0.246667 |
| CELSR1 | 0.336 | -1.57344 | 0.008439 | 0.029038 | yes | down | 0.32 | 0.33 | 0.14 | 0.21 | 0.14 | 0.14 | 0.263333 | 0.163333 |
| PKD1L1 | 56.166 | 5.811616 | 0.008485 | 0.029192 | yes | up | 0 | 0 | 0 | 0.18 | 0 | 0.34 | 0 | 0.173333 |
| VIT | 2.306 | 1.205489 | 0.008494 | 0.029217 | yes | up | 1.3 | 0.7 | 0.29 | 4.17 | 3.99 | 4.02 | 0.763333 | 4.06 |
| SPC25 | 2.854 | 1.512884 | 0.008517 | 0.029282 | yes | up | 1.14 | 0.49 | 0.51 | 4.89 | 6.38 | 3.23 | 0.713333 | 4.833333 |
| LOC100343111 | 0.174 | -2.52315 | 0.008632 | 0.029606 | yes | down | 0.98 | 0.47 | 0.54 | 0.67 | 0.08 | 0.1 | 0.663333 | 0.283333 |
| CEP128 | 2.124 | 1.087029 | 0.008694 | 0.029793 | yes | up | 0.19 | 0.38 | 0.19 | 1.21 | 1.31 | 1.36 | 0.253333 | 1.293333 |
| DAO | 19.062 | 4.252648 | 0.008734 | 0.029895 | yes | up | 0 | 0.04 | 0 | 0.58 | 1.25 | 0.34 | 0.013333 | 0.723333 |
| CAMK1G | 0.18 | -2.47678 | 0.008749 | 0.029938 | yes | down | 0.18 | 0.1 | 0.27 | 0.11 | 0.09 | 0.05 | 0.183333 | 0.083333 |
| GLI2 | 2.118 | 1.082407 | 0.008751 | 0.02994 | yes | up | 0.3 | 0.21 | 0.16 | 1.55 | 0.7 | 1.11 | 0.223333 | 1.12 |
| LOC103351338 | 16.957 | 4.083837 | 0.008774 | 0.030004 | yes | up | 0 | 0.11 | 0 | 1.67 | 1.73 | 1.11 | 0.036667 | 1.503333 |
| AMT | 0.382 | -1.38858 | 0.008818 | 0.030132 | yes | down | 2.23 | 2.02 | 5.12 | 3.21 | 1.67 | 4.17 | 3.123333 | 3.016667 |
| LOC103349185 | 2.669 | 1.416319 | 0.008837 | 0.030176 | yes | up | 0.94 | 0.46 | 0.14 | 3.85 | 3.55 | 2.04 | 0.513333 | 3.146667 |
| LOC100359251 | 24.693 | 4.626003 | 0.008884 | 0.030281 | yes | up | 0 | 0 | 0 | 0.16 | 0.13 | 0.21 | 0 | 0.166667 |
| ABHD1 | 0.172 | -2.54181 | 0.00891 | 0.030351 | yes | down | 0.28 | 0.26 | 0.53 | 0.23 | 0.12 | 0.11 | 0.356667 | 0.153333 |
| LOC100351810 | 24.572 | 4.618937 | 0.008912 | 0.030351 | yes | up | 0 | 0 | 0 | 8.08 | 4.71 | 5.55 | 0 | 6.113333 |
| LOC103351388 | 0.045 | -4.45851 | 0.009007 | 0.030636 | yes | down | 2.09 | 3.61 | 3.38 | 0 | 1.17 | 0 | 3.026667 | 0.39 |
| LOC100340502 | 0.402 | -1.31616 | 0.009009 | 0.030637 | yes | down | 0.72 | 0.33 | 0.45 | 0.45 | 0.42 | 0.6 | 0.5 | 0.49 |
| CDCA3 | 2.768 | 1.469033 | 0.009036 | 0.030716 | yes | up | 1.73 | 1.47 | 1.08 | 12.51 | 12.71 | 3.14 | 1.426667 | 9.453333 |
| LOC100358934 | 13.8 | 3.786573 | 0.009054 | 0.03076 | yes | up | 0 | 0.06 | 0.06 | 0.41 | 2.37 | 1.27 | 0.04 | 1.35 |
| ABCC4 | 0.433 | -1.20738 | 0.009063 | 0.030776 | yes | down | 2.66 | 4.36 | 2.1 | 2.69 | 1.41 | 5.26 | 3.04 | 3.12 |
| LOC103350544 | 7.237 | 2.855419 | 0.009074 | 0.030802 | yes | up | 0.02 | 0.04 | 0 | 0.43 | 0.3 | 0.25 | 0.02 | 0.326667 |
| LOC103347592 | 0.248 | -2.00926 | 0.00912 | 0.030945 | yes | down | 1.57 | 0.6 | 0.71 | 0.8 | 0.43 | 0.48 | 0.96 | 0.57 |
| GRIK1 | 14.444 | 3.852399 | 0.009246 | 0.031323 | yes | up | 0.02 | 0.02 | 0 | 0.69 | 0.29 | 0.08 | 0.013333 | 0.353333 |
| ARNTL | 2.233 | 1.159098 | 0.009354 | 0.031648 | yes | up | 1.07 | 1.26 | 2.34 | 9.08 | 5.5 | 11.58 | 1.556667 | 8.72 |
| CCRL2 | 0.348 | -1.52388 | 0.009365 | 0.031672 | yes | down | 1.02 | 2.67 | 3.9 | 2.53 | 2.52 | 1.55 | 2.53 | 2.2 |
| MAL2 | 2.292 | 1.196517 | 0.00937 | 0.031683 | yes | up | 3.69 | 3.61 | 8.2 | 46.78 | 14.44 | 27.5 | 5.166667 | 29.57333 |
| NPY4R | 12.033 | 3.588865 | 0.009375 | 0.031692 | yes | up | 0 | 0.01 | 0.05 | 0.43 | 0.15 | 0.32 | 0.02 | 0.3 |
| RRAGD | 0.389 | -1.36101 | 0.00949 | 0.032019 | yes | down | 6.53 | 5.73 | 3.04 | 1.9 | 8.45 | 3.28 | 5.1 | 4.543333 |
| LOC103351973 | 2.584 | 1.369388 | 0.009507 | 0.032069 | yes | up | 0.2 | 0.24 | 0.17 | 1.83 | 0.75 | 1.23 | 0.203333 | 1.27 |
| KLKB1 | 18.117 | 4.179299 | 0.009518 | 0.032093 | yes | up | 0.03 | 0 | 0 | 0.48 | 0.52 | 0.15 | 0.01 | 0.383333 |
| LOC103347521 | 21.602 | 4.433075 | 0.009531 | 0.032126 | yes | up | 0.05 | 0 | 0 | 2.15 | 0.24 | 2.51 | 0.016667 | 1.633333 |
| CDK18 | 0.412 | -1.28014 | 0.009552 | 0.032181 | yes | down | 1.09 | 1.81 | 1.28 | 2.48 | 0.99 | 0.76 | 1.393333 | 1.41 |
| CDC25B | 2.42 | 1.27476 | 0.009568 | 0.032229 | yes | up | 7.58 | 3.25 | 2.8 | 27.24 | 29.7 | 11.01 | 4.543333 | 22.65 |
| LOC100338530 | 3.618 | 1.855313 | 0.009579 | 0.032252 | yes | up | 0.1 | 0.2 | 0.27 | 3.71 | 1.38 | 0.28 | 0.19 | 1.79 |
| MLLT3 | 0.361 | -1.47157 | 0.009642 | 0.032423 | yes | down | 5.69 | 1.52 | 0.79 | 2.04 | 1.48 | 2.29 | 2.666667 | 1.936667 |
| SYNC | 0.3 | -1.73516 | 0.009667 | 0.032497 | yes | down | 4.66 | 1.64 | 0.52 | 1.11 | 2.01 | 1.5 | 2.273333 | 1.54 |
| PAWR | 2.18 | 1.124432 | 0.009708 | 0.032614 | yes | up | 0.56 | 0.44 | 0.41 | 3.64 | 1.83 | 1.99 | 0.47 | 2.486667 |
| LOC103352342 | 4.489 | 2.166338 | 0.009714 | 0.032628 | yes | up | 0.02 | 0.01 | 0.03 | 0.28 | 0.16 | 0.23 | 0.02 | 0.223333 |
| LOC100350471 | 0.026 | -5.23862 | 0.009729 | 0.03267 | yes | down | 0 | 0.26 | 0.43 | 0 | 0 | 0.04 | 0.23 | 0.013333 |
| WNT2 | 3.005 | 1.587303 | 0.009772 | 0.032781 | yes | up | 1.08 | 0.38 | 0.27 | 2.03 | 3.84 | 6.06 | 0.576667 | 3.976667 |
| LOC108178426 | 27.743 | 4.794041 | 0.009824 | 0.032942 | yes | up | 0 | 0 | 0 | 1.85 | 0.37 | 0.75 | 0 | 0.99 |
| COL2A1 | 0.279 | -1.84007 | 0.009829 | 0.032948 | yes | down | 0.54 | 0.36 | 0.09 | 0.23 | 0.3 | 0.11 | 0.33 | 0.213333 |
| SLC1A1 | 3.456 | 1.789274 | 0.009868 | 0.033071 | yes | up | 0.07 | 0.08 | 0.06 | 0.72 | 0.35 | 0.64 | 0.07 | 0.57 |
| LOC103351044 | 6.112 | 2.611531 | 0.009882 | 0.033109 | yes | up | 1.79 | 1.35 | 1.31 | 18.98 | 6.98 | 27.14 | 1.483333 | 17.7 |
| CACNA1A | 4.594 | 2.199694 | 0.009919 | 0.033215 | yes | up | 0.07 | 0.07 | 0 | 0.73 | 0.38 | 0.39 | 0.046667 | 0.5 |
| LOC103352385 | 0.235 | -2.08943 | 0.009943 | 0.033281 | yes | down | 0.52 | 1.85 | 1.17 | 0.81 | 0.1 | 1.53 | 1.18 | 0.813333 |
| RPL15 | 0.454 | -1.13908 | 0.010052 | 0.033619 | yes | down | 648.01 | 651.69 | 551.13 | 1007.52 | 155.6 | 792.23 | 616.9433 | 651.7833 |
| GPR61 | 0.491 | -1.02512 | 0.010109 | 0.033797 | yes | down | 0.96 | 0.54 | 0.74 | 1.05 | 0.59 | 1.01 | 0.746667 | 0.883333 |
| APCDD1 | 2.143 | 1.099324 | 0.010205 | 0.034062 | yes | up | 3.33 | 1.47 | 4.15 | 14.9 | 12.04 | 20.44 | 2.983333 | 15.79333 |
| LOC100348076 | 0.229 | -2.1287 | 0.010222 | 0.034104 | yes | down | 0.98 | 2.2 | 2.85 | 0.48 | 0.69 | 1.42 | 2.01 | 0.863333 |
| LOC100357816 | 0.311 | -1.68323 | 0.010351 | 0.034485 | yes | down | 4.01 | 4.03 | 2.82 | 1.1 | 2.26 | 4.47 | 3.62 | 2.61 |
| RAB20 | 0.113 | -3.14185 | 0.010361 | 0.034505 | yes | down | 0.42 | 0.76 | 0.63 | 0.25 | 0 | 0.25 | 0.603333 | 0.166667 |
| LOC100344012 | 3.552 | 1.828443 | 0.010373 | 0.034532 | yes | up | 0.12 | 0.14 | 0.08 | 0.97 | 0.68 | 1.26 | 0.113333 | 0.97 |
| LOC100341431 | 4.665 | 2.221836 | 0.010397 | 0.034585 | yes | up | 0.03 | 0.04 | 0.03 | 0.46 | 0.41 | 0.23 | 0.033333 | 0.366667 |
| CXADR | 0.17 | -2.55669 | 0.010399 | 0.034585 | yes | down | 0.35 | 0.36 | 0.34 | 0.12 | 0.26 | 0.06 | 0.35 | 0.146667 |
| NPY5R | 3.724 | 1.896966 | 0.010407 | 0.034603 | yes | up | 0.39 | 0.04 | 0.19 | 1.38 | 1.42 | 1.49 | 0.206667 | 1.43 |
| LOC103346509 | 0.403 | -1.30938 | 0.010411 | 0.034611 | yes | down | 2.26 | 2.86 | 4.18 | 5.71 | 1.73 | 2.1 | 3.1 | 3.18 |
| APOA2 | 0.06 | -4.06051 | 0.01043 | 0.034659 | yes | down | 903.34 | 3.6 | 1082.22 | 190.16 | 47 | 72.41 | 663.0533 | 103.19 |
| LOC100346276 | 0.355 | -1.49403 | 0.010463 | 0.034762 | yes | down | 15.15 | 19.23 | 16.57 | 3.46 | 14.2 | 25.24 | 16.98333 | 14.3 |
| LOC103347211 | 2.802 | 1.486452 | 0.010518 | 0.034903 | yes | up | 0.55 | 0.34 | 0.39 | 5.08 | 1.18 | 3.43 | 0.426667 | 3.23 |
| TEX33 | 0.025 | -5.33762 | 0.010524 | 0.034912 | yes | down | 8.01 | 1.48 | 0 | 0 | 0.54 | 0 | 3.163333 | 0.18 |
| CKMT2 | 0.047 | -4.41575 | 0.010562 | 0.035021 | yes | down | 192.49 | 35.04 | 54.08 | 0.06 | 24.36 | 2.55 | 93.87 | 8.99 |
| IGSF1 | 0.335 | -1.57709 | 0.010571 | 0.035043 | yes | down | 2.31 | 0.55 | 0.42 | 0.61 | 0.69 | 0.5 | 1.093333 | 0.6 |
| LOC100347178 | 12.468 | 3.640135 | 0.01059 | 0.0351 | yes | up | 0.06 | 0 | 0.1 | 1.12 | 2.8 | 0.54 | 0.053333 | 1.486667 |
| CDCA2 | 2.961 | 1.566289 | 0.010677 | 0.035335 | yes | up | 0.5 | 0.29 | 0.1 | 2.52 | 2.95 | 0.98 | 0.296667 | 2.15 |
| ENPP6 | 0.329 | -1.60415 | 0.010698 | 0.035387 | yes | down | 2.63 | 2.23 | 0.31 | 1.67 | 1.4 | 1.02 | 1.723333 | 1.363333 |
| LOC103348798 | 4.605 | 2.203193 | 0.010759 | 0.035575 | yes | up | 0.04 | 0.02 | 0.08 | 0.61 | 0.36 | 0.59 | 0.046667 | 0.52 |
| ST8SIA2 | 0.176 | -2.50883 | 0.010772 | 0.035603 | yes | down | 0.15 | 0.19 | 0.11 | 0 | 0.1 | 0.09 | 0.15 | 0.063333 |
| TRNAF-GAA | 3.84 | 1.941061 | 0.010928 | 0.036069 | yes | up | 0.42 | 0.22 | 0.13 | 4.24 | 0.44 | 3.51 | 0.256667 | 2.73 |
| LOC108176455 | 6.079 | 2.603949 | 0.010937 | 0.036082 | yes | up | 0.16 | 0.05 | 0.14 | 1.98 | 0.42 | 2.86 | 0.116667 | 1.753333 |
| WDR63 | 17.652 | 4.141751 | 0.010966 | 0.036143 | yes | up | 0 | 0.02 | 0 | 0.21 | 0.17 | 0.51 | 0.006667 | 0.296667 |
| IL17RB | 0.254 | -1.97568 | 0.010994 | 0.036228 | yes | down | 0.95 | 0.3 | 0.23 | 0.36 | 0.35 | 0.17 | 0.493333 | 0.293333 |
| KCNK12 | 4.098 | 2.034855 | 0.011031 | 0.036328 | yes | up | 0.07 | 0.04 | 0.02 | 0.16 | 0.59 | 0.57 | 0.043333 | 0.44 |
| LVRN | 2.178 | 1.122791 | 0.011053 | 0.036381 | yes | up | 2.95 | 1.52 | 0.75 | 6.55 | 9.02 | 20.64 | 1.74 | 12.07 |
| LOC103348206 | 5.373 | 2.425682 | 0.011068 | 0.036408 | yes | up | 0.04 | 0.07 | 0.04 | 1.08 | 0.11 | 0.64 | 0.05 | 0.61 |
| NXPH3 | 0.358 | -1.48333 | 0.011079 | 0.036427 | yes | down | 0.83 | 1.14 | 2.13 | 1.65 | 0.69 | 1.37 | 1.366667 | 1.236667 |
| NDNF | 0.142 | -2.81854 | 0.011092 | 0.036458 | yes | down | 0.16 | 0.12 | 0.15 | 0.09 | 0.03 | 0.03 | 0.143333 | 0.05 |
| LOC100349085 | 2.732 | 1.44973 | 0.011129 | 0.036546 | yes | up | 9.46 | 24.35 | 11 | 115.05 | 24.8 | 155.31 | 14.93667 | 98.38667 |
| CHRNG | 0.042 | -4.58793 | 0.01113 | 0.036546 | yes | down | 0.75 | 0.06 | 0 | 0 | 0.03 | 0.05 | 0.27 | 0.026667 |
| TLCD2 | 2.996 | 1.583103 | 0.011159 | 0.036632 | yes | up | 0.07 | 0.12 | 0.07 | 0.69 | 0.48 | 0.72 | 0.086667 | 0.63 |
| SERF1A | 2.257 | 1.174658 | 0.011225 | 0.036837 | yes | up | 4.63 | 2.5 | 2.06 | 12.32 | 17.73 | 18.91 | 3.063333 | 16.32 |
| SLC48A1 | 2.005 | 1.003715 | 0.011237 | 0.036867 | yes | up | 9.65 | 4.93 | 6.34 | 37.25 | 16.02 | 40.52 | 6.973333 | 31.26333 |
| LOC100350165 | 0.043 | -4.52425 | 0.011283 | 0.036991 | yes | down | 0.16 | 0.45 | 0.09 | 0 | 0 | 0.06 | 0.233333 | 0.02 |
| FAM196A | 0.237 | -2.07837 | 0.01132 | 0.037095 | yes | down | 0.08 | 0.22 | 0.04 | 0.04 | 0.05 | 0.09 | 0.113333 | 0.06 |
| LOC108177738 | 0.082 | -3.60665 | 0.01136 | 0.037196 | yes | down | 0.16 | 0.83 | 0.28 | 0.19 | 0.01 | 0.08 | 0.423333 | 0.093333 |
| CLHC1 | 2.267 | 1.180497 | 0.011407 | 0.037313 | yes | up | 0.39 | 0.33 | 0.32 | 1.78 | 1.7 | 2.22 | 0.346667 | 1.9 |
| CD46 | 8.019 | 3.00339 | 0.01142 | 0.037342 | yes | up | 0.11 | 0.05 | 0 | 1.11 | 0.74 | 1.21 | 0.053333 | 1.02 |
| LOC103350869 | 16.209 | 4.018765 | 0.011454 | 0.03744 | yes | up | 0.04 | 0 | 0 | 1.12 | 1.09 | 2.74 | 0.013333 | 1.65 |
| SCN3B | 0.485 | -1.04464 | 0.011457 | 0.037441 | yes | down | 4.1 | 2.39 | 1.12 | 3.27 | 2.98 | 3.21 | 2.536667 | 3.153333 |
| KCNMA1 | 0.266 | -1.9085 | 0.011471 | 0.03748 | yes | down | 4.63 | 0.86 | 0.63 | 0.44 | 2.02 | 1.2 | 2.04 | 1.22 |
| ARHGAP11A | 2.845 | 1.508417 | 0.011474 | 0.037483 | yes | up | 0.69 | 0.58 | 0.16 | 3.3 | 4.79 | 1.21 | 0.476667 | 3.1 |
| GTSF1 | 0.035 | -4.8447 | 0.011525 | 0.037627 | yes | down | 0 | 0.11 | 0.61 | 0 | 0.07 | 0 | 0.24 | 0.023333 |
| TMEM262 | 23.804 | 4.573125 | 0.011574 | 0.037764 | yes | up | 0 | 0 | 0 | 1.3 | 1.39 | 2.53 | 0 | 1.74 |
| SPP1 | 0.129 | -2.95656 | 0.011818 | 0.038477 | yes | down | 22.78 | 22.2 | 5.31 | 1.42 | 12.03 | 1.02 | 16.76333 | 4.823333 |
| LOC108177642 | 2.013 | 1.009588 | 0.011958 | 0.038879 | yes | up | 0.49 | 0.44 | 0.38 | 2.19 | 2.28 | 1.13 | 0.436667 | 1.866667 |
| SUSD4 | 3.774 | 1.916202 | 0.011989 | 0.038972 | yes | up | 0.06 | 0.13 | 0.11 | 0.42 | 1.4 | 0.85 | 0.1 | 0.89 |
| LOC100342885 | 187.98 | 7.554435 | 0.012058 | 0.039144 | yes | up | 0 | 0.14 | 0 | 40.08 | 40.35 | 0 | 0.046667 | 26.81 |
| ERMAP | 0.119 | -3.07419 | 0.012074 | 0.039188 | yes | down | 0.1 | 0.19 | 0.1 | 0.02 | 0 | 0.08 | 0.13 | 0.033333 |
| LOC100338913 | 3.219 | 1.686718 | 0.012089 | 0.039228 | yes | up | 0.24 | 1.02 | 0.49 | 7.35 | 3.15 | 2.88 | 0.583333 | 4.46 |
| TRIM14 | 3.695 | 1.885689 | 0.012114 | 0.039292 | yes | up | 0.08 | 0.14 | 0.01 | 0.71 | 0.43 | 0.88 | 0.076667 | 0.673333 |
| PSMB9 | 2.605 | 1.381499 | 0.012118 | 0.039292 | yes | up | 1.1 | 1.27 | 0.52 | 4.96 | 7.9 | 4.8 | 0.963333 | 5.886667 |
| CDCA8 | 2.895 | 1.533553 | 0.012129 | 0.039305 | yes | up | 1.36 | 1.12 | 0.32 | 7.28 | 9.04 | 2.42 | 0.933333 | 6.246667 |
| LOC108176201 | 3.156 | 1.657963 | 0.01216 | 0.039367 | yes | up | 0.18 | 0.37 | 0.36 | 1.12 | 2.58 | 2.95 | 0.303333 | 2.216667 |
| DOCK3 | 0.472 | -1.0839 | 0.012183 | 0.03941 | yes | down | 0.22 | 0.57 | 0.41 | 0.42 | 0.41 | 0.54 | 0.4 | 0.456667 |
| LETM2 | 2.361 | 1.239293 | 0.012187 | 0.039413 | yes | up | 0.27 | 0.46 | 0.2 | 1.36 | 1.53 | 1.07 | 0.31 | 1.32 |
| LOC100340177 | 0.363 | -1.46193 | 0.012207 | 0.03947 | yes | down | 1.01 | 8.01 | 3.84 | 2.84 | 4.67 | 6.93 | 4.286667 | 4.813333 |
| SLC12A3 | 4.363 | 2.125383 | 0.012262 | 0.039627 | yes | up | 0.14 | 0.12 | 0.04 | 1.54 | 1.18 | 0.79 | 0.1 | 1.17 |
| SGO1 | 2.997 | 1.583708 | 0.012273 | 0.039655 | yes | up | 0.34 | 0.33 | 0.15 | 2.43 | 2.7 | 0.62 | 0.273333 | 1.916667 |
| LOC108176549 | 3.836 | 1.939684 | 0.012356 | 0.039892 | yes | up | 0.09 | 0.03 | 0.11 | 1.15 | 0.37 | 0.65 | 0.076667 | 0.723333 |
| PAK3 | 2.694 | 1.429809 | 0.01238 | 0.039944 | yes | up | 0.09 | 0.04 | 0.03 | 0.42 | 0.31 | 0.29 | 0.053333 | 0.34 |
| CNNM1 | 3.017 | 1.593214 | 0.012392 | 0.039961 | yes | up | 0.1 | 0.04 | 0.04 | 0.57 | 0.44 | 0.24 | 0.06 | 0.416667 |
| KCNC2 | 0.049 | -4.33914 | 0.012417 | 0.040025 | yes | down | 0.15 | 0.11 | 0.03 | 0.04 | 0 | 0 | 0.096667 | 0.013333 |
| PTPRZ1 | 0.382 | -1.38896 | 0.012538 | 0.040375 | yes | down | 0.41 | 0.51 | 0.11 | 0.23 | 0.28 | 0.39 | 0.343333 | 0.3 |
| NUSAP1 | 2.665 | 1.414057 | 0.012595 | 0.040543 | yes | up | 3.35 | 3.08 | 1.25 | 23.32 | 21.42 | 4.44 | 2.56 | 16.39333 |
| MCF2L2 | 2.191 | 1.131545 | 0.012618 | 0.040595 | yes | up | 0.48 | 0.3 | 0.35 | 2.89 | 1.06 | 2.06 | 0.376667 | 2.003333 |
| LOC100341997 | 2.357 | 1.23682 | 0.012629 | 0.040623 | yes | up | 1.13 | 2.37 | 0.78 | 7.94 | 11.24 | 4.46 | 1.426667 | 7.88 |
| STEAP3 | 3.034 | 1.60102 | 0.012649 | 0.04067 | yes | up | 0.65 | 0.19 | 0.23 | 1.48 | 2.51 | 3.52 | 0.356667 | 2.503333 |
| LOC108178202 | 23.111 | 4.530532 | 0.012707 | 0.040809 | yes | up | 0 | 0 | 0 | 0.76 | 0.81 | 0.41 | 0 | 0.66 |
| NETO2 | 2.159 | 1.110532 | 0.012717 | 0.040833 | yes | up | 0.73 | 0.93 | 0.72 | 5.07 | 5.63 | 1.63 | 0.793333 | 4.11 |
| DUOX2 | 0.229 | -2.12624 | 0.012727 | 0.040859 | yes | down | 0.44 | 1.03 | 1.33 | 1.1 | 0.04 | 0.52 | 0.933333 | 0.553333 |
| LOC100348485 | 0.134 | -2.89498 | 0.012734 | 0.040874 | yes | down | 0.42 | 0.81 | 1.1 | 0.51 | 0.22 | 0.17 | 0.776667 | 0.3 |
| PFN2 | 0.388 | -1.36622 | 0.012776 | 0.040969 | yes | down | 54.71 | 17.16 | 8.87 | 17.95 | 26.49 | 13.93 | 26.91333 | 19.45667 |
| LOC103347176 | 23.5 | 4.554587 | 0.012815 | 0.041085 | yes | up | 0 | 0 | 0 | 2.9 | 5.57 | 2.96 | 0 | 3.81 |
| NINL | 0.422 | -1.24379 | 0.012961 | 0.041504 | yes | down | 0.51 | 0.23 | 0.26 | 0.33 | 0.23 | 0.43 | 0.333333 | 0.33 |
| LOC103352020 | 0.472 | -1.08342 | 0.013006 | 0.041607 | yes | down | 0.88 | 1.37 | 0.78 | 0.99 | 1.42 | 0.96 | 1.01 | 1.123333 |
| LOC108176841 | 2.633 | 1.396958 | 0.013095 | 0.041835 | yes | up | 0.14 | 0.04 | 0.04 | 0.49 | 0.48 | 0.4 | 0.073333 | 0.456667 |
| LOC108176834 | 16.923 | 4.080894 | 0.013107 | 0.041868 | yes | up | 0 | 0 | 0.33 | 1.4 | 3.44 | 5.63 | 0.11 | 3.49 |
| LOC108178280 | 12.129 | 3.60044 | 0.01314 | 0.041963 | yes | up | 0.14 | 0 | 0 | 1.73 | 1.57 | 0.58 | 0.046667 | 1.293333 |
| STC1 | 0.49 | -1.02952 | 0.01315 | 0.041988 | yes | down | 3.93 | 3.45 | 2.61 | 4.2 | 5.64 | 1.75 | 3.33 | 3.863333 |
| SAPCD2 | 2.556 | 1.353869 | 0.013189 | 0.042095 | yes | up | 0.69 | 0.9 | 0.51 | 5.42 | 5.82 | 1.49 | 0.7 | 4.243333 |
| CYP2B5 | 5.463 | 2.449576 | 0.013211 | 0.04215 | yes | up | 0.12 | 0 | 0.5 | 1.7 | 3.21 | 3.71 | 0.206667 | 2.873333 |
| HK3 | 0.397 | -1.33245 | 0.013222 | 0.042176 | yes | down | 0.28 | 0.4 | 0.49 | 0.47 | 0.3 | 0.41 | 0.39 | 0.393333 |
| HYAL3 | 2.213 | 1.146219 | 0.01325 | 0.042259 | yes | up | 0.74 | 1.41 | 0.88 | 7.84 | 3.07 | 5.32 | 1.01 | 5.41 |
| LOC100338461 | 18.121 | 4.179566 | 0.013331 | 0.042476 | yes | up | 0 | 0 | 0.02 | 0.57 | 0.08 | 0.26 | 0.006667 | 0.303333 |
| ITIH4 | 0.328 | -1.60761 | 0.013363 | 0.042562 | yes | down | 0.47 | 0.24 | 0.65 | 0.36 | 0.45 | 0.38 | 0.453333 | 0.396667 |
| KIF4A | 2.424 | 1.277453 | 0.013375 | 0.042591 | yes | up | 0.54 | 0.43 | 0.46 | 3.37 | 4.08 | 1.04 | 0.476667 | 2.83 |
| SLC26A9 | 0.488 | -1.03397 | 0.013395 | 0.042633 | yes | down | 3.63 | 8.09 | 2.39 | 6.05 | 6.87 | 5.19 | 4.703333 | 6.036667 |
| THPO | 6.759 | 2.756778 | 0.013417 | 0.042684 | yes | up | 0.03 | 0 | 0.02 | 0.2 | 0.22 | 0.37 | 0.016667 | 0.263333 |
| LOC103348167 | 17.679 | 4.143954 | 0.013428 | 0.042703 | yes | up | 0.04 | 0 | 0 | 0.25 | 1.24 | 0.46 | 0.013333 | 0.65 |
| LOC103348624 | 0.439 | -1.18772 | 0.013464 | 0.042808 | yes | down | 0.55 | 0.48 | 0.33 | 0.64 | 0.32 | 0.46 | 0.453333 | 0.473333 |
| ARG1 | 15.721 | 3.974606 | 0.013489 | 0.042856 | yes | up | 0.05 | 0 | 0 | 0.46 | 0.98 | 0.48 | 0.016667 | 0.64 |
| LOC100346811 | 2.824 | 1.497825 | 0.013622 | 0.043208 | yes | up | 0.09 | 0.28 | 3.76 | 1.24 | 4.34 | 1.38 | 1.376667 | 2.32 |
| LOC108178087 | 14.98 | 3.90494 | 0.013623 | 0.043208 | yes | up | 0.09 | 0 | 0 | 0.93 | 1.22 | 0.9 | 0.03 | 1.016667 |
| ADH2-2 | 3.846 | 1.943287 | 0.013661 | 0.043317 | yes | up | 1.39 | 0.42 | 0.87 | 6.97 | 1.35 | 16.58 | 0.893333 | 8.3 |
| LOC100339296 | 50.806 | 5.666935 | 0.013663 | 0.043318 | yes | up | 0 | 0 | 0 | 0.53 | 0.13 | 0 | 0 | 0.22 |
| LOC103345438 | 3.733 | 1.900474 | 0.013742 | 0.04351 | yes | up | 0.09 | 0.13 | 0.1 | 0.7 | 0.64 | 1.52 | 0.106667 | 0.953333 |
| KLHDC7A | 0.192 | -2.38197 | 0.013769 | 0.043587 | yes | down | 0.1 | 0.12 | 0.02 | 0.03 | 0.02 | 0.05 | 0.08 | 0.033333 |
| LOC100343144 | 2.011 | 1.008165 | 0.013777 | 0.043596 | yes | up | 4.4 | 3.25 | 7.51 | 32.17 | 25 | 19.28 | 5.053333 | 25.48333 |
| EFNA4 | 3.235 | 1.693682 | 0.013847 | 0.043775 | yes | up | 0.43 | 0.22 | 0.2 | 2.86 | 0.98 | 2.7 | 0.283333 | 2.18 |
| HMCN2 | 0.348 | -1.52345 | 0.013945 | 0.044049 | yes | down | 5.38 | 1.28 | 0.73 | 1.31 | 2.5 | 2.05 | 2.463333 | 1.953333 |
| LRRC7 | 15.088 | 3.915354 | 0.013969 | 0.044093 | yes | up | 0.01 | 0 | 0 | 0.16 | 0.08 | 0.15 | 0.003333 | 0.13 |
| LOC103345491 | 2.555 | 1.353081 | 0.013984 | 0.044114 | yes | up | 0.28 | 0.31 | 0.38 | 2.8 | 1.35 | 1.93 | 0.323333 | 2.026667 |
| LOC100339454 | 0.319 | -1.64741 | 0.014027 | 0.044237 | yes | down | 6.7 | 12.93 | 10.84 | 9.35 | 4.2 | 11.37 | 10.15667 | 8.306667 |
| ALDOB | 0.457 | -1.12952 | 0.014028 | 0.044237 | yes | down | 1.33 | 2.63 | 3.09 | 2.71 | 3.87 | 1.78 | 2.35 | 2.786667 |
| LOC103351689 | 7.584 | 2.922891 | 0.014044 | 0.044271 | yes | up | 0.06 | 0.1 | 0 | 0.94 | 0.79 | 1.03 | 0.053333 | 0.92 |
| DAB1 | 4.674 | 2.224743 | 0.014059 | 0.044309 | yes | up | 0.07 | 0.02 | 0.03 | 0.79 | 0.13 | 0.46 | 0.04 | 0.46 |
| LOC100350196 | 0.085 | -3.56262 | 0.014129 | 0.044505 | yes | down | 0.27 | 0.45 | 0.24 | 0.2 | 0 | 0 | 0.32 | 0.066667 |
| TSPYL4 | 2.158 | 1.109673 | 0.014149 | 0.04455 | yes | up | 0.22 | 0.19 | 0.11 | 1.06 | 0.82 | 0.75 | 0.173333 | 0.876667 |
| LOC103345755 | 6.071 | 2.601847 | 0.014183 | 0.04464 | yes | up | 0.03 | 0.02 | 0.01 | 0.22 | 0.29 | 0.36 | 0.02 | 0.29 |
| KCNQ5 | 0.135 | -2.89126 | 0.014226 | 0.044751 | yes | down | 1.22 | 0.15 | 0.05 | 0.05 | 0.35 | 0.02 | 0.473333 | 0.14 |
| FANCD2 | 2.116 | 1.081541 | 0.014247 | 0.044807 | yes | up | 1.89 | 2.76 | 0.7 | 9.07 | 11.44 | 5.64 | 1.783333 | 8.716667 |
| TEX12 | 0.38 | -1.39468 | 0.01425 | 0.044808 | yes | down | 2.04 | 1.68 | 0.62 | 1.28 | 1.37 | 1.09 | 1.446667 | 1.246667 |
| SEC14L5 | 0.118 | -3.07865 | 0.014291 | 0.044913 | yes | down | 0.91 | 0.11 | 0.01 | 0.08 | 0.15 | 0.04 | 0.343333 | 0.09 |
| LOC100346096 | 0.129 | -2.95298 | 0.014321 | 0.044972 | yes | down | 0.95 | 2.59 | 1.68 | 1.03 | 0.6 | 0.5 | 1.74 | 0.71 |
| LOC100342242 | 3.143 | 1.652298 | 0.014395 | 0.045128 | yes | up | 0.23 | 0.12 | 0.21 | 1.75 | 2.07 | 0.46 | 0.186667 | 1.426667 |
| NLRC3 | 2.239 | 1.163088 | 0.014428 | 0.045214 | yes | up | 0.95 | 1.11 | 1.03 | 7.31 | 7.38 | 2.7 | 1.03 | 5.796667 |
| GPR63 | 2.606 | 1.381964 | 0.014517 | 0.045442 | yes | up | 0.43 | 0.14 | 0.12 | 1.33 | 0.89 | 1.91 | 0.23 | 1.376667 |
| SULT4A1 | 0.219 | -2.19405 | 0.014557 | 0.045551 | yes | down | 0.28 | 0.23 | 0.27 | 0.15 | 0.04 | 0.22 | 0.26 | 0.136667 |
| LOC100344919 | 8.51 | 3.089111 | 0.014677 | 0.045874 | yes | up | 0.04 | 0.24 | 0.1 | 0.05 | 4.57 | 2.75 | 0.126667 | 2.456667 |
| SLC2A9 | 17.857 | 4.158392 | 0.014682 | 0.045879 | yes | up | 0.04 | 0 | 0 | 0.98 | 0.31 | 0.15 | 0.013333 | 0.48 |
| RNF222 | 21.025 | 4.394044 | 0.014761 | 0.046093 | yes | up | 0.02 | 0 | 0 | 0.48 | 0.03 | 0.59 | 0.006667 | 0.366667 |
| CDC25C | 4.594 | 2.199691 | 0.014802 | 0.046201 | yes | up | 0.25 | 0.18 | 0 | 1.86 | 1.34 | 0.56 | 0.143333 | 1.253333 |
| LOC100343611 | 0.497 | -1.00751 | 0.014944 | 0.046575 | yes | down | 0.97 | 1.25 | 1.01 | 2.08 | 0.91 | 0.9 | 1.076667 | 1.296667 |
| LOC103345228 | 0.345 | -1.53526 | 0.01507 | 0.046889 | yes | down | 0.39 | 0.19 | 0.24 | 0.42 | 0.11 | 0.16 | 0.273333 | 0.23 |
| MFSD7 | 2.065 | 1.045928 | 0.015095 | 0.046959 | yes | up | 0.4 | 0.44 | 0.28 | 1.99 | 1.21 | 2.05 | 0.373333 | 1.75 |
| KCNA6 | 3.286 | 1.716224 | 0.015117 | 0.047018 | yes | up | 0.07 | 0.14 | 0.02 | 0.49 | 0.44 | 0.83 | 0.076667 | 0.586667 |
| KIAA0895L | 2.171 | 1.118037 | 0.01513 | 0.04705 | yes | up | 0.19 | 0.38 | 0.2 | 1.58 | 0.97 | 1.38 | 0.256667 | 1.31 |
| POLE | 2.12 | 1.084015 | 0.01516 | 0.047099 | yes | up | 0.63 | 0.34 | 0.19 | 2.43 | 2.18 | 1.17 | 0.386667 | 1.926667 |
| RNF122 | 2.067 | 1.047606 | 0.015187 | 0.047174 | yes | up | 3.39 | 1.78 | 1.01 | 9.49 | 7.1 | 13.2 | 2.06 | 9.93 |
| LOC108176550 | 3.656 | 1.870278 | 0.015206 | 0.047207 | yes | up | 0.04 | 0.11 | 0.03 | 0.49 | 0.26 | 0.76 | 0.06 | 0.503333 |
| FZD9 | 0.076 | -3.71953 | 0.015221 | 0.047244 | yes | down | 2.23 | 0.1 | 0 | 0.08 | 0.23 | 0.08 | 0.776667 | 0.13 |
| LOC103351221 | 3.185 | 1.671158 | 0.015264 | 0.047351 | yes | up | 0.28 | 0.38 | 0.24 | 1.58 | 2.5 | 2.65 | 0.3 | 2.243333 |
| APOD | 2.351 | 1.23306 | 0.015279 | 0.047382 | yes | up | 106.66 | 169.18 | 115.38 | 586.5 | 1324.93 | 253.76 | 130.4067 | 721.73 |
| LOC103348766 | 21.654 | 4.436563 | 0.015303 | 0.047435 | yes | up | 0 | 0 | 0 | 0.55 | 0.37 | 0.59 | 0 | 0.503333 |
| GPRC5D | 4.351 | 2.121495 | 0.015328 | 0.047487 | yes | up | 0.07 | 0.12 | 0.03 | 0.77 | 0.64 | 0.82 | 0.073333 | 0.743333 |
| ITPKA | 0.261 | -1.93897 | 0.015343 | 0.047525 | yes | down | 0.42 | 0.39 | 0.68 | 0.45 | 0.27 | 0.25 | 0.496667 | 0.323333 |
| LOC100346803 | 2.107 | 1.075272 | 0.015375 | 0.047606 | yes | up | 0.51 | 0.69 | 0.46 | 2.75 | 2.71 | 3.45 | 0.553333 | 2.97 |
| SUGCT | 0.358 | -1.48336 | 0.015399 | 0.047672 | yes | down | 1.18 | 0.54 | 0.63 | 0.46 | 0.28 | 1.44 | 0.783333 | 0.726667 |
| LOC103348671 | 0.341 | -1.55283 | 0.015478 | 0.047897 | yes | down | 0.8 | 0.69 | 0.54 | 0.76 | 0.25 | 0.68 | 0.676667 | 0.563333 |
| DLX4 | 0.424 | -1.23888 | 0.015497 | 0.047919 | yes | down | 0.57 | 1.09 | 1.06 | 0.8 | 1.24 | 0.77 | 0.906667 | 0.936667 |
| LOC100339781 | 29.297 | 4.872693 | 0.015553 | 0.048085 | yes | up | 0 | 0 | 0.05 | 1.27 | 0 | 2.07 | 0.016667 | 1.113333 |
| SBK1 | 0.104 | -3.27046 | 0.015727 | 0.048559 | yes | down | 1.05 | 2.08 | 1.86 | 0.91 | 0.29 | 0 | 1.663333 | 0.4 |
| LOC103351088 | 0.13 | -2.94393 | 0.015749 | 0.048618 | yes | down | 4.23 | 3.32 | 1.93 | 0.6 | 0 | 0.93 | 3.16 | 0.51 |
| LOC103348499 | 0.048 | -4.36867 | 0.015812 | 0.048778 | yes | down | 0.08 | 0.12 | 0.26 | 0 | 0 | 0.05 | 0.153333 | 0.016667 |
| RNF43 | 2.9 | 1.53594 | 0.015963 | 0.049199 | yes | up | 0.06 | 0.16 | 0.04 | 0.5 | 0.56 | 0.71 | 0.086667 | 0.59 |
| LOC100353136 | 0.038 | -4.70095 | 0.016034 | 0.049379 | yes | down | 0 | 1.14 | 0.4 | 0 | 0 | 0.13 | 0.513333 | 0.043333 |
| LOC100355223 | 0.1 | -3.32723 | 0.016038 | 0.049381 | yes | down | 1.3 | 0 | 0.24 | 0.11 | 0.12 | 0.11 | 0.513333 | 0.113333 |
| GJB5 | 0.437 | -1.19325 | 0.016046 | 0.049396 | yes | down | 4.13 | 4.37 | 9.1 | 9.31 | 7.45 | 3.53 | 5.866667 | 6.763333 |
| EGR3 | 3.75 | 1.906789 | 0.016068 | 0.049438 | yes | up | 0.15 | 0.04 | 0.03 | 0.48 | 0.91 | 0.5 | 0.073333 | 0.63 |
| LOC103346470 | 0.277 | -1.85071 | 0.016104 | 0.049537 | yes | down | 0.98 | 2.06 | 2.91 | 2 | 1.51 | 0.61 | 1.983333 | 1.373333 |
| PRG3 | 0.034 | -4.89136 | 0.016125 | 0.049566 | yes | down | 0.27 | 0.26 | 0.24 | 0 | 0 | 0 | 0.256667 | 0 |
| ARL9 | 2.381 | 1.2513 | 0.016201 | 0.049754 | yes | up | 1.16 | 0.45 | 0.48 | 3.65 | 4.05 | 2.9 | 0.696667 | 3.533333 |
| LOC100354896 | 0.187 | -2.41592 | 0.016263 | 0.049926 | yes | down | 0.13 | 0.16 | 0.06 | 0.05 | 0.07 | 0.04 | 0.116667 | 0.053333 |

iBAT_D1_VS_ingWAT_M4

| Gene name | FC(ingWAT_M4/iBAT_D1) | Log2FC(ingWAT_M4/iBAT_D1) | Pvalue | Padjust | Significant | Regulate | D1iBAT1 | D1iBAT2 | D1iBAT3 | M4ingWAT1 | M4ingWAT2 | M4ingWAT3 | iBAT_D1 | ingWAT_M4 |
| --- | --- | --- | --- | --- | --- | --- | --- | --- | --- | --- | --- | --- | --- | --- |
| QPCT | 0.024 | -5.40686 | 2.4E-235 | 3.9E-231 | yes | down | 361.16 | 337.63 | 321.24 | 17.47 | 21.93 | 16.11 | 340.01 | 18.50333 |
| KCNK3 | 0.003 | -8.22008 | 2.2E-205 | 1.8E-201 | yes | down | 95.58 | 118.79 | 82.3 | 0.49 | 1.1 | 0.71 | 98.89 | 0.766667 |
| LOC100328910 | 155.615 | 7.28184 | 3.8E-161 | 2.1E-157 | yes | up | 9.52 | 4.67 | 4.48 | 2216.18 | 2217.82 | 2038.47 | 6.223333 | 2157.49 |
| DMGDH | 0.001 | -10.4881 | 5.4E-153 | 2.2E-149 | yes | down | 232.71 | 120.15 | 133.51 | 0.58 | 0.73 | 0.38 | 162.1233 | 0.563333 |
| GMPR | 0.005 | -7.77067 | 1.1E-142 | 3.5E-139 | yes | down | 488.21 | 895.55 | 491.59 | 6.33 | 5.67 | 7.87 | 625.1167 | 6.623333 |
| LOC100346046 | 1108.513 | 10.11441 | 7.4E-141 | 2E-137 | yes | up | 0.59 | 0.62 | 0.53 | 1552.06 | 690.37 | 2042.46 | 0.58 | 1428.297 |
| FABP3 | 0.007 | -7.24318 | 2.2E-120 | 5.2E-117 | yes | down | 1717.61 | 1888.73 | 1516.43 | 21.19 | 17.91 | 37.09 | 1707.59 | 25.39667 |
| GPAT4 | 0.057 | -4.13694 | 1.1E-119 | 2.3E-116 | yes | down | 271.67 | 347.89 | 285.74 | 40.54 | 42.95 | 38.16 | 301.7667 | 40.55 |
| COX2 | 0.04 | -4.65797 | 4.9E-116 | 9.1E-113 | yes | down | 67579.39 | 75887.18 | 77144.5 | 6664.31 | 8253.6 | 5503.44 | 73537.02 | 6807.117 |
| PPP1R1B | 0.004 | -7.95281 | 8.8E-108 | 1.4E-104 | yes | down | 53.32 | 70.74 | 51.32 | 0.27 | 0.55 | 0.89 | 58.46 | 0.57 |
| GPD2 | 0.031 | -5.02274 | 7.5E-105 | 1.1E-101 | yes | down | 103.06 | 78.28 | 108.95 | 7.71 | 7.98 | 6.75 | 96.76333 | 7.48 |
| LOC100341175 | 0.061 | -4.0236 | 2E-103 | 2.7E-100 | yes | down | 690.28 | 539.94 | 592.45 | 76.67 | 95.74 | 85.41 | 607.5567 | 85.94 |
| AQP8 | 359.552 | 8.490057 | 6.3E-101 | 8E-98 | yes | up | 4.88 | 2.08 | 1.02 | 1744.95 | 2717.69 | 1918.54 | 2.66 | 2127.06 |
| SUCLA2 | 0.05 | -4.31727 | 2E-100 | 2.4E-97 | yes | down | 408.77 | 464 | 396.18 | 46.29 | 64.61 | 37.26 | 422.9833 | 49.38667 |
| HP | 82.699 | 6.369805 | 1.21E-95 | 1.33E-92 | yes | up | 19.3 | 9.9 | 13.8 | 2250.34 | 2395.79 | 3502.84 | 14.33333 | 2716.323 |
| MC5R | 81.623 | 6.350901 | 2E-94 | 2.06E-91 | yes | up | 2.39 | 3.05 | 1.49 | 150.96 | 320.73 | 224.47 | 2.31 | 232.0533 |
| APOE | 124.706 | 6.962388 | 1.19E-92 | 1.15E-89 | yes | up | 31.49 | 17.76 | 12.08 | 3632.16 | 8111.29 | 6315.51 | 20.44333 | 6019.653 |
| LOC100009551 | 58.426 | 5.868531 | 8.35E-89 | 7.65E-86 | yes | up | 7.13 | 14.39 | 10.17 | 1475.23 | 1098.01 | 1417.48 | 10.56333 | 1330.24 |
| ND4L | 0.063 | -3.98376 | 3.06E-86 | 2.65E-83 | yes | down | 17325.17 | 20918.76 | 16409.85 | 2954.3 | 2858.89 | 2304.21 | 18217.93 | 2705.8 |
| ND2 | 0.062 | -4.01608 | 3.04E-85 | 2.5E-82 | yes | down | 16686.18 | 22555.63 | 16117.67 | 2770.19 | 2427.01 | 2578.26 | 18453.16 | 2591.82 |
| PDK1 | 0.035 | -4.84214 | 8.02E-85 | 6.29E-82 | yes | down | 43.65 | 59.17 | 49.51 | 4.85 | 4.37 | 2.79 | 50.77667 | 4.003333 |
| LOC100350716 | 0.06 | -4.04709 | 5.12E-84 | 3.84E-81 | yes | down | 18.65 | 14.79 | 12.07 | 1.88 | 2.41 | 2 | 15.17 | 2.096667 |
| DIO2 | 0.011 | -6.49388 | 1.14E-83 | 8.17E-81 | yes | down | 5.85 | 6.29 | 7.31 | 0.15 | 0.31 | 0.08 | 6.483333 | 0.18 |
| CPT1B | 0.089 | -3.49151 | 2.72E-80 | 1.87E-77 | yes | down | 127.28 | 107.72 | 92.56 | 24.39 | 21.97 | 18.93 | 109.1867 | 21.76333 |
| SLC4A4 | 0.045 | -4.48626 | 2.86E-78 | 1.89E-75 | yes | down | 22.45 | 28.8 | 23.72 | 3.18 | 2.4 | 2.01 | 24.99 | 2.53 |
| ETFDH | 0.12 | -3.05755 | 4.11E-78 | 2.61E-75 | yes | down | 239.9 | 219.11 | 179.47 | 56.15 | 67.2 | 55.46 | 212.8267 | 59.60333 |
| ND5 | 0.065 | -3.93292 | 2.44E-77 | 1.49E-74 | yes | down | 63007.72 | 62554.36 | 62343.01 | 12051.46 | 10575.15 | 7781.25 | 62635.03 | 10135.95 |
| KCNH1 | 0.012 | -6.38254 | 2.53E-76 | 1.49E-73 | yes | down | 7.55 | 8.32 | 4.29 | 0.11 | 0.18 | 0.26 | 6.72 | 0.183333 |
| IDH3A | 0.075 | -3.73117 | 4.71E-75 | 2.68E-72 | yes | down | 406.87 | 575.78 | 477.51 | 80.66 | 97.2 | 75.09 | 486.72 | 84.31667 |
| ACADVL | 0.066 | -3.92628 | 2.42E-73 | 1.33E-70 | yes | down | 786.87 | 941.08 | 939.41 | 125.36 | 175.39 | 110.81 | 889.12 | 137.1867 |
| BHMT2 | 0.006 | -7.28474 | 1.28E-71 | 6.8E-69 | yes | down | 31.13 | 38.13 | 40.26 | 0.62 | 0.4 | 0.43 | 36.50667 | 0.483333 |
| ND4 | 0.064 | -3.95479 | 3.67E-71 | 1.89E-68 | yes | down | 12347.23 | 16522.07 | 14129.99 | 2476.79 | 2094.26 | 1802.18 | 14333.1 | 2124.41 |
| ITIH3 | 169.334 | 7.403725 | 4.65E-70 | 2.32E-67 | yes | up | 0.17 | 0.19 | 0.07 | 39.43 | 79.92 | 49.14 | 0.143333 | 56.16333 |
| PPIF | 0.054 | -4.19815 | 5.27E-70 | 2.56E-67 | yes | down | 82.23 | 104.32 | 91.24 | 12.76 | 10.01 | 11.81 | 92.59667 | 11.52667 |
| SLC16A7 | 0.008 | -6.89646 | 2.18E-69 | 1.03E-66 | yes | down | 12.47 | 11.44 | 24.03 | 0.36 | 0.38 | 0.18 | 15.98 | 0.306667 |
| UCP1 | 0 | -18.4588 | 1.32E-68 | 6.06E-66 | yes | down | 4146.2 | 7225.82 | 4811.64 | 0.03 | 0 | 0.06 | 5394.553 | 0.03 |
| SNCG | 131.584 | 7.039842 | 1.92E-68 | 8.54E-66 | yes | up | 15.48 | 12.76 | 14.11 | 4409.11 | 1951.02 | 6077.41 | 14.11667 | 4145.847 |
| TM7SF2 | 40.564 | 5.342126 | 1.24E-67 | 5.36E-65 | yes | up | 2.49 | 1.77 | 0.85 | 160.83 | 122.76 | 147.85 | 1.703333 | 143.8133 |
| ADRA1A | 0.071 | -3.80851 | 5.9E-66 | 2.49E-63 | yes | down | 13.84 | 16.71 | 14 | 2.13 | 3.02 | 1.46 | 14.85 | 2.203333 |
| ATP6 | 0.063 | -3.98097 | 1.18E-65 | 4.87E-63 | yes | down | 30410.82 | 32018.67 | 37755.09 | 5056.12 | 6001.7 | 3850.54 | 33394.86 | 4969.453 |
| HSPA9 | 0.102 | -3.28941 | 3.52E-65 | 1.42E-62 | yes | down | 263.87 | 273.67 | 278.08 | 67.06 | 61.56 | 62.86 | 271.8733 | 63.82667 |
| ACADM | 0.033 | -4.90813 | 4.79E-65 | 1.88E-62 | yes | down | 333.02 | 302.41 | 405.62 | 28.56 | 36.4 | 17.02 | 347.0167 | 27.32667 |
| LOC103348133 | 0.044 | -4.50805 | 2.28E-64 | 8.73E-62 | yes | down | 19.07 | 22.31 | 15.42 | 2.06 | 1.45 | 1.77 | 18.93333 | 1.76 |
| ND3 | 0.064 | -3.97144 | 8.76E-64 | 3.28E-61 | yes | down | 27501.35 | 42960.9 | 30782.62 | 5383.9 | 5014.1 | 4640.26 | 33748.29 | 5012.753 |
| COL5A3 | 14.049 | 3.812415 | 2.51E-62 | 9.18E-60 | yes | up | 6.87 | 5.19 | 3.32 | 148.36 | 180.18 | 158.76 | 5.126667 | 162.4333 |
| GK | 0.041 | -4.60509 | 5.59E-61 | 2E-58 | yes | down | 28.02 | 41.45 | 27.18 | 2.82 | 3.61 | 2.27 | 32.21667 | 2.9 |
| ND6 | 0.039 | -4.677 | 2.64E-59 | 9.28E-57 | yes | down | 8343.68 | 5444.94 | 4871.46 | 777.47 | 504.79 | 407.78 | 6220.027 | 563.3467 |
| ADCK3 | 0.143 | -2.80915 | 4.06E-58 | 1.39E-55 | yes | down | 320.57 | 351.02 | 314.19 | 92.58 | 119.94 | 109.75 | 328.5933 | 107.4233 |
| LOC103348029 | 0.002 | -8.90235 | 9.95E-57 | 3.35E-54 | yes | down | 40.84 | 61.22 | 63.14 | 0.42 | 0.57 | 0 | 55.06667 | 0.33 |
| TFB2M | 0.087 | -3.52361 | 1.34E-56 | 4.41E-54 | yes | down | 38.79 | 42.95 | 39.91 | 7.8 | 10.73 | 6.17 | 40.55 | 8.233333 |
| CYTB | 0.095 | -3.38962 | 1.74E-56 | 5.62E-54 | yes | down | 20607.73 | 18026.4 | 22767.76 | 4757.55 | 4836.64 | 4022 | 20467.3 | 4538.73 |
| HIGD1A | 0.097 | -3.36141 | 4.22E-56 | 1.34E-53 | yes | down | 105.55 | 89.01 | 101.38 | 22.88 | 21.01 | 22.22 | 98.64667 | 22.03667 |
| ABCD2 | 44.292 | 5.468967 | 8.13E-55 | 2.53E-52 | yes | up | 0.54 | 1.21 | 0.3 | 83.83 | 72.24 | 55.81 | 0.683333 | 70.62667 |
| FRRS1 | 0.065 | -3.95406 | 9.14E-55 | 2.79E-52 | yes | down | 8.22 | 7.86 | 7.78 | 1.04 | 1.45 | 1.15 | 7.953333 | 1.213333 |
| COX3 | 0.1 | -3.31933 | 1.43E-54 | 4.29E-52 | yes | down | 64268.61 | 68943.2 | 77249 | 17575.01 | 16848.59 | 14532.27 | 70153.6 | 16318.62 |
| S100B | 31.885 | 4.994825 | 6.11E-53 | 1.8E-50 | yes | up | 12.48 | 9.86 | 3.63 | 569.89 | 685.76 | 633.64 | 8.656667 | 629.7633 |
| HK1 | 0.13 | -2.94111 | 1.5E-52 | 4.35E-50 | yes | down | 86.26 | 101.96 | 97.03 | 30.03 | 29.75 | 25.52 | 95.08333 | 28.43333 |
| PCK2 | 13.333 | 3.736973 | 7.03E-52 | 2E-49 | yes | up | 19.27 | 32.69 | 19.94 | 750.5 | 690.41 | 760.51 | 23.96667 | 733.8067 |
| FH | 0.113 | -3.14711 | 1.16E-51 | 3.24E-49 | yes | down | 145.51 | 138.28 | 143.65 | 29.25 | 46.89 | 36.02 | 142.48 | 37.38667 |
| MRPL39 | 0.121 | -3.05286 | 1.08E-48 | 2.96E-46 | yes | down | 119.67 | 117.28 | 107.33 | 36.45 | 35.61 | 23.62 | 114.76 | 31.89333 |
| LOC100009353 | 7.549 | 2.916296 | 4.13E-48 | 1.12E-45 | yes | up | 91.94 | 85.06 | 64.44 | 1483.99 | 1225.98 | 1392.23 | 80.48 | 1367.4 |
| HADHA | 0.121 | -3.04758 | 1.52E-47 | 4.04E-45 | yes | down | 601.24 | 519.6 | 629.27 | 166.52 | 159.14 | 162.06 | 583.37 | 162.5733 |
| LOC100359087 | 61.129 | 5.933775 | 3.46E-47 | 9.05E-45 | yes | up | 1.28 | 0.62 | 0.28 | 68.37 | 75.41 | 107.05 | 0.726667 | 83.61 |
| ACAT1 | 0.139 | -2.85087 | 4.96E-47 | 1.28E-44 | yes | down | 443.03 | 336.09 | 383.83 | 117.47 | 125.56 | 126.47 | 387.65 | 123.1667 |
| ALOX15 | 0 | -11.7292 | 5.85E-46 | 1.48E-43 | yes | down | 24.96 | 66.63 | 52.88 | 0.05 | 0.03 | 0 | 48.15667 | 0.026667 |
| EXTL1 | 0.051 | -4.30565 | 1.39E-44 | 3.48E-42 | yes | down | 8.74 | 13.22 | 7.34 | 1.4 | 0.9 | 1.03 | 9.766667 | 1.11 |
| HADHB | 0.036 | -4.79317 | 3.7E-44 | 9.11E-42 | yes | down | 1037.1 | 975.66 | 1224.12 | 84.06 | 146.79 | 47.17 | 1078.96 | 92.67333 |
| LRPPRC | 0.173 | -2.53343 | 1.12E-43 | 2.68E-41 | yes | down | 57.93 | 50.28 | 41.92 | 24.22 | 25.61 | 17.3 | 50.04333 | 22.37667 |
| IGSF8 | 8.769 | 3.132367 | 1.12E-43 | 2.68E-41 | yes | up | 4.85 | 3.65 | 3.63 | 63.88 | 103.53 | 77.6 | 4.043333 | 81.67 |
| SOD2 | 0.233 | -2.10107 | 1.91E-43 | 4.48E-41 | yes | down | 338.17 | 351.73 | 300.93 | 160.67 | 212.38 | 158.99 | 330.2767 | 177.3467 |
| PRR5L | 18.215 | 4.187054 | 1.93E-43 | 4.48E-41 | yes | up | 0.56 | 0.46 | 0.42 | 18.43 | 28.85 | 14.07 | 0.48 | 20.45 |
| LOC100347143 | 0.164 | -2.6044 | 5.35E-43 | 1.23E-40 | yes | down | 493.49 | 493.04 | 467.93 | 209.63 | 185.82 | 153.71 | 484.82 | 183.0533 |
| ST13 | 0.228 | -2.13202 | 7.78E-43 | 1.76E-40 | yes | down | 85.69 | 87.25 | 75.19 | 48.2 | 45.86 | 41.58 | 82.71 | 45.21333 |
| AIFM1 | 0.175 | -2.51793 | 1.19E-42 | 2.65E-40 | yes | down | 84.09 | 89 | 76.22 | 37.65 | 33.85 | 28.09 | 83.10333 | 33.19667 |
| SFRP5 | 24.605 | 4.620863 | 1.87E-42 | 4.1E-40 | yes | up | 1.69 | 0.79 | 0.46 | 54.23 | 77.56 | 39.8 | 0.98 | 57.19667 |
| IVNS1ABP | 0.108 | -3.21325 | 3.16E-42 | 6.85E-40 | yes | down | 100.61 | 164.78 | 94.97 | 28.37 | 35.23 | 25.05 | 120.12 | 29.55 |
| LOC100351163 | 9.881 | 3.304666 | 5.75E-42 | 1.23E-39 | yes | up | 3.2 | 3.28 | 4.04 | 74.74 | 94.21 | 78.68 | 3.506667 | 82.54333 |
| TRABD2B | 19.516 | 4.286586 | 1.35E-41 | 2.85E-39 | yes | up | 0.63 | 0.39 | 0.44 | 16.55 | 18.58 | 26.89 | 0.486667 | 20.67333 |
| FFAR2 | 0.089 | -3.49406 | 1.49E-41 | 3.11E-39 | yes | down | 23.76 | 28.54 | 25.67 | 4.29 | 5.33 | 6.44 | 25.99 | 5.353333 |
| ATP8 | 0.093 | -3.4279 | 1.76E-41 | 3.63E-39 | yes | down | 173724.6 | 147645.2 | 203919.5 | 43179.26 | 44272.08 | 33340.84 | 175096.4 | 40264.06 |
| PDHA1 | 0.207 | -2.27134 | 1.98E-41 | 4.03E-39 | yes | down | 187.67 | 180.23 | 175.65 | 87.17 | 101.29 | 72.81 | 181.1833 | 87.09 |
| DHRS11 | 0.163 | -2.61425 | 2.05E-41 | 4.12E-39 | yes | down | 107.95 | 111.63 | 113.85 | 41.49 | 41.49 | 42.41 | 111.1433 | 41.79667 |
| LIAS | 0.165 | -2.60183 | 2.96E-41 | 5.88E-39 | yes | down | 16.81 | 17.5 | 14.18 | 6.14 | 8.09 | 6.81 | 16.16333 | 7.013333 |
| LOC108176503 | 0.092 | -3.43577 | 3.66E-41 | 7.18E-39 | yes | down | 12.41 | 16.05 | 15.81 | 2.83 | 2.28 | 1.48 | 14.75667 | 2.196667 |
| LOC100339305 | 91.926 | 6.522404 | 4.5E-41 | 8.74E-39 | yes | up | 0.88 | 0.21 | 0.16 | 68.07 | 82.99 | 105.77 | 0.416667 | 85.61 |
| SIRPA | 5.789 | 2.533287 | 1.34E-40 | 2.56E-38 | yes | up | 8.52 | 7.97 | 5.36 | 83.19 | 105.78 | 97.28 | 7.283333 | 95.41667 |
| SLIT3 | 6.774 | 2.759973 | 2.22E-40 | 4.21E-38 | yes | up | 2.84 | 3.93 | 2.46 | 41.49 | 51.52 | 49.13 | 3.076667 | 47.38 |
| LOC100347571 | 0.141 | -2.82595 | 6.91E-40 | 1.29E-37 | yes | down | 529.32 | 497.38 | 593.46 | 180.78 | 172.11 | 174.68 | 540.0533 | 175.8567 |
| ACO2 | 0.176 | -2.5037 | 1.58E-39 | 2.92E-37 | yes | down | 773.84 | 692.34 | 801.18 | 287.99 | 339.94 | 297.38 | 755.7867 | 308.4367 |
| NDUFS3 | 0.224 | -2.16083 | 1.8E-39 | 3.29E-37 | yes | down | 380.65 | 377.25 | 345.37 | 183.6 | 189.72 | 191.5 | 367.7567 | 188.2733 |
| MICU3 | 0.088 | -3.50446 | 1.85E-39 | 3.35E-37 | yes | down | 6.37 | 9.69 | 8.19 | 1.57 | 1.97 | 1.3 | 8.083333 | 1.613333 |
| NRG4 | 0.076 | -3.71971 | 2.59E-39 | 4.63E-37 | yes | down | 22.44 | 30.91 | 30.68 | 2.06 | 2.94 | 3.19 | 28.01 | 2.73 |
| SRPX | 11.062 | 3.46757 | 2.68E-39 | 4.75E-37 | yes | up | 6.84 | 5.65 | 3.1 | 139.9 | 118.34 | 125.66 | 5.196667 | 127.9667 |
| LOC100338015 | 4.806 | 2.264688 | 2.97E-39 | 5.21E-37 | yes | up | 34.36 | 28.22 | 24.3 | 339.35 | 336.94 | 284.58 | 28.96 | 320.29 |
| RDH8 | 26.809 | 4.744661 | 3.71E-39 | 6.43E-37 | yes | up | 0.58 | 0.42 | 0.48 | 28.28 | 26.94 | 34.32 | 0.493333 | 29.84667 |
| AFG3L2 | 0.222 | -2.17275 | 4.46E-39 | 7.66E-37 | yes | down | 41.84 | 47.4 | 40.3 | 21.03 | 25.67 | 19.49 | 43.18 | 22.06333 |
| PLTP | 6.635 | 2.730105 | 5.6E-39 | 9.52E-37 | yes | up | 15.55 | 17.47 | 15.81 | 222.76 | 236.18 | 277.73 | 16.27667 | 245.5567 |
| APLP2 | 4.018 | 2.006353 | 8.08E-39 | 1.36E-36 | yes | up | 79.84 | 88.01 | 74.71 | 701.45 | 809.77 | 760.04 | 80.85333 | 757.0867 |
| MPC2 | 0.159 | -2.65307 | 8.68E-39 | 1.45E-36 | yes | down | 348.32 | 346.18 | 389.13 | 136.26 | 135.31 | 127.21 | 361.21 | 132.9267 |
| GNAS | 0.119 | -3.07138 | 1.04E-38 | 1.71E-36 | yes | down | 4031.76 | 4571.39 | 5277.49 | 1404.73 | 1202.83 | 1172.26 | 4626.88 | 1259.94 |
| ACAA2 | 0.122 | -3.03104 | 1.07E-38 | 1.74E-36 | yes | down | 1237.23 | 1235.06 | 1573.12 | 396.69 | 442.29 | 320.25 | 1348.47 | 386.41 |
| MTFP1 | 0.071 | -3.82378 | 1.1E-38 | 1.78E-36 | yes | down | 18.64 | 16.63 | 16.77 | 3.05 | 3.69 | 1.59 | 17.34667 | 2.776667 |
| PLXNA4 | 0.103 | -3.28087 | 1.11E-38 | 1.78E-36 | yes | down | 5.72 | 7.1 | 6.19 | 1.91 | 1.64 | 1.05 | 6.336667 | 1.533333 |
| MYLK | 4.666 | 2.222205 | 1.25E-38 | 1.98E-36 | yes | up | 10.04 | 9.89 | 6.83 | 87.82 | 87.97 | 91.37 | 8.92 | 89.05333 |
| NDUFS1 | 0.167 | -2.58613 | 1.43E-38 | 2.24E-36 | yes | down | 191.76 | 177.2 | 163.09 | 79.32 | 69.65 | 53.08 | 177.35 | 67.35 |
| ITGBL1 | 38.944 | 5.283332 | 1.88E-38 | 2.93E-36 | yes | up | 1.06 | 0.51 | 0.33 | 40.69 | 76.03 | 69.51 | 0.633333 | 62.07667 |
| ARRB1 | 6.301 | 2.655535 | 6.77E-38 | 1.04E-35 | yes | up | 6.6 | 7.28 | 5.99 | 70.52 | 122.15 | 80.44 | 6.623333 | 91.03667 |
| ACAD9 | 0.191 | -2.38593 | 1.02E-37 | 1.56E-35 | yes | down | 22.49 | 24.34 | 20.79 | 8.68 | 9.44 | 9.49 | 22.54 | 9.203333 |
| LOC100354254 | 0.048 | -4.36874 | 1.28E-37 | 1.93E-35 | yes | down | 875.68 | 1200.27 | 1287.57 | 110.34 | 89.41 | 177.28 | 1121.173 | 125.6767 |
| LOC100337966 | 0.16 | -2.64352 | 2.86E-37 | 4.29E-35 | yes | down | 635.09 | 603.45 | 680.17 | 244.69 | 219.03 | 233.36 | 639.57 | 232.36 |
| PHKA2 | 5.373 | 2.425761 | 2.91E-37 | 4.32E-35 | yes | up | 1.89 | 2.16 | 1.42 | 20.47 | 25.09 | 21.48 | 1.823333 | 22.34667 |
| LOC100350787 | 0.172 | -2.53716 | 3.53E-37 | 5.2E-35 | yes | down | 756.27 | 750.88 | 651.09 | 317.75 | 249.56 | 277.37 | 719.4133 | 281.56 |
| GFPT2 | 15.437 | 3.948313 | 4.86E-37 | 7.09E-35 | yes | up | 3.47 | 2.19 | 1.5 | 84.04 | 65.07 | 96.14 | 2.386667 | 81.75 |
| SCTR | 0.042 | -4.57055 | 5.43E-37 | 7.85E-35 | yes | down | 8.65 | 12.65 | 5.89 | 0.78 | 0.79 | 0.57 | 9.063333 | 0.713333 |
| SDHA | 0.199 | -2.32626 | 6.82E-37 | 9.78E-35 | yes | down | 706.37 | 607.87 | 678.16 | 327.72 | 329.11 | 288.29 | 664.1333 | 315.04 |
| GSN | 9.606 | 3.263905 | 7.45E-37 | 1.06E-34 | yes | up | 138.85 | 112.15 | 77.06 | 2290.36 | 1931.96 | 2778.63 | 109.3533 | 2333.65 |
| CST6 | 60.525 | 5.919467 | 9.93E-37 | 1.39E-34 | yes | up | 4.74 | 3.1 | 1.54 | 188.67 | 431.87 | 639.05 | 3.126667 | 419.8633 |
| LOC100353463 | 7.114 | 2.830661 | 9.95E-37 | 1.39E-34 | yes | up | 24.82 | 32.31 | 25.29 | 353.37 | 500.08 | 493.75 | 27.47333 | 449.0667 |
| LEP | 2706.307 | 11.40211 | 1.29E-36 | 1.78E-34 | yes | up | 0.05 | 0 | 0 | 53.72 | 148.14 | 90.61 | 0.016667 | 97.49 |
| LOC100345019 | 6.232 | 2.639761 | 3.9E-36 | 5.35E-34 | yes | up | 12.11 | 9.72 | 9.29 | 134.95 | 143.97 | 162.44 | 10.37333 | 147.12 |
| GPD1 | 11.798 | 3.56049 | 4.76E-36 | 6.48E-34 | yes | up | 151.8 | 100.13 | 65.22 | 2985.4 | 2420.23 | 2997.13 | 105.7167 | 2800.92 |
| C2 | 5.352 | 2.420123 | 5.92E-36 | 7.99E-34 | yes | up | 4.12 | 3.31 | 2.79 | 38.28 | 44.99 | 41.27 | 3.406667 | 41.51333 |
| CLPX | 0.173 | -2.53217 | 6.46E-36 | 8.65E-34 | yes | down | 55.53 | 59.94 | 44.98 | 19.71 | 28.36 | 15.78 | 53.48333 | 21.28333 |
| IL1RN | 0.03 | -5.04657 | 7.84E-36 | 1.04E-33 | yes | down | 36.87 | 101.32 | 43.36 | 3.28 | 3.42 | 5.23 | 60.51667 | 3.976667 |
| ALDH1L1 | 80.047 | 6.322778 | 9.11E-36 | 1.2E-33 | yes | up | 0.9 | 0.14 | 0.17 | 84.2 | 61.27 | 63.76 | 0.403333 | 69.74333 |
| ACKR2 | 10.172 | 3.346501 | 9.22E-36 | 1.21E-33 | yes | up | 1.05 | 0.78 | 0.49 | 14.63 | 20.62 | 17.01 | 0.773333 | 17.42 |
| CHCHD3 | 0.119 | -3.07569 | 1.11E-35 | 1.44E-33 | yes | down | 229.83 | 357.33 | 292.38 | 88.6 | 73.67 | 76.75 | 293.18 | 79.67333 |
| GMPS | 0.104 | -3.26456 | 1.32E-35 | 1.7E-33 | yes | down | 19.5 | 33.31 | 26.69 | 6.83 | 7.22 | 5.1 | 26.5 | 6.383333 |
| ACSS2 | 16.485 | 4.043079 | 1.82E-35 | 2.33E-33 | yes | up | 19.89 | 14.77 | 18.46 | 935.01 | 442.24 | 614.39 | 17.70667 | 663.88 |
| HYAL1 | 8.398 | 3.069972 | 3.47E-35 | 4.38E-33 | yes | up | 8.38 | 8.48 | 7.56 | 136.42 | 117.77 | 169.26 | 8.14 | 141.15 |
| PPARGC1A | 0.013 | -6.30516 | 3.48E-35 | 4.38E-33 | yes | down | 4.93 | 18.09 | 4.2 | 0.3 | 0.32 | 0.16 | 9.073333 | 0.26 |
| DECR1 | 0.15 | -2.73568 | 3.53E-35 | 4.41E-33 | yes | down | 424.16 | 560.36 | 554.25 | 170.62 | 214.2 | 152.53 | 512.9233 | 179.1167 |
| PPTC7 | 0.237 | -2.07816 | 1.27E-34 | 1.58E-32 | yes | down | 11.49 | 12.25 | 9.6 | 6 | 6.22 | 5.76 | 11.11333 | 5.993333 |
| TIMM44 | 0.238 | -2.07028 | 3.93E-34 | 4.84E-32 | yes | down | 55.27 | 62.39 | 49.71 | 31.58 | 31.02 | 28.39 | 55.79 | 30.33 |
| FMO1 | 38.916 | 5.282277 | 4.8E-34 | 5.86E-32 | yes | up | 0.55 | 0.3 | 0.26 | 29.25 | 22.53 | 43.96 | 0.37 | 31.91333 |
| ATP5B | 0.17 | -2.55927 | 5.89E-34 | 7.14E-32 | yes | down | 2240.99 | 2222.67 | 2497.12 | 972.17 | 863.02 | 884.61 | 2320.26 | 906.6 |
| SEC11C | 0.131 | -2.93273 | 6.35E-34 | 7.65E-32 | yes | down | 222.27 | 364.94 | 279.03 | 92.01 | 91.74 | 77.74 | 288.7467 | 87.16333 |
| IDH3B | 0.164 | -2.611 | 6.82E-34 | 8.15E-32 | yes | down | 412.69 | 458.76 | 468.68 | 165.62 | 161.34 | 187.05 | 446.71 | 171.3367 |
| IMMT | 0.24 | -2.05642 | 6.88E-34 | 8.16E-32 | yes | down | 146.73 | 136.6 | 125.74 | 74.28 | 99.45 | 67.32 | 136.3567 | 80.35 |
| SH3PXD2B | 7.822 | 2.96753 | 6.96E-34 | 8.19E-32 | yes | up | 1.07 | 0.83 | 0.52 | 13.93 | 14.21 | 14.13 | 0.806667 | 14.09 |
| DHCR24 | 6.972 | 2.801619 | 7.25E-34 | 8.48E-32 | yes | up | 4.19 | 2.92 | 2.85 | 61.21 | 50.42 | 46.09 | 3.32 | 52.57333 |
| ACOT13 | 0.165 | -2.603 | 1.26E-33 | 1.46E-31 | yes | down | 121.21 | 118.48 | 105.96 | 41.05 | 39.57 | 49.36 | 115.2167 | 43.32667 |
| FADS3 | 5.596 | 2.484454 | 1.27E-33 | 1.46E-31 | yes | up | 24.26 | 22.47 | 23.95 | 303.72 | 281.94 | 319.71 | 23.56 | 301.79 |
| CPNE4 | 0.014 | -6.15149 | 2.37E-33 | 2.71E-31 | yes | down | 3.88 | 5.23 | 2.1 | 0.16 | 0.06 | 0.13 | 3.736667 | 0.116667 |
| FAM105A | 0.078 | -3.67616 | 2.54E-33 | 2.89E-31 | yes | down | 4.89 | 7.8 | 6.61 | 0.98 | 1.66 | 0.92 | 6.433333 | 1.186667 |
| MIPEP | 0.191 | -2.3859 | 3.11E-33 | 3.51E-31 | yes | down | 22.42 | 25.39 | 23.11 | 11 | 10.15 | 9.94 | 23.64 | 10.36333 |
| MOGAT1 | 0.035 | -4.82507 | 3.93E-33 | 4.39E-31 | yes | down | 2.19 | 1.87 | 2.63 | 0.18 | 0.21 | 0.13 | 2.23 | 0.173333 |
| IGF2BP3 | 0.072 | -3.79098 | 3.94E-33 | 4.39E-31 | yes | down | 3.88 | 3.03 | 2.82 | 0.43 | 0.77 | 0.42 | 3.243333 | 0.54 |
| CRAT | 0.234 | -2.0941 | 4.26E-33 | 4.72E-31 | yes | down | 122.83 | 120.22 | 108.75 | 66.25 | 60.96 | 59.5 | 117.2667 | 62.23667 |
| DLST | 0.225 | -2.15252 | 1.51E-32 | 1.66E-30 | yes | down | 281.34 | 270.81 | 265.83 | 151.72 | 159.94 | 113.26 | 272.66 | 141.64 |
| LOC100350920 | 0.016 | -5.98237 | 2.04E-32 | 2.23E-30 | yes | down | 2.32 | 0.81 | 1.19 | 0.49 | 0.03 | 0.53 | 1.44 | 0.35 |
| GFM1 | 0.205 | -2.28395 | 2.27E-32 | 2.46E-30 | yes | down | 49.56 | 41.64 | 32.97 | 21.65 | 19.53 | 17.18 | 41.39 | 19.45333 |
| LOC100328948 | 36.481 | 5.189066 | 2.69E-32 | 2.9E-30 | yes | up | 1.54 | 1.06 | 2.2 | 157.65 | 90.78 | 171.34 | 1.6 | 139.9233 |
| ADORA1 | 38.208 | 5.255817 | 2.72E-32 | 2.91E-30 | yes | up | 0.33 | 0.58 | 0.14 | 18.46 | 27.04 | 39.36 | 0.35 | 28.28667 |
| TPT1 | 0.301 | -1.73374 | 2.91E-32 | 3.09E-30 | yes | down | 1492.88 | 1489.7 | 1229.62 | 976.49 | 982 | 928.69 | 1404.067 | 962.3933 |
| PPA1 | 0.195 | -2.36068 | 3.07E-32 | 3.24E-30 | yes | down | 203.27 | 265.13 | 215.7 | 111.85 | 105.22 | 89.27 | 228.0333 | 102.1133 |
| CRABP2 | 8.092 | 3.016507 | 3.29E-32 | 3.45E-30 | yes | up | 5.89 | 3.7 | 3.29 | 76.91 | 74.78 | 82.95 | 4.293333 | 78.21333 |
| PDHB | 0.249 | -2.00628 | 3.64E-32 | 3.8E-30 | yes | down | 252.08 | 225.67 | 217.11 | 142.56 | 141.59 | 113.01 | 231.62 | 132.3867 |
| CCND3 | 4.66 | 2.220352 | 3.8E-32 | 3.94E-30 | yes | up | 10.51 | 9 | 7.44 | 77.44 | 94.14 | 95.87 | 8.983333 | 89.15 |
| HTRA1 | 4.632 | 2.211641 | 7.63E-32 | 7.86E-30 | yes | up | 124.08 | 109.58 | 107.47 | 1187.66 | 1130.31 | 1275.64 | 113.71 | 1197.87 |
| ATP5F1 | 0.184 | -2.4402 | 8.52E-32 | 8.72E-30 | yes | down | 575.7 | 583.25 | 496.33 | 272.63 | 255.95 | 170.38 | 551.76 | 232.9867 |
| ECH1 | 0.138 | -2.85522 | 1.44E-31 | 1.46E-29 | yes | down | 423.64 | 402.33 | 538.56 | 126.01 | 191.51 | 135.56 | 454.8433 | 151.0267 |
| RNF152 | 0.134 | -2.89854 | 1.63E-31 | 1.64E-29 | yes | down | 10.5 | 11.15 | 8.44 | 3.75 | 3.5 | 1.98 | 10.03 | 3.076667 |
| DLD | 0.108 | -3.20857 | 1.71E-31 | 1.72E-29 | yes | down | 144.02 | 111.29 | 153.8 | 42.04 | 37.15 | 23.95 | 136.37 | 34.38 |
| MRPS5 | 0.204 | -2.29531 | 2.31E-31 | 2.3E-29 | yes | down | 40.84 | 47.19 | 41.65 | 22.58 | 22.13 | 16.22 | 43.22667 | 20.31 |
| MRPS35 | 0.176 | -2.50745 | 5.01E-31 | 4.97E-29 | yes | down | 48.58 | 55.93 | 57.28 | 20.94 | 23.33 | 21.6 | 53.93 | 21.95667 |
| VDAC2 | 0.265 | -1.9159 | 5.25E-31 | 5.18E-29 | yes | down | 287.01 | 266.32 | 224.84 | 172.11 | 161.27 | 136.4 | 259.39 | 156.5933 |
| PDE4D | 0.084 | -3.56679 | 6.18E-31 | 6.06E-29 | yes | down | 17.34 | 26.12 | 9.41 | 3.6 | 6.91 | 2.67 | 17.62333 | 4.393333 |
| PHKG1 | 0.021 | -5.54609 | 7.44E-31 | 7.26E-29 | yes | down | 77.3 | 40.37 | 64.92 | 2.59 | 0.98 | 4.65 | 60.86333 | 2.74 |
| CA5B | 6.965 | 2.800033 | 7.52E-31 | 7.29E-29 | yes | up | 2.63 | 2.24 | 1.33 | 29.02 | 34.23 | 26.07 | 2.066667 | 29.77333 |
| HSDL2 | 0.108 | -3.21312 | 1.16E-30 | 1.12E-28 | yes | down | 224.46 | 180.35 | 180.2 | 53.23 | 66.21 | 27.85 | 195.0033 | 49.09667 |
| C1QC | 4.35 | 2.121043 | 1.83E-30 | 1.75E-28 | yes | up | 35.84 | 27.89 | 25.88 | 301.58 | 286.26 | 290.81 | 29.87 | 292.8833 |
| PPA2 | 0.18 | -2.47452 | 2.51E-30 | 2.39E-28 | yes | down | 70.64 | 80.18 | 71.6 | 35.1 | 28.05 | 28.34 | 74.14 | 30.49667 |
| INMT | 41.288 | 5.367659 | 3.57E-30 | 3.38E-28 | yes | up | 0.62 | 0.54 | 0.08 | 36.21 | 26.54 | 48.05 | 0.413333 | 36.93333 |
| EBP | 10.883 | 3.443991 | 3.95E-30 | 3.72E-28 | yes | up | 24.39 | 25.94 | 23.97 | 786.94 | 402.44 | 635.09 | 24.76667 | 608.1567 |
| IDH3G | 0.199 | -2.33201 | 4.11E-30 | 3.85E-28 | yes | down | 365.31 | 361.58 | 397.81 | 188.7 | 170.39 | 154.51 | 374.9 | 171.2 |
| COQ5 | 0.253 | -1.98491 | 4.42E-30 | 4.11E-28 | yes | down | 38.3 | 32.31 | 30.25 | 15.24 | 15.28 | 14.15 | 33.62 | 14.89 |
| C19H17orf50 | 5.517 | 2.463777 | 4.49E-30 | 4.16E-28 | yes | up | 3.07 | 4.93 | 3.54 | 46.77 | 56.92 | 35.91 | 3.846667 | 46.53333 |
| ATP5A1 | 0.188 | -2.40977 | 4.94E-30 | 4.55E-28 | yes | down | 1403.47 | 1568.38 | 1548.89 | 758.97 | 635.23 | 563.86 | 1506.913 | 652.6867 |
| C3 | 7.683 | 2.94176 | 6.73E-30 | 6.16E-28 | yes | up | 10.59 | 9.5 | 5.94 | 126 | 136.45 | 182.44 | 8.676667 | 148.2967 |
| GNAT3 | 0.024 | -5.38507 | 8.15E-30 | 7.42E-28 | yes | down | 6.95 | 10.77 | 16.33 | 0.51 | 1.64 | 0.81 | 11.35 | 0.986667 |
| CHCHD10 | 0.115 | -3.1253 | 1.98E-29 | 1.79E-27 | yes | down | 84 | 85.2 | 112.75 | 21.57 | 26.02 | 29.1 | 93.98333 | 25.56333 |
| PXDN | 4.158 | 2.055916 | 2.09E-29 | 1.88E-27 | yes | up | 21.92 | 17.39 | 17.95 | 191.73 | 175.08 | 188.47 | 19.08667 | 185.0933 |
| THY1 | 11.084 | 3.47042 | 2.18E-29 | 1.95E-27 | yes | up | 18.43 | 20.09 | 8.34 | 385.31 | 311.84 | 445.46 | 15.62 | 380.87 |
| BDH1 | 0.17 | -2.55235 | 2.38E-29 | 2.12E-27 | yes | down | 213.64 | 193.34 | 165.86 | 64.65 | 69.33 | 96.46 | 190.9467 | 76.81333 |
| LOC100328613 | 0.032 | -4.96161 | 2.93E-29 | 2.6E-27 | yes | down | 308.91 | 818.45 | 410.3 | 30.07 | 21.07 | 57.67 | 512.5533 | 36.27 |
| LOC100345021 | 0.15 | -2.74084 | 3.01E-29 | 2.66E-27 | yes | down | 45.59 | 56.94 | 37.85 | 18.91 | 13.28 | 14.36 | 46.79333 | 15.51667 |
| MFN1 | 0.153 | -2.70416 | 3.44E-29 | 3E-27 | yes | down | 33.55 | 29.39 | 28.92 | 9.42 | 12.73 | 6.07 | 30.62 | 9.406667 |
| CPXM1 | 19.462 | 4.282566 | 3.44E-29 | 3E-27 | yes | up | 1.36 | 1.17 | 0.32 | 44.59 | 35.08 | 41.37 | 0.95 | 40.34667 |
| LOC100359306 | 9.232 | 3.206604 | 3.58E-29 | 3.09E-27 | yes | up | 2.61 | 1.76 | 3.09 | 58.61 | 59.61 | 45.7 | 2.486667 | 54.64 |
| NDUFA5 | 0.111 | -3.17357 | 3.58E-29 | 3.09E-27 | yes | down | 181 | 161 | 210.02 | 67.68 | 63.88 | 31.72 | 184.0067 | 54.42667 |
| ADAMTS2 | 5.094 | 2.348671 | 4.26E-29 | 3.66E-27 | yes | up | 8.44 | 6.2 | 5.16 | 64.36 | 83.71 | 80.1 | 6.6 | 76.05667 |
| C20H14orf142 | 0.067 | -3.90193 | 4.6E-29 | 3.93E-27 | yes | down | 59.98 | 68.68 | 105.99 | 8.16 | 14.65 | 14.11 | 78.21667 | 12.30667 |
| CPEB1 | 10.694 | 3.418666 | 5.57E-29 | 4.73E-27 | yes | up | 0.45 | 0.36 | 0.25 | 7.3 | 9.79 | 8.11 | 0.353333 | 8.4 |
| LOXL3 | 4.266 | 2.092831 | 6.19E-29 | 5.23E-27 | yes | up | 3.46 | 3.48 | 2.86 | 33.59 | 34.37 | 31.21 | 3.266667 | 33.05667 |
| LOC100358333 | 0.008 | -6.88553 | 8.6E-29 | 7.23E-27 | yes | down | 2.41 | 4.19 | 5.27 | 0.09 | 0.05 | 0.06 | 3.956667 | 0.066667 |
| MED4 | 0.152 | -2.71689 | 8.66E-29 | 7.24E-27 | yes | down | 31.04 | 37.12 | 40.54 | 13.08 | 14.35 | 11.08 | 36.23333 | 12.83667 |
| NSA2 | 0.26 | -1.94551 | 9.96E-29 | 8.29E-27 | yes | down | 47.75 | 50.14 | 43.35 | 28.46 | 29.71 | 26 | 47.08 | 28.05667 |
| SAMM50 | 0.258 | -1.95287 | 1E-28 | 8.32E-27 | yes | down | 117.8 | 111.39 | 100.02 | 70.61 | 63.68 | 59.81 | 109.7367 | 64.7 |
| ITGA11 | 13.294 | 3.732666 | 1.09E-28 | 8.96E-27 | yes | up | 1.24 | 0.9 | 0.39 | 23.29 | 22.49 | 27.85 | 0.843333 | 24.54333 |
| ALDH2 | 6.776 | 2.760538 | 1.16E-28 | 9.52E-27 | yes | up | 48.4 | 27.58 | 35.87 | 563.22 | 565.3 | 601.64 | 37.28333 | 576.72 |
| SNRPB2 | 0.2 | -2.32435 | 1.37E-28 | 1.12E-26 | yes | down | 64.6 | 71.04 | 60.19 | 33.96 | 32.77 | 23.1 | 65.27667 | 29.94333 |
| ANKRD31 | 0.132 | -2.9236 | 1.82E-28 | 1.48E-26 | yes | down | 3.92 | 4.59 | 4.35 | 1.21 | 1.31 | 1.6 | 4.286667 | 1.373333 |
| ATP1B1 | 0.085 | -3.55579 | 2.03E-28 | 1.64E-26 | yes | down | 54.53 | 54.87 | 72.52 | 11.1 | 9.11 | 15.44 | 60.64 | 11.88333 |
| COX1 | 0.12 | -3.05497 | 2.23E-28 | 1.79E-26 | yes | down | 29853.53 | 22157.19 | 34005.72 | 9802.06 | 8403.58 | 6407.68 | 28672.15 | 8204.44 |
| LOC100343982 | 0.219 | -2.19209 | 2.34E-28 | 1.87E-26 | yes | down | 1355.42 | 1545.25 | 1393.32 | 801.76 | 685.41 | 672.12 | 1431.33 | 719.7633 |
| CD248 | 7.903 | 2.982328 | 2.43E-28 | 1.93E-26 | yes | up | 6.59 | 4.97 | 5.15 | 92.55 | 81.02 | 123.23 | 5.57 | 98.93333 |
| DLAT | 0.177 | -2.49586 | 2.97E-28 | 2.35E-26 | yes | down | 48.28 | 37.06 | 39.02 | 19.09 | 19.66 | 12.11 | 41.45333 | 16.95333 |
| CCDC58 | 0.144 | -2.79905 | 3.2E-28 | 2.52E-26 | yes | down | 47.25 | 27.76 | 28.53 | 16.7 | 11.44 | 11.16 | 34.51333 | 13.1 |
| LOC100356940 | 6.366 | 2.670495 | 3.44E-28 | 2.7E-26 | yes | up | 1.55 | 1.64 | 0.79 | 28.59 | 35.18 | 24.71 | 1.326667 | 29.49333 |
| LOC100358789 | 0.091 | -3.4611 | 3.63E-28 | 2.84E-26 | yes | down | 49.9 | 35.68 | 37.88 | 9.41 | 12.27 | 4.49 | 41.15333 | 8.723333 |
| LOC100350311 | 0.083 | -3.58293 | 3.73E-28 | 2.9E-26 | yes | down | 79 | 70.63 | 54.01 | 9.66 | 19.38 | 6.56 | 67.88 | 11.86667 |
| GABBR1 | 5.724 | 2.517082 | 4.03E-28 | 3.12E-26 | yes | up | 0.8 | 0.63 | 0.53 | 8.46 | 12.77 | 8.54 | 0.653333 | 9.923333 |
| PRELID3B | 0.183 | -2.45268 | 5.05E-28 | 3.89E-26 | yes | down | 105.59 | 158.26 | 124.63 | 60.57 | 57.03 | 49.47 | 129.4933 | 55.69 |
| NDUFB6 | 0.255 | -1.97173 | 9.14E-28 | 7.01E-26 | yes | down | 335.18 | 334.89 | 335.58 | 199.22 | 166.35 | 170.51 | 335.2167 | 178.6933 |
| CKB | 14.751 | 3.88272 | 1.07E-27 | 8.2E-26 | yes | up | 18.58 | 18.53 | 12.82 | 575.02 | 298.97 | 749.68 | 16.64333 | 541.2233 |
| AEBP1 | 9.92 | 3.310411 | 1.16E-27 | 8.79E-26 | yes | up | 12.37 | 8.32 | 7.37 | 150.52 | 186.65 | 281.54 | 9.353333 | 206.2367 |
| KRT23 | 0 | -12.3229 | 1.23E-27 | 9.33E-26 | yes | down | 35.96 | 118.79 | 25.03 | 0 | 0.04 | 0.05 | 59.92667 | 0.03 |
| COL15A1 | 4.892 | 2.290527 | 1.9E-27 | 1.43E-25 | yes | up | 34.85 | 25.55 | 22.08 | 348.32 | 321.75 | 246.92 | 27.49333 | 305.6633 |
| HSPD1 | 0.174 | -2.52578 | 2.13E-27 | 1.59E-25 | yes | down | 291.93 | 271.12 | 281.26 | 139.34 | 115.06 | 82.29 | 281.4367 | 112.23 |
| SLC25A20 | 0.223 | -2.1645 | 2.24E-27 | 1.67E-25 | yes | down | 255.75 | 316.72 | 307.15 | 148.07 | 173.64 | 133.88 | 293.2067 | 151.8633 |
| PPARGC1B | 0.189 | -2.40233 | 2.54E-27 | 1.89E-25 | yes | down | 11.81 | 11.73 | 7.68 | 3.65 | 6.11 | 3.67 | 10.40667 | 4.476667 |
| WSCD2 | 21.683 | 4.43851 | 2.78E-27 | 2.05E-25 | yes | up | 0.19 | 0.15 | 0.09 | 7.52 | 5.55 | 6.76 | 0.143333 | 6.61 |
| SDK1 | 5.015 | 2.32634 | 3.69E-27 | 2.71E-25 | yes | up | 2.91 | 2.21 | 1.63 | 23.32 | 30.48 | 23.31 | 2.25 | 25.70333 |
| LOC108176518 | 0.1 | -3.32264 | 3.89E-27 | 2.85E-25 | yes | down | 3.86 | 2.94 | 3.21 | 0.64 | 1.09 | 0.65 | 3.336667 | 0.793333 |
| EGR1 | 21.679 | 4.438197 | 4.32E-27 | 3.15E-25 | yes | up | 5.58 | 2.09 | 2.06 | 191.45 | 95.85 | 171.12 | 3.243333 | 152.8067 |
| LRRC52 | 0.013 | -6.28084 | 4.7E-27 | 3.41E-25 | yes | down | 3.99 | 2.6 | 3.62 | 0.05 | 0.05 | 0.23 | 3.403333 | 0.11 |
| ABR | 3.42 | 1.774081 | 4.78E-27 | 3.46E-25 | yes | up | 5.33 | 4.72 | 5.57 | 32.88 | 40.97 | 36.48 | 5.206667 | 36.77667 |
| ADAMTS4 | 7.175 | 2.843011 | 4.88E-27 | 3.51E-25 | yes | up | 0.74 | 0.96 | 0.78 | 15.21 | 15.6 | 10.2 | 0.826667 | 13.67 |
| PDGFRL | 5.678 | 2.505445 | 5.3E-27 | 3.8E-25 | yes | up | 7.66 | 8.54 | 5.75 | 85.83 | 86.29 | 108.58 | 7.316667 | 93.56667 |
| COQ3 | 0.071 | -3.81282 | 5.71E-27 | 4.08E-25 | yes | down | 11.51 | 12.02 | 10.3 | 2.35 | 2.42 | 0.78 | 11.27667 | 1.85 |
| NPC2 | 5.036 | 2.332264 | 6.53E-27 | 4.64E-25 | yes | up | 32.93 | 35.08 | 35.72 | 394.3 | 367.9 | 434.82 | 34.57667 | 399.0067 |
| PDK2 | 0.249 | -2.00414 | 6.63E-27 | 4.69E-25 | yes | down | 54.55 | 57.21 | 51.94 | 26.39 | 34.39 | 32.97 | 54.56667 | 31.25 |
| LOC100355688 | 0.305 | -1.71398 | 8.82E-27 | 6.21E-25 | yes | down | 50.65 | 54.94 | 42.27 | 33.35 | 35.86 | 33.61 | 49.28667 | 34.27333 |
| IL10RA | 9.814 | 3.294838 | 9.32E-27 | 6.54E-25 | yes | up | 0.69 | 0.44 | 0.34 | 9.45 | 14.31 | 9.12 | 0.49 | 10.96 |
| PARM1 | 4.329 | 2.11409 | 1.12E-26 | 7.79E-25 | yes | up | 1.72 | 1.52 | 1.33 | 15.86 | 15.34 | 13.84 | 1.523333 | 15.01333 |
| NAT8L | 7.013 | 2.810036 | 1.18E-26 | 8.22E-25 | yes | up | 2.03 | 1.84 | 2.04 | 22.23 | 38.49 | 30.86 | 1.97 | 30.52667 |
| ADAMTS16 | 52.509 | 5.714502 | 1.23E-26 | 8.53E-25 | yes | up | 0.2 | 0.25 | 0.24 | 9.03 | 57.56 | 18.74 | 0.23 | 28.44333 |
| MRPS30 | 0.196 | -2.35297 | 1.33E-26 | 9.2E-25 | yes | down | 17.03 | 20.14 | 19.56 | 8.16 | 10.02 | 7.59 | 18.91 | 8.59 |
| KAZALD1 | 8.083 | 3.014947 | 1.39E-26 | 9.57E-25 | yes | up | 2.77 | 2.08 | 1.93 | 36.16 | 38.04 | 44.23 | 2.26 | 39.47667 |
| MIF | 0.223 | -2.16404 | 1.52E-26 | 1.04E-24 | yes | down | 400.96 | 424.4 | 393.65 | 180.76 | 207.93 | 236.92 | 406.3367 | 208.5367 |
| MFSD8 | 0.199 | -2.3298 | 1.88E-26 | 1.28E-24 | yes | down | 4.87 | 4.87 | 4.01 | 2.24 | 2.38 | 1.85 | 4.583333 | 2.156667 |
| OLFML1 | 9.207 | 3.202661 | 2.02E-26 | 1.37E-24 | yes | up | 6.54 | 3.75 | 2.34 | 85.81 | 83.47 | 89.18 | 4.21 | 86.15333 |
| PTCD3 | 0.212 | -2.23502 | 2.25E-26 | 1.52E-24 | yes | down | 23.71 | 27.37 | 20.52 | 10.45 | 14.1 | 8.27 | 23.86667 | 10.94 |
| FAM149A | 4.617 | 2.206941 | 2.49E-26 | 1.67E-24 | yes | up | 3.42 | 3.25 | 2.36 | 30.03 | 41.69 | 35.02 | 3.01 | 35.58 |
| PDE3B | 0.113 | -3.1457 | 2.6E-26 | 1.74E-24 | yes | down | 31.76 | 38.13 | 33.91 | 10.5 | 11.14 | 4.74 | 34.6 | 8.793333 |
| CIB4 | 0.016 | -6.00323 | 2.66E-26 | 1.78E-24 | yes | down | 4.01 | 6.2 | 4.6 | 0.06 | 0.34 | 0.25 | 4.936667 | 0.216667 |
| PARN | 0.243 | -2.04208 | 2.98E-26 | 1.98E-24 | yes | down | 22.76 | 27.32 | 24.43 | 16.09 | 15.64 | 12.54 | 24.83667 | 14.75667 |
| ALDH5A1 | 0.225 | -2.1521 | 3.2E-26 | 2.12E-24 | yes | down | 22.81 | 27.52 | 25.12 | 15.36 | 17.47 | 12.77 | 25.15 | 15.2 |
| SUCLG1 | 0.267 | -1.90351 | 3.3E-26 | 2.18E-24 | yes | down | 448.69 | 450.62 | 416.1 | 300.56 | 268.21 | 235.84 | 438.47 | 268.2033 |
| ACAD8 | 0.193 | -2.37285 | 3.37E-26 | 2.21E-24 | yes | down | 22.92 | 32.96 | 27.02 | 12.1 | 13.43 | 11.36 | 27.63333 | 12.29667 |
| LOC100356756 | 0.044 | -4.50248 | 3.42E-26 | 2.24E-24 | yes | down | 993.67 | 866.09 | 977.06 | 60.61 | 54.01 | 172.03 | 945.6067 | 95.55 |
| CLUH | 0.238 | -2.07226 | 4.64E-26 | 3.03E-24 | yes | down | 49.63 | 61.19 | 56.17 | 26.63 | 36.41 | 28.75 | 55.66333 | 30.59667 |
| IARS2 | 0.163 | -2.61416 | 5.3E-26 | 3.44E-24 | yes | down | 41.07 | 33.96 | 37.66 | 13.51 | 16.3 | 8.13 | 37.56333 | 12.64667 |
| NWD2 | 0.039 | -4.68564 | 5.52E-26 | 3.57E-24 | yes | down | 3.04 | 7.33 | 1.81 | 2.2 | 1.04 | 2 | 4.06 | 1.746667 |
| TMEM37 | 0.122 | -3.02947 | 7.68E-26 | 4.94E-24 | yes | down | 29 | 39.32 | 24.47 | 6.26 | 8.26 | 11.17 | 30.93 | 8.563333 |
| ADRB1 | 0.109 | -3.20182 | 8.39E-26 | 5.38E-24 | yes | down | 3.98 | 4.72 | 4.32 | 0.78 | 1.47 | 1.05 | 4.34 | 1.1 |
| MMP2 | 5.871 | 2.553617 | 9.57E-26 | 6.11E-24 | yes | up | 35.66 | 38.13 | 23.28 | 377.38 | 380.01 | 515.23 | 32.35667 | 424.2067 |
| ACADS | 0.172 | -2.53799 | 1.15E-25 | 7.34E-24 | yes | down | 299.48 | 290.92 | 395.04 | 118.85 | 154.05 | 116.18 | 328.48 | 129.6933 |
| SLC25A12 | 0.194 | -2.36291 | 1.31E-25 | 8.28E-24 | yes | down | 21.97 | 16.5 | 11.7 | 7.18 | 8.07 | 6.76 | 16.72333 | 7.336667 |
| TSKU | 6.361 | 2.669257 | 1.51E-25 | 9.55E-24 | yes | up | 3.11 | 21.09 | 12.96 | 87.77 | 59.87 | 41.82 | 12.38667 | 63.15333 |
| FAM96A | 0.27 | -1.88884 | 1.58E-25 | 9.93E-24 | yes | down | 59.02 | 56.54 | 46.08 | 34.69 | 38.21 | 27.26 | 53.88 | 33.38667 |
| LGMN | 3.401 | 1.766053 | 1.84E-25 | 1.16E-23 | yes | up | 51.72 | 61.16 | 44.84 | 389.24 | 416.9 | 414.06 | 52.57333 | 406.7333 |
| TBC1D9B | 3.197 | 1.676514 | 1.85E-25 | 1.16E-23 | yes | up | 13.44 | 13.17 | 10.56 | 79.56 | 108.31 | 82.39 | 12.39 | 90.08667 |
| HOXB8 | 16.974 | 4.085283 | 1.99E-25 | 1.24E-23 | yes | up | 0.4 | 0.34 | 0.19 | 12.81 | 10.19 | 11.46 | 0.31 | 11.48667 |
| NDUFA9 | 0.23 | -2.12009 | 2.39E-25 | 1.48E-23 | yes | down | 131.15 | 128.38 | 115.41 | 76.43 | 58.89 | 60.9 | 124.98 | 65.40667 |
| MRPL1 | 0.176 | -2.50342 | 2.48E-25 | 1.53E-23 | yes | down | 23.68 | 27.8 | 22.57 | 9.84 | 9.69 | 6.69 | 24.68333 | 8.74 |
| ERRFI1 | 4.04 | 2.014485 | 2.61E-25 | 1.61E-23 | yes | up | 2.52 | 2.65 | 2.37 | 23.04 | 21.39 | 24.75 | 2.513333 | 23.06 |
| LOC100345908 | 0.013 | -6.22102 | 2.73E-25 | 1.67E-23 | yes | down | 7.57 | 5.45 | 12.66 | 0.15 | 0.65 | 0.13 | 8.56 | 0.31 |
| NAMPT | 0.105 | -3.25841 | 3.52E-25 | 2.15E-23 | yes | down | 26.15 | 30.82 | 45.95 | 8.92 | 10.34 | 6.34 | 34.30667 | 8.533333 |
| PNPT1 | 0.148 | -2.75292 | 3.74E-25 | 2.28E-23 | yes | down | 11.78 | 13.53 | 13.86 | 5.45 | 6.32 | 3.25 | 13.05667 | 5.006667 |
| BHMT | 0 | -12.67 | 3.81E-25 | 2.31E-23 | yes | down | 28.03 | 28.35 | 41.67 | 0 | 0 | 0 | 32.68333 | 0 |
| COL6A1 | 6.021 | 2.590071 | 3.93E-25 | 2.37E-23 | yes | up | 180.58 | 106.2 | 91.41 | 1282.43 | 1722.31 | 1338.93 | 126.0633 | 1447.89 |
| MRPS18B | 0.169 | -2.56567 | 4.01E-25 | 2.41E-23 | yes | down | 72.24 | 72.41 | 64.77 | 25.01 | 22.25 | 32.95 | 69.80667 | 26.73667 |
| LOC103346593 | 0.121 | -3.05173 | 4.04E-25 | 2.42E-23 | yes | down | 19.32 | 25.73 | 21.63 | 3.87 | 8.87 | 6.02 | 22.22667 | 6.253333 |
| EMILIN1 | 5.037 | 2.332528 | 4.66E-25 | 2.79E-23 | yes | up | 2.58 | 2.53 | 1.53 | 26.18 | 24.15 | 24.7 | 2.213333 | 25.01 |
| DPT | 8.364 | 3.064195 | 4.76E-25 | 2.84E-23 | yes | up | 54.92 | 35.01 | 24.21 | 505.88 | 918.1 | 766.9 | 38.04667 | 730.2933 |
| PDIA6 | 3.776 | 1.916897 | 5.8E-25 | 3.44E-23 | yes | up | 56.55 | 47.95 | 33.92 | 388.56 | 460.88 | 339.16 | 46.14 | 396.2 |
| LOC100345097 | 0 | -12.472 | 8.8E-25 | 5.2E-23 | yes | down | 88.41 | 134.84 | 112.91 | 0 | 0 | 0 | 112.0533 | 0 |
| LOC100353768 | 6.336 | 2.663498 | 8.9E-25 | 5.24E-23 | yes | up | 2.67 | 3.53 | 2.68 | 33.77 | 43.6 | 49.82 | 2.96 | 42.39667 |
| KLB | 8.052 | 3.009346 | 9.11E-25 | 5.35E-23 | yes | up | 5.91 | 7.16 | 8.27 | 94.81 | 196.83 | 115.72 | 7.113333 | 135.7867 |
| PRRG4 | 0.115 | -3.1165 | 1.18E-24 | 6.92E-23 | yes | down | 7.14 | 7.8 | 3.46 | 1.23 | 1.9 | 1.69 | 6.133333 | 1.606667 |
| LOC100359112 | 7.649 | 2.935268 | 1.27E-24 | 7.41E-23 | yes | up | 1.71 | 1.76 | 1.29 | 21.56 | 32.53 | 29.58 | 1.586667 | 27.89 |
| MRPL47 | 0.206 | -2.28016 | 1.28E-24 | 7.44E-23 | yes | down | 41.93 | 41.45 | 38.15 | 20.98 | 18.3 | 15.4 | 40.51 | 18.22667 |
| DNAJA3 | 0.331 | -1.59645 | 1.47E-24 | 8.48E-23 | yes | down | 50.05 | 46.79 | 40.98 | 36.11 | 36.44 | 31.4 | 45.94 | 34.65 |
| TUFM | 0.235 | -2.08729 | 1.8E-24 | 1.04E-22 | yes | down | 148.51 | 160.02 | 158.87 | 96.76 | 84.61 | 75.71 | 155.8 | 85.69333 |
| SDHB | 0.235 | -2.08683 | 1.84E-24 | 1.06E-22 | yes | down | 455.21 | 443.04 | 464.43 | 267.73 | 227.48 | 241.33 | 454.2267 | 245.5133 |
| CTNNBL1 | 0.306 | -1.70628 | 2.09E-24 | 1.2E-22 | yes | down | 64.28 | 76.11 | 58.52 | 47.32 | 49.94 | 43.63 | 66.30333 | 46.96333 |
| FAM180B | 13.057 | 3.706766 | 2.22E-24 | 1.26E-22 | yes | up | 1.08 | 1.91 | 0.64 | 26.21 | 49.4 | 35.01 | 1.21 | 36.87333 |
| CS | 0.274 | -1.86836 | 2.44E-24 | 1.38E-22 | yes | down | 605.22 | 598.42 | 629.22 | 408.83 | 418.96 | 332.96 | 610.9533 | 386.9167 |
| ADCYAP1R1 | 6.641 | 2.731428 | 2.61E-24 | 1.48E-22 | yes | up | 0.48 | 0.45 | 0.35 | 6.8 | 8.01 | 4.7 | 0.426667 | 6.503333 |
| COL6A2 | 5.855 | 2.549655 | 2.64E-24 | 1.49E-22 | yes | up | 117.98 | 72.97 | 56.46 | 1258.17 | 1158.59 | 970.09 | 82.47 | 1128.95 |
| MRPL35 | 0.25 | -2.00249 | 2.85E-24 | 1.6E-22 | yes | down | 58.66 | 64.51 | 52.44 | 33.89 | 38.85 | 27.63 | 58.53667 | 33.45667 |
| GOT1 | 0.134 | -2.89482 | 4.34E-24 | 2.43E-22 | yes | down | 116.87 | 61.3 | 66.23 | 25.58 | 21.26 | 27.05 | 81.46667 | 24.63 |
| NDUFA6 | 0.199 | -2.32803 | 4.9E-24 | 2.74E-22 | yes | down | 513.36 | 593.03 | 536.36 | 263.18 | 211.24 | 273.69 | 547.5833 | 249.37 |
| RDH13 | 0.142 | -2.81141 | 5.71E-24 | 3.18E-22 | yes | down | 9.79 | 7.47 | 8.3 | 2.86 | 2.54 | 2.81 | 8.52 | 2.736667 |
| MPI | 0.199 | -2.32766 | 6.32E-24 | 3.51E-22 | yes | down | 20.08 | 14.44 | 16.88 | 7.67 | 8.89 | 7.25 | 17.13333 | 7.936667 |
| DNAJC25 | 0.135 | -2.8837 | 6.95E-24 | 3.85E-22 | yes | down | 14.28 | 21.74 | 15.45 | 5.32 | 7.31 | 4.53 | 17.15667 | 5.72 |
| TBRG4 | 0.221 | -2.17795 | 7.22E-24 | 3.98E-22 | yes | down | 39.02 | 33.62 | 31.79 | 14.68 | 17.68 | 20.32 | 34.81 | 17.56 |
| LOC108178477 | 0.101 | -3.30234 | 7.68E-24 | 4.22E-22 | yes | down | 2.4 | 2.37 | 1.59 | 0.52 | 0.46 | 0.46 | 2.12 | 0.48 |
| OAF | 7.995 | 2.999065 | 8.08E-24 | 4.42E-22 | yes | up | 5.61 | 5.06 | 4.24 | 84.26 | 82.19 | 104.13 | 4.97 | 90.19333 |
| LOC108177279 | 11.2 | 3.485421 | 1.1E-23 | 6E-22 | yes | up | 1.9 | 1.9 | 2.5 | 49.49 | 43.63 | 70.32 | 2.1 | 54.48 |
| LOC100348443 | 4549.174 | 12.15139 | 1.13E-23 | 6.16E-22 | yes | up | 0 | 0 | 0 | 54.09 | 36.32 | 40.36 | 0 | 43.59 |
| OLFML3 | 9.121 | 3.189256 | 1.14E-23 | 6.19E-22 | yes | up | 11.97 | 10.97 | 5.82 | 185.84 | 141.71 | 249.23 | 9.586667 | 192.26 |
| MYO5B | 0.059 | -4.084 | 1.17E-23 | 6.33E-22 | yes | down | 1.22 | 1.85 | 1.89 | 0.18 | 0.22 | 0.32 | 1.653333 | 0.24 |
| MRPS22 | 0.255 | -1.97238 | 1.19E-23 | 6.42E-22 | yes | down | 37.1 | 47.28 | 35.17 | 25.47 | 24.44 | 21.27 | 39.85 | 23.72667 |
| EEPD1 | 4.591 | 2.198723 | 1.35E-23 | 7.27E-22 | yes | up | 9.81 | 12.18 | 8.1 | 96.91 | 97.9 | 118.56 | 10.03 | 104.4567 |
| GGA2 | 4.676 | 2.225423 | 1.43E-23 | 7.65E-22 | yes | up | 15.03 | 18.41 | 10.36 | 169.72 | 143.89 | 150.97 | 14.6 | 154.86 |
| LOC100343793 | 0.24 | -2.05927 | 1.74E-23 | 9.26E-22 | yes | down | 2048.69 | 2190.49 | 2234.3 | 1294.02 | 1128.85 | 1161.09 | 2157.827 | 1194.653 |
| LOC100345961 | 0.188 | -2.40986 | 2.39E-23 | 1.27E-21 | yes | down | 6.66 | 7.55 | 5.06 | 2.98 | 2.7 | 2.52 | 6.423333 | 2.733333 |
| MOCS2 | 0.227 | -2.13839 | 2.41E-23 | 1.28E-21 | yes | down | 47.31 | 50.18 | 57.69 | 22.86 | 25.1 | 22.83 | 51.72667 | 23.59667 |
| COQ9 | 0.269 | -1.89245 | 2.73E-23 | 1.44E-21 | yes | down | 140.06 | 112.04 | 100.98 | 80.85 | 71.27 | 64.13 | 117.6933 | 72.08333 |
| EGLN3 | 0.068 | -3.875 | 3.74E-23 | 1.97E-21 | yes | down | 41.25 | 80.09 | 65.01 | 13.05 | 5.3 | 10.33 | 62.11667 | 9.56 |
| GRIN2C | 0.011 | -6.55726 | 3.85E-23 | 2.02E-21 | yes | down | 1.71 | 2.36 | 1.84 | 0.06 | 0.13 | 0 | 1.97 | 0.063333 |
| ADAMTSL3 | 4.926 | 2.300502 | 4.26E-23 | 2.23E-21 | yes | up | 1.47 | 1.35 | 1.37 | 13.02 | 22.65 | 14.23 | 1.396667 | 16.63333 |
| RBM3 | 0.125 | -3.00555 | 4.52E-23 | 2.36E-21 | yes | down | 166.91 | 235.97 | 165.1 | 40.33 | 67.29 | 25.66 | 189.3267 | 44.42667 |
| ACKR1 | 5.678 | 2.505307 | 4.64E-23 | 2.42E-21 | yes | up | 3.28 | 3.02 | 2.03 | 31.82 | 31.45 | 42.56 | 2.776667 | 35.27667 |
| MRPL19 | 0.283 | -1.82141 | 4.7E-23 | 2.44E-21 | yes | down | 36.8 | 38.07 | 35.68 | 23.03 | 23.92 | 20.19 | 36.85 | 22.38 |
| C7 | 13.758 | 3.7822 | 4.88E-23 | 2.52E-21 | yes | up | 3.05 | 1.65 | 0.78 | 43.82 | 81.33 | 47.36 | 1.826667 | 57.50333 |
| HOXC6 | 12.31 | 3.621757 | 5.81E-23 | 3E-21 | yes | up | 1.61 | 0.65 | 0.41 | 21.67 | 25.3 | 24.56 | 0.89 | 23.84333 |
| HMGCS2 | 0.053 | -4.23163 | 5.91E-23 | 3.04E-21 | yes | down | 158.94 | 203.07 | 313.49 | 23.75 | 51.44 | 13.56 | 225.1667 | 29.58333 |
| GPLD1 | 0.034 | -4.86802 | 7.68E-23 | 3.93E-21 | yes | down | 2.43 | 2.69 | 2.78 | 0.22 | 0.34 | 0.03 | 2.633333 | 0.196667 |
| ITGA7 | 10.303 | 3.365053 | 1.07E-22 | 5.48E-21 | yes | up | 25.86 | 11.07 | 8.3 | 324.77 | 337.05 | 368.61 | 15.07667 | 343.4767 |
| GPX3 | 0.111 | -3.17226 | 1.11E-22 | 5.66E-21 | yes | down | 478.69 | 471.59 | 611.6 | 81.3 | 143.45 | 176.26 | 520.6267 | 133.67 |
| CNTN1 | 6.984 | 2.804071 | 1.12E-22 | 5.69E-21 | yes | up | 1.21 | 1.01 | 1.24 | 14.89 | 17.11 | 23.2 | 1.153333 | 18.4 |
| TIMM8A | 0.217 | -2.20327 | 1.22E-22 | 6.19E-21 | yes | down | 27.01 | 32.41 | 25.55 | 12.01 | 14.56 | 15.71 | 28.32333 | 14.09333 |
| RASSF2 | 5.193 | 2.3765 | 1.27E-22 | 6.38E-21 | yes | up | 3.65 | 3.11 | 2.03 | 35.28 | 42.98 | 24.96 | 2.93 | 34.40667 |
| CSF1R | 3.513 | 1.812661 | 1.3E-22 | 6.53E-21 | yes | up | 6.51 | 5.55 | 4.77 | 47.6 | 45.04 | 41.74 | 5.61 | 44.79333 |
| PLEKHO2 | 3.251 | 1.700929 | 1.37E-22 | 6.86E-21 | yes | up | 4.08 | 4.22 | 3.66 | 28.78 | 34.86 | 25.88 | 3.986667 | 29.84 |
| CD5L | 91.808 | 6.520555 | 1.38E-22 | 6.9E-21 | yes | up | 0.14 | 0.25 | 0.06 | 38.24 | 20.94 | 32.84 | 0.15 | 30.67333 |
| PHB2 | 0.331 | -1.59646 | 1.65E-22 | 8.2E-21 | yes | down | 212.4 | 219.22 | 199.79 | 150.81 | 165.5 | 162.17 | 210.47 | 159.4933 |
| PMPCB | 0.299 | -1.74036 | 1.65E-22 | 8.2E-21 | yes | down | 83.05 | 91.66 | 82.93 | 63.74 | 60.29 | 53.05 | 85.88 | 59.02667 |
| TIMM9 | 0.134 | -2.89972 | 1.69E-22 | 8.35E-21 | yes | down | 39.29 | 47.99 | 33.41 | 10.32 | 11.5 | 15.96 | 40.23 | 12.59333 |
| PDIA4 | 3.068 | 1.617372 | 2.12E-22 | 1.05E-20 | yes | up | 42.79 | 43.15 | 35.34 | 299.54 | 321.32 | 229.58 | 40.42667 | 283.48 |
| SAR1B | 0.273 | -1.8711 | 2.16E-22 | 1.06E-20 | yes | down | 27.9 | 21.15 | 20.96 | 14.86 | 15.45 | 11.52 | 23.33667 | 13.94333 |
| PAPSS2 | 4.804 | 2.264087 | 2.23E-22 | 1.1E-20 | yes | up | 1.19 | 1.23 | 0.8 | 10.82 | 12.61 | 8.85 | 1.073333 | 10.76 |
| TMED3 | 4.421 | 2.144491 | 2.53E-22 | 1.24E-20 | yes | up | 22.53 | 19.29 | 12.39 | 160.34 | 184.96 | 183.37 | 18.07 | 176.2233 |
| LOC103349956 | 0.066 | -3.92098 | 3.09E-22 | 1.51E-20 | yes | down | 7.95 | 3.72 | 4.29 | 0.72 | 0.8 | 0.54 | 5.32 | 0.686667 |
| ATP5C1 | 0.26 | -1.94096 | 3.18E-22 | 1.54E-20 | yes | down | 524.06 | 418.33 | 340.07 | 286.44 | 258.78 | 204.02 | 427.4867 | 249.7467 |
| CTSH | 4.219 | 2.077049 | 3.31E-22 | 1.6E-20 | yes | up | 34.16 | 42.38 | 38.52 | 278.37 | 433.5 | 357.3 | 38.35333 | 356.39 |
| HACL1 | 0.141 | -2.82114 | 3.36E-22 | 1.62E-20 | yes | down | 75.98 | 88.98 | 106.21 | 27.28 | 43.89 | 20.52 | 90.39 | 30.56333 |
| LOC100358892 | 0.095 | -3.39716 | 3.84E-22 | 1.85E-20 | yes | down | 16.64 | 17.31 | 27.03 | 5.06 | 5.87 | 2.83 | 20.32667 | 4.586667 |
| ABHD18 | 0.107 | -3.22886 | 4.03E-22 | 1.94E-20 | yes | down | 12.86 | 9.73 | 8.95 | 3.34 | 2.84 | 1.19 | 10.51333 | 2.456667 |
| SERPINF1 | 9.6 | 3.263004 | 4.4E-22 | 2.11E-20 | yes | up | 90.88 | 52.9 | 32.8 | 1063.8 | 1104.83 | 1624.09 | 58.86 | 1264.24 |
| NDUFB5 | 0.284 | -1.81809 | 4.8E-22 | 2.3E-20 | yes | down | 172.21 | 169.03 | 143.2 | 118.69 | 99.9 | 94.69 | 161.48 | 104.4267 |
| LAPTM5 | 4.085 | 2.030235 | 5.09E-22 | 2.43E-20 | yes | up | 7.48 | 6.61 | 6.32 | 64.78 | 57.93 | 66.81 | 6.803333 | 63.17333 |
| CCL14 | 5.265 | 2.396509 | 5.12E-22 | 2.43E-20 | yes | up | 13.15 | 15.26 | 9.48 | 148.79 | 141.71 | 161.7 | 12.63 | 150.7333 |
| RALGPS2 | 0.175 | -2.5157 | 5.18E-22 | 2.45E-20 | yes | down | 3.66 | 4.16 | 2.1 | 1.24 | 1.58 | 1.09 | 3.306667 | 1.303333 |
| NDUFS4 | 0.264 | -1.91982 | 5.73E-22 | 2.71E-20 | yes | down | 267.73 | 265.82 | 268.08 | 181.04 | 163.83 | 141.8 | 267.21 | 162.2233 |
| RTN4IP1 | 0.199 | -2.33105 | 5.86E-22 | 2.76E-20 | yes | down | 13.16 | 11 | 9.43 | 4.84 | 4.78 | 5.55 | 11.19667 | 5.056667 |
| HSPA12A | 4.908 | 2.295275 | 5.9E-22 | 2.77E-20 | yes | up | 2.49 | 2.18 | 2.37 | 17.07 | 29.62 | 28.01 | 2.346667 | 24.9 |
| LOC100340040 | 86.675 | 6.437536 | 6.67E-22 | 3.13E-20 | yes | up | 0.2 | 0 | 0.03 | 12.33 | 13.46 | 14.13 | 0.076667 | 13.30667 |
| CADM3 | 7.363 | 2.88027 | 7.01E-22 | 3.27E-20 | yes | up | 18.59 | 14.48 | 7.31 | 204.83 | 199.69 | 256.51 | 13.46 | 220.3433 |
| MRPL2 | 0.208 | -2.26214 | 8.53E-22 | 3.97E-20 | yes | down | 52.98 | 40.55 | 51.1 | 23.71 | 22.61 | 23.14 | 48.21 | 23.15333 |
| ARPC1B | 3.118 | 1.640545 | 1.08E-21 | 5.02E-20 | yes | up | 23.15 | 27.8 | 21.49 | 176.44 | 183.11 | 161.77 | 24.14667 | 173.7733 |
| NDUFA4 | 0.304 | -1.71595 | 1.08E-21 | 5.02E-20 | yes | down | 1073.81 | 1124.88 | 1040.87 | 818.99 | 762.32 | 688.95 | 1079.853 | 756.7533 |
| LOC100357711 | 4.68 | 2.226607 | 1.1E-21 | 5.09E-20 | yes | up | 4.54 | 4.46 | 2.84 | 34.74 | 51.4 | 41.38 | 3.946667 | 42.50667 |
| SLC15A3 | 5.733 | 2.519328 | 1.37E-21 | 6.32E-20 | yes | up | 1.22 | 1.6 | 1.37 | 15.37 | 22.73 | 17.53 | 1.396667 | 18.54333 |
| SORD | 3.642 | 1.86478 | 1.41E-21 | 6.47E-20 | yes | up | 3.7 | 4.01 | 3.54 | 28.02 | 34.18 | 31.81 | 3.75 | 31.33667 |
| BNIP3 | 0.334 | -1.5811 | 1.47E-21 | 6.72E-20 | yes | down | 192.61 | 176.19 | 159.78 | 125.26 | 135.32 | 140.03 | 176.1933 | 133.5367 |
| LOC100341993 | 0.2 | -2.32153 | 1.52E-21 | 6.94E-20 | yes | down | 84.08 | 75.38 | 67.81 | 43.22 | 31.53 | 29.44 | 75.75667 | 34.73 |
| TKT | 4.837 | 2.274178 | 1.58E-21 | 7.19E-20 | yes | up | 46.84 | 36.15 | 38.01 | 555.69 | 396.4 | 383.6 | 40.33333 | 445.23 |
| LRRC75A | 4.987 | 2.318139 | 1.76E-21 | 7.99E-20 | yes | up | 2.92 | 2 | 2.04 | 23.09 | 31.81 | 24.59 | 2.32 | 26.49667 |
| GHITM | 0.118 | -3.08521 | 1.78E-21 | 8.04E-20 | yes | down | 377.1 | 469.36 | 175.99 | 110.38 | 70.73 | 82.78 | 340.8167 | 87.96333 |
| CYP2A11 | 133.752 | 7.063416 | 1.89E-21 | 8.56E-20 | yes | up | 3.17 | 2.26 | 1.24 | 1253.24 | 283.57 | 427.97 | 2.223333 | 654.9267 |
| MYH11 | 4.958 | 2.309664 | 2E-21 | 9.01E-20 | yes | up | 20.51 | 21.48 | 16.13 | 224.83 | 229.92 | 367.8 | 19.37333 | 274.1833 |
| ATPAF1 | 0.279 | -1.84067 | 2.24E-21 | 1.01E-19 | yes | down | 26.46 | 23.76 | 23.33 | 13.54 | 20.19 | 13.11 | 24.51667 | 15.61333 |
| SEPT10 | 0.212 | -2.2352 | 2.31E-21 | 1.04E-19 | yes | down | 133.79 | 198.65 | 159.43 | 81.32 | 102.83 | 56.39 | 163.9567 | 80.18 |
| TXN2 | 0.243 | -2.03912 | 2.66E-21 | 1.19E-19 | yes | down | 160.26 | 164.68 | 134.74 | 82.94 | 76.48 | 98.51 | 153.2267 | 85.97667 |
| DEFB1 | 10.351 | 3.371648 | 2.71E-21 | 1.21E-19 | yes | up | 19.13 | 29.71 | 23.17 | 432.04 | 436.68 | 838.1 | 24.00333 | 568.94 |
| RSL24D1 | 0.222 | -2.17075 | 2.87E-21 | 1.27E-19 | yes | down | 74.43 | 64.75 | 44.06 | 31.53 | 37.67 | 23.47 | 61.08 | 30.89 |
| ZFAND4 | 0.174 | -2.52072 | 2.97E-21 | 1.31E-19 | yes | down | 6.34 | 7.66 | 6.65 | 1.83 | 2.59 | 2.1 | 6.883333 | 2.173333 |
| GOT2 | 0.275 | -1.86208 | 3.28E-21 | 1.45E-19 | yes | down | 123.77 | 87.1 | 87.13 | 63.93 | 62.74 | 59.86 | 99.33333 | 62.17667 |
| ORAI2 | 4.961 | 2.31049 | 3.39E-21 | 1.5E-19 | yes | up | 3.12 | 3.94 | 2.38 | 34.49 | 43.11 | 45.54 | 3.146667 | 41.04667 |
| PCOLCE | 6.44 | 2.686964 | 4.07E-21 | 1.79E-19 | yes | up | 35.78 | 20.73 | 15.56 | 289.34 | 359.07 | 389.53 | 24.02333 | 345.98 |
| GALNT12 | 4.089 | 2.031765 | 4.11E-21 | 1.8E-19 | yes | up | 3.76 | 3.91 | 2.61 | 31.07 | 32.57 | 31.93 | 3.426667 | 31.85667 |
| LOC100353508 | 3.48 | 1.798911 | 4.15E-21 | 1.81E-19 | yes | up | 17.22 | 13.74 | 10.29 | 98.78 | 113.98 | 114.13 | 13.75 | 108.9633 |
| LAMA4 | 3.567 | 1.834842 | 4.2E-21 | 1.83E-19 | yes | up | 19.72 | 20.56 | 13.33 | 137.43 | 188.23 | 114.09 | 17.87 | 146.5833 |
| LOC100348135 | 0.195 | -2.35892 | 5.05E-21 | 2.2E-19 | yes | down | 24.08 | 24.57 | 26.24 | 11.16 | 10.44 | 11.99 | 24.96333 | 11.19667 |
| TBC1D15 | 0.202 | -2.3047 | 5.73E-21 | 2.48E-19 | yes | down | 12.7 | 13.07 | 9.58 | 5.82 | 7.04 | 3.57 | 11.78333 | 5.476667 |
| CIB2 | 0.183 | -2.44776 | 6.02E-21 | 2.61E-19 | yes | down | 167.87 | 180.08 | 238.74 | 80.12 | 83.71 | 87.03 | 195.5633 | 83.62 |
| LASP1 | 2.886 | 1.529237 | 6.17E-21 | 2.66E-19 | yes | up | 17.32 | 19.59 | 17.28 | 122.13 | 134.44 | 108.56 | 18.06333 | 121.71 |
| KIRREL | 3.409 | 1.769376 | 6.26E-21 | 2.69E-19 | yes | up | 3.78 | 3.91 | 2.7 | 23.28 | 37.05 | 19.94 | 3.463333 | 26.75667 |
| OSGEPL1 | 0.238 | -2.07239 | 6.65E-21 | 2.85E-19 | yes | down | 11.16 | 12.22 | 9.65 | 5.74 | 7.75 | 4.69 | 11.01 | 6.06 |
| SSPO | 14.015 | 3.808924 | 6.67E-21 | 2.85E-19 | yes | up | 0.12 | 0.07 | 0.04 | 1.57 | 3.39 | 1.93 | 0.076667 | 2.296667 |
| ARSE | 4.662 | 2.220824 | 7.05E-21 | 3.01E-19 | yes | up | 2.54 | 1.67 | 1.25 | 19.93 | 24.76 | 20.33 | 1.82 | 21.67333 |
| USP15 | 0.228 | -2.1314 | 7.11E-21 | 3.03E-19 | yes | down | 8.38 | 6.57 | 6.25 | 3.77 | 4.61 | 2.67 | 7.066667 | 3.683333 |
| FAM65B | 0.235 | -2.08825 | 7.42E-21 | 3.15E-19 | yes | down | 10.61 | 13.67 | 11.01 | 6.16 | 8.21 | 4.89 | 11.76333 | 6.42 |
| CPQ | 3.684 | 1.881196 | 8.84E-21 | 3.75E-19 | yes | up | 9 | 8.45 | 6.78 | 64.69 | 64.03 | 73.17 | 8.076667 | 67.29667 |
| PRKAR2B | 0.226 | -2.14291 | 9.77E-21 | 4.13E-19 | yes | down | 237.03 | 243.8 | 261.67 | 136.68 | 166.19 | 94.68 | 247.5 | 132.5167 |
| FBLN2 | 4.154 | 2.05433 | 9.98E-21 | 4.21E-19 | yes | up | 28.64 | 21.02 | 18.42 | 172.41 | 230.51 | 236.6 | 22.69333 | 213.1733 |
| SMTN | 3.793 | 1.92343 | 1E-20 | 4.22E-19 | yes | up | 16.91 | 14.37 | 10.73 | 103.97 | 125.75 | 111.52 | 14.00333 | 113.7467 |
| ADH1A | 5.573 | 2.478417 | 1.24E-20 | 5.18E-19 | yes | up | 9.29 | 13.89 | 8.71 | 99.39 | 145.79 | 153.43 | 10.63 | 132.87 |
| DSG2 | 0.023 | -5.46332 | 1.38E-20 | 5.77E-19 | yes | down | 3.14 | 4.66 | 2.59 | 0.06 | 0.07 | 0.4 | 3.463333 | 0.176667 |
| SLIRP | 0.258 | -1.95612 | 1.49E-20 | 6.21E-19 | yes | down | 227.52 | 220.75 | 216.64 | 155.88 | 145.44 | 125.08 | 221.6367 | 142.1333 |
| MTCH2 | 0.32 | -1.64319 | 1.57E-20 | 6.52E-19 | yes | down | 199.74 | 183.79 | 161.74 | 145.56 | 127.15 | 127.09 | 181.7567 | 133.2667 |
| OGDHL | 6.759 | 2.756838 | 1.57E-20 | 6.53E-19 | yes | up | 1.88 | 2.93 | 3.52 | 40 | 53.43 | 39.66 | 2.776667 | 44.36333 |
| IMPDH1 | 0.279 | -1.83947 | 1.79E-20 | 7.41E-19 | yes | down | 86.78 | 103.77 | 80.47 | 51.26 | 76.59 | 47.51 | 90.34 | 58.45333 |
| NFU1 | 0.283 | -1.82001 | 1.81E-20 | 7.48E-19 | yes | down | 47.74 | 58.5 | 44.09 | 32.97 | 32.34 | 32.19 | 50.11 | 32.5 |
| VIT | 13.407 | 3.74487 | 1.84E-20 | 7.6E-19 | yes | up | 1.3 | 0.7 | 0.29 | 25.03 | 18.8 | 22.6 | 0.763333 | 22.14333 |
| DACT2 | 7.981 | 2.996603 | 2.44E-20 | 1E-18 | yes | up | 1.32 | 0.86 | 0.58 | 16.76 | 13.01 | 17.56 | 0.92 | 15.77667 |
| C1S | 6.12 | 2.613573 | 2.58E-20 | 1.06E-18 | yes | up | 11.85 | 9.65 | 6.48 | 94.97 | 120.51 | 164.88 | 9.326667 | 126.7867 |
| LOC100340711 | 5.223 | 2.384939 | 3.02E-20 | 1.24E-18 | yes | up | 4.95 | 8.65 | 6.87 | 73.98 | 101.22 | 84.85 | 6.823333 | 86.68333 |
| CTSK | 4.192 | 2.067497 | 4E-20 | 1.63E-18 | yes | up | 33.86 | 34.44 | 20.66 | 278.68 | 251.04 | 302.09 | 29.65333 | 277.27 |
| NPC1 | 3.534 | 1.821317 | 4.02E-20 | 1.64E-18 | yes | up | 5.67 | 6.33 | 6.2 | 42.12 | 59.33 | 47.08 | 6.066667 | 49.51 |
| ECI1 | 0.23 | -2.12111 | 4.21E-20 | 1.71E-18 | yes | down | 187.4 | 235.64 | 250.07 | 121.86 | 123.06 | 113.67 | 224.37 | 119.53 |
| PEA15 | 2.76 | 1.464594 | 5.07E-20 | 2.06E-18 | yes | up | 39.68 | 38.73 | 29.77 | 221.04 | 227.63 | 227.11 | 36.06 | 225.26 |
| PCDH10 | 20.878 | 4.383903 | 5.1E-20 | 2.06E-18 | yes | up | 0.17 | 0.04 | 0.03 | 3.96 | 3.63 | 3.21 | 0.08 | 3.6 |
| ELOVL1 | 3.589 | 1.843745 | 5.18E-20 | 2.09E-18 | yes | up | 11.14 | 11.18 | 9.34 | 97.53 | 82.08 | 81.11 | 10.55333 | 86.90667 |
| POLR3C | 0.221 | -2.17856 | 5.2E-20 | 2.09E-18 | yes | down | 29.56 | 43.66 | 39.71 | 19.57 | 22.48 | 15.93 | 37.64333 | 19.32667 |
| SPARC | 3.944 | 1.979589 | 5.54E-20 | 2.22E-18 | yes | up | 1315.97 | 1360.04 | 1120.34 | 10065.75 | 10238.11 | 13480.43 | 1265.45 | 11261.43 |
| ETFBKMT | 0.118 | -3.0788 | 5.7E-20 | 2.28E-18 | yes | down | 12.3 | 16.32 | 15.45 | 3.81 | 5.65 | 2.03 | 14.69 | 3.83 |
| LRP2 | 0.079 | -3.66703 | 5.7E-20 | 2.28E-18 | yes | down | 0.42 | 0.49 | 0.65 | 0.07 | 0.13 | 0.09 | 0.52 | 0.096667 |
| ATP5L | 0.284 | -1.81599 | 6.55E-20 | 2.61E-18 | yes | down | 976.85 | 993.97 | 879.49 | 711.42 | 575.48 | 571.71 | 950.1033 | 619.5367 |
| CIAPIN1 | 0.227 | -2.13678 | 6.86E-20 | 2.73E-18 | yes | down | 55.21 | 68.41 | 66.59 | 32.53 | 30.58 | 36.17 | 63.40333 | 33.09333 |
| DDAH2 | 3.61 | 1.851821 | 7.38E-20 | 2.92E-18 | yes | up | 25.4 | 24.05 | 15.6 | 175.41 | 171.64 | 182.88 | 21.68333 | 176.6433 |
| CREB3L1 | 6.677 | 2.739302 | 7.57E-20 | 2.99E-18 | yes | up | 2.04 | 1.5 | 1 | 21.32 | 19.57 | 26.46 | 1.513333 | 22.45 |
| CCL19 | 23.092 | 4.52931 | 8.07E-20 | 3.18E-18 | yes | up | 0.96 | 0.54 | 0.93 | 34.73 | 33.08 | 59.51 | 0.81 | 42.44 |
| LOC100009483 | 27.053 | 4.757697 | 9.13E-20 | 3.59E-18 | yes | up | 1.1 | 0.66 | 0.39 | 36.34 | 22.06 | 68.47 | 0.716667 | 42.29 |
| EFHD1 | 6.223 | 2.637669 | 9.54E-20 | 3.74E-18 | yes | up | 3.77 | 3.11 | 2.04 | 49.39 | 34.18 | 40.68 | 2.973333 | 41.41667 |
| CA8 | 0.055 | -4.18192 | 1.02E-19 | 3.99E-18 | yes | down | 6.19 | 7.74 | 9.21 | 0.76 | 1.67 | 0.58 | 7.713333 | 1.003333 |
| LRP1 | 4.234 | 2.082068 | 1.14E-19 | 4.44E-18 | yes | up | 13.75 | 9.89 | 8.36 | 83.03 | 130.45 | 99.05 | 10.66667 | 104.1767 |
| CHRDL1 | 3.514 | 1.813136 | 1.14E-19 | 4.46E-18 | yes | up | 21.79 | 20.47 | 23.55 | 166.58 | 195.8 | 175.19 | 21.93667 | 179.19 |
| SUPV3L1 | 0.306 | -1.71042 | 1.15E-19 | 4.47E-18 | yes | down | 17.07 | 19.66 | 15.98 | 11.38 | 13.79 | 12.49 | 17.57 | 12.55333 |
| CAMK1 | 4.341 | 2.117895 | 1.2E-19 | 4.64E-18 | yes | up | 12.21 | 9.25 | 6.41 | 92.91 | 89.49 | 93.27 | 9.29 | 91.89 |
| TFAM | 0.163 | -2.62072 | 1.22E-19 | 4.72E-18 | yes | down | 10.7 | 12.49 | 8.31 | 4.17 | 5.25 | 2.34 | 10.5 | 3.92 |
| ZYX | 3.564 | 1.833541 | 1.28E-19 | 4.93E-18 | yes | up | 17.9 | 19.85 | 14.04 | 157.34 | 127.12 | 133.69 | 17.26333 | 139.3833 |
| MPC1 | 0.179 | -2.4803 | 1.31E-19 | 5.03E-18 | yes | down | 432.73 | 323.74 | 304.46 | 168.9 | 102.36 | 157.28 | 353.6433 | 142.8467 |
| LOC100355256 | 0.191 | -2.38862 | 1.32E-19 | 5.08E-18 | yes | down | 9.66 | 9.37 | 8.09 | 4.94 | 3.63 | 3.13 | 9.04 | 3.9 |
| HOXB6 | 15.017 | 3.908547 | 1.33E-19 | 5.12E-18 | yes | up | 0.18 | 0.53 | 0.58 | 14.26 | 11.47 | 14.01 | 0.43 | 13.24667 |
| OPA1 | 0.179 | -2.48156 | 1.48E-19 | 5.67E-18 | yes | down | 11.4 | 8.89 | 9.61 | 4.72 | 5.26 | 2.26 | 9.966667 | 4.08 |
| ISCA1 | 0.384 | -1.38265 | 1.61E-19 | 6.15E-18 | yes | down | 74.9 | 79.36 | 69.24 | 61.27 | 73.3 | 62.4 | 74.5 | 65.65667 |
| U2AF2 | 0.13 | -2.94752 | 2E-19 | 7.61E-18 | yes | down | 7.45 | 5.97 | 6.11 | 2.23 | 1.9 | 1.64 | 6.51 | 1.923333 |
| CLIC1 | 3.065 | 1.616067 | 2.03E-19 | 7.69E-18 | yes | up | 55.45 | 63.41 | 50.65 | 410.44 | 385.09 | 314.24 | 56.50333 | 369.9233 |
| LOC108176650 | 0.146 | -2.77651 | 2.11E-19 | 8.01E-18 | yes | down | 4.18 | 4.93 | 2.42 | 0.8 | 1.08 | 0.86 | 3.843333 | 0.913333 |
| AVP | 0.007 | -7.26322 | 2.13E-19 | 8.04E-18 | yes | down | 10.55 | 17.95 | 26.7 | 0.06 | 0.25 | 0.28 | 18.4 | 0.196667 |
| PRLR | 0.09 | -3.46896 | 2.13E-19 | 8.04E-18 | yes | down | 18.53 | 23.67 | 34.36 | 5.7 | 9.75 | 4.05 | 25.52 | 6.5 |
| FAM210A | 0.292 | -1.77828 | 2.24E-19 | 8.44E-18 | yes | down | 14.27 | 10.67 | 9.48 | 7.2 | 8.24 | 7.63 | 11.47333 | 7.69 |
| MFSD2A | 0.028 | -5.16214 | 2.36E-19 | 8.88E-18 | yes | down | 2.12 | 8.83 | 4.87 | 0.4 | 0.15 | 0.45 | 5.273333 | 0.333333 |
| APMAP | 3.075 | 1.620407 | 2.65E-19 | 9.93E-18 | yes | up | 15.82 | 18.6 | 14.97 | 106.88 | 119.45 | 120.8 | 16.46333 | 115.71 |
| GRIK3 | 0.048 | -4.37432 | 2.69E-19 | 1.01E-17 | yes | down | 1.95 | 1.54 | 1.44 | 0.04 | 0.2 | 0.28 | 1.643333 | 0.173333 |
| SEPT8 | 2.842 | 1.507013 | 2.79E-19 | 1.04E-17 | yes | up | 11.04 | 8.74 | 8.83 | 73.1 | 81.84 | 54.35 | 9.536667 | 69.76333 |
| PFKFB3 | 3.156 | 1.658082 | 3.02E-19 | 1.12E-17 | yes | up | 46.8 | 58.77 | 41.6 | 362.63 | 330.87 | 341.31 | 49.05667 | 344.9367 |
| MMP19 | 4.781 | 2.257402 | 3.12E-19 | 1.16E-17 | yes | up | 2.5 | 2.15 | 1.73 | 23.75 | 19.6 | 22.55 | 2.126667 | 21.96667 |
| LOC103349801 | 0.233 | -2.10032 | 3.14E-19 | 1.16E-17 | yes | down | 154.27 | 189.95 | 176.76 | 108.46 | 90.24 | 81.16 | 173.66 | 93.28667 |
| S100A10 | 3.296 | 1.720583 | 3.43E-19 | 1.27E-17 | yes | up | 185.31 | 161.48 | 108.29 | 1093.34 | 1039.4 | 1085.77 | 151.6933 | 1072.837 |
| ROR2 | 3.806 | 1.928137 | 3.5E-19 | 1.29E-17 | yes | up | 1.37 | 1.45 | 1.13 | 11.74 | 11.73 | 10.83 | 1.316667 | 11.43333 |
| VSIG4 | 3.64 | 1.863927 | 4.11E-19 | 1.51E-17 | yes | up | 6.61 | 5.99 | 4.15 | 46.24 | 47.93 | 50.27 | 5.583333 | 48.14667 |
| HMGB3 | 0.099 | -3.33029 | 4.96E-19 | 1.82E-17 | yes | down | 60.11 | 72.68 | 52.81 | 14.28 | 16.75 | 4.15 | 61.86667 | 11.72667 |
| LOXL2 | 4.562 | 2.189576 | 5.6E-19 | 2.05E-17 | yes | up | 42.65 | 26.3 | 21.02 | 292.59 | 382.61 | 260.84 | 29.99 | 312.0133 |
| PRRT4 | 741.6 | 9.534497 | 5.72E-19 | 2.09E-17 | yes | up | 0.05 | 0 | 0 | 13.57 | 26.43 | 22.72 | 0.016667 | 20.90667 |
| GLRX | 0.258 | -1.95606 | 5.77E-19 | 2.1E-17 | yes | down | 61.42 | 52.21 | 51.85 | 39.03 | 35.95 | 24.96 | 55.16 | 33.31333 |
| QSOX1 | 4.143 | 2.050783 | 5.85E-19 | 2.13E-17 | yes | up | 4 | 3.57 | 2.86 | 25.27 | 36.63 | 36.35 | 3.476667 | 32.75 |
| SOWAHC | 0.103 | -3.28101 | 5.91E-19 | 2.15E-17 | yes | down | 20.97 | 41.69 | 11.36 | 5.72 | 6.44 | 4.47 | 24.67333 | 5.543333 |
| PALD1 | 4.875 | 2.285466 | 5.93E-19 | 2.15E-17 | yes | up | 11.32 | 13.75 | 6.28 | 97.63 | 145.17 | 109.59 | 10.45 | 117.4633 |
| LOC100358417 | 0.159 | -2.65142 | 6.68E-19 | 2.42E-17 | yes | down | 14.23 | 11.87 | 9.54 | 3.75 | 3.49 | 5.97 | 11.88 | 4.403333 |
| LOC100340645 | 0.323 | -1.63227 | 7.43E-19 | 2.68E-17 | yes | down | 1430.86 | 1181.06 | 1093.36 | 990.72 | 876.25 | 862.72 | 1235.093 | 909.8967 |
| NDUFA12 | 0.246 | -2.0205 | 8.61E-19 | 3.1E-17 | yes | down | 383.08 | 307.07 | 264.11 | 192.24 | 149.84 | 195.84 | 318.0867 | 179.3067 |
| ABI3BP | 7.362 | 2.880063 | 8.89E-19 | 3.19E-17 | yes | up | 5.35 | 3.23 | 1.83 | 48.15 | 51.77 | 61.19 | 3.47 | 53.70333 |
| ST6GALNAC6 | 4.641 | 2.214332 | 9.66E-19 | 3.46E-17 | yes | up | 12.73 | 8.01 | 9.83 | 97.73 | 101.45 | 123.61 | 10.19 | 107.5967 |
| SEPN1 | 2.717 | 1.442067 | 1.07E-18 | 3.82E-17 | yes | up | 19.92 | 20.33 | 16.22 | 112.68 | 120.18 | 110.72 | 18.82333 | 114.5267 |
| FAM124A | 0.266 | -1.91221 | 1.1E-18 | 3.91E-17 | yes | down | 6.56 | 7.38 | 6.15 | 3.39 | 4.33 | 4.5 | 6.696667 | 4.073333 |
| GYPA | 0.035 | -4.84545 | 1.16E-18 | 4.14E-17 | yes | down | 5.58 | 11.47 | 7.72 | 1.12 | 0.2 | 0.47 | 8.256667 | 0.596667 |
| PRKD3 | 0.22 | -2.1845 | 1.26E-18 | 4.49E-17 | yes | down | 30.27 | 26.42 | 25.62 | 14.75 | 18.55 | 8.82 | 27.43667 | 14.04 |
| LOC103344972 | 14.255 | 3.833435 | 1.34E-18 | 4.74E-17 | yes | up | 20.87 | 17.83 | 5.14 | 521.22 | 286.93 | 533.39 | 14.61333 | 447.18 |
| ID3 | 0.247 | -2.01779 | 1.35E-18 | 4.78E-17 | yes | down | 491.74 | 527.12 | 400.77 | 218.55 | 247.93 | 326.55 | 473.21 | 264.3433 |
| TNC | 6.95 | 2.796988 | 1.36E-18 | 4.79E-17 | yes | up | 2.47 | 2.17 | 1.4 | 25.75 | 23.85 | 43.33 | 2.013333 | 30.97667 |
| LOC108176558 | 0.076 | -3.71029 | 1.49E-18 | 5.23E-17 | yes | down | 5.05 | 8.54 | 4.46 | 0.67 | 1.08 | 0.84 | 6.016667 | 0.863333 |
| TMEM132A | 3.858 | 1.947944 | 1.57E-18 | 5.52E-17 | yes | up | 1.69 | 1.48 | 1.54 | 12.56 | 15.11 | 14.05 | 1.57 | 13.90667 |
| TMBIM1 | 4.105 | 2.037267 | 1.84E-18 | 6.46E-17 | yes | up | 33.35 | 25.32 | 19.47 | 192.82 | 256.26 | 274.31 | 26.04667 | 241.13 |
| CCDC80 | 3.399 | 1.765112 | 1.86E-18 | 6.5E-17 | yes | up | 66.31 | 51.02 | 49.05 | 354.82 | 488.5 | 456.22 | 55.46 | 433.18 |
| CALU | 2.549 | 1.350126 | 1.92E-18 | 6.69E-17 | yes | up | 52.65 | 46.78 | 35.98 | 262.14 | 274.3 | 223.88 | 45.13667 | 253.44 |
| NDUFC2 | 0.24 | -2.05966 | 1.94E-18 | 6.78E-17 | yes | down | 495.33 | 620.83 | 501.21 | 342.37 | 247.88 | 292.82 | 539.1233 | 294.3567 |
| DOCK11 | 0.293 | -1.77016 | 2.05E-18 | 7.11E-17 | yes | down | 9.85 | 9.68 | 8.82 | 6.22 | 8.23 | 4.91 | 9.45 | 6.453333 |
| C18H10orf2 | 0.249 | -2.0068 | 2.43E-18 | 8.43E-17 | yes | down | 8.6 | 8.07 | 8.59 | 4.04 | 5.15 | 5.28 | 8.42 | 4.823333 |
| RNF19B | 3.029 | 1.598735 | 2.45E-18 | 8.48E-17 | yes | up | 9.12 | 9.9 | 8.88 | 66.75 | 72.32 | 67.83 | 9.3 | 68.96667 |
| MRC2 | 5.623 | 2.491459 | 2.47E-18 | 8.53E-17 | yes | up | 6.27 | 4.39 | 2.34 | 48.84 | 60.7 | 53.65 | 4.333333 | 54.39667 |
| C5AR1 | 4.214 | 2.075274 | 2.54E-18 | 8.76E-17 | yes | up | 2.34 | 2.83 | 2.08 | 24.19 | 23.52 | 21.73 | 2.416667 | 23.14667 |
| RFWD2 | 0.279 | -1.84274 | 2.68E-18 | 9.23E-17 | yes | down | 19.8 | 26.73 | 22.58 | 15.36 | 17.19 | 13.7 | 23.03667 | 15.41667 |
| SVEP1 | 3.791 | 1.922408 | 2.78E-18 | 9.54E-17 | yes | up | 6.24 | 4.54 | 4.3 | 32.99 | 53.46 | 43.75 | 5.026667 | 43.4 |
| SEC24D | 2.65 | 1.406 | 2.78E-18 | 9.54E-17 | yes | up | 6.61 | 6.42 | 5.21 | 36.28 | 41.21 | 34.16 | 6.08 | 37.21667 |
| LIPA | 4.164 | 2.057901 | 2.88E-18 | 9.83E-17 | yes | up | 18.92 | 24.97 | 18.87 | 138.58 | 259.66 | 179.69 | 20.92 | 192.6433 |
| ABAT | 8.234 | 3.041547 | 3.01E-18 | 1.03E-16 | yes | up | 0.47 | 0.24 | 0.43 | 8.14 | 7.79 | 5.78 | 0.38 | 7.236667 |
| CA12 | 0.016 | -5.95628 | 3.19E-18 | 1.09E-16 | yes | down | 1.22 | 4.38 | 1.46 | 0.17 | 0.08 | 0.18 | 2.353333 | 0.143333 |
| NDUFAF4 | 0.134 | -2.89548 | 3.51E-18 | 1.19E-16 | yes | down | 6.21 | 7.69 | 3.53 | 1.48 | 2.22 | 1.58 | 5.81 | 1.76 |
| GLDN | 0.047 | -4.41938 | 3.83E-18 | 1.3E-16 | yes | down | 11.71 | 23.37 | 8.13 | 0.79 | 0.88 | 2.74 | 14.40333 | 1.47 |
| EMILIN2 | 4.004 | 2.001411 | 3.91E-18 | 1.32E-16 | yes | up | 9.76 | 6.95 | 5.17 | 56.5 | 71.38 | 68.95 | 7.293333 | 65.61 |
| ANXA2 | 5.601 | 2.4857 | 4.03E-18 | 1.36E-16 | yes | up | 168.69 | 114.45 | 75.7 | 1772.15 | 1220.8 | 1437.25 | 119.6133 | 1476.733 |
| LOC103345217 | 0.044 | -4.49719 | 4.04E-18 | 1.36E-16 | yes | down | 6.05 | 9.34 | 3.96 | 0.72 | 0.53 | 0.67 | 6.45 | 0.64 |
| VASN | 6.621 | 2.727075 | 4.08E-18 | 1.37E-16 | yes | up | 2.07 | 1.42 | 1.69 | 24.46 | 20.02 | 32.73 | 1.726667 | 25.73667 |
| NDUFAB1 | 0.308 | -1.69684 | 4.4E-18 | 1.48E-16 | yes | down | 297.78 | 292.35 | 255.72 | 205.45 | 177.86 | 209.95 | 281.95 | 197.7533 |
| DARS | 0.276 | -1.85627 | 4.58E-18 | 1.54E-16 | yes | down | 38.56 | 33.48 | 24.63 | 21.15 | 24.69 | 15.2 | 32.22333 | 20.34667 |
| CCDC141 | 0.12 | -3.06261 | 4.68E-18 | 1.57E-16 | yes | down | 1.44 | 3.03 | 1.85 | 0.52 | 0.76 | 0.62 | 2.106667 | 0.633333 |
| COL6A3 | 4.935 | 2.30311 | 4.95E-18 | 1.65E-16 | yes | up | 67.97 | 36.66 | 28.74 | 380.74 | 575.29 | 396.35 | 44.45667 | 450.7933 |
| FZD4 | 4.357 | 2.1232 | 5.07E-18 | 1.69E-16 | yes | up | 21.94 | 26.08 | 23.69 | 183.65 | 348.66 | 199.65 | 23.90333 | 243.9867 |
| LOC108176227 | 0.029 | -5.08693 | 5.2E-18 | 1.73E-16 | yes | down | 6.84 | 3.39 | 0.98 | 0.23 | 0.18 | 0.3 | 3.736667 | 0.236667 |
| TENM4 | 5.681 | 2.506102 | 5.53E-18 | 1.84E-16 | yes | up | 1 | 1.13 | 1.24 | 20.98 | 15.74 | 11.44 | 1.123333 | 16.05333 |
| CATSPER1 | 13.989 | 3.806203 | 5.95E-18 | 1.97E-16 | yes | up | 0.54 | 0.23 | 0.15 | 9.55 | 7.5 | 10.48 | 0.306667 | 9.176667 |
| MYO19 | 0.208 | -2.26452 | 6.17E-18 | 2.04E-16 | yes | down | 8.93 | 13.37 | 7.31 | 5.05 | 5.36 | 3.4 | 9.87 | 4.603333 |
| PLA2G16 | 2.784 | 1.476994 | 6.21E-18 | 2.05E-16 | yes | up | 252.49 | 251.54 | 237.93 | 1521.65 | 1578.57 | 1625.58 | 247.32 | 1575.267 |
| CCND2 | 4.718 | 2.238055 | 7.39E-18 | 2.43E-16 | yes | up | 4.13 | 3.32 | 2.56 | 31.07 | 30.76 | 43.81 | 3.336667 | 35.21333 |
| CD82 | 3.907 | 1.965972 | 8.02E-18 | 2.63E-16 | yes | up | 15.82 | 19.84 | 13.64 | 117.56 | 163.54 | 161.98 | 16.43333 | 147.6933 |
| ARL6IP1 | 3.505 | 1.809599 | 8.33E-18 | 2.73E-16 | yes | up | 40.93 | 47.29 | 42.48 | 414.69 | 347.9 | 289.46 | 43.56667 | 350.6833 |
| MYL9 | 3.686 | 1.882009 | 8.48E-18 | 2.77E-16 | yes | up | 95.27 | 99.11 | 75.96 | 762.78 | 631.81 | 846.78 | 90.11333 | 747.1233 |
| LRRC8B | 7.534 | 2.913395 | 8.56E-18 | 2.79E-16 | yes | up | 0.62 | 0.55 | 0.57 | 11.69 | 6.45 | 11.39 | 0.58 | 9.843333 |
| GREM2 | 4.889 | 2.289599 | 8.97E-18 | 2.92E-16 | yes | up | 1.74 | 1.04 | 1.5 | 17.89 | 18.63 | 16.07 | 1.426667 | 17.53 |
| TIMM17A | 0.237 | -2.07749 | 9.04E-18 | 2.94E-16 | yes | down | 97.33 | 156.79 | 103.4 | 69.52 | 61.58 | 61.51 | 119.1733 | 64.20333 |
| IRF4 | 10.213 | 3.352389 | 9.13E-18 | 2.96E-16 | yes | up | 0.17 | 0.08 | 0.13 | 3.05 | 2.78 | 3.31 | 0.126667 | 3.046667 |
| OSBPL7 | 0.24 | -2.05951 | 9.35E-18 | 3.03E-16 | yes | down | 10.5 | 12.66 | 12.77 | 6.11 | 8.62 | 5.41 | 11.97667 | 6.713333 |
| MRPS36 | 0.272 | -1.88059 | 9.41E-18 | 3.04E-16 | yes | down | 71.79 | 54.1 | 51.75 | 44.13 | 37.14 | 30.59 | 59.21333 | 37.28667 |
| PTGES2 | 0.192 | -2.38047 | 9.43E-18 | 3.04E-16 | yes | down | 66.13 | 63.75 | 72.29 | 35.73 | 22.91 | 30.61 | 67.39 | 29.75 |
| LOC100346411 | 0.231 | -2.11544 | 9.96E-18 | 3.21E-16 | yes | down | 1168.72 | 1214.95 | 1181.15 | 761.15 | 506.24 | 611.52 | 1188.273 | 626.3033 |
| OXNAD1 | 0.223 | -2.16341 | 1.08E-17 | 3.47E-16 | yes | down | 17.66 | 21.76 | 24.43 | 10.82 | 11.63 | 10.2 | 21.28333 | 10.88333 |
| TSFM | 0.275 | -1.85988 | 1.08E-17 | 3.47E-16 | yes | down | 63.16 | 67.86 | 62.5 | 45.87 | 34.88 | 36.01 | 64.50667 | 38.92 |
| FTH1 | 3.32 | 1.731192 | 1.11E-17 | 3.57E-16 | yes | up | 1091.47 | 1281.14 | 1048.35 | 7874.43 | 8214.06 | 9815.17 | 1140.32 | 8634.553 |
| TRIM37 | 0.271 | -1.88463 | 1.18E-17 | 3.77E-16 | yes | down | 540.56 | 593.9 | 624.69 | 370.93 | 307.78 | 299.02 | 586.3833 | 325.91 |
| CLSTN2 | 0.013 | -6.32192 | 1.48E-17 | 4.72E-16 | yes | down | 0.36 | 0.3 | 0.51 | 0.01 | 0.02 | 0.01 | 0.39 | 0.013333 |
| NLGN2 | 4.194 | 2.068317 | 1.54E-17 | 4.9E-16 | yes | up | 1.2 | 0.92 | 0.79 | 8.16 | 9.87 | 9.77 | 0.97 | 9.266667 |
| NFIL3 | 4.338 | 2.116915 | 1.64E-17 | 5.19E-16 | yes | up | 4.41 | 5.82 | 5.8 | 60.63 | 58.97 | 42.04 | 5.343333 | 53.88 |
| LETMD1 | 0.299 | -1.74165 | 1.68E-17 | 5.34E-16 | yes | down | 50.31 | 64.01 | 58.58 | 37.44 | 46.01 | 36.58 | 57.63333 | 40.01 |
| MARVELD1 | 4.235 | 2.082215 | 1.81E-17 | 5.71E-16 | yes | up | 9.14 | 7.74 | 5.58 | 74.97 | 59 | 78.53 | 7.486667 | 70.83333 |
| ACTA2 | 5.351 | 2.419877 | 1.94E-17 | 6.12E-16 | yes | up | 158.09 | 186.22 | 129.67 | 2396.38 | 1307.76 | 1968.94 | 157.9933 | 1891.027 |
| LOC100351248 | 0.001 | -10.7552 | 2.1E-17 | 6.61E-16 | yes | down | 20.66 | 9.51 | 23.36 | 0 | 0 | 0 | 17.84333 | 0 |
| LOC103345437 | 0.261 | -1.93849 | 2.27E-17 | 7.15E-16 | yes | down | 49.27 | 51.07 | 58.21 | 39.62 | 33.12 | 31.49 | 52.85 | 34.74333 |
| NEO1 | 2.676 | 1.420238 | 2.28E-17 | 7.17E-16 | yes | up | 12.24 | 10.29 | 8.5 | 59.82 | 73.36 | 58.11 | 10.34333 | 63.76333 |
| NSUN4 | 0.336 | -1.57461 | 2.52E-17 | 7.88E-16 | yes | down | 15.08 | 13.04 | 12.21 | 10.22 | 11.86 | 10.75 | 13.44333 | 10.94333 |
| CPT2 | 0.321 | -1.63938 | 2.7E-17 | 8.45E-16 | yes | down | 53.97 | 69.28 | 60.92 | 45.77 | 48.25 | 42.13 | 61.39 | 45.38333 |
| IDH2 | 0.204 | -2.29133 | 2.87E-17 | 8.97E-16 | yes | down | 460.51 | 232.15 | 303.35 | 162.5 | 147.96 | 153.83 | 332.0033 | 154.7633 |
| CD74 | 8.482 | 3.08444 | 3.04E-17 | 9.47E-16 | yes | up | 8.4 | 9.16 | 13.97 | 179.16 | 156.68 | 283.6 | 10.51 | 206.48 |
| CRYM | 0.046 | -4.43515 | 3.15E-17 | 9.8E-16 | yes | down | 16.35 | 70.73 | 64.68 | 3.13 | 9.25 | 4.38 | 50.58667 | 5.586667 |
| LRRC3 | 6.874 | 2.781099 | 3.22E-17 | 9.97E-16 | yes | up | 1.35 | 1.56 | 1.51 | 15.24 | 27.57 | 27.52 | 1.473333 | 23.44333 |
| PAM | 3.111 | 1.637205 | 3.22E-17 | 9.97E-16 | yes | up | 28.3 | 24.05 | 18.38 | 165.28 | 155.88 | 171.99 | 23.57667 | 164.3833 |
| PRDX3 | 0.286 | -1.80378 | 3.26E-17 | 1.01E-15 | yes | down | 317.34 | 302.63 | 289.05 | 190.56 | 186.13 | 229.58 | 303.0067 | 202.09 |
| RNPEP | 0.246 | -2.02507 | 3.41E-17 | 1.05E-15 | yes | down | 27.54 | 42.03 | 25.03 | 20.55 | 20.81 | 14.8 | 31.53333 | 18.72 |
| ACACA | 6.793 | 2.764028 | 3.73E-17 | 1.15E-15 | yes | up | 3.28 | 2.9 | 3.33 | 66.67 | 30.63 | 49.27 | 3.17 | 48.85667 |
| NDUFAF6 | 0.275 | -1.86178 | 3.79E-17 | 1.17E-15 | yes | down | 20.72 | 20.3 | 19.11 | 13.52 | 10.65 | 12.01 | 20.04333 | 12.06 |
| MRPS24 | 0.344 | -1.54129 | 3.93E-17 | 1.21E-15 | yes | down | 56.45 | 55.03 | 48.92 | 42.64 | 48.67 | 35.52 | 53.46667 | 42.27667 |
| ADGRE5 | 4.259 | 2.090479 | 4.04E-17 | 1.24E-15 | yes | up | 2.95 | 3.81 | 3.69 | 25.15 | 39.45 | 26.79 | 3.483333 | 30.46333 |
| CD276 | 4.114 | 2.040435 | 4.2E-17 | 1.29E-15 | yes | up | 5.95 | 4.34 | 3.96 | 44.99 | 41.06 | 49.73 | 4.75 | 45.26 |
| PDSS2 | 0.266 | -1.90862 | 4.34E-17 | 1.32E-15 | yes | down | 10.56 | 9.68 | 9.01 | 6.86 | 6.19 | 4.77 | 9.75 | 5.94 |
| LACTB2 | 0.208 | -2.26221 | 4.9E-17 | 1.49E-15 | yes | down | 32.21 | 26.49 | 36.28 | 15.19 | 20.43 | 11.99 | 31.66 | 15.87 |
| APOOL | 0.222 | -2.17191 | 4.98E-17 | 1.51E-15 | yes | down | 36.05 | 41.14 | 32.46 | 23.21 | 20.16 | 12.4 | 36.55 | 18.59 |
| NDUFV2 | 0.316 | -1.6604 | 5.44E-17 | 1.65E-15 | yes | down | 211.27 | 204.5 | 149.03 | 154.88 | 128.2 | 117.45 | 188.2667 | 133.51 |
| MGP | 5.62 | 2.490504 | 5.89E-17 | 1.78E-15 | yes | up | 436.58 | 241.48 | 157.08 | 3227.19 | 4059.74 | 3251.98 | 278.38 | 3512.97 |
| LOC108177262 | 0.249 | -2.00783 | 6.11E-17 | 1.85E-15 | yes | down | 1668.78 | 1675.01 | 2020.05 | 1185.62 | 1036.84 | 891.19 | 1787.947 | 1037.883 |
| LOC108177453 | 3.032 | 1.600287 | 6.22E-17 | 1.88E-15 | yes | up | 637.15 | 802.61 | 620.47 | 5183.31 | 4902.6 | 4291.91 | 686.7433 | 4792.607 |
| GM2A | 2.984 | 1.577187 | 6.53E-17 | 1.97E-15 | yes | up | 35.29 | 34.79 | 27.64 | 203.25 | 212.53 | 244.36 | 32.57333 | 220.0467 |
| ABCA3 | 3.346 | 1.742291 | 6.64E-17 | 2E-15 | yes | up | 1.75 | 1.36 | 1.46 | 10.22 | 13.19 | 11.56 | 1.523333 | 11.65667 |
| LRRN4CL | 1177.68 | 10.20173 | 6.74E-17 | 2.02E-15 | yes | up | 0 | 0 | 0 | 18.08 | 13.94 | 11.95 | 0 | 14.65667 |
| NTRK2 | 13.649 | 3.770693 | 6.77E-17 | 2.03E-15 | yes | up | 0.8 | 0.99 | 0.15 | 18.63 | 24.29 | 15.92 | 0.646667 | 19.61333 |
| NDUFB4 | 0.311 | -1.68337 | 7.19E-17 | 2.15E-15 | yes | down | 467.2 | 507.4 | 439.22 | 388.64 | 332.37 | 290.02 | 471.2733 | 337.01 |
| FUCA1 | 2.633 | 1.396675 | 7.19E-17 | 2.15E-15 | yes | up | 14.09 | 16.51 | 12.82 | 82.74 | 94.52 | 84.24 | 14.47333 | 87.16667 |
| FKBP10 | 4.725 | 2.240234 | 7.51E-17 | 2.24E-15 | yes | up | 7.05 | 5.95 | 3.1 | 63.52 | 54.56 | 50.91 | 5.366667 | 56.33 |
| RLA-DRB1 | 10.092 | 3.335195 | 8.01E-17 | 2.38E-15 | yes | up | 1.63 | 2.83 | 3.65 | 50.7 | 52.82 | 81.94 | 2.703333 | 61.82 |
| LOC100348124 | 0.277 | -1.85184 | 8.2E-17 | 2.44E-15 | yes | down | 110.38 | 80.42 | 82.83 | 58.83 | 51.28 | 61.76 | 91.21 | 57.29 |
| HSF5 | 0.103 | -3.28429 | 8.56E-17 | 2.53E-15 | yes | down | 2.53 | 3.5 | 1.72 | 0.6 | 0.4 | 0.8 | 2.583333 | 0.6 |
| FCGRT | 3.15 | 1.655541 | 8.56E-17 | 2.53E-15 | yes | up | 55.7 | 60.32 | 53.61 | 391.11 | 379.8 | 448.53 | 56.54333 | 406.48 |
| TMEM161B | 0.199 | -2.32633 | 8.58E-17 | 2.54E-15 | yes | down | 5.5 | 7.18 | 6.73 | 3.34 | 2.91 | 2.96 | 6.47 | 3.07 |
| PLP2 | 5.33 | 2.414207 | 9.15E-17 | 2.7E-15 | yes | up | 14.4 | 10.49 | 8.11 | 153.56 | 112.93 | 128.27 | 11 | 131.5867 |
| IGFBP5 | 7.174 | 2.842801 | 9.54E-17 | 2.81E-15 | yes | up | 16.78 | 10.08 | 6.73 | 165.52 | 131.29 | 231.62 | 11.19667 | 176.1433 |
| RHOC | 2.787 | 1.478784 | 9.88E-17 | 2.9E-15 | yes | up | 44.59 | 48.27 | 39.05 | 289.31 | 268.4 | 279.01 | 43.97 | 278.9067 |
| LOC100338849 | 0.228 | -2.1314 | 9.91E-17 | 2.91E-15 | yes | down | 7.49 | 6.55 | 6.37 | 3.3 | 4.02 | 3.32 | 6.803333 | 3.546667 |
| DDX21 | 0.352 | -1.50796 | 9.99E-17 | 2.92E-15 | yes | down | 13.41 | 16.73 | 11.88 | 10.35 | 12.44 | 10.96 | 14.00667 | 11.25 |
| PANK1 | 0.177 | -2.4997 | 1.04E-16 | 3.04E-15 | yes | down | 12.51 | 14.53 | 17.55 | 5.92 | 10.48 | 5.6 | 14.86333 | 7.333333 |
| SCARB1 | 4.125 | 2.044334 | 1.09E-16 | 3.17E-15 | yes | up | 18.9 | 18.04 | 17.07 | 216.77 | 145.39 | 145.06 | 18.00333 | 169.0733 |
| FAM162A | 0.317 | -1.65775 | 1.15E-16 | 3.36E-15 | yes | down | 62.18 | 55.55 | 55.9 | 43.93 | 38.73 | 42.93 | 57.87667 | 41.86333 |
| ADRB3 | 0.196 | -2.35405 | 1.34E-16 | 3.91E-15 | yes | down | 9.11 | 12.19 | 6.46 | 3.58 | 4.02 | 3.11 | 9.253333 | 3.57 |
| LOC103352280 | 0.186 | -2.42292 | 1.35E-16 | 3.93E-15 | yes | down | 1.58 | 1.7 | 1.73 | 1 | 0.9 | 0.77 | 1.67 | 0.89 |
| COL1A2 | 7.905 | 2.982741 | 1.37E-16 | 3.96E-15 | yes | up | 460.78 | 250.11 | 129.76 | 4083.1 | 4835.84 | 5695.7 | 280.2167 | 4871.547 |
| CNPY3 | 3.001 | 1.585453 | 1.38E-16 | 4E-15 | yes | up | 7.23 | 7.67 | 6.63 | 51.58 | 51.66 | 43.33 | 7.176667 | 48.85667 |
| NDUFA10 | 0.246 | -2.02042 | 1.43E-16 | 4.11E-15 | yes | down | 264.6 | 282.52 | 335.43 | 183.23 | 150.48 | 168.27 | 294.1833 | 167.3267 |
| ETFA | 0.296 | -1.7552 | 1.43E-16 | 4.11E-15 | yes | down | 420.31 | 419.64 | 473.49 | 316.41 | 286.81 | 296.62 | 437.8133 | 299.9467 |
| SLC35F6 | 0.339 | -1.56261 | 1.45E-16 | 4.16E-15 | yes | down | 13.66 | 14.07 | 13.25 | 8.63 | 11.81 | 10.86 | 13.66 | 10.43333 |
| PTMS | 3.586 | 1.842389 | 1.61E-16 | 4.62E-15 | yes | up | 87.68 | 96.12 | 71.09 | 844.72 | 630.99 | 583.8 | 84.96333 | 686.5033 |
| BGN | 2.619 | 1.389153 | 1.62E-16 | 4.63E-15 | yes | up | 71.49 | 68.97 | 51.27 | 367.32 | 377.99 | 389.9 | 63.91 | 378.4033 |
| ZNF292 | 0.234 | -2.09363 | 1.64E-16 | 4.7E-15 | yes | down | 13.8 | 18.2 | 20.62 | 7.4 | 8.12 | 13.18 | 17.54 | 9.566667 |
| IL34 | 3.908 | 1.966322 | 1.65E-16 | 4.73E-15 | yes | up | 2.59 | 2.57 | 1.86 | 22.71 | 27.87 | 21.39 | 2.34 | 23.99 |
| ADAMTS18 | 7.465 | 2.900059 | 1.69E-16 | 4.83E-15 | yes | up | 0.53 | 0.63 | 0.43 | 8.98 | 13.89 | 4.91 | 0.53 | 9.26 |
| BTC | 12.697 | 3.666391 | 1.76E-16 | 5.02E-15 | yes | up | 0.28 | 0.37 | 0.35 | 4.87 | 16.27 | 8.57 | 0.333333 | 9.903333 |
| ACADL | 0.154 | -2.69952 | 1.78E-16 | 5.05E-15 | yes | down | 723.7 | 460.67 | 936.24 | 294.44 | 289.64 | 188.63 | 706.87 | 257.57 |
| CEL | 0.001 | -10.7847 | 1.85E-16 | 5.24E-15 | yes | down | 1.77 | 6.37 | 7.31 | 0 | 0 | 0 | 5.15 | 0 |
| SEMA5A | 3.199 | 1.677654 | 1.88E-16 | 5.33E-15 | yes | up | 2.38 | 1.84 | 1.74 | 15.71 | 16.15 | 11.57 | 1.986667 | 14.47667 |
| CSF1 | 3.659 | 1.871377 | 2.05E-16 | 5.79E-15 | yes | up | 3.24 | 3.13 | 2.61 | 23.54 | 22.27 | 28.43 | 2.993333 | 24.74667 |
| LOC100342069 | 0.082 | -3.61644 | 2.08E-16 | 5.88E-15 | yes | down | 15.36 | 40.43 | 22.38 | 6.9 | 2.51 | 5.38 | 26.05667 | 4.93 |
| ATP5H | 0.327 | -1.6146 | 2.11E-16 | 5.95E-15 | yes | down | 712.84 | 816.15 | 751.73 | 618.34 | 544.23 | 547.14 | 760.24 | 569.9033 |
| LPL | 2.792 | 1.481065 | 2.3E-16 | 6.46E-15 | yes | up | 976.79 | 1172.04 | 962.92 | 6711.32 | 7939.1 | 5332.45 | 1037.25 | 6660.957 |
| HOXC10 | 398.031 | 8.636738 | 2.42E-16 | 6.79E-15 | yes | up | 0 | 0.03 | 0.03 | 14.44 | 18.23 | 16.62 | 0.02 | 16.43 |
| KDELC2 | 3.1 | 1.63211 | 2.49E-16 | 6.97E-15 | yes | up | 6.74 | 5.3 | 4.67 | 36.17 | 35.61 | 29.16 | 5.57 | 33.64667 |
| COL1A1 | 6.659 | 2.735386 | 2.57E-16 | 7.19E-15 | yes | up | 737.6 | 405.68 | 247 | 5647.26 | 7256.16 | 8019.56 | 463.4267 | 6974.327 |
| AHR | 0.264 | -1.92221 | 2.61E-16 | 7.31E-15 | yes | down | 19.37 | 27.1 | 19.08 | 14.94 | 15.08 | 9.69 | 21.85 | 13.23667 |
| LOC100354074 | 0.183 | -2.45019 | 2.67E-16 | 7.44E-15 | yes | down | 48.81 | 52.03 | 48.45 | 18.65 | 15.34 | 27.93 | 49.76333 | 20.64 |
| SOSTDC1 | 0.022 | -5.50213 | 2.99E-16 | 8.32E-15 | yes | down | 1.49 | 7.84 | 2.73 | 0.11 | 0.18 | 0.31 | 4.02 | 0.2 |
| ADM | 4.164 | 2.058014 | 3.37E-16 | 9.37E-15 | yes | up | 8.85 | 6.92 | 7.56 | 54.92 | 103.21 | 64.42 | 7.776667 | 74.18333 |
| AGTR1 | 2.954 | 1.56256 | 3.51E-16 | 9.75E-15 | yes | up | 14.41 | 12.8 | 8.73 | 91.01 | 95.26 | 84.85 | 11.98 | 90.37333 |
| RA_M006_JSM7BED4F | 3.774 | 1.915998 | 3.6E-16 | 9.97E-15 | yes | up | 21.62 | 21.44 | 16.48 | 198.97 | 133.78 | 182.64 | 19.84667 | 171.7967 |
| CD83 | 24.964 | 4.641768 | 3.75E-16 | 1.04E-14 | yes | up | 0.24 | 0.22 | 0.11 | 17.03 | 5.97 | 8.38 | 0.19 | 10.46 |
| VWA8 | 0.288 | -1.79414 | 3.81E-16 | 1.05E-14 | yes | down | 10.94 | 10.39 | 10.44 | 7.46 | 10.18 | 5.49 | 10.59 | 7.71 |
| ARHGAP1 | 3.179 | 1.668381 | 3.87E-16 | 1.07E-14 | yes | up | 7.74 | 7.6 | 6.43 | 46.15 | 50.79 | 59.98 | 7.256667 | 52.30667 |
| LOC100339133 | 0.452 | -1.14696 | 3.93E-16 | 1.08E-14 | yes | down | 315.61 | 315.88 | 269.75 | 299.63 | 339.95 | 293.9 | 300.4133 | 311.16 |
| LOC100347623 | 976.921 | 9.932097 | 3.93E-16 | 1.08E-14 | yes | up | 0 | 0 | 0 | 21.83 | 26.99 | 16.08 | 0 | 21.63333 |
| SLC25A13 | 0.356 | -1.49164 | 4.08E-16 | 1.12E-14 | yes | down | 21.37 | 21.79 | 21.58 | 18.4 | 17.87 | 16.14 | 21.58 | 17.47 |
| PGER4 | 0.22 | -2.18178 | 4.3E-16 | 1.18E-14 | yes | down | 112.29 | 159.2 | 151.71 | 83.94 | 60.76 | 69.43 | 141.0667 | 71.37667 |
| CREB5 | 4.101 | 2.03585 | 4.74E-16 | 1.29E-14 | yes | up | 1.55 | 1.06 | 0.74 | 11.42 | 10.14 | 8.24 | 1.116667 | 9.933333 |
| VWF | 3.178 | 1.668227 | 4.81E-16 | 1.31E-14 | yes | up | 19.38 | 18.14 | 19.29 | 113.52 | 150.98 | 150.5 | 18.93667 | 138.3333 |
| LOC100356693 | 0.295 | -1.75912 | 4.83E-16 | 1.32E-14 | yes | down | 55.32 | 75.82 | 61.39 | 46.43 | 48.67 | 36.12 | 64.17667 | 43.74 |
| MAOB | 4.617 | 2.20692 | 4.92E-16 | 1.34E-14 | yes | up | 5.69 | 7.29 | 5.5 | 70.69 | 48.51 | 72.76 | 6.16 | 63.98667 |
| CLU | 9.395 | 3.231933 | 5.14E-16 | 1.4E-14 | yes | up | 14.1 | 7.06 | 4.85 | 127.15 | 143.73 | 257.09 | 8.67 | 175.99 |
| RGS6 | 0.298 | -1.7474 | 5.27E-16 | 1.43E-14 | yes | down | 447.46 | 504.63 | 501.41 | 350.34 | 285.72 | 300.98 | 484.5 | 312.3467 |
| EHD4 | 2.896 | 1.534225 | 5.35E-16 | 1.45E-14 | yes | up | 7.08 | 7.06 | 6.27 | 50.5 | 44.25 | 40.02 | 6.803333 | 44.92333 |
| ACSS3 | 0.231 | -2.11427 | 5.36E-16 | 1.45E-14 | yes | down | 31.97 | 41.32 | 36.82 | 22.62 | 23.19 | 12.6 | 36.70333 | 19.47 |
| CKMT1B | 0.001 | -9.43606 | 5.38E-16 | 1.45E-14 | yes | down | 2.97 | 13.03 | 10.66 | 0 | 0.1 | 0 | 8.886667 | 0.033333 |
| HSD17B10 | 0.293 | -1.76862 | 5.58E-16 | 1.5E-14 | yes | down | 219.08 | 248.75 | 241.03 | 181.29 | 145.38 | 150.57 | 236.2867 | 159.08 |
| FASTKD3 | 0.186 | -2.42939 | 5.97E-16 | 1.61E-14 | yes | down | 3.44 | 4.65 | 4.46 | 1.55 | 1.96 | 1.81 | 4.183333 | 1.773333 |
| ATP2B4 | 2.45 | 1.292578 | 6.43E-16 | 1.73E-14 | yes | up | 7.9 | 7.48 | 6.83 | 39.74 | 43.35 | 40.89 | 7.403333 | 41.32667 |
| STARD7 | 0.407 | -1.29642 | 7.02E-16 | 1.88E-14 | yes | down | 55 | 51.98 | 50.13 | 45.86 | 52.72 | 48.16 | 52.37 | 48.91333 |
| PTPRF | 3.489 | 1.80297 | 7.41E-16 | 1.98E-14 | yes | up | 4.26 | 5.2 | 3.8 | 29.15 | 48.94 | 29.13 | 4.42 | 35.74 |
| NFS1 | 0.337 | -1.56839 | 7.52E-16 | 2.01E-14 | yes | down | 43.9 | 54.37 | 45.44 | 37.44 | 37.87 | 36.19 | 47.90333 | 37.16667 |
| NPC1L1 | 47.726 | 5.576703 | 8.27E-16 | 2.21E-14 | yes | up | 0.02 | 0.07 | 0.01 | 3.67 | 4.43 | 2.86 | 0.033333 | 3.653333 |
| FGF14 | 14.33 | 3.841013 | 8.35E-16 | 2.22E-14 | yes | up | 0.08 | 0.06 | 0.03 | 1.59 | 2.53 | 1.72 | 0.056667 | 1.946667 |
| SLC25A42 | 0.107 | -3.22852 | 8.39E-16 | 2.23E-14 | yes | down | 42.47 | 39.37 | 31.24 | 5.11 | 12.09 | 11.35 | 37.69333 | 9.516667 |
| PGER6 | 0.111 | -3.17617 | 8.9E-16 | 2.36E-14 | yes | down | 21.23 | 51.68 | 30.89 | 6.47 | 7.18 | 12.36 | 34.6 | 8.67 |
| MDH2 | 0.256 | -1.9631 | 8.95E-16 | 2.37E-14 | yes | down | 465.74 | 340.92 | 420.39 | 268.51 | 205.3 | 248.11 | 409.0167 | 240.64 |
| CCDC59 | 0.246 | -2.02407 | 9.29E-16 | 2.46E-14 | yes | down | 11.32 | 12.47 | 9.78 | 7.7 | 8.04 | 4.24 | 11.19 | 6.66 |
| TGOLN2 | 2.8 | 1.485504 | 9.43E-16 | 2.49E-14 | yes | up | 27.24 | 31.27 | 28.36 | 168.42 | 229.39 | 166.17 | 28.95667 | 187.9933 |
| ELOVL2 | 0.109 | -3.19385 | 9.82E-16 | 2.59E-14 | yes | down | 4.16 | 7.08 | 5.31 | 1 | 2.48 | 0.8 | 5.516667 | 1.426667 |
| GPX7 | 3.22 | 1.686956 | 1.01E-15 | 2.66E-14 | yes | up | 20.78 | 18.41 | 11.36 | 118.52 | 131.24 | 115.96 | 16.85 | 121.9067 |
| LOC103351933 | 5.091 | 2.347904 | 1.08E-15 | 2.83E-14 | yes | up | 1.1 | 1.69 | 1.03 | 13.77 | 15.16 | 15.7 | 1.273333 | 14.87667 |
| STUM | 4.415 | 2.142299 | 1.14E-15 | 2.98E-14 | yes | up | 0.46 | 0.7 | 0.55 | 4.96 | 7.29 | 5.21 | 0.57 | 5.82 |
| LOC103345926 | 4.912 | 2.296169 | 1.19E-15 | 3.12E-14 | yes | up | 104.09 | 58.64 | 45.73 | 872.64 | 694.82 | 704.88 | 69.48667 | 757.4467 |
| ICAM1 | 3.763 | 1.911821 | 1.36E-15 | 3.55E-14 | yes | up | 2.87 | 2.11 | 2.54 | 18.33 | 20.91 | 24.33 | 2.506667 | 21.19 |
| CMTM3 | 3.609 | 1.851753 | 1.37E-15 | 3.57E-14 | yes | up | 8.45 | 8.28 | 5.85 | 73.89 | 56.38 | 55.36 | 7.526667 | 61.87667 |
| FASTKD2 | 0.252 | -1.98878 | 1.5E-15 | 3.9E-14 | yes | down | 4.02 | 5 | 3.99 | 2.62 | 2.5 | 2.22 | 4.336667 | 2.446667 |
| RNF115 | 0.26 | -1.94292 | 1.52E-15 | 3.95E-14 | yes | down | 59.24 | 65.52 | 71.56 | 34.27 | 32.44 | 43.24 | 65.44 | 36.65 |
| MYOCD | 6.965 | 2.800142 | 1.56E-15 | 4.07E-14 | yes | up | 0.39 | 0.32 | 0.22 | 3.84 | 3.81 | 6.49 | 0.31 | 4.713333 |
| ARL4C | 6.976 | 2.802433 | 1.71E-15 | 4.44E-14 | yes | up | 0.26 | 0.23 | 0.26 | 3.98 | 4.54 | 3.49 | 0.25 | 4.003333 |
| LOC100340191 | 0.36 | -1.47249 | 1.75E-15 | 4.53E-14 | yes | down | 25.74 | 31.47 | 25.59 | 23.29 | 26.57 | 18.91 | 27.6 | 22.92333 |
| LOC103347528 | 0.348 | -1.5232 | 1.75E-15 | 4.54E-14 | yes | down | 36.73 | 37.01 | 32.93 | 27.35 | 33.48 | 24.73 | 35.55667 | 28.52 |
| DBN1 | 4.41 | 2.140722 | 1.76E-15 | 4.55E-14 | yes | up | 12.56 | 8.45 | 5.23 | 82.86 | 84.33 | 90.05 | 8.746667 | 85.74667 |
| NDUFS6 | 0.344 | -1.53956 | 1.87E-15 | 4.82E-14 | yes | down | 368.05 | 350.18 | 388.64 | 305.67 | 291.39 | 303.74 | 368.9567 | 300.2667 |
| P2RX1 | 21.149 | 4.402517 | 1.94E-15 | 4.99E-14 | yes | up | 0.27 | 0.22 | 0.15 | 10.61 | 4.35 | 14.64 | 0.213333 | 9.866667 |
| ATP6V1E1 | 0.326 | -1.61611 | 1.96E-15 | 5.04E-14 | yes | down | 169.73 | 185.27 | 155.11 | 143.52 | 112.68 | 121.85 | 170.0367 | 126.0167 |
| ACSL5 | 3.366 | 1.751069 | 2.02E-15 | 5.17E-14 | yes | up | 1.77 | 2.23 | 1.74 | 14.84 | 17.84 | 13.28 | 1.913333 | 15.32 |
| EPHX1 | 4.543 | 2.183552 | 2.05E-15 | 5.26E-14 | yes | up | 37.48 | 56.84 | 51.44 | 372.11 | 602.78 | 574.32 | 48.58667 | 516.4033 |
| LOC100354753 | 2.739 | 1.453571 | 2.32E-15 | 5.95E-14 | yes | up | 7 | 7.86 | 6.95 | 45.64 | 53.09 | 40.03 | 7.27 | 46.25333 |
| GPAT3 | 11.901 | 3.573064 | 2.4E-15 | 6.13E-14 | yes | up | 7.82 | 5 | 1.25 | 123.38 | 157.38 | 89.01 | 4.69 | 123.2567 |
| SYNJ2BP | 0.259 | -1.94766 | 2.4E-15 | 6.13E-14 | yes | down | 8.96 | 7.43 | 8.33 | 5.22 | 6.47 | 3.41 | 8.24 | 5.033333 |
| PIPOX | 12.626 | 3.658316 | 2.44E-15 | 6.22E-14 | yes | up | 1.73 | 0.35 | 0.54 | 21.04 | 25.59 | 26.38 | 0.873333 | 24.33667 |
| TSC22D3 | 2.702 | 1.433772 | 2.52E-15 | 6.42E-14 | yes | up | 33.59 | 39.23 | 29.06 | 187.75 | 214.15 | 223.24 | 33.96 | 208.38 |
| LOC100009097 | 466.784 | 8.866611 | 2.58E-15 | 6.56E-14 | yes | up | 0 | 0.12 | 0 | 17.96 | 51.28 | 54.15 | 0.04 | 41.13 |
| RASL11A | 8.612 | 3.106331 | 2.59E-15 | 6.57E-14 | yes | up | 1.29 | 1.12 | 1.67 | 30.82 | 18.98 | 30.65 | 1.36 | 26.81667 |
| CSRP1 | 3.592 | 1.844977 | 2.74E-15 | 6.93E-14 | yes | up | 33.63 | 51.63 | 41.38 | 327.08 | 345.16 | 370.27 | 42.21333 | 347.5033 |
| INSIG2 | 0.268 | -1.89831 | 2.75E-15 | 6.94E-14 | yes | down | 13.34 | 17.74 | 13.17 | 10.15 | 10.51 | 6.44 | 14.75 | 9.033333 |
| IGFBP7 | 3.285 | 1.715957 | 2.78E-15 | 7.02E-14 | yes | up | 277.06 | 376.51 | 320.17 | 2657.45 | 2384.99 | 2313.07 | 324.58 | 2451.837 |
| GALNT16 | 3.339 | 1.739502 | 2.81E-15 | 7.09E-14 | yes | up | 9.54 | 8.56 | 6.23 | 49.31 | 64.39 | 69.66 | 8.11 | 61.12 |
| COL4A1 | 3.43 | 1.778154 | 2.91E-15 | 7.32E-14 | yes | up | 252.86 | 232.06 | 257.25 | 1996.72 | 2783.61 | 1406.91 | 247.39 | 2062.413 |
| MTHFD1 | 0.273 | -1.87312 | 2.99E-15 | 7.52E-14 | yes | down | 37.09 | 39.64 | 39.76 | 23.85 | 33.33 | 17.32 | 38.83 | 24.83333 |
| HOXA10 | 18.202 | 4.186035 | 3.02E-15 | 7.57E-14 | yes | up | 0.05 | 0.09 | 0.2 | 4.35 | 5.84 | 5.23 | 0.113333 | 5.14 |
| GSS | 2.769 | 1.46917 | 3.17E-15 | 7.93E-14 | yes | up | 20.64 | 19.13 | 19.02 | 112.55 | 128.62 | 131.81 | 19.59667 | 124.3267 |
| STK17B | 0.149 | -2.74696 | 3.34E-15 | 8.35E-14 | yes | down | 13.19 | 19.27 | 17.18 | 6.99 | 7.43 | 2.71 | 16.54667 | 5.71 |
| SLC7A6 | 5.345 | 2.41812 | 3.38E-15 | 8.44E-14 | yes | up | 4.26 | 7.22 | 3.17 | 32.57 | 78.45 | 43.32 | 4.883333 | 51.44667 |
| TRAP1 | 0.283 | -1.82056 | 3.48E-15 | 8.67E-14 | yes | down | 93.6 | 90.99 | 109.75 | 67.03 | 61.99 | 63.75 | 98.11333 | 64.25667 |
| PREX2 | 0.214 | -2.22158 | 3.67E-15 | 9.14E-14 | yes | down | 10.06 | 10.57 | 10.06 | 4.87 | 7.58 | 3.02 | 10.23 | 5.156667 |
| GBAS | 0.164 | -2.61188 | 3.73E-15 | 9.28E-14 | yes | down | 102.83 | 50.86 | 34.49 | 24.71 | 23.79 | 20.06 | 62.72667 | 22.85333 |
| KCTD6 | 0.282 | -1.82705 | 3.79E-15 | 9.4E-14 | yes | down | 12.47 | 12.97 | 12.92 | 7.4 | 10.77 | 7.9 | 12.78667 | 8.69 |
| C1R | 7.611 | 2.92816 | 3.79E-15 | 9.4E-14 | yes | up | 23.71 | 12.8 | 10.39 | 208.06 | 190.51 | 385.11 | 15.63333 | 261.2267 |
| OXCT1 | 0.231 | -2.11275 | 3.91E-15 | 9.68E-14 | yes | down | 116.21 | 82.15 | 102.41 | 60.35 | 67.26 | 34.31 | 100.2567 | 53.97333 |
| TIMP1 | 3.787 | 1.921026 | 4.01E-15 | 9.9E-14 | yes | up | 38.36 | 28.29 | 23.17 | 261.18 | 215.46 | 285.09 | 29.94 | 253.91 |
| HEBP1 | 2.733 | 1.450568 | 4.45E-15 | 1.1E-13 | yes | up | 19.88 | 21.49 | 16.75 | 126.03 | 118.08 | 117.59 | 19.37333 | 120.5667 |
| SUN2 | 2.491 | 1.316612 | 4.58E-15 | 1.13E-13 | yes | up | 17.46 | 16.31 | 14.2 | 81.26 | 107.18 | 86.43 | 15.99 | 91.62333 |
| NDUFS2 | 0.401 | -1.31768 | 5.03E-15 | 1.24E-13 | yes | down | 328.36 | 290.22 | 295.94 | 293.11 | 302.61 | 247.75 | 304.84 | 281.1567 |
| AVPR1A | 5.725 | 2.517232 | 5.1E-15 | 1.25E-13 | yes | up | 1.16 | 1.01 | 1.23 | 21.04 | 11.94 | 11.67 | 1.133333 | 14.88333 |
| UGT1-6 | 5.624 | 2.491691 | 5.15E-15 | 1.26E-13 | yes | up | 9.54 | 21.09 | 13.06 | 125.84 | 173.94 | 189.09 | 14.56333 | 162.9567 |
| MYOF | 4.172 | 2.060637 | 5.17E-15 | 1.27E-13 | yes | up | 2.07 | 1.25 | 0.97 | 14.03 | 14.78 | 11.57 | 1.43 | 13.46 |
| P3H1 | 2.67 | 1.417096 | 5.76E-15 | 1.41E-13 | yes | up | 8.18 | 8.98 | 6.82 | 46.71 | 59.81 | 40.7 | 7.993333 | 49.07333 |
| GAL3ST4 | 5.345 | 2.418175 | 5.87E-15 | 1.43E-13 | yes | up | 1.91 | 1.16 | 0.82 | 13.7 | 18.9 | 14.11 | 1.296667 | 15.57 |
| MSN | 2.447 | 1.291259 | 6.21E-15 | 1.51E-13 | yes | up | 38.3 | 42.71 | 36.24 | 234.6 | 230.57 | 192.6 | 39.08333 | 219.2567 |
| ESRRA | 0.183 | -2.45131 | 6.24E-15 | 1.52E-13 | yes | down | 16.94 | 20 | 16.06 | 6.3 | 10.53 | 5.38 | 17.66667 | 7.403333 |
| BMP1 | 2.834 | 1.502694 | 6.64E-15 | 1.61E-13 | yes | up | 17.14 | 12.36 | 11.31 | 81.53 | 93.24 | 88.31 | 13.60333 | 87.69333 |
| FAM214A | 0.351 | -1.51164 | 6.82E-15 | 1.66E-13 | yes | down | 19.5 | 20.12 | 15.76 | 14.78 | 18.81 | 11.2 | 18.46 | 14.93 |
| SFXN1 | 4.301 | 2.104594 | 6.94E-15 | 1.68E-13 | yes | up | 4.15 | 6.84 | 4.23 | 51.1 | 62.1 | 34.52 | 5.073333 | 49.24 |
| POLN | 13.456 | 3.750205 | 6.96E-15 | 1.68E-13 | yes | up | 0.13 | 0.11 | 0.07 | 8.47 | 6 | 5.13 | 0.103333 | 6.533333 |
| SLC40A1 | 0.216 | -2.2097 | 7.8E-15 | 1.89E-13 | yes | down | 44.46 | 50.96 | 68.14 | 23.92 | 37.3 | 22.74 | 54.52 | 27.98667 |
| ETFB | 0.343 | -1.54312 | 8.52E-15 | 2.06E-13 | yes | down | 254.95 | 307.48 | 310.64 | 217.92 | 223.05 | 209.66 | 291.0233 | 216.8767 |
| SAMHD1 | 3.039 | 1.603371 | 8.67E-15 | 2.09E-13 | yes | up | 7.85 | 6.38 | 4.81 | 37.93 | 45.41 | 47.05 | 6.346667 | 43.46333 |
| TMSB10 | 2.83 | 1.500785 | 9.36E-15 | 2.25E-13 | yes | up | 955.87 | 1223.37 | 928.2 | 7032.13 | 7732.19 | 5665.74 | 1035.813 | 6810.02 |
| MSANTD4 | 0.235 | -2.08714 | 9.43E-15 | 2.27E-13 | yes | down | 5.84 | 7.63 | 5.09 | 3.48 | 4.27 | 2.28 | 6.186667 | 3.343333 |
| MPHOSPH10 | 0.303 | -1.72286 | 1.08E-14 | 2.6E-13 | yes | down | 9.19 | 9.66 | 7.79 | 6.13 | 7.8 | 4.66 | 8.88 | 6.196667 |
| FOSB | 18.235 | 4.188603 | 1.09E-14 | 2.62E-13 | yes | up | 0.26 | 0.22 | 0.09 | 6.33 | 4.41 | 12.23 | 0.19 | 7.656667 |
| HBS1L | 0.337 | -1.57115 | 1.11E-14 | 2.67E-13 | yes | down | 25.57 | 22.45 | 22.25 | 17.61 | 23.18 | 14.04 | 23.42333 | 18.27667 |
| MRPL37 | 0.335 | -1.57962 | 1.13E-14 | 2.69E-13 | yes | down | 102.92 | 141.19 | 117.04 | 94.93 | 94.35 | 88.21 | 120.3833 | 92.49667 |
| AOX3 | 13.506 | 3.755517 | 1.15E-14 | 2.74E-13 | yes | up | 0.41 | 2.29 | 0.49 | 23.89 | 40.13 | 25.85 | 1.063333 | 29.95667 |
| EPRS | 0.346 | -1.53 | 1.2E-14 | 2.87E-13 | yes | down | 36.18 | 29.13 | 26.77 | 23.72 | 29.77 | 18.24 | 30.69333 | 23.91 |
| BMPER | 5.51 | 2.462102 | 1.24E-14 | 2.95E-13 | yes | up | 0.43 | 0.44 | 0.37 | 4.14 | 5.86 | 5.57 | 0.413333 | 5.19 |
| PRKRA | 0.362 | -1.46626 | 1.28E-14 | 3.05E-13 | yes | down | 33.61 | 28.62 | 26.08 | 22.57 | 24.74 | 25.66 | 29.43667 | 24.32333 |
| LOC108178113 | 0.312 | -1.67915 | 1.37E-14 | 3.24E-13 | yes | down | 2307.77 | 2401.5 | 2625.26 | 1816.15 | 1750.86 | 1884.74 | 2444.843 | 1817.25 |
| NOTCH2 | 3.476 | 1.797408 | 1.45E-14 | 3.43E-13 | yes | up | 3.58 | 2.8 | 2.02 | 18.34 | 21.23 | 24.55 | 2.8 | 21.37333 |
| COL4A2 | 2.863 | 1.517523 | 1.45E-14 | 3.43E-13 | yes | up | 93.8 | 89.81 | 102.45 | 607.49 | 754.94 | 539.93 | 95.35333 | 634.12 |
| ADAMTS12 | 4.249 | 2.087093 | 1.46E-14 | 3.46E-13 | yes | up | 7.13 | 6.91 | 4.32 | 78.42 | 60.48 | 39.89 | 6.12 | 59.59667 |
| TAP1 | 4.241 | 2.084537 | 1.47E-14 | 3.48E-13 | yes | up | 0.8 | 0.96 | 0.81 | 7.84 | 9.34 | 7.93 | 0.856667 | 8.37 |
| ENPP3 | 10.991 | 3.458227 | 1.49E-14 | 3.51E-13 | yes | up | 1.82 | 1.34 | 0.31 | 26.94 | 23.01 | 32.45 | 1.156667 | 27.46667 |
| SBDS | 0.375 | -1.41507 | 1.54E-14 | 3.63E-13 | yes | down | 35.73 | 31.61 | 33.11 | 29.15 | 32.09 | 24.56 | 33.48333 | 28.6 |
| LOC103346332 | 5.853 | 2.549159 | 1.56E-14 | 3.66E-13 | yes | up | 16.88 | 15.51 | 9.82 | 180.77 | 126.24 | 242.29 | 14.07 | 183.1 |
| RLA-DR-ALPHA | 7.012 | 2.809785 | 1.62E-14 | 3.79E-13 | yes | up | 3.35 | 6.2 | 7.85 | 81.94 | 90.45 | 113.31 | 5.8 | 95.23333 |
| BBS10 | 0.185 | -2.43719 | 1.64E-14 | 3.85E-13 | yes | down | 4.09 | 3.78 | 4.14 | 1.8 | 2.36 | 0.98 | 4.003333 | 1.713333 |
| SGPL1 | 0.42 | -1.25075 | 1.81E-14 | 4.24E-13 | yes | down | 12.49 | 12.98 | 9.67 | 10.59 | 12.86 | 10.27 | 11.71333 | 11.24 |
| MTIF2 | 0.201 | -2.31502 | 1.82E-14 | 4.25E-13 | yes | down | 8.94 | 6.95 | 6.84 | 3.19 | 3.94 | 1.81 | 7.576667 | 2.98 |
| RNASE4 | 2.714 | 1.440282 | 1.88E-14 | 4.38E-13 | yes | up | 80.65 | 61.47 | 56.73 | 418.47 | 402.18 | 409.43 | 66.28333 | 410.0267 |
| MEIS3 | 4.082 | 2.029146 | 1.99E-14 | 4.63E-13 | yes | up | 0.95 | 1.12 | 0.84 | 9.99 | 9.38 | 7.05 | 0.97 | 8.806667 |
| RABGGTB | 0.316 | -1.66234 | 2.22E-14 | 5.16E-13 | yes | down | 31.67 | 22.46 | 24.32 | 18.48 | 21.29 | 15.16 | 26.15 | 18.31 |
| LOC100353852 | 4.094 | 2.033613 | 2.49E-14 | 5.79E-13 | yes | up | 1.83 | 2.86 | 1.64 | 18.04 | 18.25 | 22.01 | 2.11 | 19.43333 |
| LOC100355548 | 0.23 | -2.12233 | 2.65E-14 | 6.15E-13 | yes | down | 33.19 | 38.56 | 33.01 | 21.99 | 20.61 | 12.81 | 34.92 | 18.47 |
| GGT5 | 5.304 | 2.407138 | 2.65E-14 | 6.15E-13 | yes | up | 0.43 | 0.48 | 0.45 | 4.94 | 5.91 | 5.64 | 0.453333 | 5.496667 |
| CSPG4 | 5.264 | 2.396261 | 2.81E-14 | 6.49E-13 | yes | up | 1.74 | 1.01 | 1.41 | 11.54 | 17.71 | 20.93 | 1.386667 | 16.72667 |
| LARP4 | 0.296 | -1.75476 | 2.81E-14 | 6.49E-13 | yes | down | 8.9 | 10.37 | 7.02 | 11.62 | 12.36 | 5.06 | 8.763333 | 9.68 |
| RASGRF2 | 4.173 | 2.061089 | 2.91E-14 | 6.71E-13 | yes | up | 0.64 | 0.86 | 0.82 | 6.54 | 8.85 | 7.47 | 0.773333 | 7.62 |
| DHRS7 | 3.48 | 1.799288 | 2.94E-14 | 6.77E-13 | yes | up | 30.57 | 22.55 | 27.94 | 170.44 | 269.29 | 219.04 | 27.02 | 219.59 |
| HS3ST1 | 6.25 | 2.64397 | 2.96E-14 | 6.8E-13 | yes | up | 1.3 | 1 | 0.72 | 13.49 | 11.71 | 17.32 | 1.006667 | 14.17333 |
| COX6A1 | 0.244 | -2.03518 | 3.06E-14 | 7.02E-13 | yes | down | 2349.02 | 3093.1 | 3630.38 | 1925.59 | 1724.16 | 1547.58 | 3024.167 | 1732.443 |
| PEG3 | 0.106 | -3.24014 | 3.25E-14 | 7.45E-13 | yes | down | 40.44 | 27.96 | 24.31 | 7.59 | 13.09 | 2.5 | 30.90333 | 7.726667 |
| CDKN3 | 3.83 | 1.937389 | 3.37E-14 | 7.71E-13 | yes | up | 6.91 | 8.63 | 4.63 | 56.28 | 73.54 | 64.05 | 6.723333 | 64.62333 |
| BAIAP2L1 | 4.414 | 2.142013 | 3.42E-14 | 7.83E-13 | yes | up | 3.44 | 5.9 | 3.2 | 31.51 | 53.27 | 41.84 | 4.18 | 42.20667 |
| LOC100346670 | 0.31 | -1.69132 | 3.44E-14 | 7.85E-13 | yes | down | 1374.26 | 1549.24 | 1611.56 | 1216.81 | 1025.23 | 1017.47 | 1511.687 | 1086.503 |
| LOC100350701 | 3.82 | 1.933398 | 3.44E-14 | 7.85E-13 | yes | up | 9.78 | 11.93 | 10.05 | 97.44 | 90.4 | 97.14 | 10.58667 | 94.99333 |
| NR2F1 | 5.233 | 2.387558 | 3.45E-14 | 7.85E-13 | yes | up | 2.4 | 3.4 | 2.82 | 39.61 | 28.33 | 34.77 | 2.873333 | 34.23667 |
| CHCHD2 | 0.216 | -2.20819 | 3.48E-14 | 7.91E-13 | yes | down | 981.03 | 591.85 | 1043.5 | 353.35 | 502.94 | 472.39 | 872.1267 | 442.8933 |
| PDHX | 0.286 | -1.80346 | 3.51E-14 | 7.97E-13 | yes | down | 34.82 | 24.72 | 17.92 | 17.73 | 16.61 | 15.51 | 25.82 | 16.61667 |
| S100A6 | 4.897 | 2.291837 | 3.55E-14 | 8.06E-13 | yes | up | 120.5 | 133.84 | 54.64 | 1133.96 | 974.92 | 1273.3 | 102.9933 | 1127.393 |
| EIF2A | 0.32 | -1.6429 | 3.61E-14 | 8.17E-13 | yes | down | 16.77 | 18.12 | 15.03 | 13.57 | 14.35 | 8.96 | 16.64 | 12.29333 |
| ZNF770 | 0.256 | -1.96638 | 3.76E-14 | 8.5E-13 | yes | down | 4.61 | 3.71 | 3.33 | 2.62 | 2.61 | 1.58 | 3.883333 | 2.27 |
| MMADHC | 0.258 | -1.95503 | 3.79E-14 | 8.56E-13 | yes | down | 40.39 | 40.23 | 38.64 | 29.81 | 24.41 | 15.87 | 39.75333 | 23.36333 |
| LOC100337961 | 8.76 | 3.130902 | 3.87E-14 | 8.72E-13 | yes | up | 1.24 | 0.83 | 1.25 | 16.93 | 23.82 | 25.9 | 1.106667 | 22.21667 |
| TST | 4.445 | 2.15214 | 3.97E-14 | 8.94E-13 | yes | up | 14.48 | 7.95 | 8.41 | 119.51 | 89.89 | 98.51 | 10.28 | 102.6367 |
| ISLR | 5.845 | 2.547267 | 4.08E-14 | 9.18E-13 | yes | up | 8.87 | 4.38 | 3.92 | 60.32 | 67.33 | 95.45 | 5.723333 | 74.36667 |
| MRPS10 | 0.378 | -1.40493 | 4.14E-14 | 9.31E-13 | yes | down | 30.11 | 37.56 | 33.05 | 19.52 | 24.82 | 18.23 | 33.57333 | 20.85667 |
| USP16 | 0.279 | -1.84166 | 4.2E-14 | 9.42E-13 | yes | down | 9.38 | 10.23 | 8.96 | 5.76 | 8.56 | 4.36 | 9.523333 | 6.226667 |
| UNC119 | 3.25 | 1.700222 | 4.35E-14 | 9.74E-13 | yes | up | 6.52 | 7.51 | 5.92 | 46.94 | 50.09 | 50.68 | 6.65 | 49.23667 |
| PRG4 | 0.078 | -3.67125 | 4.44E-14 | 9.93E-13 | yes | down | 20.97 | 6.57 | 4.12 | 1.87 | 1.84 | 1.49 | 10.55333 | 1.733333 |
| CTSZ | 0.235 | -2.08662 | 4.58E-14 | 1.02E-12 | yes | down | 176.6 | 311.48 | 284.91 | 126.18 | 148.93 | 147.14 | 257.6633 | 140.75 |
| OS9 | 2.54 | 1.344926 | 4.68E-14 | 1.04E-12 | yes | up | 27.14 | 28.26 | 28.25 | 140.07 | 197.57 | 145.39 | 27.88333 | 161.01 |
| MDFIC | 0.283 | -1.82036 | 4.72E-14 | 1.05E-12 | yes | down | 45.24 | 49.26 | 43.12 | 33.63 | 46.76 | 21.76 | 45.87333 | 34.05 |
| ITPK1 | 0.336 | -1.57446 | 4.98E-14 | 1.11E-12 | yes | down | 33.15 | 38.95 | 30.55 | 21.11 | 28.64 | 29.38 | 34.21667 | 26.37667 |
| HIBCH | 0.288 | -1.7969 | 5.12E-14 | 1.14E-12 | yes | down | 53.89 | 61.46 | 46.48 | 35.08 | 42.53 | 21.8 | 53.94333 | 33.13667 |
| TNN | 0.08 | -3.63996 | 5.29E-14 | 1.17E-12 | yes | down | 13.07 | 4.22 | 4.54 | 2.13 | 1.15 | 0.7 | 7.276667 | 1.326667 |
| ANKRD37 | 0.225 | -2.15323 | 5.57E-14 | 1.23E-12 | yes | down | 9.88 | 14.3 | 13.57 | 6.41 | 5.41 | 7.11 | 12.58333 | 6.31 |
| EPB42 | 0.092 | -3.44141 | 5.6E-14 | 1.24E-12 | yes | down | 1.82 | 3.58 | 3.15 | 0.77 | 0.35 | 0.68 | 2.85 | 0.6 |
| TPM4 | 3.835 | 1.939256 | 5.78E-14 | 1.28E-12 | yes | up | 31.15 | 43.94 | 32.45 | 404.05 | 298.32 | 239.11 | 35.84667 | 313.8267 |
| CYGB | 3.444 | 1.784251 | 5.86E-14 | 1.29E-12 | yes | up | 4.35 | 5.89 | 5.19 | 42.18 | 39.08 | 41.33 | 5.143333 | 40.86333 |
| SNCA | 0.113 | -3.15022 | 5.97E-14 | 1.32E-12 | yes | down | 25.06 | 61.62 | 44.5 | 13.52 | 5.75 | 12.31 | 43.72667 | 10.52667 |
| EGFLAM | 2.69 | 1.427715 | 5.98E-14 | 1.32E-12 | yes | up | 7.05 | 8.43 | 6.91 | 49.05 | 54.2 | 38.6 | 7.463333 | 47.28333 |
| MAP7 | 0.176 | -2.50343 | 6.12E-14 | 1.34E-12 | yes | down | 2.84 | 5.28 | 3.36 | 1.9 | 1.56 | 1.16 | 3.826667 | 1.54 |
| COL5A1 | 4.569 | 2.192028 | 6.13E-14 | 1.35E-12 | yes | up | 45.92 | 28.22 | 20.93 | 340.23 | 257.73 | 366.7 | 31.69 | 321.5533 |
| ZNF330 | 0.23 | -2.11756 | 6.15E-14 | 1.35E-12 | yes | down | 25.37 | 22.47 | 35.24 | 15.12 | 16.62 | 13.98 | 27.69333 | 15.24 |
| MMRN2 | 3.923 | 1.971925 | 6.17E-14 | 1.35E-12 | yes | up | 5.9 | 7.99 | 8.65 | 61.32 | 67.58 | 75.62 | 7.513333 | 68.17333 |
| SYN2 | 0.231 | -2.11448 | 6.25E-14 | 1.37E-12 | yes | down | 7.48 | 7.78 | 9.84 | 5.17 | 4.68 | 4.12 | 8.366667 | 4.656667 |
| LOC108176315 | 0.334 | -1.58154 | 6.33E-14 | 1.38E-12 | yes | down | 13.5 | 13 | 11.1 | 9.96 | 9.25 | 8.33 | 12.53333 | 9.18 |
| AGTR2 | 0.04 | -4.63421 | 6.38E-14 | 1.39E-12 | yes | down | 1.63 | 1.31 | 0.69 | 0.09 | 0.17 | 0.06 | 1.21 | 0.106667 |
| MTERF2 | 0.202 | -2.31064 | 6.56E-14 | 1.43E-12 | yes | down | 5.95 | 4.24 | 4.87 | 4.16 | 3.55 | 3.21 | 5.02 | 3.64 |
| SLC25A33 | 0.191 | -2.38628 | 6.7E-14 | 1.46E-12 | yes | down | 41.83 | 32.23 | 17.29 | 12.07 | 11.05 | 15.53 | 30.45 | 12.88333 |
| SMIM4 | 0.273 | -1.87352 | 6.8E-14 | 1.48E-12 | yes | down | 30.26 | 36.22 | 39.74 | 22.62 | 22 | 25.5 | 35.40667 | 23.37333 |
| SUCLG2 | 0.325 | -1.62259 | 6.84E-14 | 1.48E-12 | yes | down | 134.54 | 114.99 | 100.32 | 92.38 | 109.22 | 59.94 | 116.6167 | 87.18 |
| EPB41L3 | 4.657 | 2.219475 | 6.87E-14 | 1.49E-12 | yes | up | 2.57 | 1.47 | 1.02 | 18.48 | 18.67 | 16 | 1.686667 | 17.71667 |
| ATP5J2 | 0.339 | -1.56265 | 6.88E-14 | 1.49E-12 | yes | down | 789.29 | 817.51 | 761.93 | 661.59 | 538.83 | 633.12 | 789.5767 | 611.18 |
| NDUFB9 | 0.389 | -1.36118 | 7E-14 | 1.51E-12 | yes | down | 471.98 | 533.77 | 492.27 | 471.43 | 438.51 | 396 | 499.34 | 435.3133 |
| XBP1 | 3.252 | 1.701361 | 7.01E-14 | 1.51E-12 | yes | up | 41.27 | 44.83 | 27.81 | 295.53 | 237.6 | 291.07 | 37.97 | 274.7333 |
| EFEMP1 | 4.257 | 2.089699 | 7.25E-14 | 1.56E-12 | yes | up | 43.89 | 28.81 | 18.27 | 320.72 | 264.02 | 274.55 | 30.32333 | 286.43 |
| FAM107A | 3.926 | 1.973094 | 7.57E-14 | 1.63E-12 | yes | up | 8.4 | 9.65 | 10.21 | 63.04 | 90.42 | 101.03 | 9.42 | 84.83 |
| C13H1orf162 | 9.793 | 3.291819 | 7.75E-14 | 1.66E-12 | yes | up | 0.57 | 0.83 | 1.16 | 28.93 | 17.23 | 13.3 | 0.853333 | 19.82 |
| RRBP1 | 2.413 | 1.270854 | 7.75E-14 | 1.66E-12 | yes | up | 19.84 | 16.72 | 17.03 | 94.99 | 113.48 | 86.8 | 17.86333 | 98.42333 |
| P4HA2 | 4.663 | 2.221389 | 8.29E-14 | 1.78E-12 | yes | up | 17.19 | 11.15 | 6.59 | 108.82 | 117.5 | 134.96 | 11.64333 | 120.4267 |
| KIAA0040 | 3.027 | 1.59788 | 8.31E-14 | 1.78E-12 | yes | up | 4.25 | 4.55 | 3.75 | 31.65 | 30.22 | 21.44 | 4.183333 | 27.77 |
| KDELR2 | 2.578 | 1.366495 | 8.55E-14 | 1.83E-12 | yes | up | 20.16 | 20.27 | 17.9 | 121.38 | 109.34 | 112.03 | 19.44333 | 114.25 |
| LGALS1 | 5.36 | 2.422149 | 8.58E-14 | 1.83E-12 | yes | up | 390.45 | 234.52 | 130.52 | 3419.19 | 3171.15 | 2405.98 | 251.83 | 2998.773 |
| EIF3E | 0.341 | -1.55258 | 8.73E-14 | 1.86E-12 | yes | down | 140.43 | 135.04 | 127.85 | 114.33 | 128.16 | 76.05 | 134.44 | 106.18 |
| LOC100351488 | 3.72 | 1.895118 | 8.99E-14 | 1.91E-12 | yes | up | 14.11 | 15.56 | 13.33 | 172.87 | 160.14 | 74.84 | 14.33333 | 135.95 |
| MARS2 | 0.148 | -2.75447 | 9.16E-14 | 1.95E-12 | yes | down | 4.29 | 5.38 | 2.14 | 1.4 | 1.01 | 1.46 | 3.936667 | 1.29 |
| LOC100343920 | 21.918 | 4.454033 | 9.46E-14 | 2.01E-12 | yes | up | 1.16 | 0.62 | 0.36 | 72.18 | 14.25 | 17.31 | 0.713333 | 34.58 |
| TINAGL1 | 3.302 | 1.723279 | 9.58E-14 | 2.03E-12 | yes | up | 67.05 | 107.6 | 72.36 | 518.97 | 717.07 | 633.59 | 82.33667 | 623.21 |
| FBLN1 | 4.21 | 2.073968 | 9.66E-14 | 2.05E-12 | yes | up | 26.01 | 20.79 | 12.11 | 170.19 | 161.57 | 218.1 | 19.63667 | 183.2867 |
| COL16A1 | 3.993 | 1.997597 | 9.74E-14 | 2.06E-12 | yes | up | 8.19 | 5.99 | 3.64 | 42.26 | 60.81 | 56.86 | 5.94 | 53.31 |
| LIMA1 | 3.422 | 1.77487 | 1.05E-13 | 2.22E-12 | yes | up | 8.04 | 6.12 | 4.67 | 50.79 | 42.45 | 50.93 | 6.276667 | 48.05667 |
| MBIP | 0.324 | -1.62582 | 1.06E-13 | 2.23E-12 | yes | down | 7.63 | 8.54 | 7.13 | 5.61 | 7.15 | 4.73 | 7.766667 | 5.83 |
| LOC100346169 | 0.224 | -2.16 | 1.11E-13 | 2.33E-12 | yes | down | 29.14 | 48.9 | 34.35 | 13.64 | 25.99 | 18.82 | 37.46333 | 19.48333 |
| CAPN5 | 3.469 | 1.794663 | 1.14E-13 | 2.4E-12 | yes | up | 2.1 | 1.76 | 1.11 | 11.67 | 14.42 | 12.85 | 1.656667 | 12.98 |
| NUCB1 | 2.941 | 1.55632 | 1.16E-13 | 2.44E-12 | yes | up | 28.33 | 26.7 | 27.69 | 161.86 | 185.48 | 209.82 | 27.57333 | 185.72 |
| CDH13 | 2.961 | 1.566178 | 1.36E-13 | 2.86E-12 | yes | up | 32.13 | 22.26 | 18.18 | 144.31 | 181.68 | 160.74 | 24.19 | 162.2433 |
| GSTO1 | 0.223 | -2.16222 | 1.39E-13 | 2.91E-12 | yes | down | 174.2 | 310.02 | 195.45 | 126.86 | 90.37 | 124.62 | 226.5567 | 113.95 |
| ND1 | 0.216 | -2.21406 | 1.39E-13 | 2.92E-12 | yes | down | 3389.71 | 2811.48 | 4453.02 | 1756.31 | 1485.66 | 2075 | 3551.403 | 1772.323 |
| SDHD | 0.219 | -2.18802 | 1.48E-13 | 3.09E-12 | yes | down | 360.27 | 231.37 | 192.57 | 170.11 | 130.61 | 86.23 | 261.4033 | 128.9833 |
| DMBT1 | 0.054 | -4.22123 | 1.49E-13 | 3.1E-12 | yes | down | 8.34 | 7.09 | 5.29 | 1.06 | 0.65 | 0.16 | 6.906667 | 0.623333 |
| POLR2M | 0.417 | -1.26101 | 1.55E-13 | 3.22E-12 | yes | down | 36.43 | 29.59 | 26.74 | 28.34 | 32.03 | 28.1 | 30.92 | 29.49 |
| DNAJA2 | 0.432 | -1.20955 | 1.59E-13 | 3.31E-12 | yes | down | 40.1 | 45.35 | 39.05 | 41.68 | 43.82 | 38.01 | 41.5 | 41.17 |
| CTSB | 2.804 | 1.487591 | 1.59E-13 | 3.31E-12 | yes | up | 200.19 | 227.81 | 213.07 | 1194.09 | 1413.03 | 1517.28 | 213.69 | 1374.8 |
| SEMA7A | 0.245 | -2.02978 | 1.59E-13 | 3.31E-12 | yes | down | 216.83 | 203.56 | 147.66 | 95.46 | 161.04 | 66.7 | 189.35 | 107.7333 |
| ANKRD29 | 3.386 | 1.759538 | 1.64E-13 | 3.4E-12 | yes | up | 2.32 | 1.78 | 1.49 | 14.09 | 13.68 | 14.79 | 1.863333 | 14.18667 |
| CX3CL1 | 6.171 | 2.625498 | 1.67E-13 | 3.46E-12 | yes | up | 0.22 | 0.48 | 0.32 | 4.53 | 5.3 | 4.44 | 0.34 | 4.756667 |
| RSPO3 | 16.925 | 4.081116 | 1.72E-13 | 3.56E-12 | yes | up | 1.66 | 0.28 | 0.2 | 26.45 | 24.66 | 16.65 | 0.713333 | 22.58667 |
| FOLR2 | 3.17 | 1.664701 | 1.75E-13 | 3.61E-12 | yes | up | 8.68 | 7.47 | 5.45 | 45.66 | 48.61 | 47.88 | 7.2 | 47.38333 |
| PITRM1 | 0.322 | -1.63657 | 1.77E-13 | 3.65E-12 | yes | down | 19.37 | 18.71 | 17.31 | 14.1 | 17.68 | 9.56 | 18.46333 | 13.78 |
| HOXA9 | 51.684 | 5.691638 | 1.82E-13 | 3.75E-12 | yes | up | 0 | 0 | 0.24 | 4.18 | 3.89 | 3.71 | 0.08 | 3.926667 |
| NMNAT2 | 4.421 | 2.144483 | 1.83E-13 | 3.77E-12 | yes | up | 1.49 | 1.33 | 0.78 | 11.74 | 12.49 | 12.22 | 1.2 | 12.15 |
| ARHGDIB | 2.818 | 1.49449 | 1.84E-13 | 3.79E-12 | yes | up | 22.97 | 24.09 | 24.72 | 166.5 | 174.94 | 127.57 | 23.92667 | 156.3367 |
| AARS2 | 0.359 | -1.47624 | 1.86E-13 | 3.82E-12 | yes | down | 6.87 | 6.89 | 6.61 | 5.88 | 5.84 | 5.02 | 6.79 | 5.58 |
| CRTAP | 3.562 | 1.83284 | 1.86E-13 | 3.83E-12 | yes | up | 13.06 | 12.5 | 16.16 | 103.22 | 115.94 | 125.77 | 13.90667 | 114.9767 |
| RASL12 | 2.957 | 1.564067 | 1.88E-13 | 3.85E-12 | yes | up | 6.04 | 6.66 | 6 | 45.95 | 38.55 | 41.73 | 6.233333 | 42.07667 |
| UBXN4 | 0.382 | -1.3899 | 1.89E-13 | 3.88E-12 | yes | down | 26.47 | 27.4 | 21.46 | 23.06 | 26.36 | 16.61 | 25.11 | 22.01 |
| CCNI | 0.384 | -1.37916 | 1.91E-13 | 3.91E-12 | yes | down | 162.35 | 216.66 | 173.24 | 167.01 | 174.33 | 146.18 | 184.0833 | 162.5067 |
| C9H3orf18 | 0.256 | -1.96632 | 1.92E-13 | 3.93E-12 | yes | down | 18.46 | 23.65 | 15.08 | 8.41 | 13.25 | 13.86 | 19.06333 | 11.84 |
| LHFPL2 | 3.206 | 1.680959 | 1.97E-13 | 4.03E-12 | yes | up | 4.97 | 5 | 5.14 | 38.49 | 32.64 | 39.42 | 5.036667 | 36.85 |
| SCARA5 | 5.052 | 2.336737 | 1.98E-13 | 4.03E-12 | yes | up | 9.43 | 4.96 | 3.71 | 54.23 | 70.51 | 78.94 | 6.033333 | 67.89333 |
| AP4E1 | 0.373 | -1.42466 | 1.98E-13 | 4.03E-12 | yes | down | 3.88 | 3.36 | 2.84 | 2.97 | 3.04 | 2.75 | 3.36 | 2.92 |
| HFM1 | 0.131 | -2.93326 | 2.06E-13 | 4.19E-12 | yes | down | 1.12 | 1.38 | 0.91 | 0.47 | 0.33 | 0.26 | 1.136667 | 0.353333 |
| REM1 | 4.747 | 2.246923 | 2.09E-13 | 4.25E-12 | yes | up | 1.86 | 1.86 | 2.26 | 26.57 | 16.81 | 19.93 | 1.993333 | 21.10333 |
| COL3A1 | 6.444 | 2.688057 | 2.22E-13 | 4.5E-12 | yes | up | 1067.4 | 563.07 | 308.18 | 7146.48 | 9530.57 | 9949.44 | 646.2167 | 8875.497 |
| LTN1 | 0.255 | -1.97136 | 2.28E-13 | 4.62E-12 | yes | down | 3.98 | 3.98 | 3.79 | 2.25 | 3.25 | 1.48 | 3.916667 | 2.326667 |
| RHOU | 3.456 | 1.788988 | 2.32E-13 | 4.7E-12 | yes | up | 2.95 | 1.82 | 1.72 | 16.76 | 18.18 | 15.94 | 2.163333 | 16.96 |
| TOMM20 | 0.396 | -1.33663 | 2.34E-13 | 4.72E-12 | yes | down | 49 | 50.38 | 44.16 | 47.5 | 49.15 | 34.14 | 47.84667 | 43.59667 |
| ABCC9 | 0.375 | -1.41462 | 2.34E-13 | 4.72E-12 | yes | down | 13.21 | 11.45 | 8.36 | 9.87 | 10.59 | 7.79 | 11.00667 | 9.416667 |
| NAV1 | 2.199 | 1.136752 | 2.36E-13 | 4.76E-12 | yes | up | 4.16 | 3.2 | 2.89 | 14.8 | 17.41 | 13.54 | 3.416667 | 15.25 |
| TOMM70 | 0.389 | -1.36107 | 2.39E-13 | 4.81E-12 | yes | down | 21.56 | 20.7 | 19.1 | 19.66 | 21.05 | 14.32 | 20.45333 | 18.34333 |
| MANF | 3.257 | 1.703468 | 2.44E-13 | 4.91E-12 | yes | up | 21.14 | 28.23 | 21.52 | 206.33 | 181.1 | 142.28 | 23.63 | 176.57 |
| LOC100328967 | 3.408 | 1.769022 | 2.45E-13 | 4.92E-12 | yes | up | 34.65 | 33.45 | 32.82 | 199.28 | 389.7 | 309.18 | 33.64 | 299.3867 |
| FAM171A1 | 2.536 | 1.342376 | 2.53E-13 | 5.08E-12 | yes | up | 7.79 | 8.47 | 7.61 | 47.13 | 44.84 | 46.11 | 7.956667 | 46.02667 |
| B4GALT1 | 2.271 | 1.183034 | 2.53E-13 | 5.08E-12 | yes | up | 23.06 | 24.44 | 19.85 | 116.26 | 139.67 | 96.91 | 22.45 | 117.6133 |
| TRMT10C | 0.267 | -1.90776 | 2.57E-13 | 5.14E-12 | yes | down | 10.38 | 11.8 | 8.08 | 6.62 | 7.98 | 4.09 | 10.08667 | 6.23 |
| MMP28 | 3.763 | 1.912049 | 2.62E-13 | 5.23E-12 | yes | up | 4.19 | 3.05 | 3.64 | 28.81 | 50.02 | 27.97 | 3.626667 | 35.6 |
| HBQ1 | 5.094 | 2.348767 | 2.66E-13 | 5.32E-12 | yes | up | 8.59 | 14.18 | 22.47 | 48.11 | 47.92 | 66.63 | 15.08 | 54.22 |
| SLC25A1 | 8.652 | 3.112966 | 2.8E-13 | 5.57E-12 | yes | up | 22.18 | 27.96 | 32.09 | 592.75 | 241.8 | 774.31 | 27.41 | 536.2867 |
| DPYSL2 | 2.216 | 1.148139 | 2.85E-13 | 5.67E-12 | yes | up | 13.83 | 12.58 | 9.9 | 59.21 | 70.95 | 53.68 | 12.10333 | 61.28 |
| CCKBR | 0.015 | -6.08221 | 2.87E-13 | 5.71E-12 | yes | down | 0.5 | 0.67 | 1.5 | 0.03 | 0.01 | 0.06 | 0.89 | 0.033333 |
| DNAJC27 | 0.426 | -1.22997 | 2.98E-13 | 5.91E-12 | yes | down | 9.56 | 8.98 | 7.23 | 7.98 | 8.87 | 7.81 | 8.59 | 8.22 |
| SEMA4F | 4.082 | 2.029386 | 3.09E-13 | 6.13E-12 | yes | up | 0.67 | 0.58 | 0.42 | 5.13 | 5.42 | 5.35 | 0.556667 | 5.3 |
| MDH1 | 0.196 | -2.35387 | 3.15E-13 | 6.23E-12 | yes | down | 633.87 | 643.26 | 589 | 400.95 | 176.22 | 239.84 | 622.0433 | 272.3367 |
| ANKRD13C | 0.4 | -1.32155 | 3.37E-13 | 6.66E-12 | yes | down | 8.78 | 9 | 7.4 | 7.84 | 8.98 | 6.35 | 8.393333 | 7.723333 |
| NFATC4 | 3.233 | 1.692827 | 3.43E-13 | 6.79E-12 | yes | up | 2.72 | 2.36 | 2.11 | 20.51 | 18.86 | 13.7 | 2.396667 | 17.69 |
| TAX1BP1 | 0.329 | -1.60487 | 3.44E-13 | 6.8E-12 | yes | down | 37.15 | 38.55 | 36.06 | 31.92 | 35.41 | 20.23 | 37.25333 | 29.18667 |
| FLOT2 | 3.387 | 1.75999 | 3.61E-13 | 7.12E-12 | yes | up | 35.15 | 22.3 | 16.55 | 175.6 | 207.5 | 184.09 | 24.66667 | 189.0633 |
| AASDHPPT | 0.325 | -1.62113 | 3.65E-13 | 7.19E-12 | yes | down | 7.63 | 7.02 | 6.02 | 5.56 | 5.97 | 3.86 | 6.89 | 5.13 |
| IL16 | 2.61 | 1.383864 | 3.69E-13 | 7.25E-12 | yes | up | 4.25 | 5.21 | 3.53 | 22.72 | 26.77 | 21.06 | 4.33 | 23.51667 |
| LOC100352363 | 0.184 | -2.44457 | 3.85E-13 | 7.56E-12 | yes | down | 294.16 | 686.41 | 553.05 | 239.24 | 208.25 | 209.86 | 511.2067 | 219.1167 |
| MTX2 | 0.39 | -1.35933 | 4.11E-13 | 8.06E-12 | yes | down | 25.33 | 29.6 | 22.64 | 23.52 | 25.13 | 20.56 | 25.85667 | 23.07 |
| GALE | 3.683 | 1.8807 | 4.16E-13 | 8.16E-12 | yes | up | 3.5 | 4.31 | 4.51 | 33.95 | 33.3 | 37.25 | 4.106667 | 34.83333 |
| LOC100356833 | 0.272 | -1.87691 | 4.29E-13 | 8.39E-12 | yes | down | 7.95 | 8.16 | 5.88 | 4.11 | 3.85 | 5.51 | 7.33 | 4.49 |
| FOSL2 | 3.596 | 1.846289 | 4.37E-13 | 8.54E-12 | yes | up | 4.35 | 6.07 | 6.6 | 50.04 | 51.28 | 41.55 | 5.673333 | 47.62333 |
| STYXL1 | 0.154 | -2.69964 | 4.42E-13 | 8.63E-12 | yes | down | 6.45 | 7.63 | 5.78 | 2.15 | 1.73 | 3.23 | 6.62 | 2.37 |
| HYOU1 | 3.277 | 1.712585 | 4.7E-13 | 9.17E-12 | yes | up | 4.62 | 4.42 | 3.1 | 31.21 | 38.77 | 20.66 | 4.046667 | 30.21333 |
| SLC26A10 | 0.129 | -2.95017 | 4.73E-13 | 9.23E-12 | yes | down | 20.78 | 27.65 | 44.23 | 5.27 | 13 | 11.45 | 30.88667 | 9.906667 |
| PXMP2 | 3.067 | 1.617021 | 4.87E-13 | 9.48E-12 | yes | up | 8.79 | 7.56 | 8.15 | 64.92 | 57.03 | 64.19 | 8.166667 | 62.04667 |
| SAV1 | 0.304 | -1.71992 | 4.89E-13 | 9.5E-12 | yes | down | 16.55 | 21.77 | 18.51 | 14.02 | 16.49 | 9.59 | 18.94333 | 13.36667 |
| SLC12A2 | 0.269 | -1.89512 | 4.89E-13 | 9.5E-12 | yes | down | 10.28 | 12.7 | 12.94 | 5.46 | 10.09 | 7.1 | 11.97333 | 7.55 |
| PARVA | 2.227 | 1.155162 | 4.96E-13 | 9.63E-12 | yes | up | 23.98 | 24.71 | 22.15 | 122.56 | 135.33 | 102.81 | 23.61333 | 120.2333 |
| RAP1GAP2 | 0.056 | -4.15484 | 5.15E-13 | 9.98E-12 | yes | down | 3.91 | 4.99 | 1.17 | 0.42 | 0.21 | 0.59 | 3.356667 | 0.406667 |
| DOK4 | 2.803 | 1.486825 | 5.46E-13 | 1.06E-11 | yes | up | 5.46 | 5.54 | 4.14 | 34.92 | 34.5 | 27.27 | 5.046667 | 32.23 |
| SEMA3B | 6.024 | 2.590756 | 5.54E-13 | 1.07E-11 | yes | up | 4.19 | 2.32 | 1.44 | 28.37 | 31.55 | 47.03 | 2.65 | 35.65 |
| RAB11FIP5 | 2.274 | 1.185001 | 5.54E-13 | 1.07E-11 | yes | up | 5.65 | 5.04 | 4.43 | 24.18 | 26.97 | 25.74 | 5.04 | 25.63 |
| HOXC9 | 14.118 | 3.819455 | 6.01E-13 | 1.16E-11 | yes | up | 0.3 | 0.09 | 0.15 | 5.48 | 5.16 | 6.39 | 0.18 | 5.676667 |
| DCLK2 | 4.065 | 2.023232 | 6.02E-13 | 1.16E-11 | yes | up | 0.8 | 0.62 | 0.75 | 5.37 | 8.13 | 5.74 | 0.723333 | 6.413333 |
| MAB21L1 | 5.489 | 2.456453 | 6.17E-13 | 1.19E-11 | yes | up | 0.4 | 0.4 | 0.5 | 5.88 | 6.51 | 4.25 | 0.433333 | 5.546667 |
| COL14A1 | 4.019 | 2.006935 | 6.63E-13 | 1.27E-11 | yes | up | 33.21 | 22.73 | 12.73 | 184.95 | 244.56 | 192.6 | 22.89 | 207.37 |
| CNTNAP5 | 0.255 | -1.97115 | 6.85E-13 | 1.31E-11 | yes | down | 7.77 | 7.24 | 7.09 | 3.73 | 5.43 | 3.93 | 7.366667 | 4.363333 |
| SCUBE3 | 6.006 | 2.586358 | 7.21E-13 | 1.38E-11 | yes | up | 0.22 | 0.32 | 0.28 | 2.44 | 4.59 | 4.39 | 0.273333 | 3.806667 |
| MCFD2 | 2.553 | 1.352029 | 7.36E-13 | 1.41E-11 | yes | up | 28.45 | 29.95 | 29.49 | 167.53 | 170.95 | 176.34 | 29.29667 | 171.6067 |
| COL27A1 | 3.536 | 1.822147 | 7.57E-13 | 1.45E-11 | yes | up | 2.18 | 1.52 | 0.97 | 14.24 | 10.76 | 11.94 | 1.556667 | 12.31333 |
| LOC100353574 | 4.842 | 2.275538 | 7.57E-13 | 1.45E-11 | yes | up | 10.95 | 10.6 | 5.94 | 49.51 | 49.97 | 88.85 | 9.163333 | 62.77667 |
| EIF1AX | 0.333 | -1.58687 | 7.58E-13 | 1.45E-11 | yes | down | 10.93 | 10.32 | 10.69 | 9.24 | 9.21 | 6.13 | 10.64667 | 8.193333 |
| PEX3 | 0.324 | -1.62596 | 8.21E-13 | 1.56E-11 | yes | down | 12.99 | 13.51 | 11.18 | 10.69 | 10.51 | 6.79 | 12.56 | 9.33 |
| VASP | 3.022 | 1.595699 | 8.27E-13 | 1.57E-11 | yes | up | 8.02 | 11.77 | 9.28 | 66.27 | 72.73 | 63.25 | 9.69 | 67.41667 |
| VCL | 2.482 | 1.311416 | 8.36E-13 | 1.59E-11 | yes | up | 18.36 | 18.02 | 13.24 | 106.68 | 98.9 | 76.55 | 16.54 | 94.04333 |
| CDH23 | 0.146 | -2.78081 | 8.51E-13 | 1.62E-11 | yes | down | 2.79 | 2.04 | 1.92 | 0.54 | 1.44 | 0.66 | 2.25 | 0.88 |
| MPPE1 | 0.242 | -2.04921 | 8.64E-13 | 1.64E-11 | yes | down | 17.59 | 23.28 | 28.27 | 11.73 | 17.63 | 11.33 | 23.04667 | 13.56333 |
| MTUS2 | 5.832 | 2.544023 | 8.71E-13 | 1.65E-11 | yes | up | 0.73 | 1.61 | 0.72 | 10.33 | 18.72 | 11.02 | 1.02 | 13.35667 |
| NARS2 | 0.331 | -1.59364 | 8.93E-13 | 1.69E-11 | yes | down | 8.19 | 11.14 | 7.48 | 6.46 | 6.84 | 5.6 | 8.936667 | 6.3 |
| YEATS4 | 0.21 | -2.25136 | 9.27E-13 | 1.75E-11 | yes | down | 11.16 | 7.06 | 10.46 | 4.93 | 6.18 | 3.13 | 9.56 | 4.746667 |
| METTL17 | 0.362 | -1.46639 | 9.43E-13 | 1.78E-11 | yes | down | 16.96 | 21.01 | 13.25 | 16.06 | 14.53 | 13.78 | 17.07333 | 14.79 |
| TLR5 | 11.173 | 3.481993 | 9.92E-13 | 1.87E-11 | yes | up | 0.15 | 0.12 | 0.07 | 3.51 | 2.72 | 2.19 | 0.113333 | 2.806667 |
| AACS | 6.361 | 2.669283 | 9.94E-13 | 1.87E-11 | yes | up | 3.85 | 4.04 | 3.22 | 68.97 | 26.65 | 60.97 | 3.703333 | 52.19667 |
| MRPL42 | 0.281 | -1.83 | 1.07E-12 | 2.02E-11 | yes | down | 26.31 | 29.35 | 29.6 | 22.69 | 17.76 | 15 | 28.42 | 18.48333 |
| TIMM8B | 0.304 | -1.71678 | 1.11E-12 | 2.08E-11 | yes | down | 162.93 | 208.3 | 187.76 | 146.38 | 115.84 | 128.45 | 186.33 | 130.2233 |
| PLCG2 | 3.08 | 1.622936 | 1.15E-12 | 2.16E-11 | yes | up | 0.67 | 0.79 | 0.68 | 4.31 | 6.11 | 4.83 | 0.713333 | 5.083333 |
| ATP8B2 | 2.405 | 1.266 | 1.22E-12 | 2.3E-11 | yes | up | 8.64 | 7.05 | 5.38 | 36.67 | 43.42 | 35.66 | 7.023333 | 38.58333 |
| HIKESHI | 0.374 | -1.41697 | 1.24E-12 | 2.32E-11 | yes | down | 40.49 | 47.41 | 39.89 | 36.29 | 37.86 | 28.48 | 42.59667 | 34.21 |
| TAGLN | 3.904 | 1.965097 | 1.28E-12 | 2.39E-11 | yes | up | 107.38 | 126.81 | 99.81 | 1009.02 | 704.17 | 1215.74 | 111.3333 | 976.31 |
| LMBRD1 | 0.397 | -1.33224 | 1.32E-12 | 2.47E-11 | yes | down | 15.82 | 16.45 | 13.16 | 13.67 | 16.91 | 11.33 | 15.14333 | 13.97 |
| ADSL | 0.32 | -1.64535 | 1.4E-12 | 2.61E-11 | yes | down | 27.96 | 20.54 | 14.99 | 16.09 | 13.7 | 14.41 | 21.16333 | 14.73333 |
| STEAP1 | 6.338 | 2.663988 | 1.43E-12 | 2.67E-11 | yes | up | 1.02 | 2.18 | 2.29 | 27.67 | 24.4 | 29.41 | 1.83 | 27.16 |
| LOC108176047 | 0.273 | -1.87116 | 1.43E-12 | 2.67E-11 | yes | down | 7.65 | 10.64 | 8.29 | 5.98 | 5.83 | 3.19 | 8.86 | 5 |
| C1QTNF1 | 3.311 | 1.727408 | 1.5E-12 | 2.79E-11 | yes | up | 7.66 | 8.91 | 8.06 | 60.26 | 54.03 | 71.35 | 8.21 | 61.88 |
| NDE1 | 2.566 | 1.359271 | 1.52E-12 | 2.82E-11 | yes | up | 6.25 | 6.42 | 4.72 | 31.33 | 43.24 | 30.59 | 5.796667 | 35.05333 |
| RB1CC1 | 0.239 | -2.06492 | 1.53E-12 | 2.85E-11 | yes | down | 7.01 | 6.31 | 8.33 | 4.51 | 5.08 | 2.64 | 7.216667 | 4.076667 |
| BMP2 | 9.524 | 3.251605 | 1.54E-12 | 2.86E-11 | yes | up | 0.26 | 0.64 | 0.54 | 11.35 | 7.57 | 12.46 | 0.48 | 10.46 |
| PRPSAP2 | 2.771 | 1.470446 | 1.54E-12 | 2.86E-11 | yes | up | 3.9 | 3.57 | 3.33 | 23.58 | 24.04 | 21.07 | 3.6 | 22.89667 |
| RIN3 | 2.976 | 1.573569 | 1.55E-12 | 2.86E-11 | yes | up | 2.95 | 3.39 | 2.45 | 19.63 | 24.78 | 15.83 | 2.93 | 20.08 |
| LDLR | 4.804 | 2.264268 | 1.56E-12 | 2.89E-11 | yes | up | 2.55 | 2.06 | 3.26 | 34.97 | 23.11 | 29.21 | 2.623333 | 29.09667 |
| MMP14 | 2.701 | 1.433538 | 1.6E-12 | 2.96E-11 | yes | up | 22.4 | 23.77 | 21.16 | 160.06 | 112.43 | 177.26 | 22.44333 | 149.9167 |
| KANK1 | 3.27 | 1.70946 | 1.63E-12 | 3E-11 | yes | up | 4.37 | 3.03 | 3.83 | 25.28 | 27.68 | 31.09 | 3.743333 | 28.01667 |
| CSGALNACT1 | 2.327 | 1.21845 | 1.64E-12 | 3.02E-11 | yes | up | 4.68 | 4.31 | 3.82 | 22.28 | 25.55 | 20.52 | 4.27 | 22.78333 |
| AKAP1 | 0.3 | -1.73786 | 1.67E-12 | 3.07E-11 | yes | down | 45.33 | 71.22 | 45.91 | 31.8 | 38.55 | 42.59 | 54.15333 | 37.64667 |
| IPO7 | 0.323 | -1.62926 | 1.69E-12 | 3.11E-11 | yes | down | 31.69 | 24.54 | 23.5 | 20.92 | 24.83 | 13.5 | 26.57667 | 19.75 |
| TCN2 | 2.658 | 1.410357 | 1.76E-12 | 3.23E-11 | yes | up | 27.18 | 25.38 | 26.44 | 141.77 | 166.03 | 174.42 | 26.33333 | 160.74 |
| LOC108176153 | 0.216 | -2.21395 | 1.76E-12 | 3.23E-11 | yes | down | 57.78 | 70.26 | 78.62 | 26.77 | 32.97 | 44.2 | 68.88667 | 34.64667 |
| OTUD6B | 0.259 | -1.94834 | 1.78E-12 | 3.26E-11 | yes | down | 7.51 | 10.91 | 8.18 | 5.44 | 7.01 | 3.5 | 8.866667 | 5.316667 |
| TMEM126A | 0.339 | -1.55971 | 1.83E-12 | 3.35E-11 | yes | down | 75.59 | 65.42 | 65.5 | 61.24 | 59.96 | 40.77 | 68.83667 | 53.99 |
| ADGRD1 | 4.821 | 2.269211 | 1.86E-12 | 3.4E-11 | yes | up | 3.41 | 1.96 | 1.05 | 23.17 | 19.38 | 20.65 | 2.14 | 21.06667 |
| LOC100355623 | 3.399 | 1.765055 | 1.91E-12 | 3.49E-11 | yes | up | 4.04 | 3.75 | 2.84 | 34.75 | 25.5 | 22.39 | 3.543333 | 27.54667 |
| ARAP1 | 2.738 | 1.453042 | 1.92E-12 | 3.51E-11 | yes | up | 8.86 | 7.67 | 6.14 | 38.23 | 52.61 | 48.55 | 7.556667 | 46.46333 |
| MFN2 | 0.411 | -1.28167 | 1.97E-12 | 3.59E-11 | yes | down | 71.86 | 60.51 | 61.18 | 47.23 | 77.1 | 57.74 | 64.51667 | 60.69 |
| PRSS23 | 3.12 | 1.641575 | 2.02E-12 | 3.67E-11 | yes | up | 22.85 | 18.62 | 15.29 | 132.06 | 112.5 | 152.74 | 18.92 | 132.4333 |
| ACTN4 | 2.325 | 1.21695 | 2.03E-12 | 3.69E-11 | yes | up | 34.94 | 40.07 | 35.6 | 199.1 | 217.68 | 156.55 | 36.87 | 191.11 |
| LYPLA1 | 0.302 | -1.72644 | 2.03E-12 | 3.69E-11 | yes | down | 20.89 | 20.66 | 15.98 | 15.83 | 15.32 | 8.68 | 19.17667 | 13.27667 |
| BCAT2 | 5.691 | 2.5086 | 2.05E-12 | 3.71E-11 | yes | up | 8.53 | 3.81 | 3.2 | 56.17 | 97.11 | 49.43 | 5.18 | 67.57 |
| EGR2 | 11.178 | 3.48257 | 2.08E-12 | 3.77E-11 | yes | up | 0.38 | 0.14 | 0.11 | 6.39 | 4.26 | 5.07 | 0.21 | 5.24 |
| CAPN8 | 8.561 | 3.097856 | 2.09E-12 | 3.79E-11 | yes | up | 0.48 | 1.02 | 0.21 | 9.06 | 6.91 | 9.86 | 0.57 | 8.61 |
| PTGS1 | 3.805 | 1.92782 | 2.12E-12 | 3.84E-11 | yes | up | 5.72 | 5.53 | 3.92 | 40.65 | 34.37 | 53.8 | 5.056667 | 42.94 |
| RDH5 | 9.522 | 3.251232 | 2.14E-12 | 3.86E-11 | yes | up | 0.51 | 0.24 | 0.21 | 6.81 | 6.95 | 11.05 | 0.32 | 8.27 |
| NID1 | 2.577 | 1.365664 | 2.22E-12 | 4.01E-11 | yes | up | 48.9 | 51.07 | 47.67 | 320.32 | 337.55 | 222.08 | 49.21333 | 293.3167 |
| MSRB1 | 2.991 | 1.580734 | 2.3E-12 | 4.15E-11 | yes | up | 82.28 | 63.3 | 61.43 | 549.39 | 408.68 | 454.46 | 69.00333 | 470.8433 |
| HEXA | 2.935 | 1.553295 | 2.31E-12 | 4.15E-11 | yes | up | 36.32 | 39.21 | 33.15 | 185.65 | 258.2 | 282.96 | 36.22667 | 242.27 |
| NEURL1B | 3.923 | 1.972112 | 2.32E-12 | 4.18E-11 | yes | up | 2.46 | 2.95 | 2.03 | 20.65 | 31.6 | 15.5 | 2.48 | 22.58333 |
| TSHZ2 | 3.144 | 1.652476 | 2.33E-12 | 4.18E-11 | yes | up | 1.39 | 1.6 | 1.17 | 9.44 | 13.45 | 7.47 | 1.386667 | 10.12 |
| GNAI2 | 2.483 | 1.312213 | 2.35E-12 | 4.21E-11 | yes | up | 52.59 | 62.14 | 55.92 | 335.8 | 377.43 | 265.69 | 56.88333 | 326.3067 |
| AR | 7.61 | 2.927887 | 2.39E-12 | 4.28E-11 | yes | up | 0.53 | 0.43 | 0.16 | 6.21 | 8.68 | 4.33 | 0.373333 | 6.406667 |
| PRICKLE2 | 3.709 | 1.891177 | 2.58E-12 | 4.62E-11 | yes | up | 0.66 | 0.68 | 0.4 | 3.84 | 6.33 | 4.41 | 0.58 | 4.86 |
| TIMP3 | 3.213 | 1.683866 | 2.79E-12 | 4.99E-11 | yes | up | 26.58 | 28.73 | 28.26 | 176.7 | 199.22 | 241.35 | 27.85667 | 205.7567 |
| DKK2 | 3.645 | 1.866055 | 2.8E-12 | 5E-11 | yes | up | 2.84 | 2.11 | 1.37 | 17.63 | 20.46 | 13.99 | 2.106667 | 17.36 |
| ENPP2 | 3.425 | 1.776022 | 2.82E-12 | 5.03E-11 | yes | up | 3.03 | 2.07 | 1.57 | 15.15 | 19.22 | 17.64 | 2.223333 | 17.33667 |
| KDELR3 | 2.56 | 1.356012 | 2.88E-12 | 5.13E-11 | yes | up | 18.66 | 17.62 | 11.28 | 90.72 | 97.39 | 86.57 | 15.85333 | 91.56 |
| WEE1 | 4.705 | 2.234195 | 2.94E-12 | 5.23E-11 | yes | up | 2.25 | 2.26 | 0.91 | 22.17 | 18.14 | 16.68 | 1.806667 | 18.99667 |
| TPST1 | 2.546 | 1.348187 | 3E-12 | 5.33E-11 | yes | up | 10.75 | 10.65 | 8.61 | 62.75 | 54.76 | 55.8 | 10.00333 | 57.77 |
| CCL21 | 5.146 | 2.363373 | 3.01E-12 | 5.34E-11 | yes | up | 29.27 | 29.12 | 11.24 | 247.18 | 215.41 | 323.64 | 23.21 | 262.0767 |
| SERPING1 | 3.989 | 1.996058 | 3.24E-12 | 5.75E-11 | yes | up | 20.57 | 19.74 | 14.09 | 143.11 | 125.28 | 215.31 | 18.13333 | 161.2333 |
| PCDH18 | 5.803 | 2.536869 | 3.33E-12 | 5.89E-11 | yes | up | 0.97 | 0.63 | 0.31 | 10.56 | 7.16 | 6.81 | 0.636667 | 8.176667 |
| PRKD2 | 3.145 | 1.653096 | 3.38E-12 | 5.98E-11 | yes | up | 2.51 | 2.67 | 2.07 | 16.75 | 19.16 | 14.97 | 2.416667 | 16.96 |
| DLG4 | 3.598 | 1.847082 | 3.42E-12 | 6.06E-11 | yes | up | 0.82 | 0.85 | 0.68 | 6.36 | 7.42 | 5.41 | 0.783333 | 6.396667 |
| BCAM | 5.836 | 2.544946 | 3.44E-12 | 6.08E-11 | yes | up | 42.32 | 41.74 | 44.45 | 363.97 | 428.07 | 900.72 | 42.83667 | 564.2533 |
| TSNAX | 0.266 | -1.91076 | 3.47E-12 | 6.12E-11 | yes | down | 23.57 | 26.04 | 21.25 | 16.41 | 18.25 | 7.85 | 23.62 | 14.17 |
| SLC6A8 | 0.212 | -2.23982 | 3.49E-12 | 6.15E-11 | yes | down | 42.81 | 56.33 | 45.92 | 12.64 | 32.54 | 25.38 | 48.35333 | 23.52 |
| EXTL3 | 2.379 | 1.250332 | 3.53E-12 | 6.22E-11 | yes | up | 3.49 | 2.79 | 2.21 | 15.46 | 16.79 | 15.02 | 2.83 | 15.75667 |
| LRRK1 | 2.916 | 1.544195 | 3.66E-12 | 6.44E-11 | yes | up | 2.77 | 2.58 | 2.56 | 15.18 | 24.19 | 14.41 | 2.636667 | 17.92667 |
| LOC103352261 | 3.814 | 1.931423 | 3.68E-12 | 6.47E-11 | yes | up | 7.99 | 5.04 | 6.34 | 70.9 | 52.63 | 45.47 | 6.456667 | 56.33333 |
| NECTIN2 | 2.699 | 1.432683 | 3.82E-12 | 6.7E-11 | yes | up | 7.82 | 9.6 | 8.46 | 51.82 | 51.52 | 50.4 | 8.626667 | 51.24667 |
| ISOC2 | 0.34 | -1.55546 | 3.84E-12 | 6.73E-11 | yes | down | 87.07 | 81.39 | 97.5 | 76.53 | 73.33 | 59.98 | 88.65333 | 69.94667 |
| MRM3 | 0.377 | -1.40808 | 4.19E-12 | 7.35E-11 | yes | down | 9.68 | 10.76 | 10.35 | 8 | 10.49 | 8.38 | 10.26333 | 8.956667 |
| GCLM | 0.296 | -1.75623 | 4.25E-12 | 7.44E-11 | yes | down | 4.03 | 5.9 | 4.85 | 3.7 | 3.43 | 2.92 | 4.926667 | 3.35 |
| RBM47 | 12.836 | 3.682168 | 4.28E-12 | 7.48E-11 | yes | up | 0.04 | 0.1 | 0.11 | 2.82 | 1.82 | 2.91 | 0.083333 | 2.516667 |
| CCBE1 | 0.234 | -2.09435 | 4.44E-12 | 7.75E-11 | yes | down | 2.59 | 3.53 | 1.6 | 1.19 | 1.59 | 1.29 | 2.573333 | 1.356667 |
| NLN | 0.352 | -1.50771 | 4.5E-12 | 7.86E-11 | yes | down | 29.79 | 23.99 | 17.02 | 18.43 | 23.52 | 14.53 | 23.6 | 18.82667 |
| LYRM5 | 0.407 | -1.29758 | 4.56E-12 | 7.94E-11 | yes | down | 46.11 | 45.05 | 38.31 | 41.64 | 44.31 | 30.14 | 43.15667 | 38.69667 |
| SLC25A11 | 0.34 | -1.55621 | 4.71E-12 | 8.19E-11 | yes | down | 90.59 | 64.45 | 62.74 | 56.06 | 50.46 | 60.89 | 72.59333 | 55.80333 |
| FKBP14 | 3.123 | 1.642906 | 4.76E-12 | 8.28E-11 | yes | up | 3.57 | 3.23 | 3.09 | 28.32 | 31.5 | 26.44 | 3.296667 | 28.75333 |
| RARRES1 | 7.462 | 2.899528 | 4.79E-12 | 8.32E-11 | yes | up | 0.91 | 0.88 | 0.49 | 14.69 | 9.95 | 13.22 | 0.76 | 12.62 |
| TAGLN2 | 2.284 | 1.191828 | 4.82E-12 | 8.36E-11 | yes | up | 110.94 | 119.98 | 89.29 | 596.95 | 542.93 | 521.48 | 106.7367 | 553.7867 |
| GLG1 | 2.126 | 1.0879 | 4.93E-12 | 8.55E-11 | yes | up | 10.75 | 9.68 | 9.45 | 46.75 | 52.46 | 46.49 | 9.96 | 48.56667 |
| SUCNR1 | 0.098 | -3.35397 | 5.07E-12 | 8.78E-11 | yes | down | 15.06 | 26.94 | 27.05 | 7.24 | 7.61 | 1.47 | 23.01667 | 5.44 |
| CLEC4G | 6.763 | 2.757758 | 5.14E-12 | 8.89E-11 | yes | up | 1.16 | 0.55 | 0.76 | 11.46 | 10.59 | 15.63 | 0.823333 | 12.56 |
| C1H11orf80 | 0.112 | -3.15923 | 5.14E-12 | 8.89E-11 | yes | down | 3.45 | 7.18 | 2.6 | 0.59 | 1.08 | 1.77 | 4.41 | 1.146667 |
| RCC1L | 0.308 | -1.69862 | 5.2E-12 | 8.98E-11 | yes | down | 24.53 | 23.92 | 21.98 | 18.54 | 14.06 | 18.13 | 23.47667 | 16.91 |
| CSK | 2.549 | 1.349881 | 5.23E-12 | 9.02E-11 | yes | up | 15 | 16 | 12.43 | 87.76 | 85.04 | 79.59 | 14.47667 | 84.13 |
| LOC100354303 | 0.198 | -2.33327 | 5.3E-12 | 9.14E-11 | yes | down | 20.39 | 27.36 | 26.34 | 14.45 | 11.87 | 7.71 | 24.69667 | 11.34333 |
| LRIG1 | 3.401 | 1.76613 | 5.32E-12 | 9.16E-11 | yes | up | 8.37 | 4.63 | 4.65 | 49.79 | 46.64 | 39.52 | 5.883333 | 45.31667 |
| ESRRG | 0.251 | -1.9938 | 5.36E-12 | 9.21E-11 | yes | down | 4.28 | 2.89 | 1.9 | 1.74 | 1.61 | 1.41 | 3.023333 | 1.586667 |
| NUPR1 | 5.311 | 2.408849 | 5.4E-12 | 9.27E-11 | yes | up | 16.17 | 17.06 | 19.28 | 128.12 | 206.92 | 307.15 | 17.50333 | 214.0633 |
| LOC103346901 | 46.904 | 5.551649 | 5.44E-12 | 9.33E-11 | yes | up | 0.21 | 0.94 | 0.87 | 16.74 | 104.39 | 101.07 | 0.673333 | 74.06667 |
| HOXC8 | 425.558 | 8.733211 | 5.44E-12 | 9.33E-11 | yes | up | 0 | 0 | 0 | 7.41 | 4.88 | 8.02 | 0 | 6.77 |
| PRELP | 5.756 | 2.5251 | 5.54E-12 | 9.48E-11 | yes | up | 7.44 | 5.19 | 3.88 | 53.74 | 48.8 | 106.51 | 5.503333 | 69.68333 |
| PRR13 | 3.9 | 1.963584 | 5.7E-12 | 9.75E-11 | yes | up | 4.83 | 2.67 | 2.65 | 30.47 | 33.63 | 26.55 | 3.383333 | 30.21667 |
| DDX1 | 0.46 | -1.12172 | 5.89E-12 | 1.01E-10 | yes | down | 33.38 | 28.68 | 24.97 | 29.26 | 35.62 | 26.78 | 29.01 | 30.55333 |
| MUT | 0.343 | -1.54491 | 6.38E-12 | 1.09E-10 | yes | down | 43.01 | 57.3 | 45.92 | 38.52 | 49.53 | 28.34 | 48.74333 | 38.79667 |
| NR4A1 | 11.107 | 3.473457 | 6.51E-12 | 1.11E-10 | yes | up | 2.78 | 5.12 | 2.05 | 69.15 | 32.2 | 154.74 | 3.316667 | 85.36333 |
| PDIA3 | 2.394 | 1.259434 | 6.52E-12 | 1.11E-10 | yes | up | 63.63 | 56.08 | 44.43 | 307.83 | 359.1 | 233.27 | 54.71333 | 300.0667 |
| C1D | 0.289 | -1.79039 | 6.72E-12 | 1.14E-10 | yes | down | 15.1 | 10.16 | 8.53 | 8.32 | 8 | 5.82 | 11.26333 | 7.38 |
| PARL | 0.422 | -1.24523 | 7.01E-12 | 1.19E-10 | yes | down | 36.75 | 40.16 | 35.57 | 35.77 | 36.8 | 36.06 | 37.49333 | 36.21 |
| GFM2 | 0.176 | -2.50803 | 7.06E-12 | 1.2E-10 | yes | down | 18.15 | 15.33 | 12.24 | 7.24 | 9 | 2.61 | 15.24 | 6.283333 |
| DUSP4 | 0.073 | -3.77098 | 7.16E-12 | 1.21E-10 | yes | down | 3.7 | 8.9 | 1.59 | 0.92 | 0.67 | 0.68 | 4.73 | 0.756667 |
| USMG5 | 0.37 | -1.43344 | 7.27E-12 | 1.23E-10 | yes | down | 687.08 | 653.95 | 670.79 | 641.56 | 536.26 | 548.19 | 670.6067 | 575.3367 |
| ZNF862 | 3.349 | 1.743592 | 7.3E-12 | 1.23E-10 | yes | up | 2.41 | 1.77 | 1.28 | 14.69 | 9.66 | 8.14 | 1.82 | 10.83 |
| RECK | 2.408 | 1.267868 | 7.36E-12 | 1.24E-10 | yes | up | 2.76 | 2.54 | 1.99 | 12.74 | 15.5 | 12.45 | 2.43 | 13.56333 |
| RHBDF1 | 2.584 | 1.369617 | 7.44E-12 | 1.26E-10 | yes | up | 5.01 | 5.16 | 4.45 | 25.66 | 28.63 | 33 | 4.873333 | 29.09667 |
| LOC103351259 | 0.199 | -2.33092 | 7.47E-12 | 1.26E-10 | yes | down | 10.31 | 11.57 | 10.69 | 4.01 | 7.63 | 3.12 | 10.85667 | 4.92 |
| CASC1 | 0.191 | -2.38532 | 7.52E-12 | 1.27E-10 | yes | down | 2.33 | 2.23 | 1.92 | 0.9 | 1.31 | 0.78 | 2.16 | 0.996667 |
| FYN | 2.258 | 1.174735 | 7.87E-12 | 1.32E-10 | yes | up | 11.09 | 10.07 | 8.42 | 44.41 | 61.19 | 47.95 | 9.86 | 51.18333 |
| DNAJC2 | 0.331 | -1.59579 | 8.3E-12 | 1.4E-10 | yes | down | 7.42 | 6.51 | 6.28 | 5.46 | 5.68 | 4.22 | 6.736667 | 5.12 |
| FDXR | 0.228 | -2.13066 | 8.31E-12 | 1.4E-10 | yes | down | 19.15 | 18.44 | 24.13 | 9.67 | 10.35 | 12.77 | 20.57333 | 10.93 |
| CHST14 | 2.749 | 1.459009 | 8.43E-12 | 1.42E-10 | yes | up | 4.78 | 4.93 | 4.79 | 29.62 | 31.27 | 30.61 | 4.833333 | 30.5 |
| SOD3 | 7.868 | 2.976073 | 8.71E-12 | 1.46E-10 | yes | up | 6.39 | 7.09 | 9.85 | 61.95 | 139.43 | 222.44 | 7.776667 | 141.2733 |
| CWC15 | 0.403 | -1.30967 | 8.72E-12 | 1.46E-10 | yes | down | 46.44 | 46.4 | 37.28 | 44.23 | 43.5 | 32.28 | 43.37333 | 40.00333 |
| COPS2 | 0.318 | -1.65173 | 9.27E-12 | 1.55E-10 | yes | down | 34.84 | 33.03 | 28.44 | 26.17 | 30.86 | 15.46 | 32.10333 | 24.16333 |
| SHCBP1L | 0.002 | -9.29037 | 9.3E-12 | 1.56E-10 | yes | down | 2.4 | 0.43 | 1.98 | 0 | 0 | 0 | 1.603333 | 0 |
| YWHAH | 2.386 | 1.254797 | 9.48E-12 | 1.58E-10 | yes | up | 42.02 | 40.21 | 33.54 | 235.03 | 209.12 | 230.76 | 38.59 | 224.97 |
| PROS1 | 0.409 | -1.28846 | 9.64E-12 | 1.61E-10 | yes | down | 13.26 | 14.39 | 12.41 | 12.25 | 15.16 | 10.42 | 13.35333 | 12.61 |
| EIF3M | 0.439 | -1.18776 | 9.77E-12 | 1.63E-10 | yes | down | 148.28 | 152.03 | 140.4 | 161.16 | 153.74 | 129.41 | 146.9033 | 148.1033 |
| NPM1 | 0.306 | -1.70958 | 1.06E-11 | 1.77E-10 | yes | down | 227.51 | 262.02 | 229.64 | 199.36 | 198.51 | 108 | 239.7233 | 168.6233 |
| LCAT | 399.507 | 8.642076 | 1.07E-11 | 1.79E-10 | yes | up | 0 | 0 | 0 | 10.38 | 5.94 | 6.5 | 0 | 7.606667 |
| LOC103350039 | 4.126 | 2.044894 | 1.08E-11 | 1.79E-10 | yes | up | 0.78 | 1.02 | 0.74 | 8.09 | 15.25 | 8.39 | 0.846667 | 10.57667 |
| DSG1 | 0.009 | -6.77462 | 1.09E-11 | 1.8E-10 | yes | down | 0.28 | 0.53 | 0.25 | 0.02 | 0.11 | 0 | 0.353333 | 0.043333 |
| NCOA1 | 0.413 | -1.27575 | 1.12E-11 | 1.86E-10 | yes | down | 13.98 | 17.83 | 13.17 | 14.11 | 17.13 | 11.5 | 14.99333 | 14.24667 |
| NIPSNAP1 | 0.209 | -2.25753 | 1.13E-11 | 1.86E-10 | yes | down | 50.9 | 56.56 | 101.52 | 33.98 | 38.29 | 32.11 | 69.66 | 34.79333 |
| CDKN1A | 5.662 | 2.501379 | 1.16E-11 | 1.92E-10 | yes | up | 8.34 | 9.09 | 7.07 | 112.53 | 54.06 | 140.85 | 8.166667 | 102.48 |
| C4A | 8.967 | 3.164648 | 1.17E-11 | 1.93E-10 | yes | up | 12.37 | 8.2 | 3.75 | 101.4 | 97.36 | 269.22 | 8.106667 | 155.9933 |
| HSPA5 | 3.626 | 1.858421 | 1.18E-11 | 1.95E-10 | yes | up | 31.65 | 38.56 | 19.91 | 305.54 | 267.06 | 166.31 | 30.04 | 246.3033 |
| CPLX1 | 0.002 | -9.03363 | 1.21E-11 | 1.99E-10 | yes | down | 1.19 | 2.05 | 3.23 | 0 | 0 | 0 | 2.156667 | 0 |
| PSMD12 | 0.457 | -1.13015 | 1.21E-11 | 1.99E-10 | yes | down | 38.59 | 40.4 | 33.85 | 39.31 | 46.11 | 33.18 | 37.61333 | 39.53333 |
| TAF1D | 0.187 | -2.41646 | 1.26E-11 | 2.07E-10 | yes | down | 53.96 | 55.86 | 74.55 | 30.59 | 25.56 | 12.71 | 61.45667 | 22.95333 |
| ANGPT4 | 6.815 | 2.768787 | 1.26E-11 | 2.07E-10 | yes | up | 0.34 | 0.27 | 0.14 | 2.8 | 5.11 | 3.76 | 0.25 | 3.89 |
| LOC100353067 | 2.453 | 1.294699 | 1.26E-11 | 2.08E-10 | yes | up | 6.77 | 6.39 | 5.81 | 32.82 | 38.75 | 33.87 | 6.323333 | 35.14667 |
| LOC100340211 | 3.587 | 1.842672 | 1.33E-11 | 2.18E-10 | yes | up | 1.45 | 5.13 | 2.15 | 28.82 | 25.9 | 39.79 | 2.91 | 31.50333 |
| STAC | 4.141 | 2.050036 | 1.34E-11 | 2.2E-10 | yes | up | 1.55 | 1.22 | 0.79 | 12.67 | 9.44 | 10.81 | 1.186667 | 10.97333 |
| VAMP5 | 2.872 | 1.521967 | 1.4E-11 | 2.3E-10 | yes | up | 10.85 | 12.99 | 10.99 | 76.59 | 88.87 | 65.74 | 11.61 | 77.06667 |
| C4H12orf45 | 0.402 | -1.31492 | 1.41E-11 | 2.31E-10 | yes | down | 5.52 | 6.25 | 5.28 | 4.72 | 6.45 | 4.33 | 5.683333 | 5.166667 |
| CSNK1A1 | 0.454 | -1.13978 | 1.43E-11 | 2.34E-10 | yes | down | 74.52 | 78.22 | 64.83 | 82.28 | 93.11 | 62.45 | 72.52333 | 79.28 |
| CLSTN3 | 0.227 | -2.14224 | 1.45E-11 | 2.37E-10 | yes | down | 9.34 | 13.12 | 13.51 | 5.06 | 3.67 | 3.18 | 11.99 | 3.97 |
| ITIH5 | 3.591 | 1.844441 | 1.46E-11 | 2.38E-10 | yes | up | 19.54 | 12.03 | 9 | 90.83 | 118.05 | 123.87 | 13.52333 | 110.9167 |
| BSCL2 | 2.879 | 1.525318 | 1.5E-11 | 2.44E-10 | yes | up | 20.59 | 24.58 | 22.99 | 96.63 | 74.78 | 82.33 | 22.72 | 84.58 |
| THNSL2 | 9.159 | 3.195134 | 1.52E-11 | 2.48E-10 | yes | up | 0.82 | 0.27 | 0.19 | 7.04 | 11.01 | 5.35 | 0.426667 | 7.8 |
| SNX24 | 0.299 | -1.74311 | 1.55E-11 | 2.52E-10 | yes | down | 15.26 | 11.65 | 15.63 | 10.67 | 10.91 | 7.95 | 14.18 | 9.843333 |
| NOL7 | 0.275 | -1.86292 | 1.56E-11 | 2.54E-10 | yes | down | 16.32 | 20.13 | 16.6 | 13.32 | 13.25 | 7.12 | 17.68333 | 11.23 |
| LOC100341224 | 0.204 | -2.29564 | 1.57E-11 | 2.55E-10 | yes | down | 5.18 | 3.86 | 3.34 | 1.83 | 1.61 | 2.23 | 4.126667 | 1.89 |
| TRIO | 2.189 | 1.130093 | 1.57E-11 | 2.55E-10 | yes | up | 6.23 | 5.74 | 4.2 | 29.77 | 33.2 | 26.37 | 5.39 | 29.78 |
| MRPS9 | 0.393 | -1.34566 | 1.58E-11 | 2.56E-10 | yes | down | 51.98 | 58.73 | 50.75 | 49.29 | 60.45 | 37.6 | 53.82 | 49.11333 |
| PLVAP | 2.996 | 1.583052 | 1.66E-11 | 2.7E-10 | yes | up | 36.69 | 47.76 | 50.82 | 324.71 | 348.88 | 271.76 | 45.09 | 315.1167 |
| ADAMTS7 | 2.838 | 1.504682 | 1.67E-11 | 2.71E-10 | yes | up | 2.07 | 2.03 | 1.8 | 12.61 | 16.64 | 9.53 | 1.966667 | 12.92667 |
| JDP2 | 3.708 | 1.890673 | 1.68E-11 | 2.72E-10 | yes | up | 8.17 | 6.74 | 3.7 | 50.49 | 47.27 | 55.64 | 6.203333 | 51.13333 |
| ARHGEF37 | 0.367 | -1.44721 | 1.69E-11 | 2.72E-10 | yes | down | 16.35 | 23.27 | 15.77 | 15.24 | 18.9 | 12.79 | 18.46333 | 15.64333 |
| LOC108176462 | 0.002 | -8.6862 | 1.75E-11 | 2.82E-10 | yes | down | 3.89 | 2.83 | 3.36 | 0 | 0 | 0 | 3.36 | 0 |
| SMAD1 | 2.754 | 1.461751 | 1.75E-11 | 2.82E-10 | yes | up | 5.14 | 4.19 | 3.79 | 31.42 | 26.66 | 25.05 | 4.373333 | 27.71 |
| MFAP5 | 3.386 | 1.759471 | 1.78E-11 | 2.86E-10 | yes | up | 79.78 | 51.6 | 34.3 | 441.81 | 422.85 | 385.86 | 55.22667 | 416.84 |
| MECR | 0.315 | -1.66681 | 1.8E-11 | 2.89E-10 | yes | down | 40.9 | 38.77 | 43.4 | 30.59 | 26.82 | 35.59 | 41.02333 | 31 |
| ATP5O | 0.389 | -1.3618 | 1.8E-11 | 2.9E-10 | yes | down | 1021.47 | 1334.39 | 1077.47 | 1095.18 | 979.67 | 985.62 | 1144.443 | 1020.157 |
| LOC103352357 | 0.011 | -6.46482 | 1.8E-11 | 2.9E-10 | yes | down | 0.36 | 0.31 | 0.3 | 0 | 0.01 | 0.17 | 0.323333 | 0.06 |
| BCLAF1 | 0.308 | -1.70111 | 1.85E-11 | 2.97E-10 | yes | down | 13.87 | 12.94 | 12.72 | 10.46 | 11.74 | 5.83 | 13.17667 | 9.343333 |
| LAMTOR3 | 0.341 | -1.5531 | 1.88E-11 | 3.02E-10 | yes | down | 23.17 | 29.09 | 24.2 | 21.16 | 24.2 | 14.92 | 25.48667 | 20.09333 |
| FAM213A | 2.34 | 1.226284 | 1.93E-11 | 3.1E-10 | yes | up | 77.93 | 60.69 | 64.14 | 324.43 | 404.56 | 359.07 | 67.58667 | 362.6867 |
| MPHOSPH8 | 0.374 | -1.41877 | 1.94E-11 | 3.1E-10 | yes | down | 7.03 | 7.8 | 5.87 | 5.51 | 7.23 | 4.05 | 6.9 | 5.596667 |
| ACAD10 | 0.354 | -1.49688 | 2E-11 | 3.2E-10 | yes | down | 14.84 | 14.76 | 17.36 | 11.52 | 14.07 | 13.05 | 15.65333 | 12.88 |
| LOC108178553 | 172.281 | 7.428619 | 2.09E-11 | 3.33E-10 | yes | up | 0 | 0.03 | 0 | 4.42 | 5.51 | 2.3 | 0.01 | 4.076667 |
| DCN | 3.309 | 1.726465 | 2.11E-11 | 3.37E-10 | yes | up | 288.23 | 193.71 | 126.3 | 1479.21 | 1420.72 | 1513.96 | 202.7467 | 1471.297 |
| PSMC6 | 0.348 | -1.52259 | 2.12E-11 | 3.38E-10 | yes | down | 39.74 | 32.38 | 30.65 | 30.56 | 32.68 | 19.16 | 34.25667 | 27.46667 |
| CRISPLD2 | 5.942 | 2.570945 | 2.16E-11 | 3.44E-10 | yes | up | 0.85 | 2.61 | 1.84 | 22.27 | 24.81 | 25.81 | 1.766667 | 24.29667 |
| MTRF1 | 0.201 | -2.31559 | 2.17E-11 | 3.46E-10 | yes | down | 3.49 | 3.15 | 3.27 | 1.71 | 1.39 | 1.44 | 3.303333 | 1.513333 |
| LOC100358143 | 27.964 | 4.805477 | 2.3E-11 | 3.66E-10 | yes | up | 0 | 0.27 | 0.16 | 9.12 | 5.6 | 10.2 | 0.143333 | 8.306667 |
| COQ6 | 0.327 | -1.61069 | 2.31E-11 | 3.67E-10 | yes | down | 30.22 | 29.34 | 27.44 | 21.01 | 18.35 | 25.47 | 29 | 21.61 |
| HSF4 | 14.737 | 3.881372 | 2.32E-11 | 3.68E-10 | yes | up | 0.18 | 0.07 | 0.07 | 3.27 | 4.55 | 3.75 | 0.106667 | 3.856667 |
| DOCK3 | 0.067 | -3.89519 | 2.43E-11 | 3.85E-10 | yes | down | 0.22 | 0.57 | 0.41 | 0.04 | 0.1 | 0.05 | 0.4 | 0.063333 |
| CGRRF1 | 2.778 | 1.474114 | 2.46E-11 | 3.9E-10 | yes | up | 1.27 | 1.47 | 1.32 | 9.57 | 11.69 | 8.45 | 1.353333 | 9.903333 |
| APOO | 0.287 | -1.80018 | 2.5E-11 | 3.95E-10 | yes | down | 48.26 | 39.46 | 30.79 | 32.51 | 21.21 | 22.44 | 39.50333 | 25.38667 |
| LACTB | 0.35 | -1.51625 | 2.54E-11 | 4.01E-10 | yes | down | 26.75 | 28.58 | 21.57 | 21.41 | 25.36 | 13.9 | 25.63333 | 20.22333 |
| LVRN | 6.695 | 2.743087 | 2.62E-11 | 4.14E-10 | yes | up | 2.95 | 1.52 | 0.75 | 21.3 | 30.82 | 39.08 | 1.74 | 30.4 |
| NDUFB8 | 0.417 | -1.26341 | 2.67E-11 | 4.2E-10 | yes | down | 396.86 | 388.1 | 385.57 | 403.62 | 357.6 | 357.57 | 390.1767 | 372.93 |
| LUM | 3.418 | 1.773155 | 2.68E-11 | 4.22E-10 | yes | up | 134.46 | 106.38 | 57.74 | 725.77 | 975.78 | 605.83 | 99.52667 | 769.1267 |
| MSI2 | 0.376 | -1.41036 | 2.7E-11 | 4.25E-10 | yes | down | 12.26 | 9.35 | 10.15 | 8 | 10.57 | 6.48 | 10.58667 | 8.35 |
| RAB32 | 2.338 | 1.225125 | 2.73E-11 | 4.29E-10 | yes | up | 12.01 | 13.36 | 10.57 | 62.53 | 67.26 | 63.27 | 11.98 | 64.35333 |
| G0S2 | 5.507 | 2.461259 | 2.76E-11 | 4.33E-10 | yes | up | 59.72 | 44.94 | 26.3 | 409.91 | 316.44 | 757.25 | 43.65333 | 494.5333 |
| SLC6A17 | 0.099 | -3.34057 | 2.77E-11 | 4.34E-10 | yes | down | 0.87 | 1.6 | 1.72 | 0.21 | 0.27 | 0.49 | 1.396667 | 0.323333 |
| SNX2 | 0.319 | -1.64671 | 2.81E-11 | 4.4E-10 | yes | down | 38.51 | 44.75 | 41.18 | 32.35 | 38.78 | 19.66 | 41.48 | 30.26333 |
| CXCL10 | 13.455 | 3.750095 | 2.86E-11 | 4.47E-10 | yes | up | 0.5 | 0.34 | 0.11 | 11.24 | 7.67 | 9.2 | 0.316667 | 9.37 |
| LAMA3 | 3.412 | 1.770552 | 2.95E-11 | 4.61E-10 | yes | up | 1.06 | 1.4 | 1.41 | 10.75 | 12.94 | 8.01 | 1.29 | 10.56667 |
| GCSH | 0.297 | -1.75357 | 3.01E-11 | 4.7E-10 | yes | down | 17.87 | 16.65 | 18.84 | 14.86 | 13.15 | 8.58 | 17.78667 | 12.19667 |
| COMTD1 | 0.145 | -2.78651 | 3.01E-11 | 4.7E-10 | yes | down | 8.26 | 9.28 | 7.06 | 1.24 | 3.43 | 3.6 | 8.2 | 2.756667 |
| LOC103349390 | 0.048 | -4.3675 | 3.01E-11 | 4.7E-10 | yes | down | 2.07 | 4.58 | 1.21 | 0.48 | 0.24 | 0.1 | 2.62 | 0.273333 |
| EIF2S2 | 0.421 | -1.24656 | 3.08E-11 | 4.8E-10 | yes | down | 58.59 | 52.2 | 45.47 | 53.94 | 57.82 | 39.36 | 52.08667 | 50.37333 |
| LOC100347496 | 0.163 | -2.62057 | 3.23E-11 | 5.03E-10 | yes | down | 160.23 | 246.49 | 293.27 | 106.08 | 48.18 | 105.91 | 233.33 | 86.72333 |
| VAT1 | 2.198 | 1.136199 | 3.26E-11 | 5.07E-10 | yes | up | 21.31 | 23.96 | 21.28 | 112.91 | 115.58 | 107 | 22.18333 | 111.83 |
| PFN1 | 2.635 | 1.397735 | 3.29E-11 | 5.1E-10 | yes | up | 144.66 | 147.68 | 106 | 952.72 | 743.72 | 710.85 | 132.78 | 802.43 |
| ZNF146 | 0.297 | -1.75089 | 3.29E-11 | 5.1E-10 | yes | down | 13.93 | 12.8 | 10.54 | 8.54 | 11.75 | 5.32 | 12.42333 | 8.536667 |
| BPNT1 | 0.419 | -1.25478 | 3.31E-11 | 5.13E-10 | yes | down | 25.71 | 27.47 | 24.93 | 26.01 | 29.06 | 20.39 | 26.03667 | 25.15333 |
| DMRT2 | 7.954 | 2.991707 | 3.32E-11 | 5.14E-10 | yes | up | 0.79 | 0.24 | 0.16 | 7.9 | 7.28 | 6.15 | 0.396667 | 7.11 |
| CKS1B | 3.78 | 1.918357 | 3.32E-11 | 5.15E-10 | yes | up | 11.84 | 10.95 | 7.76 | 111.51 | 95.45 | 58.12 | 10.18333 | 88.36 |
| SLC25A46 | 0.331 | -1.59422 | 3.34E-11 | 5.17E-10 | yes | down | 14.11 | 15.44 | 14.73 | 10.47 | 14.45 | 6.88 | 14.76 | 10.6 |
| TENM3 | 0.318 | -1.65286 | 3.39E-11 | 5.23E-10 | yes | down | 2.1 | 1.76 | 1.61 | 1.32 | 1.13 | 1.5 | 1.823333 | 1.316667 |
| FAM189B | 3.589 | 1.843652 | 3.54E-11 | 5.47E-10 | yes | up | 2.33 | 1.31 | 1.19 | 12.83 | 13.22 | 13.01 | 1.61 | 13.02 |
| SH2D4A | 5.226 | 2.385606 | 3.77E-11 | 5.81E-10 | yes | up | 0.63 | 0.53 | 0.37 | 4.98 | 6.31 | 6.7 | 0.51 | 5.996667 |
| PRUNE2 | 8.669 | 3.115853 | 3.84E-11 | 5.92E-10 | yes | up | 0.78 | 0.47 | 0.13 | 10.96 | 6.13 | 8.79 | 0.46 | 8.626667 |
| LOC103346838 | 3.107 | 1.635297 | 3.91E-11 | 6.01E-10 | yes | up | 15.99 | 10.9 | 11.81 | 73.15 | 100.7 | 101.35 | 12.9 | 91.73333 |
| ABCA5 | 0.302 | -1.72835 | 3.96E-11 | 6.09E-10 | yes | down | 3.21 | 3 | 2.49 | 2.09 | 2.83 | 1.51 | 2.9 | 2.143333 |
| TRUB1 | 0.236 | -2.08379 | 4.08E-11 | 6.26E-10 | yes | down | 3.97 | 5.64 | 6.18 | 2.85 | 3.44 | 2.32 | 5.263333 | 2.87 |
| HR | 0.105 | -3.24548 | 4.1E-11 | 6.29E-10 | yes | down | 4.28 | 2.19 | 1.45 | 0.31 | 0.52 | 1.01 | 2.64 | 0.613333 |
| CERK | 0.283 | -1.82297 | 4.13E-11 | 6.34E-10 | yes | down | 36.53 | 39.63 | 17.13 | 16.44 | 22.19 | 20.31 | 31.09667 | 19.64667 |
| FBN2 | 0.232 | -2.10668 | 4.14E-11 | 6.35E-10 | yes | down | 1.2 | 1.05 | 0.68 | 0.38 | 0.51 | 0.64 | 0.976667 | 0.51 |
| PGF | 8.535 | 3.093323 | 4.27E-11 | 6.54E-10 | yes | up | 1.8 | 1.1 | 0.29 | 15.56 | 19.22 | 24.29 | 1.063333 | 19.69 |
| DNAJC11 | 0.439 | -1.18692 | 4.28E-11 | 6.54E-10 | yes | down | 30.36 | 34.91 | 27.85 | 28.07 | 32.5 | 32.79 | 31.04 | 31.12 |
| PRKCZ | 7.364 | 2.880519 | 4.32E-11 | 6.6E-10 | yes | up | 1.75 | 2.1 | 1.33 | 26.06 | 36.02 | 25.87 | 1.726667 | 29.31667 |
| ABHD14B | 3.508 | 1.810695 | 4.35E-11 | 6.63E-10 | yes | up | 7.26 | 6.39 | 6.36 | 59.31 | 50.55 | 68.96 | 6.67 | 59.60667 |
| ATP13A3 | 0.324 | -1.62405 | 4.39E-11 | 6.69E-10 | yes | down | 5 | 7.07 | 6.65 | 4.21 | 5.95 | 4.02 | 6.24 | 4.726667 |
| TROAP | 11.917 | 3.574901 | 4.43E-11 | 6.75E-10 | yes | up | 0.4 | 0.46 | 0.16 | 10.13 | 13.68 | 3.14 | 0.34 | 8.983333 |
| ZNF518A | 0.233 | -2.09937 | 4.67E-11 | 7.1E-10 | yes | down | 0.98 | 0.99 | 0.75 | 0.45 | 0.68 | 0.32 | 0.906667 | 0.483333 |
| PGM5 | 3.72 | 1.895258 | 4.7E-11 | 7.15E-10 | yes | up | 9.21 | 6.3 | 4.32 | 41.31 | 57.51 | 66.08 | 6.61 | 54.96667 |
| FDX1 | 0.369 | -1.43641 | 4.81E-11 | 7.31E-10 | yes | down | 34.21 | 36.75 | 31.63 | 31 | 32.55 | 21.48 | 34.19667 | 28.34333 |
| TGFBI | 2.905 | 1.538409 | 5.11E-11 | 7.76E-10 | yes | up | 48.33 | 32.48 | 24.84 | 197.03 | 259.47 | 240.14 | 35.21667 | 232.2133 |
| TSPO | 3.122 | 1.642325 | 5.12E-11 | 7.76E-10 | yes | up | 80.68 | 88.65 | 84.15 | 637.82 | 486.08 | 694.97 | 84.49333 | 606.29 |
| PHB | 0.395 | -1.33825 | 5.17E-11 | 7.84E-10 | yes | down | 219.4 | 210.39 | 233.74 | 203.71 | 190.66 | 210.67 | 221.1767 | 201.68 |
| PDGFRA | 4.026 | 2.009411 | 5.29E-11 | 8.02E-10 | yes | up | 4.8 | 2.7 | 2.37 | 21.87 | 30.69 | 37.16 | 3.29 | 29.90667 |
| TNFAIP3 | 2.698 | 1.431945 | 5.46E-11 | 8.25E-10 | yes | up | 3.86 | 3.26 | 3.73 | 21.6 | 27.82 | 18.08 | 3.616667 | 22.5 |
| NBN | 0.245 | -2.02839 | 5.48E-11 | 8.28E-10 | yes | down | 11.86 | 13.2 | 9.87 | 8.47 | 7.81 | 3.57 | 11.64333 | 6.616667 |
| MRPL3 | 0.418 | -1.26 | 5.55E-11 | 8.37E-10 | yes | down | 28.36 | 33.03 | 30.69 | 32.62 | 33.93 | 27.41 | 30.69333 | 31.32 |
| C18H10orf54 | 2.798 | 1.484529 | 5.58E-11 | 8.41E-10 | yes | up | 7.92 | 9.08 | 10.08 | 43.82 | 69.41 | 41.6 | 9.026667 | 51.61 |
| CCDC73 | 0.124 | -3.00655 | 5.87E-11 | 8.84E-10 | yes | down | 2.87 | 1.96 | 1.23 | 0.85 | 0.36 | 0.43 | 2.02 | 0.546667 |
| CBS | 6.602 | 2.722891 | 5.99E-11 | 9.02E-10 | yes | up | 5.78 | 1.73 | 1.82 | 37.01 | 47.98 | 51.35 | 3.11 | 45.44667 |
| OGDH | 0.343 | -1.54283 | 6.1E-11 | 9.18E-10 | yes | down | 219.25 | 260.52 | 293.33 | 199.43 | 256.76 | 167.05 | 257.7 | 207.7467 |
| LOC100345696 | 5.041 | 2.333593 | 6.13E-11 | 9.21E-10 | yes | up | 6.29 | 4.61 | 2.59 | 40.51 | 38.52 | 62.5 | 4.496667 | 47.17667 |
| LOC100338675 | 0.072 | -3.80191 | 6.22E-11 | 9.33E-10 | yes | down | 17.79 | 25.2 | 28.96 | 1.86 | 0.56 | 4.58 | 23.98333 | 2.333333 |
| EMC2 | 0.305 | -1.71217 | 6.37E-11 | 9.56E-10 | yes | down | 58.84 | 55.09 | 53.97 | 44.74 | 45.74 | 23.9 | 55.96667 | 38.12667 |
| PPAT | 0.232 | -2.10935 | 6.4E-11 | 9.6E-10 | yes | down | 2.15 | 1.93 | 1.6 | 0.84 | 1.09 | 1.08 | 1.893333 | 1.003333 |
| LOC100341357 | 0.357 | -1.48724 | 6.48E-11 | 9.7E-10 | yes | down | 15.83 | 27.68 | 17.47 | 20.86 | 33.86 | 17.29 | 20.32667 | 24.00333 |
| RPP40 | 0.393 | -1.34826 | 6.5E-11 | 9.72E-10 | yes | down | 29.46 | 27.9 | 22.56 | 24.2 | 21.65 | 25.26 | 26.64 | 23.70333 |
| COL7A1 | 0.158 | -2.66062 | 6.51E-11 | 9.72E-10 | yes | down | 0.63 | 0.61 | 0.63 | 0.15 | 0.38 | 0.16 | 0.623333 | 0.23 |
| DOC2B | 5.346 | 2.418526 | 6.63E-11 | 9.89E-10 | yes | up | 1.37 | 0.69 | 1.39 | 11.53 | 20.76 | 12.46 | 1.15 | 14.91667 |
| LOC103345308 | 0.379 | -1.39863 | 6.66E-11 | 9.94E-10 | yes | down | 10.77 | 10.65 | 9.5 | 8.72 | 9.75 | 8.44 | 10.30667 | 8.97 |
| NDUFAF5 | 0.301 | -1.73083 | 6.71E-11 | 1E-09 | yes | down | 8.62 | 9.5 | 9.64 | 6.83 | 6.57 | 5.65 | 9.253333 | 6.35 |
| GCNT4 | 396.409 | 8.630847 | 6.78E-11 | 1.01E-09 | yes | up | 0 | 0 | 0 | 4.31 | 1.51 | 4.31 | 0 | 3.376667 |
| SLC30A9 | 0.419 | -1.25391 | 6.9E-11 | 1.03E-09 | yes | down | 13.66 | 14.16 | 14.13 | 13.42 | 15.78 | 11.53 | 13.98333 | 13.57667 |
| PTGER4 | 2.774 | 1.471766 | 6.91E-11 | 1.03E-09 | yes | up | 2 | 2.02 | 2.23 | 12.66 | 15.22 | 12.29 | 2.083333 | 13.39 |
| CWC27 | 0.359 | -1.47729 | 7.11E-11 | 1.06E-09 | yes | down | 8.39 | 8 | 8.16 | 7.22 | 7.5 | 5.61 | 8.183333 | 6.776667 |
| SHOX2 | 14.828 | 3.890281 | 7.15E-11 | 1.06E-09 | yes | up | 0.32 | 0.13 | 0.12 | 7.52 | 2.68 | 8.03 | 0.19 | 6.076667 |
| COTL1 | 2.889 | 1.530784 | 7.27E-11 | 1.08E-09 | yes | up | 11.7 | 17.57 | 12.04 | 100.67 | 85.58 | 85.18 | 13.77 | 90.47667 |
| RPF1 | 0.378 | -1.40307 | 7.38E-11 | 1.09E-09 | yes | down | 16.44 | 17.96 | 12.83 | 14.3 | 13.1 | 11.5 | 15.74333 | 12.96667 |
| ILK | 2.139 | 1.096797 | 7.55E-11 | 1.12E-09 | yes | up | 62.69 | 59.33 | 43.98 | 278.19 | 270.49 | 248.72 | 55.33333 | 265.8 |
| TSPAN11 | 3.108 | 1.635935 | 7.67E-11 | 1.13E-09 | yes | up | 1.46 | 1.24 | 0.84 | 8.34 | 7.99 | 8.4 | 1.18 | 8.243333 |
| RBM45 | 0.384 | -1.38228 | 7.7E-11 | 1.14E-09 | yes | down | 9.7 | 9.97 | 7.51 | 7.55 | 8.96 | 7.42 | 9.06 | 7.976667 |
| GABRB3 | 0.022 | -5.49094 | 7.85E-11 | 1.16E-09 | yes | down | 0.44 | 0.71 | 0.26 | 0.05 | 0 | 0.01 | 0.47 | 0.02 |
| SELPLG | 3.375 | 1.755027 | 8.15E-11 | 1.2E-09 | yes | up | 1.43 | 1.25 | 1.19 | 10.44 | 9.18 | 10.08 | 1.29 | 9.9 |
| RXRG | 0.118 | -3.08822 | 8.29E-11 | 1.22E-09 | yes | down | 35.23 | 28.39 | 43.41 | 9.86 | 3.41 | 14.72 | 35.67667 | 9.33 |
| RALB | 2.384 | 1.253337 | 8.47E-11 | 1.25E-09 | yes | up | 15.72 | 19.91 | 14.53 | 96.7 | 101.23 | 75.08 | 16.72 | 91.00333 |
| THBS2 | 2.887 | 1.529608 | 8.49E-11 | 1.25E-09 | yes | up | 7.52 | 5.36 | 4.22 | 36.71 | 35.17 | 41.42 | 5.7 | 37.76667 |
| SRF | 3.065 | 1.61579 | 8.57E-11 | 1.26E-09 | yes | up | 5.33 | 4.57 | 3.08 | 35.64 | 25.69 | 28.56 | 4.326667 | 29.96333 |
| PGER5 | 2.098 | 1.068678 | 8.64E-11 | 1.27E-09 | yes | up | 213.95 | 235.44 | 175.62 | 917.52 | 1026.81 | 1036.1 | 208.3367 | 993.4767 |
| ADGRG6 | 3.481 | 1.799354 | 8.8E-11 | 1.29E-09 | yes | up | 1.94 | 1.74 | 1 | 12.63 | 15.1 | 9.06 | 1.56 | 12.26333 |
| CES4A | 0.159 | -2.64873 | 8.8E-11 | 1.29E-09 | yes | down | 3.02 | 3.3 | 2.35 | 0.59 | 1.07 | 1.52 | 2.89 | 1.06 |
| LPAR6 | 0.264 | -1.92051 | 9.13E-11 | 1.34E-09 | yes | down | 16.42 | 17.38 | 16.47 | 14.23 | 9.37 | 6.83 | 16.75667 | 10.14333 |
| LOC100328912 | 3.339 | 1.739617 | 9.21E-11 | 1.35E-09 | yes | up | 77.65 | 74.12 | 90.95 | 653.76 | 502.08 | 693.77 | 80.90667 | 616.5367 |
| MSMO1 | 6.169 | 2.625071 | 9.25E-11 | 1.35E-09 | yes | up | 6.87 | 5.96 | 5.32 | 142.67 | 47.67 | 60.18 | 6.05 | 83.50667 |
| NID2 | 2.394 | 1.259245 | 9.42E-11 | 1.38E-09 | yes | up | 24.9 | 32.69 | 24.39 | 158.89 | 176.99 | 120.71 | 27.32667 | 152.1967 |
| ECHDC3 | 0.438 | -1.1901 | 9.56E-11 | 1.39E-09 | yes | down | 66.6 | 73.45 | 69.56 | 63.43 | 72.06 | 66.46 | 69.87 | 67.31667 |
| SERPINE1 | 3.916 | 1.969507 | 9.8E-11 | 1.43E-09 | yes | up | 6.68 | 4.46 | 3.7 | 57.38 | 33.22 | 39.19 | 4.946667 | 43.26333 |
| SLC6A6 | 0.244 | -2.03601 | 9.89E-11 | 1.44E-09 | yes | down | 14.35 | 23.96 | 21.27 | 10.22 | 16.19 | 7.08 | 19.86 | 11.16333 |
| PSRC1 | 7.162 | 2.840358 | 1.02E-10 | 1.48E-09 | yes | up | 0.66 | 0.71 | 0.67 | 15.56 | 12.87 | 5.89 | 0.68 | 11.44 |
| RCN3 | 3.555 | 1.829825 | 1.03E-10 | 1.49E-09 | yes | up | 14.91 | 11.22 | 6.02 | 77.82 | 85.74 | 90.81 | 10.71667 | 84.79 |
| MTO1 | 0.315 | -1.66553 | 1.03E-10 | 1.5E-09 | yes | down | 8.51 | 7.95 | 5.98 | 8.46 | 6.23 | 4.56 | 7.48 | 6.416667 |
| ATAD1 | 0.31 | -1.68974 | 1.04E-10 | 1.51E-09 | yes | down | 13.66 | 13.67 | 15.86 | 10.35 | 13.92 | 7.22 | 14.39667 | 10.49667 |
| PERM1 | 0.08 | -3.63742 | 1.07E-10 | 1.55E-09 | yes | down | 6.74 | 3.6 | 1.91 | 0.52 | 0.3 | 1.26 | 4.083333 | 0.693333 |
| ZDHHC14 | 3.542 | 1.824701 | 1.07E-10 | 1.55E-09 | yes | up | 1.81 | 1.75 | 1.37 | 9.89 | 15.84 | 14.96 | 1.643333 | 13.56333 |
| TMEM63B | 2.204 | 1.140248 | 1.08E-10 | 1.56E-09 | yes | up | 7.83 | 8.32 | 5.56 | 34.53 | 39.69 | 34.41 | 7.236667 | 36.21 |
| HGSNAT | 2.067 | 1.047458 | 1.09E-10 | 1.58E-09 | yes | up | 17.24 | 17.44 | 13.39 | 76.97 | 77.95 | 70.25 | 16.02333 | 75.05667 |
| MTG2 | 0.428 | -1.22574 | 1.12E-10 | 1.61E-09 | yes | down | 13.39 | 14.94 | 12.76 | 12.26 | 14.79 | 12.98 | 13.69667 | 13.34333 |
| GULP1 | 0.299 | -1.74267 | 1.13E-10 | 1.63E-09 | yes | down | 6.24 | 7.27 | 5.34 | 4.52 | 5.6 | 2.78 | 6.283333 | 4.3 |
| PGGT1B | 0.395 | -1.33936 | 1.15E-10 | 1.66E-09 | yes | down | 9.96 | 10.78 | 9.5 | 9.08 | 11.59 | 7.41 | 10.08 | 9.36 |
| LYRM2 | 0.317 | -1.65767 | 1.15E-10 | 1.66E-09 | yes | down | 9.23 | 9.74 | 8.04 | 9.51 | 6.07 | 6.07 | 9.003333 | 7.216667 |
| ATXN7L2 | 0.167 | -2.58354 | 1.16E-10 | 1.68E-09 | yes | down | 4.49 | 3.68 | 1.88 | 2.92 | 1.59 | 2.09 | 3.35 | 2.2 |
| ITGA5 | 2.332 | 1.221776 | 1.19E-10 | 1.71E-09 | yes | up | 15.55 | 12.7 | 10.12 | 73.95 | 74.18 | 56.95 | 12.79 | 68.36 |
| FYTTD1 | 0.358 | -1.48086 | 1.2E-10 | 1.72E-09 | yes | down | 23.13 | 20.54 | 15.55 | 16.83 | 20.62 | 11.13 | 19.74 | 16.19333 |
| C8H12orf40 | 322.57 | 8.33347 | 1.2E-10 | 1.72E-09 | yes | up | 0 | 0.02 | 0 | 8.55 | 8.95 | 2.41 | 0.006667 | 6.636667 |
| PSD4 | 2.793 | 1.481799 | 1.21E-10 | 1.74E-09 | yes | up | 1.14 | 1.12 | 0.89 | 5.72 | 7.14 | 7.05 | 1.05 | 6.636667 |
| GLT8D2 | 3.43 | 1.778215 | 1.22E-10 | 1.75E-09 | yes | up | 2.84 | 2.75 | 1.5 | 19.58 | 17.48 | 17.36 | 2.363333 | 18.14 |
| HBB2 | 0.123 | -3.02839 | 1.23E-10 | 1.76E-09 | yes | down | 7092.06 | 18788.27 | 12438.18 | 4938.61 | 1465.77 | 4100.95 | 12772.84 | 3501.777 |
| LOC108177673 | 6.444 | 2.687968 | 1.25E-10 | 1.79E-09 | yes | up | 1.62 | 1.04 | 1.24 | 20.67 | 13.19 | 20.87 | 1.3 | 18.24333 |
| MAN1C1 | 2.99 | 1.580007 | 1.32E-10 | 1.89E-09 | yes | up | 2.48 | 2.16 | 1.24 | 12.35 | 15.64 | 11.76 | 1.96 | 13.25 |
| KRR1 | 0.298 | -1.74614 | 1.38E-10 | 1.97E-09 | yes | down | 5.3 | 6.31 | 5.24 | 4.49 | 4.45 | 2.63 | 5.616667 | 3.856667 |
| C8H12orf4 | 0.333 | -1.58452 | 1.39E-10 | 1.98E-09 | yes | down | 5.48 | 7.51 | 5.79 | 4.44 | 5.42 | 4.61 | 6.26 | 4.823333 |
| CYP2B4 | 181.132 | 7.500897 | 1.39E-10 | 1.98E-09 | yes | up | 0.9 | 5.5 | 0.24 | 450.24 | 752.8 | 1322.16 | 2.213333 | 841.7333 |
| MLXIPL | 2.538 | 1.343419 | 1.4E-10 | 1.99E-09 | yes | up | 15.75 | 11.43 | 12.51 | 90 | 69.2 | 68.49 | 13.23 | 75.89667 |
| WDR12 | 0.342 | -1.54721 | 1.41E-10 | 2E-09 | yes | down | 4.95 | 5.4 | 4.23 | 3.72 | 4.33 | 3.37 | 4.86 | 3.806667 |
| ADIPOQ | 3.008 | 1.588902 | 1.41E-10 | 2.01E-09 | yes | up | 187.79 | 271.95 | 208.9 | 1429.28 | 2171.37 | 1128.24 | 222.88 | 1576.297 |
| LOC108177708 | 0.133 | -2.90825 | 1.46E-10 | 2.07E-09 | yes | down | 83.91 | 61.69 | 18.14 | 14.97 | 16.8 | 16.36 | 54.58 | 16.04333 |
| SLC46A2 | 12.292 | 3.619627 | 1.46E-10 | 2.07E-09 | yes | up | 0.22 | 0.15 | 0.1 | 2.65 | 3.81 | 6.44 | 0.156667 | 4.3 |
| CDC73 | 0.405 | -1.30488 | 1.49E-10 | 2.12E-09 | yes | down | 6.78 | 6.66 | 5.24 | 5.91 | 7.15 | 4.31 | 6.226667 | 5.79 |
| LOC100358947 | 0.009 | -6.86712 | 1.5E-10 | 2.12E-09 | yes | down | 4.39 | 1.04 | 4.33 | 0 | 0 | 0.23 | 3.253333 | 0.076667 |
| MRPL32 | 0.238 | -2.06986 | 1.5E-10 | 2.12E-09 | yes | down | 7.44 | 9.54 | 7.97 | 4.11 | 5.24 | 4.39 | 8.316667 | 4.58 |
| MANSC1 | 2.409 | 1.268172 | 1.53E-10 | 2.16E-09 | yes | up | 5.51 | 5.69 | 4.81 | 35.14 | 32.94 | 26.36 | 5.336667 | 31.48 |
| CRYGS | 0.037 | -4.73959 | 1.56E-10 | 2.2E-09 | yes | down | 8.26 | 19.17 | 9.77 | 0.13 | 2.83 | 0.38 | 12.4 | 1.113333 |
| RARG | 2.916 | 1.544161 | 1.56E-10 | 2.21E-09 | yes | up | 4.4 | 4.97 | 5.17 | 32.5 | 28.38 | 36.58 | 4.846667 | 32.48667 |
| TMEM164 | 2.794 | 1.482494 | 1.57E-10 | 2.21E-09 | yes | up | 3.88 | 2.97 | 2.45 | 16.04 | 24.11 | 18.14 | 3.1 | 19.43 |
| SERINC1 | 0.413 | -1.27725 | 1.57E-10 | 2.21E-09 | yes | down | 58.21 | 59.56 | 61.64 | 57.87 | 69.22 | 45.26 | 59.80333 | 57.45 |
| TGFB3 | 3.347 | 1.742713 | 1.57E-10 | 2.21E-09 | yes | up | 6.59 | 4.14 | 3.23 | 28.31 | 37.27 | 39.28 | 4.653333 | 34.95333 |
| RABL3 | 0.347 | -1.5253 | 1.62E-10 | 2.28E-09 | yes | down | 17.68 | 15.82 | 18.45 | 14.32 | 14.01 | 12.35 | 17.31667 | 13.56 |
| HEY1 | 0.312 | -1.68195 | 1.63E-10 | 2.29E-09 | yes | down | 8.92 | 14.47 | 9.62 | 8.07 | 7.79 | 8.26 | 11.00333 | 8.04 |
| CDC5L | 0.37 | -1.43451 | 1.64E-10 | 2.3E-09 | yes | down | 10.03 | 11.16 | 8.95 | 9.4 | 10.29 | 5.99 | 10.04667 | 8.56 |
| TGFB1I1 | 2.585 | 1.370225 | 1.71E-10 | 2.4E-09 | yes | up | 10.99 | 12.46 | 10.84 | 84.63 | 68.79 | 68.66 | 11.43 | 74.02667 |
| NAGA | 2.691 | 1.427962 | 1.71E-10 | 2.4E-09 | yes | up | 10.96 | 17.09 | 9.86 | 74.47 | 81.96 | 73.01 | 12.63667 | 76.48 |
| SYAP1 | 0.339 | -1.55981 | 1.74E-10 | 2.44E-09 | yes | down | 31.81 | 44.58 | 34.93 | 27.16 | 33.31 | 17.71 | 37.10667 | 26.06 |
| LGALS9 | 0.219 | -2.18952 | 1.77E-10 | 2.48E-09 | yes | down | 18.17 | 38.64 | 36 | 14.3 | 20.8 | 13.12 | 30.93667 | 16.07333 |
| PLIN2 | 0.351 | -1.50898 | 1.79E-10 | 2.51E-09 | yes | down | 55.13 | 86.3 | 68.76 | 54.6 | 70.28 | 47.71 | 70.06333 | 57.53 |
| FKBP4 | 2.196 | 1.134842 | 1.83E-10 | 2.55E-09 | yes | up | 33.99 | 30.56 | 22.68 | 152.85 | 144.44 | 135.94 | 29.07667 | 144.41 |
| MMACHC | 0.202 | -2.30626 | 1.83E-10 | 2.55E-09 | yes | down | 12.27 | 13.31 | 10.49 | 4.1 | 4.17 | 8.22 | 12.02333 | 5.496667 |
| SLC2A10 | 4.796 | 2.261936 | 1.84E-10 | 2.56E-09 | yes | up | 1.76 | 2.06 | 0.81 | 9.9 | 8.7 | 10.19 | 1.543333 | 9.596667 |
| DNAJC19 | 0.397 | -1.33224 | 1.84E-10 | 2.57E-09 | yes | down | 128.76 | 115.83 | 115.63 | 124.75 | 105.46 | 98.27 | 120.0733 | 109.4933 |
| EMC4 | 0.433 | -1.20916 | 1.85E-10 | 2.58E-09 | yes | down | 43.98 | 54.89 | 43.72 | 41.72 | 47.7 | 40.65 | 47.53 | 43.35667 |
| BPGM | 0.219 | -2.19199 | 1.91E-10 | 2.65E-09 | yes | down | 10 | 17.26 | 15.08 | 8.89 | 5.27 | 7.01 | 14.11333 | 7.056667 |
| KIF16B | 0.32 | -1.64191 | 1.92E-10 | 2.67E-09 | yes | down | 7.52 | 8.36 | 6.98 | 4.68 | 8.59 | 4.22 | 7.62 | 5.83 |
| ANKS6 | 2.674 | 1.419167 | 1.93E-10 | 2.68E-09 | yes | up | 1.38 | 1.5 | 1.17 | 7.78 | 8.6 | 8.27 | 1.35 | 8.216667 |
| HTRA4 | 21.55 | 4.429621 | 1.97E-10 | 2.73E-09 | yes | up | 0.24 | 0.09 | 0 | 3.21 | 6.28 | 6.12 | 0.11 | 5.203333 |
| CEBPZ | 0.307 | -1.7014 | 1.97E-10 | 2.73E-09 | yes | down | 9.96 | 9.9 | 10.27 | 7.87 | 9.26 | 4.61 | 10.04333 | 7.246667 |
| MBNL2 | 0.286 | -1.80477 | 1.98E-10 | 2.74E-09 | yes | down | 35.21 | 36.46 | 50.83 | 29.72 | 35.39 | 18.74 | 40.83333 | 27.95 |
| LOC103347285 | 3.27 | 1.709209 | 2E-10 | 2.77E-09 | yes | up | 3.24 | 4.83 | 3.99 | 26.7 | 30.48 | 33.4 | 4.02 | 30.19333 |
| HIPK3 | 0.316 | -1.66357 | 2.01E-10 | 2.77E-09 | yes | down | 35.4 | 34.81 | 37.8 | 32.82 | 29.82 | 17.17 | 36.00333 | 26.60333 |
| HAUS7 | 0.274 | -1.86861 | 2.01E-10 | 2.78E-09 | yes | down | 4.56 | 6.71 | 4.55 | 5.86 | 5.13 | 2.95 | 5.273333 | 4.646667 |
| AGPAT1 | 2.321 | 1.214789 | 2.03E-10 | 2.8E-09 | yes | up | 13.55 | 15.72 | 10.79 | 64.59 | 71.77 | 74.44 | 13.35333 | 70.26667 |
| LOC108178244 | 5.061 | 2.339561 | 2.03E-10 | 2.8E-09 | yes | up | 45.72 | 37.38 | 37.94 | 249.99 | 458.84 | 739.02 | 40.34667 | 482.6167 |
| CAMKK2 | 2.679 | 1.421872 | 2.08E-10 | 2.86E-09 | yes | up | 2.79 | 2.67 | 2.08 | 12.78 | 15.73 | 17.42 | 2.513333 | 15.31 |
| LOC100338194 | 9.736 | 3.283316 | 2.1E-10 | 2.89E-09 | yes | up | 0.67 | 0.24 | 0.88 | 9 | 16.18 | 14.24 | 0.596667 | 13.14 |
| PFKP | 2.625 | 1.392076 | 2.11E-10 | 2.9E-09 | yes | up | 3.5 | 3.89 | 3.72 | 23.41 | 21.48 | 21.89 | 3.703333 | 22.26 |
| UCHL5 | 0.302 | -1.72831 | 2.11E-10 | 2.9E-09 | yes | down | 10.54 | 7.9 | 7.69 | 7.52 | 8.38 | 4.39 | 8.71 | 6.763333 |
| DOCK8 | 0.367 | -1.44722 | 2.13E-10 | 2.92E-09 | yes | down | 2.93 | 3.33 | 2.91 | 2.26 | 2.57 | 2.85 | 3.056667 | 2.56 |
| GNE | 2.365 | 1.242132 | 2.19E-10 | 3E-09 | yes | up | 4.18 | 3.49 | 2.61 | 17.95 | 21.76 | 17.79 | 3.426667 | 19.16667 |
| NES | 3.216 | 1.68546 | 2.26E-10 | 3.1E-09 | yes | up | 6.66 | 4.74 | 3.21 | 42.07 | 35.6 | 28.5 | 4.87 | 35.39 |
| HEMGN | 0.067 | -3.89674 | 2.28E-10 | 3.12E-09 | yes | down | 1.16 | 4.35 | 3.24 | 0.23 | 0.58 | 0.58 | 2.916667 | 0.463333 |
| ACE | 0.334 | -1.58284 | 2.28E-10 | 3.12E-09 | yes | down | 22.65 | 28.44 | 28.65 | 17.52 | 21.29 | 24.17 | 26.58 | 20.99333 |
| RPS29 | 0.107 | -3.22836 | 2.3E-10 | 3.15E-09 | yes | down | 118.95 | 167.72 | 350.86 | 51.05 | 85.72 | 36.45 | 212.51 | 57.74 |
| GRSF1 | 0.379 | -1.40093 | 2.32E-10 | 3.17E-09 | yes | down | 34.86 | 21.49 | 25.59 | 23.3 | 25.98 | 20.46 | 27.31333 | 23.24667 |
| QKI | 0.336 | -1.57367 | 2.34E-10 | 3.2E-09 | yes | down | 52.68 | 51.88 | 30.52 | 36.35 | 45.19 | 23.44 | 45.02667 | 34.99333 |
| ACOT9 | 0.443 | -1.17444 | 2.4E-10 | 3.27E-09 | yes | down | 30.31 | 34.04 | 25.35 | 32.88 | 32.23 | 25.68 | 29.9 | 30.26333 |
| ST3GAL6 | 0.259 | -1.94874 | 2.4E-10 | 3.28E-09 | yes | down | 17.71 | 21.73 | 30.8 | 13.99 | 15.36 | 12.56 | 23.41333 | 13.97 |
| GATSL2 | 0.289 | -1.79097 | 2.43E-10 | 3.31E-09 | yes | down | 11.6 | 9.31 | 8.54 | 4.33 | 7.84 | 7.38 | 9.816667 | 6.516667 |
| LOC100348091 | 0.28 | -1.837 | 2.44E-10 | 3.32E-09 | yes | down | 8.47 | 11.38 | 8.22 | 5.17 | 8.24 | 8.74 | 9.356667 | 7.383333 |
| FGFR4 | 0.168 | -2.57303 | 2.51E-10 | 3.41E-09 | yes | down | 6.62 | 4.17 | 3.85 | 1.52 | 1.16 | 2.8 | 4.88 | 1.826667 |
| PDPR | 0.335 | -1.5772 | 2.65E-10 | 3.6E-09 | yes | down | 13.56 | 15.55 | 15.93 | 9.07 | 16.44 | 10.42 | 15.01333 | 11.97667 |
| CDK5RAP1 | 0.347 | -1.52788 | 2.69E-10 | 3.65E-09 | yes | down | 10.55 | 9.03 | 10.12 | 8.92 | 7.52 | 7.2 | 9.9 | 7.88 |
| FOXP2 | 0.33 | -1.60163 | 2.76E-10 | 3.74E-09 | yes | down | 1.82 | 1.86 | 1.33 | 1.65 | 1.3 | 1.1 | 1.67 | 1.35 |
| ACADSB | 0.3 | -1.7391 | 2.8E-10 | 3.79E-09 | yes | down | 60.5 | 43.85 | 67.75 | 37.97 | 52.59 | 31.97 | 57.36667 | 40.84333 |
| LOC100355645 | 0.363 | -1.4607 | 2.84E-10 | 3.85E-09 | yes | down | 22.39 | 22.75 | 19.75 | 18.84 | 15.03 | 19.56 | 21.63 | 17.81 |
| LOC100342572 | 5.32 | 2.411414 | 2.89E-10 | 3.92E-09 | yes | up | 127.38 | 56.75 | 155.57 | 1310.47 | 1971.83 | 1078.48 | 113.2333 | 1453.593 |
| MRPL51 | 0.352 | -1.50715 | 2.97E-10 | 4.02E-09 | yes | down | 78.01 | 80.35 | 69.64 | 66.33 | 49.55 | 64.65 | 76 | 60.17667 |
| COL6A6 | 6.3 | 2.655341 | 3.03E-10 | 4.09E-09 | yes | up | 2.03 | 1.45 | 0.38 | 18.99 | 17.88 | 16.24 | 1.286667 | 17.70333 |
| STAB1 | 2.792 | 1.481323 | 3.07E-10 | 4.14E-09 | yes | up | 8.75 | 7.86 | 6.39 | 50.15 | 64.93 | 39.06 | 7.666667 | 51.38 |
| ACOX2 | 5.093 | 2.3484 | 3.19E-10 | 4.31E-09 | yes | up | 3.29 | 6.73 | 3.8 | 44.44 | 37.9 | 74.68 | 4.606667 | 52.34 |
| COX4I1 | 0.419 | -1.2545 | 3.21E-10 | 4.33E-09 | yes | down | 2435.24 | 2674.56 | 2808.35 | 2680.22 | 2609.64 | 2390.05 | 2639.383 | 2559.97 |
| BCCIP | 0.453 | -1.14207 | 3.25E-10 | 4.37E-09 | yes | down | 41.97 | 43.99 | 42.97 | 42.63 | 48.12 | 40.43 | 42.97667 | 43.72667 |
| MOB3C | 2.542 | 1.345741 | 3.31E-10 | 4.46E-09 | yes | up | 3.21 | 3.4 | 2.59 | 17.29 | 16.35 | 16.36 | 3.066667 | 16.66667 |
| MAPKAPK3 | 0.422 | -1.24518 | 3.36E-10 | 4.52E-09 | yes | down | 13.82 | 15.06 | 13.73 | 12.09 | 14.68 | 14.45 | 14.20333 | 13.74 |
| LAPTM4B | 2.065 | 1.046474 | 3.4E-10 | 4.56E-09 | yes | up | 28.83 | 33.49 | 25.98 | 141.1 | 142.02 | 132.89 | 29.43333 | 138.67 |
| MRPS27 | 0.347 | -1.52601 | 3.41E-10 | 4.58E-09 | yes | down | 13.3 | 14.87 | 14.17 | 10.65 | 10.01 | 12.91 | 14.11333 | 11.19 |
| PIP5KL1 | 5.442 | 2.444069 | 3.45E-10 | 4.63E-09 | yes | up | 1.19 | 0.86 | 0.4 | 11.92 | 7.46 | 11.99 | 0.816667 | 10.45667 |
| MOSPD1 | 0.253 | -1.98477 | 3.46E-10 | 4.63E-09 | yes | down | 11.28 | 12.2 | 9.16 | 6.5 | 9.34 | 3.34 | 10.88 | 6.393333 |
| MYADM | 3.247 | 1.69895 | 3.48E-10 | 4.66E-09 | yes | up | 41.83 | 47.08 | 24.72 | 337.03 | 228.17 | 252.77 | 37.87667 | 272.6567 |
| DAP3 | 0.466 | -1.10084 | 3.49E-10 | 4.66E-09 | yes | down | 21.95 | 23.88 | 20.84 | 23.98 | 26.28 | 21.19 | 22.22333 | 23.81667 |
| SYT10 | 0.003 | -8.23362 | 3.6E-10 | 4.82E-09 | yes | down | 0.53 | 0.5 | 0.43 | 0 | 0 | 0 | 0.486667 | 0 |
| MTCH1 | 0.477 | -1.06913 | 3.61E-10 | 4.82E-09 | yes | down | 257.13 | 311.36 | 260.11 | 282.98 | 317.12 | 296.06 | 276.2 | 298.72 |
| GGT6 | 0.071 | -3.81173 | 3.67E-10 | 4.89E-09 | yes | down | 1.37 | 1.13 | 3.69 | 0.32 | 0.3 | 0.29 | 2.063333 | 0.303333 |
| IFNGR2 | 2.744 | 1.456299 | 3.75E-10 | 5.01E-09 | yes | up | 7.75 | 7.08 | 7.97 | 51.09 | 45.01 | 46.84 | 7.6 | 47.64667 |
| LOC108175950 | 0.1 | -3.316 | 3.78E-10 | 5.03E-09 | yes | down | 1.82 | 4.67 | 5.3 | 1.35 | 0.45 | 0.91 | 3.93 | 0.903333 |
| AGPAT5 | 0.39 | -1.35926 | 3.85E-10 | 5.13E-09 | yes | down | 12.97 | 10.86 | 8.98 | 10.97 | 11.75 | 7.34 | 10.93667 | 10.02 |
| CES3 | 0.277 | -1.85223 | 3.87E-10 | 5.15E-09 | yes | down | 25.58 | 23.77 | 33.86 | 18 | 14.99 | 20.33 | 27.73667 | 17.77333 |
| BANP | 2.837 | 1.504193 | 3.91E-10 | 5.19E-09 | yes | up | 3.13 | 3.26 | 2.7 | 19.73 | 17.94 | 21.25 | 3.03 | 19.64 |
| LOC100346772 | 3.379 | 1.756704 | 3.97E-10 | 5.28E-09 | yes | up | 2.46 | 2.17 | 2.91 | 17.58 | 18.36 | 22.83 | 2.513333 | 19.59 |
| LOC100328937 | 0.148 | -2.758 | 4E-10 | 5.31E-09 | yes | down | 5.43 | 7.66 | 4.94 | 2.98 | 0.89 | 1.97 | 6.01 | 1.946667 |
| PATL1 | 0.455 | -1.13738 | 4E-10 | 5.31E-09 | yes | down | 13.95 | 16.07 | 12.75 | 15.83 | 14.73 | 13.75 | 14.25667 | 14.77 |
| LOC103347318 | 4.18 | 2.063542 | 4.08E-10 | 5.4E-09 | yes | up | 8.12 | 4.68 | 4.73 | 47.95 | 79.4 | 41.73 | 5.843333 | 56.36 |
| MED24 | 2.018 | 1.012763 | 4.16E-10 | 5.51E-09 | yes | up | 7.2 | 7.1 | 6.24 | 29.84 | 36.57 | 28.68 | 6.846667 | 31.69667 |
| SELP | 6.269 | 2.648224 | 4.21E-10 | 5.57E-09 | yes | up | 0.49 | 0.36 | 0.33 | 5.57 | 3.53 | 6.72 | 0.393333 | 5.273333 |
| CXCL16 | 2.53 | 1.33914 | 4.25E-10 | 5.61E-09 | yes | up | 5.25 | 4.66 | 4.73 | 24.55 | 36.45 | 27.73 | 4.88 | 29.57667 |
| TCEA1 | 0.38 | -1.39516 | 4.38E-10 | 5.78E-09 | yes | down | 14.91 | 16.78 | 14.46 | 14.76 | 15.85 | 9.87 | 15.38333 | 13.49333 |
| IL10 | 13.767 | 3.783184 | 4.59E-10 | 6.06E-09 | yes | up | 0.47 | 0.37 | 0.05 | 9.93 | 9.75 | 5.5 | 0.296667 | 8.393333 |
| APBB1IP | 4.068 | 2.02422 | 4.76E-10 | 6.28E-09 | yes | up | 1.22 | 1.09 | 0.94 | 11.06 | 8.33 | 10.43 | 1.083333 | 9.94 |
| SESN3 | 0.301 | -1.73422 | 4.89E-10 | 6.44E-09 | yes | down | 8.79 | 9.92 | 10 | 8.58 | 7.32 | 4.2 | 9.57 | 6.7 |
| CDR2L | 2.849 | 1.510519 | 4.96E-10 | 6.53E-09 | yes | up | 2.58 | 2.03 | 2.03 | 15.42 | 13.26 | 14.22 | 2.213333 | 14.3 |
| LOC100348511 | 0.023 | -5.43484 | 5.02E-10 | 6.61E-09 | yes | down | 25.86 | 1.29 | 33.08 | 0.78 | 1.02 | 1.54 | 20.07667 | 1.113333 |
| MRPL13 | 0.334 | -1.57994 | 5.06E-10 | 6.65E-09 | yes | down | 29.8 | 32.08 | 26.33 | 28.79 | 21.51 | 19.18 | 29.40333 | 23.16 |
| OXA1L | 0.408 | -1.29219 | 5.13E-10 | 6.74E-09 | yes | down | 66.96 | 77.86 | 65.81 | 53.84 | 75.35 | 71.65 | 70.21 | 66.94667 |
| KLHL24 | 0.33 | -1.59729 | 5.23E-10 | 6.86E-09 | yes | down | 24.14 | 20.32 | 21.67 | 18.31 | 22.08 | 10.55 | 22.04333 | 16.98 |
| RNF150 | 0.416 | -1.26571 | 5.29E-10 | 6.93E-09 | yes | down | 8.18 | 8.78 | 5.69 | 5.07 | 6.95 | 5.22 | 7.55 | 5.746667 |
| LOC100352190 | 0.026 | -5.25048 | 5.33E-10 | 6.99E-09 | yes | down | 1.7 | 2.92 | 4.53 | 0.17 | 0.19 | 0.24 | 3.05 | 0.2 |
| PNPO | 0.305 | -1.71083 | 5.38E-10 | 7.04E-09 | yes | down | 16.93 | 21.07 | 14.87 | 8.92 | 11.83 | 15.79 | 17.62333 | 12.18 |
| NDUFA2 | 0.381 | -1.39106 | 5.4E-10 | 7.07E-09 | yes | down | 298.09 | 384.74 | 340.55 | 329.43 | 277.6 | 288.31 | 341.1267 | 298.4467 |
| AIFM2 | 0.425 | -1.2347 | 5.43E-10 | 7.1E-09 | yes | down | 349.06 | 407.62 | 427.71 | 398.02 | 412.23 | 353.42 | 394.7967 | 387.89 |
| LOC103348990 | 0.021 | -5.58922 | 5.73E-10 | 7.48E-09 | yes | down | 2.09 | 2.21 | 2.27 | 0.17 | 0.15 | 0 | 2.19 | 0.106667 |
| RMDN3 | 0.458 | -1.12539 | 5.74E-10 | 7.49E-09 | yes | down | 39.57 | 40.27 | 38.03 | 42.88 | 40.75 | 41.25 | 39.29 | 41.62667 |
| NDUFA1 | 0.408 | -1.29396 | 5.95E-10 | 7.76E-09 | yes | down | 305.2 | 255.47 | 280.33 | 295.71 | 265.23 | 234.2 | 280.3333 | 265.0467 |
| SH2B3 | 2.481 | 1.310971 | 6.06E-10 | 7.9E-09 | yes | up | 3.01 | 4.01 | 3.01 | 19.33 | 23.58 | 15.79 | 3.343333 | 19.56667 |
| DNAJC7 | 0.436 | -1.19845 | 6.1E-10 | 7.94E-09 | yes | down | 37.57 | 35.55 | 28.3 | 35.41 | 37.06 | 24.69 | 33.80667 | 32.38667 |
| CDKN2AIP | 0.415 | -1.26749 | 6.33E-10 | 8.23E-09 | yes | down | 9.71 | 10.29 | 6.85 | 8.05 | 7.92 | 7.08 | 8.95 | 7.683333 |
| PDSS1 | 0.257 | -1.95884 | 6.54E-10 | 8.5E-09 | yes | down | 9.03 | 7.99 | 5.81 | 3.9 | 3.57 | 5.64 | 7.61 | 4.37 |
| GALNT10 | 3.161 | 1.660266 | 6.59E-10 | 8.56E-09 | yes | up | 1.99 | 1.6 | 1.22 | 14.04 | 12.63 | 7.91 | 1.603333 | 11.52667 |
| NEDD4L | 0.288 | -1.79444 | 6.65E-10 | 8.64E-09 | yes | down | 8.05 | 6.55 | 3.09 | 3.59 | 4.05 | 3.53 | 5.896667 | 3.723333 |
| GPT2 | 0.47 | -1.09051 | 6.66E-10 | 8.64E-09 | yes | down | 71.29 | 75.71 | 57.82 | 59.85 | 88.31 | 71.09 | 68.27333 | 73.08333 |
| CDIPT | 2.247 | 1.16819 | 6.85E-10 | 8.88E-09 | yes | up | 9.68 | 10.73 | 8.18 | 50.09 | 49.56 | 49.53 | 9.53 | 49.72667 |
| LOC100339756 | 0.365 | -1.45491 | 6.86E-10 | 8.88E-09 | yes | down | 18.07 | 15.87 | 18.65 | 15.88 | 16.06 | 14.04 | 17.53 | 15.32667 |
| SLC7A10 | 5.211 | 2.381483 | 7.01E-10 | 9.07E-09 | yes | up | 1.71 | 2.32 | 1 | 24.28 | 40.37 | 11.81 | 1.676667 | 25.48667 |
| LOC100354761 | 0.443 | -1.17303 | 7.32E-10 | 9.46E-09 | yes | down | 31.05 | 31.34 | 22.61 | 28.09 | 31.62 | 31.26 | 28.33333 | 30.32333 |
| LMOD1 | 4.546 | 2.184569 | 7.34E-10 | 9.47E-09 | yes | up | 5.63 | 3.44 | 2.76 | 36.1 | 27.7 | 54.8 | 3.943333 | 39.53333 |
| LOC100357698 | 0.423 | -1.23988 | 7.35E-10 | 9.48E-09 | yes | down | 110.06 | 117.96 | 108.81 | 112.61 | 108.34 | 107.64 | 112.2767 | 109.53 |
| MYH9 | 2.684 | 1.424258 | 7.35E-10 | 9.48E-09 | yes | up | 24.96 | 29.84 | 27.52 | 166.7 | 228.35 | 121.56 | 27.44 | 172.2033 |
| GALK1 | 0.369 | -1.43832 | 7.46E-10 | 9.6E-09 | yes | down | 18.65 | 21.94 | 20.98 | 19.62 | 18.62 | 14.23 | 20.52333 | 17.49 |
| SLC25A47 | 0.179 | -2.47878 | 7.46E-10 | 9.6E-09 | yes | down | 9.31 | 8.83 | 4.21 | 1.33 | 3.57 | 2.94 | 7.45 | 2.613333 |
| LOC100354804 | 10.743 | 3.425366 | 7.47E-10 | 9.6E-09 | yes | up | 0.33 | 0.78 | 0.07 | 7.13 | 12.17 | 15.28 | 0.393333 | 11.52667 |
| PPP1R18 | 2.517 | 1.331527 | 7.49E-10 | 9.63E-09 | yes | up | 4.25 | 4.81 | 4.53 | 23.81 | 30.99 | 19.63 | 4.53 | 24.81 |
| WDR17 | 0.221 | -2.17607 | 7.51E-10 | 9.64E-09 | yes | down | 1.28 | 1.61 | 1.41 | 0.99 | 0.68 | 0.58 | 1.433333 | 0.75 |
| CCDC190 | 5.861 | 2.551033 | 7.83E-10 | 1E-08 | yes | up | 0.53 | 0.77 | 0.53 | 10.5 | 9.2 | 13.59 | 0.61 | 11.09667 |
| PCSK6 | 2.357 | 1.236937 | 8.06E-10 | 1.03E-08 | yes | up | 8.94 | 8.79 | 6.23 | 37.43 | 42.92 | 47.18 | 7.986667 | 42.51 |
| SLU7 | 0.29 | -1.78789 | 8.32E-10 | 1.06E-08 | yes | down | 18.84 | 18.18 | 18.88 | 12.68 | 17.97 | 7.4 | 18.63333 | 12.68333 |
| RHOG | 2.238 | 1.162048 | 8.32E-10 | 1.06E-08 | yes | up | 13.88 | 15.42 | 14.45 | 75.5 | 82.12 | 67.99 | 14.58333 | 75.20333 |
| NCEH1 | 0.231 | -2.11701 | 8.33E-10 | 1.07E-08 | yes | down | 5.53 | 4.96 | 7.34 | 3.61 | 4.29 | 1.85 | 5.943333 | 3.25 |
| CD53 | 3.061 | 1.613908 | 8.38E-10 | 1.07E-08 | yes | up | 3.91 | 2.95 | 2.59 | 21.99 | 19.77 | 23.58 | 3.15 | 21.78 |
| RWDD4 | 0.308 | -1.70107 | 8.44E-10 | 1.08E-08 | yes | down | 13.02 | 13.42 | 10.67 | 9.7 | 11.54 | 5.11 | 12.37 | 8.783333 |
| COQ7 | 0.351 | -1.51112 | 8.46E-10 | 1.08E-08 | yes | down | 44.92 | 39.57 | 36.99 | 37.66 | 26.71 | 32.26 | 40.49333 | 32.21 |
| ABL1 | 2.265 | 1.179353 | 8.7E-10 | 1.11E-08 | yes | up | 5.65 | 4.5 | 4.74 | 21.58 | 35.69 | 26.96 | 4.963333 | 28.07667 |
| G6PD | 5.646 | 2.497353 | 8.72E-10 | 1.11E-08 | yes | up | 5.1 | 6.92 | 7.69 | 114.75 | 40.28 | 85.57 | 6.57 | 80.2 |
| PSME4 | 0.378 | -1.40498 | 8.91E-10 | 1.13E-08 | yes | down | 11.51 | 7.83 | 6.04 | 6.6 | 7.94 | 6.64 | 8.46 | 7.06 |
| LOC103346860 | 3.521 | 1.815955 | 8.93E-10 | 1.14E-08 | yes | up | 6.98 | 6.01 | 3.77 | 48.74 | 35.3 | 47.31 | 5.586667 | 43.78333 |
| CIR1 | 0.255 | -1.97168 | 9.06E-10 | 1.15E-08 | yes | down | 10.24 | 8.2 | 11.14 | 6.68 | 7.41 | 3.58 | 9.86 | 5.89 |
| CDC123 | 0.49 | -1.02907 | 9.2E-10 | 1.17E-08 | yes | down | 31.97 | 30.64 | 26.61 | 34.15 | 35.73 | 30.38 | 29.74 | 33.42 |
| SLC25A25 | 3.27 | 1.709169 | 9.55E-10 | 1.21E-08 | yes | up | 3.08 | 2.63 | 1.35 | 16.95 | 20.41 | 14.34 | 2.353333 | 17.23333 |
| LOC108175364 | 2.699 | 1.432598 | 9.57E-10 | 1.21E-08 | yes | up | 3.9 | 3.61 | 2.8 | 24.78 | 24.53 | 28.68 | 3.436667 | 25.99667 |
| BNIP3L | 0.337 | -1.57107 | 1.01E-09 | 1.28E-08 | yes | down | 66.26 | 89.54 | 72.24 | 69.75 | 68.95 | 38.42 | 76.01333 | 59.04 |
| NELL2 | 6.683 | 2.740389 | 1.01E-09 | 1.28E-08 | yes | up | 0.3 | 0.42 | 0.25 | 5.74 | 2.95 | 5.65 | 0.323333 | 4.78 |
| UHRF1BP1L | 0.316 | -1.66174 | 1.03E-09 | 1.3E-08 | yes | down | 10.01 | 9.93 | 10.01 | 7.25 | 10.36 | 4.45 | 9.983333 | 7.353333 |
| LOC100353245 | 2.461 | 1.299047 | 1.04E-09 | 1.31E-08 | yes | up | 133.22 | 93.19 | 83.28 | 498.03 | 612.26 | 622.71 | 103.23 | 577.6667 |
| FOXM1 | 8.228 | 3.040539 | 1.04E-09 | 1.31E-08 | yes | up | 0.63 | 0.68 | 0.27 | 11.2 | 15.38 | 3.35 | 0.526667 | 9.976667 |
| LOC100340130 | 2.324 | 1.216542 | 1.04E-09 | 1.32E-08 | yes | up | 33.83 | 36.18 | 29.08 | 202.47 | 180.05 | 143.94 | 33.03 | 175.4867 |
| LOC103349113 | 0.313 | -1.67791 | 1.04E-09 | 1.32E-08 | yes | down | 17.49 | 29.77 | 16.38 | 14.12 | 14.12 | 16.63 | 21.21333 | 14.95667 |
| FAM126A | 3.603 | 1.849335 | 1.06E-09 | 1.34E-08 | yes | up | 1.77 | 1.04 | 0.85 | 11.36 | 14.02 | 7.78 | 1.22 | 11.05333 |
| STAT6 | 2.3 | 1.201704 | 1.07E-09 | 1.35E-08 | yes | up | 6.67 | 9.2 | 7.08 | 37.01 | 46.76 | 37.04 | 7.65 | 40.27 |
| C1QTNF6 | 2.236 | 1.160796 | 1.09E-09 | 1.37E-08 | yes | up | 11.98 | 11.03 | 9.43 | 46.93 | 59.64 | 59.73 | 10.81333 | 55.43333 |
| HLF | 2.732 | 1.45012 | 1.11E-09 | 1.39E-08 | yes | up | 1.98 | 2.13 | 1.28 | 11.37 | 11.65 | 10.83 | 1.796667 | 11.28333 |
| U2SURP | 0.366 | -1.45098 | 1.11E-09 | 1.4E-08 | yes | down | 5.85 | 6.11 | 4.44 | 4.34 | 6.2 | 3.1 | 5.466667 | 4.546667 |
| LOC100351437 | 3.037 | 1.602597 | 1.13E-09 | 1.42E-08 | yes | up | 1.07 | 1.1 | 0.87 | 4.95 | 9.41 | 6.92 | 1.013333 | 7.093333 |
| SPG21 | 2.17 | 1.117809 | 1.15E-09 | 1.44E-08 | yes | up | 22.23 | 26.36 | 18.29 | 91.77 | 113.87 | 94.83 | 22.29333 | 100.1567 |
| UGDH | 2.967 | 1.569176 | 1.17E-09 | 1.46E-08 | yes | up | 11.6 | 11.91 | 11.32 | 82.08 | 60.57 | 91.27 | 11.61 | 77.97333 |
| PRAP1 | 16.338 | 4.030183 | 1.18E-09 | 1.48E-08 | yes | up | 1.06 | 0.94 | 0.72 | 27.69 | 29.49 | 40.13 | 0.906667 | 32.43667 |
| C12H6orf89 | 0.443 | -1.17597 | 1.2E-09 | 1.5E-08 | yes | down | 67.49 | 79.82 | 74.57 | 75.73 | 94.43 | 95.66 | 73.96 | 88.60667 |
| CAPG | 3.816 | 1.932194 | 1.21E-09 | 1.51E-08 | yes | up | 5.62 | 4.22 | 2.61 | 31.98 | 30.4 | 42.37 | 4.15 | 34.91667 |
| PPIL4 | 0.381 | -1.39185 | 1.21E-09 | 1.51E-08 | yes | down | 3.91 | 4.7 | 3.16 | 3.39 | 4.13 | 2.74 | 3.923333 | 3.42 |
| KLHDC2 | 0.476 | -1.07068 | 1.23E-09 | 1.54E-08 | yes | down | 34.33 | 31.15 | 26.86 | 31.82 | 34.25 | 35.54 | 30.78 | 33.87 |
| RPS6KA3 | 0.305 | -1.71258 | 1.27E-09 | 1.58E-08 | yes | down | 17.37 | 16.86 | 22.14 | 14.38 | 13.35 | 7.66 | 18.79 | 11.79667 |
| CRLS1 | 0.219 | -2.19062 | 1.28E-09 | 1.59E-08 | yes | down | 58.82 | 80.95 | 73.74 | 48.96 | 19.75 | 35.55 | 71.17 | 34.75333 |
| TOB1 | 0.27 | -1.88971 | 1.29E-09 | 1.61E-08 | yes | down | 22.47 | 22.86 | 10.41 | 12.25 | 9.45 | 13.11 | 18.58 | 11.60333 |
| ANGEL1 | 2.02 | 1.014605 | 1.3E-09 | 1.61E-08 | yes | up | 7.82 | 7.23 | 6.82 | 34.32 | 35.45 | 31.43 | 7.29 | 33.73333 |
| TRAF3IP2 | 2.46 | 1.298578 | 1.31E-09 | 1.63E-08 | yes | up | 3.78 | 4.69 | 3.32 | 23.01 | 22.56 | 21.27 | 3.93 | 22.28 |
| PTGIR | 5.112 | 2.354003 | 1.32E-09 | 1.64E-08 | yes | up | 0.34 | 0.37 | 0.17 | 3.69 | 2.86 | 3.47 | 0.293333 | 3.34 |
| POLDIP2 | 0.437 | -1.19473 | 1.32E-09 | 1.64E-08 | yes | down | 73.48 | 79.03 | 74.97 | 76.45 | 71.25 | 79.5 | 75.82667 | 75.73333 |
| SLC30A1 | 0.396 | -1.3368 | 1.34E-09 | 1.66E-08 | yes | down | 4.6 | 6.26 | 4.77 | 4.72 | 5.57 | 3.95 | 5.21 | 4.746667 |
| ELF4 | 2.32 | 1.214004 | 1.34E-09 | 1.66E-08 | yes | up | 2.78 | 3.42 | 2.65 | 14.44 | 17.16 | 14.16 | 2.95 | 15.25333 |
| ARHGAP36 | 0.007 | -7.14546 | 1.35E-09 | 1.67E-08 | yes | down | 17.39 | 2.5 | 1.46 | 0.08 | 0.05 | 0.17 | 7.116667 | 0.1 |
| RRAGB | 0.205 | -2.28851 | 1.35E-09 | 1.67E-08 | yes | down | 2.52 | 2.07 | 1.74 | 0.76 | 1.39 | 0.84 | 2.11 | 0.996667 |
| LOC100353131 | 0.004 | -7.88481 | 1.38E-09 | 1.71E-08 | yes | down | 0.87 | 1.83 | 1.4 | 0 | 0 | 0.04 | 1.366667 | 0.013333 |
| NAB2 | 2.804 | 1.487603 | 1.39E-09 | 1.71E-08 | yes | up | 2.95 | 3.77 | 2.87 | 21.34 | 17.97 | 21.82 | 3.196667 | 20.37667 |
| MICAL1 | 3.131 | 1.646688 | 1.39E-09 | 1.71E-08 | yes | up | 2.7 | 2.44 | 1.34 | 15.57 | 15.22 | 15.57 | 2.16 | 15.45333 |
| GAS7 | 3.424 | 1.77549 | 1.39E-09 | 1.72E-08 | yes | up | 6.78 | 3.18 | 2.39 | 28.11 | 27.55 | 25.86 | 4.116667 | 27.17333 |
| HEPH | 0.037 | -4.7659 | 1.41E-09 | 1.73E-08 | yes | down | 0.7 | 0.37 | 0.76 | 0.03 | 0.13 | 0 | 0.61 | 0.053333 |
| KLHL42 | 2.475 | 1.3076 | 1.41E-09 | 1.74E-08 | yes | up | 1.76 | 1.55 | 1.23 | 7.03 | 10.27 | 8.23 | 1.513333 | 8.51 |
| REEP5 | 2.02 | 1.014441 | 1.42E-09 | 1.75E-08 | yes | up | 67.84 | 62.16 | 55.08 | 286.2 | 295.61 | 274.1 | 61.69333 | 285.3033 |
| ATP8A1 | 0.319 | -1.6486 | 1.44E-09 | 1.77E-08 | yes | down | 2.44 | 1.7 | 1.53 | 1.33 | 1.83 | 1 | 1.89 | 1.386667 |
| TBCCD1 | 0.199 | -2.32611 | 1.44E-09 | 1.77E-08 | yes | down | 5.78 | 6.84 | 13 | 4.08 | 4.77 | 3.67 | 8.54 | 4.173333 |
| UBE2C | 6.488 | 2.697812 | 1.45E-09 | 1.78E-08 | yes | up | 4.54 | 3.02 | 1.13 | 52.18 | 48.37 | 25.06 | 2.896667 | 41.87 |
| MRPS31 | 0.175 | -2.51427 | 1.53E-09 | 1.88E-08 | yes | down | 14.56 | 17.07 | 11.03 | 7.84 | 7.1 | 2.22 | 14.22 | 5.72 |
| CISD1 | 0.39 | -1.35829 | 1.54E-09 | 1.89E-08 | yes | down | 67.47 | 62.46 | 66.45 | 68.61 | 53.94 | 53.05 | 65.46 | 58.53333 |
| FMNL1 | 3.801 | 1.926327 | 1.54E-09 | 1.89E-08 | yes | up | 1.1 | 0.73 | 1.03 | 7.62 | 8.46 | 8.47 | 0.953333 | 8.183333 |
| LOC100349037 | 0.402 | -1.31341 | 1.54E-09 | 1.89E-08 | yes | down | 37.29 | 40.41 | 32.28 | 35.06 | 29.05 | 36.09 | 36.66 | 33.4 |
| OAT | 2.156 | 1.108426 | 1.55E-09 | 1.89E-08 | yes | up | 52.07 | 47.68 | 43.92 | 228.22 | 224.2 | 251.99 | 47.89 | 234.8033 |
| SSH3 | 2.614 | 1.386454 | 1.55E-09 | 1.9E-08 | yes | up | 2.52 | 2.1 | 2.1 | 14.64 | 15.76 | 13.48 | 2.24 | 14.62667 |
| SHMT2 | 0.378 | -1.4022 | 1.56E-09 | 1.9E-08 | yes | down | 18.8 | 22.61 | 16.78 | 20.22 | 16.78 | 13.18 | 19.39667 | 16.72667 |
| GRK3 | 2.055 | 1.03898 | 1.56E-09 | 1.9E-08 | yes | up | 10.76 | 11.61 | 10.48 | 53.3 | 62.75 | 49.95 | 10.95 | 55.33333 |
| CPXM2 | 8.95 | 3.16194 | 1.57E-09 | 1.92E-08 | yes | up | 0.57 | 0.3 | 0.06 | 3.28 | 6.97 | 6.77 | 0.31 | 5.673333 |
| MAN2B2 | 4.169 | 2.059733 | 1.59E-09 | 1.94E-08 | yes | up | 3.69 | 2.97 | 3.09 | 28.56 | 43.98 | 22.16 | 3.25 | 31.56667 |
| LOC108178466 | 29.174 | 4.866612 | 1.6E-09 | 1.94E-08 | yes | up | 0.04 | 0.06 | 0 | 2.35 | 1.85 | 2.12 | 0.033333 | 2.106667 |
| TYROBP | 3.62 | 1.855875 | 1.61E-09 | 1.96E-08 | yes | up | 6.2 | 7.94 | 7.45 | 49.99 | 53.31 | 75.68 | 7.196667 | 59.66 |
| INTS6 | 0.37 | -1.43565 | 1.63E-09 | 1.99E-08 | yes | down | 10.2 | 11.11 | 10.56 | 9.19 | 12.68 | 6.49 | 10.62333 | 9.453333 |
| NFKB2 | 2.229 | 1.156453 | 1.72E-09 | 2.09E-08 | yes | up | 5.81 | 5.64 | 4.61 | 24.45 | 28.4 | 28.66 | 5.353333 | 27.17 |
| SH3BGRL2 | 4.151 | 2.053535 | 1.72E-09 | 2.09E-08 | yes | up | 0.46 | 0.44 | 0.29 | 5.2 | 4.33 | 3.65 | 0.396667 | 4.393333 |
| LRRC32 | 3.178 | 1.668034 | 1.73E-09 | 2.1E-08 | yes | up | 2.35 | 2.19 | 2.71 | 15.32 | 16.28 | 21.32 | 2.416667 | 17.64 |
| ALDH1A1 | 2.813 | 1.492089 | 1.74E-09 | 2.11E-08 | yes | up | 115.2 | 77.84 | 55.9 | 434.05 | 695.31 | 513.25 | 82.98 | 547.5367 |
| LOC103347191 | 2.506 | 1.325131 | 1.74E-09 | 2.11E-08 | yes | up | 5.01 | 3.98 | 3.89 | 18.11 | 28.21 | 23.22 | 4.293333 | 23.18 |
| PSMA2 | 0.442 | -1.17901 | 1.76E-09 | 2.14E-08 | yes | down | 123.34 | 105.06 | 95.66 | 122.32 | 102.33 | 100.85 | 108.02 | 108.5 |
| BORCS7 | 0.343 | -1.5421 | 1.77E-09 | 2.14E-08 | yes | down | 17.72 | 18.67 | 21.4 | 14.14 | 18.57 | 7.38 | 19.26333 | 13.36333 |
| ATP1A1 | 0.479 | -1.06244 | 1.79E-09 | 2.17E-08 | yes | down | 139.13 | 118.67 | 126.72 | 136.06 | 146.33 | 140.52 | 128.1733 | 140.97 |
| LOC100341581 | 8.338 | 3.059773 | 1.81E-09 | 2.18E-08 | yes | up | 0.18 | 0.43 | 0.15 | 4.04 | 3.25 | 6.53 | 0.253333 | 4.606667 |
| BCKDHA | 0.377 | -1.40669 | 1.81E-09 | 2.19E-08 | yes | down | 123.04 | 141.92 | 167.47 | 121.65 | 133.65 | 124.37 | 144.1433 | 126.5567 |
| NXT2 | 0.238 | -2.07313 | 1.82E-09 | 2.2E-08 | yes | down | 4.98 | 5.32 | 4.35 | 2 | 2.22 | 1.5 | 4.883333 | 1.906667 |
| ADAMTS15 | 4.249 | 2.087129 | 1.83E-09 | 2.21E-08 | yes | up | 2.16 | 2.66 | 2.33 | 16.06 | 37.96 | 16.69 | 2.383333 | 23.57 |
| CACNB3 | 2.721 | 1.443948 | 1.85E-09 | 2.23E-08 | yes | up | 2.86 | 2.31 | 2.27 | 15.89 | 19.31 | 12.07 | 2.48 | 15.75667 |
| EIF3J | 0.431 | -1.21305 | 1.91E-09 | 2.29E-08 | yes | down | 25.17 | 20.73 | 16.76 | 19.7 | 25.42 | 16.77 | 20.88667 | 20.63 |
| KCNS3 | 12.498 | 3.643671 | 1.91E-09 | 2.29E-08 | yes | up | 0.17 | 0.32 | 0.04 | 7.22 | 3.77 | 3.4 | 0.176667 | 4.796667 |
| PLBD2 | 2.284 | 1.19139 | 1.91E-09 | 2.29E-08 | yes | up | 14.53 | 16.32 | 13.74 | 89.12 | 76.82 | 66.88 | 14.86333 | 77.60667 |
| PPIB | 2.144 | 1.10025 | 1.98E-09 | 2.38E-08 | yes | up | 126.13 | 148.24 | 118.93 | 710.68 | 657.78 | 558.74 | 131.1 | 642.4 |
| LOC103347198 | 21.402 | 4.419665 | 1.99E-09 | 2.38E-08 | yes | up | 0.09 | 0.4 | 0.23 | 11.46 | 6.43 | 17.03 | 0.24 | 11.64 |
| FAT1 | 2.725 | 1.446424 | 1.99E-09 | 2.38E-08 | yes | up | 4.12 | 2.61 | 2.07 | 17.99 | 21.14 | 15.28 | 2.933333 | 18.13667 |
| DNAH12 | 0.072 | -3.79899 | 1.99E-09 | 2.38E-08 | yes | down | 0.22 | 0.16 | 0.09 | 0.03 | 0.03 | 0.01 | 0.156667 | 0.023333 |
| LGALS3 | 5.087 | 2.346859 | 2.01E-09 | 2.4E-08 | yes | up | 24.92 | 15.53 | 11.25 | 139.48 | 140.47 | 299.28 | 17.23333 | 193.0767 |
| ZNF423 | 2.086 | 1.060801 | 2.01E-09 | 2.41E-08 | yes | up | 4.13 | 4.74 | 3.52 | 19.29 | 22.87 | 17.1 | 4.13 | 19.75333 |
| TPP2 | 0.303 | -1.72321 | 2.04E-09 | 2.44E-08 | yes | down | 15.15 | 8.41 | 7.71 | 7.73 | 8.76 | 5.15 | 10.42333 | 7.213333 |
| GTF2H1 | 0.365 | -1.45325 | 2.08E-09 | 2.48E-08 | yes | down | 8.63 | 10.76 | 7.28 | 6.82 | 9.21 | 5.13 | 8.89 | 7.053333 |
| SLC19A2 | 0.088 | -3.50552 | 2.1E-09 | 2.5E-08 | yes | down | 0.94 | 1.02 | 1.1 | 0.32 | 0.18 | 0.12 | 1.02 | 0.206667 |
| LOC100347948 | 0.372 | -1.42624 | 2.1E-09 | 2.51E-08 | yes | down | 1.56 | 1.8 | 1.36 | 1.14 | 1.57 | 1.05 | 1.573333 | 1.253333 |
| CBR4 | 0.256 | -1.96421 | 2.12E-09 | 2.52E-08 | yes | down | 6.14 | 5.66 | 5.99 | 2.99 | 6.14 | 1.74 | 5.93 | 3.623333 |
| LACE1 | 0.379 | -1.39804 | 2.14E-09 | 2.55E-08 | yes | down | 11.58 | 16.08 | 11.42 | 10.06 | 12.69 | 11.48 | 13.02667 | 11.41 |
| SDC2 | 2.335 | 1.223616 | 2.15E-09 | 2.56E-08 | yes | up | 13.01 | 14.83 | 15.59 | 78.31 | 82.73 | 73.68 | 14.47667 | 78.24 |
| P3H4 | 3.815 | 1.931642 | 2.16E-09 | 2.57E-08 | yes | up | 10.68 | 6.92 | 4.24 | 54.85 | 53.59 | 75.58 | 7.28 | 61.34 |
| PDP2 | 0.337 | -1.568 | 2.19E-09 | 2.6E-08 | yes | down | 12.55 | 15.02 | 16.98 | 11.44 | 15.02 | 8.41 | 14.85 | 11.62333 |
| PAIP1 | 0.456 | -1.13267 | 2.19E-09 | 2.61E-08 | yes | down | 55.36 | 47.91 | 39.74 | 54.27 | 54.83 | 38.76 | 47.67 | 49.28667 |
| SEMA3F | 2.612 | 1.384914 | 2.21E-09 | 2.62E-08 | yes | up | 4.13 | 3.46 | 2.57 | 16.73 | 26.54 | 17.57 | 3.386667 | 20.28 |
| KIAA1211 | 0.194 | -2.36705 | 2.24E-09 | 2.66E-08 | yes | down | 4.74 | 5.97 | 1.61 | 1.65 | 1.81 | 1.36 | 4.106667 | 1.606667 |
| PAK1IP1 | 0.346 | -1.53108 | 2.26E-09 | 2.67E-08 | yes | down | 6.61 | 6.9 | 7.13 | 5.6 | 5.97 | 4.93 | 6.88 | 5.5 |
| LOC100352436 | 4.565 | 2.190501 | 2.26E-09 | 2.67E-08 | yes | up | 0.38 | 0.51 | 0.45 | 5 | 4.14 | 5.12 | 0.446667 | 4.753333 |
| RNF212 | 5.327 | 2.413286 | 2.26E-09 | 2.68E-08 | yes | up | 1.25 | 1.82 | 1.55 | 22.39 | 13.37 | 20.21 | 1.54 | 18.65667 |
| LMF2 | 2.274 | 1.185057 | 2.29E-09 | 2.71E-08 | yes | up | 5.89 | 5.99 | 6.04 | 32.22 | 32.67 | 28.9 | 5.973333 | 31.26333 |
| PFDN4 | 0.274 | -1.86714 | 2.32E-09 | 2.74E-08 | yes | down | 27.79 | 22.38 | 15.97 | 16.35 | 16.53 | 8.44 | 22.04667 | 13.77333 |
| LOC100358208 | 0.438 | -1.19038 | 2.34E-09 | 2.77E-08 | yes | down | 706.5 | 701.87 | 692.76 | 747.78 | 651.57 | 717.55 | 700.3767 | 705.6333 |
| PHF19 | 3.033 | 1.600611 | 2.35E-09 | 2.78E-08 | yes | up | 3.22 | 2.74 | 1.68 | 18.77 | 24.85 | 12.89 | 2.546667 | 18.83667 |
| PRKAG2 | 0.398 | -1.32749 | 2.41E-09 | 2.84E-08 | yes | down | 20.18 | 25.56 | 13.67 | 19.09 | 18.43 | 14.23 | 19.80333 | 17.25 |
| PGRMC2 | 0.491 | -1.02722 | 2.41E-09 | 2.84E-08 | yes | down | 51.8 | 65.35 | 47.32 | 60.86 | 69.47 | 54.19 | 54.82333 | 61.50667 |
| DSP | 3.909 | 1.966782 | 2.41E-09 | 2.84E-08 | yes | up | 1.67 | 4.27 | 1.96 | 23.66 | 29.06 | 19.21 | 2.633333 | 23.97667 |
| RIC8A | 2.069 | 1.048774 | 2.43E-09 | 2.86E-08 | yes | up | 14.98 | 15.6 | 11.43 | 67.19 | 65.34 | 64.47 | 14.00333 | 65.66667 |
| NAA35 | 0.444 | -1.17167 | 2.46E-09 | 2.9E-08 | yes | down | 11.74 | 11.91 | 9.15 | 10.96 | 13.53 | 8.78 | 10.93333 | 11.09 |
| MMD | 3.875 | 1.954307 | 2.48E-09 | 2.91E-08 | yes | up | 6.84 | 10.08 | 7.42 | 96.51 | 81.84 | 38.86 | 8.113333 | 72.40333 |
| PNP | 2.908 | 1.540136 | 2.49E-09 | 2.93E-08 | yes | up | 8.98 | 11.15 | 10.3 | 100.53 | 83.58 | 67.08 | 10.14333 | 83.73 |
| NHSL2 | 2.708 | 1.436979 | 2.56E-09 | 3.01E-08 | yes | up | 1.65 | 1.72 | 1.88 | 13.54 | 13.66 | 8.83 | 1.75 | 12.01 |
| COPG1 | 2.035 | 1.024965 | 2.58E-09 | 3.03E-08 | yes | up | 20.62 | 16.59 | 15.32 | 77.58 | 84.87 | 80.95 | 17.51 | 81.13333 |
| ISCU | 0.398 | -1.32891 | 2.59E-09 | 3.03E-08 | yes | down | 234.94 | 230.8 | 218.68 | 174.87 | 195.87 | 249.33 | 228.14 | 206.69 |
| CERCAM | 3.807 | 1.928474 | 2.59E-09 | 3.03E-08 | yes | up | 4.55 | 3.82 | 1.84 | 30.36 | 22.69 | 31.64 | 3.403333 | 28.23 |
| ARF3 | 2.027 | 1.019298 | 2.61E-09 | 3.05E-08 | yes | up | 7.62 | 8.01 | 6.98 | 36.19 | 36.03 | 32.65 | 7.536667 | 34.95667 |
| HINT1 | 0.379 | -1.40128 | 2.67E-09 | 3.13E-08 | yes | down | 312.83 | 316.03 | 315.07 | 336.79 | 249.72 | 231.24 | 314.6433 | 272.5833 |
| SREBF2 | 3.152 | 1.656116 | 2.71E-09 | 3.17E-08 | yes | up | 5.87 | 3.5 | 2.55 | 30.83 | 27.85 | 25.38 | 3.973333 | 28.02 |
| RBM34 | 0.287 | -1.80289 | 2.71E-09 | 3.17E-08 | yes | down | 4.61 | 4.31 | 3.85 | 2.83 | 3.32 | 2.3 | 4.256667 | 2.816667 |
| ACAA1 | 0.259 | -1.94886 | 2.77E-09 | 3.23E-08 | yes | down | 40.11 | 43.56 | 75.27 | 31.65 | 35.4 | 30.67 | 52.98 | 32.57333 |
| ASIC2 | 0.008 | -7.02886 | 2.78E-09 | 3.24E-08 | yes | down | 1.08 | 2.17 | 0.58 | 0.03 | 0 | 0.04 | 1.276667 | 0.023333 |
| CYR61 | 6.933 | 2.793446 | 2.82E-09 | 3.28E-08 | yes | up | 24.11 | 13.93 | 18.67 | 273.33 | 114.91 | 498.38 | 18.90333 | 295.54 |
| LOC100351387 | 2.204 | 1.140402 | 2.87E-09 | 3.34E-08 | yes | up | 86.17 | 64.72 | 60.09 | 305.09 | 442.61 | 320.15 | 70.32667 | 355.95 |
| ACTB | 2.642 | 1.401792 | 2.88E-09 | 3.34E-08 | yes | up | 413.56 | 578.34 | 381.5 | 3170.3 | 2347.25 | 2673.18 | 457.8 | 2730.243 |
| SIM2 | 0.005 | -7.79465 | 2.89E-09 | 3.36E-08 | yes | down | 2.1 | 2.61 | 0.5 | 0 | 0.06 | 0 | 1.736667 | 0.02 |
| MFAP4 | 3.284 | 1.715474 | 2.9E-09 | 3.37E-08 | yes | up | 73.4 | 64.15 | 39.95 | 302.05 | 433.44 | 561.58 | 59.16667 | 432.3567 |
| NR2F2 | 2.568 | 1.360519 | 2.91E-09 | 3.37E-08 | yes | up | 11.23 | 8.19 | 8.02 | 61.76 | 48.08 | 51.37 | 9.146667 | 53.73667 |
| USP25 | 0.337 | -1.56789 | 2.94E-09 | 3.41E-08 | yes | down | 12.56 | 6.4 | 7.09 | 6.36 | 7.23 | 6.25 | 8.683333 | 6.613333 |
| SMARCA5 | 0.4 | -1.32231 | 3.07E-09 | 3.56E-08 | yes | down | 12.79 | 15.74 | 11.3 | 12.95 | 15.44 | 8.63 | 13.27667 | 12.34 |
| SMARCA2 | 2.219 | 1.149851 | 3.09E-09 | 3.57E-08 | yes | up | 18.77 | 15.31 | 16.09 | 75.06 | 96.53 | 78.4 | 16.72333 | 83.33 |
| URI1 | 0.414 | -1.27283 | 3.1E-09 | 3.58E-08 | yes | down | 15.9 | 17.35 | 13.13 | 14.45 | 18.55 | 10.79 | 15.46 | 14.59667 |
| C17H15orf41 | 0.248 | -2.00999 | 3.1E-09 | 3.58E-08 | yes | down | 3.15 | 2.35 | 2.53 | 1.71 | 1.88 | 0.99 | 2.676667 | 1.526667 |
| ABHD14A | 2.233 | 1.158908 | 3.14E-09 | 3.62E-08 | yes | up | 16.34 | 15.44 | 13.96 | 74.37 | 88.07 | 77.56 | 15.24667 | 80 |
| C9H18orf21 | 0.428 | -1.22292 | 3.14E-09 | 3.62E-08 | yes | down | 41.62 | 45.26 | 43.98 | 43.38 | 51.83 | 37.7 | 43.62 | 44.30333 |
| CYP27A1 | 0.291 | -1.78007 | 3.15E-09 | 3.63E-08 | yes | down | 77.35 | 140.87 | 80.38 | 44.2 | 81.29 | 72.97 | 99.53333 | 66.15333 |
| PTGES | 9.477 | 3.244444 | 3.15E-09 | 3.63E-08 | yes | up | 0.76 | 0.62 | 0.37 | 12.77 | 8.12 | 15.7 | 0.583333 | 12.19667 |
| ATP5E | 0.45 | -1.15265 | 3.15E-09 | 3.63E-08 | yes | down | 1756.05 | 1946.42 | 1967.74 | 2016.57 | 2021.15 | 1845 | 1890.07 | 1960.907 |
| AK6 | 0.475 | -1.07297 | 3.21E-09 | 3.69E-08 | yes | down | 24.7 | 23.27 | 20.49 | 26.94 | 29.22 | 22.11 | 22.82 | 26.09 |
| ABCB7 | 0.385 | -1.37558 | 3.23E-09 | 3.71E-08 | yes | down | 16.26 | 14.62 | 15.51 | 15.59 | 17.03 | 10.41 | 15.46333 | 14.34333 |
| ADCY5 | 4.439 | 2.150087 | 3.29E-09 | 3.78E-08 | yes | up | 1.07 | 1.16 | 0.81 | 6.72 | 8.87 | 14.55 | 1.013333 | 10.04667 |
| ME3 | 0.11 | -3.18589 | 3.3E-09 | 3.79E-08 | yes | down | 9.6 | 4.83 | 11.71 | 0.74 | 1.42 | 3.38 | 8.713333 | 1.846667 |
| SEMA3A | 7.603 | 2.926624 | 3.31E-09 | 3.8E-08 | yes | up | 0.5 | 0.42 | 0.4 | 12.93 | 4.27 | 3.44 | 0.44 | 6.88 |
| MRPL16 | 0.429 | -1.21968 | 3.33E-09 | 3.82E-08 | yes | down | 66.25 | 71.05 | 59.96 | 67.09 | 58.17 | 68.6 | 65.75333 | 64.62 |
| SAA3 | 8.787 | 3.135418 | 3.37E-09 | 3.86E-08 | yes | up | 3.35 | 4.54 | 1.71 | 99.63 | 35.69 | 52.39 | 3.2 | 62.57 |
| BMI1 | 0.326 | -1.61747 | 3.37E-09 | 3.86E-08 | yes | down | 8.92 | 11.79 | 8 | 8.31 | 8.67 | 4.55 | 9.57 | 7.176667 |
| SMC6 | 0.312 | -1.67893 | 3.42E-09 | 3.92E-08 | yes | down | 5.62 | 5.1 | 4.97 | 4.38 | 4.69 | 2.27 | 5.23 | 3.78 |
| ZNF397 | 0.318 | -1.65338 | 3.46E-09 | 3.95E-08 | yes | down | 4.93 | 5.03 | 3.67 | 3.1 | 4.8 | 2.13 | 4.543333 | 3.343333 |
| MBOAT1 | 0.373 | -1.4238 | 3.48E-09 | 3.98E-08 | yes | down | 7.17 | 6.29 | 7.66 | 5.63 | 7.02 | 5.21 | 7.04 | 5.953333 |
| ATXN1 | 0.183 | -2.45072 | 3.52E-09 | 4.02E-08 | yes | down | 9.23 | 19.37 | 4.59 | 6.11 | 5.26 | 5.22 | 11.06333 | 5.53 |
| P3H3 | 3.887 | 1.958736 | 3.52E-09 | 4.02E-08 | yes | up | 3.57 | 2.93 | 1.65 | 27.51 | 28.61 | 34.87 | 2.716667 | 30.33 |
| C1RL | 15.183 | 3.924373 | 3.52E-09 | 4.02E-08 | yes | up | 0.18 | 0.08 | 0.05 | 1.98 | 4.86 | 3.7 | 0.103333 | 3.513333 |
| RARS2 | 0.309 | -1.69292 | 3.64E-09 | 4.15E-08 | yes | down | 5.1 | 5.66 | 4.17 | 3.39 | 4.72 | 2.6 | 4.976667 | 3.57 |
| LOC100353281 | 0.426 | -1.23062 | 3.65E-09 | 4.15E-08 | yes | down | 33.42 | 33.18 | 35.4 | 36.12 | 33.96 | 28.31 | 34 | 32.79667 |
| ELAC2 | 0.374 | -1.41926 | 3.68E-09 | 4.19E-08 | yes | down | 9.49 | 11.8 | 10.55 | 7.92 | 11.74 | 9.47 | 10.61333 | 9.71 |
| RTN4 | 0.399 | -1.32504 | 3.69E-09 | 4.2E-08 | yes | down | 322.96 | 229.79 | 175.07 | 233.35 | 223.67 | 261.22 | 242.6067 | 239.4133 |
| IFT57 | 0.262 | -1.93452 | 3.75E-09 | 4.26E-08 | yes | down | 9.46 | 17.67 | 13.67 | 8.53 | 10.4 | 5.34 | 13.6 | 8.09 |
| AKNA | 3.486 | 1.801684 | 3.82E-09 | 4.34E-08 | yes | up | 0.55 | 0.41 | 0.4 | 3.64 | 4.44 | 2.51 | 0.453333 | 3.53 |
| PRDX4 | 2.687 | 1.42605 | 3.9E-09 | 4.42E-08 | yes | up | 21.48 | 19.22 | 10.94 | 112.67 | 104.42 | 95.24 | 17.21333 | 104.11 |
| SRP9 | 0.429 | -1.22002 | 3.91E-09 | 4.43E-08 | yes | down | 27.68 | 25.47 | 22.33 | 28.08 | 26.99 | 19.14 | 25.16 | 24.73667 |
| PROCR | 3.1 | 1.632037 | 3.92E-09 | 4.44E-08 | yes | up | 9.04 | 7.17 | 10.82 | 78.11 | 59.31 | 57.64 | 9.01 | 65.02 |
| VPS4B | 0.395 | -1.33926 | 3.97E-09 | 4.49E-08 | yes | down | 14.35 | 16.9 | 17.19 | 14.27 | 18.35 | 11.64 | 16.14667 | 14.75333 |
| LOC103345855 | 4.714 | 2.237002 | 4.01E-09 | 4.54E-08 | yes | up | 2.01 | 1.6 | 1.22 | 13.11 | 30.99 | 9.75 | 1.61 | 17.95 |
| TCF19 | 6.78 | 2.761183 | 4.07E-09 | 4.6E-08 | yes | up | 0.91 | 0.75 | 0.52 | 13.25 | 16.55 | 4.38 | 0.726667 | 11.39333 |
| ZFP36 | 6.067 | 2.601076 | 4.21E-09 | 4.76E-08 | yes | up | 4.23 | 3.82 | 3.58 | 45.04 | 24.66 | 86.47 | 3.876667 | 52.05667 |
| CTSS | 2.153 | 1.106605 | 4.21E-09 | 4.76E-08 | yes | up | 10.46 | 10.64 | 9.11 | 46.06 | 60.55 | 44.09 | 10.07 | 50.23333 |
| MPZL2 | 2.248 | 1.168729 | 4.25E-09 | 4.8E-08 | yes | up | 9.07 | 10.57 | 7.84 | 52.59 | 49.52 | 38.67 | 9.16 | 46.92667 |
| BMP4 | 2.727 | 1.447468 | 4.4E-09 | 4.96E-08 | yes | up | 4.26 | 3.79 | 4.21 | 25.65 | 26.36 | 24.9 | 4.086667 | 25.63667 |
| TIMM10 | 0.343 | -1.54444 | 4.41E-09 | 4.97E-08 | yes | down | 78.72 | 78.77 | 78.78 | 70.51 | 47.48 | 64.16 | 78.75667 | 60.71667 |
| LOC100339034 | 0.004 | -8.15313 | 4.42E-09 | 4.98E-08 | yes | down | 1.02 | 1.03 | 3.33 | 0 | 0 | 0 | 1.793333 | 0 |
| POFUT2 | 2.091 | 1.064342 | 4.42E-09 | 4.98E-08 | yes | up | 17.94 | 15.52 | 12.07 | 74.66 | 73.05 | 68.81 | 15.17667 | 72.17333 |
| APLNR | 2.882 | 1.526907 | 4.44E-09 | 4.99E-08 | yes | up | 22.74 | 22.23 | 30.7 | 187.67 | 189.18 | 134.81 | 25.22333 | 170.5533 |
| EIF5B | 0.383 | -1.38324 | 4.47E-09 | 5.02E-08 | yes | down | 13.4 | 11.47 | 11.69 | 12.62 | 12.19 | 7.5 | 12.18667 | 10.77 |
| RNF6 | 0.322 | -1.63319 | 4.48E-09 | 5.03E-08 | yes | down | 11.85 | 10.96 | 13.63 | 8.56 | 12.83 | 6.23 | 12.14667 | 9.206667 |
| RELB | 6.287 | 2.652376 | 4.5E-09 | 5.05E-08 | yes | up | 0.8 | 0.39 | 0.6 | 8.45 | 7.45 | 9.62 | 0.596667 | 8.506667 |
| LOC100352275 | 0.436 | -1.19627 | 4.54E-09 | 5.09E-08 | yes | down | 382.26 | 357.75 | 326.8 | 371.05 | 318.37 | 377.97 | 355.6033 | 355.7967 |
| LOC103348850 | 0.251 | -1.99305 | 4.54E-09 | 5.09E-08 | yes | down | 4.63 | 4.54 | 9.78 | 3.77 | 4.8 | 2.73 | 6.316667 | 3.766667 |
| PIK3C3 | 0.35 | -1.51624 | 4.55E-09 | 5.1E-08 | yes | down | 10.62 | 9.24 | 11.16 | 8.03 | 11.29 | 6.11 | 10.34 | 8.476667 |
| LOC100343144 | 4.52 | 2.1762 | 4.56E-09 | 5.1E-08 | yes | up | 4.4 | 3.25 | 7.51 | 46.22 | 60.96 | 55.8 | 5.053333 | 54.32667 |
| NPEPPS | 0.49 | -1.03037 | 4.57E-09 | 5.11E-08 | yes | down | 30.06 | 33.9 | 30.47 | 32.78 | 42.09 | 31.69 | 31.47667 | 35.52 |
| CCDC47 | 0.454 | -1.13997 | 4.6E-09 | 5.14E-08 | yes | down | 28.58 | 25.74 | 21.48 | 26.82 | 32.5 | 19.74 | 25.26667 | 26.35333 |
| LOC100341181 | 0.453 | -1.14145 | 4.62E-09 | 5.16E-08 | yes | down | 22.12 | 26.97 | 21.09 | 24.54 | 28.07 | 20.51 | 23.39333 | 24.37333 |
| ABCD3 | 0.344 | -1.54129 | 4.64E-09 | 5.17E-08 | yes | down | 17.14 | 16.49 | 17.08 | 14.24 | 17.85 | 8.53 | 16.90333 | 13.54 |
| INCENP | 3.946 | 1.980366 | 4.67E-09 | 5.2E-08 | yes | up | 0.96 | 0.79 | 0.68 | 8.3 | 9.92 | 3.98 | 0.81 | 7.4 |
| ENTPD1 | 2.583 | 1.369235 | 4.74E-09 | 5.28E-08 | yes | up | 2.76 | 3.1 | 1.83 | 12.94 | 13.22 | 16 | 2.563333 | 14.05333 |
| LOC108178279 | 3.486 | 1.801557 | 4.76E-09 | 5.3E-08 | yes | up | 2.15 | 1.85 | 1.52 | 14.12 | 12.73 | 16.54 | 1.84 | 14.46333 |
| AKAP12 | 2.472 | 1.305544 | 4.76E-09 | 5.3E-08 | yes | up | 12.49 | 11.15 | 6.59 | 49.39 | 70.34 | 49.6 | 10.07667 | 56.44333 |
| DDHD1 | 0.227 | -2.13665 | 4.79E-09 | 5.33E-08 | yes | down | 17.91 | 26.8 | 17.19 | 9.2 | 15.37 | 4.75 | 20.63333 | 9.773333 |
| ZC3H12A | 3.092 | 1.628722 | 4.8E-09 | 5.33E-08 | yes | up | 1.67 | 1.89 | 1 | 12.64 | 16.16 | 9.6 | 1.52 | 12.8 |
| C3H8orf59 | 0.406 | -1.30007 | 4.86E-09 | 5.4E-08 | yes | down | 72.05 | 81.44 | 63.74 | 74.73 | 72.36 | 55.33 | 72.41 | 67.47333 |
| RACGAP1 | 4.917 | 2.297711 | 4.87E-09 | 5.41E-08 | yes | up | 2.23 | 1.93 | 1.11 | 21.98 | 28.94 | 8.69 | 1.756667 | 19.87 |
| C1QTNF2 | 4.404 | 2.138678 | 4.92E-09 | 5.46E-08 | yes | up | 3.65 | 2.13 | 1.4 | 22.94 | 18.94 | 28.38 | 2.393333 | 23.42 |
| N4BP2 | 0.289 | -1.78871 | 4.94E-09 | 5.47E-08 | yes | down | 2.34 | 2.34 | 2.32 | 1.57 | 2.28 | 0.9 | 2.333333 | 1.583333 |
| RGAG1 | 0.108 | -3.21629 | 4.98E-09 | 5.52E-08 | yes | down | 0.88 | 1.12 | 0.3 | 0.22 | 0.21 | 0.11 | 0.766667 | 0.18 |
| ARHGEF25 | 2.092 | 1.064554 | 5.05E-09 | 5.59E-08 | yes | up | 8.95 | 10.65 | 10.87 | 42.78 | 48.07 | 39.12 | 10.15667 | 43.32333 |
| LOC100358544 | 0.014 | -6.11146 | 5.09E-09 | 5.63E-08 | yes | down | 0.94 | 0.36 | 0.41 | 0.08 | 0.04 | 0 | 0.57 | 0.04 |
| AEBP2 | 0.39 | -1.35667 | 5.2E-09 | 5.75E-08 | yes | down | 7.32 | 7.5 | 7.59 | 7.55 | 7.69 | 5.53 | 7.47 | 6.923333 |
| RARRES2 | 3.865 | 1.950348 | 5.24E-09 | 5.79E-08 | yes | up | 84.95 | 143.84 | 88.18 | 844.25 | 637.23 | 1243.63 | 105.6567 | 908.37 |
| LOC103346606 | 3.309 | 1.726542 | 5.24E-09 | 5.79E-08 | yes | up | 4.62 | 2.99 | 2.33 | 28.84 | 20.88 | 24.03 | 3.313333 | 24.58333 |
| ATF1 | 0.408 | -1.29228 | 5.34E-09 | 5.89E-08 | yes | down | 10.27 | 8.95 | 6.65 | 8.14 | 9.05 | 6.4 | 8.623333 | 7.863333 |
| KPNA2 | 3.224 | 1.688955 | 5.42E-09 | 5.97E-08 | yes | up | 10.2 | 7.68 | 5.08 | 58.71 | 74.14 | 36.11 | 7.653333 | 56.32 |
| CYS1 | 2.421 | 1.275714 | 5.44E-09 | 6E-08 | yes | up | 4.7 | 4.66 | 3.94 | 22.92 | 24.37 | 26.21 | 4.433333 | 24.5 |
| RADIL | 3.928 | 1.973642 | 5.57E-09 | 6.13E-08 | yes | up | 0.74 | 0.45 | 0.47 | 3.88 | 4.91 | 5.32 | 0.553333 | 4.703333 |
| LOC103348101 | 0.236 | -2.08393 | 5.62E-09 | 6.19E-08 | yes | down | 4.14 | 4.89 | 4.17 | 3.45 | 1.72 | 1.01 | 4.4 | 2.06 |
| LOC103348141 | 0.296 | -1.75446 | 5.81E-09 | 6.38E-08 | yes | down | 3.66 | 3.2 | 2.75 | 4.18 | 6.19 | 4.57 | 3.203333 | 4.98 |
| LOC100359247 | 0.21 | -2.25342 | 5.81E-09 | 6.39E-08 | yes | down | 3.76 | 4.74 | 3.3 | 1.18 | 2.12 | 2.74 | 3.933333 | 2.013333 |
| MRPL46 | 0.392 | -1.35057 | 5.95E-09 | 6.53E-08 | yes | down | 13.6 | 14.5 | 12.45 | 11.57 | 10.84 | 13.76 | 13.51667 | 12.05667 |
| MRPL50 | 0.302 | -1.72969 | 6.32E-09 | 6.93E-08 | yes | down | 14.48 | 17.62 | 13.24 | 13.45 | 9.19 | 8.35 | 15.11333 | 10.33 |
| TRIM8 | 2.322 | 1.215532 | 6.33E-09 | 6.93E-08 | yes | up | 5.23 | 4.29 | 4.19 | 19.83 | 28.25 | 24.91 | 4.57 | 24.33 |
| PRKAA1 | 0.349 | -1.51701 | 6.36E-09 | 6.97E-08 | yes | down | 6.67 | 5.84 | 4.03 | 4.65 | 5.72 | 2.9 | 5.513333 | 4.423333 |
| DCAF13 | 0.443 | -1.1749 | 6.58E-09 | 7.21E-08 | yes | down | 13.98 | 15.69 | 12.47 | 14.63 | 16.41 | 11.8 | 14.04667 | 14.28 |
| ARHGAP5 | 0.29 | -1.7877 | 6.72E-09 | 7.35E-08 | yes | down | 7.62 | 7.6 | 7.88 | 5.92 | 7.04 | 2.74 | 7.7 | 5.233333 |
| CTSE | 0.139 | -2.84189 | 6.73E-09 | 7.35E-08 | yes | down | 4.84 | 11.23 | 10.41 | 2.99 | 1.37 | 3.93 | 8.826667 | 2.763333 |
| TEFM | 0.373 | -1.4246 | 6.76E-09 | 7.38E-08 | yes | down | 8.45 | 10.96 | 9.47 | 9.2 | 8.47 | 7.06 | 9.626667 | 8.243333 |
| PRCP | 3.141 | 1.651217 | 6.77E-09 | 7.39E-08 | yes | up | 15.69 | 16.82 | 20.54 | 139.16 | 172.88 | 85.6 | 17.68333 | 132.5467 |
| GLRX3 | 0.453 | -1.14205 | 6.78E-09 | 7.39E-08 | yes | down | 90.69 | 118.27 | 98.23 | 112.71 | 113.35 | 94.25 | 102.3967 | 106.77 |
| ANKRD28 | 0.476 | -1.06966 | 6.79E-09 | 7.4E-08 | yes | down | 7.8 | 8.27 | 7.32 | 8.38 | 9.71 | 6.59 | 7.796667 | 8.226667 |
| LOC100349768 | 0.219 | -2.19141 | 6.79E-09 | 7.4E-08 | yes | down | 986.6 | 374.03 | 287.72 | 250.63 | 257.42 | 289.18 | 549.45 | 265.7433 |
| HHAT | 4.735 | 2.243365 | 6.82E-09 | 7.43E-08 | yes | up | 0.56 | 0.52 | 0.23 | 5.07 | 4.77 | 4.34 | 0.436667 | 4.726667 |
| CNTNAP1 | 6.274 | 2.649416 | 6.86E-09 | 7.47E-08 | yes | up | 0.3 | 0.14 | 0.1 | 3.11 | 2.2 | 2.3 | 0.18 | 2.536667 |
| ISM1 | 11.467 | 3.519363 | 6.86E-09 | 7.47E-08 | yes | up | 0.08 | 0.1 | 0.07 | 2.79 | 1.73 | 1.98 | 0.083333 | 2.166667 |
| OSR1 | 3.378 | 1.756085 | 6.9E-09 | 7.5E-08 | yes | up | 3.56 | 3.01 | 2.21 | 24.62 | 18.95 | 28.16 | 2.926667 | 23.91 |
| CTGF | 2.946 | 1.558953 | 6.92E-09 | 7.52E-08 | yes | up | 20.78 | 17.68 | 19.07 | 169.72 | 104.02 | 112.26 | 19.17667 | 128.6667 |
| PRC1 | 5.581 | 2.480401 | 7.05E-09 | 7.66E-08 | yes | up | 2.72 | 2.38 | 1.18 | 30.27 | 39.86 | 10.25 | 2.093333 | 26.79333 |
| MAPK8IP2 | 0.048 | -4.37979 | 7.11E-09 | 7.71E-08 | yes | down | 0.59 | 1.58 | 1.52 | 0.18 | 0.03 | 0.2 | 1.23 | 0.136667 |
| KIT | 3.483 | 1.800198 | 7.3E-09 | 7.91E-08 | yes | up | 2.09 | 2.04 | 2.12 | 19.56 | 20.95 | 9.24 | 2.083333 | 16.58333 |
| PTK2B | 3.717 | 1.893984 | 7.32E-09 | 7.93E-08 | yes | up | 0.48 | 0.42 | 0.4 | 3.24 | 3.67 | 4.18 | 0.433333 | 3.696667 |
| COASY | 0.392 | -1.34932 | 7.38E-09 | 7.98E-08 | yes | down | 16.49 | 18.78 | 19.4 | 17.51 | 15.04 | 16.44 | 18.22333 | 16.33 |
| EMP3 | 3.446 | 1.785101 | 7.48E-09 | 8.09E-08 | yes | up | 21.09 | 15.94 | 9.82 | 120.4 | 97.1 | 131.85 | 15.61667 | 116.45 |
| SRXN1 | 0.267 | -1.90582 | 7.51E-09 | 8.12E-08 | yes | down | 20.35 | 41.92 | 24.53 | 19.3 | 15.34 | 17.92 | 28.93333 | 17.52 |
| HSD17B12 | 0.281 | -1.83042 | 7.54E-09 | 8.15E-08 | yes | down | 46.73 | 103.63 | 82.34 | 54.7 | 57 | 36.47 | 77.56667 | 49.39 |
| LOC100337909 | 3.563 | 1.833185 | 7.58E-09 | 8.18E-08 | yes | up | 1.68 | 1.48 | 1.59 | 12.94 | 16.27 | 10.41 | 1.583333 | 13.20667 |
| PCCB | 0.394 | -1.34192 | 7.66E-09 | 8.27E-08 | yes | down | 59.62 | 69.77 | 78.15 | 67.28 | 63.23 | 59.27 | 69.18 | 63.26 |
| FRMD6 | 2.298 | 1.200352 | 7.73E-09 | 8.34E-08 | yes | up | 3.65 | 3.53 | 2.41 | 18.67 | 17.43 | 13.87 | 3.196667 | 16.65667 |
| RFK | 0.363 | -1.4601 | 7.74E-09 | 8.34E-08 | yes | down | 6.49 | 9.12 | 6.58 | 7.01 | 6.87 | 4.63 | 7.396667 | 6.17 |
| NDUFB3 | 0.384 | -1.37997 | 7.75E-09 | 8.34E-08 | yes | down | 202.77 | 144.26 | 137.55 | 149.05 | 120.51 | 153.7 | 161.5267 | 141.0867 |
| SH3BP5 | 2.027 | 1.019274 | 7.83E-09 | 8.43E-08 | yes | up | 19.71 | 16.53 | 13.47 | 72.74 | 77.63 | 76.59 | 16.57 | 75.65333 |
| VBP1 | 0.354 | -1.49656 | 7.96E-09 | 8.56E-08 | yes | down | 51.55 | 55.67 | 49.46 | 44.3 | 57.34 | 28.29 | 52.22667 | 43.31 |
| LOC108176569 | 33.769 | 5.077647 | 8.03E-09 | 8.63E-08 | yes | up | 0.04 | 0.02 | 0 | 3.5 | 2.47 | 1.02 | 0.02 | 2.33 |
| TRAM2 | 2.682 | 1.423128 | 8.2E-09 | 8.81E-08 | yes | up | 4.05 | 2.38 | 2.89 | 13.92 | 22.58 | 18.31 | 3.106667 | 18.27 |
| PRMT2 | 2.056 | 1.040079 | 8.39E-09 | 9E-08 | yes | up | 8.67 | 9.78 | 6.54 | 38.46 | 45.94 | 41.13 | 8.33 | 41.84333 |
| COMMD8 | 0.264 | -1.91937 | 8.51E-09 | 9.13E-08 | yes | down | 8.01 | 9.52 | 7.56 | 6.56 | 5.84 | 2.83 | 8.363333 | 5.076667 |
| FHL5 | 2.692 | 1.428434 | 8.56E-09 | 9.17E-08 | yes | up | 2.81 | 1.95 | 1.94 | 15.56 | 14.66 | 10.66 | 2.233333 | 13.62667 |
| BST1 | 3.52 | 1.815751 | 8.6E-09 | 9.21E-08 | yes | up | 5.54 | 5.58 | 2.71 | 33 | 29.97 | 43.95 | 4.61 | 35.64 |
| MRPS15 | 0.498 | -1.00692 | 8.61E-09 | 9.22E-08 | yes | down | 60.74 | 57.12 | 56.23 | 66.77 | 67.79 | 59.47 | 58.03 | 64.67667 |
| C12H6orf136 | 0.419 | -1.25433 | 9.01E-09 | 9.64E-08 | yes | down | 16.09 | 13.57 | 14.81 | 14.54 | 15.99 | 12.54 | 14.82333 | 14.35667 |
| SKIL | 0.358 | -1.4805 | 9.07E-09 | 9.7E-08 | yes | down | 2.01 | 2.72 | 1.8 | 1.73 | 2.15 | 1.31 | 2.176667 | 1.73 |
| VWCE | 0.253 | -1.98069 | 9.2E-09 | 9.83E-08 | yes | down | 1.37 | 1.5 | 1.66 | 0.72 | 0.95 | 1.04 | 1.51 | 0.903333 |
| CLCN3 | 0.368 | -1.4432 | 9.26E-09 | 9.88E-08 | yes | down | 9.2 | 8.13 | 9.97 | 8.55 | 9.27 | 5.34 | 9.1 | 7.72 |
| FES | 2.285 | 1.192214 | 9.34E-09 | 9.97E-08 | yes | up | 3.62 | 4.76 | 3.65 | 20.94 | 21.68 | 19.68 | 4.01 | 20.76667 |
| GRIA3 | 5.583 | 2.481038 | 9.35E-09 | 9.97E-08 | yes | up | 0.58 | 0.37 | 0.18 | 4.64 | 6.93 | 2.81 | 0.376667 | 4.793333 |
| NLK | 0.395 | -1.33978 | 9.43E-09 | 1E-07 | yes | down | 3.4 | 3.88 | 3.48 | 3.15 | 4 | 2.85 | 3.586667 | 3.333333 |
| C7H2orf47 | 0.352 | -1.50731 | 9.44E-09 | 1E-07 | yes | down | 11.17 | 14.25 | 11.32 | 10.87 | 10.68 | 8.71 | 12.24667 | 10.08667 |
| ENOSF1 | 3.477 | 1.798023 | 9.48E-09 | 1.01E-07 | yes | up | 2.84 | 3.71 | 1.9 | 26.2 | 17.89 | 21.31 | 2.816667 | 21.8 |
| C14H3orf70 | 2.57 | 1.361692 | 9.49E-09 | 1.01E-07 | yes | up | 2.43 | 2.31 | 1.83 | 14.01 | 15.88 | 9.01 | 2.19 | 12.96667 |
| ACTN1 | 2.249 | 1.1691 | 9.63E-09 | 1.02E-07 | yes | up | 9.63 | 10.78 | 11.25 | 55.45 | 57.83 | 50.58 | 10.55333 | 54.62 |
| GTF2B | 0.33 | -1.59815 | 9.74E-09 | 1.03E-07 | yes | down | 26.62 | 23.18 | 35.51 | 20.83 | 25.11 | 20.3 | 28.43667 | 22.08 |
| TADA3 | 2.871 | 1.521642 | 9.75E-09 | 1.04E-07 | yes | up | 12.08 | 9.47 | 6.37 | 70.15 | 54.49 | 54.79 | 9.306667 | 59.81 |
| LOC100349757 | 2.36 | 1.238626 | 9.8E-09 | 1.04E-07 | yes | up | 6.78 | 8.33 | 8.14 | 41.19 | 44.5 | 41.5 | 7.75 | 42.39667 |
| EXOSC9 | 0.363 | -1.46306 | 9.84E-09 | 1.04E-07 | yes | down | 8.79 | 10.76 | 8.85 | 8.04 | 10.32 | 5.78 | 9.466667 | 8.046667 |
| NDRG3 | 2.034 | 1.024566 | 9.93E-09 | 1.05E-07 | yes | up | 7.71 | 8.96 | 6.79 | 36.77 | 37.1 | 34.86 | 7.82 | 36.24333 |
| VDAC1 | 0.404 | -1.30776 | 1E-08 | 1.06E-07 | yes | down | 377.7 | 236.89 | 227.28 | 280.39 | 297.19 | 283.93 | 280.6233 | 287.17 |
| LOC100349830 | 0.49 | -1.03062 | 1E-08 | 1.06E-07 | yes | down | 91.48 | 85.02 | 88.71 | 110.05 | 107.25 | 84.3 | 88.40333 | 100.5333 |
| LOC108176510 | 2.232 | 1.158319 | 1.03E-08 | 1.09E-07 | yes | up | 189.61 | 194.93 | 170.4 | 1015.68 | 849.91 | 967.98 | 184.98 | 944.5233 |
| UBXN2B | 0.297 | -1.75302 | 1.03E-08 | 1.09E-07 | yes | down | 4.2 | 5.21 | 4.57 | 3.92 | 4.43 | 1.92 | 4.66 | 3.423333 |
| RASGRP2 | 3.626 | 1.858319 | 1.03E-08 | 1.09E-07 | yes | up | 2.32 | 4.94 | 2.96 | 27.94 | 49.46 | 23.1 | 3.406667 | 33.5 |
| CMIP | 2.333 | 1.222298 | 1.04E-08 | 1.1E-07 | yes | up | 4.31 | 4.98 | 4.74 | 27.49 | 27.96 | 20.3 | 4.676667 | 25.25 |
| METAP2 | 0.401 | -1.31927 | 1.04E-08 | 1.1E-07 | yes | down | 30.43 | 24.28 | 24.29 | 26.72 | 32.65 | 17.38 | 26.33333 | 25.58333 |
| MARCKSL1 | 2.57 | 1.361765 | 1.05E-08 | 1.1E-07 | yes | up | 22.98 | 25.73 | 22.07 | 168.43 | 152.12 | 98.49 | 23.59333 | 139.68 |
| SERPINB5 | 0.009 | -6.80105 | 1.06E-08 | 1.12E-07 | yes | down | 0.61 | 1.14 | 1.32 | 0.03 | 0.03 | 0 | 1.023333 | 0.02 |
| PPP1R1A | 0.326 | -1.61776 | 1.1E-08 | 1.16E-07 | yes | down | 142.31 | 163.75 | 96.22 | 72.28 | 78.68 | 132.65 | 134.0933 | 94.53667 |
| TMEM100 | 3.308 | 1.726102 | 1.1E-08 | 1.16E-07 | yes | up | 10.16 | 11.64 | 9.42 | 54.12 | 127.54 | 62.21 | 10.40667 | 81.29 |
| LOC100348739 | 3.892 | 1.960587 | 1.17E-08 | 1.23E-07 | yes | up | 2.05 | 3.75 | 1.83 | 21.37 | 19.76 | 25.65 | 2.543333 | 22.26 |
| SLC25A15 | 0.444 | -1.17079 | 1.18E-08 | 1.24E-07 | yes | down | 11.04 | 8.12 | 8.39 | 8.65 | 10.63 | 8.79 | 9.183333 | 9.356667 |
| LOC100008822 | 2.117 | 1.082211 | 1.2E-08 | 1.26E-07 | yes | up | 153.16 | 209.29 | 146.23 | 757.6 | 873.71 | 826.69 | 169.56 | 819.3333 |
| TRAM1L1 | 0.043 | -4.54082 | 1.2E-08 | 1.26E-07 | yes | down | 0.88 | 1.12 | 0.69 | 0.18 | 0.07 | 0 | 0.896667 | 0.083333 |
| MTMR10 | 0.435 | -1.19982 | 1.21E-08 | 1.27E-07 | yes | down | 7.78 | 7.48 | 6.8 | 6.28 | 10.46 | 6.78 | 7.353333 | 7.84 |
| LOC108176505 | 0.332 | -1.59237 | 1.23E-08 | 1.29E-07 | yes | down | 90.33 | 112.22 | 107.78 | 67.7 | 117.6 | 55.23 | 103.4433 | 80.17667 |
| DGKA | 2.487 | 1.314592 | 1.25E-08 | 1.3E-07 | yes | up | 3.11 | 3.05 | 2.28 | 12.79 | 17.77 | 16.88 | 2.813333 | 15.81333 |
| LOC103350250 | 0.078 | -3.67939 | 1.25E-08 | 1.31E-07 | yes | down | 1.16 | 2.02 | 2.3 | 0.24 | 0.17 | 0.6 | 1.826667 | 0.336667 |
| CMC1 | 0.398 | -1.33032 | 1.25E-08 | 1.31E-07 | yes | down | 12.8 | 11.37 | 11.81 | 11.16 | 9.79 | 10.33 | 11.99333 | 10.42667 |
| KIFC1 | 4.996 | 2.320643 | 1.26E-08 | 1.31E-07 | yes | up | 1.16 | 1.36 | 0.76 | 14.54 | 18.09 | 5.33 | 1.093333 | 12.65333 |
| NEK2 | 7.015 | 2.810461 | 1.27E-08 | 1.33E-07 | yes | up | 1.15 | 1.33 | 0.34 | 17.25 | 20.86 | 6.45 | 0.94 | 14.85333 |
| NUAK2 | 6.618 | 2.726376 | 1.28E-08 | 1.34E-07 | yes | up | 0.41 | 0.3 | 0.22 | 6.34 | 3.06 | 4.73 | 0.31 | 4.71 |
| NCSTN | 2.446 | 1.290158 | 1.3E-08 | 1.35E-07 | yes | up | 14.99 | 12.12 | 13.12 | 85.6 | 65.94 | 72.73 | 13.41 | 74.75667 |
| NDUFAF1 | 0.403 | -1.31114 | 1.3E-08 | 1.36E-07 | yes | down | 27.48 | 25.03 | 19.97 | 24.96 | 18.54 | 19.64 | 24.16 | 21.04667 |
| ADAMTS1 | 2.633 | 1.396475 | 1.36E-08 | 1.42E-07 | yes | up | 7.05 | 6.73 | 7.57 | 46.54 | 35.38 | 46.92 | 7.116667 | 42.94667 |
| SMARCD2 | 2.005 | 1.003743 | 1.37E-08 | 1.43E-07 | yes | up | 11.89 | 14.22 | 12.09 | 56.35 | 66.17 | 53.25 | 12.73333 | 58.59 |
| INSR | 0.405 | -1.30301 | 1.39E-08 | 1.45E-07 | yes | down | 18.27 | 20.7 | 15.46 | 12.78 | 23.79 | 14.47 | 18.14333 | 17.01333 |
| KIF20A | 5.271 | 2.398212 | 1.4E-08 | 1.46E-07 | yes | up | 1.73 | 1.4 | 0.82 | 19.24 | 22.1 | 6.23 | 1.316667 | 15.85667 |
| SEPT11 | 2.176 | 1.121667 | 1.41E-08 | 1.47E-07 | yes | up | 12.72 | 9.21 | 5.84 | 40.94 | 46.02 | 37.33 | 9.256667 | 41.43 |
| ARHGEF6 | 0.429 | -1.21975 | 1.41E-08 | 1.47E-07 | yes | down | 15.68 | 16.25 | 13.83 | 15.6 | 19.18 | 10.52 | 15.25333 | 15.1 |
| SERTAD3 | 2.533 | 1.341055 | 1.43E-08 | 1.48E-07 | yes | up | 7.38 | 7.59 | 6.29 | 47.87 | 37.17 | 37.34 | 7.086667 | 40.79333 |
| M1AP | 227.205 | 7.827848 | 1.43E-08 | 1.48E-07 | yes | up | 0 | 0 | 0 | 3.15 | 0.99 | 3.58 | 0 | 2.573333 |
| FSTL1 | 2.149 | 1.103688 | 1.43E-08 | 1.48E-07 | yes | up | 195.96 | 171.42 | 110.64 | 730.09 | 825.43 | 759.63 | 159.34 | 771.7167 |
| LOC103346561 | 5.216 | 2.382832 | 1.44E-08 | 1.49E-07 | yes | up | 1.53 | 0.74 | 0.61 | 9.81 | 12.98 | 6.52 | 0.96 | 9.77 |
| SELM | 3.814 | 1.931301 | 1.44E-08 | 1.49E-07 | yes | up | 12.1 | 7.44 | 5.25 | 65.19 | 58.26 | 86.3 | 8.263333 | 69.91667 |
| UCHL3 | 0.413 | -1.27551 | 1.44E-08 | 1.49E-07 | yes | down | 20.75 | 25.31 | 18.65 | 24.12 | 23.99 | 19.11 | 21.57 | 22.40667 |
| RAB3IL1 | 2.236 | 1.16094 | 1.46E-08 | 1.51E-07 | yes | up | 6.63 | 7.31 | 5.06 | 28.42 | 33.54 | 34.48 | 6.333333 | 32.14667 |
| GBA | 2.525 | 1.33616 | 1.47E-08 | 1.52E-07 | yes | up | 3.15 | 4.18 | 3.02 | 20.03 | 19.18 | 20.24 | 3.45 | 19.81667 |
| FN1 | 3.078 | 1.621984 | 1.55E-08 | 1.6E-07 | yes | up | 96.58 | 65.24 | 36.18 | 385.76 | 532.55 | 448.54 | 66 | 455.6167 |
| DAAM2 | 2.915 | 1.543434 | 1.57E-08 | 1.62E-07 | yes | up | 4.25 | 7.44 | 3.26 | 31.5 | 37.78 | 28.59 | 4.983333 | 32.62333 |
| ATP5G1 | 0.269 | -1.89239 | 1.58E-08 | 1.63E-07 | yes | down | 427.15 | 234.16 | 173.7 | 196.13 | 128.56 | 184.1 | 278.3367 | 169.5967 |
| DPYD | 0.267 | -1.90548 | 1.59E-08 | 1.64E-07 | yes | down | 5.04 | 4.89 | 5.71 | 2.73 | 4.12 | 1.68 | 5.213333 | 2.843333 |
| CHSY1 | 2.311 | 1.208642 | 1.61E-08 | 1.66E-07 | yes | up | 3.52 | 3.22 | 2.43 | 17.12 | 15.22 | 16.53 | 3.056667 | 16.29 |
| PPL | 4.9 | 2.292847 | 1.62E-08 | 1.67E-07 | yes | up | 1.58 | 0.73 | 0.81 | 7.97 | 8.76 | 17.17 | 1.04 | 11.3 |
| KYAT3 | 0.39 | -1.35941 | 1.65E-08 | 1.7E-07 | yes | down | 56.1 | 52.77 | 54.37 | 61.14 | 41.95 | 42.79 | 54.41333 | 48.62667 |
| PLEKHA5 | 0.354 | -1.49978 | 1.66E-08 | 1.7E-07 | yes | down | 4.65 | 5.35 | 3.64 | 3.05 | 5.29 | 2.78 | 4.546667 | 3.706667 |
| SLC25A4 | 0.218 | -2.20085 | 1.68E-08 | 1.73E-07 | yes | down | 652.87 | 244 | 177.25 | 178.87 | 160.56 | 175.31 | 358.04 | 171.58 |
| MTMR6 | 0.351 | -1.51025 | 1.68E-08 | 1.73E-07 | yes | down | 7.76 | 7.4 | 7.09 | 6.13 | 8.29 | 3.77 | 7.416667 | 6.063333 |
| NTN4 | 3.766 | 1.912965 | 1.69E-08 | 1.73E-07 | yes | up | 6.37 | 3.81 | 2 | 32.01 | 31.98 | 36.92 | 4.06 | 33.63667 |
| RARA | 2.608 | 1.382994 | 1.72E-08 | 1.76E-07 | yes | up | 4.76 | 4.34 | 4.45 | 26.63 | 23.4 | 32.67 | 4.516667 | 27.56667 |
| YKT6 | 0.476 | -1.07209 | 1.72E-08 | 1.76E-07 | yes | down | 33.86 | 37.17 | 35.54 | 33.96 | 39.22 | 39.49 | 35.52333 | 37.55667 |
| MATR3 | 0.378 | -1.40271 | 1.75E-08 | 1.79E-07 | yes | down | 68.3 | 68.11 | 55.19 | 60.7 | 76.23 | 36.29 | 63.86667 | 57.74 |
| RGS4 | 5.545 | 2.47113 | 1.75E-08 | 1.79E-07 | yes | up | 2.75 | 2.99 | 2.08 | 23.37 | 26.28 | 49.62 | 2.606667 | 33.09 |
| KCTD10 | 2.079 | 1.056042 | 1.78E-08 | 1.82E-07 | yes | up | 11.87 | 14.54 | 9.57 | 52.92 | 68.81 | 49.44 | 11.99333 | 57.05667 |
| LOC100346685 | 269.65 | 8.074945 | 1.8E-08 | 1.84E-07 | yes | up | 0 | 0 | 0 | 7.71 | 1 | 2.6 | 0 | 3.77 |
| SDAD1 | 0.384 | -1.37964 | 1.83E-08 | 1.87E-07 | yes | down | 5.05 | 5.07 | 3.95 | 3.82 | 5.74 | 2.93 | 4.69 | 4.163333 |
| COG6 | 0.376 | -1.41059 | 1.83E-08 | 1.87E-07 | yes | down | 9.34 | 10.04 | 10.2 | 9.76 | 10.01 | 6.08 | 9.86 | 8.616667 |
| CD34 | 2.393 | 1.258535 | 1.87E-08 | 1.9E-07 | yes | up | 54.61 | 53.81 | 49.35 | 286.25 | 238.35 | 325.16 | 52.59 | 283.2533 |
| AHNAK | 2.455 | 1.295598 | 1.94E-08 | 1.98E-07 | yes | up | 62.52 | 46.41 | 42.63 | 213.23 | 365.96 | 284.98 | 50.52 | 288.0567 |
| PAQR4 | 2.249 | 1.169082 | 1.97E-08 | 2.01E-07 | yes | up | 4.09 | 3.5 | 2.9 | 16.75 | 21.22 | 16.82 | 3.496667 | 18.26333 |
| HPRT1 | 0.358 | -1.48156 | 1.98E-08 | 2.01E-07 | yes | down | 23.09 | 19.86 | 13.58 | 18.24 | 12.94 | 14.14 | 18.84333 | 15.10667 |
| GUCY1B3 | 3.151 | 1.655603 | 1.99E-08 | 2.03E-07 | yes | up | 4.01 | 3.43 | 2.76 | 33.58 | 20.73 | 18.26 | 3.4 | 24.19 |
| ADPGK | 2.23 | 1.156872 | 2.06E-08 | 2.09E-07 | yes | up | 6.97 | 8.64 | 6.41 | 37.73 | 38.19 | 41.17 | 7.34 | 39.03 |
| LOC100340718 | 2.654 | 1.408154 | 2.11E-08 | 2.14E-07 | yes | up | 10.62 | 9.51 | 6.87 | 57.26 | 43.44 | 59.64 | 9 | 53.44667 |
| ITGB4 | 3.832 | 1.938023 | 2.12E-08 | 2.15E-07 | yes | up | 2.53 | 1.44 | 1.15 | 13.04 | 13.91 | 22.74 | 1.706667 | 16.56333 |
| BROX | 0.405 | -1.30519 | 2.13E-08 | 2.15E-07 | yes | down | 17.33 | 20.66 | 16.12 | 18.79 | 20.75 | 11.38 | 18.03667 | 16.97333 |
| LOC100351490 | 0.281 | -1.83134 | 2.13E-08 | 2.16E-07 | yes | down | 3.05 | 4.78 | 3.82 | 1.6 | 2.7 | 1.33 | 3.883333 | 1.876667 |
| HSPA2 | 3.193 | 1.67508 | 2.14E-08 | 2.16E-07 | yes | up | 1.7 | 1.58 | 1.02 | 11.55 | 8.57 | 10.62 | 1.433333 | 10.24667 |
| USP19 | 0.494 | -1.01667 | 2.19E-08 | 2.22E-07 | yes | down | 26.46 | 33.11 | 22.02 | 27.13 | 35.78 | 29.03 | 27.19667 | 30.64667 |
| SEMA3C | 2.935 | 1.553518 | 2.19E-08 | 2.22E-07 | yes | up | 9.38 | 5.95 | 3.99 | 37.34 | 54.98 | 35.5 | 6.44 | 42.60667 |
| LOC100342996 | 0.44 | -1.18426 | 2.2E-08 | 2.22E-07 | yes | down | 725.52 | 666.5 | 499.56 | 737.05 | 574.41 | 574.01 | 630.5267 | 628.49 |
| TRIM36 | 0.214 | -2.22373 | 2.21E-08 | 2.24E-07 | yes | down | 1.5 | 2.53 | 2.89 | 1.73 | 0.88 | 1.43 | 2.306667 | 1.346667 |
| NNT | 0.359 | -1.47978 | 2.25E-08 | 2.27E-07 | yes | down | 104.07 | 56.48 | 50.51 | 58.39 | 66.27 | 48.9 | 70.35333 | 57.85333 |
| LOC103350566 | 0.119 | -3.07658 | 2.29E-08 | 2.31E-07 | yes | down | 2.5 | 1.4 | 3.24 | 1.12 | 0.47 | 0.73 | 2.38 | 0.773333 |
| PKN2 | 0.379 | -1.40124 | 2.31E-08 | 2.33E-07 | yes | down | 5.79 | 7 | 6.78 | 6.8 | 7.19 | 4.51 | 6.523333 | 6.166667 |
| P2RX4 | 2.668 | 1.41575 | 2.31E-08 | 2.33E-07 | yes | up | 3.25 | 4.99 | 3.06 | 20.43 | 25.99 | 22.46 | 3.766667 | 22.96 |
| SMCHD1 | 0.403 | -1.3099 | 2.34E-08 | 2.36E-07 | yes | down | 4.12 | 4.21 | 3.66 | 3.85 | 5.1 | 2.63 | 3.996667 | 3.86 |
| CEP70 | 0.261 | -1.93791 | 2.37E-08 | 2.38E-07 | yes | down | 6.05 | 3.35 | 3.86 | 2.14 | 3.96 | 1.86 | 4.42 | 2.653333 |
| SIRT3 | 0.474 | -1.07719 | 2.38E-08 | 2.4E-07 | yes | down | 22.35 | 26.14 | 23.09 | 26.46 | 26.01 | 25.47 | 23.86 | 25.98 |
| SPSB1 | 0.333 | -1.58648 | 2.41E-08 | 2.42E-07 | yes | down | 17.7 | 12.43 | 8.96 | 7.63 | 11.79 | 10.08 | 13.03 | 9.833333 |
| NCL | 0.495 | -1.01319 | 2.42E-08 | 2.43E-07 | yes | down | 125.43 | 127.08 | 126.21 | 148.17 | 168.62 | 117.39 | 126.24 | 144.7267 |
| RNASET2 | 2.898 | 1.534817 | 2.47E-08 | 2.48E-07 | yes | up | 10.76 | 16.16 | 13.75 | 80.09 | 80.92 | 108.13 | 13.55667 | 89.71333 |
| DHX40 | 0.443 | -1.17529 | 2.48E-08 | 2.49E-07 | yes | down | 13.56 | 12.95 | 11.51 | 13.04 | 16.48 | 9.42 | 12.67333 | 12.98 |
| EIF2AK3 | 2.095 | 1.066896 | 2.49E-08 | 2.5E-07 | yes | up | 3.22 | 2.88 | 2.64 | 13.77 | 16.61 | 11.71 | 2.913333 | 14.03 |
| SUGT1 | 0.422 | -1.24441 | 2.5E-08 | 2.5E-07 | yes | down | 17.3 | 15.14 | 14.59 | 17.16 | 17.12 | 11.37 | 15.67667 | 15.21667 |
| UBE2D1 | 0.291 | -1.78251 | 2.52E-08 | 2.52E-07 | yes | down | 14.54 | 8.02 | 13.73 | 8.28 | 7.94 | 8.2 | 12.09667 | 8.14 |
| LOC100345682 | 0.346 | -1.52992 | 2.52E-08 | 2.52E-07 | yes | down | 2.22 | 1.37 | 1.57 | 1.24 | 1.75 | 1.14 | 1.72 | 1.376667 |
| NDUFS5 | 0.456 | -1.13347 | 2.57E-08 | 2.57E-07 | yes | down | 229.65 | 210.34 | 189.78 | 249.79 | 200.1 | 204.26 | 209.9233 | 218.05 |
| FRG1 | 0.41 | -1.28465 | 2.64E-08 | 2.64E-07 | yes | down | 25.43 | 26.17 | 24.57 | 28.73 | 25.27 | 19.94 | 25.39 | 24.64667 |
| STXBP3 | 0.405 | -1.30533 | 2.69E-08 | 2.68E-07 | yes | down | 7.01 | 7.49 | 6.17 | 6.65 | 7.98 | 4.64 | 6.89 | 6.423333 |
| IMP4 | 0.424 | -1.23934 | 2.69E-08 | 2.69E-07 | yes | down | 15.62 | 16.96 | 16.86 | 16.49 | 14.7 | 16.77 | 16.48 | 15.98667 |
| PPP1R9B | 0.456 | -1.13382 | 2.71E-08 | 2.7E-07 | yes | down | 77.92 | 106.62 | 65.42 | 79.19 | 89.66 | 89.62 | 83.32 | 86.15667 |
| CD68 | 6.181 | 2.627761 | 2.76E-08 | 2.75E-07 | yes | up | 1.37 | 1.26 | 1.22 | 16.54 | 8.93 | 26.86 | 1.283333 | 17.44333 |
| KCTD11 | 2.499 | 1.321384 | 2.77E-08 | 2.76E-07 | yes | up | 3.48 | 3 | 2.57 | 17.2 | 15.38 | 18.54 | 3.016667 | 17.04 |
| UBLCP1 | 0.366 | -1.45084 | 2.78E-08 | 2.76E-07 | yes | down | 6.94 | 7.58 | 6.67 | 6.35 | 7.79 | 4.03 | 7.063333 | 6.056667 |
| SMIM10L1 | 0.423 | -1.24197 | 2.8E-08 | 2.78E-07 | yes | down | 31.65 | 32.75 | 36.12 | 32.21 | 41.76 | 25.48 | 33.50667 | 33.15 |
| CKS2 | 4.749 | 2.247746 | 2.8E-08 | 2.79E-07 | yes | up | 11.02 | 12.54 | 4.18 | 122.75 | 116.21 | 56.37 | 9.246667 | 98.44333 |
| LOC103349531 | 45.139 | 5.496297 | 2.81E-08 | 2.79E-07 | yes | up | 0.03 | 0.02 | 0 | 1.04 | 2.41 | 1.29 | 0.016667 | 1.58 |
| USP38 | 0.489 | -1.03199 | 2.85E-08 | 2.83E-07 | yes | down | 5.47 | 4.92 | 3.74 | 4.92 | 5.8 | 4.62 | 4.71 | 5.113333 |
| AGL | 0.146 | -2.77256 | 2.86E-08 | 2.84E-07 | yes | down | 21.03 | 5.34 | 4.4 | 4.49 | 2.66 | 2.7 | 10.25667 | 3.283333 |
| PIGV | 2.008 | 1.005699 | 2.87E-08 | 2.84E-07 | yes | up | 3.84 | 4.02 | 3.16 | 16.3 | 18.28 | 15.84 | 3.673333 | 16.80667 |
| LOC100351224 | 0.192 | -2.38183 | 2.9E-08 | 2.88E-07 | yes | down | 1.57 | 1.5 | 1.19 | 0.4 | 0.88 | 0.62 | 1.42 | 0.633333 |
| INPP4A | 2.287 | 1.19357 | 2.95E-08 | 2.92E-07 | yes | up | 2.72 | 2.31 | 1.97 | 11.63 | 15.14 | 9.21 | 2.333333 | 11.99333 |
| LOC103348641 | 0.233 | -2.09878 | 2.96E-08 | 2.93E-07 | yes | down | 5.41 | 7.77 | 7.03 | 2.31 | 3.49 | 1.75 | 6.736667 | 2.516667 |
| C2H2orf73 | 0.145 | -2.78691 | 2.96E-08 | 2.93E-07 | yes | down | 2.95 | 2.23 | 3.03 | 1.14 | 1.09 | 0.54 | 2.736667 | 0.923333 |
| LOC108176538 | 4.523 | 2.177399 | 2.97E-08 | 2.93E-07 | yes | up | 1.94 | 2.77 | 0.49 | 16.36 | 16.94 | 16.43 | 1.733333 | 16.57667 |
| DMXL1 | 0.441 | -1.18074 | 3E-08 | 2.96E-07 | yes | down | 5.83 | 6.16 | 4.79 | 6.18 | 6 | 3.64 | 5.593333 | 5.273333 |
| LOC100355158 | 2.184 | 1.127239 | 3.01E-08 | 2.97E-07 | yes | up | 40.57 | 44.07 | 39.02 | 196.41 | 187.62 | 224.29 | 41.22 | 202.7733 |
| GPC6 | 2.824 | 1.497923 | 3.01E-08 | 2.97E-07 | yes | up | 1.12 | 0.92 | 0.57 | 6.01 | 6.35 | 4.31 | 0.87 | 5.556667 |
| VMP1 | 0.457 | -1.12883 | 3.04E-08 | 3E-07 | yes | down | 15.19 | 20.37 | 17.11 | 18.14 | 19.64 | 17.61 | 17.55667 | 18.46333 |
| PLEKHF2 | 0.326 | -1.61799 | 3.09E-08 | 3.04E-07 | yes | down | 6.23 | 5.97 | 5.83 | 4.87 | 4.6 | 3.93 | 6.01 | 4.466667 |
| STMN1 | 5.344 | 2.417948 | 3.18E-08 | 3.12E-07 | yes | up | 11.06 | 18.81 | 5.47 | 211.73 | 124.7 | 80.79 | 11.78 | 139.0733 |
| NUDCD1 | 0.311 | -1.68661 | 3.23E-08 | 3.18E-07 | yes | down | 2.42 | 2.62 | 2.62 | 1.35 | 2.55 | 1.67 | 2.553333 | 1.856667 |
| SGMS2 | 3.071 | 1.618818 | 3.24E-08 | 3.19E-07 | yes | up | 1.37 | 0.7 | 0.78 | 7.03 | 6.88 | 5.63 | 0.95 | 6.513333 |
| TRIM23 | 0.355 | -1.495 | 3.27E-08 | 3.21E-07 | yes | down | 5.06 | 4.01 | 4.66 | 3.76 | 4.96 | 2.62 | 4.576667 | 3.78 |
| GABARAPL2 | 0.487 | -1.0372 | 3.33E-08 | 3.26E-07 | yes | down | 129.69 | 154.63 | 129.63 | 143.44 | 154.66 | 161.5 | 137.9833 | 153.2 |
| PRKCH | 2.093 | 1.065615 | 3.35E-08 | 3.28E-07 | yes | up | 6.69 | 8.27 | 5.67 | 35.05 | 34.14 | 29.12 | 6.876667 | 32.77 |
| IFT81 | 0.302 | -1.72564 | 3.41E-08 | 3.33E-07 | yes | down | 4.18 | 3.05 | 2.18 | 2.12 | 2.71 | 1.59 | 3.136667 | 2.14 |
| LMO1 | 4.362 | 2.12513 | 3.41E-08 | 3.34E-07 | yes | up | 1.92 | 3.36 | 2.78 | 19.79 | 34.88 | 27.64 | 2.686667 | 27.43667 |
| ARID5A | 3.804 | 1.927344 | 3.42E-08 | 3.34E-07 | yes | up | 2.52 | 2.66 | 1.86 | 18.98 | 13.08 | 26.65 | 2.346667 | 19.57 |
| TBC1D30 | 0.222 | -2.174 | 3.46E-08 | 3.38E-07 | yes | down | 0.74 | 0.94 | 1 | 0.39 | 0.36 | 0.56 | 0.893333 | 0.436667 |
| MTX1 | 0.369 | -1.43934 | 3.47E-08 | 3.39E-07 | yes | down | 35.53 | 47.75 | 45.49 | 42.28 | 31.28 | 35.34 | 42.92333 | 36.3 |
| LOC100355902 | 0.18 | -2.47685 | 3.48E-08 | 3.39E-07 | yes | down | 0.93 | 1 | 0.73 | 0.21 | 0.69 | 0.23 | 0.886667 | 0.376667 |
| PIGBOS1 | 0.419 | -1.25423 | 3.55E-08 | 3.47E-07 | yes | down | 16.13 | 19.03 | 15.52 | 17.07 | 16.14 | 14.55 | 16.89333 | 15.92 |
| VCAN | 3.638 | 1.862992 | 3.56E-08 | 3.47E-07 | yes | up | 8.15 | 5.72 | 2.68 | 52.39 | 37.72 | 44.82 | 5.516667 | 44.97667 |
| HECW2 | 0.453 | -1.14113 | 3.59E-08 | 3.49E-07 | yes | down | 2.06 | 2.35 | 1.93 | 2.15 | 2.72 | 1.76 | 2.113333 | 2.21 |
| UBR3 | 0.321 | -1.63929 | 3.66E-08 | 3.56E-07 | yes | down | 18.46 | 8.9 | 7.93 | 7.93 | 10.22 | 7.22 | 11.76333 | 8.456667 |
| MXD4 | 2.055 | 1.038892 | 3.69E-08 | 3.58E-07 | yes | up | 16.8 | 18 | 16.41 | 71.46 | 83.47 | 86.67 | 17.07 | 80.53333 |
| GTF2A1 | 0.388 | -1.36519 | 3.7E-08 | 3.59E-07 | yes | down | 4.28 | 4.71 | 3.7 | 4.12 | 4.64 | 2.52 | 4.23 | 3.76 |
| LOC100352119 | 4.831 | 2.272236 | 3.74E-08 | 3.63E-07 | yes | up | 4.14 | 3.44 | 2.34 | 59.26 | 28.1 | 21.06 | 3.306667 | 36.14 |
| ZNF277 | 0.38 | -1.39697 | 3.76E-08 | 3.64E-07 | yes | down | 30.55 | 36.66 | 33.01 | 33.75 | 34.23 | 19.17 | 33.40667 | 29.05 |
| TSHZ3 | 2.369 | 1.244052 | 3.87E-08 | 3.75E-07 | yes | up | 1.33 | 1.61 | 1.02 | 7.27 | 7.15 | 6.84 | 1.32 | 7.086667 |
| ZKSCAN5 | 0.475 | -1.07325 | 3.91E-08 | 3.78E-07 | yes | down | 7.24 | 6.53 | 6.8 | 7.59 | 8.47 | 8.59 | 6.856667 | 8.216667 |
| FAM13C | 0.311 | -1.68625 | 3.91E-08 | 3.78E-07 | yes | down | 8.3 | 8.33 | 9.26 | 7.94 | 6.28 | 4.01 | 8.63 | 6.076667 |
| HCAR2 | 7.169 | 2.841694 | 4.02E-08 | 3.89E-07 | yes | up | 0.96 | 0.77 | 0.34 | 10.84 | 5.13 | 16 | 0.69 | 10.65667 |
| FAM161A | 0.324 | -1.62377 | 4.03E-08 | 3.9E-07 | yes | down | 3.63 | 3.08 | 2.4 | 2.25 | 3.14 | 1.39 | 3.036667 | 2.26 |
| PODN | 4.696 | 2.231295 | 4.13E-08 | 3.99E-07 | yes | up | 5.16 | 11.43 | 4.06 | 44.22 | 69.82 | 101.73 | 6.883333 | 71.92333 |
| APPL1 | 0.417 | -1.26212 | 4.2E-08 | 4.05E-07 | yes | down | 13.18 | 11.77 | 8.95 | 10.35 | 13.57 | 7.5 | 11.3 | 10.47333 |
| KLHL22 | 2.22 | 1.150563 | 4.23E-08 | 4.08E-07 | yes | up | 10.11 | 9 | 7.18 | 43.07 | 43.1 | 46.27 | 8.763333 | 44.14667 |
| ARHGAP21 | 2.308 | 1.206894 | 4.3E-08 | 4.15E-07 | yes | up | 5.09 | 4.27 | 3.68 | 18.11 | 27.94 | 19.06 | 4.346667 | 21.70333 |
| LOC103352519 | 0.044 | -4.50682 | 4.38E-08 | 4.22E-07 | yes | down | 0.44 | 0.99 | 0.58 | 0.07 | 0.11 | 0 | 0.67 | 0.06 |
| CPM | 2.833 | 1.502182 | 4.4E-08 | 4.23E-07 | yes | up | 2.17 | 1.45 | 2.1 | 12.32 | 14.68 | 10.67 | 1.906667 | 12.55667 |
| DDO | 0.261 | -1.94035 | 4.41E-08 | 4.24E-07 | yes | down | 5.66 | 7.33 | 7.22 | 2.61 | 3.09 | 6.43 | 6.736667 | 4.043333 |
| LOC103349164 | 0.005 | -7.72492 | 4.44E-08 | 4.26E-07 | yes | down | 0.82 | 1.21 | 1.75 | 0 | 0 | 0 | 1.26 | 0 |
| MRPL38 | 0.352 | -1.50521 | 4.63E-08 | 4.44E-07 | yes | down | 30.09 | 26.72 | 29.86 | 18.91 | 21.27 | 29.71 | 28.89 | 23.29667 |
| AUH | 0.461 | -1.11688 | 4.63E-08 | 4.44E-07 | yes | down | 47.8 | 40.55 | 46.14 | 47.22 | 48.87 | 38.66 | 44.83 | 44.91667 |
| LOC100358198 | 0.058 | -4.11229 | 4.64E-08 | 4.44E-07 | yes | down | 0.54 | 1.84 | 0.79 | 0.26 | 0.02 | 0.12 | 1.056667 | 0.133333 |
| MTAP | 0.431 | -1.21429 | 4.74E-08 | 4.54E-07 | yes | down | 9.55 | 7.6 | 8.33 | 8.59 | 9.52 | 7.19 | 8.493333 | 8.433333 |
| RDH11 | 2.598 | 1.377236 | 4.9E-08 | 4.69E-07 | yes | up | 2.91 | 3.04 | 3.33 | 20.28 | 18.52 | 16.3 | 3.093333 | 18.36667 |
| TRAPPC9 | 2.098 | 1.069306 | 4.91E-08 | 4.69E-07 | yes | up | 4.19 | 3.37 | 2.98 | 17.28 | 17.37 | 15.36 | 3.513333 | 16.67 |
| LOC100353151 | 0.416 | -1.26416 | 5.02E-08 | 4.8E-07 | yes | down | 65.02 | 63.08 | 73.4 | 69.43 | 57.3 | 66.72 | 67.16667 | 64.48333 |
| ARSG | 3.871 | 1.952655 | 5.1E-08 | 4.87E-07 | yes | up | 0.51 | 0.61 | 0.47 | 4.24 | 3.92 | 5.72 | 0.53 | 4.626667 |
| CFB | 8.109 | 3.019528 | 5.13E-08 | 4.89E-07 | yes | up | 2.11 | 0.74 | 0.26 | 14.55 | 13.73 | 25.55 | 1.036667 | 17.94333 |
| KHDRBS3 | 0.306 | -1.70916 | 5.14E-08 | 4.9E-07 | yes | down | 2.08 | 2.75 | 2.59 | 1.92 | 2.08 | 1.26 | 2.473333 | 1.753333 |
| CSRP2 | 3.704 | 1.889158 | 5.16E-08 | 4.91E-07 | yes | up | 18.2 | 18.31 | 14.65 | 137.53 | 85.61 | 198.78 | 17.05333 | 140.64 |
| PLAA | 0.453 | -1.1428 | 5.16E-08 | 4.91E-07 | yes | down | 8.51 | 7.66 | 8.29 | 7.71 | 9.31 | 8.53 | 8.153333 | 8.516667 |
| C1H9orf153 | 0.232 | -2.10787 | 5.17E-08 | 4.92E-07 | yes | down | 3.49 | 5.66 | 4.54 | 3.48 | 1.55 | 2.4 | 4.563333 | 2.476667 |
| THOC7 | 0.432 | -1.21195 | 5.2E-08 | 4.95E-07 | yes | down | 25.27 | 25.49 | 19.83 | 26.24 | 25.9 | 17.51 | 23.53 | 23.21667 |
| F10 | 0.005 | -7.70453 | 5.21E-08 | 4.96E-07 | yes | down | 0.55 | 0.77 | 1.17 | 0 | 0 | 0 | 0.83 | 0 |
| NICN1 | 2.589 | 1.372419 | 5.22E-08 | 4.96E-07 | yes | up | 4.27 | 4.09 | 4.11 | 23.19 | 24.36 | 26.47 | 4.156667 | 24.67333 |
| MCP-1 | 0.029 | -5.12053 | 5.25E-08 | 4.99E-07 | yes | down | 3.98 | 13.31 | 6.16 | 1.06 | 0.26 | 0 | 7.816667 | 0.44 |
| FUS | 0.455 | -1.13557 | 5.27E-08 | 5E-07 | yes | down | 66.7 | 77.42 | 54.45 | 55.92 | 64.92 | 77.62 | 66.19 | 66.15333 |
| KLHL29 | 3.776 | 1.916903 | 5.27E-08 | 5E-07 | yes | up | 0.63 | 0.54 | 0.3 | 3.71 | 3.59 | 5.02 | 0.49 | 4.106667 |
| CWC22 | 0.38 | -1.3947 | 5.36E-08 | 5.09E-07 | yes | down | 2.12 | 2.15 | 1.76 | 1.87 | 2.06 | 1.31 | 2.01 | 1.746667 |
| ESPL1 | 4.699 | 2.232357 | 5.38E-08 | 5.1E-07 | yes | up | 0.31 | 0.18 | 0.2 | 2.23 | 3.8 | 1.45 | 0.23 | 2.493333 |
| LTV1 | 0.357 | -1.48419 | 5.4E-08 | 5.11E-07 | yes | down | 9.3 | 9.36 | 5.31 | 5.82 | 8.52 | 5.16 | 7.99 | 6.5 |
| CLTCL1 | 4.094 | 2.033649 | 5.42E-08 | 5.13E-07 | yes | up | 6.14 | 2.86 | 1.68 | 25.85 | 38.98 | 32.78 | 3.56 | 32.53667 |
| NARF | 0.494 | -1.017 | 5.44E-08 | 5.15E-07 | yes | down | 7.24 | 7.73 | 6.67 | 7.68 | 8.31 | 8.46 | 7.213333 | 8.15 |
| STOM | 2.628 | 1.393915 | 5.45E-08 | 5.16E-07 | yes | up | 18.81 | 27.43 | 17.76 | 125.76 | 105.77 | 146.69 | 21.33333 | 126.0733 |
| TPD52L1 | 0.168 | -2.57773 | 5.52E-08 | 5.21E-07 | yes | down | 21.54 | 14.67 | 19.11 | 4.46 | 5 | 13.79 | 18.44 | 7.75 |
| FGFR2 | 5.273 | 2.39852 | 5.54E-08 | 5.23E-07 | yes | up | 0.27 | 0.41 | 0.16 | 2.78 | 2.96 | 4.89 | 0.28 | 3.543333 |
| CDO1 | 2.227 | 1.155386 | 5.57E-08 | 5.25E-07 | yes | up | 151.8 | 221.26 | 128.18 | 869.54 | 877.86 | 785.93 | 167.08 | 844.4433 |
| ASB1 | 0.453 | -1.14363 | 5.67E-08 | 5.35E-07 | yes | down | 17.36 | 20.19 | 19.4 | 19.78 | 20.92 | 18.77 | 18.98333 | 19.82333 |
| ADGRF5 | 0.356 | -1.49086 | 5.7E-08 | 5.37E-07 | yes | down | 41 | 60.7 | 63.52 | 42.59 | 70.67 | 39 | 55.07333 | 50.75333 |
| AGT | 117.058 | 6.871076 | 5.74E-08 | 5.4E-07 | yes | up | 0.02 | 0.18 | 0.04 | 32.74 | 32.53 | 2.06 | 0.08 | 22.44333 |
| ADGRB2 | 4.869 | 2.283545 | 5.8E-08 | 5.46E-07 | yes | up | 0.49 | 0.24 | 0.18 | 2.78 | 4.74 | 2.57 | 0.303333 | 3.363333 |
| ESCO1 | 0.384 | -1.37913 | 5.82E-08 | 5.47E-07 | yes | down | 2.46 | 2.07 | 2.03 | 2.06 | 2.4 | 1.45 | 2.186667 | 1.97 |
| NT5C3A | 0.356 | -1.49049 | 5.84E-08 | 5.48E-07 | yes | down | 26.87 | 13.86 | 17.49 | 15.71 | 14.33 | 13.97 | 19.40667 | 14.67 |
| LOC100341136 | 5.299 | 2.405815 | 5.84E-08 | 5.48E-07 | yes | up | 0.35 | 0.71 | 0.8 | 6.13 | 12.43 | 5.55 | 0.62 | 8.036667 |
| ZNF827 | 0.362 | -1.467 | 5.86E-08 | 5.5E-07 | yes | down | 3.53 | 2.82 | 2.84 | 2.43 | 3.62 | 1.71 | 3.063333 | 2.586667 |
| SNX13 | 0.363 | -1.46172 | 5.86E-08 | 5.5E-07 | yes | down | 10.06 | 9.17 | 10.15 | 9.78 | 11.68 | 6.96 | 9.793333 | 9.473333 |
| PTGFRN | 2.336 | 1.224051 | 5.95E-08 | 5.58E-07 | yes | up | 8.96 | 6.79 | 6.34 | 29.43 | 46.46 | 41.97 | 7.363333 | 39.28667 |
| TGS1 | 0.322 | -1.63465 | 5.96E-08 | 5.59E-07 | yes | down | 1.85 | 1.59 | 1.7 | 1.22 | 1.67 | 0.95 | 1.713333 | 1.28 |
| ENY2 | 0.461 | -1.11588 | 6E-08 | 5.62E-07 | yes | down | 74.75 | 74.64 | 73.86 | 78.52 | 71.14 | 69.56 | 74.41667 | 73.07333 |
| FAM76B | 0.401 | -1.31856 | 6.04E-08 | 5.65E-07 | yes | down | 3.95 | 4.7 | 3.44 | 4.72 | 5.33 | 4.12 | 4.03 | 4.723333 |
| TNFSF12 | 3.086 | 1.625879 | 6.05E-08 | 5.66E-07 | yes | up | 3.45 | 3.47 | 2.33 | 21.42 | 18.12 | 24.42 | 3.083333 | 21.32 |
| CA2 | 0.301 | -1.73368 | 6.2E-08 | 5.79E-07 | yes | down | 13.37 | 16.11 | 18.87 | 15.34 | 10.4 | 9.81 | 16.11667 | 11.85 |
| TGFB1 | 2.81 | 1.490336 | 6.22E-08 | 5.8E-07 | yes | up | 6.04 | 9.26 | 5.79 | 50 | 48.89 | 36.93 | 7.03 | 45.27333 |
| ZNF275 | 3.372 | 1.753741 | 6.29E-08 | 5.87E-07 | yes | up | 0.45 | 0.31 | 0.3 | 2.64 | 2.38 | 3.06 | 0.353333 | 2.693333 |
| OSBPL5 | 2.97 | 1.570255 | 6.29E-08 | 5.87E-07 | yes | up | 6.51 | 5.3 | 4.45 | 24.38 | 42.38 | 43.27 | 5.42 | 36.67667 |
| CSMD2 | 0.143 | -2.80266 | 6.41E-08 | 5.98E-07 | yes | down | 0.15 | 0.26 | 0.12 | 0.05 | 0.07 | 0.05 | 0.176667 | 0.056667 |
| CD44 | 2.45 | 1.292981 | 6.43E-08 | 5.99E-07 | yes | up | 9.84 | 9.21 | 5.33 | 52.24 | 42.16 | 40.43 | 8.126667 | 44.94333 |
| PSEN2 | 0.403 | -1.31098 | 6.45E-08 | 6E-07 | yes | down | 37.91 | 49.77 | 49.04 | 47.38 | 41.22 | 41.91 | 45.57333 | 43.50333 |
| LOC108176138 | 2.908 | 1.540125 | 6.52E-08 | 6.07E-07 | yes | up | 2.55 | 4.14 | 3.28 | 20.28 | 21.48 | 24.8 | 3.323333 | 22.18667 |
| LOC103348799 | 0.044 | -4.51363 | 6.53E-08 | 6.07E-07 | yes | down | 1.15 | 2.32 | 10.03 | 0.67 | 0.51 | 0.28 | 4.5 | 0.486667 |
| SLC8B1 | 2.722 | 1.44458 | 6.53E-08 | 6.07E-07 | yes | up | 3.77 | 3.7 | 2.11 | 21.19 | 29.13 | 16.68 | 3.193333 | 22.33333 |
| IGFBP2 | 5.441 | 2.443923 | 6.6E-08 | 6.13E-07 | yes | up | 32.61 | 23.84 | 16.47 | 299.71 | 115.83 | 445.41 | 24.30667 | 286.9833 |
| ALOX5 | 4.109 | 2.038873 | 6.7E-08 | 6.21E-07 | yes | up | 2.25 | 1.64 | 2.63 | 15.07 | 34.97 | 14.37 | 2.173333 | 21.47 |
| TNXB | 3.466 | 1.793086 | 6.7E-08 | 6.21E-07 | yes | up | 57.77 | 29.17 | 17.81 | 259.05 | 197.18 | 341.41 | 34.91667 | 265.88 |
| NDUFA8 | 0.408 | -1.29399 | 6.71E-08 | 6.22E-07 | yes | down | 175.54 | 197.71 | 130.91 | 192.23 | 131.65 | 139.9 | 168.0533 | 154.5933 |
| GPR89B | 0.488 | -1.03634 | 6.74E-08 | 6.24E-07 | yes | down | 19.89 | 22.59 | 21.81 | 24.52 | 26.18 | 21.03 | 21.43 | 23.91 |
| ZMYM4 | 0.479 | -1.06323 | 6.83E-08 | 6.32E-07 | yes | down | 3.97 | 4.11 | 3.05 | 3.75 | 5.08 | 3.46 | 3.71 | 4.096667 |
| PHF20L1 | 0.427 | -1.22929 | 6.83E-08 | 6.32E-07 | yes | down | 8.45 | 9.05 | 6.37 | 7.88 | 10.07 | 5.44 | 7.956667 | 7.796667 |
| GPCPD1 | 0.407 | -1.29775 | 6.94E-08 | 6.41E-07 | yes | down | 9.72 | 6.96 | 6 | 7.97 | 6.28 | 5 | 7.56 | 6.416667 |
| FAM134B | 0.162 | -2.6265 | 6.95E-08 | 6.42E-07 | yes | down | 23.14 | 8.44 | 6.91 | 2.31 | 4.17 | 6.96 | 12.83 | 4.48 |
| L2HGDH | 0.395 | -1.34051 | 6.98E-08 | 6.45E-07 | yes | down | 5.96 | 4.18 | 3.8 | 4.61 | 4.49 | 3.4 | 4.646667 | 4.166667 |
| COPS5 | 0.479 | -1.0611 | 7.01E-08 | 6.46E-07 | yes | down | 48.84 | 41.48 | 36.64 | 49.99 | 53.44 | 36.22 | 42.32 | 46.55 |
| CHI3L1 | 41.116 | 5.361621 | 7.04E-08 | 6.5E-07 | yes | up | 0.43 | 1.45 | 1.34 | 104.28 | 205.66 | 15.86 | 1.073333 | 108.6 |
| LOC100344556 | 2.775 | 1.472459 | 7.07E-08 | 6.52E-07 | yes | up | 1.64 | 1.32 | 0.78 | 6.76 | 8.58 | 8.26 | 1.246667 | 7.866667 |
| GLI3 | 2.846 | 1.509013 | 7.09E-08 | 6.53E-07 | yes | up | 0.72 | 0.51 | 0.34 | 3.47 | 3.51 | 3.08 | 0.523333 | 3.353333 |
| LOC103346609 | 0.125 | -2.9993 | 7.14E-08 | 6.58E-07 | yes | down | 0.67 | 0.69 | 0.87 | 0.26 | 0.11 | 0.27 | 0.743333 | 0.213333 |
| LDB2 | 2.177 | 1.122623 | 7.16E-08 | 6.58E-07 | yes | up | 11.2 | 12.81 | 9.04 | 72.53 | 63.12 | 50.93 | 11.01667 | 62.19333 |
| CFL1 | 2.472 | 1.305457 | 7.17E-08 | 6.59E-07 | yes | up | 52.07 | 60.87 | 53.54 | 396.15 | 299.66 | 247.88 | 55.49333 | 314.5633 |
| HAVCR1 | 9.041 | 3.17655 | 7.2E-08 | 6.61E-07 | yes | up | 0.18 | 0.78 | 0.19 | 8.06 | 4.45 | 11.26 | 0.383333 | 7.923333 |
| LHFP | 2.016 | 1.01151 | 7.47E-08 | 6.86E-07 | yes | up | 16.66 | 15.65 | 12.99 | 73.82 | 80.72 | 54.72 | 15.1 | 69.75333 |
| TIMP2 | 2.317 | 1.212242 | 7.49E-08 | 6.87E-07 | yes | up | 55.16 | 53.54 | 36.49 | 212.66 | 241.55 | 300.42 | 48.39667 | 251.5433 |
| FAM174A | 0.411 | -1.28448 | 7.55E-08 | 6.92E-07 | yes | down | 26.46 | 17.92 | 17.34 | 19.09 | 18.75 | 19.73 | 20.57333 | 19.19 |
| PTBP2 | 0.424 | -1.23675 | 7.71E-08 | 7.07E-07 | yes | down | 6.06 | 6.54 | 3.82 | 5.31 | 5.66 | 4.77 | 5.473333 | 5.246667 |
| MRPS12 | 0.382 | -1.38954 | 7.86E-08 | 7.21E-07 | yes | down | 31.94 | 34.57 | 30.78 | 24.48 | 24.14 | 34.66 | 32.43 | 27.76 |
| MTMR4 | 2.325 | 1.217352 | 7.94E-08 | 7.27E-07 | yes | up | 3.02 | 2.65 | 2.93 | 14.05 | 20.2 | 12.16 | 2.866667 | 15.47 |
| SOGA1 | 2.285 | 1.192182 | 7.97E-08 | 7.3E-07 | yes | up | 2.28 | 2.24 | 1.51 | 8.33 | 13.9 | 9.32 | 2.01 | 10.51667 |
| CD209 | 3.959 | 1.985315 | 8.01E-08 | 7.33E-07 | yes | up | 4.74 | 2.88 | 1.58 | 19.94 | 39.02 | 23.74 | 3.066667 | 27.56667 |
| QRSL1 | 0.393 | -1.3492 | 8.07E-08 | 7.38E-07 | yes | down | 7.31 | 6.45 | 4.94 | 5.36 | 6.97 | 4.45 | 6.233333 | 5.593333 |
| LOC100344043 | 5.392 | 2.430805 | 8.14E-08 | 7.43E-07 | yes | up | 1.57 | 0.87 | 0.62 | 6.8 | 12.73 | 17.92 | 1.02 | 12.48333 |
| PLEKHF1 | 2.787 | 1.47868 | 8.15E-08 | 7.44E-07 | yes | up | 11.31 | 21.37 | 16.88 | 96.93 | 114.59 | 107.6 | 16.52 | 106.3733 |
| AHCYL2 | 2.522 | 1.334299 | 8.18E-08 | 7.46E-07 | yes | up | 3.38 | 2.38 | 1.66 | 13.83 | 16.37 | 13.45 | 2.473333 | 14.55 |
| LGALS3BP | 2.088 | 1.062438 | 8.21E-08 | 7.48E-07 | yes | up | 23.89 | 27.99 | 24.68 | 107.86 | 125.91 | 132.39 | 25.52 | 122.0533 |
| KIAA2022 | 0.034 | -4.88626 | 8.27E-08 | 7.54E-07 | yes | down | 0.09 | 0.12 | 0.45 | 0.02 | 0.02 | 0.01 | 0.22 | 0.016667 |
| LOC103347778 | 156.979 | 7.294425 | 8.34E-08 | 7.59E-07 | yes | up | 0 | 0 | 0.34 | 16.82 | 83.58 | 80.24 | 0.113333 | 60.21333 |
| CACHD1 | 3.291 | 1.718708 | 8.36E-08 | 7.6E-07 | yes | up | 2.75 | 3.74 | 1.99 | 9 | 5.96 | 4.73 | 2.826667 | 6.563333 |
| BNC2 | 3.938 | 1.977578 | 8.46E-08 | 7.69E-07 | yes | up | 0.75 | 0.49 | 0.28 | 3.56 | 6.73 | 3.69 | 0.506667 | 4.66 |
| ARHGEF17 | 2.124 | 1.086883 | 8.59E-08 | 7.81E-07 | yes | up | 2.07 | 2.18 | 2.09 | 8.71 | 12.01 | 10.35 | 2.113333 | 10.35667 |
| KIF18B | 7.394 | 2.88626 | 8.7E-08 | 7.89E-07 | yes | up | 0.32 | 0.32 | 0.1 | 4.95 | 5.84 | 1.56 | 0.246667 | 4.116667 |
| SLC38A5 | 4.57 | 2.192046 | 8.85E-08 | 8.02E-07 | yes | up | 1.07 | 1.09 | 0.32 | 7.91 | 7.96 | 8.79 | 0.826667 | 8.22 |
| CARMIL1 | 2.743 | 1.455895 | 8.85E-08 | 8.02E-07 | yes | up | 0.83 | 0.88 | 0.79 | 4.12 | 7.1 | 4.65 | 0.833333 | 5.29 |
| LOC103348123 | 0.02 | -5.6303 | 8.88E-08 | 8.04E-07 | yes | down | 0.86 | 1.2 | 0.85 | 0.52 | 0.11 | 0 | 0.97 | 0.21 |
| RIC1 | 0.469 | -1.09205 | 8.99E-08 | 8.14E-07 | yes | down | 5.23 | 5.38 | 4.75 | 5.97 | 6.48 | 4.18 | 5.12 | 5.543333 |
| LOC108175430 | 0.415 | -1.26889 | 9.05E-08 | 8.19E-07 | yes | down | 34.17 | 35.23 | 34.47 | 26.47 | 45.95 | 28.43 | 34.62333 | 33.61667 |
| YAE1D1 | 0.401 | -1.31968 | 9.28E-08 | 8.39E-07 | yes | down | 13.45 | 14.18 | 14.22 | 13.92 | 13.39 | 11.33 | 13.95 | 12.88 |
| WDR70 | 0.451 | -1.1496 | 9.28E-08 | 8.39E-07 | yes | down | 8.99 | 7.92 | 7.59 | 9.11 | 9.63 | 6.74 | 8.166667 | 8.493333 |
| CEP85L | 0.315 | -1.66866 | 9.31E-08 | 8.41E-07 | yes | down | 4.26 | 2.12 | 1.95 | 1.82 | 2.36 | 1.76 | 2.776667 | 1.98 |
| LOC100344797 | 2.985 | 1.577583 | 9.48E-08 | 8.56E-07 | yes | up | 4.1 | 2.51 | 2.32 | 14.45 | 20.7 | 21.73 | 2.976667 | 18.96 |
| UBE2L6 | 3.074 | 1.620026 | 9.66E-08 | 8.72E-07 | yes | up | 3.58 | 2.19 | 2.53 | 17.73 | 17.73 | 22.53 | 2.766667 | 19.33 |
| EBAG9 | 0.424 | -1.23704 | 9.73E-08 | 8.78E-07 | yes | down | 7.13 | 7.03 | 6.81 | 7.27 | 7.74 | 5.18 | 6.99 | 6.73 |
| NOX5 | 40.175 | 5.328232 | 9.76E-08 | 8.8E-07 | yes | up | 0.44 | 0.33 | 0.22 | 46.17 | 3.6 | 33.27 | 0.33 | 27.68 |
| BAIAP2 | 3.293 | 1.719187 | 9.83E-08 | 8.86E-07 | yes | up | 1.05 | 1.46 | 1.1 | 6.63 | 10.39 | 10.23 | 1.203333 | 9.083333 |
| ALX3 | 14.08 | 3.815562 | 1.01E-07 | 9.14E-07 | yes | up | 0.12 | 0.09 | 0 | 2.19 | 1.88 | 2.24 | 0.07 | 2.103333 |
| SLC8A1 | 2.842 | 1.506858 | 1.02E-07 | 9.16E-07 | yes | up | 0.77 | 0.58 | 0.47 | 4.29 | 4.31 | 3.63 | 0.606667 | 4.076667 |
| AADAT | 0.255 | -1.974 | 1.03E-07 | 9.23E-07 | yes | down | 9.04 | 4.92 | 3.98 | 4.61 | 3.05 | 2.55 | 5.98 | 3.403333 |
| MRPL18 | 0.405 | -1.30332 | 1.03E-07 | 9.24E-07 | yes | down | 45.02 | 46.12 | 37.83 | 35.49 | 31.71 | 44.97 | 42.99 | 37.39 |
| TTC7A | 2.462 | 1.300055 | 1.05E-07 | 9.4E-07 | yes | up | 1.67 | 1.6 | 1.32 | 7.75 | 12.06 | 7.23 | 1.53 | 9.013333 |
| SORT1 | 2.724 | 1.445689 | 1.05E-07 | 9.43E-07 | yes | up | 11 | 7.18 | 5.04 | 37.82 | 51 | 52.92 | 7.74 | 47.24667 |
| C18H10orf11 | 2.809 | 1.490033 | 1.06E-07 | 9.47E-07 | yes | up | 2.69 | 2.59 | 2.35 | 12.77 | 15.86 | 20.42 | 2.543333 | 16.35 |
| GLCE | 2.088 | 1.061972 | 1.06E-07 | 9.51E-07 | yes | up | 6.03 | 4.63 | 4.23 | 21.43 | 27.8 | 20.39 | 4.963333 | 23.20667 |
| ANKS1B | 7.462 | 2.899613 | 1.06E-07 | 9.51E-07 | yes | up | 0.06 | 1.19 | 0.03 | 1.48 | 1.38 | 1.47 | 0.426667 | 1.443333 |
| PTPN4 | 0.364 | -1.45653 | 1.07E-07 | 9.59E-07 | yes | down | 3.27 | 3.06 | 2.5 | 2.82 | 3.14 | 1.46 | 2.943333 | 2.473333 |
| IFT88 | 0.321 | -1.63913 | 1.1E-07 | 9.84E-07 | yes | down | 3.1 | 3.31 | 2.57 | 1.99 | 3.3 | 1.5 | 2.993333 | 2.263333 |
| GNAO1 | 3.262 | 1.705594 | 1.1E-07 | 9.86E-07 | yes | up | 0.94 | 0.74 | 0.98 | 5.56 | 7.6 | 5.16 | 0.886667 | 6.106667 |
| EFS | 2.804 | 1.487429 | 1.13E-07 | 1.01E-06 | yes | up | 2.27 | 1.92 | 1.43 | 12.7 | 9.45 | 13.22 | 1.873333 | 11.79 |
| LOC100356754 | 2.293 | 1.197522 | 1.14E-07 | 1.02E-06 | yes | up | 3.22 | 3.93 | 3.72 | 17.75 | 19.42 | 19.24 | 3.623333 | 18.80333 |
| SPRYD3 | 0.434 | -1.20343 | 1.15E-07 | 1.02E-06 | yes | down | 85.28 | 59.45 | 44.27 | 66.9 | 77.37 | 66.5 | 63 | 70.25667 |
| GMCL1 | 0.399 | -1.32404 | 1.15E-07 | 1.03E-06 | yes | down | 4.49 | 5.29 | 3.72 | 4.16 | 5.12 | 3.06 | 4.5 | 4.113333 |
| PGBD2 | 2.368 | 1.243549 | 1.17E-07 | 1.04E-06 | yes | up | 2.64 | 3.13 | 2.11 | 11.51 | 15.76 | 13.13 | 2.626667 | 13.46667 |
| RAB27A | 2.777 | 1.473453 | 1.17E-07 | 1.05E-06 | yes | up | 3.85 | 3.51 | 4.25 | 20.14 | 36.38 | 19.84 | 3.87 | 25.45333 |
| PLGRKT | 0.357 | -1.48519 | 1.18E-07 | 1.05E-06 | yes | down | 17.96 | 16.15 | 11.05 | 14.24 | 13.85 | 8.5 | 15.05333 | 12.19667 |
| PSPC1 | 0.45 | -1.15218 | 1.19E-07 | 1.06E-06 | yes | down | 12.33 | 10.39 | 8.72 | 11.96 | 11.32 | 8.67 | 10.48 | 10.65 |
| PAX8 | 163.556 | 7.353641 | 1.19E-07 | 1.06E-06 | yes | up | 0 | 0 | 0 | 1.33 | 1.38 | 3.64 | 0 | 2.116667 |
| PDK3 | 0.418 | -1.25974 | 1.21E-07 | 1.07E-06 | yes | down | 4.5 | 4.74 | 4.65 | 4.76 | 4.4 | 4.17 | 4.63 | 4.443333 |
| CD14 | 3.089 | 1.626941 | 1.23E-07 | 1.09E-06 | yes | up | 3.67 | 4.34 | 3.73 | 21.58 | 25.85 | 35.22 | 3.913333 | 27.55 |
| GLYAT | 3.063 | 1.614987 | 1.25E-07 | 1.11E-06 | yes | up | 10.35 | 7.08 | 5.08 | 35.75 | 62.6 | 55.49 | 7.503333 | 51.28 |
| FPGS | 0.421 | -1.24687 | 1.25E-07 | 1.11E-06 | yes | down | 8.07 | 9.82 | 8.14 | 7.42 | 8.32 | 9.26 | 8.676667 | 8.333333 |
| SUSD2 | 2.807 | 1.488931 | 1.26E-07 | 1.12E-06 | yes | up | 9 | 13.07 | 11.54 | 78.84 | 95.61 | 46.59 | 11.20333 | 73.68 |
| PDCD10 | 0.404 | -1.30659 | 1.27E-07 | 1.13E-06 | yes | down | 19.56 | 20.31 | 18.1 | 20.51 | 21.29 | 12.3 | 19.32333 | 18.03333 |
| PTPRR | 0.228 | -2.13166 | 1.28E-07 | 1.13E-06 | yes | down | 1.16 | 1.78 | 1.53 | 0.65 | 0.93 | 0.8 | 1.49 | 0.793333 |
| LOC100358870 | 3.276 | 1.71182 | 1.28E-07 | 1.14E-06 | yes | up | 3.46 | 2.6 | 3.8 | 30.95 | 25.03 | 19.9 | 3.286667 | 25.29333 |
| SH3BGRL3 | 2.099 | 1.069968 | 1.29E-07 | 1.14E-06 | yes | up | 25.39 | 28.19 | 23.28 | 126.35 | 113.55 | 127.24 | 25.62 | 122.38 |
| LOC100346827 | 0.372 | -1.42585 | 1.29E-07 | 1.14E-06 | yes | down | 10.94 | 15.24 | 9.21 | 9.77 | 10.58 | 11.93 | 11.79667 | 10.76 |
| SAFB | 2.071 | 1.050149 | 1.3E-07 | 1.15E-06 | yes | up | 14.21 | 10.77 | 12.55 | 52.3 | 72.05 | 57.72 | 12.51 | 60.69 |
| SPTA1 | 0.053 | -4.22562 | 1.31E-07 | 1.16E-06 | yes | down | 0.83 | 2.42 | 0.76 | 0.09 | 0 | 0.26 | 1.336667 | 0.116667 |
| GNAZ | 0.216 | -2.20946 | 1.31E-07 | 1.16E-06 | yes | down | 2.25 | 4.48 | 1.72 | 0.87 | 1.84 | 1.44 | 2.816667 | 1.383333 |
| TMEM45A | 2.886 | 1.529117 | 1.32E-07 | 1.16E-06 | yes | up | 2.63 | 2.01 | 1.05 | 11.67 | 15.12 | 12.76 | 1.896667 | 13.18333 |
| PDLIM2 | 2.353 | 1.234804 | 1.32E-07 | 1.16E-06 | yes | up | 7.69 | 12.13 | 8.4 | 50.54 | 54.46 | 47.02 | 9.406667 | 50.67333 |
| LOC103347061 | 0.337 | -1.5698 | 1.34E-07 | 1.18E-06 | yes | down | 30.7 | 28.48 | 24.62 | 19.11 | 30.72 | 15.74 | 27.93333 | 21.85667 |
| TRIP10 | 2.153 | 1.106217 | 1.35E-07 | 1.19E-06 | yes | up | 20.19 | 20.07 | 14.15 | 82.82 | 85.2 | 100.41 | 18.13667 | 89.47667 |
| PACS1 | 2.174 | 1.120235 | 1.35E-07 | 1.19E-06 | yes | up | 6.4 | 6.51 | 6.55 | 28.65 | 41.91 | 27.92 | 6.486667 | 32.82667 |
| CFP | 3.972 | 1.98971 | 1.36E-07 | 1.19E-06 | yes | up | 0.83 | 0.66 | 0.96 | 5.86 | 8.17 | 9.33 | 0.816667 | 7.786667 |
| C1QTNF5 | 2.585 | 1.370296 | 1.36E-07 | 1.19E-06 | yes | up | 4.51 | 5.15 | 4.02 | 29.85 | 22.96 | 27.3 | 4.56 | 26.70333 |
| LOC100346247 | 0.399 | -1.32457 | 1.37E-07 | 1.2E-06 | yes | down | 10.02 | 13.21 | 9.03 | 10.55 | 11.19 | 7.77 | 10.75333 | 9.836667 |
| NUMBL | 3.418 | 1.772948 | 1.37E-07 | 1.2E-06 | yes | up | 1.64 | 1.23 | 0.88 | 7.89 | 8.83 | 12.64 | 1.25 | 9.786667 |
| LOC100347178 | 67.338 | 6.073349 | 1.38E-07 | 1.21E-06 | yes | up | 0.06 | 0 | 0.1 | 6.97 | 5.41 | 10.23 | 0.053333 | 7.536667 |
| RCN1 | 2.356 | 1.236142 | 1.4E-07 | 1.23E-06 | yes | up | 40.57 | 33.57 | 19.35 | 162.25 | 169.68 | 160.93 | 31.16333 | 164.2867 |
| TBX3 | 0.409 | -1.29148 | 1.43E-07 | 1.25E-06 | yes | down | 6.08 | 8.01 | 7.69 | 5.16 | 9.02 | 6.09 | 7.26 | 6.756667 |
| NBEAL1 | 0.31 | -1.68747 | 1.46E-07 | 1.28E-06 | yes | down | 3.46 | 2.18 | 2.25 | 2.6 | 1.9 | 1.65 | 2.63 | 2.05 |
| LOC103347756 | 140.454 | 7.133956 | 1.47E-07 | 1.28E-06 | yes | up | 0 | 0 | 0 | 14.08 | 31.13 | 22.04 | 0 | 22.41667 |
| RPL9 | 2.481 | 1.311051 | 1.48E-07 | 1.3E-06 | yes | up | 55.43 | 80.64 | 51.21 | 283.21 | 167.84 | 167.07 | 62.42667 | 206.04 |
| LOC100344545 | 13.006 | 3.701054 | 1.49E-07 | 1.3E-06 | yes | up | 0.55 | 0.46 | 0.32 | 1.81 | 20.85 | 16.69 | 0.443333 | 13.11667 |
| SLC16A11 | 0.081 | -3.62064 | 1.49E-07 | 1.3E-06 | yes | down | 1.59 | 2.81 | 0.57 | 0.2 | 0.38 | 0.36 | 1.656667 | 0.313333 |
| CSRNP1 | 3.237 | 1.694464 | 1.49E-07 | 1.3E-06 | yes | up | 1.83 | 3.05 | 1.71 | 16.09 | 12.54 | 18.87 | 2.196667 | 15.83333 |
| FHDC1 | 3.905 | 1.965348 | 1.5E-07 | 1.31E-06 | yes | up | 0.33 | 0.27 | 0.19 | 2.09 | 2.61 | 2.42 | 0.263333 | 2.373333 |
| OSR2 | 8.603 | 3.104863 | 1.53E-07 | 1.34E-06 | yes | up | 1.99 | 0.48 | 0.18 | 18.92 | 10.02 | 20.3 | 0.883333 | 16.41333 |
| CD9 | 3.16 | 1.659946 | 1.57E-07 | 1.37E-06 | yes | up | 46.55 | 35.02 | 21.39 | 242.93 | 175.3 | 304.11 | 34.32 | 240.78 |
| LOC108176476 | 7.491 | 2.905145 | 1.58E-07 | 1.38E-06 | yes | up | 0.08 | 0.16 | 0.03 | 1.88 | 1.5 | 1.19 | 0.09 | 1.523333 |
| ADCY2 | 0.162 | -2.6227 | 1.61E-07 | 1.4E-06 | yes | down | 19.83 | 13.36 | 7.68 | 3.97 | 1.89 | 8.38 | 13.62333 | 4.746667 |
| LOC100344620 | 4.576 | 2.194046 | 1.62E-07 | 1.41E-06 | yes | up | 1.33 | 2.54 | 2.52 | 25.13 | 15.73 | 26.29 | 2.13 | 22.38333 |
| FCER1G | 3.168 | 1.663515 | 1.62E-07 | 1.41E-06 | yes | up | 7.84 | 10.24 | 9.88 | 68.44 | 53.46 | 80.45 | 9.32 | 67.45 |
| SLMAP | 0.43 | -1.21666 | 1.64E-07 | 1.43E-06 | yes | down | 20.22 | 12.37 | 13.56 | 15.69 | 18.27 | 13.53 | 15.38333 | 15.83 |
| COPS4 | 0.423 | -1.23969 | 1.66E-07 | 1.44E-06 | yes | down | 54.6 | 36.04 | 27.85 | 38.4 | 39.24 | 35.56 | 39.49667 | 37.73333 |
| EPYC | 9.954 | 3.315249 | 1.67E-07 | 1.45E-06 | yes | up | 0.23 | 0.31 | 0.07 | 3.71 | 6.86 | 2.54 | 0.203333 | 4.37 |
| TMEM185A | 2.061 | 1.043309 | 1.68E-07 | 1.46E-06 | yes | up | 5.7 | 6.58 | 5.88 | 27.9 | 28.94 | 28.92 | 6.053333 | 28.58667 |
| DNAJC15 | 0.386 | -1.37347 | 1.69E-07 | 1.46E-06 | yes | down | 39.14 | 40.06 | 37.26 | 40.76 | 39.25 | 28.45 | 38.82 | 36.15333 |
| GPR162 | 4.756 | 2.249897 | 1.69E-07 | 1.46E-06 | yes | up | 0.79 | 1.05 | 0.25 | 6.19 | 9.84 | 6.22 | 0.696667 | 7.416667 |
| NCAPD2 | 3.061 | 1.614178 | 1.7E-07 | 1.47E-06 | yes | up | 6.21 | 7.2 | 1.45 | 13.96 | 28.09 | 7.62 | 4.953333 | 16.55667 |
| PRKCD | 2.371 | 1.245436 | 1.71E-07 | 1.48E-06 | yes | up | 4.41 | 4.89 | 3.62 | 17.05 | 26.4 | 24.44 | 4.306667 | 22.63 |
| GABPA | 0.332 | -1.59044 | 1.71E-07 | 1.48E-06 | yes | down | 9.87 | 11.83 | 9.12 | 8.8 | 10.83 | 4.18 | 10.27333 | 7.936667 |
| SELENBP1 | 2.418 | 1.273945 | 1.74E-07 | 1.51E-06 | yes | up | 40.41 | 48.93 | 43.15 | 202.78 | 356.98 | 191.3 | 44.16333 | 250.3533 |
| LOC108178822 | 0.22 | -2.18459 | 1.75E-07 | 1.51E-06 | yes | down | 0.88 | 0.99 | 1.3 | 0.7 | 0.51 | 0.41 | 1.056667 | 0.54 |
| LOC108178241 | 9.137 | 3.191778 | 1.76E-07 | 1.52E-06 | yes | up | 0.07 | 0.28 | 0.16 | 3.81 | 3.31 | 4.83 | 0.17 | 3.983333 |
| APBA1 | 2.77 | 1.469901 | 1.77E-07 | 1.53E-06 | yes | up | 1.01 | 1 | 0.98 | 5.64 | 5.5 | 7.69 | 0.996667 | 6.276667 |
| COL5A2 | 2.515 | 1.330774 | 1.78E-07 | 1.53E-06 | yes | up | 76.77 | 51.22 | 35.48 | 289.74 | 393.35 | 250.68 | 54.49 | 311.2567 |
| LOC103349204 | 0.059 | -4.09018 | 1.83E-07 | 1.58E-06 | yes | down | 4.19 | 5.97 | 9.33 | 1.49 | 0.25 | 0.34 | 6.496667 | 0.693333 |
| ENHO | 4.715 | 2.237391 | 1.83E-07 | 1.58E-06 | yes | up | 2.24 | 1.17 | 1.11 | 15.09 | 13.18 | 19.31 | 1.506667 | 15.86 |
| SPATA6 | 2.665 | 1.414252 | 1.84E-07 | 1.58E-06 | yes | up | 1.58 | 1.44 | 0.95 | 8.39 | 10.44 | 5.17 | 1.323333 | 8 |
| PQLC3 | 2.317 | 1.212517 | 1.84E-07 | 1.59E-06 | yes | up | 9.91 | 13.67 | 10.1 | 48.28 | 65.96 | 64.59 | 11.22667 | 59.61 |
| LOC103350994 | 2.823 | 1.497367 | 1.85E-07 | 1.59E-06 | yes | up | 2.04 | 1.93 | 1.64 | 14.36 | 11.91 | 9.87 | 1.87 | 12.04667 |
| C12H6orf132 | 11.872 | 3.569516 | 1.85E-07 | 1.59E-06 | yes | up | 0.03 | 0.06 | 0.02 | 0.6 | 1.4 | 1.14 | 0.036667 | 1.046667 |
| UTP23 | 0.34 | -1.55432 | 1.85E-07 | 1.59E-06 | yes | down | 8.31 | 7.84 | 8.52 | 5.58 | 7.74 | 6.15 | 8.223333 | 6.49 |
| AK4 | 0.4 | -1.32128 | 1.86E-07 | 1.6E-06 | yes | down | 1.17 | 1.24 | 1.05 | 0.99 | 1.14 | 1.04 | 1.153333 | 1.056667 |
| FNBP1 | 2.257 | 1.174453 | 1.87E-07 | 1.61E-06 | yes | up | 9.49 | 8.59 | 5.97 | 33.52 | 50.84 | 39.32 | 8.016667 | 41.22667 |
| LOC100337853 | 0.321 | -1.63889 | 1.9E-07 | 1.63E-06 | yes | down | 5.9 | 6.02 | 4.86 | 2.37 | 5.61 | 4.32 | 5.593333 | 4.1 |
| ENO1 | 0.476 | -1.0715 | 1.91E-07 | 1.64E-06 | yes | down | 280.72 | 319.28 | 277.13 | 293.54 | 297.22 | 359.67 | 292.3767 | 316.81 |
| FAM214B | 2.38 | 1.251079 | 1.92E-07 | 1.64E-06 | yes | up | 6.26 | 4.52 | 5.54 | 27.04 | 24.97 | 32.52 | 5.44 | 28.17667 |
| CPED1 | 0.427 | -1.22606 | 1.93E-07 | 1.65E-06 | yes | down | 10.17 | 11.93 | 12.32 | 11.44 | 16.45 | 8.8 | 11.47333 | 12.23 |
| AIMP1 | 0.467 | -1.09813 | 1.94E-07 | 1.66E-06 | yes | down | 26.81 | 23.53 | 20.89 | 25.47 | 30.9 | 18.74 | 23.74333 | 25.03667 |
| LOC100346229 | 4.6 | 2.201559 | 1.97E-07 | 1.68E-06 | yes | up | 1.17 | 0.35 | 0.65 | 7.73 | 10.11 | 5.13 | 0.723333 | 7.656667 |
| LOC100343809 | 2.117 | 1.082185 | 1.97E-07 | 1.68E-06 | yes | up | 36.29 | 39.6 | 31.68 | 187.34 | 153.08 | 175.86 | 35.85667 | 172.0933 |
| SYTL4 | 2.34 | 1.226645 | 1.99E-07 | 1.7E-06 | yes | up | 2.49 | 1.61 | 1.09 | 9.81 | 11.33 | 7.52 | 1.73 | 9.553333 |
| LOC100346561 | 4.294 | 2.102373 | 2E-07 | 1.71E-06 | yes | up | 5.74 | 4.89 | 8.31 | 89.03 | 34.12 | 63.67 | 6.313333 | 62.27333 |
| TFRC | 0.301 | -1.73221 | 2.01E-07 | 1.71E-06 | yes | down | 12.23 | 9.6 | 4.16 | 5.61 | 7.62 | 4.35 | 8.663333 | 5.86 |
| PAFAH2 | 2.227 | 1.155179 | 2.04E-07 | 1.74E-06 | yes | up | 5.03 | 4.58 | 3.27 | 18.5 | 23.36 | 24.61 | 4.293333 | 22.15667 |
| GALNS | 2.453 | 1.294768 | 2.05E-07 | 1.75E-06 | yes | up | 2.45 | 2.92 | 2.03 | 14.66 | 13.15 | 14.24 | 2.466667 | 14.01667 |
| RAB3D | 2.228 | 1.155819 | 2.06E-07 | 1.76E-06 | yes | up | 5.2 | 3.41 | 4.6 | 14.05 | 19.29 | 19.22 | 4.403333 | 17.52 |
| GRO-A | 10.424 | 3.3819 | 2.07E-07 | 1.76E-06 | yes | up | 0.14 | 0.84 | 0.52 | 5.87 | 20.9 | 11.77 | 0.5 | 12.84667 |
| MRPS7 | 0.475 | -1.07439 | 2.08E-07 | 1.77E-06 | yes | down | 53.86 | 53.12 | 53.34 | 58.74 | 57.83 | 62.12 | 53.44 | 59.56333 |
| BOC | 2.208 | 1.142516 | 2.1E-07 | 1.79E-06 | yes | up | 4.01 | 3.24 | 2.42 | 13.89 | 18.12 | 16.22 | 3.223333 | 16.07667 |
| FLNA | 2.51 | 1.327691 | 2.1E-07 | 1.79E-06 | yes | up | 17.11 | 15.86 | 13.19 | 75.58 | 136.8 | 70.39 | 15.38667 | 94.25667 |
| SCFD1 | 0.395 | -1.34035 | 2.11E-07 | 1.79E-06 | yes | down | 9.55 | 8.46 | 8.75 | 7.96 | 11.07 | 5.82 | 8.92 | 8.283333 |
| NCKAP1L | 3.04 | 1.604134 | 2.11E-07 | 1.79E-06 | yes | up | 1.44 | 2.18 | 2.16 | 11.88 | 19.76 | 9.96 | 1.926667 | 13.86667 |
| RILPL2 | 2.344 | 1.229266 | 2.11E-07 | 1.79E-06 | yes | up | 20.22 | 27.59 | 17.2 | 127.27 | 109.87 | 108.42 | 21.67 | 115.1867 |
| GDPD5 | 2.603 | 1.380425 | 2.12E-07 | 1.8E-06 | yes | up | 2.1 | 1.97 | 1.1 | 10.25 | 10 | 9.9 | 1.723333 | 10.05 |
| LRRC8D | 0.394 | -1.34247 | 2.17E-07 | 1.84E-06 | yes | down | 7.43 | 8.75 | 9.08 | 6.74 | 10.54 | 6.09 | 8.42 | 7.79 |
| MRPL43 | 0.441 | -1.18128 | 2.18E-07 | 1.85E-06 | yes | down | 26.55 | 25.99 | 26.89 | 29.12 | 25.09 | 26.16 | 26.47667 | 26.79 |
| VAMP8 | 2.074 | 1.052482 | 2.19E-07 | 1.85E-06 | yes | up | 29.1 | 34.41 | 27.1 | 140.51 | 131.84 | 142.15 | 30.20333 | 138.1667 |
| SMO | 2.304 | 1.204169 | 2.2E-07 | 1.86E-06 | yes | up | 4.36 | 3.89 | 2.51 | 17.7 | 17.87 | 19.87 | 3.586667 | 18.48 |
| LOC100349551 | 0.317 | -1.65572 | 2.23E-07 | 1.88E-06 | yes | down | 754.82 | 404.15 | 409.01 | 363.25 | 274.19 | 470.17 | 522.66 | 369.2033 |
| LRRN4 | 0.009 | -6.72374 | 2.24E-07 | 1.9E-06 | yes | down | 0.04 | 2.24 | 0.99 | 0 | 0.05 | 0.03 | 1.09 | 0.026667 |
| FAM114A1 | 2.216 | 1.147704 | 2.25E-07 | 1.9E-06 | yes | up | 10.24 | 8.51 | 6.81 | 42.54 | 54.78 | 32.7 | 8.52 | 43.34 |
| EIF2S3 | 0.379 | -1.39815 | 2.26E-07 | 1.91E-06 | yes | down | 26.09 | 27.36 | 33.83 | 28.05 | 30.81 | 17.72 | 29.09333 | 25.52667 |
| LOXL1 | 2.857 | 1.514452 | 2.26E-07 | 1.91E-06 | yes | up | 6.82 | 4.73 | 3.21 | 26.3 | 29.9 | 37.8 | 4.92 | 31.33333 |
| ECHDC2 | 0.305 | -1.71177 | 2.27E-07 | 1.92E-06 | yes | down | 23.62 | 47.51 | 32.93 | 22.31 | 19.94 | 29.91 | 34.68667 | 24.05333 |
| SELT | 0.498 | -1.00629 | 2.31E-07 | 1.95E-06 | yes | down | 9.21 | 10.31 | 7.99 | 11.57 | 11.63 | 8.18 | 9.17 | 10.46 |
| CBX6 | 2.246 | 1.167624 | 2.31E-07 | 1.95E-06 | yes | up | 5.22 | 3.93 | 4.74 | 23.46 | 29.85 | 19.89 | 4.63 | 24.4 |
| PYROXD1 | 0.395 | -1.34154 | 2.32E-07 | 1.96E-06 | yes | down | 3.89 | 3.83 | 2.82 | 3.05 | 4.22 | 2.45 | 3.513333 | 3.24 |
| LOC100352395 | 3.182 | 1.669944 | 2.36E-07 | 1.99E-06 | yes | up | 0.49 | 0.47 | 0.36 | 4.08 | 2.83 | 2.6 | 0.44 | 3.17 |
| COQ10B | 0.481 | -1.056 | 2.37E-07 | 1.99E-06 | yes | down | 15.44 | 14.81 | 13.94 | 17.84 | 16.06 | 14.72 | 14.73 | 16.20667 |
| IKZF2 | 0.207 | -2.27201 | 2.37E-07 | 1.99E-06 | yes | down | 0.63 | 0.4 | 0.87 | 0.35 | 0.36 | 0.22 | 0.633333 | 0.31 |
| CAPZA2 | 0.403 | -1.30937 | 2.41E-07 | 2.02E-06 | yes | down | 49.9 | 37.8 | 27.01 | 38.75 | 42.36 | 24.19 | 38.23667 | 35.1 |
| NR5A2 | 2.834 | 1.502625 | 2.41E-07 | 2.03E-06 | yes | up | 1.15 | 1.35 | 1.06 | 9.05 | 6.05 | 6.05 | 1.186667 | 7.05 |
| LOC100125995 | 4.948 | 2.306825 | 2.44E-07 | 2.05E-06 | yes | up | 0.32 | 0.6 | 0.23 | 4.67 | 5.16 | 3.27 | 0.383333 | 4.366667 |
| FAM198B | 0.421 | -1.24693 | 2.44E-07 | 2.05E-06 | yes | down | 43.81 | 42.96 | 41.9 | 39.92 | 56.49 | 27.17 | 42.89 | 41.19333 |
| CREB3L2 | 2.158 | 1.110021 | 2.45E-07 | 2.05E-06 | yes | up | 4.52 | 5.08 | 3.47 | 16.53 | 24.67 | 22.96 | 4.356667 | 21.38667 |
| TCF7L1 | 2.418 | 1.273637 | 2.47E-07 | 2.07E-06 | yes | up | 5.51 | 4.87 | 6.59 | 33.37 | 33.11 | 28.88 | 5.656667 | 31.78667 |
| ATF2 | 0.403 | -1.31118 | 2.48E-07 | 2.07E-06 | yes | down | 12.14 | 11.94 | 11.89 | 13.23 | 13.16 | 7.27 | 11.99 | 11.22 |
| DDR2 | 2.131 | 1.091862 | 2.49E-07 | 2.08E-06 | yes | up | 5.25 | 4.49 | 3.84 | 25.65 | 23.82 | 16.38 | 4.526667 | 21.95 |
| CUL3 | 0.433 | -1.20838 | 2.51E-07 | 2.1E-06 | yes | down | 13.88 | 14.41 | 11.43 | 14.99 | 18.15 | 8.94 | 13.24 | 14.02667 |
| SFXN4 | 0.491 | -1.02538 | 2.51E-07 | 2.1E-06 | yes | down | 9.8 | 11.23 | 9.75 | 11.08 | 11.82 | 11.14 | 10.26 | 11.34667 |
| GUF1 | 0.344 | -1.53815 | 2.52E-07 | 2.1E-06 | yes | down | 6.31 | 6.17 | 5.1 | 4.14 | 5.86 | 2.83 | 5.86 | 4.276667 |
| NKD1 | 24.834 | 4.634236 | 2.55E-07 | 2.13E-06 | yes | up | 0.06 | 0.03 | 0.03 | 1.82 | 1.8 | 2.41 | 0.04 | 2.01 |
| SRP19 | 0.455 | -1.13752 | 2.6E-07 | 2.17E-06 | yes | down | 50.23 | 38.18 | 27.77 | 37.67 | 43.81 | 35.65 | 38.72667 | 39.04333 |
| WNT2 | 9.335 | 3.222667 | 2.62E-07 | 2.18E-06 | yes | up | 1.08 | 0.38 | 0.27 | 8.8 | 4.54 | 21.02 | 0.576667 | 11.45333 |
| TMEM177 | 0.282 | -1.82631 | 2.64E-07 | 2.2E-06 | yes | down | 5.1 | 6.11 | 4.52 | 2.77 | 2.47 | 4.86 | 5.243333 | 3.366667 |
| PIK3R6 | 3.511 | 1.812087 | 2.65E-07 | 2.21E-06 | yes | up | 0.63 | 0.79 | 0.47 | 5.25 | 6.31 | 3.48 | 0.63 | 5.013333 |
| GKAP1 | 0.414 | -1.27347 | 2.65E-07 | 2.21E-06 | yes | down | 31.15 | 28.86 | 24.97 | 22.8 | 36.24 | 21.75 | 28.32667 | 26.93 |
| PLAG1 | 0.228 | -2.13581 | 2.71E-07 | 2.25E-06 | yes | down | 1.17 | 1.01 | 0.8 | 0.8 | 0.45 | 0.27 | 0.993333 | 0.506667 |
| TTC1 | 0.47 | -1.08818 | 2.72E-07 | 2.26E-06 | yes | down | 20.62 | 15.85 | 13.23 | 18.14 | 16.58 | 18.49 | 16.56667 | 17.73667 |
| GDF5 | 11.371 | 3.507309 | 2.79E-07 | 2.32E-06 | yes | up | 0.22 | 0.12 | 0.07 | 2.36 | 4.24 | 3.86 | 0.136667 | 3.486667 |
| ZNF385A | 4.945 | 2.306043 | 2.8E-07 | 2.32E-06 | yes | up | 9.94 | 2.71 | 2.88 | 40.65 | 59.44 | 70.35 | 5.176667 | 56.81333 |
| ATP6V0E2 | 20.152 | 4.332841 | 2.8E-07 | 2.33E-06 | yes | up | 2.46 | 1.13 | 3.47 | 121.57 | 33.63 | 174.3 | 2.353333 | 109.8333 |
| ADA | 3.015 | 1.592117 | 2.82E-07 | 2.34E-06 | yes | up | 3.24 | 2.53 | 2.29 | 16.11 | 27.34 | 13.33 | 2.686667 | 18.92667 |
| AHCYL1 | 0.459 | -1.12475 | 2.89E-07 | 2.4E-06 | yes | down | 56.62 | 49.34 | 63.3 | 56.89 | 70.31 | 52.67 | 56.42 | 59.95667 |
| DCLK1 | 3.047 | 1.607575 | 2.91E-07 | 2.41E-06 | yes | up | 3.22 | 1.8 | 1.23 | 14.5 | 16.66 | 10.2 | 2.083333 | 13.78667 |
| LSS | 3.286 | 1.716462 | 2.93E-07 | 2.42E-06 | yes | up | 6.13 | 6.47 | 5.08 | 67 | 29.89 | 34.32 | 5.893333 | 43.73667 |
| PHF13 | 2.135 | 1.093953 | 2.95E-07 | 2.44E-06 | yes | up | 5.11 | 4.88 | 4.17 | 19.61 | 24.15 | 25.09 | 4.72 | 22.95 |
| LMNB1 | 4.485 | 2.165257 | 2.99E-07 | 2.48E-06 | yes | up | 5.99 | 4.46 | 2.48 | 53.92 | 62.8 | 16.66 | 4.31 | 44.46 |
| LOC100337813 | 2.146 | 1.101608 | 3E-07 | 2.48E-06 | yes | up | 12.23 | 15.98 | 13.05 | 68.62 | 75.68 | 59.89 | 13.75333 | 68.06333 |
| VDAC3 | 0.342 | -1.5486 | 3E-07 | 2.48E-06 | yes | down | 205.13 | 130.52 | 89.17 | 114.07 | 95.84 | 122.52 | 141.6067 | 110.81 |
| LOC100342263 | 2.092 | 1.064946 | 3.03E-07 | 2.5E-06 | yes | up | 4.58 | 5.25 | 4.97 | 23.65 | 28.04 | 20.04 | 4.933333 | 23.91 |
| PIP4K2B | 2.016 | 1.011404 | 3.07E-07 | 2.53E-06 | yes | up | 11.95 | 11.56 | 8.2 | 40.92 | 50.76 | 52.87 | 10.57 | 48.18333 |
| MRPS28 | 0.317 | -1.65889 | 3.07E-07 | 2.53E-06 | yes | down | 37.53 | 40.03 | 25.7 | 34.25 | 21.33 | 17.91 | 34.42 | 24.49667 |
| STAM2 | 0.454 | -1.13885 | 3.07E-07 | 2.54E-06 | yes | down | 6.21 | 7.5 | 4.84 | 6.72 | 8.55 | 4.68 | 6.183333 | 6.65 |
| PEX2 | 0.47 | -1.08777 | 3.09E-07 | 2.55E-06 | yes | down | 15.69 | 14.6 | 12.72 | 15.79 | 17.89 | 11.51 | 14.33667 | 15.06333 |
| EXOC5 | 0.399 | -1.32458 | 3.12E-07 | 2.57E-06 | yes | down | 12 | 10.39 | 8.22 | 9.84 | 12.38 | 5.88 | 10.20333 | 9.366667 |
| WIPF1 | 2.128 | 1.08937 | 3.18E-07 | 2.61E-06 | yes | up | 6 | 4.82 | 4.46 | 26.58 | 29.59 | 18.52 | 5.093333 | 24.89667 |
| RIOK2 | 0.488 | -1.03496 | 3.18E-07 | 2.62E-06 | yes | down | 11.91 | 12.23 | 8.5 | 12.48 | 13.39 | 10.36 | 10.88 | 12.07667 |
| IGF2BP2 | 0.145 | -2.78839 | 3.21E-07 | 2.64E-06 | yes | down | 4.58 | 1.34 | 0.92 | 0.58 | 0.78 | 0.83 | 2.28 | 0.73 |
| XYLT1 | 2.16 | 1.110762 | 3.21E-07 | 2.64E-06 | yes | up | 0.87 | 0.74 | 0.68 | 3.52 | 4.03 | 3.71 | 0.763333 | 3.753333 |
| GMPPB | 2.032 | 1.022891 | 3.28E-07 | 2.7E-06 | yes | up | 7.88 | 7.19 | 7.18 | 35.62 | 36.78 | 30.64 | 7.416667 | 34.34667 |
| CCDC91 | 0.45 | -1.1529 | 3.28E-07 | 2.7E-06 | yes | down | 14.54 | 15.31 | 12.64 | 17.31 | 15.71 | 10.88 | 14.16333 | 14.63333 |
| LOC103349154 | 0.189 | -2.40327 | 3.29E-07 | 2.7E-06 | yes | down | 0.4 | 0.3 | 0.52 | 0.16 | 0.23 | 0.13 | 0.406667 | 0.173333 |
| BRAP | 0.475 | -1.07551 | 3.3E-07 | 2.71E-06 | yes | down | 8.51 | 8.51 | 6.76 | 7.27 | 10.57 | 8.07 | 7.926667 | 8.636667 |
| AURKA | 5.112 | 2.353827 | 3.4E-07 | 2.79E-06 | yes | up | 0.65 | 0.89 | 0.39 | 8.41 | 10.6 | 3.6 | 0.643333 | 7.536667 |
| LOC100353567 | 2.163 | 1.112863 | 3.42E-07 | 2.8E-06 | yes | up | 25.49 | 36.95 | 26.69 | 149.19 | 138.99 | 144.37 | 29.71 | 144.1833 |
| ARHGEF1 | 2.004 | 1.003118 | 3.43E-07 | 2.81E-06 | yes | up | 11.91 | 16.04 | 12.59 | 57.28 | 79.11 | 54.79 | 13.51333 | 63.72667 |
| RBKS | 0.4 | -1.32257 | 3.49E-07 | 2.86E-06 | yes | down | 7.38 | 9.02 | 4.55 | 7 | 7.64 | 12.28 | 6.983333 | 8.973333 |
| LOC100343299 | 0.139 | -2.85168 | 3.5E-07 | 2.86E-06 | yes | down | 23.64 | 33.37 | 101.68 | 19.18 | 17.02 | 18.29 | 52.89667 | 18.16333 |
| LOC108176024 | 0.286 | -1.80757 | 3.51E-07 | 2.87E-06 | yes | down | 13.9 | 6.21 | 9.98 | 6.82 | 6.74 | 5.31 | 10.03 | 6.29 |
| NEK7 | 0.444 | -1.17162 | 3.53E-07 | 2.89E-06 | yes | down | 22.73 | 25.38 | 13.85 | 19.82 | 26.32 | 16.44 | 20.65333 | 20.86 |
| ALKBH7 | 0.367 | -1.44588 | 3.54E-07 | 2.89E-06 | yes | down | 12.48 | 16.4 | 14.73 | 11.94 | 10.76 | 13.98 | 14.53667 | 12.22667 |
| MAP7D3 | 0.346 | -1.53272 | 3.55E-07 | 2.9E-06 | yes | down | 32.5 | 42.48 | 35.28 | 30 | 42.83 | 16.57 | 36.75333 | 29.8 |
| TUBB | 2.638 | 1.399241 | 3.57E-07 | 2.91E-06 | yes | up | 80.58 | 66.32 | 49.61 | 453.98 | 504.03 | 232.35 | 65.50333 | 396.7867 |
| NOP58 | 0.361 | -1.47156 | 3.59E-07 | 2.93E-06 | yes | down | 10.34 | 11.98 | 6.65 | 9.28 | 9.23 | 5.15 | 9.656667 | 7.886667 |
| TNFRSF1A | 2.135 | 1.093919 | 3.62E-07 | 2.95E-06 | yes | up | 14.54 | 13.99 | 14.85 | 65.62 | 68.29 | 78.33 | 14.46 | 70.74667 |
| LOC100341037 | 2.393 | 1.25894 | 3.63E-07 | 2.96E-06 | yes | up | 99.89 | 139.06 | 87.73 | 711.1 | 497.44 | 552.75 | 108.8933 | 587.0967 |
| KTN1 | 0.44 | -1.18323 | 3.63E-07 | 2.96E-06 | yes | down | 23.31 | 22.16 | 19.15 | 23.54 | 27.97 | 14.38 | 21.54 | 21.96333 |
| FKBP3 | 0.341 | -1.55282 | 3.79E-07 | 3.09E-06 | yes | down | 115.47 | 56.22 | 48.78 | 61.41 | 59.81 | 47.92 | 73.49 | 56.38 |
| FZD1 | 2.457 | 1.297043 | 3.82E-07 | 3.11E-06 | yes | up | 2.56 | 3.12 | 2.56 | 16.29 | 14.91 | 15.05 | 2.746667 | 15.41667 |
| TREX2 | 0.227 | -2.13644 | 3.86E-07 | 3.14E-06 | yes | down | 3.04 | 4.36 | 3.17 | 2.42 | 1.64 | 1.48 | 3.523333 | 1.846667 |
| ACYP1 | 3.07 | 1.618229 | 3.89E-07 | 3.16E-06 | yes | up | 14.11 | 10.41 | 9.26 | 50.99 | 63.14 | 89.51 | 11.26 | 67.88 |
| NDUFA7 | 0.388 | -1.36469 | 3.9E-07 | 3.16E-06 | yes | down | 317.19 | 267.5 | 333.93 | 338.21 | 231.06 | 257.27 | 306.2067 | 275.5133 |
| CIT | 5.935 | 2.569348 | 3.9E-07 | 3.16E-06 | yes | up | 0.28 | 0.18 | 0.09 | 2.72 | 3.85 | 0.96 | 0.183333 | 2.51 |
| ATG4D | 0.428 | -1.22435 | 3.9E-07 | 3.17E-06 | yes | down | 10.81 | 11.87 | 11.74 | 10.59 | 11.2 | 12.14 | 11.47333 | 11.31 |
| LOC108178475 | 0.061 | -4.03505 | 3.91E-07 | 3.17E-06 | yes | down | 0.32 | 0.3 | 0.28 | 0.03 | 0.08 | 0.02 | 0.3 | 0.043333 |
| PPP4R2 | 0.429 | -1.21951 | 3.91E-07 | 3.17E-06 | yes | down | 12.38 | 15.1 | 10.93 | 13.91 | 15.68 | 8.43 | 12.80333 | 12.67333 |
| LGR4 | 0.496 | -1.01158 | 3.94E-07 | 3.2E-06 | yes | down | 6.07 | 5.33 | 4.71 | 6.06 | 7.45 | 4.81 | 5.37 | 6.106667 |
| THEM4 | 0.406 | -1.29931 | 3.98E-07 | 3.22E-06 | yes | down | 15.84 | 23.49 | 20.59 | 18.22 | 22.78 | 17.34 | 19.97333 | 19.44667 |
| FABP9 | 6.128 | 2.615422 | 4E-07 | 3.24E-06 | yes | up | 0.42 | 0.28 | 0.11 | 10.12 | 7.08 | 2.31 | 0.27 | 6.503333 |
| TAF2 | 0.449 | -1.15495 | 4.04E-07 | 3.27E-06 | yes | down | 5.74 | 5.76 | 4.28 | 5.6 | 7.07 | 3.94 | 5.26 | 5.536667 |
| KIAA1143 | 0.404 | -1.30583 | 4.07E-07 | 3.29E-06 | yes | down | 16.58 | 21.09 | 16.99 | 15.12 | 22.89 | 14.04 | 18.22 | 17.35 |
| NAPEPLD | 0.33 | -1.60108 | 4.09E-07 | 3.3E-06 | yes | down | 3.32 | 2.19 | 1.98 | 1.5 | 2.02 | 2.15 | 2.496667 | 1.89 |
| CHPF | 2.509 | 1.327203 | 4.1E-07 | 3.31E-06 | yes | up | 7.54 | 8.73 | 7.08 | 34.7 | 41.84 | 56.31 | 7.783333 | 44.28333 |
| AASS | 0.386 | -1.37261 | 4.12E-07 | 3.32E-06 | yes | down | 6.63 | 6.63 | 7.42 | 7.32 | 7.3 | 4.1 | 6.893333 | 6.24 |
| LOC103352377 | 0.219 | -2.18899 | 4.14E-07 | 3.34E-06 | yes | down | 14.63 | 29.42 | 15.86 | 11.43 | 10.55 | 8.1 | 19.97 | 10.02667 |
| LSM4 | 2.575 | 1.364692 | 4.14E-07 | 3.34E-06 | yes | up | 14.75 | 15.79 | 13.73 | 65.46 | 70.22 | 96.94 | 14.75667 | 77.54 |
| IL6R | 3.031 | 1.599862 | 4.14E-07 | 3.34E-06 | yes | up | 0.64 | 0.86 | 0.71 | 3.45 | 6.17 | 5.83 | 0.736667 | 5.15 |
| ZC3H8 | 0.354 | -1.49745 | 4.17E-07 | 3.36E-06 | yes | down | 4.24 | 3.93 | 4.27 | 3.74 | 3.71 | 2.84 | 4.146667 | 3.43 |
| NUDCD2 | 0.444 | -1.17165 | 4.18E-07 | 3.36E-06 | yes | down | 7.55 | 6.08 | 5.3 | 6.63 | 7.88 | 4.76 | 6.31 | 6.423333 |
| SCRN3 | 0.347 | -1.52705 | 4.19E-07 | 3.37E-06 | yes | down | 3.71 | 3.49 | 3.9 | 3.5 | 2.89 | 2.48 | 3.7 | 2.956667 |
| LOC100008716 | 8.78 | 3.134208 | 4.26E-07 | 3.43E-06 | yes | up | 0.51 | 0.28 | 0.04 | 5.6 | 4.97 | 5.41 | 0.276667 | 5.326667 |
| PRRX2 | 6.65 | 2.73328 | 4.26E-07 | 3.43E-06 | yes | up | 1.31 | 2.09 | 0.66 | 18.36 | 12.24 | 28.66 | 1.353333 | 19.75333 |
| RNF187 | 2.57 | 1.361819 | 4.29E-07 | 3.45E-06 | yes | up | 22.41 | 16.57 | 15.35 | 103.95 | 83.38 | 126.15 | 18.11 | 104.4933 |
| SNAI1 | 7.214 | 2.850745 | 4.3E-07 | 3.45E-06 | yes | up | 0.71 | 0.26 | 0.16 | 7.46 | 5.26 | 5.1 | 0.376667 | 5.94 |
| CXCL12 | 0.266 | -1.90954 | 4.31E-07 | 3.46E-06 | yes | down | 21.54 | 31.29 | 45.18 | 17.36 | 16.24 | 27.05 | 32.67 | 20.21667 |
| CHST10 | 2.496 | 1.319904 | 4.31E-07 | 3.46E-06 | yes | up | 2.36 | 2.06 | 1.34 | 10.8 | 10.3 | 11.27 | 1.92 | 10.79 |
| ARHGEF40 | 2.2 | 1.137232 | 4.33E-07 | 3.47E-06 | yes | up | 10.21 | 7.66 | 5.31 | 32.74 | 44.98 | 36.86 | 7.726667 | 38.19333 |
| AP1G2 | 4.72 | 2.238918 | 4.34E-07 | 3.47E-06 | yes | up | 0.35 | 0.26 | 0.2 | 2.25 | 3 | 2.89 | 0.27 | 2.713333 |
| CECR5 | 0.471 | -1.08546 | 4.4E-07 | 3.52E-06 | yes | down | 8.66 | 8.19 | 7.56 | 6.83 | 6.1 | 6.22 | 8.136667 | 6.383333 |
| LOC108177440 | 0.101 | -3.30851 | 4.4E-07 | 3.52E-06 | yes | down | 1.36 | 2.26 | 2.24 | 0.52 | 0.81 | 0.06 | 1.953333 | 0.463333 |
| TESK1 | 2.138 | 1.09608 | 4.43E-07 | 3.54E-06 | yes | up | 10.28 | 10.48 | 8.29 | 44.93 | 42.58 | 53.26 | 9.683333 | 46.92333 |
| CDK15 | 5.419 | 2.437992 | 4.43E-07 | 3.54E-06 | yes | up | 0.17 | 0.21 | 0.07 | 1.91 | 2.38 | 1.25 | 0.15 | 1.846667 |
| ZBED5 | 0.376 | -1.40979 | 4.45E-07 | 3.56E-06 | yes | down | 6.17 | 6.87 | 4.4 | 4.67 | 7.04 | 3.39 | 5.813333 | 5.033333 |
| LLPH | 0.423 | -1.24273 | 4.46E-07 | 3.56E-06 | yes | down | 29.47 | 23.85 | 27.75 | 24.09 | 34.3 | 17.38 | 27.02333 | 25.25667 |
| C12H6orf203 | 0.388 | -1.36506 | 4.47E-07 | 3.57E-06 | yes | down | 15.79 | 12.92 | 11.68 | 14.1 | 12.25 | 9.41 | 13.46333 | 11.92 |
| PLXNA3 | 4.073 | 2.026073 | 4.48E-07 | 3.58E-06 | yes | up | 0.67 | 0.31 | 0.18 | 3.31 | 4.24 | 3.02 | 0.386667 | 3.523333 |
| CEBPD | 2.76 | 1.464664 | 4.5E-07 | 3.59E-06 | yes | up | 11.9 | 14.95 | 10.17 | 58.15 | 73.97 | 99.24 | 12.34 | 77.12 |
| NTMT1 | 0.492 | -1.02238 | 4.51E-07 | 3.59E-06 | yes | down | 63.05 | 56.56 | 48.6 | 54.32 | 66.02 | 68.99 | 56.07 | 63.11 |
| METAP1D | 0.389 | -1.36307 | 4.58E-07 | 3.64E-06 | yes | down | 5.92 | 7.7 | 5.51 | 5.65 | 5.77 | 5.47 | 6.376667 | 5.63 |
| FBXO8 | 0.422 | -1.24352 | 4.58E-07 | 3.65E-06 | yes | down | 16.13 | 18.46 | 16.44 | 18.54 | 20.29 | 11.79 | 17.01 | 16.87333 |
| TMEM41B | 0.379 | -1.40135 | 4.64E-07 | 3.69E-06 | yes | down | 9.54 | 11.75 | 7.69 | 9.98 | 9.51 | 5.31 | 9.66 | 8.266667 |
| OPN3 | 3.439 | 1.782038 | 4.65E-07 | 3.69E-06 | yes | up | 1.21 | 0.74 | 0.48 | 6.05 | 6.22 | 4.95 | 0.81 | 5.74 |
| DYNLL1 | 2.079 | 1.055823 | 4.65E-07 | 3.7E-06 | yes | up | 122.13 | 133.76 | 109 | 680.03 | 555.11 | 499.93 | 121.63 | 578.3567 |
| PPWD1 | 0.447 | -1.16195 | 4.67E-07 | 3.71E-06 | yes | down | 6.91 | 6.01 | 5.29 | 5.5 | 6.35 | 3.48 | 6.07 | 5.11 |
| LRRC58 | 0.316 | -1.66366 | 4.68E-07 | 3.71E-06 | yes | down | 6.44 | 10.41 | 6.93 | 5.51 | 8.86 | 3.21 | 7.926667 | 5.86 |
| LHX6 | 3.419 | 1.773486 | 4.71E-07 | 3.73E-06 | yes | up | 1.24 | 1.14 | 1.12 | 13.42 | 7.83 | 6.05 | 1.166667 | 9.1 |
| THBS3 | 2.948 | 1.559847 | 4.74E-07 | 3.75E-06 | yes | up | 13.65 | 7.77 | 5.08 | 54.57 | 55.63 | 63.09 | 8.833333 | 57.76333 |
| GTPBP6 | 0.438 | -1.18965 | 4.75E-07 | 3.76E-06 | yes | down | 35.95 | 35.53 | 33.63 | 35.36 | 28.98 | 40.09 | 35.03667 | 34.81 |
| PCOLCE2 | 3.403 | 1.766807 | 4.8E-07 | 3.8E-06 | yes | up | 24.95 | 36.8 | 28.95 | 258.53 | 134.06 | 301.08 | 30.23333 | 231.2233 |
| TOMM6 | 0.466 | -1.10296 | 4.84E-07 | 3.82E-06 | yes | down | 195.07 | 256.37 | 189.53 | 207.97 | 158.8 | 202.1 | 213.6567 | 189.6233 |
| WSCD1 | 2.331 | 1.220676 | 4.88E-07 | 3.85E-06 | yes | up | 4.56 | 4.31 | 3.5 | 17.27 | 30.56 | 18.95 | 4.123333 | 22.26 |
| PPP4R3B | 0.403 | -1.31058 | 4.93E-07 | 3.89E-06 | yes | down | 13.32 | 16.12 | 13.22 | 12.9 | 18.32 | 8.33 | 14.22 | 13.18333 |
| DHX36 | 0.371 | -1.43215 | 4.94E-07 | 3.89E-06 | yes | down | 4.04 | 4.2 | 4.09 | 3.37 | 5.15 | 2.2 | 4.11 | 3.573333 |
| CLMP | 0.417 | -1.26149 | 5.02E-07 | 3.95E-06 | yes | down | 24.22 | 22.6 | 27.39 | 24.22 | 38.66 | 21.28 | 24.73667 | 28.05333 |
| FUNDC1 | 0.37 | -1.43499 | 5.03E-07 | 3.96E-06 | yes | down | 14.09 | 13.77 | 10.66 | 12.49 | 14 | 6.33 | 12.84 | 10.94 |
| AGFG2 | 2.287 | 1.193371 | 5.17E-07 | 4.06E-06 | yes | up | 3.22 | 3.72 | 4.07 | 18.13 | 22.46 | 17.86 | 3.67 | 19.48333 |
| CCL7 | 9.053 | 3.17843 | 5.19E-07 | 4.07E-06 | yes | up | 3.68 | 2.25 | 0.03 | 30.71 | 32.78 | 50.18 | 1.986667 | 37.89 |
| UCK2 | 0.429 | -1.22011 | 5.19E-07 | 4.07E-06 | yes | down | 8.44 | 6.64 | 7.47 | 7.13 | 6.45 | 8.34 | 7.516667 | 7.306667 |
| CENPA | 6.241 | 2.641808 | 5.4E-07 | 4.24E-06 | yes | up | 1.79 | 1.69 | 0.56 | 19.46 | 30.52 | 6.6 | 1.346667 | 18.86 |
| SAPCD1 | 4.615 | 2.206471 | 5.41E-07 | 4.24E-06 | yes | up | 1.82 | 1.45 | 0.52 | 15.41 | 11.55 | 11.77 | 1.263333 | 12.91 |
| UBR7 | 0.379 | -1.40123 | 5.41E-07 | 4.24E-06 | yes | down | 20.07 | 24.87 | 30.49 | 19.86 | 24.58 | 18.09 | 25.14333 | 20.84333 |
| RASSF6 | 0.238 | -2.0695 | 5.44E-07 | 4.26E-06 | yes | down | 1.47 | 1.06 | 1.43 | 0.95 | 0.74 | 0.51 | 1.32 | 0.733333 |
| C3H8orf22 | 0.029 | -5.1133 | 5.46E-07 | 4.27E-06 | yes | down | 4.6 | 1.98 | 0.34 | 0.15 | 0 | 0.29 | 2.306667 | 0.146667 |
| DPY19L4 | 0.396 | -1.33479 | 5.47E-07 | 4.28E-06 | yes | down | 6.1 | 6.9 | 5 | 5.59 | 7.6 | 3.4 | 6 | 5.53 |
| LOC108178486 | 0.241 | -2.05166 | 5.51E-07 | 4.3E-06 | yes | down | 1.48 | 3.29 | 2.11 | 1.64 | 1.17 | 1.19 | 2.293333 | 1.333333 |
| LOC103345647 | 2.287 | 1.193677 | 5.53E-07 | 4.32E-06 | yes | up | 41.15 | 58.12 | 53.67 | 300.02 | 259.6 | 246.48 | 50.98 | 268.7 |
| CNRIP1 | 2.338 | 1.225042 | 5.62E-07 | 4.39E-06 | yes | up | 7.45 | 7.46 | 6.98 | 47.44 | 34.13 | 34.99 | 7.296667 | 38.85333 |
| WDR36 | 0.444 | -1.16989 | 5.68E-07 | 4.43E-06 | yes | down | 3.03 | 3.7 | 2.57 | 3.05 | 4.01 | 2.43 | 3.1 | 3.163333 |
| LOC103346787 | 0.338 | -1.56441 | 5.77E-07 | 4.5E-06 | yes | down | 17.31 | 28.98 | 23.86 | 17.7 | 17.25 | 19.76 | 23.38333 | 18.23667 |
| DCHS2 | 4.273 | 2.095394 | 5.88E-07 | 4.58E-06 | yes | up | 0.21 | 0.08 | 0.09 | 1.06 | 1.63 | 1.03 | 0.126667 | 1.24 |
| AGA | 0.383 | -1.38643 | 5.9E-07 | 4.59E-06 | yes | down | 10.44 | 12.57 | 9.64 | 11.11 | 12.75 | 6.18 | 10.88333 | 10.01333 |
| PARP12 | 2.167 | 1.115578 | 5.94E-07 | 4.62E-06 | yes | up | 2.85 | 2.72 | 2.06 | 11.7 | 12.71 | 12.96 | 2.543333 | 12.45667 |
| DPH6 | 0.414 | -1.27395 | 5.96E-07 | 4.63E-06 | yes | down | 1.43 | 1.48 | 1.39 | 1.85 | 2.7 | 2.48 | 1.433333 | 2.343333 |
| LOC103349541 | 0.221 | -2.18034 | 5.97E-07 | 4.64E-06 | yes | down | 2.16 | 2.39 | 1.53 | 1.27 | 0.95 | 1.25 | 2.026667 | 1.156667 |
| CDKL2 | 6.322 | 2.660352 | 6E-07 | 4.66E-06 | yes | up | 0.14 | 0.11 | 0.04 | 1.39 | 1.84 | 1.5 | 0.096667 | 1.576667 |
| TP53I11 | 2.484 | 1.312707 | 6.04E-07 | 4.69E-06 | yes | up | 9.96 | 17.23 | 13.44 | 80 | 94.29 | 60.23 | 13.54333 | 78.17333 |
| PLD3 | 2.456 | 1.296086 | 6.1E-07 | 4.72E-06 | yes | up | 23.05 | 20.03 | 19.25 | 94.15 | 103.33 | 148.98 | 20.77667 | 115.4867 |
| TSPAN8 | 0.084 | -3.57206 | 6.1E-07 | 4.72E-06 | yes | down | 0.39 | 1.91 | 0.92 | 0.31 | 0.13 | 0.17 | 1.073333 | 0.203333 |
| ABCE1 | 0.398 | -1.32869 | 6.18E-07 | 4.79E-06 | yes | down | 11.75 | 12.19 | 9.92 | 11.12 | 13.92 | 6.23 | 11.28667 | 10.42333 |
| SUMF2 | 2.692 | 1.428876 | 6.37E-07 | 4.93E-06 | yes | up | 5.6 | 7.9 | 5.67 | 37.04 | 33.07 | 46.15 | 6.39 | 38.75333 |
| JUN | 3.748 | 1.906181 | 6.57E-07 | 5.09E-06 | yes | up | 24.57 | 19.02 | 17.52 | 169.22 | 88.72 | 248.95 | 20.37 | 168.9633 |
| CCPG1 | 0.459 | -1.12241 | 6.58E-07 | 5.09E-06 | yes | down | 11.9 | 9.86 | 10.19 | 10.74 | 15.04 | 8.55 | 10.65 | 11.44333 |
| TMEM70 | 0.408 | -1.29224 | 6.62E-07 | 5.11E-06 | yes | down | 13.21 | 12.83 | 11.5 | 14.24 | 12.33 | 8.57 | 12.51333 | 11.71333 |
| LOC100348947 | 2.946 | 1.558958 | 6.65E-07 | 5.13E-06 | yes | up | 0.69 | 0.62 | 0.62 | 4.43 | 4.13 | 4.41 | 0.643333 | 4.323333 |
| FAM135A | 0.393 | -1.3481 | 6.71E-07 | 5.18E-06 | yes | down | 2.74 | 3.43 | 1.78 | 3.01 | 3.71 | 1.52 | 2.65 | 2.746667 |
| LOC103350989 | 0.334 | -1.58158 | 6.74E-07 | 5.2E-06 | yes | down | 3.18 | 3.13 | 2.81 | 1.98 | 1.79 | 2.53 | 3.04 | 2.1 |
| TMEM200A | 0.143 | -2.8066 | 6.79E-07 | 5.23E-06 | yes | down | 0.82 | 1.1 | 0.59 | 0.16 | 0.45 | 0.23 | 0.836667 | 0.28 |
| NEDD9 | 2.321 | 1.214544 | 6.81E-07 | 5.24E-06 | yes | up | 6.26 | 8.68 | 5.5 | 39.3 | 31.26 | 38.24 | 6.813333 | 36.26667 |
| SLITRK2 | 0.244 | -2.03719 | 6.81E-07 | 5.24E-06 | yes | down | 0.4 | 0.59 | 0.3 | 0.2 | 0.31 | 0.21 | 0.43 | 0.24 |
| HSPA13 | 0.347 | -1.52817 | 6.85E-07 | 5.27E-06 | yes | down | 6.6 | 6.97 | 8.69 | 6.2 | 8.32 | 3.77 | 7.42 | 6.096667 |
| BAZ2B | 0.423 | -1.24111 | 6.9E-07 | 5.31E-06 | yes | down | 2.16 | 1.89 | 2.14 | 2.1 | 2.41 | 1.38 | 2.063333 | 1.963333 |
| SNX14 | 0.451 | -1.14837 | 6.93E-07 | 5.33E-06 | yes | down | 12.05 | 11.32 | 9.93 | 11.84 | 14.97 | 7.93 | 11.1 | 11.58 |
| SSB | 0.359 | -1.47603 | 7.09E-07 | 5.44E-06 | yes | down | 12.66 | 11.79 | 11.32 | 12.28 | 12.02 | 5.49 | 11.92333 | 9.93 |
| DHX29 | 0.356 | -1.49024 | 7.1E-07 | 5.45E-06 | yes | down | 7.55 | 4.91 | 3.88 | 4.41 | 6.02 | 2.9 | 5.446667 | 4.443333 |
| PDK4 | 0.047 | -4.42637 | 7.15E-07 | 5.49E-06 | yes | down | 452.22 | 343.59 | 563.72 | 27.66 | 117.1 | 12.08 | 453.1767 | 52.28 |
| CCNB1 | 4.992 | 2.319572 | 7.16E-07 | 5.49E-06 | yes | up | 4.56 | 4.16 | 1.59 | 49.42 | 52.57 | 14.63 | 3.436667 | 38.87333 |
| PGER8 | 0.316 | -1.66294 | 7.22E-07 | 5.53E-06 | yes | down | 4.61 | 7.98 | 5.29 | 4.99 | 4.77 | 3.17 | 5.96 | 4.31 |
| PDZD8 | 0.474 | -1.07625 | 7.26E-07 | 5.56E-06 | yes | down | 3.34 | 3.37 | 3.28 | 3.58 | 4.44 | 2.78 | 3.33 | 3.6 |
| FBXO27 | 2.352 | 1.233831 | 7.26E-07 | 5.56E-06 | yes | up | 7.94 | 9.46 | 6.13 | 43.15 | 36.59 | 45.14 | 7.843333 | 41.62667 |
| C2H2orf49 | 0.437 | -1.19497 | 7.27E-07 | 5.56E-06 | yes | down | 11.32 | 12.31 | 9.99 | 12.36 | 14.47 | 7.97 | 11.20667 | 11.6 |
| KIF23 | 5.644 | 2.496806 | 7.28E-07 | 5.56E-06 | yes | up | 1.43 | 0.97 | 0.43 | 14.07 | 18.5 | 4.09 | 0.943333 | 12.22 |
| AK9 | 0.091 | -3.45576 | 7.29E-07 | 5.57E-06 | yes | down | 0.17 | 0.25 | 0.27 | 0.05 | 0.07 | 0.03 | 0.23 | 0.05 |
| FBXO30 | 0.288 | -1.79578 | 7.33E-07 | 5.6E-06 | yes | down | 6.43 | 5.27 | 10.51 | 5.78 | 5.96 | 3.5 | 7.403333 | 5.08 |
| ARPC5 | 2.037 | 1.026734 | 7.35E-07 | 5.61E-06 | yes | up | 23.94 | 29.61 | 21.51 | 131.83 | 113.65 | 109.56 | 25.02 | 118.3467 |
| CDC40 | 0.429 | -1.21992 | 7.37E-07 | 5.62E-06 | yes | down | 7.6 | 7.13 | 7.8 | 7.46 | 9.53 | 5.15 | 7.51 | 7.38 |
| ESM1 | 3.821 | 1.933947 | 7.39E-07 | 5.63E-06 | yes | up | 1.16 | 0.86 | 0.39 | 7.9 | 5.89 | 6.54 | 0.803333 | 6.776667 |
| DIS3 | 0.359 | -1.47929 | 7.46E-07 | 5.68E-06 | yes | down | 3.88 | 4.19 | 4 | 2.95 | 4.77 | 1.71 | 4.023333 | 3.143333 |
| HPCA | 0.008 | -6.95121 | 7.5E-07 | 5.71E-06 | yes | down | 0.47 | 1.42 | 1.34 | 0.05 | 0 | 0 | 1.076667 | 0.016667 |
| ALKBH3 | 0.444 | -1.17222 | 7.5E-07 | 5.71E-06 | yes | down | 11.53 | 8.69 | 7.08 | 8.24 | 9.68 | 8.83 | 9.1 | 8.916667 |
| LOC103351480 | 2.831 | 1.50156 | 7.71E-07 | 5.87E-06 | yes | up | 2.18 | 3.09 | 2.51 | 18.29 | 18.94 | 13.69 | 2.593333 | 16.97333 |
| LOC108176506 | 0.016 | -5.97013 | 7.73E-07 | 5.88E-06 | yes | down | 5.26 | 3.92 | 2.44 | 0.09 | 0 | 0.4 | 3.873333 | 0.163333 |
| LOC103352131 | 4.232 | 2.081299 | 7.75E-07 | 5.89E-06 | yes | up | 2.39 | 1.65 | 1.6 | 21.58 | 14.34 | 23.29 | 1.88 | 19.73667 |
| SHMT1 | 0.403 | -1.31196 | 7.82E-07 | 5.94E-06 | yes | down | 69.39 | 76.33 | 60.31 | 83.07 | 48.09 | 55.35 | 68.67667 | 62.17 |
| TATDN1 | 0.316 | -1.66194 | 7.89E-07 | 5.99E-06 | yes | down | 10.17 | 11.39 | 8.29 | 9.4 | 8.43 | 4.14 | 9.95 | 7.323333 |
| CIB1 | 2.418 | 1.273689 | 7.96E-07 | 6.04E-06 | yes | up | 29.29 | 37.75 | 37.81 | 218.48 | 156.79 | 193.02 | 34.95 | 189.43 |
| TMEM53 | 3.375 | 1.754846 | 8E-07 | 6.07E-06 | yes | up | 1.18 | 1.94 | 0.9 | 7.96 | 10.94 | 11.75 | 1.34 | 10.21667 |
| JADE1 | 0.415 | -1.2691 | 8.12E-07 | 6.16E-06 | yes | down | 4.14 | 4.36 | 3.24 | 3.79 | 5 | 2.43 | 3.913333 | 3.74 |
| PYCR1 | 4.721 | 2.239068 | 8.19E-07 | 6.2E-06 | yes | up | 2.05 | 1.53 | 0.51 | 11.63 | 12.51 | 17.82 | 1.363333 | 13.98667 |
| NDUFC1 | 0.446 | -1.16402 | 8.23E-07 | 6.23E-06 | yes | down | 108.61 | 98.22 | 90.02 | 115.01 | 89.91 | 115.94 | 98.95 | 106.9533 |
| FBLIM1 | 3.301 | 1.722978 | 8.32E-07 | 6.3E-06 | yes | up | 1.29 | 1.25 | 0.78 | 10.33 | 6.44 | 7.68 | 1.106667 | 8.15 |
| TBC1D23 | 0.444 | -1.17172 | 8.41E-07 | 6.36E-06 | yes | down | 7.22 | 7.62 | 5.68 | 8.2 | 7.68 | 4.92 | 6.84 | 6.933333 |
| VNN1 | 4.78 | 2.25705 | 8.47E-07 | 6.4E-06 | yes | up | 0.51 | 0.34 | 0.35 | 3.17 | 4.87 | 5.11 | 0.4 | 4.383333 |
| NUDT12 | 0.334 | -1.58277 | 8.52E-07 | 6.44E-06 | yes | down | 6.09 | 6.55 | 6.57 | 5.12 | 7.13 | 2.92 | 6.403333 | 5.056667 |
| LOC103351786 | 0.042 | -4.55917 | 8.55E-07 | 6.46E-06 | yes | down | 1.66 | 0.5 | 0.36 | 0.14 | 0.09 | 0 | 0.84 | 0.076667 |
| KCNMB1 | 3.347 | 1.743063 | 8.63E-07 | 6.52E-06 | yes | up | 1.55 | 1.71 | 1.34 | 9.84 | 10.21 | 14.75 | 1.533333 | 11.6 |
| TBC1D4 | 0.464 | -1.10873 | 8.64E-07 | 6.52E-06 | yes | down | 7.85 | 10.26 | 9.59 | 9.38 | 11.71 | 9.79 | 9.233333 | 10.29333 |
| LOC108175976 | 5.679 | 2.505761 | 8.74E-07 | 6.59E-06 | yes | up | 1.38 | 1.77 | 1.51 | 15.19 | 21.03 | 24.99 | 1.553333 | 20.40333 |
| NDUFAF7 | 0.446 | -1.16623 | 8.78E-07 | 6.62E-06 | yes | down | 10.83 | 10.46 | 7.72 | 11.04 | 10.56 | 7.73 | 9.67 | 9.776667 |
| DBT | 0.374 | -1.41886 | 8.84E-07 | 6.66E-06 | yes | down | 15.02 | 11.03 | 11.36 | 14.12 | 10.99 | 6.93 | 12.47 | 10.68 |
| LOC100352595 | 0.132 | -2.92141 | 8.85E-07 | 6.66E-06 | yes | down | 1.91 | 1.16 | 2.06 | 0.14 | 0.7 | 1.06 | 1.71 | 0.633333 |
| TLL1 | 4.942 | 2.304968 | 8.92E-07 | 6.71E-06 | yes | up | 0.17 | 0.24 | 0.13 | 1.68 | 2.39 | 2 | 0.18 | 2.023333 |
| LOC103347346 | 5.419 | 2.438082 | 8.95E-07 | 6.74E-06 | yes | up | 2.37 | 3.05 | 0.87 | 17.3 | 28.21 | 30.93 | 2.096667 | 25.48 |
| SLCO5A1 | 0.241 | -2.05147 | 8.96E-07 | 6.74E-06 | yes | down | 2.89 | 1.7 | 3.55 | 1.06 | 2.61 | 1.08 | 2.713333 | 1.583333 |
| LOC100348918 | 2.4 | 1.263028 | 8.96E-07 | 6.74E-06 | yes | up | 6.96 | 7.66 | 6.34 | 43.26 | 34.85 | 36.14 | 6.986667 | 38.08333 |
| LCK | 9.984 | 3.319646 | 9E-07 | 6.76E-06 | yes | up | 0.12 | 0.08 | 0.13 | 1.67 | 2.24 | 3.53 | 0.11 | 2.48 |
| OLFML2B | 3.161 | 1.660227 | 9.05E-07 | 6.8E-06 | yes | up | 8.76 | 5.56 | 2.76 | 35.97 | 38.02 | 44.93 | 5.693333 | 39.64 |
| ZNF629 | 0.454 | -1.13912 | 9.08E-07 | 6.82E-06 | yes | down | 3.19 | 3.61 | 2.76 | 2.59 | 4.58 | 2.82 | 3.186667 | 3.33 |
| VIL1 | 0.155 | -2.68883 | 9.22E-07 | 6.92E-06 | yes | down | 1.68 | 2.86 | 2.61 | 0.52 | 0.19 | 0.58 | 2.383333 | 0.43 |
| LOC103352391 | 0.444 | -1.17183 | 9.23E-07 | 6.92E-06 | yes | down | 144.95 | 192.77 | 149.66 | 130.22 | 181.52 | 225.52 | 162.46 | 179.0867 |
| HRH2 | 4.401 | 2.137728 | 9.27E-07 | 6.95E-06 | yes | up | 0.94 | 0.39 | 0.36 | 5.05 | 4.14 | 7.4 | 0.563333 | 5.53 |
| ACOT11 | 0.299 | -1.74052 | 9.29E-07 | 6.96E-06 | yes | down | 7.44 | 3.63 | 8.26 | 3 | 5.37 | 3.32 | 6.443333 | 3.896667 |
| TUBGCP5 | 0.396 | -1.33492 | 9.31E-07 | 6.97E-06 | yes | down | 1.94 | 2.39 | 1.77 | 1.7 | 2.22 | 1.63 | 2.033333 | 1.85 |
| HIPK2 | 2.176 | 1.121774 | 9.33E-07 | 6.99E-06 | yes | up | 2.13 | 1.7 | 1.42 | 6.71 | 11.55 | 7.86 | 1.75 | 8.706667 |
| NYAP1 | 4.785 | 2.258391 | 9.37E-07 | 7.01E-06 | yes | up | 0.15 | 0.16 | 0.13 | 1.67 | 1.49 | 1.56 | 0.146667 | 1.573333 |
| ZSCAN23 | 0.274 | -1.8695 | 9.41E-07 | 7.04E-06 | yes | down | 1.03 | 1.31 | 1.13 | 0.96 | 0.76 | 0.64 | 1.156667 | 0.786667 |
| SNAPC1 | 0.438 | -1.18958 | 9.41E-07 | 7.04E-06 | yes | down | 6.57 | 8.92 | 6.38 | 7.31 | 8.64 | 6.17 | 7.29 | 7.373333 |
| THAP5 | 0.378 | -1.40438 | 9.45E-07 | 7.06E-06 | yes | down | 3.2 | 3.53 | 2.52 | 2.28 | 3.53 | 1.59 | 3.083333 | 2.466667 |
| HSPE1 | 0.495 | -1.01367 | 9.57E-07 | 7.15E-06 | yes | down | 93.85 | 95.51 | 86.06 | 138.19 | 93.94 | 116.77 | 91.80667 | 116.3 |
| CDK2AP1 | 2.387 | 1.255315 | 9.61E-07 | 7.17E-06 | yes | up | 16.53 | 13 | 8.92 | 72.29 | 62.9 | 72.7 | 12.81667 | 69.29667 |
| SLIT2 | 3.545 | 1.825701 | 9.67E-07 | 7.21E-06 | yes | up | 0.65 | 0.48 | 0.28 | 3.29 | 2.85 | 4.83 | 0.47 | 3.656667 |
| NAA25 | 0.451 | -1.14793 | 9.74E-07 | 7.26E-06 | yes | down | 3.64 | 3.25 | 2.54 | 2.87 | 4.21 | 2.53 | 3.143333 | 3.203333 |
| UBE2V2 | 0.455 | -1.13608 | 9.75E-07 | 7.27E-06 | yes | down | 16.47 | 15.29 | 12.2 | 17.39 | 17.25 | 10.44 | 14.65333 | 15.02667 |
| CUL5 | 0.336 | -1.57239 | 1E-06 | 7.44E-06 | yes | down | 5.93 | 3.03 | 3.49 | 3.59 | 3.96 | 2.07 | 4.15 | 3.206667 |
| VKORC1 | 2.306 | 1.205154 | 1E-06 | 7.46E-06 | yes | up | 44.18 | 38.63 | 44.52 | 197.04 | 203.83 | 269.35 | 42.44333 | 223.4067 |
| PSMB9 | 5.294 | 2.404358 | 1.01E-06 | 7.47E-06 | yes | up | 1.1 | 1.27 | 0.52 | 12.09 | 14.52 | 8.08 | 0.963333 | 11.56333 |
| WBSCR27 | 3.089 | 1.627085 | 1.01E-06 | 7.53E-06 | yes | up | 7.73 | 4.03 | 7.1 | 32.14 | 49.03 | 56.05 | 6.286667 | 45.74 |
| PAIP2 | 0.458 | -1.12527 | 1.02E-06 | 7.58E-06 | yes | down | 86.98 | 79.84 | 77.35 | 100.41 | 99.96 | 59.04 | 81.39 | 86.47 |
| KLHL2 | 2.381 | 1.25146 | 1.03E-06 | 7.66E-06 | yes | up | 6.74 | 6.32 | 4.77 | 39.12 | 36.65 | 21.31 | 5.943333 | 32.36 |
| INVS | 2.025 | 1.018054 | 1.06E-06 | 7.83E-06 | yes | up | 2.03 | 2.03 | 1.56 | 8.02 | 9.37 | 7.73 | 1.873333 | 8.373333 |
| ESF1 | 0.383 | -1.384 | 1.06E-06 | 7.86E-06 | yes | down | 3.95 | 3 | 3.48 | 3.22 | 3.26 | 1.89 | 3.476667 | 2.79 |
| PSMB8 | 2.986 | 1.578046 | 1.06E-06 | 7.86E-06 | yes | up | 1.98 | 3.09 | 2.55 | 18.83 | 14.73 | 18.42 | 2.54 | 17.32667 |
| RENBP | 2.645 | 1.403378 | 1.07E-06 | 7.91E-06 | yes | up | 3.02 | 4.98 | 2.19 | 19.13 | 22.09 | 18.65 | 3.396667 | 19.95667 |
| ENTPD3 | 0.247 | -2.01653 | 1.07E-06 | 7.91E-06 | yes | down | 1.94 | 3.07 | 3.92 | 1.92 | 1.26 | 2.39 | 2.976667 | 1.856667 |
| YARS2 | 0.484 | -1.04831 | 1.07E-06 | 7.94E-06 | yes | down | 13.2 | 15.94 | 13.54 | 16.34 | 15.34 | 15.55 | 14.22667 | 15.74333 |
| CHST12 | 2.765 | 1.46736 | 1.08E-06 | 7.94E-06 | yes | up | 1.7 | 1.62 | 1.08 | 8.51 | 8.06 | 10.69 | 1.466667 | 9.086667 |
| LRRC3C | 0.171 | -2.54964 | 1.08E-06 | 7.95E-06 | yes | down | 0.91 | 2.26 | 1.01 | 0.53 | 0.44 | 0.8 | 1.393333 | 0.59 |
| LOC100342097 | 3.211 | 1.682917 | 1.08E-06 | 7.96E-06 | yes | up | 6.6 | 6.6 | 5.17 | 48.59 | 45.18 | 41.11 | 6.123333 | 44.96 |
| ADH2-2 | 0.029 | -5.1015 | 1.08E-06 | 7.98E-06 | yes | down | 1.39 | 0.42 | 0.87 | 0.12 | 0.04 | 0 | 0.893333 | 0.053333 |
| TMEM43 | 2.482 | 1.311551 | 1.08E-06 | 7.99E-06 | yes | up | 20.73 | 22.92 | 18.77 | 128.19 | 84 | 135.83 | 20.80667 | 116.0067 |
| EPC2 | 0.47 | -1.09048 | 1.09E-06 | 8.03E-06 | yes | down | 4.56 | 3.85 | 3.36 | 4.38 | 5.09 | 3.33 | 3.923333 | 4.266667 |
| PSKH2 | 0.493 | -1.01894 | 1.09E-06 | 8.05E-06 | yes | down | 41.02 | 41.13 | 36.79 | 41.82 | 58.97 | 35.45 | 39.64667 | 45.41333 |
| LOC100344629 | 2.772 | 1.470975 | 1.1E-06 | 8.12E-06 | yes | up | 1.22 | 1.12 | 0.7 | 6.81 | 5.99 | 6.05 | 1.013333 | 6.283333 |
| LOC100354703 | 0.188 | -2.41406 | 1.1E-06 | 8.13E-06 | yes | down | 0.98 | 1.32 | 0.92 | 0.38 | 0.72 | 0.29 | 1.073333 | 0.463333 |
| PENK | 49.696 | 5.635071 | 1.11E-06 | 8.14E-06 | yes | up | 0.79 | 0.67 | 0.13 | 31.03 | 10.06 | 122.53 | 0.53 | 54.54 |
| PARP3 | 2.698 | 1.431951 | 1.11E-06 | 8.17E-06 | yes | up | 3.62 | 3.15 | 2.44 | 17.53 | 14.68 | 23.33 | 3.07 | 18.51333 |
| MXI1 | 0.438 | -1.19106 | 1.11E-06 | 8.19E-06 | yes | down | 13.32 | 15.4 | 17.35 | 19.09 | 16.61 | 14.95 | 15.35667 | 16.88333 |
| LTBP1 | 2.026 | 1.018657 | 1.13E-06 | 8.32E-06 | yes | up | 9.08 | 8.37 | 5.07 | 32.39 | 36.12 | 33.8 | 7.506667 | 34.10333 |
| LOC100338469 | 0.473 | -1.07984 | 1.14E-06 | 8.4E-06 | yes | down | 13.57 | 10.32 | 11.3 | 11.63 | 16.71 | 9.32 | 11.73 | 12.55333 |
| BHLHE22 | 9.428 | 3.236966 | 1.15E-06 | 8.41E-06 | yes | up | 0.03 | 0.07 | 0.05 | 1.31 | 0.97 | 1.05 | 0.05 | 1.11 |
| UNC5C | 26.294 | 4.716657 | 1.17E-06 | 8.57E-06 | yes | up | 0.04 | 0 | 0.01 | 0.38 | 2.11 | 0.52 | 0.016667 | 1.003333 |
| MYO1D | 2.045 | 1.032087 | 1.18E-06 | 8.66E-06 | yes | up | 2.02 | 2.02 | 1.96 | 8.32 | 10.26 | 9.78 | 2 | 9.453333 |
| BDP1 | 0.402 | -1.31302 | 1.19E-06 | 8.7E-06 | yes | down | 3.51 | 3.17 | 3.06 | 3.64 | 4.21 | 2.15 | 3.246667 | 3.333333 |
| LOC100351453 | 0.323 | -1.6325 | 1.19E-06 | 8.73E-06 | yes | down | 2.72 | 4.6 | 2.95 | 2.7 | 3.42 | 1.55 | 3.423333 | 2.556667 |
| DLGAP2 | 0.008 | -7.01149 | 1.21E-06 | 8.86E-06 | yes | down | 0.1 | 0.44 | 0.46 | 0 | 0.02 | 0 | 0.333333 | 0.006667 |
| LOC108177718 | 0.296 | -1.75623 | 1.21E-06 | 8.88E-06 | yes | down | 1.8 | 2.46 | 2.69 | 1.33 | 2.34 | 1.2 | 2.316667 | 1.623333 |
| LOC100352411 | 0.392 | -1.35043 | 1.22E-06 | 8.9E-06 | yes | down | 3.22 | 4.41 | 3.69 | 3.3 | 3.53 | 3.38 | 3.773333 | 3.403333 |
| SEPT6 | 2.614 | 1.386222 | 1.22E-06 | 8.9E-06 | yes | up | 1 | 0.79 | 0.85 | 4.72 | 4.96 | 5.65 | 0.88 | 5.11 |
| NOC3L | 0.472 | -1.0838 | 1.22E-06 | 8.9E-06 | yes | down | 2.25 | 2.21 | 1.87 | 2.15 | 2.72 | 1.72 | 2.11 | 2.196667 |
| UBE3A | 0.402 | -1.31549 | 1.24E-06 | 9.02E-06 | yes | down | 12.37 | 8.78 | 8.44 | 9.95 | 11.61 | 5.76 | 9.863333 | 9.106667 |
| WDFY4 | 3.024 | 1.596259 | 1.24E-06 | 9.06E-06 | yes | up | 0.15 | 0.17 | 0.17 | 1.25 | 1.23 | 0.96 | 0.163333 | 1.146667 |
| KLF10 | 2.034 | 1.024302 | 1.25E-06 | 9.1E-06 | yes | up | 10.75 | 11.23 | 10.49 | 59.45 | 50.91 | 41.27 | 10.82333 | 50.54333 |
| KIZ | 0.445 | -1.16883 | 1.25E-06 | 9.14E-06 | yes | down | 6.35 | 7.43 | 6.9 | 7.5 | 8.25 | 5.53 | 6.893333 | 7.093333 |
| LOC103345420 | 5.252 | 2.392883 | 1.29E-06 | 9.39E-06 | yes | up | 41.62 | 14.74 | 6.74 | 249.77 | 182.75 | 280.07 | 21.03333 | 237.53 |
| NFE2 | 0.152 | -2.71606 | 1.31E-06 | 9.57E-06 | yes | down | 1.67 | 5.07 | 5.16 | 1.49 | 0.61 | 1.89 | 3.966667 | 1.33 |
| TTC39B | 0.354 | -1.49688 | 1.32E-06 | 9.6E-06 | yes | down | 1.99 | 2.47 | 2.18 | 1.89 | 2.79 | 1.42 | 2.213333 | 2.033333 |
| LOC100356012 | 2.819 | 1.495257 | 1.32E-06 | 9.63E-06 | yes | up | 3.93 | 7.56 | 3.95 | 26.71 | 47.36 | 26.32 | 5.146667 | 33.46333 |
| IQGAP3 | 5.265 | 2.396307 | 1.33E-06 | 9.64E-06 | yes | up | 0.97 | 0.73 | 0.3 | 7.96 | 13.51 | 2.81 | 0.666667 | 8.093333 |
| FAM110C | 0.359 | -1.47836 | 1.34E-06 | 9.7E-06 | yes | down | 1.61 | 1.77 | 1.71 | 1.63 | 1.08 | 1.19 | 1.696667 | 1.3 |
| CNBD2 | 0.166 | -2.58988 | 1.34E-06 | 9.71E-06 | yes | down | 0.97 | 1.36 | 1.32 | 0.33 | 0.64 | 0.46 | 1.216667 | 0.476667 |
| CDCA3 | 4.901 | 2.293079 | 1.35E-06 | 9.78E-06 | yes | up | 1.73 | 1.47 | 1.08 | 19.17 | 23.09 | 6.39 | 1.426667 | 16.21667 |
| ADRA1B | 0.247 | -2.01792 | 1.36E-06 | 9.87E-06 | yes | down | 1.84 | 2.21 | 2.34 | 1.42 | 0.96 | 1.22 | 2.13 | 1.2 |
| CCNG1 | 0.433 | -1.20698 | 1.38E-06 | 9.99E-06 | yes | down | 178.39 | 103.46 | 123.67 | 148.31 | 149.09 | 102.71 | 135.1733 | 133.37 |
| USP53 | 0.357 | -1.48604 | 1.38E-06 | 1E-05 | yes | down | 1.79 | 1.46 | 1.69 | 1.25 | 1.97 | 0.9 | 1.646667 | 1.373333 |
| CDC20 | 5.003 | 2.322901 | 1.38E-06 | 1E-05 | yes | up | 3.25 | 3.17 | 1.08 | 35.95 | 38.48 | 10.42 | 2.5 | 28.28333 |
| SEMA6D | 0.376 | -1.40928 | 1.39E-06 | 1.01E-05 | yes | down | 2.55 | 4.07 | 3.81 | 3.55 | 3.22 | 2.35 | 3.476667 | 3.04 |
| FAM117A | 2.43 | 1.281236 | 1.41E-06 | 1.02E-05 | yes | up | 4.27 | 5.22 | 2.99 | 20.6 | 23.35 | 29.4 | 4.16 | 24.45 |
| CCDC3 | 3.227 | 1.6901 | 1.44E-06 | 1.04E-05 | yes | up | 0.74 | 1.35 | 0.97 | 5.43 | 10.12 | 7.44 | 1.02 | 7.663333 |
| KDM5A | 0.484 | -1.04625 | 1.45E-06 | 1.05E-05 | yes | down | 4.81 | 4.49 | 4.78 | 4.67 | 6.65 | 4.09 | 4.693333 | 5.136667 |
| TIMM50 | 0.432 | -1.2123 | 1.48E-06 | 1.06E-05 | yes | down | 42.8 | 42.5 | 46.2 | 51.51 | 36.7 | 41.77 | 43.83333 | 43.32667 |
| ITPRIP | 2.973 | 1.5721 | 1.48E-06 | 1.07E-05 | yes | up | 1.7 | 2.86 | 3 | 18.29 | 13.22 | 16.15 | 2.52 | 15.88667 |
| GAPDH | 0.311 | -1.68624 | 1.5E-06 | 1.08E-05 | yes | down | 2408.9 | 885.42 | 936.56 | 870.04 | 982.6 | 1084.92 | 1410.293 | 979.1867 |
| EFCAB7 | 0.337 | -1.5691 | 1.5E-06 | 1.08E-05 | yes | down | 2.13 | 2 | 2 | 1.86 | 1.95 | 1.14 | 2.043333 | 1.65 |
| UPF2 | 0.47 | -1.08895 | 1.51E-06 | 1.08E-05 | yes | down | 5.14 | 3.83 | 4.19 | 5.02 | 5.46 | 3.81 | 4.386667 | 4.763333 |
| UBA3 | 0.419 | -1.25328 | 1.52E-06 | 1.09E-05 | yes | down | 20.49 | 19.84 | 14.99 | 20.26 | 22.06 | 10.95 | 18.44 | 17.75667 |
| FAM177A1 | 0.467 | -1.09922 | 1.53E-06 | 1.1E-05 | yes | down | 13.3 | 11.75 | 10.14 | 15.72 | 12.11 | 9.97 | 11.73 | 12.6 |
| SCYL2 | 0.438 | -1.19197 | 1.55E-06 | 1.11E-05 | yes | down | 8.62 | 8.58 | 7.35 | 9.66 | 9.74 | 5.36 | 8.183333 | 8.253333 |
| SDF2L1 | 3.361 | 1.749078 | 1.55E-06 | 1.11E-05 | yes | up | 1.71 | 3.03 | 2.21 | 19.93 | 20.22 | 13.95 | 2.316667 | 18.03333 |
| CDC25B | 3.394 | 1.762973 | 1.56E-06 | 1.12E-05 | yes | up | 7.58 | 3.25 | 2.8 | 28.18 | 40.5 | 19.2 | 4.543333 | 29.29333 |
| TRAPPC13 | 0.424 | -1.23742 | 1.59E-06 | 1.14E-05 | yes | down | 10.25 | 10.76 | 6.87 | 9.78 | 10.36 | 5.7 | 9.293333 | 8.613333 |
| ECI2 | 0.498 | -1.00707 | 1.59E-06 | 1.14E-05 | yes | down | 15.67 | 20.58 | 12.98 | 15.79 | 19.29 | 19.26 | 16.41 | 18.11333 |
| ADAM23 | 6.164 | 2.623951 | 1.59E-06 | 1.14E-05 | yes | up | 0.87 | 0.26 | 0.1 | 5.57 | 8.72 | 6.51 | 0.41 | 6.933333 |
| VEZT | 0.467 | -1.09866 | 1.59E-06 | 1.14E-05 | yes | down | 6.69 | 5.59 | 4.07 | 5.93 | 6.37 | 4.79 | 5.45 | 5.696667 |
| AFAP1L2 | 2.271 | 1.183136 | 1.61E-06 | 1.16E-05 | yes | up | 4.16 | 4.37 | 1.94 | 14.07 | 23 | 17.98 | 3.49 | 18.35 |
| TRIM47 | 2.529 | 1.338833 | 1.62E-06 | 1.16E-05 | yes | up | 6.41 | 7.65 | 5.18 | 44.99 | 27.64 | 36.52 | 6.413333 | 36.38333 |
| MRPL44 | 0.477 | -1.06805 | 1.62E-06 | 1.16E-05 | yes | down | 23.4 | 30.39 | 25.65 | 31.72 | 28.37 | 26.9 | 26.48 | 28.99667 |
| WARS2 | 0.409 | -1.28931 | 1.64E-06 | 1.17E-05 | yes | down | 3.6 | 4.16 | 3.61 | 3.62 | 4.59 | 2.92 | 3.79 | 3.71 |
| RGL1 | 2.722 | 1.444854 | 1.64E-06 | 1.17E-05 | yes | up | 5.5 | 5.59 | 5.85 | 30.43 | 41.58 | 35.49 | 5.646667 | 35.83333 |
| OSBPL8 | 0.396 | -1.33758 | 1.64E-06 | 1.17E-05 | yes | down | 7.87 | 6.49 | 5.92 | 6.65 | 8.41 | 3.58 | 6.76 | 6.213333 |
| LOC100354647 | 2.134 | 1.093248 | 1.64E-06 | 1.17E-05 | yes | up | 6.62 | 6.37 | 5.23 | 28.25 | 26.39 | 32.83 | 6.073333 | 29.15667 |
| TRAPPC8 | 0.419 | -1.25452 | 1.65E-06 | 1.17E-05 | yes | down | 7.16 | 7.57 | 6.34 | 7.16 | 8.44 | 4.25 | 7.023333 | 6.616667 |
| NT5C3B | 0.356 | -1.48926 | 1.66E-06 | 1.19E-05 | yes | down | 31.38 | 21.16 | 39.14 | 25.98 | 30.82 | 20.39 | 30.56 | 25.73 |
| PALMD | 0.398 | -1.32821 | 1.66E-06 | 1.19E-05 | yes | down | 15.78 | 20.06 | 12.17 | 16.92 | 17.97 | 8.95 | 16.00333 | 14.61333 |
| PDCD5 | 0.433 | -1.20749 | 1.67E-06 | 1.19E-05 | yes | down | 48.13 | 34.17 | 36.54 | 41.88 | 39.92 | 29.67 | 39.61333 | 37.15667 |
| LOC100356847 | 0.432 | -1.21126 | 1.71E-06 | 1.22E-05 | yes | down | 2.56 | 2.78 | 2.22 | 2.95 | 3.03 | 1.92 | 2.52 | 2.633333 |
| LOC100358742 | 4.177 | 2.062626 | 1.73E-06 | 1.23E-05 | yes | up | 2.24 | 2.79 | 2.51 | 16.92 | 33.08 | 23.91 | 2.513333 | 24.63667 |
| PLXDC1 | 4.906 | 2.294421 | 1.74E-06 | 1.24E-05 | yes | up | 2.58 | 1.43 | 0.46 | 10.28 | 23.84 | 16.5 | 1.49 | 16.87333 |
| ITCH | 0.484 | -1.04733 | 1.77E-06 | 1.25E-05 | yes | down | 8.71 | 8.09 | 7.19 | 8.2 | 12.03 | 6.58 | 7.996667 | 8.936667 |
| BIVM | 0.485 | -1.04352 | 1.78E-06 | 1.26E-05 | yes | down | 11.46 | 9.84 | 9.21 | 13.39 | 12.93 | 8.51 | 10.17 | 11.61 |
| SAMD15 | 0.207 | -2.26992 | 1.79E-06 | 1.27E-05 | yes | down | 1.25 | 3.56 | 1.25 | 1.05 | 1.24 | 0.65 | 2.02 | 0.98 |
| LRRC17 | 4.559 | 2.188601 | 1.79E-06 | 1.27E-05 | yes | up | 8.78 | 4.22 | 1.51 | 50.53 | 40.36 | 52.41 | 4.836667 | 47.76667 |
| LOXHD1 | 7.669 | 2.939025 | 1.81E-06 | 1.28E-05 | yes | up | 4.24 | 4.61 | 1.8 | 30.29 | 14.91 | 24.92 | 3.55 | 23.37333 |
| MLYCD | 0.491 | -1.02508 | 1.81E-06 | 1.29E-05 | yes | down | 27.46 | 21.06 | 20.94 | 22.92 | 24.42 | 27.96 | 23.15333 | 25.1 |
| LOC108176490 | 2.36 | 1.238641 | 1.82E-06 | 1.29E-05 | yes | up | 10.5 | 7.48 | 7.79 | 47.13 | 37.71 | 52.36 | 8.59 | 45.73333 |
| CHCHD5 | 0.392 | -1.35027 | 1.84E-06 | 1.3E-05 | yes | down | 23.31 | 27.22 | 23.37 | 28.4 | 19.72 | 18.01 | 24.63333 | 22.04333 |
| D2HGDH | 0.477 | -1.06841 | 1.85E-06 | 1.31E-05 | yes | down | 14.74 | 14.96 | 18.09 | 17.62 | 17.78 | 15.63 | 15.93 | 17.01 |
| RPF2 | 0.468 | -1.09488 | 1.85E-06 | 1.31E-05 | yes | down | 5.35 | 6.53 | 5.03 | 5.64 | 7.6 | 4.95 | 5.636667 | 6.063333 |
| ZRANB2 | 0.368 | -1.44262 | 1.85E-06 | 1.31E-05 | yes | down | 22.59 | 22.58 | 18.68 | 21.99 | 30.24 | 11.76 | 21.28333 | 21.33 |
| LOC100338378 | 0.499 | -1.00347 | 1.85E-06 | 1.31E-05 | yes | down | 10.57 | 10.96 | 9.38 | 10.33 | 13.92 | 10.56 | 10.30333 | 11.60333 |
| ADH2-1 | 0.158 | -2.66399 | 1.85E-06 | 1.31E-05 | yes | down | 2.3 | 2.25 | 4.08 | 1.36 | 1.5 | 0.38 | 2.876667 | 1.08 |
| SLC4A1 | 0.075 | -3.74497 | 1.9E-06 | 1.34E-05 | yes | down | 0.16 | 0.93 | 0.83 | 0.17 | 0.05 | 0.12 | 0.64 | 0.113333 |
| IL17RE | 16.828 | 4.072813 | 1.91E-06 | 1.34E-05 | yes | up | 0.02 | 0.07 | 0.02 | 1.49 | 1.29 | 1.37 | 0.036667 | 1.383333 |
| NAAA | 2.258 | 1.17515 | 1.91E-06 | 1.35E-05 | yes | up | 3.19 | 4.34 | 3.53 | 18.17 | 19.18 | 19.87 | 3.686667 | 19.07333 |
| BUB1 | 5.085 | 2.346352 | 1.94E-06 | 1.36E-05 | yes | up | 0.6 | 0.55 | 0.22 | 6.9 | 6.76 | 2.16 | 0.456667 | 5.273333 |
| EBF2 | 0.41 | -1.28795 | 1.95E-06 | 1.37E-05 | yes | down | 10.77 | 12.71 | 15.04 | 11.63 | 16.44 | 9.2 | 12.84 | 12.42333 |
| SYCE3 | 0.103 | -3.28627 | 1.95E-06 | 1.37E-05 | yes | down | 0.69 | 1.52 | 2.36 | 0.17 | 0.16 | 0.12 | 1.523333 | 0.15 |
| ECM1 | 3.068 | 1.617293 | 1.96E-06 | 1.38E-05 | yes | up | 2.9 | 2.75 | 2.46 | 19.59 | 12.06 | 23.99 | 2.703333 | 18.54667 |
| NFX1 | 0.445 | -1.16712 | 1.96E-06 | 1.38E-05 | yes | down | 8.07 | 12.36 | 8.46 | 8.53 | 12.97 | 8.24 | 9.63 | 9.913333 |
| SLC25A5 | 0.364 | -1.45839 | 1.97E-06 | 1.38E-05 | yes | down | 701.85 | 505.62 | 910.98 | 707.64 | 649.91 | 458.66 | 706.15 | 605.4033 |
| LOC100356168 | 0.257 | -1.95842 | 1.98E-06 | 1.39E-05 | yes | down | 6.31 | 6.41 | 5.38 | 1.37 | 5.04 | 4.3 | 6.033333 | 3.57 |
| MRPL4 | 0.319 | -1.6501 | 1.98E-06 | 1.39E-05 | yes | down | 17.27 | 16.69 | 24.72 | 12.78 | 14.94 | 16.11 | 19.56 | 14.61 |
| LOC100352044 | 2.179 | 1.123459 | 1.99E-06 | 1.4E-05 | yes | up | 33.84 | 45.18 | 36.15 | 142.43 | 220.29 | 212.16 | 38.39 | 191.6267 |
| MYLK3 | 0.037 | -4.7478 | 2.03E-06 | 1.42E-05 | yes | down | 22.25 | 3.79 | 1.47 | 0.78 | 0.91 | 0.64 | 9.17 | 0.776667 |
| PPP2R2A | 0.467 | -1.09779 | 2.03E-06 | 1.43E-05 | yes | down | 29.04 | 25.45 | 28.69 | 34.58 | 35.53 | 23.04 | 27.72667 | 31.05 |
| PDGFRB | 2.116 | 1.081031 | 2.05E-06 | 1.44E-05 | yes | up | 20.88 | 22.39 | 27.08 | 104.52 | 136.68 | 106.88 | 23.45 | 116.0267 |
| PXDC1 | 2.301 | 1.202282 | 2.06E-06 | 1.44E-05 | yes | up | 11.25 | 13.76 | 8.41 | 56.32 | 49.53 | 66.54 | 11.14 | 57.46333 |
| S1PR2 | 2.384 | 1.253134 | 2.07E-06 | 1.45E-05 | yes | up | 1.75 | 1.48 | 1.15 | 8.78 | 7.93 | 6.87 | 1.46 | 7.86 |
| LOC108175372 | 4.938 | 2.304025 | 2.08E-06 | 1.45E-05 | yes | up | 0.93 | 0.84 | 0.35 | 6.7 | 7.54 | 9.79 | 0.706667 | 8.01 |
| IL33 | 2.243 | 1.165252 | 2.09E-06 | 1.46E-05 | yes | up | 5.34 | 3.11 | 2.66 | 17.72 | 20.06 | 14.97 | 3.703333 | 17.58333 |
| BMT2 | 0.429 | -1.22111 | 2.1E-06 | 1.46E-05 | yes | down | 3.51 | 3.35 | 3.33 | 3.9 | 3.74 | 2.43 | 3.396667 | 3.356667 |
| RNF122 | 4.198 | 2.069562 | 2.11E-06 | 1.47E-05 | yes | up | 3.39 | 1.78 | 1.01 | 25.49 | 12.63 | 18.64 | 2.06 | 18.92 |
| SLC17A9 | 4.965 | 2.311926 | 2.13E-06 | 1.48E-05 | yes | up | 0.4 | 0.26 | 0.19 | 3.07 | 4.26 | 2.02 | 0.283333 | 3.116667 |
| LOC100358318 | 0.374 | -1.41832 | 2.14E-06 | 1.49E-05 | yes | down | 38.06 | 43.14 | 50.87 | 27.13 | 38.16 | 48.91 | 44.02333 | 38.06667 |
| SHB | 2.99 | 1.580027 | 2.16E-06 | 1.51E-05 | yes | up | 2.57 | 2.69 | 1.45 | 13.31 | 12.14 | 19.02 | 2.236667 | 14.82333 |
| SLC6A2 | 3.368 | 1.751809 | 2.18E-06 | 1.52E-05 | yes | up | 0.21 | 0.28 | 0.15 | 1.46 | 1.9 | 1.57 | 0.213333 | 1.643333 |
| NOS2 | 4.049 | 2.017575 | 2.22E-06 | 1.54E-05 | yes | up | 0.34 | 0.15 | 0.2 | 2.17 | 2.21 | 2.22 | 0.23 | 2.2 |
| FAM129A | 2.471 | 1.304956 | 2.23E-06 | 1.55E-05 | yes | up | 3.95 | 4.06 | 2.48 | 12.33 | 23.08 | 18.13 | 3.496667 | 17.84667 |
| LOC100347603 | 0.438 | -1.19169 | 2.26E-06 | 1.57E-05 | yes | down | 6.43 | 7.32 | 8.08 | 7.79 | 9.33 | 5.6 | 7.276667 | 7.573333 |
| LOC108177728 | 86.309 | 6.431434 | 2.26E-06 | 1.57E-05 | yes | up | 0.19 | 0 | 0 | 3.75 | 7.66 | 11.17 | 0.063333 | 7.526667 |
| DUOX2 | 0.069 | -3.85175 | 2.29E-06 | 1.59E-05 | yes | down | 0.44 | 1.03 | 1.33 | 0.37 | 0.02 | 0.05 | 0.933333 | 0.146667 |
| TK1 | 5.518 | 2.464231 | 2.3E-06 | 1.6E-05 | yes | up | 3.73 | 3.07 | 1.27 | 43.12 | 48.25 | 9.92 | 2.69 | 33.76333 |
| UTP11 | 0.449 | -1.15369 | 2.34E-06 | 1.62E-05 | yes | down | 32.54 | 29.22 | 19.65 | 30.52 | 30.5 | 21.9 | 27.13667 | 27.64 |
| GABRD | 3.469 | 1.794335 | 2.36E-06 | 1.63E-05 | yes | up | 27.46 | 79.57 | 42.63 | 384.84 | 305.16 | 470.83 | 49.88667 | 386.9433 |
| TOP2B | 0.413 | -1.27686 | 2.38E-06 | 1.65E-05 | yes | down | 20.07 | 15.37 | 16.9 | 18.45 | 21.7 | 10.19 | 17.44667 | 16.78 |
| GPATCH4 | 0.404 | -1.30912 | 2.39E-06 | 1.66E-05 | yes | down | 6.12 | 8.93 | 5.61 | 5.47 | 6.19 | 7.4 | 6.886667 | 6.353333 |
| LOC103346934 | 0.238 | -2.07307 | 2.4E-06 | 1.66E-05 | yes | down | 2.88 | 3.25 | 1.1 | 1.34 | 1.12 | 1.39 | 2.41 | 1.283333 |
| MRPS23 | 0.469 | -1.09235 | 2.42E-06 | 1.68E-05 | yes | down | 52.21 | 55.15 | 40.33 | 63.44 | 46.92 | 46.26 | 49.23 | 52.20667 |
| CACNB4 | 0.268 | -1.90103 | 2.47E-06 | 1.71E-05 | yes | down | 1.01 | 1.89 | 0.7 | 7.61 | 6.14 | 5.03 | 1.2 | 6.26 |
| RELL1 | 2.07 | 1.049354 | 2.51E-06 | 1.74E-05 | yes | up | 6.61 | 4.63 | 4.12 | 23.24 | 23.81 | 24.11 | 5.12 | 23.72 |
| TBK1 | 0.476 | -1.072 | 2.51E-06 | 1.74E-05 | yes | down | 6.93 | 7.35 | 6.58 | 7.37 | 9.73 | 5.47 | 6.953333 | 7.523333 |
| CCDC85C | 2.352 | 1.233672 | 2.57E-06 | 1.78E-05 | yes | up | 2.01 | 2.16 | 2.62 | 11.29 | 13.04 | 12.74 | 2.263333 | 12.35667 |
| LOC100345822 | 0.362 | -1.46568 | 2.59E-06 | 1.79E-05 | yes | down | 13.18 | 13.59 | 14.29 | 11.35 | 16.87 | 6.81 | 13.68667 | 11.67667 |
| IKBKE | 2.653 | 1.407472 | 2.6E-06 | 1.79E-05 | yes | up | 1.34 | 1.57 | 0.76 | 6.34 | 8.9 | 6.66 | 1.223333 | 7.3 |
| LOC100348760 | 0.351 | -1.51205 | 2.62E-06 | 1.8E-05 | yes | down | 393.64 | 190.53 | 212.14 | 214.84 | 159.13 | 249.03 | 265.4367 | 207.6667 |
| FCHO2 | 0.456 | -1.13215 | 2.7E-06 | 1.86E-05 | yes | down | 5.81 | 7.42 | 6.32 | 6.99 | 8.89 | 4.92 | 6.516667 | 6.933333 |
| DLEU7 | 0.01 | -6.62683 | 2.72E-06 | 1.88E-05 | yes | down | 0.98 | 1.43 | 0.42 | 0.05 | 0 | 0 | 0.943333 | 0.016667 |
| GOT1L1 | 0.008 | -6.9854 | 2.73E-06 | 1.88E-05 | yes | down | 0.43 | 0.7 | 0.62 | 0 | 0 | 0 | 0.583333 | 0 |
| LOC100357816 | 0.081 | -3.63223 | 2.74E-06 | 1.88E-05 | yes | down | 4.01 | 4.03 | 2.82 | 0.84 | 0.47 | 0.77 | 3.62 | 0.693333 |
| NAA40 | 0.455 | -1.13681 | 2.76E-06 | 1.9E-05 | yes | down | 6.49 | 7.57 | 4.71 | 5.1 | 8.18 | 6.23 | 6.256667 | 6.503333 |
| ACAT2 | 3.73 | 1.898994 | 2.8E-06 | 1.93E-05 | yes | up | 21.02 | 27.61 | 27.62 | 359.54 | 120.47 | 167.38 | 25.41667 | 215.7967 |
| LOC103350429 | 123.618 | 6.94974 | 2.82E-06 | 1.94E-05 | yes | up | 0 | 0 | 0 | 1.46 | 0.68 | 2.89 | 0 | 1.676667 |
| SPP1 | 0.237 | -2.07964 | 2.82E-06 | 1.94E-05 | yes | down | 22.78 | 22.2 | 5.31 | 7.13 | 8.32 | 10.64 | 16.76333 | 8.696667 |
| PFDN6 | 0.497 | -1.00938 | 2.85E-06 | 1.96E-05 | yes | down | 60.92 | 57.46 | 47.54 | 63.19 | 52.54 | 59.04 | 55.30667 | 58.25667 |
| FN3K | 0.389 | -1.36337 | 2.87E-06 | 1.97E-05 | yes | down | 11.2 | 14.29 | 17.6 | 13.49 | 13.8 | 11 | 14.36333 | 12.76333 |
| ERN2 | 6.279 | 2.65051 | 2.89E-06 | 1.98E-05 | yes | up | 0.14 | 0.09 | 0.17 | 1.54 | 1.68 | 2.47 | 0.133333 | 1.896667 |
| NOV | 3.388 | 1.760548 | 2.92E-06 | 2E-05 | yes | up | 9.97 | 8.08 | 2.94 | 52.42 | 40.54 | 61.62 | 6.996667 | 51.52667 |
| FXR1 | 0.387 | -1.37 | 2.92E-06 | 2E-05 | yes | down | 103.34 | 51.19 | 51.88 | 66.53 | 71.25 | 52.76 | 68.80333 | 63.51333 |
| CD37 | 4.07 | 2.025175 | 2.93E-06 | 2.01E-05 | yes | up | 1.12 | 1.35 | 1.57 | 11.44 | 8.9 | 16.97 | 1.346667 | 12.43667 |
| CALD1 | 2.216 | 1.148031 | 2.95E-06 | 2.02E-05 | yes | up | 18.86 | 20.3 | 21.59 | 125.15 | 119.38 | 74.3 | 20.25 | 106.2767 |
| SPRTN | 0.443 | -1.1746 | 2.97E-06 | 2.03E-05 | yes | down | 5.06 | 4.86 | 4.48 | 5.7 | 6.01 | 3.44 | 4.8 | 5.05 |
| SMAD7 | 0.442 | -1.17942 | 2.97E-06 | 2.03E-05 | yes | down | 13.64 | 14.7 | 9.85 | 11.18 | 10.89 | 14.43 | 12.73 | 12.16667 |
| ORC4 | 0.356 | -1.48877 | 2.99E-06 | 2.04E-05 | yes | down | 2.78 | 3.85 | 3.23 | 2.6 | 3.84 | 1.71 | 3.286667 | 2.716667 |
| GALNTL5 | 0.031 | -5.0138 | 3.02E-06 | 2.06E-05 | yes | down | 0.58 | 0.27 | 0.79 | 0 | 0.12 | 0 | 0.546667 | 0.04 |
| ANXA4 | 3.337 | 1.738611 | 3.11E-06 | 2.12E-05 | yes | up | 7.72 | 4.53 | 2.34 | 32.54 | 31.32 | 39.38 | 4.863333 | 34.41333 |
| EXT1 | 2.216 | 1.147643 | 3.11E-06 | 2.12E-05 | yes | up | 7.44 | 5.04 | 4.59 | 31.32 | 28.79 | 37.22 | 5.69 | 32.44333 |
| ZDHHC17 | 0.443 | -1.17305 | 3.12E-06 | 2.13E-05 | yes | down | 5.68 | 5.23 | 5.07 | 5.5 | 7.32 | 3.82 | 5.326667 | 5.546667 |
| LOC103347367 | 0.476 | -1.06962 | 3.12E-06 | 2.13E-05 | yes | down | 58.55 | 73.31 | 76.2 | 70.72 | 86.1 | 74.11 | 69.35333 | 76.97667 |
| RBP4 | 4.628 | 2.210489 | 3.14E-06 | 2.14E-05 | yes | up | 64.82 | 75.27 | 67.81 | 355.25 | 401.86 | 1309.62 | 69.3 | 688.91 |
| CREBRF | 0.402 | -1.31588 | 3.15E-06 | 2.14E-05 | yes | down | 3.14 | 2.71 | 2.91 | 2.4 | 2.93 | 1.41 | 2.92 | 2.246667 |
| SMOC1 | 0.311 | -1.68715 | 3.2E-06 | 2.18E-05 | yes | down | 27.17 | 39.52 | 12.34 | 12.88 | 25.36 | 17.12 | 26.34333 | 18.45333 |
| LOC100354345 | 11.885 | 3.57104 | 3.21E-06 | 2.18E-05 | yes | up | 0.04 | 0.03 | 0.02 | 0.5 | 0.71 | 1.3 | 0.03 | 0.836667 |
| TMEM64 | 0.439 | -1.18888 | 3.21E-06 | 2.18E-05 | yes | down | 7.83 | 4.98 | 7.18 | 6.94 | 7.81 | 5.55 | 6.663333 | 6.766667 |
| RSPH3 | 2.06 | 1.042492 | 3.22E-06 | 2.19E-05 | yes | up | 3.47 | 3.14 | 2.53 | 14.45 | 14.63 | 13.7 | 3.046667 | 14.26 |
| NAA15 | 0.435 | -1.2011 | 3.22E-06 | 2.19E-05 | yes | down | 4.96 | 4.21 | 2.65 | 4.06 | 4.69 | 2.9 | 3.94 | 3.883333 |
| LMX1A | 5.002 | 2.322602 | 3.26E-06 | 2.21E-05 | yes | up | 0.31 | 0.17 | 0.1 | 2.43 | 2.03 | 1.9 | 0.193333 | 2.12 |
| ABHD5 | 0.361 | -1.46867 | 3.26E-06 | 2.21E-05 | yes | down | 48.11 | 52.34 | 66.79 | 49.64 | 72.34 | 25.3 | 55.74667 | 49.09333 |
| SLC30A7 | 0.346 | -1.53069 | 3.26E-06 | 2.21E-05 | yes | down | 5 | 7.71 | 3.87 | 3.52 | 6.95 | 2.98 | 5.526667 | 4.483333 |
| ASB4 | 0.109 | -3.19421 | 3.32E-06 | 2.25E-05 | yes | down | 7.41 | 1.08 | 1.19 | 0.76 | 1.33 | 0.3 | 3.226667 | 0.796667 |
| NIPSNAP3A | 0.386 | -1.37517 | 3.32E-06 | 2.25E-05 | yes | down | 35.79 | 34.25 | 36.98 | 36.71 | 41.96 | 18.02 | 35.67333 | 32.23 |
| PID1 | 2.189 | 1.130187 | 3.35E-06 | 2.27E-05 | yes | up | 4.86 | 3.89 | 2.84 | 17.69 | 18.15 | 20.66 | 3.863333 | 18.83333 |
| ARSB | 3.28 | 1.713557 | 3.38E-06 | 2.28E-05 | yes | up | 5.22 | 3.67 | 3.5 | 35.14 | 37.34 | 20.56 | 4.13 | 31.01333 |
| MYCT1 | 0.364 | -1.45751 | 3.38E-06 | 2.29E-05 | yes | down | 20.24 | 27.4 | 22.98 | 20.17 | 29.39 | 10.93 | 23.54 | 20.16333 |
| TMEM233 | 46.918 | 5.552068 | 3.39E-06 | 2.29E-05 | yes | up | 1.41 | 0.18 | 0.05 | 48.74 | 37.37 | 96.93 | 0.546667 | 61.01333 |
| NGFR | 0.404 | -1.30852 | 3.41E-06 | 2.3E-05 | yes | down | 3.79 | 3.5 | 2.89 | 2.08 | 4.03 | 2.98 | 3.393333 | 3.03 |
| DLGAP4 | 2.463 | 1.300244 | 3.41E-06 | 2.3E-05 | yes | up | 9.67 | 7.38 | 4.5 | 33.04 | 37.83 | 46.18 | 7.183333 | 39.01667 |
| ERAL1 | 0.462 | -1.11479 | 3.42E-06 | 2.31E-05 | yes | down | 11.67 | 10.89 | 9.17 | 12.61 | 9.6 | 10.96 | 10.57667 | 11.05667 |
| LOC103350239 | 2.747 | 1.457848 | 3.42E-06 | 2.31E-05 | yes | up | 1.37 | 1.21 | 1.07 | 7.19 | 10.62 | 5.48 | 1.216667 | 7.763333 |
| LOC100357076 | 0.387 | -1.37123 | 3.42E-06 | 2.31E-05 | yes | down | 2.74 | 2.71 | 1.9 | 1.4 | 3.2 | 2.09 | 2.45 | 2.23 |
| GRPEL2 | 0.418 | -1.25723 | 3.43E-06 | 2.31E-05 | yes | down | 4.3 | 3.85 | 2.64 | 3.16 | 4.06 | 2.36 | 3.596667 | 3.193333 |
| DOK1 | 2.461 | 1.299136 | 3.43E-06 | 2.31E-05 | yes | up | 1.64 | 1.59 | 1.77 | 8.9 | 9.37 | 10.5 | 1.666667 | 9.59 |
| SLC25A26 | 0.428 | -1.22401 | 3.45E-06 | 2.32E-05 | yes | down | 6.79 | 7.26 | 6.79 | 8.6 | 6.71 | 6.03 | 6.946667 | 7.113333 |
| ACSF2 | 0.473 | -1.07895 | 3.45E-06 | 2.33E-05 | yes | down | 237.09 | 247.04 | 300.26 | 314.78 | 310.25 | 239.38 | 261.4633 | 288.1367 |
| XPO4 | 0.459 | -1.12361 | 3.5E-06 | 2.36E-05 | yes | down | 29 | 17.38 | 10.64 | 11.23 | 16.72 | 8.88 | 19.00667 | 12.27667 |
| C6H16orf52 | 0.429 | -1.22134 | 3.54E-06 | 2.38E-05 | yes | down | 6.43 | 6.68 | 5.83 | 7.72 | 8.51 | 4.88 | 6.313333 | 7.036667 |
| LOC108176745 | 0.416 | -1.26599 | 3.54E-06 | 2.38E-05 | yes | down | 18.96 | 17.07 | 16.36 | 11.64 | 16.95 | 19.34 | 17.46333 | 15.97667 |
| MYL10 | 0.006 | -7.4485 | 3.58E-06 | 2.41E-05 | yes | down | 12.46 | 2.46 | 0.27 | 0.16 | 0 | 0 | 5.063333 | 0.053333 |
| STAG2 | 0.407 | -1.2965 | 3.6E-06 | 2.42E-05 | yes | down | 13.19 | 10.09 | 10.93 | 11.69 | 14.21 | 6.38 | 11.40333 | 10.76 |
| OTUD7A | 7.61 | 2.927926 | 3.62E-06 | 2.43E-05 | yes | up | 0.31 | 0.39 | 0.03 | 4.03 | 4.1 | 8.26 | 0.243333 | 5.463333 |
| CHD7 | 0.495 | -1.01507 | 3.63E-06 | 2.44E-05 | yes | down | 3.03 | 2.81 | 3.08 | 3.22 | 4.18 | 2.74 | 2.973333 | 3.38 |
| PI16 | 3.153 | 1.656915 | 3.64E-06 | 2.45E-05 | yes | up | 16.29 | 13.65 | 14.1 | 71.46 | 115.73 | 137.11 | 14.68 | 108.1 |
| WBP4 | 0.382 | -1.38743 | 3.65E-06 | 2.45E-05 | yes | down | 12.69 | 11.46 | 9.74 | 12.53 | 11.51 | 5.84 | 11.29667 | 9.96 |
| MFAP3 | 0.405 | -1.30297 | 3.74E-06 | 2.5E-05 | yes | down | 7.5 | 9.12 | 7.64 | 7.09 | 11.03 | 4.78 | 8.086667 | 7.633333 |
| MYL4 | 0.014 | -6.138 | 3.74E-06 | 2.5E-05 | yes | down | 56.03 | 5.65 | 1.32 | 0.59 | 0.75 | 0.6 | 21 | 0.646667 |
| TRPV2 | 2.127 | 1.088548 | 3.76E-06 | 2.51E-05 | yes | up | 1.32 | 1.17 | 1.06 | 6 | 6.35 | 4.7 | 1.183333 | 5.683333 |
| RNF13 | 0.482 | -1.05262 | 3.8E-06 | 2.54E-05 | yes | down | 22.59 | 25.11 | 22.97 | 29.03 | 27.53 | 19.01 | 23.55667 | 25.19 |
| PARS2 | 0.429 | -1.21992 | 3.86E-06 | 2.58E-05 | yes | down | 2.94 | 2.87 | 2.59 | 2.3 | 2.61 | 2.78 | 2.8 | 2.563333 |
| DPYSL3 | 2.881 | 1.526713 | 3.91E-06 | 2.61E-05 | yes | up | 17.56 | 11.28 | 5.29 | 65.92 | 81.68 | 70.69 | 11.37667 | 72.76333 |
| VPS50 | 0.49 | -1.02884 | 3.92E-06 | 2.61E-05 | yes | down | 6.04 | 5.18 | 4.03 | 5.69 | 6.95 | 4.37 | 5.083333 | 5.67 |
| FNIP1 | 0.419 | -1.25369 | 3.94E-06 | 2.62E-05 | yes | down | 7.64 | 8.71 | 5.84 | 7.62 | 10.39 | 4.63 | 7.396667 | 7.546667 |
| IQUB | 0.266 | -1.90955 | 4E-06 | 2.66E-05 | yes | down | 3.89 | 3.35 | 4.4 | 2.35 | 4.13 | 0.99 | 3.88 | 2.49 |
| QSER1 | 0.497 | -1.01007 | 4.02E-06 | 2.67E-05 | yes | down | 4.54 | 3.9 | 2.95 | 4.59 | 5.15 | 3.16 | 3.796667 | 4.3 |
| MEF2A | 0.488 | -1.03375 | 4.02E-06 | 2.67E-05 | yes | down | 9.81 | 11.54 | 9.32 | 12.76 | 13.67 | 8.05 | 10.22333 | 11.49333 |
| CSF2RA | 3.091 | 1.627947 | 4.02E-06 | 2.67E-05 | yes | up | 1.25 | 1.64 | 0.76 | 9.3 | 8.13 | 7.86 | 1.216667 | 8.43 |
| LOC100346271 | 3.099 | 1.631623 | 4.03E-06 | 2.67E-05 | yes | up | 0.81 | 0.71 | 0.51 | 3.77 | 6.71 | 3.12 | 0.676667 | 4.533333 |
| LOC100340803 | 2.058 | 1.041563 | 4.04E-06 | 2.68E-05 | yes | up | 14.54 | 16.76 | 14 | 83.45 | 67.15 | 62.3 | 15.1 | 70.96667 |
| BCORL1 | 2.069 | 1.049227 | 4.04E-06 | 2.68E-05 | yes | up | 2.95 | 1.97 | 2.01 | 6.2 | 10.53 | 8.29 | 2.31 | 8.34 |
| ADCYAP1 | 0.043 | -4.53393 | 4.05E-06 | 2.68E-05 | yes | down | 0.51 | 0.93 | 0.27 | 0.03 | 0.14 | 0 | 0.57 | 0.056667 |
| LOC100345757 | 75.287 | 6.234338 | 4.09E-06 | 2.71E-05 | yes | up | 0 | 0 | 0.06 | 0.47 | 7.77 | 5.16 | 0.02 | 4.466667 |
| PIKFYVE | 0.42 | -1.25222 | 4.1E-06 | 2.72E-05 | yes | down | 4.22 | 4.23 | 3.27 | 3.65 | 5.58 | 2.46 | 3.906667 | 3.896667 |
| TTC32 | 0.32 | -1.64246 | 4.11E-06 | 2.72E-05 | yes | down | 23.72 | 25.22 | 25.13 | 17.79 | 13.71 | 23.71 | 24.69 | 18.40333 |
| CEBPG | 0.449 | -1.15674 | 4.12E-06 | 2.72E-05 | yes | down | 9.69 | 10.24 | 6.13 | 9.17 | 11.27 | 6.06 | 8.686667 | 8.833333 |
| WNT10B | 6.365 | 2.670174 | 4.15E-06 | 2.74E-05 | yes | up | 0.5 | 0.38 | 0.25 | 5.61 | 2.21 | 7.88 | 0.376667 | 5.233333 |
| GLB1 | 2.237 | 1.161762 | 4.15E-06 | 2.74E-05 | yes | up | 10.6 | 11.8 | 8.21 | 56.11 | 41.23 | 56.04 | 10.20333 | 51.12667 |
| LARP7 | 0.327 | -1.61078 | 4.17E-06 | 2.75E-05 | yes | down | 12.45 | 9.5 | 9.21 | 9.69 | 10.29 | 3.7 | 10.38667 | 7.893333 |
| SERINC2 | 2.977 | 1.574028 | 4.17E-06 | 2.76E-05 | yes | up | 10.34 | 9.31 | 7.81 | 60.5 | 37.83 | 83.47 | 9.153333 | 60.6 |
| ZBTB8OS | 0.497 | -1.00835 | 4.19E-06 | 2.76E-05 | yes | down | 14.5 | 16.81 | 13.77 | 17.79 | 16.9 | 16.08 | 15.02667 | 16.92333 |
| KIF2C | 4.853 | 2.278783 | 4.19E-06 | 2.77E-05 | yes | up | 1 | 0.96 | 0.43 | 10.12 | 13.19 | 3.15 | 0.796667 | 8.82 |
| LOC100341667 | 2.559 | 1.355538 | 4.22E-06 | 2.78E-05 | yes | up | 9.03 | 9.12 | 10.07 | 71.41 | 51.6 | 43.17 | 9.406667 | 55.39333 |
| CARNMT1 | 0.335 | -1.57651 | 4.24E-06 | 2.79E-05 | yes | down | 12.89 | 6.33 | 5.58 | 7.16 | 5.82 | 5.52 | 8.266667 | 6.166667 |
| CALML4 | 3.851 | 1.945365 | 4.26E-06 | 2.8E-05 | yes | up | 3.95 | 1.53 | 1.77 | 26.5 | 23.97 | 25.13 | 2.416667 | 25.2 |
| ERGIC1 | 2.093 | 1.065696 | 4.26E-06 | 2.81E-05 | yes | up | 25.72 | 17.76 | 17.42 | 89.34 | 96.9 | 103.78 | 20.3 | 96.67333 |
| MBTD1 | 0.428 | -1.22485 | 4.27E-06 | 2.81E-05 | yes | down | 6.17 | 7.02 | 5.07 | 5.98 | 7.87 | 3.85 | 6.086667 | 5.9 |
| LOC100008826 | 7.847 | 2.972112 | 4.27E-06 | 2.81E-05 | yes | up | 1.55 | 0.56 | 0.27 | 14.55 | 7.75 | 17.78 | 0.793333 | 13.36 |
| CDHR5 | 46.424 | 5.536795 | 4.31E-06 | 2.83E-05 | yes | up | 0 | 0.13 | 0 | 3.61 | 3.39 | 5.53 | 0.043333 | 4.176667 |
| HYAL3 | 4.111 | 2.039646 | 4.32E-06 | 2.84E-05 | yes | up | 0.74 | 1.41 | 0.88 | 8.07 | 6.99 | 12.89 | 1.01 | 9.316667 |
| CHRM4 | 0.011 | -6.56013 | 4.34E-06 | 2.85E-05 | yes | down | 0.55 | 0.63 | 1.18 | 0.05 | 0 | 0 | 0.786667 | 0.016667 |
| LDLRAD3 | 2.118 | 1.082898 | 4.35E-06 | 2.85E-05 | yes | up | 3.34 | 3.37 | 3.28 | 12.45 | 18.57 | 17.71 | 3.33 | 16.24333 |
| IL17RC | 2.156 | 1.108645 | 4.36E-06 | 2.86E-05 | yes | up | 3.24 | 3.17 | 2.7 | 12.42 | 17.44 | 17.87 | 3.036667 | 15.91 |
| NUDT5 | 0.427 | -1.2266 | 4.37E-06 | 2.86E-05 | yes | down | 24.41 | 32.94 | 25.92 | 33.06 | 30.42 | 19.94 | 27.75667 | 27.80667 |
| ADORA2A | 0.259 | -1.94825 | 4.37E-06 | 2.86E-05 | yes | down | 4.57 | 6.22 | 9.52 | 6.07 | 3.53 | 2.82 | 6.77 | 4.14 |
| DAAM1 | 0.45 | -1.15169 | 4.38E-06 | 2.87E-05 | yes | down | 7.2 | 6.12 | 8.32 | 8.15 | 8.49 | 6.12 | 7.213333 | 7.586667 |
| KCNK6 | 3.472 | 1.795576 | 4.39E-06 | 2.88E-05 | yes | up | 0.52 | 0.42 | 0.2 | 2.14 | 3.28 | 3.41 | 0.38 | 2.943333 |
| SLC27A2 | 0.025 | -5.31274 | 4.4E-06 | 2.88E-05 | yes | down | 0.43 | 0.14 | 1.17 | 0.05 | 0 | 0.06 | 0.58 | 0.036667 |
| B3GALT2 | 0.082 | -3.6146 | 4.47E-06 | 2.93E-05 | yes | down | 1.67 | 0.43 | 0.18 | 0.17 | 0.1 | 0.13 | 0.76 | 0.133333 |
| FKTN | 0.492 | -1.02355 | 4.49E-06 | 2.94E-05 | yes | down | 4.75 | 3.83 | 3.05 | 3.55 | 5.65 | 3.45 | 3.876667 | 4.216667 |
| TMEM33 | 0.488 | -1.0364 | 4.53E-06 | 2.96E-05 | yes | down | 6.46 | 7.03 | 6.78 | 8.35 | 9.02 | 6.06 | 6.756667 | 7.81 |
| LOC100353664 | 2.215 | 1.147463 | 4.54E-06 | 2.96E-05 | yes | up | 3.8 | 3.96 | 3.53 | 16.93 | 20.1 | 20.3 | 3.763333 | 19.11 |
| MARCH7 | 0.473 | -1.07874 | 4.54E-06 | 2.96E-05 | yes | down | 15.94 | 17.86 | 13.7 | 15.36 | 22.3 | 11.32 | 15.83333 | 16.32667 |
| ONECUT2 | 0.038 | -4.70318 | 4.55E-06 | 2.97E-05 | yes | down | 0.06 | 0.07 | 0.07 | 0.01 | 0 | 0 | 0.066667 | 0.003333 |
| PCBD1 | 2.409 | 1.268526 | 4.56E-06 | 2.97E-05 | yes | up | 15.04 | 13.08 | 11.29 | 80.22 | 55.29 | 77.44 | 13.13667 | 70.98333 |
| CDK7 | 0.432 | -1.20928 | 4.59E-06 | 2.99E-05 | yes | down | 12.51 | 11.78 | 11.57 | 12.23 | 15.97 | 8.06 | 11.95333 | 12.08667 |
| LOC103352385 | 0.155 | -2.68898 | 4.64E-06 | 3.03E-05 | yes | down | 0.52 | 1.85 | 1.17 | 0.5 | 0.7 | 0.7 | 1.18 | 0.633333 |
| LOC100358368 | 0.36 | -1.47246 | 4.68E-06 | 3.05E-05 | yes | down | 642.79 | 321.97 | 309.65 | 340.07 | 264.85 | 415.1 | 424.8033 | 340.0067 |
| LOC100345975 | 2.637 | 1.398939 | 4.72E-06 | 3.07E-05 | yes | up | 1.13 | 2.15 | 4.2 | 10.32 | 11.46 | 18.02 | 2.493333 | 13.26667 |
| LOC100343913 | 25.064 | 4.647522 | 4.72E-06 | 3.07E-05 | yes | up | 0.38 | 0 | 0.08 | 11.35 | 2.42 | 10.81 | 0.153333 | 8.193333 |
| NRM | 4.228 | 2.080131 | 4.76E-06 | 3.1E-05 | yes | up | 0.78 | 0.63 | 0.65 | 8.93 | 8.24 | 3.28 | 0.686667 | 6.816667 |
| ATF3 | 23.506 | 4.554939 | 4.78E-06 | 3.1E-05 | yes | up | 2.5 | 1.74 | 1.19 | 74.21 | 13.83 | 183.06 | 1.81 | 90.36667 |
| PLAU | 2.091 | 1.064459 | 4.78E-06 | 3.1E-05 | yes | up | 20.43 | 24.35 | 20.4 | 123.13 | 88.08 | 98.6 | 21.72667 | 103.27 |
| CNOT7 | 0.411 | -1.28172 | 4.81E-06 | 3.12E-05 | yes | down | 25.49 | 28.13 | 20.37 | 28.88 | 27.64 | 13.52 | 24.66333 | 23.34667 |
| SMPDL3A | 0.436 | -1.19828 | 4.84E-06 | 3.14E-05 | yes | down | 7.02 | 10.03 | 9.41 | 8.14 | 10.95 | 7.79 | 8.82 | 8.96 |
| LOC100351642 | 2.839 | 1.505161 | 4.84E-06 | 3.14E-05 | yes | up | 22.24 | 19.52 | 13.2 | 93.6 | 84.74 | 166.66 | 18.32 | 115 |
| ARL13B | 0.38 | -1.39466 | 4.86E-06 | 3.15E-05 | yes | down | 2.99 | 2.58 | 3.29 | 2.57 | 3.33 | 1.95 | 2.953333 | 2.616667 |
| AURKB | 5.405 | 2.434221 | 4.9E-06 | 3.18E-05 | yes | up | 1.57 | 1.22 | 0.49 | 15.68 | 19.75 | 5.15 | 1.093333 | 13.52667 |
| FOXN2 | 0.428 | -1.2254 | 4.91E-06 | 3.18E-05 | yes | down | 4.88 | 4.28 | 3.58 | 6.25 | 7.7 | 6.52 | 4.246667 | 6.823333 |
| SP4 | 0.439 | -1.18911 | 4.94E-06 | 3.2E-05 | yes | down | 1.36 | 1.42 | 0.95 | 1.1 | 1.52 | 1.19 | 1.243333 | 1.27 |
| PLK1 | 2.637 | 1.39868 | 4.95E-06 | 3.2E-05 | yes | up | 1.76 | 1.84 | 1.12 | 13.16 | 12.63 | 5.96 | 1.573333 | 10.58333 |
| MRPS18C | 0.451 | -1.14731 | 4.97E-06 | 3.21E-05 | yes | down | 31.69 | 24.3 | 20.26 | 27 | 30.03 | 22.17 | 25.41667 | 26.4 |
| MCP2 | 0.08 | -3.63832 | 5E-06 | 3.23E-05 | yes | down | 2.09 | 9.29 | 5.82 | 0.53 | 1.38 | 1.22 | 5.733333 | 1.043333 |
| DDX52 | 0.472 | -1.08364 | 5.01E-06 | 3.23E-05 | yes | down | 4.75 | 5.97 | 3.78 | 5.07 | 6.24 | 4.17 | 4.833333 | 5.16 |
| SH3RF2 | 0.164 | -2.60971 | 5.02E-06 | 3.24E-05 | yes | down | 1.75 | 2.21 | 0.37 | 0.39 | 0.46 | 0.66 | 1.443333 | 0.503333 |
| CRACR2A | 2.452 | 1.294037 | 5.07E-06 | 3.27E-05 | yes | up | 0.97 | 1.13 | 0.69 | 4.54 | 6.82 | 4.31 | 0.93 | 5.223333 |
| SENP6 | 0.443 | -1.17346 | 5.07E-06 | 3.27E-05 | yes | down | 8.19 | 7.18 | 7.45 | 9.61 | 10.39 | 5.44 | 7.606667 | 8.48 |
| BCAS2 | 0.48 | -1.0598 | 5.08E-06 | 3.27E-05 | yes | down | 33.2 | 34.36 | 24.44 | 39.57 | 34.96 | 25.8 | 30.66667 | 33.44333 |
| LOC100358736 | 0.256 | -1.96805 | 5.12E-06 | 3.3E-05 | yes | down | 0.86 | 0.86 | 0.62 | 0.49 | 0.48 | 0.38 | 0.78 | 0.45 |
| LSG1 | 0.494 | -1.01759 | 5.17E-06 | 3.33E-05 | yes | down | 10.28 | 14.88 | 11.65 | 13.93 | 15.13 | 12.88 | 12.27 | 13.98 |
| RLIM | 0.441 | -1.18135 | 5.18E-06 | 3.34E-05 | yes | down | 4.37 | 5.8 | 5.41 | 6.23 | 5.87 | 3.81 | 5.193333 | 5.303333 |
| MRPS25 | 0.478 | -1.06402 | 5.18E-06 | 3.34E-05 | yes | down | 63.75 | 81.44 | 60.14 | 80.09 | 60.12 | 72.26 | 68.44333 | 70.82333 |
| ERBIN | 0.471 | -1.08756 | 5.22E-06 | 3.36E-05 | yes | down | 8.91 | 8.16 | 6.36 | 9.66 | 10.61 | 5.93 | 7.81 | 8.733333 |
| B3GALT1 | 0.097 | -3.36287 | 5.24E-06 | 3.37E-05 | yes | down | 0.8 | 0.18 | 0.27 | 0.11 | 0.08 | 0.07 | 0.416667 | 0.086667 |
| COPZ2 | 2.05 | 1.035526 | 5.24E-06 | 3.37E-05 | yes | up | 25.99 | 36 | 19.64 | 126.46 | 106.52 | 90.54 | 27.21 | 107.84 |
| HEMK1 | 0.484 | -1.04756 | 5.28E-06 | 3.4E-05 | yes | down | 4.41 | 4.56 | 3.85 | 4.53 | 5.24 | 4.41 | 4.273333 | 4.726667 |
| RORC | 7.165 | 2.841007 | 5.31E-06 | 3.41E-05 | yes | up | 0.14 | 0.7 | 0.49 | 6.84 | 4.55 | 14.16 | 0.443333 | 8.516667 |
| IL1R2 | 3.672 | 1.87656 | 5.36E-06 | 3.44E-05 | yes | up | 2.02 | 1.94 | 3.69 | 13.9 | 25.54 | 26.72 | 2.55 | 22.05333 |
| GFOD1 | 0.4 | -1.32028 | 5.37E-06 | 3.45E-05 | yes | down | 9.56 | 19.2 | 9.41 | 10.92 | 11.16 | 12.28 | 12.72333 | 11.45333 |
| SLC12A3 | 10.897 | 3.445858 | 5.43E-06 | 3.48E-05 | yes | up | 0.14 | 0.12 | 0.04 | 2.9 | 2.25 | 3.05 | 0.1 | 2.733333 |
| CCDC25 | 0.402 | -1.31525 | 5.44E-06 | 3.49E-05 | yes | down | 15.01 | 13.15 | 12.81 | 15.02 | 16.24 | 7.29 | 13.65667 | 12.85 |
| KIAA1211L | 3.063 | 1.614811 | 5.46E-06 | 3.5E-05 | yes | up | 0.71 | 0.53 | 0.33 | 3.92 | 3.2 | 3.66 | 0.523333 | 3.593333 |
| ADAMTS17 | 0.307 | -1.70346 | 5.5E-06 | 3.52E-05 | yes | down | 3.35 | 3.4 | 1.34 | 1.18 | 2.24 | 2.13 | 2.696667 | 1.85 |
| UTP3 | 0.385 | -1.37546 | 5.5E-06 | 3.52E-05 | yes | down | 4.55 | 4.55 | 3.07 | 4.61 | 3.17 | 2.77 | 4.056667 | 3.516667 |
| PHLDA3 | 4.368 | 2.126826 | 5.53E-06 | 3.54E-05 | yes | up | 11.29 | 3.49 | 2.32 | 53.65 | 46.36 | 62.45 | 5.7 | 54.15333 |
| CHM | 0.427 | -1.22631 | 5.59E-06 | 3.58E-05 | yes | down | 3.9 | 4.92 | 5.45 | 6.52 | 5.99 | 5.45 | 4.756667 | 5.986667 |
| TUBA1B | 2.606 | 1.381651 | 5.6E-06 | 3.58E-05 | yes | up | 205.61 | 157.38 | 90.5 | 1051.09 | 1075.19 | 547.79 | 151.1633 | 891.3567 |
| CCDC93 | 0.452 | -1.14676 | 5.62E-06 | 3.59E-05 | yes | down | 9.63 | 14.1 | 8.29 | 9.66 | 14.43 | 7.6 | 10.67333 | 10.56333 |
| GLI2 | 3.185 | 1.671126 | 5.65E-06 | 3.61E-05 | yes | up | 0.3 | 0.21 | 0.16 | 1.85 | 1.51 | 1.37 | 0.223333 | 1.576667 |
| TAPBP | 2.479 | 1.309622 | 5.7E-06 | 3.64E-05 | yes | up | 3.28 | 2.92 | 4.03 | 15.95 | 20.86 | 22.21 | 3.41 | 19.67333 |
| LOC103348556 | 0.309 | -1.69498 | 5.7E-06 | 3.64E-05 | yes | down | 8.42 | 14.78 | 6.13 | 4.99 | 9.49 | 6.74 | 9.776667 | 7.073333 |
| NEMF | 0.433 | -1.20755 | 5.71E-06 | 3.64E-05 | yes | down | 4.04 | 3.61 | 3.51 | 3.74 | 5.02 | 2.49 | 3.72 | 3.75 |
| HAS2 | 4.09 | 2.03207 | 5.79E-06 | 3.69E-05 | yes | up | 0.43 | 0.34 | 0.25 | 3.58 | 2.48 | 2.65 | 0.34 | 2.903333 |
| LOC100341595 | 47.602 | 5.572951 | 5.81E-06 | 3.7E-05 | yes | up | 0 | 0.11 | 0 | 2.02 | 6.3 | 4.27 | 0.036667 | 4.196667 |
| TARS | 0.47 | -1.08845 | 5.81E-06 | 3.7E-05 | yes | down | 19.88 | 13.71 | 13.65 | 18.13 | 20.61 | 12.34 | 15.74667 | 17.02667 |
| ARID5B | 2.263 | 1.178503 | 5.82E-06 | 3.7E-05 | yes | up | 4.86 | 3.02 | 2.92 | 22.47 | 17.6 | 15.47 | 3.6 | 18.51333 |
| SNED1 | 2.119 | 1.083316 | 5.91E-06 | 3.76E-05 | yes | up | 4.67 | 3.94 | 2.68 | 16.37 | 21.34 | 24.88 | 3.763333 | 20.86333 |
| LOC108178409 | 0.365 | -1.45484 | 5.95E-06 | 3.79E-05 | yes | down | 5.3 | 8.06 | 6.47 | 6.92 | 5.31 | 4.35 | 6.61 | 5.526667 |
| TIPRL | 0.448 | -1.15786 | 5.98E-06 | 3.81E-05 | yes | down | 14.15 | 13.84 | 14.01 | 15.78 | 18.65 | 9.43 | 14 | 14.62 |
| PAM16 | 0.385 | -1.37873 | 6.02E-06 | 3.83E-05 | yes | down | 38.14 | 73.09 | 53.23 | 49.77 | 45.69 | 47.4 | 54.82 | 47.62 |
| RUNX2 | 3.975 | 1.99081 | 6.12E-06 | 3.89E-05 | yes | up | 0.19 | 0.14 | 0.1 | 1.43 | 1.47 | 1.09 | 0.143333 | 1.33 |
| VPS37A | 0.46 | -1.11976 | 6.12E-06 | 3.89E-05 | yes | down | 2.84 | 2.41 | 2.02 | 2.75 | 2.84 | 2.02 | 2.423333 | 2.536667 |
| PLK3 | 3.678 | 1.87909 | 6.13E-06 | 3.89E-05 | yes | up | 0.67 | 0.96 | 0.49 | 7.74 | 4.17 | 5.42 | 0.706667 | 5.776667 |
| TRPT1 | 0.445 | -1.16768 | 6.17E-06 | 3.91E-05 | yes | down | 14.59 | 16.99 | 17.47 | 15.84 | 15.12 | 17.21 | 16.35 | 16.05667 |
| EPHB2 | 3.283 | 1.714823 | 6.18E-06 | 3.92E-05 | yes | up | 2.17 | 0.96 | 0.46 | 8.3 | 12.87 | 8.85 | 1.196667 | 10.00667 |
| MOXD1 | 5.788 | 2.53295 | 6.2E-06 | 3.93E-05 | yes | up | 1.61 | 0.31 | 0.34 | 8.56 | 7.64 | 12.25 | 0.753333 | 9.483333 |
| CHST7 | 2.369 | 1.244357 | 6.26E-06 | 3.96E-05 | yes | up | 5.56 | 7.64 | 6.68 | 26.61 | 39.88 | 42.54 | 6.626667 | 36.34333 |
| MRAS | 2.169 | 1.117103 | 6.28E-06 | 3.98E-05 | yes | up | 3.89 | 4.69 | 2.62 | 15.79 | 18.31 | 20.53 | 3.733333 | 18.21 |
| CARHSP1 | 2.481 | 1.310677 | 6.34E-06 | 4.01E-05 | yes | up | 3.55 | 7.05 | 4.97 | 28.87 | 29.3 | 28.65 | 5.19 | 28.94 |
| LOC103351279 | 0.378 | -1.40173 | 6.43E-06 | 4.06E-05 | yes | down | 2.36 | 2 | 1.37 | 1.79 | 1.78 | 1.17 | 1.91 | 1.58 |
| IDE | 0.496 | -1.01049 | 6.44E-06 | 4.07E-05 | yes | down | 6.17 | 4.49 | 4.43 | 5.63 | 7.16 | 4.57 | 5.03 | 5.786667 |
| SAMD10 | 5.826 | 2.542529 | 6.51E-06 | 4.11E-05 | yes | up | 0.25 | 0.33 | 0.26 | 4.11 | 4.05 | 3.13 | 0.28 | 3.763333 |
| NAV3 | 2.522 | 1.334822 | 6.52E-06 | 4.12E-05 | yes | up | 0.75 | 1.1 | 0.95 | 6.71 | 6.09 | 3.68 | 0.933333 | 5.493333 |
| HDAC7 | 2.28 | 1.188754 | 6.53E-06 | 4.12E-05 | yes | up | 11.43 | 7.95 | 8.32 | 55.45 | 37.07 | 47.84 | 9.233333 | 46.78667 |
| ARL14EP | 0.409 | -1.29137 | 6.57E-06 | 4.14E-05 | yes | down | 7.99 | 8.71 | 6.77 | 8.21 | 9.66 | 4.58 | 7.823333 | 7.483333 |
| PIK3C2A | 0.395 | -1.3394 | 6.63E-06 | 4.18E-05 | yes | down | 3.6 | 3.76 | 3.49 | 3.36 | 4.8 | 1.88 | 3.616667 | 3.346667 |
| SHROOM1 | 2.327 | 1.21877 | 6.65E-06 | 4.19E-05 | yes | up | 1.84 | 2.9 | 1.8 | 10.97 | 10.8 | 12.66 | 2.18 | 11.47667 |
| APH1B | 0.463 | -1.11137 | 6.67E-06 | 4.2E-05 | yes | down | 10.3 | 14.69 | 13.96 | 11.73 | 16.89 | 13.45 | 12.98333 | 14.02333 |
| APLN | 2.846 | 1.508864 | 6.73E-06 | 4.23E-05 | yes | up | 5.15 | 4.22 | 1.68 | 20.17 | 24.09 | 25.27 | 3.683333 | 23.17667 |
| ERN1 | 2.515 | 1.330628 | 6.83E-06 | 4.29E-05 | yes | up | 0.69 | 0.66 | 0.58 | 2.56 | 5.08 | 3.59 | 0.643333 | 3.743333 |
| SOX9 | 5.799 | 2.535776 | 6.84E-06 | 4.3E-05 | yes | up | 0.18 | 0.2 | 0.08 | 2.44 | 1.25 | 2.14 | 0.153333 | 1.943333 |
| ITGA3 | 2.361 | 1.23947 | 6.95E-06 | 4.36E-05 | yes | up | 1.07 | 1.11 | 0.66 | 4.74 | 4.78 | 5.5 | 0.946667 | 5.006667 |
| BDKRB2 | 3.82 | 1.933601 | 7.03E-06 | 4.41E-05 | yes | up | 0.75 | 0.77 | 0.57 | 5.49 | 3.26 | 8.6 | 0.696667 | 5.783333 |
| ONECUT1 | 0.015 | -6.07747 | 7.07E-06 | 4.43E-05 | yes | down | 0.32 | 0.1 | 0.05 | 0 | 0.02 | 0 | 0.156667 | 0.006667 |
| ORAI3 | 2.168 | 1.116168 | 7.1E-06 | 4.45E-05 | yes | up | 3.53 | 4.26 | 4.02 | 18.79 | 19.23 | 19.08 | 3.936667 | 19.03333 |
| GABRB2 | 139.371 | 7.122784 | 7.12E-06 | 4.46E-05 | yes | up | 0 | 0 | 0 | 5.81 | 1.58 | 14.26 | 0 | 7.216667 |
| LOC100353012 | 87.459 | 6.450537 | 7.16E-06 | 4.48E-05 | yes | up | 0 | 0 | 0 | 0.36 | 0.54 | 0.23 | 0 | 0.376667 |
| SUN3 | 4.619 | 2.207463 | 7.21E-06 | 4.51E-05 | yes | up | 1.86 | 1.48 | 1.17 | 22.43 | 11.87 | 12.12 | 1.503333 | 15.47333 |
| KDR | 0.458 | -1.12697 | 7.22E-06 | 4.51E-05 | yes | down | 40.78 | 68.23 | 58.09 | 53.78 | 72.39 | 46.18 | 55.7 | 57.45 |
| TEX10 | 0.46 | -1.12128 | 7.28E-06 | 4.55E-05 | yes | down | 3.22 | 3.1 | 2.77 | 3.5 | 4.01 | 2.61 | 3.03 | 3.373333 |
| ZNF672 | 0.309 | -1.69279 | 7.33E-06 | 4.58E-05 | yes | down | 2.64 | 1.94 | 3.25 | 1.52 | 2.86 | 1.32 | 2.61 | 1.9 |
| BCR | 2.172 | 1.118784 | 7.43E-06 | 4.64E-05 | yes | up | 4.05 | 5.18 | 4.83 | 27.23 | 19.81 | 26.78 | 4.686667 | 24.60667 |
| LOC103347974 | 0.173 | -2.53399 | 7.43E-06 | 4.64E-05 | yes | down | 2.16 | 2.25 | 2.4 | 0.42 | 0.91 | 1.47 | 2.27 | 0.933333 |
| RP9 | 0.439 | -1.18722 | 7.52E-06 | 4.69E-05 | yes | down | 7.27 | 6.9 | 6.54 | 6.6 | 6.62 | 4.5 | 6.903333 | 5.906667 |
| LOC108177164 | 2.291 | 1.195992 | 7.53E-06 | 4.7E-05 | yes | up | 27.59 | 46.13 | 27.03 | 190.42 | 154.96 | 169.79 | 33.58333 | 171.7233 |
| PGAM1 | 0.495 | -1.01514 | 7.58E-06 | 4.72E-05 | yes | down | 148.36 | 172.57 | 169.71 | 218.81 | 170.98 | 167.79 | 163.5467 | 185.86 |
| RND2 | 3.027 | 1.597777 | 7.64E-06 | 4.76E-05 | yes | up | 0.89 | 1.02 | 0.65 | 5.74 | 7.7 | 4.38 | 0.853333 | 5.94 |
| VPS26A | 0.436 | -1.19673 | 7.66E-06 | 4.77E-05 | yes | down | 24.41 | 38.26 | 28.57 | 36.19 | 34.82 | 20.86 | 30.41333 | 30.62333 |
| GPR1 | 5.076 | 2.343833 | 7.66E-06 | 4.77E-05 | yes | up | 0.97 | 0.43 | 0.12 | 5.9 | 4.96 | 5.59 | 0.506667 | 5.483333 |
| LOC100349989 | 7.137 | 2.835285 | 7.82E-06 | 4.86E-05 | yes | up | 0.12 | 0.1 | 0.05 | 0.84 | 1.79 | 1.66 | 0.09 | 1.43 |
| CCNT2 | 0.404 | -1.30879 | 7.83E-06 | 4.87E-05 | yes | down | 8.6 | 10.58 | 5.83 | 7.91 | 11.53 | 4.9 | 8.336667 | 8.113333 |
| MBNL3 | 3.073 | 1.619638 | 7.85E-06 | 4.88E-05 | yes | up | 0.3 | 0.15 | 0.17 | 2.95 | 1.98 | 2.59 | 0.206667 | 2.506667 |
| DNAJC13 | 0.445 | -1.16681 | 7.86E-06 | 4.88E-05 | yes | down | 6.33 | 6.6 | 6.25 | 9.8 | 12.19 | 6.99 | 6.393333 | 9.66 |
| IGSF10 | 3.072 | 1.618957 | 7.9E-06 | 4.91E-05 | yes | up | 1.45 | 1.28 | 0.45 | 5.27 | 9.09 | 7.38 | 1.06 | 7.246667 |
| GSTM2 | 4.847 | 2.276974 | 7.92E-06 | 4.92E-05 | yes | up | 23.03 | 5.78 | 3.57 | 105.57 | 113.14 | 123.09 | 10.79333 | 113.9333 |
| LOC100345427 | 3.069 | 1.617553 | 7.93E-06 | 4.92E-05 | yes | up | 1.07 | 1.26 | 1.03 | 10.3 | 7.33 | 5.66 | 1.12 | 7.763333 |
| SHISA3 | 4.118 | 2.042034 | 8.06E-06 | 5E-05 | yes | up | 0.53 | 0.47 | 0.15 | 2.98 | 4 | 3.51 | 0.383333 | 3.496667 |
| LOC100346308 | 6.446 | 2.688353 | 8.18E-06 | 5.07E-05 | yes | up | 0.88 | 0.29 | 0.24 | 6.32 | 3.68 | 9.62 | 0.47 | 6.54 |
| NSUN3 | 0.244 | -2.03246 | 8.28E-06 | 5.13E-05 | yes | down | 0.85 | 0.97 | 1.11 | 0.55 | 0.77 | 0.36 | 0.976667 | 0.56 |
| HOXB9 | 40.12 | 5.326251 | 8.3E-06 | 5.14E-05 | yes | up | 0.05 | 0 | 0 | 1.44 | 1.98 | 1.12 | 0.016667 | 1.513333 |
| THOC1 | 0.493 | -1.01954 | 8.31E-06 | 5.14E-05 | yes | down | 4.73 | 5.59 | 3.71 | 4.98 | 5.68 | 3.49 | 4.676667 | 4.716667 |
| FST | 2.866 | 1.519168 | 8.32E-06 | 5.14E-05 | yes | up | 7.16 | 4.27 | 10.13 | 54.53 | 50.94 | 40.42 | 7.186667 | 48.63 |
| LMBRD2 | 0.414 | -1.272 | 8.37E-06 | 5.17E-05 | yes | down | 2.27 | 2.05 | 1.8 | 2.14 | 2.55 | 1.16 | 2.04 | 1.95 |
| CCDC82 | 0.447 | -1.16312 | 8.47E-06 | 5.23E-05 | yes | down | 4.44 | 4.92 | 4.5 | 4.75 | 6.06 | 3.53 | 4.62 | 4.78 |
| TAF13 | 0.347 | -1.52674 | 8.5E-06 | 5.25E-05 | yes | down | 7.91 | 6.37 | 5.55 | 7.37 | 4.56 | 3.61 | 6.61 | 5.18 |
| HS3ST6 | 10.046 | 3.328518 | 8.5E-06 | 5.25E-05 | yes | up | 0.03 | 0.14 | 0.11 | 3.42 | 1.6 | 1.33 | 0.093333 | 2.116667 |
| BLZF1 | 0.398 | -1.32805 | 8.55E-06 | 5.28E-05 | yes | down | 9 | 9.35 | 11.4 | 9.41 | 11.52 | 5.94 | 9.916667 | 8.956667 |
| BCL2 | 0.45 | -1.15246 | 8.57E-06 | 5.29E-05 | yes | down | 8.54 | 9.37 | 12.11 | 9.5 | 10.83 | 8.11 | 10.00667 | 9.48 |
| KEL | 0.088 | -3.51031 | 8.62E-06 | 5.32E-05 | yes | down | 0.27 | 0.94 | 0.64 | 0.19 | 0.09 | 0.11 | 0.616667 | 0.13 |
| ARGLU1 | 0.432 | -1.20961 | 8.82E-06 | 5.44E-05 | yes | down | 25.97 | 35.78 | 18.19 | 34.61 | 38.97 | 27.01 | 26.64667 | 33.53 |
| LOC100357801 | 0.241 | -2.05197 | 8.84E-06 | 5.44E-05 | yes | down | 0.81 | 2.69 | 1.37 | 1.18 | 1.01 | 0.5 | 1.623333 | 0.896667 |
| MMAA | 0.446 | -1.16519 | 8.89E-06 | 5.48E-05 | yes | down | 5.84 | 6.38 | 5.06 | 5.63 | 7.95 | 4.52 | 5.76 | 6.033333 |
| IRAK2 | 2.488 | 1.314955 | 8.93E-06 | 5.5E-05 | yes | up | 1.32 | 2.04 | 1.83 | 8 | 11.01 | 11.29 | 1.73 | 10.1 |
| ARHGEF10 | 2.599 | 1.378079 | 8.96E-06 | 5.51E-05 | yes | up | 4.47 | 4.55 | 3.97 | 20.71 | 23.33 | 32.66 | 4.33 | 25.56667 |
| CCDC90B | 0.41 | -1.28741 | 9.02E-06 | 5.55E-05 | yes | down | 13.45 | 21.69 | 10.89 | 17.09 | 14.67 | 10.46 | 15.34333 | 14.07333 |
| LOC100359308 | 0.457 | -1.12841 | 9.12E-06 | 5.61E-05 | yes | down | 4.65 | 4.41 | 3.31 | 4.06 | 4.75 | 3.64 | 4.123333 | 4.15 |
| LOC103346740 | 0.351 | -1.51124 | 9.14E-06 | 5.62E-05 | yes | down | 114.36 | 167.55 | 160.17 | 141.56 | 130.27 | 98.85 | 147.36 | 123.56 |
| TIMP4 | 0.358 | -1.48129 | 9.19E-06 | 5.65E-05 | yes | down | 27.46 | 41.78 | 27.32 | 28.32 | 51.88 | 39.74 | 32.18667 | 39.98 |
| HIVEP3 | 3.514 | 1.813314 | 9.23E-06 | 5.67E-05 | yes | up | 0.13 | 0.07 | 0.09 | 0.61 | 0.86 | 0.85 | 0.096667 | 0.773333 |
| PTAR1 | 0.499 | -1.00161 | 9.32E-06 | 5.72E-05 | yes | down | 2.45 | 2.27 | 1.82 | 2.22 | 3.4 | 1.97 | 2.18 | 2.53 |
| HPDL | 0.16 | -2.64645 | 9.32E-06 | 5.72E-05 | yes | down | 0.84 | 1.19 | 0.54 | 0.33 | 0.32 | 0.27 | 0.856667 | 0.306667 |
| NR4A3 | 16.091 | 4.008172 | 9.41E-06 | 5.77E-05 | yes | up | 0.11 | 0.62 | 0.16 | 9.4 | 3.67 | 16.78 | 0.296667 | 9.95 |
| REEP3 | 0.444 | -1.17146 | 9.47E-06 | 5.81E-05 | yes | down | 16.71 | 20.61 | 16.01 | 17.78 | 24.39 | 10.5 | 17.77667 | 17.55667 |
| TEAD3 | 2.453 | 1.294616 | 9.49E-06 | 5.82E-05 | yes | up | 1.88 | 1.47 | 1.47 | 8.5 | 7.83 | 10.43 | 1.606667 | 8.92 |
| JMJD1C | 0.421 | -1.24973 | 9.51E-06 | 5.83E-05 | yes | down | 5.88 | 4.83 | 6.53 | 6.1 | 7.2 | 3.68 | 5.746667 | 5.66 |
| PLA2G12A | 0.34 | -1.55546 | 9.6E-06 | 5.88E-05 | yes | down | 43.19 | 20.98 | 14.63 | 18.21 | 25.74 | 15.86 | 26.26667 | 19.93667 |
| LOC100338343 | 0.479 | -1.06126 | 9.6E-06 | 5.88E-05 | yes | down | 9.66 | 7.31 | 6.85 | 9.21 | 8.56 | 8.16 | 7.94 | 8.643333 |
| RMDN1 | 0.385 | -1.37545 | 9.61E-06 | 5.88E-05 | yes | down | 7.31 | 8.71 | 5.86 | 6.52 | 5.18 | 7.34 | 7.293333 | 6.346667 |
| HAL | 20.39 | 4.349763 | 9.64E-06 | 5.9E-05 | yes | up | 0.02 | 0.02 | 0.05 | 0.71 | 3.07 | 0.65 | 0.03 | 1.476667 |
| LOC100358539 | 2.597 | 1.376886 | 9.69E-06 | 5.93E-05 | yes | up | 0.68 | 0.81 | 0.72 | 4.42 | 5 | 3.64 | 0.736667 | 4.353333 |
| ANGPT1 | 0.255 | -1.96964 | 9.75E-06 | 5.96E-05 | yes | down | 4.59 | 4.54 | 10.13 | 4.47 | 5.27 | 2.02 | 6.42 | 3.92 |
| SNTB1 | 0.396 | -1.33601 | 9.76E-06 | 5.96E-05 | yes | down | 24.13 | 16.28 | 16.64 | 22.95 | 19.78 | 10.31 | 19.01667 | 17.68 |
| PHOSPHO1 | 0.087 | -3.5308 | 9.79E-06 | 5.97E-05 | yes | down | 36.4 | 49.82 | 25.31 | 4.03 | 2.46 | 14.78 | 37.17667 | 7.09 |
| LOC100345095 | 0.088 | -3.51199 | 9.79E-06 | 5.97E-05 | yes | down | 1.25 | 0.77 | 4.61 | 0.55 | 0.73 | 0.17 | 2.21 | 0.483333 |
| LOC103351843 | 2.121 | 1.084562 | 9.79E-06 | 5.97E-05 | yes | up | 7.28 | 6.47 | 6.04 | 40.21 | 39.64 | 32.86 | 6.596667 | 37.57 |
| NUDT7 | 0.473 | -1.08159 | 9.99E-06 | 6.09E-05 | yes | down | 23.07 | 22.36 | 22.48 | 27.74 | 22.93 | 22.87 | 22.63667 | 24.51333 |
| TYMS | 3.126 | 1.644511 | 1E-05 | 6.12E-05 | yes | up | 2.85 | 2.88 | 2.46 | 24.35 | 25.5 | 9.61 | 2.73 | 19.82 |
| WWOX | 2.089 | 1.063135 | 1.01E-05 | 6.13E-05 | yes | up | 7 | 5.14 | 5.02 | 27.95 | 25.36 | 26.81 | 5.72 | 26.70667 |
| TRIM33 | 0.482 | -1.05363 | 1.01E-05 | 6.14E-05 | yes | down | 3.31 | 3.36 | 2.91 | 3.65 | 4.63 | 2.42 | 3.193333 | 3.566667 |
| RUSC2 | 2.047 | 1.03349 | 1.01E-05 | 6.16E-05 | yes | up | 6 | 3.73 | 3.51 | 18.74 | 21.89 | 20.79 | 4.413333 | 20.47333 |
| INPP5D | 2.283 | 1.191173 | 1.01E-05 | 6.17E-05 | yes | up | 0.83 | 1.05 | 0.81 | 4.06 | 5.45 | 4.62 | 0.896667 | 4.71 |
| STAT2 | 2.054 | 1.038343 | 1.02E-05 | 6.21E-05 | yes | up | 4.23 | 3.43 | 3.93 | 18.01 | 22.27 | 17.46 | 3.863333 | 19.24667 |
| UGT1-4 | 2.591 | 1.373481 | 1.02E-05 | 6.21E-05 | yes | up | 1.02 | 1.41 | 1.25 | 6.44 | 8.12 | 7.45 | 1.226667 | 7.336667 |
| ERCC8 | 0.414 | -1.27097 | 1.02E-05 | 6.22E-05 | yes | down | 2.63 | 2.97 | 3.12 | 2.87 | 2.91 | 2.67 | 2.906667 | 2.816667 |
| ELMOD1 | 0.038 | -4.70253 | 1.03E-05 | 6.25E-05 | yes | down | 0.18 | 0.64 | 0.8 | 0 | 0.06 | 0.11 | 0.54 | 0.056667 |
| RBM14 | 2.381 | 1.251743 | 1.03E-05 | 6.25E-05 | yes | up | 3.25 | 6.02 | 5.94 | 28.85 | 29.03 | 28.38 | 5.07 | 28.75333 |
| TRERF1 | 2.16 | 1.111092 | 1.04E-05 | 6.3E-05 | yes | up | 0.81 | 0.84 | 0.6 | 3.9 | 4.28 | 2.7 | 0.75 | 3.626667 |
| ZC3H15 | 0.441 | -1.17953 | 1.04E-05 | 6.32E-05 | yes | down | 17.74 | 16.69 | 14.47 | 19.42 | 20.33 | 10.03 | 16.3 | 16.59333 |
| CTAGE5 | 0.468 | -1.09477 | 1.04E-05 | 6.33E-05 | yes | down | 7.9 | 6.08 | 6.56 | 8.04 | 8.7 | 5.42 | 6.846667 | 7.386667 |
| HSP90AA1 | 2.367 | 1.242949 | 1.05E-05 | 6.35E-05 | yes | up | 137.29 | 116.95 | 108.37 | 671.12 | 452.23 | 812.18 | 120.87 | 645.1767 |
| RBBP9 | 0.467 | -1.09731 | 1.05E-05 | 6.36E-05 | yes | down | 3.15 | 3.52 | 2.94 | 2.95 | 4.32 | 3.1 | 3.203333 | 3.456667 |
| LOC108176788 | 0.399 | -1.32607 | 1.05E-05 | 6.38E-05 | yes | down | 3.19 | 3.9 | 3.74 | 2.65 | 3.48 | 4.52 | 3.61 | 3.55 |
| CAMKK1 | 3.551 | 1.828039 | 1.06E-05 | 6.39E-05 | yes | up | 0.61 | 0.47 | 0.3 | 2.42 | 3.88 | 4.78 | 0.46 | 3.693333 |
| FPGT | 0.382 | -1.3902 | 1.06E-05 | 6.39E-05 | yes | down | 3.65 | 3.68 | 3.02 | 3.66 | 3.09 | 2.19 | 3.45 | 2.98 |
| LOC103349708 | 2.364 | 1.241406 | 1.07E-05 | 6.47E-05 | yes | up | 4.6 | 2.85 | 2.54 | 17.6 | 20.98 | 15.14 | 3.33 | 17.90667 |
| TLDC2 | 4.886 | 2.288629 | 1.07E-05 | 6.47E-05 | yes | up | 0.18 | 0.41 | 0.31 | 3.45 | 2.52 | 4.05 | 0.3 | 3.34 |
| MAP6D1 | 2.136 | 1.09488 | 1.08E-05 | 6.51E-05 | yes | up | 0.99 | 0.92 | 0.75 | 4.5 | 5.38 | 3.87 | 0.886667 | 4.583333 |
| PABPC4 | 0.487 | -1.03924 | 1.09E-05 | 6.6E-05 | yes | down | 74.06 | 45.8 | 40.4 | 56.37 | 58.88 | 60.09 | 53.42 | 58.44667 |
| KCNAB1 | 2.786 | 1.478047 | 1.12E-05 | 6.75E-05 | yes | up | 2 | 1.51 | 1 | 7.78 | 7.58 | 12.26 | 1.503333 | 9.206667 |
| LOC100339548 | 0.053 | -4.24156 | 1.12E-05 | 6.77E-05 | yes | down | 0.33 | 0.61 | 0.41 | 0.03 | 0.1 | 0.04 | 0.45 | 0.056667 |
| RNMT | 0.459 | -1.12225 | 1.12E-05 | 6.78E-05 | yes | down | 5.2 | 4.91 | 3.94 | 5 | 6.69 | 3.2 | 4.683333 | 4.963333 |
| BEST4 | 0.051 | -4.29289 | 1.14E-05 | 6.85E-05 | yes | down | 0.21 | 0.27 | 0.44 | 0.04 | 0.04 | 0.03 | 0.306667 | 0.036667 |
| KLF15 | 2.374 | 1.24742 | 1.15E-05 | 6.91E-05 | yes | up | 2.19 | 1.1 | 2.4 | 10.69 | 9.36 | 10.83 | 1.896667 | 10.29333 |
| N4BP2L2 | 0.445 | -1.16951 | 1.15E-05 | 6.92E-05 | yes | down | 6.44 | 7.31 | 6.41 | 7.1 | 9.44 | 4.36 | 6.72 | 6.966667 |
| CLCN5 | 2.236 | 1.16069 | 1.15E-05 | 6.93E-05 | yes | up | 1.44 | 0.94 | 0.72 | 5.48 | 6.1 | 4.25 | 1.033333 | 5.276667 |
| GAREM2 | 0.161 | -2.63199 | 1.15E-05 | 6.93E-05 | yes | down | 0.36 | 1.08 | 0.81 | 0.18 | 0.43 | 0.25 | 0.75 | 0.286667 |
| SLC25A35 | 2.705 | 1.435516 | 1.17E-05 | 7.03E-05 | yes | up | 1.69 | 1.84 | 1.14 | 11.82 | 10.07 | 6.77 | 1.556667 | 9.553333 |
| TMTC1 | 2.036 | 1.025904 | 1.17E-05 | 7.03E-05 | yes | up | 1.86 | 1.39 | 1.48 | 6.64 | 7.2 | 8.14 | 1.576667 | 7.326667 |
| MAP2K4 | 0.471 | -1.08606 | 1.2E-05 | 7.2E-05 | yes | down | 7.05 | 4.57 | 4.17 | 5.9 | 6.33 | 4.68 | 5.263333 | 5.636667 |
| PARD6B | 0.245 | -2.02742 | 1.2E-05 | 7.2E-05 | yes | down | 0.85 | 0.94 | 0.81 | 0.31 | 0.53 | 0.64 | 0.866667 | 0.493333 |
| CFTR | 0.009 | -6.77019 | 1.2E-05 | 7.2E-05 | yes | down | 0.18 | 0.08 | 0.14 | 0 | 0 | 0 | 0.133333 | 0 |
| LRWD1 | 0.467 | -1.09941 | 1.2E-05 | 7.21E-05 | yes | down | 5.6 | 5.84 | 5.43 | 7.05 | 5.96 | 5 | 5.623333 | 6.003333 |
| ROBO1 | 2.468 | 1.303401 | 1.21E-05 | 7.26E-05 | yes | up | 2.39 | 3.37 | 1.74 | 5.46 | 4.98 | 4.25 | 2.5 | 4.896667 |
| DGKH | 0.345 | -1.53361 | 1.22E-05 | 7.29E-05 | yes | down | 1.6 | 1.05 | 1.2 | 0.87 | 1.62 | 0.6 | 1.283333 | 1.03 |
| LOC103347221 | 2.241 | 1.16423 | 1.22E-05 | 7.31E-05 | yes | up | 44.12 | 42.57 | 43.29 | 201.25 | 336.61 | 148.24 | 43.32667 | 228.7 |
| DMTN | 2.885 | 1.528685 | 1.22E-05 | 7.33E-05 | yes | up | 1.42 | 1.96 | 1.5 | 13.48 | 8.26 | 12.63 | 1.626667 | 11.45667 |
| AZI2 | 0.428 | -1.22366 | 1.23E-05 | 7.35E-05 | yes | down | 12.19 | 12.19 | 9.34 | 12.24 | 14.93 | 6.75 | 11.24 | 11.30667 |
| LOC100339431 | 0.498 | -1.00617 | 1.23E-05 | 7.36E-05 | yes | down | 658.96 | 665.88 | 729.54 | 835.29 | 727.24 | 777.99 | 684.7933 | 780.1733 |
| LRRC2 | 0.28 | -1.8387 | 1.23E-05 | 7.38E-05 | yes | down | 10.17 | 5.52 | 6.8 | 3.54 | 3.4 | 7.21 | 7.496667 | 4.716667 |
| LOC100355448 | 2.434 | 1.283098 | 1.24E-05 | 7.43E-05 | yes | up | 1.19 | 1.88 | 1.28 | 7.73 | 9.08 | 7.45 | 1.45 | 8.086667 |
| SH3GL2 | 2.604 | 1.380754 | 1.25E-05 | 7.45E-05 | yes | up | 1.63 | 0.89 | 1.35 | 7.03 | 8.07 | 8.06 | 1.29 | 7.72 |
| NOS3 | 2.33 | 1.220086 | 1.26E-05 | 7.54E-05 | yes | up | 3.7 | 6.37 | 5.69 | 23.49 | 34.15 | 26.59 | 5.253333 | 28.07667 |
| TDRD3 | 0.405 | -1.30243 | 1.29E-05 | 7.68E-05 | yes | down | 7.15 | 7.26 | 7.66 | 8.08 | 8.21 | 4.05 | 7.356667 | 6.78 |
| LOC100354435 | 110.274 | 6.784944 | 1.3E-05 | 7.72E-05 | yes | up | 5.07 | 6.91 | 1.45 | 717.95 | 10.55 | 2352.55 | 4.476667 | 1027.017 |
| LOC100347463 | 2.018 | 1.01279 | 1.31E-05 | 7.79E-05 | yes | up | 10.28 | 9.95 | 9.6 | 49.11 | 39.93 | 48.09 | 9.943333 | 45.71 |
| MYOC | 6.948 | 2.796547 | 1.31E-05 | 7.81E-05 | yes | up | 2.35 | 0.7 | 0.21 | 13.05 | 9.95 | 24.75 | 1.086667 | 15.91667 |
| LOC103346166 | 0.027 | -5.19323 | 1.32E-05 | 7.84E-05 | yes | down | 0.26 | 1.34 | 1.05 | 0.1 | 0 | 0.07 | 0.883333 | 0.056667 |
| TSPO2 | 0.13 | -2.94201 | 1.33E-05 | 7.89E-05 | yes | down | 9.81 | 12.06 | 16.47 | 3.92 | 4.22 | 1.73 | 12.78 | 3.29 |
| MST1 | 8.029 | 3.005233 | 1.33E-05 | 7.92E-05 | yes | up | 0.11 | 0.18 | 0.02 | 1.46 | 2.23 | 1.77 | 0.103333 | 1.82 |
| ORM1 | 143.47 | 7.164605 | 1.34E-05 | 7.98E-05 | yes | up | 0 | 0 | 0 | 5.86 | 0.4 | 11.73 | 0 | 5.996667 |
| BCL3 | 3.104 | 1.634218 | 1.35E-05 | 8.01E-05 | yes | up | 0.88 | 0.97 | 0.82 | 7.88 | 5.36 | 5.56 | 0.89 | 6.266667 |
| LOC100354037 | 68.687 | 6.101955 | 1.36E-05 | 8.07E-05 | yes | up | 24.76 | 5.12 | 11.68 | 2399.66 | 48.67 | 3681.43 | 13.85333 | 2043.253 |
| LOC100008880 | 917.396 | 9.8414 | 1.37E-05 | 8.11E-05 | yes | up | 0 | 0 | 0 | 4.46 | 208.51 | 57.91 | 0 | 90.29333 |
| NFYC | 0.483 | -1.05121 | 1.38E-05 | 8.16E-05 | yes | down | 20.95 | 27.04 | 25.46 | 25.95 | 26.42 | 31.11 | 24.48333 | 27.82667 |
| LOC100359197 | 14.128 | 3.820484 | 1.38E-05 | 8.19E-05 | yes | up | 0.06 | 0.16 | 0.14 | 1.31 | 5.95 | 4.83 | 0.12 | 4.03 |
| ACCSL | 3.91 | 1.966992 | 1.4E-05 | 8.29E-05 | yes | up | 7.74 | 6.18 | 8.88 | 55.43 | 31.87 | 113.06 | 7.6 | 66.78667 |
| SLC35G1 | 0.376 | -1.41104 | 1.4E-05 | 8.3E-05 | yes | down | 1.69 | 1.63 | 1.43 | 1.37 | 1.67 | 1.06 | 1.583333 | 1.366667 |
| PXMP4 | 2.564 | 1.35836 | 1.41E-05 | 8.31E-05 | yes | up | 3.64 | 3.29 | 3.83 | 25.29 | 15.26 | 22.24 | 3.586667 | 20.93 |
| C20H14orf1 | 2.013 | 1.009459 | 1.41E-05 | 8.33E-05 | yes | up | 11.58 | 12.36 | 12.7 | 65.49 | 56.31 | 50.2 | 12.21333 | 57.33333 |
| ARSK | 2.959 | 1.565229 | 1.41E-05 | 8.33E-05 | yes | up | 1.3 | 2.07 | 0.95 | 7.52 | 12.16 | 9.14 | 1.44 | 9.606667 |
| LOC100342182 | 0.009 | -6.77975 | 1.42E-05 | 8.39E-05 | yes | down | 2.15 | 1.42 | 3.12 | 0 | 0 | 0 | 2.23 | 0 |
| BTG3 | 0.435 | -1.20078 | 1.42E-05 | 8.4E-05 | yes | down | 12.14 | 12.96 | 14.35 | 15.49 | 14.23 | 9.38 | 13.15 | 13.03333 |
| PFKFB1 | 2.107 | 1.075291 | 1.43E-05 | 8.42E-05 | yes | up | 21.68 | 12.53 | 13.2 | 81.36 | 79.91 | 62.43 | 15.80333 | 74.56667 |
| ACSS1 | 0.265 | -1.91785 | 1.44E-05 | 8.48E-05 | yes | down | 83.24 | 20.12 | 23.21 | 25.86 | 26.34 | 22.37 | 42.19 | 24.85667 |
| PLXDC2 | 2.1 | 1.070504 | 1.44E-05 | 8.5E-05 | yes | up | 3.56 | 2.61 | 2.03 | 13.88 | 11.8 | 12.92 | 2.733333 | 12.86667 |
| TGIF1 | 2.068 | 1.048355 | 1.45E-05 | 8.57E-05 | yes | up | 5.43 | 6.53 | 6.71 | 31.86 | 26.8 | 26.05 | 6.223333 | 28.23667 |
| ADRA2A | 10.339 | 3.369976 | 1.46E-05 | 8.59E-05 | yes | up | 0.45 | 0.18 | 0 | 6.17 | 3.02 | 4.49 | 0.21 | 4.56 |
| TDP1 | 2.259 | 1.175425 | 1.47E-05 | 8.64E-05 | yes | up | 2.57 | 2.06 | 1.49 | 11.35 | 12.19 | 8.01 | 2.04 | 10.51667 |
| ECSIT | 0.472 | -1.08339 | 1.48E-05 | 8.7E-05 | yes | down | 39.67 | 39.87 | 38.48 | 43.2 | 33.21 | 48.44 | 39.34 | 41.61667 |
| GALNT18 | 2.081 | 1.057536 | 1.48E-05 | 8.73E-05 | yes | up | 6.84 | 5.78 | 4.64 | 30.42 | 22.94 | 27.4 | 5.753333 | 26.92 |
| TTC33 | 0.419 | -1.25425 | 1.48E-05 | 8.73E-05 | yes | down | 6.8 | 4.48 | 5.24 | 4.56 | 5.88 | 5.48 | 5.506667 | 5.306667 |
| RHNO1 | 2.982 | 1.576229 | 1.48E-05 | 8.73E-05 | yes | up | 1.86 | 1.2 | 1.28 | 11.31 | 9.67 | 8.57 | 1.446667 | 9.85 |
| FERMT1 | 0.094 | -3.41601 | 1.5E-05 | 8.83E-05 | yes | down | 0.58 | 0.3 | 1.04 | 0.07 | 0.27 | 0.05 | 0.64 | 0.13 |
| ALOX5AP | 2.461 | 1.299347 | 1.51E-05 | 8.84E-05 | yes | up | 4.96 | 5.89 | 3.93 | 26.82 | 23.89 | 31.2 | 4.926667 | 27.30333 |
| RASSF10 | 4.008 | 2.00277 | 1.52E-05 | 8.92E-05 | yes | up | 0.2 | 0.17 | 0.07 | 1.07 | 1.29 | 1.51 | 0.146667 | 1.29 |
| ASF1A | 0.406 | -1.29972 | 1.53E-05 | 8.97E-05 | yes | down | 8.61 | 8.53 | 8.27 | 9.88 | 10.85 | 7.24 | 8.47 | 9.323333 |
| MRVI1 | 2.161 | 1.112017 | 1.54E-05 | 9.04E-05 | yes | up | 1.25 | 1.78 | 1.79 | 7.97 | 9.69 | 7 | 1.606667 | 8.22 |
| HID1 | 2.263 | 1.178349 | 1.55E-05 | 9.07E-05 | yes | up | 3.61 | 4.52 | 4.35 | 21.05 | 28.11 | 17.56 | 4.16 | 22.24 |
| TRAPPC6B | 0.477 | -1.06938 | 1.56E-05 | 9.13E-05 | yes | down | 6.94 | 7.8 | 8.35 | 8.21 | 10.11 | 7.32 | 7.696667 | 8.546667 |
| BRIX1 | 0.42 | -1.25076 | 1.56E-05 | 9.13E-05 | yes | down | 8.79 | 11.95 | 6.63 | 9.32 | 7.76 | 8.79 | 9.123333 | 8.623333 |
| TPRA1 | 0.466 | -1.10207 | 1.57E-05 | 9.17E-05 | yes | down | 20.95 | 27.33 | 30.37 | 26.53 | 30.81 | 28.38 | 26.21667 | 28.57333 |
| NPR3 | 0.261 | -1.94005 | 1.57E-05 | 9.17E-05 | yes | down | 37.26 | 55.96 | 48.24 | 13.92 | 63.1 | 17.12 | 47.15333 | 31.38 |
| GDPD3 | 2.316 | 1.211653 | 1.58E-05 | 9.24E-05 | yes | up | 1.65 | 2.14 | 1.71 | 11.75 | 8.39 | 8.96 | 1.833333 | 9.7 |
| LOC100343529 | 0.319 | -1.64855 | 1.59E-05 | 9.27E-05 | yes | down | 13.58 | 14.57 | 13.87 | 13.66 | 9.92 | 7.18 | 14.00667 | 10.25333 |
| ABHD17B | 0.398 | -1.32876 | 1.6E-05 | 9.31E-05 | yes | down | 6.04 | 6.49 | 4.15 | 6.37 | 5.61 | 3.33 | 5.56 | 5.103333 |
| SKIV2L2 | 0.499 | -1.00266 | 1.6E-05 | 9.35E-05 | yes | down | 11.01 | 10.37 | 10.8 | 12.51 | 15.99 | 8.91 | 10.72667 | 12.47 |
| TWIST2 | 4.711 | 2.236016 | 1.62E-05 | 9.45E-05 | yes | up | 1.73 | 1.32 | 0.68 | 12.67 | 10.69 | 15.62 | 1.243333 | 12.99333 |
| MCOLN3 | 6.594 | 2.721195 | 1.64E-05 | 9.54E-05 | yes | up | 0.7 | 0.98 | 0.43 | 23.03 | 2.97 | 5.17 | 0.703333 | 10.39 |
| SETD6 | 0.277 | -1.85189 | 1.64E-05 | 9.55E-05 | yes | down | 2.45 | 1.85 | 2.4 | 1.36 | 2.51 | 0.9 | 2.233333 | 1.59 |
| LOC100352631 | 0.014 | -6.15213 | 1.64E-05 | 9.57E-05 | yes | down | 0.68 | 0.79 | 0.48 | 0 | 0.06 | 0 | 0.65 | 0.02 |
| PPP1R14A | 3.003 | 1.586504 | 1.65E-05 | 9.62E-05 | yes | up | 2.83 | 5.61 | 2.44 | 20.95 | 22.42 | 29.94 | 3.626667 | 24.43667 |
| VPS13A | 0.434 | -1.20446 | 1.65E-05 | 9.62E-05 | yes | down | 1.64 | 1.25 | 1.34 | 1.44 | 1.9 | 0.92 | 1.41 | 1.42 |
| FLCN | 0.49 | -1.02773 | 1.67E-05 | 9.73E-05 | yes | down | 10.28 | 13.03 | 12.53 | 12.31 | 13.37 | 14.72 | 11.94667 | 13.46667 |
| HSPA14 | 0.469 | -1.09348 | 1.68E-05 | 9.79E-05 | yes | down | 7.02 | 10.04 | 8.05 | 8.21 | 11.26 | 6.36 | 8.37 | 8.61 |
| METTL25 | 0.26 | -1.94128 | 1.69E-05 | 9.84E-05 | yes | down | 1.39 | 1.59 | 1.76 | 0.85 | 1.47 | 0.71 | 1.58 | 1.01 |
| P2RX7 | 2.882 | 1.527246 | 1.7E-05 | 9.86E-05 | yes | up | 0.57 | 0.43 | 0.24 | 2.25 | 3.14 | 2.62 | 0.413333 | 2.67 |
| DUSP1 | 2.973 | 1.572005 | 1.71E-05 | 9.93E-05 | yes | up | 16.19 | 10.56 | 11.45 | 62.13 | 61.94 | 132.48 | 12.73333 | 85.51667 |
| ARID4B | 0.402 | -1.31642 | 1.73E-05 | 0.0001 | yes | down | 4.65 | 4.95 | 5.01 | 4.49 | 6.54 | 2.38 | 4.87 | 4.47 |
| LOC108178230 | 0.009 | -6.77926 | 1.74E-05 | 0.000101 | yes | down | 0.55 | 0.33 | 0.13 | 0 | 0 | 0 | 0.336667 | 0 |
| MIER1 | 0.478 | -1.06626 | 1.76E-05 | 0.000102 | yes | down | 7.71 | 6.85 | 6.35 | 8.29 | 9.72 | 5.02 | 6.97 | 7.676667 |
| WWTR1 | 0.481 | -1.05609 | 1.76E-05 | 0.000102 | yes | down | 46.42 | 54.75 | 60.88 | 60.39 | 77.34 | 42.75 | 54.01667 | 60.16 |
| ZDHHC15 | 2.083 | 1.058409 | 1.76E-05 | 0.000102 | yes | up | 0.8 | 0.82 | 0.62 | 3.86 | 3.59 | 3.14 | 0.746667 | 3.53 |
| LOC100357419 | 35.878 | 5.165026 | 1.77E-05 | 0.000102 | yes | up | 0.09 | 0.03 | 0.05 | 2.63 | 0.53 | 11.29 | 0.056667 | 4.816667 |
| JUP | 2.03 | 1.021609 | 1.77E-05 | 0.000103 | yes | up | 14.53 | 16.81 | 16.45 | 69.25 | 104.13 | 56.53 | 15.93 | 76.63667 |
| LOC100343231 | 5.852 | 2.549009 | 1.78E-05 | 0.000103 | yes | up | 2.94 | 0.37 | 1.05 | 11.33 | 19.74 | 26.29 | 1.453333 | 19.12 |
| AIF1 | 2.36 | 1.239086 | 1.78E-05 | 0.000103 | yes | up | 8.62 | 7.79 | 5.58 | 37.65 | 37.08 | 42.65 | 7.33 | 39.12667 |
| JAML | 3.906 | 1.965775 | 1.79E-05 | 0.000103 | yes | up | 0.47 | 0.48 | 0.45 | 4.59 | 3.25 | 4.32 | 0.466667 | 4.053333 |
| WDR44 | 0.435 | -1.20148 | 1.79E-05 | 0.000103 | yes | down | 5.15 | 3.58 | 3.43 | 4.15 | 5.34 | 2.69 | 4.053333 | 4.06 |
| LOC100344478 | 0.409 | -1.2887 | 1.81E-05 | 0.000104 | yes | down | 9.59 | 11.4 | 9.31 | 11.99 | 9.22 | 7.3 | 10.1 | 9.503333 |
| CDH20 | 3.331 | 1.736047 | 1.81E-05 | 0.000104 | yes | up | 0.29 | 0.3 | 0.16 | 1.8 | 2.2 | 1.64 | 0.25 | 1.88 |
| HOXA11 | 93.962 | 6.554003 | 1.81E-05 | 0.000104 | yes | up | 0 | 0 | 0 | 0.4 | 2.1 | 0.56 | 0 | 1.02 |
| NCF4 | 3.205 | 1.680295 | 1.82E-05 | 0.000105 | yes | up | 0.92 | 0.87 | 0.45 | 3.86 | 5.36 | 7.19 | 0.746667 | 5.47 |
| FAM98B | 0.474 | -1.07638 | 1.82E-05 | 0.000105 | yes | down | 16.07 | 17.01 | 12.58 | 15.21 | 21.77 | 11.89 | 15.22 | 16.29 |
| SPPL2A | 0.437 | -1.19533 | 1.82E-05 | 0.000105 | yes | down | 19.79 | 15.56 | 11.06 | 13.9 | 17.59 | 8 | 15.47 | 13.16333 |
| LMBR1 | 0.478 | -1.06366 | 1.84E-05 | 0.000106 | yes | down | 5.51 | 7.49 | 6.33 | 6.96 | 9.33 | 5.09 | 6.443333 | 7.126667 |
| GATA6 | 2.661 | 1.411958 | 1.84E-05 | 0.000106 | yes | up | 1.21 | 1.7 | 1.19 | 5.99 | 9.56 | 9.16 | 1.366667 | 8.236667 |
| SPAG5 | 4.914 | 2.296966 | 1.84E-05 | 0.000106 | yes | up | 0.79 | 0.68 | 0.31 | 7.41 | 10.73 | 1.89 | 0.593333 | 6.676667 |
| FAM167B | 2.801 | 1.485768 | 1.85E-05 | 0.000106 | yes | up | 1.37 | 2.16 | 1.3 | 12.13 | 11.25 | 7.55 | 1.61 | 10.31 |
| FBN1 | 2.265 | 1.179554 | 1.86E-05 | 0.000107 | yes | up | 38.71 | 27.84 | 17.28 | 129 | 190.99 | 108.7 | 27.94333 | 142.8967 |
| NUP35 | 0.444 | -1.17049 | 1.88E-05 | 0.000108 | yes | down | 7.83 | 12.17 | 7 | 9.95 | 9.03 | 8.21 | 9 | 9.063333 |
| LOC100345907 | 29.769 | 4.895752 | 1.9E-05 | 0.000109 | yes | up | 0 | 0.05 | 0.11 | 1 | 4.17 | 5.31 | 0.053333 | 3.493333 |
| C19H17orf58 | 4.046 | 2.016355 | 1.92E-05 | 0.00011 | yes | up | 1.98 | 1.17 | 3.19 | 14.93 | 20.45 | 25.29 | 2.113333 | 20.22333 |
| DCUN1D1 | 0.432 | -1.21176 | 1.92E-05 | 0.00011 | yes | down | 10.39 | 6.68 | 5.08 | 7.66 | 8.86 | 5.16 | 7.383333 | 7.226667 |
| LOC100351973 | 2.193 | 1.13289 | 1.92E-05 | 0.00011 | yes | up | 7.9 | 11.01 | 8.85 | 43.87 | 39.9 | 51.35 | 9.253333 | 45.04 |
| CHAC2 | 0.107 | -3.23034 | 1.93E-05 | 0.000111 | yes | down | 1.2 | 1.06 | 0.96 | 0.35 | 0.43 | 0 | 1.073333 | 0.26 |
| CNTLN | 0.42 | -1.25038 | 1.93E-05 | 0.000111 | yes | down | 1.42 | 1.13 | 1.45 | 1.37 | 1.51 | 1.03 | 1.333333 | 1.303333 |
| MTM1 | 0.457 | -1.12887 | 1.94E-05 | 0.000111 | yes | down | 3.95 | 3.65 | 3.44 | 4.52 | 4.46 | 2.75 | 3.68 | 3.91 |
| PTPRZ1 | 0.171 | -2.54454 | 1.94E-05 | 0.000111 | yes | down | 0.41 | 0.51 | 0.11 | 0.09 | 0.14 | 0.16 | 0.343333 | 0.13 |
| AKR1C5 | 36.185 | 5.17731 | 1.95E-05 | 0.000111 | yes | up | 0.23 | 0 | 0 | 13.46 | 1.97 | 1.67 | 0.076667 | 5.7 |
| MAGOH | 0.466 | -1.10215 | 1.95E-05 | 0.000111 | yes | down | 18.84 | 20.09 | 13.14 | 19.86 | 22.78 | 12.8 | 17.35667 | 18.48 |
| E2F2 | 4.186 | 2.065612 | 1.95E-05 | 0.000111 | yes | up | 0.22 | 0.29 | 0.16 | 2.25 | 3.19 | 1.05 | 0.223333 | 2.163333 |
| LOC100009136 | 0.021 | -5.58627 | 1.95E-05 | 0.000111 | yes | down | 1.91 | 13.37 | 2.29 | 0.73 | 0 | 0 | 5.856667 | 0.243333 |
| NTNG2 | 3.172 | 1.665386 | 1.96E-05 | 0.000112 | yes | up | 0.74 | 0.76 | 0.46 | 3.66 | 4.76 | 5.5 | 0.653333 | 4.64 |
| LOC100353330 | 0.347 | -1.52609 | 1.96E-05 | 0.000112 | yes | down | 48.51 | 56.83 | 52.92 | 19.19 | 66.64 | 40.11 | 52.75333 | 41.98 |
| LFNG | 2.006 | 1.004392 | 1.97E-05 | 0.000112 | yes | up | 4.28 | 4.81 | 3.59 | 20.44 | 18.56 | 18.8 | 4.226667 | 19.26667 |
| RASGEF1B | 2.155 | 1.107771 | 1.97E-05 | 0.000112 | yes | up | 5.42 | 7.5 | 4.63 | 30.31 | 23.68 | 30.94 | 5.85 | 28.31 |
| GUCY1A3 | 2.367 | 1.243274 | 1.97E-05 | 0.000112 | yes | up | 6.19 | 3.42 | 3.73 | 30.54 | 22.81 | 19.85 | 4.446667 | 24.4 |
| CDH11 | 2.239 | 1.162929 | 1.98E-05 | 0.000113 | yes | up | 3.47 | 2.86 | 2.13 | 14.75 | 19.61 | 9.57 | 2.82 | 14.64333 |
| FNDC5 | 0.09 | -3.4667 | 1.99E-05 | 0.000113 | yes | down | 55.24 | 10.26 | 7.65 | 4.48 | 4.56 | 4.38 | 24.38333 | 4.473333 |
| GPR61 | 0.266 | -1.91076 | 2E-05 | 0.000114 | yes | down | 0.96 | 0.54 | 0.74 | 0.36 | 0.48 | 0.54 | 0.746667 | 0.46 |
| LOC100351302 | 2.587 | 1.371023 | 2.01E-05 | 0.000114 | yes | up | 1.71 | 1.09 | 0.93 | 5.99 | 7.3 | 7.37 | 1.243333 | 6.886667 |
| PLXNB2 | 2.011 | 1.007826 | 2.03E-05 | 0.000116 | yes | up | 8.14 | 5.3 | 4.01 | 22.22 | 32.85 | 22.98 | 5.816667 | 26.01667 |
| YPEL4 | 0.226 | -2.14701 | 2.04E-05 | 0.000116 | yes | down | 1.03 | 2.03 | 2.16 | 0.84 | 0.64 | 1.26 | 1.74 | 0.913333 |
| LOC100350949 | 0.002 | -9.37884 | 2.05E-05 | 0.000116 | yes | down | 531.34 | 0 | 491.72 | 0.72 | 1.84 | 1.08 | 341.02 | 1.213333 |
| MLPH | 51.235 | 5.679064 | 2.06E-05 | 0.000117 | yes | up | 0 | 0.05 | 0.04 | 0.68 | 3.52 | 7 | 0.03 | 3.733333 |
| RAPH1 | 2.372 | 1.245814 | 2.08E-05 | 0.000118 | yes | up | 5.71 | 3.6 | 2.28 | 15.04 | 30.88 | 16.77 | 3.863333 | 20.89667 |
| FBXL19 | 2.201 | 1.138376 | 2.08E-05 | 0.000118 | yes | up | 0.94 | 1.07 | 0.96 | 4.58 | 5.76 | 4.61 | 0.99 | 4.983333 |
| ADAM19 | 2.39 | 1.25707 | 2.08E-05 | 0.000118 | yes | up | 3.97 | 3.22 | 1.78 | 14.93 | 17.25 | 10.01 | 2.99 | 14.06333 |
| ADORA2B | 3.284 | 1.715553 | 2.09E-05 | 0.000118 | yes | up | 0.46 | 0.76 | 0.57 | 4.78 | 4.21 | 4.51 | 0.596667 | 4.5 |
| HHIP | 5.773 | 2.52935 | 2.09E-05 | 0.000118 | yes | up | 0.2 | 0.17 | 0.02 | 1.6 | 3.65 | 1.48 | 0.13 | 2.243333 |
| TRPM7 | 0.386 | -1.37302 | 2.09E-05 | 0.000118 | yes | down | 5.76 | 5.24 | 5.73 | 5.47 | 7.34 | 2.68 | 5.576667 | 5.163333 |
| VASH1 | 2.538 | 1.343585 | 2.09E-05 | 0.000119 | yes | up | 8.24 | 5.58 | 4.17 | 31.46 | 53.08 | 21.42 | 5.996667 | 35.32 |
| GTPBP8 | 0.447 | -1.16326 | 2.11E-05 | 0.00012 | yes | down | 8.57 | 7.29 | 8.24 | 7.21 | 7.78 | 8.36 | 8.033333 | 7.783333 |
| TMEM201 | 0.496 | -1.01166 | 2.12E-05 | 0.00012 | yes | down | 4.03 | 3.24 | 3.36 | 3.34 | 3.89 | 4.3 | 3.543333 | 3.843333 |
| TECRL | 0.001 | -9.51366 | 2.15E-05 | 0.000121 | yes | down | 18.04 | 2.07 | 0.17 | 0.05 | 0 | 0 | 6.76 | 0.016667 |
| LONRF1 | 2.124 | 1.086454 | 2.15E-05 | 0.000121 | yes | up | 2.7 | 2.5 | 1.61 | 12.05 | 12.59 | 8.32 | 2.27 | 10.98667 |
| FAM64A | 5.708 | 2.512888 | 2.15E-05 | 0.000121 | yes | up | 0.95 | 1.36 | 0.31 | 13.43 | 16.89 | 3.71 | 0.873333 | 11.34333 |
| MYNN | 0.446 | -1.16568 | 2.17E-05 | 0.000123 | yes | down | 5.22 | 5.09 | 3.76 | 4.81 | 6.59 | 3.09 | 4.69 | 4.83 |
| LGALS12 | 2.732 | 1.449944 | 2.18E-05 | 0.000123 | yes | up | 10.45 | 21.77 | 10.3 | 45.95 | 96.49 | 78.23 | 14.17333 | 73.55667 |
| THUMPD1 | 0.432 | -1.2119 | 2.18E-05 | 0.000123 | yes | down | 6.23 | 6.24 | 5.47 | 5.43 | 8.82 | 3.29 | 5.98 | 5.846667 |
| GMFG | 3.058 | 1.612616 | 2.2E-05 | 0.000124 | yes | up | 5.19 | 5.09 | 3.82 | 43.94 | 31.92 | 22.07 | 4.7 | 32.64333 |
| APCDD1 | 3.639 | 1.863687 | 2.2E-05 | 0.000124 | yes | up | 3.33 | 1.47 | 4.15 | 33.55 | 16.88 | 25.01 | 2.983333 | 25.14667 |
| ARL5A | 0.475 | -1.07268 | 2.21E-05 | 0.000124 | yes | down | 11.87 | 9.92 | 9.17 | 13.43 | 14.15 | 7.18 | 10.32 | 11.58667 |
| FAM184A | 0.14 | -2.84082 | 2.22E-05 | 0.000125 | yes | down | 0.47 | 0.31 | 0.22 | 0.08 | 0.13 | 0.1 | 0.333333 | 0.103333 |
| PPP1R13L | 0.302 | -1.72626 | 2.23E-05 | 0.000125 | yes | down | 1.58 | 1.75 | 1.66 | 1.09 | 0.95 | 1.43 | 1.663333 | 1.156667 |
| LOC103348580 | 187.788 | 7.552965 | 2.23E-05 | 0.000125 | yes | up | 0.06 | 0.07 | 0 | 0.66 | 15.15 | 33.4 | 0.043333 | 16.40333 |
| EFCC1 | 2.473 | 1.306531 | 2.23E-05 | 0.000125 | yes | up | 1.1 | 0.98 | 0.95 | 4.49 | 6 | 7.91 | 1.01 | 6.133333 |
| FBXO32 | 0.261 | -1.93628 | 2.24E-05 | 0.000126 | yes | down | 10.96 | 5.59 | 18.4 | 4.7 | 11.49 | 6.15 | 11.65 | 7.446667 |
| CORO1C | 2.01 | 1.007021 | 2.24E-05 | 0.000126 | yes | up | 11.56 | 17.93 | 9.48 | 61.83 | 59.99 | 54.99 | 12.99 | 58.93667 |
| TAF1 | 0.454 | -1.13868 | 2.27E-05 | 0.000128 | yes | down | 4.04 | 4.73 | 3.93 | 3.79 | 6.72 | 3.1 | 4.233333 | 4.536667 |
| BTNL9 | 0.374 | -1.42037 | 2.3E-05 | 0.000129 | yes | down | 74.79 | 92.12 | 89.49 | 68.97 | 122.18 | 39.43 | 85.46667 | 76.86 |
| APOC3 | 80.645 | 6.333519 | 2.35E-05 | 0.000132 | yes | up | 0 | 0 | 0 | 5.88 | 6.37 | 1.98 | 0 | 4.743333 |
| CDCA8 | 5.047 | 2.335561 | 2.35E-05 | 0.000132 | yes | up | 1.36 | 1.12 | 0.32 | 13.84 | 14.04 | 3.88 | 0.933333 | 10.58667 |
| TOPORS | 0.483 | -1.05135 | 2.36E-05 | 0.000132 | yes | down | 9.45 | 8.83 | 11.94 | 24.98 | 17.85 | 27.77 | 10.07333 | 23.53333 |
| ELMOD2 | 0.436 | -1.19663 | 2.38E-05 | 0.000133 | yes | down | 3.63 | 3.38 | 3.09 | 3.84 | 4.34 | 2.06 | 3.366667 | 3.413333 |
| ZC2HC1A | 0.429 | -1.22036 | 2.38E-05 | 0.000133 | yes | down | 3.86 | 3.79 | 2.76 | 3.58 | 4.34 | 2.14 | 3.47 | 3.353333 |
| KCTD15 | 2.058 | 1.041474 | 2.41E-05 | 0.000135 | yes | up | 2.76 | 2.88 | 1.7 | 11.52 | 10.43 | 11.88 | 2.446667 | 11.27667 |
| TMEM117 | 2.781 | 1.475458 | 2.41E-05 | 0.000135 | yes | up | 1.73 | 1.36 | 0.69 | 8.5 | 6.61 | 8.14 | 1.26 | 7.75 |
| DENND2D | 0.371 | -1.42956 | 2.42E-05 | 0.000135 | yes | down | 2.78 | 2.69 | 3.66 | 3.09 | 4.75 | 5.22 | 3.043333 | 4.353333 |
| LOC103346967 | 138.855 | 7.117438 | 2.42E-05 | 0.000135 | yes | up | 0 | 0 | 0 | 1.9 | 2.19 | 0.04 | 0 | 1.376667 |
| TAF9B | 0.443 | -1.17546 | 2.42E-05 | 0.000135 | yes | down | 8.75 | 11.42 | 10.12 | 11.84 | 12.64 | 6.51 | 10.09667 | 10.33 |
| LOC103347895 | 5.706 | 2.512604 | 2.43E-05 | 0.000136 | yes | up | 0.12 | 0.09 | 0.04 | 0.87 | 0.77 | 1.44 | 0.083333 | 1.026667 |
| ARL4A | 0.46 | -1.12032 | 2.44E-05 | 0.000136 | yes | down | 11.65 | 14.79 | 11.65 | 15.07 | 18.84 | 9.7 | 12.69667 | 14.53667 |
| SMPD1 | 2.092 | 1.065214 | 2.44E-05 | 0.000136 | yes | up | 7.78 | 8.57 | 9.46 | 42.25 | 36.52 | 45.23 | 8.603333 | 41.33333 |
| GGPS1 | 0.473 | -1.08044 | 2.45E-05 | 0.000137 | yes | down | 8.62 | 8.73 | 7.88 | 9.25 | 11.71 | 6.66 | 8.41 | 9.206667 |
| LOC103345948 | 0.265 | -1.91378 | 2.49E-05 | 0.000139 | yes | down | 0.99 | 1.19 | 1.64 | 0.9 | 1.08 | 0.44 | 1.273333 | 0.806667 |
| SIGLEC15 | 0.103 | -3.2749 | 2.5E-05 | 0.000139 | yes | down | 0.64 | 0.36 | 0.54 | 0.08 | 0.02 | 0.35 | 0.513333 | 0.15 |
| AGPS | 0.498 | -1.00484 | 2.52E-05 | 0.000141 | yes | down | 9.6 | 6.03 | 9.84 | 9.69 | 16.23 | 9.2 | 8.49 | 11.70667 |
| NSRP1 | 0.436 | -1.19785 | 2.54E-05 | 0.000142 | yes | down | 5.74 | 4.69 | 5.32 | 5.59 | 6.8 | 3.58 | 5.25 | 5.323333 |
| CLIP3 | 2.839 | 1.505308 | 2.56E-05 | 0.000142 | yes | up | 3.78 | 3.23 | 3.02 | 13.81 | 13.36 | 19.74 | 3.343333 | 15.63667 |
| IDO1 | 0.238 | -2.06804 | 2.57E-05 | 0.000143 | yes | down | 1.24 | 2.89 | 1.99 | 0.91 | 1.04 | 1.69 | 2.04 | 1.213333 |
| SLC37A3 | 2.084 | 1.059034 | 2.57E-05 | 0.000143 | yes | up | 3.82 | 3.23 | 2.1 | 13.97 | 13.66 | 15.08 | 3.05 | 14.23667 |
| RPL34 | 29.803 | 4.897402 | 2.59E-05 | 0.000144 | yes | up | 0.93 | 0.94 | 0.16 | 3.12 | 220.81 | 245.64 | 0.676667 | 156.5233 |
| KCNK5 | 3.011 | 1.590039 | 2.6E-05 | 0.000144 | yes | up | 1.25 | 0.67 | 1.29 | 6.62 | 6.76 | 8.29 | 1.07 | 7.223333 |
| CYP2C30 | 1057.066 | 10.04585 | 2.65E-05 | 0.000147 | yes | up | 0 | 0 | 0 | 39.1 | 0.95 | 24.06 | 0 | 21.37 |
| KCNB2 | 15.321 | 3.93743 | 2.67E-05 | 0.000148 | yes | up | 0.04 | 0.05 | 0 | 1.26 | 0.49 | 1.36 | 0.03 | 1.036667 |
| DPH5 | 0.372 | -1.42663 | 2.67E-05 | 0.000148 | yes | down | 7 | 8.17 | 9.61 | 5.83 | 9.38 | 4.95 | 8.26 | 6.72 |
| GPR173 | 2.717 | 1.441842 | 2.67E-05 | 0.000148 | yes | up | 0.95 | 0.58 | 0.34 | 3.44 | 4.16 | 3.73 | 0.623333 | 3.776667 |
| TNKS2 | 0.488 | -1.03562 | 2.67E-05 | 0.000148 | yes | down | 20.03 | 13.35 | 10.84 | 15.46 | 21.19 | 12.9 | 14.74 | 16.51667 |
| ZKSCAN8 | 0.423 | -1.24034 | 2.69E-05 | 0.000149 | yes | down | 3.39 | 3.43 | 2.7 | 2.76 | 4.68 | 1.94 | 3.173333 | 3.126667 |
| RASA2 | 0.413 | -1.27699 | 2.7E-05 | 0.000149 | yes | down | 3.14 | 2.97 | 1.99 | 2.73 | 3.49 | 1.47 | 2.7 | 2.563333 |
| COL23A1 | 3.058 | 1.61242 | 2.7E-05 | 0.00015 | yes | up | 0.69 | 0.65 | 0.53 | 3.77 | 3.43 | 5.71 | 0.623333 | 4.303333 |
| LOC103351328 | 0.119 | -3.06549 | 2.72E-05 | 0.000151 | yes | down | 1.56 | 1 | 1.89 | 0.34 | 0.74 | 0.19 | 1.483333 | 0.423333 |
| CARMIL3 | 17.069 | 4.093305 | 2.73E-05 | 0.000151 | yes | up | 0.01 | 0.03 | 0.01 | 0.42 | 1.07 | 0.57 | 0.016667 | 0.686667 |
| LOC108177695 | 0.455 | -1.13639 | 2.73E-05 | 0.000151 | yes | down | 12.67 | 16.3 | 12.8 | 16.32 | 24 | 9.94 | 13.92333 | 16.75333 |
| HSPG2 | 2.28 | 1.189159 | 2.77E-05 | 0.000153 | yes | up | 69.59 | 75.85 | 77.89 | 366.74 | 586.45 | 371.17 | 74.44333 | 441.4533 |
| SPRYD7 | 0.486 | -1.04078 | 2.78E-05 | 0.000153 | yes | down | 4.29 | 4.9 | 3.68 | 5.79 | 4.49 | 3.92 | 4.29 | 4.733333 |
| GRAMD1B | 0.361 | -1.47157 | 2.79E-05 | 0.000154 | yes | down | 2.1 | 1.32 | 1.89 | 1.46 | 1.16 | 1.75 | 1.77 | 1.456667 |
| LOC108178691 | 0.039 | -4.68695 | 2.8E-05 | 0.000154 | yes | down | 0.3 | 0.06 | 0.34 | 0 | 0.05 | 0.02 | 0.233333 | 0.023333 |
| LOC100355990 | 3.021 | 1.594925 | 2.81E-05 | 0.000155 | yes | up | 0.8 | 1.14 | 0.98 | 6.05 | 8.61 | 5.19 | 0.973333 | 6.616667 |
| BCL6B | 2.227 | 1.155412 | 2.81E-05 | 0.000155 | yes | up | 5.6 | 7.89 | 7.84 | 39.24 | 46.35 | 25.92 | 7.11 | 37.17 |
| YBX2 | 0.331 | -1.59492 | 2.81E-05 | 0.000155 | yes | down | 7.76 | 6.5 | 8.33 | 2.37 | 7.31 | 6.3 | 7.53 | 5.326667 |
| RLF | 0.451 | -1.14819 | 2.83E-05 | 0.000156 | yes | down | 3.35 | 3.57 | 2.92 | 3.29 | 4.88 | 2.16 | 3.28 | 3.443333 |
| CCDC68 | 0.169 | -2.56594 | 2.86E-05 | 0.000157 | yes | down | 1.86 | 1.1 | 2.59 | 0.39 | 1.41 | 0.48 | 1.85 | 0.76 |
| CDKN2C | 2.292 | 1.196463 | 2.91E-05 | 0.00016 | yes | up | 13.67 | 10.3 | 14.63 | 68.02 | 73.94 | 37.01 | 12.86667 | 59.65667 |
| IL18BP | 3.226 | 1.689856 | 2.91E-05 | 0.00016 | yes | up | 1.35 | 1.42 | 1.45 | 11.56 | 7.69 | 9.99 | 1.406667 | 9.746667 |
| C19H17orf53 | 0.327 | -1.61384 | 2.92E-05 | 0.00016 | yes | down | 2.71 | 3.13 | 2.49 | 2.19 | 3.18 | 0.98 | 2.776667 | 2.116667 |
| KIF20B | 0.069 | -3.85935 | 2.94E-05 | 0.000161 | yes | down | 10.71 | 11.86 | 29.26 | 2.98 | 2.79 | 0.38 | 17.27667 | 2.05 |
| FSIP1 | 70.882 | 6.147342 | 2.98E-05 | 0.000163 | yes | up | 0 | 0 | 0 | 0.86 | 0.79 | 0.54 | 0 | 0.73 |
| ANG | 4.344 | 2.119099 | 3.01E-05 | 0.000165 | yes | up | 2.77 | 4.79 | 3.2 | 27.36 | 18.67 | 58.08 | 3.586667 | 34.70333 |
| FAM208B | 0.417 | -1.26226 | 3.02E-05 | 0.000165 | yes | down | 6.09 | 6.45 | 4.85 | 5.71 | 7.7 | 2.9 | 5.796667 | 5.436667 |
| TRDMT1 | 0.44 | -1.18279 | 3.03E-05 | 0.000165 | yes | down | 2.74 | 4.1 | 3.38 | 4.3 | 3.95 | 5.31 | 3.406667 | 4.52 |
| BZW2 | 0.364 | -1.45839 | 3.06E-05 | 0.000167 | yes | down | 72.55 | 36.8 | 19.01 | 40.2 | 35.8 | 34.88 | 42.78667 | 36.96 |
| RBM27 | 0.481 | -1.05644 | 3.07E-05 | 0.000168 | yes | down | 2.41 | 2.45 | 2.17 | 2.8 | 3.3 | 1.76 | 2.343333 | 2.62 |
| EGR3 | 8.235 | 3.041811 | 3.08E-05 | 0.000168 | yes | up | 0.15 | 0.04 | 0.03 | 1.77 | 0.76 | 1.38 | 0.073333 | 1.303333 |
| LHX2 | 51.949 | 5.699019 | 3.08E-05 | 0.000168 | yes | up | 0 | 0.03 | 0.03 | 4.3 | 0.35 | 3.32 | 0.02 | 2.656667 |
| LOC108178624 | 0.37 | -1.43343 | 3.1E-05 | 0.000169 | yes | down | 11.24 | 15.38 | 11.26 | 15.24 | 15.92 | 10.77 | 12.62667 | 13.97667 |
| TMEM14A | 2.7 | 1.432881 | 3.11E-05 | 0.000169 | yes | up | 4.89 | 7.93 | 6.5 | 37.06 | 28.09 | 32.4 | 6.44 | 32.51667 |
| CTSD | 0.476 | -1.06965 | 3.12E-05 | 0.00017 | yes | down | 372.45 | 573.45 | 437.87 | 399.14 | 510.6 | 596.7 | 461.2567 | 502.1467 |
| LOC103352014 | 4.535 | 2.180984 | 3.13E-05 | 0.00017 | yes | up | 0.37 | 0.63 | 0.67 | 7.2 | 3.73 | 6.26 | 0.556667 | 5.73 |
| TMEM241 | 2.181 | 1.125157 | 3.15E-05 | 0.000171 | yes | up | 1.55 | 1.57 | 1.48 | 6.88 | 7.62 | 5.19 | 1.533333 | 6.563333 |
| FITM2 | 2.229 | 1.156314 | 3.15E-05 | 0.000172 | yes | up | 28.36 | 38.99 | 39.86 | 130.76 | 248.9 | 181.45 | 35.73667 | 187.0367 |
| SMPD3 | 61.975 | 5.953624 | 3.16E-05 | 0.000172 | yes | up | 0.08 | 0.04 | 0.01 | 1.57 | 0.95 | 16.47 | 0.043333 | 6.33 |
| SPOPL | 0.396 | -1.33668 | 3.17E-05 | 0.000172 | yes | down | 5.97 | 5.97 | 5.59 | 6.49 | 7.06 | 2.7 | 5.843333 | 5.416667 |
| RHOB | 2.152 | 1.10594 | 3.17E-05 | 0.000172 | yes | up | 57.12 | 75.84 | 46.72 | 249.08 | 249.21 | 367.5 | 59.89333 | 288.5967 |
| SPAG4 | 0.205 | -2.28595 | 3.18E-05 | 0.000173 | yes | down | 1.15 | 1.6 | 1.95 | 0.68 | 0.92 | 0.66 | 1.566667 | 0.753333 |
| RPE | 0.463 | -1.11213 | 3.18E-05 | 0.000173 | yes | down | 7.28 | 9.18 | 7.89 | 10.17 | 9.8 | 6.01 | 8.116667 | 8.66 |
| SERPIND1 | 15.601 | 3.963602 | 3.19E-05 | 0.000173 | yes | up | 0.03 | 0.03 | 0.05 | 0.95 | 1.5 | 1.32 | 0.036667 | 1.256667 |
| RASSF8 | 0.488 | -1.03415 | 3.19E-05 | 0.000173 | yes | down | 8.59 | 8.4 | 9.73 | 8.42 | 13.92 | 8.32 | 8.906667 | 10.22 |
| ZNF451 | 0.462 | -1.11465 | 3.19E-05 | 0.000173 | yes | down | 3.97 | 3.61 | 3.31 | 4.22 | 4.64 | 2.44 | 3.63 | 3.766667 |
| COQ2 | 0.479 | -1.06062 | 3.2E-05 | 0.000173 | yes | down | 13.92 | 9.67 | 9.89 | 11.94 | 11.76 | 13.2 | 11.16 | 12.3 |
| LOC103352092 | 3.755 | 1.908903 | 3.21E-05 | 0.000174 | yes | up | 0.32 | 0.16 | 0.25 | 2.09 | 2.92 | 1.33 | 0.243333 | 2.113333 |
| CA1 | 0.173 | -2.53299 | 3.21E-05 | 0.000174 | yes | down | 3.71 | 7.57 | 5.21 | 2.31 | 0.66 | 3.48 | 5.496667 | 2.15 |
| LOC100009451 | 4.859 | 2.28079 | 3.26E-05 | 0.000177 | yes | up | 1.03 | 0.55 | 0.26 | 3.6 | 7.57 | 8.59 | 0.613333 | 6.586667 |
| TYW3 | 0.354 | -1.4985 | 3.27E-05 | 0.000177 | yes | down | 3.53 | 3.96 | 4.62 | 3.32 | 3.84 | 1.83 | 4.036667 | 2.996667 |
| LOC100340939 | 5.081 | 2.34502 | 3.3E-05 | 0.000178 | yes | up | 1.41 | 1.52 | 0.44 | 14.62 | 20.34 | 4.13 | 1.123333 | 13.03 |
| CCDC8 | 4.138 | 2.048776 | 3.3E-05 | 0.000178 | yes | up | 1.62 | 0.64 | 0.28 | 7.92 | 7.17 | 7.78 | 0.846667 | 7.623333 |
| ST8SIA4 | 0.431 | -1.21588 | 3.3E-05 | 0.000178 | yes | down | 5.25 | 6.4 | 4.29 | 4.99 | 7.77 | 3.16 | 5.313333 | 5.306667 |
| BID | 2.96 | 1.565626 | 3.31E-05 | 0.000179 | yes | up | 1 | 0.85 | 0.86 | 5.21 | 4.34 | 7.23 | 0.903333 | 5.593333 |
| LOC100357259 | 0.437 | -1.19326 | 3.37E-05 | 0.000182 | yes | down | 1.82 | 1.91 | 1.31 | 2.07 | 1.8 | 1.14 | 1.68 | 1.67 |
| UFL1 | 0.391 | -1.35625 | 3.37E-05 | 0.000182 | yes | down | 5.41 | 6.04 | 4.95 | 5.3 | 7.04 | 2.48 | 5.466667 | 4.94 |
| ARRDC1 | 2.049 | 1.035178 | 3.38E-05 | 0.000183 | yes | up | 2.82 | 3.31 | 3.89 | 17.28 | 19.13 | 13.82 | 3.34 | 16.74333 |
| FAM49B | 2.069 | 1.048747 | 3.4E-05 | 0.000183 | yes | up | 7.22 | 6.06 | 3.77 | 30.4 | 24.83 | 21.67 | 5.683333 | 25.63333 |
| IL17RD | 4.346 | 2.119847 | 3.4E-05 | 0.000183 | yes | up | 0.72 | 0.23 | 0.13 | 3.41 | 4.51 | 2.52 | 0.36 | 3.48 |
| RASGRP3 | 0.472 | -1.08263 | 3.42E-05 | 0.000184 | yes | down | 8.39 | 6.64 | 9.33 | 8.42 | 11.21 | 9.41 | 8.12 | 9.68 |
| ATG4C | 0.477 | -1.06928 | 3.48E-05 | 0.000187 | yes | down | 2.23 | 2.06 | 1.81 | 2.13 | 2.83 | 1.71 | 2.033333 | 2.223333 |
| PRAF2 | 2.154 | 1.106783 | 3.54E-05 | 0.00019 | yes | up | 5 | 6.56 | 6.33 | 34.18 | 27.01 | 27.34 | 5.963333 | 29.51 |
| RSF1 | 0.454 | -1.13965 | 3.61E-05 | 0.000194 | yes | down | 2.58 | 2.59 | 2.33 | 2.51 | 3.82 | 1.63 | 2.5 | 2.653333 |
| SUSD4 | 5.886 | 2.557217 | 3.65E-05 | 0.000196 | yes | up | 0.06 | 0.13 | 0.11 | 1.09 | 1.41 | 1.54 | 0.1 | 1.346667 |
| ACP5 | 4.907 | 2.294849 | 3.66E-05 | 0.000197 | yes | up | 1.64 | 1.18 | 1.55 | 17.8 | 10.19 | 20.55 | 1.456667 | 16.18 |
| PLOD1 | 2.176 | 1.121909 | 3.66E-05 | 0.000197 | yes | up | 15.44 | 23.19 | 16.73 | 116.27 | 98.31 | 62.69 | 18.45333 | 92.42333 |
| LOC108177032 | 2.852 | 1.511994 | 3.68E-05 | 0.000197 | yes | up | 12.58 | 10.95 | 10.86 | 82.99 | 86.75 | 64.88 | 11.46333 | 78.20667 |
| SCN7A | 0.308 | -1.69691 | 3.7E-05 | 0.000198 | yes | down | 1.94 | 2.87 | 0.9 | 0.77 | 1.85 | 1.35 | 1.903333 | 1.323333 |
| FMO5 | 0.344 | -1.53804 | 3.7E-05 | 0.000198 | yes | down | 2.23 | 1.55 | 2.34 | 1.82 | 2.35 | 1.19 | 2.04 | 1.786667 |
| SLC25A16 | 0.446 | -1.16516 | 3.71E-05 | 0.000198 | yes | down | 4.7 | 5.03 | 6.61 | 8.24 | 8.57 | 11.73 | 5.446667 | 9.513333 |
| ZNF26 | 0.449 | -1.15412 | 3.72E-05 | 0.000199 | yes | down | 2.54 | 2.09 | 1.66 | 2.12 | 2.6 | 1.69 | 2.096667 | 2.136667 |
| GLIPR2 | 2.599 | 1.377788 | 3.79E-05 | 0.000203 | yes | up | 11.37 | 5.86 | 3.95 | 42.93 | 38.06 | 40.93 | 7.06 | 40.64 |
| PRR11 | 5.747 | 2.522934 | 3.86E-05 | 0.000206 | yes | up | 0.2 | 0.29 | 0.08 | 3.22 | 3.45 | 0.76 | 0.19 | 2.476667 |
| LOC100340198 | 0.449 | -1.15593 | 3.89E-05 | 0.000208 | yes | down | 120.61 | 175.85 | 167.77 | 177.4 | 155.74 | 155.56 | 154.7433 | 162.9 |
| RHOV | 12.566 | 3.651476 | 3.9E-05 | 0.000208 | yes | up | 0.04 | 0.21 | 0.07 | 1.59 | 1.66 | 5.63 | 0.106667 | 2.96 |
| LOC103349960 | 50.125 | 5.647454 | 3.91E-05 | 0.000209 | yes | up | 0 | 0.05 | 0 | 1.74 | 2.26 | 1.77 | 0.016667 | 1.923333 |
| BTG1 | 2.22 | 1.150414 | 3.92E-05 | 0.000209 | yes | up | 46.34 | 84.46 | 78.13 | 366.89 | 384.05 | 328.32 | 69.64333 | 359.7533 |
| NIPBL | 0.419 | -1.25578 | 3.92E-05 | 0.000209 | yes | down | 4.34 | 3.86 | 4.72 | 4.32 | 6.01 | 2.45 | 4.306667 | 4.26 |
| GRM8 | 0.114 | -3.13748 | 3.93E-05 | 0.000209 | yes | down | 0.29 | 0.25 | 0.36 | 0.1 | 0.06 | 0.07 | 0.3 | 0.076667 |
| GLMN | 0.275 | -1.86455 | 3.94E-05 | 0.00021 | yes | down | 2.15 | 2.05 | 1.2 | 1.11 | 1.58 | 0.57 | 1.8 | 1.086667 |
| LOC108177714 | 0.325 | -1.62285 | 3.94E-05 | 0.00021 | yes | down | 10.43 | 21.41 | 10.34 | 11.07 | 23.66 | 8.65 | 14.06 | 14.46 |
| VOPP1 | 2.281 | 1.189675 | 3.94E-05 | 0.00021 | yes | up | 3.87 | 2.42 | 1.74 | 13.19 | 13.53 | 14.24 | 2.676667 | 13.65333 |
| SMC5 | 0.458 | -1.12774 | 3.97E-05 | 0.000211 | yes | down | 4.43 | 4.03 | 3.4 | 3.55 | 5.93 | 2.67 | 3.953333 | 4.05 |
| HOPX | 3.487 | 1.802057 | 3.98E-05 | 0.000212 | yes | up | 1.86 | 0.74 | 0.86 | 8.63 | 11.12 | 7.55 | 1.153333 | 9.1 |
| CCL11 | 9.286 | 3.214998 | 4.02E-05 | 0.000214 | yes | up | 0.15 | 0.18 | 0.04 | 2.98 | 2.07 | 2.63 | 0.123333 | 2.56 |
| LOC100355134 | 0.391 | -1.35568 | 4.06E-05 | 0.000216 | yes | down | 5.36 | 4.07 | 2.44 | 3.96 | 3.33 | 3.07 | 3.956667 | 3.453333 |
| SORBS2 | 2.306 | 1.205179 | 4.07E-05 | 0.000216 | yes | up | 3.2 | 2.95 | 3.81 | 16.06 | 15.48 | 22.2 | 3.32 | 17.91333 |
| NFKBIE | 2.87 | 1.521274 | 4.08E-05 | 0.000217 | yes | up | 0.95 | 1.77 | 1.14 | 10.12 | 7.32 | 7.69 | 1.286667 | 8.376667 |
| ZNF268 | 0.462 | -1.1152 | 4.08E-05 | 0.000217 | yes | down | 2.75 | 3.93 | 2.07 | 2.77 | 3.05 | 3.3 | 2.916667 | 3.04 |
| NAP1L1 | 0.491 | -1.02708 | 4.08E-05 | 0.000217 | yes | down | 64.4 | 58.41 | 48.18 | 82.37 | 79.7 | 43.93 | 56.99667 | 68.66667 |
| LOC103348079 | 2.214 | 1.146451 | 4.19E-05 | 0.000222 | yes | up | 1.29 | 1.22 | 1 | 4.24 | 7.31 | 6.21 | 1.17 | 5.92 |
| KNSTRN | 3.624 | 1.857568 | 4.27E-05 | 0.000226 | yes | up | 2.31 | 2.12 | 1.22 | 19.32 | 20.9 | 6.05 | 1.883333 | 15.42333 |
| LOC100351040 | 0.008 | -7.01974 | 4.32E-05 | 0.000229 | yes | down | 0.1 | 0.64 | 0.05 | 0 | 0 | 0 | 0.263333 | 0 |
| R3HCC1L | 0.488 | -1.03429 | 4.32E-05 | 0.000229 | yes | down | 2.87 | 3.71 | 3.34 | 3.37 | 4.56 | 3.22 | 3.306667 | 3.716667 |
| SATB2 | 2.875 | 1.523663 | 4.35E-05 | 0.00023 | yes | up | 0.4 | 0.45 | 0.34 | 2.03 | 3.9 | 2.47 | 0.396667 | 2.8 |
| NPR1 | 2.041 | 1.02909 | 4.36E-05 | 0.000231 | yes | up | 22.56 | 28.78 | 25.43 | 98.71 | 174.58 | 95.02 | 25.59 | 122.77 |
| LOC100358489 | 73.557 | 6.200795 | 4.39E-05 | 0.000232 | yes | up | 0 | 0 | 0 | 1.44 | 1.06 | 0.46 | 0 | 0.986667 |
| CD36 | 0.38 | -1.39529 | 4.45E-05 | 0.000235 | yes | down | 945.54 | 1323.53 | 1375.83 | 1117.59 | 1669.09 | 572.67 | 1214.967 | 1119.783 |
| LOC108178465 | 0.265 | -1.91606 | 4.45E-05 | 0.000235 | yes | down | 23.45 | 11.74 | 10.98 | 6.92 | 17.46 | 4.18 | 15.39 | 9.52 |
| TRNAK-CUU | 0.038 | -4.72004 | 4.47E-05 | 0.000236 | yes | down | 1.49 | 2.29 | 2.3 | 0.31 | 0 | 0.22 | 2.026667 | 0.176667 |
| SEMA4B | 2.169 | 1.117311 | 4.49E-05 | 0.000237 | yes | up | 3.11 | 2.39 | 3.5 | 12.42 | 17.73 | 15.46 | 3 | 15.20333 |
| MDM1 | 0.353 | -1.50163 | 4.5E-05 | 0.000237 | yes | down | 2.25 | 3.38 | 1.83 | 2.09 | 2.8 | 1.16 | 2.486667 | 2.016667 |
| NKAIN1 | 6.707 | 2.745721 | 4.5E-05 | 0.000238 | yes | up | 0.18 | 0.12 | 0.04 | 1.95 | 1.26 | 1.75 | 0.113333 | 1.653333 |
| ADAMTS9 | 2.03 | 1.021401 | 4.57E-05 | 0.000241 | yes | up | 3.73 | 4.25 | 3.09 | 16.4 | 24.65 | 11.42 | 3.69 | 17.49 |
| FHL1 | 0.356 | -1.49065 | 4.57E-05 | 0.000241 | yes | down | 1585.86 | 934.61 | 506.07 | 655.3 | 621.15 | 1067.58 | 1008.847 | 781.3433 |
| ARHGEF28 | 0.37 | -1.43619 | 4.58E-05 | 0.000241 | yes | down | 2.94 | 4.75 | 5.08 | 2.93 | 5.47 | 2.53 | 4.256667 | 3.643333 |
| ZNF644 | 0.446 | -1.16349 | 4.64E-05 | 0.000244 | yes | down | 4.66 | 3.76 | 4.14 | 6.1 | 4.87 | 2.63 | 4.186667 | 4.533333 |
| LOC100351380 | 0.487 | -1.03882 | 4.66E-05 | 0.000245 | yes | down | 2.43 | 1.84 | 1.43 | 2.03 | 2.91 | 2 | 1.9 | 2.313333 |
| PARP4 | 2.138 | 1.09635 | 4.68E-05 | 0.000246 | yes | up | 3.35 | 1.81 | 1.74 | 10.75 | 11.56 | 10.82 | 2.3 | 11.04333 |
| LOC103348533 | 13.003 | 3.700767 | 4.71E-05 | 0.000247 | yes | up | 0 | 0.44 | 0.27 | 6.97 | 7.6 | 7.11 | 0.236667 | 7.226667 |
| SRSF5 | 0.405 | -1.30328 | 4.8E-05 | 0.000252 | yes | down | 41.12 | 54.55 | 37.64 | 36.25 | 50.95 | 19.4 | 44.43667 | 35.53333 |
| SLC41A3 | 4.246 | 2.086157 | 4.83E-05 | 0.000254 | yes | up | 1.88 | 0.59 | 0.44 | 10.5 | 12.22 | 5.22 | 0.97 | 9.313333 |
| CD1E | 67.421 | 6.075133 | 4.85E-05 | 0.000254 | yes | up | 0 | 0 | 0 | 0.71 | 1.16 | 1.29 | 0 | 1.053333 |
| EFHD2 | 2.044 | 1.031077 | 4.85E-05 | 0.000254 | yes | up | 11.65 | 8.24 | 10.22 | 56.44 | 44.5 | 40.19 | 10.03667 | 47.04333 |
| PIK3CA | 0.438 | -1.19141 | 4.87E-05 | 0.000255 | yes | down | 4.56 | 3.15 | 4.25 | 4.35 | 5.34 | 2.57 | 3.986667 | 4.086667 |
| AAED1 | 0.473 | -1.07925 | 4.92E-05 | 0.000258 | yes | down | 8.82 | 13.73 | 8.85 | 11.92 | 14.47 | 8.46 | 10.46667 | 11.61667 |
| TMED5 | 0.391 | -1.35604 | 4.93E-05 | 0.000258 | yes | down | 18.03 | 24.34 | 16.63 | 22.19 | 22.12 | 8.4 | 19.66667 | 17.57 |
| LCP1 | 2.087 | 1.061226 | 4.94E-05 | 0.000258 | yes | up | 2.02 | 2.12 | 1.17 | 6.81 | 9.45 | 8.76 | 1.77 | 8.34 |
| RASD2 | 3.829 | 1.936804 | 4.96E-05 | 0.000259 | yes | up | 1.07 | 0.59 | 0.88 | 9.6 | 3.63 | 8.52 | 0.846667 | 7.25 |
| LOC100338099 | 0.396 | -1.33602 | 4.97E-05 | 0.00026 | yes | down | 3.4 | 3.84 | 2.61 | 3.6 | 3.73 | 1.68 | 3.283333 | 3.003333 |
| POT1 | 0.459 | -1.12216 | 4.97E-05 | 0.00026 | yes | down | 4.79 | 4.63 | 4.87 | 5 | 6.72 | 3.38 | 4.763333 | 5.033333 |
| CSF2RB | 2.732 | 1.449694 | 5.02E-05 | 0.000262 | yes | up | 0.44 | 0.85 | 0.72 | 3.66 | 5.39 | 3.62 | 0.67 | 4.223333 |
| LYRM7 | 0.36 | -1.47374 | 5.04E-05 | 0.000263 | yes | down | 11.81 | 9.06 | 5.3 | 7.27 | 6.36 | 5.04 | 8.723333 | 6.223333 |
| SSTR4 | 0.024 | -5.36579 | 5.08E-05 | 0.000265 | yes | down | 0.25 | 0.44 | 0.28 | 0.05 | 0 | 0 | 0.323333 | 0.016667 |
| P2RY14 | 0.367 | -1.44707 | 5.11E-05 | 0.000266 | yes | down | 2.53 | 2.69 | 3.62 | 2.04 | 3.71 | 1.93 | 2.946667 | 2.56 |
| SPACA9 | 0.128 | -2.96417 | 5.15E-05 | 0.000268 | yes | down | 0.69 | 1.23 | 1.35 | 0.19 | 0.27 | 0.27 | 1.09 | 0.243333 |
| LOC100344587 | 0.16 | -2.64002 | 5.16E-05 | 0.000269 | yes | down | 0.61 | 1.6 | 1.74 | 0.55 | 0.55 | 0.37 | 1.316667 | 0.49 |
| SLC2A9 | 50.617 | 5.661538 | 5.23E-05 | 0.000272 | yes | up | 0.04 | 0 | 0 | 1.08 | 0.95 | 1.78 | 0.013333 | 1.27 |
| NALCN | 34.473 | 5.107403 | 5.25E-05 | 0.000273 | yes | up | 0.02 | 0 | 0 | 0.25 | 0.76 | 0.42 | 0.006667 | 0.476667 |
| SAPCD2 | 4.082 | 2.029109 | 5.31E-05 | 0.000276 | yes | up | 0.69 | 0.9 | 0.51 | 7.78 | 9.77 | 2.26 | 0.7 | 6.603333 |
| RAD54L | 3.589 | 1.843751 | 5.4E-05 | 0.000281 | yes | up | 0.45 | 0.37 | 0.35 | 3.1 | 4.6 | 1.78 | 0.39 | 3.16 |
| DYRK3 | 3.886 | 1.958311 | 5.41E-05 | 0.000281 | yes | up | 0.36 | 0.47 | 0.45 | 4.22 | 2.44 | 4.58 | 0.426667 | 3.746667 |
| ERP27 | 14.439 | 3.851912 | 5.46E-05 | 0.000283 | yes | up | 0.04 | 0 | 0.02 | 0.29 | 1.08 | 0.35 | 0.02 | 0.573333 |
| PHGDH | 2.048 | 1.033879 | 5.51E-05 | 0.000286 | yes | up | 32.74 | 56.37 | 41.08 | 204.15 | 259.25 | 158.92 | 43.39667 | 207.44 |
| NECTIN1 | 2.556 | 1.353831 | 5.52E-05 | 0.000286 | yes | up | 0.55 | 0.44 | 0.38 | 2.68 | 2.2 | 2.97 | 0.456667 | 2.616667 |
| GAS1 | 2.606 | 1.382038 | 5.53E-05 | 0.000286 | yes | up | 10.1 | 6.3 | 6 | 34.04 | 33.81 | 62.19 | 7.466667 | 43.34667 |
| TMEM38B | 0.27 | -1.89062 | 5.53E-05 | 0.000286 | yes | down | 10.58 | 4.77 | 2.91 | 3.31 | 2.79 | 4.68 | 6.086667 | 3.593333 |
| LOC100349270 | 64.148 | 6.003336 | 5.55E-05 | 0.000287 | yes | up | 0 | 0 | 0 | 0.29 | 0.39 | 0.31 | 0 | 0.33 |
| DOK5 | 0.15 | -2.73742 | 5.58E-05 | 0.000288 | yes | down | 18.24 | 3.67 | 2.94 | 2.1 | 1.26 | 4.29 | 8.283333 | 2.55 |
| N4BP3 | 2.076 | 1.053943 | 5.59E-05 | 0.000289 | yes | up | 5.17 | 6.26 | 6.98 | 25.21 | 37.42 | 27.63 | 6.136667 | 30.08667 |
| OAS2 | 7.404 | 2.888209 | 5.59E-05 | 0.000289 | yes | up | 0.53 | 0.13 | 0.05 | 3.32 | 1.92 | 5.76 | 0.236667 | 3.666667 |
| LRRK2 | 2.078 | 1.054957 | 5.6E-05 | 0.000289 | yes | up | 1.26 | 1.1 | 1.03 | 5.29 | 7.19 | 3.56 | 1.13 | 5.346667 |
| TBC1D32 | 0.334 | -1.581 | 5.6E-05 | 0.000289 | yes | down | 0.69 | 0.95 | 0.53 | 0.41 | 0.76 | 0.66 | 0.723333 | 0.61 |
| FAT3 | 14.01 | 3.80836 | 5.62E-05 | 0.00029 | yes | up | 0.01 | 0 | 0 | 39.76 | 0.2 | 0.1 | 0.003333 | 13.35333 |
| TMEM110 | 2.055 | 1.039094 | 5.63E-05 | 0.00029 | yes | up | 2.5 | 2.34 | 1.63 | 9.88 | 10.25 | 10.75 | 2.156667 | 10.29333 |
| MYH3 | 0.006 | -7.28729 | 5.66E-05 | 0.000292 | yes | down | 86.32 | 12.24 | 0.45 | 0.31 | 0.02 | 0.94 | 33.00333 | 0.423333 |
| PVR | 2.683 | 1.424065 | 5.72E-05 | 0.000295 | yes | up | 1.41 | 1.82 | 1.87 | 9.22 | 10.96 | 11.52 | 1.7 | 10.56667 |
| ADAL | 0.359 | -1.47872 | 5.76E-05 | 0.000297 | yes | down | 2.08 | 1.92 | 2 | 1.76 | 1.81 | 1.59 | 2 | 1.72 |
| CNTFR | 3.833 | 1.938631 | 5.76E-05 | 0.000297 | yes | up | 2.64 | 2.62 | 0.9 | 8.26 | 30.91 | 14.3 | 2.053333 | 17.82333 |
| S1PR3 | 0.364 | -1.45689 | 5.87E-05 | 0.000302 | yes | down | 4.76 | 7.54 | 6.54 | 5.17 | 8.29 | 2.8 | 6.28 | 5.42 |
| GPR21 | 4.507 | 2.172301 | 5.91E-05 | 0.000304 | yes | up | 0.36 | 0.22 | 0.19 | 2.87 | 1.52 | 3.33 | 0.256667 | 2.573333 |
| LOC100342167 | 2.027 | 1.019145 | 5.92E-05 | 0.000304 | yes | up | 3.75 | 4.3 | 2.81 | 11.42 | 17.64 | 16.89 | 3.62 | 15.31667 |
| ANKRD55 | 2.949 | 1.56024 | 5.93E-05 | 0.000305 | yes | up | 0.42 | 0.59 | 0.62 | 4.3 | 3.99 | 2.92 | 0.543333 | 3.736667 |
| CLPP | 0.477 | -1.06858 | 6E-05 | 0.000308 | yes | down | 15.47 | 21.49 | 18.95 | 19.27 | 20.9 | 19.42 | 18.63667 | 19.86333 |
| PCM1 | 0.457 | -1.12855 | 6E-05 | 0.000308 | yes | down | 7.55 | 4.95 | 4.47 | 5.9 | 8.33 | 3.76 | 5.656667 | 5.996667 |
| FAM117B | 0.401 | -1.31782 | 6E-05 | 0.000308 | yes | down | 2.54 | 3.27 | 3.99 | 3.56 | 3.39 | 2.25 | 3.266667 | 3.066667 |
| COQ10A | 0.33 | -1.60131 | 6.04E-05 | 0.00031 | yes | down | 20.64 | 8.46 | 6.37 | 6.38 | 8.61 | 9.23 | 11.82333 | 8.073333 |
| RCOR2 | 0.309 | -1.6942 | 6.11E-05 | 0.000314 | yes | down | 7.82 | 6.96 | 2.27 | 3.85 | 4.56 | 3.28 | 5.683333 | 3.896667 |
| TUBB6 | 2.603 | 1.379898 | 6.14E-05 | 0.000315 | yes | up | 25.08 | 15.52 | 7.37 | 107.9 | 102.34 | 69.37 | 15.99 | 93.20333 |
| CD302 | 0.467 | -1.09807 | 6.2E-05 | 0.000318 | yes | down | 32.26 | 40.74 | 35.08 | 29.76 | 58 | 30.68 | 36.02667 | 39.48 |
| ZCCHC11 | 0.484 | -1.04679 | 6.2E-05 | 0.000318 | yes | down | 4.62 | 3.35 | 3.13 | 4.34 | 5.22 | 2.77 | 3.7 | 4.11 |
| PSD2 | 0.017 | -5.87456 | 6.22E-05 | 0.000318 | yes | down | 0.15 | 0.14 | 0.13 | 0 | 0.01 | 0 | 0.14 | 0.003333 |
| LOC108176222 | 0.011 | -6.49645 | 6.22E-05 | 0.000319 | yes | down | 0.93 | 0.35 | 0.87 | 0 | 0 | 0 | 0.716667 | 0 |
| LOC100339259 | 0.371 | -1.4287 | 6.26E-05 | 0.00032 | yes | down | 47.81 | 38.66 | 39.9 | 55.01 | 21.64 | 29.6 | 42.12333 | 35.41667 |
| ZBTB43 | 0.483 | -1.05099 | 6.27E-05 | 0.000321 | yes | down | 2.49 | 2.77 | 1.51 | 2.07 | 2.55 | 2.76 | 2.256667 | 2.46 |
| ZSWIM7 | 2.733 | 1.450263 | 6.34E-05 | 0.000324 | yes | up | 3.05 | 1.71 | 2.21 | 14.29 | 15.21 | 12.84 | 2.323333 | 14.11333 |
| LOC100355766 | 39.637 | 5.308783 | 6.34E-05 | 0.000324 | yes | up | 0 | 0.04 | 0.04 | 0.56 | 3.95 | 3.53 | 0.026667 | 2.68 |
| MCCC1 | 0.442 | -1.17652 | 6.39E-05 | 0.000326 | yes | down | 14.32 | 12.59 | 16.18 | 15.47 | 20.01 | 9.45 | 14.36333 | 14.97667 |
| PIGL | 2.367 | 1.243306 | 6.45E-05 | 0.000329 | yes | up | 1.1 | 1.08 | 0.99 | 4.81 | 6.11 | 4.83 | 1.056667 | 5.25 |
| CNTN4 | 0.192 | -2.3837 | 6.57E-05 | 0.000335 | yes | down | 0.28 | 0.52 | 0.22 | 0.09 | 0.15 | 0.21 | 0.34 | 0.15 |
| FAM3D | 19.927 | 4.316662 | 6.57E-05 | 0.000335 | yes | up | 0.03 | 0.15 | 0 | 4.05 | 2.39 | 4.14 | 0.06 | 3.526667 |
| LOC100338511 | 0.309 | -1.69378 | 6.63E-05 | 0.000338 | yes | down | 14.61 | 16.92 | 18.37 | 14.23 | 5.4 | 15.2 | 16.63333 | 11.61 |
| HCLS1 | 2.698 | 1.432058 | 6.7E-05 | 0.000341 | yes | up | 0.76 | 1.47 | 0.86 | 5.43 | 6.7 | 6.87 | 1.03 | 6.333333 |
| LOC100356307 | 2.977 | 1.573841 | 6.7E-05 | 0.000341 | yes | up | 131.83 | 49.97 | 47.68 | 445.51 | 541.07 | 348.31 | 76.49333 | 444.9633 |
| PUS3 | 0.437 | -1.19266 | 6.72E-05 | 0.000342 | yes | down | 6.24 | 5.78 | 5.16 | 5.89 | 8.23 | 3.6 | 5.726667 | 5.906667 |
| LOC100354670 | 0.077 | -3.69662 | 6.94E-05 | 0.000352 | yes | down | 0.4 | 2.49 | 0.82 | 0.07 | 0.08 | 0.51 | 1.236667 | 0.22 |
| DRD1 | 6.387 | 2.675048 | 6.94E-05 | 0.000352 | yes | up | 0.29 | 0.06 | 0.08 | 1.83 | 0.89 | 2.33 | 0.143333 | 1.683333 |
| DIRAS2 | 5.816 | 2.54 | 6.96E-05 | 0.000353 | yes | up | 0.11 | 0.1 | 0.05 | 0.9 | 0.81 | 1.67 | 0.086667 | 1.126667 |
| CAPN12 | 11.049 | 3.465793 | 7.01E-05 | 0.000355 | yes | up | 0.78 | 0.02 | 0 | 5.54 | 7.83 | 8.4 | 0.266667 | 7.256667 |
| LPAR1 | 2.542 | 1.345729 | 7.02E-05 | 0.000355 | yes | up | 5.58 | 3.03 | 1.81 | 21.09 | 20.28 | 17.26 | 3.473333 | 19.54333 |
| CISH | 0.317 | -1.65597 | 7.03E-05 | 0.000356 | yes | down | 32.58 | 32.26 | 36.82 | 36.73 | 27.03 | 9.14 | 33.88667 | 24.3 |
| OSGIN2 | 0.367 | -1.44596 | 7.11E-05 | 0.00036 | yes | down | 1.05 | 1.09 | 1.05 | 1.1 | 0.9 | 0.67 | 1.063333 | 0.89 |
| PSMD14 | 0.493 | -1.0197 | 7.12E-05 | 0.00036 | yes | down | 20.08 | 12.03 | 13.86 | 17.92 | 17.21 | 16.53 | 15.32333 | 17.22 |
| LOC100358241 | 3.473 | 1.796126 | 7.13E-05 | 0.00036 | yes | up | 19.72 | 11.66 | 8.91 | 91.47 | 194.92 | 40.89 | 13.43 | 109.0933 |
| LOC100341772 | 0.238 | -2.07161 | 7.16E-05 | 0.000361 | yes | down | 10.54 | 12.15 | 11.65 | 11.17 | 6.12 | 1.59 | 11.44667 | 6.293333 |
| CDKN2D | 2.205 | 1.140644 | 7.17E-05 | 0.000362 | yes | up | 5.08 | 3.66 | 3.92 | 22.61 | 20.46 | 20.59 | 4.22 | 21.22 |
| NCAPG2 | 3.097 | 1.631072 | 7.19E-05 | 0.000363 | yes | up | 11.15 | 9.42 | 6.48 | 52.2 | 60.44 | 46.26 | 9.016667 | 52.96667 |
| DNAJC21 | 0.443 | -1.17518 | 7.2E-05 | 0.000363 | yes | down | 8.47 | 4.62 | 5 | 6.02 | 5.99 | 6.13 | 6.03 | 6.046667 |
| GIN1 | 0.257 | -1.95827 | 7.22E-05 | 0.000364 | yes | down | 1.26 | 1.03 | 1.91 | 0.99 | 0.78 | 0.74 | 1.4 | 0.836667 |
| SYTL2 | 2.424 | 1.27762 | 7.25E-05 | 0.000365 | yes | up | 1.14 | 0.92 | 0.86 | 7.16 | 5.39 | 3.63 | 0.973333 | 5.393333 |
| MOSPD2 | 0.457 | -1.13054 | 7.28E-05 | 0.000367 | yes | down | 11.33 | 11.96 | 9.06 | 24.24 | 21.33 | 17.02 | 10.78333 | 20.86333 |
| MEIOB | 0.192 | -2.38075 | 7.29E-05 | 0.000367 | yes | down | 0.94 | 1.03 | 0.79 | 0.36 | 0.43 | 0.48 | 0.92 | 0.423333 |
| ZC4H2 | 2.546 | 1.348119 | 7.32E-05 | 0.000368 | yes | up | 0.66 | 0.98 | 0.63 | 5.34 | 4.53 | 3.41 | 0.756667 | 4.426667 |
| LOC103348108 | 0.298 | -1.74758 | 7.36E-05 | 0.00037 | yes | down | 0.83 | 1.14 | 0.69 | 0.68 | 0.4 | 0.73 | 0.886667 | 0.603333 |
| LOC103351504 | 0.494 | -1.01801 | 7.38E-05 | 0.000371 | yes | down | 8.11 | 12.59 | 10.98 | 9.41 | 10 | 8.13 | 10.56 | 9.18 |
| KIN | 0.418 | -1.25753 | 7.48E-05 | 0.000376 | yes | down | 3.08 | 4.12 | 2.65 | 3.23 | 4.33 | 1.95 | 3.283333 | 3.17 |
| CMC2 | 0.491 | -1.02512 | 7.49E-05 | 0.000376 | yes | down | 19.82 | 18.62 | 9.92 | 24.36 | 21.92 | 19.7 | 16.12 | 21.99333 |
| SEMA4A | 3.377 | 1.755901 | 7.51E-05 | 0.000377 | yes | up | 0.18 | 0.27 | 0.19 | 1.72 | 1.97 | 1.57 | 0.213333 | 1.753333 |
| ICE2 | 0.456 | -1.13189 | 7.54E-05 | 0.000379 | yes | down | 9.42 | 8 | 8.76 | 10.79 | 12.72 | 5.69 | 8.726667 | 9.733333 |
| LOC100355643 | 0.334 | -1.58423 | 7.68E-05 | 0.000385 | yes | down | 3.41 | 2.67 | 2.27 | 2.13 | 2 | 2.19 | 2.783333 | 2.106667 |
| LOC100342853 | 0.082 | -3.60608 | 7.72E-05 | 0.000387 | yes | down | 1.19 | 3.61 | 3.56 | 0.77 | 0.17 | 0.66 | 2.786667 | 0.533333 |
| TMEM136 | 3.275 | 1.711526 | 7.73E-05 | 0.000387 | yes | up | 0.77 | 0.68 | 0.18 | 3.75 | 4.63 | 3.5 | 0.543333 | 3.96 |
| SGCB | 2.154 | 1.107323 | 7.73E-05 | 0.000387 | yes | up | 19.34 | 8.98 | 13.45 | 61.48 | 82.48 | 65.85 | 13.92333 | 69.93667 |
| SERTAD4 | 3.867 | 1.951269 | 7.76E-05 | 0.000389 | yes | up | 0.79 | 0.36 | 0.18 | 6.5 | 3.54 | 2.96 | 0.443333 | 4.333333 |
| SCP2 | 0.417 | -1.26217 | 7.77E-05 | 0.000389 | yes | down | 70.84 | 153.92 | 69.14 | 86.23 | 125.42 | 69.42 | 97.96667 | 93.69 |
| UCP3 | 0.12 | -3.06447 | 7.85E-05 | 0.000392 | yes | down | 9.82 | 1.2 | 0.63 | 1.5 | 0.54 | 0.89 | 3.883333 | 0.976667 |
| CHUK | 0.425 | -1.23603 | 7.91E-05 | 0.000395 | yes | down | 4.42 | 4.74 | 4.48 | 4.33 | 6.69 | 2.78 | 4.546667 | 4.6 |
| AMIGO2 | 2.796 | 1.48335 | 7.93E-05 | 0.000396 | yes | up | 0.3 | 0.25 | 0.22 | 1.15 | 1.71 | 2.04 | 0.256667 | 1.633333 |
| C18H10orf35 | 4.524 | 2.177556 | 8.14E-05 | 0.000406 | yes | up | 1.31 | 0.97 | 1.13 | 10.33 | 5.35 | 17.62 | 1.136667 | 11.1 |
| PKM | 0.345 | -1.53678 | 8.21E-05 | 0.000409 | yes | down | 183.68 | 144.58 | 132.04 | 121.36 | 55.46 | 162.1 | 153.4333 | 112.9733 |
| LITAF | 2.34 | 1.226602 | 8.26E-05 | 0.000411 | yes | up | 4.54 | 6.91 | 4.95 | 39.14 | 25.1 | 29.41 | 5.466667 | 31.21667 |
| C4H12orf66 | 2.044 | 1.031731 | 8.31E-05 | 0.000413 | yes | up | 2.6 | 2.38 | 2.17 | 8.34 | 12.25 | 12.82 | 2.383333 | 11.13667 |
| ACAD11 | 0.499 | -1.00219 | 8.33E-05 | 0.000414 | yes | down | 4.35 | 4.51 | 5.27 | 5.4 | 6.58 | 4.49 | 4.71 | 5.49 |
| LOC103349650 | 2.43 | 1.281228 | 8.33E-05 | 0.000414 | yes | up | 9.31 | 11.88 | 12.22 | 72.9 | 50.31 | 63.69 | 11.13667 | 62.3 |
| LOC100347604 | 0.382 | -1.38651 | 8.39E-05 | 0.000417 | yes | down | 2.17 | 3.24 | 1.93 | 2.18 | 3.08 | 1.14 | 2.446667 | 2.133333 |
| HIGD2A | 0.475 | -1.07258 | 8.43E-05 | 0.000419 | yes | down | 44.2 | 53.4 | 37.24 | 50.02 | 38.7 | 56.09 | 44.94667 | 48.27 |
| MMP16 | 0.466 | -1.10275 | 8.45E-05 | 0.000419 | yes | down | 1.38 | 1.16 | 1.12 | 1.58 | 1.43 | 0.89 | 1.22 | 1.3 |
| MID1IP1 | 3.628 | 1.859136 | 8.67E-05 | 0.000429 | yes | up | 3.6 | 4.08 | 4.8 | 48.01 | 12.38 | 40.49 | 4.16 | 33.62667 |
| CCDC92 | 2.572 | 1.362879 | 8.89E-05 | 0.00044 | yes | up | 2.43 | 1.69 | 1 | 9.6 | 11.03 | 8.33 | 1.706667 | 9.653333 |
| LOC103346930 | 12.155 | 3.603491 | 8.94E-05 | 0.000442 | yes | up | 0.09 | 0.05 | 0.52 | 4.26 | 2.01 | 12.64 | 0.22 | 6.303333 |
| NKAP | 0.489 | -1.03079 | 8.95E-05 | 0.000442 | yes | down | 2.87 | 3.38 | 2.78 | 3.55 | 3.97 | 2.65 | 3.01 | 3.39 |
| CNST | 0.493 | -1.01902 | 8.96E-05 | 0.000443 | yes | down | 6.4 | 7.67 | 5.16 | 8.35 | 8.27 | 5.15 | 6.41 | 7.256667 |
| NR2C1 | 0.489 | -1.03221 | 9.04E-05 | 0.000446 | yes | down | 4.51 | 5.82 | 3.58 | 5.22 | 6.74 | 3.63 | 4.636667 | 5.196667 |
| ARMC5 | 2.398 | 1.261701 | 9.09E-05 | 0.000449 | yes | up | 1.45 | 0.69 | 0.92 | 5.13 | 5.94 | 5.66 | 1.02 | 5.576667 |
| COMMD7 | 2.082 | 1.058025 | 9.11E-05 | 0.00045 | yes | up | 6 | 4.39 | 3.65 | 25.5 | 18.56 | 21.81 | 4.68 | 21.95667 |
| MRPL48 | 0.437 | -1.19359 | 9.15E-05 | 0.000451 | yes | down | 25.7 | 16.58 | 16.71 | 20.29 | 15.51 | 23.4 | 19.66333 | 19.73333 |
| NUP54 | 0.467 | -1.09941 | 9.16E-05 | 0.000452 | yes | down | 6.03 | 6.74 | 5.47 | 6.95 | 8.3 | 4.17 | 6.08 | 6.473333 |
| DTL | 2.544 | 1.347288 | 9.21E-05 | 0.000454 | yes | up | 0.86 | 1.12 | 0.62 | 5.53 | 6.7 | 3.23 | 0.866667 | 5.153333 |
| LOC103349169 | 0.402 | -1.31356 | 9.24E-05 | 0.000455 | yes | down | 3.06 | 5.53 | 5.09 | 5.69 | 5.97 | 4.1 | 4.56 | 5.253333 |
| EMCN | 0.486 | -1.04043 | 9.3E-05 | 0.000458 | yes | down | 23.49 | 35.34 | 26.75 | 37.51 | 43.53 | 21.55 | 28.52667 | 34.19667 |
| MAP3K7 | 0.486 | -1.04032 | 9.34E-05 | 0.00046 | yes | down | 4.35 | 4.49 | 3.42 | 4.92 | 6.4 | 2.95 | 4.086667 | 4.756667 |
| SH3TC2 | 3.204 | 1.679726 | 9.35E-05 | 0.00046 | yes | up | 0.84 | 0.94 | 0.23 | 4.27 | 6.73 | 3.34 | 0.67 | 4.78 |
| ZNF654 | 0.397 | -1.33256 | 9.44E-05 | 0.000464 | yes | down | 2.78 | 3.36 | 2.63 | 3.34 | 3.32 | 1.29 | 2.923333 | 2.65 |
| LOC100342322 | 0.118 | -3.08304 | 9.45E-05 | 0.000464 | yes | down | 1.14 | 0.63 | 3.66 | 1.75 | 3.55 | 0.69 | 1.81 | 1.996667 |
| TAF3 | 0.46 | -1.12169 | 9.49E-05 | 0.000466 | yes | down | 3.07 | 3.13 | 3.7 | 3.7 | 4.58 | 2.4 | 3.3 | 3.56 |
| SMIM3 | 2.312 | 1.209093 | 9.55E-05 | 0.000469 | yes | up | 1.91 | 1.34 | 0.83 | 14.62 | 8.91 | 13.24 | 1.36 | 12.25667 |
| MMGT1 | 0.483 | -1.04859 | 9.68E-05 | 0.000475 | yes | down | 5.34 | 6.69 | 5.71 | 7.71 | 7.93 | 4.24 | 5.913333 | 6.626667 |
| LOC103351904 | 0.441 | -1.18101 | 9.73E-05 | 0.000477 | yes | down | 7.67 | 8.18 | 8.83 | 10.79 | 13.42 | 6.66 | 8.226667 | 10.29 |
| PBLD | 0.409 | -1.29154 | 9.75E-05 | 0.000478 | yes | down | 4.82 | 4.93 | 5.15 | 3.28 | 5.24 | 3.25 | 4.966667 | 3.923333 |
| ARHGAP44 | 2.768 | 1.468661 | 9.85E-05 | 0.000483 | yes | up | 0.38 | 0.35 | 0.34 | 1.72 | 2.44 | 2.44 | 0.356667 | 2.2 |
| C16H1orf27 | 0.5 | -1.00062 | 9.92E-05 | 0.000486 | yes | down | 3.11 | 3.25 | 3.43 | 4.41 | 4.94 | 2.77 | 3.263333 | 4.04 |
| LOC100343412 | 0.434 | -1.20263 | 9.93E-05 | 0.000486 | yes | down | 2.18 | 3.01 | 1.99 | 2.54 | 3.14 | 1.79 | 2.393333 | 2.49 |
| PRR15 | 13.977 | 3.80499 | 9.94E-05 | 0.000486 | yes | up | 0.02 | 0.03 | 0.01 | 0.51 | 0.72 | 0.81 | 0.02 | 0.68 |
| PAFAH1B3 | 2.087 | 1.061274 | 9.97E-05 | 0.000488 | yes | up | 15.03 | 15.83 | 19.72 | 91.62 | 74.22 | 78.05 | 16.86 | 81.29667 |
| MTRR | 0.431 | -1.21516 | 1E-04 | 0.000489 | yes | down | 1.86 | 1.23 | 1.45 | 1.24 | 1.79 | 1.74 | 1.513333 | 1.59 |
| LOC100340125 | 2.021 | 1.015115 | 0.0001 | 0.00049 | yes | up | 7.8 | 13.54 | 9.74 | 48.36 | 50.44 | 45.4 | 10.36 | 48.06667 |
| LOC108178630 | 0.018 | -5.78394 | 0.000101 | 0.000496 | yes | down | 3.04 | 4.53 | 3 | 0 | 0.49 | 0 | 3.523333 | 0.163333 |
| SFRP2 | 5.117 | 2.355182 | 0.000102 | 0.000498 | yes | up | 1.98 | 1.64 | 3.26 | 5.65 | 44.93 | 34.51 | 2.293333 | 28.36333 |
| HPSE | 3.042 | 1.605012 | 0.000103 | 0.000501 | yes | up | 0.48 | 0.36 | 0.2 | 2.28 | 2.6 | 2.17 | 0.346667 | 2.35 |
| FAM65C | 4.13 | 2.046232 | 0.000103 | 0.000501 | yes | up | 0.18 | 0.07 | 0.14 | 1.1 | 1.14 | 1.46 | 0.13 | 1.233333 |
| CYTH4 | 2.139 | 1.096775 | 0.000103 | 0.000503 | yes | up | 0.96 | 1 | 0.84 | 4.46 | 4.76 | 4.45 | 0.933333 | 4.556667 |
| KLHL28 | 0.402 | -1.31346 | 0.000103 | 0.000503 | yes | down | 1.95 | 1.57 | 1.37 | 1.69 | 2.09 | 0.8 | 1.63 | 1.526667 |
| ADTRP | 2.319 | 1.213532 | 0.000104 | 0.000506 | yes | up | 8.18 | 15.79 | 11.56 | 57.01 | 63.3 | 85.31 | 11.84333 | 68.54 |
| ARSA | 2.062 | 1.044217 | 0.000105 | 0.00051 | yes | up | 7.99 | 9.96 | 8.39 | 39.04 | 33.82 | 50.34 | 8.78 | 41.06667 |
| HOXD10 | 17.934 | 4.164664 | 0.000105 | 0.00051 | yes | up | 0.07 | 0.06 | 0.06 | 3.31 | 2.83 | 1.85 | 0.063333 | 2.663333 |
| CYP4B1 | 0.356 | -1.49084 | 0.000106 | 0.000514 | yes | down | 13.29 | 10.38 | 10.15 | 4.02 | 11.87 | 11.81 | 11.27333 | 9.233333 |
| KPNA5 | 0.474 | -1.07628 | 0.000106 | 0.000516 | yes | down | 1.66 | 1.74 | 1.19 | 1.76 | 1.45 | 1.35 | 1.53 | 1.52 |
| TLX3 | 0.011 | -6.5477 | 0.000106 | 0.000516 | yes | down | 0.12 | 0.32 | 0.57 | 0 | 0 | 0 | 0.336667 | 0 |
| SRGAP1 | 2.198 | 1.136143 | 0.000107 | 0.000518 | yes | up | 1.28 | 0.92 | 0.68 | 4.94 | 6.35 | 3.21 | 0.96 | 4.833333 |
| ASAH1 | 0.498 | -1.00591 | 0.000107 | 0.000519 | yes | down | 47.89 | 67.54 | 58.95 | 63 | 93.08 | 47.63 | 58.12667 | 67.90333 |
| LOC108176480 | 2.389 | 1.256237 | 0.000107 | 0.00052 | yes | up | 1.03 | 1.11 | 0.8 | 3.74 | 4.61 | 7.62 | 0.98 | 5.323333 |
| AGTPBP1 | 0.424 | -1.23896 | 0.000108 | 0.000522 | yes | down | 2.71 | 1.18 | 1.65 | 1.92 | 2.31 | 1.32 | 1.846667 | 1.85 |
| NFAM1 | 2.684 | 1.424245 | 0.000108 | 0.000525 | yes | up | 0.21 | 0.21 | 0.15 | 1.19 | 1.27 | 1.03 | 0.19 | 1.163333 |
| TESMIN | 0.036 | -4.78109 | 0.000109 | 0.000527 | yes | down | 3.4 | 9.5 | 5.54 | 0 | 0.07 | 0 | 6.146667 | 0.023333 |
| LOC108176489 | 4.486 | 2.16543 | 0.000109 | 0.000529 | yes | up | 0.17 | 0.26 | 0.08 | 1.4 | 2.16 | 1.59 | 0.17 | 1.716667 |
| LOC100355642 | 0.01 | -6.63554 | 0.00011 | 0.000531 | yes | down | 1.59 | 1.42 | 4.12 | 0 | 0 | 0.13 | 2.376667 | 0.043333 |
| NPHP3 | 0.444 | -1.17211 | 0.00011 | 0.000534 | yes | down | 2.71 | 3.56 | 1.8 | 2.35 | 3.91 | 1.96 | 2.69 | 2.74 |
| IRS2 | 0.291 | -1.77868 | 0.00011 | 0.000534 | yes | down | 4.04 | 5.58 | 4.98 | 2.49 | 5.39 | 2.15 | 4.866667 | 3.343333 |
| REEP4 | 2.388 | 1.255554 | 0.000111 | 0.000536 | yes | up | 3.46 | 2.99 | 3.14 | 21.99 | 18.42 | 12.23 | 3.196667 | 17.54667 |
| SLC35G2 | 0.479 | -1.06052 | 0.000112 | 0.000539 | yes | down | 4.03 | 5.06 | 4.3 | 4.02 | 5.7 | 4.9 | 4.463333 | 4.873333 |
| LOC100343223 | 3.173 | 1.666039 | 0.000112 | 0.000543 | yes | up | 5.27 | 11.25 | 3.61 | 62.01 | 29.48 | 49.02 | 6.71 | 46.83667 |
| ABCC3 | 2.903 | 1.53738 | 0.000113 | 0.000545 | yes | up | 0.21 | 0.19 | 0.22 | 1.12 | 1.72 | 1.41 | 0.206667 | 1.416667 |
| SEC63 | 0.48 | -1.05789 | 0.000114 | 0.000549 | yes | down | 17.07 | 16.62 | 17.32 | 19.39 | 26.22 | 11.84 | 17.00333 | 19.15 |
| PACRGL | 0.37 | -1.43581 | 0.000114 | 0.000549 | yes | down | 2.64 | 3.07 | 3.8 | 4.14 | 3.22 | 1.96 | 3.17 | 3.106667 |
| PLEK2 | 0.143 | -2.80258 | 0.000114 | 0.000551 | yes | down | 2.43 | 2 | 0.34 | 0.57 | 0.72 | 0.19 | 1.59 | 0.493333 |
| LOC108176548 | 4.516 | 2.175012 | 0.000115 | 0.000553 | yes | up | 0.16 | 0.17 | 0.14 | 2.81 | 1.34 | 3.55 | 0.156667 | 2.566667 |
| PQLC1 | 2.372 | 1.246276 | 0.000115 | 0.000553 | yes | up | 15.46 | 8.98 | 5.06 | 42.78 | 61.02 | 51.77 | 9.833333 | 51.85667 |
| MYC | 2.106 | 1.074219 | 0.000115 | 0.000553 | yes | up | 6.77 | 6.29 | 5.66 | 26.34 | 22.3 | 35.03 | 6.24 | 27.89 |
| FAR1 | 0.442 | -1.17837 | 0.000115 | 0.000553 | yes | down | 7.95 | 7.63 | 5.17 | 6.93 | 8.4 | 3.56 | 6.916667 | 6.296667 |
| LOC108178461 | 3.358 | 1.747606 | 0.000116 | 0.000557 | yes | up | 0.57 | 0.37 | 0.65 | 5.22 | 6.41 | 3.19 | 0.53 | 4.94 |
| IQSEC2 | 2.1 | 1.070597 | 0.000116 | 0.000558 | yes | up | 3.83 | 1.08 | 0.98 | 5.79 | 7.31 | 7.11 | 1.963333 | 6.736667 |
| CCNB2 | 3.878 | 1.955257 | 0.000116 | 0.000558 | yes | up | 3.33 | 2.96 | 1.08 | 25.89 | 31.83 | 7.35 | 2.456667 | 21.69 |
| LOC103346472 | 82.88 | 6.372953 | 0.000116 | 0.00056 | yes | up | 0 | 0 | 0 | 0.82 | 0.1 | 1.13 | 0 | 0.683333 |
| C1H11orf87 | 11.737 | 3.552939 | 0.000116 | 0.00056 | yes | up | 0.02 | 0.02 | 0.01 | 0.38 | 0.53 | 0.22 | 0.016667 | 0.376667 |
| PSME1 | 2 | 1.000315 | 0.000117 | 0.000561 | yes | up | 26.57 | 19.83 | 13.77 | 92.14 | 81.67 | 99.47 | 20.05667 | 91.09333 |
| PTPRB | 0.446 | -1.16615 | 0.000118 | 0.000565 | yes | down | 16.16 | 21.71 | 21.55 | 18.13 | 33.38 | 14.28 | 19.80667 | 21.93 |
| ANKRA2 | 0.495 | -1.01491 | 0.000118 | 0.000565 | yes | down | 4.96 | 5.44 | 4.4 | 3.92 | 5.98 | 5.65 | 4.933333 | 5.183333 |
| MTERF3 | 0.47 | -1.08959 | 0.000118 | 0.000567 | yes | down | 5.23 | 5 | 5.12 | 5.8 | 6.32 | 4.53 | 5.116667 | 5.55 |
| MYSM1 | 0.421 | -1.2479 | 0.000118 | 0.000567 | yes | down | 3.28 | 3.16 | 2.25 | 2.52 | 4.34 | 1.63 | 2.896667 | 2.83 |
| FAM49A | 2.195 | 1.133954 | 0.000118 | 0.000567 | yes | up | 2.51 | 1.62 | 1.08 | 9.58 | 8.93 | 7.15 | 1.736667 | 8.553333 |
| SMG9 | 2.074 | 1.05231 | 0.000119 | 0.000569 | yes | up | 2.4 | 2.03 | 2.4 | 10.11 | 10.41 | 11.96 | 2.276667 | 10.82667 |
| TTC14 | 0.387 | -1.36776 | 0.000119 | 0.000572 | yes | down | 4.64 | 4.8 | 4.05 | 4.08 | 5.52 | 1.97 | 4.496667 | 3.856667 |
| TRIM46 | 4.305 | 2.106081 | 0.00012 | 0.000573 | yes | up | 0.2 | 0.13 | 0.14 | 1.39 | 1.06 | 1.82 | 0.156667 | 1.423333 |
| PIK3R1 | 2.601 | 1.379017 | 0.00012 | 0.000577 | yes | up | 7.22 | 6.03 | 4.31 | 19.91 | 60.73 | 23.88 | 5.853333 | 34.84 |
| RMI1 | 0.368 | -1.44159 | 0.000121 | 0.000578 | yes | down | 2.96 | 3.21 | 2.34 | 2.92 | 3.33 | 1.25 | 2.836667 | 2.5 |
| GDF9 | 3.16 | 1.65982 | 0.000121 | 0.000578 | yes | up | 0.71 | 0.79 | 0.77 | 6.11 | 3.85 | 5.91 | 0.756667 | 5.29 |
| LOC100355489 | 0.498 | -1.0062 | 0.000123 | 0.000586 | yes | down | 29.45 | 23.64 | 17.08 | 28.74 | 33.25 | 17.89 | 23.39 | 26.62667 |
| STEAP3 | 4.28 | 2.097679 | 0.000123 | 0.000587 | yes | up | 0.65 | 0.19 | 0.23 | 2.75 | 4.01 | 3.48 | 0.356667 | 3.413333 |
| BLVRA | 4.026 | 2.009491 | 0.000123 | 0.000588 | yes | up | 2.18 | 1.33 | 1.65 | 17.08 | 6.27 | 22.68 | 1.72 | 15.34333 |
| EML1 | 2.105 | 1.073723 | 0.000123 | 0.00059 | yes | up | 5.46 | 3.7 | 2.34 | 16.91 | 17.86 | 19.19 | 3.833333 | 17.98667 |
| COL21A1 | 3.589 | 1.843678 | 0.000124 | 0.000591 | yes | up | 2.53 | 1.06 | 0.61 | 12.95 | 12.68 | 6.99 | 1.4 | 10.87333 |
| PKHD1L1 | 5.18 | 2.372928 | 0.000124 | 0.000591 | yes | up | 0.02 | 0.04 | 0.01 | 0.25 | 0.26 | 0.31 | 0.023333 | 0.273333 |
| B3GNT7 | 0.342 | -1.54746 | 0.000125 | 0.000598 | yes | down | 2.51 | 2.56 | 2.33 | 1.38 | 1.51 | 2.87 | 2.466667 | 1.92 |
| NIPAL2 | 0.394 | -1.34415 | 0.000126 | 0.000599 | yes | down | 7.8 | 5.37 | 3.68 | 6.35 | 5.77 | 2.72 | 5.616667 | 4.946667 |
| LOC100358069 | 0.459 | -1.12388 | 0.000126 | 0.000601 | yes | down | 138.1 | 131.49 | 112.65 | 162.04 | 90.68 | 142.75 | 127.4133 | 131.8233 |
| FAM46B | 20.082 | 4.327806 | 0.000126 | 0.000602 | yes | up | 0.02 | 0.04 | 0 | 0.69 | 0.75 | 1.56 | 0.02 | 1 |
| MATN4 | 4.777 | 2.256094 | 0.000127 | 0.000604 | yes | up | 0.25 | 0.34 | 0.35 | 3.79 | 2.18 | 4.57 | 0.313333 | 3.513333 |
| EXOSC8 | 0.44 | -1.18393 | 0.000127 | 0.000604 | yes | down | 5.3 | 4.35 | 4.62 | 5.3 | 5.88 | 3.38 | 4.756667 | 4.853333 |
| LOC100341997 | 3.477 | 1.797936 | 0.000128 | 0.000606 | yes | up | 1.13 | 2.37 | 0.78 | 14.16 | 14.25 | 5.46 | 1.426667 | 11.29 |
| LOC100353286 | 0.452 | -1.14681 | 0.000128 | 0.000607 | yes | down | 6.68 | 4.54 | 3.89 | 4.32 | 7.52 | 3.91 | 5.036667 | 5.25 |
| GCNT2 | 2.343 | 1.228126 | 0.000128 | 0.000607 | yes | up | 0.84 | 1.57 | 0.81 | 5.12 | 5.62 | 6.32 | 1.073333 | 5.686667 |
| NGB | 0.045 | -4.4591 | 0.000129 | 0.000612 | yes | down | 0.47 | 0.4 | 0.47 | 0.07 | 0.07 | 0.09 | 0.446667 | 0.076667 |
| SEZ6L | 0.051 | -4.28945 | 0.000129 | 0.000613 | yes | down | 0.07 | 0.24 | 0.24 | 0.03 | 0.02 | 0.02 | 0.183333 | 0.023333 |
| LOC108177268 | 0.259 | -1.94699 | 0.00013 | 0.000616 | yes | down | 2.15 | 2.31 | 1.86 | 1.08 | 1.47 | 0.92 | 2.106667 | 1.156667 |
| RIMS4 | 4.089 | 2.031701 | 0.00013 | 0.000617 | yes | up | 0.12 | 0.12 | 0.07 | 0.65 | 1.17 | 1.05 | 0.103333 | 0.956667 |
| LOC108176329 | 2.499 | 1.321247 | 0.00013 | 0.000618 | yes | up | 0.96 | 1.93 | 0.91 | 5.96 | 11.28 | 8.88 | 1.266667 | 8.706667 |
| IMMP2L | 0.436 | -1.19598 | 0.00013 | 0.000618 | yes | down | 7.57 | 6.67 | 5.11 | 6.48 | 4.77 | 6.98 | 6.45 | 6.076667 |
| PHACTR2 | 0.425 | -1.23347 | 0.000131 | 0.000618 | yes | down | 7.31 | 8.08 | 7.07 | 6.84 | 11.84 | 3.89 | 7.486667 | 7.523333 |
| TOMM22 | 0.457 | -1.13082 | 0.000132 | 0.000626 | yes | down | 31.18 | 56.22 | 47.96 | 49.93 | 45.63 | 47.26 | 45.12 | 47.60667 |
| LOC103346247 | 6.166 | 2.624298 | 0.000133 | 0.00063 | yes | up | 0.15 | 0.18 | 0.3 | 1.92 | 3.42 | 3.88 | 0.21 | 3.073333 |
| LOC100356569 | 2.47 | 1.304649 | 0.000133 | 0.000631 | yes | up | 0.81 | 0.57 | 0.61 | 3.92 | 5.1 | 2.39 | 0.663333 | 3.803333 |
| LOC100353215 | 0.365 | -1.45483 | 0.000134 | 0.000634 | yes | down | 1.71 | 1.52 | 1.74 | 1.93 | 1.46 | 0.8 | 1.656667 | 1.396667 |
| EEF2KMT | 0.315 | -1.66553 | 0.000135 | 0.000638 | yes | down | 2.43 | 3.95 | 2.5 | 2.37 | 2.76 | 1.29 | 2.96 | 2.14 |
| TMEM119 | 2.234 | 1.159424 | 0.000136 | 0.000641 | yes | up | 3.77 | 2.81 | 2.03 | 11.37 | 13.87 | 16.29 | 2.87 | 13.84333 |
| PRPF40A | 0.4 | -1.32321 | 0.000136 | 0.000642 | yes | down | 12.79 | 11.88 | 11.22 | 9.94 | 17.91 | 5.4 | 11.96333 | 11.08333 |
| ARHGEF39 | 5.971 | 2.578037 | 0.000137 | 0.000648 | yes | up | 0.52 | 0.41 | 0.23 | 7.06 | 7.29 | 1.34 | 0.386667 | 5.23 |
| RPP30 | 0.49 | -1.03016 | 0.000138 | 0.000648 | yes | down | 16.67 | 20.31 | 19.11 | 25.52 | 20.63 | 17.05 | 18.69667 | 21.06667 |
| LOC100356702 | 0.056 | -4.16025 | 0.000138 | 0.00065 | yes | down | 0.27 | 0.23 | 0.16 | 0.04 | 0.04 | 0 | 0.22 | 0.026667 |
| PPFIA4 | 0.083 | -3.58871 | 0.000139 | 0.000654 | yes | down | 3.01 | 0.37 | 0.17 | 0.13 | 0.07 | 0.36 | 1.183333 | 0.186667 |
| L3MBTL1 | 4.662 | 2.221097 | 0.00014 | 0.000656 | yes | up | 0.12 | 0.2 | 0.07 | 0.8 | 1.78 | 1.06 | 0.13 | 1.213333 |
| CCNF | 4.143 | 2.050611 | 0.000141 | 0.000664 | yes | up | 0.53 | 0.5 | 0.19 | 4.39 | 5.75 | 1.37 | 0.406667 | 3.836667 |
| LOC100355311 | 0.452 | -1.14632 | 0.000144 | 0.000676 | yes | down | 5.84 | 4.54 | 4.17 | 5.87 | 4.55 | 4.51 | 4.85 | 4.976667 |
| LOC103345867 | 0.316 | -1.66324 | 0.000144 | 0.000677 | yes | down | 0.71 | 0.68 | 0.43 | 0.27 | 0.69 | 0.36 | 0.606667 | 0.44 |
| DNAJB14 | 0.467 | -1.0975 | 0.000145 | 0.000678 | yes | down | 3.54 | 3.58 | 3.62 | 4.16 | 5.24 | 2.32 | 3.58 | 3.906667 |
| MYO7A | 3.189 | 1.673321 | 0.000145 | 0.000681 | yes | up | 0.66 | 0.46 | 0.28 | 4.42 | 4.27 | 1.47 | 0.466667 | 3.386667 |
| LOC103349335 | 0.084 | -3.56773 | 0.000146 | 0.000682 | yes | down | 0.79 | 2.13 | 2.61 | 0.15 | 0.28 | 0.36 | 1.843333 | 0.263333 |
| IER3 | 2.709 | 1.437875 | 0.000146 | 0.000683 | yes | up | 7.54 | 5.15 | 2.55 | 33.1 | 23.42 | 34.03 | 5.08 | 30.18333 |
| TMEM65 | 0.33 | -1.59755 | 0.000146 | 0.000685 | yes | down | 11.59 | 5.24 | 4.09 | 6.45 | 6.02 | 3.05 | 6.973333 | 5.173333 |
| LOC100338396 | 7.611 | 2.927992 | 0.000147 | 0.000688 | yes | up | 0.22 | 0.33 | 0.06 | 3.56 | 3.15 | 3.79 | 0.203333 | 3.5 |
| LOC103351772 | 0.221 | -2.17613 | 0.000148 | 0.00069 | yes | down | 64.03 | 78.18 | 119.96 | 41.91 | 49.27 | 56.85 | 87.39 | 49.34333 |
| SCN5A | 0.04 | -4.63324 | 0.000148 | 0.000691 | yes | down | 0.25 | 0.47 | 0.02 | 0.02 | 0 | 0.04 | 0.246667 | 0.02 |
| ELMO1 | 2.023 | 1.016635 | 0.000148 | 0.000694 | yes | up | 4.68 | 4.63 | 2.34 | 16.45 | 18.5 | 18.31 | 3.883333 | 17.75333 |
| LOC103350570 | 18.319 | 4.1953 | 0.00015 | 0.000701 | yes | up | 0.01 | 0.02 | 0 | 0.35 | 0.58 | 0.25 | 0.01 | 0.393333 |
| NUSAP1 | 3.763 | 1.911828 | 0.00015 | 0.000702 | yes | up | 3.35 | 3.08 | 1.25 | 31.45 | 29.05 | 6.61 | 2.56 | 22.37 |
| LOC103349692 | 0.079 | -3.65751 | 0.000151 | 0.000703 | yes | down | 0.1 | 0.42 | 0.17 | 0.03 | 0.05 | 0.04 | 0.23 | 0.04 |
| LOC100338650 | 0.01 | -6.57964 | 0.000151 | 0.000705 | yes | down | 0.09 | 0.84 | 0.3 | 0 | 0 | 0 | 0.41 | 0 |
| LOC108178205 | 0.303 | -1.72394 | 0.000152 | 0.000707 | yes | down | 4.85 | 8.26 | 7 | 7.65 | 2.19 | 3.85 | 6.703333 | 4.563333 |
| PANX1 | 2.722 | 1.444853 | 0.000152 | 0.000708 | yes | up | 0.99 | 0.7 | 0.71 | 6.34 | 4.48 | 3.91 | 0.8 | 4.91 |
| NHSL1 | 2.33 | 1.220569 | 0.000153 | 0.000711 | yes | up | 1.32 | 1.1 | 1.88 | 6.2 | 9.13 | 7.75 | 1.433333 | 7.693333 |
| LOC100349285 | 3.286 | 1.716195 | 0.000154 | 0.000715 | yes | up | 84.3 | 112.43 | 163.21 | 493.3 | 688.09 | 1548.69 | 119.98 | 910.0267 |
| CNNM4 | 2.289 | 1.194441 | 0.000157 | 0.000729 | yes | up | 1.54 | 0.9 | 1.51 | 6.69 | 6.59 | 7.56 | 1.316667 | 6.946667 |
| RIF1 | 0.387 | -1.37123 | 0.000157 | 0.00073 | yes | down | 2.17 | 1.94 | 1.87 | 1.77 | 2.83 | 0.86 | 1.993333 | 1.82 |
| LOC108176477 | 2.458 | 1.297531 | 0.000158 | 0.000732 | yes | up | 1.15 | 0.91 | 0.79 | 3.59 | 5.01 | 5.93 | 0.95 | 4.843333 |
| CHN1 | 2.27 | 1.182436 | 0.000158 | 0.000734 | yes | up | 4.17 | 4.23 | 3 | 20.86 | 25.25 | 11.21 | 3.8 | 19.10667 |
| TRUB2 | 0.396 | -1.33734 | 0.000159 | 0.000736 | yes | down | 2.79 | 4.04 | 2.82 | 1.74 | 3.36 | 3.66 | 3.216667 | 2.92 |
| LOC108176107 | 0.439 | -1.18747 | 0.000159 | 0.000738 | yes | down | 4.09 | 2.61 | 2.23 | 2.41 | 3.09 | 3.35 | 2.976667 | 2.95 |
| DNTTIP2 | 0.493 | -1.02138 | 0.00016 | 0.000743 | yes | down | 4.11 | 4.6 | 2.64 | 4.08 | 5.5 | 3.05 | 3.783333 | 4.21 |
| SEC62 | 0.466 | -1.1006 | 0.000161 | 0.000744 | yes | down | 20.29 | 26.34 | 24.3 | 27.08 | 35.41 | 15.24 | 23.64333 | 25.91 |
| NLRP3 | 2.191 | 1.131306 | 0.000161 | 0.000746 | yes | up | 0.56 | 0.62 | 0.46 | 2.29 | 3.47 | 2.39 | 0.546667 | 2.716667 |
| LOC103350421 | 0.47 | -1.08945 | 0.000162 | 0.000748 | yes | down | 2.21 | 2.95 | 3.19 | 1.81 | 2.71 | 3.5 | 2.783333 | 2.673333 |
| MDGA1 | 6.263 | 2.646811 | 0.000162 | 0.000751 | yes | up | 0.06 | 0.14 | 0.03 | 0.83 | 1.4 | 1.07 | 0.076667 | 1.1 |
| LOC100338043 | 2.006 | 1.004594 | 0.000162 | 0.000751 | yes | up | 11.03 | 14.1 | 12.64 | 67.8 | 48.44 | 56.91 | 12.59 | 57.71667 |
| SQLE | 2.566 | 1.359685 | 0.000163 | 0.000753 | yes | up | 1.58 | 1.9 | 1.2 | 13.25 | 7.55 | 6.29 | 1.56 | 9.03 |
| LOC103346668 | 2.757 | 1.462841 | 0.000163 | 0.000754 | yes | up | 1 | 1.53 | 0.58 | 6.48 | 5.84 | 8.31 | 1.036667 | 6.876667 |
| ABHD11 | 0.469 | -1.09238 | 0.000164 | 0.000756 | yes | down | 13.66 | 11.21 | 12.19 | 13.51 | 9.57 | 14.49 | 12.35333 | 12.52333 |
| ARL9 | 3.8 | 1.926071 | 0.000167 | 0.000771 | yes | up | 1.16 | 0.45 | 0.48 | 5.91 | 4.66 | 5.4 | 0.696667 | 5.323333 |
| SUV39H1 | 2.791 | 1.480999 | 0.000168 | 0.000776 | yes | up | 0.49 | 0.5 | 0.47 | 3.9 | 3.51 | 1.98 | 0.486667 | 3.13 |
| KCNC4 | 0.064 | -3.97419 | 0.000169 | 0.000779 | yes | down | 6.27 | 6.37 | 3.23 | 0.42 | 0.24 | 2.34 | 5.29 | 1 |
| LOC100343274 | 0.478 | -1.06361 | 0.000169 | 0.00078 | yes | down | 5.08 | 7.42 | 5.41 | 5.09 | 7.9 | 6.76 | 5.97 | 6.583333 |
| C1H9orf72 | 0.468 | -1.09486 | 0.00017 | 0.000781 | yes | down | 13.13 | 15.84 | 15.39 | 19.54 | 18.02 | 9.45 | 14.78667 | 15.67 |
| GNPDA2 | 0.448 | -1.15768 | 0.000171 | 0.000787 | yes | down | 2.99 | 2.6 | 2.91 | 3.35 | 3.81 | 2.34 | 2.833333 | 3.166667 |
| TPBG | 3.13 | 1.646326 | 0.000172 | 0.000793 | yes | up | 0.82 | 0.42 | 0.44 | 4.73 | 3.25 | 3.83 | 0.56 | 3.936667 |
| NAGPA | 2.255 | 1.172822 | 0.000173 | 0.000796 | yes | up | 2.71 | 2.27 | 2.83 | 11.01 | 12.44 | 16.93 | 2.603333 | 13.46 |
| TRMT6 | 0.391 | -1.35656 | 0.000174 | 0.000801 | yes | down | 1.31 | 1.52 | 0.9 | 1.05 | 1.13 | 1.09 | 1.243333 | 1.09 |
| SVIP | 0.459 | -1.12337 | 0.000175 | 0.000802 | yes | down | 2.14 | 2.12 | 2.1 | 1.73 | 2.58 | 2.43 | 2.12 | 2.246667 |
| LOC100352730 | 2.248 | 1.168607 | 0.000175 | 0.000803 | yes | up | 1.71 | 1.72 | 1.55 | 11.44 | 10.09 | 7.96 | 1.66 | 9.83 |
| CALB1 | 0.011 | -6.47846 | 0.000176 | 0.000807 | yes | down | 0.07 | 0.39 | 0.43 | 0 | 0 | 0 | 0.296667 | 0 |
| MET | 0.334 | -1.5822 | 0.000177 | 0.00081 | yes | down | 3.74 | 2.66 | 7.58 | 3.57 | 3.57 | 4.01 | 4.66 | 3.716667 |
| WFS1 | 0.443 | -1.17411 | 0.000177 | 0.000812 | yes | down | 7.77 | 5.33 | 8.26 | 5.98 | 9.6 | 6.67 | 7.12 | 7.416667 |
| TRIM10 | 0.09 | -3.47179 | 0.000177 | 0.000812 | yes | down | 0.36 | 0.43 | 0.38 | 0.19 | 0.03 | 0.04 | 0.39 | 0.086667 |
| SCARF2 | 3.331 | 1.735834 | 0.000177 | 0.000812 | yes | up | 1.19 | 1.5 | 1.22 | 9.38 | 10.52 | 10.12 | 1.303333 | 10.00667 |
| MXD3 | 5.206 | 2.380234 | 0.000179 | 0.000818 | yes | up | 1.35 | 0.82 | 0.29 | 13.46 | 12.21 | 3.05 | 0.82 | 9.573333 |
| TGFA | 11.421 | 3.513674 | 0.000179 | 0.000819 | yes | up | 0.07 | 0.02 | 0.05 | 1.48 | 0.15 | 1.8 | 0.046667 | 1.143333 |
| TC2N | 2.698 | 1.431826 | 0.000179 | 0.000819 | yes | up | 0.92 | 0.57 | 0.36 | 3.98 | 4.29 | 2.98 | 0.616667 | 3.75 |
| LOC103347250 | 2.296 | 1.199322 | 0.00018 | 0.000824 | yes | up | 5.49 | 6.54 | 5.28 | 22.29 | 43.38 | 27.04 | 5.77 | 30.90333 |
| DNAJC28 | 0.327 | -1.61287 | 0.000181 | 0.000825 | yes | down | 2.19 | 1.72 | 1.27 | 1.15 | 2.12 | 0.64 | 1.726667 | 1.303333 |
| PRDM16 | 0.283 | -1.82302 | 0.000182 | 0.000831 | yes | down | 1.34 | 1.42 | 0.65 | 0.49 | 0.77 | 0.91 | 1.136667 | 0.723333 |
| PRKCDBP | 0.403 | -1.31283 | 0.000183 | 0.000832 | yes | down | 90.17 | 176.24 | 189.37 | 145.32 | 115.75 | 163.84 | 151.9267 | 141.6367 |
| PLOD2 | 2.037 | 1.026655 | 0.000183 | 0.000836 | yes | up | 2.95 | 2.17 | 2.97 | 14.08 | 15.04 | 9.33 | 2.696667 | 12.81667 |
| RNF217 | 0.446 | -1.16405 | 0.000184 | 0.000836 | yes | down | 1.41 | 1.02 | 1.5 | 1.5 | 1.3 | 1.25 | 1.31 | 1.35 |
| SLC38A10 | 2.268 | 1.181185 | 0.000184 | 0.000836 | yes | up | 19.04 | 23.09 | 21.13 | 67.99 | 117.61 | 146.2 | 21.08667 | 110.6 |
| UBXN7 | 0.466 | -1.10072 | 0.000185 | 0.000843 | yes | down | 6.64 | 5.94 | 9.07 | 9.25 | 10.36 | 6.76 | 7.216667 | 8.79 |
| DCAF6 | 0.382 | -1.38877 | 0.000185 | 0.000843 | yes | down | 37.21 | 15.69 | 11.12 | 17.37 | 21.24 | 17.05 | 21.34 | 18.55333 |
| HMGCR | 2.056 | 1.039632 | 0.000186 | 0.000847 | yes | up | 3.84 | 4.57 | 3.32 | 24.25 | 14.7 | 15.46 | 3.91 | 18.13667 |
| SAMD13 | 0.291 | -1.78076 | 0.000188 | 0.000856 | yes | down | 4.87 | 4.55 | 2.99 | 5.88 | 10.88 | 7.7 | 4.136667 | 8.153333 |
| ST6GALNAC4 | 2.426 | 1.278519 | 0.000188 | 0.000856 | yes | up | 8.41 | 4.23 | 3.34 | 30.94 | 23.32 | 31.06 | 5.326667 | 28.44 |
| PNRC2 | 0.47 | -1.08856 | 0.000188 | 0.000856 | yes | down | 56.13 | 60.29 | 60.29 | 63.4 | 95.33 | 37.34 | 58.90333 | 65.35667 |
| SENP7 | 0.456 | -1.13283 | 0.00019 | 0.000861 | yes | down | 2.6 | 1.82 | 2.16 | 2.66 | 2.41 | 1.48 | 2.193333 | 2.183333 |
| LOC103348279 | 42.317 | 5.403159 | 0.00019 | 0.000861 | yes | up | 0 | 0 | 0.19 | 8.06 | 4.01 | 8.38 | 0.063333 | 6.816667 |
| TNK2 | 2.525 | 1.336063 | 0.00019 | 0.000863 | yes | up | 10.45 | 11 | 3.82 | 31.92 | 50.97 | 56.79 | 8.423333 | 46.56 |
| ID1 | 0.44 | -1.18523 | 0.000192 | 0.000871 | yes | down | 43.2 | 53.86 | 39.08 | 34.61 | 35.84 | 64.2 | 45.38 | 44.88333 |
| LCORL | 0.438 | -1.19177 | 0.000193 | 0.000878 | yes | down | 3.39 | 3.67 | 2.79 | 4.6 | 4.42 | 2.12 | 3.283333 | 3.713333 |
| LOC103347553 | 9.796 | 3.292203 | 0.000194 | 0.000879 | yes | up | 0.36 | 0.11 | 0.32 | 3.27 | 4.94 | 9.06 | 0.263333 | 5.756667 |
| DNM1 | 3.095 | 1.630064 | 0.000194 | 0.000881 | yes | up | 3.29 | 2.28 | 0.76 | 11.03 | 19.14 | 12.7 | 2.11 | 14.29 |
| WDR72 | 0.421 | -1.24859 | 0.000202 | 0.000914 | yes | down | 3.39 | 2.24 | 2.69 | 2.35 | 1.73 | 3.17 | 2.773333 | 2.416667 |
| LIF | 4.437 | 2.149486 | 0.000204 | 0.000921 | yes | up | 0.58 | 0.1 | 0.07 | 0.89 | 0.95 | 1.18 | 0.25 | 1.006667 |
| KIF3C | 2.037 | 1.026187 | 0.000205 | 0.000926 | yes | up | 3.03 | 1.81 | 1.67 | 7.84 | 11.67 | 10.54 | 2.17 | 10.01667 |
| LOC100355630 | 54.016 | 5.755324 | 0.000205 | 0.000926 | yes | up | 0 | 0 | 0 | 1.15 | 0.84 | 0.86 | 0 | 0.95 |
| ING3 | 0.483 | -1.05027 | 0.000208 | 0.000938 | yes | down | 4.94 | 5.49 | 4.53 | 6.12 | 6.73 | 3.73 | 4.986667 | 5.526667 |
| PLEKHA6 | 2.915 | 1.54363 | 0.00021 | 0.000949 | yes | up | 0.28 | 0.17 | 0.24 | 1.02 | 2.33 | 1.36 | 0.23 | 1.57 |
| SNX16 | 0.42 | -1.25016 | 0.000211 | 0.00095 | yes | down | 2.13 | 2.05 | 1.51 | 2.35 | 1.96 | 1.12 | 1.896667 | 1.81 |
| SLC37A1 | 0.239 | -2.06759 | 0.000211 | 0.000951 | yes | down | 1.41 | 1.52 | 0.54 | 0.4 | 0.52 | 0.92 | 1.156667 | 0.613333 |
| RUNDC3A | 0.074 | -3.76203 | 0.000211 | 0.000952 | yes | down | 0.44 | 1.09 | 0.59 | 0.29 | 0 | 0.05 | 0.706667 | 0.113333 |
| VPS54 | 0.484 | -1.04794 | 0.000211 | 0.000952 | yes | down | 3.73 | 4.04 | 4.37 | 5.05 | 5.65 | 2.98 | 4.046667 | 4.56 |
| TRIM25 | 2.079 | 1.05558 | 0.000211 | 0.000952 | yes | up | 3.45 | 4.36 | 3.22 | 12.81 | 26.93 | 13.77 | 3.676667 | 17.83667 |
| PCYOX1L | 2.09 | 1.063338 | 0.000212 | 0.000952 | yes | up | 2.53 | 3.06 | 1.89 | 15.29 | 18.49 | 9.2 | 2.493333 | 14.32667 |
| FAM120C | 2.004 | 1.002885 | 0.000212 | 0.000955 | yes | up | 1.25 | 0.78 | 0.97 | 3.85 | 5.72 | 4.11 | 1 | 4.56 |
| IL2RG | 10.514 | 3.39421 | 0.000214 | 0.000962 | yes | up | 0.19 | 0.09 | 0.25 | 3.21 | 2.88 | 6.64 | 0.176667 | 4.243333 |
| PPP1R9A | 2.225 | 1.153613 | 0.000215 | 0.000965 | yes | up | 1.92 | 1.44 | 0.72 | 6.55 | 8.52 | 5.27 | 1.36 | 6.78 |
| HPS6 | 2.292 | 1.196451 | 0.000215 | 0.000965 | yes | up | 1.02 | 1.13 | 1.07 | 5.57 | 7.64 | 4.04 | 1.073333 | 5.75 |
| TMF1 | 0.442 | -1.1788 | 0.000215 | 0.000966 | yes | down | 4.26 | 3.79 | 3.5 | 4.06 | 5.76 | 2.09 | 3.85 | 3.97 |
| GDF10 | 4.965 | 2.311671 | 0.000217 | 0.000974 | yes | up | 0.3 | 0.53 | 0.17 | 1.99 | 2.44 | 6.48 | 0.333333 | 3.636667 |
| KLF4 | 2.209 | 1.14307 | 0.000217 | 0.000974 | yes | up | 8.81 | 12.86 | 9.15 | 48.62 | 37.94 | 66.38 | 10.27333 | 50.98 |
| FAM81A | 2.8 | 1.485672 | 0.000218 | 0.000979 | yes | up | 0.61 | 0.39 | 0.23 | 3.13 | 2.59 | 2.38 | 0.41 | 2.7 |
| IGSF22 | 0.294 | -1.76426 | 0.000218 | 0.00098 | yes | down | 0.59 | 1.28 | 1.06 | 0.46 | 0.77 | 0.35 | 0.976667 | 0.526667 |
| COQ4 | 0.412 | -1.2803 | 0.00022 | 0.000985 | yes | down | 7.4 | 7.12 | 6.39 | 7.55 | 4.29 | 7.55 | 6.97 | 6.463333 |
| LOC100350798 | 81.156 | 6.342628 | 0.00022 | 0.000986 | yes | up | 0 | 0 | 0 | 0.37 | 7.66 | 19.87 | 0 | 9.3 |
| RWDD1 | 0.456 | -1.13242 | 0.000225 | 0.001006 | yes | down | 29.41 | 29.84 | 27.54 | 41.16 | 32.88 | 17.36 | 28.93 | 30.46667 |
| CCDC50 | 0.494 | -1.01664 | 0.000227 | 0.001019 | yes | down | 6.77 | 6.94 | 6.46 | 7.98 | 10.96 | 4.61 | 6.723333 | 7.85 |
| LOC100342508 | 0.343 | -1.54186 | 0.000231 | 0.001033 | yes | down | 2.41 | 3.24 | 1.87 | 3.16 | 1.47 | 1.15 | 2.506667 | 1.926667 |
| CNTNAP4 | 0.03 | -5.06847 | 0.000231 | 0.001034 | yes | down | 0.07 | 0.12 | 0.22 | 0 | 0.01 | 0.02 | 0.136667 | 0.01 |
| TENM2 | 5.747 | 2.522776 | 0.000232 | 0.001036 | yes | up | 0.05 | 0.09 | 0.03 | 0.21 | 1.18 | 0.83 | 0.056667 | 0.74 |
| LRRC40 | 0.444 | -1.17237 | 0.000232 | 0.001036 | yes | down | 4.69 | 3.43 | 4.98 | 9.78 | 9.95 | 5.07 | 4.366667 | 8.266667 |
| ORC3 | 0.408 | -1.29336 | 0.000233 | 0.00104 | yes | down | 3.7 | 3.56 | 2.85 | 3.99 | 3.82 | 1.64 | 3.37 | 3.15 |
| CACNA1C | 2.301 | 1.201985 | 0.000234 | 0.001044 | yes | up | 1.41 | 3.8 | 0.69 | 14.43 | 13.3 | 11.79 | 1.966667 | 13.17333 |
| LOC100339055 | 2.446 | 1.290484 | 0.000234 | 0.001044 | yes | up | 0.64 | 0.89 | 0.46 | 4.17 | 3.94 | 2.62 | 0.663333 | 3.576667 |
| LOC100342660 | 0.051 | -4.30213 | 0.000235 | 0.001049 | yes | down | 0.16 | 0.35 | 0.23 | 0 | 0.05 | 0.02 | 0.246667 | 0.023333 |
| CCSAP | 2.492 | 1.317316 | 0.000236 | 0.001052 | yes | up | 0.42 | 0.38 | 0.25 | 2.27 | 2.04 | 1.58 | 0.35 | 1.963333 |
| KIF21B | 0.327 | -1.6119 | 0.000236 | 0.001052 | yes | down | 1.11 | 1.98 | 0.46 | 0.82 | 1.02 | 0.76 | 1.183333 | 0.866667 |
| LOC108175448 | 0.2 | -2.3202 | 0.000237 | 0.001058 | yes | down | 1.15 | 1.97 | 3.52 | 1.39 | 1.27 | 0.32 | 2.213333 | 0.993333 |
| ADCY3 | 0.424 | -1.23904 | 0.000239 | 0.001064 | yes | down | 20.21 | 35.2 | 27.59 | 19.7 | 44.89 | 18.94 | 27.66667 | 27.84333 |
| NTN1 | 2.186 | 1.128104 | 0.000239 | 0.001066 | yes | up | 2.48 | 1.77 | 2.15 | 10.17 | 9.48 | 12.22 | 2.133333 | 10.62333 |
| SLF2 | 0.495 | -1.0142 | 0.000241 | 0.001071 | yes | down | 8.27 | 6.21 | 6.8 | 7.3 | 11.99 | 5.28 | 7.093333 | 8.19 |
| CYP4A5 | 0.06 | -4.05002 | 0.000241 | 0.001074 | yes | down | 0.34 | 0.37 | 0.34 | 0.07 | 0 | 0.08 | 0.35 | 0.05 |
| EXOSC5 | 0.437 | -1.19277 | 0.000248 | 0.001103 | yes | down | 12.84 | 17.29 | 17.79 | 11.72 | 16.68 | 20.24 | 15.97333 | 16.21333 |
| C19H17orf75 | 0.498 | -1.00486 | 0.000252 | 0.001118 | yes | down | 4.54 | 4.83 | 3.81 | 4.27 | 8.03 | 4.08 | 4.393333 | 5.46 |
| CHSY3 | 2.431 | 1.281713 | 0.000252 | 0.00112 | yes | up | 0.5 | 0.5 | 0.36 | 2.62 | 2.32 | 2.52 | 0.453333 | 2.486667 |
| ODC1 | 0.403 | -1.30986 | 0.000253 | 0.001123 | yes | down | 78.18 | 183.49 | 105.57 | 146 | 80.41 | 106.76 | 122.4133 | 111.0567 |
| GRIN3A | 2.297 | 1.199561 | 0.000254 | 0.001124 | yes | up | 0.56 | 0.29 | 0.34 | 2.29 | 2.3 | 1.65 | 0.396667 | 2.08 |
| PCBP3 | 3.325 | 1.733375 | 0.000255 | 0.00113 | yes | up | 0.44 | 0.6 | 0.43 | 2.29 | 4.15 | 4.87 | 0.49 | 3.77 |
| HTR4 | 5.614 | 2.489011 | 0.000255 | 0.00113 | yes | up | 0.05 | 0.04 | 0.03 | 0.5 | 0.56 | 0.43 | 0.04 | 0.496667 |
| RLA-A3 | 2.151 | 1.10482 | 0.000256 | 0.001133 | yes | up | 29.24 | 33.95 | 33.97 | 125.98 | 120.25 | 207.25 | 32.38667 | 151.16 |
| LOC103348828 | 0.352 | -1.50626 | 0.000256 | 0.001133 | yes | down | 0.89 | 0.83 | 0.69 | 0.67 | 0.84 | 0.65 | 0.803333 | 0.72 |
| MT3 | 0.356 | -1.49071 | 0.000257 | 0.001136 | yes | down | 70.08 | 41.41 | 37.82 | 39.06 | 25.28 | 55.33 | 49.77 | 39.89 |
| TIMELESS | 2.919 | 1.545522 | 0.000257 | 0.001136 | yes | up | 1.18 | 0.8 | 0.52 | 5.59 | 8.12 | 2.61 | 0.833333 | 5.44 |
| STARD4 | 3.198 | 1.677195 | 0.000258 | 0.001141 | yes | up | 0.99 | 1.46 | 1.52 | 16.81 | 7.64 | 4.81 | 1.323333 | 9.753333 |
| ORC2 | 0.471 | -1.08704 | 0.000259 | 0.001145 | yes | down | 2.89 | 2.89 | 3.02 | 3.58 | 4.05 | 1.98 | 2.933333 | 3.203333 |
| DDAH1 | 0.324 | -1.62798 | 0.000261 | 0.001151 | yes | down | 14.17 | 4.66 | 4.03 | 5.38 | 3.74 | 6.84 | 7.62 | 5.32 |
| LOC100345438 | 0.331 | -1.59319 | 0.000262 | 0.001157 | yes | down | 5.11 | 3.12 | 2.05 | 2.77 | 2.02 | 2.77 | 3.426667 | 2.52 |
| IER2 | 2.609 | 1.383372 | 0.000262 | 0.001157 | yes | up | 1.87 | 1.66 | 1.88 | 12.4 | 7.42 | 12.16 | 1.803333 | 10.66 |
| LOC103350124 | 7.379 | 2.883381 | 0.000263 | 0.001158 | yes | up | 0.37 | 0.14 | 0 | 3.15 | 2.31 | 1.7 | 0.17 | 2.386667 |
| SEZ6L2 | 8.789 | 3.135625 | 0.000263 | 0.00116 | yes | up | 0.19 | 0.05 | 0.02 | 3.41 | 0.98 | 0.48 | 0.086667 | 1.623333 |
| GTF3C3 | 0.49 | -1.02823 | 0.000265 | 0.001168 | yes | down | 5.89 | 5.57 | 5.15 | 6.26 | 8.96 | 3.8 | 5.536667 | 6.34 |
| HN1 | 2.492 | 1.317112 | 0.000266 | 0.001171 | yes | up | 22.8 | 10.25 | 6.22 | 68.62 | 67.28 | 58.13 | 13.09 | 64.67667 |
| PPM1L | 0.474 | -1.07659 | 0.000267 | 0.001175 | yes | down | 1.55 | 1.11 | 1.5 | 1.54 | 1.83 | 1.19 | 1.386667 | 1.52 |
| FXYD3 | 25.777 | 4.687999 | 0.000271 | 0.001191 | yes | up | 0.07 | 0.07 | 0 | 4 | 1.72 | 2.15 | 0.046667 | 2.623333 |
| PPM1E | 0.283 | -1.823 | 0.000271 | 0.001191 | yes | down | 0.33 | 0.36 | 0.26 | 0.28 | 0.16 | 0.16 | 0.316667 | 0.2 |
| ZNF322 | 0.49 | -1.02904 | 0.000272 | 0.001195 | yes | down | 6.42 | 7.8 | 6.42 | 8.14 | 10.6 | 4.6 | 6.88 | 7.78 |
| LOC100359245 | 2.551 | 1.351182 | 0.000272 | 0.001195 | yes | up | 103.17 | 47.73 | 45.98 | 467.87 | 468.26 | 207.19 | 65.62667 | 381.1067 |
| ZDBF2 | 0.109 | -3.20081 | 0.000274 | 0.001201 | yes | down | 0.78 | 0.12 | 0.04 | 0.11 | 0.06 | 0.06 | 0.313333 | 0.076667 |
| SLC16A3 | 0.166 | -2.59308 | 0.000275 | 0.001208 | yes | down | 8.02 | 1.79 | 1 | 1.44 | 0.78 | 1.65 | 3.603333 | 1.29 |
| LOC100338913 | 5.475 | 2.45295 | 0.000277 | 0.001216 | yes | up | 0.24 | 1.02 | 0.49 | 11.98 | 5.77 | 3.24 | 0.583333 | 6.996667 |
| LOC100343291 | 2.197 | 1.135355 | 0.000279 | 0.001221 | yes | up | 0.59 | 0.83 | 0.65 | 3.79 | 3.53 | 3.09 | 0.69 | 3.47 |
| CMTM7 | 2.247 | 1.167712 | 0.00028 | 0.001228 | yes | up | 4.57 | 6.85 | 3.94 | 22.95 | 23.83 | 31.16 | 5.12 | 25.98 |
| RANBP3L | 4.612 | 2.205536 | 0.000283 | 0.001239 | yes | up | 0.14 | 0.14 | 0.32 | 2.83 | 2.16 | 0.95 | 0.2 | 1.98 |
| ITGB2 | 2.237 | 1.161513 | 0.000284 | 0.001243 | yes | up | 3.35 | 3.22 | 2.86 | 11.34 | 16.24 | 23.75 | 3.143333 | 17.11 |
| LOC108177483 | 50.609 | 5.661309 | 0.000289 | 0.001263 | yes | up | 0 | 0 | 0 | 4.65 | 4.87 | 5.16 | 0 | 4.893333 |
| B4GAT1 | 2.098 | 1.069346 | 0.00029 | 0.001267 | yes | up | 6.59 | 4.53 | 4.93 | 24.95 | 20.35 | 30.7 | 5.35 | 25.33333 |
| CST3 | 3.038 | 1.60326 | 0.000292 | 0.001276 | yes | up | 286.88 | 305.13 | 484.78 | 1492.35 | 1781.49 | 4304.03 | 358.93 | 2525.957 |
| CD40 | 2.098 | 1.069346 | 0.000293 | 0.001281 | yes | up | 2.02 | 2.22 | 2.26 | 11.28 | 13.18 | 7.33 | 2.166667 | 10.59667 |
| CAST | 2.073 | 1.051623 | 0.000294 | 0.001286 | yes | up | 28.7 | 20.91 | 13.17 | 81.76 | 92.35 | 121.96 | 20.92667 | 98.69 |
| PTPRT | 0.299 | -1.74136 | 0.000296 | 0.001291 | yes | down | 0.28 | 0.41 | 0.5 | 0.13 | 0.4 | 0.31 | 0.396667 | 0.28 |
| IFT74 | 0.354 | -1.49734 | 0.0003 | 0.00131 | yes | down | 1.17 | 1.34 | 1.92 | 1.13 | 1.58 | 0.91 | 1.476667 | 1.206667 |
| IL32 | 2.862 | 1.516811 | 0.000301 | 0.001312 | yes | up | 2.14 | 2.11 | 2.08 | 9.88 | 13.15 | 18.39 | 2.11 | 13.80667 |
| PLXNA1 | 2.26 | 1.17629 | 0.000301 | 0.001314 | yes | up | 4.24 | 3.24 | 1.97 | 11.19 | 25.18 | 12.66 | 3.15 | 16.34333 |
| FMOD | 3.642 | 1.86477 | 0.000306 | 0.001332 | yes | up | 2.63 | 1.66 | 0.87 | 7.78 | 6.87 | 22.64 | 1.72 | 12.43 |
| PTPN6 | 2.059 | 1.041779 | 0.000307 | 0.001337 | yes | up | 1.65 | 1.94 | 1.29 | 6.85 | 7.33 | 8.56 | 1.626667 | 7.58 |
| FUCA2 | 2.192 | 1.132037 | 0.000308 | 0.001339 | yes | up | 1.38 | 0.84 | 0.68 | 4.89 | 4.4 | 5.47 | 0.966667 | 4.92 |
| LOC100343802 | 0.414 | -1.2738 | 0.000309 | 0.001343 | yes | down | 179.99 | 300.58 | 118.51 | 195.08 | 133.35 | 222.79 | 199.6933 | 183.74 |
| TMEM51 | 2.707 | 1.436761 | 0.000309 | 0.001344 | yes | up | 0.99 | 0.77 | 0.66 | 3.79 | 5.69 | 5.63 | 0.806667 | 5.036667 |
| LOC108178025 | 3.062 | 1.614355 | 0.000309 | 0.001344 | yes | up | 1.64 | 1.7 | 1.46 | 7.22 | 9.23 | 16.78 | 1.6 | 11.07667 |
| LOC100340053 | 0.206 | -2.28144 | 0.000311 | 0.00135 | yes | down | 0.74 | 0.71 | 0.74 | 0.41 | 0.31 | 0.34 | 0.73 | 0.353333 |
| TMEM35B | 2.95 | 1.560539 | 0.000311 | 0.00135 | yes | up | 3.24 | 6.45 | 1.85 | 22.72 | 20.53 | 30.93 | 3.846667 | 24.72667 |
| MEP1B | 6.093 | 2.607235 | 0.000312 | 0.001354 | yes | up | 0.1 | 0.09 | 0.04 | 1.39 | 0.81 | 0.92 | 0.076667 | 1.04 |
| KCNMB3 | 0.336 | -1.57524 | 0.000314 | 0.001362 | yes | down | 1.38 | 2.14 | 2.22 | 1.92 | 1.96 | 0.73 | 1.913333 | 1.536667 |
| PIBF1 | 0.427 | -1.22859 | 0.000315 | 0.001364 | yes | down | 2.98 | 2.97 | 3.67 | 3.31 | 4.47 | 1.91 | 3.206667 | 3.23 |
| LOC100353477 | 0.461 | -1.11769 | 0.000315 | 0.001365 | yes | down | 4.88 | 4.53 | 3.13 | 5.09 | 5.01 | 3.01 | 4.18 | 4.37 |
| LOC100353320 | 0.015 | -6.02055 | 0.000317 | 0.001373 | yes | down | 405.02 | 452.44 | 608.22 | 32.09 | 0 | 18.42 | 488.56 | 16.83667 |
| MANBA | 2.836 | 1.503924 | 0.000318 | 0.001377 | yes | up | 0.45 | 0.51 | 0.14 | 2.58 | 2.25 | 2.66 | 0.366667 | 2.496667 |
| KCNQ1 | 0.491 | -1.02721 | 0.000321 | 0.001389 | yes | down | 0.67 | 0.79 | 0.59 | 1.59 | 1.85 | 1.32 | 0.683333 | 1.586667 |
| TPRKB | 0.477 | -1.06673 | 0.000323 | 0.001398 | yes | down | 10.59 | 10.32 | 7.44 | 13.05 | 10.24 | 6.55 | 9.45 | 9.946667 |
| NDUFA4L2 | 2.167 | 1.115788 | 0.000324 | 0.001401 | yes | up | 10.55 | 10.29 | 10.09 | 71.93 | 43.4 | 40.12 | 10.31 | 51.81667 |
| RAD51B | 4.267 | 2.093317 | 0.000325 | 0.001405 | yes | up | 5.49 | 4.68 | 1.48 | 20.59 | 13.74 | 57.4 | 3.883333 | 30.57667 |
| MYH13 | 0.027 | -5.23324 | 0.000326 | 0.00141 | yes | down | 247.41 | 46.22 | 8.12 | 2.41 | 0.3 | 10.78 | 100.5833 | 4.496667 |
| PTTG1 | 2.232 | 1.158604 | 0.000327 | 0.00141 | yes | up | 6.78 | 6.71 | 5.18 | 36.9 | 39.48 | 19.62 | 6.223333 | 32 |
| DIXDC1 | 2.707 | 1.436569 | 0.000327 | 0.001412 | yes | up | 1.37 | 0.69 | 0.31 | 5.82 | 3.88 | 4.93 | 0.79 | 4.876667 |
| PXDNL | 7.312 | 2.870314 | 0.000332 | 0.001429 | yes | up | 0.1 | 0.16 | 0.24 | 5.9 | 0.39 | 1.95 | 0.166667 | 2.746667 |
| LOC100340177 | 0.2 | -2.32033 | 0.000334 | 0.001439 | yes | down | 1.01 | 8.01 | 3.84 | 2.33 | 4.52 | 0.91 | 4.286667 | 2.586667 |
| MEGF9 | 0.465 | -1.10438 | 0.000334 | 0.00144 | yes | down | 17.37 | 26.1 | 12.43 | 20.57 | 27.36 | 12.36 | 18.63333 | 20.09667 |
| BUB1B | 3.557 | 1.830804 | 0.000336 | 0.001446 | yes | up | 1.23 | 0.82 | 0.29 | 7.39 | 8.57 | 2.65 | 0.78 | 6.203333 |
| IL1R1 | 2.031 | 1.022006 | 0.000339 | 0.001456 | yes | up | 5.02 | 5.05 | 4.35 | 19.55 | 34.84 | 14.07 | 4.806667 | 22.82 |
| MZT1 | 0.432 | -1.21148 | 0.000339 | 0.001457 | yes | down | 9 | 5.92 | 3.55 | 6.36 | 7.27 | 4.02 | 6.156667 | 5.883333 |
| PIN4 | 0.482 | -1.0517 | 0.000341 | 0.001464 | yes | down | 43.3 | 30.5 | 25.32 | 42.79 | 40.3 | 26.34 | 33.04 | 36.47667 |
| SNAI2 | 2.2 | 1.137281 | 0.000343 | 0.001471 | yes | up | 4.32 | 3.66 | 2.35 | 22.69 | 13.84 | 13.95 | 3.443333 | 16.82667 |
| FBXL3 | 0.445 | -1.16672 | 0.000343 | 0.001474 | yes | down | 8.74 | 8.43 | 11.35 | 11.37 | 12.88 | 5.7 | 9.506667 | 9.983333 |
| GORAB | 0.484 | -1.0482 | 0.000345 | 0.00148 | yes | down | 6.37 | 4.55 | 3.6 | 8.85 | 5.02 | 5.87 | 4.84 | 6.58 |
| LOC100340124 | 2.855 | 1.51374 | 0.000347 | 0.001488 | yes | up | 28.29 | 14.98 | 10.78 | 70.06 | 209.04 | 78.72 | 18.01667 | 119.2733 |
| SPOCK2 | 2.277 | 1.186952 | 0.000347 | 0.001488 | yes | up | 2.23 | 1.37 | 1.12 | 6.13 | 7.56 | 10.26 | 1.573333 | 7.983333 |
| ABCC4 | 0.435 | -1.20211 | 0.000347 | 0.001488 | yes | down | 2.66 | 4.36 | 2.1 | 2.39 | 2.59 | 3.87 | 3.04 | 2.95 |
| ESRRB | 0.045 | -4.4633 | 0.000354 | 0.001515 | yes | down | 1.82 | 0.23 | 0.01 | 0.03 | 0.01 | 0.16 | 0.686667 | 0.066667 |
| PHF3 | 0.453 | -1.14318 | 0.000355 | 0.001519 | yes | down | 5.99 | 5.29 | 8.13 | 7.34 | 9.28 | 4.41 | 6.47 | 7.01 |
| MTHFS | 2.003 | 1.002268 | 0.000356 | 0.001523 | yes | up | 4.66 | 6.57 | 4.4 | 26.38 | 21.06 | 22.3 | 5.21 | 23.24667 |
| LOC100350067 | 0.022 | -5.52059 | 0.000358 | 0.00153 | yes | down | 0.42 | 0.5 | 0.63 | 0 | 0.07 | 0 | 0.516667 | 0.023333 |
| LOC100347254 | 4.93 | 2.301588 | 0.000364 | 0.001551 | yes | up | 0.64 | 0.63 | 0.12 | 2.6 | 4.98 | 7.39 | 0.463333 | 4.99 |
| KCNS1 | 19.842 | 4.310449 | 0.000367 | 0.001563 | yes | up | 0.02 | 0.02 | 0.02 | 0.43 | 0.35 | 1.69 | 0.02 | 0.823333 |
| TCTEX1D2 | 0.385 | -1.37692 | 0.000368 | 0.001565 | yes | down | 4.94 | 6.21 | 3.92 | 4.2 | 3.28 | 5.56 | 5.023333 | 4.346667 |
| LOC103349722 | 0.491 | -1.02552 | 0.00037 | 0.001577 | yes | down | 48.56 | 86.06 | 57.82 | 71.28 | 67.33 | 79.46 | 64.14667 | 72.69 |
| LOC100349217 | 0.032 | -4.95818 | 0.000372 | 0.001581 | yes | down | 0.25 | 0.59 | 0.62 | 0.05 | 0.05 | 0 | 0.486667 | 0.033333 |
| SLC19A3 | 44.705 | 5.48235 | 0.000374 | 0.00159 | yes | up | 0.02 | 0 | 0 | 0.26 | 0.48 | 1.48 | 0.006667 | 0.74 |
| JCHAIN | 3.634 | 1.861701 | 0.000377 | 0.001602 | yes | up | 2.16 | 0.61 | 0.64 | 5.58 | 14.08 | 9.59 | 1.136667 | 9.75 |
| NIPAL4 | 3.906 | 1.965825 | 0.000379 | 0.001608 | yes | up | 0.18 | 0.17 | 0.11 | 1.4 | 1.88 | 0.82 | 0.153333 | 1.366667 |
| MYBL2 | 4.029 | 2.010523 | 0.000381 | 0.001615 | yes | up | 0.54 | 0.5 | 0.39 | 5.39 | 6.8 | 1.24 | 0.476667 | 4.476667 |
| CADPS2 | 0.492 | -1.02307 | 0.000383 | 0.00162 | yes | down | 3.71 | 3.75 | 3.8 | 5.29 | 4.71 | 2.72 | 3.753333 | 4.24 |
| KCNE4 | 2.33 | 1.220611 | 0.000391 | 0.001653 | yes | up | 3.63 | 5.24 | 1.76 | 15.08 | 12 | 18.49 | 3.543333 | 15.19 |
| DOCK2 | 2.161 | 1.11191 | 0.000392 | 0.001654 | yes | up | 0.37 | 0.44 | 0.26 | 1.57 | 2.02 | 1.65 | 0.356667 | 1.746667 |
| SRD5A3 | 2.126 | 1.088058 | 0.000395 | 0.001668 | yes | up | 2.84 | 3.42 | 2.99 | 18.1 | 14.28 | 12.65 | 3.083333 | 15.01 |
| TMTC2 | 0.465 | -1.10522 | 0.000397 | 0.001677 | yes | down | 13.29 | 9.45 | 7.76 | 9.65 | 10.34 | 4.12 | 10.16667 | 8.036667 |
| PLAUR | 2.749 | 1.45902 | 0.000404 | 0.001703 | yes | up | 2.5 | 1.14 | 1.9 | 11.92 | 13.98 | 7.21 | 1.846667 | 11.03667 |
| PPM1F | 2.073 | 1.051995 | 0.000413 | 0.00174 | yes | up | 9.64 | 10.07 | 9.28 | 36.26 | 75.43 | 30.24 | 9.663333 | 47.31 |
| KIAA0895 | 4.426 | 2.145985 | 0.000414 | 0.001743 | yes | up | 0.12 | 0.09 | 0.05 | 0.75 | 1.01 | 0.87 | 0.086667 | 0.876667 |
| HJURP | 3.489 | 1.802757 | 0.000416 | 0.001748 | yes | up | 0.46 | 0.58 | 0.17 | 2.85 | 5.08 | 1.07 | 0.403333 | 3 |
| ABCC6 | 2.998 | 1.58393 | 0.000417 | 0.001752 | yes | up | 0.19 | 0.15 | 0.09 | 0.87 | 1.05 | 0.99 | 0.143333 | 0.97 |
| MX1 | 2.149 | 1.103394 | 0.000418 | 0.001756 | yes | up | 22.32 | 19.1 | 13.13 | 69.56 | 68.84 | 120.07 | 18.18333 | 86.15667 |
| MTR | 0.496 | -1.01061 | 0.000419 | 0.001759 | yes | down | 1.75 | 1.39 | 1.43 | 1.15 | 2.56 | 1.52 | 1.523333 | 1.743333 |
| GINS2 | 3.329 | 1.735096 | 0.000419 | 0.001759 | yes | up | 2.23 | 1.6 | 0.72 | 11.44 | 15.74 | 7.1 | 1.516667 | 11.42667 |
| LOC103345228 | 0.199 | -2.33123 | 0.000426 | 0.001788 | yes | down | 0.39 | 0.19 | 0.24 | 0.12 | 0.21 | 0.05 | 0.273333 | 0.126667 |
| CBX2 | 2.487 | 1.314337 | 0.000427 | 0.001789 | yes | up | 0.52 | 0.41 | 0.32 | 2.05 | 3.34 | 1.82 | 0.416667 | 2.403333 |
| DBR1 | 0.492 | -1.022 | 0.000427 | 0.001791 | yes | down | 1.89 | 2.4 | 1.58 | 2.06 | 2.43 | 2.11 | 1.956667 | 2.2 |
| ZNF471 | 0.417 | -1.2623 | 0.000429 | 0.001798 | yes | down | 1.24 | 1.34 | 1.37 | 1.45 | 1.99 | 0.84 | 1.316667 | 1.426667 |
| SLC2A12 | 3.928 | 1.9737 | 0.000429 | 0.001798 | yes | up | 0.6 | 0.14 | 0.44 | 3.2 | 5.78 | 1.97 | 0.393333 | 3.65 |
| LOC103351277 | 0.406 | -1.3017 | 0.00043 | 0.001801 | yes | down | 4.86 | 2.69 | 2.06 | 3.97 | 2.92 | 2.47 | 3.203333 | 3.12 |
| DCLRE1A | 0.49 | -1.03035 | 0.000431 | 0.001804 | yes | down | 3.39 | 2.89 | 3.57 | 4.25 | 4.66 | 2.49 | 3.283333 | 3.8 |
| CRABP1 | 2.351 | 1.233458 | 0.000431 | 0.001804 | yes | up | 7.22 | 5.62 | 5.62 | 27.1 | 27.26 | 43.91 | 6.153333 | 32.75667 |
| LOC100347331 | 4.185 | 2.065396 | 0.000433 | 0.001809 | yes | up | 0.4 | 0.31 | 0.4 | 3.64 | 2.86 | 4.05 | 0.37 | 3.516667 |
| LOC100350390 | 0.368 | -1.44358 | 0.000433 | 0.00181 | yes | down | 1.11 | 1.04 | 1.25 | 1.01 | 0.83 | 1.03 | 1.133333 | 0.956667 |
| IRX1 | 35.14 | 5.135032 | 0.000434 | 0.001813 | yes | up | 0.05 | 0 | 0 | 1.59 | 0.99 | 1.41 | 0.016667 | 1.33 |
| METTL21C | 0.066 | -3.92127 | 0.000434 | 0.001813 | yes | down | 2.53 | 0.54 | 0.06 | 0.09 | 0.05 | 0.35 | 1.043333 | 0.163333 |
| B3GALNT2 | 2.013 | 1.009383 | 0.000437 | 0.001823 | yes | up | 10 | 5.15 | 4.86 | 26.69 | 33.84 | 30.99 | 6.67 | 30.50667 |
| FIGF | 0.139 | -2.84693 | 0.000442 | 0.001844 | yes | down | 1.2 | 0.79 | 0.43 | 0.33 | 0.43 | 0 | 0.806667 | 0.253333 |
| STYX | 0.49 | -1.02967 | 0.000444 | 0.001853 | yes | down | 3.58 | 2.8 | 1.96 | 3.76 | 3.86 | 2.34 | 2.78 | 3.32 |
| IMPA1 | 0.482 | -1.05313 | 0.000445 | 0.001854 | yes | down | 6.75 | 7.71 | 8.42 | 10.59 | 9.91 | 5.49 | 7.626667 | 8.663333 |
| FBLN7 | 2.909 | 1.540419 | 0.000448 | 0.001866 | yes | up | 0.29 | 0.17 | 0.2 | 1.93 | 1.53 | 0.95 | 0.22 | 1.47 |
| HEATR4 | 0.409 | -1.28876 | 0.000449 | 0.001869 | yes | down | 1.47 | 2.11 | 0.98 | 1.06 | 1.7 | 1.61 | 1.52 | 1.456667 |
| DACT3 | 3.609 | 1.851532 | 0.00045 | 0.001874 | yes | up | 1.62 | 1.08 | 0.51 | 8.72 | 6.38 | 10.38 | 1.07 | 8.493333 |
| LOC108177116 | 0.279 | -1.8394 | 0.000451 | 0.001876 | yes | down | 0.68 | 1.59 | 0.99 | 0.65 | 1.28 | 0.59 | 1.086667 | 0.84 |
| LOC100358731 | 13.218 | 3.724473 | 0.000452 | 0.001879 | yes | up | 0 | 0.04 | 0.16 | 3.26 | 0.89 | 1.77 | 0.066667 | 1.973333 |
| HPSE2 | 9.365 | 3.227283 | 0.000452 | 0.00188 | yes | up | 8.83 | 0.01 | 0.02 | 2.45 | 3.12 | 0.7 | 2.953333 | 2.09 |
| TREX1 | 2.494 | 1.318315 | 0.000453 | 0.001881 | yes | up | 1.95 | 1.52 | 1.89 | 8.65 | 8.98 | 12.71 | 1.786667 | 10.11333 |
| RHAG | 0.014 | -6.14471 | 0.000454 | 0.001887 | yes | down | 0.12 | 0.26 | 0.43 | 0 | 0 | 0 | 0.27 | 0 |
| KCNA3 | 0.179 | -2.48046 | 0.00046 | 0.001907 | yes | down | 0.1 | 0.22 | 0.08 | 0.12 | 0.09 | 0.05 | 0.133333 | 0.086667 |
| LOC108178304 | 0.109 | -3.19254 | 0.00046 | 0.001907 | yes | down | 0.56 | 0.64 | 0.76 | 0.29 | 0.05 | 0.14 | 0.653333 | 0.16 |
| LOC108178580 | 26.919 | 4.750569 | 0.000461 | 0.00191 | yes | up | 0 | 0.08 | 0.08 | 0.86 | 5.14 | 4.55 | 0.053333 | 3.516667 |
| LOC103347403 | 2.912 | 1.541766 | 0.000461 | 0.00191 | yes | up | 0.79 | 1.08 | 0.49 | 5.53 | 4.07 | 6.25 | 0.786667 | 5.283333 |
| COL4A6 | 2.726 | 1.446716 | 0.000471 | 0.001951 | yes | up | 0.15 | 0.22 | 0.16 | 0.94 | 0.99 | 1.34 | 0.176667 | 1.09 |
| ZNF24 | 0.492 | -1.02434 | 0.000471 | 0.001951 | yes | down | 6.52 | 6.09 | 4.38 | 6.44 | 9.23 | 3.66 | 5.663333 | 6.443333 |
| GDF11 | 2.484 | 1.312382 | 0.000471 | 0.001951 | yes | up | 1.23 | 0.66 | 0.51 | 5.61 | 3.88 | 4.19 | 0.8 | 4.56 |
| AGPAT4 | 3.744 | 1.904396 | 0.000472 | 0.001953 | yes | up | 0.07 | 0.09 | 0.03 | 0.58 | 0.43 | 0.49 | 0.063333 | 0.5 |
| LOC100351250 | 2.354 | 1.234957 | 0.00048 | 0.001985 | yes | up | 2.03 | 1.88 | 1.1 | 9.71 | 10.51 | 6.55 | 1.67 | 8.923333 |
| LOC103346344 | 2.381 | 1.251617 | 0.00048 | 0.001986 | yes | up | 5.59 | 5.98 | 4.55 | 31.54 | 15.46 | 35.99 | 5.373333 | 27.66333 |
| SLC1A2 | 0.011 | -6.5108 | 0.000482 | 0.00199 | yes | down | 0.01 | 0.29 | 0.06 | 0 | 0 | 0 | 0.12 | 0 |
| LOC100357574 | 0.251 | -1.99462 | 0.000482 | 0.001992 | yes | down | 1.7 | 1.43 | 0.84 | 0.84 | 0.86 | 0.54 | 1.323333 | 0.746667 |
| LOC103351479 | 2.655 | 1.408533 | 0.000485 | 0.002001 | yes | up | 1.19 | 1.82 | 1.02 | 6.69 | 12.27 | 6.4 | 1.343333 | 8.453333 |
| LOC108176565 | 0.031 | -5.02943 | 0.000485 | 0.002001 | yes | down | 0.06 | 0.3 | 0.21 | 0.02 | 0.02 | 0 | 0.19 | 0.013333 |
| SELE | 6.458 | 2.691175 | 0.000485 | 0.002001 | yes | up | 0.09 | 0.14 | 0.02 | 1.07 | 0.83 | 1.64 | 0.083333 | 1.18 |
| RUFY4 | 0.315 | -1.66511 | 0.000485 | 0.002002 | yes | down | 0.89 | 1 | 0.55 | 0.52 | 0.84 | 1.01 | 0.813333 | 0.79 |
| LPAR5 | 3.226 | 1.689677 | 0.000487 | 0.002007 | yes | up | 0.21 | 0.28 | 0.11 | 1.17 | 1.57 | 1.56 | 0.2 | 1.433333 |
| RET | 4.159 | 2.056089 | 0.000491 | 0.002022 | yes | up | 0.56 | 0.1 | 0.17 | 1.94 | 2.16 | 3.49 | 0.276667 | 2.53 |
| NOL8 | 0.492 | -1.02212 | 0.000492 | 0.002025 | yes | down | 2.98 | 3.13 | 2.62 | 3.32 | 5.24 | 2.31 | 2.91 | 3.623333 |
| HES1 | 2.185 | 1.127955 | 0.000494 | 0.002034 | yes | up | 4.81 | 4.81 | 3.57 | 21.1 | 15.3 | 25.39 | 4.396667 | 20.59667 |
| LOC100348097 | 4.523 | 2.177385 | 0.000494 | 0.002034 | yes | up | 0.06 | 0.34 | 0.29 | 2.12 | 1.65 | 3.52 | 0.23 | 2.43 |
| SOCS4 | 0.456 | -1.13209 | 0.000496 | 0.00204 | yes | down | 2.53 | 3.14 | 2.21 | 3.49 | 3.29 | 1.49 | 2.626667 | 2.756667 |
| TXNRD3 | 0.496 | -1.01228 | 0.0005 | 0.002055 | yes | down | 2.63 | 3.81 | 3.31 | 3.75 | 4.08 | 3.53 | 3.25 | 3.786667 |
| ARNTL2 | 18.546 | 4.213068 | 0.000502 | 0.00206 | yes | up | 0.02 | 0.43 | 0.06 | 7.32 | 11.3 | 2.72 | 0.17 | 7.113333 |
| CCL22 | 17.521 | 4.130996 | 0.000507 | 0.002084 | yes | up | 0 | 0.03 | 0.05 | 0.69 | 0.72 | 2.01 | 0.026667 | 1.14 |
| ZDHHC23 | 4.156 | 2.055082 | 0.000508 | 0.002087 | yes | up | 0.19 | 0.25 | 0.1 | 2.89 | 0.74 | 1.24 | 0.18 | 1.623333 |
| TNFRSF11B | 3.52 | 1.81554 | 0.000511 | 0.002098 | yes | up | 0.81 | 0.5 | 0.3 | 6.12 | 2.56 | 3.71 | 0.536667 | 4.13 |
| MAP1A | 2.922 | 1.546977 | 0.000511 | 0.002098 | yes | up | 1.14 | 0.38 | 0.27 | 3.16 | 4.04 | 4.3 | 0.596667 | 3.833333 |
| NAA50 | 0.485 | -1.04451 | 0.000512 | 0.002099 | yes | down | 38.24 | 20.29 | 15.76 | 26.22 | 25.21 | 28.72 | 24.76333 | 26.71667 |
| LOC108177737 | 14.689 | 3.876683 | 0.000512 | 0.002099 | yes | up | 0.03 | 0.03 | 0.02 | 1.07 | 0.62 | 0.92 | 0.026667 | 0.87 |
| SGF29 | 2.025 | 1.017747 | 0.000512 | 0.002099 | yes | up | 3.63 | 3.49 | 2.52 | 14.33 | 14.13 | 15.69 | 3.213333 | 14.71667 |
| MMP12 | 0.076 | -3.71723 | 0.000512 | 0.002099 | yes | down | 0.35 | 1.26 | 0.15 | 0.04 | 0.16 | 0.1 | 0.586667 | 0.1 |
| ANKRD42 | 0.471 | -1.08733 | 0.000518 | 0.002124 | yes | down | 3.03 | 3.34 | 3.39 | 3.03 | 4.88 | 2.56 | 3.253333 | 3.49 |
| LOC108176648 | 2.912 | 1.542183 | 0.000522 | 0.002137 | yes | up | 0.85 | 1.21 | 0.63 | 5.13 | 6.14 | 6.47 | 0.896667 | 5.913333 |
| LOC108175785 | 4.723 | 2.239621 | 0.000523 | 0.00214 | yes | up | 0.37 | 1.7 | 0.16 | 10.24 | 10.98 | 17.89 | 0.743333 | 13.03667 |
| ACKR4 | 2.448 | 1.291379 | 0.000525 | 0.002147 | yes | up | 0.93 | 0.68 | 0.65 | 2.96 | 4.2 | 5.33 | 0.753333 | 4.163333 |
| SLF1 | 0.486 | -1.04246 | 0.000525 | 0.002147 | yes | down | 0.86 | 0.62 | 0.71 | 0.81 | 1.08 | 0.65 | 0.73 | 0.846667 |
| HAUS2 | 0.482 | -1.05426 | 0.000525 | 0.002147 | yes | down | 4.67 | 5.91 | 2.98 | 5.1 | 4.98 | 3.53 | 4.52 | 4.536667 |
| LRIF1 | 0.469 | -1.09373 | 0.000525 | 0.002147 | yes | down | 1.8 | 2.3 | 1.78 | 2.63 | 2.43 | 1.58 | 1.96 | 2.213333 |
| LOC103347586 | 5.863 | 2.551608 | 0.000525 | 0.002147 | yes | up | 16.18 | 4.47 | 2.55 | 170.38 | 164.94 | 54.54 | 7.733333 | 129.9533 |
| LOC103349265 | 0.435 | -1.20114 | 0.000526 | 0.002149 | yes | down | 11.52 | 15.33 | 7.75 | 11.35 | 9.19 | 13.19 | 11.53333 | 11.24333 |
| DNAJC24 | 0.429 | -1.21989 | 0.000533 | 0.002175 | yes | down | 5.45 | 4.1 | 4.48 | 5.11 | 5.36 | 4.08 | 4.676667 | 4.85 |
| RNF148 | 0.115 | -3.12271 | 0.000534 | 0.002178 | yes | down | 1.03 | 0.79 | 1.02 | 0.33 | 0.18 | 0.23 | 0.946667 | 0.246667 |
| TSTD1 | 0.298 | -1.74429 | 0.000537 | 0.002191 | yes | down | 4.89 | 6.67 | 8.54 | 5.12 | 6.45 | 2.75 | 6.7 | 4.773333 |
| LOC100346439 | 0.141 | -2.8302 | 0.000539 | 0.002194 | yes | down | 6.63 | 8.13 | 2.82 | 0.55 | 4.49 | 0.69 | 5.86 | 1.91 |
| BPI | 0.051 | -4.30394 | 0.000541 | 0.002201 | yes | down | 0.23 | 0.52 | 0.42 | 0.08 | 0 | 0.06 | 0.39 | 0.046667 |
| LOC100343709 | 0.489 | -1.03176 | 0.000547 | 0.002223 | yes | down | 4.53 | 6.31 | 4.17 | 6.01 | 7.33 | 3.35 | 5.003333 | 5.563333 |
| NUDT18 | 2.484 | 1.312433 | 0.000547 | 0.002225 | yes | up | 2.28 | 2.41 | 2.59 | 10.7 | 12.05 | 18.67 | 2.426667 | 13.80667 |
| MAP4K1 | 4.47 | 2.160436 | 0.000549 | 0.00223 | yes | up | 0.14 | 0.16 | 0.23 | 1.78 | 2.15 | 1.59 | 0.176667 | 1.84 |
| LOC103347295 | 6.059 | 2.598988 | 0.000549 | 0.002233 | yes | up | 0.03 | 0.25 | 0.09 | 1.58 | 1.77 | 1.69 | 0.123333 | 1.68 |
| EMB | 0.363 | -1.46055 | 0.000551 | 0.00224 | yes | down | 3.48 | 6.07 | 3 | 2.55 | 5.89 | 1.7 | 4.183333 | 3.38 |
| ZNF543 | 0.484 | -1.04659 | 0.000557 | 0.002261 | yes | down | 1.8 | 1.68 | 1.36 | 1.63 | 2.39 | 1.32 | 1.613333 | 1.78 |
| LOC103349006 | 0.121 | -3.04134 | 0.00056 | 0.002271 | yes | down | 0.37 | 0.23 | 0.57 | 0.11 | 0.2 | 0.04 | 0.39 | 0.116667 |
| MUM1L1 | 0.153 | -2.70428 | 0.000562 | 0.002279 | yes | down | 2.26 | 0.42 | 0.16 | 0.29 | 0.31 | 0.33 | 0.946667 | 0.31 |
| MCM3 | 2.45 | 1.292781 | 0.000566 | 0.002293 | yes | up | 4.71 | 2.72 | 2.06 | 16.68 | 27.16 | 9.45 | 3.163333 | 17.76333 |
| LOC103347364 | 0.291 | -1.78177 | 0.00057 | 0.002309 | yes | down | 2 | 1.72 | 2.12 | 1.5 | 1.78 | 0.67 | 1.946667 | 1.316667 |
| ALAS2 | 0.306 | -1.70822 | 0.000575 | 0.002328 | yes | down | 26.38 | 65.65 | 70.75 | 49.1 | 16.01 | 48.45 | 54.26 | 37.85333 |
| SCLT1 | 0.324 | -1.62432 | 0.000576 | 0.00233 | yes | down | 0.87 | 1.01 | 1.48 | 1 | 1.1 | 0.48 | 1.12 | 0.86 |
| AQP4 | 8.804 | 3.138162 | 0.000577 | 0.002333 | yes | up | 6.11 | 0.94 | 0.78 | 69.24 | 50.03 | 31.25 | 2.61 | 50.17333 |
| ARHGAP4 | 2.344 | 1.228731 | 0.000583 | 0.002354 | yes | up | 0.81 | 0.52 | 0.5 | 3.12 | 2.95 | 3.36 | 0.61 | 3.143333 |
| SH3PXD2A | 2.155 | 1.107835 | 0.000583 | 0.002356 | yes | up | 5.89 | 3.73 | 3.28 | 13.87 | 33.86 | 16.76 | 4.3 | 21.49667 |
| LOC103349362 | 679.055 | 9.407384 | 0.000586 | 0.002365 | yes | up | 0 | 0 | 0 | 0.17 | 16.43 | 37.35 | 0 | 17.98333 |
| CDC42EP2 | 2.218 | 1.149292 | 0.000587 | 0.002371 | yes | up | 10.85 | 10.3 | 4.93 | 49.67 | 30.66 | 46.84 | 8.693333 | 42.39 |
| PLCB1 | 0.499 | -1.00205 | 0.000593 | 0.002393 | yes | down | 2.64 | 2.77 | 1.37 | 2.45 | 3.36 | 1.85 | 2.26 | 2.553333 |
| COL9A1 | 0.015 | -6.09648 | 0.000595 | 0.0024 | yes | down | 0.16 | 0.07 | 0.28 | 0 | 0 | 0 | 0.17 | 0 |
| LOC100351865 | 2.391 | 1.257494 | 0.000595 | 0.002402 | yes | up | 0.41 | 0.43 | 0.41 | 2.37 | 1.98 | 2.62 | 0.416667 | 2.323333 |
| BTNL10 | 0.176 | -2.50269 | 0.000596 | 0.002403 | yes | down | 1.15 | 4.98 | 2.82 | 2.58 | 0.23 | 1.51 | 2.983333 | 1.44 |
| LOC103348003 | 3.546 | 1.826211 | 0.000597 | 0.002408 | yes | up | 1.06 | 0.97 | 0.84 | 4.34 | 9.59 | 9.49 | 0.956667 | 7.806667 |
| ANPEP | 2.405 | 1.266056 | 0.000598 | 0.00241 | yes | up | 1.69 | 1.24 | 0.64 | 5.22 | 7.09 | 6.89 | 1.19 | 6.4 |
| LOC100347480 | 0.081 | -3.62227 | 0.0006 | 0.002419 | yes | down | 9.94 | 27.68 | 11.09 | 2.2 | 0.38 | 5.85 | 16.23667 | 2.81 |
| GGACT | 0.389 | -1.36148 | 0.000604 | 0.002434 | yes | down | 2.7 | 2.18 | 2.83 | 2.31 | 2.12 | 2.4 | 2.57 | 2.276667 |
| FANCD2 | 2.987 | 1.578891 | 0.000612 | 0.002461 | yes | up | 1.89 | 2.76 | 0.7 | 12.74 | 18.43 | 5.6 | 1.783333 | 12.25667 |
| MTFR2 | 3.63 | 1.860118 | 0.000612 | 0.002461 | yes | up | 0.9 | 0.83 | 0.25 | 6.27 | 8.75 | 2.25 | 0.66 | 5.756667 |
| RRAGD | 0.424 | -1.23819 | 0.000614 | 0.00247 | yes | down | 6.53 | 5.73 | 3.04 | 3.42 | 3.95 | 6.91 | 5.1 | 4.76 |
| TRAIP | 3.923 | 1.97211 | 0.000616 | 0.002475 | yes | up | 0.51 | 0.28 | 0.08 | 2.58 | 3.54 | 1.49 | 0.29 | 2.536667 |
| LOC100339625 | 2.051 | 1.036415 | 0.000619 | 0.002488 | yes | up | 2.85 | 1.91 | 2.33 | 12.74 | 12.81 | 8.03 | 2.363333 | 11.19333 |
| LOC100357462 | 23.704 | 4.567054 | 0.000621 | 0.002493 | yes | up | 0 | 0 | 0.69 | 6.05 | 16.7 | 19.29 | 0.23 | 14.01333 |
| LOC103349339 | 0.013 | -6.2667 | 0.000624 | 0.002503 | yes | down | 0.42 | 0.45 | 0.03 | 0 | 0 | 0 | 0.3 | 0 |
| MCAT | 0.471 | -1.08761 | 0.000627 | 0.002515 | yes | down | 4.33 | 4.44 | 4.94 | 4.98 | 3.96 | 5.88 | 4.57 | 4.94 |
| KIF14 | 4.556 | 2.187768 | 0.000628 | 0.002518 | yes | up | 0.34 | 0.21 | 0.07 | 2.46 | 3.28 | 0.54 | 0.206667 | 2.093333 |
| FAM83D | 3.911 | 1.96754 | 0.000628 | 0.00252 | yes | up | 0.47 | 0.23 | 0.12 | 2.49 | 3.12 | 1.53 | 0.273333 | 2.38 |
| LOC108177670 | 4.377 | 2.129998 | 0.000632 | 0.002533 | yes | up | 0.61 | 0.61 | 0.77 | 7.4 | 9.79 | 3.05 | 0.663333 | 6.746667 |
| CMTR2 | 0.48 | -1.05925 | 0.000634 | 0.002538 | yes | down | 1.52 | 1.62 | 1.27 | 1.91 | 1.42 | 1.47 | 1.47 | 1.6 |
| GDPD1 | 0.284 | -1.81627 | 0.000639 | 0.002555 | yes | down | 1.42 | 0.87 | 2.37 | 1.11 | 1.19 | 0.88 | 1.553333 | 1.06 |
| KBTBD11 | 2.143 | 1.099763 | 0.000639 | 0.002555 | yes | up | 1.26 | 1.51 | 1.07 | 4.09 | 7.43 | 7.11 | 1.28 | 6.21 |
| LOC100353821 | 18.882 | 4.238964 | 0.000642 | 0.002567 | yes | up | 0 | 0.07 | 0.03 | 1.46 | 0.37 | 2.24 | 0.033333 | 1.356667 |
| ARG1 | 32.276 | 5.012389 | 0.000643 | 0.002571 | yes | up | 0.05 | 0 | 0 | 1.13 | 1.71 | 1.03 | 0.016667 | 1.29 |
| BAMBI | 3.6 | 1.848133 | 0.000646 | 0.002579 | yes | up | 0.81 | 1.34 | 0.52 | 5.18 | 3.79 | 11.87 | 0.89 | 6.946667 |
| LOC100358590 | 0.339 | -1.56054 | 0.000662 | 0.002637 | yes | down | 1.42 | 4.73 | 1.49 | 2.44 | 1.87 | 1.48 | 2.546667 | 1.93 |
| PTX4 | 0.23 | -2.12304 | 0.000666 | 0.00265 | yes | down | 0.75 | 1.22 | 0.4 | 0.33 | 0.54 | 0.36 | 0.79 | 0.41 |
| TNF | 21.452 | 4.423015 | 0.000668 | 0.002657 | yes | up | 0.04 | 0 | 0.03 | 1.63 | 0.78 | 0.99 | 0.023333 | 1.133333 |
| ARRDC3 | 0.471 | -1.08668 | 0.000669 | 0.002661 | yes | down | 21.95 | 24.31 | 33.29 | 34.02 | 36.76 | 17.95 | 26.51667 | 29.57667 |
| LOC103351453 | 0.186 | -2.42459 | 0.00067 | 0.002666 | yes | down | 2.15 | 3.13 | 1.94 | 1.53 | 1.34 | 0.09 | 2.406667 | 0.986667 |
| SPINT1 | 4.242 | 2.084588 | 0.000671 | 0.002669 | yes | up | 0.29 | 0.18 | 0.12 | 2.04 | 1.07 | 2.35 | 0.196667 | 1.82 |
| BBOX1 | 0.201 | -2.31828 | 0.000673 | 0.002675 | yes | down | 1.07 | 1.45 | 1.26 | 1.2 | 0.24 | 0.26 | 1.26 | 0.566667 |
| FMO2 | 2.577 | 1.365744 | 0.000676 | 0.002686 | yes | up | 3.57 | 1.62 | 2.28 | 8.33 | 19.36 | 7.16 | 2.49 | 11.61667 |
| RHOQ | 0.449 | -1.1554 | 0.000676 | 0.002686 | yes | down | 32.58 | 29.53 | 27.01 | 22.16 | 51.19 | 20.95 | 29.70667 | 31.43333 |
| CCDC126 | 0.496 | -1.01204 | 0.000679 | 0.002696 | yes | down | 10.27 | 11.17 | 10.29 | 14.71 | 16.73 | 7.06 | 10.57667 | 12.83333 |
| LSAMP | 3.52 | 1.815393 | 0.000679 | 0.002697 | yes | up | 0.96 | 0.27 | 0.13 | 3.56 | 1.34 | 1.27 | 0.453333 | 2.056667 |
| RASGRP1 | 0.355 | -1.49334 | 0.00068 | 0.002698 | yes | down | 0.48 | 0.31 | 0.36 | 0.32 | 0.38 | 0.19 | 0.383333 | 0.296667 |
| TMEM120A | 0.477 | -1.0665 | 0.000688 | 0.00273 | yes | down | 23.19 | 21.48 | 18.04 | 19.36 | 31.95 | 18.49 | 20.90333 | 23.26667 |
| LOC100356864 | 0.224 | -2.16113 | 0.000692 | 0.002743 | yes | down | 7.6 | 7.2 | 9.28 | 5.13 | 0.73 | 6.17 | 8.026667 | 4.01 |
| DOCK5 | 2.049 | 1.035208 | 0.000704 | 0.002787 | yes | up | 0.36 | 0.31 | 0.19 | 3.24 | 2.19 | 3.45 | 0.286667 | 2.96 |
| ALCAM | 0.41 | -1.28727 | 0.000712 | 0.002814 | yes | down | 0.74 | 0.93 | 0.42 | 0.57 | 0.74 | 0.62 | 0.696667 | 0.643333 |
| LOC100009135 | 0.046 | -4.43245 | 0.000717 | 0.002833 | yes | down | 0.24 | 8.56 | 2.37 | 0.24 | 0.27 | 0.7 | 3.723333 | 0.403333 |
| ARHGAP33 | 2.654 | 1.408403 | 0.000718 | 0.002837 | yes | up | 0.33 | 0.32 | 0.23 | 1.7 | 2.57 | 1.15 | 0.293333 | 1.806667 |
| BSP1 | 0.411 | -1.28195 | 0.000722 | 0.002849 | yes | down | 509.83 | 538.72 | 433.07 | 753.07 | 306.08 | 321.58 | 493.8733 | 460.2433 |
| BOLA3 | 0.492 | -1.02204 | 0.000723 | 0.002852 | yes | down | 98.4 | 101.25 | 91.4 | 116.77 | 78.02 | 132.14 | 97.01667 | 108.9767 |
| CDH19 | 0.331 | -1.59489 | 0.000725 | 0.002858 | yes | down | 1.87 | 1.19 | 0.49 | 0.6 | 1.09 | 1.05 | 1.183333 | 0.913333 |
| PON3 | 2.751 | 1.460198 | 0.000728 | 0.00287 | yes | up | 2.3 | 1.79 | 1.53 | 9 | 19.75 | 7.41 | 1.873333 | 12.05333 |
| EIF2AK2 | 0.455 | -1.13619 | 0.00073 | 0.002876 | yes | down | 2.37 | 1.55 | 1.72 | 1.51 | 2.29 | 1.81 | 1.88 | 1.87 |
| LPAR4 | 0.349 | -1.5178 | 0.000733 | 0.002887 | yes | down | 2.68 | 5.42 | 7.22 | 6.12 | 5.5 | 2.56 | 5.106667 | 4.726667 |
| CEP57 | 0.474 | -1.07829 | 0.000735 | 0.002894 | yes | down | 3.23 | 3.07 | 2.97 | 3.82 | 4.51 | 1.89 | 3.09 | 3.406667 |
| ZFHX2 | 3.607 | 1.850818 | 0.000736 | 0.002894 | yes | up | 0.05 | 0.06 | 0.04 | 0.3 | 0.7 | 0.32 | 0.05 | 0.44 |
| TRIM14 | 5.449 | 2.446096 | 0.00074 | 0.002912 | yes | up | 0.08 | 0.14 | 0.01 | 0.82 | 0.72 | 1.24 | 0.076667 | 0.926667 |
| TDO2 | 0.016 | -5.93235 | 0.000743 | 0.00292 | yes | down | 0.18 | 0.17 | 0.29 | 0 | 0 | 0 | 0.213333 | 0 |
| BTG2 | 2.525 | 1.33622 | 0.000745 | 0.002929 | yes | up | 11.94 | 6.74 | 14.77 | 78.34 | 41.58 | 74.48 | 11.15 | 64.8 |
| PEMT | 2.74 | 1.454105 | 0.000747 | 0.002934 | yes | up | 3.47 | 1.88 | 1.33 | 16.18 | 10.67 | 13.35 | 2.226667 | 13.4 |
| GMDS | 2.207 | 1.14228 | 0.000748 | 0.002937 | yes | up | 3.23 | 3.25 | 1.92 | 16.5 | 12.35 | 12.74 | 2.8 | 13.86333 |
| PLEKHG3 | 2.076 | 1.053529 | 0.00075 | 0.002942 | yes | up | 3.53 | 4.13 | 2.17 | 10.07 | 22.44 | 14.94 | 3.276667 | 15.81667 |
| CD1B | 30.854 | 4.947369 | 0.00075 | 0.002942 | yes | up | 0.07 | 0 | 0 | 0.74 | 1.43 | 1.13 | 0.023333 | 1.1 |
| LOC100349036 | 0.23 | -2.12066 | 0.00075 | 0.002942 | yes | down | 0.56 | 0.83 | 0.46 | 0.22 | 0.37 | 0.39 | 0.616667 | 0.326667 |
| MICU2 | 0.496 | -1.0104 | 0.000752 | 0.002948 | yes | down | 15.16 | 10.05 | 7.66 | 14.13 | 14.86 | 8.37 | 10.95667 | 12.45333 |
| DTX4 | 2.087 | 1.061765 | 0.000753 | 0.002952 | yes | up | 2.28 | 1.46 | 0.95 | 6.9 | 9.79 | 5.54 | 1.563333 | 7.41 |
| OLFM2 | 2.194 | 1.133677 | 0.000754 | 0.002954 | yes | up | 1.11 | 1.18 | 0.87 | 4.89 | 5.41 | 5.45 | 1.053333 | 5.25 |
| LOC100339996 | 2.052 | 1.037244 | 0.000754 | 0.002956 | yes | up | 13.72 | 8.92 | 6.67 | 51.31 | 56.91 | 28.87 | 9.77 | 45.69667 |
| SNX20 | 2.96 | 1.565594 | 0.000758 | 0.002969 | yes | up | 0.35 | 0.65 | 0.51 | 3.13 | 3.95 | 3.26 | 0.503333 | 3.446667 |
| GADD45G | 0.374 | -1.41709 | 0.000761 | 0.002977 | yes | down | 30.19 | 52 | 55.04 | 17.9 | 53.09 | 50.43 | 45.74333 | 40.47333 |
| RAB15 | 2.705 | 1.435644 | 0.000766 | 0.002994 | yes | up | 0.74 | 1.06 | 0.36 | 4.18 | 6.27 | 2.84 | 0.72 | 4.43 |
| KCNA4 | 0.013 | -6.22276 | 0.000769 | 0.003005 | yes | down | 0.17 | 0.12 | 0.01 | 0 | 0 | 0 | 0.1 | 0 |
| DNASE1L1 | 2.156 | 1.108154 | 0.000775 | 0.003025 | yes | up | 17.37 | 8.17 | 6.16 | 51.25 | 53.16 | 48.07 | 10.56667 | 50.82667 |
| ME2 | 0.468 | -1.09609 | 0.000775 | 0.003026 | yes | down | 3.7 | 2.38 | 1.99 | 2.29 | 4.27 | 2.13 | 2.69 | 2.896667 |
| PAPLN | 3.201 | 1.678456 | 0.000779 | 0.003039 | yes | up | 0.73 | 0.26 | 0.19 | 1.95 | 2.88 | 3.52 | 0.393333 | 2.783333 |
| LOC103350281 | 0.399 | -1.32418 | 0.00078 | 0.003042 | yes | down | 217.37 | 214.51 | 209.55 | 326.76 | 124.31 | 128.93 | 213.81 | 193.3333 |
| PKMYT1 | 3.143 | 1.652338 | 0.00078 | 0.003042 | yes | up | 0.44 | 0.46 | 0.25 | 2.13 | 3.21 | 2.24 | 0.383333 | 2.526667 |
| C17H14orf28 | 0.448 | -1.15821 | 0.000785 | 0.003058 | yes | down | 2.97 | 1.83 | 1.48 | 2.62 | 2.07 | 1.82 | 2.093333 | 2.17 |
| PLEKHB1 | 2.72 | 1.443537 | 0.000788 | 0.00307 | yes | up | 5.13 | 3.12 | 1.75 | 11.89 | 16.86 | 30.19 | 3.333333 | 19.64667 |
| CD2 | 53.771 | 5.748763 | 0.000791 | 0.00308 | yes | up | 0 | 0 | 0 | 0.52 | 0.29 | 1.7 | 0 | 0.836667 |
| LOC100358934 | 23.865 | 4.576825 | 0.000792 | 0.003083 | yes | up | 0 | 0.06 | 0.06 | 0.62 | 3.95 | 2.49 | 0.04 | 2.353333 |
| DPP6 | 0.063 | -3.98512 | 0.000803 | 0.003122 | yes | down | 0.06 | 0.15 | 0.15 | 0.01 | 0 | 0.05 | 0.12 | 0.02 |
| LOC100357313 | 6.876 | 2.78158 | 0.000806 | 0.003132 | yes | up | 0.03 | 0.07 | 0.01 | 0.52 | 0.45 | 0.68 | 0.036667 | 0.55 |
| DYNLRB2 | 0.472 | -1.08371 | 0.000807 | 0.003138 | yes | down | 17.17 | 21.7 | 17.15 | 18.64 | 16.32 | 25.35 | 18.67333 | 20.10333 |
| C4H12orf73 | 0.496 | -1.01023 | 0.000808 | 0.00314 | yes | down | 15.85 | 10.19 | 8.1 | 16.47 | 16.13 | 15.06 | 11.38 | 15.88667 |
| LOC103347314 | 8.292 | 3.051787 | 0.000809 | 0.003144 | yes | up | 0.05 | 0.07 | 0.02 | 0.6 | 1.09 | 1.15 | 0.046667 | 0.946667 |
| DEF6 | 2.809 | 1.48982 | 0.00081 | 0.003145 | yes | up | 0.35 | 0.49 | 0.3 | 2.02 | 2.76 | 2.49 | 0.38 | 2.423333 |
| TANK | 0.489 | -1.03151 | 0.000817 | 0.003171 | yes | down | 6.29 | 9.53 | 6.09 | 9.1 | 10.3 | 5.06 | 7.303333 | 8.153333 |
| C14H3orf38 | 0.476 | -1.07171 | 0.000819 | 0.003177 | yes | down | 4.94 | 4.99 | 4.32 | 4.97 | 6.89 | 2.64 | 4.75 | 4.833333 |
| SNCB | 7.081 | 2.824002 | 0.000819 | 0.003178 | yes | up | 1.08 | 0.1 | 0.05 | 8.75 | 5.03 | 4.65 | 0.41 | 6.143333 |
| LRRC39 | 0.034 | -4.86646 | 0.00082 | 0.00318 | yes | down | 33.67 | 4.46 | 0.57 | 0.73 | 0.28 | 1.73 | 12.9 | 0.913333 |
| FLT1 | 0.471 | -1.08646 | 0.00082 | 0.00318 | yes | down | 11.17 | 18.47 | 22.73 | 16.18 | 20.68 | 21.01 | 17.45667 | 19.29 |
| HSPB1 | 2.957 | 1.564055 | 0.000822 | 0.003185 | yes | up | 185.38 | 141.6 | 141.07 | 695.72 | 456.03 | 1900.59 | 156.0167 | 1017.447 |
| LOC100338714 | 0.487 | -1.03904 | 0.000832 | 0.003221 | yes | down | 396.41 | 708.29 | 660.4 | 728.96 | 590.53 | 703.51 | 588.3667 | 674.3333 |
| BIRC3 | 2.14 | 1.097535 | 0.00084 | 0.003251 | yes | up | 1.23 | 1.66 | 1.99 | 10.02 | 8.85 | 5.62 | 1.626667 | 8.163333 |
| MTERF1 | 0.352 | -1.50763 | 0.000859 | 0.00332 | yes | down | 1.23 | 1.93 | 1.25 | 0.87 | 1.72 | 0.97 | 1.47 | 1.186667 |
| SULF2 | 2.417 | 1.273348 | 0.000863 | 0.003332 | yes | up | 0.95 | 1.29 | 1.23 | 4.65 | 5.32 | 9.15 | 1.156667 | 6.373333 |
| TRMT10A | 0.467 | -1.09945 | 0.000863 | 0.003332 | yes | down | 2.21 | 2.7 | 1.62 | 2.53 | 3.13 | 1.49 | 2.176667 | 2.383333 |
| LOC100348075 | 2.578 | 1.366484 | 0.000864 | 0.003333 | yes | up | 1.06 | 0.77 | 0.95 | 5.54 | 7.55 | 3.64 | 0.926667 | 5.576667 |
| GRO-B | 16.726 | 4.064046 | 0.000864 | 0.003334 | yes | up | 1.24 | 0.86 | 0 | 20.8 | 23.14 | 23.36 | 0.7 | 22.43333 |
| LOC103350962 | 22.513 | 4.492658 | 0.000867 | 0.003344 | yes | up | 0 | 0.03 | 0.04 | 1.44 | 1.94 | 0.42 | 0.023333 | 1.266667 |
| LOC100353293 | 2.864 | 1.517943 | 0.000867 | 0.003344 | yes | up | 0.6 | 0.85 | 0.69 | 5.31 | 4.59 | 4.2 | 0.713333 | 4.7 |
| SFRP1 | 8.15 | 3.026804 | 0.000867 | 0.003344 | yes | up | 4.38 | 1.19 | 0.57 | 40.47 | 16.28 | 48.31 | 2.046667 | 35.02 |
| LGI3 | 4.514 | 2.17434 | 0.000874 | 0.003363 | yes | up | 0.39 | 0.17 | 0.13 | 3.86 | 0.9 | 2 | 0.23 | 2.253333 |
| ST6GAL1 | 2.023 | 1.016172 | 0.000878 | 0.003379 | yes | up | 5.07 | 3.33 | 5.22 | 19.82 | 18.17 | 25.19 | 4.54 | 21.06 |
| RSPH14 | 0.049 | -4.36344 | 0.000883 | 0.003396 | yes | down | 1.52 | 1.18 | 0.91 | 0.18 | 0 | 0 | 1.203333 | 0.06 |
| B9D1 | 2.006 | 1.004457 | 0.000884 | 0.0034 | yes | up | 2.7 | 2.5 | 1.83 | 9.65 | 9.21 | 10.84 | 2.343333 | 9.9 |
| LOC100346553 | 0.274 | -1.86986 | 0.000887 | 0.003408 | yes | down | 0.81 | 0.98 | 0.78 | 0.15 | 0.87 | 0.63 | 0.856667 | 0.55 |
| ARL6 | 0.432 | -1.21062 | 0.000888 | 0.003411 | yes | down | 2.56 | 2.7 | 2.47 | 2.84 | 2.6 | 2.2 | 2.576667 | 2.546667 |
| WNT16 | 0.011 | -6.526 | 0.00089 | 0.003419 | yes | down | 0.48 | 0.03 | 0.02 | 0 | 0 | 0 | 0.176667 | 0 |
| RPS6KB1 | 0.497 | -1.00931 | 0.000891 | 0.003422 | yes | down | 11.4 | 7.93 | 6.62 | 10.81 | 12.84 | 6.1 | 8.65 | 9.916667 |
| LOC103346937 | 0.233 | -2.10282 | 0.000901 | 0.003456 | yes | down | 1.4 | 0.71 | 0.84 | 0.51 | 0.56 | 0.5 | 0.983333 | 0.523333 |
| LOC100351672 | 2.255 | 1.173076 | 0.000906 | 0.003475 | yes | up | 10.24 | 11.94 | 18.64 | 91.34 | 55.76 | 67.51 | 13.60667 | 71.53667 |
| SUSD5 | 3.033 | 1.600525 | 0.000907 | 0.003476 | yes | up | 0.51 | 0.53 | 0.69 | 2.49 | 2.73 | 6.69 | 0.576667 | 3.97 |
| WTIP | 2.327 | 1.218224 | 0.00091 | 0.003489 | yes | up | 1.29 | 1.15 | 1.47 | 6.78 | 6.66 | 7.59 | 1.303333 | 7.01 |
| LOC108177001 | 0.086 | -3.53207 | 0.000917 | 0.003512 | yes | down | 0.4 | 0.17 | 0.23 | 0.06 | 0.06 | 0.04 | 0.266667 | 0.053333 |
| CDK5RAP2 | 2.015 | 1.010805 | 0.000918 | 0.003514 | yes | up | 1.07 | 1.1 | 0.77 | 4.29 | 6.55 | 2.88 | 0.98 | 4.573333 |
| LOC103345952 | 0.482 | -1.05352 | 0.000918 | 0.003514 | yes | down | 3.07 | 5.2 | 3.75 | 3.4 | 5.73 | 3.24 | 4.006667 | 4.123333 |
| ASTN2 | 3.877 | 1.955001 | 0.00092 | 0.003519 | yes | up | 0.2 | 0.15 | 0.1 | 1.4 | 0.65 | 1.8 | 0.15 | 1.283333 |
| TKTL1 | 0.017 | -5.9131 | 0.000924 | 0.003532 | yes | down | 0.08 | 0.19 | 0.18 | 0 | 0 | 0 | 0.15 | 0 |
| CDK8 | 0.493 | -1.02074 | 0.000932 | 0.00356 | yes | down | 2.64 | 2.4 | 1.76 | 2.14 | 3.66 | 1.91 | 2.266667 | 2.57 |
| LOC103348254 | 14.345 | 3.842482 | 0.000933 | 0.00356 | yes | up | 0 | 0.26 | 0 | 3.42 | 4.22 | 0.63 | 0.086667 | 2.756667 |
| LOC100344910 | 0.397 | -1.3321 | 0.000935 | 0.003568 | yes | down | 506.18 | 507.63 | 540.82 | 512.1 | 207.26 | 660.95 | 518.21 | 460.1033 |
| CCNG2 | 0.444 | -1.17052 | 0.00095 | 0.003617 | yes | down | 23.15 | 22.73 | 44.17 | 30.83 | 40.99 | 24.7 | 30.01667 | 32.17333 |
| RTN4RL1 | 2.28 | 1.188858 | 0.000955 | 0.003633 | yes | up | 1.47 | 0.65 | 1.18 | 5.71 | 5.17 | 6.48 | 1.1 | 5.786667 |
| T | 0.021 | -5.55964 | 0.000956 | 0.003635 | yes | down | 0.82 | 0.18 | 0.1 | 0.04 | 0 | 0 | 0.366667 | 0.013333 |
| LMX1B | 8.423 | 3.074339 | 0.00096 | 0.003647 | yes | up | 0.07 | 0.03 | 0.05 | 1.08 | 0.16 | 1.46 | 0.05 | 0.9 |
| MCUB | 2.495 | 1.319192 | 0.000962 | 0.003653 | yes | up | 1.51 | 2.47 | 0.95 | 9.57 | 7.66 | 10.14 | 1.643333 | 9.123333 |
| LTBP2 | 2.584 | 1.369797 | 0.00097 | 0.003683 | yes | up | 1.1 | 2.05 | 1.09 | 3.81 | 13.56 | 8.22 | 1.413333 | 8.53 |
| NT5M | 0.315 | -1.66441 | 0.000974 | 0.003694 | yes | down | 3.26 | 2.04 | 2.28 | 1.28 | 2.37 | 2.49 | 2.526667 | 2.046667 |
| LOC100346672 | 10.721 | 3.422417 | 0.000974 | 0.003697 | yes | up | 0 | 0.04 | 0.01 | 0.42 | 0.25 | 0.53 | 0.016667 | 0.4 |
| CASP8AP2 | 0.325 | -1.62032 | 0.000975 | 0.003697 | yes | down | 1.07 | 0.87 | 1.27 | 1 | 1.22 | 0.26 | 1.07 | 0.826667 |
| LOC108176025 | 0.039 | -4.67248 | 0.000978 | 0.003708 | yes | down | 1.54 | 0.12 | 0.06 | 0.07 | 0.07 | 0 | 0.573333 | 0.046667 |
| ORMDL1 | 0.429 | -1.21969 | 0.000983 | 0.003723 | yes | down | 2.21 | 2.54 | 3.5 | 2.75 | 3.24 | 2.35 | 2.75 | 2.78 |
| LOC108178892 | 3.944 | 1.979671 | 0.000986 | 0.003732 | yes | up | 0.84 | 0.76 | 1.04 | 9.02 | 11.1 | 4.43 | 0.88 | 8.183333 |
| LOC100351134 | 2.978 | 1.57432 | 0.000999 | 0.003778 | yes | up | 0.75 | 0.75 | 0.61 | 3.96 | 8.59 | 2.08 | 0.703333 | 4.876667 |
| CSDC2 | 5.737 | 2.520183 | 0.000999 | 0.00378 | yes | up | 0.16 | 0.07 | 0.09 | 1.09 | 0.81 | 2.12 | 0.106667 | 1.34 |
| METRNL | 2.327 | 1.218249 | 0.001006 | 0.003801 | yes | up | 3.36 | 3.59 | 3.22 | 16.06 | 11.87 | 25.27 | 3.39 | 17.73333 |
| TBX5 | 0.494 | -1.01794 | 0.001008 | 0.00381 | yes | down | 2.04 | 2.72 | 2.95 | 3 | 2.55 | 2.85 | 2.57 | 2.8 |
| DSG4 | 0.046 | -4.45064 | 0.001008 | 0.00381 | yes | down | 0.31 | 0.11 | 0.09 | 0 | 0.05 | 0 | 0.17 | 0.016667 |
| LOC100354564 | 0.178 | -2.49353 | 0.001012 | 0.00382 | yes | down | 3.19 | 2.02 | 0.73 | 0.78 | 1.21 | 0.42 | 1.98 | 0.803333 |
| SLCO2B1 | 2.739 | 1.453578 | 0.001013 | 0.003823 | yes | up | 0.5 | 0.69 | 0.77 | 4.11 | 2.47 | 5.56 | 0.653333 | 4.046667 |
| TROVE2 | 0.442 | -1.17728 | 0.001014 | 0.003828 | yes | down | 3.98 | 4.27 | 4.36 | 3.5 | 6.96 | 2.19 | 4.203333 | 4.216667 |
| LOC100340458 | 4.284 | 2.098853 | 0.001017 | 0.003837 | yes | up | 0.89 | 0.33 | 0.09 | 5.71 | 3.49 | 8.49 | 0.436667 | 5.896667 |
| CARF | 0.458 | -1.12741 | 0.001018 | 0.00384 | yes | down | 1.33 | 1.78 | 1.26 | 1.63 | 2.15 | 1.05 | 1.456667 | 1.61 |
| LOC108178072 | 0.369 | -1.43996 | 0.001022 | 0.003853 | yes | down | 3.67 | 6.25 | 3.57 | 2.78 | 3.51 | 5.04 | 4.496667 | 3.776667 |
| SYN3 | 0.368 | -1.44297 | 0.001024 | 0.00386 | yes | down | 0.32 | 0.55 | 0.71 | 0.46 | 0.49 | 0.35 | 0.526667 | 0.433333 |
| FAM131B | 4.24 | 2.084044 | 0.001025 | 0.00386 | yes | up | 0.13 | 0.1 | 0.04 | 0.78 | 1.21 | 0.58 | 0.09 | 0.856667 |
| ASB14 | 0.16 | -2.64148 | 0.001029 | 0.003873 | yes | down | 0.57 | 0.28 | 0.08 | 0.16 | 0.1 | 0.13 | 0.31 | 0.13 |
| AKAP5 | 2.507 | 1.326079 | 0.001029 | 0.003873 | yes | up | 0.32 | 0.47 | 0.3 | 2.45 | 2.34 | 1.41 | 0.363333 | 2.066667 |
| HAND2 | 22.372 | 4.483601 | 0.001031 | 0.003878 | yes | up | 0 | 0 | 0.13 | 1.69 | 1.51 | 4.06 | 0.043333 | 2.42 |
| IRF5 | 2.23 | 1.157038 | 0.001036 | 0.003896 | yes | up | 1.35 | 1.48 | 0.82 | 4.76 | 5.7 | 7.43 | 1.216667 | 5.963333 |
| LOC103345230 | 0.342 | -1.54666 | 0.00104 | 0.003908 | yes | down | 0.79 | 1.42 | 1.41 | 0.45 | 1.39 | 0.48 | 1.206667 | 0.773333 |
| CXHXorf23 | 0.482 | -1.05417 | 0.001041 | 0.00391 | yes | down | 1.32 | 1.75 | 1.45 | 1.92 | 1.89 | 1.34 | 1.506667 | 1.716667 |
| TMEM74B | 0.386 | -1.3733 | 0.001046 | 0.003927 | yes | down | 0.18 | 0.18 | 0.16 | 0.16 | 0.14 | 0.15 | 0.173333 | 0.15 |
| NEPRO | 0.419 | -1.2566 | 0.001046 | 0.003927 | yes | down | 0.85 | 0.98 | 0.74 | 0.91 | 0.96 | 0.58 | 0.856667 | 0.816667 |
| BCHE | 0.176 | -2.50652 | 0.001051 | 0.003943 | yes | down | 2.71 | 1 | 0.43 | 1 | 0.23 | 0.33 | 1.38 | 0.52 |
| MAP9 | 0.433 | -1.20643 | 0.001056 | 0.003958 | yes | down | 1.42 | 1.49 | 1.86 | 2.04 | 1.94 | 0.87 | 1.59 | 1.616667 |
| UBE2T | 3.629 | 1.859542 | 0.001064 | 0.003987 | yes | up | 0.98 | 1.16 | 0.78 | 9.52 | 12.31 | 2.96 | 0.973333 | 8.263333 |
| ZWILCH | 3.134 | 1.647999 | 0.001065 | 0.003989 | yes | up | 1.17 | 0.75 | 0.52 | 6.17 | 9.16 | 1.95 | 0.813333 | 5.76 |
| ZSCAN16 | 0.381 | -1.39128 | 0.001068 | 0.003997 | yes | down | 0.83 | 1.4 | 0.63 | 0.68 | 1.64 | 0.53 | 0.953333 | 0.95 |
| KRT2 | 43.652 | 5.44797 | 0.001069 | 0.003998 | yes | up | 0 | 0 | 0 | 0.33 | 0.23 | 0.56 | 0 | 0.373333 |
| ZFAT | 2.275 | 1.186167 | 0.001074 | 0.004016 | yes | up | 1.19 | 1.2 | 0.75 | 5.13 | 6.86 | 2.99 | 1.046667 | 4.993333 |
| LOC100351528 | 4.58 | 2.195362 | 0.001081 | 0.00404 | yes | up | 0.21 | 0.12 | 0.14 | 1.64 | 2.14 | 0.9 | 0.156667 | 1.56 |
| MLLT3 | 0.295 | -1.75992 | 0.001085 | 0.004053 | yes | down | 5.69 | 1.52 | 0.79 | 1.51 | 1.6 | 1.24 | 2.666667 | 1.45 |
| CDHR1 | 0.049 | -4.34326 | 0.001086 | 0.004057 | yes | down | 9.03 | 10.92 | 0.16 | 0.62 | 0.32 | 1.11 | 6.703333 | 0.683333 |
| ENPP1 | 2.492 | 1.317141 | 0.001091 | 0.004072 | yes | up | 5.01 | 4.28 | 2.72 | 16.11 | 42.13 | 11.82 | 4.003333 | 23.35333 |
| SASH3 | 2.345 | 1.229369 | 0.001103 | 0.004113 | yes | up | 0.73 | 0.58 | 0.51 | 2.43 | 3.56 | 3.71 | 0.606667 | 3.233333 |
| CLEC4F | 2.325 | 1.217446 | 0.001104 | 0.004117 | yes | up | 2.72 | 1.83 | 1.11 | 8.03 | 9.32 | 11.89 | 1.886667 | 9.746667 |
| PRKG2 | 5.75 | 2.523641 | 0.001107 | 0.004127 | yes | up | 0.02 | 0.09 | 0.03 | 0.48 | 0.48 | 0.57 | 0.046667 | 0.51 |
| LOC108176481 | 2.34 | 1.226271 | 0.001128 | 0.004205 | yes | up | 14.43 | 6.04 | 6.94 | 31.78 | 57.91 | 56.3 | 9.136667 | 48.66333 |
| LOC100337875 | 0.427 | -1.22891 | 0.001131 | 0.004215 | yes | down | 2.49 | 2.32 | 3.11 | 1.84 | 3.48 | 2.76 | 2.64 | 2.693333 |
| EDN1 | 2.016 | 1.011629 | 0.001135 | 0.004227 | yes | up | 3.81 | 3.73 | 2.59 | 16.12 | 12.12 | 17.48 | 3.376667 | 15.24 |
| USP45 | 0.405 | -1.30472 | 0.001137 | 0.004233 | yes | down | 1.79 | 1.76 | 1.27 | 1.37 | 2.39 | 0.76 | 1.606667 | 1.506667 |
| BCAR3 | 2.166 | 1.115139 | 0.00114 | 0.004241 | yes | up | 2.94 | 3.26 | 2.01 | 13.92 | 7.75 | 15.15 | 2.736667 | 12.27333 |
| PHEX | 0.08 | -3.64568 | 0.001143 | 0.004252 | yes | down | 0.14 | 0.11 | 0.05 | 0.03 | 0.02 | 0 | 0.1 | 0.016667 |
| ENOX1 | 0.457 | -1.1306 | 0.001145 | 0.004257 | yes | down | 4.27 | 6.16 | 4.17 | 5.47 | 7.7 | 2.59 | 4.866667 | 5.253333 |
| OXSR1 | 0.48 | -1.05971 | 0.001149 | 0.004273 | yes | down | 15.66 | 12.03 | 11.06 | 10.1 | 21.49 | 12.25 | 12.91667 | 14.61333 |
| MECOM | 0.498 | -1.00541 | 0.001156 | 0.004295 | yes | down | 5.96 | 7.04 | 8.92 | 9.76 | 10.73 | 5.51 | 7.306667 | 8.666667 |
| CDC45 | 4.023 | 2.008372 | 0.00116 | 0.004306 | yes | up | 14.41 | 9.98 | 16.43 | 34.78 | 66.22 | 39.11 | 13.60667 | 46.70333 |
| MTMR11 | 2.001 | 1.001044 | 0.001162 | 0.004314 | yes | up | 0.82 | 0.97 | 0.73 | 4.07 | 3.65 | 3.74 | 0.84 | 3.82 |
| LOC103345145 | 18.736 | 4.227757 | 0.001175 | 0.004361 | yes | up | 0 | 0.07 | 0.03 | 1.57 | 1.34 | 0.98 | 0.033333 | 1.296667 |
| TMEM223 | 0.477 | -1.06719 | 0.001179 | 0.004374 | yes | down | 17.62 | 19.16 | 17.82 | 23.3 | 13.96 | 21.82 | 18.2 | 19.69333 |
| CEBPB | 0.483 | -1.04934 | 0.001184 | 0.004392 | yes | down | 19.31 | 23.46 | 19.65 | 19.5 | 17.04 | 32.13 | 20.80667 | 22.89 |
| C1GALT1 | 0.478 | -1.06435 | 0.001185 | 0.004394 | yes | down | 3.24 | 4.55 | 3.19 | 4.78 | 5.25 | 2.13 | 3.66 | 4.053333 |
| LOC100338274 | 0.106 | -3.23896 | 0.001194 | 0.004423 | yes | down | 1.67 | 0.21 | 0.05 | 0.07 | 0.22 | 0.16 | 0.643333 | 0.15 |
| MSX2 | 2.908 | 1.539979 | 0.001196 | 0.004429 | yes | up | 0.34 | 0.6 | 0.72 | 4.29 | 2.86 | 4.01 | 0.553333 | 3.72 |
| FANCI | 2.988 | 1.579266 | 0.001209 | 0.004472 | yes | up | 0.7 | 0.65 | 0.29 | 4.18 | 5.6 | 1.45 | 0.546667 | 3.743333 |
| LOC103347148 | 6.873 | 2.78101 | 0.001217 | 0.004494 | yes | up | 2.24 | 0.25 | 0.61 | 9.57 | 21.31 | 20.28 | 1.033333 | 17.05333 |
| EPHB3 | 2.157 | 1.109182 | 0.001217 | 0.004496 | yes | up | 1.71 | 0.98 | 0.68 | 4.37 | 6.36 | 5.58 | 1.123333 | 5.436667 |
| CCL5 | 28.446 | 4.830133 | 0.001219 | 0.004499 | yes | up | 0.1 | 0 | 0 | 1.7 | 2.1 | 2.4 | 0.033333 | 2.066667 |
| SLC9A3R1 | 2.014 | 1.010082 | 0.001223 | 0.004508 | yes | up | 1.97 | 2.2 | 2.5 | 11.2 | 9.21 | 10.52 | 2.223333 | 10.31 |
| DIAPH3 | 3.397 | 1.764303 | 0.001223 | 0.004508 | yes | up | 0.37 | 0.44 | 0.17 | 2.56 | 4.35 | 0.88 | 0.326667 | 2.596667 |
| LOC103347176 | 42.199 | 5.399137 | 0.001224 | 0.004509 | yes | up | 0 | 0 | 0 | 3.29 | 9.87 | 7.21 | 0 | 6.79 |
| MECP2 | 2.988 | 1.579181 | 0.001226 | 0.004514 | yes | up | 0.78 | 0.6 | 1.61 | 7.32 | 7.51 | 6.49 | 0.996667 | 7.106667 |
| COL11A1 | 0.296 | -1.75749 | 0.001238 | 0.004555 | yes | down | 0.8 | 0.33 | 0.2 | 0.23 | 0.35 | 0.29 | 0.443333 | 0.29 |
| HRASLS5 | 0.343 | -1.54229 | 0.001246 | 0.00458 | yes | down | 0.74 | 2.35 | 1.22 | 0.51 | 1.19 | 1.58 | 1.436667 | 1.093333 |
| C4H12orf29 | 0.35 | -1.51483 | 0.001254 | 0.004609 | yes | down | 3.09 | 2.34 | 3.23 | 2.61 | 3.25 | 1.17 | 2.886667 | 2.343333 |
| LOC108175367 | 11.665 | 3.5441 | 0.001256 | 0.004615 | yes | up | 0 | 0.22 | 0.63 | 7.32 | 5.09 | 11.68 | 0.283333 | 8.03 |
| PRR5 | 4.227 | 2.079568 | 0.001261 | 0.004631 | yes | up | 0.4 | 0.14 | 0.22 | 4.06 | 1.69 | 2.1 | 0.253333 | 2.616667 |
| CDT1 | 2.796 | 1.483296 | 0.001273 | 0.004667 | yes | up | 0.72 | 0.98 | 0.45 | 4.31 | 6.48 | 2.97 | 0.716667 | 4.586667 |
| LOC103347325 | 3.921 | 1.971294 | 0.001292 | 0.004727 | yes | up | 0.23 | 0.52 | 0.13 | 1.97 | 3 | 2.86 | 0.293333 | 2.61 |
| ERFE | 4.236 | 2.082607 | 0.0013 | 0.004754 | yes | up | 0.2 | 0.18 | 0.11 | 0.82 | 1.76 | 2.13 | 0.163333 | 1.57 |
| CD6 | 33.944 | 5.085079 | 0.0013 | 0.004754 | yes | up | 0.02 | 0 | 0 | 0.23 | 0.29 | 0.97 | 0.006667 | 0.496667 |
| DNAJC1 | 0.475 | -1.07267 | 0.001305 | 0.004771 | yes | down | 6.38 | 7.62 | 10.51 | 10.39 | 10.81 | 6.38 | 8.17 | 9.193333 |
| MYEF2 | 0.479 | -1.06115 | 0.001308 | 0.004781 | yes | down | 5.66 | 5.01 | 5.05 | 6.14 | 7.86 | 3.01 | 5.24 | 5.67 |
| SULT2B1 | 8.036 | 3.006525 | 0.001309 | 0.004781 | yes | up | 0.48 | 0.25 | 0.24 | 8.41 | 0.55 | 7.54 | 0.323333 | 5.5 |
| LOC103349243 | 0.28 | -1.83424 | 0.001324 | 0.004831 | yes | down | 1.22 | 1.65 | 1.24 | 1.3 | 0.81 | 0.47 | 1.37 | 0.86 |
| FAM198A | 3.924 | 1.972292 | 0.001338 | 0.004878 | yes | up | 0.63 | 0.16 | 0.12 | 2.64 | 2.63 | 2.54 | 0.303333 | 2.603333 |
| COL8A2 | 2.315 | 1.211038 | 0.00134 | 0.004882 | yes | up | 0.57 | 0.45 | 0.26 | 1.67 | 2.64 | 2.41 | 0.426667 | 2.24 |
| NUBPL | 0.401 | -1.3181 | 0.001346 | 0.004897 | yes | down | 5.85 | 4.66 | 7.53 | 7.61 | 5.72 | 3.56 | 6.013333 | 5.63 |
| LOC103350737 | 0.014 | -6.18174 | 0.001351 | 0.004917 | yes | down | 4.84 | 0.96 | 0.23 | 0 | 0 | 0 | 2.01 | 0 |
| MAP6 | 2.351 | 1.23324 | 0.001355 | 0.004926 | yes | up | 0.64 | 0.29 | 0.54 | 1.99 | 2.16 | 2.52 | 0.49 | 2.223333 |
| TNNT2 | 0.07 | -3.83156 | 0.001356 | 0.00493 | yes | down | 9.24 | 1.15 | 0.24 | 0.19 | 0.08 | 1.25 | 3.543333 | 0.506667 |
| CYTL1 | 10.084 | 3.334022 | 0.001359 | 0.00494 | yes | up | 0.18 | 0.24 | 0.15 | 4.09 | 0.72 | 7.84 | 0.19 | 4.216667 |
| FAM102A | 2.145 | 1.101309 | 0.00136 | 0.004942 | yes | up | 2.22 | 1.86 | 2.56 | 8.65 | 11.59 | 12.81 | 2.213333 | 11.01667 |
| PLAT | 2.451 | 1.293527 | 0.00136 | 0.004943 | yes | up | 5.18 | 3.96 | 2.88 | 22.85 | 11.1 | 30.59 | 4.006667 | 21.51333 |
| SLC9A9 | 2.391 | 1.257422 | 0.001376 | 0.004995 | yes | up | 1.87 | 1.39 | 0.46 | 6.39 | 6.01 | 6.27 | 1.24 | 6.223333 |
| PORCN | 2.262 | 1.177397 | 0.001381 | 0.005009 | yes | up | 1.01 | 1.65 | 1.14 | 6.56 | 5.44 | 7.64 | 1.266667 | 6.546667 |
| C2H2orf40 | 9.746 | 3.284753 | 0.001383 | 0.005015 | yes | up | 0.12 | 0.06 | 0.05 | 1.82 | 1.65 | 1.64 | 0.076667 | 1.703333 |
| CCDC138 | 0.41 | -1.28796 | 0.001391 | 0.005041 | yes | down | 1.15 | 2.51 | 1.67 | 1.7 | 1.95 | 1.25 | 1.776667 | 1.633333 |
| LOC103345297 | 2.334 | 1.222812 | 0.001394 | 0.005047 | yes | up | 0.81 | 0.91 | 0.73 | 4.93 | 4.49 | 3.47 | 0.816667 | 4.296667 |
| LRRN1 | 3.223 | 1.68821 | 0.001395 | 0.005051 | yes | up | 0.4 | 0.11 | 0.15 | 1.55 | 1.56 | 1.67 | 0.22 | 1.593333 |
| SLC38A3 | 2.336 | 1.224298 | 0.0014 | 0.005066 | yes | up | 0.87 | 0.91 | 0.64 | 2.85 | 6.27 | 3.95 | 0.806667 | 4.356667 |
| KLHL6 | 0.421 | -1.24779 | 0.001402 | 0.005073 | yes | down | 1.16 | 2.14 | 1.73 | 1.3 | 1.98 | 1.66 | 1.676667 | 1.646667 |
| LOC100358696 | 2.391 | 1.257493 | 0.001431 | 0.005169 | yes | up | 0.76 | 0.78 | 0.99 | 4.01 | 5.11 | 5.11 | 0.843333 | 4.743333 |
| RTL1 | 0.059 | -4.08665 | 0.001435 | 0.00518 | yes | down | 2.54 | 0.21 | 0.04 | 0 | 0.08 | 0.1 | 0.93 | 0.06 |
| FAR2 | 3.03 | 1.599149 | 0.001437 | 0.005187 | yes | up | 1.03 | 0.42 | 0.24 | 4.87 | 3.89 | 2.45 | 0.563333 | 3.736667 |
| LOC103349635 | 0.156 | -2.68162 | 0.001437 | 0.005187 | yes | down | 0.16 | 0.15 | 0.29 | 0.08 | 0.1 | 0.04 | 0.2 | 0.073333 |
| FAM175A | 0.423 | -1.24157 | 0.001441 | 0.005201 | yes | down | 4.66 | 3.24 | 3.31 | 4.35 | 4.58 | 1.8 | 3.736667 | 3.576667 |
| LOC103345583 | 0.019 | -5.73857 | 0.001453 | 0.005238 | yes | down | 0.33 | 0.35 | 0.19 | 0 | 0 | 0 | 0.29 | 0 |
| LOC108178220 | 3.28 | 1.71367 | 0.001458 | 0.005252 | yes | up | 0.19 | 0.35 | 0.15 | 1.37 | 1.95 | 1.83 | 0.23 | 1.716667 |
| ANXA8 | 2.761 | 1.464945 | 0.001472 | 0.005298 | yes | up | 12.53 | 8.29 | 3.37 | 53.43 | 25.97 | 64.13 | 8.063333 | 47.84333 |
| DYNLT3 | 0.453 | -1.14233 | 0.001475 | 0.005306 | yes | down | 10.61 | 9.02 | 8.63 | 13.9 | 10.86 | 4.73 | 9.42 | 9.83 |
| KIF15 | 3.131 | 1.646662 | 0.001476 | 0.005308 | yes | up | 0.91 | 0.69 | 0.37 | 5.08 | 7.78 | 1.45 | 0.656667 | 4.77 |
| EXOC3L1 | 2.177 | 1.122397 | 0.001477 | 0.005308 | yes | up | 1.73 | 2.34 | 2.49 | 12.03 | 13.46 | 6.26 | 2.186667 | 10.58333 |
| ZNF540 | 0.294 | -1.76374 | 0.001489 | 0.005349 | yes | down | 0.78 | 0.77 | 0.49 | 0.56 | 0.51 | 0.17 | 0.68 | 0.413333 |
| CDRT1 | 2.554 | 1.352818 | 0.001505 | 0.005405 | yes | up | 0.28 | 0.29 | 0.35 | 1.55 | 2.7 | 1.69 | 0.306667 | 1.98 |
| NPY1R | 2.949 | 1.560083 | 0.001524 | 0.005473 | yes | up | 0.81 | 0.35 | 0.6 | 3.35 | 3.21 | 4.2 | 0.586667 | 3.586667 |
| LOC100352672 | 8.725 | 3.125194 | 0.001526 | 0.005478 | yes | up | 0 | 0.04 | 0.1 | 1.21 | 0.66 | 1.08 | 0.046667 | 0.983333 |
| SERGEF | 2.208 | 1.142791 | 0.001532 | 0.005495 | yes | up | 2.15 | 1.27 | 1.27 | 6.62 | 7.17 | 8.88 | 1.563333 | 7.556667 |
| LIPT1 | 0.343 | -1.54289 | 0.00154 | 0.005523 | yes | down | 1.65 | 1.82 | 2.14 | 2.04 | 1.4 | 1 | 1.87 | 1.48 |
| GIMAP1 | 2.936 | 1.553777 | 0.001542 | 0.005524 | yes | up | 0.41 | 0.67 | 0.17 | 3.12 | 3.11 | 2.05 | 0.416667 | 2.76 |
| LOC100346512 | 3.687 | 1.882441 | 0.001545 | 0.005536 | yes | up | 0.15 | 0.09 | 0.2 | 1.54 | 1.11 | 1.05 | 0.146667 | 1.233333 |
| LOC108176161 | 43.066 | 5.428491 | 0.001548 | 0.005543 | yes | up | 0 | 0 | 0 | 2.42 | 12.65 | 5.94 | 0 | 7.003333 |
| PTX3 | 0.299 | -1.74035 | 0.001573 | 0.005626 | yes | down | 0.72 | 0.8 | 1.34 | 0.79 | 0.77 | 0.52 | 0.953333 | 0.693333 |
| RPGR | 0.301 | -1.73092 | 0.00158 | 0.005649 | yes | down | 1.19 | 0.46 | 0.93 | 0.74 | 0.55 | 0.47 | 0.86 | 0.586667 |
| MCM4 | 2.629 | 1.39458 | 0.001581 | 0.005651 | yes | up | 2.24 | 1.59 | 0.65 | 10.08 | 12.14 | 4.37 | 1.493333 | 8.863333 |
| CCNC | 0.48 | -1.0576 | 0.001596 | 0.005695 | yes | down | 6.2 | 6.92 | 7.19 | 9.63 | 9.15 | 4.24 | 6.77 | 7.673333 |
| G3BP1 | 0.494 | -1.0173 | 0.001614 | 0.005753 | yes | down | 16.33 | 16.64 | 9.44 | 20.04 | 19.07 | 8.57 | 14.13667 | 15.89333 |
| FNDC1 | 2.065 | 1.046473 | 0.001617 | 0.005762 | yes | up | 15.87 | 9.02 | 5.99 | 43.17 | 39.23 | 57.8 | 10.29333 | 46.73333 |
| DCLRE1C | 0.409 | -1.28914 | 0.001621 | 0.005775 | yes | down | 0.9 | 1.08 | 1.16 | 0.9 | 1.36 | 0.72 | 1.046667 | 0.993333 |
| HDC | 0.118 | -3.08891 | 0.001635 | 0.005822 | yes | down | 0.37 | 0.2 | 0.37 | 0.03 | 0.06 | 0.18 | 0.313333 | 0.09 |
| FAM81B | 0.025 | -5.32917 | 0.001638 | 0.005831 | yes | down | 0.64 | 0.31 | 0.07 | 0 | 0 | 0.06 | 0.34 | 0.02 |
| SCUBE1 | 0.368 | -1.44318 | 0.001643 | 0.005843 | yes | down | 1.76 | 2.28 | 0.69 | 1.1 | 1.18 | 1.53 | 1.576667 | 1.27 |
| NEDD1 | 0.476 | -1.07077 | 0.001646 | 0.005854 | yes | down | 3.87 | 2.1 | 1.93 | 2.7 | 3.86 | 1.96 | 2.633333 | 2.84 |
| PLA2G15 | 2.044 | 1.031509 | 0.001652 | 0.005873 | yes | up | 4.54 | 2.34 | 2.16 | 10.26 | 20.02 | 12.06 | 3.013333 | 14.11333 |
| AZIN2 | 2.6 | 1.378584 | 0.001654 | 0.005877 | yes | up | 0.52 | 0.4 | 1.19 | 2.58 | 3.23 | 3.45 | 0.703333 | 3.086667 |
| NLRC5 | 5.746 | 2.522569 | 0.001656 | 0.005883 | yes | up | 0.05 | 0.07 | 0 | 0.34 | 0.7 | 0.47 | 0.04 | 0.503333 |
| SH3GL3 | 2.298 | 1.200304 | 0.001658 | 0.00589 | yes | up | 1.6 | 2.63 | 1.51 | 13.78 | 9.8 | 8.1 | 1.913333 | 10.56 |
| MICAL2 | 2.179 | 1.123984 | 0.001672 | 0.005933 | yes | up | 1.29 | 1.09 | 0.45 | 3.61 | 4.23 | 5.59 | 0.943333 | 4.476667 |
| CD1C | 4.691 | 2.229859 | 0.001674 | 0.005937 | yes | up | 0.7 | 0.56 | 0.14 | 2.54 | 3.66 | 8.36 | 0.466667 | 4.853333 |
| CLDN7 | 5.892 | 2.558824 | 0.001695 | 0.006005 | yes | up | 0.16 | 0.1 | 0.14 | 1.61 | 1.84 | 1.87 | 0.133333 | 1.773333 |
| LOC100357862 | 17.527 | 4.131484 | 0.001704 | 0.006033 | yes | up | 0 | 0.08 | 0.07 | 2.37 | 3.5 | 5.56 | 0.05 | 3.81 |
| CHRD | 3.033 | 1.600943 | 0.001708 | 0.006048 | yes | up | 1.12 | 0.35 | 0.29 | 3.83 | 3.4 | 4.44 | 0.586667 | 3.89 |
| TET1 | 0.444 | -1.17296 | 0.001711 | 0.006054 | yes | down | 0.46 | 0.27 | 0.27 | 0.36 | 0.41 | 0.24 | 0.333333 | 0.336667 |
| VCAM1 | 2.048 | 1.034143 | 0.001711 | 0.006056 | yes | up | 2.4 | 5.53 | 4.11 | 18.86 | 24.69 | 14.71 | 4.013333 | 19.42 |
| SOCS3 | 2.63 | 1.395305 | 0.001733 | 0.006129 | yes | up | 2.7 | 1.38 | 1.3 | 13.46 | 6.1 | 11.64 | 1.793333 | 10.4 |
| LOC100349247 | 2.158 | 1.109809 | 0.001733 | 0.006129 | yes | up | 12.75 | 14.05 | 15.68 | 58.38 | 46.04 | 99.31 | 14.16 | 67.91 |
| FAM185A | 0.41 | -1.28686 | 0.001738 | 0.006143 | yes | down | 2.29 | 1.93 | 2.47 | 1.45 | 1.68 | 3.14 | 2.23 | 2.09 |
| LOC103345486 | 15.78 | 3.979989 | 0.001745 | 0.006165 | yes | up | 0.89 | 0 | 0 | 4.34 | 7.48 | 15.53 | 0.296667 | 9.116667 |
| PIEZO2 | 2.368 | 1.243598 | 0.001746 | 0.006167 | yes | up | 0.43 | 0.19 | 0.31 | 2.17 | 1.66 | 1.19 | 0.31 | 1.673333 |
| VAV1 | 2.163 | 1.112868 | 0.001749 | 0.006172 | yes | up | 0.93 | 0.7 | 0.78 | 3.28 | 5.12 | 3.88 | 0.803333 | 4.093333 |
| CCDC142 | 2.314 | 1.210563 | 0.001752 | 0.006184 | yes | up | 0.41 | 0.42 | 0.41 | 1.92 | 2.83 | 2.36 | 0.413333 | 2.37 |
| LOC100346527 | 0.429 | -1.2224 | 0.001755 | 0.006192 | yes | down | 1.26 | 1.48 | 1.75 | 1.19 | 2.19 | 1.11 | 1.496667 | 1.496667 |
| SGK2 | 17.099 | 4.095846 | 0.001773 | 0.006249 | yes | up | 0.01 | 0.01 | 0 | 0.28 | 0.33 | 0.3 | 0.006667 | 0.303333 |
| CDH24 | 6.924 | 2.791524 | 0.001797 | 0.006326 | yes | up | 0.02 | 0.1 | 0.03 | 0.57 | 1.48 | 0.38 | 0.05 | 0.81 |
| MPP7 | 0.361 | -1.46905 | 0.00181 | 0.006368 | yes | down | 4.1 | 1.65 | 0.85 | 1.51 | 1.98 | 1.79 | 2.2 | 1.76 |
| NMB | 8.306 | 3.054112 | 0.001826 | 0.006421 | yes | up | 0.17 | 0.5 | 0.44 | 4.56 | 3.18 | 13.27 | 0.37 | 7.003333 |
| LOC103352076 | 15.17 | 3.923161 | 0.001835 | 0.006445 | yes | up | 0.08 | 0 | 0.03 | 0.73 | 0.54 | 2.38 | 0.036667 | 1.216667 |
| GC | 42.121 | 5.396476 | 0.001837 | 0.006453 | yes | up | 0 | 0 | 0 | 0.79 | 0.23 | 0.87 | 0 | 0.63 |
| IRX3 | 0.333 | -1.58848 | 0.001844 | 0.006475 | yes | down | 3.89 | 1.81 | 2.68 | 1.89 | 2.49 | 2.03 | 2.793333 | 2.136667 |
| CFAP43 | 0.367 | -1.44517 | 0.001845 | 0.006475 | yes | down | 1.07 | 1.44 | 0.83 | 1.7 | 0.85 | 0.59 | 1.113333 | 1.046667 |
| DNAAF2 | 0.445 | -1.1683 | 0.001846 | 0.006477 | yes | down | 4.97 | 4.85 | 4.66 | 8.08 | 7.31 | 2.19 | 4.826667 | 5.86 |
| SLC9A2 | 0.085 | -3.56227 | 0.001847 | 0.006481 | yes | down | 2.04 | 0.14 | 0.03 | 0.14 | 0.05 | 0.2 | 0.736667 | 0.13 |
| CFAP36 | 0.431 | -1.21473 | 0.00185 | 0.006487 | yes | down | 5.07 | 3.6 | 4.34 | 5.96 | 4.08 | 2.78 | 4.336667 | 4.273333 |
| LOC108178421 | 0.029 | -5.08801 | 0.001852 | 0.006492 | yes | down | 0.11 | 0.18 | 0.13 | 0 | 0.02 | 0 | 0.14 | 0.006667 |
| LOC100359295 | 0.323 | -1.63116 | 0.001866 | 0.006536 | yes | down | 0.42 | 0.77 | 0.89 | 0.42 | 0.74 | 0.44 | 0.693333 | 0.533333 |
| ZNF214 | 0.38 | -1.39761 | 0.001885 | 0.006591 | yes | down | 1.1 | 1.5 | 1.2 | 1.34 | 1.56 | 0.51 | 1.266667 | 1.136667 |
| LOC103347362 | 2.017 | 1.012278 | 0.001893 | 0.006619 | yes | up | 7.44 | 4.3 | 4.57 | 19.85 | 32.72 | 23.13 | 5.436667 | 25.23333 |
| SLC16A6 | 0.26 | -1.94409 | 0.001895 | 0.006624 | yes | down | 0.56 | 0.35 | 0.26 | 0.14 | 0.36 | 0.19 | 0.39 | 0.23 |
| LOC100356339 | 54.847 | 5.777344 | 0.001904 | 0.006654 | yes | up | 0 | 0 | 0 | 0.06 | 1.62 | 1.23 | 0 | 0.97 |
| AGO3 | 0.461 | -1.11715 | 0.001907 | 0.006664 | yes | down | 0.57 | 0.5 | 0.58 | 0.43 | 0.7 | 0.59 | 0.55 | 0.573333 |
| LOC100351416 | 9.683 | 3.275498 | 0.00191 | 0.00667 | yes | up | 0 | 0.05 | 0.17 | 1.05 | 1.74 | 2.44 | 0.073333 | 1.743333 |
| DSN1 | 3.155 | 1.657622 | 0.001914 | 0.006684 | yes | up | 1.08 | 1.07 | 0.4 | 7.6 | 9.95 | 2.48 | 0.85 | 6.676667 |
| TTC39C | 0.488 | -1.03601 | 0.001926 | 0.00672 | yes | down | 2.46 | 3.2 | 3.49 | 4.06 | 4.16 | 2.57 | 3.05 | 3.596667 |
| KRT18 | 0.029 | -5.09395 | 0.001937 | 0.006754 | yes | down | 0.48 | 0.26 | 0.21 | 0 | 0.05 | 0 | 0.316667 | 0.016667 |
| CA13 | 0.488 | -1.03507 | 0.001947 | 0.006785 | yes | down | 13.33 | 15.25 | 19.25 | 24.02 | 26.55 | 12.08 | 15.94333 | 20.88333 |
| FAM162B | 16.015 | 4.001329 | 0.001953 | 0.006807 | yes | up | 0.14 | 0 | 0.23 | 5.37 | 0.94 | 7.18 | 0.123333 | 4.496667 |
| GPC4 | 2.074 | 1.052369 | 0.001956 | 0.00681 | yes | up | 7.23 | 3.06 | 2.92 | 21.63 | 25.12 | 15.87 | 4.403333 | 20.87333 |
| SERPINI1 | 2.071 | 1.050155 | 0.001956 | 0.00681 | yes | up | 1.92 | 1.92 | 1.37 | 10.09 | 6.49 | 7.79 | 1.736667 | 8.123333 |
| LOC100359206 | 0.021 | -5.58063 | 0.001976 | 0.006874 | yes | down | 0.05 | 0.29 | 0.01 | 0 | 0 | 0.02 | 0.116667 | 0.006667 |
| CDCA5 | 2.516 | 1.331292 | 0.001981 | 0.006887 | yes | up | 1.69 | 1.34 | 0.65 | 7.26 | 10.11 | 3.8 | 1.226667 | 7.056667 |
| LURAP1L | 2.531 | 1.339863 | 0.001981 | 0.006887 | yes | up | 1.2 | 0.64 | 0.55 | 4.55 | 3.82 | 5.1 | 0.796667 | 4.49 |
| KIAA1107 | 0.369 | -1.43968 | 0.001993 | 0.006922 | yes | down | 0.23 | 0.42 | 0.3 | 0.27 | 0.3 | 0.23 | 0.316667 | 0.266667 |
| LOC103350087 | 0.079 | -3.67105 | 0.001994 | 0.006925 | yes | down | 0.19 | 0.64 | 0.4 | 0.1 | 0.05 | 0.07 | 0.41 | 0.073333 |
| LOC100352110 | 2.634 | 1.397507 | 0.001995 | 0.006927 | yes | up | 3.55 | 2.16 | 1.71 | 15.33 | 10.66 | 16.22 | 2.473333 | 14.07 |
| SLC6A9 | 2.218 | 1.149325 | 0.002009 | 0.00697 | yes | up | 0.57 | 0.91 | 0.69 | 3.61 | 3.15 | 4.2 | 0.723333 | 3.653333 |
| S100A9 | 0.257 | -1.96159 | 0.002018 | 0.006998 | yes | down | 10.75 | 49.85 | 62.01 | 16.43 | 14.28 | 43.23 | 40.87 | 24.64667 |
| SEMA3G | 0.443 | -1.17522 | 0.002028 | 0.007027 | yes | down | 17.89 | 24.41 | 34.07 | 18.05 | 44.86 | 19.31 | 25.45667 | 27.40667 |
| LOC100351810 | 37.321 | 5.22191 | 0.002034 | 0.007047 | yes | up | 0 | 0 | 0 | 5.25 | 10.43 | 10.42 | 0 | 8.7 |
| KIAA1324 | 3.903 | 1.964658 | 0.002049 | 0.007097 | yes | up | 0.37 | 0.17 | 0.17 | 1.86 | 1.23 | 2.26 | 0.236667 | 1.783333 |
| NAPSA | 6.58 | 2.718093 | 0.002061 | 0.007131 | yes | up | 0.28 | 0.5 | 0.24 | 3.61 | 3.52 | 7.99 | 0.34 | 5.04 |
| CD320 | 2.08 | 1.056828 | 0.002071 | 0.007164 | yes | up | 5.75 | 5.23 | 3.98 | 20.57 | 18.22 | 30.96 | 4.986667 | 23.25 |
| IRAK1BP1 | 0.416 | -1.26397 | 0.002072 | 0.007165 | yes | down | 2.96 | 2.57 | 2.72 | 3.02 | 2.79 | 2.07 | 2.75 | 2.626667 |
| LOC108178968 | 0.238 | -2.07278 | 0.002107 | 0.007269 | yes | down | 0.66 | 0.4 | 0.82 | 0.51 | 0.38 | 0.18 | 0.626667 | 0.356667 |
| ARHGAP22 | 3.151 | 1.655608 | 0.002115 | 0.007293 | yes | up | 0.54 | 0.43 | 0.13 | 2.49 | 3.54 | 3.28 | 0.366667 | 3.103333 |
| LOC100347628 | 0.26 | -1.94073 | 0.002118 | 0.007302 | yes | down | 1.94 | 2.35 | 4.04 | 0.58 | 1.38 | 3.11 | 2.776667 | 1.69 |
| FUT1 | 5.057 | 2.338352 | 0.002121 | 0.007309 | yes | up | 0.05 | 0.09 | 0.06 | 0.91 | 0.56 | 1.12 | 0.066667 | 0.863333 |
| GFAP | 5.229 | 2.38642 | 0.002127 | 0.007327 | yes | up | 0.2 | 0.06 | 0.06 | 0.59 | 1.28 | 1.82 | 0.106667 | 1.23 |
| TRPM1 | 6.399 | 2.677949 | 0.002144 | 0.007378 | yes | up | 0.1 | 0.26 | 0.05 | 0.13 | 3.48 | 2.32 | 0.136667 | 1.976667 |
| LRRC6 | 0.031 | -5.03479 | 0.002144 | 0.007378 | yes | down | 0.26 | 0.24 | 0.3 | 0 | 0 | 0.06 | 0.266667 | 0.02 |
| RBM41 | 0.377 | -1.40681 | 0.002148 | 0.007389 | yes | down | 1.04 | 1.26 | 1.36 | 1.1 | 1.58 | 0.52 | 1.22 | 1.066667 |
| COL8A1 | 2.361 | 1.239251 | 0.002154 | 0.007407 | yes | up | 4.26 | 2.93 | 1.31 | 10.62 | 14.75 | 21.81 | 2.833333 | 15.72667 |
| LOC103346963 | 2.798 | 1.484517 | 0.00216 | 0.007427 | yes | up | 2.04 | 1.34 | 1.2 | 7.23 | 15.57 | 6.9 | 1.526667 | 9.9 |
| LOC100343315 | 0.463 | -1.1102 | 0.002164 | 0.007439 | yes | down | 5.21 | 5.11 | 3.43 | 4.65 | 6.77 | 3.24 | 4.583333 | 4.886667 |
| BNIPL | 37.036 | 5.210861 | 0.00218 | 0.007489 | yes | up | 0 | 0 | 0 | 0.5 | 0.36 | 0.6 | 0 | 0.486667 |
| GCHFR | 0.477 | -1.0671 | 0.002184 | 0.007503 | yes | down | 9.83 | 18.69 | 15.99 | 19.34 | 19.24 | 11.02 | 14.83667 | 16.53333 |
| KIF4A | 3.224 | 1.688735 | 0.002188 | 0.007513 | yes | up | 0.54 | 0.43 | 0.46 | 4.2 | 5.98 | 0.9 | 0.476667 | 3.693333 |
| GTPBP10 | 0.344 | -1.54 | 0.002191 | 0.007519 | yes | down | 1.73 | 2.64 | 5.07 | 2.49 | 2.99 | 2.34 | 3.146667 | 2.606667 |
| LOC100350375 | 0.121 | -3.05093 | 0.002196 | 0.007533 | yes | down | 0.91 | 0.63 | 0.4 | 0 | 1.23 | 0.13 | 0.646667 | 0.453333 |
| FGF1 | 2.665 | 1.413992 | 0.002203 | 0.007554 | yes | up | 5.77 | 3.23 | 1.79 | 14.58 | 13.4 | 27.42 | 3.596667 | 18.46667 |
| LOC100358827 | 2.01 | 1.007358 | 0.002211 | 0.007575 | yes | up | 4.01 | 5.21 | 4.49 | 14.76 | 20.06 | 27.98 | 4.57 | 20.93333 |
| PRDM8 | 2.412 | 1.269971 | 0.002226 | 0.00762 | yes | up | 0.6 | 0.46 | 0.24 | 1.97 | 2.06 | 1.83 | 0.433333 | 1.953333 |
| MCM5 | 2.288 | 1.193905 | 0.002239 | 0.007657 | yes | up | 3.83 | 2.37 | 1.78 | 16.13 | 19 | 6.65 | 2.66 | 13.92667 |
| LOC103347017 | 2.722 | 1.444425 | 0.002239 | 0.007657 | yes | up | 10.66 | 3.16 | 6.03 | 40.12 | 41.19 | 41.86 | 6.616667 | 41.05667 |
| ACLY | 2.425 | 1.277693 | 0.002241 | 0.007663 | yes | up | 33.11 | 21.6 | 42.36 | 287.34 | 107.35 | 142.94 | 32.35667 | 179.21 |
| BRINP3 | 4.246 | 2.086254 | 0.002243 | 0.007667 | yes | up | 0.04 | 0.06 | 0.04 | 0.28 | 0.57 | 0.44 | 0.046667 | 0.43 |
| LOC108176678 | 5.344 | 2.417946 | 0.002244 | 0.007672 | yes | up | 0.05 | 0.23 | 0.09 | 0.77 | 1.23 | 2.45 | 0.123333 | 1.483333 |
| FAS | 2.058 | 1.040898 | 0.002245 | 0.007673 | yes | up | 3.15 | 3.38 | 2.48 | 19.86 | 13.41 | 7.61 | 3.003333 | 13.62667 |
| NXPH2 | 2.615 | 1.386981 | 0.002267 | 0.007737 | yes | up | 0.62 | 0.44 | 0.38 | 4.06 | 2.54 | 1.9 | 0.48 | 2.833333 |
| PDCD2L | 0.492 | -1.02249 | 0.002272 | 0.007753 | yes | down | 4.71 | 5.58 | 3.39 | 4.8 | 4 | 5.73 | 4.56 | 4.843333 |
| LOC100343510 | 2.126 | 1.087977 | 0.002285 | 0.00779 | yes | up | 4.34 | 3.84 | 2.97 | 25.63 | 13.39 | 10.44 | 3.716667 | 16.48667 |
| L3HYPDH | 2.571 | 1.362367 | 0.002302 | 0.00784 | yes | up | 1.46 | 0.97 | 0.52 | 4.86 | 5.75 | 6.27 | 0.983333 | 5.626667 |
| C1QTNF7 | 2.299 | 1.200736 | 0.002318 | 0.00789 | yes | up | 1.16 | 0.9 | 0.72 | 3.46 | 7.32 | 4.43 | 0.926667 | 5.07 |
| LOC108176754 | 0.054 | -4.20371 | 0.002362 | 0.008029 | yes | down | 8.66 | 10.49 | 6.82 | 0 | 1.44 | 1.75 | 8.656667 | 1.063333 |
| RASSF9 | 0.31 | -1.69071 | 0.002363 | 0.008032 | yes | down | 1.37 | 2.7 | 2.91 | 2.01 | 2.72 | 0.48 | 2.326667 | 1.736667 |
| LOC108176507 | 8.479 | 3.083885 | 0.002365 | 0.008038 | yes | up | 0.02 | 0.04 | 0.01 | 0.36 | 0.31 | 0.64 | 0.023333 | 0.436667 |
| EDN3 | 0.06 | -4.04925 | 0.002372 | 0.008057 | yes | down | 1.3 | 0.09 | 0.12 | 0.03 | 0 | 0.18 | 0.503333 | 0.07 |
| TP53I3 | 2.191 | 1.131804 | 0.002375 | 0.008066 | yes | up | 1.14 | 1.99 | 1.44 | 6.59 | 8.33 | 8.14 | 1.523333 | 7.686667 |
| CDC25A | 4.309 | 2.107315 | 0.002375 | 0.008066 | yes | up | 0.24 | 0.22 | 0.03 | 1.44 | 2.6 | 0.76 | 0.163333 | 1.6 |
| LOC108177034 | 0.181 | -2.46628 | 0.002377 | 0.008071 | yes | down | 0.71 | 0.59 | 1.16 | 0.19 | 0.2 | 0.33 | 0.82 | 0.24 |
| ADAMTS6 | 0.306 | -1.70947 | 0.002379 | 0.008072 | yes | down | 0.41 | 0.52 | 0.11 | 0.25 | 0.31 | 0.16 | 0.346667 | 0.24 |
| TNNI3K | 10.224 | 3.353828 | 0.002385 | 0.008091 | yes | up | 0.02 | 0.05 | 0.02 | 0.72 | 0.86 | 0.21 | 0.03 | 0.596667 |
| LOC103350977 | 0.116 | -3.10371 | 0.00239 | 0.008108 | yes | down | 0.47 | 1.52 | 0.64 | 0.08 | 0.17 | 0.49 | 0.876667 | 0.246667 |
| RSAD1 | 0.363 | -1.46152 | 0.002393 | 0.008115 | yes | down | 1.72 | 1.88 | 1.04 | 1.05 | 0.91 | 1.81 | 1.546667 | 1.256667 |
| LOC103346188 | 2.927 | 1.549211 | 0.002395 | 0.008122 | yes | up | 0.76 | 1.29 | 0.58 | 1.32 | 2.64 | 1.58 | 0.876667 | 1.846667 |
| LOC100347994 | 6.052 | 2.597383 | 0.002402 | 0.00814 | yes | up | 0.63 | 0.02 | 0.05 | 3.72 | 2.14 | 3.33 | 0.233333 | 3.063333 |
| TRIM66 | 0.276 | -1.85882 | 0.002409 | 0.008163 | yes | down | 0.26 | 0.4 | 0.4 | 0.12 | 0.25 | 0.33 | 0.353333 | 0.233333 |
| ULK4 | 2.294 | 1.19778 | 0.002422 | 0.008196 | yes | up | 2.96 | 5.4 | 2.15 | 7.76 | 4.07 | 2.73 | 3.503333 | 4.853333 |
| TRMT11 | 0.418 | -1.25762 | 0.002423 | 0.0082 | yes | down | 1.78 | 1.37 | 2.18 | 1.94 | 2.03 | 1.2 | 1.776667 | 1.723333 |
| LIPJ | 0.3 | -1.73913 | 0.002438 | 0.008248 | yes | down | 0.89 | 1.27 | 0.84 | 0.51 | 0.83 | 1.12 | 1 | 0.82 |
| LOC100348192 | 0.44 | -1.18454 | 0.002456 | 0.008302 | yes | down | 3.25 | 3.88 | 1.86 | 3.27 | 4.01 | 1.7 | 2.996667 | 2.993333 |
| METTL15 | 0.464 | -1.10668 | 0.002465 | 0.008327 | yes | down | 1.51 | 1.65 | 2.19 | 2.06 | 2.72 | 1.65 | 1.783333 | 2.143333 |
| STXBP5L | 0.2 | -2.32366 | 0.002472 | 0.008346 | yes | down | 0.09 | 0.07 | 0.2 | 0.07 | 0.08 | 0.03 | 0.12 | 0.06 |
| NFKBIZ | 2.508 | 1.326732 | 0.002497 | 0.008412 | yes | up | 0.51 | 0.89 | 0.35 | 3.46 | 3.89 | 2.59 | 0.583333 | 3.313333 |
| LOC100342797 | 0.225 | -2.15249 | 0.002519 | 0.008478 | yes | down | 3.29 | 4.5 | 1.63 | 0.88 | 2.65 | 1.45 | 3.14 | 1.66 |
| SLC35A3 | 0.374 | -1.42021 | 0.002524 | 0.00849 | yes | down | 1.01 | 1.42 | 1 | 1.02 | 1.37 | 0.56 | 1.143333 | 0.983333 |
| CCNA2 | 3.016 | 1.5925 | 0.002533 | 0.008518 | yes | up | 1.86 | 1.77 | 0.74 | 10.78 | 15.61 | 3.79 | 1.456667 | 10.06 |
| LOC103351551 | 46.104 | 5.526835 | 0.002537 | 0.008529 | yes | up | 0 | 0 | 0 | 0.06 | 0.81 | 0.19 | 0 | 0.353333 |
| TMEM63C | 2.461 | 1.299348 | 0.00254 | 0.008537 | yes | up | 0.26 | 0.25 | 0.18 | 0.89 | 1.87 | 1.18 | 0.23 | 1.313333 |
| CNNM1 | 3.908 | 1.966304 | 0.002549 | 0.008565 | yes | up | 0.1 | 0.04 | 0.04 | 0.71 | 0.55 | 0.28 | 0.06 | 0.513333 |
| TRIP13 | 2.704 | 1.435353 | 0.002586 | 0.008679 | yes | up | 0.76 | 1.2 | 0.48 | 4.91 | 7.55 | 2.7 | 0.813333 | 5.053333 |
| MAD1L1 | 2.112 | 1.078284 | 0.002586 | 0.008679 | yes | up | 2.33 | 2.04 | 1.94 | 9.98 | 12.49 | 8.24 | 2.103333 | 10.23667 |
| APOA1 | 17.15 | 4.100111 | 0.002588 | 0.008684 | yes | up | 0 | 0.15 | 0 | 1.58 | 1.92 | 2.01 | 0.05 | 1.836667 |
| CSPG5 | 2.108 | 1.075869 | 0.002598 | 0.008711 | yes | up | 1.99 | 1.81 | 1.31 | 6.48 | 12.64 | 6.12 | 1.703333 | 8.413333 |
| GATM | 0.324 | -1.62551 | 0.002606 | 0.008732 | yes | down | 10.1 | 4.25 | 1.48 | 2.69 | 3.53 | 4.91 | 5.276667 | 3.71 |
| LOC103352055 | 0.497 | -1.00948 | 0.002606 | 0.008733 | yes | down | 23.29 | 32.87 | 27.25 | 35.13 | 24.6 | 35.26 | 27.80333 | 31.66333 |
| LOC108178915 | 8.46 | 3.080649 | 0.002622 | 0.008779 | yes | up | 0.12 | 0.45 | 0 | 2.34 | 5.24 | 2.46 | 0.19 | 3.346667 |
| LOC103349247 | 3.795 | 1.923927 | 0.002628 | 0.008798 | yes | up | 0.64 | 0.57 | 0.9 | 2.59 | 11.59 | 2.85 | 0.703333 | 5.676667 |
| LOC108177081 | 0.21 | -2.25052 | 0.002658 | 0.008891 | yes | down | 0.7 | 0.46 | 0.4 | 0.14 | 0.42 | 0.2 | 0.52 | 0.253333 |
| GABRE | 0.329 | -1.60204 | 0.002685 | 0.008974 | yes | down | 0.51 | 0.97 | 0.39 | 0.36 | 0.63 | 0.41 | 0.623333 | 0.466667 |
| MKI67 | 3.12 | 1.641385 | 0.002707 | 0.00904 | yes | up | 1.88 | 0.93 | 0.69 | 9.27 | 13.55 | 1.74 | 1.166667 | 8.186667 |
| SOX6 | 0.349 | -1.51874 | 0.00273 | 0.009107 | yes | down | 1.65 | 0.6 | 0.41 | 0.49 | 0.58 | 0.98 | 0.886667 | 0.683333 |
| LOC103345242 | 13.285 | 3.731764 | 0.002735 | 0.009125 | yes | up | 0 | 0.09 | 0.25 | 1.03 | 1.55 | 6.91 | 0.113333 | 3.163333 |
| DZIP1L | 2.746 | 1.457337 | 0.002768 | 0.009231 | yes | up | 0.4 | 0.32 | 0.14 | 1.18 | 2.37 | 1.8 | 0.286667 | 1.783333 |
| BHLHE40 | 2.306 | 1.205115 | 0.002778 | 0.009259 | yes | up | 8.43 | 10.2 | 6.06 | 44.05 | 19.81 | 59.92 | 8.23 | 41.26 |
| CENPL | 4.175 | 2.061805 | 0.002795 | 0.009312 | yes | up | 0.22 | 0.14 | 0.22 | 2.42 | 2.27 | 0.98 | 0.193333 | 1.89 |
| LOC100353783 | 74.165 | 6.21267 | 0.002843 | 0.009456 | yes | up | 0.72 | 0.11 | 0 | 46.04 | 1.18 | 95.32 | 0.276667 | 47.51333 |
| OSBPL3 | 2.441 | 1.287261 | 0.002872 | 0.009544 | yes | up | 0.41 | 0.27 | 0.11 | 1.29 | 1.81 | 1.28 | 0.263333 | 1.46 |
| GXYLT2 | 4.167 | 2.058917 | 0.002886 | 0.009588 | yes | up | 1 | 0.47 | 0.06 | 2.67 | 5.5 | 5.62 | 0.51 | 4.596667 |
| RBP5 | 5.571 | 2.478 | 0.002891 | 0.0096 | yes | up | 0.14 | 0.38 | 0.16 | 3.01 | 1.7 | 3.29 | 0.226667 | 2.666667 |
| SLC9B1 | 0.298 | -1.74784 | 0.002906 | 0.009639 | yes | down | 0.63 | 1.76 | 1 | 1.11 | 1.02 | 0.55 | 1.13 | 0.893333 |
| LOC100345224 | 0.309 | -1.69658 | 0.002913 | 0.009655 | yes | down | 0.22 | 0.88 | 0.51 | 0.51 | 0.37 | 0.26 | 0.536667 | 0.38 |
| LOC103345487 | 2.866 | 1.518911 | 0.002919 | 0.009671 | yes | up | 0.89 | 1.51 | 1.02 | 6.28 | 9.53 | 6.97 | 1.14 | 7.593333 |
| CDHR4 | 6.027 | 2.591381 | 0.002921 | 0.009677 | yes | up | 0.09 | 0.02 | 0.06 | 0.83 | 1.12 | 0.48 | 0.056667 | 0.81 |
| NXPE3 | 2.06 | 1.042396 | 0.00297 | 0.009815 | yes | up | 1.1 | 0.52 | 0.76 | 3.57 | 4.4 | 3.25 | 0.793333 | 3.74 |
| LOC103348554 | 0.085 | -3.56097 | 0.002972 | 0.009821 | yes | down | 0.17 | 1.73 | 2.1 | 0.42 | 0 | 0.38 | 1.333333 | 0.266667 |
| RRM2 | 2.962 | 1.566403 | 0.002985 | 0.009858 | yes | up | 2.74 | 2.63 | 1.16 | 18.83 | 21.62 | 3.84 | 2.176667 | 14.76333 |
| PSMC3IP | 3.176 | 1.667195 | 0.002987 | 0.009859 | yes | up | 0.76 | 0.42 | 0.26 | 4.55 | 4.11 | 1.53 | 0.48 | 3.396667 |
| DPEP3 | 36.261 | 5.180349 | 0.003007 | 0.009915 | yes | up | 0 | 0 | 0 | 0.24 | 0.66 | 0.72 | 0 | 0.54 |
| LOC108178044 | 0.019 | -5.71015 | 0.003013 | 0.009934 | yes | down | 0.1 | 0.04 | 0.21 | 0 | 0 | 0 | 0.116667 | 0 |
| ARHGEF19 | 3.309 | 1.726447 | 0.003023 | 0.009963 | yes | up | 0.22 | 0.16 | 0.09 | 1.01 | 1.44 | 1.09 | 0.156667 | 1.18 |
| LOC103345244 | 8.356 | 3.062793 | 0.003026 | 0.009971 | yes | up | 0.6 | 0.09 | 0.13 | 1.34 | 1.9 | 10.73 | 0.273333 | 4.656667 |
| ERBB4 | 0.089 | -3.49441 | 0.003032 | 0.009988 | yes | down | 0.24 | 0.05 | 0 | 0.02 | 0.01 | 0.02 | 0.096667 | 0.016667 |
| TEX12 | 0.212 | -2.23602 | 0.003053 | 0.010052 | yes | down | 2.04 | 1.68 | 0.62 | 0.53 | 0.34 | 1.23 | 1.446667 | 0.7 |
| APOA2 | 0.039 | -4.67957 | 0.003054 | 0.010053 | yes | down | 903.34 | 3.6 | 1082.22 | 104.54 | 45.9 | 33.56 | 663.0533 | 61.33333 |
| LGI4 | 2.726 | 1.446554 | 0.003061 | 0.010073 | yes | up | 1 | 0.73 | 0.41 | 4.18 | 2.37 | 6.14 | 0.713333 | 4.23 |
| LOC103346961 | 3.983 | 1.993978 | 0.003072 | 0.010108 | yes | up | 0.19 | 0.39 | 0.24 | 1.44 | 2.78 | 3.32 | 0.273333 | 2.513333 |
| LOC103347389 | 24.403 | 4.60897 | 0.00308 | 0.010128 | yes | up | 0 | 0 | 0.07 | 3.78 | 6.46 | 7.35 | 0.023333 | 5.863333 |
| PLA2R1 | 2.224 | 1.15295 | 0.00309 | 0.010155 | yes | up | 1 | 0.41 | 0.61 | 2.65 | 4.23 | 2.49 | 0.673333 | 3.123333 |
| PRR32 | 0.022 | -5.49276 | 0.003106 | 0.010203 | yes | down | 25.83 | 4.52 | 0.36 | 0.43 | 0 | 0.95 | 10.23667 | 0.46 |
| APOC1 | 23.821 | 4.574149 | 0.003123 | 0.010255 | yes | up | 0.31 | 0 | 0 | 6.89 | 4.94 | 4.57 | 0.103333 | 5.466667 |
| CDK1 | 3.156 | 1.65808 | 0.003126 | 0.010261 | yes | up | 3.87 | 3.96 | 1.17 | 25.83 | 32.95 | 5.67 | 3 | 21.48333 |
| ZNF660 | 0.412 | -1.27996 | 0.003144 | 0.010305 | yes | down | 1.69 | 1.52 | 2.15 | 2.08 | 2.4 | 0.83 | 1.786667 | 1.77 |
| RAPGEF4 | 0.489 | -1.03198 | 0.00315 | 0.01032 | yes | down | 1.44 | 1.58 | 2.38 | 2.06 | 2.21 | 1.99 | 1.8 | 2.086667 |
| NFATC2 | 2.244 | 1.166332 | 0.003167 | 0.010366 | yes | up | 0.93 | 0.58 | 0.44 | 2.93 | 2.26 | 4.44 | 0.65 | 3.21 |
| ABCB9 | 0.303 | -1.72053 | 0.003177 | 0.010394 | yes | down | 0.47 | 0.33 | 0.39 | 0.33 | 0.21 | 0.29 | 0.396667 | 0.276667 |
| LOC100353731 | 0.396 | -1.33517 | 0.003179 | 0.010398 | yes | down | 61.24 | 87.05 | 45.43 | 25.98 | 47.26 | 98.57 | 64.57333 | 57.27 |
| RIIAD1 | 0.193 | -2.37038 | 0.003182 | 0.010405 | yes | down | 2.39 | 4.06 | 3.01 | 1.08 | 2.47 | 0.71 | 3.153333 | 1.42 |
| DLK1 | 2.701 | 1.43339 | 0.003199 | 0.010447 | yes | up | 72.94 | 26.99 | 10.8 | 250.04 | 195.63 | 203.54 | 36.91 | 216.4033 |
| LOC100350196 | 0.033 | -4.93386 | 0.003213 | 0.010488 | yes | down | 0.27 | 0.45 | 0.24 | 0.06 | 0 | 0 | 0.32 | 0.02 |
| LOC100356618 | 0.416 | -1.26472 | 0.00322 | 0.010509 | yes | down | 6.92 | 10.92 | 5.59 | 9.08 | 10.89 | 3.38 | 7.81 | 7.783333 |
| TNFAIP8L2 | 2.261 | 1.177195 | 0.00324 | 0.010574 | yes | up | 0.97 | 1.54 | 0.55 | 4.43 | 7.03 | 6.42 | 1.02 | 5.96 |
| ST6GALNAC3 | 0.445 | -1.16839 | 0.003241 | 0.010574 | yes | down | 2.12 | 1.59 | 1.47 | 1.97 | 2.8 | 0.74 | 1.726667 | 1.836667 |
| DDN | 0.064 | -3.97327 | 0.003253 | 0.010604 | yes | down | 1.15 | 0.18 | 0.1 | 0 | 0 | 0.2 | 0.476667 | 0.066667 |
| GCNT1 | 2.413 | 1.271029 | 0.003257 | 0.010616 | yes | up | 0.34 | 0.26 | 0.22 | 1.32 | 1.51 | 1.62 | 0.273333 | 1.483333 |
| XKR8 | 2.137 | 1.095848 | 0.003258 | 0.010616 | yes | up | 1.22 | 0.91 | 0.86 | 4.7 | 4.04 | 5.71 | 0.996667 | 4.816667 |
| BRWD3 | 0.426 | -1.22964 | 0.003265 | 0.010639 | yes | down | 1.45 | 1.15 | 1.36 | 1.22 | 2.09 | 0.6 | 1.32 | 1.303333 |
| POLE | 2.804 | 1.487317 | 0.003277 | 0.010671 | yes | up | 0.63 | 0.34 | 0.19 | 2.7 | 3.83 | 0.94 | 0.386667 | 2.49 |
| LOC103347283 | 4.122 | 2.043206 | 0.003279 | 0.010674 | yes | up | 0.29 | 1.62 | 0.81 | 12.32 | 11.02 | 5.71 | 0.906667 | 9.683333 |
| CD4 | 17.756 | 4.150215 | 0.003299 | 0.010735 | yes | up | 0 | 0.04 | 0.04 | 0.32 | 1.3 | 1.65 | 0.026667 | 1.09 |
| NACAD | 3.915 | 1.969174 | 0.003305 | 0.010745 | yes | up | 0.16 | 0.18 | 0.02 | 0.63 | 1.01 | 1.4 | 0.12 | 1.013333 |
| ATOH8 | 2.035 | 1.024801 | 0.003307 | 0.010748 | yes | up | 1.43 | 1.58 | 1.3 | 4.1 | 7.97 | 8.08 | 1.436667 | 6.716667 |
| CD8A | 19.348 | 4.27408 | 0.003331 | 0.010819 | yes | up | 0.06 | 0 | 0 | 0.41 | 0.45 | 1.54 | 0.02 | 0.8 |
| LOC108176233 | 20.898 | 4.385279 | 0.003341 | 0.01085 | yes | up | 0.18 | 0 | 0 | 1.74 | 0.71 | 5.01 | 0.06 | 2.486667 |
| APITD1 | 2.495 | 1.319153 | 0.003349 | 0.010868 | yes | up | 2.73 | 3.97 | 1.93 | 25.99 | 21.68 | 13.5 | 2.876667 | 20.39 |
| MXRA7 | 0.456 | -1.13283 | 0.003362 | 0.010904 | yes | down | 45.76 | 30.29 | 27.72 | 45.3 | 49.18 | 14.44 | 34.59 | 36.30667 |
| SLC22A23 | 0.439 | -1.18684 | 0.003376 | 0.010945 | yes | down | 3.07 | 2.01 | 2.14 | 1.21 | 2.92 | 2.84 | 2.406667 | 2.323333 |
| LOC100008796 | 47.286 | 5.563341 | 0.003397 | 0.011009 | yes | up | 0 | 0 | 0 | 0.28 | 9.72 | 5.67 | 0 | 5.223333 |
| TOP2A | 2.634 | 1.397405 | 0.003398 | 0.011009 | yes | up | 3.77 | 3.08 | 1.6 | 22.72 | 26.59 | 5.85 | 2.816667 | 18.38667 |
| FGG | 11.836 | 3.565119 | 0.003401 | 0.011016 | yes | up | 0 | 0 | 0.15 | 1.88 | 0.66 | 1.62 | 0.05 | 1.386667 |
| IRX5 | 0.328 | -1.61022 | 0.003405 | 0.011029 | yes | down | 2.19 | 0.97 | 0.97 | 1.15 | 1.47 | 0.47 | 1.376667 | 1.03 |
| SUGCT | 0.331 | -1.59395 | 0.003416 | 0.011058 | yes | down | 1.18 | 0.54 | 0.63 | 0.73 | 0.38 | 0.65 | 0.783333 | 0.586667 |
| KCNQ5 | 0.136 | -2.87741 | 0.003423 | 0.011079 | yes | down | 1.22 | 0.15 | 0.05 | 0.14 | 0.04 | 0.22 | 0.473333 | 0.133333 |
| EPHA6 | 2.131 | 1.091216 | 0.003462 | 0.011185 | yes | up | 0.76 | 0.7 | 0.62 | 2.84 | 3.57 | 1.75 | 0.693333 | 2.72 |
| CORO2A | 3.89 | 1.959662 | 0.003466 | 0.011192 | yes | up | 0.12 | 0.4 | 0.06 | 0.82 | 2.05 | 2.16 | 0.193333 | 1.676667 |
| LOC103348900 | 3.528 | 1.818839 | 0.003468 | 0.011194 | yes | up | 0.25 | 0.05 | 0.05 | 0.9 | 0.79 | 1.05 | 0.116667 | 0.913333 |
| EFHC2 | 0.389 | -1.36127 | 0.003477 | 0.011216 | yes | down | 0.66 | 0.93 | 0.88 | 0.74 | 1.05 | 0.46 | 0.823333 | 0.75 |
| TRPV4 | 2.787 | 1.47876 | 0.003483 | 0.011234 | yes | up | 0.16 | 0.53 | 0.34 | 2.27 | 2.43 | 1.97 | 0.343333 | 2.223333 |
| ARNTL | 2.356 | 1.23647 | 0.00351 | 0.011313 | yes | up | 1.07 | 1.26 | 2.34 | 9.33 | 6.6 | 9.9 | 1.556667 | 8.61 |
| DERL3 | 0.326 | -1.61523 | 0.003522 | 0.011348 | yes | down | 2.83 | 4.37 | 3.08 | 2.85 | 3.93 | 0.97 | 3.426667 | 2.583333 |
| FGF7 | 4.23 | 2.080782 | 0.003538 | 0.011393 | yes | up | 0.3 | 0.31 | 0.14 | 3.64 | 1.58 | 1.24 | 0.25 | 2.153333 |
| FBN3 | 0.04 | -4.63622 | 0.003542 | 0.011398 | yes | down | 0.15 | 0.01 | 0.02 | 0.01 | 0 | 0.01 | 0.06 | 0.006667 |
| LOC103345866 | 40.368 | 5.335147 | 0.003552 | 0.011427 | yes | up | 0.02 | 0 | 0 | 0.08 | 0.13 | 4.97 | 0.006667 | 1.726667 |
| ATG16L2 | 2.444 | 1.289173 | 0.003564 | 0.011461 | yes | up | 0.84 | 0.47 | 0.46 | 2.79 | 3.54 | 3.48 | 0.59 | 3.27 |
| GRP | 0.014 | -6.16703 | 0.003574 | 0.011489 | yes | down | 0 | 1.85 | 1.13 | 0 | 0 | 0 | 0.993333 | 0 |
| OAS3 | 2.145 | 1.100763 | 0.003575 | 0.01149 | yes | up | 0.9 | 0.44 | 0.41 | 2.76 | 2.48 | 3.15 | 0.583333 | 2.796667 |
| PLEKHD1 | 0.024 | -5.36903 | 0.003586 | 0.01152 | yes | down | 0.74 | 0.1 | 0 | 0.04 | 0 | 0 | 0.28 | 0.013333 |
| LOC103348671 | 0.22 | -2.1838 | 0.003626 | 0.01164 | yes | down | 0.8 | 0.69 | 0.54 | 0.33 | 0.57 | 0.13 | 0.676667 | 0.343333 |
| LOC100339846 | 0.168 | -2.5748 | 0.00366 | 0.011735 | yes | down | 1.09 | 0.12 | 0.11 | 0.1 | 0.5 | 0.42 | 0.44 | 0.34 |
| SLC18A2 | 13.395 | 3.743663 | 0.003661 | 0.011735 | yes | up | 0 | 0 | 0.09 | 0.61 | 1.85 | 0.57 | 0.03 | 1.01 |
| NPY4R | 16.512 | 4.045453 | 0.003677 | 0.011781 | yes | up | 0 | 0.01 | 0.05 | 0.32 | 0.19 | 0.59 | 0.02 | 0.366667 |
| RNF112 | 18.112 | 4.178898 | 0.003681 | 0.011789 | yes | up | 0.03 | 0 | 1.7 | 4.24 | 5.36 | 40.12 | 0.576667 | 16.57333 |
| LOC100344509 | 0.469 | -1.09272 | 0.003681 | 0.011789 | yes | down | 4.77 | 7.22 | 4.3 | 6.36 | 2.99 | 6.24 | 5.43 | 5.196667 |
| PIK3R5 | 2.422 | 1.276367 | 0.003684 | 0.011794 | yes | up | 0.9 | 0.85 | 0.44 | 2.66 | 7.13 | 2.5 | 0.73 | 4.096667 |
| LOC100343001 | 0.269 | -1.89324 | 0.003694 | 0.01182 | yes | down | 1.26 | 2.8 | 0.5 | 1.01 | 1.24 | 0.94 | 1.52 | 1.063333 |
| TRIM58 | 0.297 | -1.74944 | 0.003728 | 0.011918 | yes | down | 1.39 | 4.28 | 4.16 | 1.8 | 0.79 | 4.28 | 3.276667 | 2.29 |
| LOC103347033 | 7.954 | 2.991592 | 0.003739 | 0.011947 | yes | up | 0.28 | 0.51 | 0 | 7.2 | 2.94 | 3.55 | 0.263333 | 4.563333 |
| NHEJ1 | 2.203 | 1.139738 | 0.003763 | 0.012016 | yes | up | 1.08 | 1.42 | 0.66 | 6.41 | 4.87 | 5.79 | 1.053333 | 5.69 |
| SPATA5 | 0.447 | -1.16019 | 0.003781 | 0.012067 | yes | down | 1.17 | 0.73 | 1.16 | 1.05 | 1.35 | 0.79 | 1.02 | 1.063333 |
| PRG2 | 0.022 | -5.48026 | 0.003796 | 0.012109 | yes | down | 0.45 | 0.33 | 0.39 | 0 | 0 | 0 | 0.39 | 0 |
| BTBD11 | 2.301 | 1.202111 | 0.003829 | 0.012204 | yes | up | 0.39 | 0.52 | 0.34 | 3.5 | 4.06 | 8.84 | 0.416667 | 5.466667 |
| GPRC5D | 6.763 | 2.757755 | 0.003848 | 0.012256 | yes | up | 0.07 | 0.12 | 0.03 | 1.52 | 0.54 | 1.15 | 0.073333 | 1.07 |
| S100A3 | 24.55 | 4.617665 | 0.003849 | 0.012256 | yes | up | 0.11 | 0 | 0 | 0.89 | 1.27 | 2.77 | 0.036667 | 1.643333 |
| LOC108175458 | 0.495 | -1.01534 | 0.003874 | 0.012326 | yes | down | 6.11 | 4.66 | 3.51 | 5.08 | 7.75 | 3.38 | 4.76 | 5.403333 |
| PCSK2 | 12.84 | 3.682573 | 0.003876 | 0.012331 | yes | up | 0.03 | 0.01 | 0 | 0.73 | 0.17 | 0.28 | 0.013333 | 0.393333 |
| CAMKMT | 2.28 | 1.189175 | 0.003882 | 0.012347 | yes | up | 0.76 | 1.19 | 0.49 | 3.5 | 5.52 | 3.67 | 0.813333 | 4.23 |
| LOC103350892 | 0.432 | -1.21096 | 0.003925 | 0.012465 | yes | down | 2.63 | 3.71 | 2.08 | 5.28 | 4.91 | 3.23 | 2.806667 | 4.473333 |
| RAB11FIP4 | 0.3 | -1.73902 | 0.003983 | 0.012639 | yes | down | 2.56 | 1.19 | 0.77 | 0.46 | 1.1 | 1.43 | 1.506667 | 0.996667 |
| LONP1 | 0.271 | -1.8859 | 0.003989 | 0.012652 | yes | down | 11.31 | 11.39 | 6.17 | 4.55 | 8.46 | 5.13 | 9.623333 | 6.046667 |
| EHF | 15.963 | 3.996638 | 0.004055 | 0.012847 | yes | up | 0 | 0.02 | 0 | 0.23 | 0.32 | 0.17 | 0.006667 | 0.24 |
| LOC108177736 | 0.159 | -2.65535 | 0.004108 | 0.013012 | yes | down | 0.16 | 0.25 | 0.13 | 0.06 | 0.08 | 0.05 | 0.18 | 0.063333 |
| EPHA3 | 2.504 | 1.32397 | 0.004127 | 0.013069 | yes | up | 1.46 | 1.08 | 0.42 | 4.76 | 9.45 | 2.74 | 0.986667 | 5.65 |
| NR2C2AP | 0.418 | -1.25899 | 0.004143 | 0.013115 | yes | down | 3.07 | 6.16 | 5.11 | 4.35 | 6.64 | 7.24 | 4.78 | 6.076667 |
| LOC103350176 | 0.244 | -2.0368 | 0.004154 | 0.013138 | yes | down | 1.43 | 0.59 | 1.7 | 0.43 | 0.81 | 0.89 | 1.24 | 0.71 |
| MCM10 | 3.969 | 1.988933 | 0.004188 | 0.013231 | yes | up | 0.11 | 0.11 | 0.06 | 0.94 | 1.31 | 0.3 | 0.093333 | 0.85 |
| PPP2R2B | 3.813 | 1.930752 | 0.004191 | 0.013237 | yes | up | 0.37 | 0.4 | 0.15 | 2.99 | 1.09 | 3.51 | 0.306667 | 2.53 |
| FAM71F1 | 0.246 | -2.0215 | 0.004194 | 0.013245 | yes | down | 0.67 | 0.48 | 0.68 | 0.43 | 0.22 | 0.38 | 0.61 | 0.343333 |
| LOC100345842 | 0.115 | -3.12292 | 0.004228 | 0.013343 | yes | down | 0.89 | 0.98 | 0.79 | 0.04 | 0 | 0.63 | 0.886667 | 0.223333 |
| LOC100101568 | 0.187 | -2.4152 | 0.004232 | 0.013353 | yes | down | 0.55 | 0.55 | 0.56 | 0.31 | 0.17 | 0.22 | 0.553333 | 0.233333 |
| LOC103346185 | 21.855 | 4.449882 | 0.004254 | 0.013405 | yes | up | 0 | 0 | 0.05 | 0.99 | 1.09 | 0.87 | 0.016667 | 0.983333 |
| STC2 | 0.283 | -1.81945 | 0.004377 | 0.013759 | yes | down | 1.7 | 1.41 | 0.85 | 0.78 | 0.19 | 1.46 | 1.32 | 0.81 |
| LOC100345894 | 2.151 | 1.104747 | 0.004377 | 0.013759 | yes | up | 1.06 | 0.79 | 0.53 | 2.84 | 3.26 | 5.29 | 0.793333 | 3.796667 |
| CEP97 | 0.487 | -1.03686 | 0.004407 | 0.01384 | yes | down | 0.85 | 0.69 | 0.53 | 0.83 | 0.98 | 0.5 | 0.69 | 0.77 |
| FABP7 | 10.792 | 3.431844 | 0.004498 | 0.014102 | yes | up | 6.75 | 4.6 | 12.19 | 488.47 | 24.95 | 72.56 | 7.846667 | 195.3267 |
| EFHC1 | 2.917 | 1.544638 | 0.004499 | 0.014102 | yes | up | 0.21 | 0.83 | 0.79 | 5.56 | 4.75 | 2.93 | 0.61 | 4.413333 |
| LOC108176493 | 0.067 | -3.89629 | 0.004513 | 0.014138 | yes | down | 0.52 | 0.17 | 0.11 | 0 | 0.04 | 0.09 | 0.266667 | 0.043333 |
| BMP3 | 2.402 | 1.264373 | 0.004517 | 0.014148 | yes | up | 0.42 | 0.41 | 0.15 | 1.99 | 1.3 | 1.81 | 0.326667 | 1.7 |
| SERPINA12 | 4.252 | 2.088118 | 0.004519 | 0.014149 | yes | up | 0.41 | 0.57 | 0.38 | 9.29 | 2.21 | 1.36 | 0.453333 | 4.286667 |
| SLC7A8 | 0.46 | -1.11938 | 0.00452 | 0.014149 | yes | down | 9.11 | 8 | 2.69 | 5 | 6.51 | 8.42 | 6.6 | 6.643333 |
| BEST1 | 3.959 | 1.985185 | 0.004565 | 0.014282 | yes | up | 0.18 | 0.14 | 0.04 | 0.9 | 0.93 | 1.32 | 0.12 | 1.05 |
| ANKRD13D | 2.282 | 1.190187 | 0.004613 | 0.014418 | yes | up | 0.82 | 1.45 | 0.59 | 5.27 | 4.69 | 3.47 | 0.953333 | 4.476667 |
| HOXD3 | 2.166 | 1.114853 | 0.004615 | 0.014422 | yes | up | 0.83 | 0.68 | 0.61 | 3.54 | 6.04 | 1.81 | 0.706667 | 3.796667 |
| KIAA0101 | 3.333 | 1.736614 | 0.004619 | 0.01443 | yes | up | 3.63 | 2.26 | 1.66 | 26.47 | 26.72 | 3.77 | 2.516667 | 18.98667 |
| PRKG1 | 0.386 | -1.37469 | 0.004626 | 0.014447 | yes | down | 8.97 | 2.91 | 1.69 | 3.73 | 4.46 | 3.72 | 4.523333 | 3.97 |
| LOC100353065 | 0.35 | -1.5137 | 0.004636 | 0.014472 | yes | down | 1.61 | 1.26 | 0.51 | 0.53 | 1.04 | 1.11 | 1.126667 | 0.893333 |
| LOC100345795 | 0.387 | -1.37115 | 0.004642 | 0.014488 | yes | down | 8.02 | 6.06 | 7.56 | 10.8 | 6.02 | 2.46 | 7.213333 | 6.426667 |
| JMJD7 | 0.24 | -2.05727 | 0.004645 | 0.014496 | yes | down | 3.27 | 1.65 | 1.49 | 1.95 | 0.32 | 1 | 2.136667 | 1.09 |
| SYNGAP1 | 2.534 | 1.341183 | 0.004648 | 0.014503 | yes | up | 0.2 | 0.18 | 0.09 | 0.99 | 0.74 | 0.94 | 0.156667 | 0.89 |
| LOC100343611 | 0.461 | -1.11856 | 0.004653 | 0.014509 | yes | down | 0.97 | 1.25 | 1.01 | 0.7 | 1.53 | 1.08 | 1.076667 | 1.103333 |
| ADAM8 | 0.414 | -1.27067 | 0.004671 | 0.014557 | yes | down | 0.59 | 0.99 | 0.91 | 0.57 | 1.15 | 0.51 | 0.83 | 0.743333 |
| LOC100341622 | 0.489 | -1.03259 | 0.004704 | 0.014645 | yes | down | 5.39 | 5.4 | 6.84 | 4.32 | 7.52 | 8.27 | 5.876667 | 6.703333 |
| RASD1 | 0.409 | -1.28986 | 0.004748 | 0.014773 | yes | down | 7.67 | 11.7 | 14.66 | 6 | 8.8 | 17.37 | 11.34333 | 10.72333 |
| RAB8A | 2.02 | 1.014606 | 0.004797 | 0.014899 | yes | up | 1.6 | 2.24 | 1.8 | 7.51 | 8.26 | 10.33 | 1.88 | 8.7 |
| GAS2L1 | 2.993 | 1.581715 | 0.004801 | 0.014907 | yes | up | 1.54 | 2.75 | 1.75 | 13.13 | 15.03 | 13.7 | 2.013333 | 13.95333 |
| SLC7A1 | 2.291 | 1.195793 | 0.004802 | 0.01491 | yes | up | 0.49 | 0.45 | 0.19 | 1.57 | 2.24 | 1.98 | 0.376667 | 1.93 |
| GPER1 | 2.654 | 1.408131 | 0.004805 | 0.014915 | yes | up | 0.16 | 0.41 | 0.48 | 2.26 | 2.36 | 1.97 | 0.35 | 2.196667 |
| LOC100341028 | 0.488 | -1.03508 | 0.004823 | 0.014958 | yes | down | 10.17 | 10.19 | 8.83 | 8.69 | 11.01 | 13.14 | 9.73 | 10.94667 |
| LOC100344228 | 0.163 | -2.618 | 0.004853 | 0.015038 | yes | down | 0.92 | 0.74 | 0.58 | 0.36 | 0.08 | 0.4 | 0.746667 | 0.28 |
| LOC103349209 | 0.089 | -3.48795 | 0.004968 | 0.015377 | yes | down | 0.2 | 0.1 | 0.11 | 0.04 | 0.04 | 0 | 0.136667 | 0.026667 |
| LOC100340649 | 2.469 | 1.303882 | 0.005018 | 0.015522 | yes | up | 0.52 | 0.71 | 0.57 | 4.91 | 3.06 | 2.25 | 0.6 | 3.406667 |
| RLA-DMA | 2.605 | 1.381454 | 0.005024 | 0.015536 | yes | up | 0.74 | 0.81 | 1.15 | 5.06 | 5.92 | 5.57 | 0.9 | 5.516667 |
| LOC108177244 | 0.38 | -1.3953 | 0.005072 | 0.015676 | yes | down | 1.92 | 1.91 | 1.73 | 1.74 | 1.73 | 1.27 | 1.853333 | 1.58 |
| TTPA | 0.287 | -1.79983 | 0.005095 | 0.015738 | yes | down | 0.32 | 0.53 | 0.79 | 0.35 | 0.81 | 0.22 | 0.546667 | 0.46 |
| DENND1C | 2.245 | 1.166659 | 0.005147 | 0.015884 | yes | up | 0.56 | 0.54 | 0.47 | 2.55 | 3.2 | 2.37 | 0.523333 | 2.706667 |
| LOC100354896 | 0.056 | -4.15865 | 0.005147 | 0.015884 | yes | down | 0.13 | 0.16 | 0.06 | 0 | 0.02 | 0.03 | 0.116667 | 0.016667 |
| BTK | 2.143 | 1.099553 | 0.005166 | 0.015935 | yes | up | 0.46 | 0.54 | 0.37 | 2.18 | 2.31 | 2.2 | 0.456667 | 2.23 |
| C1H9orf85 | 0.472 | -1.08275 | 0.005229 | 0.01611 | yes | down | 5.26 | 6.06 | 4 | 6.99 | 5.19 | 3.17 | 5.106667 | 5.116667 |
| ACSL6 | 0.167 | -2.58546 | 0.005254 | 0.016177 | yes | down | 2.83 | 0.24 | 0.44 | 0.63 | 0.12 | 0.59 | 1.17 | 0.446667 |
| MSH5 | 6.729 | 2.75048 | 0.005257 | 0.016184 | yes | up | 0.08 | 0 | 0.1 | 1.08 | 0.83 | 0.8 | 0.06 | 0.903333 |
| NFASC | 2.227 | 1.155239 | 0.005258 | 0.016184 | yes | up | 0.83 | 0.32 | 0.26 | 1.53 | 3.8 | 1.82 | 0.47 | 2.383333 |
| PRR16 | 6.732 | 2.751018 | 0.00526 | 0.016188 | yes | up | 0.23 | 0.02 | 0 | 1.54 | 0.93 | 1.12 | 0.083333 | 1.196667 |
| LOC100348940 | 52.778 | 5.721874 | 0.005286 | 0.016256 | yes | up | 0 | 0 | 0 | 0.96 | 1.29 | 0 | 0 | 0.75 |
| CXADR | 0.078 | -3.68127 | 0.005303 | 0.016296 | yes | down | 0.35 | 0.36 | 0.34 | 0 | 0.06 | 0.15 | 0.35 | 0.07 |
| ATP1B2 | 2.05 | 1.035444 | 0.005323 | 0.016354 | yes | up | 2.26 | 3.56 | 3.43 | 15.48 | 12.55 | 14.69 | 3.083333 | 14.24 |
| LOC100356734 | 2.059 | 1.042164 | 0.005326 | 0.016361 | yes | up | 3 | 3.06 | 3.4 | 7.02 | 19.09 | 15.62 | 3.153333 | 13.91 |
| GPR88 | 4.083 | 2.029497 | 0.005338 | 0.01639 | yes | up | 0.04 | 0.05 | 0.07 | 0.39 | 0.67 | 0.42 | 0.053333 | 0.493333 |
| KCTD14 | 0.163 | -2.61397 | 0.005358 | 0.016443 | yes | down | 0.73 | 0.97 | 0.44 | 0.31 | 0.51 | 0.11 | 0.713333 | 0.31 |
| FAM217A | 0.424 | -1.23647 | 0.005366 | 0.01646 | yes | down | 1.56 | 2.6 | 1.09 | 1.54 | 2.15 | 1.08 | 1.75 | 1.59 |
| ADAMTSL4 | 2.267 | 1.180758 | 0.005383 | 0.016499 | yes | up | 21.48 | 5.9 | 10.95 | 27.89 | 27.19 | 40.36 | 12.77667 | 31.81333 |
| TCEAL7 | 0.015 | -6.02382 | 0.005396 | 0.016532 | yes | down | 93.55 | 13.97 | 0.54 | 0.47 | 0 | 3.06 | 36.02 | 1.176667 |
| DDX28 | 0.447 | -1.16136 | 0.005404 | 0.016552 | yes | down | 2.63 | 4.04 | 2.96 | 3.13 | 2.81 | 3.94 | 3.21 | 3.293333 |
| ALKBH8 | 0.444 | -1.17229 | 0.005409 | 0.016567 | yes | down | 1.34 | 0.98 | 1.17 | 1.31 | 1.5 | 0.69 | 1.163333 | 1.166667 |
| AGBL1 | 0.307 | -1.70221 | 0.005425 | 0.016601 | yes | down | 4.29 | 1.55 | 0.98 | 2.41 | 0.85 | 1.39 | 2.273333 | 1.55 |
| CD247 | 13.019 | 3.702504 | 0.005428 | 0.016608 | yes | up | 1.75 | 2.3 | 2.21 | 85.32 | 1.2 | 114.47 | 2.086667 | 66.99667 |
| EPHA1 | 4.096 | 2.034156 | 0.005481 | 0.016758 | yes | up | 0.04 | 0.12 | 0.11 | 0.51 | 1.25 | 0.87 | 0.09 | 0.876667 |
| LOC100346546 | 0.28 | -1.83841 | 0.005498 | 0.016799 | yes | down | 0.18 | 0.11 | 0.2 | 0.13 | 0.12 | 0.06 | 0.163333 | 0.103333 |
| LOC103351304 | 2.804 | 1.487697 | 0.005502 | 0.016808 | yes | up | 0.23 | 0.31 | 0.13 | 1.55 | 1.74 | 1 | 0.223333 | 1.43 |
| NLRC3 | 2.419 | 1.274231 | 0.005505 | 0.016811 | yes | up | 0.95 | 1.11 | 1.03 | 5.47 | 10.5 | 2.6 | 1.03 | 6.19 |
| TFAP2A | 0.109 | -3.19542 | 0.005506 | 0.016814 | yes | down | 0.19 | 0.09 | 0.21 | 0.02 | 0.06 | 0.03 | 0.163333 | 0.036667 |
| ACAN | 3.806 | 1.928378 | 0.005512 | 0.016829 | yes | up | 0.08 | 0.17 | 0.19 | 0.3 | 2.8 | 1.09 | 0.146667 | 1.396667 |
| LOC100343081 | 0.261 | -1.94044 | 0.00554 | 0.016898 | yes | down | 1.32 | 2.69 | 2.69 | 1.7 | 1.35 | 0.24 | 2.233333 | 1.096667 |
| TNFRSF9 | 8.743 | 3.128069 | 0.005565 | 0.016957 | yes | up | 0.06 | 0.05 | 0 | 0.6 | 0.49 | 0.96 | 0.036667 | 0.683333 |
| HCN1 | 2.765 | 1.46707 | 0.005565 | 0.016957 | yes | up | 0.14 | 0.19 | 0.12 | 1.17 | 0.71 | 0.99 | 0.15 | 0.956667 |
| PIF1 | 9.27 | 3.212506 | 0.005567 | 0.01696 | yes | up | 0.02 | 0.07 | 0 | 0.8 | 0.82 | 0.29 | 0.03 | 0.636667 |
| FCER2 | 14.718 | 3.879535 | 0.005577 | 0.01698 | yes | up | 0.06 | 0.05 | 0 | 0.62 | 0.86 | 1.74 | 0.036667 | 1.073333 |
| LOC108175449 | 3.387 | 1.760058 | 0.005624 | 0.017106 | yes | up | 10.31 | 0.54 | 5 | 15.29 | 14.42 | 7.24 | 5.283333 | 12.31667 |
| RNF207 | 0.09 | -3.47406 | 0.005672 | 0.017228 | yes | down | 0.57 | 0.12 | 0.21 | 0 | 0.03 | 0.16 | 0.3 | 0.063333 |
| PEAK1 | 2.023 | 1.016261 | 0.005747 | 0.017437 | yes | up | 1.98 | 1.27 | 1.99 | 6.9 | 12.26 | 8.35 | 1.746667 | 9.17 |
| SPI1 | 3.022 | 1.595722 | 0.005754 | 0.017454 | yes | up | 0.54 | 0.37 | 0.64 | 3.79 | 3.56 | 3.44 | 0.516667 | 3.596667 |
| LUZP2 | 6.75 | 2.754809 | 0.005775 | 0.017509 | yes | up | 0.02 | 0.02 | 0.01 | 0.25 | 0.31 | 0.18 | 0.016667 | 0.246667 |
| SP6 | 5.982 | 2.580579 | 0.005815 | 0.017615 | yes | up | 0.08 | 0.04 | 0.14 | 1.37 | 1.59 | 0.63 | 0.086667 | 1.196667 |
| SLC26A9 | 0.368 | -1.44142 | 0.005871 | 0.017773 | yes | down | 3.63 | 8.09 | 2.39 | 3.96 | 9.32 | 2.34 | 4.703333 | 5.206667 |
| LOC100352745 | 2.31 | 1.207641 | 0.005898 | 0.017845 | yes | up | 0.31 | 0.56 | 0.26 | 2.35 | 1.99 | 1.63 | 0.376667 | 1.99 |
| LOC100353317 | 4.289 | 2.100581 | 0.005902 | 0.017852 | yes | up | 0.09 | 0.13 | 0.07 | 0.87 | 1 | 0.63 | 0.096667 | 0.833333 |
| CHML | 0.43 | -1.21729 | 0.005911 | 0.017872 | yes | down | 1.14 | 1.52 | 1.45 | 1.4 | 2.26 | 0.54 | 1.37 | 1.4 |
| SPTBN4 | 0.335 | -1.57723 | 0.005913 | 0.017874 | yes | down | 0.25 | 0.23 | 0.32 | 0.1 | 0.38 | 0.17 | 0.266667 | 0.216667 |
| LOC103349856 | 0.271 | -1.88499 | 0.005917 | 0.017882 | yes | down | 0.98 | 0.75 | 0.75 | 0.43 | 0.67 | 0.41 | 0.826667 | 0.503333 |
| DCDC2C | 0.023 | -5.41431 | 0.005925 | 0.017897 | yes | down | 0.66 | 42.49 | 4.28 | 0 | 1.95 | 0.7 | 15.81 | 0.883333 |
| LOC100345090 | 0.437 | -1.19489 | 0.005962 | 0.017996 | yes | down | 0.77 | 0.54 | 0.6 | 0.46 | 0.74 | 0.73 | 0.636667 | 0.643333 |
| PMP2 | 0.307 | -1.70426 | 0.005973 | 0.018024 | yes | down | 9.32 | 6.51 | 1.92 | 1.8 | 2.29 | 7.52 | 5.916667 | 3.87 |
| PRND | 4.995 | 2.320463 | 0.005986 | 0.018057 | yes | up | 1.04 | 0.13 | 0.02 | 3.73 | 5.27 | 3.8 | 0.396667 | 4.266667 |
| TRIM77 | 3.814 | 1.931187 | 0.005986 | 0.018057 | yes | up | 0.06 | 0.26 | 0.28 | 1.96 | 2.56 | 1.03 | 0.2 | 1.85 |
| DMRT3 | 11.832 | 3.564683 | 0.006019 | 0.018153 | yes | up | 0.02 | 0 | 0.04 | 0.2 | 0.53 | 1.1 | 0.02 | 0.61 |
| LOC103346857 | 2.301 | 1.202504 | 0.006045 | 0.018218 | yes | up | 0.66 | 1.02 | 0.89 | 3.53 | 5.18 | 5 | 0.856667 | 4.57 |
| LOC103349432 | 8.659 | 3.114222 | 0.006046 | 0.018218 | yes | up | 0.1 | 0 | 0.25 | 3.19 | 1.91 | 2.16 | 0.116667 | 2.42 |
| CP | 2.723 | 1.445276 | 0.006097 | 0.018362 | yes | up | 0.61 | 0.22 | 0.33 | 2.9 | 3.06 | 1.23 | 0.386667 | 2.396667 |
| LOC108176869 | 2.541 | 1.345284 | 0.006105 | 0.018378 | yes | up | 0.32 | 0.24 | 0.1 | 1.49 | 1.27 | 0.94 | 0.22 | 1.233333 |
| COL9A3 | 2.656 | 1.409181 | 0.006108 | 0.018384 | yes | up | 2.23 | 2.7 | 1.04 | 4.51 | 13.81 | 17.09 | 1.99 | 11.80333 |
| ERICH3 | 0.125 | -2.99763 | 0.006129 | 0.018444 | yes | down | 0.14 | 0.06 | 0.14 | 0.02 | 0.02 | 0.06 | 0.113333 | 0.033333 |
| PWWP2B | 2.249 | 1.169517 | 0.006138 | 0.018467 | yes | up | 0.58 | 0.58 | 0.42 | 2.24 | 2.4 | 3.37 | 0.526667 | 2.67 |
| LOC103349679 | 2.645 | 1.403033 | 0.006158 | 0.018521 | yes | up | 0.74 | 0.54 | 0.65 | 4.08 | 2.07 | 3.28 | 0.643333 | 3.143333 |
| LOC103348323 | 0.18 | -2.47707 | 0.006162 | 0.018527 | yes | down | 0.91 | 0.99 | 0.71 | 0.29 | 0.52 | 0.27 | 0.87 | 0.36 |
| MRAP | 0.422 | -1.2435 | 0.006229 | 0.018702 | yes | down | 7.7 | 10.3 | 12.7 | 12.69 | 14.07 | 4.12 | 10.23333 | 10.29333 |
| STIL | 4.37 | 2.127708 | 0.006255 | 0.018765 | yes | up | 0.16 | 0.09 | 0.04 | 1.16 | 1.57 | 0.19 | 0.096667 | 0.973333 |
| LOC103345512 | 3.209 | 1.682316 | 0.006298 | 0.01888 | yes | up | 0.26 | 0.26 | 0.22 | 1.01 | 3.38 | 1.15 | 0.246667 | 1.846667 |
| KIAA0408 | 0.414 | -1.27347 | 0.006312 | 0.018921 | yes | down | 5.97 | 5.22 | 3.12 | 2.12 | 8.63 | 2.13 | 4.77 | 4.293333 |
| LOC108175719 | 0.058 | -4.11457 | 0.006323 | 0.018949 | yes | down | 0.58 | 0.78 | 1.24 | 0.15 | 0 | 0.21 | 0.866667 | 0.12 |
| LOC103349979 | 0.024 | -5.3931 | 0.006324 | 0.01895 | yes | down | 0.31 | 0.28 | 0.63 | 0 | 0 | 0 | 0.406667 | 0 |
| AK5 | 7.135 | 2.834862 | 0.006336 | 0.018982 | yes | up | 0.12 | 0.02 | 0 | 0.91 | 0.73 | 0.39 | 0.046667 | 0.676667 |
| GPR34 | 0.428 | -1.22398 | 0.006361 | 0.019048 | yes | down | 1.5 | 1.43 | 0.98 | 1.05 | 1.92 | 0.88 | 1.303333 | 1.283333 |
| DEPDC1B | 3.819 | 1.933225 | 0.006392 | 0.01914 | yes | up | 0.19 | 0.23 | 0.09 | 1.78 | 2.18 | 0.42 | 0.17 | 1.46 |
| STXBP2 | 2.315 | 1.211207 | 0.006401 | 0.019157 | yes | up | 0.45 | 0.76 | 0.9 | 3.36 | 4.7 | 3.51 | 0.703333 | 3.856667 |
| LOC100337703 | 0.399 | -1.32402 | 0.00642 | 0.01921 | yes | down | 55.28 | 18.25 | 27.34 | 25.91 | 15.4 | 47.33 | 33.62333 | 29.54667 |
| LOC103346986 | 3.051 | 1.609129 | 0.006426 | 0.019224 | yes | up | 0.32 | 0.2 | 0.14 | 1.5 | 2.21 | 1.11 | 0.22 | 1.606667 |
| NEK5 | 0.358 | -1.48089 | 0.006467 | 0.019339 | yes | down | 0.52 | 1.22 | 1.27 | 1.3 | 0.68 | 0.52 | 1.003333 | 0.833333 |
| VAMP1 | 0.443 | -1.17491 | 0.006492 | 0.019407 | yes | down | 1.69 | 1.3 | 0.64 | 1.12 | 1.57 | 0.91 | 1.21 | 1.2 |
| ERCC6L | 3.611 | 1.852534 | 0.006493 | 0.019407 | yes | up | 0.14 | 0.08 | 0.12 | 1.29 | 1.26 | 0.24 | 0.113333 | 0.93 |
| CKMT2 | 0.024 | -5.39026 | 0.006543 | 0.019545 | yes | down | 192.49 | 35.04 | 54.08 | 1.65 | 0 | 11.92 | 93.87 | 4.523333 |
| MPP6 | 0.374 | -1.41818 | 0.006549 | 0.019555 | yes | down | 2.88 | 1.08 | 0.65 | 1.54 | 1.02 | 1.12 | 1.536667 | 1.226667 |
| IBTK | 0.49 | -1.02966 | 0.006569 | 0.019602 | yes | down | 7.14 | 2.97 | 2.31 | 5.03 | 5.23 | 3.39 | 4.14 | 4.55 |
| ADAMTS8 | 4.245 | 2.085843 | 0.006583 | 0.019637 | yes | up | 0.87 | 0.22 | 0.04 | 3.02 | 2.16 | 4.91 | 0.376667 | 3.363333 |
| ARPP21 | 0.045 | -4.47747 | 0.006587 | 0.019644 | yes | down | 8.58 | 0.53 | 0.13 | 0.13 | 0.06 | 0.71 | 3.08 | 0.3 |
| KIF1A | 4.241 | 2.084275 | 0.006609 | 0.019699 | yes | up | 0.5 | 0.46 | 0.17 | 2.94 | 5.34 | 2.66 | 0.376667 | 3.646667 |
| LOC108176550 | 4.008 | 2.002759 | 0.006639 | 0.019783 | yes | up | 0.04 | 0.11 | 0.03 | 0.36 | 0.73 | 0.51 | 0.06 | 0.533333 |
| TRPC3 | 0.282 | -1.82605 | 0.006641 | 0.019783 | yes | down | 0.46 | 0.56 | 0.26 | 0.15 | 0.59 | 0.12 | 0.426667 | 0.286667 |
| LOC103349156 | 0.277 | -1.85225 | 0.006656 | 0.01982 | yes | down | 0.39 | 0.69 | 0.53 | 0.49 | 0.16 | 0.45 | 0.536667 | 0.366667 |
| LOC100350165 | 0.023 | -5.42775 | 0.006669 | 0.019855 | yes | down | 0.16 | 0.45 | 0.09 | 0 | 0 | 0 | 0.233333 | 0 |
| LIN7A | 3.074 | 1.620116 | 0.006681 | 0.01989 | yes | up | 0.11 | 0.07 | 0.07 | 0.8 | 0.38 | 0.54 | 0.083333 | 0.573333 |
| TLR2 | 0.454 | -1.13893 | 0.0067 | 0.019936 | yes | down | 0.83 | 0.81 | 1.52 | 1.32 | 1.52 | 0.95 | 1.053333 | 1.263333 |
| SCAI | 0.389 | -1.36249 | 0.006703 | 0.019936 | yes | down | 0.42 | 0.36 | 0.29 | 0.35 | 0.39 | 0.2 | 0.356667 | 0.313333 |
| YBX3 | 0.473 | -1.08113 | 0.006712 | 0.019952 | yes | down | 261.1 | 114.07 | 81.32 | 137.4 | 113.03 | 205.24 | 152.1633 | 151.89 |
| ZMYM5 | 0.475 | -1.07341 | 0.006718 | 0.019967 | yes | down | 1.43 | 1.4 | 1.16 | 1.71 | 1.87 | 0.67 | 1.33 | 1.416667 |
| IGDCC4 | 2.353 | 1.234721 | 0.006763 | 0.020069 | yes | up | 4.07 | 1.53 | 0.74 | 8.87 | 13.58 | 10.77 | 2.113333 | 11.07333 |
| MTHFD2L | 0.447 | -1.16099 | 0.006802 | 0.020171 | yes | down | 1.36 | 1.41 | 1.41 | 2.5 | 1.88 | 0.86 | 1.393333 | 1.746667 |
| RNF135 | 2.072 | 1.051063 | 0.006803 | 0.020171 | yes | up | 1.31 | 2.33 | 1.42 | 7.91 | 8.18 | 7.9 | 1.686667 | 7.996667 |
| TPX2 | 2.321 | 1.214924 | 0.006854 | 0.020306 | yes | up | 2.74 | 2.86 | 1.66 | 14.46 | 20.44 | 4.27 | 2.42 | 13.05667 |
| GNRH1 | 20.881 | 4.384145 | 0.006881 | 0.020379 | yes | up | 0 | 0.05 | 0 | 1.57 | 1.31 | 0.53 | 0.016667 | 1.136667 |
| CAMK1D | 2.636 | 1.398266 | 0.006945 | 0.020546 | yes | up | 0.92 | 0.24 | 0.29 | 2.88 | 2.32 | 3.2 | 0.483333 | 2.8 |
| PREPL | 0.371 | -1.4287 | 0.006965 | 0.020601 | yes | down | 20.4 | 5.38 | 2.75 | 7.71 | 9.3 | 7.24 | 9.51 | 8.083333 |
| NPAS4 | 22.606 | 4.498605 | 0.006982 | 0.020642 | yes | up | 0.01 | 0 | 0 | 0.22 | 0.09 | 0.43 | 0.003333 | 0.246667 |
| MLF1 | 0.074 | -3.75441 | 0.007012 | 0.020719 | yes | down | 89.08 | 10.91 | 2.23 | 6.48 | 1.19 | 10.16 | 34.07333 | 5.943333 |
| TLR6 | 2.014 | 1.010387 | 0.007028 | 0.020765 | yes | up | 0.68 | 0.57 | 0.3 | 2.32 | 2.17 | 2.3 | 0.516667 | 2.263333 |
| LOC103351100 | 0.014 | -6.12219 | 0.007046 | 0.020807 | yes | down | 0.83 | 0.08 | 0 | 0 | 0 | 0 | 0.303333 | 0 |
| LAMP5 | 23.318 | 4.543367 | 0.007072 | 0.020876 | yes | up | 0.03 | 0 | 0 | 0.46 | 0.13 | 0.89 | 0.01 | 0.493333 |
| ACTG2 | 2.126 | 1.087809 | 0.007075 | 0.020881 | yes | up | 97.43 | 37.18 | 25.33 | 287.83 | 212.61 | 244.04 | 53.31333 | 248.16 |
| POMC | 0.426 | -1.23232 | 0.007121 | 0.021001 | yes | down | 4.2 | 4.54 | 6.16 | 4.74 | 3.02 | 6.82 | 4.966667 | 4.86 |
| LOC100346173 | 8.105 | 3.018812 | 0.007128 | 0.021019 | yes | up | 0.04 | 0 | 0.06 | 0.86 | 0.24 | 0.49 | 0.033333 | 0.53 |
| IL12RB2 | 3.231 | 1.691959 | 0.00716 | 0.021106 | yes | up | 0.15 | 0.11 | 0.08 | 1.1 | 0.7 | 0.79 | 0.113333 | 0.863333 |
| BIRC7 | 0.455 | -1.13571 | 0.007202 | 0.021218 | yes | down | 1.39 | 2.08 | 1.27 | 2.46 | 2.18 | 1.76 | 1.58 | 2.133333 |
| FOS | 5.281 | 2.400898 | 0.00721 | 0.021238 | yes | up | 17.32 | 4.47 | 3.7 | 68.88 | 60.86 | 189.69 | 8.496667 | 106.4767 |
| PRIMPOL | 3.349 | 1.743608 | 0.007236 | 0.021303 | yes | up | 0.49 | 0.5 | 0.18 | 3.34 | 4.72 | 1.01 | 0.39 | 3.023333 |
| LOC108176093 | 12.919 | 3.691376 | 0.007238 | 0.021303 | yes | up | 0.01 | 0 | 0.01 | 0.29 | 0.24 | 0.19 | 0.006667 | 0.24 |
| EDAR | 0.24 | -2.05781 | 0.00729 | 0.02144 | yes | down | 0.28 | 0.37 | 0.34 | 0.09 | 0.1 | 0.38 | 0.33 | 0.19 |
| SP110 | 2.657 | 1.409587 | 0.007304 | 0.021472 | yes | up | 0.64 | 0.8 | 1.28 | 1.61 | 8.74 | 2.67 | 0.906667 | 4.34 |
| ZBTB20 | 0.345 | -1.536 | 0.007337 | 0.021561 | yes | down | 0.52 | 0.32 | 0.65 | 0.31 | 0.51 | 0.32 | 0.496667 | 0.38 |
| AWAT2 | 20.182 | 4.335022 | 0.007379 | 0.021677 | yes | up | 0 | 0.05 | 0 | 0.84 | 0.46 | 0.95 | 0.016667 | 0.75 |
| RAC2 | 2.787 | 1.478764 | 0.007423 | 0.021778 | yes | up | 0.46 | 1.94 | 1.2 | 6.06 | 5.86 | 10.9 | 1.2 | 7.606667 |
| LOC103350510 | 0.457 | -1.1291 | 0.007477 | 0.02191 | yes | down | 3.55 | 5.13 | 4.1 | 4.96 | 4.72 | 3.75 | 4.26 | 4.476667 |
| TMEM138 | 2.093 | 1.065349 | 0.007477 | 0.02191 | yes | up | 1.14 | 1.71 | 1.27 | 7.56 | 5.96 | 6.16 | 1.373333 | 6.56 |
| LOC103350040 | 11.418 | 3.513199 | 0.007479 | 0.021912 | yes | up | 0 | 0.08 | 0.04 | 3.12 | 2.55 | 0.4 | 0.04 | 2.023333 |
| NSUN6 | 0.408 | -1.2921 | 0.00756 | 0.022123 | yes | down | 1.65 | 1.1 | 1.16 | 1.01 | 1.94 | 0.75 | 1.303333 | 1.233333 |
| PPBP | 0.054 | -4.20266 | 0.007624 | 0.022266 | yes | down | 1.77 | 2.83 | 1.8 | 0 | 0.82 | 0 | 2.133333 | 0.273333 |
| NRL | 4.147 | 2.0522 | 0.007626 | 0.022268 | yes | up | 0.08 | 0.13 | 0.08 | 0.89 | 0.72 | 0.84 | 0.096667 | 0.816667 |
| LOC100341069 | 0.165 | -2.59973 | 0.007631 | 0.02228 | yes | down | 0.74 | 0.89 | 0.61 | 0.1 | 0.25 | 0.28 | 0.746667 | 0.21 |
| SLC7A5 | 2.485 | 1.313065 | 0.007633 | 0.02228 | yes | up | 1.51 | 0.83 | 0.46 | 7.03 | 2.83 | 5.22 | 0.933333 | 5.026667 |
| LOC108175930 | 47.476 | 5.569132 | 0.007644 | 0.022301 | yes | up | 0 | 0 | 0 | 9.5 | 10 | 0 | 0 | 6.5 |
| ABCC2 | 3.005 | 1.587413 | 0.007654 | 0.022327 | yes | up | 0.12 | 0.14 | 0.09 | 0.43 | 0.75 | 1.17 | 0.116667 | 0.783333 |
| IKZF1 | 3.208 | 1.681623 | 0.007682 | 0.022399 | yes | up | 0.12 | 0.12 | 0.26 | 0.86 | 1.04 | 1.76 | 0.166667 | 1.22 |
| SPOCK3 | 0.465 | -1.10551 | 0.007731 | 0.022529 | yes | down | 6.4 | 4.72 | 4.56 | 4.58 | 10.04 | 2.56 | 5.226667 | 5.726667 |
| LOC108176091 | 3.12 | 1.641648 | 0.007739 | 0.022551 | yes | up | 0.09 | 0.07 | 0.08 | 0.64 | 0.39 | 0.6 | 0.08 | 0.543333 |
| LOC103349946 | 0.048 | -4.38493 | 0.007741 | 0.022552 | yes | down | 2.92 | 0.66 | 0 | 0 | 0.09 | 0.76 | 1.193333 | 0.283333 |
| CXCL9 | 21.633 | 4.435193 | 0.007757 | 0.022586 | yes | up | 0.02 | 0 | 0 | 0.13 | 0.75 | 0.35 | 0.006667 | 0.41 |
| LOC103345416 | 0.036 | -4.80491 | 0.007805 | 0.022695 | yes | down | 9.45 | 1.48 | 2.85 | 0.85 | 0 | 0 | 4.593333 | 0.283333 |
| TMEM163 | 19.192 | 4.262458 | 0.007805 | 0.022695 | yes | up | 0 | 0.03 | 0 | 0.41 | 0.57 | 0.57 | 0.01 | 0.516667 |
| CDKL4 | 0.218 | -2.19496 | 0.007867 | 0.022846 | yes | down | 0.17 | 0.3 | 0.26 | 0.09 | 0.13 | 0.16 | 0.243333 | 0.126667 |
| EFNA4 | 3.299 | 1.722058 | 0.007873 | 0.02286 | yes | up | 0.43 | 0.22 | 0.2 | 2.03 | 1.85 | 2.35 | 0.283333 | 2.076667 |
| KCNK12 | 4.22 | 2.077321 | 0.007902 | 0.022927 | yes | up | 0.07 | 0.04 | 0.02 | 0.17 | 0.69 | 0.49 | 0.043333 | 0.45 |
| LOC100355665 | 4.691 | 2.229789 | 0.007969 | 0.023109 | yes | up | 0.14 | 0.12 | 0.35 | 1.56 | 2.5 | 2.79 | 0.203333 | 2.283333 |
| SPAG1 | 2.036 | 1.025423 | 0.008009 | 0.023208 | yes | up | 0.42 | 0.47 | 0.31 | 1.63 | 2.01 | 1.91 | 0.4 | 1.85 |
| LOC103350594 | 0.352 | -1.50442 | 0.008016 | 0.023222 | yes | down | 1.46 | 1.82 | 1.06 | 1.28 | 0.84 | 0.95 | 1.446667 | 1.023333 |
| CD86 | 2.623 | 1.391311 | 0.008026 | 0.023241 | yes | up | 0.2 | 0.27 | 0.17 | 1.18 | 1.57 | 1.11 | 0.213333 | 1.286667 |
| PCDH20 | 0.399 | -1.32581 | 0.008043 | 0.023287 | yes | down | 0.74 | 1.02 | 0.73 | 0.31 | 1.31 | 0.72 | 0.83 | 0.78 |
| DLGAP1 | 2.643 | 1.402026 | 0.008107 | 0.023459 | yes | up | 0.52 | 0.4 | 0.16 | 3.3 | 2.78 | 2.66 | 0.36 | 2.913333 |
| LOC103346040 | 2.477 | 1.308836 | 0.008148 | 0.023553 | yes | up | 0.52 | 0.45 | 0.57 | 1.52 | 4.8 | 2.7 | 0.513333 | 3.006667 |
| AKR1B10 | 2.086 | 1.061024 | 0.008157 | 0.023576 | yes | up | 8.48 | 4.25 | 3.21 | 34.59 | 23.48 | 16.18 | 5.313333 | 24.75 |
| TMEM40 | 0.331 | -1.59304 | 0.00816 | 0.023581 | yes | down | 3.8 | 2.89 | 1.78 | 4.67 | 1.19 | 5.54 | 2.823333 | 3.8 |
| MILR1 | 3.94 | 1.978237 | 0.008172 | 0.023608 | yes | up | 0.2 | 0.47 | 0.23 | 4.14 | 1.48 | 2.85 | 0.3 | 2.823333 |
| FGD2 | 4.216 | 2.075895 | 0.008177 | 0.023616 | yes | up | 0.09 | 0.04 | 0.1 | 0.6 | 1.03 | 0.67 | 0.076667 | 0.766667 |
| DAO | 19.711 | 4.300899 | 0.008182 | 0.023628 | yes | up | 0 | 0.04 | 0 | 0.51 | 1.23 | 0.5 | 0.013333 | 0.746667 |
| LOC100347161 | 0.08 | -3.65177 | 0.008215 | 0.023713 | yes | down | 0.56 | 1.51 | 0.72 | 0.29 | 0 | 0.21 | 0.93 | 0.166667 |
| ST8SIA5 | 0.432 | -1.20987 | 0.008221 | 0.023727 | yes | down | 3.21 | 2.33 | 3.27 | 1.69 | 6.01 | 1.53 | 2.936667 | 3.076667 |
| GINS3 | 4.166 | 2.058648 | 0.008259 | 0.023833 | yes | up | 0.1 | 0.14 | 0.04 | 0.88 | 1.25 | 0.55 | 0.093333 | 0.893333 |
| CCDC71L | 2.009 | 1.006652 | 0.008266 | 0.023838 | yes | up | 1.16 | 1.18 | 0.69 | 6.28 | 5.09 | 2.44 | 1.01 | 4.603333 |
| SCN3B | 0.481 | -1.05729 | 0.008331 | 0.023981 | yes | down | 4.1 | 2.39 | 1.12 | 2.59 | 3.16 | 3.32 | 2.536667 | 3.023333 |
| ZNF583 | 0.448 | -1.1579 | 0.008379 | 0.024107 | yes | down | 1.6 | 1.05 | 0.79 | 1.15 | 1.45 | 0.94 | 1.146667 | 1.18 |
| KCNE3 | 3.042 | 1.605028 | 0.008422 | 0.024219 | yes | up | 0.28 | 0.29 | 0.21 | 2.1 | 1.75 | 1.53 | 0.26 | 1.793333 |
| OCLN | 0.493 | -1.02101 | 0.008429 | 0.024231 | yes | down | 2.08 | 3.57 | 3.01 | 4.48 | 2.67 | 2.78 | 2.886667 | 3.31 |
| SRRM3 | 2.776 | 1.472905 | 0.008438 | 0.024251 | yes | up | 0.71 | 0.43 | 0.19 | 2.48 | 2.51 | 3.23 | 0.443333 | 2.74 |
| PCDHB2 | 5.751 | 2.523866 | 0.008453 | 0.024269 | yes | up | 0.04 | 0.07 | 0.03 | 0.34 | 1.25 | 0.3 | 0.046667 | 0.63 |
| LOC100343263 | 13.059 | 3.706933 | 0.008464 | 0.024296 | yes | up | 0 | 0.04 | 0.04 | 0.47 | 1.15 | 0.48 | 0.026667 | 0.7 |
| THBS4 | 0.121 | -3.05103 | 0.008547 | 0.024509 | yes | down | 10.66 | 1.91 | 0.71 | 0.39 | 0.91 | 2.09 | 4.426667 | 1.13 |
| LOC100009166 | 0.367 | -1.44655 | 0.008549 | 0.024511 | yes | down | 2.68 | 4.97 | 6.58 | 5.85 | 3.05 | 3.95 | 4.743333 | 4.283333 |
| KNTC1 | 2.441 | 1.287754 | 0.008573 | 0.024575 | yes | up | 0.73 | 0.72 | 0.29 | 3.54 | 5.11 | 1.1 | 0.58 | 3.25 |
| LOC100352880 | 5.604 | 2.486523 | 0.008579 | 0.024589 | yes | up | 8.28 | 1.91 | 0.84 | 26.08 | 84.48 | 28.85 | 3.676667 | 46.47 |
| CCR2 | 3.027 | 1.598099 | 0.008581 | 0.024591 | yes | up | 0.12 | 0.27 | 0.11 | 0.83 | 1.45 | 1.01 | 0.166667 | 1.096667 |
| LOC100346306 | 33.108 | 5.049098 | 0.008593 | 0.024615 | yes | up | 0 | 0 | 0 | 3.13 | 1.53 | 11.4 | 0 | 5.353333 |
| ADAMTS20 | 0.286 | -1.80485 | 0.008602 | 0.024633 | yes | down | 0.82 | 0.43 | 0.08 | 0.19 | 0.3 | 0.34 | 0.443333 | 0.276667 |
| CDC25C | 5.244 | 2.390739 | 0.008641 | 0.024736 | yes | up | 0.25 | 0.18 | 0 | 1.53 | 2.01 | 0.62 | 0.143333 | 1.386667 |
| HOXB5 | 0.436 | -1.19614 | 0.008653 | 0.024756 | yes | down | 2.93 | 3.91 | 3.7 | 2.05 | 4.78 | 5.38 | 3.513333 | 4.07 |
| INF2 | 2.08 | 1.056414 | 0.00869 | 0.02485 | yes | up | 1.69 | 0.81 | 1.65 | 6.68 | 6.32 | 6.94 | 1.383333 | 6.646667 |
| SLC48A1 | 2.347 | 1.230905 | 0.008707 | 0.024896 | yes | up | 9.65 | 4.93 | 6.34 | 31.18 | 16.76 | 61.13 | 6.973333 | 36.35667 |
| LOC103346060 | 0.335 | -1.57849 | 0.008721 | 0.024925 | yes | down | 0.57 | 0.68 | 0.35 | 0.37 | 0.69 | 0.18 | 0.533333 | 0.413333 |
| CELSR1 | 2.338 | 1.225234 | 0.008756 | 0.02501 | yes | up | 0.32 | 0.33 | 0.14 | 1.1 | 1.61 | 1.6 | 0.263333 | 1.436667 |
| LOC103350109 | 0.426 | -1.23036 | 0.008762 | 0.025023 | yes | down | 4.96 | 5.14 | 6.15 | 2.68 | 4.86 | 3.45 | 5.416667 | 3.663333 |
| PRSS35 | 2.414 | 1.271244 | 0.008841 | 0.025229 | yes | up | 0.49 | 0.84 | 0.32 | 3.08 | 2.28 | 3.48 | 0.55 | 2.946667 |
| WNT8A | 4.566 | 2.191046 | 0.008846 | 0.025241 | yes | up | 0.08 | 0.07 | 0.07 | 0.55 | 0.66 | 0.95 | 0.073333 | 0.72 |
| INHBB | 3.268 | 1.708577 | 0.008877 | 0.025311 | yes | up | 0.22 | 0.23 | 0.12 | 2.22 | 0.85 | 1.02 | 0.19 | 1.363333 |
| LOC103346792 | 36.062 | 5.172425 | 0.008881 | 0.025319 | yes | up | 0 | 0 | 0 | 0.3 | 4.9 | 7.48 | 0 | 4.226667 |
| POLR3G | 2.401 | 1.263465 | 0.008885 | 0.025321 | yes | up | 0.31 | 0.22 | 0.28 | 1.35 | 2.02 | 1.16 | 0.27 | 1.51 |
| HOXD1 | 2.87 | 1.521026 | 0.008905 | 0.025372 | yes | up | 0.54 | 0.34 | 0.56 | 3.63 | 2.37 | 3.48 | 0.48 | 3.16 |
| LOC100350471 | 0.027 | -5.18693 | 0.008917 | 0.025398 | yes | down | 0 | 0.26 | 0.43 | 0 | 0 | 0.05 | 0.23 | 0.016667 |
| KLHDC7A | 0.07 | -3.84655 | 0.008958 | 0.02549 | yes | down | 0.1 | 0.12 | 0.02 | 0 | 0 | 0.04 | 0.08 | 0.013333 |
| MSR1 | 0.461 | -1.11865 | 0.008991 | 0.025565 | yes | down | 0.64 | 0.8 | 0.47 | 0.98 | 0.85 | 0.66 | 0.636667 | 0.83 |
| SLC16A2 | 0.477 | -1.06873 | 0.009081 | 0.02578 | yes | down | 9.42 | 24.62 | 6.05 | 14.99 | 15.72 | 11.28 | 13.36333 | 13.99667 |
| LOC108177096 | 0.379 | -1.39929 | 0.009103 | 0.025827 | yes | down | 0.8 | 1.58 | 1.19 | 1.16 | 1.17 | 0.78 | 1.19 | 1.036667 |
| GREM1 | 9.646 | 3.269921 | 0.009199 | 0.026076 | yes | up | 0.06 | 0.03 | 0 | 0.42 | 0.46 | 0.83 | 0.03 | 0.57 |
| LOC108176197 | 0.197 | -2.34163 | 0.009201 | 0.026076 | yes | down | 0.46 | 0.52 | 0.54 | 0.11 | 0.3 | 0.3 | 0.506667 | 0.236667 |
| LOC103345386 | 3.552 | 1.828447 | 0.009212 | 0.026104 | yes | up | 0.69 | 0.51 | 0.26 | 5.82 | 4.82 | 2.22 | 0.486667 | 4.286667 |
| SEZ6 | 29.054 | 4.86067 | 0.009264 | 0.026234 | yes | up | 0 | 0 | 0 | 0.06 | 0.19 | 0.22 | 0 | 0.156667 |
| PDZD4 | 2.086 | 1.06102 | 0.009299 | 0.026315 | yes | up | 0.42 | 0.32 | 0.52 | 2.13 | 2.16 | 1.82 | 0.42 | 2.036667 |
| PDGFA | 3.085 | 1.625415 | 0.009354 | 0.02645 | yes | up | 2.7 | 1.31 | 2.61 | 14.92 | 18.49 | 18.32 | 2.206667 | 17.24333 |
| LOC103350426 | 0.053 | -4.23548 | 0.009358 | 0.026457 | yes | down | 79.89 | 9.71 | 1.32 | 3.17 | 0.24 | 6.37 | 30.30667 | 3.26 |
| DPYSL5 | 0.074 | -3.75674 | 0.009437 | 0.026664 | yes | down | 0.28 | 0.14 | 0.32 | 0 | 0 | 0.14 | 0.246667 | 0.046667 |
| HDX | 0.267 | -1.90525 | 0.009444 | 0.026678 | yes | down | 0.17 | 0.13 | 0.18 | 0.17 | 0.06 | 0.05 | 0.16 | 0.093333 |
| SPX | 10.101 | 3.336484 | 0.009466 | 0.026726 | yes | up | 0.03 | 0.03 | 0.03 | 0.16 | 0.95 | 0.82 | 0.03 | 0.643333 |
| RNASE6 | 2.606 | 1.38201 | 0.009476 | 0.026749 | yes | up | 1.46 | 2.07 | 0.96 | 8.84 | 6.21 | 9.79 | 1.496667 | 8.28 |
| LOC100353969 | 0.04 | -4.63944 | 0.009501 | 0.026816 | yes | down | 0.6 | 0.52 | 1.04 | 0.03 | 0 | 0 | 0.72 | 0.01 |
| EPM2AIP1 | 0.347 | -1.52664 | 0.009516 | 0.026855 | yes | down | 1.78 | 1.87 | 1.91 | 1.47 | 2.62 | 0.47 | 1.853333 | 1.52 |
| ART5 | 0.144 | -2.79523 | 0.009534 | 0.0269 | yes | down | 3.24 | 0.29 | 0.08 | 0.39 | 0.14 | 0.55 | 1.203333 | 0.36 |
| ZWINT | 3.185 | 1.671487 | 0.009543 | 0.026921 | yes | up | 0.31 | 0.65 | 0.45 | 4.4 | 5.78 | 1.09 | 0.47 | 3.756667 |
| TIMD4 | 31.294 | 4.967835 | 0.009545 | 0.026923 | yes | up | 0 | 0 | 0 | 1.19 | 0.51 | 0.15 | 0 | 0.616667 |
| CEP55 | 3.159 | 1.659399 | 0.009573 | 0.026996 | yes | up | 0.73 | 0.69 | 0.23 | 5.04 | 5.92 | 0.9 | 0.55 | 3.953333 |
| GPR19 | 2.883 | 1.527442 | 0.009628 | 0.027133 | yes | up | 0.63 | 0.55 | 0.46 | 5.21 | 4.31 | 1.03 | 0.546667 | 3.516667 |
| TSPAN1 | 7.622 | 2.93013 | 0.009646 | 0.02717 | yes | up | 0.15 | 0 | 0.18 | 0.64 | 1.05 | 4.01 | 0.11 | 1.9 |
| LOC100347945 | 0.467 | -1.09938 | 0.009674 | 0.027229 | yes | down | 0.95 | 1.15 | 1.12 | 1.42 | 1.35 | 0.68 | 1.073333 | 1.15 |
| MKRN2OS | 0.409 | -1.29149 | 0.009687 | 0.027262 | yes | down | 0.97 | 1.78 | 1.84 | 1.46 | 1.49 | 1.42 | 1.53 | 1.456667 |
| LOC103347584 | 8.086 | 3.01546 | 0.009747 | 0.027406 | yes | up | 0 | 0.49 | 1.02 | 7.96 | 13.18 | 4.77 | 0.503333 | 8.636667 |
| GK5 | 0.498 | -1.00607 | 0.009767 | 0.027459 | yes | down | 1.36 | 1.21 | 0.57 | 1.47 | 0.82 | 1.22 | 1.046667 | 1.17 |
| SLC35F2 | 3.86 | 1.948466 | 0.009787 | 0.027502 | yes | up | 0.18 | 0.13 | 0.1 | 1.39 | 1.35 | 0.86 | 0.136667 | 1.2 |
| LOC108176663 | 18.483 | 4.208142 | 0.009812 | 0.027557 | yes | up | 0.12 | 0 | 0 | 2.72 | 1.61 | 1.3 | 0.04 | 1.876667 |
| LOC103347610 | 32.765 | 5.034103 | 0.009855 | 0.027662 | yes | up | 0 | 0 | 0 | 1.23 | 0.31 | 3.29 | 0 | 1.61 |
| KERA | 8.585 | 3.101846 | 0.009886 | 0.027732 | yes | up | 1.23 | 0.51 | 0.03 | 5.12 | 21.44 | 8.37 | 0.59 | 11.64333 |
| RGS10 | 0.488 | -1.03636 | 0.009917 | 0.027814 | yes | down | 11.37 | 22.63 | 7.18 | 14.36 | 14.13 | 16.25 | 13.72667 | 14.91333 |
| LOC100341660 | 0.113 | -3.15097 | 0.009979 | 0.02797 | yes | down | 0.29 | 0.44 | 0.23 | 0.02 | 0.08 | 0.11 | 0.32 | 0.07 |
| LOC100344091 | 0.428 | -1.22521 | 0.010006 | 0.028039 | yes | down | 1.52 | 1.23 | 1.72 | 1.91 | 1.93 | 0.63 | 1.49 | 1.49 |
| LOC108176634 | 0.254 | -1.9745 | 0.01002 | 0.028076 | yes | down | 4.98 | 13.86 | 3.01 | 8.57 | 2.06 | 1.48 | 7.283333 | 4.036667 |
| LOC103350144 | 0.39 | -1.35787 | 0.010026 | 0.028086 | yes | down | 1.88 | 4.6 | 2.19 | 2.22 | 4.78 | 3.44 | 2.89 | 3.48 |
| LOC100303760 | 0.025 | -5.3145 | 0.010049 | 0.028147 | yes | down | 505.1 | 49.49 | 7.22 | 5.23 | 0.02 | 23.31 | 187.27 | 9.52 |
| STRA6 | 3.644 | 1.865368 | 0.010051 | 0.028147 | yes | up | 0.48 | 0.18 | 0.05 | 1.39 | 1.72 | 2.67 | 0.236667 | 1.926667 |
| PVALB | 37.425 | 5.225942 | 0.010054 | 0.028151 | yes | up | 1.37 | 0.13 | 0 | 28.62 | 0.65 | 83.2 | 0.5 | 37.49 |
| SCRT1 | 0.089 | -3.49395 | 0.01008 | 0.028217 | yes | down | 1.61 | 0.11 | 0.03 | 0.17 | 0.05 | 0.12 | 0.583333 | 0.113333 |
| LOC100344884 | 18.085 | 4.176727 | 0.010092 | 0.028249 | yes | up | 0.42 | 0.18 | 0.14 | 25.57 | 0.07 | 8.48 | 0.246667 | 11.37333 |
| LOC108178545 | 0.238 | -2.07009 | 0.010097 | 0.028253 | yes | down | 2.89 | 2.96 | 3.61 | 1.88 | 6.5 | 0.74 | 3.153333 | 3.04 |
| TMEM215 | 0.024 | -5.3724 | 0.010153 | 0.028384 | yes | down | 0.02 | 0 | 0.22 | 0 | 0.01 | 0 | 0.08 | 0.003333 |
| ANKRD45 | 2.593 | 1.374725 | 0.010185 | 0.028464 | yes | up | 0.31 | 0.35 | 0.24 | 1.89 | 1.92 | 1.69 | 0.3 | 1.833333 |
| DNAH7 | 0.027 | -5.22184 | 0.010232 | 0.028584 | yes | down | 0.02 | 0.02 | 0.01 | 0 | 0 | 0 | 0.016667 | 0 |
| CAMP | 0.117 | -3.09724 | 0.010277 | 0.028696 | yes | down | 0.37 | 1.28 | 1.72 | 0.57 | 0.22 | 0 | 1.123333 | 0.263333 |
| LOC103351740 | 9.295 | 3.216468 | 0.01029 | 0.028719 | yes | up | 0 | 0.02 | 0.01 | 0.23 | 0.17 | 0.3 | 0.01 | 0.233333 |
| LOC100344012 | 4.624 | 2.209286 | 0.010308 | 0.028764 | yes | up | 0.12 | 0.14 | 0.08 | 0.42 | 1.17 | 1.98 | 0.113333 | 1.19 |
| LOC100349428 | 0.454 | -1.13831 | 0.010355 | 0.028881 | yes | down | 15.51 | 18.86 | 14.48 | 5.75 | 8.85 | 31.78 | 16.28333 | 15.46 |
| CCDC146 | 0.22 | -2.18564 | 0.010381 | 0.028938 | yes | down | 0.22 | 0.17 | 0.25 | 0.07 | 0.06 | 0.2 | 0.213333 | 0.11 |
| ABCA6 | 2.057 | 1.040735 | 0.010405 | 0.028994 | yes | up | 1.15 | 1.12 | 0.74 | 3.66 | 8.4 | 2.2 | 1.003333 | 4.753333 |
| DAGLA | 2.127 | 1.08886 | 0.010467 | 0.029134 | yes | up | 1.24 | 0.49 | 0.29 | 2.33 | 4.47 | 3.15 | 0.673333 | 3.316667 |
| SLC2A4RG | 4.174 | 2.061574 | 0.010485 | 0.029167 | yes | up | 1.23 | 1.09 | 0.22 | 7.16 | 9.25 | 8.42 | 0.846667 | 8.276667 |
| LOC103346470 | 0.226 | -2.14766 | 0.010486 | 0.029167 | yes | down | 0.98 | 2.06 | 2.91 | 1.59 | 0.35 | 1.04 | 1.983333 | 0.993333 |
| C1QL4 | 3.909 | 1.966923 | 0.010496 | 0.029186 | yes | up | 0.26 | 0.18 | 0.14 | 3.1 | 0.83 | 1.1 | 0.193333 | 1.676667 |
| LOC108176666 | 3.881 | 1.956358 | 0.010499 | 0.029189 | yes | up | 0.7 | 0.23 | 0.32 | 4.51 | 4.02 | 1.53 | 0.416667 | 3.353333 |
| RGS20 | 2.326 | 1.217544 | 0.010504 | 0.029196 | yes | up | 10.39 | 15.98 | 8.68 | 31.24 | 41.8 | 19.59 | 11.68333 | 30.87667 |
| PBK | 2.851 | 1.511515 | 0.010534 | 0.02927 | yes | up | 0.97 | 0.61 | 0.33 | 4.76 | 6.32 | 1.35 | 0.636667 | 4.143333 |
| SIX4 | 0.339 | -1.55988 | 0.010565 | 0.02934 | yes | down | 1.89 | 0.82 | 0.39 | 0.81 | 0.33 | 1.11 | 1.033333 | 0.75 |
| SLC6A4 | 3.874 | 1.953774 | 0.01057 | 0.029345 | yes | up | 0.17 | 0.1 | 0.05 | 1.23 | 0.55 | 0.97 | 0.106667 | 0.916667 |
| TRMT61B | 0.319 | -1.64818 | 0.010582 | 0.029369 | yes | down | 1.35 | 0.74 | 0.75 | 0.95 | 0.93 | 0.19 | 0.946667 | 0.69 |
| ADAM18 | 6.748 | 2.754466 | 0.010587 | 0.029379 | yes | up | 0.05 | 0.13 | 0.02 | 1.29 | 0.59 | 0.55 | 0.066667 | 0.81 |
| SPRY3 | 3.415 | 1.771903 | 0.010592 | 0.029388 | yes | up | 0.15 | 0.22 | 0.11 | 1.9 | 0.53 | 0.59 | 0.16 | 1.006667 |
| CATSPER2 | 3.246 | 1.698668 | 0.010615 | 0.029446 | yes | up | 0.32 | 0.12 | 0.08 | 0.81 | 1.36 | 1.04 | 0.173333 | 1.07 |
| BATF | 3.061 | 1.614047 | 0.01063 | 0.029484 | yes | up | 0.33 | 0.58 | 0.89 | 5.17 | 4.21 | 3.6 | 0.6 | 4.326667 |
| LOC103347896 | 3.496 | 1.80566 | 0.010672 | 0.029573 | yes | up | 0.08 | 0.06 | 0.08 | 0.43 | 0.85 | 0.57 | 0.073333 | 0.616667 |
| PCDH7 | 3.133 | 1.647724 | 0.010802 | 0.02992 | yes | up | 0.5 | 0.21 | 0.14 | 2.23 | 1 | 2.36 | 0.283333 | 1.863333 |
| LOC103348167 | 25.725 | 4.685073 | 0.010829 | 0.029984 | yes | up | 0.04 | 0 | 0 | 0.1 | 2.76 | 0.24 | 0.013333 | 1.033333 |
| IFIT2 | 0.456 | -1.13206 | 0.010904 | 0.030146 | yes | down | 2.13 | 1.1 | 0.91 | 1.36 | 1.99 | 0.95 | 1.38 | 1.433333 |
| LOC103348222 | 0.121 | -3.04354 | 0.010919 | 0.030177 | yes | down | 0.34 | 0.26 | 0.27 | 0.03 | 0 | 0.18 | 0.29 | 0.07 |
| PRKAA2 | 0.207 | -2.26948 | 0.011023 | 0.03041 | yes | down | 3.56 | 0.3 | 0.33 | 0.6 | 0.32 | 0.92 | 1.396667 | 0.613333 |
| LOC103350987 | 4.083 | 2.029503 | 0.011055 | 0.030487 | yes | up | 0.01 | 0.02 | 0.05 | 0.2 | 0.52 | 0.22 | 0.026667 | 0.313333 |
| LOC103350198 | 26.479 | 4.726788 | 0.011073 | 0.030531 | yes | up | 0 | 0 | 0 | 0.25 | 0.46 | 0.53 | 0 | 0.413333 |
| PAPOLB | 6.549 | 2.711272 | 0.011095 | 0.030571 | yes | up | 0.03 | 0.04 | 0 | 0.3 | 0.28 | 0.34 | 0.023333 | 0.306667 |
| LOC108176252 | 0.132 | -2.91595 | 0.011146 | 0.030697 | yes | down | 0.57 | 0.87 | 0.49 | 0.1 | 0.22 | 0.28 | 0.643333 | 0.2 |
| MUTYH | 2.178 | 1.122776 | 0.011148 | 0.030697 | yes | up | 0.84 | 0.7 | 0.58 | 3.48 | 4.65 | 2.5 | 0.706667 | 3.543333 |
| ASB15 | 0.035 | -4.82036 | 0.011162 | 0.030725 | yes | down | 8.9 | 1.02 | 0.17 | 0.31 | 0 | 0.43 | 3.363333 | 0.246667 |
| FAM227B | 44.578 | 5.478256 | 0.011165 | 0.030728 | yes | up | 0 | 0 | 0 | 0 | 6.2 | 3.93 | 0 | 3.376667 |
| LOC103347448 | 45.457 | 5.506417 | 0.011191 | 0.030785 | yes | up | 0 | 0 | 0 | 0.7 | 0 | 0.32 | 0 | 0.34 |
| LOC100352425 | 0.102 | -3.29617 | 0.011204 | 0.030817 | yes | down | 0.66 | 0.51 | 0.65 | 0.29 | 0.11 | 0 | 0.606667 | 0.133333 |
| LOC100342885 | 171.236 | 7.419838 | 0.011207 | 0.030818 | yes | up | 0 | 0.14 | 0 | 34.07 | 37.34 | 0 | 0.046667 | 23.80333 |
| LOC100343607 | 72.341 | 6.176743 | 0.011218 | 0.030844 | yes | up | 0 | 0.05 | 0.09 | 11.28 | 0 | 13.02 | 0.046667 | 8.1 |
| LOC103348624 | 0.345 | -1.53683 | 0.011277 | 0.030997 | yes | down | 0.55 | 0.48 | 0.33 | 0.46 | 0.48 | 0.14 | 0.453333 | 0.36 |
| LOC108177265 | 19.455 | 4.282082 | 0.011282 | 0.031 | yes | up | 0.1 | 0 | 0 | 0.52 | 1.37 | 2.48 | 0.033333 | 1.456667 |
| LOC103350181 | 22.831 | 4.512947 | 0.011326 | 0.031115 | yes | up | 0 | 0.08 | 0 | 0.18 | 1.21 | 3.09 | 0.026667 | 1.493333 |
| SLITRK5 | 0.308 | -1.69729 | 0.011342 | 0.031153 | yes | down | 0.16 | 0.16 | 0.08 | 0.1 | 0.06 | 0.11 | 0.133333 | 0.09 |
| LOC100341951 | 14.27 | 3.834911 | 0.011344 | 0.031153 | yes | up | 0 | 0.32 | 0 | 5.41 | 1.52 | 1.94 | 0.106667 | 2.956667 |
| TICRR | 6.106 | 2.610324 | 0.011357 | 0.031185 | yes | up | 0.08 | 0.02 | 0.05 | 0.51 | 1.53 | 0.18 | 0.05 | 0.74 |
| LOC100357628 | 15.798 | 3.981636 | 0.011391 | 0.031263 | yes | up | 0.08 | 0.07 | 0 | 4 | 0.54 | 0.46 | 0.05 | 1.666667 |
| LOC103351002 | 0.105 | -3.25825 | 0.011448 | 0.031408 | yes | down | 0.78 | 0.71 | 0.13 | 0.16 | 0.09 | 0.11 | 0.54 | 0.12 |
| LOC108175429 | 6.168 | 2.624915 | 0.011484 | 0.031497 | yes | up | 0.12 | 0.05 | 0 | 0.44 | 0.64 | 1.1 | 0.056667 | 0.726667 |
| LOC103349122 | 0.411 | -1.28402 | 0.011496 | 0.031525 | yes | down | 2.17 | 1.66 | 1.78 | 1.2 | 3.38 | 0.9 | 1.87 | 1.826667 |
| LOC100353287 | 28.546 | 4.835221 | 0.011617 | 0.031836 | yes | up | 0 | 0 | 0 | 0.14 | 0.27 | 0.7 | 0 | 0.37 |
| PELI3 | 0.317 | -1.65736 | 0.01167 | 0.031974 | yes | down | 0.31 | 0.56 | 0.44 | 0.4 | 0.32 | 0.22 | 0.436667 | 0.313333 |
| SPATA6L | 2.12 | 1.083828 | 0.01169 | 0.032018 | yes | up | 1.24 | 1.47 | 1.1 | 2.69 | 4.23 | 2.12 | 1.27 | 3.013333 |
| CXCL13 | 29.93 | 4.903532 | 0.011712 | 0.032063 | yes | up | 0 | 0 | 0 | 0.11 | 2.51 | 0.72 | 0 | 1.113333 |
| LOC103352413 | 0.043 | -4.5248 | 0.011718 | 0.032073 | yes | down | 1.14 | 0.51 | 0.81 | 0 | 0.22 | 0 | 0.82 | 0.073333 |
| PCK1 | 0.203 | -2.30118 | 0.011767 | 0.032188 | yes | down | 898.83 | 1267.15 | 675.53 | 79.19 | 1062.9 | 241.2 | 947.17 | 461.0967 |
| DCUN1D2 | 0.462 | -1.11356 | 0.011825 | 0.032336 | yes | down | 4.63 | 2.57 | 1.86 | 2.24 | 2.3 | 4.66 | 3.02 | 3.066667 |
| DLX4 | 0.403 | -1.30961 | 0.011903 | 0.032537 | yes | down | 0.57 | 1.09 | 1.06 | 0.85 | 1.07 | 0.66 | 0.906667 | 0.86 |
| CARD11 | 6.985 | 2.804247 | 0.011967 | 0.032663 | yes | up | 0.02 | 0.03 | 0.01 | 0.21 | 0.38 | 0.39 | 0.02 | 0.326667 |
| SMARCA1 | 0.466 | -1.10198 | 0.011993 | 0.032724 | yes | down | 2.89 | 1.47 | 0.86 | 1.85 | 2.48 | 1.12 | 1.74 | 1.816667 |
| LOC108176680 | 0.403 | -1.31251 | 0.011999 | 0.032727 | yes | down | 0.94 | 1.22 | 0.93 | 0.68 | 1.54 | 0.69 | 1.03 | 0.97 |
| LOC100349542 | 0.413 | -1.27428 | 0.012 | 0.032727 | yes | down | 1.68 | 1.52 | 0.72 | 0.96 | 0.76 | 1.87 | 1.306667 | 1.196667 |
| INHBA | 2.367 | 1.243076 | 0.012025 | 0.032788 | yes | up | 0.36 | 0.24 | 0.19 | 1.89 | 1.4 | 0.95 | 0.263333 | 1.413333 |
| LOC103352476 | 0.347 | -1.52545 | 0.012055 | 0.032853 | yes | down | 1.47 | 1.83 | 0.75 | 1.54 | 0.94 | 0.64 | 1.35 | 1.04 |
| TECTB | 0.094 | -3.41354 | 0.012071 | 0.032883 | yes | down | 0.68 | 0.25 | 0.1 | 0.04 | 0 | 0.17 | 0.343333 | 0.07 |
| LOC100355748 | 11.227 | 3.488952 | 0.012087 | 0.032921 | yes | up | 0.08 | 0.07 | 0.07 | 0.67 | 0.46 | 4.32 | 0.073333 | 1.816667 |
| SACS | 0.436 | -1.19852 | 0.012104 | 0.032955 | yes | down | 3.71 | 1.02 | 0.84 | 1.81 | 2.48 | 1.08 | 1.856667 | 1.79 |
| AP3B2 | 3.21 | 1.682462 | 0.012116 | 0.032978 | yes | up | 0.07 | 0.24 | 0.11 | 1.46 | 0.74 | 0.86 | 0.14 | 1.02 |
| FCMR | 7.425 | 2.892435 | 0.012138 | 0.033033 | yes | up | 0.1 | 0.03 | 0 | 0.71 | 0.75 | 0.57 | 0.043333 | 0.676667 |
| PKHD1 | 0.081 | -3.62519 | 0.012155 | 0.033071 | yes | down | 0.18 | 0.01 | 0 | 0 | 1.08 | 0.01 | 0.063333 | 0.363333 |
| CAPRIN2 | 2.104 | 1.073379 | 0.012245 | 0.033307 | yes | up | 0.49 | 0.28 | 0.26 | 1.34 | 2.01 | 1.56 | 0.343333 | 1.636667 |
| SHCBP1 | 3.018 | 1.59368 | 0.012321 | 0.033486 | yes | up | 0.96 | 0.58 | 0.36 | 4.82 | 7.55 | 0.92 | 0.633333 | 4.43 |
| SERPINB10 | 0.03 | -5.05182 | 0.012327 | 0.033497 | yes | down | 0 | 0.28 | 0.49 | 0.06 | 0 | 0 | 0.256667 | 0.02 |
| LOC100008921 | 7.078 | 2.823392 | 0.012423 | 0.033719 | yes | up | 0.2 | 0.18 | 0.35 | 5.82 | 3.48 | 2.68 | 0.243333 | 3.993333 |
| RBBP8 | 0.426 | -1.2306 | 0.012437 | 0.033745 | yes | down | 2.06 | 2.71 | 1.85 | 2.1 | 3.87 | 0.7 | 2.206667 | 2.223333 |
| LBX1 | 0.056 | -4.15932 | 0.012456 | 0.033787 | yes | down | 1.07 | 0.09 | 0.03 | 0.13 | 0 | 0 | 0.396667 | 0.043333 |
| LOC100346276 | 0.434 | -1.20411 | 0.012519 | 0.033946 | yes | down | 15.15 | 19.23 | 16.57 | 5.48 | 18.41 | 26.89 | 16.98333 | 16.92667 |
| PDPN | 3.459 | 1.790549 | 0.012531 | 0.033973 | yes | up | 1.47 | 0.58 | 0.05 | 4.77 | 5.01 | 5.83 | 0.7 | 5.203333 |
| LOC103347127 | 0.357 | -1.486 | 0.012631 | 0.034215 | yes | down | 10.79 | 11.98 | 5.53 | 6.16 | 10.79 | 6.35 | 9.433333 | 7.766667 |
| GJD4 | 0.13 | -2.94318 | 0.012717 | 0.034415 | yes | down | 0.1 | 0.15 | 0.23 | 0.03 | 0.06 | 0.07 | 0.16 | 0.053333 |
| PLA2G2D | 7.432 | 2.893656 | 0.012749 | 0.03449 | yes | up | 3.86 | 0.3 | 3.98 | 61.95 | 11.42 | 64.14 | 2.713333 | 45.83667 |
| PRICKLE3 | 2.344 | 1.229047 | 0.012758 | 0.034507 | yes | up | 0.69 | 0.63 | 0.8 | 4.36 | 3.93 | 3.21 | 0.706667 | 3.833333 |
| RASSF5 | 2.404 | 1.265253 | 0.012791 | 0.034585 | yes | up | 0.18 | 0.38 | 0.21 | 1.44 | 1.18 | 1.57 | 0.256667 | 1.396667 |
| LEF1 | 3.109 | 1.636681 | 0.012794 | 0.03459 | yes | up | 0.15 | 0.08 | 0.15 | 0.98 | 0.77 | 0.91 | 0.126667 | 0.886667 |
| LOC100352842 | 49.143 | 5.618912 | 0.012829 | 0.034672 | yes | up | 0 | 0 | 0 | 0.04 | 0.75 | 0 | 0 | 0.263333 |
| SFRP4 | 4.075 | 2.026965 | 0.01285 | 0.034722 | yes | up | 22.57 | 11.78 | 4.09 | 74.89 | 64.4 | 193.04 | 12.81333 | 110.7767 |
| ART3 | 0.142 | -2.82066 | 0.012883 | 0.034806 | yes | down | 23.6 | 3.35 | 1.29 | 2.38 | 1.4 | 4.23 | 9.413333 | 2.67 |
| FANCE | 2.055 | 1.039251 | 0.012916 | 0.03489 | yes | up | 0.79 | 0.68 | 0.9 | 4.42 | 4.68 | 2.28 | 0.79 | 3.793333 |
| PRIMA1 | 3.984 | 1.994301 | 0.012933 | 0.034927 | yes | up | 0.1 | 0.4 | 0.13 | 1.85 | 1.3 | 2.43 | 0.21 | 1.86 |
| PKP2 | 0.459 | -1.12417 | 0.012942 | 0.034939 | yes | down | 0.6 | 0.87 | 0.95 | 0.89 | 0.9 | 0.79 | 0.806667 | 0.86 |
| SPINK8 | 16.541 | 4.047975 | 0.013032 | 0.035162 | yes | up | 0.11 | 0.1 | 0 | 2.37 | 0.13 | 4.59 | 0.07 | 2.363333 |
| AZGP1 | 0.376 | -1.41048 | 0.013044 | 0.03519 | yes | down | 1.24 | 0.94 | 0.85 | 0.91 | 0.76 | 0.68 | 1.01 | 0.783333 |
| LYSMD2 | 2.055 | 1.039348 | 0.013125 | 0.035362 | yes | up | 1.52 | 1.24 | 0.81 | 5.77 | 4.54 | 6 | 1.19 | 5.436667 |
| PRTFDC1 | 2.706 | 1.436346 | 0.013156 | 0.035428 | yes | up | 1.24 | 0.62 | 0.2 | 3.44 | 3.7 | 4.95 | 0.686667 | 4.03 |
| LOC100339454 | 0.335 | -1.57758 | 0.013184 | 0.035497 | yes | down | 6.7 | 12.93 | 10.84 | 7.86 | 6.48 | 13.28 | 10.15667 | 9.206667 |
| MUC19 | 6.975 | 2.802228 | 0.013192 | 0.035513 | yes | up | 0.01 | 1.84 | 1.82 | 13.13 | 18.42 | 28.86 | 1.223333 | 20.13667 |
| RMI2 | 3.224 | 1.6888 | 0.013251 | 0.035643 | yes | up | 0.32 | 0.55 | 0.31 | 4.33 | 3.55 | 0.88 | 0.393333 | 2.92 |
| LOC103347239 | 4.996 | 2.320672 | 0.013301 | 0.035748 | yes | up | 0.39 | 0.35 | 0.11 | 3.38 | 2.4 | 3.65 | 0.283333 | 3.143333 |
| IPP | 0.494 | -1.01659 | 0.013334 | 0.035829 | yes | down | 1.79 | 0.79 | 1.13 | 1.44 | 1.71 | 1.07 | 1.236667 | 1.406667 |
| NTSR2 | 0.028 | -5.15724 | 0.013335 | 0.035829 | yes | down | 0.12 | 0.07 | 0.21 | 0 | 0 | 0 | 0.133333 | 0 |
| LOC103350569 | 3.245 | 1.698047 | 0.013363 | 0.035885 | yes | up | 1.81 | 0.45 | 0.24 | 4.18 | 8.65 | 5.36 | 0.833333 | 6.063333 |
| LOC100338167 | 0.012 | -6.42141 | 0.013382 | 0.035926 | yes | down | 0 | 5.23 | 0 | 0 | 0 | 0 | 1.743333 | 0 |
| CD3E | 11.983 | 3.582937 | 0.013445 | 0.036073 | yes | up | 0.1 | 0.18 | 0 | 0.52 | 0.92 | 5.68 | 0.093333 | 2.373333 |
| HTR1B | 5.422 | 2.438889 | 0.01349 | 0.036157 | yes | up | 0.18 | 0.05 | 0.15 | 1.3 | 0.88 | 2.51 | 0.126667 | 1.563333 |
| ATP2A2 | 0.459 | -1.12498 | 0.01349 | 0.036157 | yes | down | 117.72 | 42.21 | 23.26 | 64.26 | 58.57 | 68.5 | 61.06333 | 63.77667 |
| LOC103348750 | 0.335 | -1.57911 | 0.013509 | 0.036188 | yes | down | 1.17 | 3.33 | 1.44 | 2.18 | 1.33 | 1.42 | 1.98 | 1.643333 |
| LOC100341994 | 3.404 | 1.767031 | 0.013511 | 0.036188 | yes | up | 0.11 | 0.09 | 0.1 | 0.47 | 0.84 | 1.02 | 0.1 | 0.776667 |
| LOC100343032 | 0.438 | -1.19131 | 0.013536 | 0.036244 | yes | down | 2.67 | 3.36 | 3.36 | 3.29 | 3.13 | 3.02 | 3.13 | 3.146667 |
| TBX1 | 2.879 | 1.525479 | 0.013547 | 0.036261 | yes | up | 3.89 | 1.82 | 1.98 | 15.2 | 5.7 | 24.5 | 2.563333 | 15.13333 |
| LOC103346853 | 2.927 | 1.549221 | 0.013581 | 0.036322 | yes | up | 0.33 | 0.2 | 0.24 | 0.98 | 1.51 | 2.6 | 0.256667 | 1.696667 |
| FCRL5 | 7.579 | 2.921925 | 0.013595 | 0.036356 | yes | up | 0.02 | 0 | 0.02 | 0.24 | 0.43 | 0.12 | 0.013333 | 0.263333 |
| GGN | 25.031 | 4.645626 | 0.013604 | 0.036366 | yes | up | 0 | 0 | 0 | 0.39 | 0.29 | 0.23 | 0 | 0.303333 |
| SLC16A14 | 4.002 | 2.000897 | 0.01362 | 0.036404 | yes | up | 0.05 | 0.06 | 0.03 | 0.31 | 0.74 | 0.24 | 0.046667 | 0.43 |
| FRMPD4 | 0.386 | -1.37397 | 0.01366 | 0.036487 | yes | down | 0.16 | 0.23 | 0.22 | 0.12 | 0.17 | 0.26 | 0.203333 | 0.183333 |
| MAB21L3 | 0.333 | -1.58832 | 0.013679 | 0.036533 | yes | down | 0.99 | 0.35 | 0.73 | 0.3 | 0.74 | 0.55 | 0.69 | 0.53 |
| TRPM6 | 0.31 | -1.68883 | 0.013752 | 0.03669 | yes | down | 0.2 | 0.12 | 0.1 | 0.14 | 0.06 | 0.08 | 0.14 | 0.093333 |
| EME1 | 2.315 | 1.210898 | 0.013797 | 0.036787 | yes | up | 1.25 | 0.99 | 0.34 | 4.53 | 6.51 | 2.49 | 0.86 | 4.51 |
| LOC100338778 | 2.669 | 1.416172 | 0.013901 | 0.037046 | yes | up | 0.09 | 0.21 | 0.11 | 0.74 | 1.06 | 0.75 | 0.136667 | 0.85 |
| LOC108178767 | 0.288 | -1.79498 | 0.013908 | 0.037059 | yes | down | 0.91 | 0.85 | 0.24 | 0.34 | 0.85 | 0.13 | 0.666667 | 0.44 |
| MC2R | 33.68 | 5.07381 | 0.013959 | 0.037183 | yes | up | 0 | 0 | 0 | 0.08 | 0.66 | 0.03 | 0 | 0.256667 |
| TP63 | 0.319 | -1.64665 | 0.014044 | 0.037386 | yes | down | 1.6 | 0.23 | 0.17 | 0.19 | 0.49 | 0.4 | 0.666667 | 0.36 |
| ZNF782 | 0.397 | -1.33429 | 0.014063 | 0.03743 | yes | down | 0.47 | 0.47 | 0.5 | 0.47 | 0.57 | 0.28 | 0.48 | 0.44 |
| LOC100353203 | 47.793 | 5.578742 | 0.014078 | 0.037459 | yes | up | 0 | 0 | 0 | 2.31 | 0 | 0.21 | 0 | 0.84 |
| CIITA | 2.716 | 1.441674 | 0.014107 | 0.037518 | yes | up | 0.04 | 0.15 | 0.13 | 0.72 | 0.66 | 0.62 | 0.106667 | 0.666667 |
| FAM47E | 0.045 | -4.48124 | 0.014129 | 0.03757 | yes | down | 0.17 | 0.22 | 0.06 | 0 | 0 | 0.05 | 0.15 | 0.016667 |
| LOC103349783 | 0.464 | -1.10856 | 0.014155 | 0.037633 | yes | down | 0.96 | 0.4 | 0.43 | 0.72 | 0.88 | 0.47 | 0.596667 | 0.69 |
| GNA14 | 3.567 | 1.834738 | 0.014234 | 0.03783 | yes | up | 0.48 | 0.31 | 0.03 | 1.49 | 1.75 | 1.71 | 0.273333 | 1.65 |
| LOC103350276 | 0.283 | -1.82161 | 0.014255 | 0.037869 | yes | down | 7.97 | 2.27 | 0.92 | 0.97 | 1.93 | 3.99 | 3.72 | 2.296667 |
| EMX2 | 4.397 | 2.136623 | 0.014275 | 0.037914 | yes | up | 0.63 | 0.16 | 0.12 | 3.12 | 0.46 | 4.79 | 0.303333 | 2.79 |
| CACNA1A | 5.198 | 2.377848 | 0.014277 | 0.037914 | yes | up | 0.07 | 0.07 | 0 | 0.35 | 1.07 | 0.23 | 0.046667 | 0.55 |
| TSPYL5 | 2.737 | 1.452522 | 0.01438 | 0.038127 | yes | up | 0.31 | 0.16 | 0.09 | 1 | 0.95 | 1.45 | 0.186667 | 1.133333 |
| SPATA20 | 2.584 | 1.369434 | 0.014407 | 0.038179 | yes | up | 0.25 | 0.19 | 0.14 | 1.12 | 1.17 | 1.11 | 0.193333 | 1.133333 |
| LOC100348203 | 13.034 | 3.704233 | 0.01457 | 0.038566 | yes | up | 0 | 0.02 | 0.02 | 0.11 | 0.44 | 0.76 | 0.013333 | 0.436667 |
| LOC108178938 | 0.218 | -2.19938 | 0.014572 | 0.038566 | yes | down | 0.18 | 0.17 | 0.19 | 0.06 | 0.09 | 0.12 | 0.18 | 0.09 |
| C1QTNF3 | 0.37 | -1.43569 | 0.014598 | 0.03863 | yes | down | 6.91 | 1.92 | 1.17 | 1.76 | 2.18 | 3.99 | 3.333333 | 2.643333 |
| LGI2 | 6.732 | 2.751029 | 0.014671 | 0.038793 | yes | up | 0 | 0.04 | 0.05 | 0.76 | 0.47 | 0.24 | 0.03 | 0.49 |
| GPRIN1 | 4.456 | 2.155601 | 0.014703 | 0.03887 | yes | up | 0.37 | 0.08 | 0.32 | 1.75 | 3.01 | 3.29 | 0.256667 | 2.683333 |
| KLHL32 | 0.411 | -1.28444 | 0.014758 | 0.039003 | yes | down | 1.96 | 3.15 | 2.9 | 2.58 | 3.2 | 2.22 | 2.67 | 2.666667 |
| LOC108176549 | 3.752 | 1.907798 | 0.014779 | 0.039046 | yes | up | 0.09 | 0.03 | 0.11 | 0.64 | 1.02 | 0.39 | 0.076667 | 0.683333 |
| LOC100342521 | 0.123 | -3.02845 | 0.014843 | 0.039197 | yes | down | 0.16 | 0.43 | 0.23 | 0 | 0.19 | 0.05 | 0.273333 | 0.08 |
| FOXF2 | 8.606 | 3.105289 | 0.01486 | 0.03923 | yes | up | 0.09 | 0 | 0.04 | 0.77 | 0.58 | 1.16 | 0.043333 | 0.836667 |
| LOC108175795 | 3.664 | 1.873431 | 0.014867 | 0.039241 | yes | up | 0.31 | 0.12 | 0.19 | 1.93 | 0.98 | 2.23 | 0.206667 | 1.713333 |
| LOC100347617 | 0.105 | -3.24725 | 0.014905 | 0.039327 | yes | down | 0.7 | 0.5 | 0.09 | 0 | 0.3 | 0 | 0.43 | 0.1 |
| S100A12 | 0.273 | -1.87353 | 0.014941 | 0.039403 | yes | down | 2.98 | 20.81 | 24.55 | 7.88 | 4.16 | 18.88 | 16.11333 | 10.30667 |
| DNAJC5B | 26.48 | 4.726843 | 0.015011 | 0.039563 | yes | up | 0 | 0 | 0 | 0.36 | 0.39 | 0.08 | 0 | 0.276667 |
| NEURL2 | 0.359 | -1.47847 | 0.015048 | 0.039655 | yes | down | 7.84 | 3.03 | 1.85 | 2.24 | 2.38 | 5.31 | 4.24 | 3.31 |
| WDR89 | 0.47 | -1.08785 | 0.015079 | 0.039725 | yes | down | 1.71 | 2.45 | 1.87 | 2.3 | 3.04 | 1.18 | 2.01 | 2.173333 |
| PITPNM3 | 4.838 | 2.274352 | 0.015114 | 0.039795 | yes | up | 0.04 | 0.03 | 0 | 0.2 | 0.36 | 0.22 | 0.023333 | 0.26 |
| CETP | 0.237 | -2.07494 | 0.015305 | 0.040248 | yes | down | 0.33 | 0.61 | 0.43 | 0.43 | 0.27 | 0.05 | 0.456667 | 0.25 |
| HYI | 2.584 | 1.369862 | 0.015318 | 0.040274 | yes | up | 2.09 | 2.34 | 2.1 | 24.77 | 4 | 8.21 | 2.176667 | 12.32667 |
| ATP5I | 0.422 | -1.24441 | 0.01532 | 0.040274 | yes | down | 2.56 | 5.12 | 5.83 | 4.6 | 3.43 | 5.28 | 4.503333 | 4.436667 |
| C14H21orf58 | 3.672 | 1.876721 | 0.015364 | 0.040372 | yes | up | 0.12 | 0.32 | 0.3 | 1.89 | 1.52 | 2.81 | 0.246667 | 2.073333 |
| ADAM33 | 2.231 | 1.157979 | 0.015432 | 0.040525 | yes | up | 0.43 | 0.51 | 0.17 | 1.26 | 1.96 | 2.23 | 0.37 | 1.816667 |
| P2RY8 | 9.708 | 3.279247 | 0.015476 | 0.040621 | yes | up | 0 | 0.06 | 0 | 0.26 | 0.49 | 0.59 | 0.02 | 0.446667 |
| MKRN3 | 0.167 | -2.58384 | 0.015529 | 0.040734 | yes | down | 0.2 | 0.1 | 0.27 | 0.02 | 0.08 | 0.13 | 0.19 | 0.076667 |
| DIS3L | 2.149 | 1.103739 | 0.015556 | 0.04079 | yes | up | 0.41 | 0.27 | 0.51 | 2.26 | 2.4 | 1.33 | 0.396667 | 1.996667 |
| FGF18 | 4.151 | 2.053551 | 0.015568 | 0.040816 | yes | up | 0.62 | 0.09 | 0.36 | 4.42 | 2.86 | 2.74 | 0.356667 | 3.34 |
| FAM83H | 24.645 | 4.623227 | 0.015574 | 0.040827 | yes | up | 0 | 0 | 0 | 0.45 | 0.88 | 1.13 | 0 | 0.82 |
| SLC25A21 | 0.429 | -1.22246 | 0.015601 | 0.040877 | yes | down | 1.67 | 1.76 | 1.97 | 1.72 | 1.13 | 0.9 | 1.8 | 1.25 |
| SYT2 | 4.409 | 2.140523 | 0.015617 | 0.040911 | yes | up | 0.17 | 0.18 | 0.18 | 1.68 | 0.07 | 3.24 | 0.176667 | 1.663333 |
| SLITRK4 | 0.133 | -2.91102 | 0.015694 | 0.041089 | yes | down | 0.21 | 0.03 | 0.03 | 0.01 | 0.02 | 0.05 | 0.09 | 0.026667 |
| SLC19A1 | 2.265 | 1.179399 | 0.015723 | 0.041137 | yes | up | 3.24 | 4.09 | 1.35 | 10.17 | 9.36 | 24.91 | 2.893333 | 14.81333 |
| LOC103350952 | 0.295 | -1.7613 | 0.015723 | 0.041137 | yes | down | 1.13 | 1.02 | 0.66 | 0.74 | 1.15 | 0.05 | 0.936667 | 0.646667 |
| LOC108175489 | 16.324 | 4.028904 | 0.015744 | 0.041174 | yes | up | 0 | 0 | 0.13 | 2.58 | 1.55 | 1.8 | 0.043333 | 1.976667 |
| CXCR1 | 2.138 | 1.096352 | 0.015773 | 0.041229 | yes | up | 0.44 | 0.54 | 0.66 | 2.03 | 3.58 | 2.98 | 0.546667 | 2.863333 |
| LOC103346936 | 0.42 | -1.25156 | 0.015868 | 0.041451 | yes | down | 3.29 | 2.96 | 4.41 | 3.57 | 7.76 | 4.55 | 3.553333 | 5.293333 |
| TMEM200C | 0.218 | -2.19937 | 0.015985 | 0.041718 | yes | down | 0.19 | 0.32 | 0.07 | 0.08 | 0.06 | 0.14 | 0.193333 | 0.093333 |
| PDLIM5 | 0.426 | -1.22987 | 0.015988 | 0.041718 | yes | down | 134.61 | 25.17 | 9.45 | 29.52 | 20.88 | 53.89 | 56.41 | 34.76333 |
| LOC108177035 | 0.274 | -1.86989 | 0.016054 | 0.041857 | yes | down | 0.18 | 0.31 | 0.15 | 0.11 | 0.19 | 0.1 | 0.213333 | 0.133333 |
| LOC103351959 | 2.675 | 1.419724 | 0.016136 | 0.042038 | yes | up | 0.08 | 0.2 | 0.15 | 0.62 | 1.09 | 0.64 | 0.143333 | 0.783333 |
| LOC100358160 | 23.995 | 4.584642 | 0.016141 | 0.042039 | yes | up | 0 | 0 | 0 | 1.14 | 0.76 | 1.12 | 0 | 1.006667 |
| CENPW | 2.012 | 1.008375 | 0.016157 | 0.042073 | yes | up | 3.06 | 3.95 | 3.51 | 14.66 | 23.67 | 11.39 | 3.506667 | 16.57333 |
| PAWR | 2.247 | 1.168317 | 0.016235 | 0.042233 | yes | up | 0.56 | 0.44 | 0.41 | 3.39 | 2.31 | 1.52 | 0.47 | 2.406667 |
| PABPC4L | 0.495 | -1.01517 | 0.016237 | 0.042233 | yes | down | 1.01 | 1.13 | 1.58 | 1.59 | 1.74 | 1 | 1.24 | 1.443333 |
| LOC100348276 | 0.347 | -1.52728 | 0.016286 | 0.042328 | yes | down | 8.09 | 3.33 | 11.34 | 5.16 | 12.13 | 0.8 | 7.586667 | 6.03 |
| MRLN | 0.054 | -4.20301 | 0.016342 | 0.04244 | yes | down | 41.62 | 7.45 | 1.01 | 3.41 | 0 | 2.11 | 16.69333 | 1.84 |
| CRLF2 | 6.592 | 2.720754 | 0.016354 | 0.042466 | yes | up | 0.02 | 0.2 | 0.18 | 1.68 | 1.4 | 2.41 | 0.133333 | 1.83 |
| BASP1 | 2.204 | 1.140217 | 0.016589 | 0.04302 | yes | up | 3.05 | 2.44 | 1.59 | 10.23 | 5.64 | 18.16 | 2.36 | 11.34333 |
| HCRTR1 | 6.437 | 2.686391 | 0.016612 | 0.04306 | yes | up | 0.03 | 0.02 | 0.04 | 0.49 | 0.39 | 0.53 | 0.03 | 0.47 |
| CMTM5 | 3.772 | 1.915168 | 0.016651 | 0.043156 | yes | up | 0.75 | 1.06 | 0.43 | 12.57 | 1.72 | 4.02 | 0.746667 | 6.103333 |
| TMEM92 | 18.263 | 4.190834 | 0.016674 | 0.043207 | yes | up | 0.02 | 0 | 0 | 0.48 | 0.08 | 0.32 | 0.006667 | 0.293333 |
| LOC100349085 | 4.159 | 2.056114 | 0.01669 | 0.04323 | yes | up | 9.46 | 24.35 | 11 | 136.42 | 33.58 | 233.59 | 14.93667 | 134.53 |
| FBP2 | 0.103 | -3.27896 | 0.016737 | 0.043323 | yes | down | 41.8 | 11.38 | 2.93 | 3.76 | 0.29 | 7.79 | 18.70333 | 3.946667 |
| LOC103348256 | 0.045 | -4.47289 | 0.016797 | 0.043444 | yes | down | 0.07 | 0.27 | 0.09 | 0.03 | 0 | 0 | 0.143333 | 0.01 |
| SDHAF4 | 0.5 | -1.0013 | 0.016869 | 0.043609 | yes | down | 11.92 | 4.86 | 7.69 | 12.67 | 9.43 | 7.15 | 8.156667 | 9.75 |
| CNTNAP2 | 0.307 | -1.70585 | 0.016908 | 0.043692 | yes | down | 0.12 | 0.2 | 0.08 | 0.05 | 0.09 | 0.14 | 0.133333 | 0.093333 |
| LOC100349763 | 24.035 | 4.587038 | 0.016983 | 0.043844 | yes | up | 0 | 0 | 0 | 0.4 | 0.18 | 0.35 | 0 | 0.31 |
| LOC103348008 | 2.16 | 1.111206 | 0.017222 | 0.044384 | yes | up | 1.09 | 0.71 | 1.17 | 4.65 | 5.4 | 4.81 | 0.99 | 4.953333 |
| UBXN11 | 0.452 | -1.1453 | 0.017285 | 0.044531 | yes | down | 1.15 | 2.5 | 2.18 | 1.65 | 2.24 | 2.49 | 1.943333 | 2.126667 |
| ALOX12B | 0.047 | -4.39706 | 0.017313 | 0.044598 | yes | down | 0.05 | 0.15 | 0.12 | 0 | 0 | 0.04 | 0.106667 | 0.013333 |
| ITGAX | 4.161 | 2.056951 | 0.0174 | 0.04478 | yes | up | 0.07 | 0.03 | 0.05 | 0.27 | 0.44 | 0.66 | 0.05 | 0.456667 |
| LOC108178504 | 0.031 | -4.99685 | 0.017503 | 0.044988 | yes | down | 0.1 | 0.09 | 0.11 | 0 | 0 | 0 | 0.1 | 0 |
| LOC103347750 | 25.051 | 4.646795 | 0.017562 | 0.045097 | yes | up | 0 | 0 | 0 | 0.71 | 3.48 | 2.7 | 0 | 2.296667 |
| SPTBN2 | 2.132 | 1.092346 | 0.017673 | 0.04534 | yes | up | 0.24 | 0.1 | 0.09 | 0.65 | 0.83 | 0.58 | 0.143333 | 0.686667 |
| A4GALT | 3.365 | 1.750762 | 0.017682 | 0.045349 | yes | up | 0.14 | 0.13 | 0.03 | 0.46 | 0.66 | 1.04 | 0.1 | 0.72 |
| LOC108176426 | 2.536 | 1.34246 | 0.017698 | 0.04537 | yes | up | 1.33 | 2.4 | 1.3 | 10.6 | 10.51 | 8.16 | 1.676667 | 9.756667 |
| NCAPG | 2.878 | 1.525171 | 0.017793 | 0.045578 | yes | up | 0.64 | 0.38 | 0.25 | 3.93 | 3.84 | 0.56 | 0.423333 | 2.776667 |
| CD8B | 15.53 | 3.956987 | 0.017925 | 0.045895 | yes | up | 0 | 0 | 0.1 | 1.08 | 0.98 | 1.24 | 0.033333 | 1.1 |
| TCF7 | 2.497 | 1.320451 | 0.017974 | 0.045997 | yes | up | 0.59 | 0.33 | 0.59 | 2.23 | 2.51 | 5.29 | 0.503333 | 3.343333 |
| LOC103352485 | 17.495 | 4.128882 | 0.017986 | 0.046016 | yes | up | 0 | 0.02 | 0 | 0.2 | 0.09 | 0.44 | 0.006667 | 0.243333 |
| LOC100342163 | 0.338 | -1.56288 | 0.018123 | 0.046316 | yes | down | 0.2 | 0.32 | 0.27 | 0.29 | 0.18 | 0.13 | 0.263333 | 0.2 |
| DNAH11 | 2.771 | 1.470162 | 0.018148 | 0.046372 | yes | up | 0.06 | 0.02 | 0.02 | 0.19 | 0.2 | 0.23 | 0.033333 | 0.206667 |
| SEC16B | 2.659 | 1.410979 | 0.018185 | 0.04646 | yes | up | 0.14 | 0.13 | 0.16 | 0.49 | 1.12 | 1.03 | 0.143333 | 0.88 |
| LOC100009134 | 0.054 | -4.21958 | 0.018225 | 0.046547 | yes | down | 0.25 | 3.57 | 0.85 | 0.51 | 0 | 0 | 1.556667 | 0.17 |
| CENPM | 2.43 | 1.280751 | 0.01839 | 0.046952 | yes | up | 1.61 | 1.01 | 0.9 | 8.58 | 11.38 | 2.22 | 1.173333 | 7.393333 |
| RAD51 | 2.602 | 1.379366 | 0.018456 | 0.047113 | yes | up | 1.39 | 1.1 | 0.45 | 7.26 | 8.48 | 2.09 | 0.98 | 5.943333 |
| LOC103349207 | 2.322 | 1.215184 | 0.018599 | 0.047413 | yes | up | 0.41 | 0.25 | 0.44 | 1.76 | 1.51 | 2.58 | 0.366667 | 1.95 |
| MEIG1 | 0.153 | -2.70732 | 0.018782 | 0.04779 | yes | down | 0.61 | 0.31 | 0.42 | 0.13 | 0.1 | 0.38 | 0.446667 | 0.203333 |
| LOC100353156 | 2.982 | 1.576243 | 0.018802 | 0.047828 | yes | up | 0.11 | 0.14 | 0.17 | 0.99 | 1.4 | 0.5 | 0.14 | 0.963333 |
| LOC100355380 | 4.468 | 2.159666 | 0.018829 | 0.047881 | yes | up | 0.07 | 0.06 | 0.15 | 1.43 | 0.56 | 0.89 | 0.093333 | 0.96 |
| ZNF804A | 0.032 | -4.98738 | 0.018879 | 0.047994 | yes | down | 0.03 | 0.05 | 0.05 | 0 | 0 | 0 | 0.043333 | 0 |
| MCF2L2 | 2.014 | 1.01038 | 0.018911 | 0.048067 | yes | up | 0.48 | 0.3 | 0.35 | 2.15 | 1.35 | 1.64 | 0.376667 | 1.713333 |
| CENPI | 2.191 | 1.131897 | 0.018993 | 0.048238 | yes | up | 0.52 | 0.59 | 0.24 | 2.27 | 3.18 | 1.21 | 0.45 | 2.22 |
| DKKL1 | 16.49 | 4.043488 | 0.019028 | 0.048306 | yes | up | 0 | 0.1 | 0 | 0.56 | 1.25 | 2.23 | 0.033333 | 1.346667 |
| MIS18A | 2.15 | 1.104141 | 0.019058 | 0.048366 | yes | up | 1.88 | 1.06 | 1 | 8.95 | 6.13 | 4.06 | 1.313333 | 6.38 |
| LOC108176529 | 2.107 | 1.075512 | 0.019088 | 0.048428 | yes | up | 0.44 | 0.61 | 0.6 | 3.14 | 3.02 | 1.88 | 0.55 | 2.68 |
| ABLIM2 | 0.245 | -2.03166 | 0.019097 | 0.048436 | yes | down | 11.01 | 2.9 | 1.89 | 0.42 | 2.52 | 5.09 | 5.266667 | 2.676667 |
| DNA2 | 3.81 | 1.929647 | 0.019126 | 0.048494 | yes | up | 0.05 | 0.07 | 0.05 | 0.69 | 0.68 | 0.16 | 0.056667 | 0.51 |
| CAMK1G | 0.153 | -2.7073 | 0.019141 | 0.048509 | yes | down | 0.18 | 0.1 | 0.27 | 0.12 | 0.08 | 0 | 0.183333 | 0.066667 |
| HAAO | 3.908 | 1.966291 | 0.019147 | 0.048518 | yes | up | 0.19 | 0.13 | 0.24 | 2.5 | 1.9 | 0.64 | 0.186667 | 1.68 |
| LOC108177715 | 17.15 | 4.100145 | 0.019271 | 0.048791 | yes | up | 0 | 0 | 0.09 | 0.53 | 0.82 | 2.55 | 0.03 | 1.3 |
| AGBL3 | 0.337 | -1.56959 | 0.019273 | 0.048791 | yes | down | 0.16 | 0.8 | 0.85 | 0.55 | 0.59 | 0.49 | 0.603333 | 0.543333 |
| LOC100356510 | 0.198 | -2.3333 | 0.019281 | 0.048803 | yes | down | 0.99 | 1.72 | 0.47 | 0.64 | 0 | 0.7 | 1.06 | 0.446667 |
| CENPF | 2.572 | 1.362824 | 0.019285 | 0.048803 | yes | up | 0.67 | 0.53 | 0.22 | 3.1 | 4.72 | 0.59 | 0.473333 | 2.803333 |
| LOC100355689 | 4.243 | 2.085058 | 0.019369 | 0.048997 | yes | up | 0.04 | 0.06 | 0.04 | 0.12 | 0.62 | 0.66 | 0.046667 | 0.466667 |
| LOC100346096 | 0.095 | -3.39644 | 0.019378 | 0.049005 | yes | down | 0.95 | 2.59 | 1.68 | 0.43 | 0.72 | 0 | 1.74 | 0.383333 |
| LOC103352191 | 2.805 | 1.488229 | 0.019411 | 0.04908 | yes | up | 0.09 | 0.07 | 0.04 | 0.47 | 0.51 | 0.3 | 0.066667 | 0.426667 |
| ARMC2 | 2.706 | 1.436026 | 0.019416 | 0.04908 | yes | up | 1.23 | 0.13 | 0.11 | 3.84 | 3.99 | 3.85 | 0.49 | 3.893333 |
| LOC108176880 | 3.064 | 1.615525 | 0.019416 | 0.04908 | yes | up | 0.27 | 0.42 | 0.47 | 2.46 | 4.02 | 2.85 | 0.386667 | 3.11 |
| CENPT | 3.039 | 1.603438 | 0.019607 | 0.049523 | yes | up | 0.58 | 0.42 | 0.23 | 3.67 | 4.15 | 0.79 | 0.41 | 2.87 |
| SPC25 | 2.776 | 1.472874 | 0.019671 | 0.049664 | yes | up | 1.14 | 0.49 | 0.51 | 5.57 | 5.76 | 2.19 | 0.713333 | 4.506667 |
| LOC100008885 | 0.047 | -4.40062 | 0.019688 | 0.049699 | yes | down | 0.36 | 0.13 | 0.07 | 0.05 | 0 | 0 | 0.186667 | 0.016667 |
| LOC100338677 | 0.022 | -5.48473 | 0.019713 | 0.049754 | yes | down | 0 | 0.99 | 1.32 | 0 | 0 | 0 | 0.77 | 0 |
| VWA3B | 0.026 | -5.25554 | 0.019759 | 0.049853 | yes | down | 0.15 | 0.01 | 0.01 | 0 | 0 | 0 | 0.056667 | 0 |
| LOC103349303 | 0.078 | -3.68576 | 0.019796 | 0.049931 | yes | down | 4.25 | 0.54 | 0.1 | 0.42 | 0.02 | 0.36 | 1.63 | 0.266667 |
| STAC2 | 6.089 | 2.606247 | 0.019802 | 0.049931 | yes | up | 0.1 | 0.04 | 0 | 0.52 | 0.24 | 1.02 | 0.046667 | 0.593333 |

iBAT_D14_VS_iBAT_M1

| Gene name | FC(iBAT_M1/iBAT_D14) | Log2FC(iBAT_M1/iBAT_D14) | Pvalue | Padjust | Significant | Regulate | D14iBAT1 | D14iBAT2 | D14iBAT3 | M1iBAT1 | M1iBAT2 | M1iBAT3 | iBAT_D14 | iBAT_M1 |
| --- | --- | --- | --- | --- | --- | --- | --- | --- | --- | --- | --- | --- | --- | --- |
| LOC108177974 | 29.397 | 4.877579 | 1.7E-177 | 2.6E-173 | yes | up | 56.72 | 55.02 | 54.18 | 2350.55 | 2144.07 | 3123.81 | 55.30667 | 2539.477 |
| PMEPA1 | 0.037 | -4.73729 | 3.64E-79 | 2.76E-75 | yes | down | 17.91 | 27.98 | 21.58 | 0.95 | 0.89 | 1.16 | 22.49 | 1 |
| LOC100352536 | 0.005 | -7.62612 | 7.74E-75 | 3.91E-71 | yes | down | 639.39 | 801.98 | 627.65 | 1.33 | 8.31 | 6.29 | 689.6733 | 5.31 |
| PPIF | 0.116 | -3.11153 | 1.32E-66 | 5E-63 | yes | down | 85.94 | 113.18 | 102.96 | 15.78 | 15.66 | 22.9 | 100.6933 | 18.11333 |
| IDH2 | 0.2 | -2.3225 | 1.26E-60 | 3.81E-57 | yes | down | 546.34 | 714.26 | 624.53 | 192.16 | 207.04 | 182.79 | 628.3767 | 193.9967 |
| KCNK3 | 0.042 | -4.57263 | 4.96E-53 | 1.25E-49 | yes | down | 35.85 | 76.05 | 46.92 | 2.68 | 4.37 | 3.04 | 52.94 | 3.363333 |
| TIMM8A | 0.166 | -2.58881 | 3.97E-52 | 8.61E-49 | yes | down | 31.12 | 38.54 | 38.9 | 9.17 | 9.17 | 9.52 | 36.18667 | 9.286667 |
| UCP1 | 0.032 | -4.94529 | 1.18E-48 | 2.24E-45 | yes | down | 1895.23 | 3480.95 | 2355.41 | 78.55 | 187.12 | 112.87 | 2577.197 | 126.18 |
| MMP11 | 10.05 | 3.329149 | 5.37E-46 | 9.05E-43 | yes | up | 1.35 | 2.37 | 2.08 | 29.12 | 26.25 | 34.83 | 1.933333 | 30.06667 |
| SEMA7A | 0.131 | -2.93679 | 1.89E-45 | 2.87E-42 | yes | down | 225.56 | 377.91 | 370.73 | 69.01 | 56.6 | 70.21 | 324.7333 | 65.27333 |
| PRELID3B | 0.175 | -2.51326 | 2.87E-45 | 3.95E-42 | yes | down | 121.54 | 135.07 | 140.22 | 45.89 | 36.8 | 30.65 | 132.2767 | 37.78 |
| LOC100352363 | 238.297 | 7.896619 | 3.15E-43 | 3.98E-40 | yes | up | 2.14 | 0.7 | 1.32 | 216.98 | 637.93 | 591.35 | 1.386667 | 482.0867 |
| GNAS | 0.12 | -3.06425 | 4.73E-43 | 5.52E-40 | yes | down | 4680.31 | 6057.31 | 5802.71 | 913.76 | 1261.56 | 826.04 | 5513.443 | 1000.453 |
| LOC100358417 | 0.113 | -3.1451 | 1.12E-39 | 1.22E-36 | yes | down | 12.39 | 19.85 | 12.86 | 3.02 | 2.6 | 2.3 | 15.03333 | 2.64 |
| KYAT3 | 0.236 | -2.08122 | 3.11E-39 | 3.14E-36 | yes | down | 91.33 | 112.15 | 108.52 | 35.75 | 42.42 | 35.93 | 104 | 38.03333 |
| LOC100341175 | 0.251 | -1.99303 | 2.21E-38 | 2.1E-35 | yes | down | 908.93 | 1017.78 | 846.44 | 321.05 | 395.14 | 359.13 | 924.3833 | 358.44 |
| G0S2 | 0.048 | -4.38009 | 5.2E-37 | 4.39E-34 | yes | down | 166.83 | 199.61 | 285.03 | 7.82 | 15.95 | 20.97 | 217.1567 | 14.91333 |
| OASL | 48.527 | 5.600716 | 5.21E-37 | 4.39E-34 | yes | up | 0.67 | 0.48 | 0.87 | 74.46 | 51.84 | 25.76 | 0.673333 | 50.68667 |
| ACSL1 | 0.185 | -2.43339 | 1.78E-35 | 1.42E-32 | yes | down | 255.54 | 338.39 | 372.77 | 86.08 | 99 | 80.53 | 322.2333 | 88.53667 |
| FAM84B | 20.984 | 4.391219 | 3.26E-34 | 2.47E-31 | yes | up | 0.84 | 0.65 | 0.69 | 13.41 | 20.11 | 36.79 | 0.726667 | 23.43667 |
| EGLN3 | 0.134 | -2.90111 | 2.86E-33 | 2.07E-30 | yes | down | 29.68 | 47.94 | 30.92 | 5.63 | 8.69 | 7.91 | 36.18 | 7.41 |
| ISCA1 | 0.361 | -1.47098 | 4.22E-33 | 2.91E-30 | yes | down | 102.49 | 125.65 | 116.34 | 58.47 | 62.83 | 71.16 | 114.8267 | 64.15333 |
| MED9 | 0.115 | -3.12396 | 1.11E-32 | 7.32E-30 | yes | down | 13.11 | 14.79 | 11.87 | 1.78 | 2.33 | 3.01 | 13.25667 | 2.373333 |
| PDE4D | 0.248 | -2.01258 | 1.66E-32 | 1.05E-29 | yes | down | 31.17 | 31.43 | 28.08 | 10.81 | 15.62 | 22.39 | 30.22667 | 16.27333 |
| AKT2 | 0.201 | -2.31485 | 4.26E-32 | 2.58E-29 | yes | down | 128.7 | 184.64 | 175.74 | 43.52 | 57.82 | 45.94 | 163.0267 | 49.09333 |
| TBRG4 | 0.285 | -1.80869 | 9.02E-31 | 5.26E-28 | yes | down | 43.65 | 62.78 | 51.3 | 20.33 | 23.68 | 24.62 | 52.57667 | 22.87667 |
| RTP4 | 10.822 | 3.435911 | 2.57E-30 | 1.44E-27 | yes | up | 5.34 | 6.82 | 4.12 | 111.12 | 105.79 | 66.51 | 5.426667 | 94.47333 |
| LOC100347175 | 0.314 | -1.66993 | 5.61E-30 | 3.04E-27 | yes | down | 62.78 | 81.42 | 67.81 | 30.8 | 36.05 | 35.89 | 70.67 | 34.24667 |
| ACAD9 | 0.198 | -2.3359 | 5.97E-30 | 3.12E-27 | yes | down | 16.46 | 28.43 | 19.58 | 6.85 | 6.82 | 6.86 | 21.49 | 6.843333 |
| KCTD7 | 6.436 | 2.686181 | 9.53E-30 | 4.82E-27 | yes | up | 2.04 | 1.62 | 1.91 | 20.38 | 19.8 | 16.25 | 1.856667 | 18.81 |
| HK1 | 0.242 | -2.04836 | 1.5E-29 | 7.35E-27 | yes | down | 43.4 | 73.26 | 57.24 | 21.79 | 21.69 | 21.08 | 57.96667 | 21.52 |
| GPT2 | 0.249 | -2.0044 | 1.95E-29 | 9.23E-27 | yes | down | 57.89 | 94.36 | 83.74 | 29.38 | 31.04 | 30.33 | 78.66333 | 30.25 |
| SDHD | 0.428 | -1.22281 | 2.58E-29 | 1.19E-26 | yes | down | 350.97 | 399.84 | 351.93 | 229.14 | 241.22 | 264.05 | 367.58 | 244.8033 |
| TRIM25 | 7.859 | 2.974347 | 4.25E-29 | 1.9E-26 | yes | up | 2.07 | 1.52 | 1.45 | 24.61 | 19.73 | 17.24 | 1.68 | 20.52667 |
| EXTL1 | 0.204 | -2.29315 | 6.02E-29 | 2.61E-26 | yes | down | 11.57 | 15.35 | 12.5 | 4.66 | 4.31 | 3.36 | 13.14 | 4.11 |
| ST6GALNAC6 | 3.121 | 1.642194 | 1.04E-28 | 4.38E-26 | yes | up | 9.91 | 12.69 | 10.49 | 48.13 | 51.06 | 61.19 | 11.03 | 53.46 |
| LOC100358333 | 7.118 | 2.831569 | 1.77E-28 | 7.17E-26 | yes | up | 4.45 | 3.29 | 2.57 | 21.35 | 31.78 | 21.36 | 3.436667 | 24.83 |
| MRPS12 | 0.239 | -2.06589 | 1.8E-28 | 7.17E-26 | yes | down | 51.6 | 51.87 | 62.98 | 21.17 | 21.22 | 20.64 | 55.48333 | 21.01 |
| TMEM140 | 5.185 | 2.374247 | 1.94E-28 | 7.54E-26 | yes | up | 21.72 | 18.25 | 21.08 | 133.07 | 174.9 | 187.83 | 20.35 | 165.2667 |
| ADORA1 | 0.057 | -4.12791 | 2.69E-28 | 1.02E-25 | yes | down | 6.65 | 12.44 | 16.74 | 0.99 | 1.08 | 0.85 | 11.94333 | 0.973333 |
| MRPL18 | 0.307 | -1.7042 | 4.67E-28 | 1.73E-25 | yes | down | 40.58 | 47.74 | 46.36 | 21.26 | 22.76 | 19.05 | 44.89333 | 21.02333 |
| LOC100356386 | 0.001 | -9.66648 | 8.85E-28 | 3.2E-25 | yes | down | 24.95 | 20.82 | 32.64 | 0.1 | 0.05 | 0 | 26.13667 | 0.05 |
| IRF9 | 9.108 | 3.18711 | 1.28E-27 | 4.51E-25 | yes | up | 3.35 | 3.17 | 4.39 | 65.61 | 52.24 | 36.54 | 3.636667 | 51.46333 |
| ADH2-2 | 7.863 | 2.975087 | 1.58E-27 | 5.46E-25 | yes | up | 1.3 | 2.54 | 2.29 | 20.15 | 25.3 | 28.87 | 2.043333 | 24.77333 |
| GOT1 | 0.236 | -2.08188 | 4.51E-27 | 1.52E-24 | yes | down | 65.44 | 101.13 | 72.82 | 24.1 | 31.38 | 31.33 | 79.79667 | 28.93667 |
| MRPL2 | 0.263 | -1.92835 | 8.12E-27 | 2.68E-24 | yes | down | 35.67 | 54.3 | 47 | 17.31 | 18.41 | 19.66 | 45.65667 | 18.46 |
| RMDN3 | 0.282 | -1.82464 | 1.36E-26 | 4.38E-24 | yes | down | 71.11 | 81.17 | 94.86 | 30.89 | 33.84 | 38.44 | 82.38 | 34.39 |
| TP53INP1 | 9.524 | 3.251516 | 2.15E-26 | 6.78E-24 | yes | up | 10.3 | 7.84 | 9.83 | 74.27 | 131.09 | 176.05 | 9.323333 | 127.1367 |
| ATXN7L2 | 0.125 | -3.00146 | 2.29E-26 | 7.08E-24 | yes | down | 4 | 7.74 | 6.23 | 2.12 | 2.5 | 2.33 | 5.99 | 2.316667 |
| PPTC7 | 0.304 | -1.71973 | 3.49E-26 | 1.06E-23 | yes | down | 16.37 | 17.29 | 16.38 | 8.56 | 7.87 | 7.06 | 16.68 | 7.83 |
| SMIM4 | 0.237 | -2.07968 | 5.43E-26 | 1.62E-23 | yes | down | 38.31 | 59.49 | 46.23 | 13.21 | 16.51 | 16.72 | 48.01 | 15.48 |
| KIFC3 | 0.166 | -2.58758 | 9.73E-26 | 2.84E-23 | yes | down | 5.88 | 9.11 | 9.48 | 2.38 | 1.73 | 1.92 | 8.156667 | 2.01 |
| RCOR2 | 0.123 | -3.02173 | 1.11E-25 | 3.18E-23 | yes | down | 11.33 | 11.58 | 16.62 | 2.21 | 2.77 | 2.58 | 13.17667 | 2.52 |
| COQ3 | 0.142 | -2.81645 | 1.29E-25 | 3.61E-23 | yes | down | 5.94 | 8.51 | 6.38 | 1.37 | 1.39 | 1.84 | 6.943333 | 1.533333 |
| LOC103345923 | 0.237 | -2.07569 | 2.6E-25 | 7.18E-23 | yes | down | 216 | 276.45 | 281.72 | 72.18 | 98.05 | 114.33 | 258.0567 | 94.85333 |
| LOC100356572 | 0.136 | -2.87998 | 5.08E-25 | 1.38E-22 | yes | down | 238.26 | 221.84 | 259.44 | 69.31 | 46.91 | 35.1 | 239.8467 | 50.44 |
| TMEM177 | 0.15 | -2.73281 | 7.77E-25 | 2.07E-22 | yes | down | 6.59 | 11.59 | 11.08 | 2.32 | 2.3 | 2.07 | 9.753333 | 2.23 |
| LOC100349792 | 0.331 | -1.59507 | 8.04E-25 | 2.1E-22 | yes | down | 35.93 | 48.98 | 45.56 | 20.42 | 21.14 | 25.29 | 43.49 | 22.28333 |
| LOC103350421 | 5.018 | 2.327118 | 9.3E-25 | 2.36E-22 | yes | up | 0.89 | 1.25 | 0.94 | 7.96 | 6.84 | 4.09 | 1.026667 | 6.296667 |
| ALOX15 | 0.02 | -5.63121 | 9.33E-25 | 2.36E-22 | yes | down | 11.63 | 29.94 | 37.81 | 1.58 | 0.24 | 0.67 | 26.46 | 0.83 |
| IRF4 | 33.539 | 5.067751 | 1.19E-24 | 2.95E-22 | yes | up | 0.77 | 0.37 | 0.25 | 11.74 | 18.56 | 39.75 | 0.463333 | 23.35 |
| FAM222A | 0.074 | -3.76082 | 1.52E-24 | 3.72E-22 | yes | down | 3.92 | 5.71 | 6.95 | 0.72 | 0.76 | 0.38 | 5.526667 | 0.62 |
| KIF20B | 0.169 | -2.56551 | 1.67E-24 | 4.03E-22 | yes | down | 5.94 | 8.36 | 7.2 | 1.42 | 1.79 | 1.32 | 7.166667 | 1.51 |
| LOC100346448 | 2.405 | 1.26614 | 2.41E-24 | 5.71E-22 | yes | up | 17.06 | 19.01 | 19.74 | 68.85 | 64.45 | 76.2 | 18.60333 | 69.83333 |
| WFS1 | 0.273 | -1.87363 | 2.98E-24 | 6.96E-22 | yes | down | 14.27 | 14.71 | 15.13 | 6.78 | 5.84 | 6.04 | 14.70333 | 6.22 |
| MB | 0.161 | -2.63486 | 4.72E-24 | 1.09E-21 | yes | down | 286.16 | 327.71 | 190.13 | 46.54 | 61.85 | 84.63 | 268 | 64.34 |
| FFAR2 | 0.091 | -3.45185 | 5.7E-24 | 1.29E-21 | yes | down | 23.26 | 37.7 | 38.34 | 2.71 | 4.35 | 7.45 | 33.1 | 4.836667 |
| INSIG1 | 5.937 | 2.569825 | 7.72E-24 | 1.72E-21 | yes | up | 11.15 | 10.66 | 7.64 | 83.29 | 73.52 | 135.47 | 9.816667 | 97.42667 |
| DNAJC11 | 0.322 | -1.6358 | 1.58E-23 | 3.48E-21 | yes | down | 34.12 | 49.68 | 48.84 | 22.44 | 20.58 | 22.9 | 44.21333 | 21.97333 |
| LOC100351672 | 6.55 | 2.711586 | 4.96E-23 | 1.08E-20 | yes | up | 7.13 | 7.14 | 9.52 | 59.44 | 70.4 | 114.91 | 7.93 | 81.58333 |
| LOC108176503 | 0.217 | -2.20119 | 5.61E-23 | 1.2E-20 | yes | down | 14.37 | 23.62 | 15.33 | 4.99 | 3.21 | 6.07 | 17.77333 | 4.756667 |
| SOD2 | 0.37 | -1.43319 | 7.26E-23 | 1.53E-20 | yes | down | 316.82 | 447.24 | 393.85 | 198.06 | 211.74 | 252.23 | 385.97 | 220.6767 |
| XPNPEP1 | 0.415 | -1.26856 | 1.08E-22 | 2.24E-20 | yes | down | 60.02 | 79.5 | 66.35 | 42.26 | 38.84 | 50.12 | 68.62333 | 43.74 |
| MRPL46 | 0.279 | -1.83943 | 1.1E-22 | 2.25E-20 | yes | down | 11.88 | 17.21 | 14.74 | 6.32 | 5.49 | 7.13 | 14.61 | 6.313333 |
| DLAT | 0.281 | -1.83351 | 1.35E-22 | 2.73E-20 | yes | down | 29.51 | 38.97 | 33.24 | 14.6 | 16.71 | 12.45 | 33.90667 | 14.58667 |
| IL6R | 6.834 | 2.772797 | 1.77E-22 | 3.53E-20 | yes | up | 0.49 | 0.56 | 0.66 | 7.5 | 4.55 | 5.94 | 0.57 | 5.996667 |
| C1H9orf153 | 0.24 | -2.06088 | 3.46E-22 | 6.81E-20 | yes | down | 5.02 | 6.5 | 6.03 | 2.16 | 1.77 | 2.6 | 5.85 | 2.176667 |
| PPP1R1B | 0.266 | -1.90977 | 4.6E-22 | 8.95E-20 | yes | down | 95.94 | 111.72 | 135.67 | 43.32 | 40.67 | 58.28 | 114.4433 | 47.42333 |
| TTC25 | 0.055 | -4.18781 | 6.02E-22 | 1.16E-19 | yes | down | 5.53 | 12.33 | 9.34 | 0.35 | 1.25 | 0.7 | 9.066667 | 0.766667 |
| LOC103346281 | 0.003 | -8.53646 | 6.34E-22 | 1.2E-19 | yes | down | 3.69 | 3.15 | 4.47 | 0 | 0.01 | 0.02 | 3.77 | 0.01 |
| DLST | 0.347 | -1.52579 | 6.96E-22 | 1.3E-19 | yes | down | 234.26 | 325.78 | 296.05 | 131.71 | 162.73 | 163.43 | 285.3633 | 152.6233 |
| TMEM120B | 0.198 | -2.33635 | 7.71E-22 | 1.43E-19 | yes | down | 16.16 | 26.1 | 23.31 | 6.83 | 6.54 | 6.29 | 21.85667 | 6.553333 |
| MX1 | 9.963 | 3.316597 | 8.36E-22 | 1.53E-19 | yes | up | 8.85 | 9.01 | 9.38 | 190.01 | 159.64 | 72.62 | 9.08 | 140.7567 |
| TIMM10 | 0.345 | -1.53586 | 9.5E-22 | 1.72E-19 | yes | down | 76.51 | 82.46 | 89.64 | 43.73 | 43.46 | 45.5 | 82.87 | 44.23 |
| YWHAG | 0.346 | -1.53236 | 9.84E-22 | 1.76E-19 | yes | down | 54.83 | 81.84 | 69.54 | 33.19 | 38.61 | 37.74 | 68.73667 | 36.51333 |
| TNN | 0.074 | -3.75587 | 1.16E-21 | 2.04E-19 | yes | down | 87.34 | 36.22 | 83.24 | 5.41 | 9.51 | 9.92 | 68.93333 | 8.28 |
| IQUB | 0.335 | -1.57678 | 1.66E-21 | 2.9E-19 | yes | down | 4.6 | 5.2 | 5.03 | 2.33 | 2.82 | 2.56 | 4.943333 | 2.57 |
| CEL | 0.029 | -5.12255 | 2.11E-21 | 3.63E-19 | yes | down | 5.36 | 3.81 | 3.51 | 0.26 | 0.25 | 0.04 | 4.226667 | 0.183333 |
| PTRF | 0.456 | -1.13344 | 2.96E-21 | 5.05E-19 | yes | down | 258.99 | 313.76 | 262.73 | 181.38 | 187.3 | 221.36 | 278.4933 | 196.68 |
| EHD3 | 0.19 | -2.39849 | 3.62E-21 | 6.1E-19 | yes | down | 15.09 | 31.5 | 29.94 | 7.15 | 7.3 | 8.14 | 25.51 | 7.53 |
| IFI44 | 11.148 | 3.478652 | 4.1E-21 | 6.84E-19 | yes | up | 2.02 | 1.89 | 1.33 | 40.45 | 32.56 | 16.82 | 1.746667 | 29.94333 |
| SESTD1 | 2.671 | 1.417461 | 4.45E-21 | 7.34E-19 | yes | up | 6.66 | 7.3 | 6.44 | 25.21 | 20.43 | 25.52 | 6.8 | 23.72 |
| PSMC3 | 0.39 | -1.35901 | 4.87E-21 | 7.95E-19 | yes | down | 90.39 | 123.75 | 112.89 | 61.39 | 60.12 | 75.36 | 109.01 | 65.62333 |
| GAB2 | 2.505 | 1.324598 | 5.7E-21 | 9.2E-19 | yes | up | 7.47 | 9.64 | 7.92 | 30.28 | 29.86 | 36.88 | 8.343333 | 32.34 |
| ACLY | 0.197 | -2.3461 | 7.16E-21 | 1.14E-18 | yes | down | 70.64 | 114.91 | 89.89 | 21.48 | 35.37 | 25.45 | 91.81333 | 27.43333 |
| AIFM1 | 0.327 | -1.61393 | 1.06E-20 | 1.67E-18 | yes | down | 74.93 | 101.67 | 86.88 | 39.62 | 50.41 | 42.18 | 87.82667 | 44.07 |
| FAM19A3 | 32.567 | 5.025343 | 1.31E-20 | 2.05E-18 | yes | up | 0.04 | 0.01 | 0.11 | 2.88 | 2.46 | 3.36 | 0.053333 | 2.9 |
| SNTB1 | 0.37 | -1.43297 | 1.67E-20 | 2.58E-18 | yes | down | 23.45 | 35.94 | 28.78 | 15.11 | 15.25 | 18.51 | 29.39 | 16.29 |
| LOC100328910 | 9.735 | 3.283144 | 1.73E-20 | 2.66E-18 | yes | up | 50.82 | 59.83 | 102.16 | 867.44 | 722.46 | 1669.67 | 70.93667 | 1086.523 |
| NDUFAB1 | 0.378 | -1.40321 | 2.03E-20 | 3.07E-18 | yes | down | 337.38 | 427.55 | 379.73 | 208.29 | 244.01 | 213.51 | 381.5533 | 221.9367 |
| N4BP2L1 | 4.24 | 2.083963 | 2.13E-20 | 3.21E-18 | yes | up | 4.31 | 3.94 | 5.64 | 28.8 | 21.38 | 30.58 | 4.63 | 26.92 |
| ITPK1 | 0.337 | -1.56999 | 2.33E-20 | 3.47E-18 | yes | down | 24 | 36.01 | 28.56 | 14.03 | 14.18 | 17.92 | 29.52333 | 15.37667 |
| SMOC1 | 0.322 | -1.63446 | 2.44E-20 | 3.57E-18 | yes | down | 22.89 | 37.82 | 31.78 | 15.82 | 16.33 | 18.31 | 30.83 | 16.82 |
| ABCC5 | 7.008 | 2.809086 | 2.44E-20 | 3.57E-18 | yes | up | 3.25 | 4.47 | 2.76 | 22.8 | 32.65 | 53.1 | 3.493333 | 36.18333 |
| SH3D19 | 3.447 | 1.78528 | 2.85E-20 | 4.11E-18 | yes | up | 5.79 | 4.67 | 5.21 | 26.57 | 23.61 | 32.67 | 5.223333 | 27.61667 |
| AHCYL1 | 0.412 | -1.27765 | 3.23E-20 | 4.63E-18 | yes | down | 233.97 | 278.01 | 295.83 | 159.79 | 177.38 | 176.59 | 269.27 | 171.2533 |
| HSPA9 | 0.351 | -1.50978 | 3.41E-20 | 4.84E-18 | yes | down | 116.42 | 179.63 | 142.23 | 76.76 | 83.13 | 75.95 | 146.0933 | 78.61333 |
| SLC46A3 | 8.612 | 3.10631 | 4.84E-20 | 6.79E-18 | yes | up | 0.48 | 0.35 | 0.44 | 4.94 | 5.17 | 7.39 | 0.423333 | 5.833333 |
| LOC103351504 | 0.178 | -2.48692 | 6.8E-20 | 9.47E-18 | yes | down | 27.19 | 14.73 | 20.97 | 3.24 | 4.58 | 4.71 | 20.96333 | 4.176667 |
| SEPT10 | 0.351 | -1.50844 | 1.04E-19 | 1.44E-17 | yes | down | 189.88 | 220.85 | 239.56 | 105.23 | 105.56 | 143.61 | 216.7633 | 118.1333 |
| NEDD4L | 0.231 | -2.11411 | 1.06E-19 | 1.44E-17 | yes | down | 4.63 | 7.26 | 8.14 | 2.26 | 2.48 | 2.28 | 6.676667 | 2.34 |
| EPRS | 0.375 | -1.41396 | 1.15E-19 | 1.56E-17 | yes | down | 24.78 | 40.35 | 30.56 | 18.97 | 17.03 | 16.8 | 31.89667 | 17.6 |
| DGAT1 | 0.366 | -1.45192 | 1.43E-19 | 1.91E-17 | yes | down | 49.65 | 74.55 | 65.19 | 31.96 | 33.89 | 38.3 | 63.13 | 34.71667 |
| ANTXR2 | 3.516 | 1.814013 | 2.47E-19 | 3.29E-17 | yes | up | 20.98 | 22.03 | 20.63 | 88.25 | 110.82 | 149.64 | 21.21333 | 116.2367 |
| DGAT2 | 0.185 | -2.43786 | 2.83E-19 | 3.73E-17 | yes | down | 652.41 | 1161.48 | 1320.29 | 249.03 | 317.05 | 373.26 | 1044.727 | 313.1133 |
| IDH3A | 0.162 | -2.62679 | 3.5E-19 | 4.58E-17 | yes | down | 273.99 | 431.96 | 282.62 | 52.49 | 109.1 | 80.41 | 329.5233 | 80.66667 |
| SRP9 | 0.437 | -1.19397 | 3.98E-19 | 5.17E-17 | yes | down | 33.24 | 37.64 | 36.37 | 24.49 | 21.96 | 26.32 | 35.75 | 24.25667 |
| HMGCS1 | 0.432 | -1.20988 | 4.98E-19 | 6.41E-17 | yes | down | 8.54 | 12.21 | 10.21 | 6.75 | 5.93 | 6.83 | 10.32 | 6.503333 |
| NDUFAF4 | 0.229 | -2.12438 | 5.26E-19 | 6.71E-17 | yes | down | 7.19 | 8.64 | 10.54 | 3.06 | 3.2 | 3.09 | 8.79 | 3.116667 |
| MRS2 | 0.397 | -1.33324 | 6.03E-19 | 7.62E-17 | yes | down | 31.64 | 38.38 | 40.47 | 21.78 | 19.44 | 22.72 | 36.83 | 21.31333 |
| UBA7 | 11.458 | 3.518284 | 6.54E-19 | 8.2E-17 | yes | up | 0.86 | 1.08 | 1.11 | 26.67 | 17.65 | 9.18 | 1.016667 | 17.83333 |
| PHB | 0.351 | -1.50889 | 7.11E-19 | 8.84E-17 | yes | down | 201.28 | 288.62 | 240.64 | 126.3 | 145.86 | 123.73 | 243.5133 | 131.9633 |
| CLUH | 0.27 | -1.88896 | 8.8E-19 | 1.09E-16 | yes | down | 48.6 | 79.35 | 70.57 | 24.77 | 32.12 | 25.11 | 66.17333 | 27.33333 |
| LOC103347528 | 0.285 | -1.81122 | 1.06E-18 | 1.3E-16 | yes | down | 30.04 | 44.2 | 42.63 | 17.07 | 17.69 | 16.39 | 38.95667 | 17.05 |
| KCNQ1 | 0.275 | -1.86452 | 1.08E-18 | 1.31E-16 | yes | down | 1.97 | 2.1 | 1.86 | 0.64 | 0.93 | 1.22 | 1.976667 | 0.93 |
| C7 | 5.561 | 2.475243 | 1.18E-18 | 1.42E-16 | yes | up | 2.99 | 4.89 | 2.99 | 44.4 | 22.93 | 28.41 | 3.623333 | 31.91333 |
| C4H20orf27 | 0.356 | -1.49094 | 1.99E-18 | 2.38E-16 | yes | down | 65.51 | 102.79 | 90.46 | 47.98 | 46 | 48.8 | 86.25333 | 47.59333 |
| BAG1 | 0.416 | -1.26504 | 2.08E-18 | 2.47E-16 | yes | down | 70.4 | 112.16 | 90.02 | 56.62 | 59.87 | 55.08 | 90.86 | 57.19 |
| LOC100328613 | 18.86 | 4.237263 | 2.3E-18 | 2.71E-16 | yes | up | 0.55 | 0.85 | 2.65 | 37.88 | 46.66 | 33.96 | 1.35 | 39.5 |
| HMGB3 | 0.175 | -2.51326 | 2.33E-18 | 2.72E-16 | yes | down | 48.18 | 81 | 85.36 | 23.22 | 18.5 | 11.32 | 71.51333 | 17.68 |
| MCAM | 0.264 | -1.92069 | 2.43E-18 | 2.82E-16 | yes | down | 127.99 | 218.3 | 187.52 | 50.99 | 69.33 | 83.89 | 177.9367 | 68.07 |
| SGIP1 | 5.974 | 2.578643 | 2.84E-18 | 3.26E-16 | yes | up | 0.29 | 0.41 | 0.29 | 2.97 | 2.94 | 3.3 | 0.33 | 3.07 |
| MDH1 | 0.325 | -1.62076 | 2.88E-18 | 3.29E-16 | yes | down | 428.34 | 614.43 | 493.01 | 204.48 | 252.55 | 312.1 | 511.9267 | 256.3767 |
| SLC25A10 | 0.338 | -1.56588 | 3.31E-18 | 3.75E-16 | yes | down | 26 | 25.84 | 25.09 | 13.15 | 11.49 | 15.14 | 25.64333 | 13.26 |
| MIEF2 | 0.233 | -2.10383 | 3.96E-18 | 4.45E-16 | yes | down | 7.06 | 10.2 | 8.21 | 2.39 | 2.75 | 3.73 | 8.49 | 2.956667 |
| HSD17B10 | 0.374 | -1.4205 | 6.28E-18 | 7E-16 | yes | down | 155.1 | 208.72 | 163.16 | 87.78 | 110.58 | 104.46 | 175.66 | 100.94 |
| NOTCH2 | 2.161 | 1.112007 | 6.63E-18 | 7.34E-16 | yes | up | 3.36 | 3.74 | 3.21 | 11.66 | 10.4 | 12.51 | 3.436667 | 11.52333 |
| IFI44L | 9.461 | 3.241974 | 6.91E-18 | 7.54E-16 | yes | up | 1.74 | 2.56 | 2.04 | 47.3 | 33.87 | 16.43 | 2.113333 | 32.53333 |
| MRPL41 | 0.264 | -1.92128 | 6.91E-18 | 7.54E-16 | yes | down | 81.35 | 92.82 | 61.37 | 30.16 | 27.7 | 38.83 | 78.51333 | 32.23 |
| ETFDH | 0.314 | -1.67134 | 1E-17 | 1.09E-15 | yes | down | 172.44 | 226.51 | 183.17 | 93.57 | 124.24 | 139.77 | 194.04 | 119.1933 |
| ZNF503 | 3.281 | 1.714038 | 1.02E-17 | 1.1E-15 | yes | up | 1.5 | 1.99 | 1.74 | 7.82 | 8.89 | 9.87 | 1.743333 | 8.86 |
| MXRA7 | 0.134 | -2.89748 | 1.22E-17 | 1.3E-15 | yes | down | 28.63 | 65.25 | 66.21 | 7.75 | 13.6 | 11.81 | 53.36333 | 11.05333 |
| SLC25A11 | 0.349 | -1.51925 | 1.44E-17 | 1.53E-15 | yes | down | 60.26 | 85.34 | 67.45 | 34.44 | 43 | 36.55 | 71.01667 | 37.99667 |
| ECHDC1 | 0.298 | -1.74898 | 1.71E-17 | 1.81E-15 | yes | down | 17.87 | 25.67 | 19.97 | 11.24 | 9.23 | 7.61 | 21.17 | 9.36 |
| TP53I11 | 0.345 | -1.53455 | 1.93E-17 | 2.02E-15 | yes | down | 27.81 | 35.55 | 32.7 | 13.71 | 18.44 | 19.02 | 32.02 | 17.05667 |
| LYPLA1 | 0.416 | -1.26638 | 2E-17 | 2.08E-15 | yes | down | 16.91 | 23.82 | 20.68 | 12.63 | 13.23 | 13.45 | 20.47 | 13.10333 |
| IARS2 | 0.402 | -1.31357 | 2.25E-17 | 2.32E-15 | yes | down | 26.5 | 35.64 | 33.11 | 20.93 | 18.25 | 22.13 | 31.75 | 20.43667 |
| LBP | 4.737 | 2.244079 | 2.28E-17 | 2.34E-15 | yes | up | 9.88 | 14.21 | 15.32 | 77.91 | 87.96 | 148.03 | 13.13667 | 104.6333 |
| ECSIT | 0.386 | -1.37244 | 2.42E-17 | 2.47E-15 | yes | down | 41.73 | 54.49 | 41.21 | 25.43 | 28.96 | 26.99 | 45.81 | 27.12667 |
| RFK | 0.287 | -1.79904 | 2.59E-17 | 2.62E-15 | yes | down | 16.9 | 16.8 | 23.23 | 9.01 | 7.42 | 9.08 | 18.97667 | 8.503333 |
| TSFM | 0.318 | -1.65472 | 3.5E-17 | 3.51E-15 | yes | down | 58.15 | 72.59 | 61.35 | 27.94 | 38.23 | 28.21 | 64.03 | 31.46 |
| PDZK1 | 0.098 | -3.35219 | 4.15E-17 | 4.14E-15 | yes | down | 3.34 | 6.31 | 8.21 | 1.21 | 0.87 | 0.7 | 5.953333 | 0.926667 |
| SLC4A5 | 13.452 | 3.749796 | 4.74E-17 | 4.67E-15 | yes | up | 0.14 | 0.09 | 0.05 | 2.06 | 1.6 | 2.04 | 0.093333 | 1.9 |
| GPRIN3 | 4.332 | 2.114998 | 5.39E-17 | 5.27E-15 | yes | up | 0.29 | 0.26 | 0.28 | 1.69 | 1.78 | 2.16 | 0.276667 | 1.876667 |
| TMEM37 | 0.102 | -3.29854 | 5.57E-17 | 5.42E-15 | yes | down | 50.59 | 75.19 | 90.72 | 4.93 | 13.38 | 15.79 | 72.16667 | 11.36667 |
| FEZ1 | 0.273 | -1.87466 | 5.82E-17 | 5.63E-15 | yes | down | 56.97 | 63.49 | 86.52 | 24.69 | 26.89 | 34.7 | 68.99333 | 28.76 |
| LOC100345217 | 0.001 | -10.2147 | 6.24E-17 | 5.99E-15 | yes | down | 27.47 | 35.66 | 24.13 | 0 | 0 | 0 | 29.08667 | 0 |
| HSPD1 | 0.384 | -1.38058 | 9.15E-17 | 8.73E-15 | yes | down | 166.71 | 220.74 | 196.04 | 118.88 | 124.77 | 100.73 | 194.4967 | 114.7933 |
| MC5R | 9.954 | 3.315232 | 9.94E-17 | 9.42E-15 | yes | up | 6.3 | 5.28 | 8.12 | 39.26 | 93.46 | 81.54 | 6.566667 | 71.42 |
| SAMM50 | 0.275 | -1.86162 | 1.1E-16 | 1.04E-14 | yes | down | 114.98 | 157.47 | 140.26 | 48.43 | 73.32 | 52.14 | 137.57 | 57.96333 |
| NDUFS3 | 0.331 | -1.5959 | 1.28E-16 | 1.2E-14 | yes | down | 386.55 | 538.05 | 439.78 | 189.45 | 271 | 232.34 | 454.7933 | 230.93 |
| ND5 | 0.409 | -1.28983 | 1.54E-16 | 1.44E-14 | yes | down | 65596.28 | 62584.24 | 67298.96 | 38445.16 | 42171.02 | 38570.36 | 65159.83 | 39728.85 |
| MYO19 | 0.263 | -1.92543 | 1.61E-16 | 1.49E-14 | yes | down | 11.52 | 17.33 | 15.93 | 5.81 | 7.19 | 5.08 | 14.92667 | 6.026667 |
| GPD2 | 0.435 | -1.20162 | 1.63E-16 | 1.5E-14 | yes | down | 140.08 | 166.79 | 168.88 | 108.05 | 116.56 | 100.19 | 158.5833 | 108.2667 |
| BPNT1 | 0.377 | -1.40864 | 1.7E-16 | 1.56E-14 | yes | down | 25.83 | 37.1 | 36.74 | 20.15 | 18.01 | 19.64 | 33.22333 | 19.26667 |
| ACOT11 | 0.221 | -2.17571 | 1.77E-16 | 1.61E-14 | yes | down | 13.57 | 24.4 | 24.39 | 5.48 | 7.92 | 6.66 | 20.78667 | 6.686667 |
| MARS2 | 0.122 | -3.04039 | 1.9E-16 | 1.71E-14 | yes | down | 2.27 | 5.76 | 5.23 | 0.85 | 0.83 | 0.77 | 4.42 | 0.816667 |
| GSN | 2.836 | 1.503853 | 1.98E-16 | 1.78E-14 | yes | up | 209.4 | 224.58 | 163.73 | 761.48 | 830.5 | 1043.04 | 199.2367 | 878.34 |
| INSR | 2.247 | 1.167771 | 2.32E-16 | 2.07E-14 | yes | up | 7.99 | 8.59 | 8.39 | 25.99 | 27.81 | 33.45 | 8.323333 | 29.08333 |
| HSPH1 | 0.315 | -1.66477 | 2.36E-16 | 2.09E-14 | yes | down | 12.99 | 17.82 | 16.19 | 9.36 | 6.72 | 6.9 | 15.66667 | 7.66 |
| LOC100350045 | 0.301 | -1.73084 | 2.37E-16 | 2.09E-14 | yes | down | 69.23 | 88.08 | 67.66 | 33.69 | 41.52 | 28.77 | 74.99 | 34.66 |
| SUPV3L1 | 0.416 | -1.26428 | 2.8E-16 | 2.46E-14 | yes | down | 14.19 | 19.24 | 16.5 | 10.62 | 10.83 | 10.94 | 16.64333 | 10.79667 |
| SLC25A13 | 0.417 | -1.26056 | 3.02E-16 | 2.63E-14 | yes | down | 20.02 | 25.78 | 20.86 | 12.45 | 14.84 | 14.98 | 22.22 | 14.09 |
| TNFAIP3 | 4.896 | 2.291509 | 3.06E-16 | 2.65E-14 | yes | up | 5.61 | 5.46 | 2.88 | 39.99 | 30.37 | 36.22 | 4.65 | 35.52667 |
| DHRS11 | 0.178 | -2.49196 | 4.01E-16 | 3.46E-14 | yes | down | 161.46 | 230.9 | 194.89 | 58.33 | 68.56 | 31.66 | 195.75 | 52.85 |
| TUBB4A | 0.345 | -1.53342 | 4.18E-16 | 3.59E-14 | yes | down | 30.23 | 47.65 | 41.99 | 22.64 | 20.64 | 19.42 | 39.95667 | 20.9 |
| EZH1 | 2.443 | 1.288603 | 4.41E-16 | 3.76E-14 | yes | up | 11.12 | 10.01 | 11.39 | 39.77 | 37.05 | 46.15 | 10.84 | 40.99 |
| LOC100344545 | 0.4 | -1.32279 | 5.36E-16 | 4.54E-14 | yes | down | 22.57 | 25.52 | 21.96 | 14.53 | 12.36 | 16.65 | 23.35 | 14.51333 |
| SLC22A3 | 0.2 | -2.32362 | 5.67E-16 | 4.78E-14 | yes | down | 18.57 | 32.96 | 24.31 | 4.89 | 7.22 | 10.25 | 25.28 | 7.453333 |
| GPX1 | 2.3 | 1.201388 | 8.39E-16 | 7.03E-14 | yes | up | 197.83 | 257.1 | 191.66 | 821.89 | 659.12 | 815.21 | 215.53 | 765.4067 |
| ID3 | 0.315 | -1.66442 | 9.19E-16 | 7.66E-14 | yes | down | 315.64 | 313.72 | 247.18 | 114.38 | 137.78 | 179.38 | 292.18 | 143.8467 |
| FGF10 | 3.123 | 1.643001 | 9.29E-16 | 7.71E-14 | yes | up | 1.79 | 2.13 | 1.7 | 10.05 | 7.34 | 9.91 | 1.873333 | 9.1 |
| IGFBP5 | 3.808 | 1.928948 | 1.05E-15 | 8.63E-14 | yes | up | 21.17 | 36.86 | 25.7 | 131.31 | 140.38 | 219.82 | 27.91 | 163.8367 |
| LOC100357567 | 0.35 | -1.51626 | 1.07E-15 | 8.76E-14 | yes | down | 27.03 | 41.89 | 32.61 | 19.99 | 17.59 | 16.82 | 33.84333 | 18.13333 |
| LOC100356413 | 0.425 | -1.2333 | 1.11E-15 | 9.04E-14 | yes | down | 45.83 | 66.36 | 56.2 | 37.02 | 34.85 | 35.07 | 56.13 | 35.64667 |
| APLN | 0.048 | -4.38098 | 1.18E-15 | 9.55E-14 | yes | down | 1.56 | 7.76 | 5.46 | 0.47 | 0.34 | 0.25 | 4.926667 | 0.353333 |
| CITED2 | 3.278 | 1.712935 | 1.3E-15 | 1.05E-13 | yes | up | 14.57 | 15.41 | 11.07 | 56.33 | 72.61 | 80.07 | 13.68333 | 69.67 |
| GTPBP2 | 2.273 | 1.184379 | 1.69E-15 | 1.35E-13 | yes | up | 13.85 | 13.96 | 13.86 | 45.1 | 51.31 | 50.78 | 13.89 | 49.06333 |
| CRAT | 0.306 | -1.70862 | 1.87E-15 | 1.49E-13 | yes | down | 97.25 | 133.74 | 102.71 | 38 | 59.52 | 60.68 | 111.2333 | 52.73333 |
| TRIB3 | 0.145 | -2.78184 | 2.07E-15 | 1.64E-13 | yes | down | 4.17 | 7.31 | 9.17 | 1.38 | 1.41 | 1.86 | 6.883333 | 1.55 |
| EDAR | 0.051 | -4.29462 | 2.33E-15 | 1.84E-13 | yes | down | 1.77 | 1.32 | 1.5 | 0.05 | 0.11 | 0.22 | 1.53 | 0.126667 |
| HIGD2A | 0.403 | -1.30949 | 2.4E-15 | 1.89E-13 | yes | down | 62.88 | 78.36 | 69.55 | 43.08 | 45.5 | 42.32 | 70.26333 | 43.63333 |
| GALK1 | 0.258 | -1.95568 | 2.58E-15 | 2.02E-13 | yes | down | 16.13 | 25.34 | 17.19 | 8.57 | 5.74 | 9.03 | 19.55333 | 7.78 |
| IDH3B | 0.406 | -1.30202 | 2.7E-15 | 2.1E-13 | yes | down | 377.88 | 558.6 | 494.99 | 260.29 | 302.59 | 354.89 | 477.1567 | 305.9233 |
| MITF | 3.173 | 1.665811 | 2.98E-15 | 2.31E-13 | yes | up | 2.69 | 2.07 | 2.51 | 11.41 | 12.27 | 12.73 | 2.423333 | 12.13667 |
| ACAD10 | 0.343 | -1.54575 | 3.25E-15 | 2.5E-13 | yes | down | 16.31 | 21.23 | 20.07 | 9.32 | 11.98 | 9.05 | 19.20333 | 10.11667 |
| PRKAR2B | 0.363 | -1.46279 | 3.46E-15 | 2.66E-13 | yes | down | 247.78 | 348.71 | 348.88 | 145.61 | 180.27 | 206.46 | 315.1233 | 177.4467 |
| GPD1 | 0.215 | -2.2191 | 4.15E-15 | 3.16E-13 | yes | down | 344.3 | 492.62 | 600.79 | 102.29 | 205.82 | 158.5 | 479.2367 | 155.5367 |
| VWA8 | 0.473 | -1.0799 | 5.43E-15 | 4.12E-13 | yes | down | 9.72 | 11.57 | 10.21 | 8.78 | 7.08 | 8.23 | 10.5 | 8.03 |
| MFN2 | 0.425 | -1.23544 | 5.51E-15 | 4.16E-13 | yes | down | 56.31 | 74.35 | 75.49 | 44.08 | 51.23 | 39.8 | 68.71667 | 45.03667 |
| TOMM40L | 0.353 | -1.50307 | 5.89E-15 | 4.43E-13 | yes | down | 17.19 | 22.23 | 24.74 | 11.74 | 11.8 | 10.55 | 21.38667 | 11.36333 |
| CA8 | 0.142 | -2.81566 | 7.12E-15 | 5.32E-13 | yes | down | 7.62 | 11.85 | 7.35 | 2.57 | 1.31 | 1.97 | 8.94 | 1.95 |
| GGT6 | 0.159 | -2.65353 | 7.3E-15 | 5.43E-13 | yes | down | 3.88 | 3.98 | 3.33 | 0.95 | 0.5 | 0.94 | 3.73 | 0.796667 |
| UBAP1L | 0.094 | -3.41226 | 9.29E-15 | 6.85E-13 | yes | down | 2.5 | 3.41 | 4.08 | 0.34 | 0.53 | 0.57 | 3.33 | 0.48 |
| ATG4D | 0.353 | -1.50258 | 9.39E-15 | 6.89E-13 | yes | down | 11.72 | 16.14 | 12.85 | 7.3 | 7.63 | 7.12 | 13.57 | 7.35 |
| HLX | 2.581 | 1.36808 | 9.84E-15 | 7.18E-13 | yes | up | 3.4 | 4.34 | 4.34 | 15.94 | 16.08 | 16.15 | 4.026667 | 16.05667 |
| LOC103349764 | 0.329 | -1.60564 | 1.12E-14 | 8.16E-13 | yes | down | 44.9 | 54.56 | 51.42 | 19.03 | 12.32 | 18.55 | 50.29333 | 16.63333 |
| LOC100338849 | 0.215 | -2.21455 | 1.15E-14 | 8.34E-13 | yes | down | 7.23 | 5.86 | 6.68 | 2.45 | 2.11 | 2.04 | 6.59 | 2.2 |
| SLIT3 | 2.36 | 1.238889 | 1.34E-14 | 9.67E-13 | yes | up | 3.62 | 3.62 | 4.37 | 13.92 | 13.42 | 15.33 | 3.87 | 14.22333 |
| SESN3 | 2.612 | 1.385004 | 1.36E-14 | 9.73E-13 | yes | up | 5.6 | 5.17 | 4.53 | 18.63 | 21.7 | 22.34 | 5.1 | 20.89 |
| STARD7 | 0.47 | -1.09004 | 1.59E-14 | 1.13E-12 | yes | down | 56.97 | 71.24 | 73.28 | 49.13 | 42.46 | 54.26 | 67.16333 | 48.61667 |
| LOC108176200 | 0.058 | -4.10216 | 1.65E-14 | 1.17E-12 | yes | down | 1.79 | 2.13 | 1.77 | 0.22 | 0.05 | 0.26 | 1.896667 | 0.176667 |
| EI24 | 0.392 | -1.35142 | 2.37E-14 | 1.67E-12 | yes | down | 32.39 | 49.67 | 45.53 | 24.14 | 23.36 | 29.92 | 42.53 | 25.80667 |
| PHOSPHO1 | 0.126 | -2.99111 | 2.5E-14 | 1.75E-12 | yes | down | 19.96 | 36.67 | 21.15 | 4.02 | 7.39 | 2.31 | 25.92667 | 4.573333 |
| C7H2orf88 | 0.158 | -2.65993 | 2.71E-14 | 1.9E-12 | yes | down | 10.96 | 23.96 | 28.84 | 4.56 | 3.59 | 6.31 | 21.25333 | 4.82 |
| SDHC | 0.44 | -1.18567 | 2.8E-14 | 1.95E-12 | yes | down | 254.65 | 331.4 | 278.98 | 174.63 | 217.03 | 193.47 | 288.3433 | 195.0433 |
| CHCHD4 | 0.336 | -1.57542 | 2.81E-14 | 1.95E-12 | yes | down | 14.19 | 23.02 | 16.72 | 9.21 | 7.14 | 9.59 | 17.97667 | 8.646667 |
| HIGD1A | 0.233 | -2.10022 | 2.91E-14 | 2E-12 | yes | down | 35.51 | 83.08 | 45.17 | 19.83 | 18.21 | 19.05 | 54.58667 | 19.03 |
| CBX7 | 2.892 | 1.531822 | 3.33E-14 | 2.29E-12 | yes | up | 3.27 | 3.12 | 3.6 | 13.41 | 13.66 | 18.3 | 3.33 | 15.12333 |
| LOC108176024 | 0.261 | -1.9405 | 3.39E-14 | 2.32E-12 | yes | down | 18.64 | 21.06 | 29.82 | 8.38 | 10.22 | 8.91 | 23.17333 | 9.17 |
| FBXL3 | 2.471 | 1.305365 | 3.59E-14 | 2.44E-12 | yes | up | 6.43 | 5.94 | 5.96 | 23.37 | 20.49 | 26.83 | 6.11 | 23.56333 |
| MRPS5 | 0.386 | -1.37396 | 3.63E-14 | 2.46E-12 | yes | down | 19.46 | 29.4 | 24.47 | 13.13 | 13.75 | 16.84 | 24.44333 | 14.57333 |
| LOC100352100 | 0.451 | -1.14723 | 3.71E-14 | 2.5E-12 | yes | down | 80.12 | 106.28 | 88.23 | 57.82 | 66.13 | 67.16 | 91.54333 | 63.70333 |
| ACSS3 | 2.015 | 1.01053 | 3.83E-14 | 2.57E-12 | yes | up | 8 | 9.26 | 8.09 | 26.18 | 26.38 | 25.89 | 8.45 | 26.15 |
| PPARA | 0.337 | -1.57117 | 3.98E-14 | 2.66E-12 | yes | down | 20.13 | 23.51 | 25.47 | 9.24 | 14.03 | 12.61 | 23.03667 | 11.96 |
| LHX8 | 4.352 | 2.1218 | 4.09E-14 | 2.72E-12 | yes | up | 3.53 | 3.18 | 1.6 | 20.45 | 14.77 | 17.05 | 2.77 | 17.42333 |
| ABR | 2.387 | 1.255174 | 4.41E-14 | 2.92E-12 | yes | up | 5.63 | 6.15 | 5.79 | 24.37 | 20.44 | 24.1 | 5.856667 | 22.97 |
| HRASLS5 | 6.036 | 2.593475 | 4.56E-14 | 3.01E-12 | yes | up | 1.02 | 1.54 | 2.06 | 14.61 | 15.37 | 25.72 | 1.54 | 18.56667 |
| PLXNB3 | 0.239 | -2.06279 | 5.19E-14 | 3.41E-12 | yes | down | 2.32 | 2.24 | 2.31 | 0.59 | 0.92 | 1.05 | 2.29 | 0.853333 |
| ACO2 | 0.314 | -1.6692 | 5.23E-14 | 3.42E-12 | yes | down | 900.28 | 1276.14 | 1044.6 | 509.99 | 629.48 | 407.37 | 1073.673 | 515.6133 |
| ND6 | 0.167 | -2.58008 | 5.25E-14 | 3.42E-12 | yes | down | 9677.91 | 7210.54 | 8334.84 | 968.47 | 2443.26 | 2982.3 | 8407.763 | 2131.343 |
| HACD2 | 0.363 | -1.46121 | 5.49E-14 | 3.56E-12 | yes | down | 44.66 | 62.55 | 72.44 | 34.99 | 38.35 | 37.96 | 59.88333 | 37.1 |
| MRPL35 | 0.355 | -1.49282 | 6.42E-14 | 4.14E-12 | yes | down | 67.46 | 92.18 | 77.07 | 35.59 | 47.99 | 47.47 | 78.90333 | 43.68333 |
| LOC100345657 | 31.392 | 4.972331 | 6.47E-14 | 4.16E-12 | yes | up | 0.08 | 0.15 | 0.12 | 11.75 | 3.15 | 1.64 | 0.116667 | 5.513333 |
| NDUFAF1 | 0.433 | -1.20845 | 7.16E-14 | 4.59E-12 | yes | down | 22.76 | 31.43 | 26.97 | 15.25 | 16.3 | 20.1 | 27.05333 | 17.21667 |
| COQ5 | 0.431 | -1.21293 | 7.44E-14 | 4.74E-12 | yes | down | 27.37 | 40.35 | 38.19 | 19.33 | 19.88 | 19.63 | 35.30333 | 19.61333 |
| FAM210A | 0.395 | -1.34065 | 7.65E-14 | 4.86E-12 | yes | down | 11.68 | 16.7 | 14.94 | 7.59 | 9.65 | 9.43 | 14.44 | 8.89 |
| SRPX | 3.044 | 1.605865 | 8.28E-14 | 5.23E-12 | yes | up | 6.89 | 10.68 | 8.08 | 34.51 | 35.66 | 50.56 | 8.55 | 40.24333 |
| COX2 | 0.36 | -1.47304 | 8.36E-14 | 5.26E-12 | yes | down | 70879.59 | 55173.47 | 66291.15 | 31781.27 | 37955.82 | 38572.65 | 64114.74 | 36103.25 |
| PDPR | 0.356 | -1.48849 | 8.39E-14 | 5.26E-12 | yes | down | 20.49 | 29.13 | 25.96 | 16.32 | 13.13 | 12.47 | 25.19333 | 13.97333 |
| TFRC | 0.088 | -3.50328 | 9.62E-14 | 6.01E-12 | yes | down | 35.71 | 110.65 | 39.99 | 13.86 | 5.28 | 5.46 | 62.11667 | 8.2 |
| OPA1 | 0.463 | -1.11241 | 9.77E-14 | 6.07E-12 | yes | down | 10.06 | 11.01 | 12.12 | 8.33 | 7.31 | 7.21 | 11.06333 | 7.616667 |
| ATP5F1 | 0.466 | -1.10027 | 1.03E-13 | 6.35E-12 | yes | down | 388.38 | 562.19 | 458.45 | 296.61 | 330.37 | 378.63 | 469.6733 | 335.2033 |
| SAR1B | 0.468 | -1.09413 | 1.05E-13 | 6.51E-12 | yes | down | 23.56 | 25.77 | 26.76 | 16.41 | 19.78 | 20.52 | 25.36333 | 18.90333 |
| BDH1 | 0.385 | -1.37567 | 1.06E-13 | 6.53E-12 | yes | down | 87.58 | 120.21 | 86.39 | 52.48 | 67.38 | 68.45 | 98.06 | 62.77 |
| TMEM159 | 6.167 | 2.624616 | 1.19E-13 | 7.3E-12 | yes | up | 30.46 | 17.22 | 19.33 | 202.51 | 135.36 | 322.83 | 22.33667 | 220.2333 |
| LOC100356626 | 3.481 | 1.799372 | 1.2E-13 | 7.3E-12 | yes | up | 1.71 | 1.47 | 1.42 | 6.19 | 9.21 | 9.64 | 1.533333 | 8.346667 |
| ATP1A1 | 0.419 | -1.25538 | 1.59E-13 | 9.64E-12 | yes | down | 141.64 | 224.13 | 186.78 | 106.88 | 124.32 | 125.12 | 184.1833 | 118.7733 |
| PLEKHF1 | 5.363 | 2.422955 | 1.66E-13 | 1E-11 | yes | up | 9.07 | 11.94 | 5.72 | 50.42 | 66.99 | 104.9 | 8.91 | 74.10333 |
| PDHB | 0.386 | -1.37261 | 1.67E-13 | 1E-11 | yes | down | 165.91 | 242.37 | 206.68 | 107.94 | 140.36 | 115.67 | 204.9867 | 121.3233 |
| SLC39A13 | 0.296 | -1.75605 | 1.87E-13 | 1.12E-11 | yes | down | 20.23 | 37.96 | 28.82 | 14.85 | 13.49 | 11.33 | 29.00333 | 13.22333 |
| LOC103347403 | 18.417 | 4.202989 | 2.04E-13 | 1.21E-11 | yes | up | 0.59 | 0.21 | 0.2 | 14.16 | 10.91 | 5.31 | 0.333333 | 10.12667 |
| OXNAD1 | 0.33 | -1.59825 | 2.16E-13 | 1.27E-11 | yes | down | 22.66 | 26.35 | 32.26 | 13.45 | 15.27 | 12.05 | 27.09 | 13.59 |
| SP1 | 2.168 | 1.116296 | 2.19E-13 | 1.29E-11 | yes | up | 5.46 | 6.33 | 5.28 | 16.96 | 18.2 | 22.02 | 5.69 | 19.06 |
| MGEA5 | 2.141 | 1.098604 | 2.3E-13 | 1.35E-11 | yes | up | 14.83 | 15.45 | 16.46 | 52.39 | 44.61 | 61.02 | 15.58 | 52.67333 |
| TIMM17A | 0.441 | -1.18247 | 2.38E-13 | 1.39E-11 | yes | down | 76.25 | 98.6 | 76.75 | 52.36 | 50.45 | 68.54 | 83.86667 | 57.11667 |
| AARS2 | 0.38 | -1.39625 | 2.43E-13 | 1.41E-11 | yes | down | 4.77 | 6.06 | 5.54 | 2.99 | 3.39 | 3 | 5.456667 | 3.126667 |
| CRLS1 | 0.301 | -1.73379 | 2.46E-13 | 1.42E-11 | yes | down | 91.2 | 120.63 | 123.55 | 36.47 | 59.34 | 58.29 | 111.7933 | 51.36667 |
| MRPL39 | 0.385 | -1.37629 | 2.46E-13 | 1.42E-11 | yes | down | 85.25 | 119.17 | 99.71 | 50.24 | 68.94 | 60.32 | 101.3767 | 59.83333 |
| MRPS7 | 0.429 | -1.22237 | 2.54E-13 | 1.46E-11 | yes | down | 58.63 | 75.42 | 60.5 | 37.93 | 47.59 | 39.29 | 64.85 | 41.60333 |
| HDHD2 | 0.43 | -1.21602 | 2.63E-13 | 1.5E-11 | yes | down | 29.08 | 31.95 | 31.7 | 14.5 | 20.48 | 19.15 | 30.91 | 18.04333 |
| ADSL | 0.369 | -1.43649 | 3.02E-13 | 1.72E-11 | yes | down | 18.47 | 31.23 | 24.51 | 14.26 | 13.85 | 12.28 | 24.73667 | 13.46333 |
| LRPPRC | 0.417 | -1.26151 | 3.31E-13 | 1.87E-11 | yes | down | 30.55 | 48.71 | 37.29 | 23.71 | 27.81 | 26.84 | 38.85 | 26.12 |
| LOC103345217 | 0.048 | -4.37386 | 3.78E-13 | 2.13E-11 | yes | down | 4.62 | 4.49 | 6.6 | 0.42 | 0.62 | 0.09 | 5.236667 | 0.376667 |
| MRPL1 | 0.444 | -1.17177 | 3.87E-13 | 2.18E-11 | yes | down | 16.78 | 23.37 | 21.75 | 13.91 | 13.77 | 14.36 | 20.63333 | 14.01333 |
| NFYC | 0.437 | -1.19497 | 4.3E-13 | 2.4E-11 | yes | down | 23.5 | 30.59 | 26.67 | 20.95 | 16.23 | 18.26 | 26.92 | 18.48 |
| SPON1 | 2.358 | 1.237705 | 4.43E-13 | 2.46E-11 | yes | up | 8.95 | 10.63 | 9.22 | 31.79 | 29.92 | 43.95 | 9.6 | 35.22 |
| ATG14 | 3.861 | 1.948835 | 4.53E-13 | 2.51E-11 | yes | up | 1.64 | 1.32 | 2 | 7.98 | 10.13 | 11.93 | 1.653333 | 10.01333 |
| UMPS | 0.239 | -2.06378 | 4.6E-13 | 2.54E-11 | yes | down | 9.62 | 13.83 | 16.57 | 6.15 | 3.79 | 4.73 | 13.34 | 4.89 |
| MMACHC | 0.217 | -2.20193 | 4.69E-13 | 2.58E-11 | yes | down | 11.85 | 14 | 21.39 | 4.56 | 5.61 | 5.77 | 15.74667 | 5.313333 |
| CHAC1 | 0.042 | -4.56009 | 4.81E-13 | 2.63E-11 | yes | down | 1.9 | 0.96 | 1.56 | 0.06 | 0.13 | 0.11 | 1.473333 | 0.1 |
| ABCB8 | 0.271 | -1.88344 | 5.35E-13 | 2.92E-11 | yes | down | 3.06 | 5.55 | 5.09 | 1.93 | 2.03 | 1.72 | 4.566667 | 1.893333 |
| ISG15 | 15.362 | 3.941323 | 5.41E-13 | 2.94E-11 | yes | up | 3.84 | 3.19 | 3.3 | 124.51 | 94.23 | 23.48 | 3.443333 | 80.74 |
| SDC2 | 2.954 | 1.562426 | 5.43E-13 | 2.94E-11 | yes | up | 13.23 | 13.19 | 13.19 | 44.6 | 63.04 | 74.83 | 13.20333 | 60.82333 |
| ALDH5A1 | 0.463 | -1.11193 | 5.75E-13 | 3.11E-11 | yes | down | 30.43 | 38.97 | 36.1 | 23.7 | 26.74 | 28.93 | 35.16667 | 26.45667 |
| C12H6orf47 | 0.374 | -1.4177 | 6.09E-13 | 3.27E-11 | yes | down | 14.99 | 20.28 | 22.55 | 10.77 | 11.26 | 11.39 | 19.27333 | 11.14 |
| MRPL51 | 0.412 | -1.27883 | 6.11E-13 | 3.27E-11 | yes | down | 60.71 | 85.04 | 68.37 | 47.93 | 43.68 | 42.24 | 71.37333 | 44.61667 |
| SNX33 | 3.531 | 1.819969 | 6.76E-13 | 3.6E-11 | yes | up | 2.2 | 2.88 | 3.41 | 13.4 | 18.89 | 13.83 | 2.83 | 15.37333 |
| NTRK2 | 2.637 | 1.398773 | 6.77E-13 | 3.6E-11 | yes | up | 1.4 | 1.75 | 2.07 | 6.39 | 7.14 | 7.74 | 1.74 | 7.09 |
| NDUFC2 | 0.382 | -1.38659 | 7.03E-13 | 3.71E-11 | yes | down | 432.31 | 615.72 | 510.31 | 264.4 | 357.74 | 290.86 | 519.4467 | 304.3333 |
| NDUFA9 | 0.367 | -1.44757 | 7.42E-13 | 3.89E-11 | yes | down | 94.49 | 150.6 | 125.24 | 62.02 | 80.35 | 65.22 | 123.4433 | 69.19667 |
| PRDX3 | 0.466 | -1.10289 | 7.65E-13 | 4E-11 | yes | down | 238.71 | 313 | 266.37 | 175.95 | 209.08 | 189.38 | 272.6933 | 191.47 |
| VDR | 8.268 | 3.047591 | 8.38E-13 | 4.37E-11 | yes | up | 0.97 | 1.34 | 1.27 | 8.24 | 17.72 | 18.81 | 1.193333 | 14.92333 |
| DCPS | 508.754 | 8.990824 | 1.01E-12 | 5.25E-11 | yes | up | 0 | 0 | 0 | 9.69 | 8.15 | 5.47 | 0 | 7.77 |
| GAB1 | 2.392 | 1.258109 | 1.01E-12 | 5.25E-11 | yes | up | 5.6 | 7.21 | 5.25 | 18.55 | 22.06 | 24.58 | 6.02 | 21.73 |
| PRPF19 | 0.497 | -1.00911 | 1.11E-12 | 5.72E-11 | yes | down | 24.79 | 28.95 | 25.04 | 21.45 | 17.55 | 21.68 | 26.26 | 20.22667 |
| PTGES2 | 0.271 | -1.88249 | 1.12E-12 | 5.76E-11 | yes | down | 48.52 | 78.64 | 60.05 | 25.45 | 31.9 | 19.52 | 62.40333 | 25.62333 |
| LOC100008606 | 14.973 | 3.904273 | 1.14E-12 | 5.85E-11 | yes | up | 5.69 | 6 | 4.87 | 228.7 | 87.14 | 36.6 | 5.52 | 117.48 |
| ATP5B | 0.392 | -1.35244 | 1.17E-12 | 6E-11 | yes | down | 2483.27 | 3047.96 | 2409.3 | 1442.05 | 1902.88 | 1432.62 | 2646.843 | 1592.517 |
| YEATS4 | 0.342 | -1.54961 | 1.23E-12 | 6.26E-11 | yes | down | 10.11 | 13.69 | 10.67 | 7.84 | 6.01 | 6.72 | 11.49 | 6.856667 |
| C14H3orf58 | 4.475 | 2.161753 | 1.27E-12 | 6.45E-11 | yes | up | 1.32 | 1.16 | 1.2 | 7.1 | 6.28 | 12.64 | 1.226667 | 8.673333 |
| PERM1 | 0.08 | -3.64343 | 1.29E-12 | 6.51E-11 | yes | down | 7.65 | 9.56 | 6.36 | 0.47 | 1.88 | 0.51 | 7.856667 | 0.953333 |
| PDSS1 | 0.317 | -1.6592 | 1.31E-12 | 6.62E-11 | yes | down | 6.63 | 9.98 | 7.52 | 4 | 3.73 | 3.84 | 8.043333 | 3.856667 |
| WDR19 | 3.122 | 1.642576 | 1.32E-12 | 6.65E-11 | yes | up | 1.86 | 1.51 | 1.62 | 9.38 | 7.35 | 7.61 | 1.663333 | 8.113333 |
| LOC100342996 | 0.49 | -1.0287 | 1.39E-12 | 6.95E-11 | yes | down | 773.4 | 1029.47 | 833.01 | 589.04 | 696.86 | 701.94 | 878.6267 | 662.6133 |
| USP18 | 13.288 | 3.732081 | 1.77E-12 | 8.86E-11 | yes | up | 0.42 | 0.47 | 0.55 | 13.7 | 10.11 | 3.67 | 0.48 | 9.16 |
| ZNF423 | 0.358 | -1.48108 | 1.78E-12 | 8.86E-11 | yes | down | 5.7 | 8.68 | 6.99 | 3.51 | 4.5 | 3.71 | 7.123333 | 3.906667 |
| ACAA2 | 0.387 | -1.36793 | 1.79E-12 | 8.9E-11 | yes | down | 1437.23 | 1792.79 | 1516.86 | 732.92 | 1104.79 | 997.28 | 1582.293 | 944.9967 |
| PARP8 | 2.823 | 1.497062 | 1.84E-12 | 9.07E-11 | yes | up | 1.02 | 1.34 | 1.22 | 4.55 | 5.99 | 4.98 | 1.193333 | 5.173333 |
| NDUFA5 | 0.424 | -1.23739 | 1.94E-12 | 9.58E-11 | yes | down | 129.23 | 156 | 137.69 | 95.82 | 107.15 | 89.75 | 140.9733 | 97.57333 |
| ISOC2 | 0.481 | -1.05447 | 1.99E-12 | 9.78E-11 | yes | down | 62.21 | 70.3 | 59.03 | 42.41 | 47.92 | 51.75 | 63.84667 | 47.36 |
| CRYL1 | 0.392 | -1.35119 | 2.12E-12 | 1.04E-10 | yes | down | 41 | 62.11 | 56.2 | 30.14 | 35.48 | 30.07 | 53.10333 | 31.89667 |
| MRPL47 | 0.465 | -1.10357 | 2.23E-12 | 1.09E-10 | yes | down | 38.38 | 45.6 | 46.46 | 28.74 | 31.98 | 31.96 | 43.48 | 30.89333 |
| MIGA2 | 0.28 | -1.8388 | 2.26E-12 | 1.1E-10 | yes | down | 28.07 | 33.2 | 46.3 | 12.89 | 20.27 | 17.64 | 35.85667 | 16.93333 |
| MIEF1 | 0.405 | -1.30469 | 2.45E-12 | 1.19E-10 | yes | down | 18.96 | 30.41 | 27.71 | 14.89 | 16.11 | 16.68 | 25.69333 | 15.89333 |
| SEMA4B | 4.336 | 2.116517 | 2.46E-12 | 1.19E-10 | yes | up | 6.2 | 3.91 | 3.86 | 27.62 | 25.45 | 42.95 | 4.656667 | 32.00667 |
| MRPS36 | 0.429 | -1.21968 | 2.47E-12 | 1.19E-10 | yes | down | 56.33 | 73.76 | 64.86 | 35.37 | 44.89 | 49.46 | 64.98333 | 43.24 |
| SNCA | 0.372 | -1.42643 | 2.69E-12 | 1.29E-10 | yes | down | 31.17 | 30.67 | 26.82 | 15.59 | 12.81 | 20.26 | 29.55333 | 16.22 |
| AOX3 | 5.339 | 2.416695 | 2.71E-12 | 1.3E-10 | yes | up | 1.81 | 1.08 | 1.16 | 7.22 | 15.04 | 14.74 | 1.35 | 12.33333 |
| ACSF2 | 0.346 | -1.53297 | 2.8E-12 | 1.34E-10 | yes | down | 88.64 | 98.12 | 126.72 | 44.25 | 58.81 | 66.59 | 104.4933 | 56.55 |
| SLC19A1 | 0.135 | -2.89264 | 2.82E-12 | 1.34E-10 | yes | down | 3.74 | 5.33 | 7.75 | 0.96 | 0.7 | 1.91 | 5.606667 | 1.19 |
| CAD | 0.372 | -1.42766 | 3.15E-12 | 1.49E-10 | yes | down | 3.06 | 2.87 | 3.23 | 1.88 | 1.72 | 1.57 | 3.053333 | 1.723333 |
| KIAA0100 | 0.476 | -1.07129 | 3.19E-12 | 1.51E-10 | yes | down | 24.96 | 31.95 | 29.78 | 23.18 | 20.1 | 19.07 | 28.89667 | 20.78333 |
| INMT | 9.493 | 3.246834 | 3.29E-12 | 1.55E-10 | yes | up | 0.62 | 0.47 | 1.06 | 13.29 | 5.48 | 13.6 | 0.716667 | 10.79 |
| NDUFA12 | 0.426 | -1.23043 | 3.36E-12 | 1.58E-10 | yes | down | 276.75 | 365.12 | 291.45 | 177.41 | 229.79 | 206.2 | 311.1067 | 204.4667 |
| PLSCR4 | 3.264 | 1.706738 | 3.97E-12 | 1.84E-10 | yes | up | 5 | 6.38 | 5.99 | 25.01 | 20.09 | 38.04 | 5.79 | 27.71333 |
| CCL21 | 2.943 | 1.557118 | 4.36E-12 | 2E-10 | yes | up | 11.3 | 18.73 | 10.94 | 58.63 | 60.49 | 65.59 | 13.65667 | 61.57 |
| DLD | 0.395 | -1.3388 | 4.55E-12 | 2.09E-10 | yes | down | 145.49 | 184.4 | 179.45 | 92.02 | 123.81 | 93.65 | 169.78 | 103.16 |
| NDUFA10 | 0.385 | -1.37715 | 5.32E-12 | 2.43E-10 | yes | down | 324.7 | 387.97 | 313.79 | 177.27 | 246.92 | 183.18 | 342.1533 | 202.4567 |
| LOC103345082 | 0.188 | -2.41069 | 5.64E-12 | 2.57E-10 | yes | down | 8.36 | 15.33 | 10.73 | 3.92 | 2.92 | 1.99 | 11.47333 | 2.943333 |
| DNAJA3 | 0.473 | -1.08028 | 6.09E-12 | 2.76E-10 | yes | down | 36.49 | 49.38 | 44.32 | 27.59 | 33.68 | 33.79 | 43.39667 | 31.68667 |
| RNF115 | 0.376 | -1.41073 | 6.1E-12 | 2.76E-10 | yes | down | 84.68 | 101.35 | 93.13 | 39.17 | 58.65 | 65.53 | 93.05333 | 54.45 |
| ADCY10 | 4.57 | 2.192332 | 6.19E-12 | 2.8E-10 | yes | up | 0.6 | 0.4 | 0.53 | 3.24 | 3.14 | 5.12 | 0.51 | 3.833333 |
| PRMT1 | 0.469 | -1.09223 | 6.42E-12 | 2.89E-10 | yes | down | 92.65 | 134.2 | 104.96 | 70.93 | 83.18 | 85.64 | 110.6033 | 79.91667 |
| NDUFB3 | 0.446 | -1.16532 | 6.67E-12 | 2.99E-10 | yes | down | 195.3 | 233.41 | 204.42 | 126.45 | 164.47 | 143.78 | 211.0433 | 144.9 |
| EFEMP1 | 2.078 | 1.055021 | 6.88E-12 | 3.08E-10 | yes | up | 30.7 | 41.6 | 30.13 | 118.08 | 97.91 | 112.42 | 34.14333 | 109.47 |
| PDSS2 | 0.401 | -1.31998 | 6.99E-12 | 3.12E-10 | yes | down | 7.2 | 9.32 | 8.38 | 4.72 | 5.56 | 5.09 | 8.3 | 5.123333 |
| CADPS2 | 0.396 | -1.33702 | 7.41E-12 | 3.3E-10 | yes | down | 6.13 | 6.6 | 6.62 | 3.71 | 4.54 | 3.59 | 6.45 | 3.946667 |
| CIAPIN1 | 0.412 | -1.27983 | 7.57E-12 | 3.35E-10 | yes | down | 33.04 | 44.48 | 40.7 | 20.63 | 23.98 | 30.86 | 39.40667 | 25.15667 |
| MTFP1 | 0.247 | -2.01846 | 7.83E-12 | 3.45E-10 | yes | down | 18.45 | 26.02 | 26.48 | 9.49 | 11 | 6.06 | 23.65 | 8.85 |
| NDUFV2 | 0.441 | -1.18199 | 7.93E-12 | 3.49E-10 | yes | down | 176.7 | 237.97 | 187.18 | 112.44 | 146.97 | 150.09 | 200.6167 | 136.5 |
| ENO1 | 0.424 | -1.23921 | 8.03E-12 | 3.51E-10 | yes | down | 269.69 | 380.38 | 353.93 | 203.98 | 248.23 | 200.18 | 334.6667 | 217.4633 |
| MTCH2 | 0.382 | -1.3888 | 8.18E-12 | 3.57E-10 | yes | down | 194.45 | 271.13 | 206.79 | 103.31 | 152.26 | 142.82 | 224.1233 | 132.7967 |
| SCTR | 0.072 | -3.80047 | 8.77E-12 | 3.81E-10 | yes | down | 5.01 | 14.84 | 22.01 | 0.57 | 2.76 | 2.81 | 13.95333 | 2.046667 |
| TIMM44 | 0.333 | -1.58669 | 9.34E-12 | 4.05E-10 | yes | down | 46.94 | 67.8 | 47.35 | 23.03 | 34.33 | 25.11 | 54.03 | 27.49 |
| COX3 | 0.453 | -1.1438 | 1.11E-11 | 4.77E-10 | yes | down | 67862.42 | 63526.96 | 71579.65 | 46936.04 | 51062.66 | 44483.44 | 67656.34 | 47494.05 |
| PTX3 | 37.136 | 5.214734 | 1.18E-11 | 5.06E-10 | yes | up | 5.48 | 2.81 | 1.82 | 182.48 | 55.43 | 388.45 | 3.37 | 208.7867 |
| MPP2 | 0.227 | -2.1399 | 1.21E-11 | 5.18E-10 | yes | down | 5.76 | 7.64 | 12.42 | 2.57 | 3.2 | 3.44 | 8.606667 | 3.07 |
| CISD1 | 0.404 | -1.30918 | 1.25E-11 | 5.32E-10 | yes | down | 85.6 | 94.03 | 112.2 | 52.33 | 67.62 | 62.4 | 97.27667 | 60.78333 |
| LOC100353245 | 5.076 | 2.343766 | 1.32E-11 | 5.61E-10 | yes | up | 97.82 | 90.51 | 82.5 | 1020.11 | 696.89 | 393.51 | 90.27667 | 703.5033 |
| AKAP1 | 0.441 | -1.18132 | 1.4E-11 | 5.92E-10 | yes | down | 30.43 | 47.33 | 41.13 | 22.09 | 28.07 | 28.55 | 39.63 | 26.23667 |
| FAH | 0.373 | -1.42196 | 1.49E-11 | 6.29E-10 | yes | down | 52.71 | 74.14 | 66.74 | 28.33 | 38.84 | 44.29 | 64.53 | 37.15333 |
| LOC100346985 | 21.83 | 4.44826 | 1.55E-11 | 6.52E-10 | yes | up | 0.56 | 0.47 | 0.64 | 43.43 | 8.05 | 6.31 | 0.556667 | 19.26333 |
| YPEL3 | 2.681 | 1.422643 | 1.67E-11 | 7.02E-10 | yes | up | 31.14 | 25.92 | 27.86 | 96.7 | 98.29 | 137.87 | 28.30667 | 110.9533 |
| CYTB | 0.347 | -1.52558 | 1.78E-11 | 7.4E-10 | yes | down | 19576.98 | 17131.39 | 20389.01 | 8584.96 | 12681.94 | 9498.33 | 19032.46 | 10255.08 |
| ABCA8 | 2.356 | 1.236107 | 1.78E-11 | 7.4E-10 | yes | up | 12.42 | 10.62 | 13.76 | 43.22 | 41.81 | 49.98 | 12.26667 | 45.00333 |
| GMPR | 0.263 | -1.92519 | 1.83E-11 | 7.58E-10 | yes | down | 93.83 | 206.85 | 106.37 | 41.97 | 58.43 | 60.55 | 135.6833 | 53.65 |
| UBE2L6 | 9.362 | 3.226829 | 2.12E-11 | 8.76E-10 | yes | up | 1.89 | 1.84 | 2.21 | 52.09 | 19.6 | 14.26 | 1.98 | 28.65 |
| LOC103348748 | 0.411 | -1.28268 | 2.14E-11 | 8.81E-10 | yes | down | 18.25 | 21.58 | 16 | 11.19 | 10.93 | 13.44 | 18.61 | 11.85333 |
| GLTSCR2 | 2.1 | 1.070131 | 2.17E-11 | 8.88E-10 | yes | up | 17.81 | 23.32 | 20.34 | 60.55 | 62.26 | 77.12 | 20.49 | 66.64333 |
| MTMR14 | 2.174 | 1.120179 | 2.37E-11 | 9.7E-10 | yes | up | 8.84 | 8.56 | 7.68 | 23.52 | 24.26 | 27.3 | 8.36 | 25.02667 |
| LARP4 | 0.441 | -1.18032 | 2.43E-11 | 9.9E-10 | yes | down | 12.38 | 16.75 | 19.8 | 11.76 | 14.02 | 11.57 | 16.31 | 12.45 |
| PHB2 | 0.416 | -1.2646 | 2.59E-11 | 1.05E-09 | yes | down | 143.17 | 219.5 | 180 | 97.41 | 107.48 | 143.86 | 180.89 | 116.25 |
| BMP4 | 3.511 | 1.811843 | 2.59E-11 | 1.05E-09 | yes | up | 6.08 | 4.26 | 4.06 | 23.48 | 24.69 | 31.12 | 4.8 | 26.43 |
| PM20D2 | 3.642 | 1.864569 | 2.63E-11 | 1.06E-09 | yes | up | 9.43 | 6.47 | 6.62 | 30.95 | 49.03 | 45.6 | 7.506667 | 41.86 |
| ANG | 6.014 | 2.588369 | 2.65E-11 | 1.07E-09 | yes | up | 4.58 | 4.25 | 6.02 | 65.45 | 26.54 | 46.9 | 4.95 | 46.29667 |
| C1H11orf54 | 0.354 | -1.4999 | 2.72E-11 | 1.09E-09 | yes | down | 17.21 | 26.8 | 24.16 | 9.66 | 13.26 | 14.39 | 22.72333 | 12.43667 |
| TXNDC5 | 0.448 | -1.15927 | 2.73E-11 | 1.09E-09 | yes | down | 41.13 | 51.72 | 60.33 | 37.18 | 36.15 | 34.75 | 51.06 | 36.02667 |
| BEND7 | 3.2 | 1.677957 | 2.94E-11 | 1.18E-09 | yes | up | 3.59 | 4.07 | 5.42 | 16.88 | 17.1 | 27.66 | 4.36 | 20.54667 |
| MRPL43 | 0.462 | -1.11479 | 3.47E-11 | 1.38E-09 | yes | down | 20.47 | 26.4 | 24.41 | 16.29 | 16.33 | 18.29 | 23.76 | 16.97 |
| ANXA6 | 0.482 | -1.05174 | 3.48E-11 | 1.39E-09 | yes | down | 90.52 | 133.7 | 113.78 | 76.65 | 89.46 | 84.35 | 112.6667 | 83.48667 |
| PDE7A | 2.766 | 1.467979 | 3.54E-11 | 1.4E-09 | yes | up | 5.72 | 5.42 | 5.52 | 19.2 | 25.41 | 31.19 | 5.553333 | 25.26667 |
| IRF7 | 42.083 | 5.395177 | 3.54E-11 | 1.4E-09 | yes | up | 2.36 | 1.83 | 1.82 | 273.4 | 70.67 | 47.95 | 2.003333 | 130.6733 |
| LOC100345038 | 2.98 | 1.575309 | 3.55E-11 | 1.4E-09 | yes | up | 1.26 | 1.89 | 2.04 | 7.98 | 7.81 | 8.97 | 1.73 | 8.253333 |
| TXN2 | 0.436 | -1.19846 | 3.65E-11 | 1.44E-09 | yes | down | 88.13 | 103.52 | 81.76 | 55.92 | 64.48 | 82.51 | 91.13667 | 67.63667 |
| FAM13A | 3.629 | 1.859446 | 3.72E-11 | 1.46E-09 | yes | up | 58.85 | 34.03 | 47.17 | 214.49 | 286.24 | 341.86 | 46.68333 | 280.8633 |
| VARS | 0.462 | -1.11271 | 3.76E-11 | 1.47E-09 | yes | down | 5.46 | 7.52 | 6.08 | 4.56 | 4.52 | 4.48 | 6.353333 | 4.52 |
| ND3 | 0.45 | -1.15056 | 3.77E-11 | 1.47E-09 | yes | down | 30865.46 | 26765.75 | 27132.97 | 20418.57 | 20002.31 | 18339.04 | 28254.73 | 19586.64 |
| LOC100357099 | 2.527 | 1.337518 | 3.86E-11 | 1.51E-09 | yes | up | 0.8 | 0.81 | 0.78 | 3.25 | 3.01 | 3.1 | 0.796667 | 3.12 |
| DRAP1 | 0.46 | -1.11988 | 3.94E-11 | 1.53E-09 | yes | down | 50.4 | 65.21 | 55.33 | 35.29 | 40.42 | 45.96 | 56.98 | 40.55667 |
| COPS4 | 0.461 | -1.11656 | 3.97E-11 | 1.54E-09 | yes | down | 35.68 | 55.9 | 41.12 | 29.63 | 30.59 | 33.55 | 44.23333 | 31.25667 |
| APOO | 0.447 | -1.16015 | 4.36E-11 | 1.69E-09 | yes | down | 34.89 | 50.38 | 39.34 | 25.81 | 30.82 | 29.91 | 41.53667 | 28.84667 |
| CABLES1 | 2.9 | 1.535841 | 4.47E-11 | 1.73E-09 | yes | up | 4.45 | 4.36 | 4.49 | 15.56 | 13.91 | 24.2 | 4.433333 | 17.89 |
| TIMM50 | 0.39 | -1.3592 | 4.72E-11 | 1.81E-09 | yes | down | 31.69 | 53.32 | 40.45 | 22.37 | 27.03 | 25.38 | 41.82 | 24.92667 |
| GPR88 | 6.456 | 2.690684 | 4.79E-11 | 1.83E-09 | yes | up | 0.28 | 0.18 | 0.22 | 1.81 | 1.86 | 3.18 | 0.226667 | 2.283333 |
| LOC100355134 | 0.203 | -2.30113 | 4.83E-11 | 1.84E-09 | yes | down | 2.42 | 5.14 | 4.23 | 1.31 | 1.08 | 1.26 | 3.93 | 1.216667 |
| FST | 3.773 | 1.915658 | 5.16E-11 | 1.97E-09 | yes | up | 5.78 | 5.17 | 4.79 | 25.15 | 27.2 | 45.17 | 5.246667 | 32.50667 |
| LOC100345202 | 0.172 | -2.54086 | 5.22E-11 | 1.99E-09 | yes | down | 6.19 | 7 | 12.31 | 1.64 | 2.53 | 2.69 | 8.5 | 2.286667 |
| RGS3 | 3.542 | 1.824693 | 5.34E-11 | 2.03E-09 | yes | up | 45.32 | 49.74 | 50.73 | 207.5 | 266.68 | 489.64 | 48.59667 | 321.2733 |
| CLMP | 0.482 | -1.05141 | 5.51E-11 | 2.08E-09 | yes | down | 34.14 | 36.96 | 40.97 | 29.63 | 28.04 | 36.32 | 37.35667 | 31.33 |
| SOWAHC | 0.247 | -2.01819 | 5.77E-11 | 2.17E-09 | yes | down | 15.54 | 21.29 | 32.79 | 7.09 | 7.62 | 11.64 | 23.20667 | 8.783333 |
| HSD11B1 | 0.307 | -1.70214 | 6.08E-11 | 2.28E-09 | yes | down | 20.62 | 18.51 | 24.86 | 13.22 | 8.1 | 9.33 | 21.33 | 10.21667 |
| NMNAT3 | 0.28 | -1.83897 | 6.5E-11 | 2.44E-09 | yes | down | 25.82 | 26.73 | 29.11 | 7.86 | 15.54 | 11.67 | 27.22 | 11.69 |
| ULK1 | 3.186 | 1.671712 | 6.68E-11 | 2.49E-09 | yes | up | 7.9 | 6.13 | 8.07 | 27.71 | 39.31 | 43.58 | 7.366667 | 36.86667 |
| RASGRP3 | 2.868 | 1.520129 | 6.68E-11 | 2.49E-09 | yes | up | 5.96 | 4.6 | 3.9 | 21.46 | 24.1 | 29.76 | 4.82 | 25.10667 |
| ST8SIA5 | 0.187 | -2.41624 | 6.86E-11 | 2.55E-09 | yes | down | 2.45 | 3.76 | 4.22 | 0.52 | 1.18 | 1.32 | 3.476667 | 1.006667 |
| MLANA | 7.535 | 2.913572 | 6.99E-11 | 2.59E-09 | yes | up | 0.98 | 0.8 | 1.6 | 13.05 | 12.98 | 16.68 | 1.126667 | 14.23667 |
| ADAMTS16 | 7.724 | 2.949432 | 7.28E-11 | 2.69E-09 | yes | up | 0.18 | 0.14 | 0.19 | 2.21 | 2.58 | 1.31 | 0.17 | 2.033333 |
| SLC40A1 | 2.692 | 1.428866 | 7.42E-11 | 2.73E-09 | yes | up | 18.56 | 17.83 | 14.44 | 56.87 | 67.52 | 91.56 | 16.94333 | 71.98333 |
| DDB2 | 2.316 | 1.211517 | 7.5E-11 | 2.74E-09 | yes | up | 4.79 | 4.97 | 5.75 | 15.66 | 18 | 24.33 | 5.17 | 19.33 |
| ELAC2 | 0.394 | -1.34279 | 7.51E-11 | 2.74E-09 | yes | down | 10.47 | 14.4 | 13.4 | 6.65 | 8.85 | 8.16 | 12.75667 | 7.886667 |
| MTX1 | 0.412 | -1.27997 | 7.88E-11 | 2.87E-09 | yes | down | 30.32 | 43.88 | 36.13 | 20.34 | 26.9 | 22.41 | 36.77667 | 23.21667 |
| AK2 | 0.359 | -1.47873 | 8.5E-11 | 3.09E-09 | yes | down | 212.89 | 230.42 | 309.33 | 156.94 | 151 | 120.3 | 250.88 | 142.7467 |
| KIAA1462 | 0.489 | -1.03218 | 8.92E-11 | 3.23E-09 | yes | down | 21.57 | 22.24 | 17.36 | 14.64 | 13.94 | 17.02 | 20.39 | 15.2 |
| NUPR1 | 3.239 | 1.695622 | 9.95E-11 | 3.59E-09 | yes | up | 14.72 | 19.83 | 17.21 | 66.57 | 71.99 | 122.89 | 17.25333 | 87.15 |
| DPF3 | 3.229 | 1.691284 | 1.01E-10 | 3.65E-09 | yes | up | 0.64 | 0.63 | 0.57 | 2.91 | 3.4 | 2.77 | 0.613333 | 3.026667 |
| ACP6 | 0.237 | -2.07866 | 1.03E-10 | 3.7E-09 | yes | down | 6.55 | 8.21 | 7.86 | 2.36 | 3.79 | 2.02 | 7.54 | 2.723333 |
| GYPC | 2.545 | 1.347399 | 1.03E-10 | 3.7E-09 | yes | up | 22.04 | 21.21 | 19.05 | 66.12 | 77.96 | 103.58 | 20.76667 | 82.55333 |
| ATP5A1 | 0.413 | -1.27626 | 1.08E-10 | 3.87E-09 | yes | down | 1280.97 | 1701.01 | 1445.72 | 762.33 | 1130.58 | 914.94 | 1475.9 | 935.95 |
| LOC100356833 | 0.327 | -1.61068 | 1.09E-10 | 3.87E-09 | yes | down | 7.58 | 9.39 | 6.4 | 4.76 | 3.86 | 3.12 | 7.79 | 3.913333 |
| FAM126A | 3.067 | 1.617039 | 1.09E-10 | 3.87E-09 | yes | up | 1.99 | 1.45 | 1.43 | 5.27 | 7.62 | 8.48 | 1.623333 | 7.123333 |
| DACT1 | 2.907 | 1.539437 | 1.09E-10 | 3.87E-09 | yes | up | 0.89 | 1.69 | 1.29 | 5.56 | 5.55 | 7.22 | 1.29 | 6.11 |
| KIAA0430 | 2.291 | 1.196 | 1.14E-10 | 4.03E-09 | yes | up | 2.94 | 2.66 | 3.36 | 11.13 | 10.02 | 11.04 | 2.986667 | 10.73 |
| LOC100348091 | 0.324 | -1.62757 | 1.27E-10 | 4.44E-09 | yes | down | 7.58 | 11.03 | 11.94 | 5.27 | 4.21 | 6.35 | 10.18333 | 5.276667 |
| AEBP1 | 2.073 | 1.051473 | 1.27E-10 | 4.44E-09 | yes | up | 10.22 | 15.77 | 14.22 | 44.32 | 40.98 | 44.5 | 13.40333 | 43.26667 |
| ARMC6 | 0.227 | -2.13645 | 1.27E-10 | 4.45E-09 | yes | down | 2.93 | 5.21 | 4.66 | 1.6 | 1.65 | 1.36 | 4.266667 | 1.536667 |
| CKMT2 | 0.239 | -2.06491 | 1.43E-10 | 4.97E-09 | yes | down | 61.52 | 89.48 | 28.57 | 15.96 | 33.31 | 21.91 | 59.85667 | 23.72667 |
| PRPSAP2 | 2.713 | 1.439767 | 1.51E-10 | 5.24E-09 | yes | up | 3.32 | 3.2 | 3.34 | 11.55 | 14.47 | 15.8 | 3.286667 | 13.94 |
| C18H10orf2 | 0.361 | -1.47182 | 1.52E-10 | 5.26E-09 | yes | down | 6.44 | 7.23 | 9.88 | 4.26 | 4.05 | 4.84 | 7.85 | 4.383333 |
| DNAJB12 | 0.496 | -1.01146 | 1.57E-10 | 5.44E-09 | yes | down | 13.69 | 18.09 | 18.43 | 12.62 | 12.48 | 13.29 | 16.73667 | 12.79667 |
| LOC103345926 | 4.024 | 2.008637 | 1.58E-10 | 5.44E-09 | yes | up | 99.68 | 64.96 | 97.2 | 523.09 | 365.31 | 789.36 | 87.28 | 559.2533 |
| GNPAT | 0.487 | -1.03725 | 1.68E-10 | 5.78E-09 | yes | down | 49.48 | 60.04 | 64.67 | 39.38 | 42.58 | 51.98 | 58.06333 | 44.64667 |
| DDHD1 | 3.92 | 1.970781 | 1.75E-10 | 6.02E-09 | yes | up | 4.75 | 6.79 | 5.44 | 20.74 | 25.72 | 50.68 | 5.66 | 32.38 |
| SLIRP | 0.398 | -1.33084 | 1.77E-10 | 6.07E-09 | yes | down | 183.54 | 222.58 | 173.03 | 90.17 | 116.92 | 136.72 | 193.05 | 114.6033 |
| PDHA1 | 0.465 | -1.1062 | 1.8E-10 | 6.17E-09 | yes | down | 169.86 | 234.01 | 209.59 | 121.71 | 162.59 | 154.78 | 204.4867 | 146.36 |
| LOC100354761 | 0.458 | -1.12671 | 1.89E-10 | 6.45E-09 | yes | down | 24.76 | 37.35 | 32.09 | 20.19 | 24.08 | 23.37 | 31.4 | 22.54667 |
| LIX1 | 6.108 | 2.610703 | 1.96E-10 | 6.66E-09 | yes | up | 0.84 | 0.93 | 0.85 | 4.38 | 7.68 | 13.02 | 0.873333 | 8.36 |
| LOC100345779 | 12.767 | 3.674312 | 2.08E-10 | 7.06E-09 | yes | up | 0.28 | 0.29 | 0.29 | 11.17 | 3.78 | 2.15 | 0.286667 | 5.7 |
| IFIT3 | 9.359 | 3.226382 | 2.1E-10 | 7.13E-09 | yes | up | 3.55 | 2.43 | 2.67 | 72.62 | 38.41 | 14.36 | 2.883333 | 41.79667 |
| COQ9 | 0.411 | -1.2842 | 2.12E-10 | 7.16E-09 | yes | down | 118.18 | 169.16 | 143.95 | 76.17 | 108.45 | 86.29 | 143.7633 | 90.30333 |
| SDHA | 0.392 | -1.35109 | 2.15E-10 | 7.27E-09 | yes | down | 640.03 | 846.96 | 685.12 | 375.77 | 544.28 | 389.3 | 724.0367 | 436.45 |
| LOC103349253 | 3.688 | 1.88266 | 2.24E-10 | 7.54E-09 | yes | up | 4.09 | 8.67 | 6.63 | 18.94 | 26.07 | 16.37 | 6.463333 | 20.46 |
| NAMPT | 2.298 | 1.200359 | 2.25E-10 | 7.54E-09 | yes | up | 23.45 | 23.39 | 21.02 | 72.65 | 67.72 | 103.36 | 22.62 | 81.24333 |
| ITPR1 | 0.321 | -1.63955 | 2.26E-10 | 7.56E-09 | yes | down | 8.2 | 9.81 | 8.7 | 4.8 | 5.32 | 3 | 8.903333 | 4.373333 |
| MRPS18B | 0.373 | -1.42208 | 2.55E-10 | 8.49E-09 | yes | down | 31.85 | 52.27 | 39.79 | 20.33 | 27.38 | 22.93 | 41.30333 | 23.54667 |
| VCAM1 | 6.704 | 2.744995 | 2.59E-10 | 8.62E-09 | yes | up | 1.12 | 3.04 | 1.87 | 33.91 | 10.95 | 17.21 | 2.01 | 20.69 |
| IL33 | 2.628 | 1.393835 | 2.63E-10 | 8.74E-09 | yes | up | 2.37 | 2.5 | 2.75 | 12.43 | 8.6 | 10.45 | 2.54 | 10.49333 |
| GHITM | 0.296 | -1.75652 | 2.66E-10 | 8.82E-09 | yes | down | 260.27 | 286.76 | 380.58 | 108.16 | 112.57 | 209.54 | 309.2033 | 143.4233 |
| LRP3 | 0.267 | -1.90422 | 2.73E-10 | 9.02E-09 | yes | down | 38.11 | 62.91 | 62.05 | 13.27 | 23.12 | 28.41 | 54.35667 | 21.6 |
| NKIRAS2 | 0.463 | -1.11117 | 2.86E-10 | 9.44E-09 | yes | down | 45.17 | 50.17 | 54.28 | 29.03 | 39.03 | 36.95 | 49.87333 | 35.00333 |
| LOC103349807 | 315.002 | 8.299215 | 2.89E-10 | 9.52E-09 | yes | up | 0 | 0 | 0 | 20.34 | 24.73 | 11.04 | 0 | 18.70333 |
| REC8 | 4.007 | 2.002508 | 2.92E-10 | 9.59E-09 | yes | up | 0.8 | 0.83 | 0.7 | 6.04 | 6.23 | 4.46 | 0.776667 | 5.576667 |
| ZNF281 | 2.61 | 1.384226 | 3.04E-10 | 9.98E-09 | yes | up | 2.81 | 2.35 | 1.86 | 8.94 | 7.49 | 9.5 | 2.34 | 8.643333 |
| MCM7 | 2.755 | 1.461897 | 3.5E-10 | 1.14E-08 | yes | up | 4.57 | 4.73 | 3.53 | 21.9 | 14.47 | 19.03 | 4.276667 | 18.46667 |
| NDUFS6 | 0.468 | -1.09642 | 4.03E-10 | 1.3E-08 | yes | down | 407.42 | 483.12 | 441.84 | 289.71 | 378.76 | 313.95 | 444.1267 | 327.4733 |
| NDUFB1 | 0.255 | -1.97062 | 4.08E-10 | 1.32E-08 | yes | down | 55.41 | 58.87 | 53.73 | 22.68 | 17.89 | 25.63 | 56.00333 | 22.06667 |
| IMPDH1 | 0.495 | -1.01384 | 4.59E-10 | 1.48E-08 | yes | down | 266.77 | 353.18 | 325.91 | 201.23 | 237.91 | 285.6 | 315.2867 | 241.58 |
| ECI1 | 0.4 | -1.32342 | 4.81E-10 | 1.54E-08 | yes | down | 264.48 | 292.72 | 238.79 | 131.33 | 199.89 | 158.52 | 265.33 | 163.2467 |
| LOC100359112 | 2.932 | 1.551654 | 4.95E-10 | 1.58E-08 | yes | up | 5.03 | 5.66 | 5.43 | 27.86 | 17.26 | 28.56 | 5.373333 | 24.56 |
| LOC100358069 | 2.324 | 1.216719 | 5.14E-10 | 1.64E-08 | yes | up | 43.85 | 46.04 | 32.96 | 146.76 | 130.76 | 165 | 40.95 | 147.5067 |
| CCDC28A | 6.341 | 2.664786 | 5.21E-10 | 1.65E-08 | yes | up | 1.42 | 0.59 | 0.71 | 7.9 | 8.53 | 10.75 | 0.906667 | 9.06 |
| GOT2 | 0.361 | -1.47056 | 5.44E-10 | 1.72E-08 | yes | down | 58.22 | 95.28 | 61.04 | 37.4 | 47.23 | 33.17 | 71.51333 | 39.26667 |
| LOC108176462 | 0.034 | -4.88214 | 5.6E-10 | 1.77E-08 | yes | down | 5.89 | 4.98 | 1.88 | 0.31 | 0.16 | 0.2 | 4.25 | 0.223333 |
| PALMD | 0.353 | -1.50033 | 5.64E-10 | 1.78E-08 | yes | down | 16.3 | 22.56 | 16.58 | 7.12 | 10.25 | 12.98 | 18.48 | 10.11667 |
| SLC25A1 | 0.44 | -1.18356 | 5.98E-10 | 1.88E-08 | yes | down | 54.44 | 79.42 | 75.64 | 41.26 | 51.44 | 49.42 | 69.83333 | 47.37333 |
| MRPL4 | 0.367 | -1.44588 | 6.02E-10 | 1.88E-08 | yes | down | 20.54 | 23.61 | 20.22 | 11.1 | 12.85 | 13 | 21.45667 | 12.31667 |
| CES4A | 0.234 | -2.0966 | 6.03E-10 | 1.88E-08 | yes | down | 3.89 | 5.11 | 4.24 | 1.48 | 2.12 | 1.1 | 4.413333 | 1.566667 |
| MTIF2 | 0.45 | -1.15253 | 6.23E-10 | 1.94E-08 | yes | down | 7 | 8.86 | 9.64 | 5.91 | 5.29 | 5.32 | 8.5 | 5.506667 |
| LOC103347146 | 0.321 | -1.63936 | 6.48E-10 | 2.01E-08 | yes | down | 9.57 | 6.93 | 8.85 | 4.79 | 4.04 | 3.97 | 8.45 | 4.266667 |
| ZFYVE1 | 2.469 | 1.303935 | 7.02E-10 | 2.17E-08 | yes | up | 6.57 | 6.16 | 5.04 | 18.06 | 23.35 | 26.68 | 5.923333 | 22.69667 |
| NDUFS1 | 0.403 | -1.31024 | 7.06E-10 | 2.17E-08 | yes | down | 134.26 | 190.66 | 156.1 | 86.22 | 122.64 | 87.81 | 160.34 | 98.89 |
| GCHFR | 0.333 | -1.58487 | 7.12E-10 | 2.19E-08 | yes | down | 9.92 | 14.91 | 14.42 | 5.5 | 7.04 | 7.66 | 13.08333 | 6.733333 |
| ABCD3 | 0.489 | -1.03079 | 7.46E-10 | 2.28E-08 | yes | down | 11.92 | 18.27 | 14.93 | 10.74 | 10.73 | 12.8 | 15.04 | 11.42333 |
| ZFAND4 | 0.282 | -1.82683 | 8.06E-10 | 2.45E-08 | yes | down | 5.58 | 7.6 | 11.5 | 2.93 | 2.97 | 3.8 | 8.226667 | 3.233333 |
| IFIH1 | 8.235 | 3.041849 | 8.12E-10 | 2.46E-08 | yes | up | 0.61 | 0.51 | 0.72 | 14.16 | 5.91 | 3.57 | 0.613333 | 7.88 |
| COQ6 | 0.329 | -1.60454 | 8.15E-10 | 2.47E-08 | yes | down | 40.47 | 57.09 | 37.26 | 19.75 | 29.19 | 18.78 | 44.94 | 22.57333 |
| ARL4D | 2.887 | 1.529434 | 8.24E-10 | 2.49E-08 | yes | up | 3.8 | 4.35 | 3.43 | 14.39 | 15.64 | 21.99 | 3.86 | 17.34 |
| COQ7 | 0.436 | -1.19886 | 8.4E-10 | 2.53E-08 | yes | down | 35.06 | 52.24 | 42.66 | 29.88 | 30.53 | 26.25 | 43.32 | 28.88667 |
| G6PC3 | 0.499 | -1.00277 | 8.64E-10 | 2.6E-08 | yes | down | 23.36 | 32.63 | 26.92 | 19.74 | 22.27 | 21.57 | 27.63667 | 21.19333 |
| GALE | 0.345 | -1.53587 | 8.7E-10 | 2.62E-08 | yes | down | 8.9 | 16.2 | 14.37 | 6.8 | 6.15 | 8.07 | 13.15667 | 7.006667 |
| UNC5B | 0.297 | -1.75257 | 8.76E-10 | 2.63E-08 | yes | down | 6.37 | 9.82 | 12.32 | 3.25 | 5.03 | 4.75 | 9.503333 | 4.343333 |
| LIFR | 3.154 | 1.657402 | 8.84E-10 | 2.65E-08 | yes | up | 10.56 | 6.03 | 6.67 | 39.31 | 35.89 | 40.84 | 7.753333 | 38.68 |
| TMEM143 | 0.361 | -1.46954 | 8.88E-10 | 2.65E-08 | yes | down | 22.54 | 25.63 | 29.6 | 13.55 | 16.95 | 11.1 | 25.92333 | 13.86667 |
| LOC100353747 | 0.003 | -8.38244 | 9.03E-10 | 2.69E-08 | yes | down | 1.73 | 0.91 | 2.55 | 0 | 0 | 0 | 1.73 | 0 |
| GRN | 2.248 | 1.168806 | 9.14E-10 | 2.72E-08 | yes | up | 25.31 | 28.14 | 26.36 | 114.1 | 77.98 | 85.8 | 26.60333 | 92.62667 |
| PHKA2 | 3.737 | 1.901986 | 9.44E-10 | 2.8E-08 | yes | up | 1.03 | 1.83 | 2 | 6.47 | 9.95 | 11.49 | 1.62 | 9.303333 |
| GSE1 | 0.458 | -1.12668 | 9.88E-10 | 2.93E-08 | yes | down | 10.78 | 15.01 | 10.59 | 8.52 | 7.29 | 10.06 | 12.12667 | 8.623333 |
| PDE1A | 2.456 | 1.296175 | 1.08E-09 | 3.2E-08 | yes | up | 8.68 | 8.29 | 6.61 | 20.75 | 15.78 | 25.46 | 7.86 | 20.66333 |
| HSPBP1 | 0.357 | -1.48712 | 1.15E-09 | 3.37E-08 | yes | down | 7.9 | 11.22 | 7.16 | 5.03 | 4.79 | 4.54 | 8.76 | 4.786667 |
| CCDC71L | 4.337 | 2.116806 | 1.16E-09 | 3.4E-08 | yes | up | 1.57 | 1.72 | 1.5 | 7.22 | 7.89 | 17.62 | 1.596667 | 10.91 |
| QPCT | 0.39 | -1.35928 | 1.16E-09 | 3.4E-08 | yes | down | 147.76 | 204.14 | 228.05 | 92.04 | 109.99 | 149.03 | 193.3167 | 117.02 |
| OLFML3 | 2.365 | 1.241616 | 1.2E-09 | 3.5E-08 | yes | up | 11.57 | 19.48 | 13.41 | 60.17 | 46.5 | 54.56 | 14.82 | 53.74333 |
| LOC100357076 | 0.363 | -1.46394 | 1.22E-09 | 3.57E-08 | yes | down | 2.71 | 2.94 | 3.85 | 1.74 | 1.44 | 2.3 | 3.166667 | 1.826667 |
| SSPO | 4.659 | 2.220087 | 1.24E-09 | 3.62E-08 | yes | up | 0.18 | 0.11 | 0.1 | 1.01 | 0.69 | 0.87 | 0.13 | 0.856667 |
| LOC100356803 | 0.489 | -1.03195 | 1.32E-09 | 3.84E-08 | yes | down | 421.76 | 570.58 | 483.88 | 311.83 | 406.95 | 391.84 | 492.0733 | 370.2067 |
| PDHX | 0.47 | -1.08912 | 1.37E-09 | 3.98E-08 | yes | down | 22.09 | 28.69 | 24.72 | 15.68 | 20.77 | 18.16 | 25.16667 | 18.20333 |
| DPT | 3.015 | 1.592264 | 1.38E-09 | 3.99E-08 | yes | up | 40.59 | 69.1 | 34.6 | 230.25 | 260.99 | 183.13 | 48.09667 | 224.79 |
| LOC100358008 | 0.404 | -1.30934 | 1.48E-09 | 4.27E-08 | yes | down | 21.69 | 26.42 | 24.45 | 13.32 | 15.44 | 16.58 | 24.18667 | 15.11333 |
| STIP1 | 0.419 | -1.25616 | 1.49E-09 | 4.29E-08 | yes | down | 35.2 | 56.35 | 45.76 | 32.94 | 29.23 | 25.03 | 45.77 | 29.06667 |
| FPGS | 0.445 | -1.16842 | 1.57E-09 | 4.51E-08 | yes | down | 12.28 | 14.95 | 15.48 | 8.92 | 10.96 | 9.44 | 14.23667 | 9.773333 |
| CHCHD2 | 0.333 | -1.58754 | 1.59E-09 | 4.56E-08 | yes | down | 698 | 533.43 | 422.7 | 220.56 | 304.55 | 332.19 | 551.3767 | 285.7667 |
| LOC100356224 | 0.004 | -8.03926 | 1.61E-09 | 4.6E-08 | yes | down | 2.35 | 2.34 | 1.58 | 0 | 0 | 0 | 2.09 | 0 |
| KCTD21 | 2.299 | 1.200695 | 1.67E-09 | 4.76E-08 | yes | up | 2.36 | 2.99 | 3.57 | 9.17 | 9.23 | 11.72 | 2.973333 | 10.04 |
| MXI1 | 2.685 | 1.424763 | 1.69E-09 | 4.81E-08 | yes | up | 9.39 | 6.71 | 7.28 | 28.56 | 31.6 | 41.12 | 7.793333 | 33.76 |
| NEXN | 0.303 | -1.72483 | 1.73E-09 | 4.92E-08 | yes | down | 43.07 | 53.89 | 34.55 | 14.19 | 27.29 | 18.53 | 43.83667 | 20.00333 |
| MRPS15 | 0.461 | -1.11611 | 1.74E-09 | 4.94E-08 | yes | down | 57.2 | 84.97 | 79.42 | 46.25 | 55.86 | 53.53 | 73.86333 | 51.88 |
| BPGM | 0.374 | -1.4203 | 1.77E-09 | 5E-08 | yes | down | 9.95 | 8.51 | 10.4 | 4.85 | 5.5 | 6.37 | 9.62 | 5.573333 |
| RGS10 | 3.444 | 1.784089 | 1.82E-09 | 5.11E-08 | yes | up | 7.95 | 7.8 | 7.32 | 31.93 | 33.6 | 59.29 | 7.69 | 41.60667 |
| SLC35A4 | 0.436 | -1.19847 | 1.83E-09 | 5.13E-08 | yes | down | 15.64 | 25.22 | 24.01 | 13.93 | 12.87 | 15.17 | 21.62333 | 13.99 |
| LOC100340865 | 12.98 | 3.698198 | 1.83E-09 | 5.13E-08 | yes | up | 1.21 | 1.61 | 0.97 | 52.81 | 8.1 | 12.36 | 1.263333 | 24.42333 |
| MPC1 | 0.313 | -1.67765 | 1.84E-09 | 5.16E-08 | yes | down | 390.43 | 565.28 | 406.9 | 148.34 | 294.83 | 194.63 | 454.2033 | 212.6 |
| PPARGC1B | 0.363 | -1.46328 | 1.85E-09 | 5.18E-08 | yes | down | 12.76 | 16.77 | 18.26 | 7.53 | 11.41 | 7.53 | 15.93 | 8.823333 |
| ATP6V1A | 0.44 | -1.18592 | 1.92E-09 | 5.36E-08 | yes | down | 22.66 | 36.41 | 31.78 | 22.75 | 16.13 | 20.97 | 30.28333 | 19.95 |
| EXOSC4 | 0.375 | -1.41512 | 1.95E-09 | 5.44E-08 | yes | down | 7.27 | 10.24 | 7.75 | 4.61 | 4.48 | 5.21 | 8.42 | 4.766667 |
| DTX3L | 5.864 | 2.551807 | 2E-09 | 5.56E-08 | yes | up | 1.22 | 1.28 | 1.48 | 19.69 | 10.53 | 5.87 | 1.326667 | 12.03 |
| CMC2 | 0.466 | -1.10208 | 2.06E-09 | 5.7E-08 | yes | down | 14.11 | 17.42 | 16.84 | 12.73 | 12.1 | 14.23 | 16.12333 | 13.02 |
| SFXN1 | 2.972 | 1.571248 | 2.06E-09 | 5.7E-08 | yes | up | 2.31 | 3.73 | 3.44 | 12.53 | 14.63 | 20.04 | 3.16 | 15.73333 |
| LOC100358789 | 0.314 | -1.66887 | 2.16E-09 | 5.96E-08 | yes | down | 23.42 | 45.78 | 32.64 | 13.89 | 20.74 | 13.91 | 33.94667 | 16.18 |
| NTMT1 | 0.416 | -1.26598 | 2.17E-09 | 5.98E-08 | yes | down | 47.94 | 64.27 | 47.26 | 29.69 | 38.92 | 33.09 | 53.15667 | 33.9 |
| LOC100351933 | 0.008 | -6.93487 | 2.2E-09 | 6.05E-08 | yes | down | 10.17 | 20.66 | 9.4 | 0 | 0 | 0.59 | 13.41 | 0.196667 |
| FBXL4 | 2.6 | 1.378459 | 2.37E-09 | 6.53E-08 | yes | up | 3.39 | 3.13 | 3.51 | 11.01 | 14.85 | 14.92 | 3.343333 | 13.59333 |
| PPA1 | 0.363 | -1.4619 | 2.38E-09 | 6.53E-08 | yes | down | 75.16 | 157.97 | 91.38 | 59.69 | 55.39 | 66.77 | 108.17 | 60.61667 |
| LOC103347227 | 2.512 | 1.328801 | 2.4E-09 | 6.57E-08 | yes | up | 8.56 | 9.56 | 9.72 | 31.2 | 36.9 | 46.04 | 9.28 | 38.04667 |
| GLRX5 | 0.395 | -1.34185 | 2.45E-09 | 6.68E-08 | yes | down | 44.84 | 45.93 | 45.07 | 21.4 | 32.15 | 28.98 | 45.28 | 27.51 |
| LOC103348123 | 0.058 | -4.11779 | 2.48E-09 | 6.75E-08 | yes | down | 1.32 | 3.14 | 2.86 | 0.19 | 0.08 | 0.21 | 2.44 | 0.16 |
| TBC1D25 | 0.424 | -1.2382 | 2.63E-09 | 7.14E-08 | yes | down | 4.91 | 6.72 | 6.14 | 4.1 | 3.91 | 3.57 | 5.923333 | 3.86 |
| CRYM | 3.803 | 1.92707 | 2.8E-09 | 7.58E-08 | yes | up | 19.19 | 16.42 | 13.91 | 88.26 | 134.21 | 65.84 | 16.50667 | 96.10333 |
| TKT | 0.354 | -1.49743 | 2.95E-09 | 7.95E-08 | yes | down | 119.12 | 171.96 | 184.53 | 74.36 | 110.64 | 73.05 | 158.5367 | 86.01667 |
| LOC103347061 | 0.285 | -1.81049 | 2.95E-09 | 7.95E-08 | yes | down | 23.02 | 33.42 | 36.51 | 14.82 | 14.44 | 11.26 | 30.98333 | 13.50667 |
| LOC100347571 | 0.373 | -1.42244 | 3.07E-09 | 8.23E-08 | yes | down | 467.17 | 570.33 | 442.28 | 242.76 | 369.7 | 233.2 | 493.26 | 281.8867 |
| ECH1 | 0.418 | -1.25743 | 3.07E-09 | 8.23E-08 | yes | down | 424.82 | 485.36 | 374.96 | 229 | 343.87 | 268.1 | 428.38 | 280.3233 |
| ADAMTS17 | 0.381 | -1.39232 | 3.2E-09 | 8.53E-08 | yes | down | 3.04 | 3.77 | 3.63 | 1.59 | 2.42 | 2.12 | 3.48 | 2.043333 |
| GALM | 0.3 | -1.73526 | 3.33E-09 | 8.87E-08 | yes | down | 9.26 | 9.17 | 14.6 | 4.13 | 5.92 | 5.38 | 11.01 | 5.143333 |
| LACE1 | 0.366 | -1.4489 | 3.49E-09 | 9.23E-08 | yes | down | 15.35 | 21.35 | 22.54 | 10.57 | 13.3 | 9.39 | 19.74667 | 11.08667 |
| IDH3G | 0.423 | -1.24297 | 3.49E-09 | 9.23E-08 | yes | down | 395.08 | 502.89 | 407.32 | 249.54 | 350.51 | 243.54 | 435.0967 | 281.1967 |
| RANGAP1 | 0.386 | -1.37153 | 3.5E-09 | 9.23E-08 | yes | down | 9.62 | 13.66 | 13.13 | 8.78 | 5.38 | 7.64 | 12.13667 | 7.266667 |
| L2HGDH | 0.485 | -1.04365 | 3.72E-09 | 9.8E-08 | yes | down | 5.99 | 6.8 | 7.17 | 5.19 | 5.01 | 4.75 | 6.653333 | 4.983333 |
| CYP2A11 | 40.842 | 5.351966 | 3.76E-09 | 9.87E-08 | yes | up | 7.55 | 1.29 | 1.26 | 128.53 | 254.65 | 308.49 | 3.366667 | 230.5567 |
| STEAP2 | 0.232 | -2.10904 | 3.76E-09 | 9.87E-08 | yes | down | 1.15 | 1.39 | 1.68 | 0.39 | 0.58 | 0.54 | 1.406667 | 0.503333 |
| TOMM22 | 0.409 | -1.28872 | 3.82E-09 | 1E-07 | yes | down | 39.14 | 48.95 | 48.73 | 22.63 | 27.93 | 36.57 | 45.60667 | 29.04333 |
| MBOAT1 | 3.205 | 1.680247 | 3.89E-09 | 1.02E-07 | yes | up | 0.97 | 1.65 | 1.68 | 7.7 | 5.56 | 7.38 | 1.433333 | 6.88 |
| GNG2 | 2.526 | 1.337077 | 4.03E-09 | 1.05E-07 | yes | up | 7.2 | 7.38 | 7.45 | 34.94 | 20.06 | 33.26 | 7.343333 | 29.42 |
| CTPS1 | 0.152 | -2.71639 | 4.03E-09 | 1.05E-07 | yes | down | 3.1 | 5.67 | 3.77 | 1.68 | 0.59 | 0.63 | 4.18 | 0.966667 |
| FH | 0.404 | -1.30652 | 4.04E-09 | 1.05E-07 | yes | down | 122.58 | 151.42 | 124.68 | 73.14 | 104.52 | 69.44 | 132.8933 | 82.36667 |
| GHR | 2.766 | 1.467682 | 4.05E-09 | 1.05E-07 | yes | up | 45.22 | 30.06 | 35.73 | 144 | 138.51 | 203.11 | 37.00333 | 161.8733 |
| CHCHD3 | 0.353 | -1.50365 | 4.11E-09 | 1.06E-07 | yes | down | 149.7 | 209.53 | 167.44 | 69.09 | 126 | 89.39 | 175.5567 | 94.82667 |
| DERL3 | 41.647 | 5.380144 | 4.17E-09 | 1.08E-07 | yes | up | 0.09 | 0.09 | 0.19 | 4.28 | 6.55 | 12.34 | 0.123333 | 7.723333 |
| MAT2A | 0.497 | -1.00904 | 4.25E-09 | 1.1E-07 | yes | down | 49.45 | 57.8 | 47.91 | 42.98 | 30.08 | 42.16 | 51.72 | 38.40667 |
| GEMIN4 | 0.324 | -1.62707 | 4.32E-09 | 1.11E-07 | yes | down | 3.34 | 5.97 | 4.45 | 1.74 | 2.55 | 2.52 | 4.586667 | 2.27 |
| LOC100343255 | 0.482 | -1.05167 | 4.37E-09 | 1.12E-07 | yes | down | 602.86 | 816.67 | 718.62 | 499.71 | 598.9 | 481.53 | 712.7167 | 526.7133 |
| PHKG1 | 0.351 | -1.50997 | 4.5E-09 | 1.15E-07 | yes | down | 71.99 | 75.01 | 54.77 | 25.65 | 44.78 | 33.48 | 67.25667 | 34.63667 |
| NNT | 0.418 | -1.25841 | 4.75E-09 | 1.21E-07 | yes | down | 73.34 | 105.43 | 77.96 | 46.24 | 67.91 | 51.76 | 85.57667 | 55.30333 |
| VDAC1 | 0.486 | -1.04135 | 5.01E-09 | 1.27E-07 | yes | down | 305.64 | 424.13 | 306.56 | 219.4 | 304.77 | 252.91 | 345.4433 | 259.0267 |
| HBP1 | 2.33 | 1.220424 | 5.17E-09 | 1.31E-07 | yes | up | 36.51 | 26.43 | 30.62 | 107.87 | 105.11 | 129.29 | 31.18667 | 114.09 |
| PRRG4 | 0.389 | -1.36067 | 5.18E-09 | 1.31E-07 | yes | down | 4.11 | 6.93 | 4.76 | 2.7 | 2.8 | 3.89 | 5.266667 | 3.13 |
| CKB | 0.331 | -1.59498 | 5.33E-09 | 1.35E-07 | yes | down | 45.46 | 96.68 | 71.69 | 29.12 | 42.67 | 35.74 | 71.27667 | 35.84333 |
| FICD | 0.281 | -1.83365 | 5.67E-09 | 1.43E-07 | yes | down | 4.23 | 2.92 | 3.71 | 1.74 | 1.2 | 1.87 | 3.62 | 1.603333 |
| ACVR2A | 2.695 | 1.430341 | 5.78E-09 | 1.45E-07 | yes | up | 1.71 | 1.8 | 1.73 | 5.25 | 8.03 | 8.65 | 1.746667 | 7.31 |
| BYSL | 0.31 | -1.69079 | 5.91E-09 | 1.48E-07 | yes | down | 3.94 | 7 | 5.32 | 2.93 | 1.97 | 2.85 | 5.42 | 2.583333 |
| OXA1L | 0.362 | -1.46574 | 6.02E-09 | 1.5E-07 | yes | down | 70.95 | 98 | 89.27 | 32.15 | 55.16 | 58.58 | 86.07333 | 48.63 |
| DDX58 | 4.468 | 2.159666 | 6.85E-09 | 1.7E-07 | yes | up | 7.56 | 7.11 | 5.34 | 75.89 | 35.69 | 28.94 | 6.67 | 46.84 |
| GATSL3 | 0.45 | -1.15193 | 7.03E-09 | 1.75E-07 | yes | down | 45.49 | 42.38 | 47.36 | 28.84 | 26.72 | 40.03 | 45.07667 | 31.86333 |
| MARS | 0.43 | -1.2176 | 7.13E-09 | 1.77E-07 | yes | down | 8.21 | 13.67 | 10.55 | 7.67 | 7.15 | 6.51 | 10.81 | 7.11 |
| LOC100337780 | 0.017 | -5.85296 | 7.18E-09 | 1.78E-07 | yes | down | 0.97 | 0.62 | 0.8 | 0.04 | 0.02 | 0 | 0.796667 | 0.02 |
| IFI35 | 6.657 | 2.734963 | 7.22E-09 | 1.78E-07 | yes | up | 1.21 | 1.69 | 1.11 | 18.76 | 14.76 | 6.81 | 1.336667 | 13.44333 |
| LOC100009451 | 14.009 | 3.808328 | 7.64E-09 | 1.88E-07 | yes | up | 0.76 | 0.58 | 1.46 | 38.34 | 17.08 | 5.29 | 0.933333 | 20.23667 |
| LOC103352162 | 0.035 | -4.85565 | 7.65E-09 | 1.88E-07 | yes | down | 2.13 | 2.27 | 1.91 | 0.07 | 0.2 | 0.09 | 2.103333 | 0.12 |
| PEX16 | 0.455 | -1.13687 | 7.83E-09 | 1.92E-07 | yes | down | 30.16 | 30.61 | 40.15 | 20.01 | 22.22 | 26.3 | 33.64 | 22.84333 |
| ABI3BP | 2.099 | 1.06955 | 7.85E-09 | 1.92E-07 | yes | up | 4.9 | 7.85 | 6.06 | 22.2 | 19.17 | 19.91 | 6.27 | 20.42667 |
| OGDH | 0.471 | -1.0849 | 8.32E-09 | 2.04E-07 | yes | down | 298.83 | 374.53 | 390.81 | 296.16 | 256.03 | 221.75 | 354.7233 | 257.98 |
| HP | 20.296 | 4.343155 | 8.49E-09 | 2.07E-07 | yes | up | 116.38 | 33.99 | 35.41 | 1542.44 | 1538.42 | 3022.6 | 61.92667 | 2034.487 |
| CCDC86 | 0.168 | -2.57577 | 8.52E-09 | 2.08E-07 | yes | down | 14.66 | 9.29 | 5.34 | 3.49 | 1.91 | 2.32 | 9.763333 | 2.573333 |
| FAM162A | 0.48 | -1.05896 | 9.19E-09 | 2.23E-07 | yes | down | 42.25 | 56.84 | 45.01 | 28.62 | 38.18 | 39.9 | 48.03333 | 35.56667 |
| FAM65C | 4.943 | 2.305277 | 9.47E-09 | 2.3E-07 | yes | up | 0.34 | 0.17 | 0.29 | 1.7 | 2.2 | 2.27 | 0.266667 | 2.056667 |
| LOC100340645 | 0.438 | -1.19117 | 9.54E-09 | 2.31E-07 | yes | down | 1648.46 | 1708.27 | 1324.83 | 1033.83 | 1215.76 | 899.51 | 1560.52 | 1049.7 |
| RDH13 | 0.257 | -1.95979 | 1.05E-08 | 2.53E-07 | yes | down | 5.03 | 10.85 | 12.15 | 3.79 | 3.56 | 3.81 | 9.343333 | 3.72 |
| TPD52L1 | 0.403 | -1.31065 | 1.11E-08 | 2.67E-07 | yes | down | 17.92 | 29.41 | 24.92 | 12.54 | 15.78 | 15.97 | 24.08333 | 14.76333 |
| HAL | 93.472 | 6.546468 | 1.13E-08 | 2.71E-07 | yes | up | 0.02 | 0 | 0.02 | 1.34 | 1.25 | 2.77 | 0.013333 | 1.786667 |
| C4H12orf73 | 0.416 | -1.2667 | 1.15E-08 | 2.76E-07 | yes | down | 17.55 | 25.86 | 25.69 | 12.74 | 14.39 | 11.3 | 23.03333 | 12.81 |
| MTHFD1 | 0.478 | -1.06487 | 1.21E-08 | 2.88E-07 | yes | down | 39.53 | 45.44 | 46.06 | 25.23 | 34.13 | 37.84 | 43.67667 | 32.4 |
| LOC108178771 | 122.506 | 6.936712 | 1.22E-08 | 2.91E-07 | yes | up | 0.01 | 0 | 0.02 | 0.47 | 10.8 | 8.66 | 0.01 | 6.643333 |
| FURIN | 0.347 | -1.52673 | 1.23E-08 | 2.93E-07 | yes | down | 63.33 | 73.82 | 112.87 | 54.97 | 33.76 | 44.26 | 83.34 | 44.33 |
| LOC100355902 | 0.26 | -1.94292 | 1.32E-08 | 3.13E-07 | yes | down | 1.2 | 1.28 | 1.67 | 0.36 | 0.67 | 0.65 | 1.383333 | 0.56 |
| DSP | 0.431 | -1.21544 | 1.33E-08 | 3.16E-07 | yes | down | 10.73 | 11.37 | 13.35 | 8.14 | 9.11 | 6.79 | 11.81667 | 8.013333 |
| JAGN1 | 0.398 | -1.32829 | 1.33E-08 | 3.16E-07 | yes | down | 34.8 | 46.21 | 45.96 | 24.16 | 25.18 | 37.9 | 42.32333 | 29.08 |
| NXPH3 | 4.719 | 2.238558 | 1.36E-08 | 3.21E-07 | yes | up | 0.49 | 0.62 | 0.63 | 3.48 | 4.95 | 4.22 | 0.58 | 4.216667 |
| TECR | 0.382 | -1.38657 | 1.44E-08 | 3.39E-07 | yes | down | 101.54 | 179.48 | 123.94 | 72.17 | 91.8 | 71.23 | 134.9867 | 78.4 |
| CNNM3 | 2.432 | 1.282067 | 1.45E-08 | 3.41E-07 | yes | up | 6.71 | 6.9 | 7.81 | 20.68 | 30.3 | 26.08 | 7.14 | 25.68667 |
| P2RX4 | 2.566 | 1.359244 | 1.49E-08 | 3.49E-07 | yes | up | 3.87 | 4.77 | 3.97 | 20.43 | 13.11 | 16.5 | 4.203333 | 16.68 |
| METTL1 | 0.298 | -1.74553 | 1.59E-08 | 3.7E-07 | yes | down | 3.58 | 5.47 | 4.49 | 2.63 | 1.61 | 1.96 | 4.513333 | 2.066667 |
| LOC100343291 | 3.822 | 1.934186 | 1.59E-08 | 3.71E-07 | yes | up | 0.48 | 0.44 | 0.44 | 3.37 | 2.09 | 2.66 | 0.453333 | 2.706667 |
| MPP7 | 0.377 | -1.40749 | 1.6E-08 | 3.72E-07 | yes | down | 2.23 | 3.08 | 2.02 | 1.36 | 1.23 | 1.73 | 2.443333 | 1.44 |
| TYMS | 3.439 | 1.782111 | 1.63E-08 | 3.78E-07 | yes | up | 1.15 | 1.37 | 1.58 | 8.64 | 6.16 | 7.15 | 1.366667 | 7.316667 |
| LOC100355688 | 0.484 | -1.04555 | 1.7E-08 | 3.94E-07 | yes | down | 48.99 | 68.32 | 58.95 | 52.02 | 40.83 | 38.94 | 58.75333 | 43.93 |
| IFIT5 | 9.222 | 3.205068 | 1.77E-08 | 4.09E-07 | yes | up | 1.85 | 1.37 | 0.69 | 31.54 | 14.18 | 5.82 | 1.303333 | 17.18 |
| TMEM201 | 0.362 | -1.46407 | 1.79E-08 | 4.11E-07 | yes | down | 3.56 | 5.78 | 6.06 | 2.53 | 3.64 | 2.48 | 5.133333 | 2.883333 |
| LOC100340718 | 0.5 | -1.00043 | 1.8E-08 | 4.15E-07 | yes | down | 12.06 | 16.8 | 15.67 | 12.46 | 11.12 | 10.82 | 14.84333 | 11.46667 |
| LOC103349363 | 0.057 | -4.12543 | 1.93E-08 | 4.43E-07 | yes | down | 1.91 | 2.12 | 2.19 | 0 | 0.24 | 0.19 | 2.073333 | 0.143333 |
| LAMA3 | 2.495 | 1.318922 | 2.07E-08 | 4.74E-07 | yes | up | 0.86 | 0.93 | 1.2 | 4.26 | 3.44 | 4.14 | 0.996667 | 3.946667 |
| CHKB | 0.5 | -1.00055 | 2.11E-08 | 4.81E-07 | yes | down | 22.9 | 29.29 | 29.77 | 20.21 | 21.16 | 21.99 | 27.32 | 21.12 |
| LOC108178465 | 0.146 | -2.77819 | 2.18E-08 | 4.96E-07 | yes | down | 18.39 | 17.69 | 16.75 | 6.09 | 4.34 | 1.24 | 17.61 | 3.89 |
| FABP3 | 0.204 | -2.2924 | 2.22E-08 | 5.05E-07 | yes | down | 392.79 | 434.21 | 301.49 | 49.14 | 180.84 | 124.1 | 376.1633 | 118.0267 |
| LRWD1 | 0.35 | -1.51418 | 2.34E-08 | 5.32E-07 | yes | down | 4.3 | 6.87 | 4.27 | 2.35 | 2.84 | 3.1 | 5.146667 | 2.763333 |
| TFAP4 | 2.915 | 1.543642 | 2.45E-08 | 5.53E-07 | yes | up | 1.49 | 1.51 | 2.07 | 6.2 | 7.95 | 9.04 | 1.69 | 7.73 |
| TMX1 | 2.076 | 1.053597 | 2.46E-08 | 5.54E-07 | yes | up | 4.92 | 5.67 | 5.28 | 19.67 | 14.63 | 16.74 | 5.29 | 17.01333 |
| MRPS28 | 0.417 | -1.26283 | 2.51E-08 | 5.64E-07 | yes | down | 25.45 | 38.16 | 28.71 | 17.66 | 20.82 | 20.61 | 30.77333 | 19.69667 |
| PRSS23 | 2.563 | 1.357747 | 2.53E-08 | 5.67E-07 | yes | up | 23.84 | 25.61 | 14.95 | 71.19 | 83.1 | 102.15 | 21.46667 | 85.48 |
| CAPN15 | 0.38 | -1.39759 | 2.55E-08 | 5.71E-07 | yes | down | 6.03 | 8.69 | 9.36 | 5.16 | 5.14 | 3.67 | 8.026667 | 4.656667 |
| LOC100358892 | 2.456 | 1.296167 | 2.59E-08 | 5.79E-07 | yes | up | 13.06 | 11.93 | 10.72 | 39.32 | 37.4 | 60.93 | 11.90333 | 45.88333 |
| GRSF1 | 0.446 | -1.16522 | 2.7E-08 | 6.03E-07 | yes | down | 23.2 | 35.43 | 29.53 | 14.7 | 20.79 | 23.33 | 29.38667 | 19.60667 |
| KLHL21 | 3.263 | 1.70629 | 2.71E-08 | 6.03E-07 | yes | up | 9.48 | 5.52 | 8.39 | 29.03 | 40.64 | 51.15 | 7.796667 | 40.27333 |
| OAS3 | 10.197 | 3.350043 | 2.71E-08 | 6.03E-07 | yes | up | 0.36 | 0.37 | 0.54 | 13.12 | 4.99 | 2 | 0.423333 | 6.703333 |
| G3BP1 | 0.472 | -1.0829 | 2.77E-08 | 6.14E-07 | yes | down | 15.56 | 22.68 | 18.8 | 16.45 | 11.59 | 13.39 | 19.01333 | 13.81 |
| HMGB2 | 2.6 | 1.378777 | 2.83E-08 | 6.26E-07 | yes | up | 34.73 | 34.45 | 21.74 | 110.97 | 103.04 | 155.54 | 30.30667 | 123.1833 |
| MRPS10 | 0.468 | -1.09391 | 2.91E-08 | 6.45E-07 | yes | down | 28.96 | 31.28 | 34.06 | 21.8 | 18.1 | 15.97 | 31.43333 | 18.62333 |
| CREG1 | 2.165 | 1.114442 | 2.96E-08 | 6.54E-07 | yes | up | 16.94 | 18.39 | 19.14 | 49.09 | 55.44 | 77.97 | 18.15667 | 60.83333 |
| MDH2 | 0.376 | -1.41213 | 3.02E-08 | 6.63E-07 | yes | down | 505.29 | 630.23 | 487.28 | 289.38 | 409.67 | 234 | 540.9333 | 311.0167 |
| LOC100348135 | 0.448 | -1.15888 | 3.03E-08 | 6.64E-07 | yes | down | 39.41 | 42.85 | 28.6 | 24 | 25.32 | 27.69 | 36.95333 | 25.67 |
| LOC100342335 | 2.422 | 1.276409 | 3.04E-08 | 6.66E-07 | yes | up | 2.02 | 2.64 | 2.52 | 11.64 | 7.73 | 6.25 | 2.393333 | 8.54 |
| NRIP3 | 0.254 | -1.97706 | 3.42E-08 | 7.44E-07 | yes | down | 1.44 | 1.27 | 1 | 0.42 | 0.45 | 0.6 | 1.236667 | 0.49 |
| DIO2 | 0.267 | -1.90307 | 3.69E-08 | 8.01E-07 | yes | down | 7.21 | 6.61 | 4.76 | 1.84 | 3.18 | 1.82 | 6.193333 | 2.28 |
| LOC100352395 | 3.265 | 1.707197 | 3.88E-08 | 8.37E-07 | yes | up | 0.35 | 0.39 | 0.43 | 1.47 | 2.09 | 2.35 | 0.39 | 1.97 |
| VDAC3 | 0.445 | -1.16697 | 3.91E-08 | 8.43E-07 | yes | down | 114.91 | 178.03 | 115.09 | 85.01 | 111.19 | 99.04 | 136.01 | 98.41333 |
| NSDHL | 0.457 | -1.13014 | 3.94E-08 | 8.49E-07 | yes | down | 24.12 | 34.18 | 29.64 | 25.43 | 15.56 | 20.26 | 29.31333 | 20.41667 |
| LOC108175456 | 0.005 | -7.67489 | 3.96E-08 | 8.51E-07 | yes | down | 0.47 | 1.22 | 0.99 | 0 | 0 | 0 | 0.893333 | 0 |
| TUFM | 0.44 | -1.18595 | 4.05E-08 | 8.67E-07 | yes | down | 148.71 | 184.36 | 142.9 | 92.18 | 135.86 | 98.95 | 158.6567 | 108.9967 |
| HHIP | 3.216 | 1.685198 | 4.11E-08 | 8.8E-07 | yes | up | 0.3 | 0.61 | 0.43 | 1.88 | 1.65 | 2.42 | 0.446667 | 1.983333 |
| MIPEP | 0.473 | -1.08033 | 4.16E-08 | 8.88E-07 | yes | down | 13.52 | 19.95 | 17.24 | 13.96 | 12.05 | 10.75 | 16.90333 | 12.25333 |
| SNX6 | 2.003 | 1.002313 | 4.19E-08 | 8.94E-07 | yes | up | 8.4 | 12.47 | 11.19 | 37.24 | 32.25 | 30.6 | 10.68667 | 33.36333 |
| STYXL1 | 0.326 | -1.61665 | 4.35E-08 | 9.25E-07 | yes | down | 5.95 | 7.28 | 9.37 | 4.27 | 3.46 | 4.09 | 7.533333 | 3.94 |
| SATB2 | 2.636 | 1.398492 | 4.44E-08 | 9.44E-07 | yes | up | 1.6 | 1.31 | 1.9 | 5.87 | 6.67 | 7.12 | 1.603333 | 6.553333 |
| TBC1D14 | 0.456 | -1.13294 | 4.55E-08 | 9.65E-07 | yes | down | 20.22 | 24.73 | 24.84 | 17.81 | 14.92 | 13.24 | 23.26333 | 15.32333 |
| YBX2 | 0.313 | -1.67499 | 4.56E-08 | 9.65E-07 | yes | down | 6.21 | 5.42 | 5.58 | 3.21 | 2.62 | 1.94 | 5.736667 | 2.59 |
| ATP5G1 | 0.303 | -1.72411 | 4.6E-08 | 9.74E-07 | yes | down | 235.18 | 366.38 | 200.61 | 81.43 | 164.54 | 126.59 | 267.39 | 124.1867 |
| TTC19 | 0.453 | -1.14347 | 4.77E-08 | 1.01E-06 | yes | down | 11.45 | 15.58 | 15.46 | 11.71 | 8.31 | 9.77 | 14.16333 | 9.93 |
| TXNIP | 2.351 | 1.233311 | 4.78E-08 | 1.01E-06 | yes | up | 712.95 | 792.23 | 585.91 | 2142.29 | 2049.8 | 3488.18 | 697.03 | 2560.09 |
| GLYCTK | 0.344 | -1.53782 | 4.8E-08 | 1.01E-06 | yes | down | 356.36 | 488.77 | 593.05 | 191.59 | 314.63 | 208.46 | 479.3933 | 238.2267 |
| LOC103347346 | 4.493 | 2.167743 | 4.8E-08 | 1.01E-06 | yes | up | 2.17 | 3.06 | 4.06 | 25.96 | 19.76 | 18.84 | 3.096667 | 21.52 |
| VDAC2 | 0.487 | -1.03782 | 4.85E-08 | 1.02E-06 | yes | down | 258.81 | 319.93 | 265.69 | 162.48 | 236.35 | 239.6 | 281.4767 | 212.81 |
| LDLRAD3 | 2.772 | 1.471149 | 4.94E-08 | 1.03E-06 | yes | up | 3.49 | 3.77 | 4.17 | 11.98 | 14.17 | 23.46 | 3.81 | 16.53667 |
| LOC100348853 | 0.277 | -1.84961 | 4.97E-08 | 1.04E-06 | yes | down | 23.83 | 18.17 | 28.75 | 12.61 | 10.57 | 6.74 | 23.58333 | 9.973333 |
| SLC25A15 | 0.46 | -1.11969 | 5.03E-08 | 1.05E-06 | yes | down | 5.86 | 8.19 | 8.04 | 4.41 | 5.26 | 6.08 | 7.363333 | 5.25 |
| ZNF70 | 2.465 | 1.301325 | 5.06E-08 | 1.06E-06 | yes | up | 1.25 | 1.43 | 1.35 | 5.71 | 3.86 | 5.89 | 1.343333 | 5.153333 |
| MRPS26 | 0.476 | -1.07182 | 5.11E-08 | 1.06E-06 | yes | down | 55.17 | 58.69 | 51.3 | 34.26 | 47.11 | 40.01 | 55.05333 | 40.46 |
| CPT1B | 0.401 | -1.31872 | 5.24E-08 | 1.09E-06 | yes | down | 185.88 | 246.03 | 223.69 | 118.45 | 174.44 | 106.32 | 218.5333 | 133.07 |
| LIAS | 0.495 | -1.01576 | 5.3E-08 | 1.1E-06 | yes | down | 13.81 | 17.09 | 19.4 | 12.15 | 13.37 | 13.41 | 16.76667 | 12.97667 |
| MTFMT | 0.368 | -1.44132 | 5.31E-08 | 1.1E-06 | yes | down | 4.9 | 5.75 | 6.75 | 3.2 | 3.23 | 3.51 | 5.8 | 3.313333 |
| LOC108178244 | 0.454 | -1.13829 | 5.53E-08 | 1.14E-06 | yes | down | 64.48 | 67.16 | 50.01 | 43.67 | 37.54 | 49.86 | 60.55 | 43.69 |
| LOC100351828 | 0.425 | -1.23353 | 5.54E-08 | 1.14E-06 | yes | down | 12.28 | 22.36 | 17.64 | 10.37 | 11.2 | 12.39 | 17.42667 | 11.32 |
| PEBP1 | 0.438 | -1.19038 | 5.61E-08 | 1.15E-06 | yes | down | 229.34 | 388.27 | 299.62 | 159.12 | 217.78 | 240.17 | 305.7433 | 205.69 |
| ZFP36L1 | 2.643 | 1.402003 | 5.89E-08 | 1.2E-06 | yes | up | 41.24 | 38.8 | 33.57 | 104.38 | 158.85 | 206.45 | 37.87 | 156.56 |
| HARS | 0.456 | -1.13236 | 5.89E-08 | 1.2E-06 | yes | down | 11.34 | 16.53 | 15.25 | 11.8 | 9.12 | 9.32 | 14.37333 | 10.08 |
| SREBF1 | 0.325 | -1.62046 | 5.92E-08 | 1.21E-06 | yes | down | 11.61 | 16.46 | 12.25 | 4.37 | 8.97 | 6.74 | 13.44 | 6.693333 |
| MRPS21 | 0.425 | -1.23291 | 6.03E-08 | 1.23E-06 | yes | down | 19.76 | 31.8 | 27.41 | 14.24 | 17.62 | 18.6 | 26.32333 | 16.82 |
| AKAP7 | 2.584 | 1.369807 | 6.05E-08 | 1.23E-06 | yes | up | 6.68 | 6.52 | 7.18 | 25.51 | 17.01 | 29.79 | 6.793333 | 24.10333 |
| PLEKHA5 | 2.432 | 1.282226 | 6.06E-08 | 1.23E-06 | yes | up | 1.85 | 1.92 | 1.95 | 6.55 | 5.77 | 9.4 | 1.906667 | 7.24 |
| HADHA | 0.335 | -1.57617 | 6.43E-08 | 1.3E-06 | yes | down | 599.23 | 730.92 | 634.7 | 300.03 | 477.04 | 228.94 | 654.95 | 335.3367 |
| LOC108175916 | 0.006 | -7.47686 | 6.49E-08 | 1.31E-06 | yes | down | 1.86 | 1.82 | 1.65 | 0 | 0 | 0 | 1.776667 | 0 |
| IL1RN | 6.238 | 2.641164 | 6.6E-08 | 1.33E-06 | yes | up | 10.76 | 6.77 | 8.82 | 111.42 | 26.07 | 134.26 | 8.783333 | 90.58333 |
| SPSB1 | 0.324 | -1.62805 | 7.06E-08 | 1.41E-06 | yes | down | 11.53 | 25.72 | 22.84 | 8.18 | 10.3 | 11.25 | 20.03 | 9.91 |
| CDK16 | 0.478 | -1.06628 | 7.63E-08 | 1.51E-06 | yes | down | 37.72 | 56.09 | 38.17 | 28.09 | 37.15 | 31.82 | 43.99333 | 32.35333 |
| METTL17 | 0.48 | -1.05879 | 7.67E-08 | 1.52E-06 | yes | down | 17.25 | 27.85 | 17.88 | 16.46 | 14.24 | 16.66 | 20.99333 | 15.78667 |
| FASTKD2 | 0.364 | -1.45971 | 7.98E-08 | 1.58E-06 | yes | down | 3.59 | 5.03 | 3.7 | 2.23 | 1.64 | 2.96 | 4.106667 | 2.276667 |
| LOC100337966 | 0.44 | -1.18337 | 8.04E-08 | 1.59E-06 | yes | down | 673.77 | 764.3 | 694.08 | 352.85 | 613.16 | 469.13 | 710.7167 | 478.38 |
| APH1B | 2.999 | 1.584427 | 8.29E-08 | 1.63E-06 | yes | up | 12.53 | 8.57 | 6.21 | 45.25 | 34.91 | 47.62 | 9.103333 | 42.59333 |
| KYAT1 | 0.466 | -1.1016 | 8.45E-08 | 1.66E-06 | yes | down | 13.82 | 18.35 | 17.26 | 9.31 | 11.96 | 13.63 | 16.47667 | 11.63333 |
| LOC100338525 | 2.075 | 1.053058 | 8.52E-08 | 1.67E-06 | yes | up | 40.22 | 32.92 | 41.48 | 124.48 | 109.27 | 138.69 | 38.20667 | 124.1467 |
| AOX1 | 2.275 | 1.185791 | 8.72E-08 | 1.7E-06 | yes | up | 13.17 | 15.35 | 15.97 | 43.93 | 46.35 | 77.76 | 14.83 | 56.01333 |
| CCNJ | 0.406 | -1.29893 | 8.9E-08 | 1.74E-06 | yes | down | 2.03 | 3.07 | 2.85 | 1.8 | 1.4 | 1.78 | 2.65 | 1.66 |
| AR | 3.277 | 1.712526 | 9E-08 | 1.75E-06 | yes | up | 0.46 | 0.55 | 0.56 | 2.41 | 2.58 | 2.99 | 0.523333 | 2.66 |
| CD5L | 22.725 | 4.506233 | 9.07E-08 | 1.76E-06 | yes | up | 0.21 | 0.07 | 0.3 | 13.2 | 3.01 | 4.38 | 0.193333 | 6.863333 |
| FAHD1 | 0.252 | -1.99001 | 9.18E-08 | 1.78E-06 | yes | down | 9.83 | 11.37 | 10.04 | 2.26 | 4.28 | 5.88 | 10.41333 | 4.14 |
| LOC103349243 | 174.009 | 7.443019 | 9.19E-08 | 1.78E-06 | yes | up | 0 | 0 | 0 | 1.07 | 2.53 | 3.22 | 0 | 2.273333 |
| ALKBH7 | 0.337 | -1.56995 | 9.35E-08 | 1.81E-06 | yes | down | 12.8 | 16.03 | 9.88 | 5.68 | 8.15 | 6.13 | 12.90333 | 6.653333 |
| CHP1 | 0.424 | -1.23737 | 9.37E-08 | 1.81E-06 | yes | down | 32.09 | 57.45 | 43.01 | 26.93 | 24.09 | 35.62 | 44.18333 | 28.88 |
| LOC108177718 | 4.036 | 2.01302 | 9.38E-08 | 1.81E-06 | yes | up | 0.79 | 0.48 | 1.15 | 4.58 | 4.52 | 6.4 | 0.806667 | 5.166667 |
| FAM63A | 2.387 | 1.255466 | 1.01E-07 | 1.94E-06 | yes | up | 2.38 | 3.57 | 3.49 | 9.22 | 11.12 | 13.74 | 3.146667 | 11.36 |
| ACADS | 0.466 | -1.10092 | 1.03E-07 | 1.97E-06 | yes | down | 379.11 | 428.91 | 395.91 | 224.83 | 352.89 | 282.42 | 401.31 | 286.7133 |
| KITLG | 0.495 | -1.01442 | 1.03E-07 | 1.98E-06 | yes | down | 13.32 | 10.82 | 11.31 | 9.64 | 8.47 | 9.24 | 11.81667 | 9.116667 |
| LOC100344750 | 2.182 | 1.125809 | 1.05E-07 | 2E-06 | yes | up | 2.83 | 2.75 | 3.73 | 9.91 | 9.71 | 11.56 | 3.103333 | 10.39333 |
| CLPB | 0.466 | -1.1011 | 1.06E-07 | 2.02E-06 | yes | down | 9.06 | 13.25 | 12.19 | 8.04 | 9.37 | 7.24 | 11.5 | 8.216667 |
| TRAPPC4 | 0.451 | -1.15037 | 1.07E-07 | 2.05E-06 | yes | down | 34.2 | 50.76 | 57.96 | 30.56 | 31.06 | 35.88 | 47.64 | 32.5 |
| ADRB3 | 0.196 | -2.34914 | 1.17E-07 | 2.23E-06 | yes | down | 5.6 | 12.75 | 12.45 | 1.67 | 2.54 | 2.65 | 10.26667 | 2.286667 |
| TCF7L2 | 2.103 | 1.072343 | 1.19E-07 | 2.26E-06 | yes | up | 3.94 | 5.19 | 4.75 | 15.86 | 11.63 | 17.94 | 4.626667 | 15.14333 |
| C1QTNF7 | 3.18 | 1.668933 | 1.2E-07 | 2.28E-06 | yes | up | 1.9 | 1.18 | 1.56 | 6.84 | 8.36 | 6.6 | 1.546667 | 7.266667 |
| VKORC1L1 | 0.489 | -1.03325 | 1.21E-07 | 2.29E-06 | yes | down | 31.59 | 34.24 | 42.49 | 22.71 | 27.4 | 32.18 | 36.10667 | 27.43 |
| LOC100009247 | 0.474 | -1.0764 | 1.21E-07 | 2.29E-06 | yes | down | 14.58 | 21.51 | 21 | 12.7 | 15.61 | 13.79 | 19.03 | 14.03333 |
| NUS1 | 0.457 | -1.13069 | 1.22E-07 | 2.31E-06 | yes | down | 12.62 | 11.83 | 12.67 | 10.47 | 8.51 | 7.3 | 12.37333 | 8.76 |
| TST | 3.129 | 1.645846 | 1.22E-07 | 2.31E-06 | yes | up | 12.9 | 14.13 | 11.72 | 40.14 | 55.68 | 96.02 | 12.91667 | 63.94667 |
| BCL2L11 | 3.821 | 1.934123 | 1.34E-07 | 2.53E-06 | yes | up | 2.3 | 1.14 | 2.34 | 15.12 | 8.25 | 11.79 | 1.926667 | 11.72 |
| CCNG2 | 2.487 | 1.314331 | 1.35E-07 | 2.53E-06 | yes | up | 13.6 | 15.12 | 10.8 | 39.07 | 44.26 | 70.3 | 13.17333 | 51.21 |
| ITGBL1 | 2.95 | 1.560635 | 1.43E-07 | 2.67E-06 | yes | up | 1.54 | 2.91 | 2.52 | 8.26 | 14.39 | 12.65 | 2.323333 | 11.76667 |
| LOC100353852 | 5.247 | 2.391436 | 1.43E-07 | 2.67E-06 | yes | up | 1.84 | 1.27 | 0.88 | 17.56 | 5.8 | 8.78 | 1.33 | 10.71333 |
| CLSTN3 | 0.218 | -2.19994 | 1.47E-07 | 2.75E-06 | yes | down | 20.71 | 12.82 | 35.51 | 8.52 | 9.23 | 3.96 | 23.01333 | 7.236667 |
| LOC103345437 | 0.464 | -1.10829 | 1.51E-07 | 2.8E-06 | yes | down | 33.71 | 46.36 | 36.36 | 27.69 | 33.7 | 24.56 | 38.81 | 28.65 |
| LOC100342572 | 13.191 | 3.721511 | 1.53E-07 | 2.84E-06 | yes | up | 28 | 25.92 | 15.32 | 130.62 | 546.29 | 760.48 | 23.08 | 479.13 |
| CEP104 | 0.429 | -1.22012 | 1.54E-07 | 2.85E-06 | yes | down | 9.22 | 9.72 | 12.3 | 7.43 | 6.99 | 5.75 | 10.41333 | 6.723333 |
| BTG1 | 3.137 | 1.649399 | 1.55E-07 | 2.87E-06 | yes | up | 78.32 | 57.4 | 38.52 | 230.88 | 243.97 | 388.59 | 58.08 | 287.8133 |
| LOC100353320 | 0.42 | -1.25308 | 1.58E-07 | 2.92E-06 | yes | down | 249.86 | 231.89 | 228.27 | 117.4 | 190.93 | 152.19 | 236.6733 | 153.5067 |
| CERK | 0.431 | -1.21264 | 1.6E-07 | 2.95E-06 | yes | down | 18.02 | 35.18 | 23.68 | 14.5 | 17.12 | 19.07 | 25.62667 | 16.89667 |
| CLK1 | 3.104 | 1.634079 | 1.61E-07 | 2.97E-06 | yes | up | 27.67 | 45.48 | 67.22 | 167.54 | 180.46 | 245.08 | 46.79 | 197.6933 |
| UGT1-6 | 2.98 | 1.575147 | 1.68E-07 | 3.09E-06 | yes | up | 7.47 | 7.03 | 7.04 | 25.97 | 24.69 | 51.66 | 7.18 | 34.10667 |
| PRELID3A | 5.784 | 2.532164 | 1.69E-07 | 3.1E-06 | yes | up | 0.39 | 0.33 | 0.34 | 1.51 | 3.33 | 4.23 | 0.353333 | 3.023333 |
| FBXO48 | 2.706 | 1.435999 | 1.7E-07 | 3.12E-06 | yes | up | 0.67 | 1.2 | 0.49 | 3.61 | 3.9 | 5.01 | 0.786667 | 4.173333 |
| IMMT | 0.472 | -1.08451 | 1.79E-07 | 3.27E-06 | yes | down | 122.77 | 159.62 | 154.94 | 80.6 | 127.83 | 120 | 145.7767 | 109.4767 |
| SSX2IP | 0.322 | -1.63553 | 1.8E-07 | 3.29E-06 | yes | down | 1.33 | 1.95 | 3.34 | 1.25 | 0.83 | 0.74 | 2.206667 | 0.94 |
| LOC103347240 | 7.781 | 2.959885 | 1.81E-07 | 3.32E-06 | yes | up | 0.66 | 0.7 | 0.94 | 16.15 | 8.68 | 2.59 | 0.766667 | 9.14 |
| FAM184B | 2.278 | 1.187799 | 1.87E-07 | 3.4E-06 | yes | up | 3.03 | 2.77 | 2.44 | 8.09 | 7.78 | 8.43 | 2.746667 | 8.1 |
| DNAAF2 | 0.312 | -1.67964 | 1.88E-07 | 3.42E-06 | yes | down | 8.23 | 9.36 | 12.58 | 6.48 | 3.54 | 3.31 | 10.05667 | 4.443333 |
| KLHL24 | 2.599 | 1.377885 | 1.91E-07 | 3.47E-06 | yes | up | 15.58 | 9.22 | 10.76 | 50.6 | 42.59 | 53.11 | 11.85333 | 48.76667 |
| C13H1orf105 | 0.378 | -1.40376 | 1.93E-07 | 3.51E-06 | yes | down | 4.8 | 5.32 | 7.39 | 3.54 | 3.68 | 3.27 | 5.836667 | 3.496667 |
| LOC100339499 | 2.776 | 1.472817 | 1.98E-07 | 3.58E-06 | yes | up | 6.38 | 7.4 | 5.78 | 17.1 | 24.86 | 32.66 | 6.52 | 24.87333 |
| BTBD3 | 2.477 | 1.308776 | 1.98E-07 | 3.58E-06 | yes | up | 4.73 | 3.43 | 2.97 | 14.21 | 13.95 | 14.22 | 3.71 | 14.12667 |
| ATXN7L1 | 2.043 | 1.030406 | 2.13E-07 | 3.84E-06 | yes | up | 3.58 | 4.7 | 4.65 | 13.36 | 9.97 | 11.71 | 4.31 | 11.68 |
| LOC108176045 | 0.006 | -7.4195 | 2.15E-07 | 3.88E-06 | yes | down | 0.21 | 0.16 | 0.33 | 0 | 0 | 0 | 0.233333 | 0 |
| SLC7A8 | 3.131 | 1.64649 | 2.16E-07 | 3.9E-06 | yes | up | 5.43 | 7.36 | 4.69 | 37.75 | 15.91 | 31.28 | 5.826667 | 28.31333 |
| MRPS31 | 0.362 | -1.46416 | 2.21E-07 | 3.97E-06 | yes | down | 13.24 | 19.24 | 12.93 | 10.15 | 6.26 | 9.03 | 15.13667 | 8.48 |
| FIGF | 5.272 | 2.39829 | 2.25E-07 | 4.04E-06 | yes | up | 1.18 | 0.64 | 0.37 | 4.69 | 5.57 | 8.05 | 0.73 | 6.103333 |
| LOC103346967 | 0.073 | -3.76648 | 2.25E-07 | 4.04E-06 | yes | down | 4.32 | 3.92 | 2.09 | 0.62 | 0.04 | 0.57 | 3.443333 | 0.41 |
| RHOF | 0.433 | -1.20697 | 2.29E-07 | 4.1E-06 | yes | down | 11.66 | 15.61 | 17.63 | 14.62 | 11.05 | 11.08 | 14.96667 | 12.25 |
| LOC100359087 | 5.243 | 2.390335 | 2.3E-07 | 4.11E-06 | yes | up | 1.45 | 0.47 | 1.3 | 7.65 | 7.63 | 6.86 | 1.073333 | 7.38 |
| LOC108178323 | 0.003 | -8.62924 | 2.33E-07 | 4.15E-06 | yes | down | 4.25 | 7.28 | 0.12 | 0.03 | 0 | 0 | 3.883333 | 0.01 |
| SLC25A20 | 0.471 | -1.08686 | 2.33E-07 | 4.15E-06 | yes | down | 227.53 | 354.97 | 286.12 | 165.04 | 241.21 | 218.68 | 289.54 | 208.31 |
| SMUG1 | 2.393 | 1.258521 | 2.35E-07 | 4.18E-06 | yes | up | 4.98 | 7.83 | 8.54 | 20.33 | 24.15 | 18.94 | 7.116667 | 21.14 |
| FITM2 | 0.461 | -1.116 | 2.38E-07 | 4.22E-06 | yes | down | 66.18 | 60.73 | 74.86 | 41.8 | 40.81 | 63.53 | 67.25667 | 48.71333 |
| LYRM2 | 0.408 | -1.29429 | 2.38E-07 | 4.23E-06 | yes | down | 5.31 | 9.81 | 8.92 | 5.44 | 5.62 | 6.57 | 8.013333 | 5.876667 |
| NFXL1 | 2.046 | 1.033157 | 2.43E-07 | 4.3E-06 | yes | up | 1.3 | 1.77 | 1.43 | 4.64 | 4.55 | 5.02 | 1.5 | 4.736667 |
| FTSJ3 | 0.466 | -1.10301 | 2.45E-07 | 4.34E-06 | yes | down | 9.91 | 14.58 | 12.43 | 10.07 | 6.61 | 9.91 | 12.30667 | 8.863333 |
| LOC100356012 | 3.721 | 1.895823 | 2.49E-07 | 4.39E-06 | yes | up | 3.25 | 2.93 | 3.63 | 30.7 | 13.25 | 13.74 | 3.27 | 19.23 |
| NDUFA6 | 0.499 | -1.00189 | 2.57E-07 | 4.53E-06 | yes | down | 350.6 | 491 | 375.9 | 265.95 | 362.96 | 302.3 | 405.8333 | 310.4033 |
| GFM1 | 0.408 | -1.29511 | 2.62E-07 | 4.6E-06 | yes | down | 17.53 | 36.59 | 25.74 | 16.6 | 17.44 | 14.35 | 26.62 | 16.13 |
| ZFAND2B | 2.631 | 1.395641 | 2.66E-07 | 4.66E-06 | yes | up | 7.73 | 6.29 | 9.23 | 25.47 | 30.3 | 40.02 | 7.75 | 31.93 |
| ACADVL | 0.449 | -1.1551 | 2.66E-07 | 4.66E-06 | yes | down | 454.1 | 664.12 | 550.58 | 283.82 | 464.34 | 400.23 | 556.2667 | 382.7967 |
| LOC103347841 | 5.316 | 2.41024 | 2.76E-07 | 4.82E-06 | yes | up | 0.11 | 0.13 | 0.06 | 0.94 | 0.73 | 0.8 | 0.1 | 0.823333 |
| HDAC5 | 2.512 | 1.328594 | 2.77E-07 | 4.84E-06 | yes | up | 11.49 | 9.87 | 13.86 | 33.23 | 50.91 | 55.83 | 11.74 | 46.65667 |
| AFF1 | 2.983 | 1.5766 | 2.78E-07 | 4.85E-06 | yes | up | 3.19 | 2.4 | 2.05 | 15.97 | 8.09 | 11.71 | 2.546667 | 11.92333 |
| ETFA | 0.415 | -1.26787 | 2.79E-07 | 4.86E-06 | yes | down | 577.27 | 679.47 | 591.01 | 307.18 | 528.15 | 344.6 | 615.9167 | 393.31 |
| EIF2B1 | 0.466 | -1.10079 | 2.9E-07 | 5.03E-06 | yes | down | 11.81 | 19.17 | 14.16 | 10.96 | 8.85 | 12.41 | 15.04667 | 10.74 |
| LOC103347778 | 27.719 | 4.79279 | 2.95E-07 | 5.12E-06 | yes | up | 0.4 | 1.73 | 1.8 | 119.68 | 7.25 | 16.14 | 1.31 | 47.69 |
| PAK1IP1 | 0.367 | -1.44617 | 3.02E-07 | 5.22E-06 | yes | down | 6.14 | 11.48 | 7.21 | 5.16 | 4.51 | 4.2 | 8.276667 | 4.623333 |
| ADK | 0.465 | -1.10525 | 3.04E-07 | 5.26E-06 | yes | down | 12.68 | 20.82 | 16.93 | 13.47 | 13.26 | 12.24 | 16.81 | 12.99 |
| C17H14orf93 | 2.205 | 1.140667 | 3.11E-07 | 5.37E-06 | yes | up | 2.26 | 2.25 | 2.45 | 7.53 | 6.94 | 9.47 | 2.32 | 7.98 |
| LOC103347486 | 2.381 | 1.251561 | 3.14E-07 | 5.43E-06 | yes | up | 76.54 | 57.63 | 52.07 | 278.05 | 205.21 | 210.18 | 62.08 | 231.1467 |
| LOC103347367 | 0.362 | -1.46521 | 3.22E-07 | 5.55E-06 | yes | down | 53.38 | 79.13 | 73.5 | 42.41 | 45.61 | 25.47 | 68.67 | 37.83 |
| HPRT1 | 0.49 | -1.02791 | 3.22E-07 | 5.55E-06 | yes | down | 16.66 | 21.89 | 16.07 | 15.52 | 11.23 | 14.64 | 18.20667 | 13.79667 |
| SP100 | 5.899 | 2.560355 | 3.24E-07 | 5.57E-06 | yes | up | 1.34 | 1.02 | 1.53 | 20.98 | 8.76 | 5.94 | 1.296667 | 11.89333 |
| YWHAB | 0.45 | -1.15169 | 3.26E-07 | 5.6E-06 | yes | down | 61.79 | 120.42 | 96.25 | 72.02 | 56.68 | 61.72 | 92.82 | 63.47333 |
| CREBRF | 2.825 | 1.498107 | 3.31E-07 | 5.68E-06 | yes | up | 5.03 | 1.24 | 3.3 | 8.26 | 11.46 | 12.67 | 3.19 | 10.79667 |
| RAP2B | 2.183 | 1.126543 | 3.34E-07 | 5.71E-06 | yes | up | 3.76 | 3.93 | 4.42 | 12.99 | 12.36 | 16.12 | 4.036667 | 13.82333 |
| FARSB | 0.419 | -1.25525 | 3.34E-07 | 5.71E-06 | yes | down | 9.3 | 17.7 | 10.86 | 7.63 | 7.61 | 9.01 | 12.62 | 8.083333 |
| LOC103351664 | 0.498 | -1.00647 | 3.4E-07 | 5.8E-06 | yes | down | 16.18 | 19.99 | 13.79 | 13.81 | 12.63 | 12.2 | 16.65333 | 12.88 |
| KHDRBS3 | 0.337 | -1.56854 | 3.45E-07 | 5.87E-06 | yes | down | 2.44 | 2.88 | 2.68 | 1.13 | 1.83 | 1.18 | 2.666667 | 1.38 |
| MICALL1 | 0.384 | -1.38188 | 3.49E-07 | 5.93E-06 | yes | down | 8.31 | 10.67 | 11.92 | 7.7 | 6.09 | 4.42 | 10.3 | 6.07 |
| PSTK | 2.046 | 1.032895 | 3.54E-07 | 6E-06 | yes | up | 11.45 | 11.46 | 9.82 | 14.7 | 16.13 | 20.59 | 10.91 | 17.14 |
| SCRN1 | 0.377 | -1.40854 | 3.55E-07 | 6E-06 | yes | down | 4.31 | 6.82 | 4.93 | 2.09 | 3.52 | 3.73 | 5.353333 | 3.113333 |
| LOC100356242 | 41.006 | 5.357779 | 3.57E-07 | 6.04E-06 | yes | up | 0.55 | 0.16 | 0.25 | 42.98 | 15.59 | 3.86 | 0.32 | 20.81 |
| ABHD11 | 0.35 | -1.51534 | 3.69E-07 | 6.22E-06 | yes | down | 5.8 | 9.15 | 7.64 | 3.65 | 4.74 | 3.42 | 7.53 | 3.936667 |
| SAMHD1 | 3.913 | 1.968383 | 3.69E-07 | 6.22E-06 | yes | up | 6.12 | 9.04 | 7.74 | 73.86 | 22.2 | 39.04 | 7.633333 | 45.03333 |
| CPEB1 | 3.914 | 1.968678 | 3.73E-07 | 6.27E-06 | yes | up | 0.54 | 0.59 | 0.53 | 2.15 | 3.23 | 4.8 | 0.553333 | 3.393333 |
| PPARGC1A | 0.39 | -1.35753 | 3.76E-07 | 6.31E-06 | yes | down | 3.02 | 3.95 | 2.03 | 1.84 | 1.71 | 1.89 | 3 | 1.813333 |
| ISM1 | 6.33 | 2.662101 | 3.84E-07 | 6.43E-06 | yes | up | 0.25 | 0.22 | 0.33 | 3.94 | 1.37 | 2.62 | 0.266667 | 2.643333 |
| GNPTAB | 2.037 | 1.026589 | 3.88E-07 | 6.5E-06 | yes | up | 2.69 | 2.36 | 3.08 | 8.84 | 7.62 | 9.43 | 2.71 | 8.63 |
| BATF2 | 5.563 | 2.475734 | 3.93E-07 | 6.56E-06 | yes | up | 1.25 | 1.11 | 0.95 | 17.02 | 5.58 | 5.95 | 1.103333 | 9.516667 |
| FRMD8 | 0.456 | -1.1339 | 3.96E-07 | 6.6E-06 | yes | down | 14.35 | 18.9 | 18.75 | 14.53 | 9.29 | 11.51 | 17.33333 | 11.77667 |
| CDC42EP1 | 0.484 | -1.04791 | 4.03E-07 | 6.69E-06 | yes | down | 16.07 | 22.73 | 21.44 | 12.46 | 14.04 | 18.67 | 20.08 | 15.05667 |
| SLC5A6 | 0.484 | -1.04579 | 4.06E-07 | 6.73E-06 | yes | down | 5.28 | 4.93 | 5.54 | 3.44 | 3.59 | 4.61 | 5.25 | 3.88 |
| CH25H | 4.294 | 2.102375 | 4.07E-07 | 6.74E-06 | yes | up | 1.04 | 0.89 | 0.58 | 4 | 4.42 | 8.48 | 0.836667 | 5.633333 |
| CIDEC | 0.47 | -1.08794 | 4.11E-07 | 6.8E-06 | yes | down | 629.11 | 763.04 | 793.54 | 381.5 | 558.58 | 655.13 | 728.5633 | 531.7367 |
| HOGA1 | 0.399 | -1.32699 | 4.15E-07 | 6.86E-06 | yes | down | 13.02 | 12.69 | 14.35 | 10.27 | 7.84 | 6.29 | 13.35333 | 8.133333 |
| ADAM23 | 6.411 | 2.680647 | 4.18E-07 | 6.89E-06 | yes | up | 0.25 | 0.47 | 0.15 | 1.46 | 3.79 | 4.15 | 0.29 | 3.133333 |
| SLC22A4 | 0.251 | -1.99533 | 4.2E-07 | 6.93E-06 | yes | down | 2.75 | 2.34 | 2.48 | 0.75 | 0.76 | 1.32 | 2.523333 | 0.943333 |
| SYCE3 | 0.126 | -2.99264 | 4.35E-07 | 7.15E-06 | yes | down | 4.16 | 2.14 | 5.24 | 3.03 | 10.9 | 1.33 | 3.846667 | 5.086667 |
| TXLNG | 0.424 | -1.23949 | 4.72E-07 | 7.73E-06 | yes | down | 5.52 | 9.49 | 9.82 | 5.36 | 4.47 | 6.33 | 8.276667 | 5.386667 |
| ANKS1A | 2.098 | 1.068848 | 4.75E-07 | 7.77E-06 | yes | up | 27.18 | 21.61 | 25.88 | 66.64 | 83.83 | 93.85 | 24.89 | 81.44 |
| PCK1 | 0.343 | -1.54512 | 4.75E-07 | 7.77E-06 | yes | down | 1139.11 | 1416.21 | 998.55 | 539.27 | 380.08 | 979.58 | 1184.623 | 632.9767 |
| CACNB4 | 0.222 | -2.1737 | 4.76E-07 | 7.77E-06 | yes | down | 1.83 | 1.52 | 1.61 | 1.21 | 3.72 | 1.48 | 1.653333 | 2.136667 |
| SIAH2 | 2.812 | 1.491711 | 4.79E-07 | 7.81E-06 | yes | up | 130.45 | 86.78 | 86.75 | 334.8 | 390.1 | 629.99 | 101.3267 | 451.63 |
| C14H3orf33 | 0.251 | -1.99383 | 4.91E-07 | 8.01E-06 | yes | down | 2.26 | 3.12 | 2.74 | 0.83 | 1.06 | 1.28 | 2.706667 | 1.056667 |
| MGLL | 0.438 | -1.19053 | 4.96E-07 | 8.08E-06 | yes | down | 178.47 | 184.18 | 212.51 | 96.86 | 163.74 | 128.55 | 191.72 | 129.7167 |
| LOC103346717 | 0.163 | -2.61987 | 4.97E-07 | 8.08E-06 | yes | down | 0.72 | 0.42 | 1.03 | 0.12 | 0.22 | 0.22 | 0.723333 | 0.186667 |
| TRAF4 | 0.395 | -1.34053 | 5.02E-07 | 8.15E-06 | yes | down | 14.18 | 20.67 | 20.15 | 7.66 | 11.7 | 14.33 | 18.33333 | 11.23 |
| LOC100339318 | 8.306 | 3.05422 | 5.13E-07 | 8.31E-06 | yes | up | 2 | 2.12 | 2.88 | 84.99 | 8.61 | 20.79 | 2.333333 | 38.13 |
| MRPS30 | 0.487 | -1.03871 | 5.16E-07 | 8.34E-06 | yes | down | 14.55 | 19.81 | 14.64 | 10.32 | 11.24 | 15.4 | 16.33333 | 12.32 |
| GDPGP1 | 0.489 | -1.03226 | 5.39E-07 | 8.68E-06 | yes | down | 5.09 | 5.81 | 6.46 | 4.56 | 3.48 | 5.16 | 5.786667 | 4.4 |
| NDN | 0.362 | -1.46675 | 5.44E-07 | 8.73E-06 | yes | down | 21.73 | 54.68 | 34.85 | 18.12 | 18.67 | 24.54 | 37.08667 | 20.44333 |
| SMAD7 | 0.38 | -1.39736 | 5.56E-07 | 8.9E-06 | yes | down | 9.32 | 13.7 | 12.21 | 5 | 5.75 | 10.2 | 11.74333 | 6.983333 |
| LARP6 | 2.838 | 1.504678 | 5.58E-07 | 8.9E-06 | yes | up | 1.13 | 2.02 | 1.69 | 6.64 | 6.04 | 8.56 | 1.613333 | 7.08 |
| PTPA | 0.457 | -1.12895 | 5.68E-07 | 9.04E-06 | yes | down | 18.02 | 31.01 | 26.47 | 19.7 | 16.41 | 17.04 | 25.16667 | 17.71667 |
| BOLA3 | 0.383 | -1.38388 | 5.7E-07 | 9.06E-06 | yes | down | 64.87 | 113.09 | 66.29 | 45.28 | 52.13 | 42.6 | 81.41667 | 46.67 |
| LOC100342321 | 81.337 | 6.345837 | 5.81E-07 | 9.23E-06 | yes | up | 0.09 | 0.11 | 0.05 | 25.23 | 4.96 | 1.16 | 0.083333 | 10.45 |
| GPATCH4 | 0.395 | -1.34161 | 6.01E-07 | 9.51E-06 | yes | down | 4.65 | 8.48 | 6.08 | 4.13 | 3.14 | 4.47 | 6.403333 | 3.913333 |
| LOC100341136 | 5.503 | 2.460231 | 6.52E-07 | 1.03E-05 | yes | up | 0.57 | 0.82 | 1.54 | 13.64 | 5.46 | 4.7 | 0.976667 | 7.933333 |
| PC | 0.433 | -1.2077 | 6.64E-07 | 1.05E-05 | yes | down | 8.22 | 13.1 | 11.42 | 6.41 | 8.54 | 6.72 | 10.91333 | 7.223333 |
| NAGLU | 0.449 | -1.15528 | 6.71E-07 | 1.06E-05 | yes | down | 6.6 | 10.52 | 9.58 | 5.91 | 5.59 | 8.01 | 8.9 | 6.503333 |
| RPL36AL | 0.498 | -1.00453 | 6.72E-07 | 1.06E-05 | yes | down | 70.37 | 84.82 | 78.47 | 56.38 | 47.53 | 77.87 | 77.88667 | 60.59333 |
| XAF1 | 8.381 | 3.067094 | 6.77E-07 | 1.06E-05 | yes | up | 2.91 | 1.59 | 2.3 | 59.41 | 19.75 | 9.51 | 2.266667 | 29.55667 |
| C8H12orf4 | 2.596 | 1.376273 | 6.87E-07 | 1.08E-05 | yes | up | 1.99 | 2.46 | 2.07 | 10.74 | 7.36 | 7.49 | 2.173333 | 8.53 |
| RENBP | 3.678 | 1.879044 | 6.94E-07 | 1.09E-05 | yes | up | 2.49 | 3.19 | 3.79 | 26.72 | 13.02 | 12.26 | 3.156667 | 17.33333 |
| NME4 | 0.415 | -1.26874 | 7.05E-07 | 1.1E-05 | yes | down | 64.5 | 97.57 | 59.25 | 30.99 | 54.63 | 49.91 | 73.77333 | 45.17667 |
| LOC103346040 | 3.25 | 1.700628 | 7.06E-07 | 1.1E-05 | yes | up | 1.34 | 1.07 | 1.64 | 4.68 | 9.55 | 8.73 | 1.35 | 7.653333 |
| APOD | 2.304 | 1.204036 | 7.07E-07 | 1.1E-05 | yes | up | 196.09 | 178.62 | 266.02 | 608.77 | 739.71 | 981.09 | 213.5767 | 776.5233 |
| BCAM | 2.559 | 1.355599 | 7.14E-07 | 1.11E-05 | yes | up | 29.44 | 34.71 | 25.75 | 78.07 | 111.57 | 169 | 29.96667 | 119.5467 |
| LOC108178486 | 3.737 | 1.901793 | 7.29E-07 | 1.14E-05 | yes | up | 0.4 | 0.3 | 0.78 | 3.69 | 2.32 | 3.15 | 0.493333 | 3.053333 |
| LOC103346740 | 0.328 | -1.60667 | 7.4E-07 | 1.15E-05 | yes | down | 119.38 | 133.48 | 150.3 | 68.51 | 80.06 | 50.35 | 134.3867 | 66.30667 |
| LOC100345908 | 4.519 | 2.175928 | 7.64E-07 | 1.18E-05 | yes | up | 3.24 | 7.94 | 5.17 | 15.55 | 34.17 | 57.22 | 5.45 | 35.64667 |
| GPR85 | 0.316 | -1.6625 | 7.66E-07 | 1.19E-05 | yes | down | 2.2 | 2.78 | 3.18 | 1.13 | 1.76 | 2.1 | 2.72 | 1.663333 |
| SLC24A3 | 0.424 | -1.23894 | 7.83E-07 | 1.21E-05 | yes | down | 16.83 | 14.54 | 21.65 | 9.7 | 10.45 | 15.12 | 17.67333 | 11.75667 |
| ARHGEF37 | 0.5 | -1.00053 | 7.86E-07 | 1.21E-05 | yes | down | 8.88 | 15.27 | 13.84 | 9.12 | 9.79 | 10.09 | 12.66333 | 9.666667 |
| NYNRIN | 2.196 | 1.135085 | 8.15E-07 | 1.25E-05 | yes | up | 0.81 | 1.11 | 1.17 | 3.84 | 3.62 | 2.97 | 1.03 | 3.476667 |
| MME | 0.416 | -1.26413 | 8.16E-07 | 1.26E-05 | yes | down | 32.76 | 28.02 | 27.2 | 17.34 | 23.65 | 14.82 | 29.32667 | 18.60333 |
| LOC100345689 | 59.064 | 5.884199 | 8.54E-07 | 1.31E-05 | yes | up | 0 | 0 | 0.05 | 2.21 | 1.46 | 1.1 | 0.016667 | 1.59 |
| ROGDI | 0.414 | -1.27183 | 8.73E-07 | 1.33E-05 | yes | down | 8.5 | 8.96 | 10.79 | 5.17 | 5.28 | 7.88 | 9.416667 | 6.11 |
| CCL19 | 16.012 | 4.001111 | 8.8E-07 | 1.34E-05 | yes | up | 0.59 | 0.23 | 0.52 | 21.95 | 4.3 | 7.29 | 0.446667 | 11.18 |
| RARG | 2.385 | 1.253784 | 8.86E-07 | 1.35E-05 | yes | up | 2.83 | 5.09 | 5.29 | 13.44 | 14.44 | 20.74 | 4.403333 | 16.20667 |
| SAT1 | 2.264 | 1.179188 | 8.94E-07 | 1.36E-05 | yes | up | 225.11 | 167.97 | 178.26 | 699.74 | 664.87 | 810.96 | 190.4467 | 725.19 |
| BARD1 | 2.443 | 1.288721 | 9.06E-07 | 1.38E-05 | yes | up | 1.44 | 1.15 | 1.25 | 4 | 4.83 | 5.65 | 1.28 | 4.826667 |
| TET2 | 2.077 | 1.054731 | 9.24E-07 | 1.4E-05 | yes | up | 5.21 | 4.77 | 3.79 | 11.8 | 15.37 | 14.94 | 4.59 | 14.03667 |
| LGALS3BP | 3.372 | 1.753615 | 9.43E-07 | 1.43E-05 | yes | up | 16.55 | 18.45 | 14.16 | 116.2 | 94.25 | 43.07 | 16.38667 | 84.50667 |
| RASA3 | 0.475 | -1.07399 | 9.59E-07 | 1.45E-05 | yes | down | 10.62 | 18.43 | 14.94 | 12.15 | 9.36 | 10.52 | 14.66333 | 10.67667 |
| NPR3 | 3.063 | 1.615052 | 9.94E-07 | 1.5E-05 | yes | up | 18.03 | 26.3 | 25.86 | 124.48 | 133.74 | 58.58 | 23.39667 | 105.6 |
| PIK3IP1 | 3.032 | 1.600298 | 1E-06 | 1.51E-05 | yes | up | 9.16 | 4.92 | 7.58 | 25.98 | 33.86 | 45.63 | 7.22 | 35.15667 |
| MAMLD1 | 2.29 | 1.195644 | 1.01E-06 | 1.52E-05 | yes | up | 2.31 | 2.15 | 2.98 | 10.04 | 8.34 | 7.79 | 2.48 | 8.723333 |
| LOC103351994 | 2.243 | 1.165299 | 1.04E-06 | 1.56E-05 | yes | up | 1.07 | 1.68 | 1.64 | 4.55 | 4.84 | 5.87 | 1.463333 | 5.086667 |
| FRY | 2.05 | 1.035512 | 1.06E-06 | 1.59E-05 | yes | up | 5.99 | 4.74 | 4.04 | 17.91 | 15.74 | 21.15 | 4.923333 | 18.26667 |
| FLRT2 | 2.286 | 1.192563 | 1.07E-06 | 1.6E-05 | yes | up | 1.2 | 1.48 | 0.92 | 3.43 | 4.42 | 4.84 | 1.2 | 4.23 |
| MDK | 5.449 | 2.445923 | 1.11E-06 | 1.66E-05 | yes | up | 3.91 | 8.57 | 3.42 | 74.54 | 24.36 | 32.09 | 5.3 | 43.66333 |
| LOC108175423 | 0.389 | -1.36234 | 1.12E-06 | 1.67E-05 | yes | down | 12.18 | 21.09 | 20.32 | 10.11 | 12.45 | 9.44 | 17.86333 | 10.66667 |
| IGSF8 | 2.583 | 1.369286 | 1.21E-06 | 1.8E-05 | yes | up | 4 | 4.85 | 5.74 | 14.58 | 16.68 | 27.74 | 4.863333 | 19.66667 |
| CNNM4 | 2.373 | 1.246769 | 1.22E-06 | 1.81E-05 | yes | up | 1.64 | 1.51 | 2.3 | 6.32 | 7.4 | 6.35 | 1.816667 | 6.69 |
| WNT5B | 0.302 | -1.72785 | 1.23E-06 | 1.82E-05 | yes | down | 5.9 | 4.97 | 8.46 | 2.24 | 3.14 | 3.7 | 6.443333 | 3.026667 |
| REPS1 | 2.205 | 1.140862 | 1.23E-06 | 1.82E-05 | yes | up | 5.87 | 5.08 | 5 | 16.46 | 15.02 | 23.54 | 5.316667 | 18.34 |
| ADM | 2.768 | 1.468599 | 1.31E-06 | 1.93E-05 | yes | up | 23.12 | 23.25 | 18.82 | 56.3 | 92.99 | 135.49 | 21.73 | 94.92667 |
| LOC100342015 | 2.58 | 1.367455 | 1.32E-06 | 1.93E-05 | yes | up | 0.34 | 0.44 | 0.51 | 1.64 | 1.58 | 1.54 | 0.43 | 1.586667 |
| DHX58 | 3.095 | 1.629743 | 1.33E-06 | 1.95E-05 | yes | up | 3.61 | 4.71 | 5.57 | 25.57 | 27.04 | 13.06 | 4.63 | 21.89 |
| RET | 0.327 | -1.61252 | 1.35E-06 | 1.98E-05 | yes | down | 0.99 | 1.16 | 0.84 | 0.55 | 0.5 | 0.47 | 0.996667 | 0.506667 |
| ATPAF1 | 0.488 | -1.03586 | 1.38E-06 | 2.01E-05 | yes | down | 8.8 | 16.26 | 11.89 | 9.26 | 9.02 | 9 | 12.31667 | 9.093333 |
| TGFBR3 | 2.081 | 1.057568 | 1.38E-06 | 2.03E-05 | yes | up | 23.97 | 19.29 | 21.02 | 66.43 | 56.35 | 90.46 | 21.42667 | 71.08 |
| SLC43A1 | 2.377 | 1.249341 | 1.4E-06 | 2.05E-05 | yes | up | 8.18 | 7.96 | 7.9 | 20.26 | 24.38 | 38.03 | 8.013333 | 27.55667 |
| LOC100338010 | 0.493 | -1.02163 | 1.43E-06 | 2.08E-05 | yes | down | 16.49 | 26.8 | 21.19 | 16.67 | 13.86 | 15.79 | 21.49333 | 15.44 |
| RRAGB | 0.357 | -1.48463 | 1.49E-06 | 2.16E-05 | yes | down | 2.9 | 3.96 | 2.84 | 1.78 | 2.09 | 1.43 | 3.233333 | 1.766667 |
| NIPSNAP1 | 0.263 | -1.92718 | 1.51E-06 | 2.18E-05 | yes | down | 64.77 | 69.95 | 61.53 | 17.06 | 46.96 | 18.09 | 65.41667 | 27.37 |
| CP | 9.854 | 3.300639 | 1.53E-06 | 2.22E-05 | yes | up | 0.8 | 0.41 | 0.47 | 18.47 | 2.22 | 4.73 | 0.56 | 8.473333 |
| LOC108177287 | 0.197 | -2.34164 | 1.56E-06 | 2.26E-05 | yes | down | 18.01 | 20.34 | 12.26 | 2.22 | 7.12 | 6.05 | 16.87 | 5.13 |
| SLC1A4 | 0.268 | -1.89903 | 1.63E-06 | 2.34E-05 | yes | down | 2.7 | 4.14 | 4.9 | 2.4 | 0.88 | 1.59 | 3.913333 | 1.623333 |
| LOC108177747 | 27.793 | 4.796644 | 1.67E-06 | 2.41E-05 | yes | up | 0.01 | 0.01 | 0.01 | 0.58 | 0.43 | 0.54 | 0.01 | 0.516667 |
| TMEM120A | 0.209 | -2.25908 | 1.68E-06 | 2.42E-05 | yes | down | 16.98 | 29.6 | 25.58 | 8.18 | 12.41 | 3.89 | 24.05333 | 8.16 |
| ELOVL6 | 0.468 | -1.0945 | 1.71E-06 | 2.46E-05 | yes | down | 27.38 | 33.17 | 29.68 | 16.28 | 19.41 | 29.66 | 30.07667 | 21.78333 |
| CHCHD5 | 0.446 | -1.16341 | 1.72E-06 | 2.47E-05 | yes | down | 12.91 | 18.83 | 16.24 | 10.23 | 12.04 | 10.6 | 15.99333 | 10.95667 |
| CNTROB | 2.231 | 1.157946 | 1.72E-06 | 2.47E-05 | yes | up | 2.13 | 1.85 | 2.12 | 6.88 | 7.33 | 6.44 | 2.033333 | 6.883333 |
| HOXA6 | 2.777 | 1.473634 | 1.73E-06 | 2.48E-05 | yes | up | 2.98 | 2.37 | 4.27 | 10.99 | 14.75 | 16.19 | 3.206667 | 13.97667 |
| NAB2 | 2.013 | 1.009099 | 1.74E-06 | 2.49E-05 | yes | up | 2.52 | 3.12 | 3.5 | 10.55 | 8.33 | 9.5 | 3.046667 | 9.46 |
| LOC103344945 | 2.844 | 1.50812 | 1.78E-06 | 2.55E-05 | yes | up | 34.32 | 22.33 | 24.9 | 137.31 | 71.78 | 159.4 | 27.18333 | 122.83 |
| TTC39C | 2.446 | 1.290324 | 1.81E-06 | 2.58E-05 | yes | up | 1.13 | 1.49 | 1.75 | 5.57 | 4.74 | 6.28 | 1.456667 | 5.53 |
| ST3GAL3 | 0.485 | -1.04352 | 1.87E-06 | 2.67E-05 | yes | down | 10.79 | 16.04 | 10.36 | 9.19 | 9.75 | 9.4 | 12.39667 | 9.446667 |
| UCK2 | 0.418 | -1.25781 | 1.93E-06 | 2.75E-05 | yes | down | 9.56 | 10.94 | 9.02 | 5.57 | 4.47 | 9.33 | 9.84 | 6.456667 |
| LOC103346934 | 0.382 | -1.38959 | 1.95E-06 | 2.77E-05 | yes | down | 2.81 | 4.25 | 3.55 | 1.73 | 1.97 | 2.67 | 3.536667 | 2.123333 |
| SLC30A2 | 55.884 | 5.804363 | 1.96E-06 | 2.78E-05 | yes | up | 0.02 | 0 | 0.01 | 1.48 | 0.65 | 0.55 | 0.01 | 0.893333 |
| SFMBT2 | 2.084 | 1.059404 | 2.03E-06 | 2.86E-05 | yes | up | 1.89 | 1.42 | 1.59 | 5.53 | 5.09 | 5.41 | 1.633333 | 5.343333 |
| NWD2 | 0.381 | -1.39289 | 2.03E-06 | 2.87E-05 | yes | down | 6.04 | 6.74 | 9.13 | 2.74 | 3.51 | 4.35 | 7.303333 | 3.533333 |
| LOC108176153 | 0.419 | -1.25631 | 2.05E-06 | 2.89E-05 | yes | down | 39.33 | 52.22 | 47.73 | 21.09 | 31.99 | 37.37 | 46.42667 | 30.15 |
| PDGFD | 3.023 | 1.59577 | 2.07E-06 | 2.91E-05 | yes | up | 29 | 18.88 | 24.6 | 73.02 | 105.74 | 140.97 | 24.16 | 106.5767 |
| PNO1 | 115.3 | 6.849251 | 2.1E-06 | 2.94E-05 | yes | up | 0 | 0 | 0 | 2.53 | 1.79 | 1.03 | 0 | 1.783333 |
| TUBG1 | 0.487 | -1.03682 | 2.12E-06 | 2.96E-05 | yes | down | 12.31 | 21.39 | 15.01 | 10.84 | 12.05 | 13.55 | 16.23667 | 12.14667 |
| LOC103352291 | 2.692 | 1.428539 | 2.13E-06 | 2.97E-05 | yes | up | 10.43 | 12.54 | 11.68 | 57.76 | 32.31 | 59.46 | 11.55 | 49.84333 |
| ASNS | 0.434 | -1.20505 | 2.17E-06 | 3.02E-05 | yes | down | 5.62 | 7.74 | 8.64 | 5.33 | 5.03 | 4.31 | 7.333333 | 4.89 |
| IRGM | 80.492 | 6.330766 | 2.22E-06 | 3.1E-05 | yes | up | 0.07 | 0.18 | 0.1 | 84.72 | 1.68 | 3.58 | 0.116667 | 29.99333 |
| PCOLCE | 2.379 | 1.250109 | 2.23E-06 | 3.11E-05 | yes | up | 26.79 | 43.48 | 33.96 | 149.83 | 138.41 | 88.97 | 34.74333 | 125.7367 |
| TRUB1 | 0.426 | -1.2301 | 2.24E-06 | 3.11E-05 | yes | down | 8.37 | 7.4 | 8.2 | 6.65 | 4.44 | 4.42 | 7.99 | 5.17 |
| BMPER | 3.804 | 1.927621 | 2.31E-06 | 3.21E-05 | yes | up | 0.28 | 0.52 | 0.42 | 1.56 | 2.89 | 2.67 | 0.406667 | 2.373333 |
| NAALADL2 | 2.332 | 1.221747 | 2.33E-06 | 3.23E-05 | yes | up | 0.44 | 0.44 | 0.48 | 1.31 | 1.76 | 1.84 | 0.453333 | 1.636667 |
| NFS1 | 0.477 | -1.06936 | 2.35E-06 | 3.26E-05 | yes | down | 26.21 | 42.97 | 36.96 | 21.2 | 23.2 | 32.18 | 35.38 | 25.52667 |
| DUOX2 | 0.208 | -2.26493 | 2.43E-06 | 3.36E-05 | yes | down | 3.16 | 5.69 | 1.45 | 0.62 | 1.32 | 1.32 | 3.433333 | 1.086667 |
| CDH18 | 3.696 | 1.885782 | 2.45E-06 | 3.38E-05 | yes | up | 0.75 | 0.46 | 0.96 | 3.83 | 4.78 | 2.58 | 0.723333 | 3.73 |
| PARP12 | 4.003 | 2.001172 | 2.45E-06 | 3.39E-05 | yes | up | 2.35 | 3.21 | 2.05 | 26.69 | 9.98 | 9.94 | 2.536667 | 15.53667 |
| HSPB1 | 0.46 | -1.12162 | 2.46E-06 | 3.4E-05 | yes | down | 274.65 | 324.24 | 188.18 | 150.47 | 206.63 | 200.69 | 262.3567 | 185.93 |
| KIAA1551 | 2.97 | 1.570223 | 2.48E-06 | 3.42E-05 | yes | up | 2.75 | 1.62 | 1.38 | 11.07 | 9.09 | 8.21 | 1.916667 | 9.456667 |
| CISD3 | 0.468 | -1.09533 | 2.5E-06 | 3.43E-05 | yes | down | 217.65 | 290.69 | 251.37 | 159.1 | 232.35 | 153.51 | 253.2367 | 181.6533 |
| LOC100357878 | 6.525 | 2.706037 | 2.57E-06 | 3.51E-05 | yes | up | 0.09 | 0.26 | 0.15 | 1.91 | 1.45 | 1.63 | 0.166667 | 1.663333 |
| DCLRE1B | 2.498 | 1.320913 | 2.62E-06 | 3.59E-05 | yes | up | 0.88 | 1.32 | 1.42 | 5.23 | 4.31 | 4.4 | 1.206667 | 4.646667 |
| SLC15A3 | 5.307 | 2.407837 | 2.64E-06 | 3.6E-05 | yes | up | 1.37 | 1.77 | 1.78 | 25.73 | 6.46 | 8.13 | 1.64 | 13.44 |
| IL15RA | 4.145 | 2.051453 | 2.67E-06 | 3.64E-05 | yes | up | 1.01 | 0.37 | 0.6 | 12.51 | 2.56 | 4.36 | 0.66 | 6.476667 |
| LOC103348556 | 0.385 | -1.37604 | 2.74E-06 | 3.73E-05 | yes | down | 14.2 | 13.07 | 21.12 | 6.42 | 11.79 | 12.99 | 16.13 | 10.4 |
| PPP1R3E | 2.813 | 1.492102 | 2.79E-06 | 3.79E-05 | yes | up | 9.68 | 8.61 | 14.85 | 30.54 | 53.85 | 61.58 | 11.04667 | 48.65667 |
| ACO1 | 0.484 | -1.04721 | 2.85E-06 | 3.86E-05 | yes | down | 51.58 | 88.51 | 68.96 | 39.1 | 51.18 | 61.97 | 69.68333 | 50.75 |
| SLCO2B1 | 3.857 | 1.947663 | 3E-06 | 4.04E-05 | yes | up | 0.34 | 0.54 | 0.26 | 1.69 | 2.88 | 2.07 | 0.38 | 2.213333 |
| HSDL2 | 0.498 | -1.00697 | 3.05E-06 | 4.1E-05 | yes | down | 91.21 | 161.85 | 131.09 | 80.09 | 103.02 | 110.68 | 128.05 | 97.93 |
| SLC25A33 | 0.282 | -1.8278 | 3.07E-06 | 4.12E-05 | yes | down | 37.84 | 44.91 | 48.28 | 8.85 | 17.09 | 32.07 | 43.67667 | 19.33667 |
| PTER | 0.471 | -1.08472 | 3.17E-06 | 4.25E-05 | yes | down | 7.51 | 9.44 | 12.17 | 7.75 | 6.98 | 6.49 | 9.706667 | 7.073333 |
| GRTP1 | 0.351 | -1.51034 | 3.29E-06 | 4.39E-05 | yes | down | 3.61 | 3.99 | 3.23 | 1.83 | 2.17 | 1.93 | 3.61 | 1.976667 |
| COL25A1 | 5.925 | 2.566735 | 3.29E-06 | 4.39E-05 | yes | up | 0.23 | 0.22 | 0.18 | 3.36 | 1.09 | 1.07 | 0.21 | 1.84 |
| RORC | 4.945 | 2.306061 | 3.31E-06 | 4.41E-05 | yes | up | 3.26 | 1.5 | 2.16 | 10.87 | 11.32 | 26.9 | 2.306667 | 16.36333 |
| METTL25 | 0.359 | -1.47796 | 3.32E-06 | 4.43E-05 | yes | down | 2.15 | 2.48 | 2.45 | 1.24 | 1.49 | 1.29 | 2.36 | 1.34 |
| SHCBP1L | 0.198 | -2.33546 | 3.42E-06 | 4.54E-05 | yes | down | 2.51 | 2.4 | 2.25 | 1.45 | 0.39 | 0.43 | 2.386667 | 0.756667 |
| ND1 | 0.489 | -1.03196 | 3.56E-06 | 4.71E-05 | yes | down | 8712.85 | 7446.45 | 8051.58 | 7322.61 | 6115.41 | 4937.21 | 8070.293 | 6125.077 |
| MOV10 | 2.451 | 1.293142 | 3.75E-06 | 4.95E-05 | yes | up | 1.66 | 1.79 | 1.81 | 8.83 | 5.86 | 5.26 | 1.753333 | 6.65 |
| LOC100349976 | 0.307 | -1.70497 | 3.76E-06 | 4.97E-05 | yes | down | 3.49 | 8.88 | 5.83 | 3.49 | 2.45 | 2.53 | 6.066667 | 2.823333 |
| ACOX2 | 0.36 | -1.47323 | 3.86E-06 | 5.09E-05 | yes | down | 7.93 | 7.89 | 13.15 | 4.94 | 4.07 | 7.44 | 9.656667 | 5.483333 |
| ATP5SL | 0.498 | -1.00469 | 3.86E-06 | 5.09E-05 | yes | down | 13.11 | 13.78 | 13.11 | 8.38 | 7.92 | 12.45 | 13.33333 | 9.583333 |
| TTC4 | 0.439 | -1.18888 | 3.89E-06 | 5.13E-05 | yes | down | 4.31 | 6.27 | 6.08 | 3.73 | 2.83 | 4.88 | 5.553333 | 3.813333 |
| B3GNT4 | 3.676 | 1.878014 | 4E-06 | 5.26E-05 | yes | up | 1.16 | 0.54 | 0.88 | 4.59 | 4.8 | 5.57 | 0.86 | 4.986667 |
| AVEN | 0.452 | -1.1455 | 4.06E-06 | 5.33E-05 | yes | down | 6.35 | 9.5 | 6.59 | 5.61 | 4.59 | 5.53 | 7.48 | 5.243333 |
| DMBT1 | 0.017 | -5.85369 | 4.13E-06 | 5.43E-05 | yes | down | 1.37 | 4.47 | 7.38 | 0.05 | 0.07 | 0.29 | 4.406667 | 0.136667 |
| HSF4 | 4.703 | 2.233442 | 4.16E-06 | 5.46E-05 | yes | up | 0.23 | 0.45 | 0.53 | 2 | 2.52 | 3.72 | 0.403333 | 2.746667 |
| YPEL1 | 2.071 | 1.05025 | 4.4E-06 | 5.75E-05 | yes | up | 3.04 | 3.05 | 3.52 | 8.27 | 11.05 | 11.65 | 3.203333 | 10.32333 |
| ARMCX6 | 2.111 | 1.077805 | 4.46E-06 | 5.8E-05 | yes | up | 2.61 | 3.84 | 3.05 | 8.43 | 11 | 11.12 | 3.166667 | 10.18333 |
| PLA2G12A | 0.444 | -1.17212 | 4.46E-06 | 5.8E-05 | yes | down | 16.75 | 22.2 | 12.19 | 10.68 | 11 | 13.82 | 17.04667 | 11.83333 |
| SPTA1 | 0.213 | -2.2295 | 4.46E-06 | 5.8E-05 | yes | down | 0.88 | 0.94 | 0.6 | 0.45 | 0.42 | 0.56 | 0.806667 | 0.476667 |
| CAMKK2 | 2.054 | 1.038711 | 4.47E-06 | 5.8E-05 | yes | up | 2.25 | 3.08 | 3.46 | 7.76 | 8.88 | 11.28 | 2.93 | 9.306667 |
| LOC100346269 | 2.272 | 1.183813 | 4.51E-06 | 5.85E-05 | yes | up | 0.65 | 1.14 | 1.23 | 3.09 | 2.53 | 3.3 | 1.006667 | 2.973333 |
| RSAD2 | 5.131 | 2.359139 | 4.56E-06 | 5.91E-05 | yes | up | 2.83 | 1.27 | 1.25 | 21.82 | 13.39 | 6.35 | 1.783333 | 13.85333 |
| NUP35 | 0.379 | -1.39801 | 4.59E-06 | 5.95E-05 | yes | down | 5.4 | 11.46 | 9.88 | 4.51 | 5.77 | 5.2 | 8.913333 | 5.16 |
| PTGR1 | 0.419 | -1.25392 | 4.61E-06 | 5.96E-05 | yes | down | 211.64 | 350.59 | 286.86 | 136.77 | 240.28 | 158.72 | 283.03 | 178.59 |
| ZNF74 | 2.911 | 1.541708 | 4.61E-06 | 5.96E-05 | yes | up | 1.12 | 1.37 | 1.76 | 4.26 | 7.22 | 7.87 | 1.416667 | 6.45 |
| PGAM1 | 0.482 | -1.05304 | 4.75E-06 | 6.13E-05 | yes | down | 73.58 | 119.75 | 84.09 | 85.91 | 59.11 | 59.47 | 92.47333 | 68.16333 |
| DCLK1 | 2.039 | 1.02795 | 4.79E-06 | 6.17E-05 | yes | up | 2.3 | 3.21 | 1.85 | 8.31 | 6.16 | 8.1 | 2.453333 | 7.523333 |
| LOC100338456 | 0.178 | -2.49239 | 4.86E-06 | 6.27E-05 | yes | down | 4.71 | 9.41 | 4.62 | 0.66 | 0.54 | 2.78 | 6.246667 | 1.326667 |
| MRPL50 | 0.477 | -1.0688 | 4.89E-06 | 6.3E-05 | yes | down | 9.42 | 11.64 | 9.72 | 8.25 | 6.65 | 7.71 | 10.26 | 7.536667 |
| ITM2C | 0.44 | -1.1828 | 5.02E-06 | 6.45E-05 | yes | down | 3.89 | 4.72 | 4.43 | 2.86 | 3.12 | 2.89 | 4.346667 | 2.956667 |
| ENHO | 5.07 | 2.342053 | 5.03E-06 | 6.46E-05 | yes | up | 0.6 | 1.07 | 0.9 | 4.9 | 5.43 | 9.98 | 0.856667 | 6.77 |
| GMNN | 2.23 | 1.157105 | 5.04E-06 | 6.47E-05 | yes | up | 4.92 | 5.53 | 5.09 | 18.89 | 15.62 | 22.36 | 5.18 | 18.95667 |
| S100B | 2.64 | 1.400763 | 5.08E-06 | 6.51E-05 | yes | up | 9.31 | 14.46 | 15.42 | 44.86 | 48.55 | 75.27 | 13.06333 | 56.22667 |
| B3GNT7 | 0.301 | -1.73302 | 5.2E-06 | 6.66E-05 | yes | down | 1.71 | 3.67 | 2.57 | 1.17 | 1.48 | 0.96 | 2.65 | 1.203333 |
| GADD45GIP1 | 0.414 | -1.27132 | 5.3E-06 | 6.77E-05 | yes | down | 15.71 | 16.21 | 15.79 | 11.07 | 7.49 | 12.34 | 15.90333 | 10.3 |
| ZBTB7C | 0.381 | -1.39339 | 5.45E-06 | 6.95E-05 | yes | down | 2.51 | 2.12 | 1.63 | 1 | 1.25 | 1.46 | 2.086667 | 1.236667 |
| LOC100340255 | 5.41 | 2.435594 | 5.65E-06 | 7.18E-05 | yes | up | 1.28 | 1.53 | 1.58 | 23.65 | 9.06 | 3.94 | 1.463333 | 12.21667 |
| PDE3A | 2.023 | 1.016289 | 5.73E-06 | 7.27E-05 | yes | up | 0.86 | 1.02 | 0.77 | 2.58 | 2.39 | 3.38 | 0.883333 | 2.783333 |
| MECP2 | 3.144 | 1.652498 | 5.79E-06 | 7.33E-05 | yes | up | 1.33 | 1.2 | 0.85 | 5.03 | 4.61 | 6.77 | 1.126667 | 5.47 |
| SAMD9 | 4.293 | 2.102115 | 5.9E-06 | 7.43E-05 | yes | up | 0.83 | 0.8 | 0.65 | 8.7 | 4.07 | 2.32 | 0.76 | 5.03 |
| SLC25A42 | 0.254 | -1.97747 | 6.27E-06 | 7.88E-05 | yes | down | 20.67 | 23.19 | 23.86 | 5.1 | 10.6 | 11.03 | 22.57333 | 8.91 |
| LOC100348760 | 0.434 | -1.20326 | 6.33E-06 | 7.95E-05 | yes | down | 197.57 | 308.75 | 166.21 | 121.02 | 185.39 | 138.4 | 224.1767 | 148.27 |
| LOC103349284 | 94.7 | 6.565296 | 6.36E-06 | 7.98E-05 | yes | up | 0 | 0 | 0 | 1.52 | 0.68 | 1.24 | 0 | 1.146667 |
| ARHGDIB | 2.546 | 1.348481 | 6.37E-06 | 7.98E-05 | yes | up | 18.57 | 17.65 | 20.73 | 104.99 | 47.17 | 74.33 | 18.98333 | 75.49667 |
| FTL | 2.306 | 1.205691 | 6.4E-06 | 8.01E-05 | yes | up | 498.55 | 474 | 653.98 | 1548.77 | 1540.42 | 2822.52 | 542.1767 | 1970.57 |
| SARS2 | 0.424 | -1.23745 | 6.57E-06 | 8.2E-05 | yes | down | 11.21 | 19.06 | 16.09 | 7.61 | 10.9 | 7.5 | 15.45333 | 8.67 |
| SUN2 | 2.246 | 1.16714 | 6.93E-06 | 8.63E-05 | yes | up | 15.26 | 18 | 15.99 | 39.29 | 52.12 | 81.08 | 16.41667 | 57.49667 |
| CCDC190 | 2.974 | 1.572166 | 7.03E-06 | 8.74E-05 | yes | up | 0.89 | 0.95 | 1.14 | 5.65 | 6.28 | 10.47 | 0.993333 | 7.466667 |
| DCAF4 | 2.256 | 1.17367 | 7.23E-06 | 8.96E-05 | yes | up | 1.63 | 1.78 | 1.74 | 5.61 | 5.38 | 7.08 | 1.716667 | 6.023333 |
| SOX4 | 3.074 | 1.620005 | 7.25E-06 | 8.97E-05 | yes | up | 1.02 | 1.24 | 0.79 | 3.01 | 4.32 | 7.3 | 1.016667 | 4.876667 |
| SKIL | 0.417 | -1.26297 | 7.27E-06 | 8.98E-05 | yes | down | 3.54 | 2.98 | 3.7 | 2.82 | 1.56 | 2.01 | 3.406667 | 2.13 |
| STAT6 | 2.038 | 1.027371 | 7.29E-06 | 9E-05 | yes | up | 5.82 | 7.44 | 7.16 | 27.77 | 17.86 | 19 | 6.806667 | 21.54333 |
| ESRRA | 0.426 | -1.23102 | 7.38E-06 | 9.09E-05 | yes | down | 12.2 | 14.44 | 16 | 8.14 | 10.41 | 8.8 | 14.21333 | 9.116667 |
| LOC108176449 | 7.601 | 2.926262 | 7.6E-06 | 9.34E-05 | yes | up | 0.14 | 0.02 | 0.18 | 1.11 | 1.23 | 2.31 | 0.113333 | 1.55 |
| ADPRHL1 | 0.111 | -3.17093 | 8.03E-06 | 9.8E-05 | yes | down | 8.15 | 11.88 | 4.2 | 0.15 | 2.1 | 1.97 | 8.076667 | 1.406667 |
| NEMP1 | 2.34 | 1.22653 | 8.06E-06 | 9.83E-05 | yes | up | 1.01 | 0.76 | 0.71 | 2.9 | 3.09 | 2.66 | 0.826667 | 2.883333 |
| PTRH1 | 0.454 | -1.13773 | 8.12E-06 | 9.89E-05 | yes | down | 4.49 | 6.68 | 5.88 | 4.66 | 3.23 | 4.25 | 5.683333 | 4.046667 |
| TTC9 | 0.463 | -1.11193 | 8.2E-06 | 9.97E-05 | yes | down | 56.35 | 83.32 | 80.42 | 42.99 | 39.97 | 72.91 | 73.36333 | 51.95667 |
| ABHD6 | 2.466 | 1.302115 | 8.21E-06 | 9.98E-05 | yes | up | 1.24 | 2.33 | 1.23 | 6.13 | 5.76 | 6.19 | 1.6 | 6.026667 |
| ASB18 | 0.266 | -1.9112 | 8.26E-06 | 0.0001 | yes | down | 1.72 | 1.83 | 2.29 | 0.73 | 0.87 | 0.75 | 1.946667 | 0.783333 |
| ADAMTSL1 | 2.687 | 1.425876 | 8.44E-06 | 0.000102 | yes | up | 7.68 | 3.87 | 6.84 | 22.5 | 29.71 | 12.33 | 6.13 | 21.51333 |
| RNF144B | 2.545 | 1.347766 | 8.46E-06 | 0.000102 | yes | up | 0.81 | 1.28 | 1.22 | 4.09 | 3.65 | 3.06 | 1.103333 | 3.6 |
| NIP7 | 0.434 | -1.20412 | 8.58E-06 | 0.000103 | yes | down | 5.62 | 9.01 | 8.72 | 5.66 | 4.14 | 5.25 | 7.783333 | 5.016667 |
| ST6GALNAC2 | 0.462 | -1.11384 | 8.83E-06 | 0.000106 | yes | down | 4.79 | 5.49 | 5.84 | 3.77 | 3.05 | 4.38 | 5.373333 | 3.733333 |
| SUOX | 0.02 | -5.66117 | 8.84E-06 | 0.000106 | yes | down | 1.85 | 11.45 | 8.07 | 0.16 | 0 | 0.11 | 7.123333 | 0.09 |
| GLRX | 0.417 | -1.26336 | 8.87E-06 | 0.000107 | yes | down | 46.7 | 52.6 | 92.25 | 42.47 | 40.36 | 41.68 | 63.85 | 41.50333 |
| C16H1orf131 | 0.409 | -1.29127 | 8.89E-06 | 0.000107 | yes | down | 9.35 | 10.15 | 13.96 | 6.01 | 8.24 | 6.23 | 11.15333 | 6.826667 |
| MRPL17 | 0.393 | -1.34908 | 8.97E-06 | 0.000108 | yes | down | 161.69 | 144.33 | 106.31 | 119.97 | 59.53 | 63.92 | 137.4433 | 81.14 |
| MAGIX | 0.198 | -2.33556 | 9.38E-06 | 0.000112 | yes | down | 5.13 | 4.13 | 1.79 | 0.94 | 1.43 | 0.97 | 3.683333 | 1.113333 |
| SUGP2 | 0.406 | -1.29918 | 9.48E-06 | 0.000113 | yes | down | 4.64 | 5.39 | 6.14 | 3.66 | 3.94 | 2.47 | 5.39 | 3.356667 |
| LOC100358208 | 0.472 | -1.08198 | 9.73E-06 | 0.000116 | yes | down | 833.78 | 948 | 840.73 | 489.27 | 836.74 | 573.04 | 874.17 | 633.0167 |
| CCSAP | 2.727 | 1.447414 | 1.07E-05 | 0.000127 | yes | up | 0.35 | 0.31 | 0.36 | 1.46 | 1.24 | 1.65 | 0.34 | 1.45 |
| MTERF3 | 0.442 | -1.17687 | 1.1E-05 | 0.00013 | yes | down | 5.33 | 8.59 | 8.53 | 5.35 | 4.48 | 5.5 | 7.483333 | 5.11 |
| PTPRD | 2.578 | 1.366031 | 1.13E-05 | 0.000134 | yes | up | 1.2 | 1.25 | 0.98 | 4.09 | 3.02 | 7.05 | 1.143333 | 4.72 |
| TXNRD2 | 0.455 | -1.13497 | 1.15E-05 | 0.000135 | yes | down | 7.18 | 9.27 | 9.67 | 5.45 | 7.53 | 5.27 | 8.706667 | 6.083333 |
| LOC100337961 | 2.922 | 1.546981 | 1.15E-05 | 0.000135 | yes | up | 2.78 | 4.43 | 3.36 | 15.99 | 10.55 | 21.37 | 3.523333 | 15.97 |
| DENND5B | 0.459 | -1.12219 | 1.15E-05 | 0.000135 | yes | down | 1.28 | 1.87 | 1.21 | 0.96 | 1.17 | 0.93 | 1.453333 | 1.02 |
| CCL14 | 2.094 | 1.066009 | 1.17E-05 | 0.000137 | yes | up | 12.27 | 17.92 | 14.74 | 54.69 | 38.98 | 51.79 | 14.97667 | 48.48667 |
| OLFML1 | 2.069 | 1.049228 | 1.17E-05 | 0.000137 | yes | up | 2.72 | 4.64 | 4.22 | 10.58 | 13.57 | 12.66 | 3.86 | 12.27 |
| NDUFAF7 | 0.38 | -1.39499 | 1.18E-05 | 0.000137 | yes | down | 7.13 | 8.09 | 10.83 | 3.5 | 5.54 | 6.67 | 8.683333 | 5.236667 |
| ADCK1 | 0.403 | -1.31285 | 1.18E-05 | 0.000138 | yes | down | 1.93 | 2.27 | 2.35 | 1.28 | 1.33 | 1.74 | 2.183333 | 1.45 |
| LOC103345854 | 0.01 | -6.71159 | 1.19E-05 | 0.000138 | yes | down | 2.04 | 1.66 | 2.8 | 0 | 0 | 0 | 2.166667 | 0 |
| OSBPL11 | 0.4 | -1.32157 | 1.21E-05 | 0.00014 | yes | down | 24.36 | 19.15 | 25.37 | 19.14 | 8.75 | 15.33 | 22.96 | 14.40667 |
| PXYLP1 | 0.415 | -1.26712 | 1.21E-05 | 0.000141 | yes | down | 3.44 | 4.98 | 3.96 | 1.84 | 3.24 | 2.82 | 4.126667 | 2.633333 |
| CD247 | 3.224 | 1.688966 | 1.22E-05 | 0.000142 | yes | up | 4.54 | 3.1 | 7.99 | 28.89 | 22.71 | 26.08 | 5.21 | 25.89333 |
| LOC100354703 | 0.307 | -1.70579 | 1.24E-05 | 0.000143 | yes | down | 1.2 | 1.35 | 1.36 | 0.67 | 0.69 | 0.48 | 1.303333 | 0.613333 |
| LOC103348242 | 0.182 | -2.46085 | 1.24E-05 | 0.000143 | yes | down | 19.6 | 31.45 | 61.92 | 6.51 | 6.3 | 24.22 | 37.65667 | 12.34333 |
| KIAA0408 | 0.457 | -1.12848 | 1.25E-05 | 0.000144 | yes | down | 17.21 | 14.72 | 14.17 | 10.37 | 12.13 | 11.74 | 15.36667 | 11.41333 |
| IKZF2 | 2.841 | 1.506155 | 1.27E-05 | 0.000147 | yes | up | 1.13 | 0.56 | 0.67 | 3.31 | 3.98 | 3.39 | 0.786667 | 3.56 |
| FCHSD1 | 2.228 | 1.155791 | 1.29E-05 | 0.000147 | yes | up | 1.08 | 2.04 | 1.47 | 5.27 | 5.03 | 5.16 | 1.53 | 5.153333 |
| AGT | 0.115 | -3.12333 | 1.37E-05 | 0.000156 | yes | down | 0.39 | 0.52 | 0.71 | 0.11 | 0.09 | 0.09 | 0.54 | 0.096667 |
| LOC100349709 | 0.033 | -4.93604 | 1.38E-05 | 0.000156 | yes | down | 0.43 | 0.05 | 0.35 | 0.03 | 0.02 | 0 | 0.276667 | 0.016667 |
| SCUBE1 | 0.291 | -1.78188 | 1.38E-05 | 0.000157 | yes | down | 1.61 | 1.78 | 2.42 | 0.72 | 0.45 | 0.91 | 1.936667 | 0.693333 |
| SGTB | 2.73 | 1.449016 | 1.39E-05 | 0.000157 | yes | up | 4.82 | 4.05 | 2.69 | 9.53 | 10.78 | 9.08 | 3.853333 | 9.796667 |
| HPSE2 | 6.183 | 2.628363 | 1.39E-05 | 0.000158 | yes | up | 6.56 | 2.04 | 10.74 | 3.37 | 3.05 | 5.59 | 6.446667 | 4.003333 |
| BRIX1 | 0.488 | -1.03527 | 1.41E-05 | 0.00016 | yes | down | 10 | 12.92 | 12.17 | 10.66 | 6.87 | 8.95 | 11.69667 | 8.826667 |
| TNFAIP8 | 2.316 | 1.211356 | 1.44E-05 | 0.000162 | yes | up | 8.83 | 6.41 | 7.33 | 31.06 | 19.48 | 31.78 | 7.523333 | 27.44 |
| LOC100342112 | 0.013 | -6.26338 | 1.49E-05 | 0.000167 | yes | down | 0.69 | 1.04 | 1.78 | 0.06 | 0 | 0 | 1.17 | 0.02 |
| CCDC58 | 0.434 | -1.20294 | 1.49E-05 | 0.000167 | yes | down | 22.73 | 34.11 | 24.65 | 23.13 | 17.24 | 16.34 | 27.16333 | 18.90333 |
| CBL | 0.424 | -1.23621 | 1.49E-05 | 0.000167 | yes | down | 8.66 | 12.74 | 14.79 | 10.44 | 7.2 | 5.85 | 12.06333 | 7.83 |
| SNRNP25 | 0.476 | -1.0721 | 1.58E-05 | 0.000176 | yes | down | 19.94 | 34.41 | 25.11 | 15.27 | 17.94 | 23.72 | 26.48667 | 18.97667 |
| CLK4 | 2.184 | 1.126852 | 1.59E-05 | 0.000177 | yes | up | 6.03 | 4.01 | 6.68 | 15.21 | 18.56 | 20.66 | 5.573333 | 18.14333 |
| TUBA4A | 0.263 | -1.92602 | 1.59E-05 | 0.000177 | yes | down | 28.86 | 61.68 | 13.81 | 15.64 | 14.67 | 10.77 | 34.78333 | 13.69333 |
| LOC108177431 | 54.831 | 5.776931 | 1.59E-05 | 0.000177 | yes | up | 0 | 1.05 | 5.01 | 142.68 | 145.46 | 222.07 | 2.02 | 170.07 |
| C17H14orf37 | 0.389 | -1.36367 | 1.6E-05 | 0.000178 | yes | down | 8.64 | 4.33 | 7.97 | 15.03 | 6.46 | 14.07 | 6.98 | 11.85333 |
| SRD5A1 | 0.341 | -1.55127 | 1.6E-05 | 0.000178 | yes | down | 10.11 | 13.45 | 16.44 | 10.64 | 5.07 | 4.97 | 13.33333 | 6.893333 |
| CLIP4 | 0.413 | -1.27668 | 1.62E-05 | 0.000179 | yes | down | 3.23 | 3.39 | 2.72 | 1.47 | 2.54 | 1.94 | 3.113333 | 1.983333 |
| LOC103345230 | 2.295 | 1.198541 | 1.62E-05 | 0.00018 | yes | up | 1.72 | 1.54 | 2.25 | 4.87 | 6.03 | 6.9 | 1.836667 | 5.933333 |
| MRPL38 | 0.474 | -1.07677 | 1.63E-05 | 0.00018 | yes | down | 17.43 | 27 | 21.04 | 12.33 | 14.31 | 21.45 | 21.82333 | 16.03 |
| MAP2K3 | 0.487 | -1.03725 | 1.63E-05 | 0.00018 | yes | down | 23.77 | 38.09 | 26.22 | 18.88 | 26.71 | 18.81 | 29.36 | 21.46667 |
| LOC100343018 | 0.212 | -2.24023 | 1.65E-05 | 0.000181 | yes | down | 2.51 | 2.71 | 1.96 | 1.03 | 0.6 | 0.78 | 2.393333 | 0.803333 |
| ABCA6 | 2.996 | 1.583145 | 1.71E-05 | 0.000188 | yes | up | 2.59 | 1.73 | 2.82 | 17.01 | 6.93 | 9.92 | 2.38 | 11.28667 |
| ABCA1 | 2.734 | 1.45109 | 1.74E-05 | 0.00019 | yes | up | 6.46 | 4.4 | 3.92 | 16.49 | 16.83 | 19.54 | 4.926667 | 17.62 |
| SDC1 | 0.252 | -1.98735 | 1.75E-05 | 0.000191 | yes | down | 9.82 | 27.12 | 22.99 | 4.55 | 4.98 | 13.9 | 19.97667 | 7.81 |
| LOC100350311 | 0.291 | -1.77902 | 1.77E-05 | 0.000193 | yes | down | 14.98 | 27.58 | 9.23 | 11.35 | 4.95 | 7.78 | 17.26333 | 8.026667 |
| LOC100345757 | 0.01 | -6.60266 | 1.77E-05 | 0.000193 | yes | down | 2.13 | 0.04 | 2.62 | 0 | 0.04 | 0.05 | 1.596667 | 0.03 |
| AP1B1 | 0.41 | -1.28605 | 1.77E-05 | 0.000193 | yes | down | 21.43 | 31.79 | 25.73 | 24.68 | 16.41 | 10.93 | 26.31667 | 17.34 |
| PRKACA | 0.324 | -1.6243 | 1.78E-05 | 0.000193 | yes | down | 31.4 | 41.73 | 45.02 | 19.56 | 18.72 | 19.55 | 39.38333 | 19.27667 |
| LOC103347127 | 0.161 | -2.63294 | 1.81E-05 | 0.000197 | yes | down | 15.68 | 17.06 | 10.14 | 2.43 | 5.29 | 2.67 | 14.29333 | 3.463333 |
| NFKB2 | 2.249 | 1.169185 | 1.83E-05 | 0.000199 | yes | up | 4.93 | 5.13 | 4.95 | 23.93 | 14.28 | 14.02 | 5.003333 | 17.41 |
| PRDM16 | 0.297 | -1.75216 | 1.83E-05 | 0.000199 | yes | down | 1.53 | 2.37 | 2.22 | 1.04 | 1.15 | 0.56 | 2.04 | 0.916667 |
| TRMT61A | 0.377 | -1.4071 | 1.84E-05 | 0.0002 | yes | down | 2.43 | 5.83 | 5.78 | 3.04 | 2.45 | 2.6 | 4.68 | 2.696667 |
| HADHB | 0.439 | -1.18608 | 1.87E-05 | 0.000202 | yes | down | 788.8 | 1054.53 | 836.65 | 508.96 | 837.75 | 444.31 | 893.3267 | 597.0067 |
| TMEM139 | 0.205 | -2.28671 | 2.06E-05 | 0.000222 | yes | down | 5.56 | 2.76 | 8.42 | 2.22 | 1.55 | 1.28 | 5.58 | 1.683333 |
| AIF1L | 0.484 | -1.04682 | 2.08E-05 | 0.000223 | yes | down | 14.16 | 17.85 | 14.65 | 7.87 | 13.08 | 14.11 | 15.55333 | 11.68667 |
| LOC103345465 | 5.262 | 2.395497 | 2.17E-05 | 0.000232 | yes | up | 0.26 | 0.29 | 0.18 | 1.66 | 1.26 | 1.55 | 0.243333 | 1.49 |
| GK | 0.497 | -1.00893 | 2.23E-05 | 0.000238 | yes | down | 16.45 | 31.02 | 18.44 | 10.11 | 14.8 | 18.91 | 21.97 | 14.60667 |
| CD38 | 5.54 | 2.469891 | 2.24E-05 | 0.000238 | yes | up | 0.53 | 0.93 | 0.46 | 11.56 | 2.42 | 2.93 | 0.64 | 5.636667 |
| MPI | 0.399 | -1.32513 | 2.25E-05 | 0.000239 | yes | down | 7.6 | 10.02 | 6.19 | 4.38 | 6.3 | 3.81 | 7.936667 | 4.83 |
| NARF | 0.493 | -1.01897 | 2.26E-05 | 0.00024 | yes | down | 5.03 | 7.67 | 5.61 | 3.5 | 5.61 | 4.85 | 6.103333 | 4.653333 |
| TRIM14 | 6.501 | 2.70074 | 2.27E-05 | 0.000241 | yes | up | 0.19 | 0.12 | 0.13 | 2.8 | 0.86 | 0.88 | 0.146667 | 1.513333 |
| BMF | 2.925 | 1.548632 | 2.29E-05 | 0.000243 | yes | up | 0.96 | 0.45 | 1.55 | 5.68 | 4.42 | 4.49 | 0.986667 | 4.863333 |
| LOC100338821 | 77.977 | 6.284978 | 2.3E-05 | 0.000244 | yes | up | 0 | 0 | 0 | 0.53 | 0.57 | 0.98 | 0 | 0.693333 |
| RTN4IP1 | 0.403 | -1.30962 | 2.36E-05 | 0.00025 | yes | down | 10.85 | 11.61 | 17.74 | 7.86 | 10.41 | 6.72 | 13.4 | 8.33 |
| LOC100353768 | 2.415 | 1.272066 | 2.36E-05 | 0.00025 | yes | up | 2.65 | 2.31 | 2.01 | 9.2 | 6.34 | 11.54 | 2.323333 | 9.026667 |
| LOC108176749 | 92.924 | 6.537981 | 2.41E-05 | 0.000254 | yes | up | 0 | 0 | 0 | 0.24 | 0.83 | 0.33 | 0 | 0.466667 |
| LOC103346478 | 0.307 | -1.70255 | 2.45E-05 | 0.000258 | yes | down | 0.7 | 0.66 | 0.58 | 0.32 | 0.35 | 0.25 | 0.646667 | 0.306667 |
| MTRF1 | 0.351 | -1.51167 | 2.47E-05 | 0.000259 | yes | down | 2.45 | 4.72 | 4.12 | 2.53 | 1.68 | 1.84 | 3.763333 | 2.016667 |
| EPHA1 | 0.229 | -2.12524 | 2.59E-05 | 0.00027 | yes | down | 0.59 | 0.58 | 0.62 | 0.22 | 0.24 | 0.17 | 0.596667 | 0.21 |
| PIK3CD | 6.506 | 2.701717 | 2.62E-05 | 0.000273 | yes | up | 0.31 | 0.34 | 0.46 | 7.98 | 1.65 | 1.51 | 0.37 | 3.713333 |
| CDH19 | 2.215 | 1.147189 | 2.68E-05 | 0.000278 | yes | up | 1.14 | 1.29 | 0.9 | 4.42 | 3.04 | 4.28 | 1.11 | 3.913333 |
| SERPINE2 | 2.184 | 1.126847 | 2.71E-05 | 0.000281 | yes | up | 2.77 | 2.78 | 2.39 | 7.9 | 7.3 | 11.83 | 2.646667 | 9.01 |
| HMOX1 | 3.015 | 1.592391 | 2.75E-05 | 0.000285 | yes | up | 16.18 | 13.42 | 7.76 | 77.51 | 32.15 | 67.46 | 12.45333 | 59.04 |
| POLR3B | 0.465 | -1.10479 | 2.77E-05 | 0.000287 | yes | down | 1.55 | 2.59 | 2.53 | 1.55 | 1.59 | 1.69 | 2.223333 | 1.61 |
| OAS2 | 50.452 | 5.656848 | 2.82E-05 | 0.000292 | yes | up | 0.12 | 0.27 | 0.1 | 32.84 | 2.75 | 1.13 | 0.163333 | 12.24 |
| ARHGAP36 | 0.099 | -3.33795 | 2.94E-05 | 0.000303 | yes | down | 1.53 | 2.69 | 0.2 | 0.19 | 0.24 | 0.22 | 1.473333 | 0.216667 |
| POMK | 0.489 | -1.03334 | 3.06E-05 | 0.000314 | yes | down | 2.91 | 4.2 | 3.12 | 2.24 | 2.15 | 3.42 | 3.41 | 2.603333 |
| PLTP | 2.093 | 1.065788 | 3.11E-05 | 0.000319 | yes | up | 23.71 | 31.88 | 36.58 | 126.23 | 67.94 | 109.15 | 30.72333 | 101.1067 |
| KIAA1257 | 0.122 | -3.03471 | 3.17E-05 | 0.000323 | yes | down | 0.59 | 0.49 | 0.83 | 0.06 | 0.23 | 0.05 | 0.636667 | 0.113333 |
| LOC103346609 | 0.325 | -1.6195 | 3.25E-05 | 0.000331 | yes | down | 1.71 | 2.7 | 1.21 | 0.79 | 0.94 | 0.9 | 1.873333 | 0.876667 |
| LOC100358368 | 0.462 | -1.11487 | 3.26E-05 | 0.000332 | yes | down | 312.2 | 474.14 | 253.24 | 184.63 | 299.54 | 248.86 | 346.5267 | 244.3433 |
| ATP2A2 | 0.332 | -1.58862 | 3.26E-05 | 0.000332 | yes | down | 62.9 | 87.85 | 27.52 | 29.23 | 24.78 | 41.37 | 59.42333 | 31.79333 |
| KLF15 | 3.776 | 1.916854 | 3.27E-05 | 0.000332 | yes | up | 2.66 | 1.9 | 0.97 | 12.56 | 9.16 | 9.56 | 1.843333 | 10.42667 |
| SOCS2 | 2.649 | 1.405324 | 3.49E-05 | 0.000352 | yes | up | 4.67 | 7.64 | 5.06 | 15.66 | 19.03 | 36.74 | 5.79 | 23.81 |
| LOC100353469 | 0.44 | -1.186 | 3.5E-05 | 0.000353 | yes | down | 16.84 | 31.48 | 21.7 | 14.94 | 14.95 | 17.28 | 23.34 | 15.72333 |
| OSBPL7 | 0.367 | -1.44505 | 3.57E-05 | 0.000359 | yes | down | 12.17 | 17.84 | 17.9 | 7.82 | 12.81 | 5.37 | 15.97 | 8.666667 |
| VWCE | 2.869 | 1.520377 | 3.62E-05 | 0.000364 | yes | up | 0.55 | 0.78 | 0.46 | 2 | 3.51 | 2.46 | 0.596667 | 2.656667 |
| YPEL2 | 2.113 | 1.079137 | 3.69E-05 | 0.00037 | yes | up | 14.84 | 9.16 | 12.17 | 35.28 | 36.56 | 49.04 | 12.05667 | 40.29333 |
| LOC100355223 | 101.573 | 6.666368 | 3.71E-05 | 0.000372 | yes | up | 0 | 0 | 0 | 0.21 | 1.3 | 1.59 | 0 | 1.033333 |
| JADE2 | 2.705 | 1.435441 | 3.83E-05 | 0.000383 | yes | up | 2.21 | 1.2 | 1.36 | 8.59 | 4.78 | 7.02 | 1.59 | 6.796667 |
| LOC100337853 | 0.449 | -1.15519 | 3.85E-05 | 0.000385 | yes | down | 4.8 | 8.85 | 6.58 | 5.45 | 3.42 | 5.24 | 6.743333 | 4.703333 |
| ADPRHL2 | 0.49 | -1.02785 | 3.88E-05 | 0.000387 | yes | down | 10.39 | 17.36 | 10.86 | 7.18 | 9.09 | 12.55 | 12.87 | 9.606667 |
| ENAH | 0.462 | -1.11295 | 3.9E-05 | 0.000388 | yes | down | 11.63 | 16.65 | 10.63 | 8.18 | 10.27 | 9.47 | 12.97 | 9.306667 |
| LOC103348916 | 2.903 | 1.53756 | 3.94E-05 | 0.000392 | yes | up | 1 | 1.38 | 1.27 | 4.44 | 5.39 | 6.38 | 1.216667 | 5.403333 |
| FBXO32 | 9.168 | 3.196589 | 3.98E-05 | 0.000395 | yes | up | 5.21 | 1.85 | 1.03 | 27.03 | 40.79 | 51.66 | 2.696667 | 39.82667 |
| SPATA6L | 2.901 | 1.536443 | 4.06E-05 | 0.000402 | yes | up | 1.29 | 0.82 | 1.96 | 4.87 | 6.3 | 5.56 | 1.356667 | 5.576667 |
| PTPN5 | 5.735 | 2.519767 | 4.07E-05 | 0.000403 | yes | up | 0.92 | 0.4 | 0.26 | 2.69 | 4.13 | 7.75 | 0.526667 | 4.856667 |
| HAS2 | 3.396 | 1.763633 | 4.09E-05 | 0.000405 | yes | up | 0.99 | 0.4 | 0.57 | 3.86 | 2.8 | 3.76 | 0.653333 | 3.473333 |
| CCDC192 | 3.135 | 1.648417 | 4.13E-05 | 0.000408 | yes | up | 1.26 | 1.1 | 1.18 | 4.52 | 4.73 | 8.4 | 1.18 | 5.883333 |
| HSPA4L | 0.401 | -1.31846 | 4.18E-05 | 0.000412 | yes | down | 0.72 | 1.01 | 0.91 | 0.54 | 0.51 | 0.6 | 0.88 | 0.55 |
| PARP9 | 3.581 | 1.840257 | 4.21E-05 | 0.000416 | yes | up | 2.47 | 1.72 | 2.35 | 21.05 | 10.41 | 5.62 | 2.18 | 12.36 |
| VNN1 | 5.286 | 2.402086 | 4.25E-05 | 0.000418 | yes | up | 0.37 | 0.14 | 0.23 | 1.57 | 1.97 | 2.35 | 0.246667 | 1.963333 |
| ZDHHC14 | 2.438 | 1.285793 | 4.29E-05 | 0.000422 | yes | up | 2.37 | 2.38 | 2.23 | 9.89 | 5.8 | 5.53 | 2.326667 | 7.073333 |
| C12H6orf136 | 0.474 | -1.07848 | 4.31E-05 | 0.000423 | yes | down | 15.84 | 21.91 | 22.56 | 11.39 | 18.6 | 13.8 | 20.10333 | 14.59667 |
| P2RX7 | 2.468 | 1.303486 | 4.35E-05 | 0.000427 | yes | up | 0.4 | 0.61 | 0.45 | 2.25 | 1.58 | 1.71 | 0.486667 | 1.846667 |
| SMPD3 | 64.527 | 6.011832 | 4.36E-05 | 0.000427 | yes | up | 0.04 | 0.01 | 0.06 | 0.13 | 2.7 | 10.17 | 0.036667 | 4.333333 |
| LOC100344547 | 0.338 | -1.56661 | 4.38E-05 | 0.000428 | yes | down | 1.87 | 3.1 | 2.58 | 1.16 | 1.52 | 1.22 | 2.516667 | 1.3 |
| C14H21orf91 | 2.581 | 1.368039 | 4.38E-05 | 0.000428 | yes | up | 1.51 | 0.85 | 0.91 | 5.34 | 3.67 | 4.29 | 1.09 | 4.433333 |
| LIMD2 | 4.632 | 2.21152 | 4.39E-05 | 0.000428 | yes | up | 1.69 | 2.23 | 2.23 | 27.63 | 7.78 | 8.33 | 2.05 | 14.58 |
| LOC100337800 | 0.487 | -1.03825 | 4.4E-05 | 0.00043 | yes | down | 4.44 | 6.36 | 4.57 | 3.85 | 4.02 | 3.33 | 5.123333 | 3.733333 |
| LOC103351772 | 0.266 | -1.9104 | 4.48E-05 | 0.000436 | yes | down | 180.65 | 135.7 | 95.68 | 49.15 | 68.89 | 45.2 | 137.3433 | 54.41333 |
| LOC100348276 | 5.126 | 2.357928 | 4.57E-05 | 0.000443 | yes | up | 1.17 | 0.71 | 1.85 | 3.16 | 19.15 | 25.49 | 1.243333 | 15.93333 |
| C13H1orf162 | 4.644 | 2.215454 | 4.61E-05 | 0.000447 | yes | up | 5.08 | 1.59 | 2.28 | 43.29 | 7.81 | 14.37 | 2.983333 | 21.82333 |
| ANKRD34A | 3.579 | 1.83971 | 4.66E-05 | 0.000451 | yes | up | 0.23 | 0.61 | 0.82 | 1.65 | 1.96 | 2.48 | 0.553333 | 2.03 |
| FAM180B | 0.324 | -1.62412 | 4.73E-05 | 0.000457 | yes | down | 4.3 | 3.04 | 6.67 | 2.42 | 1.94 | 2.74 | 4.67 | 2.366667 |
| C4A | 7.829 | 2.968918 | 4.74E-05 | 0.000458 | yes | up | 14.87 | 13.55 | 13.91 | 341.65 | 88.81 | 83.54 | 14.11 | 171.3333 |
| TSPAN33 | 3.051 | 1.609122 | 4.77E-05 | 0.00046 | yes | up | 0.95 | 0.75 | 0.93 | 3.15 | 3.4 | 6.11 | 0.876667 | 4.22 |
| PAK5 | 151.657 | 7.244666 | 4.8E-05 | 0.000463 | yes | up | 0 | 0.01 | 0 | 2.84 | 0.2 | 0.03 | 0.003333 | 1.023333 |
| PENK | 2.46 | 1.298424 | 4.82E-05 | 0.000464 | yes | up | 3.07 | 5.46 | 2.67 | 11.56 | 12.97 | 17.8 | 3.733333 | 14.11 |
| RTL1 | 0.015 | -6.06406 | 4.93E-05 | 0.000473 | yes | down | 0.83 | 2.74 | 0.08 | 0 | 0.04 | 0 | 1.216667 | 0.013333 |
| TRAP1 | 0.492 | -1.024 | 4.98E-05 | 0.000477 | yes | down | 70.82 | 93.4 | 67.21 | 44.33 | 75.16 | 54.79 | 77.14333 | 58.09333 |
| LOC100354911 | 2.014 | 1.009787 | 5.11E-05 | 0.000489 | yes | up | 3.82 | 4.71 | 3.52 | 9.5 | 11.16 | 16.1 | 4.016667 | 12.25333 |
| INIP | 0.497 | -1.00912 | 5.13E-05 | 0.00049 | yes | down | 8.02 | 11.4 | 11.89 | 9.27 | 7.35 | 7.32 | 10.43667 | 7.98 |
| HK2 | 0.435 | -1.20117 | 5.23E-05 | 0.000498 | yes | down | 11.75 | 10.43 | 6 | 5.42 | 6.05 | 7.69 | 9.393333 | 6.386667 |
| FOXP1 | 2.267 | 1.180849 | 5.33E-05 | 0.000507 | yes | up | 5.3 | 4.1 | 2.81 | 11.45 | 12.73 | 16.88 | 4.07 | 13.68667 |
| FAM110B | 2.817 | 1.494247 | 5.36E-05 | 0.000509 | yes | up | 0.38 | 0.67 | 0.73 | 1.8 | 2.98 | 3.16 | 0.593333 | 2.646667 |
| LOC100346439 | 69.762 | 6.124364 | 5.42E-05 | 0.000513 | yes | up | 0 | 0 | 0 | 2.56 | 4.81 | 4.44 | 0 | 3.936667 |
| LOC103351083 | 2.757 | 1.463104 | 5.51E-05 | 0.000521 | yes | up | 3.2 | 1.45 | 1.79 | 12.01 | 7.31 | 5.69 | 2.146667 | 8.336667 |
| CDK8 | 0.427 | -1.22816 | 5.52E-05 | 0.000522 | yes | down | 2.56 | 3.65 | 4.6 | 1.84 | 2.49 | 2.81 | 3.603333 | 2.38 |
| LOC100340875 | 0.435 | -1.20039 | 5.6E-05 | 0.000527 | yes | down | 37.03 | 47.07 | 34.49 | 23.39 | 34.55 | 20.64 | 39.53 | 26.19333 |
| WDR72 | 3.046 | 1.607113 | 5.67E-05 | 0.000532 | yes | up | 1.36 | 2.9 | 0.87 | 6.96 | 6.47 | 8.74 | 1.71 | 7.39 |
| RRS1 | 0.306 | -1.7077 | 5.68E-05 | 0.000533 | yes | down | 3.42 | 6.56 | 6.02 | 4.17 | 1.7 | 1.71 | 5.333333 | 2.526667 |
| PDE12 | 0.428 | -1.22441 | 5.68E-05 | 0.000533 | yes | down | 2.9 | 3.25 | 3.17 | 2.89 | 1.57 | 1.71 | 3.106667 | 2.056667 |
| PSME1 | 4.066 | 2.023449 | 5.69E-05 | 0.000533 | yes | up | 12.15 | 15.63 | 16.51 | 194.49 | 46.7 | 49.79 | 14.76333 | 96.99333 |
| AIFM2 | 0.489 | -1.03231 | 5.7E-05 | 0.000534 | yes | down | 381.75 | 507.28 | 483.21 | 223.15 | 407.58 | 415.49 | 457.4133 | 348.74 |
| STAC | 2.053 | 1.037755 | 5.81E-05 | 0.000543 | yes | up | 1.66 | 1.51 | 1.92 | 5.38 | 4.49 | 6.51 | 1.696667 | 5.46 |
| SLC25A12 | 0.452 | -1.14499 | 5.85E-05 | 0.000546 | yes | down | 9.19 | 14.58 | 9.14 | 5.63 | 9.84 | 7.21 | 10.97 | 7.56 |
| SNN | 2.215 | 1.147269 | 6.04E-05 | 0.000563 | yes | up | 1.49 | 1.98 | 2.3 | 4.93 | 8.26 | 7.81 | 1.923333 | 7 |
| RTCB | 0.433 | -1.20876 | 6.18E-05 | 0.000575 | yes | down | 28.11 | 65.58 | 68.73 | 36.5 | 36.07 | 34.61 | 54.14 | 35.72667 |
| APCDD1 | 2.769 | 1.46919 | 6.22E-05 | 0.000579 | yes | up | 4.92 | 4.93 | 10.85 | 26.38 | 22.49 | 41.35 | 6.9 | 30.07333 |
| CIB2 | 0.435 | -1.19935 | 6.63E-05 | 0.000613 | yes | down | 97.93 | 135.54 | 103.93 | 56.56 | 108 | 59.08 | 112.4667 | 74.54667 |
| LOC100355114 | 0.412 | -1.28037 | 6.68E-05 | 0.000617 | yes | down | 3.25 | 4.74 | 3.75 | 3.5 | 1.83 | 2.09 | 3.913333 | 2.473333 |
| TGDS | 2.073 | 1.051476 | 6.73E-05 | 0.00062 | yes | up | 3.46 | 2.65 | 3.22 | 9.05 | 8.15 | 9.44 | 3.11 | 8.88 |
| PCF11 | 2.059 | 1.041845 | 6.76E-05 | 0.000622 | yes | up | 7.16 | 4.83 | 6.31 | 14.93 | 17.62 | 20.63 | 6.1 | 17.72667 |
| LOC100346046 | 2.502 | 1.32299 | 6.85E-05 | 0.000628 | yes | up | 1.71 | 1.59 | 1.28 | 3.58 | 5.98 | 8.33 | 1.526667 | 5.963333 |
| LOC100343802 | 2.636 | 1.398079 | 6.89E-05 | 0.000631 | yes | up | 552.68 | 246.94 | 332.47 | 1118.8 | 1634.71 | 1982.51 | 377.3633 | 1578.673 |
| GCG | 4.496 | 2.168513 | 6.93E-05 | 0.000634 | yes | up | 2.46 | 3.05 | 4.82 | 13.44 | 11.85 | 48.64 | 3.443333 | 24.64333 |
| ALDH1A1 | 2.151 | 1.104809 | 7.02E-05 | 0.000641 | yes | up | 65.98 | 103.86 | 57.26 | 170.19 | 258.25 | 303.28 | 75.7 | 243.9067 |
| LOC100357219 | 3.028 | 1.598294 | 7.07E-05 | 0.000644 | yes | up | 30.29 | 14.21 | 11.11 | 85.48 | 78.24 | 103.39 | 18.53667 | 89.03667 |
| CLEC4F | 4.302 | 2.10495 | 7.21E-05 | 0.000656 | yes | up | 1.59 | 1.51 | 1.61 | 19.06 | 7.45 | 4.65 | 1.57 | 10.38667 |
| SLC43A2 | 2.422 | 1.276357 | 7.61E-05 | 0.000689 | yes | up | 2.55 | 4.5 | 2.56 | 9.24 | 12.23 | 20.85 | 3.203333 | 14.10667 |
| HDC | 0.087 | -3.51958 | 7.68E-05 | 0.000694 | yes | down | 0.52 | 0.36 | 0.37 | 0.09 | 0.02 | 0.06 | 0.416667 | 0.056667 |
| GATSL2 | 0.419 | -1.25435 | 7.84E-05 | 0.000708 | yes | down | 5.67 | 10.16 | 7.81 | 3.66 | 6.58 | 4.85 | 7.88 | 5.03 |
| KBTBD11 | 2.472 | 1.305743 | 7.87E-05 | 0.00071 | yes | up | 1.88 | 2.82 | 1.45 | 5.21 | 8.13 | 9.8 | 2.05 | 7.713333 |
| LOC108177361 | 0.269 | -1.89562 | 7.99E-05 | 0.00072 | yes | down | 0.46 | 0.46 | 0.29 | 0.16 | 0.14 | 0.21 | 0.403333 | 0.17 |
| BHMT | 6.151 | 2.620719 | 8.31E-05 | 0.000745 | yes | up | 2.51 | 1.43 | 1.06 | 7.01 | 6.66 | 36.28 | 1.666667 | 16.65 |
| ARSE | 2.159 | 1.110088 | 8.39E-05 | 0.000751 | yes | up | 1.26 | 2.17 | 1.86 | 6.98 | 7.94 | 4.8 | 1.763333 | 6.573333 |
| LOC103350464 | 0.379 | -1.39909 | 8.42E-05 | 0.000753 | yes | down | 4.27 | 7.18 | 8.48 | 4.41 | 3.16 | 4.1 | 6.643333 | 3.89 |
| LOC108178205 | 2.222 | 1.151673 | 8.43E-05 | 0.000753 | yes | up | 26.57 | 14.41 | 24.17 | 72.49 | 64.55 | 91.64 | 21.71667 | 76.22667 |
| DOLPP1 | 0.322 | -1.63564 | 8.49E-05 | 0.000758 | yes | down | 4.43 | 3.71 | 4.06 | 2.16 | 1.81 | 2.09 | 4.066667 | 2.02 |
| TP53INP2 | 0.496 | -1.01176 | 8.53E-05 | 0.000761 | yes | down | 20.21 | 17.7 | 12.26 | 12.61 | 14.52 | 11.47 | 16.72333 | 12.86667 |
| PLCXD1 | 3.293 | 1.719434 | 8.55E-05 | 0.000762 | yes | up | 0.32 | 0.44 | 0.45 | 1.72 | 1.5 | 2.22 | 0.403333 | 1.813333 |
| ST6GAL1 | 2.21 | 1.143937 | 8.72E-05 | 0.000776 | yes | up | 2.86 | 3.03 | 2.57 | 12.57 | 6.02 | 10.45 | 2.82 | 9.68 |
| SNPH | 0.215 | -2.21654 | 8.78E-05 | 0.000781 | yes | down | 0.36 | 1.97 | 1.19 | 0.29 | 0.45 | 0.38 | 1.173333 | 0.373333 |
| LOC103351035 | 4.153 | 2.05425 | 9.06E-05 | 0.000805 | yes | up | 1.35 | 2.17 | 2.12 | 21.02 | 4.45 | 7.05 | 1.88 | 10.84 |
| HS3ST1 | 2.346 | 1.230444 | 9.18E-05 | 0.000814 | yes | up | 2.9 | 2.72 | 2.33 | 11.56 | 6.26 | 11.59 | 2.65 | 9.803333 |
| DENND6B | 2.085 | 1.059937 | 9.38E-05 | 0.000832 | yes | up | 4.54 | 4.99 | 5.94 | 11.62 | 17.31 | 17.23 | 5.156667 | 15.38667 |
| USP54 | 2.527 | 1.337631 | 9.44E-05 | 0.000836 | yes | up | 2.34 | 2.51 | 1.62 | 11.22 | 4.46 | 9.68 | 2.156667 | 8.453333 |
| LOC103351715 | 144.781 | 7.177727 | 9.54E-05 | 0.000844 | yes | up | 0 | 0 | 0 | 8.82 | 0.66 | 0.29 | 0 | 3.256667 |
| EMP1 | 0.457 | -1.12889 | 9.56E-05 | 0.000845 | yes | down | 81.87 | 65.15 | 46.55 | 56.21 | 38.57 | 43.15 | 64.52333 | 45.97667 |
| RXRG | 0.498 | -1.00521 | 9.57E-05 | 0.000846 | yes | down | 77.05 | 77.02 | 96.99 | 45.72 | 69.07 | 47.55 | 83.68667 | 54.11333 |
| ZNF660 | 2.812 | 1.491593 | 9.76E-05 | 0.000861 | yes | up | 0.48 | 0.57 | 0.56 | 2.41 | 2.59 | 3 | 0.536667 | 2.666667 |
| LOC100349768 | 0.484 | -1.04663 | 9.76E-05 | 0.000861 | yes | down | 380.33 | 515.55 | 299.43 | 210.37 | 368.79 | 307.95 | 398.4367 | 295.7033 |
| LYRM7 | 0.446 | -1.16346 | 9.94E-05 | 0.000875 | yes | down | 6.78 | 10.74 | 8.1 | 4.71 | 5.79 | 4.91 | 8.54 | 5.136667 |
| ANKEF1 | 2.438 | 1.285809 | 0.000102 | 0.000891 | yes | up | 1.06 | 0.64 | 0.92 | 3.41 | 3.13 | 3.46 | 0.873333 | 3.333333 |
| PHYHD1 | 2.257 | 1.174503 | 0.000103 | 0.000901 | yes | up | 13.14 | 15.92 | 16.89 | 32.51 | 56.27 | 72.84 | 15.31667 | 53.87333 |
| ZC3HAV1 | 2.784 | 1.476973 | 0.000103 | 0.000904 | yes | up | 2.01 | 2.01 | 2.24 | 13.08 | 8.88 | 4.89 | 2.086667 | 8.95 |
| TSEN2 | 0.477 | -1.0677 | 0.000103 | 0.000905 | yes | down | 3.06 | 4.71 | 4.53 | 2.94 | 2.43 | 3.96 | 4.1 | 3.11 |
| CES3 | 0.454 | -1.13945 | 0.000105 | 0.000917 | yes | down | 73.06 | 94.39 | 77.88 | 61.42 | 73.11 | 35.24 | 81.77667 | 56.59 |
| BCAT1 | 29.214 | 4.868603 | 0.000105 | 0.000918 | yes | up | 0.15 | 0.01 | 0 | 2.25 | 1 | 1.76 | 0.053333 | 1.67 |
| PPM1K | 2.104 | 1.073395 | 0.000108 | 0.000938 | yes | up | 4.03 | 4.41 | 2.79 | 16.01 | 8.68 | 11.96 | 3.743333 | 12.21667 |
| ATAD2 | 2.392 | 1.258172 | 0.00011 | 0.000959 | yes | up | 0.65 | 0.72 | 0.62 | 3.29 | 2.14 | 1.89 | 0.663333 | 2.44 |
| FMO5 | 2.397 | 1.261202 | 0.000112 | 0.000975 | yes | up | 0.48 | 0.85 | 1.11 | 2.85 | 2.22 | 3.3 | 0.813333 | 2.79 |
| LOC103346332 | 2.096 | 1.067937 | 0.000113 | 0.000978 | yes | up | 13.35 | 17.17 | 14.3 | 57.39 | 32.47 | 56.08 | 14.94 | 48.64667 |
| LOC108176887 | 0.042 | -4.5654 | 0.000113 | 0.000979 | yes | down | 28.87 | 14.98 | 7.35 | 0 | 0 | 3.9 | 17.06667 | 1.3 |
| VIPR1 | 3.779 | 1.917865 | 0.000113 | 0.00098 | yes | up | 0.38 | 0.31 | 0.24 | 1.17 | 1.92 | 2.39 | 0.31 | 1.826667 |
| SLC38A9 | 2.216 | 1.148237 | 0.000114 | 0.000986 | yes | up | 1 | 0.6 | 0.81 | 2.91 | 2.4 | 2.8 | 0.803333 | 2.703333 |
| SCARA3 | 2.102 | 1.071736 | 0.000117 | 0.001008 | yes | up | 7.11 | 10.08 | 6.86 | 17.7 | 24.07 | 36.68 | 8.016667 | 26.15 |
| EPB41L1 | 0.489 | -1.03147 | 0.00012 | 0.001033 | yes | down | 15.31 | 23.77 | 19.45 | 10.22 | 19.17 | 14.51 | 19.51 | 14.63333 |
| SYCE1L | 3.18 | 1.668905 | 0.00012 | 0.001035 | yes | up | 0.28 | 0.36 | 0.85 | 2.28 | 2.62 | 2.45 | 0.496667 | 2.45 |
| BCAT2 | 2.097 | 1.068653 | 0.000125 | 0.001073 | yes | up | 2.94 | 4.29 | 2.31 | 9.56 | 9.55 | 13.59 | 3.18 | 10.9 |
| LGALS9 | 4.469 | 2.159974 | 0.000127 | 0.001089 | yes | up | 6.88 | 11.06 | 11.41 | 140.96 | 32.23 | 27.36 | 9.783333 | 66.85 |
| LOC100346827 | 0.475 | -1.07379 | 0.000127 | 0.001089 | yes | down | 6.31 | 11.77 | 9.26 | 5.77 | 6.55 | 8.94 | 9.113333 | 7.086667 |
| FFAR4 | 0.095 | -3.3932 | 0.000128 | 0.001092 | yes | down | 0.09 | 0.55 | 1.17 | 0.12 | 0.05 | 0.09 | 0.603333 | 0.086667 |
| EEF2KMT | 0.233 | -2.10466 | 0.000129 | 0.001104 | yes | down | 1.65 | 1.53 | 1.95 | 0.82 | 0.63 | 0.37 | 1.71 | 0.606667 |
| LOC100339259 | 0.365 | -1.45385 | 0.00013 | 0.001107 | yes | down | 29.16 | 34.15 | 54.47 | 18.76 | 32.72 | 14.39 | 39.26 | 21.95667 |
| MC2R | 36.782 | 5.200918 | 0.000131 | 0.001113 | yes | up | 0.04 | 0 | 0 | 0.2 | 0.9 | 0.9 | 0.013333 | 0.666667 |
| SLC20A2 | 0.448 | -1.15731 | 0.000133 | 0.001129 | yes | down | 8.99 | 8.18 | 4.86 | 4.03 | 4.49 | 6.22 | 7.343333 | 4.913333 |
| TAF5 | 2.124 | 1.086752 | 0.000133 | 0.001132 | yes | up | 0.85 | 0.94 | 1.11 | 2.66 | 2.91 | 4.07 | 0.966667 | 3.213333 |
| ADAR | 2.031 | 1.022124 | 0.000134 | 0.00114 | yes | up | 12.81 | 17.73 | 17.13 | 65.4 | 48.41 | 34.43 | 15.89 | 49.41333 |
| AQP4 | 2.739 | 1.453398 | 0.000137 | 0.001159 | yes | up | 2.05 | 2.9 | 2.02 | 6.78 | 15.51 | 6.63 | 2.323333 | 9.64 |
| HYLS1 | 2.26 | 1.176403 | 0.000137 | 0.001159 | yes | up | 1.27 | 1.02 | 1.26 | 4 | 3.33 | 4.1 | 1.183333 | 3.81 |
| TTPA | 2.125 | 1.087405 | 0.000138 | 0.001164 | yes | up | 2.1 | 1.52 | 1.47 | 4.86 | 5.09 | 5.35 | 1.696667 | 5.1 |
| ANKRD33B | 2.982 | 1.576329 | 0.000138 | 0.001166 | yes | up | 0.58 | 0.25 | 0.26 | 1.8 | 1.55 | 1.82 | 0.363333 | 1.723333 |
| PPP1R9A | 2.255 | 1.172982 | 0.000138 | 0.001168 | yes | up | 3.65 | 2.57 | 4.16 | 8.26 | 11.43 | 16.44 | 3.46 | 12.04333 |
| ENDOG | 0.382 | -1.38729 | 0.000139 | 0.00117 | yes | down | 12.07 | 14.83 | 12.92 | 4.1 | 7.98 | 11.7 | 13.27333 | 7.926667 |
| HCAR2 | 0.233 | -2.10203 | 0.00014 | 0.001176 | yes | down | 7.56 | 3.45 | 3.76 | 3.23 | 1.12 | 1.07 | 4.923333 | 1.806667 |
| LOC100347785 | 2.692 | 1.428857 | 0.00014 | 0.001181 | yes | up | 0.6 | 0.69 | 1.03 | 5.62 | 2.67 | 3.24 | 0.773333 | 3.843333 |
| LOC103346561 | 3.213 | 1.683965 | 0.000141 | 0.001183 | yes | up | 0.34 | 0.33 | 0.57 | 4.17 | 2.54 | 2.82 | 0.413333 | 3.176667 |
| PPEF1 | 4.816 | 2.267889 | 0.000141 | 0.001184 | yes | up | 0.1 | 0.21 | 0.07 | 1.08 | 0.6 | 1.09 | 0.126667 | 0.923333 |
| LOC100359197 | 7.506 | 2.90804 | 0.000142 | 0.001191 | yes | up | 0.34 | 0.49 | 0.2 | 7.58 | 1.71 | 2.47 | 0.343333 | 3.92 |
| LOC100355642 | 0.428 | -1.22325 | 0.000143 | 0.001192 | yes | down | 4.21 | 3.63 | 2.36 | 2.07 | 2.98 | 2.04 | 3.4 | 2.363333 |
| LOC103346806 | 2.231 | 1.157671 | 0.000144 | 0.001202 | yes | up | 83.45 | 57.67 | 62.04 | 246.55 | 212.06 | 224.77 | 67.72 | 227.7933 |
| LOC100344012 | 10.397 | 3.378129 | 0.000146 | 0.001214 | yes | up | 0.12 | 0.18 | 0.19 | 6.04 | 0.98 | 0.78 | 0.163333 | 2.6 |
| LOC100341914 | 4.387 | 2.133354 | 0.000146 | 0.001215 | yes | up | 0.34 | 0.21 | 0.33 | 2.02 | 1.11 | 3.06 | 0.293333 | 2.063333 |
| LOC103350870 | 2.915 | 1.543425 | 0.000147 | 0.001221 | yes | up | 0.18 | 0.31 | 0.29 | 1.22 | 0.97 | 1.35 | 0.26 | 1.18 |
| CNTNAP5 | 0.45 | -1.15333 | 0.000149 | 0.001235 | yes | down | 9.79 | 7.59 | 7.62 | 4.47 | 6.86 | 6.21 | 8.333333 | 5.846667 |
| FAXDC2 | 2.022 | 1.01566 | 0.000149 | 0.001237 | yes | up | 8.02 | 5.95 | 7.19 | 26 | 18.6 | 33.12 | 7.053333 | 25.90667 |
| ENPP3 | 3.989 | 1.99598 | 0.00015 | 0.00124 | yes | up | 1.4 | 1.7 | 1.02 | 2.63 | 9.21 | 13.82 | 1.373333 | 8.553333 |
| CLU | 2.88 | 1.526227 | 0.000153 | 0.001262 | yes | up | 21.58 | 36.75 | 14.37 | 57.98 | 96.79 | 168.22 | 24.23333 | 107.6633 |
| SUN3 | 0.278 | -1.84921 | 0.000154 | 0.001266 | yes | down | 13.09 | 5.36 | 9.46 | 3.86 | 2.49 | 5.57 | 9.303333 | 3.973333 |
| CXCR1 | 3.966 | 1.987535 | 0.000156 | 0.001281 | yes | up | 0.72 | 0.48 | 0.26 | 3.79 | 2.18 | 2.55 | 0.486667 | 2.84 |
| IRF8 | 5.588 | 2.482269 | 0.000158 | 0.001294 | yes | up | 0.81 | 1.51 | 1.62 | 22.92 | 7.88 | 2.52 | 1.313333 | 11.10667 |
| RUFY4 | 0.389 | -1.36082 | 0.000158 | 0.001298 | yes | down | 2.4 | 2.23 | 2.99 | 1.93 | 1.24 | 1.44 | 2.54 | 1.536667 |
| LOC103352078 | 3.159 | 1.659277 | 0.000162 | 0.001325 | yes | up | 0.39 | 0.3 | 0.38 | 1.29 | 1.5 | 2.56 | 0.356667 | 1.783333 |
| RDH8 | 7.742 | 2.952666 | 0.000163 | 0.001333 | yes | up | 0.09 | 0.5 | 0.28 | 1.04 | 4.85 | 4.21 | 0.29 | 3.366667 |
| METAP1D | 0.382 | -1.3869 | 0.000164 | 0.001337 | yes | down | 2.59 | 3.74 | 3.21 | 1.94 | 2.2 | 1.4 | 3.18 | 1.846667 |
| RNF24 | 0.322 | -1.63696 | 0.000164 | 0.001337 | yes | down | 4.24 | 6.84 | 7.88 | 3.81 | 3.7 | 1.55 | 6.32 | 3.02 |
| LOC108177708 | 0.409 | -1.2908 | 0.000166 | 0.001354 | yes | down | 37.61 | 23.97 | 32.21 | 23.75 | 18.78 | 17.05 | 31.26333 | 19.86 |
| SLC17A9 | 0.332 | -1.59077 | 0.000167 | 0.001356 | yes | down | 2.28 | 1.72 | 1.87 | 1.44 | 0.94 | 0.82 | 1.956667 | 1.066667 |
| BLM | 0.449 | -1.15654 | 0.000168 | 0.001365 | yes | down | 10.19 | 7.7 | 13.5 | 3.56 | 2.14 | 3.45 | 10.46333 | 3.05 |
| BHLHE22 | 10.369 | 3.374193 | 0.000171 | 0.001388 | yes | up | 0.06 | 0.01 | 0.01 | 0.57 | 0.35 | 0.51 | 0.026667 | 0.476667 |
| ALPK1 | 4.562 | 2.189625 | 0.000175 | 0.001413 | yes | up | 0.23 | 0.16 | 0.46 | 3.42 | 1.03 | 1.68 | 0.283333 | 2.043333 |
| LOC108178692 | 4.256 | 2.089634 | 0.00018 | 0.001451 | yes | up | 0.18 | 0.14 | 1.73 | 0.98 | 1.09 | 0.99 | 0.683333 | 1.02 |
| LOC100358663 | 533.007 | 9.05801 | 0.000182 | 0.001461 | yes | up | 0 | 0 | 0 | 18.5 | 3.3 | 0.37 | 0 | 7.39 |
| LOC108176811 | 8.555 | 3.096781 | 0.000184 | 0.00148 | yes | up | 0.15 | 0.22 | 0.07 | 1.58 | 1.66 | 2.03 | 0.146667 | 1.756667 |
| LOC103347691 | 0.109 | -3.19293 | 0.000185 | 0.001485 | yes | down | 0.55 | 0.36 | 0.23 | 0.08 | 0.04 | 0.08 | 0.38 | 0.066667 |
| LOC108175372 | 0.147 | -2.76284 | 0.000185 | 0.001485 | yes | down | 1.06 | 1.48 | 1.57 | 0.17 | 0.1 | 0.09 | 1.37 | 0.12 |
| HOXB2 | 2.218 | 1.149568 | 0.000186 | 0.001488 | yes | up | 1.63 | 1.93 | 1.91 | 4.48 | 6.17 | 6.72 | 1.823333 | 5.79 |
| LOC100345918 | 14.501 | 3.858097 | 0.000187 | 0.001497 | yes | up | 3.4 | 0.7 | 0.85 | 67.86 | 36.06 | 10.62 | 1.65 | 38.18 |
| FZD7 | 2.259 | 1.175503 | 0.000189 | 0.001512 | yes | up | 1.01 | 1.17 | 1.34 | 3.25 | 4.01 | 5.17 | 1.173333 | 4.143333 |
| LOC103347986 | 10.873 | 3.442642 | 0.000195 | 0.00155 | yes | up | 0.14 | 0.35 | 0.18 | 6.71 | 3.09 | 1.44 | 0.223333 | 3.746667 |
| SAMD9L | 9.141 | 3.192355 | 0.000195 | 0.001552 | yes | up | 1.3 | 1.22 | 0.83 | 33.81 | 8.94 | 4.6 | 1.116667 | 15.78333 |
| IKBKE | 3.712 | 1.89218 | 0.000196 | 0.00156 | yes | up | 1.65 | 1.41 | 1.68 | 17.07 | 5.58 | 4.84 | 1.58 | 9.163333 |
| LOC100346767 | 0.014 | -6.20028 | 0.000198 | 0.001571 | yes | down | 0.19 | 0.58 | 0.32 | 0 | 0 | 0 | 0.363333 | 0 |
| HSD11B2 | 0.297 | -1.75157 | 0.000198 | 0.001574 | yes | down | 1.13 | 1.7 | 1.68 | 0.56 | 0.59 | 0.94 | 1.503333 | 0.696667 |
| TMA16 | 0.485 | -1.0442 | 0.0002 | 0.001585 | yes | down | 6.11 | 8.57 | 7.87 | 6.99 | 4.14 | 5.65 | 7.516667 | 5.593333 |
| GPRC5B | 2.344 | 1.228682 | 0.000205 | 0.00162 | yes | up | 4.07 | 8.62 | 5.72 | 13.42 | 22.02 | 30.77 | 6.136667 | 22.07 |
| KLHL25 | 0.414 | -1.27107 | 0.000205 | 0.001622 | yes | down | 3.19 | 5.9 | 6.53 | 2.2 | 3.86 | 3.89 | 5.206667 | 3.316667 |
| RGS20 | 2.39 | 1.256813 | 0.000209 | 0.00165 | yes | up | 17.95 | 15.1 | 20.03 | 25.83 | 21.72 | 40.26 | 17.69333 | 29.27 |
| MRPL32 | 0.467 | -1.09701 | 0.000211 | 0.001657 | yes | down | 10.76 | 10.66 | 8.61 | 5.98 | 6.45 | 9.52 | 10.01 | 7.316667 |
| DHTKD1 | 2.252 | 1.171423 | 0.000211 | 0.00166 | yes | up | 1.82 | 1.84 | 1.1 | 4 | 5.57 | 7.13 | 1.586667 | 5.566667 |
| PLEKHO2 | 2.161 | 1.111437 | 0.000215 | 0.001688 | yes | up | 3.22 | 4.18 | 3.72 | 17.84 | 9.83 | 9.32 | 3.706667 | 12.33 |
| TMEM173 | 5.058 | 2.338522 | 0.000218 | 0.001714 | yes | up | 0.91 | 1.41 | 1.19 | 19.14 | 4.32 | 3.12 | 1.17 | 8.86 |
| FAM196A | 0.126 | -2.98302 | 0.000219 | 0.001714 | yes | down | 0.09 | 0.19 | 0.51 | 0.04 | 0.08 | 0.03 | 0.263333 | 0.05 |
| SH3BP2 | 2.009 | 1.006516 | 0.00022 | 0.001722 | yes | up | 4.67 | 5.44 | 6.43 | 21.52 | 11.81 | 18.42 | 5.513333 | 17.25 |
| LOC103348240 | 0.08 | -3.65064 | 0.000222 | 0.001735 | yes | down | 0.48 | 5.12 | 8.27 | 0.22 | 0.62 | 0.83 | 4.623333 | 0.556667 |
| MYL10 | 0.03 | -5.06439 | 0.000222 | 0.001738 | yes | down | 1.7 | 3.14 | 1.23 | 0 | 0.26 | 0 | 2.023333 | 0.086667 |
| ZNF250 | 2.094 | 1.066336 | 0.000226 | 0.001765 | yes | up | 1.87 | 1.48 | 2.13 | 5.36 | 6.33 | 6.21 | 1.826667 | 5.966667 |
| LOC100342463 | 0.409 | -1.29068 | 0.000227 | 0.001767 | yes | down | 2.32 | 2.45 | 2.91 | 1.42 | 1.67 | 1.73 | 2.56 | 1.606667 |
| RGN | 0.238 | -2.06821 | 0.000227 | 0.001769 | yes | down | 1.59 | 1.12 | 1.74 | 0.32 | 0.61 | 0.69 | 1.483333 | 0.54 |
| LOC108176507 | 58.74 | 5.876277 | 0.000229 | 0.001779 | yes | up | 0 | 0 | 0 | 0.36 | 0.16 | 0.19 | 0 | 0.236667 |
| SLC16A1 | 0.467 | -1.0995 | 0.000238 | 0.001844 | yes | down | 38.57 | 45.97 | 32.26 | 16.23 | 33.12 | 35.03 | 38.93333 | 28.12667 |
| MALL | 0.45 | -1.15334 | 0.000238 | 0.001844 | yes | down | 19.13 | 29.34 | 12.21 | 11.17 | 13.72 | 17.03 | 20.22667 | 13.97333 |
| L3MBTL2 | 0.45 | -1.15271 | 0.000239 | 0.001845 | yes | down | 2.29 | 3.81 | 3.19 | 2.25 | 1.38 | 2.88 | 3.096667 | 2.17 |
| CHI3L1 | 5.741 | 2.521238 | 0.00024 | 0.00185 | yes | up | 0.65 | 0.51 | 0.84 | 12.22 | 1.62 | 4.13 | 0.666667 | 5.99 |
| CHST11 | 0.34 | -1.55738 | 0.000245 | 0.001884 | yes | down | 1.63 | 3.83 | 2.7 | 1.3 | 0.99 | 1.9 | 2.72 | 1.396667 |
| BORA | 2.007 | 1.005051 | 0.000246 | 0.001893 | yes | up | 0.99 | 1.38 | 1.46 | 4.14 | 3.41 | 4.34 | 1.276667 | 3.963333 |
| MYL4 | 0.096 | -3.38264 | 0.000249 | 0.001911 | yes | down | 11.71 | 6.12 | 0.77 | 0.22 | 1.24 | 1.39 | 6.2 | 0.95 |
| LOC108175810 | 18.228 | 4.188084 | 0.000251 | 0.001921 | yes | up | 0.33 | 0 | 0.15 | 2.42 | 5.75 | 5.45 | 0.16 | 4.54 |
| LOC103346593 | 0.289 | -1.79002 | 0.000253 | 0.001935 | yes | down | 9.2 | 16.33 | 4.38 | 5.33 | 2.12 | 5.85 | 9.97 | 4.433333 |
| OPN3 | 2.26 | 1.176218 | 0.000256 | 0.001952 | yes | up | 1.39 | 1.29 | 1.16 | 3.13 | 4.77 | 5.38 | 1.28 | 4.426667 |
| AGBL3 | 4.138 | 2.048865 | 0.000256 | 0.001955 | yes | up | 0.12 | 0.32 | 0.34 | 2 | 2.35 | 1.46 | 0.26 | 1.936667 |
| ARSG | 3.547 | 1.826463 | 0.00026 | 0.001979 | yes | up | 0.22 | 0.26 | 0.27 | 0.76 | 1.51 | 1.79 | 0.25 | 1.353333 |
| LOC100008980 | 0.015 | -6.09279 | 0.00026 | 0.001979 | yes | down | 0.6 | 0.5 | 0.3 | 0 | 0 | 0 | 0.466667 | 0 |
| CASP4 | 4.028 | 2.010067 | 0.000262 | 0.001992 | yes | up | 1.11 | 1.59 | 1.36 | 17.66 | 4.53 | 6.08 | 1.353333 | 9.423333 |
| OPHN1 | 2.243 | 1.165722 | 0.000262 | 0.001995 | yes | up | 2.14 | 1.97 | 2.93 | 6.59 | 6.43 | 11.87 | 2.346667 | 8.296667 |
| GLIS2 | 0.495 | -1.01433 | 0.000263 | 0.001996 | yes | down | 2.89 | 5.44 | 5.37 | 3.88 | 3.34 | 3.13 | 4.566667 | 3.45 |
| TRIM69 | 94.958 | 6.569218 | 0.000266 | 0.002013 | yes | up | 0 | 0 | 0 | 2.09 | 0.21 | 0.19 | 0 | 0.83 |
| HSF5 | 0.349 | -1.51834 | 0.00028 | 0.002113 | yes | down | 1.69 | 2.76 | 1.74 | 1.43 | 1.25 | 0.71 | 2.063333 | 1.13 |
| LOC103350962 | 5.465 | 2.450187 | 0.000288 | 0.002169 | yes | up | 0.15 | 0.18 | 0.16 | 1.65 | 0.88 | 1.75 | 0.163333 | 1.426667 |
| LOC108178220 | 3.333 | 1.736943 | 0.000292 | 0.002193 | yes | up | 0.45 | 0.23 | 0.3 | 1.98 | 1.38 | 1.78 | 0.326667 | 1.713333 |
| DTHD1 | 15.195 | 3.925531 | 0.000296 | 0.002219 | yes | up | 0.01 | 0.03 | 0.06 | 1.99 | 0.33 | 0.12 | 0.033333 | 0.813333 |
| LOC108177008 | 2.632 | 1.396258 | 0.000297 | 0.002222 | yes | up | 0.78 | 0.62 | 0.57 | 2.51 | 2.72 | 2.94 | 0.656667 | 2.723333 |
| LOC100348108 | 5.84 | 2.545855 | 0.000298 | 0.00223 | yes | up | 1.07 | 1.05 | 0.86 | 20.3 | 2.59 | 3.32 | 0.993333 | 8.736667 |
| LOC108177118 | 44.552 | 5.477403 | 0.000299 | 0.00224 | yes | up | 0 | 0 | 0.07 | 0.62 | 2.32 | 2.35 | 0.023333 | 1.763333 |
| SETD6 | 0.424 | -1.23877 | 0.0003 | 0.00224 | yes | down | 1.78 | 3.37 | 3.58 | 2.02 | 1.94 | 2.06 | 2.91 | 2.006667 |
| FSTL4 | 4.939 | 2.304225 | 0.000303 | 0.002264 | yes | up | 0.09 | 0.11 | 0.03 | 0.31 | 0.55 | 0.84 | 0.076667 | 0.566667 |
| SHB | 0.484 | -1.04554 | 0.000306 | 0.002281 | yes | down | 5.12 | 8.1 | 9 | 4.51 | 4.9 | 7.3 | 7.406667 | 5.57 |
| CD274 | 4.633 | 2.212081 | 0.000312 | 0.002316 | yes | up | 1.78 | 0.5 | 0.51 | 11.47 | 4.04 | 4.17 | 0.93 | 6.56 |
| APOE | 2.54 | 1.344624 | 0.000314 | 0.002332 | yes | up | 26.17 | 57.45 | 44.18 | 239.02 | 124.64 | 127.72 | 42.6 | 163.7933 |
| EPT1 | 0.44 | -1.18281 | 0.000323 | 0.002391 | yes | down | 0.97 | 1.44 | 1.31 | 0.96 | 0.76 | 0.81 | 1.24 | 0.843333 |
| GCA | 3.776 | 1.916956 | 0.000324 | 0.002392 | yes | up | 1.14 | 1.19 | 0.96 | 9.16 | 2.57 | 7.89 | 1.096667 | 6.54 |
| ERAP1 | 2.859 | 1.515409 | 0.000328 | 0.002415 | yes | up | 10.69 | 7.34 | 5.95 | 45.95 | 19.06 | 17.23 | 7.993333 | 27.41333 |
| HTR7 | 2.602 | 1.379734 | 0.000335 | 0.002465 | yes | up | 0.51 | 0.52 | 0.54 | 2.88 | 1.25 | 2.24 | 0.523333 | 2.123333 |
| TIFA | 3.369 | 1.752515 | 0.000336 | 0.002467 | yes | up | 1.09 | 1.15 | 1.17 | 10.18 | 3.86 | 3.68 | 1.136667 | 5.906667 |
| IL1R1 | 2.076 | 1.053719 | 0.000336 | 0.002467 | yes | up | 6.72 | 4.2 | 3.9 | 12.88 | 16.33 | 18.58 | 4.94 | 15.93 |
| RRP12 | 0.467 | -1.09699 | 0.00034 | 0.00249 | yes | down | 3.99 | 5.85 | 3.34 | 3.85 | 3.3 | 2.23 | 4.393333 | 3.126667 |
| RARRES1 | 3.162 | 1.660989 | 0.00035 | 0.002553 | yes | up | 1.4 | 2.08 | 0.94 | 8.97 | 3.96 | 8.68 | 1.473333 | 7.203333 |
| LOC103350057 | 2.75 | 1.45919 | 0.000357 | 0.002599 | yes | up | 0.75 | 1.05 | 0.95 | 3.12 | 4.6 | 2.51 | 0.916667 | 3.41 |
| KLHDC7A | 0.047 | -4.41716 | 0.000358 | 0.002602 | yes | down | 0.11 | 0.06 | 0.18 | 0.01 | 0 | 0.02 | 0.116667 | 0.01 |
| TIGAR | 0.295 | -1.76272 | 0.000358 | 0.002605 | yes | down | 0.72 | 1.56 | 1.25 | 0.73 | 0.48 | 0.36 | 1.176667 | 0.523333 |
| LOC103346635 | 3.026 | 1.597607 | 0.000359 | 0.002606 | yes | up | 0.44 | 0.48 | 0.53 | 1.71 | 2.17 | 2.78 | 0.483333 | 2.22 |
| ABCC10 | 2.135 | 1.094013 | 0.000359 | 0.00261 | yes | up | 2.14 | 1.41 | 1.61 | 4.22 | 5.72 | 6.39 | 1.72 | 5.443333 |
| SLC25A4 | 0.48 | -1.05965 | 0.00036 | 0.002611 | yes | down | 261.14 | 394.27 | 203.69 | 138.93 | 254.77 | 237.22 | 286.3667 | 210.3067 |
| MYLK3 | 0.262 | -1.93307 | 0.000361 | 0.002619 | yes | down | 4.5 | 5.18 | 2.33 | 0.67 | 2.7 | 1.42 | 4.003333 | 1.596667 |
| LOC103348798 | 0.099 | -3.33094 | 0.000372 | 0.002681 | yes | down | 0.11 | 0.38 | 0.72 | 0.07 | 0.03 | 0.09 | 0.403333 | 0.063333 |
| RGAG1 | 0.24 | -2.0593 | 0.000374 | 0.002692 | yes | down | 0.21 | 0.41 | 0.26 | 0.09 | 0.09 | 0.14 | 0.293333 | 0.106667 |
| LOC100343655 | 53.349 | 5.737384 | 0.000375 | 0.002703 | yes | up | 0 | 0 | 0 | 0.5 | 0.74 | 1.11 | 0 | 0.783333 |
| LOC103347627 | 17.839 | 4.156935 | 0.000381 | 0.002743 | yes | up | 0.47 | 0.19 | 0.34 | 24.35 | 3.18 | 1.92 | 0.333333 | 9.816667 |
| DCAF16 | 2.018 | 1.013142 | 0.000384 | 0.002757 | yes | up | 3.5 | 2.32 | 3.72 | 9.78 | 8.43 | 12.22 | 3.18 | 10.14333 |
| GLRB | 0.452 | -1.14416 | 0.000385 | 0.002764 | yes | down | 2.07 | 3.8 | 5.55 | 2.42 | 2.57 | 2.38 | 3.806667 | 2.456667 |
| TP53I3 | 2.48 | 1.310279 | 0.000386 | 0.00277 | yes | up | 1.54 | 1.3 | 1.32 | 5 | 4.19 | 6.99 | 1.386667 | 5.393333 |
| MYPOP | 0.29 | -1.78447 | 0.000389 | 0.002788 | yes | down | 1.2 | 1.65 | 2.04 | 0.46 | 0.76 | 1.01 | 1.63 | 0.743333 |
| TAP2 | 10.153 | 3.34382 | 0.000391 | 0.002801 | yes | up | 1.35 | 1.73 | 1.52 | 58.13 | 7.55 | 7.81 | 1.533333 | 24.49667 |
| ITIH3 | 6.487 | 2.697603 | 0.000394 | 0.002821 | yes | up | 0.06 | 0.2 | 0.13 | 1.31 | 2.06 | 0.46 | 0.13 | 1.276667 |
| RNF212 | 0.296 | -1.75473 | 0.000395 | 0.002824 | yes | down | 3.06 | 3.08 | 5.51 | 1.27 | 1.6 | 2.9 | 3.883333 | 1.923333 |
| NDUFAF5 | 0.484 | -1.04629 | 0.000397 | 0.002836 | yes | down | 7.65 | 13.17 | 7.36 | 5.88 | 6.8 | 7.43 | 9.393333 | 6.703333 |
| SNAPC5 | 0.469 | -1.09118 | 0.000397 | 0.002836 | yes | down | 6.99 | 11.6 | 11.46 | 7.26 | 7.21 | 8.11 | 10.01667 | 7.526667 |
| KTI12 | 0.442 | -1.17896 | 0.000408 | 0.002905 | yes | down | 6.33 | 9.66 | 5.37 | 6.56 | 3.38 | 4.53 | 7.12 | 4.823333 |
| TPPP | 0.456 | -1.13351 | 0.000411 | 0.00292 | yes | down | 8.44 | 19.47 | 8.57 | 9.74 | 6.82 | 8.67 | 12.16 | 8.41 |
| ZNF526 | 2.034 | 1.024503 | 0.000413 | 0.002937 | yes | up | 0.96 | 1.19 | 1.25 | 3.83 | 2.73 | 4.3 | 1.133333 | 3.62 |
| MYH3 | 0.013 | -6.24153 | 0.000426 | 0.003016 | yes | down | 5.29 | 22.44 | 0.1 | 0.01 | 0.32 | 0.21 | 9.276667 | 0.18 |
| LOC103351157 | 7.76 | 2.95606 | 0.000428 | 0.003028 | yes | up | 0.02 | 0.03 | 0.07 | 0.38 | 0.59 | 0.44 | 0.04 | 0.47 |
| LOC103349722 | 2.061 | 1.043152 | 0.000438 | 0.00309 | yes | up | 27.16 | 22.55 | 22.22 | 61.33 | 60.41 | 110.55 | 23.97667 | 77.43 |
| HERC5 | 3.79 | 1.922085 | 0.000449 | 0.003156 | yes | up | 1.06 | 1.13 | 1.2 | 8.49 | 3.45 | 2.76 | 1.13 | 4.9 |
| SP110 | 3.472 | 1.795816 | 0.00045 | 0.003158 | yes | up | 0.91 | 0.37 | 0.62 | 5.14 | 3.05 | 2.67 | 0.633333 | 3.62 |
| LOC100339314 | 2.47 | 1.304423 | 0.000451 | 0.003162 | yes | up | 288.56 | 332.91 | 267.04 | 498.99 | 1166.05 | 1769.86 | 296.17 | 1144.967 |
| TLR5 | 3.054 | 1.61073 | 0.000453 | 0.003173 | yes | up | 0.26 | 0.22 | 0.31 | 1.68 | 0.93 | 1.16 | 0.263333 | 1.256667 |
| C17H15orf41 | 0.363 | -1.46371 | 0.000457 | 0.003199 | yes | down | 1.71 | 1.81 | 2.89 | 1.62 | 1.11 | 0.87 | 2.136667 | 1.2 |
| TRMT6 | 0.413 | -1.27671 | 0.000464 | 0.003245 | yes | down | 1.6 | 2.12 | 2.07 | 1.74 | 0.84 | 1.18 | 1.93 | 1.253333 |
| GREM2 | 2.017 | 1.011923 | 0.00047 | 0.00328 | yes | up | 0.59 | 1.07 | 0.59 | 1.87 | 1.97 | 3.36 | 0.75 | 2.4 |
| LOC103346165 | 2.162 | 1.11213 | 0.000472 | 0.003283 | yes | up | 0.58 | 0.61 | 1.07 | 1.46 | 1.95 | 1.54 | 0.753333 | 1.65 |
| SSH2 | 2.077 | 1.054482 | 0.000478 | 0.003322 | yes | up | 5.75 | 4.08 | 2.76 | 15.76 | 11.55 | 15.51 | 4.196667 | 14.27333 |
| S100A8 | 2.916 | 1.544203 | 0.000479 | 0.003328 | yes | up | 12.26 | 16.58 | 6.48 | 60.22 | 28.37 | 71.19 | 11.77333 | 53.26 |
| NOTO | 0.38 | -1.39621 | 0.000483 | 0.003355 | yes | down | 4.64 | 3.26 | 2.67 | 1.22 | 1.9 | 2.44 | 3.523333 | 1.853333 |
| LOC103349229 | 2.231 | 1.15758 | 0.000487 | 0.003371 | yes | up | 1.1 | 1.36 | 1.93 | 4.59 | 3.63 | 3.91 | 1.463333 | 4.043333 |
| LOC100351756 | 0.008 | -6.92594 | 0.00049 | 0.003388 | yes | down | 0.16 | 4.71 | 6.51 | 0 | 0.11 | 0 | 3.793333 | 0.036667 |
| CA4 | 0.482 | -1.05318 | 0.000491 | 0.003399 | yes | down | 76.75 | 50.34 | 42.73 | 51.68 | 33.56 | 42.65 | 56.60667 | 42.63 |
| PRDM1 | 4.208 | 2.073246 | 0.000498 | 0.003438 | yes | up | 0.25 | 0.29 | 0.15 | 2.72 | 0.72 | 0.98 | 0.23 | 1.473333 |
| LOC100350373 | 56.809 | 5.828041 | 0.000501 | 0.003449 | yes | up | 0 | 0 | 0 | 0.29 | 0.61 | 1.31 | 0 | 0.736667 |
| LOC108176463 | 4.941 | 2.304869 | 0.000503 | 0.00346 | yes | up | 0.26 | 0.32 | 0.39 | 4.83 | 1.09 | 1.43 | 0.323333 | 2.45 |
| MAML3 | 2.556 | 1.353708 | 0.000515 | 0.003535 | yes | up | 0.32 | 0.45 | 0.8 | 1.59 | 2.09 | 2.59 | 0.523333 | 2.09 |
| NSUN4 | 0.425 | -1.23418 | 0.000522 | 0.003574 | yes | down | 11.92 | 11.59 | 20.94 | 6.8 | 7.38 | 14.27 | 14.81667 | 9.483333 |
| LOC108176201 | 4.642 | 2.21486 | 0.000523 | 0.003585 | yes | up | 0.51 | 0.3 | 0.66 | 3.05 | 1.52 | 3.34 | 0.49 | 2.636667 |
| DYNLRB2 | 2.394 | 1.25914 | 0.000525 | 0.003594 | yes | up | 12.57 | 5.91 | 10.52 | 34.81 | 27.59 | 33.76 | 9.666667 | 32.05333 |
| DCDC2C | 13.767 | 3.783094 | 0.000526 | 0.003598 | yes | up | 0 | 0.13 | 0.06 | 1.1 | 1.16 | 2.91 | 0.063333 | 1.723333 |
| LOC100348005 | 2.215 | 1.147364 | 0.000528 | 0.003607 | yes | up | 1.25 | 1.35 | 2.16 | 5.03 | 4.86 | 6.67 | 1.586667 | 5.52 |
| LOC100352301 | 2.424 | 1.27756 | 0.000538 | 0.003673 | yes | up | 0.47 | 0.63 | 0.58 | 1.87 | 1.87 | 1.63 | 0.56 | 1.79 |
| LOC100355679 | 0.016 | -5.97493 | 0.000541 | 0.003687 | yes | down | 0.21 | 1.12 | 0.39 | 0 | 0 | 0 | 0.573333 | 0 |
| SLC1A5 | 0.407 | -1.29534 | 0.000544 | 0.003711 | yes | down | 22.58 | 34.67 | 42.87 | 10.52 | 22.16 | 28.36 | 33.37333 | 20.34667 |
| STAT2 | 3.501 | 1.807901 | 0.000554 | 0.003767 | yes | up | 3.11 | 4.6 | 4.45 | 42.45 | 13.6 | 10 | 4.053333 | 22.01667 |
| HMGCS2 | 0.33 | -1.59784 | 0.000563 | 0.003827 | yes | down | 39.25 | 21.93 | 15.03 | 6.38 | 13.21 | 20.34 | 25.40333 | 13.31 |
| ELOVL2 | 0.325 | -1.61956 | 0.000564 | 0.00383 | yes | down | 13.56 | 13.39 | 16.35 | 3.57 | 13.38 | 4.57 | 14.43333 | 7.173333 |
| RLA-A3 | 6.601 | 2.722787 | 0.000566 | 0.003843 | yes | up | 28.22 | 34.67 | 35.11 | 729.7 | 113.58 | 123.93 | 32.66667 | 322.4033 |
| PVR | 0.442 | -1.17753 | 0.000569 | 0.003856 | yes | down | 5.34 | 4.23 | 3.94 | 3.41 | 2.44 | 3.51 | 4.503333 | 3.12 |
| CTSK | 3.343 | 1.741105 | 0.000584 | 0.003942 | yes | up | 24.53 | 31.24 | 28.29 | 290.4 | 53.45 | 90.47 | 28.02 | 144.7733 |
| DHRS3 | 0.398 | -1.33046 | 0.000586 | 0.003954 | yes | down | 7.6 | 17.2 | 21.73 | 7.13 | 10.28 | 9.31 | 15.51 | 8.906667 |
| LOC103348694 | 2.573 | 1.363209 | 0.00059 | 0.003978 | yes | up | 0.73 | 0.61 | 0.72 | 2.07 | 2.5 | 4.03 | 0.686667 | 2.866667 |
| LOC100351224 | 0.273 | -1.87159 | 0.000594 | 0.003999 | yes | down | 0.78 | 2.37 | 0.91 | 0.61 | 0.61 | 0.43 | 1.353333 | 0.55 |
| PDXP | 0.434 | -1.20391 | 0.000597 | 0.004022 | yes | down | 2.74 | 4.91 | 4.66 | 2.5 | 2.12 | 3.75 | 4.103333 | 2.79 |
| SLC16A11 | 3.544 | 1.825175 | 0.000598 | 0.004026 | yes | up | 0.32 | 0.34 | 0.61 | 1.73 | 2.15 | 3.12 | 0.423333 | 2.333333 |
| LOC100339055 | 2.621 | 1.390053 | 0.000615 | 0.004117 | yes | up | 0.59 | 0.79 | 0.43 | 1.56 | 2.9 | 2.96 | 0.603333 | 2.473333 |
| LOC103350952 | 0.398 | -1.3284 | 0.000619 | 0.004142 | yes | down | 0.9 | 0.97 | 0.89 | 0.78 | 0.38 | 0.55 | 0.92 | 0.57 |
| LOC100349198 | 2.041 | 1.029023 | 0.000636 | 0.004235 | yes | up | 0.66 | 0.73 | 0.62 | 2.52 | 1.73 | 2.46 | 0.67 | 2.236667 |
| LOC100344620 | 2.623 | 1.391477 | 0.00064 | 0.004261 | yes | up | 2.44 | 2.59 | 3.21 | 16.58 | 7.28 | 9.73 | 2.746667 | 11.19667 |
| SH3GL2 | 0.44 | -1.18415 | 0.000642 | 0.004269 | yes | down | 3.98 | 7.49 | 8.43 | 3.9 | 5.87 | 3.55 | 6.633333 | 4.44 |
| LOC103347003 | 8.414 | 3.072821 | 0.000645 | 0.004288 | yes | up | 0.29 | 0.14 | 0.34 | 6.23 | 3.45 | 0.28 | 0.256667 | 3.32 |
| BBOX1 | 0.292 | -1.77494 | 0.000652 | 0.004326 | yes | down | 1.36 | 1.52 | 3.17 | 0.87 | 0.55 | 1.33 | 2.016667 | 0.916667 |
| IL27RA | 3.391 | 1.761903 | 0.000656 | 0.004345 | yes | up | 0.42 | 0.68 | 0.55 | 4.83 | 2.1 | 1.48 | 0.55 | 2.803333 |
| TYROBP | 3.978 | 1.992081 | 0.000656 | 0.004345 | yes | up | 7.2 | 9.43 | 10.43 | 116.89 | 20.37 | 28.37 | 9.02 | 55.21 |
| AP5Z1 | 2.001 | 1.000734 | 0.000659 | 0.004359 | yes | up | 1.12 | 1.71 | 1.42 | 4.78 | 3.15 | 5.28 | 1.416667 | 4.403333 |
| ND4L | 0.479 | -1.06202 | 0.000669 | 0.004411 | yes | down | 12525.82 | 10445.8 | 13117.1 | 12661.41 | 7484.3 | 6137.8 | 12029.57 | 8761.17 |
| AMT | 0.375 | -1.41614 | 0.000691 | 0.004545 | yes | down | 2.43 | 5.12 | 4.18 | 2.62 | 2.74 | 1.25 | 3.91 | 2.203333 |
| NAGPA | 2.576 | 1.365295 | 0.000702 | 0.004615 | yes | up | 1.2 | 1.74 | 1.78 | 9.6 | 3.96 | 5.22 | 1.573333 | 6.26 |
| LOC100339419 | 4.495 | 2.168323 | 0.000708 | 0.004651 | yes | up | 0.1 | 0.04 | 0.11 | 0.5 | 0.55 | 0.76 | 0.083333 | 0.603333 |
| LOC100347711 | 4.748 | 2.247458 | 0.000712 | 0.004674 | yes | up | 1.05 | 1.77 | 1.43 | 20.03 | 2.86 | 7.64 | 1.416667 | 10.17667 |
| CCNA2 | 7.773 | 2.958429 | 0.000727 | 0.004753 | yes | up | 0.19 | 0.04 | 0.37 | 4.63 | 1.87 | 1.24 | 0.2 | 2.58 |
| RASGRP2 | 2.185 | 1.127813 | 0.000729 | 0.004759 | yes | up | 2.38 | 3.48 | 4.23 | 10.59 | 10.31 | 9.26 | 3.363333 | 10.05333 |
| MANBA | 2.201 | 1.138108 | 0.000737 | 0.004804 | yes | up | 0.48 | 0.56 | 0.32 | 1.36 | 1.58 | 1.38 | 0.453333 | 1.44 |
| LOC100343635 | 0.176 | -2.50807 | 0.000745 | 0.004847 | yes | down | 0.24 | 0.48 | 0.35 | 0.04 | 0.16 | 0.08 | 0.356667 | 0.093333 |
| NUAK2 | 4.226 | 2.079414 | 0.000758 | 0.004918 | yes | up | 0.39 | 0.36 | 0.22 | 3.69 | 0.91 | 2.22 | 0.323333 | 2.273333 |
| LOC100349551 | 0.465 | -1.1059 | 0.000764 | 0.004952 | yes | down | 395.83 | 631 | 291.59 | 248.99 | 430.94 | 247.82 | 439.4733 | 309.25 |
| SFTPD | 7.304 | 2.868722 | 0.000765 | 0.004956 | yes | up | 0.21 | 0.2 | 0.18 | 3.6 | 1.01 | 2.08 | 0.196667 | 2.23 |
| P2RY4 | 0.026 | -5.28849 | 0.000765 | 0.004956 | yes | down | 0.28 | 0.66 | 0.6 | 0 | 0.05 | 0 | 0.513333 | 0.016667 |
| TRAM1L1 | 0.248 | -2.01245 | 0.000774 | 0.005006 | yes | down | 0.59 | 0.85 | 0.47 | 0.23 | 0.21 | 0.29 | 0.636667 | 0.243333 |
| BBC3 | 2.647 | 1.404613 | 0.000776 | 0.005015 | yes | up | 0.54 | 1.39 | 1.22 | 4.16 | 4.01 | 4.63 | 1.05 | 4.266667 |
| CX3CL1 | 4.583 | 2.196226 | 0.000778 | 0.005028 | yes | up | 0.83 | 1.21 | 0.8 | 14.56 | 2.17 | 2.8 | 0.946667 | 6.51 |
| RASD2 | 0.245 | -2.03136 | 0.000788 | 0.005081 | yes | down | 6.65 | 2.37 | 3.24 | 0.52 | 1.78 | 2.54 | 4.086667 | 1.613333 |
| DUSP4 | 0.343 | -1.54289 | 0.000788 | 0.005083 | yes | down | 5.8 | 3.68 | 2.17 | 1.7 | 1.62 | 3.05 | 3.883333 | 2.123333 |
| HOMER2 | 0.167 | -2.58618 | 0.000793 | 0.005108 | yes | down | 9.12 | 10.64 | 1 | 0.55 | 1.88 | 2.95 | 6.92 | 1.793333 |
| SIGLEC1 | 3.058 | 1.612693 | 0.000796 | 0.005126 | yes | up | 1.56 | 1.89 | 1.9 | 15.72 | 5.98 | 3.67 | 1.783333 | 8.456667 |
| LOC100340649 | 4.252 | 2.088272 | 0.000801 | 0.005155 | yes | up | 0.36 | 0.25 | 0.45 | 4.17 | 1.44 | 1.47 | 0.353333 | 2.36 |
| IL10RA | 4.305 | 2.105885 | 0.000802 | 0.005158 | yes | up | 1.74 | 1.67 | 1.17 | 22.49 | 3.25 | 4.84 | 1.526667 | 10.19333 |
| C2H16orf91 | 0.078 | -3.6784 | 0.000803 | 0.005159 | yes | down | 3.08 | 2.96 | 1.64 | 0.21 | 0.43 | 0.28 | 2.56 | 0.306667 |
| BRI3BP | 0.457 | -1.13067 | 0.000829 | 0.00529 | yes | down | 4.01 | 4.97 | 6.72 | 6.64 | 2.6 | 3.53 | 5.233333 | 4.256667 |
| NAAA | 2.114 | 1.079675 | 0.000831 | 0.005297 | yes | up | 3.43 | 4.01 | 5.22 | 15.78 | 8.5 | 17.6 | 4.22 | 13.96 |
| STK17B | 0.499 | -1.00198 | 0.000841 | 0.00535 | yes | down | 20.5 | 17.26 | 14 | 18.26 | 12.08 | 9.93 | 17.25333 | 13.42333 |
| LOC103345896 | 4.536 | 2.181294 | 0.000844 | 0.005366 | yes | up | 0.16 | 0.14 | 0.11 | 1.61 | 0.68 | 0.61 | 0.136667 | 0.966667 |
| ZNFX1 | 2.116 | 1.081215 | 0.000847 | 0.005387 | yes | up | 9.27 | 8.87 | 8.5 | 42.77 | 26.34 | 17.73 | 8.88 | 28.94667 |
| MYO16 | 0.147 | -2.76646 | 0.00085 | 0.005403 | yes | down | 0.18 | 0.15 | 0.15 | 0.38 | 0.39 | 0.39 | 0.16 | 0.386667 |
| SLC7A10 | 0.33 | -1.60053 | 0.000854 | 0.005421 | yes | down | 1.32 | 3.72 | 2.49 | 0.79 | 1.42 | 2.02 | 2.51 | 1.41 |
| LOC103346641 | 0.268 | -1.8986 | 0.00086 | 0.005451 | yes | down | 0.84 | 2.16 | 1.32 | 0.5 | 0.44 | 0.85 | 1.44 | 0.596667 |
| TMEM35B | 2.431 | 1.281519 | 0.000861 | 0.005457 | yes | up | 1.42 | 3.23 | 2.36 | 9.63 | 8.86 | 7.5 | 2.336667 | 8.663333 |
| ESPL1 | 3.138 | 1.64987 | 0.000875 | 0.005526 | yes | up | 0.11 | 0.18 | 0.2 | 1.17 | 0.67 | 0.52 | 0.163333 | 0.786667 |
| MICAL1 | 2.328 | 1.21889 | 0.000881 | 0.005551 | yes | up | 2.03 | 2.36 | 1.71 | 10.96 | 4.77 | 6.54 | 2.033333 | 7.423333 |
| C3H8orf22 | 0.326 | -1.61688 | 0.000895 | 0.005629 | yes | down | 6.1 | 6.3 | 3.44 | 1.63 | 4.31 | 1.96 | 5.28 | 2.633333 |
| TLR3 | 3.427 | 1.776852 | 0.000895 | 0.005629 | yes | up | 0.88 | 0.3 | 0.57 | 4.71 | 2.94 | 1.65 | 0.583333 | 3.1 |
| HAGH | 0.497 | -1.00734 | 0.0009 | 0.005649 | yes | down | 6.49 | 6.86 | 6.5 | 3.44 | 6.39 | 5.45 | 6.616667 | 5.093333 |
| LOC103346476 | 0.166 | -2.58978 | 0.0009 | 0.00565 | yes | down | 2.7 | 5.05 | 2.97 | 0.6 | 0.14 | 0.03 | 3.573333 | 0.256667 |
| HDHD3 | 0.315 | -1.66587 | 0.000903 | 0.00566 | yes | down | 1.63 | 3.11 | 4.25 | 1.11 | 1.71 | 1.54 | 2.996667 | 1.453333 |
| LOC100352996 | 66.014 | 6.044709 | 0.000908 | 0.005692 | yes | up | 0 | 0 | 0.28 | 28.48 | 2.23 | 0.77 | 0.093333 | 10.49333 |
| IGFN1 | 14.479 | 3.855841 | 0.000912 | 0.005715 | yes | up | 0.08 | 0.12 | 0.07 | 0.05 | 7.07 | 0.52 | 0.09 | 2.546667 |
| LOC100349247 | 7.583 | 2.922817 | 0.00093 | 0.005809 | yes | up | 9.89 | 13.48 | 14.58 | 337.25 | 44.29 | 62.56 | 12.65 | 148.0333 |
| LOC103345165 | 9.491 | 3.246493 | 0.000963 | 0.005983 | yes | up | 0.17 | 0.22 | 0.05 | 0.52 | 2.45 | 3.5 | 0.146667 | 2.156667 |
| BMP3 | 0.419 | -1.25594 | 0.000975 | 0.006051 | yes | down | 0.59 | 0.96 | 1.16 | 0.69 | 0.42 | 0.66 | 0.903333 | 0.59 |
| LOC100345136 | 5.66 | 2.500728 | 0.000992 | 0.006146 | yes | up | 0.52 | 0.81 | 0.63 | 14.76 | 1.59 | 2.02 | 0.653333 | 6.123333 |
| ANKRD44 | 2.055 | 1.039453 | 0.001002 | 0.006202 | yes | up | 2.17 | 1.69 | 1.72 | 8.17 | 4.39 | 4.98 | 1.86 | 5.846667 |
| ADAT1 | 2.444 | 1.289423 | 0.00101 | 0.006245 | yes | up | 0.32 | 0.37 | 0.53 | 1.23 | 1.44 | 1.87 | 0.406667 | 1.513333 |
| LOC108176028 | 0.496 | -1.01211 | 0.001036 | 0.006375 | yes | down | 3.01 | 5 | 5.04 | 3.83 | 3.39 | 2.85 | 4.35 | 3.356667 |
| INCENP | 2.278 | 1.188043 | 0.001037 | 0.006375 | yes | up | 0.37 | 0.41 | 0.52 | 1.99 | 1.35 | 1.24 | 0.433333 | 1.526667 |
| LOC108178739 | 0.017 | -5.91138 | 0.001048 | 0.006425 | yes | down | 0.07 | 0.02 | 0.06 | 0 | 0 | 0 | 0.05 | 0 |
| PLAUR | 0.322 | -1.63362 | 0.001049 | 0.00643 | yes | down | 11.46 | 4.89 | 5.24 | 4.66 | 3.47 | 4.05 | 7.196667 | 4.06 |
| EVA1C | 0.478 | -1.06468 | 0.001052 | 0.006449 | yes | down | 2.2 | 3.6 | 3.45 | 2.46 | 1.5 | 2.85 | 3.083333 | 2.27 |
| DRAM1 | 3.412 | 1.770695 | 0.001057 | 0.006465 | yes | up | 2.56 | 1.67 | 1.47 | 19.11 | 4.09 | 7.75 | 1.9 | 10.31667 |
| ZC3H12B | 2.34 | 1.226403 | 0.001058 | 0.006473 | yes | up | 10.24 | 4.49 | 0.99 | 22.47 | 21.85 | 28.92 | 5.24 | 24.41333 |
| LRIG1 | 2.092 | 1.065205 | 0.00106 | 0.006478 | yes | up | 8.36 | 6.1 | 4.15 | 15.33 | 18.45 | 27.71 | 6.203333 | 20.49667 |
| CCDC77 | 2.743 | 1.455686 | 0.00106 | 0.006478 | yes | up | 0.46 | 0.37 | 0.54 | 2.39 | 1.46 | 2.05 | 0.456667 | 1.966667 |
| ZNF583 | 0.477 | -1.06785 | 0.001076 | 0.006567 | yes | down | 1.6 | 2.15 | 1.68 | 1.45 | 1.25 | 1.21 | 1.81 | 1.303333 |
| IL10 | 3.438 | 1.781571 | 0.001078 | 0.006572 | yes | up | 0.44 | 0.59 | 0.67 | 2.32 | 2.24 | 3.99 | 0.566667 | 2.85 |
| LOC108176036 | 12.701 | 3.666834 | 0.001078 | 0.006572 | yes | up | 0 | 0.14 | 0.11 | 3.38 | 0.48 | 0.76 | 0.083333 | 1.54 |
| DOCK3 | 2.383 | 1.252937 | 0.001079 | 0.006572 | yes | up | 0.18 | 0.14 | 0.25 | 0.69 | 0.77 | 0.64 | 0.19 | 0.7 |
| TBC1D30 | 0.472 | -1.08321 | 0.001084 | 0.006602 | yes | down | 0.57 | 1.13 | 0.73 | 0.51 | 0.58 | 0.67 | 0.81 | 0.586667 |
| SYNE2 | 2.319 | 1.213248 | 0.001097 | 0.006664 | yes | up | 4.8 | 5.57 | 4.49 | 16.62 | 20.24 | 10.45 | 4.953333 | 15.77 |
| PLA2R1 | 2.288 | 1.194041 | 0.001124 | 0.006792 | yes | up | 1.16 | 0.92 | 1.81 | 6.06 | 2.73 | 5.39 | 1.296667 | 4.726667 |
| TAPBP | 2.754 | 1.461647 | 0.001129 | 0.006818 | yes | up | 3.59 | 4.36 | 4.1 | 30.96 | 10.45 | 9.71 | 4.016667 | 17.04 |
| LOC103351283 | 2.988 | 1.579304 | 0.001144 | 0.006895 | yes | up | 0.49 | 0.24 | 0.28 | 2.11 | 1.16 | 0.8 | 0.336667 | 1.356667 |
| PTK2B | 5.668 | 2.502885 | 0.001144 | 0.006895 | yes | up | 0.34 | 0.31 | 0.31 | 7.04 | 0.87 | 0.97 | 0.32 | 2.96 |
| DNAJC28 | 0.489 | -1.03238 | 0.001153 | 0.006936 | yes | down | 1.47 | 1.33 | 1.57 | 1.14 | 1.14 | 1.02 | 1.456667 | 1.1 |
| KCNG4 | 0.166 | -2.58861 | 0.001162 | 0.006985 | yes | down | 0.07 | 0.25 | 0.16 | 0.03 | 0.06 | 0.03 | 0.16 | 0.04 |
| LOC100346561 | 2.162 | 1.112517 | 0.001165 | 0.007001 | yes | up | 5.73 | 4.86 | 5.36 | 11.86 | 26.98 | 14.43 | 5.316667 | 17.75667 |
| SSBP2 | 2.226 | 1.154462 | 0.001196 | 0.007151 | yes | up | 8.65 | 4.32 | 7.82 | 18.92 | 21.65 | 33.28 | 6.93 | 24.61667 |
| KLF9 | 2.178 | 1.122993 | 0.001198 | 0.007161 | yes | up | 30.5 | 18.8 | 13.6 | 69.43 | 56.35 | 96.16 | 20.96667 | 73.98 |
| AGTR2 | 0.285 | -1.81237 | 0.001205 | 0.007199 | yes | down | 0.64 | 0.7 | 0.38 | 0.25 | 0.27 | 0.22 | 0.573333 | 0.246667 |
| RPS3A | 2.173 | 1.119511 | 0.001218 | 0.007254 | yes | up | 197.26 | 228.01 | 90.99 | 427.31 | 495.37 | 863.53 | 172.0867 | 595.4033 |
| ADAMTS8 | 0.235 | -2.09079 | 0.001222 | 0.007275 | yes | down | 0.88 | 1.59 | 0.35 | 0.24 | 0.43 | 0.33 | 0.94 | 0.333333 |
| DOK1 | 2.31 | 1.207726 | 0.001228 | 0.007296 | yes | up | 0.82 | 1.85 | 1.64 | 6.05 | 5.03 | 4.22 | 1.436667 | 5.1 |
| AGAP2 | 2.07 | 1.049909 | 0.001238 | 0.007345 | yes | up | 1.03 | 1.91 | 0.84 | 3.29 | 4.19 | 3.6 | 1.26 | 3.693333 |
| PEBP4 | 0.407 | -1.29551 | 0.001242 | 0.007364 | yes | down | 65.55 | 70.84 | 109.6 | 31.87 | 81.19 | 40.7 | 81.99667 | 51.25333 |
| CSF2RB | 4.926 | 2.300299 | 0.001256 | 0.007434 | yes | up | 0.65 | 0.81 | 0.53 | 11.47 | 1.28 | 2.12 | 0.663333 | 4.956667 |
| LOC108176104 | 0.215 | -2.21976 | 0.001259 | 0.007445 | yes | down | 1.16 | 5.38 | 3.85 | 1.25 | 0.44 | 1.75 | 3.463333 | 1.146667 |
| ABCC4 | 0.281 | -1.83191 | 0.00128 | 0.007556 | yes | down | 7.26 | 2.34 | 2.71 | 2.97 | 0.75 | 1.87 | 4.103333 | 1.863333 |
| TNFSF10 | 3.045 | 1.606488 | 0.001289 | 0.007599 | yes | up | 11.47 | 7.72 | 9.68 | 88.91 | 25.01 | 23.29 | 9.623333 | 45.73667 |
| SESN2 | 2.487 | 1.314384 | 0.001363 | 0.007984 | yes | up | 0.39 | 0.66 | 0.45 | 2.66 | 1.46 | 1.61 | 0.5 | 1.91 |
| PALB2 | 0.478 | -1.06573 | 0.001373 | 0.008026 | yes | down | 1.15 | 1.27 | 1.25 | 1.34 | 0.97 | 1.29 | 1.223333 | 1.2 |
| RBM43 | 2.567 | 1.359987 | 0.001399 | 0.008176 | yes | up | 1.22 | 1.21 | 0.58 | 5.34 | 3.03 | 3.61 | 1.003333 | 3.993333 |
| MBLAC1 | 0.33 | -1.59767 | 0.001403 | 0.008184 | yes | down | 2.06 | 2.93 | 1.78 | 1.75 | 0.6 | 1.07 | 2.256667 | 1.14 |
| LOC100352436 | 4.27 | 2.09435 | 0.001411 | 0.008215 | yes | up | 0.6 | 0.61 | 0.48 | 7.04 | 0.9 | 3.22 | 0.563333 | 3.72 |
| LOC103348029 | 0.473 | -1.07915 | 0.001434 | 0.008327 | yes | down | 4.52 | 7.18 | 4.75 | 4.24 | 3.05 | 4.65 | 5.483333 | 3.98 |
| MROH2A | 31.561 | 4.980064 | 0.001445 | 0.008376 | yes | up | 0 | 0 | 0.01 | 0.07 | 0.14 | 0.24 | 0.003333 | 0.15 |
| LOC103349237 | 0.07 | -3.8366 | 0.001445 | 0.008376 | yes | down | 2.27 | 4.45 | 0.04 | 0.04 | 0.32 | 0.37 | 2.253333 | 0.243333 |
| LOC108178313 | 2.986 | 1.577983 | 0.00149 | 0.0086 | yes | up | 1.69 | 1.24 | 2 | 5.83 | 5.87 | 11.76 | 1.643333 | 7.82 |
| SNX20 | 5.648 | 2.497688 | 0.001492 | 0.008609 | yes | up | 0.28 | 0.42 | 0.17 | 5.01 | 1 | 1.39 | 0.29 | 2.466667 |
| PLXNC1 | 3.91 | 1.967046 | 0.001512 | 0.008704 | yes | up | 0.1 | 0.11 | 0.07 | 0.94 | 1.26 | 1.13 | 0.093333 | 1.11 |
| CPLX1 | 0.109 | -3.19754 | 0.001531 | 0.008809 | yes | down | 0.61 | 1.44 | 0.5 | 0.12 | 0.28 | 0 | 0.85 | 0.133333 |
| GBA | 2.5 | 1.321721 | 0.001539 | 0.008851 | yes | up | 2.56 | 2.47 | 2.55 | 16.36 | 6.38 | 6.67 | 2.526667 | 9.803333 |
| COQ4 | 0.48 | -1.05879 | 0.001564 | 0.00898 | yes | down | 3.59 | 4.23 | 4.54 | 3.62 | 3.19 | 2.29 | 4.12 | 3.033333 |
| HGH1 | 0.345 | -1.53608 | 0.001566 | 0.008987 | yes | down | 1.96 | 2.05 | 1.7 | 0.95 | 1.05 | 1.16 | 1.903333 | 1.053333 |
| TRPV1 | 4.009 | 2.003092 | 0.00157 | 0.009004 | yes | up | 0.05 | 0.17 | 0.21 | 0.62 | 0.81 | 1.29 | 0.143333 | 0.906667 |
| FCER1G | 4.426 | 2.146008 | 0.001579 | 0.009048 | yes | up | 8.47 | 11.78 | 12.53 | 166.81 | 20.85 | 27.48 | 10.92667 | 71.71333 |
| ZNF862 | 2.226 | 1.154469 | 0.001582 | 0.009052 | yes | up | 1.28 | 1.87 | 1.74 | 4.44 | 3.23 | 4.13 | 1.63 | 3.933333 |
| SELP | 2.337 | 1.224388 | 0.001582 | 0.009052 | yes | up | 0.55 | 0.88 | 0.52 | 1.63 | 2.32 | 3.06 | 0.65 | 2.336667 |
| LOC100353012 | 66.124 | 6.047092 | 0.00159 | 0.009094 | yes | up | 0 | 0 | 0 | 0.26 | 0.01 | 0.35 | 0 | 0.206667 |
| SERPINI1 | 2.043 | 1.030759 | 0.001592 | 0.0091 | yes | up | 1.14 | 1.15 | 1.01 | 3.69 | 3.5 | 3.24 | 1.1 | 3.476667 |
| CASQ2 | 0.1 | -3.31851 | 0.001597 | 0.009122 | yes | down | 40.38 | 53.95 | 2.83 | 2.81 | 4.55 | 7.72 | 32.38667 | 5.026667 |
| LOC100341315 | 3.186 | 1.671889 | 0.001613 | 0.009198 | yes | up | 0.24 | 0.21 | 0.28 | 1.35 | 0.91 | 1.39 | 0.243333 | 1.216667 |
| SMAD6 | 0.457 | -1.13043 | 0.001624 | 0.009244 | yes | down | 2.33 | 5.57 | 5.59 | 2.87 | 2.79 | 3.81 | 4.496667 | 3.156667 |
| PGER8 | 2.823 | 1.497213 | 0.001627 | 0.009257 | yes | up | 1.34 | 3.3 | 1.84 | 5.47 | 6.87 | 16.21 | 2.16 | 9.516667 |
| LOC100347239 | 3.292 | 1.718957 | 0.001671 | 0.009457 | yes | up | 2.37 | 1.94 | 3.09 | 9.57 | 8.08 | 20.92 | 2.466667 | 12.85667 |
| C16H1orf106 | 0.093 | -3.42606 | 0.001678 | 0.009488 | yes | down | 1.17 | 0.53 | 1.86 | 0.37 | 0 | 0.16 | 1.186667 | 0.176667 |
| ENPP1 | 2.167 | 1.115788 | 0.001691 | 0.009555 | yes | up | 3.27 | 3.48 | 3.09 | 5.93 | 14.85 | 12.19 | 3.28 | 10.99 |
| TVP23A | 36.056 | 5.172168 | 0.001713 | 0.009644 | yes | up | 0 | 0.03 | 0 | 1.35 | 0.32 | 0.23 | 0.01 | 0.633333 |
| SLC16A12 | 0.2 | -2.31929 | 0.001722 | 0.00969 | yes | down | 0.22 | 0.22 | 0.1 | 0.06 | 0.05 | 0.06 | 0.18 | 0.056667 |
| DACH1 | 0.48 | -1.05801 | 0.001725 | 0.009703 | yes | down | 2.53 | 6.06 | 2.97 | 2.12 | 3.17 | 3.12 | 3.853333 | 2.803333 |
| CEP152 | 2.133 | 1.092918 | 0.001735 | 0.009753 | yes | up | 0.35 | 0.31 | 0.4 | 1.36 | 1.17 | 0.92 | 0.353333 | 1.15 |
| ARID5B | 2.376 | 1.248262 | 0.001756 | 0.009852 | yes | up | 8.89 | 4.97 | 2.75 | 19.27 | 18.4 | 24.97 | 5.536667 | 20.88 |
| BZW2 | 0.466 | -1.10263 | 0.001759 | 0.009861 | yes | down | 36.2 | 63.39 | 25.96 | 19.62 | 39.88 | 33.33 | 41.85 | 30.94333 |
| RSPO3 | 3.206 | 1.680623 | 0.001766 | 0.009889 | yes | up | 0.54 | 0.63 | 0.18 | 1.66 | 1.48 | 2.88 | 0.45 | 2.006667 |
| UNC5D | 0.455 | -1.13545 | 0.00177 | 0.009906 | yes | down | 12.52 | 10.38 | 8.65 | 6.99 | 7.11 | 8.1 | 10.51667 | 7.4 |
| LSM3 | 2.223 | 1.152765 | 0.001788 | 0.009994 | yes | up | 8.89 | 13.29 | 11.29 | 20.16 | 41.04 | 54.33 | 11.15667 | 38.51 |
| LOC100351810 | 48.498 | 5.599845 | 0.001791 | 0.010009 | yes | up | 0 | 0.56 | 0 | 36.26 | 5.3 | 1.43 | 0.186667 | 14.33 |
| LOC100349085 | 15.776 | 3.979643 | 0.001794 | 0.010021 | yes | up | 15.71 | 25.18 | 29.34 | 1486.61 | 40.35 | 171.62 | 23.41 | 566.1933 |
| PML | 2.536 | 1.34259 | 0.001804 | 0.010069 | yes | up | 2.01 | 3.68 | 2.93 | 18.98 | 7.85 | 6.5 | 2.873333 | 11.11 |
| GRO-A | 2.482 | 1.311748 | 0.001841 | 0.010257 | yes | up | 2.19 | 2.61 | 2.32 | 11.67 | 4.51 | 9.67 | 2.373333 | 8.616667 |
| PTP4A3 | 0.147 | -2.76577 | 0.001857 | 0.010335 | yes | down | 126.23 | 156.58 | 96.59 | 5.4 | 57.39 | 21.66 | 126.4667 | 28.15 |
| SMTNL2 | 0.275 | -1.86013 | 0.001881 | 0.01045 | yes | down | 5.32 | 7.31 | 5.09 | 0.58 | 4.72 | 2.19 | 5.906667 | 2.496667 |
| LOC100343456 | 6.657 | 2.734848 | 0.001923 | 0.010663 | yes | up | 0.04 | 0.08 | 0.22 | 1.55 | 1.32 | 0.74 | 0.113333 | 1.203333 |
| LOC100348918 | 2.127 | 1.088625 | 0.001957 | 0.010815 | yes | up | 4.11 | 6.98 | 3.39 | 12.25 | 14.23 | 21.02 | 4.826667 | 15.83333 |
| MCM5 | 2.302 | 1.202593 | 0.001967 | 0.010863 | yes | up | 0.95 | 2.07 | 1.27 | 6.96 | 3.36 | 4.75 | 1.43 | 5.023333 |
| C1H11orf1 | 2.209 | 1.143349 | 0.001975 | 0.010894 | yes | up | 1.13 | 0.57 | 0.99 | 3.23 | 3.05 | 2.93 | 0.896667 | 3.07 |
| CPXM1 | 2.338 | 1.225044 | 0.00198 | 0.010922 | yes | up | 1.05 | 3.43 | 2.27 | 7.68 | 9.55 | 6.5 | 2.25 | 7.91 |
| ADRA1B | 0.314 | -1.67339 | 0.001992 | 0.010973 | yes | down | 1.06 | 3.3 | 2.34 | 1.33 | 1.14 | 0.6 | 2.233333 | 1.023333 |
| TCTEX1D4 | 0.313 | -1.67775 | 0.001996 | 0.010987 | yes | down | 1.9 | 1.17 | 1.18 | 0.63 | 0.77 | 0.67 | 1.416667 | 0.69 |
| LOC100351055 | 5.038 | 2.33295 | 0.002015 | 0.011072 | yes | up | 0.07 | 1.9 | 0.93 | 1.9 | 5.18 | 2.13 | 0.966667 | 3.07 |
| LPXN | 3.619 | 1.855656 | 0.002019 | 0.011089 | yes | up | 0.62 | 0.86 | 1.06 | 9.02 | 1.96 | 2.77 | 0.846667 | 4.583333 |
| GPR182 | 2.065 | 1.045929 | 0.002034 | 0.011158 | yes | up | 1.73 | 2.07 | 2.02 | 3.8 | 7.1 | 7.59 | 1.94 | 6.163333 |
| KCNQ4 | 0.342 | -1.54689 | 0.002077 | 0.011364 | yes | down | 9.72 | 9.74 | 2.15 | 3.1 | 4.31 | 4.79 | 7.203333 | 4.066667 |
| LOC103350467 | 0.448 | -1.15932 | 0.002096 | 0.011454 | yes | down | 2.18 | 2.76 | 2.94 | 1.09 | 2.27 | 2.1 | 2.626667 | 1.82 |
| ALKBH8 | 0.439 | -1.18913 | 0.002122 | 0.011562 | yes | down | 1.13 | 1.44 | 1.13 | 1.07 | 0.54 | 0.84 | 1.233333 | 0.816667 |
| RNF148 | 0.21 | -2.25041 | 0.002184 | 0.011872 | yes | down | 1.02 | 2.13 | 1.38 | 0.79 | 0.29 | 0.36 | 1.51 | 0.48 |
| LOC100353664 | 2.138 | 1.096395 | 0.00219 | 0.011899 | yes | up | 1.48 | 3.47 | 3.71 | 9.11 | 9.08 | 10.21 | 2.886667 | 9.466667 |
| TBC1D10C | 7.184 | 2.844799 | 0.002202 | 0.011952 | yes | up | 0.07 | 0.18 | 0.13 | 2.93 | 0.76 | 0.57 | 0.126667 | 1.42 |
| IL18R1 | 65.076 | 6.024047 | 0.002216 | 0.012012 | yes | up | 0 | 0 | 0 | 0.65 | 0.03 | 0.08 | 0 | 0.253333 |
| SLAMF6 | 22.299 | 4.478891 | 0.002252 | 0.012148 | yes | up | 0.03 | 0.05 | 0.02 | 2.93 | 0.05 | 0.2 | 0.033333 | 1.06 |
| LCAT | 18.657 | 4.221623 | 0.002252 | 0.012148 | yes | up | 0 | 0.15 | 0 | 0.3 | 1.38 | 2.29 | 0.05 | 1.323333 |
| LOC100341303 | 0.436 | -1.19602 | 0.00226 | 0.01218 | yes | down | 2.78 | 3.25 | 3.03 | 1.62 | 2.19 | 2.33 | 3.02 | 2.046667 |
| MYT1L | 0.046 | -4.4344 | 0.002285 | 0.012299 | yes | down | 0.06 | 0.15 | 0.04 | 0.01 | 0.01 | 0 | 0.083333 | 0.006667 |
| LOC100344240 | 0.473 | -1.07888 | 0.002301 | 0.012374 | yes | down | 1.06 | 4.27 | 1.39 | 0.96 | 3.68 | 0.84 | 2.24 | 1.826667 |
| DUSP6 | 0.439 | -1.18852 | 0.002318 | 0.012446 | yes | down | 54.69 | 31.22 | 18.57 | 21.43 | 21.63 | 30.73 | 34.82667 | 24.59667 |
| NFAM1 | 6.327 | 2.661443 | 0.002318 | 0.012446 | yes | up | 0.14 | 0.2 | 0.1 | 3.41 | 0.23 | 0.5 | 0.146667 | 1.38 |
| LOC100353758 | 0.431 | -1.21524 | 0.002348 | 0.01257 | yes | down | 4.43 | 4.2 | 4.21 | 1.54 | 3.15 | 3.98 | 4.28 | 2.89 |
| SPIRE2 | 0.472 | -1.08291 | 0.002368 | 0.012667 | yes | down | 1.29 | 1.96 | 1.91 | 1 | 1.27 | 1.5 | 1.72 | 1.256667 |
| NRK | 0.5 | -1.00137 | 0.002384 | 0.012742 | yes | down | 2.43 | 4.03 | 1.74 | 1.95 | 2.53 | 1.74 | 2.733333 | 2.073333 |
| C8H12orf40 | 11.486 | 3.521791 | 0.002389 | 0.012759 | yes | up | 0 | 0.03 | 0.16 | 0.44 | 2.3 | 0.61 | 0.063333 | 1.116667 |
| PRAM1 | 8.321 | 3.056736 | 0.002391 | 0.012767 | yes | up | 0.12 | 0.06 | 0.11 | 3.11 | 0.3 | 0.38 | 0.096667 | 1.263333 |
| LOC100353330 | 0.441 | -1.18023 | 0.002394 | 0.01277 | yes | down | 19.77 | 44.82 | 45.71 | 15.56 | 16.36 | 11.92 | 36.76667 | 14.61333 |
| LOC100351979 | 0.448 | -1.15944 | 0.002435 | 0.01297 | yes | down | 3.05 | 5.15 | 7.1 | 4.38 | 3.31 | 2.28 | 5.1 | 3.323333 |
| EIF2AK2 | 3.054 | 1.610835 | 0.002435 | 0.01297 | yes | up | 1.22 | 1.33 | 1.44 | 10.34 | 5.45 | 2.37 | 1.33 | 6.053333 |
| FPR1 | 7.289 | 2.86581 | 0.00244 | 0.01299 | yes | up | 0.63 | 0.46 | 0.33 | 13.4 | 0.87 | 1.84 | 0.473333 | 5.37 |
| LOC103351365 | 26.376 | 4.721143 | 0.002446 | 0.013013 | yes | up | 0 | 0.06 | 0 | 1.1 | 0.73 | 0.53 | 0.02 | 0.786667 |
| LOC103345755 | 4.211 | 2.074118 | 0.002455 | 0.013046 | yes | up | 0.1 | 0.05 | 0.09 | 0.38 | 0.53 | 0.59 | 0.08 | 0.5 |
| LOC100353293 | 5.82 | 2.540969 | 0.002466 | 0.013097 | yes | up | 0 | 0.29 | 0.49 | 2.23 | 1.77 | 3.42 | 0.26 | 2.473333 |
| LOC108177439 | 49.051 | 5.616211 | 0.002483 | 0.013173 | yes | up | 0 | 0 | 0 | 2.01 | 1.34 | 0.21 | 0 | 1.186667 |
| IER2 | 0.197 | -2.34546 | 0.002488 | 0.013191 | yes | down | 21.93 | 3.98 | 1.86 | 4.04 | 1.98 | 2.91 | 9.256667 | 2.976667 |
| LOC108178461 | 2.455 | 1.295873 | 0.002491 | 0.013203 | yes | up | 0.51 | 0.77 | 0.49 | 2.36 | 1.84 | 3.23 | 0.59 | 2.476667 |
| SLC4A1 | 0.207 | -2.27458 | 0.002505 | 0.013254 | yes | down | 0.11 | 0.34 | 0.29 | 0.08 | 0.05 | 0.13 | 0.246667 | 0.086667 |
| HES1 | 0.34 | -1.55663 | 0.002524 | 0.013339 | yes | down | 28.1 | 12.64 | 6.54 | 6.11 | 9.06 | 10.81 | 15.76 | 8.66 |
| SEMA4A | 6.937 | 2.794412 | 0.002529 | 0.013354 | yes | up | 0.08 | 0.22 | 0.15 | 3.72 | 0.4 | 0.49 | 0.15 | 1.536667 |
| LOC103349936 | 0.209 | -2.25651 | 0.002546 | 0.013438 | yes | down | 4.86 | 10.65 | 3.58 | 3.15 | 2.54 | 0.21 | 6.363333 | 1.966667 |
| CENPW | 2.648 | 1.404705 | 0.002552 | 0.01344 | yes | up | 3.74 | 1.67 | 2.51 | 9.54 | 11.35 | 12.06 | 2.64 | 10.98333 |
| SAP30 | 2.094 | 1.065967 | 0.002552 | 0.01344 | yes | up | 2.03 | 2.19 | 1.75 | 4.42 | 7.93 | 6.97 | 1.99 | 6.44 |
| MYOD1 | 0.08 | -3.63505 | 0.002557 | 0.013456 | yes | down | 1.15 | 1.36 | 0.09 | 0 | 0.13 | 0.2 | 0.866667 | 0.11 |
| PDCD2L | 0.479 | -1.06045 | 0.002562 | 0.013476 | yes | down | 5.2 | 5.26 | 4.56 | 3.31 | 2.64 | 5.04 | 5.006667 | 3.663333 |
| ANGPTL4 | 2.012 | 1.008911 | 0.002585 | 0.013579 | yes | up | 219.56 | 160.08 | 136.46 | 308.46 | 582.3 | 738.74 | 172.0333 | 543.1667 |
| SNCG | 2.007 | 1.005257 | 0.002612 | 0.013688 | yes | up | 11.92 | 17.7 | 15.7 | 27.01 | 53.14 | 62.04 | 15.10667 | 47.39667 |
| LOC103352375 | 4.602 | 2.202283 | 0.002629 | 0.013765 | yes | up | 0.26 | 0.25 | 0.23 | 1.83 | 2.5 | 1.28 | 0.246667 | 1.87 |
| PIK3R5 | 3.315 | 1.728954 | 0.002712 | 0.014129 | yes | up | 0.66 | 0.48 | 0.6 | 5.86 | 1.46 | 1.67 | 0.58 | 2.996667 |
| LOC103346901 | 17.793 | 4.153236 | 0.002717 | 0.014141 | yes | up | 4.46 | 3.23 | 2.27 | 251.62 | 19.17 | 7.1 | 3.32 | 92.63 |
| GPR65 | 15.668 | 3.969778 | 0.002726 | 0.014175 | yes | up | 0.04 | 0.11 | 0.03 | 3.77 | 0.21 | 0.31 | 0.06 | 1.43 |
| CPN2 | 0.199 | -2.33043 | 0.002728 | 0.014178 | yes | down | 0.64 | 0.27 | 0.37 | 0.13 | 0.06 | 0.23 | 0.426667 | 0.14 |
| LOC100328967 | 6.679 | 2.739554 | 0.002734 | 0.014204 | yes | up | 52.48 | 82.71 | 89.76 | 1876.93 | 375.33 | 236.23 | 74.98333 | 829.4967 |
| ZSWIM7 | 2.025 | 1.017696 | 0.002742 | 0.014239 | yes | up | 2.11 | 3.99 | 3.25 | 8.73 | 9.37 | 8.74 | 3.116667 | 8.946667 |
| MFAP3L | 2.396 | 1.260792 | 0.002757 | 0.0143 | yes | up | 2.71 | 1.67 | 3.21 | 4.59 | 10.19 | 14.28 | 2.53 | 9.686667 |
| CCDC141 | 3.258 | 1.704004 | 0.002764 | 0.014328 | yes | up | 0.4 | 0.49 | 0.44 | 4.57 | 1.07 | 1.16 | 0.443333 | 2.266667 |
| MKI67 | 4.404 | 2.138844 | 0.00278 | 0.014387 | yes | up | 0.24 | 0.48 | 0.22 | 4.59 | 1.16 | 0.39 | 0.313333 | 2.046667 |
| PABPC1L | 0.079 | -3.66688 | 0.002785 | 0.014405 | yes | down | 0.2 | 0.53 | 0.43 | 0.13 | 0.03 | 0.03 | 0.386667 | 0.063333 |
| LOC108177094 | 3.31 | 1.7267 | 0.002788 | 0.014413 | yes | up | 0.19 | 0.38 | 0.23 | 1.3 | 1.33 | 1.38 | 0.266667 | 1.336667 |
| TRIM5 | 2.237 | 1.16176 | 0.002793 | 0.014432 | yes | up | 4.02 | 4.31 | 3.57 | 21.1 | 9.87 | 8.94 | 3.966667 | 13.30333 |
| BARX1 | 0.206 | -2.28125 | 0.002812 | 0.014513 | yes | down | 1.07 | 1.21 | 1.49 | 0.36 | 0.1 | 0.79 | 1.256667 | 0.416667 |
| BID | 4.269 | 2.093738 | 0.002831 | 0.014608 | yes | up | 0.51 | 1.02 | 0.4 | 11.29 | 1.04 | 1.7 | 0.643333 | 4.676667 |
| LOC108176881 | 5.008 | 2.324172 | 0.002837 | 0.014626 | yes | up | 0.07 | 0.07 | 0.17 | 0.4 | 0.76 | 0.72 | 0.103333 | 0.626667 |
| IL34 | 2.116 | 1.081313 | 0.002842 | 0.014643 | yes | up | 1.66 | 2.92 | 2.14 | 10.03 | 4.14 | 10.24 | 2.24 | 8.136667 |
| TAPBPL | 3.251 | 1.701071 | 0.002878 | 0.0148 | yes | up | 2.4 | 3.95 | 3.7 | 34.65 | 7.12 | 8.24 | 3.35 | 16.67 |
| SNTA1 | 0.499 | -1.00377 | 0.002899 | 0.014889 | yes | down | 32.12 | 44.68 | 39.75 | 16.03 | 42.37 | 30.66 | 38.85 | 29.68667 |
| FAM111A | 3.149 | 1.655085 | 0.002917 | 0.014967 | yes | up | 1.02 | 1.12 | 0.82 | 10.06 | 2.25 | 2.61 | 0.986667 | 4.973333 |
| LOC100349257 | 14.928 | 3.899929 | 0.002933 | 0.01504 | yes | up | 1.84 | 0.74 | 0.72 | 66.56 | 5.33 | 3.04 | 1.1 | 24.97667 |
| CRACR2A | 3.923 | 1.97214 | 0.002943 | 0.015073 | yes | up | 0.53 | 0.81 | 0.58 | 8.55 | 1.34 | 1.72 | 0.64 | 3.87 |
| FUT4 | 0.48 | -1.05842 | 0.002956 | 0.015137 | yes | down | 1.54 | 1.49 | 2.09 | 1.78 | 0.85 | 1.21 | 1.706667 | 1.28 |
| ZFAND5 | 2.295 | 1.198631 | 0.002958 | 0.015143 | yes | up | 57.73 | 33.34 | 25.3 | 116.86 | 137.84 | 232.99 | 38.79 | 162.5633 |
| HPSE | 4.99 | 2.319056 | 0.00297 | 0.015194 | yes | up | 0.14 | 0.2 | 0.28 | 3.37 | 0.83 | 0.52 | 0.206667 | 1.573333 |
| DAPP1 | 15.278 | 3.93339 | 0.002974 | 0.015207 | yes | up | 0 | 0.12 | 0.13 | 4.84 | 0.37 | 0.25 | 0.083333 | 1.82 |
| ANKRD31 | 0.131 | -2.93084 | 0.003033 | 0.015465 | yes | down | 15.99 | 8.32 | 11.31 | 6 | 1.03 | 0.54 | 11.87333 | 2.523333 |
| NCF4 | 4.945 | 2.305988 | 0.003041 | 0.015495 | yes | up | 0.65 | 0.65 | 0.74 | 12.27 | 1.34 | 1.73 | 0.68 | 5.113333 |
| CYTIP | 4.568 | 2.191483 | 0.003043 | 0.015498 | yes | up | 0.58 | 0.61 | 0.72 | 9.88 | 0.96 | 2.71 | 0.636667 | 4.516667 |
| UPP1 | 3.526 | 1.818125 | 0.003052 | 0.015537 | yes | up | 1.39 | 3.13 | 1.12 | 3.84 | 8.54 | 20.56 | 1.88 | 10.98 |
| PRG4 | 0.264 | -1.92295 | 0.00306 | 0.015562 | yes | down | 10.78 | 4.05 | 2.68 | 0.65 | 3.1 | 3.3 | 5.836667 | 2.35 |
| LOC100357462 | 58.999 | 5.882622 | 0.003068 | 0.015579 | yes | up | 0 | 0 | 0 | 15.91 | 1.07 | 1.4 | 0 | 6.126667 |
| LOC100359306 | 3.158 | 1.6588 | 0.003068 | 0.015579 | yes | up | 5.51 | 7.73 | 8.33 | 71.62 | 12.99 | 18.3 | 7.19 | 34.30333 |
| TOB1 | 0.437 | -1.19537 | 0.003068 | 0.015579 | yes | down | 30.87 | 11.06 | 12.37 | 11.79 | 10.25 | 14.05 | 18.1 | 12.03 |
| INHBB | 0.286 | -1.8062 | 0.003117 | 0.015786 | yes | down | 4.93 | 1.84 | 1.14 | 1 | 1.72 | 0.85 | 2.636667 | 1.19 |
| LOC100340502 | 0.373 | -1.42304 | 0.003128 | 0.015835 | yes | down | 0.44 | 0.78 | 0.75 | 0.45 | 0.35 | 0.3 | 0.656667 | 0.366667 |
| HHIPL2 | 13.972 | 3.804479 | 0.003149 | 0.015922 | yes | up | 0.04 | 0 | 0.04 | 0.08 | 0.76 | 0.91 | 0.026667 | 0.583333 |
| FBN2 | 0.427 | -1.22816 | 0.003187 | 0.01608 | yes | down | 0.49 | 0.76 | 0.27 | 0.37 | 0.28 | 0.34 | 0.506667 | 0.33 |
| LOC103351959 | 4.303 | 2.105366 | 0.003192 | 0.016099 | yes | up | 0.01 | 0.21 | 0.17 | 0.89 | 0.6 | 0.68 | 0.13 | 0.723333 |
| LOC108176735 | 2.283 | 1.191029 | 0.003194 | 0.016107 | yes | up | 1.34 | 1.47 | 1.98 | 15.93 | 2.87 | 6.45 | 1.596667 | 8.416667 |
| DIXDC1 | 2.26 | 1.176354 | 0.003196 | 0.016111 | yes | up | 2.48 | 0.52 | 1.92 | 3.83 | 6.16 | 6.55 | 1.64 | 5.513333 |
| TCEAL2 | 3.429 | 1.777833 | 0.003217 | 0.016197 | yes | up | 1.05 | 0.47 | 0.53 | 1.93 | 5 | 4.61 | 0.683333 | 3.846667 |
| CCDC30 | 2.635 | 1.397645 | 0.00322 | 0.016203 | yes | up | 0.27 | 0.27 | 0.35 | 1.24 | 1.28 | 1.25 | 0.296667 | 1.256667 |
| CHAC2 | 0.2 | -2.31853 | 0.003224 | 0.016218 | yes | down | 0.49 | 0.6 | 0.83 | 0.3 | 0.19 | 0.09 | 0.64 | 0.193333 |
| COLQ | 0.221 | -2.17989 | 0.003233 | 0.016256 | yes | down | 3.3 | 2.31 | 0.41 | 0.33 | 0.67 | 1.12 | 2.006667 | 0.706667 |
| SLITRK6 | 2.202 | 1.138931 | 0.00325 | 0.016322 | yes | up | 0.41 | 0.62 | 0.8 | 1.65 | 2.71 | 1.81 | 0.61 | 2.056667 |
| LOC103350570 | 5.818 | 2.540496 | 0.003289 | 0.0165 | yes | up | 0.08 | 0.02 | 0.05 | 0.14 | 0.48 | 0.81 | 0.05 | 0.476667 |
| NRL | 11.398 | 3.510716 | 0.003304 | 0.016559 | yes | up | 0 | 0.03 | 0.05 | 0.34 | 0.42 | 0.43 | 0.026667 | 0.396667 |
| LOC108176193 | 2.4 | 1.263048 | 0.003312 | 0.016594 | yes | up | 0.44 | 0.57 | 0.51 | 1.62 | 2.1 | 1.51 | 0.506667 | 1.743333 |
| XKR5 | 5.406 | 2.434565 | 0.003313 | 0.016594 | yes | up | 0.11 | 0.24 | 0.04 | 0.38 | 1.37 | 1.5 | 0.13 | 1.083333 |
| LOC103346738 | 0.427 | -1.22854 | 0.003322 | 0.016632 | yes | down | 2.19 | 1.63 | 2.57 | 1.09 | 2.28 | 0.84 | 2.13 | 1.403333 |
| CYP2B4 | 2.687 | 1.425811 | 0.003324 | 0.016636 | yes | up | 1.24 | 7.07 | 5.56 | 15.49 | 20.75 | 19.36 | 4.623333 | 18.53333 |
| MBOAT2 | 0.405 | -1.30418 | 0.003387 | 0.016919 | yes | down | 2.28 | 2.05 | 0.86 | 0.74 | 1.23 | 1.53 | 1.73 | 1.166667 |
| LOC100342826 | 6.264 | 2.647064 | 0.003396 | 0.01695 | yes | up | 6.64 | 1.85 | 1.43 | 55.41 | 35.25 | 13.51 | 3.306667 | 34.72333 |
| IL18RAP | 35.758 | 5.160191 | 0.003418 | 0.017042 | yes | up | 0 | 0.03 | 0 | 1.42 | 0.04 | 0.09 | 0.01 | 0.516667 |
| METTL21B | 0.402 | -1.31584 | 0.00343 | 0.017094 | yes | down | 2.66 | 1.67 | 4.33 | 1.96 | 2.18 | 1.32 | 2.886667 | 1.82 |
| LOC100341088 | 15.533 | 3.957229 | 0.003435 | 0.01711 | yes | up | 0.11 | 0 | 0.03 | 10.9 | 0.72 | 0.44 | 0.046667 | 4.02 |
| LOC103348554 | 34.465 | 5.107078 | 0.003511 | 0.017387 | yes | up | 0 | 0.08 | 0.07 | 3.14 | 4.45 | 0 | 0.05 | 2.53 |
| LOC100342121 | 0.067 | -3.8891 | 0.003533 | 0.017475 | yes | down | 1.51 | 9.3 | 2.65 | 0 | 1.27 | 0 | 4.486667 | 0.423333 |
| LOC100338018 | 0.026 | -5.26361 | 0.003543 | 0.017517 | yes | down | 2.34 | 2.32 | 0.17 | 0 | 0 | 0.23 | 1.61 | 0.076667 |
| NECAB3 | 3.982 | 1.993621 | 0.0036 | 0.017765 | yes | up | 0.13 | 0.19 | 0.12 | 0.79 | 0.82 | 1.11 | 0.146667 | 0.906667 |
| PRC1 | 2.294 | 1.197809 | 0.003619 | 0.017845 | yes | up | 0.88 | 1.15 | 0.42 | 2.87 | 3.22 | 2.52 | 0.816667 | 2.87 |
| ACAP1 | 3.336 | 1.737914 | 0.003626 | 0.017877 | yes | up | 0.38 | 0.51 | 0.31 | 3.71 | 1.23 | 1.1 | 0.4 | 2.013333 |
| KCNE5 | 0.036 | -4.78036 | 0.003637 | 0.017912 | yes | down | 0.99 | 0.35 | 0.04 | 0 | 0.04 | 0.05 | 0.46 | 0.03 |
| LOC108175950 | 0.469 | -1.09153 | 0.003653 | 0.017977 | yes | down | 1.47 | 1.51 | 2.07 | 1.32 | 1.13 | 1.29 | 1.683333 | 1.246667 |
| LOC108176622 | 4.865 | 2.282401 | 0.003672 | 0.018061 | yes | up | 0.69 | 0.31 | 0.18 | 3.83 | 3.46 | 3.71 | 0.393333 | 3.666667 |
| HYKK | 2.085 | 1.060305 | 0.003681 | 0.018099 | yes | up | 0.58 | 1.52 | 1.13 | 3.85 | 5.36 | 2.97 | 1.076667 | 4.06 |
| LOC108176880 | 2.893 | 1.532757 | 0.003683 | 0.018099 | yes | up | 1.04 | 1.71 | 1.39 | 3.39 | 7.58 | 4.97 | 1.38 | 5.313333 |
| SGK1 | 0.223 | -2.16545 | 0.003686 | 0.018105 | yes | down | 286.46 | 115.49 | 58.09 | 60.68 | 38.33 | 79.85 | 153.3467 | 59.62 |
| LOC100345090 | 0.408 | -1.294 | 0.003697 | 0.018155 | yes | down | 0.45 | 0.67 | 0.61 | 0.37 | 0.26 | 0.46 | 0.576667 | 0.363333 |
| JAML | 3.481 | 1.799418 | 0.003709 | 0.018193 | yes | up | 0.67 | 0.75 | 0.68 | 7.75 | 2.06 | 2.5 | 0.7 | 4.103333 |
| IL17RA | 2.29 | 1.195136 | 0.003739 | 0.018308 | yes | up | 1.38 | 1.52 | 1.58 | 8.61 | 3.44 | 3.81 | 1.493333 | 5.286667 |
| POLR1A | 0.475 | -1.07516 | 0.003739 | 0.018308 | yes | down | 1.51 | 2.43 | 1.92 | 2.21 | 1 | 1.03 | 1.953333 | 1.413333 |
| ZNF597 | 2.539 | 1.344271 | 0.003748 | 0.018345 | yes | up | 0.46 | 0.54 | 0.28 | 1.71 | 1.18 | 1.98 | 0.426667 | 1.623333 |
| LRRN3 | 3.709 | 1.890942 | 0.003768 | 0.01843 | yes | up | 0.87 | 0.21 | 0.16 | 2.72 | 1.56 | 3.17 | 0.413333 | 2.483333 |
| SELPLG | 3.904 | 1.964895 | 0.00378 | 0.018484 | yes | up | 1.2 | 1.36 | 2.04 | 20.94 | 2.73 | 4.07 | 1.533333 | 9.246667 |
| C1S | 5.079 | 2.344489 | 0.003797 | 0.018547 | yes | up | 16.77 | 12.95 | 17.08 | 258.33 | 66.12 | 46.7 | 15.6 | 123.7167 |
| SLC35G2 | 0.46 | -1.11888 | 0.003811 | 0.018598 | yes | down | 2.55 | 3.88 | 5.53 | 2.05 | 3.45 | 2.99 | 3.986667 | 2.83 |
| FHL1 | 0.496 | -1.01154 | 0.003832 | 0.018684 | yes | down | 851.03 | 1434.04 | 508.44 | 464.35 | 748.82 | 885.11 | 931.17 | 699.4267 |
| LOC103347072 | 68.027 | 6.088032 | 0.003844 | 0.018732 | yes | up | 0 | 0 | 0 | 14.36 | 0.35 | 0.99 | 0 | 5.233333 |
| TMPRSS13 | 2.809 | 1.489964 | 0.003856 | 0.018782 | yes | up | 0.23 | 0.3 | 0.69 | 2.13 | 1.33 | 1.86 | 0.406667 | 1.773333 |
| SOX17 | 0.415 | -1.26879 | 0.003875 | 0.018863 | yes | down | 23.36 | 15.03 | 6.29 | 9.42 | 9.89 | 9.83 | 14.89333 | 9.713333 |
| AIF1 | 4.469 | 2.159842 | 0.003902 | 0.01898 | yes | up | 5.44 | 7.68 | 6.21 | 105.24 | 9.36 | 17.37 | 6.443333 | 43.99 |
| KCTD14 | 0.166 | -2.59147 | 0.003922 | 0.019057 | yes | down | 0.51 | 0.92 | 0.9 | 0.2 | 0.27 | 0.17 | 0.776667 | 0.213333 |
| GPRIN1 | 7.952 | 2.991281 | 0.003926 | 0.019066 | yes | up | 0.09 | 0.18 | 0.16 | 0.84 | 2.44 | 2.04 | 0.143333 | 1.773333 |
| ONECUT2 | 0.319 | -1.64937 | 0.003946 | 0.019146 | yes | down | 0.11 | 0.1 | 0.17 | 0.1 | 0.04 | 0.05 | 0.126667 | 0.063333 |
| C1R | 4.283 | 2.098784 | 0.003953 | 0.019175 | yes | up | 28.4 | 23.89 | 39.68 | 413.54 | 89.3 | 111.21 | 30.65667 | 204.6833 |
| LOC108176506 | 0.191 | -2.38729 | 0.003962 | 0.019211 | yes | down | 0.82 | 2.58 | 1.35 | 0.11 | 0.47 | 0.46 | 1.583333 | 0.346667 |
| LOC100328948 | 8.591 | 3.102858 | 0.00399 | 0.019325 | yes | up | 2.56 | 0.47 | 0.16 | 11.21 | 15.44 | 25.33 | 1.063333 | 17.32667 |
| DUSP3 | 0.456 | -1.13418 | 0.003998 | 0.019342 | yes | down | 3.25 | 4.48 | 2.98 | 2.42 | 3.04 | 1.97 | 3.57 | 2.476667 |
| LOC100343223 | 2.193 | 1.133157 | 0.004006 | 0.019372 | yes | up | 1.96 | 3.68 | 5.46 | 10.78 | 10.67 | 16.3 | 3.7 | 12.58333 |
| LOC100338822 | 13.459 | 3.75046 | 0.004012 | 0.019389 | yes | up | 2.24 | 2.58 | 2.83 | 125.04 | 3.34 | 18.41 | 2.55 | 48.93 |
| LOC100356524 | 4.657 | 2.21941 | 0.004037 | 0.019478 | yes | up | 0.2 | 0.19 | 0.06 | 0.75 | 1.08 | 0.94 | 0.15 | 0.923333 |
| LOC100354610 | 2.221 | 1.151068 | 0.004047 | 0.019512 | yes | up | 0.72 | 0.6 | 1.12 | 2.28 | 3.05 | 3.28 | 0.813333 | 2.87 |
| SEPT6 | 2.045 | 1.031861 | 0.0041 | 0.019713 | yes | up | 1.1 | 0.83 | 0.9 | 3.26 | 1.84 | 2.58 | 0.943333 | 2.56 |
| LOC108177148 | 2.323 | 1.216087 | 0.004118 | 0.019778 | yes | up | 1.19 | 1.26 | 1.39 | 4.56 | 4.16 | 5.51 | 1.28 | 4.743333 |
| LOC103347049 | 12.931 | 3.692745 | 0.004165 | 0.019943 | yes | up | 0.4 | 0.39 | 0.34 | 2.14 | 5.38 | 15.97 | 0.376667 | 7.83 |
| LOC103345623 | 2.806 | 1.488634 | 0.004178 | 0.019992 | yes | up | 0.59 | 0.49 | 0.51 | 2.44 | 2.05 | 3.22 | 0.53 | 2.57 |
| PGPEP1 | 2.066 | 1.047132 | 0.004213 | 0.020136 | yes | up | 3.3 | 3.65 | 3.92 | 16.36 | 11.73 | 6.05 | 3.623333 | 11.38 |
| MYH13 | 0.026 | -5.23929 | 0.004232 | 0.020211 | yes | down | 26.81 | 150.18 | 3.36 | 0.01 | 3.85 | 1.06 | 60.11667 | 1.64 |
| LOC100343999 | 2.679 | 1.421895 | 0.004242 | 0.020252 | yes | up | 0.3 | 0.47 | 0.96 | 3.12 | 1.97 | 2.26 | 0.576667 | 2.45 |
| LOC103346238 | 7.316 | 2.871044 | 0.004247 | 0.020266 | yes | up | 0.09 | 0.14 | 0.45 | 6.85 | 0.37 | 1.27 | 0.226667 | 2.83 |
| TMCC3 | 2.39 | 1.256995 | 0.004313 | 0.020519 | yes | up | 8.97 | 5.05 | 3.41 | 11.87 | 21.41 | 31.08 | 5.81 | 21.45333 |
| SERPING1 | 4.476 | 2.162147 | 0.00437 | 0.020747 | yes | up | 26.14 | 28.15 | 30.17 | 411.85 | 76.94 | 95.45 | 28.15333 | 194.7467 |
| XIRP1 | 0.027 | -5.2378 | 0.004398 | 0.020866 | yes | down | 27.6 | 18.08 | 0.1 | 0.02 | 0.75 | 1.17 | 15.26 | 0.646667 |
| LOC103351338 | 4.147 | 2.051954 | 0.004421 | 0.020942 | yes | up | 0.35 | 0.75 | 0.63 | 2 | 4.41 | 4.55 | 0.576667 | 3.653333 |
| LOC108176740 | 8.185 | 3.032974 | 0.004426 | 0.020952 | yes | up | 0.06 | 0.24 | 0.05 | 0.68 | 0.96 | 2.87 | 0.116667 | 1.503333 |
| FMNL1 | 4.064 | 2.02286 | 0.004508 | 0.021291 | yes | up | 0.52 | 1.24 | 1.69 | 17.82 | 2.27 | 3.11 | 1.15 | 7.733333 |
| DCUN1D2 | 0.428 | -1.22274 | 0.004574 | 0.021575 | yes | down | 2.52 | 6.41 | 6.13 | 2.98 | 4.56 | 3.12 | 5.02 | 3.553333 |
| LOC103351328 | 0.212 | -2.23631 | 0.004593 | 0.021642 | yes | down | 0.9 | 1.05 | 0.56 | 0.29 | 0.23 | 0.3 | 0.836667 | 0.273333 |
| MZB1 | 57.514 | 5.84585 | 0.004615 | 0.021726 | yes | up | 0 | 0 | 0 | 3.67 | 0.42 | 0.11 | 0 | 1.4 |
| RNF19B | 2.559 | 1.35577 | 0.004638 | 0.021799 | yes | up | 11.76 | 9.54 | 14.65 | 90.53 | 26.87 | 27.93 | 11.98333 | 48.44333 |
| LOC103346372 | 0.42 | -1.25193 | 0.004666 | 0.02192 | yes | down | 126.07 | 113.41 | 172.11 | 40.05 | 131.29 | 79.93 | 137.1967 | 83.75667 |
| RIMS4 | 0.26 | -1.94509 | 0.004672 | 0.021938 | yes | down | 0.13 | 0.31 | 0.35 | 0.06 | 0.14 | 0.11 | 0.263333 | 0.103333 |
| N6AMT1 | 2.689 | 1.426981 | 0.004686 | 0.021993 | yes | up | 2.82 | 2.33 | 1 | 7.67 | 5.75 | 12.48 | 2.05 | 8.633333 |
| LOC100358934 | 23.416 | 4.549441 | 0.00473 | 0.022178 | yes | up | 0 | 0 | 0.06 | 1.29 | 0.48 | 0.67 | 0.02 | 0.813333 |
| LOC108177659 | 3.402 | 1.766497 | 0.004736 | 0.022198 | yes | up | 0.31 | 0.34 | 0.75 | 2.14 | 2.67 | 2.76 | 0.466667 | 2.523333 |
| TENM2 | 3.084 | 1.624631 | 0.004749 | 0.022237 | yes | up | 0.04 | 0.08 | 0.06 | 0.2 | 0.42 | 0.28 | 0.06 | 0.3 |
| GPSM3 | 2.402 | 1.264202 | 0.00484 | 0.022572 | yes | up | 2.81 | 3.74 | 2.09 | 18.19 | 6.42 | 7.54 | 2.88 | 10.71667 |
| HK3 | 8.653 | 3.11318 | 0.004841 | 0.022572 | yes | up | 0.22 | 0.07 | 0.25 | 6.64 | 0.19 | 0.54 | 0.18 | 2.456667 |
| LOC100357816 | 0.317 | -1.65891 | 0.004841 | 0.022572 | yes | down | 3.03 | 6.16 | 4.88 | 3.1 | 2.11 | 1.52 | 4.69 | 2.243333 |
| SPZ1 | 0.423 | -1.23967 | 0.004876 | 0.022699 | yes | down | 1.45 | 1.68 | 2.16 | 1.5 | 0.96 | 1.15 | 1.763333 | 1.203333 |
| ATP8B4 | 5.992 | 2.582952 | 0.004956 | 0.022989 | yes | up | 0.08 | 0.07 | 0.11 | 2.04 | 0.2 | 0.23 | 0.086667 | 0.823333 |
| PKDREJ | 0.448 | -1.15997 | 0.004961 | 0.023004 | yes | down | 0.31 | 0.57 | 0.47 | 0.34 | 0.34 | 0.23 | 0.45 | 0.303333 |
| FOXO6 | 0.118 | -3.0826 | 0.005099 | 0.023565 | yes | down | 0.23 | 0.14 | 0.18 | 0.02 | 0.06 | 0.02 | 0.183333 | 0.033333 |
| SKAP1 | 3.676 | 1.877994 | 0.005101 | 0.023569 | yes | up | 1.22 | 2.84 | 3.22 | 6.47 | 6.07 | 3.45 | 2.426667 | 5.33 |
| TMEM86A | 2.437 | 1.284924 | 0.005118 | 0.023638 | yes | up | 10.18 | 3.7 | 4.41 | 18.11 | 17.8 | 36.16 | 6.096667 | 24.02333 |
| LOC103352385 | 2.008 | 1.005461 | 0.005136 | 0.023709 | yes | up | 0.75 | 1.17 | 0.89 | 2.65 | 2.29 | 3.84 | 0.936667 | 2.926667 |
| TMEM220 | 3.452 | 1.787589 | 0.005164 | 0.023829 | yes | up | 1.2 | 0.96 | 1.04 | 5.96 | 5.77 | 5.37 | 1.066667 | 5.7 |
| SH3BGRL2 | 2.371 | 1.245204 | 0.005179 | 0.023892 | yes | up | 0.63 | 0.67 | 0.47 | 1.64 | 2.13 | 1.77 | 0.59 | 1.846667 |
| LOC108178892 | 3.51 | 1.811457 | 0.005248 | 0.024165 | yes | up | 0.6 | 0.81 | 0.73 | 5.09 | 2.49 | 4.02 | 0.713333 | 3.866667 |
| LOC108178530 | 0.394 | -1.34401 | 0.005304 | 0.024358 | yes | down | 2.88 | 1.84 | 0.96 | 1.24 | 1.19 | 1.07 | 1.893333 | 1.166667 |
| LOC100355689 | 7.005 | 2.808333 | 0.005317 | 0.024411 | yes | up | 0.19 | 0 | 0.03 | 0.39 | 0.79 | 1.1 | 0.073333 | 0.76 |
| CD68 | 11.66 | 3.543516 | 0.005363 | 0.02459 | yes | up | 1.4 | 1.62 | 2.07 | 81.43 | 2.91 | 7.06 | 1.696667 | 30.46667 |
| LOC103348003 | 3.184 | 1.670832 | 0.005372 | 0.024623 | yes | up | 0.35 | 1.36 | 1.61 | 5.04 | 6.79 | 4.27 | 1.106667 | 5.366667 |
| DHDH | 0.31 | -1.68807 | 0.005385 | 0.024674 | yes | down | 1.16 | 1.31 | 1.64 | 0.41 | 0.82 | 0.75 | 1.37 | 0.66 |
| MYCL | 0.203 | -2.30251 | 0.005386 | 0.024674 | yes | down | 0.52 | 0.29 | 0.46 | 0.27 | 0.03 | 0.1 | 0.423333 | 0.133333 |
| IL17RB | 0.236 | -2.0855 | 0.005409 | 0.024729 | yes | down | 0.94 | 0.4 | 0.44 | 0.33 | 0.14 | 0.21 | 0.593333 | 0.226667 |
| DPP6 | 0.258 | -1.95291 | 0.005438 | 0.024833 | yes | down | 0.61 | 0.21 | 1.11 | 2.56 | 0.31 | 0.12 | 0.643333 | 0.996667 |
| RGS4 | 2.874 | 1.52312 | 0.005442 | 0.024842 | yes | up | 0.87 | 4.1 | 3.33 | 7.92 | 14.54 | 13.77 | 2.766667 | 12.07667 |
| C1H11orf80 | 2.556 | 1.353681 | 0.005483 | 0.025022 | yes | up | 0.44 | 0.53 | 0.81 | 1.22 | 3.12 | 3.01 | 0.593333 | 2.45 |
| TNFAIP8L2 | 4.819 | 2.268786 | 0.005514 | 0.025145 | yes | up | 0.93 | 1.21 | 1.28 | 13 | 1.38 | 2.1 | 1.14 | 5.493333 |
| BTBD19 | 0.496 | -1.01187 | 0.005529 | 0.02521 | yes | down | 2.24 | 1.45 | 1.4 | 1.08 | 1.79 | 1.34 | 1.696667 | 1.403333 |
| RNASEL | 2.268 | 1.181536 | 0.005541 | 0.025256 | yes | up | 0.31 | 0.34 | 0.29 | 1.09 | 1.07 | 1.11 | 0.313333 | 1.09 |
| SLA | 12.939 | 3.693616 | 0.005577 | 0.025411 | yes | up | 0 | 0.15 | 0.14 | 4.58 | 0.1 | 0.36 | 0.096667 | 1.68 |
| EME1 | 2.376 | 1.248583 | 0.005591 | 0.025468 | yes | up | 0.26 | 0.53 | 0.55 | 1.87 | 1.48 | 1.54 | 0.446667 | 1.63 |
| AZGP1 | 2.329 | 1.219543 | 0.005609 | 0.025533 | yes | up | 0.75 | 0.87 | 1.3 | 4.85 | 2.67 | 3.51 | 0.973333 | 3.676667 |
| LOC100347041 | 5.014 | 2.326033 | 0.005668 | 0.025747 | yes | up | 0.12 | 0.35 | 0.5 | 5.58 | 1.02 | 1.5 | 0.323333 | 2.7 |
| GJA3 | 10.778 | 3.429989 | 0.005691 | 0.0258 | yes | up | 0.09 | 0.04 | 0.04 | 2.64 | 0.19 | 0.13 | 0.056667 | 0.986667 |
| SSC4D | 0.145 | -2.78157 | 0.005705 | 0.025852 | yes | down | 0.58 | 0.61 | 0.26 | 0.09 | 0.22 | 0 | 0.483333 | 0.103333 |
| LOC100339846 | 12.092 | 3.595942 | 0.005706 | 0.025852 | yes | up | 0.19 | 0.01 | 0.17 | 5.3 | 5.38 | 0.01 | 0.123333 | 3.563333 |
| LOC103349385 | 0.168 | -2.57567 | 0.005762 | 0.02607 | yes | down | 1.12 | 2 | 0.67 | 0.4 | 0.3 | 0.25 | 1.263333 | 0.316667 |
| LOC100342769 | 0.265 | -1.91437 | 0.005788 | 0.02615 | yes | down | 1.83 | 0.53 | 1.39 | 0.56 | 0.47 | 0.58 | 1.25 | 0.536667 |
| LOC108176465 | 2.316 | 1.211517 | 0.005793 | 0.02616 | yes | up | 1.58 | 2.24 | 0.79 | 4.5 | 6.32 | 5.35 | 1.536667 | 5.39 |
| SPRY4 | 0.198 | -2.33844 | 0.005859 | 0.026394 | yes | down | 55.87 | 14.64 | 7.68 | 8.65 | 7.54 | 8.87 | 26.06333 | 8.353333 |
| ATG16L2 | 2.579 | 1.366972 | 0.005919 | 0.026595 | yes | up | 0.91 | 0.62 | 0.85 | 4.83 | 2.3 | 1.96 | 0.793333 | 3.03 |
| ENOSF1 | 4.354 | 2.122204 | 0.005936 | 0.026657 | yes | up | 0.27 | 0.46 | 3 | 5.64 | 8.96 | 10.58 | 1.243333 | 8.393333 |
| TLDC2 | 3.002 | 1.585938 | 0.005952 | 0.026717 | yes | up | 0.26 | 0.39 | 0.67 | 3.34 | 1.33 | 1.69 | 0.44 | 2.12 |
| LOC103346325 | 0.405 | -1.30569 | 0.005962 | 0.026754 | yes | down | 7.17 | 12.75 | 11.66 | 9.63 | 5.64 | 4.07 | 10.52667 | 6.446667 |
| VAMP1 | 2.582 | 1.368529 | 0.005971 | 0.026787 | yes | up | 0.76 | 1.03 | 0.58 | 5.22 | 2.03 | 1.9 | 0.79 | 3.05 |
| CCDC39 | 2.333 | 1.221978 | 0.005974 | 0.026792 | yes | up | 0.31 | 0.54 | 1.15 | 1.83 | 2.58 | 2.89 | 0.666667 | 2.433333 |
| LMCD1 | 0.202 | -2.30495 | 0.005985 | 0.026821 | yes | down | 108.07 | 101.61 | 12.74 | 18.43 | 20.11 | 31.9 | 74.14 | 23.48 |
| FGD3 | 6.962 | 2.799559 | 0.006 | 0.026861 | yes | up | 0.18 | 0.09 | 0.07 | 3.08 | 0.36 | 0.26 | 0.113333 | 1.233333 |
| C1RL | 6.473 | 2.694331 | 0.006102 | 0.027227 | yes | up | 0.09 | 0.23 | 0.28 | 4.86 | 0.51 | 0.54 | 0.2 | 1.97 |
| LOC108175669 | 4.115 | 2.040921 | 0.006116 | 0.027255 | yes | up | 0.09 | 0.31 | 0.13 | 0.95 | 1.19 | 1.09 | 0.176667 | 1.076667 |
| SPA17 | 2.243 | 1.165406 | 0.006116 | 0.027255 | yes | up | 2.14 | 2.41 | 3.11 | 9.55 | 7.4 | 6.84 | 2.553333 | 7.93 |
| KIAA1107 | 2.079 | 1.056159 | 0.00614 | 0.027344 | yes | up | 0.41 | 0.24 | 0.36 | 1.13 | 1.25 | 0.91 | 0.336667 | 1.096667 |
| LOC103349006 | 0.186 | -2.42931 | 0.00619 | 0.027546 | yes | down | 0.19 | 0.54 | 1.22 | 0.27 | 0.22 | 0.06 | 0.65 | 0.183333 |
| CYTH4 | 3.946 | 1.980525 | 0.006191 | 0.027546 | yes | up | 0.61 | 0.87 | 0.84 | 10.84 | 1.44 | 1.66 | 0.773333 | 4.646667 |
| CCDC191 | 2.22 | 1.150734 | 0.006197 | 0.027564 | yes | up | 0.29 | 0.33 | 0.51 | 0.98 | 1.13 | 1.75 | 0.376667 | 1.286667 |
| LOC103350985 | 0.296 | -1.75545 | 0.006269 | 0.027821 | yes | down | 0.98 | 4.03 | 1.64 | 1.72 | 0.46 | 0.79 | 2.216667 | 0.99 |
| LOC103345532 | 3.397 | 1.764182 | 0.006294 | 0.027909 | yes | up | 0.3 | 0.34 | 0.22 | 1.33 | 1.19 | 2.03 | 0.286667 | 1.516667 |
| LOC100358230 | 0.446 | -1.16387 | 0.006297 | 0.027913 | yes | down | 5.52 | 4.77 | 5.81 | 5.39 | 2.82 | 2.49 | 5.366667 | 3.566667 |
| PTCHD1 | 2.148 | 1.103274 | 0.006325 | 0.028019 | yes | up | 0.31 | 0.27 | 0.14 | 0.72 | 0.92 | 0.76 | 0.24 | 0.8 |
| LOC100356727 | 0.077 | -3.69048 | 0.006367 | 0.028175 | yes | down | 0.06 | 0.07 | 0.74 | 0 | 0.04 | 0.07 | 0.29 | 0.036667 |
| SERPINB5 | 0.226 | -2.14315 | 0.006392 | 0.028269 | yes | down | 0.97 | 1.24 | 1.95 | 0.43 | 0.91 | 0.06 | 1.386667 | 0.466667 |
| LOC100350375 | 6.511 | 2.702965 | 0.006411 | 0.028342 | yes | up | 0.12 | 0.06 | 0.26 | 1.17 | 0.21 | 4.59 | 0.146667 | 1.99 |
| CFTR | 0.295 | -1.76169 | 0.006416 | 0.028356 | yes | down | 0.65 | 0.35 | 0.82 | 0.43 | 0.29 | 0.1 | 0.606667 | 0.273333 |
| SHANK2 | 0.098 | -3.35712 | 0.006547 | 0.028878 | yes | down | 0.2 | 0.03 | 0.08 | 0 | 0.02 | 0.03 | 0.103333 | 0.016667 |
| ICAM1 | 3.298 | 1.721615 | 0.006582 | 0.028974 | yes | up | 2.98 | 3.31 | 2.91 | 32.67 | 4.2 | 6.62 | 3.066667 | 14.49667 |
| LTB4R | 0.289 | -1.79303 | 0.006673 | 0.029313 | yes | down | 0.37 | 0.39 | 0.41 | 0.24 | 0.2 | 0.1 | 0.39 | 0.18 |
| LOC100343557 | 3.994 | 1.99787 | 0.006682 | 0.029334 | yes | up | 324.92 | 69 | 86.83 | 741.86 | 1030.82 | 1258.37 | 160.25 | 1010.35 |
| LOC108176066 | 9.284 | 3.214696 | 0.006696 | 0.029383 | yes | up | 0 | 0.11 | 0.1 | 0.4 | 1.29 | 1.19 | 0.07 | 0.96 |
| LOC103350039 | 2.81 | 1.49033 | 0.006704 | 0.029408 | yes | up | 1.35 | 2.22 | 1.91 | 21.58 | 1.57 | 5.95 | 1.826667 | 9.7 |
| LOC100358198 | 0.352 | -1.50822 | 0.006752 | 0.029593 | yes | down | 0.64 | 0.28 | 0.62 | 0.33 | 0.2 | 0.33 | 0.513333 | 0.286667 |
| LOC100346939 | 10.674 | 3.415976 | 0.006789 | 0.029739 | yes | up | 0.04 | 0.04 | 0.04 | 1.09 | 0.24 | 0.7 | 0.04 | 0.676667 |
| LOC103346127 | 2.374 | 1.247081 | 0.006805 | 0.029783 | yes | up | 0.4 | 0.38 | 0.64 | 2 | 1.85 | 1.76 | 0.473333 | 1.87 |
| GFI1 | 65.318 | 6.029406 | 0.006846 | 0.029925 | yes | up | 0 | 0 | 0 | 1.13 | 0.02 | 0.03 | 0 | 0.393333 |
| GMFG | 3.348 | 1.743482 | 0.006855 | 0.029959 | yes | up | 4.1 | 5.23 | 4.47 | 49.52 | 9.15 | 12.09 | 4.6 | 23.58667 |
| BARX2 | 0.319 | -1.65043 | 0.006876 | 0.030031 | yes | down | 2.43 | 6.29 | 1.63 | 0.75 | 1.68 | 2.31 | 3.45 | 1.58 |
| MYO1F | 3.411 | 1.770211 | 0.006878 | 0.030033 | yes | up | 0.55 | 0.71 | 0.9 | 13.28 | 0.97 | 1.37 | 0.72 | 5.206667 |
| LOC108176666 | 3.19 | 1.67336 | 0.006948 | 0.030285 | yes | up | 0.5 | 0.5 | 0.96 | 4.75 | 3.16 | 2.33 | 0.653333 | 3.413333 |
| LOC103346274 | 8.319 | 3.056398 | 0.006953 | 0.030296 | yes | up | 0 | 0.1 | 0.05 | 1.5 | 0.2 | 0.59 | 0.05 | 0.763333 |
| CSF3R | 2.545 | 1.347884 | 0.007065 | 0.030734 | yes | up | 0.42 | 0.43 | 0.29 | 0.81 | 1.64 | 2.07 | 0.38 | 1.506667 |
| DEF6 | 4.124 | 2.043903 | 0.007071 | 0.030749 | yes | up | 0.42 | 0.39 | 0.37 | 5.51 | 0.73 | 1.32 | 0.393333 | 2.52 |
| CLVS1 | 2.174 | 1.120498 | 0.007125 | 0.030904 | yes | up | 0.79 | 1.5 | 1.09 | 2.2 | 3.39 | 5.8 | 1.126667 | 3.796667 |
| LOC100345833 | 0.426 | -1.23233 | 0.00713 | 0.030919 | yes | down | 1.23 | 3.71 | 2.93 | 1.37 | 1.83 | 1.92 | 2.623333 | 1.706667 |
| LOC108178915 | 3.655 | 1.869771 | 0.007185 | 0.031102 | yes | up | 0.64 | 0.62 | 0.96 | 1.7 | 6.18 | 5 | 0.74 | 4.293333 |
| RUNX2 | 2.756 | 1.462606 | 0.007196 | 0.031131 | yes | up | 0.27 | 0.16 | 0.42 | 1.65 | 0.71 | 0.98 | 0.283333 | 1.113333 |
| RACGAP1 | 2.342 | 1.227635 | 0.007197 | 0.031131 | yes | up | 0.67 | 1.31 | 0.55 | 4.14 | 2.93 | 1.85 | 0.843333 | 2.973333 |
| LOC100344252 | 9.592 | 3.261825 | 0.007213 | 0.03117 | yes | up | 0.15 | 0 | 0.15 | 3.49 | 0.64 | 0.41 | 0.1 | 1.513333 |
| NCF2 | 5.56 | 2.475211 | 0.007215 | 0.03117 | yes | up | 0.36 | 0.63 | 0.81 | 12.16 | 0.71 | 1.42 | 0.6 | 4.763333 |
| FADS6 | 0.155 | -2.68974 | 0.007239 | 0.03124 | yes | down | 0.22 | 0.11 | 0.34 | 0.08 | 0.02 | 0.06 | 0.223333 | 0.053333 |
| CHST1 | 0.467 | -1.09729 | 0.007313 | 0.031466 | yes | down | 1.33 | 3.56 | 2.82 | 1.56 | 1.89 | 2.05 | 2.57 | 1.833333 |
| ATP6AP1L | 2.306 | 1.205539 | 0.007321 | 0.031478 | yes | up | 0.31 | 0.31 | 0.42 | 0.91 | 1.49 | 1.37 | 0.346667 | 1.256667 |
| MSTN | 12.72 | 3.66906 | 0.007345 | 0.031552 | yes | up | 0 | 0.06 | 0.11 | 0.45 | 2.24 | 0.57 | 0.056667 | 1.086667 |
| LOC103347389 | 13.86 | 3.792864 | 0.007377 | 0.031671 | yes | up | 0.08 | 0.08 | 0.21 | 18.23 | 0.68 | 2.66 | 0.123333 | 7.19 |
| KLHL33 | 2.858 | 1.515245 | 0.007409 | 0.031784 | yes | up | 1.99 | 2.09 | 1.05 | 8.1 | 16.12 | 8.22 | 1.71 | 10.81333 |
| NDUFA13 | 0.442 | -1.17797 | 0.007444 | 0.031905 | yes | down | 6.99 | 6.19 | 7.05 | 3.58 | 5.46 | 4.84 | 6.743333 | 4.626667 |
| XCR1 | 56.243 | 5.8136 | 0.007459 | 0.031963 | yes | up | 0 | 0 | 0 | 2.08 | 0.09 | 0.06 | 0 | 0.743333 |
| DQX1 | 3.135 | 1.648669 | 0.007462 | 0.031965 | yes | up | 0.56 | 0.46 | 1.2 | 1.78 | 2.58 | 4.88 | 0.74 | 3.08 |
| PTDSS2 | 0.49 | -1.02866 | 0.007467 | 0.031977 | yes | down | 8.04 | 16 | 18.29 | 9.22 | 11.73 | 10.9 | 14.11 | 10.61667 |
| BTBD11 | 2.204 | 1.140188 | 0.007499 | 0.032068 | yes | up | 0.65 | 0.37 | 0.61 | 4.71 | 1.16 | 3.63 | 0.543333 | 3.166667 |
| PLCH1 | 3.394 | 1.763058 | 0.007586 | 0.032405 | yes | up | 0.03 | 0.06 | 0.07 | 0.24 | 0.27 | 0.31 | 0.053333 | 0.273333 |
| ANKDD1B | 3.308 | 1.726142 | 0.007599 | 0.032439 | yes | up | 0.56 | 0.51 | 0.67 | 0.91 | 4.26 | 3.45 | 0.58 | 2.873333 |
| FOLR2 | 2.785 | 1.477621 | 0.007604 | 0.032443 | yes | up | 5.45 | 9.66 | 10.86 | 75.04 | 15.81 | 22.38 | 8.656667 | 37.74333 |
| B2M | 5.77 | 2.528579 | 0.007607 | 0.032449 | yes | up | 128.57 | 141.52 | 151.87 | 2887.63 | 387.76 | 485.36 | 140.6533 | 1253.583 |
| LOC103345542 | 3.458 | 1.789796 | 0.007618 | 0.032477 | yes | up | 0.46 | 0.2 | 0.22 | 1.03 | 2.17 | 1.59 | 0.293333 | 1.596667 |
| PNMT | 0.071 | -3.8173 | 0.007702 | 0.03279 | yes | down | 4.13 | 1.49 | 0 | 0.09 | 0.52 | 0.06 | 1.873333 | 0.223333 |
| ITGB7 | 3.777 | 1.917327 | 0.007707 | 0.032793 | yes | up | 0.48 | 0.68 | 0.67 | 7.6 | 1.18 | 1.34 | 0.61 | 3.373333 |
| MFSD2A | 0.393 | -1.34886 | 0.007707 | 0.032793 | yes | down | 0.94 | 1.73 | 0.93 | 0.38 | 1.03 | 0.74 | 1.2 | 0.716667 |
| LOC100346112 | 0.456 | -1.13305 | 0.007738 | 0.032878 | yes | down | 181.71 | 210.94 | 55.37 | 108.41 | 76.57 | 132.04 | 149.34 | 105.6733 |
| LOC100343554 | 2.779 | 1.474663 | 0.007949 | 0.033658 | yes | up | 0.78 | 3.18 | 0.82 | 3.45 | 8.43 | 7.9 | 1.593333 | 6.593333 |
| TMEM27 | 5.18 | 2.37309 | 0.00795 | 0.033658 | yes | up | 0.18 | 0.04 | 0.15 | 0.69 | 0.89 | 1.47 | 0.123333 | 1.016667 |
| LOC100358379 | 16.338 | 4.030131 | 0.00799 | 0.033811 | yes | up | 0 | 0.39 | 0 | 7.58 | 0.64 | 0.93 | 0.13 | 3.05 |
| LOC108177909 | 19.727 | 4.302101 | 0.008035 | 0.03396 | yes | up | 0.04 | 0 | 0 | 0.74 | 0.49 | 0.43 | 0.013333 | 0.553333 |
| LOC103350037 | 3.744 | 1.904599 | 0.008044 | 0.033979 | yes | up | 0.34 | 0.88 | 0.9 | 8.7 | 1.36 | 1.99 | 0.706667 | 4.016667 |
| MMS22L | 2.725 | 1.446471 | 0.008068 | 0.03406 | yes | up | 0.16 | 0.34 | 0.23 | 1.56 | 0.76 | 0.66 | 0.243333 | 0.993333 |
| LDHA | 0.451 | -1.15014 | 0.008109 | 0.034183 | yes | down | 351.75 | 363.39 | 187.53 | 114.98 | 352.53 | 153.07 | 300.89 | 206.86 |
| KRT17 | 24.264 | 4.600766 | 0.00813 | 0.034236 | yes | up | 0 | 0 | 0.07 | 2.38 | 0.08 | 0.44 | 0.023333 | 0.966667 |
| THBS4 | 0.168 | -2.57479 | 0.008159 | 0.03434 | yes | down | 13.68 | 6.49 | 1.35 | 0.97 | 3.11 | 1.6 | 7.173333 | 1.893333 |
| TNF | 33.706 | 5.074924 | 0.008197 | 0.034454 | yes | up | 0 | 0.04 | 0 | 1.78 | 0.11 | 0.09 | 0.013333 | 0.66 |
| LOC103348580 | 6.183 | 2.628287 | 0.008213 | 0.034491 | yes | up | 0.36 | 0.49 | 0.34 | 0.26 | 6.4 | 4.86 | 0.396667 | 3.84 |
| ABCC3 | 4.853 | 2.278874 | 0.008229 | 0.03454 | yes | up | 0.22 | 0.18 | 0.17 | 3.57 | 0.39 | 0.35 | 0.19 | 1.436667 |
| LOC103351245 | 0.232 | -2.10925 | 0.00826 | 0.034594 | yes | down | 0.12 | 0.27 | 0.09 | 0.07 | 0.06 | 0.03 | 0.16 | 0.053333 |
| PDLIM5 | 0.399 | -1.32566 | 0.008264 | 0.034598 | yes | down | 70.32 | 79.75 | 8.03 | 12.1 | 31.13 | 25.44 | 52.7 | 22.89 |
| ZNF300 | 3.277 | 1.712509 | 0.008346 | 0.034893 | yes | up | 0.21 | 0.36 | 0.52 | 2.36 | 1.1 | 0.77 | 0.363333 | 1.41 |
| NOCT | 0.486 | -1.03997 | 0.008394 | 0.035076 | yes | down | 22.35 | 12.28 | 10.1 | 8.06 | 9.88 | 17.14 | 14.91 | 11.69333 |
| TACSTD2 | 6.56 | 2.713591 | 0.008405 | 0.035105 | yes | up | 0.02 | 0.1 | 0.02 | 0.68 | 0.25 | 0.48 | 0.046667 | 0.47 |
| TRPV4 | 0.443 | -1.17444 | 0.008414 | 0.03513 | yes | down | 1.49 | 1.25 | 0.73 | 0.87 | 0.82 | 0.69 | 1.156667 | 0.793333 |
| LOC103350544 | 5.955 | 2.573992 | 0.008442 | 0.035217 | yes | up | 0.03 | 0 | 0.12 | 0.45 | 0.41 | 0.61 | 0.05 | 0.49 |
| FNDC5 | 0.148 | -2.7556 | 0.008473 | 0.035309 | yes | down | 13.84 | 23.63 | 1.19 | 2.05 | 4.04 | 3.53 | 12.88667 | 3.206667 |
| HNMT | 0.487 | -1.03874 | 0.008502 | 0.035403 | yes | down | 13 | 17.25 | 17.08 | 19.56 | 7.13 | 8.53 | 15.77667 | 11.74 |
| ACSL5 | 2.477 | 1.308369 | 0.008583 | 0.035708 | yes | up | 1.7 | 1.99 | 2.19 | 14.18 | 3.1 | 5.59 | 1.96 | 7.623333 |
| IL21R | 29.906 | 4.902373 | 0.008588 | 0.03571 | yes | up | 0.03 | 0 | 0 | 1.26 | 0.06 | 0.18 | 0.01 | 0.5 |
| CBR1 | 2.436 | 1.284581 | 0.008596 | 0.035732 | yes | up | 1.62 | 3.08 | 2.17 | 4.78 | 8.74 | 14.1 | 2.29 | 9.206667 |
| THAP9 | 2.29 | 1.195441 | 0.008603 | 0.035741 | yes | up | 0.27 | 0.57 | 0.63 | 1.06 | 1.74 | 2.38 | 0.49 | 1.726667 |
| LOC100352024 | 0.147 | -2.76257 | 0.008642 | 0.03584 | yes | down | 0.09 | 1.41 | 1.73 | 0.05 | 0.49 | 0.16 | 1.076667 | 0.233333 |
| C14H3orf80 | 0.331 | -1.59304 | 0.008647 | 0.035846 | yes | down | 0.34 | 0.53 | 0.27 | 0.12 | 0.2 | 0.25 | 0.38 | 0.19 |
| LOC100352023 | 0.337 | -1.56878 | 0.008674 | 0.035939 | yes | down | 0.57 | 0.61 | 0.52 | 0.36 | 0.31 | 0.19 | 0.566667 | 0.286667 |
| SPHK1 | 4.421 | 2.144458 | 0.008687 | 0.035974 | yes | up | 0.21 | 0.16 | 0.14 | 2.26 | 0.5 | 0.81 | 0.17 | 1.19 |
| PVALB | 50.7 | 5.663915 | 0.008725 | 0.036102 | yes | up | 0.54 | 3.4 | 0 | 0.88 | 235.94 | 44.87 | 1.313333 | 93.89667 |
| TMEM55A | 3.404 | 1.767114 | 0.008737 | 0.036123 | yes | up | 0.17 | 0.13 | 0.24 | 0.9 | 0.75 | 1.26 | 0.18 | 0.97 |
| PLPP6 | 2.032 | 1.023132 | 0.008787 | 0.0363 | yes | up | 1.22 | 0.65 | 0.76 | 3.08 | 2.41 | 2.95 | 0.876667 | 2.813333 |
| LNX1 | 4.614 | 2.205879 | 0.00879 | 0.0363 | yes | up | 0.35 | 0.25 | 0.31 | 0.26 | 4.13 | 3.63 | 0.303333 | 2.673333 |
| TRNAK-CUU | 0.19 | -2.39586 | 0.008798 | 0.036322 | yes | down | 3.82 | 0.85 | 1.87 | 0.96 | 0.53 | 0.52 | 2.18 | 0.67 |
| SLC7A7 | 2.781 | 1.475371 | 0.008814 | 0.036372 | yes | up | 1.04 | 1.26 | 1.19 | 9.7 | 2.14 | 2.94 | 1.163333 | 4.926667 |
| CARD11 | 15.685 | 3.971278 | 0.008837 | 0.036443 | yes | up | 0 | 0.02 | 0.04 | 1.29 | 0.09 | 0.06 | 0.02 | 0.48 |
| KCNC4 | 0.08 | -3.64144 | 0.008912 | 0.036682 | yes | down | 7.06 | 6.46 | 5.75 | 0.02 | 2.04 | 1.01 | 6.423333 | 1.023333 |
| LOC100350907 | 5.611 | 2.488295 | 0.008943 | 0.036764 | yes | up | 0.04 | 0.17 | 0.2 | 2.64 | 0.27 | 0.68 | 0.136667 | 1.196667 |
| AURKA | 3.48 | 1.799247 | 0.008949 | 0.036779 | yes | up | 0.29 | 0.32 | 0.3 | 2.94 | 1.05 | 0.77 | 0.303333 | 1.586667 |
| CD83 | 4.543 | 2.183549 | 0.008969 | 0.036849 | yes | up | 1.21 | 0.68 | 0.8 | 15.7 | 1.11 | 2.31 | 0.896667 | 6.373333 |
| LOC100340711 | 2.124 | 1.087052 | 0.00898 | 0.036874 | yes | up | 4.98 | 7.78 | 5.03 | 10.36 | 16.28 | 37.59 | 5.93 | 21.41 |
| EFCAB2 | 2.125 | 1.08729 | 0.008985 | 0.036879 | yes | up | 5.38 | 3.6 | 1.98 | 18.89 | 27.76 | 32.15 | 3.653333 | 26.26667 |
| PPAT | 0.428 | -1.22538 | 0.008986 | 0.036879 | yes | down | 0.83 | 1.55 | 0.83 | 0.85 | 0.79 | 0.44 | 1.07 | 0.693333 |
| EPSTI1 | 2.136 | 1.094665 | 0.009007 | 0.036907 | yes | up | 5.82 | 5.67 | 8.15 | 39.18 | 12.24 | 18.08 | 6.546667 | 23.16667 |
| KLHL34 | 0.192 | -2.38076 | 0.009007 | 0.036907 | yes | down | 3.49 | 2.62 | 0.13 | 0.22 | 0.55 | 1.14 | 2.08 | 0.636667 |
| ZC3H12D | 11.623 | 3.53889 | 0.009019 | 0.036938 | yes | up | 0 | 0.03 | 0.03 | 0.48 | 0.18 | 0.17 | 0.02 | 0.276667 |
| LOC108178049 | 10.619 | 3.408537 | 0.009032 | 0.036977 | yes | up | 0.1 | 0.28 | 0.09 | 5.97 | 1.07 | 0.34 | 0.156667 | 2.46 |
| LOC108177987 | 10.838 | 3.438074 | 0.009043 | 0.036983 | yes | up | 0.07 | 0 | 0.49 | 4.4 | 0.3 | 0.47 | 0.186667 | 1.723333 |
| LOC103349017 | 5.512 | 2.462568 | 0.009072 | 0.037071 | yes | up | 0.14 | 0.27 | 0.06 | 0.83 | 1.54 | 1.25 | 0.156667 | 1.206667 |
| LOC100355766 | 7.037 | 2.814882 | 0.009075 | 0.037074 | yes | up | 0.05 | 0.14 | 0.25 | 3.45 | 0.34 | 0.91 | 0.146667 | 1.566667 |
| ARNTL2 | 9.512 | 3.249685 | 0.009079 | 0.037075 | yes | up | 0.04 | 0.05 | 0.03 | 0.05 | 0.09 | 1.44 | 0.04 | 0.526667 |
| SCN5A | 0.196 | -2.35292 | 0.009115 | 0.037193 | yes | down | 0.31 | 0.73 | 0.27 | 0.01 | 0.26 | 0.11 | 0.436667 | 0.126667 |
| NUBPL | 0.458 | -1.12797 | 0.00916 | 0.03732 | yes | down | 4.21 | 5.95 | 2.14 | 2.73 | 2.68 | 2.89 | 4.1 | 2.766667 |
| LOC100101600 | 79.149 | 6.306501 | 0.009184 | 0.037397 | yes | up | 0 | 0 | 0 | 4.84 | 0 | 0.18 | 0 | 1.673333 |
| ELMOD1 | 5.083 | 2.345821 | 0.009346 | 0.037974 | yes | up | 0.05 | 0.14 | 0.02 | 0.7 | 0.39 | 0.47 | 0.07 | 0.52 |
| LOC108176315 | 0.498 | -1.00568 | 0.009377 | 0.038063 | yes | down | 11.29 | 17.24 | 22.71 | 6.76 | 12.58 | 17.34 | 17.08 | 12.22667 |
| CEBPA | 0.48 | -1.05786 | 0.009452 | 0.038345 | yes | down | 81.8 | 118.3 | 138.64 | 37.72 | 96.42 | 144.58 | 112.9133 | 92.90667 |
| IL1B | 3.654 | 1.869589 | 0.009454 | 0.038345 | yes | up | 0.34 | 0.16 | 0.4 | 1.13 | 1.54 | 2.54 | 0.3 | 1.736667 |
| LOC100339073 | 11.052 | 3.46623 | 0.009457 | 0.038345 | yes | up | 0.02 | 0 | 0.04 | 0.59 | 0.11 | 0.31 | 0.02 | 0.336667 |
| LOC100346219 | 13.167 | 3.718844 | 0.009477 | 0.038396 | yes | up | 0.73 | 0 | 0 | 10.43 | 4.76 | 0.5 | 0.243333 | 5.23 |
| LOC103346961 | 6.433 | 2.685381 | 0.009481 | 0.038404 | yes | up | 0.05 | 0.09 | 0.08 | 0.73 | 0.94 | 0.49 | 0.073333 | 0.72 |
| CEBPD | 2.22 | 1.150261 | 0.009513 | 0.038501 | yes | up | 58.34 | 32.25 | 16.69 | 136.44 | 81.06 | 161.86 | 35.76 | 126.4533 |
| RCAN2 | 0.339 | -1.56085 | 0.009518 | 0.038512 | yes | down | 3.92 | 4.48 | 0.59 | 0.93 | 1.18 | 2.54 | 2.996667 | 1.55 |
| LOC103350201 | 2.827 | 1.499265 | 0.009571 | 0.038665 | yes | up | 0.36 | 0.35 | 1.06 | 1.45 | 2.64 | 3.85 | 0.59 | 2.646667 |
| ANKFN1 | 3.16 | 1.659924 | 0.009577 | 0.038678 | yes | up | 0.02 | 0.06 | 0.05 | 0.25 | 0.15 | 0.25 | 0.043333 | 0.216667 |
| RIOK1 | 0.485 | -1.04343 | 0.009597 | 0.038736 | yes | down | 3.25 | 3.36 | 3.19 | 3.43 | 2.57 | 1.28 | 3.266667 | 2.426667 |
| LOC108178448 | 5.685 | 2.507097 | 0.009608 | 0.038761 | yes | up | 0.2 | 0.43 | 0.06 | 4.37 | 1 | 0.51 | 0.23 | 1.96 |
| IGSF22 | 2.461 | 1.29953 | 0.009654 | 0.038919 | yes | up | 1.65 | 0.43 | 1.24 | 3.41 | 3.44 | 5.36 | 1.106667 | 4.07 |
| RAP1GAP2 | 0.386 | -1.37286 | 0.009655 | 0.038919 | yes | down | 0.68 | 1.16 | 0.92 | 0.81 | 0.27 | 0.6 | 0.92 | 0.56 |
| LOC103352041 | 6.711 | 2.74652 | 0.009745 | 0.039251 | yes | up | 0.09 | 0.04 | 0 | 0.29 | 0.48 | 0.63 | 0.043333 | 0.466667 |
| LMO7 | 0.345 | -1.53402 | 0.009769 | 0.039306 | yes | down | 8.6 | 5.08 | 1.12 | 2.22 | 3.23 | 3.36 | 4.933333 | 2.936667 |
| SELL | 4.404 | 2.138824 | 0.009865 | 0.03962 | yes | up | 0.34 | 0.26 | 0.43 | 5.07 | 0.62 | 1.4 | 0.343333 | 2.363333 |
| SARM1 | 2.432 | 1.282165 | 0.009878 | 0.039647 | yes | up | 0.25 | 0.15 | 0.17 | 0.73 | 0.73 | 0.71 | 0.19 | 0.723333 |
| CENPM | 3.449 | 1.786101 | 0.009927 | 0.039819 | yes | up | 0.73 | 0.22 | 0.28 | 4.34 | 2.26 | 1.38 | 0.41 | 2.66 |
| PLCB2 | 4.547 | 2.184795 | 0.00993 | 0.039819 | yes | up | 0.15 | 0.12 | 0.36 | 3.55 | 0.38 | 0.68 | 0.21 | 1.536667 |
| ARG1 | 9.11 | 3.187414 | 0.009965 | 0.039917 | yes | up | 0.36 | 0.05 | 0.05 | 5.68 | 0.25 | 1.09 | 0.153333 | 2.34 |
| AURKB | 3.477 | 1.797686 | 0.009985 | 0.039975 | yes | up | 0.46 | 0.53 | 0.5 | 4.88 | 1.75 | 1.24 | 0.496667 | 2.623333 |
| LOC103346516 | 2.007 | 1.004943 | 0.010002 | 0.040023 | yes | up | 1.75 | 0.79 | 2.55 | 6.61 | 6.57 | 4.65 | 1.696667 | 5.943333 |
| LRRC4 | 3.918 | 1.970071 | 0.010103 | 0.040352 | yes | up | 0.04 | 0.09 | 0.18 | 0.51 | 0.81 | 0.54 | 0.103333 | 0.62 |
| ESRRB | 0.105 | -3.25432 | 0.010103 | 0.040352 | yes | down | 0.63 | 1 | 0.02 | 0 | 0.12 | 0.15 | 0.55 | 0.09 |
| LOC100341366 | 2.543 | 1.346451 | 0.010143 | 0.040479 | yes | up | 0.46 | 0.46 | 0.58 | 2.6 | 1.64 | 1.38 | 0.5 | 1.873333 |
| SLC44A3 | 0.348 | -1.52473 | 0.010156 | 0.040511 | yes | down | 6.2 | 4.91 | 5.2 | 5.64 | 2.28 | 0.78 | 5.436667 | 2.9 |
| PARVG | 5.801 | 2.53623 | 0.010201 | 0.040666 | yes | up | 0.33 | 0.94 | 0.47 | 10.42 | 1.62 | 2.03 | 0.58 | 4.69 |
| LOC103346185 | 7.094 | 2.826581 | 0.01021 | 0.040694 | yes | up | 0.18 | 0 | 0.1 | 1.18 | 1.23 | 0.68 | 0.093333 | 1.03 |
| SUV39H1 | 2.027 | 1.019068 | 0.010216 | 0.040707 | yes | up | 0.58 | 0.42 | 0.36 | 1.36 | 1.3 | 1.64 | 0.453333 | 1.433333 |
| STAT1 | 4.452 | 2.154482 | 0.010235 | 0.040747 | yes | up | 10.27 | 15.8 | 15.13 | 199.44 | 50.6 | 29.6 | 13.73333 | 93.21333 |
| TNNT2 | 0.121 | -3.04264 | 0.010327 | 0.041063 | yes | down | 4.49 | 3.73 | 0.25 | 0 | 0.75 | 0.92 | 2.823333 | 0.556667 |
| LOC103351405 | 9.583 | 3.260463 | 0.010375 | 0.04121 | yes | up | 0 | 0.02 | 0.04 | 0.41 | 0.18 | 0.3 | 0.02 | 0.296667 |
| ANKRD45 | 3.054 | 1.610775 | 0.010483 | 0.041552 | yes | up | 0.5 | 0.24 | 0.43 | 2.61 | 1.55 | 1.09 | 0.39 | 1.75 |
| SYBU | 0.479 | -1.06046 | 0.010504 | 0.041624 | yes | down | 4.34 | 6.74 | 4.74 | 5.09 | 1.64 | 5.06 | 5.273333 | 3.93 |
| TPX2 | 2.05 | 1.035804 | 0.010546 | 0.041746 | yes | up | 1.21 | 1.62 | 1.41 | 6.9 | 3.83 | 2.61 | 1.413333 | 4.446667 |
| APBB1IP | 2.872 | 1.522145 | 0.010566 | 0.041792 | yes | up | 0.82 | 1.08 | 1.16 | 8.78 | 2.01 | 2.73 | 1.02 | 4.506667 |
| SGSM1 | 2.101 | 1.071305 | 0.010612 | 0.041932 | yes | up | 0.29 | 0.43 | 0.47 | 1.11 | 1.74 | 0.94 | 0.396667 | 1.263333 |
| GSDMD | 2.793 | 1.481907 | 0.010637 | 0.041988 | yes | up | 3.16 | 3.29 | 2.18 | 25.45 | 5.48 | 6.45 | 2.876667 | 12.46 |
| TP63 | 0.423 | -1.24083 | 0.010652 | 0.042033 | yes | down | 0.66 | 0.96 | 0.42 | 0.21 | 0.5 | 0.48 | 0.68 | 0.396667 |
| LOC100352110 | 4.535 | 2.181101 | 0.010661 | 0.04206 | yes | up | 1.29 | 2.12 | 2.37 | 32.11 | 3.51 | 3.29 | 1.926667 | 12.97 |
| LOC108177586 | 81.215 | 6.343669 | 0.010901 | 0.042839 | yes | up | 0 | 0 | 0 | 22.6 | 0 | 0.43 | 0 | 7.676667 |
| LOC103351558 | 2.179 | 1.123686 | 0.010937 | 0.042956 | yes | up | 1.05 | 1.43 | 2.05 | 2.71 | 6.26 | 5.77 | 1.51 | 4.913333 |
| COL9A3 | 2.134 | 1.093334 | 0.01109 | 0.043459 | yes | up | 0.76 | 2.48 | 1.35 | 5.58 | 4.56 | 4.69 | 1.53 | 4.943333 |
| GPR156 | 3.034 | 1.601434 | 0.011106 | 0.043505 | yes | up | 0.2 | 0.08 | 0.11 | 0.84 | 0.41 | 0.57 | 0.13 | 0.606667 |
| WDSUB1 | 2.154 | 1.107013 | 0.011124 | 0.043546 | yes | up | 0.63 | 1.15 | 0.97 | 3.28 | 1.98 | 3.91 | 0.916667 | 3.056667 |
| OAS1 | 3.011 | 1.590331 | 0.011147 | 0.043626 | yes | up | 3.01 | 1.58 | 2.6 | 22.3 | 7.4 | 3.86 | 2.396667 | 11.18667 |
| LOC103350566 | 0.106 | -3.23665 | 0.011232 | 0.0439 | yes | down | 11.01 | 15.61 | 14.18 | 4.87 | 0.42 | 0.42 | 13.6 | 1.903333 |
| LOC108178928 | 0.151 | -2.72442 | 0.01127 | 0.044027 | yes | down | 0.35 | 0.54 | 0.54 | 0.32 | 0.06 | 0 | 0.476667 | 0.126667 |
| SAPCD2 | 3.398 | 1.764829 | 0.011317 | 0.044166 | yes | up | 0.12 | 0.26 | 0.09 | 1.03 | 0.83 | 0.54 | 0.156667 | 0.8 |
| GATA3 | 3.64 | 1.864029 | 0.011382 | 0.044374 | yes | up | 0.06 | 0.07 | 0.13 | 0.77 | 0.46 | 0.28 | 0.086667 | 0.503333 |
| P2RY6 | 8.835 | 3.143225 | 0.01143 | 0.044538 | yes | up | 0.02 | 0.09 | 0.08 | 2.27 | 0.07 | 0.36 | 0.063333 | 0.9 |
| HPDL | 0.282 | -1.82742 | 0.01158 | 0.045041 | yes | down | 1.02 | 0.87 | 1.17 | 0.09 | 0.59 | 0.68 | 1.02 | 0.453333 |
| ADH2-1 | 2.051 | 1.036232 | 0.011612 | 0.045155 | yes | up | 4.29 | 3.58 | 4.93 | 6.71 | 14.43 | 20.2 | 4.266667 | 13.78 |
| LOC100354921 | 0.093 | -3.42104 | 0.011635 | 0.045209 | yes | down | 0.52 | 0.47 | 0.37 | 0.21 | 0 | 0 | 0.453333 | 0.07 |
| MTHFD2 | 0.466 | -1.1018 | 0.011713 | 0.045432 | yes | down | 7.07 | 12.63 | 5.69 | 9.85 | 4.29 | 3.8 | 8.463333 | 5.98 |
| CD37 | 3.458 | 1.790012 | 0.01175 | 0.045527 | yes | up | 1.64 | 1.95 | 2.81 | 25.69 | 3.29 | 5.27 | 2.133333 | 11.41667 |
| LOC103348754 | 4.976 | 2.314909 | 0.011809 | 0.045697 | yes | up | 0.05 | 0.13 | 0.02 | 0.4 | 0.38 | 0.65 | 0.066667 | 0.476667 |
| AKNA | 3.727 | 1.897913 | 0.011821 | 0.045718 | yes | up | 0.33 | 0.7 | 0.64 | 6.84 | 0.81 | 0.86 | 0.556667 | 2.836667 |
| HDX | 0.373 | -1.42436 | 0.011823 | 0.045718 | yes | down | 0.22 | 0.28 | 0.2 | 0.17 | 0.15 | 0.07 | 0.233333 | 0.13 |
| LYSMD2 | 3.13 | 1.646372 | 0.011833 | 0.045735 | yes | up | 0.98 | 1.21 | 0.55 | 8.05 | 1.76 | 3.37 | 0.913333 | 4.393333 |
| LOC108176227 | 0.124 | -3.01324 | 0.011843 | 0.045759 | yes | down | 5.27 | 18.98 | 6.28 | 0.19 | 4.46 | 0.89 | 10.17667 | 1.846667 |
| LOC100345097 | 0.001 | -9.83215 | 0.011863 | 0.045815 | yes | down | 56.25 | 0 | 4.66 | 0 | 0 | 0 | 20.30333 | 0 |
| LOC100348052 | 904.6 | 9.821136 | 0.011949 | 0.046062 | yes | up | 0 | 0 | 0 | 0 | 20.94 | 45.98 | 0 | 22.30667 |
| PAQR9 | 0.28 | -1.83766 | 0.012046 | 0.046388 | yes | down | 0.29 | 0.4 | 0.65 | 0.26 | 0.03 | 0.29 | 0.446667 | 0.193333 |
| CDCA2 | 4.977 | 2.315305 | 0.012088 | 0.046505 | yes | up | 0.07 | 0.08 | 0.06 | 0.89 | 0.57 | 0.12 | 0.07 | 0.526667 |
| LOC103350415 | 6.067 | 2.600943 | 0.01215 | 0.04673 | yes | up | 0.13 | 0.2 | 0.07 | 1.28 | 1 | 1.04 | 0.133333 | 1.106667 |
| CCR1 | 4.667 | 2.222442 | 0.012258 | 0.047075 | yes | up | 0.41 | 0.38 | 0.29 | 5.68 | 0.77 | 0.63 | 0.36 | 2.36 |
| DNAH2 | 0.111 | -3.16918 | 0.012303 | 0.047198 | yes | down | 0.02 | 0.03 | 0.03 | 0 | 0.01 | 0 | 0.026667 | 0.003333 |
| ARHGAP15 | 4.07 | 2.025157 | 0.01244 | 0.047675 | yes | up | 0.28 | 0.28 | 0.31 | 3.92 | 0.72 | 0.87 | 0.29 | 1.836667 |
| LOC100348403 | 0.067 | -3.90458 | 0.012457 | 0.04773 | yes | down | 0.09 | 0.33 | 0.05 | 0.02 | 0.02 | 0 | 0.156667 | 0.013333 |
| STAT4 | 7.016 | 2.810617 | 0.012521 | 0.047937 | yes | up | 0.12 | 0.05 | 0.11 | 1.97 | 0.3 | 0.16 | 0.093333 | 0.81 |
| MERTK | 2.218 | 1.149032 | 0.012529 | 0.047956 | yes | up | 3.61 | 3.1 | 2.48 | 18.99 | 4.95 | 7.86 | 3.063333 | 10.6 |
| LOC100338194 | 0.319 | -1.64846 | 0.012544 | 0.047977 | yes | down | 1.45 | 2.93 | 1.26 | 0.45 | 0.52 | 1.53 | 1.88 | 0.833333 |
| LOC100351298 | 3.353 | 1.745509 | 0.01257 | 0.048064 | yes | up | 0.24 | 0.32 | 0.7 | 3.81 | 0.76 | 2.15 | 0.42 | 2.24 |
| GAS2L3 | 0.444 | -1.1724 | 0.012666 | 0.048348 | yes | down | 0.57 | 0.34 | 0.33 | 0.35 | 0.26 | 0.26 | 0.413333 | 0.29 |
| LOC108175843 | 15.488 | 3.953067 | 0.012705 | 0.048484 | yes | up | 0 | 0.15 | 0.27 | 8.67 | 0.28 | 1.07 | 0.14 | 3.34 |
| SIRPB2 | 2.103 | 1.07258 | 0.012788 | 0.048751 | yes | up | 0.32 | 0.66 | 0.62 | 2.03 | 1.42 | 1.71 | 0.533333 | 1.72 |
| CACNA1G | 2.668 | 1.415602 | 0.012912 | 0.049114 | yes | up | 0.06 | 0.11 | 0.09 | 0.25 | 0.5 | 0.31 | 0.086667 | 0.353333 |
| DACH2 | 2.473 | 1.305971 | 0.012922 | 0.049137 | yes | up | 0.15 | 0.2 | 0.6 | 0.93 | 1.89 | 1.04 | 0.316667 | 1.286667 |
| LOC103351520 | 3.125 | 1.644075 | 0.012972 | 0.049278 | yes | up | 0.91 | 1.23 | 4.61 | 11.08 | 15.05 | 14.84 | 2.25 | 13.65667 |
| CD14 | 2.816 | 1.49389 | 0.012999 | 0.049334 | yes | up | 9.25 | 7.7 | 5.23 | 69.01 | 11.85 | 16.21 | 7.393333 | 32.35667 |
| DNAH12 | 0.275 | -1.8647 | 0.013068 | 0.049533 | yes | down | 0.15 | 0.22 | 0.13 | 0.09 | 0.11 | 0.01 | 0.166667 | 0.07 |
| LOC103346390 | 0.275 | -1.8612 | 0.013072 | 0.049535 | yes | down | 0.31 | 0.4 | 0.06 | 0.1 | 0.12 | 0.12 | 0.256667 | 0.113333 |

iBAT_D14_VS_iBAT_M4

| Gene name | FC(iBAT_M4/iBAT_D14) | Log2FC(iBAT_M4/iBAT_D14) | Pvalue | Padjust | Significant | Regulate | D14iBAT1 | D14iBAT2 | D14iBAT3 | M4iBAT1 | M4iBAT2 | M4iBAT3 | iBAT_D14 | iBAT_M4 |
| --- | --- | --- | --- | --- | --- | --- | --- | --- | --- | --- | --- | --- | --- | --- |
| SNCG | 73.337 | 6.196474 | 8.9E-195 | 1.5E-190 | yes | up | 11.92 | 17.7 | 15.7 | 3445.74 | 2070.04 | 2401.75 | 15.10667 | 2639.177 |
| GNAS | 0.088 | -3.50576 | 9.4E-174 | 7.8E-170 | yes | down | 4680.31 | 6057.31 | 5802.71 | 1240.08 | 1064.67 | 1018.94 | 5513.443 | 1107.897 |
| PPIF | 0.062 | -4.0175 | 1.1E-141 | 6.3E-138 | yes | down | 85.94 | 113.18 | 102.96 | 14.16 | 12.75 | 16.4 | 100.6933 | 14.43667 |
| GPAT4 | 0.1 | -3.31713 | 1.9E-119 | 7.7E-116 | yes | down | 138.28 | 142.78 | 128.72 | 33.39 | 28.38 | 34.76 | 136.5933 | 32.17667 |
| RNF115 | 0.169 | -2.56676 | 1E-109 | 3.5E-106 | yes | down | 84.68 | 101.35 | 93.13 | 33.64 | 31.09 | 35.99 | 93.05333 | 33.57333 |
| COX1 | 0.149 | -2.74752 | 5.44E-98 | 1.5E-94 | yes | down | 37581.46 | 39557.49 | 39085 | 12894.12 | 13430.43 | 13536.51 | 38741.32 | 13287.02 |
| ND5 | 0.112 | -3.16319 | 6.88E-97 | 1.63E-93 | yes | down | 65596.28 | 62584.24 | 67298.96 | 15982.22 | 15887.78 | 17702.12 | 65159.83 | 16524.04 |
| ITGA7 | 8.232 | 3.041295 | 2.08E-95 | 4.3E-92 | yes | up | 19.6 | 24.28 | 16.72 | 421.7 | 360.25 | 368.75 | 20.2 | 383.5667 |
| MPC1 | 0.125 | -2.99987 | 4.41E-94 | 8.1E-91 | yes | down | 390.43 | 565.28 | 406.9 | 141.56 | 131.43 | 126.7 | 454.2033 | 133.23 |
| SDHB | 0.218 | -2.19637 | 1.16E-93 | 1.92E-90 | yes | down | 505.63 | 646.86 | 558.91 | 306.98 | 267.46 | 295.63 | 570.4667 | 290.0233 |
| LOC100346046 | 334.739 | 8.386894 | 3.28E-93 | 4.94E-90 | yes | up | 1.71 | 1.59 | 1.28 | 1681.04 | 423.03 | 1524.45 | 1.526667 | 1209.507 |
| LOC100342572 | 42.618 | 5.413394 | 1.26E-91 | 1.74E-88 | yes | up | 28 | 25.92 | 15.32 | 2340.98 | 2664.43 | 1896.7 | 23.08 | 2300.703 |
| AQP4 | 19.935 | 4.317219 | 2.34E-88 | 2.98E-85 | yes | up | 2.05 | 2.9 | 2.02 | 139.5 | 79.99 | 103.53 | 2.323333 | 107.6733 |
| ACADS | 0.18 | -2.47286 | 1.03E-85 | 1.21E-82 | yes | down | 379.11 | 428.91 | 395.91 | 161.68 | 149.26 | 179.79 | 401.31 | 163.5767 |
| COX3 | 0.129 | -2.95742 | 5.92E-84 | 6.53E-81 | yes | down | 67862.42 | 63526.96 | 71579.65 | 19581.63 | 20356.52 | 21467.15 | 67656.34 | 20468.43 |
| LOC100359087 | 57.447 | 5.844169 | 2.39E-83 | 2.48E-80 | yes | up | 1.45 | 0.47 | 1.3 | 84.97 | 90.12 | 91.84 | 1.073333 | 88.97667 |
| NDUFA10 | 0.211 | -2.24425 | 3.44E-83 | 3.35E-80 | yes | down | 324.7 | 387.97 | 313.79 | 176.19 | 155.04 | 172.06 | 342.1533 | 167.7633 |
| LOC100347571 | 0.172 | -2.53581 | 3.89E-82 | 3.58E-79 | yes | down | 467.17 | 570.33 | 442.28 | 220.17 | 173.17 | 201.23 | 493.26 | 198.19 |
| DHRS11 | 0.09 | -3.48028 | 4.41E-82 | 3.84E-79 | yes | down | 161.46 | 230.9 | 194.89 | 42.07 | 32.1 | 47.94 | 195.75 | 40.70333 |
| LEP | 155.926 | 7.284719 | 6.43E-81 | 5.32E-78 | yes | up | 0.34 | 0.16 | 0.64 | 75.37 | 130.73 | 82.93 | 0.38 | 96.34333 |
| NDUFS3 | 0.19 | -2.39367 | 3E-80 | 2.37E-77 | yes | down | 386.55 | 538.05 | 439.78 | 207.19 | 194.61 | 201.86 | 454.7933 | 201.22 |
| MDH2 | 0.206 | -2.28194 | 3.23E-79 | 2.43E-76 | yes | down | 505.29 | 630.23 | 487.28 | 276.01 | 248.94 | 252.14 | 540.9333 | 259.03 |
| HADHB | 0.075 | -3.73702 | 1.09E-78 | 7.87E-76 | yes | down | 788.8 | 1054.53 | 836.65 | 127.97 | 144.98 | 194.96 | 893.3267 | 155.97 |
| ND6 | 0.04 | -4.62724 | 1.38E-74 | 9.53E-72 | yes | down | 9677.91 | 7210.54 | 8334.84 | 936.7 | 914.38 | 463.62 | 8407.763 | 771.5667 |
| MRPL39 | 0.179 | -2.48334 | 1.26E-73 | 8.32E-71 | yes | down | 85.25 | 119.17 | 99.71 | 44.75 | 40.73 | 40.73 | 101.3767 | 42.07 |
| SDHD | 0.242 | -2.04419 | 2.32E-71 | 1.48E-68 | yes | down | 350.97 | 399.84 | 351.93 | 229.13 | 204.44 | 192.28 | 367.58 | 208.6167 |
| PDE4D | 0.111 | -3.17034 | 5.19E-71 | 3.18E-68 | yes | down | 31.17 | 31.43 | 28.08 | 13.73 | 5.57 | 15.47 | 30.22667 | 11.59 |
| TNN | 0.004 | -8.03071 | 7.37E-71 | 4.36E-68 | yes | down | 87.34 | 36.22 | 83.24 | 0.92 | 0.25 | 0.63 | 68.93333 | 0.6 |
| ECI1 | 0.196 | -2.35379 | 1.97E-68 | 1.13E-65 | yes | down | 264.48 | 292.72 | 238.79 | 120.1 | 119.69 | 123.39 | 265.33 | 121.06 |
| MC5R | 36.235 | 5.179295 | 5.29E-66 | 2.86E-63 | yes | up | 6.3 | 5.28 | 8.12 | 269.99 | 409.68 | 375.87 | 6.566667 | 351.8467 |
| LOC100339305 | 54.739 | 5.77449 | 5.36E-66 | 2.86E-63 | yes | up | 0.53 | 0.74 | 0.2 | 65.59 | 71.52 | 61.63 | 0.49 | 66.24667 |
| ECH1 | 0.161 | -2.6331 | 6.08E-66 | 3.14E-63 | yes | down | 424.82 | 485.36 | 374.96 | 171.61 | 137.92 | 182.39 | 428.38 | 163.9733 |
| PDK1 | 0.124 | -3.00907 | 1.54E-65 | 7.72E-63 | yes | down | 22.84 | 25.25 | 20.93 | 6.26 | 5.63 | 7.65 | 23.00667 | 6.513333 |
| LOC100337966 | 0.301 | -1.73326 | 4.74E-65 | 2.31E-62 | yes | down | 673.77 | 764.3 | 694.08 | 511.39 | 444.31 | 481.23 | 710.7167 | 478.9767 |
| ATP5A1 | 0.217 | -2.20169 | 2.59E-63 | 1.22E-60 | yes | down | 1280.97 | 1701.01 | 1445.72 | 841.67 | 633.31 | 763.74 | 1475.9 | 746.24 |
| AOX3 | 19.348 | 4.274116 | 2.23E-62 | 1.02E-59 | yes | up | 1.81 | 1.08 | 1.16 | 39.48 | 45.49 | 55.76 | 1.35 | 46.91 |
| CST6 | 28.803 | 4.848127 | 2.96E-62 | 1.33E-59 | yes | up | 7.9 | 4.67 | 3.65 | 340.08 | 369.52 | 391.59 | 5.406667 | 367.0633 |
| ALDH5A1 | 0.21 | -2.25073 | 3.67E-62 | 1.6E-59 | yes | down | 30.43 | 38.97 | 36.1 | 20.84 | 17.12 | 16.36 | 35.16667 | 18.10667 |
| PRELID3B | 0.142 | -2.81146 | 3.16E-60 | 1.34E-57 | yes | down | 121.54 | 135.07 | 140.22 | 48.22 | 49.24 | 39.09 | 132.2767 | 45.51667 |
| LOC100328910 | 22.11 | 4.466639 | 8.66E-60 | 3.58E-57 | yes | up | 50.82 | 59.83 | 102.16 | 3518.64 | 4070.66 | 3360.8 | 70.93667 | 3650.033 |
| ATP5B | 0.172 | -2.53852 | 5.23E-59 | 2.11E-56 | yes | down | 2483.27 | 3047.96 | 2409.3 | 1057.96 | 895.79 | 1222.51 | 2646.843 | 1058.753 |
| SUCLG1 | 0.314 | -1.6689 | 5.75E-59 | 2.26E-56 | yes | down | 424.03 | 535.19 | 468.39 | 379.38 | 324.01 | 339.9 | 475.87 | 347.7633 |
| LOC100350787 | 0.212 | -2.24028 | 9.83E-59 | 3.78E-56 | yes | down | 733.28 | 927.74 | 890.63 | 423.81 | 369.19 | 467.77 | 850.55 | 420.2567 |
| MTCH1 | 0.321 | -1.64147 | 1.12E-58 | 4.22E-56 | yes | down | 264.99 | 324.56 | 293.92 | 224.96 | 214.87 | 212.96 | 294.49 | 217.5967 |
| TUFM | 0.241 | -2.05047 | 1.2E-58 | 4.41E-56 | yes | down | 148.71 | 184.36 | 142.9 | 101.21 | 83.76 | 86.9 | 158.6567 | 90.62333 |
| ACAA2 | 0.137 | -2.87155 | 2.86E-58 | 1.03E-55 | yes | down | 1437.23 | 1792.79 | 1516.86 | 448.63 | 442.11 | 612.65 | 1582.293 | 501.13 |
| LOC108178113 | 0.287 | -1.80319 | 1.47E-57 | 5.19E-55 | yes | down | 3176.02 | 3413.65 | 2880.88 | 2294.87 | 2073.17 | 2314.43 | 3156.85 | 2227.49 |
| TBRG4 | 0.137 | -2.8657 | 5.91E-57 | 2.04E-54 | yes | down | 43.65 | 62.78 | 51.3 | 14.38 | 15.24 | 18.51 | 52.57667 | 16.04333 |
| DLD | 0.232 | -2.10861 | 7.13E-57 | 2.41E-54 | yes | down | 145.49 | 184.4 | 179.45 | 93.15 | 81.59 | 99.36 | 169.78 | 91.36667 |
| CRAT | 0.228 | -2.13288 | 2.27E-56 | 7.53E-54 | yes | down | 97.25 | 133.74 | 102.71 | 64.69 | 55.93 | 55.62 | 111.2333 | 58.74667 |
| HMGB3 | 0.073 | -3.77542 | 1.05E-55 | 3.41E-53 | yes | down | 48.18 | 81 | 85.36 | 11.57 | 14.29 | 11.46 | 71.51333 | 12.44 |
| NDUFS6 | 0.305 | -1.71195 | 2.19E-55 | 6.95E-53 | yes | down | 407.42 | 483.12 | 441.84 | 355.68 | 311.03 | 334.54 | 444.1267 | 333.75 |
| SAMM50 | 0.225 | -2.14979 | 3.35E-55 | 1.05E-52 | yes | down | 114.98 | 157.47 | 140.26 | 79.17 | 62.77 | 74.47 | 137.57 | 72.13667 |
| TM7SF2 | 13.528 | 3.757873 | 4.06E-55 | 1.24E-52 | yes | up | 2.62 | 3.23 | 2.24 | 112.49 | 87.66 | 68.73 | 2.696667 | 89.62667 |
| QPCT | 0.061 | -4.03953 | 9.23E-55 | 2.78E-52 | yes | down | 147.76 | 204.14 | 228.05 | 22.38 | 22.08 | 37.19 | 193.3167 | 27.21667 |
| AIFM1 | 0.208 | -2.2646 | 1.38E-54 | 4.09E-52 | yes | down | 74.93 | 101.67 | 86.88 | 47.26 | 35.28 | 44.83 | 87.82667 | 42.45667 |
| COX2 | 0.111 | -3.16817 | 2.34E-54 | 6.8E-52 | yes | down | 70879.59 | 55173.47 | 66291.15 | 14877.61 | 17603.19 | 18024.67 | 64114.74 | 16835.16 |
| LOC100352363 | 99.079 | 6.630509 | 7.54E-54 | 2.15E-51 | yes | up | 2.14 | 0.7 | 1.32 | 309.92 | 301.86 | 304.68 | 1.386667 | 305.4867 |
| LOC100343793 | 0.293 | -1.77001 | 2.79E-52 | 7.83E-50 | yes | down | 1944.09 | 2140.07 | 1837.31 | 1531.06 | 1226.48 | 1363.03 | 1973.823 | 1373.523 |
| ATP6V1E1 | 0.295 | -1.76222 | 3.12E-52 | 8.62E-50 | yes | down | 187.83 | 224.08 | 218.95 | 151.15 | 131.06 | 151.07 | 210.2867 | 144.4267 |
| ACO2 | 0.167 | -2.57944 | 7.7E-52 | 2.09E-49 | yes | down | 900.28 | 1276.14 | 1044.6 | 380.38 | 375.93 | 486.5 | 1073.673 | 414.27 |
| SDHA | 0.249 | -2.00582 | 1.25E-51 | 3.33E-49 | yes | down | 640.03 | 846.96 | 685.12 | 420.16 | 383.85 | 455.69 | 724.0367 | 419.9 |
| GREM2 | 11.119 | 3.475014 | 1.48E-51 | 3.9E-49 | yes | up | 0.59 | 1.07 | 0.59 | 19.27 | 18.5 | 14.36 | 0.75 | 17.37667 |
| IDH3G | 0.204 | -2.29177 | 2.19E-51 | 5.65E-49 | yes | down | 395.08 | 502.89 | 407.32 | 217.79 | 168.89 | 231.08 | 435.0967 | 205.92 |
| AHCYL1 | 0.113 | -3.14499 | 5.88E-51 | 1.5E-48 | yes | down | 233.97 | 278.01 | 295.83 | 64 | 55.12 | 89.43 | 269.27 | 69.51667 |
| APOE | 21.09 | 4.398516 | 1.66E-50 | 4.17E-48 | yes | up | 26.17 | 57.45 | 44.18 | 1839.82 | 2731.43 | 1839.31 | 42.6 | 2136.853 |
| ITIH3 | 113.378 | 6.824999 | 3.42E-50 | 8.46E-48 | yes | up | 0.06 | 0.2 | 0.13 | 23.91 | 46.14 | 32.33 | 0.13 | 34.12667 |
| IDH3A | 0.143 | -2.80138 | 9.4E-50 | 2.29E-47 | yes | down | 273.99 | 431.96 | 282.62 | 99.18 | 107.64 | 117.7 | 329.5233 | 108.1733 |
| PDHA1 | 0.238 | -2.06972 | 1.18E-49 | 2.84E-47 | yes | down | 169.86 | 234.01 | 209.59 | 118.56 | 97.65 | 122.44 | 204.4867 | 112.8833 |
| CHCHD10 | 0.199 | -2.32597 | 1.97E-49 | 4.66E-47 | yes | down | 57.9 | 75.18 | 56.85 | 28.61 | 32.33 | 28.63 | 63.31 | 29.85667 |
| LOC100347143 | 0.256 | -1.96315 | 1.34E-48 | 3.13E-46 | yes | down | 395.97 | 556.31 | 420.06 | 284.64 | 250.96 | 279.66 | 457.4467 | 271.7533 |
| PPA2 | 0.249 | -2.00467 | 2.07E-48 | 4.76E-46 | yes | down | 78.08 | 77.72 | 73.08 | 49.12 | 41.74 | 43.67 | 76.29333 | 44.84333 |
| ND4 | 0.117 | -3.09079 | 3.22E-48 | 7.3E-46 | yes | down | 14882.97 | 12381.68 | 15275.94 | 3334.01 | 3704.94 | 4650.12 | 14180.2 | 3896.357 |
| IMPDH1 | 0.114 | -3.13919 | 5.45E-48 | 1.22E-45 | yes | down | 266.77 | 353.18 | 325.91 | 71.48 | 68.21 | 108.49 | 315.2867 | 82.72667 |
| ATP5H | 0.325 | -1.62369 | 6.3E-48 | 1.39E-45 | yes | down | 803.39 | 1019.93 | 827.55 | 694.8 | 636.67 | 680.61 | 883.6233 | 670.6933 |
| MPC2 | 0.276 | -1.85919 | 7.36E-48 | 1.6E-45 | yes | down | 323.91 | 337.55 | 340.86 | 223.16 | 221.15 | 203.57 | 334.1067 | 215.96 |
| SEPT10 | 0.25 | -2.00079 | 8.29E-48 | 1.78E-45 | yes | down | 189.88 | 220.85 | 239.56 | 114.13 | 114.36 | 127.48 | 216.7633 | 118.6567 |
| FAM222A | 0.033 | -4.90254 | 1.23E-47 | 2.61E-45 | yes | down | 3.92 | 5.71 | 6.95 | 0.42 | 0.43 | 0.43 | 5.526667 | 0.426667 |
| INMT | 24.565 | 4.618536 | 4.61E-47 | 9.66E-45 | yes | up | 0.62 | 0.47 | 1.06 | 46.91 | 31.38 | 46.91 | 0.716667 | 41.73333 |
| ATP6 | 0.12 | -3.06262 | 5.01E-47 | 1.04E-44 | yes | down | 39607.67 | 30054.45 | 41171.62 | 9492.52 | 10250.74 | 11643.64 | 36944.58 | 10462.3 |
| PCK2 | 15.674 | 3.970281 | 1.39E-46 | 2.83E-44 | yes | up | 12.37 | 19.9 | 29.45 | 892.11 | 671.73 | 710.56 | 20.57333 | 758.1333 |
| LOC100349830 | 0.365 | -1.45345 | 1.45E-46 | 2.93E-44 | yes | down | 140.03 | 172.25 | 150.11 | 148.92 | 120.41 | 133.18 | 154.13 | 134.17 |
| CHCHD3 | 0.28 | -1.83473 | 1.8E-46 | 3.58E-44 | yes | down | 149.7 | 209.53 | 167.44 | 121.12 | 105.98 | 115.58 | 175.5567 | 114.2267 |
| RDH8 | 53.615 | 5.744554 | 1.08E-45 | 2.14E-43 | yes | up | 0.09 | 0.5 | 0.28 | 32.65 | 33 | 41.33 | 0.29 | 35.66 |
| ADAMTS16 | 35.474 | 5.148696 | 3.21E-45 | 6.25E-43 | yes | up | 0.18 | 0.14 | 0.19 | 10.49 | 11.84 | 18.91 | 0.17 | 13.74667 |
| DEFB1 | 34.151 | 5.093835 | 4.34E-45 | 8.35E-43 | yes | up | 11.4 | 6 | 6.61 | 470.86 | 578.58 | 912.09 | 8.003333 | 653.8433 |
| ADAMTS18 | 10.961 | 3.454305 | 6.78E-45 | 1.29E-42 | yes | up | 0.33 | 0.66 | 0.38 | 12.86 | 11.05 | 10.08 | 0.456667 | 11.33 |
| IDH2 | 0.081 | -3.62943 | 6.99E-45 | 1.31E-42 | yes | down | 546.34 | 714.26 | 624.53 | 94.48 | 160.68 | 94.3 | 628.3767 | 116.4867 |
| NDUFAB1 | 0.276 | -1.85803 | 9.86E-45 | 1.83E-42 | yes | down | 337.38 | 427.55 | 379.73 | 286.61 | 211.58 | 243.46 | 381.5533 | 247.2167 |
| PRKAG2 | 0.242 | -2.04456 | 1.26E-43 | 2.32E-41 | yes | down | 29.75 | 38.99 | 35.13 | 18.79 | 18.03 | 20.69 | 34.62333 | 19.17 |
| PDE3B | 0.281 | -1.83002 | 1.3E-43 | 2.36E-41 | yes | down | 42.82 | 45.99 | 51.38 | 31.75 | 28.15 | 30.12 | 46.73 | 30.00667 |
| FAM126A | 5.529 | 2.467006 | 4.26E-43 | 7.67E-41 | yes | up | 1.99 | 1.45 | 1.43 | 20.1 | 19.16 | 18.75 | 1.623333 | 19.33667 |
| ETFDH | 0.218 | -2.20054 | 7.63E-43 | 1.36E-40 | yes | down | 172.44 | 226.51 | 183.17 | 95.88 | 101.6 | 140.36 | 194.04 | 112.6133 |
| LOC100346670 | 0.33 | -1.59792 | 8.58E-43 | 1.51E-40 | yes | down | 1590.44 | 1656.48 | 1455.14 | 1284.35 | 1183.89 | 1223.98 | 1567.353 | 1230.74 |
| MRPS12 | 0.171 | -2.54895 | 1.08E-42 | 1.88E-40 | yes | down | 51.6 | 51.87 | 62.98 | 20.59 | 21.1 | 22.34 | 55.48333 | 21.34333 |
| ACAD9 | 0.154 | -2.70222 | 1.15E-42 | 1.97E-40 | yes | down | 16.46 | 28.43 | 19.58 | 7.31 | 7.13 | 7.3 | 21.49 | 7.246667 |
| ATP8 | 0.151 | -2.72792 | 1.2E-42 | 2.04E-40 | yes | down | 199788.1 | 168629 | 216032 | 68818.54 | 75201.95 | 86905.65 | 194816.4 | 76975.38 |
| MTCH2 | 0.245 | -2.02636 | 1.32E-42 | 2.24E-40 | yes | down | 194.45 | 271.13 | 206.79 | 129.16 | 131.95 | 124.44 | 224.1233 | 128.5167 |
| GABRD | 8.163 | 3.029021 | 2.4E-42 | 4.02E-40 | yes | up | 12.22 | 14.6 | 18.61 | 259.1 | 273.35 | 328.98 | 15.14333 | 287.1433 |
| OXNAD1 | 0.173 | -2.53459 | 2.5E-42 | 4.14E-40 | yes | down | 22.66 | 26.35 | 32.26 | 10.97 | 10.3 | 10.57 | 27.09 | 10.61333 |
| MYO19 | 0.169 | -2.56705 | 2.8E-42 | 4.58E-40 | yes | down | 11.52 | 17.33 | 15.93 | 7 | 4.86 | 5.84 | 14.92667 | 5.9 |
| CYTB | 0.13 | -2.94833 | 3.57E-42 | 5.79E-40 | yes | down | 19576.98 | 17131.39 | 20389.01 | 4859.1 | 5264.82 | 7198.4 | 19032.46 | 5774.107 |
| DLST | 0.234 | -2.09715 | 5.21E-42 | 8.37E-40 | yes | down | 234.26 | 325.78 | 296.05 | 186.69 | 131.46 | 146.96 | 285.3633 | 155.0367 |
| IMMT | 0.294 | -1.76396 | 1.27E-41 | 2.02E-39 | yes | down | 122.77 | 159.62 | 154.94 | 100.94 | 104.43 | 103.03 | 145.7767 | 102.8 |
| ISCA1 | 0.237 | -2.07442 | 1.32E-41 | 2.08E-39 | yes | down | 102.49 | 125.65 | 116.34 | 56.82 | 67.64 | 64.66 | 114.8267 | 63.04 |
| PDPR | 0.25 | -2.00047 | 1.44E-41 | 2.25E-39 | yes | down | 20.49 | 29.13 | 25.96 | 15.37 | 13.49 | 15.01 | 25.19333 | 14.62333 |
| ACSS2 | 13.958 | 3.803043 | 1.57E-41 | 2.4E-39 | yes | up | 14.42 | 21.91 | 19.46 | 853.64 | 347.55 | 621.29 | 18.59667 | 607.4933 |
| ST6GALNAC6 | 3.412 | 1.770455 | 1.57E-41 | 2.4E-39 | yes | up | 9.91 | 12.69 | 10.49 | 94.99 | 76.93 | 88 | 11.03 | 86.64 |
| ACADVL | 0.149 | -2.74455 | 4.08E-41 | 6.19E-39 | yes | down | 454.1 | 664.12 | 550.58 | 174.28 | 157.85 | 242.58 | 556.2667 | 191.57 |
| COX6A1 | 0.353 | -1.50191 | 5.06E-41 | 7.61E-39 | yes | down | 2301.77 | 2461.12 | 2262.99 | 2187.05 | 1845.83 | 1852.01 | 2341.96 | 1961.63 |
| LOC100341175 | 0.093 | -3.42647 | 2.03E-40 | 3.03E-38 | yes | down | 908.93 | 1017.78 | 846.44 | 132.59 | 218.1 | 244.21 | 924.3833 | 198.3 |
| OGDH | 0.31 | -1.69191 | 3.25E-40 | 4.8E-38 | yes | down | 298.83 | 374.53 | 390.81 | 262.22 | 246.96 | 251.97 | 354.7233 | 253.7167 |
| NDUFB3 | 0.357 | -1.48579 | 5.24E-40 | 7.67E-38 | yes | down | 195.3 | 233.41 | 204.42 | 179.63 | 167.42 | 181.65 | 211.0433 | 176.2333 |
| NRIP1 | 3.578 | 1.838995 | 6.37E-40 | 9.25E-38 | yes | up | 3.11 | 3.94 | 3.76 | 29.44 | 30.83 | 28.57 | 3.603333 | 29.61333 |
| MTFP1 | 0.096 | -3.3853 | 6.86E-40 | 9.86E-38 | yes | down | 18.45 | 26.02 | 26.48 | 6.38 | 3.38 | 5.95 | 23.65 | 5.236667 |
| FADS3 | 3.212 | 1.683478 | 8.3E-40 | 1.18E-37 | yes | up | 32.16 | 42.52 | 33.94 | 269.23 | 265.3 | 273.61 | 36.20667 | 269.38 |
| COQ9 | 0.24 | -2.05667 | 9.58E-40 | 1.35E-37 | yes | down | 118.18 | 169.16 | 143.95 | 90.01 | 81.31 | 68.93 | 143.7633 | 80.08333 |
| NDUFS1 | 0.267 | -1.90752 | 1.13E-39 | 1.58E-37 | yes | down | 134.26 | 190.66 | 156.1 | 108.21 | 83.32 | 105.07 | 160.34 | 98.86667 |
| NDUFA9 | 0.236 | -2.08514 | 2.08E-39 | 2.89E-37 | yes | down | 94.49 | 150.6 | 125.24 | 72.58 | 61.96 | 67.14 | 123.4433 | 67.22667 |
| HK1 | 0.162 | -2.62368 | 4E-39 | 5.51E-37 | yes | down | 43.4 | 73.26 | 57.24 | 25.43 | 16.98 | 23.11 | 57.96667 | 21.84 |
| IDH3B | 0.178 | -2.49129 | 4.57E-39 | 6.26E-37 | yes | down | 377.88 | 558.6 | 494.99 | 187.64 | 160.33 | 246.72 | 477.1567 | 198.23 |
| PREX2 | 0.301 | -1.73161 | 1.42E-38 | 1.93E-36 | yes | down | 12.23 | 14.94 | 11.89 | 9.66 | 9.23 | 8.36 | 13.02 | 9.083333 |
| LOC100356012 | 7.195 | 2.846897 | 6.4E-38 | 8.6E-36 | yes | up | 3.25 | 2.93 | 3.63 | 53.92 | 64.38 | 46.4 | 3.27 | 54.9 |
| ADCK3 | 0.141 | -2.82899 | 6.52E-38 | 8.7E-36 | yes | down | 442.39 | 470.67 | 390.87 | 117.4 | 122.75 | 184.25 | 434.6433 | 141.4667 |
| ND3 | 0.139 | -2.84637 | 7.57E-38 | 1E-35 | yes | down | 30865.46 | 26765.75 | 27132.97 | 7487.68 | 9202.21 | 11850.8 | 28254.73 | 9513.563 |
| BCKDHA | 0.361 | -1.46887 | 8.95E-38 | 1.18E-35 | yes | down | 146.03 | 177.38 | 167.76 | 145.38 | 123.2 | 144.41 | 163.7233 | 137.6633 |
| CIAPIN1 | 0.263 | -1.92499 | 2.12E-37 | 2.76E-35 | yes | down | 33.04 | 44.48 | 40.7 | 23.3 | 23.1 | 25.72 | 39.40667 | 24.04 |
| ISCU | 0.327 | -1.6132 | 2.32E-37 | 3E-35 | yes | down | 287.83 | 288.14 | 270.08 | 224.59 | 201.67 | 221.84 | 282.0167 | 216.0333 |
| LOC100358417 | 0.107 | -3.22698 | 3.3E-37 | 4.24E-35 | yes | down | 12.39 | 19.85 | 12.86 | 3.77 | 3 | 5.02 | 15.03333 | 3.93 |
| LOC100355688 | 0.252 | -1.98728 | 5.46E-37 | 6.95E-35 | yes | down | 48.99 | 68.32 | 58.95 | 33.11 | 36.66 | 34.13 | 58.75333 | 34.63333 |
| UGT1-6 | 6.844 | 2.77486 | 8.12E-37 | 1.03E-34 | yes | up | 7.47 | 7.03 | 7.04 | 87.67 | 112.21 | 118.72 | 7.18 | 106.2 |
| SLC4A4 | 0.122 | -3.03993 | 8.48E-37 | 1.06E-34 | yes | down | 12.2 | 16.89 | 11.69 | 4.4 | 2.49 | 4.54 | 13.59333 | 3.81 |
| AQP8 | 500.565 | 8.967413 | 1.56E-36 | 1.94E-34 | yes | up | 0.44 | 1.33 | 0.28 | 748.58 | 468.03 | 1121.34 | 0.683333 | 779.3167 |
| ALDH1L1 | 48.39 | 5.596632 | 1.83E-36 | 2.26E-34 | yes | up | 0.38 | 0.51 | 0.11 | 56.45 | 32.95 | 23.58 | 0.333333 | 37.66 |
| GOT1 | 0.124 | -3.01379 | 3.64E-36 | 4.46E-34 | yes | down | 65.44 | 101.13 | 72.82 | 21.89 | 28.76 | 17.42 | 79.79667 | 22.69 |
| SDHC | 0.33 | -1.59776 | 3.78E-36 | 4.6E-34 | yes | down | 254.65 | 331.4 | 278.98 | 217.03 | 227.39 | 218.1 | 288.3433 | 220.84 |
| ACOT13 | 0.204 | -2.29061 | 6.52E-36 | 7.88E-34 | yes | down | 119.96 | 154.94 | 139.9 | 60.89 | 58.02 | 79.73 | 138.2667 | 66.21333 |
| PRDX3 | 0.317 | -1.65892 | 1.37E-35 | 1.64E-33 | yes | down | 238.71 | 313 | 266.37 | 206.2 | 188.72 | 220.08 | 272.6933 | 205 |
| ATF6 | 0.237 | -2.07749 | 1.44E-35 | 1.72E-33 | yes | down | 20.99 | 21.14 | 22.35 | 10.84 | 11.51 | 13.17 | 21.49333 | 11.84 |
| COL5A3 | 8.681 | 3.117906 | 1.71E-35 | 2.02E-33 | yes | up | 6.98 | 8.68 | 4.42 | 147.97 | 107.04 | 151.46 | 6.693333 | 135.49 |
| ND4L | 0.177 | -2.50045 | 2.37E-35 | 2.79E-33 | yes | down | 12525.82 | 10445.8 | 13117.1 | 4643.3 | 4910.35 | 6162.44 | 12029.57 | 5238.697 |
| LETMD1 | 0.323 | -1.62872 | 1.28E-34 | 1.49E-32 | yes | down | 68.11 | 74.19 | 73.46 | 54.03 | 48.62 | 58.98 | 71.92 | 53.87667 |
| MCOLN3 | 41.88 | 5.388192 | 1.88E-34 | 2.18E-32 | yes | up | 0.17 | 0.22 | 0.14 | 49.31 | 13.56 | 20.3 | 0.176667 | 27.72333 |
| PRLR | 0.263 | -1.9289 | 2.25E-34 | 2.58E-32 | yes | down | 28.09 | 33.98 | 23.62 | 16.42 | 13.29 | 19.03 | 28.56333 | 16.24667 |
| NDUFC2 | 0.294 | -1.76654 | 2.28E-34 | 2.6E-32 | yes | down | 432.31 | 615.72 | 510.31 | 394.81 | 307.06 | 373.37 | 519.4467 | 358.4133 |
| TBC1D10A | 0.221 | -2.17895 | 3.62E-34 | 4.1E-32 | yes | down | 36.81 | 43.98 | 42.75 | 24.39 | 17.28 | 17.48 | 41.18 | 19.71667 |
| MFN2 | 0.355 | -1.49352 | 4.04E-34 | 4.55E-32 | yes | down | 56.31 | 74.35 | 75.49 | 64.03 | 51.98 | 65.94 | 68.71667 | 60.65 |
| IGSF8 | 6.021 | 2.590001 | 1.42E-33 | 1.59E-31 | yes | up | 4 | 4.85 | 5.74 | 60.98 | 78.3 | 64.02 | 4.863333 | 67.76667 |
| LOC100347175 | 0.323 | -1.6298 | 1.9E-33 | 2.12E-31 | yes | down | 62.78 | 81.42 | 67.81 | 53.35 | 54.79 | 50.76 | 70.67 | 52.96667 |
| LOC100358208 | 0.388 | -1.3676 | 3.08E-33 | 3.4E-31 | yes | down | 833.78 | 948 | 840.73 | 868.54 | 796.34 | 748.62 | 874.17 | 804.5 |
| NIPSNAP1 | 0.229 | -2.12722 | 4.95E-33 | 5.43E-31 | yes | down | 64.77 | 69.95 | 61.53 | 47.67 | 27.62 | 30.85 | 65.41667 | 35.38 |
| KCNH1 | 0.089 | -3.49276 | 5.5E-33 | 5.99E-31 | yes | down | 2.07 | 2.25 | 1.34 | 0.4 | 0.36 | 0.42 | 1.886667 | 0.393333 |
| TSFM | 0.275 | -1.86152 | 6.88E-33 | 7.44E-31 | yes | down | 58.15 | 72.59 | 61.35 | 47.78 | 33.38 | 43.09 | 64.03 | 41.41667 |
| TRABD2B | 7.254 | 2.858732 | 8.64E-33 | 9.29E-31 | yes | up | 0.79 | 1.37 | 1.3 | 16.24 | 19.69 | 21.12 | 1.153333 | 19.01667 |
| VDAC2 | 0.31 | -1.68795 | 9E-33 | 9.61E-31 | yes | down | 258.81 | 319.93 | 265.69 | 209.94 | 217.68 | 180.19 | 281.4767 | 202.6033 |
| ND2 | 0.112 | -3.16194 | 1.54E-32 | 1.63E-30 | yes | down | 17417.47 | 12295.92 | 16240.55 | 3159.24 | 3684.37 | 5237.32 | 15317.98 | 4026.977 |
| SEMA7A | 0.195 | -2.35645 | 1.65E-32 | 1.74E-30 | yes | down | 225.56 | 377.91 | 370.73 | 169.06 | 130.45 | 142.94 | 324.7333 | 147.4833 |
| SMIM4 | 0.222 | -2.17202 | 2.02E-32 | 2.11E-30 | yes | down | 38.31 | 59.49 | 46.23 | 25.55 | 23.82 | 26.65 | 48.01 | 25.34 |
| LOC100348135 | 0.168 | -2.57708 | 2.06E-32 | 2.14E-30 | yes | down | 39.41 | 42.85 | 28.6 | 15 | 14.89 | 13.41 | 36.95333 | 14.43333 |
| LOC100346411 | 0.295 | -1.75884 | 2.27E-32 | 2.35E-30 | yes | down | 1053.84 | 1370.1 | 1039.96 | 941.13 | 677.13 | 804.33 | 1154.633 | 807.53 |
| FAM105A | 0.043 | -4.5394 | 2.45E-32 | 2.52E-30 | yes | down | 18.79 | 24.68 | 11.86 | 1.63 | 1.19 | 3.19 | 18.44333 | 2.003333 |
| DECR1 | 0.265 | -1.91528 | 8.03E-32 | 8.2E-30 | yes | down | 503.2 | 601.3 | 594.22 | 333.46 | 306.98 | 415.35 | 566.24 | 351.93 |
| LOC108178465 | 0.158 | -2.66506 | 1.27E-31 | 1.29E-29 | yes | down | 18.39 | 17.69 | 16.75 | 6.45 | 7.29 | 5.81 | 17.61 | 6.516667 |
| NFYC | 0.348 | -1.52347 | 1.51E-31 | 1.52E-29 | yes | down | 23.5 | 30.59 | 26.67 | 23.62 | 21.08 | 22.78 | 26.92 | 22.49333 |
| RSPO3 | 29.418 | 4.878612 | 1.82E-31 | 1.83E-29 | yes | up | 0.54 | 0.63 | 0.18 | 27.05 | 35.76 | 17.37 | 0.45 | 26.72667 |
| ETFB | 0.377 | -1.40718 | 2.87E-31 | 2.86E-29 | yes | down | 301.17 | 300.69 | 269.64 | 249.26 | 232.94 | 256.37 | 290.5 | 246.19 |
| FFAR2 | 0.056 | -4.15588 | 3.96E-31 | 3.93E-29 | yes | down | 23.26 | 37.7 | 38.34 | 3.3 | 2.63 | 6.66 | 33.1 | 4.196667 |
| TMEM233 | 22.671 | 4.502783 | 4.01E-31 | 3.95E-29 | yes | up | 0.43 | 1.4 | 0.37 | 45.21 | 46.88 | 61.61 | 0.733333 | 51.23333 |
| MRPL35 | 0.301 | -1.73338 | 4.04E-31 | 3.95E-29 | yes | down | 67.46 | 92.18 | 77.07 | 56.86 | 52.73 | 57.99 | 78.90333 | 55.86 |
| ADAM23 | 17.606 | 4.138022 | 4.79E-31 | 4.66E-29 | yes | up | 0.25 | 0.47 | 0.15 | 11.9 | 16.07 | 12.9 | 0.29 | 13.62333 |
| ORAI2 | 4.064 | 2.02286 | 6.26E-31 | 6.05E-29 | yes | up | 2.29 | 3.23 | 1.98 | 28.31 | 32.82 | 30.45 | 2.5 | 30.52667 |
| DNAJA3 | 0.36 | -1.47477 | 1.03E-30 | 9.91E-29 | yes | down | 36.49 | 49.38 | 44.32 | 37.5 | 33.4 | 37.31 | 43.39667 | 36.07 |
| COX4I1 | 0.411 | -1.28217 | 1.17E-30 | 1.12E-28 | yes | down | 2887.75 | 3415.24 | 2802.94 | 3232.93 | 2678.02 | 2872.07 | 3035.31 | 2927.673 |
| LPL | 4.265 | 2.092655 | 1.28E-30 | 1.22E-28 | yes | up | 794.75 | 1175.65 | 726.83 | 8689.72 | 8316.34 | 9381.38 | 899.0767 | 8795.813 |
| GATSL3 | 0.222 | -2.16948 | 1.31E-30 | 1.23E-28 | yes | down | 45.49 | 42.38 | 47.36 | 27.73 | 23.92 | 19.36 | 45.07667 | 23.67 |
| LOC100009353 | 3.003 | 1.586572 | 2.72E-30 | 2.55E-28 | yes | up | 95.66 | 131.32 | 118.8 | 808.43 | 733.08 | 858.3 | 115.26 | 799.9367 |
| NDUFB8 | 0.388 | -1.36769 | 2.99E-30 | 2.79E-28 | yes | down | 401.31 | 488.7 | 412.32 | 438.99 | 382.52 | 361.74 | 434.11 | 394.4167 |
| LOC108176503 | 0.209 | -2.25729 | 3.73E-30 | 3.46E-28 | yes | down | 14.37 | 23.62 | 15.33 | 6.78 | 5.84 | 7.61 | 17.77333 | 6.743333 |
| TIMM10 | 0.288 | -1.79659 | 4.11E-30 | 3.8E-28 | yes | down | 76.51 | 82.46 | 89.64 | 62.41 | 50.26 | 53.72 | 82.87 | 55.46333 |
| PHB | 0.325 | -1.62027 | 6.06E-30 | 5.57E-28 | yes | down | 201.28 | 288.62 | 240.64 | 213.7 | 160.46 | 180.07 | 243.5133 | 184.7433 |
| MTHFD1 | 0.331 | -1.59407 | 7.25E-30 | 6.63E-28 | yes | down | 39.53 | 45.44 | 46.06 | 32.47 | 35.57 | 32.51 | 43.67667 | 33.51667 |
| SLC25A33 | 0.138 | -2.85422 | 8.94E-30 | 8.12E-28 | yes | down | 37.84 | 44.91 | 48.28 | 10.17 | 13.82 | 17.95 | 43.67667 | 13.98 |
| IVNS1ABP | 0.175 | -2.51438 | 1.16E-29 | 1.05E-27 | yes | down | 153.06 | 114.07 | 137.15 | 49.77 | 50.5 | 66.25 | 134.76 | 55.50667 |
| TIMM17A | 0.317 | -1.6586 | 1.32E-29 | 1.19E-27 | yes | down | 76.25 | 98.6 | 76.75 | 63.81 | 64.15 | 57.33 | 83.86667 | 61.76333 |
| HSPA9 | 0.262 | -1.93499 | 1.5E-29 | 1.35E-27 | yes | down | 116.42 | 179.63 | 142.23 | 86.76 | 77.01 | 100.47 | 146.0933 | 88.08 |
| MRPL18 | 0.339 | -1.5597 | 2.09E-29 | 1.86E-27 | yes | down | 40.58 | 47.74 | 46.36 | 37.09 | 28.28 | 35.57 | 44.89333 | 33.64667 |
| PIPOX | 11.703 | 3.548861 | 2.28E-29 | 2.02E-27 | yes | up | 1.3 | 2.15 | 1.06 | 40.93 | 29.93 | 51.05 | 1.503333 | 40.63667 |
| ADAMTS15 | 6.378 | 2.673132 | 2.49E-29 | 2.19E-27 | yes | up | 1.98 | 1.64 | 1.83 | 30.75 | 30.41 | 22.24 | 1.816667 | 27.8 |
| ECSIT | 0.326 | -1.61683 | 3.71E-29 | 3.25E-27 | yes | down | 41.73 | 54.49 | 41.21 | 38.28 | 31.39 | 33.54 | 45.81 | 34.40333 |
| CS | 0.321 | -1.64011 | 4.37E-29 | 3.81E-27 | yes | down | 589.92 | 831.02 | 703.27 | 526.66 | 462.83 | 586.12 | 708.07 | 525.2033 |
| SFXN1 | 6.565 | 2.714839 | 4.97E-29 | 4.3E-27 | yes | up | 2.31 | 3.73 | 3.44 | 51.41 | 58.06 | 37.35 | 3.16 | 48.94 |
| WFS1 | 0.165 | -2.59861 | 6.17E-29 | 5.32E-27 | yes | down | 14.27 | 14.71 | 15.13 | 7.68 | 4.17 | 5.28 | 14.70333 | 5.71 |
| TIMM44 | 0.281 | -1.82942 | 7.82E-29 | 6.7E-27 | yes | down | 46.94 | 67.8 | 47.35 | 39.74 | 33.21 | 32.7 | 54.03 | 35.21667 |
| GPAT3 | 6.566 | 2.71497 | 1.17E-28 | 1E-26 | yes | up | 7.06 | 5.51 | 4.79 | 80.73 | 102.88 | 80.48 | 5.786667 | 88.03 |
| ACP6 | 0.189 | -2.40663 | 1.53E-28 | 1.3E-26 | yes | down | 6.55 | 8.21 | 7.86 | 3.91 | 2.8 | 3.23 | 7.54 | 3.313333 |
| COQ6 | 0.239 | -2.06244 | 1.94E-28 | 1.64E-26 | yes | down | 40.47 | 57.09 | 37.26 | 24.13 | 23.25 | 27.18 | 44.94 | 24.85333 |
| NDUFA3 | 0.4 | -1.32147 | 2.05E-28 | 1.72E-26 | yes | down | 495.86 | 569.66 | 483.71 | 521.86 | 481.25 | 492.7 | 516.41 | 498.6033 |
| PTGES2 | 0.202 | -2.30772 | 3.13E-28 | 2.61E-26 | yes | down | 48.52 | 78.64 | 60.05 | 33.82 | 22.59 | 30.92 | 62.40333 | 29.11 |
| NDUFB5 | 0.349 | -1.51754 | 3.33E-28 | 2.77E-26 | yes | down | 173.09 | 189.95 | 177.53 | 163.19 | 150.09 | 128.96 | 180.19 | 147.4133 |
| DLAT | 0.335 | -1.57942 | 4.78E-28 | 3.96E-26 | yes | down | 29.51 | 38.97 | 33.24 | 30.95 | 24.83 | 23.25 | 33.90667 | 26.34333 |
| ADSL | 0.229 | -2.12416 | 5.52E-28 | 4.54E-26 | yes | down | 18.47 | 31.23 | 24.51 | 13.81 | 13.46 | 11.82 | 24.73667 | 13.03 |
| NDUFA12 | 0.318 | -1.6542 | 5.86E-28 | 4.8E-26 | yes | down | 276.75 | 365.12 | 291.45 | 268.78 | 223.61 | 203.77 | 311.1067 | 232.0533 |
| PAOX | 0.37 | -1.43409 | 5.99E-28 | 4.89E-26 | yes | down | 37.77 | 43.97 | 37.82 | 38.71 | 30.4 | 34.48 | 39.85333 | 34.53 |
| MRPS35 | 0.329 | -1.60251 | 6.91E-28 | 5.61E-26 | yes | down | 32.71 | 40.64 | 40.1 | 29.56 | 28.62 | 28.86 | 37.81667 | 29.01333 |
| NDUFA6 | 0.356 | -1.48887 | 7.75E-28 | 6.26E-26 | yes | down | 350.6 | 491 | 375.9 | 357.48 | 327.22 | 334.23 | 405.8333 | 339.6433 |
| NDUFA7 | 0.33 | -1.60131 | 1.18E-27 | 9.51E-26 | yes | down | 372.25 | 447.19 | 343.52 | 352.9 | 291.34 | 280.12 | 387.6533 | 308.12 |
| STARD7 | 0.276 | -1.85982 | 1.3E-27 | 1.04E-25 | yes | down | 56.97 | 71.24 | 73.28 | 37.67 | 42.28 | 47.91 | 67.16333 | 42.62 |
| ADAMTS17 | 0.229 | -2.1287 | 2.18E-27 | 1.74E-25 | yes | down | 3.04 | 3.77 | 3.63 | 1.71 | 1.69 | 2.11 | 3.48 | 1.836667 |
| ETFA | 0.294 | -1.76612 | 2.23E-27 | 1.77E-25 | yes | down | 577.27 | 679.47 | 591.01 | 421.08 | 347.07 | 501.88 | 615.9167 | 423.3433 |
| PMEPA1 | 0.158 | -2.66405 | 2.35E-27 | 1.85E-25 | yes | down | 17.91 | 27.98 | 21.58 | 5.59 | 5.15 | 9.55 | 22.49 | 6.763333 |
| SMARCA2 | 3.078 | 1.621791 | 3.05E-27 | 2.39E-25 | yes | up | 16.08 | 17.17 | 17.3 | 105.35 | 118.05 | 117.13 | 16.85 | 113.51 |
| ACAT1 | 0.279 | -1.84368 | 3.59E-27 | 2.8E-25 | yes | down | 239.85 | 294.18 | 226.71 | 149.32 | 150.12 | 191.94 | 253.58 | 163.7933 |
| TFRC | 0.05 | -4.31779 | 5.09E-27 | 3.95E-25 | yes | down | 35.71 | 110.65 | 39.99 | 5.72 | 9.62 | 5.5 | 62.11667 | 6.946667 |
| LOC100356756 | 0.084 | -3.57021 | 5.24E-27 | 4.05E-25 | yes | down | 1340.88 | 1431.09 | 1179.73 | 141.5 | 267.63 | 385.67 | 1317.233 | 264.9333 |
| SLC16A7 | 0.078 | -3.68271 | 5.34E-27 | 4.11E-25 | yes | down | 24.67 | 18.01 | 17.48 | 2.79 | 2.07 | 5.29 | 20.05333 | 3.383333 |
| S100B | 9.156 | 3.194746 | 5.97E-27 | 4.57E-25 | yes | up | 9.31 | 14.46 | 15.42 | 312.54 | 354.23 | 191.97 | 13.06333 | 286.2467 |
| ACADM | 0.139 | -2.85145 | 6.48E-27 | 4.94E-25 | yes | down | 201.72 | 223.07 | 367.28 | 74.38 | 80.29 | 100.43 | 264.0233 | 85.03333 |
| MRPL2 | 0.217 | -2.20295 | 8.01E-27 | 6.08E-25 | yes | down | 35.67 | 54.3 | 47 | 22.69 | 18.84 | 27.48 | 45.65667 | 23.00333 |
| SLC25A11 | 0.286 | -1.80746 | 1.12E-26 | 8.44E-25 | yes | down | 60.26 | 85.34 | 67.45 | 47.2 | 51.82 | 41.52 | 71.01667 | 46.84667 |
| C20H14orf142 | 0.216 | -2.21374 | 1.27E-26 | 9.58E-25 | yes | down | 42.85 | 43.13 | 33.64 | 18.09 | 21.24 | 20.7 | 39.87333 | 20.01 |
| PHKA2 | 8.473 | 3.082897 | 1.41E-26 | 1.06E-24 | yes | up | 1.03 | 1.83 | 2 | 30.6 | 22.93 | 40.36 | 1.62 | 31.29667 |
| NDUFB6 | 0.444 | -1.17242 | 1.47E-26 | 1.09E-24 | yes | down | 228.66 | 291.49 | 239.37 | 250.53 | 230.39 | 211.02 | 253.1733 | 230.6467 |
| OGDHL | 5.113 | 2.354088 | 1.55E-26 | 1.15E-24 | yes | up | 3.08 | 3.29 | 4.33 | 41.53 | 48.71 | 36.05 | 3.566667 | 42.09667 |
| PSMC3 | 0.333 | -1.58851 | 1.66E-26 | 1.22E-24 | yes | down | 90.39 | 123.75 | 112.89 | 92.38 | 85.96 | 73.49 | 109.01 | 83.94333 |
| VWA8 | 0.363 | -1.46304 | 1.7E-26 | 1.25E-24 | yes | down | 9.72 | 11.57 | 10.21 | 8.78 | 9.72 | 9.34 | 10.5 | 9.28 |
| FURIN | 0.177 | -2.50019 | 1.71E-26 | 1.25E-24 | yes | down | 63.33 | 73.82 | 112.87 | 31.52 | 32.33 | 36.82 | 83.34 | 33.55667 |
| KYAT3 | 0.299 | -1.74018 | 2.18E-26 | 1.59E-24 | yes | down | 91.33 | 112.15 | 108.52 | 90.96 | 60.18 | 67.51 | 104 | 72.88333 |
| SLC25A20 | 0.325 | -1.6201 | 2.31E-26 | 1.67E-24 | yes | down | 227.53 | 354.97 | 286.12 | 228.29 | 198.38 | 223.13 | 289.54 | 216.6 |
| MRPS22 | 0.358 | -1.48217 | 2.38E-26 | 1.72E-24 | yes | down | 27.4 | 34.35 | 32.14 | 27.92 | 24.11 | 26.45 | 31.29667 | 26.16 |
| ELAC2 | 0.293 | -1.77071 | 3.05E-26 | 2.2E-24 | yes | down | 10.47 | 14.4 | 13.4 | 9.14 | 9.16 | 9.17 | 12.75667 | 9.156667 |
| HYAL1 | 3.063 | 1.61516 | 3.62E-26 | 2.59E-24 | yes | up | 11.73 | 13.99 | 12.13 | 90.2 | 71.71 | 94.04 | 12.61667 | 85.31667 |
| MLYCD | 0.379 | -1.39944 | 3.99E-26 | 2.84E-24 | yes | down | 26.84 | 32.39 | 31.22 | 26.51 | 23.92 | 26.98 | 30.15 | 25.80333 |
| ND1 | 0.103 | -3.2808 | 4.89E-26 | 3.47E-24 | yes | down | 8712.85 | 7446.45 | 8051.58 | 1309.84 | 1569.25 | 2954.95 | 8070.293 | 1944.68 |
| MRPL16 | 0.37 | -1.43512 | 6.23E-26 | 4.41E-24 | yes | down | 65.88 | 84.2 | 79.58 | 63.82 | 59.74 | 68.56 | 76.55333 | 64.04 |
| GSN | 3.168 | 1.663583 | 1.05E-25 | 7.37E-24 | yes | up | 209.4 | 224.58 | 163.73 | 1517.61 | 1397.54 | 1493.06 | 199.2367 | 1469.403 |
| MIEF2 | 0.217 | -2.20215 | 1.07E-25 | 7.53E-24 | yes | down | 7.06 | 10.2 | 8.21 | 3.86 | 4.07 | 4.56 | 8.49 | 4.163333 |
| SOWAHC | 0.135 | -2.88747 | 1.32E-25 | 9.18E-24 | yes | down | 15.54 | 21.29 | 32.79 | 7.9 | 5.87 | 8.27 | 23.20667 | 7.346667 |
| AFG3L2 | 0.346 | -1.53226 | 1.56E-25 | 1.08E-23 | yes | down | 29.46 | 35.89 | 34 | 24.49 | 26.32 | 28.68 | 33.11667 | 26.49667 |
| PRR5L | 6.869 | 2.780133 | 1.79E-25 | 1.24E-23 | yes | up | 2.11 | 1.56 | 2.54 | 30.96 | 40.68 | 28.17 | 2.07 | 33.27 |
| MRPL46 | 0.28 | -1.83624 | 1.81E-25 | 1.25E-23 | yes | down | 11.88 | 17.21 | 14.74 | 9.24 | 9.21 | 9.93 | 14.61 | 9.46 |
| SNRPB2 | 0.33 | -1.60151 | 2.18E-25 | 1.49E-23 | yes | down | 71.59 | 70.46 | 71.38 | 53.08 | 52.51 | 59.2 | 71.14333 | 54.93 |
| RBM3 | 0.311 | -1.6857 | 2.58E-25 | 1.77E-23 | yes | down | 109.99 | 151.46 | 104.35 | 76.13 | 75.96 | 58.5 | 121.9333 | 70.19667 |
| GPX3 | 0.084 | -3.57507 | 2.85E-25 | 1.94E-23 | yes | down | 802.22 | 880.23 | 952.83 | 99.11 | 261.7 | 142.24 | 878.4267 | 167.6833 |
| RASGRF2 | 3.507 | 1.810091 | 3E-25 | 2.03E-23 | yes | up | 1.31 | 1.07 | 1.18 | 10.14 | 7.73 | 9.26 | 1.186667 | 9.043333 |
| HSD17B10 | 0.322 | -1.63303 | 3.71E-25 | 2.51E-23 | yes | down | 155.1 | 208.72 | 163.16 | 157.71 | 109.27 | 130.03 | 175.66 | 132.3367 |
| BCAM | 4.286 | 2.099515 | 4.06E-25 | 2.73E-23 | yes | up | 29.44 | 34.71 | 25.75 | 248.04 | 289.05 | 354.24 | 29.96667 | 297.11 |
| OLFML1 | 6.562 | 2.714229 | 5.1E-25 | 3.41E-23 | yes | up | 2.72 | 4.64 | 4.22 | 49.21 | 72.65 | 51.05 | 3.86 | 57.63667 |
| LOC100338015 | 5.092 | 2.348369 | 5.44E-25 | 3.63E-23 | yes | up | 13.75 | 26.33 | 21.88 | 290.86 | 234.19 | 203.61 | 20.65333 | 242.8867 |
| ATP5J2 | 0.387 | -1.37015 | 5.62E-25 | 3.74E-23 | yes | down | 709.54 | 896.5 | 709.69 | 789.75 | 606.33 | 729.49 | 771.91 | 708.5233 |
| LOC103346934 | 0.124 | -3.00724 | 8.54E-25 | 5.65E-23 | yes | down | 2.81 | 4.25 | 3.55 | 1.21 | 0.94 | 1 | 3.536667 | 1.05 |
| PHB2 | 0.352 | -1.50588 | 1.12E-24 | 7.41E-23 | yes | down | 143.17 | 219.5 | 180 | 154.33 | 141.67 | 145.78 | 180.89 | 147.26 |
| EIF2B3 | 0.332 | -1.58948 | 1.15E-24 | 7.53E-23 | yes | down | 19.02 | 21.28 | 20.92 | 15.35 | 14.84 | 16.98 | 20.40667 | 15.72333 |
| TOMM40L | 0.296 | -1.75482 | 1.15E-24 | 7.53E-23 | yes | down | 17.19 | 22.23 | 24.74 | 16.2 | 13.77 | 16.71 | 21.38667 | 15.56 |
| NMNAT3 | 0.291 | -1.78111 | 1.45E-24 | 9.43E-23 | yes | down | 25.82 | 26.73 | 29.11 | 20.5 | 15.64 | 19.82 | 27.22 | 18.65333 |
| ALOX15 | 0 | -12.6735 | 1.92E-24 | 1.24E-22 | yes | down | 11.63 | 29.94 | 37.81 | 0.03 | 0 | 0 | 26.46 | 0.01 |
| OXA1L | 0.301 | -1.73447 | 2.21E-24 | 1.43E-22 | yes | down | 70.95 | 98 | 89.27 | 54.28 | 62.04 | 65.01 | 86.07333 | 60.44333 |
| GPD2 | 0.065 | -3.95051 | 2.23E-24 | 1.44E-22 | yes | down | 140.08 | 166.79 | 168.88 | 14.7 | 16 | 40.98 | 158.5833 | 23.89333 |
| ATP5F1 | 0.281 | -1.82948 | 2.54E-24 | 1.63E-22 | yes | down | 388.38 | 562.19 | 458.45 | 343.75 | 327.23 | 245.73 | 469.6733 | 305.57 |
| GK | 0.142 | -2.81283 | 3.06E-24 | 1.96E-22 | yes | down | 16.45 | 31.02 | 18.44 | 5.02 | 4.39 | 7.86 | 21.97 | 5.756667 |
| SSPO | 14.305 | 3.838439 | 4.09E-24 | 2.6E-22 | yes | up | 0.18 | 0.11 | 0.1 | 3.79 | 2.51 | 6.22 | 0.13 | 4.173333 |
| LOC103350566 | 0.05 | -4.32399 | 4.28E-24 | 2.71E-22 | yes | down | 11.01 | 15.61 | 14.18 | 3.02 | 0.46 | 1.26 | 13.6 | 1.58 |
| MRPS7 | 0.355 | -1.49316 | 5.39E-24 | 3.4E-22 | yes | down | 58.63 | 75.42 | 60.5 | 64.07 | 50.62 | 45.97 | 64.85 | 53.55333 |
| BTC | 36.648 | 5.195678 | 5.66E-24 | 3.56E-22 | yes | up | 0.09 | 0.17 | 0.08 | 6.77 | 12.52 | 12.35 | 0.113333 | 10.54667 |
| COQ7 | 0.298 | -1.74616 | 6.91E-24 | 4.33E-22 | yes | down | 35.06 | 52.24 | 42.66 | 34.07 | 27.37 | 28.82 | 43.32 | 30.08667 |
| SLC25A13 | 0.341 | -1.5505 | 7.34E-24 | 4.59E-22 | yes | down | 20.02 | 25.78 | 20.86 | 19.77 | 14.41 | 18.27 | 22.22 | 17.48333 |
| ENO1 | 0.331 | -1.59483 | 1.04E-23 | 6.44E-22 | yes | down | 269.69 | 380.38 | 353.93 | 255.04 | 224.54 | 290.44 | 334.6667 | 256.6733 |
| MRPS36 | 0.337 | -1.56717 | 1.33E-23 | 8.26E-22 | yes | down | 56.33 | 73.76 | 64.86 | 54.31 | 52.96 | 42.47 | 64.98333 | 49.91333 |
| HIF3A | 0.13 | -2.94685 | 1.37E-23 | 8.47E-22 | yes | down | 5.47 | 3.45 | 4.88 | 1.92 | 1.32 | 1.4 | 4.6 | 1.546667 |
| LOC100340645 | 0.349 | -1.51733 | 1.51E-23 | 9.31E-22 | yes | down | 1648.46 | 1708.27 | 1324.83 | 1447.61 | 1237.04 | 1206.12 | 1560.52 | 1296.923 |
| IGF1 | 3.19 | 1.673565 | 1.54E-23 | 9.44E-22 | yes | up | 18.48 | 17.73 | 17.49 | 123.5 | 145.41 | 126.21 | 17.9 | 131.7067 |
| COQ5 | 0.329 | -1.60168 | 2.03E-23 | 1.24E-21 | yes | down | 27.37 | 40.35 | 38.19 | 17.85 | 17.89 | 18.81 | 35.30333 | 18.18333 |
| LOC100356386 | 0.001 | -10.5226 | 2.74E-23 | 1.66E-21 | yes | down | 24.95 | 20.82 | 32.64 | 0.13 | 0 | 0 | 26.13667 | 0.043333 |
| ANKRD31 | 0.102 | -3.28973 | 3.21E-23 | 1.95E-21 | yes | down | 15.99 | 8.32 | 11.31 | 2.71 | 2.32 | 3.9 | 11.87333 | 2.976667 |
| TIMM8A | 0.178 | -2.48828 | 4.73E-23 | 2.86E-21 | yes | down | 31.12 | 38.54 | 38.9 | 10.74 | 17.84 | 16.08 | 36.18667 | 14.88667 |
| CLPX | 0.364 | -1.45616 | 5.35E-23 | 3.22E-21 | yes | down | 39.28 | 54.91 | 50.01 | 37.78 | 37.12 | 40.66 | 48.06667 | 38.52 |
| FAM149A | 4.623 | 2.208884 | 6.17E-23 | 3.7E-21 | yes | up | 3.07 | 4.59 | 4.08 | 37.22 | 52.66 | 44.26 | 3.913333 | 44.71333 |
| EBP | 5.45 | 2.446311 | 6.45E-23 | 3.86E-21 | yes | up | 30.36 | 45.91 | 34.82 | 630.56 | 304.05 | 483.85 | 37.03 | 472.82 |
| MRPS18B | 0.263 | -1.92863 | 7.29E-23 | 4.34E-21 | yes | down | 31.85 | 52.27 | 39.79 | 25.63 | 26.62 | 22.94 | 41.30333 | 25.06333 |
| DDAH2 | 2.481 | 1.310666 | 9.8E-23 | 5.81E-21 | yes | up | 19.08 | 26.12 | 19.72 | 134.33 | 118.35 | 126.19 | 21.64 | 126.29 |
| PLXNB3 | 0.246 | -2.0262 | 9.89E-23 | 5.85E-21 | yes | down | 2.32 | 2.24 | 2.31 | 1.53 | 1.1 | 1.31 | 2.29 | 1.313333 |
| APOO | 0.326 | -1.6188 | 1.12E-22 | 6.6E-21 | yes | down | 34.89 | 50.38 | 39.34 | 32.17 | 32.31 | 30.23 | 41.53667 | 31.57 |
| NDUFB9 | 0.473 | -1.07972 | 1.36E-22 | 7.97E-21 | yes | down | 474.99 | 599.99 | 524.46 | 610.54 | 533.62 | 534.92 | 533.1467 | 559.6933 |
| IL1RN | 0.094 | -3.41729 | 1.47E-22 | 8.57E-21 | yes | down | 10.76 | 6.77 | 8.82 | 1.68 | 1.53 | 2.65 | 8.783333 | 1.953333 |
| ITPK1 | 0.319 | -1.64738 | 1.51E-22 | 8.82E-21 | yes | down | 24 | 36.01 | 28.56 | 21.07 | 19.98 | 24.23 | 29.52333 | 21.76 |
| TMBIM1 | 2.645 | 1.4031 | 1.84E-22 | 1.07E-20 | yes | up | 28.75 | 33.74 | 27.92 | 173.01 | 189.21 | 191.65 | 30.13667 | 184.6233 |
| LOC108176024 | 0.17 | -2.5573 | 1.88E-22 | 1.09E-20 | yes | down | 18.64 | 21.06 | 29.82 | 7.87 | 7.24 | 11.04 | 23.17333 | 8.716667 |
| CIB2 | 0.375 | -1.41455 | 2.5E-22 | 1.44E-20 | yes | down | 97.93 | 135.54 | 103.93 | 97 | 93.68 | 103.28 | 112.4667 | 97.98667 |
| MIPEP | 0.3 | -1.73693 | 3.78E-22 | 2.17E-20 | yes | down | 13.52 | 19.95 | 17.24 | 10.7 | 12.1 | 12.22 | 16.90333 | 11.67333 |
| ATP5O | 0.468 | -1.09626 | 5.42E-22 | 3.11E-20 | yes | down | 1027.04 | 1338.36 | 1094.39 | 1312.11 | 1178.59 | 1301.97 | 1153.263 | 1264.223 |
| SMOC1 | 0.223 | -2.1622 | 8.24E-22 | 4.7E-20 | yes | down | 22.89 | 37.82 | 31.78 | 12.16 | 17.4 | 16.11 | 30.83 | 15.22333 |
| POLN | 22.585 | 4.497268 | 8.73E-22 | 4.96E-20 | yes | up | 0.13 | 0.13 | 0.11 | 23.09 | 8.75 | 10.79 | 0.123333 | 14.21 |
| NPC2 | 3.614 | 1.85345 | 1.14E-21 | 6.44E-20 | yes | up | 40.16 | 32.39 | 44.5 | 329.96 | 326.49 | 338.08 | 39.01667 | 331.51 |
| NDUFV2 | 0.38 | -1.3969 | 1.25E-21 | 7.07E-20 | yes | down | 176.7 | 237.97 | 187.18 | 187.34 | 184.57 | 159.83 | 200.6167 | 177.2467 |
| PPTC7 | 0.232 | -2.10992 | 1.38E-21 | 7.76E-20 | yes | down | 16.37 | 17.29 | 16.38 | 7.37 | 7.87 | 11.62 | 16.68 | 8.953333 |
| MRPL49 | 0.459 | -1.12304 | 1.68E-21 | 9.4E-20 | yes | down | 54.18 | 59.34 | 54.86 | 66.31 | 57.23 | 56.86 | 56.12667 | 60.13333 |
| LOC100345430 | 0.003 | -8.3291 | 2.02E-21 | 1.12E-19 | yes | down | 22.13 | 21.74 | 19.2 | 0.5 | 0 | 0 | 21.02333 | 0.166667 |
| RFK | 0.204 | -2.29221 | 2.02E-21 | 1.12E-19 | yes | down | 16.9 | 16.8 | 23.23 | 7.31 | 9.15 | 10.52 | 18.97667 | 8.993333 |
| MRPL43 | 0.34 | -1.55629 | 2.48E-21 | 1.38E-19 | yes | down | 20.47 | 26.4 | 24.41 | 20.12 | 18.22 | 18.18 | 23.76 | 18.84 |
| SRPX | 3.887 | 1.95858 | 2.5E-21 | 1.38E-19 | yes | up | 6.89 | 10.68 | 8.08 | 69.52 | 87.1 | 72.66 | 8.55 | 76.42667 |
| IARS2 | 0.392 | -1.34964 | 2.56E-21 | 1.41E-19 | yes | down | 26.5 | 35.64 | 33.11 | 27.13 | 27.38 | 26.58 | 31.75 | 27.03 |
| NDUFS2 | 0.395 | -1.33894 | 2.93E-21 | 1.61E-19 | yes | down | 265.06 | 400.28 | 312.05 | 319.75 | 281.02 | 292.72 | 325.7967 | 297.83 |
| ECHS1 | 0.475 | -1.07451 | 3.01E-21 | 1.65E-19 | yes | down | 481.97 | 533.4 | 455.16 | 593.93 | 516.07 | 522.05 | 490.1767 | 544.0167 |
| BDH1 | 0.323 | -1.63017 | 3.14E-21 | 1.71E-19 | yes | down | 87.58 | 120.21 | 86.39 | 78.91 | 62.5 | 91.34 | 98.06 | 77.58333 |
| RCOR2 | 0.138 | -2.85566 | 3.26E-21 | 1.77E-19 | yes | down | 11.33 | 11.58 | 16.62 | 3.12 | 4.45 | 5.12 | 13.17667 | 4.23 |
| MRPL42 | 0.4 | -1.32073 | 3.56E-21 | 1.93E-19 | yes | down | 27.75 | 33.36 | 30.93 | 31.26 | 25.84 | 29.09 | 30.68 | 28.73 |
| MFN1 | 0.319 | -1.6491 | 4.41E-21 | 2.38E-19 | yes | down | 27.52 | 33.92 | 32.88 | 19.35 | 24.66 | 23.72 | 31.44 | 22.57667 |
| SIRT3 | 0.399 | -1.326 | 6.31E-21 | 3.4E-19 | yes | down | 30.17 | 36.92 | 31.61 | 32.55 | 28.67 | 34.56 | 32.9 | 31.92667 |
| PRKAG1 | 0.414 | -1.27281 | 7.62E-21 | 4.09E-19 | yes | down | 36.54 | 48.16 | 40.14 | 40.41 | 39.84 | 38.37 | 41.61333 | 39.54 |
| ELOVL2 | 0.098 | -3.35761 | 7.8E-21 | 4.16E-19 | yes | down | 13.56 | 13.39 | 16.35 | 3.83 | 4.48 | 1.52 | 14.43333 | 3.276667 |
| LOC108177262 | 0.417 | -1.2627 | 7.8E-21 | 4.16E-19 | yes | down | 1413.12 | 1845.65 | 1423.56 | 1714.22 | 1512.29 | 1409.53 | 1560.777 | 1545.347 |
| PRRG4 | 0.171 | -2.54585 | 7.83E-21 | 4.17E-19 | yes | down | 4.11 | 6.93 | 4.76 | 1.83 | 1.64 | 2.77 | 5.266667 | 2.08 |
| NKIRAS2 | 0.309 | -1.69469 | 8.7E-21 | 4.62E-19 | yes | down | 45.17 | 50.17 | 54.28 | 37.05 | 28.38 | 40.24 | 49.87333 | 35.22333 |
| TRAP1 | 0.356 | -1.48848 | 9.12E-21 | 4.82E-19 | yes | down | 70.82 | 93.4 | 67.21 | 63.78 | 65.45 | 62.35 | 77.14333 | 63.86 |
| CA5B | 4.341 | 2.118049 | 9.4E-21 | 4.96E-19 | yes | up | 2.88 | 5.36 | 4.17 | 35.36 | 44.61 | 41.59 | 4.136667 | 40.52 |
| DNAJC11 | 0.323 | -1.6294 | 9.7E-21 | 5.1E-19 | yes | down | 34.12 | 49.68 | 48.84 | 30.65 | 33.06 | 35.09 | 44.21333 | 32.93333 |
| LRPPRC | 0.352 | -1.50752 | 1.05E-20 | 5.51E-19 | yes | down | 30.55 | 48.71 | 37.29 | 34.55 | 35.18 | 36.28 | 38.85 | 35.33667 |
| ATP5E | 0.452 | -1.1471 | 1.3E-20 | 6.79E-19 | yes | down | 1908.24 | 2195.82 | 1805.37 | 2273.88 | 2136.25 | 1956.72 | 1969.81 | 2122.283 |
| GLRX | 0.16 | -2.6463 | 1.45E-20 | 7.55E-19 | yes | down | 46.7 | 52.6 | 92.25 | 25.47 | 26.19 | 20.95 | 63.85 | 24.20333 |
| HIGD2A | 0.284 | -1.81624 | 1.47E-20 | 7.62E-19 | yes | down | 62.88 | 78.36 | 69.55 | 51.58 | 35.41 | 54.11 | 70.26333 | 47.03333 |
| LOC103346165 | 3.676 | 1.878124 | 1.51E-20 | 7.78E-19 | yes | up | 0.58 | 0.61 | 1.07 | 3.88 | 3.53 | 3.57 | 0.753333 | 3.66 |
| FUS | 0.398 | -1.32871 | 1.59E-20 | 8.17E-19 | yes | down | 75.18 | 80.84 | 79.12 | 65.52 | 55.81 | 70.86 | 78.38 | 64.06333 |
| MRPS30 | 0.347 | -1.52886 | 1.61E-20 | 8.28E-19 | yes | down | 14.55 | 19.81 | 14.64 | 13.75 | 12.47 | 13.11 | 16.33333 | 13.11 |
| CPT1B | 0.143 | -2.80476 | 2.02E-20 | 1.03E-18 | yes | down | 185.88 | 246.03 | 223.69 | 68.77 | 39.99 | 107.13 | 218.5333 | 71.96333 |
| LOC100342996 | 0.423 | -1.24267 | 2.02E-20 | 1.03E-18 | yes | down | 773.4 | 1029.47 | 833.01 | 1016.78 | 798.14 | 827.12 | 878.6267 | 880.68 |
| SOD2 | 0.282 | -1.82499 | 2.37E-20 | 1.21E-18 | yes | down | 316.82 | 447.24 | 393.85 | 202.32 | 268.22 | 279.72 | 385.97 | 250.0867 |
| SUPV3L1 | 0.331 | -1.59719 | 2.58E-20 | 1.31E-18 | yes | down | 14.19 | 19.24 | 16.5 | 12.37 | 13.95 | 11.94 | 16.64333 | 12.75333 |
| FAM210A | 0.316 | -1.66034 | 2.62E-20 | 1.33E-18 | yes | down | 11.68 | 16.7 | 14.94 | 9.38 | 11.11 | 11.55 | 14.44 | 10.68 |
| CD5L | 46.465 | 5.538059 | 3.39E-20 | 1.71E-18 | yes | up | 0.21 | 0.07 | 0.3 | 24.16 | 13.57 | 26.18 | 0.193333 | 21.30333 |
| ACAA1 | 0.349 | -1.51959 | 3.63E-20 | 1.83E-18 | yes | down | 34.58 | 50.23 | 44.25 | 35.79 | 36.82 | 31.5 | 43.02 | 34.70333 |
| MRPS9 | 0.343 | -1.54482 | 4.01E-20 | 2.01E-18 | yes | down | 78.09 | 115.69 | 84.84 | 68.7 | 74.41 | 76.96 | 92.87333 | 73.35667 |
| ACSS1 | 0.187 | -2.4177 | 4.53E-20 | 2.27E-18 | yes | down | 62.78 | 57.54 | 34.87 | 26.42 | 23.38 | 18.21 | 51.73 | 22.67 |
| MRPL41 | 0.261 | -1.93517 | 5.84E-20 | 2.91E-18 | yes | down | 81.35 | 92.82 | 61.37 | 57.47 | 43.94 | 44.14 | 78.51333 | 48.51667 |
| HADHA | 0.175 | -2.51417 | 6.1E-20 | 3.03E-18 | yes | down | 599.23 | 730.92 | 634.7 | 254.33 | 164.82 | 381.33 | 654.95 | 266.8267 |
| TENM2 | 18.915 | 4.241473 | 6.32E-20 | 3.13E-18 | yes | up | 0.04 | 0.08 | 0.06 | 1.83 | 2.12 | 4.21 | 0.06 | 2.72 |
| HBQ1 | 6.476 | 2.695186 | 6.33E-20 | 3.13E-18 | yes | up | 6.26 | 11.96 | 8.09 | 59.8 | 41.7 | 34.89 | 8.77 | 45.46333 |
| C7 | 7.888 | 2.979606 | 6.78E-20 | 3.33E-18 | yes | up | 2.99 | 4.89 | 2.99 | 51.95 | 100.68 | 51.76 | 3.623333 | 68.13 |
| LOC100345711 | 0.446 | -1.16447 | 6.78E-20 | 3.33E-18 | yes | down | 56.89 | 72.69 | 63.7 | 73.54 | 62.77 | 65.09 | 64.42667 | 67.13333 |
| LOC100343982 | 0.372 | -1.42706 | 7.36E-20 | 3.6E-18 | yes | down | 967.68 | 1196.65 | 829.19 | 963.09 | 857 | 798.97 | 997.84 | 873.02 |
| MIF | 0.438 | -1.19039 | 8.21E-20 | 4.01E-18 | yes | down | 129.39 | 166.23 | 147.16 | 161.11 | 149.84 | 145.86 | 147.5933 | 152.27 |
| PDHB | 0.401 | -1.32007 | 8.4E-20 | 4.09E-18 | yes | down | 165.91 | 242.37 | 206.68 | 198.48 | 194.8 | 176.35 | 204.9867 | 189.8767 |
| PANK3 | 2.511 | 1.328184 | 8.7E-20 | 4.22E-18 | yes | up | 3.22 | 3.69 | 3.82 | 19.92 | 20.33 | 22.15 | 3.576667 | 20.8 |
| INSIG1 | 9.427 | 3.236849 | 1.05E-19 | 5.09E-18 | yes | up | 11.15 | 10.66 | 7.64 | 254.44 | 105.32 | 324.82 | 9.816667 | 228.1933 |
| SUCLA2 | 0.197 | -2.34104 | 1.09E-19 | 5.27E-18 | yes | down | 335.46 | 485.89 | 389.75 | 130.98 | 170.39 | 247.99 | 403.7 | 183.12 |
| DPP4 | 0.22 | -2.18718 | 1.12E-19 | 5.41E-18 | yes | down | 15.53 | 20.42 | 22.67 | 7.66 | 9.66 | 12.33 | 19.54 | 9.883333 |
| CYP2B4 | 81.426 | 6.347415 | 1.39E-19 | 6.68E-18 | yes | up | 1.24 | 7.07 | 5.56 | 510.43 | 893.72 | 1084.18 | 4.623333 | 829.4433 |
| ALDH2 | 4.703 | 2.233555 | 1.42E-19 | 6.8E-18 | yes | up | 47.96 | 70.41 | 32.39 | 579.19 | 507.22 | 549.66 | 50.25333 | 545.3567 |
| NDUFA4 | 0.434 | -1.20452 | 1.7E-19 | 8.13E-18 | yes | down | 987.09 | 1145.86 | 923.43 | 1030.2 | 1082.81 | 1012.86 | 1018.793 | 1041.957 |
| ATP1A1 | 0.315 | -1.66574 | 2.33E-19 | 1.11E-17 | yes | down | 141.64 | 224.13 | 186.78 | 123.23 | 122.72 | 155.1 | 184.1833 | 133.6833 |
| GSTP1 | 2.265 | 1.179709 | 2.38E-19 | 1.13E-17 | yes | up | 77.48 | 79.93 | 80.89 | 452.12 | 424.81 | 390.12 | 79.43333 | 422.35 |
| NAT8L | 7.198 | 2.847583 | 2.81E-19 | 1.33E-17 | yes | up | 1.03 | 1.68 | 2.36 | 26.58 | 23.48 | 32.45 | 1.69 | 27.50333 |
| PSPC1 | 0.339 | -1.55959 | 2.96E-19 | 1.39E-17 | yes | down | 16.46 | 20.48 | 14.99 | 14.91 | 11.34 | 14.21 | 17.31 | 13.48667 |
| LOC103347528 | 0.293 | -1.77048 | 2.98E-19 | 1.4E-17 | yes | down | 30.04 | 44.2 | 42.63 | 25.98 | 24.02 | 29.71 | 38.95667 | 26.57 |
| TMEM143 | 0.268 | -1.89836 | 3.01E-19 | 1.41E-17 | yes | down | 22.54 | 25.63 | 29.6 | 14.92 | 12.55 | 19.82 | 25.92333 | 15.76333 |
| CPEB1 | 6.219 | 2.6367 | 3.46E-19 | 1.62E-17 | yes | up | 0.54 | 0.59 | 0.53 | 6.18 | 8.52 | 9.13 | 0.553333 | 7.943333 |
| MRPL47 | 0.359 | -1.47963 | 3.79E-19 | 1.77E-17 | yes | down | 38.38 | 45.6 | 46.46 | 34.97 | 37.67 | 35.03 | 43.48 | 35.89 |
| TMEM177 | 0.154 | -2.69815 | 3.97E-19 | 1.84E-17 | yes | down | 6.59 | 11.59 | 11.08 | 3.15 | 2.5 | 4.79 | 9.753333 | 3.48 |
| GABARAPL2 | 0.338 | -1.56676 | 4.13E-19 | 1.91E-17 | yes | down | 164.81 | 184.93 | 198.47 | 123.52 | 153.55 | 151.33 | 182.7367 | 142.8 |
| EPHX2 | 0.356 | -1.48942 | 4.14E-19 | 1.91E-17 | yes | down | 245.88 | 363.84 | 312.57 | 244.16 | 224.78 | 290.01 | 307.43 | 252.9833 |
| KCNQ1 | 0.162 | -2.62539 | 4.34E-19 | 2E-17 | yes | down | 1.97 | 2.1 | 1.86 | 1.46 | 0.87 | 1.06 | 1.976667 | 1.13 |
| ANK2 | 4.131 | 2.046487 | 5.13E-19 | 2.36E-17 | yes | up | 8.77 | 9.7 | 7.56 | 76.38 | 126.38 | 92.57 | 8.676667 | 98.44333 |
| RRAGB | 0.176 | -2.50492 | 5.17E-19 | 2.37E-17 | yes | down | 2.9 | 3.96 | 2.84 | 1.45 | 1.23 | 1.28 | 3.233333 | 1.32 |
| MRPS10 | 0.346 | -1.53074 | 6.07E-19 | 2.78E-17 | yes | down | 28.96 | 31.28 | 34.06 | 20.87 | 16.75 | 22.89 | 31.43333 | 20.17 |
| RMDN3 | 0.343 | -1.54211 | 7.05E-19 | 3.21E-17 | yes | down | 71.11 | 81.17 | 94.86 | 73.34 | 54.73 | 72.3 | 82.38 | 66.79 |
| KIRREL | 2.623 | 1.390944 | 7.22E-19 | 3.28E-17 | yes | up | 3.32 | 4.21 | 3.42 | 27.16 | 18.38 | 23.54 | 3.65 | 23.02667 |
| SEC11C | 0.421 | -1.2484 | 7.31E-19 | 3.31E-17 | yes | down | 104.9 | 146.82 | 113.33 | 122.5 | 115.85 | 119.38 | 121.6833 | 119.2433 |
| RDH13 | 0.096 | -3.38199 | 7.54E-19 | 3.41E-17 | yes | down | 5.03 | 10.85 | 12.15 | 2.91 | 1.71 | 1.54 | 9.343333 | 2.053333 |
| MRPL4 | 0.217 | -2.20273 | 7.67E-19 | 3.46E-17 | yes | down | 20.54 | 23.61 | 20.22 | 13.76 | 9.54 | 9.68 | 21.45667 | 10.99333 |
| SERPINF1 | 3.864 | 1.950257 | 8.3E-19 | 3.73E-17 | yes | up | 76.44 | 102.64 | 69.31 | 600.69 | 878.44 | 761.43 | 82.79667 | 746.8533 |
| FEZ1 | 0.139 | -2.8453 | 8.84E-19 | 3.96E-17 | yes | down | 56.97 | 63.49 | 86.52 | 16.03 | 16.45 | 32.91 | 68.99333 | 21.79667 |
| PCCB | 0.445 | -1.16967 | 9.67E-19 | 4.33E-17 | yes | down | 66.77 | 82.3 | 72.71 | 81.84 | 65.1 | 82.46 | 73.92667 | 76.46667 |
| TOMM22 | 0.331 | -1.5948 | 9.93E-19 | 4.43E-17 | yes | down | 39.14 | 48.95 | 48.73 | 32.66 | 34.12 | 39.05 | 45.60667 | 35.27667 |
| KDELC2 | 2.429 | 1.280388 | 1.28E-18 | 5.69E-17 | yes | up | 3.83 | 5.6 | 4.36 | 28.15 | 22.37 | 26.52 | 4.596667 | 25.68 |
| RDH5 | 12.603 | 3.655698 | 1.4E-18 | 6.2E-17 | yes | up | 0.22 | 0.21 | 0.3 | 7.36 | 11.43 | 8.23 | 0.243333 | 9.006667 |
| MRPL1 | 0.385 | -1.37649 | 1.48E-18 | 6.56E-17 | yes | down | 16.78 | 23.37 | 21.75 | 20.09 | 17.89 | 17.35 | 20.63333 | 18.44333 |
| PPARGC1A | 0.158 | -2.66312 | 1.52E-18 | 6.72E-17 | yes | down | 3.02 | 3.95 | 2.03 | 0.88 | 1.07 | 1.32 | 3 | 1.09 |
| MRPL3 | 0.455 | -1.13616 | 1.59E-18 | 7.01E-17 | yes | down | 31.05 | 36.66 | 33.73 | 35.25 | 34.43 | 38.62 | 33.81333 | 36.1 |
| LOC100356833 | 0.211 | -2.24262 | 1.64E-18 | 7.19E-17 | yes | down | 7.58 | 9.39 | 6.4 | 2.89 | 3.84 | 4.64 | 7.79 | 3.79 |
| ATP6V0E2 | 15.242 | 3.930025 | 1.71E-18 | 7.5E-17 | yes | up | 1.13 | 1.79 | 2.07 | 60.61 | 24.18 | 103.82 | 1.663333 | 62.87 |
| NWD2 | 0.102 | -3.29001 | 1.76E-18 | 7.69E-17 | yes | down | 6.04 | 6.74 | 9.13 | 3.83 | 1.23 | 3.39 | 7.303333 | 2.816667 |
| MDH1 | 0.268 | -1.89772 | 1.99E-18 | 8.68E-17 | yes | down | 428.34 | 614.43 | 493.01 | 432.34 | 231.73 | 294.52 | 511.9267 | 319.53 |
| FST | 3.7 | 1.887392 | 2.31E-18 | 1E-16 | yes | up | 5.78 | 5.17 | 4.79 | 50.36 | 49.24 | 40.26 | 5.246667 | 46.62 |
| MPPE1 | 0.344 | -1.53767 | 2.33E-18 | 1.01E-16 | yes | down | 15.02 | 20.85 | 16.25 | 13.24 | 14.67 | 11.56 | 17.37333 | 13.15667 |
| NDUFB11 | 0.463 | -1.11035 | 2.35E-18 | 1.02E-16 | yes | down | 185.75 | 238.19 | 185.01 | 230.06 | 215.52 | 212.47 | 202.9833 | 219.35 |
| FLOT2 | 2.717 | 1.441983 | 2.56E-18 | 1.1E-16 | yes | up | 19.35 | 29.37 | 20.37 | 147.15 | 155.63 | 138.43 | 23.03 | 147.07 |
| PHOSPHO1 | 0.069 | -3.84993 | 2.75E-18 | 1.18E-16 | yes | down | 19.96 | 36.67 | 21.15 | 2.34 | 7.41 | 2.88 | 25.92667 | 4.21 |
| LOC100340711 | 3.765 | 1.912637 | 2.81E-18 | 1.21E-16 | yes | up | 4.98 | 7.78 | 5.03 | 57.55 | 57.56 | 45.17 | 5.93 | 53.42667 |
| LOC100347623 | 13.563 | 3.76159 | 2.89E-18 | 1.24E-16 | yes | up | 0.61 | 0.76 | 2.26 | 36.23 | 43.5 | 35.84 | 1.21 | 38.52333 |
| GRIK3 | 0.034 | -4.88373 | 3.07E-18 | 1.31E-16 | yes | down | 1.28 | 0.73 | 3.24 | 0.12 | 0.13 | 0.17 | 1.75 | 0.14 |
| MTUS2 | 5.099 | 2.350272 | 3.26E-18 | 1.39E-16 | yes | up | 0.97 | 2.13 | 1.84 | 16.58 | 20.52 | 18.42 | 1.646667 | 18.50667 |
| ALKBH3 | 0.385 | -1.37525 | 3.36E-18 | 1.42E-16 | yes | down | 10.78 | 13.77 | 11.44 | 11.15 | 9.64 | 9.17 | 11.99667 | 9.986667 |
| DUSP4 | 0.054 | -4.21817 | 3.4E-18 | 1.44E-16 | yes | down | 5.8 | 3.68 | 2.17 | 0.63 | 0.34 | 0.51 | 3.883333 | 0.493333 |
| TXN2 | 0.298 | -1.74779 | 3.63E-18 | 1.53E-16 | yes | down | 88.13 | 103.52 | 81.76 | 94.56 | 58.41 | 61.38 | 91.13667 | 71.45 |
| PPARGC1B | 0.199 | -2.32564 | 3.63E-18 | 1.53E-16 | yes | down | 12.76 | 16.77 | 18.26 | 6.14 | 5.45 | 10.48 | 15.93 | 7.356667 |
| EPB41L3 | 3.801 | 1.926314 | 3.92E-18 | 1.65E-16 | yes | up | 1.5 | 2.66 | 1.83 | 20 | 17.9 | 14.92 | 1.996667 | 17.60667 |
| GLDN | 0.061 | -4.03446 | 3.94E-18 | 1.65E-16 | yes | down | 2.67 | 7.03 | 3.05 | 0.32 | 0.88 | 0.54 | 4.25 | 0.58 |
| CES3 | 0.144 | -2.79144 | 4.27E-18 | 1.79E-16 | yes | down | 73.06 | 94.39 | 77.88 | 25.12 | 15.15 | 42.14 | 81.77667 | 27.47 |
| NDUFA5 | 0.272 | -1.87862 | 4.59E-18 | 1.91E-16 | yes | down | 129.23 | 156 | 137.69 | 148.6 | 124.21 | 111.61 | 140.9733 | 128.14 |
| FAM162A | 0.421 | -1.24771 | 4.82E-18 | 2E-16 | yes | down | 42.25 | 56.84 | 45.01 | 50.25 | 47.73 | 42.58 | 48.03333 | 46.85333 |
| ATP5G1 | 0.198 | -2.33336 | 4.88E-18 | 2.03E-16 | yes | down | 235.18 | 366.38 | 200.61 | 128.65 | 151.32 | 93.97 | 267.39 | 124.6467 |
| MRM3 | 0.41 | -1.28761 | 4.94E-18 | 2.04E-16 | yes | down | 8.66 | 10.34 | 9.91 | 9.93 | 7.99 | 9.62 | 9.636667 | 9.18 |
| LOC100343920 | 2.971 | 1.570878 | 5.4E-18 | 2.23E-16 | yes | up | 5.33 | 8.48 | 8.65 | 59.21 | 46.46 | 49.99 | 7.486667 | 51.88667 |
| C4H12orf73 | 0.294 | -1.76597 | 5.53E-18 | 2.28E-16 | yes | down | 17.55 | 25.86 | 25.69 | 18.94 | 22.7 | 21.55 | 23.03333 | 21.06333 |
| D2HGDH | 0.378 | -1.40442 | 6.24E-18 | 2.56E-16 | yes | down | 17.26 | 25.61 | 21.31 | 18.87 | 15.51 | 17.66 | 21.39333 | 17.34667 |
| MRPS26 | 0.34 | -1.55846 | 6.89E-18 | 2.82E-16 | yes | down | 55.17 | 58.69 | 51.3 | 39.18 | 48.56 | 42.85 | 55.05333 | 43.53 |
| AIFM2 | 0.441 | -1.18177 | 7.08E-18 | 2.89E-16 | yes | down | 381.75 | 507.28 | 483.21 | 503.66 | 410.11 | 485.44 | 457.4133 | 466.4033 |
| BNIP3 | 0.429 | -1.22194 | 8.05E-18 | 3.28E-16 | yes | down | 164.56 | 178.38 | 158.36 | 153.62 | 164.93 | 174.94 | 167.1 | 164.4967 |
| NDUFS5 | 0.459 | -1.12245 | 8.47E-18 | 3.44E-16 | yes | down | 212.29 | 279.18 | 245.9 | 301 | 246.42 | 250.3 | 245.79 | 265.9067 |
| ELN | 0.21 | -2.25025 | 1.09E-17 | 4.42E-16 | yes | down | 114.95 | 129.19 | 89.8 | 73.34 | 34.34 | 58.47 | 111.3133 | 55.38333 |
| LOC108178477 | 0.195 | -2.36185 | 1.33E-17 | 5.36E-16 | yes | down | 1.97 | 2.8 | 2.61 | 1.42 | 1.1 | 0.84 | 2.46 | 1.12 |
| OPA1 | 0.459 | -1.12299 | 1.38E-17 | 5.56E-16 | yes | down | 10.06 | 11.01 | 12.12 | 13.36 | 11.35 | 11.19 | 11.06333 | 11.96667 |
| CGRRF1 | 2.762 | 1.465939 | 1.65E-17 | 6.62E-16 | yes | up | 1.81 | 2.04 | 1.96 | 14.09 | 15.69 | 13.78 | 1.936667 | 14.52 |
| GRIA3 | 4.646 | 2.215961 | 1.67E-17 | 6.71E-16 | yes | up | 0.51 | 0.67 | 0.37 | 6.43 | 4.75 | 5.53 | 0.516667 | 5.57 |
| LRP2 | 0.181 | -2.46433 | 1.68E-17 | 6.71E-16 | yes | down | 2.27 | 1.66 | 3.07 | 1.1 | 0.76 | 1.14 | 2.333333 | 1 |
| CEL | 0.001 | -10.4724 | 1.73E-17 | 6.89E-16 | yes | down | 5.36 | 3.81 | 3.51 | 0 | 0 | 0 | 4.226667 | 0 |
| LOC100354254 | 0.139 | -2.85118 | 1.79E-17 | 7.13E-16 | yes | down | 704.32 | 1083.94 | 1003.36 | 184.2 | 256.4 | 471.84 | 930.54 | 304.1467 |
| LOC103345217 | 0.05 | -4.31487 | 2.14E-17 | 8.48E-16 | yes | down | 4.62 | 4.49 | 6.6 | 1.02 | 0.4 | 0.45 | 5.236667 | 0.623333 |
| LOC103348133 | 0.081 | -3.6177 | 2.15E-17 | 8.51E-16 | yes | down | 4.71 | 6.78 | 4.39 | 1.17 | 1.27 | 0.39 | 5.293333 | 0.943333 |
| ID3 | 0.252 | -1.9896 | 2.17E-17 | 8.57E-16 | yes | down | 315.64 | 313.72 | 247.18 | 166.34 | 127.43 | 224.63 | 292.18 | 172.8 |
| ECHDC3 | 0.492 | -1.02403 | 2.2E-17 | 8.65E-16 | yes | down | 69.54 | 86.32 | 80.34 | 81.71 | 83.66 | 88.35 | 78.73333 | 84.57333 |
| PDK4 | 0.096 | -3.38588 | 2.45E-17 | 9.62E-16 | yes | down | 613.98 | 372.79 | 166.78 | 82.81 | 87.36 | 90.05 | 384.5167 | 86.74 |
| ACACA | 7.021 | 2.811734 | 2.54E-17 | 9.97E-16 | yes | up | 1.8 | 2.6 | 2.74 | 57.19 | 19.37 | 41.09 | 2.38 | 39.21667 |
| NDUFA11 | 0.406 | -1.30164 | 2.82E-17 | 1.1E-15 | yes | down | 124.93 | 139.78 | 119.42 | 143.78 | 100.3 | 122.34 | 128.0433 | 122.14 |
| ZFAND4 | 0.181 | -2.46258 | 3.3E-17 | 1.29E-15 | yes | down | 5.58 | 7.6 | 11.5 | 3.04 | 3.81 | 3.51 | 8.226667 | 3.453333 |
| CLUH | 0.271 | -1.88616 | 3.54E-17 | 1.38E-15 | yes | down | 48.6 | 79.35 | 70.57 | 39.5 | 32.03 | 51.59 | 66.17333 | 41.04 |
| TST | 3.301 | 1.722718 | 3.67E-17 | 1.42E-15 | yes | up | 12.9 | 14.13 | 11.72 | 127.64 | 78.7 | 93.66 | 12.91667 | 100 |
| AR | 5.591 | 2.483032 | 3.67E-17 | 1.42E-15 | yes | up | 0.46 | 0.55 | 0.56 | 8.47 | 5.46 | 6.59 | 0.523333 | 6.84 |
| GHITM | 0.192 | -2.38131 | 4.35E-17 | 1.68E-15 | yes | down | 260.27 | 286.76 | 380.58 | 158.7 | 81.18 | 176.62 | 309.2033 | 138.8333 |
| SCUBE1 | 0.056 | -4.14587 | 4.54E-17 | 1.75E-15 | yes | down | 1.61 | 1.78 | 2.42 | 0.18 | 0.52 | 0.1 | 1.936667 | 0.266667 |
| METTL17 | 0.324 | -1.62555 | 4.94E-17 | 1.9E-15 | yes | down | 17.25 | 27.85 | 17.88 | 16.32 | 15.2 | 17.11 | 20.99333 | 16.21 |
| BPGM | 0.242 | -2.0473 | 5.13E-17 | 1.97E-15 | yes | down | 9.95 | 8.51 | 10.4 | 4.84 | 6.12 | 5.21 | 9.62 | 5.39 |
| PRPSAP2 | 3.054 | 1.610885 | 5.34E-17 | 2.04E-15 | yes | up | 3.32 | 3.2 | 3.34 | 27.51 | 20.89 | 22.31 | 3.286667 | 23.57 |
| MTMR4 | 2.77 | 1.469966 | 5.39E-17 | 2.06E-15 | yes | up | 2.07 | 3.15 | 2.44 | 16.68 | 14.19 | 17.92 | 2.553333 | 16.26333 |
| SLC22A4 | 0.048 | -4.38479 | 5.64E-17 | 2.15E-15 | yes | down | 2.75 | 2.34 | 2.48 | 0.09 | 0.35 | 0.41 | 2.523333 | 0.283333 |
| FH | 0.23 | -2.11884 | 6.33E-17 | 2.41E-15 | yes | down | 122.58 | 151.42 | 124.68 | 47.61 | 78.82 | 84.52 | 132.8933 | 70.31667 |
| OSBPL7 | 0.275 | -1.85989 | 6.53E-17 | 2.47E-15 | yes | down | 12.17 | 17.84 | 17.9 | 10.3 | 7.34 | 11.9 | 15.97 | 9.846667 |
| LOC103345437 | 0.39 | -1.35689 | 6.53E-17 | 2.47E-15 | yes | down | 33.71 | 46.36 | 36.36 | 40.16 | 32.37 | 40.37 | 38.81 | 37.63333 |
| MRC2 | 2.922 | 1.546847 | 7E-17 | 2.64E-15 | yes | up | 3.78 | 5.34 | 3.35 | 27.35 | 27.65 | 28.89 | 4.156667 | 27.96333 |
| IL15RA | 6.665 | 2.736519 | 7.2E-17 | 2.71E-15 | yes | up | 1.01 | 0.37 | 0.6 | 9.66 | 6.75 | 11.84 | 0.66 | 9.416667 |
| TRIM25 | 3.757 | 1.909648 | 7.24E-17 | 2.72E-15 | yes | up | 2.07 | 1.52 | 1.45 | 15.82 | 13.14 | 15.38 | 1.68 | 14.78 |
| PLSCR4 | 2.249 | 1.169193 | 7.8E-17 | 2.93E-15 | yes | up | 5 | 6.38 | 5.99 | 30.04 | 31.39 | 29.57 | 5.79 | 30.33333 |
| DHCR24 | 2.891 | 1.531451 | 8.16E-17 | 3.06E-15 | yes | up | 4.75 | 7.29 | 6.62 | 49.73 | 34.43 | 41 | 6.22 | 41.72 |
| BCKDK | 0.437 | -1.1944 | 8.66E-17 | 3.24E-15 | yes | down | 42.06 | 59.38 | 53.82 | 55.09 | 52.82 | 49.28 | 51.75333 | 52.39667 |
| EFEMP1 | 2.897 | 1.534513 | 8.79E-17 | 3.28E-15 | yes | up | 30.7 | 41.6 | 30.13 | 210.64 | 202.27 | 271.8 | 34.14333 | 228.2367 |
| PLEKHF1 | 4.719 | 2.23855 | 1.07E-16 | 3.98E-15 | yes | up | 9.07 | 11.94 | 5.72 | 103.78 | 107.61 | 80.09 | 8.91 | 97.16 |
| CPT2 | 0.385 | -1.3777 | 1.11E-16 | 4.12E-15 | yes | down | 48.07 | 56.36 | 64.67 | 51.27 | 44.11 | 56.05 | 56.36667 | 50.47667 |
| MYLK3 | 0.082 | -3.60263 | 1.27E-16 | 4.72E-15 | yes | down | 4.5 | 5.18 | 2.33 | 0.69 | 1.19 | 0.45 | 4.003333 | 0.776667 |
| FMO1 | 8.357 | 3.063058 | 1.36E-16 | 5.02E-15 | yes | up | 2.4 | 3.84 | 4.37 | 52.89 | 43.83 | 109.48 | 3.536667 | 68.73333 |
| LOC103344988 | 0.228 | -2.13184 | 1.36E-16 | 5.02E-15 | yes | down | 182.65 | 182.24 | 132.96 | 109.53 | 57.44 | 105.24 | 165.95 | 90.73667 |
| RFWD2 | 0.482 | -1.05144 | 1.37E-16 | 5.02E-15 | yes | down | 16.56 | 18.64 | 17.29 | 20.56 | 20.17 | 18.87 | 17.49667 | 19.86667 |
| POLDIP2 | 0.436 | -1.19716 | 1.57E-16 | 5.78E-15 | yes | down | 53.4 | 74.98 | 66.01 | 62.7 | 65.04 | 68.04 | 64.79667 | 65.26 |
| PRDM16 | 0.117 | -3.09523 | 1.9E-16 | 6.95E-15 | yes | down | 1.53 | 2.37 | 2.22 | 0.69 | 0.37 | 0.6 | 2.04 | 0.553333 |
| MKI67 | 14.569 | 3.86483 | 1.91E-16 | 6.97E-15 | yes | up | 0.24 | 0.48 | 0.22 | 14.43 | 12.12 | 3.52 | 0.313333 | 10.02333 |
| LOC100338714 | 0.458 | -1.1267 | 1.98E-16 | 7.21E-15 | yes | down | 461.14 | 597.69 | 476.72 | 596.32 | 562.28 | 538.88 | 511.85 | 565.8267 |
| ABAT | 7.564 | 2.919152 | 2.08E-16 | 7.55E-15 | yes | up | 0.21 | 0.4 | 0.28 | 6.86 | 4.88 | 3.94 | 0.296667 | 5.226667 |
| ADPRHL1 | 0.041 | -4.61248 | 2.08E-16 | 7.56E-15 | yes | down | 8.15 | 11.88 | 4.2 | 0.83 | 1.32 | 0.22 | 8.076667 | 0.79 |
| PEG3 | 0.312 | -1.68046 | 2.1E-16 | 7.59E-15 | yes | down | 42.49 | 55.07 | 58.07 | 37.04 | 29.11 | 46.62 | 51.87667 | 37.59 |
| PTX3 | 0.068 | -3.88401 | 2.24E-16 | 8.09E-15 | yes | down | 5.48 | 2.81 | 1.82 | 0.82 | 0.37 | 0.53 | 3.37 | 0.573333 |
| CERK | 0.258 | -1.95211 | 2.29E-16 | 8.25E-15 | yes | down | 18.02 | 35.18 | 23.68 | 17.85 | 11.68 | 16.22 | 25.62667 | 15.25 |
| TFB2M | 0.381 | -1.3911 | 2.54E-16 | 9.13E-15 | yes | down | 21.94 | 24.27 | 23.54 | 19.02 | 22.9 | 20.38 | 23.25 | 20.76667 |
| ATG4D | 0.296 | -1.75533 | 2.59E-16 | 9.28E-15 | yes | down | 11.72 | 16.14 | 12.85 | 9.11 | 7.67 | 11.16 | 13.57 | 9.313333 |
| NDUFAF4 | 0.223 | -2.16668 | 2.59E-16 | 9.29E-15 | yes | down | 7.19 | 8.64 | 10.54 | 3.6 | 4.54 | 5.48 | 8.79 | 4.54 |
| GGA2 | 3.811 | 1.930324 | 2.92E-16 | 1.05E-14 | yes | up | 15.77 | 16.1 | 24.07 | 193.93 | 126.66 | 183.6 | 18.64667 | 168.0633 |
| TSPAN5 | 0.267 | -1.903 | 3.52E-16 | 1.26E-14 | yes | down | 6.97 | 8.24 | 6.97 | 5.03 | 5.39 | 3.33 | 7.393333 | 4.583333 |
| IGF2BP3 | 0.199 | -2.33111 | 3.95E-16 | 1.41E-14 | yes | down | 2.23 | 1.9 | 2 | 1.11 | 0.99 | 0.76 | 2.043333 | 0.953333 |
| KLB | 4.554 | 2.187018 | 4.24E-16 | 1.5E-14 | yes | up | 14.71 | 12.57 | 17.79 | 112.87 | 170.66 | 192.27 | 15.02333 | 158.6 |
| NOVA1 | 2.36 | 1.238963 | 4.47E-16 | 1.58E-14 | yes | up | 2.48 | 2.54 | 2.96 | 17.35 | 12.18 | 15.13 | 2.66 | 14.88667 |
| POR | 0.333 | -1.5867 | 5.01E-16 | 1.77E-14 | yes | down | 104.12 | 87.73 | 85.7 | 88.27 | 60.04 | 69.86 | 92.51667 | 72.72333 |
| EEPD1 | 2.657 | 1.40991 | 5.39E-16 | 1.9E-14 | yes | up | 17.11 | 18.59 | 18.57 | 135.03 | 86.33 | 118.71 | 18.09 | 113.3567 |
| GOT2 | 0.315 | -1.66436 | 5.45E-16 | 1.92E-14 | yes | down | 58.22 | 95.28 | 61.04 | 49.4 | 57.86 | 47.83 | 71.51333 | 51.69667 |
| ABR | 2.623 | 1.390968 | 5.56E-16 | 1.95E-14 | yes | up | 5.63 | 6.15 | 5.79 | 34.15 | 28.25 | 35.17 | 5.856667 | 32.52333 |
| HSDL2 | 0.349 | -1.51769 | 5.56E-16 | 1.95E-14 | yes | down | 91.21 | 161.85 | 131.09 | 103.83 | 104.69 | 99.62 | 128.05 | 102.7133 |
| OS9 | 2.142 | 1.099121 | 5.71E-16 | 2E-14 | yes | up | 23.09 | 31.85 | 22.63 | 133.06 | 131.98 | 124.83 | 25.85667 | 129.9567 |
| FN3K | 0.346 | -1.53306 | 6.35E-16 | 2.22E-14 | yes | down | 15.17 | 21.89 | 20.04 | 17.43 | 13.67 | 12.82 | 19.03333 | 14.64 |
| MTX1 | 0.4 | -1.32243 | 6.6E-16 | 2.3E-14 | yes | down | 30.32 | 43.88 | 36.13 | 40.12 | 29.1 | 33.34 | 36.77667 | 34.18667 |
| UCK2 | 0.303 | -1.72312 | 6.79E-16 | 2.36E-14 | yes | down | 9.56 | 10.94 | 9.02 | 6.43 | 5.4 | 8.53 | 9.84 | 6.786667 |
| MIGA2 | 0.235 | -2.08832 | 7.67E-16 | 2.66E-14 | yes | down | 28.07 | 33.2 | 46.3 | 17.32 | 17.6 | 25.96 | 35.85667 | 20.29333 |
| LIAS | 0.358 | -1.48182 | 7.8E-16 | 2.7E-14 | yes | down | 13.81 | 17.09 | 19.4 | 12.93 | 13.65 | 14.67 | 16.76667 | 13.75 |
| CTSE | 0.125 | -3.00378 | 8.17E-16 | 2.82E-14 | yes | down | 4.53 | 4.35 | 4.47 | 0.81 | 1.2 | 1.84 | 4.45 | 1.283333 |
| ALDH1A1 | 4.94 | 2.304542 | 8.62E-16 | 2.97E-14 | yes | up | 65.98 | 103.86 | 57.26 | 628.01 | 869.95 | 1110.68 | 75.7 | 869.5467 |
| ARL6IP1 | 3.243 | 1.697332 | 9.56E-16 | 3.29E-14 | yes | up | 39.44 | 50.79 | 59.65 | 492.27 | 326.02 | 318.22 | 49.96 | 378.8367 |
| MRPS25 | 0.428 | -1.22426 | 9.71E-16 | 3.33E-14 | yes | down | 59.23 | 73.59 | 59.08 | 68.01 | 61.46 | 52.26 | 63.96667 | 60.57667 |
| GALK1 | 0.217 | -2.20458 | 1.05E-15 | 3.61E-14 | yes | down | 16.13 | 25.34 | 17.19 | 12.83 | 10.12 | 6.59 | 19.55333 | 9.846667 |
| EPRS | 0.437 | -1.19401 | 1.1E-15 | 3.76E-14 | yes | down | 24.78 | 40.35 | 30.56 | 34.58 | 32.81 | 35.34 | 31.89667 | 34.24333 |
| GDPGP1 | 0.36 | -1.47533 | 1.11E-15 | 3.79E-14 | yes | down | 5.09 | 5.81 | 6.46 | 5.13 | 4.39 | 5.09 | 5.786667 | 4.87 |
| RNF152 | 0.341 | -1.5535 | 1.13E-15 | 3.86E-14 | yes | down | 6.91 | 8.51 | 7.96 | 7.3 | 4.45 | 6.82 | 7.793333 | 6.19 |
| UQCRQ | 0.463 | -1.11056 | 1.15E-15 | 3.92E-14 | yes | down | 1155.25 | 1505.01 | 1233.03 | 1400.43 | 1364.16 | 1570 | 1297.763 | 1444.863 |
| LSM3 | 2.801 | 1.485773 | 1.24E-15 | 4.19E-14 | yes | up | 8.89 | 13.29 | 11.29 | 80.29 | 68.86 | 70.08 | 11.15667 | 73.07667 |
| LOC103346787 | 0.306 | -1.70839 | 1.34E-15 | 4.53E-14 | yes | down | 21.8 | 27.01 | 24.52 | 17.81 | 14.71 | 20.12 | 24.44333 | 17.54667 |
| DPT | 6.72 | 2.748511 | 1.34E-15 | 4.54E-14 | yes | up | 40.59 | 69.1 | 34.6 | 441.1 | 900.48 | 874.53 | 48.09667 | 738.7033 |
| MRPL22 | 0.373 | -1.42436 | 1.41E-15 | 4.75E-14 | yes | down | 46.87 | 70.64 | 70.99 | 56.43 | 52.24 | 55.61 | 62.83333 | 54.76 |
| ATP5L | 0.455 | -1.13532 | 1.43E-15 | 4.81E-14 | yes | down | 615.37 | 796.7 | 601.33 | 829.41 | 693.88 | 660.91 | 671.1333 | 728.0667 |
| FPGS | 0.301 | -1.73142 | 1.46E-15 | 4.89E-14 | yes | down | 12.28 | 14.95 | 15.48 | 9.61 | 7.84 | 12.42 | 14.23667 | 9.956667 |
| PDIA6 | 2.441 | 1.287527 | 1.59E-15 | 5.32E-14 | yes | up | 33.75 | 51.05 | 39.2 | 232.96 | 246.45 | 218.5 | 41.33333 | 232.6367 |
| LOC100009483 | 5.813 | 2.539384 | 1.61E-15 | 5.38E-14 | yes | up | 1.1 | 1.93 | 2.05 | 26.62 | 17.42 | 25.55 | 1.693333 | 23.19667 |
| HACL1 | 0.436 | -1.1984 | 1.68E-15 | 5.62E-14 | yes | down | 37.11 | 55.08 | 49.5 | 49.73 | 47.72 | 45.77 | 47.23 | 47.74 |
| APLP2 | 2.216 | 1.147878 | 1.77E-15 | 5.9E-14 | yes | up | 119.31 | 164.51 | 154.2 | 733.91 | 745.47 | 790.46 | 146.0067 | 756.6133 |
| SEMA3A | 9.099 | 3.185647 | 1.95E-15 | 6.48E-14 | yes | up | 0.34 | 0.63 | 0.3 | 14.14 | 5.01 | 5.75 | 0.423333 | 8.3 |
| MRPS15 | 0.407 | -1.29515 | 2.11E-15 | 7.01E-14 | yes | down | 57.2 | 84.97 | 79.42 | 69.47 | 66.76 | 65.3 | 73.86333 | 67.17667 |
| LOC103348029 | 0.078 | -3.6774 | 2.13E-15 | 7.06E-14 | yes | down | 4.52 | 7.18 | 4.75 | 0.93 | 1.1 | 1.3 | 5.483333 | 1.11 |
| LOC100350311 | 0.15 | -2.73477 | 2.33E-15 | 7.69E-14 | yes | down | 14.98 | 27.58 | 9.23 | 7.61 | 6.37 | 5.81 | 17.26333 | 6.596667 |
| CCDC58 | 0.342 | -1.54904 | 2.38E-15 | 7.86E-14 | yes | down | 22.73 | 34.11 | 24.65 | 22.61 | 25.15 | 27.61 | 27.16333 | 25.12333 |
| NID2 | 2.377 | 1.24912 | 3.32E-15 | 1.09E-13 | yes | up | 19.52 | 23.58 | 22.27 | 134.52 | 128.99 | 99.58 | 21.79 | 121.03 |
| MMACHC | 0.175 | -2.51295 | 3.6E-15 | 1.18E-13 | yes | down | 11.85 | 14 | 21.39 | 4.95 | 6.36 | 7.92 | 15.74667 | 6.41 |
| LOC103350570 | 14.996 | 3.906498 | 3.81E-15 | 1.25E-13 | yes | up | 0.08 | 0.02 | 0.05 | 2 | 1.72 | 1.65 | 0.05 | 1.79 |
| RRP36 | 0.419 | -1.25529 | 3.91E-15 | 1.28E-13 | yes | down | 18.16 | 20.97 | 19.25 | 18.87 | 16.82 | 18.03 | 19.46 | 17.90667 |
| GATSL2 | 0.247 | -2.01658 | 4.25E-15 | 1.39E-13 | yes | down | 5.67 | 10.16 | 7.81 | 3.94 | 4.54 | 4.93 | 7.88 | 4.47 |
| PCDH18 | 3.768 | 1.913687 | 4.39E-15 | 1.43E-13 | yes | up | 0.61 | 0.65 | 0.65 | 6.27 | 4.07 | 6.48 | 0.636667 | 5.606667 |
| MECR | 0.345 | -1.53416 | 4.77E-15 | 1.55E-13 | yes | down | 28.59 | 36.93 | 46.34 | 34.94 | 29.66 | 32.17 | 37.28667 | 32.25667 |
| BCAT2 | 7 | 2.80733 | 4.92E-15 | 1.6E-13 | yes | up | 2.94 | 4.29 | 2.31 | 42.73 | 76.48 | 34.24 | 3.18 | 51.15 |
| DEPTOR | 2.074 | 1.05213 | 5.08E-15 | 1.64E-13 | yes | up | 2.61 | 2.8 | 2.77 | 14.08 | 12.74 | 12.63 | 2.726667 | 13.15 |
| SIDT2 | 0.374 | -1.41853 | 5.35E-15 | 1.73E-13 | yes | down | 28.2 | 26.34 | 34.77 | 28.43 | 25.99 | 23.25 | 29.77 | 25.89 |
| CTPS1 | 0.204 | -2.29257 | 5.55E-15 | 1.79E-13 | yes | down | 3.1 | 5.67 | 3.77 | 1.65 | 2.2 | 2.01 | 4.18 | 1.953333 |
| CRABP2 | 6.815 | 2.768749 | 7.18E-15 | 2.31E-13 | yes | up | 1.82 | 4.7 | 1.64 | 40.84 | 46.4 | 38.92 | 2.72 | 42.05333 |
| LACE1 | 0.31 | -1.68824 | 7.25E-15 | 2.33E-13 | yes | down | 15.35 | 21.35 | 22.54 | 14.32 | 11.45 | 17.17 | 19.74667 | 14.31333 |
| RASGRP2 | 4.697 | 2.231592 | 8.13E-15 | 2.6E-13 | yes | up | 2.38 | 3.48 | 4.23 | 36.59 | 36.79 | 32.49 | 3.363333 | 35.29 |
| RAB27A | 3.8 | 1.926046 | 8.21E-15 | 2.62E-13 | yes | up | 7.32 | 6.02 | 9.94 | 59.6 | 71.04 | 74.18 | 7.76 | 68.27333 |
| FIBIN | 0.157 | -2.67289 | 9.3E-15 | 2.96E-13 | yes | down | 16.79 | 18.92 | 17.93 | 8.26 | 2.97 | 8.57 | 17.88 | 6.6 |
| IGFBP2 | 2.647 | 1.404263 | 1.05E-14 | 3.32E-13 | yes | up | 24.61 | 39.43 | 28.75 | 195.36 | 168.51 | 206.44 | 30.93 | 190.1033 |
| SNCA | 0.119 | -3.06688 | 1.05E-14 | 3.33E-13 | yes | down | 31.17 | 30.67 | 26.82 | 3.34 | 8.35 | 12.1 | 29.55333 | 7.93 |
| LOC100356413 | 0.445 | -1.16828 | 1.09E-14 | 3.44E-13 | yes | down | 45.83 | 66.36 | 56.2 | 62.29 | 50.23 | 61.48 | 56.13 | 58 |
| DHRS7 | 3.352 | 1.744889 | 1.12E-14 | 3.52E-13 | yes | up | 35.2 | 26.76 | 30.32 | 215.8 | 283.4 | 225.49 | 30.76 | 241.5633 |
| CA8 | 0.149 | -2.74369 | 1.12E-14 | 3.52E-13 | yes | down | 7.62 | 11.85 | 7.35 | 3.83 | 1.71 | 3.77 | 8.94 | 3.103333 |
| SGMS2 | 3.106 | 1.634833 | 1.15E-14 | 3.61E-13 | yes | up | 1.49 | 1.37 | 1.1 | 10.79 | 7.94 | 9.66 | 1.32 | 9.463333 |
| LOC100345908 | 0.087 | -3.52361 | 1.21E-14 | 3.79E-13 | yes | down | 3.24 | 7.94 | 5.17 | 0.55 | 1.2 | 1.24 | 5.45 | 0.996667 |
| PDSS1 | 0.29 | -1.78702 | 1.24E-14 | 3.86E-13 | yes | down | 6.63 | 9.98 | 7.52 | 4.93 | 5.6 | 5.3 | 8.043333 | 5.276667 |
| MMADHC | 0.406 | -1.30108 | 1.29E-14 | 4.02E-13 | yes | down | 41.18 | 46.61 | 40.94 | 40.76 | 45.4 | 34.74 | 42.91 | 40.3 |
| RBP4 | 10.135 | 3.341324 | 1.42E-14 | 4.42E-13 | yes | up | 16.01 | 29.84 | 42.45 | 379.22 | 481.12 | 1119.11 | 29.43333 | 659.8167 |
| FGF1 | 4.25 | 2.087608 | 1.44E-14 | 4.48E-13 | yes | up | 3 | 2.45 | 1.92 | 21.24 | 18.42 | 30.92 | 2.456667 | 23.52667 |
| DDAH1 | 0.125 | -2.9951 | 1.48E-14 | 4.59E-13 | yes | down | 8.61 | 10.71 | 8.12 | 2.13 | 4.42 | 1.41 | 9.146667 | 2.653333 |
| STYXL1 | 0.188 | -2.40774 | 1.66E-14 | 5.12E-13 | yes | down | 5.95 | 7.28 | 9.37 | 3.13 | 2.81 | 4.14 | 7.533333 | 3.36 |
| MRPL37 | 0.336 | -1.57215 | 1.81E-14 | 5.58E-13 | yes | down | 85.52 | 117.08 | 70.75 | 81.67 | 71.73 | 59.83 | 91.11667 | 71.07667 |
| MRPS5 | 0.406 | -1.30188 | 1.83E-14 | 5.64E-13 | yes | down | 19.46 | 29.4 | 24.47 | 23.04 | 23.34 | 21.25 | 24.44333 | 22.54333 |
| ZNF672 | 0.303 | -1.72219 | 1.84E-14 | 5.64E-13 | yes | down | 3.6 | 3.56 | 3.51 | 2.04 | 2.85 | 2.33 | 3.556667 | 2.406667 |
| C12H6orf89 | 0.373 | -1.42439 | 1.94E-14 | 5.95E-13 | yes | down | 69.28 | 73.27 | 81.95 | 73.85 | 68.06 | 75.95 | 74.83333 | 72.62 |
| ZHX1 | 2.002 | 1.001802 | 2E-14 | 6.1E-13 | yes | up | 5.07 | 5.86 | 5.64 | 24.53 | 23.78 | 26.05 | 5.523333 | 24.78667 |
| ST6GALNAC2 | 0.118 | -3.08543 | 2.03E-14 | 6.2E-13 | yes | down | 4.79 | 5.49 | 5.84 | 1 | 1 | 2.51 | 5.373333 | 1.503333 |
| OBSL1 | 0.296 | -1.75486 | 2.17E-14 | 6.6E-13 | yes | down | 63.3 | 60.11 | 82.94 | 38.16 | 44.23 | 60.27 | 68.78333 | 47.55333 |
| PAM | 2.07 | 1.049797 | 2.22E-14 | 6.74E-13 | yes | up | 23.69 | 28.4 | 21.74 | 117.08 | 117.01 | 119.37 | 24.61 | 117.82 |
| ARRB1 | 3.324 | 1.733127 | 2.25E-14 | 6.83E-13 | yes | up | 9.81 | 14.61 | 18.29 | 119.63 | 88.77 | 119.56 | 14.23667 | 109.32 |
| EGFLAM | 2.837 | 1.504204 | 2.31E-14 | 6.98E-13 | yes | up | 5.18 | 6.23 | 6.18 | 43.43 | 30.31 | 30.68 | 5.863333 | 34.80667 |
| ABCF3 | 0.482 | -1.05265 | 2.69E-14 | 8.12E-13 | yes | down | 19.99 | 26.32 | 24.51 | 25.98 | 24.41 | 28.1 | 23.60667 | 26.16333 |
| ACOT11 | 0.227 | -2.1397 | 2.74E-14 | 8.25E-13 | yes | down | 13.57 | 24.4 | 24.39 | 9.79 | 5.92 | 13.82 | 20.78667 | 9.843333 |
| NFXL1 | 2.653 | 1.407812 | 2.84E-14 | 8.54E-13 | yes | up | 1.3 | 1.77 | 1.43 | 9.2 | 8.97 | 9.35 | 1.5 | 9.173333 |
| SAR1B | 0.412 | -1.27962 | 3.07E-14 | 9.22E-13 | yes | down | 23.56 | 25.77 | 26.76 | 20.93 | 25.51 | 19.95 | 25.36333 | 22.13 |
| SLC24A2 | 14.973 | 3.904298 | 3.37E-14 | 1.01E-12 | yes | up | 0.32 | 0.09 | 0.07 | 5.79 | 6.19 | 4.9 | 0.16 | 5.626667 |
| SIRPA | 2.843 | 1.507591 | 3.38E-14 | 1.01E-12 | yes | up | 8.03 | 9.98 | 12.94 | 67.2 | 70.47 | 66.16 | 10.31667 | 67.94333 |
| LOC100338675 | 0.095 | -3.39395 | 3.39E-14 | 1.01E-12 | yes | down | 47.91 | 51.92 | 53.26 | 4.69 | 3.66 | 20.67 | 51.03 | 9.673333 |
| RALGPS2 | 0.372 | -1.42708 | 3.7E-14 | 1.1E-12 | yes | down | 2.05 | 2.53 | 2.07 | 1.74 | 1.93 | 2.05 | 2.216667 | 1.906667 |
| NUDT5 | 0.349 | -1.51736 | 3.77E-14 | 1.12E-12 | yes | down | 30.66 | 37.66 | 42.14 | 36.2 | 28.42 | 25.56 | 36.82 | 30.06 |
| COQ3 | 0.192 | -2.37869 | 3.9E-14 | 1.16E-12 | yes | down | 5.94 | 8.51 | 6.38 | 2.29 | 4.2 | 2.71 | 6.943333 | 3.066667 |
| SAA3 | 9.762 | 3.287235 | 3.91E-14 | 1.16E-12 | yes | up | 3.7 | 1.42 | 2.34 | 59.66 | 42.11 | 79.39 | 2.486667 | 60.38667 |
| EXTL1 | 0.109 | -3.19189 | 4.06E-14 | 1.2E-12 | yes | down | 11.57 | 15.35 | 12.5 | 3.85 | 1.1 | 5.12 | 13.14 | 3.356667 |
| EI24 | 0.424 | -1.23896 | 4.13E-14 | 1.22E-12 | yes | down | 32.39 | 49.67 | 45.53 | 42.38 | 42.44 | 40.61 | 42.53 | 41.81 |
| DOC2B | 5.74 | 2.52096 | 4.24E-14 | 1.25E-12 | yes | up | 1.04 | 1.1 | 0.69 | 18.56 | 10.88 | 8.86 | 0.943333 | 12.76667 |
| PHF19 | 3.011 | 1.590132 | 4.24E-14 | 1.25E-12 | yes | up | 1.83 | 2.56 | 2.49 | 17.44 | 18.91 | 13.69 | 2.293333 | 16.68 |
| KIFC3 | 0.31 | -1.69079 | 4.41E-14 | 1.3E-12 | yes | down | 5.88 | 9.11 | 9.48 | 7.18 | 4.7 | 5.27 | 8.156667 | 5.716667 |
| GGT6 | 0.087 | -3.52973 | 4.49E-14 | 1.32E-12 | yes | down | 3.88 | 3.98 | 3.33 | 0.3 | 0.44 | 1.14 | 3.73 | 0.626667 |
| HIGD1A | 0.241 | -2.05001 | 4.64E-14 | 1.36E-12 | yes | down | 35.51 | 83.08 | 45.17 | 28.31 | 30.56 | 30.53 | 54.58667 | 29.8 |
| LOC103345647 | 2.026 | 1.018403 | 5.24E-14 | 1.53E-12 | yes | up | 42.46 | 44.33 | 48.63 | 233.12 | 191.65 | 217.69 | 45.14 | 214.1533 |
| WDR41 | 0.428 | -1.22568 | 5.38E-14 | 1.57E-12 | yes | down | 29.33 | 40.77 | 33.4 | 33.33 | 35.96 | 33.24 | 34.5 | 34.17667 |
| LOC103349531 | 29.16 | 4.865931 | 5.52E-14 | 1.61E-12 | yes | up | 0.06 | 0 | 0.17 | 4.2 | 3.69 | 7.26 | 0.076667 | 5.05 |
| C12H6orf136 | 0.35 | -1.51607 | 5.87E-14 | 1.7E-12 | yes | down | 15.84 | 21.91 | 22.56 | 15.48 | 17.93 | 15.41 | 20.10333 | 16.27333 |
| BPNT1 | 0.412 | -1.27967 | 5.91E-14 | 1.71E-12 | yes | down | 25.83 | 37.1 | 36.74 | 30.31 | 31.56 | 32.92 | 33.22333 | 31.59667 |
| PLOD2 | 3.22 | 1.687008 | 6.17E-14 | 1.78E-12 | yes | up | 2.82 | 3.27 | 2.09 | 18.18 | 22.4 | 20.19 | 2.726667 | 20.25667 |
| CHL1 | 0.311 | -1.68526 | 6.25E-14 | 1.81E-12 | yes | down | 1.92 | 2.78 | 3.08 | 1.06 | 3.19 | 0.82 | 2.593333 | 1.69 |
| C13H1orf105 | 0.285 | -1.81226 | 6.29E-14 | 1.81E-12 | yes | down | 4.8 | 5.32 | 7.39 | 4.08 | 4.11 | 3.72 | 5.836667 | 3.97 |
| LOC100353320 | 0.199 | -2.32719 | 6.49E-14 | 1.87E-12 | yes | down | 249.86 | 231.89 | 228.27 | 136.59 | 57.78 | 146.75 | 236.6733 | 113.7067 |
| LOC100350957 | 9.049 | 3.17775 | 6.71E-14 | 1.92E-12 | yes | up | 0.6 | 1.4 | 0.34 | 12.15 | 22.12 | 17.59 | 0.78 | 17.28667 |
| L2HGDH | 0.432 | -1.21209 | 6.76E-14 | 1.93E-12 | yes | down | 5.99 | 6.8 | 7.17 | 6.68 | 6.49 | 6.8 | 6.653333 | 6.656667 |
| RILPL2 | 2.26 | 1.176036 | 6.93E-14 | 1.98E-12 | yes | up | 22 | 21.63 | 21.63 | 127.09 | 109.91 | 110.12 | 21.75333 | 115.7067 |
| NNT | 0.416 | -1.26521 | 7.03E-14 | 2.01E-12 | yes | down | 73.34 | 105.43 | 77.96 | 78.37 | 88.35 | 81.09 | 85.57667 | 82.60333 |
| NQO1 | 3.609 | 1.85169 | 7.54E-14 | 2.15E-12 | yes | up | 3.8 | 4.44 | 5.08 | 69.05 | 29.37 | 48.23 | 4.44 | 48.88333 |
| WDR45 | 0.458 | -1.12517 | 7.87E-14 | 2.24E-12 | yes | down | 24.81 | 29.4 | 27.98 | 31.85 | 28.93 | 25.95 | 27.39667 | 28.91 |
| PRKD3 | 0.409 | -1.28845 | 8.43E-14 | 2.39E-12 | yes | down | 21.53 | 28.54 | 32.55 | 26.67 | 23.83 | 27.81 | 27.54 | 26.10333 |
| LBP | 3.533 | 1.820972 | 8.86E-14 | 2.51E-12 | yes | up | 9.88 | 14.21 | 15.32 | 130.28 | 95.19 | 165.63 | 13.13667 | 130.3667 |
| PPARA | 0.373 | -1.4245 | 9.98E-14 | 2.82E-12 | yes | down | 20.13 | 23.51 | 25.47 | 16.65 | 20.28 | 22.66 | 23.03667 | 19.86333 |
| TOP2A | 10.319 | 3.367227 | 1.09E-13 | 3.07E-12 | yes | up | 0.84 | 1.12 | 0.79 | 28.26 | 32.88 | 7.39 | 0.916667 | 22.84333 |
| LOC100355645 | 0.426 | -1.23161 | 1.12E-13 | 3.16E-12 | yes | down | 13.85 | 18.42 | 17.89 | 18.03 | 16.63 | 14.96 | 16.72 | 16.54 |
| ME1 | 5.729 | 2.518394 | 1.14E-13 | 3.21E-12 | yes | up | 1 | 2.42 | 1.38 | 25.06 | 12.93 | 25.22 | 1.6 | 21.07 |
| DNAJC25 | 0.377 | -1.40673 | 1.21E-13 | 3.4E-12 | yes | down | 14.77 | 17.41 | 15.84 | 12.07 | 10.76 | 15.89 | 16.00667 | 12.90667 |
| TMEM201 | 0.316 | -1.66187 | 1.22E-13 | 3.4E-12 | yes | down | 3.56 | 5.78 | 6.06 | 3.53 | 3.77 | 3.79 | 5.133333 | 3.696667 |
| NDUFA1 | 0.499 | -1.00407 | 1.24E-13 | 3.45E-12 | yes | down | 296.52 | 321.69 | 272.46 | 356.93 | 361.25 | 336.68 | 296.89 | 351.62 |
| LOC100345019 | 2.591 | 1.373611 | 1.29E-13 | 3.61E-12 | yes | up | 12.56 | 13.89 | 16.29 | 92.08 | 70.91 | 96.3 | 14.24667 | 86.43 |
| LOC100328613 | 13.377 | 3.741723 | 1.42E-13 | 3.94E-12 | yes | up | 0.55 | 0.85 | 2.65 | 31.54 | 32.21 | 62 | 1.35 | 41.91667 |
| SLC31A1 | 0.464 | -1.10706 | 1.42E-13 | 3.94E-12 | yes | down | 9.65 | 12.53 | 10.83 | 11.9 | 10.48 | 13.44 | 11.00333 | 11.94 |
| PTCD3 | 0.423 | -1.242 | 1.45E-13 | 4.03E-12 | yes | down | 14.78 | 22.37 | 19.45 | 17.38 | 18.55 | 16.42 | 18.86667 | 17.45 |
| MTG2 | 0.43 | -1.21654 | 1.49E-13 | 4.13E-12 | yes | down | 15.87 | 22.39 | 18.95 | 15.2 | 12.52 | 14.09 | 19.07 | 13.93667 |
| TRUB1 | 0.285 | -1.81065 | 1.53E-13 | 4.22E-12 | yes | down | 8.37 | 7.4 | 8.2 | 4.31 | 5.56 | 5.9 | 7.99 | 5.256667 |
| YEATS4 | 0.371 | -1.42945 | 1.54E-13 | 4.26E-12 | yes | down | 10.11 | 13.69 | 10.67 | 9.48 | 10.84 | 9.61 | 11.49 | 9.976667 |
| ACSM5 | 3.56 | 1.831901 | 1.65E-13 | 4.54E-12 | yes | up | 8.83 | 14.75 | 9.47 | 124.76 | 67.63 | 80.98 | 11.01667 | 91.12333 |
| BMPER | 5.395 | 2.431556 | 1.66E-13 | 4.56E-12 | yes | up | 0.28 | 0.52 | 0.42 | 4.52 | 6.04 | 4.55 | 0.406667 | 5.036667 |
| COL5A2 | 2.394 | 1.259649 | 1.66E-13 | 4.56E-12 | yes | up | 47.09 | 56.95 | 38.24 | 305.94 | 240.76 | 244.55 | 47.42667 | 263.75 |
| C1H9orf153 | 0.262 | -1.93479 | 1.89E-13 | 5.16E-12 | yes | down | 5.02 | 6.5 | 6.03 | 3.81 | 2.32 | 4.54 | 5.85 | 3.556667 |
| DCLK2 | 2.741 | 1.45489 | 2.02E-13 | 5.51E-12 | yes | up | 1.07 | 1.16 | 1.23 | 8.14 | 5.68 | 7.78 | 1.153333 | 7.2 |
| G6PD | 6.233 | 2.639854 | 2.06E-13 | 5.61E-12 | yes | up | 3.77 | 6.06 | 4.73 | 104.85 | 34.18 | 87.63 | 4.853333 | 75.55333 |
| MRPL19 | 0.461 | -1.11654 | 2.06E-13 | 5.62E-12 | yes | down | 31.11 | 35.32 | 37.19 | 36.69 | 28.41 | 34.72 | 34.54 | 33.27333 |
| PSMF1 | 0.407 | -1.29661 | 2.07E-13 | 5.63E-12 | yes | down | 14.73 | 23.28 | 20.55 | 17.71 | 17.88 | 19.29 | 19.52 | 18.29333 |
| VARS | 0.44 | -1.18344 | 2.2E-13 | 5.95E-12 | yes | down | 5.46 | 7.52 | 6.08 | 6.48 | 6.15 | 6.75 | 6.353333 | 6.46 |
| ENPP1 | 3.985 | 1.99467 | 2.2E-13 | 5.95E-12 | yes | up | 3.27 | 3.48 | 3.09 | 27.34 | 40.36 | 22.8 | 3.28 | 30.16667 |
| MED4 | 0.455 | -1.13669 | 2.32E-13 | 6.28E-12 | yes | down | 22.74 | 26.64 | 26.68 | 26.39 | 25.67 | 29.27 | 25.35333 | 27.11 |
| WDR83 | 0.365 | -1.45391 | 2.41E-13 | 6.51E-12 | yes | down | 22.46 | 21.97 | 20.38 | 21.36 | 14.8 | 20.14 | 21.60333 | 18.76667 |
| NTRK2 | 2.783 | 1.476658 | 2.5E-13 | 6.74E-12 | yes | up | 1.4 | 1.75 | 2.07 | 12.32 | 11.86 | 9.41 | 1.74 | 11.19667 |
| LOC100341136 | 5.07 | 2.341969 | 2.58E-13 | 6.93E-12 | yes | up | 0.57 | 0.82 | 1.54 | 12.79 | 11.17 | 10.16 | 0.976667 | 11.37333 |
| CHAC1 | 0.035 | -4.85502 | 2.58E-13 | 6.94E-12 | yes | down | 1.9 | 0.96 | 1.56 | 0.08 | 0.03 | 0.24 | 1.473333 | 0.116667 |
| ME3 | 0.161 | -2.63295 | 3.1E-13 | 8.31E-12 | yes | down | 8.91 | 8 | 7.69 | 0.95 | 2.2 | 2.44 | 8.2 | 1.863333 |
| CTNNBL1 | 0.457 | -1.12888 | 3.12E-13 | 8.35E-12 | yes | down | 36.57 | 52.38 | 46.78 | 53.25 | 40.89 | 49.26 | 45.24333 | 47.8 |
| VDAC3 | 0.328 | -1.60655 | 3.2E-13 | 8.54E-12 | yes | down | 114.91 | 178.03 | 115.09 | 102.84 | 119.74 | 102.4 | 136.01 | 108.3267 |
| LOC108176153 | 0.259 | -1.95005 | 3.26E-13 | 8.7E-12 | yes | down | 39.33 | 52.22 | 47.73 | 20.8 | 28.86 | 34.87 | 46.42667 | 28.17667 |
| GAL3ST4 | 2.987 | 1.5788 | 3.58E-13 | 9.52E-12 | yes | up | 1.9 | 1.89 | 1.6 | 12.53 | 12.87 | 11.42 | 1.796667 | 12.27333 |
| SYN2 | 0.345 | -1.53631 | 3.61E-13 | 9.58E-12 | yes | down | 6.87 | 8.86 | 7.15 | 7.73 | 4.86 | 5.99 | 7.626667 | 6.193333 |
| SLIRP | 0.38 | -1.39448 | 4.51E-13 | 1.19E-11 | yes | down | 183.54 | 222.58 | 173.03 | 185.47 | 174.75 | 149.56 | 193.05 | 169.9267 |
| KYAT1 | 0.392 | -1.34953 | 4.67E-13 | 1.23E-11 | yes | down | 13.82 | 18.35 | 17.26 | 15.08 | 16.07 | 12.53 | 16.47667 | 14.56 |
| GALNT16 | 2.408 | 1.268002 | 4.71E-13 | 1.24E-11 | yes | up | 5.2 | 8.23 | 5.89 | 36.45 | 32.16 | 38.55 | 6.44 | 35.72 |
| C18H10orf2 | 0.297 | -1.7491 | 4.72E-13 | 1.24E-11 | yes | down | 6.44 | 7.23 | 9.88 | 5.25 | 4.56 | 6.44 | 7.85 | 5.416667 |
| VGLL3 | 4.257 | 2.089803 | 4.77E-13 | 1.25E-11 | yes | up | 0.55 | 0.75 | 0.37 | 6.13 | 4.16 | 6.28 | 0.556667 | 5.523333 |
| ESRRA | 0.259 | -1.95156 | 4.77E-13 | 1.25E-11 | yes | down | 12.2 | 14.44 | 16 | 7.72 | 9.87 | 8.01 | 14.21333 | 8.533333 |
| ENDOG | 0.356 | -1.48838 | 4.78E-13 | 1.25E-11 | yes | down | 12.07 | 14.83 | 12.92 | 10.15 | 10.11 | 12.72 | 13.27333 | 10.99333 |
| NSUN2 | 0.478 | -1.06517 | 4.83E-13 | 1.26E-11 | yes | down | 18.17 | 26.16 | 21.43 | 24.28 | 23.89 | 24.37 | 21.92 | 24.18 |
| KIF16B | 0.472 | -1.08443 | 5.06E-13 | 1.32E-11 | yes | down | 7.14 | 9.68 | 9.34 | 9.53 | 9.66 | 9.69 | 8.72 | 9.626667 |
| LOC100345021 | 0.241 | -2.05336 | 5.1E-13 | 1.33E-11 | yes | down | 31.37 | 42.57 | 41.4 | 25.59 | 12.31 | 26.96 | 38.44667 | 21.62 |
| LOC108177279 | 3.659 | 1.871325 | 5.44E-13 | 1.41E-11 | yes | up | 6.05 | 7 | 8.38 | 58.39 | 46.04 | 78.73 | 7.143333 | 61.05333 |
| LOC108176315 | 0.242 | -2.04798 | 5.45E-13 | 1.41E-11 | yes | down | 11.29 | 17.24 | 22.71 | 10.81 | 10.51 | 14.06 | 17.08 | 11.79333 |
| LOC100346561 | 6.468 | 2.693431 | 5.51E-13 | 1.43E-11 | yes | up | 5.73 | 4.86 | 5.36 | 131.88 | 35.39 | 79.38 | 5.316667 | 82.21667 |
| NEU1 | 0.423 | -1.24097 | 5.83E-13 | 1.51E-11 | yes | down | 18.45 | 20.94 | 18.47 | 16.49 | 17.64 | 18.89 | 19.28667 | 17.67333 |
| CBS | 6.478 | 2.695658 | 5.84E-13 | 1.51E-11 | yes | up | 3.75 | 2.46 | 1.3 | 32.42 | 41.97 | 39.4 | 2.503333 | 37.93 |
| RIDA | 2.389 | 1.256266 | 6.32E-13 | 1.63E-11 | yes | up | 11.33 | 11.41 | 10.59 | 70.66 | 53.31 | 62.91 | 11.11 | 62.29333 |
| SLC30A9 | 0.45 | -1.1534 | 6.67E-13 | 1.72E-11 | yes | down | 16.95 | 19.1 | 15.11 | 17.33 | 18.9 | 17.06 | 17.05333 | 17.76333 |
| CISD1 | 0.461 | -1.11682 | 6.76E-13 | 1.74E-11 | yes | down | 85.6 | 94.03 | 112.2 | 111.41 | 96.85 | 106.56 | 97.27667 | 104.94 |
| NAA40 | 0.383 | -1.38329 | 8.6E-13 | 2.21E-11 | yes | down | 4.85 | 7.43 | 6.68 | 5.81 | 5.68 | 5.3 | 6.32 | 5.596667 |
| ACAD8 | 0.439 | -1.18609 | 9.13E-13 | 2.34E-11 | yes | down | 11.7 | 13.96 | 14.28 | 13.14 | 13.12 | 14.51 | 13.31333 | 13.59 |
| MRPS31 | 0.289 | -1.78957 | 9.62E-13 | 2.46E-11 | yes | down | 13.24 | 19.24 | 12.93 | 12.23 | 9.6 | 8.73 | 15.13667 | 10.18667 |
| FBN2 | 0.111 | -3.17487 | 9.77E-13 | 2.5E-11 | yes | down | 0.49 | 0.76 | 0.27 | 0.13 | 0.11 | 0.14 | 0.506667 | 0.126667 |
| SPAG4 | 0.096 | -3.37498 | 1.01E-12 | 2.57E-11 | yes | down | 2.33 | 2.94 | 1.84 | 0.61 | 0.52 | 0.47 | 2.37 | 0.533333 |
| VDAC1 | 0.424 | -1.2383 | 1.02E-12 | 2.59E-11 | yes | down | 305.64 | 424.13 | 306.56 | 329.53 | 386.7 | 369.18 | 345.4433 | 361.8033 |
| LOC103346740 | 0.291 | -1.78086 | 1.09E-12 | 2.76E-11 | yes | down | 119.38 | 133.48 | 150.3 | 106.29 | 88.73 | 96.47 | 134.3867 | 97.16333 |
| LOC100008743 | 2.063 | 1.044961 | 1.16E-12 | 2.94E-11 | yes | up | 7.01 | 9.34 | 8.9 | 37.49 | 36.25 | 39.82 | 8.416667 | 37.85333 |
| LRRK2 | 2.166 | 1.114815 | 1.23E-12 | 3.1E-11 | yes | up | 1.56 | 1.53 | 1.69 | 8.62 | 7.6 | 7.52 | 1.593333 | 7.913333 |
| CTSK | 2.034 | 1.024341 | 1.32E-12 | 3.32E-11 | yes | up | 24.53 | 31.24 | 28.29 | 124.63 | 137.56 | 134.02 | 28.02 | 132.07 |
| PDK2 | 0.339 | -1.55981 | 1.39E-12 | 3.5E-11 | yes | down | 42.96 | 45.52 | 46.7 | 30.9 | 44.65 | 30.54 | 45.06 | 35.36333 |
| SPSB1 | 0.222 | -2.17024 | 1.45E-12 | 3.65E-11 | yes | down | 11.53 | 25.72 | 22.84 | 8.51 | 9.69 | 12.35 | 20.03 | 10.18333 |
| SYNJ2BP | 0.476 | -1.07105 | 1.57E-12 | 3.93E-11 | yes | down | 8.67 | 12.51 | 9.84 | 11.51 | 10.22 | 11.56 | 10.34 | 11.09667 |
| LOC100348435 | 2.044 | 1.031621 | 1.57E-12 | 3.94E-11 | yes | up | 33.84 | 40.17 | 44.45 | 212.29 | 163.27 | 187.14 | 39.48667 | 187.5667 |
| ADRA1A | 0.157 | -2.67576 | 1.69E-12 | 4.23E-11 | yes | down | 15.04 | 21.27 | 19.47 | 4.67 | 2.66 | 9.6 | 18.59333 | 5.643333 |
| FGF10 | 2.699 | 1.432633 | 1.72E-12 | 4.3E-11 | yes | up | 1.79 | 2.13 | 1.7 | 11.61 | 13.23 | 10.34 | 1.873333 | 11.72667 |
| SUOX | 0.121 | -3.04339 | 1.74E-12 | 4.34E-11 | yes | down | 1.85 | 11.45 | 8.07 | 0.48 | 1.05 | 0.9 | 7.123333 | 0.81 |
| LOC103349390 | 0.073 | -3.78102 | 1.83E-12 | 4.55E-11 | yes | down | 1.18 | 2.3 | 1.63 | 0.42 | 0.27 | 0.16 | 1.703333 | 0.283333 |
| CLU | 4.223 | 2.078398 | 1.87E-12 | 4.64E-11 | yes | up | 21.58 | 36.75 | 14.37 | 229.37 | 254.35 | 211.56 | 24.23333 | 231.76 |
| LOC103349363 | 0.108 | -3.21701 | 1.91E-12 | 4.74E-11 | yes | down | 1.91 | 2.12 | 2.19 | 0.43 | 0.33 | 0.58 | 2.073333 | 0.446667 |
| NDUFAF1 | 0.418 | -1.26014 | 1.99E-12 | 4.94E-11 | yes | down | 22.76 | 31.43 | 26.97 | 22.01 | 26.63 | 26.22 | 27.05333 | 24.95333 |
| TMX1 | 2.402 | 1.264462 | 2.01E-12 | 4.98E-11 | yes | up | 4.92 | 5.67 | 5.28 | 35.44 | 28.65 | 24.9 | 5.29 | 29.66333 |
| MRPL38 | 0.404 | -1.30901 | 2.03E-12 | 5.01E-11 | yes | down | 17.43 | 27 | 21.04 | 19.85 | 19.37 | 21.86 | 21.82333 | 20.36 |
| NPC1L1 | 20.166 | 4.333831 | 2.07E-12 | 5.09E-11 | yes | up | 0.1 | 0.02 | 0.04 | 2.91 | 2.36 | 1.93 | 0.053333 | 2.4 |
| AVP | 0.052 | -4.25945 | 2.08E-12 | 5.11E-11 | yes | down | 11.2 | 4.7 | 4.93 | 0.42 | 0.89 | 1.02 | 6.943333 | 0.776667 |
| ARSE | 2.763 | 1.466451 | 2.11E-12 | 5.18E-11 | yes | up | 1.26 | 2.17 | 1.86 | 13.68 | 13.02 | 12.24 | 1.763333 | 12.98 |
| LOC100351672 | 3.927 | 1.973479 | 2.16E-12 | 5.3E-11 | yes | up | 7.13 | 7.14 | 9.52 | 108.03 | 50.92 | 62.32 | 7.93 | 73.75667 |
| PERM1 | 0.07 | -3.84592 | 2.18E-12 | 5.34E-11 | yes | down | 7.65 | 9.56 | 6.36 | 0.37 | 2.52 | 0.8 | 7.856667 | 1.23 |
| SLC25A47 | 0.157 | -2.67527 | 2.53E-12 | 6.16E-11 | yes | down | 11.45 | 5.97 | 5.86 | 2.47 | 3.12 | 2.34 | 7.76 | 2.643333 |
| COMTD1 | 0.142 | -2.81355 | 2.62E-12 | 6.38E-11 | yes | down | 4.05 | 5.58 | 3.95 | 1.08 | 1.74 | 1.65 | 4.526667 | 1.49 |
| SORD | 2.261 | 1.17679 | 2.68E-12 | 6.5E-11 | yes | up | 4.39 | 5.37 | 4.17 | 25.68 | 25.16 | 22.14 | 4.643333 | 24.32667 |
| PDSS2 | 0.448 | -1.15975 | 2.87E-12 | 6.97E-11 | yes | down | 7.2 | 9.32 | 8.38 | 8.56 | 8.36 | 8.91 | 8.3 | 8.61 |
| RABL3 | 0.453 | -1.14318 | 3.01E-12 | 7.3E-11 | yes | down | 17.61 | 22.71 | 22.22 | 23.29 | 20.87 | 23.31 | 20.84667 | 22.49 |
| STUM | 3.183 | 1.670589 | 3.19E-12 | 7.73E-11 | yes | up | 0.59 | 0.61 | 0.42 | 4.59 | 3.76 | 3.59 | 0.54 | 3.98 |
| CA2 | 0.359 | -1.47649 | 3.4E-12 | 8.21E-11 | yes | down | 14.39 | 19.68 | 16.95 | 19.55 | 11.96 | 12.45 | 17.00667 | 14.65333 |
| P4HA1 | 2.405 | 1.265754 | 3.41E-12 | 8.22E-11 | yes | up | 11.22 | 15.1 | 10.15 | 64.34 | 62.1 | 76.29 | 12.15667 | 67.57667 |
| GABBR1 | 2.518 | 1.332311 | 3.55E-12 | 8.55E-11 | yes | up | 1.51 | 2.23 | 1.75 | 12.51 | 9.86 | 11.27 | 1.83 | 11.21333 |
| EGLN3 | 0.214 | -2.22756 | 3.84E-12 | 9.22E-11 | yes | down | 29.68 | 47.94 | 30.92 | 25.89 | 9.31 | 18.91 | 36.18 | 18.03667 |
| PPM1H | 2.671 | 1.417458 | 3.92E-12 | 9.38E-11 | yes | up | 1.96 | 2.95 | 3.39 | 18.41 | 15.59 | 17.26 | 2.766667 | 17.08667 |
| SLC25A10 | 0.475 | -1.07276 | 4.01E-12 | 9.58E-11 | yes | down | 26 | 25.84 | 25.09 | 31.68 | 26.23 | 26.22 | 25.64333 | 28.04333 |
| ROBO1 | 4.78 | 2.256891 | 4.09E-12 | 9.76E-11 | yes | up | 0.77 | 2.32 | 1.14 | 6.02 | 7.95 | 6.51 | 1.41 | 6.826667 |
| MUL1 | 0.492 | -1.02467 | 4.48E-12 | 1.07E-10 | yes | down | 11.13 | 14.6 | 14.06 | 14.96 | 14.75 | 15.26 | 13.26333 | 14.99 |
| LGALS1 | 3.934 | 1.976041 | 4.49E-12 | 1.07E-10 | yes | up | 175.25 | 264.92 | 128.14 | 2052.8 | 1920.25 | 1189.55 | 189.4367 | 1720.867 |
| CLTCL1 | 4.519 | 2.175879 | 4.5E-12 | 1.07E-10 | yes | up | 2.91 | 3.8 | 1.47 | 25.38 | 31.69 | 28.66 | 2.726667 | 28.57667 |
| PCSK6 | 2.047 | 1.033383 | 4.68E-12 | 1.11E-10 | yes | up | 6.35 | 7.25 | 5.48 | 32.51 | 29.01 | 29.14 | 6.36 | 30.22 |
| NDUFAF3 | 0.432 | -1.21018 | 4.9E-12 | 1.16E-10 | yes | down | 32.27 | 38.09 | 30.46 | 33.79 | 35.36 | 28.22 | 33.60667 | 32.45667 |
| CKS2 | 5.97 | 2.577664 | 5.12E-12 | 1.21E-10 | yes | up | 3.73 | 4.94 | 4.46 | 70.98 | 76.94 | 36 | 4.376667 | 61.30667 |
| SRP9 | 0.486 | -1.03964 | 5.41E-12 | 1.28E-10 | yes | down | 33.24 | 37.64 | 36.37 | 38.4 | 43.22 | 39.56 | 35.75 | 40.39333 |
| HTRA1 | 2.616 | 1.38725 | 5.93E-12 | 1.4E-10 | yes | up | 91.97 | 140.04 | 133.09 | 806.23 | 816.2 | 585.01 | 121.7 | 735.8133 |
| ACAD10 | 0.351 | -1.51197 | 6.11E-12 | 1.44E-10 | yes | down | 16.31 | 21.23 | 20.07 | 15.75 | 11.3 | 19.9 | 19.20333 | 15.65 |
| ARSG | 5.781 | 2.531369 | 6.25E-12 | 1.47E-10 | yes | up | 0.22 | 0.26 | 0.27 | 3.91 | 2.66 | 3.67 | 0.25 | 3.413333 |
| EPYC | 9.663 | 3.272436 | 6.28E-12 | 1.47E-10 | yes | up | 0.26 | 1.53 | 0.46 | 16.91 | 16.14 | 14.66 | 0.75 | 15.90333 |
| MMD | 3.533 | 1.82089 | 6.34E-12 | 1.48E-10 | yes | up | 6.36 | 9.74 | 7.62 | 76 | 74.75 | 41.16 | 7.906667 | 63.97 |
| PRAP1 | 36.864 | 5.204142 | 6.35E-12 | 1.48E-10 | yes | up | 0.48 | 0.46 | 0.3 | 41.29 | 53 | 34.75 | 0.413333 | 43.01333 |
| INPP4A | 2.221 | 1.151395 | 6.41E-12 | 1.5E-10 | yes | up | 1.74 | 2.09 | 1.89 | 11.42 | 9.25 | 8.86 | 1.906667 | 9.843333 |
| LOC100342335 | 2.652 | 1.407017 | 7.16E-12 | 1.67E-10 | yes | up | 2.02 | 2.64 | 2.52 | 14.38 | 13.28 | 13.7 | 2.393333 | 13.78667 |
| SUCLG2 | 0.477 | -1.06746 | 7.65E-12 | 1.78E-10 | yes | down | 147.08 | 158.8 | 122.41 | 154.01 | 160.95 | 165.44 | 142.7633 | 160.1333 |
| GMPR | 0.037 | -4.76098 | 7.85E-12 | 1.82E-10 | yes | down | 93.83 | 206.85 | 106.37 | 3.54 | 13.4 | 16.08 | 135.6833 | 11.00667 |
| FGFR4 | 0.243 | -2.0425 | 7.92E-12 | 1.84E-10 | yes | down | 3.78 | 3.56 | 2.86 | 1.39 | 2.22 | 2.13 | 3.4 | 1.913333 |
| THEM4 | 0.3 | -1.73897 | 7.93E-12 | 1.84E-10 | yes | down | 19.52 | 28.18 | 26.48 | 15.89 | 22.17 | 14.81 | 24.72667 | 17.62333 |
| RPL9 | 2.213 | 1.145961 | 8.31E-12 | 1.92E-10 | yes | up | 46.48 | 60.76 | 51.17 | 180.64 | 191.76 | 150.78 | 52.80333 | 174.3933 |
| ADRB1 | 0.221 | -2.17641 | 8.31E-12 | 1.92E-10 | yes | down | 6.36 | 4.05 | 3.71 | 2.91 | 2.42 | 2.06 | 4.706667 | 2.463333 |
| MRPL51 | 0.476 | -1.07217 | 8.92E-12 | 2.05E-10 | yes | down | 60.71 | 85.04 | 68.37 | 88.56 | 73.02 | 75.54 | 71.37333 | 79.04 |
| CRYL1 | 0.295 | -1.76084 | 9.42E-12 | 2.16E-10 | yes | down | 41 | 62.11 | 56.2 | 52.99 | 25.96 | 31.2 | 53.10333 | 36.71667 |
| NFS1 | 0.438 | -1.1909 | 9.43E-12 | 2.16E-10 | yes | down | 26.21 | 42.97 | 36.96 | 37.3 | 35.63 | 33.33 | 35.38 | 35.42 |
| SYCE3 | 0.091 | -3.46444 | 9.86E-12 | 2.26E-10 | yes | down | 4.16 | 2.14 | 5.24 | 0.43 | 0.14 | 2.25 | 3.846667 | 0.94 |
| LOC100343291 | 4.171 | 2.060408 | 1.03E-11 | 2.35E-10 | yes | up | 0.48 | 0.44 | 0.44 | 3.88 | 4.96 | 4.36 | 0.453333 | 4.4 |
| MAP6D1 | 4.994 | 2.320148 | 1.05E-11 | 2.39E-10 | yes | up | 0.43 | 0.65 | 0.74 | 5.98 | 7.41 | 7.82 | 0.606667 | 7.07 |
| GTF2B | 0.302 | -1.72773 | 1.11E-11 | 2.53E-10 | yes | down | 53.33 | 36.12 | 33.84 | 28.3 | 27.98 | 31.56 | 41.09667 | 29.28 |
| LOC108176887 | 0.002 | -9.12699 | 1.15E-11 | 2.61E-10 | yes | down | 28.87 | 14.98 | 7.35 | 0 | 0 | 0 | 17.06667 | 0 |
| MPI | 0.317 | -1.65536 | 1.28E-11 | 2.9E-10 | yes | down | 7.6 | 10.02 | 6.19 | 7.04 | 5.99 | 5 | 7.936667 | 6.01 |
| MRPS24 | 0.367 | -1.44581 | 1.33E-11 | 3.01E-10 | yes | down | 55.67 | 47.1 | 57.27 | 43.59 | 52.1 | 42.39 | 53.34667 | 46.02667 |
| CLSTN2 | 0.04 | -4.6437 | 1.34E-11 | 3.03E-10 | yes | down | 0.23 | 0.13 | 0.2 | 0.01 | 0.02 | 0.02 | 0.186667 | 0.016667 |
| STK17B | 0.366 | -1.44898 | 1.34E-11 | 3.03E-10 | yes | down | 20.5 | 17.26 | 14 | 15.57 | 13.56 | 15.59 | 17.25333 | 14.90667 |
| APMAP | 2.043 | 1.030685 | 1.35E-11 | 3.05E-10 | yes | up | 15.92 | 23.56 | 21.44 | 98.73 | 96.8 | 91.96 | 20.30667 | 95.83 |
| TRPT1 | 0.421 | -1.24644 | 1.36E-11 | 3.08E-10 | yes | down | 16.55 | 23.14 | 19.45 | 22.44 | 15.86 | 18.9 | 19.71333 | 19.06667 |
| CFTR | 0.014 | -6.11381 | 1.37E-11 | 3.09E-10 | yes | down | 0.65 | 0.35 | 0.82 | 0.03 | 0 | 0.03 | 0.606667 | 0.02 |
| RASSF2 | 2.546 | 1.34851 | 1.41E-11 | 3.17E-10 | yes | up | 3.48 | 4.55 | 3.24 | 27.21 | 20.15 | 18.49 | 3.756667 | 21.95 |
| ATP5S | 0.418 | -1.25684 | 1.45E-11 | 3.27E-10 | yes | down | 23.52 | 27.34 | 26.82 | 21.25 | 25.71 | 28.39 | 25.89333 | 25.11667 |
| LOC100347480 | 0.102 | -3.28951 | 1.48E-11 | 3.31E-10 | yes | down | 41.36 | 44.03 | 44.76 | 4.37 | 5.94 | 20.53 | 43.38333 | 10.28 |
| LRRC8B | 3.175 | 1.666781 | 1.5E-11 | 3.36E-10 | yes | up | 1.37 | 1.61 | 1.57 | 11.03 | 8.02 | 14.52 | 1.516667 | 11.19 |
| FAM213A | 2.129 | 1.090408 | 1.52E-11 | 3.39E-10 | yes | up | 122.53 | 134.01 | 130.82 | 567.91 | 620.47 | 732.24 | 129.12 | 640.2067 |
| MRS2 | 0.486 | -1.03986 | 1.55E-11 | 3.47E-10 | yes | down | 31.64 | 38.38 | 40.47 | 45.9 | 38.45 | 34.6 | 36.83 | 39.65 |
| VCP | 0.48 | -1.05862 | 1.67E-11 | 3.72E-10 | yes | down | 79.52 | 111.68 | 103.04 | 101.01 | 104.41 | 120.17 | 98.08 | 108.53 |
| S100A11 | 0.394 | -1.34196 | 1.76E-11 | 3.89E-10 | yes | down | 608.76 | 753.74 | 723.08 | 583.14 | 782 | 555.23 | 695.1933 | 640.1233 |
| TMEM139 | 0.068 | -3.87908 | 1.87E-11 | 4.14E-10 | yes | down | 5.56 | 2.76 | 8.42 | 0.51 | 0.99 | 1.18 | 5.58 | 0.893333 |
| C17H14orf37 | 0.201 | -2.31203 | 2E-11 | 4.42E-10 | yes | down | 8.64 | 4.33 | 7.97 | 14.22 | 16.45 | 18.14 | 6.98 | 16.27 |
| LOC100009551 | 23.807 | 4.573306 | 2E-11 | 4.42E-10 | yes | up | 38.89 | 10.6 | 14.41 | 1534.89 | 923.33 | 1170.32 | 21.3 | 1209.513 |
| MTG1 | 0.463 | -1.1116 | 2.01E-11 | 4.43E-10 | yes | down | 18.71 | 25.49 | 20.01 | 25.62 | 20.71 | 22.3 | 21.40333 | 22.87667 |
| CCNA2 | 22.07 | 4.463985 | 2.29E-11 | 5.04E-10 | yes | up | 0.19 | 0.04 | 0.37 | 13.11 | 14.87 | 5.12 | 0.2 | 11.03333 |
| ADIPOQ | 3.232 | 1.692244 | 2.38E-11 | 5.23E-10 | yes | up | 151.88 | 228.7 | 207.21 | 1375.9 | 1883.85 | 1088.34 | 195.93 | 1449.363 |
| LOC103347534 | 0.261 | -1.93737 | 2.48E-11 | 5.44E-10 | yes | down | 19.29 | 22.66 | 25.11 | 17.9 | 13.65 | 9.98 | 22.35333 | 13.84333 |
| LOC103347061 | 0.288 | -1.79385 | 2.53E-11 | 5.52E-10 | yes | down | 23.02 | 33.42 | 36.51 | 24.73 | 17 | 21.81 | 30.98333 | 21.18 |
| SLC25A12 | 0.279 | -1.8438 | 2.53E-11 | 5.53E-10 | yes | down | 9.19 | 14.58 | 9.14 | 6.6 | 9.08 | 5.28 | 10.97 | 6.986667 |
| MYO5B | 0.118 | -3.07929 | 2.56E-11 | 5.57E-10 | yes | down | 1.25 | 0.88 | 1.2 | 0.21 | 0.2 | 0.51 | 1.11 | 0.306667 |
| GDF5 | 9.737 | 3.283468 | 2.62E-11 | 5.7E-10 | yes | up | 0.09 | 0.33 | 0.42 | 6.96 | 5.22 | 6.92 | 0.28 | 6.366667 |
| TYMS | 4.829 | 2.271699 | 2.63E-11 | 5.7E-10 | yes | up | 1.15 | 1.37 | 1.58 | 17.45 | 18.6 | 10.24 | 1.366667 | 15.43 |
| ZNF827 | 0.409 | -1.29151 | 2.66E-11 | 5.77E-10 | yes | down | 3.54 | 4.33 | 4.85 | 4.86 | 3.27 | 3.97 | 4.24 | 4.033333 |
| CDH23 | 0.26 | -1.94083 | 2.73E-11 | 5.9E-10 | yes | down | 2.95 | 2.47 | 3.97 | 2.29 | 1.22 | 2.47 | 3.13 | 1.993333 |
| CLPB | 0.439 | -1.18749 | 2.85E-11 | 6.15E-10 | yes | down | 9.06 | 13.25 | 12.19 | 12.89 | 9.61 | 12.59 | 11.5 | 11.69667 |
| EDIL3 | 4.779 | 2.256729 | 3.06E-11 | 6.57E-10 | yes | up | 0.24 | 0.36 | 0.38 | 4.83 | 3.58 | 2.63 | 0.326667 | 3.68 |
| ABCD2 | 3.423 | 1.775265 | 3.13E-11 | 6.72E-10 | yes | up | 7.35 | 13.56 | 14.11 | 112.53 | 62.87 | 76.14 | 11.67333 | 83.84667 |
| LOC103348123 | 0.016 | -6.0081 | 3.18E-11 | 6.81E-10 | yes | down | 1.32 | 3.14 | 2.86 | 0.59 | 0 | 0.13 | 2.44 | 0.24 |
| TMEM161B | 0.335 | -1.57914 | 3.21E-11 | 6.86E-10 | yes | down | 6 | 7.89 | 7.39 | 4.9 | 5.02 | 7.1 | 7.093333 | 5.673333 |
| HSF4 | 5.944 | 2.571538 | 3.35E-11 | 7.16E-10 | yes | up | 0.23 | 0.45 | 0.53 | 5.23 | 5.06 | 5.24 | 0.403333 | 5.176667 |
| MARCKS | 2.555 | 1.353245 | 3.5E-11 | 7.47E-10 | yes | up | 6.11 | 10.67 | 9.83 | 59.46 | 51.99 | 46.42 | 8.87 | 52.62333 |
| IMP4 | 0.463 | -1.11225 | 3.57E-11 | 7.62E-10 | yes | down | 10.96 | 15.49 | 13.78 | 14.42 | 12.77 | 15.87 | 13.41 | 14.35333 |
| GPX7 | 2.67 | 1.41695 | 3.63E-11 | 7.73E-10 | yes | up | 10.16 | 15.14 | 10.16 | 69.92 | 83.13 | 65.02 | 11.82 | 72.69 |
| LACTB2 | 0.355 | -1.49356 | 3.9E-11 | 8.28E-10 | yes | down | 47.2 | 35.18 | 46.89 | 32.82 | 35.81 | 38.9 | 43.09 | 35.84333 |
| ATAD2 | 3.843 | 1.942228 | 3.98E-11 | 8.44E-10 | yes | up | 0.65 | 0.72 | 0.62 | 7.34 | 6.53 | 3.94 | 0.663333 | 5.936667 |
| LOC100348091 | 0.297 | -1.75377 | 4.12E-11 | 8.72E-10 | yes | down | 7.58 | 11.03 | 11.94 | 5.95 | 8.78 | 8.52 | 10.18333 | 7.75 |
| PLBD1 | 2.796 | 1.483239 | 4.12E-11 | 8.72E-10 | yes | up | 9.35 | 13.5 | 10.56 | 71.01 | 87.63 | 56.39 | 11.13667 | 71.67667 |
| LOC100356693 | 0.463 | -1.11193 | 4.27E-11 | 9.01E-10 | yes | down | 40.62 | 49.3 | 51.1 | 51.93 | 55.07 | 44.92 | 47.00667 | 50.64 |
| KNL1 | 13.639 | 3.769659 | 4.43E-11 | 9.34E-10 | yes | up | 0.05 | 0.08 | 0.06 | 2.24 | 3 | 0.69 | 0.063333 | 1.976667 |
| TRIM77 | 6.741 | 2.753068 | 4.51E-11 | 9.5E-10 | yes | up | 0.45 | 0.19 | 0.39 | 5.72 | 5.42 | 5.14 | 0.343333 | 5.426667 |
| SOD3 | 3.324 | 1.732906 | 4.56E-11 | 9.57E-10 | yes | up | 17.07 | 21.06 | 18.44 | 87.24 | 154.17 | 140.17 | 18.85667 | 127.1933 |
| NOTCH2 | 2.387 | 1.255392 | 4.8E-11 | 1E-09 | yes | up | 3.36 | 3.74 | 3.21 | 18.08 | 15.59 | 23.44 | 3.436667 | 19.03667 |
| KIF23 | 15.085 | 3.915052 | 4.98E-11 | 1.04E-09 | yes | up | 0.25 | 0.48 | 0.09 | 9.7 | 13.22 | 3.37 | 0.273333 | 8.763333 |
| DLGAP5 | 13.344 | 3.738162 | 5.02E-11 | 1.05E-09 | yes | up | 0.13 | 0.23 | 0.11 | 6.58 | 8.17 | 2.32 | 0.156667 | 5.69 |
| FKBP10 | 2.397 | 1.260986 | 5.33E-11 | 1.11E-09 | yes | up | 3.7 | 5.68 | 3.68 | 26.54 | 23.39 | 22.64 | 4.353333 | 24.19 |
| VNN1 | 16.904 | 4.079267 | 5.44E-11 | 1.13E-09 | yes | up | 0.37 | 0.14 | 0.23 | 5.37 | 16.23 | 6.13 | 0.246667 | 9.243333 |
| BCL2L1 | 0.378 | -1.40271 | 5.8E-11 | 1.21E-09 | yes | down | 43.13 | 47.35 | 34.95 | 32.79 | 35.55 | 47.36 | 41.81 | 38.56667 |
| KIF2C | 19.587 | 4.291793 | 5.89E-11 | 1.22E-09 | yes | up | 0.1 | 0.29 | 0.04 | 7.64 | 8.27 | 2.65 | 0.143333 | 6.186667 |
| ITGBL1 | 5.171 | 2.370404 | 5.99E-11 | 1.24E-09 | yes | up | 1.54 | 2.91 | 2.52 | 18.5 | 43.13 | 21.17 | 2.323333 | 27.6 |
| ETFBKMT | 0.322 | -1.63656 | 6.05E-11 | 1.25E-09 | yes | down | 6.46 | 8.17 | 7.25 | 5.17 | 3.79 | 3.6 | 7.293333 | 4.186667 |
| TMEM37 | 0.203 | -2.30294 | 6.07E-11 | 1.25E-09 | yes | down | 50.59 | 75.19 | 90.72 | 23.66 | 23.81 | 53.76 | 72.16667 | 33.74333 |
| SORT1 | 2.006 | 1.004034 | 6.22E-11 | 1.29E-09 | yes | up | 10.18 | 11.48 | 10.88 | 45.73 | 48.25 | 56.63 | 10.84667 | 50.20333 |
| TIMM50 | 0.363 | -1.46092 | 6.49E-11 | 1.34E-09 | yes | down | 31.69 | 53.32 | 40.45 | 44.13 | 33.76 | 27.81 | 41.82 | 35.23333 |
| CENPF | 11.094 | 3.471679 | 6.73E-11 | 1.39E-09 | yes | up | 0.22 | 0.2 | 0.08 | 6.3 | 5.25 | 1.5 | 0.166667 | 4.35 |
| CATSPER1 | 4.914 | 2.296864 | 6.84E-11 | 1.41E-09 | yes | up | 0.49 | 1.01 | 1.15 | 12.48 | 6.83 | 11.07 | 0.883333 | 10.12667 |
| G6PC3 | 0.498 | -1.00482 | 7.13E-11 | 1.47E-09 | yes | down | 23.36 | 32.63 | 26.92 | 35.22 | 27.75 | 32.99 | 27.63667 | 31.98667 |
| ALKBH7 | 0.313 | -1.6753 | 7.41E-11 | 1.52E-09 | yes | down | 12.8 | 16.03 | 9.88 | 10.65 | 9.77 | 7.8 | 12.90333 | 9.406667 |
| ATP6V1A | 0.436 | -1.19777 | 7.52E-11 | 1.54E-09 | yes | down | 22.66 | 36.41 | 31.78 | 32.95 | 25.68 | 34.9 | 30.28333 | 31.17667 |
| RTN4IP1 | 0.234 | -2.09703 | 7.53E-11 | 1.54E-09 | yes | down | 10.85 | 11.61 | 17.74 | 6.65 | 5.12 | 10.17 | 13.4 | 7.313333 |
| AADAT | 0.388 | -1.36561 | 7.73E-11 | 1.58E-09 | yes | down | 6.65 | 9.98 | 9.4 | 9.63 | 6.71 | 6.96 | 8.676667 | 7.766667 |
| APOOL | 0.46 | -1.12051 | 7.82E-11 | 1.6E-09 | yes | down | 26.12 | 29.46 | 31.15 | 36.14 | 30.67 | 26.52 | 28.91 | 31.11 |
| SET | 2.545 | 1.34778 | 7.97E-11 | 1.62E-09 | yes | up | 9.43 | 9.82 | 8.97 | 73.7 | 49.14 | 43.77 | 9.406667 | 55.53667 |
| PECR | 0.468 | -1.09403 | 8E-11 | 1.63E-09 | yes | down | 12.11 | 14.24 | 13.56 | 16.43 | 12.56 | 14.67 | 13.30333 | 14.55333 |
| CDKL4 | 0.129 | -2.95776 | 8.42E-11 | 1.71E-09 | yes | down | 1.01 | 0.97 | 0.88 | 0.39 | 0.2 | 0.28 | 0.953333 | 0.29 |
| COL12A1 | 0.384 | -1.38054 | 8.51E-11 | 1.73E-09 | yes | down | 14.27 | 13.39 | 12.17 | 12.31 | 9.25 | 14.66 | 13.27667 | 12.07333 |
| RPS6KA6 | 2.325 | 1.21694 | 8.76E-11 | 1.77E-09 | yes | up | 1.07 | 1.13 | 0.93 | 6.69 | 5.26 | 7.18 | 1.043333 | 6.376667 |
| GSE1 | 0.474 | -1.07671 | 8.91E-11 | 1.8E-09 | yes | down | 10.78 | 15.01 | 10.59 | 14.83 | 12.8 | 12.21 | 12.12667 | 13.28 |
| FABP7 | 41.799 | 5.385414 | 9.56E-11 | 1.93E-09 | yes | up | 5.65 | 4.14 | 4.47 | 907.09 | 89.54 | 458.69 | 4.753333 | 485.1067 |
| LOC100354074 | 0.32 | -1.64502 | 9.62E-11 | 1.94E-09 | yes | down | 31.38 | 51.01 | 40.75 | 25.81 | 24.76 | 40.36 | 41.04667 | 30.31 |
| NSUN4 | 0.284 | -1.81475 | 9.95E-11 | 2E-09 | yes | down | 11.92 | 11.59 | 20.94 | 10.04 | 10 | 11.4 | 14.81667 | 10.48 |
| ADTRP | 8.962 | 3.163832 | 1.01E-10 | 2.03E-09 | yes | up | 3.94 | 1.07 | 2.49 | 45.55 | 44.1 | 87.45 | 2.5 | 59.03333 |
| PRKCZ | 10.183 | 3.348062 | 1.07E-10 | 2.14E-09 | yes | up | 1.34 | 0.74 | 1 | 24.47 | 29.43 | 20.09 | 1.026667 | 24.66333 |
| AARS2 | 0.452 | -1.14482 | 1.17E-10 | 2.33E-09 | yes | down | 4.77 | 6.06 | 5.54 | 6.71 | 4.61 | 5.79 | 5.456667 | 5.703333 |
| LOC103351772 | 0.093 | -3.42216 | 1.27E-10 | 2.54E-09 | yes | down | 180.65 | 135.7 | 95.68 | 35.6 | 16.68 | 51.18 | 137.3433 | 34.48667 |
| CAMK1 | 2.143 | 1.099802 | 1.28E-10 | 2.55E-09 | yes | up | 10.34 | 14.03 | 11.01 | 61.01 | 60.51 | 56.43 | 11.79333 | 59.31667 |
| OPN3 | 2.937 | 1.554164 | 1.37E-10 | 2.72E-09 | yes | up | 1.39 | 1.29 | 1.16 | 8.67 | 8.1 | 8.61 | 1.28 | 8.46 |
| LOC103352357 | 0.211 | -2.24735 | 1.42E-10 | 2.81E-09 | yes | down | 2.09 | 1.6 | 3.13 | 1.17 | 0.59 | 1.36 | 2.273333 | 1.04 |
| ADAMTS7 | 2.835 | 1.503106 | 1.48E-10 | 2.93E-09 | yes | up | 1.26 | 1.85 | 1.52 | 13.38 | 9.22 | 7.93 | 1.543333 | 10.17667 |
| SCN5A | 0.032 | -4.98268 | 1.52E-10 | 3E-09 | yes | down | 0.31 | 0.73 | 0.27 | 0.05 | 0 | 0.05 | 0.436667 | 0.033333 |
| LOC100358789 | 0.3 | -1.73694 | 1.55E-10 | 3.07E-09 | yes | down | 23.42 | 45.78 | 32.64 | 23.07 | 29.15 | 17.5 | 33.94667 | 23.24 |
| LOC103347367 | 0.42 | -1.25152 | 1.57E-10 | 3.1E-09 | yes | down | 53.38 | 79.13 | 73.5 | 80.11 | 52.52 | 69.84 | 68.67 | 67.49 |
| LOC100343255 | 0.471 | -1.08493 | 1.71E-10 | 3.38E-09 | yes | down | 602.86 | 816.67 | 718.62 | 950.54 | 616.57 | 823.11 | 712.7167 | 796.74 |
| LRRC8D | 0.461 | -1.11753 | 1.73E-10 | 3.4E-09 | yes | down | 6.62 | 9.21 | 7.36 | 8.9 | 8.59 | 7.22 | 7.73 | 8.236667 |
| PSRC1 | 7.462 | 2.899567 | 1.79E-10 | 3.53E-09 | yes | up | 0.55 | 0.38 | 0.52 | 12.25 | 8.01 | 5.09 | 0.483333 | 8.45 |
| LOC103346593 | 0.122 | -3.0376 | 1.82E-10 | 3.57E-09 | yes | down | 9.2 | 16.33 | 4.38 | 2.38 | 4.17 | 1.72 | 9.97 | 2.756667 |
| OLFML3 | 2.398 | 1.262043 | 1.86E-10 | 3.65E-09 | yes | up | 11.57 | 19.48 | 13.41 | 78.88 | 74.73 | 91.8 | 14.82 | 81.80333 |
| LOC108176462 | 0.021 | -5.58554 | 1.89E-10 | 3.7E-09 | yes | down | 5.89 | 4.98 | 1.88 | 0.1 | 0 | 0.51 | 4.25 | 0.203333 |
| CYP2A11 | 59.308 | 5.890164 | 1.91E-10 | 3.74E-09 | yes | up | 7.55 | 1.29 | 1.26 | 818.14 | 414.89 | 293.83 | 3.366667 | 508.9533 |
| LOC108176518 | 0.289 | -1.79297 | 1.95E-10 | 3.82E-09 | yes | down | 5.4 | 5.81 | 5.88 | 5.06 | 2.1 | 2.8 | 5.696667 | 3.32 |
| ECHDC2 | 0.419 | -1.2563 | 2.04E-10 | 3.97E-09 | yes | down | 35.01 | 32.39 | 36.64 | 35.48 | 27.26 | 39.63 | 34.68 | 34.12333 |
| MRPL13 | 0.447 | -1.1606 | 2.17E-10 | 4.23E-09 | yes | down | 25.84 | 33.95 | 30.69 | 38.61 | 26.4 | 30.75 | 30.16 | 31.92 |
| CD38 | 3.005 | 1.587482 | 2.31E-10 | 4.48E-09 | yes | up | 0.53 | 0.93 | 0.46 | 4.68 | 3.54 | 5.05 | 0.64 | 4.423333 |
| LOC103348242 | 0.1 | -3.31786 | 2.4E-10 | 4.65E-09 | yes | down | 19.6 | 31.45 | 61.92 | 15.18 | 14.96 | 3.24 | 37.65667 | 11.12667 |
| COL15A1 | 3.745 | 1.905147 | 2.43E-10 | 4.7E-09 | yes | up | 22.25 | 32.38 | 14.12 | 233.88 | 220.09 | 139.14 | 22.91667 | 197.7033 |
| CCDC190 | 4.094 | 2.033507 | 2.46E-10 | 4.75E-09 | yes | up | 0.89 | 0.95 | 1.14 | 13.75 | 11.18 | 19.43 | 0.993333 | 14.78667 |
| LUM | 2.558 | 1.354851 | 2.47E-10 | 4.77E-09 | yes | up | 79.38 | 120.57 | 69.2 | 474.39 | 524.62 | 582.63 | 89.71667 | 527.2133 |
| LMF2 | 2.204 | 1.140336 | 2.54E-10 | 4.9E-09 | yes | up | 3.34 | 5 | 3.76 | 22.93 | 19.36 | 19.52 | 4.033333 | 20.60333 |
| IFT57 | 0.402 | -1.31646 | 2.56E-10 | 4.93E-09 | yes | down | 22.9 | 20.85 | 22.79 | 17.51 | 21.1 | 19.02 | 22.18 | 19.21 |
| C19H17orf50 | 2.882 | 1.527135 | 2.59E-10 | 4.97E-09 | yes | up | 5.33 | 7 | 10.59 | 53.64 | 40.77 | 51.17 | 7.64 | 48.52667 |
| LOC100338849 | 0.342 | -1.54793 | 2.61E-10 | 5.02E-09 | yes | down | 7.23 | 5.86 | 6.68 | 5.26 | 5.35 | 5.27 | 6.59 | 5.293333 |
| SDC2 | 2.711 | 1.438603 | 2.62E-10 | 5.03E-09 | yes | up | 13.23 | 13.19 | 13.19 | 62.52 | 86.23 | 99.81 | 13.20333 | 82.85333 |
| PNPO | 0.32 | -1.64527 | 2.7E-10 | 5.17E-09 | yes | down | 11.36 | 13.13 | 16.52 | 8 | 9.14 | 13.21 | 13.67 | 10.11667 |
| LOC100341581 | 4.046 | 2.016426 | 2.74E-10 | 5.24E-09 | yes | up | 0.36 | 0.54 | 0.35 | 3.69 | 3.43 | 4.18 | 0.416667 | 3.766667 |
| TANC2 | 2.338 | 1.225518 | 2.77E-10 | 5.29E-09 | yes | up | 1.34 | 1.17 | 0.98 | 6.94 | 5.36 | 6.28 | 1.163333 | 6.193333 |
| MIEF1 | 0.399 | -1.32495 | 2.83E-10 | 5.41E-09 | yes | down | 18.96 | 30.41 | 27.71 | 20.71 | 18.14 | 25.38 | 25.69333 | 21.41 |
| KCTD6 | 0.278 | -1.84643 | 2.88E-10 | 5.48E-09 | yes | down | 37.03 | 31.67 | 46.61 | 18.99 | 24.18 | 34.09 | 38.43667 | 25.75333 |
| TROAP | 12.292 | 3.61966 | 2.99E-10 | 5.68E-09 | yes | up | 0.1 | 0.37 | 0.29 | 8.79 | 9.05 | 2.83 | 0.253333 | 6.89 |
| EMC4 | 0.457 | -1.12832 | 3.05E-10 | 5.79E-09 | yes | down | 50.98 | 65.62 | 65.44 | 54.37 | 61.94 | 58.36 | 60.68 | 58.22333 |
| SNTA1 | 0.418 | -1.25713 | 3.11E-10 | 5.9E-09 | yes | down | 32.12 | 44.68 | 39.75 | 31.4 | 43.39 | 37.2 | 38.85 | 37.33 |
| C8H12orf40 | 109.788 | 6.778576 | 3.13E-10 | 5.93E-09 | yes | up | 0 | 0.03 | 0.16 | 27.41 | 13.14 | 9.3 | 0.063333 | 16.61667 |
| LOC100354761 | 0.464 | -1.10892 | 3.14E-10 | 5.94E-09 | yes | down | 24.76 | 37.35 | 32.09 | 32.1 | 35.3 | 35.37 | 31.4 | 34.25667 |
| SLC35A4 | 0.447 | -1.16277 | 3.2E-10 | 6.04E-09 | yes | down | 15.64 | 25.22 | 24.01 | 22.65 | 20.96 | 18.93 | 21.62333 | 20.84667 |
| TTC9 | 0.336 | -1.57302 | 3.22E-10 | 6.08E-09 | yes | down | 56.35 | 83.32 | 80.42 | 47.23 | 74.42 | 54.08 | 73.36333 | 58.57667 |
| LOC103350039 | 2.107 | 1.074872 | 3.41E-10 | 6.42E-09 | yes | up | 1.35 | 2.22 | 1.91 | 13.18 | 9.79 | 10.01 | 1.826667 | 10.99333 |
| PRC1 | 8.565 | 3.09852 | 3.48E-10 | 6.54E-09 | yes | up | 0.88 | 1.15 | 0.42 | 20.13 | 22.02 | 6.28 | 0.816667 | 16.14333 |
| ERN2 | 18.266 | 4.191107 | 3.54E-10 | 6.65E-09 | yes | up | 0.08 | 0.11 | 0.09 | 2.99 | 1.61 | 7.03 | 0.093333 | 3.876667 |
| NDUFA8 | 0.472 | -1.08324 | 3.55E-10 | 6.65E-09 | yes | down | 147.87 | 169.74 | 140.45 | 214.23 | 148.08 | 146.53 | 152.6867 | 169.6133 |
| LOC100353852 | 4.027 | 2.009695 | 3.58E-10 | 6.71E-09 | yes | up | 1.84 | 1.27 | 0.88 | 15.13 | 12.1 | 9.86 | 1.33 | 12.36333 |
| MPP2 | 0.273 | -1.87201 | 3.58E-10 | 6.71E-09 | yes | down | 5.76 | 7.64 | 12.42 | 6.03 | 4.37 | 6.17 | 8.606667 | 5.523333 |
| LIX1L | 2.099 | 1.069968 | 3.7E-10 | 6.92E-09 | yes | up | 3.54 | 4.41 | 3.97 | 21.77 | 19.62 | 16.69 | 3.973333 | 19.36 |
| DCLK1 | 2.374 | 1.247056 | 3.83E-10 | 7.14E-09 | yes | up | 2.3 | 3.21 | 1.85 | 13.68 | 12.4 | 13.5 | 2.453333 | 13.19333 |
| LOC100348443 | 80.024 | 6.322366 | 3.91E-10 | 7.29E-09 | yes | up | 0.08 | 0.46 | 0.02 | 49.36 | 18.95 | 27.88 | 0.186667 | 32.06333 |
| SDC1 | 0.182 | -2.45513 | 3.99E-10 | 7.42E-09 | yes | down | 9.82 | 27.12 | 22.99 | 6.15 | 6.19 | 12.5 | 19.97667 | 8.28 |
| MT3 | 0.22 | -2.18661 | 4.07E-10 | 7.55E-09 | yes | down | 24.87 | 36.44 | 34.62 | 20.1 | 18.75 | 11.79 | 31.97667 | 16.88 |
| SLC38A1 | 0.387 | -1.37047 | 4.2E-10 | 7.77E-09 | yes | down | 8.72 | 8.44 | 9.98 | 7.48 | 8.14 | 6.88 | 9.046667 | 7.5 |
| ESPL1 | 4.927 | 2.300747 | 4.51E-10 | 8.34E-09 | yes | up | 0.11 | 0.18 | 0.2 | 2.38 | 1.87 | 1.39 | 0.163333 | 1.88 |
| GCNT4 | 336.917 | 8.396248 | 4.64E-10 | 8.57E-09 | yes | up | 0 | 0 | 0 | 4.24 | 0.97 | 4.13 | 0 | 3.113333 |
| MMRN1 | 0.339 | -1.56105 | 4.71E-10 | 8.69E-09 | yes | down | 7.4 | 7.24 | 6.23 | 7.38 | 5.36 | 3.86 | 6.956667 | 5.533333 |
| OXTR | 0.024 | -5.39897 | 4.96E-10 | 9.13E-09 | yes | down | 1.47 | 0.55 | 0.45 | 0.06 | 0.08 | 0 | 0.823333 | 0.046667 |
| PRKAR2B | 0.499 | -1.00396 | 5.32E-10 | 9.79E-09 | yes | down | 247.78 | 348.71 | 348.88 | 350.26 | 353.4 | 399.98 | 315.1233 | 367.88 |
| SFRP4 | 3.039 | 1.603427 | 5.43E-10 | 9.97E-09 | yes | up | 6.22 | 9.57 | 7.14 | 45.43 | 42.85 | 72.26 | 7.643333 | 53.51333 |
| FOXM1 | 12.296 | 3.620096 | 5.48E-10 | 1E-08 | yes | up | 0.21 | 0.31 | 0.16 | 7.48 | 9.75 | 1.89 | 0.226667 | 6.373333 |
| ATXN7L2 | 0.186 | -2.42286 | 5.63E-10 | 1.03E-08 | yes | down | 4 | 7.74 | 6.23 | 2.82 | 2.83 | 4.77 | 5.99 | 3.473333 |
| RACGAP1 | 6.677 | 2.7393 | 5.66E-10 | 1.03E-08 | yes | up | 0.67 | 1.31 | 0.55 | 15.14 | 17.19 | 6.12 | 0.843333 | 12.81667 |
| LOC103352162 | 0.064 | -3.97383 | 5.67E-10 | 1.04E-08 | yes | down | 2.13 | 2.27 | 1.91 | 0.18 | 0.48 | 0.34 | 2.103333 | 0.333333 |
| ADAMTS20 | 0.162 | -2.6249 | 5.73E-10 | 1.05E-08 | yes | down | 0.43 | 0.78 | 0.41 | 0.15 | 0.23 | 0.22 | 0.54 | 0.2 |
| NEDD4L | 0.398 | -1.32797 | 5.75E-10 | 1.05E-08 | yes | down | 4.63 | 7.26 | 8.14 | 6.1 | 6.18 | 5.93 | 6.676667 | 6.07 |
| RANGAP1 | 0.487 | -1.03946 | 5.8E-10 | 1.06E-08 | yes | down | 9.62 | 13.66 | 13.13 | 13.08 | 14.74 | 13.54 | 12.13667 | 13.78667 |
| SMAD7 | 0.404 | -1.30829 | 6.05E-10 | 1.1E-08 | yes | down | 9.32 | 13.7 | 12.21 | 9.41 | 9.34 | 12.93 | 11.74333 | 10.56 |
| ZNF330 | 0.455 | -1.13574 | 6.22E-10 | 1.13E-08 | yes | down | 20.85 | 23.9 | 20.73 | 19.91 | 24.32 | 24.83 | 21.82667 | 23.02 |
| UBAP1L | 0.063 | -3.9942 | 6.38E-10 | 1.15E-08 | yes | down | 2.5 | 3.41 | 4.08 | 1.08 | 0.27 | 0.1 | 3.33 | 0.483333 |
| PHGDH | 2.279 | 1.188226 | 6.42E-10 | 1.16E-08 | yes | up | 34.09 | 49.81 | 49.18 | 274.96 | 238.94 | 188.16 | 44.36 | 234.02 |
| FAM49B | 2.266 | 1.180413 | 7E-10 | 1.26E-08 | yes | up | 5.07 | 7.37 | 5.08 | 27.32 | 29.68 | 32.9 | 5.84 | 29.96667 |
| VRK3 | 0.457 | -1.12974 | 7.05E-10 | 1.27E-08 | yes | down | 15.38 | 17.87 | 19.49 | 19.34 | 15.06 | 18.94 | 17.58 | 17.78 |
| THBS2 | 2.145 | 1.10095 | 7.4E-10 | 1.33E-08 | yes | up | 6.84 | 5.5 | 5.83 | 31.77 | 29.06 | 30.53 | 6.056667 | 30.45333 |
| FASTKD2 | 0.389 | -1.36304 | 7.52E-10 | 1.35E-08 | yes | down | 3.59 | 5.03 | 3.7 | 4.38 | 3.35 | 3.24 | 4.106667 | 3.656667 |
| FAM124A | 0.273 | -1.8726 | 7.52E-10 | 1.35E-08 | yes | down | 7.43 | 7.68 | 6.52 | 3.71 | 3.18 | 6.83 | 7.21 | 4.573333 |
| LOC100344139 | 224.69 | 7.811789 | 7.56E-10 | 1.35E-08 | yes | up | 0 | 0 | 0 | 6.73 | 8.82 | 8.43 | 0 | 7.993333 |
| MITF | 2.624 | 1.391728 | 8.02E-10 | 1.44E-08 | yes | up | 2.69 | 2.07 | 2.51 | 18.76 | 12.47 | 14.31 | 2.423333 | 15.18 |
| ANKS1B | 6.631 | 2.729213 | 8.2E-10 | 1.46E-08 | yes | up | 0.07 | 0.44 | 0.15 | 1.89 | 2.07 | 3.49 | 0.22 | 2.483333 |
| BDNF | 6.689 | 2.741873 | 8.33E-10 | 1.49E-08 | yes | up | 0.28 | 0.21 | 0.21 | 2.37 | 3.1 | 5.31 | 0.233333 | 3.593333 |
| CLPP | 0.439 | -1.18676 | 8.47E-10 | 1.51E-08 | yes | down | 17.87 | 19.59 | 16.59 | 19.79 | 17.52 | 18.64 | 18.01667 | 18.65 |
| HBB2 | 0.161 | -2.6325 | 8.7E-10 | 1.55E-08 | yes | down | 5879.16 | 5595.18 | 5414.91 | 808.83 | 1950.5 | 3563.21 | 5629.75 | 2107.513 |
| ACOX3 | 0.364 | -1.45719 | 8.95E-10 | 1.59E-08 | yes | down | 28.24 | 25.2 | 27.95 | 19.31 | 27.34 | 22.67 | 27.13 | 23.10667 |
| JAGN1 | 0.36 | -1.47213 | 9.32E-10 | 1.66E-08 | yes | down | 34.8 | 46.21 | 45.96 | 33.55 | 33.46 | 49.54 | 42.32333 | 38.85 |
| COL23A1 | 3.328 | 1.73471 | 9.62E-10 | 1.7E-08 | yes | up | 0.56 | 0.79 | 0.6 | 5.12 | 5.5 | 4.48 | 0.65 | 5.033333 |
| TENM4 | 2.961 | 1.566296 | 1.01E-09 | 1.78E-08 | yes | up | 2.25 | 2.49 | 1.95 | 9.55 | 10.98 | 7.92 | 2.23 | 9.483333 |
| AEBP1 | 2.46 | 1.29882 | 1.01E-09 | 1.79E-08 | yes | up | 10.22 | 15.77 | 14.22 | 61.83 | 86.12 | 78.6 | 13.40333 | 75.51667 |
| LOC100346229 | 3.78 | 1.91829 | 1.02E-09 | 1.81E-08 | yes | up | 0.78 | 0.89 | 1.01 | 10.45 | 7.8 | 5.2 | 0.893333 | 7.816667 |
| LOC100341772 | 0.182 | -2.45735 | 1.07E-09 | 1.88E-08 | yes | down | 11.71 | 15.68 | 13.93 | 10.11 | 5.07 | 2.55 | 13.77333 | 5.91 |
| FAM111A | 3.897 | 1.962436 | 1.07E-09 | 1.88E-08 | yes | up | 1.02 | 1.12 | 0.82 | 13.59 | 10.66 | 6.35 | 0.986667 | 10.2 |
| ASPM | 7.975 | 2.995398 | 1.08E-09 | 1.9E-08 | yes | up | 0.09 | 0.21 | 0.16 | 3.08 | 3.83 | 1.12 | 0.153333 | 2.676667 |
| MSMO1 | 3.185 | 1.671461 | 1.09E-09 | 1.91E-08 | yes | up | 10.05 | 15.06 | 13.86 | 122.35 | 57.07 | 110.39 | 12.99 | 96.60333 |
| CCSAP | 3.351 | 1.744412 | 1.11E-09 | 1.94E-08 | yes | up | 0.35 | 0.31 | 0.36 | 2.85 | 2.5 | 2.61 | 0.34 | 2.653333 |
| HARS | 0.482 | -1.05329 | 1.13E-09 | 1.99E-08 | yes | down | 11.34 | 16.53 | 15.25 | 17.78 | 15.66 | 14.73 | 14.37333 | 16.05667 |
| COL7A1 | 0.275 | -1.8637 | 1.14E-09 | 1.99E-08 | yes | down | 1.2 | 0.77 | 0.96 | 0.71 | 0.49 | 0.69 | 0.976667 | 0.63 |
| FKBP5 | 0.226 | -2.14842 | 1.2E-09 | 2.09E-08 | yes | down | 31.34 | 16.91 | 26.26 | 11.06 | 18.71 | 9.55 | 24.83667 | 13.10667 |
| DNAAF2 | 0.407 | -1.29555 | 1.25E-09 | 2.17E-08 | yes | down | 8.23 | 9.36 | 12.58 | 9.98 | 7.9 | 5.34 | 10.05667 | 7.74 |
| SSH1 | 0.417 | -1.26355 | 1.28E-09 | 2.22E-08 | yes | down | 6.5 | 6.12 | 8.71 | 8.58 | 6.65 | 6.95 | 7.11 | 7.393333 |
| FABP3 | 0.043 | -4.53829 | 1.29E-09 | 2.24E-08 | yes | down | 392.79 | 434.21 | 301.49 | 9.28 | 60.7 | 40.76 | 376.1633 | 36.91333 |
| CHCHD2 | 0.33 | -1.59783 | 1.31E-09 | 2.27E-08 | yes | down | 698 | 533.43 | 422.7 | 395.68 | 521.84 | 374.53 | 551.3767 | 430.6833 |
| MRRF | 0.483 | -1.05073 | 1.35E-09 | 2.33E-08 | yes | down | 11.64 | 14.09 | 12.97 | 13.04 | 14.11 | 15.82 | 12.9 | 14.32333 |
| NAMPT | 0.434 | -1.20353 | 1.35E-09 | 2.33E-08 | yes | down | 23.45 | 23.39 | 21.02 | 21 | 19.17 | 28.25 | 22.62 | 22.80667 |
| SCTR | 0.134 | -2.90192 | 1.35E-09 | 2.33E-08 | yes | down | 5.01 | 14.84 | 22.01 | 3.67 | 2.46 | 5.72 | 13.95333 | 3.95 |
| GFM1 | 0.383 | -1.38496 | 1.39E-09 | 2.39E-08 | yes | down | 17.53 | 36.59 | 25.74 | 23.52 | 22.5 | 24.81 | 26.62 | 23.61 |
| IAH1 | 0.485 | -1.04258 | 1.39E-09 | 2.39E-08 | yes | down | 18.77 | 21.92 | 22.36 | 26.56 | 20.8 | 22.7 | 21.01667 | 23.35333 |
| CERCAM | 2.908 | 1.540153 | 1.46E-09 | 2.51E-08 | yes | up | 2.06 | 3.44 | 2.23 | 19.96 | 12.45 | 19.43 | 2.576667 | 17.28 |
| LOC100353574 | 3.197 | 1.676901 | 1.49E-09 | 2.56E-08 | yes | up | 17.3 | 18.86 | 17.51 | 56.8 | 54.96 | 106.69 | 17.89 | 72.81667 |
| ASIC2 | 0.015 | -6.07203 | 1.51E-09 | 2.58E-08 | yes | down | 0.83 | 0.65 | 1.32 | 0.07 | 0 | 0.03 | 0.933333 | 0.033333 |
| GBAS | 0.302 | -1.72741 | 1.52E-09 | 2.59E-08 | yes | down | 50.31 | 74.93 | 41.65 | 36.24 | 50.95 | 28.2 | 55.63 | 38.46333 |
| SLC22A23 | 0.328 | -1.6096 | 1.61E-09 | 2.75E-08 | yes | down | 3.34 | 3.83 | 2.38 | 2.28 | 1.94 | 2.71 | 3.183333 | 2.31 |
| ZMIZ1 | 0.485 | -1.04385 | 1.63E-09 | 2.78E-08 | yes | down | 10.07 | 15.52 | 14.24 | 15.7 | 12.57 | 15.7 | 13.27667 | 14.65667 |
| MBNL3 | 4.547 | 2.184786 | 1.67E-09 | 2.83E-08 | yes | up | 0.22 | 0.24 | 0.2 | 3.57 | 1.68 | 2.93 | 0.22 | 2.726667 |
| COL6A1 | 2.13 | 1.09074 | 1.79E-09 | 3.03E-08 | yes | up | 143.81 | 179.61 | 127.88 | 784.05 | 679.92 | 694.64 | 150.4333 | 719.5367 |
| SPATA6 | 2.863 | 1.517301 | 1.82E-09 | 3.08E-08 | yes | up | 1.92 | 1.72 | 2.86 | 12.99 | 16.14 | 15.15 | 2.166667 | 14.76 |
| CAPN15 | 0.462 | -1.1125 | 1.84E-09 | 3.11E-08 | yes | down | 6.03 | 8.69 | 9.36 | 9.43 | 7.98 | 8.4 | 8.026667 | 8.603333 |
| GLRX5 | 0.403 | -1.31061 | 1.84E-09 | 3.11E-08 | yes | down | 44.84 | 45.93 | 45.07 | 48.87 | 48.47 | 30.97 | 45.28 | 42.77 |
| MICU3 | 0.263 | -1.92835 | 1.87E-09 | 3.16E-08 | yes | down | 11.47 | 5.29 | 8.44 | 4.51 | 4.99 | 6.01 | 8.4 | 5.17 |
| SPAG5 | 11.951 | 3.579031 | 2.08E-09 | 3.51E-08 | yes | up | 0.18 | 0.25 | 0.09 | 6.04 | 6.96 | 1.56 | 0.173333 | 4.853333 |
| CARHSP1 | 2.481 | 1.310724 | 2.11E-09 | 3.55E-08 | yes | up | 2.73 | 4.61 | 3.06 | 22.86 | 18.49 | 18.97 | 3.466667 | 20.10667 |
| ADAM12 | 2.944 | 1.557603 | 2.33E-09 | 3.91E-08 | yes | up | 0.98 | 1.5 | 0.71 | 7.02 | 7.12 | 7.26 | 1.063333 | 7.133333 |
| CADM4 | 0.395 | -1.34069 | 2.34E-09 | 3.91E-08 | yes | down | 6.62 | 9.09 | 9.01 | 9.33 | 8.59 | 6.21 | 8.24 | 8.043333 |
| OSGEPL1 | 0.426 | -1.23089 | 2.34E-09 | 3.91E-08 | yes | down | 10.86 | 11.26 | 12.85 | 12.49 | 12.95 | 10.71 | 11.65667 | 12.05 |
| PAPSS2 | 3.561 | 1.832288 | 2.36E-09 | 3.94E-08 | yes | up | 0.81 | 1.66 | 0.86 | 7.71 | 6.84 | 10.43 | 1.11 | 8.326667 |
| TPST2 | 2.194 | 1.133476 | 2.38E-09 | 3.96E-08 | yes | up | 7.5 | 8.92 | 8.86 | 53.96 | 39.36 | 36.09 | 8.426667 | 43.13667 |
| FER | 2.187 | 1.128766 | 2.39E-09 | 3.99E-08 | yes | up | 1.58 | 1.77 | 1.47 | 8.14 | 6.89 | 10.06 | 1.606667 | 8.363333 |
| RASA3 | 0.418 | -1.25858 | 2.48E-09 | 4.13E-08 | yes | down | 10.62 | 18.43 | 14.94 | 17.17 | 12.18 | 13.19 | 14.66333 | 14.18 |
| BLM | 0.302 | -1.72718 | 2.48E-09 | 4.13E-08 | yes | down | 10.19 | 7.7 | 13.5 | 2.88 | 3.81 | 2.56 | 10.46333 | 3.083333 |
| SLC25A42 | 0.203 | -2.29731 | 2.48E-09 | 4.13E-08 | yes | down | 20.67 | 23.19 | 23.86 | 8.16 | 11.95 | 12.95 | 22.57333 | 11.02 |
| SNX33 | 2.307 | 1.206004 | 2.68E-09 | 4.43E-08 | yes | up | 2.2 | 2.88 | 3.41 | 16.68 | 12.75 | 15.69 | 2.83 | 15.04 |
| INCENP | 4.65 | 2.217375 | 2.75E-09 | 4.55E-08 | yes | up | 0.37 | 0.41 | 0.52 | 5.86 | 5.6 | 2.65 | 0.433333 | 4.703333 |
| LOC108175950 | 0.1 | -3.32232 | 2.77E-09 | 4.57E-08 | yes | down | 1.47 | 1.51 | 2.07 | 0.35 | 0.16 | 0.6 | 1.683333 | 0.37 |
| ZBTB14 | 2.126 | 1.088257 | 2.89E-09 | 4.76E-08 | yes | up | 1.71 | 2.19 | 2.21 | 11.48 | 9.15 | 9.92 | 2.036667 | 10.18333 |
| LOC100344910 | 0.251 | -1.99488 | 2.9E-09 | 4.78E-08 | yes | down | 492.69 | 1153.31 | 388.2 | 367.72 | 417.58 | 372.42 | 678.0667 | 385.9067 |
| LOC103346609 | 0.136 | -2.87623 | 2.99E-09 | 4.92E-08 | yes | down | 1.71 | 2.7 | 1.21 | 0.85 | 0.17 | 0.61 | 1.873333 | 0.543333 |
| KIT | 3.929 | 1.974194 | 3.06E-09 | 5.02E-08 | yes | up | 1.77 | 1.95 | 2.53 | 28.3 | 15.33 | 11.44 | 2.083333 | 18.35667 |
| PHYHD1 | 2.258 | 1.175328 | 3.09E-09 | 5.07E-08 | yes | up | 13.14 | 15.92 | 16.89 | 96.84 | 62.66 | 84.3 | 15.31667 | 81.26667 |
| MID1IP1 | 5.17 | 2.370085 | 3.14E-09 | 5.14E-08 | yes | up | 3.19 | 3.09 | 2.48 | 44.61 | 14.44 | 49.15 | 2.92 | 36.06667 |
| SAMD9 | 2.562 | 1.357209 | 3.17E-09 | 5.18E-08 | yes | up | 0.83 | 0.8 | 0.65 | 5.28 | 3.68 | 4.69 | 0.76 | 4.55 |
| LOC103346277 | 0.145 | -2.78709 | 3.25E-09 | 5.31E-08 | yes | down | 3.9 | 1.82 | 3.71 | 1.51 | 0.64 | 1.23 | 3.143333 | 1.126667 |
| LMNB1 | 5.551 | 2.472838 | 3.27E-09 | 5.34E-08 | yes | up | 2.13 | 2.92 | 1.67 | 32.63 | 41.87 | 12.56 | 2.24 | 29.02 |
| LOC100353245 | 2.43 | 1.281111 | 3.32E-09 | 5.41E-08 | yes | up | 97.82 | 90.51 | 82.5 | 413.8 | 547.02 | 586.54 | 90.27667 | 515.7867 |
| CDK1 | 10.016 | 3.324187 | 3.4E-09 | 5.54E-08 | yes | up | 0.71 | 1.33 | 0.68 | 22.08 | 36.3 | 7.06 | 0.906667 | 21.81333 |
| NEURL1B | 4.126 | 2.04481 | 3.51E-09 | 5.71E-08 | yes | up | 1.35 | 2.6 | 1.74 | 27.93 | 13.08 | 13.62 | 1.896667 | 18.21 |
| TMEM45B | 0.033 | -4.93537 | 3.62E-09 | 5.87E-08 | yes | down | 1.78 | 0.57 | 2.21 | 0.22 | 0.1 | 0.04 | 1.52 | 0.12 |
| FNDC3A | 2.029 | 1.020934 | 3.72E-09 | 6.03E-08 | yes | up | 6.11 | 6.52 | 6.19 | 37.41 | 26.47 | 25.76 | 6.273333 | 29.88 |
| ARHGAP36 | 0.013 | -6.23801 | 3.96E-09 | 6.4E-08 | yes | down | 1.53 | 2.69 | 0.2 | 0.03 | 0.11 | 0 | 1.473333 | 0.046667 |
| MSRB1 | 2.195 | 1.13425 | 4.03E-09 | 6.51E-08 | yes | up | 62.13 | 84.73 | 61.91 | 446.1 | 341.52 | 286.17 | 69.59 | 357.93 |
| LOC103349801 | 0.454 | -1.13971 | 4.05E-09 | 6.53E-08 | yes | down | 85.64 | 130.52 | 104.97 | 141.1 | 109.61 | 94.84 | 107.0433 | 115.1833 |
| LOC103346606 | 2.902 | 1.537152 | 4.3E-09 | 6.92E-08 | yes | up | 1.57 | 2.54 | 1.78 | 16.75 | 11.85 | 10.76 | 1.963333 | 13.12 |
| FRRS1 | 0.334 | -1.58203 | 4.4E-09 | 7.07E-08 | yes | down | 9.28 | 12.74 | 12.6 | 9.43 | 5.86 | 11.84 | 11.54 | 9.043333 |
| ARHGAP11A | 9.706 | 3.278932 | 4.4E-09 | 7.07E-08 | yes | up | 0.16 | 0.18 | 0.08 | 3.3 | 4.79 | 1.21 | 0.14 | 3.1 |
| LOC100358143 | 7.082 | 2.824245 | 4.46E-09 | 7.15E-08 | yes | up | 0.21 | 0.37 | 0.58 | 6.39 | 5.48 | 7.22 | 0.386667 | 6.363333 |
| METTL1 | 0.355 | -1.49288 | 4.55E-09 | 7.27E-08 | yes | down | 3.58 | 5.47 | 4.49 | 3.91 | 2.98 | 4.19 | 4.513333 | 3.693333 |
| BHMT2 | 0.046 | -4.44603 | 4.84E-09 | 7.74E-08 | yes | down | 96.28 | 79.58 | 67.09 | 4.99 | 1.97 | 17.32 | 80.98333 | 8.093333 |
| BAG1 | 0.444 | -1.17184 | 4.88E-09 | 7.79E-08 | yes | down | 70.4 | 112.16 | 90.02 | 83.97 | 98.34 | 70.15 | 90.86 | 84.15333 |
| LOC100337875 | 0.341 | -1.5518 | 4.91E-09 | 7.82E-08 | yes | down | 3.17 | 5 | 4.61 | 3.69 | 2.54 | 3.86 | 4.26 | 3.363333 |
| SKA3 | 3.036 | 1.602258 | 4.94E-09 | 7.86E-08 | yes | up | 0.27 | 0.5 | 0.32 | 3.96 | 3.51 | 2.33 | 0.363333 | 3.266667 |
| HPRT1 | 0.449 | -1.15502 | 4.97E-09 | 7.91E-08 | yes | down | 16.66 | 21.89 | 16.07 | 23.31 | 15.27 | 18.64 | 18.20667 | 19.07333 |
| TTC4 | 0.341 | -1.5522 | 4.98E-09 | 7.91E-08 | yes | down | 4.31 | 6.27 | 6.08 | 3.4 | 4.27 | 5.29 | 5.553333 | 4.32 |
| AGXT2 | 0.246 | -2.0206 | 5E-09 | 7.93E-08 | yes | down | 1.34 | 0.93 | 1.14 | 0.5 | 0.61 | 0.46 | 1.136667 | 0.523333 |
| SNN | 3.664 | 1.8735 | 5E-09 | 7.94E-08 | yes | up | 1.49 | 1.98 | 2.3 | 24.72 | 16.27 | 10.26 | 1.923333 | 17.08333 |
| DDB2 | 2.105 | 1.073929 | 5.11E-09 | 8.1E-08 | yes | up | 4.79 | 4.97 | 5.75 | 26.4 | 24.69 | 20.99 | 5.17 | 24.02667 |
| MEIS3 | 2.861 | 1.516581 | 5.17E-09 | 8.17E-08 | yes | up | 0.86 | 1.31 | 1.09 | 8.85 | 5.51 | 6 | 1.086667 | 6.786667 |
| GTDC1 | 2.171 | 1.118366 | 5.17E-09 | 8.17E-08 | yes | up | 2.09 | 2.2 | 2.11 | 10.15 | 11.4 | 10.33 | 2.133333 | 10.62667 |
| LOC100341181 | 0.466 | -1.1006 | 5.28E-09 | 8.33E-08 | yes | down | 32.5 | 32.42 | 36.06 | 32.02 | 35.98 | 41.66 | 33.66 | 36.55333 |
| GSTM2 | 3.917 | 1.969879 | 5.63E-09 | 8.87E-08 | yes | up | 6.86 | 12.1 | 4.93 | 69.82 | 91.47 | 51.99 | 7.963333 | 71.09333 |
| PIK3R1 | 2.922 | 1.547013 | 5.8E-09 | 9.12E-08 | yes | up | 6.58 | 9.32 | 8.25 | 37.39 | 70.77 | 52.3 | 8.05 | 53.48667 |
| CDC20 | 10.099 | 3.336079 | 6.05E-09 | 9.47E-08 | yes | up | 0.55 | 1.42 | 0.35 | 22.04 | 24.58 | 6.54 | 0.773333 | 17.72 |
| LOC100358198 | 0.078 | -3.67207 | 6.08E-09 | 9.52E-08 | yes | down | 0.64 | 0.28 | 0.62 | 0.08 | 0.09 | 0.11 | 0.513333 | 0.093333 |
| ZFP36L1 | 2.134 | 1.093453 | 6.16E-09 | 9.64E-08 | yes | up | 41.24 | 38.8 | 33.57 | 205.41 | 205.22 | 156.11 | 37.87 | 188.9133 |
| LOC100008606 | 3.754 | 1.908458 | 6.92E-09 | 1.08E-07 | yes | up | 5.69 | 6 | 4.87 | 60.6 | 25.7 | 49.6 | 5.52 | 45.3 |
| HDAC6 | 2.084 | 1.05939 | 6.98E-09 | 1.09E-07 | yes | up | 1.66 | 1.96 | 1.76 | 10.23 | 7.76 | 8.07 | 1.793333 | 8.686667 |
| LOC100349056 | 0.005 | -7.72076 | 7.11E-09 | 1.11E-07 | yes | down | 2.53 | 2.5 | 2.54 | 0 | 0 | 0 | 2.523333 | 0 |
| MYBBP1A | 0.49 | -1.02832 | 7.31E-09 | 1.14E-07 | yes | down | 9.2 | 15.09 | 11.98 | 14.86 | 14.15 | 18.27 | 12.09 | 15.76 |
| LOC100353012 | 178.028 | 7.475957 | 7.44E-09 | 1.16E-07 | yes | up | 0 | 0 | 0 | 1 | 0.54 | 0.85 | 0 | 0.796667 |
| MRPS28 | 0.442 | -1.17686 | 7.47E-09 | 1.16E-07 | yes | down | 25.45 | 38.16 | 28.71 | 32.82 | 29.17 | 33.53 | 30.77333 | 31.84 |
| FAM214B | 2.01 | 1.007061 | 7.49E-09 | 1.16E-07 | yes | up | 4.07 | 4.25 | 4.61 | 22.49 | 18.99 | 23.72 | 4.31 | 21.73333 |
| ADH1A | 0.381 | -1.39314 | 7.5E-09 | 1.16E-07 | yes | down | 337.12 | 337.2 | 412.88 | 284.6 | 241.67 | 424.92 | 362.4 | 317.0633 |
| LDHD | 0.383 | -1.3859 | 7.6E-09 | 1.18E-07 | yes | down | 23.28 | 17.11 | 16.68 | 16.99 | 14.39 | 20.39 | 19.02333 | 17.25667 |
| C7H2orf47 | 0.463 | -1.10942 | 7.7E-09 | 1.19E-07 | yes | down | 11.07 | 14.28 | 12.37 | 14.21 | 14.34 | 13.94 | 12.57333 | 14.16333 |
| LOC100347349 | 2.301 | 1.202558 | 7.94E-09 | 1.23E-07 | yes | up | 1.19 | 1.74 | 1.22 | 9.14 | 6.07 | 6.93 | 1.383333 | 7.38 |
| BST1 | 2.493 | 1.317776 | 8.01E-09 | 1.24E-07 | yes | up | 4.25 | 6.33 | 5.33 | 27.04 | 26.89 | 37.19 | 5.303333 | 30.37333 |
| CENPV | 0.359 | -1.47638 | 8.03E-09 | 1.24E-07 | yes | down | 9.45 | 9.6 | 9.18 | 6.5 | 7.77 | 8.51 | 9.41 | 7.593333 |
| ADCYAP1R1 | 4.202 | 2.071235 | 8.16E-09 | 1.26E-07 | yes | up | 0.35 | 0.52 | 0.48 | 6.9 | 3.56 | 2.84 | 0.45 | 4.433333 |
| LOC100358892 | 0.4 | -1.32156 | 8.5E-09 | 1.31E-07 | yes | down | 13.06 | 11.93 | 10.72 | 10.22 | 9.49 | 13.68 | 11.90333 | 11.13 |
| LOC100349768 | 0.375 | -1.41542 | 8.56E-09 | 1.31E-07 | yes | down | 380.33 | 515.55 | 299.43 | 300.69 | 425.97 | 303.46 | 398.4367 | 343.3733 |
| BUB1 | 10.511 | 3.393793 | 8.74E-09 | 1.34E-07 | yes | up | 0.14 | 0.18 | 0.11 | 4.74 | 4.33 | 1.26 | 0.143333 | 3.443333 |
| LOC100355548 | 0.414 | -1.27276 | 8.79E-09 | 1.35E-07 | yes | down | 33.76 | 36.79 | 34.72 | 28.28 | 34.5 | 39.41 | 35.09 | 34.06333 |
| MAGI3 | 2.197 | 1.135627 | 9.09E-09 | 1.39E-07 | yes | up | 0.98 | 1.17 | 1.07 | 6.74 | 5 | 4.89 | 1.073333 | 5.543333 |
| NEK2 | 14.88 | 3.89529 | 9.16E-09 | 1.4E-07 | yes | up | 0.15 | 0.52 | 0.13 | 10.43 | 13.89 | 2.68 | 0.266667 | 9 |
| FAM19A3 | 18.114 | 4.179042 | 9.17E-09 | 1.4E-07 | yes | up | 0.04 | 0.01 | 0.11 | 1.83 | 3.97 | 1.31 | 0.053333 | 2.37 |
| ANGPTL2 | 0.43 | -1.216 | 9.2E-09 | 1.4E-07 | yes | down | 49.8 | 63.23 | 63.61 | 45.34 | 63.39 | 66.2 | 58.88 | 58.31 |
| ELOVL1 | 2.05 | 1.035775 | 9.3E-09 | 1.42E-07 | yes | up | 8.51 | 12.56 | 11.1 | 60.37 | 49.21 | 45.15 | 10.72333 | 51.57667 |
| CCNB1 | 9.582 | 3.260285 | 9.46E-09 | 1.44E-07 | yes | up | 0.72 | 1.73 | 0.59 | 25.82 | 32.84 | 7.48 | 1.013333 | 22.04667 |
| PIP5KL1 | 3.284 | 1.71544 | 9.63E-09 | 1.47E-07 | yes | up | 0.67 | 1.02 | 0.61 | 7.78 | 5.57 | 5.83 | 0.766667 | 6.393333 |
| MARS | 0.463 | -1.11145 | 9.7E-09 | 1.47E-07 | yes | down | 8.21 | 13.67 | 10.55 | 11.49 | 10.47 | 12.58 | 10.81 | 11.51333 |
| SOX4 | 2.749 | 1.459145 | 9.71E-09 | 1.47E-07 | yes | up | 1.02 | 1.24 | 0.79 | 7.88 | 6.38 | 5.22 | 1.016667 | 6.493333 |
| LOC100355623 | 2.248 | 1.168705 | 9.93E-09 | 1.51E-07 | yes | up | 4.84 | 7.74 | 4.5 | 31.32 | 27.61 | 29.61 | 5.693333 | 29.51333 |
| KNSTRN | 6.911 | 2.788978 | 1.02E-08 | 1.54E-07 | yes | up | 0.68 | 1.19 | 0.62 | 14.85 | 18.1 | 6.04 | 0.83 | 12.99667 |
| LOC100343802 | 0.225 | -2.15228 | 1.02E-08 | 1.55E-07 | yes | down | 552.68 | 246.94 | 332.47 | 206.55 | 128.88 | 287.36 | 377.3633 | 207.5967 |
| PAX8 | 155.92 | 7.284662 | 1.03E-08 | 1.56E-07 | yes | up | 0 | 0 | 0.03 | 3.41 | 4.61 | 7.39 | 0.01 | 5.136667 |
| PRX | 0.218 | -2.19853 | 1.04E-08 | 1.57E-07 | yes | down | 4.7 | 7.61 | 9.66 | 3.22 | 5.56 | 2.14 | 7.323333 | 3.64 |
| TSHZ2 | 2.491 | 1.316833 | 1.05E-08 | 1.59E-07 | yes | up | 1.76 | 2.99 | 2.54 | 17.13 | 10.19 | 13.98 | 2.43 | 13.76667 |
| PIGV | 2.07 | 1.049306 | 1.06E-08 | 1.59E-07 | yes | up | 4.12 | 4.23 | 4.92 | 22.66 | 18.15 | 22.97 | 4.423333 | 21.26 |
| CHI3L1 | 11.752 | 3.554781 | 1.1E-08 | 1.65E-07 | yes | up | 0.65 | 0.51 | 0.84 | 19.94 | 30.78 | 3.92 | 0.666667 | 18.21333 |
| NARF | 0.488 | -1.03436 | 1.11E-08 | 1.66E-07 | yes | down | 5.03 | 7.67 | 5.61 | 7.77 | 5.8 | 6.72 | 6.103333 | 6.763333 |
| SLCO2A1 | 0.263 | -1.92705 | 1.16E-08 | 1.74E-07 | yes | down | 2.8 | 3.77 | 2.07 | 1.25 | 2.19 | 1.71 | 2.88 | 1.716667 |
| ATP1B1 | 0.198 | -2.33929 | 1.17E-08 | 1.76E-07 | yes | down | 34.48 | 36.71 | 13.78 | 9.93 | 18.27 | 10.52 | 28.32333 | 12.90667 |
| SNX21 | 0.471 | -1.08698 | 1.19E-08 | 1.78E-07 | yes | down | 27.25 | 35.54 | 38.41 | 29.4 | 29.8 | 37.49 | 33.73333 | 32.23 |
| SLC25A30 | 0.474 | -1.07749 | 1.21E-08 | 1.81E-07 | yes | down | 12.13 | 8.4 | 9.6 | 12.82 | 13.67 | 17.3 | 10.04333 | 14.59667 |
| KIAA0101 | 12.789 | 3.676804 | 1.23E-08 | 1.83E-07 | yes | up | 0.87 | 0.99 | 0.34 | 25.35 | 26.43 | 5.43 | 0.733333 | 19.07 |
| LOC100346467 | 2.869 | 1.520354 | 1.23E-08 | 1.84E-07 | yes | up | 1.57 | 1.37 | 1.54 | 8.77 | 8.91 | 11.62 | 1.493333 | 9.766667 |
| FAM117A | 2.457 | 1.296956 | 1.27E-08 | 1.89E-07 | yes | up | 2.68 | 4.91 | 3.17 | 20.78 | 21.65 | 21.17 | 3.586667 | 21.2 |
| CDK5RAP1 | 0.415 | -1.26911 | 1.29E-08 | 1.92E-07 | yes | down | 7.1 | 9.08 | 9.65 | 10.43 | 6.32 | 8.38 | 8.61 | 8.376667 |
| IRAK4 | 2.174 | 1.12055 | 1.33E-08 | 1.97E-07 | yes | up | 4.88 | 6.15 | 6.32 | 27 | 32 | 27.67 | 5.783333 | 28.89 |
| ASS1 | 3.783 | 1.919696 | 1.37E-08 | 2.03E-07 | yes | up | 2.87 | 3.89 | 2.41 | 37.86 | 15.64 | 27.84 | 3.056667 | 27.11333 |
| COMMD5 | 0.457 | -1.12925 | 1.41E-08 | 2.08E-07 | yes | down | 10.43 | 13.69 | 11.14 | 13.28 | 11.69 | 12.58 | 11.75333 | 12.51667 |
| VSIG10 | 2.13 | 1.090788 | 1.45E-08 | 2.14E-07 | yes | up | 1.62 | 1.89 | 2.04 | 10.64 | 7.68 | 8.44 | 1.85 | 8.92 |
| C1QTNF9 | 2.867 | 1.519303 | 1.47E-08 | 2.17E-07 | yes | up | 12.55 | 19.48 | 12.24 | 79.09 | 70.63 | 112.52 | 14.75667 | 87.41333 |
| CES4A | 0.291 | -1.77895 | 1.5E-08 | 2.2E-07 | yes | down | 3.89 | 5.11 | 4.24 | 2.65 | 2.07 | 4.2 | 4.413333 | 2.973333 |
| CRY2 | 0.483 | -1.0492 | 1.51E-08 | 2.21E-07 | yes | down | 11.19 | 11.44 | 9.69 | 12.88 | 9.8 | 13.82 | 10.77333 | 12.16667 |
| C19H17orf53 | 0.338 | -1.56418 | 1.53E-08 | 2.25E-07 | yes | down | 2.02 | 2.6 | 2.22 | 1.86 | 2.08 | 1.42 | 2.28 | 1.786667 |
| TPD52L1 | 0.164 | -2.60762 | 1.57E-08 | 2.31E-07 | yes | down | 17.92 | 29.41 | 24.92 | 2.29 | 11.6 | 10.39 | 24.08333 | 8.093333 |
| CPXM1 | 3.764 | 1.9121 | 1.63E-08 | 2.39E-07 | yes | up | 1.05 | 3.43 | 2.27 | 22.85 | 17.11 | 18.13 | 2.25 | 19.36333 |
| NCKAP1L | 2.023 | 1.016179 | 1.64E-08 | 2.39E-07 | yes | up | 1.73 | 1.95 | 1.78 | 10.21 | 7.57 | 7.95 | 1.82 | 8.576667 |
| LOC108176678 | 7.774 | 2.958581 | 1.66E-08 | 2.42E-07 | yes | up | 0.05 | 0.2 | 0.2 | 2.58 | 2.44 | 3.05 | 0.15 | 2.69 |
| STMN1 | 4.623 | 2.208849 | 1.67E-08 | 2.43E-07 | yes | up | 7.02 | 11.06 | 5.78 | 119.28 | 100.42 | 41.16 | 7.953333 | 86.95333 |
| SEPT11 | 2.185 | 1.127768 | 1.72E-08 | 2.5E-07 | yes | up | 6.6 | 11.1 | 7.32 | 31.15 | 30.34 | 35.58 | 8.34 | 32.35667 |
| CDCA2 | 12.635 | 3.659302 | 1.75E-08 | 2.54E-07 | yes | up | 0.07 | 0.08 | 0.06 | 2.52 | 2.95 | 0.98 | 0.07 | 2.15 |
| LOXHD1 | 12.181 | 3.606584 | 1.8E-08 | 2.62E-07 | yes | up | 0.88 | 1.84 | 2.27 | 63.71 | 22.8 | 45.97 | 1.663333 | 44.16 |
| BUB1B | 8.815 | 3.139951 | 1.8E-08 | 2.62E-07 | yes | up | 0.12 | 0.34 | 0.14 | 4.91 | 5.9 | 1.73 | 0.2 | 4.18 |
| LOC108176749 | 163.049 | 7.349165 | 1.81E-08 | 2.62E-07 | yes | up | 0 | 0 | 0 | 1.72 | 1.08 | 0.98 | 0 | 1.26 |
| LOC100346985 | 4.376 | 2.129571 | 1.87E-08 | 2.71E-07 | yes | up | 0.56 | 0.47 | 0.64 | 5.76 | 3.86 | 7.67 | 0.556667 | 5.763333 |
| TLR3 | 5.729 | 2.518397 | 1.88E-08 | 2.72E-07 | yes | up | 0.88 | 0.3 | 0.57 | 4.87 | 8.42 | 9.93 | 0.583333 | 7.74 |
| HDAC5 | 2.305 | 1.204953 | 1.92E-08 | 2.78E-07 | yes | up | 11.49 | 9.87 | 13.86 | 57.66 | 60.39 | 73.11 | 11.74 | 63.72 |
| CPN2 | 0.004 | -7.80687 | 1.92E-08 | 2.78E-07 | yes | down | 0.64 | 0.27 | 0.37 | 0 | 0 | 0 | 0.426667 | 0 |
| KIFC1 | 7.242 | 2.856384 | 1.94E-08 | 2.8E-07 | yes | up | 0.35 | 0.73 | 0.4 | 10.33 | 11.2 | 2.96 | 0.493333 | 8.163333 |
| ATP5SL | 0.475 | -1.07437 | 1.94E-08 | 2.8E-07 | yes | down | 13.11 | 13.78 | 13.11 | 19.54 | 13.12 | 12.11 | 13.33333 | 14.92333 |
| ASMTL | 0.374 | -1.42069 | 2.15E-08 | 3.09E-07 | yes | down | 6.99 | 7.01 | 5.59 | 5.15 | 3.54 | 2.29 | 6.53 | 3.66 |
| PMP22 | 0.272 | -1.87941 | 2.19E-08 | 3.14E-07 | yes | down | 59.69 | 104.65 | 112.26 | 39.78 | 92.91 | 52.05 | 92.2 | 61.58 |
| POSTN | 0.44 | -1.18378 | 2.24E-08 | 3.21E-07 | yes | down | 70.9 | 113.41 | 91.19 | 74.52 | 99.56 | 102.2 | 91.83333 | 92.09333 |
| TPX2 | 3.997 | 1.999018 | 2.25E-08 | 3.23E-07 | yes | up | 1.21 | 1.62 | 1.41 | 14.19 | 17.9 | 7.04 | 1.413333 | 13.04333 |
| NCAPD2 | 3.39 | 1.761132 | 2.28E-08 | 3.26E-07 | yes | up | 2.94 | 4.9 | 7.65 | 19.42 | 17.21 | 7.3 | 5.163333 | 14.64333 |
| PELI3 | 0.097 | -3.36998 | 2.28E-08 | 3.26E-07 | yes | down | 0.88 | 1.01 | 0.65 | 0.09 | 0.3 | 0.18 | 0.846667 | 0.19 |
| LAMA4 | 2.268 | 1.18114 | 2.36E-08 | 3.37E-07 | yes | up | 20.22 | 33.31 | 20.02 | 124.76 | 140.47 | 120.07 | 24.51667 | 128.4333 |
| PI15 | 0.186 | -2.42988 | 2.36E-08 | 3.37E-07 | yes | down | 1.23 | 1.57 | 1.8 | 0.71 | 0.6 | 0.68 | 1.533333 | 0.663333 |
| LOC100351488 | 2.83 | 1.500821 | 2.41E-08 | 3.43E-07 | yes | up | 8.92 | 11.71 | 12.47 | 112.88 | 73.97 | 61.75 | 11.03333 | 82.86667 |
| PTEN | 2.049 | 1.035027 | 2.41E-08 | 3.44E-07 | yes | up | 8.95 | 9.64 | 10.31 | 44.55 | 54.26 | 40.54 | 9.633333 | 46.45 |
| HSD11B1 | 0.151 | -2.73062 | 2.42E-08 | 3.44E-07 | yes | down | 20.62 | 18.51 | 24.86 | 2.47 | 6.35 | 13.43 | 21.33 | 7.416667 |
| ITM2C | 0.341 | -1.55123 | 2.53E-08 | 3.59E-07 | yes | down | 3.89 | 4.72 | 4.43 | 4.1 | 3.69 | 2.59 | 4.346667 | 3.46 |
| TMEM64 | 0.492 | -1.02412 | 2.7E-08 | 3.81E-07 | yes | down | 9.52 | 8.03 | 8.25 | 9.91 | 9.67 | 10.1 | 8.6 | 9.893333 |
| RUFY4 | 0.279 | -1.84116 | 2.72E-08 | 3.84E-07 | yes | down | 2.4 | 2.23 | 2.99 | 1.02 | 1.42 | 1.98 | 2.54 | 1.473333 |
| PINK1 | 0.307 | -1.70421 | 2.73E-08 | 3.85E-07 | yes | down | 141.43 | 155.25 | 153.13 | 59.76 | 127.67 | 129.4 | 149.9367 | 105.61 |
| LOC103346372 | 0.182 | -2.46176 | 2.77E-08 | 3.9E-07 | yes | down | 126.07 | 113.41 | 172.11 | 47.46 | 33.1 | 111.21 | 137.1967 | 63.92333 |
| DOCK8 | 0.42 | -1.24985 | 2.77E-08 | 3.9E-07 | yes | down | 2.25 | 3.43 | 2.91 | 3.6 | 2.14 | 2.71 | 2.863333 | 2.816667 |
| VCAM1 | 3.543 | 1.825125 | 2.78E-08 | 3.91E-07 | yes | up | 1.12 | 3.04 | 1.87 | 23.28 | 12.94 | 14.15 | 2.01 | 16.79 |
| AMPD3 | 0.285 | -1.81175 | 2.84E-08 | 3.99E-07 | yes | down | 2.4 | 2.27 | 1.26 | 1.12 | 1.38 | 1.41 | 1.976667 | 1.303333 |
| LOC103347146 | 0.325 | -1.62015 | 2.99E-08 | 4.2E-07 | yes | down | 9.57 | 6.93 | 8.85 | 8.42 | 6.74 | 4.6 | 8.45 | 6.586667 |
| BNC2 | 2.825 | 1.498281 | 3.13E-08 | 4.38E-07 | yes | up | 0.6 | 1.13 | 1 | 4.45 | 5.47 | 4.94 | 0.91 | 4.953333 |
| FTL | 3.043 | 1.605304 | 3.16E-08 | 4.42E-07 | yes | up | 498.55 | 474 | 653.98 | 2594.29 | 3712.8 | 5264.87 | 542.1767 | 3857.32 |
| B3GNT7 | 0.242 | -2.0458 | 3.3E-08 | 4.59E-07 | yes | down | 1.71 | 3.67 | 2.57 | 1.36 | 1.09 | 1.96 | 2.65 | 1.47 |
| EPHX1 | 2.664 | 1.413683 | 3.31E-08 | 4.6E-07 | yes | up | 98.79 | 71.33 | 116.23 | 501.44 | 579.29 | 673.66 | 95.45 | 584.7967 |
| LOC100352880 | 5.444 | 2.444708 | 3.39E-08 | 4.71E-07 | yes | up | 3.5 | 3.66 | 1.66 | 26.89 | 59.9 | 23.2 | 2.94 | 36.66333 |
| SLC2A4 | 2.163 | 1.113064 | 3.41E-08 | 4.73E-07 | yes | up | 22.16 | 17.45 | 15.36 | 90.56 | 81.03 | 102.42 | 18.32333 | 91.33667 |
| MRPS27 | 0.459 | -1.12464 | 3.61E-08 | 4.99E-07 | yes | down | 8.41 | 12.29 | 8.91 | 9.67 | 9.52 | 12.13 | 9.87 | 10.44 |
| LOC100359306 | 2.199 | 1.136696 | 3.7E-08 | 5.12E-07 | yes | up | 5.51 | 7.73 | 8.33 | 42.46 | 37.65 | 30.92 | 7.19 | 37.01 |
| ATXN2 | 0.436 | -1.19845 | 3.76E-08 | 5.18E-07 | yes | down | 19.23 | 26.71 | 25.8 | 22.22 | 20.17 | 30.3 | 23.91333 | 24.23 |
| ZNF367 | 5.922 | 2.566068 | 3.82E-08 | 5.27E-07 | yes | up | 0.3 | 0.4 | 0.19 | 3.47 | 6.07 | 2.5 | 0.296667 | 4.013333 |
| CRYM | 0.255 | -1.97068 | 3.86E-08 | 5.31E-07 | yes | down | 19.19 | 16.42 | 13.91 | 5.31 | 11.66 | 12.15 | 16.50667 | 9.706667 |
| TMEM132A | 2.762 | 1.465822 | 3.9E-08 | 5.36E-07 | yes | up | 1.64 | 1.58 | 1.39 | 13.69 | 7.91 | 8.35 | 1.536667 | 9.983333 |
| PLEKHO2 | 2.185 | 1.127919 | 4.02E-08 | 5.51E-07 | yes | up | 3.22 | 4.18 | 3.72 | 24.44 | 16.85 | 15.41 | 3.706667 | 18.9 |
| CA1 | 0.134 | -2.90435 | 4.1E-08 | 5.62E-07 | yes | down | 3.42 | 5.86 | 5.99 | 0.69 | 1.38 | 2.58 | 5.09 | 1.55 |
| CKAP2L | 10.053 | 3.329586 | 4.15E-08 | 5.67E-07 | yes | up | 0.17 | 0.43 | 0.06 | 6.18 | 6.44 | 2.36 | 0.22 | 4.993333 |
| CCNB2 | 9.718 | 3.280688 | 4.15E-08 | 5.67E-07 | yes | up | 0.64 | 1.29 | 0.26 | 18.53 | 23.79 | 6.16 | 0.73 | 16.16 |
| YARS2 | 0.495 | -1.01391 | 4.18E-08 | 5.71E-07 | yes | down | 10.1 | 15.7 | 12.78 | 15.28 | 14.78 | 14.43 | 12.86 | 14.83 |
| CNBD2 | 0.196 | -2.35012 | 4.18E-08 | 5.71E-07 | yes | down | 1.37 | 1.44 | 1.24 | 0.75 | 0.49 | 0.61 | 1.35 | 0.616667 |
| EXOSC4 | 0.436 | -1.19657 | 4.24E-08 | 5.77E-07 | yes | down | 7.27 | 10.24 | 7.75 | 9.64 | 8.46 | 8.25 | 8.42 | 8.783333 |
| SLC22A3 | 0.493 | -1.02135 | 4.36E-08 | 5.93E-07 | yes | down | 18.57 | 32.96 | 24.31 | 29.58 | 25.97 | 27.86 | 25.28 | 27.80333 |
| LARP4 | 0.439 | -1.18803 | 4.36E-08 | 5.93E-07 | yes | down | 12.38 | 16.75 | 19.8 | 17.94 | 20.73 | 19.15 | 16.31 | 19.27333 |
| LRRC3 | 3.213 | 1.683955 | 4.43E-08 | 6.02E-07 | yes | up | 1.62 | 2.57 | 1.75 | 12.52 | 18.72 | 12.51 | 1.98 | 14.58333 |
| LOC108177673 | 3.162 | 1.660824 | 4.46E-08 | 6.06E-07 | yes | up | 2.71 | 2.17 | 1.41 | 15.89 | 11.34 | 20.22 | 2.096667 | 15.81667 |
| HJURP | 7.11 | 2.829856 | 4.71E-08 | 6.37E-07 | yes | up | 0.19 | 0.22 | 0.11 | 3.67 | 2.51 | 1.13 | 0.173333 | 2.436667 |
| ARAP2 | 2.267 | 1.180828 | 4.77E-08 | 6.45E-07 | yes | up | 0.94 | 0.78 | 0.94 | 5.65 | 4.17 | 4.44 | 0.886667 | 4.753333 |
| LOC100358241 | 7.616 | 2.929045 | 4.78E-08 | 6.45E-07 | yes | up | 2.54 | 6.25 | 3.58 | 54.74 | 130.52 | 26.31 | 4.123333 | 70.52333 |
| COL4A1 | 2.184 | 1.127009 | 4.81E-08 | 6.49E-07 | yes | up | 243.65 | 308.89 | 223.03 | 1796.38 | 1327.47 | 1071.68 | 258.5233 | 1398.51 |
| FA2H | 0.143 | -2.8059 | 4.88E-08 | 6.58E-07 | yes | down | 0.75 | 1.31 | 1.84 | 0.38 | 0.55 | 0.38 | 1.3 | 0.436667 |
| ECT2 | 8.803 | 3.138062 | 4.93E-08 | 6.64E-07 | yes | up | 0.07 | 0.26 | 0.1 | 3.36 | 3.86 | 1.27 | 0.143333 | 2.83 |
| CCL14 | 2.248 | 1.168699 | 5.11E-08 | 6.87E-07 | yes | up | 12.27 | 17.92 | 14.74 | 74.37 | 81.18 | 81.09 | 14.97667 | 78.88 |
| PCK1 | 0.23 | -2.11876 | 5.23E-08 | 7.02E-07 | yes | down | 1139.11 | 1416.21 | 998.55 | 261.11 | 812.71 | 785.75 | 1184.623 | 619.8567 |
| ODC1 | 0.491 | -1.02681 | 5.32E-08 | 7.14E-07 | yes | down | 56.51 | 95.03 | 65.66 | 90.6 | 68.93 | 86.49 | 72.4 | 82.00667 |
| IFI44L | 2.859 | 1.515409 | 5.35E-08 | 7.17E-07 | yes | up | 1.74 | 2.56 | 2.04 | 19.66 | 9.02 | 17.4 | 2.113333 | 15.36 |
| KIF20A | 5.783 | 2.531888 | 5.37E-08 | 7.18E-07 | yes | up | 0.49 | 1.05 | 0.58 | 11.36 | 12.94 | 3.83 | 0.706667 | 9.376667 |
| HEY1 | 0.425 | -1.23438 | 5.37E-08 | 7.19E-07 | yes | down | 6.56 | 11.41 | 7.13 | 8.71 | 8.16 | 7.83 | 8.366667 | 8.233333 |
| PDZK1 | 0.118 | -3.08038 | 5.44E-08 | 7.27E-07 | yes | down | 3.34 | 6.31 | 8.21 | 2.87 | 0.38 | 2.22 | 5.953333 | 1.823333 |
| GPD1 | 2.065 | 1.046322 | 5.67E-08 | 7.56E-07 | yes | up | 344.3 | 492.62 | 600.79 | 2850.45 | 2119.34 | 2316.5 | 479.2367 | 2428.763 |
| SAPCD2 | 11.93 | 3.576556 | 5.73E-08 | 7.63E-07 | yes | up | 0.12 | 0.26 | 0.09 | 5.42 | 5.82 | 1.49 | 0.156667 | 4.243333 |
| NCAPG2 | 3.503 | 1.808642 | 5.78E-08 | 7.69E-07 | yes | up | 7.6 | 8.37 | 6.88 | 43.64 | 43.32 | 34.43 | 7.616667 | 40.46333 |
| BZW2 | 0.306 | -1.70838 | 5.87E-08 | 7.79E-07 | yes | down | 36.2 | 63.39 | 25.96 | 26.53 | 36.66 | 25 | 41.85 | 29.39667 |
| S100A6 | 2.177 | 1.122544 | 6.09E-08 | 8.07E-07 | yes | up | 136.65 | 164.47 | 101.23 | 675.23 | 753.03 | 655.8 | 134.1167 | 694.6867 |
| SLC25A1 | 2.914 | 1.542926 | 6.17E-08 | 8.17E-07 | yes | up | 54.44 | 79.42 | 75.64 | 579.64 | 266.95 | 588.62 | 69.83333 | 478.4033 |
| MTFMT | 0.392 | -1.3499 | 6.23E-08 | 8.24E-07 | yes | down | 4.9 | 5.75 | 6.75 | 5.15 | 4.92 | 5.84 | 5.8 | 5.303333 |
| GPLD1 | 0.164 | -2.61167 | 6.23E-08 | 8.24E-07 | yes | down | 0.63 | 0.42 | 0.74 | 0.23 | 0.24 | 0.18 | 0.596667 | 0.216667 |
| UBE2C | 8.378 | 3.066577 | 6.27E-08 | 8.28E-07 | yes | up | 1.2 | 1.45 | 0.87 | 29.31 | 31.25 | 8.15 | 1.173333 | 22.90333 |
| TMEM150A | 0.438 | -1.192 | 6.29E-08 | 8.31E-07 | yes | down | 9.97 | 8.84 | 10.34 | 10.75 | 10.19 | 8.74 | 9.716667 | 9.893333 |
| ENHO | 4.95 | 2.307542 | 6.35E-08 | 8.38E-07 | yes | up | 0.6 | 1.07 | 0.9 | 9.88 | 8.47 | 11.27 | 0.856667 | 9.873333 |
| COL27A1 | 3.622 | 1.856915 | 6.52E-08 | 8.6E-07 | yes | up | 1.83 | 1.8 | 1.99 | 23.19 | 9.15 | 17.34 | 1.873333 | 16.56 |
| AS3MT | 0.359 | -1.47987 | 6.53E-08 | 8.6E-07 | yes | down | 19.31 | 13.54 | 22.8 | 14.98 | 17.53 | 14.47 | 18.55 | 15.66 |
| BTNL10 | 0.148 | -2.76096 | 6.63E-08 | 8.73E-07 | yes | down | 2.86 | 1.6 | 2.76 | 0.42 | 0.56 | 1.57 | 2.406667 | 0.85 |
| IGFBP7 | 2.373 | 1.246959 | 6.76E-08 | 8.89E-07 | yes | up | 247.68 | 325.31 | 176.18 | 1729.44 | 1275.68 | 1165.38 | 249.7233 | 1390.167 |
| NAB2 | 2.168 | 1.116486 | 6.8E-08 | 8.92E-07 | yes | up | 2.52 | 3.12 | 3.5 | 14.94 | 16.92 | 13.87 | 3.046667 | 15.24333 |
| MCM3 | 2.63 | 1.394961 | 7.29E-08 | 9.52E-07 | yes | up | 1.52 | 2.13 | 1.85 | 10.12 | 13.64 | 9.11 | 1.833333 | 10.95667 |
| LOC100343510 | 2.84 | 1.505692 | 7.48E-08 | 9.77E-07 | yes | up | 4.4 | 8.81 | 4.91 | 46.88 | 27.79 | 39.79 | 6.04 | 38.15333 |
| LOC100339499 | 2.549 | 1.349985 | 7.67E-08 | 1E-06 | yes | up | 6.38 | 7.4 | 5.78 | 42.01 | 27.14 | 37.54 | 6.52 | 35.56333 |
| LTBP2 | 0.435 | -1.20052 | 7.78E-08 | 1.01E-06 | yes | down | 1.02 | 1.6 | 1.13 | 1.77 | 1.13 | 1.34 | 1.25 | 1.413333 |
| KNTC1 | 4.874 | 2.285202 | 7.82E-08 | 1.02E-06 | yes | up | 0.21 | 0.28 | 0.2 | 3.49 | 3.13 | 1.21 | 0.23 | 2.61 |
| HSPBP1 | 0.431 | -1.21424 | 8.02E-08 | 1.04E-06 | yes | down | 7.9 | 11.22 | 7.16 | 8.85 | 9.17 | 8.16 | 8.76 | 8.726667 |
| ROGDI | 0.423 | -1.23981 | 8.12E-08 | 1.06E-06 | yes | down | 8.5 | 8.96 | 10.79 | 10.56 | 9.8 | 7.62 | 9.416667 | 9.326667 |
| LOC103350535 | 0.399 | -1.32623 | 8.2E-08 | 1.06E-06 | yes | down | 18.85 | 20.61 | 16.9 | 17.27 | 13.06 | 22.3 | 18.78667 | 17.54333 |
| NUPR1 | 2.418 | 1.273689 | 8.2E-08 | 1.06E-06 | yes | up | 14.72 | 19.83 | 17.21 | 75.55 | 108.69 | 105.73 | 17.25333 | 96.65667 |
| CTSH | 2.169 | 1.116809 | 8.24E-08 | 1.07E-06 | yes | up | 43.15 | 60.74 | 58.75 | 223.03 | 294.45 | 252.73 | 54.21333 | 256.7367 |
| KCNJ8 | 3.059 | 1.613265 | 8.27E-08 | 1.07E-06 | yes | up | 4.95 | 9.48 | 5.93 | 71.06 | 36.84 | 36.8 | 6.786667 | 48.23333 |
| ACOX2 | 2.759 | 1.464184 | 8.34E-08 | 1.08E-06 | yes | up | 7.93 | 7.89 | 13.15 | 51.01 | 67.94 | 66.28 | 9.656667 | 61.74333 |
| STARD4 | 3.48 | 1.799006 | 8.73E-08 | 1.13E-06 | yes | up | 1.67 | 2.99 | 2.49 | 26.08 | 9.99 | 21.82 | 2.383333 | 19.29667 |
| PLP2 | 3.267 | 1.707821 | 8.87E-08 | 1.14E-06 | yes | up | 16.52 | 22.15 | 8.27 | 125.35 | 110.5 | 122.88 | 15.64667 | 119.5767 |
| FAHD1 | 0.451 | -1.14913 | 9.05E-08 | 1.17E-06 | yes | down | 9.83 | 11.37 | 10.04 | 10.62 | 11.33 | 11.54 | 10.41333 | 11.16333 |
| PLTP | 2.227 | 1.154913 | 9.14E-08 | 1.18E-06 | yes | up | 23.71 | 31.88 | 36.58 | 132.48 | 175.54 | 161.55 | 30.72333 | 156.5233 |
| LOC103347329 | 0.105 | -3.25492 | 9.15E-08 | 1.18E-06 | yes | down | 12.2 | 8.86 | 4.2 | 0.95 | 3.98 | 1.2 | 8.42 | 2.043333 |
| LOC103349888 | 3.35 | 1.744057 | 9.18E-08 | 1.18E-06 | yes | up | 0.62 | 1.78 | 1.05 | 9.32 | 7.79 | 5.82 | 1.15 | 7.643333 |
| RARRES2 | 2.213 | 1.14597 | 9.24E-08 | 1.19E-06 | yes | up | 137.14 | 142.48 | 155.86 | 625.98 | 678.41 | 947.5 | 145.16 | 750.63 |
| LOC100340040 | 8.907 | 3.155017 | 9.26E-08 | 1.19E-06 | yes | up | 0.05 | 0.16 | 0.35 | 3.82 | 4.65 | 4.31 | 0.186667 | 4.26 |
| AURKA | 7.642 | 2.933857 | 9.29E-08 | 1.19E-06 | yes | up | 0.29 | 0.32 | 0.3 | 6.7 | 6.9 | 2.2 | 0.303333 | 5.266667 |
| LOC103344972 | 2.586 | 1.370633 | 9.43E-08 | 1.21E-06 | yes | up | 17.71 | 27.46 | 15.93 | 111.83 | 142.74 | 111.96 | 20.36667 | 122.1767 |
| CXCL12 | 0.381 | -1.39032 | 9.5E-08 | 1.22E-06 | yes | down | 12.09 | 20.35 | 10.69 | 10.49 | 13.03 | 14.05 | 14.37667 | 12.52333 |
| HOXD4 | 2.411 | 1.269684 | 9.5E-08 | 1.22E-06 | yes | up | 2.49 | 3.18 | 2.27 | 16.67 | 12.67 | 11.52 | 2.646667 | 13.62 |
| RNF170 | 2.3 | 1.201718 | 9.53E-08 | 1.22E-06 | yes | up | 2.47 | 2.57 | 2.91 | 18.21 | 12.86 | 11.47 | 2.65 | 14.18 |
| PENK | 5.338 | 2.416333 | 9.54E-08 | 1.22E-06 | yes | up | 3.07 | 5.46 | 2.67 | 22.31 | 37.34 | 76.48 | 3.733333 | 45.37667 |
| U2AF2 | 0.398 | -1.32812 | 9.57E-08 | 1.22E-06 | yes | down | 4.37 | 6 | 5.3 | 4.89 | 4.63 | 4.98 | 5.223333 | 4.833333 |
| THRSP | 2.484 | 1.312824 | 9.82E-08 | 1.25E-06 | yes | up | 220.78 | 451.31 | 340.72 | 2004.82 | 1174.32 | 1378.17 | 337.6033 | 1519.103 |
| TFB1M | 0.495 | -1.01546 | 1.03E-07 | 1.31E-06 | yes | down | 12.56 | 15.74 | 16.67 | 18.54 | 17.96 | 15.54 | 14.99 | 17.34667 |
| MFAP3L | 2.87 | 1.521157 | 1.03E-07 | 1.31E-06 | yes | up | 2.71 | 1.67 | 3.21 | 15.79 | 16.82 | 18.51 | 2.53 | 17.04 |
| GNAT3 | 0.029 | -5.13189 | 1.03E-07 | 1.32E-06 | yes | down | 28.88 | 31.86 | 28.13 | 2.36 | 4.06 | 0.72 | 29.62333 | 2.38 |
| LOC100341883 | 4.996 | 2.320635 | 1.04E-07 | 1.32E-06 | yes | up | 0.18 | 0.24 | 0.24 | 2.55 | 2.13 | 3.12 | 0.22 | 2.6 |
| METTL25 | 0.359 | -1.47914 | 1.06E-07 | 1.34E-06 | yes | down | 2.15 | 2.48 | 2.45 | 2.15 | 1.77 | 2.01 | 2.36 | 1.976667 |
| COQ10B | 0.416 | -1.26678 | 1.09E-07 | 1.38E-06 | yes | down | 30.31 | 23.38 | 20.82 | 21.48 | 24.75 | 26.28 | 24.83667 | 24.17 |
| ISOC2 | 0.433 | -1.20687 | 1.1E-07 | 1.39E-06 | yes | down | 62.21 | 70.3 | 59.03 | 84.95 | 63.19 | 45.61 | 63.84667 | 64.58333 |
| BHLHE22 | 16.925 | 4.081099 | 1.1E-07 | 1.4E-06 | yes | up | 0.06 | 0.01 | 0.01 | 1.39 | 1.17 | 0.91 | 0.026667 | 1.156667 |
| SHCBP1 | 10.288 | 3.362888 | 1.13E-07 | 1.43E-06 | yes | up | 0.12 | 0.36 | 0.12 | 5.09 | 7.04 | 1.8 | 0.2 | 4.643333 |
| IQGAP3 | 7.515 | 2.909717 | 1.14E-07 | 1.44E-06 | yes | up | 0.42 | 0.46 | 0.17 | 8.16 | 8.11 | 1.92 | 0.35 | 6.063333 |
| CYP2C30 | 118.685 | 6.89099 | 1.17E-07 | 1.47E-06 | yes | up | 0.08 | 0 | 0 | 22.95 | 1.07 | 10.84 | 0.026667 | 11.62 |
| ARHGAP1 | 2.145 | 1.101117 | 1.17E-07 | 1.47E-06 | yes | up | 8.72 | 8.29 | 7.06 | 39.63 | 32.17 | 48.78 | 8.023333 | 40.19333 |
| AP4B1 | 0.488 | -1.03425 | 1.2E-07 | 1.51E-06 | yes | down | 8.37 | 9.42 | 10.15 | 10.97 | 8.74 | 12.68 | 9.313333 | 10.79667 |
| IRF9 | 3.096 | 1.63029 | 1.21E-07 | 1.52E-06 | yes | up | 3.35 | 3.17 | 4.39 | 23.41 | 18.66 | 37.13 | 3.636667 | 26.4 |
| SCRN1 | 0.473 | -1.07952 | 1.25E-07 | 1.56E-06 | yes | down | 4.31 | 6.82 | 4.93 | 5.49 | 6.48 | 5.39 | 5.353333 | 5.786667 |
| LOC100345427 | 3.404 | 1.767288 | 1.25E-07 | 1.57E-06 | yes | up | 0.99 | 1.34 | 0.95 | 13.21 | 7.23 | 6.83 | 1.093333 | 9.09 |
| GPC3 | 0.317 | -1.65863 | 1.26E-07 | 1.57E-06 | yes | down | 11.97 | 21.8 | 14.32 | 18.23 | 8.66 | 8.56 | 16.03 | 11.81667 |
| LOC100353131 | 0.255 | -1.97198 | 1.26E-07 | 1.57E-06 | yes | down | 2.25 | 1.92 | 3.17 | 1.25 | 1.28 | 1.82 | 2.446667 | 1.45 |
| CDCA8 | 8.387 | 3.068187 | 1.27E-07 | 1.58E-06 | yes | up | 0.34 | 0.39 | 0.24 | 7.28 | 9.04 | 2.42 | 0.323333 | 6.246667 |
| CCDC59 | 0.475 | -1.073 | 1.33E-07 | 1.66E-06 | yes | down | 10.58 | 10.33 | 13.1 | 13.03 | 13.28 | 14.28 | 11.33667 | 13.53 |
| CKS1B | 3.201 | 1.678568 | 1.35E-07 | 1.68E-06 | yes | up | 6 | 8.45 | 7.61 | 64.7 | 66.73 | 33.74 | 7.353333 | 55.05667 |
| MARS2 | 0.218 | -2.19729 | 1.36E-07 | 1.69E-06 | yes | down | 2.27 | 5.76 | 5.23 | 1.34 | 2.01 | 3.2 | 4.42 | 2.183333 |
| CBR1 | 3.151 | 1.656036 | 1.36E-07 | 1.69E-06 | yes | up | 1.62 | 3.08 | 2.17 | 18.3 | 17.3 | 19.51 | 2.29 | 18.37 |
| LOC100351490 | 0.433 | -1.20628 | 1.36E-07 | 1.69E-06 | yes | down | 5.07 | 6.39 | 7.14 | 4.4 | 3.25 | 5.64 | 6.2 | 4.43 |
| LIFR | 2.72 | 1.443542 | 1.37E-07 | 1.7E-06 | yes | up | 10.56 | 6.03 | 6.67 | 50.21 | 42.02 | 58.02 | 7.753333 | 50.08333 |
| FGF14 | 6.697 | 2.743606 | 1.37E-07 | 1.7E-06 | yes | up | 0.11 | 0.11 | 0.15 | 1.17 | 3.14 | 1.44 | 0.123333 | 1.916667 |
| KPNA2 | 2.877 | 1.52477 | 1.41E-07 | 1.74E-06 | yes | up | 3.94 | 7.71 | 6.66 | 36.76 | 51.47 | 31.87 | 6.103333 | 40.03333 |
| TCF19 | 9.731 | 3.282628 | 1.41E-07 | 1.75E-06 | yes | up | 0.15 | 0.56 | 0.13 | 6.78 | 8.72 | 2.83 | 0.28 | 6.11 |
| LIMD2 | 3.072 | 1.619274 | 1.47E-07 | 1.81E-06 | yes | up | 1.69 | 2.23 | 2.23 | 19.31 | 13.11 | 11.89 | 2.05 | 14.77 |
| PGBD2 | 2.432 | 1.282102 | 1.47E-07 | 1.82E-06 | yes | up | 1.65 | 2.38 | 2.58 | 11.88 | 10.54 | 13.68 | 2.203333 | 12.03333 |
| SNRNP25 | 0.461 | -1.11838 | 1.48E-07 | 1.82E-06 | yes | down | 19.94 | 34.41 | 25.11 | 30.05 | 27.46 | 25.71 | 26.48667 | 27.74 |
| PLXND1 | 2.162 | 1.11242 | 1.52E-07 | 1.87E-06 | yes | up | 13.44 | 19.9 | 15.63 | 105.54 | 58.89 | 79.3 | 16.32333 | 81.24333 |
| AGTR2 | 0.105 | -3.24711 | 1.54E-07 | 1.9E-06 | yes | down | 0.64 | 0.7 | 0.38 | 0.13 | 0.14 | 0.15 | 0.573333 | 0.14 |
| BCL6 | 2.496 | 1.319506 | 1.55E-07 | 1.91E-06 | yes | up | 11.56 | 11.56 | 11.35 | 45.08 | 48.78 | 82.13 | 11.49 | 58.66333 |
| NFATC4 | 3.608 | 1.851065 | 1.55E-07 | 1.91E-06 | yes | up | 0.83 | 1.96 | 1.14 | 15.79 | 8.74 | 7.53 | 1.31 | 10.68667 |
| MFSD2A | 0.092 | -3.44285 | 1.56E-07 | 1.92E-06 | yes | down | 0.94 | 1.73 | 0.93 | 0.16 | 0.52 | 0.08 | 1.2 | 0.253333 |
| LOC100349105 | 0.481 | -1.05632 | 1.57E-07 | 1.93E-06 | yes | down | 45.78 | 46.34 | 45.02 | 44.46 | 56.18 | 54.01 | 45.71333 | 51.55 |
| TRAK1 | 2.057 | 1.040369 | 1.61E-07 | 1.97E-06 | yes | up | 21.98 | 23.95 | 26.2 | 104.82 | 71.53 | 118.76 | 24.04333 | 98.37 |
| ARHGDIB | 2.369 | 1.244446 | 1.62E-07 | 1.98E-06 | yes | up | 18.57 | 17.65 | 20.73 | 120.17 | 120.7 | 75.23 | 18.98333 | 105.3667 |
| SLC25A4 | 0.351 | -1.51151 | 1.63E-07 | 1.99E-06 | yes | down | 261.14 | 394.27 | 203.69 | 193.22 | 305.01 | 192.46 | 286.3667 | 230.23 |
| FRMD8 | 0.475 | -1.07278 | 1.63E-07 | 1.99E-06 | yes | down | 14.35 | 18.9 | 18.75 | 22.33 | 13.76 | 18.68 | 17.33333 | 18.25667 |
| NDUFAF5 | 0.353 | -1.50313 | 1.64E-07 | 2E-06 | yes | down | 7.65 | 13.17 | 7.36 | 6.33 | 7.25 | 6.59 | 9.393333 | 6.723333 |
| KIF18B | 12.562 | 3.651007 | 1.66E-07 | 2.02E-06 | yes | up | 0.13 | 0.1 | 0.07 | 4.11 | 4.01 | 0.72 | 0.1 | 2.946667 |
| LOC100353654 | 0.358 | -1.48166 | 1.69E-07 | 2.05E-06 | yes | down | 80.23 | 69.12 | 72.06 | 42.78 | 86.7 | 60.34 | 73.80333 | 63.27333 |
| ATG14 | 2.34 | 1.226591 | 1.7E-07 | 2.07E-06 | yes | up | 1.64 | 1.32 | 2 | 9.76 | 8.46 | 9.02 | 1.653333 | 9.08 |
| SH3PXD2B | 2.401 | 1.263624 | 1.7E-07 | 2.07E-06 | yes | up | 1.91 | 1.61 | 1.27 | 9.51 | 7.1 | 10.31 | 1.596667 | 8.973333 |
| PCOLCE | 2.192 | 1.132537 | 1.72E-07 | 2.09E-06 | yes | up | 26.79 | 43.48 | 33.96 | 147.54 | 203.71 | 172.88 | 34.74333 | 174.71 |
| CIT | 8.492 | 3.086149 | 1.74E-07 | 2.12E-06 | yes | up | 0.05 | 0.15 | 0.06 | 2.61 | 1.92 | 0.59 | 0.086667 | 1.706667 |
| MTRF1 | 0.303 | -1.72029 | 1.79E-07 | 2.18E-06 | yes | down | 2.45 | 4.72 | 4.12 | 2.6 | 2.07 | 3.11 | 3.763333 | 2.593333 |
| ADRB3 | 0.335 | -1.57644 | 1.8E-07 | 2.19E-06 | yes | down | 5.6 | 12.75 | 12.45 | 7.09 | 6.13 | 6.06 | 10.26667 | 6.426667 |
| SEC22C | 2.027 | 1.019576 | 1.86E-07 | 2.25E-06 | yes | up | 1 | 1.43 | 1.3 | 5.87 | 5.09 | 6.28 | 1.243333 | 5.746667 |
| FAM46C | 2.245 | 1.166808 | 1.88E-07 | 2.27E-06 | yes | up | 0.92 | 0.75 | 0.96 | 4.75 | 4.49 | 4.56 | 0.876667 | 4.6 |
| PALD1 | 2.086 | 1.060684 | 1.92E-07 | 2.31E-06 | yes | up | 27.04 | 35.2 | 41.76 | 162.11 | 147.9 | 201.62 | 34.66667 | 170.5433 |
| EMC9 | 0.483 | -1.04894 | 1.93E-07 | 2.32E-06 | yes | down | 33.16 | 30.94 | 24.78 | 35.89 | 32.45 | 29.06 | 29.62667 | 32.46667 |
| SFXN4 | 0.468 | -1.09507 | 2.03E-07 | 2.44E-06 | yes | down | 11.26 | 13.77 | 14.94 | 11.73 | 11.62 | 14.94 | 13.32333 | 12.76333 |
| LOC100343081 | 0.355 | -1.49498 | 2.03E-07 | 2.44E-06 | yes | down | 5.57 | 5.21 | 6.46 | 4.01 | 3.51 | 3.58 | 5.746667 | 3.7 |
| PPM1K | 2.143 | 1.099853 | 2.08E-07 | 2.49E-06 | yes | up | 4.03 | 4.41 | 2.79 | 20.69 | 15.72 | 19.17 | 3.743333 | 18.52667 |
| LOC100353463 | 2.369 | 1.244269 | 2.08E-07 | 2.49E-06 | yes | up | 65.81 | 83.22 | 98.12 | 368.89 | 424.64 | 584.52 | 82.38333 | 459.35 |
| DPP6 | 0.098 | -3.35119 | 2.12E-07 | 2.53E-06 | yes | down | 0.61 | 0.21 | 1.11 | 0.13 | 0.16 | 0.16 | 0.643333 | 0.15 |
| SHCBP1L | 0.055 | -4.1841 | 2.16E-07 | 2.59E-06 | yes | down | 2.51 | 2.4 | 2.25 | 0.15 | 0.05 | 0.87 | 2.386667 | 0.356667 |
| KALRN | 0.427 | -1.22645 | 2.2E-07 | 2.63E-06 | yes | down | 2.97 | 2.54 | 2.03 | 2.75 | 1.73 | 1.33 | 2.513333 | 1.936667 |
| GADD45G | 0.066 | -3.92979 | 2.21E-07 | 2.63E-06 | yes | down | 279.65 | 209.13 | 51.73 | 16.08 | 37.17 | 29.33 | 180.17 | 27.52667 |
| ZFP90 | 2.007 | 1.00505 | 2.21E-07 | 2.63E-06 | yes | up | 2.65 | 3.83 | 2.84 | 15.8 | 14.13 | 12.95 | 3.106667 | 14.29333 |
| IMMP2L | 0.382 | -1.38913 | 2.25E-07 | 2.68E-06 | yes | down | 6.97 | 6.44 | 8.28 | 5.61 | 5.51 | 7.63 | 7.23 | 6.25 |
| CARMIL1 | 2.312 | 1.208924 | 2.25E-07 | 2.68E-06 | yes | up | 0.85 | 0.87 | 0.76 | 4.48 | 3.88 | 5.12 | 0.826667 | 4.493333 |
| ARMCX6 | 2.229 | 1.156314 | 2.26E-07 | 2.69E-06 | yes | up | 2.61 | 3.84 | 3.05 | 19.1 | 16.62 | 13.19 | 3.166667 | 16.30333 |
| WSCD2 | 3.065 | 1.615798 | 2.31E-07 | 2.75E-06 | yes | up | 0.36 | 0.45 | 0.34 | 3.08 | 2.17 | 2.61 | 0.383333 | 2.62 |
| LOC100353349 | 0.362 | -1.4656 | 2.31E-07 | 2.75E-06 | yes | down | 3.03 | 5.22 | 3.6 | 2.67 | 4.1 | 3.04 | 3.95 | 3.27 |
| CDKN2C | 2.284 | 1.191558 | 2.33E-07 | 2.76E-06 | yes | up | 12.17 | 12.13 | 10.92 | 74.22 | 57.94 | 46.33 | 11.74 | 59.49667 |
| HP | 16.741 | 4.065273 | 2.36E-07 | 2.79E-06 | yes | up | 116.38 | 33.99 | 35.41 | 1265.74 | 3076.44 | 3076.01 | 61.92667 | 2472.73 |
| DENND5B | 0.377 | -1.40579 | 2.37E-07 | 2.8E-06 | yes | down | 1.28 | 1.87 | 1.21 | 1.02 | 1.51 | 1.24 | 1.453333 | 1.256667 |
| PAFAH1B3 | 2.468 | 1.303327 | 2.41E-07 | 2.85E-06 | yes | up | 7.45 | 11.17 | 8.34 | 61.63 | 54.29 | 38.36 | 8.986667 | 51.42667 |
| NDC80 | 12.76 | 3.673516 | 2.48E-07 | 2.93E-06 | yes | up | 0.22 | 0.16 | 0.05 | 5.2 | 5.94 | 1.45 | 0.143333 | 4.196667 |
| PRR11 | 17.227 | 4.106597 | 2.48E-07 | 2.93E-06 | yes | up | 0.03 | 0.08 | 0.04 | 2.78 | 2.56 | 0.48 | 0.05 | 1.94 |
| ABCD1 | 2.17 | 1.117561 | 2.53E-07 | 2.98E-06 | yes | up | 2.79 | 2.62 | 2.44 | 14.25 | 9.52 | 12.29 | 2.616667 | 12.02 |
| CENPA | 9.871 | 3.303175 | 2.7E-07 | 3.18E-06 | yes | up | 0.66 | 0.42 | 0.57 | 13.49 | 19.7 | 2.42 | 0.55 | 11.87 |
| WARS2 | 0.457 | -1.12951 | 2.8E-07 | 3.28E-06 | yes | down | 3.26 | 4.67 | 3.6 | 4.06 | 4.34 | 4.1 | 3.843333 | 4.166667 |
| LOC100355311 | 0.429 | -1.22204 | 2.8E-07 | 3.28E-06 | yes | down | 4.79 | 6.22 | 5.95 | 5.92 | 4.58 | 6.28 | 5.653333 | 5.593333 |
| NT5M | 0.256 | -1.96427 | 2.81E-07 | 3.29E-06 | yes | down | 4.44 | 3.3 | 3.29 | 2.34 | 2.12 | 3.13 | 3.676667 | 2.53 |
| ITPR1 | 0.393 | -1.34735 | 2.84E-07 | 3.32E-06 | yes | down | 8.2 | 9.81 | 8.7 | 8.64 | 4.98 | 10.87 | 8.903333 | 8.163333 |
| LSS | 3.495 | 1.805257 | 2.84E-07 | 3.33E-06 | yes | up | 4.92 | 7.08 | 6.72 | 74.43 | 22.54 | 54.44 | 6.24 | 50.47 |
| KIF11 | 6.649 | 2.733176 | 2.86E-07 | 3.34E-06 | yes | up | 0.31 | 0.56 | 0.17 | 5.79 | 7.85 | 2.17 | 0.346667 | 5.27 |
| LCAT | 35.394 | 5.145422 | 2.87E-07 | 3.35E-06 | yes | up | 0 | 0.15 | 0 | 4.39 | 4.38 | 2.65 | 0.05 | 3.806667 |
| OSBPL11 | 0.355 | -1.49426 | 2.97E-07 | 3.46E-06 | yes | down | 24.36 | 19.15 | 25.37 | 12.8 | 19.06 | 24.88 | 22.96 | 18.91333 |
| TMEM245 | 2 | 1.00027 | 2.98E-07 | 3.48E-06 | yes | up | 5.77 | 8.13 | 5.08 | 30.62 | 31.03 | 25.35 | 6.326667 | 29 |
| ZWILCH | 4.683 | 2.227577 | 3.06E-07 | 3.56E-06 | yes | up | 0.29 | 0.47 | 0.36 | 4.72 | 5.25 | 2.16 | 0.373333 | 4.043333 |
| TRAM1L1 | 0.086 | -3.53743 | 3.07E-07 | 3.57E-06 | yes | down | 0.59 | 0.85 | 0.47 | 0.17 | 0.11 | 0.1 | 0.636667 | 0.126667 |
| TNNI3K | 9.023 | 3.173602 | 3.09E-07 | 3.59E-06 | yes | up | 0.04 | 0.13 | 0.1 | 2.63 | 1.05 | 1.78 | 0.09 | 1.82 |
| KIF21B | 0.203 | -2.30383 | 3.1E-07 | 3.6E-06 | yes | down | 0.32 | 0.95 | 0.97 | 0.46 | 0.25 | 0.34 | 0.746667 | 0.35 |
| PER1 | 0.429 | -1.22222 | 3.14E-07 | 3.63E-06 | yes | down | 33.24 | 18.31 | 28.29 | 31.11 | 37.64 | 23.52 | 26.61333 | 30.75667 |
| HYLS1 | 3.129 | 1.645566 | 3.16E-07 | 3.65E-06 | yes | up | 1.27 | 1.02 | 1.26 | 8.85 | 11.96 | 6.93 | 1.183333 | 9.246667 |
| COL6A3 | 2.242 | 1.1646 | 3.17E-07 | 3.66E-06 | yes | up | 54.67 | 52.72 | 33.49 | 345.66 | 212.15 | 274.47 | 46.96 | 277.4267 |
| IGFBP5 | 2.133 | 1.092658 | 3.17E-07 | 3.66E-06 | yes | up | 21.17 | 36.86 | 25.7 | 116.36 | 147.92 | 143 | 27.91 | 135.76 |
| WDR12 | 0.401 | -1.31864 | 3.29E-07 | 3.79E-06 | yes | down | 5.51 | 7.09 | 6 | 4.43 | 6.86 | 5.89 | 6.2 | 5.726667 |
| CCKBR | 0.007 | -7.22568 | 3.35E-07 | 3.86E-06 | yes | down | 0.19 | 0.14 | 0.23 | 0 | 0 | 0 | 0.186667 | 0 |
| KIF20B | 0.276 | -1.85561 | 3.35E-07 | 3.86E-06 | yes | down | 5.94 | 8.36 | 7.2 | 4.31 | 3.88 | 1.65 | 7.166667 | 3.28 |
| ID2 | 0.275 | -1.86111 | 3.36E-07 | 3.86E-06 | yes | down | 5.46 | 4.65 | 5 | 3.48 | 2.16 | 4.16 | 5.036667 | 3.266667 |
| NCAPG | 8.383 | 3.067436 | 3.6E-07 | 4.12E-06 | yes | up | 0.13 | 0.28 | 0.1 | 3.53 | 4.76 | 1.25 | 0.17 | 3.18 |
| LOC100328948 | 47.79 | 5.578634 | 3.62E-07 | 4.14E-06 | yes | up | 2.56 | 0.47 | 0.16 | 177.83 | 106.24 | 148.22 | 1.063333 | 144.0967 |
| HPSE2 | 8.752 | 3.129592 | 3.64E-07 | 4.16E-06 | yes | up | 6.56 | 2.04 | 10.74 | 26.92 | 6.85 | 31.53 | 6.446667 | 21.76667 |
| USP24 | 0.415 | -1.26836 | 3.67E-07 | 4.18E-06 | yes | down | 25.2 | 27.73 | 15.21 | 19.33 | 21.46 | 24.58 | 22.71333 | 21.79 |
| LOC103345926 | 2.427 | 1.278996 | 3.87E-07 | 4.39E-06 | yes | up | 99.68 | 64.96 | 97.2 | 479.07 | 546.07 | 490.24 | 87.28 | 505.1267 |
| RARG | 2.171 | 1.118625 | 3.9E-07 | 4.42E-06 | yes | up | 2.83 | 5.09 | 5.29 | 22.07 | 18.76 | 22.69 | 4.403333 | 21.17333 |
| SQLE | 2.181 | 1.125238 | 3.96E-07 | 4.48E-06 | yes | up | 1.97 | 2.98 | 1.97 | 12.66 | 12.05 | 10.11 | 2.306667 | 11.60667 |
| LOC100349353 | 0.477 | -1.0683 | 4E-07 | 4.52E-06 | yes | down | 27.66 | 29.86 | 23 | 27 | 29.26 | 35.16 | 26.84 | 30.47333 |
| SPTA1 | 0.095 | -3.39204 | 4.03E-07 | 4.56E-06 | yes | down | 0.88 | 0.94 | 0.6 | 0.01 | 0.51 | 0.3 | 0.806667 | 0.273333 |
| SGO2 | 11.613 | 3.537697 | 4.06E-07 | 4.59E-06 | yes | up | 0.04 | 0.18 | 0.04 | 3.06 | 2.74 | 0.87 | 0.086667 | 2.223333 |
| TMED4 | 0.487 | -1.03708 | 4.16E-07 | 4.68E-06 | yes | down | 157.03 | 138.07 | 176.35 | 153.91 | 177.53 | 204.59 | 157.15 | 178.6767 |
| WNK4 | 0.414 | -1.27367 | 4.19E-07 | 4.71E-06 | yes | down | 1.87 | 2.91 | 2.28 | 2.92 | 1.69 | 2.26 | 2.353333 | 2.29 |
| CEP55 | 10.449 | 3.385357 | 4.19E-07 | 4.71E-06 | yes | up | 0.22 | 0.35 | 0.14 | 5.57 | 9.86 | 1.51 | 0.236667 | 5.646667 |
| KIAA0408 | 0.315 | -1.66861 | 4.36E-07 | 4.89E-06 | yes | down | 17.21 | 14.72 | 14.17 | 6.9 | 14.14 | 14.26 | 15.36667 | 11.76667 |
| RELN | 0.382 | -1.38742 | 4.54E-07 | 5.08E-06 | yes | down | 0.57 | 0.75 | 0.45 | 0.48 | 0.52 | 0.56 | 0.59 | 0.52 |
| LOC100358539 | 4.803 | 2.263926 | 4.58E-07 | 5.11E-06 | yes | up | 0.39 | 0.74 | 0.61 | 4.56 | 4.21 | 10.44 | 0.58 | 6.403333 |
| PHIP | 2.177 | 1.122552 | 4.71E-07 | 5.26E-06 | yes | up | 2.19 | 1.66 | 1.97 | 12.11 | 8.26 | 9.13 | 1.94 | 9.833333 |
| CSPG4 | 2.506 | 1.325167 | 4.86E-07 | 5.42E-06 | yes | up | 1.72 | 1.49 | 1.01 | 10.29 | 7.63 | 6.9 | 1.406667 | 8.273333 |
| CNTROB | 2.1 | 1.070455 | 4.87E-07 | 5.42E-06 | yes | up | 2.13 | 1.85 | 2.12 | 10.7 | 9.04 | 8.59 | 2.033333 | 9.443333 |
| DDX58 | 2.125 | 1.08755 | 4.89E-07 | 5.44E-06 | yes | up | 7.56 | 7.11 | 5.34 | 33.23 | 27.97 | 39.03 | 6.67 | 33.41 |
| NEMP1 | 2.351 | 1.233039 | 4.9E-07 | 5.45E-06 | yes | up | 1.01 | 0.76 | 0.71 | 5.09 | 4.16 | 4.28 | 0.826667 | 4.51 |
| FHL1 | 0.333 | -1.58845 | 4.95E-07 | 5.5E-06 | yes | down | 851.03 | 1434.04 | 508.44 | 591.69 | 738.28 | 761.26 | 931.17 | 697.0767 |
| F3 | 0.326 | -1.61505 | 4.96E-07 | 5.51E-06 | yes | down | 132.11 | 73.41 | 82.72 | 53.57 | 82.03 | 85.7 | 96.08 | 73.76667 |
| LOC100349037 | 0.232 | -2.10861 | 5.04E-07 | 5.58E-06 | yes | down | 52.85 | 36.55 | 28.79 | 34.73 | 21.25 | 9.54 | 39.39667 | 21.84 |
| SRD5A1 | 0.402 | -1.31432 | 5.05E-07 | 5.58E-06 | yes | down | 10.11 | 13.45 | 16.44 | 14.3 | 10 | 11.05 | 13.33333 | 11.78333 |
| GCHFR | 0.355 | -1.49238 | 5.14E-07 | 5.68E-06 | yes | down | 9.92 | 14.91 | 14.42 | 13.41 | 12.06 | 7.02 | 13.08333 | 10.83 |
| LOC103345364 | 0.373 | -1.4215 | 5.22E-07 | 5.76E-06 | yes | down | 25.56 | 28.61 | 20.71 | 18.25 | 18.54 | 28.76 | 24.96 | 21.85 |
| EPB42 | 0.167 | -2.5838 | 5.35E-07 | 5.89E-06 | yes | down | 1.46 | 1.15 | 1.1 | 0.25 | 0.37 | 0.83 | 1.236667 | 0.483333 |
| LSM11 | 2.669 | 1.416257 | 5.43E-07 | 5.97E-06 | yes | up | 0.29 | 0.43 | 0.48 | 2.81 | 2.28 | 2.32 | 0.4 | 2.47 |
| LOC103349956 | 0.25 | -1.99717 | 5.45E-07 | 5.99E-06 | yes | down | 3.54 | 6.13 | 2.29 | 2.04 | 1.15 | 2.35 | 3.986667 | 1.846667 |
| LOC100354564 | 0.273 | -1.8744 | 5.49E-07 | 6.03E-06 | yes | down | 4.16 | 4.55 | 4.29 | 3.47 | 2.16 | 2.86 | 4.333333 | 2.83 |
| LOXL2 | 2.894 | 1.532938 | 5.61E-07 | 6.15E-06 | yes | up | 28.73 | 37.27 | 16.56 | 172.75 | 231.53 | 143.58 | 27.52 | 182.62 |
| LOC100352785 | 4.991 | 2.319297 | 5.65E-07 | 6.19E-06 | yes | up | 0.41 | 0.74 | 0.27 | 5.84 | 6.73 | 3.52 | 0.473333 | 5.363333 |
| LOC100339548 | 0.139 | -2.84321 | 5.66E-07 | 6.2E-06 | yes | down | 0.77 | 0.77 | 0.87 | 0.13 | 0.32 | 0.32 | 0.803333 | 0.256667 |
| GNPTAB | 2.944 | 1.557942 | 5.68E-07 | 6.21E-06 | yes | up | 2.69 | 2.36 | 3.08 | 11.48 | 20.63 | 23.2 | 2.71 | 18.43667 |
| N4BP2L1 | 2.07 | 1.049402 | 5.69E-07 | 6.22E-06 | yes | up | 4.31 | 3.94 | 5.64 | 19.76 | 18.65 | 19.93 | 4.63 | 19.44667 |
| AGK | 0.496 | -1.0111 | 5.79E-07 | 6.33E-06 | yes | down | 11.48 | 15.78 | 11.33 | 15.73 | 14.79 | 13.95 | 12.86333 | 14.82333 |
| MTO1 | 0.484 | -1.04573 | 5.96E-07 | 6.48E-06 | yes | down | 6.09 | 7.78 | 6.9 | 10.03 | 7.08 | 7.75 | 6.923333 | 8.286667 |
| CCL19 | 8.421 | 3.074056 | 5.97E-07 | 6.48E-06 | yes | up | 0.59 | 0.23 | 0.52 | 7.82 | 9.77 | 8.97 | 0.446667 | 8.853333 |
| HOGA1 | 0.471 | -1.08668 | 6.01E-07 | 6.52E-06 | yes | down | 13.02 | 12.69 | 14.35 | 14.44 | 11.11 | 17.37 | 13.35333 | 14.30667 |
| SLC44A1 | 2.051 | 1.036454 | 6.03E-07 | 6.53E-06 | yes | up | 10.84 | 11.35 | 10.68 | 61.68 | 82 | 49.68 | 10.95667 | 64.45333 |
| LOC103347657 | 6.136 | 2.617319 | 6.05E-07 | 6.56E-06 | yes | up | 1.54 | 1.96 | 1.18 | 23.07 | 28.27 | 16 | 1.56 | 22.44667 |
| USH2A | 8.07 | 3.012652 | 6.08E-07 | 6.58E-06 | yes | up | 0.09 | 0.04 | 0.07 | 2.52 | 1.03 | 2.57 | 0.066667 | 2.04 |
| UBE2L6 | 3.213 | 1.683872 | 6.16E-07 | 6.66E-06 | yes | up | 1.89 | 1.84 | 2.21 | 13.49 | 10.36 | 20.92 | 1.98 | 14.92333 |
| ADA | 3.904 | 1.964974 | 6.22E-07 | 6.72E-06 | yes | up | 2.29 | 1.5 | 1.9 | 17.4 | 25.31 | 10.95 | 1.896667 | 17.88667 |
| NARS2 | 0.459 | -1.12372 | 6.29E-07 | 6.79E-06 | yes | down | 6.13 | 9.21 | 8.08 | 7.84 | 6.75 | 9.1 | 7.806667 | 7.896667 |
| SLC7A8 | 0.364 | -1.45904 | 6.42E-07 | 6.92E-06 | yes | down | 5.43 | 7.36 | 4.69 | 4.82 | 3.21 | 6.69 | 5.826667 | 4.906667 |
| TANGO2 | 0.435 | -1.20001 | 6.45E-07 | 6.95E-06 | yes | down | 23.09 | 23.18 | 26.7 | 19.44 | 24.36 | 31.97 | 24.32333 | 25.25667 |
| SPATA6L | 3.355 | 1.746157 | 6.51E-07 | 7.01E-06 | yes | up | 1.29 | 0.82 | 1.96 | 9.9 | 6.48 | 9.61 | 1.356667 | 8.663333 |
| LOC103348240 | 0.009 | -6.74545 | 6.57E-07 | 7.05E-06 | yes | down | 0.48 | 5.12 | 8.27 | 0.14 | 0.17 | 0 | 4.623333 | 0.103333 |
| UGT1-4 | 3.198 | 1.677272 | 6.8E-07 | 7.29E-06 | yes | up | 0.44 | 0.78 | 0.64 | 5.22 | 4.48 | 4.16 | 0.62 | 4.62 |
| LOC103349113 | 0.409 | -1.29136 | 6.84E-07 | 7.33E-06 | yes | down | 20.9 | 16.33 | 12.47 | 14.88 | 17.3 | 15.34 | 16.56667 | 15.84 |
| SMAD6 | 0.305 | -1.7146 | 6.91E-07 | 7.4E-06 | yes | down | 2.33 | 5.57 | 5.59 | 3.74 | 2.66 | 3.07 | 4.496667 | 3.156667 |
| CBR4 | 0.494 | -1.01862 | 6.96E-07 | 7.45E-06 | yes | down | 4.73 | 5.28 | 5.63 | 6.25 | 7.3 | 4.72 | 5.213333 | 6.09 |
| POLR2I | 0.459 | -1.12423 | 7.07E-07 | 7.56E-06 | yes | down | 44.28 | 45.47 | 48.91 | 60.84 | 50.64 | 40.39 | 46.22 | 50.62333 |
| BARX1 | 0.047 | -4.4198 | 7.31E-07 | 7.8E-06 | yes | down | 1.07 | 1.21 | 1.49 | 0.07 | 0 | 0.33 | 1.256667 | 0.133333 |
| TP53INP1 | 2.499 | 1.321334 | 7.44E-07 | 7.93E-06 | yes | up | 10.3 | 7.84 | 9.83 | 67.14 | 45.23 | 39.3 | 9.323333 | 50.55667 |
| CRLS1 | 0.284 | -1.8178 | 7.66E-07 | 8.15E-06 | yes | down | 91.2 | 120.63 | 123.55 | 81 | 30.97 | 107.7 | 111.7933 | 73.22333 |
| LOC100350716 | 0.272 | -1.87683 | 7.72E-07 | 8.2E-06 | yes | down | 1.82 | 2.96 | 1.16 | 1.6 | 1.2 | 0.92 | 1.98 | 1.24 |
| MYCL | 0.092 | -3.44142 | 7.84E-07 | 8.32E-06 | yes | down | 0.52 | 0.29 | 0.46 | 0.15 | 0.07 | 0.06 | 0.423333 | 0.093333 |
| C7H2orf88 | 0.35 | -1.51631 | 7.92E-07 | 8.38E-06 | yes | down | 10.96 | 23.96 | 28.84 | 16.54 | 13.85 | 21.37 | 21.25333 | 17.25333 |
| TTK | 7.592 | 2.924418 | 8.17E-07 | 8.63E-06 | yes | up | 0.1 | 0.22 | 0.09 | 3.39 | 2.92 | 0.89 | 0.136667 | 2.4 |
| GPT2 | 0.491 | -1.02484 | 8.18E-07 | 8.63E-06 | yes | down | 57.89 | 94.36 | 83.74 | 74.19 | 93.69 | 97.4 | 78.66333 | 88.42667 |
| EMILIN1 | 2.722 | 1.444441 | 8.24E-07 | 8.69E-06 | yes | up | 2.14 | 2.51 | 1.18 | 15.21 | 10.36 | 11.32 | 1.943333 | 12.29667 |
| CD82 | 2.21 | 1.144347 | 8.49E-07 | 8.95E-06 | yes | up | 23.9 | 22.26 | 15.96 | 139.63 | 94.21 | 94.66 | 20.70667 | 109.5 |
| PRRT4 | 21.959 | 4.456758 | 8.55E-07 | 9E-06 | yes | up | 0.05 | 0.34 | 0.79 | 14.16 | 22.03 | 17.84 | 0.393333 | 18.01 |
| CCND2 | 2.65 | 1.405875 | 8.57E-07 | 9.03E-06 | yes | up | 2.5 | 4.66 | 5.14 | 20.81 | 22.17 | 31.71 | 4.1 | 24.89667 |
| CLSPN | 9.056 | 3.178946 | 8.74E-07 | 9.19E-06 | yes | up | 0.05 | 0.13 | 0.05 | 1.74 | 2.22 | 0.65 | 0.076667 | 1.536667 |
| C1H11orf1 | 3.371 | 1.753163 | 8.88E-07 | 9.33E-06 | yes | up | 1.13 | 0.57 | 0.99 | 8.15 | 5.37 | 8.73 | 0.896667 | 7.416667 |
| LOC103347974 | 0.172 | -2.54261 | 9.09E-07 | 9.53E-06 | yes | down | 1.29 | 2.77 | 3.58 | 0.8 | 0.91 | 1.28 | 2.546667 | 0.996667 |
| TCEAL9 | 2.006 | 1.004077 | 9.14E-07 | 9.58E-06 | yes | up | 9.71 | 12.19 | 9.46 | 53.52 | 51.37 | 41.82 | 10.45333 | 48.90333 |
| PAMR1 | 0.449 | -1.15525 | 9.21E-07 | 9.64E-06 | yes | down | 5.25 | 5.21 | 3.66 | 5.76 | 4.24 | 4.86 | 4.706667 | 4.953333 |
| SERPINB5 | 0.172 | -2.53539 | 9.22E-07 | 9.65E-06 | yes | down | 0.97 | 1.24 | 1.95 | 0.71 | 0.25 | 0.72 | 1.386667 | 0.56 |
| LOC100351707 | 4.135 | 2.047816 | 9.36E-07 | 9.79E-06 | yes | up | 0.73 | 0.36 | 0.38 | 5.07 | 4.94 | 4.22 | 0.49 | 4.743333 |
| PXDNL | 2.961 | 1.566311 | 9.59E-07 | 1E-05 | yes | up | 0.34 | 0.32 | 0.29 | 1.96 | 2.05 | 2.53 | 0.316667 | 2.18 |
| LOC103351259 | 0.364 | -1.45827 | 9.68E-07 | 1.01E-05 | yes | down | 22.49 | 15.75 | 24.34 | 13.6 | 18.15 | 13.23 | 20.86 | 14.99333 |
| ACKR2 | 2.27 | 1.182843 | 9.82E-07 | 1.02E-05 | yes | up | 1.79 | 1.42 | 1.35 | 6.8 | 8.3 | 7.63 | 1.52 | 7.576667 |
| NTMT1 | 0.405 | -1.3055 | 1.02E-06 | 1.06E-05 | yes | down | 47.94 | 64.27 | 47.26 | 44.05 | 66.22 | 39.32 | 53.15667 | 49.86333 |
| RDH10 | 0.462 | -1.11277 | 1.02E-06 | 1.06E-05 | yes | down | 3.95 | 3.91 | 4.27 | 3.97 | 4.25 | 4.82 | 4.043333 | 4.346667 |
| LRIG3 | 2.294 | 1.197745 | 1.03E-06 | 1.07E-05 | yes | up | 4.28 | 2.83 | 3.12 | 18.06 | 14.93 | 20.68 | 3.41 | 17.89 |
| WNT11 | 0.312 | -1.67841 | 1.05E-06 | 1.09E-05 | yes | down | 2.63 | 3.48 | 3.89 | 2.71 | 1.33 | 3.48 | 3.333333 | 2.506667 |
| MXRA7 | 0.195 | -2.362 | 1.06E-06 | 1.09E-05 | yes | down | 28.63 | 65.25 | 66.21 | 29.09 | 36.2 | 7.68 | 53.36333 | 24.32333 |
| LOC108178197 | 0.456 | -1.13366 | 1.06E-06 | 1.09E-05 | yes | down | 5.31 | 5.59 | 6.37 | 6.09 | 4.56 | 5.82 | 5.756667 | 5.49 |
| LOC108177164 | 2.376 | 1.248454 | 1.09E-06 | 1.12E-05 | yes | up | 14.07 | 28.97 | 15.89 | 124.9 | 97.48 | 94 | 19.64333 | 105.46 |
| LOC100340939 | 8.766 | 3.131938 | 1.12E-06 | 1.15E-05 | yes | up | 0.3 | 0.37 | 0.22 | 6.15 | 9.53 | 2.24 | 0.296667 | 5.973333 |
| ALDOC | 2.643 | 1.40227 | 1.12E-06 | 1.16E-05 | yes | up | 133.38 | 304.2 | 154.78 | 1002.52 | 1112.36 | 1443.47 | 197.4533 | 1186.117 |
| MARC2 | 2.069 | 1.048888 | 1.13E-06 | 1.16E-05 | yes | up | 18.75 | 21.11 | 27.36 | 115.78 | 111.54 | 91.35 | 22.40667 | 106.2233 |
| LOC108178466 | 5.247 | 2.391438 | 1.14E-06 | 1.17E-05 | yes | up | 0.19 | 0.14 | 0.25 | 3.45 | 1.76 | 2.03 | 0.193333 | 2.413333 |
| CNTN1 | 2.179 | 1.123776 | 1.17E-06 | 1.2E-05 | yes | up | 3.04 | 3.85 | 4.29 | 15 | 17.9 | 22.98 | 3.726667 | 18.62667 |
| SAMHD1 | 2.161 | 1.111649 | 1.18E-06 | 1.21E-05 | yes | up | 6.12 | 9.04 | 7.74 | 29.23 | 35.24 | 47.27 | 7.633333 | 37.24667 |
| COL21A1 | 2.233 | 1.158984 | 1.19E-06 | 1.22E-05 | yes | up | 2.05 | 2.47 | 1.55 | 10.23 | 11.26 | 7.98 | 2.023333 | 9.823333 |
| RNF19B | 2.191 | 1.131418 | 1.2E-06 | 1.23E-05 | yes | up | 11.76 | 9.54 | 14.65 | 63.21 | 70.33 | 55.07 | 11.98333 | 62.87 |
| LOC100338511 | 0.237 | -2.07615 | 1.23E-06 | 1.25E-05 | yes | down | 17.77 | 21.29 | 33.94 | 15.97 | 5.35 | 19.29 | 24.33333 | 13.53667 |
| RFXAP | 2.08 | 1.056497 | 1.23E-06 | 1.25E-05 | yes | up | 3.25 | 3.73 | 4.83 | 17.8 | 17.13 | 18.65 | 3.936667 | 17.86 |
| ARNTL | 3.546 | 1.826096 | 1.25E-06 | 1.27E-05 | yes | up | 1.32 | 1.13 | 0.69 | 9.08 | 5.5 | 11.58 | 1.046667 | 8.72 |
| EHD3 | 0.361 | -1.47097 | 1.26E-06 | 1.28E-05 | yes | down | 15.09 | 31.5 | 29.94 | 25.22 | 12.99 | 25.84 | 25.51 | 21.35 |
| THY1 | 2.049 | 1.034913 | 1.26E-06 | 1.28E-05 | yes | up | 20.57 | 37.67 | 25.03 | 132.92 | 111.38 | 148.25 | 27.75667 | 130.85 |
| VCAN | 2.121 | 1.084545 | 1.29E-06 | 1.31E-05 | yes | up | 4.57 | 6.37 | 3.56 | 23.54 | 20.79 | 24.44 | 4.833333 | 22.92333 |
| PAK1IP1 | 0.406 | -1.30135 | 1.3E-06 | 1.32E-05 | yes | down | 6.14 | 11.48 | 7.21 | 6.86 | 8.25 | 7.96 | 8.276667 | 7.69 |
| KLHL2 | 2.076 | 1.053623 | 1.3E-06 | 1.32E-05 | yes | up | 5.4 | 5.88 | 5.19 | 33.66 | 26.39 | 19.84 | 5.49 | 26.63 |
| SARS2 | 0.437 | -1.19558 | 1.3E-06 | 1.32E-05 | yes | down | 11.21 | 19.06 | 16.09 | 14.99 | 9.88 | 15.74 | 15.45333 | 13.53667 |
| LOC108176507 | 95.242 | 6.573532 | 1.33E-06 | 1.34E-05 | yes | up | 0 | 0 | 0 | 0.62 | 0.62 | 0.49 | 0 | 0.576667 |
| IGIP | 2.314 | 1.210252 | 1.33E-06 | 1.35E-05 | yes | up | 2.01 | 2.85 | 3.82 | 15.3 | 16.05 | 15.17 | 2.893333 | 15.50667 |
| NELL2 | 4.89 | 2.289867 | 1.35E-06 | 1.37E-05 | yes | up | 0.32 | 0.59 | 0.43 | 3.86 | 2.83 | 8.29 | 0.446667 | 4.993333 |
| LOC100349542 | 0.267 | -1.90305 | 1.37E-06 | 1.38E-05 | yes | down | 1.46 | 1.47 | 2.11 | 1.84 | 0.82 | 0.63 | 1.68 | 1.096667 |
| LOC108176569 | 10.782 | 3.430569 | 1.39E-06 | 1.4E-05 | yes | up | 0.1 | 0.07 | 0.18 | 5.45 | 1.97 | 1.1 | 0.116667 | 2.84 |
| GMPS | 0.376 | -1.40992 | 1.4E-06 | 1.41E-05 | yes | down | 6.76 | 15.52 | 7.22 | 8.98 | 7.24 | 9.06 | 9.833333 | 8.426667 |
| LOC100343223 | 3.629 | 1.859666 | 1.42E-06 | 1.43E-05 | yes | up | 1.96 | 3.68 | 5.46 | 38.6 | 19.49 | 35.95 | 3.7 | 31.34667 |
| CREB3L1 | 2.007 | 1.004985 | 1.43E-06 | 1.44E-05 | yes | up | 2.48 | 3.46 | 2.68 | 12.27 | 14.34 | 13.21 | 2.873333 | 13.27333 |
| LOC108176227 | 0.038 | -4.72931 | 1.43E-06 | 1.44E-05 | yes | down | 5.27 | 18.98 | 6.28 | 0.37 | 0.27 | 1.94 | 10.17667 | 0.86 |
| CALM3 | 2.395 | 1.25982 | 1.45E-06 | 1.46E-05 | yes | up | 39.34 | 62.86 | 37.88 | 243.13 | 297.38 | 185.85 | 46.69333 | 242.12 |
| LOC103350421 | 2.078 | 1.055502 | 1.45E-06 | 1.46E-05 | yes | up | 0.89 | 1.25 | 0.94 | 3.24 | 3.25 | 3.82 | 1.026667 | 3.436667 |
| EPHB2 | 2.051 | 1.036047 | 1.46E-06 | 1.46E-05 | yes | up | 0.96 | 1.23 | 1.09 | 6.89 | 4.33 | 5.72 | 1.093333 | 5.646667 |
| CORO2A | 3.732 | 1.899994 | 1.47E-06 | 1.47E-05 | yes | up | 0.18 | 0.27 | 0.25 | 1.68 | 1.88 | 2.49 | 0.233333 | 2.016667 |
| ZNF862 | 3.17 | 1.664371 | 1.47E-06 | 1.47E-05 | yes | up | 1.28 | 1.87 | 1.74 | 7.78 | 6.99 | 10.42 | 1.63 | 8.396667 |
| NAGS | 0.281 | -1.83177 | 1.47E-06 | 1.48E-05 | yes | down | 1.3 | 2.96 | 2.37 | 1.37 | 1.2 | 1.7 | 2.21 | 1.423333 |
| MYL4 | 0.063 | -3.97846 | 1.51E-06 | 1.52E-05 | yes | down | 11.71 | 6.12 | 0.77 | 0.59 | 1.36 | 0.85 | 6.2 | 0.933333 |
| LY96 | 0.439 | -1.18633 | 1.55E-06 | 1.55E-05 | yes | down | 7.25 | 7.99 | 6.48 | 13.22 | 9.62 | 10.74 | 7.24 | 11.19333 |
| ONECUT2 | 0.182 | -2.45643 | 1.57E-06 | 1.57E-05 | yes | down | 0.11 | 0.1 | 0.17 | 0.06 | 0.04 | 0.07 | 0.126667 | 0.056667 |
| LOC100356940 | 2.581 | 1.368075 | 1.58E-06 | 1.58E-05 | yes | up | 1.76 | 3.35 | 3.11 | 24.48 | 25.08 | 11.86 | 2.74 | 20.47333 |
| RTCB | 0.383 | -1.38638 | 1.61E-06 | 1.6E-05 | yes | down | 28.11 | 65.58 | 68.73 | 51.32 | 43.59 | 47.85 | 54.14 | 47.58667 |
| KCNC4 | 0.03 | -5.04923 | 1.62E-06 | 1.61E-05 | yes | down | 7.06 | 6.46 | 5.75 | 0.1 | 1.68 | 0.22 | 6.423333 | 0.666667 |
| MSTO1 | 0.483 | -1.05083 | 1.66E-06 | 1.64E-05 | yes | down | 4.54 | 7.53 | 6.25 | 7.17 | 5.99 | 6.86 | 6.106667 | 6.673333 |
| ACAT2 | 3.258 | 1.704002 | 1.66E-06 | 1.64E-05 | yes | up | 24.24 | 36.79 | 29.18 | 396.95 | 125.08 | 173.31 | 30.07 | 231.78 |
| DCBLD1 | 2.47 | 1.304686 | 1.73E-06 | 1.71E-05 | yes | up | 2.11 | 1.28 | 1.47 | 11.21 | 7.93 | 9.49 | 1.62 | 9.543333 |
| LOC100357099 | 2.11 | 1.077192 | 1.78E-06 | 1.76E-05 | yes | up | 0.8 | 0.81 | 0.78 | 4.7 | 3.07 | 4.02 | 0.796667 | 3.93 |
| LOC100342463 | 0.269 | -1.89564 | 1.81E-06 | 1.79E-05 | yes | down | 2.32 | 2.45 | 2.91 | 1.33 | 1.26 | 2.32 | 2.56 | 1.636667 |
| IGF2BP2 | 0.197 | -2.3452 | 1.83E-06 | 1.8E-05 | yes | down | 1.35 | 2.08 | 0.97 | 0.46 | 1.1 | 0.43 | 1.466667 | 0.663333 |
| OAS2 | 10.331 | 3.368932 | 1.87E-06 | 1.84E-05 | yes | up | 0.12 | 0.27 | 0.1 | 2.09 | 1.5 | 7.8 | 0.163333 | 3.796667 |
| NAV3 | 2.168 | 1.1165 | 1.87E-06 | 1.84E-05 | yes | up | 0.94 | 1.88 | 1.33 | 7.99 | 6.88 | 6.48 | 1.383333 | 7.116667 |
| LOC100348151 | 17.66 | 4.142414 | 1.93E-06 | 1.89E-05 | yes | up | 0.02 | 0.05 | 0.04 | 2.44 | 0.39 | 2.41 | 0.036667 | 1.746667 |
| AACS | 3.606 | 1.850441 | 1.95E-06 | 1.91E-05 | yes | up | 2.49 | 5.37 | 4.59 | 41.57 | 15.67 | 46.7 | 4.15 | 34.64667 |
| PTMS | 2.138 | 1.09646 | 1.97E-06 | 1.93E-05 | yes | up | 67.69 | 97.59 | 62.4 | 424.08 | 415.42 | 276.46 | 75.89333 | 371.9867 |
| KCNN3 | 2.351 | 1.233522 | 2E-06 | 1.96E-05 | yes | up | 0.52 | 0.6 | 0.37 | 2.47 | 2.17 | 2.86 | 0.496667 | 2.5 |
| MAL2 | 3.134 | 1.64813 | 2.01E-06 | 1.96E-05 | yes | up | 3.6 | 3.44 | 4.37 | 46.78 | 14.44 | 27.5 | 3.803333 | 29.57333 |
| ADCK1 | 0.36 | -1.47555 | 2.04E-06 | 1.99E-05 | yes | down | 1.93 | 2.27 | 2.35 | 1.99 | 2.51 | 1.48 | 2.183333 | 1.993333 |
| PLK1 | 2.548 | 1.349505 | 2.07E-06 | 2.02E-05 | yes | up | 0.74 | 1.6 | 0.89 | 10.2 | 7.82 | 5.5 | 1.076667 | 7.84 |
| MAL | 0.322 | -1.63366 | 2.1E-06 | 2.04E-05 | yes | down | 2.35 | 3.72 | 4.06 | 3.86 | 3.54 | 1.94 | 3.376667 | 3.113333 |
| MTIF2 | 0.454 | -1.1377 | 2.12E-06 | 2.06E-05 | yes | down | 7 | 8.86 | 9.64 | 10.76 | 6.33 | 7.39 | 8.5 | 8.16 |
| NDUFA13 | 0.214 | -2.22593 | 2.19E-06 | 2.12E-05 | yes | down | 6.99 | 6.19 | 7.05 | 2.78 | 2.73 | 4.73 | 6.743333 | 3.413333 |
| GFM2 | 0.488 | -1.03489 | 2.19E-06 | 2.12E-05 | yes | down | 8.6 | 11.62 | 11.41 | 14.22 | 13.04 | 8.99 | 10.54333 | 12.08333 |
| SLC27A2 | 0.018 | -5.81332 | 2.2E-06 | 2.13E-05 | yes | down | 0.53 | 0.52 | 0.23 | 0 | 0.03 | 0.03 | 0.426667 | 0.02 |
| NEIL3 | 14.42 | 3.849983 | 2.21E-06 | 2.14E-05 | yes | up | 0.14 | 0.11 | 0.02 | 3.97 | 4.22 | 0.92 | 0.09 | 3.036667 |
| ZBED6 | 4.274 | 2.095468 | 2.21E-06 | 2.14E-05 | yes | up | 0.16 | 0.63 | 0.77 | 5.82 | 4.09 | 5.39 | 0.52 | 5.1 |
| CENPE | 5.418 | 2.437815 | 2.21E-06 | 2.14E-05 | yes | up | 0.13 | 0.11 | 0.08 | 2.07 | 1.42 | 0.55 | 0.106667 | 1.346667 |
| ESRRG | 0.436 | -1.19627 | 2.22E-06 | 2.14E-05 | yes | down | 4.81 | 3.89 | 5.83 | 4.47 | 4.54 | 6.49 | 4.843333 | 5.166667 |
| KIF15 | 4.385 | 2.132588 | 2.24E-06 | 2.16E-05 | yes | up | 0.49 | 0.42 | 0.39 | 5.49 | 6.01 | 1.9 | 0.433333 | 4.466667 |
| LGALS3BP | 2.035 | 1.024708 | 2.26E-06 | 2.19E-05 | yes | up | 16.55 | 18.45 | 14.16 | 92.7 | 54.74 | 86.77 | 16.38667 | 78.07 |
| RASL11A | 3.427 | 1.776795 | 2.27E-06 | 2.19E-05 | yes | up | 0.99 | 1.3 | 1.18 | 8.86 | 6.84 | 11.91 | 1.156667 | 9.203333 |
| FAM3D | 44.671 | 5.481258 | 2.27E-06 | 2.19E-05 | yes | up | 0 | 0.16 | 0 | 6.44 | 3.53 | 4.24 | 0.053333 | 4.736667 |
| PPA1 | 0.459 | -1.12373 | 2.3E-06 | 2.21E-05 | yes | down | 75.16 | 157.97 | 91.38 | 125.88 | 108.19 | 107.94 | 108.17 | 114.0033 |
| RNF148 | 0.073 | -3.7689 | 2.3E-06 | 2.21E-05 | yes | down | 1.02 | 2.13 | 1.38 | 0.29 | 0.1 | 0.37 | 1.51 | 0.253333 |
| TMEM120A | 0.356 | -1.49087 | 2.3E-06 | 2.22E-05 | yes | down | 16.98 | 29.6 | 25.58 | 17.54 | 21.62 | 27.13 | 24.05333 | 22.09667 |
| LOC103349243 | 90.612 | 6.501638 | 2.32E-06 | 2.23E-05 | yes | up | 0 | 0 | 0 | 2.31 | 1.33 | 1.77 | 0 | 1.803333 |
| MICALL1 | 0.436 | -1.19638 | 2.36E-06 | 2.27E-05 | yes | down | 8.31 | 10.67 | 11.92 | 12.52 | 6.78 | 12.19 | 10.3 | 10.49667 |
| ITGA11 | 2.575 | 1.36435 | 2.43E-06 | 2.33E-05 | yes | up | 1.56 | 3.22 | 2.2 | 12.13 | 10.89 | 18.13 | 2.326667 | 13.71667 |
| C7H2orf72 | 0.138 | -2.85912 | 2.45E-06 | 2.35E-05 | yes | down | 1.51 | 1.03 | 1.82 | 0.24 | 0.29 | 0.87 | 1.453333 | 0.466667 |
| KIF4A | 8.329 | 3.058108 | 2.47E-06 | 2.36E-05 | yes | up | 0.22 | 0.17 | 0.05 | 3.37 | 4.08 | 1.04 | 0.146667 | 2.83 |
| ERCC8 | 0.478 | -1.06554 | 2.48E-06 | 2.37E-05 | yes | down | 3.46 | 3.72 | 3.38 | 3.64 | 3.66 | 4.45 | 3.52 | 3.916667 |
| SLC15A3 | 2.438 | 1.285536 | 2.48E-06 | 2.37E-05 | yes | up | 1.37 | 1.77 | 1.78 | 8.86 | 11.11 | 7.82 | 1.64 | 9.263333 |
| SERPINI1 | 2.968 | 1.569331 | 2.48E-06 | 2.37E-05 | yes | up | 1.14 | 1.15 | 1.01 | 10.76 | 5.71 | 6.86 | 1.1 | 7.776667 |
| LOC100359112 | 2.546 | 1.348092 | 2.5E-06 | 2.39E-05 | yes | up | 5.03 | 5.66 | 5.43 | 22.34 | 31.6 | 41.11 | 5.373333 | 31.68333 |
| NIPAL2 | 0.382 | -1.38837 | 2.58E-06 | 2.46E-05 | yes | down | 8.31 | 7.23 | 6.46 | 8.83 | 6.76 | 4.09 | 7.333333 | 6.56 |
| SAMD9L | 3.068 | 1.617305 | 2.61E-06 | 2.48E-05 | yes | up | 1.3 | 1.22 | 0.83 | 8.9 | 4.54 | 10.74 | 1.116667 | 8.06 |
| ADAM19 | 2.501 | 1.322443 | 2.72E-06 | 2.58E-05 | yes | up | 1.94 | 3.16 | 1.7 | 17.35 | 9.08 | 11.86 | 2.266667 | 12.76333 |
| LNP1 | 26.234 | 4.713352 | 2.73E-06 | 2.59E-05 | yes | up | 0.25 | 0 | 0.22 | 21.77 | 2.11 | 6.33 | 0.156667 | 10.07 |
| ADRA1B | 0.151 | -2.72814 | 2.77E-06 | 2.62E-05 | yes | down | 1.06 | 3.3 | 2.34 | 1.34 | 0.43 | 0.59 | 2.233333 | 0.786667 |
| WBSCR27 | 2.284 | 1.191438 | 2.78E-06 | 2.63E-05 | yes | up | 5.16 | 7.03 | 4.88 | 22.06 | 36.3 | 31.7 | 5.69 | 30.02 |
| NR2F2 | 2.059 | 1.04192 | 2.8E-06 | 2.65E-05 | yes | up | 5.17 | 8.75 | 8.55 | 42.52 | 32.57 | 33.37 | 7.49 | 36.15333 |
| C3H8orf37 | 2.02 | 1.014593 | 2.82E-06 | 2.67E-05 | yes | up | 1.5 | 1.61 | 1.33 | 7.8 | 6.78 | 6.25 | 1.48 | 6.943333 |
| DKK3 | 0.309 | -1.69575 | 2.86E-06 | 2.7E-05 | yes | down | 2.08 | 3.73 | 1.86 | 1.86 | 2.19 | 1.37 | 2.556667 | 1.806667 |
| NYNRIN | 3.103 | 1.633607 | 2.94E-06 | 2.77E-05 | yes | up | 0.81 | 1.11 | 1.17 | 12.28 | 5.76 | 4.49 | 1.03 | 7.51 |
| LOC100342112 | 0.011 | -6.53511 | 2.95E-06 | 2.78E-05 | yes | down | 0.69 | 1.04 | 1.78 | 0 | 0.09 | 0 | 1.17 | 0.03 |
| SLC9C2 | 4.31 | 2.107851 | 2.96E-06 | 2.78E-05 | yes | up | 0.1 | 0.07 | 0.08 | 0.92 | 0.86 | 0.81 | 0.083333 | 0.863333 |
| COL6A2 | 2.136 | 1.095011 | 3.05E-06 | 2.86E-05 | yes | up | 89.04 | 145.16 | 86.67 | 601.53 | 620.21 | 315.49 | 106.9567 | 512.41 |
| ZBTB43 | 0.395 | -1.3384 | 3.07E-06 | 2.89E-05 | yes | down | 2.25 | 2.71 | 3.25 | 2.07 | 1.98 | 3.51 | 2.736667 | 2.52 |
| NHSL2 | 2.394 | 1.259448 | 3.09E-06 | 2.9E-05 | yes | up | 1.85 | 2.01 | 1.29 | 13.98 | 9.59 | 7.88 | 1.716667 | 10.48333 |
| FAM189B | 2.068 | 1.048218 | 3.18E-06 | 2.97E-05 | yes | up | 1.36 | 2.07 | 1.31 | 8.34 | 7.45 | 6.85 | 1.58 | 7.546667 |
| ESM1 | 3.315 | 1.728925 | 3.18E-06 | 2.98E-05 | yes | up | 0.75 | 0.78 | 0.49 | 4.65 | 6.59 | 4.19 | 0.673333 | 5.143333 |
| GRID2 | 0.238 | -2.07121 | 3.19E-06 | 2.98E-05 | yes | down | 0.24 | 0.31 | 0.24 | 0.15 | 0.13 | 0.16 | 0.263333 | 0.146667 |
| LMO1 | 3.137 | 1.649568 | 3.21E-06 | 3E-05 | yes | up | 3.9 | 7.44 | 6.21 | 28.82 | 51.62 | 46.25 | 5.85 | 42.23 |
| TMED3 | 2.052 | 1.036815 | 3.23E-06 | 3.02E-05 | yes | up | 15.62 | 22.55 | 16.96 | 82.11 | 104.3 | 77.5 | 18.37667 | 87.97 |
| MIIP | 0.426 | -1.23072 | 3.31E-06 | 3.08E-05 | yes | down | 12.14 | 11.51 | 15.39 | 18.65 | 13.03 | 9.81 | 13.01333 | 13.83 |
| UNC5B | 0.461 | -1.11656 | 3.4E-06 | 3.17E-05 | yes | down | 6.37 | 9.82 | 12.32 | 10.99 | 10.44 | 8.95 | 9.503333 | 10.12667 |
| COL28A1 | 0.388 | -1.36568 | 3.42E-06 | 3.19E-05 | yes | down | 1.46 | 2.1 | 1.76 | 1.58 | 1.05 | 2.14 | 1.773333 | 1.59 |
| RCC1L | 0.453 | -1.14211 | 3.52E-06 | 3.27E-05 | yes | down | 9.99 | 13.21 | 16.18 | 16.68 | 10.16 | 14.55 | 13.12667 | 13.79667 |
| NUF2 | 5.554 | 2.4736 | 3.55E-06 | 3.29E-05 | yes | up | 0.41 | 0.31 | 0.23 | 4.38 | 6.31 | 1.59 | 0.316667 | 4.093333 |
| IL6R | 2.605 | 1.381381 | 3.83E-06 | 3.55E-05 | yes | up | 0.49 | 0.56 | 0.66 | 2.7 | 3.46 | 4.07 | 0.57 | 3.41 |
| P3H3 | 2.05 | 1.035684 | 3.84E-06 | 3.55E-05 | yes | up | 2.48 | 3.09 | 3.45 | 14.87 | 16.56 | 15.4 | 3.006667 | 15.61 |
| VIL1 | 0.202 | -2.30555 | 3.86E-06 | 3.57E-05 | yes | down | 1.1 | 0.72 | 1.52 | 0.49 | 0.26 | 0.35 | 1.113333 | 0.366667 |
| MCM5 | 2.681 | 1.42288 | 3.94E-06 | 3.64E-05 | yes | up | 0.95 | 2.07 | 1.27 | 9.68 | 9.69 | 6.97 | 1.43 | 8.78 |
| FAM84B | 2.896 | 1.533999 | 3.98E-06 | 3.67E-05 | yes | up | 0.84 | 0.65 | 0.69 | 7.19 | 3.69 | 3.41 | 0.726667 | 4.763333 |
| MNT | 2.01 | 1.006865 | 4.01E-06 | 3.69E-05 | yes | up | 1.03 | 0.92 | 1.17 | 4.98 | 4.57 | 5.09 | 1.04 | 4.88 |
| STAG3 | 0.175 | -2.51369 | 4.1E-06 | 3.77E-05 | yes | down | 0.39 | 0.34 | 0.45 | 0.24 | 0.09 | 0.16 | 0.393333 | 0.163333 |
| GATM | 0.252 | -1.99043 | 4.13E-06 | 3.79E-05 | yes | down | 3.5 | 8.19 | 3.48 | 2.01 | 4.18 | 2.17 | 5.056667 | 2.786667 |
| HPDL | 0.155 | -2.68567 | 4.13E-06 | 3.79E-05 | yes | down | 1.02 | 0.87 | 1.17 | 0.56 | 0.12 | 0.44 | 1.02 | 0.373333 |
| SSC4D | 0.043 | -4.5307 | 4.17E-06 | 3.82E-05 | yes | down | 0.58 | 0.61 | 0.26 | 0.12 | 0 | 0.03 | 0.483333 | 0.05 |
| TIMP4 | 0.417 | -1.26085 | 4.22E-06 | 3.87E-05 | yes | down | 42.64 | 49.08 | 46.86 | 34.7 | 60.94 | 40.21 | 46.19333 | 45.28333 |
| STRIP2 | 2.417 | 1.273296 | 4.24E-06 | 3.88E-05 | yes | up | 2.07 | 2.94 | 4.24 | 20.35 | 11.75 | 16.79 | 3.083333 | 16.29667 |
| ARMCX5 | 0.151 | -2.72654 | 4.33E-06 | 3.94E-05 | yes | down | 1.56 | 1.03 | 0.81 | 0.48 | 0.14 | 0.6 | 1.133333 | 0.406667 |
| UNC5D | 0.305 | -1.71446 | 4.4E-06 | 4.01E-05 | yes | down | 12.52 | 10.38 | 8.65 | 9.18 | 6.93 | 6.15 | 10.51667 | 7.42 |
| LOC103347318 | 2.727 | 1.447293 | 4.45E-06 | 4.04E-05 | yes | up | 5.29 | 7.04 | 3.58 | 31.69 | 38.19 | 30.48 | 5.303333 | 33.45333 |
| LOC100358230 | 0.34 | -1.55839 | 4.46E-06 | 4.05E-05 | yes | down | 5.52 | 4.77 | 5.81 | 5.2 | 2.92 | 5.58 | 5.366667 | 4.566667 |
| L3MBTL2 | 0.479 | -1.06161 | 4.54E-06 | 4.12E-05 | yes | down | 2.29 | 3.81 | 3.19 | 3.42 | 2.97 | 3.88 | 3.096667 | 3.423333 |
| NUSAP1 | 5.326 | 2.413053 | 4.71E-06 | 4.27E-05 | yes | up | 1.08 | 1.55 | 1.04 | 23.32 | 21.42 | 4.44 | 1.223333 | 16.39333 |
| CDC25B | 3.808 | 1.929041 | 4.77E-06 | 4.31E-05 | yes | up | 2.47 | 3.5 | 3.35 | 27.24 | 29.7 | 11.01 | 3.106667 | 22.65 |
| WHRN | 0.351 | -1.51175 | 4.78E-06 | 4.32E-05 | yes | down | 3.31 | 1.82 | 3.09 | 2.09 | 2.41 | 2.29 | 2.74 | 2.263333 |
| ARHGAP33 | 4.544 | 2.184006 | 4.79E-06 | 4.32E-05 | yes | up | 0.12 | 0.16 | 0.19 | 2.33 | 1.64 | 1.03 | 0.156667 | 1.666667 |
| ARMC6 | 0.401 | -1.31656 | 4.82E-06 | 4.35E-05 | yes | down | 2.93 | 5.21 | 4.66 | 4.5 | 2.77 | 4.23 | 4.266667 | 3.833333 |
| FAT3 | 15.523 | 3.956366 | 4.85E-06 | 4.37E-05 | yes | up | 0 | 0.01 | 0.01 | 46.78 | 0.15 | 0.12 | 0.006667 | 15.68333 |
| AURKB | 7.146 | 2.837066 | 4.88E-06 | 4.39E-05 | yes | up | 0.46 | 0.53 | 0.5 | 9.03 | 12.96 | 2.71 | 0.496667 | 8.233333 |
| LOC100348518 | 2.056 | 1.039682 | 4.9E-06 | 4.4E-05 | yes | up | 5.76 | 8.63 | 10.62 | 38.27 | 36.73 | 43.73 | 8.336667 | 39.57667 |
| LOC100338821 | 81.912 | 6.355997 | 4.93E-06 | 4.43E-05 | yes | up | 0 | 0 | 0 | 1.27 | 1.15 | 0.83 | 0 | 1.083333 |
| FAM110B | 2.465 | 1.301785 | 5.01E-06 | 4.5E-05 | yes | up | 0.38 | 0.67 | 0.73 | 4.18 | 3.39 | 3.61 | 0.593333 | 3.726667 |
| NME4 | 0.425 | -1.23455 | 5.09E-06 | 4.56E-05 | yes | down | 64.5 | 97.57 | 59.25 | 51.89 | 76.71 | 58.17 | 73.77333 | 62.25667 |
| ST3GAL6 | 0.451 | -1.14721 | 5.11E-06 | 4.58E-05 | yes | down | 28.56 | 18.73 | 25.08 | 23.8 | 25.04 | 30.7 | 24.12333 | 26.51333 |
| LOC100344884 | 79.999 | 6.321907 | 5.11E-06 | 4.58E-05 | yes | up | 0.12 | 0.08 | 0.15 | 38.14 | 0.44 | 24.14 | 0.116667 | 20.90667 |
| LOC108178461 | 3.473 | 1.79617 | 5.12E-06 | 4.58E-05 | yes | up | 0.51 | 0.77 | 0.49 | 6.11 | 6.01 | 3.91 | 0.59 | 5.343333 |
| MRPL32 | 0.447 | -1.16065 | 5.26E-06 | 4.69E-05 | yes | down | 10.76 | 10.66 | 8.61 | 9.89 | 10.04 | 11.45 | 10.01 | 10.46 |
| ZNF629 | 0.451 | -1.14726 | 5.33E-06 | 4.74E-05 | yes | down | 2.84 | 3.04 | 4.62 | 4.46 | 3.06 | 3.7 | 3.5 | 3.74 |
| TMEM223 | 0.395 | -1.33953 | 5.44E-06 | 4.84E-05 | yes | down | 11.23 | 15.94 | 14.2 | 10.17 | 15.6 | 12.33 | 13.79 | 12.7 |
| LOC100009097 | 3.547 | 1.826526 | 5.52E-06 | 4.91E-05 | yes | up | 1.14 | 1.75 | 2.45 | 10.21 | 13.07 | 18.38 | 1.78 | 13.88667 |
| PEBP4 | 0.288 | -1.79612 | 5.78E-06 | 5.12E-05 | yes | down | 65.55 | 70.84 | 109.6 | 38.56 | 34.99 | 92.92 | 81.99667 | 55.49 |
| IFIH1 | 3.343 | 1.741312 | 5.86E-06 | 5.19E-05 | yes | up | 0.61 | 0.51 | 0.72 | 4.19 | 3.81 | 7.75 | 0.613333 | 5.25 |
| PBK | 11.655 | 3.542839 | 5.87E-06 | 5.19E-05 | yes | up | 0.19 | 0.16 | 0.14 | 3.96 | 7.71 | 0.96 | 0.163333 | 4.21 |
| CENPI | 3.333 | 1.736874 | 5.95E-06 | 5.26E-05 | yes | up | 0.32 | 0.35 | 0.48 | 3.18 | 3.4 | 2.14 | 0.383333 | 2.906667 |
| LRRN4CL | 153.533 | 7.262407 | 6.05E-06 | 5.34E-05 | yes | up | 0 | 0 | 0 | 3.11 | 2.73 | 0.17 | 0 | 2.003333 |
| ALAS2 | 0.26 | -1.94406 | 6.12E-06 | 5.39E-05 | yes | down | 26 | 34.13 | 33.53 | 7.56 | 16.57 | 31.58 | 31.22 | 18.57 |
| C16H1orf106 | 0.039 | -4.68396 | 6.2E-06 | 5.46E-05 | yes | down | 1.17 | 0.53 | 1.86 | 0.17 | 0.09 | 0.08 | 1.186667 | 0.113333 |
| LOC100354753 | 2.02 | 1.014605 | 6.21E-06 | 5.47E-05 | yes | up | 4.67 | 8.54 | 7.32 | 36.63 | 32.88 | 26.68 | 6.843333 | 32.06333 |
| LOC103348850 | 0.445 | -1.16869 | 6.32E-06 | 5.56E-05 | yes | down | 6.89 | 8.48 | 7.99 | 9.31 | 4.04 | 8.49 | 7.786667 | 7.28 |
| TMEM74B | 0.244 | -2.03444 | 6.54E-06 | 5.75E-05 | yes | down | 0.15 | 0.19 | 0.29 | 0.14 | 0.08 | 0.13 | 0.21 | 0.116667 |
| CPLX1 | 0.019 | -5.72232 | 6.72E-06 | 5.9E-05 | yes | down | 0.61 | 1.44 | 0.5 | 0 | 0.11 | 0 | 0.85 | 0.036667 |
| LOC100355134 | 0.367 | -1.44534 | 6.75E-06 | 5.92E-05 | yes | down | 2.42 | 5.14 | 4.23 | 2.81 | 3.58 | 3.51 | 3.93 | 3.3 |
| SEMA4F | 2.638 | 1.399544 | 6.99E-06 | 6.12E-05 | yes | up | 0.76 | 0.52 | 0.71 | 5.29 | 3.22 | 3.71 | 0.663333 | 4.073333 |
| TP53INP2 | 0.476 | -1.07194 | 7.07E-06 | 6.18E-05 | yes | down | 20.21 | 17.7 | 12.26 | 17.4 | 19.42 | 18.81 | 16.72333 | 18.54333 |
| COL3A1 | 2.025 | 1.018249 | 7.09E-06 | 6.2E-05 | yes | up | 743.41 | 1147.66 | 725.01 | 3075.48 | 4370.26 | 4242.49 | 872.0267 | 3896.077 |
| LOC103347259 | 0.448 | -1.15887 | 7.11E-06 | 6.21E-05 | yes | down | 10.01 | 13.34 | 12.35 | 16.9 | 9.06 | 12.57 | 11.9 | 12.84333 |
| RRS1 | 0.379 | -1.39812 | 7.28E-06 | 6.35E-05 | yes | down | 3.42 | 6.56 | 6.02 | 3.88 | 3.96 | 5.37 | 5.333333 | 4.403333 |
| DHODH | 0.495 | -1.01535 | 7.4E-06 | 6.46E-05 | yes | down | 5.63 | 9.39 | 7.25 | 8.35 | 8.76 | 7.48 | 7.423333 | 8.196667 |
| C9H3orf18 | 0.419 | -1.25334 | 7.67E-06 | 6.68E-05 | yes | down | 11.28 | 14.16 | 21.05 | 11.14 | 15.99 | 18.29 | 15.49667 | 15.14 |
| EIF2B1 | 0.494 | -1.01702 | 7.68E-06 | 6.68E-05 | yes | down | 11.81 | 19.17 | 14.16 | 14.18 | 17.92 | 18.76 | 15.04667 | 16.95333 |
| CADPS2 | 0.491 | -1.02669 | 7.7E-06 | 6.69E-05 | yes | down | 6.13 | 6.6 | 6.62 | 10.42 | 6.22 | 5.94 | 6.45 | 7.526667 |
| HHIP | 3.311 | 1.727342 | 7.83E-06 | 6.8E-05 | yes | up | 0.3 | 0.61 | 0.43 | 3.01 | 4.38 | 1.87 | 0.446667 | 3.086667 |
| FANCI | 3.772 | 1.915264 | 7.98E-06 | 6.91E-05 | yes | up | 0.43 | 0.32 | 0.28 | 4.13 | 3.35 | 1.7 | 0.343333 | 3.06 |
| M1AP | 58.714 | 5.875629 | 8.03E-06 | 6.94E-05 | yes | up | 0 | 0.03 | 0 | 1.51 | 1.21 | 1.41 | 0.01 | 1.376667 |
| ACOT8 | 0.459 | -1.12399 | 8.29E-06 | 7.15E-05 | yes | down | 12.87 | 18.6 | 21.33 | 21.25 | 15.92 | 20.41 | 17.6 | 19.19333 |
| EFCC1 | 4.341 | 2.118152 | 8.33E-06 | 7.17E-05 | yes | up | 0.39 | 0.74 | 1.41 | 7.6 | 4.31 | 13.25 | 0.846667 | 8.386667 |
| NCEH1 | 0.441 | -1.18124 | 8.36E-06 | 7.19E-05 | yes | down | 4.72 | 3.53 | 3.6 | 4.45 | 4.6 | 3.2 | 3.95 | 4.083333 |
| AGT | 23.291 | 4.541724 | 8.37E-06 | 7.19E-05 | yes | up | 0.39 | 0.52 | 0.71 | 43.88 | 41.67 | 2.79 | 0.54 | 29.44667 |
| KIAA2022 | 0.097 | -3.37211 | 8.37E-06 | 7.19E-05 | yes | down | 0.11 | 0.1 | 0.12 | 0.02 | 0.01 | 0.05 | 0.11 | 0.026667 |
| ABCB8 | 0.484 | -1.04824 | 8.4E-06 | 7.22E-05 | yes | down | 3.06 | 5.55 | 5.09 | 5.97 | 4.28 | 5.05 | 4.566667 | 5.1 |
| DSG1 | 0.144 | -2.79497 | 8.63E-06 | 7.4E-05 | yes | down | 2.06 | 1.87 | 1.91 | 0.85 | 0.05 | 1.4 | 1.946667 | 0.766667 |
| AKR1C5 | 9.609 | 3.264453 | 8.76E-06 | 7.5E-05 | yes | up | 0.19 | 1.26 | 0.6 | 32.38 | 6.73 | 6.39 | 0.683333 | 15.16667 |
| NAGPA | 2.225 | 1.153751 | 8.76E-06 | 7.5E-05 | yes | up | 1.2 | 1.74 | 1.78 | 7.38 | 8.35 | 8.6 | 1.573333 | 8.11 |
| TRMT6 | 0.407 | -1.29851 | 8.83E-06 | 7.55E-05 | yes | down | 1.6 | 2.12 | 2.07 | 1.74 | 1.49 | 2.38 | 1.93 | 1.87 |
| CAPN8 | 4.835 | 2.273589 | 8.95E-06 | 7.64E-05 | yes | up | 0.37 | 2.04 | 2.19 | 13.9 | 7.29 | 21.69 | 1.533333 | 14.29333 |
| FAM196A | 0.102 | -3.29994 | 9.06E-06 | 7.72E-05 | yes | down | 0.09 | 0.19 | 0.51 | 0.04 | 0.05 | 0.09 | 0.263333 | 0.06 |
| LRRC75A | 2.488 | 1.31511 | 9.2E-06 | 7.84E-05 | yes | up | 1.97 | 3.81 | 4.13 | 18.47 | 22 | 16.17 | 3.303333 | 18.88 |
| MRPL57 | 0.498 | -1.00474 | 9.27E-06 | 7.89E-05 | yes | down | 22.94 | 26.76 | 26.31 | 27.33 | 30.4 | 22.99 | 25.33667 | 26.90667 |
| LOC108176869 | 2.943 | 1.557237 | 9.33E-06 | 7.93E-05 | yes | up | 0.27 | 0.29 | 0.41 | 1.9 | 2.57 | 2.15 | 0.323333 | 2.206667 |
| FARSB | 0.486 | -1.0399 | 9.35E-06 | 7.94E-05 | yes | down | 9.3 | 17.7 | 10.86 | 14.32 | 12.96 | 14.96 | 12.62 | 14.08 |
| PRCP | 2.444 | 1.289378 | 9.42E-06 | 7.98E-05 | yes | up | 10.89 | 22.12 | 14.69 | 117.44 | 94.9 | 59.73 | 15.9 | 90.69 |
| PPM1L | 0.428 | -1.22272 | 9.69E-06 | 8.21E-05 | yes | down | 2.44 | 3.16 | 3.24 | 2.49 | 2.19 | 4.03 | 2.946667 | 2.903333 |
| GUCY1A3 | 2.777 | 1.473401 | 9.89E-06 | 8.36E-05 | yes | up | 2.47 | 2.84 | 2.56 | 27.25 | 10.26 | 12.83 | 2.623333 | 16.78 |
| GXYLT2 | 4.716 | 2.237716 | 1.04E-05 | 8.74E-05 | yes | up | 0.34 | 0.17 | 0.39 | 2.82 | 3.03 | 4.09 | 0.3 | 3.313333 |
| UTP18 | 0.495 | -1.01471 | 1.04E-05 | 8.77E-05 | yes | down | 8.37 | 10.91 | 9.43 | 8.83 | 10.99 | 13.32 | 9.57 | 11.04667 |
| MBP | 0.335 | -1.57912 | 1.08E-05 | 9.07E-05 | yes | down | 16.28 | 29.54 | 39.75 | 9.68 | 22.42 | 15.1 | 28.52333 | 15.73333 |
| ZNF250 | 2.328 | 1.219021 | 1.08E-05 | 9.07E-05 | yes | up | 1.87 | 1.48 | 2.13 | 11.98 | 9.69 | 8.34 | 1.826667 | 10.00333 |
| HDC | 0.051 | -4.29328 | 1.09E-05 | 9.15E-05 | yes | down | 0.52 | 0.36 | 0.37 | 0.03 | 0 | 0.12 | 0.416667 | 0.05 |
| FAM64A | 10.267 | 3.359949 | 1.11E-05 | 9.3E-05 | yes | up | 0.27 | 0.44 | 0.08 | 7.88 | 8.86 | 1.64 | 0.263333 | 6.126667 |
| SLC35G1 | 0.334 | -1.58355 | 1.11E-05 | 9.35E-05 | yes | down | 0.87 | 1.53 | 1.25 | 0.9 | 0.8 | 1.1 | 1.216667 | 0.933333 |
| LOC103345165 | 10.621 | 3.408806 | 1.12E-05 | 9.36E-05 | yes | up | 0.17 | 0.22 | 0.05 | 2.57 | 2.75 | 5.37 | 0.146667 | 3.563333 |
| LOC100349447 | 0.313 | -1.67396 | 1.12E-05 | 9.43E-05 | yes | down | 202.39 | 127.9 | 66.68 | 92.48 | 118.52 | 82.35 | 132.3233 | 97.78333 |
| LOC100351134 | 2.295 | 1.198728 | 1.13E-05 | 9.47E-05 | yes | up | 0.87 | 1 | 0.98 | 5.94 | 5.17 | 4.22 | 0.95 | 5.11 |
| LXN | 3.361 | 1.748789 | 1.14E-05 | 9.55E-05 | yes | up | 4.89 | 19.19 | 10.51 | 116.1 | 61.29 | 87.16 | 11.53 | 88.18333 |
| RASD1 | 0.074 | -3.75772 | 1.15E-05 | 9.63E-05 | yes | down | 53.01 | 17.22 | 8.42 | 2.76 | 4.5 | 6.67 | 26.21667 | 4.643333 |
| POLE | 2.902 | 1.537137 | 1.16E-05 | 9.72E-05 | yes | up | 0.25 | 0.32 | 0.29 | 2.43 | 2.18 | 1.17 | 0.286667 | 1.926667 |
| LOC100349081 | 80.01 | 6.322105 | 1.17E-05 | 9.73E-05 | yes | up | 0 | 0 | 0 | 0.88 | 1.36 | 2.02 | 0 | 1.42 |
| MAP7 | 0.408 | -1.29319 | 1.17E-05 | 9.73E-05 | yes | down | 1.53 | 2.25 | 1.56 | 2.25 | 1.21 | 1.6 | 1.78 | 1.686667 |
| DAAM2 | 2.048 | 1.03403 | 1.17E-05 | 9.75E-05 | yes | up | 4.3 | 8.55 | 6.41 | 34.87 | 23.25 | 31.68 | 6.42 | 29.93333 |
| GPATCH4 | 0.411 | -1.28124 | 1.18E-05 | 9.78E-05 | yes | down | 4.65 | 8.48 | 6.08 | 4.58 | 6.59 | 7.16 | 6.403333 | 6.11 |
| NRL | 22.671 | 4.502805 | 1.18E-05 | 9.78E-05 | yes | up | 0 | 0.03 | 0.05 | 1.35 | 0.94 | 1.39 | 0.026667 | 1.226667 |
| SLC46A2 | 8.135 | 3.024105 | 1.19E-05 | 9.86E-05 | yes | up | 0.07 | 0.05 | 0.17 | 1.74 | 1.12 | 2.66 | 0.096667 | 1.84 |
| PHKG1 | 0.064 | -3.96212 | 1.21E-05 | 0.0001 | yes | down | 71.99 | 75.01 | 54.77 | 1.19 | 12.5 | 14.68 | 67.25667 | 9.456667 |
| CCDC86 | 0.238 | -2.07371 | 1.21E-05 | 0.0001 | yes | down | 14.66 | 9.29 | 5.34 | 3.97 | 3.6 | 8.85 | 9.763333 | 5.473333 |
| COQ10A | 0.342 | -1.54662 | 1.22E-05 | 0.000101 | yes | down | 11.01 | 14.03 | 8.12 | 4.68 | 12.86 | 7.09 | 11.05333 | 8.21 |
| LDLRAD3 | 2.404 | 1.265608 | 1.23E-05 | 0.000102 | yes | up | 3.49 | 3.77 | 4.17 | 15.08 | 18.57 | 29.86 | 3.81 | 21.17 |
| LOC100349551 | 0.356 | -1.49194 | 1.24E-05 | 0.000102 | yes | down | 395.83 | 631 | 291.59 | 279.1 | 519.11 | 268.02 | 439.4733 | 355.41 |
| FAM185A | 0.405 | -1.3043 | 1.27E-05 | 0.000105 | yes | down | 2.62 | 2.3 | 3.16 | 2 | 2.53 | 3.08 | 2.693333 | 2.536667 |
| LOC103346850 | 0.33 | -1.60126 | 1.28E-05 | 0.000106 | yes | down | 3.09 | 3.25 | 2.36 | 2.46 | 1.59 | 2.62 | 2.9 | 2.223333 |
| LOC103351001 | 2.759 | 1.464295 | 1.3E-05 | 0.000107 | yes | up | 0.41 | 0.44 | 0.39 | 2.4 | 3.03 | 2.52 | 0.413333 | 2.65 |
| ZNF286A | 2.067 | 1.047797 | 1.3E-05 | 0.000107 | yes | up | 1.19 | 1.24 | 1.24 | 6.37 | 3.94 | 5.52 | 1.223333 | 5.276667 |
| ADAMTS12 | 2.154 | 1.10734 | 1.3E-05 | 0.000107 | yes | up | 8.35 | 8.73 | 7.41 | 57.64 | 35.55 | 29.57 | 8.163333 | 40.92 |
| MC2R | 36.547 | 5.191664 | 1.35E-05 | 0.000111 | yes | up | 0.04 | 0 | 0 | 0.78 | 1.3 | 0.81 | 0.013333 | 0.963333 |
| CKAP2 | 2.856 | 1.514049 | 1.37E-05 | 0.000112 | yes | up | 1.06 | 1.09 | 1.65 | 10.21 | 9.89 | 5.38 | 1.266667 | 8.493333 |
| ZNF660 | 3.252 | 1.701321 | 1.41E-05 | 0.000116 | yes | up | 0.48 | 0.57 | 0.56 | 6.15 | 2.71 | 3.39 | 0.536667 | 4.083333 |
| BTG1 | 2.38 | 1.251075 | 1.43E-05 | 0.000117 | yes | up | 78.32 | 57.4 | 38.52 | 318.91 | 367.91 | 287.08 | 58.08 | 324.6333 |
| UCP1 | 0.003 | -8.22076 | 1.43E-05 | 0.000117 | yes | down | 1895.23 | 3480.95 | 2355.41 | 0.1 | 0.67 | 58.09 | 2577.197 | 19.62 |
| MMRN2 | 2.045 | 1.032267 | 1.43E-05 | 0.000117 | yes | up | 10.73 | 8.8 | 7.31 | 55.72 | 36.52 | 37.29 | 8.946667 | 43.17667 |
| LOC100355902 | 0.377 | -1.4078 | 1.45E-05 | 0.000118 | yes | down | 1.2 | 1.28 | 1.67 | 0.83 | 1.2 | 1.59 | 1.383333 | 1.206667 |
| DMGDH | 0.03 | -5.04917 | 1.45E-05 | 0.000118 | yes | down | 89.41 | 58.47 | 14.68 | 3.26 | 0.86 | 9.28 | 54.18667 | 4.466667 |
| RTL1 | 0.026 | -5.2776 | 1.46E-05 | 0.000119 | yes | down | 0.83 | 2.74 | 0.08 | 0.03 | 0.09 | 0 | 1.216667 | 0.04 |
| CHAF1B | 0.363 | -1.46082 | 1.47E-05 | 0.00012 | yes | down | 3.31 | 5.31 | 3.22 | 3.05 | 3.9 | 3.05 | 3.946667 | 3.333333 |
| MTHFSD | 0.451 | -1.14918 | 1.48E-05 | 0.000121 | yes | down | 4.67 | 4 | 4.63 | 3.56 | 4.19 | 5.76 | 4.433333 | 4.503333 |
| ANKRD34A | 3.5 | 1.807337 | 1.48E-05 | 0.000121 | yes | up | 0.23 | 0.61 | 0.82 | 2.3 | 2.58 | 2.84 | 0.553333 | 2.573333 |
| FAR2 | 2.853 | 1.512597 | 1.48E-05 | 0.000121 | yes | up | 0.32 | 0.66 | 0.33 | 2.8 | 2.8 | 2.99 | 0.436667 | 2.863333 |
| DEPDC1B | 17.45 | 4.125182 | 1.51E-05 | 0.000122 | yes | up | 0.04 | 0.08 | 0 | 1.89 | 2.32 | 0.5 | 0.04 | 1.57 |
| C19H17orf58 | 3.049 | 1.608149 | 1.53E-05 | 0.000124 | yes | up | 2.3 | 3.88 | 3.37 | 14.69 | 27.24 | 24.98 | 3.183333 | 22.30333 |
| FHL2 | 0.22 | -2.18432 | 1.58E-05 | 0.000127 | yes | down | 8.92 | 6.62 | 2.69 | 2.94 | 4.46 | 1.96 | 6.076667 | 3.12 |
| LOC100350390 | 0.35 | -1.51369 | 1.6E-05 | 0.000129 | yes | down | 1.01 | 1.68 | 1.23 | 1.06 | 0.88 | 1.21 | 1.306667 | 1.05 |
| LOC108177728 | 21.532 | 4.428392 | 1.63E-05 | 0.000131 | yes | up | 0 | 0 | 0.39 | 4.21 | 9.31 | 7.66 | 0.13 | 7.06 |
| TMEM35B | 2.722 | 1.444549 | 1.64E-05 | 0.000132 | yes | up | 1.42 | 3.23 | 2.36 | 16.26 | 12.06 | 15.35 | 2.336667 | 14.55667 |
| GLB1L | 2.012 | 1.00858 | 1.64E-05 | 0.000132 | yes | up | 1.63 | 2.82 | 2.36 | 11.6 | 8.84 | 9.71 | 2.27 | 10.05 |
| GDF9 | 2.581 | 1.367657 | 1.65E-05 | 0.000133 | yes | up | 1.56 | 1.26 | 1.79 | 10.78 | 6.8 | 12.17 | 1.536667 | 9.916667 |
| ARSK | 3.348 | 1.743346 | 1.65E-05 | 0.000133 | yes | up | 0.58 | 1.32 | 2.04 | 9.91 | 11.32 | 9.08 | 1.313333 | 10.10333 |
| FASTKD3 | 0.456 | -1.13429 | 1.66E-05 | 0.000133 | yes | down | 2.32 | 3 | 2.42 | 2.53 | 2.05 | 3.11 | 2.58 | 2.563333 |
| CCDC85C | 2.662 | 1.412431 | 1.67E-05 | 0.000134 | yes | up | 0.74 | 2.21 | 1.4 | 9.54 | 8.41 | 8.47 | 1.45 | 8.806667 |
| ENPP3 | 2.302 | 1.202776 | 1.68E-05 | 0.000134 | yes | up | 1.4 | 1.7 | 1.02 | 8.17 | 5.6 | 8.36 | 1.373333 | 7.376667 |
| MX1 | 2.731 | 1.44963 | 1.68E-05 | 0.000134 | yes | up | 8.85 | 9.01 | 9.38 | 50.46 | 33.96 | 90.01 | 9.08 | 58.14333 |
| LOC100351302 | 2.525 | 1.336124 | 1.69E-05 | 0.000135 | yes | up | 1.84 | 1.63 | 2.02 | 12.6 | 6.3 | 12.53 | 1.83 | 10.47667 |
| LOC100349976 | 0.352 | -1.50445 | 1.69E-05 | 0.000135 | yes | down | 3.49 | 8.88 | 5.83 | 3.9 | 5.01 | 5.66 | 6.066667 | 4.856667 |
| LOC103352092 | 4.511 | 2.173546 | 1.72E-05 | 0.000138 | yes | up | 0.13 | 0.13 | 0.34 | 2.86 | 2 | 1.49 | 0.2 | 2.116667 |
| SAPCD1 | 3.51 | 1.81132 | 1.74E-05 | 0.000139 | yes | up | 0.69 | 1.13 | 1.15 | 7.67 | 9.91 | 6.65 | 0.99 | 8.076667 |
| ADGRE5 | 2.323 | 1.215872 | 1.75E-05 | 0.00014 | yes | up | 2.69 | 4.79 | 4.85 | 22.26 | 15.42 | 15.22 | 4.11 | 17.63333 |
| HHAT | 3.044 | 1.606161 | 1.8E-05 | 0.000143 | yes | up | 0.34 | 0.48 | 0.42 | 3.74 | 2.59 | 2.53 | 0.413333 | 2.953333 |
| C15H4orf32 | 2.718 | 1.442444 | 1.8E-05 | 0.000143 | yes | up | 2.47 | 2.57 | 2.58 | 15.95 | 10.61 | 21.97 | 2.54 | 16.17667 |
| KCNK3 | 0.028 | -5.17327 | 1.82E-05 | 0.000144 | yes | down | 35.85 | 76.05 | 46.92 | 0.93 | 0.33 | 8.74 | 52.94 | 3.333333 |
| NR1H3 | 2.138 | 1.096196 | 1.82E-05 | 0.000145 | yes | up | 20.01 | 16.97 | 23.91 | 138.51 | 80.29 | 84.75 | 20.29667 | 101.1833 |
| KCNIP3 | 3.449 | 1.786025 | 1.84E-05 | 0.000146 | yes | up | 0.32 | 0.38 | 0.23 | 2.25 | 2.95 | 2.28 | 0.31 | 2.493333 |
| LOC108178580 | 25.348 | 4.663772 | 1.88E-05 | 0.000149 | yes | up | 0 | 0 | 0.24 | 2.97 | 5.19 | 6.6 | 0.08 | 4.92 |
| ABCA1 | 2.689 | 1.427294 | 1.96E-05 | 0.000154 | yes | up | 6.46 | 4.4 | 3.92 | 31.32 | 21.46 | 22.76 | 4.926667 | 25.18 |
| TCTEX1D4 | 0.206 | -2.27623 | 2.02E-05 | 0.000159 | yes | down | 1.9 | 1.17 | 1.18 | 0.75 | 0.53 | 0.69 | 1.416667 | 0.656667 |
| PLK4 | 4.639 | 2.213712 | 2.03E-05 | 0.00016 | yes | up | 0.24 | 0.52 | 0.24 | 4.03 | 4.94 | 1.56 | 0.333333 | 3.51 |
| AP1G2 | 2.536 | 1.342527 | 2.04E-05 | 0.00016 | yes | up | 0.83 | 0.64 | 1.11 | 5.09 | 4.12 | 5.04 | 0.86 | 4.75 |
| NIPA1 | 0.404 | -1.30855 | 2.05E-05 | 0.000161 | yes | down | 0.92 | 0.87 | 1.09 | 0.92 | 0.67 | 1.11 | 0.96 | 0.9 |
| ZNF74 | 2.401 | 1.263359 | 2.08E-05 | 0.000163 | yes | up | 1.12 | 1.37 | 1.76 | 10.39 | 7.1 | 6.31 | 1.416667 | 7.933333 |
| OAF | 2.256 | 1.173647 | 2.08E-05 | 0.000163 | yes | up | 5.37 | 8.95 | 7.37 | 35.72 | 37.57 | 41.2 | 7.23 | 38.16333 |
| TNFAIP8 | 2.009 | 1.006534 | 2.11E-05 | 0.000165 | yes | up | 8.83 | 6.41 | 7.33 | 38.85 | 36.95 | 30.41 | 7.523333 | 35.40333 |
| CASP3 | 2.88 | 1.526123 | 2.11E-05 | 0.000165 | yes | up | 0.62 | 0.8 | 0.89 | 5.84 | 4.21 | 5.1 | 0.77 | 5.05 |
| REEP4 | 2.75 | 1.459531 | 2.13E-05 | 0.000166 | yes | up | 1.59 | 1.89 | 1.92 | 14.29 | 12.53 | 8.01 | 1.8 | 11.61 |
| MCM7 | 2.057 | 1.040857 | 2.13E-05 | 0.000166 | yes | up | 4.57 | 4.73 | 3.53 | 23.22 | 22.8 | 15.29 | 4.276667 | 20.43667 |
| SDSL | 2.85 | 1.510892 | 2.14E-05 | 0.000168 | yes | up | 1.75 | 3.28 | 2.32 | 22.26 | 16.86 | 10.67 | 2.45 | 16.59667 |
| SLC7A6 | 2.147 | 1.102298 | 2.15E-05 | 0.000168 | yes | up | 15.71 | 13.09 | 17.44 | 65.47 | 87.48 | 72.37 | 15.41333 | 75.10667 |
| TRUB2 | 0.422 | -1.24566 | 2.16E-05 | 0.000168 | yes | down | 2.34 | 3.01 | 3.59 | 2.36 | 2.64 | 3.7 | 2.98 | 2.9 |
| C3H8orf22 | 0.17 | -2.55363 | 2.2E-05 | 0.000171 | yes | down | 6.1 | 6.3 | 3.44 | 0.52 | 3.59 | 1.96 | 5.28 | 2.023333 |
| TRAIP | 5.969 | 2.577441 | 2.2E-05 | 0.000171 | yes | up | 0.07 | 0.19 | 0.08 | 1.38 | 2.21 | 1.06 | 0.113333 | 1.55 |
| GRIN3A | 2.171 | 1.118355 | 2.25E-05 | 0.000175 | yes | up | 0.58 | 0.69 | 0.47 | 2.54 | 3.39 | 2.74 | 0.58 | 2.89 |
| SCARB1 | 2.748 | 1.458294 | 2.25E-05 | 0.000175 | yes | up | 15.8 | 17.9 | 14.6 | 178.37 | 70.78 | 65.82 | 16.1 | 104.99 |
| LOC100353768 | 2.439 | 1.286032 | 2.27E-05 | 0.000177 | yes | up | 2.65 | 2.31 | 2.01 | 10.23 | 17.25 | 13.31 | 2.323333 | 13.59667 |
| KIAA1257 | 0.261 | -1.93608 | 2.31E-05 | 0.000179 | yes | down | 0.59 | 0.49 | 0.83 | 0.46 | 0.41 | 0.3 | 0.636667 | 0.39 |
| KCNMB3 | 0.426 | -1.23274 | 2.31E-05 | 0.000179 | yes | down | 2.1 | 2.99 | 3.38 | 2.62 | 3.07 | 1.76 | 2.823333 | 2.483333 |
| SLC37A1 | 0.273 | -1.87552 | 2.32E-05 | 0.00018 | yes | down | 1.79 | 2.11 | 2.27 | 0.76 | 0.98 | 2.14 | 2.056667 | 1.293333 |
| CHP2 | 0.43 | -1.2189 | 2.32E-05 | 0.00018 | yes | down | 18.4 | 12.3 | 17.55 | 21.7 | 19.24 | 16.98 | 16.08333 | 19.30667 |
| LOC100344673 | 0.358 | -1.48376 | 2.33E-05 | 0.00018 | yes | down | 2.62 | 1.58 | 1.93 | 2.61 | 1.47 | 1.14 | 2.043333 | 1.74 |
| BCAT1 | 34.734 | 5.118265 | 2.34E-05 | 0.000181 | yes | up | 0.15 | 0.01 | 0 | 2.27 | 2.56 | 3.43 | 0.053333 | 2.753333 |
| LOC100358489 | 8.526 | 3.091947 | 2.36E-05 | 0.000183 | yes | up | 0.16 | 0.16 | 0 | 2.3 | 2.22 | 2.03 | 0.106667 | 2.183333 |
| EDAR | 0.129 | -2.95347 | 2.37E-05 | 0.000183 | yes | down | 1.77 | 1.32 | 1.5 | 0.32 | 0.07 | 1.02 | 1.53 | 0.47 |
| RELL1 | 2.238 | 1.162388 | 2.42E-05 | 0.000187 | yes | up | 5.31 | 4.54 | 2.84 | 20.3 | 23.49 | 22.21 | 4.23 | 22 |
| FAM134B | 0.189 | -2.4014 | 2.48E-05 | 0.00019 | yes | down | 23.02 | 12.27 | 3.89 | 2.95 | 8.22 | 5.83 | 13.06 | 5.666667 |
| PGAP1 | 2.051 | 1.036165 | 2.48E-05 | 0.000191 | yes | up | 0.32 | 0.47 | 0.45 | 2.45 | 1.52 | 1.97 | 0.413333 | 1.98 |
| ADCY2 | 0.215 | -2.21725 | 2.49E-05 | 0.000192 | yes | down | 7.24 | 11.17 | 5.56 | 1.51 | 7.48 | 2.54 | 7.99 | 3.843333 |
| LOC108176565 | 0.057 | -4.12188 | 2.5E-05 | 0.000192 | yes | down | 0.21 | 0.37 | 0.62 | 0.02 | 0 | 0.16 | 0.4 | 0.06 |
| LOC100341622 | 0.377 | -1.40866 | 2.53E-05 | 0.000194 | yes | down | 5.86 | 8.32 | 5.17 | 4.24 | 4.94 | 7.52 | 6.45 | 5.566667 |
| PRUNE2 | 10.928 | 3.449919 | 2.54E-05 | 0.000194 | yes | up | 0.74 | 1.18 | 0.27 | 22.58 | 5.17 | 27.95 | 0.73 | 18.56667 |
| COQ4 | 0.445 | -1.16822 | 2.55E-05 | 0.000195 | yes | down | 3.59 | 4.23 | 4.54 | 5.19 | 3.57 | 4.11 | 4.12 | 4.29 |
| MOGAT2 | 8.897 | 3.153363 | 2.56E-05 | 0.000196 | yes | up | 0.48 | 0.11 | 0.16 | 8.58 | 1.13 | 6.75 | 0.25 | 5.486667 |
| CDC25C | 17.257 | 4.109087 | 2.59E-05 | 0.000198 | yes | up | 0.04 | 0.05 | 0.02 | 1.86 | 1.34 | 0.56 | 0.036667 | 1.253333 |
| NDN | 0.436 | -1.19787 | 2.67E-05 | 0.000204 | yes | down | 21.73 | 54.68 | 34.85 | 44.34 | 29.66 | 37.29 | 37.08667 | 37.09667 |
| RGS2 | 0.314 | -1.67183 | 2.69E-05 | 0.000205 | yes | down | 26.08 | 18.78 | 8 | 13.75 | 13.15 | 12.25 | 17.62 | 13.05 |
| SETD6 | 0.39 | -1.35762 | 2.69E-05 | 0.000205 | yes | down | 1.78 | 3.37 | 3.58 | 2.84 | 2.13 | 2.9 | 2.91 | 2.623333 |
| SAMD10 | 4.517 | 2.175406 | 2.7E-05 | 0.000206 | yes | up | 0.3 | 0.25 | 0.58 | 3.94 | 3.9 | 4.16 | 0.376667 | 4 |
| DIAPH3 | 7.494 | 2.905654 | 2.72E-05 | 0.000207 | yes | up | 0.05 | 0.36 | 0.07 | 2.64 | 4.03 | 1.28 | 0.16 | 2.65 |
| SPX | 66.513 | 6.055574 | 2.73E-05 | 0.000207 | yes | up | 0 | 0 | 0 | 0.71 | 1 | 0.65 | 0 | 0.786667 |
| C12H6orf47 | 0.435 | -1.20152 | 2.77E-05 | 0.00021 | yes | down | 14.99 | 20.28 | 22.55 | 15.86 | 14.8 | 27.53 | 19.27333 | 19.39667 |
| LOC103352280 | 0.392 | -1.3499 | 2.86E-05 | 0.000216 | yes | down | 0.71 | 0.67 | 0.96 | 0.81 | 0.74 | 0.76 | 0.78 | 0.77 |
| IL1R1 | 2.833 | 1.502574 | 2.91E-05 | 0.00022 | yes | up | 6.72 | 4.2 | 3.9 | 25.2 | 46.56 | 23.01 | 4.94 | 31.59 |
| LOC100341993 | 0.414 | -1.27392 | 2.95E-05 | 0.000222 | yes | down | 50.95 | 28.17 | 50.71 | 46.95 | 36.26 | 45.02 | 43.27667 | 42.74333 |
| ANGPTL4 | 0.31 | -1.6885 | 3E-05 | 0.000226 | yes | down | 219.56 | 160.08 | 136.46 | 117.02 | 196.02 | 58.31 | 172.0333 | 123.7833 |
| PDCD2L | 0.453 | -1.14292 | 3.01E-05 | 0.000226 | yes | down | 5.2 | 5.26 | 4.56 | 4.82 | 5.89 | 5.55 | 5.006667 | 5.42 |
| MTHFD2 | 0.402 | -1.3137 | 3.01E-05 | 0.000226 | yes | down | 7.07 | 12.63 | 5.69 | 8.53 | 8.88 | 5.98 | 8.463333 | 7.796667 |
| MXD3 | 8.781 | 3.134328 | 3.05E-05 | 0.000229 | yes | up | 0.45 | 0.16 | 0.2 | 9.2 | 5.84 | 1.84 | 0.27 | 5.626667 |
| PRND | 9.486 | 3.245735 | 3.12E-05 | 0.000234 | yes | up | 0.1 | 0.27 | 0 | 3.36 | 2.97 | 1.45 | 0.123333 | 2.593333 |
| ATP5I | 0.337 | -1.56819 | 3.16E-05 | 0.000237 | yes | down | 5.67 | 6.98 | 7.84 | 4.97 | 7.31 | 3.84 | 6.83 | 5.373333 |
| LOC103349247 | 5.226 | 2.385776 | 3.22E-05 | 0.000241 | yes | up | 0.2 | 0.5 | 1.04 | 8.79 | 7.89 | 6.73 | 0.58 | 7.803333 |
| FABP9 | 4.584 | 2.196663 | 3.23E-05 | 0.000241 | yes | up | 0.22 | 3.05 | 1.8 | 34.93 | 12.76 | 8.98 | 1.69 | 18.89 |
| ANLN | 6.515 | 2.703792 | 3.23E-05 | 0.000241 | yes | up | 0.18 | 0.37 | 0.06 | 3.41 | 4.28 | 1.26 | 0.203333 | 2.983333 |
| SCN3B | 0.403 | -1.31284 | 3.23E-05 | 0.000241 | yes | down | 2.64 | 4.69 | 1.91 | 3.27 | 2.98 | 3.21 | 3.08 | 3.153333 |
| HEPACAM | 0.286 | -1.80378 | 3.25E-05 | 0.000242 | yes | down | 0.52 | 0.84 | 0.61 | 0.55 | 0.34 | 0.42 | 0.656667 | 0.436667 |
| ZCCHC5 | 4.471 | 2.160565 | 3.29E-05 | 0.000245 | yes | up | 0.52 | 3.66 | 1.81 | 21.86 | 12.28 | 26.08 | 1.996667 | 20.07333 |
| LOC100340458 | 5.147 | 2.36367 | 3.31E-05 | 0.000246 | yes | up | 0.38 | 1.1 | 0.13 | 4.37 | 1.91 | 8.93 | 0.536667 | 5.07 |
| KHDRBS3 | 0.425 | -1.23447 | 3.34E-05 | 0.000248 | yes | down | 2.44 | 2.88 | 2.68 | 3.34 | 2.91 | 1.67 | 2.666667 | 2.64 |
| LOC108176621 | 0.092 | -3.4425 | 3.45E-05 | 0.000256 | yes | down | 14.99 | 12.51 | 18.13 | 2.55 | 0.72 | 7.23 | 15.21 | 3.5 |
| LOC100345038 | 2.049 | 1.035197 | 3.54E-05 | 0.000262 | yes | up | 1.26 | 1.89 | 2.04 | 8.28 | 8.42 | 9.16 | 1.73 | 8.62 |
| EPHA6 | 4.076 | 2.027177 | 3.63E-05 | 0.000269 | yes | up | 1.02 | 0.3 | 0.29 | 8.42 | 12.25 | 7.19 | 0.536667 | 9.286667 |
| PTP4A3 | 0.088 | -3.507 | 3.64E-05 | 0.000269 | yes | down | 126.23 | 156.58 | 96.59 | 9.76 | 54.87 | 10.39 | 126.4667 | 25.00667 |
| CDCA3 | 6.986 | 2.804514 | 3.66E-05 | 0.00027 | yes | up | 0.53 | 1.04 | 0.21 | 12.51 | 12.71 | 3.14 | 0.593333 | 9.453333 |
| LOC108176017 | 0.033 | -4.90275 | 3.66E-05 | 0.00027 | yes | down | 2.62 | 3.74 | 0.36 | 0.38 | 0 | 0.12 | 2.24 | 0.166667 |
| ESCO2 | 7.751 | 2.954373 | 3.76E-05 | 0.000276 | yes | up | 0.07 | 0.11 | 0.13 | 2.59 | 2.52 | 0.54 | 0.103333 | 1.883333 |
| LOC100339055 | 2.991 | 1.580723 | 3.76E-05 | 0.000276 | yes | up | 0.59 | 0.79 | 0.43 | 5.1 | 4.96 | 2.56 | 0.603333 | 4.206667 |
| LOC100349792 | 0.382 | -1.38775 | 3.77E-05 | 0.000277 | yes | down | 35.93 | 48.98 | 45.56 | 27.15 | 59.25 | 27.41 | 43.49 | 37.93667 |
| LOC100340053 | 0.213 | -2.23134 | 3.83E-05 | 0.000281 | yes | down | 0.98 | 0.96 | 0.69 | 0.42 | 0.45 | 0.47 | 0.876667 | 0.446667 |
| LOC108178822 | 0.372 | -1.4264 | 3.86E-05 | 0.000283 | yes | down | 1.73 | 1.71 | 1.09 | 1.61 | 1.45 | 0.87 | 1.51 | 1.31 |
| XYLB | 0.476 | -1.07241 | 3.89E-05 | 0.000284 | yes | down | 11.08 | 17.24 | 15.8 | 12.34 | 20.27 | 15.21 | 14.70667 | 15.94 |
| LOC100357076 | 0.43 | -1.21839 | 4.02E-05 | 0.000294 | yes | down | 2.71 | 2.94 | 3.85 | 2.21 | 3.92 | 3.24 | 3.166667 | 3.123333 |
| PLXNA3 | 2.115 | 1.080694 | 4.06E-05 | 0.000296 | yes | up | 0.41 | 0.52 | 0.35 | 2.4 | 1.76 | 2.1 | 0.426667 | 2.086667 |
| LOC103350429 | 33.099 | 5.048725 | 4.08E-05 | 0.000297 | yes | up | 0.07 | 0 | 0.06 | 2.01 | 0.75 | 2.4 | 0.043333 | 1.72 |
| LOC108176489 | 4.587 | 2.197465 | 4.08E-05 | 0.000297 | yes | up | 0.11 | 0.19 | 0.25 | 2.89 | 1.5 | 1.5 | 0.183333 | 1.963333 |
| GALNTL5 | 0.038 | -4.70939 | 4.15E-05 | 0.000302 | yes | down | 1.5 | 0.04 | 0.39 | 0.05 | 0.06 | 0.07 | 0.643333 | 0.06 |
| METTL21B | 0.289 | -1.79051 | 4.18E-05 | 0.000304 | yes | down | 2.66 | 1.67 | 4.33 | 1.96 | 1.32 | 2.63 | 2.886667 | 1.97 |
| LOC108177122 | 0.221 | -2.17694 | 4.19E-05 | 0.000305 | yes | down | 1.18 | 1.8 | 1.58 | 3.39 | 0.88 | 1.53 | 1.52 | 1.933333 |
| DHTKD1 | 2.042 | 1.030006 | 4.2E-05 | 0.000305 | yes | up | 1.82 | 1.84 | 1.1 | 7.93 | 7.33 | 7.33 | 1.586667 | 7.53 |
| APBB1IP | 2.409 | 1.26833 | 4.21E-05 | 0.000306 | yes | up | 0.82 | 1.08 | 1.16 | 5.75 | 4.79 | 6.59 | 1.02 | 5.71 |
| LOC103346561 | 3.724 | 1.896925 | 4.37E-05 | 0.000316 | yes | up | 0.34 | 0.33 | 0.57 | 6.05 | 6 | 3.05 | 0.413333 | 5.033333 |
| MPP7 | 0.407 | -1.29599 | 4.39E-05 | 0.000317 | yes | down | 2.23 | 3.08 | 2.02 | 1.51 | 2.99 | 2.35 | 2.443333 | 2.283333 |
| AMT | 0.334 | -1.58012 | 4.44E-05 | 0.000321 | yes | down | 2.43 | 5.12 | 4.18 | 3.21 | 1.67 | 4.17 | 3.91 | 3.016667 |
| SCARF1 | 2.028 | 1.020308 | 4.59E-05 | 0.000331 | yes | up | 3.75 | 5.97 | 5.17 | 34.99 | 23.12 | 18.48 | 4.963333 | 25.53 |
| FICD | 0.342 | -1.54925 | 4.59E-05 | 0.000331 | yes | down | 4.23 | 2.92 | 3.71 | 2.01 | 2.24 | 4.44 | 3.62 | 2.896667 |
| MYH13 | 0.01 | -6.68265 | 4.7E-05 | 0.000338 | yes | down | 26.81 | 150.18 | 3.36 | 0.06 | 3.19 | 0.12 | 60.11667 | 1.123333 |
| VASH1 | 2.031 | 1.022348 | 4.83E-05 | 0.000346 | yes | up | 4.55 | 8.47 | 5.46 | 36.76 | 28.16 | 21.37 | 6.16 | 28.76333 |
| C2H2orf73 | 0.337 | -1.56997 | 4.84E-05 | 0.000347 | yes | down | 2.42 | 2.68 | 2.43 | 1.85 | 1.57 | 2.53 | 2.51 | 1.983333 |
| LOC108178630 | 0.067 | -3.89745 | 4.84E-05 | 0.000347 | yes | down | 4.32 | 5.2 | 2.21 | 1.03 | 0.55 | 1 | 3.91 | 0.86 |
| SIGLEC15 | 0.2 | -2.3246 | 4.86E-05 | 0.000348 | yes | down | 0.54 | 1.01 | 0.83 | 0.3 | 0.12 | 0.63 | 0.793333 | 0.35 |
| KIAA1211 | 0.394 | -1.34385 | 4.86E-05 | 0.000348 | yes | down | 1.54 | 1.24 | 2.44 | 1.97 | 0.98 | 1.39 | 1.74 | 1.446667 |
| NTN4 | 2.074 | 1.052369 | 4.89E-05 | 0.00035 | yes | up | 4.28 | 6.89 | 4.08 | 19.28 | 28.71 | 24.09 | 5.083333 | 24.02667 |
| GJB2 | 0.011 | -6.475 | 4.93E-05 | 0.000352 | yes | down | 0.18 | 0.56 | 0.22 | 0 | 0 | 0 | 0.32 | 0 |
| MCAT | 0.451 | -1.14974 | 4.98E-05 | 0.000355 | yes | down | 4.27 | 5.02 | 6.52 | 4.91 | 3.8 | 6.1 | 5.27 | 4.936667 |
| SH3RF2 | 0.237 | -2.07463 | 5.04E-05 | 0.000359 | yes | down | 0.93 | 0.66 | 0.39 | 0.33 | 0.34 | 0.33 | 0.66 | 0.333333 |
| NETO2 | 3.835 | 1.939269 | 5.08E-05 | 0.000361 | yes | up | 0.42 | 0.67 | 0.32 | 5.07 | 5.63 | 1.63 | 0.47 | 4.11 |
| ACADL | 0.464 | -1.10749 | 5.16E-05 | 0.000367 | yes | down | 824.64 | 827.12 | 740.36 | 779.48 | 572.27 | 1246.79 | 797.3733 | 866.18 |
| TP53I3 | 2.421 | 1.275329 | 5.2E-05 | 0.000369 | yes | up | 1.54 | 1.3 | 1.32 | 7.79 | 7.41 | 8.31 | 1.386667 | 7.836667 |
| GLYCTK | 0.399 | -1.32376 | 5.25E-05 | 0.000372 | yes | down | 356.36 | 488.77 | 593.05 | 575.94 | 214.43 | 483.53 | 479.3933 | 424.6333 |
| SMC2 | 2.485 | 1.312978 | 5.26E-05 | 0.000373 | yes | up | 0.8 | 1.02 | 1.11 | 6.44 | 7.34 | 3.42 | 0.976667 | 5.733333 |
| NYX | 10.133 | 3.341053 | 5.27E-05 | 0.000374 | yes | up | 0 | 0.02 | 0.05 | 0.72 | 0.49 | 0.44 | 0.023333 | 0.55 |
| LOC100358947 | 0.011 | -6.44581 | 5.3E-05 | 0.000375 | yes | down | 0.64 | 0.3 | 0.31 | 0 | 0 | 0 | 0.416667 | 0 |
| SGO1 | 6.945 | 2.795978 | 5.31E-05 | 0.000376 | yes | up | 0.14 | 0.16 | 0.06 | 2.43 | 2.7 | 0.62 | 0.12 | 1.916667 |
| LOC103346472 | 57.929 | 5.856202 | 5.37E-05 | 0.00038 | yes | up | 0.02 | 0.02 | 0 | 2.06 | 0.09 | 3.62 | 0.013333 | 1.923333 |
| IL1RAPL1 | 3.087 | 1.626222 | 5.4E-05 | 0.000382 | yes | up | 0.19 | 0.09 | 0.2 | 1.34 | 1.23 | 0.98 | 0.16 | 1.183333 |
| IL34 | 2.083 | 1.058509 | 5.6E-05 | 0.000395 | yes | up | 1.66 | 2.92 | 2.14 | 13.28 | 12.06 | 10.08 | 2.24 | 11.80667 |
| LIPA | 2.313 | 1.209941 | 5.64E-05 | 0.000398 | yes | up | 43.45 | 33.52 | 55.53 | 160.23 | 266.37 | 279.11 | 44.16667 | 235.2367 |
| CARMIL3 | 4.464 | 2.158378 | 5.69E-05 | 0.000401 | yes | up | 0.08 | 0.07 | 0.11 | 1.07 | 0.76 | 0.89 | 0.086667 | 0.906667 |
| MLPH | 14.793 | 3.886796 | 5.99E-05 | 0.000421 | yes | up | 0.06 | 0.17 | 0.1 | 0.85 | 4.17 | 5.04 | 0.11 | 3.353333 |
| PROX1 | 0.228 | -2.13577 | 6.05E-05 | 0.000424 | yes | down | 0.78 | 0.64 | 0.21 | 0.27 | 0.37 | 0.22 | 0.543333 | 0.286667 |
| KRT23 | 0.011 | -6.56488 | 6.09E-05 | 0.000427 | yes | down | 13.82 | 4.3 | 14.03 | 0.04 | 0 | 0.74 | 10.71667 | 0.26 |
| SLC7A10 | 2.695 | 1.43012 | 6.21E-05 | 0.000435 | yes | up | 1.32 | 3.72 | 2.49 | 16.06 | 26.03 | 15.48 | 2.51 | 19.19 |
| EHF | 18.675 | 4.223058 | 6.22E-05 | 0.000435 | yes | up | 0.01 | 0.01 | 0.01 | 0.48 | 0.57 | 0.26 | 0.01 | 0.436667 |
| RAD54L | 4.436 | 2.149166 | 6.27E-05 | 0.000438 | yes | up | 0.12 | 0.26 | 0.36 | 3.21 | 3.25 | 1.69 | 0.246667 | 2.716667 |
| KTI12 | 0.416 | -1.26569 | 6.31E-05 | 0.000441 | yes | down | 6.33 | 9.66 | 5.37 | 5.12 | 6.16 | 9.07 | 7.12 | 6.783333 |
| NEURL2 | 0.155 | -2.68541 | 6.35E-05 | 0.000443 | yes | down | 2.85 | 6.11 | 0.96 | 1.52 | 1.15 | 0.86 | 3.306667 | 1.176667 |
| MCM6 | 2.75 | 1.459501 | 6.41E-05 | 0.000447 | yes | up | 1.15 | 1.95 | 0.95 | 9.72 | 10.48 | 5.27 | 1.35 | 8.49 |
| GCA | 3.047 | 1.607366 | 6.51E-05 | 0.000454 | yes | up | 1.14 | 1.19 | 0.96 | 4.46 | 8.39 | 9.19 | 1.096667 | 7.346667 |
| LOC108176754 | 0.216 | -2.20873 | 6.52E-05 | 0.000454 | yes | down | 7.67 | 5.63 | 2.44 | 4.29 | 1.06 | 2.85 | 5.246667 | 2.733333 |
| TMEM14A | 2.212 | 1.145426 | 6.52E-05 | 0.000454 | yes | up | 6.17 | 5.36 | 6.29 | 29.73 | 26.77 | 29.96 | 5.94 | 28.82 |
| CST3 | 0.493 | -1.02131 | 6.53E-05 | 0.000454 | yes | down | 1649.69 | 1508.29 | 1758.22 | 1317.18 | 2066.1 | 2320.48 | 1638.733 | 1901.253 |
| ZNF583 | 0.44 | -1.18529 | 6.59E-05 | 0.000458 | yes | down | 1.6 | 2.15 | 1.68 | 1.98 | 1.73 | 1.68 | 1.81 | 1.796667 |
| RYR2 | 2.927 | 1.549617 | 6.67E-05 | 0.000462 | yes | up | 0.21 | 0.15 | 0.17 | 1.93 | 0.73 | 1.02 | 0.176667 | 1.226667 |
| ZWINT | 7.75 | 2.954277 | 6.7E-05 | 0.000464 | yes | up | 0.19 | 0.12 | 0.15 | 3.21 | 4.05 | 1.09 | 0.153333 | 2.783333 |
| LSAMP | 3.679 | 1.879375 | 6.7E-05 | 0.000464 | yes | up | 0.51 | 0.4 | 0.8 | 8.98 | 3.61 | 2.45 | 0.57 | 5.013333 |
| BRCA1 | 5.299 | 2.405643 | 6.73E-05 | 0.000465 | yes | up | 0.06 | 0.13 | 0.11 | 1.49 | 1.49 | 0.43 | 0.1 | 1.136667 |
| ZNF667 | 2.364 | 1.241401 | 6.74E-05 | 0.000466 | yes | up | 0.8 | 0.52 | 0.77 | 4.59 | 3.26 | 3.82 | 0.696667 | 3.89 |
| NEK8 | 2.09 | 1.063819 | 6.79E-05 | 0.000469 | yes | up | 1.46 | 1.64 | 1.79 | 10.22 | 5.47 | 8.83 | 1.63 | 8.173333 |
| PRG4 | 0.243 | -2.0398 | 6.79E-05 | 0.000469 | yes | down | 10.78 | 4.05 | 2.68 | 3.47 | 3.95 | 2.27 | 5.836667 | 3.23 |
| CCDC30 | 3.291 | 1.71844 | 6.9E-05 | 0.000476 | yes | up | 0.27 | 0.27 | 0.35 | 2.11 | 2.08 | 2.68 | 0.296667 | 2.29 |
| IGSF10 | 2.071 | 1.050568 | 6.95E-05 | 0.000479 | yes | up | 0.99 | 1.4 | 1.01 | 5.34 | 3.84 | 7.16 | 1.133333 | 5.446667 |
| CDHR5 | 43.467 | 5.441855 | 6.96E-05 | 0.00048 | yes | up | 0 | 0 | 0.05 | 2.77 | 1.72 | 2.7 | 0.016667 | 2.396667 |
| LOC103345512 | 6.094 | 2.60735 | 7.02E-05 | 0.000484 | yes | up | 0.05 | 0.1 | 0.24 | 2.07 | 1.18 | 2.14 | 0.13 | 1.796667 |
| LOC108176107 | 0.474 | -1.07608 | 7.06E-05 | 0.000486 | yes | down | 1.52 | 2.49 | 2.26 | 2.36 | 2.39 | 2.12 | 2.09 | 2.29 |
| LOC100347239 | 7.095 | 2.826897 | 7.09E-05 | 0.000488 | yes | up | 2.37 | 1.94 | 3.09 | 91.41 | 8.87 | 28.13 | 2.466667 | 42.80333 |
| CCDC102A | 2.343 | 1.228374 | 7.16E-05 | 0.000492 | yes | up | 1.63 | 2.52 | 1.77 | 15.06 | 9.45 | 7.85 | 1.973333 | 10.78667 |
| FANCD2 | 2.247 | 1.168133 | 7.2E-05 | 0.000495 | yes | up | 1.68 | 1.62 | 1.8 | 9.07 | 11.44 | 5.64 | 1.7 | 8.716667 |
| RELB | 2.679 | 1.421929 | 7.24E-05 | 0.000497 | yes | up | 0.74 | 1.23 | 0.98 | 6.4 | 5.61 | 6.36 | 0.983333 | 6.123333 |
| LOC100352317 | 0.017 | -5.87608 | 7.28E-05 | 0.0005 | yes | down | 0.96 | 1.32 | 0.42 | 0 | 0.1 | 0 | 0.9 | 0.033333 |
| GALNT15 | 0.375 | -1.41349 | 7.39E-05 | 0.000506 | yes | down | 12.48 | 6.68 | 7.58 | 11.76 | 5.71 | 6.65 | 8.913333 | 8.04 |
| LOC108177714 | 0.364 | -1.45744 | 7.61E-05 | 0.00052 | yes | down | 22.42 | 10.6 | 16.32 | 16.68 | 20.83 | 16.16 | 16.44667 | 17.89 |
| EME1 | 3.142 | 1.651822 | 7.63E-05 | 0.000522 | yes | up | 0.26 | 0.53 | 0.55 | 4.01 | 3.33 | 2.44 | 0.446667 | 3.26 |
| PCYOX1L | 2.045 | 1.032341 | 7.65E-05 | 0.000523 | yes | up | 1.34 | 1.88 | 1.45 | 10.83 | 9.37 | 6.23 | 1.556667 | 8.81 |
| LOC100356864 | 0.179 | -2.48138 | 7.69E-05 | 0.000525 | yes | down | 11.35 | 7.98 | 23.33 | 8.25 | 1.51 | 8.5 | 14.22 | 6.086667 |
| POLQ | 7.823 | 2.967779 | 7.74E-05 | 0.000528 | yes | up | 0.02 | 0.04 | 0.02 | 0.68 | 0.57 | 0.16 | 0.026667 | 0.47 |
| MCM4 | 2.952 | 1.561851 | 7.75E-05 | 0.000529 | yes | up | 0.86 | 0.91 | 0.6 | 6.1 | 7.09 | 2.98 | 0.79 | 5.39 |
| BTNL2 | 7.704 | 2.945698 | 7.79E-05 | 0.000531 | yes | up | 0.07 | 0.48 | 0.06 | 5.5 | 2.31 | 2.62 | 0.203333 | 3.476667 |
| GDF11 | 2.208 | 1.142998 | 7.81E-05 | 0.000532 | yes | up | 0.62 | 0.86 | 0.64 | 5.62 | 3.28 | 3.4 | 0.706667 | 4.1 |
| ENC1 | 0.493 | -1.0203 | 7.9E-05 | 0.000538 | yes | down | 14.49 | 9.77 | 11.06 | 14.51 | 11.51 | 16.7 | 11.77333 | 14.24 |
| HOXB2 | 2.06 | 1.042664 | 7.91E-05 | 0.000538 | yes | up | 1.63 | 1.93 | 1.91 | 9.28 | 7.79 | 7.1 | 1.823333 | 8.056667 |
| IFRD1 | 0.448 | -1.15895 | 7.96E-05 | 0.000541 | yes | down | 10.23 | 11.97 | 6.51 | 7.78 | 11.54 | 10.19 | 9.57 | 9.836667 |
| LOC103347198 | 4.443 | 2.151533 | 8.08E-05 | 0.000548 | yes | up | 1.31 | 0.59 | 0.46 | 9.64 | 5.85 | 9.42 | 0.786667 | 8.303333 |
| VWF | 2.066 | 1.046653 | 8.08E-05 | 0.000548 | yes | up | 17.15 | 14.03 | 10.75 | 89.74 | 48.03 | 67.2 | 13.97667 | 68.32333 |
| LOC108176070 | 0.077 | -3.69957 | 8.09E-05 | 0.000549 | yes | down | 4.15 | 2.92 | 0.53 | 0.62 | 0.33 | 0.41 | 2.533333 | 0.453333 |
| KLF4 | 0.379 | -1.40058 | 8.11E-05 | 0.00055 | yes | down | 61.6 | 39.09 | 29.6 | 26.95 | 36.83 | 52.13 | 43.43 | 38.63667 |
| LOC100356307 | 2.423 | 1.276999 | 8.2E-05 | 0.000555 | yes | up | 40.99 | 86.6 | 48.01 | 297.76 | 367.95 | 151.8 | 58.53333 | 272.5033 |
| CEP152 | 2.185 | 1.12739 | 8.22E-05 | 0.000557 | yes | up | 0.35 | 0.31 | 0.4 | 1.95 | 1.63 | 1.74 | 0.353333 | 1.773333 |
| PTTG1 | 2.649 | 1.40535 | 8.25E-05 | 0.000558 | yes | up | 3.85 | 4.72 | 2.97 | 25.78 | 29.42 | 15.81 | 3.846667 | 23.67 |
| LOC108176515 | 2.192 | 1.131956 | 8.37E-05 | 0.000566 | yes | up | 1.04 | 1.01 | 1.34 | 6.15 | 5.61 | 5.6 | 1.13 | 5.786667 |
| GPC6 | 2.051 | 1.036565 | 8.6E-05 | 0.00058 | yes | up | 1.02 | 1.54 | 0.88 | 5.87 | 4.09 | 6.33 | 1.146667 | 5.43 |
| LOC100347331 | 4.249 | 2.087014 | 8.76E-05 | 0.00059 | yes | up | 0.22 | 0.83 | 0.4 | 4.06 | 5.05 | 4.93 | 0.483333 | 4.68 |
| NEGR1 | 2.509 | 1.327393 | 8.82E-05 | 0.000594 | yes | up | 0.58 | 0.71 | 0.43 | 3.58 | 2.95 | 3.45 | 0.573333 | 3.326667 |
| CRYGS | 0.171 | -2.5478 | 9.11E-05 | 0.000612 | yes | down | 5.34 | 3.11 | 2.21 | 0.74 | 1.43 | 2.01 | 3.553333 | 1.393333 |
| ZNF385A | 2.606 | 1.381983 | 9.12E-05 | 0.000612 | yes | up | 5.39 | 10.58 | 4.27 | 28.9 | 51.62 | 39.18 | 6.746667 | 39.9 |
| MEST | 2.297 | 1.199897 | 9.21E-05 | 0.000618 | yes | up | 2.09 | 3.29 | 1.34 | 13.48 | 10.46 | 11.63 | 2.24 | 11.85667 |
| LOC100348760 | 0.388 | -1.3668 | 9.39E-05 | 0.000629 | yes | down | 197.57 | 308.75 | 166.21 | 144 | 304.72 | 143.88 | 224.1767 | 197.5333 |
| CLSTN3 | 0.344 | -1.53832 | 9.42E-05 | 0.00063 | yes | down | 20.71 | 12.82 | 35.51 | 16.93 | 11.81 | 25.15 | 23.01333 | 17.96333 |
| PANK1 | 0.392 | -1.35103 | 9.48E-05 | 0.000634 | yes | down | 23.71 | 17.5 | 22.76 | 14.14 | 17.51 | 29.45 | 21.32333 | 20.36667 |
| ST8SIA5 | 0.402 | -1.31523 | 9.66E-05 | 0.000644 | yes | down | 2.45 | 3.76 | 4.22 | 2.57 | 4.66 | 2.32 | 3.476667 | 3.183333 |
| NUPR2 | 0.226 | -2.14736 | 9.67E-05 | 0.000645 | yes | down | 3.37 | 4.38 | 3.22 | 1.86 | 1.55 | 2.4 | 3.656667 | 1.936667 |
| LOC100347617 | 0.192 | -2.38173 | 9.73E-05 | 0.000648 | yes | down | 0.62 | 0.68 | 0.93 | 0.53 | 0.1 | 0.42 | 0.743333 | 0.35 |
| TMEM56 | 2.111 | 1.078056 | 9.94E-05 | 0.000662 | yes | up | 0.81 | 0.61 | 1.01 | 4.74 | 3.28 | 3.97 | 0.81 | 3.996667 |
| RPS3A | 2.167 | 1.115594 | 0.0001 | 0.000667 | yes | up | 197.26 | 228.01 | 90.99 | 907.77 | 883.88 | 851.89 | 172.0867 | 881.18 |
| PRR16 | 6.935 | 2.793962 | 0.0001 | 0.000668 | yes | up | 0 | 0.27 | 0.17 | 2.9 | 1.38 | 2.6 | 0.146667 | 2.293333 |
| LOC100348403 | 6.427 | 2.684106 | 0.000101 | 0.00067 | yes | up | 0.09 | 0.33 | 0.05 | 3.12 | 3.61 | 1.91 | 0.156667 | 2.88 |
| DDX28 | 0.36 | -1.47283 | 0.000101 | 0.000672 | yes | down | 4.06 | 4.25 | 3.15 | 3.75 | 1.97 | 3.9 | 3.82 | 3.206667 |
| PCLO | 2.412 | 1.270023 | 0.000101 | 0.000673 | yes | up | 0.25 | 0.56 | 0.99 | 4.4 | 2.42 | 3.89 | 0.6 | 3.57 |
| LOC100341595 | 27.312 | 4.771448 | 0.000103 | 0.000682 | yes | up | 0 | 0.12 | 0 | 2.55 | 1.77 | 3.05 | 0.04 | 2.456667 |
| AKAP5 | 2.843 | 1.507548 | 0.000105 | 0.000694 | yes | up | 0.25 | 0.41 | 0.37 | 2.31 | 2.81 | 1.6 | 0.343333 | 2.24 |
| MARCO | 0.061 | -4.033 | 0.000107 | 0.000703 | yes | down | 0.25 | 0.6 | 0.41 | 0.1 | 0.05 | 0.05 | 0.42 | 0.066667 |
| LOC100344547 | 0.406 | -1.30179 | 0.000108 | 0.000711 | yes | down | 1.87 | 3.1 | 2.58 | 2.34 | 2.63 | 2.11 | 2.516667 | 2.36 |
| LOC103347691 | 0.131 | -2.93173 | 0.000108 | 0.000712 | yes | down | 0.55 | 0.36 | 0.23 | 0.08 | 0.11 | 0.15 | 0.38 | 0.113333 |
| HPS6 | 2.104 | 1.073076 | 0.000108 | 0.000713 | yes | up | 0.71 | 1.22 | 1 | 5.16 | 4.54 | 4.56 | 0.976667 | 4.753333 |
| HOXD3 | 2.728 | 1.447882 | 0.000111 | 0.000728 | yes | up | 0.42 | 0.61 | 0.54 | 5.77 | 3.18 | 2.12 | 0.523333 | 3.69 |
| ARMC9 | 0.487 | -1.03939 | 0.000111 | 0.000728 | yes | down | 5.46 | 5.61 | 7.44 | 9.14 | 6.78 | 5.39 | 6.17 | 7.103333 |
| NMNAT2 | 2.152 | 1.10593 | 0.000112 | 0.000735 | yes | up | 1.45 | 1.74 | 1.34 | 10.01 | 6.13 | 7.05 | 1.51 | 7.73 |
| LOC100348097 | 4.819 | 2.268641 | 0.000112 | 0.000737 | yes | up | 0.17 | 0.25 | 0.12 | 2.13 | 0.94 | 2.93 | 0.18 | 2 |
| CHST11 | 0.321 | -1.64143 | 0.000113 | 0.000738 | yes | down | 1.63 | 3.83 | 2.7 | 1.66 | 1.42 | 2.77 | 2.72 | 1.95 |
| RORC | 9.876 | 3.303909 | 0.000113 | 0.00074 | yes | up | 3.26 | 1.5 | 2.16 | 30.23 | 14.69 | 89.7 | 2.306667 | 44.87333 |
| MEP1B | 3.436 | 1.780638 | 0.000113 | 0.000743 | yes | up | 0.31 | 0.26 | 0.27 | 3.26 | 1.46 | 1.96 | 0.28 | 2.226667 |
| CDCA5 | 2.886 | 1.528825 | 0.000114 | 0.000743 | yes | up | 0.55 | 0.73 | 0.73 | 5.15 | 5.25 | 2.92 | 0.67 | 4.44 |
| BRIP1 | 6.125 | 2.614712 | 0.000115 | 0.000753 | yes | up | 0.05 | 0.07 | 0.07 | 1.17 | 1.18 | 0.42 | 0.063333 | 0.923333 |
| THBD | 0.432 | -1.21095 | 0.000117 | 0.000762 | yes | down | 27.74 | 26.01 | 15.53 | 24.24 | 29.62 | 15.78 | 23.09333 | 23.21333 |
| RARRES1 | 3.084 | 1.624766 | 0.000117 | 0.000766 | yes | up | 1.4 | 2.08 | 0.94 | 11.88 | 6.33 | 13.46 | 1.473333 | 10.55667 |
| LOC103348672 | 0.136 | -2.87802 | 0.000118 | 0.000769 | yes | down | 3.6 | 2.68 | 1.66 | 0.7 | 1.24 | 0.45 | 2.646667 | 0.796667 |
| LOC100352639 | 4.304 | 2.1057 | 0.000118 | 0.000769 | yes | up | 0.32 | 0.76 | 0.21 | 5.22 | 6.29 | 2.76 | 0.43 | 4.756667 |
| LOC108178884 | 0.438 | -1.19151 | 0.000118 | 0.000769 | yes | down | 5.26 | 5.83 | 9.07 | 6.22 | 5.88 | 7.07 | 6.72 | 6.39 |
| TEX12 | 0.271 | -1.88605 | 0.000118 | 0.000771 | yes | down | 2.53 | 1.58 | 1.77 | 1.28 | 1.37 | 1.09 | 1.96 | 1.246667 |
| COASY | 0.498 | -1.00574 | 0.000122 | 0.000794 | yes | down | 15.82 | 20.13 | 21.42 | 21.7 | 14.62 | 29.07 | 19.12333 | 21.79667 |
| RENBP | 2.077 | 1.054611 | 0.000124 | 0.000803 | yes | up | 2.49 | 3.19 | 3.79 | 15.58 | 16.36 | 11.72 | 3.156667 | 14.55333 |
| CNTNAP5 | 0.448 | -1.15715 | 0.000127 | 0.000822 | yes | down | 9.79 | 7.59 | 7.62 | 6.86 | 10.76 | 8.5 | 8.333333 | 8.706667 |
| LZTS1 | 2.075 | 1.052775 | 0.000128 | 0.000831 | yes | up | 1.36 | 1.45 | 1.76 | 10.22 | 5.74 | 6.29 | 1.523333 | 7.416667 |
| LOC100351437 | 2.049 | 1.035017 | 0.000129 | 0.000837 | yes | up | 1.29 | 1.21 | 1.59 | 6.73 | 4.5 | 8.24 | 1.363333 | 6.49 |
| ACKR3 | 0.39 | -1.3569 | 0.000131 | 0.000849 | yes | down | 44.78 | 34.77 | 15.75 | 31.2 | 30.77 | 25.46 | 31.76667 | 29.14333 |
| SLC4A1 | 0.14 | -2.84011 | 0.000131 | 0.00085 | yes | down | 0.11 | 0.34 | 0.29 | 0.07 | 0.05 | 0.13 | 0.246667 | 0.083333 |
| ENDOU | 0.203 | -2.30234 | 0.000132 | 0.000856 | yes | down | 0.32 | 0.16 | 0.39 | 0.16 | 0.1 | 0.16 | 0.29 | 0.14 |
| LOC100357878 | 5.733 | 2.519232 | 0.000133 | 0.000857 | yes | up | 0.09 | 0.26 | 0.15 | 2.74 | 0.99 | 2.89 | 0.166667 | 2.206667 |
| MEX3B | 2.291 | 1.195972 | 0.000134 | 0.000865 | yes | up | 0.6 | 0.49 | 0.45 | 3.07 | 2.32 | 2.83 | 0.513333 | 2.74 |
| MROH2A | 42.422 | 5.406734 | 0.000134 | 0.000867 | yes | up | 0 | 0 | 0.01 | 0.46 | 0.16 | 0.26 | 0.003333 | 0.293333 |
| ALDH4A1 | 2.228 | 1.155631 | 0.000135 | 0.000869 | yes | up | 5.64 | 7.6 | 3.61 | 38.94 | 27.26 | 21.07 | 5.616667 | 29.09 |
| GABRB2 | 63.348 | 5.98523 | 0.000135 | 0.000869 | yes | up | 0 | 0 | 0 | 6.48 | 1.12 | 4.35 | 0 | 3.983333 |
| TSTD1 | 0.345 | -1.53415 | 0.000135 | 0.00087 | yes | down | 10.61 | 6.61 | 7.45 | 8.81 | 5.64 | 6.09 | 8.223333 | 6.846667 |
| ERMP1 | 2.045 | 1.032032 | 0.000135 | 0.00087 | yes | up | 1.72 | 1.55 | 1.45 | 9.15 | 8.19 | 5.22 | 1.573333 | 7.52 |
| DFFB | 0.302 | -1.72637 | 0.000136 | 0.000876 | yes | down | 16.6 | 15.36 | 22.48 | 12.67 | 6.87 | 24.59 | 18.14667 | 14.71 |
| LOC108176104 | 0.207 | -2.26883 | 0.000136 | 0.000877 | yes | down | 1.16 | 5.38 | 3.85 | 2.17 | 1.79 | 0.97 | 3.463333 | 1.643333 |
| ARNTL2 | 13.829 | 3.789625 | 0.000136 | 0.000877 | yes | up | 0.04 | 0.05 | 0.03 | 1.95 | 1.42 | 0.16 | 0.04 | 1.176667 |
| CYP2B5 | 11.573 | 3.5327 | 0.000137 | 0.000879 | yes | up | 0.3 | 0.04 | 0 | 1.7 | 3.21 | 3.71 | 0.113333 | 2.873333 |
| LOC103352131 | 2.229 | 1.156622 | 0.000137 | 0.000879 | yes | up | 5.62 | 4.1 | 3.65 | 26.32 | 19.99 | 21.4 | 4.456667 | 22.57 |
| LOC100337961 | 2.898 | 1.535195 | 0.000137 | 0.000879 | yes | up | 2.78 | 4.43 | 3.36 | 13.33 | 28.32 | 28.52 | 3.523333 | 23.39 |
| POLR3G | 2.498 | 1.320694 | 0.000138 | 0.000887 | yes | up | 0.38 | 0.47 | 0.36 | 2.34 | 2.25 | 2.43 | 0.403333 | 2.34 |
| QSOX1 | 2.06 | 1.04257 | 0.000139 | 0.000888 | yes | up | 2.99 | 3.75 | 3.74 | 12.93 | 21.77 | 14.84 | 3.493333 | 16.51333 |
| PLPPR1 | 10.132 | 3.340818 | 0.00014 | 0.000893 | yes | up | 0.03 | 0.05 | 0.05 | 1.1 | 0.81 | 1.23 | 0.043333 | 1.046667 |
| SPTB | 0.245 | -2.0263 | 0.000141 | 0.000899 | yes | down | 4.86 | 5.2 | 2.09 | 2.6 | 6.15 | 3.32 | 4.05 | 4.023333 |
| FMO2 | 3.429 | 1.777586 | 0.000142 | 0.000905 | yes | up | 7.34 | 2.07 | 1.54 | 27.18 | 21.18 | 27.06 | 3.65 | 25.14 |
| SREBF1 | 2.298 | 1.200306 | 0.000143 | 0.000911 | yes | up | 11.61 | 16.46 | 12.25 | 73.08 | 39.11 | 103.08 | 13.44 | 71.75667 |
| SLC6A1 | 0.1 | -3.31615 | 0.000144 | 0.000917 | yes | down | 0.21 | 0.36 | 0.5 | 0.04 | 0.18 | 0.02 | 0.356667 | 0.08 |
| SLC45A4 | 0.397 | -1.33252 | 0.000145 | 0.000925 | yes | down | 1.27 | 0.82 | 0.89 | 1.09 | 0.88 | 0.81 | 0.993333 | 0.926667 |
| KATNAL1 | 3.031 | 1.599655 | 0.000146 | 0.000927 | yes | up | 0.36 | 0.34 | 0.62 | 3.37 | 3.23 | 3.32 | 0.44 | 3.306667 |
| CNTNAP1 | 3.073 | 1.619742 | 0.000146 | 0.000929 | yes | up | 0.27 | 0.33 | 0.28 | 2.81 | 1.11 | 2.42 | 0.293333 | 2.113333 |
| SLC22A1 | 0.051 | -4.29487 | 0.000147 | 0.000935 | yes | down | 0.2 | 0.32 | 0.18 | 0.03 | 0.03 | 0.03 | 0.233333 | 0.03 |
| PSMC3IP | 3.633 | 1.861213 | 0.000149 | 0.000946 | yes | up | 0.56 | 0.31 | 0.3 | 3.85 | 3.93 | 2.23 | 0.39 | 3.336667 |
| LOC100342015 | 2.038 | 1.02699 | 0.000149 | 0.000947 | yes | up | 0.34 | 0.44 | 0.51 | 1.95 | 1.82 | 1.93 | 0.43 | 1.9 |
| PCSK7 | 0.487 | -1.03735 | 0.00015 | 0.000953 | yes | down | 19.59 | 12.95 | 11.58 | 17.67 | 16.73 | 16.92 | 14.70667 | 17.10667 |
| TSPAN11 | 2.277 | 1.186823 | 0.00015 | 0.000953 | yes | up | 0.56 | 1.08 | 0.74 | 5.58 | 3.71 | 3.26 | 0.793333 | 4.183333 |
| SLC26A2 | 0.424 | -1.23742 | 0.000156 | 0.000989 | yes | down | 1.18 | 1.41 | 1.07 | 1.17 | 0.88 | 1.53 | 1.22 | 1.193333 |
| LOC108177638 | 0.027 | -5.22629 | 0.000158 | 0.000997 | yes | down | 0.75 | 0.29 | 0.18 | 0.04 | 0 | 0.04 | 0.406667 | 0.026667 |
| NR5A2 | 2.454 | 1.295141 | 0.000158 | 0.000999 | yes | up | 1.07 | 0.93 | 0.5 | 4.43 | 3.68 | 6.4 | 0.833333 | 4.836667 |
| DNAJC5B | 37.116 | 5.213953 | 0.00016 | 0.00101 | yes | up | 0 | 0 | 0.02 | 0.86 | 0.68 | 0.77 | 0.006667 | 0.77 |
| NAALADL2 | 2.053 | 1.037846 | 0.000161 | 0.001012 | yes | up | 0.44 | 0.44 | 0.48 | 1.88 | 2.66 | 1.92 | 0.453333 | 2.153333 |
| NDUFAF7 | 0.495 | -1.01323 | 0.000161 | 0.001013 | yes | down | 7.13 | 8.09 | 10.83 | 12.16 | 9.96 | 7.7 | 8.683333 | 9.94 |
| ZRANB3 | 2.621 | 1.390318 | 0.000162 | 0.001018 | yes | up | 0.23 | 0.25 | 0.36 | 1.96 | 1.65 | 1.52 | 0.28 | 1.71 |
| RIMKLB | 0.066 | -3.91151 | 0.000163 | 0.001025 | yes | down | 0.11 | 0.05 | 0.1 | 0.02 | 0.03 | 0 | 0.086667 | 0.016667 |
| LOC103348324 | 8.743 | 3.128168 | 0.000165 | 0.00104 | yes | up | 0.13 | 0 | 0.35 | 4.17 | 2.57 | 3.4 | 0.16 | 3.38 |
| TACC2 | 0.347 | -1.52866 | 0.000166 | 0.001041 | yes | down | 19.23 | 26.1 | 11.55 | 13.93 | 24.31 | 15.59 | 18.96 | 17.94333 |
| SLC6A8 | 0.468 | -1.09507 | 0.000169 | 0.001059 | yes | down | 31.58 | 34.3 | 36.36 | 23.22 | 45.97 | 38.78 | 34.08 | 35.99 |
| ID1 | 0.471 | -1.08488 | 0.000171 | 0.001072 | yes | down | 30.9 | 31.72 | 30.59 | 22.65 | 33.43 | 46.01 | 31.07 | 34.03 |
| ABI3BP | 2.015 | 1.011043 | 0.000172 | 0.001079 | yes | up | 4.9 | 7.85 | 6.06 | 21.11 | 27.2 | 37.67 | 6.27 | 28.66 |
| LOC103347537 | 0.094 | -3.4141 | 0.000172 | 0.001079 | yes | down | 1.34 | 2.16 | 1.56 | 0.37 | 0.39 | 0.35 | 1.686667 | 0.37 |
| EPHA3 | 2.013 | 1.009311 | 0.000173 | 0.001083 | yes | up | 0.76 | 1.04 | 0.68 | 4.94 | 3.63 | 3.02 | 0.826667 | 3.863333 |
| LSR | 2.08 | 1.056264 | 0.000173 | 0.001083 | yes | up | 1.47 | 1.11 | 1.35 | 6.94 | 5.05 | 7.06 | 1.31 | 6.35 |
| NLRC5 | 5.78 | 2.531123 | 0.000174 | 0.001086 | yes | up | 0.02 | 0.04 | 0.06 | 0.46 | 0.4 | 0.72 | 0.04 | 0.526667 |
| ANKEF1 | 2.973 | 1.572102 | 0.000176 | 0.001097 | yes | up | 1.06 | 0.64 | 0.92 | 9.17 | 3.27 | 6.1 | 0.873333 | 6.18 |
| TRNAK-CUU | 0.079 | -3.67031 | 0.000176 | 0.001097 | yes | down | 3.82 | 0.85 | 1.87 | 0.54 | 0.17 | 0.52 | 2.18 | 0.41 |
| FERMT1 | 0.259 | -1.94841 | 0.000176 | 0.0011 | yes | down | 1.05 | 0.94 | 1.91 | 1.02 | 0.32 | 1.04 | 1.3 | 0.793333 |
| GLIS3 | 0.343 | -1.54387 | 0.000177 | 0.001102 | yes | down | 0.41 | 0.41 | 0.41 | 0.22 | 0.36 | 0.39 | 0.41 | 0.323333 |
| DNER | 0.084 | -3.5693 | 0.000182 | 0.001132 | yes | down | 0.21 | 0.28 | 0.22 | 0.07 | 0 | 0.07 | 0.236667 | 0.046667 |
| LOC103347403 | 4.709 | 2.235316 | 0.000183 | 0.001138 | yes | up | 0.59 | 0.21 | 0.2 | 5.65 | 2.23 | 4.11 | 0.333333 | 3.996667 |
| GMNN | 2.305 | 1.204743 | 0.000184 | 0.001142 | yes | up | 4.92 | 5.53 | 5.09 | 35.87 | 33.56 | 16.98 | 5.18 | 28.80333 |
| SORCS1 | 0.304 | -1.71861 | 0.000184 | 0.001147 | yes | down | 0.36 | 0.29 | 0.33 | 0.17 | 0.33 | 0.2 | 0.326667 | 0.233333 |
| PYCR1 | 2.263 | 1.177962 | 0.000185 | 0.001148 | yes | up | 1.43 | 2.47 | 1.67 | 11.38 | 7.6 | 10.22 | 1.856667 | 9.733333 |
| LAMA3 | 2.312 | 1.208926 | 0.000185 | 0.001149 | yes | up | 0.86 | 0.93 | 1.2 | 8.08 | 4.23 | 3.97 | 0.996667 | 5.426667 |
| PGM2L1 | 2.692 | 1.428785 | 0.000187 | 0.00116 | yes | up | 0.21 | 0.25 | 0.3 | 1.67 | 1.56 | 1.54 | 0.253333 | 1.59 |
| RNF24 | 0.403 | -1.31292 | 0.000189 | 0.001173 | yes | down | 4.24 | 6.84 | 7.88 | 6.54 | 3.56 | 7.78 | 6.32 | 5.96 |
| RXRG | 0.358 | -1.48303 | 0.00019 | 0.001175 | yes | down | 77.05 | 77.02 | 96.99 | 64.6 | 20.74 | 82.88 | 83.68667 | 56.07333 |
| LOC103348410 | 2.841 | 1.50644 | 0.000191 | 0.001185 | yes | up | 0.41 | 0.35 | 0.32 | 2.74 | 1.62 | 2.32 | 0.36 | 2.226667 |
| PIM1 | 0.351 | -1.50931 | 0.000194 | 0.001201 | yes | down | 20.94 | 9.32 | 11.55 | 7.67 | 15.79 | 11.08 | 13.93667 | 11.51333 |
| MRPL48 | 0.401 | -1.32001 | 0.000196 | 0.001211 | yes | down | 9.78 | 27.74 | 22.79 | 15.83 | 16.58 | 23.15 | 20.10333 | 18.52 |
| LOC103349185 | 2.056 | 1.039717 | 0.000198 | 0.001223 | yes | up | 0.55 | 0.73 | 0.69 | 3.85 | 3.55 | 2.04 | 0.656667 | 3.146667 |
| PKDCC | 2.067 | 1.047821 | 0.000198 | 0.001225 | yes | up | 3.48 | 8.34 | 6.69 | 32.3 | 24.13 | 31.5 | 6.17 | 29.31 |
| DOK4 | 2.418 | 1.273588 | 0.000201 | 0.00124 | yes | up | 2.51 | 6.15 | 5.24 | 35.25 | 24.68 | 17.69 | 4.633333 | 25.87333 |
| CCDC28A | 3.247 | 1.699027 | 0.000204 | 0.001259 | yes | up | 1.42 | 0.59 | 0.71 | 7.79 | 7.56 | 5.55 | 0.906667 | 6.966667 |
| ARHGEF39 | 6.115 | 2.612296 | 0.000205 | 0.001262 | yes | up | 0.18 | 0.21 | 0.27 | 4.45 | 3.73 | 1.17 | 0.22 | 3.116667 |
| PPFIA4 | 0.088 | -3.50897 | 0.000206 | 0.001268 | yes | down | 0.58 | 0.82 | 0.09 | 0.11 | 0.18 | 0.02 | 0.496667 | 0.103333 |
| LOC100351785 | 2.845 | 1.508579 | 0.000208 | 0.001276 | yes | up | 0.36 | 1.06 | 0.92 | 7.09 | 4.1 | 4.29 | 0.78 | 5.16 |
| VAMP5 | 2.386 | 1.254307 | 0.000209 | 0.001283 | yes | up | 5.48 | 11.26 | 7.7 | 52.62 | 52.55 | 29.84 | 8.146667 | 45.00333 |
| PDE6A | 0.085 | -3.56082 | 0.000212 | 0.001301 | yes | down | 0.06 | 0.33 | 0.33 | 0.05 | 0.02 | 0.07 | 0.24 | 0.046667 |
| KRTCAP3 | 0.021 | -5.53973 | 0.000213 | 0.001307 | yes | down | 0.66 | 1.04 | 0.51 | 0 | 0.11 | 0 | 0.736667 | 0.036667 |
| LOC103345465 | 4.383 | 2.131768 | 0.000213 | 0.001307 | yes | up | 0.26 | 0.29 | 0.18 | 2.52 | 1.24 | 1.73 | 0.243333 | 1.83 |
| E2F7 | 4.953 | 2.308355 | 0.000214 | 0.001311 | yes | up | 0.08 | 0.08 | 0.02 | 0.72 | 0.76 | 0.52 | 0.06 | 0.666667 |
| LOC100348052 | 671.662 | 9.391592 | 0.000214 | 0.001314 | yes | up | 0 | 0 | 0 | 38.6 | 0.38 | 36.39 | 0 | 25.12333 |
| LOC103347778 | 6.535 | 2.708203 | 0.000217 | 0.001328 | yes | up | 0.4 | 1.73 | 1.8 | 13.85 | 25.89 | 27.41 | 1.31 | 22.38333 |
| LOC103350763 | 19.137 | 4.258288 | 0.000218 | 0.001331 | yes | up | 0.04 | 0.07 | 0.06 | 2.75 | 3.29 | 0.13 | 0.056667 | 2.056667 |
| HMMR | 7.869 | 2.976233 | 0.000223 | 0.001358 | yes | up | 0 | 0.18 | 0.17 | 2.88 | 2.59 | 0.86 | 0.116667 | 2.11 |
| NXPH2 | 2.898 | 1.535035 | 0.000224 | 0.001368 | yes | up | 0.46 | 0.36 | 0.39 | 3.92 | 2.17 | 2.16 | 0.403333 | 2.75 |
| SUV39H2 | 3.062 | 1.614702 | 0.000226 | 0.001375 | yes | up | 0.27 | 0.3 | 0.5 | 2.87 | 2.93 | 1.78 | 0.356667 | 2.526667 |
| VDR | 3.517 | 1.814229 | 0.000229 | 0.001394 | yes | up | 0.97 | 1.34 | 1.27 | 6.67 | 6.13 | 15.78 | 1.193333 | 9.526667 |
| C11H5orf30 | 2.007 | 1.005221 | 0.000232 | 0.001408 | yes | up | 1.83 | 2.25 | 1.88 | 9.1 | 6.74 | 11.96 | 1.986667 | 9.266667 |
| C1S | 2.238 | 1.162428 | 0.000232 | 0.001409 | yes | up | 16.77 | 12.95 | 17.08 | 52.25 | 75.98 | 115.44 | 15.6 | 81.22333 |
| ERCC6L | 6.47 | 2.693763 | 0.000232 | 0.00141 | yes | up | 0.02 | 0.06 | 0.07 | 1 | 1.03 | 0.29 | 0.05 | 0.773333 |
| C14H3orf33 | 0.417 | -1.26342 | 0.000233 | 0.00141 | yes | down | 2.26 | 3.12 | 2.74 | 2.15 | 2.96 | 2.7 | 2.706667 | 2.603333 |
| MLANA | 5.105 | 2.351895 | 0.000233 | 0.001414 | yes | up | 0.98 | 0.8 | 1.6 | 5.34 | 22.48 | 14.35 | 1.126667 | 14.05667 |
| YPEL4 | 0.298 | -1.74559 | 0.000234 | 0.001416 | yes | down | 0.48 | 0.77 | 0.72 | 0.51 | 0.38 | 0.53 | 0.656667 | 0.473333 |
| LOC103350569 | 4.102 | 2.036378 | 0.000238 | 0.001439 | yes | up | 0.63 | 0.61 | 0.43 | 4.47 | 7.43 | 4 | 0.556667 | 5.3 |
| PLLP | 0.223 | -2.16298 | 0.000243 | 0.001464 | yes | down | 1.1 | 1.68 | 2.5 | 0.59 | 1.51 | 0.62 | 1.76 | 0.906667 |
| LOC100340502 | 0.321 | -1.63897 | 0.000244 | 0.001469 | yes | down | 0.44 | 0.78 | 0.75 | 0.45 | 0.42 | 0.6 | 0.656667 | 0.49 |
| LOC103347364 | 0.435 | -1.20075 | 0.000245 | 0.001473 | yes | down | 2.44 | 3.19 | 3.59 | 3.19 | 2.89 | 3.28 | 3.073333 | 3.12 |
| LOC108178739 | 0.014 | -6.18166 | 0.000245 | 0.001474 | yes | down | 0.07 | 0.02 | 0.06 | 0 | 0 | 0 | 0.05 | 0 |
| TLR5 | 3.004 | 1.586978 | 0.000247 | 0.001484 | yes | up | 0.26 | 0.22 | 0.31 | 1.81 | 2.36 | 1.37 | 0.263333 | 1.846667 |
| USP2 | 0.218 | -2.19709 | 0.000252 | 0.001512 | yes | down | 6.14 | 5.52 | 1.27 | 1.43 | 3.94 | 1.43 | 4.31 | 2.266667 |
| ABCC2 | 3.642 | 1.864622 | 0.000254 | 0.001524 | yes | up | 0.17 | 0.1 | 0.07 | 0.87 | 1.06 | 0.94 | 0.113333 | 0.956667 |
| SFRP2 | 5.601 | 2.485773 | 0.000259 | 0.001552 | yes | up | 0.37 | 0.32 | 0.37 | 3.71 | 8.21 | 1.82 | 0.353333 | 4.58 |
| LOC103346775 | 2.994 | 1.582132 | 0.000262 | 0.001565 | yes | up | 0.5 | 0.87 | 1.28 | 8.65 | 7.25 | 4.2 | 0.883333 | 6.7 |
| TIMELESS | 2.245 | 1.166582 | 0.000265 | 0.001579 | yes | up | 0.5 | 1.03 | 0.81 | 4.37 | 4.26 | 2.79 | 0.78 | 3.806667 |
| SEZ6L2 | 9.102 | 3.18619 | 0.000266 | 0.001583 | yes | up | 0.11 | 0.08 | 0.08 | 4.85 | 0.55 | 0.47 | 0.09 | 1.956667 |
| LOC100346342 | 3.093 | 1.628904 | 0.000266 | 0.001583 | yes | up | 0.24 | 0.23 | 0.83 | 2.99 | 3.2 | 2.34 | 0.433333 | 2.843333 |
| SFRP5 | 6.275 | 2.649559 | 0.000266 | 0.001583 | yes | up | 2.04 | 2.39 | 4.08 | 35.35 | 74.29 | 13.88 | 2.836667 | 41.17333 |
| KIF5C | 0.352 | -1.50481 | 0.000267 | 0.001588 | yes | down | 0.34 | 0.47 | 0.59 | 0.38 | 0.46 | 0.3 | 0.466667 | 0.38 |
| IFI44 | 2.943 | 1.557239 | 0.00027 | 0.001604 | yes | up | 2.02 | 1.89 | 1.33 | 11.91 | 5.46 | 17.85 | 1.746667 | 11.74 |
| TECRL | 0.004 | -7.81711 | 0.000274 | 0.001629 | yes | down | 6.75 | 4.83 | 0.08 | 0 | 0.13 | 0 | 3.886667 | 0.043333 |
| RBPMS2 | 0.477 | -1.0668 | 0.000275 | 0.00163 | yes | down | 15.87 | 11.3 | 8.33 | 14.25 | 11.47 | 14.72 | 11.83333 | 13.48 |
| PAQR7 | 2.143 | 1.099668 | 0.00028 | 0.00166 | yes | up | 0.77 | 1.32 | 1.17 | 5.44 | 5.99 | 4.73 | 1.086667 | 5.386667 |
| LOC103349409 | 31.12 | 4.959761 | 0.000284 | 0.001679 | yes | up | 0 | 0.08 | 0.1 | 5.6 | 0.16 | 6.18 | 0.06 | 3.98 |
| LOC100342121 | 0.046 | -4.44357 | 0.000284 | 0.001681 | yes | down | 1.51 | 9.3 | 2.65 | 0 | 1.28 | 0 | 4.486667 | 0.426667 |
| LOC100344587 | 0.216 | -2.21128 | 0.000284 | 0.001681 | yes | down | 1.14 | 3.58 | 0.91 | 0.74 | 1.18 | 0.79 | 1.876667 | 0.903333 |
| LOC100345657 | 5.181 | 2.373155 | 0.000285 | 0.001684 | yes | up | 0.08 | 0.15 | 0.12 | 1.12 | 0.43 | 2.64 | 0.116667 | 1.396667 |
| ADAL | 0.439 | -1.18771 | 0.00029 | 0.001709 | yes | down | 1.87 | 3.18 | 2.05 | 2.09 | 2.37 | 2.45 | 2.366667 | 2.303333 |
| BMP3 | 0.293 | -1.77297 | 0.00029 | 0.001712 | yes | down | 0.59 | 0.96 | 1.16 | 0.27 | 0.87 | 0.67 | 0.903333 | 0.603333 |
| HS3ST6 | 3.767 | 1.913454 | 0.0003 | 0.001767 | yes | up | 0.15 | 0.27 | 0.29 | 3.02 | 1.31 | 1.91 | 0.236667 | 2.08 |
| LOC103346961 | 10.695 | 3.418907 | 0.000303 | 0.001781 | yes | up | 0.05 | 0.09 | 0.08 | 0.91 | 2.45 | 2.04 | 0.073333 | 1.8 |
| LOC103348141 | 0.483 | -1.04927 | 0.000306 | 0.001795 | yes | down | 4.04 | 2.86 | 4.18 | 5.54 | 3.48 | 5.42 | 3.693333 | 4.813333 |
| UGT8 | 0.149 | -2.74928 | 0.000306 | 0.001797 | yes | down | 0.74 | 1.74 | 2.1 | 0.17 | 1.18 | 0.13 | 1.526667 | 0.493333 |
| LOC108177708 | 0.396 | -1.33717 | 0.000311 | 0.001826 | yes | down | 37.61 | 23.97 | 32.21 | 19.24 | 32.48 | 36.8 | 31.26333 | 29.50667 |
| SBSPON | 0.479 | -1.06066 | 0.000312 | 0.001826 | yes | down | 2.86 | 4.63 | 3.21 | 3.19 | 4.88 | 3.66 | 3.566667 | 3.91 |
| SLC9A3R1 | 2.376 | 1.248365 | 0.000316 | 0.00185 | yes | up | 0.93 | 1.31 | 1.63 | 9.07 | 4.84 | 7.29 | 1.29 | 7.066667 |
| LOC100352781 | 0.015 | -6.10747 | 0.000319 | 0.001867 | yes | down | 1.12 | 0.48 | 1.23 | 0 | 0 | 0.01 | 0.943333 | 0.003333 |
| CENPM | 4.469 | 2.159939 | 0.00032 | 0.001869 | yes | up | 0.73 | 0.22 | 0.28 | 6.81 | 5.11 | 3.05 | 0.41 | 4.99 |
| LOC100345961 | 0.496 | -1.01165 | 0.000321 | 0.001872 | yes | down | 3.43 | 2.76 | 4.59 | 4.46 | 4.11 | 4 | 3.593333 | 4.19 |
| OASL | 4.416 | 2.142816 | 0.000321 | 0.001872 | yes | up | 0.67 | 0.48 | 0.87 | 7 | 2.06 | 12.03 | 0.673333 | 7.03 |
| AMOTL2 | 0.471 | -1.08576 | 0.000321 | 0.001874 | yes | down | 24.72 | 15.54 | 21.84 | 17.96 | 19.55 | 31.22 | 20.7 | 22.91 |
| LOC103348853 | 8.442 | 3.077511 | 0.000321 | 0.001876 | yes | up | 0.11 | 0.04 | 0.07 | 1.45 | 0.97 | 1.79 | 0.073333 | 1.403333 |
| BCORL1 | 2.23 | 1.156979 | 0.000327 | 0.001905 | yes | up | 0.74 | 2.06 | 2.53 | 10.73 | 4.67 | 10.22 | 1.776667 | 8.54 |
| FASTKD5 | 0.476 | -1.07228 | 0.000327 | 0.001905 | yes | down | 2.48 | 5.12 | 4.38 | 3.55 | 4.17 | 5.21 | 3.993333 | 4.31 |
| MCM10 | 6.77 | 2.759108 | 0.000327 | 0.001905 | yes | up | 0.04 | 0.05 | 0.07 | 0.9 | 1.35 | 0.32 | 0.053333 | 0.856667 |
| TP63 | 0.334 | -1.58058 | 0.000327 | 0.001907 | yes | down | 0.66 | 0.96 | 0.42 | 0.39 | 0.38 | 0.29 | 0.68 | 0.353333 |
| LOC103346296 | 32.38 | 5.017036 | 0.000329 | 0.001915 | yes | up | 0.07 | 0 | 0.04 | 8.07 | 0.04 | 2.02 | 0.036667 | 3.376667 |
| CDH20 | 4.917 | 2.29769 | 0.000332 | 0.001933 | yes | up | 0.17 | 0.04 | 0.08 | 1.19 | 0.72 | 1.51 | 0.096667 | 1.14 |
| LOC108175394 | 3.106 | 1.634889 | 0.000333 | 0.001934 | yes | up | 0.6 | 0.46 | 0.31 | 6.78 | 1.88 | 5.71 | 0.456667 | 4.79 |
| DTWD1 | 2.048 | 1.033989 | 0.000334 | 0.001941 | yes | up | 6.14 | 7.24 | 6.3 | 32 | 20.32 | 23.27 | 6.56 | 25.19667 |
| DDIT4 | 0.487 | -1.03848 | 0.000342 | 0.00198 | yes | down | 102.2 | 179 | 80.24 | 182.61 | 115.62 | 109.01 | 120.48 | 135.7467 |
| BAG3 | 0.472 | -1.0842 | 0.000345 | 0.001996 | yes | down | 42.44 | 40.03 | 27.98 | 28.44 | 35.88 | 56.6 | 36.81667 | 40.30667 |
| LOC103348580 | 12.031 | 3.588722 | 0.000349 | 0.002021 | yes | up | 0.36 | 0.49 | 0.34 | 0.31 | 11.78 | 21.05 | 0.396667 | 11.04667 |
| SH3BGRL2 | 3.039 | 1.603804 | 0.000351 | 0.002028 | yes | up | 0.63 | 0.67 | 0.47 | 4.98 | 3.49 | 2.08 | 0.59 | 3.516667 |
| SPC25 | 4.346 | 2.119617 | 0.000352 | 0.002032 | yes | up | 0.53 | 0.59 | 0.33 | 4.89 | 6.38 | 3.23 | 0.483333 | 4.833333 |
| FAM110C | 0.462 | -1.11263 | 0.000352 | 0.002034 | yes | down | 2.55 | 2.32 | 4.08 | 2.99 | 2.54 | 3.88 | 2.983333 | 3.136667 |
| EMID1 | 3.538 | 1.823109 | 0.000356 | 0.002052 | yes | up | 0.66 | 0.84 | 1.18 | 10.89 | 11.47 | 5.25 | 0.893333 | 9.203333 |
| CXCL10 | 4.397 | 2.136368 | 0.00036 | 0.002075 | yes | up | 2.67 | 2.84 | 0.49 | 16.17 | 11.71 | 33.44 | 2 | 20.44 |
| LOC100358368 | 0.409 | -1.29063 | 0.000363 | 0.002087 | yes | down | 312.2 | 474.14 | 253.24 | 232.34 | 509.23 | 223.85 | 346.5267 | 321.8067 |
| DYRK3 | 3.646 | 1.86623 | 0.00037 | 0.002124 | yes | up | 0.25 | 0.22 | 0.18 | 2.39 | 1.59 | 1.51 | 0.216667 | 1.83 |
| LOC103348990 | 0.166 | -2.58878 | 0.00037 | 0.002125 | yes | down | 1.14 | 1.02 | 2.06 | 0.79 | 0.67 | 0.2 | 1.406667 | 0.553333 |
| CDC25A | 5.569 | 2.477446 | 0.000373 | 0.002138 | yes | up | 0.09 | 0.07 | 0.08 | 1.36 | 1.32 | 0.51 | 0.08 | 1.063333 |
| LOC103352286 | 5.367 | 2.423998 | 0.000378 | 0.002164 | yes | up | 0.12 | 0.13 | 0.81 | 4.34 | 4.96 | 3.99 | 0.353333 | 4.43 |
| SDC4 | 0.369 | -1.4385 | 0.000379 | 0.002171 | yes | down | 116.54 | 49.25 | 43.34 | 46.12 | 64.75 | 72.32 | 69.71 | 61.06333 |
| MMP28 | 2.228 | 1.15565 | 0.00038 | 0.002172 | yes | up | 4.81 | 7.59 | 9.9 | 49.72 | 22.14 | 42.93 | 7.433333 | 38.26333 |
| PGER8 | 2.196 | 1.134686 | 0.000383 | 0.00219 | yes | up | 1.34 | 3.3 | 1.84 | 12.58 | 9.78 | 11.02 | 2.16 | 11.12667 |
| CAND2 | 0.275 | -1.86407 | 0.000383 | 0.002191 | yes | down | 2.88 | 2.99 | 1.11 | 1.09 | 2.52 | 0.79 | 2.326667 | 1.466667 |
| APITD1 | 3.726 | 1.897516 | 0.000386 | 0.002208 | yes | up | 1.01 | 2.05 | 1.2 | 11.07 | 17.89 | 5.94 | 1.42 | 11.63333 |
| CASQ2 | 0.059 | -4.07942 | 0.000392 | 0.002236 | yes | down | 40.38 | 53.95 | 2.83 | 1.57 | 7.53 | 3.79 | 32.38667 | 4.296667 |
| RPS29 | 0.36 | -1.47208 | 0.000395 | 0.00225 | yes | down | 119.51 | 58.79 | 84.01 | 99.29 | 83.59 | 53.73 | 87.43667 | 78.87 |
| LOC108175609 | 3.777 | 1.917248 | 0.000397 | 0.00226 | yes | up | 0.19 | 0.27 | 0.43 | 4.38 | 1.45 | 2.18 | 0.296667 | 2.67 |
| GEN1 | 4.52 | 2.176317 | 0.000401 | 0.002281 | yes | up | 0.09 | 0.05 | 0.1 | 0.97 | 1.01 | 0.48 | 0.08 | 0.82 |
| TBC1D30 | 0.344 | -1.54122 | 0.000402 | 0.002285 | yes | down | 0.57 | 1.13 | 0.73 | 0.7 | 0.31 | 0.92 | 0.81 | 0.643333 |
| PRDM1 | 3.373 | 1.754118 | 0.000403 | 0.002293 | yes | up | 0.25 | 0.29 | 0.15 | 2.25 | 0.81 | 2.32 | 0.23 | 1.793333 |
| CCNF | 4.197 | 2.069464 | 0.000412 | 0.002333 | yes | up | 0.17 | 0.35 | 0.4 | 4.7 | 3.27 | 1.01 | 0.306667 | 2.993333 |
| LOC103346901 | 3.519 | 1.815153 | 0.000412 | 0.002335 | yes | up | 4.46 | 3.23 | 2.27 | 18.05 | 29.02 | 37.64 | 3.32 | 28.23667 |
| IL17RD | 2.254 | 1.172619 | 0.000413 | 0.002342 | yes | up | 0.37 | 0.52 | 0.27 | 2.48 | 1.75 | 1.85 | 0.386667 | 2.026667 |
| MANBA | 2.131 | 1.091575 | 0.000419 | 0.002368 | yes | up | 0.48 | 0.56 | 0.32 | 2.23 | 2.01 | 2.15 | 0.453333 | 2.13 |
| HAS1 | 0.044 | -4.51273 | 0.000421 | 0.00238 | yes | down | 2.2 | 0.21 | 0.12 | 0.11 | 0.12 | 0.05 | 0.843333 | 0.093333 |
| NUP35 | 0.496 | -1.01142 | 0.000424 | 0.002394 | yes | down | 5.4 | 11.46 | 9.88 | 9.76 | 11.52 | 9.1 | 8.913333 | 10.12667 |
| LOC100350502 | 0.371 | -1.42989 | 0.000425 | 0.002399 | yes | down | 1.15 | 0.92 | 1.23 | 1.04 | 0.87 | 0.97 | 1.1 | 0.96 |
| LOC103349250 | 0.094 | -3.41677 | 0.000427 | 0.002408 | yes | down | 0.4 | 0.37 | 0.31 | 0.07 | 0.12 | 0.14 | 0.36 | 0.11 |
| MAGI2 | 0.365 | -1.45518 | 0.000427 | 0.002409 | yes | down | 0.37 | 0.41 | 0.64 | 0.5 | 0.31 | 0.39 | 0.473333 | 0.4 |
| FAAP24 | 3.051 | 1.609401 | 0.00043 | 0.002422 | yes | up | 0.6 | 0.38 | 0.36 | 3.65 | 3.44 | 2.25 | 0.446667 | 3.113333 |
| NPL | 0.363 | -1.46302 | 0.00043 | 0.002423 | yes | down | 7.78 | 7.17 | 11.71 | 5.43 | 4.42 | 10.7 | 8.886667 | 6.85 |
| SLCO2B1 | 3.76 | 1.91088 | 0.000431 | 0.002428 | yes | up | 0.34 | 0.54 | 0.26 | 5.56 | 1.19 | 3.3 | 0.38 | 3.35 |
| RBM47 | 3.924 | 1.972248 | 0.000432 | 0.002431 | yes | up | 0.12 | 0.3 | 0.17 | 1.54 | 0.83 | 2.9 | 0.196667 | 1.756667 |
| LOC103346127 | 3.59 | 1.844115 | 0.00044 | 0.002473 | yes | up | 0.4 | 0.38 | 0.64 | 7.17 | 3.08 | 2.17 | 0.473333 | 4.14 |
| PHLDA1 | 0.39 | -1.35972 | 0.000441 | 0.002476 | yes | down | 9.67 | 4.63 | 3.7 | 5.68 | 4.46 | 6.91 | 6 | 5.683333 |
| LOC103347914 | 16.46 | 4.040931 | 0.000447 | 0.002508 | yes | up | 0.11 | 0 | 0.19 | 3.57 | 1.03 | 4.23 | 0.1 | 2.943333 |
| LOC100359146 | 0.3 | -1.7387 | 0.000447 | 0.002508 | yes | down | 0.67 | 0.89 | 0.62 | 0.49 | 0.35 | 0.66 | 0.726667 | 0.5 |
| APOLD1 | 0.121 | -3.04694 | 0.000449 | 0.002516 | yes | down | 319.75 | 106.1 | 34.92 | 51.74 | 37.41 | 52.46 | 153.59 | 47.20333 |
| LOC103345596 | 48.844 | 5.610102 | 0.000453 | 0.002536 | yes | up | 0 | 0 | 0 | 1.28 | 2.12 | 0.61 | 0 | 1.336667 |
| LOC103350250 | 0.186 | -2.42571 | 0.00046 | 0.002576 | yes | down | 0.44 | 0.65 | 0.49 | 0.27 | 0.19 | 0.22 | 0.526667 | 0.226667 |
| SGK1 | 0.124 | -3.01728 | 0.000461 | 0.002579 | yes | down | 286.46 | 115.49 | 58.09 | 83.76 | 23.79 | 37.67 | 153.3467 | 48.40667 |
| PAQR9 | 0.217 | -2.204 | 0.000463 | 0.002586 | yes | down | 0.29 | 0.4 | 0.65 | 0.17 | 0.08 | 0.42 | 0.446667 | 0.223333 |
| PGR | 5.421 | 2.438615 | 0.000465 | 0.002594 | yes | up | 0.04 | 0.17 | 0.05 | 1.51 | 0.76 | 0.98 | 0.086667 | 1.083333 |
| CLK1 | 2.168 | 1.1166 | 0.000479 | 0.002667 | yes | up | 27.67 | 45.48 | 67.22 | 159.1 | 248.64 | 239.44 | 46.79 | 215.7267 |
| UBXN11 | 0.432 | -1.21186 | 0.000488 | 0.002716 | yes | down | 2.04 | 2.46 | 3.4 | 2.6 | 2.83 | 2.76 | 2.633333 | 2.73 |
| CENPN | 2.546 | 1.348318 | 0.000496 | 0.002754 | yes | up | 1.44 | 1.69 | 1.1 | 8.78 | 11.19 | 4.88 | 1.41 | 8.283333 |
| B3GALNT1 | 2.96 | 1.565527 | 0.000498 | 0.002766 | yes | up | 0.61 | 1.66 | 1.87 | 5.61 | 9.68 | 12.07 | 1.38 | 9.12 |
| LOC103349541 | 0.424 | -1.23931 | 0.000501 | 0.002779 | yes | down | 2.39 | 1.88 | 2.01 | 1.96 | 2.26 | 2.5 | 2.093333 | 2.24 |
| MYF5 | 0.037 | -4.76194 | 0.000502 | 0.002781 | yes | down | 1.57 | 0.87 | 0.11 | 0 | 0.22 | 0 | 0.85 | 0.073333 |
| LOC108177072 | 6.898 | 2.786243 | 0.000502 | 0.002783 | yes | up | 0.16 | 0.05 | 0.04 | 2.4 | 1.5 | 2.43 | 0.083333 | 2.11 |
| ABLIM2 | 0.144 | -2.79722 | 0.000503 | 0.002786 | yes | down | 7.89 | 4.02 | 2.65 | 0.51 | 3.8 | 1.15 | 4.853333 | 1.82 |
| ACOT7 | 2.03 | 1.021829 | 0.00051 | 0.002819 | yes | up | 5.73 | 5.04 | 4.1 | 29.94 | 24.54 | 16.16 | 4.956667 | 23.54667 |
| FANCE | 2.843 | 1.507606 | 0.000515 | 0.002841 | yes | up | 0.49 | 0.53 | 0.48 | 4.52 | 3.4 | 2 | 0.5 | 3.306667 |
| PIK3R6 | 3.277 | 1.712529 | 0.000521 | 0.002866 | yes | up | 0.23 | 0.74 | 0.87 | 7.25 | 3.58 | 3.79 | 0.613333 | 4.873333 |
| CYTIP | 2.328 | 1.219151 | 0.000522 | 0.002876 | yes | up | 0.58 | 0.61 | 0.72 | 4.18 | 3.5 | 2.82 | 0.636667 | 3.5 |
| RRM2 | 4.105 | 2.037389 | 0.000525 | 0.002887 | yes | up | 0.86 | 1.48 | 0.42 | 9.77 | 13.08 | 2.9 | 0.92 | 8.583333 |
| REM1 | 2.207 | 1.141996 | 0.000527 | 0.002895 | yes | up | 1.69 | 2.64 | 1.59 | 15.32 | 7.75 | 9.17 | 1.973333 | 10.74667 |
| LOC103348241 | 0.056 | -4.16093 | 0.000535 | 0.002934 | yes | down | 118.62 | 7.79 | 122.26 | 12.64 | 2.58 | 19.34 | 82.89 | 11.52 |
| MSR1 | 0.39 | -1.35764 | 0.000537 | 0.002946 | yes | down | 1.63 | 1.54 | 1.42 | 2.06 | 0.86 | 2.01 | 1.53 | 1.643333 |
| IDO1 | 0.37 | -1.43613 | 0.000538 | 0.002951 | yes | down | 5.64 | 4.28 | 3.11 | 2.61 | 3.17 | 5.73 | 4.343333 | 3.836667 |
| PHEX | 0.241 | -2.05362 | 0.000544 | 0.002982 | yes | down | 0.23 | 0.15 | 0.16 | 0.09 | 0.12 | 0.1 | 0.18 | 0.103333 |
| TRMT61A | 0.462 | -1.11422 | 0.000545 | 0.002986 | yes | down | 2.43 | 5.83 | 5.78 | 4.72 | 4.13 | 6 | 4.68 | 4.95 |
| DSN1 | 4.405 | 2.139214 | 0.000552 | 0.00302 | yes | up | 0.42 | 0.84 | 0.18 | 5.76 | 4.94 | 1.65 | 0.48 | 4.116667 |
| LPXN | 2.358 | 1.237826 | 0.000559 | 0.003055 | yes | up | 0.62 | 0.86 | 1.06 | 4.9 | 3.51 | 4.95 | 0.846667 | 4.453333 |
| LOC103345583 | 0.017 | -5.84757 | 0.000564 | 0.003082 | yes | down | 0.22 | 0.37 | 0.33 | 0 | 0 | 0 | 0.306667 | 0 |
| ALOX5 | 2.018 | 1.012826 | 0.000569 | 0.003105 | yes | up | 3.08 | 4.52 | 2.46 | 15.28 | 19.38 | 11.86 | 3.353333 | 15.50667 |
| LOC108178917 | 0.325 | -1.62347 | 0.000571 | 0.003109 | yes | down | 1.2 | 1.46 | 1.1 | 0.85 | 0.46 | 0.85 | 1.253333 | 0.72 |
| NOX5 | 4.009 | 2.003065 | 0.000584 | 0.003179 | yes | up | 0.44 | 0.85 | 0.59 | 5.77 | 1.92 | 10.28 | 0.626667 | 5.99 |
| MTHFS | 2.137 | 1.095715 | 0.000593 | 0.003221 | yes | up | 2.49 | 5.2 | 3.32 | 19.23 | 20.55 | 13.87 | 3.67 | 17.88333 |
| LOC100339259 | 0.441 | -1.17984 | 0.000599 | 0.003252 | yes | down | 29.16 | 34.15 | 54.47 | 61.92 | 24.19 | 37.1 | 39.26 | 41.07 |
| KIAA1524 | 3.961 | 1.985862 | 0.0006 | 0.003259 | yes | up | 0.12 | 0.08 | 0.26 | 1.55 | 1.59 | 1.14 | 0.153333 | 1.426667 |
| KLHDC7A | 0.126 | -2.988 | 0.000601 | 0.003263 | yes | down | 0.11 | 0.06 | 0.18 | 0.03 | 0.02 | 0.05 | 0.116667 | 0.033333 |
| SLC2A12 | 2.449 | 1.291971 | 0.000603 | 0.00327 | yes | up | 0.74 | 1.14 | 1.79 | 5.4 | 8.86 | 6.44 | 1.223333 | 6.9 |
| ABCC6 | 3.75 | 1.906902 | 0.000606 | 0.003286 | yes | up | 0.09 | 0.07 | 0.09 | 1.14 | 0.48 | 0.66 | 0.083333 | 0.76 |
| LOC100348276 | 3.519 | 1.815255 | 0.000611 | 0.003307 | yes | up | 1.17 | 0.71 | 1.85 | 18.79 | 22.54 | 3.58 | 1.243333 | 14.97 |
| LOC103345827 | 47.892 | 5.58171 | 0.000611 | 0.003309 | yes | up | 0 | 0 | 0 | 0.4 | 0.11 | 0.46 | 0 | 0.323333 |
| ADCY10 | 2.23 | 1.157124 | 0.000618 | 0.003338 | yes | up | 0.6 | 0.4 | 0.53 | 2.59 | 2.16 | 3.69 | 0.51 | 2.813333 |
| DOK1 | 2.138 | 1.096187 | 0.00062 | 0.003346 | yes | up | 0.82 | 1.85 | 1.64 | 7.44 | 6.06 | 7.55 | 1.436667 | 7.016667 |
| QRSL1 | 0.494 | -1.01666 | 0.000623 | 0.00336 | yes | down | 4.55 | 7.57 | 4.75 | 6.26 | 7.93 | 4.94 | 5.623333 | 6.376667 |
| STIL | 7.535 | 2.913683 | 0.000629 | 0.003388 | yes | up | 0.01 | 0.12 | 0.04 | 1.43 | 1.14 | 0.26 | 0.056667 | 0.943333 |
| MMS22L | 2.793 | 1.481617 | 0.000631 | 0.003396 | yes | up | 0.16 | 0.34 | 0.23 | 1.83 | 1.71 | 1.1 | 0.243333 | 1.546667 |
| THAP9 | 2.334 | 1.222864 | 0.000631 | 0.003396 | yes | up | 0.27 | 0.57 | 0.63 | 2.42 | 2.42 | 3.01 | 0.49 | 2.616667 |
| LNX1 | 4.273 | 2.095187 | 0.000632 | 0.003402 | yes | up | 0.35 | 0.25 | 0.31 | 7.06 | 1.61 | 2.54 | 0.303333 | 3.736667 |
| TAF1D | 0.479 | -1.06198 | 0.000633 | 0.003405 | yes | down | 44.69 | 35.35 | 33.48 | 65.84 | 41.04 | 34.6 | 37.84 | 47.16 |
| CHRD | 2.26 | 1.176272 | 0.000633 | 0.003405 | yes | up | 0.75 | 0.93 | 0.66 | 3.13 | 3.91 | 5.36 | 0.78 | 4.133333 |
| DUOX2 | 0.068 | -3.87621 | 0.000645 | 0.003461 | yes | down | 3.16 | 5.69 | 1.45 | 1.1 | 0.04 | 0.52 | 3.433333 | 0.553333 |
| LOC108176529 | 2.997 | 1.583551 | 0.00065 | 0.003488 | yes | up | 0.37 | 0.79 | 0.64 | 5.1 | 4.99 | 2.35 | 0.6 | 4.146667 |
| DCUN1D2 | 0.337 | -1.5691 | 0.000652 | 0.003495 | yes | down | 2.52 | 6.41 | 6.13 | 2.35 | 6.28 | 3.15 | 5.02 | 3.926667 |
| LOC100344919 | 30.879 | 4.948575 | 0.000656 | 0.003517 | yes | up | 0.04 | 0.07 | 0 | 0.05 | 4.57 | 2.75 | 0.036667 | 2.456667 |
| TK1 | 3.943 | 1.979154 | 0.000656 | 0.003517 | yes | up | 2.29 | 2.02 | 0.83 | 20.2 | 22.06 | 5.03 | 1.713333 | 15.76333 |
| NOS2 | 2.636 | 1.398587 | 0.000659 | 0.003528 | yes | up | 0.35 | 0.43 | 0.52 | 3.91 | 1.5 | 2.63 | 0.433333 | 2.68 |
| CCDC77 | 2.568 | 1.360376 | 0.000663 | 0.003547 | yes | up | 0.46 | 0.37 | 0.54 | 2.43 | 2.89 | 2.87 | 0.456667 | 2.73 |
| LOC108178025 | 2.511 | 1.328277 | 0.000673 | 0.003595 | yes | up | 2.77 | 1.52 | 2.29 | 10.18 | 11.44 | 17.33 | 2.193333 | 12.98333 |
| ARHGAP22 | 3.299 | 1.722056 | 0.000679 | 0.003625 | yes | up | 0.98 | 0.42 | 0.42 | 3.54 | 5.55 | 4.58 | 0.606667 | 4.556667 |
| CDK15 | 3.081 | 1.623471 | 0.000681 | 0.003633 | yes | up | 0.11 | 0.35 | 0.29 | 1.31 | 1.65 | 2.3 | 0.25 | 1.753333 |
| GRIP1 | 7.86 | 2.974493 | 0.000684 | 0.003646 | yes | up | 0.05 | 0.03 | 0.05 | 0.63 | 1.41 | 0.19 | 0.043333 | 0.743333 |
| LOC100346306 | 40.412 | 5.336718 | 0.000684 | 0.003647 | yes | up | 0 | 0.41 | 0 | 6.05 | 4.08 | 30.45 | 0.136667 | 13.52667 |
| SDK2 | 0.363 | -1.46257 | 0.000693 | 0.003689 | yes | down | 8.8 | 3.96 | 7.03 | 11.08 | 5.13 | 3.31 | 6.596667 | 6.506667 |
| TLR6 | 2.257 | 1.174448 | 0.000697 | 0.003709 | yes | up | 0.58 | 0.51 | 0.51 | 2.86 | 1.92 | 3.52 | 0.533333 | 2.766667 |
| RLA-DMB | 0.288 | -1.79383 | 0.000703 | 0.003735 | yes | down | 3.3 | 5.24 | 5.39 | 2.27 | 5.5 | 1.42 | 4.643333 | 3.063333 |
| HEPH | 0.098 | -3.3522 | 0.000706 | 0.003746 | yes | down | 0.09 | 0.2 | 0.2 | 0.06 | 0.05 | 0 | 0.163333 | 0.036667 |
| LOC100337703 | 0.387 | -1.37084 | 0.000716 | 0.003795 | yes | down | 31.48 | 38.66 | 24.76 | 17.51 | 47.2 | 19.06 | 31.63333 | 27.92333 |
| LOC100351224 | 0.321 | -1.64089 | 0.000731 | 0.003869 | yes | down | 0.78 | 2.37 | 0.91 | 1.01 | 1 | 0.93 | 1.353333 | 0.98 |
| LOC100348995 | 2.974 | 1.572527 | 0.00074 | 0.00391 | yes | up | 1.16 | 1.27 | 1.01 | 6.86 | 7.37 | 6.94 | 1.146667 | 7.056667 |
| FADS6 | 0.114 | -3.13142 | 0.00074 | 0.003911 | yes | down | 0.22 | 0.11 | 0.34 | 0.04 | 0.05 | 0.09 | 0.223333 | 0.06 |
| LOC103349966 | 0.016 | -5.98929 | 0.000758 | 0.003997 | yes | down | 0.69 | 0.6 | 0.14 | 0 | 0 | 0 | 0.476667 | 0 |
| C17H15orf41 | 0.466 | -1.10078 | 0.000764 | 0.004025 | yes | down | 1.71 | 1.81 | 2.89 | 2.39 | 2.46 | 2.13 | 2.136667 | 2.326667 |
| IRS2 | 0.286 | -1.80586 | 0.000769 | 0.004049 | yes | down | 5.35 | 3.96 | 4.77 | 3.11 | 1.19 | 5.26 | 4.693333 | 3.186667 |
| LOC100358277 | 49.405 | 5.626591 | 0.000778 | 0.004092 | yes | up | 0 | 0 | 0 | 6.52 | 1.01 | 2.2 | 0 | 3.243333 |
| LOC100009332 | 5.604 | 2.486489 | 0.000782 | 0.004113 | yes | up | 0.82 | 1.52 | 0.82 | 1.96 | 15.82 | 22.93 | 1.053333 | 13.57 |
| PKHD1 | 0.034 | -4.86035 | 0.000784 | 0.004121 | yes | down | 0.7 | 0.52 | 0.02 | 1.58 | 0.65 | 0 | 0.413333 | 0.743333 |
| HELLS | 3.077 | 1.621757 | 0.00079 | 0.004144 | yes | up | 0.34 | 0.41 | 0.33 | 2.72 | 3.64 | 1.31 | 0.36 | 2.556667 |
| LOC103352496 | 2.391 | 1.257778 | 0.000792 | 0.004152 | yes | up | 1.83 | 1.67 | 1 | 7.03 | 5.98 | 12.07 | 1.5 | 8.36 |
| CEBPB | 0.46 | -1.12009 | 0.000793 | 0.004154 | yes | down | 21.81 | 22.24 | 20 | 12.76 | 25.53 | 31.06 | 21.35 | 23.11667 |
| LOC100344189 | 2.298 | 1.200263 | 0.000801 | 0.004198 | yes | up | 1.22 | 0.95 | 1.97 | 12.41 | 7.04 | 6.62 | 1.38 | 8.69 |
| LOC108177738 | 0.059 | -4.07231 | 0.000812 | 0.004251 | yes | down | 0.42 | 0.87 | 0.53 | 0.19 | 0.01 | 0.08 | 0.606667 | 0.093333 |
| LOC103348265 | 0.147 | -2.7697 | 0.000813 | 0.004251 | yes | down | 0.24 | 0.38 | 0.48 | 0.07 | 0.16 | 0.14 | 0.366667 | 0.123333 |
| LOC103347127 | 0.33 | -1.59937 | 0.000814 | 0.004255 | yes | down | 15.68 | 17.06 | 10.14 | 14.8 | 11.52 | 7.8 | 14.29333 | 11.37333 |
| APLNR | 2.433 | 1.282695 | 0.000817 | 0.004269 | yes | up | 20.3 | 18.87 | 16 | 187.47 | 79.65 | 52.55 | 18.39 | 106.5567 |
| ATP2A2 | 0.37 | -1.43444 | 0.000827 | 0.004318 | yes | down | 62.9 | 87.85 | 27.52 | 38.6 | 77.18 | 39.79 | 59.42333 | 51.85667 |
| AK9 | 0.358 | -1.48001 | 0.000827 | 0.004318 | yes | down | 0.3 | 0.36 | 0.68 | 0.47 | 0.33 | 0.33 | 0.446667 | 0.376667 |
| RGS4 | 3.535 | 1.821526 | 0.00083 | 0.004332 | yes | up | 0.87 | 4.1 | 3.33 | 25.79 | 11.4 | 31.7 | 2.766667 | 22.96333 |
| LOC108176834 | 0.235 | -2.09209 | 0.00083 | 0.004332 | yes | down | 5.11 | 4.01 | 6.13 | 1.4 | 3.44 | 5.63 | 5.083333 | 3.49 |
| LOC100353046 | 239.009 | 7.90092 | 0.000831 | 0.004334 | yes | up | 0.08 | 0 | 0 | 32.89 | 12.13 | 0.09 | 0.026667 | 15.03667 |
| FAM20A | 0.392 | -1.35209 | 0.000837 | 0.004359 | yes | down | 0.82 | 1.18 | 0.56 | 0.89 | 0.68 | 0.74 | 0.853333 | 0.77 |
| LOC100342321 | 12.091 | 3.595891 | 0.000845 | 0.004396 | yes | up | 0.09 | 0.11 | 0.05 | 1.74 | 0.16 | 5.21 | 0.083333 | 2.37 |
| DEPDC7 | 2.869 | 1.520438 | 0.000846 | 0.004399 | yes | up | 0.3 | 0.51 | 0.49 | 3.58 | 2.72 | 2.36 | 0.433333 | 2.886667 |
| MDK | 2.297 | 1.199638 | 0.000847 | 0.004404 | yes | up | 3.91 | 8.57 | 3.42 | 30.4 | 28.63 | 25.06 | 5.3 | 28.03 |
| TMEM26 | 0.241 | -2.05309 | 0.000849 | 0.004409 | yes | down | 2.43 | 0.62 | 0.89 | 0.7 | 1.13 | 0.44 | 1.313333 | 0.756667 |
| SUV39H1 | 2.669 | 1.416217 | 0.000852 | 0.004427 | yes | up | 0.58 | 0.42 | 0.36 | 2.82 | 3.71 | 1.91 | 0.453333 | 2.813333 |
| SYCE1L | 2.687 | 1.426044 | 0.000858 | 0.004455 | yes | up | 0.28 | 0.36 | 0.85 | 4.05 | 2.49 | 2.85 | 0.496667 | 3.13 |
| SPACA9 | 0.177 | -2.5011 | 0.000864 | 0.004482 | yes | down | 0.46 | 0.76 | 0.69 | 0.76 | 0.12 | 0.27 | 0.636667 | 0.383333 |
| LOC103350112 | 0.153 | -2.70527 | 0.000871 | 0.004517 | yes | down | 0.41 | 0.58 | 0.22 | 0.15 | 0.16 | 0.11 | 0.403333 | 0.14 |
| TCEAL7 | 0.039 | -4.68166 | 0.000882 | 0.004566 | yes | down | 16.73 | 37.67 | 0.35 | 1.08 | 2.83 | 0.74 | 18.25 | 1.55 |
| KRT5 | 0.014 | -6.119 | 0.000882 | 0.004568 | yes | down | 0.38 | 0.11 | 0.05 | 0 | 0 | 0 | 0.18 | 0 |
| RANBP3L | 2.305 | 1.20462 | 0.000883 | 0.004568 | yes | up | 0.77 | 0.97 | 1.14 | 7.76 | 3.4 | 4.09 | 0.96 | 5.083333 |
| C2H2orf15 | 0.043 | -4.53642 | 0.000884 | 0.004571 | yes | down | 0.39 | 0.67 | 0.8 | 0.2 | 0 | 0.19 | 0.62 | 0.13 |
| LOC100343299 | 0.094 | -3.40613 | 0.000886 | 0.004584 | yes | down | 215.57 | 26.56 | 27.14 | 16.03 | 27.05 | 18.24 | 89.75667 | 20.44 |
| LOC100341303 | 0.327 | -1.61232 | 0.000901 | 0.00465 | yes | down | 2.78 | 3.25 | 3.03 | 1.88 | 3.66 | 1.32 | 3.02 | 2.286667 |
| LOC100340280 | 2.107 | 1.075315 | 0.00091 | 0.004686 | yes | up | 1.74 | 1.87 | 1.04 | 7 | 8.97 | 6.62 | 1.55 | 7.53 |
| MICAL2 | 0.468 | -1.09418 | 0.000913 | 0.004702 | yes | down | 1.2 | 1.62 | 0.7 | 1.49 | 1.08 | 1.25 | 1.173333 | 1.273333 |
| LOC103351304 | 2.89 | 1.531166 | 0.000914 | 0.004703 | yes | up | 0.25 | 0.31 | 0.21 | 1.72 | 2.08 | 1.32 | 0.256667 | 1.706667 |
| RAD54B | 5.558 | 2.474447 | 0.000917 | 0.004716 | yes | up | 0.11 | 0.12 | 0.05 | 1.88 | 1.25 | 0.42 | 0.093333 | 1.183333 |
| LOC100357462 | 39.627 | 5.308416 | 0.000925 | 0.00475 | yes | up | 0 | 0 | 0 | 4.57 | 6.83 | 8.21 | 0 | 6.536667 |
| CORO6 | 0.441 | -1.1803 | 0.00093 | 0.004773 | yes | down | 16.76 | 15.46 | 6.98 | 8.22 | 16.63 | 10.29 | 13.06667 | 11.71333 |
| GRIA1 | 57.904 | 5.855596 | 0.000934 | 0.00479 | yes | up | 0 | 0 | 0.01 | 0.02 | 1.16 | 0.13 | 0.003333 | 0.436667 |
| REEP2 | 0.219 | -2.18801 | 0.000937 | 0.004806 | yes | down | 1 | 0.54 | 0.73 | 0.25 | 0.63 | 0.28 | 0.756667 | 0.386667 |
| CNGB1 | 0.206 | -2.27747 | 0.000939 | 0.004809 | yes | down | 0.36 | 0.17 | 0.3 | 0.05 | 0.13 | 0.22 | 0.276667 | 0.133333 |
| GPSM2 | 2.455 | 1.295431 | 0.000948 | 0.004851 | yes | up | 1.43 | 1.51 | 1.2 | 10.29 | 8.56 | 3.64 | 1.38 | 7.496667 |
| LOC100356734 | 2.358 | 1.237863 | 0.000952 | 0.004868 | yes | up | 1.62 | 2.43 | 1.82 | 5.18 | 13.31 | 8.44 | 1.956667 | 8.976667 |
| CCDC142 | 2.601 | 1.379097 | 0.000958 | 0.004895 | yes | up | 0.27 | 0.34 | 0.44 | 2.16 | 1.6 | 1.84 | 0.35 | 1.866667 |
| LOC108177747 | 12.59 | 3.654194 | 0.000982 | 0.005004 | yes | up | 0.01 | 0.01 | 0.01 | 0.38 | 0.2 | 0.47 | 0.01 | 0.35 |
| NFE2 | 0.251 | -1.9916 | 0.000983 | 0.005009 | yes | down | 1.53 | 0.87 | 1.14 | 0.34 | 0.62 | 1.07 | 1.18 | 0.676667 |
| ZNF697 | 0.446 | -1.16442 | 0.000988 | 0.005032 | yes | down | 3.85 | 1.68 | 3.36 | 3.54 | 2.6 | 3.27 | 2.963333 | 3.136667 |
| ANPEP | 2.459 | 1.29789 | 0.000994 | 0.005056 | yes | up | 0.36 | 0.77 | 0.6 | 3.71 | 2.33 | 3.82 | 0.576667 | 3.286667 |
| TNNT2 | 0.06 | -4.05559 | 0.000995 | 0.005056 | yes | down | 4.49 | 3.73 | 0.25 | 0.11 | 1.31 | 0 | 2.823333 | 0.473333 |
| LOC100346672 | 9.356 | 3.225924 | 0.000998 | 0.005071 | yes | up | 0.01 | 0.02 | 0.03 | 0.37 | 0.16 | 0.8 | 0.02 | 0.443333 |
| DNAJB5 | 0.313 | -1.67611 | 0.001 | 0.005078 | yes | down | 16.12 | 13.97 | 3.94 | 5.84 | 11.76 | 7.22 | 11.34333 | 8.273333 |
| LOC100347161 | 0.019 | -5.75301 | 0.001009 | 0.00512 | yes | down | 0.41 | 0.8 | 0.96 | 0 | 0 | 0 | 0.723333 | 0 |
| ZBED8 | 2.769 | 1.469211 | 0.001023 | 0.005181 | yes | up | 0.2 | 0.24 | 0.47 | 2.13 | 1.74 | 1.29 | 0.303333 | 1.72 |
| LOC100338113 | 7.059 | 2.819444 | 0.001033 | 0.005222 | yes | up | 0.05 | 0.14 | 0.04 | 2.21 | 1.03 | 0.46 | 0.076667 | 1.233333 |
| ANKRD45 | 3 | 1.584906 | 0.001035 | 0.005228 | yes | up | 0.5 | 0.24 | 0.43 | 2.56 | 2.56 | 3.09 | 0.39 | 2.736667 |
| HAS2 | 2.536 | 1.342612 | 0.001036 | 0.005232 | yes | up | 0.99 | 0.4 | 0.57 | 4.34 | 3.99 | 3.58 | 0.653333 | 3.97 |
| SLC46A3 | 2.719 | 1.443148 | 0.001042 | 0.005253 | yes | up | 0.48 | 0.35 | 0.44 | 1.83 | 2.76 | 3.45 | 0.423333 | 2.68 |
| IL17RB | 0.207 | -2.27525 | 0.001049 | 0.005282 | yes | down | 0.94 | 0.4 | 0.44 | 0.36 | 0.35 | 0.17 | 0.593333 | 0.293333 |
| CHRNG | 0.03 | -5.07566 | 0.001053 | 0.005298 | yes | down | 0.53 | 0.57 | 0 | 0 | 0.03 | 0.05 | 0.366667 | 0.026667 |
| ST3GAL5 | 0.468 | -1.09669 | 0.001064 | 0.005351 | yes | down | 2.67 | 2.9 | 2.56 | 2.01 | 3.35 | 3.36 | 2.71 | 2.906667 |
| DSG4 | 0.031 | -4.99056 | 0.00107 | 0.005374 | yes | down | 2.11 | 1.03 | 2.92 | 0 | 0.04 | 0.41 | 2.02 | 0.15 |
| B3GNTL1 | 0.488 | -1.03566 | 0.001091 | 0.005465 | yes | down | 2.81 | 3.01 | 3.01 | 4.44 | 2.6 | 3.84 | 2.943333 | 3.626667 |
| SGIP1 | 3.739 | 1.902759 | 0.001096 | 0.005482 | yes | up | 0.29 | 0.41 | 0.29 | 2.2 | 1.18 | 5.58 | 0.33 | 2.986667 |
| LMCD1 | 0.151 | -2.7237 | 0.001097 | 0.005482 | yes | down | 108.07 | 101.61 | 12.74 | 25.15 | 31.25 | 21.96 | 74.14 | 26.12 |
| GPR162 | 2.569 | 1.361283 | 0.0011 | 0.005498 | yes | up | 0.54 | 0.51 | 0.63 | 2.66 | 4.65 | 2.68 | 0.56 | 3.33 |
| ARSB | 2.495 | 1.318815 | 0.001103 | 0.005508 | yes | up | 2.06 | 3.23 | 3.26 | 21.69 | 13.97 | 13.71 | 2.85 | 16.45667 |
| LOC103352375 | 4.732 | 2.242587 | 0.001109 | 0.005539 | yes | up | 0.26 | 0.25 | 0.23 | 3.59 | 2.92 | 1.9 | 0.246667 | 2.803333 |
| THNSL2 | 2.025 | 1.017618 | 0.001113 | 0.005551 | yes | up | 1.49 | 2.13 | 2.29 | 9.08 | 10.18 | 6.13 | 1.97 | 8.463333 |
| LOC108177094 | 4.76 | 2.251105 | 0.001124 | 0.005597 | yes | up | 0.19 | 0.38 | 0.23 | 4.2 | 0.92 | 3.75 | 0.266667 | 2.956667 |
| OTOG | 0.063 | -3.99053 | 0.001127 | 0.005613 | yes | down | 0.02 | 0.05 | 0.13 | 0.01 | 0 | 0.02 | 0.066667 | 0.01 |
| LOC103352463 | 0.456 | -1.13311 | 0.001154 | 0.005733 | yes | down | 2.15 | 3.17 | 2.14 | 2.58 | 2.36 | 3.23 | 2.486667 | 2.723333 |
| PPP1R14A | 2.168 | 1.116368 | 0.001155 | 0.005735 | yes | up | 1.77 | 4.56 | 2.36 | 15.48 | 12.76 | 14.96 | 2.896667 | 14.4 |
| LOC100353664 | 2.148 | 1.103001 | 0.001155 | 0.005735 | yes | up | 1.48 | 3.47 | 3.71 | 16.84 | 12.7 | 13.54 | 2.886667 | 14.36 |
| HSF5 | 0.419 | -1.25456 | 0.001163 | 0.005768 | yes | down | 1.69 | 2.76 | 1.74 | 1.34 | 1.58 | 2.94 | 2.063333 | 1.953333 |
| CCRL2 | 0.267 | -1.90709 | 0.001172 | 0.005801 | yes | down | 5.98 | 3.33 | 1.22 | 2.53 | 2.52 | 1.55 | 3.51 | 2.2 |
| LOC100355642 | 0.076 | -3.71368 | 0.001174 | 0.005809 | yes | down | 4.21 | 3.63 | 2.36 | 0.32 | 0.04 | 1.21 | 3.4 | 0.523333 |
| ZNF436 | 2.215 | 1.147218 | 0.001181 | 0.005834 | yes | up | 0.91 | 1.36 | 1.85 | 10.61 | 4.94 | 5.76 | 1.373333 | 7.103333 |
| SAMD15 | 3.417 | 1.772802 | 0.001183 | 0.005842 | yes | up | 0.06 | 0.27 | 0.24 | 1.34 | 1.24 | 1.55 | 0.19 | 1.376667 |
| TSPAN15 | 2.45 | 1.292957 | 0.001199 | 0.005908 | yes | up | 5.09 | 14.15 | 13.04 | 73.17 | 33.07 | 81.85 | 10.76 | 62.69667 |
| CACHD1 | 2.073 | 1.051536 | 0.001235 | 0.006074 | yes | up | 1.25 | 0.73 | 0.9 | 6.33 | 4.34 | 3.53 | 0.96 | 4.733333 |
| GPR19 | 4.13 | 2.046087 | 0.001245 | 0.006117 | yes | up | 0.23 | 0.52 | 0.22 | 3.28 | 5.6 | 1.15 | 0.323333 | 3.343333 |
| TC2N | 3.68 | 1.879772 | 0.001254 | 0.006153 | yes | up | 0.51 | 0.39 | 0.34 | 7.34 | 1.64 | 2.42 | 0.413333 | 3.8 |
| KCNE5 | 0.03 | -5.06485 | 0.001276 | 0.006254 | yes | down | 0.99 | 0.35 | 0.04 | 0.05 | 0.05 | 0 | 0.46 | 0.033333 |
| DUSP6 | 0.378 | -1.40386 | 0.001285 | 0.006292 | yes | down | 54.69 | 31.22 | 18.57 | 50.82 | 22 | 21.62 | 34.82667 | 31.48 |
| SUSD2 | 2.005 | 1.003466 | 0.001303 | 0.006372 | yes | up | 6.95 | 9.01 | 8.01 | 48.45 | 43.41 | 19.75 | 7.99 | 37.20333 |
| GPRIN3 | 2.049 | 1.035115 | 0.001322 | 0.006454 | yes | up | 0.29 | 0.26 | 0.28 | 1.74 | 1.33 | 0.95 | 0.276667 | 1.34 |
| HOXC6 | 2.912 | 1.541804 | 0.001336 | 0.006517 | yes | up | 0.29 | 0.82 | 0.52 | 3.52 | 2.5 | 3.32 | 0.543333 | 3.113333 |
| MIS18BP1 | 3.628 | 1.859363 | 0.001341 | 0.006533 | yes | up | 0.13 | 0.39 | 0.29 | 3.27 | 2.59 | 0.83 | 0.27 | 2.23 |
| LOC103346515 | 0.146 | -2.77757 | 0.00135 | 0.006573 | yes | down | 1.75 | 0.33 | 0.45 | 0.26 | 0.28 | 0.35 | 0.843333 | 0.296667 |
| FGF7 | 4.321 | 2.111494 | 0.001351 | 0.006575 | yes | up | 0.42 | 0.12 | 0.28 | 3.54 | 2.58 | 1.65 | 0.273333 | 2.59 |
| FGF11 | 3.072 | 1.619267 | 0.00137 | 0.006662 | yes | up | 0.17 | 0.22 | 0.08 | 1.29 | 1.13 | 0.94 | 0.156667 | 1.12 |
| LOC108176252 | 0.279 | -1.8438 | 0.001382 | 0.006715 | yes | down | 2.37 | 1.94 | 3.07 | 1.97 | 2.1 | 0.78 | 2.46 | 1.616667 |
| LOC103350276 | 0.353 | -1.50406 | 0.001403 | 0.00681 | yes | down | 2.13 | 3.6 | 1.35 | 1.43 | 2.15 | 2.19 | 2.36 | 1.923333 |
| F10 | 0.017 | -5.85608 | 0.001407 | 0.006821 | yes | down | 0.42 | 0.2 | 0.07 | 0 | 0 | 0 | 0.23 | 0 |
| LOC103348239 | 0.029 | -5.09966 | 0.001409 | 0.006826 | yes | down | 0.42 | 0.62 | 1.02 | 0.13 | 0 | 0 | 0.686667 | 0.043333 |
| TRIM58 | 0.212 | -2.23892 | 0.001418 | 0.006862 | yes | down | 2.37 | 1.08 | 3.57 | 0.39 | 0.88 | 2.49 | 2.34 | 1.253333 |
| BHMT | 0.074 | -3.75588 | 0.001423 | 0.006883 | yes | down | 2.51 | 1.43 | 1.06 | 0 | 0 | 0.86 | 1.666667 | 0.286667 |
| LRRC2 | 0.344 | -1.53756 | 0.001425 | 0.006891 | yes | down | 7.79 | 5.69 | 3.36 | 3.18 | 7.23 | 3.07 | 5.613333 | 4.493333 |
| MRAP2 | 0.252 | -1.98597 | 0.001431 | 0.006917 | yes | down | 0.73 | 1.09 | 0.75 | 0.9 | 0.35 | 0.27 | 0.856667 | 0.506667 |
| LOC103346040 | 2.245 | 1.166739 | 0.001432 | 0.006919 | yes | up | 1.34 | 1.07 | 1.64 | 4.2 | 7.05 | 9.94 | 1.35 | 7.063333 |
| KIF14 | 2.655 | 1.408602 | 0.001432 | 0.006919 | yes | up | 0.57 | 0.49 | 0.48 | 4.05 | 4.24 | 1.33 | 0.513333 | 3.206667 |
| SPDL1 | 4.189 | 2.06645 | 0.001445 | 0.006978 | yes | up | 0.09 | 0.46 | 0.18 | 2.72 | 2.97 | 1.07 | 0.243333 | 2.253333 |
| RNF212 | 2.28 | 1.188788 | 0.001448 | 0.006988 | yes | up | 3.06 | 3.08 | 5.51 | 28.24 | 12.79 | 22.54 | 3.883333 | 21.19 |
| DLGAP2 | 0.042 | -4.56253 | 0.001451 | 0.006996 | yes | down | 0.08 | 0.23 | 0.05 | 0 | 0.02 | 0.02 | 0.12 | 0.013333 |
| LRRC4 | 5.722 | 2.516537 | 0.001451 | 0.006996 | yes | up | 0.04 | 0.09 | 0.18 | 2.61 | 0.92 | 0.65 | 0.103333 | 1.393333 |
| CFB | 2.955 | 1.563026 | 0.001457 | 0.007018 | yes | up | 1.47 | 0.85 | 1.34 | 4.5 | 5.72 | 15.11 | 1.22 | 8.443333 |
| EDNRA | 2.26 | 1.176256 | 0.001479 | 0.007119 | yes | up | 1.92 | 1.64 | 1.53 | 13.54 | 4.82 | 7.18 | 1.696667 | 8.513333 |
| S1PR2 | 2.089 | 1.062717 | 0.001503 | 0.00722 | yes | up | 0.82 | 2.08 | 0.78 | 5.46 | 4.71 | 5.56 | 1.226667 | 5.243333 |
| UBALD1 | 0.491 | -1.02686 | 0.001505 | 0.007226 | yes | down | 13.28 | 12.03 | 10.7 | 12.32 | 13.06 | 20.16 | 12.00333 | 15.18 |
| LOC100342182 | 0.016 | -5.96473 | 0.001513 | 0.00726 | yes | down | 1.54 | 2.26 | 0.19 | 0 | 0 | 0 | 1.33 | 0 |
| CACNB4 | 0.466 | -1.1013 | 0.001519 | 0.007285 | yes | down | 1.83 | 1.52 | 1.61 | 12.84 | 16.16 | 7.05 | 1.653333 | 12.01667 |
| RAD51AP1 | 4.92 | 2.298547 | 0.001524 | 0.00731 | yes | up | 0.16 | 0.18 | 0.04 | 1.84 | 1.88 | 0.63 | 0.126667 | 1.45 |
| LOC103346705 | 0.21 | -2.25216 | 0.001527 | 0.007316 | yes | down | 2.23 | 0.93 | 0.26 | 0.52 | 0.56 | 0.77 | 1.14 | 0.616667 |
| KIF24 | 3.182 | 1.670098 | 0.001527 | 0.007316 | yes | up | 0.18 | 0.15 | 0.1 | 0.86 | 1.49 | 0.77 | 0.143333 | 1.04 |
| S100A9 | 0.353 | -1.50031 | 0.001533 | 0.007337 | yes | down | 18.08 | 22.01 | 9.67 | 8.29 | 10.78 | 21.97 | 16.58667 | 13.68 |
| CENPJ | 3.175 | 1.666704 | 0.001533 | 0.007337 | yes | up | 0.1 | 0.09 | 0.09 | 0.75 | 0.74 | 0.63 | 0.093333 | 0.706667 |
| ARHGAP44 | 2.789 | 1.479881 | 0.001548 | 0.0074 | yes | up | 0.2 | 0.18 | 0.13 | 0.93 | 1.05 | 1.2 | 0.17 | 1.06 |
| GNRH1 | 20.719 | 4.37289 | 0.001552 | 0.007416 | yes | up | 0 | 0 | 0.11 | 2.84 | 2.82 | 0.77 | 0.036667 | 2.143333 |
| LOC108176823 | 0.277 | -1.85154 | 0.001562 | 0.007456 | yes | down | 2.07 | 0.98 | 0.48 | 0.74 | 0.66 | 0.92 | 1.176667 | 0.773333 |
| CCDC146 | 0.297 | -1.75102 | 0.001577 | 0.007521 | yes | down | 0.27 | 0.31 | 0.46 | 0.27 | 0.2 | 0.26 | 0.346667 | 0.243333 |
| KCNMA1 | 0.296 | -1.75513 | 0.0016 | 0.007624 | yes | down | 2.31 | 2.19 | 0.93 | 0.44 | 2.02 | 1.2 | 1.81 | 1.22 |
| TIMM9 | 0.421 | -1.24712 | 0.001612 | 0.007675 | yes | down | 16.64 | 34.88 | 16.33 | 16.05 | 16.65 | 31.36 | 22.61667 | 21.35333 |
| MMP12 | 0.031 | -5.00825 | 0.001614 | 0.007685 | yes | down | 0.88 | 0.07 | 0.25 | 0 | 0.09 | 0 | 0.4 | 0.03 |
| CDKL2 | 3.402 | 1.766468 | 0.00165 | 0.007823 | yes | up | 0.4 | 0.07 | 0.34 | 1.55 | 2.5 | 2.25 | 0.27 | 2.1 |
| COL6A6 | 2.68 | 1.422059 | 0.001661 | 0.007864 | yes | up | 1.2 | 2.83 | 0.57 | 10.96 | 10.14 | 6.9 | 1.533333 | 9.333333 |
| LOC100357816 | 0.246 | -2.0208 | 0.001669 | 0.0079 | yes | down | 3.03 | 6.16 | 4.88 | 1.1 | 2.26 | 4.47 | 4.69 | 2.61 |
| TOB1 | 0.402 | -1.31577 | 0.001686 | 0.00797 | yes | down | 30.87 | 11.06 | 12.37 | 13.27 | 21.83 | 15.86 | 18.1 | 16.98667 |
| CXCR4 | 2.261 | 1.176834 | 0.001688 | 0.007975 | yes | up | 2.76 | 2.87 | 1.8 | 10.17 | 9.33 | 19.5 | 2.476667 | 13 |
| TNFSF13B | 3.999 | 1.999488 | 0.001689 | 0.007979 | yes | up | 0.27 | 0.18 | 0.28 | 1.82 | 3.07 | 1.86 | 0.243333 | 2.25 |
| LOC100357209 | 5.036 | 2.332325 | 0.001695 | 0.008005 | yes | up | 0.26 | 0.15 | 0.46 | 6.56 | 2.1 | 1.89 | 0.29 | 3.516667 |
| KEL | 0.056 | -4.1505 | 0.001709 | 0.008066 | yes | down | 0.12 | 0.26 | 0.31 | 0 | 0 | 0.09 | 0.23 | 0.03 |
| FANCM | 5.433 | 2.441859 | 0.001716 | 0.008091 | yes | up | 0.02 | 0.03 | 0.02 | 0.31 | 0.35 | 0.13 | 0.023333 | 0.263333 |
| GABRB3 | 5.598 | 2.484933 | 0.001725 | 0.008129 | yes | up | 0.05 | 0.2 | 0.03 | 1.68 | 0.28 | 1.66 | 0.093333 | 1.206667 |
| PLXNA4 | 2.588 | 1.371584 | 0.001737 | 0.008176 | yes | up | 0.39 | 0.4 | 0.38 | 4.31 | 1.23 | 1.6 | 0.39 | 2.38 |
| CCDC17 | 2.384 | 1.253208 | 0.001758 | 0.008267 | yes | up | 0.35 | 0.4 | 0.4 | 2.51 | 2.04 | 1.68 | 0.383333 | 2.076667 |
| HDX | 0.368 | -1.44337 | 0.001769 | 0.008302 | yes | down | 0.22 | 0.28 | 0.2 | 0.23 | 0.17 | 0.19 | 0.233333 | 0.196667 |
| UBE2T | 4.709 | 2.235518 | 0.001776 | 0.008325 | yes | up | 0.43 | 0.47 | 0.3 | 5.72 | 6.25 | 1.49 | 0.4 | 4.486667 |
| CSPG5 | 2.296 | 1.19928 | 0.001786 | 0.00836 | yes | up | 1.74 | 1.48 | 1.64 | 6.09 | 12.34 | 7.86 | 1.62 | 8.763333 |
| PROSER2 | 0.409 | -1.2903 | 0.001797 | 0.008402 | yes | down | 0.49 | 0.43 | 0.49 | 0.51 | 0.37 | 0.47 | 0.47 | 0.45 |
| CENPP | 4.488 | 2.166229 | 0.001804 | 0.008428 | yes | up | 0.15 | 0.18 | 0.2 | 3.17 | 1.52 | 1.55 | 0.176667 | 2.08 |
| LOC100348391 | 4.835 | 2.273633 | 0.001812 | 0.008457 | yes | up | 0.25 | 0.15 | 0.18 | 3.5 | 2.53 | 0.83 | 0.193333 | 2.286667 |
| SKA1 | 8.893 | 3.152655 | 0.001835 | 0.008541 | yes | up | 0.12 | 0.22 | 0.06 | 2.92 | 4.37 | 0.79 | 0.133333 | 2.693333 |
| BBOX1 | 0.342 | -1.54857 | 0.001836 | 0.008542 | yes | down | 1.36 | 1.52 | 3.17 | 2.42 | 0.82 | 1.57 | 2.016667 | 1.603333 |
| RAP1GAP2 | 0.279 | -1.8435 | 0.00187 | 0.008678 | yes | down | 0.68 | 1.16 | 0.92 | 0.84 | 0.17 | 0.8 | 0.92 | 0.603333 |
| TMEM254 | 2.026 | 1.018838 | 0.001873 | 0.008687 | yes | up | 1.09 | 0.83 | 3.52 | 6.27 | 12.71 | 7.01 | 1.813333 | 8.663333 |
| LOC100342534 | 0.405 | -1.30311 | 0.001875 | 0.008693 | yes | down | 10.51 | 10.82 | 16.2 | 12.83 | 8.19 | 15.3 | 12.51 | 12.10667 |
| DOK3 | 0.366 | -1.45088 | 0.001886 | 0.008733 | yes | down | 0.82 | 0.62 | 0.7 | 0.55 | 0.66 | 0.62 | 0.713333 | 0.61 |
| KCNJ15 | 2.696 | 1.430967 | 0.00189 | 0.008748 | yes | up | 0.24 | 0.19 | 0.32 | 1.61 | 1.9 | 1.23 | 0.25 | 1.58 |
| LRRN1 | 2.465 | 1.301498 | 0.001891 | 0.008751 | yes | up | 0.22 | 0.56 | 0.29 | 1.71 | 2.39 | 1.87 | 0.356667 | 1.99 |
| FXYD3 | 9.785 | 3.290506 | 0.00193 | 0.008913 | yes | up | 0.08 | 0.35 | 0 | 2.88 | 1.53 | 1.91 | 0.143333 | 2.106667 |
| TUFT1 | 0.441 | -1.18112 | 0.001944 | 0.00897 | yes | down | 2.51 | 2.31 | 2.19 | 3.26 | 2.78 | 1.24 | 2.336667 | 2.426667 |
| LOC108178486 | 2.507 | 1.32621 | 0.001947 | 0.008983 | yes | up | 0.4 | 0.3 | 0.78 | 3.75 | 2.18 | 4.37 | 0.493333 | 3.433333 |
| RNASEL | 2.571 | 1.362187 | 0.001954 | 0.009006 | yes | up | 0.31 | 0.34 | 0.29 | 2.66 | 1.47 | 1.49 | 0.313333 | 1.873333 |
| LOC103346704 | 0.18 | -2.47175 | 0.001968 | 0.00906 | yes | down | 2.35 | 0.59 | 0.35 | 0.35 | 0.31 | 0.77 | 1.096667 | 0.476667 |
| LOC100354784 | 6.289 | 2.652765 | 0.001995 | 0.009169 | yes | up | 0.5 | 0.48 | 0.43 | 7.95 | 6.21 | 6.84 | 0.47 | 7 |
| WISP1 | 0.326 | -1.61751 | 0.002003 | 0.009203 | yes | down | 0.76 | 0.48 | 0.26 | 0.41 | 0.44 | 0.31 | 0.5 | 0.386667 |
| LOC103347060 | 0.195 | -2.35629 | 0.002026 | 0.0093 | yes | down | 0.71 | 1.54 | 0.45 | 0.24 | 0.21 | 0.75 | 0.9 | 0.4 |
| METTL21C | 0.009 | -6.78478 | 0.002042 | 0.009364 | yes | down | 56.13 | 4.14 | 0.03 | 0.14 | 1.63 | 0.02 | 20.1 | 0.596667 |
| YBX3 | 0.465 | -1.10336 | 0.00206 | 0.009434 | yes | down | 157.8 | 212.3 | 109.7 | 116.25 | 255.03 | 119.08 | 159.9333 | 163.4533 |
| GCG | 0.098 | -3.35632 | 0.002061 | 0.009434 | yes | down | 2.46 | 3.05 | 4.82 | 0.08 | 2.16 | 0 | 3.443333 | 0.746667 |
| SLC16A10 | 0.29 | -1.78651 | 0.002071 | 0.009472 | yes | down | 1.68 | 2.45 | 0.61 | 0.65 | 1.57 | 0.9 | 1.58 | 1.04 |
| LOC108176506 | 0.176 | -2.50806 | 0.002072 | 0.009474 | yes | down | 0.82 | 2.58 | 1.35 | 0.54 | 0.12 | 0.27 | 1.583333 | 0.31 |
| LOC100358749 | 0.438 | -1.18971 | 0.002079 | 0.009498 | yes | down | 7.35 | 5.47 | 7.47 | 9.08 | 9.11 | 6.89 | 6.763333 | 8.36 |
| APBA2 | 0.395 | -1.33976 | 0.002089 | 0.009535 | yes | down | 0.52 | 0.47 | 0.6 | 0.44 | 0.4 | 0.63 | 0.53 | 0.49 |
| SPATA2 | 0.452 | -1.14588 | 0.002095 | 0.009552 | yes | down | 1.57 | 2.15 | 2.26 | 2.6 | 1.94 | 1.8 | 1.993333 | 2.113333 |
| C14H21orf58 | 10.386 | 3.376524 | 0.002095 | 0.009552 | yes | up | 0.06 | 0.06 | 0.05 | 1.34 | 1.21 | 1.4 | 0.056667 | 1.316667 |
| LOC103351388 | 0.019 | -5.74023 | 0.002117 | 0.009636 | yes | down | 0.14 | 13.85 | 9.81 | 0 | 1.17 | 0 | 7.933333 | 0.39 |
| LAYN | 2.638 | 1.399521 | 0.00213 | 0.00969 | yes | up | 2.08 | 2.94 | 2.45 | 29.26 | 11.41 | 7.1 | 2.49 | 15.92333 |
| CDRT1 | 2.316 | 1.211403 | 0.002139 | 0.009727 | yes | up | 0.7 | 0.41 | 0.9 | 3.55 | 3.32 | 4.5 | 0.67 | 3.79 |
| LOC100351364 | 0.033 | -4.91241 | 0.00214 | 0.00973 | yes | down | 0.38 | 0.37 | 0.33 | 0 | 0 | 0.07 | 0.36 | 0.023333 |
| SMIM5 | 0.156 | -2.67937 | 0.002147 | 0.009756 | yes | down | 0.3 | 0.64 | 0.42 | 0.11 | 0.23 | 0.15 | 0.453333 | 0.163333 |
| CBX2 | 2.954 | 1.562808 | 0.00215 | 0.009766 | yes | up | 0.18 | 0.38 | 0.33 | 3.15 | 1.92 | 1 | 0.296667 | 2.023333 |
| LOC100346597 | 2.026 | 1.018366 | 0.002159 | 0.009803 | yes | up | 1.98 | 1.69 | 2.64 | 12.95 | 11.58 | 5.81 | 2.103333 | 10.11333 |
| LOC103349807 | 283.442 | 8.146912 | 0.002201 | 0.009983 | yes | up | 0 | 0 | 0 | 42.49 | 36.25 | 0.17 | 0 | 26.30333 |
| ABCC4 | 0.316 | -1.66423 | 0.002203 | 0.009986 | yes | down | 7.26 | 2.34 | 2.71 | 2.69 | 1.41 | 5.26 | 4.103333 | 3.12 |
| LOC100346112 | 0.421 | -1.24663 | 0.002215 | 0.010032 | yes | down | 181.71 | 210.94 | 55.37 | 129.66 | 164.68 | 143.35 | 149.34 | 145.8967 |
| SPTBN2 | 0.488 | -1.03538 | 0.002234 | 0.010108 | yes | down | 0.52 | 0.41 | 0.34 | 0.42 | 0.51 | 0.51 | 0.423333 | 0.48 |
| MAB21L1 | 2.089 | 1.062497 | 0.002247 | 0.010147 | yes | up | 0.72 | 1.27 | 1.9 | 5.71 | 6.13 | 6.92 | 1.296667 | 6.253333 |
| TRIM46 | 3.525 | 1.817546 | 0.002247 | 0.010147 | yes | up | 0.06 | 0.16 | 0.21 | 1.55 | 0.76 | 1.14 | 0.143333 | 1.15 |
| HMCN2 | 0.18 | -2.47219 | 0.002259 | 0.010186 | yes | down | 7.6 | 5.31 | 1.06 | 1.31 | 2.5 | 2.05 | 4.656667 | 1.953333 |
| LOC103351328 | 0.175 | -2.51367 | 0.002277 | 0.010257 | yes | down | 0.9 | 1.05 | 0.56 | 0.15 | 0.25 | 0.6 | 0.836667 | 0.333333 |
| MND1 | 4.68 | 2.226518 | 0.002281 | 0.01027 | yes | up | 0.47 | 0.32 | 0.36 | 4.24 | 5.43 | 2.45 | 0.383333 | 4.04 |
| RNF135 | 2.189 | 1.130523 | 0.002289 | 0.010296 | yes | up | 1.3 | 1.14 | 1.77 | 8.03 | 7.24 | 6.45 | 1.403333 | 7.24 |
| IL27RA | 2.231 | 1.157574 | 0.002296 | 0.010323 | yes | up | 0.42 | 0.68 | 0.55 | 3.86 | 2.75 | 1.91 | 0.55 | 2.84 |
| LOC108176398 | 3.787 | 1.921002 | 0.002301 | 0.010339 | yes | up | 0.08 | 0.09 | 0.34 | 1.44 | 1.22 | 2.68 | 0.17 | 1.78 |
| LOC103349284 | 33.377 | 5.060788 | 0.002345 | 0.010515 | yes | up | 0 | 0 | 0 | 0.44 | 0.59 | 0.77 | 0 | 0.6 |
| KRT8 | 0.337 | -1.57126 | 0.002361 | 0.010575 | yes | down | 2.52 | 3.08 | 0.83 | 2.15 | 1.27 | 1.61 | 2.143333 | 1.676667 |
| RUNX2 | 2.803 | 1.48676 | 0.002368 | 0.0106 | yes | up | 0.27 | 0.16 | 0.42 | 2.52 | 1.38 | 1.19 | 0.283333 | 1.696667 |
| KCNQ4 | 0.367 | -1.44726 | 0.002377 | 0.01063 | yes | down | 9.72 | 9.74 | 2.15 | 7.05 | 6.33 | 5.12 | 7.203333 | 6.166667 |
| LOC108176476 | 4.013 | 2.004555 | 0.00238 | 0.010636 | yes | up | 0.23 | 0.15 | 0.03 | 1.1 | 1.3 | 1.42 | 0.136667 | 1.273333 |
| WDR4 | 0.424 | -1.23867 | 0.002382 | 0.010642 | yes | down | 1.5 | 2.88 | 1.69 | 2.01 | 1.52 | 2.36 | 2.023333 | 1.963333 |
| NALCN | 8.686 | 3.118613 | 0.002387 | 0.01066 | yes | up | 0.02 | 0.02 | 0 | 0.23 | 0.24 | 0.27 | 0.013333 | 0.246667 |
| LOC100341893 | 0.404 | -1.30699 | 0.002388 | 0.010665 | yes | down | 0.98 | 0.65 | 1.03 | 1.06 | 0.7 | 0.78 | 0.886667 | 0.846667 |
| PRIMPOL | 4.607 | 2.203771 | 0.002394 | 0.010685 | yes | up | 0.22 | 0.57 | 0.09 | 3.75 | 4.25 | 1.38 | 0.293333 | 3.126667 |
| GYPA | 0.255 | -1.973 | 0.002408 | 0.010736 | yes | down | 1.79 | 1.92 | 1.98 | 0.29 | 0.76 | 2.26 | 1.896667 | 1.103333 |
| KCNC2 | 0.033 | -4.93904 | 0.002427 | 0.0108 | yes | down | 0.24 | 0.17 | 0.1 | 0.04 | 0 | 0 | 0.17 | 0.013333 |
| LOC108177422 | 0.025 | -5.32807 | 0.002427 | 0.0108 | yes | down | 0.17 | 0.11 | 1.01 | 0 | 0.08 | 0 | 0.43 | 0.026667 |
| THBS1 | 0.155 | -2.68576 | 0.002463 | 0.010942 | yes | down | 157.41 | 48.35 | 20.72 | 23.64 | 23.77 | 37.52 | 75.49333 | 28.31 |
| TKTL1 | 0.022 | -5.51685 | 0.002472 | 0.010971 | yes | down | 0.13 | 0.08 | 0.14 | 0 | 0 | 0 | 0.116667 | 0 |
| LOC103346325 | 0.446 | -1.16523 | 0.002477 | 0.010991 | yes | down | 7.17 | 12.75 | 11.66 | 12.86 | 7 | 13.35 | 10.52667 | 11.07 |
| TPGS1 | 0.486 | -1.04162 | 0.002503 | 0.011091 | yes | down | 5.01 | 4.8 | 3 | 5.28 | 4.19 | 5.1 | 4.27 | 4.856667 |
| DNAH14 | 5.781 | 2.53143 | 0.002514 | 0.011134 | yes | up | 0 | 0.01 | 0.02 | 0.26 | 0.09 | 0.12 | 0.01 | 0.156667 |
| MAD2L1 | 2.011 | 1.008141 | 0.002533 | 0.011201 | yes | up | 4.28 | 4.12 | 3.9 | 21.55 | 24.68 | 11.47 | 4.1 | 19.23333 |
| RAB11FIP4 | 0.332 | -1.58882 | 0.002538 | 0.011222 | yes | down | 0.71 | 1.34 | 1.44 | 0.51 | 0.81 | 1.32 | 1.163333 | 0.88 |
| LOC103350977 | 0.117 | -3.09233 | 0.002544 | 0.011237 | yes | down | 0.73 | 0.65 | 0.49 | 0.09 | 0.35 | 0.1 | 0.623333 | 0.18 |
| MYH3 | 0.004 | -8.12789 | 0.002561 | 0.0113 | yes | down | 5.29 | 22.44 | 0.1 | 0 | 0.21 | 0 | 9.276667 | 0.07 |
| LOC103347148 | 14.873 | 3.894635 | 0.002563 | 0.011305 | yes | up | 0 | 0.13 | 0.12 | 6.21 | 8.58 | 7 | 0.083333 | 7.263333 |
| HPSE | 2.817 | 1.493956 | 0.002591 | 0.011416 | yes | up | 0.14 | 0.2 | 0.28 | 1.4 | 1.6 | 1.07 | 0.206667 | 1.356667 |
| LOC103346717 | 0.207 | -2.26947 | 0.002594 | 0.011423 | yes | down | 0.72 | 0.42 | 1.03 | 0.05 | 0.29 | 0.7 | 0.723333 | 0.346667 |
| RASGRP1 | 0.475 | -1.07387 | 0.002623 | 0.011542 | yes | down | 0.39 | 0.45 | 0.35 | 0.37 | 0.52 | 0.43 | 0.396667 | 0.44 |
| EPHB1 | 4.341 | 2.118164 | 0.002671 | 0.011729 | yes | up | 0.1 | 0.13 | 0.03 | 1.16 | 0.33 | 1.13 | 0.086667 | 0.873333 |
| MYBL1 | 3.228 | 1.690645 | 0.00268 | 0.011765 | yes | up | 0.24 | 0.23 | 0.08 | 1.28 | 2 | 0.77 | 0.183333 | 1.35 |
| DTX3L | 2.113 | 1.079006 | 0.002706 | 0.011865 | yes | up | 1.22 | 1.28 | 1.48 | 4.96 | 4.52 | 10.41 | 1.326667 | 6.63 |
| ACYP1 | 2.262 | 1.17731 | 0.002723 | 0.011929 | yes | up | 21.81 | 8.79 | 18.66 | 67.1 | 75.75 | 95.15 | 16.42 | 79.33333 |
| E2F2 | 3.664 | 1.873556 | 0.002744 | 0.012004 | yes | up | 0.16 | 0.22 | 0.14 | 1.91 | 2.1 | 0.46 | 0.173333 | 1.49 |
| LOC100342769 | 0.259 | -1.94872 | 0.002756 | 0.012048 | yes | down | 1.83 | 0.53 | 1.39 | 0.81 | 0.58 | 0.94 | 1.25 | 0.776667 |
| GPRC5A | 0.351 | -1.50866 | 0.002791 | 0.012179 | yes | down | 39.89 | 15.06 | 8.95 | 17.76 | 22.54 | 13.52 | 21.3 | 17.94 |
| LOC100356727 | 0.106 | -3.24025 | 0.002825 | 0.01231 | yes | down | 0.06 | 0.07 | 0.74 | 0.07 | 0.05 | 0.09 | 0.29 | 0.07 |
| LOC108178220 | 2.741 | 1.454515 | 0.002828 | 0.012321 | yes | up | 0.45 | 0.23 | 0.3 | 2.95 | 1.75 | 1.69 | 0.326667 | 2.13 |
| LOC100347628 | 0.291 | -1.77903 | 0.002833 | 0.012332 | yes | down | 2.98 | 1.65 | 1.33 | 2.31 | 0.48 | 1.41 | 1.986667 | 1.4 |
| FRK | 4.822 | 2.269531 | 0.002862 | 0.01244 | yes | up | 0.03 | 0.07 | 0.02 | 0.56 | 0.42 | 0.37 | 0.04 | 0.45 |
| MPZ | 0.12 | -3.0529 | 0.002866 | 0.012454 | yes | down | 98.43 | 210.08 | 232.64 | 14.97 | 111.56 | 17.97 | 180.3833 | 48.16667 |
| LGALS2 | 0.089 | -3.48433 | 0.002867 | 0.012454 | yes | down | 2.24 | 0.89 | 1.42 | 0.47 | 0 | 0.61 | 1.516667 | 0.36 |
| HDHD3 | 0.384 | -1.37931 | 0.002878 | 0.01249 | yes | down | 1.63 | 3.11 | 4.25 | 2.87 | 1.82 | 3.35 | 2.996667 | 2.68 |
| LOC100346811 | 3.91 | 1.967051 | 0.002882 | 0.012501 | yes | up | 0.18 | 0.06 | 1.92 | 1.24 | 4.34 | 1.38 | 0.72 | 2.32 |
| GPRIN1 | 6.91 | 2.788719 | 0.002889 | 0.012528 | yes | up | 0.09 | 0.18 | 0.16 | 2.48 | 1.81 | 2.73 | 0.143333 | 2.34 |
| LOC103346166 | 0.035 | -4.82084 | 0.002913 | 0.012616 | yes | down | 0.24 | 0.29 | 0.26 | 0.06 | 0 | 0 | 0.263333 | 0.02 |
| LOC100341997 | 2.522 | 1.334569 | 0.002914 | 0.012619 | yes | up | 0.72 | 1.66 | 1.68 | 7.94 | 11.24 | 4.46 | 1.353333 | 7.88 |
| LOC103349747 | 0.449 | -1.15649 | 0.002926 | 0.012663 | yes | down | 6.76 | 4.67 | 6.02 | 5.27 | 4.97 | 11.4 | 5.816667 | 7.213333 |
| LOC100343447 | 3.144 | 1.652649 | 0.002934 | 0.012681 | yes | up | 0.13 | 0.2 | 0.22 | 1.98 | 1.31 | 0.93 | 0.183333 | 1.406667 |
| PARPBP | 4.093 | 2.033252 | 0.002934 | 0.012681 | yes | up | 0.16 | 0.18 | 0.17 | 1.78 | 2.43 | 0.82 | 0.17 | 1.676667 |
| LOC100355644 | 0.358 | -1.48008 | 0.002934 | 0.012681 | yes | down | 1.05 | 0.94 | 0.67 | 0.97 | 0.33 | 0.94 | 0.886667 | 0.746667 |
| COL8A1 | 0.412 | -1.28093 | 0.002945 | 0.012713 | yes | down | 4.46 | 2.97 | 1.32 | 2.29 | 2.97 | 3.06 | 2.916667 | 2.773333 |
| LOC100351250 | 2.344 | 1.228815 | 0.002976 | 0.012832 | yes | up | 0.71 | 1.33 | 0.75 | 6.22 | 5.09 | 3.99 | 0.93 | 5.1 |
| ZBTB20 | 0.394 | -1.34547 | 0.003012 | 0.012951 | yes | down | 0.69 | 0.63 | 0.52 | 0.49 | 0.48 | 0.7 | 0.613333 | 0.556667 |
| TRIM10 | 0.123 | -3.02103 | 0.003014 | 0.012959 | yes | down | 0.2 | 0.25 | 0.23 | 0.04 | 0.04 | 0.12 | 0.226667 | 0.066667 |
| LOC100342069 | 0.201 | -2.31449 | 0.003051 | 0.013105 | yes | down | 18.4 | 15.66 | 15.87 | 3.33 | 3.25 | 17.67 | 16.64333 | 8.083333 |
| GRO-B | 0.329 | -1.60566 | 0.003071 | 0.013177 | yes | down | 30.14 | 11.53 | 4.84 | 9.26 | 15.91 | 14.33 | 15.50333 | 13.16667 |
| KCNC1 | 0.153 | -2.70913 | 0.003073 | 0.013185 | yes | down | 0.34 | 0.86 | 0.09 | 0.03 | 0.31 | 0.09 | 0.43 | 0.143333 |
| LOC100353330 | 0.429 | -1.22102 | 0.003088 | 0.013245 | yes | down | 19.77 | 44.82 | 45.71 | 16.57 | 51.33 | 36.04 | 36.76667 | 34.64667 |
| LOC103350861 | 0.016 | -6.00086 | 0.003113 | 0.013338 | yes | down | 0.62 | 0.04 | 0.07 | 0 | 0 | 0 | 0.243333 | 0 |
| PCDH8 | 4.098 | 2.034957 | 0.003127 | 0.013389 | yes | up | 0.04 | 0.07 | 0.05 | 0.71 | 0.28 | 0.49 | 0.053333 | 0.493333 |
| KLF9 | 0.492 | -1.02315 | 0.003137 | 0.013425 | yes | down | 30.5 | 18.8 | 13.6 | 28.09 | 28.19 | 18.26 | 20.96667 | 24.84667 |
| SIM2 | 0.43 | -1.21686 | 0.003158 | 0.013498 | yes | down | 1.07 | 1.37 | 0.66 | 1.49 | 1.13 | 1.16 | 1.033333 | 1.26 |
| FZD9 | 0.111 | -3.1691 | 0.003166 | 0.013517 | yes | down | 0.72 | 0.74 | 0.06 | 0.08 | 0.23 | 0.08 | 0.506667 | 0.13 |
| NFATC2 | 0.42 | -1.25161 | 0.003169 | 0.013526 | yes | down | 3.07 | 1.83 | 1.04 | 1.44 | 2.26 | 2.16 | 1.98 | 1.953333 |
| LOC103349679 | 2.147 | 1.10257 | 0.003175 | 0.013541 | yes | up | 1.02 | 0.93 | 1.25 | 7.28 | 3.33 | 4.27 | 1.066667 | 4.96 |
| LOC103346522 | 22.603 | 4.498423 | 0.003198 | 0.013624 | yes | up | 0.06 | 0 | 0 | 0.79 | 0.77 | 1.6 | 0.02 | 1.053333 |
| HAS3 | 3.223 | 1.688324 | 0.003215 | 0.013687 | yes | up | 0.45 | 0.22 | 0.31 | 4.44 | 1.31 | 2.13 | 0.326667 | 2.626667 |
| CDHR4 | 6.285 | 2.651879 | 0.003231 | 0.013748 | yes | up | 0 | 0.09 | 0.08 | 1.23 | 0.78 | 0.48 | 0.056667 | 0.83 |
| FOXC1 | 0.445 | -1.16864 | 0.003236 | 0.013762 | yes | down | 2.6 | 1.87 | 1.27 | 1.91 | 2 | 2.07 | 1.913333 | 1.993333 |
| MAPT | 2.535 | 1.341736 | 0.003236 | 0.013762 | yes | up | 3.91 | 3 | 4.53 | 13.18 | 9.64 | 34.74 | 3.813333 | 19.18667 |
| LOC103348254 | 8.796 | 3.136867 | 0.003237 | 0.013762 | yes | up | 0.19 | 0.33 | 0 | 2.21 | 7.41 | 0.77 | 0.173333 | 3.463333 |
| LOC103348003 | 4.077 | 2.027659 | 0.003239 | 0.013766 | yes | up | 0.35 | 1.36 | 1.61 | 7.07 | 4.82 | 19.47 | 1.106667 | 10.45333 |
| TYRO3 | 2.071 | 1.050196 | 0.003241 | 0.013772 | yes | up | 0.48 | 1.37 | 0.8 | 4.14 | 3.51 | 4.83 | 0.883333 | 4.16 |
| LOC103348828 | 0.474 | -1.07652 | 0.003242 | 0.013772 | yes | down | 0.62 | 1.49 | 1.04 | 1.15 | 1.29 | 0.76 | 1.05 | 1.066667 |
| LOC103351479 | 2.05 | 1.035849 | 0.003244 | 0.013779 | yes | up | 1.01 | 2.18 | 1.75 | 9.65 | 6.66 | 7.25 | 1.646667 | 7.853333 |
| LOC103351283 | 2.252 | 1.171433 | 0.003302 | 0.013985 | yes | up | 0.49 | 0.24 | 0.28 | 1.72 | 1.11 | 1.37 | 0.336667 | 1.4 |
| ARG2 | 0.171 | -2.54775 | 0.003348 | 0.014148 | yes | down | 6.73 | 2.75 | 0.3 | 0.43 | 2.2 | 0.6 | 3.26 | 1.076667 |
| LOC103347896 | 5.346 | 2.418375 | 0.003357 | 0.014182 | yes | up | 0.03 | 0.03 | 0.04 | 0.47 | 0.42 | 0.44 | 0.033333 | 0.443333 |
| TMEM45A | 2.08 | 1.05658 | 0.003367 | 0.014212 | yes | up | 0.94 | 1.62 | 0.55 | 6.26 | 4.81 | 5.27 | 1.036667 | 5.446667 |
| CDK18 | 0.367 | -1.44544 | 0.003389 | 0.014297 | yes | down | 2.01 | 1.2 | 1.59 | 2.48 | 0.99 | 0.76 | 1.6 | 1.41 |
| SLITRK6 | 2.249 | 1.169017 | 0.003415 | 0.014394 | yes | up | 0.41 | 0.62 | 0.8 | 5 | 2.25 | 2.4 | 0.61 | 3.216667 |
| HOXC5 | 6.808 | 2.767134 | 0.003419 | 0.014403 | yes | up | 0.19 | 0.09 | 0.17 | 2.61 | 1.78 | 2.99 | 0.15 | 2.46 |
| LOC100358934 | 25.957 | 4.69807 | 0.003432 | 0.01445 | yes | up | 0 | 0 | 0.06 | 0.41 | 2.37 | 1.27 | 0.02 | 1.35 |
| FOXO6 | 0.121 | -3.04302 | 0.003455 | 0.014527 | yes | down | 0.23 | 0.14 | 0.18 | 0.03 | 0.11 | 0.02 | 0.183333 | 0.053333 |
| KIAA0895L | 2.735 | 1.4518 | 0.003471 | 0.01458 | yes | up | 0.1 | 0.31 | 0.2 | 1.58 | 0.97 | 1.38 | 0.203333 | 1.31 |
| TLDC2 | 2.558 | 1.355238 | 0.003516 | 0.014733 | yes | up | 0.26 | 0.39 | 0.67 | 2.24 | 3.14 | 2.84 | 0.44 | 2.74 |
| LOC100356472 | 45.457 | 5.506427 | 0.003518 | 0.014737 | yes | up | 0 | 0 | 0 | 2.41 | 0.18 | 4.9 | 0 | 2.496667 |
| KLK7 | 0.048 | -4.38077 | 0.003524 | 0.014757 | yes | down | 0.16 | 0.48 | 0.63 | 0 | 0.14 | 0 | 0.423333 | 0.046667 |
| RHOV | 5.397 | 2.432266 | 0.003577 | 0.014933 | yes | up | 0.23 | 0.15 | 0.1 | 0.99 | 1.11 | 3.94 | 0.16 | 2.013333 |
| ZNF275 | 2.069 | 1.048801 | 0.003589 | 0.014969 | yes | up | 0.19 | 0.49 | 0.37 | 2.04 | 1.35 | 1.61 | 0.35 | 1.666667 |
| NDUFB1 | 0.479 | -1.06323 | 0.003603 | 0.01502 | yes | down | 55.41 | 58.87 | 53.73 | 50.84 | 46.82 | 98.46 | 56.00333 | 65.37333 |
| PHACTR1 | 0.496 | -1.01202 | 0.0037 | 0.015392 | yes | down | 16.58 | 7.51 | 8.88 | 11.6 | 15.61 | 12.68 | 10.99 | 13.29667 |
| SERPINA12 | 9.745 | 3.284697 | 0.003704 | 0.015401 | yes | up | 0.07 | 0.11 | 0.06 | 4.32 | 1.06 | 0.17 | 0.08 | 1.85 |
| TMEM98 | 3.539 | 1.82321 | 0.003706 | 0.015404 | yes | up | 0.14 | 0.27 | 0.25 | 2.79 | 2.23 | 0.42 | 0.22 | 1.813333 |
| BID | 2.249 | 1.169166 | 0.003713 | 0.015431 | yes | up | 0.51 | 1.02 | 0.4 | 3.02 | 2.36 | 3.39 | 0.643333 | 2.923333 |
| LOC103347420 | 0.1 | -3.31967 | 0.003722 | 0.015462 | yes | down | 0.51 | 0.66 | 0.4 | 0 | 0.29 | 0.07 | 0.523333 | 0.12 |
| LOC100353317 | 8.664 | 3.115085 | 0.003725 | 0.015469 | yes | up | 0 | 0.08 | 0.02 | 0.61 | 0.76 | 0.48 | 0.033333 | 0.616667 |
| LOC100346271 | 2.142 | 1.09928 | 0.003737 | 0.01551 | yes | up | 0.7 | 0.63 | 1.43 | 5.44 | 5.38 | 3.88 | 0.92 | 4.9 |
| VSTM2B | 0.036 | -4.79231 | 0.003788 | 0.015688 | yes | down | 0.14 | 0.27 | 0.33 | 0 | 0.06 | 0 | 0.246667 | 0.02 |
| RAD51C | 2.072 | 1.05075 | 0.003799 | 0.015728 | yes | up | 1.71 | 1.04 | 1.96 | 8.7 | 3.97 | 6.82 | 1.57 | 6.496667 |
| MYOD1 | 0.062 | -4.00366 | 0.003818 | 0.015789 | yes | down | 1.15 | 1.36 | 0.09 | 0 | 0.36 | 0 | 0.866667 | 0.12 |
| LOC108178854 | 7.417 | 2.890841 | 0.003821 | 0.015795 | yes | up | 0.07 | 0.05 | 0.02 | 1.65 | 0.36 | 0.57 | 0.046667 | 0.86 |
| LOC103347164 | 0.054 | -4.20329 | 0.003822 | 0.015797 | yes | down | 0.08 | 0.2 | 0.06 | 0 | 0.02 | 0.02 | 0.113333 | 0.013333 |
| CNTN4 | 0.393 | -1.34667 | 0.003869 | 0.01596 | yes | down | 0.33 | 0.65 | 0.36 | 0.26 | 0.35 | 0.52 | 0.446667 | 0.376667 |
| MAGIX | 0.339 | -1.55949 | 0.003892 | 0.016026 | yes | down | 5.13 | 4.13 | 1.79 | 5.02 | 1.94 | 1.9 | 3.683333 | 2.953333 |
| PLK3 | 0.287 | -1.7994 | 0.003983 | 0.016351 | yes | down | 10.44 | 2.17 | 1.91 | 2.75 | 3.13 | 4.25 | 4.84 | 3.376667 |
| ECE2 | 2.839 | 1.505242 | 0.003986 | 0.016361 | yes | up | 0.51 | 0.38 | 0.24 | 3.23 | 2.13 | 2.08 | 0.376667 | 2.48 |
| LOC100358696 | 2.03 | 1.0218 | 0.003989 | 0.016368 | yes | up | 0.95 | 0.67 | 0.96 | 3.82 | 3.99 | 4.55 | 0.86 | 4.12 |
| ABCG4 | 0.273 | -1.87237 | 0.003992 | 0.016376 | yes | down | 2.26 | 0.68 | 0.72 | 0.97 | 0.29 | 1.39 | 1.22 | 0.883333 |
| TRIP13 | 2.806 | 1.488737 | 0.004041 | 0.016543 | yes | up | 0.56 | 0.53 | 0.41 | 3.66 | 4.41 | 1.64 | 0.5 | 3.236667 |
| LOC108177684 | 0.058 | -4.11086 | 0.004042 | 0.016544 | yes | down | 3.11 | 3.23 | 0 | 0.15 | 0.32 | 0.14 | 2.113333 | 0.203333 |
| LOC103351558 | 2.038 | 1.027184 | 0.004062 | 0.016604 | yes | up | 1.05 | 1.43 | 2.05 | 9.65 | 6.07 | 5.29 | 1.51 | 7.003333 |
| XIRP1 | 0.018 | -5.79571 | 0.004072 | 0.016633 | yes | down | 27.6 | 18.08 | 0.1 | 0.06 | 1.83 | 0.03 | 15.26 | 0.64 |
| C9H18orf54 | 2.259 | 1.1758 | 0.004094 | 0.016711 | yes | up | 0.69 | 0.52 | 0.76 | 4.72 | 3.57 | 2.14 | 0.656667 | 3.476667 |
| TSPAN8 | 0.167 | -2.58404 | 0.004122 | 0.016801 | yes | down | 0.38 | 0.15 | 0.58 | 0.06 | 0.09 | 0.29 | 0.37 | 0.146667 |
| CDC45 | 2.851 | 1.511621 | 0.00413 | 0.016827 | yes | up | 17.38 | 26.19 | 20.57 | 44.62 | 25.81 | 58.67 | 21.38 | 43.03333 |
| MAG | 0.101 | -3.31153 | 0.004148 | 0.016886 | yes | down | 0.36 | 0.39 | 0.98 | 0 | 0.35 | 0.05 | 0.576667 | 0.133333 |
| DOK5 | 0.214 | -2.2273 | 0.004151 | 0.016896 | yes | down | 3.36 | 6.98 | 0.57 | 1.21 | 2.68 | 1.56 | 3.636667 | 1.816667 |
| OAS1 | 2.805 | 1.488018 | 0.004183 | 0.01701 | yes | up | 3.01 | 1.58 | 2.6 | 31.63 | 12.15 | 7.17 | 2.396667 | 16.98333 |
| SLC2A9 | 35.357 | 5.143934 | 0.004188 | 0.017025 | yes | up | 0 | 0 | 0 | 0.98 | 0.31 | 0.15 | 0 | 0.48 |
| LOC100348108 | 2.389 | 1.25663 | 0.004209 | 0.017099 | yes | up | 1.07 | 1.05 | 0.86 | 7.46 | 5.59 | 2.46 | 0.993333 | 5.17 |
| LOC103351453 | 0.378 | -1.40467 | 0.004213 | 0.017109 | yes | down | 3.95 | 2 | 3.08 | 3.34 | 1.91 | 1.78 | 3.01 | 2.343333 |
| LOC100345425 | 35.977 | 5.168997 | 0.004234 | 0.017179 | yes | up | 0 | 0 | 0 | 1.41 | 0.28 | 0.27 | 0 | 0.653333 |
| HYAL3 | 2.42 | 1.275098 | 0.004246 | 0.017218 | yes | up | 0.62 | 1.15 | 1.09 | 7.84 | 3.07 | 5.32 | 0.953333 | 5.41 |
| LOC100358590 | 2.03 | 1.021835 | 0.004251 | 0.017231 | yes | up | 0.62 | 0.42 | 0.37 | 2.42 | 2.56 | 1.71 | 0.47 | 2.23 |
| LOC103348702 | 7.436 | 2.894546 | 0.004258 | 0.017248 | yes | up | 0.02 | 0 | 0.34 | 1.27 | 2.97 | 1.96 | 0.12 | 2.066667 |
| PCYT1B | 2.664 | 1.413496 | 0.004269 | 0.017285 | yes | up | 0.1 | 0.15 | 0.11 | 0.82 | 0.51 | 0.91 | 0.12 | 0.746667 |
| SLC25A37 | 2.07 | 1.049533 | 0.004296 | 0.017377 | yes | up | 3.94 | 5.19 | 5.25 | 11.77 | 26.88 | 36.77 | 4.793333 | 25.14 |
| ADGRG3 | 0.098 | -3.34535 | 0.004302 | 0.017396 | yes | down | 0.2 | 0.15 | 0.07 | 0.05 | 0.03 | 0.02 | 0.14 | 0.033333 |
| LOC103345394 | 3.342 | 1.740702 | 0.004317 | 0.017438 | yes | up | 2.08 | 2.25 | 0.61 | 16.24 | 10.88 | 18.58 | 1.646667 | 15.23333 |
| TMEM255A | 2.089 | 1.062596 | 0.004337 | 0.017505 | yes | up | 0.87 | 1.48 | 1.57 | 9.6 | 5.25 | 3.9 | 1.306667 | 6.25 |
| FBXO43 | 26.618 | 4.734354 | 0.004418 | 0.017786 | yes | up | 0 | 0.02 | 0 | 0.82 | 0.39 | 0.07 | 0.006667 | 0.426667 |
| CAMKMT | 2.407 | 1.267113 | 0.004419 | 0.017786 | yes | up | 0.69 | 0.98 | 1.03 | 3.22 | 7.41 | 4.22 | 0.9 | 4.95 |
| CAPN12 | 2.712 | 1.439281 | 0.004439 | 0.017864 | yes | up | 0.42 | 0.74 | 1.28 | 7.59 | 5.92 | 3.69 | 0.813333 | 5.733333 |
| MCF2L2 | 2.394 | 1.259601 | 0.004444 | 0.017881 | yes | up | 0.33 | 0.36 | 0.38 | 2.89 | 1.06 | 2.06 | 0.356667 | 2.003333 |
| LOC103344962 | 0.291 | -1.78335 | 0.004479 | 0.017993 | yes | down | 0.45 | 0.42 | 0.56 | 0.37 | 0.31 | 0.31 | 0.476667 | 0.33 |
| CAMKK1 | 2.127 | 1.088819 | 0.004486 | 0.018009 | yes | up | 0.25 | 0.74 | 0.47 | 2.48 | 2.27 | 2.38 | 0.486667 | 2.376667 |
| HCAR1 | 2.018 | 1.013028 | 0.004515 | 0.018105 | yes | up | 3.6 | 3.57 | 4.78 | 22.83 | 9.09 | 25.43 | 3.983333 | 19.11667 |
| LOC100356870 | 0.037 | -4.75352 | 0.004532 | 0.018165 | yes | down | 0.6 | 0.69 | 0.29 | 0 | 0.17 | 0 | 0.526667 | 0.056667 |
| GPR63 | 2.633 | 1.396471 | 0.004535 | 0.018172 | yes | up | 0.1 | 0.29 | 0.3 | 1.33 | 0.89 | 1.91 | 0.23 | 1.376667 |
| LOC103351959 | 3.827 | 1.936212 | 0.004538 | 0.018178 | yes | up | 0.01 | 0.21 | 0.17 | 1.3 | 0.77 | 1.03 | 0.13 | 1.033333 |
| SRRM5 | 3.512 | 1.812331 | 0.004543 | 0.018196 | yes | up | 0.07 | 0.04 | 0.12 | 0.96 | 0.45 | 0.47 | 0.076667 | 0.626667 |
| SLC6A17 | 0.21 | -2.25072 | 0.004553 | 0.018225 | yes | down | 0.2 | 0.51 | 0.33 | 0.19 | 0.02 | 0.29 | 0.346667 | 0.166667 |
| ENOSF1 | 7.496 | 2.906026 | 0.004569 | 0.018278 | yes | up | 0.27 | 0.46 | 3 | 30.04 | 14.42 | 20.83 | 1.243333 | 21.76333 |
| LOC108178915 | 3.278 | 1.712843 | 0.004569 | 0.018278 | yes | up | 0.64 | 0.62 | 0.96 | 9.05 | 5.49 | 3.14 | 0.74 | 5.893333 |
| PTPRR | 0.385 | -1.37685 | 0.004615 | 0.018421 | yes | down | 0.92 | 0.62 | 0.81 | 0.6 | 0.99 | 0.53 | 0.783333 | 0.706667 |
| IL15 | 0.438 | -1.19211 | 0.004638 | 0.018501 | yes | down | 2.12 | 4.42 | 4.25 | 2.37 | 1.92 | 1.59 | 3.596667 | 1.96 |
| PPAT | 0.405 | -1.30481 | 0.004648 | 0.018532 | yes | down | 0.83 | 1.55 | 0.83 | 0.59 | 1.04 | 1.33 | 1.07 | 0.986667 |
| SGK2 | 3.195 | 1.675601 | 0.004767 | 0.018957 | yes | up | 0.12 | 0.09 | 0.13 | 0.67 | 0.54 | 1.35 | 0.113333 | 0.853333 |
| GRIN2C | 0.168 | -2.57517 | 0.004773 | 0.018977 | yes | down | 0.25 | 0.35 | 0.25 | 0.13 | 0.13 | 0 | 0.283333 | 0.086667 |
| LOC103350917 | 15.838 | 3.985342 | 0.004779 | 0.018996 | yes | up | 0 | 0.06 | 0 | 1.25 | 0.48 | 0.38 | 0.02 | 0.703333 |
| IL17RE | 2.589 | 1.372271 | 0.004818 | 0.019122 | yes | up | 0.28 | 0.23 | 0.25 | 1.92 | 1.43 | 1.16 | 0.253333 | 1.503333 |
| CR1 | 0.191 | -2.39086 | 0.004821 | 0.019128 | yes | down | 0.09 | 0.12 | 0.07 | 0.03 | 0.08 | 0.02 | 0.093333 | 0.043333 |
| SHISA3 | 2.358 | 1.237278 | 0.004825 | 0.019136 | yes | up | 0.31 | 0.89 | 0.66 | 3.75 | 3.99 | 2.29 | 0.62 | 3.343333 |
| LOC103346986 | 2.345 | 1.229409 | 0.004852 | 0.019209 | yes | up | 0.53 | 0.63 | 0.29 | 3.07 | 2.52 | 2.22 | 0.483333 | 2.603333 |
| RAB20 | 0.1 | -3.32596 | 0.004852 | 0.019209 | yes | down | 0.62 | 0.7 | 0.82 | 0.25 | 0 | 0.25 | 0.713333 | 0.166667 |
| ADAMTS8 | 0.294 | -1.7653 | 0.004872 | 0.019278 | yes | down | 0.88 | 1.59 | 0.35 | 0.51 | 0.95 | 0.42 | 0.94 | 0.626667 |
| LOC100303760 | 0.02 | -5.61225 | 0.004915 | 0.019393 | yes | down | 37.15 | 116.57 | 4.26 | 0.01 | 6.72 | 0.25 | 52.66 | 2.326667 |
| STC2 | 0.44 | -1.18329 | 0.004918 | 0.019399 | yes | down | 1.3 | 0.81 | 0.86 | 0.87 | 0.77 | 1.43 | 0.99 | 1.023333 |
| WFIKKN2 | 0.427 | -1.22684 | 0.004941 | 0.019485 | yes | down | 1.03 | 0.63 | 0.49 | 0.72 | 0.82 | 0.62 | 0.716667 | 0.72 |
| XKR7 | 0.117 | -3.09479 | 0.004962 | 0.019557 | yes | down | 0.06 | 0.28 | 0.21 | 0 | 0.12 | 0.02 | 0.183333 | 0.046667 |
| EPSTI1 | 0.443 | -1.17428 | 0.004963 | 0.019557 | yes | down | 5.82 | 5.67 | 8.15 | 5.27 | 4.32 | 11.57 | 6.546667 | 7.053333 |
| LCN2 | 14.221 | 3.82996 | 0.004995 | 0.019672 | yes | up | 0.16 | 0.23 | 0 | 0.1 | 6.51 | 5.6 | 0.13 | 4.07 |
| LOC100339919 | 0.346 | -1.53285 | 0.00502 | 0.019763 | yes | down | 0.38 | 0.34 | 0.56 | 0.5 | 0.31 | 0.22 | 0.426667 | 0.343333 |
| DSG2 | 0.461 | -1.11821 | 0.005043 | 0.019838 | yes | down | 1.14 | 1.96 | 1.59 | 1.59 | 0.84 | 2.55 | 1.563333 | 1.66 |
| LOC103344968 | 0.021 | -5.54515 | 0.005045 | 0.019839 | yes | down | 0.04 | 0.38 | 0.14 | 0 | 0 | 0 | 0.186667 | 0 |
| KCTD14 | 0.229 | -2.12425 | 0.00516 | 0.020228 | yes | down | 0.51 | 0.92 | 0.9 | 0.44 | 0.38 | 0.37 | 0.776667 | 0.396667 |
| LOC103350201 | 2.886 | 1.529153 | 0.005176 | 0.020278 | yes | up | 0.36 | 0.35 | 1.06 | 6.13 | 3.86 | 2.55 | 0.59 | 4.18 |
| NIPAL1 | 0.033 | -4.90643 | 0.005181 | 0.020283 | yes | down | 0.17 | 0.06 | 0.03 | 0 | 0.02 | 0 | 0.086667 | 0.006667 |
| SPOCK1 | 3.797 | 1.924988 | 0.005186 | 0.020296 | yes | up | 0.26 | 0.1 | 0.23 | 3.73 | 0.64 | 1.13 | 0.196667 | 1.833333 |
| STXBP5L | 3.482 | 1.799823 | 0.005191 | 0.020312 | yes | up | 0.03 | 0.04 | 0.04 | 0.5 | 0.17 | 0.26 | 0.036667 | 0.31 |
| LOC103345403 | 2.332 | 1.221841 | 0.005199 | 0.020339 | yes | up | 0.36 | 0.73 | 0.4 | 2.62 | 2.4 | 2.27 | 0.496667 | 2.43 |
| CHRDL2 | 0.046 | -4.4506 | 0.0052 | 0.020339 | yes | down | 0.2 | 0.58 | 0.03 | 0 | 0.04 | 0.04 | 0.27 | 0.026667 |
| ADAM2 | 0.329 | -1.60413 | 0.005215 | 0.020378 | yes | down | 0.44 | 0.81 | 1.59 | 0.99 | 0.9 | 0.46 | 0.946667 | 0.783333 |
| AZIN2 | 2.373 | 1.246992 | 0.005245 | 0.020477 | yes | up | 0.44 | 0.28 | 0.61 | 2.42 | 2.51 | 2.52 | 0.443333 | 2.483333 |
| CEP112 | 2.134 | 1.093725 | 0.005295 | 0.020647 | yes | up | 0.64 | 0.31 | 0.62 | 2.38 | 2.63 | 2.86 | 0.523333 | 2.623333 |
| LOC103349621 | 3.57 | 1.835894 | 0.005301 | 0.02066 | yes | up | 0.18 | 0.07 | 0.18 | 1.12 | 1.2 | 1.21 | 0.143333 | 1.176667 |
| LOC100343001 | 0.474 | -1.07844 | 0.005301 | 0.02066 | yes | down | 1.6 | 4.15 | 2.2 | 3.63 | 2.48 | 3.8 | 2.65 | 3.303333 |
| SPRY4 | 0.17 | -2.55637 | 0.005308 | 0.020681 | yes | down | 55.87 | 14.64 | 7.68 | 12.26 | 5.5 | 14.81 | 26.06333 | 10.85667 |
| C4A | 2.147 | 1.102508 | 0.005326 | 0.020731 | yes | up | 14.87 | 13.55 | 13.91 | 54.14 | 34.71 | 123.57 | 14.11 | 70.80667 |
| LOC103348533 | 14.294 | 3.8373 | 0.005345 | 0.020792 | yes | up | 0.31 | 0 | 0 | 3.02 | 4.87 | 1.89 | 0.103333 | 3.26 |
| LOC103348061 | 11.598 | 3.535823 | 0.005357 | 0.020827 | yes | up | 0.02 | 0 | 0.04 | 0.48 | 0.23 | 1.19 | 0.02 | 0.633333 |
| LOC108178938 | 0.385 | -1.37845 | 0.005378 | 0.020899 | yes | down | 0.42 | 0.43 | 0.46 | 0.46 | 0.29 | 0.42 | 0.436667 | 0.39 |
| FAM221B | 0.211 | -2.24615 | 0.005419 | 0.021046 | yes | down | 0.34 | 0.23 | 0.52 | 0.32 | 0.07 | 0.17 | 0.363333 | 0.186667 |
| LOC103349209 | 0.141 | -2.82211 | 0.005476 | 0.021241 | yes | down | 0.07 | 0.2 | 0.21 | 0.05 | 0.02 | 0.09 | 0.16 | 0.053333 |
| PPP1R1B | 0.027 | -5.2032 | 0.005483 | 0.021264 | yes | down | 95.94 | 111.72 | 135.67 | 0.21 | 0.06 | 21.4 | 114.4433 | 7.223333 |
| ZNF385D | 0.47 | -1.08808 | 0.005517 | 0.021379 | yes | down | 0.93 | 0.82 | 0.47 | 0.87 | 0.81 | 0.8 | 0.74 | 0.826667 |
| KCNJ5 | 40.097 | 5.32543 | 0.00552 | 0.021387 | yes | up | 0 | 0 | 0.01 | 0.36 | 0 | 0.87 | 0.003333 | 0.41 |
| LOC108177741 | 2.533 | 1.340914 | 0.005526 | 0.021404 | yes | up | 0.51 | 0.24 | 0.47 | 2.87 | 1.7 | 2.83 | 0.406667 | 2.466667 |
| MEOX1 | 2.293 | 1.19712 | 0.005555 | 0.021502 | yes | up | 0.44 | 1.51 | 0.82 | 6.94 | 3.96 | 3.7 | 0.923333 | 4.866667 |
| LOC100339353 | 0.038 | -4.70928 | 0.005574 | 0.021572 | yes | down | 0.28 | 0.82 | 0.33 | 0 | 0 | 0.11 | 0.476667 | 0.036667 |
| LGI1 | 0.457 | -1.13051 | 0.005602 | 0.02167 | yes | down | 0.87 | 0.98 | 0.48 | 0.68 | 0.69 | 1.1 | 0.776667 | 0.823333 |
| MYBL2 | 7.595 | 2.925016 | 0.005607 | 0.021683 | yes | up | 0.05 | 0.34 | 0 | 3.08 | 2.94 | 0.5 | 0.13 | 2.173333 |
| SLC26A9 | 0.399 | -1.32543 | 0.005619 | 0.021727 | yes | down | 1.71 | 5.38 | 7.78 | 6.05 | 6.87 | 5.19 | 4.956667 | 6.036667 |
| EEF2KMT | 0.365 | -1.4538 | 0.005634 | 0.021768 | yes | down | 1.65 | 1.53 | 1.95 | 2.22 | 0.65 | 1.57 | 1.71 | 1.48 |
| TTLL8 | 0.062 | -4.01658 | 0.005646 | 0.021809 | yes | down | 0.09 | 0.13 | 0.15 | 0.03 | 0 | 0.03 | 0.123333 | 0.02 |
| GUCY2C | 13.378 | 3.741826 | 0.005647 | 0.021809 | yes | up | 0 | 0.04 | 0.01 | 1.43 | 0.11 | 0.15 | 0.016667 | 0.563333 |
| MSTN | 9.856 | 3.300964 | 0.005692 | 0.02195 | yes | up | 0 | 0.06 | 0.11 | 0.82 | 1.68 | 1.37 | 0.056667 | 1.29 |
| LOC100350920 | 0.087 | -3.52501 | 0.005707 | 0.021978 | yes | down | 5.21 | 2.11 | 2.73 | 0.25 | 0.03 | 1.41 | 3.35 | 0.563333 |
| TMEM86A | 0.422 | -1.24382 | 0.00571 | 0.021984 | yes | down | 10.18 | 3.7 | 4.41 | 8.75 | 4.75 | 5.29 | 6.096667 | 6.263333 |
| PODXL2 | 0.319 | -1.64859 | 0.005723 | 0.02203 | yes | down | 1.58 | 2.44 | 1.33 | 2.29 | 0.62 | 0.97 | 1.783333 | 1.293333 |
| COL25A1 | 3.028 | 1.598465 | 0.005757 | 0.022144 | yes | up | 0.23 | 0.22 | 0.18 | 1.29 | 0.5 | 2.39 | 0.21 | 1.393333 |
| MZF1 | 3.273 | 1.710447 | 0.005821 | 0.022374 | yes | up | 0.29 | 0.24 | 0.15 | 2.07 | 1.63 | 1.47 | 0.226667 | 1.723333 |
| KIAA1324 | 3.319 | 1.730636 | 0.005826 | 0.022389 | yes | up | 0.1 | 0.22 | 0.37 | 1.86 | 0.56 | 4.16 | 0.23 | 2.193333 |
| CTHRC1 | 3.378 | 1.756004 | 0.005848 | 0.022453 | yes | up | 1.13 | 3.89 | 1 | 19.96 | 20.58 | 5.57 | 2.006667 | 15.37 |
| RMI2 | 3.629 | 1.859737 | 0.005867 | 0.022517 | yes | up | 0.24 | 0.27 | 0.14 | 2.52 | 1.97 | 0.98 | 0.216667 | 1.823333 |
| ZFP3 | 0.384 | -1.3791 | 0.005877 | 0.022547 | yes | down | 1.13 | 3.02 | 3.07 | 2.63 | 1.54 | 1.98 | 2.406667 | 2.05 |
| CH25H | 0.407 | -1.29554 | 0.005891 | 0.022593 | yes | down | 1.04 | 0.89 | 0.58 | 0.64 | 0.78 | 0.95 | 0.836667 | 0.79 |
| LOC103350157 | 0.29 | -1.78384 | 0.005957 | 0.02282 | yes | down | 3.2 | 1.67 | 1.08 | 2.44 | 0.89 | 0.81 | 1.983333 | 1.38 |
| SEC14L5 | 0.172 | -2.53537 | 0.006103 | 0.023308 | yes | down | 0.22 | 0.41 | 0.07 | 0.08 | 0.15 | 0.04 | 0.233333 | 0.09 |
| PCDH10 | 3.617 | 1.854733 | 0.006112 | 0.02333 | yes | up | 0.03 | 0.05 | 0.05 | 0.66 | 0.2 | 0.18 | 0.043333 | 0.346667 |
| LOC103347240 | 2.23 | 1.157253 | 0.006127 | 0.023379 | yes | up | 0.66 | 0.7 | 0.94 | 4.06 | 2.01 | 5.95 | 0.766667 | 4.006667 |
| LOC103346242 | 0.039 | -4.67556 | 0.006172 | 0.023527 | yes | down | 2.3 | 0.85 | 0.11 | 0 | 0 | 0.29 | 1.086667 | 0.096667 |
| TCFL5 | 0.129 | -2.95065 | 0.006219 | 0.023678 | yes | down | 0.3 | 0.29 | 0.23 | 0.12 | 0.13 | 0 | 0.273333 | 0.083333 |
| LOC100356510 | 53.749 | 5.748154 | 0.006312 | 0.02399 | yes | up | 0 | 0 | 0 | 1.41 | 0 | 1.83 | 0 | 1.08 |
| LOC100338734 | 0.096 | -3.37892 | 0.006373 | 0.024203 | yes | down | 0.27 | 0.42 | 0.16 | 0.04 | 0.14 | 0 | 0.283333 | 0.06 |
| TMEM173 | 2.096 | 1.067632 | 0.006374 | 0.024203 | yes | up | 0.91 | 1.41 | 1.19 | 8.85 | 4.15 | 4.34 | 1.17 | 5.78 |
| GAREM2 | 0.301 | -1.73296 | 0.006386 | 0.024243 | yes | down | 0.18 | 0.33 | 0.3 | 0.12 | 0.2 | 0.24 | 0.27 | 0.186667 |
| KCNB2 | 4.137 | 2.048679 | 0.006438 | 0.024408 | yes | up | 0.02 | 0.16 | 0.14 | 1.11 | 0.44 | 1.68 | 0.106667 | 1.076667 |
| LMO7 | 0.201 | -2.31625 | 0.006526 | 0.024684 | yes | down | 8.6 | 5.08 | 1.12 | 2.32 | 3.43 | 1.9 | 4.933333 | 2.55 |
| DIO2 | 0.156 | -2.68323 | 0.00656 | 0.02479 | yes | down | 7.21 | 6.61 | 4.76 | 0.96 | 0.15 | 3.43 | 6.193333 | 1.513333 |
| HBEGF | 0.369 | -1.43947 | 0.006571 | 0.024819 | yes | down | 8.3 | 3.72 | 1.67 | 3.44 | 4.63 | 3.91 | 4.563333 | 3.993333 |
| LOC108176477 | 2.048 | 1.034044 | 0.006573 | 0.024821 | yes | up | 1.03 | 1.18 | 0.62 | 4.44 | 3.5 | 5.65 | 0.943333 | 4.53 |
| LOC108178071 | 4.923 | 2.299541 | 0.006587 | 0.024863 | yes | up | 0.06 | 0.05 | 0.07 | 0.89 | 0.63 | 0.63 | 0.06 | 0.716667 |
| GINS2 | 2.112 | 1.078929 | 0.006598 | 0.024896 | yes | up | 2.2 | 1.34 | 2.58 | 10.35 | 10.6 | 9.68 | 2.04 | 10.21 |
| ST8SIA3 | 0.115 | -3.11593 | 0.006626 | 0.024984 | yes | down | 0.08 | 0.19 | 0.25 | 0.01 | 0.13 | 0 | 0.173333 | 0.046667 |
| LOC108176491 | 5.1 | 2.350385 | 0.006626 | 0.024984 | yes | up | 0.03 | 0.06 | 0.21 | 1.64 | 0.73 | 0.77 | 0.1 | 1.046667 |
| CTGF | 0.407 | -1.29524 | 0.006719 | 0.025264 | yes | down | 47.01 | 27.72 | 18.84 | 17.87 | 18.39 | 53.72 | 31.19 | 29.99333 |
| LOC103347358 | 0.103 | -3.27848 | 0.006743 | 0.025326 | yes | down | 0.35 | 0.23 | 0.41 | 0 | 0.15 | 0.11 | 0.33 | 0.086667 |
| COLQ | 0.281 | -1.83028 | 0.006756 | 0.025353 | yes | down | 3.3 | 2.31 | 0.41 | 1.55 | 1.77 | 0.73 | 2.006667 | 1.35 |
| LOC100340649 | 2.834 | 1.50264 | 0.006772 | 0.025401 | yes | up | 0.36 | 0.25 | 0.45 | 3.99 | 1.78 | 1.49 | 0.353333 | 2.42 |
| TRPV4 | 0.46 | -1.12094 | 0.00678 | 0.025422 | yes | down | 1.49 | 1.25 | 0.73 | 1.12 | 1.14 | 1.46 | 1.156667 | 1.24 |
| LOC108176656 | 2.567 | 1.360082 | 0.006817 | 0.025534 | yes | up | 0.24 | 0.49 | 0.56 | 1.93 | 3.08 | 2.62 | 0.43 | 2.543333 |
| LOC103350873 | 0.064 | -3.95654 | 0.006862 | 0.025684 | yes | down | 0.69 | 0.44 | 0.05 | 0 | 0.08 | 0.12 | 0.393333 | 0.066667 |
| LOC100346600 | 2.634 | 1.397109 | 0.006866 | 0.025692 | yes | up | 0.19 | 0.37 | 0.34 | 3.31 | 1.83 | 1.54 | 0.3 | 2.226667 |
| CENPT | 4.366 | 2.126475 | 0.006927 | 0.025897 | yes | up | 0.34 | 0.15 | 0.1 | 3.1 | 2.26 | 0.77 | 0.196667 | 2.043333 |
| LOC100343469 | 3.398 | 1.764861 | 0.006956 | 0.025991 | yes | up | 0.06 | 0.12 | 0.15 | 0.78 | 0.64 | 1.16 | 0.11 | 0.86 |
| CENPW | 2.423 | 1.276728 | 0.007007 | 0.026144 | yes | up | 3.74 | 1.67 | 2.51 | 13.23 | 19.26 | 12.99 | 2.64 | 15.16 |
| CLVS2 | 0.122 | -3.03891 | 0.00717 | 0.026638 | yes | down | 0.04 | 0.06 | 0.1 | 0.01 | 0.04 | 0.01 | 0.066667 | 0.02 |
| CPVL | 0.294 | -1.7657 | 0.007182 | 0.026672 | yes | down | 0.96 | 0.65 | 0.39 | 0.35 | 0.79 | 0.3 | 0.666667 | 0.48 |
| RP1L1 | 24.134 | 4.592984 | 0.007195 | 0.0267 | yes | up | 0.01 | 0 | 0 | 0.47 | 0.13 | 0.04 | 0.003333 | 0.213333 |
| PON3 | 2.101 | 1.071142 | 0.007216 | 0.026767 | yes | up | 2.79 | 2.9 | 4.23 | 18.11 | 21.45 | 9.21 | 3.306667 | 16.25667 |
| CCDC92 | 2.218 | 1.148949 | 0.007241 | 0.026844 | yes | up | 0.65 | 1.75 | 0.9 | 5.54 | 6.47 | 4.56 | 1.1 | 5.523333 |
| PLA2G12A | 0.497 | -1.00816 | 0.007274 | 0.026947 | yes | down | 16.75 | 22.2 | 12.19 | 16.67 | 30.29 | 11.88 | 17.04667 | 19.61333 |
| LRRC52 | 0.077 | -3.70437 | 0.00729 | 0.026995 | yes | down | 6.87 | 6.94 | 6.86 | 0.48 | 0.03 | 3.18 | 6.89 | 1.23 |
| PSD2 | 0.131 | -2.93258 | 0.007295 | 0.027005 | yes | down | 0.11 | 0.07 | 0.07 | 0.03 | 0.03 | 0.01 | 0.083333 | 0.023333 |
| LOC100342729 | 0.176 | -2.50739 | 0.007302 | 0.02702 | yes | down | 0.31 | 1.52 | 0.82 | 0.19 | 0.31 | 0.55 | 0.883333 | 0.35 |
| NPY5R | 3.666 | 1.874277 | 0.007356 | 0.027204 | yes | up | 0.05 | 0.26 | 0.25 | 1.38 | 1.42 | 1.49 | 0.186667 | 1.43 |
| ANKRD66 | 17.201 | 4.104451 | 0.007358 | 0.027204 | yes | up | 0 | 0.92 | 0.11 | 9.58 | 22.04 | 0.88 | 0.343333 | 10.83333 |
| LOC100343456 | 5.023 | 2.328593 | 0.007367 | 0.027231 | yes | up | 0.04 | 0.08 | 0.22 | 2.17 | 0.89 | 1.15 | 0.113333 | 1.403333 |
| LOC100339707 | 0.131 | -2.93552 | 0.00741 | 0.027376 | yes | down | 0.22 | 0.21 | 0.19 | 0.11 | 0.04 | 0.04 | 0.206667 | 0.063333 |
| CENPK | 2.848 | 1.510194 | 0.007412 | 0.027377 | yes | up | 0.36 | 0.59 | 0.9 | 4.46 | 5.96 | 2.02 | 0.616667 | 4.146667 |
| LOC100345261 | 2.28 | 1.189145 | 0.007428 | 0.027406 | yes | up | 2.01 | 3.5 | 2.4 | 13.87 | 11.01 | 17.41 | 2.636667 | 14.09667 |
| LOC103351973 | 3.422 | 1.774757 | 0.007457 | 0.027491 | yes | up | 0.04 | 0.19 | 0.24 | 1.83 | 0.75 | 1.23 | 0.156667 | 1.27 |
| LOC103347295 | 4.071 | 2.025428 | 0.007503 | 0.027635 | yes | up | 0.18 | 0.14 | 0.03 | 1.33 | 1.29 | 1.01 | 0.116667 | 1.21 |
| C1RL | 4.102 | 2.036187 | 0.007503 | 0.027635 | yes | up | 0.09 | 0.23 | 0.28 | 0.6 | 2.79 | 2.16 | 0.2 | 1.85 |
| LOC108178472 | 0.04 | -4.6515 | 0.007518 | 0.027664 | yes | down | 0.21 | 0.32 | 0.21 | 0 | 0.05 | 0 | 0.246667 | 0.016667 |
| MAFF | 0.242 | -2.04953 | 0.007538 | 0.027724 | yes | down | 7.15 | 4.21 | 1.55 | 1.22 | 2.61 | 3.55 | 4.303333 | 2.46 |
| RCAN2 | 0.351 | -1.51159 | 0.007589 | 0.027884 | yes | down | 3.92 | 4.48 | 0.59 | 1.36 | 3.95 | 1.84 | 2.996667 | 2.383333 |
| LOC100350067 | 0.08 | -3.64851 | 0.007627 | 0.028004 | yes | down | 0.24 | 0.81 | 0.21 | 0 | 0.16 | 0.07 | 0.42 | 0.076667 |
| DRP2 | 0.139 | -2.85096 | 0.007683 | 0.028171 | yes | down | 1.28 | 1.96 | 2.39 | 0.17 | 1.44 | 0.13 | 1.876667 | 0.58 |
| LHX6 | 2.219 | 1.149609 | 0.007688 | 0.028178 | yes | up | 0.67 | 1.83 | 1.44 | 11.2 | 4.39 | 4.68 | 1.313333 | 6.756667 |
| IMPG2 | 3.622 | 1.856748 | 0.007688 | 0.028178 | yes | up | 0.07 | 0.08 | 0.04 | 0.71 | 0.33 | 0.56 | 0.063333 | 0.533333 |
| LOC103351528 | 0.492 | -1.02366 | 0.00772 | 0.028262 | yes | down | 0.48 | 0.84 | 0.64 | 0.88 | 0.69 | 0.45 | 0.653333 | 0.673333 |
| LOC100356881 | 7.845 | 2.971807 | 0.007729 | 0.02829 | yes | up | 0.83 | 1.98 | 0 | 21.56 | 25.72 | 3.51 | 0.936667 | 16.93 |
| LOC108177970 | 0.451 | -1.14868 | 0.007737 | 0.028306 | yes | down | 1.29 | 1.88 | 0.81 | 1.41 | 1.29 | 1.44 | 1.326667 | 1.38 |
| LOC103347207 | 3.548 | 1.826945 | 0.007746 | 0.028326 | yes | up | 0.18 | 0.07 | 0.32 | 2.65 | 1.14 | 1.08 | 0.19 | 1.623333 |
| FSIP1 | 28.077 | 4.811307 | 0.007754 | 0.028349 | yes | up | 0 | 0 | 0 | 0.13 | 0.45 | 0.31 | 0 | 0.296667 |
| WDHD1 | 2.294 | 1.198171 | 0.007758 | 0.028349 | yes | up | 0.23 | 0.44 | 0.31 | 2.19 | 1.95 | 1.01 | 0.326667 | 1.716667 |
| LOC108178530 | 0.415 | -1.27017 | 0.007761 | 0.028349 | yes | down | 2.88 | 1.84 | 0.96 | 2.05 | 2.23 | 1.28 | 1.893333 | 1.853333 |
| PIF1 | 36.731 | 5.198914 | 0.007813 | 0.028515 | yes | up | 0 | 0 | 0 | 0.77 | 0.32 | 0.03 | 0 | 0.373333 |
| ZYG11A | 26.638 | 4.735389 | 0.007918 | 0.028862 | yes | up | 0 | 0 | 0 | 0.09 | 0.19 | 0.19 | 0 | 0.156667 |
| SYT10 | 0.028 | -5.17701 | 0.00795 | 0.028952 | yes | down | 0 | 0.18 | 0.17 | 0 | 0 | 0.02 | 0.116667 | 0.006667 |
| SCUBE3 | 2.311 | 1.208611 | 0.00796 | 0.028972 | yes | up | 0.16 | 0.22 | 0.15 | 1.07 | 0.54 | 1.23 | 0.176667 | 0.946667 |
| LOC103348228 | 0.041 | -4.60245 | 0.007968 | 0.028991 | yes | down | 0.13 | 0.5 | 0.28 | 0 | 0.08 | 0 | 0.303333 | 0.026667 |
| LOC100341069 | 0.191 | -2.39147 | 0.007994 | 0.029061 | yes | down | 0.72 | 0.4 | 0.88 | 0.16 | 0.15 | 0.33 | 0.666667 | 0.213333 |
| FAM110D | 2.269 | 1.181931 | 0.007999 | 0.029066 | yes | up | 1.37 | 1.42 | 1.78 | 11.3 | 7.55 | 5.62 | 1.523333 | 8.156667 |
| L3HYPDH | 2.237 | 1.161322 | 0.008022 | 0.029142 | yes | up | 0.65 | 1.12 | 0.53 | 3.78 | 4.12 | 3.89 | 0.766667 | 3.93 |
| ZCWPW1 | 0.39 | -1.35664 | 0.008046 | 0.029216 | yes | down | 0.54 | 0.42 | 0.74 | 0.56 | 0.51 | 0.57 | 0.566667 | 0.546667 |
| GATA3 | 3.694 | 1.885023 | 0.008048 | 0.029219 | yes | up | 0.06 | 0.07 | 0.13 | 1.31 | 0.37 | 0.69 | 0.086667 | 0.79 |
| RAD51 | 2.908 | 1.540141 | 0.008091 | 0.029329 | yes | up | 0.56 | 0.73 | 0.48 | 4.59 | 5.25 | 1.81 | 0.59 | 3.883333 |
| HSBP1L1 | 0.221 | -2.178 | 0.008136 | 0.029474 | yes | down | 0.89 | 1.13 | 2.23 | 1 | 0.49 | 0.7 | 1.416667 | 0.73 |
| HMGCLL1 | 8.831 | 3.142497 | 0.008184 | 0.029628 | yes | up | 0 | 0.11 | 0.05 | 0.88 | 1.07 | 0.85 | 0.053333 | 0.933333 |
| LOC103345342 | 3.529 | 1.819211 | 0.008206 | 0.029694 | yes | up | 0.03 | 0.09 | 0.08 | 0.68 | 0.42 | 0.48 | 0.066667 | 0.526667 |
| NCAM2 | 3.938 | 1.977501 | 0.008414 | 0.030362 | yes | up | 0.05 | 0.01 | 0.04 | 0.45 | 0.38 | 0.16 | 0.033333 | 0.33 |
| CSRNP1 | 0.252 | -1.98728 | 0.008486 | 0.030601 | yes | down | 32.25 | 10.8 | 6.17 | 8.7 | 12.61 | 8.45 | 16.40667 | 9.92 |
| LOC103346677 | 7.951 | 2.991132 | 0.008527 | 0.030727 | yes | up | 0.03 | 0.02 | 0.02 | 0.71 | 0.13 | 0.37 | 0.023333 | 0.403333 |
| LOC103347791 | 3.037 | 1.602851 | 0.008618 | 0.031038 | yes | up | 3.21 | 2.84 | 0.6 | 10.15 | 16.05 | 11.3 | 2.216667 | 12.5 |
| LOC100347994 | 4.972 | 2.313892 | 0.008673 | 0.031175 | yes | up | 0.79 | 0.09 | 0.04 | 5.95 | 2.13 | 3.19 | 0.306667 | 3.756667 |
| NHEJ1 | 2.154 | 1.107008 | 0.008678 | 0.031178 | yes | up | 0.8 | 0.94 | 0.8 | 4.53 | 2.51 | 5.51 | 0.846667 | 4.183333 |
| LOC108177155 | 0.219 | -2.18791 | 0.008685 | 0.031194 | yes | down | 0.08 | 0.08 | 0.08 | 0.05 | 0.02 | 0.06 | 0.08 | 0.043333 |
| KIAA1549L | 2.853 | 1.512456 | 0.008716 | 0.0313 | yes | up | 0.06 | 0.09 | 0.06 | 0.76 | 0.24 | 0.3 | 0.07 | 0.433333 |
| APOC3 | 21.61 | 4.433601 | 0.008737 | 0.031369 | yes | up | 0 | 0.14 | 0 | 2.35 | 4.64 | 0.53 | 0.046667 | 2.506667 |
| WDSUB1 | 2.13 | 1.091081 | 0.008785 | 0.031521 | yes | up | 0.63 | 1.15 | 0.97 | 5.27 | 5.29 | 2.96 | 0.916667 | 4.506667 |
| MTFR2 | 2.78 | 1.475204 | 0.008832 | 0.031661 | yes | up | 0.75 | 0.52 | 0.33 | 3.15 | 5.54 | 1.63 | 0.533333 | 3.44 |
| LOC100353758 | 0.441 | -1.18013 | 0.008888 | 0.031813 | yes | down | 4.43 | 4.2 | 4.21 | 6.38 | 1.65 | 5.44 | 4.28 | 4.49 |
| CCDC157 | 0.221 | -2.18004 | 0.008999 | 0.032179 | yes | down | 0.23 | 0.22 | 0.26 | 0.13 | 0.07 | 0.18 | 0.236667 | 0.126667 |
| DNAH7 | 0.088 | -3.50986 | 0.009077 | 0.032427 | yes | down | 0.01 | 0.03 | 0.04 | 0.01 | 0.01 | 0 | 0.026667 | 0.006667 |
| LOC100350038 | 21.015 | 4.393364 | 0.009087 | 0.032456 | yes | up | 0 | 0.07 | 0 | 1.98 | 0.98 | 0.23 | 0.023333 | 1.063333 |
| LOC108176201 | 3.485 | 1.801017 | 0.009129 | 0.032573 | yes | up | 0.51 | 0.3 | 0.66 | 1.12 | 2.58 | 2.95 | 0.49 | 2.216667 |
| PGF | 2.076 | 1.053788 | 0.009148 | 0.032631 | yes | up | 3.73 | 1.61 | 2.35 | 9.76 | 15.85 | 11.84 | 2.563333 | 12.48333 |
| S1PR3 | 2.287 | 1.193712 | 0.009216 | 0.032838 | yes | up | 1.63 | 1.93 | 1.1 | 11.87 | 9.87 | 3.07 | 1.553333 | 8.27 |
| LOC103351410 | 2.817 | 1.493909 | 0.009245 | 0.032913 | yes | up | 0.16 | 0.07 | 0.16 | 1.52 | 0.47 | 0.71 | 0.13 | 0.9 |
| LOC100338530 | 3.517 | 1.814543 | 0.009255 | 0.032941 | yes | up | 0.2 | 0.32 | 0.1 | 3.71 | 1.38 | 0.28 | 0.206667 | 1.79 |
| LOC100339296 | 51.759 | 5.69373 | 0.009265 | 0.032963 | yes | up | 0 | 0 | 0 | 0.53 | 0.13 | 0 | 0 | 0.22 |
| TTC39A | 2.427 | 1.279133 | 0.00934 | 0.033194 | yes | up | 0.25 | 0.35 | 0.21 | 1.61 | 1.61 | 1.13 | 0.27 | 1.45 |
| LOC100358731 | 4.085 | 2.030386 | 0.009391 | 0.033339 | yes | up | 0.43 | 0.04 | 0.46 | 5.47 | 2.03 | 1.8 | 0.31 | 3.1 |
| CEP128 | 2.163 | 1.113233 | 0.009419 | 0.033428 | yes | up | 0.27 | 0.16 | 0.33 | 1.21 | 1.31 | 1.36 | 0.253333 | 1.293333 |
| IFIT3 | 2.486 | 1.314031 | 0.00942 | 0.033428 | yes | up | 3.55 | 2.43 | 2.67 | 12.51 | 6.54 | 31.7 | 2.883333 | 16.91667 |
| CTXN1 | 0.177 | -2.49912 | 0.009422 | 0.033428 | yes | down | 0.29 | 0.71 | 0.78 | 0.18 | 0.29 | 0.26 | 0.593333 | 0.243333 |
| SKIDA1 | 4.843 | 2.275944 | 0.009427 | 0.03344 | yes | up | 0 | 0.09 | 0.21 | 1.55 | 0.95 | 0.96 | 0.1 | 1.153333 |
| LOC100348715 | 0.353 | -1.50227 | 0.009452 | 0.033507 | yes | down | 1.16 | 1.32 | 1.22 | 0.7 | 1.05 | 1.2 | 1.233333 | 0.983333 |
| LOC100343032 | 0.405 | -1.30266 | 0.009638 | 0.034093 | yes | down | 2.55 | 3.75 | 2.02 | 3.08 | 3.13 | 1.92 | 2.773333 | 2.71 |
| LOC100345097 | 0.001 | -10.1056 | 0.009705 | 0.034308 | yes | down | 56.25 | 0 | 4.66 | 0 | 0 | 0 | 20.30333 | 0 |
| UBE2U | 0.126 | -2.98597 | 0.009775 | 0.03454 | yes | down | 0.57 | 0.42 | 0.15 | 0.15 | 0 | 0.17 | 0.38 | 0.106667 |
| LOC103350094 | 2.512 | 1.328856 | 0.00982 | 0.034677 | yes | up | 0.51 | 0.6 | 0.45 | 3.89 | 3.23 | 2.06 | 0.52 | 3.06 |
| DNHD1 | 2.754 | 1.4613 | 0.009837 | 0.034732 | yes | up | 0.05 | 0.02 | 0.04 | 0.34 | 0.15 | 0.28 | 0.036667 | 0.256667 |
| ST6GALNAC3 | 2.144 | 1.100475 | 0.009849 | 0.034767 | yes | up | 0.59 | 0.27 | 0.43 | 2.41 | 1.7 | 1.46 | 0.43 | 1.856667 |
| GRIP2 | 2.463 | 1.300695 | 0.010007 | 0.035248 | yes | up | 1.13 | 1.27 | 1.21 | 16.29 | 5.41 | 4.67 | 1.203333 | 8.79 |
| FBN3 | 0.297 | -1.75071 | 0.010018 | 0.035276 | yes | down | 0.28 | 0.33 | 0.06 | 0.25 | 0.11 | 0.11 | 0.223333 | 0.156667 |
| FGF16 | 0.295 | -1.76272 | 0.010021 | 0.035276 | yes | down | 1.87 | 1.36 | 0.47 | 1 | 0.44 | 1.13 | 1.233333 | 0.856667 |
| LOC100341774 | 2.25 | 1.169632 | 0.010054 | 0.035383 | yes | up | 2.43 | 1.52 | 2.36 | 10.18 | 12.96 | 10.4 | 2.103333 | 11.18 |
| TMEM35A | 5.286 | 2.402157 | 0.010109 | 0.035532 | yes | up | 0.16 | 0.12 | 0.04 | 1.2 | 2.09 | 0.58 | 0.106667 | 1.29 |
| GIPR | 3.486 | 1.801464 | 0.010148 | 0.035653 | yes | up | 0.39 | 0.12 | 0.14 | 3.55 | 0.63 | 1.85 | 0.216667 | 2.01 |
| DFNB59 | 2.128 | 1.089173 | 0.010209 | 0.035818 | yes | up | 0.31 | 0.32 | 0.19 | 1.36 | 1.1 | 1.52 | 0.273333 | 1.326667 |
| LOC108175923 | 0.232 | -2.109 | 0.010258 | 0.03595 | yes | down | 0.54 | 0.52 | 1.21 | 0.84 | 0.19 | 0.23 | 0.756667 | 0.42 |
| GABRE | 0.342 | -1.54971 | 0.010272 | 0.035989 | yes | down | 0.84 | 1.02 | 1.76 | 0.78 | 0.32 | 1.78 | 1.206667 | 0.96 |
| LOC103346094 | 13.264 | 3.729407 | 0.010298 | 0.036065 | yes | up | 0 | 0 | 0.03 | 0.53 | 0.14 | 0.39 | 0.01 | 0.353333 |
| MELK | 4.877 | 2.28604 | 0.010324 | 0.03612 | yes | up | 0.2 | 0.19 | 0 | 2.2 | 1.55 | 0.67 | 0.13 | 1.473333 |
| PRRX2 | 2.359 | 1.238435 | 0.010367 | 0.036245 | yes | up | 0.97 | 1.26 | 1.04 | 5.56 | 5.67 | 6.79 | 1.09 | 6.006667 |
| CHEK2 | 2.529 | 1.338376 | 0.010393 | 0.036315 | yes | up | 0.35 | 0.44 | 0.3 | 1.9 | 2.51 | 1.65 | 0.363333 | 2.02 |
| CAPS2 | 0.348 | -1.52112 | 0.010429 | 0.036411 | yes | down | 0.43 | 0.23 | 0.38 | 0.2 | 0.32 | 0.37 | 0.346667 | 0.296667 |
| SPIN4 | 2.646 | 1.403755 | 0.010479 | 0.036577 | yes | up | 0.13 | 0.12 | 0.18 | 1.07 | 1.06 | 0.52 | 0.143333 | 0.883333 |
| MCM2 | 2.225 | 1.153839 | 0.010535 | 0.036741 | yes | up | 0.79 | 2.76 | 1.08 | 8.95 | 9.62 | 4.66 | 1.543333 | 7.743333 |
| CDA | 0.373 | -1.42114 | 0.010603 | 0.036939 | yes | down | 1.48 | 0.98 | 1.2 | 0.8 | 1.01 | 1.4 | 1.22 | 1.07 |
| FAM180B | 2.044 | 1.031281 | 0.010625 | 0.036992 | yes | up | 4.3 | 3.04 | 6.67 | 14.2 | 29.76 | 21.42 | 4.67 | 21.79333 |
| LOC108176449 | 4.44 | 2.150681 | 0.010656 | 0.037072 | yes | up | 0.14 | 0.02 | 0.18 | 2.38 | 0.43 | 1.31 | 0.113333 | 1.373333 |
| WDR63 | 17.848 | 4.157703 | 0.010671 | 0.037114 | yes | up | 0.02 | 0 | 0 | 0.21 | 0.17 | 0.51 | 0.006667 | 0.296667 |
| GRIA4 | 3.879 | 1.95552 | 0.010706 | 0.037222 | yes | up | 0.06 | 0.03 | 0.08 | 0.79 | 0.51 | 0.32 | 0.056667 | 0.54 |
| LOC100345689 | 20.636 | 4.367119 | 0.010721 | 0.03724 | yes | up | 0 | 0 | 0.05 | 0.03 | 1.67 | 0.53 | 0.016667 | 0.743333 |
| GDNF | 0.129 | -2.95774 | 0.01074 | 0.037283 | yes | down | 0.26 | 0.21 | 0.01 | 0.04 | 0.08 | 0.03 | 0.16 | 0.05 |
| LOC103352483 | 4.025 | 2.008928 | 0.010749 | 0.037306 | yes | up | 0.16 | 0.41 | 0.23 | 2.05 | 1.84 | 3.28 | 0.266667 | 2.39 |
| NOTO | 0.455 | -1.13463 | 0.01083 | 0.037556 | yes | down | 4.64 | 3.26 | 2.67 | 4.84 | 4.59 | 1.59 | 3.523333 | 3.673333 |
| LOC103351405 | 9.154 | 3.194461 | 0.01097 | 0.037961 | yes | up | 0 | 0.02 | 0.04 | 0.63 | 0.48 | 0.18 | 0.02 | 0.43 |
| LOC103346853 | 0.392 | -1.35225 | 0.011039 | 0.038136 | yes | down | 1.3 | 0.71 | 1.03 | 0.99 | 0.46 | 1.37 | 1.013333 | 0.94 |
| MEX3A | 16.003 | 4.000254 | 0.011187 | 0.038632 | yes | up | 0 | 0 | 0.01 | 0.2 | 0.16 | 0.16 | 0.003333 | 0.173333 |
| PTPRT | 0.389 | -1.36264 | 0.011206 | 0.038667 | yes | down | 0.72 | 0.38 | 0.77 | 0.28 | 0.37 | 1.04 | 0.623333 | 0.563333 |
| LOC108178005 | 0.402 | -1.31622 | 0.011211 | 0.038667 | yes | down | 0.28 | 0.2 | 0.29 | 0.27 | 0.16 | 0.31 | 0.256667 | 0.246667 |
| IL6 | 0.068 | -3.88145 | 0.011297 | 0.038929 | yes | down | 2.58 | 0.18 | 0 | 0.15 | 0.24 | 0.07 | 0.92 | 0.153333 |
| RERGL | 2.011 | 1.007673 | 0.011456 | 0.039396 | yes | up | 1.71 | 1.03 | 1.77 | 6.2 | 8.26 | 6.82 | 1.503333 | 7.093333 |
| UNC13D | 0.338 | -1.56537 | 0.011524 | 0.039614 | yes | down | 1.99 | 2.08 | 1.52 | 2.85 | 0.91 | 0.74 | 1.863333 | 1.5 |
| TMEM144 | 0.48 | -1.05937 | 0.011681 | 0.040046 | yes | down | 0.76 | 0.66 | 0.95 | 1.06 | 0.58 | 1.09 | 0.79 | 0.91 |
| MYOM3 | 0.051 | -4.28185 | 0.01169 | 0.040068 | yes | down | 13.37 | 13.34 | 0.82 | 0.07 | 2.97 | 0.09 | 9.176667 | 1.043333 |
| SLC6A4 | 5.614 | 2.489083 | 0.011738 | 0.040192 | yes | up | 0.03 | 0.02 | 0.08 | 0.91 | 0.32 | 0.63 | 0.043333 | 0.62 |
| LOC103345228 | 0.352 | -1.50784 | 0.011767 | 0.040272 | yes | down | 0.29 | 0.24 | 0.29 | 0.42 | 0.11 | 0.16 | 0.273333 | 0.23 |
| LOC103345317 | 0.362 | -1.46544 | 0.011771 | 0.04028 | yes | down | 27.3 | 25.31 | 16.89 | 32.44 | 3.12 | 25.65 | 23.16667 | 20.40333 |
| NRCAM | 0.246 | -2.02178 | 0.011777 | 0.040289 | yes | down | 0.17 | 0.32 | 0.09 | 0.1 | 0.19 | 0.03 | 0.193333 | 0.106667 |
| CKMT2 | 0.057 | -4.14415 | 0.011799 | 0.040358 | yes | down | 61.52 | 89.48 | 28.57 | 0.06 | 24.36 | 2.55 | 59.85667 | 8.99 |
| BMPR1B | 2.62 | 1.389695 | 0.011924 | 0.040718 | yes | up | 0.32 | 0.34 | 0.06 | 1.52 | 1.62 | 1.13 | 0.24 | 1.423333 |
| TRPM3 | 0.317 | -1.65651 | 0.012039 | 0.04106 | yes | down | 0.14 | 0.09 | 0.04 | 0.09 | 0.05 | 0.06 | 0.09 | 0.066667 |
| CFL2 | 0.49 | -1.02917 | 0.012103 | 0.041253 | yes | down | 43.41 | 58.2 | 15.37 | 37.78 | 56.49 | 36.73 | 38.99333 | 43.66667 |
| DNA2 | 3.322 | 1.732259 | 0.012112 | 0.041267 | yes | up | 0.09 | 0.09 | 0.07 | 0.92 | 0.78 | 0.26 | 0.083333 | 0.653333 |
| TRIM59 | 2.246 | 1.167186 | 0.012117 | 0.041276 | yes | up | 0.35 | 0.71 | 0.75 | 3.73 | 3.79 | 1.94 | 0.603333 | 3.153333 |
| LOC103347841 | 2.476 | 1.308269 | 0.012224 | 0.041597 | yes | up | 0.11 | 0.13 | 0.06 | 0.73 | 0.44 | 0.57 | 0.1 | 0.58 |
| GARNL3 | 3.236 | 1.694381 | 0.012231 | 0.04161 | yes | up | 0.18 | 0.18 | 0.15 | 2.59 | 0.76 | 0.57 | 0.17 | 1.306667 |
| LOC100340204 | 2.656 | 1.409288 | 0.012244 | 0.041648 | yes | up | 0.68 | 0.64 | 0.37 | 4.59 | 1.7 | 3.49 | 0.563333 | 3.26 |
| LEKR1 | 5.526 | 2.466137 | 0.012265 | 0.04171 | yes | up | 0.16 | 0.15 | 0.03 | 0.63 | 0.36 | 0.45 | 0.113333 | 0.48 |
| LOC100357219 | 2.616 | 1.387205 | 0.012287 | 0.04176 | yes | up | 30.29 | 14.21 | 11.11 | 236.19 | 58.08 | 66.97 | 18.53667 | 120.4133 |
| ITIH4 | 0.236 | -2.08594 | 0.01233 | 0.041896 | yes | down | 0.92 | 1.58 | 0.14 | 0.36 | 0.45 | 0.38 | 0.88 | 0.396667 |
| OAS3 | 2.237 | 1.161562 | 0.012351 | 0.041959 | yes | up | 0.36 | 0.37 | 0.54 | 2.29 | 1.07 | 3.32 | 0.423333 | 2.226667 |
| LOC100339419 | 3.592 | 1.844847 | 0.012395 | 0.042077 | yes | up | 0.1 | 0.04 | 0.11 | 0.55 | 0.39 | 1.21 | 0.083333 | 0.716667 |
| LOC100351002 | 0.089 | -3.49405 | 0.012398 | 0.042077 | yes | down | 0.11 | 0.37 | 0.48 | 0.13 | 0 | 0.06 | 0.32 | 0.063333 |
| LOC103349420 | 3.787 | 1.920877 | 0.01248 | 0.04231 | yes | up | 0.43 | 0.99 | 1.62 | 9.06 | 10.33 | 11.34 | 1.013333 | 10.24333 |
| CISH | 0.283 | -1.8189 | 0.012538 | 0.04249 | yes | down | 57.96 | 60.67 | 48.87 | 58.99 | 37.91 | 8.16 | 55.83333 | 35.02 |
| LOC108175669 | 3.678 | 1.879018 | 0.012548 | 0.042502 | yes | up | 0.09 | 0.31 | 0.13 | 2.1 | 1.26 | 0.86 | 0.176667 | 1.406667 |
| DBF4B | 2.068 | 1.048509 | 0.012554 | 0.042511 | yes | up | 0.38 | 0.37 | 0.62 | 2.97 | 1.57 | 2.01 | 0.456667 | 2.183333 |
| LOC100341143 | 0.023 | -5.41132 | 0.012664 | 0.042828 | yes | down | 0.04 | 0 | 0.64 | 0 | 0.05 | 0 | 0.226667 | 0.016667 |
| LOC103349385 | 0.223 | -2.16705 | 0.012672 | 0.042843 | yes | down | 1.12 | 2 | 0.67 | 0.27 | 1 | 0.69 | 1.263333 | 0.653333 |
| CNNM1 | 2.725 | 1.446046 | 0.012732 | 0.042998 | yes | up | 0.06 | 0.08 | 0.06 | 0.57 | 0.44 | 0.24 | 0.066667 | 0.416667 |
| FAM81B | 0.046 | -4.4388 | 0.012737 | 0.043005 | yes | down | 0.17 | 0.29 | 0.08 | 0 | 0 | 0.05 | 0.18 | 0.016667 |
| RGAG1 | 0.446 | -1.16559 | 0.012755 | 0.043031 | yes | down | 0.21 | 0.41 | 0.26 | 0.32 | 0.25 | 0.33 | 0.293333 | 0.3 |
| GINS1 | 3.128 | 1.645019 | 0.012769 | 0.043045 | yes | up | 0.61 | 0.94 | 0.39 | 5.27 | 5.04 | 2.07 | 0.646667 | 4.126667 |
| LOC100357965 | 2.837 | 1.504505 | 0.01283 | 0.043222 | yes | up | 0.29 | 0.29 | 0.13 | 15.39 | 3.3 | 5.07 | 0.236667 | 7.92 |
| NRIP3 | 0.489 | -1.03276 | 0.01295 | 0.043565 | yes | down | 1.44 | 1.27 | 1 | 1.74 | 1.79 | 0.72 | 1.236667 | 1.416667 |
| LOC100352436 | 2.062 | 1.043799 | 0.012967 | 0.043615 | yes | up | 0.6 | 0.61 | 0.48 | 2.55 | 1.7 | 3.8 | 0.563333 | 2.683333 |
| SOX17 | 0.46 | -1.12008 | 0.013017 | 0.043755 | yes | down | 23.36 | 15.03 | 6.29 | 23.66 | 13.21 | 12.35 | 14.89333 | 16.40667 |
| LOC103349783 | 0.486 | -1.04149 | 0.013038 | 0.043799 | yes | down | 1.94 | 1.13 | 0.66 | 1.42 | 1.35 | 1.66 | 1.243333 | 1.476667 |
| IGSF11 | 0.311 | -1.68316 | 0.013127 | 0.044053 | yes | down | 0.26 | 0.4 | 0.4 | 0.32 | 0.07 | 0.37 | 0.353333 | 0.253333 |
| EGR2 | 0.337 | -1.57071 | 0.01323 | 0.044364 | yes | down | 6.15 | 1.27 | 1.08 | 2.64 | 2.17 | 2.05 | 2.833333 | 2.286667 |
| RIPPLY3 | 4.972 | 2.313777 | 0.013291 | 0.04455 | yes | up | 0.12 | 0.04 | 0.07 | 1.15 | 0.68 | 0.95 | 0.076667 | 0.926667 |
| LOC100358333 | 0.351 | -1.51071 | 0.013367 | 0.044744 | yes | down | 4.45 | 3.29 | 2.57 | 0.91 | 1.62 | 3.48 | 3.436667 | 2.003333 |
| LOC108175372 | 2.341 | 1.22697 | 0.013368 | 0.044744 | yes | up | 1.06 | 1.48 | 1.57 | 5.89 | 10.51 | 7.74 | 1.37 | 8.046667 |
| KIF18A | 2.038 | 1.027395 | 0.013384 | 0.04478 | yes | up | 0.62 | 0.51 | 0.32 | 2.68 | 2.64 | 1.62 | 0.483333 | 2.313333 |
| LOC100345090 | 0.499 | -1.00287 | 0.013496 | 0.045054 | yes | down | 0.45 | 0.67 | 0.61 | 0.52 | 0.63 | 0.82 | 0.576667 | 0.656667 |
| BCAS1 | 0.15 | -2.7367 | 0.013499 | 0.045054 | yes | down | 0.45 | 0.55 | 1.07 | 0 | 0.62 | 0.06 | 0.69 | 0.226667 |
| LOC100352631 | 0.094 | -3.4036 | 0.013531 | 0.045116 | yes | down | 0.18 | 0.1 | 0.5 | 0.05 | 0.06 | 0.05 | 0.26 | 0.053333 |
| LOC100348238 | 2.352 | 1.233871 | 0.013605 | 0.045318 | yes | up | 0.29 | 0.11 | 0.34 | 1.37 | 1 | 1.77 | 0.246667 | 1.38 |
| MBLAC1 | 0.38 | -1.39586 | 0.013661 | 0.045475 | yes | down | 2.06 | 2.93 | 1.78 | 2.92 | 0.54 | 2.57 | 2.256667 | 2.01 |
| ERRFI1 | 0.13 | -2.94797 | 0.013675 | 0.045515 | yes | down | 96.3 | 11.99 | 4.81 | 11.22 | 12.78 | 14.35 | 37.7 | 12.78333 |
| LOC103347756 | 7.053 | 2.818337 | 0.013737 | 0.045691 | yes | up | 0 | 0.76 | 0.68 | 4.74 | 11.26 | 7.27 | 0.48 | 7.756667 |
| STC1 | 0.368 | -1.44241 | 0.01374 | 0.045692 | yes | down | 7.83 | 4 | 1.47 | 4.2 | 5.64 | 1.75 | 4.433333 | 3.863333 |
| OLFM2 | 2.255 | 1.173014 | 0.013776 | 0.045776 | yes | up | 1.06 | 0.9 | 0.34 | 4.94 | 3.05 | 4.18 | 0.766667 | 4.056667 |
| LOC100345861 | 0.452 | -1.14412 | 0.013779 | 0.045776 | yes | down | 7.01 | 10.73 | 4.94 | 8.76 | 11.55 | 3.22 | 7.56 | 7.843333 |
| CELSR1 | 0.345 | -1.53596 | 0.013854 | 0.045971 | yes | down | 0.22 | 0.39 | 0.15 | 0.21 | 0.14 | 0.14 | 0.253333 | 0.163333 |
| PPP1R13L | 0.496 | -1.01255 | 0.013886 | 0.046058 | yes | down | 0.66 | 1.12 | 1.09 | 1.18 | 0.95 | 1.17 | 0.956667 | 1.1 |
| S100A12 | 0.344 | -1.54032 | 0.013958 | 0.04623 | yes | down | 13.62 | 12.29 | 3.99 | 3.6 | 7.98 | 12.5 | 9.966667 | 8.026667 |
| LOC103349960 | 5.08 | 2.344861 | 0.014066 | 0.046517 | yes | up | 0.15 | 0.05 | 0.09 | 1.69 | 0.99 | 0.77 | 0.096667 | 1.15 |
| CCDC89 | 0.346 | -1.53217 | 0.014077 | 0.046535 | yes | down | 0.89 | 1.17 | 0.47 | 0.54 | 0.52 | 0.95 | 0.843333 | 0.67 |
| LOC100352190 | 0.151 | -2.72611 | 0.014077 | 0.046535 | yes | down | 0.64 | 0.46 | 1.27 | 0.64 | 0.21 | 0 | 0.79 | 0.283333 |
| RASEF | 5.465 | 2.450222 | 0.014134 | 0.046713 | yes | up | 0.06 | 0.01 | 0.02 | 0.51 | 0.11 | 0.63 | 0.03 | 0.416667 |
| LOC100349989 | 2.523 | 1.334962 | 0.014232 | 0.04699 | yes | up | 0.26 | 0.15 | 0.2 | 1.14 | 0.76 | 1.76 | 0.203333 | 1.22 |
| LOC103351895 | 3.782 | 1.919181 | 0.014267 | 0.047087 | yes | up | 0.15 | 0.07 | 0.06 | 1.11 | 0.42 | 1.01 | 0.093333 | 0.846667 |
| LGR6 | 0.443 | -1.17553 | 0.014287 | 0.047129 | yes | down | 0.22 | 0.28 | 0.23 | 0.19 | 0.25 | 0.29 | 0.243333 | 0.243333 |
| LOC103346668 | 2.095 | 1.067241 | 0.014288 | 0.047129 | yes | up | 0.72 | 1.08 | 0.29 | 4.84 | 2.9 | 3.24 | 0.696667 | 3.66 |
| PRR32 | 0.027 | -5.19286 | 0.014371 | 0.047356 | yes | down | 1.46 | 7.4 | 0.36 | 0 | 0.54 | 0 | 3.073333 | 0.18 |
| RNF43 | 2.75 | 1.459535 | 0.014394 | 0.047407 | yes | up | 0.1 | 0.14 | 0.04 | 0.5 | 0.56 | 0.71 | 0.093333 | 0.59 |
| FAM25A | 15.271 | 3.932704 | 0.0144 | 0.047411 | yes | up | 0.32 | 0 | 0 | 1.98 | 3.16 | 0.87 | 0.106667 | 2.003333 |
| LOC100339447 | 0.045 | -4.4846 | 0.014437 | 0.047515 | yes | down | 0.06 | 0.32 | 0.43 | 0 | 0 | 0.07 | 0.27 | 0.023333 |
| TLL1 | 2.097 | 1.068437 | 0.014453 | 0.047556 | yes | up | 0.42 | 0.34 | 0.43 | 2.58 | 1.15 | 2.21 | 0.396667 | 1.98 |
| LOC100355611 | 0.481 | -1.05477 | 0.014464 | 0.047576 | yes | down | 37.14 | 39.06 | 15.76 | 25.37 | 29.33 | 49.91 | 30.65333 | 34.87 |
| LOC100353731 | 0.421 | -1.24796 | 0.014499 | 0.047671 | yes | down | 67.82 | 29.71 | 63.09 | 28.98 | 33.28 | 100.01 | 53.54 | 54.09 |
| LRRC7 | 15.289 | 3.934411 | 0.01451 | 0.047698 | yes | up | 0 | 0 | 0.01 | 0.16 | 0.08 | 0.15 | 0.003333 | 0.13 |
| CENPL | 3.228 | 1.690717 | 0.014519 | 0.047719 | yes | up | 0.15 | 0.25 | 0.19 | 2.42 | 1.15 | 0.91 | 0.196667 | 1.493333 |
| PAWR | 2.072 | 1.05098 | 0.014541 | 0.047772 | yes | up | 0.5 | 0.51 | 0.51 | 3.64 | 1.83 | 1.99 | 0.506667 | 2.486667 |
| LOC108176074 | 0.2 | -2.32152 | 0.014574 | 0.047851 | yes | down | 1.74 | 1.46 | 1.32 | 0.8 | 0.57 | 0.78 | 1.506667 | 0.716667 |
| PDE6H | 2.185 | 1.127421 | 0.014637 | 0.048029 | yes | up | 0.56 | 0.78 | 0.67 | 4.37 | 3.08 | 2.42 | 0.67 | 3.29 |
| ADAM18 | 2.682 | 1.423239 | 0.014715 | 0.048246 | yes | up | 0.17 | 0.24 | 0.36 | 2.58 | 1.17 | 1.54 | 0.256667 | 1.763333 |
| LOC103350176 | 0.472 | -1.08367 | 0.014732 | 0.048284 | yes | down | 1.14 | 1.81 | 1.61 | 1.92 | 1.77 | 1.38 | 1.52 | 1.69 |
| KRBOX1 | 0.335 | -1.57626 | 0.014747 | 0.048323 | yes | down | 2.68 | 4 | 2.81 | 0.88 | 1.85 | 5.85 | 3.163333 | 2.86 |
| EXO5 | 2.171 | 1.118439 | 0.014874 | 0.048691 | yes | up | 0.31 | 0.57 | 0.64 | 2.73 | 2.22 | 2.71 | 0.506667 | 2.553333 |
| LOC103351933 | 2.633 | 1.396461 | 0.014973 | 0.048949 | yes | up | 2.65 | 0.49 | 3.04 | 11.73 | 15.88 | 10.18 | 2.06 | 12.59667 |
| OMP | 11.202 | 3.485644 | 0.015124 | 0.049412 | yes | up | 0 | 0 | 0.02 | 0.23 | 0.12 | 0.22 | 0.006667 | 0.19 |
| KCNH2 | 0.264 | -1.91873 | 0.01517 | 0.049535 | yes | down | 1.35 | 0.46 | 0.24 | 0.24 | 0.42 | 0.57 | 0.683333 | 0.41 |
| PLB1 | 0.272 | -1.88001 | 0.015239 | 0.04968 | yes | down | 0.11 | 0.35 | 1.05 | 0.27 | 0.38 | 0.39 | 0.503333 | 0.346667 |
| CFAP126 | 0.484 | -1.04587 | 0.015254 | 0.049708 | yes | down | 0.94 | 1.19 | 1.52 | 1.03 | 1.59 | 1.65 | 1.216667 | 1.423333 |
| LOC103351899 | 0.044 | -4.49376 | 0.015261 | 0.049721 | yes | down | 0.15 | 0.1 | 0.02 | 0 | 0.03 | 0 | 0.09 | 0.01 |
| KCNS3 | 7.306 | 2.868985 | 0.0153 | 0.049784 | yes | up | 0.16 | 0.12 | 0 | 3.32 | 0.21 | 0.65 | 0.093333 | 1.393333 |

iBAT_D14_VS_ingWAT_M4

| Gene name | FC(ingWAT_M4/iBAT_D14) | Log2FC(ingWAT_M4/iBAT_D14) | Pvalue | Padjust | Significant | Regulate | D14iBAT1 | D14iBAT2 | D14iBAT3 | M4ingWAT1 | M4ingWAT2 | M4ingWAT3 | iBAT_D14 | ingWAT_M4 |
| --- | --- | --- | --- | --- | --- | --- | --- | --- | --- | --- | --- | --- | --- | --- |
| GPD2 | 0.02 | -5.65492 | 2.7E-282 | 4.4E-278 | yes | down | 140.08 | 166.79 | 168.88 | 7.71 | 7.98 | 6.75 | 158.5833 | 7.48 |
| LOC100341175 | 0.042 | -4.57655 | 6.6E-193 | 5.5E-189 | yes | down | 908.93 | 1017.78 | 846.44 | 76.67 | 95.74 | 85.41 | 924.3833 | 85.94 |
| AQP8 | 1420.887 | 10.47258 | 7.6E-178 | 4.2E-174 | yes | up | 0.44 | 1.33 | 0.28 | 1744.95 | 2717.69 | 1918.54 | 0.683333 | 2127.06 |
| CPT1B | 0.046 | -4.45218 | 2.5E-142 | 1E-138 | yes | down | 185.88 | 246.03 | 223.69 | 24.39 | 21.97 | 18.93 | 218.5333 | 21.76333 |
| PPP1R1B | 0.002 | -8.87726 | 5.3E-140 | 1.8E-136 | yes | down | 95.94 | 111.72 | 135.67 | 0.27 | 0.55 | 0.89 | 114.4433 | 0.57 |
| SLC16A7 | 0.007 | -7.09046 | 2.1E-135 | 5.9E-132 | yes | down | 24.67 | 18.01 | 17.48 | 0.36 | 0.38 | 0.18 | 20.05333 | 0.306667 |
| LOC100346046 | 435.996 | 8.76817 | 3.1E-115 | 7.3E-112 | yes | up | 1.71 | 1.59 | 1.28 | 1552.06 | 690.37 | 2042.46 | 1.526667 | 1428.297 |
| KCNK3 | 0.007 | -7.25207 | 5E-114 | 1E-110 | yes | down | 35.85 | 76.05 | 46.92 | 0.49 | 1.1 | 0.71 | 52.94 | 0.766667 |
| GNAT3 | 0.01 | -6.71446 | 6.2E-114 | 1.1E-110 | yes | down | 28.88 | 31.86 | 28.13 | 0.51 | 1.64 | 0.81 | 29.62333 | 0.986667 |
| BHMT2 | 0.003 | -8.19754 | 1.5E-112 | 2.5E-109 | yes | down | 96.28 | 79.58 | 67.09 | 0.62 | 0.4 | 0.43 | 80.98333 | 0.483333 |
| QPCT | 0.043 | -4.53445 | 6.2E-112 | 9.3E-109 | yes | down | 147.76 | 204.14 | 228.05 | 17.47 | 21.93 | 16.11 | 193.3167 | 18.50333 |
| PDE4D | 0.06 | -4.04957 | 9.2E-103 | 1.3E-99 | yes | down | 31.17 | 31.43 | 28.08 | 3.6 | 6.91 | 2.67 | 30.22667 | 4.393333 |
| COX2 | 0.047 | -4.41598 | 4.6E-99 | 5.88E-96 | yes | down | 70879.59 | 55173.47 | 66291.15 | 6664.31 | 8253.6 | 5503.44 | 64114.74 | 6807.117 |
| AHCYL1 | 0.102 | -3.29991 | 1.01E-96 | 1.19E-93 | yes | down | 233.97 | 278.01 | 295.83 | 56.89 | 70.31 | 52.67 | 269.27 | 59.95667 |
| SUCLA2 | 0.055 | -4.18709 | 2.3E-95 | 2.53E-92 | yes | down | 335.46 | 485.89 | 389.75 | 46.29 | 64.61 | 37.26 | 403.7 | 49.38667 |
| ACAA2 | 0.11 | -3.18424 | 4.59E-95 | 4.73E-92 | yes | down | 1437.23 | 1792.79 | 1516.86 | 396.69 | 442.29 | 320.25 | 1582.293 | 386.41 |
| S100B | 21.129 | 4.40118 | 1.87E-94 | 1.82E-91 | yes | up | 9.31 | 14.46 | 15.42 | 569.89 | 685.76 | 633.64 | 13.06333 | 629.7633 |
| PPIF | 0.052 | -4.25984 | 1.13E-92 | 1.03E-89 | yes | down | 85.94 | 113.18 | 102.96 | 12.76 | 10.01 | 11.81 | 100.6933 | 11.52667 |
| HADHA | 0.113 | -3.14679 | 2.65E-90 | 2.3E-87 | yes | down | 599.23 | 730.92 | 634.7 | 166.52 | 159.14 | 162.06 | 654.95 | 162.5733 |
| ADRA1A | 0.057 | -4.1365 | 8.95E-86 | 7.39E-83 | yes | down | 15.04 | 21.27 | 19.47 | 2.13 | 3.02 | 1.46 | 18.59333 | 2.203333 |
| ND3 | 0.078 | -3.68247 | 1.1E-85 | 8.66E-83 | yes | down | 30865.46 | 26765.75 | 27132.97 | 5383.9 | 5014.1 | 4640.26 | 28254.73 | 5012.753 |
| GPAT4 | 0.133 | -2.91383 | 1E-83 | 7.53E-81 | yes | down | 138.28 | 142.78 | 128.72 | 40.54 | 42.95 | 38.16 | 136.5933 | 40.55 |
| DHRS11 | 0.097 | -3.36394 | 8.54E-83 | 6.13E-80 | yes | down | 161.46 | 230.9 | 194.89 | 41.49 | 41.49 | 42.41 | 195.75 | 41.79667 |
| APOE | 61.267 | 5.937046 | 1.6E-81 | 1.1E-78 | yes | up | 26.17 | 57.45 | 44.18 | 3632.16 | 8111.29 | 6315.51 | 42.6 | 6019.653 |
| ACO2 | 0.13 | -2.93845 | 2E-81 | 1.32E-78 | yes | down | 900.28 | 1276.14 | 1044.6 | 287.99 | 339.94 | 297.38 | 1073.673 | 308.4367 |
| IMPDH1 | 0.083 | -3.58928 | 4.24E-81 | 2.69E-78 | yes | down | 266.77 | 353.18 | 325.91 | 51.26 | 76.59 | 47.51 | 315.2867 | 58.45333 |
| IDH2 | 0.112 | -3.1549 | 1.3E-79 | 7.97E-77 | yes | down | 546.34 | 714.26 | 624.53 | 162.5 | 147.96 | 153.83 | 628.3767 | 154.7633 |
| ADCK3 | 0.111 | -3.17415 | 2.64E-78 | 1.56E-75 | yes | down | 442.39 | 470.67 | 390.87 | 92.58 | 119.94 | 109.75 | 434.6433 | 107.4233 |
| ACADS | 0.149 | -2.74489 | 4.53E-78 | 2.58E-75 | yes | down | 379.11 | 428.91 | 395.91 | 118.85 | 154.05 | 116.18 | 401.31 | 129.6933 |
| EXTL1 | 0.039 | -4.68456 | 2.01E-76 | 1.11E-73 | yes | down | 11.57 | 15.35 | 12.5 | 1.4 | 0.9 | 1.03 | 13.14 | 1.11 |
| LEP | 161.13 | 7.332085 | 2.38E-75 | 1.27E-72 | yes | up | 0.34 | 0.16 | 0.64 | 53.72 | 148.14 | 90.61 | 0.38 | 97.49 |
| TM7SF2 | 23.121 | 4.53114 | 4.78E-75 | 2.47E-72 | yes | up | 2.62 | 3.23 | 2.24 | 160.83 | 122.76 | 147.85 | 2.696667 | 143.8133 |
| ETFDH | 0.115 | -3.11755 | 5.05E-75 | 2.52E-72 | yes | down | 172.44 | 226.51 | 183.17 | 56.15 | 67.2 | 55.46 | 194.04 | 59.60333 |
| COX3 | 0.108 | -3.21091 | 2.59E-74 | 1.26E-71 | yes | down | 67862.42 | 63526.96 | 71579.65 | 17575.01 | 16848.59 | 14532.27 | 67656.34 | 16318.62 |
| GNAS | 0.105 | -3.24617 | 3E-74 | 1.42E-71 | yes | down | 4680.31 | 6057.31 | 5802.71 | 1404.73 | 1202.83 | 1172.26 | 5513.443 | 1259.94 |
| ND4 | 0.067 | -3.89537 | 2.69E-73 | 1.23E-70 | yes | down | 14882.97 | 12381.68 | 15275.94 | 2476.79 | 2094.26 | 1802.18 | 14180.2 | 2124.41 |
| ND5 | 0.071 | -3.81953 | 3.44E-73 | 1.53E-70 | yes | down | 65596.28 | 62584.24 | 67298.96 | 12051.46 | 10575.15 | 7781.25 | 65159.83 | 10135.95 |
| FRRS1 | 0.046 | -4.45252 | 5.35E-73 | 2.33E-70 | yes | down | 9.28 | 12.74 | 12.6 | 1.04 | 1.45 | 1.15 | 11.54 | 1.213333 |
| SNCG | 126.448 | 6.982406 | 7.34E-73 | 3.11E-70 | yes | up | 11.92 | 17.7 | 15.7 | 4409.11 | 1951.02 | 6077.41 | 15.10667 | 4145.847 |
| DECR1 | 0.14 | -2.83486 | 1.26E-72 | 5.19E-70 | yes | down | 503.2 | 601.3 | 594.22 | 170.62 | 214.2 | 152.53 | 566.24 | 179.1167 |
| ATP6 | 0.059 | -4.07597 | 1.17E-71 | 4.71E-69 | yes | down | 39607.67 | 30054.45 | 41171.62 | 5056.12 | 6001.7 | 3850.54 | 36944.58 | 4969.453 |
| GMPR | 0.022 | -5.48206 | 1.36E-70 | 5.33E-68 | yes | down | 93.83 | 206.85 | 106.37 | 6.33 | 5.67 | 7.87 | 135.6833 | 6.623333 |
| ALDH1L1 | 96.195 | 6.587886 | 2.08E-70 | 7.97E-68 | yes | up | 0.38 | 0.51 | 0.11 | 84.2 | 61.27 | 63.76 | 0.333333 | 69.74333 |
| ITIH3 | 189.981 | 7.56971 | 2.17E-70 | 8.15E-68 | yes | up | 0.06 | 0.2 | 0.13 | 39.43 | 79.92 | 49.14 | 0.13 | 56.16333 |
| CYTB | 0.107 | -3.22839 | 4.48E-70 | 1.64E-67 | yes | down | 19576.98 | 17131.39 | 20389.01 | 4757.55 | 4836.64 | 4022 | 19032.46 | 4538.73 |
| ND6 | 0.031 | -5.02174 | 4.58E-68 | 1.64E-65 | yes | down | 9677.91 | 7210.54 | 8334.84 | 777.47 | 504.79 | 407.78 | 8407.763 | 563.3467 |
| COX1 | 0.093 | -3.42402 | 1.04E-67 | 3.66E-65 | yes | down | 37581.46 | 39557.49 | 39085 | 9802.06 | 8403.58 | 6407.68 | 38741.32 | 8204.44 |
| LOC100342572 | 27.808 | 4.797411 | 8.41E-67 | 2.89E-64 | yes | up | 28 | 25.92 | 15.32 | 1310.47 | 1971.83 | 1078.48 | 23.08 | 1453.593 |
| LOC100339305 | 73.131 | 6.192405 | 1.31E-65 | 4.42E-63 | yes | up | 0.53 | 0.74 | 0.2 | 68.07 | 82.99 | 105.77 | 0.49 | 85.61 |
| GK | 0.061 | -4.04421 | 2.39E-65 | 7.89E-63 | yes | down | 16.45 | 31.02 | 18.44 | 2.82 | 3.61 | 2.27 | 21.97 | 2.9 |
| ACADVL | 0.111 | -3.17281 | 2.89E-64 | 9.36E-62 | yes | down | 454.1 | 664.12 | 550.58 | 125.36 | 175.39 | 110.81 | 556.2667 | 137.1867 |
| DIO2 | 0.017 | -5.88854 | 8.03E-63 | 2.55E-60 | yes | down | 7.21 | 6.61 | 4.76 | 0.15 | 0.31 | 0.08 | 6.193333 | 0.18 |
| UCP1 | 0 | -17.3285 | 1.61E-61 | 5.01E-59 | yes | down | 1895.23 | 3480.95 | 2355.41 | 0.03 | 0 | 0.06 | 2577.197 | 0.03 |
| FAM105A | 0.027 | -5.1981 | 5.27E-61 | 1.61E-58 | yes | down | 18.79 | 24.68 | 11.86 | 0.98 | 1.66 | 0.92 | 18.44333 | 1.186667 |
| PDK1 | 0.08 | -3.64452 | 2.24E-60 | 6.73E-58 | yes | down | 22.84 | 25.25 | 20.93 | 4.85 | 4.37 | 2.79 | 23.00667 | 4.003333 |
| LRP2 | 0.018 | -5.77753 | 6.17E-60 | 1.82E-57 | yes | down | 2.27 | 1.66 | 3.07 | 0.07 | 0.13 | 0.09 | 2.333333 | 0.096667 |
| IDH3A | 0.117 | -3.10085 | 1.74E-59 | 5.05E-57 | yes | down | 273.99 | 431.96 | 282.62 | 80.66 | 97.2 | 75.09 | 329.5233 | 84.31667 |
| LOC100350920 | 0.007 | -7.19474 | 2.34E-59 | 6.67E-57 | yes | down | 5.21 | 2.11 | 2.73 | 0.49 | 0.03 | 0.53 | 3.35 | 0.35 |
| TNN | 0.009 | -6.86194 | 5.76E-58 | 1.61E-55 | yes | down | 87.34 | 36.22 | 83.24 | 2.13 | 1.15 | 0.7 | 68.93333 | 1.326667 |
| LOC100337966 | 0.154 | -2.70281 | 6.58E-58 | 1.81E-55 | yes | down | 673.77 | 764.3 | 694.08 | 244.69 | 219.03 | 233.36 | 710.7167 | 232.36 |
| PPTC7 | 0.162 | -2.62187 | 1.13E-57 | 3.07E-55 | yes | down | 16.37 | 17.29 | 16.38 | 6 | 6.22 | 5.76 | 16.68 | 5.993333 |
| ND2 | 0.076 | -3.72162 | 2.25E-57 | 5.98E-55 | yes | down | 17417.47 | 12295.92 | 16240.55 | 2770.19 | 2427.01 | 2578.26 | 15317.98 | 2591.82 |
| FABP3 | 0.031 | -5.0186 | 4.45E-57 | 1.17E-54 | yes | down | 392.79 | 434.21 | 301.49 | 21.19 | 17.91 | 37.09 | 376.1633 | 25.39667 |
| FH | 0.126 | -2.98502 | 3.92E-56 | 1.01E-53 | yes | down | 122.58 | 151.42 | 124.68 | 29.25 | 46.89 | 36.02 | 132.8933 | 37.38667 |
| IVNS1ABP | 0.098 | -3.34852 | 8.6E-56 | 2.18E-53 | yes | down | 153.06 | 114.07 | 137.15 | 28.37 | 35.23 | 25.05 | 134.76 | 29.55 |
| ND4L | 0.098 | -3.35694 | 9.7E-56 | 2.42E-53 | yes | down | 12525.82 | 10445.8 | 13117.1 | 2954.3 | 2858.89 | 2304.21 | 12029.57 | 2705.8 |
| LOC100347571 | 0.162 | -2.62558 | 1.33E-55 | 3.27E-53 | yes | down | 467.17 | 570.33 | 442.28 | 180.78 | 172.11 | 174.68 | 493.26 | 175.8567 |
| MPC2 | 0.179 | -2.48277 | 1.45E-55 | 3.52E-53 | yes | down | 323.91 | 337.55 | 340.86 | 136.26 | 135.31 | 127.21 | 334.1067 | 132.9267 |
| ATP8 | 0.087 | -3.52 | 2.24E-55 | 5.36E-53 | yes | down | 199788.1 | 168629 | 216032 | 43179.26 | 44272.08 | 33340.84 | 194816.4 | 40264.06 |
| ITGA7 | 7.724 | 2.949289 | 2.55E-55 | 6E-53 | yes | up | 19.6 | 24.28 | 16.72 | 324.77 | 337.05 | 368.61 | 20.2 | 343.4767 |
| ATP5B | 0.156 | -2.68133 | 9.35E-55 | 2.17E-52 | yes | down | 2483.27 | 3047.96 | 2409.3 | 972.17 | 863.02 | 884.61 | 2646.843 | 906.6 |
| SDHA | 0.196 | -2.3505 | 1E-54 | 2.29E-52 | yes | down | 640.03 | 846.96 | 685.12 | 327.72 | 329.11 | 288.29 | 724.0367 | 315.04 |
| OLFML1 | 10.262 | 3.359287 | 3.17E-53 | 7.16E-51 | yes | up | 2.72 | 4.64 | 4.22 | 85.81 | 83.47 | 89.18 | 3.86 | 86.15333 |
| DLD | 0.091 | -3.45087 | 3.31E-53 | 7.39E-51 | yes | down | 145.49 | 184.4 | 179.45 | 42.04 | 37.15 | 23.95 | 169.78 | 34.38 |
| CES3 | 0.099 | -3.32954 | 6.45E-53 | 1.42E-50 | yes | down | 73.06 | 94.39 | 77.88 | 18 | 14.99 | 20.33 | 81.77667 | 17.77333 |
| LOC103350566 | 0.022 | -5.48935 | 5.77E-52 | 1.25E-49 | yes | down | 11.01 | 15.61 | 14.18 | 1.12 | 0.47 | 0.73 | 13.6 | 0.773333 |
| SLC4A4 | 0.086 | -3.54412 | 1.89E-51 | 4.05E-49 | yes | down | 12.2 | 16.89 | 11.69 | 3.18 | 2.4 | 2.01 | 13.59333 | 2.53 |
| LOC108176503 | 0.073 | -3.78166 | 1.5E-50 | 3.17E-48 | yes | down | 14.37 | 23.62 | 15.33 | 2.83 | 2.28 | 1.48 | 17.77333 | 2.196667 |
| LOC100359087 | 56.6 | 5.822718 | 2.77E-50 | 5.79E-48 | yes | up | 1.45 | 0.47 | 1.3 | 68.37 | 75.41 | 107.05 | 1.073333 | 83.61 |
| GREM2 | 11.305 | 3.498863 | 3.76E-50 | 7.76E-48 | yes | up | 0.59 | 1.07 | 0.59 | 17.89 | 18.63 | 16.07 | 0.75 | 17.53 |
| IDH3G | 0.179 | -2.48315 | 2.69E-49 | 5.48E-47 | yes | down | 395.08 | 502.89 | 407.32 | 188.7 | 170.39 | 154.51 | 435.0967 | 171.2 |
| ACADM | 0.046 | -4.44402 | 4.45E-49 | 8.95E-47 | yes | down | 201.72 | 223.07 | 367.28 | 28.56 | 36.4 | 17.02 | 264.0233 | 27.32667 |
| UGT1-6 | 11.377 | 3.50801 | 1.35E-48 | 2.69E-46 | yes | up | 7.47 | 7.03 | 7.04 | 125.84 | 173.94 | 189.09 | 7.18 | 162.9567 |
| MTFP1 | 0.054 | -4.21196 | 2.1E-48 | 4.13E-46 | yes | down | 18.45 | 26.02 | 26.48 | 3.05 | 3.69 | 1.59 | 23.65 | 2.776667 |
| PDHA1 | 0.192 | -2.37789 | 5.21E-48 | 1.01E-45 | yes | down | 169.86 | 234.01 | 209.59 | 87.17 | 101.29 | 72.81 | 204.4867 | 87.09 |
| MC5R | 24.78 | 4.631092 | 5.86E-48 | 1.12E-45 | yes | up | 6.3 | 5.28 | 8.12 | 150.96 | 320.73 | 224.47 | 6.566667 | 232.0533 |
| NDUFS3 | 0.188 | -2.41161 | 7.19E-48 | 1.36E-45 | yes | down | 386.55 | 538.05 | 439.78 | 183.6 | 189.72 | 191.5 | 454.7933 | 188.2733 |
| LRRC52 | 0.006 | -7.26984 | 8.37E-47 | 1.57E-44 | yes | down | 6.87 | 6.94 | 6.86 | 0.05 | 0.05 | 0.23 | 6.89 | 0.11 |
| TMEM37 | 0.054 | -4.20884 | 8.87E-47 | 1.64E-44 | yes | down | 50.59 | 75.19 | 90.72 | 6.26 | 8.26 | 11.17 | 72.16667 | 8.563333 |
| ECH1 | 0.155 | -2.68661 | 3.02E-46 | 5.54E-44 | yes | down | 424.82 | 485.36 | 374.96 | 126.01 | 191.51 | 135.56 | 428.38 | 151.0267 |
| PCDH10 | 40.052 | 5.323811 | 3.91E-46 | 7.1E-44 | yes | up | 0.03 | 0.05 | 0.05 | 3.96 | 3.63 | 3.21 | 0.043333 | 3.6 |
| ND1 | 0.099 | -3.33042 | 4.72E-46 | 8.47E-44 | yes | down | 8712.85 | 7446.45 | 8051.58 | 1756.31 | 1485.66 | 2075 | 8070.293 | 1772.323 |
| COL5A3 | 10.952 | 3.453098 | 1.48E-45 | 2.63E-43 | yes | up | 6.98 | 8.68 | 4.42 | 148.36 | 180.18 | 158.76 | 6.693333 | 162.4333 |
| ECI1 | 0.203 | -2.30136 | 2.87E-45 | 5.05E-43 | yes | down | 264.48 | 292.72 | 238.79 | 121.86 | 123.06 | 113.67 | 265.33 | 119.53 |
| HADHB | 0.046 | -4.44104 | 5.26E-45 | 9.14E-43 | yes | down | 788.8 | 1054.53 | 836.65 | 84.06 | 146.79 | 47.17 | 893.3267 | 92.67333 |
| AOX3 | 13.08 | 3.70929 | 1.54E-44 | 2.64E-42 | yes | up | 1.81 | 1.08 | 1.16 | 23.89 | 40.13 | 25.85 | 1.35 | 29.95667 |
| AIFM1 | 0.172 | -2.5418 | 5.78E-44 | 9.83E-42 | yes | down | 74.93 | 101.67 | 86.88 | 37.65 | 33.85 | 28.09 | 87.82667 | 33.19667 |
| SOD2 | 0.208 | -2.26871 | 6.57E-44 | 1.11E-41 | yes | down | 316.82 | 447.24 | 393.85 | 160.67 | 212.38 | 158.99 | 385.97 | 177.3467 |
| PRELID3B | 0.19 | -2.39855 | 7.02E-44 | 1.17E-41 | yes | down | 121.54 | 135.07 | 140.22 | 60.57 | 57.03 | 49.47 | 132.2767 | 55.69 |
| LOC100328910 | 13.751 | 3.78142 | 7.74E-44 | 1.28E-41 | yes | up | 50.82 | 59.83 | 102.16 | 2216.18 | 2217.82 | 2038.47 | 70.93667 | 2157.49 |
| LOC100350787 | 0.151 | -2.73121 | 8.22E-44 | 1.34E-41 | yes | down | 733.28 | 927.74 | 890.63 | 317.75 | 249.56 | 277.37 | 850.55 | 281.56 |
| IDH3B | 0.164 | -2.61056 | 1.13E-43 | 1.83E-41 | yes | down | 377.88 | 558.6 | 494.99 | 165.62 | 161.34 | 187.05 | 477.1567 | 171.3367 |
| PCK2 | 16.087 | 4.007821 | 1.51E-43 | 2.42E-41 | yes | up | 12.37 | 19.9 | 29.45 | 750.5 | 690.41 | 760.51 | 20.57333 | 733.8067 |
| ALDH5A1 | 0.178 | -2.48868 | 3.92E-43 | 6.22E-41 | yes | down | 30.43 | 38.97 | 36.1 | 15.36 | 17.47 | 12.77 | 35.16667 | 15.2 |
| ISCA1 | 0.258 | -1.95384 | 4.09E-43 | 6.37E-41 | yes | down | 102.49 | 125.65 | 116.34 | 61.27 | 73.3 | 62.4 | 114.8267 | 65.65667 |
| KCTD6 | 0.085 | -3.56144 | 4.09E-43 | 6.37E-41 | yes | down | 37.03 | 31.67 | 46.61 | 7.4 | 10.77 | 7.9 | 38.43667 | 8.69 |
| FFAR2 | 0.075 | -3.73382 | 1.43E-42 | 2.21E-40 | yes | down | 23.26 | 37.7 | 38.34 | 4.29 | 5.33 | 6.44 | 33.1 | 5.353333 |
| ACADL | 0.145 | -2.78958 | 2.83E-42 | 4.33E-40 | yes | down | 824.64 | 827.12 | 740.36 | 294.44 | 289.64 | 188.63 | 797.3733 | 257.57 |
| GPX3 | 0.069 | -3.85617 | 3.44E-42 | 5.2E-40 | yes | down | 802.22 | 880.23 | 952.83 | 81.3 | 143.45 | 176.26 | 878.4267 | 133.67 |
| PPARGC1B | 0.126 | -2.99015 | 5.37E-42 | 8.06E-40 | yes | down | 12.76 | 16.77 | 18.26 | 3.65 | 6.11 | 3.67 | 15.93 | 4.476667 |
| LOC108176518 | 0.063 | -3.99326 | 5.85E-42 | 8.7E-40 | yes | down | 5.4 | 5.81 | 5.88 | 0.64 | 1.09 | 0.65 | 5.696667 | 0.793333 |
| ACOT13 | 0.142 | -2.82034 | 1.22E-41 | 1.8E-39 | yes | down | 119.96 | 154.94 | 139.9 | 41.05 | 39.57 | 49.36 | 138.2667 | 43.32667 |
| PLVAP | 4.323 | 2.112127 | 1.9E-41 | 2.77E-39 | yes | up | 31.09 | 36.51 | 30.89 | 324.71 | 348.88 | 271.76 | 32.83 | 315.1167 |
| ANKRD31 | 0.049 | -4.36025 | 1.98E-41 | 2.87E-39 | yes | down | 15.99 | 8.32 | 11.31 | 1.21 | 1.31 | 1.6 | 11.87333 | 1.373333 |
| MRPL39 | 0.141 | -2.82264 | 2.11E-41 | 3.03E-39 | yes | down | 85.25 | 119.17 | 99.71 | 36.45 | 35.61 | 23.62 | 101.3767 | 31.89333 |
| PPARGC1A | 0.039 | -4.68599 | 3.58E-41 | 5.1E-39 | yes | down | 3.02 | 3.95 | 2.03 | 0.3 | 0.32 | 0.16 | 3 | 0.26 |
| LOC100352363 | 75.423 | 6.236926 | 5.33E-41 | 7.51E-39 | yes | up | 2.14 | 0.7 | 1.32 | 239.24 | 208.25 | 209.86 | 1.386667 | 219.1167 |
| LOC108176227 | 0.011 | -6.5092 | 8.05E-41 | 1.13E-38 | yes | down | 5.27 | 18.98 | 6.28 | 0.23 | 0.18 | 0.3 | 10.17667 | 0.236667 |
| LOC100354254 | 0.061 | -4.03198 | 2E-40 | 2.78E-38 | yes | down | 704.32 | 1083.94 | 1003.36 | 110.34 | 89.41 | 177.28 | 930.54 | 125.6767 |
| ACSS2 | 16.52 | 4.046133 | 2.14E-40 | 2.95E-38 | yes | up | 14.42 | 21.91 | 19.46 | 935.01 | 442.24 | 614.39 | 18.59667 | 663.88 |
| ETFA | 0.22 | -2.18229 | 4E-40 | 5.45E-38 | yes | down | 577.27 | 679.47 | 591.01 | 316.41 | 286.81 | 296.62 | 615.9167 | 299.9467 |
| ACAT1 | 0.22 | -2.18127 | 5.17E-40 | 6.99E-38 | yes | down | 239.85 | 294.18 | 226.71 | 117.47 | 125.56 | 126.47 | 253.58 | 123.1667 |
| ELOVL2 | 0.043 | -4.52612 | 6.64E-40 | 8.91E-38 | yes | down | 13.56 | 13.39 | 16.35 | 1 | 2.48 | 0.8 | 14.43333 | 1.426667 |
| LETMD1 | 0.253 | -1.98388 | 1.03E-39 | 1.37E-37 | yes | down | 68.11 | 74.19 | 73.46 | 37.44 | 46.01 | 36.58 | 71.92 | 40.01 |
| SDHB | 0.196 | -2.3518 | 1.42E-39 | 1.88E-37 | yes | down | 505.63 | 646.86 | 558.91 | 267.73 | 227.48 | 241.33 | 570.4667 | 245.5133 |
| SLIT3 | 5.521 | 2.464935 | 4.69E-39 | 6.14E-37 | yes | up | 3.62 | 3.62 | 4.37 | 41.49 | 51.52 | 49.13 | 3.87 | 47.38 |
| ALOX15 | 0.001 | -10.7608 | 5.87E-39 | 7.63E-37 | yes | down | 11.63 | 29.94 | 37.81 | 0.05 | 0.03 | 0 | 26.46 | 0.026667 |
| NDUFA5 | 0.113 | -3.14516 | 6.07E-39 | 7.83E-37 | yes | down | 129.23 | 156 | 137.69 | 67.68 | 63.88 | 31.72 | 140.9733 | 54.42667 |
| PHKG1 | 0.02 | -5.64506 | 7.29E-39 | 9.33E-37 | yes | down | 71.99 | 75.01 | 54.77 | 2.59 | 0.98 | 4.65 | 67.25667 | 2.74 |
| SRPX | 6.849 | 2.775886 | 7.64E-39 | 9.7E-37 | yes | up | 6.89 | 10.68 | 8.08 | 139.9 | 118.34 | 125.66 | 8.55 | 127.9667 |
| LOC100347143 | 0.182 | -2.45849 | 2.23E-38 | 2.81E-36 | yes | down | 395.97 | 556.31 | 420.06 | 209.63 | 185.82 | 153.71 | 457.4467 | 183.0533 |
| MRC2 | 5.95 | 2.572868 | 3.08E-38 | 3.86E-36 | yes | up | 3.78 | 5.34 | 3.35 | 48.84 | 60.7 | 53.65 | 4.156667 | 54.39667 |
| IGSF8 | 7.514 | 2.909656 | 3.68E-38 | 4.57E-36 | yes | up | 4 | 4.85 | 5.74 | 63.88 | 103.53 | 77.6 | 4.863333 | 81.67 |
| ACOT11 | 0.1 | -3.3261 | 5.17E-38 | 6.37E-36 | yes | down | 13.57 | 24.4 | 24.39 | 3 | 5.37 | 3.32 | 20.78667 | 3.896667 |
| DEFB1 | 31.739 | 4.988166 | 9.3E-38 | 1.14E-35 | yes | up | 11.4 | 6 | 6.61 | 432.04 | 436.68 | 838.1 | 8.003333 | 568.94 |
| COL14A1 | 4.44 | 2.150592 | 1.1E-37 | 1.33E-35 | yes | up | 18.18 | 25.76 | 19.3 | 184.95 | 244.56 | 192.6 | 21.08 | 207.37 |
| LOC100358333 | 0.007 | -7.15431 | 1.58E-37 | 1.9E-35 | yes | down | 4.45 | 3.29 | 2.57 | 0.09 | 0.05 | 0.06 | 3.436667 | 0.066667 |
| RDH8 | 47.337 | 5.564888 | 1.61E-37 | 1.93E-35 | yes | up | 0.09 | 0.5 | 0.28 | 28.28 | 26.94 | 34.32 | 0.29 | 29.84667 |
| GPAT3 | 9.498 | 3.247645 | 2.14E-37 | 2.54E-35 | yes | up | 7.06 | 5.51 | 4.79 | 123.38 | 157.38 | 89.01 | 5.786667 | 123.2567 |
| RSPO3 | 25.732 | 4.68551 | 2.2E-37 | 2.59E-35 | yes | up | 0.54 | 0.63 | 0.18 | 26.45 | 24.66 | 16.65 | 0.45 | 22.58667 |
| NIPSNAP1 | 0.238 | -2.07023 | 3.81E-37 | 4.47E-35 | yes | down | 64.77 | 69.95 | 61.53 | 33.98 | 38.29 | 32.11 | 65.41667 | 34.79333 |
| NAMPT | 0.169 | -2.56325 | 6.5E-37 | 7.55E-35 | yes | down | 23.45 | 23.39 | 21.02 | 8.92 | 10.34 | 6.34 | 22.62 | 8.533333 |
| TBRG4 | 0.155 | -2.69429 | 7.24E-37 | 8.32E-35 | yes | down | 43.65 | 62.78 | 51.3 | 14.68 | 17.68 | 20.32 | 52.57667 | 17.56 |
| COL6A1 | 4.964 | 2.311502 | 7.25E-37 | 8.32E-35 | yes | up | 143.81 | 179.61 | 127.88 | 1282.43 | 1722.31 | 1338.93 | 150.4333 | 1447.89 |
| TBC1D10A | 0.231 | -2.116 | 1.34E-36 | 1.53E-34 | yes | down | 36.81 | 43.98 | 42.75 | 20.08 | 23.35 | 21.72 | 41.18 | 21.71667 |
| TFB2M | 0.158 | -2.66606 | 1.79E-36 | 2.03E-34 | yes | down | 21.94 | 24.27 | 23.54 | 7.8 | 10.73 | 6.17 | 23.25 | 8.233333 |
| ITGBL1 | 11.077 | 3.469525 | 2.93E-36 | 3.29E-34 | yes | up | 1.54 | 2.91 | 2.52 | 40.69 | 76.03 | 69.51 | 2.323333 | 62.07667 |
| GABRD | 12.023 | 3.587781 | 4.23E-36 | 4.71E-34 | yes | up | 12.22 | 14.6 | 18.61 | 384.84 | 305.16 | 470.83 | 15.14333 | 386.9433 |
| MYO19 | 0.143 | -2.81075 | 4.91E-36 | 5.44E-34 | yes | down | 11.52 | 17.33 | 15.93 | 5.05 | 5.36 | 3.4 | 14.92667 | 4.603333 |
| GOT1 | 0.142 | -2.81739 | 5.5E-36 | 6.05E-34 | yes | down | 65.44 | 101.13 | 72.82 | 25.58 | 21.26 | 27.05 | 79.79667 | 24.63 |
| HOXC6 | 23.971 | 4.583201 | 5.97E-36 | 6.53E-34 | yes | up | 0.29 | 0.82 | 0.52 | 21.67 | 25.3 | 24.56 | 0.543333 | 23.84333 |
| SAMM50 | 0.214 | -2.22588 | 6.81E-36 | 7.39E-34 | yes | down | 114.98 | 157.47 | 140.26 | 70.61 | 63.68 | 59.81 | 137.57 | 64.7 |
| ATP5A1 | 0.201 | -2.31368 | 7.53E-36 | 8.12E-34 | yes | down | 1280.97 | 1701.01 | 1445.72 | 758.97 | 635.23 | 563.86 | 1475.9 | 652.6867 |
| LOC100338015 | 7.092 | 2.82618 | 8.37E-36 | 8.97E-34 | yes | up | 13.75 | 26.33 | 21.88 | 339.35 | 336.94 | 284.58 | 20.65333 | 320.29 |
| NDUFAF4 | 0.09 | -3.47136 | 9.23E-36 | 9.82E-34 | yes | down | 7.19 | 8.64 | 10.54 | 1.48 | 2.22 | 1.58 | 8.79 | 1.76 |
| PDE3B | 0.086 | -3.53789 | 1.1E-35 | 1.16E-33 | yes | down | 42.82 | 45.99 | 51.38 | 10.5 | 11.14 | 4.74 | 46.73 | 8.793333 |
| NWD2 | 0.034 | -4.8753 | 1.55E-35 | 1.62E-33 | yes | down | 6.04 | 6.74 | 9.13 | 2.2 | 1.04 | 2 | 7.303333 | 1.746667 |
| MRPL35 | 0.191 | -2.38949 | 1.55E-35 | 1.62E-33 | yes | down | 67.46 | 92.18 | 77.07 | 33.89 | 38.85 | 27.63 | 78.90333 | 33.45667 |
| CS | 0.248 | -2.01221 | 2.8E-35 | 2.9E-33 | yes | down | 589.92 | 831.02 | 703.27 | 408.83 | 418.96 | 332.96 | 708.07 | 386.9167 |
| TFRC | 0.043 | -4.52553 | 3.6E-35 | 3.72E-33 | yes | down | 35.71 | 110.65 | 39.99 | 5.61 | 7.62 | 4.35 | 62.11667 | 5.86 |
| HSPA9 | 0.2 | -2.32184 | 3.78E-35 | 3.88E-33 | yes | down | 116.42 | 179.63 | 142.23 | 67.06 | 61.56 | 62.86 | 146.0933 | 63.82667 |
| LOC100009353 | 5.411 | 2.435855 | 4.3E-35 | 4.38E-33 | yes | up | 95.66 | 131.32 | 118.8 | 1483.99 | 1225.98 | 1392.23 | 115.26 | 1367.4 |
| ATP5S | 0.233 | -2.10369 | 6.22E-35 | 6.29E-33 | yes | down | 23.52 | 27.34 | 26.82 | 12.65 | 15.73 | 11.79 | 25.89333 | 13.39 |
| MFN1 | 0.135 | -2.88637 | 7.24E-35 | 7.29E-33 | yes | down | 27.52 | 33.92 | 32.88 | 9.42 | 12.73 | 6.07 | 31.44 | 9.406667 |
| SEPT10 | 0.175 | -2.51261 | 1.24E-34 | 1.24E-32 | yes | down | 189.88 | 220.85 | 239.56 | 81.32 | 102.83 | 56.39 | 216.7633 | 80.18 |
| LOC100356756 | 0.033 | -4.93942 | 1.65E-34 | 1.64E-32 | yes | down | 1340.88 | 1431.09 | 1179.73 | 60.61 | 54.01 | 172.03 | 1317.233 | 95.55 |
| IMMT | 0.238 | -2.06915 | 1.84E-34 | 1.81E-32 | yes | down | 122.77 | 159.62 | 154.94 | 74.28 | 99.45 | 67.32 | 145.7767 | 80.35 |
| NMNAT3 | 0.183 | -2.44837 | 4.23E-34 | 4.16E-32 | yes | down | 25.82 | 26.73 | 29.11 | 12.16 | 11.89 | 9.92 | 27.22 | 11.32333 |
| VDAC2 | 0.252 | -1.98818 | 5.96E-34 | 5.83E-32 | yes | down | 258.81 | 319.93 | 265.69 | 172.11 | 161.27 | 136.4 | 281.4767 | 156.5933 |
| DLST | 0.224 | -2.15547 | 8.9E-34 | 8.64E-32 | yes | down | 234.26 | 325.78 | 296.05 | 151.72 | 159.94 | 113.26 | 285.3633 | 141.64 |
| PDIA6 | 4.337 | 2.11673 | 1.29E-33 | 1.25E-31 | yes | up | 33.75 | 51.05 | 39.2 | 388.56 | 460.88 | 339.16 | 41.33333 | 396.2 |
| PPA2 | 0.18 | -2.47165 | 1.37E-33 | 1.32E-31 | yes | down | 78.08 | 77.72 | 73.08 | 35.1 | 28.05 | 28.34 | 76.29333 | 30.49667 |
| SLC25A33 | 0.134 | -2.89823 | 1.57E-33 | 1.5E-31 | yes | down | 37.84 | 44.91 | 48.28 | 12.07 | 11.05 | 15.53 | 43.67667 | 12.88333 |
| LIAS | 0.164 | -2.60976 | 2.51E-33 | 2.38E-31 | yes | down | 13.81 | 17.09 | 19.4 | 6.14 | 8.09 | 6.81 | 16.76667 | 7.013333 |
| KCNH1 | 0.043 | -4.52571 | 2.67E-33 | 2.52E-31 | yes | down | 2.07 | 2.25 | 1.34 | 0.11 | 0.18 | 0.26 | 1.886667 | 0.183333 |
| C2 | 5.107 | 2.352491 | 3.17E-33 | 2.97E-31 | yes | up | 2.84 | 4.3 | 3.94 | 38.28 | 44.99 | 41.27 | 3.693333 | 41.51333 |
| ORAI2 | 5.629 | 2.492978 | 4.88E-33 | 4.55E-31 | yes | up | 2.29 | 3.23 | 1.98 | 34.49 | 43.11 | 45.54 | 2.5 | 41.04667 |
| NDUFS1 | 0.189 | -2.40169 | 6.93E-33 | 6.43E-31 | yes | down | 134.26 | 190.66 | 156.1 | 79.32 | 69.65 | 53.08 | 160.34 | 67.35 |
| TIMM8A | 0.176 | -2.50901 | 1.43E-32 | 1.32E-30 | yes | down | 31.12 | 38.54 | 38.9 | 12.01 | 14.56 | 15.71 | 36.18667 | 14.09333 |
| LOC100340711 | 6.063 | 2.599967 | 1.59E-32 | 1.46E-30 | yes | up | 4.98 | 7.78 | 5.03 | 73.98 | 101.22 | 84.85 | 5.93 | 86.68333 |
| CHCHD3 | 0.207 | -2.27181 | 2.01E-32 | 1.83E-30 | yes | down | 149.7 | 209.53 | 167.44 | 88.6 | 73.67 | 76.75 | 175.5567 | 79.67333 |
| LOC100355688 | 0.263 | -1.92624 | 2.16E-32 | 1.95E-30 | yes | down | 48.99 | 68.32 | 58.95 | 33.35 | 35.86 | 33.61 | 58.75333 | 34.27333 |
| OGDHL | 5.58 | 2.480216 | 3.89E-32 | 3.5E-30 | yes | up | 3.08 | 3.29 | 4.33 | 40 | 53.43 | 39.66 | 3.566667 | 44.36333 |
| LOC108178113 | 0.252 | -1.9909 | 3.9E-32 | 3.5E-30 | yes | down | 3176.02 | 3413.65 | 2880.88 | 1816.15 | 1750.86 | 1884.74 | 3156.85 | 1817.25 |
| SCTR | 0.023 | -5.42034 | 4.87E-32 | 4.34E-30 | yes | down | 5.01 | 14.84 | 22.01 | 0.78 | 0.79 | 0.57 | 13.95333 | 0.713333 |
| RNF115 | 0.187 | -2.41861 | 5.31E-32 | 4.71E-30 | yes | down | 84.68 | 101.35 | 93.13 | 34.27 | 32.44 | 43.24 | 93.05333 | 36.65 |
| OXNAD1 | 0.185 | -2.43272 | 7.61E-32 | 6.72E-30 | yes | down | 22.66 | 26.35 | 32.26 | 10.82 | 11.63 | 10.2 | 27.09 | 10.88333 |
| SLC25A20 | 0.239 | -2.06524 | 8.46E-32 | 7.43E-30 | yes | down | 227.53 | 354.97 | 286.12 | 148.07 | 173.64 | 133.88 | 289.54 | 151.8633 |
| HOXB6 | 42.467 | 5.408278 | 1.72E-31 | 1.5E-29 | yes | up | 0.04 | 0.23 | 0.05 | 14.26 | 11.47 | 14.01 | 0.106667 | 13.24667 |
| INMT | 23.404 | 4.548657 | 1.78E-31 | 1.54E-29 | yes | up | 0.62 | 0.47 | 1.06 | 36.21 | 26.54 | 48.05 | 0.716667 | 36.93333 |
| SNRPB2 | 0.189 | -2.40629 | 2.16E-31 | 1.87E-29 | yes | down | 71.59 | 70.46 | 71.38 | 33.96 | 32.77 | 23.1 | 71.14333 | 29.94333 |
| KCNQ1 | 0.142 | -2.81356 | 3.31E-31 | 2.85E-29 | yes | down | 1.97 | 2.1 | 1.86 | 1.59 | 1.85 | 1.32 | 1.976667 | 1.586667 |
| FAM210A | 0.238 | -2.07257 | 3.34E-31 | 2.86E-29 | yes | down | 11.68 | 16.7 | 14.94 | 7.2 | 8.24 | 7.63 | 14.44 | 7.69 |
| SUCLG1 | 0.256 | -1.96713 | 5.08E-31 | 4.33E-29 | yes | down | 424.03 | 535.19 | 468.39 | 300.56 | 268.21 | 235.84 | 475.87 | 268.2033 |
| LOC100348135 | 0.137 | -2.8682 | 6.49E-31 | 5.49E-29 | yes | down | 39.41 | 42.85 | 28.6 | 11.16 | 10.44 | 11.99 | 36.95333 | 11.19667 |
| ADAMTS16 | 73.188 | 6.193535 | 8.06E-31 | 6.79E-29 | yes | up | 0.18 | 0.14 | 0.19 | 9.03 | 57.56 | 18.74 | 0.17 | 28.44333 |
| COQ9 | 0.228 | -2.13286 | 8.35E-31 | 7E-29 | yes | down | 118.18 | 169.16 | 143.95 | 80.85 | 71.27 | 64.13 | 143.7633 | 72.08333 |
| LOC100349830 | 0.288 | -1.79506 | 8.88E-31 | 7.4E-29 | yes | down | 140.03 | 172.25 | 150.11 | 110.05 | 107.25 | 84.3 | 154.13 | 100.5333 |
| SOWAHC | 0.109 | -3.19701 | 1.07E-30 | 8.85E-29 | yes | down | 15.54 | 21.29 | 32.79 | 5.72 | 6.44 | 4.47 | 23.20667 | 5.543333 |
| CST6 | 34.615 | 5.11331 | 1.08E-30 | 8.88E-29 | yes | up | 7.9 | 4.67 | 3.65 | 188.67 | 431.87 | 639.05 | 5.406667 | 419.8633 |
| RMDN3 | 0.221 | -2.17572 | 1.09E-30 | 8.92E-29 | yes | down | 71.11 | 81.17 | 94.86 | 42.88 | 40.75 | 41.25 | 82.38 | 41.62667 |
| FKBP10 | 5.873 | 2.554132 | 1.19E-30 | 9.73E-29 | yes | up | 3.7 | 5.68 | 3.68 | 63.52 | 54.56 | 50.91 | 4.353333 | 56.33 |
| COL6A2 | 4.893 | 2.290604 | 1.36E-30 | 1.11E-28 | yes | up | 89.04 | 145.16 | 86.67 | 1258.17 | 1158.59 | 970.09 | 106.9567 | 1128.95 |
| GGT6 | 0.043 | -4.55166 | 1.39E-30 | 1.12E-28 | yes | down | 3.88 | 3.98 | 3.33 | 0.32 | 0.3 | 0.29 | 3.73 | 0.303333 |
| MED4 | 0.227 | -2.14107 | 1.57E-30 | 1.26E-28 | yes | down | 22.74 | 26.64 | 26.68 | 13.08 | 14.35 | 11.08 | 25.35333 | 12.83667 |
| ACKR2 | 5.421 | 2.438602 | 2.12E-30 | 1.7E-28 | yes | up | 1.79 | 1.42 | 1.35 | 14.63 | 20.62 | 17.01 | 1.52 | 17.42 |
| NDUFA10 | 0.223 | -2.16399 | 2.29E-30 | 1.82E-28 | yes | down | 324.7 | 387.97 | 313.79 | 183.23 | 150.48 | 168.27 | 342.1533 | 167.3267 |
| SAR1B | 0.26 | -1.94461 | 2.84E-30 | 2.25E-28 | yes | down | 23.56 | 25.77 | 26.76 | 14.86 | 15.45 | 11.52 | 25.36333 | 13.94333 |
| LOC100353768 | 8.104 | 3.018682 | 3.02E-30 | 2.38E-28 | yes | up | 2.65 | 2.31 | 2.01 | 33.77 | 43.6 | 49.82 | 2.323333 | 42.39667 |
| PRKAG2 | 0.223 | -2.16335 | 3.32E-30 | 2.61E-28 | yes | down | 29.75 | 38.99 | 35.13 | 19.09 | 18.43 | 14.23 | 34.62333 | 17.25 |
| CLUH | 0.211 | -2.24789 | 4.23E-30 | 3.3E-28 | yes | down | 48.6 | 79.35 | 70.57 | 26.63 | 36.41 | 28.75 | 66.17333 | 30.59667 |
| SFXN1 | 6.924 | 2.791599 | 4.24E-30 | 3.3E-28 | yes | up | 2.31 | 3.73 | 3.44 | 51.1 | 62.1 | 34.52 | 3.16 | 49.24 |
| LOC108176024 | 0.135 | -2.88699 | 4.76E-30 | 3.69E-28 | yes | down | 18.64 | 21.06 | 29.82 | 6.82 | 6.74 | 5.31 | 23.17333 | 6.29 |
| GHITM | 0.13 | -2.94147 | 6.13E-30 | 4.73E-28 | yes | down | 260.27 | 286.76 | 380.58 | 110.38 | 70.73 | 82.78 | 309.2033 | 87.96333 |
| RFK | 0.146 | -2.77665 | 6.85E-30 | 5.26E-28 | yes | down | 16.9 | 16.8 | 23.23 | 7.01 | 6.87 | 4.63 | 18.97667 | 6.17 |
| ATF6 | 0.271 | -1.88246 | 7.77E-30 | 5.94E-28 | yes | down | 20.99 | 21.14 | 22.35 | 12.06 | 14.58 | 12.27 | 21.49333 | 12.97 |
| GPX7 | 4.684 | 2.227672 | 9.92E-30 | 7.55E-28 | yes | up | 10.16 | 15.14 | 10.16 | 118.52 | 131.24 | 115.96 | 11.82 | 121.9067 |
| NDUFS6 | 0.294 | -1.7651 | 1.34E-29 | 1.02E-27 | yes | down | 407.42 | 483.12 | 441.84 | 305.67 | 291.39 | 303.74 | 444.1267 | 300.2667 |
| AFG3L2 | 0.3 | -1.73595 | 1.5E-29 | 1.13E-27 | yes | down | 29.46 | 35.89 | 34 | 21.03 | 25.67 | 19.49 | 33.11667 | 22.06333 |
| ADH1A | 0.166 | -2.58694 | 1.68E-29 | 1.26E-27 | yes | down | 337.12 | 337.2 | 412.88 | 99.39 | 145.79 | 153.43 | 362.4 | 132.87 |
| AQP4 | 9.834 | 3.297706 | 1.91E-29 | 1.43E-27 | yes | up | 2.05 | 2.9 | 2.02 | 69.24 | 50.03 | 31.25 | 2.323333 | 50.17333 |
| PRKAR2B | 0.185 | -2.43359 | 1.92E-29 | 1.43E-27 | yes | down | 247.78 | 348.71 | 348.88 | 136.68 | 166.19 | 94.68 | 315.1233 | 132.5167 |
| CHCHD10 | 0.181 | -2.46688 | 2.22E-29 | 1.64E-27 | yes | down | 57.9 | 75.18 | 56.85 | 21.57 | 26.02 | 29.1 | 63.31 | 25.56333 |
| CTSK | 4.511 | 2.173528 | 2.37E-29 | 1.74E-27 | yes | up | 24.53 | 31.24 | 28.29 | 278.68 | 251.04 | 302.09 | 28.02 | 277.27 |
| CLPX | 0.201 | -2.31802 | 2.58E-29 | 1.9E-27 | yes | down | 39.28 | 54.91 | 50.01 | 19.71 | 28.36 | 15.78 | 48.06667 | 21.28333 |
| TUFM | 0.241 | -2.0546 | 2.77E-29 | 2.02E-27 | yes | down | 148.71 | 184.36 | 142.9 | 96.76 | 84.61 | 75.71 | 158.6567 | 85.69333 |
| ENPP3 | 9.183 | 3.198964 | 3.43E-29 | 2.49E-27 | yes | up | 1.4 | 1.7 | 1.02 | 26.94 | 23.01 | 32.45 | 1.373333 | 27.46667 |
| CCL21 | 8.847 | 3.145237 | 3.9E-29 | 2.82E-27 | yes | up | 11.3 | 18.73 | 10.94 | 247.18 | 215.41 | 323.64 | 13.65667 | 262.0767 |
| PRRG4 | 0.137 | -2.87121 | 5.29E-29 | 3.81E-27 | yes | down | 4.11 | 6.93 | 4.76 | 1.23 | 1.9 | 1.69 | 5.266667 | 1.606667 |
| BCAT2 | 9.383 | 3.230068 | 8.13E-29 | 5.84E-27 | yes | up | 2.94 | 4.29 | 2.31 | 56.17 | 97.11 | 49.43 | 3.18 | 67.57 |
| MDH2 | 0.202 | -2.30561 | 1.06E-28 | 7.54E-27 | yes | down | 505.29 | 630.23 | 487.28 | 268.51 | 205.3 | 248.11 | 540.9333 | 240.64 |
| NTRK2 | 5.067 | 2.341173 | 1.1E-28 | 7.8E-27 | yes | up | 1.4 | 1.75 | 2.07 | 18.63 | 24.29 | 15.92 | 1.74 | 19.61333 |
| SERPINF1 | 6.896 | 2.785698 | 2.09E-28 | 1.48E-26 | yes | up | 76.44 | 102.64 | 69.31 | 1063.8 | 1104.83 | 1624.09 | 82.79667 | 1264.24 |
| LOC108178477 | 0.09 | -3.48143 | 2.16E-28 | 1.52E-26 | yes | down | 1.97 | 2.8 | 2.61 | 0.52 | 0.46 | 0.46 | 2.46 | 0.48 |
| FAM222A | 0.06 | -4.07045 | 2.72E-28 | 1.91E-26 | yes | down | 3.92 | 5.71 | 6.95 | 0.65 | 0.51 | 1.02 | 5.526667 | 0.726667 |
| COQ5 | 0.247 | -2.01892 | 2.73E-28 | 1.91E-26 | yes | down | 27.37 | 40.35 | 38.19 | 15.24 | 15.28 | 14.15 | 35.30333 | 14.89 |
| OSBPL7 | 0.192 | -2.38128 | 3.19E-28 | 2.22E-26 | yes | down | 12.17 | 17.84 | 17.9 | 6.11 | 8.62 | 5.41 | 15.97 | 6.713333 |
| NDUFAB1 | 0.235 | -2.08692 | 3.44E-28 | 2.38E-26 | yes | down | 337.38 | 427.55 | 379.73 | 205.45 | 177.86 | 209.95 | 381.5533 | 197.7533 |
| CCDC58 | 0.167 | -2.57901 | 4.37E-28 | 3.02E-26 | yes | down | 22.73 | 34.11 | 24.65 | 16.7 | 11.44 | 11.16 | 27.16333 | 13.1 |
| CRAT | 0.256 | -1.96788 | 5.15E-28 | 3.54E-26 | yes | down | 97.25 | 133.74 | 102.71 | 66.25 | 60.96 | 59.5 | 111.2333 | 62.23667 |
| PRLR | 0.091 | -3.45542 | 6.09E-28 | 4.17E-26 | yes | down | 28.09 | 33.98 | 23.62 | 5.7 | 9.75 | 4.05 | 28.56333 | 6.5 |
| LGALS1 | 7.244 | 2.856808 | 8.05E-28 | 5.49E-26 | yes | up | 175.25 | 264.92 | 128.14 | 3419.19 | 3171.15 | 2405.98 | 189.4367 | 2998.773 |
| HK1 | 0.224 | -2.15929 | 8.57E-28 | 5.82E-26 | yes | down | 43.4 | 73.26 | 57.24 | 30.03 | 29.75 | 25.52 | 57.96667 | 28.43333 |
| CRABP2 | 13.359 | 3.739765 | 1.3E-27 | 8.8E-26 | yes | up | 1.82 | 4.7 | 1.64 | 76.91 | 74.78 | 82.95 | 2.72 | 78.21333 |
| LARP4 | 0.167 | -2.58597 | 1.71E-27 | 1.15E-25 | yes | down | 12.38 | 16.75 | 19.8 | 11.62 | 12.36 | 5.06 | 16.31 | 9.68 |
| MRPL47 | 0.197 | -2.34202 | 1.93E-27 | 1.3E-25 | yes | down | 38.38 | 45.6 | 46.46 | 20.98 | 18.3 | 15.4 | 43.48 | 18.22667 |
| C7 | 6.667 | 2.737047 | 2.19E-27 | 1.47E-25 | yes | up | 2.99 | 4.89 | 2.99 | 43.82 | 81.33 | 47.36 | 3.623333 | 57.50333 |
| EGFLAM | 4.156 | 2.055296 | 2.81E-27 | 1.87E-25 | yes | up | 5.18 | 6.23 | 6.18 | 49.05 | 54.2 | 38.6 | 5.863333 | 47.28333 |
| LOC100008606 | 5.206 | 2.380301 | 4.47E-27 | 2.97E-25 | yes | up | 5.69 | 6 | 4.87 | 61.3 | 58.07 | 51.07 | 5.52 | 56.81333 |
| TMED3 | 4.32 | 2.110957 | 4.5E-27 | 2.97E-25 | yes | up | 15.62 | 22.55 | 16.96 | 160.34 | 184.96 | 183.37 | 18.37667 | 176.2233 |
| MPC1 | 0.142 | -2.81453 | 4.76E-27 | 3.13E-25 | yes | down | 390.43 | 565.28 | 406.9 | 168.9 | 102.36 | 157.28 | 454.2033 | 142.8467 |
| MICU3 | 0.085 | -3.56033 | 5.58E-27 | 3.66E-25 | yes | down | 11.47 | 5.29 | 8.44 | 1.57 | 1.97 | 1.3 | 8.4 | 1.613333 |
| OGDH | 0.263 | -1.92925 | 6.16E-27 | 4.02E-25 | yes | down | 298.83 | 374.53 | 390.81 | 199.43 | 256.76 | 167.05 | 354.7233 | 207.7467 |
| LOC100343793 | 0.272 | -1.87892 | 6.23E-27 | 4.05E-25 | yes | down | 1944.09 | 2140.07 | 1837.31 | 1294.02 | 1128.85 | 1161.09 | 1973.823 | 1194.653 |
| TRUB1 | 0.162 | -2.62389 | 6.7E-27 | 4.34E-25 | yes | down | 8.37 | 7.4 | 8.2 | 2.85 | 3.44 | 2.32 | 7.99 | 2.87 |
| NQO1 | 4.389 | 2.134 | 7.9E-27 | 5.09E-25 | yes | up | 3.8 | 4.44 | 5.08 | 62.08 | 52.53 | 65.74 | 4.44 | 60.11667 |
| MRPS35 | 0.262 | -1.93141 | 8.54E-27 | 5.48E-25 | yes | down | 32.71 | 40.64 | 40.1 | 20.94 | 23.33 | 21.6 | 37.81667 | 21.95667 |
| DNAJC25 | 0.155 | -2.69206 | 8.75E-27 | 5.6E-25 | yes | down | 14.77 | 17.41 | 15.84 | 5.32 | 7.31 | 4.53 | 16.00667 | 5.72 |
| PLEKHO2 | 3.629 | 1.859391 | 8.83E-27 | 5.62E-25 | yes | up | 3.22 | 4.18 | 3.72 | 28.78 | 34.86 | 25.88 | 3.706667 | 29.84 |
| LOC100345019 | 4.674 | 2.224786 | 9.83E-27 | 6.24E-25 | yes | up | 12.56 | 13.89 | 16.29 | 134.95 | 143.97 | 162.44 | 14.24667 | 147.12 |
| HTRA1 | 4.503 | 2.170985 | 1.04E-26 | 6.56E-25 | yes | up | 91.97 | 140.04 | 133.09 | 1187.66 | 1130.31 | 1275.64 | 121.7 | 1197.87 |
| SMIM4 | 0.204 | -2.29436 | 1.87E-26 | 1.18E-24 | yes | down | 38.31 | 59.49 | 46.23 | 22.62 | 22 | 25.5 | 48.01 | 23.37333 |
| FLOT2 | 3.674 | 1.87735 | 2.02E-26 | 1.27E-24 | yes | up | 19.35 | 29.37 | 20.37 | 175.6 | 207.5 | 184.09 | 23.03 | 189.0633 |
| SDHD | 0.158 | -2.66236 | 2.46E-26 | 1.54E-24 | yes | down | 350.97 | 399.84 | 351.93 | 170.11 | 130.61 | 86.23 | 367.58 | 128.9833 |
| PHKA2 | 6.253 | 2.644631 | 2.58E-26 | 1.61E-24 | yes | up | 1.03 | 1.83 | 2 | 20.47 | 25.09 | 21.48 | 1.62 | 22.34667 |
| ST6GALNAC6 | 4.44 | 2.150622 | 2.65E-26 | 1.64E-24 | yes | up | 9.91 | 12.69 | 10.49 | 97.73 | 101.45 | 123.61 | 11.03 | 107.5967 |
| C20H14orf142 | 0.14 | -2.83812 | 3.79E-26 | 2.35E-24 | yes | down | 42.85 | 43.13 | 33.64 | 8.16 | 14.65 | 14.11 | 39.87333 | 12.30667 |
| ATXN7L2 | 0.09 | -3.47797 | 4.21E-26 | 2.59E-24 | yes | down | 4 | 7.74 | 6.23 | 2.92 | 1.59 | 2.09 | 5.99 | 2.2 |
| SUOX | 0.048 | -4.38349 | 4.77E-26 | 2.92E-24 | yes | down | 1.85 | 11.45 | 8.07 | 0.2 | 0.36 | 0.37 | 7.123333 | 0.31 |
| NDUFB5 | 0.261 | -1.9366 | 6E-26 | 3.67E-24 | yes | down | 173.09 | 189.95 | 177.53 | 118.69 | 99.9 | 94.69 | 180.19 | 104.4267 |
| ACAD9 | 0.203 | -2.29938 | 6.04E-26 | 3.68E-24 | yes | down | 16.46 | 28.43 | 19.58 | 8.68 | 9.44 | 9.49 | 21.49 | 9.203333 |
| LOC103348850 | 0.214 | -2.22582 | 7.7E-26 | 4.67E-24 | yes | down | 6.89 | 8.48 | 7.99 | 3.77 | 4.8 | 2.73 | 7.786667 | 3.766667 |
| ABAT | 11.052 | 3.466292 | 7.75E-26 | 4.69E-24 | yes | up | 0.21 | 0.4 | 0.28 | 8.14 | 7.79 | 5.78 | 0.296667 | 7.236667 |
| BCKDHA | 0.35 | -1.51517 | 9.19E-26 | 5.54E-24 | yes | down | 146.03 | 177.38 | 167.76 | 121.65 | 133.65 | 124.37 | 163.7233 | 126.5567 |
| DMGDH | 0.002 | -8.81523 | 1.03E-25 | 6.2E-24 | yes | down | 89.41 | 58.47 | 14.68 | 0.58 | 0.73 | 0.38 | 54.18667 | 0.563333 |
| GBAS | 0.187 | -2.41585 | 1.11E-25 | 6.65E-24 | yes | down | 50.31 | 74.93 | 41.65 | 24.71 | 23.79 | 20.06 | 55.63 | 22.85333 |
| RCOR2 | 0.133 | -2.91473 | 1.18E-25 | 7.01E-24 | yes | down | 11.33 | 11.58 | 16.62 | 3.85 | 4.56 | 3.28 | 13.17667 | 3.896667 |
| TIMM44 | 0.256 | -1.96735 | 1.21E-25 | 7.21E-24 | yes | down | 46.94 | 67.8 | 47.35 | 31.58 | 31.02 | 28.39 | 54.03 | 30.33 |
| ADAMTS2 | 4.074 | 2.026316 | 1.32E-25 | 7.81E-24 | yes | up | 7.21 | 10.4 | 8.13 | 64.36 | 83.71 | 80.1 | 8.58 | 76.05667 |
| COL1A1 | 4.68 | 2.22639 | 1.39E-25 | 8.17E-24 | yes | up | 543.61 | 817.36 | 645.46 | 5647.26 | 7256.16 | 8019.56 | 668.81 | 6974.327 |
| FADS3 | 3.806 | 1.92814 | 1.58E-25 | 9.26E-24 | yes | up | 32.16 | 42.52 | 33.94 | 303.72 | 281.94 | 319.71 | 36.20667 | 301.79 |
| LRPPRC | 0.233 | -2.10254 | 1.73E-25 | 1.01E-23 | yes | down | 30.55 | 48.71 | 37.29 | 24.22 | 25.61 | 17.3 | 38.85 | 22.37667 |
| COL1A2 | 5.192 | 2.376154 | 1.92E-25 | 1.12E-23 | yes | up | 332.66 | 560.67 | 401.93 | 4083.1 | 4835.84 | 5695.7 | 431.7533 | 4871.547 |
| HYAL1 | 5.391 | 2.430459 | 2.2E-25 | 1.28E-23 | yes | up | 11.73 | 13.99 | 12.13 | 136.42 | 117.77 | 169.26 | 12.61667 | 141.15 |
| NSA2 | 0.297 | -1.75267 | 2.36E-25 | 1.37E-23 | yes | down | 39.86 | 46.26 | 41.69 | 28.46 | 29.71 | 26 | 42.60333 | 28.05667 |
| ESRRG | 0.147 | -2.7684 | 2.71E-25 | 1.56E-23 | yes | down | 4.81 | 3.89 | 5.83 | 1.74 | 1.61 | 1.41 | 4.843333 | 1.586667 |
| DLAT | 0.226 | -2.14805 | 2.88E-25 | 1.66E-23 | yes | down | 29.51 | 38.97 | 33.24 | 19.09 | 19.66 | 12.11 | 33.90667 | 16.95333 |
| MRPS30 | 0.238 | -2.0725 | 3.38E-25 | 1.94E-23 | yes | down | 14.55 | 19.81 | 14.64 | 8.16 | 10.02 | 7.59 | 16.33333 | 8.59 |
| LOC100345021 | 0.185 | -2.43368 | 3.79E-25 | 2.17E-23 | yes | down | 31.37 | 42.57 | 41.4 | 18.91 | 13.28 | 14.36 | 38.44667 | 15.51667 |
| LACTB2 | 0.163 | -2.62082 | 3.82E-25 | 2.17E-23 | yes | down | 47.2 | 35.18 | 46.89 | 15.19 | 20.43 | 11.99 | 43.09 | 15.87 |
| TMEM233 | 27.688 | 4.79117 | 5.55E-25 | 3.15E-23 | yes | up | 0.43 | 1.4 | 0.37 | 48.74 | 37.37 | 96.93 | 0.733333 | 61.01333 |
| SFRP5 | 8.867 | 3.14841 | 6.83E-25 | 3.86E-23 | yes | up | 2.04 | 2.39 | 4.08 | 54.23 | 77.56 | 39.8 | 2.836667 | 57.19667 |
| TRABD2B | 8.418 | 3.073445 | 7.15E-25 | 4.03E-23 | yes | up | 0.79 | 1.37 | 1.3 | 16.55 | 18.58 | 26.89 | 1.153333 | 20.67333 |
| CPEB1 | 6.804 | 2.766463 | 7.49E-25 | 4.2E-23 | yes | up | 0.54 | 0.59 | 0.53 | 7.3 | 9.79 | 8.11 | 0.553333 | 8.4 |
| LOC103352357 | 0.002 | -9.2229 | 7.91E-25 | 4.43E-23 | yes | down | 2.09 | 1.6 | 3.13 | 0 | 0.01 | 0.17 | 2.273333 | 0.06 |
| CA8 | 0.051 | -4.3008 | 7.95E-25 | 4.43E-23 | yes | down | 7.62 | 11.85 | 7.35 | 0.76 | 1.67 | 0.58 | 8.94 | 1.003333 |
| TF | 3.701 | 1.887992 | 8.04E-25 | 4.47E-23 | yes | up | 17.9 | 18.09 | 17.44 | 139.25 | 140.54 | 156.5 | 17.81 | 145.43 |
| MRPL2 | 0.231 | -2.11486 | 1.13E-24 | 6.27E-23 | yes | down | 35.67 | 54.3 | 47 | 23.71 | 22.61 | 23.14 | 45.65667 | 23.15333 |
| DDAH2 | 3.654 | 1.869299 | 1.19E-24 | 6.57E-23 | yes | up | 19.08 | 26.12 | 19.72 | 175.41 | 171.64 | 182.88 | 21.64 | 176.6433 |
| ATP5H | 0.292 | -1.77513 | 1.22E-24 | 6.7E-23 | yes | down | 803.39 | 1019.93 | 827.55 | 618.34 | 544.23 | 547.14 | 883.6233 | 569.9033 |
| LOC100340040 | 29.873 | 4.900762 | 1.28E-24 | 7.04E-23 | yes | up | 0.05 | 0.16 | 0.35 | 12.33 | 13.46 | 14.13 | 0.186667 | 13.30667 |
| LOC103344972 | 10.171 | 3.346334 | 1.6E-24 | 8.72E-23 | yes | up | 17.71 | 27.46 | 15.93 | 521.22 | 286.93 | 533.39 | 20.36667 | 447.18 |
| MRPS9 | 0.238 | -2.06914 | 1.65E-24 | 8.99E-23 | yes | down | 78.09 | 115.69 | 84.84 | 49.29 | 60.45 | 37.6 | 92.87333 | 49.11333 |
| DSG1 | 0.002 | -8.97662 | 1.98E-24 | 1.07E-22 | yes | down | 2.06 | 1.87 | 1.91 | 0.02 | 0.11 | 0 | 1.946667 | 0.043333 |
| THY1 | 6.361 | 2.669227 | 2E-24 | 1.08E-22 | yes | up | 20.57 | 37.67 | 25.03 | 385.31 | 311.84 | 445.46 | 27.75667 | 380.87 |
| PCOLCE | 4.548 | 2.185298 | 2.22E-24 | 1.2E-22 | yes | up | 26.79 | 43.48 | 33.96 | 289.34 | 359.07 | 389.53 | 34.74333 | 345.98 |
| RNF152 | 0.178 | -2.48819 | 2.23E-24 | 1.2E-22 | yes | down | 6.91 | 8.51 | 7.96 | 3.75 | 3.5 | 1.98 | 7.793333 | 3.076667 |
| SBDS | 0.309 | -1.69326 | 2.35E-24 | 1.26E-22 | yes | down | 39.37 | 44.93 | 42.57 | 29.15 | 32.09 | 24.56 | 42.29 | 28.6 |
| LOC100356386 | 0 | -12.3158 | 3.19E-24 | 1.7E-22 | yes | down | 24.95 | 20.82 | 32.64 | 0.01 | 0 | 0 | 26.13667 | 0.003333 |
| ACACA | 9.512 | 3.249808 | 3.23E-24 | 1.72E-22 | yes | up | 1.8 | 2.6 | 2.74 | 66.67 | 30.63 | 49.27 | 2.38 | 48.85667 |
| CD5L | 72.09 | 6.171727 | 3.31E-24 | 1.76E-22 | yes | up | 0.21 | 0.07 | 0.3 | 38.24 | 20.94 | 32.84 | 0.193333 | 30.67333 |
| PERM1 | 0.042 | -4.58728 | 3.33E-24 | 1.76E-22 | yes | down | 7.65 | 9.56 | 6.36 | 0.52 | 0.3 | 1.26 | 7.856667 | 0.693333 |
| LOC100340645 | 0.261 | -1.93747 | 3.41E-24 | 1.8E-22 | yes | down | 1648.46 | 1708.27 | 1324.83 | 990.72 | 876.25 | 862.72 | 1560.52 | 909.8967 |
| TMEM161B | 0.186 | -2.42614 | 3.51E-24 | 1.85E-22 | yes | down | 6 | 7.89 | 7.39 | 3.34 | 2.91 | 2.96 | 7.093333 | 3.07 |
| COL3A1 | 4.865 | 2.282499 | 3.59E-24 | 1.88E-22 | yes | up | 743.41 | 1147.66 | 725.01 | 7146.48 | 9530.57 | 9949.44 | 872.0267 | 8875.497 |
| ATP5F1 | 0.224 | -2.15793 | 3.94E-24 | 2.06E-22 | yes | down | 388.38 | 562.19 | 458.45 | 272.63 | 255.95 | 170.38 | 469.6733 | 232.9867 |
| PANK1 | 0.119 | -3.06766 | 4.03E-24 | 2.1E-22 | yes | down | 23.71 | 17.5 | 22.76 | 5.92 | 10.48 | 5.6 | 21.32333 | 7.333333 |
| APLNR | 4.158 | 2.055894 | 4.31E-24 | 2.24E-22 | yes | up | 20.3 | 18.87 | 16 | 187.67 | 189.18 | 134.81 | 18.39 | 170.5533 |
| PDPR | 0.21 | -2.25257 | 4.48E-24 | 2.32E-22 | yes | down | 20.49 | 29.13 | 25.96 | 9.07 | 16.44 | 10.42 | 25.19333 | 11.97667 |
| MTCH2 | 0.269 | -1.8922 | 5.08E-24 | 2.62E-22 | yes | down | 194.45 | 271.13 | 206.79 | 145.56 | 127.15 | 127.09 | 224.1233 | 133.2667 |
| GSTP1 | 3.175 | 1.666627 | 5.4E-24 | 2.77E-22 | yes | up | 77.48 | 79.93 | 80.89 | 573.11 | 556.11 | 551.54 | 79.43333 | 560.2533 |
| ACAD10 | 0.304 | -1.71829 | 5.74E-24 | 2.94E-22 | yes | down | 16.31 | 21.23 | 20.07 | 11.52 | 14.07 | 13.05 | 19.20333 | 12.88 |
| KYAT3 | 0.212 | -2.23762 | 6.88E-24 | 3.52E-22 | yes | down | 91.33 | 112.15 | 108.52 | 61.14 | 41.95 | 42.79 | 104 | 48.62667 |
| MOCS2 | 0.308 | -1.6974 | 6.9E-24 | 3.52E-22 | yes | down | 38.31 | 38.25 | 39.49 | 22.86 | 25.1 | 22.83 | 38.68333 | 23.59667 |
| GSN | 5.367 | 2.424201 | 8.95E-24 | 4.55E-22 | yes | up | 209.4 | 224.58 | 163.73 | 2290.36 | 1931.96 | 2778.63 | 199.2367 | 2333.65 |
| LOC100345430 | 0.004 | -7.83802 | 9.68E-24 | 4.9E-22 | yes | down | 22.13 | 21.74 | 19.2 | 0 | 0 | 0.79 | 21.02333 | 0.263333 |
| PDHB | 0.293 | -1.77101 | 9.85E-24 | 4.97E-22 | yes | down | 165.91 | 242.37 | 206.68 | 142.56 | 141.59 | 113.01 | 204.9867 | 132.3867 |
| IFT57 | 0.166 | -2.58645 | 1.42E-23 | 7.14E-22 | yes | down | 22.9 | 20.85 | 22.79 | 8.53 | 10.4 | 5.34 | 22.18 | 8.09 |
| AEBP1 | 7.064 | 2.820408 | 1.47E-23 | 7.39E-22 | yes | up | 10.22 | 15.77 | 14.22 | 150.52 | 186.65 | 281.54 | 13.40333 | 206.2367 |
| ADCYAP1R1 | 6.498 | 2.699964 | 1.58E-23 | 7.88E-22 | yes | up | 0.35 | 0.52 | 0.48 | 6.8 | 8.01 | 4.7 | 0.45 | 6.503333 |
| ARNTL2 | 88.121 | 6.461407 | 1.7E-23 | 8.48E-22 | yes | up | 0.04 | 0.05 | 0.03 | 7.32 | 11.3 | 2.72 | 0.04 | 7.113333 |
| NKIRAS2 | 0.285 | -1.81247 | 1.73E-23 | 8.61E-22 | yes | down | 45.17 | 50.17 | 54.28 | 33.48 | 35.45 | 37.52 | 49.87333 | 35.48333 |
| STARD7 | 0.33 | -1.59997 | 2.03E-23 | 1.01E-21 | yes | down | 56.97 | 71.24 | 73.28 | 45.86 | 52.72 | 48.16 | 67.16333 | 48.91333 |
| OPA1 | 0.164 | -2.60964 | 2.21E-23 | 1.09E-21 | yes | down | 10.06 | 11.01 | 12.12 | 4.72 | 5.26 | 2.26 | 11.06333 | 4.08 |
| PDHX | 0.3 | -1.73585 | 2.79E-23 | 1.38E-21 | yes | down | 22.09 | 28.69 | 24.72 | 17.73 | 16.61 | 15.51 | 25.16667 | 16.61667 |
| EPHX2 | 0.309 | -1.6938 | 2.99E-23 | 1.47E-21 | yes | down | 245.88 | 363.84 | 312.57 | 212.08 | 211.49 | 201.15 | 307.43 | 208.24 |
| EBP | 7.601 | 2.926147 | 3.7E-23 | 1.81E-21 | yes | up | 30.36 | 45.91 | 34.82 | 786.94 | 402.44 | 635.09 | 37.03 | 608.1567 |
| MRPL1 | 0.186 | -2.42484 | 3.96E-23 | 1.93E-21 | yes | down | 16.78 | 23.37 | 21.75 | 9.84 | 9.69 | 6.69 | 20.63333 | 8.74 |
| ARSE | 4.612 | 2.205482 | 4.28E-23 | 2.09E-21 | yes | up | 1.26 | 2.17 | 1.86 | 19.93 | 24.76 | 20.33 | 1.763333 | 21.67333 |
| ETFB | 0.353 | -1.50346 | 5.19E-23 | 2.52E-21 | yes | down | 301.17 | 300.69 | 269.64 | 217.92 | 223.05 | 209.66 | 290.5 | 216.8767 |
| COL4A2 | 3.066 | 1.616278 | 6.58E-23 | 3.19E-21 | yes | up | 88.09 | 109.9 | 82.02 | 607.49 | 754.94 | 539.93 | 93.33667 | 634.12 |
| EFEMP1 | 3.838 | 1.940501 | 7.87E-23 | 3.8E-21 | yes | up | 30.7 | 41.6 | 30.13 | 320.72 | 264.02 | 274.55 | 34.14333 | 286.43 |
| LOC100358417 | 0.131 | -2.93564 | 1.2E-22 | 5.77E-21 | yes | down | 12.39 | 19.85 | 12.86 | 3.75 | 3.49 | 5.97 | 15.03333 | 4.403333 |
| ZNF330 | 0.315 | -1.66608 | 1.36E-22 | 6.51E-21 | yes | down | 20.85 | 23.9 | 20.73 | 15.12 | 16.62 | 13.98 | 21.82667 | 15.24 |
| NID2 | 3.115 | 1.639348 | 1.37E-22 | 6.54E-21 | yes | up | 19.52 | 23.58 | 22.27 | 158.89 | 176.99 | 120.71 | 21.79 | 152.1967 |
| NDUFB6 | 0.331 | -1.59529 | 1.51E-22 | 7.2E-21 | yes | down | 228.66 | 291.49 | 239.37 | 199.22 | 166.35 | 170.51 | 253.1733 | 178.6933 |
| OBSL1 | 0.228 | -2.1345 | 1.56E-22 | 7.42E-21 | yes | down | 63.3 | 60.11 | 82.94 | 28.94 | 46.03 | 33.74 | 68.78333 | 36.23667 |
| MRPS36 | 0.26 | -1.94118 | 1.58E-22 | 7.49E-21 | yes | down | 56.33 | 73.76 | 64.86 | 44.13 | 37.14 | 30.59 | 64.98333 | 37.28667 |
| ATP6V0E2 | 29.096 | 4.862764 | 1.61E-22 | 7.61E-21 | yes | up | 1.13 | 1.79 | 2.07 | 121.57 | 33.63 | 174.3 | 1.663333 | 109.8333 |
| HMGB3 | 0.076 | -3.72532 | 2.27E-22 | 1.07E-20 | yes | down | 48.18 | 81 | 85.36 | 14.28 | 16.75 | 4.15 | 71.51333 | 11.72667 |
| IARS2 | 0.205 | -2.28907 | 3.22E-22 | 1.51E-20 | yes | down | 26.5 | 35.64 | 33.11 | 13.51 | 16.3 | 8.13 | 31.75 | 12.64667 |
| NNT | 0.306 | -1.70627 | 3.38E-22 | 1.59E-20 | yes | down | 73.34 | 105.43 | 77.96 | 58.39 | 66.27 | 48.9 | 85.57667 | 57.85333 |
| EMILIN2 | 3.432 | 1.779045 | 3.77E-22 | 1.76E-20 | yes | up | 7.49 | 10.54 | 8.13 | 56.5 | 71.38 | 68.95 | 8.72 | 65.61 |
| PEG3 | 0.065 | -3.94222 | 3.88E-22 | 1.81E-20 | yes | down | 42.49 | 55.07 | 58.07 | 7.59 | 13.09 | 2.5 | 51.87667 | 7.726667 |
| KDELR3 | 3.113 | 1.638088 | 4.17E-22 | 1.94E-20 | yes | up | 12.15 | 15.68 | 12.17 | 90.72 | 97.39 | 86.57 | 13.33333 | 91.56 |
| MYLK3 | 0.084 | -3.56569 | 4.25E-22 | 1.97E-20 | yes | down | 4.5 | 5.18 | 2.33 | 0.78 | 0.91 | 0.64 | 4.003333 | 0.776667 |
| DPT | 6.826 | 2.771118 | 4.53E-22 | 2.09E-20 | yes | up | 40.59 | 69.1 | 34.6 | 505.88 | 918.1 | 766.9 | 48.09667 | 730.2933 |
| WFS1 | 0.225 | -2.15189 | 4.89E-22 | 2.25E-20 | yes | down | 14.27 | 14.71 | 15.13 | 5.98 | 9.6 | 6.67 | 14.70333 | 7.416667 |
| UBE2C | 16.056 | 4.005004 | 4.92E-22 | 2.26E-20 | yes | up | 1.2 | 1.45 | 0.87 | 52.18 | 48.37 | 25.06 | 1.173333 | 41.87 |
| ARHGDIB | 3.682 | 1.88035 | 6.59E-22 | 3.02E-20 | yes | up | 18.57 | 17.65 | 20.73 | 166.5 | 174.94 | 127.57 | 18.98333 | 156.3367 |
| ATP6V1E1 | 0.273 | -1.87367 | 7.03E-22 | 3.22E-20 | yes | down | 187.83 | 224.08 | 218.95 | 143.52 | 112.68 | 121.85 | 210.2867 | 126.0167 |
| BTC | 35.149 | 5.1354 | 7.69E-22 | 3.51E-20 | yes | up | 0.09 | 0.17 | 0.08 | 4.87 | 16.27 | 8.57 | 0.113333 | 9.903333 |
| LOC100345908 | 0.024 | -5.39696 | 9E-22 | 4.09E-20 | yes | down | 3.24 | 7.94 | 5.17 | 0.15 | 0.65 | 0.13 | 5.45 | 0.31 |
| ZFAND4 | 0.127 | -2.97919 | 9.38E-22 | 4.25E-20 | yes | down | 5.58 | 7.6 | 11.5 | 1.83 | 2.59 | 2.1 | 8.226667 | 2.173333 |
| CCL19 | 43.044 | 5.42774 | 1.06E-21 | 4.78E-20 | yes | up | 0.59 | 0.23 | 0.52 | 34.73 | 33.08 | 59.51 | 0.446667 | 42.44 |
| LOC100358789 | 0.115 | -3.11415 | 1.21E-21 | 5.46E-20 | yes | down | 23.42 | 45.78 | 32.64 | 9.41 | 12.27 | 4.49 | 33.94667 | 8.723333 |
| SLC15A3 | 5.068 | 2.341466 | 1.23E-21 | 5.53E-20 | yes | up | 1.37 | 1.77 | 1.78 | 15.37 | 22.73 | 17.53 | 1.64 | 18.54333 |
| PDGFRB | 2.713 | 1.439713 | 1.28E-21 | 5.76E-20 | yes | up | 17.3 | 22.08 | 18.55 | 104.52 | 136.68 | 106.88 | 19.31 | 116.0267 |
| NPC2 | 4.606 | 2.20341 | 1.47E-21 | 6.55E-20 | yes | up | 40.16 | 32.39 | 44.5 | 394.3 | 367.9 | 434.82 | 39.01667 | 399.0067 |
| SIRPA | 4.18 | 2.06334 | 2.02E-21 | 9.03E-20 | yes | up | 8.03 | 9.98 | 12.94 | 83.19 | 105.78 | 97.28 | 10.31667 | 95.41667 |
| WSCD2 | 8.175 | 3.031206 | 2.14E-21 | 9.51E-20 | yes | up | 0.36 | 0.45 | 0.34 | 7.52 | 5.55 | 6.76 | 0.383333 | 6.61 |
| SEMA7A | 0.147 | -2.7632 | 2.34E-21 | 1.04E-19 | yes | down | 225.56 | 377.91 | 370.73 | 95.46 | 161.04 | 66.7 | 324.7333 | 107.7333 |
| CALM3 | 3.522 | 1.816533 | 2.93E-21 | 1.3E-19 | yes | up | 39.34 | 62.86 | 37.88 | 352.09 | 352.04 | 300.49 | 46.69333 | 334.8733 |
| NDUFA4 | 0.333 | -1.58542 | 4.54E-21 | 2E-19 | yes | down | 987.09 | 1145.86 | 923.43 | 818.99 | 762.32 | 688.95 | 1018.793 | 756.7533 |
| VCAN | 4.483 | 2.164585 | 5.18E-21 | 2.28E-19 | yes | up | 4.57 | 6.37 | 3.56 | 52.39 | 37.72 | 44.82 | 4.833333 | 44.97667 |
| ABR | 3.12 | 1.641552 | 5.3E-21 | 2.33E-19 | yes | up | 5.63 | 6.15 | 5.79 | 32.88 | 40.97 | 36.48 | 5.856667 | 36.77667 |
| GALNT16 | 4.346 | 2.119673 | 5.64E-21 | 2.47E-19 | yes | up | 5.2 | 8.23 | 5.89 | 49.31 | 64.39 | 69.66 | 6.44 | 61.12 |
| SCARB1 | 4.781 | 2.257283 | 5.78E-21 | 2.53E-19 | yes | up | 15.8 | 17.9 | 14.6 | 216.77 | 145.39 | 145.06 | 16.1 | 169.0733 |
| CKS2 | 10.033 | 3.326672 | 5.83E-21 | 2.54E-19 | yes | up | 3.73 | 4.94 | 4.46 | 122.75 | 116.21 | 56.37 | 4.376667 | 98.44333 |
| AK3 | 0.333 | -1.58606 | 5.88E-21 | 2.55E-19 | yes | down | 249.84 | 328.89 | 313.19 | 214.37 | 274.68 | 183.85 | 297.3067 | 224.3 |
| C3H8orf22 | 0.012 | -6.40115 | 6.09E-21 | 2.64E-19 | yes | down | 6.1 | 6.3 | 3.44 | 0.15 | 0 | 0.29 | 5.28 | 0.146667 |
| P4HA2 | 3.967 | 1.98809 | 6.12E-21 | 2.64E-19 | yes | up | 12.73 | 17.3 | 11.68 | 108.82 | 117.5 | 134.96 | 13.90333 | 120.4267 |
| MAN1C1 | 3.682 | 1.880539 | 6.41E-21 | 2.76E-19 | yes | up | 1.57 | 1.63 | 1.65 | 12.35 | 15.64 | 11.76 | 1.616667 | 13.25 |
| SYNJ2BP | 0.222 | -2.1727 | 6.44E-21 | 2.77E-19 | yes | down | 8.67 | 12.51 | 9.84 | 5.22 | 6.47 | 3.41 | 10.34 | 5.033333 |
| PLEKHF1 | 5.429 | 2.440751 | 6.47E-21 | 2.77E-19 | yes | up | 9.07 | 11.94 | 5.72 | 96.93 | 114.59 | 107.6 | 8.91 | 106.3733 |
| YEATS4 | 0.186 | -2.42972 | 6.53E-21 | 2.79E-19 | yes | down | 10.11 | 13.69 | 10.67 | 4.93 | 6.18 | 3.13 | 11.49 | 4.746667 |
| ACADSB | 0.289 | -1.78897 | 7.31E-21 | 3.12E-19 | yes | down | 57.57 | 66.21 | 65.53 | 37.97 | 52.59 | 31.97 | 63.10333 | 40.84333 |
| CCL14 | 4.55 | 2.185872 | 8.59E-21 | 3.65E-19 | yes | up | 12.27 | 17.92 | 14.74 | 148.79 | 141.71 | 161.7 | 14.97667 | 150.7333 |
| RCN3 | 3.822 | 1.934511 | 9.17E-21 | 3.89E-19 | yes | up | 8.49 | 12.83 | 9.04 | 77.82 | 85.74 | 90.81 | 10.12 | 84.79 |
| LGMN | 2.833 | 1.502521 | 9.89E-21 | 4.19E-19 | yes | up | 65.47 | 65.49 | 63.48 | 389.24 | 416.9 | 414.06 | 64.81333 | 406.7333 |
| IGFBP7 | 4.47 | 2.160171 | 1.03E-20 | 4.36E-19 | yes | up | 247.68 | 325.31 | 176.18 | 2657.45 | 2384.99 | 2313.07 | 249.7233 | 2451.837 |
| OSGEPL1 | 0.212 | -2.23661 | 1.04E-20 | 4.38E-19 | yes | down | 10.86 | 11.26 | 12.85 | 5.74 | 7.75 | 4.69 | 11.65667 | 6.06 |
| COL15A1 | 6.08 | 2.604122 | 1.13E-20 | 4.73E-19 | yes | up | 22.25 | 32.38 | 14.12 | 348.32 | 321.75 | 246.92 | 22.91667 | 305.6633 |
| PDK2 | 0.312 | -1.67853 | 1.14E-20 | 4.76E-19 | yes | down | 42.96 | 45.52 | 46.7 | 26.39 | 34.39 | 32.97 | 45.06 | 31.25 |
| PIPOX | 7.405 | 2.888474 | 1.18E-20 | 4.93E-19 | yes | up | 1.3 | 2.15 | 1.06 | 21.04 | 25.59 | 26.38 | 1.503333 | 24.33667 |
| NDUFA9 | 0.243 | -2.04113 | 1.19E-20 | 4.97E-19 | yes | down | 94.49 | 150.6 | 125.24 | 76.43 | 58.89 | 60.9 | 123.4433 | 65.40667 |
| TSFM | 0.272 | -1.87602 | 1.22E-20 | 5.07E-19 | yes | down | 58.15 | 72.59 | 61.35 | 45.87 | 34.88 | 36.01 | 64.03 | 38.92 |
| COX6A1 | 0.331 | -1.59492 | 1.27E-20 | 5.25E-19 | yes | down | 2301.77 | 2461.12 | 2262.99 | 1925.59 | 1724.16 | 1547.58 | 2341.96 | 1732.443 |
| PCDH18 | 5.833 | 2.544205 | 1.69E-20 | 6.97E-19 | yes | up | 0.61 | 0.65 | 0.65 | 10.56 | 7.16 | 6.81 | 0.636667 | 8.176667 |
| MIPEP | 0.28 | -1.83737 | 1.8E-20 | 7.44E-19 | yes | down | 13.52 | 19.95 | 17.24 | 11 | 10.15 | 9.94 | 16.90333 | 10.36333 |
| LOC103346606 | 5.803 | 2.536731 | 1.95E-20 | 8.02E-19 | yes | up | 1.57 | 2.54 | 1.78 | 28.84 | 20.88 | 24.03 | 1.963333 | 24.58333 |
| RASSF2 | 4.15 | 2.053111 | 1.95E-20 | 8.02E-19 | yes | up | 3.48 | 4.55 | 3.24 | 35.28 | 42.98 | 24.96 | 3.756667 | 34.40667 |
| TMBIM1 | 3.628 | 1.859372 | 2.02E-20 | 8.29E-19 | yes | up | 28.75 | 33.74 | 27.92 | 192.82 | 256.26 | 274.31 | 30.13667 | 241.13 |
| FUCA1 | 3.193 | 1.675092 | 2.18E-20 | 8.89E-19 | yes | up | 11.79 | 14.96 | 10.37 | 82.74 | 94.52 | 84.24 | 12.37333 | 87.16667 |
| LOC103351259 | 0.098 | -3.34852 | 2.18E-20 | 8.89E-19 | yes | down | 22.49 | 15.75 | 24.34 | 4.01 | 7.63 | 3.12 | 20.86 | 4.92 |
| RASL11A | 10.687 | 3.41783 | 2.33E-20 | 9.48E-19 | yes | up | 0.99 | 1.3 | 1.18 | 30.82 | 18.98 | 30.65 | 1.156667 | 26.81667 |
| PREX2 | 0.176 | -2.50794 | 2.34E-20 | 9.51E-19 | yes | down | 12.23 | 14.94 | 11.89 | 4.87 | 7.58 | 3.02 | 13.02 | 5.156667 |
| TMEM132A | 4.082 | 2.029426 | 2.35E-20 | 9.52E-19 | yes | up | 1.64 | 1.58 | 1.39 | 12.56 | 15.11 | 14.05 | 1.536667 | 13.90667 |
| GSTM2 | 6.591 | 2.720551 | 2.68E-20 | 1.08E-18 | yes | up | 6.86 | 12.1 | 4.93 | 105.57 | 113.14 | 123.09 | 7.963333 | 113.9333 |
| QSOX1 | 4.244 | 2.085384 | 2.86E-20 | 1.15E-18 | yes | up | 2.99 | 3.75 | 3.74 | 25.27 | 36.63 | 36.35 | 3.493333 | 32.75 |
| ABI3BP | 4.037 | 2.013182 | 3.25E-20 | 1.31E-18 | yes | up | 4.9 | 7.85 | 6.06 | 48.15 | 51.77 | 61.19 | 6.27 | 53.70333 |
| PPM1L | 0.234 | -2.09644 | 3.33E-20 | 1.33E-18 | yes | down | 2.44 | 3.16 | 3.24 | 1.54 | 1.83 | 1.19 | 2.946667 | 1.52 |
| SDHC | 0.332 | -1.58859 | 3.72E-20 | 1.49E-18 | yes | down | 254.65 | 331.4 | 278.98 | 229.52 | 201.38 | 201.33 | 288.3433 | 210.7433 |
| PMEPA1 | 0.198 | -2.33665 | 3.83E-20 | 1.53E-18 | yes | down | 17.91 | 27.98 | 21.58 | 4.97 | 10.91 | 10.79 | 22.49 | 8.89 |
| DMRT2 | 7.569 | 2.92004 | 4.08E-20 | 1.62E-18 | yes | up | 0.21 | 0.58 | 0.49 | 7.9 | 7.28 | 6.15 | 0.426667 | 7.11 |
| GATSL3 | 0.295 | -1.76208 | 4.13E-20 | 1.64E-18 | yes | down | 45.49 | 42.38 | 47.36 | 25.71 | 35.64 | 28.47 | 45.07667 | 29.94 |
| FAM124A | 0.255 | -1.97059 | 4.23E-20 | 1.68E-18 | yes | down | 7.43 | 7.68 | 6.52 | 3.39 | 4.33 | 4.5 | 7.21 | 4.073333 |
| FTH1 | 3.231 | 1.691852 | 4.47E-20 | 1.77E-18 | yes | up | 1123.38 | 1303.17 | 1200.75 | 7874.43 | 8214.06 | 9815.17 | 1209.1 | 8634.553 |
| MMP2 | 4.134 | 2.0474 | 4.99E-20 | 1.96E-18 | yes | up | 39.51 | 55.08 | 46.83 | 377.38 | 380.01 | 515.23 | 47.14 | 424.2067 |
| SEC11C | 0.324 | -1.62531 | 5.35E-20 | 2.1E-18 | yes | down | 104.9 | 146.82 | 113.33 | 92.01 | 91.74 | 77.74 | 121.6833 | 87.16333 |
| LOC100346670 | 0.31 | -1.68923 | 5.6E-20 | 2.19E-18 | yes | down | 1590.44 | 1656.48 | 1455.14 | 1216.81 | 1025.23 | 1017.47 | 1567.353 | 1086.503 |
| KIRREL | 3.186 | 1.67173 | 6.04E-20 | 2.36E-18 | yes | up | 3.32 | 4.21 | 3.42 | 23.28 | 37.05 | 19.94 | 3.65 | 26.75667 |
| ADRB1 | 0.103 | -3.28153 | 6.11E-20 | 2.38E-18 | yes | down | 6.36 | 4.05 | 3.71 | 0.78 | 1.47 | 1.05 | 4.706667 | 1.1 |
| NAT8L | 8.269 | 3.047777 | 6.11E-20 | 2.38E-18 | yes | up | 1.03 | 1.68 | 2.36 | 22.23 | 38.49 | 30.86 | 1.69 | 30.52667 |
| FAM149A | 3.647 | 1.866837 | 6.29E-20 | 2.44E-18 | yes | up | 3.07 | 4.59 | 4.08 | 30.03 | 41.69 | 35.02 | 3.913333 | 35.58 |
| BGN | 5.381 | 2.427938 | 6.6E-20 | 2.56E-18 | yes | up | 29.26 | 46.65 | 20.98 | 367.32 | 377.99 | 389.9 | 32.29667 | 378.4033 |
| LGALS3BP | 3.379 | 1.756726 | 6.75E-20 | 2.61E-18 | yes | up | 16.55 | 18.45 | 14.16 | 107.86 | 125.91 | 132.39 | 16.38667 | 122.0533 |
| SCUBE3 | 9.928 | 3.311439 | 6.9E-20 | 2.66E-18 | yes | up | 0.16 | 0.22 | 0.15 | 2.44 | 4.59 | 4.39 | 0.176667 | 3.806667 |
| COX4I1 | 0.38 | -1.39424 | 6.96E-20 | 2.68E-18 | yes | down | 2887.75 | 3415.24 | 2802.94 | 2680.22 | 2609.64 | 2390.05 | 3035.31 | 2559.97 |
| LOC108177262 | 0.298 | -1.74733 | 7.54E-20 | 2.9E-18 | yes | down | 1413.12 | 1845.65 | 1423.56 | 1185.62 | 1036.84 | 891.19 | 1560.777 | 1037.883 |
| ALDH2 | 5.261 | 2.395253 | 7.59E-20 | 2.91E-18 | yes | up | 47.96 | 70.41 | 32.39 | 563.22 | 565.3 | 601.64 | 50.25333 | 576.72 |
| NDUFB9 | 0.37 | -1.43563 | 7.92E-20 | 3.03E-18 | yes | down | 474.99 | 599.99 | 524.46 | 471.43 | 438.51 | 396 | 533.1467 | 435.3133 |
| FIBIN | 0.163 | -2.61529 | 8.13E-20 | 3.1E-18 | yes | down | 16.79 | 18.92 | 17.93 | 6.28 | 4.69 | 8.21 | 17.88 | 6.393333 |
| CYP2B4 | 87.398 | 6.449522 | 8.49E-20 | 3.23E-18 | yes | up | 1.24 | 7.07 | 5.56 | 450.24 | 752.8 | 1322.16 | 4.623333 | 841.7333 |
| TEAD2 | 3.147 | 1.653907 | 8.75E-20 | 3.32E-18 | yes | up | 7.35 | 7.71 | 6.92 | 46.74 | 56.29 | 52.13 | 7.326667 | 51.72 |
| MTHFD1 | 0.253 | -1.98017 | 9.32E-20 | 3.53E-18 | yes | down | 39.53 | 45.44 | 46.06 | 23.85 | 33.33 | 17.32 | 43.67667 | 24.83333 |
| PLXND1 | 3.203 | 1.679389 | 9.93E-20 | 3.75E-18 | yes | up | 13.44 | 19.9 | 15.63 | 102.04 | 142.14 | 96.27 | 16.32333 | 113.4833 |
| LMF2 | 3.525 | 1.817424 | 1.06E-19 | 3.99E-18 | yes | up | 3.34 | 5 | 3.76 | 32.22 | 32.67 | 28.9 | 4.033333 | 31.26333 |
| OS9 | 2.789 | 1.479694 | 1.19E-19 | 4.49E-18 | yes | up | 23.09 | 31.85 | 22.63 | 140.07 | 197.57 | 145.39 | 25.85667 | 161.01 |
| EMILIN1 | 5.883 | 2.556461 | 1.2E-19 | 4.51E-18 | yes | up | 2.14 | 2.51 | 1.18 | 26.18 | 24.15 | 24.7 | 1.943333 | 25.01 |
| LOC103349956 | 0.096 | -3.38019 | 1.24E-19 | 4.66E-18 | yes | down | 3.54 | 6.13 | 2.29 | 0.72 | 0.8 | 0.54 | 3.986667 | 0.686667 |
| DHCR24 | 3.86 | 1.948782 | 1.31E-19 | 4.89E-18 | yes | up | 4.75 | 7.29 | 6.62 | 61.21 | 50.42 | 46.09 | 6.22 | 52.57333 |
| EMC4 | 0.349 | -1.52044 | 1.31E-19 | 4.89E-18 | yes | down | 50.98 | 65.62 | 65.44 | 41.72 | 47.7 | 40.65 | 60.68 | 43.35667 |
| LOC100358892 | 0.172 | -2.53891 | 1.35E-19 | 5.03E-18 | yes | down | 13.06 | 11.93 | 10.72 | 5.06 | 5.87 | 2.83 | 11.90333 | 4.586667 |
| COTL1 | 3.884 | 1.957461 | 1.36E-19 | 5.06E-18 | yes | up | 8.55 | 13.62 | 9.82 | 100.67 | 85.58 | 85.18 | 10.66333 | 90.47667 |
| LOC100351488 | 4.899 | 2.292417 | 1.48E-19 | 5.48E-18 | yes | up | 8.92 | 11.71 | 12.47 | 172.87 | 160.14 | 74.84 | 11.03333 | 135.95 |
| LOC100347496 | 0.119 | -3.07578 | 1.5E-19 | 5.53E-18 | yes | down | 349.54 | 287.72 | 369.87 | 106.08 | 48.18 | 105.91 | 335.71 | 86.72333 |
| MRPL19 | 0.307 | -1.70155 | 1.52E-19 | 5.59E-18 | yes | down | 31.11 | 35.32 | 37.19 | 23.03 | 23.92 | 20.19 | 34.54 | 22.38 |
| FPGS | 0.265 | -1.91665 | 1.76E-19 | 6.47E-18 | yes | down | 12.28 | 14.95 | 15.48 | 7.42 | 8.32 | 9.26 | 14.23667 | 8.333333 |
| TTC9 | 0.205 | -2.28817 | 2.01E-19 | 7.39E-18 | yes | down | 56.35 | 83.32 | 80.42 | 33.7 | 50.68 | 25.67 | 73.36333 | 36.68333 |
| BNIP3 | 0.363 | -1.46157 | 2.09E-19 | 7.63E-18 | yes | down | 164.56 | 178.38 | 158.36 | 125.26 | 135.32 | 140.03 | 167.1 | 133.5367 |
| MYLK | 3.949 | 1.981451 | 2.18E-19 | 7.95E-18 | yes | up | 8.89 | 15.14 | 8.37 | 87.82 | 87.97 | 91.37 | 10.8 | 89.05333 |
| VDAC1 | 0.353 | -1.50376 | 2.31E-19 | 8.43E-18 | yes | down | 305.64 | 424.13 | 306.56 | 280.39 | 297.19 | 283.93 | 345.4433 | 287.17 |
| RXRG | 0.066 | -3.92806 | 2.43E-19 | 8.84E-18 | yes | down | 77.05 | 77.02 | 96.99 | 9.86 | 3.41 | 14.72 | 83.68667 | 9.33 |
| IGF2BP3 | 0.118 | -3.08913 | 2.5E-19 | 9.08E-18 | yes | down | 2.23 | 1.9 | 2 | 0.43 | 0.77 | 0.42 | 2.043333 | 0.54 |
| DNAJA3 | 0.364 | -1.45885 | 2.72E-19 | 9.85E-18 | yes | down | 36.49 | 49.38 | 44.32 | 36.11 | 36.44 | 31.4 | 43.39667 | 34.65 |
| RALGPS2 | 0.264 | -1.92096 | 2.87E-19 | 1.04E-17 | yes | down | 2.05 | 2.53 | 2.07 | 1.24 | 1.58 | 1.09 | 2.216667 | 1.303333 |
| C1D | 0.267 | -1.90269 | 2.95E-19 | 1.06E-17 | yes | down | 12.25 | 13.23 | 11.87 | 8.32 | 8 | 5.82 | 12.45 | 7.38 |
| ACKR1 | 4.255 | 2.089284 | 3.06E-19 | 1.1E-17 | yes | up | 3.48 | 4.39 | 3.53 | 31.82 | 31.45 | 42.56 | 3.8 | 35.27667 |
| LOC100358870 | 7.23 | 2.853963 | 3.11E-19 | 1.12E-17 | yes | up | 1.12 | 1.76 | 1.93 | 30.95 | 25.03 | 19.9 | 1.603333 | 25.29333 |
| ELOVL1 | 3.656 | 1.870246 | 3.37E-19 | 1.21E-17 | yes | up | 8.51 | 12.56 | 11.1 | 97.53 | 82.08 | 81.11 | 10.72333 | 86.90667 |
| ATP5C1 | 0.323 | -1.63229 | 3.46E-19 | 1.24E-17 | yes | down | 331.93 | 403.02 | 332.74 | 286.44 | 258.78 | 204.02 | 355.8967 | 249.7467 |
| SDC2 | 2.67 | 1.417072 | 3.48E-19 | 1.24E-17 | yes | up | 13.23 | 13.19 | 13.19 | 78.31 | 82.73 | 73.68 | 13.20333 | 78.24 |
| CPXM1 | 8.358 | 3.063118 | 3.67E-19 | 1.3E-17 | yes | up | 1.05 | 3.43 | 2.27 | 44.59 | 35.08 | 41.37 | 2.25 | 40.34667 |
| COL6A3 | 4.097 | 2.034699 | 3.67E-19 | 1.3E-17 | yes | up | 54.67 | 52.72 | 33.49 | 380.74 | 575.29 | 396.35 | 46.96 | 450.7933 |
| C1QC | 3.142 | 1.651472 | 3.69E-19 | 1.31E-17 | yes | up | 33.12 | 45.85 | 48.68 | 301.58 | 286.26 | 290.81 | 42.55 | 292.8833 |
| LACE1 | 0.259 | -1.94842 | 3.75E-19 | 1.33E-17 | yes | down | 15.35 | 21.35 | 22.54 | 10.06 | 12.69 | 11.48 | 19.74667 | 11.41 |
| NDUFS4 | 0.327 | -1.61327 | 3.82E-19 | 1.35E-17 | yes | down | 211.06 | 251.96 | 205.59 | 181.04 | 163.83 | 141.8 | 222.87 | 162.2233 |
| ROR2 | 3.729 | 1.898932 | 3.9E-19 | 1.37E-17 | yes | up | 1.28 | 1.61 | 1.3 | 11.74 | 11.73 | 10.83 | 1.396667 | 11.43333 |
| C1QTNF6 | 3.451 | 1.787123 | 4.65E-19 | 1.63E-17 | yes | up | 6.04 | 8.79 | 6.83 | 46.93 | 59.64 | 59.73 | 7.22 | 55.43333 |
| OLFML3 | 6.031 | 2.592475 | 4.97E-19 | 1.74E-17 | yes | up | 11.57 | 19.48 | 13.41 | 185.84 | 141.71 | 249.23 | 14.82 | 192.26 |
| P2RX1 | 66.963 | 6.065285 | 5.12E-19 | 1.79E-17 | yes | up | 0.05 | 0.07 | 0.09 | 10.61 | 4.35 | 14.64 | 0.07 | 9.866667 |
| LOXL3 | 2.979 | 1.575014 | 5.43E-19 | 1.9E-17 | yes | up | 4.26 | 5.8 | 5.21 | 33.59 | 34.37 | 31.21 | 5.09 | 33.05667 |
| AIFM2 | 0.381 | -1.39159 | 5.56E-19 | 1.94E-17 | yes | down | 381.75 | 507.28 | 483.21 | 398.02 | 412.23 | 353.42 | 457.4133 | 387.89 |
| EPB41L3 | 4.035 | 2.012721 | 5.68E-19 | 1.97E-17 | yes | up | 1.5 | 2.66 | 1.83 | 18.48 | 18.67 | 16 | 1.996667 | 17.71667 |
| NFATC4 | 6.263 | 2.646774 | 5.77E-19 | 2E-17 | yes | up | 0.83 | 1.96 | 1.14 | 20.51 | 18.86 | 13.7 | 1.31 | 17.69 |
| BCAM | 8.702 | 3.121371 | 5.95E-19 | 2.06E-17 | yes | up | 29.44 | 34.71 | 25.75 | 363.97 | 428.07 | 900.72 | 29.96667 | 564.2533 |
| CAMK1 | 3.548 | 1.827143 | 6E-19 | 2.07E-17 | yes | up | 10.34 | 14.03 | 11.01 | 92.91 | 89.49 | 93.27 | 11.79333 | 91.89 |
| FBLN1 | 4.458 | 2.156483 | 6.4E-19 | 2.21E-17 | yes | up | 14.16 | 20.85 | 21.52 | 170.19 | 161.57 | 218.1 | 18.84333 | 183.2867 |
| RTN4IP1 | 0.17 | -2.55311 | 6.65E-19 | 2.29E-17 | yes | down | 10.85 | 11.61 | 17.74 | 4.84 | 4.78 | 5.55 | 13.4 | 5.056667 |
| NDUFA12 | 0.262 | -1.93172 | 6.74E-19 | 2.31E-17 | yes | down | 276.75 | 365.12 | 291.45 | 192.24 | 149.84 | 195.84 | 311.1067 | 179.3067 |
| MTIF2 | 0.179 | -2.48478 | 7.37E-19 | 2.52E-17 | yes | down | 7 | 8.86 | 9.64 | 3.19 | 3.94 | 1.81 | 8.5 | 2.98 |
| STUM | 4.863 | 2.281982 | 7.68E-19 | 2.63E-17 | yes | up | 0.59 | 0.61 | 0.42 | 4.96 | 7.29 | 5.21 | 0.54 | 5.82 |
| KRT23 | 0.001 | -9.92514 | 7.7E-19 | 2.63E-17 | yes | down | 13.82 | 4.3 | 14.03 | 0 | 0.04 | 0.05 | 10.71667 | 0.03 |
| NDUFV2 | 0.303 | -1.7236 | 9.26E-19 | 3.15E-17 | yes | down | 176.7 | 237.97 | 187.18 | 154.88 | 128.2 | 117.45 | 200.6167 | 133.51 |
| HSPD1 | 0.262 | -1.93225 | 1E-18 | 3.4E-17 | yes | down | 166.71 | 220.74 | 196.04 | 139.34 | 115.06 | 82.29 | 194.4967 | 112.23 |
| RNF6 | 0.208 | -2.26696 | 1.01E-18 | 3.42E-17 | yes | down | 20.37 | 17.35 | 21.56 | 8.56 | 12.83 | 6.23 | 19.76 | 9.206667 |
| COQ6 | 0.22 | -2.18245 | 1.05E-18 | 3.56E-17 | yes | down | 40.47 | 57.09 | 37.26 | 21.01 | 18.35 | 25.47 | 44.94 | 21.61 |
| KCNJ8 | 4.638 | 2.213408 | 1.08E-18 | 3.65E-17 | yes | up | 4.95 | 9.48 | 5.93 | 69.56 | 62.47 | 72.57 | 6.786667 | 68.2 |
| NDUFC2 | 0.258 | -1.95403 | 1.12E-18 | 3.75E-17 | yes | down | 432.31 | 615.72 | 510.31 | 342.37 | 247.88 | 292.82 | 519.4467 | 294.3567 |
| ADAM23 | 9.085 | 3.183472 | 1.12E-18 | 3.75E-17 | yes | up | 0.25 | 0.47 | 0.15 | 5.57 | 8.72 | 6.51 | 0.29 | 6.933333 |
| RECK | 3.152 | 1.656395 | 1.21E-18 | 4.07E-17 | yes | up | 1.88 | 2.12 | 1.73 | 12.74 | 15.5 | 12.45 | 1.91 | 13.56333 |
| KDELC2 | 3.1 | 1.632369 | 1.41E-18 | 4.71E-17 | yes | up | 3.83 | 5.6 | 4.36 | 36.17 | 35.61 | 29.16 | 4.596667 | 33.64667 |
| ACAA1 | 0.343 | -1.54244 | 1.43E-18 | 4.76E-17 | yes | down | 34.58 | 50.23 | 44.25 | 31.65 | 35.4 | 30.67 | 43.02 | 32.57333 |
| PAM | 3.055 | 1.611385 | 1.5E-18 | 5.01E-17 | yes | up | 23.69 | 28.4 | 21.74 | 165.28 | 155.88 | 171.99 | 24.61 | 164.3833 |
| MOXD1 | 9.946 | 3.31408 | 1.59E-18 | 5.29E-17 | yes | up | 0.41 | 0.54 | 0.36 | 8.56 | 7.64 | 12.25 | 0.436667 | 9.483333 |
| DOC2B | 7.15 | 2.838029 | 1.64E-18 | 5.43E-17 | yes | up | 1.04 | 1.1 | 0.69 | 11.53 | 20.76 | 12.46 | 0.943333 | 14.91667 |
| LAMA3 | 4.618 | 2.207399 | 1.72E-18 | 5.7E-17 | yes | up | 0.86 | 0.93 | 1.2 | 10.75 | 12.94 | 8.01 | 0.996667 | 10.56667 |
| LYRM5 | 0.303 | -1.72203 | 1.83E-18 | 6.04E-17 | yes | down | 57.7 | 60.48 | 67.66 | 41.64 | 44.31 | 30.14 | 61.94667 | 38.69667 |
| VWF | 4.444 | 2.151731 | 1.89E-18 | 6.24E-17 | yes | up | 17.15 | 14.03 | 10.75 | 113.52 | 150.98 | 150.5 | 13.97667 | 138.3333 |
| P3H1 | 3.062 | 1.61443 | 1.91E-18 | 6.29E-17 | yes | up | 6.42 | 8.53 | 6.71 | 46.71 | 59.81 | 40.7 | 7.22 | 49.07333 |
| LOC100350957 | 14.113 | 3.818951 | 2E-18 | 6.59E-17 | yes | up | 0.6 | 1.4 | 0.34 | 17.11 | 35.99 | 26.45 | 0.78 | 26.51667 |
| LOC100341181 | 0.324 | -1.62383 | 2.09E-18 | 6.85E-17 | yes | down | 32.5 | 32.42 | 36.06 | 24.54 | 28.07 | 20.51 | 33.66 | 24.37333 |
| SPARC | 3.795 | 1.924077 | 2.21E-18 | 7.23E-17 | yes | up | 1115.68 | 1643.31 | 1339.62 | 10065.75 | 10238.11 | 13480.43 | 1366.203 | 11261.43 |
| LOC100353852 | 6.753 | 2.755466 | 2.22E-18 | 7.25E-17 | yes | up | 1.84 | 1.27 | 0.88 | 18.04 | 18.25 | 22.01 | 1.33 | 19.43333 |
| CES4A | 0.107 | -3.2294 | 2.24E-18 | 7.3E-17 | yes | down | 3.89 | 5.11 | 4.24 | 0.59 | 1.07 | 1.52 | 4.413333 | 1.06 |
| ZNF521 | 2.972 | 1.571404 | 2.28E-18 | 7.41E-17 | yes | up | 2.18 | 2.82 | 2.41 | 16.35 | 16.52 | 15.56 | 2.47 | 16.14333 |
| HSDL2 | 0.173 | -2.53324 | 2.49E-18 | 8.08E-17 | yes | down | 91.21 | 161.85 | 131.09 | 53.23 | 66.21 | 27.85 | 128.05 | 49.09667 |
| SLC25A13 | 0.363 | -1.46262 | 2.66E-18 | 8.61E-17 | yes | down | 20.02 | 25.78 | 20.86 | 18.4 | 17.87 | 16.14 | 22.22 | 17.47 |
| NEO1 | 2.659 | 1.410996 | 2.75E-18 | 8.9E-17 | yes | up | 10.22 | 12.44 | 9.81 | 59.82 | 73.36 | 58.11 | 10.82333 | 63.76333 |
| ADSL | 0.275 | -1.86163 | 2.84E-18 | 9.16E-17 | yes | down | 18.47 | 31.23 | 24.51 | 16.09 | 13.7 | 14.41 | 24.73667 | 14.73333 |
| PSPC1 | 0.281 | -1.83289 | 2.86E-18 | 9.22E-17 | yes | down | 16.46 | 20.48 | 14.99 | 11.96 | 11.32 | 8.67 | 17.31 | 10.65 |
| ADAMTS18 | 9.305 | 3.217951 | 2.88E-18 | 9.25E-17 | yes | up | 0.33 | 0.66 | 0.38 | 8.98 | 13.89 | 4.91 | 0.456667 | 9.26 |
| SIRT3 | 0.355 | -1.4922 | 3E-18 | 9.63E-17 | yes | down | 30.17 | 36.92 | 31.61 | 26.46 | 26.01 | 25.47 | 32.9 | 25.98 |
| CIB2 | 0.338 | -1.56591 | 3.03E-18 | 9.72E-17 | yes | down | 97.93 | 135.54 | 103.93 | 80.12 | 83.71 | 87.03 | 112.4667 | 83.62 |
| HBQ1 | 7.545 | 2.915579 | 3.22E-18 | 1.03E-16 | yes | up | 6.26 | 11.96 | 8.09 | 48.11 | 47.92 | 66.63 | 8.77 | 54.22 |
| DOCK11 | 0.262 | -1.93441 | 3.23E-18 | 1.03E-16 | yes | down | 10.42 | 9.49 | 12.34 | 6.22 | 8.23 | 4.91 | 10.75 | 6.453333 |
| EGLN3 | 0.123 | -3.01756 | 3.43E-18 | 1.09E-16 | yes | down | 29.68 | 47.94 | 30.92 | 13.05 | 5.3 | 10.33 | 36.18 | 9.56 |
| ACSS1 | 0.216 | -2.21016 | 3.46E-18 | 1.1E-16 | yes | down | 62.78 | 57.54 | 34.87 | 25.86 | 26.34 | 22.37 | 51.73 | 24.85667 |
| RFWD2 | 0.378 | -1.40191 | 3.59E-18 | 1.14E-16 | yes | down | 16.56 | 18.64 | 17.29 | 15.36 | 17.19 | 13.7 | 17.49667 | 15.41667 |
| OAF | 5.655 | 2.499626 | 3.87E-18 | 1.22E-16 | yes | up | 5.37 | 8.95 | 7.37 | 84.26 | 82.19 | 104.13 | 7.23 | 90.19333 |
| L2HGDH | 0.281 | -1.82909 | 4.35E-18 | 1.38E-16 | yes | down | 5.99 | 6.8 | 7.17 | 4.61 | 4.49 | 3.4 | 6.653333 | 4.166667 |
| HIBADH | 0.37 | -1.43416 | 4.87E-18 | 1.54E-16 | yes | down | 147.2 | 158.47 | 179.03 | 126.23 | 138.51 | 132.55 | 161.5667 | 132.43 |
| PTMS | 4.169 | 2.059648 | 4.88E-18 | 1.54E-16 | yes | up | 67.69 | 97.59 | 62.4 | 844.72 | 630.99 | 583.8 | 75.89333 | 686.5033 |
| CCDC80 | 3.583 | 1.841096 | 5.05E-18 | 1.59E-16 | yes | up | 44.17 | 72.82 | 51.75 | 354.82 | 488.5 | 456.22 | 56.24667 | 433.18 |
| SH3PXD2B | 3.986 | 1.994914 | 5.07E-18 | 1.59E-16 | yes | up | 1.91 | 1.61 | 1.27 | 13.93 | 14.21 | 14.13 | 1.596667 | 14.09 |
| BMP1 | 2.58 | 1.367556 | 5.1E-18 | 1.6E-16 | yes | up | 13.26 | 17.68 | 14.89 | 81.53 | 93.24 | 88.31 | 15.27667 | 87.69333 |
| NEK2 | 25.105 | 4.649898 | 5.7E-18 | 1.78E-16 | yes | up | 0.15 | 0.52 | 0.13 | 17.25 | 20.86 | 6.45 | 0.266667 | 14.85333 |
| ITPR1 | 0.387 | -1.37005 | 5.83E-18 | 1.82E-16 | yes | down | 8.2 | 9.81 | 8.7 | 6.71 | 9.08 | 7.12 | 8.903333 | 7.636667 |
| DFFB | 0.162 | -2.62615 | 5.91E-18 | 1.84E-16 | yes | down | 16.6 | 15.36 | 22.48 | 7.11 | 6.84 | 10.7 | 18.14667 | 8.216667 |
| IL34 | 4.263 | 2.091831 | 6.36E-18 | 1.98E-16 | yes | up | 1.66 | 2.92 | 2.14 | 22.71 | 27.87 | 21.39 | 2.24 | 23.99 |
[truncated: 1,282,803 more chars]
