# Supplementary material for: Comparative transcriptomic analysis of rabbit interscapular brown adipose tissue whitening under physiological conditions
Source: Adipocyte. 2022 Aug 29;11(1):529–49. doi: 10.1080/21623945.2022.2111053 (PMC9427046; doi:10.1080/21623945.2022.2111053)
Supplement: Supplemental Material [file KADI_A_2111053_SM2303.zip › Supplementary/Table S4_S9.docx]

**Table S4. Number of DEGs in different comparison groups**

| group | total DEG | up | down |
| --- | --- | --- | --- |
| iBAT_D1_vs_iBAT_D14 | 1049 | 547 | 502 |
| iBAT_D1_vs_iBAT_M1 | 2345 | 1272 | 1073 |
| iBAT_D1_vs_iBAT_M4 | 2928 | 1469 | 1459 |
| iBAT_D1_vs_ingWAT_M4 | 4323 | 2178 | 2145 |
| iBAT_D14_vs_iBAT_M1 | 1844 | 911 | 933 |
| iBAT_D14_vs_iBAT_M4 | 2610 | 1240 | 1370 |
| iBAT_D14_vs_ingWAT_M4 | 4365 | 2221 | 2144 |
| iBAT_M1_vs_iBAT_M4 | 1579 | 726 | 853 |
| iBAT_M1_vs_ingWAT_M4 | 3258 | 1498 | 1760 |
| iBAT_M4_vs_ingWAT_M4 | 1396 | 497 | 899 |

**Table S5. Statistical table of KEGG enrichment analysis of highly expressed genes for Brown Phase, Transition Phase and Whitened BAT Phase.**

iBAT_D1 and D14 (Brown Phase) High level gene

| Pathway id | Description | Database | Pvalue | Padjust |
| --- | --- | --- | --- | --- |
| map00190 | Oxidative phosphorylation | KEGG PATHWAY | 1.92E-31 | 2.78E-29 |
| map05012 | Parkinson disease | KEGG PATHWAY | 7.59E-31 | 5.51E-29 |
| map04714 | Thermogenesis | KEGG PATHWAY | 5.33E-25 | 2.58E-23 |
| map05016 | Huntington disease | KEGG PATHWAY | 2.56E-24 | 9.26E-23 |
| map05010 | Alzheimer disease | KEGG PATHWAY | 1.34E-22 | 3.89E-21 |
| map04932 | Non-alcoholic fatty liver disease (NAFLD) | KEGG PATHWAY | 1.79E-16 | 4.32E-15 |
| map04723 | Retrograde endocannabinoid signaling | KEGG PATHWAY | 1.19E-10 | 2.46E-09 |
| map04260 | Cardiac muscle contraction | KEGG PATHWAY | 1.81E-10 | 3.28E-09 |
| map00020 | Citrate cycle (TCA cycle) | KEGG PATHWAY | 2.68E-08 | 4.32E-07 |
| map00071 | Fatty acid degradation | KEGG PATHWAY | 1.82E-07 | 2.63E-06 |
| map00280 | Valine, leucine and isoleucine degradation | KEGG PATHWAY | 1.36E-06 | 1.8E-05 |
| map00360 | Phenylalanine metabolism | KEGG PATHWAY | 0.00026 | 0.003136 |
| map00620 | Pyruvate metabolism | KEGG PATHWAY | 0.000582 | 0.006494 |
| map00650 | Butanoate metabolism | KEGG PATHWAY | 0.000875 | 0.00906 |
| map00400 | Phenylalanine, tyrosine and tryptophan biosynthesis | KEGG PATHWAY | 0.001444 | 0.013961 |
| map00350 | Tyrosine metabolism | KEGG PATHWAY | 0.001574 | 0.014266 |
| map00072 | Synthesis and degradation of ketone bodies | KEGG PATHWAY | 0.002034 | 0.01735 |
| map00640 | Propanoate metabolism | KEGG PATHWAY | 0.003311 | 0.026673 |
| map00330 | Arginine and proline metabolism | KEGG PATHWAY | 0.004796 | 0.036602 |
| map00010 | Glycolysis / Gluconeogenesis | KEGG PATHWAY | 0.005531 | 0.040101 |

iBAT_M1 (Transition Phase) High level gene

| Pathway id | Description | Database | Pvalue | Padjust |
| --- | --- | --- | --- | --- |
| map05133 | Pertussis | KEGG PATHWAY | 0.000185 | 0.034706 |
| map04940 | Type I diabetes mellitus | KEGG PATHWAY | 0.000797 | 0.074916 |
| map05120 | Epithelial cell signaling in Helicobacter pylori infection | KEGG PATHWAY | 0.001697 | 0.079746 |
| map04137 | Mitophagy - animal | KEGG PATHWAY | 0.002805 | 0.087899 |
| map04621 | NOD-like receptor signaling pathway | KEGG PATHWAY | 0.003329 | 0.089394 |
| map04610 | Complement and coagulation cascades | KEGG PATHWAY | 0.001493 | 0.093556 |
| map05167 | Kaposi sarcoma-associated herpesvirus infection | KEGG PATHWAY | 0.002611 | 0.098188 |
| map04668 | TNF signaling pathway | KEGG PATHWAY | 0.004466 | 0.104957 |
| map04966 | Collecting duct acid secretion | KEGG PATHWAY | 0.015541 | 0.243478 |
| map04926 | Relaxin signaling pathway | KEGG PATHWAY | 0.013375 | 0.251454 |
| map04721 | Synaptic vesicle cycle | KEGG PATHWAY | 0.017694 | 0.255886 |
| map04624 | Toll and Imd signaling pathway | KEGG PATHWAY | 0.015332 | 0.262031 |
| map03040 | Spliceosome | KEGG PATHWAY | 0.012871 | 0.268869 |
| map04146 | Peroxisome | KEGG PATHWAY | 0.024736 | 0.290653 |
| map04727 | GABAergic synapse | KEGG PATHWAY | 0.021874 | 0.293734 |
| map05142 | Chagas disease (American trypanosomiasis) | KEGG PATHWAY | 0.023676 | 0.296744 |
| map04136 | Autophagy - other | KEGG PATHWAY | 0.044335 | 0.308701 |
| map00630 | Glyoxylate and dicarboxylate metabolism | KEGG PATHWAY | 0.036363 | 0.31074 |
| map05030 | Cocaine addiction | KEGG PATHWAY | 0.041611 | 0.312912 |
| map04212 | Longevity regulating pathway - worm | KEGG PATHWAY | 0.043496 | 0.314511 |
| map04931 | Insulin resistance | KEGG PATHWAY | 0.035187 | 0.315008 |
| map05219 | Bladder cancer | KEGG PATHWAY | 0.038953 | 0.318398 |
| map04013 | MAPK signaling pathway - fly | KEGG PATHWAY | 0.029068 | 0.321452 |
| map04210 | Apoptosis | KEGG PATHWAY | 0.041056 | 0.321605 |
| map04623 | Cytosolic DNA-sensing pathway | KEGG PATHWAY | 0.048541 | 0.325918 |
| map00071 | Fatty acid degradation | KEGG PATHWAY | 0.031396 | 0.327914 |
| map04140 | Autophagy - animal | KEGG PATHWAY | 0.035147 | 0.330379 |
| map04145 | Phagosome | KEGG PATHWAY | 0.034111 | 0.33752 |

iBAT_M4 (Whitened BAT Phase) High level gene

| Pathway id | Description | Database | Pvalue | Padjust |
| --- | --- | --- | --- | --- |
| map04141 | Protein processing in endoplasmic reticulum | KEGG PATHWAY | 2.08E-15 | 6.79E-13 |
| map04144 | Endocytosis | KEGG PATHWAY | 1.8E-12 | 2.94E-10 |
| map04142 | Lysosome | KEGG PATHWAY | 8.51E-11 | 9.27E-09 |
| map05132 | Salmonella infection | KEGG PATHWAY | 2.61E-10 | 2.13E-08 |
| map03040 | Spliceosome | KEGG PATHWAY | 6.76E-10 | 4.42E-08 |
| map04962 | Vasopressin-regulated water reabsorption | KEGG PATHWAY | 1.04E-09 | 5.69E-08 |
| map04510 | Focal adhesion | KEGG PATHWAY | 4.1E-08 | 1.91E-06 |
| map04120 | Ubiquitin mediated proteolysis | KEGG PATHWAY | 6.54E-08 | 2.67E-06 |
| map03015 | mRNA surveillance pathway | KEGG PATHWAY | 1.91E-06 | 6.95E-05 |
| map04114 | Oocyte meiosis | KEGG PATHWAY | 3.35E-06 | 0.00011 |
| map05205 | Proteoglycans in cancer | KEGG PATHWAY | 5.86E-06 | 0.000174 |
| map00510 | N-Glycan biosynthesis | KEGG PATHWAY | 1.01E-05 | 0.000276 |
| map04013 | MAPK signaling pathway - fly | KEGG PATHWAY | 1.62E-05 | 0.000407 |
| map05165 | Human papillomavirus infection | KEGG PATHWAY | 3.5E-05 | 0.000816 |
| map05110 | Vibrio cholerae infection | KEGG PATHWAY | 4.48E-05 | 0.000978 |
| map04512 | ECM-receptor interaction | KEGG PATHWAY | 4.86E-05 | 0.000994 |
| map03060 | Protein export | KEGG PATHWAY | 5.5E-05 | 0.001059 |
| map04152 | AMPK signaling pathway | KEGG PATHWAY | 7.78E-05 | 0.001413 |
| map00513 | Various types of N-glycan biosynthesis | KEGG PATHWAY | 8.86E-05 | 0.001525 |
| map04071 | Sphingolipid signaling pathway | KEGG PATHWAY | 0.00016 | 0.002486 |
| map05020 | Prion diseases | KEGG PATHWAY | 0.000157 | 0.002568 |
| map05211 | Renal cell carcinoma | KEGG PATHWAY | 0.000183 | 0.002716 |
| map05100 | Bacterial invasion of epithelial cells | KEGG PATHWAY | 0.000197 | 0.002807 |
| map05214 | Glioma | KEGG PATHWAY | 0.00036 | 0.004903 |
| map05203 | Viral carcinogenesis | KEGG PATHWAY | 0.000415 | 0.005425 |
| map00531 | Glycosaminoglycan degradation | KEGG PATHWAY | 0.000439 | 0.005521 |
| map04810 | Regulation of actin cytoskeleton | KEGG PATHWAY | 0.000656 | 0.007946 |
| map05120 | Epithelial cell signaling in Helicobacter pylori infection | KEGG PATHWAY | 0.000689 | 0.008048 |
| map04926 | Relaxin signaling pathway | KEGG PATHWAY | 0.001056 | 0.011909 |
| map05131 | Shigellosis | KEGG PATHWAY | 0.001363 | 0.014858 |
| map04979 | Cholesterol metabolism | KEGG PATHWAY | 0.001621 | 0.01656 |
| map04721 | Synaptic vesicle cycle | KEGG PATHWAY | 0.001673 | 0.016581 |
| map01522 | Endocrine resistance | KEGG PATHWAY | 0.001591 | 0.016786 |
| map05220 | Chronic myeloid leukemia | KEGG PATHWAY | 0.001835 | 0.017651 |
| map05163 | Human cytomegalovirus infection | KEGG PATHWAY | 0.00204 | 0.01803 |
| map05200 | Pathways in cancer | KEGG PATHWAY | 0.001997 | 0.018139 |
| map03013 | RNA transport | KEGG PATHWAY | 0.001953 | 0.018247 |
| map04933 | AGE-RAGE signaling pathway in diabetic complications | KEGG PATHWAY | 0.002169 | 0.018667 |
| map00604 | Glycosphingolipid biosynthesis - ganglio series | KEGG PATHWAY | 0.002325 | 0.019498 |
| map04728 | Dopaminergic synapse | KEGG PATHWAY | 0.002524 | 0.02063 |
| map05216 | Thyroid cancer | KEGG PATHWAY | 0.00326 | 0.026001 |
| map05215 | Prostate cancer | KEGG PATHWAY | 0.003371 | 0.026242 |
| map05225 | Hepatocellular carcinoma | KEGG PATHWAY | 0.003773 | 0.028695 |
| map04910 | Insulin signaling pathway | KEGG PATHWAY | 0.004007 | 0.029781 |
| map04130 | SNARE interactions in vesicular transport | KEGG PATHWAY | 0.004793 | 0.034828 |
| map04611 | Platelet activation | KEGG PATHWAY | 0.005155 | 0.036643 |
| map05160 | Hepatitis C | KEGG PATHWAY | 0.006168 | 0.042915 |

**Table S6. Results of KEGG enrichment analysis for gene set including profile 15, 16, 25, 8, 24, 22 and 13.**

| Pathway id | Description | Database | Pvalue | Padjust |
| --- | --- | --- | --- | --- |
| map04142 | Lysosome | KEGG PATHWAY | 2.13E-12 | 3.66E-10 |
| map05168 | Herpes simplex virus 1 infection | KEGG PATHWAY | 1.32E-12 | 4.54E-10 |
| map04110 | Cell cycle | KEGG PATHWAY | 4.2E-11 | 3.61E-09 |
| map04360 | Axon guidance | KEGG PATHWAY | 3.31E-11 | 3.8E-09 |
| map04070 | Phosphatidylinositol signaling system | KEGG PATHWAY | 1.77E-10 | 1.22E-08 |
| map04144 | Endocytosis | KEGG PATHWAY | 2.57E-10 | 1.47E-08 |
| map05165 | Human papillomavirus infection | KEGG PATHWAY | 1.01E-09 | 4.96E-08 |
| map05200 | Pathways in cancer | KEGG PATHWAY | 2.5E-09 | 1.08E-07 |
| map04010 | MAPK signaling pathway | KEGG PATHWAY | 6.25E-09 | 2.39E-07 |
| map05215 | Prostate cancer | KEGG PATHWAY | 1.5E-08 | 5.16E-07 |
| map00562 | Inositol phosphate metabolism | KEGG PATHWAY | 9.9E-08 | 3.1E-06 |
| map05223 | Non-small cell lung cancer | KEGG PATHWAY | 4.31E-07 | 1.23E-05 |
| map05220 | Chronic myeloid leukemia | KEGG PATHWAY | 9.43E-07 | 2.49E-05 |
| map03460 | Fanconi anemia pathway | KEGG PATHWAY | 1.54E-06 | 3.53E-05 |
| map04062 | Chemokine signaling pathway | KEGG PATHWAY | 1.48E-06 | 3.63E-05 |
| map05206 | MicroRNAs in cancer | KEGG PATHWAY | 1.71E-06 | 3.67E-05 |
| map00510 | N-Glycan biosynthesis | KEGG PATHWAY | 2.7E-06 | 5.47E-05 |
| map04611 | Platelet activation | KEGG PATHWAY | 4.15E-06 | 6.2E-05 |
| map05163 | Human cytomegalovirus infection | KEGG PATHWAY | 3.26E-06 | 6.23E-05 |
| map05214 | Glioma | KEGG PATHWAY | 3.46E-06 | 6.26E-05 |
| map00514 | Other types of O-glycan biosynthesis | KEGG PATHWAY | 4.07E-06 | 6.37E-05 |
| map04933 | AGE-RAGE signaling pathway in diabetic complications | KEGG PATHWAY | 3.97E-06 | 6.5E-05 |
| map04391 | Hippo signaling pathway - fly | KEGG PATHWAY | 3.93E-06 | 6.76E-05 |
| map04218 | Cellular senescence | KEGG PATHWAY | 8.73E-06 | 0.000125 |
| map05205 | Proteoglycans in cancer | KEGG PATHWAY | 9.17E-06 | 0.000126 |
| map01522 | Endocrine resistance | KEGG PATHWAY | 9.8E-06 | 0.00013 |
| map04390 | Hippo signaling pathway | KEGG PATHWAY | 1.02E-05 | 0.000131 |
| map04071 | Sphingolipid signaling pathway | KEGG PATHWAY | 1.1E-05 | 0.000136 |
| map04935 | Growth hormone synthesis, secretion and action | KEGG PATHWAY | 1.35E-05 | 0.000155 |
| map00513 | Various types of N-glycan biosynthesis | KEGG PATHWAY | 1.35E-05 | 0.00016 |
| map04728 | Dopaminergic synapse | KEGG PATHWAY | 1.69E-05 | 0.000187 |
| map05222 | Small cell lung cancer | KEGG PATHWAY | 2.66E-05 | 0.000286 |
| map04392 | Hippo signaling pathway - multiple species | KEGG PATHWAY | 3.74E-05 | 0.00039 |
| map04926 | Relaxin signaling pathway | KEGG PATHWAY | 3.89E-05 | 0.000394 |
| map04013 | MAPK signaling pathway - fly | KEGG PATHWAY | 4.9E-05 | 0.000481 |
| map04668 | TNF signaling pathway | KEGG PATHWAY | 4.9E-05 | 0.000481 |
| map04380 | Osteoclast differentiation | KEGG PATHWAY | 6.11E-05 | 0.000568 |
| map05225 | Hepatocellular carcinoma | KEGG PATHWAY | 6.48E-05 | 0.000587 |
| map00511 | Other glycan degradation | KEGG PATHWAY | 6.86E-05 | 0.000605 |
| map05161 | Hepatitis B | KEGG PATHWAY | 7.99E-05 | 0.000687 |
| map04512 | ECM-receptor interaction | KEGG PATHWAY | 8.25E-05 | 0.000692 |
| map00310 | Lysine degradation | KEGG PATHWAY | 8.8E-05 | 0.000721 |
| map04722 | Neurotrophin signaling pathway | KEGG PATHWAY | 9.06E-05 | 0.000724 |
| map04931 | Insulin resistance | KEGG PATHWAY | 0.000132 | 0.001035 |
| map04152 | AMPK signaling pathway | KEGG PATHWAY | 0.000141 | 0.00108 |
| map04919 | Thyroid hormone signaling pathway | KEGG PATHWAY | 0.000153 | 0.001141 |
| map04724 | Glutamatergic synapse | KEGG PATHWAY | 0.000162 | 0.001182 |
| map04916 | Melanogenesis | KEGG PATHWAY | 0.000215 | 0.001544 |
| map03030 | DNA replication | KEGG PATHWAY | 0.000248 | 0.001743 |
| map05412 | Arrhythmogenic right ventricular cardiomyopathy (ARVC) | KEGG PATHWAY | 0.000274 | 0.001886 |
| map05418 | Fluid shear stress and atherosclerosis | KEGG PATHWAY | 0.000294 | 0.001945 |
| map04350 | TGF-beta signaling pathway | KEGG PATHWAY | 0.0003 | 0.001946 |
| map04962 | Vasopressin-regulated water reabsorption | KEGG PATHWAY | 0.000288 | 0.001946 |
| map00564 | Glycerophospholipid metabolism | KEGG PATHWAY | 0.00032 | 0.002041 |
| map05160 | Hepatitis C | KEGG PATHWAY | 0.000444 | 0.002776 |
| map00531 | Glycosaminoglycan degradation | KEGG PATHWAY | 0.00051 | 0.003023 |
| map04961 | Endocrine and other factor-regulated calcium reabsorption | KEGG PATHWAY | 0.000503 | 0.003035 |
| map04114 | Oocyte meiosis | KEGG PATHWAY | 0.000499 | 0.003066 |
| map05133 | Pertussis | KEGG PATHWAY | 0.000624 | 0.003638 |
| map04068 | FoxO signaling pathway | KEGG PATHWAY | 0.000713 | 0.004089 |
| map04141 | Protein processing in endoplasmic reticulum | KEGG PATHWAY | 0.000768 | 0.004333 |
| map04120 | Ubiquitin mediated proteolysis | KEGG PATHWAY | 0.000881 | 0.004887 |
| map04914 | Progesterone-mediated oocyte maturation | KEGG PATHWAY | 0.00091 | 0.004971 |
| map00534 | Glycosaminoglycan biosynthesis - heparan sulfate / heparin | KEGG PATHWAY | 0.001142 | 0.006137 |
| map04912 | GnRH signaling pathway | KEGG PATHWAY | 0.00116 | 0.006138 |
| map05218 | Melanoma | KEGG PATHWAY | 0.001362 | 0.0071 |
| map04713 | Circadian entrainment | KEGG PATHWAY | 0.001424 | 0.007309 |
| map04330 | Notch signaling pathway | KEGG PATHWAY | 0.001714 | 0.00867 |
| map05224 | Breast cancer | KEGG PATHWAY | 0.001865 | 0.009296 |
| map05167 | Kaposi sarcoma-associated herpesvirus infection | KEGG PATHWAY | 0.002143 | 0.010381 |
| map04725 | Cholinergic synapse | KEGG PATHWAY | 0.002142 | 0.010526 |
| map05221 | Acute myeloid leukemia | KEGG PATHWAY | 0.002244 | 0.010719 |
| map04730 | Long-term depression | KEGG PATHWAY | 0.002316 | 0.010913 |
| map04929 | GnRH secretion | KEGG PATHWAY | 0.002605 | 0.012111 |
| map03440 | Homologous recombination | KEGG PATHWAY | 0.002937 | 0.013473 |
| map00532 | Glycosaminoglycan biosynthesis - chondroitin sulfate / dermatan sulfate | KEGG PATHWAY | 0.0034 | 0.015389 |
| map04361 | Axon regeneration | KEGG PATHWAY | 0.003629 | 0.016212 |
| map04370 | VEGF signaling pathway | KEGG PATHWAY | 0.00371 | 0.016363 |
| map05226 | Gastric cancer | KEGG PATHWAY | 0.003787 | 0.016488 |
| map04925 | Aldosterone synthesis and secretion | KEGG PATHWAY | 0.004255 | 0.018297 |
| map00520 | Amino sugar and nucleotide sugar metabolism | KEGG PATHWAY | 0.004416 | 0.018753 |
| map04920 | Adipocytokine signaling pathway | KEGG PATHWAY | 0.006068 | 0.025457 |
| map04012 | ErbB signaling pathway | KEGG PATHWAY | 0.006166 | 0.025556 |
| map00515 | Mannose type O-glycan biosynthesis | KEGG PATHWAY | 0.007191 | 0.029449 |
| map04270 | Vascular smooth muscle contraction | KEGG PATHWAY | 0.007375 | 0.029847 |
| map04310 | Wnt signaling pathway | KEGG PATHWAY | 0.007549 | 0.030194 |
| map04721 | Synaptic vesicle cycle | KEGG PATHWAY | 0.007772 | 0.030731 |
| map05020 | Prion diseases | KEGG PATHWAY | 0.008001 | 0.031275 |
| map04934 | Cushing syndrome | KEGG PATHWAY | 0.009285 | 0.035886 |
| map05032 | Morphine addiction | KEGG PATHWAY | 0.010047 | 0.03798 |
| map05216 | Thyroid cancer | KEGG PATHWAY | 0.009986 | 0.038167 |
| map05203 | Viral carcinogenesis | KEGG PATHWAY | 0.012223 | 0.045702 |
| map04921 | Oxytocin signaling pathway | KEGG PATHWAY | 0.012885 | 0.046658 |
| map04320 | Dorso-ventral axis formation | KEGG PATHWAY | 0.012778 | 0.046761 |
| map05213 | Endometrial cancer | KEGG PATHWAY | 0.012718 | 0.047042 |
| map04530 | Tight junction | KEGG PATHWAY | 0.013604 | 0.048748 |

**Table S7. Results of GO enrichment analysis for gene set including profile 15, 16, 25, 8, 24, 22 and 13.**

| go_id | go_type | discription | p_uncorrected | p_corrected |
| --- | --- | --- | --- | --- |
| GO:0072583 | BP | clathrin-dependent endocytosis | 3.10E-07 | 0.000304 |
| GO:0034446 | BP | substrate adhesion-dependent cell spreading | 3.70E-07 | 0.000304 |
| GO:0061572 | BP | actin filament bundle organization | 8.95E-07 | 0.000304 |
| GO:0031098 | BP | stress-activated protein kinase signaling cascade | 1.17E-06 | 0.000304 |
| GO:0001568 | BP | blood vessel development | 1.19E-06 | 0.000304 |
| GO:0032715 | BP | negative regulation of interleukin-6 production | 1.33E-06 | 0.000304 |
| GO:0006024 | BP | glycosaminoglycan biosynthetic process | 1.39E-06 | 0.000304 |
| GO:0030203 | BP | glycosaminoglycan metabolic process | 1.40E-06 | 0.000304 |
| GO:0022406 | BP | membrane docking | 1.40E-06 | 0.000304 |
| GO:0006888 | BP | endoplasmic reticulum to Golgi vesicle-mediated transport | 1.47E-06 | 0.000304 |
| GO:0031589 | BP | cell-substrate adhesion | 1.47E-06 | 0.000304 |
| GO:0048812 | BP | neuron projection morphogenesis | 1.68E-06 | 0.000304 |
| GO:0010212 | BP | response to ionizing radiation | 1.68E-06 | 0.000304 |
| GO:1902115 | BP | regulation of organelle assembly | 1.79E-06 | 0.000304 |
| GO:0006302 | BP | double-strand break repair | 1.79E-06 | 0.000304 |
| GO:0120039 | BP | plasma membrane bounded cell projection morphogenesis | 1.80E-06 | 0.000304 |
| GO:0000725 | BP | recombinational repair | 1.81E-06 | 0.000304 |
| GO:0072659 | BP | protein localization to plasma membrane | 1.86E-06 | 0.000304 |
| GO:0043062 | BP | extracellular structure organization | 1.89E-06 | 0.000304 |
| GO:0030198 | BP | extracellular matrix organization | 1.89E-06 | 0.000304 |
| GO:0051983 | BP | regulation of chromosome segregation | 1.89E-06 | 0.000304 |
| GO:0006023 | BP | aminoglycan biosynthetic process | 1.90E-06 | 0.000304 |
| GO:0007160 | BP | cell-matrix adhesion | 1.90E-06 | 0.000304 |
| GO:1903510 | BP | mucopolysaccharide metabolic process | 1.93E-06 | 0.000304 |
| GO:0140056 | BP | organelle localization by membrane tethering | 1.95E-06 | 0.000304 |
| GO:0007088 | BP | regulation of mitotic nuclear division | 2.02E-06 | 0.000304 |
| GO:0031570 | BP | DNA integrity checkpoint | 2.07E-06 | 0.000304 |
| GO:0032874 | BP | positive regulation of stress-activated MAPK cascade | 2.08E-06 | 0.000304 |
| GO:0007051 | BP | spindle organization | 2.11E-06 | 0.000304 |
| GO:0006898 | BP | receptor-mediated endocytosis | 2.11E-06 | 0.000304 |
| GO:0009100 | BP | glycoprotein metabolic process | 2.12E-06 | 0.000304 |
| GO:0043122 | BP | regulation of I-kappaB kinase/NF-kappaB signaling | 2.15E-06 | 0.000304 |
| GO:0050709 | BP | negative regulation of protein secretion | 2.18E-06 | 0.000304 |
| GO:0001525 | BP | angiogenesis | 2.19E-06 | 0.000304 |
| GO:0006022 | BP | aminoglycan metabolic process | 2.20E-06 | 0.000304 |
| GO:0006260 | BP | DNA replication | 2.22E-06 | 0.000304 |
| GO:0018209 | BP | peptidyl-serine modification | 2.28E-06 | 0.000304 |
| GO:0045931 | BP | positive regulation of mitotic cell cycle | 2.29E-06 | 0.000304 |
| GO:0007265 | BP | Ras protein signal transduction | 2.29E-06 | 0.000304 |
| GO:0043406 | BP | positive regulation of MAP kinase activity | 2.30E-06 | 0.000304 |
| GO:0048858 | BP | cell projection morphogenesis | 2.31E-06 | 0.000304 |
| GO:0016050 | BP | vesicle organization | 2.33E-06 | 0.000304 |
| GO:0018105 | BP | peptidyl-serine phosphorylation | 2.42E-06 | 0.000304 |
| GO:0007034 | BP | vacuolar transport | 2.50E-06 | 0.000304 |
| GO:0006897 | BP | endocytosis | 2.50E-06 | 0.000304 |
| GO:0030099 | BP | myeloid cell differentiation | 2.54E-06 | 0.000304 |
| GO:0016197 | BP | endosomal transport | 2.55E-06 | 0.000304 |
| GO:0034330 | BP | cell junction organization | 2.56E-06 | 0.000304 |
| GO:0046330 | BP | positive regulation of JNK cascade | 2.57E-06 | 0.000304 |
| GO:1904950 | BP | negative regulation of establishment of protein localization | 2.60E-06 | 0.000304 |
| GO:0032989 | BP | cellular component morphogenesis | 2.60E-06 | 0.000304 |
| GO:0032990 | BP | cell part morphogenesis | 2.60E-06 | 0.000304 |
| GO:0051271 | BP | negative regulation of cellular component movement | 2.62E-06 | 0.000304 |
| GO:0040013 | BP | negative regulation of locomotion | 2.62E-06 | 0.000304 |
| GO:0051056 | BP | regulation of small GTPase mediated signal transduction | 2.62E-06 | 0.000304 |
| GO:0051017 | BP | actin filament bundle assembly | 2.63E-06 | 0.000304 |
| GO:0051091 | BP | positive regulation of DNA-binding transcription factor activity | 2.63E-06 | 0.000304 |
| GO:0032886 | BP | regulation of microtubule-based process | 2.64E-06 | 0.000304 |
| GO:0050710 | BP | negative regulation of cytokine secretion | 2.66E-06 | 0.000304 |
| GO:0043405 | BP | regulation of MAP kinase activity | 2.66E-06 | 0.000304 |
| GO:0046486 | BP | glycerolipid metabolic process | 2.71E-06 | 0.000304 |
| GO:0000724 | BP | double-strand break repair via homologous recombination | 2.75E-06 | 0.000304 |
| GO:0070085 | BP | glycosylation | 2.75E-06 | 0.000304 |
| GO:0001818 | BP | negative regulation of cytokine production | 2.81E-06 | 0.000304 |
| GO:0070304 | BP | positive regulation of stress-activated protein kinase signaling cascade | 2.84E-06 | 0.000304 |
| GO:0032147 | BP | activation of protein kinase activity | 2.85E-06 | 0.000304 |
| GO:0051783 | BP | regulation of nuclear division | 2.87E-06 | 0.000304 |
| GO:0070507 | BP | regulation of microtubule cytoskeleton organization | 2.87E-06 | 0.000304 |
| GO:0071902 | BP | positive regulation of protein serine/threonine kinase activity | 2.90E-06 | 0.000304 |
| GO:0032872 | BP | regulation of stress-activated MAPK cascade | 2.91E-06 | 0.000304 |
| GO:0010769 | BP | regulation of cell morphogenesis involved in differentiation | 2.92E-06 | 0.000304 |
| GO:0010810 | BP | regulation of cell-substrate adhesion | 2.94E-06 | 0.000304 |
| GO:0032102 | BP | negative regulation of response to external stimulus | 2.94E-06 | 0.000304 |
| GO:2000146 | BP | negative regulation of cell motility | 3.00E-06 | 0.000304 |
| GO:2001020 | BP | regulation of response to DNA damage stimulus | 3.03E-06 | 0.000304 |
| GO:0031345 | BP | negative regulation of cell projection organization | 3.03E-06 | 0.000304 |
| GO:0050727 | BP | regulation of inflammatory response | 3.05E-06 | 0.000304 |
| GO:0007169 | BP | transmembrane receptor protein tyrosine kinase signaling pathway | 3.14E-06 | 0.000304 |
| GO:0006644 | BP | phospholipid metabolic process | 3.14E-06 | 0.000304 |
| GO:0035239 | BP | tube morphogenesis | 3.15E-06 | 0.000304 |
| GO:0090068 | BP | positive regulation of cell cycle process | 3.15E-06 | 0.000304 |
| GO:0120031 | BP | plasma membrane bounded cell projection assembly | 3.19E-06 | 0.000304 |
| GO:0046578 | BP | regulation of Ras protein signal transduction | 3.19E-06 | 0.000304 |
| GO:1901992 | BP | positive regulation of mitotic cell cycle phase transition | 3.19E-06 | 0.000304 |
| GO:1901987 | BP | regulation of cell cycle phase transition | 3.27E-06 | 0.000304 |
| GO:0045787 | BP | positive regulation of cell cycle | 3.27E-06 | 0.000304 |
| GO:0009314 | BP | response to radiation | 3.27E-06 | 0.000304 |
| GO:0051224 | BP | negative regulation of protein transport | 3.27E-06 | 0.000304 |
| GO:0032956 | BP | regulation of actin cytoskeleton organization | 3.28E-06 | 0.000304 |
| GO:1902903 | BP | regulation of supramolecular fiber organization | 3.28E-06 | 0.000304 |
| GO:0010975 | BP | regulation of neuron projection development | 3.32E-06 | 0.000304 |
| GO:0007015 | BP | actin filament organization | 3.33E-06 | 0.000304 |
| GO:0016569 | BP | covalent chromatin modification | 3.39E-06 | 0.000304 |
| GO:0000904 | BP | cell morphogenesis involved in differentiation | 3.41E-06 | 0.000304 |
| GO:0048193 | BP | Golgi vesicle transport | 3.46E-06 | 0.000304 |
| GO:1990778 | BP | protein localization to cell periphery | 3.48E-06 | 0.000304 |
| GO:0030335 | BP | positive regulation of cell migration | 3.48E-06 | 0.000304 |
| GO:0034329 | BP | cell junction assembly | 3.54E-06 | 0.000304 |
| GO:0009887 | BP | animal organ morphogenesis | 3.57E-06 | 0.000304 |
| GO:0070302 | BP | regulation of stress-activated protein kinase signaling cascade | 3.59E-06 | 0.000304 |
| GO:0098609 | BP | cell-cell adhesion | 3.61E-06 | 0.000304 |
| GO:0097435 | BP | supramolecular fiber organization | 3.62E-06 | 0.000304 |
| GO:0043507 | BP | positive regulation of JUN kinase activity | 3.63E-06 | 0.000304 |
| GO:0042326 | BP | negative regulation of phosphorylation | 3.63E-06 | 0.000304 |
| GO:0016570 | BP | histone modification | 3.63E-06 | 0.000304 |
| GO:2000147 | BP | positive regulation of cell motility | 3.64E-06 | 0.000304 |
| GO:0033674 | BP | positive regulation of kinase activity | 3.66E-06 | 0.000304 |
| GO:1903827 | BP | regulation of cellular protein localization | 3.73E-06 | 0.000304 |
| GO:2001252 | BP | positive regulation of chromosome organization | 3.74E-06 | 0.000304 |
| GO:0001933 | BP | negative regulation of protein phosphorylation | 3.76E-06 | 0.000304 |
| GO:0030031 | BP | cell projection assembly | 3.78E-06 | 0.000304 |
| GO:0051272 | BP | positive regulation of cellular component movement | 3.80E-06 | 0.000304 |
| GO:0060627 | BP | regulation of vesicle-mediated transport | 3.80E-06 | 0.000304 |
| GO:0043413 | BP | macromolecule glycosylation | 3.83E-06 | 0.000304 |
| GO:0006486 | BP | protein glycosylation | 3.83E-06 | 0.000304 |
| GO:0030036 | BP | actin cytoskeleton organization | 3.83E-06 | 0.000304 |
| GO:0051640 | BP | organelle localization | 3.87E-06 | 0.000304 |
| GO:0045860 | BP | positive regulation of protein kinase activity | 3.92E-06 | 0.000304 |
| GO:0007264 | BP | small GTPase mediated signal transduction | 3.93E-06 | 0.000304 |
| GO:0006887 | BP | exocytosis | 3.93E-06 | 0.000304 |
| GO:0045785 | BP | positive regulation of cell adhesion | 3.93E-06 | 0.000304 |
| GO:0051090 | BP | regulation of DNA-binding transcription factor activity | 3.94E-06 | 0.000304 |
| GO:0040017 | BP | positive regulation of locomotion | 3.98E-06 | 0.000304 |
| GO:0045786 | BP | negative regulation of cell cycle | 3.98E-06 | 0.000304 |
| GO:0000122 | BP | negative regulation of transcription by RNA polymerase II | 3.99E-06 | 0.000304 |
| GO:0010638 | BP | positive regulation of organelle organization | 4.00E-06 | 0.000304 |
| GO:0031346 | BP | positive regulation of cell projection organization | 4.05E-06 | 0.000304 |
| GO:0051347 | BP | positive regulation of transferase activity | 4.07E-06 | 0.000304 |
| GO:0044089 | BP | positive regulation of cellular component biogenesis | 4.07E-06 | 0.000304 |
| GO:1901990 | BP | regulation of mitotic cell cycle phase transition | 4.11E-06 | 0.000304 |
| GO:0007167 | BP | enzyme linked receptor protein signaling pathway | 4.15E-06 | 0.000304 |
| GO:0006650 | BP | glycerophospholipid metabolic process | 4.15E-06 | 0.000304 |
| GO:1902532 | BP | negative regulation of intracellular signal transduction | 4.15E-06 | 0.000304 |
| GO:0045321 | BP | leukocyte activation | 4.17E-06 | 0.000304 |
| GO:0008285 | BP | negative regulation of cell population proliferation | 4.18E-06 | 0.000304 |
| GO:0022604 | BP | regulation of cell morphogenesis | 4.19E-06 | 0.000304 |
| GO:2001022 | BP | positive regulation of response to DNA damage stimulus | 4.23E-06 | 0.000304 |
| GO:0048534 | BP | hematopoietic or lymphoid organ development | 4.29E-06 | 0.000304 |
| GO:0006906 | BP | vesicle fusion | 4.35E-06 | 0.000304 |
| GO:0048646 | BP | anatomical structure formation involved in morphogenesis | 4.40E-06 | 0.000304 |
| GO:0120036 | BP | plasma membrane bounded cell projection organization | 4.43E-06 | 0.000304 |
| GO:0032970 | BP | regulation of actin filament-based process | 4.46E-06 | 0.000304 |
| GO:0030029 | BP | actin filament-based process | 4.46E-06 | 0.000304 |
| GO:0071900 | BP | regulation of protein serine/threonine kinase activity | 4.47E-06 | 0.000304 |
| GO:0016477 | BP | cell migration | 4.47E-06 | 0.000304 |
| GO:0061136 | BP | regulation of proteasomal protein catabolic process | 4.49E-06 | 0.000304 |
| GO:0002683 | BP | negative regulation of immune system process | 4.52E-06 | 0.000304 |
| GO:0033045 | BP | regulation of sister chromatid segregation | 4.52E-06 | 0.000304 |
| GO:0006468 | BP | protein phosphorylation | 4.55E-06 | 0.000304 |
| GO:0051129 | BP | negative regulation of cellular component organization | 4.56E-06 | 0.000304 |
| GO:0043408 | BP | regulation of MAPK cascade | 4.57E-06 | 0.000304 |
| GO:0120035 | BP | regulation of plasma membrane bounded cell projection organization | 4.57E-06 | 0.000304 |
| GO:0030100 | BP | regulation of endocytosis | 4.57E-06 | 0.000304 |
| GO:0045936 | BP | negative regulation of phosphate metabolic process | 4.59E-06 | 0.000304 |
| GO:0010563 | BP | negative regulation of phosphorus metabolic process | 4.59E-06 | 0.000304 |
| GO:0032940 | BP | secretion by cell | 4.59E-06 | 0.000304 |
| GO:0000226 | BP | microtubule cytoskeleton organization | 4.59E-06 | 0.000304 |
| GO:0140352 | BP | export from cell | 4.61E-06 | 0.000304 |
| GO:0032446 | BP | protein modification by small protein conjugation | 4.61E-06 | 0.000304 |
| GO:0031400 | BP | negative regulation of protein modification process | 4.64E-06 | 0.000304 |
| GO:0046903 | BP | secretion | 4.68E-06 | 0.000304 |
| GO:0001775 | BP | cell activation | 4.69E-06 | 0.000304 |
| GO:0051493 | BP | regulation of cytoskeleton organization | 4.71E-06 | 0.000304 |
| GO:0048870 | BP | cell motility | 4.72E-06 | 0.000304 |
| GO:0007017 | BP | microtubule-based process | 4.72E-06 | 0.000304 |
| GO:0048731 | BP | system development | 4.73E-06 | 0.000304 |
| GO:0043410 | BP | positive regulation of MAPK cascade | 4.74E-06 | 0.000304 |
| GO:0001934 | BP | positive regulation of protein phosphorylation | 4.74E-06 | 0.000304 |
| GO:0030030 | BP | cell projection organization | 4.75E-06 | 0.000304 |
| GO:0022610 | BP | biological adhesion | 4.75E-06 | 0.000304 |
| GO:0043549 | BP | regulation of kinase activity | 4.77E-06 | 0.000304 |
| GO:0045859 | BP | regulation of protein kinase activity | 4.77E-06 | 0.000304 |
| GO:0050767 | BP | regulation of neurogenesis | 4.82E-06 | 0.000304 |
| GO:0030336 | BP | negative regulation of cell migration | 4.82E-06 | 0.000304 |
| GO:0000281 | BP | mitotic cytokinesis | 4.82E-06 | 0.000304 |
| GO:0016567 | BP | protein ubiquitination | 4.90E-06 | 0.000304 |
| GO:0080135 | BP | regulation of cellular response to stress | 4.92E-06 | 0.000304 |
| GO:0009628 | BP | response to abiotic stimulus | 4.95E-06 | 0.000304 |
| GO:0007155 | BP | cell adhesion | 5.01E-06 | 0.000304 |
| GO:0018193 | BP | peptidyl-amino acid modification | 5.01E-06 | 0.000304 |
| GO:1903531 | BP | negative regulation of secretion by cell | 5.06E-06 | 0.000304 |
| GO:0051093 | BP | negative regulation of developmental process | 5.08E-06 | 0.000304 |
| GO:0006325 | BP | chromatin organization | 5.15E-06 | 0.000304 |
| GO:0031347 | BP | regulation of defense response | 5.17E-06 | 0.000304 |
| GO:0010564 | BP | regulation of cell cycle process | 5.19E-06 | 0.000304 |
| GO:0080171 | BP | lytic vacuole organization | 5.20E-06 | 0.000304 |
| GO:0007040 | BP | lysosome organization | 5.20E-06 | 0.000304 |
| GO:0007346 | BP | regulation of mitotic cell cycle | 5.22E-06 | 0.000304 |
| GO:0007010 | BP | cytoskeleton organization | 5.23E-06 | 0.000304 |
| GO:0051338 | BP | regulation of transferase activity | 5.28E-06 | 0.000304 |
| GO:0030155 | BP | regulation of cell adhesion | 5.30E-06 | 0.000304 |
| GO:0031344 | BP | regulation of cell projection organization | 5.32E-06 | 0.000304 |
| GO:0040012 | BP | regulation of locomotion | 5.33E-06 | 0.000304 |
| GO:0060284 | BP | regulation of cell development | 5.33E-06 | 0.000304 |
| GO:0033044 | BP | regulation of chromosome organization | 5.35E-06 | 0.000304 |
| GO:1902679 | BP | negative regulation of RNA biosynthetic process | 5.38E-06 | 0.000304 |
| GO:1903507 | BP | negative regulation of nucleic acid-templated transcription | 5.38E-06 | 0.000304 |
| GO:0040011 | BP | locomotion | 5.39E-06 | 0.000304 |
| GO:0051960 | BP | regulation of nervous system development | 5.45E-06 | 0.000305 |
| GO:1903047 | BP | mitotic cell cycle process | 5.48E-06 | 0.000305 |
| GO:0061024 | BP | membrane organization | 5.50E-06 | 0.000305 |
| GO:0070647 | BP | protein modification by small protein conjugation or removal | 5.50E-06 | 0.000305 |
| GO:0016311 | BP | dephosphorylation | 5.59E-06 | 0.000305 |
| GO:0009896 | BP | positive regulation of catabolic process | 5.68E-06 | 0.000305 |
| GO:2000145 | BP | regulation of cell motility | 5.76E-06 | 0.000305 |
| GO:0042327 | BP | positive regulation of phosphorylation | 5.77E-06 | 0.000305 |
| GO:0046839 | BP | phospholipid dephosphorylation | 5.78E-06 | 0.000305 |
| GO:0051130 | BP | positive regulation of cellular component organization | 5.79E-06 | 0.000305 |
| GO:0009888 | BP | tissue development | 5.89E-06 | 0.000305 |
| GO:0032101 | BP | regulation of response to external stimulus | 5.91E-06 | 0.000305 |
| GO:0006974 | BP | cellular response to DNA damage stimulus | 5.94E-06 | 0.000305 |
| GO:0022603 | BP | regulation of anatomical structure morphogenesis | 5.96E-06 | 0.000305 |
| GO:0060341 | BP | regulation of cellular localization | 5.97E-06 | 0.000305 |
| GO:0034613 | BP | cellular protein localization | 5.99E-06 | 0.000305 |
| GO:0045597 | BP | positive regulation of cell differentiation | 6.00E-06 | 0.000305 |
| GO:0044087 | BP | regulation of cellular component biogenesis | 6.00E-06 | 0.000305 |
| GO:1902533 | BP | positive regulation of intracellular signal transduction | 6.00E-06 | 0.000305 |
| GO:0030334 | BP | regulation of cell migration | 6.01E-06 | 0.000305 |
| GO:0022402 | BP | cell cycle process | 6.06E-06 | 0.000305 |
| GO:0051092 | BP | positive regulation of NF-kappaB transcription factor activity | 6.07E-06 | 0.000305 |
| GO:0050777 | BP | negative regulation of immune response | 6.07E-06 | 0.000305 |
| GO:0043085 | BP | positive regulation of catalytic activity | 6.07E-06 | 0.000305 |
| GO:0045944 | BP | positive regulation of transcription by RNA polymerase II | 6.09E-06 | 0.000305 |
| GO:0010558 | BP | negative regulation of macromolecule biosynthetic process | 6.10E-06 | 0.000305 |
| GO:0002521 | BP | leukocyte differentiation | 6.10E-06 | 0.000305 |
| GO:0023057 | BP | negative regulation of signaling | 6.13E-06 | 0.000305 |
| GO:0051726 | BP | regulation of cell cycle | 6.17E-06 | 0.000305 |
| GO:0001932 | BP | regulation of protein phosphorylation | 6.18E-06 | 0.000305 |
| GO:0009968 | BP | negative regulation of signal transduction | 6.21E-06 | 0.000305 |
| GO:0031401 | BP | positive regulation of protein modification process | 6.23E-06 | 0.000305 |
| GO:0046328 | BP | regulation of JNK cascade | 6.24E-06 | 0.000305 |
| GO:0070925 | BP | organelle assembly | 6.27E-06 | 0.000305 |
| GO:0045937 | BP | positive regulation of phosphate metabolic process | 6.34E-06 | 0.000305 |
| GO:0010562 | BP | positive regulation of phosphorus metabolic process | 6.34E-06 | 0.000305 |
| GO:0044093 | BP | positive regulation of molecular function | 6.38E-06 | 0.000305 |
| GO:0032880 | BP | regulation of protein localization | 6.38E-06 | 0.000305 |
| GO:0009790 | BP | embryo development | 6.39E-06 | 0.000305 |
| GO:0009792 | BP | embryo development ending in birth or egg hatching | 6.39E-06 | 0.000305 |
| GO:0051270 | BP | regulation of cellular component movement | 6.39E-06 | 0.000305 |
| GO:0016192 | BP | vesicle-mediated transport | 6.40E-06 | 0.000305 |
| GO:0070727 | BP | cellular macromolecule localization | 6.42E-06 | 0.000305 |
| GO:0045892 | BP | negative regulation of transcription, DNA-templated | 6.55E-06 | 0.000305 |
| GO:0071214 | BP | cellular response to abiotic stimulus | 6.55E-06 | 0.000305 |
| GO:0104004 | BP | cellular response to environmental stimulus | 6.55E-06 | 0.000305 |
| GO:0009653 | BP | anatomical structure morphogenesis | 6.58E-06 | 0.000305 |
| GO:0098751 | BP | bone cell development | 6.68E-06 | 0.000306 |
| GO:0042127 | BP | regulation of cell population proliferation | 6.72E-06 | 0.000306 |
| GO:0006928 | BP | movement of cell or subcellular component | 6.73E-06 | 0.000306 |
| GO:0070201 | BP | regulation of establishment of protein localization | 6.80E-06 | 0.000307 |
| GO:0051253 | BP | negative regulation of RNA metabolic process | 6.82E-06 | 0.000307 |
| GO:0010720 | BP | positive regulation of cell development | 6.86E-06 | 0.000307 |
| GO:0010648 | BP | negative regulation of cell communication | 6.86E-06 | 0.000307 |
| GO:0045664 | BP | regulation of neuron differentiation | 6.88E-06 | 0.000307 |
| GO:0051241 | BP | negative regulation of multicellular organismal process | 6.91E-06 | 0.000307 |
| GO:0050728 | BP | negative regulation of inflammatory response | 6.91E-06 | 0.000307 |
| GO:0045934 | BP | negative regulation of nucleobase-containing compound metabolic process | 6.92E-06 | 0.000307 |
| GO:0045595 | BP | regulation of cell differentiation | 6.95E-06 | 0.000307 |
| GO:0110053 | BP | regulation of actin filament organization | 6.99E-06 | 0.000307 |
| GO:0009967 | BP | positive regulation of signal transduction | 7.03E-06 | 0.000307 |
| GO:0051247 | BP | positive regulation of protein metabolic process | 7.11E-06 | 0.000307 |
| GO:0033554 | BP | cellular response to stress | 7.15E-06 | 0.000307 |
| GO:0033043 | BP | regulation of organelle organization | 7.18E-06 | 0.000307 |
| GO:0045893 | BP | positive regulation of transcription, DNA-templated | 7.18E-06 | 0.000307 |
| GO:0032270 | BP | positive regulation of cellular protein metabolic process | 7.18E-06 | 0.000307 |
| GO:2000113 | BP | negative regulation of cellular macromolecule biosynthetic process | 7.20E-06 | 0.000307 |
| GO:0051223 | BP | regulation of protein transport | 7.21E-06 | 0.000307 |
| GO:0009890 | BP | negative regulation of biosynthetic process | 7.24E-06 | 0.000307 |
| GO:0080134 | BP | regulation of response to stress | 7.29E-06 | 0.000307 |
| GO:0046856 | BP | phosphatidylinositol dephosphorylation | 7.30E-06 | 0.000307 |
| GO:0150115 | BP | cell-substrate junction organization | 7.30E-06 | 0.000307 |
| GO:0031327 | BP | negative regulation of cellular biosynthetic process | 7.31E-06 | 0.000307 |
| GO:1903508 | BP | positive regulation of nucleic acid-templated transcription | 7.32E-06 | 0.000307 |
| GO:0015833 | BP | peptide transport | 7.34E-06 | 0.000307 |
| GO:1902680 | BP | positive regulation of RNA biosynthetic process | 7.36E-06 | 0.000307 |
| GO:0051094 | BP | positive regulation of developmental process | 7.37E-06 | 0.000307 |
| GO:0002682 | BP | regulation of immune system process | 7.38E-06 | 0.000307 |
| GO:0035556 | BP | intracellular signal transduction | 7.39E-06 | 0.000307 |
| GO:1902531 | BP | regulation of intracellular signal transduction | 7.41E-06 | 0.000307 |
| GO:0042886 | BP | amide transport | 7.44E-06 | 0.000307 |
| GO:0048585 | BP | negative regulation of response to stimulus | 7.45E-06 | 0.000307 |
| GO:0002009 | BP | morphogenesis of an epithelium | 7.50E-06 | 0.000307 |
| GO:0002792 | BP | negative regulation of peptide secretion | 7.60E-06 | 0.000308 |
| GO:0051240 | BP | positive regulation of multicellular organismal process | 7.66E-06 | 0.000308 |
| GO:0019220 | BP | regulation of phosphate metabolic process | 7.67E-06 | 0.000308 |
| GO:0042325 | BP | regulation of phosphorylation | 7.69E-06 | 0.000308 |
| GO:0051254 | BP | positive regulation of RNA metabolic process | 7.73E-06 | 0.000308 |
| GO:0071310 | BP | cellular response to organic substance | 7.74E-06 | 0.000308 |
| GO:0015031 | BP | protein transport | 7.75E-06 | 0.000308 |
| GO:0051649 | BP | establishment of localization in cell | 7.76E-06 | 0.000308 |
| GO:0051174 | BP | regulation of phosphorus metabolic process | 7.76E-06 | 0.000308 |
| GO:0045935 | BP | positive regulation of nucleobase-containing compound metabolic process | 7.80E-06 | 0.000308 |
| GO:0050790 | BP | regulation of catalytic activity | 7.87E-06 | 0.000308 |
| GO:0010647 | BP | positive regulation of cell communication | 7.89E-06 | 0.000308 |
| GO:0010557 | BP | positive regulation of macromolecule biosynthetic process | 7.89E-06 | 0.000308 |
| GO:0006357 | BP | regulation of transcription by RNA polymerase II | 7.89E-06 | 0.000308 |
| GO:0045184 | BP | establishment of protein localization | 7.90E-06 | 0.000308 |
| GO:0031329 | BP | regulation of cellular catabolic process | 7.91E-06 | 0.000308 |
| GO:0009891 | BP | positive regulation of biosynthetic process | 7.94E-06 | 0.000308 |
| GO:0033036 | BP | macromolecule localization | 8.01E-06 | 0.000308 |
| GO:0009894 | BP | regulation of catabolic process | 8.11E-06 | 0.00031 |
| GO:0071705 | BP | nitrogen compound transport | 8.12E-06 | 0.00031 |
| GO:0051052 | BP | regulation of DNA metabolic process | 8.15E-06 | 0.00031 |
| GO:0023056 | BP | positive regulation of signaling | 8.15E-06 | 0.00031 |
| GO:0007166 | BP | cell surface receptor signaling pathway | 8.21E-06 | 0.00031 |
| GO:0010629 | BP | negative regulation of gene expression | 8.21E-06 | 0.00031 |
| GO:0030154 | BP | cell differentiation | 8.25E-06 | 0.00031 |
| GO:0051216 | BP | cartilage development | 8.25E-06 | 0.00031 |
| GO:0016571 | BP | histone methylation | 8.25E-06 | 0.00031 |
| GO:0046907 | BP | intracellular transport | 8.26E-06 | 0.00031 |
| GO:0031399 | BP | regulation of protein modification process | 8.32E-06 | 0.00031 |
| GO:0070887 | BP | cellular response to chemical stimulus | 8.42E-06 | 0.000312 |
| GO:0051172 | BP | negative regulation of nitrogen compound metabolic process | 8.44E-06 | 0.000312 |
| GO:0031324 | BP | negative regulation of cellular metabolic process | 8.46E-06 | 0.000312 |
| GO:0050793 | BP | regulation of developmental process | 8.50E-06 | 0.000313 |
| GO:0051049 | BP | regulation of transport | 8.80E-06 | 0.000323 |
| GO:2000026 | BP | regulation of multicellular organismal development | 8.81E-06 | 0.000323 |
| GO:0071702 | BP | organic substance transport | 8.82E-06 | 0.000323 |
| GO:0032268 | BP | regulation of cellular protein metabolic process | 8.84E-06 | 0.000323 |
| GO:0006282 | BP | regulation of DNA repair | 8.87E-06 | 0.000323 |
| GO:0048584 | BP | positive regulation of response to stimulus | 8.87E-06 | 0.000323 |
| GO:0048869 | BP | cellular developmental process | 8.90E-06 | 0.000323 |
| GO:0031328 | BP | positive regulation of cellular biosynthetic process | 8.91E-06 | 0.000323 |
| GO:0065009 | BP | regulation of molecular function | 8.91E-06 | 0.000323 |
| GO:0008104 | BP | protein localization | 9.00E-06 | 0.000325 |
| GO:0033047 | BP | regulation of mitotic sister chromatid segregation | 9.10E-06 | 0.000327 |
| GO:0006996 | BP | organelle organization | 9.26E-06 | 0.00033 |
| GO:0035855 | BP | megakaryocyte development | 9.29E-06 | 0.00033 |
| GO:0036211 | BP | protein modification process | 9.34E-06 | 0.000331 |
| GO:0006464 | BP | cellular protein modification process | 9.34E-06 | 0.000331 |
| GO:0006281 | BP | DNA repair | 9.38E-06 | 0.000331 |
| GO:0046777 | BP | protein autophosphorylation | 9.42E-06 | 0.000332 |
| GO:0051128 | BP | regulation of cellular component organization | 9.44E-06 | 0.000332 |
| GO:0022607 | BP | cellular component assembly | 9.49E-06 | 0.000333 |
| GO:0051301 | BP | cell division | 9.51E-06 | 0.000333 |
| GO:0010628 | BP | positive regulation of gene expression | 9.61E-06 | 0.000334 |
| GO:0006793 | BP | phosphorus metabolic process | 9.61E-06 | 0.000334 |
| GO:0030199 | BP | collagen fibril organization | 9.66E-06 | 0.000334 |
| GO:0006270 | BP | DNA replication initiation | 9.68E-06 | 0.000334 |
| GO:0051246 | BP | regulation of protein metabolic process | 9.68E-06 | 0.000334 |
| GO:0009892 | BP | negative regulation of metabolic process | 9.70E-06 | 0.000334 |
| GO:0051641 | BP | cellular localization | 9.71E-06 | 0.000334 |
| GO:0043412 | BP | macromolecule modification | 9.95E-06 | 0.000338 |
| GO:0032879 | BP | regulation of localization | 9.95E-06 | 0.000338 |
| GO:0006950 | BP | response to stress | 1.00E-05 | 0.000338 |
| GO:0051716 | BP | cellular response to stimulus | 1.00E-05 | 0.000338 |
| GO:0048856 | BP | anatomical structure development | 1.00E-05 | 0.000338 |
| GO:0006355 | BP | regulation of transcription, DNA-templated | 1.01E-05 | 0.000338 |
| GO:0010631 | BP | epithelial cell migration | 1.01E-05 | 0.000338 |
| GO:0010605 | BP | negative regulation of macromolecule metabolic process | 1.01E-05 | 0.000338 |
| GO:0031331 | BP | positive regulation of cellular catabolic process | 1.02E-05 | 0.000341 |
| GO:0009966 | BP | regulation of signal transduction | 1.03E-05 | 0.000341 |
| GO:0051173 | BP | positive regulation of nitrogen compound metabolic process | 1.03E-05 | 0.000341 |
| GO:0045732 | BP | positive regulation of protein catabolic process | 1.03E-05 | 0.000341 |
| GO:2001141 | BP | regulation of RNA biosynthetic process | 1.05E-05 | 0.000346 |
| GO:0051252 | BP | regulation of RNA metabolic process | 1.05E-05 | 0.000346 |
| GO:0009893 | BP | positive regulation of metabolic process | 1.05E-05 | 0.000347 |
| GO:2000112 | BP | regulation of cellular macromolecule biosynthetic process | 1.06E-05 | 0.000347 |
| GO:0010243 | BP | response to organonitrogen compound | 1.06E-05 | 0.000347 |
| GO:0051239 | BP | regulation of multicellular organismal process | 1.06E-05 | 0.000347 |
| GO:0010556 | BP | regulation of macromolecule biosynthetic process | 1.07E-05 | 0.000348 |
| GO:0006796 | BP | phosphate-containing compound metabolic process | 1.07E-05 | 0.000348 |
| GO:0010646 | BP | regulation of cell communication | 1.07E-05 | 0.000348 |
| GO:0019219 | BP | regulation of nucleobase-containing compound metabolic process | 1.07E-05 | 0.000348 |
| GO:0051962 | BP | positive regulation of nervous system development | 1.10E-05 | 0.000353 |
| GO:0048705 | BP | skeletal system morphogenesis | 1.10E-05 | 0.000354 |
| GO:0031326 | BP | regulation of cellular biosynthetic process | 1.11E-05 | 0.000354 |
| GO:0048583 | BP | regulation of response to stimulus | 1.12E-05 | 0.000357 |
| GO:0044092 | BP | negative regulation of molecular function | 1.13E-05 | 0.000359 |
| GO:1903506 | BP | regulation of nucleic acid-templated transcription | 1.13E-05 | 0.000359 |
| GO:0031325 | BP | positive regulation of cellular metabolic process | 1.13E-05 | 0.000359 |
| GO:0031348 | BP | negative regulation of defense response | 1.14E-05 | 0.00036 |
| GO:0023051 | BP | regulation of signaling | 1.14E-05 | 0.00036 |
| GO:0010604 | BP | positive regulation of macromolecule metabolic process | 1.14E-05 | 0.00036 |
| GO:1901698 | BP | response to nitrogen compound | 1.15E-05 | 0.00036 |
| GO:0051234 | BP | establishment of localization | 1.16E-05 | 0.000364 |
| GO:0051179 | BP | localization | 1.17E-05 | 0.000364 |
| GO:0065008 | BP | regulation of biological quality | 1.17E-05 | 0.000364 |
| GO:0006810 | BP | transport | 1.19E-05 | 0.00037 |
| GO:0007165 | BP | signal transduction | 1.21E-05 | 0.000375 |
| GO:0010468 | BP | regulation of gene expression | 1.21E-05 | 0.000375 |
| GO:0009889 | BP | regulation of biosynthetic process | 1.22E-05 | 0.000375 |
| GO:0009411 | BP | response to UV | 1.23E-05 | 0.000377 |
| GO:0044782 | BP | cilium organization | 1.23E-05 | 0.000377 |
| GO:0050896 | BP | response to stimulus | 1.23E-05 | 0.000377 |
| GO:0016043 | BP | cellular component organization | 1.24E-05 | 0.000379 |
| GO:0048519 | BP | negative regulation of biological process | 1.26E-05 | 0.000383 |
| GO:0048523 | BP | negative regulation of cellular process | 1.26E-05 | 0.000383 |
| GO:0044839 | BP | cell cycle G2/M phase transition | 1.27E-05 | 0.000383 |
| GO:0007052 | BP | mitotic spindle organization | 1.27E-05 | 0.000383 |
| GO:0034502 | BP | protein localization to chromosome | 1.27E-05 | 0.000383 |
| GO:0051171 | BP | regulation of nitrogen compound metabolic process | 1.28E-05 | 0.000387 |
| GO:1902850 | BP | microtubule cytoskeleton organization involved in mitosis | 1.30E-05 | 0.000393 |
| GO:0032502 | BP | developmental process | 1.31E-05 | 0.000395 |
| GO:0071840 | BP | cellular component organization or biogenesis | 1.31E-05 | 0.000395 |
| GO:1901888 | BP | regulation of cell junction assembly | 1.32E-05 | 0.000396 |
| GO:0000902 | BP | cell morphogenesis | 1.33E-05 | 0.000398 |
| GO:0048522 | BP | positive regulation of cellular process | 1.35E-05 | 0.000403 |
| GO:0008150 | BP | biological_process | 1.35E-05 | 0.000405 |
| GO:0060255 | BP | regulation of macromolecule metabolic process | 1.36E-05 | 0.000405 |
| GO:0031323 | BP | regulation of cellular metabolic process | 1.36E-05 | 0.000405 |
| GO:0007275 | BP | multicellular organism development | 1.39E-05 | 0.000414 |
| GO:0019222 | BP | regulation of metabolic process | 1.40E-05 | 0.000415 |
| GO:0042176 | BP | regulation of protein catabolic process | 1.41E-05 | 0.000417 |
| GO:0048518 | BP | positive regulation of biological process | 1.42E-05 | 0.00042 |
| GO:0080090 | BP | regulation of primary metabolic process | 1.43E-05 | 0.000423 |
| GO:0007044 | BP | cell-substrate junction assembly | 1.48E-05 | 0.000433 |
| GO:0051984 | BP | positive regulation of chromosome segregation | 1.48E-05 | 0.000433 |
| GO:0008360 | BP | regulation of cell shape | 1.48E-05 | 0.000433 |
| GO:0000086 | BP | G2/M transition of mitotic cell cycle | 1.54E-05 | 0.000446 |
| GO:0007041 | BP | lysosomal transport | 1.54E-05 | 0.000447 |
| GO:0050789 | BP | regulation of biological process | 1.55E-05 | 0.000449 |
| GO:0042119 | BP | neutrophil activation | 1.56E-05 | 0.000449 |
| GO:0009987 | BP | cellular process | 1.58E-05 | 0.000453 |
| GO:0050794 | BP | regulation of cellular process | 1.58E-05 | 0.000454 |
| GO:1903362 | BP | regulation of cellular protein catabolic process | 1.60E-05 | 0.000457 |
| GO:0065007 | BP | biological regulation | 1.60E-05 | 0.000457 |
| GO:0010770 | BP | positive regulation of cell morphogenesis involved in differentiation | 1.60E-05 | 0.000457 |
| GO:1901989 | BP | positive regulation of cell cycle phase transition | 1.76E-05 | 0.000495 |
| GO:0060560 | BP | developmental growth involved in morphogenesis | 1.76E-05 | 0.000495 |
| GO:1905269 | BP | positive regulation of chromatin organization | 1.80E-05 | 0.000505 |
| GO:0010632 | BP | regulation of epithelial cell migration | 1.81E-05 | 0.000507 |
| GO:1901701 | BP | cellular response to oxygen-containing compound | 1.84E-05 | 0.000513 |
| GO:0090174 | BP | organelle membrane fusion | 1.88E-05 | 0.000525 |
| GO:1902806 | BP | regulation of cell cycle G1/S phase transition | 1.91E-05 | 0.000533 |
| GO:0002283 | BP | neutrophil activation involved in immune response | 1.95E-05 | 0.000541 |
| GO:1901342 | BP | regulation of vasculature development | 1.95E-05 | 0.000542 |
| GO:0048514 | BP | blood vessel morphogenesis | 2.01E-05 | 0.000556 |
| GO:0090087 | BP | regulation of peptide transport | 2.02E-05 | 0.000559 |
| GO:0036230 | BP | granulocyte activation | 2.05E-05 | 0.000567 |
| GO:0043123 | BP | positive regulation of I-kappaB kinase/NF-kappaB signaling | 2.07E-05 | 0.000571 |
| GO:0000075 | BP | cell cycle checkpoint | 2.18E-05 | 0.000599 |
| GO:0006629 | BP | lipid metabolic process | 2.20E-05 | 0.000604 |
| GO:0048729 | BP | tissue morphogenesis | 2.23E-05 | 0.000611 |
| GO:0045596 | BP | negative regulation of cell differentiation | 2.32E-05 | 0.000631 |
| GO:0050776 | BP | regulation of immune response | 2.43E-05 | 0.000659 |
| GO:0045992 | BP | negative regulation of embryonic development | 2.44E-05 | 0.000661 |
| GO:0007610 | BP | behavior | 2.51E-05 | 0.000678 |
| GO:0060271 | BP | cilium assembly | 2.66E-05 | 0.000719 |
| GO:0051650 | BP | establishment of vesicle localization | 2.78E-05 | 0.000746 |
| GO:0051648 | BP | vesicle localization | 2.83E-05 | 0.000756 |
| GO:0090224 | BP | regulation of spindle organization | 2.86E-05 | 0.000763 |
| GO:0051048 | BP | negative regulation of secretion | 2.97E-05 | 0.00079 |
| GO:1902117 | BP | positive regulation of organelle assembly | 2.98E-05 | 0.000793 |
| GO:0043009 | BP | chordate embryonic development | 3.14E-05 | 0.000834 |
| GO:0060325 | BP | face morphogenesis | 3.31E-05 | 0.000875 |
| GO:0006476 | BP | protein deacetylation | 3.32E-05 | 0.000876 |
| GO:0000018 | BP | regulation of DNA recombination | 3.34E-05 | 0.000882 |
| GO:0031110 | BP | regulation of microtubule polymerization or depolymerization | 3.38E-05 | 0.000889 |
| GO:0007409 | BP | axonogenesis | 3.43E-05 | 0.000901 |
| GO:1904375 | BP | regulation of protein localization to cell periphery | 3.61E-05 | 0.000945 |
| GO:0016601 | BP | Rac protein signal transduction | 3.70E-05 | 0.000963 |
| GO:0030201 | BP | heparan sulfate proteoglycan metabolic process | 3.70E-05 | 0.000963 |
| GO:0030705 | BP | cytoskeleton-dependent intracellular transport | 3.72E-05 | 0.000964 |
| GO:0007163 | BP | establishment or maintenance of cell polarity | 3.78E-05 | 0.000979 |
| GO:1900076 | BP | regulation of cellular response to insulin stimulus | 4.04E-05 | 0.001046 |
| GO:0050865 | BP | regulation of cell activation | 4.11E-05 | 0.001061 |
| GO:0016482 | BP | cytosolic transport | 4.17E-05 | 0.001072 |
| GO:0000077 | BP | DNA damage checkpoint | 4.23E-05 | 0.001086 |
| GO:0043312 | BP | neutrophil degranulation | 4.44E-05 | 0.001134 |
| GO:0034968 | BP | histone lysine methylation | 4.45E-05 | 0.001134 |
| GO:0050770 | BP | regulation of axonogenesis | 4.62E-05 | 0.001173 |
| GO:0099111 | BP | microtubule-based transport | 4.68E-05 | 0.001183 |
| GO:0035303 | BP | regulation of dephosphorylation | 4.69E-05 | 0.001185 |
| GO:0010811 | BP | positive regulation of cell-substrate adhesion | 4.72E-05 | 0.001189 |
| GO:0045598 | BP | regulation of fat cell differentiation | 4.77E-05 | 0.001199 |
| GO:0001959 | BP | regulation of cytokine-mediated signaling pathway | 4.77E-05 | 0.001199 |
| GO:2000780 | BP | negative regulation of double-strand break repair | 4.80E-05 | 0.001202 |
| GO:0045738 | BP | negative regulation of DNA repair | 4.80E-05 | 0.001202 |
| GO:0090066 | BP | regulation of anatomical structure size | 4.81E-05 | 0.001203 |
| GO:0001817 | BP | regulation of cytokine production | 4.81E-05 | 0.001203 |
| GO:1901699 | BP | cellular response to nitrogen compound | 4.87E-05 | 0.001215 |
| GO:2000045 | BP | regulation of G1/S transition of mitotic cell cycle | 4.92E-05 | 0.001227 |
| GO:0051225 | BP | spindle assembly | 5.03E-05 | 0.001251 |
| GO:0007178 | BP | transmembrane receptor protein serine/threonine kinase signaling pathway | 5.07E-05 | 0.00126 |
| GO:0060236 | BP | regulation of mitotic spindle organization | 5.15E-05 | 0.001275 |
| GO:0001701 | BP | in utero embryonic development | 5.16E-05 | 0.001276 |
| GO:0048145 | BP | regulation of fibroblast proliferation | 5.32E-05 | 0.001313 |
| GO:0050854 | BP | regulation of antigen receptor-mediated signaling pathway | 5.36E-05 | 0.001321 |
| GO:0051054 | BP | positive regulation of DNA metabolic process | 5.38E-05 | 0.001325 |
| GO:0048589 | BP | developmental growth | 5.53E-05 | 0.00136 |
| GO:0007050 | BP | cell cycle arrest | 5.59E-05 | 0.001373 |
| GO:0050808 | BP | synapse organization | 5.88E-05 | 0.001441 |
| GO:0071168 | BP | protein localization to chromatin | 5.99E-05 | 0.001462 |
| GO:0048538 | BP | thymus development | 6.01E-05 | 0.001465 |
| GO:2000008 | BP | regulation of protein localization to cell surface | 6.12E-05 | 0.001487 |
| GO:0032386 | BP | regulation of intracellular transport | 6.32E-05 | 0.001535 |
| GO:0045216 | BP | cell-cell junction organization | 6.42E-05 | 0.001556 |
| GO:0045739 | BP | positive regulation of DNA repair | 6.47E-05 | 0.001564 |
| GO:0042770 | BP | signal transduction in response to DNA damage | 6.60E-05 | 0.001591 |
| GO:0061025 | BP | membrane fusion | 6.79E-05 | 0.001632 |
| GO:0050866 | BP | negative regulation of cell activation | 6.80E-05 | 0.001632 |
| GO:0050769 | BP | positive regulation of neurogenesis | 6.88E-05 | 0.00165 |
| GO:0098742 | BP | cell-cell adhesion via plasma-membrane adhesion molecules | 6.96E-05 | 0.001667 |
| GO:0032434 | BP | regulation of proteasomal ubiquitin-dependent protein catabolic process | 6.97E-05 | 0.001668 |
| GO:0030278 | BP | regulation of ossification | 7.08E-05 | 0.001689 |
| GO:0048013 | BP | ephrin receptor signaling pathway | 7.12E-05 | 0.001693 |
| GO:0071478 | BP | cellular response to radiation | 7.19E-05 | 0.001707 |
| GO:0071479 | BP | cellular response to ionizing radiation | 7.51E-05 | 0.001776 |
| GO:0002697 | BP | regulation of immune effector process | 7.57E-05 | 0.001788 |
| GO:0016049 | BP | cell growth | 7.82E-05 | 0.001841 |
| GO:0032231 | BP | regulation of actin filament bundle assembly | 8.00E-05 | 0.00188 |
| GO:0042339 | BP | keratan sulfate metabolic process | 8.06E-05 | 0.001886 |
| GO:0002063 | BP | chondrocyte development | 8.06E-05 | 0.001886 |
| GO:0007063 | BP | regulation of sister chromatid cohesion | 8.06E-05 | 0.001886 |
| GO:0034976 | BP | response to endoplasmic reticulum stress | 8.07E-05 | 0.001887 |
| GO:0051492 | BP | regulation of stress fiber assembly | 8.37E-05 | 0.001953 |
| GO:0031122 | BP | cytoplasmic microtubule organization | 8.43E-05 | 0.001964 |
| GO:0000209 | BP | protein polyubiquitination | 8.79E-05 | 0.002046 |
| GO:1903364 | BP | positive regulation of cellular protein catabolic process | 8.80E-05 | 0.002047 |
| GO:0120034 | BP | positive regulation of plasma membrane bounded cell projection assembly | 8.93E-05 | 0.002073 |
| GO:0072698 | BP | protein localization to microtubule cytoskeleton | 9.09E-05 | 0.002105 |
| GO:0002274 | BP | myeloid leukocyte activation | 9.09E-05 | 0.002105 |
| GO:0046488 | BP | phosphatidylinositol metabolic process | 9.12E-05 | 0.002108 |
| GO:0002685 | BP | regulation of leukocyte migration | 9.13E-05 | 0.002108 |
| GO:2000779 | BP | regulation of double-strand break repair | 9.38E-05 | 0.002162 |
| GO:1902275 | BP | regulation of chromatin organization | 9.63E-05 | 0.002216 |
| GO:0002698 | BP | negative regulation of immune effector process | 9.64E-05 | 0.002216 |
| GO:0048639 | BP | positive regulation of developmental growth | 9.85E-05 | 0.002261 |
| GO:0007162 | BP | negative regulation of cell adhesion | 9.94E-05 | 0.002277 |
| GO:0010977 | BP | negative regulation of neuron projection development | 9.95E-05 | 0.002277 |
| GO:0002819 | BP | regulation of adaptive immune response | 9.97E-05 | 0.002279 |
| GO:0030010 | BP | establishment of cell polarity | 0.000101 | 0.002295 |
| GO:0030097 | BP | hemopoiesis | 0.000101 | 0.002312 |
| GO:0051656 | BP | establishment of organelle localization | 0.000101 | 0.002312 |
| GO:0043542 | BP | endothelial cell migration | 0.000103 | 0.002345 |
| GO:0031175 | BP | neuron projection development | 0.000105 | 0.002386 |
| GO:0006275 | BP | regulation of DNA replication | 0.000107 | 0.002431 |
| GO:0045765 | BP | regulation of angiogenesis | 0.000108 | 0.002445 |
| GO:0016575 | BP | histone deacetylation | 0.000109 | 0.002455 |
| GO:0040007 | BP | growth | 0.00011 | 0.002476 |
| GO:0045055 | BP | regulated exocytosis | 0.000111 | 0.002493 |
| GO:0002790 | BP | peptide secretion | 0.000111 | 0.002496 |
| GO:0009952 | BP | anterior/posterior pattern specification | 0.000111 | 0.002496 |
| GO:0050807 | BP | regulation of synapse organization | 0.000111 | 0.0025 |
| GO:0046626 | BP | regulation of insulin receptor signaling pathway | 0.000112 | 0.002513 |
| GO:1903050 | BP | regulation of proteolysis involved in cellular protein catabolic process | 0.000113 | 0.002537 |
| GO:0090169 | BP | regulation of spindle assembly | 0.000115 | 0.002577 |
| GO:0043506 | BP | regulation of JUN kinase activity | 0.000121 | 0.002688 |
| GO:0045637 | BP | regulation of myeloid cell differentiation | 0.000121 | 0.002692 |
| GO:1900024 | BP | regulation of substrate adhesion-dependent cell spreading | 0.000121 | 0.002692 |
| GO:0098732 | BP | macromolecule deacylation | 0.000121 | 0.002692 |
| GO:0035601 | BP | protein deacylation | 0.000121 | 0.002692 |
| GO:0120032 | BP | regulation of plasma membrane bounded cell projection assembly | 0.000122 | 0.002715 |
| GO:0043124 | BP | negative regulation of I-kappaB kinase/NF-kappaB signaling | 0.000123 | 0.002726 |
| GO:0033673 | BP | negative regulation of kinase activity | 0.000125 | 0.00277 |
| GO:0048598 | BP | embryonic morphogenesis | 0.000127 | 0.002788 |
| GO:0019216 | BP | regulation of lipid metabolic process | 0.000128 | 0.002812 |
| GO:0035295 | BP | tube development | 0.000134 | 0.002943 |
| GO:0044090 | BP | positive regulation of vacuole organization | 0.000135 | 0.002944 |
| GO:0032688 | BP | negative regulation of interferon-beta production | 0.000135 | 0.002944 |
| GO:0044772 | BP | mitotic cell cycle phase transition | 0.000135 | 0.002944 |
| GO:1905114 | BP | cell surface receptor signaling pathway involved in cell-cell signaling | 0.000135 | 0.002946 |
| GO:0030865 | BP | cortical cytoskeleton organization | 0.000142 | 0.003071 |
| GO:0048588 | BP | developmental cell growth | 0.000147 | 0.00317 |
| GO:0010976 | BP | positive regulation of neuron projection development | 0.000148 | 0.003187 |
| GO:0032675 | BP | regulation of interleukin-6 production | 0.000152 | 0.003267 |
| GO:0032088 | BP | negative regulation of NF-kappaB transcription factor activity | 0.000153 | 0.003292 |
| GO:0098876 | BP | vesicle-mediated transport to the plasma membrane | 0.000153 | 0.003292 |
| GO:0018022 | BP | peptidyl-lysine methylation | 0.000154 | 0.003299 |
| GO:0030217 | BP | T cell differentiation | 0.000154 | 0.003307 |
| GO:0006026 | BP | aminoglycan catabolic process | 0.00016 | 0.003423 |
| GO:0051248 | BP | negative regulation of protein metabolic process | 0.000162 | 0.003451 |
| GO:0060759 | BP | regulation of response to cytokine stimulus | 0.000171 | 0.003633 |
| GO:0045665 | BP | negative regulation of neuron differentiation | 0.000171 | 0.003634 |
| GO:0018205 | BP | peptidyl-lysine modification | 0.000173 | 0.003666 |
| GO:0034383 | BP | low-density lipoprotein particle clearance | 0.000175 | 0.003681 |
| GO:0009263 | BP | deoxyribonucleotide biosynthetic process | 0.000175 | 0.003681 |
| GO:2000042 | BP | negative regulation of double-strand break repair via homologous recombination | 0.000175 | 0.003681 |
| GO:0048468 | BP | cell development | 0.000175 | 0.003681 |
| GO:0045995 | BP | regulation of embryonic development | 0.000178 | 0.003746 |
| GO:0060491 | BP | regulation of cell projection assembly | 0.000179 | 0.003748 |
| GO:1900016 | BP | negative regulation of cytokine production involved in inflammatory response | 0.000183 | 0.003829 |
| GO:0051098 | BP | regulation of binding | 0.000184 | 0.003846 |
| GO:0030098 | BP | lymphocyte differentiation | 0.000186 | 0.003875 |
| GO:0002703 | BP | regulation of leukocyte mediated immunity | 0.000186 | 0.003876 |
| GO:0016055 | BP | Wnt signaling pathway | 0.000186 | 0.003876 |
| GO:1905508 | BP | protein localization to microtubule organizing center | 0.000188 | 0.003883 |
| GO:0016572 | BP | histone phosphorylation | 0.000188 | 0.003883 |
| GO:0071539 | BP | protein localization to centrosome | 0.000188 | 0.003883 |
| GO:0097711 | BP | ciliary basal body-plasma membrane docking | 0.000188 | 0.003883 |
| GO:0048008 | BP | platelet-derived growth factor receptor signaling pathway | 0.000188 | 0.003883 |
| GO:0050772 | BP | positive regulation of axonogenesis | 0.000188 | 0.003883 |
| GO:0070848 | BP | response to growth factor | 0.000189 | 0.003898 |
| GO:0097581 | BP | lamellipodium organization | 0.000196 | 0.004035 |
| GO:0006892 | BP | post-Golgi vesicle-mediated transport | 0.000197 | 0.004044 |
| GO:0032720 | BP | negative regulation of tumor necrosis factor production | 0.0002 | 0.004096 |
| GO:0044770 | BP | cell cycle phase transition | 0.000205 | 0.004194 |
| GO:0007219 | BP | Notch signaling pathway | 0.000206 | 0.004204 |
| GO:0051050 | BP | positive regulation of transport | 0.000208 | 0.00424 |
| GO:0043433 | BP | negative regulation of DNA-binding transcription factor activity | 0.000209 | 0.004262 |
| GO:0072401 | BP | signal transduction involved in DNA integrity checkpoint | 0.000212 | 0.00431 |
| GO:0072422 | BP | signal transduction involved in DNA damage checkpoint | 0.000212 | 0.00431 |
| GO:2001236 | BP | regulation of extrinsic apoptotic signaling pathway | 0.000212 | 0.004312 |
| GO:0045005 | BP | DNA-dependent DNA replication maintenance of fidelity | 0.000216 | 0.00437 |
| GO:0043112 | BP | receptor metabolic process | 0.000217 | 0.004387 |
| GO:0051051 | BP | negative regulation of transport | 0.000221 | 0.004461 |
| GO:1901700 | BP | response to oxygen-containing compound | 0.000222 | 0.00448 |
| GO:0010569 | BP | regulation of double-strand break repair via homologous recombination | 0.000225 | 0.004546 |
| GO:0061138 | BP | morphogenesis of a branching epithelium | 0.000235 | 0.004714 |
| GO:0030071 | BP | regulation of mitotic metaphase/anaphase transition | 0.000236 | 0.004744 |
| GO:0106118 | BP | regulation of sterol biosynthetic process | 0.000238 | 0.004756 |
| GO:0046475 | BP | glycerophospholipid catabolic process | 0.000238 | 0.004756 |
| GO:0045540 | BP | regulation of cholesterol biosynthetic process | 0.000238 | 0.004756 |
| GO:0007249 | BP | I-kappaB kinase/NF-kappaB signaling | 0.000239 | 0.004756 |
| GO:0051496 | BP | positive regulation of stress fiber assembly | 0.000239 | 0.004756 |
| GO:0032271 | BP | regulation of protein polymerization | 0.000243 | 0.004842 |
| GO:0010506 | BP | regulation of autophagy | 0.000244 | 0.004842 |
| GO:1902017 | BP | regulation of cilium assembly | 0.000249 | 0.004919 |
| GO:0071260 | BP | cellular response to mechanical stimulus | 0.000249 | 0.004919 |
| GO:0034504 | BP | protein localization to nucleus | 0.000257 | 0.005079 |
| GO:0009306 | BP | protein secretion | 0.000261 | 0.005134 |
| GO:0009719 | BP | response to endogenous stimulus | 0.000265 | 0.00522 |
| GO:0002822 | BP | regulation of adaptive immune response based on somatic recombination of immune receptors built from immunoglobulin superfamily domains | 0.000268 | 0.005254 |
| GO:0051058 | BP | negative regulation of small GTPase mediated signal transduction | 0.000282 | 0.005518 |
| GO:0010965 | BP | regulation of mitotic sister chromatid separation | 0.000291 | 0.00569 |
| GO:0032269 | BP | negative regulation of cellular protein metabolic process | 0.000293 | 0.005717 |
| GO:0002376 | BP | immune system process | 0.000297 | 0.005778 |
| GO:0071692 | BP | protein localization to extracellular region | 0.000298 | 0.005792 |
| GO:0035592 | BP | establishment of protein localization to extracellular region | 0.000298 | 0.005792 |
| GO:0050708 | BP | regulation of protein secretion | 0.000301 | 0.00582 |
| GO:0071363 | BP | cellular response to growth factor stimulus | 0.000305 | 0.005905 |
| GO:0002275 | BP | myeloid cell activation involved in immune response | 0.000305 | 0.005905 |
| GO:1903530 | BP | regulation of secretion by cell | 0.000307 | 0.005926 |
| GO:0046649 | BP | lymphocyte activation | 0.00031 | 0.005985 |
| GO:0006493 | BP | protein O-linked glycosylation | 0.000319 | 0.006152 |
| GO:0045778 | BP | positive regulation of ossification | 0.000319 | 0.006152 |
| GO:0006027 | BP | glycosaminoglycan catabolic process | 0.000321 | 0.006163 |
| GO:0045773 | BP | positive regulation of axon extension | 0.000321 | 0.006163 |
| GO:0021955 | BP | central nervous system neuron axonogenesis | 0.000321 | 0.006163 |
| GO:0043299 | BP | leukocyte degranulation | 0.000325 | 0.006232 |
| GO:0010634 | BP | positive regulation of epithelial cell migration | 0.000326 | 0.006236 |
| GO:2000786 | BP | positive regulation of autophagosome assembly | 0.000329 | 0.006257 |
| GO:0018146 | BP | keratan sulfate biosynthetic process | 0.000329 | 0.006257 |
| GO:0030953 | BP | astral microtubule organization | 0.000329 | 0.006257 |
| GO:0032501 | BP | multicellular organismal process | 0.000335 | 0.006374 |
| GO:0007411 | BP | axon guidance | 0.000346 | 0.006568 |
| GO:0099177 | BP | regulation of trans-synaptic signaling | 0.00035 | 0.006637 |
| GO:0030204 | BP | chondroitin sulfate metabolic process | 0.000353 | 0.006681 |
| GO:1903829 | BP | positive regulation of cellular protein localization | 0.000355 | 0.006699 |
| GO:0000723 | BP | telomere maintenance | 0.000357 | 0.006718 |
| GO:0001501 | BP | skeletal system development | 0.000357 | 0.006718 |
| GO:0050773 | BP | regulation of dendrite development | 0.000357 | 0.006718 |
| GO:1990138 | BP | neuron projection extension | 0.000362 | 0.006794 |
| GO:2000781 | BP | positive regulation of double-strand break repair | 0.000363 | 0.006794 |
| GO:0051403 | BP | stress-activated MAPK cascade | 0.000363 | 0.006794 |
| GO:0030501 | BP | positive regulation of bone mineralization | 0.000363 | 0.006794 |
| GO:0035307 | BP | positive regulation of protein dephosphorylation | 0.000363 | 0.006794 |
| GO:1901184 | BP | regulation of ERBB signaling pathway | 0.000363 | 0.006794 |
| GO:0010812 | BP | negative regulation of cell-substrate adhesion | 0.000365 | 0.006816 |
| GO:0060249 | BP | anatomical structure homeostasis | 0.000366 | 0.00683 |
| GO:0010970 | BP | transport along microtubule | 0.000367 | 0.006834 |
| GO:0097485 | BP | neuron projection guidance | 0.000369 | 0.006866 |
| GO:0062033 | BP | positive regulation of mitotic sister chromatid segregation | 0.000373 | 0.006918 |
| GO:0045724 | BP | positive regulation of cilium assembly | 0.000373 | 0.006918 |
| GO:0044409 | BP | entry into host | 0.000375 | 0.00695 |
| GO:0008284 | BP | positive regulation of cell population proliferation | 0.000391 | 0.007242 |
| GO:0043409 | BP | negative regulation of MAPK cascade | 0.000393 | 0.007265 |
| GO:0051046 | BP | regulation of secretion | 0.000394 | 0.007275 |
| GO:0048844 | BP | artery morphogenesis | 0.000399 | 0.007348 |
| GO:0007018 | BP | microtubule-based movement | 0.000401 | 0.007383 |
| GO:0061512 | BP | protein localization to cilium | 0.000401 | 0.007388 |
| GO:0030032 | BP | lamellipodium assembly | 0.000403 | 0.007412 |
| GO:1905897 | BP | regulation of response to endoplasmic reticulum stress | 0.000407 | 0.007461 |
| GO:0110020 | BP | regulation of actomyosin structure organization | 0.000407 | 0.007461 |
| GO:0000819 | BP | sister chromatid segregation | 0.000407 | 0.007463 |
| GO:0000187 | BP | activation of MAPK activity | 0.000408 | 0.007468 |
| GO:0015012 | BP | heparan sulfate proteoglycan biosynthetic process | 0.000414 | 0.007548 |
| GO:0032964 | BP | collagen biosynthetic process | 0.000414 | 0.007548 |
| GO:0031058 | BP | positive regulation of histone modification | 0.000421 | 0.007645 |
| GO:0000910 | BP | cytokinesis | 0.000421 | 0.007645 |
| GO:0061640 | BP | cytoskeleton-dependent cytokinesis | 0.000421 | 0.007645 |
| GO:0060348 | BP | bone development | 0.000425 | 0.0077 |
| GO:1903556 | BP | negative regulation of tumor necrosis factor superfamily cytokine production | 0.000425 | 0.0077 |
| GO:0008283 | BP | cell population proliferation | 0.00043 | 0.007782 |
| GO:0043087 | BP | regulation of GTPase activity | 0.000432 | 0.007812 |
| GO:0050804 | BP | modulation of chemical synaptic transmission | 0.000432 | 0.007812 |
| GO:0007389 | BP | pattern specification process | 0.000434 | 0.007836 |
| GO:1902099 | BP | regulation of metaphase/anaphase transition of cell cycle | 0.000437 | 0.007881 |
| GO:0045927 | BP | positive regulation of growth | 0.000438 | 0.007881 |
| GO:0030111 | BP | regulation of Wnt signaling pathway | 0.000442 | 0.007939 |
| GO:0001952 | BP | regulation of cell-matrix adhesion | 0.000452 | 0.008116 |
| GO:0048568 | BP | embryonic organ development | 0.000455 | 0.008141 |
| GO:0032535 | BP | regulation of cellular component size | 0.000456 | 0.008146 |
| GO:0050673 | BP | epithelial cell proliferation | 0.000458 | 0.008171 |
| GO:0042058 | BP | regulation of epidermal growth factor receptor signaling pathway | 0.000465 | 0.008254 |
| GO:0006890 | BP | retrograde vesicle-mediated transport, Golgi to endoplasmic reticulum | 0.000465 | 0.008254 |
| GO:0002573 | BP | myeloid leukocyte differentiation | 0.00047 | 0.008327 |
| GO:0070301 | BP | cellular response to hydrogen peroxide | 0.000473 | 0.008386 |
| GO:0002694 | BP | regulation of leukocyte activation | 0.00048 | 0.008494 |
| GO:0072089 | BP | stem cell proliferation | 0.000491 | 0.008671 |
| GO:1903706 | BP | regulation of hemopoiesis | 0.000491 | 0.008671 |
| GO:0050890 | BP | cognition | 0.000493 | 0.008686 |
| GO:1902904 | BP | negative regulation of supramolecular fiber organization | 0.000493 | 0.00869 |
| GO:1901652 | BP | response to peptide | 0.000501 | 0.008801 |
| GO:1905818 | BP | regulation of chromosome separation | 0.000501 | 0.008801 |
| GO:0060021 | BP | roof of mouth development | 0.000501 | 0.008801 |
| GO:1903076 | BP | regulation of protein localization to plasma membrane | 0.000502 | 0.008805 |
| GO:0001763 | BP | morphogenesis of a branching structure | 0.000502 | 0.008805 |
| GO:0006508 | BP | proteolysis | 0.000544 | 0.009508 |
| GO:0001954 | BP | positive regulation of cell-matrix adhesion | 0.000562 | 0.009783 |
| GO:0071901 | BP | negative regulation of protein serine/threonine kinase activity | 0.000568 | 0.009875 |
| GO:0071417 | BP | cellular response to organonitrogen compound | 0.000586 | 0.010162 |
| GO:0050663 | BP | cytokine secretion | 0.000634 | 0.010935 |
| GO:1900077 | BP | negative regulation of cellular response to insulin stimulus | 0.000634 | 0.010935 |
| GO:0010594 | BP | regulation of endothelial cell migration | 0.000653 | 0.011257 |
| GO:0050764 | BP | regulation of phagocytosis | 0.000655 | 0.01128 |
| GO:0009395 | BP | phospholipid catabolic process | 0.000674 | 0.011596 |
| GO:0090329 | BP | regulation of DNA-dependent DNA replication | 0.000674 | 0.011596 |
| GO:0032092 | BP | positive regulation of protein binding | 0.000679 | 0.011673 |
| GO:0007266 | BP | Rho protein signal transduction | 0.000686 | 0.011764 |
| GO:0048704 | BP | embryonic skeletal system morphogenesis | 0.000688 | 0.011777 |
| GO:0008593 | BP | regulation of Notch signaling pathway | 0.000688 | 0.011777 |
| GO:0048278 | BP | vesicle docking | 0.000703 | 0.011992 |
| GO:1901983 | BP | regulation of protein acetylation | 0.000703 | 0.011992 |
| GO:0035306 | BP | positive regulation of dephosphorylation | 0.000703 | 0.011992 |
| GO:1901890 | BP | positive regulation of cell junction assembly | 0.000707 | 0.01204 |
| GO:0031529 | BP | ruffle organization | 0.000711 | 0.012104 |
| GO:1904018 | BP | positive regulation of vasculature development | 0.000712 | 0.012104 |
| GO:0034097 | BP | response to cytokine | 0.000713 | 0.012118 |
| GO:0016310 | BP | phosphorylation | 0.000716 | 0.012162 |
| GO:1903900 | BP | regulation of viral life cycle | 0.000734 | 0.012439 |
| GO:0050855 | BP | regulation of B cell receptor signaling pathway | 0.000738 | 0.012479 |
| GO:2000010 | BP | positive regulation of protein localization to cell surface | 0.000738 | 0.012479 |
| GO:0106027 | BP | neuron projection organization | 0.000751 | 0.012652 |
| GO:0010971 | BP | positive regulation of G2/M transition of mitotic cell cycle | 0.000751 | 0.012652 |
| GO:0071709 | BP | membrane assembly | 0.000751 | 0.012652 |
| GO:0046434 | BP | organophosphate catabolic process | 0.00076 | 0.012793 |
| GO:1902905 | BP | positive regulation of supramolecular fiber organization | 0.000763 | 0.012799 |
| GO:0043254 | BP | regulation of protein-containing complex assembly | 0.000765 | 0.012799 |
| GO:0008654 | BP | phospholipid biosynthetic process | 0.000768 | 0.012814 |
| GO:0043393 | BP | regulation of protein binding | 0.000779 | 0.012974 |
| GO:0044380 | BP | protein localization to cytoskeleton | 0.000782 | 0.012991 |
| GO:0060688 | BP | regulation of morphogenesis of a branching structure | 0.000782 | 0.012991 |
| GO:0045807 | BP | positive regulation of endocytosis | 0.000792 | 0.013139 |
| GO:0034139 | BP | regulation of toll-like receptor 3 signaling pathway | 0.000802 | 0.013168 |
| GO:0034638 | BP | phosphatidylcholine catabolic process | 0.000802 | 0.013168 |
| GO:0006297 | BP | nucleotide-excision repair, DNA gap filling | 0.000802 | 0.013168 |
| GO:0099638 | BP | endosome to plasma membrane protein transport | 0.000802 | 0.013168 |
| GO:0048333 | BP | mesodermal cell differentiation | 0.000802 | 0.013168 |
| GO:0014909 | BP | smooth muscle cell migration | 0.000802 | 0.013168 |
| GO:0048732 | BP | gland development | 0.000806 | 0.013217 |
| GO:0034394 | BP | protein localization to cell surface | 0.000829 | 0.013577 |
| GO:0071495 | BP | cellular response to endogenous stimulus | 0.000831 | 0.013598 |
| GO:1903828 | BP | negative regulation of cellular protein localization | 0.000832 | 0.013598 |
| GO:0051495 | BP | positive regulation of cytoskeleton organization | 0.000843 | 0.013728 |
| GO:0035107 | BP | appendage morphogenesis | 0.000843 | 0.013728 |
| GO:0035108 | BP | limb morphogenesis | 0.000843 | 0.013728 |
| GO:0050792 | BP | regulation of viral process | 0.000844 | 0.013729 |
| GO:0010718 | BP | positive regulation of epithelial to mesenchymal transition | 0.000852 | 0.013817 |
| GO:0090181 | BP | regulation of cholesterol metabolic process | 0.000852 | 0.013817 |
| GO:0048762 | BP | mesenchymal cell differentiation | 0.000853 | 0.013817 |
| GO:0099518 | BP | vesicle cytoskeletal trafficking | 0.000863 | 0.013955 |
| GO:0046580 | BP | negative regulation of Ras protein signal transduction | 0.000872 | 0.014062 |
| GO:0042147 | BP | retrograde transport, endosome to Golgi | 0.000873 | 0.014062 |
| GO:0002366 | BP | leukocyte activation involved in immune response | 0.000885 | 0.014235 |
| GO:1901136 | BP | carbohydrate derivative catabolic process | 0.000909 | 0.01459 |
| GO:0051494 | BP | negative regulation of cytoskeleton organization | 0.000909 | 0.01459 |
| GO:0007223 | BP | Wnt signaling pathway, calcium modulating pathway | 0.00093 | 0.014861 |
| GO:1902993 | BP | positive regulation of amyloid precursor protein catabolic process | 0.00093 | 0.014861 |
| GO:0009221 | BP | pyrimidine deoxyribonucleotide biosynthetic process | 0.00093 | 0.014861 |
| GO:0051590 | BP | positive regulation of neurotransmitter transport | 0.00093 | 0.014861 |
| GO:0022038 | BP | corpus callosum development | 0.00093 | 0.014861 |
| GO:0007224 | BP | smoothened signaling pathway | 0.000936 | 0.014928 |
| GO:0045747 | BP | positive regulation of Notch signaling pathway | 0.000936 | 0.014928 |
| GO:0002263 | BP | cell activation involved in immune response | 0.000951 | 0.01512 |
| GO:0048511 | BP | rhythmic process | 0.00096 | 0.015218 |
| GO:0090100 | BP | positive regulation of transmembrane receptor protein serine/threonine kinase signaling pathway | 0.000976 | 0.015449 |
| GO:0030500 | BP | regulation of bone mineralization | 0.000978 | 0.01546 |
| GO:0034612 | BP | response to tumor necrosis factor | 0.000981 | 0.015484 |
| GO:0007033 | BP | vacuole organization | 0.000981 | 0.015484 |
| GO:0048638 | BP | regulation of developmental growth | 0.000989 | 0.01561 |
| GO:0006469 | BP | negative regulation of protein kinase activity | 0.000992 | 0.015647 |
| GO:0072395 | BP | signal transduction involved in cell cycle checkpoint | 0.001019 | 0.016015 |
| GO:0032648 | BP | regulation of interferon-beta production | 0.001019 | 0.016015 |
| GO:0006029 | BP | proteoglycan metabolic process | 0.001019 | 0.016015 |
| GO:0032963 | BP | collagen metabolic process | 0.001019 | 0.016015 |
| GO:0010948 | BP | negative regulation of cell cycle process | 0.00106 | 0.016587 |
| GO:0045069 | BP | regulation of viral genome replication | 0.001083 | 0.016869 |
| GO:0061387 | BP | regulation of extent of cell growth | 0.001083 | 0.016869 |
| GO:0044255 | BP | cellular lipid metabolic process | 0.001101 | 0.01714 |
| GO:0021987 | BP | cerebral cortex development | 0.001103 | 0.01714 |
| GO:0045824 | BP | negative regulation of innate immune response | 0.001103 | 0.01714 |
| GO:0045910 | BP | negative regulation of DNA recombination | 0.001128 | 0.017467 |
| GO:0002762 | BP | negative regulation of myeloid leukocyte differentiation | 0.001128 | 0.017467 |
| GO:0046627 | BP | negative regulation of insulin receptor signaling pathway | 0.00113 | 0.017467 |
| GO:0007254 | BP | JNK cascade | 0.00113 | 0.017467 |
| GO:0006998 | BP | nuclear envelope organization | 0.00113 | 0.017467 |
| GO:2000027 | BP | regulation of animal organ morphogenesis | 0.001141 | 0.017627 |
| GO:0002252 | BP | immune effector process | 0.001152 | 0.017784 |
| GO:0001667 | BP | ameboidal-type cell migration | 0.001165 | 0.017944 |
| GO:0044262 | BP | cellular carbohydrate metabolic process | 0.001165 | 0.017944 |
| GO:0009416 | BP | response to light stimulus | 0.00119 | 0.018306 |
| GO:0002695 | BP | negative regulation of leukocyte activation | 0.00122 | 0.01875 |
| GO:0002706 | BP | regulation of lymphocyte mediated immunity | 0.00122 | 0.01875 |
| GO:0000070 | BP | mitotic sister chromatid segregation | 0.001243 | 0.019048 |
| GO:0006298 | BP | mismatch repair | 0.001243 | 0.019048 |
| GO:0014812 | BP | muscle cell migration | 0.001243 | 0.019048 |
| GO:0030177 | BP | positive regulation of Wnt signaling pathway | 0.001263 | 0.019327 |
| GO:0061951 | BP | establishment of protein localization to plasma membrane | 0.001284 | 0.019616 |
| GO:0060070 | BP | canonical Wnt signaling pathway | 0.001299 | 0.019829 |
| GO:0002791 | BP | regulation of peptide secretion | 0.001305 | 0.019897 |
| GO:0061097 | BP | regulation of protein tyrosine kinase activity | 0.001321 | 0.020127 |
| GO:0023014 | BP | signal transduction by protein phosphorylation | 0.001334 | 0.020311 |
| GO:0010771 | BP | negative regulation of cell morphogenesis involved in differentiation | 0.001386 | 0.021051 |
| GO:0006517 | BP | protein deglycosylation | 0.001392 | 0.021075 |
| GO:0001935 | BP | endothelial cell proliferation | 0.001392 | 0.021075 |
| GO:0052126 | BP | movement in host environment | 0.001392 | 0.021075 |
| GO:0046718 | BP | viral entry into host cell | 0.001412 | 0.021264 |
| GO:0030220 | BP | platelet formation | 0.001412 | 0.021264 |
| GO:0035924 | BP | cellular response to vascular endothelial growth factor stimulus | 0.001412 | 0.021264 |
| GO:0001919 | BP | regulation of receptor recycling | 0.001412 | 0.021264 |
| GO:0060324 | BP | face development | 0.001412 | 0.021264 |
| GO:0048562 | BP | embryonic organ morphogenesis | 0.001432 | 0.021558 |
| GO:0007049 | BP | cell cycle | 0.001436 | 0.021602 |
| GO:2001021 | BP | negative regulation of response to DNA damage stimulus | 0.001454 | 0.021801 |
| GO:0097178 | BP | ruffle assembly | 0.001456 | 0.021801 |
| GO:0002294 | BP | CD4-positive, alpha-beta T cell differentiation involved in immune response | 0.001456 | 0.021801 |
| GO:0042093 | BP | T-helper cell differentiation | 0.001456 | 0.021801 |
| GO:0014066 | BP | regulation of phosphatidylinositol 3-kinase signaling | 0.001463 | 0.021887 |
| GO:0002011 | BP | morphogenesis of an epithelial sheet | 0.001499 | 0.022293 |
| GO:0031297 | BP | replication fork processing | 0.001499 | 0.022293 |
| GO:1902003 | BP | regulation of amyloid-beta formation | 0.001499 | 0.022293 |
| GO:1900120 | BP | regulation of receptor binding | 0.001499 | 0.022293 |
| GO:1901673 | BP | regulation of mitotic spindle assembly | 0.001499 | 0.022293 |
| GO:0002244 | BP | hematopoietic progenitor cell differentiation | 0.00151 | 0.022414 |
| GO:0051276 | BP | chromosome organization | 0.001512 | 0.022436 |
| GO:0030866 | BP | cortical actin cytoskeleton organization | 0.001594 | 0.023546 |
| GO:0033619 | BP | membrane protein proteolysis | 0.001594 | 0.023546 |
| GO:0061028 | BP | establishment of endothelial barrier | 0.001594 | 0.023546 |
| GO:0051961 | BP | negative regulation of nervous system development | 0.00162 | 0.023841 |
| GO:0009101 | BP | glycoprotein biosynthetic process | 0.001622 | 0.023841 |
| GO:0033046 | BP | negative regulation of sister chromatid segregation | 0.001622 | 0.023841 |
| GO:0051348 | BP | negative regulation of transferase activity | 0.001642 | 0.024099 |
| GO:1903052 | BP | positive regulation of proteolysis involved in cellular protein catabolic process | 0.001668 | 0.024463 |
| GO:0046503 | BP | glycerolipid catabolic process | 0.001681 | 0.02456 |
| GO:0045839 | BP | negative regulation of mitotic nuclear division | 0.001681 | 0.02456 |
| GO:0001837 | BP | epithelial to mesenchymal transition | 0.001681 | 0.02456 |
| GO:0032233 | BP | positive regulation of actin filament bundle assembly | 0.001681 | 0.02456 |
| GO:0050806 | BP | positive regulation of synaptic transmission | 0.001687 | 0.024628 |
| GO:0002889 | BP | regulation of immunoglobulin mediated immune response | 0.001719 | 0.024996 |
| GO:0002712 | BP | regulation of B cell mediated immunity | 0.001719 | 0.024996 |
| GO:0002064 | BP | epithelial cell development | 0.0018 | 0.0261 |
| GO:0045930 | BP | negative regulation of mitotic cell cycle | 0.001843 | 0.026701 |
| GO:1900087 | BP | positive regulation of G1/S transition of mitotic cell cycle | 0.001877 | 0.027155 |
| GO:0006066 | BP | alcohol metabolic process | 0.001915 | 0.027672 |
| GO:0030853 | BP | negative regulation of granulocyte differentiation | 0.001955 | 0.027958 |
| GO:0140289 | BP | protein mono-ADP-ribosylation | 0.001955 | 0.027958 |
| GO:0060100 | BP | positive regulation of phagocytosis, engulfment | 0.001955 | 0.027958 |
| GO:1905155 | BP | positive regulation of membrane invagination | 0.001955 | 0.027958 |
| GO:1903726 | BP | negative regulation of phospholipid metabolic process | 0.001955 | 0.027958 |
| GO:2001269 | BP | positive regulation of cysteine-type endopeptidase activity involved in apoptotic signaling pathway | 0.001955 | 0.027958 |
| GO:0043982 | BP | histone H4-K8 acetylation | 0.001955 | 0.027958 |
| GO:0017185 | BP | peptidyl-lysine hydroxylation | 0.001955 | 0.027958 |
| GO:0035457 | BP | cellular response to interferon-alpha | 0.001955 | 0.027958 |
| GO:0070213 | BP | protein auto-ADP-ribosylation | 0.001955 | 0.027958 |
| GO:0043981 | BP | histone H4-K5 acetylation | 0.001955 | 0.027958 |
| GO:0070940 | BP | dephosphorylation of RNA polymerase II C-terminal domain | 0.001955 | 0.027958 |
| GO:0090162 | BP | establishment of epithelial cell polarity | 0.001999 | 0.028474 |
| GO:0072413 | BP | signal transduction involved in mitotic cell cycle checkpoint | 0.001999 | 0.028474 |
| GO:1902402 | BP | signal transduction involved in mitotic DNA damage checkpoint | 0.001999 | 0.028474 |
| GO:1902403 | BP | signal transduction involved in mitotic DNA integrity checkpoint | 0.001999 | 0.028474 |
| GO:1905330 | BP | regulation of morphogenesis of an epithelium | 0.002014 | 0.028639 |
| GO:0007030 | BP | Golgi organization | 0.002014 | 0.028639 |
| GO:0061035 | BP | regulation of cartilage development | 0.002059 | 0.029201 |
| GO:0035721 | BP | intraciliary retrograde transport | 0.002074 | 0.029201 |
| GO:0050872 | BP | white fat cell differentiation | 0.002074 | 0.029201 |
| GO:0051145 | BP | smooth muscle cell differentiation | 0.002074 | 0.029201 |
| GO:0010457 | BP | centriole-centriole cohesion | 0.002074 | 0.029201 |
| GO:0045628 | BP | regulation of T-helper 2 cell differentiation | 0.002074 | 0.029201 |
| GO:0090239 | BP | regulation of histone H4 acetylation | 0.002074 | 0.029201 |
| GO:1905820 | BP | positive regulation of chromosome separation | 0.002074 | 0.029201 |
| GO:0031056 | BP | regulation of histone modification | 0.002075 | 0.029201 |
| GO:0036297 | BP | interstrand cross-link repair | 0.002094 | 0.029405 |
| GO:0043966 | BP | histone H3 acetylation | 0.002094 | 0.029405 |
| GO:0034332 | BP | adherens junction organization | 0.002101 | 0.029444 |
| GO:2000756 | BP | regulation of peptidyl-lysine acetylation | 0.002101 | 0.029444 |
| GO:0048592 | BP | eye morphogenesis | 0.002101 | 0.029444 |
| GO:0051603 | BP | proteolysis involved in cellular protein catabolic process | 0.002136 | 0.029883 |
| GO:0048284 | BP | organelle fusion | 0.002153 | 0.030077 |
| GO:0033363 | BP | secretory granule organization | 0.002168 | 0.030271 |
| GO:0032200 | BP | telomere organization | 0.002198 | 0.030602 |
| GO:0010508 | BP | positive regulation of autophagy | 0.002202 | 0.030635 |
| GO:1903725 | BP | regulation of phospholipid metabolic process | 0.002273 | 0.031588 |
| GO:0009262 | BP | deoxyribonucleotide metabolic process | 0.002308 | 0.031981 |
| GO:0006040 | BP | amino sugar metabolic process | 0.002308 | 0.031981 |
| GO:0001961 | BP | positive regulation of cytokine-mediated signaling pathway | 0.002308 | 0.031981 |
| GO:0045191 | BP | regulation of isotype switching | 0.002308 | 0.031981 |
| GO:0051235 | BP | maintenance of location | 0.002363 | 0.032701 |
| GO:0030307 | BP | positive regulation of cell growth | 0.002383 | 0.032945 |
| GO:0007009 | BP | plasma membrane organization | 0.002459 | 0.033956 |
| GO:0045599 | BP | negative regulation of fat cell differentiation | 0.002467 | 0.033969 |
| GO:0035065 | BP | regulation of histone acetylation | 0.002467 | 0.033969 |
| GO:0007156 | BP | homophilic cell adhesion via plasma membrane adhesion molecules | 0.002482 | 0.034158 |
| GO:0060828 | BP | regulation of canonical Wnt signaling pathway | 0.002496 | 0.034317 |
| GO:1905475 | BP | regulation of protein localization to membrane | 0.002527 | 0.034673 |
| GO:0032692 | BP | negative regulation of interleukin-1 production | 0.002561 | 0.034897 |
| GO:0050858 | BP | negative regulation of antigen receptor-mediated signaling pathway | 0.002561 | 0.034897 |
| GO:1904030 | BP | negative regulation of cyclin-dependent protein kinase activity | 0.002561 | 0.034897 |
| GO:0048488 | BP | synaptic vesicle endocytosis | 0.002561 | 0.034897 |
| GO:0140238 | BP | presynaptic endocytosis | 0.002561 | 0.034897 |
| GO:0045601 | BP | regulation of endothelial cell differentiation | 0.002561 | 0.034897 |
| GO:0045736 | BP | negative regulation of cyclin-dependent protein serine/threonine kinase activity | 0.002561 | 0.034897 |
| GO:0002691 | BP | regulation of cellular extravasation | 0.002561 | 0.034897 |
| GO:0007159 | BP | leukocyte cell-cell adhesion | 0.002571 | 0.034945 |
| GO:0050766 | BP | positive regulation of phagocytosis | 0.002571 | 0.034945 |
| GO:0048565 | BP | digestive tract development | 0.002571 | 0.034945 |
| GO:0043550 | BP | regulation of lipid kinase activity | 0.002571 | 0.034945 |
| GO:0000278 | BP | mitotic cell cycle | 0.002575 | 0.034971 |
| GO:0031023 | BP | microtubule organizing center organization | 0.002579 | 0.035003 |
| GO:1902808 | BP | positive regulation of cell cycle G1/S phase transition | 0.002633 | 0.035635 |
| GO:0048146 | BP | positive regulation of fibroblast proliferation | 0.002633 | 0.035635 |
| GO:1905515 | BP | non-motile cilium assembly | 0.002633 | 0.035635 |
| GO:0045185 | BP | maintenance of protein location | 0.002638 | 0.035681 |
| GO:0072384 | BP | organelle transport along microtubule | 0.002678 | 0.036148 |
| GO:0048771 | BP | tissue remodeling | 0.002678 | 0.036148 |
| GO:1902991 | BP | regulation of amyloid precursor protein catabolic process | 0.002701 | 0.036414 |
| GO:0030316 | BP | osteoclast differentiation | 0.002701 | 0.036414 |
| GO:0071359 | BP | cellular response to dsRNA | 0.002714 | 0.036429 |
| GO:1901071 | BP | glucosamine-containing compound metabolic process | 0.002714 | 0.036429 |
| GO:0099563 | BP | modification of synaptic structure | 0.002724 | 0.036429 |
| GO:0007099 | BP | centriole replication | 0.002724 | 0.036429 |
| GO:0009265 | BP | 2'-deoxyribonucleotide biosynthetic process | 0.002724 | 0.036429 |
| GO:0002053 | BP | positive regulation of mesenchymal cell proliferation | 0.002724 | 0.036429 |
| GO:0046385 | BP | deoxyribose phosphate biosynthetic process | 0.002724 | 0.036429 |
| GO:0048745 | BP | smooth muscle tissue development | 0.002724 | 0.036429 |
| GO:0060216 | BP | definitive hemopoiesis | 0.002724 | 0.036429 |
| GO:0061647 | BP | histone H3-K9 modification | 0.002724 | 0.036429 |
| GO:1903975 | BP | regulation of glial cell migration | 0.002724 | 0.036429 |
| GO:0048854 | BP | brain morphogenesis | 0.002724 | 0.036429 |
| GO:0045017 | BP | glycerolipid biosynthetic process | 0.002831 | 0.037736 |
| GO:1901653 | BP | cellular response to peptide | 0.002831 | 0.037736 |
| GO:0022407 | BP | regulation of cell-cell adhesion | 0.002836 | 0.03777 |
| GO:0048754 | BP | branching morphogenesis of an epithelial tube | 0.00285 | 0.037834 |
| GO:0099560 | BP | synaptic membrane adhesion | 0.002854 | 0.037834 |
| GO:0035909 | BP | aorta morphogenesis | 0.002854 | 0.037834 |
| GO:0006312 | BP | mitotic recombination | 0.002854 | 0.037834 |
| GO:0032331 | BP | negative regulation of chondrocyte differentiation | 0.002854 | 0.037834 |
| GO:0008589 | BP | regulation of smoothened signaling pathway | 0.00286 | 0.037884 |
| GO:0003002 | BP | regionalization | 0.002886 | 0.038187 |
| GO:0003013 | BP | circulatory system process | 0.002945 | 0.038887 |
| GO:0051668 | BP | localization within membrane | 0.002969 | 0.039173 |
| GO:2000060 | BP | positive regulation of ubiquitin-dependent protein catabolic process | 0.003016 | 0.039743 |
| GO:0022408 | BP | negative regulation of cell-cell adhesion | 0.003061 | 0.040309 |
| GO:0001764 | BP | neuron migration | 0.003085 | 0.040594 |
| GO:1900744 | BP | regulation of p38MAPK cascade | 0.003103 | 0.040695 |
| GO:0043300 | BP | regulation of leukocyte degranulation | 0.003103 | 0.040695 |
| GO:0001885 | BP | endothelial cell development | 0.003103 | 0.040695 |
| GO:0018196 | BP | peptidyl-asparagine modification | 0.003103 | 0.040695 |
| GO:0032330 | BP | regulation of chondrocyte differentiation | 0.003103 | 0.040695 |
| GO:1903305 | BP | regulation of regulated secretory pathway | 0.003113 | 0.040802 |
| GO:1902749 | BP | regulation of cell cycle G2/M phase transition | 0.003116 | 0.040817 |
| GO:0072665 | BP | protein localization to vacuole | 0.003146 | 0.041076 |
| GO:0061351 | BP | neural precursor cell proliferation | 0.003146 | 0.041076 |
| GO:0007626 | BP | locomotory behavior | 0.003161 | 0.041251 |
| GO:0062197 | BP | cellular response to chemical stress | 0.003171 | 0.0413 |
| GO:0051985 | BP | negative regulation of chromosome segregation | 0.003232 | 0.042056 |
| GO:1901800 | BP | positive regulation of proteasomal protein catabolic process | 0.003278 | 0.042629 |
| GO:1903320 | BP | regulation of protein modification by small protein conjugation or removal | 0.003284 | 0.042682 |
| GO:0006487 | BP | protein N-linked glycosylation | 0.003286 | 0.042684 |
| GO:0032436 | BP | positive regulation of proteasomal ubiquitin-dependent protein catabolic process | 0.003336 | 0.043298 |
| GO:0044057 | BP | regulation of system process | 0.003357 | 0.043539 |
| GO:0090263 | BP | positive regulation of canonical Wnt signaling pathway | 0.003378 | 0.04371 |
| GO:0033157 | BP | regulation of intracellular protein transport | 0.003383 | 0.04374 |
| GO:0071496 | BP | cellular response to external stimulus | 0.003443 | 0.044456 |
| GO:0070936 | BP | protein K48-linked ubiquitination | 0.003561 | 0.045859 |
| GO:2000401 | BP | regulation of lymphocyte migration | 0.003582 | 0.046074 |
| GO:0034121 | BP | regulation of toll-like receptor signaling pathway | 0.003582 | 0.046074 |
| GO:0007059 | BP | chromosome segregation | 0.00359 | 0.04609 |
| GO:0034614 | BP | cellular response to reactive oxygen species | 0.00361 | 0.046196 |
| GO:0030832 | BP | regulation of actin filament length | 0.00361 | 0.046196 |
| GO:0008064 | BP | regulation of actin polymerization or depolymerization | 0.00361 | 0.046196 |
| GO:0007611 | BP | learning or memory | 0.00361 | 0.046196 |
| GO:0031060 | BP | regulation of histone methylation | 0.003669 | 0.046924 |
| GO:2001032 | BP | regulation of double-strand break repair via nonhomologous end joining | 0.003742 | 0.04776 |
| GO:0007080 | BP | mitotic metaphase plate congression | 0.003742 | 0.04776 |
| GO:0000731 | BP | DNA synthesis involved in DNA repair | 0.003742 | 0.04776 |
| GO:1903708 | BP | positive regulation of hemopoiesis | 0.003778 | 0.048189 |
| GO:1901799 | BP | negative regulation of proteasomal protein catabolic process | 0.003885 | 0.049393 |
| GO:0090307 | BP | mitotic spindle assembly | 0.003941 | 0.049788 |
| GO:0048839 | BP | inner ear development | 0.003941 | 0.049788 |
| GO:0003206 | BP | cardiac chamber morphogenesis | 0.003941 | 0.049788 |
| GO:0005775 | CC | vacuolar lumen | 9.44E-07 | 0.000304 |
| GO:0099023 | CC | vesicle tethering complex | 1.56E-06 | 0.000304 |
| GO:0098791 | CC | Golgi apparatus subcompartment | 2.30E-06 | 0.000304 |
| GO:0031984 | CC | organelle subcompartment | 2.31E-06 | 0.000304 |
| GO:0005814 | CC | centriole | 2.41E-06 | 0.000304 |
| GO:0005774 | CC | vacuolar membrane | 2.50E-06 | 0.000304 |
| GO:0010008 | CC | endosome membrane | 2.54E-06 | 0.000304 |
| GO:0005876 | CC | spindle microtubule | 2.63E-06 | 0.000304 |
| GO:0055037 | CC | recycling endosome | 2.63E-06 | 0.000304 |
| GO:0045177 | CC | apical part of cell | 2.66E-06 | 0.000304 |
| GO:0005769 | CC | early endosome | 2.88E-06 | 0.000304 |
| GO:0005773 | CC | vacuole | 3.05E-06 | 0.000304 |
| GO:0005765 | CC | lysosomal membrane | 3.20E-06 | 0.000304 |
| GO:0098852 | CC | lytic vacuole membrane | 3.20E-06 | 0.000304 |
| GO:0000323 | CC | lytic vacuole | 3.28E-06 | 0.000304 |
| GO:0005764 | CC | lysosome | 3.28E-06 | 0.000304 |
| GO:0012506 | CC | vesicle membrane | 3.57E-06 | 0.000304 |
| GO:0036064 | CC | ciliary basal body | 3.61E-06 | 0.000304 |
| GO:0030659 | CC | cytoplasmic vesicle membrane | 3.71E-06 | 0.000304 |
| GO:0005911 | CC | cell-cell junction | 3.92E-06 | 0.000304 |
| GO:0000139 | CC | Golgi membrane | 3.98E-06 | 0.000304 |
| GO:0005901 | CC | caveola | 4.35E-06 | 0.000304 |
| GO:0016607 | CC | nuclear speck | 4.47E-06 | 0.000304 |
| GO:0043202 | CC | lysosomal lumen | 4.53E-06 | 0.000304 |
| GO:0005813 | CC | centrosome | 4.60E-06 | 0.000304 |
| GO:0048471 | CC | perinuclear region of cytoplasm | 4.63E-06 | 0.000304 |
| GO:0070161 | CC | anchoring junction | 4.65E-06 | 0.000304 |
| GO:0005768 | CC | endosome | 4.71E-06 | 0.000304 |
| GO:0098805 | CC | whole membrane | 4.94E-06 | 0.000304 |
| GO:0005815 | CC | microtubule organizing center | 5.22E-06 | 0.000304 |
| GO:0005802 | CC | trans-Golgi network | 5.30E-06 | 0.000304 |
| GO:1990234 | CC | transferase complex | 5.71E-06 | 0.000305 |
| GO:1904949 | CC | ATPase complex | 5.76E-06 | 0.000305 |
| GO:0000922 | CC | spindle pole | 5.83E-06 | 0.000305 |
| GO:0005856 | CC | cytoskeleton | 5.90E-06 | 0.000305 |
| GO:0098590 | CC | plasma membrane region | 5.96E-06 | 0.000305 |
| GO:0005794 | CC | Golgi apparatus | 6.15E-06 | 0.000305 |
| GO:0120025 | CC | plasma membrane bounded cell projection | 6.41E-06 | 0.000305 |
| GO:0016604 | CC | nuclear body | 6.50E-06 | 0.000305 |
| GO:0005667 | CC | transcription regulator complex | 6.57E-06 | 0.000305 |
| GO:0042995 | CC | cell projection | 6.83E-06 | 0.000307 |
| GO:0031410 | CC | cytoplasmic vesicle | 6.91E-06 | 0.000307 |
| GO:0097708 | CC | intracellular vesicle | 7.24E-06 | 0.000307 |
| GO:0005783 | CC | endoplasmic reticulum | 7.32E-06 | 0.000307 |
| GO:0098588 | CC | bounding membrane of organelle | 7.79E-06 | 0.000308 |
| GO:0030054 | CC | cell junction | 7.93E-06 | 0.000308 |
| GO:0031012 | CC | extracellular matrix | 8.16E-06 | 0.00031 |
| GO:0031982 | CC | vesicle | 8.32E-06 | 0.00031 |
| GO:0031090 | CC | organelle membrane | 9.81E-06 | 0.000336 |
| GO:0015629 | CC | actin cytoskeleton | 9.98E-06 | 0.000338 |
| GO:0005654 | CC | nucleoplasm | 1.03E-05 | 0.000341 |
| GO:0005874 | CC | microtubule | 1.05E-05 | 0.000346 |
| GO:0062023 | CC | collagen-containing extracellular matrix | 1.07E-05 | 0.000348 |
| GO:0070603 | CC | SWI/SNF superfamily-type complex | 1.15E-05 | 0.000362 |
| GO:0005829 | CC | cytosol | 1.24E-05 | 0.000378 |
| GO:0043231 | CC | intracellular membrane-bounded organelle | 1.53E-05 | 0.000445 |
| GO:0043227 | CC | membrane-bounded organelle | 1.56E-05 | 0.000449 |
| GO:0043226 | CC | organelle | 1.62E-05 | 0.000463 |
| GO:0043229 | CC | intracellular organelle | 1.64E-05 | 0.000467 |
| GO:0000145 | CC | exocyst | 1.69E-05 | 0.000478 |
| GO:0005634 | CC | nucleus | 1.99E-05 | 0.000553 |
| GO:0031901 | CC | early endosome membrane | 2.23E-05 | 0.000611 |
| GO:0005925 | CC | focal adhesion | 2.38E-05 | 0.000647 |
| GO:0030496 | CC | midbody | 2.40E-05 | 0.00065 |
| GO:0001726 | CC | ruffle | 2.78E-05 | 0.000746 |
| GO:0034451 | CC | centriolar satellite | 2.83E-05 | 0.000756 |
| GO:0030136 | CC | clathrin-coated vesicle | 3.18E-05 | 0.000842 |
| GO:0044853 | CC | plasma membrane raft | 3.51E-05 | 0.000921 |
| GO:0030055 | CC | cell-substrate junction | 3.59E-05 | 0.000942 |
| GO:0030027 | CC | lamellipodium | 3.72E-05 | 0.000964 |
| GO:0000151 | CC | ubiquitin ligase complex | 4.40E-05 | 0.001124 |
| GO:0005789 | CC | endoplasmic reticulum membrane | 5.09E-05 | 0.001263 |
| GO:0000785 | CC | chromatin | 6.03E-05 | 0.001467 |
| GO:0030135 | CC | coated vesicle | 8.01E-05 | 0.001881 |
| GO:0019898 | CC | extrinsic component of membrane | 0.000107 | 0.002422 |
| GO:0000776 | CC | kinetochore | 0.000124 | 0.002735 |
| GO:0035097 | CC | histone methyltransferase complex | 0.000147 | 0.00317 |
| GO:0031902 | CC | late endosome membrane | 0.000177 | 0.003718 |
| GO:0033116 | CC | endoplasmic reticulum-Golgi intermediate compartment membrane | 0.0002 | 0.004096 |
| GO:0005770 | CC | late endosome | 0.000226 | 0.00456 |
| GO:0005819 | CC | spindle | 0.00023 | 0.004626 |
| GO:0035869 | CC | ciliary transition zone | 0.000249 | 0.004919 |
| GO:0005604 | CC | basement membrane | 0.000272 | 0.005336 |
| GO:0061695 | CC | transferase complex, transferring phosphorus-containing groups | 0.000277 | 0.005427 |
| GO:1902494 | CC | catalytic complex | 0.000282 | 0.005513 |
| GO:0005938 | CC | cell cortex | 0.000326 | 0.006236 |
| GO:0044232 | CC | organelle membrane contact site | 0.000353 | 0.006681 |
| GO:0005912 | CC | adherens junction | 0.000364 | 0.006804 |
| GO:0042641 | CC | actomyosin | 0.000399 | 0.007348 |
| GO:0030139 | CC | endocytic vesicle | 0.000409 | 0.007479 |
| GO:0042555 | CC | MCM complex | 0.000414 | 0.007548 |
| GO:0030662 | CC | coated vesicle membrane | 0.000437 | 0.007881 |
| GO:0005694 | CC | chromosome | 0.000442 | 0.007939 |
| GO:0031227 | CC | intrinsic component of endoplasmic reticulum membrane | 0.000455 | 0.008141 |
| GO:0005793 | CC | endoplasmic reticulum-Golgi intermediate compartment | 0.000501 | 0.008801 |
| GO:0005929 | CC | cilium | 0.000544 | 0.009508 |
| GO:0072686 | CC | mitotic spindle | 0.000553 | 0.009659 |
| GO:0000123 | CC | histone acetyltransferase complex | 0.000568 | 0.009875 |
| GO:0098978 | CC | glutamatergic synapse | 0.000584 | 0.01014 |
| GO:0098589 | CC | membrane region | 0.000681 | 0.011695 |
| GO:0005581 | CC | collagen trimer | 0.000688 | 0.011777 |
| GO:0030119 | CC | AP-type membrane coat adaptor complex | 0.000782 | 0.012991 |
| GO:0017053 | CC | transcription repressor complex | 0.000792 | 0.013139 |
| GO:0140268 | CC | endoplasmic reticulum-plasma membrane contact site | 0.000802 | 0.013168 |
| GO:0005788 | CC | endoplasmic reticulum lumen | 0.000837 | 0.013653 |
| GO:0098857 | CC | membrane microdomain | 0.000849 | 0.013796 |
| GO:0098687 | CC | chromosomal region | 0.000858 | 0.013892 |
| GO:0032432 | CC | actin filament bundle | 0.000871 | 0.014057 |
| GO:0035578 | CC | azurophil granule lumen | 0.000956 | 0.015161 |
| GO:0043235 | CC | receptor complex | 0.001026 | 0.0161 |
| GO:0045121 | CC | membrane raft | 0.001051 | 0.016466 |
| GO:0044295 | CC | axonal growth cone | 0.00113 | 0.017467 |
| GO:0016514 | CC | SWI/SNF complex | 0.001392 | 0.021075 |
| GO:0030427 | CC | site of polarized growth | 0.001509 | 0.022414 |
| GO:0031965 | CC | nuclear membrane | 0.001611 | 0.023785 |
| GO:0097517 | CC | contractile actin filament bundle | 0.001622 | 0.023841 |
| GO:0001725 | CC | stress fiber | 0.001622 | 0.023841 |
| GO:0030120 | CC | vesicle coat | 0.001681 | 0.02456 |
| GO:0005798 | CC | Golgi-associated vesicle | 0.001691 | 0.024649 |
| GO:0051233 | CC | spindle midzone | 0.001877 | 0.027155 |
| GO:0031010 | CC | ISWI-type complex | 0.002074 | 0.029201 |
| GO:0030176 | CC | integral component of endoplasmic reticulum membrane | 0.002198 | 0.030602 |
| GO:0005875 | CC | microtubule associated complex | 0.002348 | 0.032509 |
| GO:0005671 | CC | Ada2/Gcn5/Ada3 transcription activator complex | 0.002854 | 0.037834 |
| GO:0000242 | CC | pericentriolar material | 0.002854 | 0.037834 |
| GO:0031461 | CC | cullin-RING ubiquitin ligase complex | 0.003004 | 0.039611 |
| GO:0070160 | CC | tight junction | 0.003378 | 0.04371 |
| GO:0005923 | CC | bicellular tight junction | 0.003506 | 0.045218 |
| GO:0012505 | CC | endomembrane system | 0.003542 | 0.045649 |
| GO:0034708 | CC | methyltransferase complex | 0.00359 | 0.04609 |
| GO:0005635 | CC | nuclear envelope | 0.00359 | 0.04609 |
| GO:0009986 | CC | cell surface | 0.0036 | 0.046185 |
| GO:0030424 | CC | axon | 0.003862 | 0.049167 |
| GO:0005801 | CC | cis-Golgi network | 0.003941 | 0.049788 |
| GO:0005088 | MF | Ras guanyl-nucleotide exchange factor activity | 1.37E-06 | 0.000304 |
| GO:0017137 | MF | Rab GTPase binding | 1.94E-06 | 0.000304 |
| GO:0005518 | MF | collagen binding | 2.12E-06 | 0.000304 |
| GO:0035091 | MF | phosphatidylinositol binding | 2.22E-06 | 0.000304 |
| GO:0030674 | MF | protein-macromolecule adaptor activity | 2.49E-06 | 0.000304 |
| GO:0061659 | MF | ubiquitin-like protein ligase activity | 2.62E-06 | 0.000304 |
| GO:0030695 | MF | GTPase regulator activity | 2.63E-06 | 0.000304 |
| GO:0042826 | MF | histone deacetylase binding | 2.72E-06 | 0.000304 |
| GO:0031267 | MF | small GTPase binding | 3.00E-06 | 0.000304 |
| GO:0061630 | MF | ubiquitin protein ligase activity | 3.02E-06 | 0.000304 |
| GO:0060090 | MF | molecular adaptor activity | 3.04E-06 | 0.000304 |
| GO:0005543 | MF | phospholipid binding | 3.10E-06 | 0.000304 |
| GO:0017016 | MF | Ras GTPase binding | 3.14E-06 | 0.000304 |
| GO:0019787 | MF | ubiquitin-like protein transferase activity | 3.29E-06 | 0.000304 |
| GO:0005096 | MF | GTPase activator activity | 3.40E-06 | 0.000304 |
| GO:0017048 | MF | Rho GTPase binding | 3.57E-06 | 0.000304 |
| GO:0060589 | MF | nucleoside-triphosphatase regulator activity | 3.58E-06 | 0.000304 |
| GO:0004709 | MF | MAP kinase kinase kinase activity | 3.81E-06 | 0.000304 |
| GO:0003779 | MF | actin binding | 3.90E-06 | 0.000304 |
| GO:0051020 | MF | GTPase binding | 4.10E-06 | 0.000304 |
| GO:0004842 | MF | ubiquitin-protein transferase activity | 4.20E-06 | 0.000304 |
| GO:0008047 | MF | enzyme activator activity | 4.35E-06 | 0.000304 |
| GO:0030276 | MF | clathrin binding | 4.53E-06 | 0.000304 |
| GO:0003712 | MF | transcription coregulator activity | 4.59E-06 | 0.000304 |
| GO:0004674 | MF | protein serine/threonine kinase activity | 4.66E-06 | 0.000304 |
| GO:0019904 | MF | protein domain specific binding | 4.79E-06 | 0.000304 |
| GO:0004713 | MF | protein tyrosine kinase activity | 4.96E-06 | 0.000304 |
| GO:0019900 | MF | kinase binding | 5.00E-06 | 0.000304 |
| GO:0019901 | MF | protein kinase binding | 5.06E-06 | 0.000304 |
| GO:0016773 | MF | phosphotransferase activity, alcohol group as acceptor | 5.11E-06 | 0.000304 |
| GO:0000976 | MF | transcription regulatory region sequence-specific DNA binding | 5.11E-06 | 0.000304 |
| GO:0008289 | MF | lipid binding | 5.27E-06 | 0.000304 |
| GO:0000978 | MF | RNA polymerase II cis-regulatory region sequence-specific DNA binding | 5.49E-06 | 0.000305 |
| GO:0005509 | MF | calcium ion binding | 5.50E-06 | 0.000305 |
| GO:0000977 | MF | RNA polymerase II transcription regulatory region sequence-specific DNA binding | 5.54E-06 | 0.000305 |
| GO:0003713 | MF | transcription coactivator activity | 5.73E-06 | 0.000305 |
| GO:0004672 | MF | protein kinase activity | 5.88E-06 | 0.000305 |
| GO:0001067 | MF | regulatory region nucleic acid binding | 6.16E-06 | 0.000305 |
| GO:0008092 | MF | cytoskeletal protein binding | 6.24E-06 | 0.000305 |
| GO:0003690 | MF | double-stranded DNA binding | 6.34E-06 | 0.000305 |
| GO:0052866 | MF | phosphatidylinositol phosphate phosphatase activity | 6.49E-06 | 0.000305 |
| GO:1990837 | MF | sequence-specific double-stranded DNA binding | 6.53E-06 | 0.000305 |
| GO:0000987 | MF | cis-regulatory region sequence-specific DNA binding | 6.60E-06 | 0.000305 |
| GO:0043565 | MF | sequence-specific DNA binding | 6.66E-06 | 0.000306 |
| GO:0016301 | MF | kinase activity | 6.72E-06 | 0.000306 |
| GO:0016772 | MF | transferase activity, transferring phosphorus-containing groups | 7.45E-06 | 0.000307 |
| GO:0015631 | MF | tubulin binding | 7.67E-06 | 0.000308 |
| GO:0003700 | MF | DNA-binding transcription factor activity | 7.68E-06 | 0.000308 |
| GO:0042802 | MF | identical protein binding | 7.84E-06 | 0.000308 |
| GO:0140110 | MF | transcription regulator activity | 7.87E-06 | 0.000308 |
| GO:0005524 | MF | ATP binding | 7.96E-06 | 0.000308 |
| GO:0042803 | MF | protein homodimerization activity | 8.20E-06 | 0.00031 |
| GO:0030554 | MF | adenyl nucleotide binding | 8.92E-06 | 0.000323 |
| GO:0019899 | MF | enzyme binding | 9.04E-06 | 0.000326 |
| GO:0032559 | MF | adenyl ribonucleotide binding | 9.23E-06 | 0.00033 |
| GO:0003677 | MF | DNA binding | 9.64E-06 | 0.000334 |
| GO:0140096 | MF | catalytic activity, acting on a protein | 9.71E-06 | 0.000334 |
| GO:0035639 | MF | purine ribonucleoside triphosphate binding | 9.87E-06 | 0.000337 |
| GO:0140297 | MF | DNA-binding transcription factor binding | 1.01E-05 | 0.000338 |
| GO:0097367 | MF | carbohydrate derivative binding | 1.01E-05 | 0.000338 |
| GO:0032553 | MF | ribonucleotide binding | 1.03E-05 | 0.000342 |
| GO:0017124 | MF | SH3 domain binding | 1.06E-05 | 0.000347 |
| GO:0016740 | MF | transferase activity | 1.08E-05 | 0.000349 |
| GO:0032555 | MF | purine ribonucleotide binding | 1.10E-05 | 0.000353 |
| GO:0140030 | MF | modification-dependent protein binding | 1.10E-05 | 0.000354 |
| GO:0000981 | MF | DNA-binding transcription factor activity, RNA polymerase II-specific | 1.11E-05 | 0.000354 |
| GO:0043168 | MF | anion binding | 1.13E-05 | 0.000359 |
| GO:0046872 | MF | metal ion binding | 1.14E-05 | 0.00036 |
| GO:0017076 | MF | purine nucleotide binding | 1.17E-05 | 0.000364 |
| GO:0044877 | MF | protein-containing complex binding | 1.21E-05 | 0.000375 |
| GO:0043169 | MF | cation binding | 1.23E-05 | 0.000378 |
| GO:0043167 | MF | ion binding | 1.45E-05 | 0.000428 |
| GO:0008134 | MF | transcription factor binding | 1.45E-05 | 0.000428 |
| GO:0003824 | MF | catalytic activity | 1.48E-05 | 0.000433 |
| GO:0005515 | MF | protein binding | 1.52E-05 | 0.000444 |
| GO:0005488 | MF | binding | 1.62E-05 | 0.000463 |
| GO:0030159 | MF | signaling receptor complex adaptor activity | 1.69E-05 | 0.000478 |
| GO:0042393 | MF | histone binding | 1.93E-05 | 0.000539 |
| GO:0005085 | MF | guanyl-nucleotide exchange factor activity | 2.26E-05 | 0.000619 |
| GO:0035591 | MF | signaling adaptor activity | 2.92E-05 | 0.000777 |
| GO:0000149 | MF | SNARE binding | 3.43E-05 | 0.000901 |
| GO:0034593 | MF | phosphatidylinositol bisphosphate phosphatase activity | 3.70E-05 | 0.000963 |
| GO:0036094 | MF | small molecule binding | 4.28E-05 | 0.001098 |
| GO:0003714 | MF | transcription corepressor activity | 4.54E-05 | 0.001156 |
| GO:0005178 | MF | integrin binding | 4.64E-05 | 0.001176 |
| GO:0019838 | MF | growth factor binding | 4.72E-05 | 0.001189 |
| GO:0000166 | MF | nucleotide binding | 5.93E-05 | 0.001452 |
| GO:0017112 | MF | Rab guanyl-nucleotide exchange factor activity | 6.01E-05 | 0.001465 |
| GO:1901265 | MF | nucleoside phosphate binding | 6.50E-05 | 0.001568 |
| GO:0019902 | MF | phosphatase binding | 7.08E-05 | 0.001689 |
| GO:1901981 | MF | phosphatidylinositol phosphate binding | 7.12E-05 | 0.001693 |
| GO:0043621 | MF | protein self-association | 0.000103 | 0.002345 |
| GO:0015923 | MF | mannosidase activity | 0.00012 | 0.002675 |
| GO:0140312 | MF | cargo adaptor activity | 0.000135 | 0.002944 |
| GO:0008017 | MF | microtubule binding | 0.00014 | 0.003035 |
| GO:0005544 | MF | calcium-dependent phospholipid binding | 0.000142 | 0.003071 |
| GO:0018024 | MF | histone-lysine N-methyltransferase activity | 0.000167 | 0.003565 |
| GO:0140457 | MF | protein demethylase activity | 0.000175 | 0.003681 |
| GO:0032452 | MF | histone demethylase activity | 0.000175 | 0.003681 |
| GO:0001216 | MF | DNA-binding transcription activator activity | 0.000181 | 0.003784 |
| GO:0070530 | MF | K63-linked polyubiquitin modification-dependent protein binding | 0.000188 | 0.003883 |
| GO:0017160 | MF | Ral GTPase binding | 0.000188 | 0.003883 |
| GO:1990782 | MF | protein tyrosine kinase binding | 0.000195 | 0.004018 |
| GO:0019199 | MF | transmembrane receptor protein kinase activity | 0.000195 | 0.004018 |
| GO:0016811 | MF | hydrolase activity, acting on carbon-nitrogen (but not peptide) bonds, in linear amides | 0.000237 | 0.004746 |
| GO:0004559 | MF | alpha-mannosidase activity | 0.000238 | 0.004756 |
| GO:0017147 | MF | Wnt-protein binding | 0.000281 | 0.0055 |
| GO:0042578 | MF | phosphoric ester hydrolase activity | 0.000298 | 0.005791 |
| GO:0003682 | MF | chromatin binding | 0.0003 | 0.005813 |
| GO:0001228 | MF | DNA-binding transcription activator activity, RNA polymerase II-specific | 0.000325 | 0.006232 |
| GO:0042285 | MF | xylosyltransferase activity | 0.000329 | 0.006257 |
| GO:0035252 | MF | UDP-xylosyltransferase activity | 0.000329 | 0.006257 |
| GO:0005102 | MF | signaling receptor binding | 0.00034 | 0.006452 |
| GO:0019905 | MF | syntaxin binding | 0.000363 | 0.006794 |
| GO:0043178 | MF | alcohol binding | 0.000437 | 0.007881 |
| GO:0016798 | MF | hydrolase activity, acting on glycosyl bonds | 0.000524 | 0.009166 |
| GO:0051213 | MF | dioxygenase activity | 0.000557 | 0.009705 |
| GO:0033558 | MF | protein deacetylase activity | 0.000634 | 0.010935 |
| GO:0004407 | MF | histone deacetylase activity | 0.000751 | 0.012652 |
| GO:0052744 | MF | phosphatidylinositol monophosphate phosphatase activity | 0.000802 | 0.013168 |
| GO:0035615 | MF | clathrin adaptor activity | 0.000802 | 0.013168 |
| GO:0005068 | MF | transmembrane receptor protein tyrosine kinase adaptor activity | 0.000802 | 0.013168 |
| GO:0030165 | MF | PDZ domain binding | 0.000853 | 0.013817 |
| GO:0048365 | MF | Rac GTPase binding | 0.000871 | 0.014057 |
| GO:0019213 | MF | deacetylase activity | 0.000871 | 0.014057 |
| GO:0005161 | MF | platelet-derived growth factor receptor binding | 0.00093 | 0.014861 |
| GO:0032266 | MF | phosphatidylinositol-3-phosphate binding | 0.000936 | 0.014928 |
| GO:0061629 | MF | RNA polymerase II-specific DNA-binding transcription factor binding | 0.000938 | 0.014933 |
| GO:0030291 | MF | protein serine/threonine kinase inhibitor activity | 0.000956 | 0.015161 |
| GO:0030971 | MF | receptor tyrosine kinase binding | 0.001026 | 0.0161 |
| GO:0008234 | MF | cysteine-type peptidase activity | 0.001071 | 0.016712 |
| GO:0019903 | MF | protein phosphatase binding | 0.001165 | 0.017944 |
| GO:0030234 | MF | enzyme regulator activity | 0.001265 | 0.019345 |
| GO:0016278 | MF | lysine N-methyltransferase activity | 0.001386 | 0.021051 |
| GO:0016279 | MF | protein-lysine N-methyltransferase activity | 0.001386 | 0.021051 |
| GO:0051015 | MF | actin filament binding | 0.001404 | 0.021228 |
| GO:0000030 | MF | mannosyltransferase activity | 0.001499 | 0.022293 |
| GO:0046527 | MF | glucosyltransferase activity | 0.001499 | 0.022293 |
| GO:0042813 | MF | Wnt-activated receptor activity | 0.001499 | 0.022293 |
| GO:0140034 | MF | methylation-dependent protein binding | 0.00153 | 0.022671 |
| GO:0035064 | MF | methylated histone binding | 0.00153 | 0.022671 |
| GO:0038024 | MF | cargo receptor activity | 0.001691 | 0.024649 |
| GO:0032934 | MF | sterol binding | 0.001719 | 0.024996 |
| GO:0051864 | MF | histone demethylase activity (H3-K36 specific) | 0.001955 | 0.027958 |
| GO:0004438 | MF | phosphatidylinositol-3-phosphatase activity | 0.001955 | 0.027958 |
| GO:0005227 | MF | calcium activated cation channel activity | 0.001999 | 0.028474 |
| GO:0031593 | MF | polyubiquitin modification-dependent protein binding | 0.002059 | 0.029201 |
| GO:0008195 | MF | phosphatidate phosphatase activity | 0.002074 | 0.029201 |
| GO:0030228 | MF | lipoprotein particle receptor activity | 0.002074 | 0.029201 |
| GO:1990404 | MF | protein ADP-ribosylase activity | 0.002074 | 0.029201 |
| GO:0008536 | MF | Ran GTPase binding | 0.002467 | 0.033969 |
| GO:0046983 | MF | protein dimerization activity | 0.002515 | 0.034539 |
| GO:0033218 | MF | amide binding | 0.002529 | 0.034673 |
| GO:0001217 | MF | DNA-binding transcription repressor activity | 0.002535 | 0.034733 |
| GO:0097159 | MF | organic cyclic compound binding | 0.002589 | 0.035111 |
| GO:0019207 | MF | kinase regulator activity | 0.003127 | 0.040936 |
| GO:0001540 | MF | amyloid-beta binding | 0.003146 | 0.041076 |
| GO:0017022 | MF | myosin binding | 0.003146 | 0.041076 |
| GO:0042054 | MF | histone methyltransferase activity | 0.003167 | 0.04129 |
| GO:0001227 | MF | DNA-binding transcription repressor activity, RNA polymerase II-specific | 0.003171 | 0.0413 |
| GO:0016706 | MF | 2-oxoglutarate-dependent dioxygenase activity | 0.003885 | 0.049393 |

**Table S8: Results of GO enrichment analysis for profile 15.**

| go_id | go_type | discription | p_uncorrected | p_corrected |
| --- | --- | --- | --- | --- |
| GO:0045824 | BP | negative regulation of innate immune response | 2.79E-07 | 0.001070103 |
| GO:0051250 | BP | negative regulation of lymphocyte activation | 5.99E-07 | 0.001070103 |
| GO:0032715 | BP | negative regulation of interleukin-6 production | 6.00E-07 | 0.001070103 |
| GO:0051607 | BP | defense response to virus | 6.02E-07 | 0.001070103 |
| GO:0050854 | BP | regulation of antigen receptor-mediated signaling pathway | 6.57E-07 | 0.001070103 |
| GO:0050670 | BP | regulation of lymphocyte proliferation | 7.42E-07 | 0.001070103 |
| GO:0001818 | BP | negative regulation of cytokine production | 8.29E-07 | 0.001070103 |
| GO:0032675 | BP | regulation of interleukin-6 production | 9.50E-07 | 0.001070103 |
| GO:0032944 | BP | regulation of mononuclear cell proliferation | 9.99E-07 | 0.001070103 |
| GO:0070663 | BP | regulation of leukocyte proliferation | 1.03E-06 | 0.001070103 |
| GO:0050777 | BP | negative regulation of immune response | 1.06E-06 | 0.001070103 |
| GO:0050855 | BP | regulation of B cell receptor signaling pathway | 1.23E-06 | 0.001070103 |
| GO:0045646 | BP | regulation of erythrocyte differentiation | 1.24E-06 | 0.001070103 |
| GO:0050866 | BP | negative regulation of cell activation | 1.36E-06 | 0.001070103 |
| GO:0050727 | BP | regulation of inflammatory response | 1.45E-06 | 0.001070103 |
| GO:0002695 | BP | negative regulation of leukocyte activation | 1.50E-06 | 0.001070103 |
| GO:0009615 | BP | response to virus | 1.67E-06 | 0.001070103 |
| GO:0045637 | BP | regulation of myeloid cell differentiation | 1.67E-06 | 0.001070103 |
| GO:0050864 | BP | regulation of B cell activation | 1.77E-06 | 0.001070103 |
| GO:0050776 | BP | regulation of immune response | 1.83E-06 | 0.001070103 |
| GO:0070664 | BP | negative regulation of leukocyte proliferation | 1.87E-06 | 0.001070103 |
| GO:0031348 | BP | negative regulation of defense response | 1.89E-06 | 0.001070103 |
| GO:0050778 | BP | positive regulation of immune response | 2.01E-06 | 0.001070103 |
| GO:0002697 | BP | regulation of immune effector process | 2.12E-06 | 0.001070103 |
| GO:0032102 | BP | negative regulation of response to external stimulus | 2.15E-06 | 0.001070103 |
| GO:0050865 | BP | regulation of cell activation | 2.16E-06 | 0.001070103 |
| GO:0002683 | BP | negative regulation of immune system process | 2.19E-06 | 0.001070103 |
| GO:0001817 | BP | regulation of cytokine production | 2.28E-06 | 0.001070103 |
| GO:0045321 | BP | leukocyte activation | 2.31E-06 | 0.001070103 |
| GO:0051249 | BP | regulation of lymphocyte activation | 2.45E-06 | 0.001070103 |
| GO:0046649 | BP | lymphocyte activation | 2.47E-06 | 0.001070103 |
| GO:0002252 | BP | immune effector process | 2.56E-06 | 0.001070103 |
| GO:0032101 | BP | regulation of response to external stimulus | 2.56E-06 | 0.001070103 |
| GO:0002694 | BP | regulation of leukocyte activation | 2.68E-06 | 0.001070103 |
| GO:0001819 | BP | positive regulation of cytokine production | 2.76E-06 | 0.001070103 |
| GO:0051707 | BP | response to other organism | 2.79E-06 | 0.001070103 |
| GO:0098542 | BP | defense response to other organism | 2.87E-06 | 0.001070103 |
| GO:0002684 | BP | positive regulation of immune system process | 2.91E-06 | 0.001070103 |
| GO:0006955 | BP | immune response | 2.95E-06 | 0.001070103 |
| GO:0031347 | BP | regulation of defense response | 2.95E-06 | 0.001070103 |
| GO:0001775 | BP | cell activation | 3.05E-06 | 0.001070103 |
| GO:0006952 | BP | defense response | 3.07E-06 | 0.001070103 |
| GO:0009607 | BP | response to biotic stimulus | 3.12E-06 | 0.001070103 |
| GO:0034097 | BP | response to cytokine | 3.19E-06 | 0.001070103 |
| GO:0009605 | BP | response to external stimulus | 3.26E-06 | 0.001070103 |
| GO:0043207 | BP | response to external biotic stimulus | 3.31E-06 | 0.001070103 |
| GO:0044419 | BP | interspecies interaction between organisms | 3.33E-06 | 0.001070103 |
| GO:0002274 | BP | myeloid leukocyte activation | 3.35E-06 | 0.001070103 |
| GO:0042127 | BP | regulation of cell population proliferation | 3.48E-06 | 0.001070103 |
| GO:1903706 | BP | regulation of hemopoiesis | 3.57E-06 | 0.001070103 |
| GO:0002682 | BP | regulation of immune system process | 3.64E-06 | 0.001070103 |
| GO:0035556 | BP | intracellular signal transduction | 3.83E-06 | 0.001070103 |
| GO:0080134 | BP | regulation of response to stress | 3.85E-06 | 0.001070103 |
| GO:0051240 | BP | positive regulation of multicellular organismal process | 3.88E-06 | 0.001070103 |
| GO:0032655 | BP | regulation of interleukin-12 production | 3.93E-06 | 0.001070103 |
| GO:0002376 | BP | immune system process | 3.94E-06 | 0.001070103 |
| GO:0007166 | BP | cell surface receptor signaling pathway | 4.18E-06 | 0.001070103 |
| GO:0032680 | BP | regulation of tumor necrosis factor production | 4.23E-06 | 0.001070103 |
| GO:0050672 | BP | negative regulation of lymphocyte proliferation | 4.26E-06 | 0.001070103 |
| GO:0006935 | BP | chemotaxis | 4.35E-06 | 0.001070103 |
| GO:0045937 | BP | positive regulation of phosphate metabolic process | 4.47E-06 | 0.001070103 |
| GO:0010562 | BP | positive regulation of phosphorus metabolic process | 4.47E-06 | 0.001070103 |
| GO:0002521 | BP | leukocyte differentiation | 4.49E-06 | 0.001070103 |
| GO:2000026 | BP | regulation of multicellular organismal development | 4.55E-06 | 0.001070103 |
| GO:0042330 | BP | taxis | 4.76E-06 | 0.001070103 |
| GO:0030098 | BP | lymphocyte differentiation | 4.77E-06 | 0.001070103 |
| GO:0051247 | BP | positive regulation of protein metabolic process | 4.80E-06 | 0.001070103 |
| GO:0009892 | BP | negative regulation of metabolic process | 4.86E-06 | 0.001070103 |
| GO:1903506 | BP | regulation of nucleic acid-templated transcription | 5.00E-06 | 0.001070103 |
| GO:0010629 | BP | negative regulation of gene expression | 5.05E-06 | 0.001070103 |
| GO:0010605 | BP | negative regulation of macromolecule metabolic process | 5.06E-06 | 0.001070103 |
| GO:2001141 | BP | regulation of RNA biosynthetic process | 5.07E-06 | 0.001070103 |
| GO:0048584 | BP | positive regulation of response to stimulus | 5.09E-06 | 0.001070103 |
| GO:0006950 | BP | response to stress | 5.12E-06 | 0.001070103 |
| GO:0050793 | BP | regulation of developmental process | 5.13E-06 | 0.001070103 |
| GO:0051252 | BP | regulation of RNA metabolic process | 5.13E-06 | 0.001070103 |
| GO:0048585 | BP | negative regulation of response to stimulus | 5.14E-06 | 0.001070103 |
| GO:0051239 | BP | regulation of multicellular organismal process | 5.14E-06 | 0.001070103 |
| GO:0051173 | BP | positive regulation of nitrogen compound metabolic process | 5.14E-06 | 0.001070103 |
| GO:0023051 | BP | regulation of signaling | 5.27E-06 | 0.001070103 |
| GO:0009966 | BP | regulation of signal transduction | 5.36E-06 | 0.001070103 |
| GO:0032945 | BP | negative regulation of mononuclear cell proliferation | 5.36E-06 | 0.001070103 |
| GO:0009893 | BP | positive regulation of metabolic process | 5.37E-06 | 0.001070103 |
| GO:0007165 | BP | signal transduction | 5.48E-06 | 0.001070103 |
| GO:2000112 | BP | regulation of cellular macromolecule biosynthetic process | 5.51E-06 | 0.001070103 |
| GO:0031326 | BP | regulation of cellular biosynthetic process | 5.57E-06 | 0.001070103 |
| GO:0010556 | BP | regulation of macromolecule biosynthetic process | 5.68E-06 | 0.001070103 |
| GO:0009889 | BP | regulation of biosynthetic process | 5.73E-06 | 0.001070103 |
| GO:1903555 | BP | regulation of tumor necrosis factor superfamily cytokine production | 5.75E-06 | 0.001070103 |
| GO:0010604 | BP | positive regulation of macromolecule metabolic process | 5.87E-06 | 0.001070103 |
| GO:0006355 | BP | regulation of transcription, DNA-templated | 5.87E-06 | 0.001070103 |
| GO:0010646 | BP | regulation of cell communication | 5.87E-06 | 0.001070103 |
| GO:0048583 | BP | regulation of response to stimulus | 5.91E-06 | 0.001070103 |
| GO:0031325 | BP | positive regulation of cellular metabolic process | 6.00E-06 | 0.001076368 |
| GO:0019219 | BP | regulation of nucleobase-containing compound metabolic process | 6.15E-06 | 0.00108778 |
| GO:1902531 | BP | regulation of intracellular signal transduction | 6.17E-06 | 0.00108778 |
| GO:0048518 | BP | positive regulation of biological process | 6.44E-06 | 0.001124552 |
| GO:0048522 | BP | positive regulation of cellular process | 6.56E-06 | 0.001134956 |
| GO:0050858 | BP | negative regulation of antigen receptor-mediated signaling pathway | 6.86E-06 | 0.001150233 |
| GO:0051171 | BP | regulation of nitrogen compound metabolic process | 6.95E-06 | 0.001150233 |
| GO:0002263 | BP | cell activation involved in immune response | 6.95E-06 | 0.001150233 |
| GO:0050896 | BP | response to stimulus | 7.10E-06 | 0.001150233 |
| GO:0019222 | BP | regulation of metabolic process | 7.11E-06 | 0.001150233 |
| GO:0002831 | BP | regulation of response to biotic stimulus | 7.16E-06 | 0.001150233 |
| GO:0045648 | BP | positive regulation of erythrocyte differentiation | 7.19E-06 | 0.001150233 |
| GO:0031323 | BP | regulation of cellular metabolic process | 7.21E-06 | 0.001150233 |
| GO:0010468 | BP | regulation of gene expression | 7.22E-06 | 0.001150233 |
| GO:0048523 | BP | negative regulation of cellular process | 7.29E-06 | 0.001150828 |
| GO:0080090 | BP | regulation of primary metabolic process | 7.38E-06 | 0.001150828 |
| GO:0051094 | BP | positive regulation of developmental process | 7.44E-06 | 0.001150828 |
| GO:0045595 | BP | regulation of cell differentiation | 7.51E-06 | 0.001150828 |
| GO:0048519 | BP | negative regulation of biological process | 7.51E-06 | 0.001150828 |
| GO:0008150 | BP | biological_process | 7.90E-06 | 0.00119445 |
| GO:0065007 | BP | biological regulation | 7.92E-06 | 0.00119445 |
| GO:0060255 | BP | regulation of macromolecule metabolic process | 8.00E-06 | 0.00119445 |
| GO:0009967 | BP | positive regulation of signal transduction | 8.10E-06 | 0.00119445 |
| GO:0050789 | BP | regulation of biological process | 8.10E-06 | 0.00119445 |
| GO:0050794 | BP | regulation of cellular process | 8.59E-06 | 0.001248067 |
| GO:0051241 | BP | negative regulation of multicellular organismal process | 8.72E-06 | 0.001253585 |
| GO:0010647 | BP | positive regulation of cell communication | 8.75E-06 | 0.001253585 |
| GO:0023056 | BP | positive regulation of signaling | 9.06E-06 | 0.00127959 |
| GO:0019220 | BP | regulation of phosphate metabolic process | 9.31E-06 | 0.001305277 |
| GO:0051174 | BP | regulation of phosphorus metabolic process | 9.39E-06 | 0.001307785 |
| GO:0002832 | BP | negative regulation of response to biotic stimulus | 1.01E-05 | 0.001403132 |
| GO:0002699 | BP | positive regulation of immune effector process | 1.03E-05 | 0.001407254 |
| GO:0002366 | BP | leukocyte activation involved in immune response | 1.04E-05 | 0.001407254 |
| GO:0002253 | BP | activation of immune response | 1.14E-05 | 0.001533509 |
| GO:0044093 | BP | positive regulation of molecular function | 1.18E-05 | 0.001571108 |
| GO:0042327 | BP | positive regulation of phosphorylation | 1.19E-05 | 0.001572312 |
| GO:0051716 | BP | cellular response to stimulus | 1.38E-05 | 0.001790492 |
| GO:0051270 | BP | regulation of cellular component movement | 1.50E-05 | 0.001926685 |
| GO:0030334 | BP | regulation of cell migration | 1.58E-05 | 0.002015544 |
| GO:0030099 | BP | myeloid cell differentiation | 1.61E-05 | 0.002038493 |
| GO:0042325 | BP | regulation of phosphorylation | 1.63E-05 | 0.002051164 |
| GO:0045088 | BP | regulation of innate immune response | 1.76E-05 | 0.00220177 |
| GO:0008284 | BP | positive regulation of cell population proliferation | 1.89E-05 | 0.002346543 |
| GO:0051091 | BP | positive regulation of DNA-binding transcription factor activity | 1.91E-05 | 0.002346543 |
| GO:0032940 | BP | secretion by cell | 2.00E-05 | 0.002440811 |
| GO:0045639 | BP | positive regulation of myeloid cell differentiation | 2.03E-05 | 0.002459218 |
| GO:0030888 | BP | regulation of B cell proliferation | 2.13E-05 | 0.002564173 |
| GO:2000145 | BP | regulation of cell motility | 2.14E-05 | 0.002564173 |
| GO:0070887 | BP | cellular response to chemical stimulus | 2.33E-05 | 0.002756563 |
| GO:0051172 | BP | negative regulation of nitrogen compound metabolic process | 2.46E-05 | 0.002884248 |
| GO:0045577 | BP | regulation of B cell differentiation | 2.46E-05 | 0.002884248 |
| GO:0071359 | BP | cellular response to dsRNA | 2.54E-05 | 0.002954674 |
| GO:0032270 | BP | positive regulation of cellular protein metabolic process | 2.81E-05 | 0.003218808 |
| GO:0065009 | BP | regulation of molecular function | 2.92E-05 | 0.003316282 |
| GO:0051246 | BP | regulation of protein metabolic process | 2.93E-05 | 0.003316282 |
| GO:0050868 | BP | negative regulation of T cell activation | 3.05E-05 | 0.003413621 |
| GO:0002275 | BP | myeloid cell activation involved in immune response | 3.07E-05 | 0.003414193 |
| GO:0019882 | BP | antigen processing and presentation | 3.14E-05 | 0.003448827 |
| GO:0034340 | BP | response to type I interferon | 3.21E-05 | 0.003511738 |
| GO:0061756 | BP | leukocyte adhesion to vascular endothelial cell | 3.24E-05 | 0.003525598 |
| GO:0030217 | BP | T cell differentiation | 3.30E-05 | 0.003550607 |
| GO:0050856 | BP | regulation of T cell receptor signaling pathway | 3.50E-05 | 0.003728073 |
| GO:0032879 | BP | regulation of localization | 3.57E-05 | 0.003783934 |
| GO:0002703 | BP | regulation of leukocyte mediated immunity | 4.19E-05 | 0.004350848 |
| GO:0040012 | BP | regulation of locomotion | 4.35E-05 | 0.004485551 |
| GO:1903530 | BP | regulation of secretion by cell | 4.51E-05 | 0.004627929 |
| GO:0042113 | BP | B cell activation | 4.72E-05 | 0.004774695 |
| GO:0002819 | BP | regulation of adaptive immune response | 5.40E-05 | 0.005383857 |
| GO:0031324 | BP | negative regulation of cellular metabolic process | 5.41E-05 | 0.005383857 |
| GO:0002822 | BP | regulation of adaptive immune response based on somatic recombination of immune receptors built from immunoglobulin superfamily domains | 5.43E-05 | 0.005383857 |
| GO:0010628 | BP | positive regulation of gene expression | 5.52E-05 | 0.005444353 |
| GO:0071345 | BP | cellular response to cytokine stimulus | 5.96E-05 | 0.005849755 |
| GO:0042221 | BP | response to chemical | 6.38E-05 | 0.00622987 |
| GO:0036230 | BP | granulocyte activation | 6.67E-05 | 0.006451715 |
| GO:0002696 | BP | positive regulation of leukocyte activation | 7.34E-05 | 0.006991658 |
| GO:0050871 | BP | positive regulation of B cell activation | 7.58E-05 | 0.007151308 |
| GO:0050867 | BP | positive regulation of cell activation | 9.40E-05 | 0.008721008 |
| GO:0042093 | BP | T-helper cell differentiation | 9.42E-05 | 0.008721008 |
| GO:0002294 | BP | CD4-positive, alpha-beta T cell differentiation involved in immune response | 9.42E-05 | 0.008721008 |
| GO:0002292 | BP | T cell differentiation involved in immune response | 9.58E-05 | 0.008721008 |
| GO:0016064 | BP | immunoglobulin mediated immune response | 9.58E-05 | 0.008721008 |
| GO:0043367 | BP | CD4-positive, alpha-beta T cell differentiation | 9.58E-05 | 0.008721008 |
| GO:0031399 | BP | regulation of protein modification process | 9.59E-05 | 0.008721008 |
| GO:0051046 | BP | regulation of secretion | 0.000105 | 0.009490924 |
| GO:0043410 | BP | positive regulation of MAPK cascade | 0.000108 | 0.009732921 |
| GO:0050663 | BP | cytokine secretion | 0.000117 | 0.010495414 |
| GO:1903038 | BP | negative regulation of leukocyte cell-cell adhesion | 0.000123 | 0.011015497 |
| GO:0001934 | BP | positive regulation of protein phosphorylation | 0.000129 | 0.011409406 |
| GO:0140352 | BP | export from cell | 0.000129 | 0.011409406 |
| GO:0008285 | BP | negative regulation of cell population proliferation | 0.000133 | 0.011755272 |
| GO:0006898 | BP | receptor-mediated endocytosis | 0.00014 | 0.012213753 |
| GO:0035710 | BP | CD4-positive, alpha-beta T cell activation | 0.00014 | 0.012213753 |
| GO:0050707 | BP | regulation of cytokine secretion | 0.000146 | 0.01262431 |
| GO:0045071 | BP | negative regulation of viral genome replication | 0.000149 | 0.012669727 |
| GO:0002293 | BP | alpha-beta T cell differentiation involved in immune response | 0.00015 | 0.012669727 |
| GO:0150077 | BP | regulation of neuroinflammatory response | 0.00015 | 0.012669727 |
| GO:0002287 | BP | alpha-beta T cell activation involved in immune response | 0.00015 | 0.012669727 |
| GO:0050863 | BP | regulation of T cell activation | 0.000154 | 0.012953203 |
| GO:0002764 | BP | immune response-regulating signaling pathway | 0.000156 | 0.013047902 |
| GO:0031401 | BP | positive regulation of protein modification process | 0.000157 | 0.013047902 |
| GO:0062197 | BP | cellular response to chemical stress | 0.000157 | 0.013057575 |
| GO:0001932 | BP | regulation of protein phosphorylation | 0.00017 | 0.013839325 |
| GO:1901136 | BP | carbohydrate derivative catabolic process | 0.000175 | 0.014164484 |
| GO:0050708 | BP | regulation of protein secretion | 0.000187 | 0.015049977 |
| GO:0032652 | BP | regulation of interleukin-1 production | 0.000188 | 0.015073483 |
| GO:0023061 | BP | signal release | 0.000191 | 0.015280345 |
| GO:0032651 | BP | regulation of interleukin-1 beta production | 0.0002 | 0.015889124 |
| GO:0034599 | BP | cellular response to oxidative stress | 0.000207 | 0.016387446 |
| GO:0050901 | BP | leukocyte tethering or rolling | 0.000214 | 0.0169425 |
| GO:0030155 | BP | regulation of cell adhesion | 0.000222 | 0.017365526 |
| GO:1901071 | BP | glucosamine-containing compound metabolic process | 0.000229 | 0.017760257 |
| GO:0002685 | BP | regulation of leukocyte migration | 0.00023 | 0.017760257 |
| GO:0002283 | BP | neutrophil activation involved in immune response | 0.000231 | 0.017776071 |
| GO:1903901 | BP | negative regulation of viral life cycle | 0.000239 | 0.018274864 |
| GO:1903037 | BP | regulation of leukocyte cell-cell adhesion | 0.000245 | 0.018715698 |
| GO:0002883 | BP | regulation of hypersensitivity | 0.000254 | 0.019311263 |
| GO:0032268 | BP | regulation of cellular protein metabolic process | 0.00026 | 0.019663318 |
| GO:0042035 | BP | regulation of cytokine biosynthetic process | 0.000262 | 0.019765581 |
| GO:0043122 | BP | regulation of I-kappaB kinase/NF-kappaB signaling | 0.00027 | 0.020254863 |
| GO:0002768 | BP | immune response-regulating cell surface receptor signaling pathway | 0.000288 | 0.021431929 |
| GO:1903708 | BP | positive regulation of hemopoiesis | 0.000288 | 0.021431929 |
| GO:0042129 | BP | regulation of T cell proliferation | 0.000291 | 0.021539063 |
| GO:0042119 | BP | neutrophil activation | 0.000295 | 0.021740217 |
| GO:0010558 | BP | negative regulation of macromolecule biosynthetic process | 0.0003 | 0.021912747 |
| GO:0010648 | BP | negative regulation of cell communication | 0.000301 | 0.021912747 |
| GO:0006954 | BP | inflammatory response | 0.000301 | 0.021912747 |
| GO:0023057 | BP | negative regulation of signaling | 0.000311 | 0.022548638 |
| GO:0002824 | BP | positive regulation of adaptive immune response based on somatic recombination of immune receptors built from immunoglobulin superfamily domains | 0.000322 | 0.023198403 |
| GO:0051253 | BP | negative regulation of RNA metabolic process | 0.00033 | 0.023750769 |
| GO:0032674 | BP | regulation of interleukin-5 production | 0.000338 | 0.023865875 |
| GO:0061912 | BP | selective autophagy | 0.000338 | 0.023865875 |
| GO:0071310 | BP | cellular response to organic substance | 0.000344 | 0.024204067 |
| GO:0045934 | BP | negative regulation of nucleobase-containing compound metabolic process | 0.000348 | 0.024417616 |
| GO:0009143 | BP | nucleoside triphosphate catabolic process | 0.000351 | 0.024432707 |
| GO:0071360 | BP | cellular response to exogenous dsRNA | 0.000351 | 0.024432707 |
| GO:0002886 | BP | regulation of myeloid leukocyte mediated immunity | 0.000372 | 0.025623357 |
| GO:0050792 | BP | regulation of viral process | 0.000372 | 0.025623357 |
| GO:0009890 | BP | negative regulation of biosynthetic process | 0.000394 | 0.027058381 |
| GO:0031327 | BP | negative regulation of cellular biosynthetic process | 0.000408 | 0.027936867 |
| GO:0002698 | BP | negative regulation of immune effector process | 0.000412 | 0.028094003 |
| GO:0002821 | BP | positive regulation of adaptive immune response | 0.000431 | 0.029229028 |
| GO:0009968 | BP | negative regulation of signal transduction | 0.000432 | 0.029229028 |
| GO:0050728 | BP | negative regulation of inflammatory response | 0.000437 | 0.029235349 |
| GO:0009896 | BP | positive regulation of catabolic process | 0.000437 | 0.029235349 |
| GO:0046631 | BP | alpha-beta T cell activation | 0.000438 | 0.029235349 |
| GO:0007159 | BP | leukocyte cell-cell adhesion | 0.000438 | 0.029235349 |
| GO:0001805 | BP | positive regulation of type III hypersensitivity | 0.000449 | 0.029515513 |
| GO:0001803 | BP | regulation of type III hypersensitivity | 0.000449 | 0.029515513 |
| GO:0002282 | BP | microglial cell activation involved in immune response | 0.000449 | 0.029515513 |
| GO:0071357 | BP | cellular response to type I interferon | 0.000454 | 0.029515513 |
| GO:0002483 | BP | antigen processing and presentation of endogenous peptide antigen | 0.000454 | 0.029515513 |
| GO:0019885 | BP | antigen processing and presentation of endogenous peptide antigen via MHC class I | 0.000454 | 0.029515513 |
| GO:0032720 | BP | negative regulation of tumor necrosis factor production | 0.000458 | 0.029639067 |
| GO:0045892 | BP | negative regulation of transcription, DNA-templated | 0.000473 | 0.030508291 |
| GO:0007033 | BP | vacuole organization | 0.00048 | 0.030813102 |
| GO:0006897 | BP | endocytosis | 0.000493 | 0.0313079 |
| GO:0043408 | BP | regulation of MAPK cascade | 0.000494 | 0.0313079 |
| GO:0046632 | BP | alpha-beta T cell differentiation | 0.000494 | 0.0313079 |
| GO:0032508 | BP | DNA duplex unwinding | 0.000494 | 0.0313079 |
| GO:0045069 | BP | regulation of viral genome replication | 0.000496 | 0.031354353 |
| GO:0043549 | BP | regulation of kinase activity | 0.000524 | 0.033012949 |
| GO:0000122 | BP | negative regulation of transcription by RNA polymerase II | 0.000527 | 0.033124924 |
| GO:0050860 | BP | negative regulation of T cell receptor signaling pathway | 0.000547 | 0.034147946 |
| GO:0051251 | BP | positive regulation of lymphocyte activation | 0.000553 | 0.034415818 |
| GO:0006357 | BP | regulation of transcription by RNA polymerase II | 0.000558 | 0.034631198 |
| GO:1903556 | BP | negative regulation of tumor necrosis factor superfamily cytokine production | 0.000561 | 0.034711238 |
| GO:1903507 | BP | negative regulation of nucleic acid-templated transcription | 0.000566 | 0.034784246 |
| GO:1902679 | BP | negative regulation of RNA biosynthetic process | 0.000566 | 0.034784246 |
| GO:0034614 | BP | cellular response to reactive oxygen species | 0.00057 | 0.034806035 |
| GO:0048525 | BP | negative regulation of viral process | 0.00057 | 0.034806035 |
| GO:0002706 | BP | regulation of lymphocyte mediated immunity | 0.000587 | 0.035751169 |
| GO:0045619 | BP | regulation of lymphocyte differentiation | 0.000618 | 0.037310631 |
| GO:0097191 | BP | extrinsic apoptotic signaling pathway | 0.00064 | 0.038520606 |
| GO:0042110 | BP | T cell activation | 0.000652 | 0.039037007 |
| GO:0032642 | BP | regulation of chemokine production | 0.000653 | 0.039037007 |
| GO:0002474 | BP | antigen processing and presentation of peptide antigen via MHC class I | 0.000682 | 0.040392305 |
| GO:0019724 | BP | B cell mediated immunity | 0.00069 | 0.040790517 |
| GO:0050764 | BP | regulation of phagocytosis | 0.000713 | 0.041870272 |
| GO:0050679 | BP | positive regulation of epithelial cell proliferation | 0.000732 | 0.04287727 |
| GO:0050678 | BP | regulation of epithelial cell proliferation | 0.000738 | 0.043076963 |
| GO:0007162 | BP | negative regulation of cell adhesion | 0.000767 | 0.044539181 |
| GO:0045087 | BP | innate immune response | 0.000816 | 0.046582637 |
| GO:0045579 | BP | positive regulation of B cell differentiation | 0.000818 | 0.046582637 |
| GO:0002864 | BP | regulation of acute inflammatory response to antigenic stimulus | 0.000818 | 0.046582637 |
| GO:0006013 | BP | mannose metabolic process | 0.000819 | 0.046582637 |
| GO:0032703 | BP | negative regulation of interleukin-2 production | 0.000819 | 0.046582637 |
| GO:0042130 | BP | negative regulation of T cell proliferation | 0.000825 | 0.046812759 |
| GO:0051092 | BP | positive regulation of NF-kappaB transcription factor activity | 0.000838 | 0.047266079 |
| GO:0043331 | BP | response to dsRNA | 0.00084 | 0.047266079 |
| GO:1902533 | BP | positive regulation of intracellular signal transduction | 0.000888 | 0.049832645 |
| GO:0000323 | CC | lytic vacuole | 1.34E-06 | 0.001070103 |
| GO:0005764 | CC | lysosome | 1.34E-06 | 0.001070103 |
| GO:0031901 | CC | early endosome membrane | 1.48E-06 | 0.001070103 |
| GO:0005773 | CC | vacuole | 1.82E-06 | 0.001070103 |
| GO:0098805 | CC | whole membrane | 1.91E-05 | 0.002346543 |
| GO:0019898 | CC | extrinsic component of membrane | 2.95E-05 | 0.003316282 |
| GO:0010008 | CC | endosome membrane | 4.02E-05 | 0.004217659 |
| GO:0044530 | CC | supraspliceosomal complex | 0.000161 | 0.013298276 |
| GO:0097629 | CC | extrinsic component of omegasome membrane | 0.000449 | 0.029515513 |
| GO:0042612 | CC | MHC class I protein complex | 0.000454 | 0.029515513 |
| GO:0043202 | CC | lysosomal lumen | 0.000458 | 0.029639067 |
| GO:0031902 | CC | late endosome membrane | 0.000655 | 0.039037007 |
| GO:0140375 | MF | immune receptor activity | 5.63E-07 | 0.001070103 |
| GO:0043565 | MF | sequence-specific DNA binding | 3.99E-06 | 0.001070103 |
| GO:0140110 | MF | transcription regulator activity | 4.40E-06 | 0.001070103 |
| GO:0004896 | MF | cytokine receptor activity | 8.47E-06 | 0.001240203 |
| GO:0003690 | MF | double-stranded DNA binding | 8.88E-06 | 0.001263192 |
| GO:0043169 | MF | cation binding | 1.04E-05 | 0.001407254 |
| GO:1990837 | MF | sequence-specific double-stranded DNA binding | 1.15E-05 | 0.001533509 |
| GO:0046872 | MF | metal ion binding | 1.40E-05 | 0.001806896 |
| GO:0000976 | MF | transcription regulatory region sequence-specific DNA binding | 2.71E-05 | 0.003135642 |
| GO:0001067 | MF | regulatory region nucleic acid binding | 2.76E-05 | 0.003181174 |
| GO:0035091 | MF | phosphatidylinositol binding | 3.09E-05 | 0.003415417 |
| GO:0003700 | MF | DNA-binding transcription factor activity | 3.29E-05 | 0.003550607 |
| GO:0005488 | MF | binding | 3.45E-05 | 0.003688427 |
| GO:0001228 | MF | DNA-binding transcription activator activity, RNA polymerase II-specific | 3.84E-05 | 0.004050021 |
| GO:0001216 | MF | DNA-binding transcription activator activity | 4.20E-05 | 0.004350848 |
| GO:0005515 | MF | protein binding | 7.46E-05 | 0.007074783 |
| GO:0015923 | MF | mannosidase activity | 0.00014 | 0.012213753 |
| GO:0005088 | MF | Ras guanyl-nucleotide exchange factor activity | 0.000147 | 0.012646684 |
| GO:0000981 | MF | DNA-binding transcription factor activity, RNA polymerase II-specific | 0.000153 | 0.01291458 |
| GO:0016798 | MF | hydrolase activity, acting on glycosyl bonds | 0.00017 | 0.013839151 |
| GO:0032266 | MF | phosphatidylinositol-3-phosphate binding | 0.000217 | 0.017107953 |
| GO:1901981 | MF | phosphatidylinositol phosphate binding | 0.000223 | 0.01740206 |
| GO:0008134 | MF | transcription factor binding | 0.000283 | 0.021148386 |
| GO:0003677 | MF | DNA binding | 0.000302 | 0.021912747 |
| GO:0043325 | MF | phosphatidylinositol-3,4-bisphosphate binding | 0.000338 | 0.023865875 |
| GO:0070530 | MF | K63-linked polyubiquitin modification-dependent protein binding | 0.000338 | 0.023865875 |
| GO:0000977 | MF | RNA polymerase II transcription regulatory region sequence-specific DNA binding | 0.000608 | 0.036889844 |
| GO:0000987 | MF | cis-regulatory region sequence-specific DNA binding | 0.000616 | 0.037272615 |
| GO:0004559 | MF | alpha-mannosidase activity | 0.000681 | 0.040392305 |
| GO:0019955 | MF | cytokine binding | 0.000698 | 0.041142861 |
| GO:0004553 | MF | hydrolase activity, hydrolyzing O-glycosyl compounds | 0.000811 | 0.046582637 |

**Table S9: Results of GO enrichment analysis for profile 16**

| go_id | go_type | discription | p_uncorrected | p_corrected |
| --- | --- | --- | --- | --- |
| GO:1902115 | BP | regulation of organelle assembly | 1.96E-06 | 0.001039 |
| GO:0007264 | BP | small GTPase mediated signal transduction | 2.13E-06 | 0.001039 |
| GO:1902905 | BP | positive regulation of supramolecular fiber organization | 2.15E-06 | 0.001039 |
| GO:0048193 | BP | Golgi vesicle transport | 2.36E-06 | 0.001039 |
| GO:0010638 | BP | positive regulation of organelle organization | 2.44E-06 | 0.001039 |
| GO:0007265 | BP | Ras protein signal transduction | 2.61E-06 | 0.001039 |
| GO:0051493 | BP | regulation of cytoskeleton organization | 2.74E-06 | 0.001039 |
| GO:1902903 | BP | regulation of supramolecular fiber organization | 2.75E-06 | 0.001039 |
| GO:1902117 | BP | positive regulation of organelle assembly | 2.86E-06 | 0.001039 |
| GO:0007017 | BP | microtubule-based process | 2.88E-06 | 0.001039 |
| GO:0016570 | BP | histone modification | 3.07E-06 | 0.001039 |
| GO:0007167 | BP | enzyme linked receptor protein signaling pathway | 3.24E-06 | 0.001039 |
| GO:0006325 | BP | chromatin organization | 3.26E-06 | 0.001039 |
| GO:0000226 | BP | microtubule cytoskeleton organization | 3.31E-06 | 0.001039 |
| GO:0070507 | BP | regulation of microtubule cytoskeleton organization | 3.31E-06 | 0.001039 |
| GO:0022402 | BP | cell cycle process | 3.37E-06 | 0.001039 |
| GO:0016567 | BP | protein ubiquitination | 3.48E-06 | 0.001039 |
| GO:0070647 | BP | protein modification by small protein conjugation or removal | 3.63E-06 | 0.001039 |
| GO:0007010 | BP | cytoskeleton organization | 3.73E-06 | 0.001039 |
| GO:0030030 | BP | cell projection organization | 3.73E-06 | 0.001039 |
| GO:0016569 | BP | covalent chromatin modification | 3.73E-06 | 0.001039 |
| GO:0033044 | BP | regulation of chromosome organization | 3.77E-06 | 0.001039 |
| GO:0120036 | BP | plasma membrane bounded cell projection organization | 3.87E-06 | 0.001039 |
| GO:0032446 | BP | protein modification by small protein conjugation | 3.95E-06 | 0.001039 |
| GO:0110053 | BP | regulation of actin filament organization | 3.98E-06 | 0.001039 |
| GO:0018193 | BP | peptidyl-amino acid modification | 4.05E-06 | 0.001039 |
| GO:1903047 | BP | mitotic cell cycle process | 4.05E-06 | 0.001039 |
| GO:0032956 | BP | regulation of actin cytoskeleton organization | 4.05E-06 | 0.001039 |
| GO:0006974 | BP | cellular response to DNA damage stimulus | 4.06E-06 | 0.001039 |
| GO:0016192 | BP | vesicle-mediated transport | 4.27E-06 | 0.001039 |
| GO:0080135 | BP | regulation of cellular response to stress | 4.41E-06 | 0.001039 |
| GO:0051130 | BP | positive regulation of cellular component organization | 4.47E-06 | 0.001039 |
| GO:0044087 | BP | regulation of cellular component biogenesis | 4.61E-06 | 0.001039 |
| GO:0033043 | BP | regulation of organelle organization | 4.75E-06 | 0.001039 |
| GO:0032886 | BP | regulation of microtubule-based process | 4.77E-06 | 0.001039 |
| GO:0045934 | BP | negative regulation of nucleobase-containing compound metabolic process | 4.79E-06 | 0.001039 |
| GO:0035556 | BP | intracellular signal transduction | 5.07E-06 | 0.001039 |
| GO:0008104 | BP | protein localization | 5.24E-06 | 0.001039 |
| GO:0033554 | BP | cellular response to stress | 5.64E-06 | 0.001039 |
| GO:0051726 | BP | regulation of cell cycle | 5.77E-06 | 0.001039 |
| GO:0033036 | BP | macromolecule localization | 5.91E-06 | 0.001039 |
| GO:0051649 | BP | establishment of localization in cell | 6.02E-06 | 0.001039 |
| GO:0006996 | BP | organelle organization | 6.04E-06 | 0.001039 |
| GO:0009966 | BP | regulation of signal transduction | 6.14E-06 | 0.001039 |
| GO:0051641 | BP | cellular localization | 6.22E-06 | 0.001039 |
| GO:0043412 | BP | macromolecule modification | 6.29E-06 | 0.001039 |
| GO:0051128 | BP | regulation of cellular component organization | 6.30E-06 | 0.001039 |
| GO:0006464 | BP | cellular protein modification process | 6.53E-06 | 0.001039 |
| GO:0036211 | BP | protein modification process | 6.53E-06 | 0.001039 |
| GO:0032970 | BP | regulation of actin filament-based process | 6.53E-06 | 0.001039 |
| GO:0120031 | BP | plasma membrane bounded cell projection assembly | 6.66E-06 | 0.001039 |
| GO:0009653 | BP | anatomical structure morphogenesis | 6.67E-06 | 0.001039 |
| GO:0045184 | BP | establishment of protein localization | 6.80E-06 | 0.001039 |
| GO:0051716 | BP | cellular response to stimulus | 6.83E-06 | 0.001039 |
| GO:0051252 | BP | regulation of RNA metabolic process | 6.99E-06 | 0.001039 |
| GO:0006355 | BP | regulation of transcription, DNA-templated | 7.20E-06 | 0.001039 |
| GO:0023051 | BP | regulation of signaling | 7.30E-06 | 0.001039 |
| GO:0031326 | BP | regulation of cellular biosynthetic process | 7.31E-06 | 0.001039 |
| GO:2000112 | BP | regulation of cellular macromolecule biosynthetic process | 7.34E-06 | 0.001039 |
| GO:2001252 | BP | positive regulation of chromosome organization | 7.36E-06 | 0.001039 |
| GO:1903506 | BP | regulation of nucleic acid-templated transcription | 7.37E-06 | 0.001039 |
| GO:0031325 | BP | positive regulation of cellular metabolic process | 7.37E-06 | 0.001039 |
| GO:0090224 | BP | regulation of spindle organization | 7.39E-06 | 0.001039 |
| GO:2001141 | BP | regulation of RNA biosynthetic process | 7.46E-06 | 0.001039 |
| GO:0051225 | BP | spindle assembly | 7.49E-06 | 0.001039 |
| GO:0051234 | BP | establishment of localization | 7.54E-06 | 0.001039 |
| GO:0009889 | BP | regulation of biosynthetic process | 7.79E-06 | 0.001039 |
| GO:0048583 | BP | regulation of response to stimulus | 7.79E-06 | 0.001039 |
| GO:0010646 | BP | regulation of cell communication | 7.81E-06 | 0.001039 |
| GO:0010556 | BP | regulation of macromolecule biosynthetic process | 7.87E-06 | 0.001039 |
| GO:0051179 | BP | localization | 7.95E-06 | 0.001039 |
| GO:0051640 | BP | organelle localization | 7.98E-06 | 0.001039 |
| GO:0019219 | BP | regulation of nucleobase-containing compound metabolic process | 8.20E-06 | 0.001042 |
| GO:0090169 | BP | regulation of spindle assembly | 8.22E-06 | 0.001042 |
| GO:0048518 | BP | positive regulation of biological process | 8.51E-06 | 0.00106 |
| GO:0048523 | BP | negative regulation of cellular process | 8.52E-06 | 0.00106 |
| GO:0046907 | BP | intracellular transport | 8.75E-06 | 0.001076 |
| GO:0048519 | BP | negative regulation of biological process | 8.85E-06 | 0.001081 |
| GO:0032271 | BP | regulation of protein polymerization | 9.05E-06 | 0.001091 |
| GO:0060255 | BP | regulation of macromolecule metabolic process | 9.09E-06 | 0.001091 |
| GO:0080090 | BP | regulation of primary metabolic process | 9.10E-06 | 0.001091 |
| GO:0048522 | BP | positive regulation of cellular process | 9.20E-06 | 0.001091 |
| GO:0051171 | BP | regulation of nitrogen compound metabolic process | 9.30E-06 | 0.001091 |
| GO:0032502 | BP | developmental process | 9.47E-06 | 0.001091 |
| GO:0031323 | BP | regulation of cellular metabolic process | 9.51E-06 | 0.001091 |
| GO:0071840 | BP | cellular component organization or biogenesis | 9.53E-06 | 0.001091 |
| GO:0016043 | BP | cellular component organization | 9.56E-06 | 0.001091 |
| GO:0031324 | BP | negative regulation of cellular metabolic process | 9.62E-06 | 0.001091 |
| GO:0044089 | BP | positive regulation of cellular component biogenesis | 9.63E-06 | 0.001091 |
| GO:0008150 | BP | biological_process | 9.64E-06 | 0.001091 |
| GO:0030031 | BP | cell projection assembly | 1.02E-05 | 0.001116 |
| GO:0009893 | BP | positive regulation of metabolic process | 1.02E-05 | 0.001116 |
| GO:0006281 | BP | DNA repair | 1.03E-05 | 0.001116 |
| GO:0019222 | BP | regulation of metabolic process | 1.04E-05 | 0.001116 |
| GO:0002064 | BP | epithelial cell development | 1.04E-05 | 0.001116 |
| GO:0050794 | BP | regulation of cellular process | 1.05E-05 | 0.001116 |
| GO:0010604 | BP | positive regulation of macromolecule metabolic process | 1.05E-05 | 0.001116 |
| GO:0045892 | BP | negative regulation of transcription, DNA-templated | 1.05E-05 | 0.001116 |
| GO:0006508 | BP | proteolysis | 1.07E-05 | 0.001122 |
| GO:0009987 | BP | cellular process | 1.07E-05 | 0.001122 |
| GO:0050789 | BP | regulation of biological process | 1.15E-05 | 0.001151 |
| GO:0065007 | BP | biological regulation | 1.15E-05 | 0.001151 |
| GO:0097435 | BP | supramolecular fiber organization | 1.17E-05 | 0.001163 |
| GO:0051173 | BP | positive regulation of nitrogen compound metabolic process | 1.17E-05 | 0.001163 |
| GO:0007165 | BP | signal transduction | 1.30E-05 | 0.001266 |
| GO:0031399 | BP | regulation of protein modification process | 1.31E-05 | 0.001267 |
| GO:0006810 | BP | transport | 1.31E-05 | 0.001267 |
| GO:0007155 | BP | cell adhesion | 1.34E-05 | 0.001292 |
| GO:2000113 | BP | negative regulation of cellular macromolecule biosynthetic process | 1.41E-05 | 0.001341 |
| GO:0051253 | BP | negative regulation of RNA metabolic process | 1.53E-05 | 0.001429 |
| GO:0022610 | BP | biological adhesion | 1.53E-05 | 0.001429 |
| GO:0033045 | BP | regulation of sister chromatid segregation | 1.56E-05 | 0.001444 |
| GO:0010468 | BP | regulation of gene expression | 1.59E-05 | 0.001467 |
| GO:0050793 | BP | regulation of developmental process | 1.63E-05 | 0.001493 |
| GO:0015031 | BP | protein transport | 1.68E-05 | 0.001511 |
| GO:0080134 | BP | regulation of response to stress | 1.68E-05 | 0.001511 |
| GO:0051603 | BP | proteolysis involved in cellular protein catabolic process | 1.71E-05 | 0.001524 |
| GO:0032535 | BP | regulation of cellular component size | 1.73E-05 | 0.001536 |
| GO:0031327 | BP | negative regulation of cellular biosynthetic process | 1.79E-05 | 0.00158 |
| GO:0031589 | BP | cell-substrate adhesion | 1.98E-05 | 0.001722 |
| GO:0051495 | BP | positive regulation of cytoskeleton organization | 2.06E-05 | 0.001782 |
| GO:0048585 | BP | negative regulation of response to stimulus | 2.09E-05 | 0.001803 |
| GO:0032879 | BP | regulation of localization | 2.33E-05 | 0.001986 |
| GO:0042886 | BP | amide transport | 2.36E-05 | 0.001997 |
| GO:0016241 | BP | regulation of macroautophagy | 2.37E-05 | 0.001997 |
| GO:0007346 | BP | regulation of mitotic cell cycle | 2.45E-05 | 0.002061 |
| GO:0048856 | BP | anatomical structure development | 2.61E-05 | 0.00217 |
| GO:0071705 | BP | nitrogen compound transport | 2.85E-05 | 0.002367 |
| GO:0030334 | BP | regulation of cell migration | 2.89E-05 | 0.002391 |
| GO:0015833 | BP | peptide transport | 2.96E-05 | 0.002435 |
| GO:2000786 | BP | positive regulation of autophagosome assembly | 3.02E-05 | 0.002456 |
| GO:0033047 | BP | regulation of mitotic sister chromatid segregation | 3.04E-05 | 0.002463 |
| GO:0009890 | BP | negative regulation of biosynthetic process | 3.34E-05 | 0.002648 |
| GO:0007051 | BP | spindle organization | 3.42E-05 | 0.002697 |
| GO:0007169 | BP | transmembrane receptor protein tyrosine kinase signaling pathway | 3.57E-05 | 0.002798 |
| GO:0051172 | BP | negative regulation of nitrogen compound metabolic process | 3.61E-05 | 0.002816 |
| GO:0010564 | BP | regulation of cell cycle process | 3.70E-05 | 0.002865 |
| GO:1903507 | BP | negative regulation of nucleic acid-templated transcription | 4.15E-05 | 0.003143 |
| GO:1902679 | BP | negative regulation of RNA biosynthetic process | 4.15E-05 | 0.003143 |
| GO:0043087 | BP | regulation of GTPase activity | 4.15E-05 | 0.003143 |
| GO:0030029 | BP | actin filament-based process | 4.15E-05 | 0.003143 |
| GO:0060236 | BP | regulation of mitotic spindle organization | 4.85E-05 | 0.003615 |
| GO:0051984 | BP | positive regulation of chromosome segregation | 5.16E-05 | 0.003836 |
| GO:0051052 | BP | regulation of DNA metabolic process | 5.58E-05 | 0.004098 |
| GO:0001525 | BP | angiogenesis | 5.97E-05 | 0.004318 |
| GO:0022603 | BP | regulation of anatomical structure morphogenesis | 5.99E-05 | 0.004318 |
| GO:0030071 | BP | regulation of mitotic metaphase/anaphase transition | 6.69E-05 | 0.004725 |
| GO:0002762 | BP | negative regulation of myeloid leukocyte differentiation | 6.81E-05 | 0.00479 |
| GO:2000785 | BP | regulation of autophagosome assembly | 7.10E-05 | 0.004958 |
| GO:0007088 | BP | regulation of mitotic nuclear division | 7.31E-05 | 0.005087 |
| GO:0040012 | BP | regulation of locomotion | 7.40E-05 | 0.005136 |
| GO:0030832 | BP | regulation of actin filament length | 7.80E-05 | 0.005357 |
| GO:0008064 | BP | regulation of actin polymerization or depolymerization | 7.80E-05 | 0.005357 |
| GO:0007166 | BP | cell surface receptor signaling pathway | 8.00E-05 | 0.005453 |
| GO:0007015 | BP | actin filament organization | 8.08E-05 | 0.005493 |
| GO:2000145 | BP | regulation of cell motility | 8.23E-05 | 0.005499 |
| GO:1901673 | BP | regulation of mitotic spindle assembly | 8.23E-05 | 0.005499 |
| GO:0051983 | BP | regulation of chromosome segregation | 8.23E-05 | 0.005499 |
| GO:0006897 | BP | endocytosis | 8.43E-05 | 0.005614 |
| GO:0048588 | BP | developmental cell growth | 8.50E-05 | 0.005638 |
| GO:0051270 | BP | regulation of cellular component movement | 8.56E-05 | 0.005663 |
| GO:1902099 | BP | regulation of metaphase/anaphase transition of cell cycle | 8.72E-05 | 0.005747 |
| GO:0090066 | BP | regulation of anatomical structure size | 8.81E-05 | 0.005771 |
| GO:0044090 | BP | positive regulation of vacuole organization | 8.84E-05 | 0.005771 |
| GO:1905820 | BP | positive regulation of chromosome separation | 8.84E-05 | 0.005771 |
| GO:0045931 | BP | positive regulation of mitotic cell cycle | 9.31E-05 | 0.006056 |
| GO:0016482 | BP | cytosolic transport | 9.49E-05 | 0.006157 |
| GO:0010558 | BP | negative regulation of macromolecule biosynthetic process | 9.58E-05 | 0.006173 |
| GO:0070727 | BP | cellular macromolecule localization | 9.83E-05 | 0.006252 |
| GO:0051261 | BP | protein depolymerization | 0.000102 | 0.006451 |
| GO:0031570 | BP | DNA integrity checkpoint | 0.000103 | 0.006506 |
| GO:0065009 | BP | regulation of molecular function | 0.000104 | 0.006512 |
| GO:0070925 | BP | organelle assembly | 0.000111 | 0.006926 |
| GO:0071168 | BP | protein localization to chromatin | 0.000111 | 0.006937 |
| GO:0071702 | BP | organic substance transport | 0.000116 | 0.007188 |
| GO:0031344 | BP | regulation of cell projection organization | 0.000122 | 0.007569 |
| GO:0034976 | BP | response to endoplasmic reticulum stress | 0.000124 | 0.007637 |
| GO:0006892 | BP | post-Golgi vesicle-mediated transport | 0.000127 | 0.007758 |
| GO:2001021 | BP | negative regulation of response to DNA damage stimulus | 0.000127 | 0.007758 |
| GO:0006511 | BP | ubiquitin-dependent protein catabolic process | 0.000129 | 0.00784 |
| GO:0000904 | BP | cell morphogenesis involved in differentiation | 0.000132 | 0.008012 |
| GO:0034613 | BP | cellular protein localization | 0.000135 | 0.008129 |
| GO:0044093 | BP | positive regulation of molecular function | 0.000141 | 0.008351 |
| GO:0019941 | BP | modification-dependent protein catabolic process | 0.000154 | 0.008998 |
| GO:1905818 | BP | regulation of chromosome separation | 0.000154 | 0.008998 |
| GO:0051053 | BP | negative regulation of DNA metabolic process | 0.000158 | 0.009154 |
| GO:0016197 | BP | endosomal transport | 0.000158 | 0.009154 |
| GO:0010965 | BP | regulation of mitotic sister chromatid separation | 0.000172 | 0.009889 |
| GO:0051246 | BP | regulation of protein metabolic process | 0.000178 | 0.010177 |
| GO:0098751 | BP | bone cell development | 0.000179 | 0.01019 |
| GO:0032231 | BP | regulation of actin filament bundle assembly | 0.000184 | 0.01038 |
| GO:0000018 | BP | regulation of DNA recombination | 0.000185 | 0.01038 |
| GO:0120035 | BP | regulation of plasma membrane bounded cell projection organization | 0.000187 | 0.010461 |
| GO:1903827 | BP | regulation of cellular protein localization | 0.00019 | 0.010652 |
| GO:0006260 | BP | DNA replication | 0.000203 | 0.011264 |
| GO:0051496 | BP | positive regulation of stress fiber assembly | 0.000209 | 0.011538 |
| GO:0035728 | BP | response to hepatocyte growth factor | 0.000213 | 0.011705 |
| GO:1905897 | BP | regulation of response to endoplasmic reticulum stress | 0.000222 | 0.012127 |
| GO:0043632 | BP | modification-dependent macromolecule catabolic process | 0.000227 | 0.01238 |
| GO:0034502 | BP | protein localization to chromosome | 0.000228 | 0.01238 |
| GO:0006357 | BP | regulation of transcription by RNA polymerase II | 0.00023 | 0.01243 |
| GO:0008154 | BP | actin polymerization or depolymerization | 0.000233 | 0.012501 |
| GO:0030833 | BP | regulation of actin filament polymerization | 0.000234 | 0.012501 |
| GO:0016049 | BP | cell growth | 0.000234 | 0.012501 |
| GO:0045786 | BP | negative regulation of cell cycle | 0.000235 | 0.012515 |
| GO:0060627 | BP | regulation of vesicle-mediated transport | 0.000235 | 0.012515 |
| GO:2000780 | BP | negative regulation of double-strand break repair | 0.00024 | 0.012675 |
| GO:0045738 | BP | negative regulation of DNA repair | 0.00024 | 0.012675 |
| GO:0035329 | BP | hippo signaling | 0.00024 | 0.012675 |
| GO:0030203 | BP | glycosaminoglycan metabolic process | 0.000252 | 0.013262 |
| GO:0031023 | BP | microtubule organizing center organization | 0.000271 | 0.014154 |
| GO:0044088 | BP | regulation of vacuole organization | 0.000276 | 0.014358 |
| GO:0006928 | BP | movement of cell or subcellular component | 0.000279 | 0.014468 |
| GO:0097581 | BP | lamellipodium organization | 0.000298 | 0.015265 |
| GO:0045910 | BP | negative regulation of DNA recombination | 0.000301 | 0.015371 |
| GO:0043507 | BP | positive regulation of JUN kinase activity | 0.000307 | 0.015619 |
| GO:0062033 | BP | positive regulation of mitotic sister chromatid segregation | 0.000313 | 0.015766 |
| GO:0031329 | BP | regulation of cellular catabolic process | 0.000314 | 0.015766 |
| GO:0009790 | BP | embryo development | 0.000314 | 0.015766 |
| GO:0009792 | BP | embryo development ending in birth or egg hatching | 0.000314 | 0.015766 |
| GO:0006302 | BP | double-strand break repair | 0.000321 | 0.016003 |
| GO:0031110 | BP | regulation of microtubule polymerization or depolymerization | 0.000325 | 0.016127 |
| GO:0006644 | BP | phospholipid metabolic process | 0.000326 | 0.016127 |
| GO:0032268 | BP | regulation of cellular protein metabolic process | 0.00033 | 0.016239 |
| GO:0018205 | BP | peptidyl-lysine modification | 0.00033 | 0.016239 |
| GO:0006022 | BP | aminoglycan metabolic process | 0.000353 | 0.017248 |
| GO:0030036 | BP | actin cytoskeleton organization | 0.000357 | 0.017428 |
| GO:0022604 | BP | regulation of cell morphogenesis | 0.000366 | 0.017821 |
| GO:0043547 | BP | positive regulation of GTPase activity | 0.000369 | 0.017901 |
| GO:0010648 | BP | negative regulation of cell communication | 0.000374 | 0.018038 |
| GO:0030301 | BP | cholesterol transport | 0.000374 | 0.018038 |
| GO:0043542 | BP | endothelial cell migration | 0.000374 | 0.018038 |
| GO:0044782 | BP | cilium organization | 0.000376 | 0.01806 |
| GO:0016050 | BP | vesicle organization | 0.00038 | 0.018242 |
| GO:0031647 | BP | regulation of protein stability | 0.000381 | 0.018252 |
| GO:0031401 | BP | positive regulation of protein modification process | 0.000382 | 0.018252 |
| GO:0032365 | BP | intracellular lipid transport | 0.000385 | 0.018322 |
| GO:0030042 | BP | actin filament depolymerization | 0.00039 | 0.018427 |
| GO:0032229 | BP | negative regulation of synaptic transmission, GABAergic | 0.000392 | 0.018427 |
| GO:0034447 | BP | very-low-density lipoprotein particle clearance | 0.000392 | 0.018427 |
| GO:1901987 | BP | regulation of cell cycle phase transition | 0.000408 | 0.01903 |
| GO:0031346 | BP | positive regulation of cell projection organization | 0.000419 | 0.019432 |
| GO:0000902 | BP | cell morphogenesis | 0.00042 | 0.01945 |
| GO:0046856 | BP | phosphatidylinositol dephosphorylation | 0.000423 | 0.019533 |
| GO:0018279 | BP | protein N-linked glycosylation via asparagine | 0.000434 | 0.019862 |
| GO:1901021 | BP | positive regulation of calcium ion transmembrane transporter activity | 0.000434 | 0.019862 |
| GO:0043085 | BP | positive regulation of catalytic activity | 0.000434 | 0.019862 |
| GO:0032880 | BP | regulation of protein localization | 0.00044 | 0.019957 |
| GO:0046488 | BP | phosphatidylinositol metabolic process | 0.000442 | 0.019957 |
| GO:1901970 | BP | positive regulation of mitotic sister chromatid separation | 0.000444 | 0.019957 |
| GO:1902101 | BP | positive regulation of metaphase/anaphase transition of cell cycle | 0.000444 | 0.019957 |
| GO:0045842 | BP | positive regulation of mitotic metaphase/anaphase transition | 0.000444 | 0.019957 |
| GO:0060560 | BP | developmental growth involved in morphogenesis | 0.000449 | 0.020089 |
| GO:0023057 | BP | negative regulation of signaling | 0.000453 | 0.02023 |
| GO:0036503 | BP | ERAD pathway | 0.000488 | 0.021571 |
| GO:1902017 | BP | regulation of cilium assembly | 0.0005 | 0.022046 |
| GO:0032233 | BP | positive regulation of actin filament bundle assembly | 0.0005 | 0.022046 |
| GO:0016571 | BP | histone methylation | 0.000503 | 0.022099 |
| GO:1905508 | BP | protein localization to microtubule organizing center | 0.00052 | 0.022714 |
| GO:0071539 | BP | protein localization to centrosome | 0.00052 | 0.022714 |
| GO:0018209 | BP | peptidyl-serine modification | 0.00053 | 0.023078 |
| GO:0051301 | BP | cell division | 0.000533 | 0.023152 |
| GO:0023056 | BP | positive regulation of signaling | 0.000534 | 0.02317 |
| GO:2001020 | BP | regulation of response to DNA damage stimulus | 0.000538 | 0.023276 |
| GO:0033046 | BP | negative regulation of sister chromatid segregation | 0.000549 | 0.023436 |
| GO:0031113 | BP | regulation of microtubule polymerization | 0.000549 | 0.023436 |
| GO:1902850 | BP | microtubule cytoskeleton organization involved in mitosis | 0.000554 | 0.023603 |
| GO:0006650 | BP | glycerophospholipid metabolic process | 0.000556 | 0.023659 |
| GO:0043254 | BP | regulation of protein-containing complex assembly | 0.00056 | 0.023793 |
| GO:0061024 | BP | membrane organization | 0.000562 | 0.023822 |
| GO:0140056 | BP | organelle localization by membrane tethering | 0.000564 | 0.023862 |
| GO:1903510 | BP | mucopolysaccharide metabolic process | 0.000576 | 0.024263 |
| GO:0051492 | BP | regulation of stress fiber assembly | 0.000576 | 0.024263 |
| GO:0016239 | BP | positive regulation of macroautophagy | 0.000591 | 0.024758 |
| GO:0030032 | BP | lamellipodium assembly | 0.000595 | 0.02489 |
| GO:0043549 | BP | regulation of kinase activity | 0.000597 | 0.024929 |
| GO:0060341 | BP | regulation of cellular localization | 0.000603 | 0.02508 |
| GO:0018196 | BP | peptidyl-asparagine modification | 0.000618 | 0.025568 |
| GO:2000026 | BP | regulation of multicellular organismal development | 0.00062 | 0.025626 |
| GO:0051239 | BP | regulation of multicellular organismal process | 0.000626 | 0.025818 |
| GO:0007178 | BP | transmembrane receptor protein serine/threonine kinase signaling pathway | 0.00066 | 0.026998 |
| GO:2000042 | BP | negative regulation of double-strand break repair via homologous recombination | 0.000684 | 0.027781 |
| GO:0090068 | BP | positive regulation of cell cycle process | 0.000695 | 0.028082 |
| GO:0050790 | BP | regulation of catalytic activity | 0.000706 | 0.028341 |
| GO:1905269 | BP | positive regulation of chromatin organization | 0.000711 | 0.028414 |
| GO:0009968 | BP | negative regulation of signal transduction | 0.000722 | 0.028812 |
| GO:0051985 | BP | negative regulation of chromosome segregation | 0.000736 | 0.029252 |
| GO:0009888 | BP | tissue development | 0.000744 | 0.029457 |
| GO:0000077 | BP | DNA damage checkpoint | 0.000754 | 0.029713 |
| GO:0032386 | BP | regulation of intracellular transport | 0.000757 | 0.029784 |
| GO:0032273 | BP | positive regulation of protein polymerization | 0.000777 | 0.03052 |
| GO:0009894 | BP | regulation of catabolic process | 0.000788 | 0.030875 |
| GO:0034446 | BP | substrate adhesion-dependent cell spreading | 0.000805 | 0.031434 |
| GO:0001932 | BP | regulation of protein phosphorylation | 0.000816 | 0.031776 |
| GO:0034453 | BP | microtubule anchoring | 0.000828 | 0.0322 |
| GO:0042147 | BP | retrograde transport, endosome to Golgi | 0.000842 | 0.032606 |
| GO:1905048 | BP | regulation of metallopeptidase activity | 0.000856 | 0.032889 |
| GO:0035855 | BP | megakaryocyte development | 0.000856 | 0.032889 |
| GO:0006303 | BP | double-strand break repair via nonhomologous end joining | 0.000863 | 0.032889 |
| GO:0008156 | BP | negative regulation of DNA replication | 0.000892 | 0.033573 |
| GO:0006298 | BP | mismatch repair | 0.000892 | 0.033573 |
| GO:0031112 | BP | positive regulation of microtubule polymerization or depolymerization | 0.000897 | 0.033573 |
| GO:0061028 | BP | establishment of endothelial barrier | 0.000897 | 0.033573 |
| GO:1904292 | BP | regulation of ERAD pathway | 0.000897 | 0.033573 |
| GO:1903508 | BP | positive regulation of nucleic acid-templated transcription | 0.0009 | 0.033573 |
| GO:0150172 | BP | regulation of phosphatidylcholine metabolic process | 0.000904 | 0.033573 |
| GO:0017185 | BP | peptidyl-lysine hydroxylation | 0.000904 | 0.033573 |
| GO:1902680 | BP | positive regulation of RNA biosynthetic process | 0.000909 | 0.033688 |
| GO:1901992 | BP | positive regulation of mitotic cell cycle phase transition | 0.000914 | 0.033774 |
| GO:0070304 | BP | positive regulation of stress-activated protein kinase signaling cascade | 0.000923 | 0.033935 |
| GO:0010647 | BP | positive regulation of cell communication | 0.000924 | 0.033935 |
| GO:0072659 | BP | protein localization to plasma membrane | 0.000926 | 0.033961 |
| GO:0048646 | BP | anatomical structure formation involved in morphogenesis | 0.000948 | 0.034652 |
| GO:0032366 | BP | intracellular sterol transport | 0.000977 | 0.03558 |
| GO:0035729 | BP | cellular response to hepatocyte growth factor stimulus | 0.000977 | 0.03558 |
| GO:0009896 | BP | positive regulation of catabolic process | 0.001013 | 0.036789 |
| GO:0006275 | BP | regulation of DNA replication | 0.001029 | 0.037062 |
| GO:0042770 | BP | signal transduction in response to DNA damage | 0.001036 | 0.037239 |
| GO:1990778 | BP | protein localization to cell periphery | 0.00105 | 0.03765 |
| GO:0045911 | BP | positive regulation of DNA recombination | 0.001083 | 0.03871 |
| GO:0045859 | BP | regulation of protein kinase activity | 0.001108 | 0.03945 |
| GO:0015918 | BP | sterol transport | 0.001118 | 0.03967 |
| GO:0007063 | BP | regulation of sister chromatid cohesion | 0.001133 | 0.040125 |
| GO:1902531 | BP | regulation of intracellular signal transduction | 0.001161 | 0.040979 |
| GO:0009314 | BP | response to radiation | 0.001165 | 0.041011 |
| GO:0010506 | BP | regulation of autophagy | 0.001166 | 0.041011 |
| GO:0000726 | BP | non-recombinational repair | 0.001184 | 0.041491 |
| GO:0046834 | BP | lipid phosphorylation | 0.001184 | 0.041491 |
| GO:0009100 | BP | glycoprotein metabolic process | 0.001188 | 0.041574 |
| GO:0045893 | BP | positive regulation of transcription, DNA-templated | 0.001202 | 0.041951 |
| GO:0010243 | BP | response to organonitrogen compound | 0.001206 | 0.041983 |
| GO:2001032 | BP | regulation of double-strand break repair via nonhomologous end joining | 0.001263 | 0.043721 |
| GO:0106118 | BP | regulation of sterol biosynthetic process | 0.001269 | 0.043721 |
| GO:0046475 | BP | glycerophospholipid catabolic process | 0.001269 | 0.043721 |
| GO:0045540 | BP | regulation of cholesterol biosynthetic process | 0.001269 | 0.043721 |
| GO:0030111 | BP | regulation of Wnt signaling pathway | 0.001336 | 0.045856 |
| GO:0008360 | BP | regulation of cell shape | 0.001385 | 0.047287 |
| GO:0045935 | BP | positive regulation of nucleobase-containing compound metabolic process | 0.001465 | 0.04968 |
| GO:0051129 | BP | negative regulation of cellular component organization | 0.001466 | 0.04968 |
| GO:0022406 | BP | membrane docking | 0.001467 | 0.04968 |
| GO:0046330 | BP | positive regulation of JNK cascade | 0.001467 | 0.04968 |
| GO:0005814 | CC | centriole | 1.08E-06 | 0.001039 |
| GO:0005813 | CC | centrosome | 2.70E-06 | 0.001039 |
| GO:0005768 | CC | endosome | 2.74E-06 | 0.001039 |
| GO:0000139 | CC | Golgi membrane | 3.10E-06 | 0.001039 |
| GO:0005856 | CC | cytoskeleton | 3.22E-06 | 0.001039 |
| GO:0005815 | CC | microtubule organizing center | 3.30E-06 | 0.001039 |
| GO:0016604 | CC | nuclear body | 3.88E-06 | 0.001039 |
| GO:0005794 | CC | Golgi apparatus | 4.13E-06 | 0.001039 |
| GO:0031410 | CC | cytoplasmic vesicle | 4.34E-06 | 0.001039 |
| GO:1990234 | CC | transferase complex | 4.54E-06 | 0.001039 |
| GO:0097708 | CC | intracellular vesicle | 4.54E-06 | 0.001039 |
| GO:0031984 | CC | organelle subcompartment | 5.14E-06 | 0.001039 |
| GO:0005769 | CC | early endosome | 5.16E-06 | 0.001039 |
| GO:0098588 | CC | bounding membrane of organelle | 5.44E-06 | 0.001039 |
| GO:0000123 | CC | histone acetyltransferase complex | 5.89E-06 | 0.001039 |
| GO:0001726 | CC | ruffle | 5.89E-06 | 0.001039 |
| GO:0031982 | CC | vesicle | 5.99E-06 | 0.001039 |
| GO:0005654 | CC | nucleoplasm | 7.04E-06 | 0.001039 |
| GO:0015629 | CC | actin cytoskeleton | 7.25E-06 | 0.001039 |
| GO:0120103 | CC | centriolar subdistal appendage | 7.65E-06 | 0.001039 |
| GO:0005829 | CC | cytosol | 8.14E-06 | 0.001042 |
| GO:0043226 | CC | organelle | 1.12E-05 | 0.001151 |
| GO:0043227 | CC | membrane-bounded organelle | 1.19E-05 | 0.001176 |
| GO:0043229 | CC | intracellular organelle | 1.20E-05 | 0.001176 |
| GO:0031090 | CC | organelle membrane | 1.65E-05 | 0.001504 |
| GO:0005773 | CC | vacuole | 1.66E-05 | 0.001506 |
| GO:0036064 | CC | ciliary basal body | 2.24E-05 | 0.001926 |
| GO:0010008 | CC | endosome membrane | 2.55E-05 | 0.002131 |
| GO:1902493 | CC | acetyltransferase complex | 2.99E-05 | 0.002443 |
| GO:0031248 | CC | protein acetyltransferase complex | 2.99E-05 | 0.002443 |
| GO:0005765 | CC | lysosomal membrane | 3.10E-05 | 0.002487 |
| GO:0098852 | CC | lytic vacuole membrane | 3.10E-05 | 0.002487 |
| GO:0005874 | CC | microtubule | 3.43E-05 | 0.002697 |
| GO:0030496 | CC | midbody | 6.25E-05 | 0.004459 |
| GO:0071782 | CC | endoplasmic reticulum tubular network | 9.61E-05 | 0.006173 |
| GO:0055037 | CC | recycling endosome | 0.000119 | 0.007409 |
| GO:0098791 | CC | Golgi apparatus subcompartment | 0.000123 | 0.007591 |
| GO:0005819 | CC | spindle | 0.000127 | 0.007758 |
| GO:0000922 | CC | spindle pole | 0.000136 | 0.008129 |
| GO:0030027 | CC | lamellipodium | 0.000141 | 0.008351 |
| GO:0016607 | CC | nuclear speck | 0.000167 | 0.009639 |
| GO:0098687 | CC | chromosomal region | 0.000184 | 0.01038 |
| GO:0000323 | CC | lytic vacuole | 0.000193 | 0.010722 |
| GO:0005764 | CC | lysosome | 0.000193 | 0.010722 |
| GO:0051233 | CC | spindle midzone | 0.000209 | 0.011543 |
| GO:0031965 | CC | nuclear membrane | 0.000233 | 0.012501 |
| GO:1902494 | CC | catalytic complex | 0.000241 | 0.012709 |
| GO:0005774 | CC | vacuolar membrane | 0.00029 | 0.014959 |
| GO:0048471 | CC | perinuclear region of cytoplasm | 0.000427 | 0.019698 |
| GO:0099023 | CC | vesicle tethering complex | 0.000478 | 0.021217 |
| GO:0005802 | CC | trans-Golgi network | 0.000513 | 0.022484 |
| GO:0000118 | CC | histone deacetylase complex | 0.000703 | 0.028292 |
| GO:0035097 | CC | histone methyltransferase complex | 0.000711 | 0.028414 |
| GO:0000776 | CC | kinetochore | 0.00083 | 0.0322 |
| GO:0000151 | CC | ubiquitin ligase complex | 0.000844 | 0.032623 |
| GO:0071339 | CC | MLL1 complex | 0.000863 | 0.032889 |
| GO:0044665 | CC | MLL1/2 complex | 0.000863 | 0.032889 |
| GO:1990752 | CC | microtubule end | 0.000897 | 0.033573 |
| GO:0032133 | CC | chromosome passenger complex | 0.000904 | 0.033573 |
| GO:0030136 | CC | clathrin-coated vesicle | 0.001018 | 0.036804 |
| GO:0005876 | CC | spindle microtubule | 0.001118 | 0.03967 |
| GO:0030139 | CC | endocytic vesicle | 0.001145 | 0.040495 |
| GO:0017137 | MF | Rab GTPase binding | 1.46E-06 | 0.001039 |
| GO:0031267 | MF | small GTPase binding | 1.61E-06 | 0.001039 |
| GO:0140030 | MF | modification-dependent protein binding | 2.09E-06 | 0.001039 |
| GO:0060090 | MF | molecular adaptor activity | 2.13E-06 | 0.001039 |
| GO:0017016 | MF | Ras GTPase binding | 2.14E-06 | 0.001039 |
| GO:0051020 | MF | GTPase binding | 2.35E-06 | 0.001039 |
| GO:0019787 | MF | ubiquitin-like protein transferase activity | 2.70E-06 | 0.001039 |
| GO:0004842 | MF | ubiquitin-protein transferase activity | 3.18E-06 | 0.001039 |
| GO:0005096 | MF | GTPase activator activity | 3.94E-06 | 0.001039 |
| GO:0005543 | MF | phospholipid binding | 6.18E-06 | 0.001039 |
| GO:0019899 | MF | enzyme binding | 6.88E-06 | 0.001039 |
| GO:0140096 | MF | catalytic activity, acting on a protein | 7.61E-06 | 0.001039 |
| GO:0016740 | MF | transferase activity | 7.78E-06 | 0.001039 |
| GO:0008092 | MF | cytoskeletal protein binding | 7.98E-06 | 0.001039 |
| GO:0043169 | MF | cation binding | 8.57E-06 | 0.00106 |
| GO:0046872 | MF | metal ion binding | 9.22E-06 | 0.001091 |
| GO:0005515 | MF | protein binding | 1.01E-05 | 0.001116 |
| GO:0043167 | MF | ion binding | 1.08E-05 | 0.001125 |
| GO:0019904 | MF | protein domain specific binding | 1.09E-05 | 0.001125 |
| GO:0005488 | MF | binding | 1.14E-05 | 0.001151 |
| GO:0140034 | MF | methylation-dependent protein binding | 1.14E-05 | 0.001151 |
| GO:0035064 | MF | methylated histone binding | 1.14E-05 | 0.001151 |
| GO:0016301 | MF | kinase activity | 1.41E-05 | 0.001341 |
| GO:0030674 | MF | protein-macromolecule adaptor activity | 1.54E-05 | 0.00143 |
| GO:0003677 | MF | DNA binding | 1.68E-05 | 0.001511 |
| GO:0042393 | MF | histone binding | 1.74E-05 | 0.001536 |
| GO:0030695 | MF | GTPase regulator activity | 2.33E-05 | 0.001986 |
| GO:0035091 | MF | phosphatidylinositol binding | 3.27E-05 | 0.002606 |
| GO:0044877 | MF | protein-containing complex binding | 3.62E-05 | 0.002816 |
| GO:0034593 | MF | phosphatidylinositol bisphosphate phosphatase activity | 4.46E-05 | 0.003366 |
| GO:0034212 | MF | peptide N-acetyltransferase activity | 4.58E-05 | 0.003444 |
| GO:0071936 | MF | coreceptor activity involved in Wnt signaling pathway | 5.52E-05 | 0.00407 |
| GO:0140110 | MF | transcription regulator activity | 5.64E-05 | 0.004125 |
| GO:0003713 | MF | transcription coactivator activity | 5.97E-05 | 0.004318 |
| GO:0061659 | MF | ubiquitin-like protein ligase activity | 6.24E-05 | 0.004459 |
| GO:0019900 | MF | kinase binding | 6.57E-05 | 0.004673 |
| GO:0016773 | MF | phosphotransferase activity, alcohol group as acceptor | 7.00E-05 | 0.004907 |
| GO:0005158 | MF | insulin receptor binding | 8.23E-05 | 0.005499 |
| GO:0070840 | MF | dynein complex binding | 9.61E-05 | 0.006173 |
| GO:0061733 | MF | peptide-lysine-N-acetyltransferase activity | 9.78E-05 | 0.006239 |
| GO:0003779 | MF | actin binding | 0.000135 | 0.008129 |
| GO:0052744 | MF | phosphatidylinositol monophosphate phosphatase activity | 0.000168 | 0.009705 |
| GO:0052866 | MF | phosphatidylinositol phosphate phosphatase activity | 0.000179 | 0.01019 |
| GO:0060589 | MF | nucleoside-triphosphatase regulator activity | 0.000212 | 0.011665 |
| GO:0004674 | MF | protein serine/threonine kinase activity | 0.000228 | 0.01238 |
| GO:0016772 | MF | transferase activity, transferring phosphorus-containing groups | 0.000256 | 0.013396 |
| GO:0016410 | MF | N-acyltransferase activity | 0.000272 | 0.014156 |
| GO:0003712 | MF | transcription coregulator activity | 0.000287 | 0.014829 |
| GO:0036033 | MF | mediator complex binding | 0.000292 | 0.015041 |
| GO:0061630 | MF | ubiquitin protein ligase activity | 0.000296 | 0.015199 |
| GO:0003682 | MF | chromatin binding | 0.000313 | 0.015766 |
| GO:0017124 | MF | SH3 domain binding | 0.000325 | 0.016127 |
| GO:0004402 | MF | histone acetyltransferase activity | 0.00039 | 0.018427 |
| GO:0017034 | MF | Rap guanyl-nucleotide exchange factor activity | 0.000392 | 0.018427 |
| GO:0042285 | MF | xylosyltransferase activity | 0.000444 | 0.019957 |
| GO:0035252 | MF | UDP-xylosyltransferase activity | 0.000444 | 0.019957 |
| GO:0008080 | MF | N-acetyltransferase activity | 0.000449 | 0.020089 |
| GO:0005085 | MF | guanyl-nucleotide exchange factor activity | 0.000455 | 0.02025 |
| GO:0018024 | MF | histone-lysine N-methyltransferase activity | 0.000588 | 0.024691 |
| GO:0003824 | MF | catalytic activity | 0.000669 | 0.027313 |
| GO:0140297 | MF | DNA-binding transcription factor binding | 0.000736 | 0.029252 |
| GO:0019905 | MF | syntaxin binding | 0.000748 | 0.029561 |
| GO:0019902 | MF | phosphatase binding | 0.0008 | 0.031285 |
| GO:0031593 | MF | polyubiquitin modification-dependent protein binding | 0.000864 | 0.032889 |
| GO:0042054 | MF | histone methyltransferase activity | 0.000874 | 0.033155 |
| GO:0004438 | MF | phosphatidylinositol-3-phosphatase activity | 0.000904 | 0.033573 |
| GO:0004672 | MF | protein kinase activity | 0.00092 | 0.033918 |
| GO:0008234 | MF | cysteine-type peptidase activity | 0.000942 | 0.034483 |
| GO:0016278 | MF | lysine N-methyltransferase activity | 0.001018 | 0.036804 |
| GO:0016279 | MF | protein-lysine N-methyltransferase activity | 0.001018 | 0.036804 |
| GO:0019901 | MF | protein kinase binding | 0.001247 | 0.043254 |
